# Improved Mass Spectrometry–Based Methods Reveal Abundant Propionylation and Tissue-Specific Histone Propionylation Profiles

**DOI:** 10.1016/j.mcpro.2024.100799

**Published:** 2024-06-11

**Authors:** Alessandro Vai, Roberta Noberini, Chiara Ghirardi, Dieggo Rodrigues de Paula, Michele Carminati, Rani Pallavi, Nathália Araújo, Patrick Varga-Weisz, Tiziana Bonaldi

**Affiliations:** 1 Department of Experimental Oncology, European Institute of Oncology (IEO) IRCSS, Milan, Italy; 2 International Laboratory for Microbiome Host Epigenetics, Department of Genetics, Evolution, Microbiology, and Immunology, Institute of Biology, University of Campinas, Campinas, São Paulo, Brazil; 3 São Paulo Excellence Chair, Department of Genetics, Evolution, Microbiology, and Immunology, Institute of Biology, University of Campinas, Campinas, São Paulo, Brazil; 4 School of Biological Sciences, University of Essex, Colchester, UK; 5 Department of Oncology and Hematology-Oncology (DIPO), University of Milan, Milan, Italy

**Keywords:** epigenetics, histone acylations, histone butyrylation, histone post-translational modifications, histone propionylation

## Abstract

Histone posttranslational modifications (PTMs) have crucial roles in a multitude of cellular processes, and their aberrant levels have been linked with numerous diseases, including cancer. Although histone PTM investigations have focused so far on methylations and acetylations, alternative long-chain acylations emerged as new dimension, as they are linked to cellular metabolic states and affect gene expression through mechanisms distinct from those regulated by acetylation. Mass spectrometry is the most powerful, comprehensive, and unbiased method to study histone PTMs. However, typical mass spectrometry–based protocols for histone PTM analysis do not allow the identification of naturally occurring propionylation and butyrylation. Here, we present improved state-of-the-art sample preparation and analysis protocols to quantitate these classes of modifications. After testing different derivatization methods coupled to protease digestion, we profiled common histone PTMs and histone acylations in seven mouse tissues and human normal and tumor breast clinical samples, obtaining a map of propionylations and butyrylations found in different tissue contexts. A quantitative histone PTM analysis also revealed a contribution of histone acylations in discriminating different tissues, also upon perturbation with antibiotics, and breast cancer samples from the normal counterpart. Our results show that profiling only classical modifications is limiting and highlight the importance of using sample preparation methods that allow the analysis of the widest possible spectrum of histone modifications, paving the way for deeper insights into their functional significance in cellular processes and disease states.

## Highlights

• Histone acylation has emerged as an important new dimension in genome regulation.
• Propionylations and butyrylations cannot be analyzed using current digestion protocols.
• We tested alternative digestion protocols to overcome this limitation.
• Histone propionylation is a frequent and abundant modification.
• Histone acylations discriminate different mouse and human tissues.

## Graphical Abstract

**Figure:**
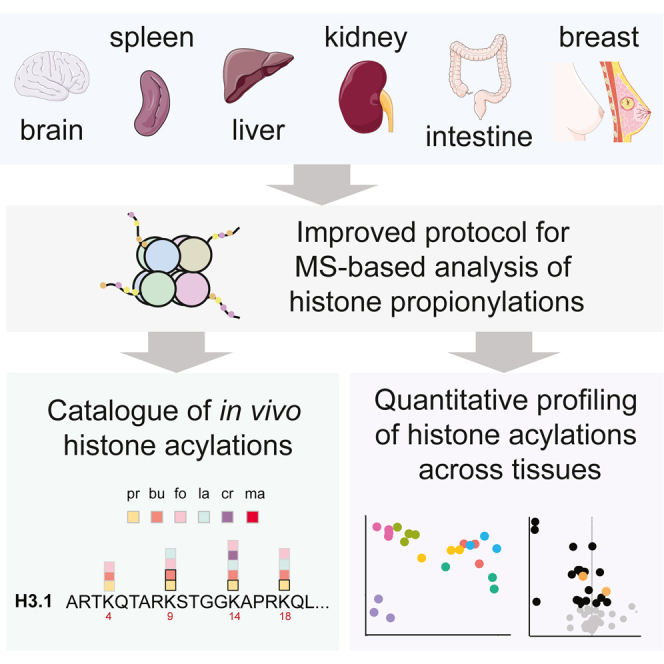

## In Brief

Optimized histone in-gel derivatization coupled to protease digestion protocols for mass spectrometry analysis of histone posttranslational modifications enabled the definition of a catalog of propionylations and butyrylations found *in vivo* in different tissue contexts. A quantitative analysis also revealed a contribution of these modifications in discriminating different mouse tissues and breast cancer samples from the normal counterpart. Our study highlights the importance of using sample preparation methods that allow the analysis of the widest possible spectrum of histone modifications.

Histone posttranslational modifications (PTMs), singly or in combination, generate a complex epigenetic code (the histone code) that defines the functional state of underlying DNA (1), directly affecting a multitude of cellular processes involving DNA, such as gene transcription, DNA replication and repair (2). Aberrant histone PTM levels have been linked with numerous diseases, including cancer (3). More than 500 different histone PTMs have been mapped over the years and new ones are continuously being discovered (4). The most common and best characterized histone PTMs comprise lysine methylations and acetylations. However, lysine residues can also be modified by other short-chain acyl groups, which comprise formylation, propionylation, butyrylation, crotonylation, β-hydroxy-butyrylation, 2-hydroxy-isobutyrylation, malonylation, succinylation, glutarylation, and lactoylation (5). Histone acetyl transferases (HATs), which catalyze the deposition of acetyl groups, have also been shown to catalyze the deposition of other acyl groups using different acyl-CoA donors that originate from metabolic intermediates in many metabolic pathways. The enzyme p300 is the HAT with the broadest acylase capability, able to catalyze the deposition of propionyl, butyryl, crotonyl, β-hydroxybutyryl, succinyl, and glutaryl groups to lysine residues (6, 7, 8, 9). However, its affinity toward acyl groups is inversely proportional to the bulkiness of the group, consequently exhibiting the highest efficiency toward acetyl-CoA (10). Additionally, acylation can occur nonenzymatically, particularly within the mitochondria, where the relatively high pH and high concentrations of acyl-CoAs favor the reaction (11). For what concerns acylation removal, class III NAD(+)-dependent deacetylases (sirtuins) have been proven to have broad-range deacylation activities (12) and class I histone deacetylases are also efficient debutyrylase and decrotonylases (13, 14).

Increasing evidence suggests that these histone acylations are closely linked to the metabolic state of a cell: a correlation between the levels of diverse acyl-CoA species and the global abundance of their respective histone acylation has been reported (10, 15, 16). In this regard, the microbiota play a pivotal role in shaping the epigenome, since it generates short-chain fatty acids (SCFAs) from the fermentation of complex carbohydrates, ensuing nutrients catabolism. In turn, SCFAs act as donors of acyl-CoA groups which can potentially react with histone residues (17). Moreover, histone acylations affect gene expression through mechanisms distinct from those regulated by acetylation (18). For instance, crotonylation is mainly enriched at active gene promoters and enhancers, associates with gene regulation and it is involved in many biological pathways that regulate diverse cellular functions, ranging from tumor immunity to telomere maintenance (16, 19, 20, 21, 22). In addition, histone propionylation and butyrylation have been shown to localize at promoter and transcription start site regions, and propionylation is able to stimulate transcription at similar levels as acetylation (23). Histone acylations play a critical role in various diseases, including cancer: for instance impairment of propionylation of histone H3 at lysine 23 has been observed in neurodevelopmental disorders and cancer (24). In addition, lysine crotonylation was shown to significantly decrease in prostate tumors, where it positively correlates with the tumor grade (25).

In the last decade, mass spectrometry (MS) has emerged as the most powerful, comprehensive, and unbiased method to study histone PTMs (26). The most frequently employed approach for histone PTMs analysis is the so-called “bottom-up” approach, where histones are digested into relatively short peptides (27). Since histones are rich in basic amino acids, a classical trypsin digestion would generate peptides that are too short for MS analysis. The usual strategy to overcome this issue consists in using the trypsin protease after chemical acylation of lysines with trypsin-blocking chemicals like deuterated acetic anhydride (28) or, more often, propionic anhydride (29). Using these approaches, trypsin will only cut at arginine residues, producing an “ArgC-like” digestion pattern, with peptides of more suitable length for MS analysis. In addition, chemical acylation, which occurs on unmodified and mono-methylated lysines, can help discriminating isobaric peptides, by determining a mass shift or by inducing a chromatographic shift that allows peptides bearing distinct arrangements of the same PTMs to elute at different retention times (28). Usually, Arg-C like digested peptides undergo a second round of derivatization at N terminus with either propionic anhydride or phenyl isocyanate (PIC). The latter strategy has proved particularly useful to improve the chromatographic retention and detectability of short and hydrophilic peptides (30). Furthermore, this protocol improves the chromatographic separation of the differentially acetylated forms of histone H3 peptide 27 to 40 and of the histone H4 tail (31).

Nonetheless, all protocols involving lysine propionylation present limitations when employed to study histone acylations, because the propionyl groups that are chemically added to the unmodified and monomethylated lysines have the same nominal mass, and are thus not distinguishable from naturally occurring propionylation and butyrylation (the latter has a mass which is the sum of a propionylation+monomethylation), respectively.

Therefore, given the growing importance attributed to histone acylations in the cross talk between epigenetic regulation of gene expression, cell metabolism, and pathological states, we sought to improve the current state-of-the-art experimental protocols for histone sample preparation prior to MS analysis in order to be able to unambiguously identify and accurately quantify this group of modifications. In particular, we set up alternative derivatization strategies, consisting in the use of deuterated anhydrides, which have different delta masses than nondeuterated counterparts, but same chemical properties, and thus allow the unambiguous detection of endogenous propionylations and butyrylations. We evaluated the different protocols and adopted the protocol that led to the highest number of identifiable and quantifiable propionylated/butyrylated peptides to profile mouse and human patient tissues. Our results provide a reference map of histone propionylations/butyrylations found *in vivo* and shows the contribution of these acylations in discriminating different tissues.

## Experimental Procedures

### Mouse Tissues

All mouse tissues with the exception of colon and cecum were obtained from 8 to 10 weeks old C57BL6 mice (Charles River Laboratories Italia), which were maintained in the animal facility (European Institute of Oncology Cogentech Facility) under specific pathogen-free conditions and fed on (VRF1 (P); Special Diet Services; #801900). Animal use was approved by the local Animal Welfare Committee and Ministerial Project no. 130/17. Mice were euthanized by exposure to CO_2_, and organs (heart, spleen, lung, liver, and kidney) were collected, washed in ice-cold 1X PBS, snap-frozen, and stored at −80 °C.

For the analysis of colon and cecum tissues, 20 to 25 weeks old C57BL/6 mice were provided by the Multidisciplinary Centre for Biological Investigation (CEMIB) and maintained in the animal facility of the Department of Genetics, Evolution, Microbiology, and Immunology at the University of Campinas. The protocols used were approved by Ethics Committee on the Use of Animals, University of Campinas (protocol number 5882-1/2021). All mice were kept in specific pathogen-free conditions and fed *ad lib*. Animals were treated with 200 μl of a mixture of antibiotics cocktail daily for 3 days by gavage. The antibiotics cocktail consisted of 5 mg/ml of gentamicin, 2.5 mg/ml of vancomycin, 5 mg/ml of ampicillin, 5 mg/ml of metronidazole, and 5 mg/ml of neomycin in distilled water. Eight hours after the treatment on the third day, the animals were euthanized by isofluorane overdose, followed by cervical dislocation. Cecum and colon from control and antibiotic- treated mice were collected and epithelial cells were extracted as described (32).

### Patient Tissues

Matched fresh-frozen breast cancer specimens and normal breast tissues surrounding the tumors were obtained from patients undergoing surgery for the removal of clinically confirmed neoplasia at the European Institute of Oncology (Milan). The samples were obtained from the Biobank for Translational Medicine Unit (B4MED) of the European Institute of Oncology. Sample collection by the Biobank, with patient consent, was approved by the Ethical Committee of the European Institute of Oncology on June 6, 2011, and the samples can be used for research purposes, including future uses, without any further approval by the Ethical Committee (33). The studies in this work abide by the Declaration of Helsinki principles. The samples were rapidly frozen in isopentane vapor using a flash-freeze apparatus. The tissues were then placed in isopentane at −120 °C for 3 min and stored in cryopreservation chambers at −80 °C. Breast cancer subtypes were defined as described (34). Samples were selected to have a tumor cellularity of at least 50%, as assessed by H&E staining. Specimens with *in situ* carcinoma areas, large necrosis areas, and massive phlogistic infiltrate were discarded.

### Histone Enrichment from Fresh and Fresh-Frozen Tissues

Approximately 20 to 70 mg of frozen tissue were thawed on ice, cut in small pieces with scissors, and homogenized in 1 ml of nuclei isolation buffer composed of PBS containing 0.1% Triton X-100 and protease inhibitors (0.5 mM PMSF, 5 μM aprotinin, 5 μM leupeptin, and 5 mM Na-butyrate) using a 1 ml Dounce homogenizer. The homogenate was filtered through a 100 μm cell strainer to remove tissue debris and pipetted several times using a 200 μl pipette tip. Nuclei were then obtained as described (35). The fresh colon and cecum of mice were opened longitudinally and gently washed in cold PBS twice to remove the feces, cut into small pieces, and incubated with 10 mM EDTA/Hank’s buffered saline solution (HBSS) for 20 min (1000 rpm, 37 °C) with a quick handshake after every 5 min. The tissue pieces were removed before centrifugation (300*g*, 5 min, 4 °C), and the pellet was washed with 0.04% bovine serum albumin (BSA)/HBSS to remove EDTA. A buffer containing 10 mM DTT/0.04% BSA/HBSS was added to the pellet under shaker agitation for 10 min to remove mucus. An equal amount of 0.04% BSA/PBS was added and the samples were filtered using a 70 μm cell strainer. Cells were centrifuged (300*g*, 5 min, 4 °C), resuspended in 1 ml of nuclei isolation buffer and pipetted vigorously through a 200 μl pipette tip several times to facilitate membrane rupture. Nuclei were isolated as described (35).

### Histone Digestion

Prior to digestion, 3 to 5 μg of histones were mixed with an equal amount of a histone super-stable isotope labeling by amino acids in cell culture (super-SILAC) mix, which was generated as previously described (36) and used as internal standard for quantification. Histones were separated on a 17% SDS-PAGE gel. Bands corresponding to the histone octamer (H3, H2A, H2B, and H4) were in-gel digested as previously described (31). Briefly, gel pieces were destained with repeated washes in 50% acetonitrile (ACN; Carlo Erba) in H_2_O and completely dehydrated in 100% ACN. Samples were then chemically acylated with propionic anhydride (Sigma-Aldrich; PRO-PIC protocol), deuterated propionic anhydride (98%, Sigma-Aldrich; D5PRO-PIC and D5PRO-D5PRO protocol), or deuterated acetic anhydride (98.5%, Thermo Fisher Scientific; D3Ac-PIC protocol). Of note, the reaction was performed without the addition of catalyzers (sodium propionate or sodium acetate), which has been added in the past (37) to facilitate the derivatization, since they represent a source of light propionyl/acetyl groups that could artifactually increase the abundance of these modifications. After shaking for 4 h at 37 °C, chemically modified gel pieces were washed in H_2_O, progressively dehydrated with increasing percentages of ACN (from 50% to 100%) and digested overnight with 100 ng/μl trypsin (Promega) in 50 mM NH_4_HCO_3_ at 37 °C. Digested peptides were extracted using 100% ACN. The peptides were concentrated to a volume below 5 μl, diluted to 9 μl (for D5PRO-D5PRO protocol) or 15 μl (for PRO-PIC, D5PRO-PIC, and D3Ac-PIC protocols) with H_2_O and N terminally derivatized with deuterated propionic anhydride (Sigma-Aldrich) or PIC (Sigma-Aldrich). The derivatization with PIC was performed by adding 1 μl of 1 M triethylammonium bicarbonate buffer (Sigma-Aldrich) and 3 μl of a freshly prepared 5% v/v PIC solution in 100% ACN, and incubating the mixture for 1.5 h at 37 °C. The samples were then acidified by adding 8 μl of 1% TFA (Life Technology). The derivatization with deuterated propionic anhydride was performed by adding 1 μl of 1 M triethylammonium bicarbonate buffer and 1 μl of a 1:100 dilution of deuterated propionic anhydride in ddH_2_O, vortexing and incubating for 2 min at room temperature. The reaction was stopped by adding 1 μl of 80 mM hydroxylamine (Sigma-Aldrich) for 20 min at room temperature and the samples were acidified with TFA, as described above. All samples were desalted on handmade C18 StageTips prior to liquid chromatography (LC)-MS/MS.

### LC-MS Analysis of Histone PTMs

Peptide mixtures were separated by reversed-phase chromatography on an EASY-nLC 1200 or a Vanquish Neo HPLC system through an EASY-Spray column (Thermo Fisher Scientific), 25 cm long (inner diameter 75 μm, PepMap C18, 2 μm particles), which was connected online to a Q Exactive PLUS or a Q Exactive HF instrument. Solvent A was 0.1% formic acid in H_2_O and solvent B was 80% ACN plus 0.1% formic acid. Three microliters of histone peptide mixture were injected in an aqueous 1% TFA solution at a flow rate of 250 nl/min and were separated with a 55-min linear gradient of 10 to 45% solvent B. The Q Exactive instruments were operated in the data-dependent acquisition mode to automatically switch between full scan MS and MS/MS acquisition. Survey full scan MS spectra (*m/z* 300–1350) were analyzed in the Orbitrap detector with a resolution of 60,000 to 70,000 at *m/z* 200. The 10 to 12 most intense peptide ions with charge state 2 to 4 were sequentially isolated to a target value for MS1 of 3 × 10^6^ and fragmented by higher-energy collision dissociation with a normalized collision energy setting of 28%. The maximum allowed ion accumulation times were 20 ms for full scans and 80 ms for MS/MS, and the target value for MS/MS was set to 1 × 10^5^. The dynamic exclusion time was set to 10 to 20 s, and the standard mass spectrometric conditions for all experiments were as follows: spray voltage of 1.7 kV, no sheath, and no auxiliary gas flow.

### Histone PTM Data Analysis

Acquired RAW data were analyzed using the MaxQuant (38, https://www.maxquant.org/) software (version 1.6.2.10) with the integrated Andromeda search engine (39). For mouse samples, the UniProt reference proteome UP000000589 (release 07/2021) filtered to retained only SwissProt histone sequences (51 entries plus common contaminants as included by default by MaxQuant) was used for peptide identification; while the UniProt reference proteome UP000005640 (release 11/21) filtered to retained only SwissProt histone peptides (64 entries), plus common contaminants, was employed for human samples. The choice of using a histone databased was dictated by the need to run multiple searches containing multiple PTMs in a reasonable amount of time. We verified that using a small database does not generate misleading assignments by comparing the results obtained with the histone database plus contaminants with the whole UniProt mouse proteome (63,367 entries) and contaminants. Only 15 spectra assigned to histones out of 3626 (0.4%) changed assignment when searched against the whole proteome, and only five of these had a better match against other proteins (Supplemental Fig. S1).

Arg10 was selected as heavy label (multiplicity of 2) in MaxQuant. Enzyme specificity was set to Arg-C. The estimated false discovery rate of all peptide identifications was set at a maximum of 1%. The mass tolerance for precursor ions was set to 20 ppm during the first search and to 4.5 ppm during the main search. The mass tolerance for fragment ions was set to 20 ppm. Two missed cleavages were allowed, and the minimum peptide length was set to four amino acids. The Match Between Runs option was enabled, with the match time window and the alignment time window of 2 min and 20 min, respectively. Variable modifications included chemical D3-acetylation/propionylation/D5-propionylation (+45.0294/+56.0262/+61.0576 Da), lysine monomethylation with D3-acetylation/propionylation/D5-propionylation (+59.0454/+70.0422/+75.0736 Da), dimethylation (+28.031 Da), trimethylation (+42.046 Da), and acetylation (+42.010 Da). Given our interest in analyzing histone acylations, lysine propionylation (+56.0262 Da), butyrylation (+70.0422 Da), crotonylation (+68.0262 Da), formylation (+27.9949 Da), malonylation (+86.0004 Da), glutarylation (+114.031694 Da), succinylation (+100.0160 Da), hydroxybutyrylation (+86.0368 Da), and lactylation (+72.021129 Da) were also included in the search as variable modifications. N-terminal D5-propionylation (+61.0576 Da) and PIC labeling (+119.0371 Da) were set as fixed modifications. To reduce the search time and the rate of false positives, which increase with increasing number of variable modifications included in the database search (40), the raw data were analyzed through multiple parallel MaxQuant jobs, searching for one different histone acylations at the time, in addition to lysine monomethylation/dimethylation/trimethylation, lysine D5-propionylation/D3-acetylation/propionylation and lysine acetylation. The results of the single searches were then merged. After merging, ambiguity for spectra that were associated to different modified sequences in different searches was resolved by retaining the peptide-to-spectrum match with the highest Andromeda score. Peptides with Andromeda score less than 40 or localization probability score less than 0.75 were removed. These cut-offs are commonly used in phopshoproteomics studies (41). Histone PTM quantitation was carried out using EpiProfile 2.0, which performs peak extraction and extracted ion chromatogram quantification in an automated manner, using *a priori* knowledge of peptides retention time (42). Acylated sites were added manually to EpiProfile, along with their retention time, as reported by MaxQuant. The program was run specifying ndebug = 1 and selecting the SILAC option. For the quantification of nonacylated sites, EpiProfile was run with default parameters (ndebug = 0). Histone modifications not quantified by EpiProfile or wrongly determined (*e.g.*, with the wrong retention time of the light and/or heavy peak) were manually validated using QualBrowser (version 4.1.31.9) (Thermo Fisher Scientific), as described (31). The % relative abundance (%RA) was estimated for the light and heavy peaks by dividing the area under the curve of each modified peptide for the sum of the areas corresponding to all the observed forms of that peptide and multiplying by 100. L/H ratios were finally calculated by dividing the light and heavy %RA values for each peptide. Because the sum of all the modified and unmodified forms of a certain peptide should be constant in all the samples, using ratios of RAs corrects for different amounts of starting material. The list of identified peptides is reported in Supplemental Tables S2 and S3, while the log2 L/H ratios for the quantified peptides for all the samples analyzed are reported in Supplemental Table S4.

### Visualization

Data display was carried out using GraphPad Prism 9.3.1 (GraphPad, https://www.graphpad.com/features), the R package ggplot2 3.4.2 (43), and the R package ComplexHeatmap 2.16.0 (44). The UpSet plot was produced with the R package ComplexUpset 1.3.5 (45). Annotation of spectra was produced with a custom python script which relies on the spectrum_utils package 0.4.2 (46, 47). Data were visualized as log2 of normalized L/H ratios using a heatmap display. The graphical abstract was generated with the aid of Smart Servier images (https://smart.servier.com/).

**Fig. 1:**
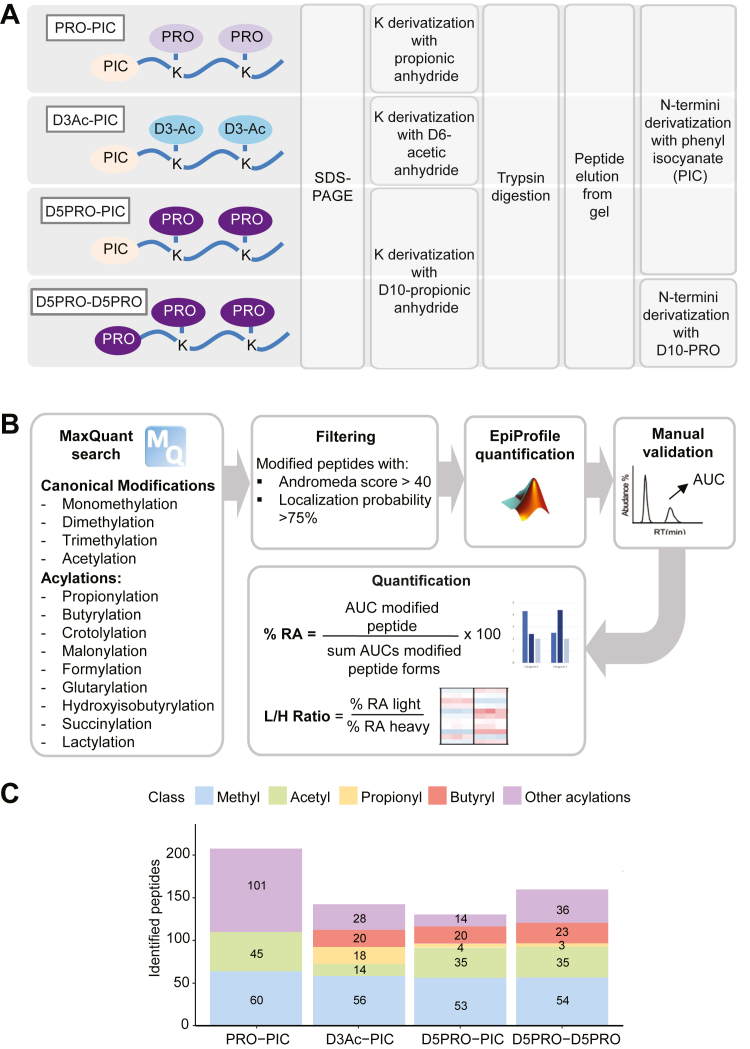
**Comparison of in-gel derivatization methods for MS-based analysis of histone acylations.** Schematic representation of the in-gel derivatization protocols (*A*) and the data analysis workflow (*B*) used in this study. *C*, *bar charts* depicting the number of differentially modified peptides identified using the different histone digestion protocols. The methyl-category contains monomethylation/dimethylation/trimethylation. Other acylations are as follow: formylation, crotonylation, succinylation, malonylation, hydroxyisobutyrylation,glutarylation, and lactylation. MS, mass spectrometry.

**Fig. 2:**
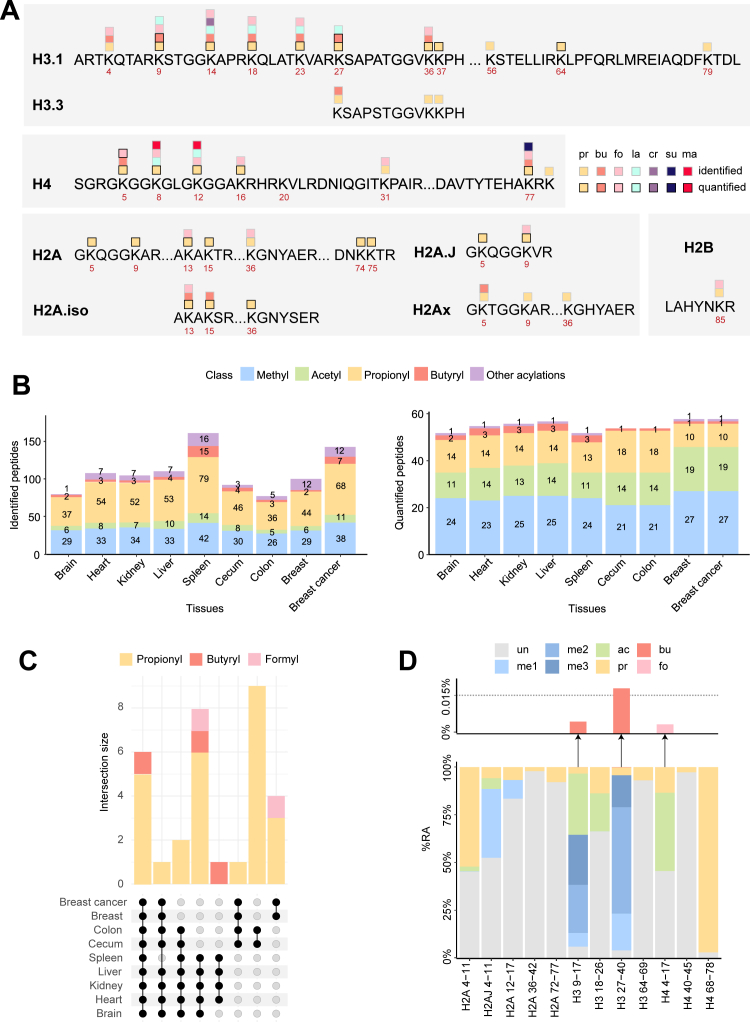
**Propionylations and butyrylation profiling of mouse and human tissues.***A*, catalog of acylated sites identified and/or quantified from mouse and human tissues using the D3Ac-PIC protocol. *B*, differentially modified peptides identified (*left*) and quantified (*right*) in different mouse (brain, heart, kidney, liver, spleen, cecum, and colon) and human (breast and breast cancer) tissues. The methyl category contains monomethylation/dimethylation/trimethylation. Other acylations are as follow: formylation (fo), crotonylation (cr), succinylation (su), malonylation (ma), and lactylation (la). For acetylations and methylations, peptides present in EpiProfile were quantified, even if the corresponding MS/MS spectrum was not identified. *C*, upset plot showing the intersection of differentially acylated peptides in different mouse and human tissues. *D*, *bar chart* showing the modification % relative abundance (%RA) for each peptide. Averages of all the tissue analyzed are displayed. For peptides modified with different modifications, the area was summed to the area of each single modification: for example, the area of a peptide carrying simultaneously a methyl group and an acetyl group was summed to both the area of the methylation and the area of acetylation. To limit the influence of the detection efficiency on %RA estimates, we computed scaling factors to have a total area of 1 × 10^9^ for all the peptide groups and then computed %RAs. D3Ac, deuterated acetyl.

### Experimental Design and Statistical Rationale

Statistical testing was performed using GraphPad Prism and the R package limma 3.56.2 (48). L/H ratios were normalized on the average value across the samples or on the average across untreated samples. Values above third quantile +1.5∗interquantile range or below first quantile − 1.5∗interquantile range were considered outliers and were discarded from the analysis. Changes in modified peptides between two groups were evaluated by two sample Student *t* test, with *p* value <0.05. Linear discriminant analysis effect size (LEfSe) was performed using the lefse bioconda package 1.1.2 with default parameters and setting the threshold of linear discriminant analysis score to 0. For the analyses that do not tolerate missing values (principal component analysis [PCA] and LEfse), these were replaced with the mean for the particular peptide across conditions. To assess differences among breast normal tissue, luminal A (LuA) and triple negative (TN) samples, the limma duplicateCorrelation function was used to account for the presence of matched and nonmatched samples. All experiments were carried out in biological replicates: three replicates in the protocol comparison experiment, three replicates for mouse pan tissues experiment, four replicates for untreated cecum and colon and five replicates for antibiotic-treated cecum and colon, six normal breast tissues, four LuA breast cancer samples (all matched), five TN breast cancer tissues (two matched and three nonmatched). The detailed composition of each analyzed experiment is described in Supplemental Table S1.

## Results

### Comparison of Novel In-Gel Histone Derivatization Protocols Suitable for Histone Acylation Analysis

Our reference protocol for in-gel derivatization coupled to protease digestion of histones (PRO-PIC protocol, (31)) involves a first round of derivatization of lysines with propionic anhydride and a second round of derivatization of the peptide N termini, which is carried out after trypsin digestion, with PIC. This protocol was optimized to be performed in-gel, since SDS-PAGE separates histones from other proteins and removes the detergents and contaminants interfering with the MS analysis often present in histone preparations (especially those from clinical samples) (49). We previously found that the PRO-PIC strategy performs better in terms of number of quantifiable peptides compared with related approaches, including the widely used PRO-PRO method, where both lysines and N termini of tryptic peptides are derivatized with propionic anhydride (29). To further improve the PRO-PIC protocol and overcome the lack of detection of endogenous lysine propionylation and butyrylation linked with the use of propionic anhydride, we replaced this reagent with two alternative trypsin-blocking chemicals: deuterated propionic anhidride in the D5PRO-PIC protocol and deuterated acetic anhydride in the D3Ac-PIC protocol (Fig. 1*A*). The rationale for using these deuterated compounds is that they are chemically and chromatographically identical to their unlabeled counterparts, but distinguishable by, respectively, a 5 and 3 Da mass difference. We also included in the comparison the D5PRO-D5PRO protocol, which has already been adopted to study histone acylations (10) and consists in the use of D10-propionic anhydride to derivatize both lysines and peptide N termini. First, we verified the lysine derivatization efficiency with the deuterated reagents, which was higher than 99% with both D3Ac and D5PRO. By performing the protocols on naked recombinant histone H3.1, we also showed that lysine derivatization does not introduce artifactual light propionylation during sample preparation (Supplemental Fig. S2*A* and *B*).

We compared the performance of the three derivatization methods applied to 5 μg of histone octamer obtained from fresh-frozen mouse spleen. To limit the search space, we searched for one different acylation at a time, in addition to classical histone PTMs (lysine monomethylation/dimethylation/trimethylation and acetylation). We then merged the results of each search, keeping the best identification according to the Andromeda score when a spectrum was interpreted differently (*i.e.*, same spectrum assigned to different modified sequences) across the various searches (Fig. 1*B*). All the protocols involving deuterated reagents allowed the identification of 20 to 24 butyrylations, with the D3Ac-PIC protocol showing the highest number of identified propionylated peptides (Fig. 1*C*, Supplemental Tables S2, and S3), which is accompanied by a lower number of acetylations. This is possibly a consequence of the “isobarization effect” of D3-acetylation, which we have previously reported (28). The addition of deuterated acetyl groups to unmodified lysines causes the isobarization of all the species bearing multiple natural acetylations, which become indistinguishable in the chromatographic separation. Oppositely, the protocols involving lysine derivatization with propionic anhydride are ideal to distinguish differentially acetylated histone peptides (31), but likely cause a similar isobarization effect for propionylations, which could explain the lack of identifications of naturally occurring propionylations in the D5PRO-PIC and D5PRO-D5PRO protocols. Interestingly, a higher number of other acylations (including formylation, crotonylation, succinylation, malonylation, hydroxyisobutyrylation, glutarylation, and lactylation) were identified in the classical PRO-PIC protocol (Fig. 1*C* and Supplemental Fig. S3*A*). Because the standard PRO-PIC approach appears to be the most appropriate protocol to detect these additional acylations, and the D3Ac-PIC is the most suitable to study propionylations and butyrylations, the two methods can be considered complementary to obtain a comprehensive profiling of common histone PTMs and histone acylations. For the purpose of this study, which is particularly focused on propionylations and butyrylations, we chose the D3Ac-PIC protocol to investigate propionylations and butyrylations in different contexts.

### Propionylation/Butyrylation Profiling of a Collection of Mouse and Human Tissues

With the goal to obtain a comprehensive catalog of propionylations/butyrylations present in vivo, we employed the D3Ac-PIC protocol to profile histone PTMs in a diverse set of samples comprising several different mouse tissues (brain, cecum, colon, heart, kidney, liver, and spleen), as well as in normal and cancerous human breast tissues (Fig. 2*A*, Supplemental Tables S2, and S3). In total, we identified 107 propionylated peptides, 18 butyrylated peptides, and 32 containing other acylations (Fig. 2*B*, left panel, Supplemental Tables S2, and S3). To assess quantitative differences among samples, we then performed peptide quantification (Fig. 2*B*, right panel). For the quantification to be accurate and robust, we relied on the use of a super-SILAC standard, which consists of a mix of heavy isotope-labeled histones purified from four different cancer cell lines, which is added to the samples prior to histone peptides gel separation and digestion (36). Acetylations and methylations were also quantified in a subset of tissues (brain, heart, kidney, liver, and spleen) processed with our reference PRO-PIC protocol, to verify that the two protocols provide the same results for classical histone PTMs (Supplemental Fig. S3*B*). We considered an acylated peptide quantifiable if: (1) it was identified in at least two replicates; (2) it was also identified in the super-SILAC spike-in used as internal standard; (3) it corresponded to a clearly resolved chromatographic peak. After applying the quantification filters, less than 20% of the acylated peptides identified was quantifiable (Fig. 2*B*, right panel), including 27 propionylated peptides, three butyrylated peptides, and two formylated peptides. Of these, six were common to all the tissues analyzed (containing propionylations on H3 9–17 and 27–40 peptides and a butyrylation on H3K9), while approximately half were found only in two tissues (Fig. 2*C*).

Importantly, we found that propionylations are not only present and detectable on peptides, but are also frequently occurring and fairly abundant. Even though RAs estimated by MS are influenced by peptide detection efficiency in bottom-up MS (50), they still provide a rough estimation of the amount of a certain modification. We observed that propionylation accounts for 2% to 52% of histone peptides (11% on average), with the propionylated version of peptide H4 68 to 78 reaching 97% (Fig. 2*D*), further indicating the potential biological impact of this PTM on genome function.

### Pan-Tissue Propionylation/Butyrylation Profiling Reveales Diversity in Mouse-Tissue Epigenetic Landscapes

The level of histone PTMs is influenced by the availability of metabolites, such as acyl-CoA species, in the cell (51). HATs catalyze histone acylation using different acyl-CoA groups, which are intermediates in many metabolic pathways. Therefore, we set to investigate quantitively histone acylation levels in the various mouse tissues analyzed, where we expected to detect changes linked to the different availability of acyl-CoA species, reflecting tissue specific metabolic profiles.

**Fig. 3:**
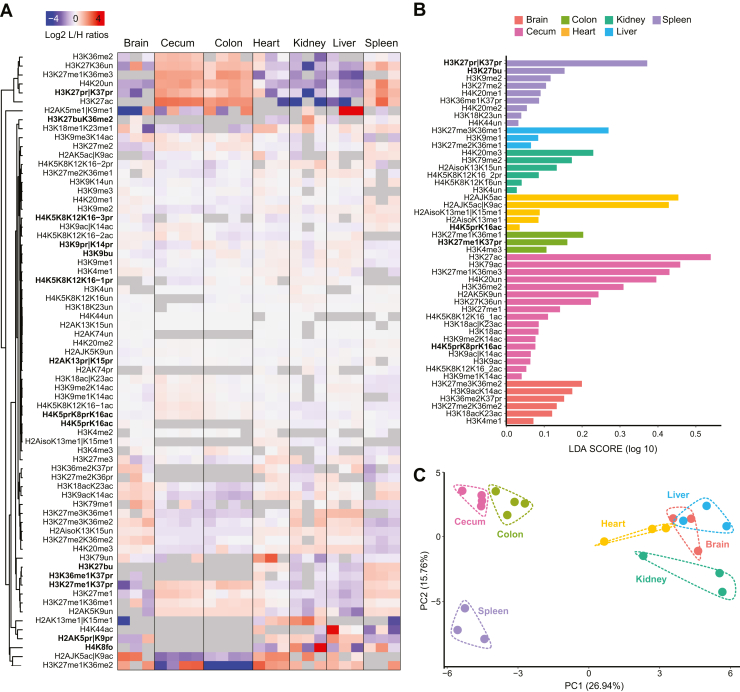
**A MS-based histone PTM map of mouse tissues.***A*, heatmap display of the log2 L/H ratios (light: mouse tissues, heavy: spike-in standard) of differentially modified histone peptides. Each row was normalized on the row mean computed across all tissues. Rows clustering was performed with Euclidean distance and complete linkage. The *gray color* represents missing values. The iso suffix means that different isoforms of the canonical histone share the same peptide sequence. *B*, *bar chart* displaying the effect size (LDA score) of the significant modified peptides in determining tissue identity according to LEfSE analysis. *C*, PCA of histone PTMs data of different mouse tissues. The data were scaled to have zero mean and unitary variance. Un, unmodified; me1, monomethylation; me2, dimethylation; me3, trimethylation; ac, acetylation; pr, propionylation; bu, butyrylation; fo, formylation. “|”= one residue or the other. LEfSe, linear discriminant analysis effect size; MS, mass spectrometry; PCA, principal component analysis; PTM, posttranslational modification.

In total, we quantified 42 differentially acetylated/methylated peptides and 18 acylated peptides (Supplemental Tables S2, and S3). Globally, there are major differences among tissues, involving both classic PTMs and newly annotated acylations (Fig. 3*A*). Some modifications are consistently identified in few tissues, with no (or sporadic) quantification in other tissues, such as H3K27bu, which is found at higher levels in spleen and heart, at lower levels in liver and kidney, and is undetectable in the other tissues. The LEfSe highlights seven propionylated/butyrylated peptides among the features most strongly discriminating the differences among tissues (Fig. 3*B*). Furthermore, spleen displayed H3K27pr|K37pr and H3K27bu as the top two most important peptides in defining tissue identity, reinforcing the idea that this class of modifications is important in regulating biological processes and determining tissue specificities. The PCA separates the different tissues based on their histone modification profiles, with spleen, cecum, and colon having the most divergent epigenetic profiles (Fig. 3*C*).

**Fig. 4:**
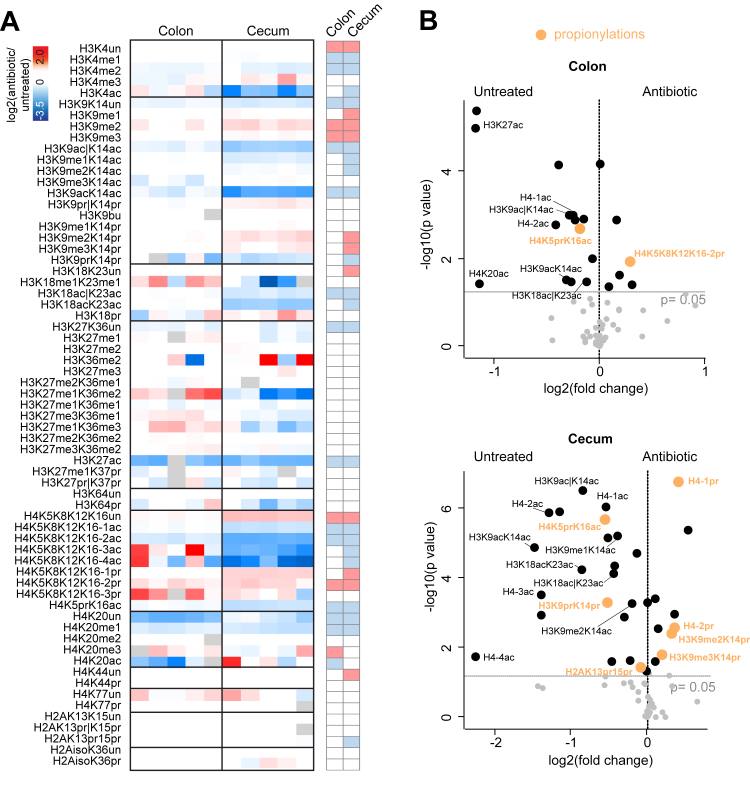
**Quantitative profiling of histone acylations levels in mouse cecum and colon tissues upon antibiotic treatment.***A*, heatmap display of the log2 L/H ratios calculated for the indicated histone PTMs for cecum and colon, normalized on the average of the untreated mice. L: light (cecum or colon), H: heavy (spike-in standard). The *gray color* indicates peptides that were not quantified. The *right panel* highlights significant changes (*p* < 0.05 by Student’s *t* test) for the comparison between antibiotic-treated and untreated mice. *B*, volcano plot showing significantly changing histone PTMs upon antibiotic treatment in cecum (*top*) and colon (*bottom*). Significant propionylations and acetylations are labeled. “|”= one residue or the other. PTM, posttranslational modification.

To understand dynamic changes in histone acylations, we also analyzed histone PTMs in cecum and colon mouse tissues after treatment with an antibiotic-cocktail, which we showed efficiently depletes the gut microbiota and leads to drastic changes in microbial derived SCFAs (13). The rationale is that these tissues are particularly rich in acyl-CoAs that directly derive from the microbiota catabolic activity, which involves breaking down complex carbohydrates such as from soluble plant fibers into SCFAs. Therefore, antibiotic treatment should decrease the formation of acyl-CoAs and, consequently, the level of histone acylations, as reported in (52, 53, 54). Antibiotic treatment caused remarkable and mostly concordant changes in the two tissues, with more marked alterations in cecum (Fig. 4*A*). According with previous findings (13), acetylations on most histone H3 and H4 lysines were significantly reduced after antibiotic treatment (Fig. 4, *A* and *B*). H2AK13prK15pr and H3K9prK14pr also decreased after antibiotic treatment in cecum, while the monopropionylated and dipropionylated histone H4 tail and H3K14pr alone increased after treatment (Fig. 4*B*).

### Histone Acylation Levels Help Discriminate Normal from Breast Cancer Patients

After the landmark discoveries of the loss of H4K16ac and of H4K20me3 in cancer (55), and the prognostic value of histone PTMs in various types of cancers (56, 57), a growing interest in studying histone PTMs in cancer clinical samples has emerged during the last years. Given the implication of histone acylations in several metabolic processes altered in cancer, we applied our novel protocol to a small set of breast cancer samples belonging to the LuA and TN subtypes, which are the most divergent breast cancer subtypes (34), along with their normal counterpart, where we previously detected changes in classical epigenetic marks (34).

**Fig. 5:**
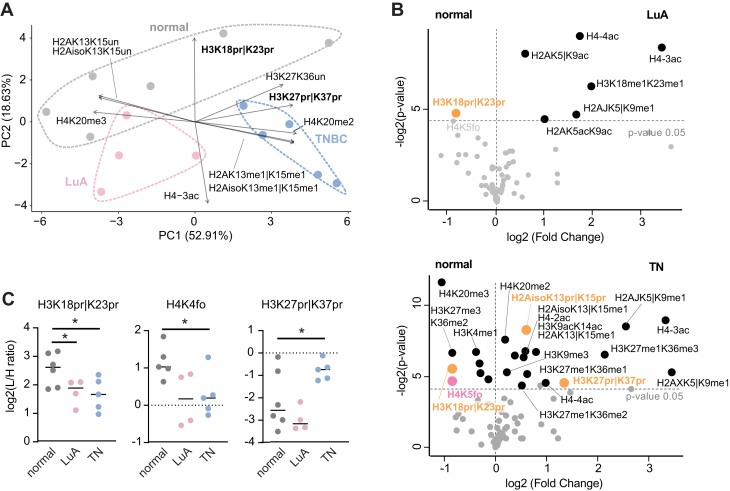
**Epigenetic profiling of normal breast tissue, luminal A, and triple negative breast cancers.***A*, biplot representing the PCA of quantitative histone PTM data for the analysis of normal breast and breast cancer tissues (six breast tissues, four LuA samples (of which three matched with normal samples), and five TN samples (two matched with normal tissue)). The ten histone PTMs with the highest loadings are shown. *B*, volcano plots showing significant changes in histone PTMs in LuA breast cancers (*top*) and TN breast cancer (*bottom*) compared with normal tissues. *C*, differences in selected histone PTMs in the analyzed normal breast and breast cancer tissues. ∗*p* < 0.05. The significance was assed using a moderated *t* test accounting for interpatient variability in the normal tissue. Significant modified acylated (including acetylated) peptides are labeled, as well as previously reported changes. “|”= one residue or the other. LuA, luminal A; MS, mass spectrometry; PCA, principal component analysis; PTM, posttranslational modification; TN, triple negative.

We observed that clusters obtained through the profiling of histone PTMs are well-defined and formed exclusively by samples belonging to the same group, with LuA tumors close in space to normal samples, consistent with their similar expression profiles (58) (Fig. 5*A*). Notably, the differentially modified peptides that most drive separation include two acylated sites, as well as H4K20me3, a reported hallmark of cancer (55). We observed a higher number of significant changes in TN compared to normal than LuA compared to normal, in line with what we reported in (34). Both LuA and TN samples showed an increase of the hyper-acetylated versions of the histone H4 tail and a decrease of H3K18pr|K23pr and H4K5fo (Fig. 5, *B* and *C*). In addition, LuA samples showed a decreased acetylation on histone H2A, while TN samples showed many additional changes in methylated peptides (Fig. 5 and Supplemental Fig. S4). These include a previously reported increase of H3K9me3 and decrease of H3K27me3 and H4K20me3 (Fig. 5*B*). Two propionylated peptides, H3K27pr|K37pr and H2AisoK13pr|K15pr, were also increased in TN tumors (Fig. 5, *B* and *C*).

## Discussion

MS-based methods offer numerous advantages over antibody-based methods for studying histone marks: (1) the simultaneous profiling of potentially all histone PTMs present in a sample, instead of only one/few; (2) the possibility to analyze in an unbiased manner uncommon and novel modifications, against which specific antibodies may be difficult to obtain; and (3) the absence of the epitope masking effect often associated with antibody-based analyses (59). MS-based methods were critical in the discovery of acylations alternative to acetylation, which emerged as an important and complex new dimension in genome regulation. However, the comprehensive analysis of these modifications requires improved technology. In this study, we implemented and tested different derivatization protocols to overcome the issue of distinguishing endogenous histone propionylation and butyrylation from those that are introduced with common protocols involving lysine derivatization with propionic anhydride, since they have the exact same chemical composition and thus the same mass. All the protocols tested allowed the identification and quantification of several propionylated peptides, and a few butyrylated peptides, with the D3Ac-PIC enabling the quantification of the highest number of propionylations. Of note, the deuterated acetic anhydride reagent is typically ∼10 time less expensive than deuterated propionic anhydride, which makes it also more suitable for large scale analyses. We found that the increased number of propionylated peptides detected with the D3Ac-PIC protocol is accompanied—and probably facilitated—by a reduction of the number of identified acetylated peptides. This is likely due to an “isobarization effect” that causes the collapse of differentially acetylated histone peptide forms into one form, thus reducing the complexity of the peptide mixture and improving sensitivity for the detection of other modifications. Along the same line, the derivatization of histones with d0-acetic anhydride has been previously shown to facilitate the detection of low-abundance histone methylations (60).

Our MS quantitative analysis revealed that histone propionylation is a rather frequent and abundant histone mark, with a %RA in some instances comparable to classical histone marks such as methylation and acetylation, reinforcing the need of suitable procedures to investigate this PTM. The real stoichiometry of this modification is, however, uncertain, as the MS estimated abundance is influenced by the physio-chemical peptide properties (50). Indeed, it is known that propionylation increases peptide hydrophobicity and induces a chromatographic shift enhancing peptide detectability. Knowing the real abundance of these modifications would represent a valuable piece of information. To obtain accurate RA estimates one should rely on a synthetic histone peptide library (50). However, the construction of such library is limited by the cost of isotopically labeled peptides and the challenges related to the synthesis of peptides containing unusual modifications/modified sites.

The majority of nonacetyl lysine acylated sites identified were propionylations and, in a minor fraction, butyrylations, which -in our experimental conditions- were much less abundant. Other acylations were identified only sporadically, possibly due to the use of a protocol that is particularly well suited for propionylated peptides, but not for other acylations. We found that only a minor fraction of the identified acylations can be quantified. One limitation of our study is the reliance, for histone peptide quantification, on a histone-focused super-SILAC internal standard. We have previously shown that the use of an internal standard leads to a much higher confidence and robustness in peptide quantification, particularly for low-abundance modifications (37). This is the case of most peptides carrying uncommon modifications, such as acylations, which are often present in very low stoichiometry, close to the limit of detection and with a low signal-to-noise ratio. However, this approach limits the analysis to the PTMs present in the cell line–derived histone standard, which may be different from tissues, since the latter may have a different metabolic complexity from the cultured cells used for the spike-in implementation. Indeed, when we inspected this issue, we observed that, of the 102 acylated sites identified in at least two replicates and in at least one mouse tissue, about 30% (30) of the sites did not have an ID counterpart in the spike-in and were, therefore, not quantified in this study. To overcome this limitation, in the future one could explore the possibility to grow the cultured cells used for the super-SILAC mix in the presence of acyl-CoA compounds, in order to boost the overall rate of *in vitro* histone acylation, leading to a wider representation of acylated peptides, eventually allowing the accurate quantification of all possible *in vivo* histone acylated sites.

Interestingly, histone propionylated and butyrylated sites displayed changes both among mouse tissues and normal breast/breast cancer tissues. In addition, these marks have also a high power, in some cases even higher than classic histone PTMs, in discriminating tissues, as indicated by both the PCA and LEfSe. Antibiotic treatment caused a general and concordant decrease of histone acetylations in mouse colon and cecum, with more marked changes in cecum, in line with the fact that cecum stores food material where bacteria break down complex carbodydrates and is, thus, more closely in contact with the microbiome than colon. Two doubly propionylated peptides (H2AK13prK15pr and H3K9prK14pr) also decreased after antibiotic treatment in cecum, while other propionylations increased. Because the increase is observed in peptides—H3 9 to 17 and the histone H4 tail—that are usually substantially acetylated, it is possible that the loss of acetylation indirectly causes an increase of propionylation, by freeing residues that are usually occupied by acetyl groups. Alternatively, antibiotic treatment may cause a decrease of “hyperpropionylated” peptides, and not of partially propionylated ones. In partial agreement with our results, in a recent paper, butyrylations and—to a lesser extent—propionylations on histone H3 were found to decrease in the mouse intestine upon antibiotic treatment (53). It must be noted that in that study MS analysis was only employed for the identifications of acylations, while the quantification of changes was performed by Western blot analysis, with potential biases due to antibody cross-reactivity and lack of site-specific resolution. Gut microbiota is associated with cancer (61); hence, future investigations in this direction of histone acylations could provide better understanding of cancer epigenetic mechanisms.

Histone propionylation has also been linked to the catabolism of isoleucine, generating nuclear propionyl-CoA pools (16). Future work should disentangle to what extent histone propionylation is linked to extracellular (*e.g.*, microbiota-derived) propionate and to intracellular generated propionyl-CoA through amino acid catabolism and how this is linked to tissue function.

In breast cancer samples, we confirmed some previously reported changes found in tumors with respect to classic histone marks (Supplemental Fig. S4), including the loss of H4K20me3 (55), the decrease of H3K27me3-containing peptides and the increase of methylated H3K9me3 (34). In addition, we highlighted a novel increase of acetylation on histone H2A in the LuA subtype and an increase of the hyperacetylated form of the histone H4 tail in both LuA and TN tumors, compared with normal breast tissues. An increase of the hyper-acetylated histone H4 was also recently observed in uveal melanoma compared with control normal uvea (62). This is coherent with the fact the TN present a more active metabolism, leading to a higher concentration of acetyl-CoA. For what concerns acylations, we observed a decrease of H3K18pr|K23pr and H4k5fo in both subtypes and an increase of few other propionylations in TN tumors only. Interestingly, H3K23 propionylation has been associated with active transcription and is catalyzed by complexes containing bromodomain- and PHD finger–containing protein 1, which is mutated in different cancer types (24).

Overall, our results point out that profiling only classical modification might be limiting, since other modifications could regulate important biological processes that might have been previously overlooked. Our study also highlights the importance of using a sample preparation method that allows the analysis of the widest possible spectrum of histone modifications.

## Data Availability

The MS proteomics data have been deposited to the ProteomeXchange Consortium *via* the PRIDE (63) partner repository with the dataset identifier PXD050362.

## Supplemental data

This article contains supplemental data.

## Conflicts of interest

A. V. and M. C. are PhD students within the European School of Molecular Medicine (SEMM). The other authors declare no competing interests.
