# Supplementary material for: Improved Mass Spectrometry–Based Methods Reveal Abundant Propionylation and Tissue-Specific Histone Propionylation Profiles
Source: Mol Cell Proteomics. 2024 Jun 11;23(7):100799. doi: 10.1016/j.mcpro.2024.100799 (PMC11277384; doi:10.1016/j.mcpro.2024.100799)
Supplement: Supplemental table figures [file mmc1.docx]

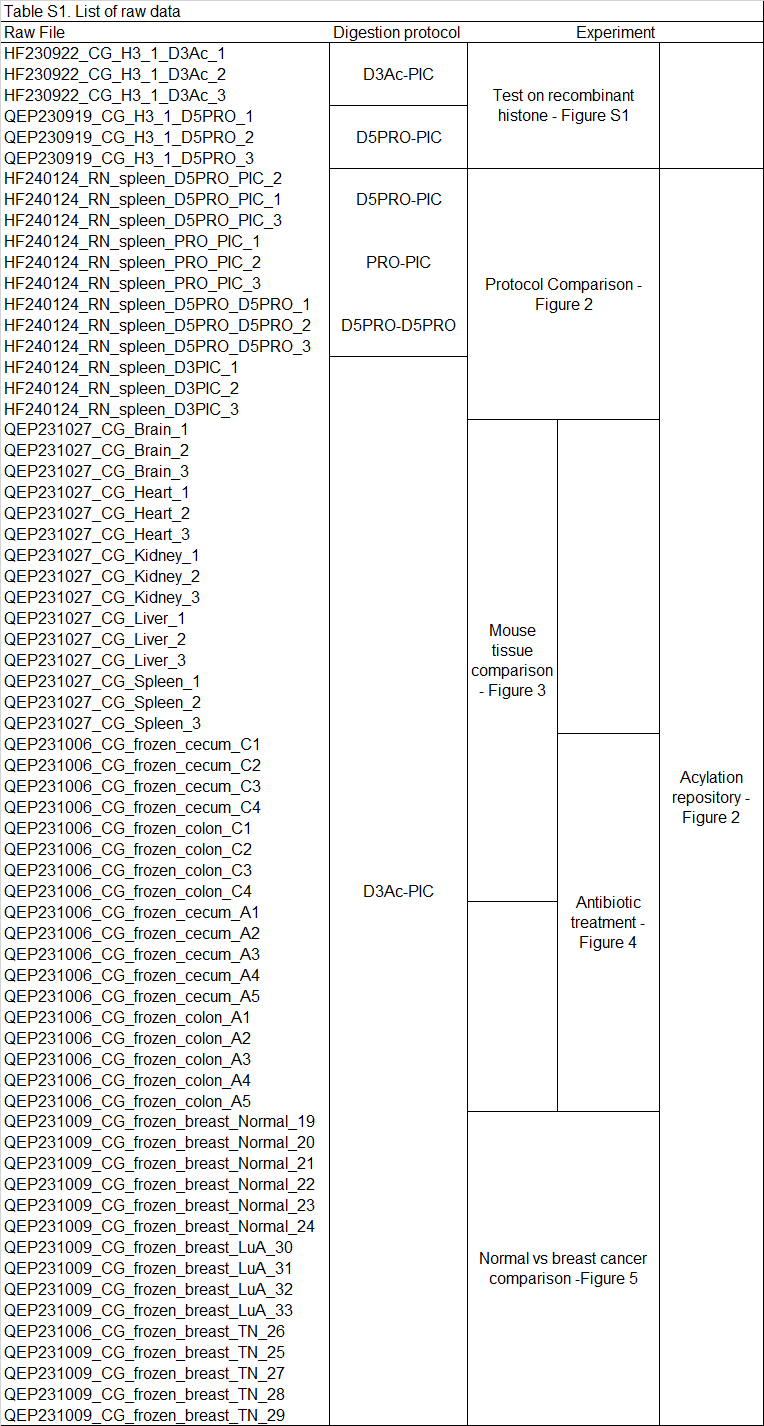


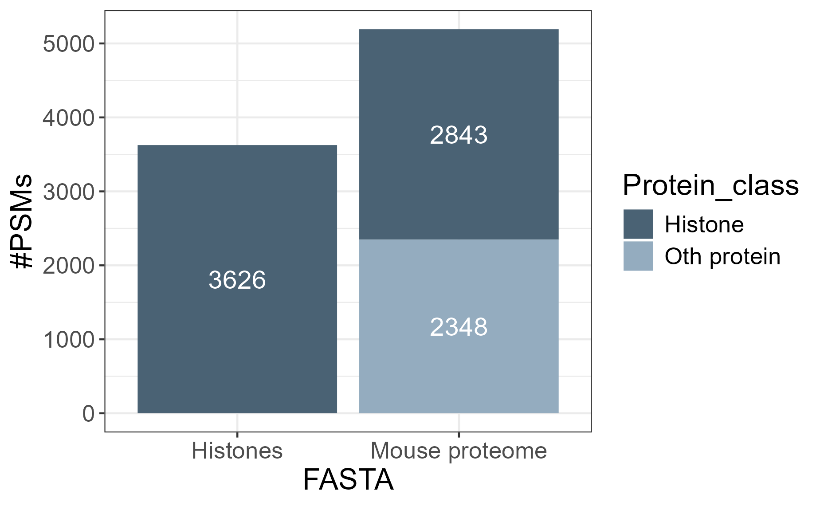

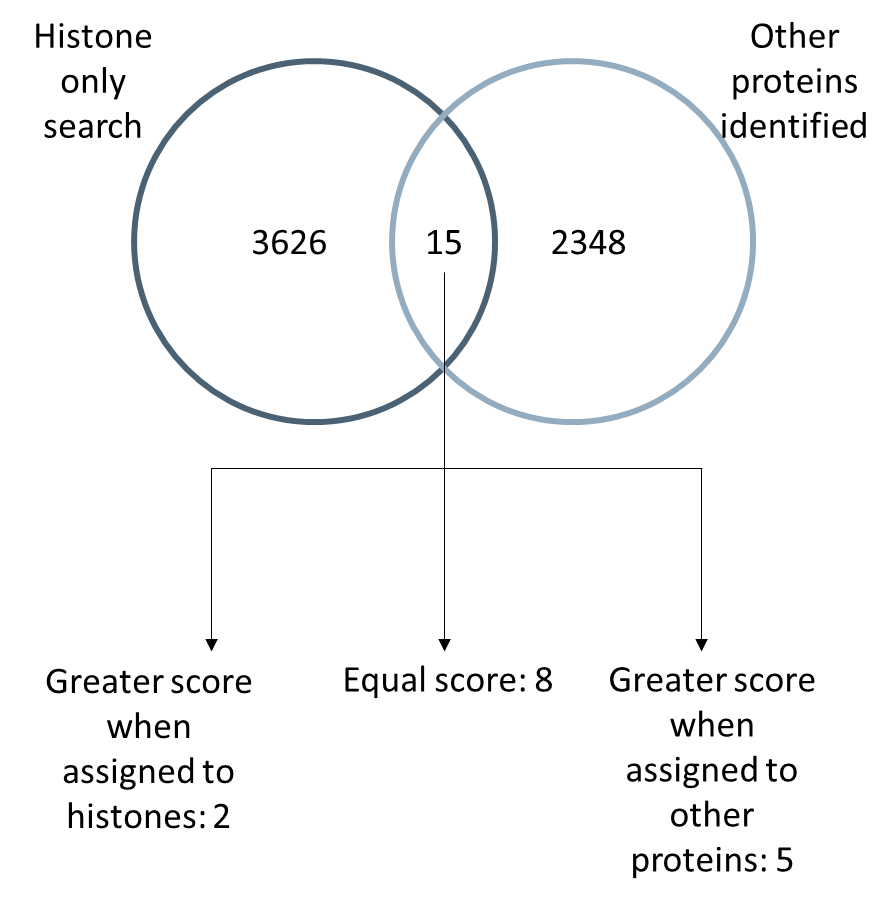


**Fig S1**. Comparison of FASTA files used in MaxQuant for the search of histone acylations. MaxQuant was run as described in the method section but in case 1) using a FASTA file containing only histone and common contaminants sequences and in case 2) the whole UniProt mouse proteome. A) Barplot showing the number of peptide-to-spectrum matches (PSMs) assigned to histone peptides (dark color) and to other non-histone proteins (light color). B) Overlap of PSMs assigned to histones in case 1) and to other proteins in case 2).


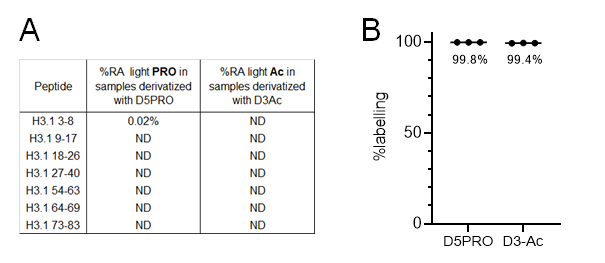


**Fig S2**. Comparison of in-gel derivatization methods for MS-based analysis of histone acylations. (A) % of light propionylation or acetylation found in recombinant histone H3.1 derivatized using D5PRO or D3Ac. ND: not detected. (B) Average % of lysine derivatization in unmodified histone H3 peptides with D5PRO or D3Ac in the spleen samples shown in Figure 1C. The amount of a derivatized peptide relative to the total amount (derivatized+non-derivatized/partially derivatized peptide) was calculated.


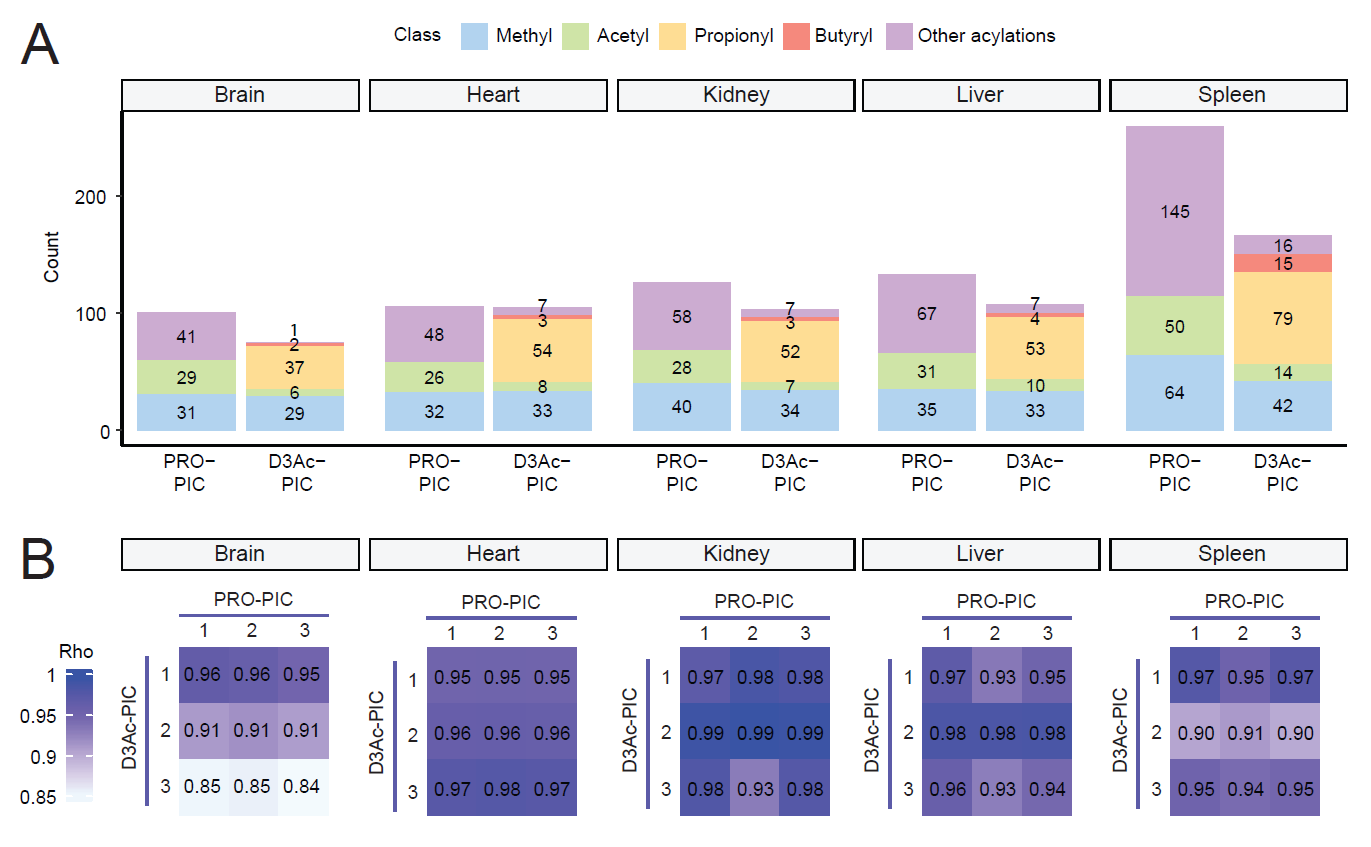


**Fig S3**. Comparison of D3Ac-PIC and PRO-PIC protocol. (A) Bar-charts depicting the number of differentially modified peptides identified using the D3Ac-PIC and PRO-PIC protocols in different mouse tissues. The methyl-category contains mono-/di-/tri-methylations. Other acylations: formylation, crotonylation, succinylation, malonylation, hydroxyisobutyrylation,glutarylation, and lactylation. (B) Correlation matrix based on Pearson correlation coefficients (rho) of L/H ratios (light channel: tissue sample; heavy channel: spike-in standard) for methylated and acetylated histone peptides quantified from tissue samples processed in technical triplicates through the D3Ac-PIC and PRO-PIC protocols.


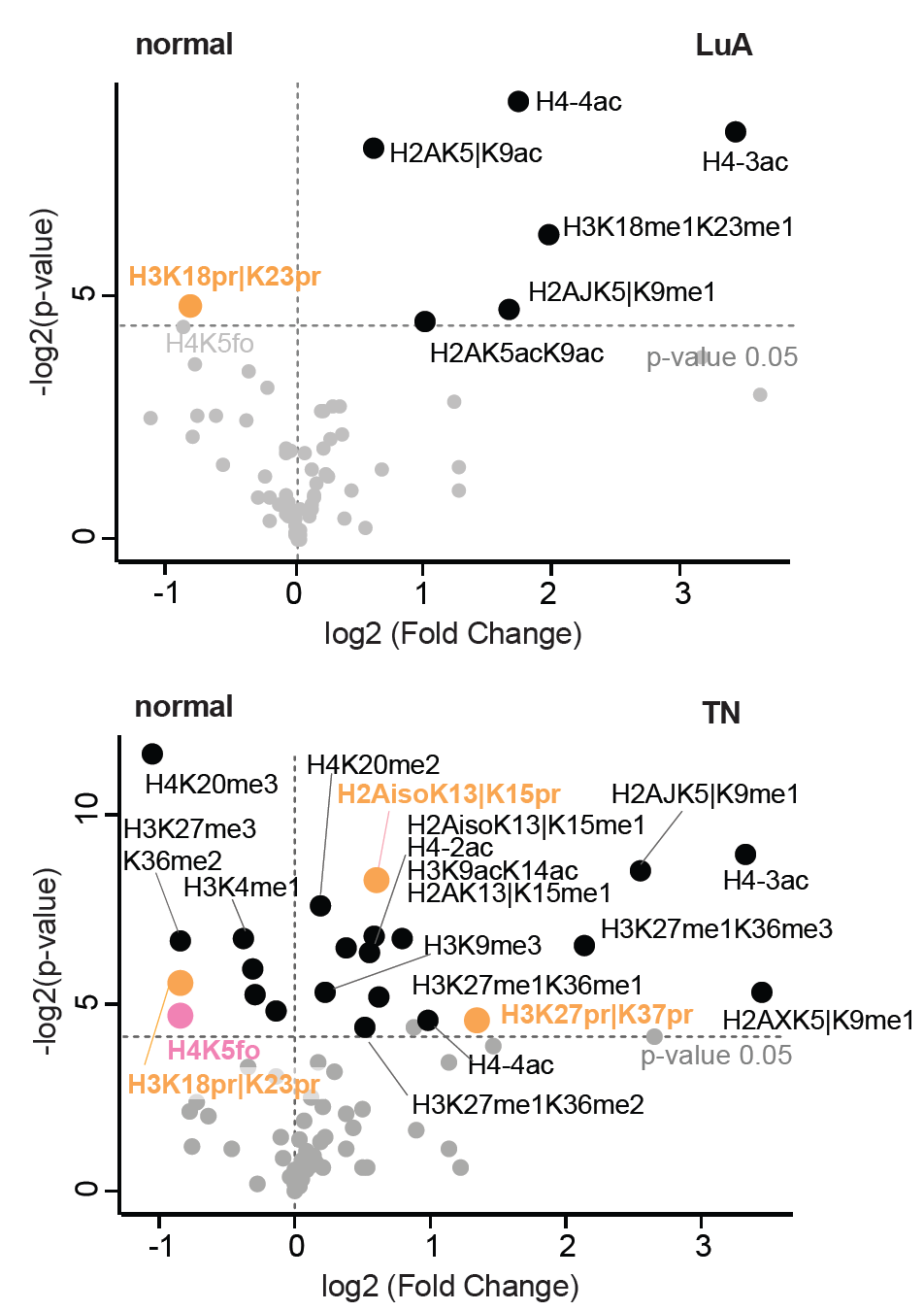


**Fig S4**. Epigenetic profiling of normal breast and breast cancer tissues. Volcano plots showing significant changes in histone PTMs in luminal A breast cancers (top) and triple negative breast cancer (bottom) compared with normal tissues. The significance was assed using a moderated t-test accounting for inter-patient variability in the normal tissue. “|”= one residue or the other.
