# Supplementary material for: Improved Mass Spectrometry–Based Methods Reveal Abundant Propionylation and Tissue-Specific Histone Propionylation Profiles
Source: Mol Cell Proteomics. 2024 Jun 11;23(7):100799. doi: 10.1016/j.mcpro.2024.100799 (PMC11277384; doi:10.1016/j.mcpro.2024.100799)
Supplement: Supplemental data [file mmc5.pdf]

Spleen_PRO-PIC

Raw file: HF240124_RN_spleen_PRO_PIC_3, Scan: 17222, m/z: 651.83806, Charge: 2, RT (min): 31.76, Score: 68.915

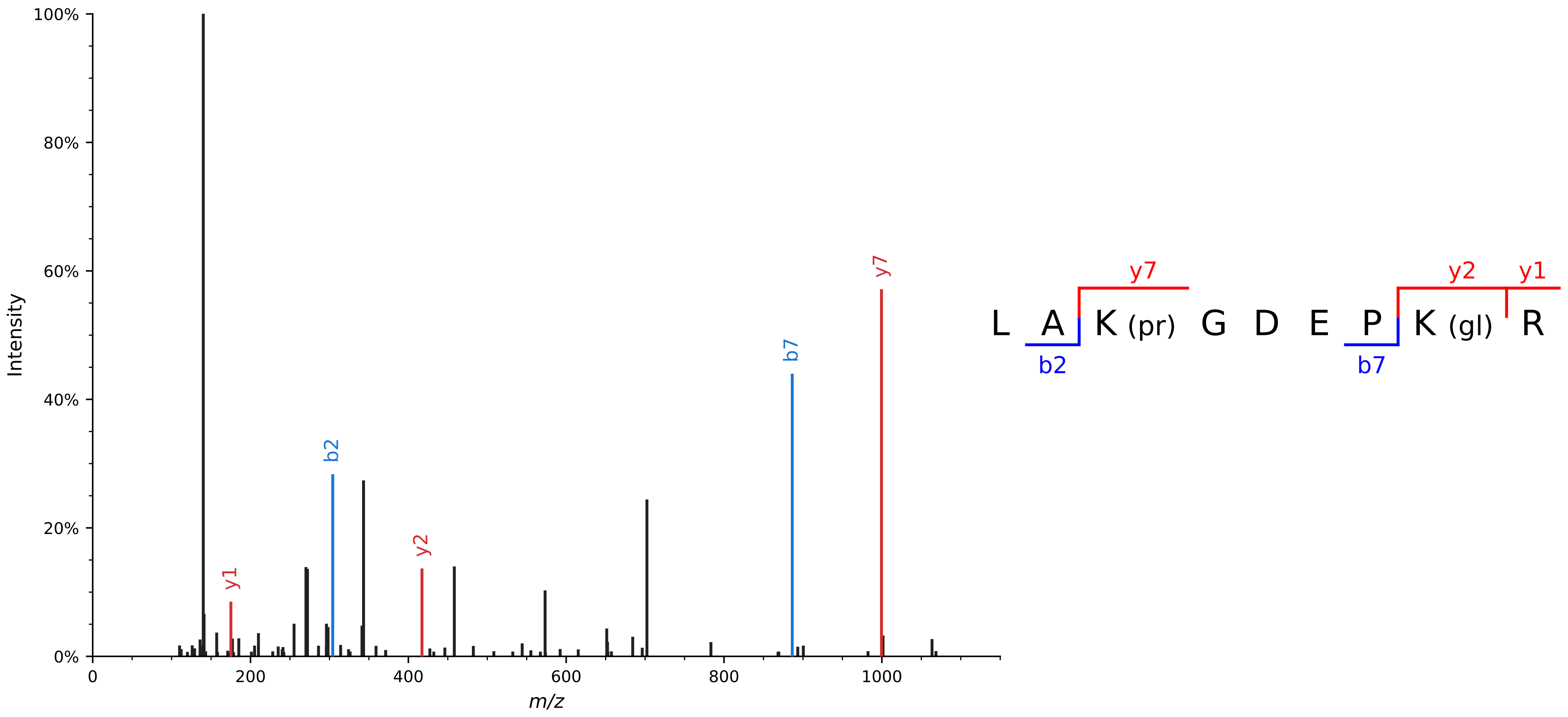

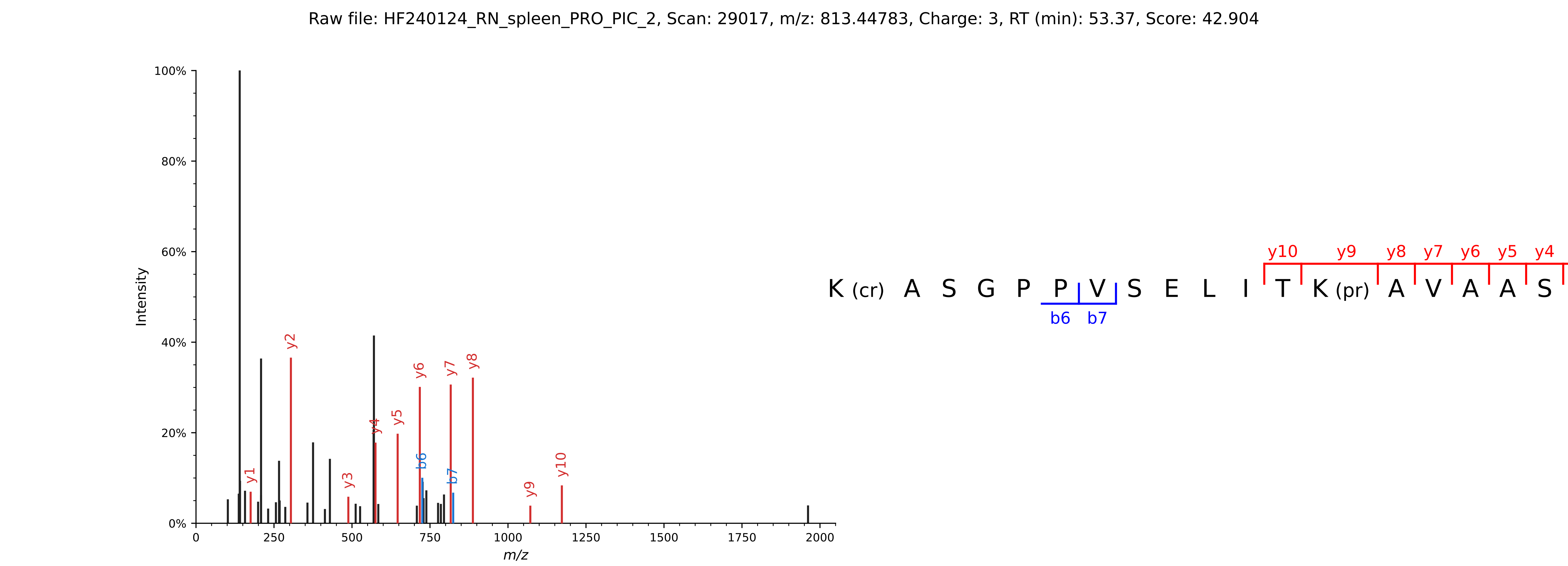

Raw file: HF240124_RN_spleen_PRO_PIC_2, Scan: 29017, m/z: 813.44783, Charge: 3, RT (min): 53.37, Score: 42.904
Intensity
m/z
y1
y2
y3
y4
y5
y6
b6
y7
b7
y8
y9
y10
K (cr) A S G P P V S E L I T K (pr) A V A A S
y10 y9 y8 y7 y6 y5 y4
b6 b7

Raw file: HF240124_RN_spleen_PRO_PIC_3, Scan: 8805, m/z: 488.27216, Charge: 2, RT (min): 16.94, Score: 56.091

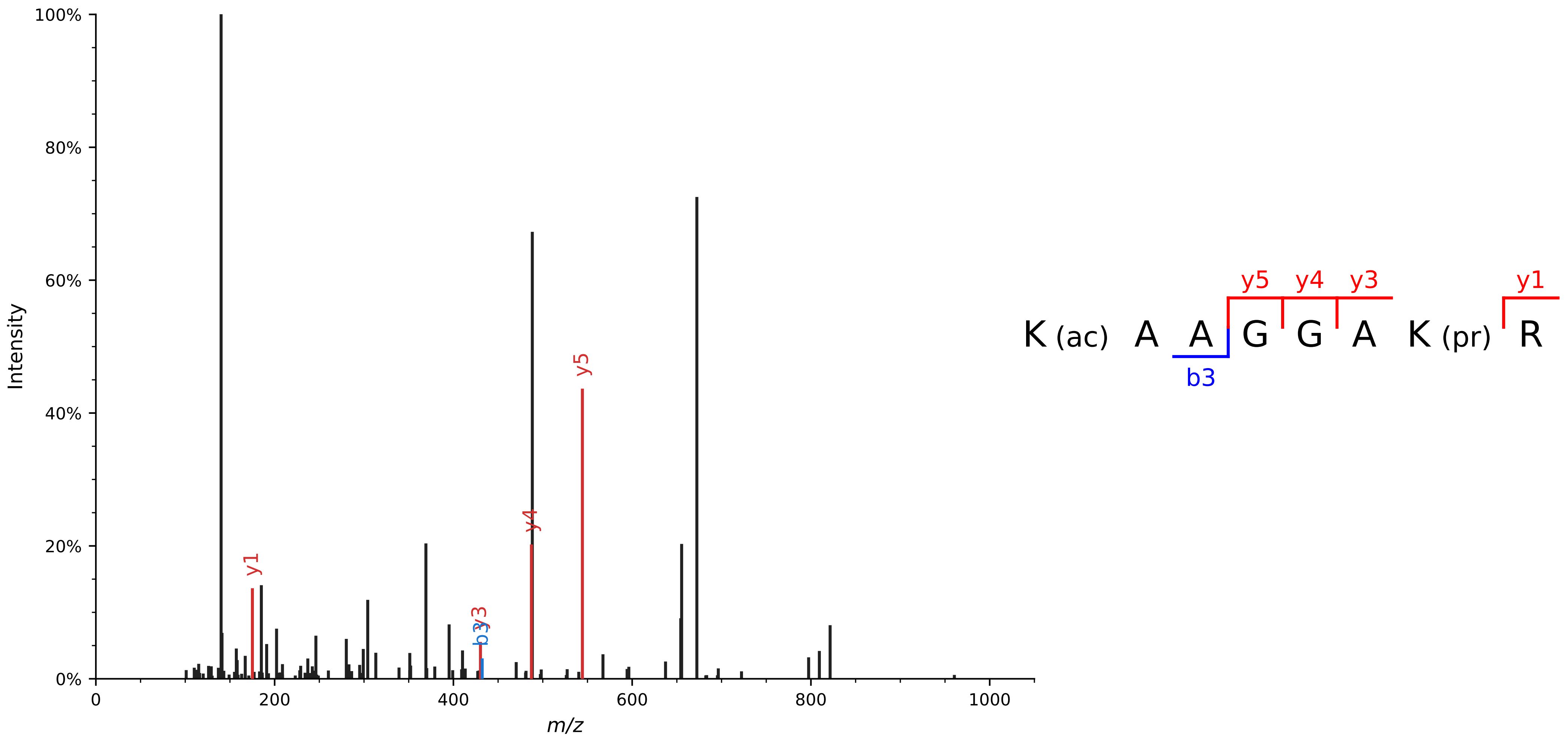

Raw file: HF240124_RN_spleen_PRO_PIC_3, Scan: 7390, m/z: 510.26707, Charge: 2, RT (min): 14.50, Score: 44.692

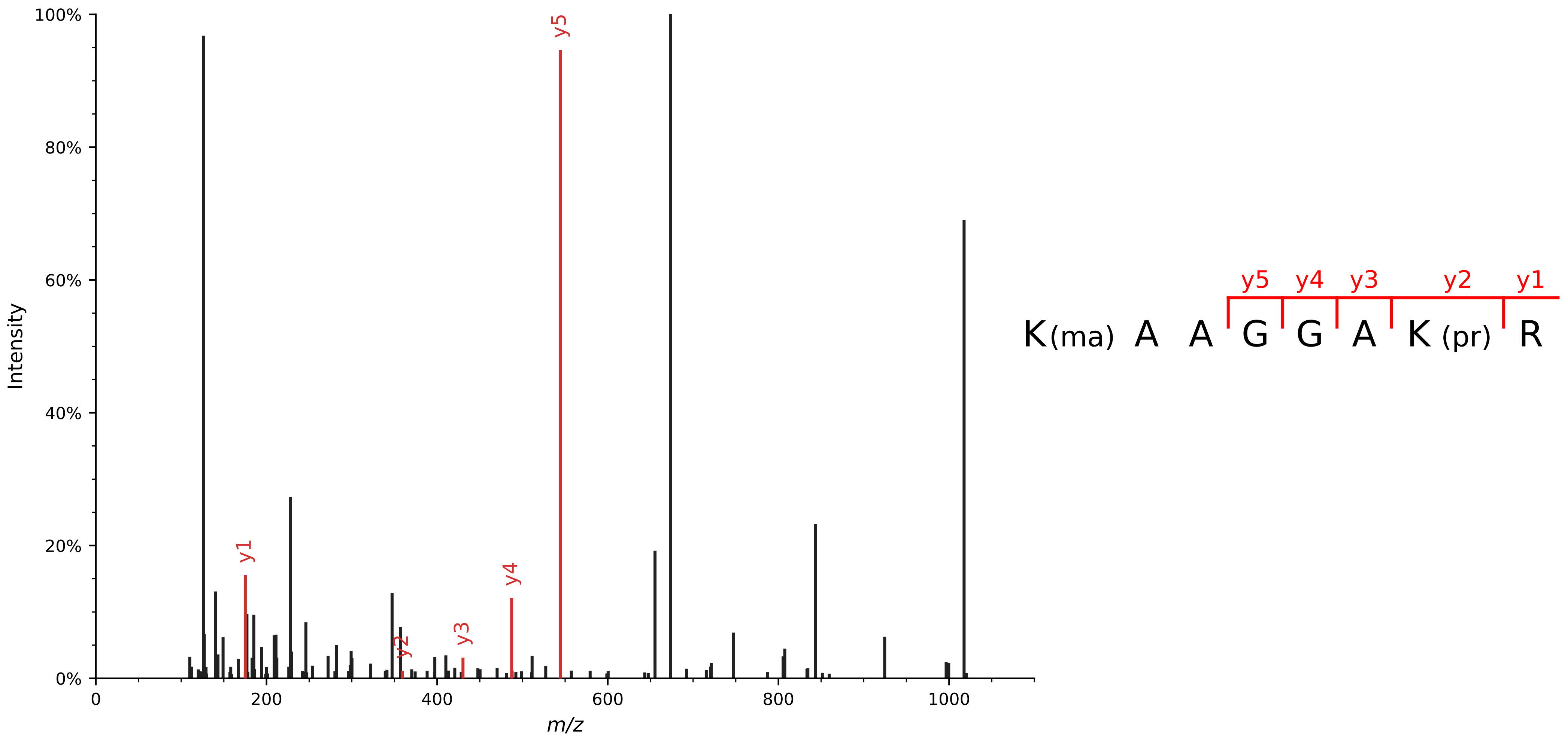

Raw file: HF240124_RN_spleen_PRO_PIC_2, Scan: 12493, m/z: 502.28781, Charge: 2, RT (min): 23.42, Score: 110.61

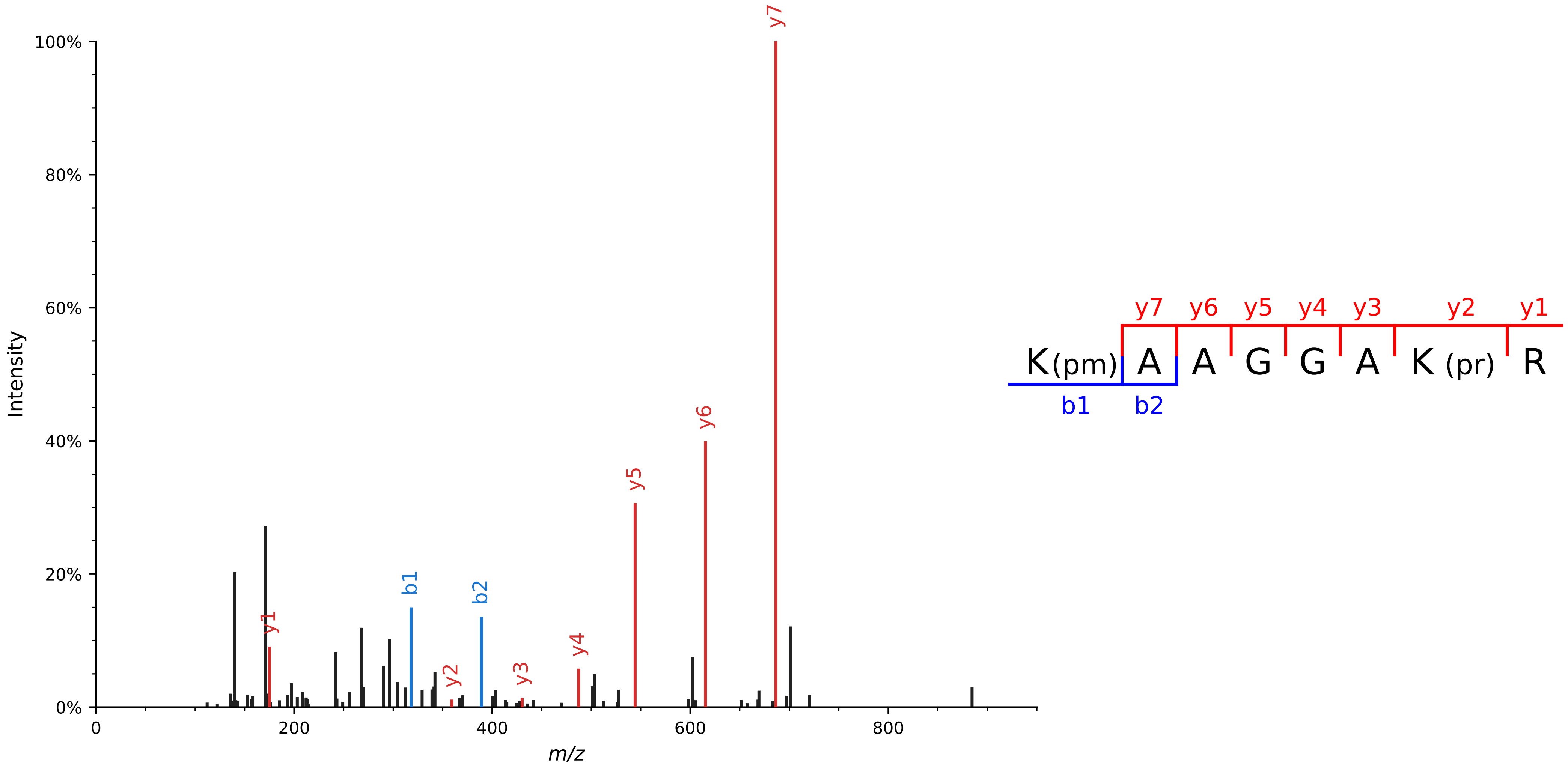

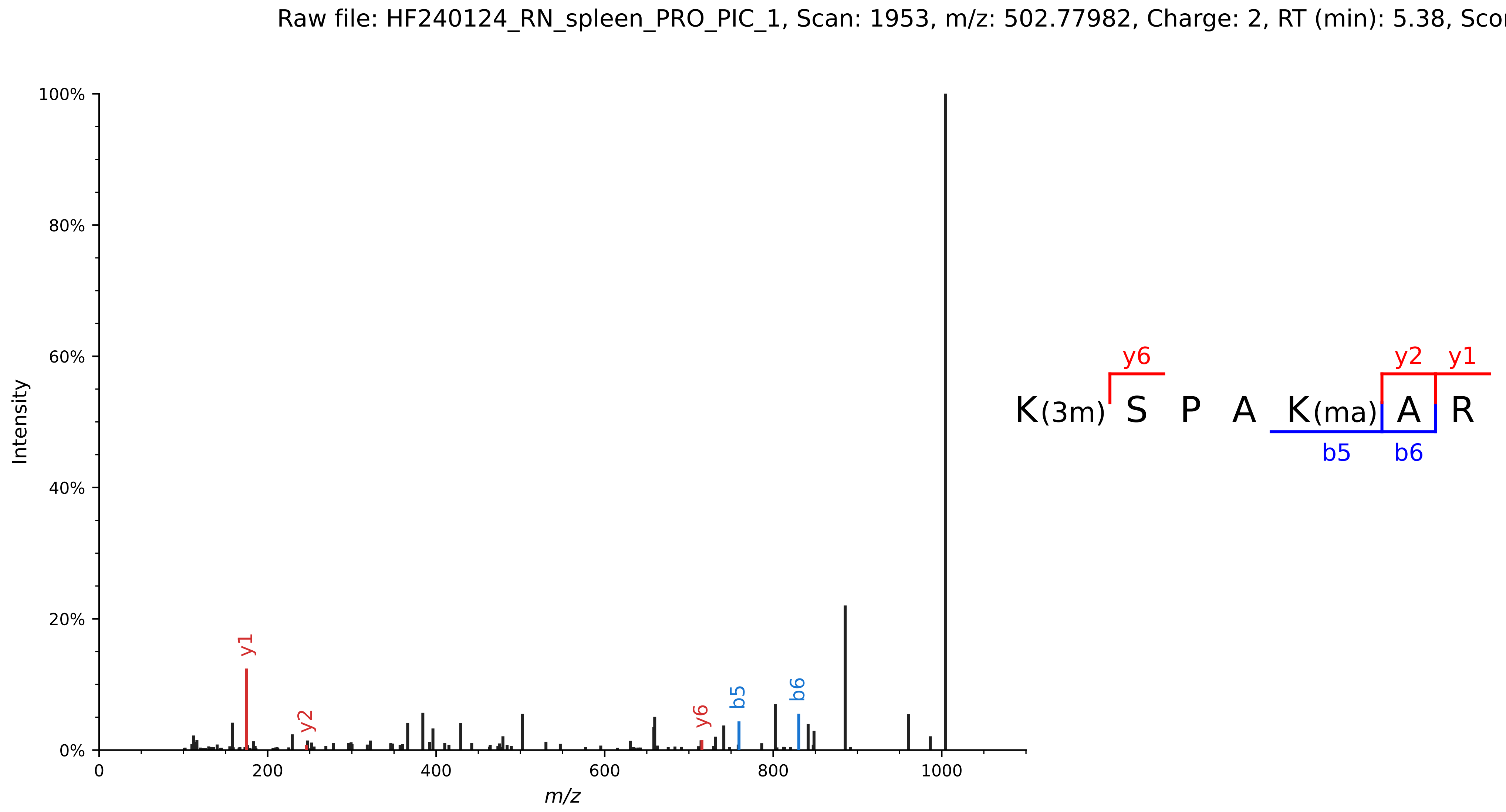

Raw file: HF240124_RN_spleen_PRO_PIC_1, Scan: 1953, m/z: 502.77982, Charge: 2, RT (min): 5.38, Score: 50.966
100%
80%
60%
40%
20%
0%
Intensity
0
200
400
600
800
1000
m/z
y1
y2
y6
b5
b6
K(3m) S P A K(ma) A R
y6
y2
y1
b5
b6

Raw file: HF240124_RN_spleen_PRO_PIC_3, Scan: 11969, m/z: 509.78764, Charge: 2, RT (min): 22.50, Score: 44.611

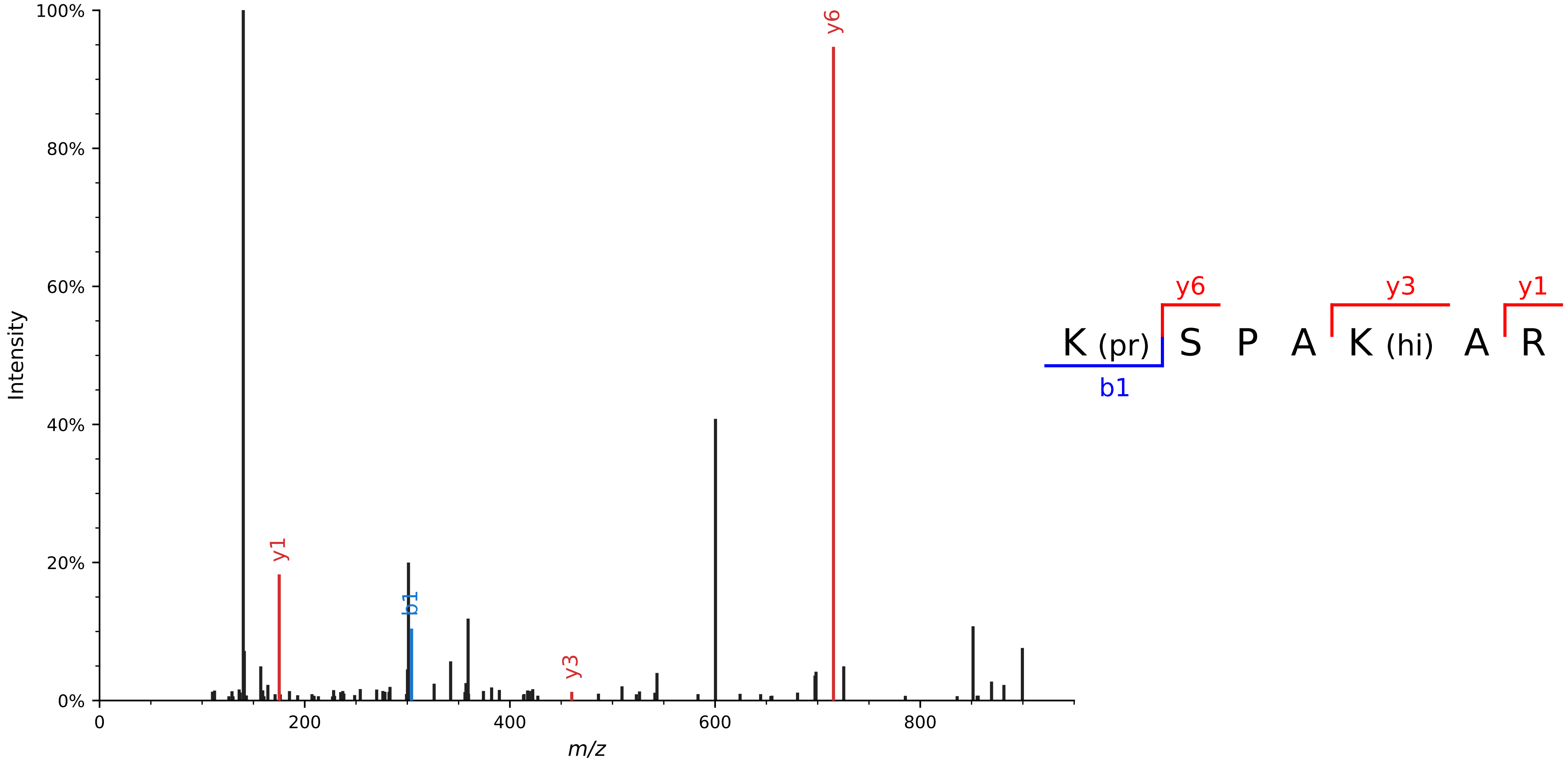

Raw file: HF240124_RN_spleen_PRO_PIC_2, Scan: 9388, m/z: 439.24815, Charge: 2, RT (min): 17.97, Score: 57.281

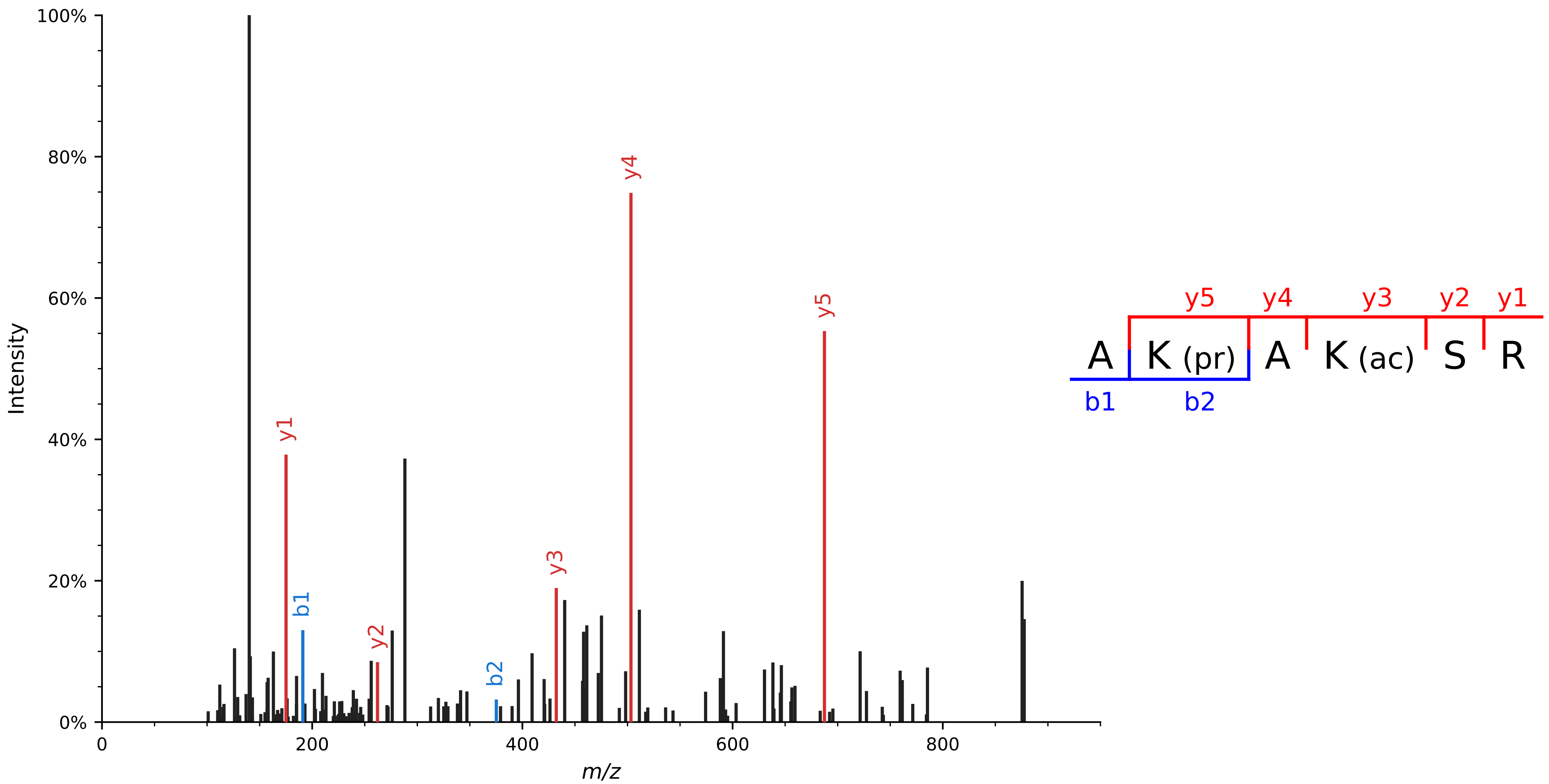

Raw file: HF240124_RN_spleen_PRO_PIC_2, Scan: 9746, m/z: 520.74343, Charge: 2, RT (min): 18.58, Score: 91.9

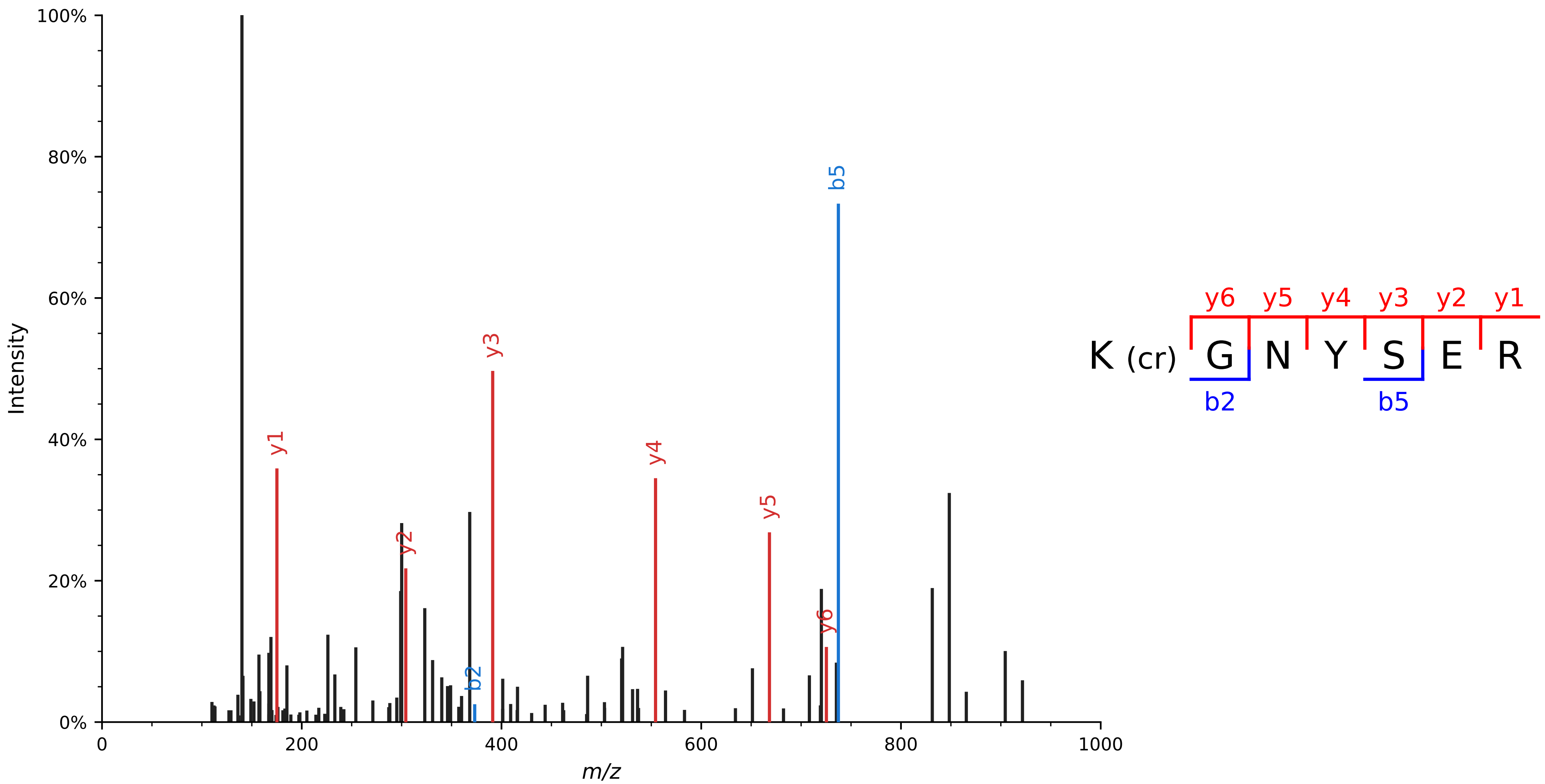

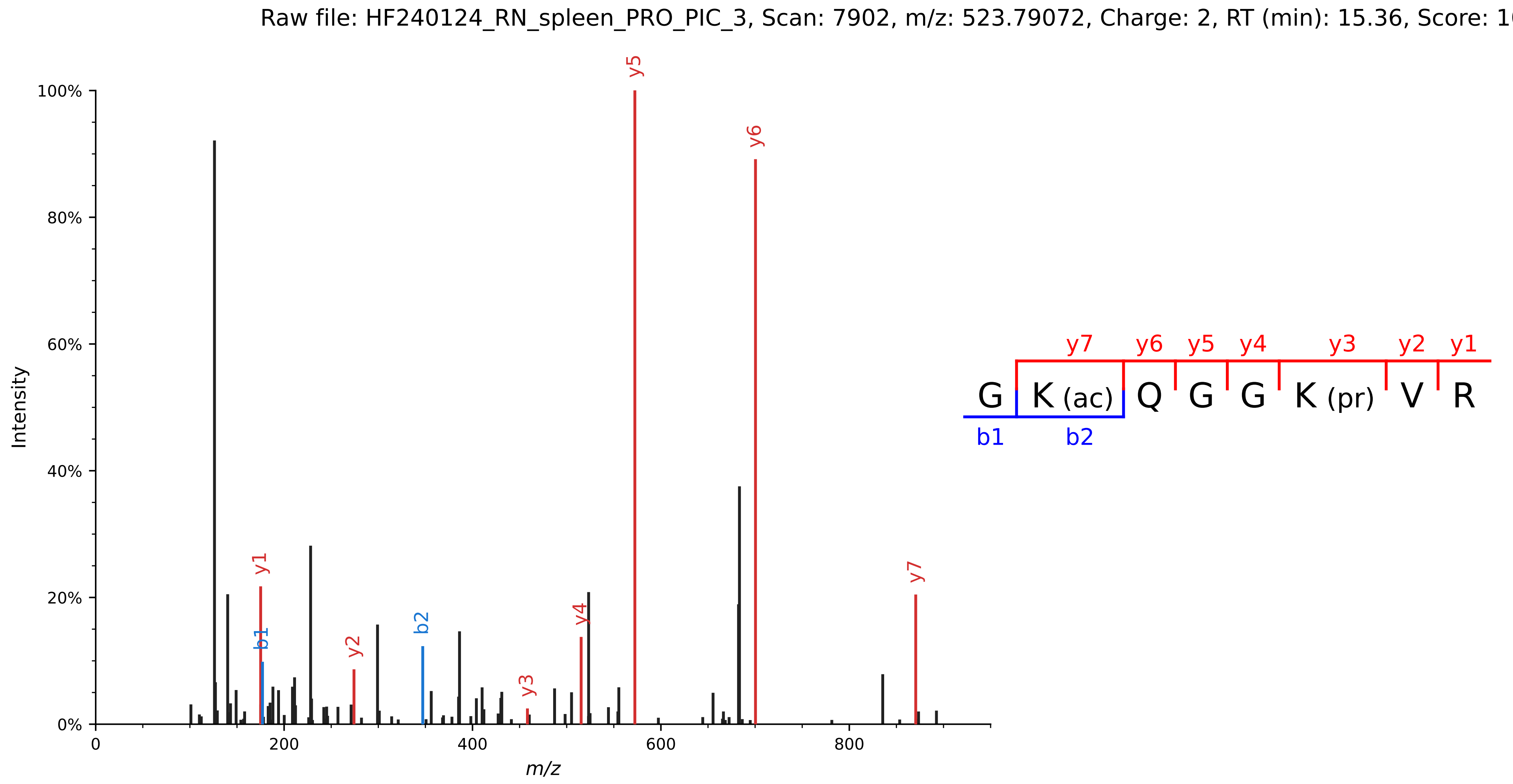

Raw file: HF240124_RN_spleen_PRO_PIC_3, Scan: 7902, m/z: 523.79072, Charge: 2, RT (min): 15.36, Score: 102.72
Intensity
m/z
0%
20%
40%
60%
80%
100%
0
200
400
600
800
y1
b1
y2
b2
y3
y4
y5
y6
y7
G K (ac) Q G G K (pr) V R
b1
b2

Raw file: HF240124_RN_spleen_PRO_PIC_3, Scan: 6382, m/z: 509.77507, Charge: 2, RT (min): 12.71, Score: 127.84

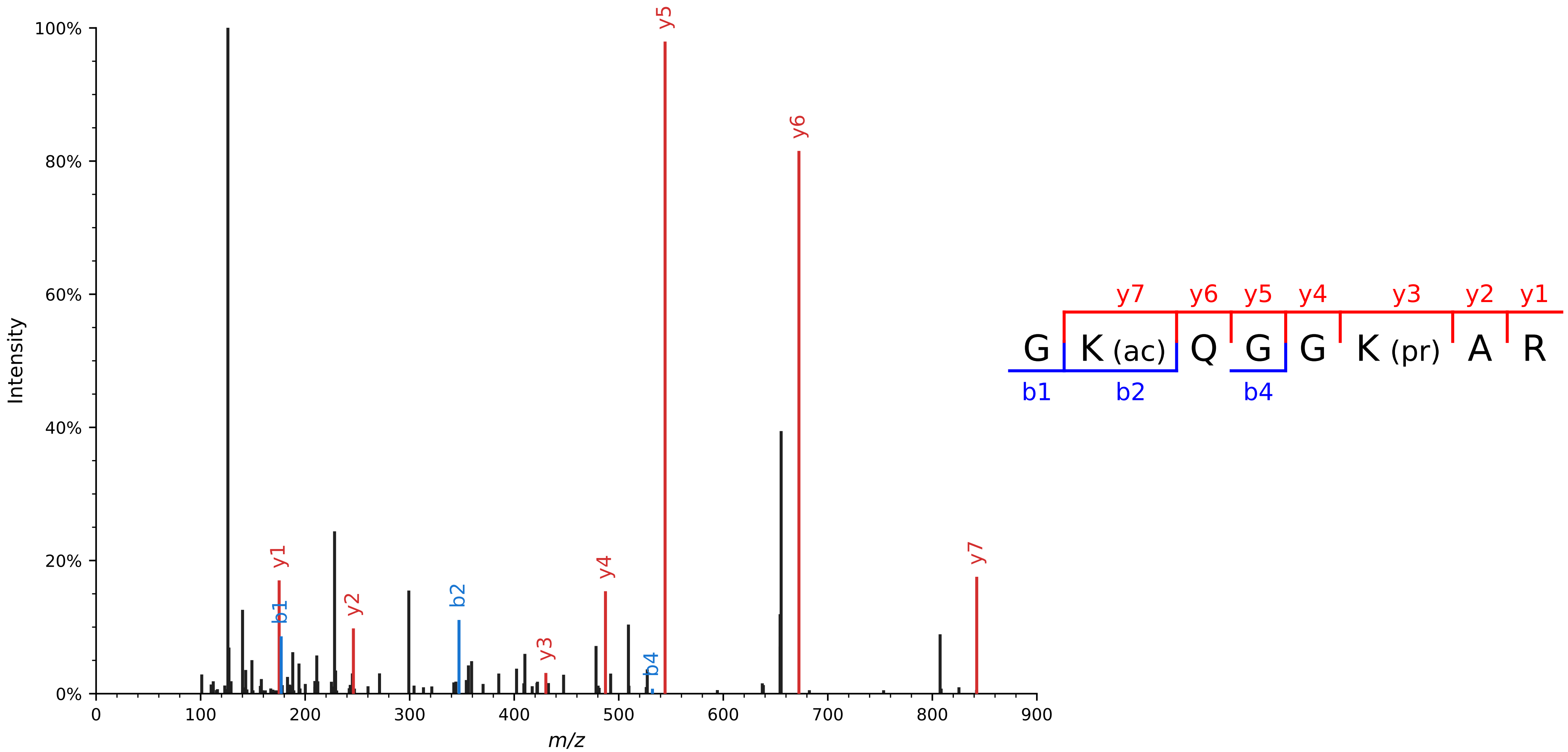

Raw file: HF240124_RN_spleen_PRO_PIC_3, Scan: 9102, m/z: 522.78289, Charge: 2, RT (min): 17.47, Score: 94.407

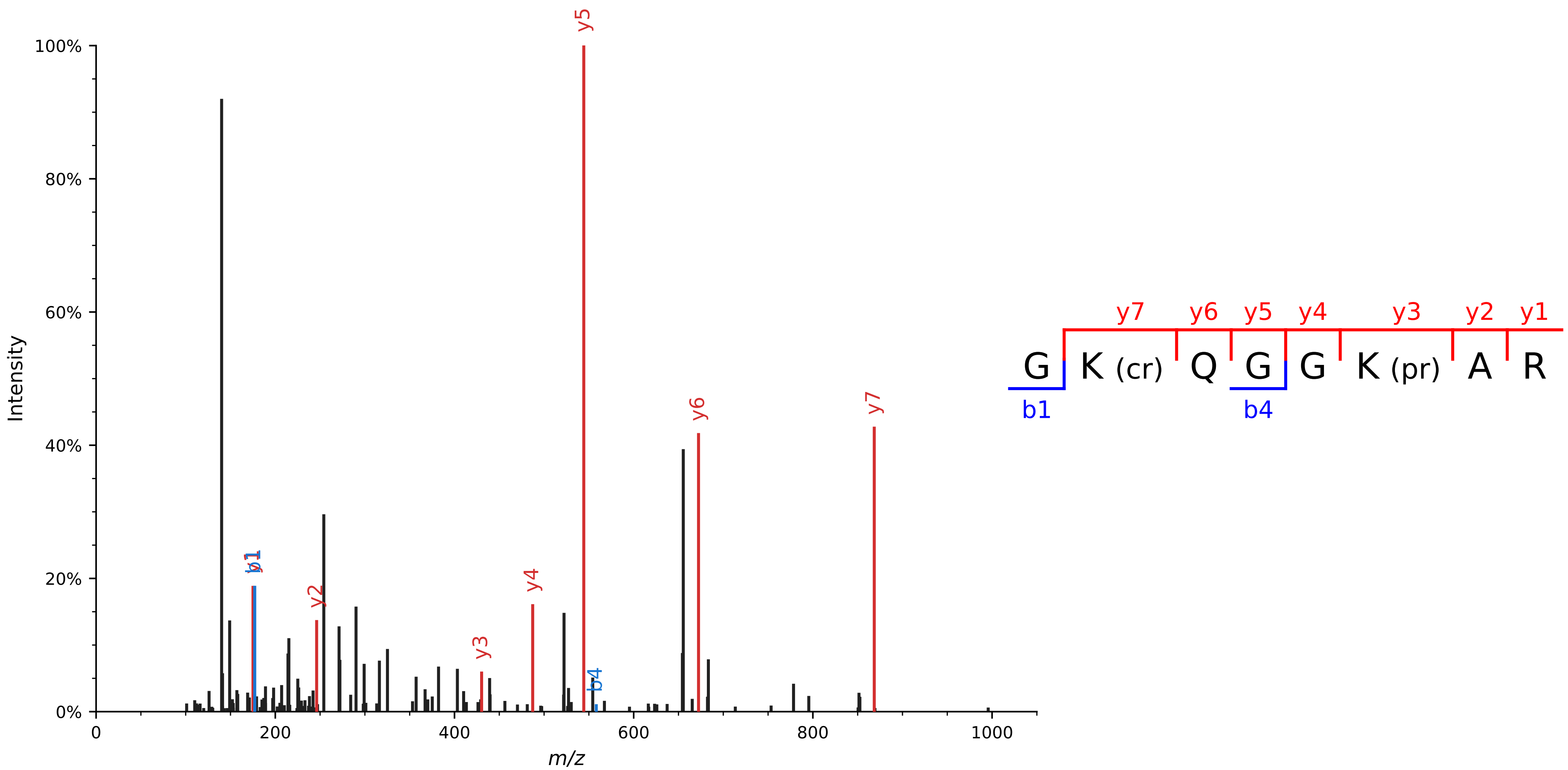

Raw file: HF240124_RN_spleen_PRO_PIC_2, Scan: 6550, m/z: 509.77507, Charge: 2, RT (min): 13.07, Score: 127.84

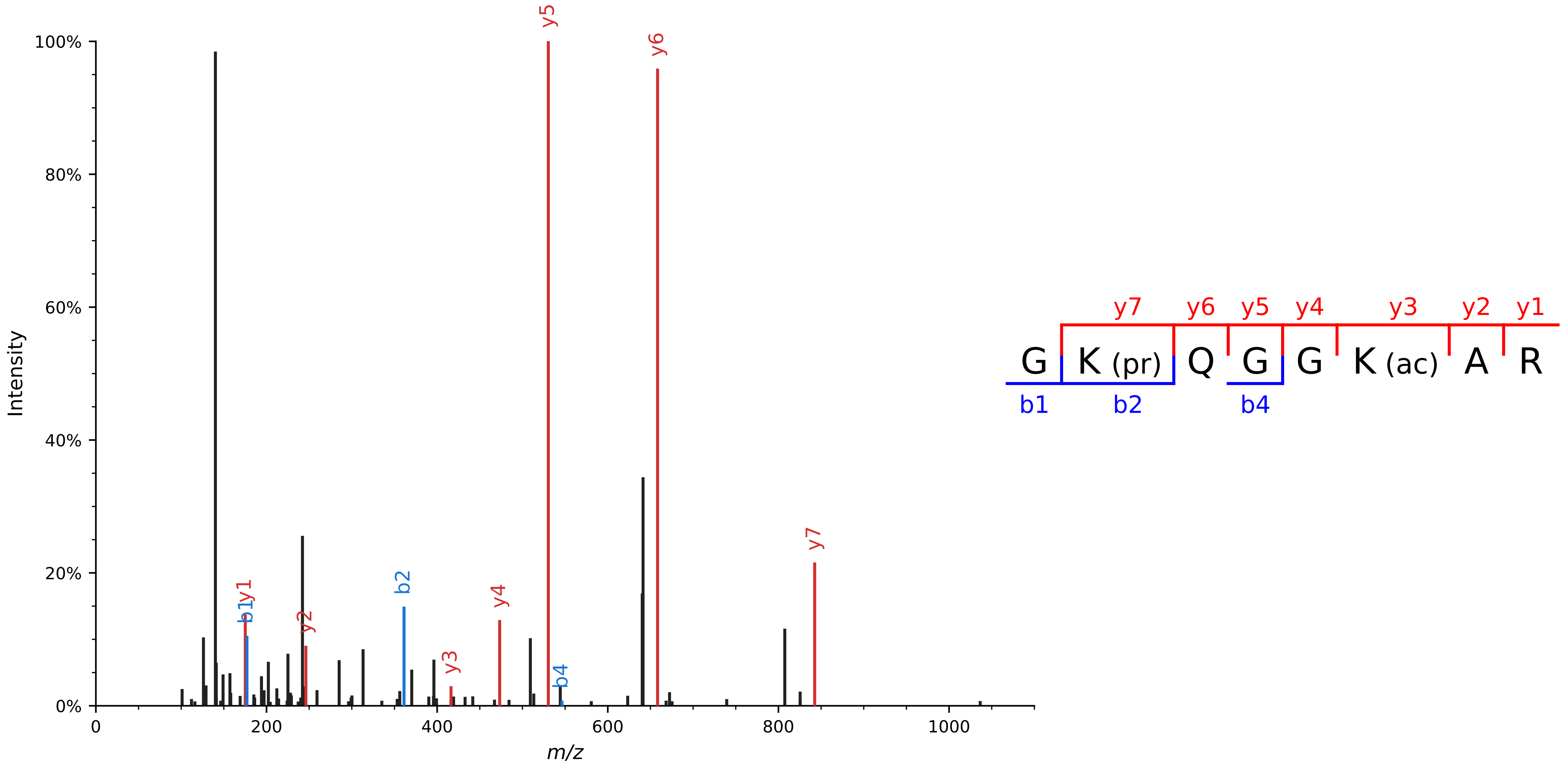

Raw file: HF240124_RN_spleen_PRO_PIC_3, Scan: 6771, m/z: 524.78035, Charge: 2, RT (min): 13.43, Score: 67.032

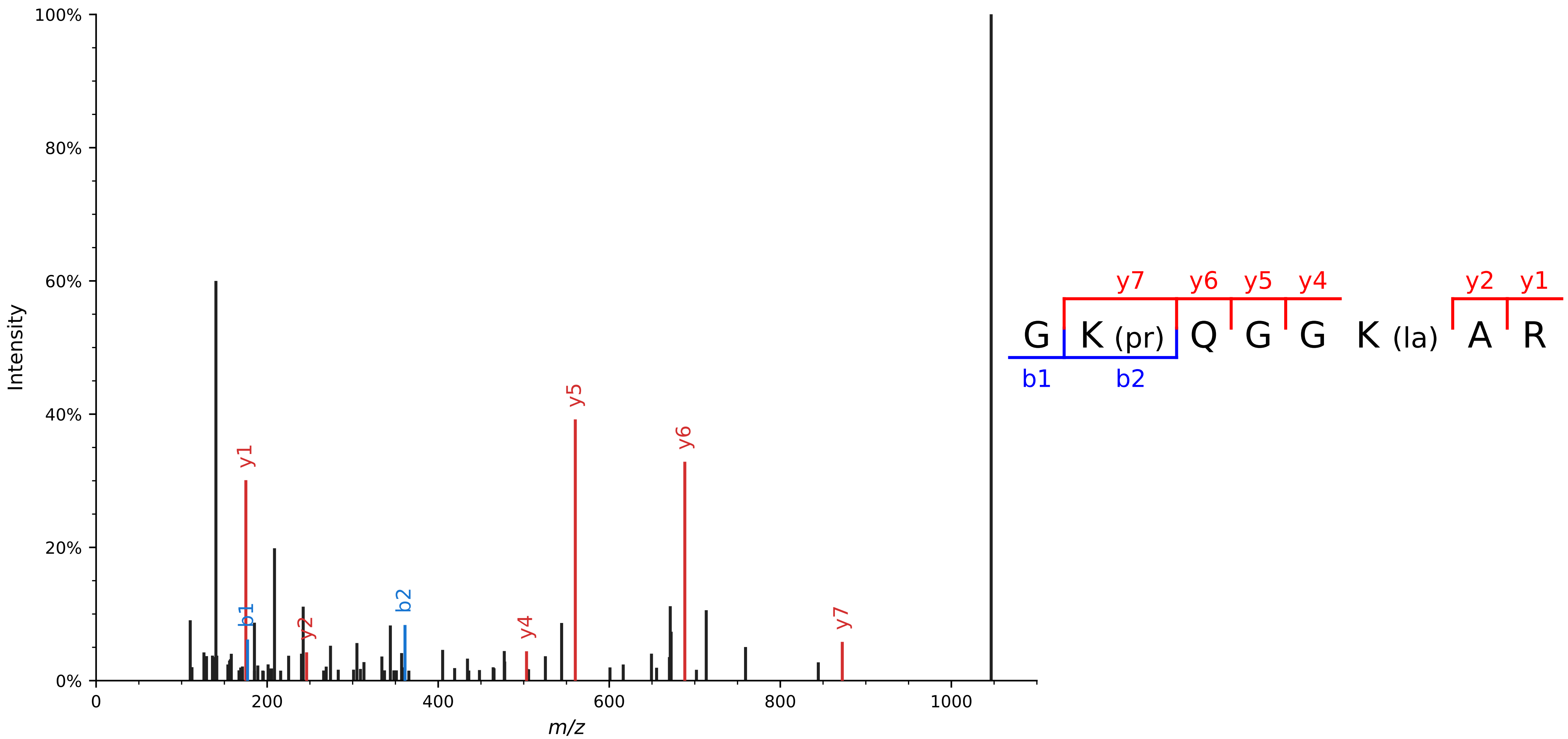

Raw file: HF240124_RN_spleen_PRO_PIC_2, Scan: 9784, m/z: 446.25598, Charge: 2, RT (min): 18.65, Score: 76.035

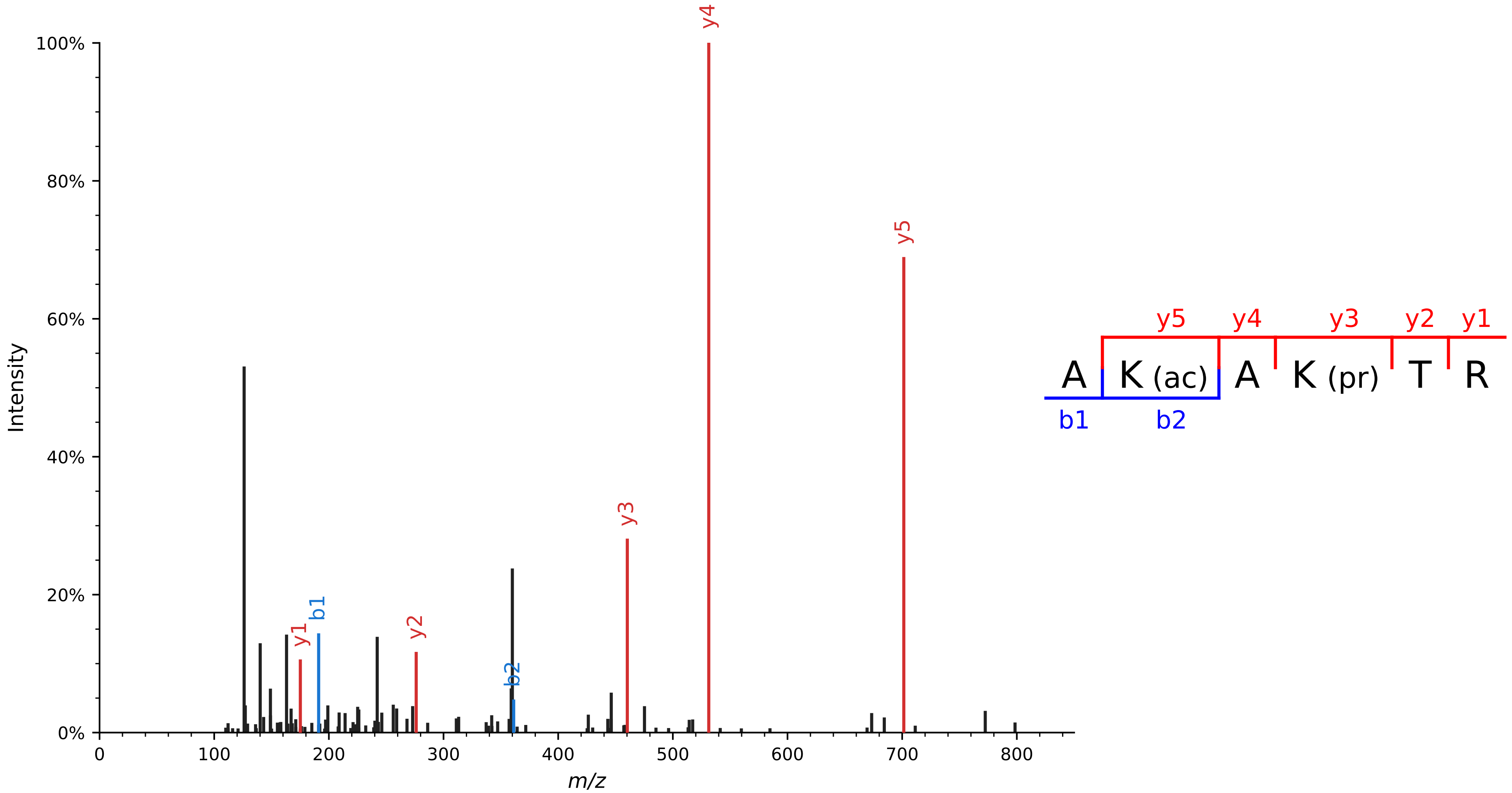

Raw file: HF240124_RN_spleen_PRO_PIC_3, Scan: 9694, m/z: 446.25598, Charge: 2, RT (min): 18.47, Score: 79.146

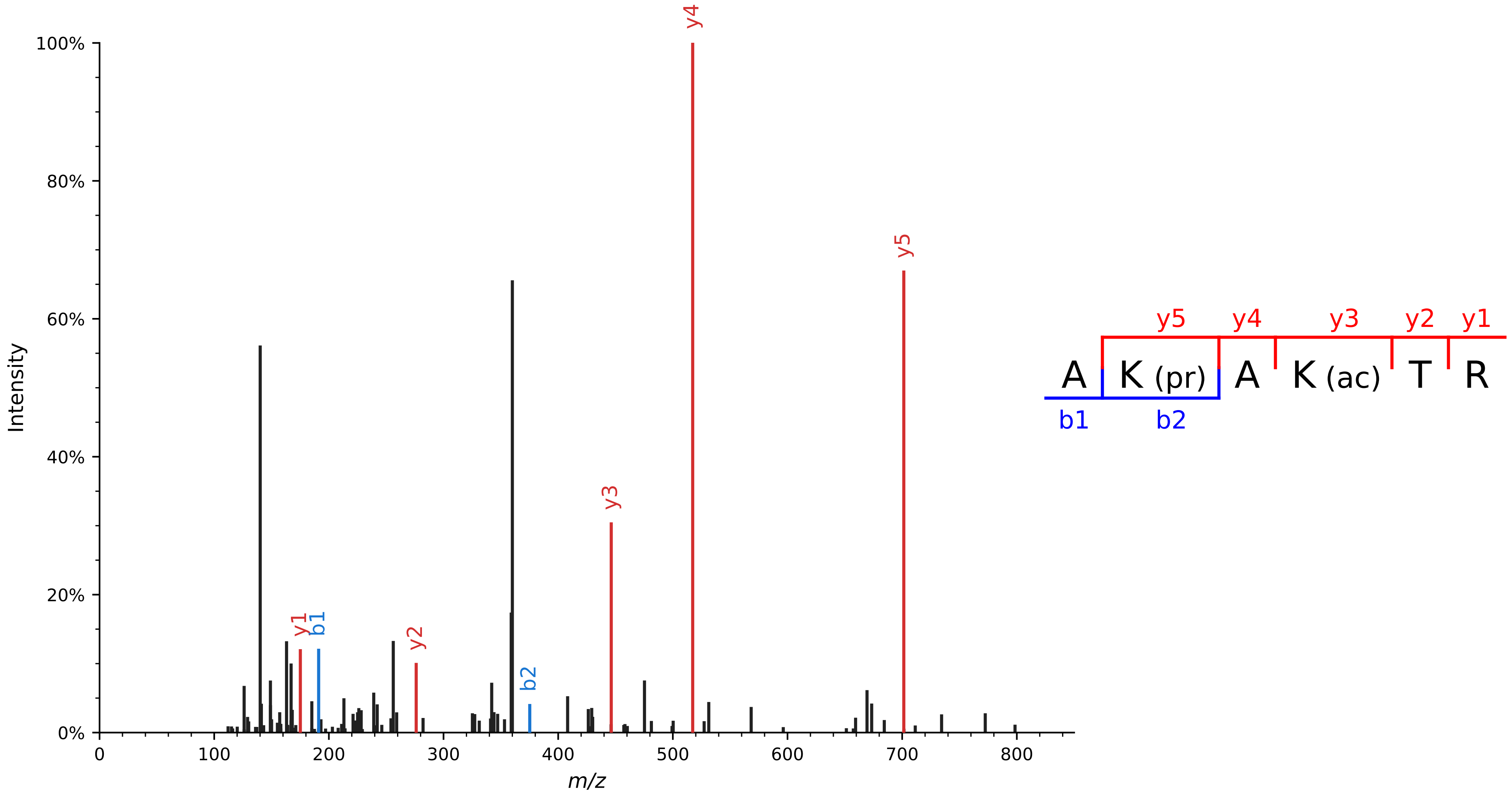

Raw file: HF240124_RN_spleen_PRO_PIC_2, Scan: 7310, m/z: 496.26962, Charge: 2, RT (min): 14.37, Score: 111.82

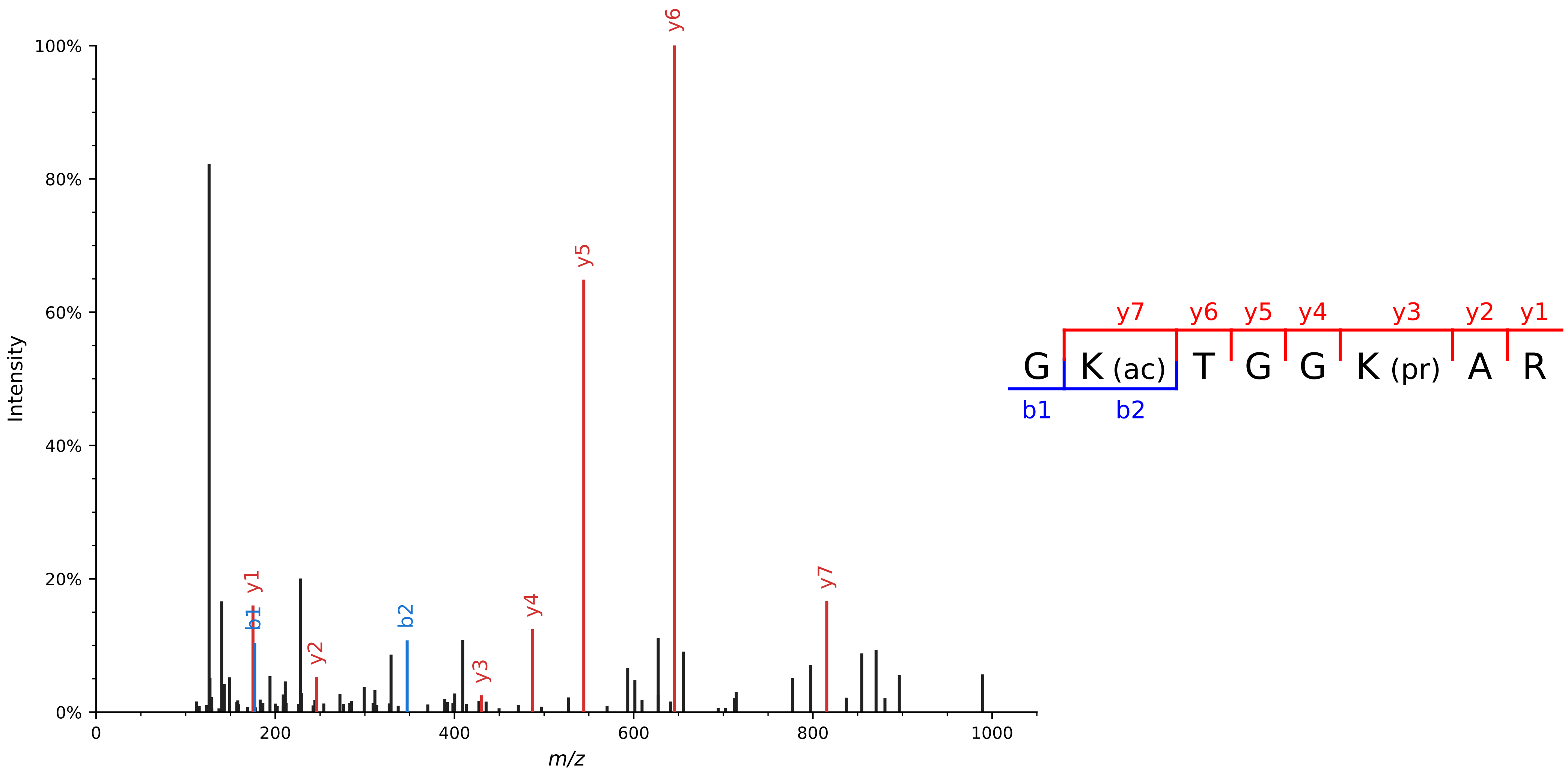

Raw file: HF240124_RN_spleen_PRO_PIC_3, Scan: 7444, m/z: 496.26962, Charge: 2, RT (min): 14.60, Score: 98.997

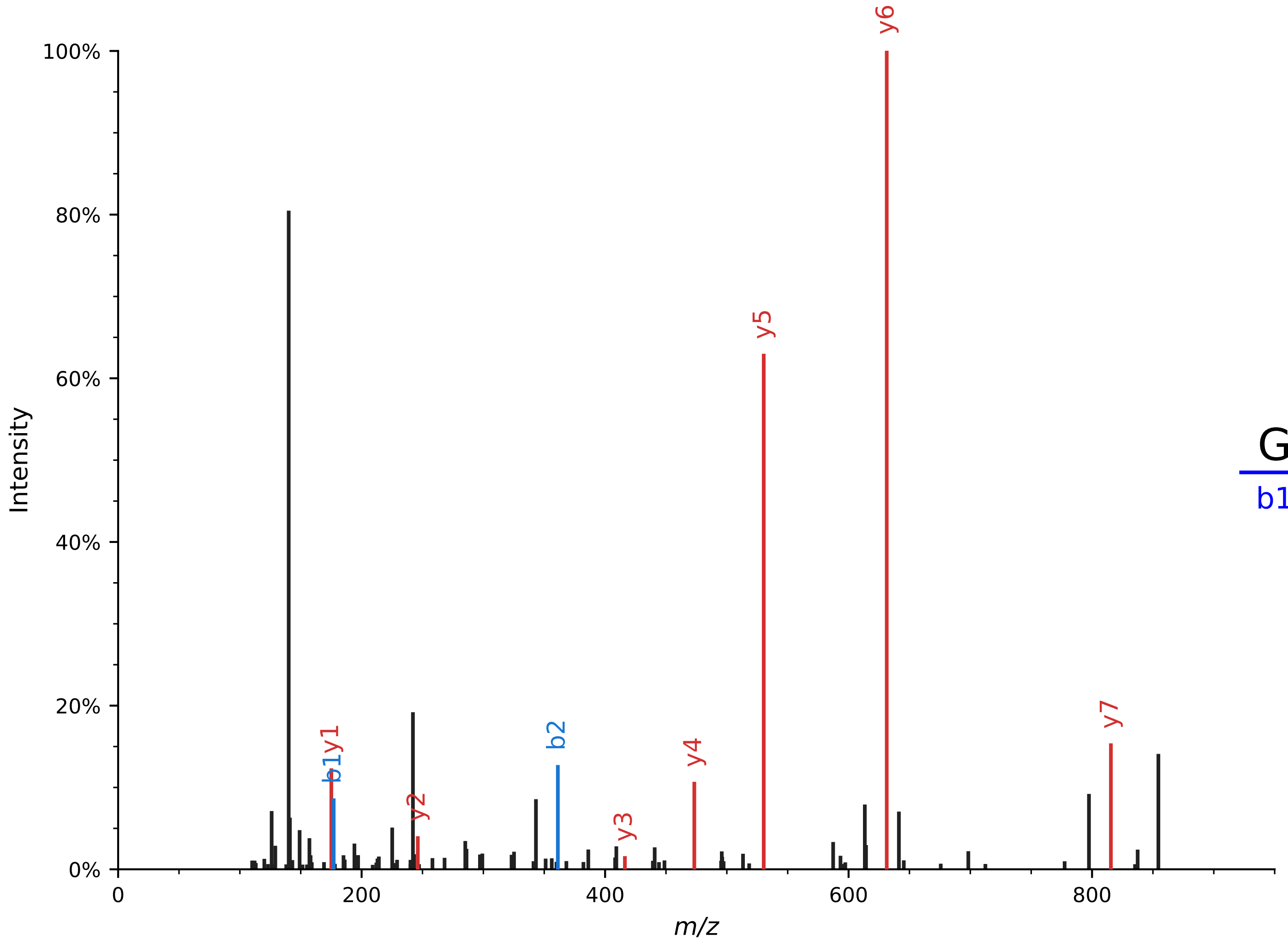

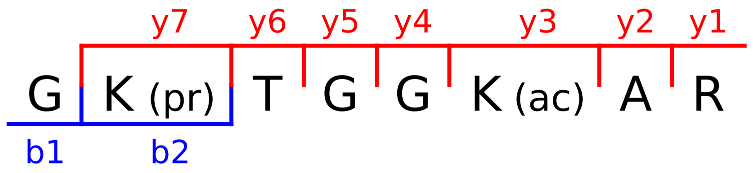

Raw file: HF240124_RN_spleen_PRO_PIC_3, Scan: 3020, m/z: 526.25142, Charge: 2, RT (min): 6.67, Score: 41.029

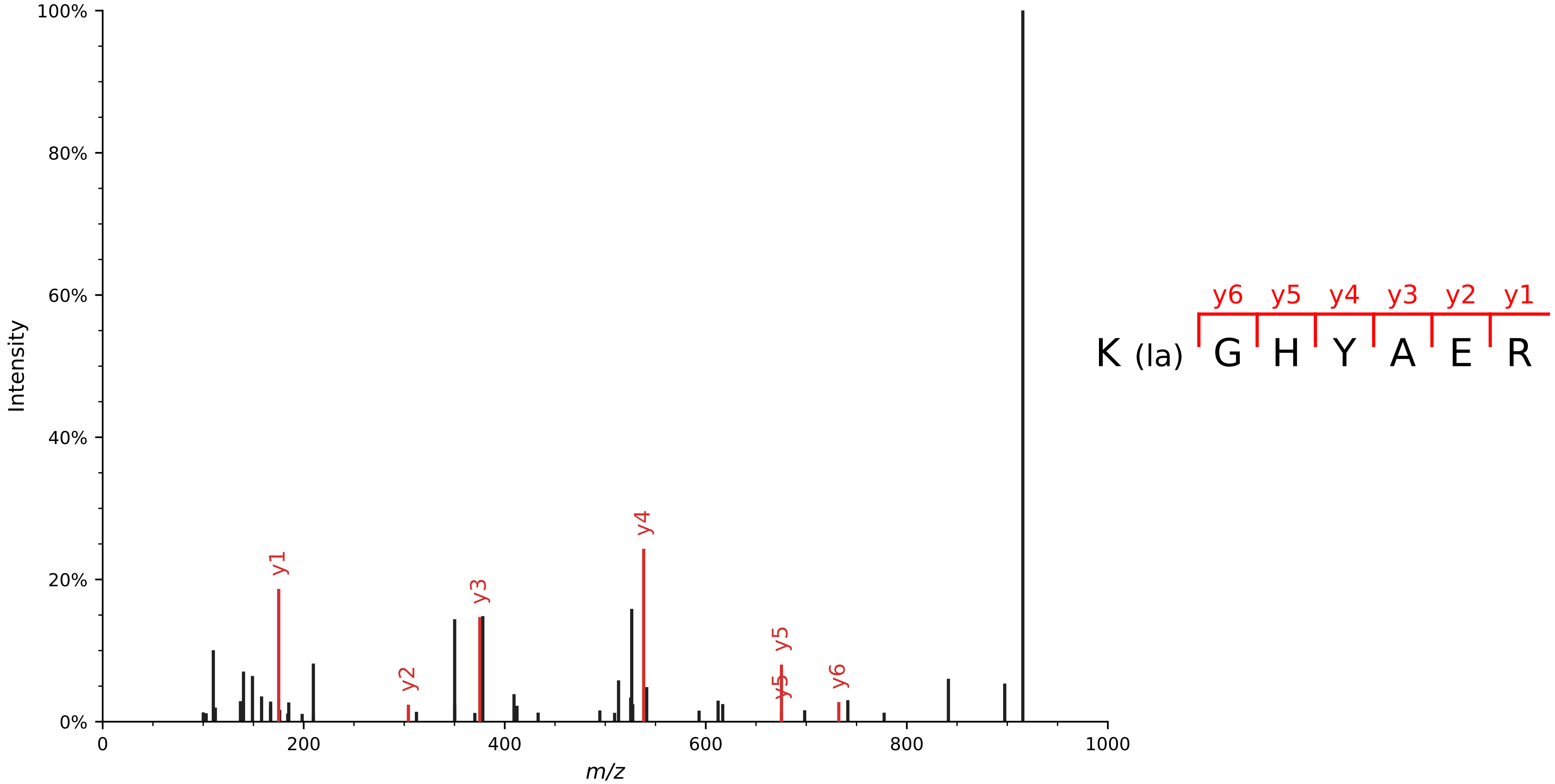

Raw file: HF240124_RN_spleen_PRO_PIC_2, Scan: 7416, m/z: 524.76978, Charge: 2, RT (min): 14.56, Score: 61.123

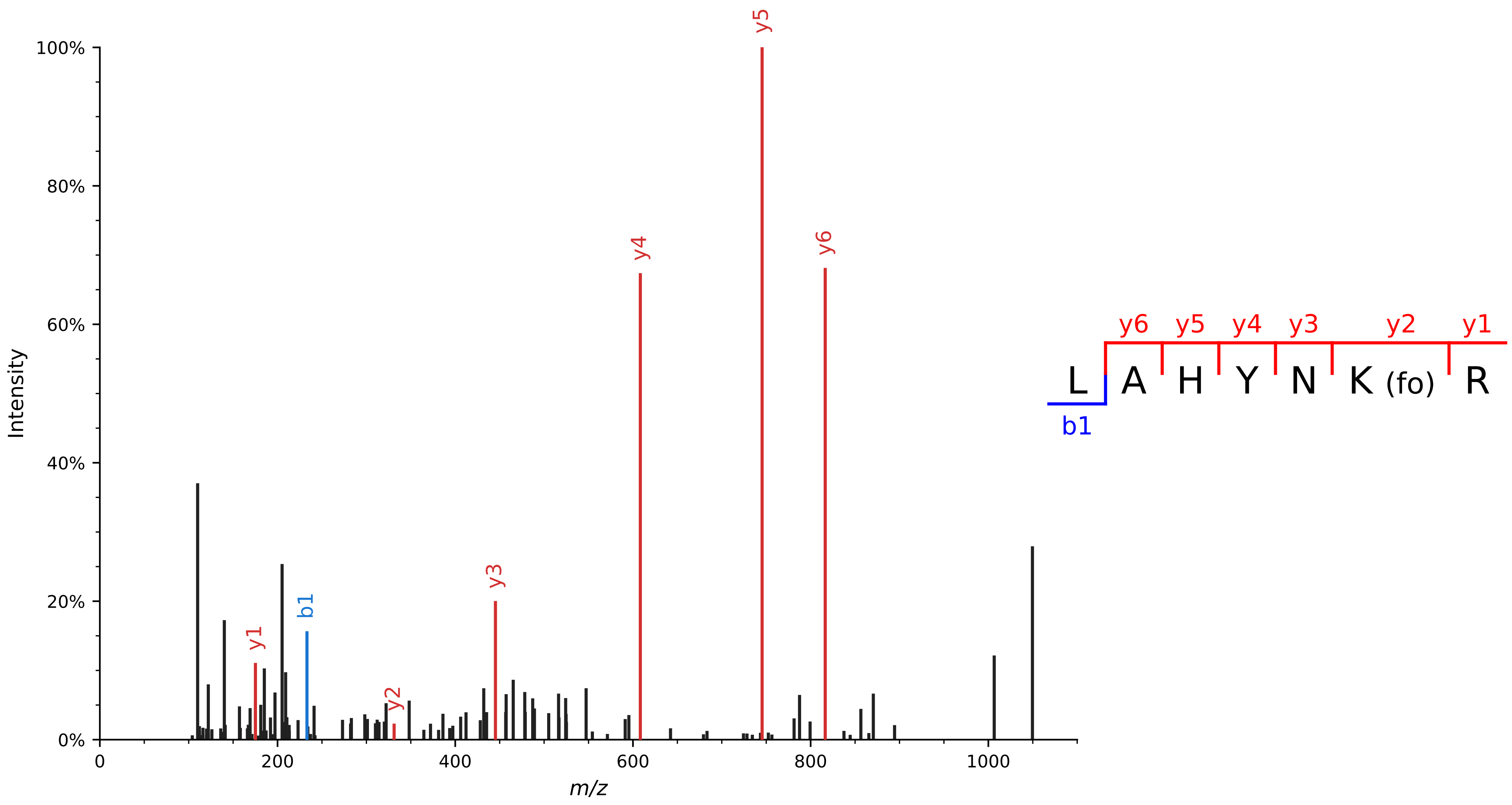

Raw file: HF240124_RN_spleen_PRO_PIC_2, Scan: 20483, m/z: 567.78817, Charge: 2, RT (min): 37.65, Score: 55.395

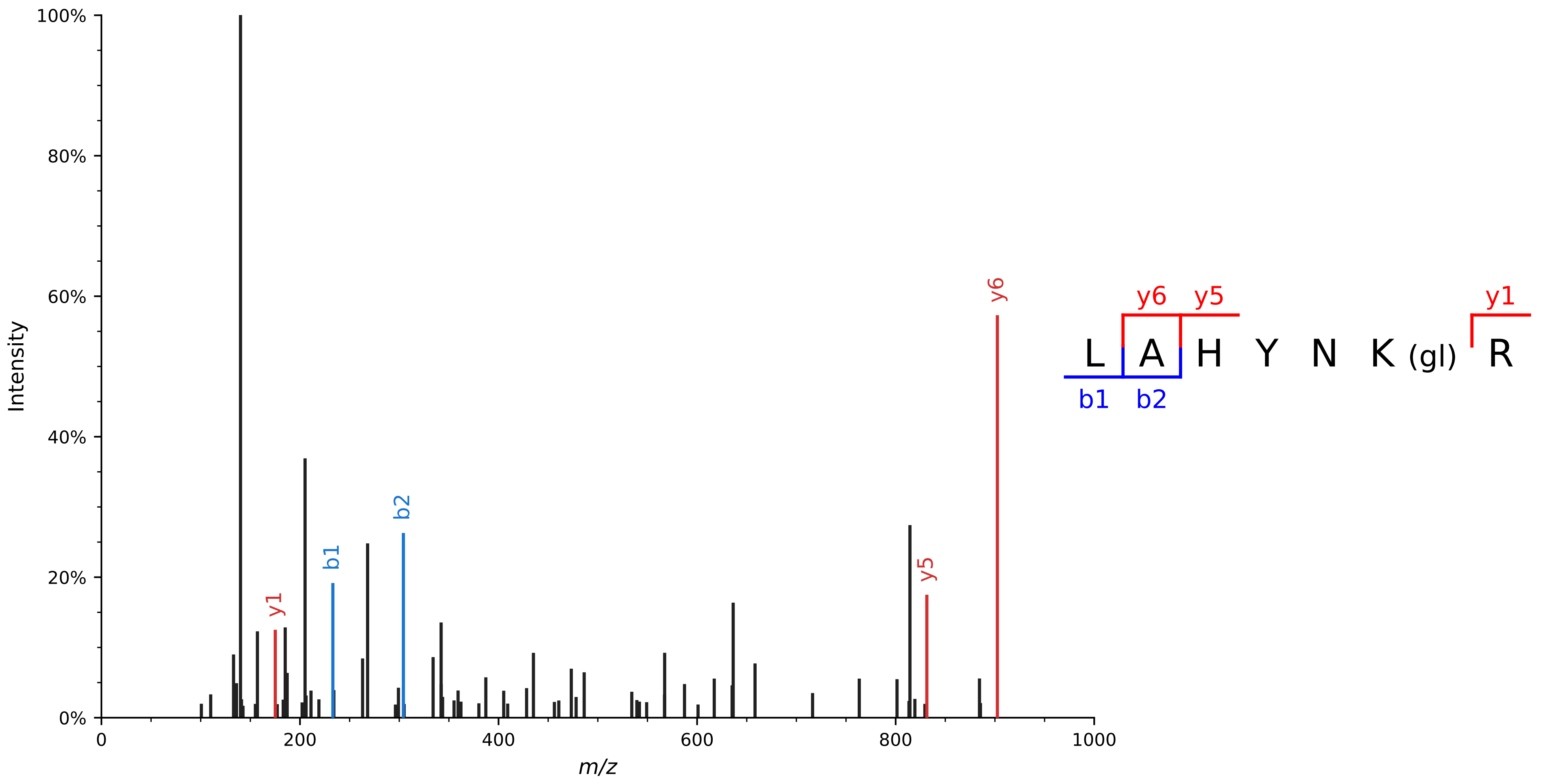

Raw file: HF240124_RN_spleen_PRO_PIC_3, Scan: 8036, m/z: 546.78289, Charge: 2, RT (min): 15.59, Score: 80.377

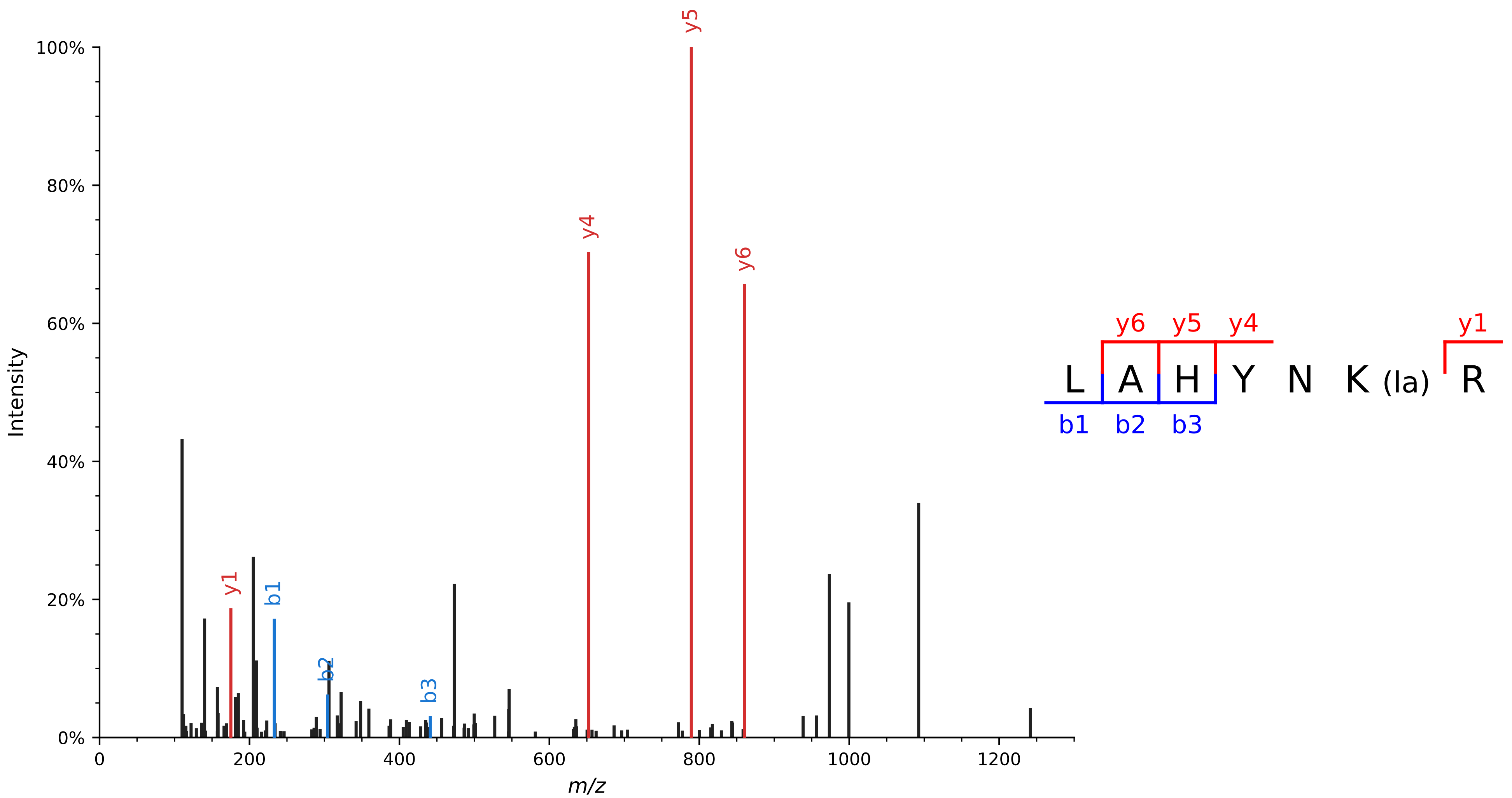

Raw file: HF240124_RN_spleen_PRO_PIC_3, Scan: 5620, m/z: 553.77252, Charge: 2, RT (min): 11.23, Score: 42.719

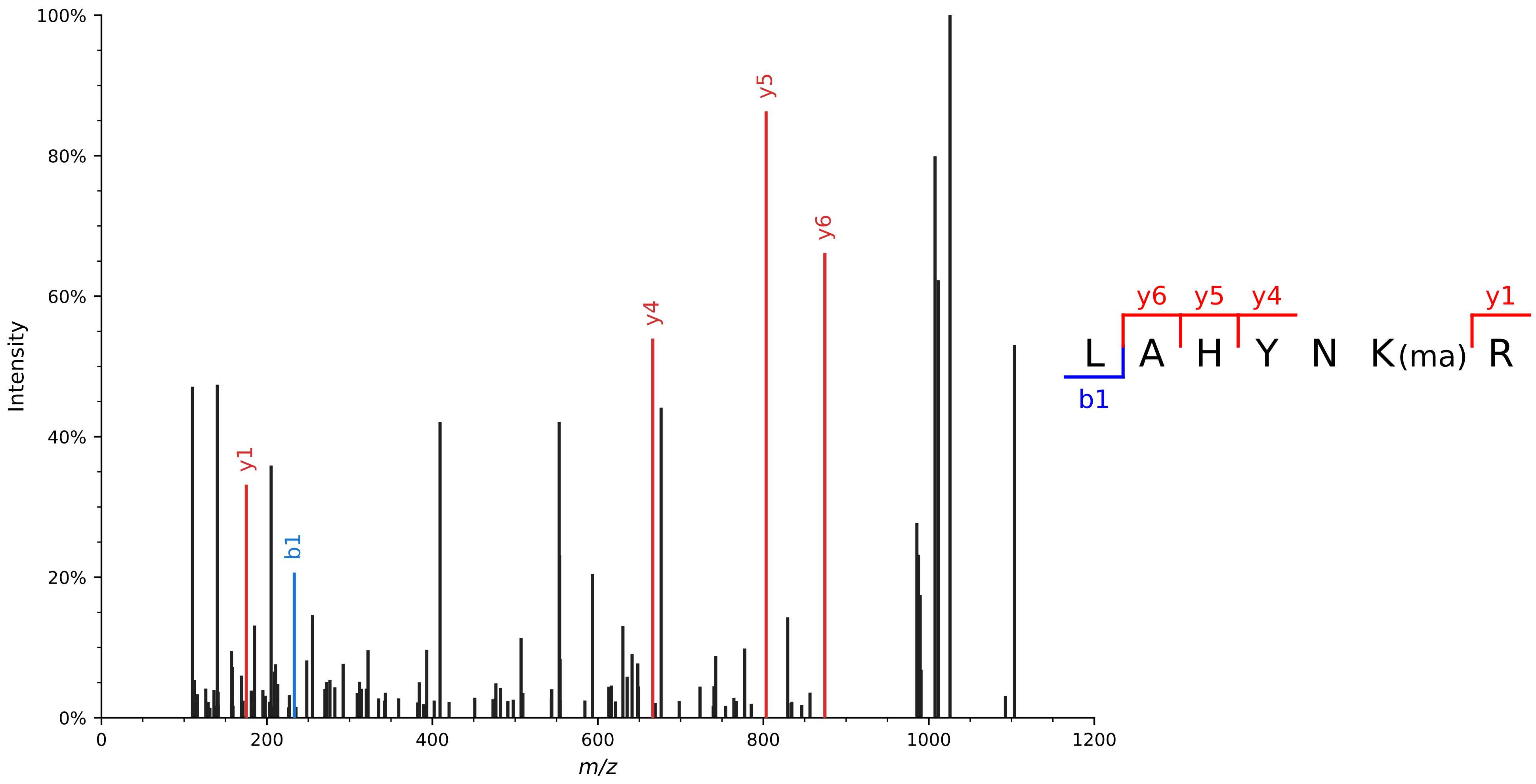

Raw file: HF240124_RN_spleen_PRO_PIC_2, Scan: 10077, m/z: 545.79326, Charge: 2, RT (min): 19.25, Score: 104.94

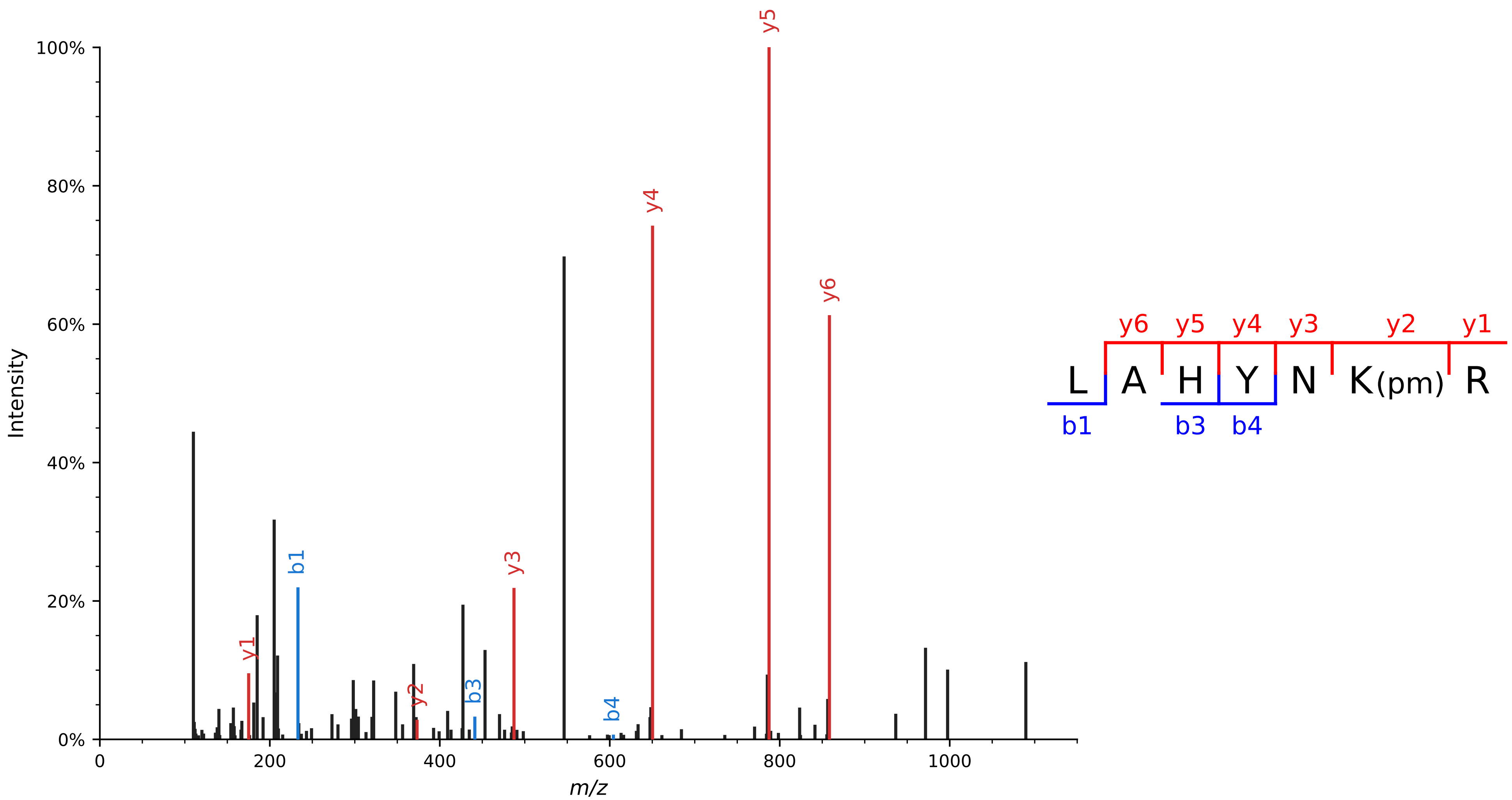

Raw file: HF240124_RN_spleen_PRO_PIC_2, Scan: 20438, m/z: 630.35896, Charge: 2, RT (min): 37.57, Score: 91.318

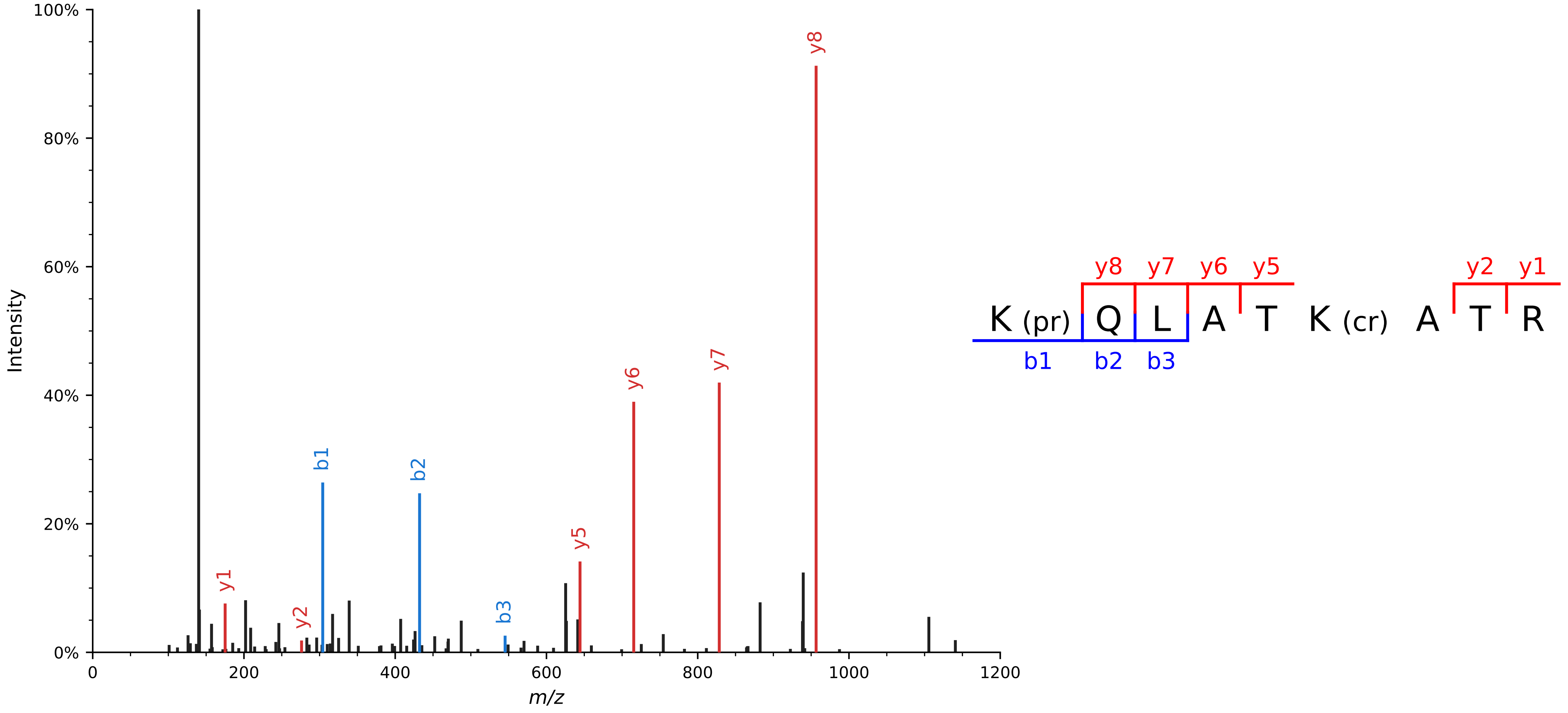

Raw file: HF240124_RN_spleen_PRO_PIC_3, Scan: 18504, m/z: 653.3617, Charge: 2, RT (min): 34.03, Score: 45.972

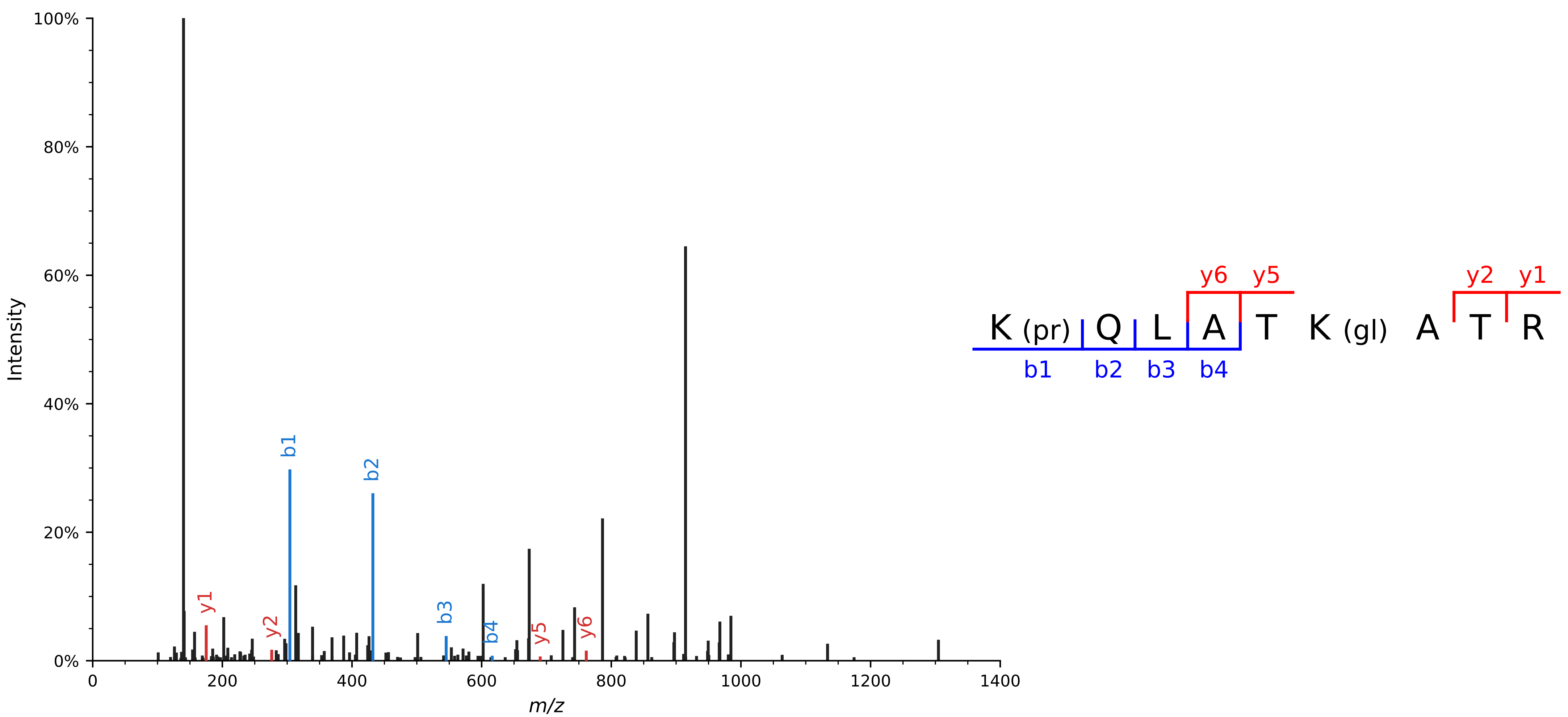

Raw file: HF240124_RN_spleen_PRO_PIC_3, Scan: 9033, m/z: 861.96793, Charge: 2, RT (min): 17.34, Score: 186.87

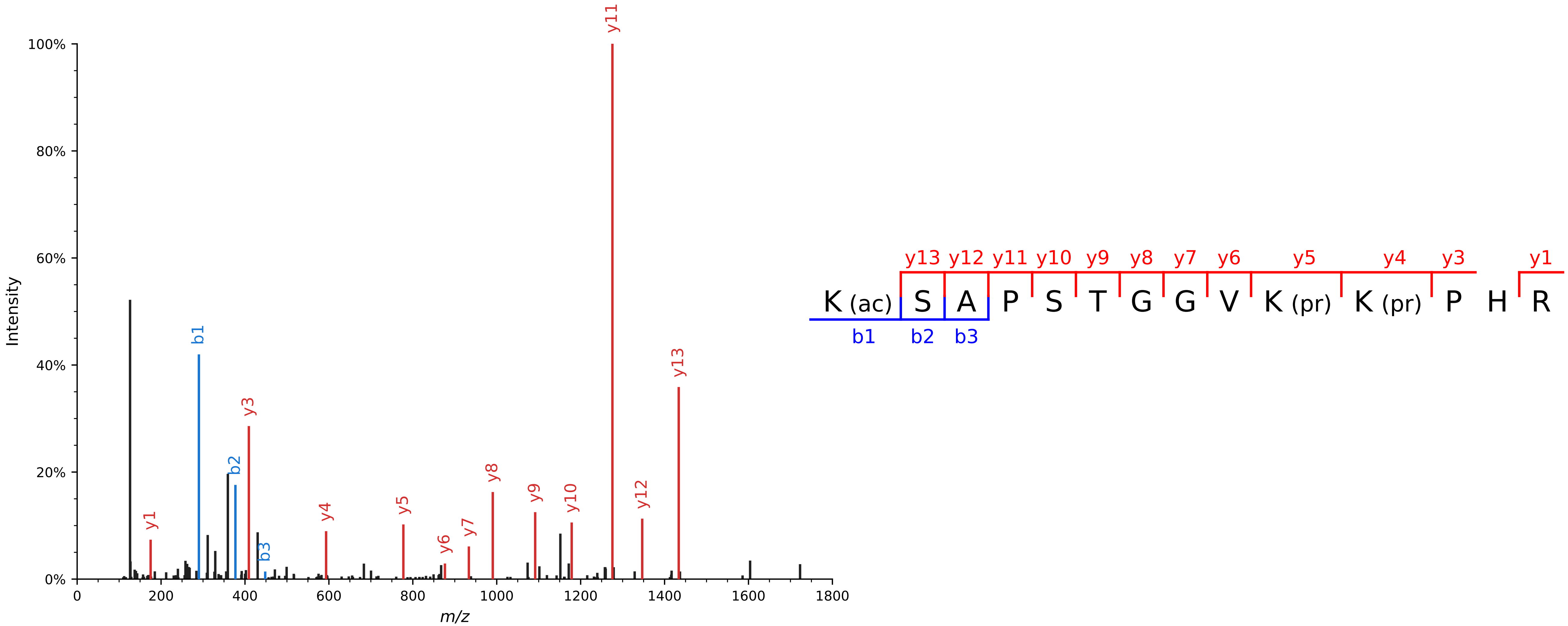

Raw file: HF240124_RN_spleen_PRO_PIC_1, Scan: 11039, m/z: 875.98358, Charge: 2, RT (min): 21.76, Score: 135.99

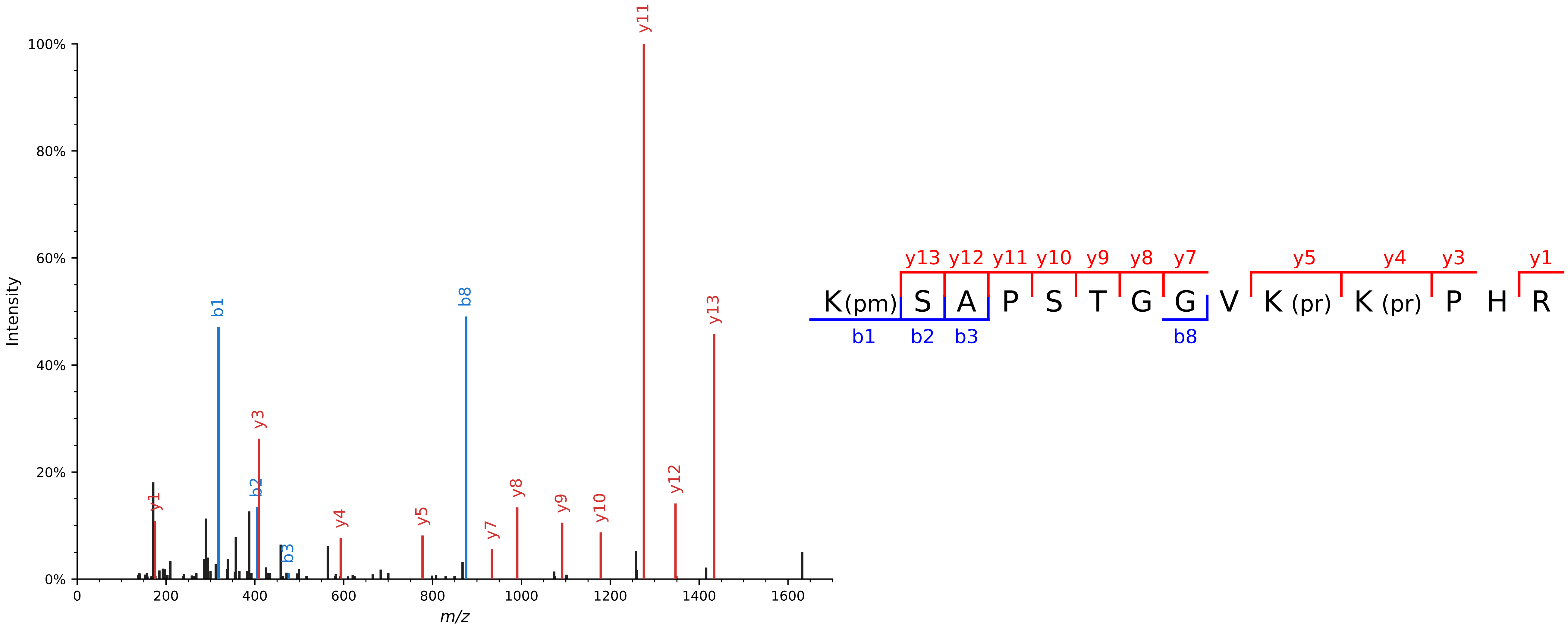

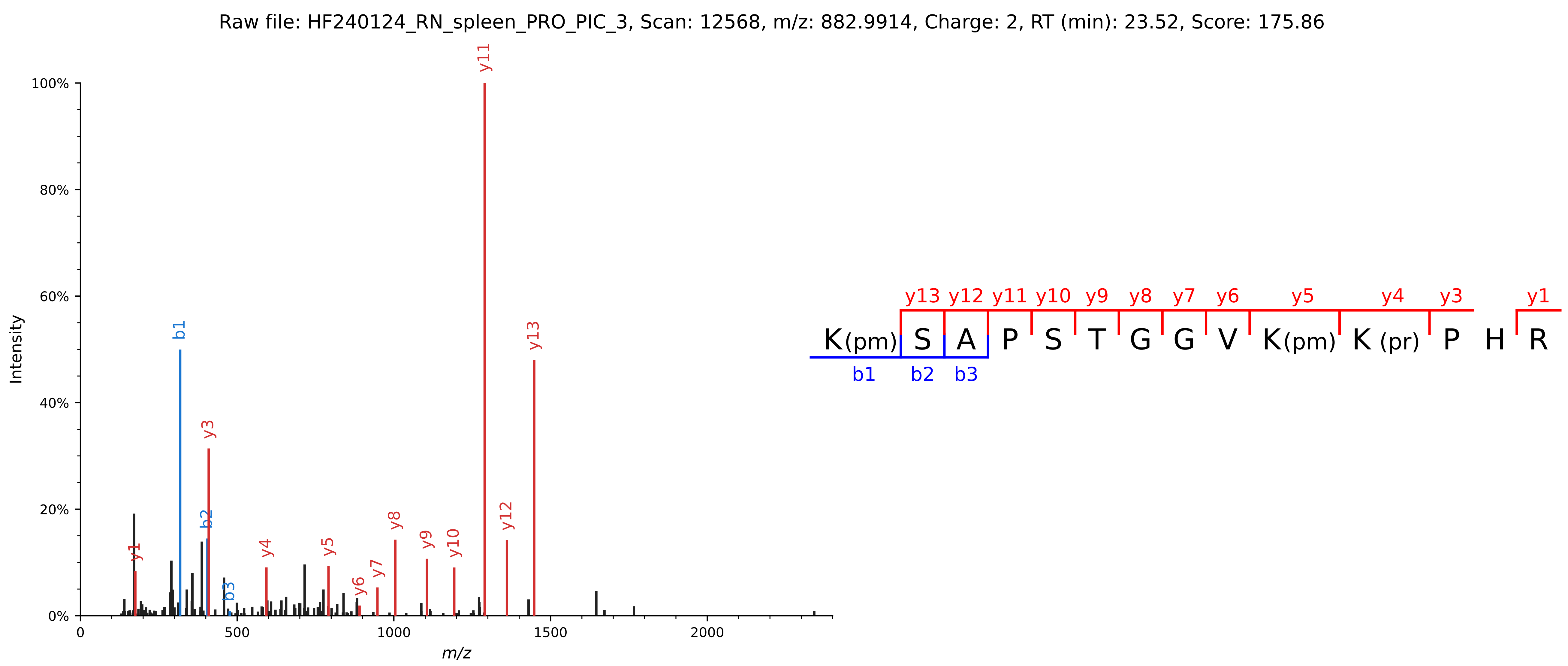

Raw file: HF240124_RN_spleen_PRO_PIC_3, Scan: 12568, m/z: 882.9914, Charge: 2, RT (min): 23.52, Score: 175.86
Intensity
m/z
K(pm) S A P S T G G V K(pm) K (pr) P H R
y13 y12 y11 y10 y9 y8 y7 y6 y5 y4 y3 y1
b1 b2 b3

Raw file: HF240124_RN_spleen_PRO_PIC_3, Scan: 8967, m/z: 889.99923, Charge: 2, RT (min): 17.21, Score: 53.237

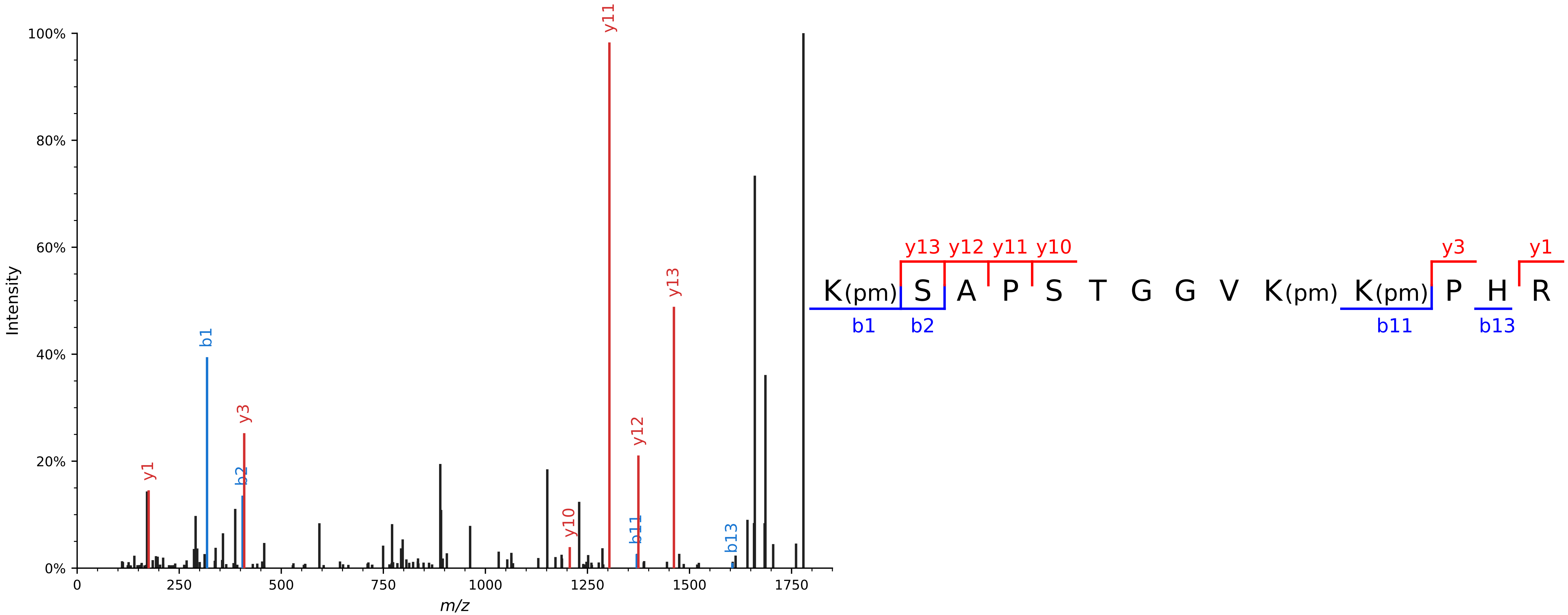

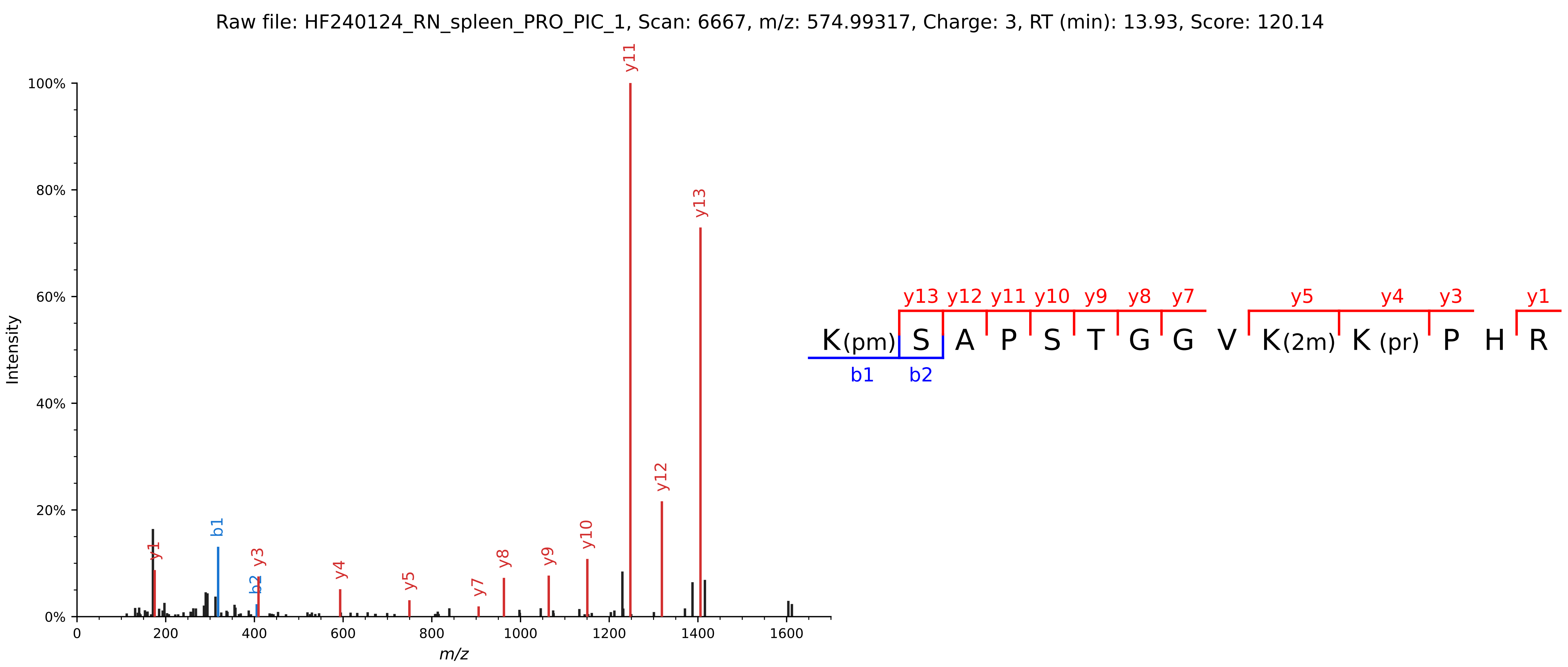

Raw file: HF240124_RN_spleen_PRO_PIC_1, Scan: 6667, m/z: 574.99317, Charge: 3, RT (min): 13.93, Score: 120.14
Intensity
m/z
0%
20%
40%
60%
80%
100%
0
200
400
600
800
1000
1200
1400
1600
y1
b1
b2
y3
y4
y5
y7
y8
y9
y10
y11
y12
y13
K(pm) S A P S T G G V K(2m) K (pr) P H R

Raw file: HF240124_RN_spleen_PRO_PIC_2, Scan: 6907, m/z: 868.99395, Charge: 2, RT (min): 13.69, Score: 72.2

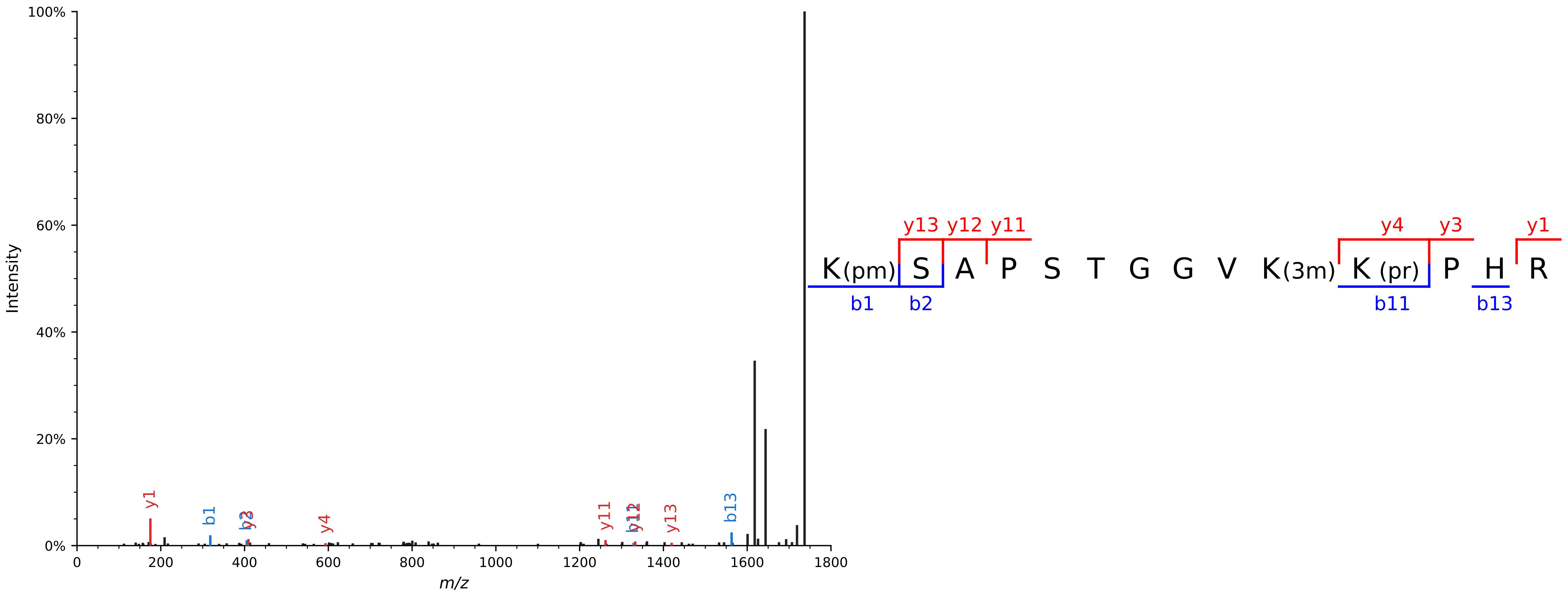

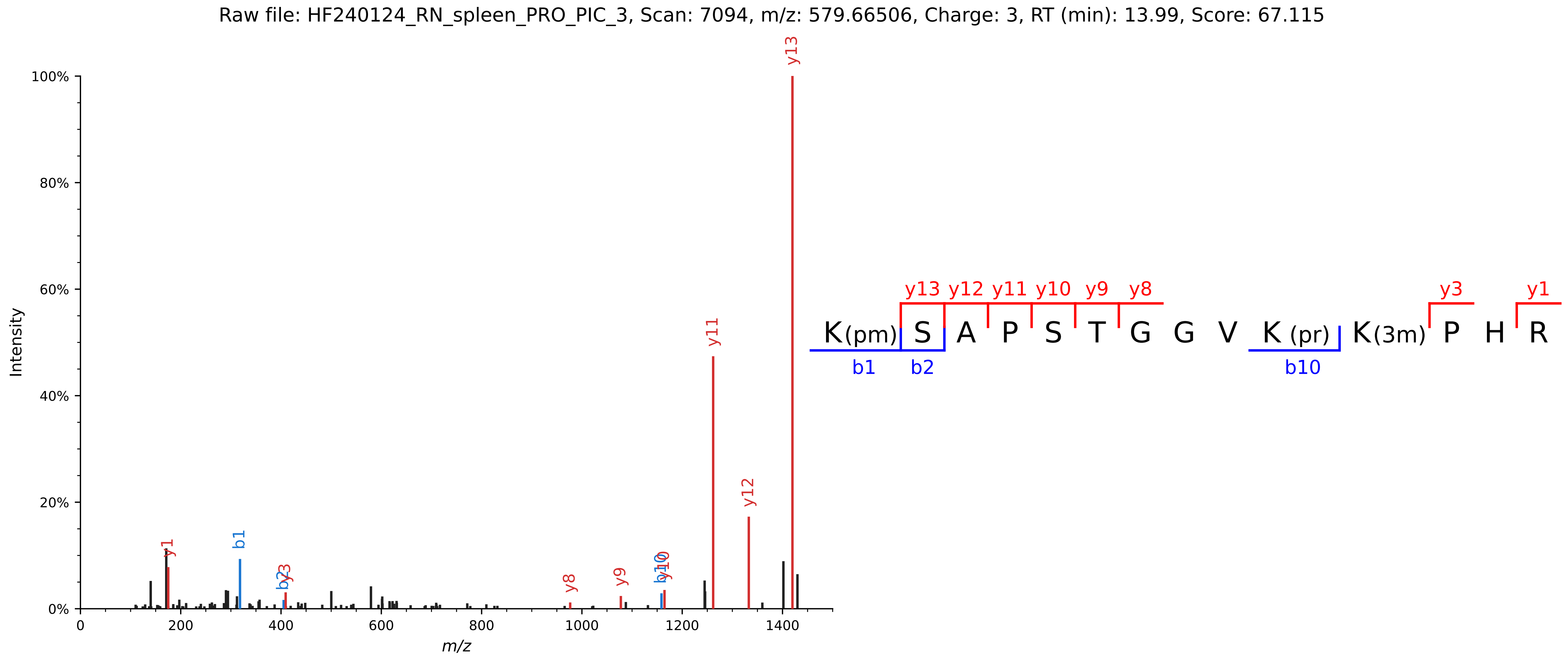

Raw file: HF240124_RN_spleen_PRO_PIC_3, Scan: 7094, m/z: 579.66506, Charge: 3, RT (min): 13.99, Score: 67.115
Intensity
m/z
y13 y12 y11 y10 y9 y8 y3 y1
K(pm) S A P S T G G V K (pr) K(3m) P H R
b1 b2 b10

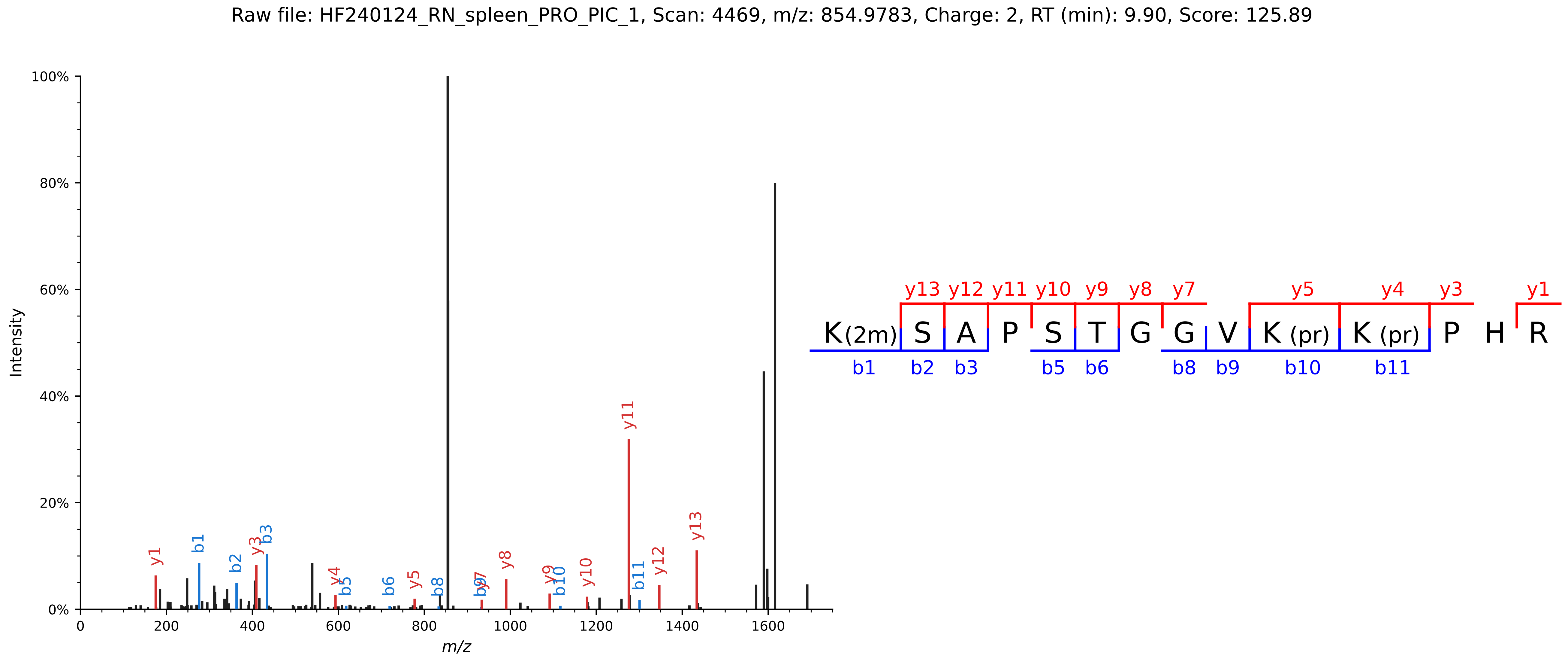

Raw file: HF240124_RN_spleen_PRO_PIC_1, Scan: 4469, m/z: 854.9783, Charge: 2, RT (min): 9.90, Score: 125.89
Intensity
m/z
K(2m) S A P S T G G V K (pr) K (pr) P H R
y13 y12 y11 y10 y9 y8 y7 y5 y4 y3 y1
b1 b2 b3 b5 b6 b8 b9 b10 b11

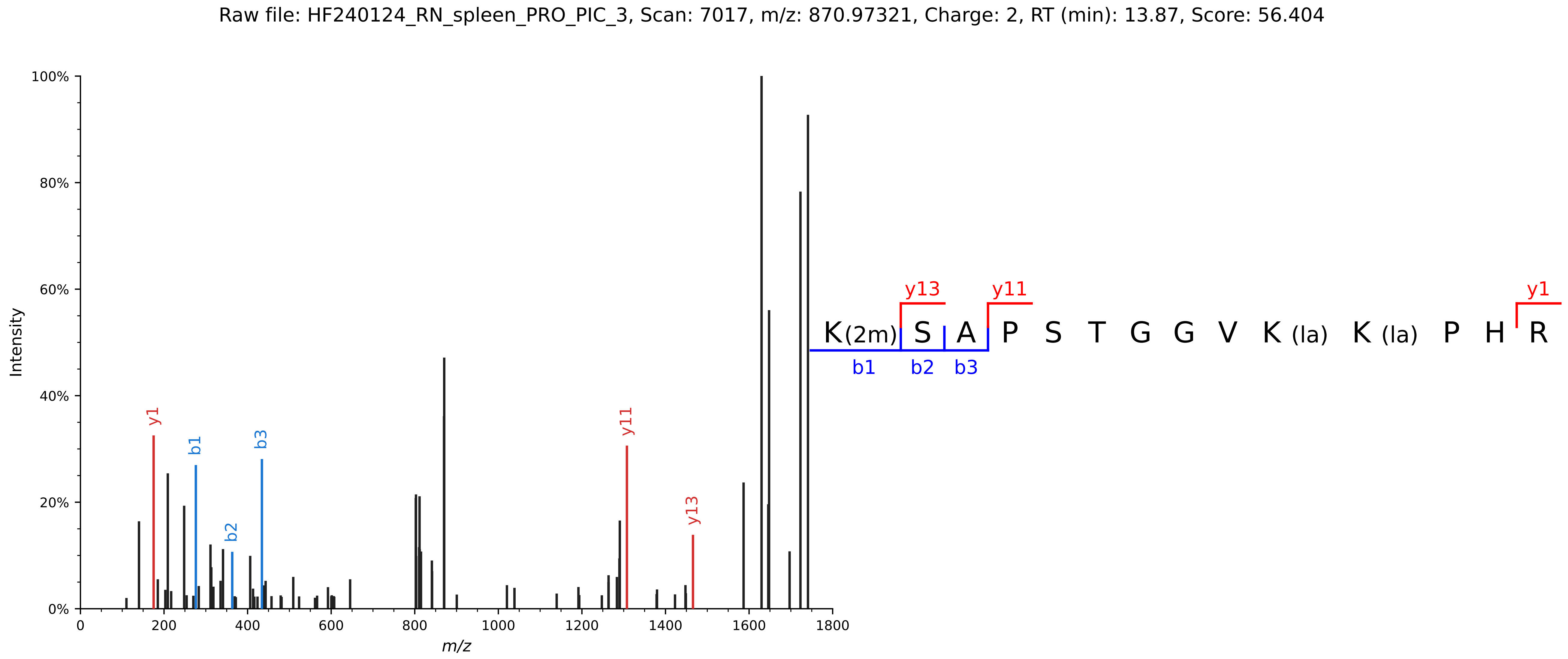
Raw file: HF240124_RN_spleen_PRO_PIC_3, Scan: 7017, m/z: 870.97321, Charge: 2, RT (min): 13.87, Score: 56.404
Intensity
m/z
y1
b1
b2
b3
y11
y13
K(2m) S A P S T G G V K (la) K (la) P H R

Raw file: HF240124_RN_spleen_PRO_PIC_2, Scan: 5783, m/z: 861.98612, Charge: 2, RT (min): 11.61, Score: 173.76

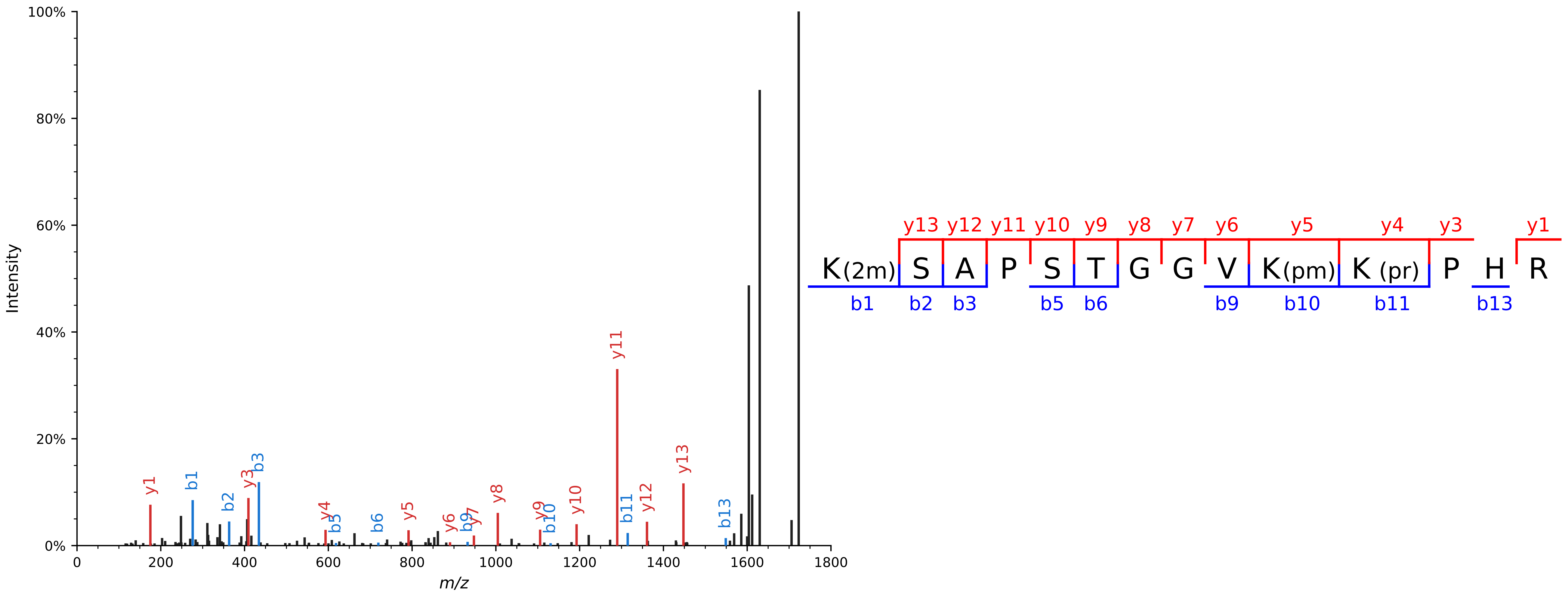

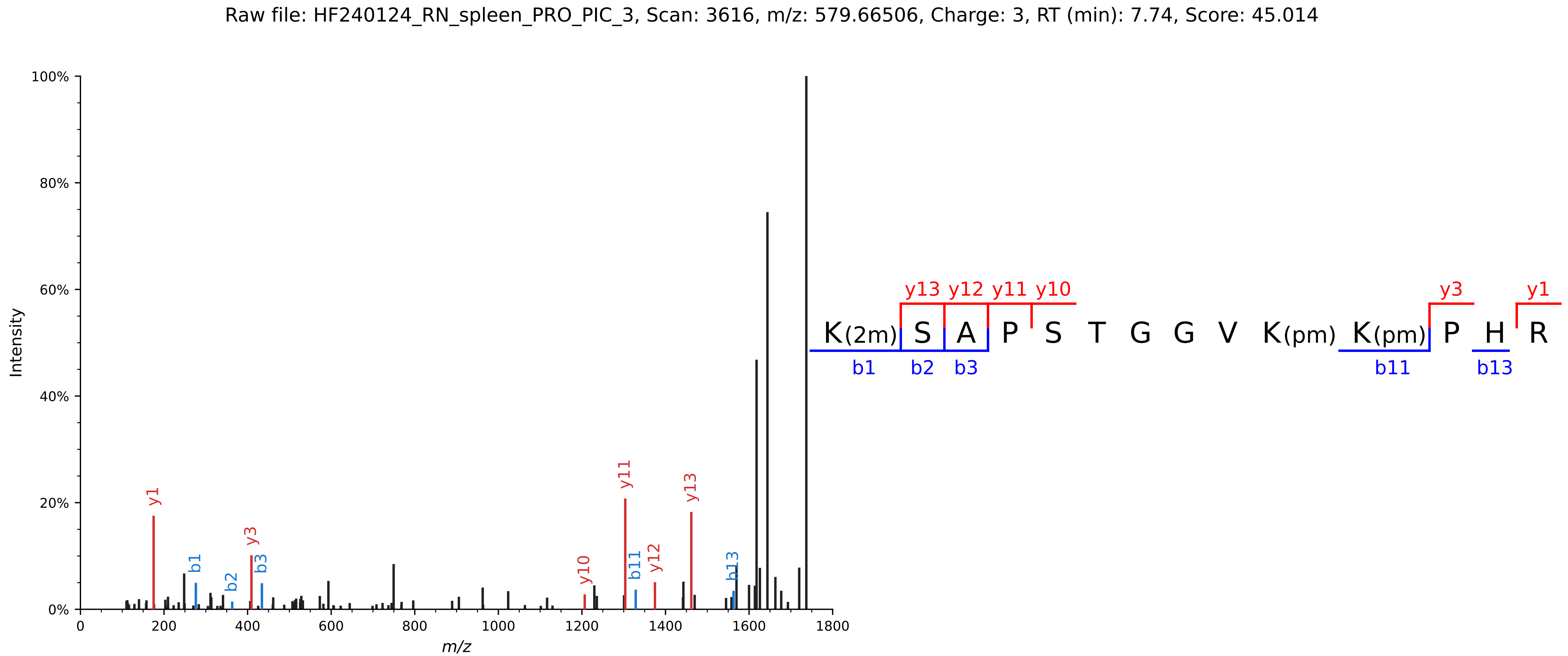
Raw file: HF240124_RN_spleen_PRO_PIC_3, Scan: 3616, m/z: 579.66506, Charge: 3, RT (min): 7.74, Score: 45.014
Intensity
m/z
y1
b1
b2
y3
b3
y10
y11
b11
y12
y13
b13
y13 y12 y11 y10
y3
y1
K(2m) S A P S T G G V K(pm) K(pm) P H R
b1
b2
b3
b11
b13

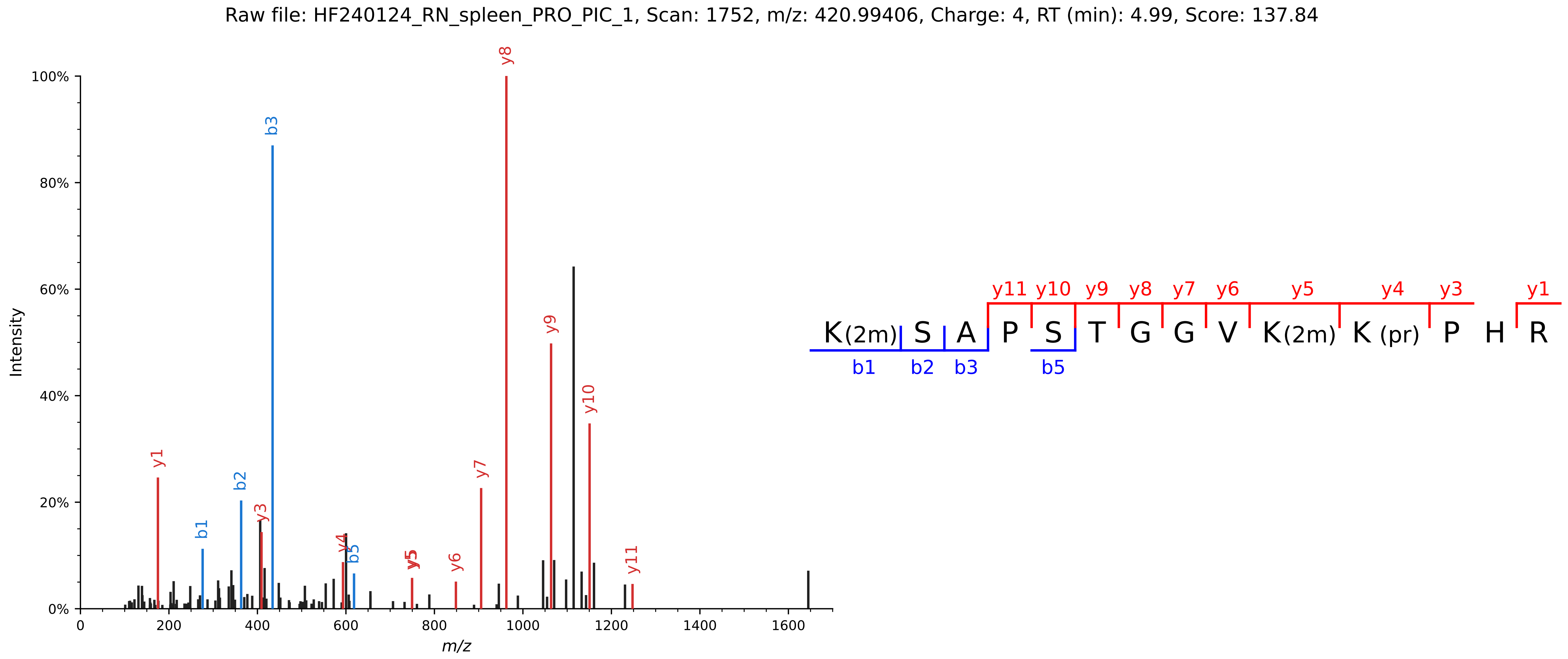

Raw file: HF240124_RN_spleen_PRO_PIC_1, Scan: 1752, m/z: 420.99406, Charge: 4, RT (min): 4.99, Score: 137.84
Intensity
m/z
y1 b1 b2 y3 b3 y4 b5 y5 y6 y7 y8 y9 y10 y11
K(2m) S A P S T G G V K(2m) K (pr) P H R
y11 y10 y9 y8 y7 y6 y5 y4 y3 y1
b1 b2 b3 b5

Raw file: HF240124_RN_spleen_PRO_PIC_1, Scan: 6310, m/z: 862.97575, Charge: 2, RT (min): 13.28, Score: 40.715

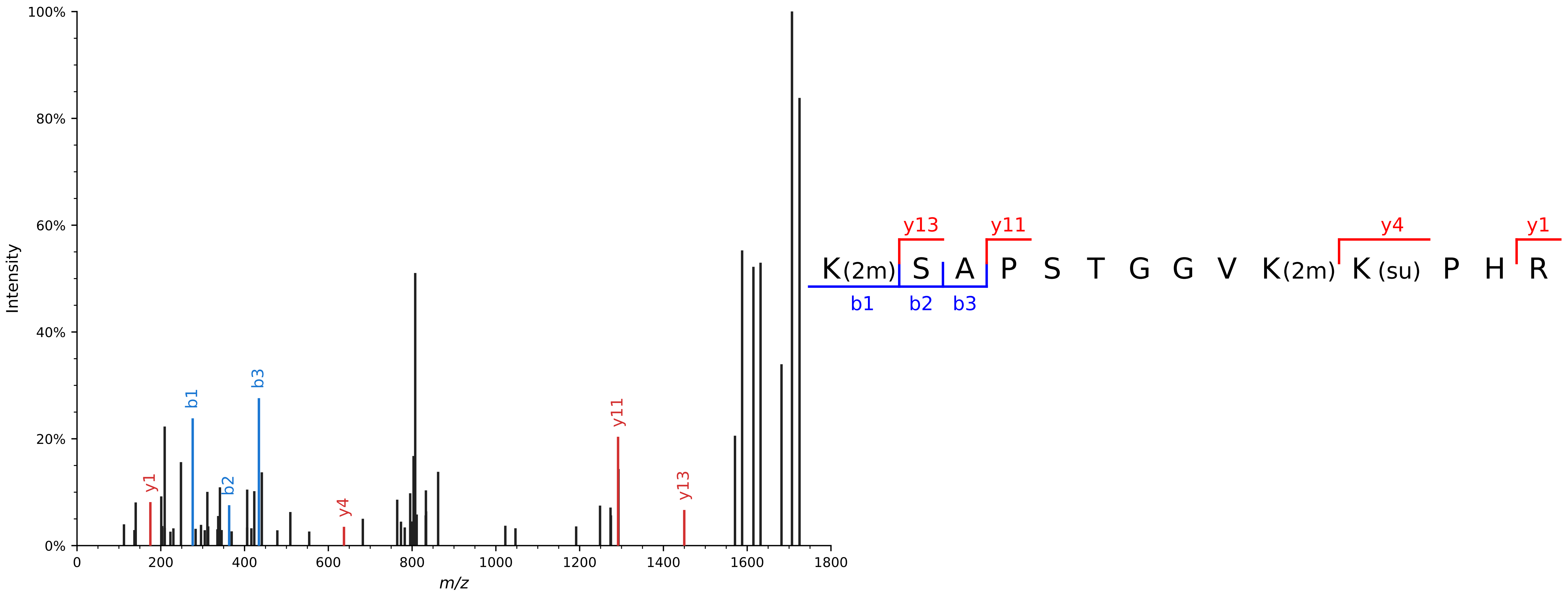

Raw file: HF240124_RN_spleen_PRO_PIC_2, Scan: 1929, m/z: 424.49797, Charge: 4, RT (min): 4.78, Score: 72.681

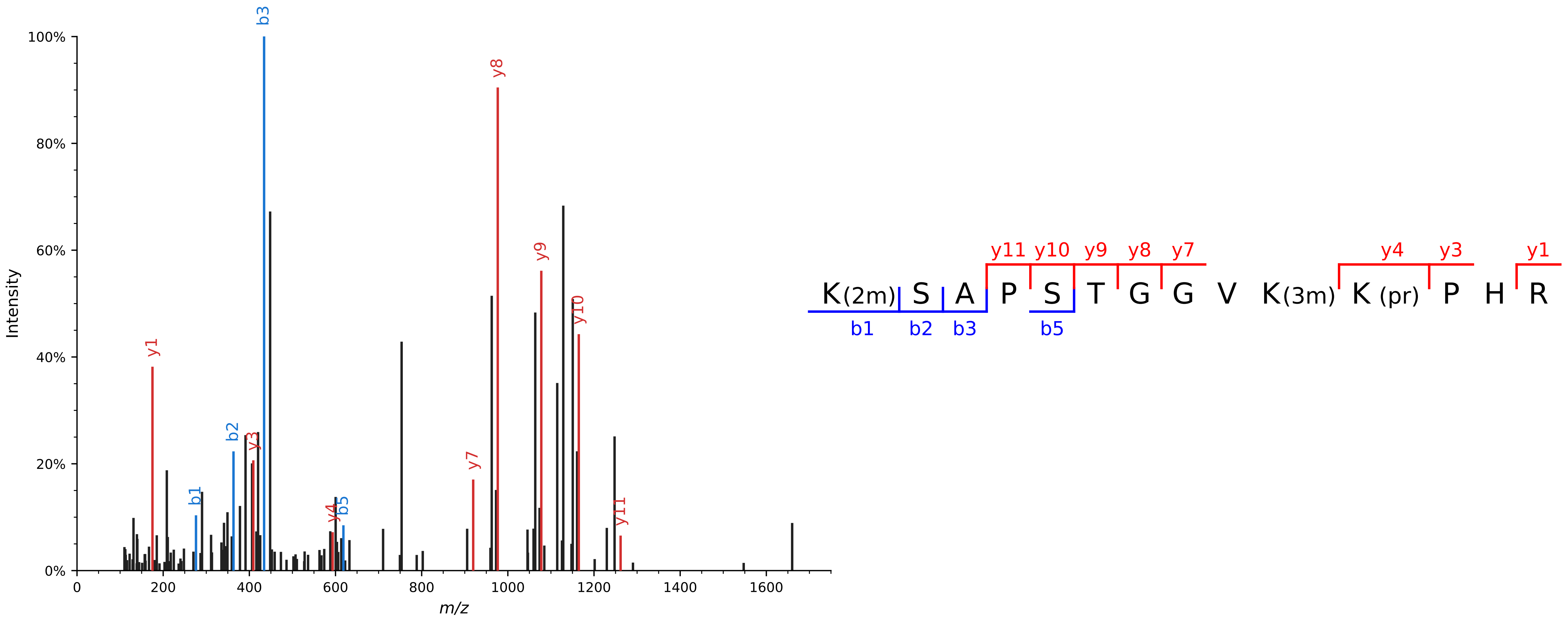

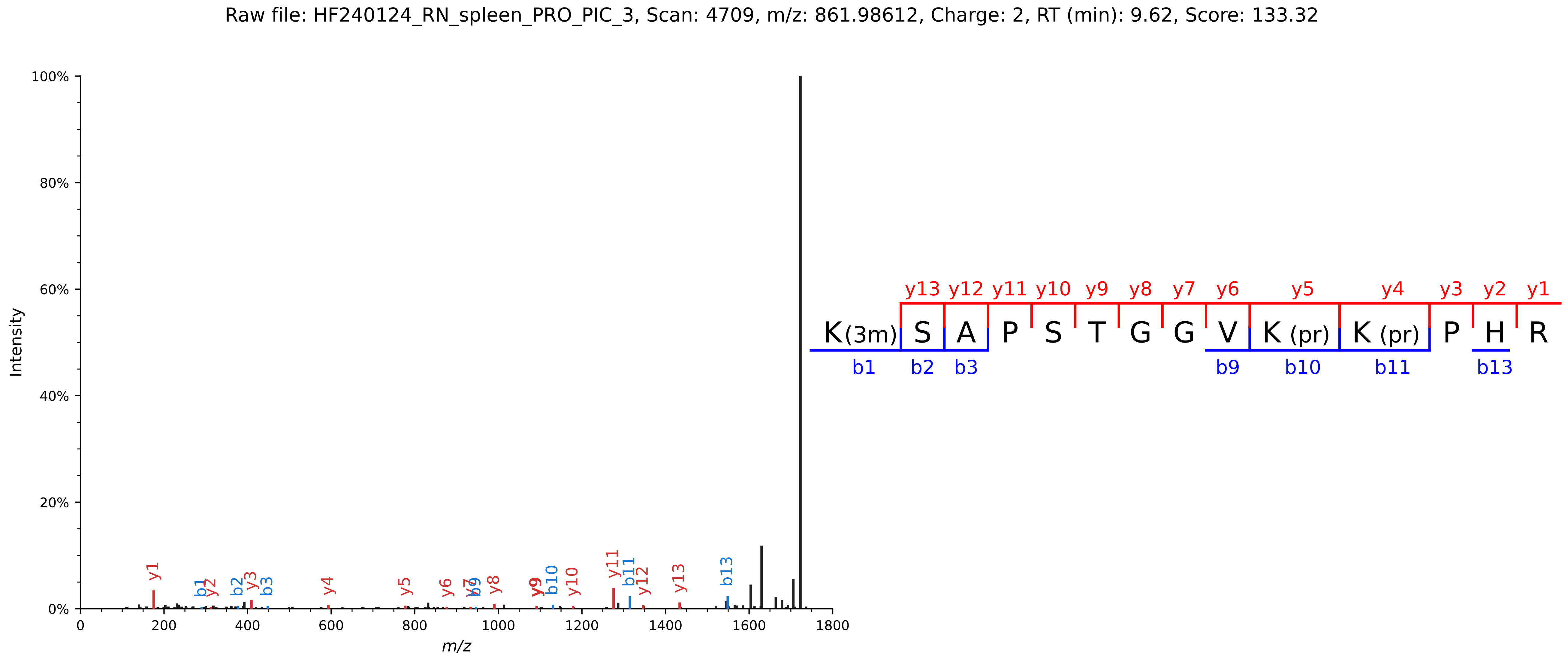

Raw file: HF240124_RN_spleen_PRO_PIC_3, Scan: 4709, m/z: 861.98612, Charge: 2, RT (min): 9.62, Score: 133.32
Intensity
m/z
K(3m) S A P S T G G V K (pr) K (pr) P H R
y13 y12 y11 y10 y9 y8 y7 y6 y5 y4 y3 y2 y1
b1 b2 b3 b9 b10 b11 b13

Raw file: HF240124_RN_spleen_PRO_PIC_1, Scan: 5385, m/z: 579.66506, Charge: 3, RT (min): 11.52, Score: 148.7

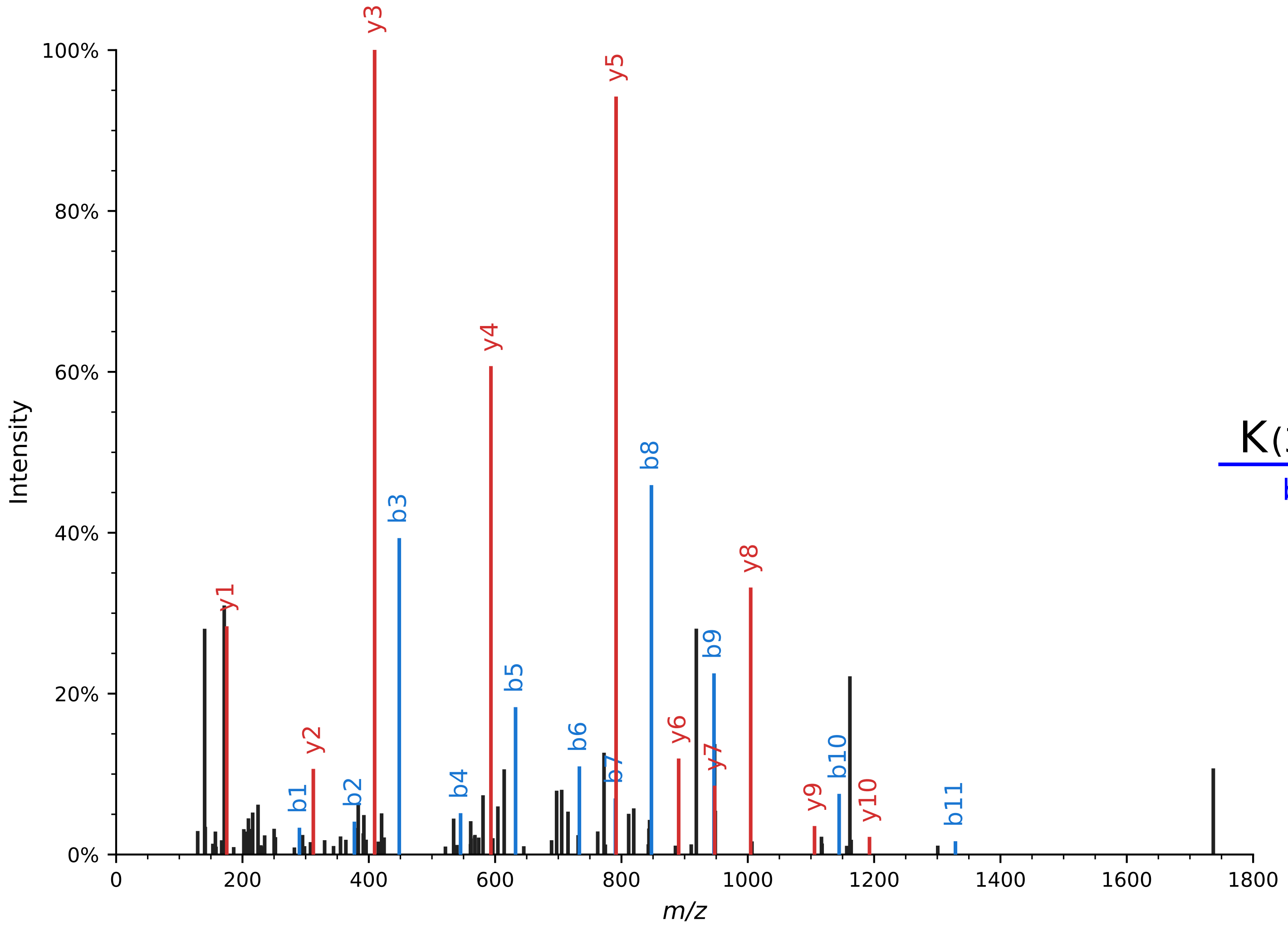

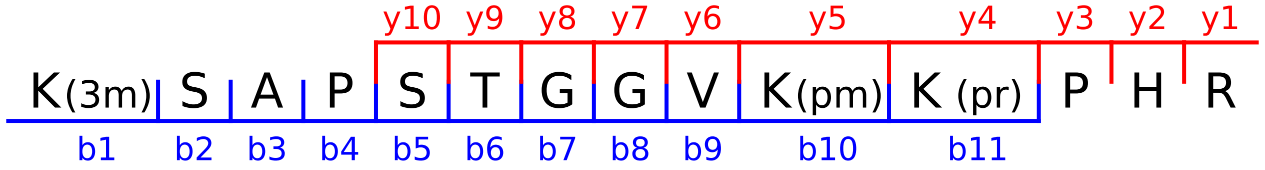

Raw file: HF240124_RN_spleen_PRO_PIC_3, Scan: 1957, m/z: 565.66153, Charge: 3, RT (min): 4.78, Score: 74.769

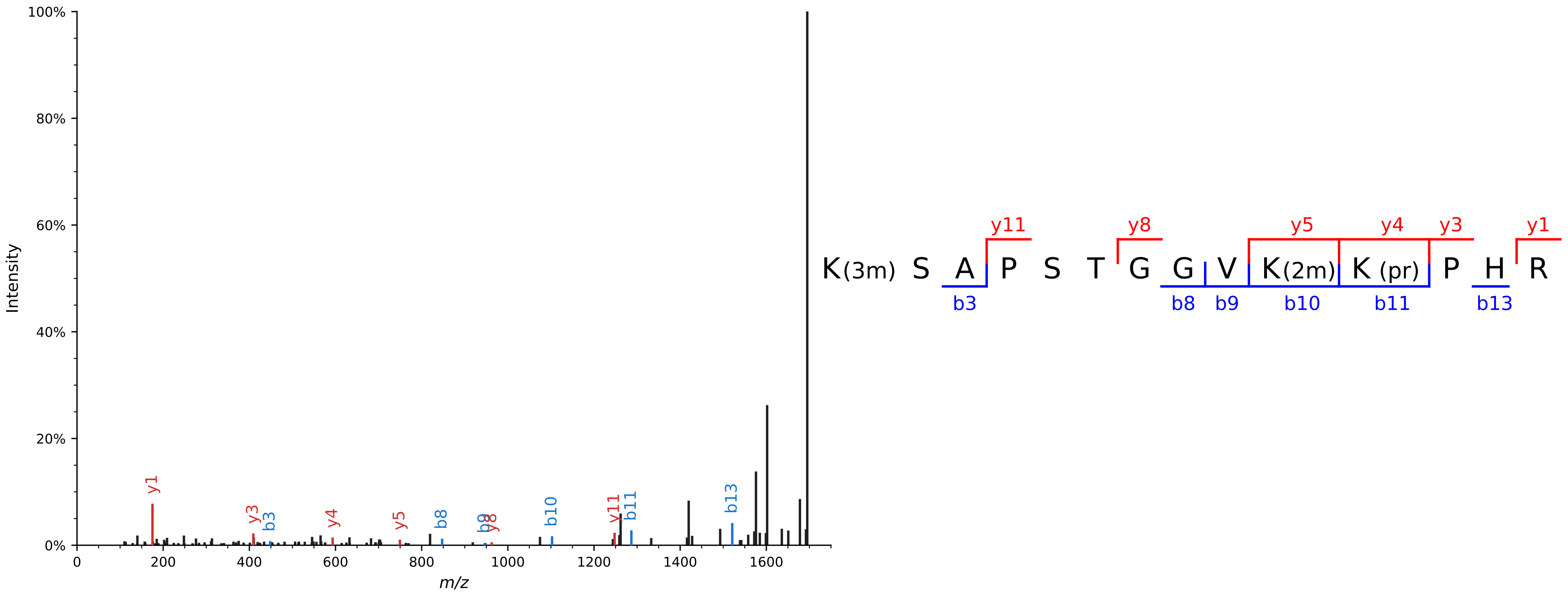

Raw file: HF240124_RN_spleen_PRO_PIC_3, Scan: 1962, m/z: 570.33342, Charge: 3, RT (min): 4.79, Score: 50.088

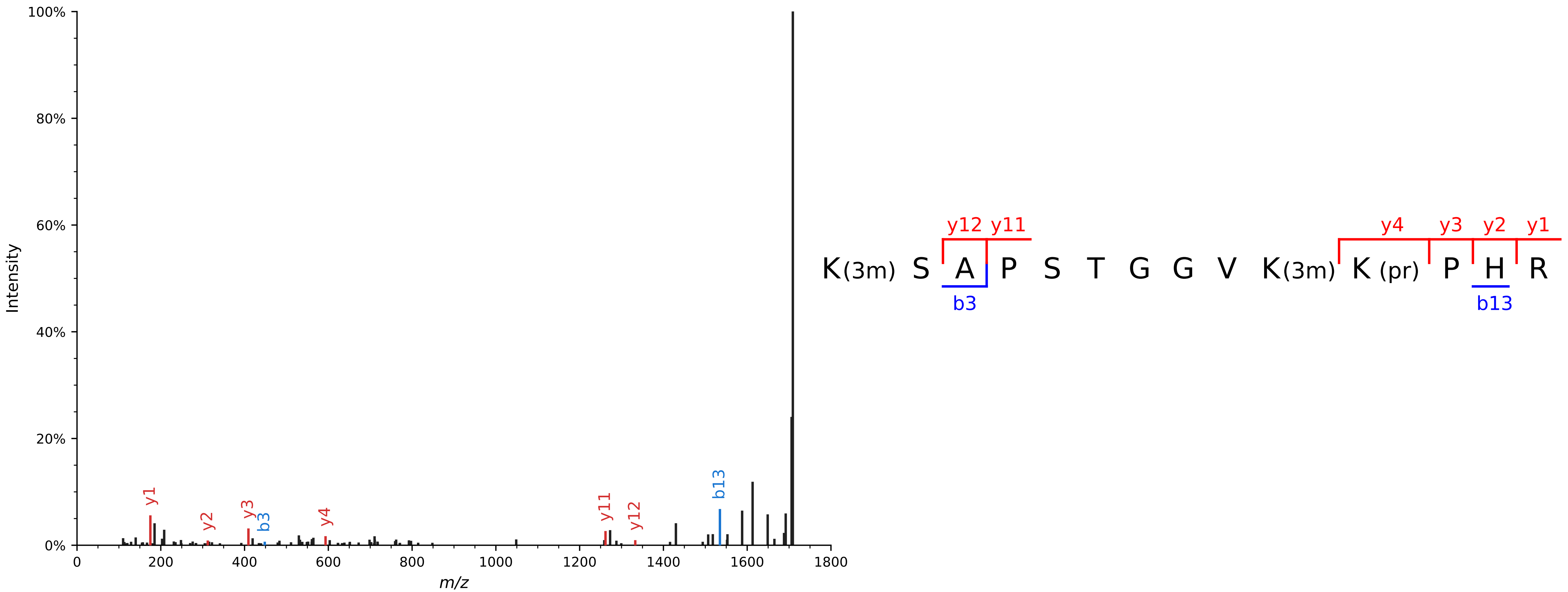

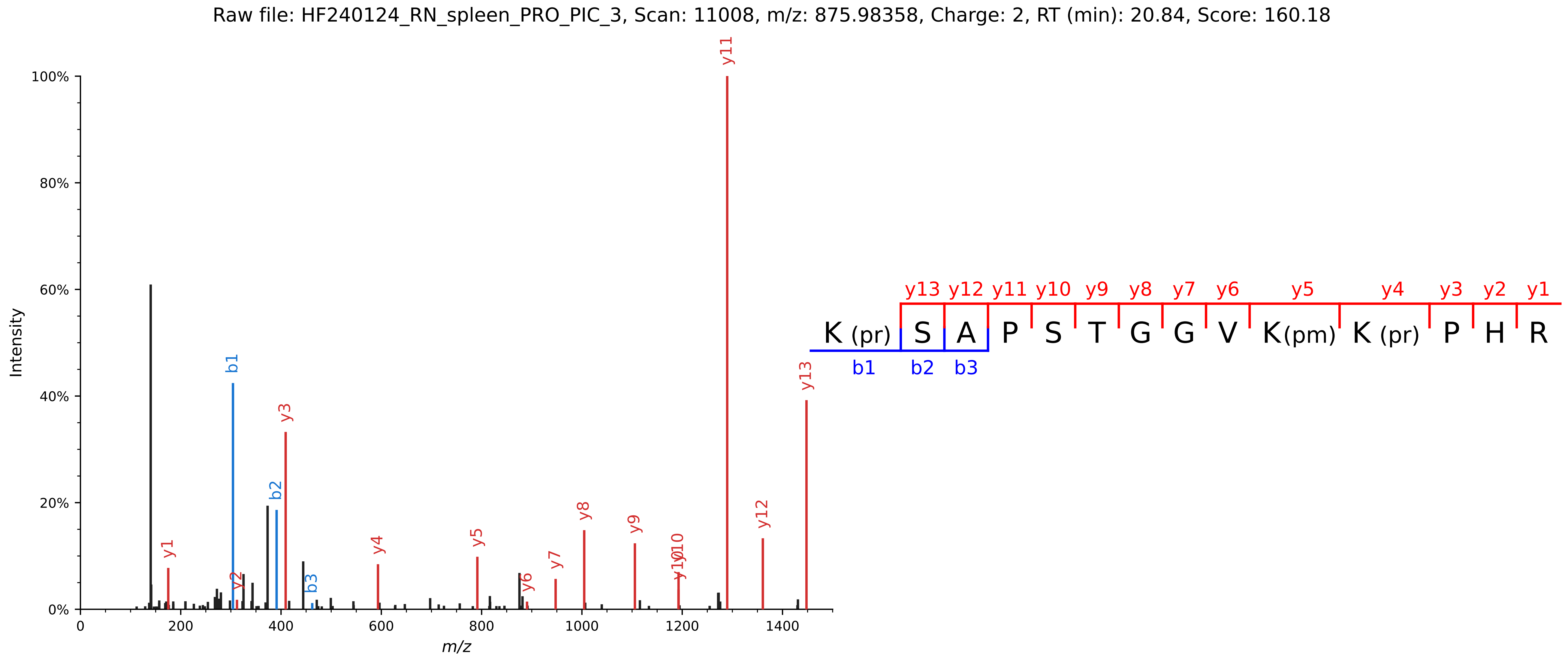

Raw file: HF240124_RN_spleen_PRO_PIC_3, Scan: 11008, m/z: 875.98358, Charge: 2, RT (min): 20.84, Score: 160.18
Intensity
m/z
K(pr) S A P S T G G V K(pm) K(pr) P H R
b1 b2 b3
y13 y12 y11 y10 y9 y8 y7 y6 y5 y4 y3 y2 y1

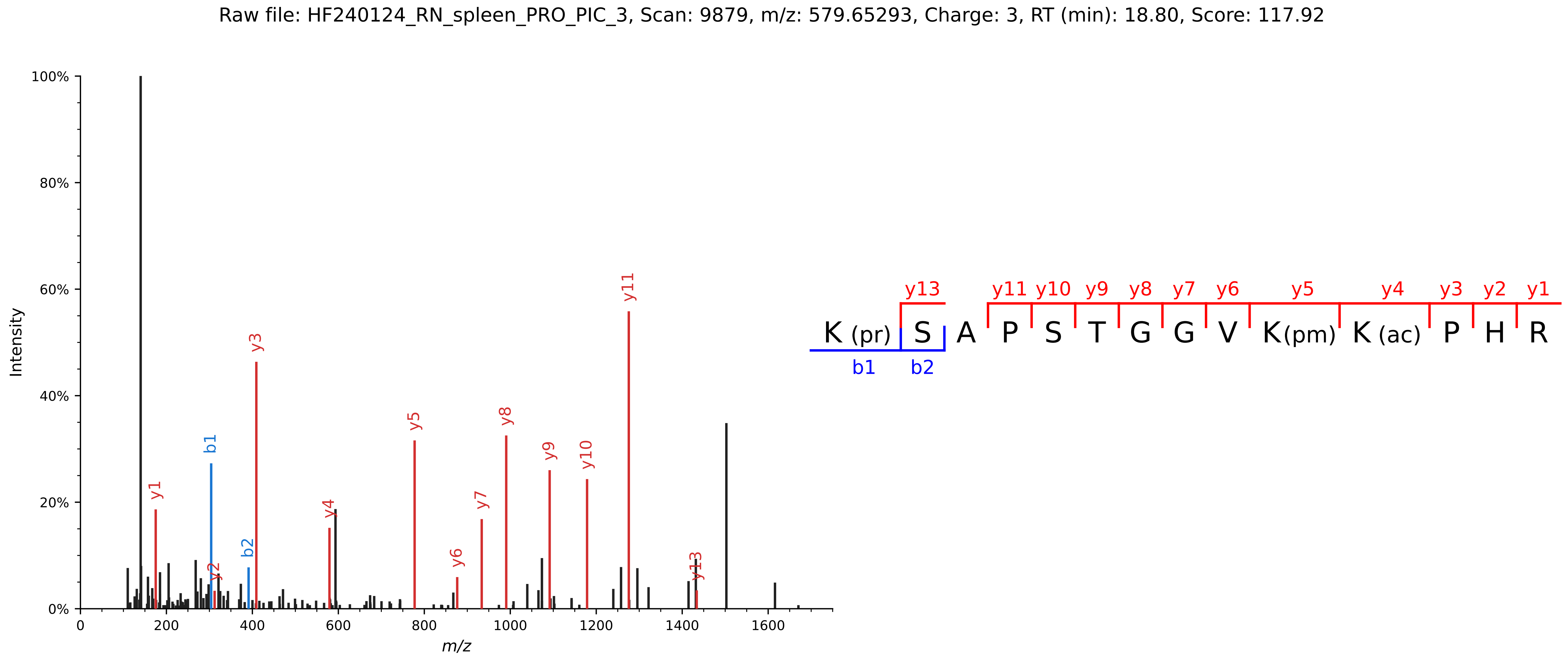
Raw file: HF240124_RN_spleen_PRO_PIC_3, Scan: 9879, m/z: 579.65293, Charge: 3, RT (min): 18.80, Score: 117.92
Intensity
m/z
100%
80%
60%
40%
20%
0%
0
200
400
600
800
1000
1200
1400
1600
y1
b1
y2
b2
y3
y4
y5
y6
y7
y8
y9
y10
y11
y13
K (pr) S A P S T G G V K(pm) K (ac) P H R
y13 y11 y10 y9 y8 y7 y6 y5 y4 y3 y2 y1
b1 b2

Raw file: HF240124_RN_spleen_PRO_PIC_2, Scan: 7717, m/z: 882.9914, Charge: 2, RT (min): 15.06, Score: 59.542

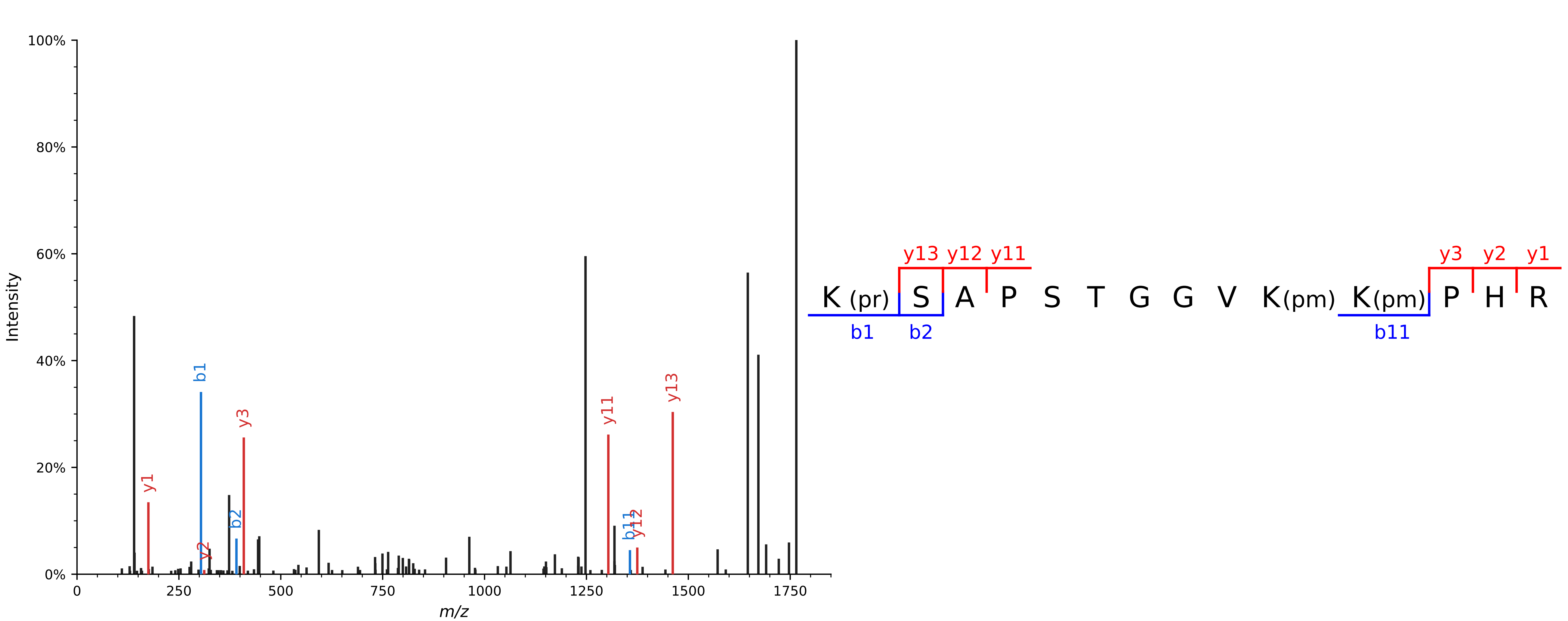

Raw file: HF240124_RN_spleen_PRO_PIC_1, Scan: 6693, m/z: 570.32129, Charge: 3, RT (min): 13.98, Score: 41.577

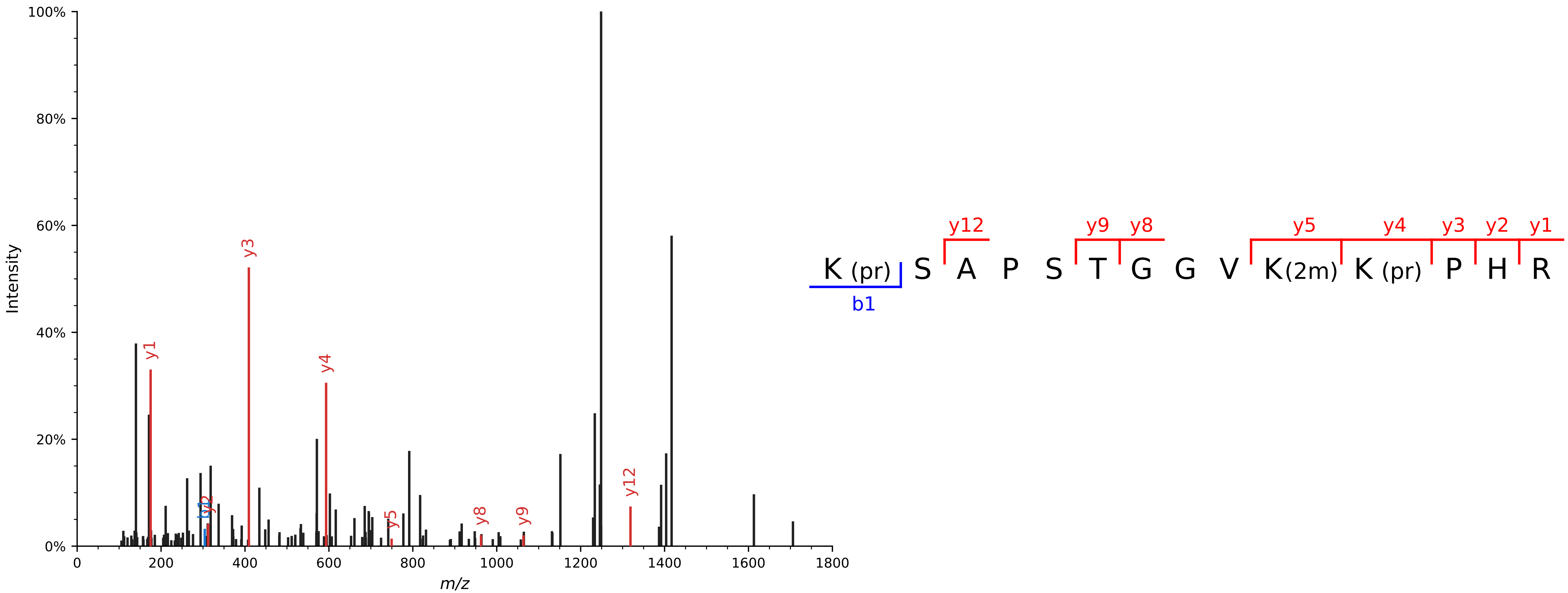

Raw file: HF240124_RN_spleen_PRO_PIC_1, Scan: 5500, m/z: 433.22996, Charge: 2, RT (min): 11.72, Score: 116.87

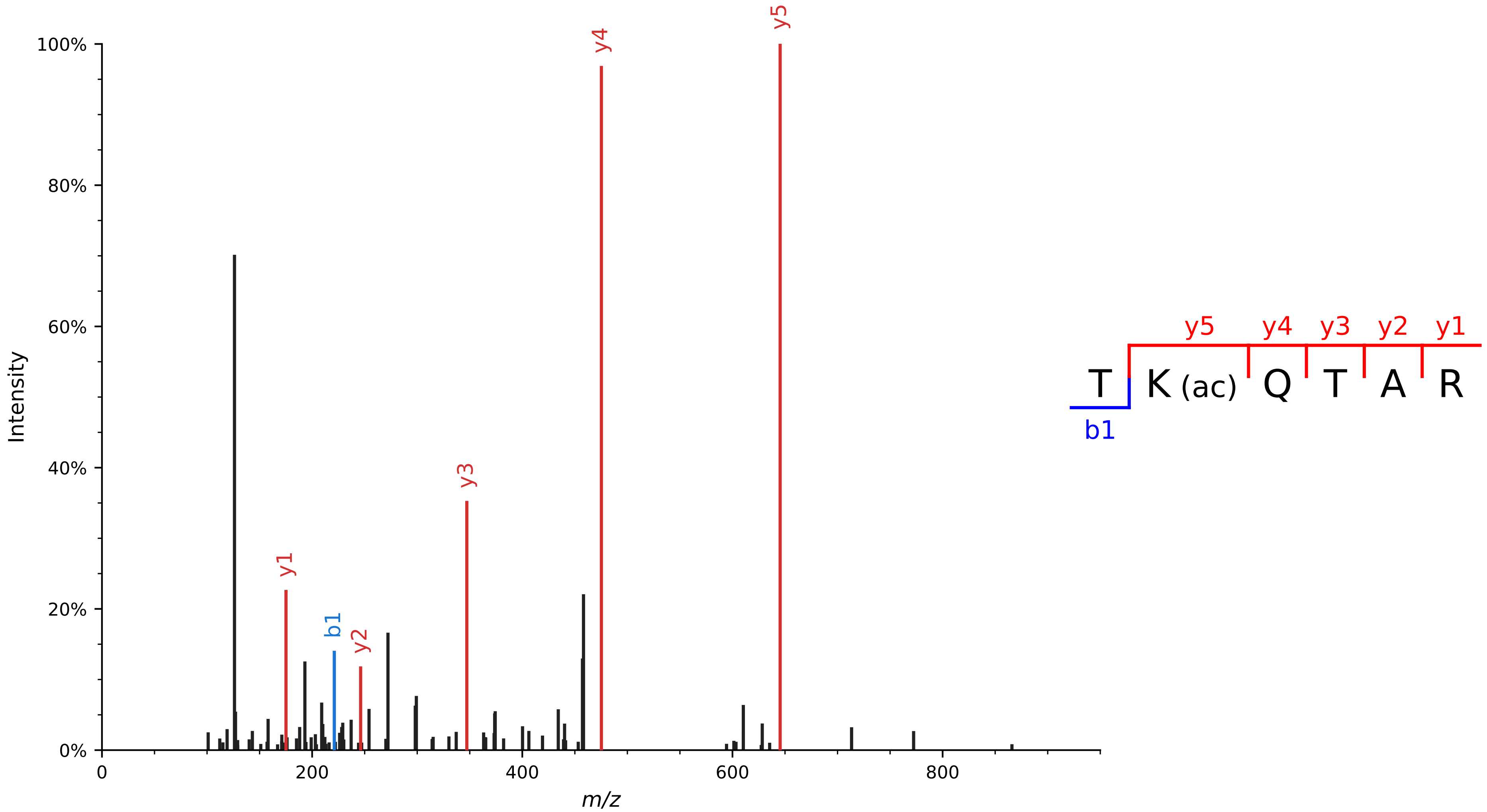

Raw file: HF240124_RN_spleen_PRO_PIC_3, Scan: 7052, m/z: 446.23778, Charge: 2, RT (min): 13.92, Score: 54.525

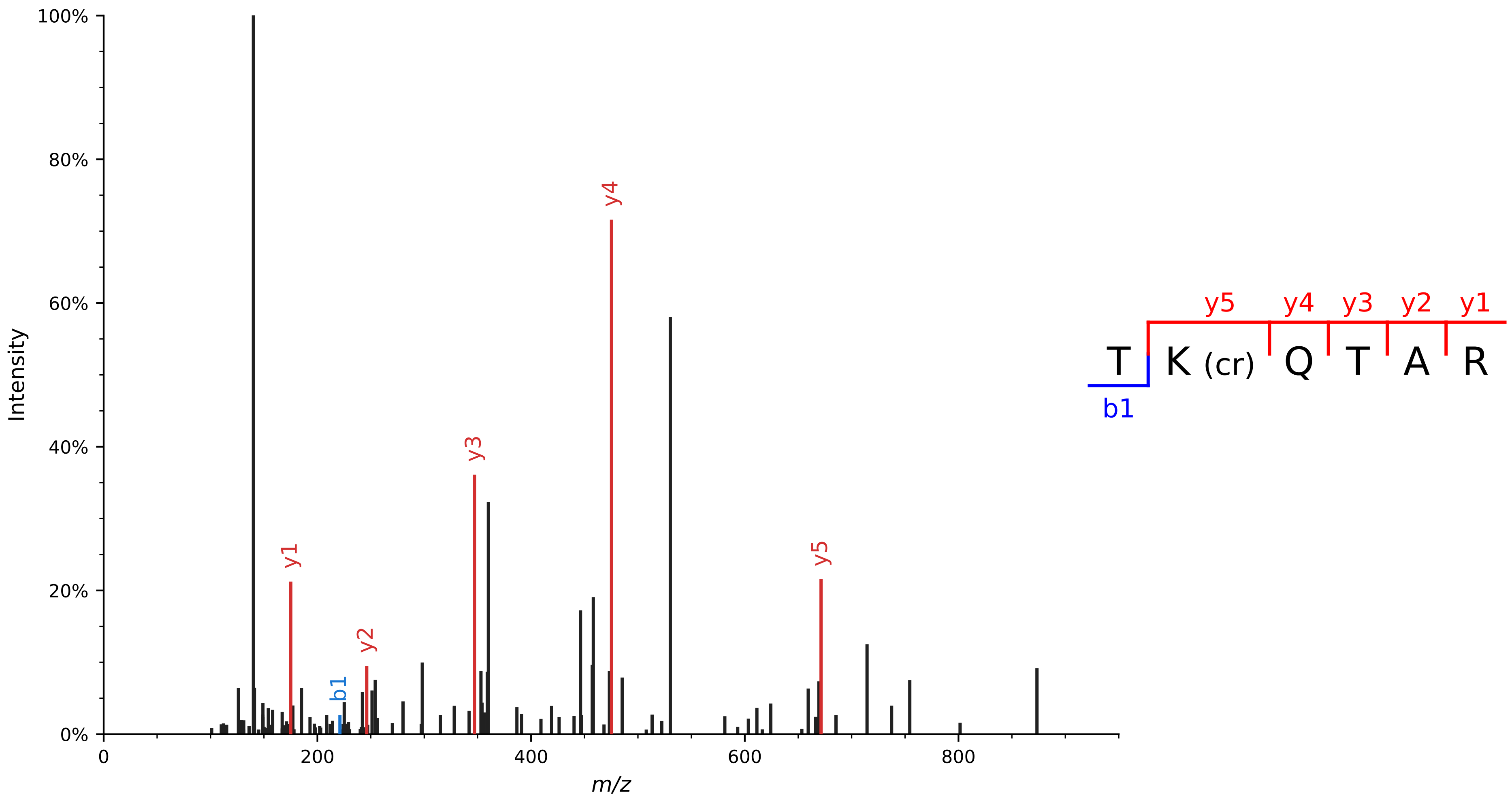

Raw file: HF240124_RN_spleen_PRO_PIC_3, Scan: 9425, m/z: 447.24561, Charge: 2, RT (min): 18.01, Score: 85.061

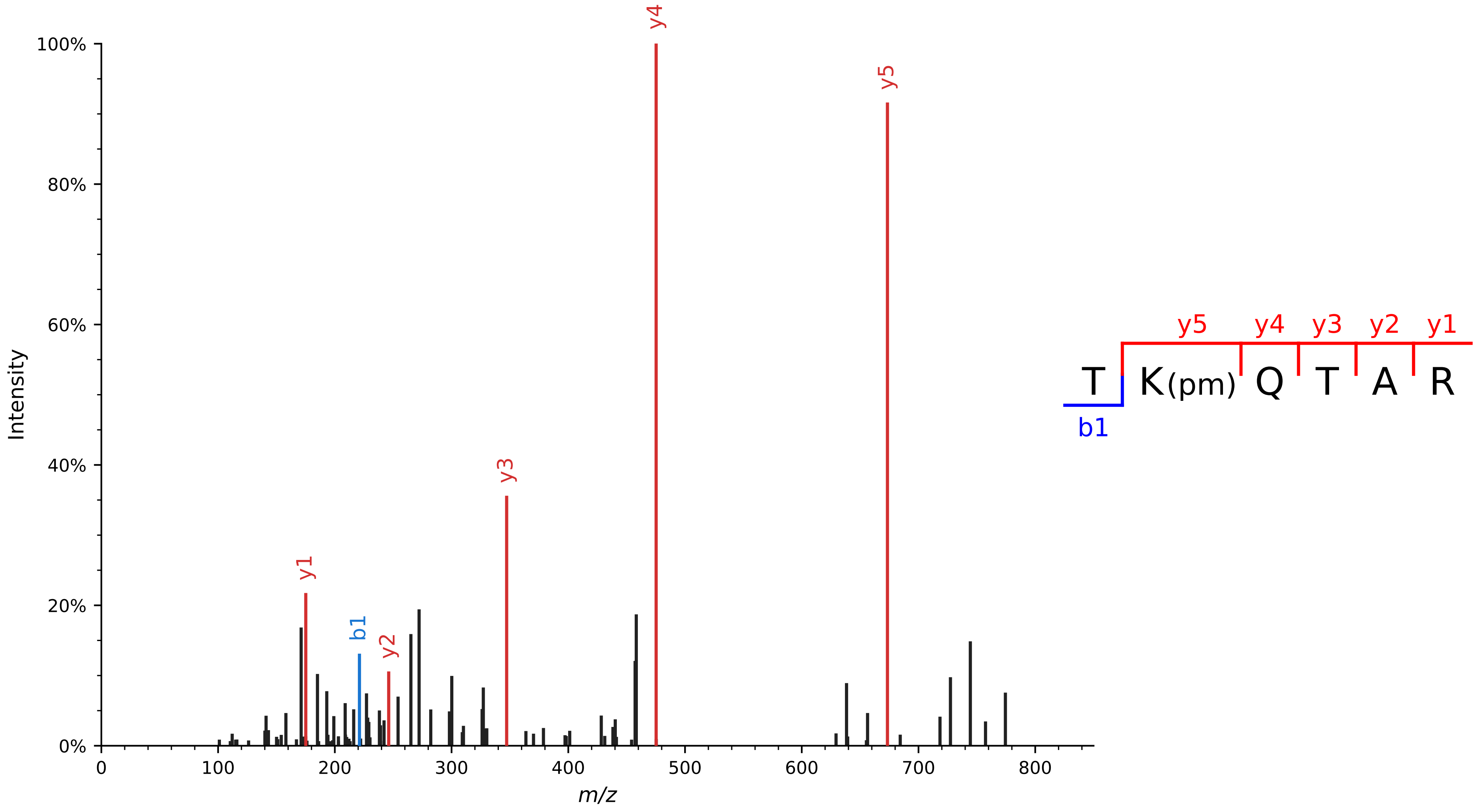

Raw file: HF240124_RN_spleen_PRO_PIC_3, Scan: 1733, m/z: 426.24033, Charge: 2, RT (min): 4.44, Score: 145.04

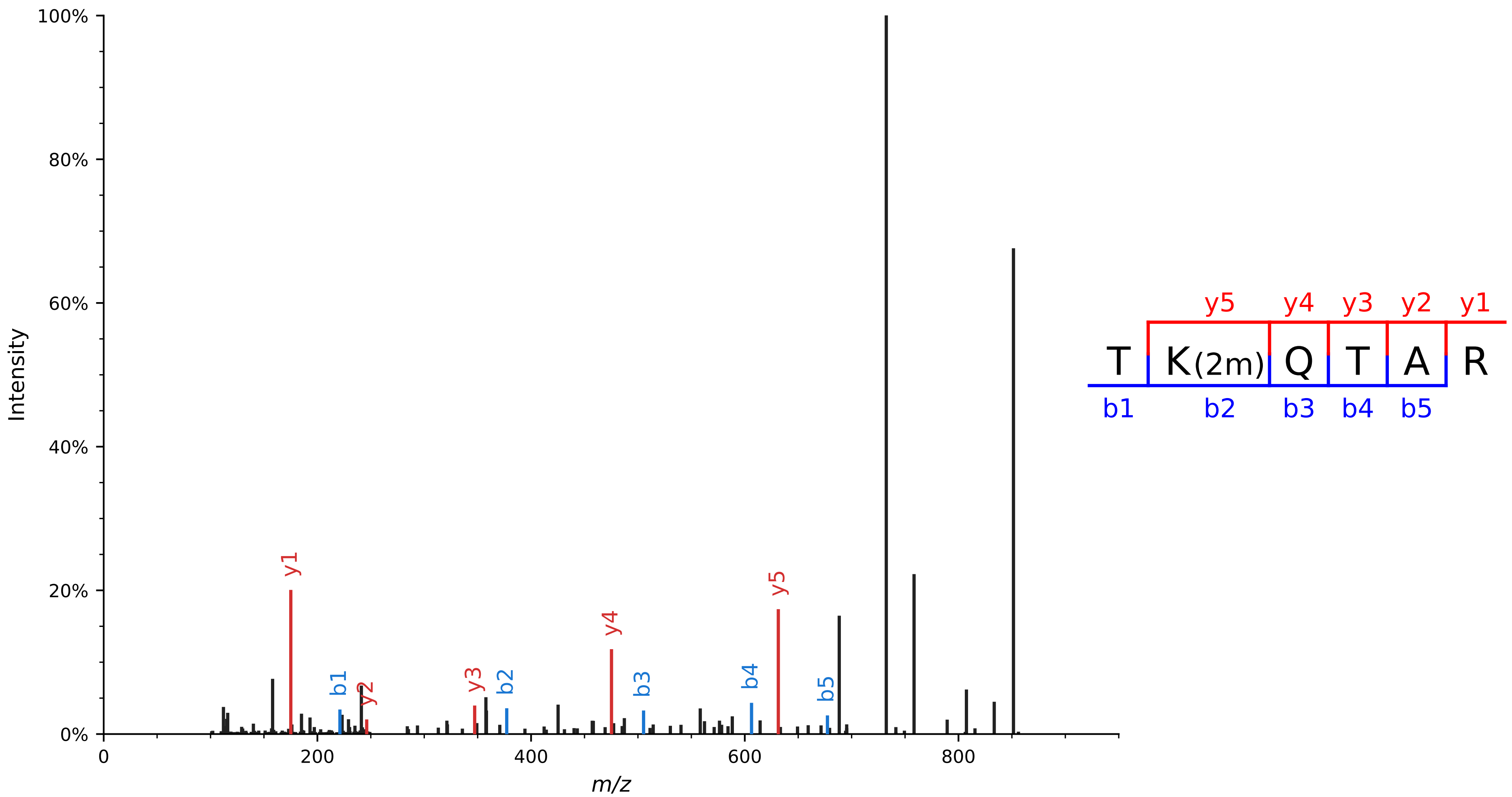

Raw file: HF240124_RN_spleen_PRO_PIC_3, Scan: 1737, m/z: 433.24815, Charge: 2, RT (min): 4.45, Score: 61.357

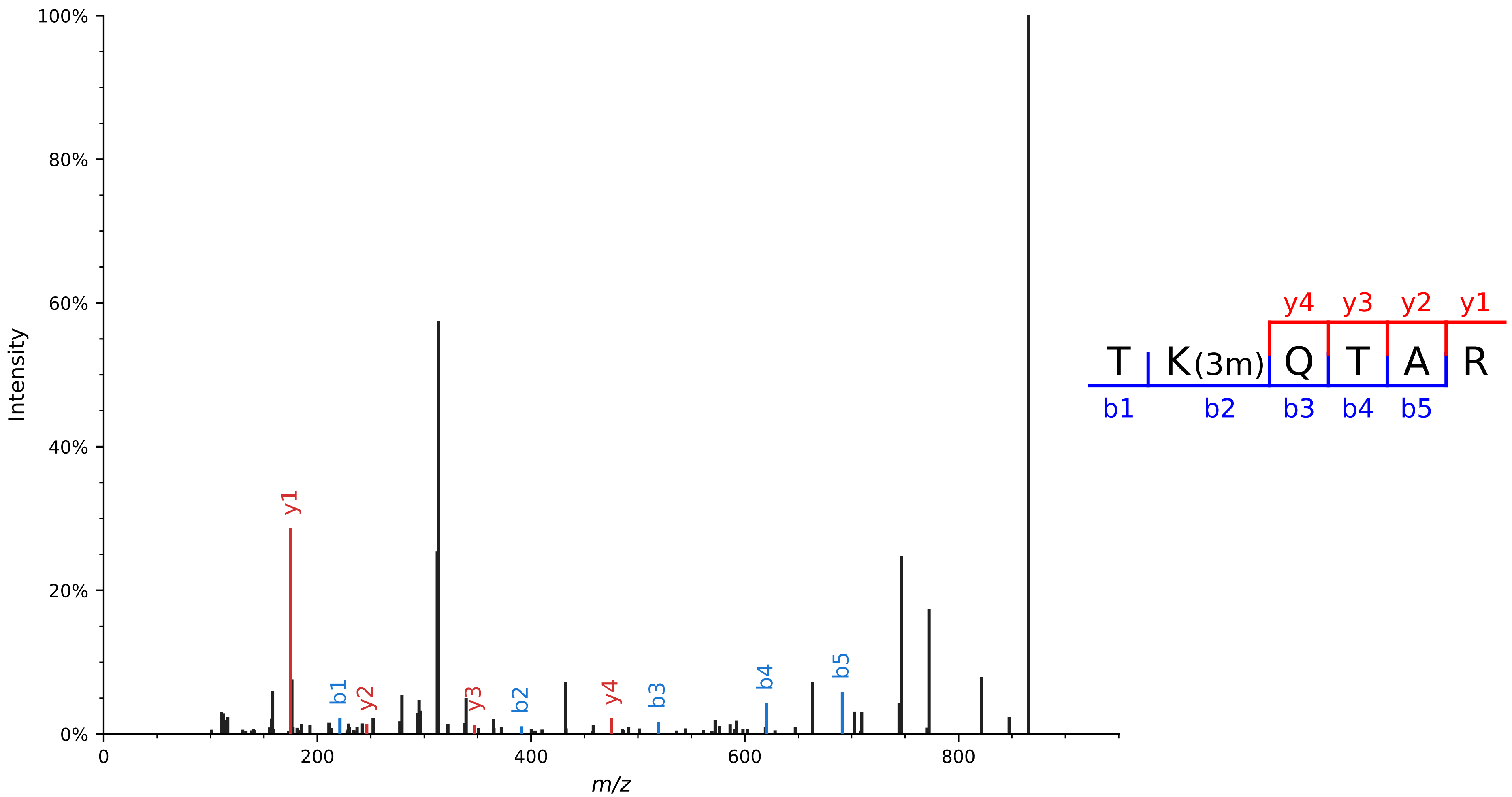

Raw file: HF240124_RN_spleen_PRO_PIC_2, Scan: 8662, m/z: 559.80128, Charge: 2, RT (min): 16.70, Score: 140.09

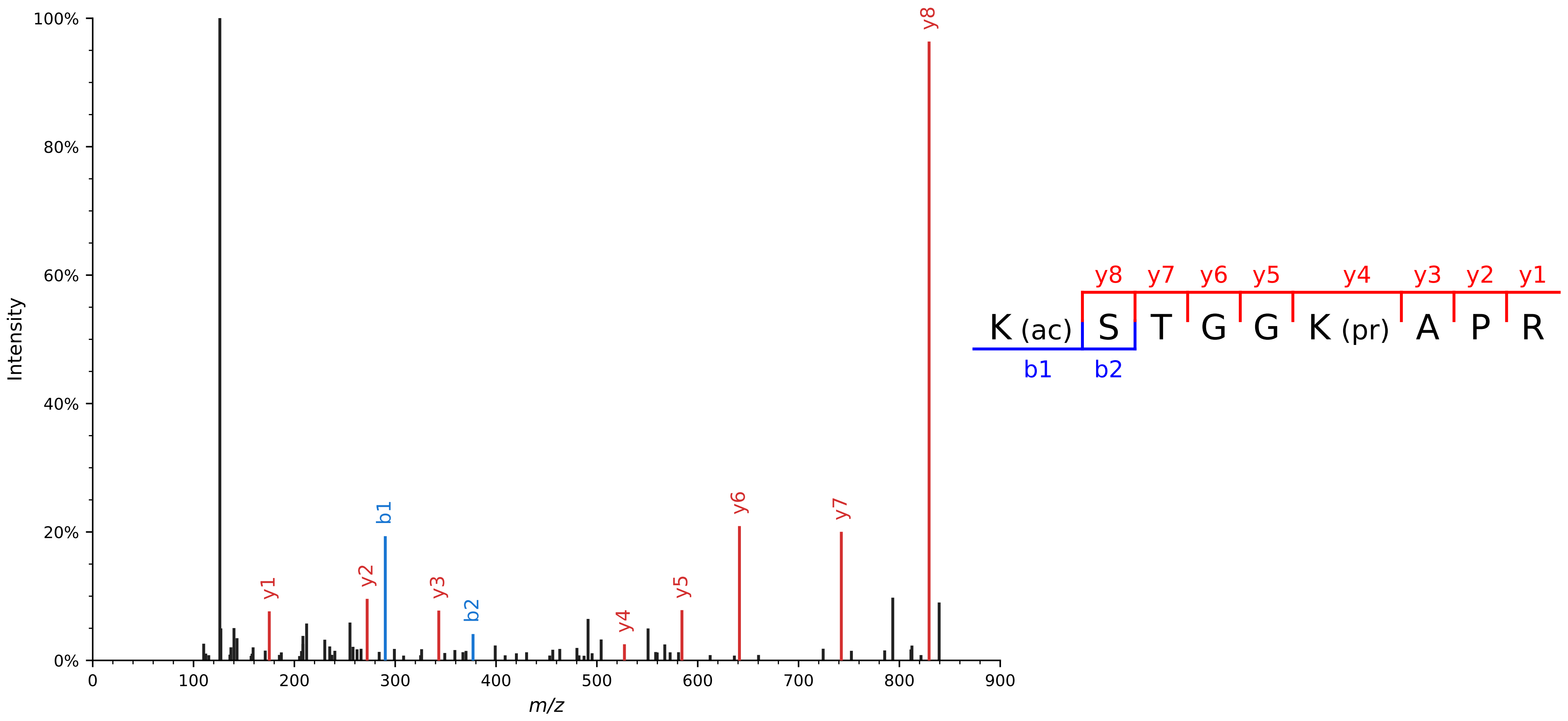

Raw file: HF240124_RN_spleen_PRO_PIC_1, Scan: 7174, m/z: 552.79345, Charge: 2, RT (min): 14.83, Score: 136.75

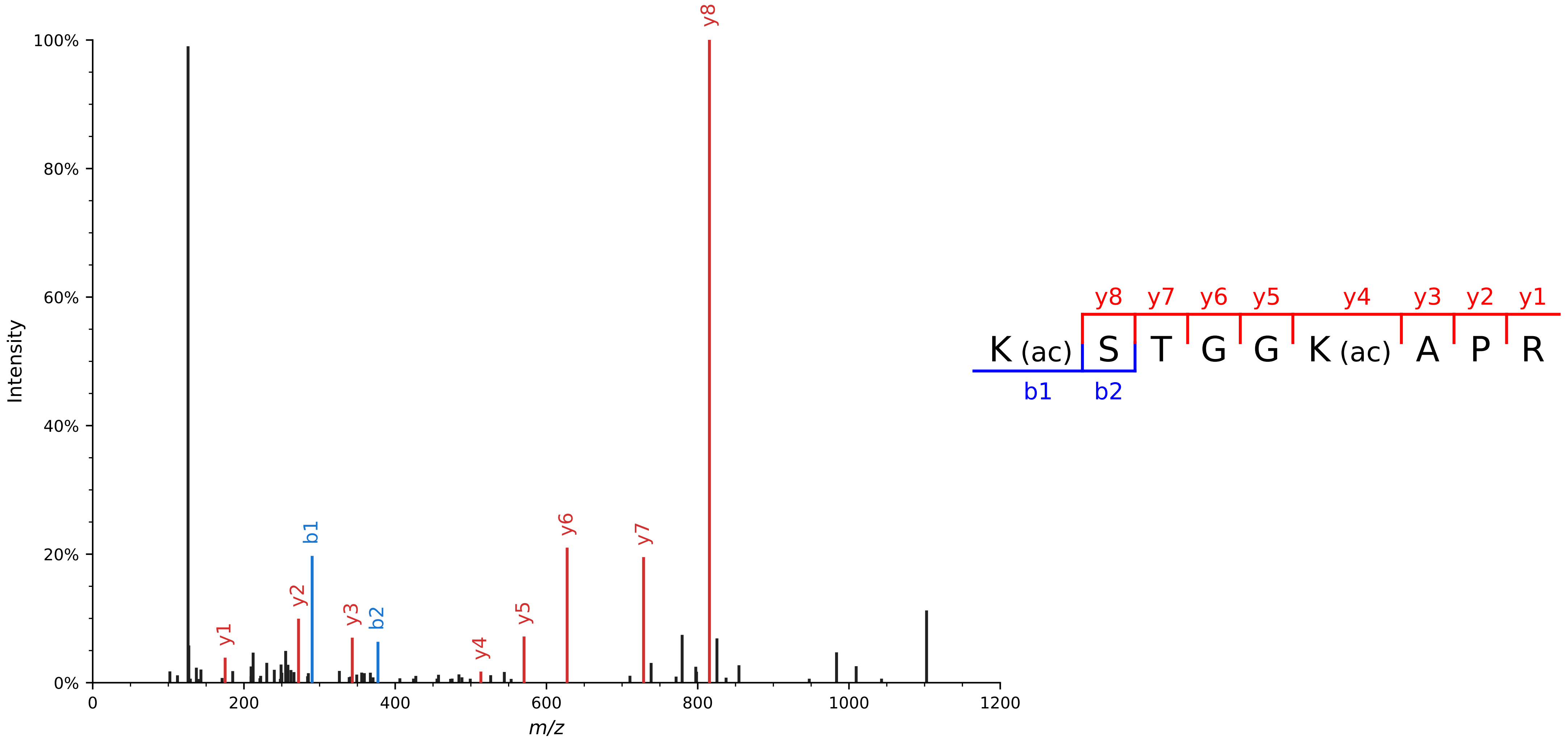

Raw file: HF240124_RN_spleen_PRO_PIC_2, Scan: 10491, m/z: 572.8091, Charge: 2, RT (min): 19.97, Score: 84.916

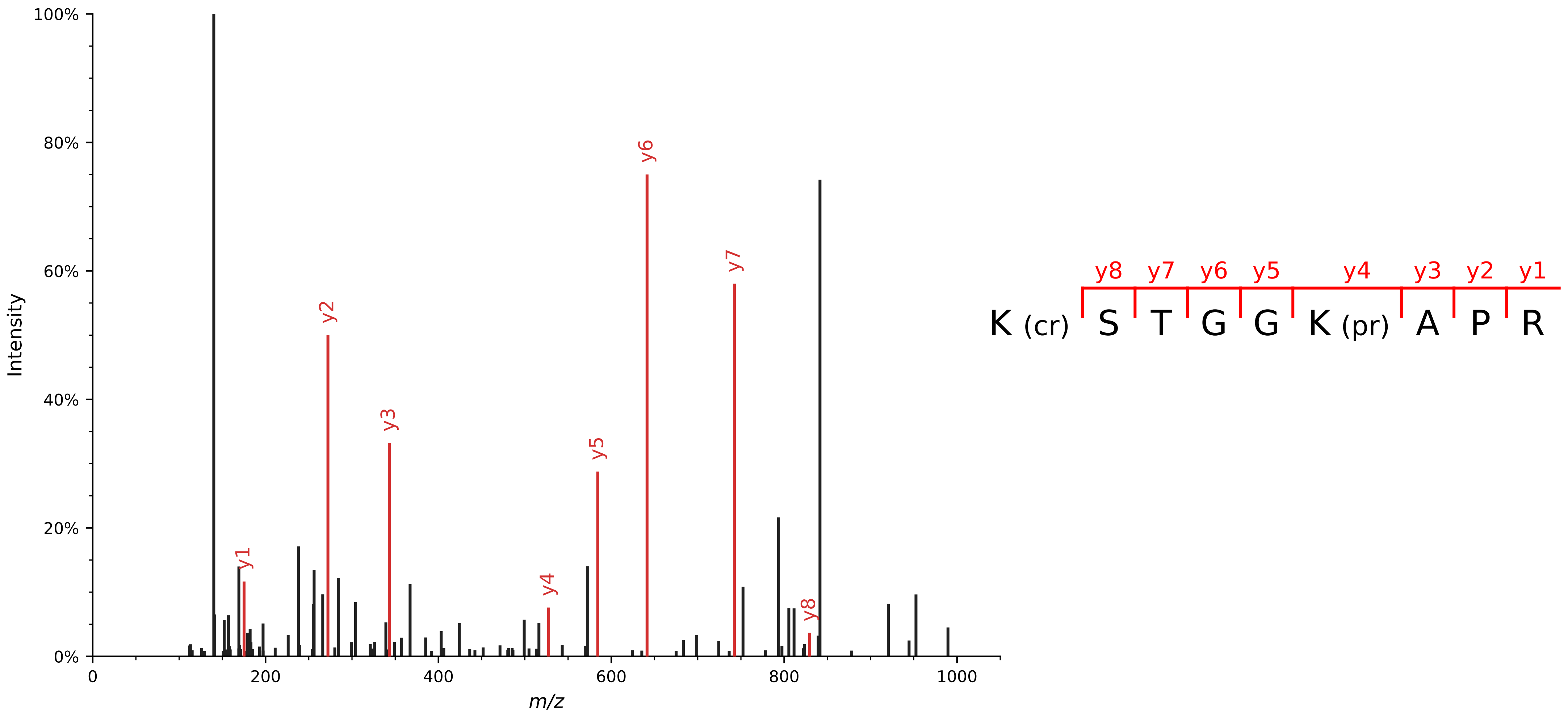

Raw file: HF240124_RN_spleen_PRO_PIC_1, Scan: 8923, m/z: 565.80128, Charge: 2, RT (min): 17.93, Score: 75.738

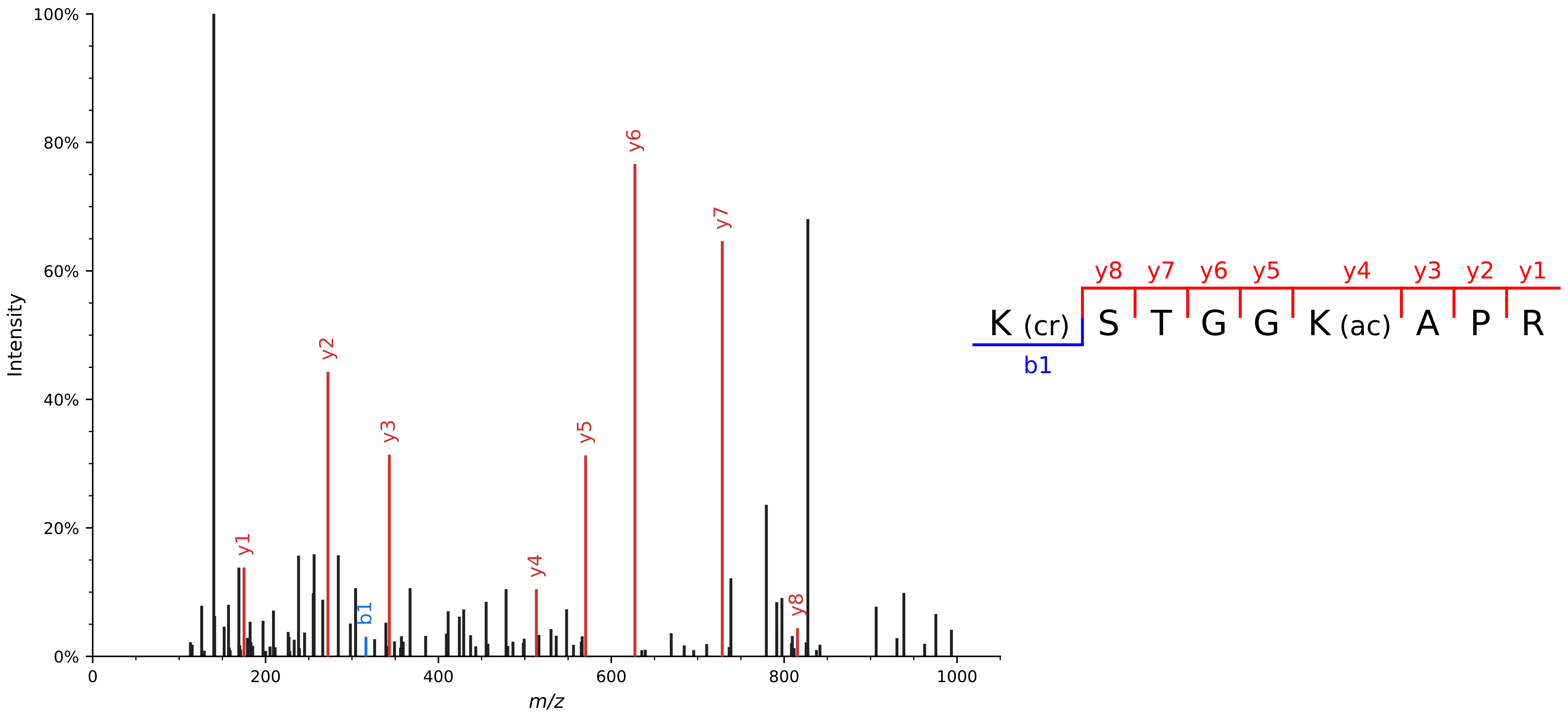

Raw file: HF240124_RN_spleen_PRO_PIC_1, Scan: 7604, m/z: 567.79874, Charge: 2, RT (min): 15.60, Score: 40.352

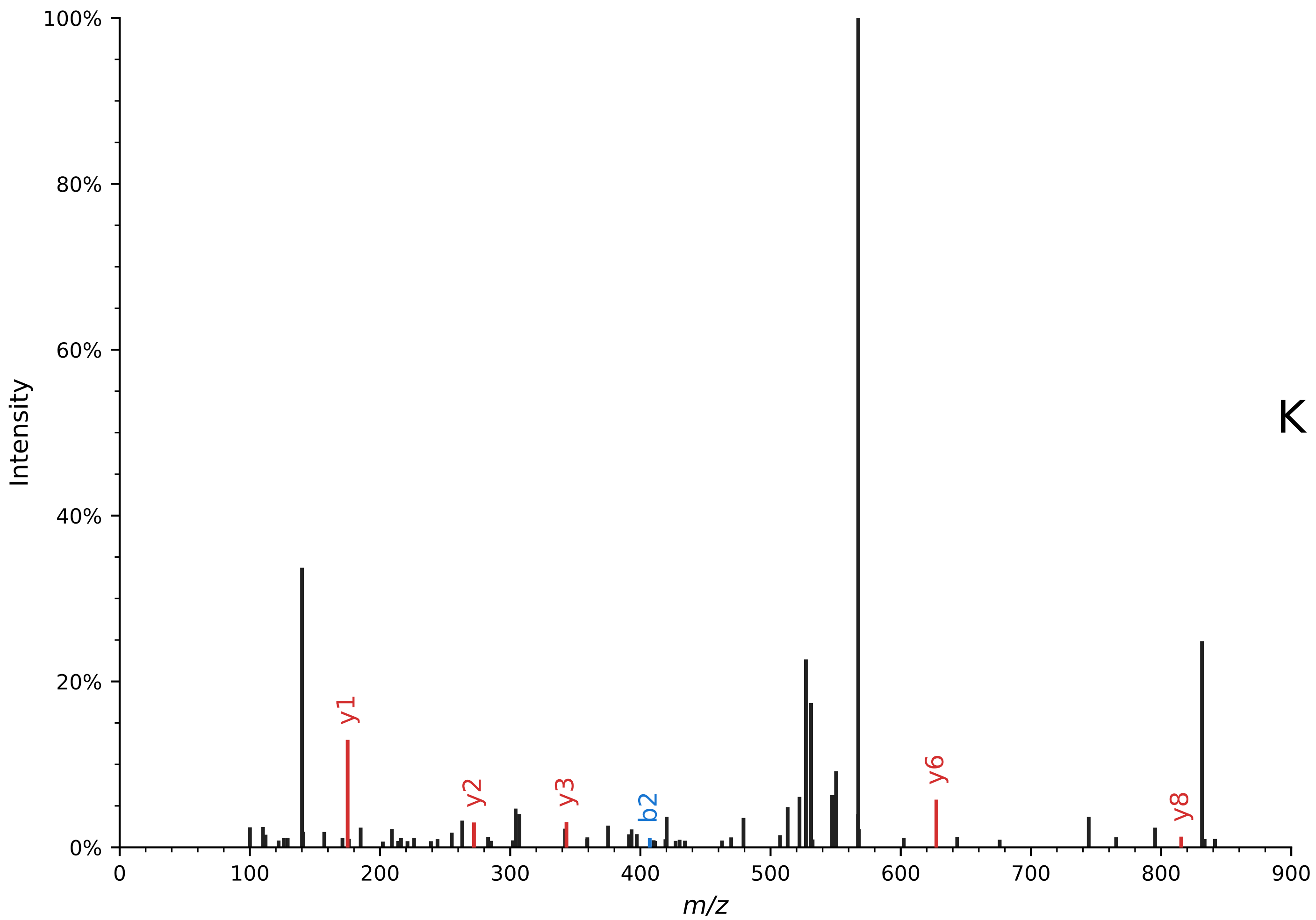

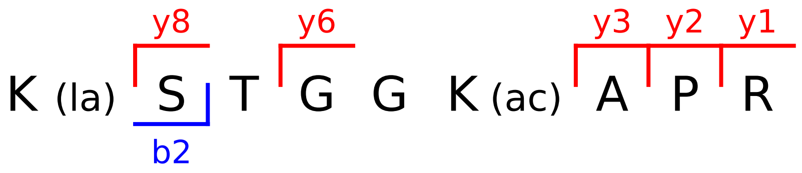

Raw file: HF240124_RN_spleen_PRO_PIC_1, Scan: 11579, m/z: 573.81693, Charge: 2, RT (min): 22.75, Score: 120.46

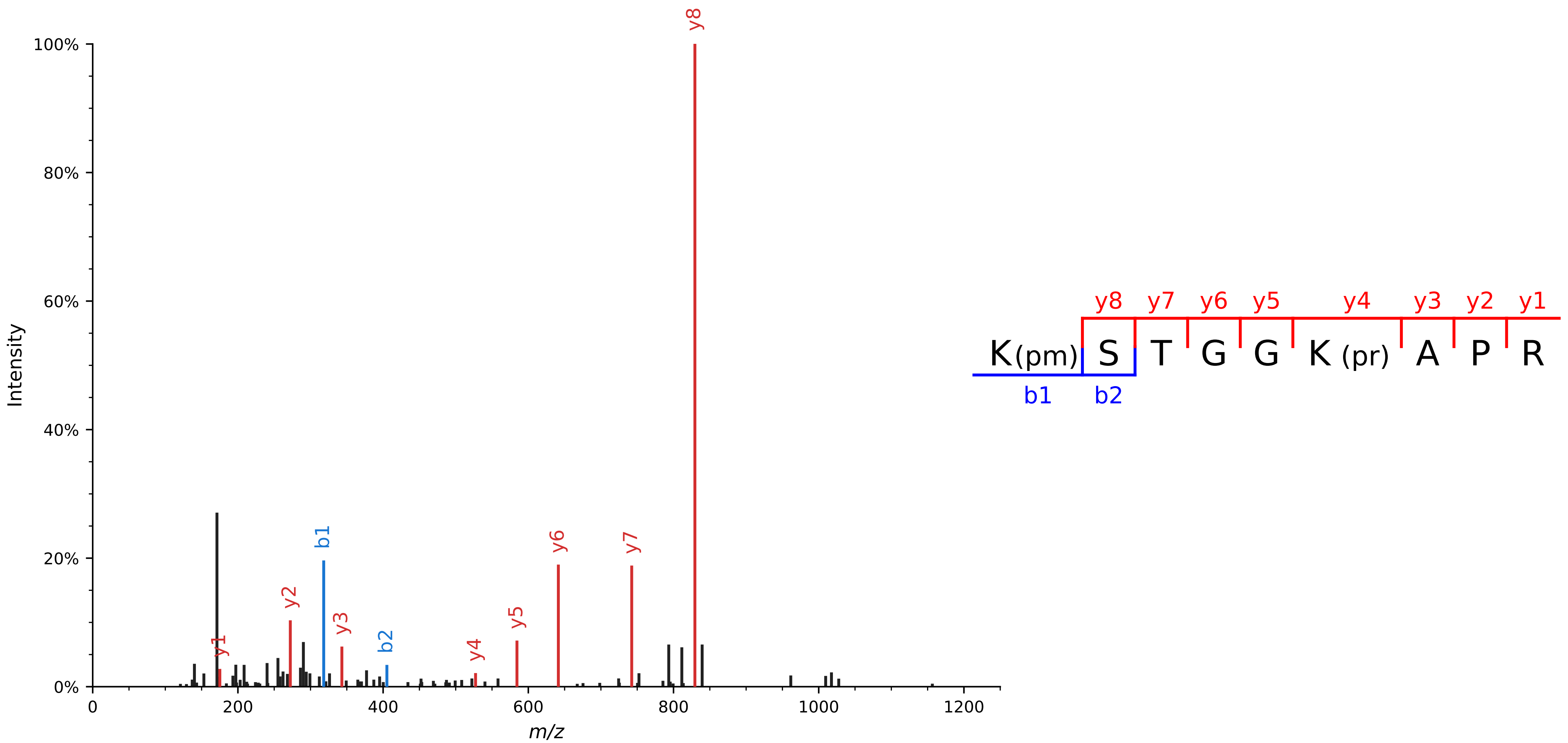

Raw file: HF240124_RN_spleen_PRO_PIC_2, Scan: 10962, m/z: 566.8091, Charge: 2, RT (min): 20.78, Score: 129.47

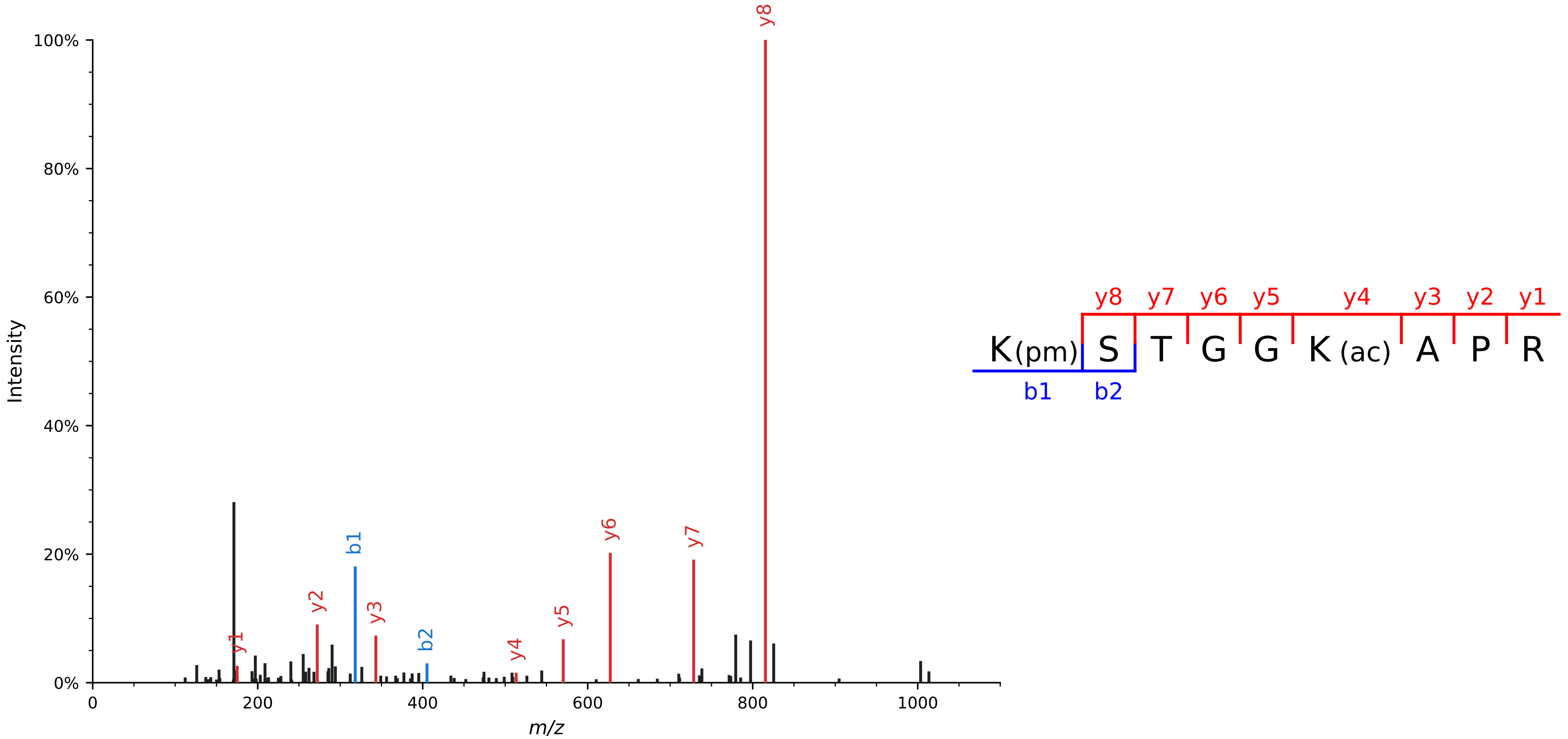

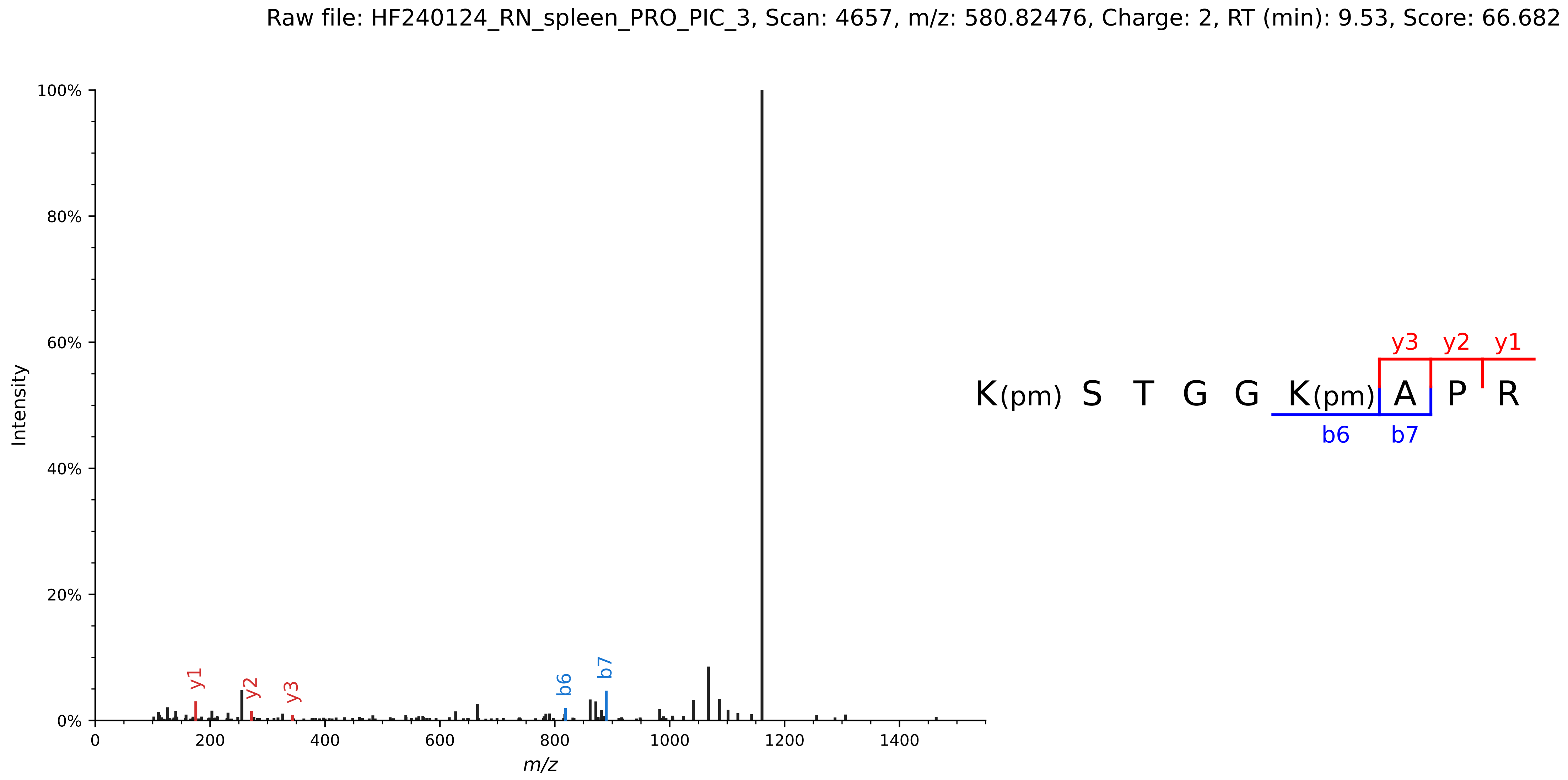
Raw file: HF240124_RN_spleen_PRO_PIC_3, Scan: 4657, m/z: 580.82476, Charge: 2, RT (min): 9.53, Score: 66.682
100%
80%
60%
40%
20%
0%
Intensity
0
200
400
600
800
1000
1200
1400
m/z
y1
y2
y3
b6
b7
K(pm) S T G G K(pm) A P R
y3 y2 y1
b6 b7

Raw file: HF240124_RN_spleen_PRO_PIC_1, Scan: 2501, m/z: 552.81165, Charge: 2, RT (min): 6.39, Score: 128.86

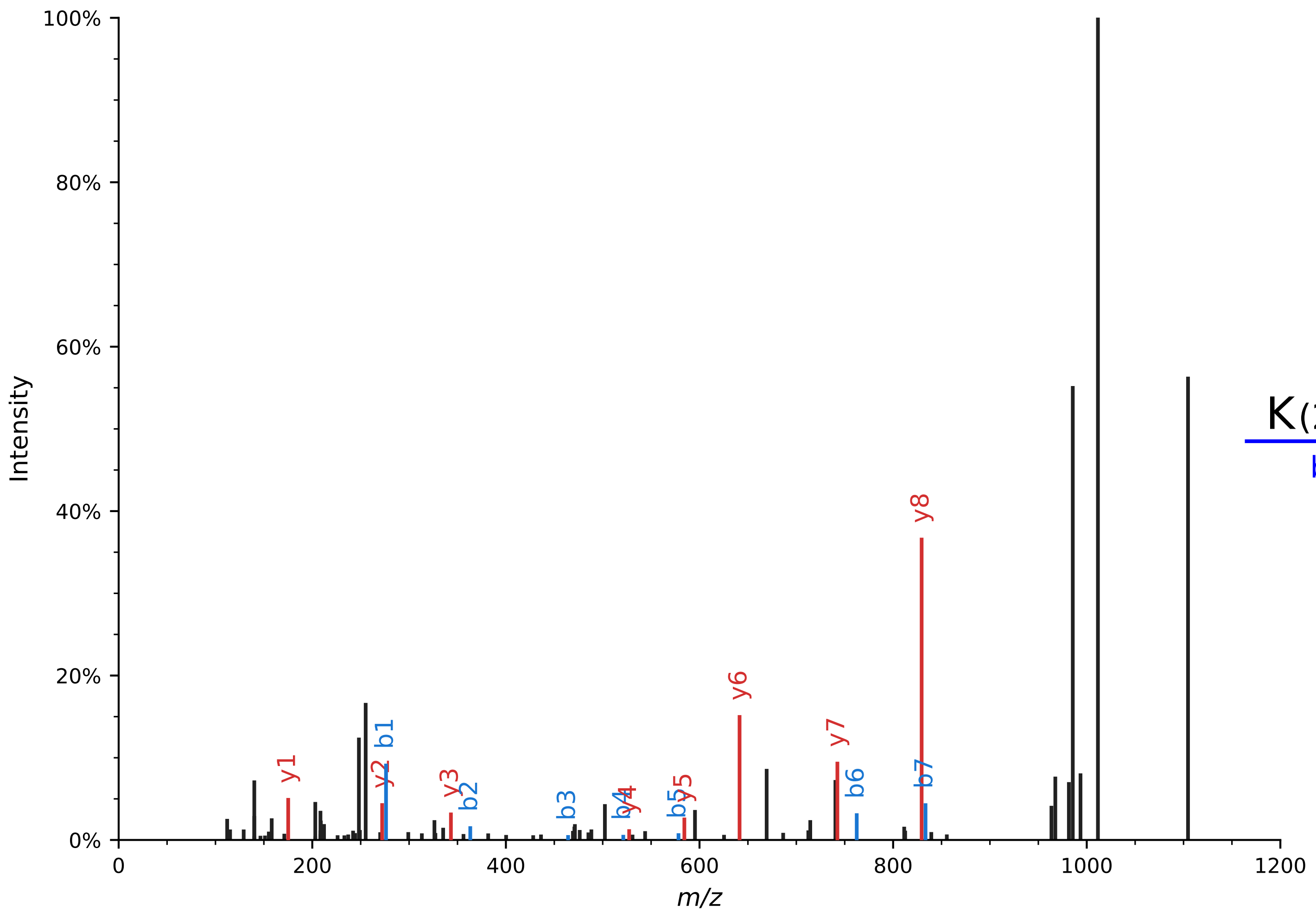

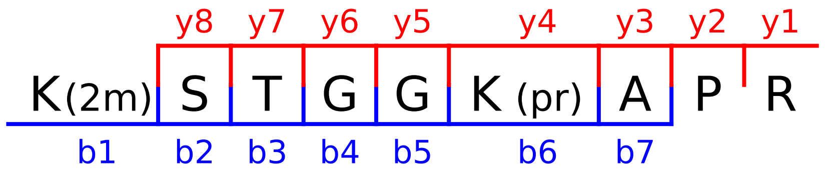

Raw file: HF240124_RN_spleen_PRO_PIC_1, Scan: 1853, m/z: 545.80382, Charge: 2, RT (min): 5.17, Score: 136.57

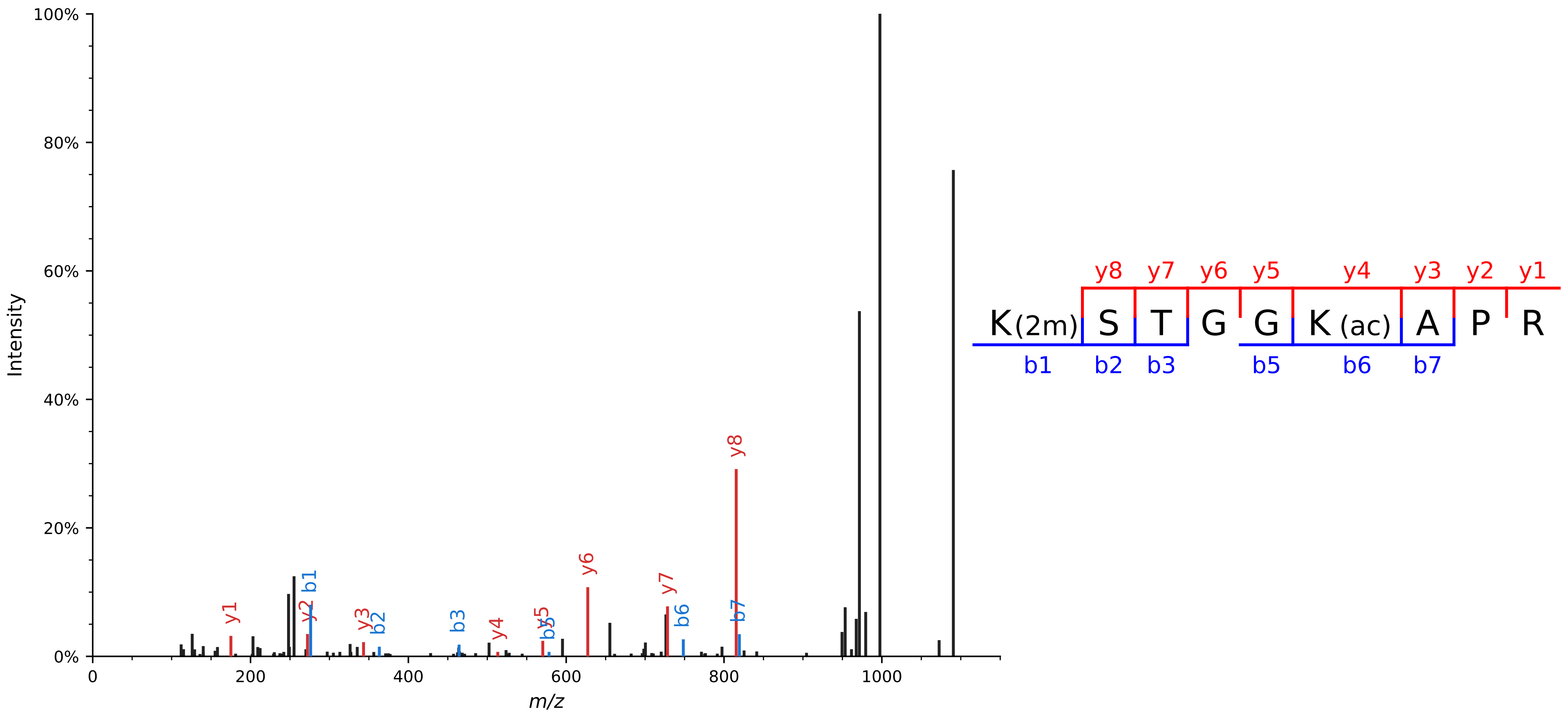

Raw file: HF240124_RN_spleen_PRO_PIC_2, Scan: 3186, m/z: 558.81165, Charge: 2, RT (min): 7.03, Score: 69.628

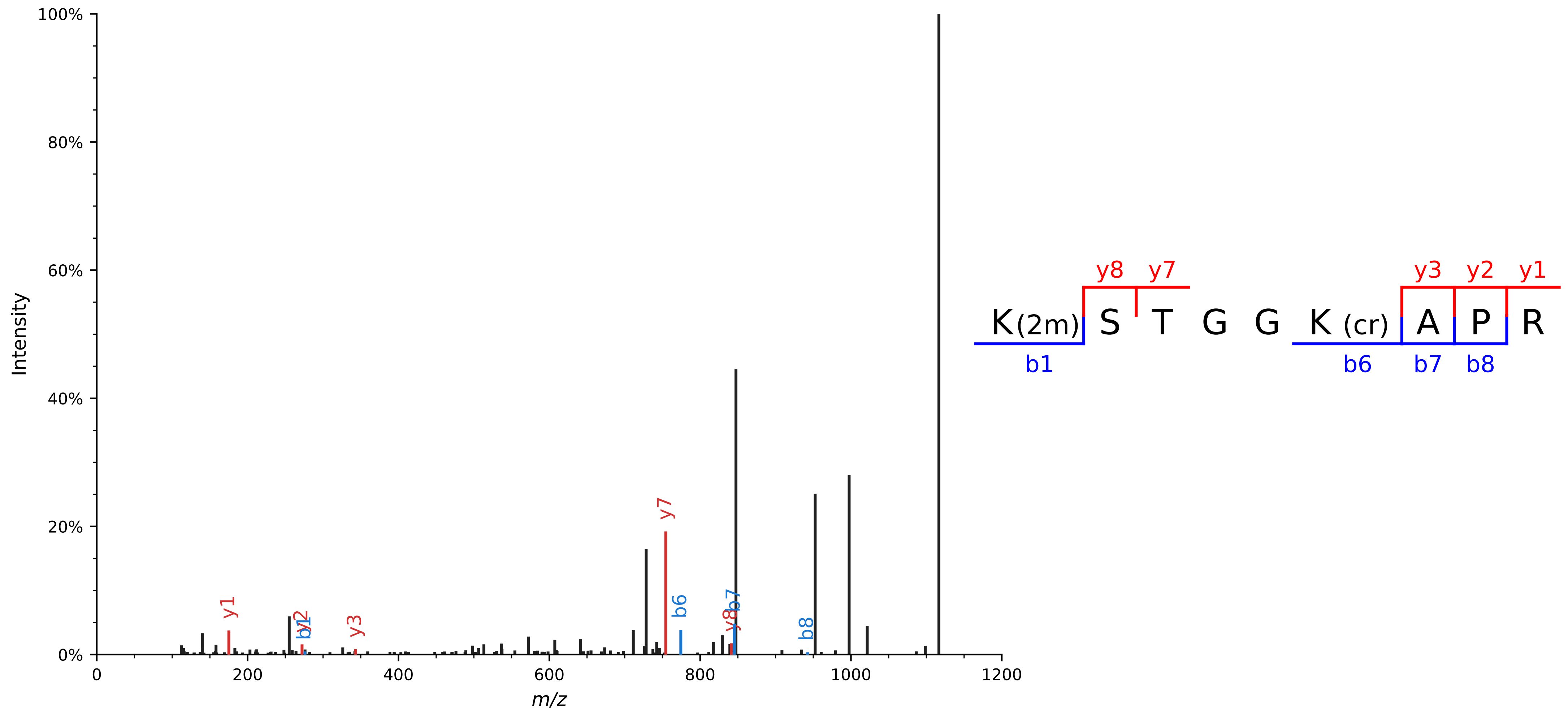

Raw file: HF240124_RN_spleen_PRO_PIC_1, Scan: 3524, m/z: 559.81947, Charge: 2, RT (min): 8.23, Score: 80.438

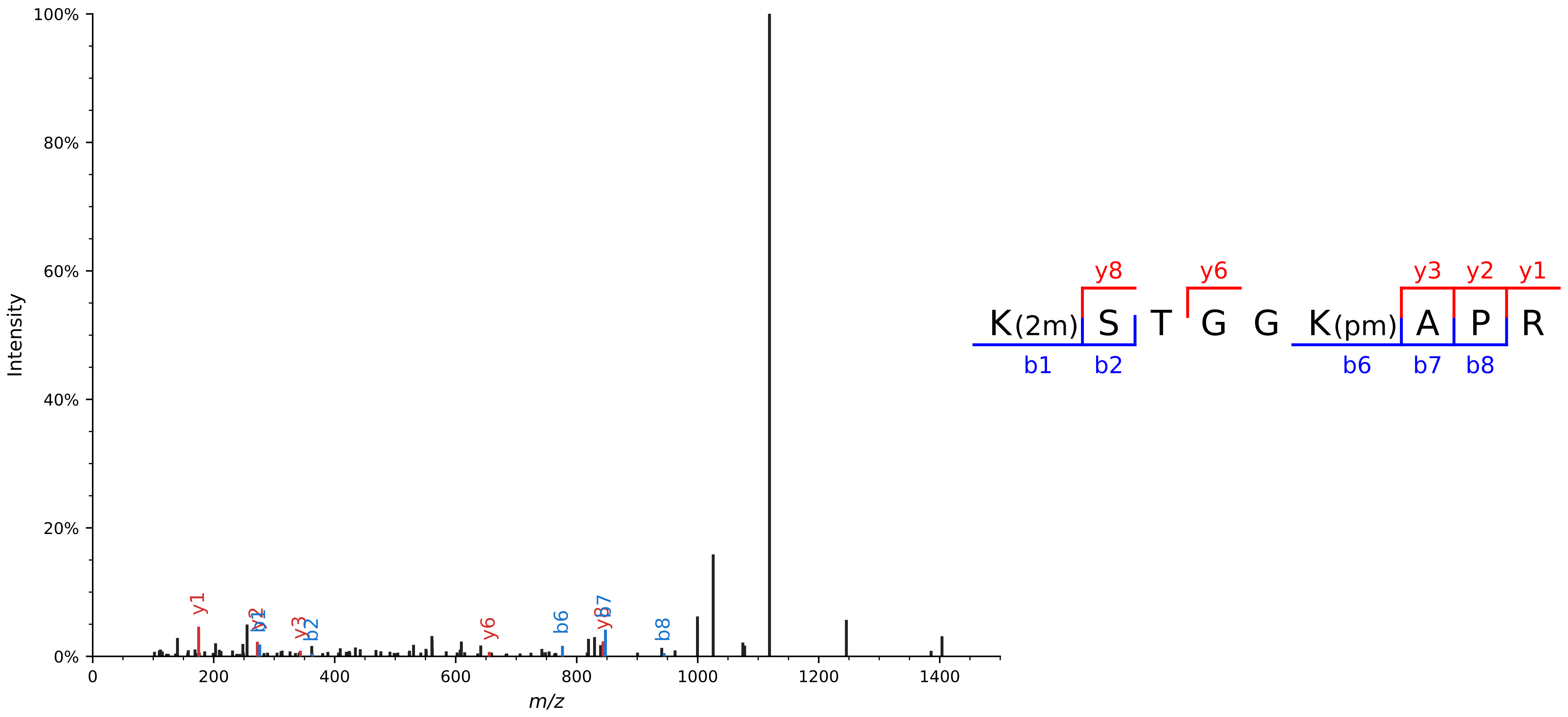

Raw file: HF240124_RN_spleen_PRO_PIC_1, Scan: 2561, m/z: 559.81947, Charge: 2, RT (min): 6.51, Score: 125.82

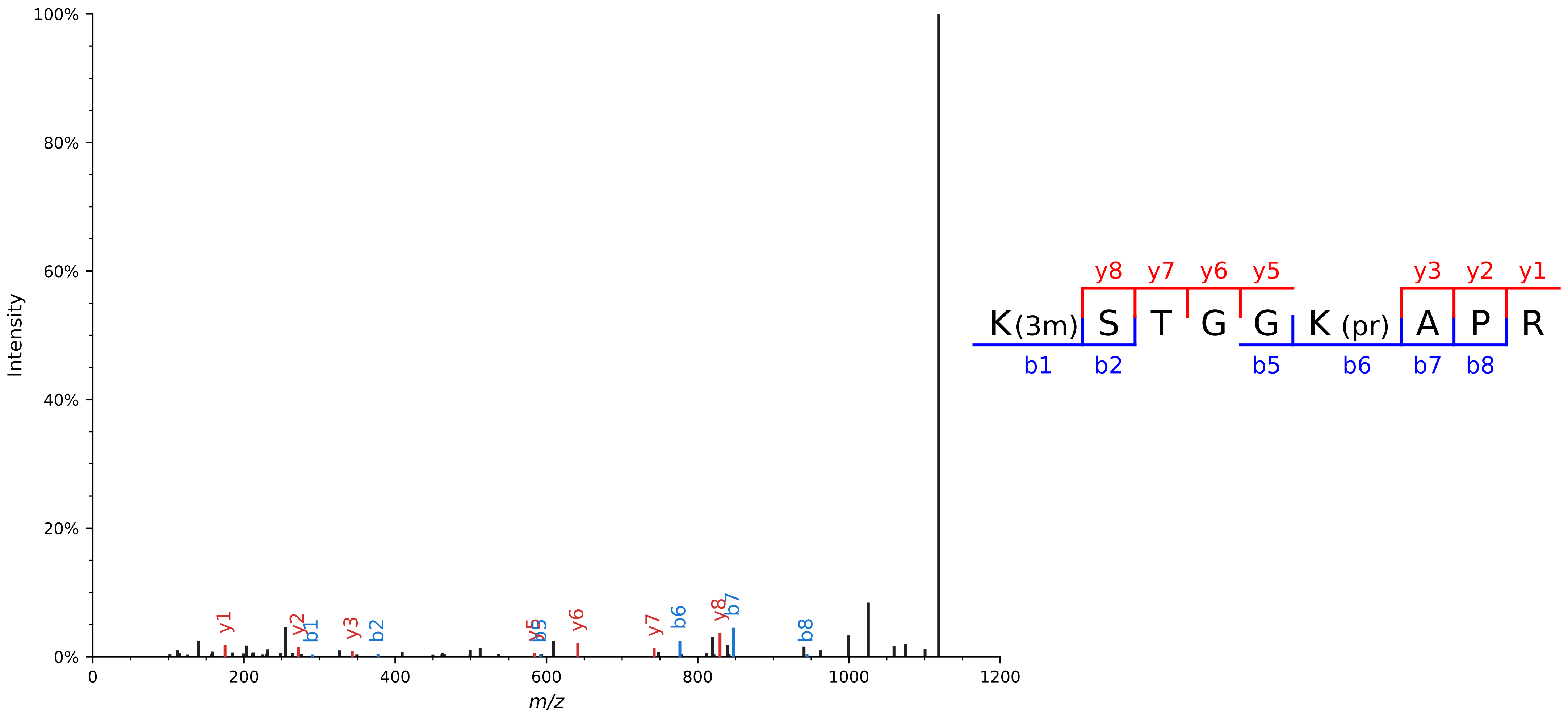

Raw file: HF240124_RN_spleen_PRO_PIC_3, Scan: 2163, m/z: 552.81165, Charge: 2, RT (min): 5.13, Score: 125.57

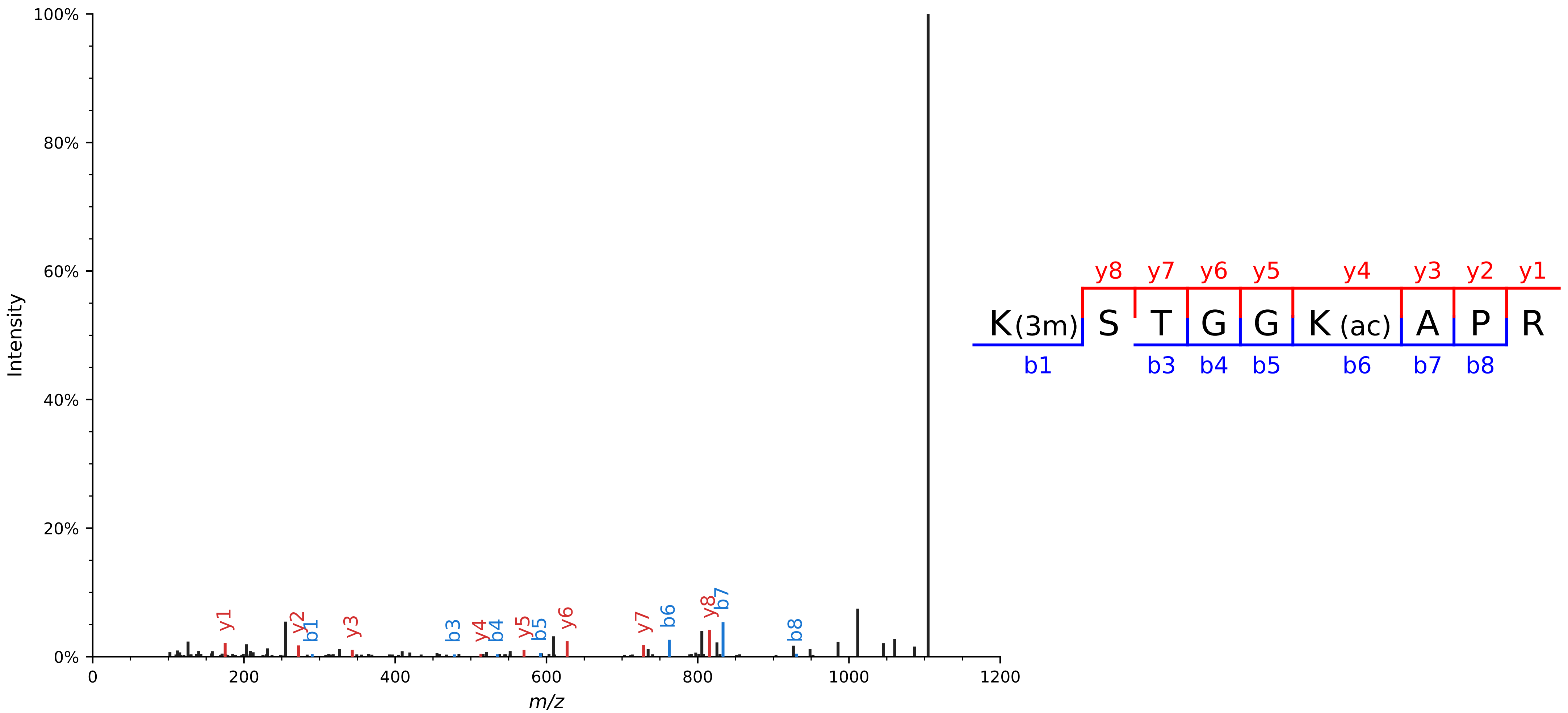

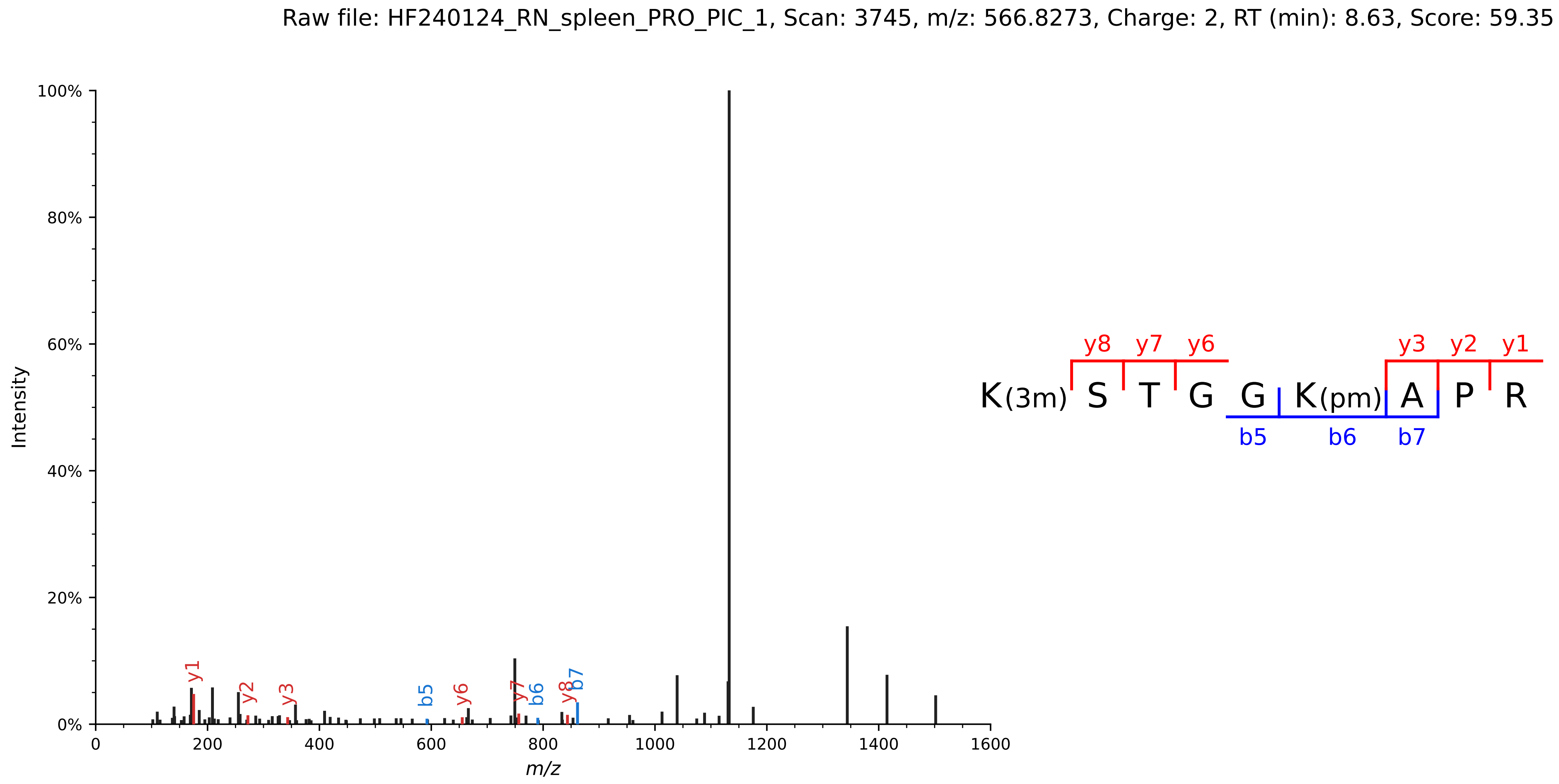
Raw file: HF240124_RN_spleen_PRO_PIC_1, Scan: 3745, m/z: 566.8273, Charge: 2, RT (min): 8.63, Score: 59.35
Intensity
m/z
y1
y2
y3
b5
y6
y7
b6
y8
b7
K(3m) S T G G K(pm) A P R
y8 y7 y6 y3 y2 y1
b5 b6 b7

Raw file: HF240124_RN_spleen_PRO_PIC_2, Scan: 8766, m/z: 559.80128, Charge: 2, RT (min): 16.90, Score: 131.06

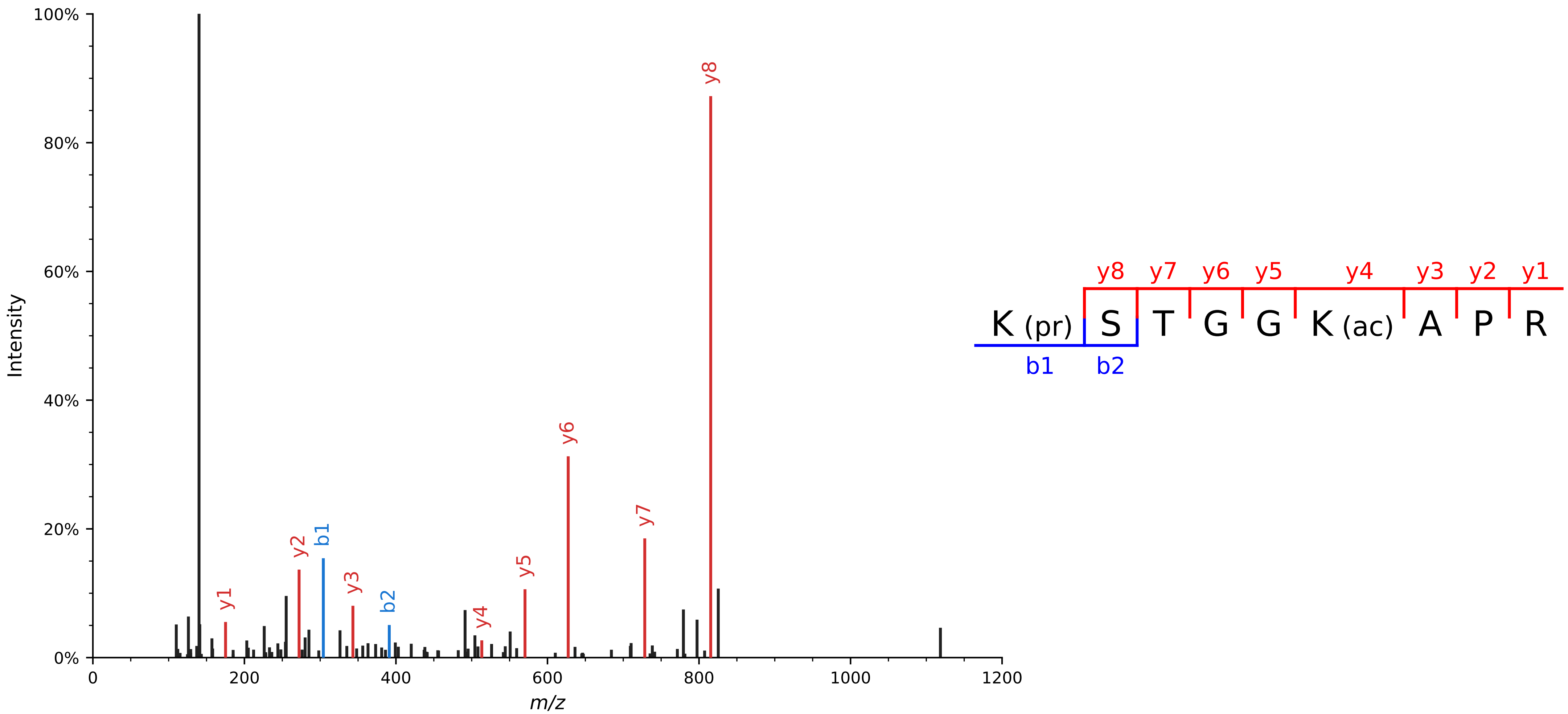

Raw file: HF240124_RN_spleen_PRO_PIC_1, Scan: 10401, m/z: 595.81184, Charge: 2, RT (min): 20.64, Score: 67.169

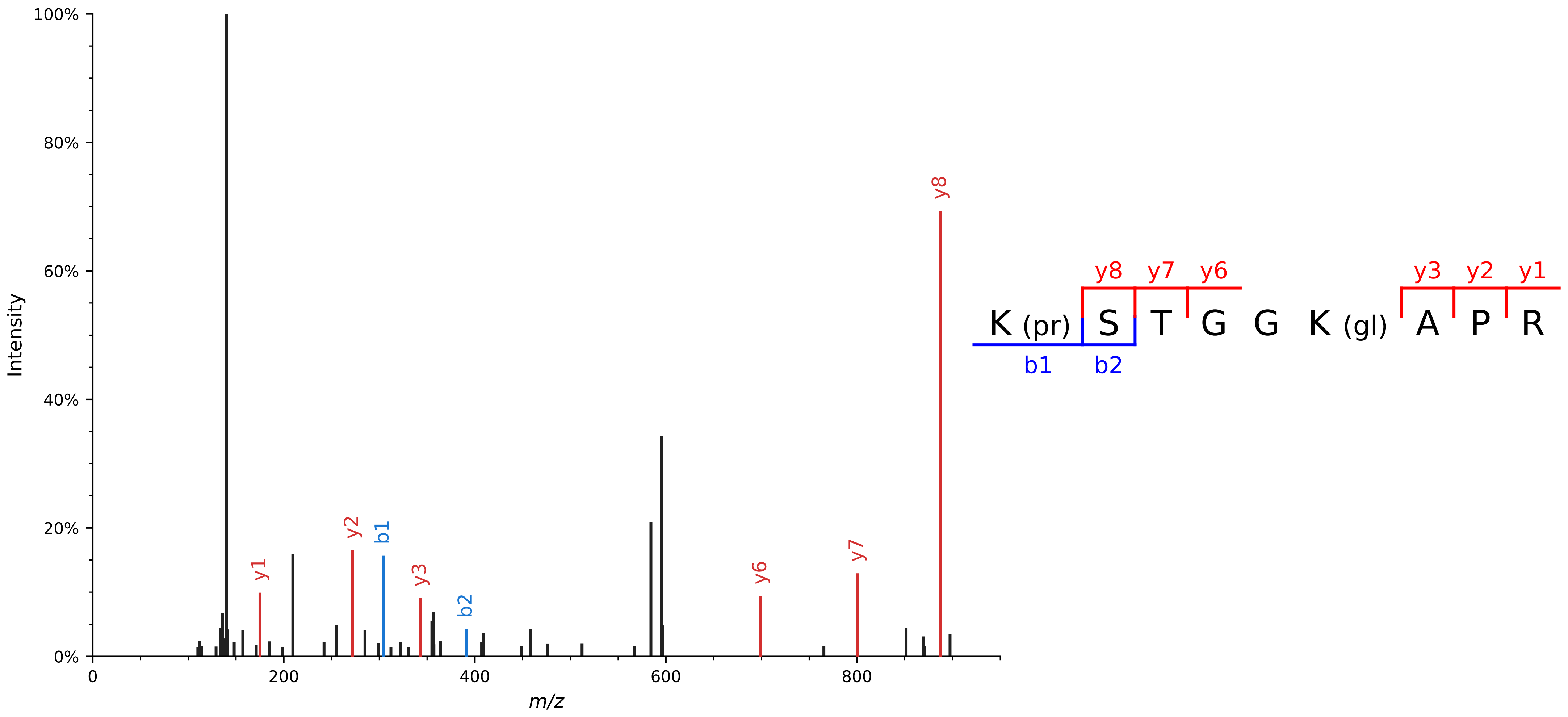

Raw file: HF240124_RN_spleen_PRO_PIC_3, Scan: 11506, m/z: 573.81693, Charge: 2, RT (min): 21.70, Score: 75.738

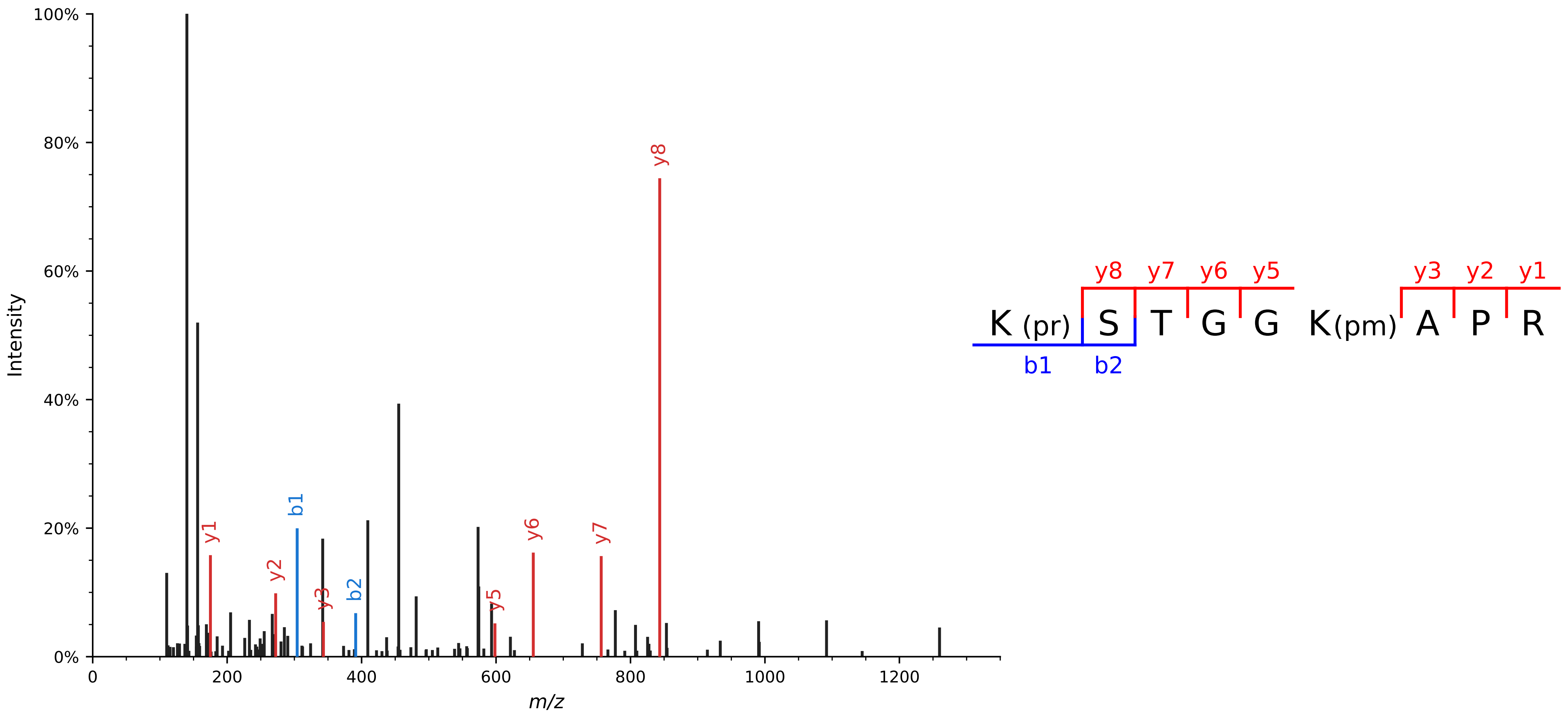

Raw file: HF240124_RN_spleen_PRO_PIC_1, Scan: 2846, m/z: 559.81947, Charge: 2, RT (min): 7.03, Score: 100.45

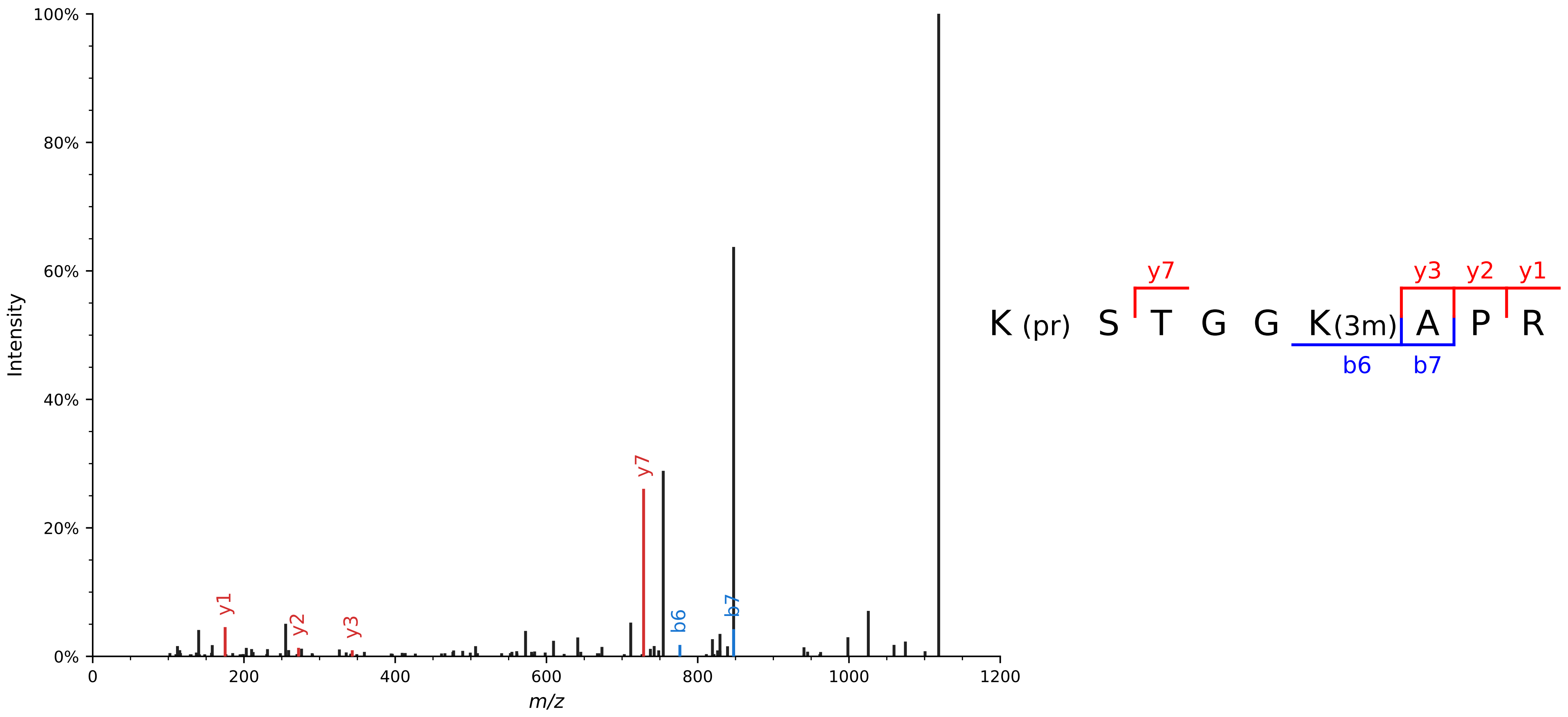

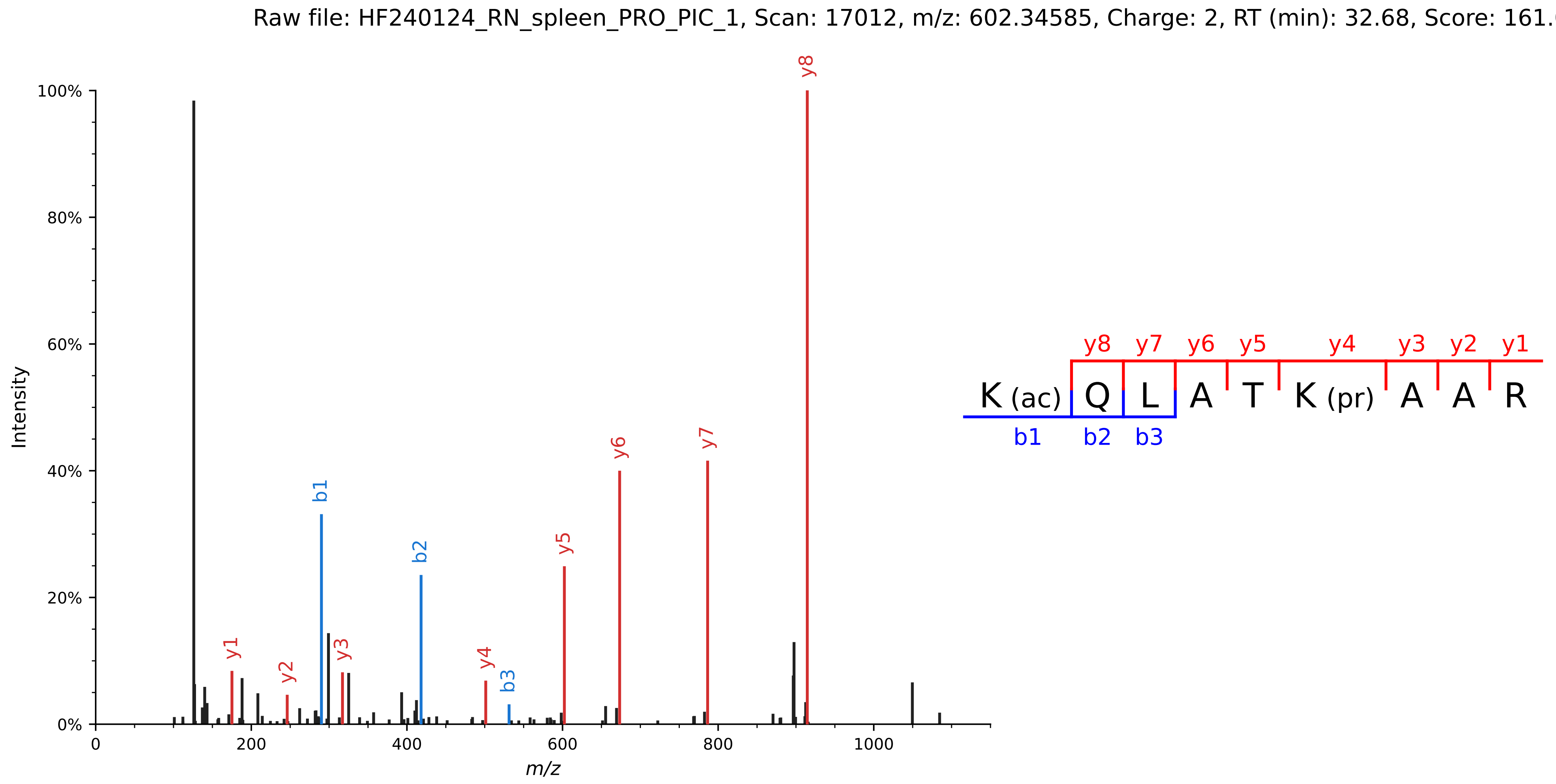

Raw file: HF240124_RN_spleen_PRO_PIC_1, Scan: 17012, m/z: 602.34585, Charge: 2, RT (min): 32.68, Score: 161.02
Intensity
m/z
0%
20%
40%
60%
80%
100%
0
200
400
600
800
1000
y1
y2
b1
y3
b2
y4
b3
y5
y6
y7
y8
y8 y7 y6 y5 y4 y3 y2 y1
K (ac) Q L A T K (pr) A A R
b1 b2 b3

Raw file: HF240124_RN_spleen_PRO_PIC_3, Scan: 16526, m/z: 595.33803, Charge: 2, RT (min): 30.53, Score: 157.68

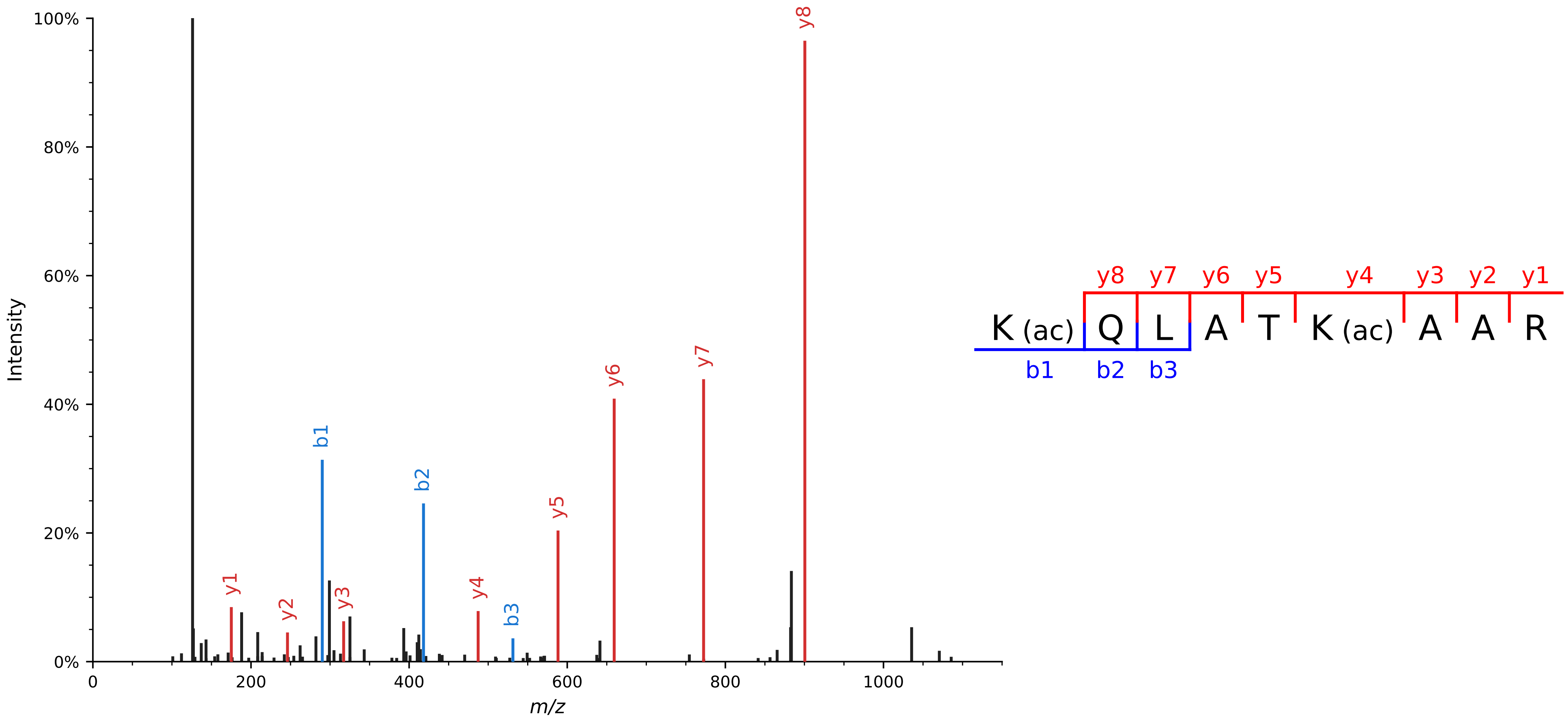

Raw file: HF240124_RN_spleen_PRO_PIC_3, Scan: 14421, m/z: 608.34585, Charge: 2, RT (min): 26.82, Score: 40.268

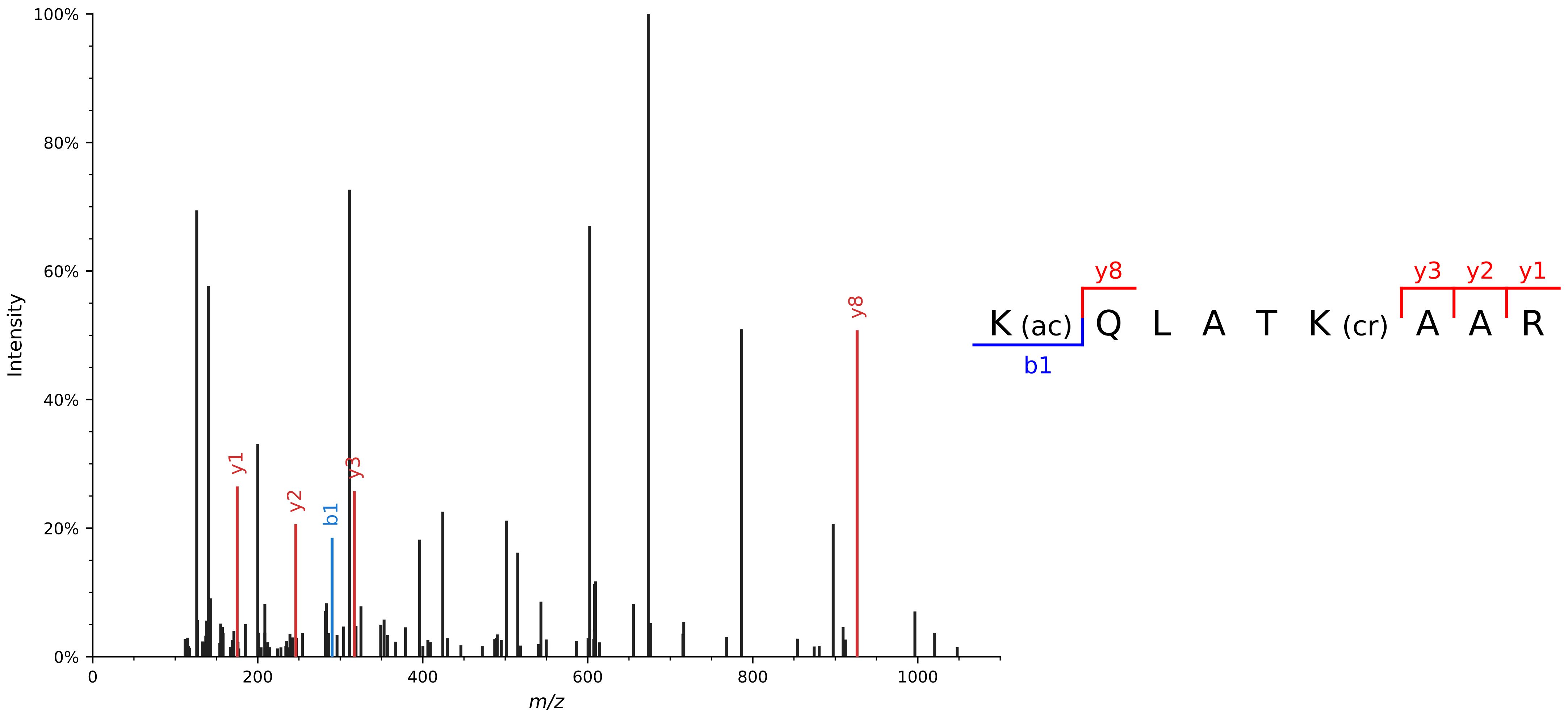

Raw file: HF240124_RN_spleen_PRO_PIC_1, Scan: 15315, m/z: 615.35368, Charge: 2, RT (min): 29.56, Score: 146.27

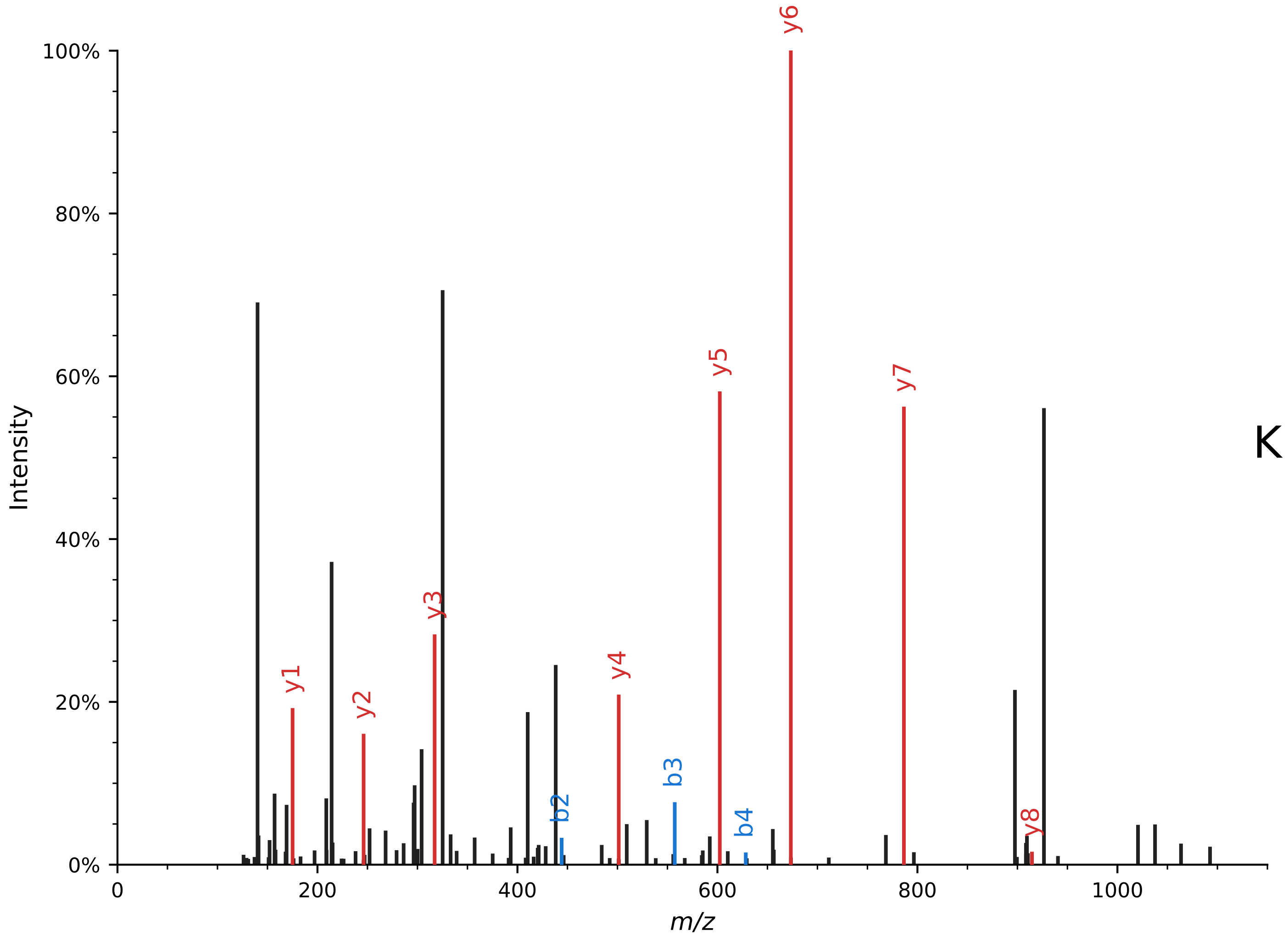

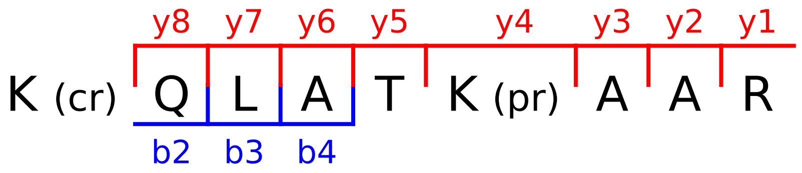

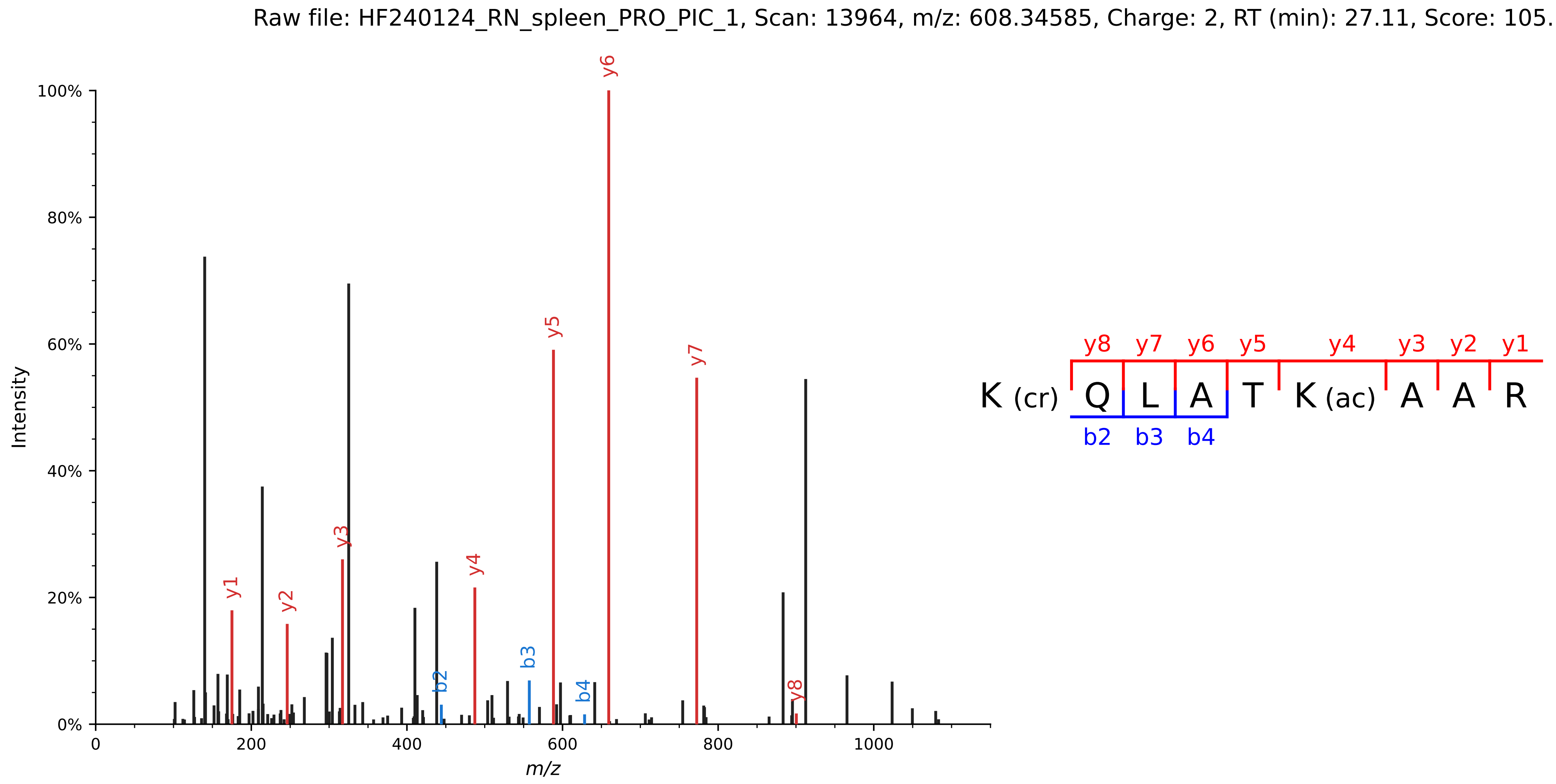

Raw file: HF240124_RN_spleen_PRO_PIC_1, Scan: 13964, m/z: 608.34585, Charge: 2, RT (min): 27.11, Score: 105.78
Intensity
m/z
y1
y2
y3
y4
y5
y6
y7
y8
b2
b3
b4
K (cr) Q L A T K (ac) A A R

Raw file: HF240124_RN_spleen_PRO_PIC_2, Scan: 17360, m/z: 595.33803, Charge: 2, RT (min): 32.04, Score: 126.52

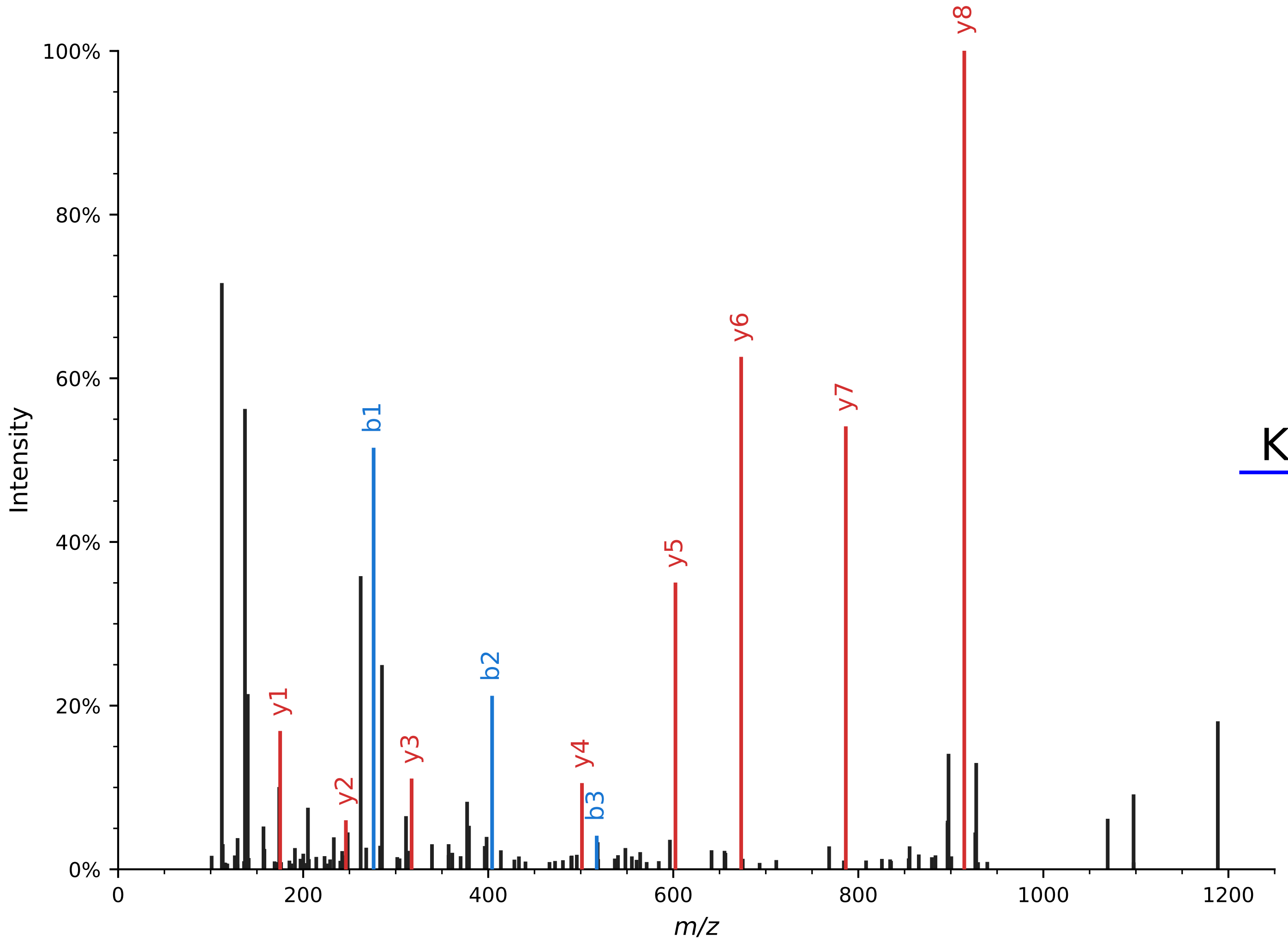

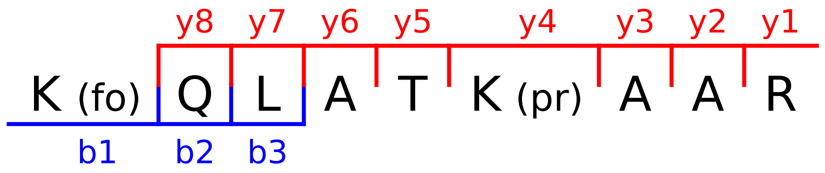

Raw file: HF240124_RN_spleen_PRO_PIC_3, Scan: 18734, m/z: 624.35896, Charge: 2, RT (min): 34.44, Score: 84.476

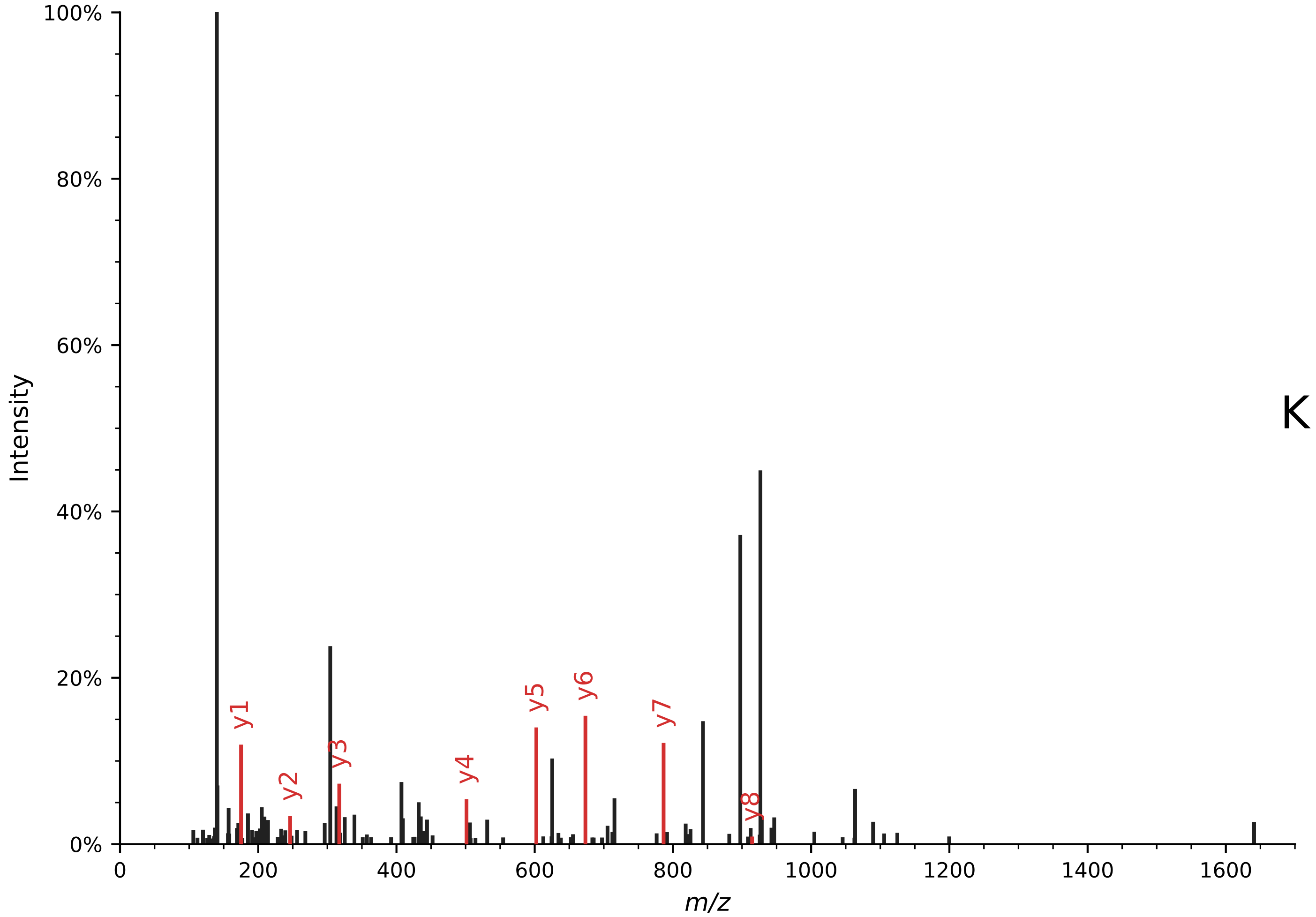

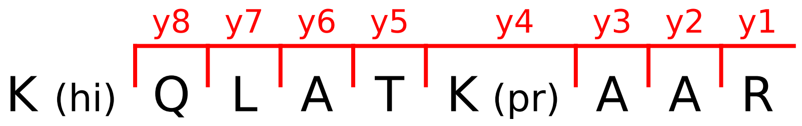

Raw file: HF240124_RN_spleen_PRO_PIC_1, Scan: 19854, m/z: 616.3615, Charge: 2, RT (min): 37.93, Score: 170.66

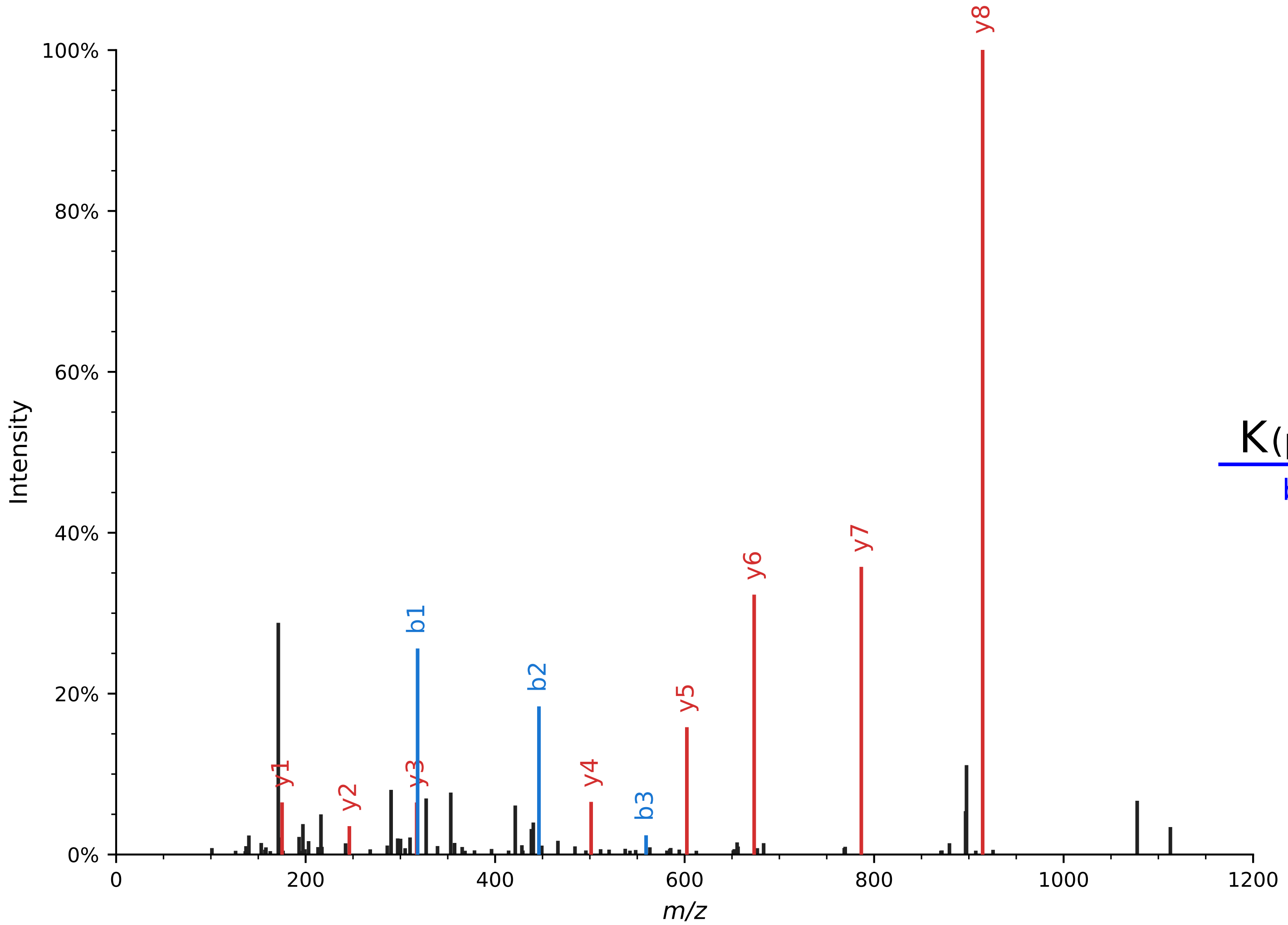

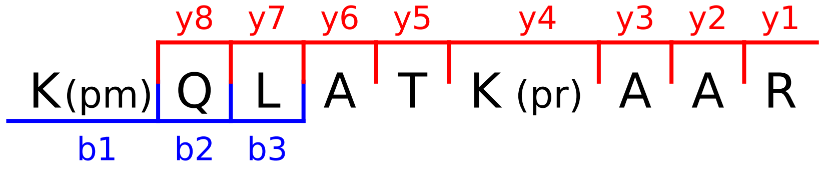

Raw file: HF240124_RN_spleen_PRO_PIC_3, Scan: 21796, m/z: 623.36933, Charge: 2, RT (min): 39.94, Score: 105.57

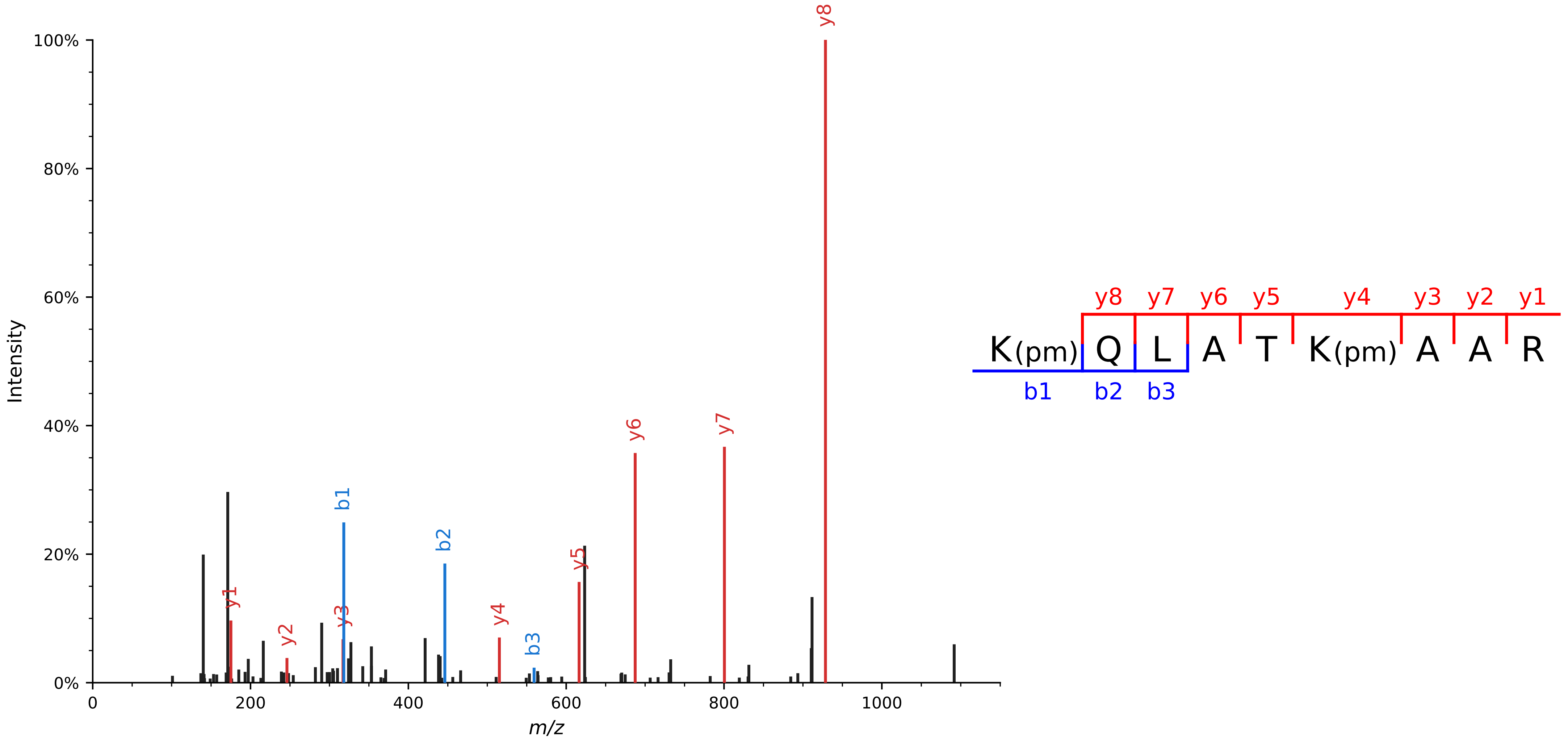

Raw file: HF240124_RN_spleen_PRO_PIC_2, Scan: 9588, m/z: 595.35622, Charge: 2, RT (min): 18.32, Score: 107.69

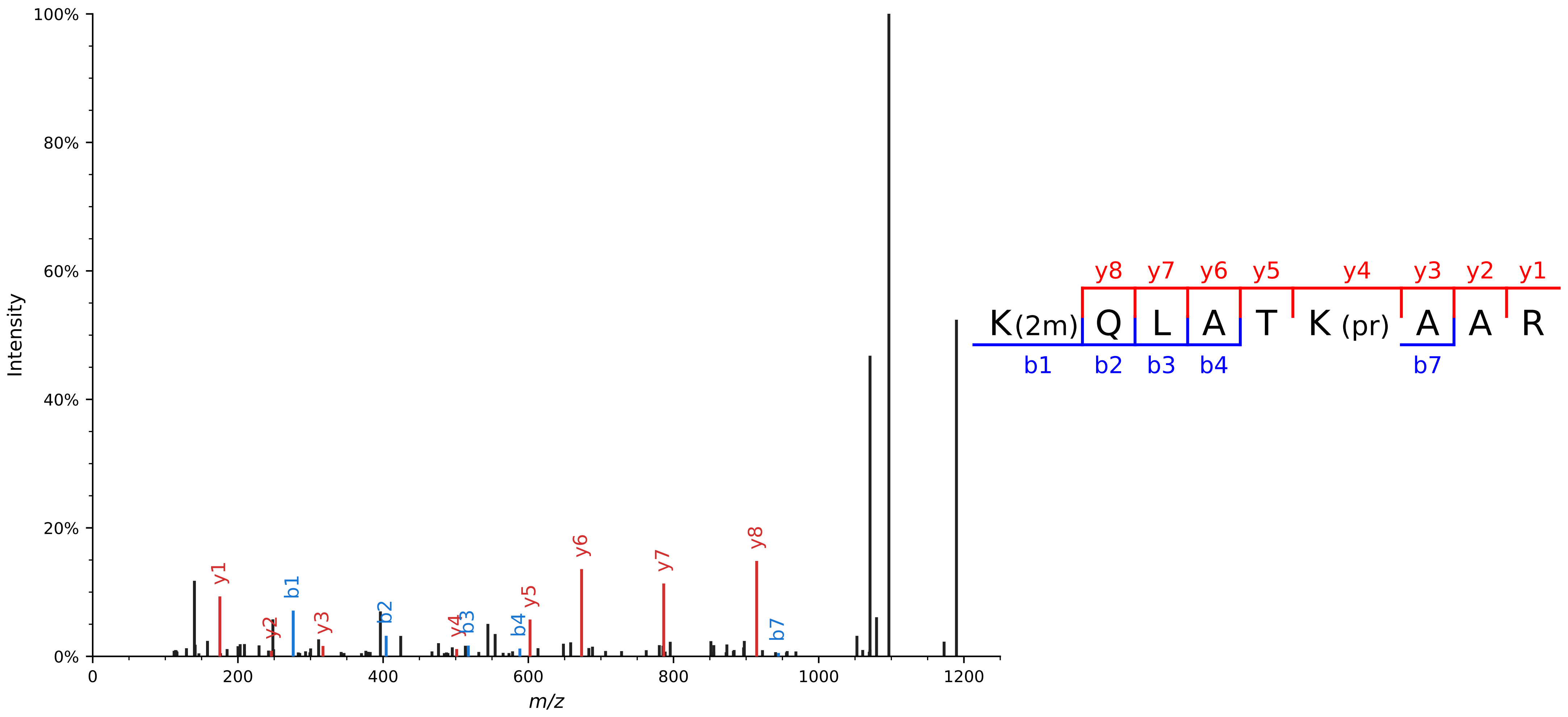

Raw file: HF240124_RN_spleen_PRO_PIC_3, Scan: 18055, m/z: 602.34585, Charge: 2, RT (min): 33.24, Score: 155.1

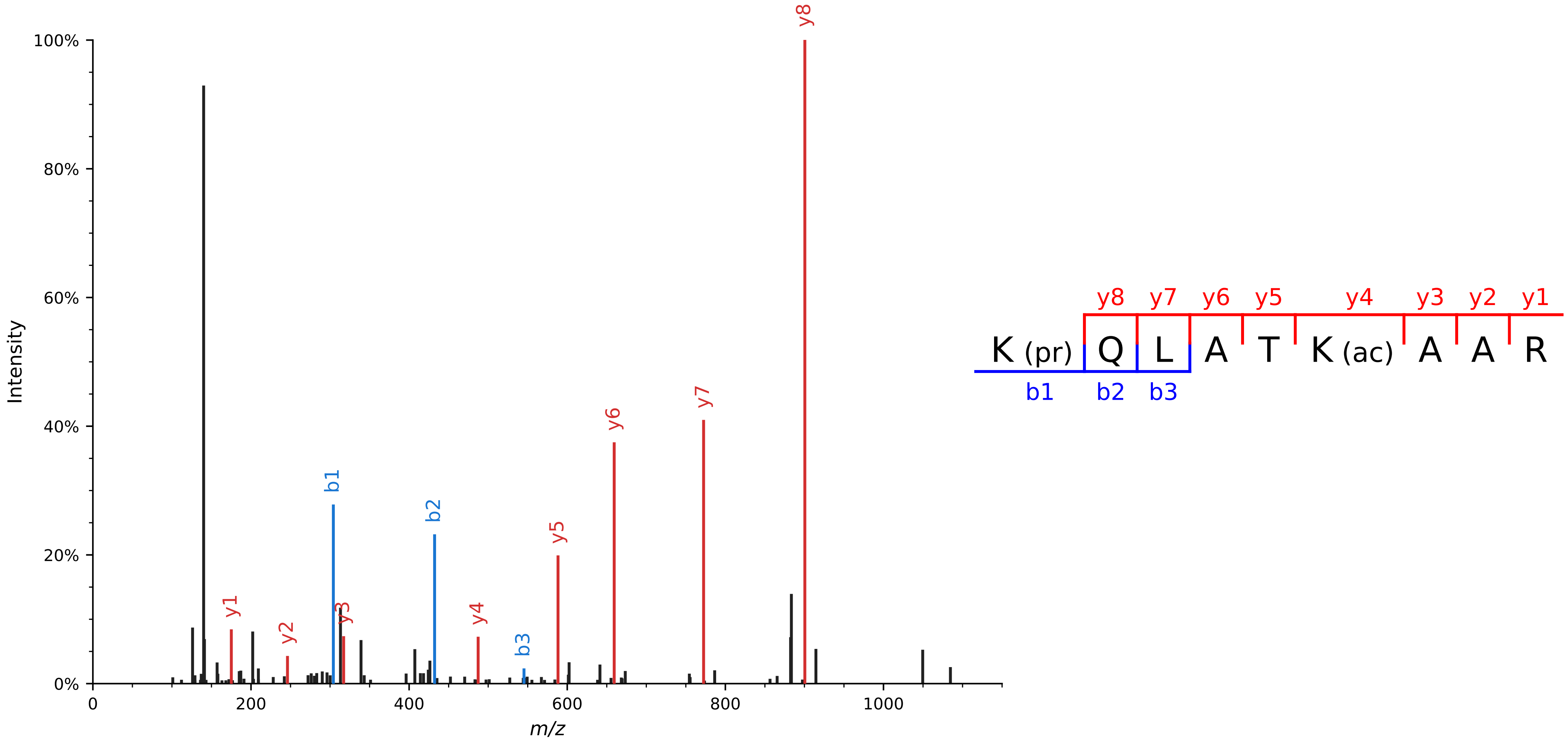

Raw file: HF240124_RN_spleen_PRO_PIC_3, Scan: 17483, m/z: 595.33803, Charge: 2, RT (min): 32.23, Score: 98.629

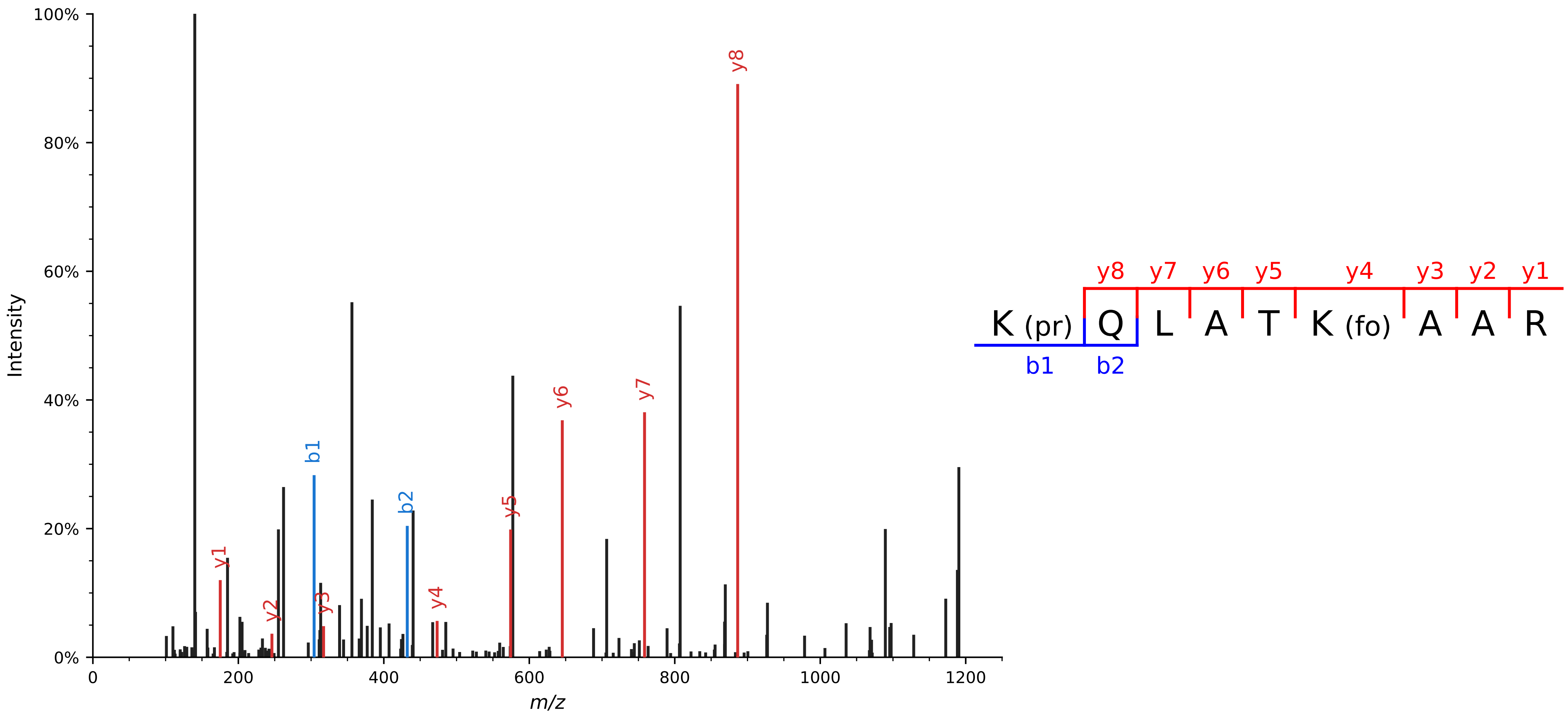

Raw file: HF240124_RN_spleen_PRO_PIC_2, Scan: 18837, m/z: 638.35642, Charge: 2, RT (min): 34.66, Score: 87.696

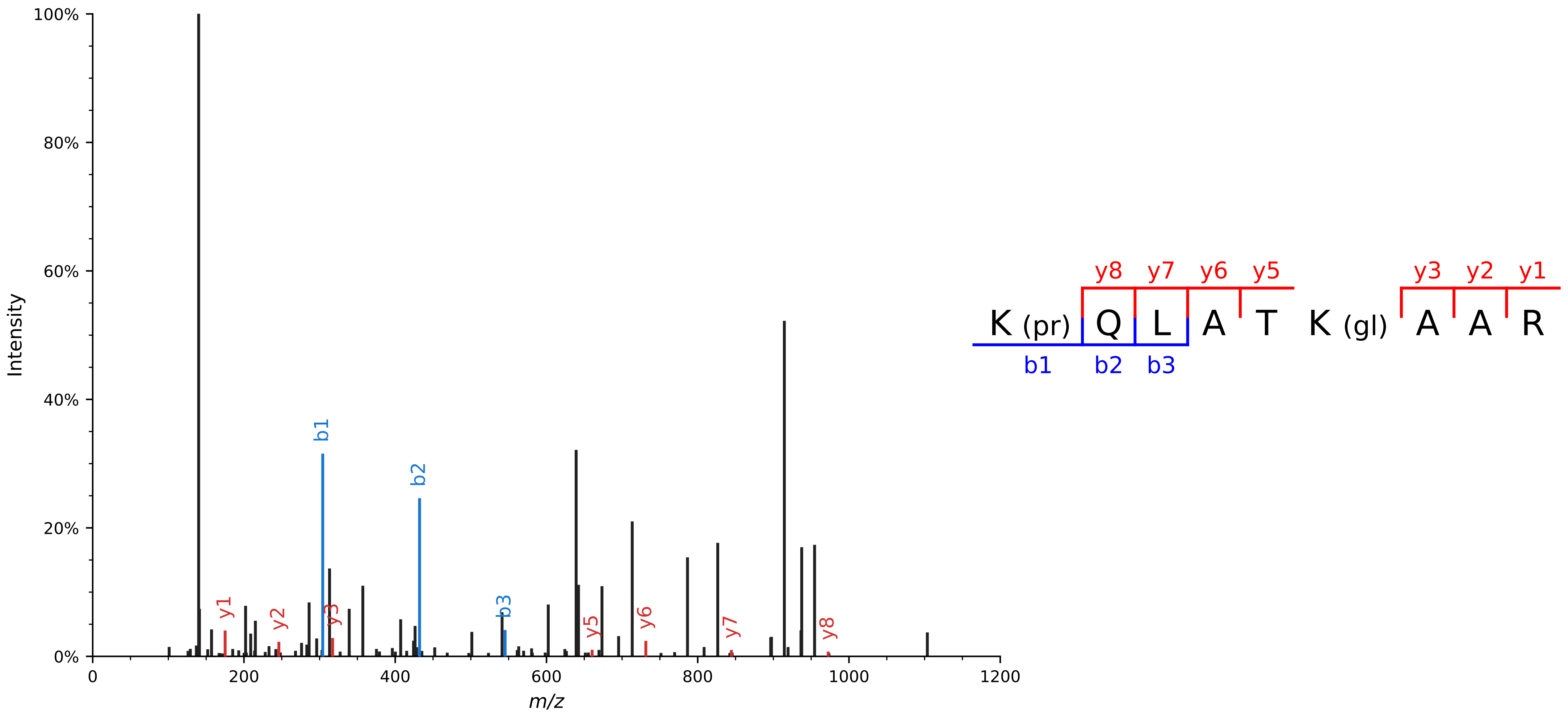

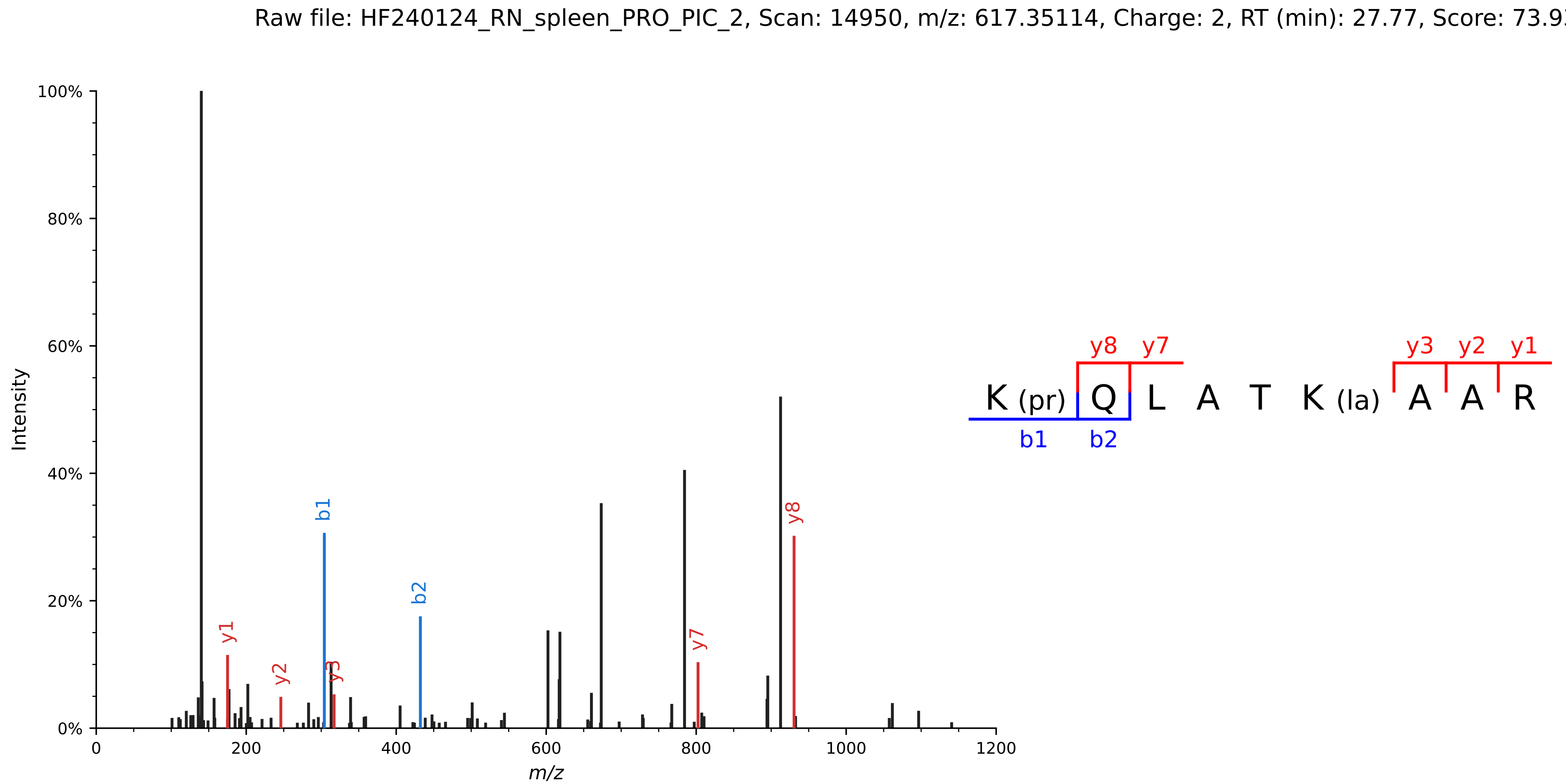
Raw file: HF240124_RN_spleen_PRO_PIC_2, Scan: 14950, m/z: 617.35114, Charge: 2, RT (min): 27.77, Score: 73.931
100%
80%
60%
40%
20%
0%
Intensity
0
200
400
600
800
1000
1200
m/z
y1
y2
y3
b1
b2
y7
y8
K (pr) Q L A T K (la) A A R
y8 y7 y3 y2 y1
b1 b2

Raw file: HF240124_RN_spleen_PRO_PIC_3, Scan: 20378, m/z: 616.3615, Charge: 2, RT (min): 37.39, Score: 176.4

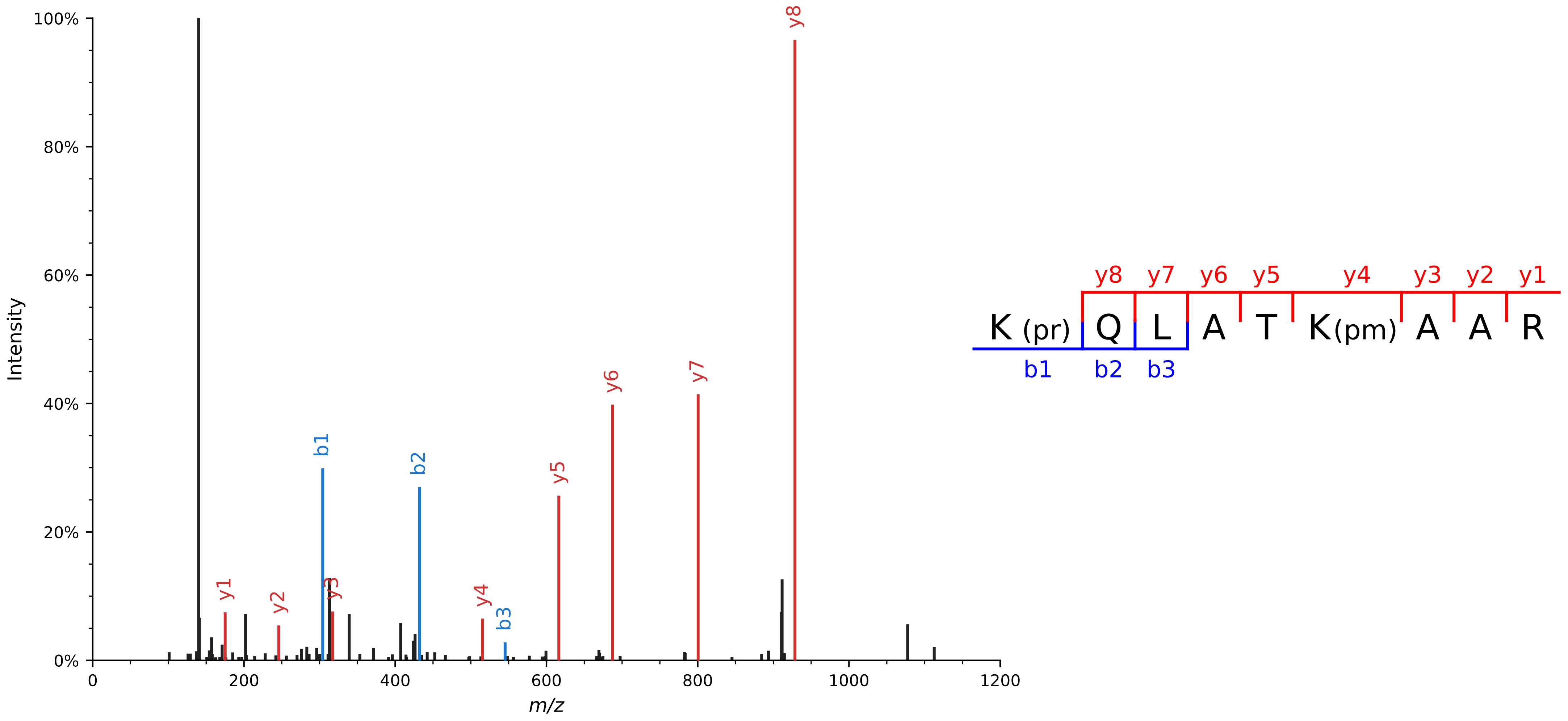

Raw file: HF240124_RN_spleen_PRO_PIC_2, Scan: 9348, m/z: 853.97047, Charge: 2, RT (min): 17.91, Score: 183.33

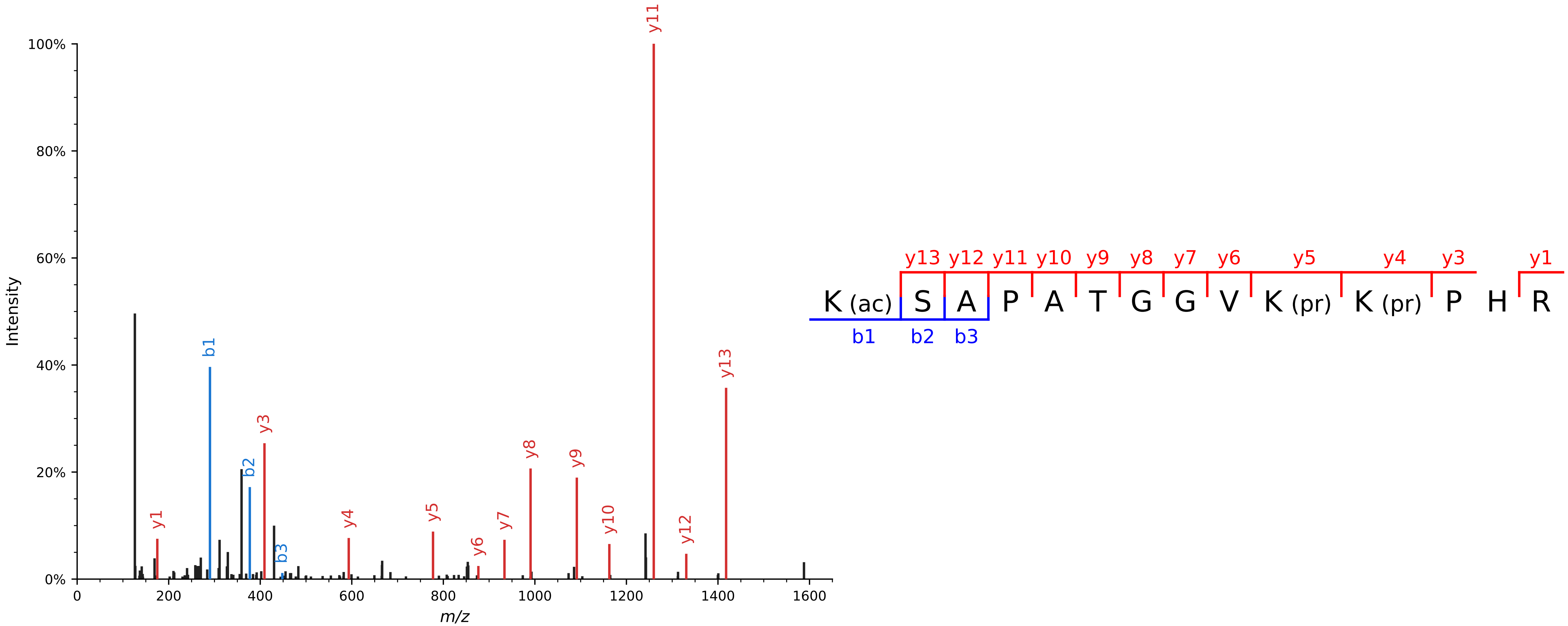

Raw file: HF240124_RN_spleen_PRO_PIC_3, Scan: 5428, m/z: 839.97301, Charge: 2, RT (min): 10.90, Score: 160.15

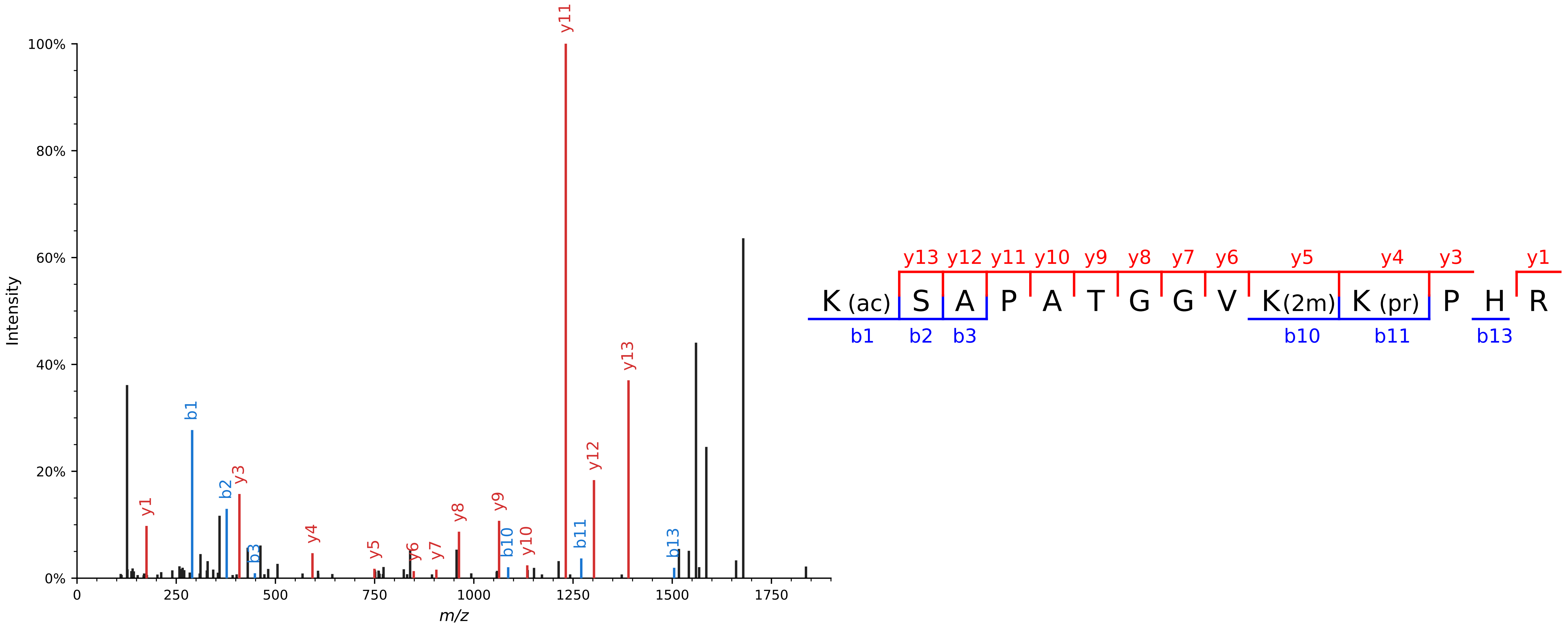

Raw file: HF240124_RN_spleen_PRO_PIC_3, Scan: 10744, m/z: 866.9783, Charge: 2, RT (min): 20.39, Score: 158.42

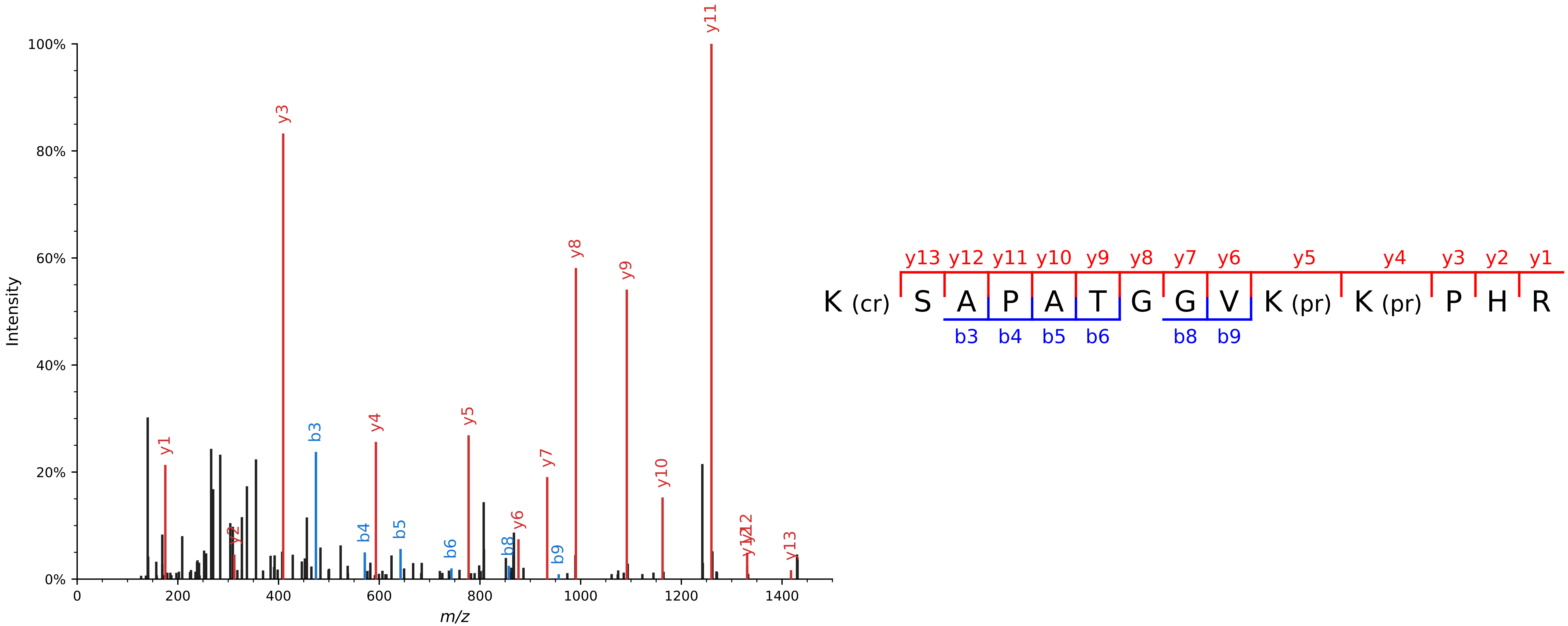

Raw file: HF240124_RN_spleen_PRO_PIC_3, Scan: 11067, m/z: 859.97047, Charge: 2, RT (min): 20.93, Score: 65.842

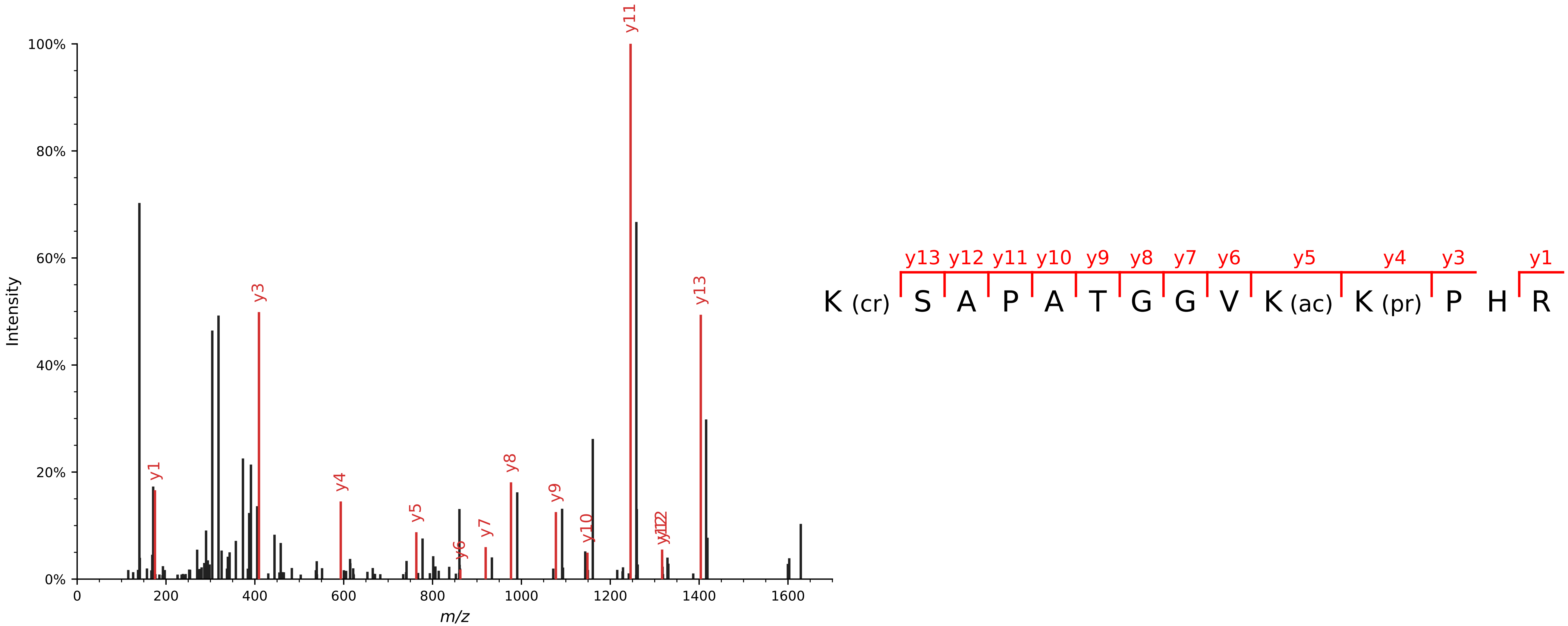

Raw file: HF240124_RN_spleen_PRO_PIC_2, Scan: 11924, m/z: 878.9783, Charge: 2, RT (min): 22.46, Score: 43.628

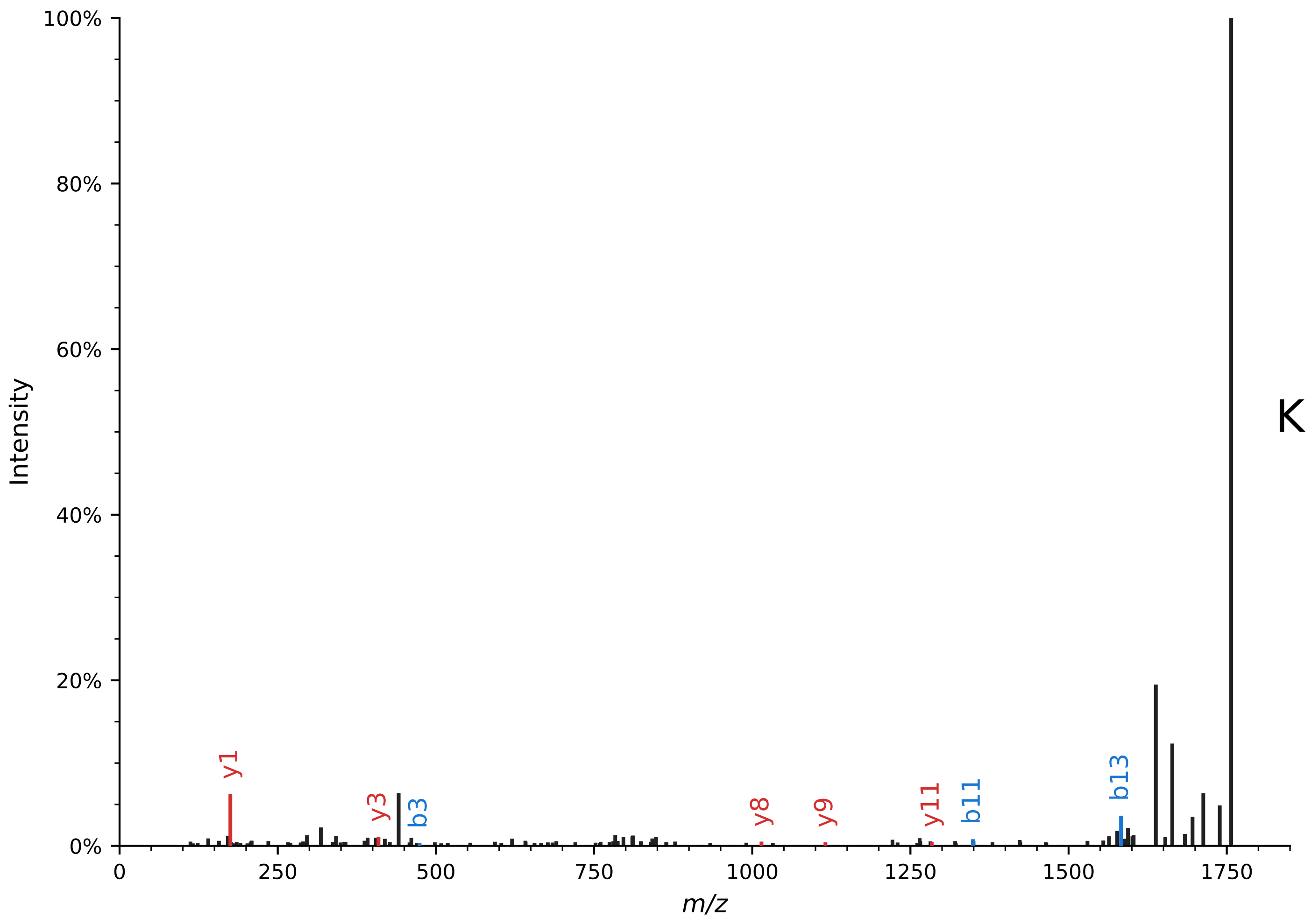

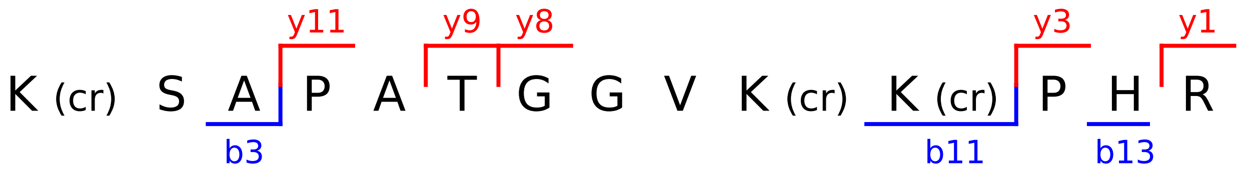

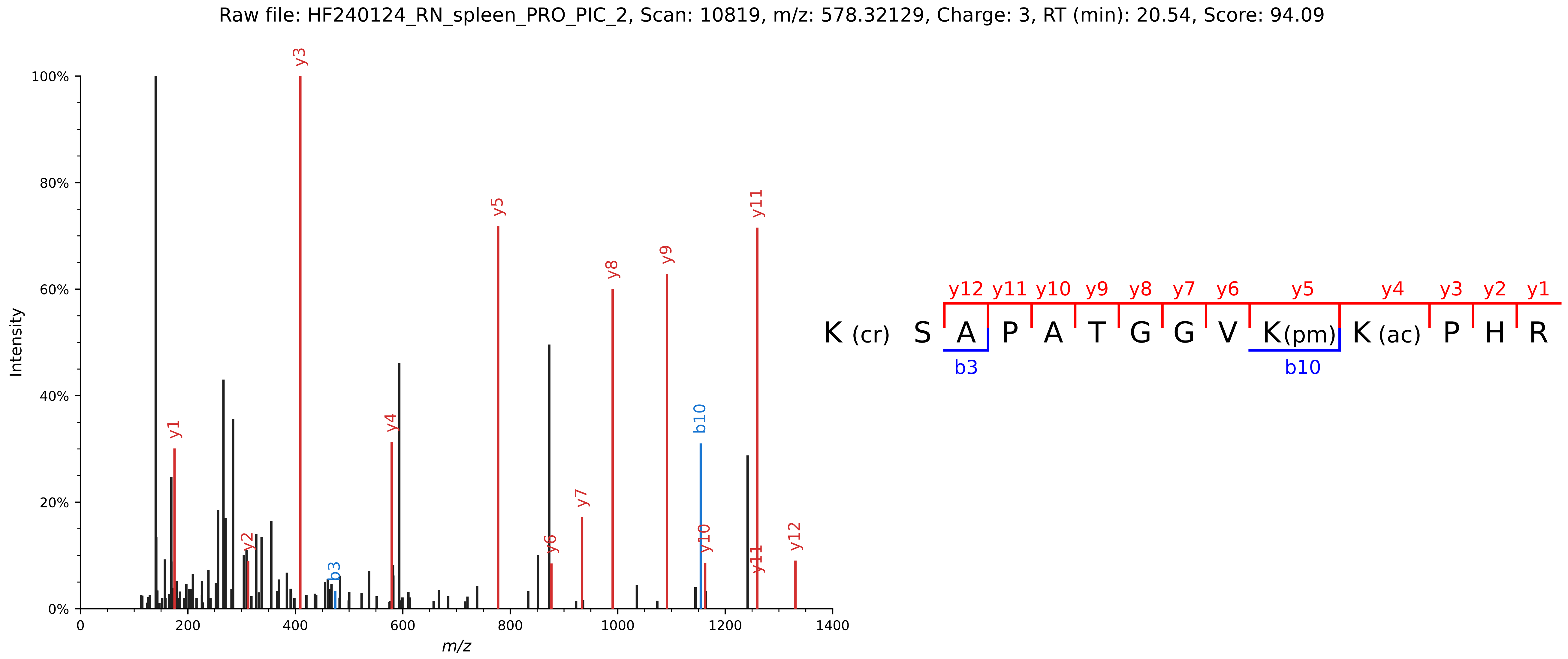

Raw file: HF240124_RN_spleen_PRO_PIC_2, Scan: 10819, m/z: 578.32129, Charge: 3, RT (min): 20.54, Score: 94.09
Intensity
m/z
y1
y2
y3
b3
y4
y5
y6
y7
y8
y9
b10
y10
y11
y12
K (cr) S A P A T G G V K(pm) K (ac) P H R

Raw file: HF240124_RN_spleen_PRO_PIC_3, Scan: 7644, m/z: 568.98965, Charge: 3, RT (min): 14.93, Score: 67.964

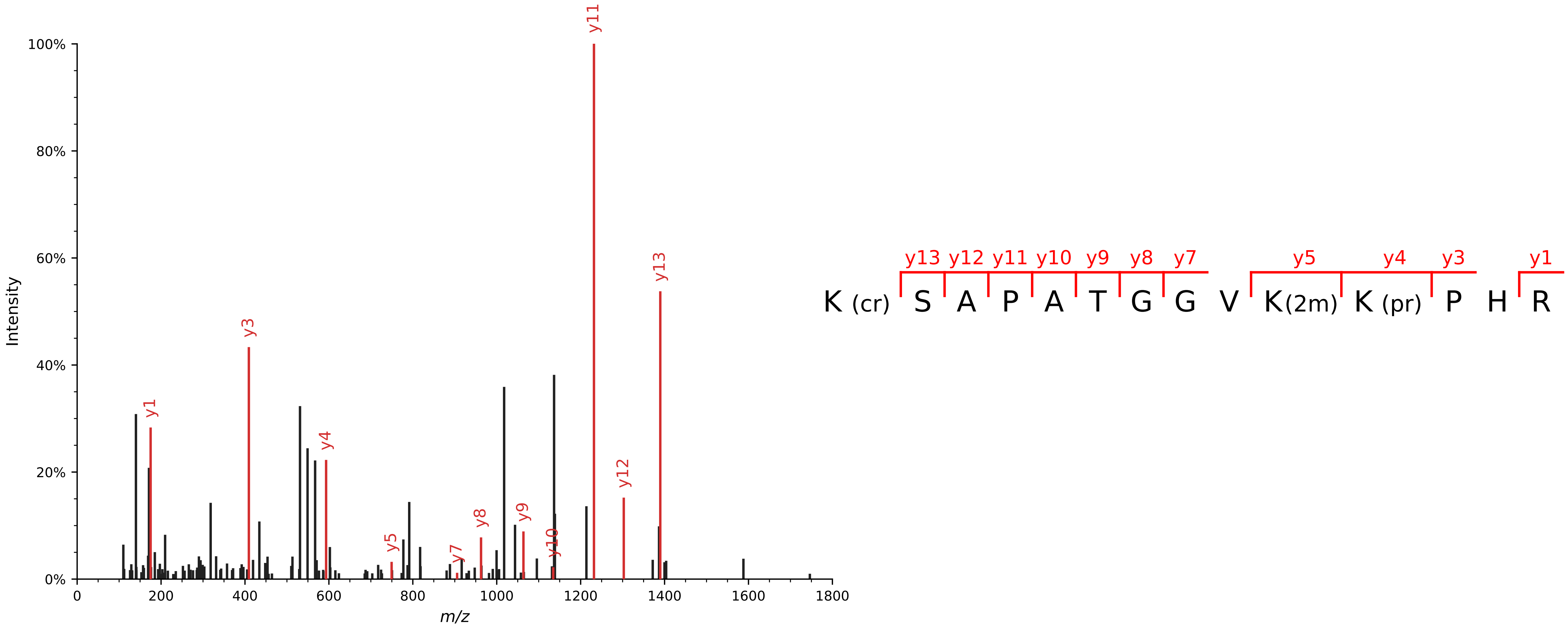

Raw file: HF240124_RN_spleen_PRO_PIC_3, Scan: 7555, m/z: 573.66153, Charge: 3, RT (min): 14.79, Score: 41.092

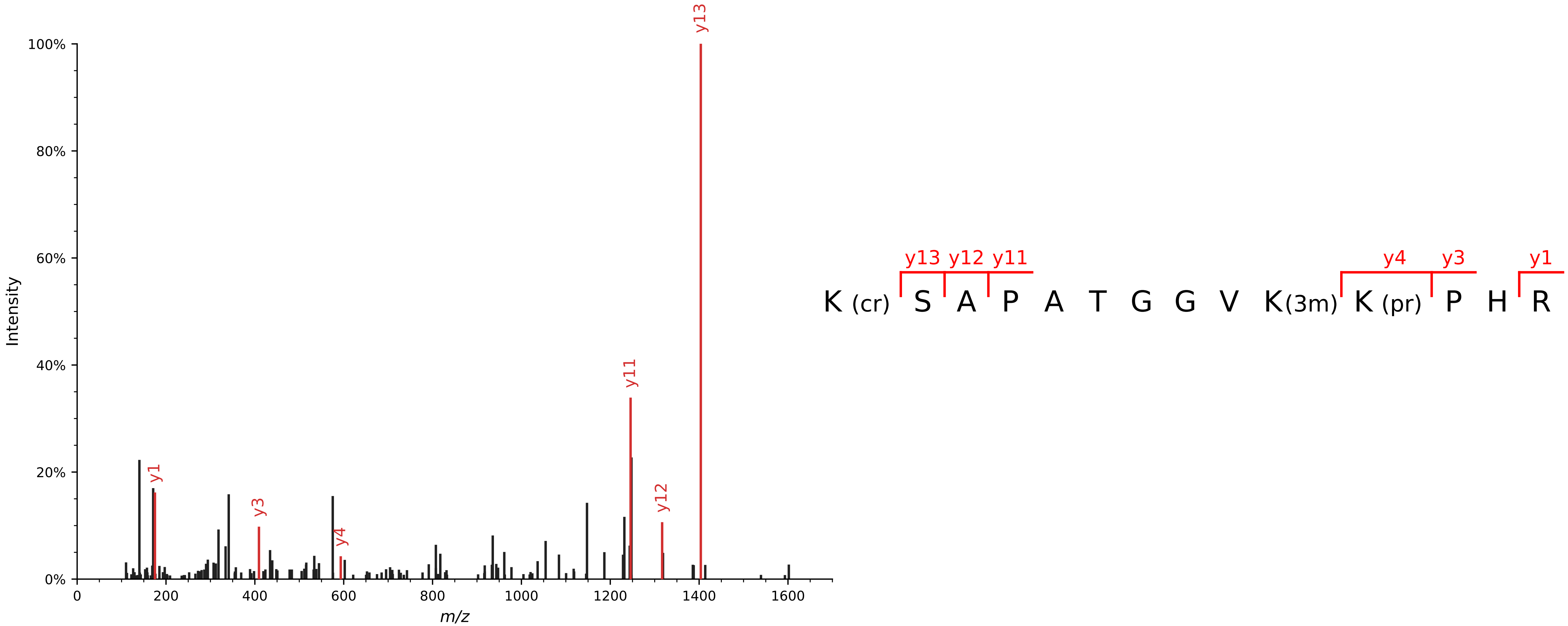

Raw file: HF240124_RN_spleen_PRO_PIC_3, Scan: 11452, m/z: 593.65645, Charge: 3, RT (min): 21.60, Score: 80.522

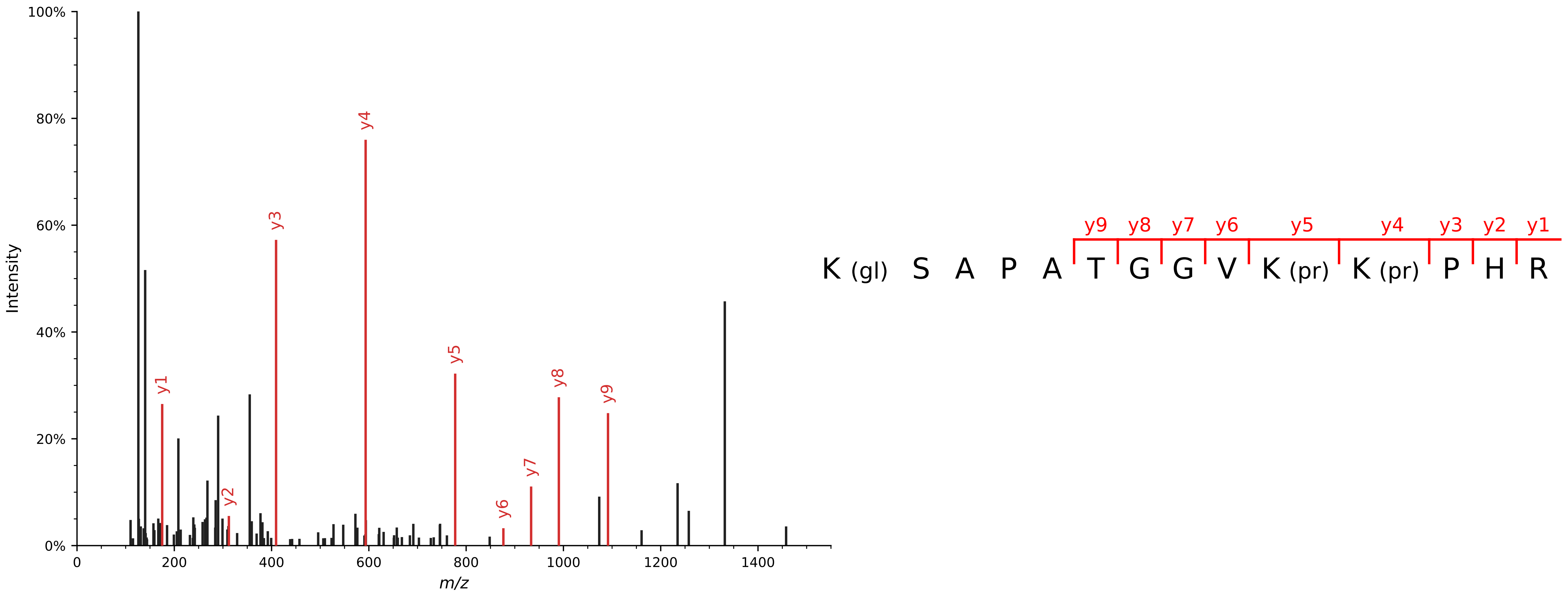

Raw file: HF240124_RN_spleen_PRO_PIC_3, Scan: 7412, m/z: 574.99317, Charge: 3, RT (min): 14.54, Score: 45.115

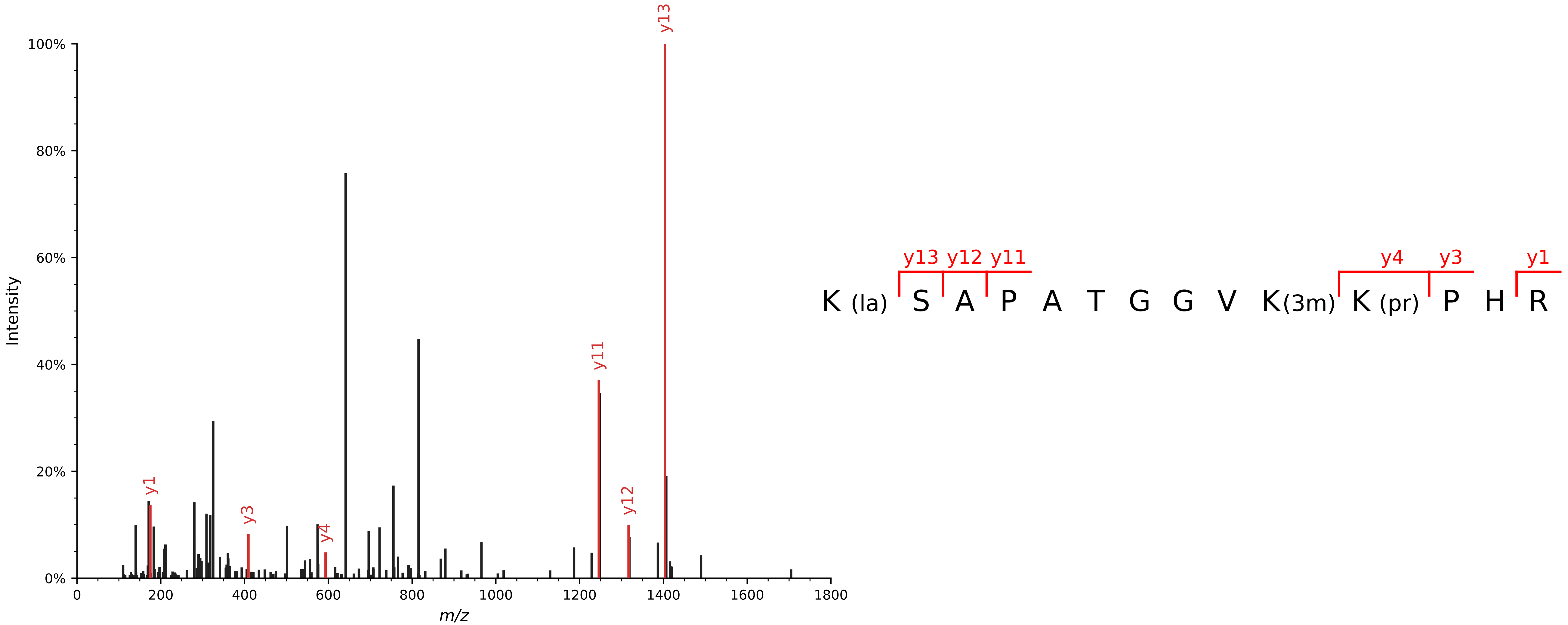

Raw file: HF240124_RN_spleen_PRO_PIC_2, Scan: 12056, m/z: 867.98612, Charge: 2, RT (min): 22.69, Score: 175.15

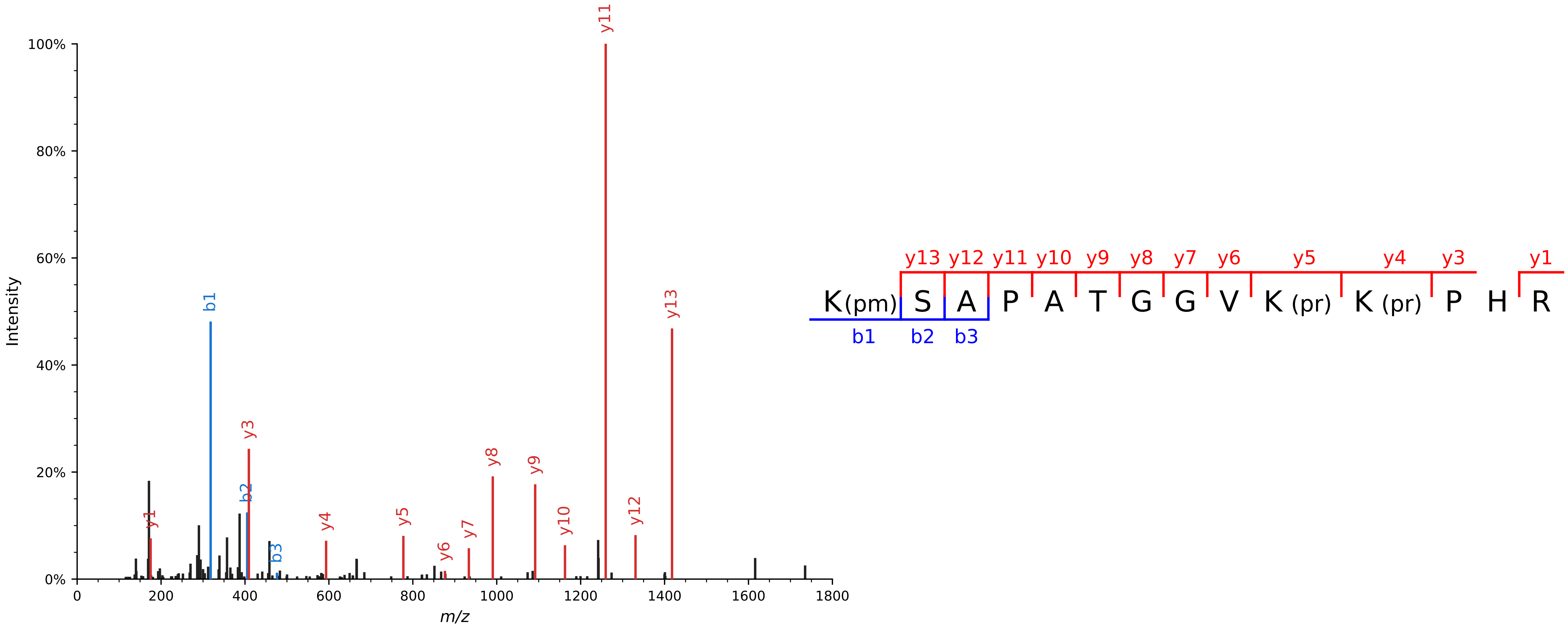

Raw file: HF240124_RN_spleen_PRO_PIC_3, Scan: 10848, m/z: 853.97047, Charge: 2, RT (min): 20.57, Score: 99.283

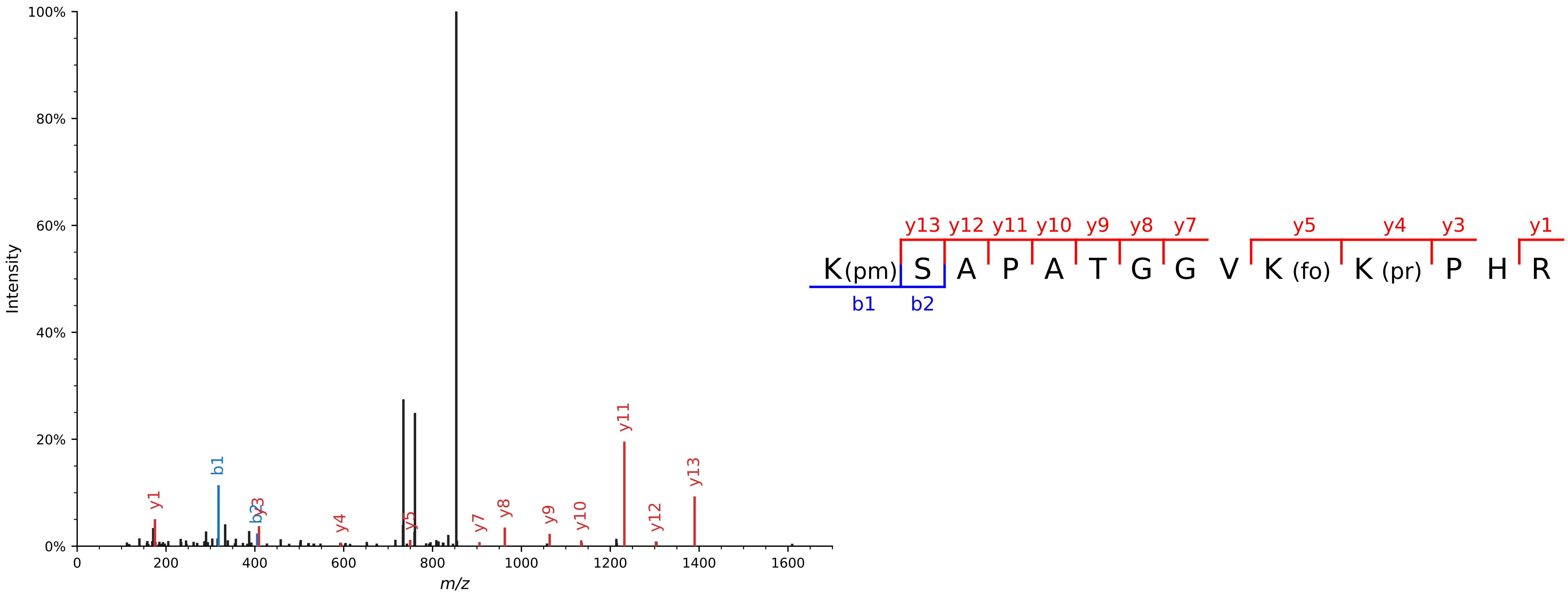

Raw file: HF240124_RN_spleen_PRO_PIC_2, Scan: 12931, m/z: 874.99395, Charge: 2, RT (min): 24.19, Score: 172.8

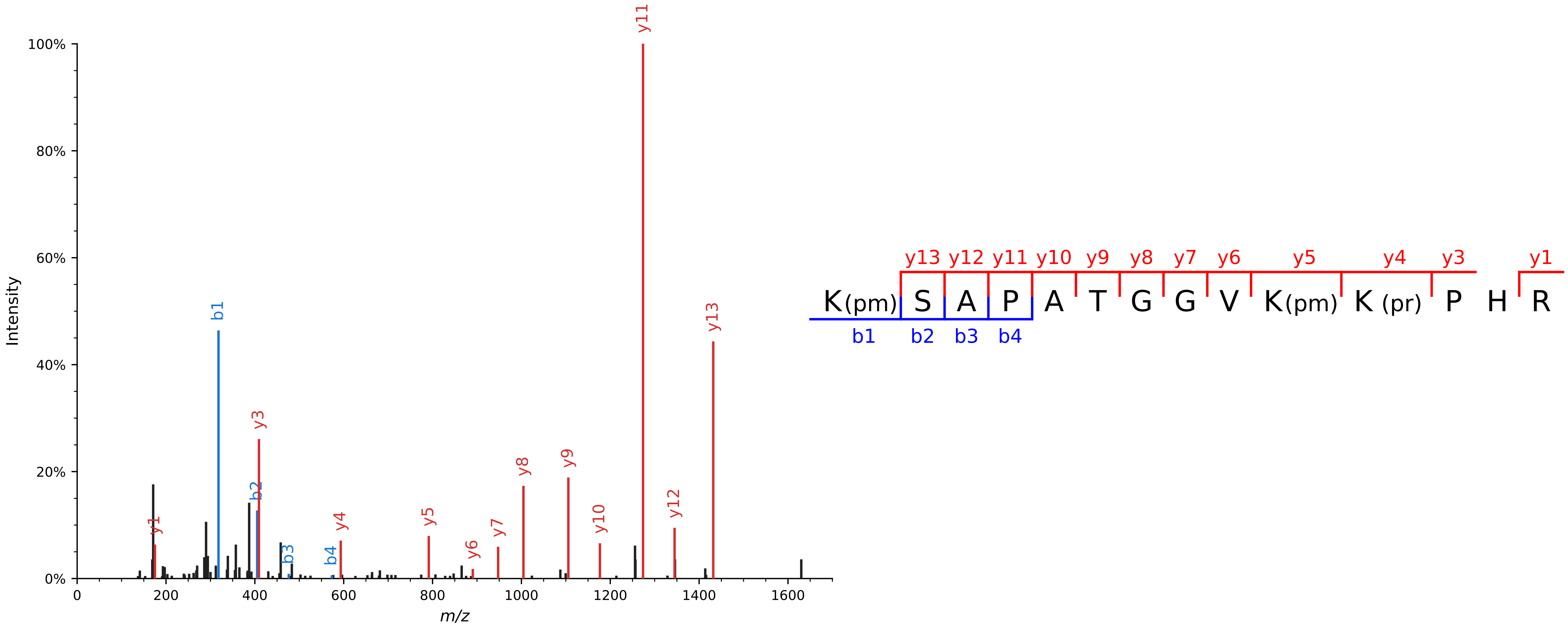

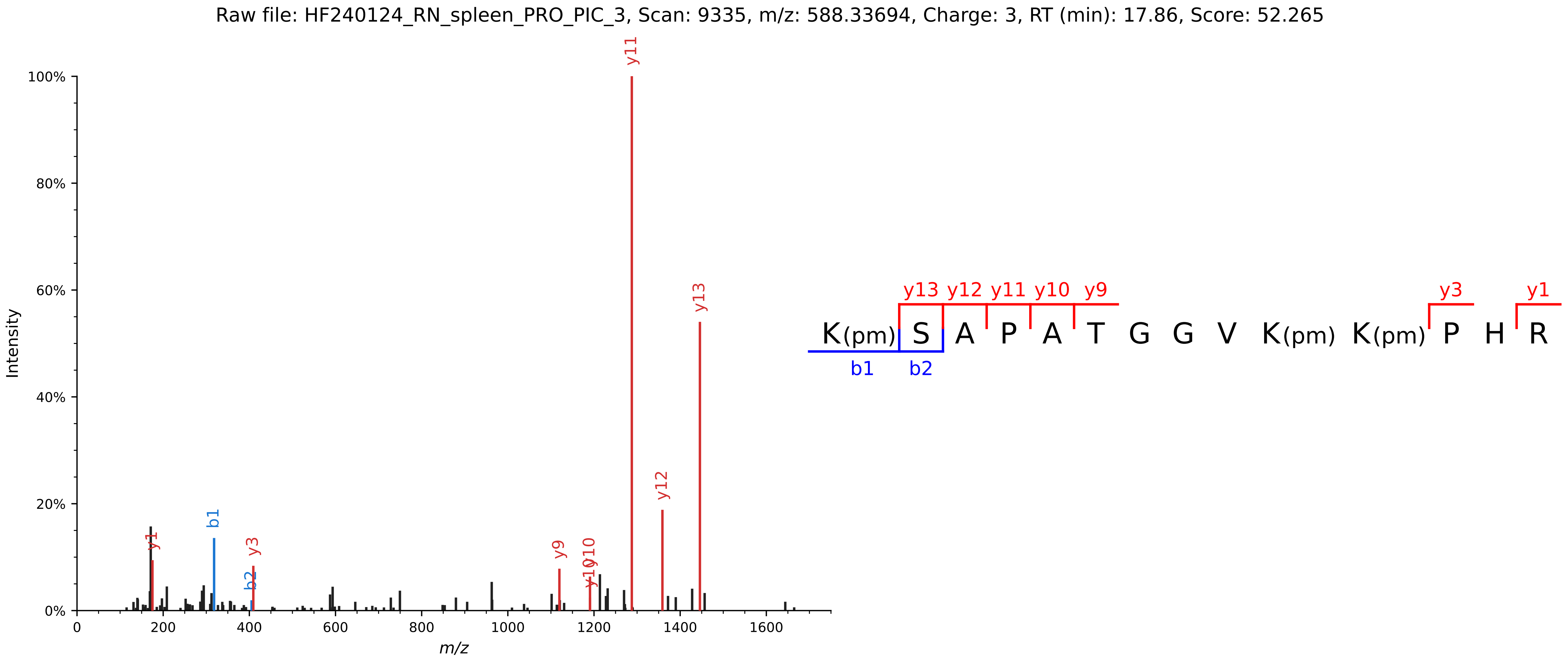

Raw file: HF240124_RN_spleen_PRO_PIC_3, Scan: 9335, m/z: 588.33694, Charge: 3, RT (min): 17.86, Score: 52.265
Intensity
m/z
0%
20%
40%
60%
80%
100%
0
200
400
600
800
1000
1200
1400
1600
y1
b1
b2
y3
y9
y10
y11
y12
y13
K(pm) S A P A T G G V K(pm) K(pm) P H R

Raw file: HF240124_RN_spleen_PRO_PIC_3, Scan: 7296, m/z: 853.98866, Charge: 2, RT (min): 14.33, Score: 168.3

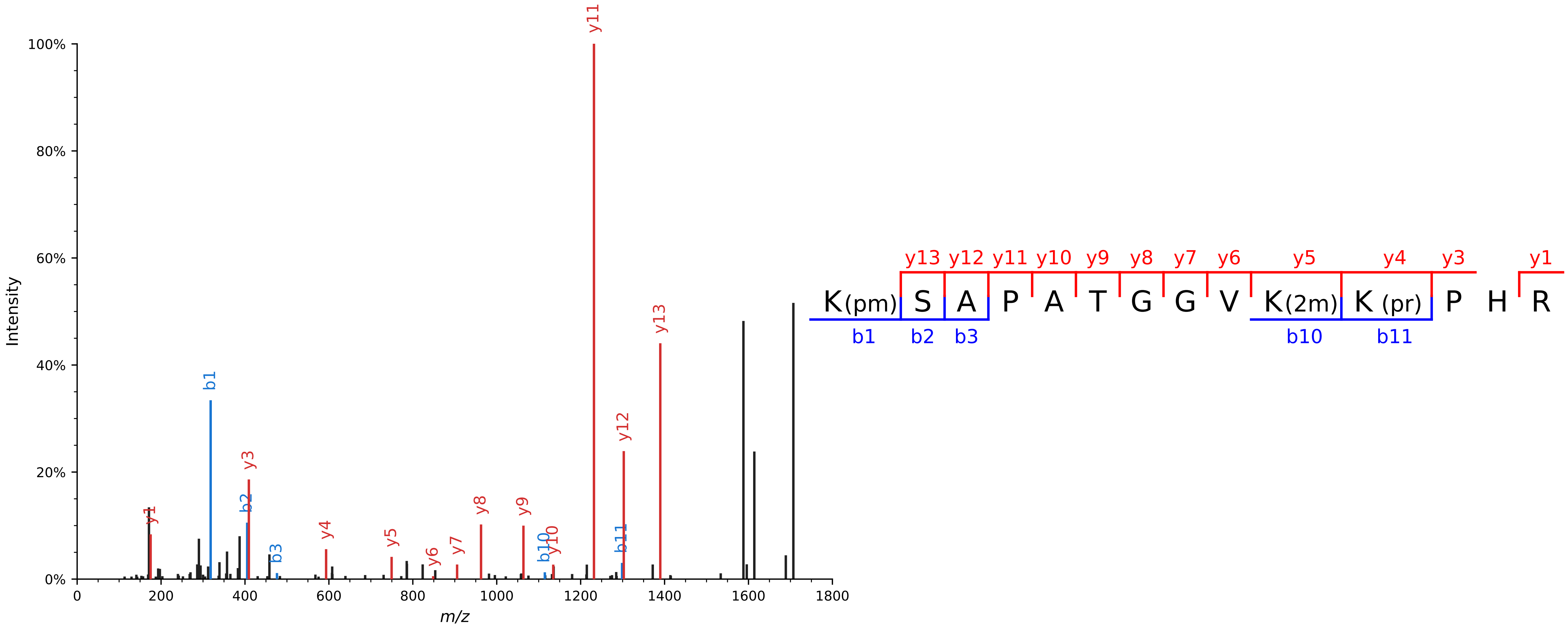

Raw file: HF240124_RN_spleen_PRO_PIC_2, Scan: 7151, m/z: 860.99649, Charge: 2, RT (min): 14.10, Score: 90.685

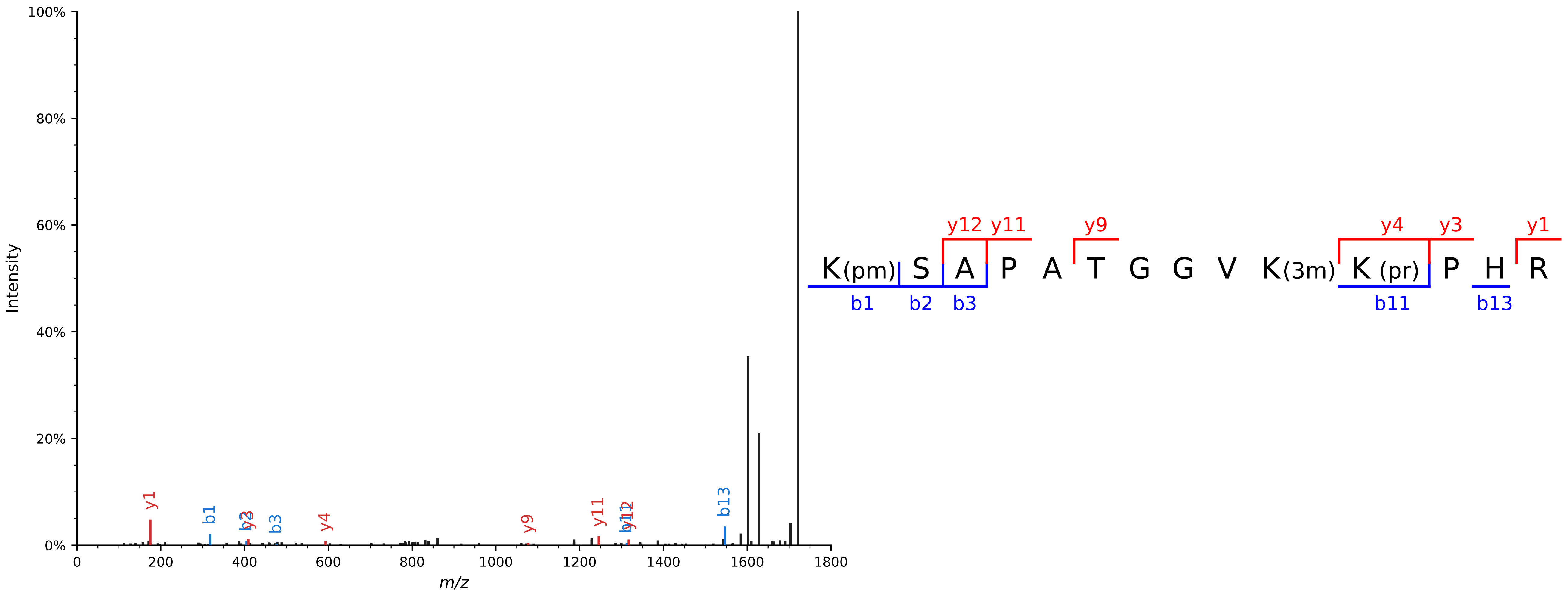

Raw file: HF240124_RN_spleen_PRO_PIC_3, Scan: 7183, m/z: 564.98965, Charge: 3, RT (min): 14.14, Score: 96.591

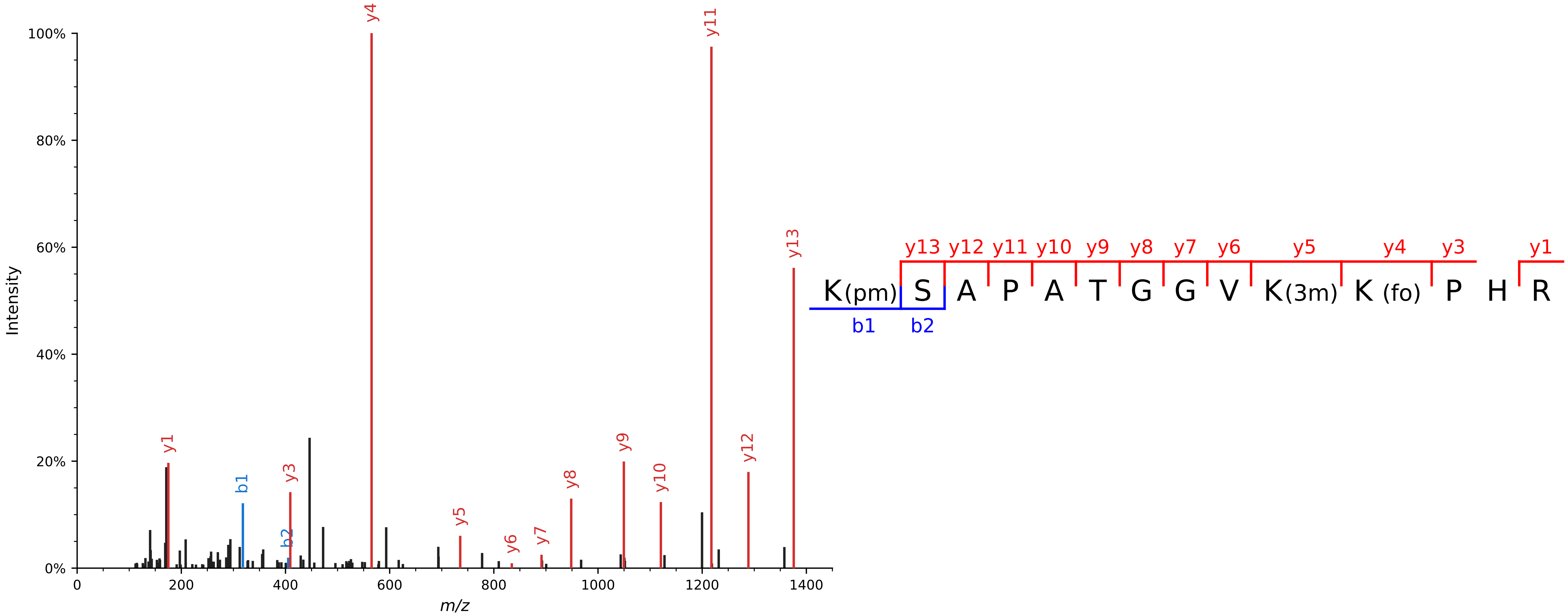

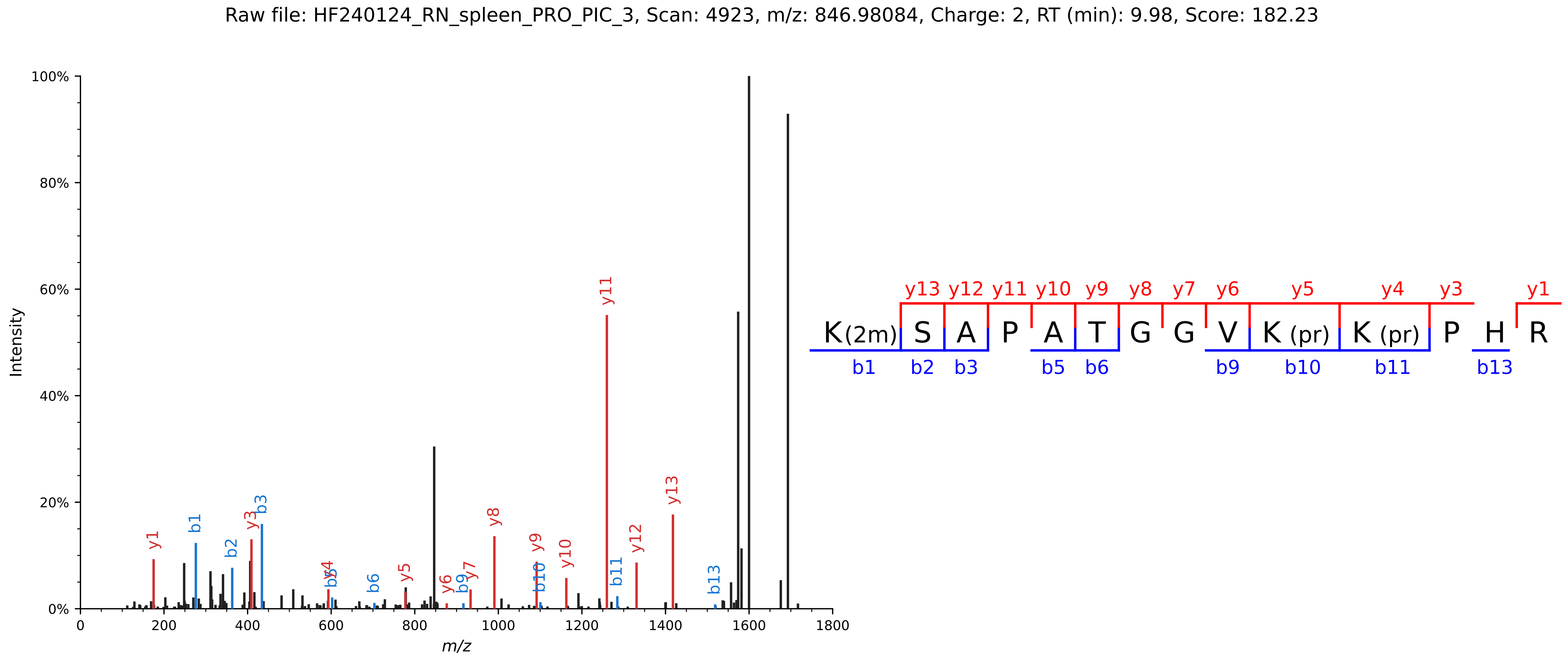

Raw file: HF240124_RN_spleen_PRO_PIC_3, Scan: 4923, m/z: 846.98084, Charge: 2, RT (min): 9.98, Score: 182.23
Intensity
m/z
K(2m) S A P A T G G V K (pr) K (pr) P H R
y13 y12 y11 y10 y9 y8 y7 y6 y5 y4 y3 y1
b1 b2 b3 b5 b6 b9 b10 b11 b13

Raw file: HF240124_RN_spleen_PRO_PIC_3, Scan: 4504, m/z: 560.31777, Charge: 3, RT (min): 9.27, Score: 121.99

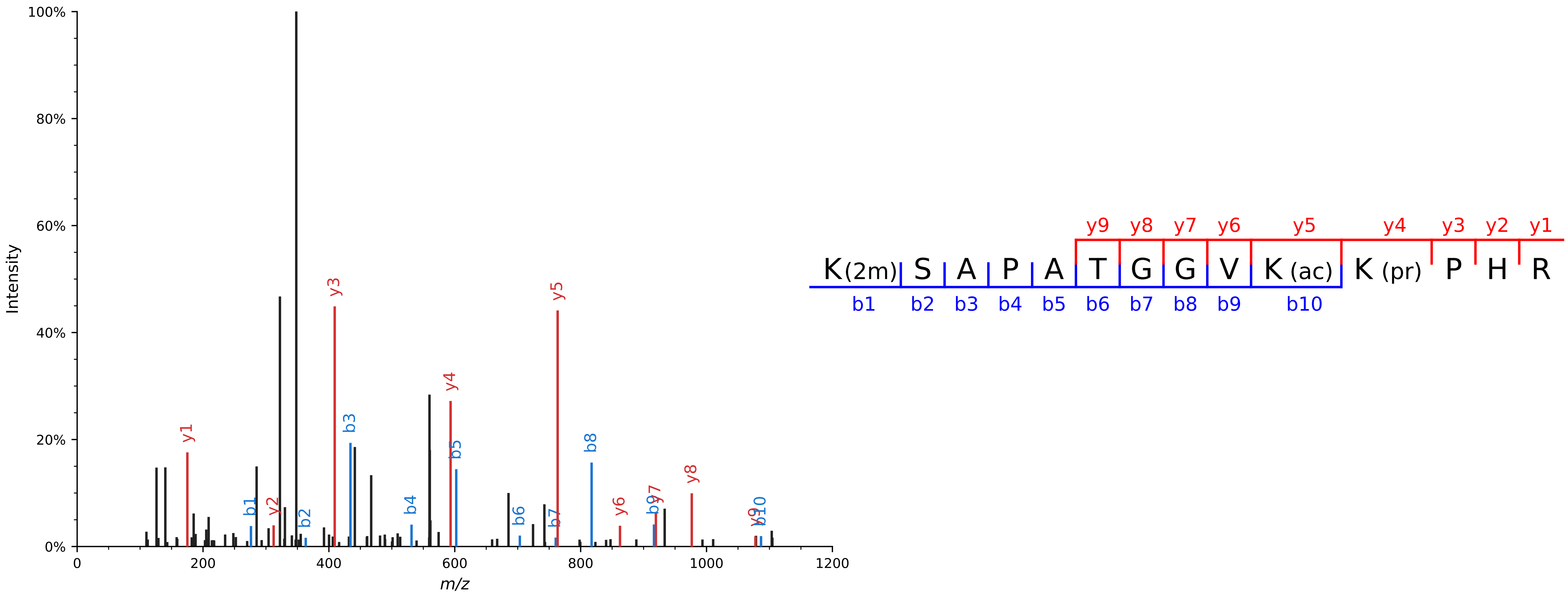

Raw file: HF240124_RN_spleen_PRO_PIC_3, Scan: 4312, m/z: 832.96519, Charge: 2, RT (min): 8.95, Score: 85.288

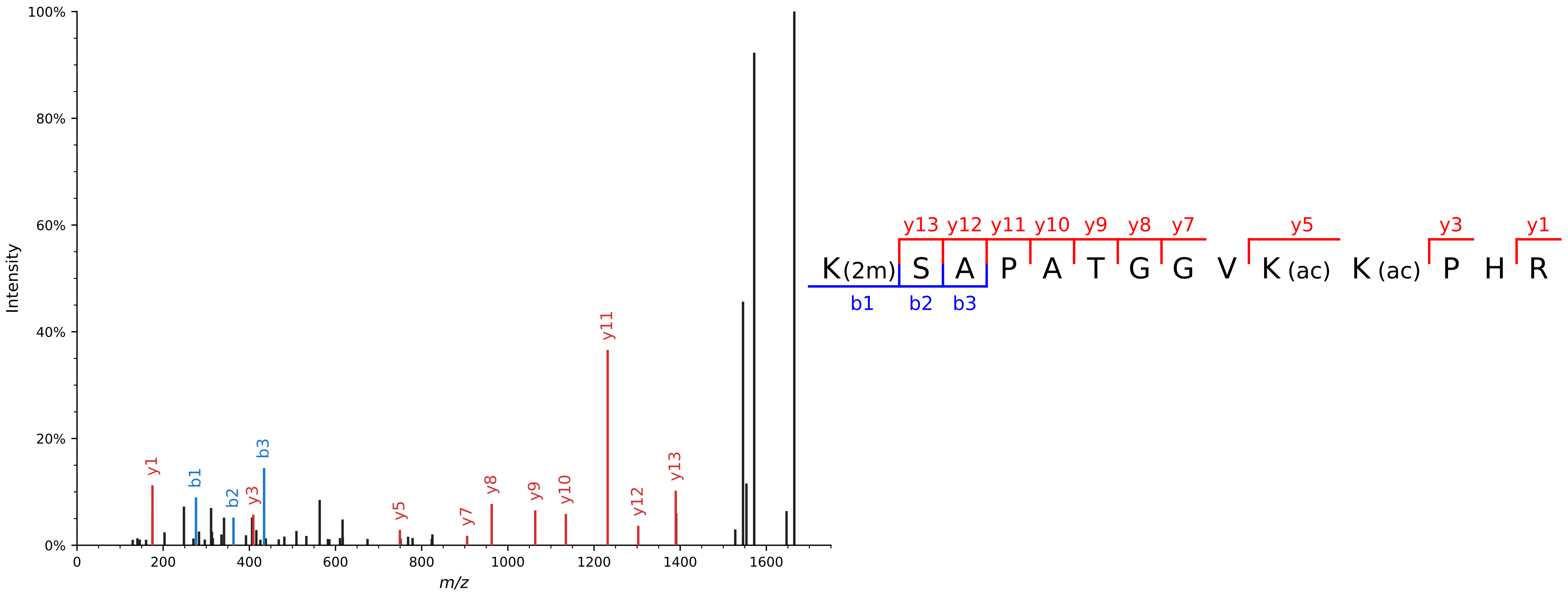

Raw file: HF240124_RN_spleen_PRO_PIC_1, Scan: 5635, m/z: 568.98965, Charge: 3, RT (min): 11.99, Score: 77.192

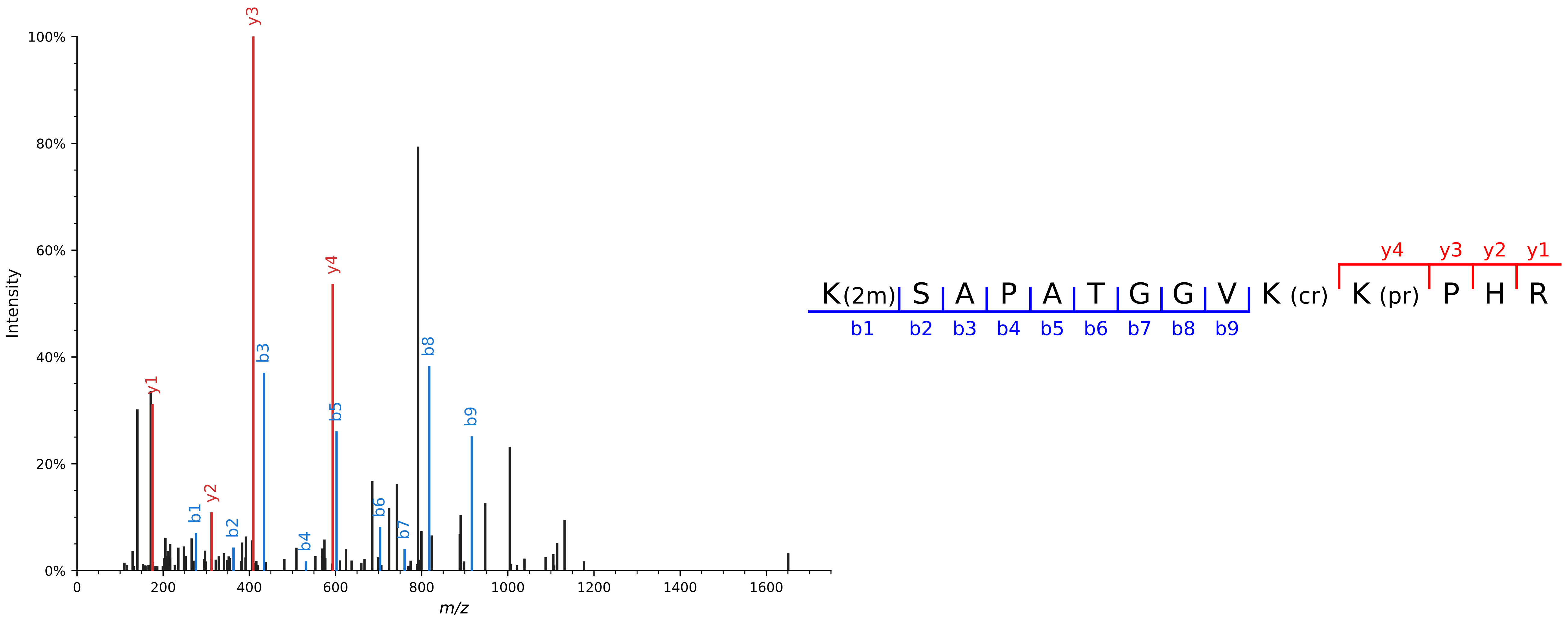

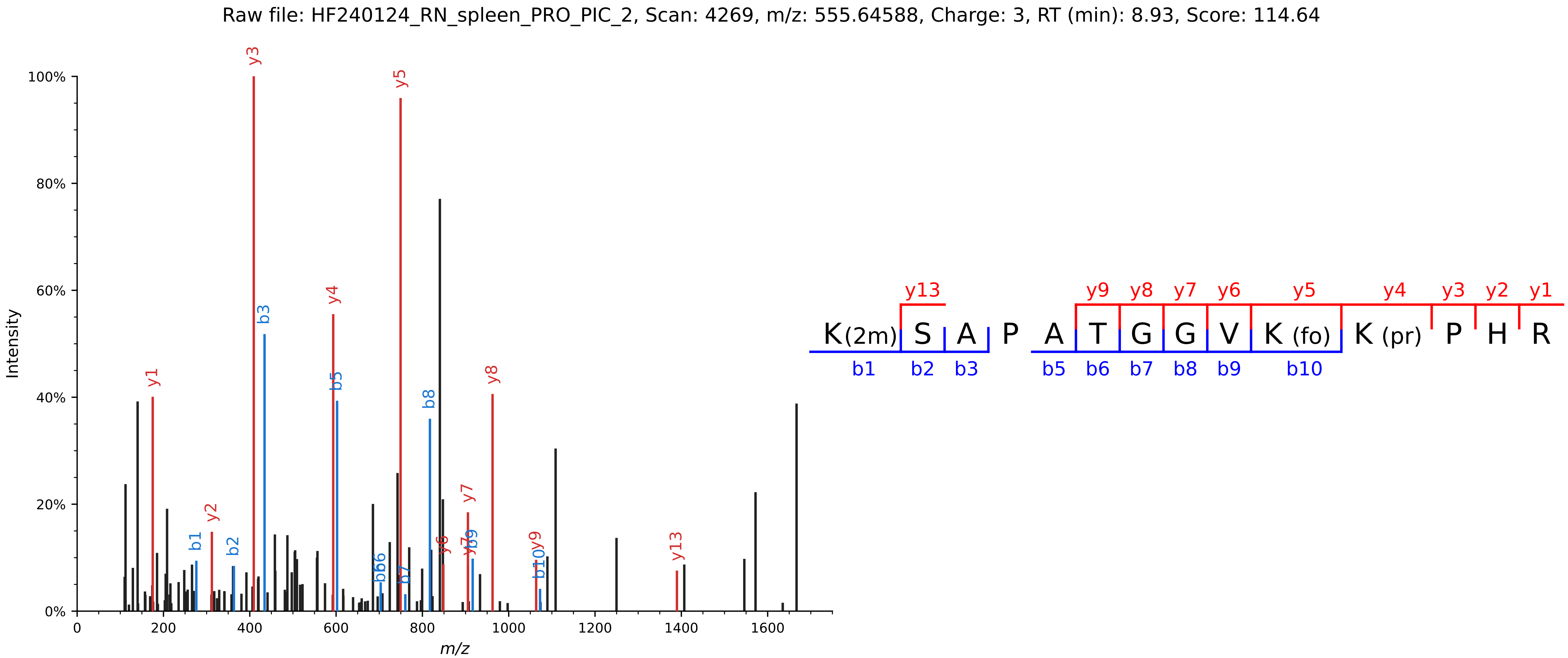

Raw file: HF240124_RN_spleen_PRO_PIC_2, Scan: 4269, m/z: 555.64588, Charge: 3, RT (min): 8.93, Score: 114.64
Intensity
m/z
100%
80%
60%
40%
20%
0%
0
200
400
600
800
1000
1200
1400
1600
y1
y2
y3
y4
y5
y6
y7
y8
y9
y13
b1
b2
b3
b5
b6
b7
b8
b9
b10
K(2m) S A P A T G G V K (fo) K (pr) P H R

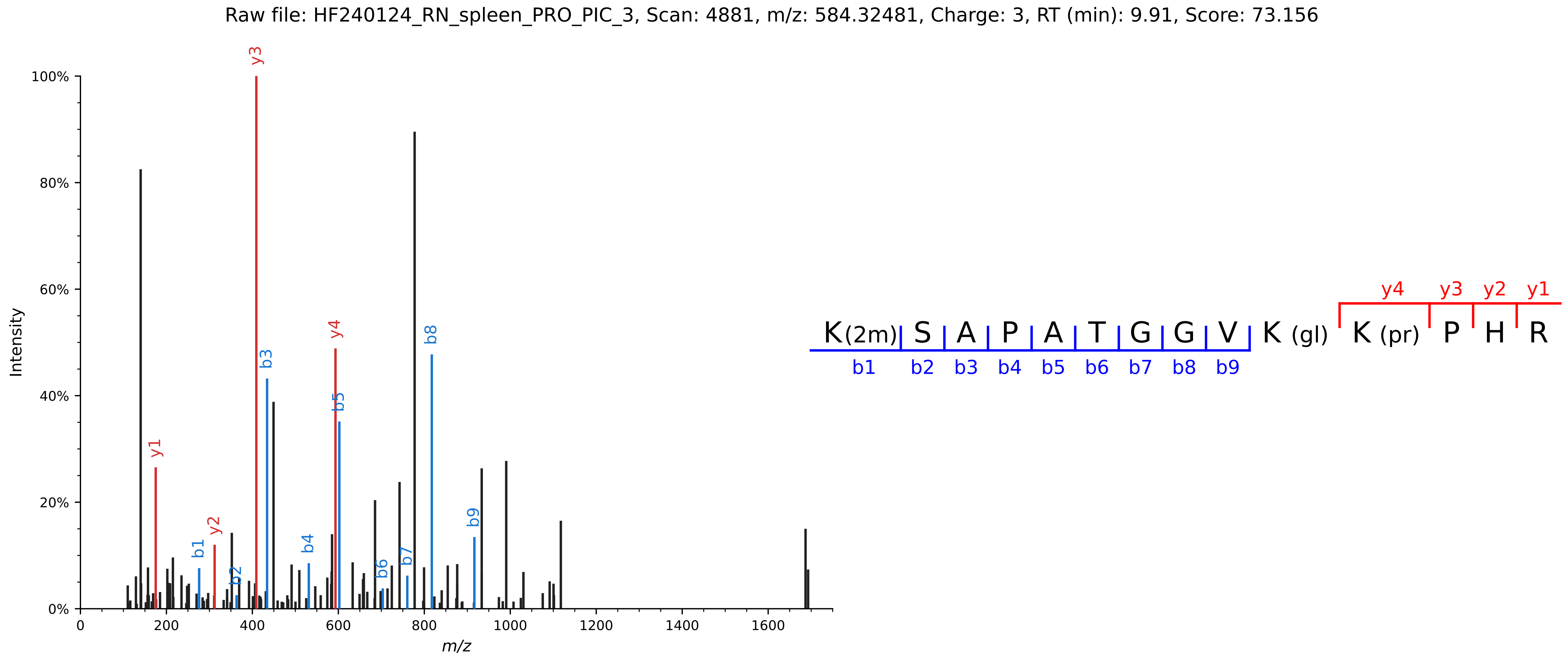

Raw file: HF240124_RN_spleen_PRO_PIC_3, Scan: 4881, m/z: 584.32481, Charge: 3, RT (min): 9.91, Score: 73.156
Intensity
m/z
100%
80%
60%
40%
20%
0%
0
200
400
600
800
1000
1200
1400
1600
y1
y2
y3
y4
b1
b2
b3
b4
b5
b6
b7
b8
b9
K(2m) S A P A T G G V K (gl) K (pr) P H R

Raw file: HF240124_RN_spleen_PRO_PIC_3, Scan: 5638, m/z: 574.99317, Charge: 3, RT (min): 11.27, Score: 57.136

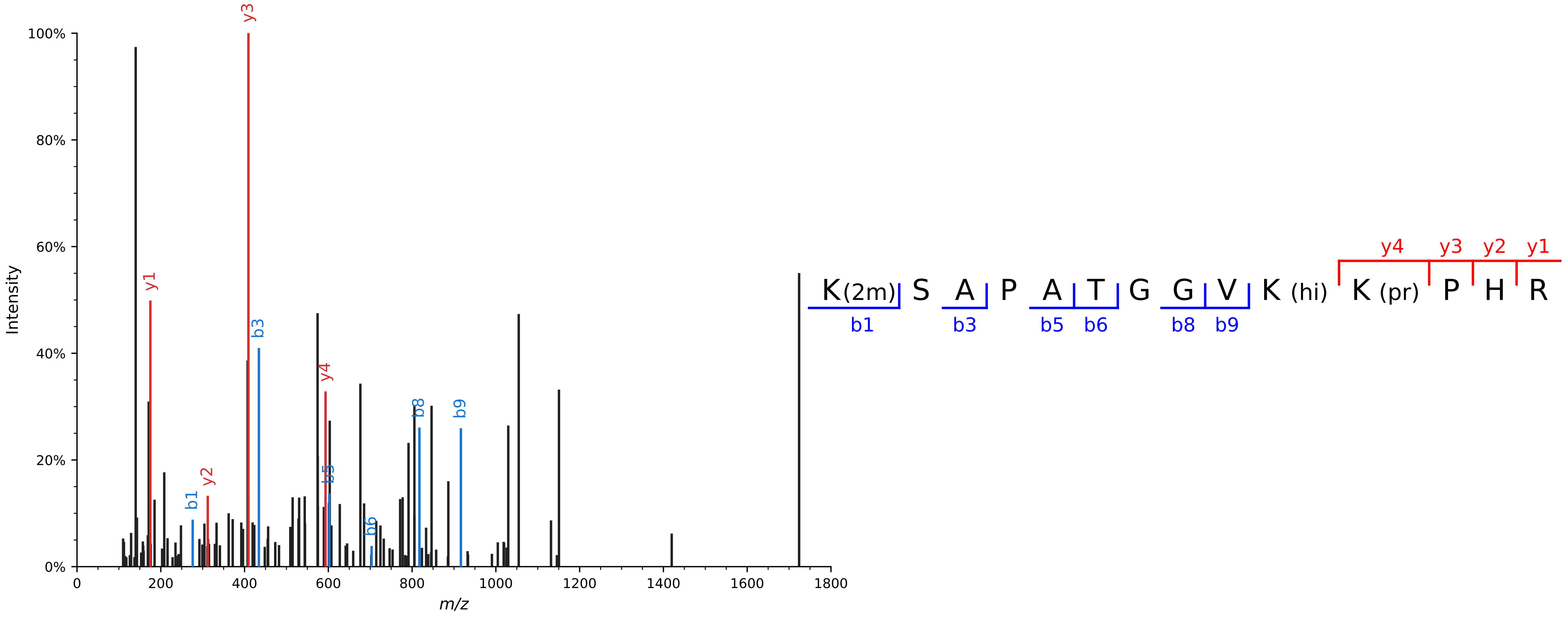

Raw file: HF240124_RN_spleen_PRO_PIC_3, Scan: 4625, m/z: 570.32129, Charge: 3, RT (min): 9.48, Score: 58.723

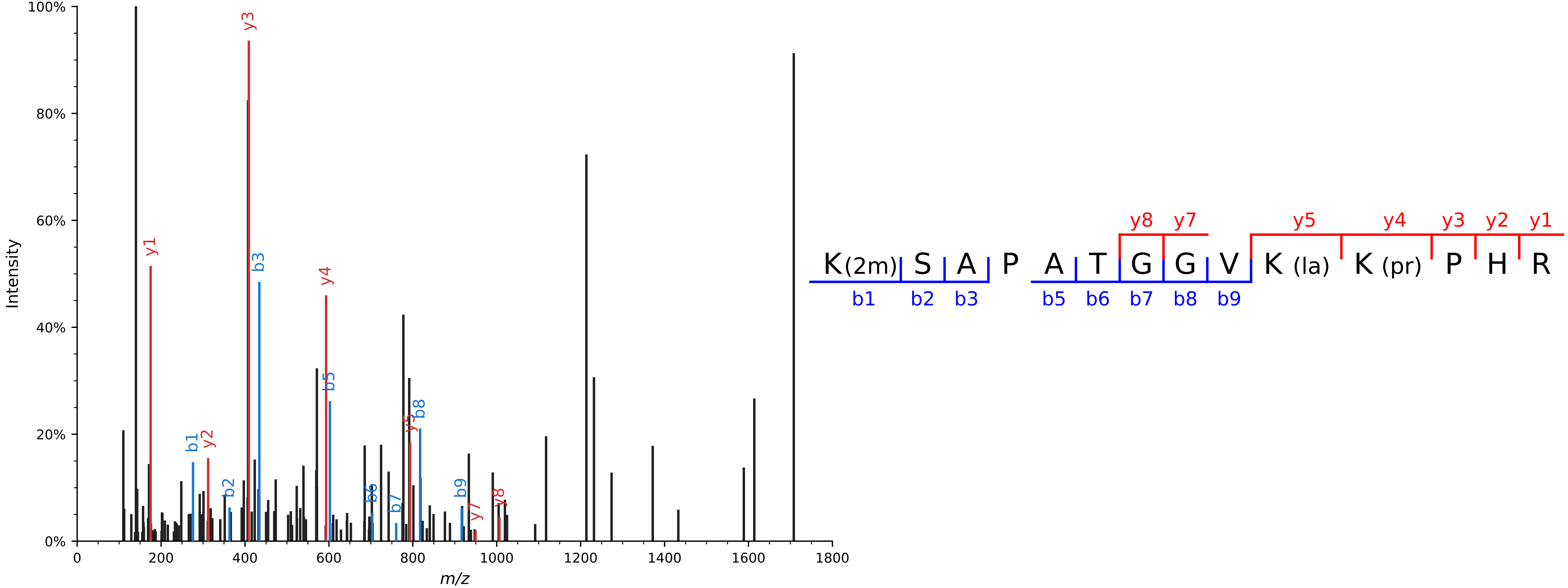

Raw file: HF240124_RN_spleen_PRO_PIC_3, Scan: 6735, m/z: 862.97575, Charge: 2, RT (min): 13.38, Score: 104.59

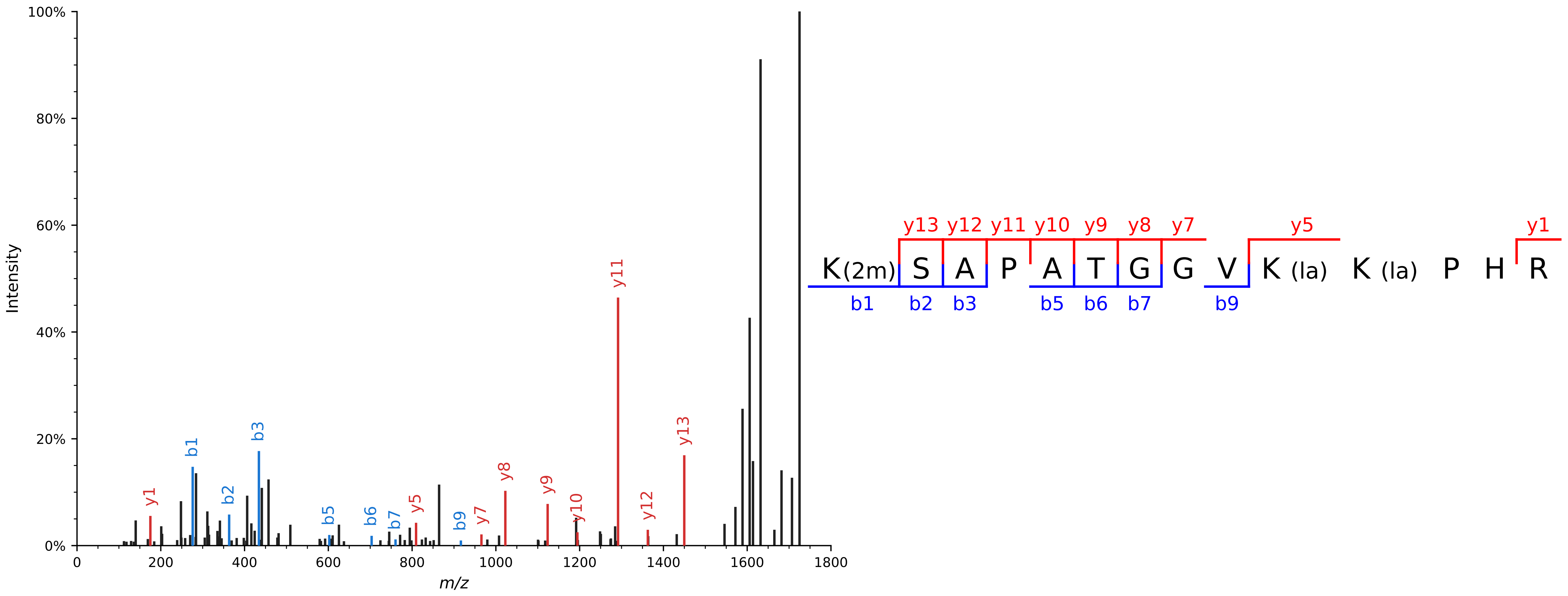

Raw file: HF240124_RN_spleen_PRO_PIC_1, Scan: 5703, m/z: 853.98866, Charge: 2, RT (min): 12.12, Score: 196.11

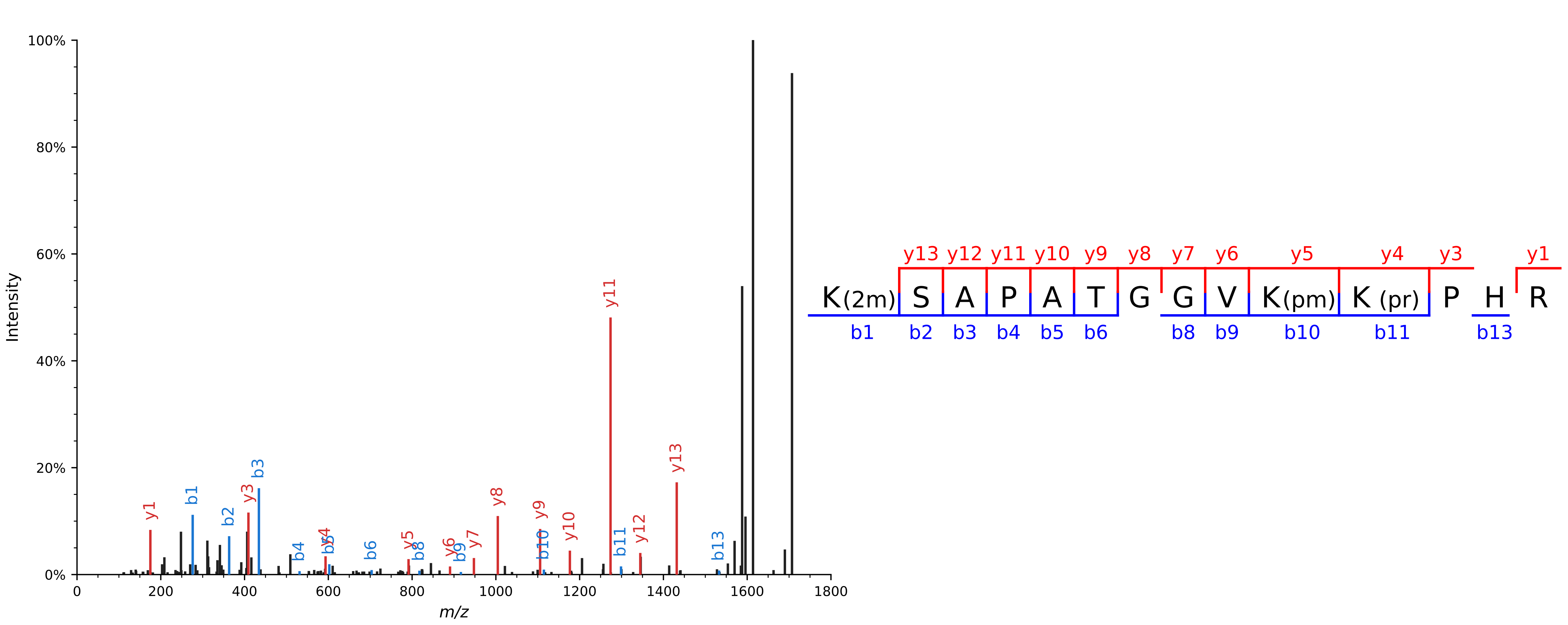

Raw file: HF240124_RN_spleen_PRO_PIC_2, Scan: 4085, m/z: 860.99649, Charge: 2, RT (min): 8.61, Score: 96.946

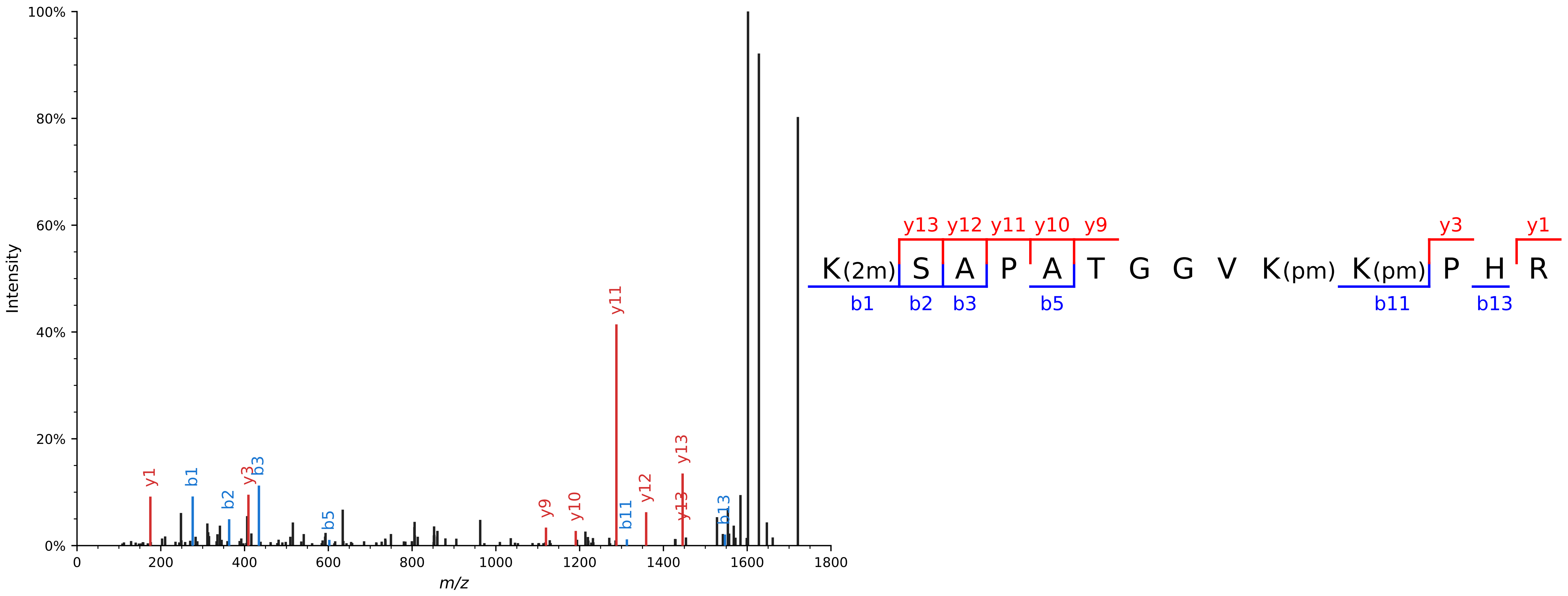

Raw file: HF240124_RN_spleen_PRO_PIC_1, Scan: 1910, m/z: 832.98338, Charge: 2, RT (min): 5.28, Score: 169.98

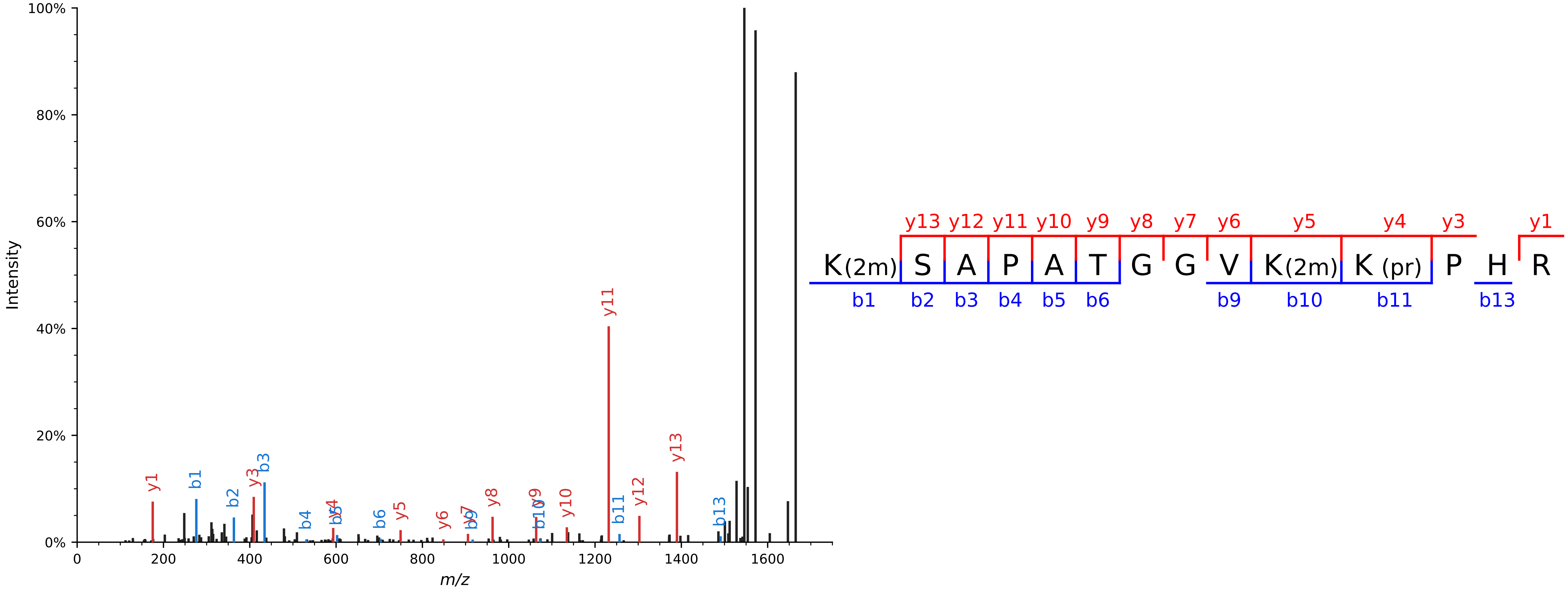

Raw file: HF240124_RN_spleen_PRO_PIC_1, Scan: 4031, m/z: 560.31777, Charge: 3, RT (min): 9.14, Score: 138.73

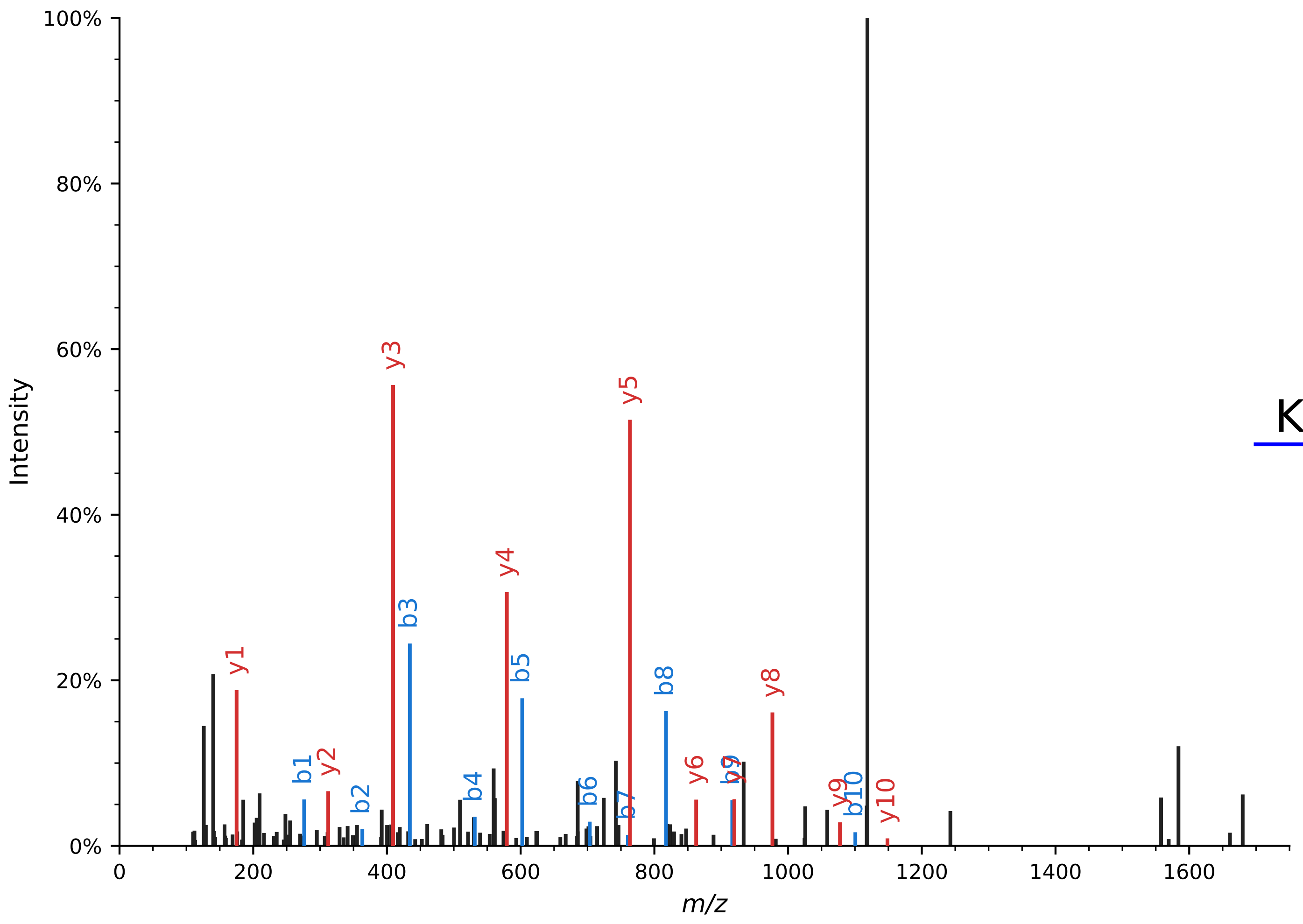

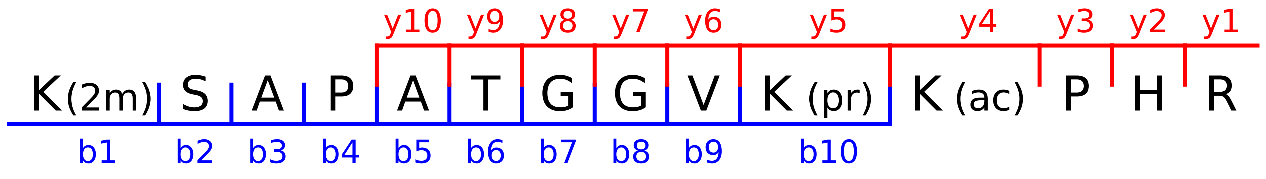

Raw file: HF240124_RN_spleen_PRO_PIC_2, Scan: 4055, m/z: 555.64588, Charge: 3, RT (min): 8.55, Score: 109.55

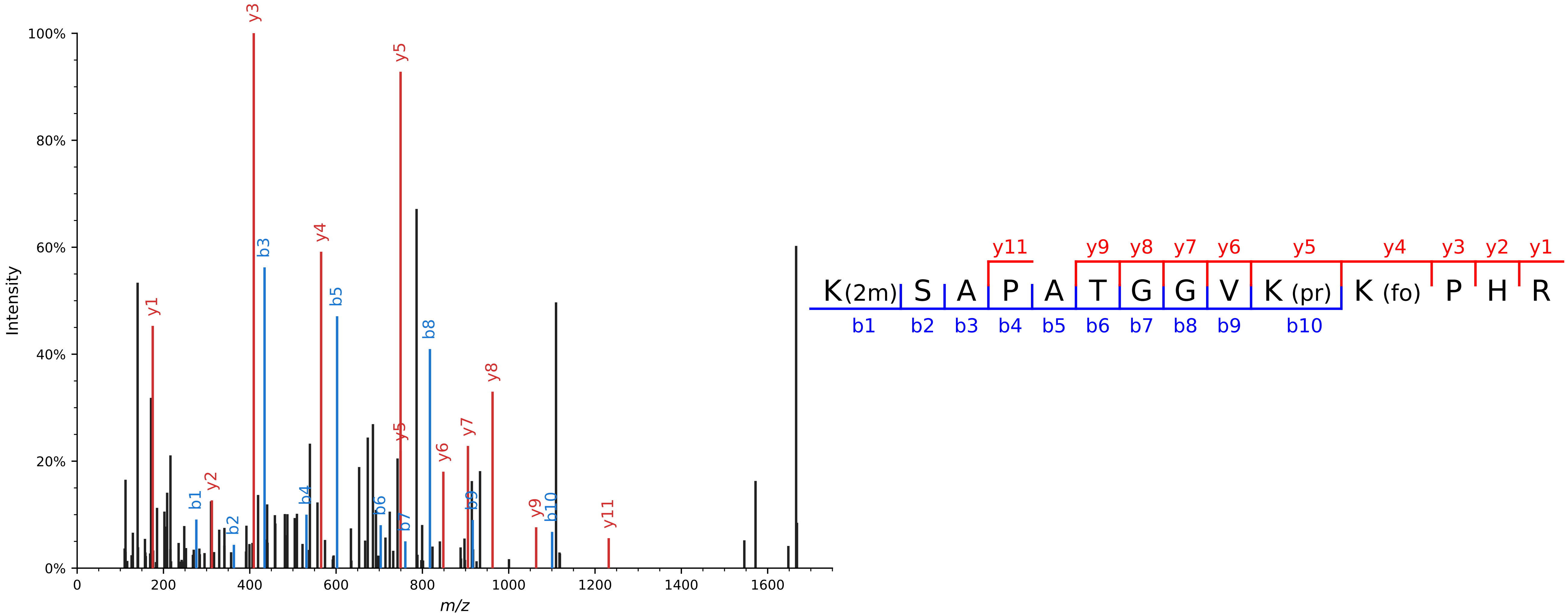

Raw file: HF240124_RN_spleen_PRO_PIC_1, Scan: 6155, m/z: 584.32481, Charge: 3, RT (min): 13.01, Score: 40.941

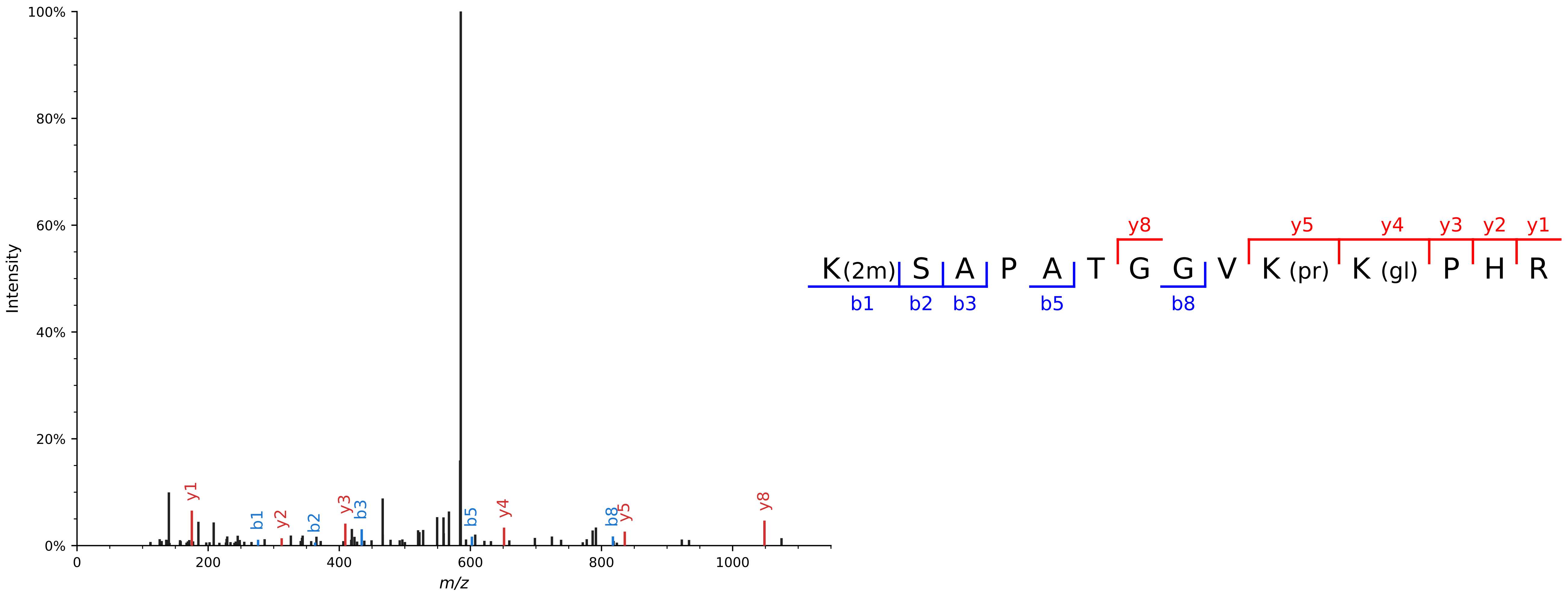

Raw file: HF240124_RN_spleen_PRO_PIC_2, Scan: 5278, m/z: 569.66153, Charge: 3, RT (min): 10.69, Score: 151.56

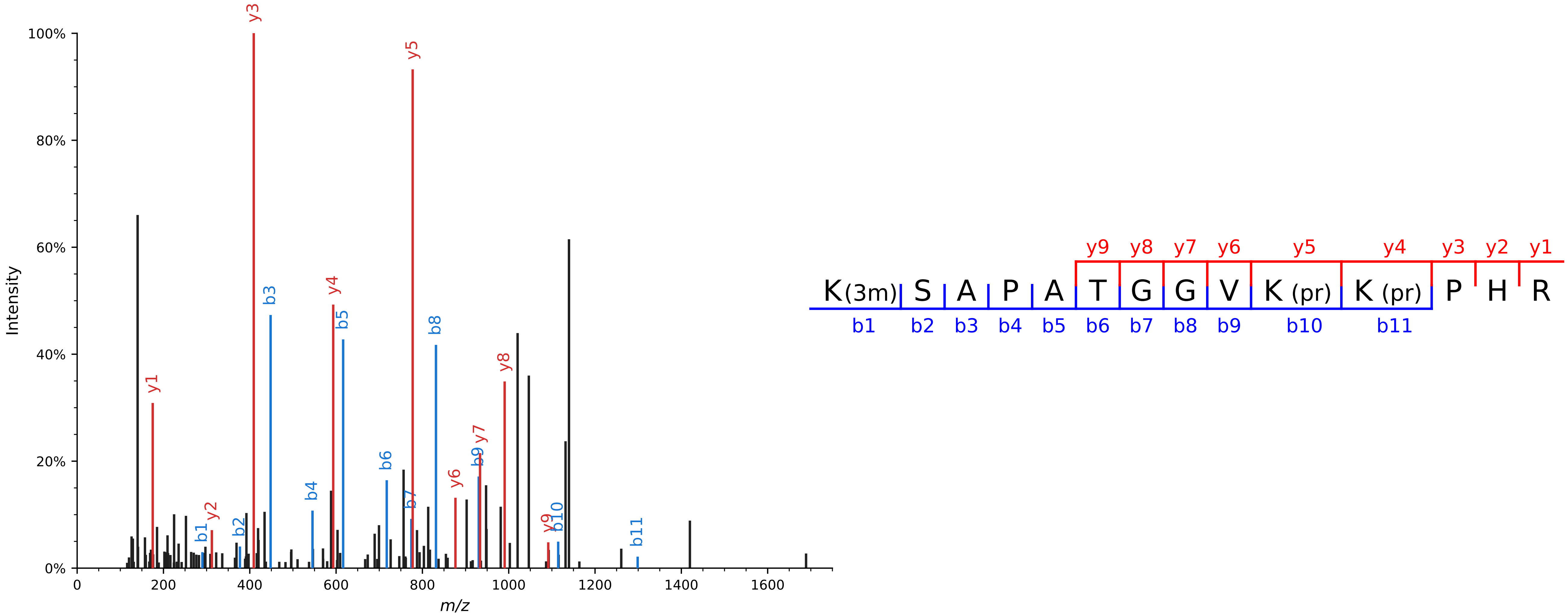

Raw file: HF240124_RN_spleen_PRO_PIC_1, Scan: 4117, m/z: 564.98965, Charge: 3, RT (min): 9.29, Score: 131.96

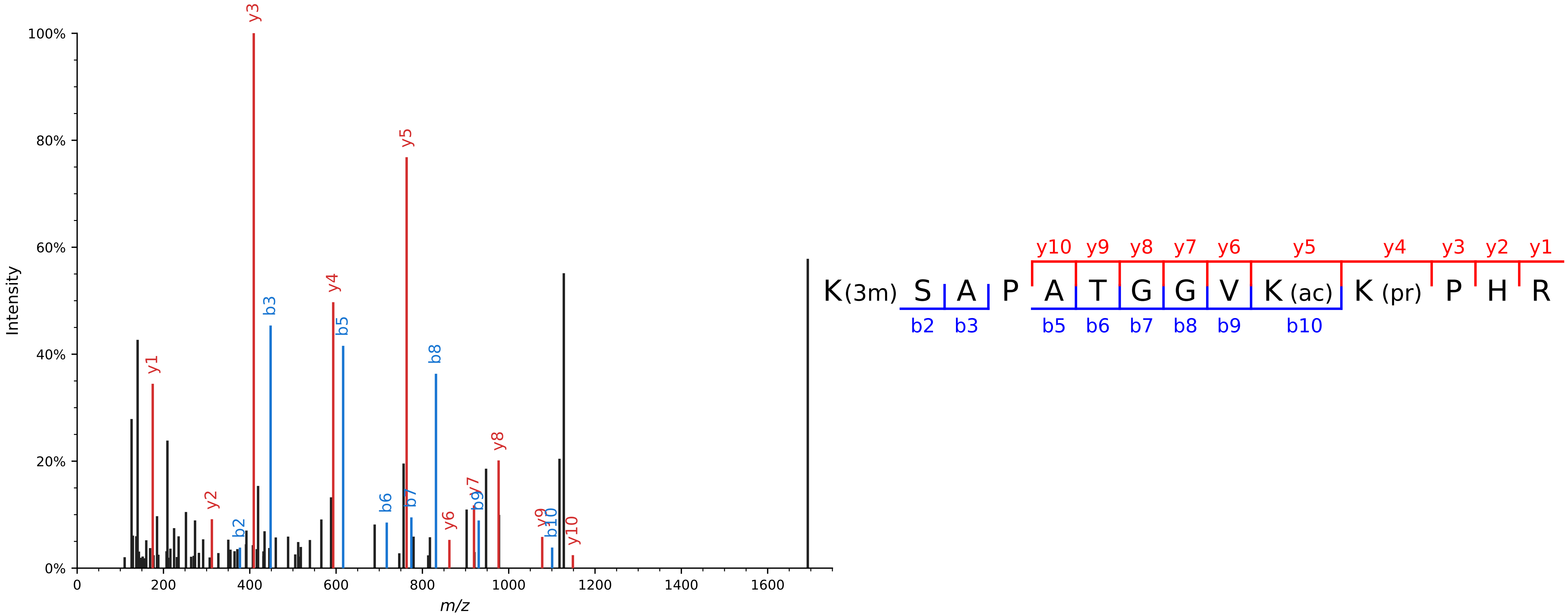

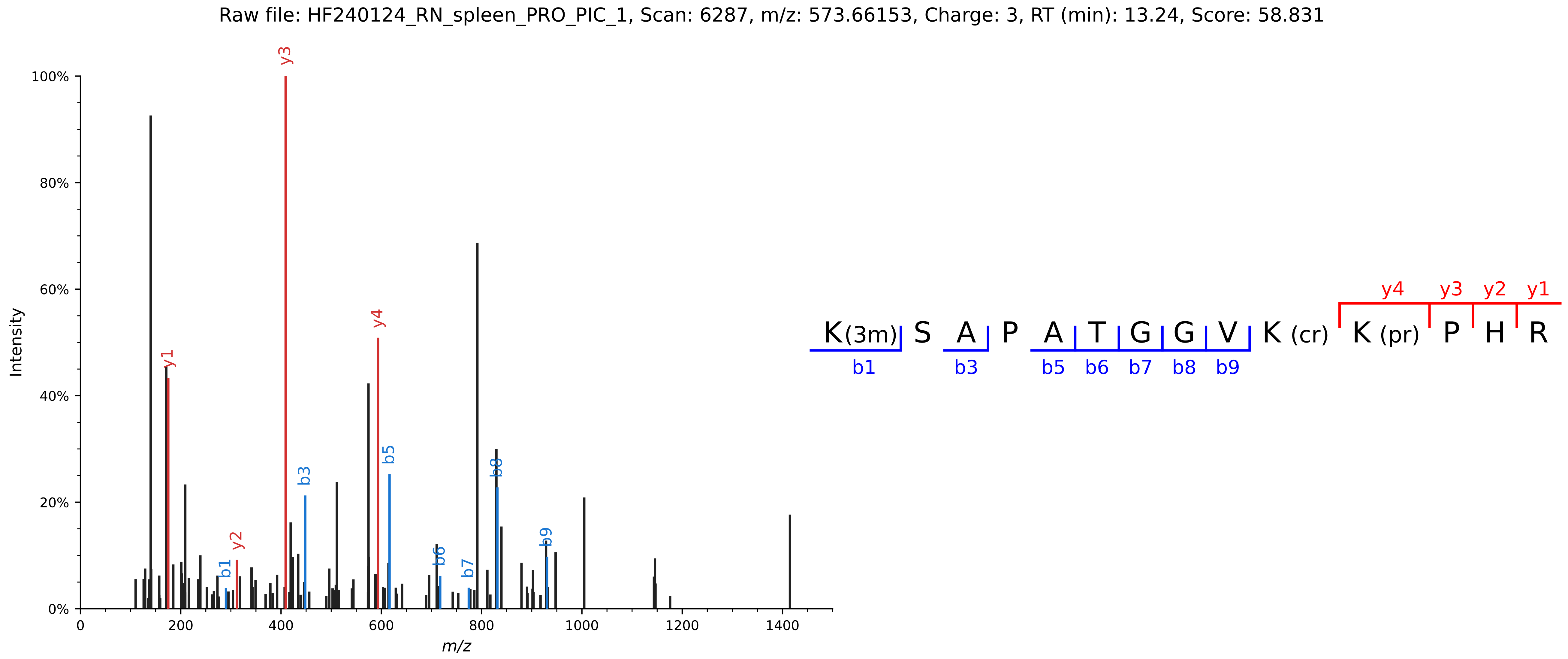
Raw file: HF240124_RN_spleen_PRO_PIC_1, Scan: 6287, m/z: 573.66153, Charge: 3, RT (min): 13.24, Score: 58.831
Intensity
m/z
y1
y2
y3
y4
b1
b3
b5
b6
b7
b8
b9
K(3m) S A P A T G G V K (cr) K (pr) P H R

Raw file: HF240124_RN_spleen_PRO_PIC_1, Scan: 3950, m/z: 560.31777, Charge: 3, RT (min): 9.00, Score: 49.188

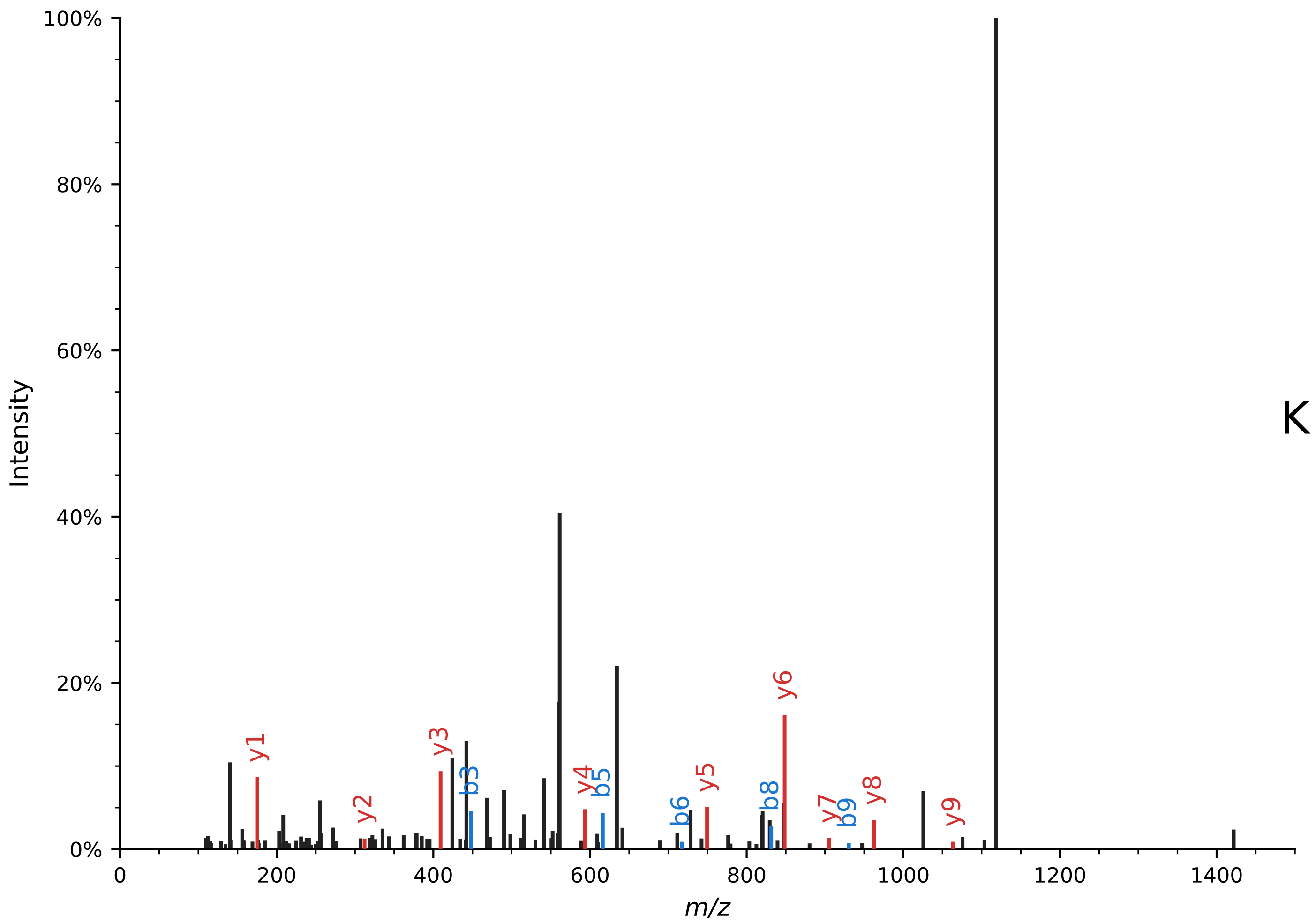

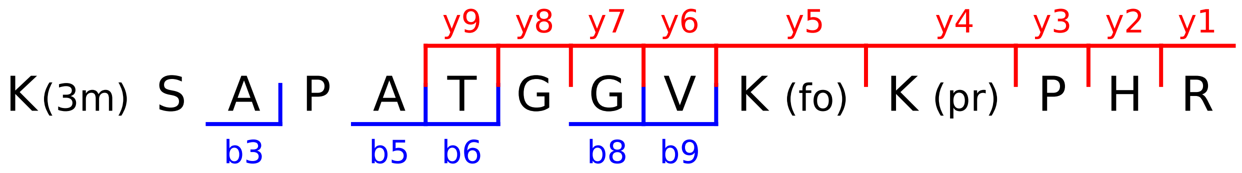

Raw file: HF240124_RN_spleen_PRO_PIC_3, Scan: 5841, m/z: 860.99649, Charge: 2, RT (min): 11.70, Score: 163.51

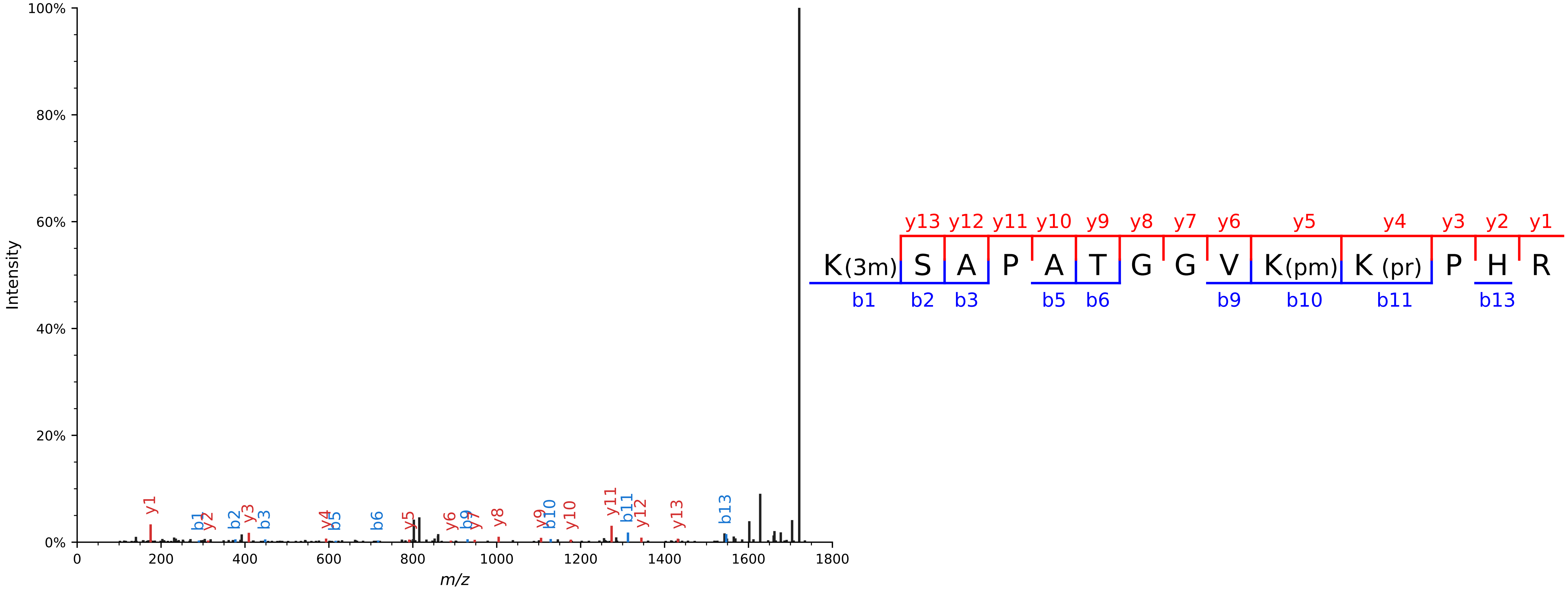

Raw file: HF240124_RN_spleen_PRO_PIC_2, Scan: 4052, m/z: 434.50579, Charge: 4, RT (min): 8.55, Score: 43.813

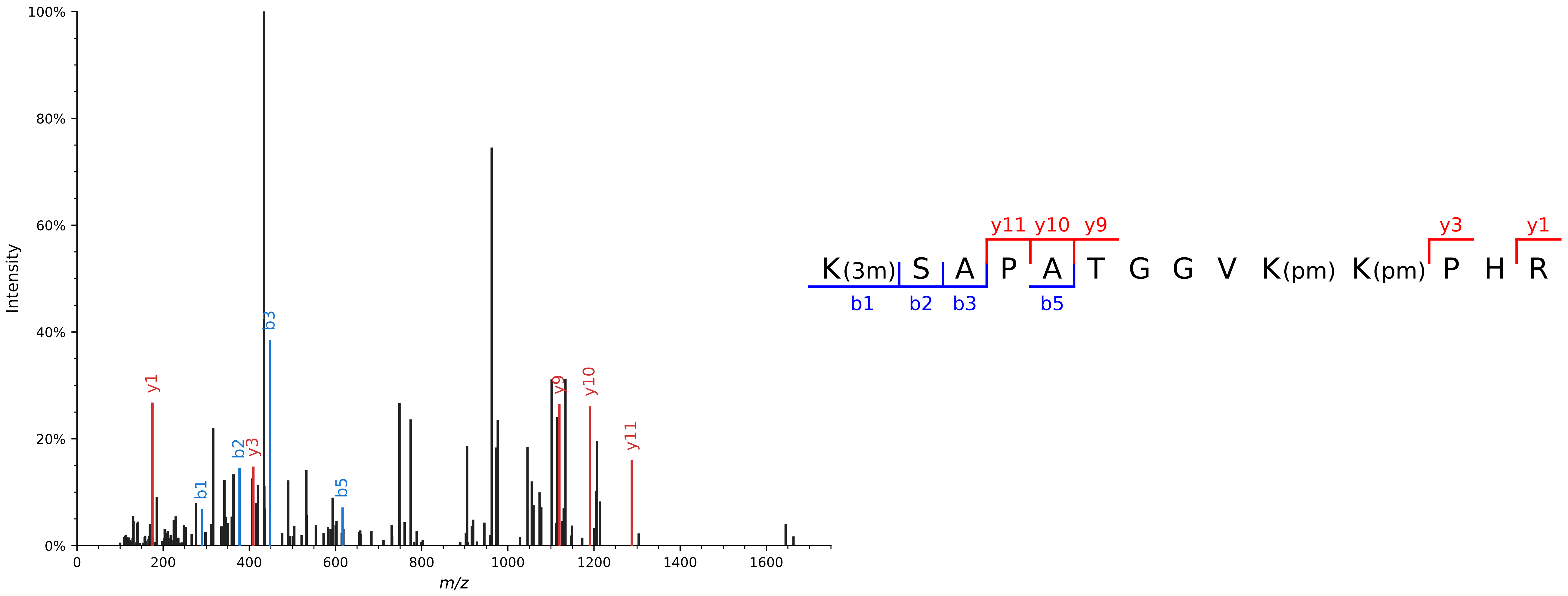

Raw file: HF240124_RN_spleen_PRO_PIC_2, Scan: 2092, m/z: 560.3299, Charge: 3, RT (min): 5.04, Score: 97.45

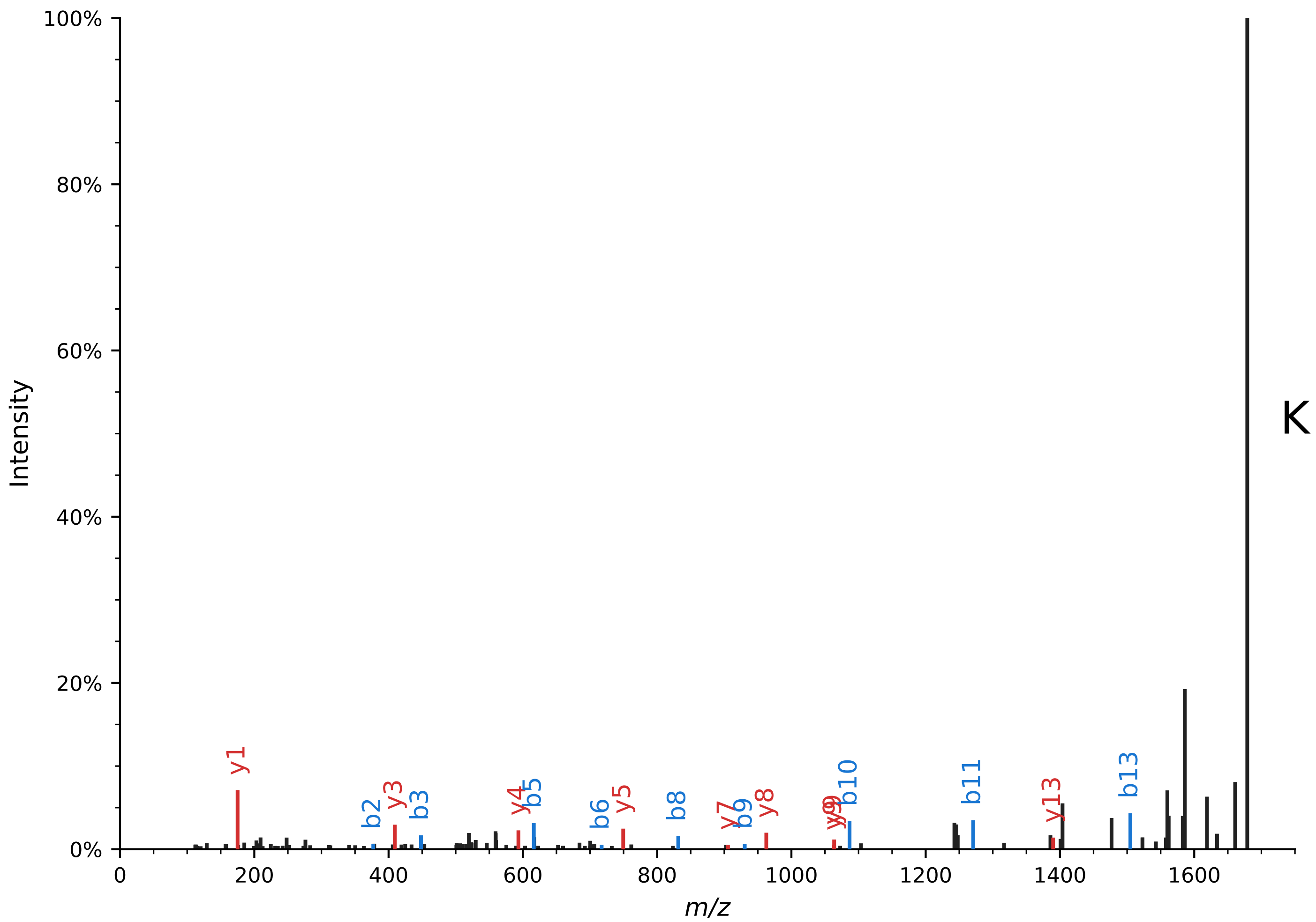

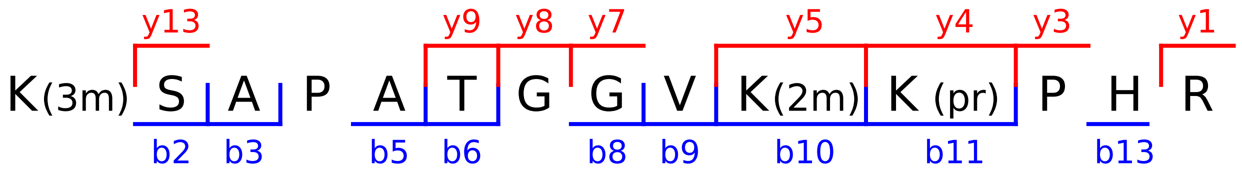

Raw file: HF240124_RN_spleen_PRO_PIC_1, Scan: 3978, m/z: 564.98965, Charge: 3, RT (min): 9.05, Score: 67.846

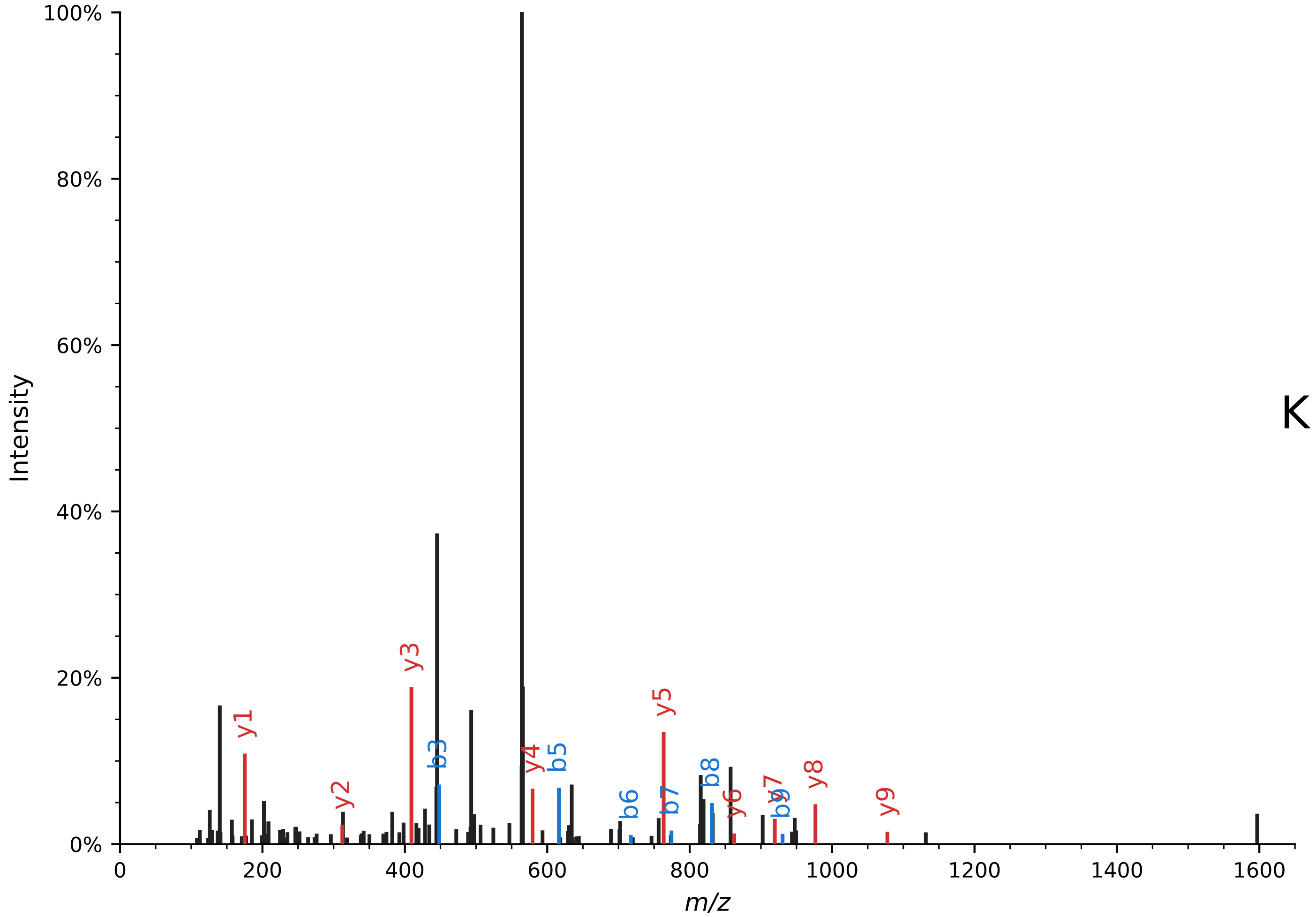

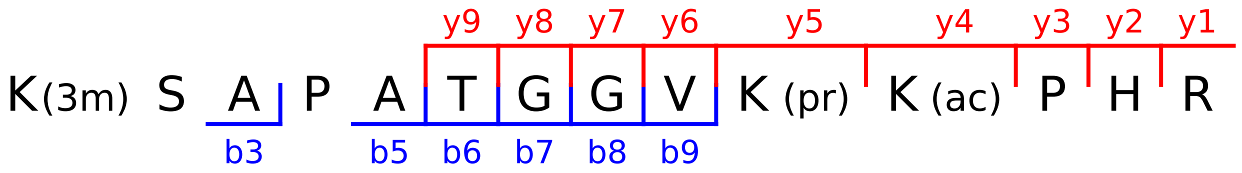

Raw file: HF240124_RN_spleen_PRO_PIC_3, Scan: 9632, m/z: 853.97047, Charge: 2, RT (min): 18.37, Score: 143.79

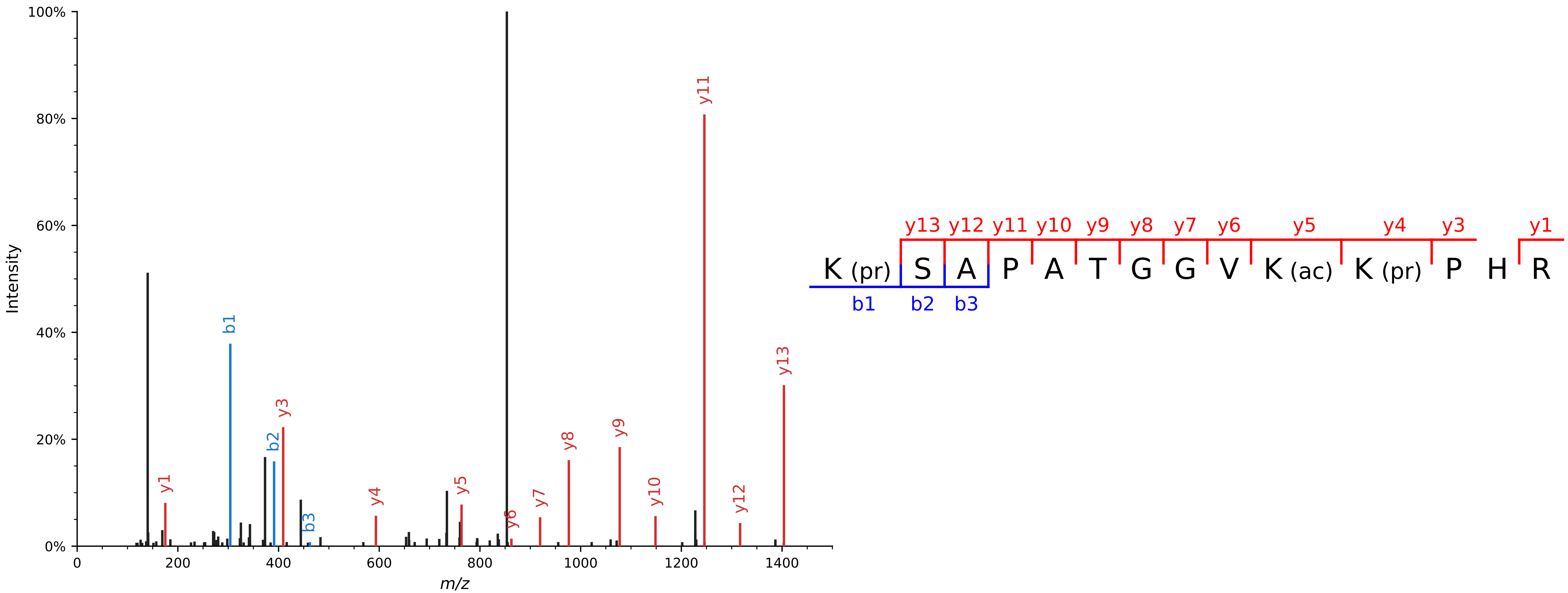

Raw file: HF240124_RN_spleen_PRO_PIC_3, Scan: 13779, m/z: 876.97321, Charge: 2, RT (min): 25.73, Score: 113.63

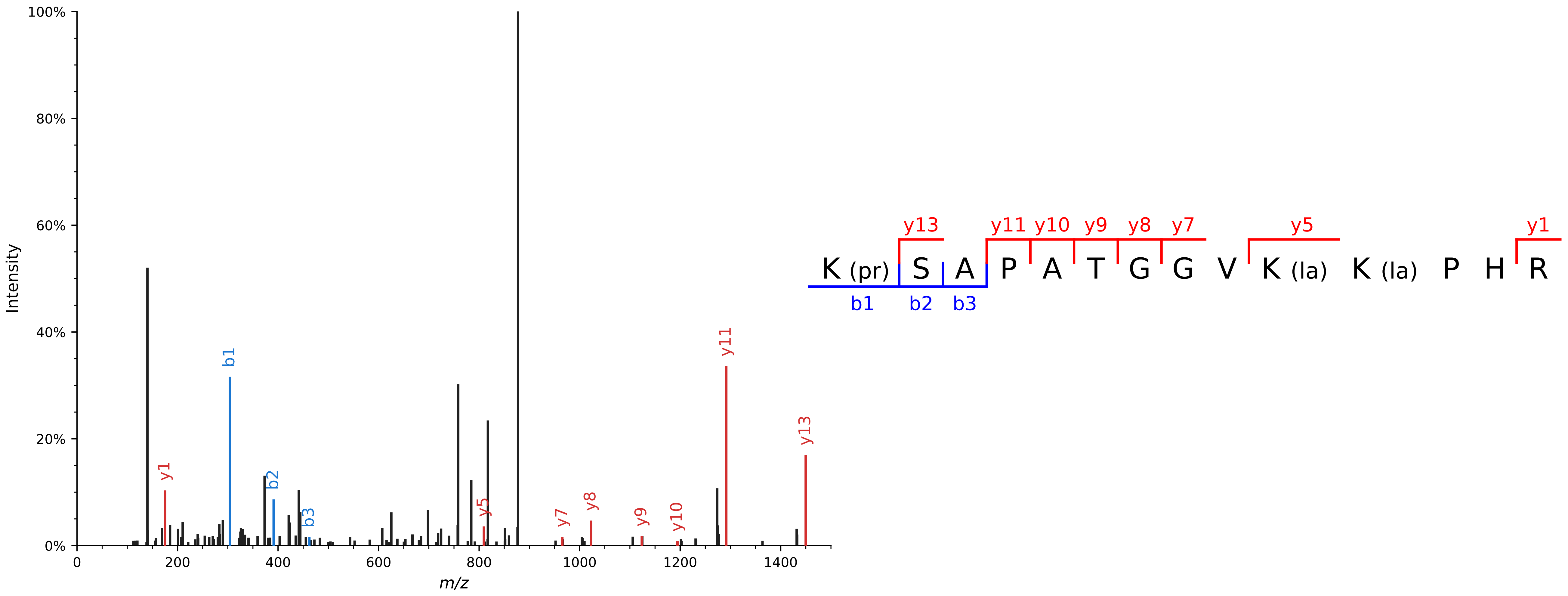

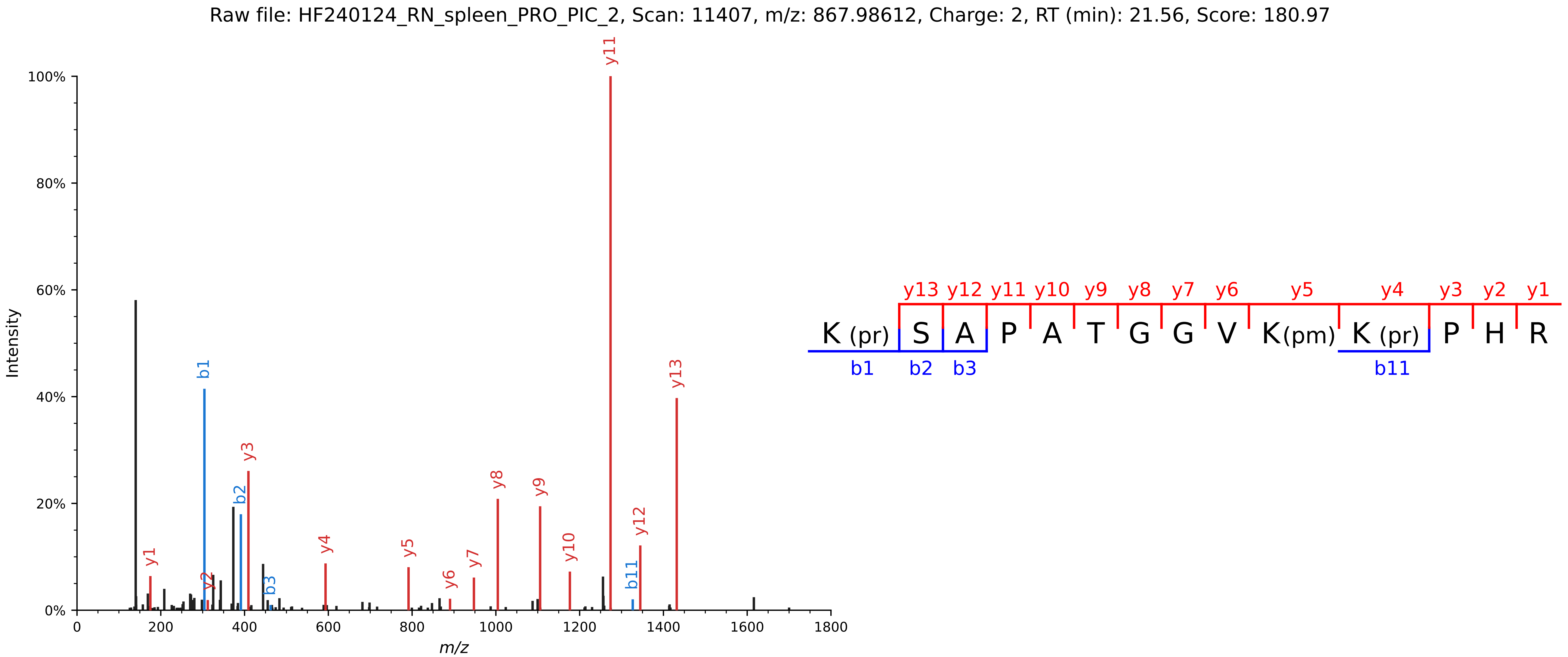
Raw file: HF240124_RN_spleen_PRO_PIC_2, Scan: 11407, m/z: 867.98612, Charge: 2, RT (min): 21.56, Score: 180.97
Intensity
m/z
0%
20%
40%
60%
80%
100%
0
200
400
600
800
1000
1200
1400
1600
1800
y1
y2
y3
y4
y5
y6
y7
y8
y9
y10
y11
y12
y13
b1
b2
b3
b11
y13 y12 y11 y10 y9 y8 y7 y6 y5 y4 y3 y2 y1
K (pr) S A P A T G G V K(pm) K (pr) P H R
b1 b2 b3 b11

Raw file: HF240124_RN_spleen_PRO_PIC_3, Scan: 10772, m/z: 874.99395, Charge: 2, RT (min): 20.44, Score: 83.397

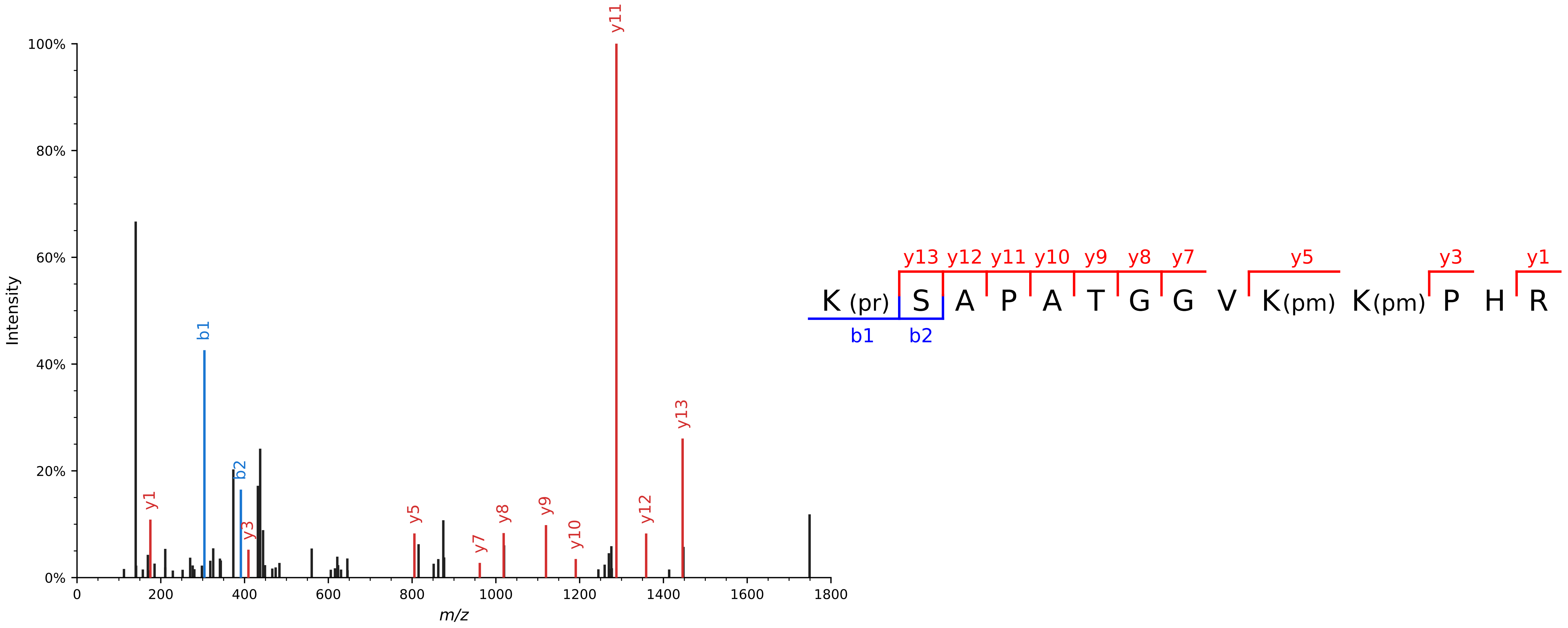

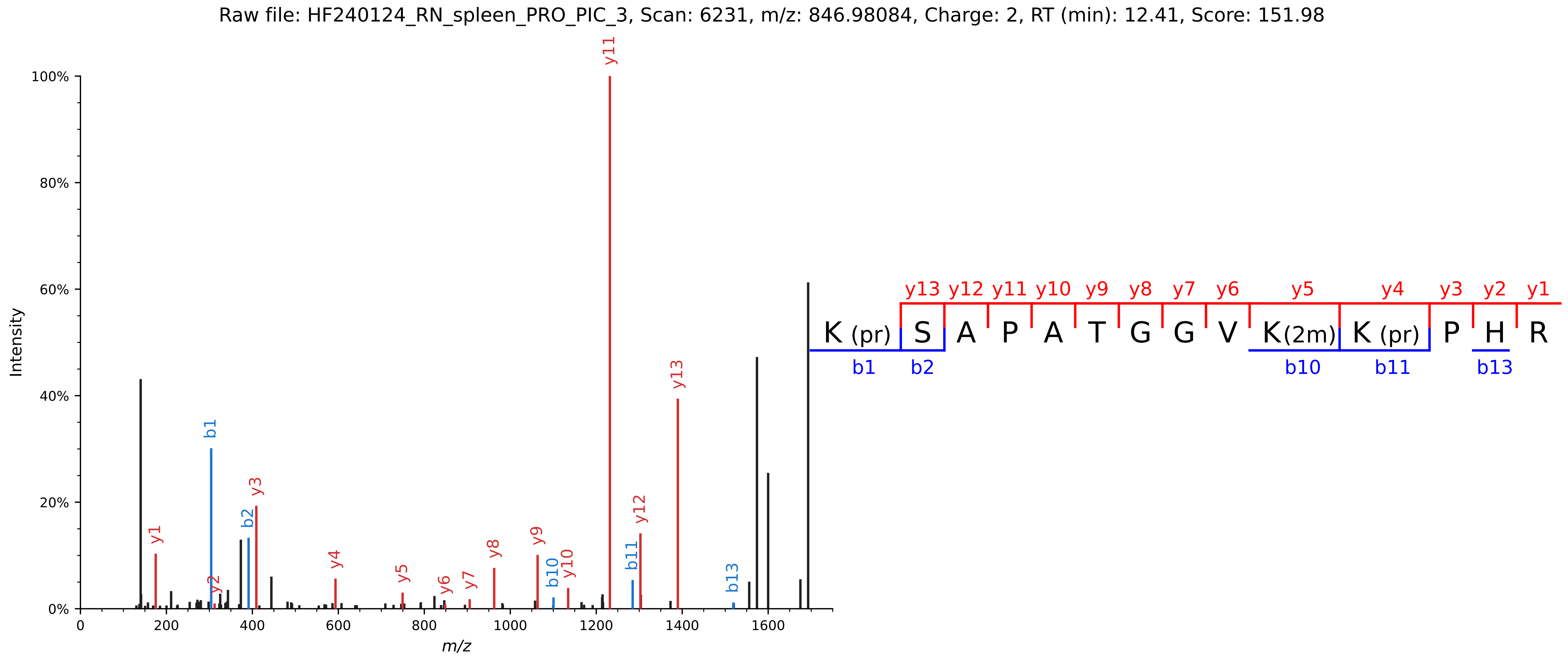
Raw file: HF240124_RN_spleen_PRO_PIC_3, Scan: 6231, m/z: 846.98084, Charge: 2, RT (min): 12.41, Score: 151.98
Intensity
m/z
K(pr) S A P A T G G V K(2m) K(pr) P H R
y13 y12 y11 y10 y9 y8 y7 y6 y5 y4 y3 y2 y1
b1 b2 b10 b11 b13

Raw file: HF240124_RN_spleen_PRO_PIC_3, Scan: 6147, m/z: 569.66153, Charge: 3, RT (min): 12.26, Score: 100.22

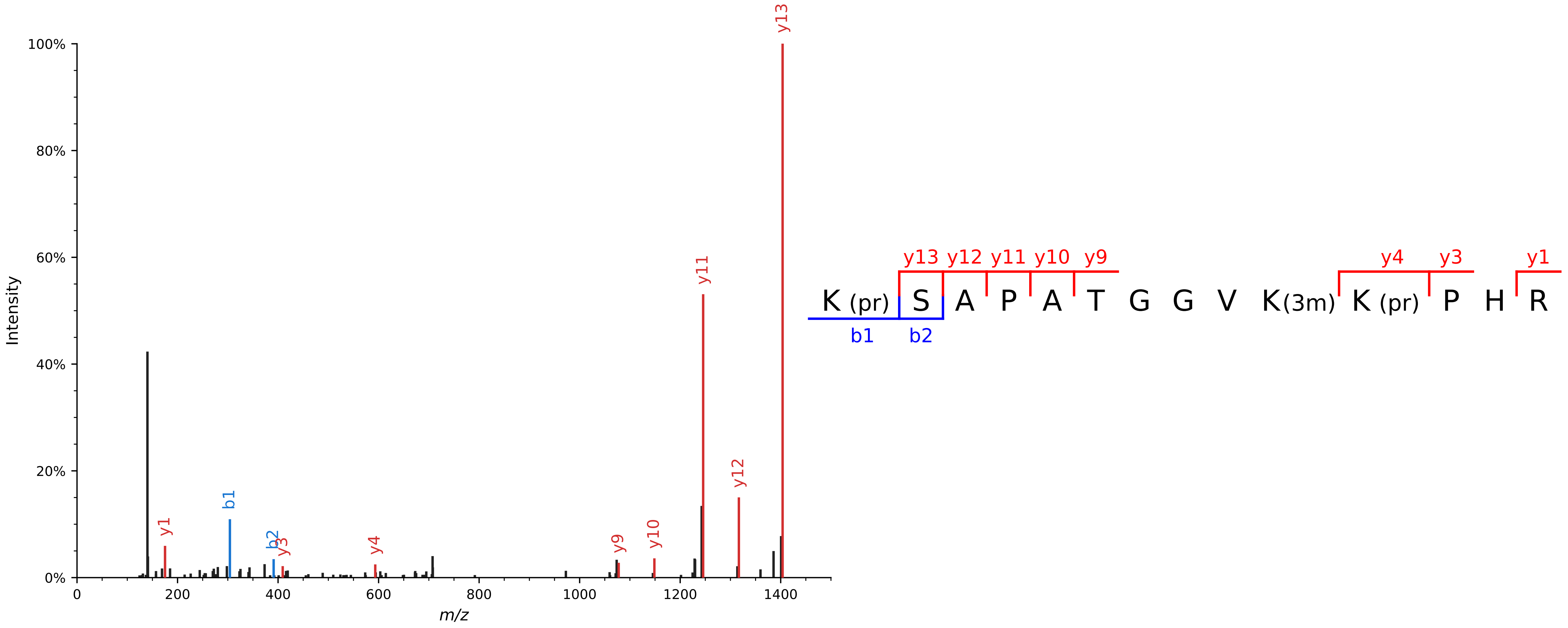

Raw file: HF240124_RN_spleen_PRO_PIC_2, Scan: 9575, m/z: 569.64941, Charge: 3, RT (min): 18.30, Score: 94.551

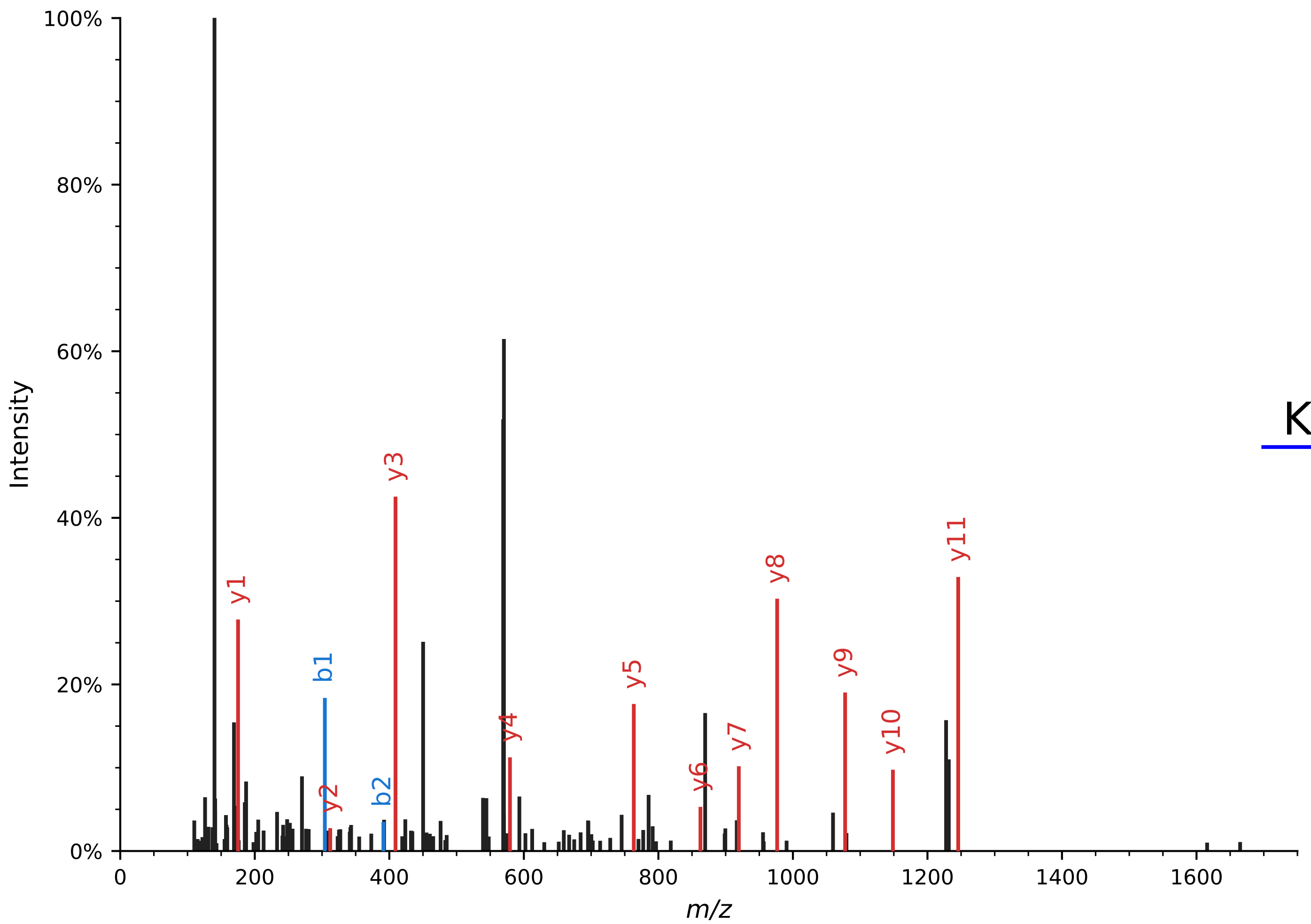

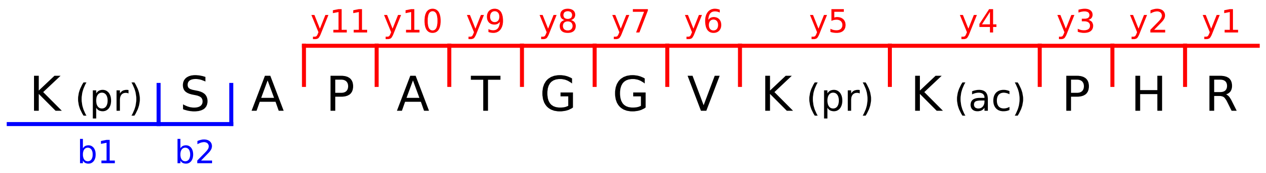

Raw file: HF240124_RN_spleen_PRO_PIC_3, Scan: 20681, m/z: 706.38263, Charge: 2, RT (min): 37.94, Score: 94.692

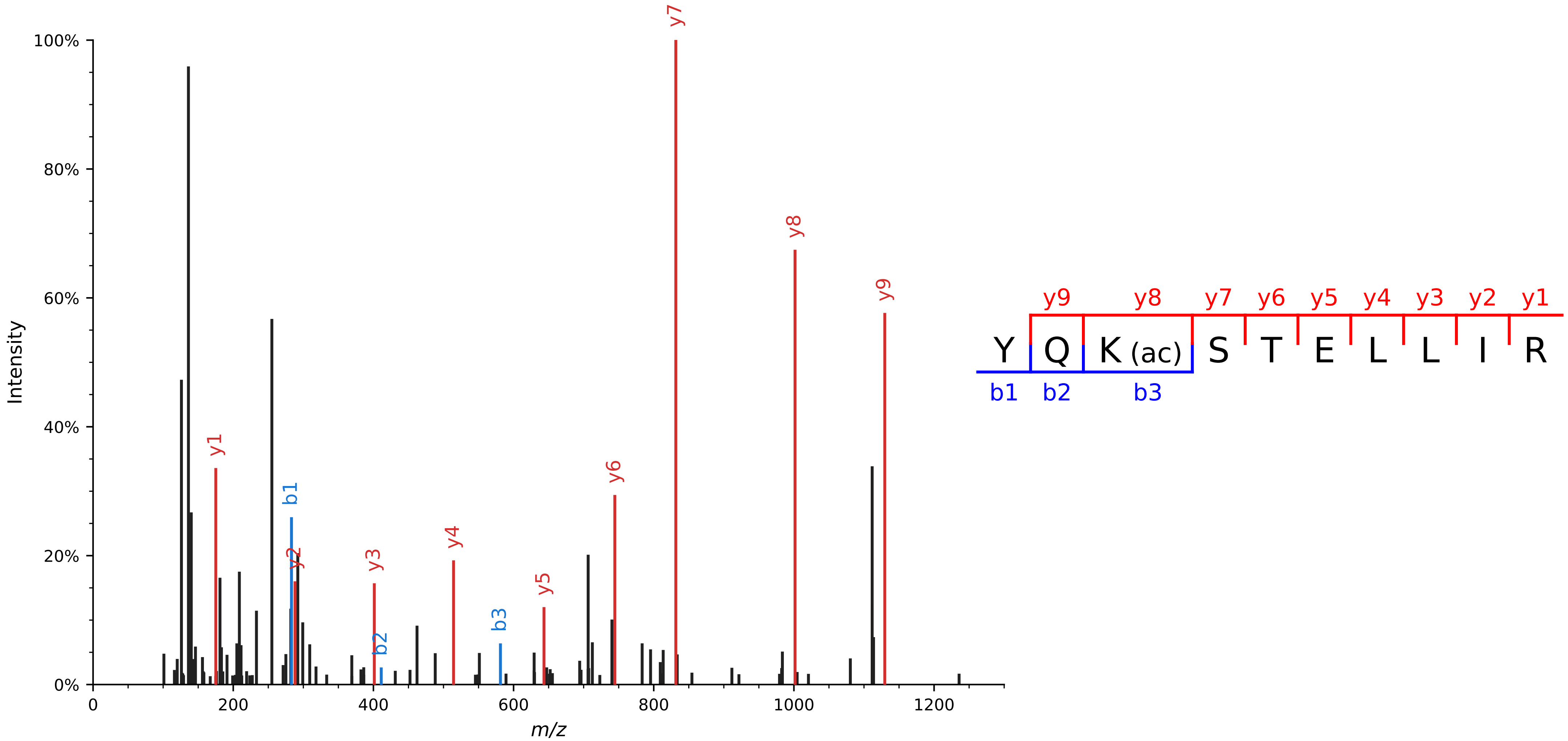

Raw file: HF240124_RN_spleen_PRO_PIC_2, Scan: 20424, m/z: 699.37481, Charge: 2, RT (min): 37.54, Score: 107.57

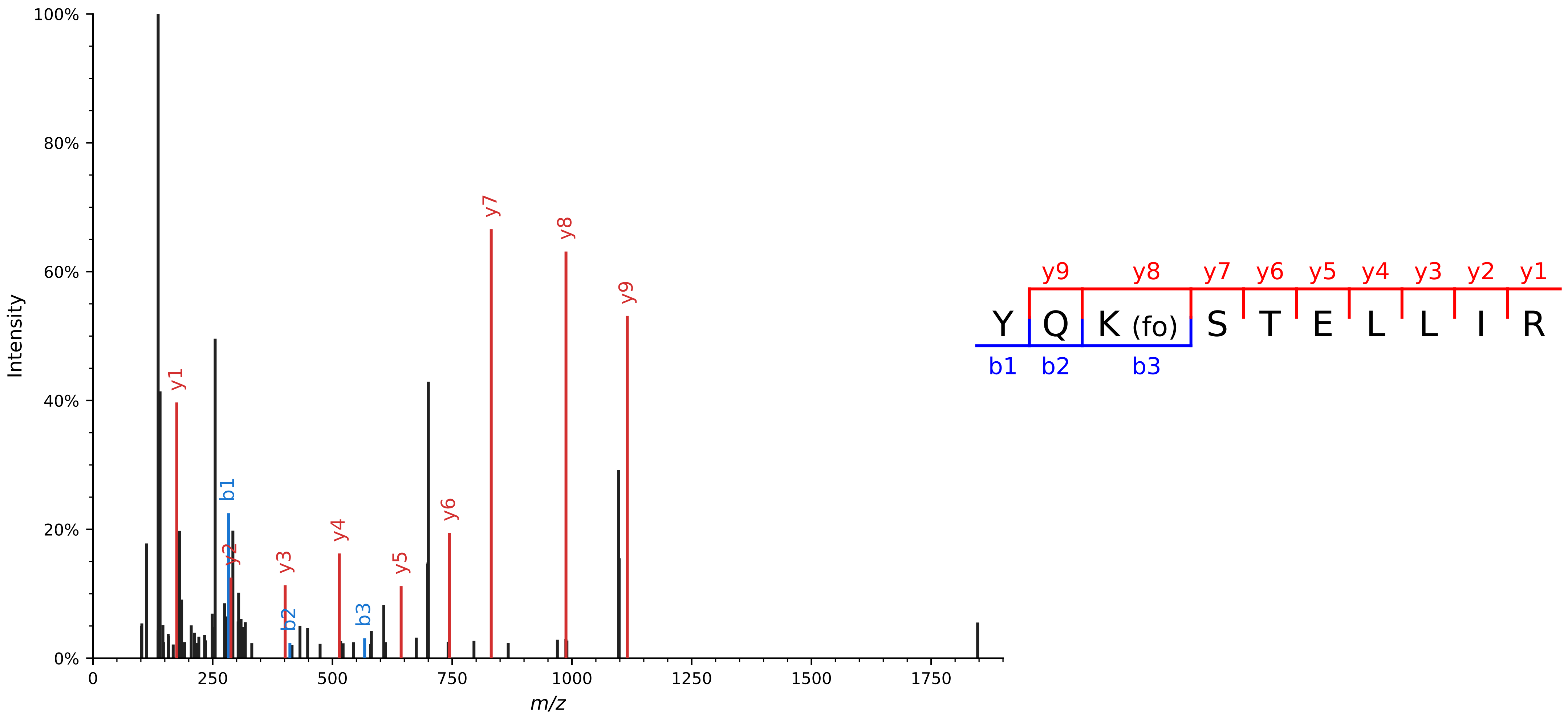

Raw file: HF240124_RN_spleen_PRO_PIC_1, Scan: 17092, m/z: 721.38792, Charge: 2, RT (min): 32.83, Score: 56.563

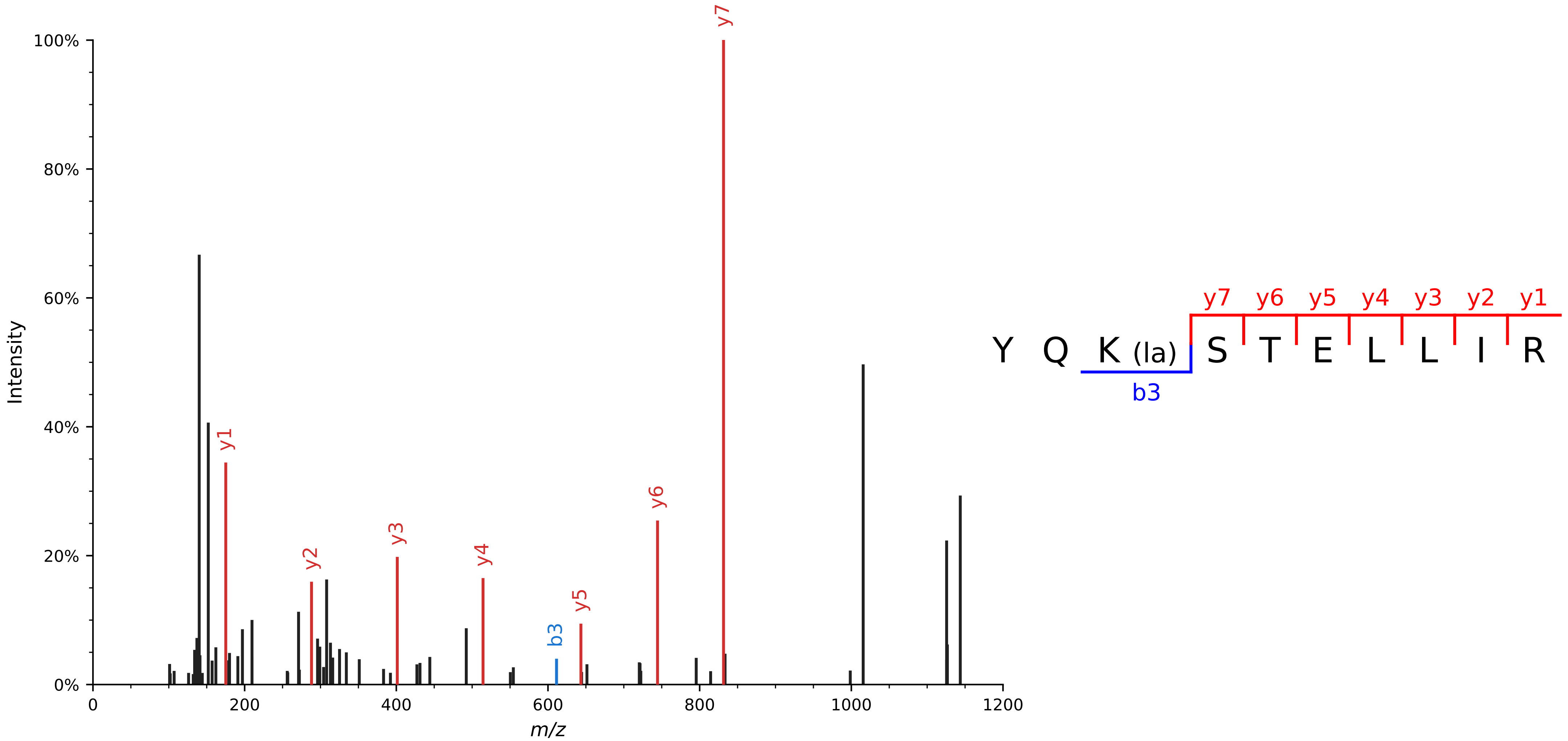

Raw file: HF240124_RN_spleen_PRO_PIC_2, Scan: 19569, m/z: 488.27417, Charge: 2, RT (min): 35.99, Score: 77.906

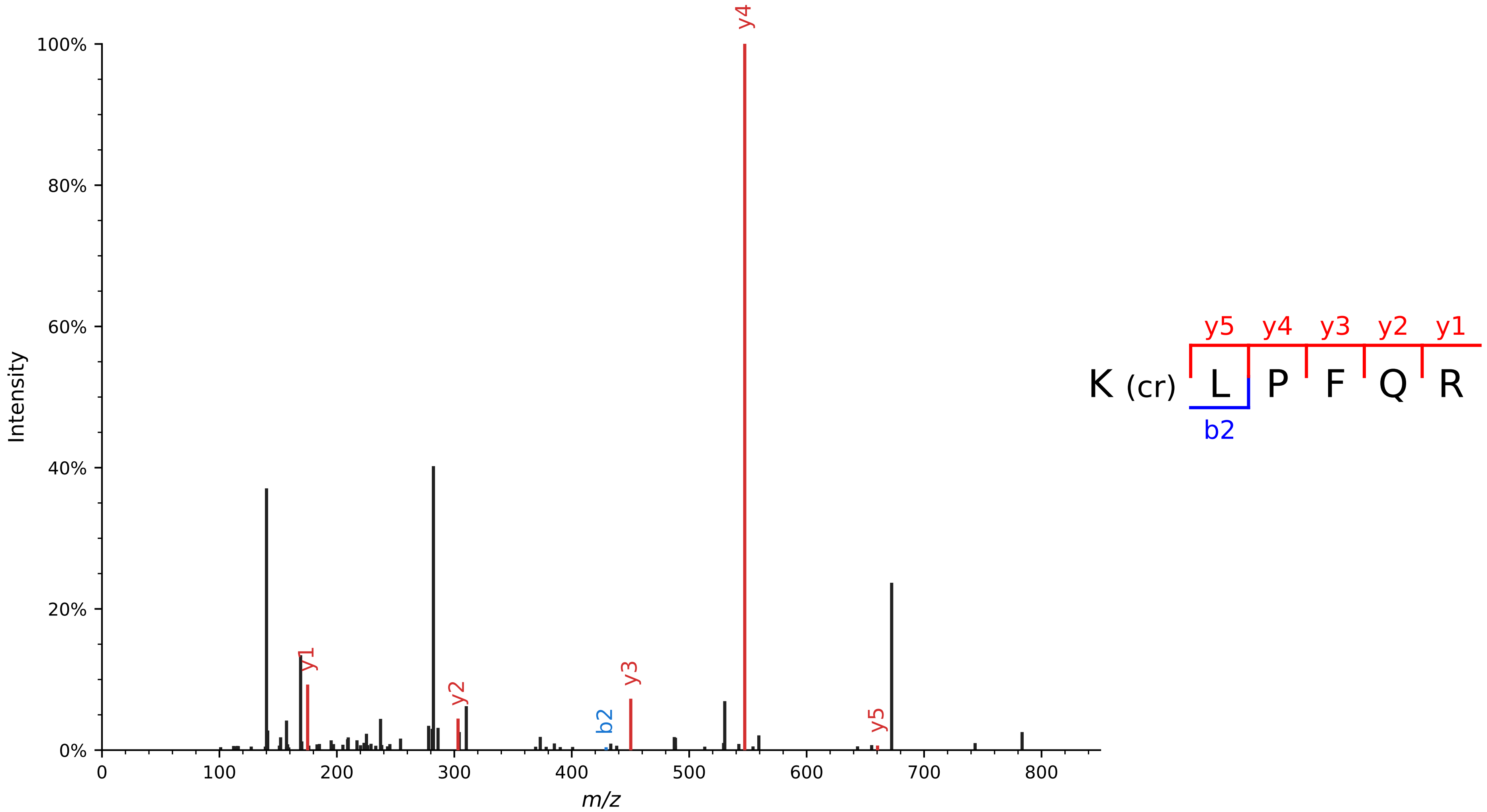

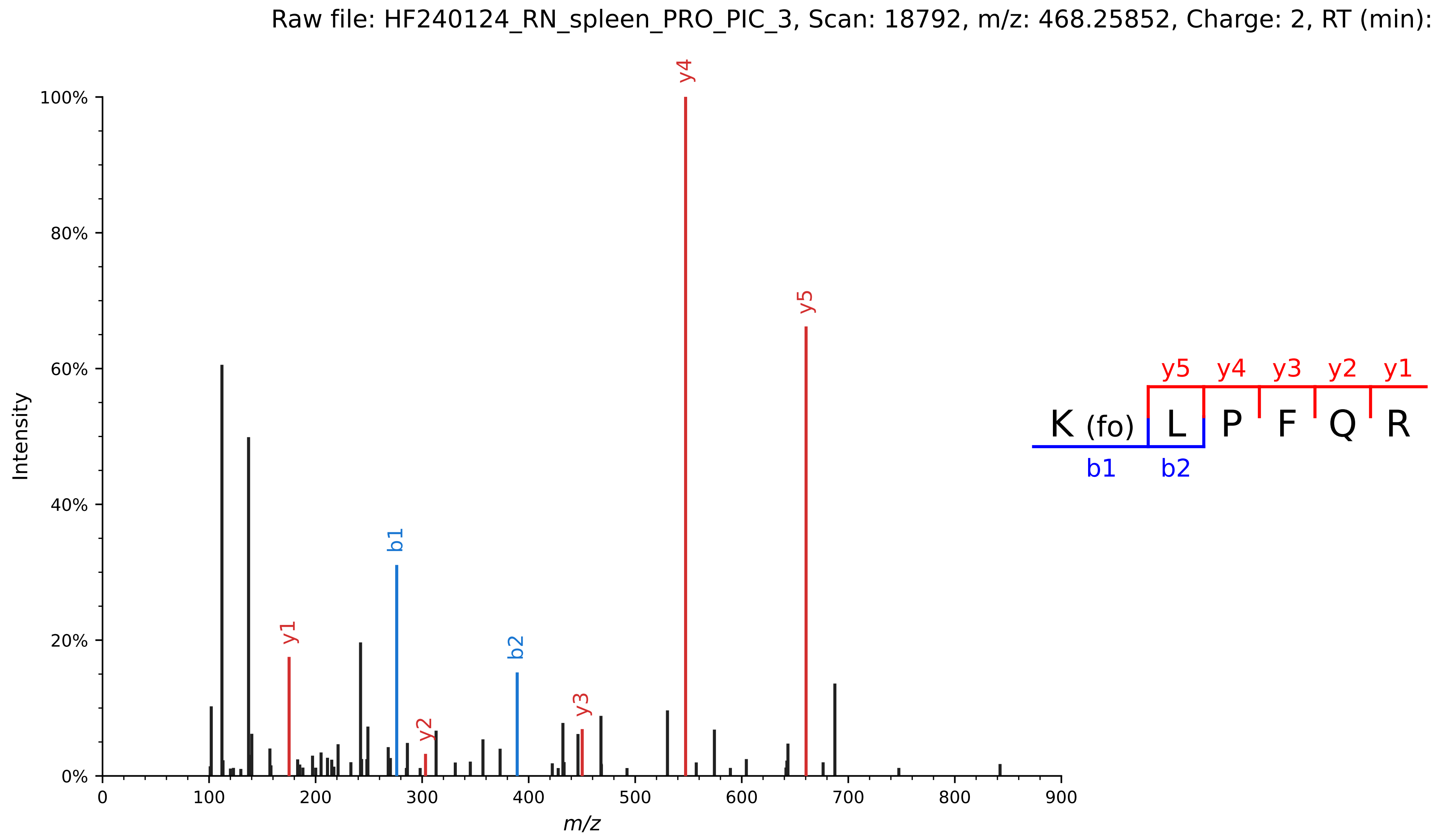

Raw file: HF240124_RN_spleen_PRO_PIC_3, Scan: 18792, m/z: 468.25852, Charge: 2, RT (min): 34.55, Score: 77.906
Intensity
m/z
y1
y2
y3
y4
y5
b1
b2
K (fo) L P F Q R

Raw file: HF240124_RN_spleen_PRO_PIC_3, Scan: 21196, m/z: 511.27691, Charge: 2, RT (min): 38.86, Score: 86.344

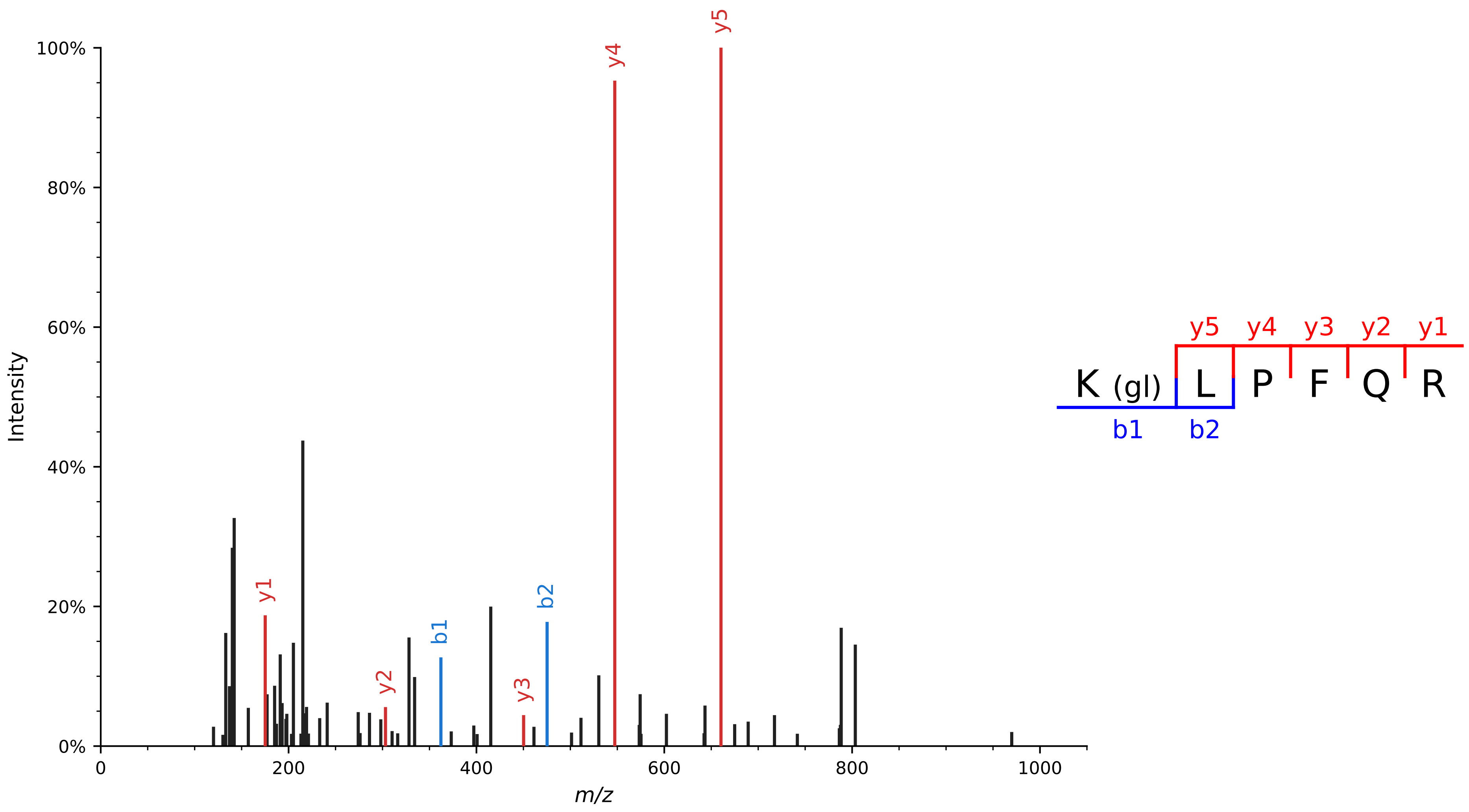

Raw file: HF240124_RN_spleen_PRO_PIC_2, Scan: 18773, m/z: 490.27163, Charge: 2, RT (min): 34.55, Score: 94.669

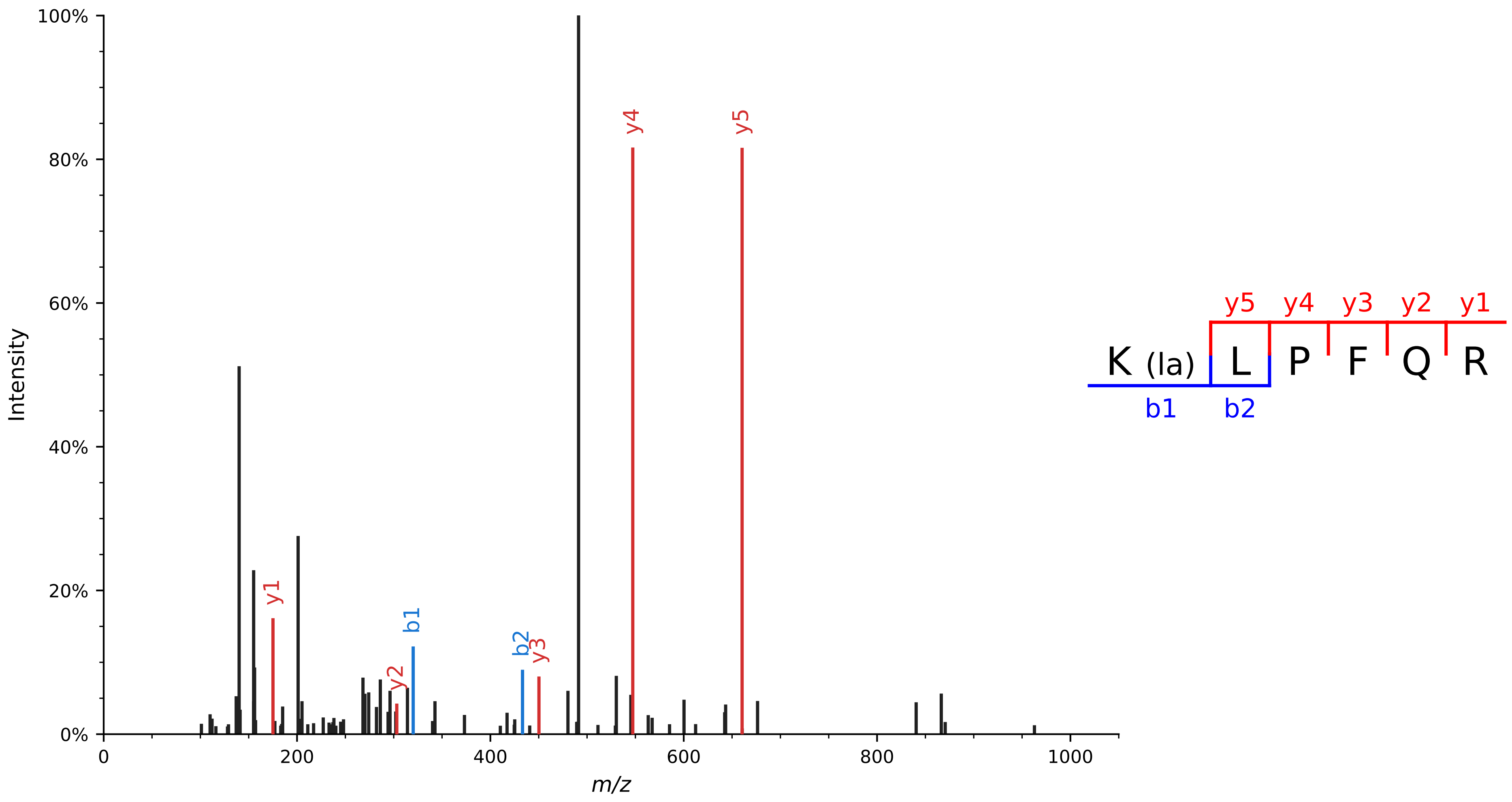

Raw file: HF240124_RN_spleen_PRO_PIC_3, Scan: 21594, m/z: 489.28199, Charge: 2, RT (min): 39.58, Score: 78.166

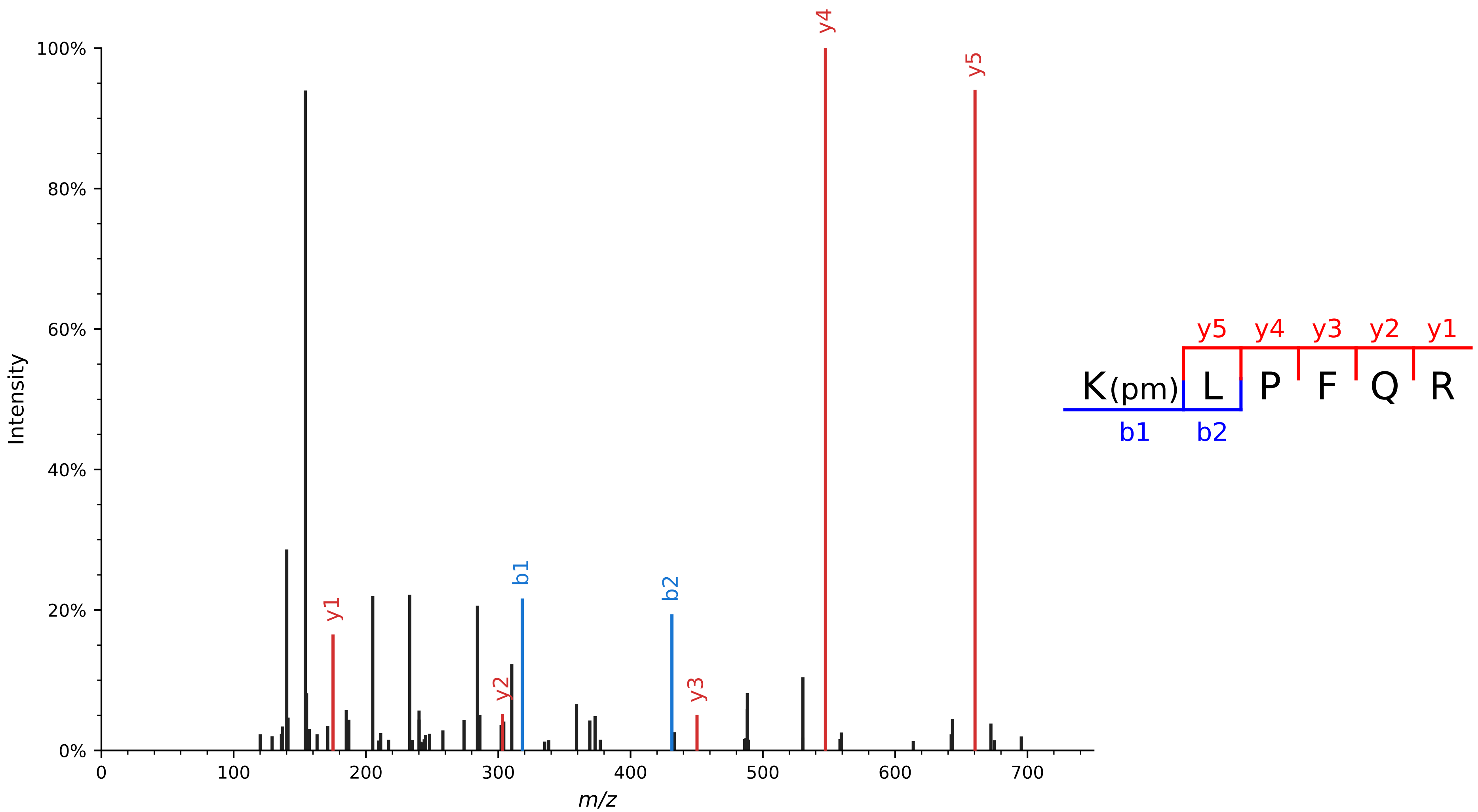

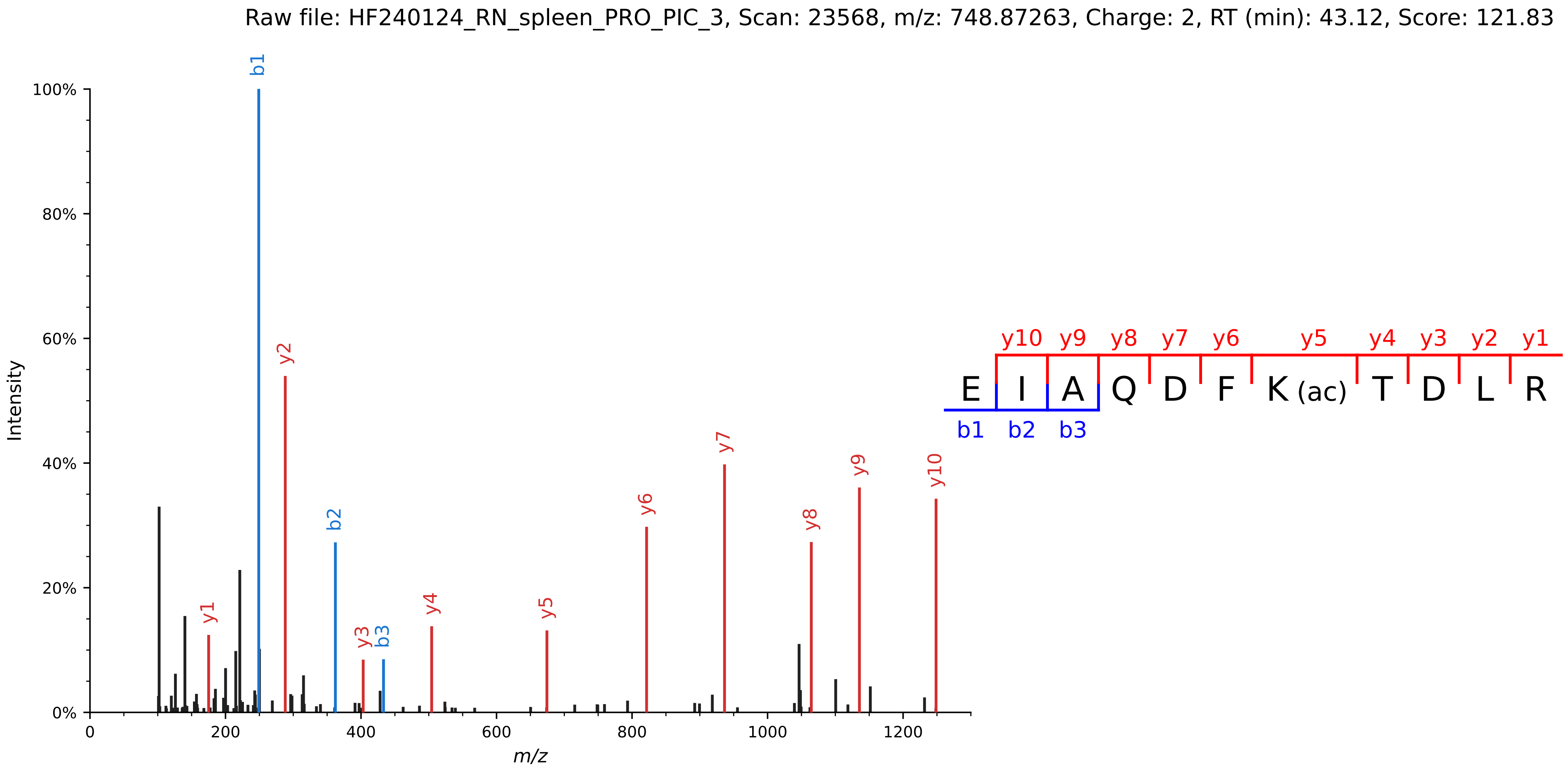

Raw file: HF240124_RN_spleen_PRO_PIC_3, Scan: 23568, m/z: 748.87263, Charge: 2, RT (min): 43.12, Score: 121.83
Intensity
m/z
0%
20%
40%
60%
80%
100%
0
200
400
600
800
1000
1200
b1
b2
b3
y1
y2
y3
y4
y5
y6
y7
y8
y9
y10
E I A Q D F K (ac) T D L R

Raw file: HF240124_RN_spleen_PRO_PIC_2, Scan: 23284, m/z: 741.86481, Charge: 2, RT (min): 42.69, Score: 125.48

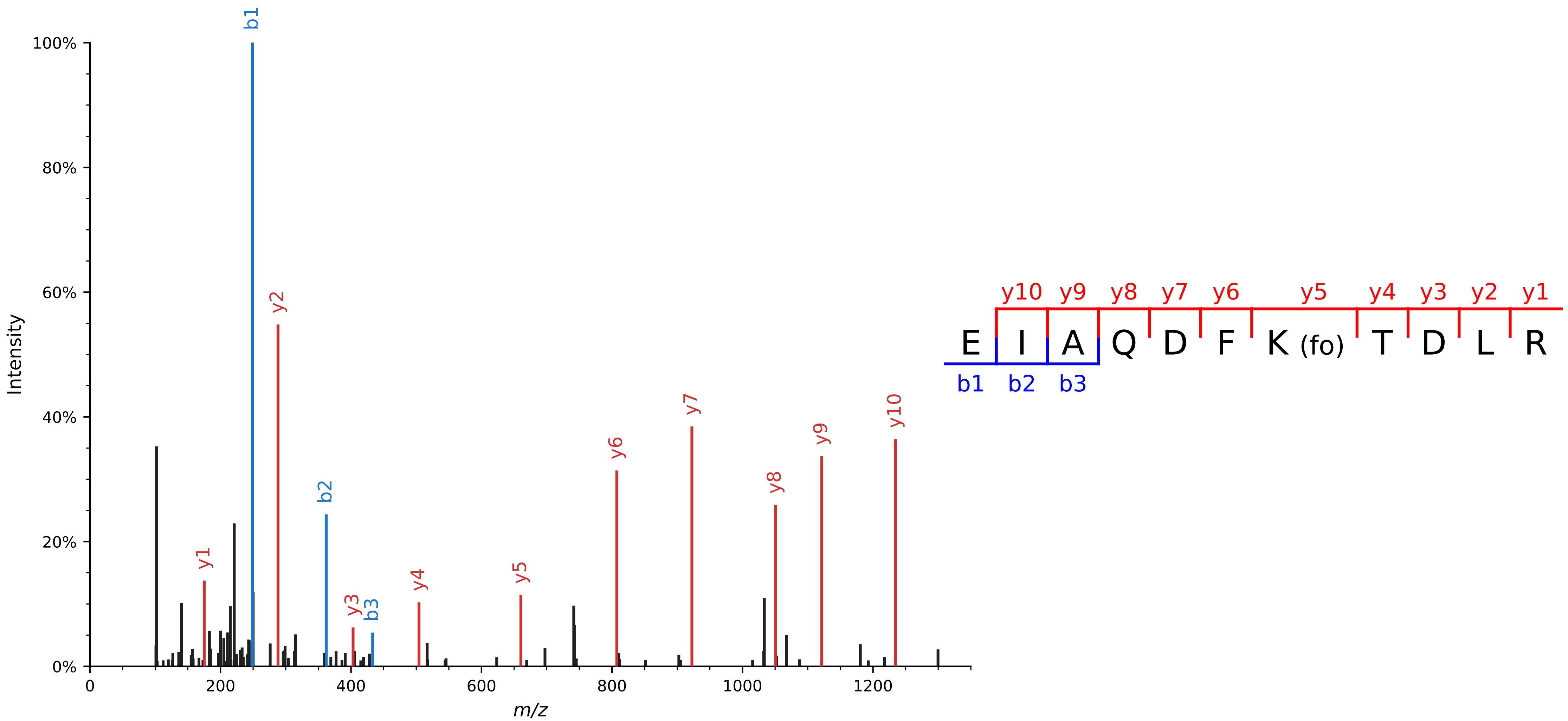

Raw file: HF240124_RN_spleen_PRO_PIC_3, Scan: 22025, m/z: 763.87791, Charge: 2, RT (min): 40.35, Score: 112.75

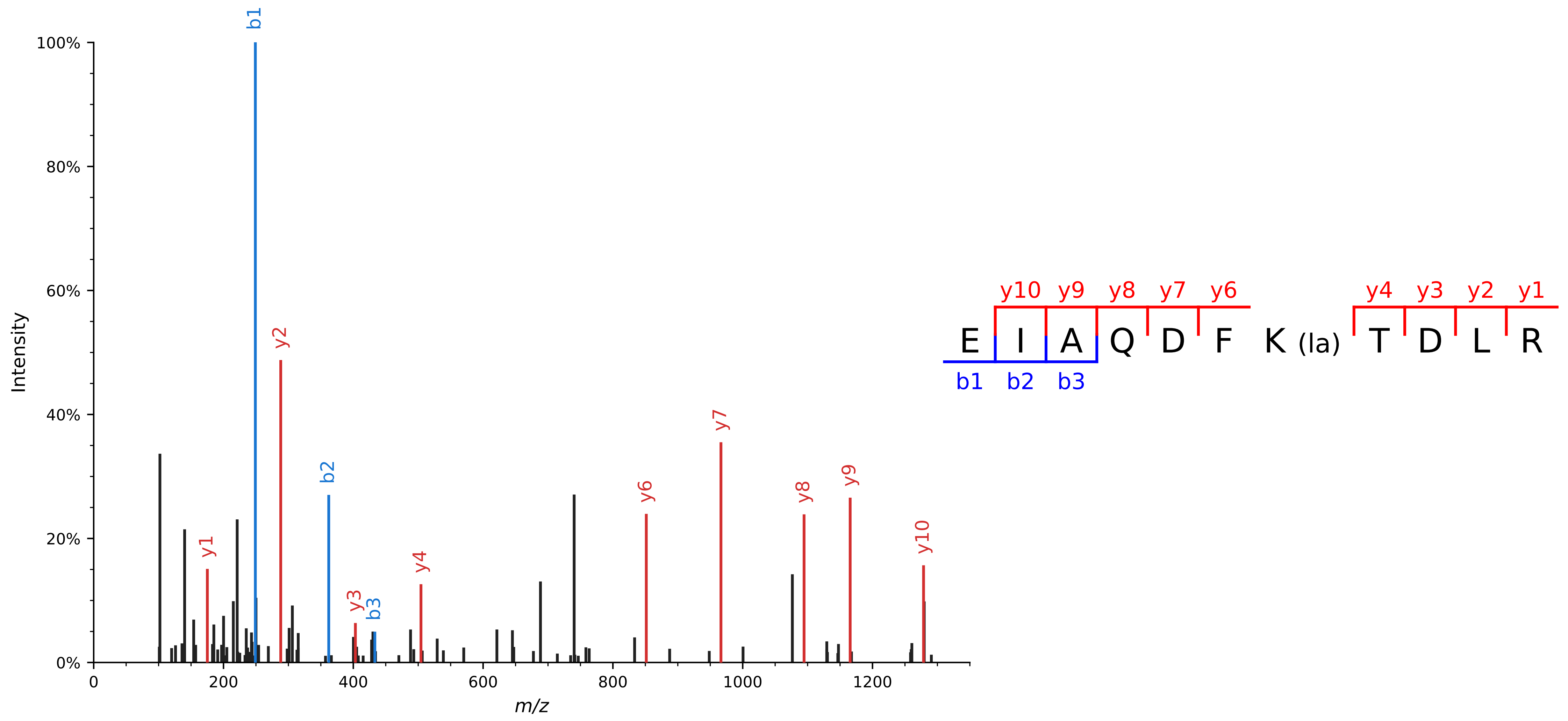

Raw file: HF240124_RN_spleen_PRO_PIC_2, Scan: 26191, m/z: 762.88828, Charge: 2, RT (min): 48.06, Score: 190.24

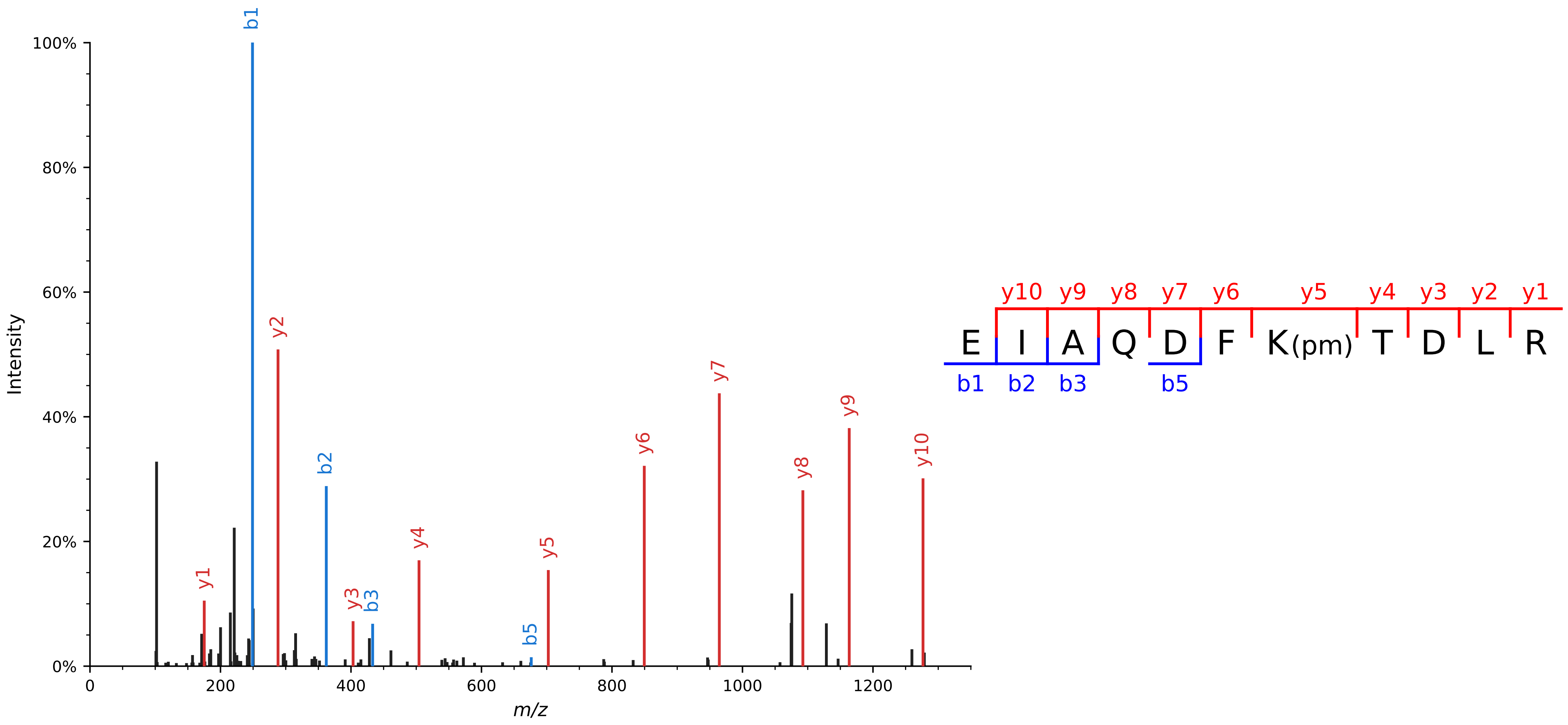

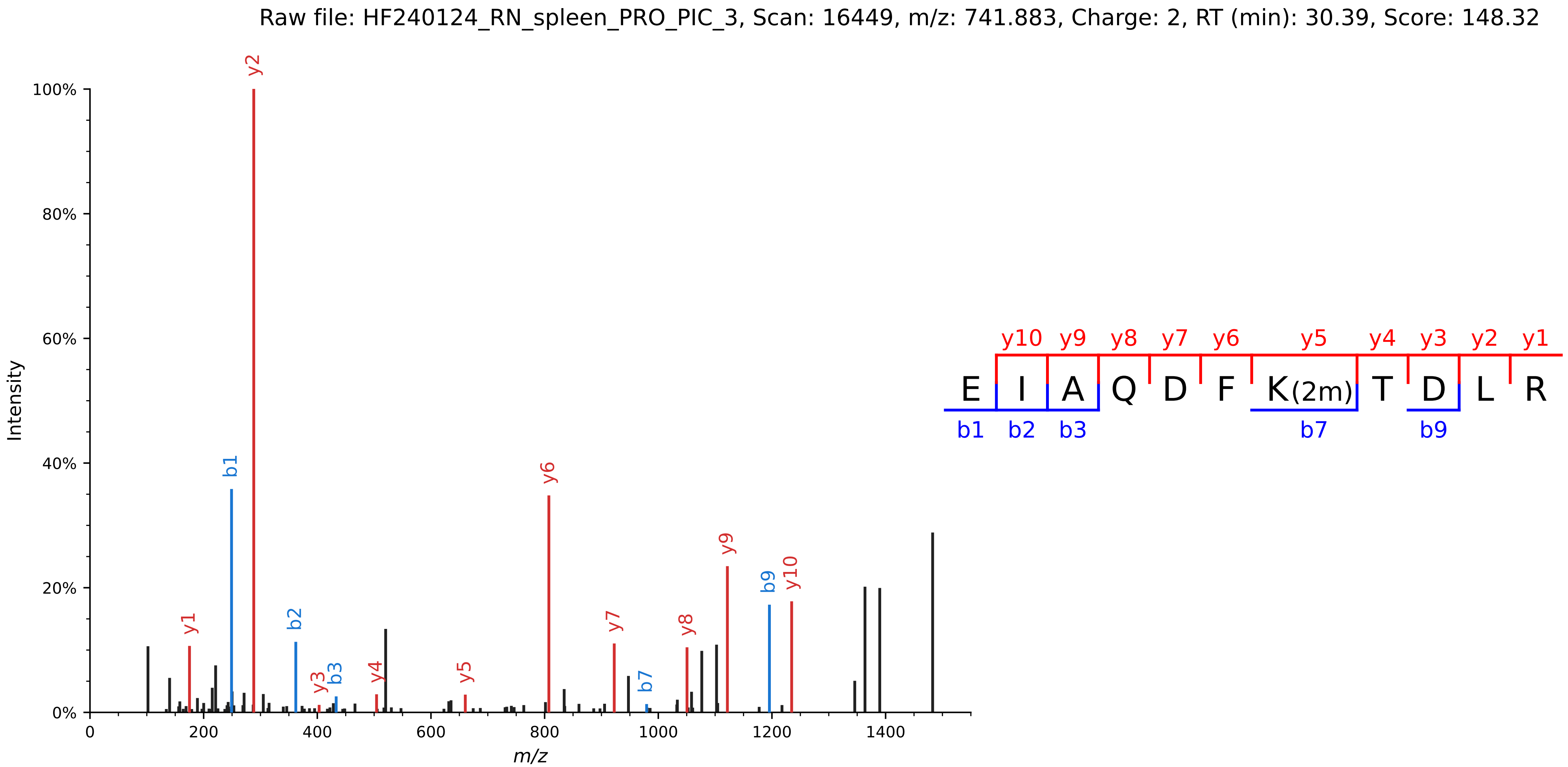

Raw file: HF240124_RN_spleen_PRO_PIC_3, Scan: 16449, m/z: 741.883, Charge: 2, RT (min): 30.39, Score: 148.32
Intensity
m/z
y10 y9 y8 y7 y6 y5 y4 y3 y2 y1
E I A Q D F K(2m) T D L R
b1 b2 b3 b7 b9

Raw file: HF240124_RN_spleen_PRO_PIC_3, Scan: 24218, m/z: 809.43708, Charge: 2, RT (min): 44.31, Score: 44.863

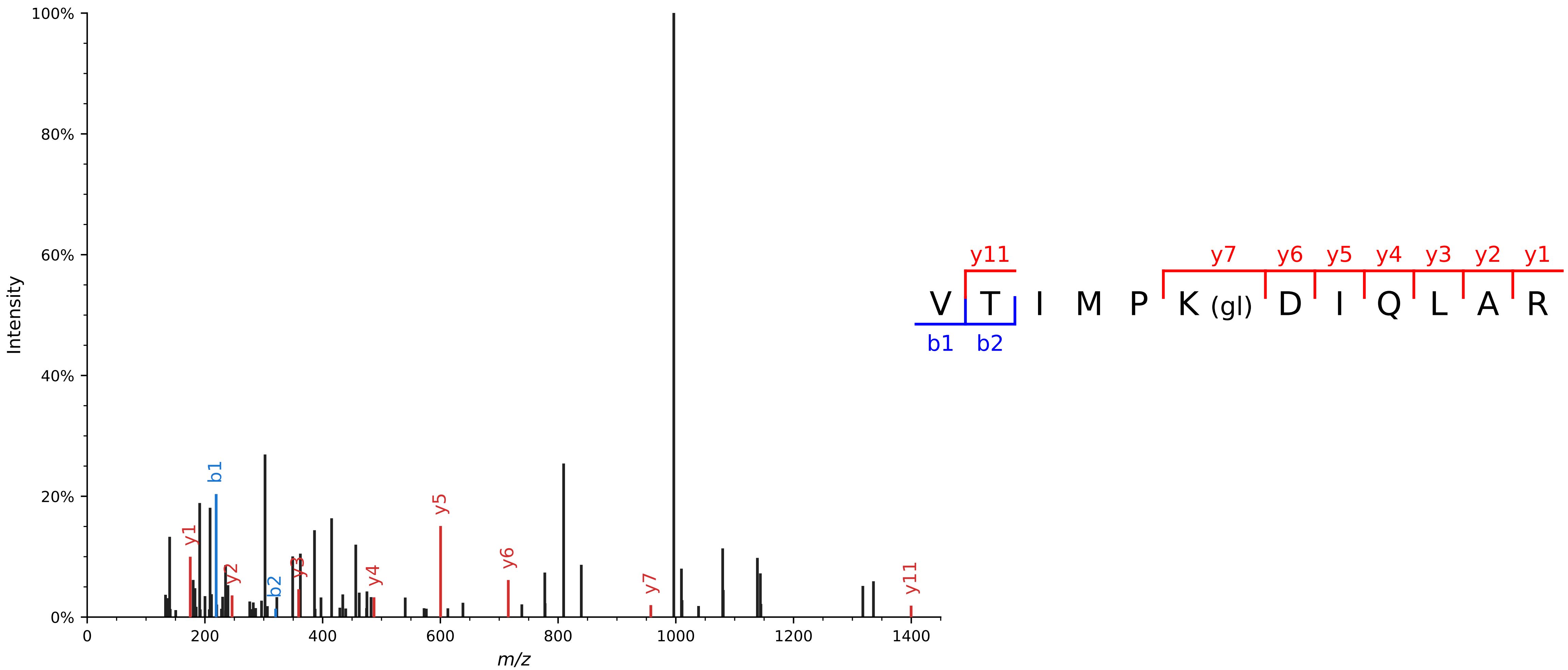

Raw file: HF240124_RN_spleen_PRO_PIC_2, Scan: 22531, m/z: 788.4318, Charge: 2, RT (min): 41.33, Score: 114.5

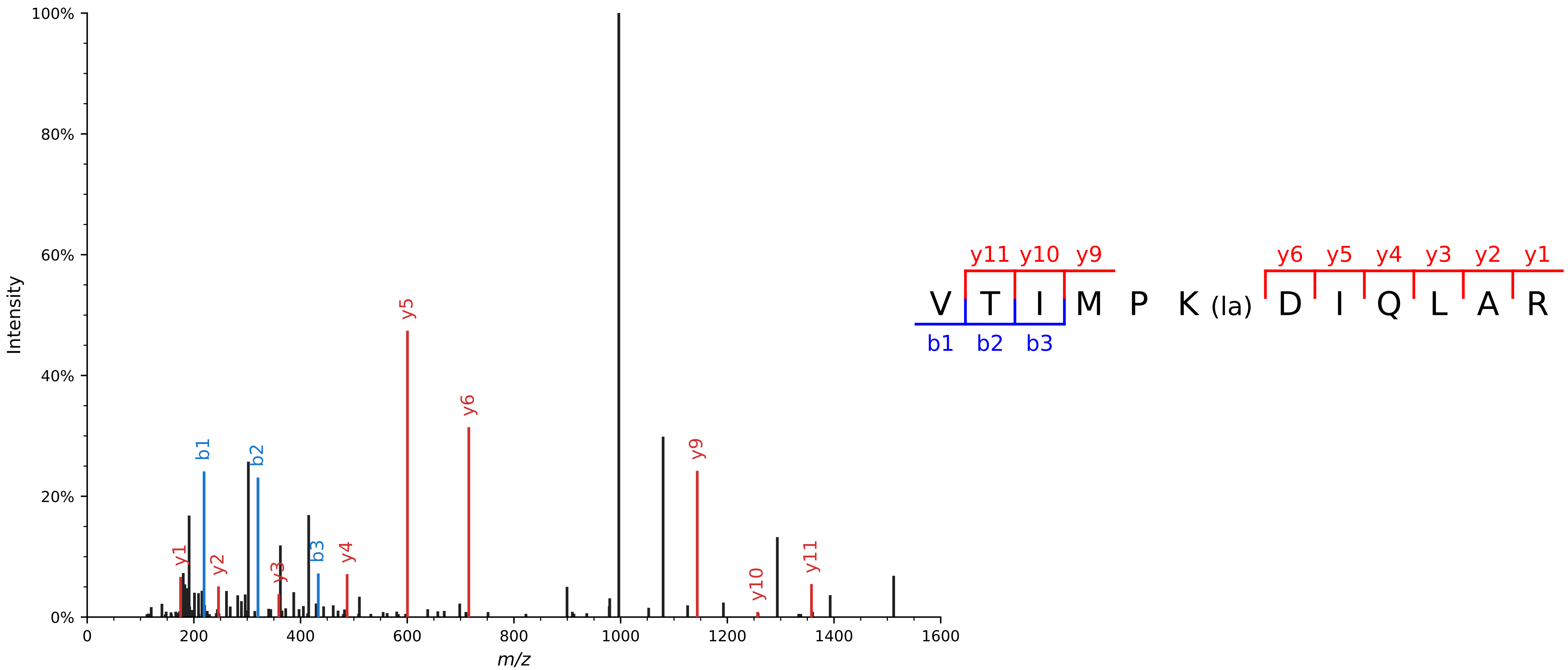

Raw file: HF240124_RN_spleen_PRO_PIC_3, Scan: 16061, m/z: 787.44217, Charge: 2, RT (min): 29.71, Score: 72.879

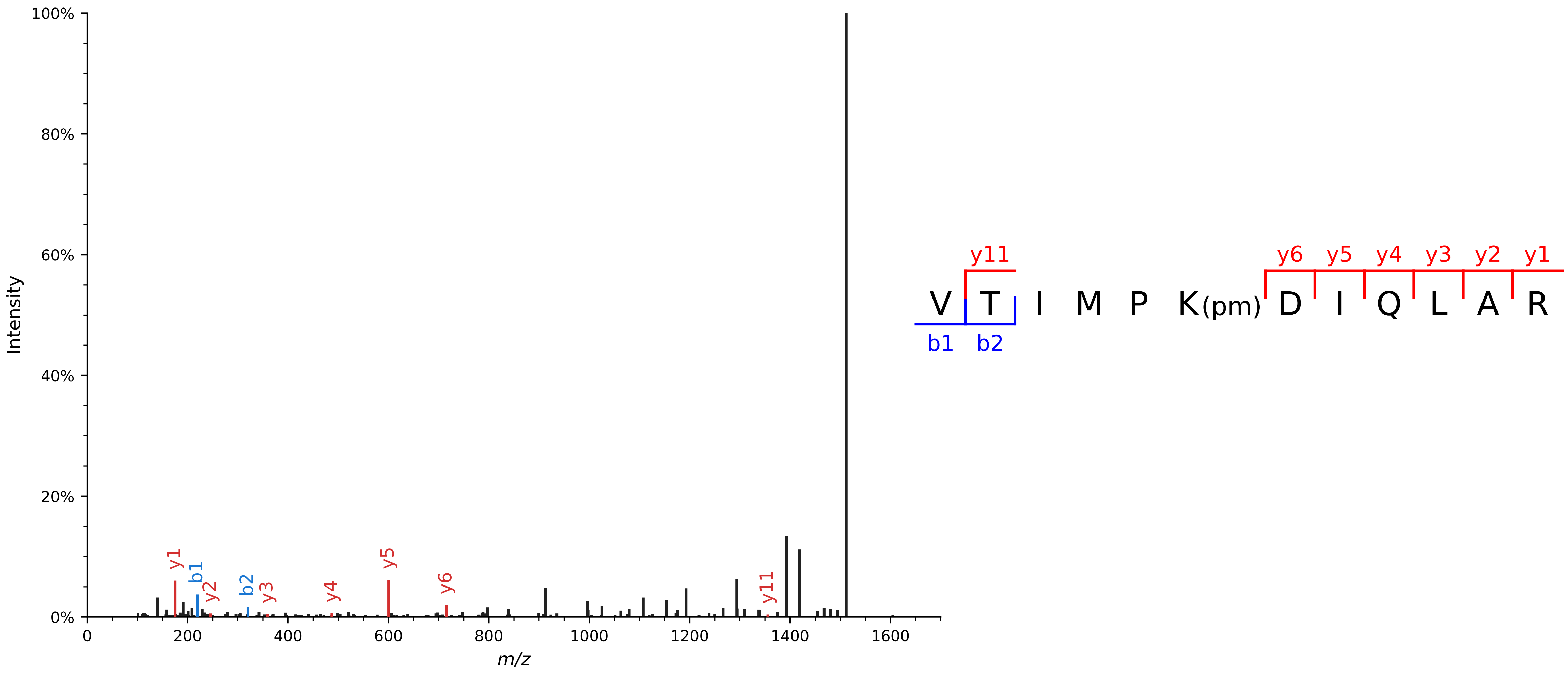

Raw file: HF240124_RN_spleen_PRO_PIC_3, Scan: 23536, m/z: 802.42926, Charge: 2, RT (min): 43.06, Score: 56.432

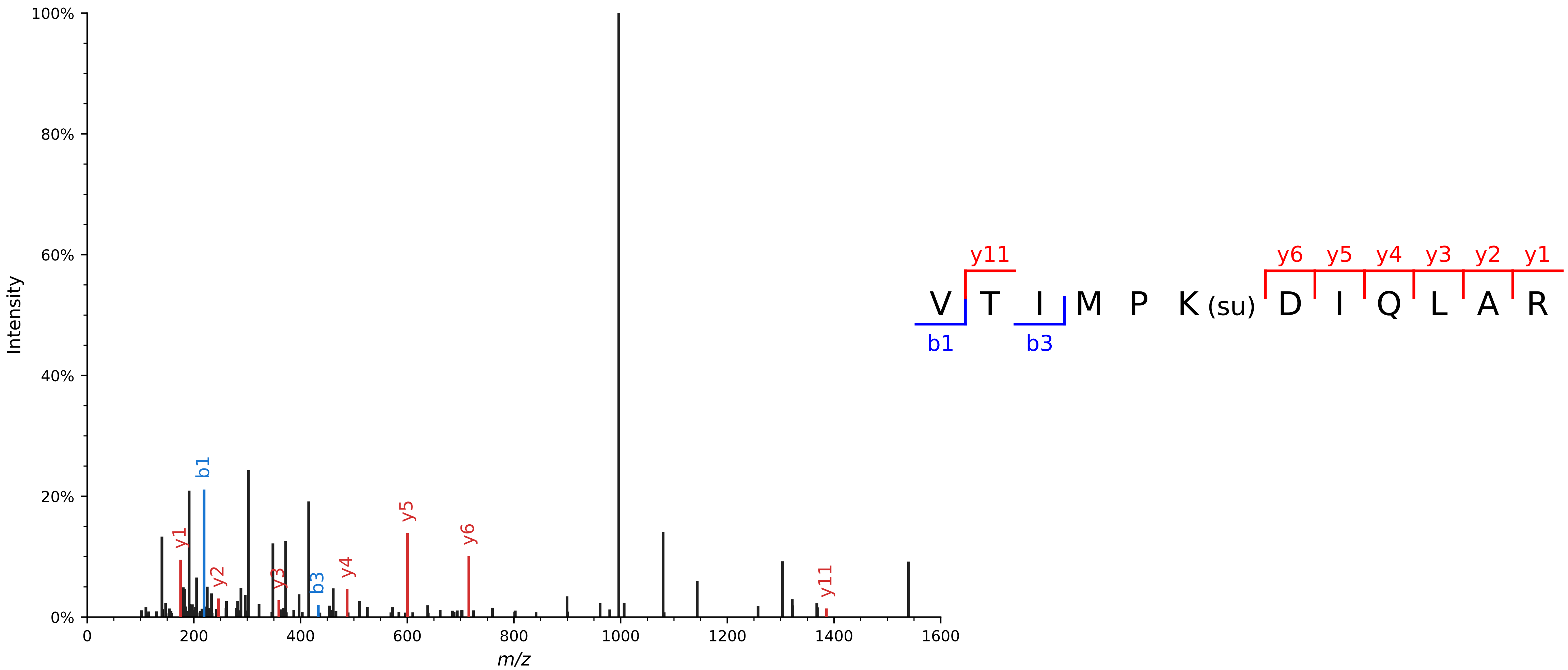

Raw file: HF240124_RN_spleen_PRO_PIC_1, Scan: 13722, m/z: 800.45191, Charge: 2, RT (min): 26.68, Score: 167.12

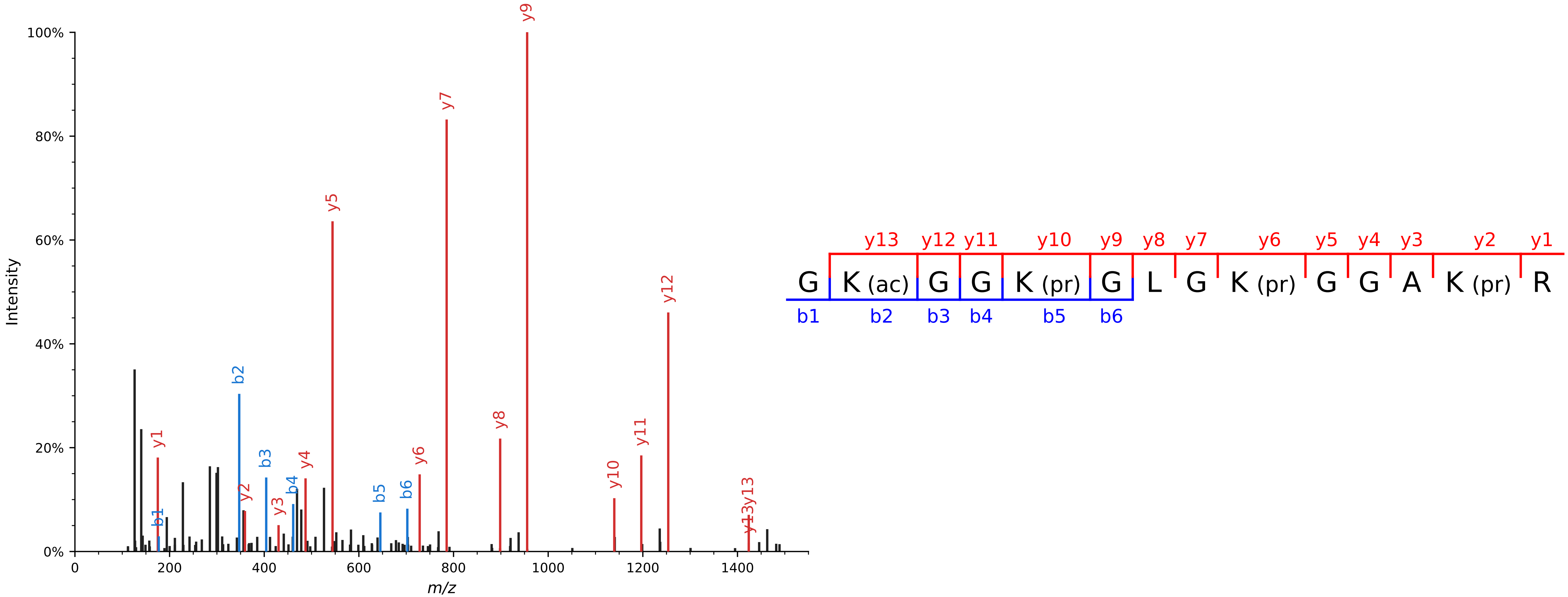

Raw file: HF240124_RN_spleen_PRO_PIC_3, Scan: 12013, m/z: 786.43626, Charge: 2, RT (min): 22.58, Score: 171.92

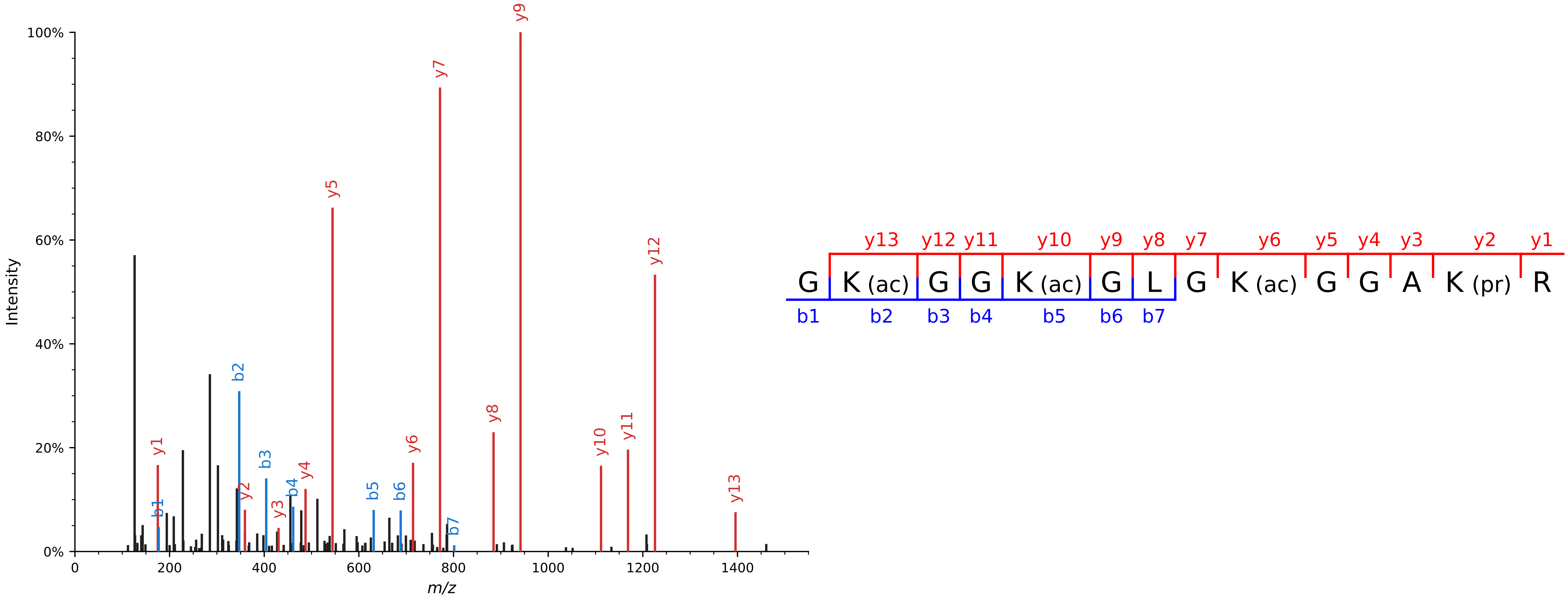

Raw file: HF240124_RN_spleen_PRO_PIC_2, Scan: 11030, m/z: 779.42844, Charge: 2, RT (min): 20.90, Score: 157.68

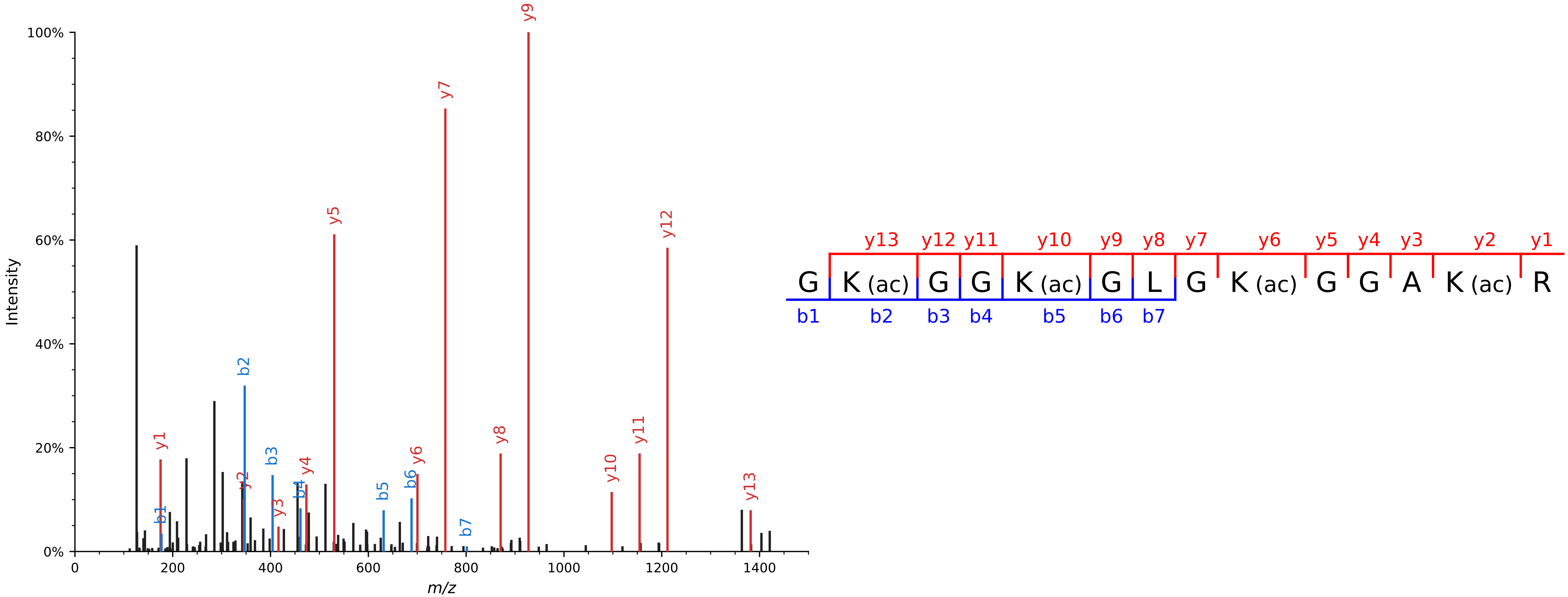

Raw file: HF240124_RN_spleen_PRO_PIC_2, Scan: 13341, m/z: 809.439, Charge: 2, RT (min): 25.00, Score: 42.475

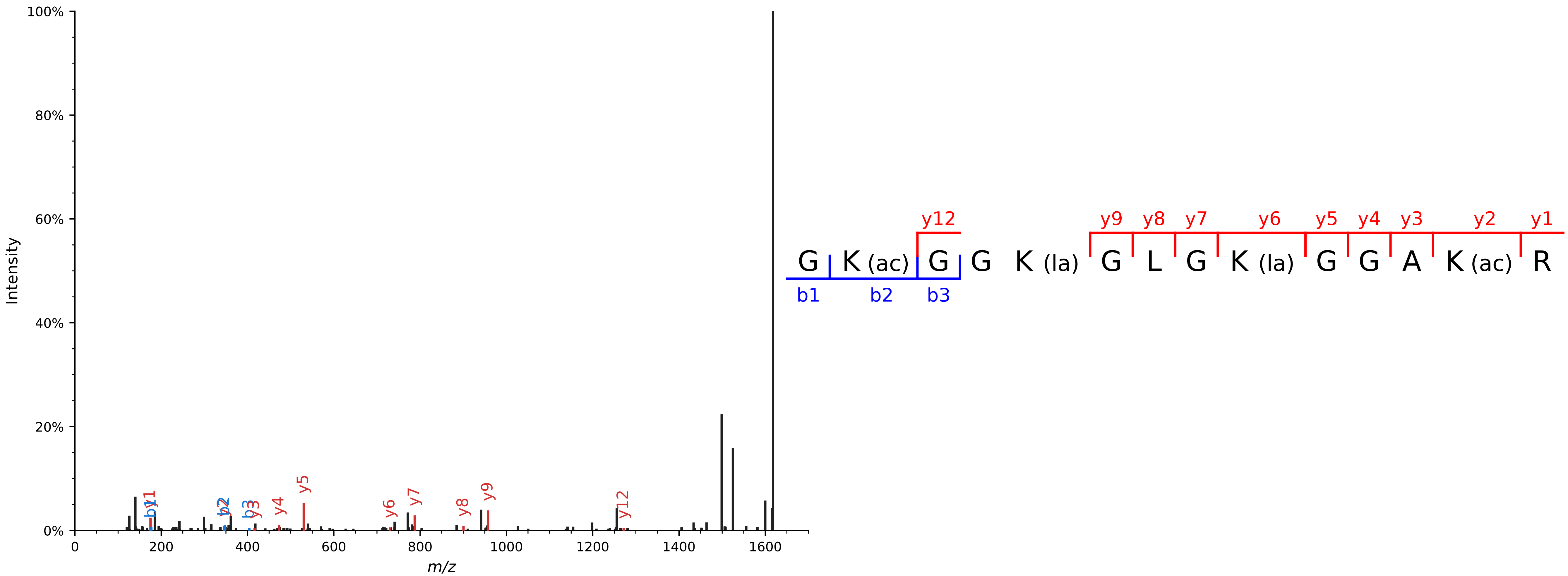

Raw file: HF240124_RN_spleen_PRO_PIC_1, Scan: 12511, m/z: 793.44409, Charge: 2, RT (min): 24.48, Score: 164.65

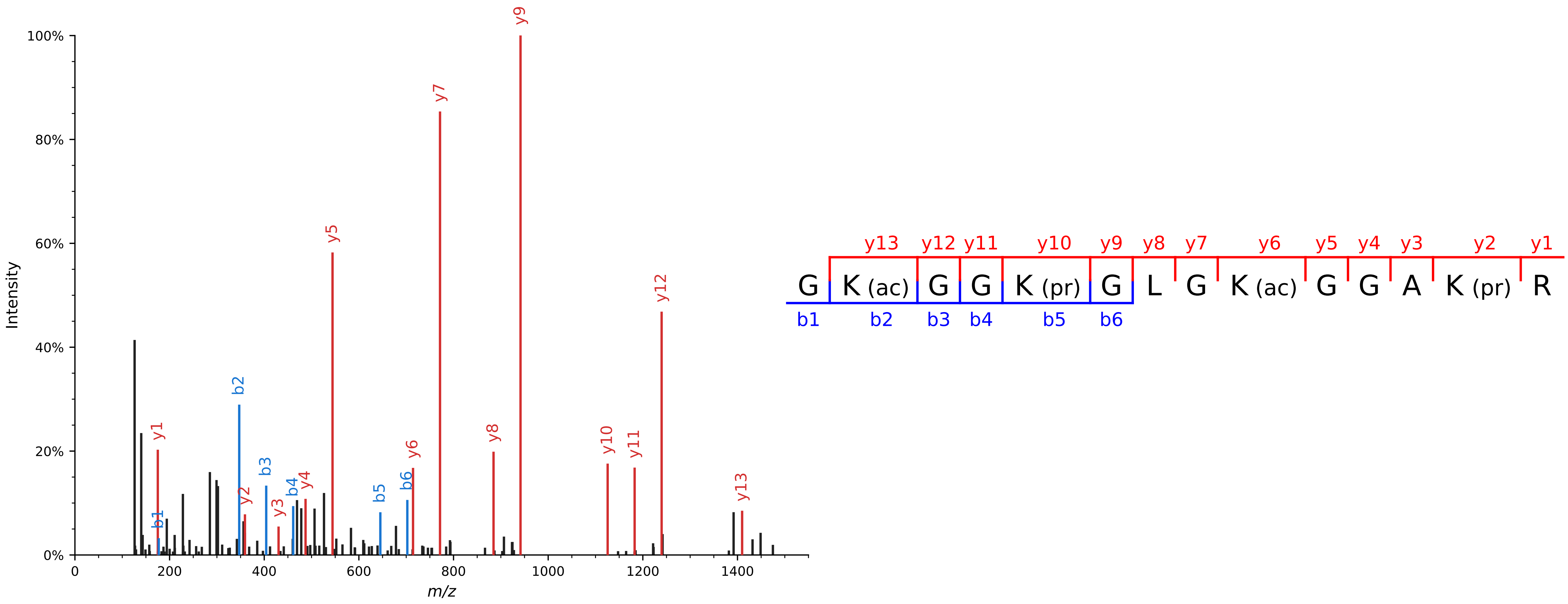

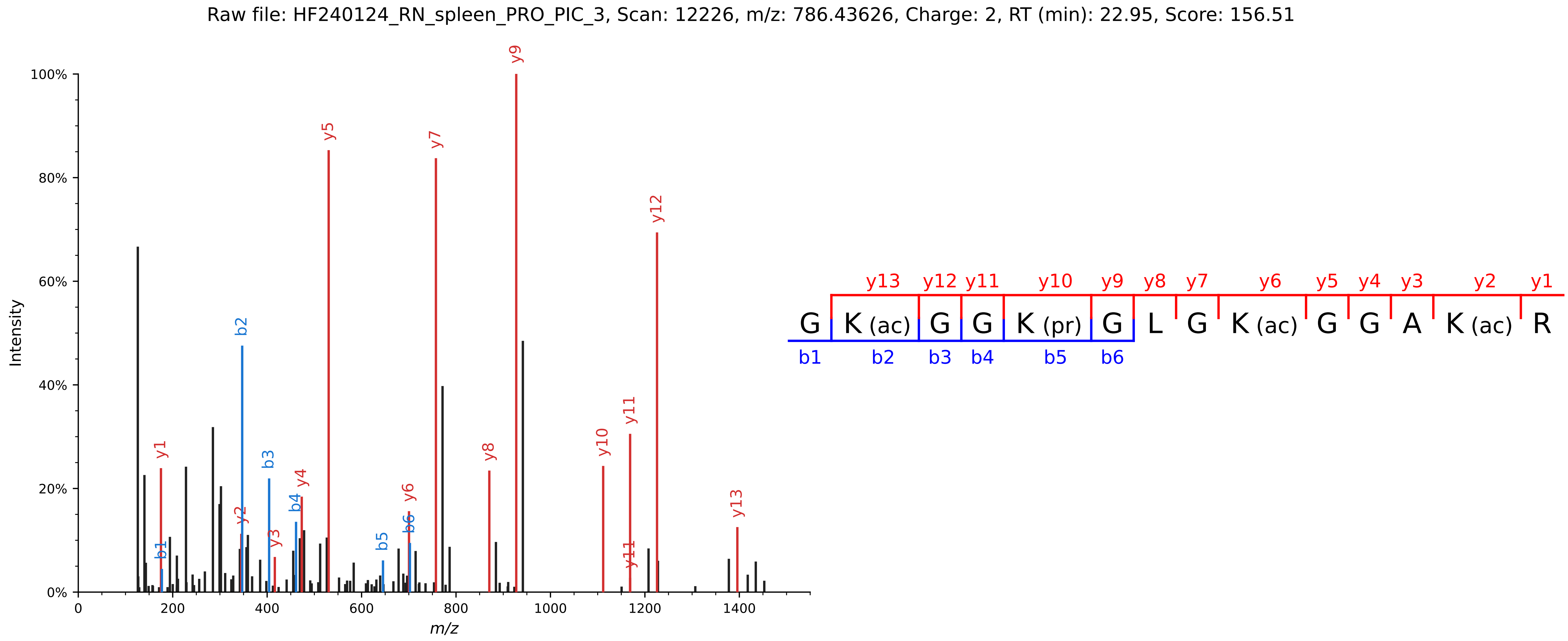
Raw file: HF240124_RN_spleen_PRO_PIC_3, Scan: 12226, m/z: 786.43626, Charge: 2, RT (min): 22.95, Score: 156.51
Intensity
m/z
G K (ac) G G K (pr) G L G K (ac) G G A K (ac) R
y13 y12 y11 y10 y9 y8 y7 y6 y5 y4 y3 y2 y1
b1 b2 b3 b4 b5 b6

Raw file: HF240124_RN_spleen_PRO_PIC_1, Scan: 12793, m/z: 793.44409, Charge: 2, RT (min): 25.02, Score: 169.58

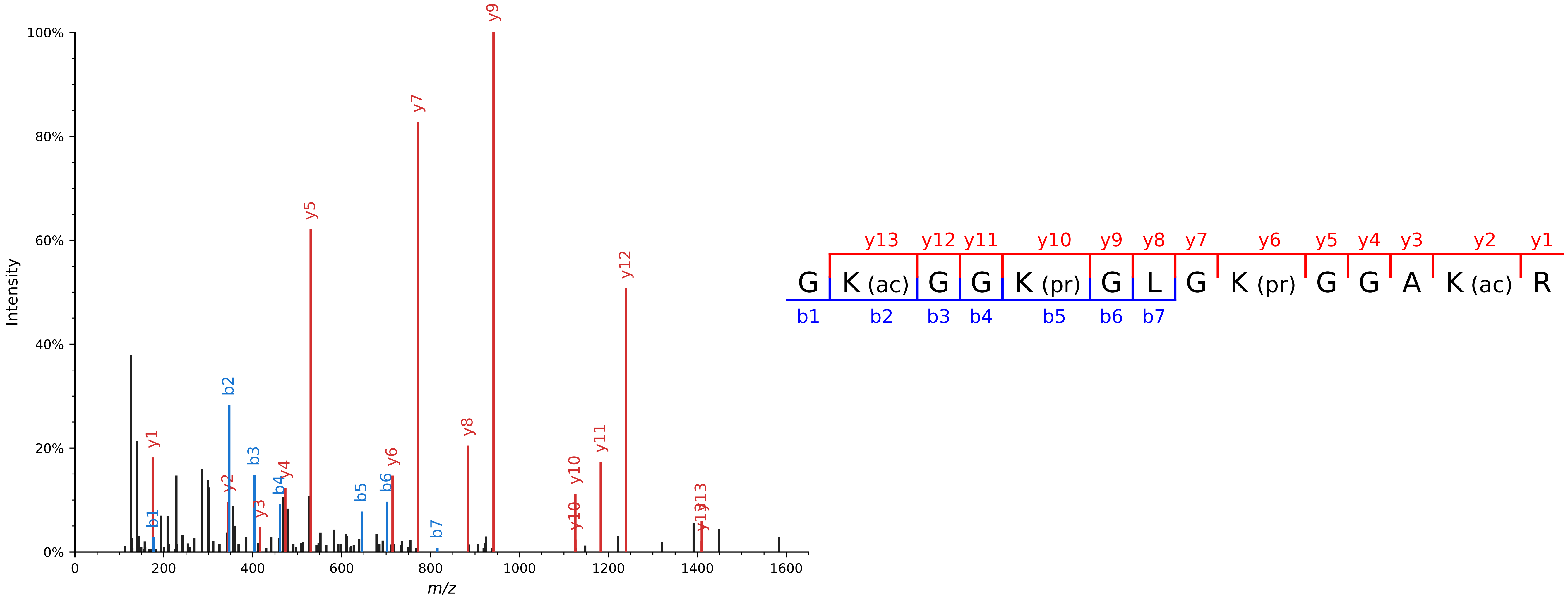

Raw file: HF240124_RN_spleen_PRO_PIC_3, Scan: 16450, m/z: 813.45974, Charge: 2, RT (min): 30.39, Score: 134.57

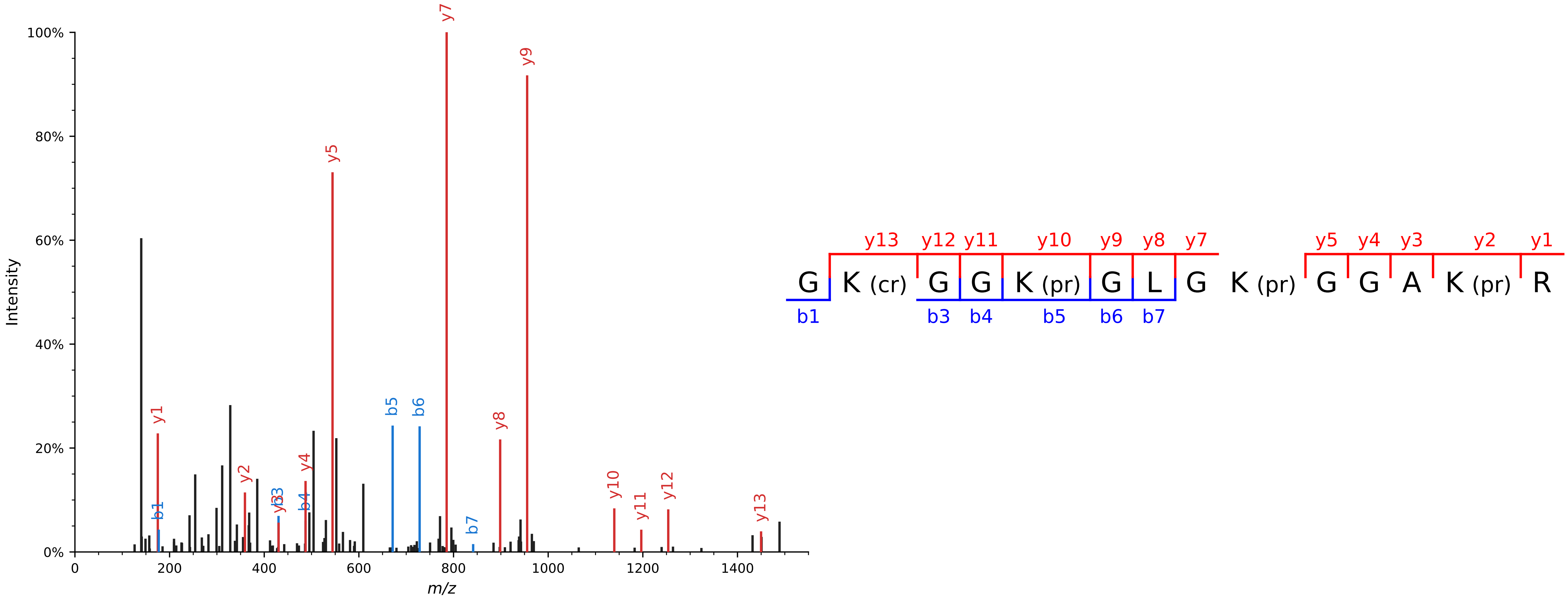

Raw file: HF240124_RN_spleen_PRO_PIC_1, Scan: 12422, m/z: 786.43626, Charge: 2, RT (min): 24.27, Score: 107.06

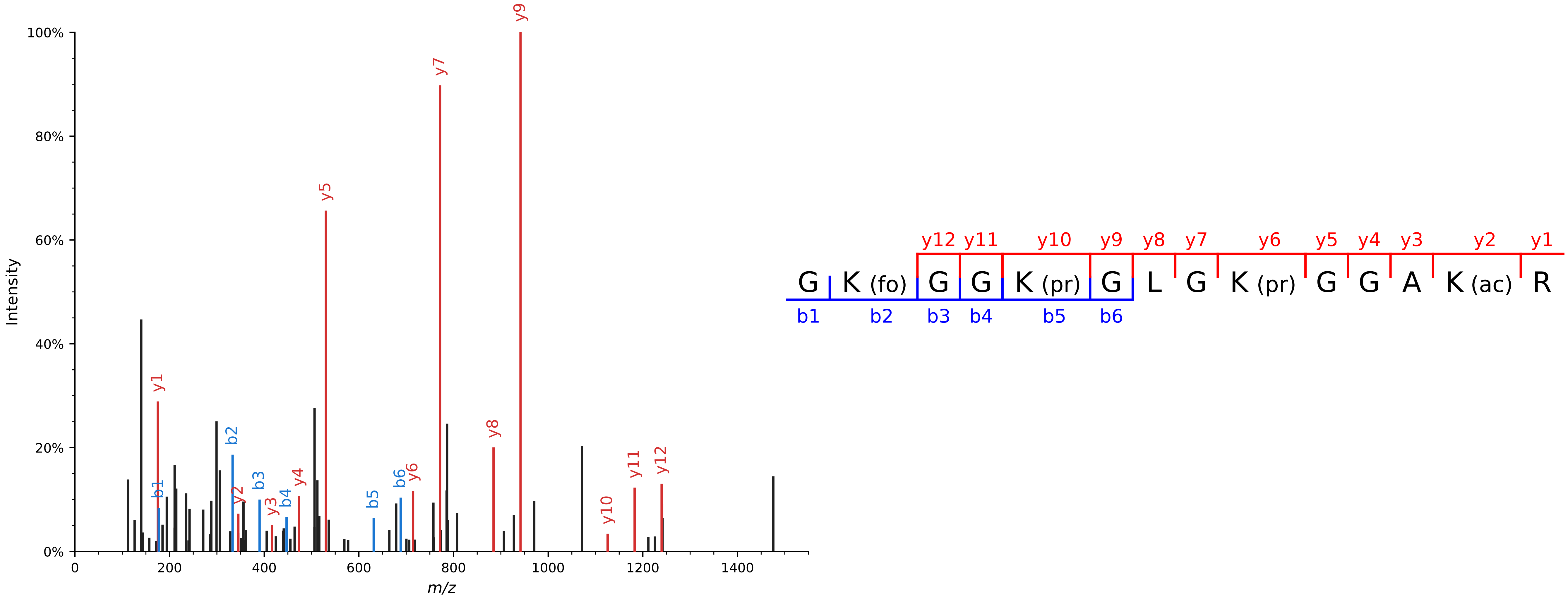

Raw file: HF240124_RN_spleen_PRO_PIC_3, Scan: 16117, m/z: 836.46248, Charge: 2, RT (min): 29.81, Score: 119.53

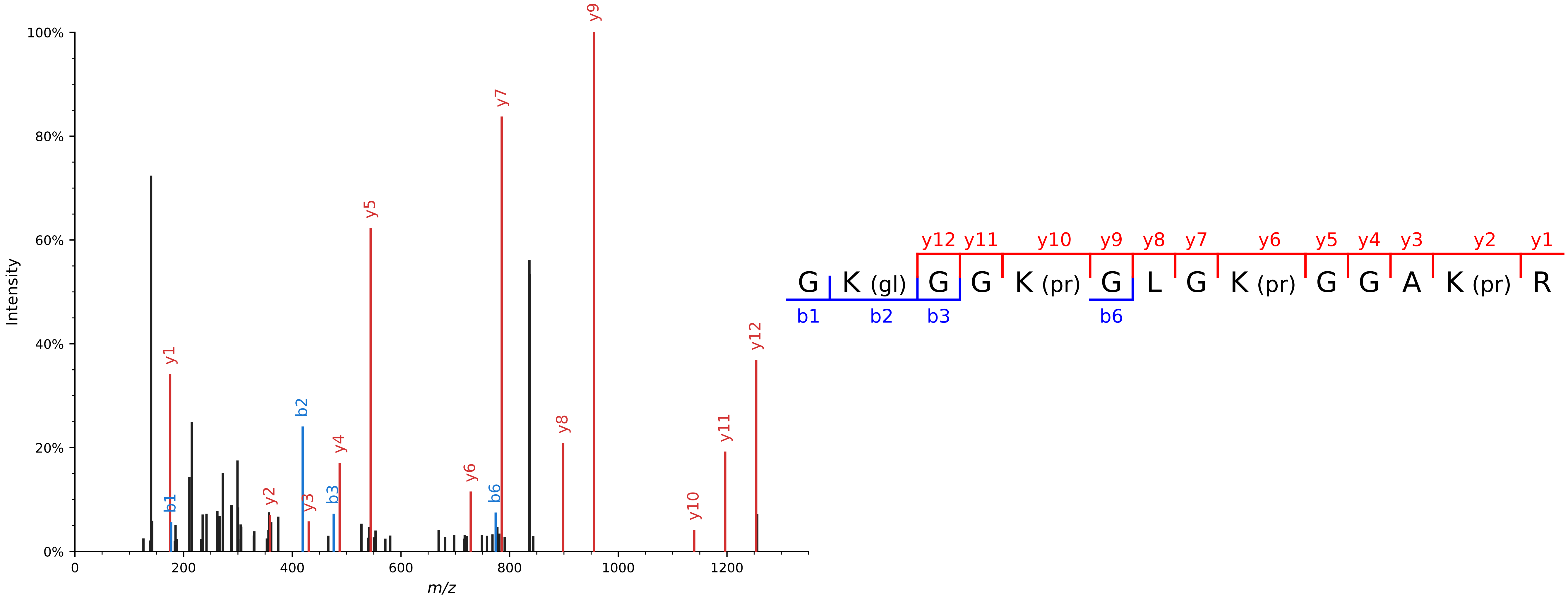

Raw file: HF240124_RN_spleen_PRO_PIC_1, Scan: 14623, m/z: 829.45465, Charge: 2, RT (min): 28.29, Score: 55.314

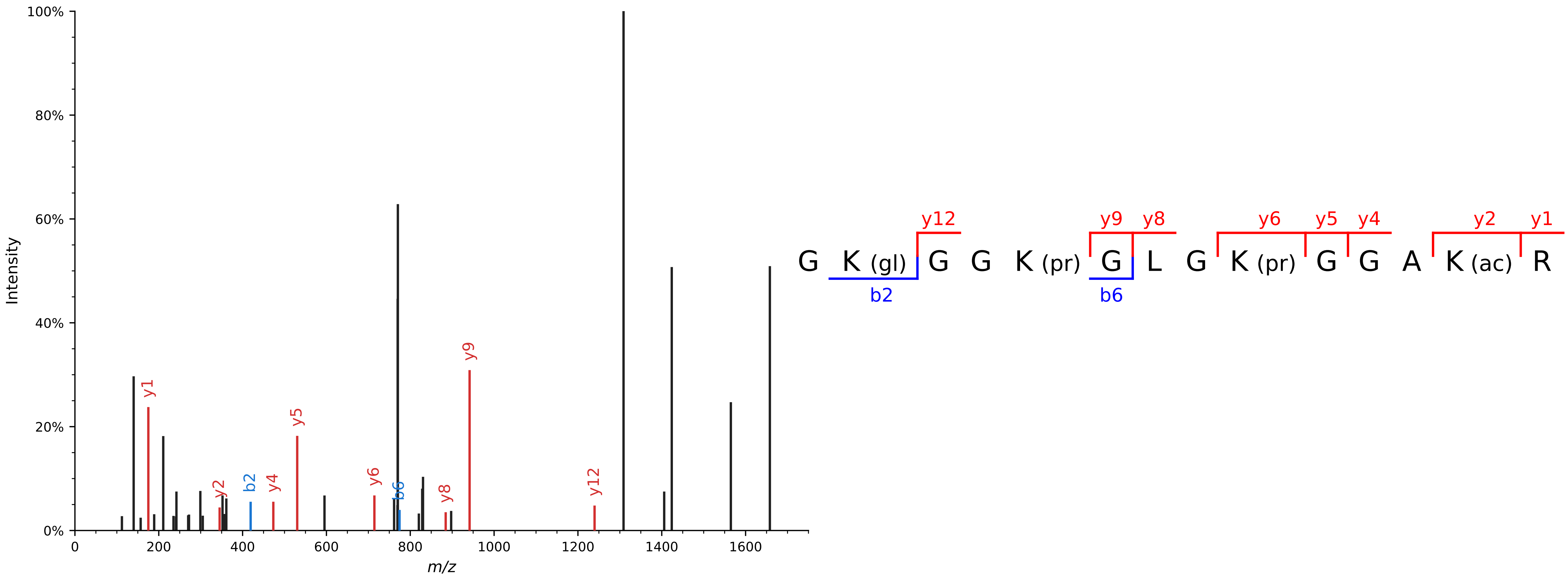

Raw file: HF240124_RN_spleen_PRO_PIC_2, Scan: 14192, m/z: 815.4572, Charge: 2, RT (min): 26.46, Score: 79.82

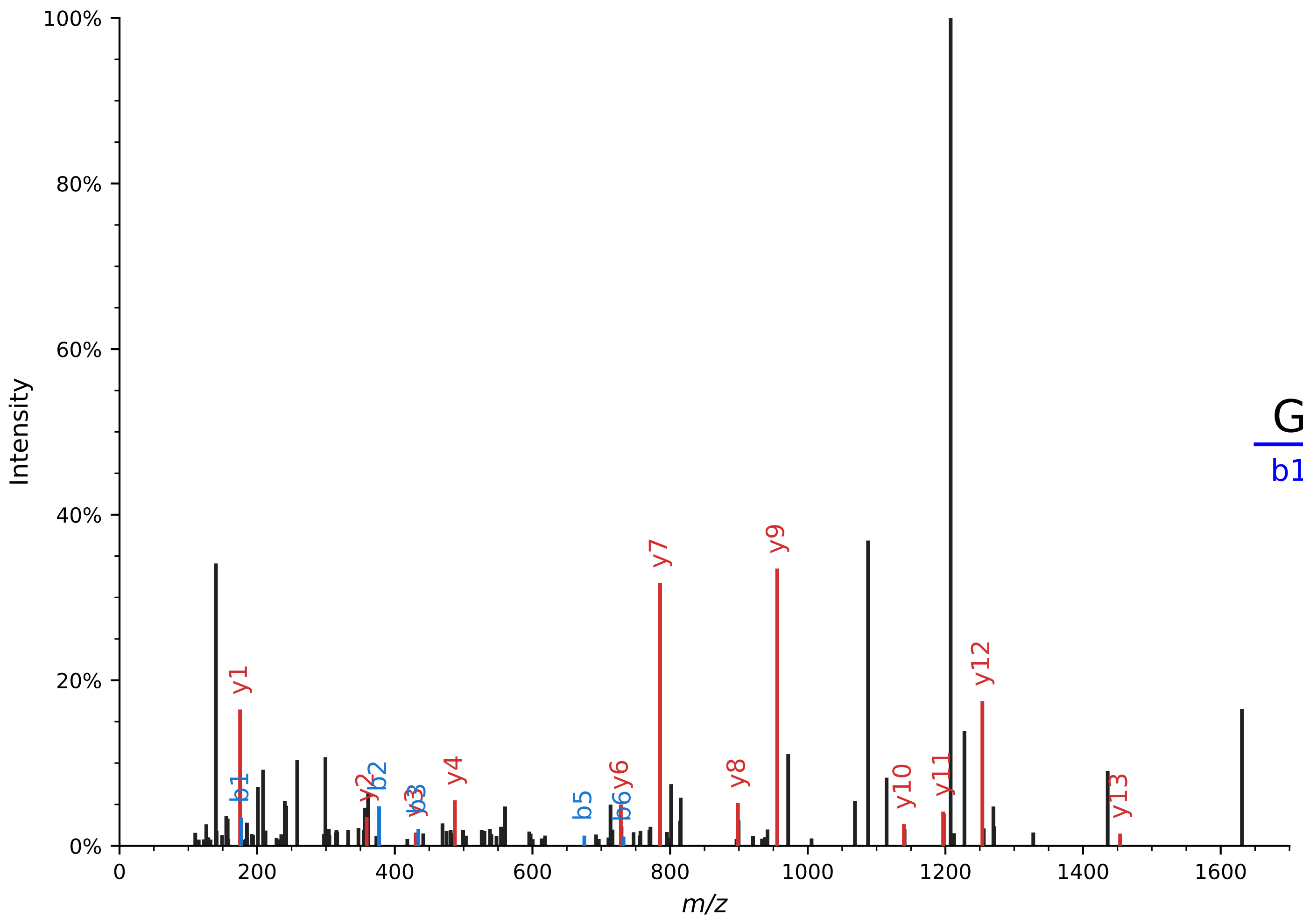

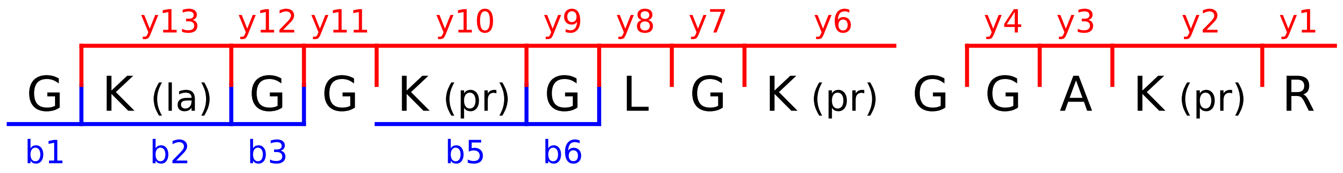

Raw file: HF240124_RN_spleen_PRO_PIC_3, Scan: 14822, m/z: 823.45465, Charge: 2, RT (min): 27.52, Score: 48.004

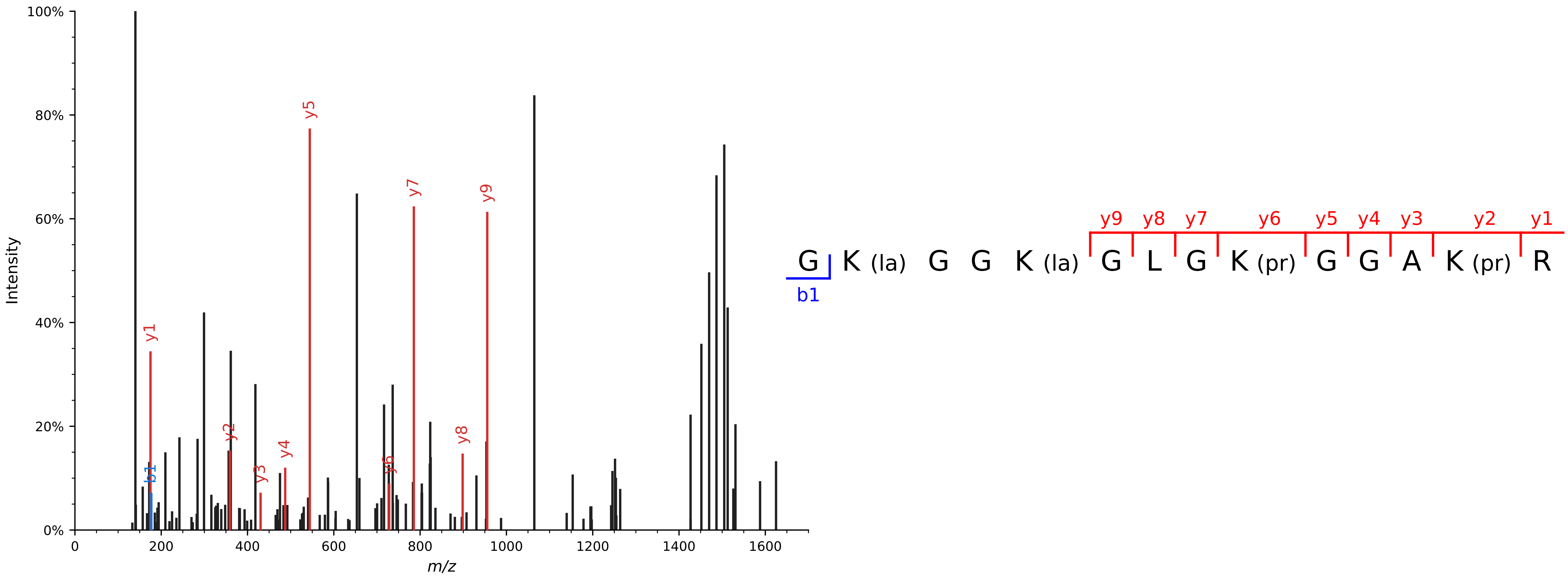

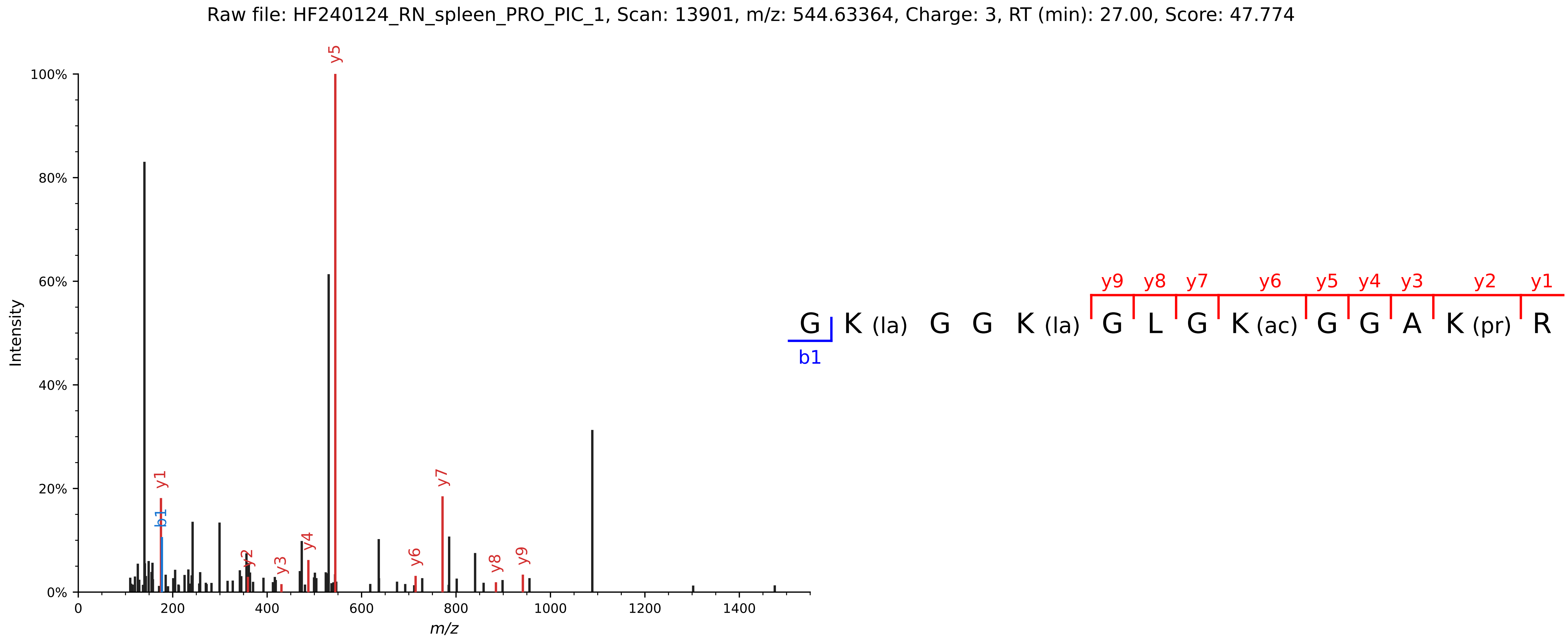

Raw file: HF240124_RN_spleen_PRO_PIC_1, Scan: 13901, m/z: 544.63364, Charge: 3, RT (min): 27.00, Score: 47.774
Intensity
m/z
y1
b1
y2
y3
y4
y5
y6
y7
y8
y9
G K (la) G G K (la) G L G K (ac) G G A K (pr) R

Raw file: HF240124_RN_spleen_PRO_PIC_3, Scan: 13882, m/z: 816.44683, Charge: 2, RT (min): 25.91, Score: 57.019

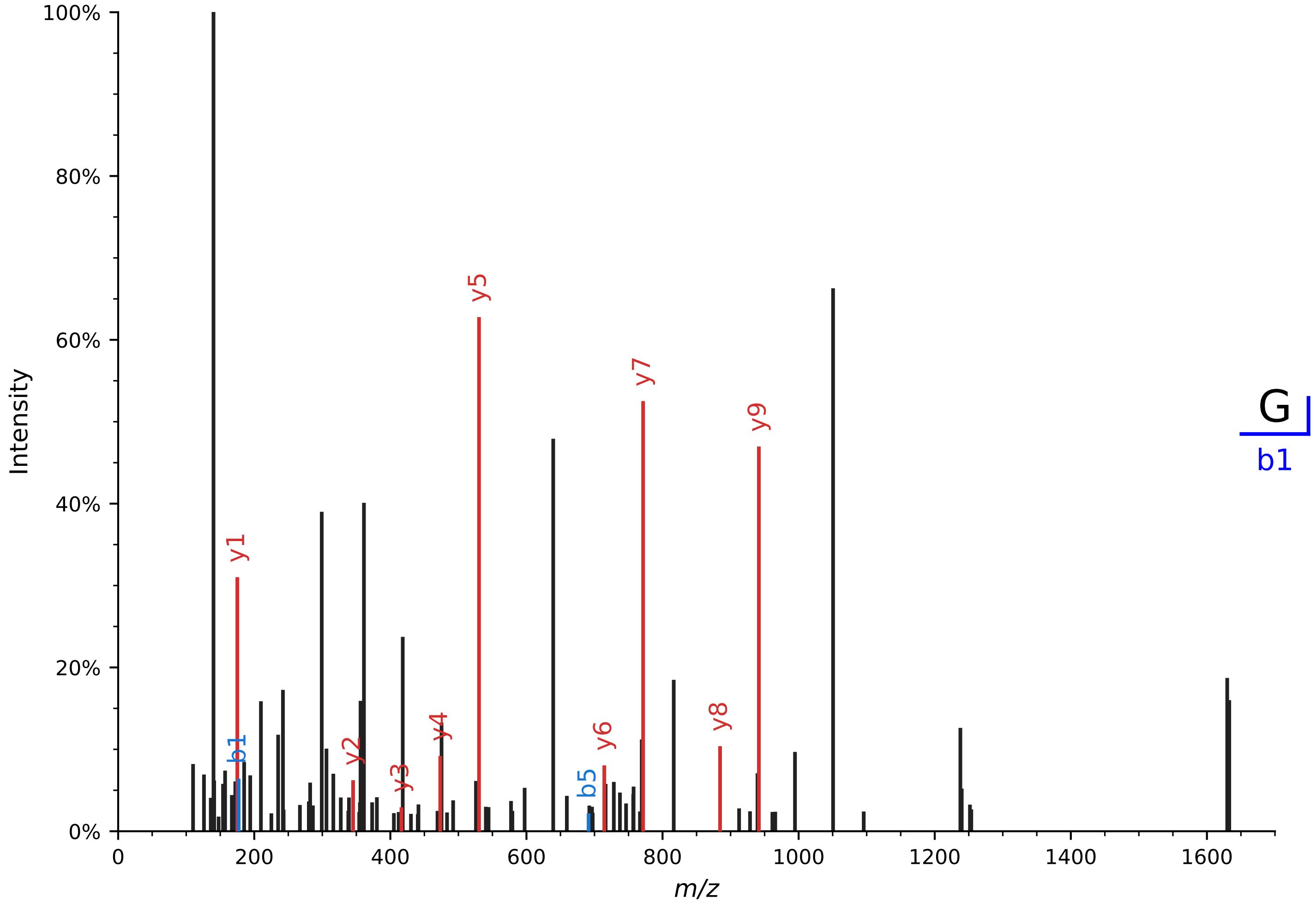

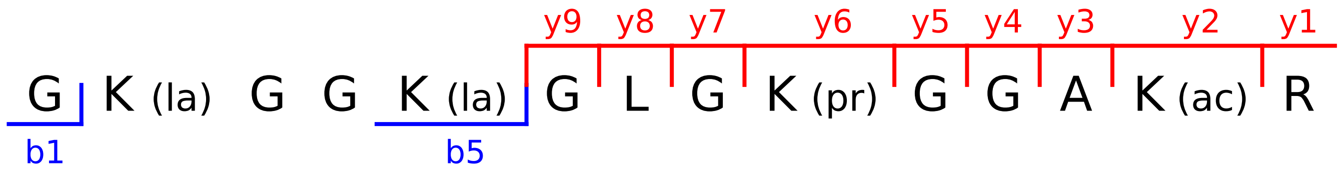

Raw file: HF240124_RN_spleen_PRO_PIC_2, Scan: 16300, m/z: 843.4703, Charge: 2, RT (min): 30.18, Score: 90.108

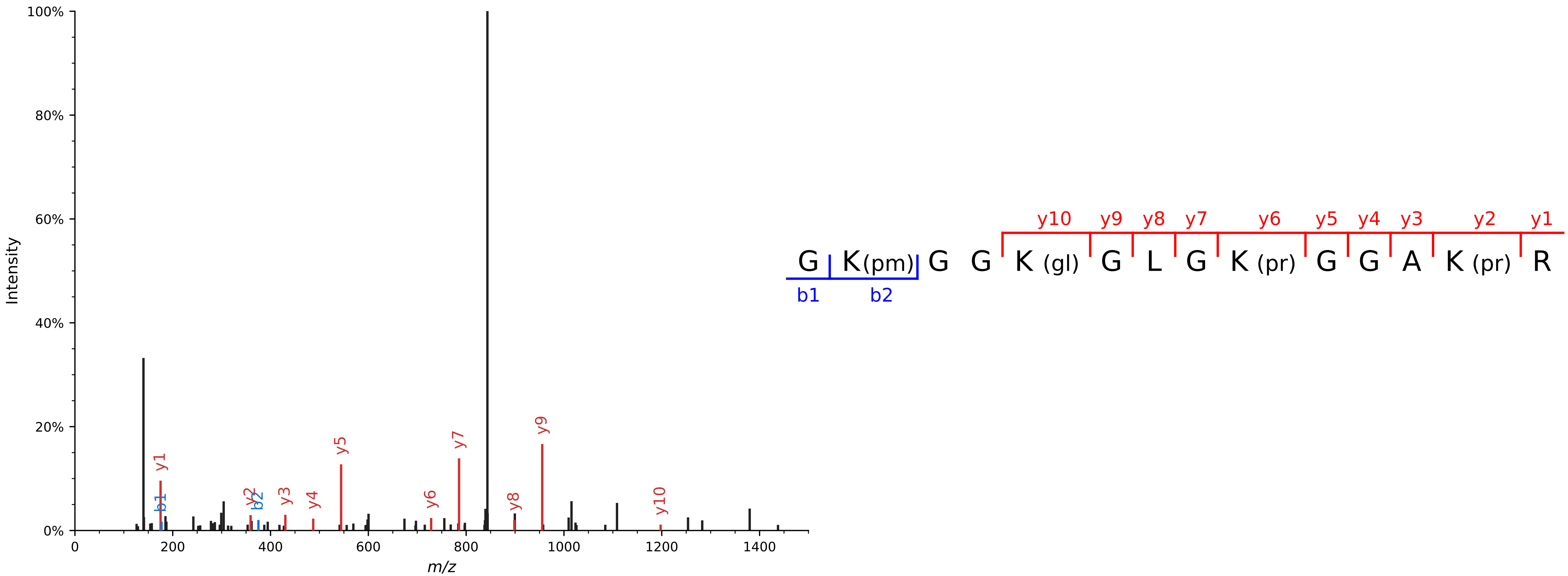

Raw file: HF240124_RN_spleen_PRO_PIC_1, Scan: 12694, m/z: 793.44409, Charge: 2, RT (min): 24.84, Score: 145.55

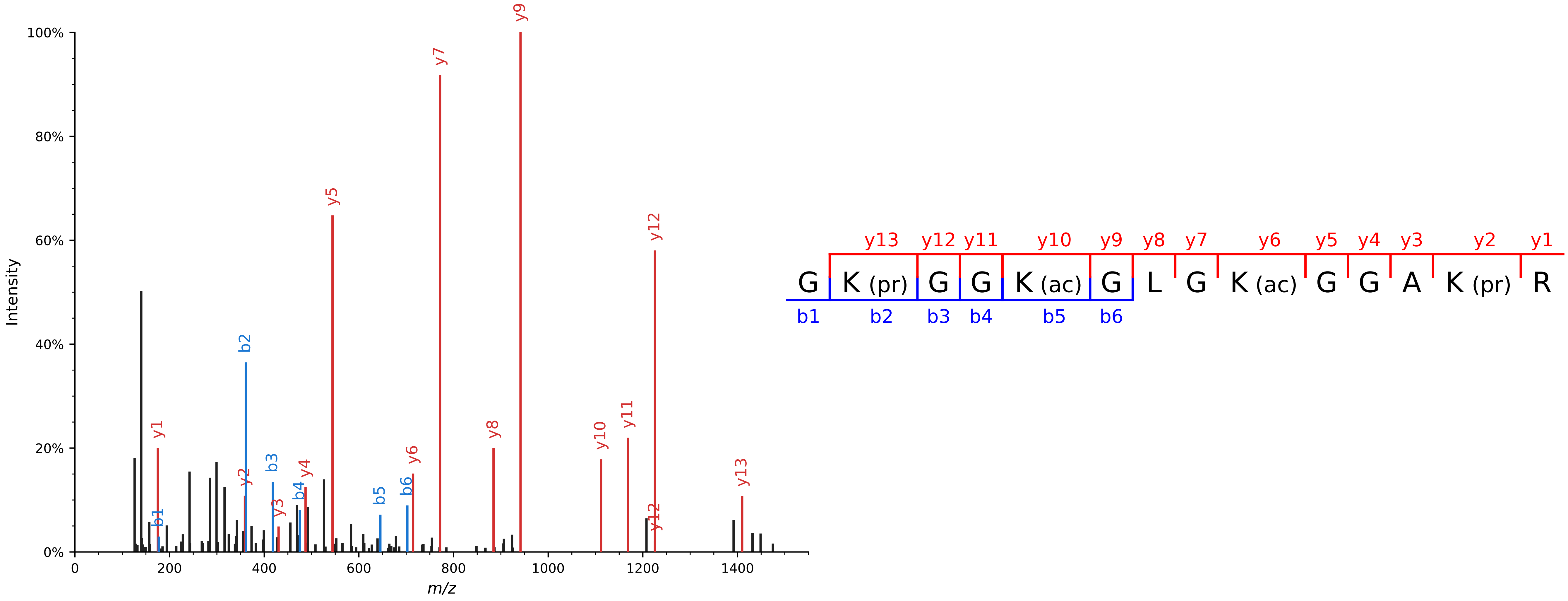

Raw file: HF240124_RN_spleen_PRO_PIC_2, Scan: 12370, m/z: 786.43626, Charge: 2, RT (min): 23.21, Score: 156.51

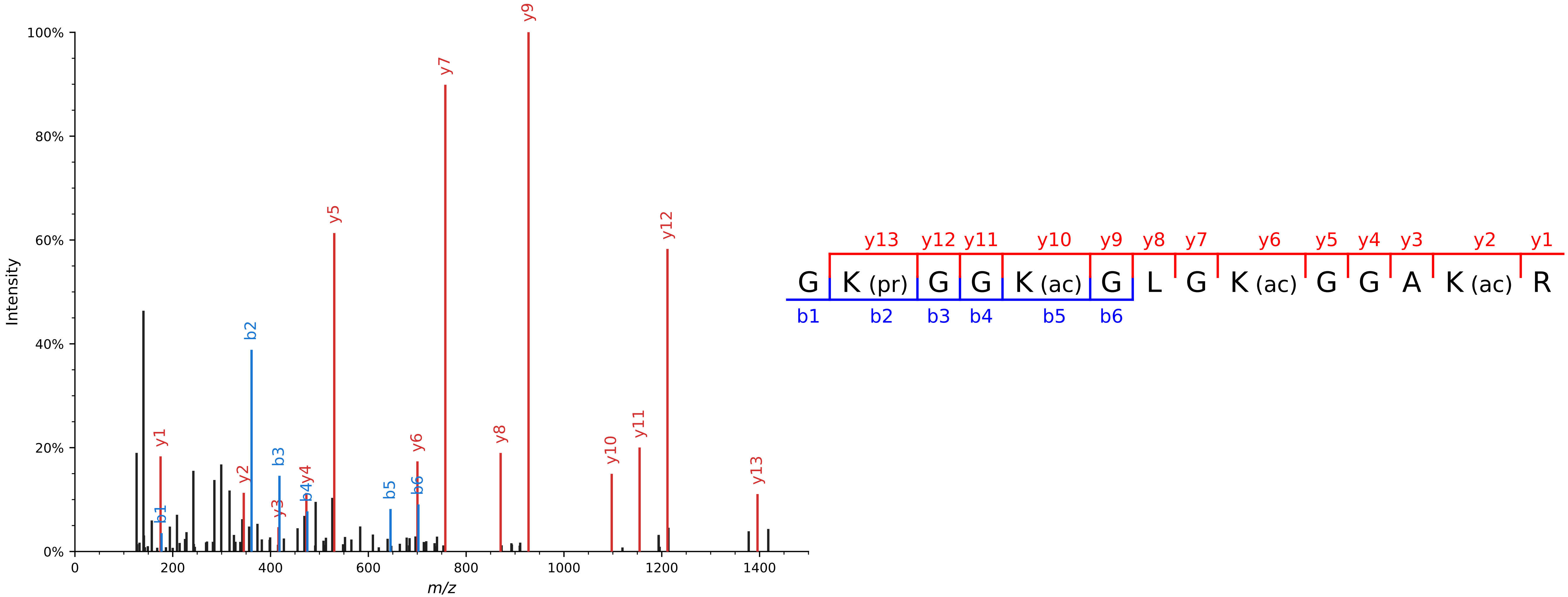

Raw file: HF240124_RN_spleen_PRO_PIC_1, Scan: 13201, m/z: 529.29848, Charge: 3, RT (min): 25.75, Score: 62.68

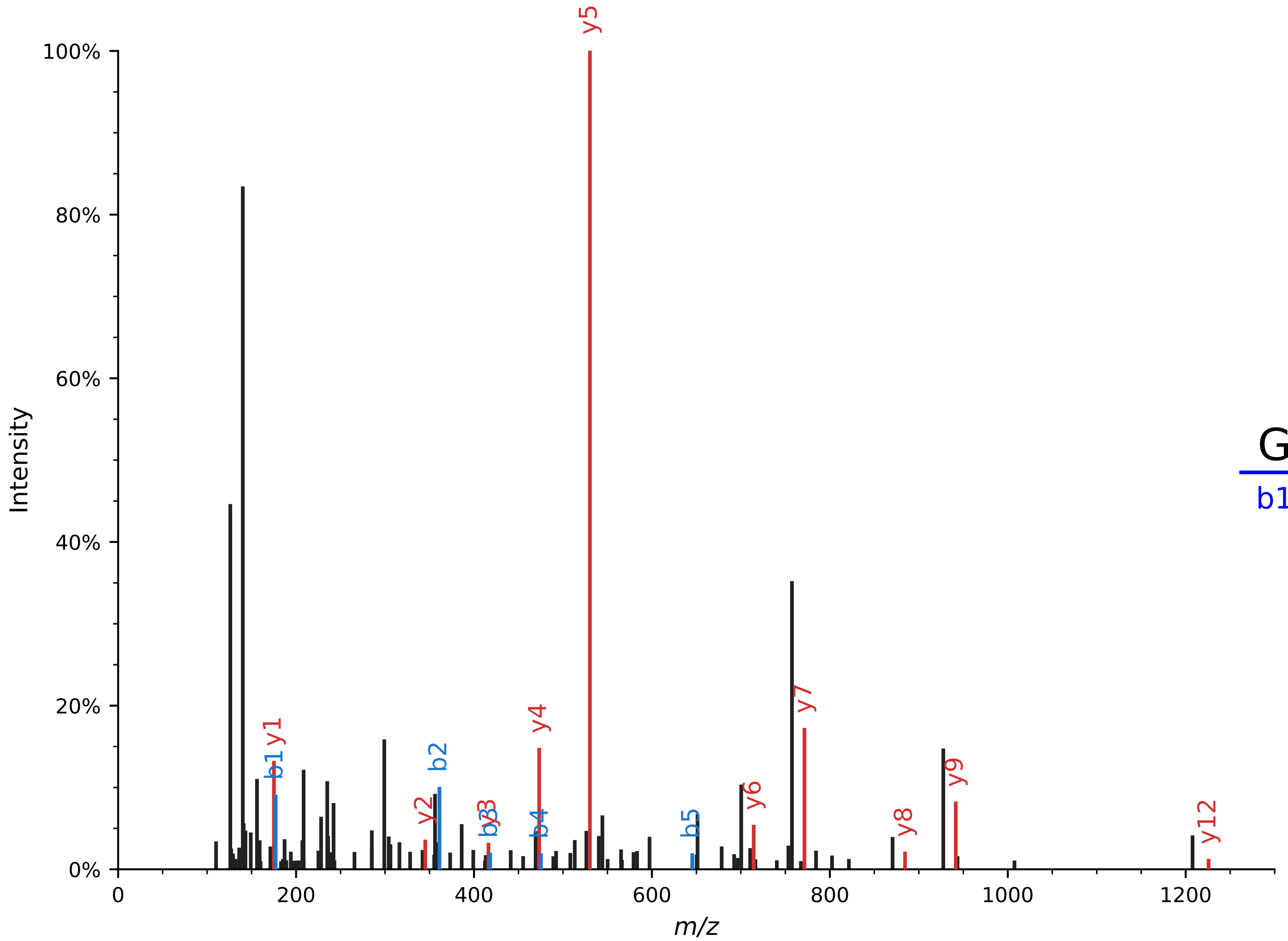

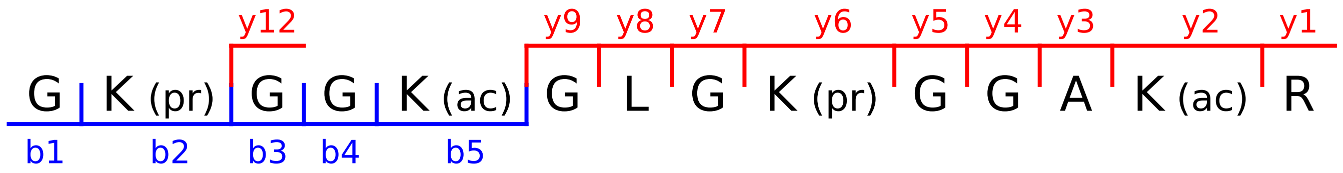

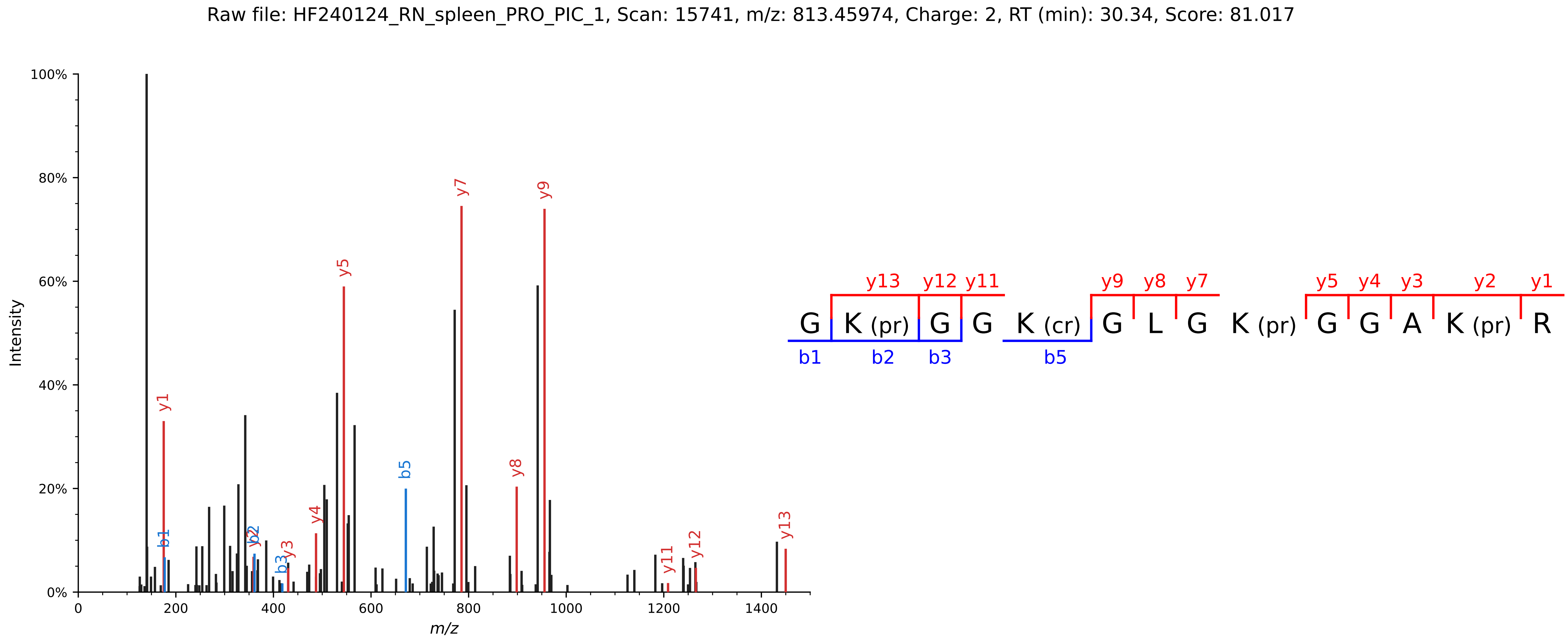

Raw file: HF240124_RN_spleen_PRO_PIC_1, Scan: 15741, m/z: 813.45974, Charge: 2, RT (min): 30.34, Score: 81.017
Intensity
m/z
G K (pr) G G K (cr) G L G K (pr) G G A K (pr) R

Raw file: HF240124_RN_spleen_PRO_PIC_3, Scan: 15248, m/z: 806.45191, Charge: 2, RT (min): 28.26, Score: 105.46

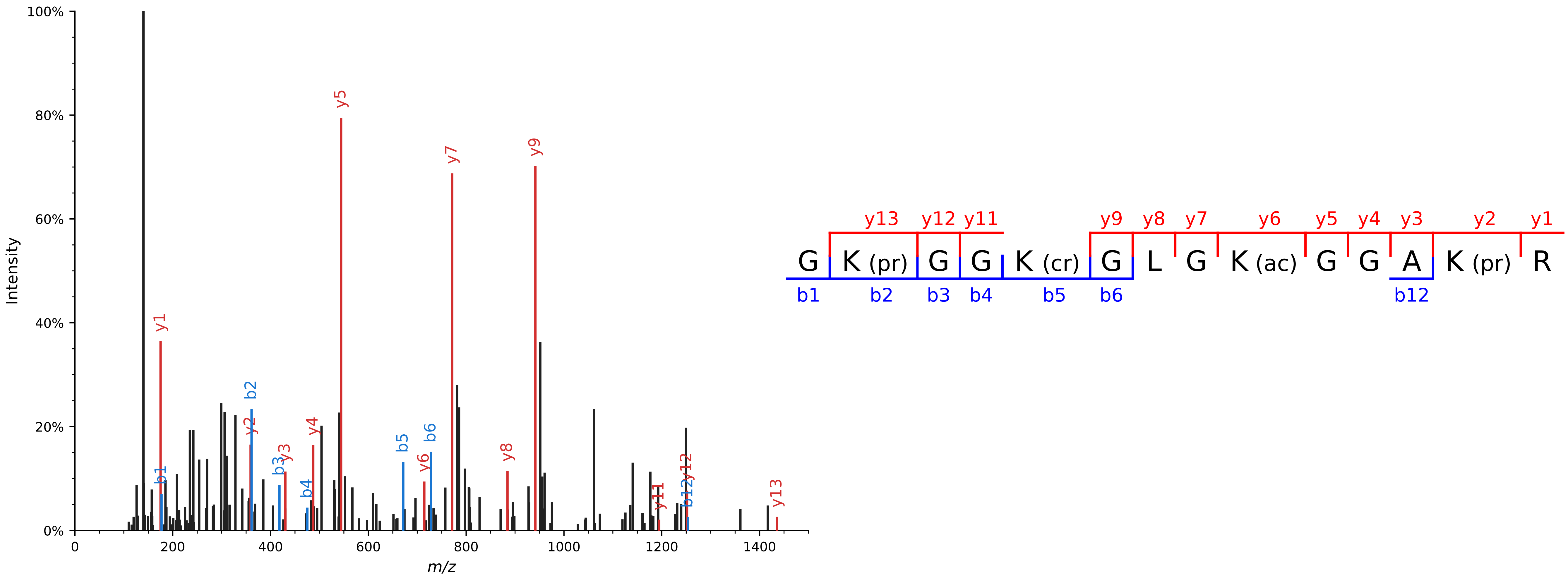

Raw file: HF240124_RN_spleen_PRO_PIC_1, Scan: 13504, m/z: 793.44409, Charge: 2, RT (min): 26.30, Score: 136.02

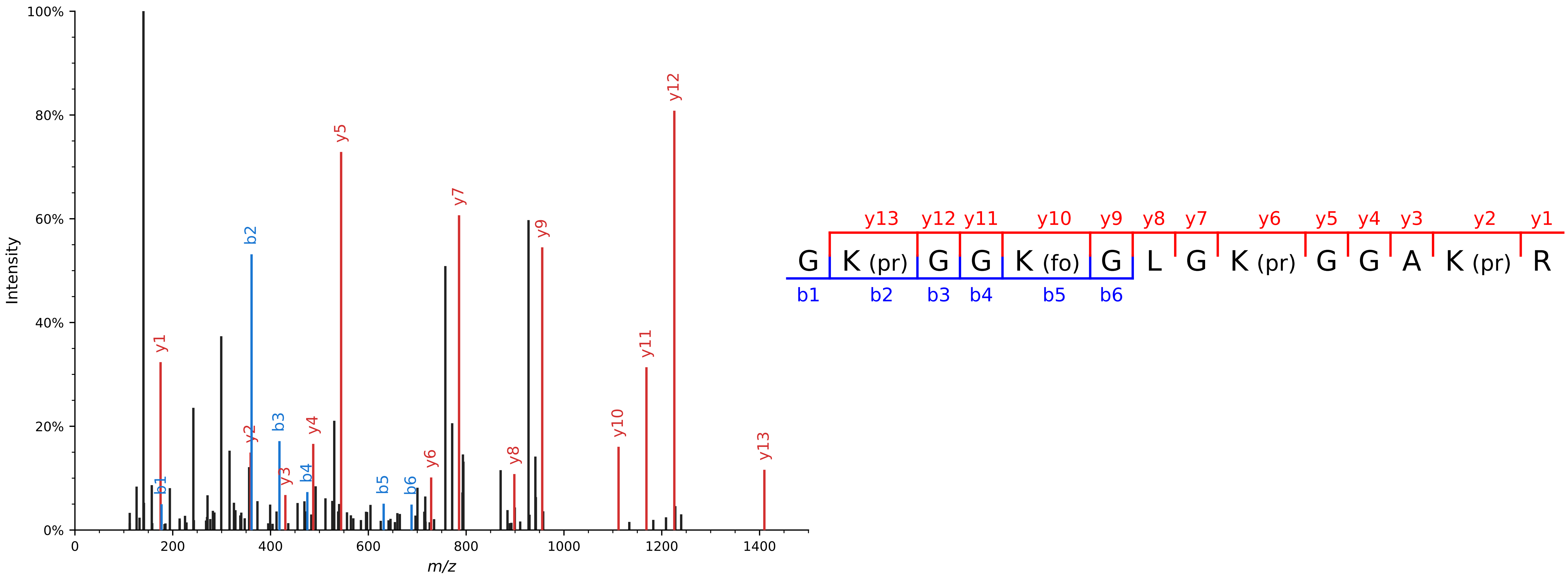

Raw file: HF240124_RN_spleen_PRO_PIC_1, Scan: 15601, m/z: 836.46248, Charge: 2, RT (min): 30.08, Score: 107.83

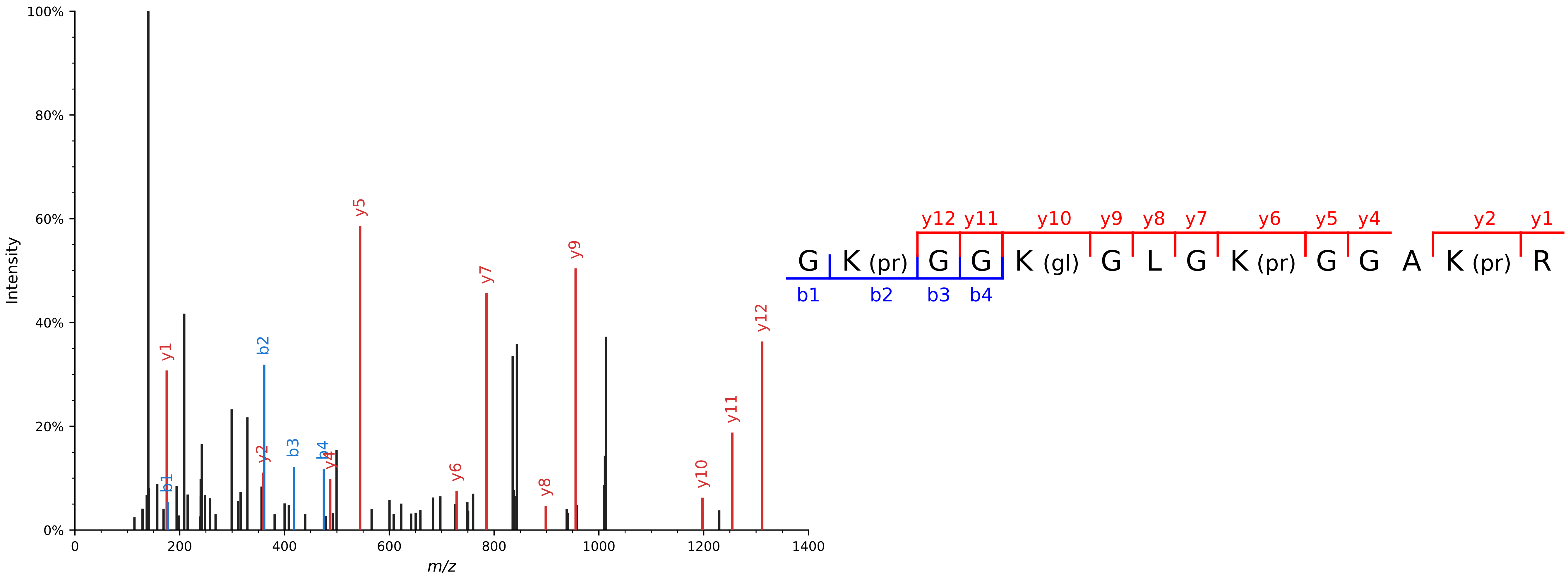

Raw file: HF240124_RN_spleen_PRO_PIC_3, Scan: 14972, m/z: 543.97389, Charge: 3, RT (min): 27.79, Score: 65.805

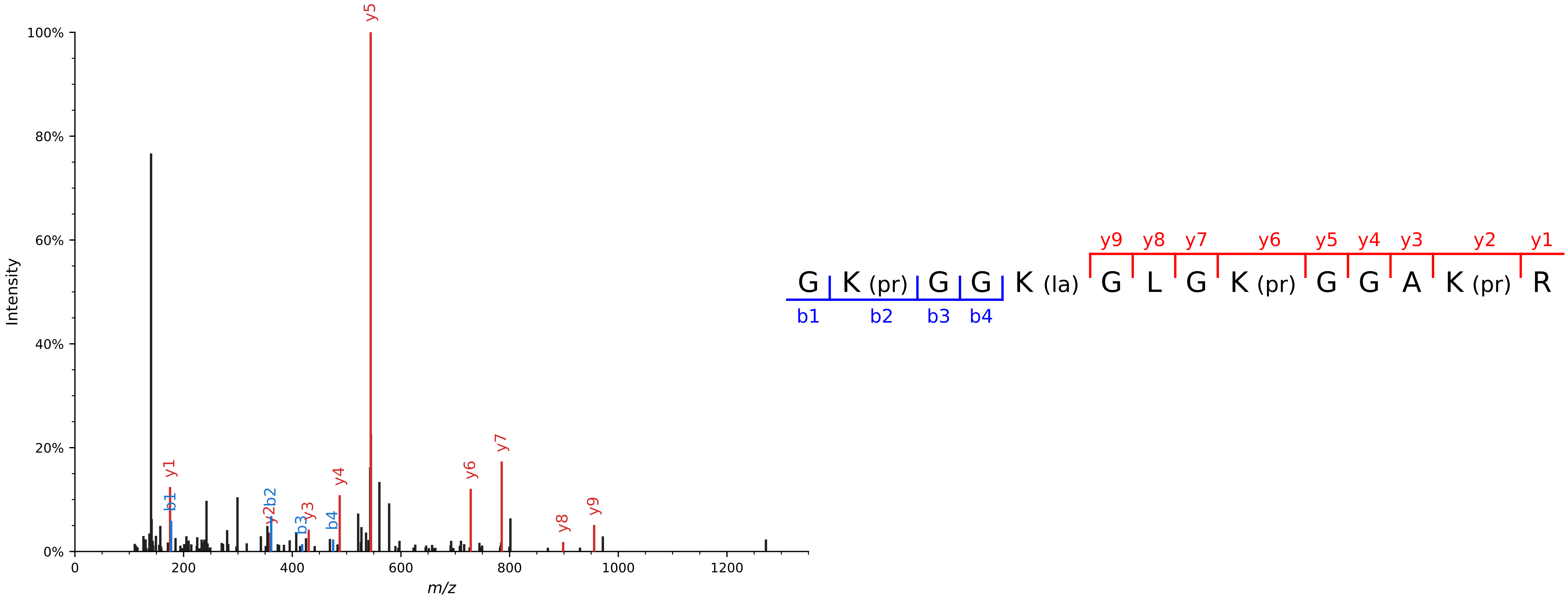

Raw file: HF240124_RN_spleen_PRO_PIC_2, Scan: 13640, m/z: 823.45465, Charge: 2, RT (min): 25.52, Score: 45.879

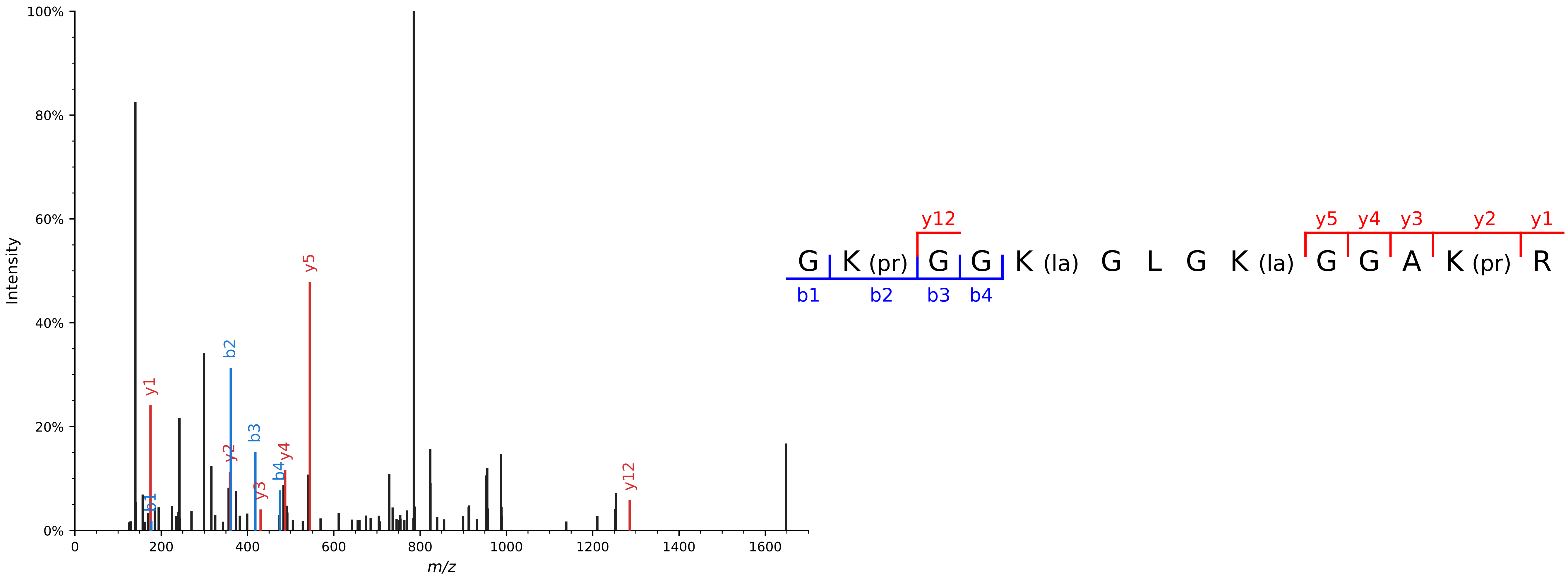

Raw file: HF240124_RN_spleen_PRO_PIC_1, Scan: 16340, m/z: 543.31413, Charge: 3, RT (min): 31.44, Score: 58.268

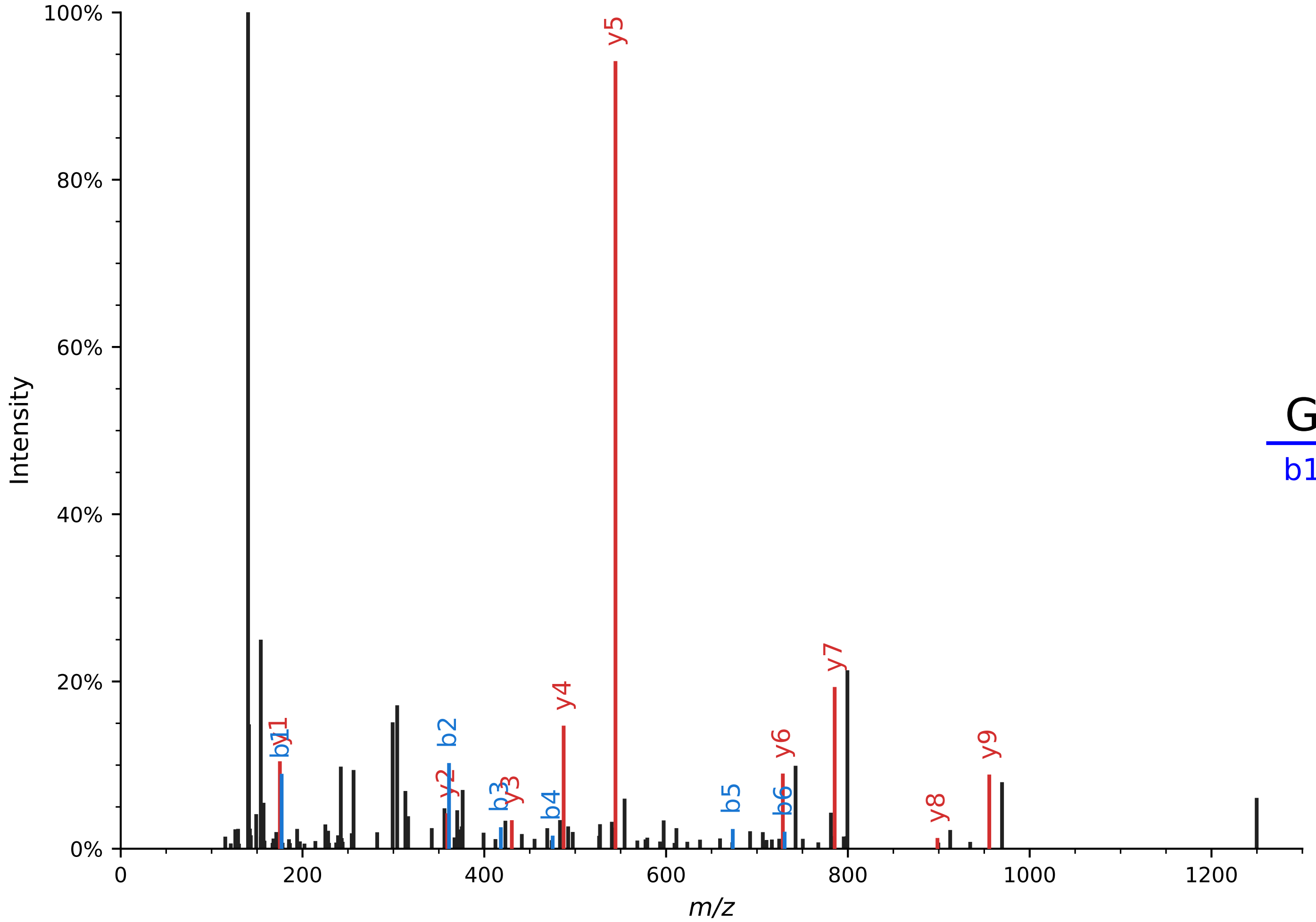

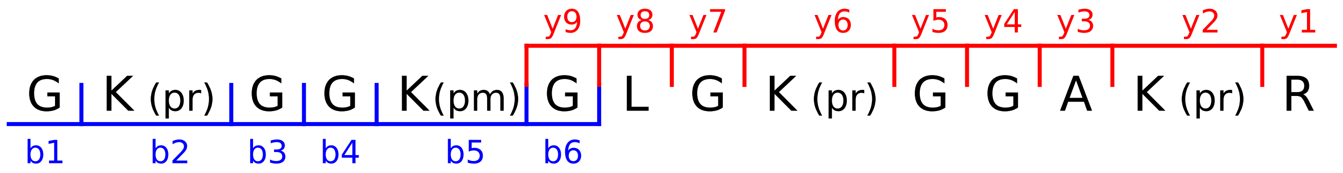

Raw file: HF240124_RN_spleen_PRO_PIC_1, Scan: 14224, m/z: 844.47813, Charge: 2, RT (min): 27.57, Score: 52.278

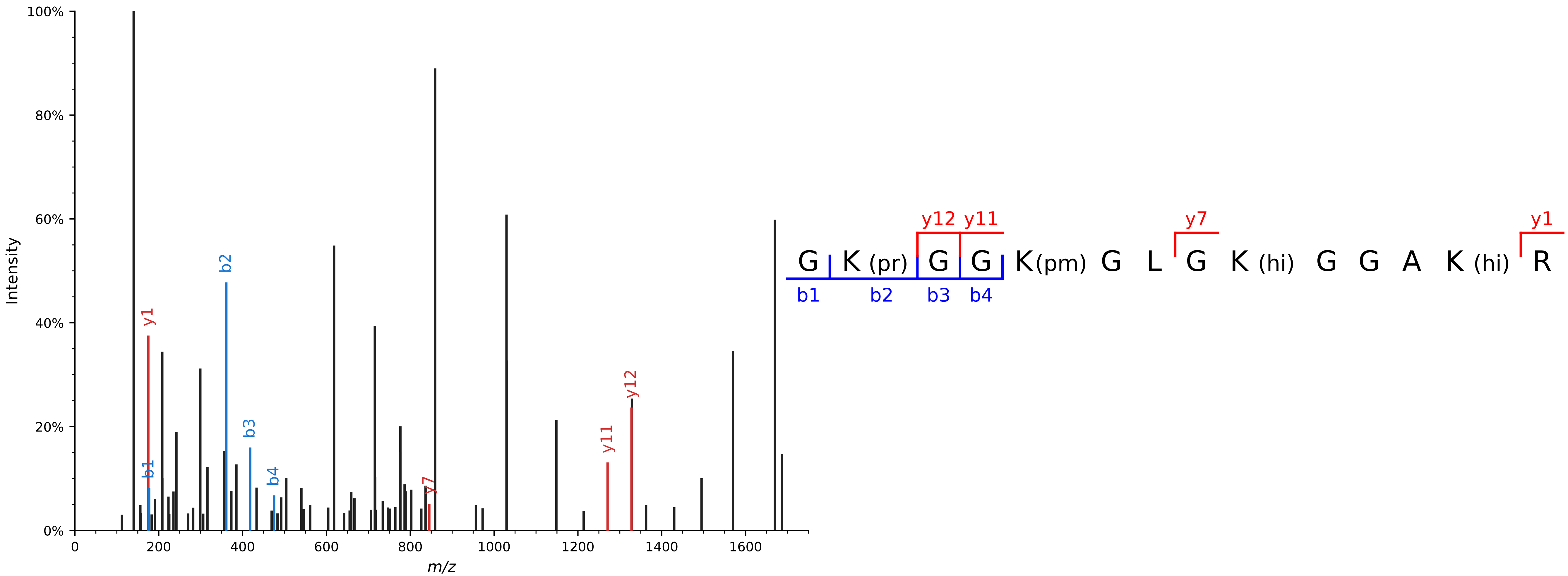

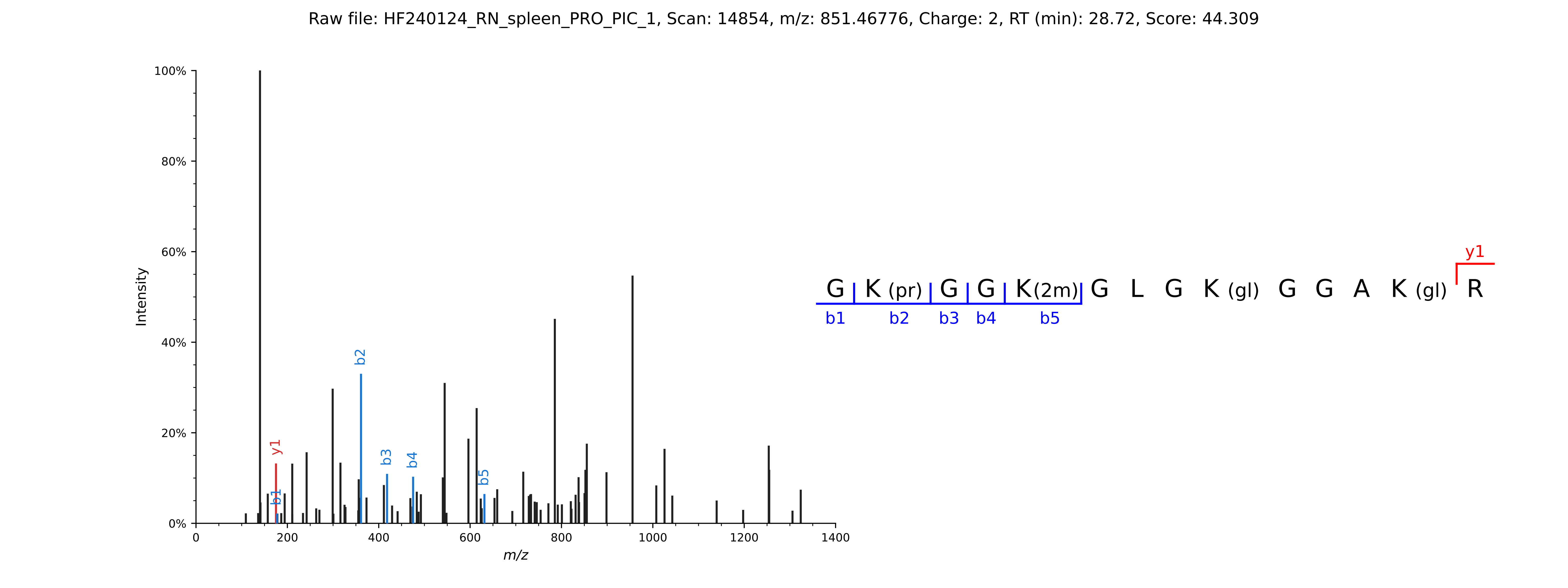

Raw file: HF240124_RN_spleen_PRO_PIC_1, Scan: 14854, m/z: 851.46776, Charge: 2, RT (min): 28.72, Score: 44.309
Intensity
m/z
y1
b1
b2
b3
b4
b5
G K (pr) G G K(2m) G L G K (gl) G G A K (gl) R

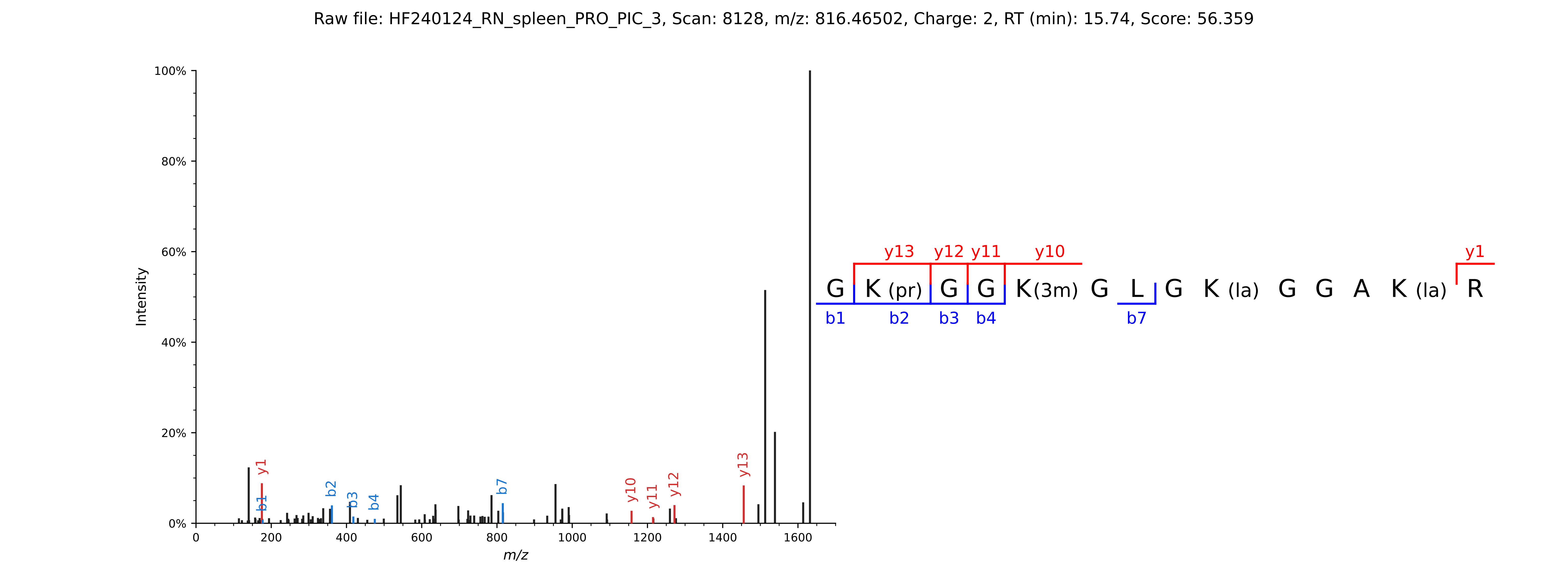

Raw file: HF240124_RN_spleen_PRO_PIC_3, Scan: 8128, m/z: 816.46502, Charge: 2, RT (min): 15.74, Score: 56.359
Intensity
m/z
y1
b1
b2
b3
b4
b7
y10
y11
y12
y13
G K (pr) G G K(3m) G L G K (la) G G A K (la) R

Raw file: HF240124_RN_spleen_PRO_PIC_1, Scan: 13821, m/z: 800.45191, Charge: 2, RT (min): 26.86, Score: 173.71

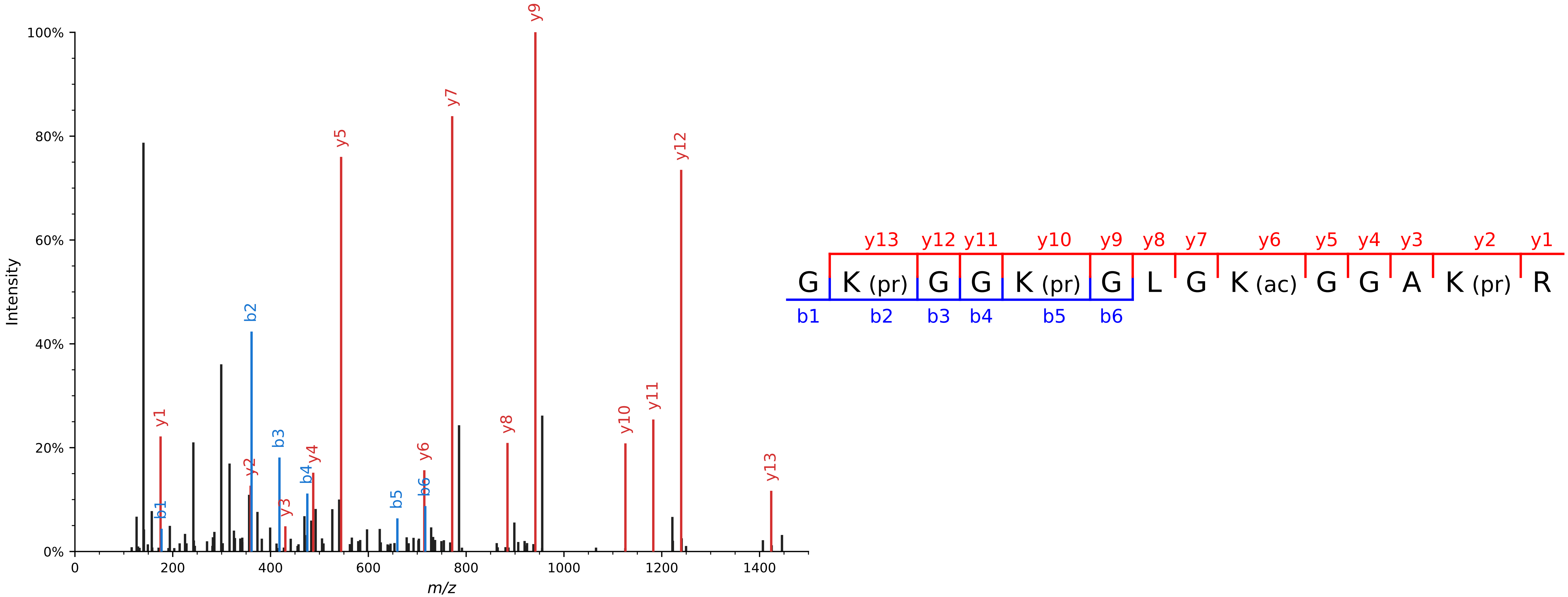

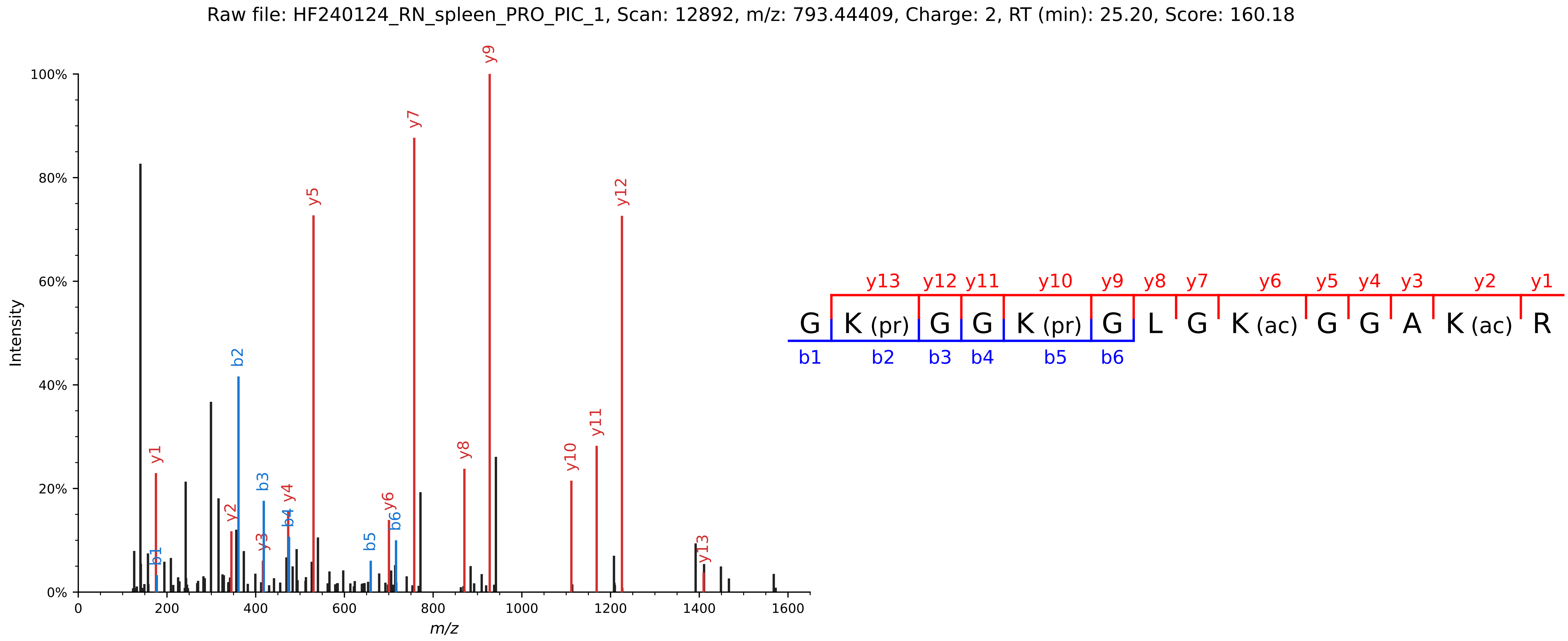
Raw file: HF240124_RN_spleen_PRO_PIC_1, Scan: 12892, m/z: 793.44409, Charge: 2, RT (min): 25.20, Score: 160.18
Intensity
m/z
G K (pr) G G K (pr) G L G K (ac) G G A K (ac) R
y13 y12 y11 y10 y9 y8 y7 y6 y5 y4 y3 y2 y1
b1 b2 b3 b4 b5 b6

Raw file: HF240124_RN_spleen_PRO_PIC_2, Scan: 16260, m/z: 836.46248, Charge: 2, RT (min): 30.10, Score: 118.4

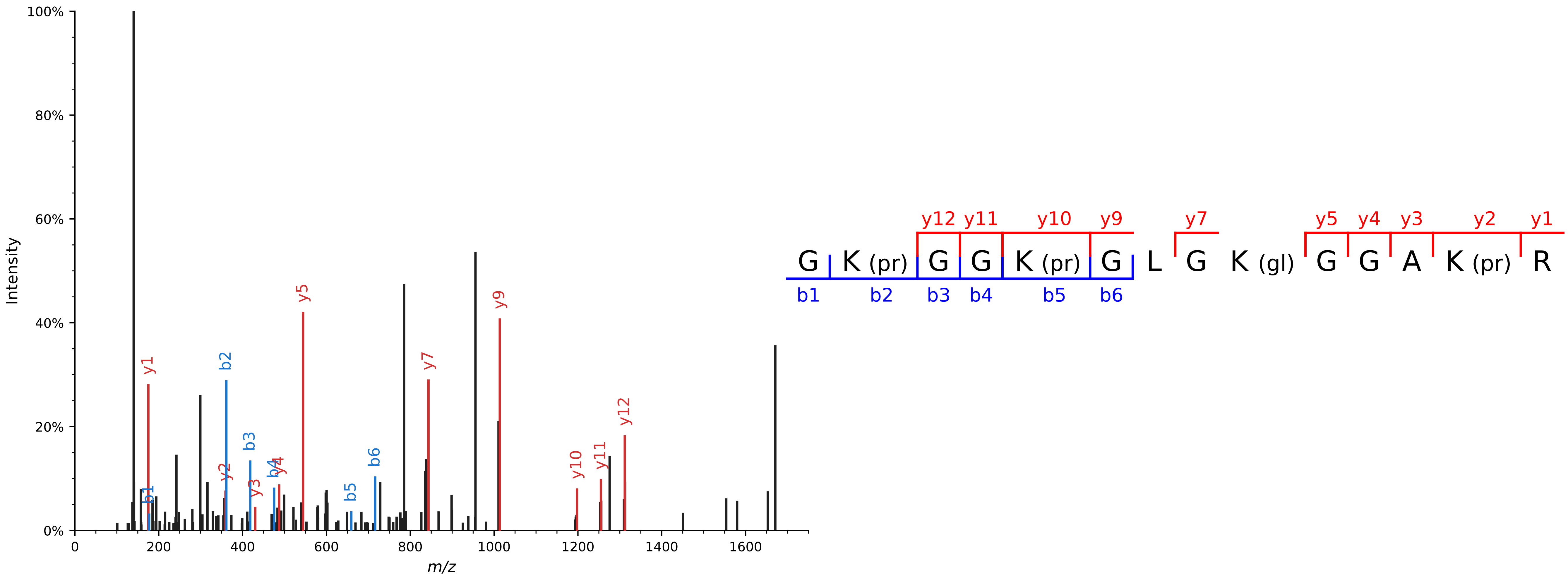

Raw file: HF240124_RN_spleen_PRO_PIC_3, Scan: 13345, m/z: 539.30201, Charge: 3, RT (min): 24.98, Score: 56.147

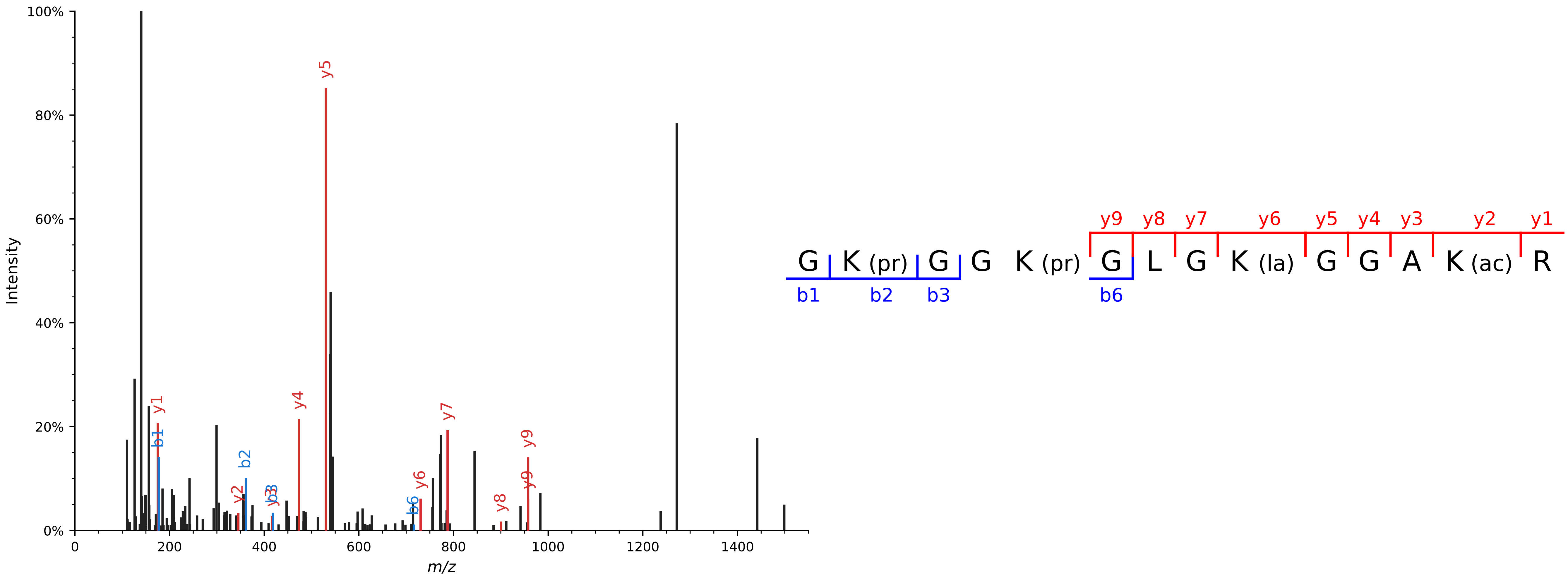

Raw file: HF240124_RN_spleen_PRO_PIC_3, Scan: 13400, m/z: 823.45465, Charge: 2, RT (min): 25.08, Score: 48.741

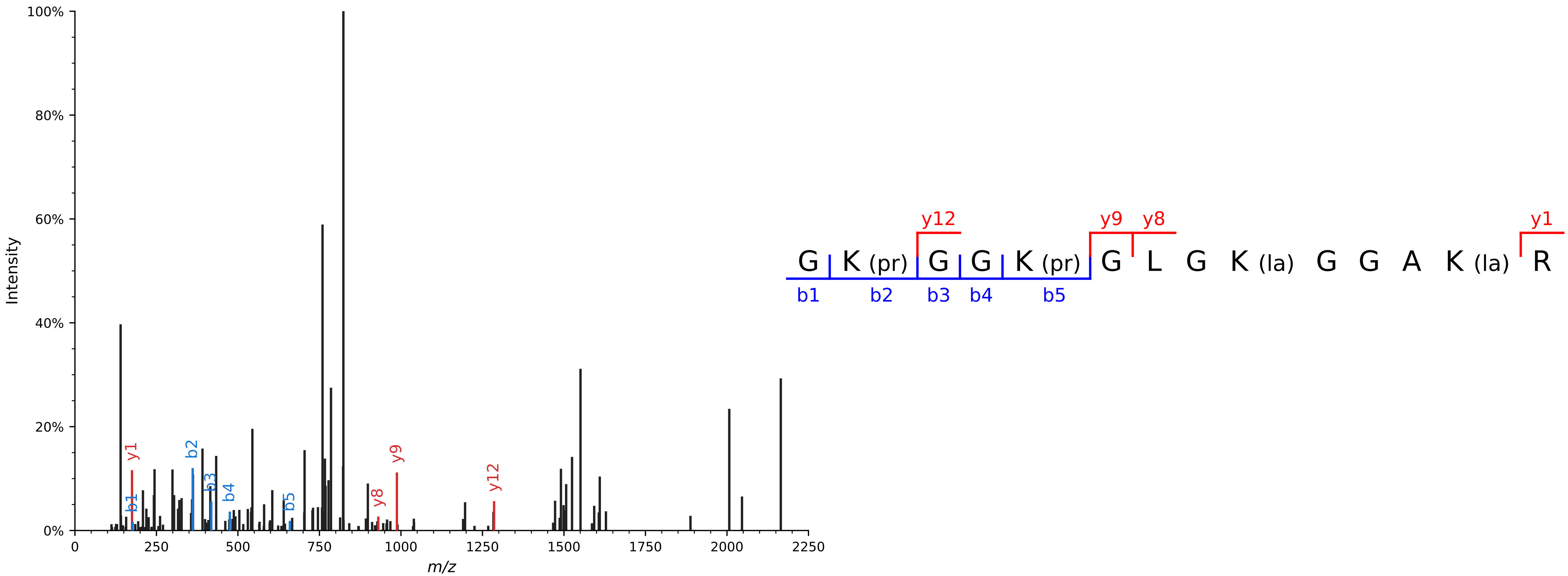

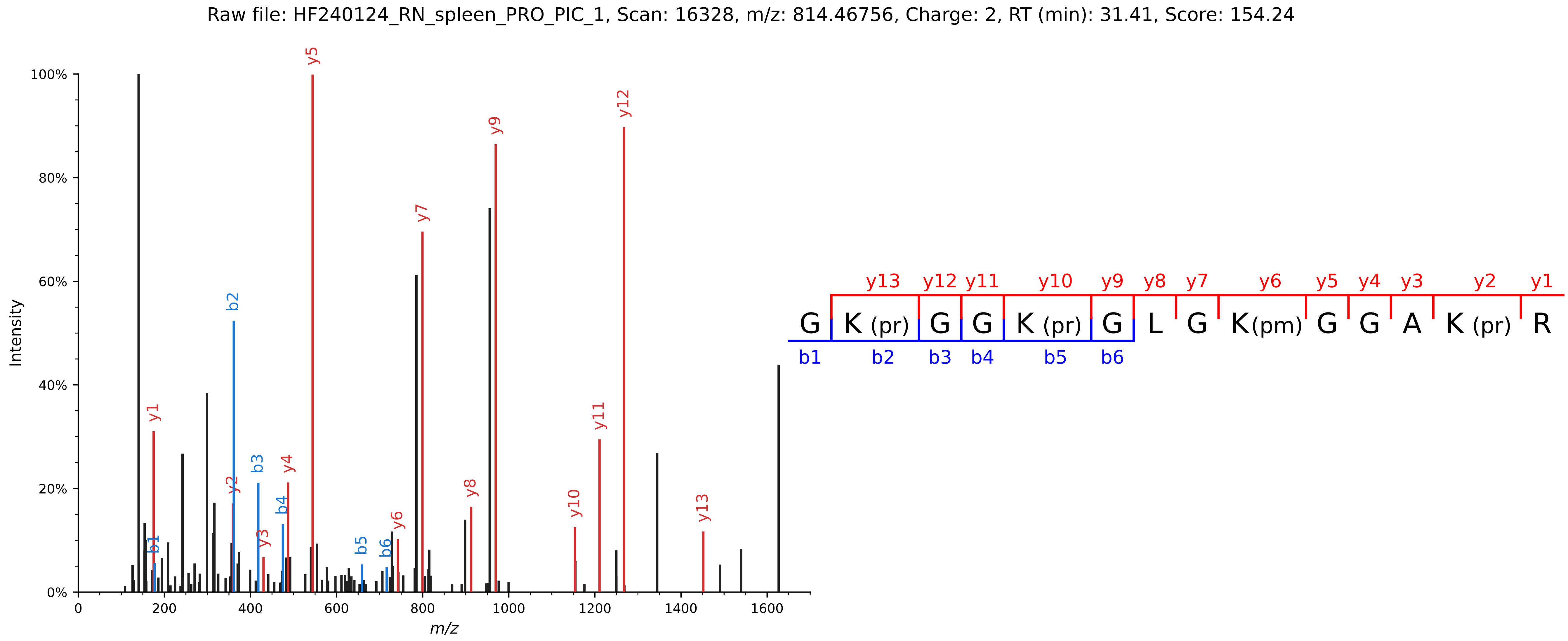

Raw file: HF240124_RN_spleen_PRO_PIC_1, Scan: 16328, m/z: 814.46756, Charge: 2, RT (min): 31.41, Score: 154.24
Intensity
m/z
y13 y12 y11 y10 y9 y8 y7 y6 y5 y4 y3 y2 y1
G K (pr) G G K (pr) G L G K(pm) G G A K (pr) R
b1 b2 b3 b4 b5 b6

Raw file: HF240124_RN_spleen_PRO_PIC_3, Scan: 19132, m/z: 843.4703, Charge: 2, RT (min): 35.16, Score: 82.417

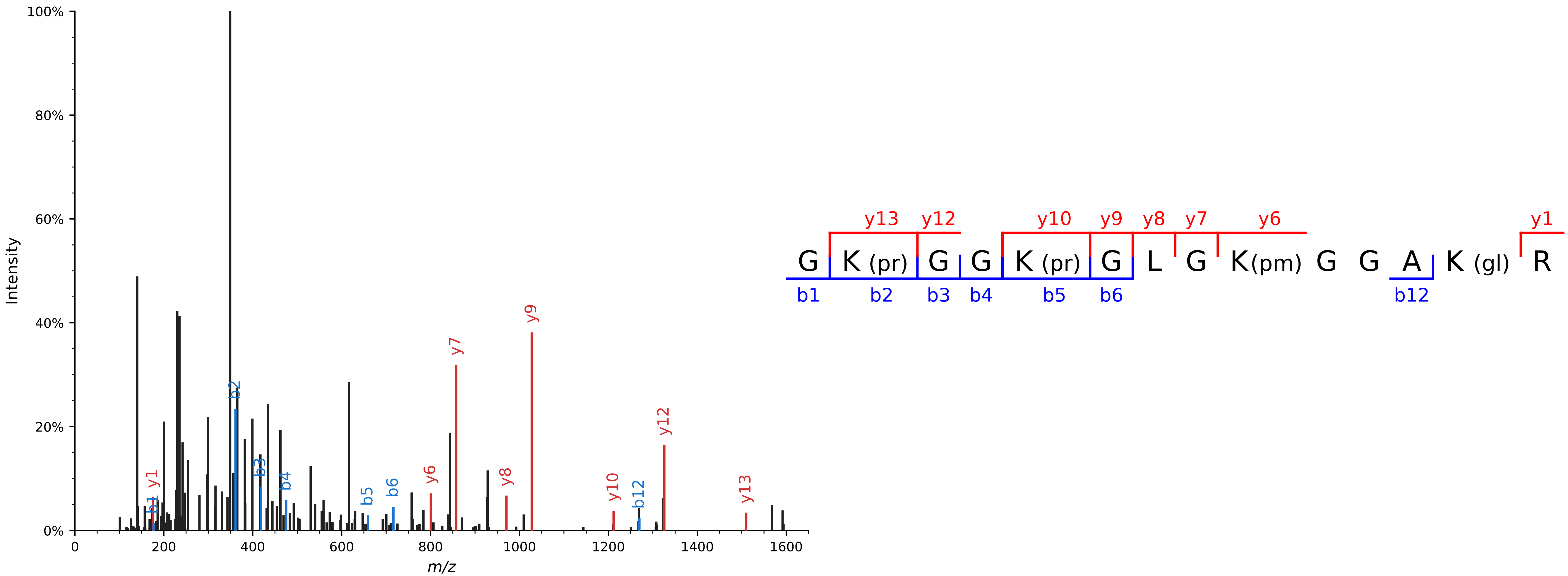

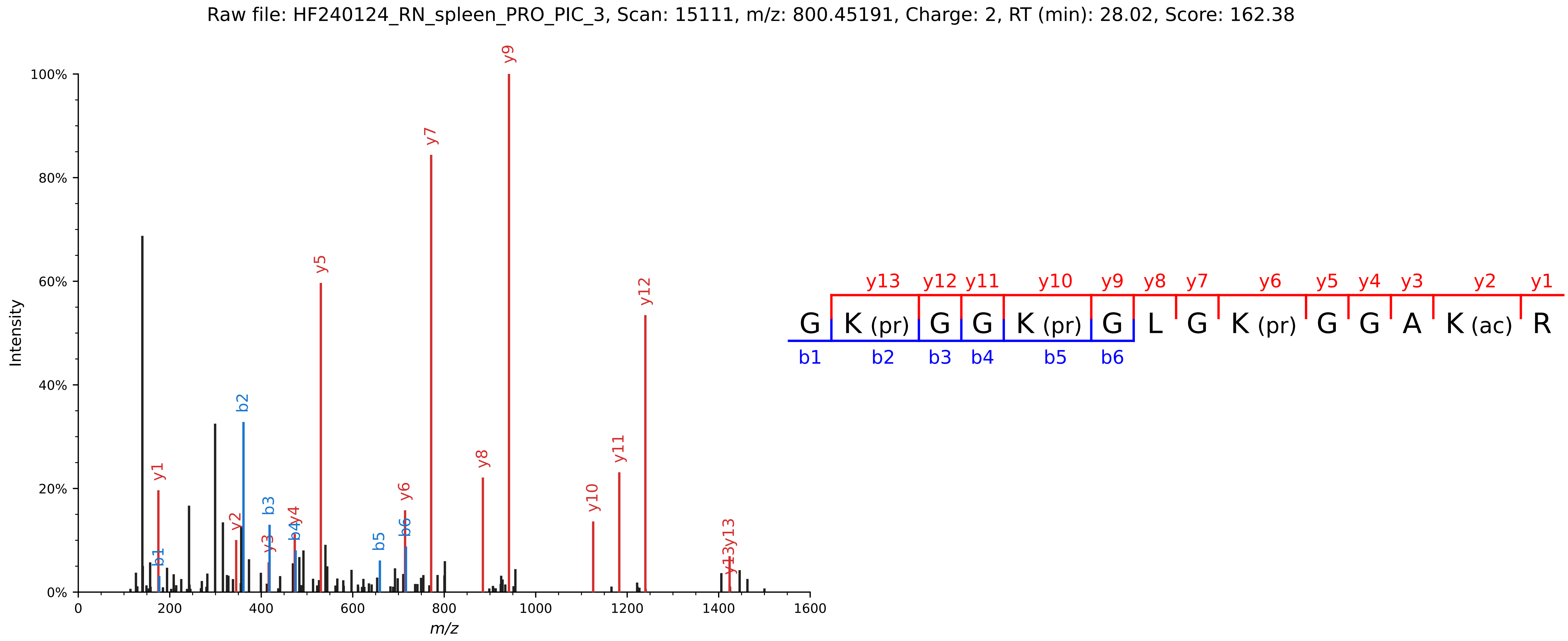

Raw file: HF240124_RN_spleen_PRO_PIC_3, Scan: 15111, m/z: 800.45191, Charge: 2, RT (min): 28.02, Score: 162.38
Intensity
m/z
0%
20%
40%
60%
80%
100%
0
200
400
600
800
1000
1200
1400
1600
y1
y2
y3
y4
y5
y6
y7
y8
y9
y10
y11
y12
y13
b1
b2
b3
b4
b5
b6
y13 y12 y11 y10 y9 y8 y7 y6 y5 y4 y3 y2 y1
G K (pr) G G K (pr) G L G K (pr) G G A K (ac) R
b1 b2 b3 b4 b5 b6

Raw file: HF240124_RN_spleen_PRO_PIC_2, Scan: 14406, m/z: 793.44409, Charge: 2, RT (min): 26.82, Score: 136.02

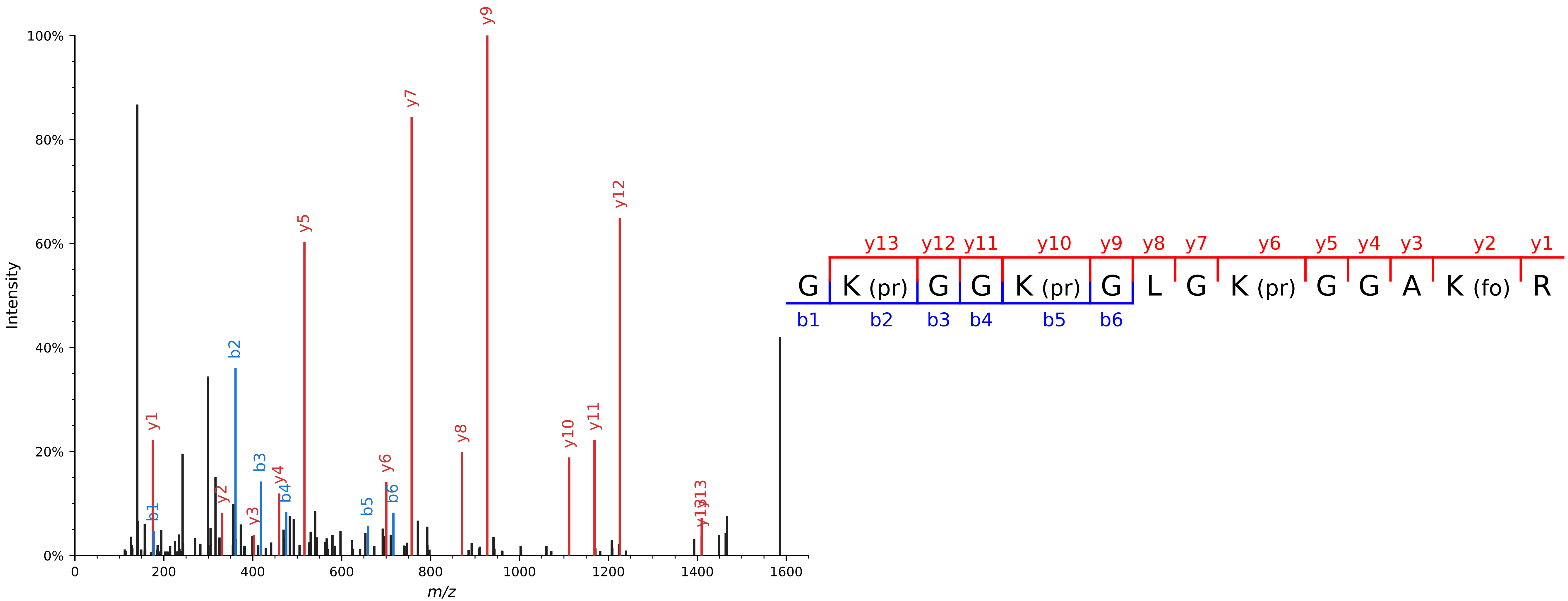

Raw file: HF240124_RN_spleen_PRO_PIC_1, Scan: 17351, m/z: 836.46248, Charge: 2, RT (min): 33.31, Score: 84.658

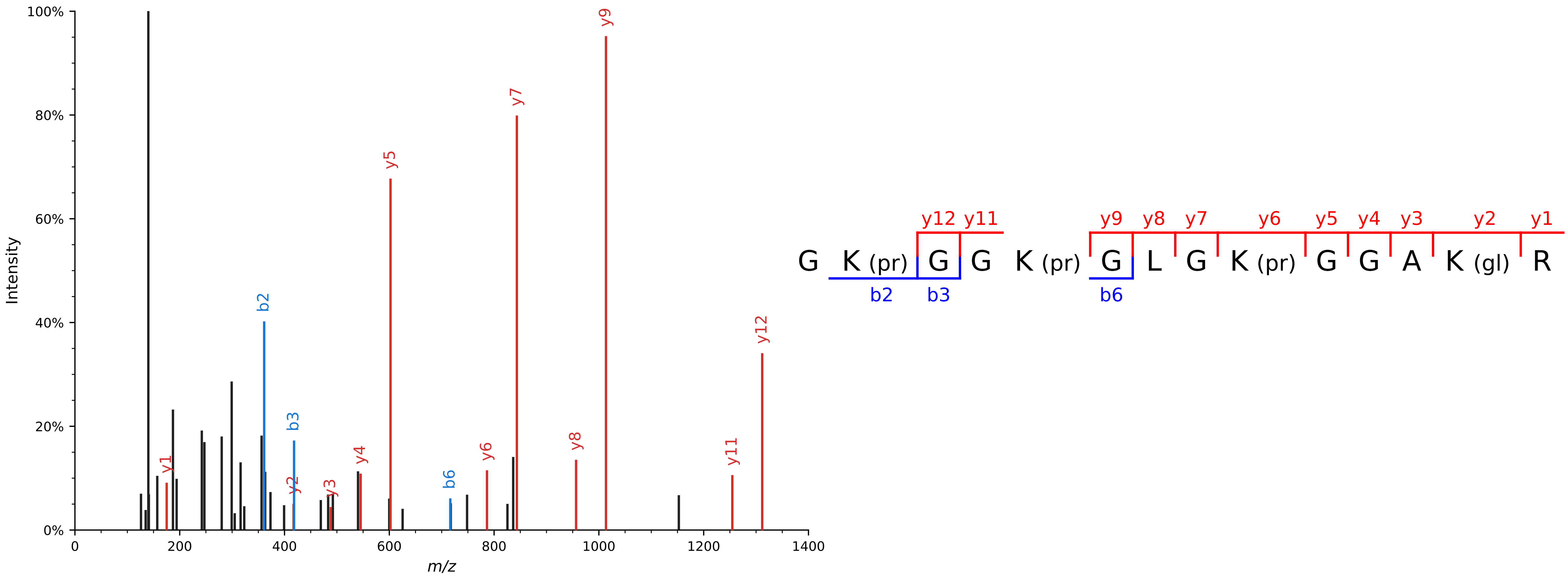

Raw file: HF240124_RN_spleen_PRO_PIC_2, Scan: 16766, m/z: 814.46756, Charge: 2, RT (min): 30.99, Score: 154.35

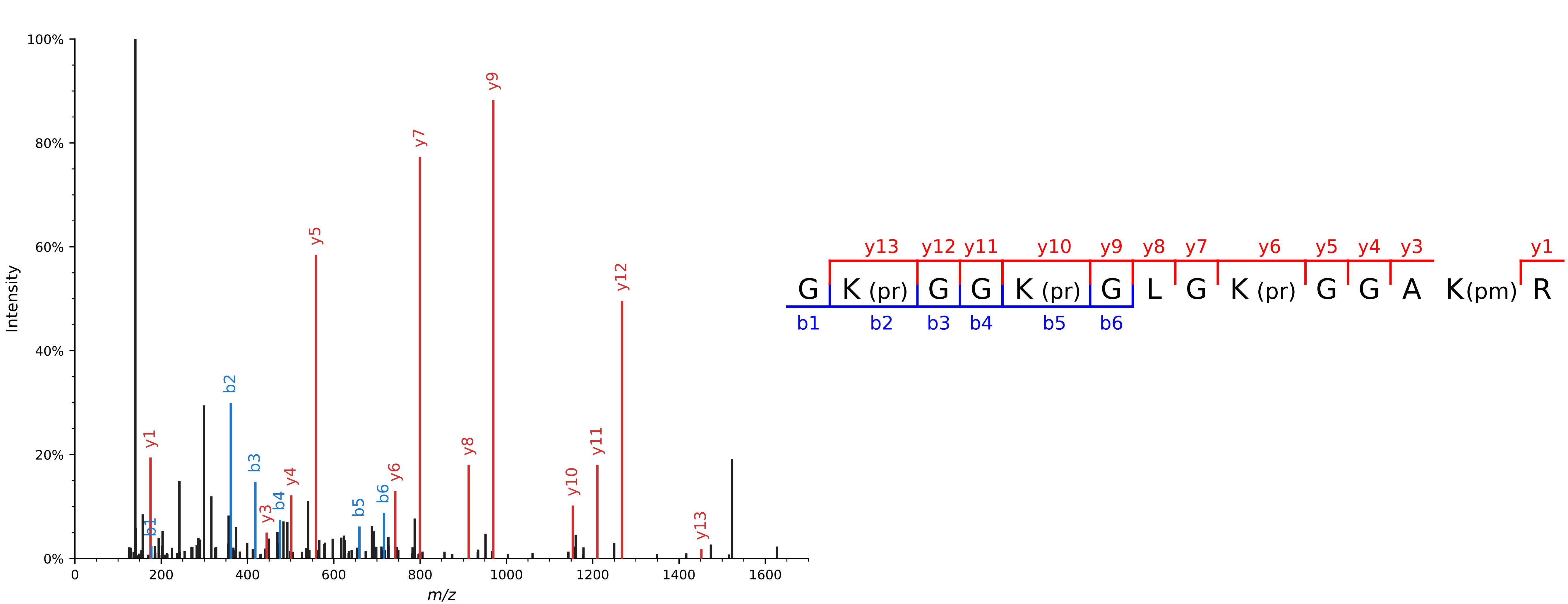

Raw file: HF240124_RN_spleen_PRO_PIC_2, Scan: 17860, m/z: 743.90426, Charge: 2, RT (min): 32.92, Score: 186.95

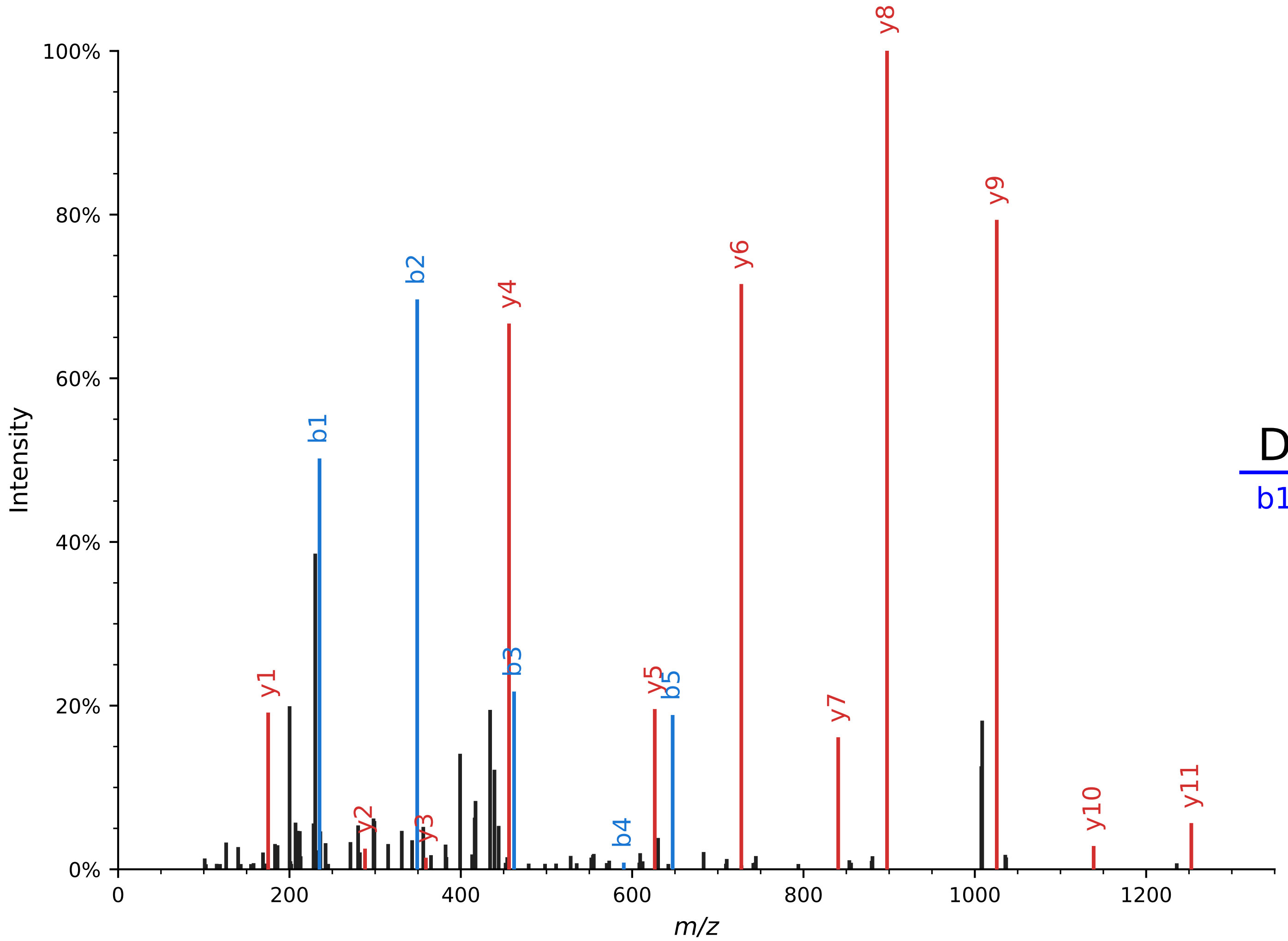

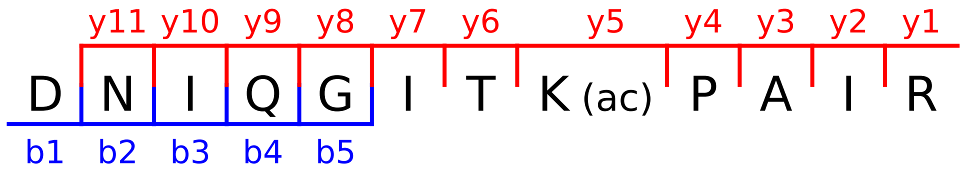

Raw file: HF240124_RN_spleen_PRO_PIC_2, Scan: 20116, m/z: 756.91209, Charge: 2, RT (min): 36.97, Score: 40.724

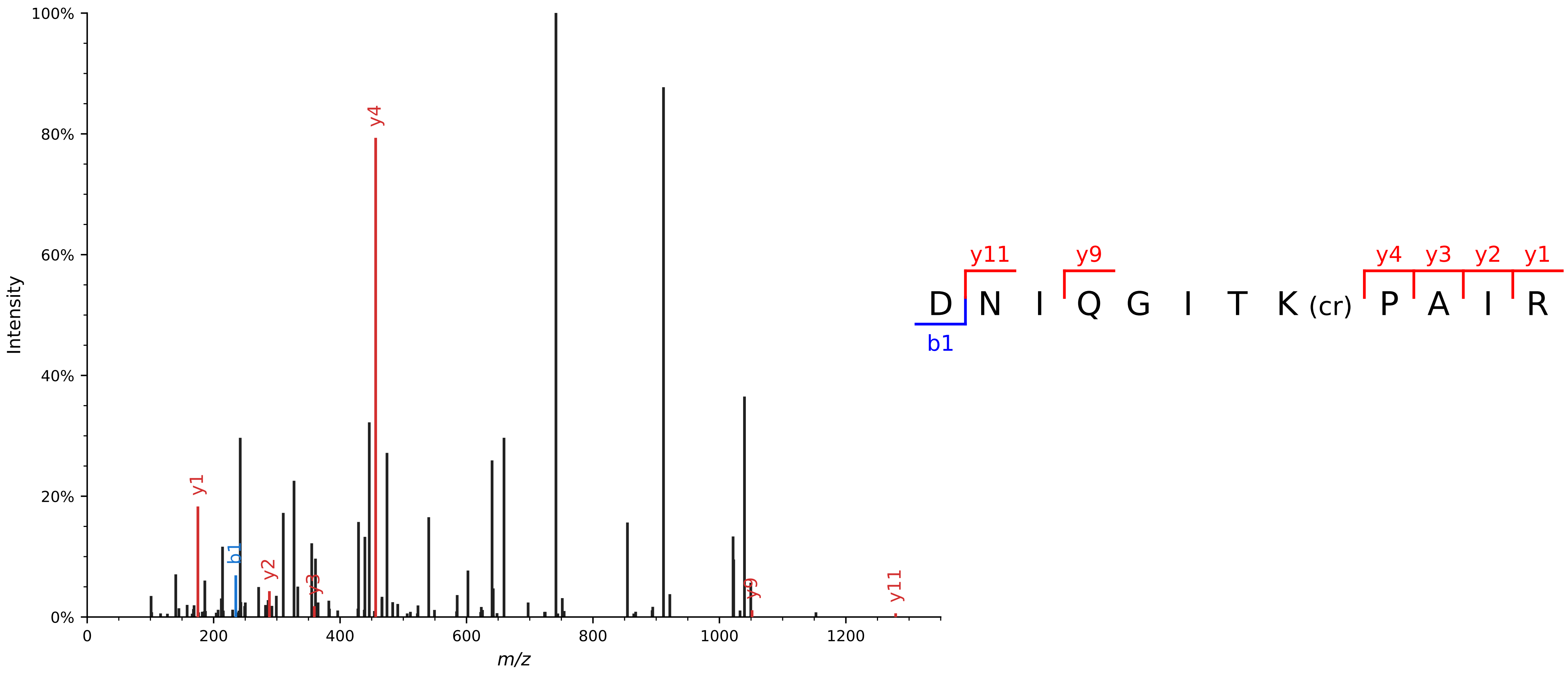

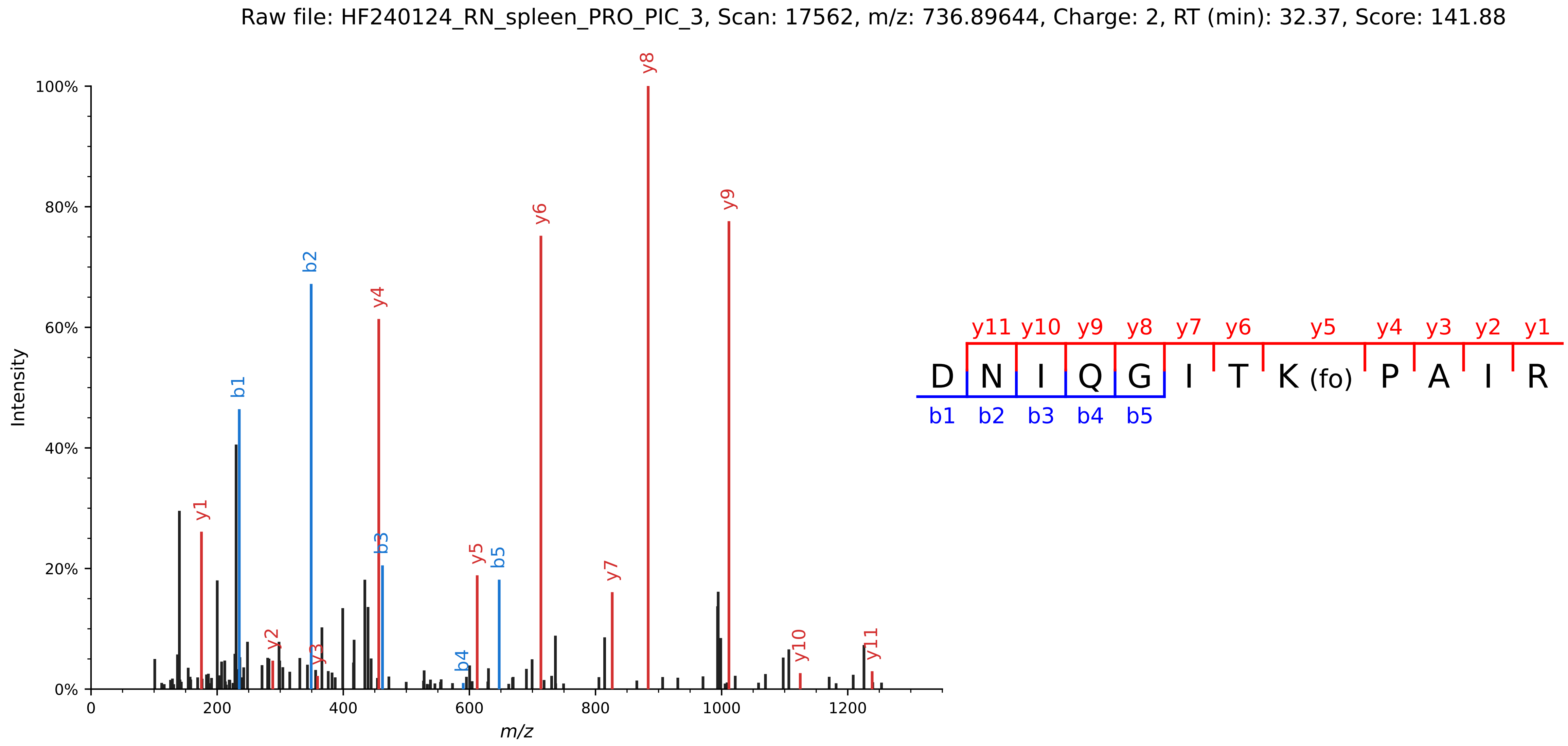

Raw file: HF240124_RN_spleen_PRO_PIC_3, Scan: 17562, m/z: 736.89644, Charge: 2, RT (min): 32.37, Score: 141.88
Intensity
m/z
D N I Q G I T K (fo) P A I R
y11 y10 y9 y8 y7 y6 y5 y4 y3 y2 y1
b1 b2 b3 b4 b5

Raw file: HF240124_RN_spleen_PRO_PIC_3, Scan: 19529, m/z: 779.91483, Charge: 2, RT (min): 35.87, Score: 91.658

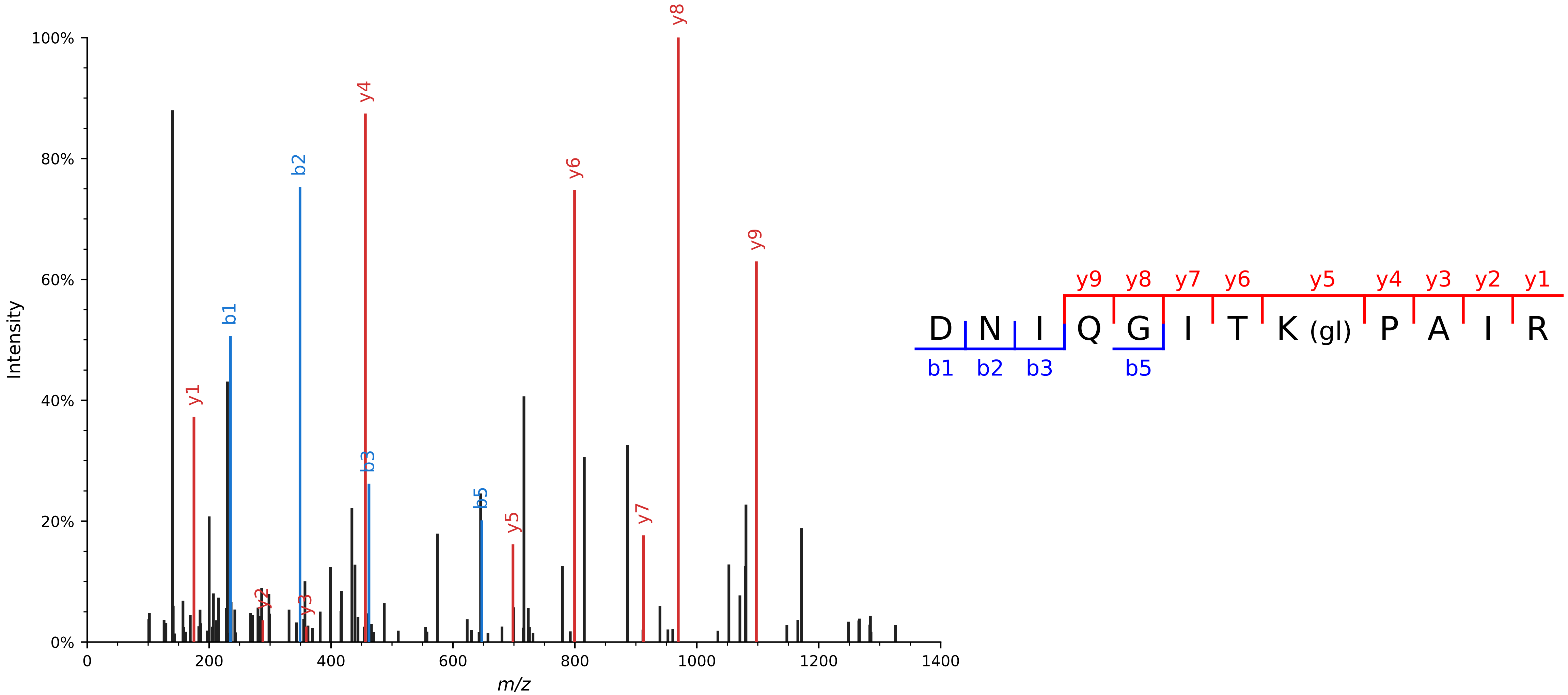

Raw file: HF240124_RN_spleen_PRO_PIC_2, Scan: 18854, m/z: 765.91737, Charge: 2, RT (min): 34.70, Score: 56.432

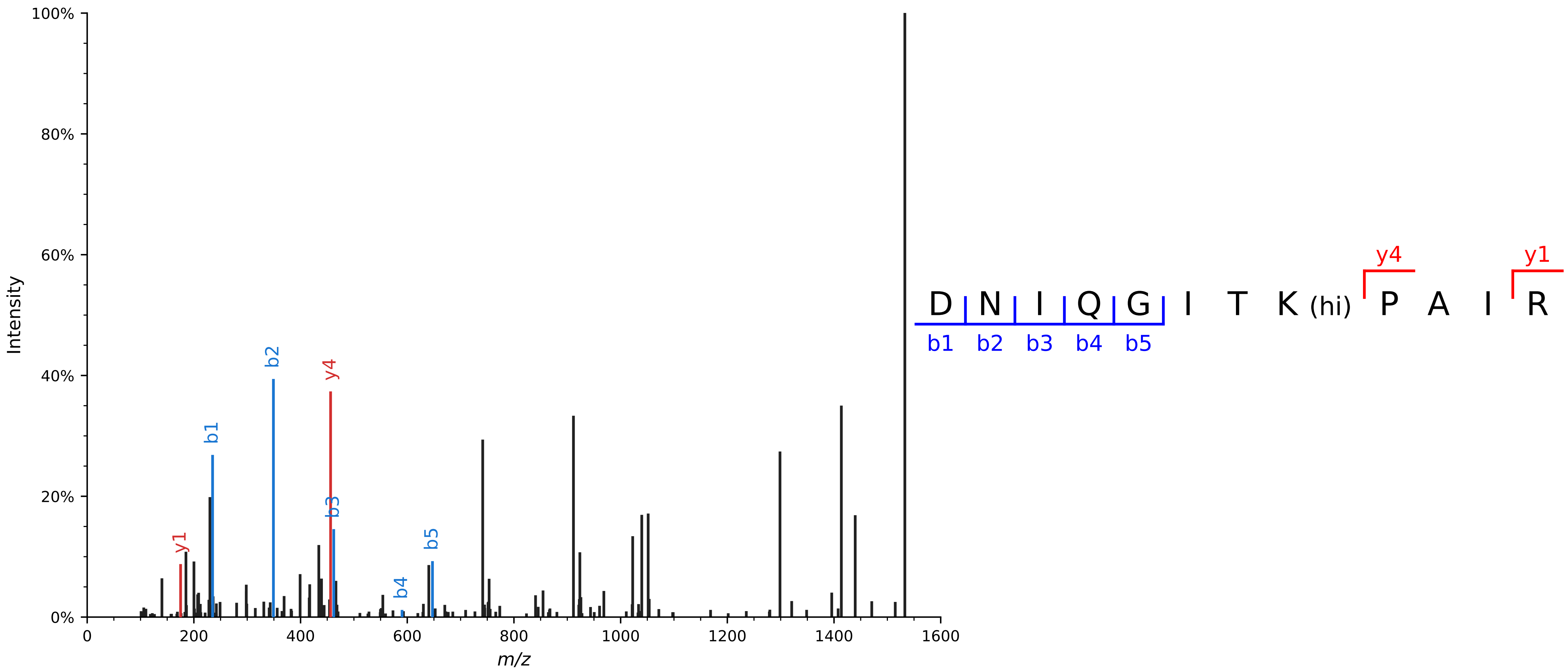

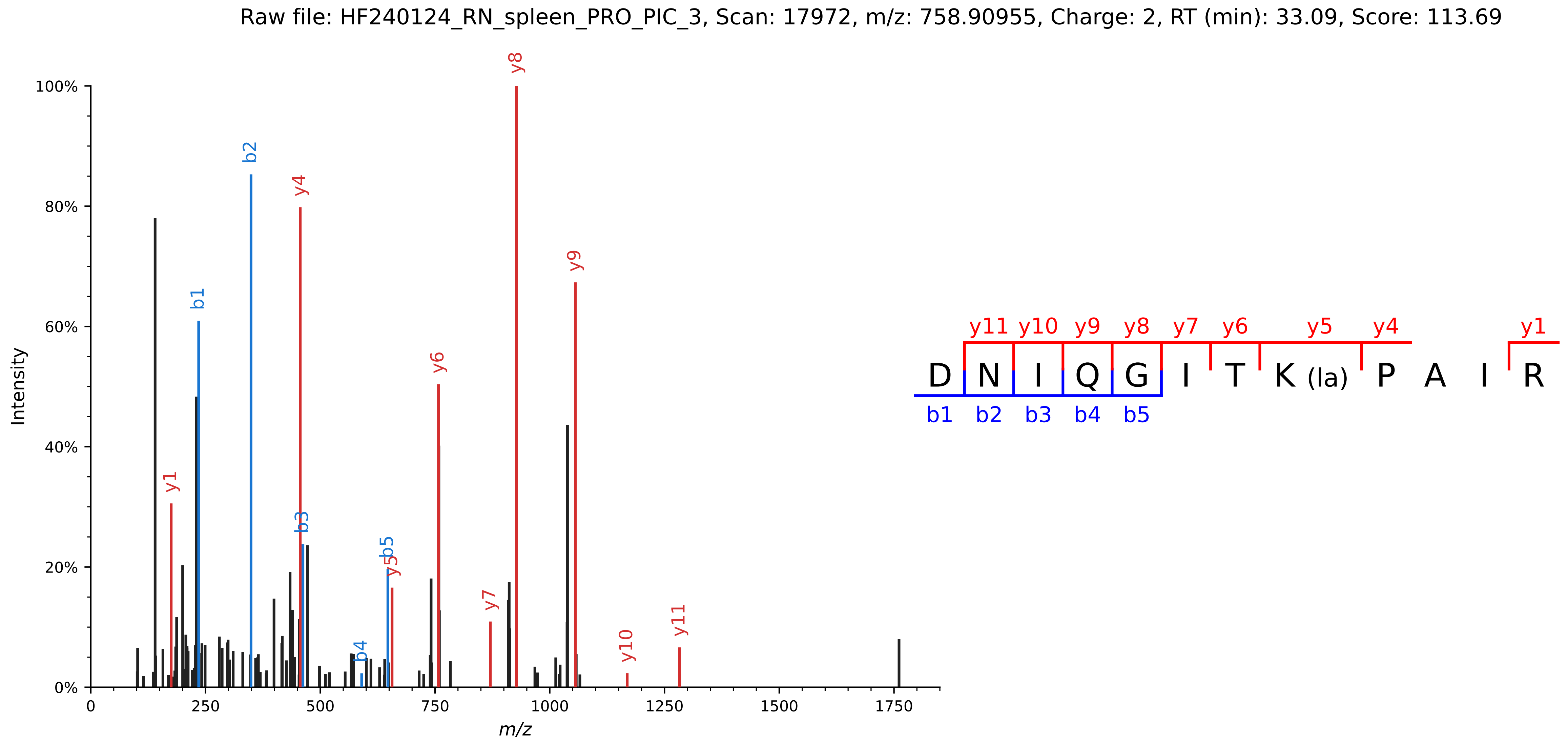

Raw file: HF240124_RN_spleen_PRO_PIC_3, Scan: 17972, m/z: 758.90955, Charge: 2, RT (min): 33.09, Score: 113.69
Intensity
m/z
y8
b2
y4
y9
b1
y6
y1
b3
b5
y5
y7
b4
y10
y11
D N I Q G I T K (la) P A I R
y11 y10 y9 y8 y7 y6 y5 y4 y1
b1 b2 b3 b4 b5

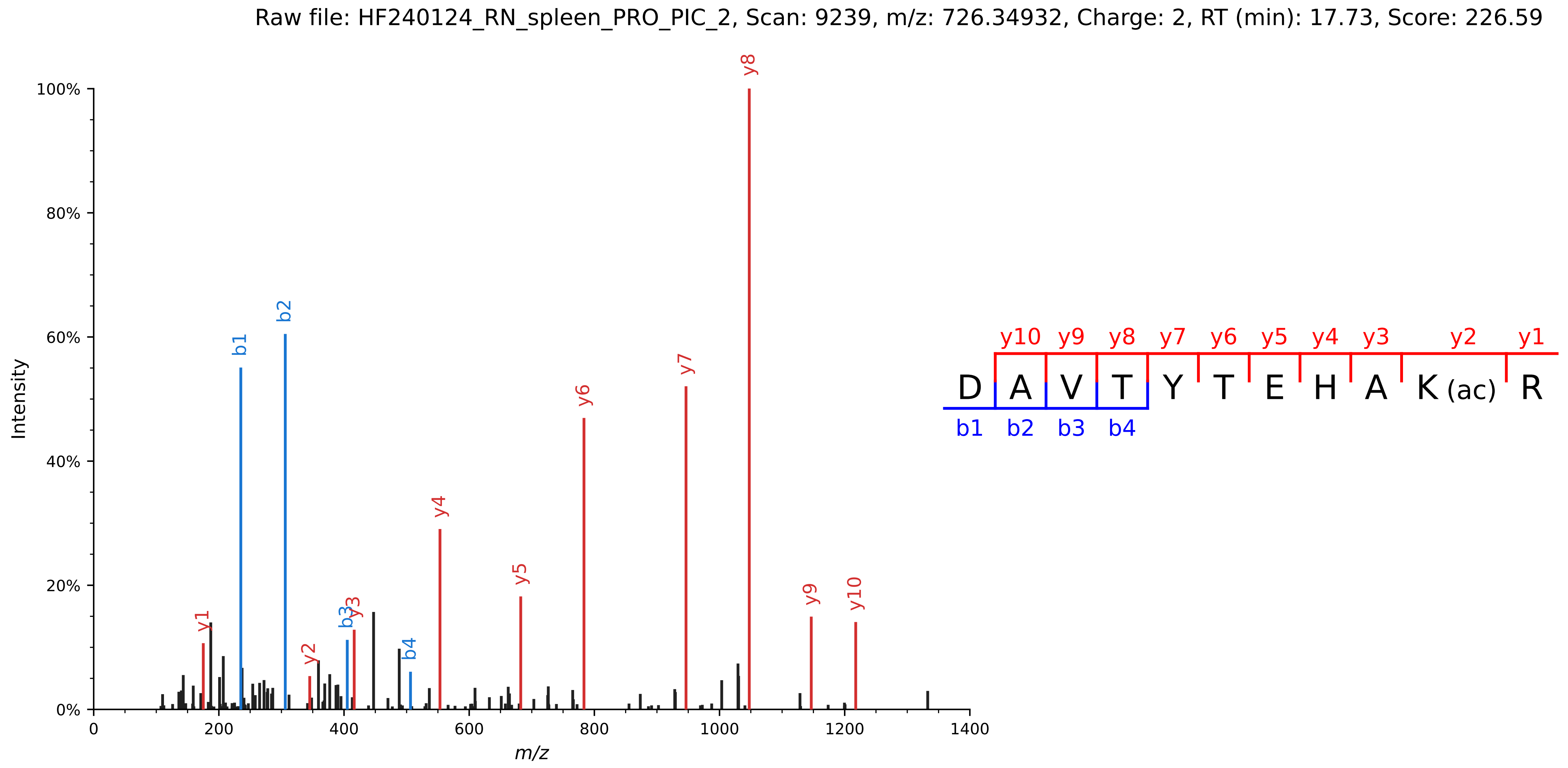

Raw file: HF240124_RN_spleen_PRO_PIC_2, Scan: 9239, m/z: 726.34932, Charge: 2, RT (min): 17.73, Score: 226.59
Intensity
m/z
y10 y9 y8 y7 y6 y5 y4 y3 y2 y1
D A V T Y T E H A K (ac) R
b1 b2 b3 b4

Raw file: HF240124_RN_spleen_PRO_PIC_2, Scan: 8895, m/z: 719.3415, Charge: 2, RT (min): 17.12, Score: 160.59

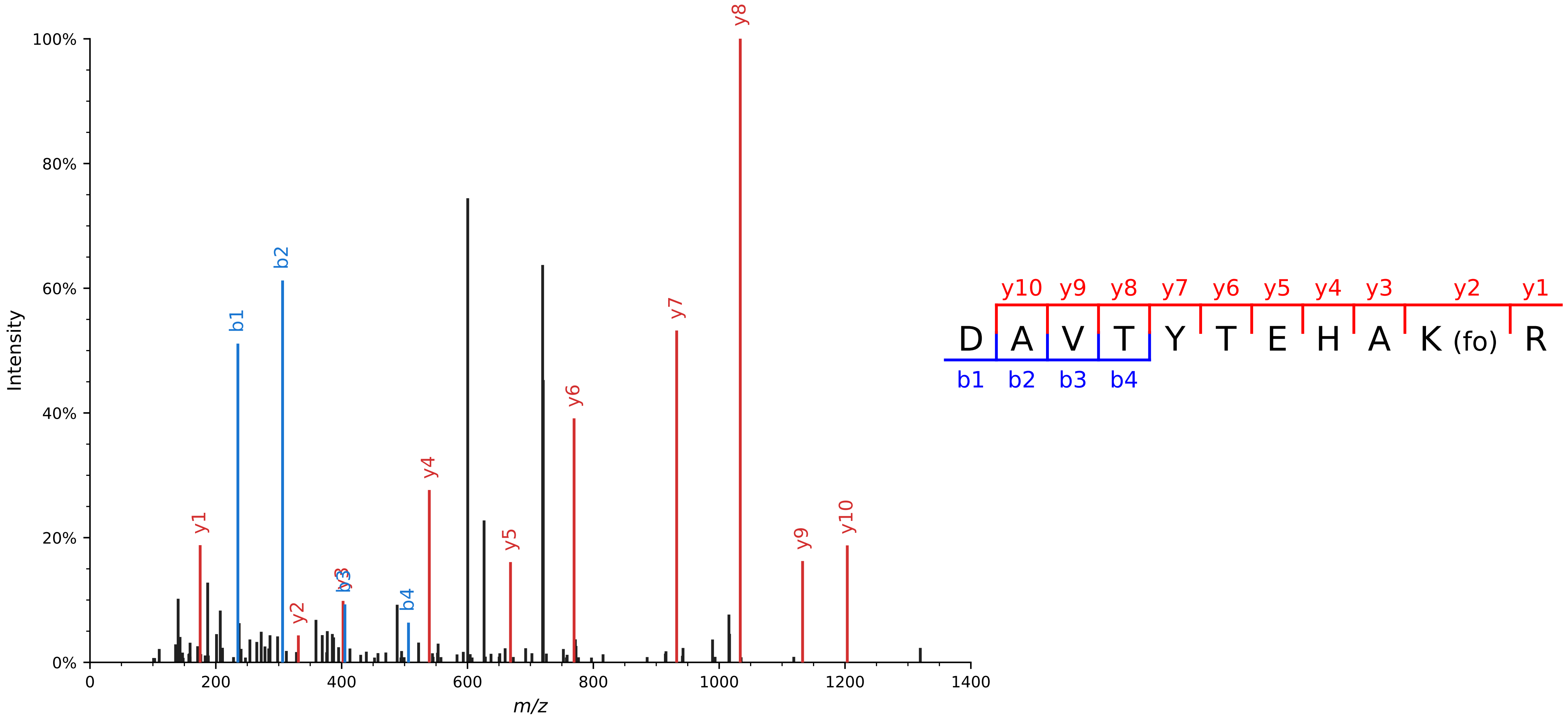

Raw file: HF240124_RN_spleen_PRO_PIC_3, Scan: 14080, m/z: 762.35989, Charge: 2, RT (min): 26.25, Score: 118.18

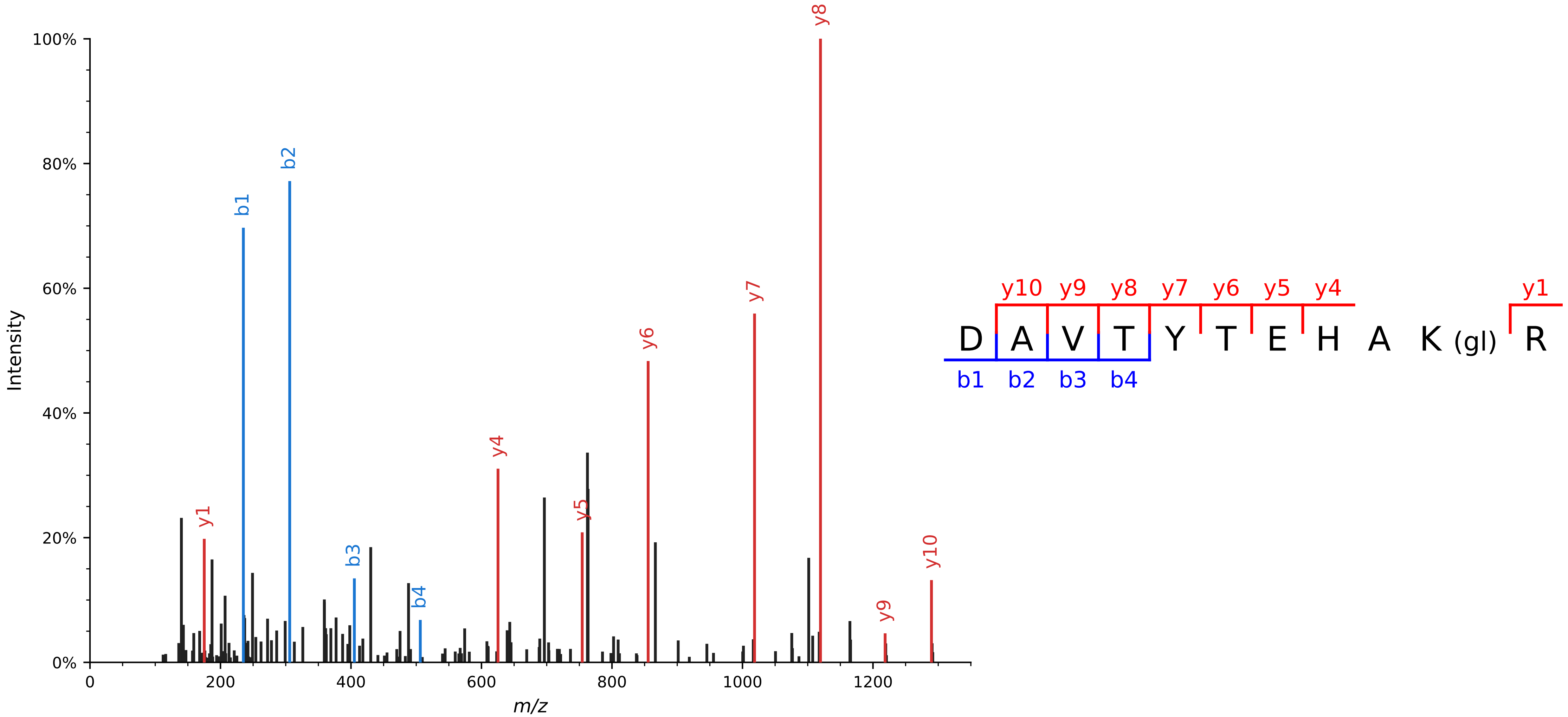

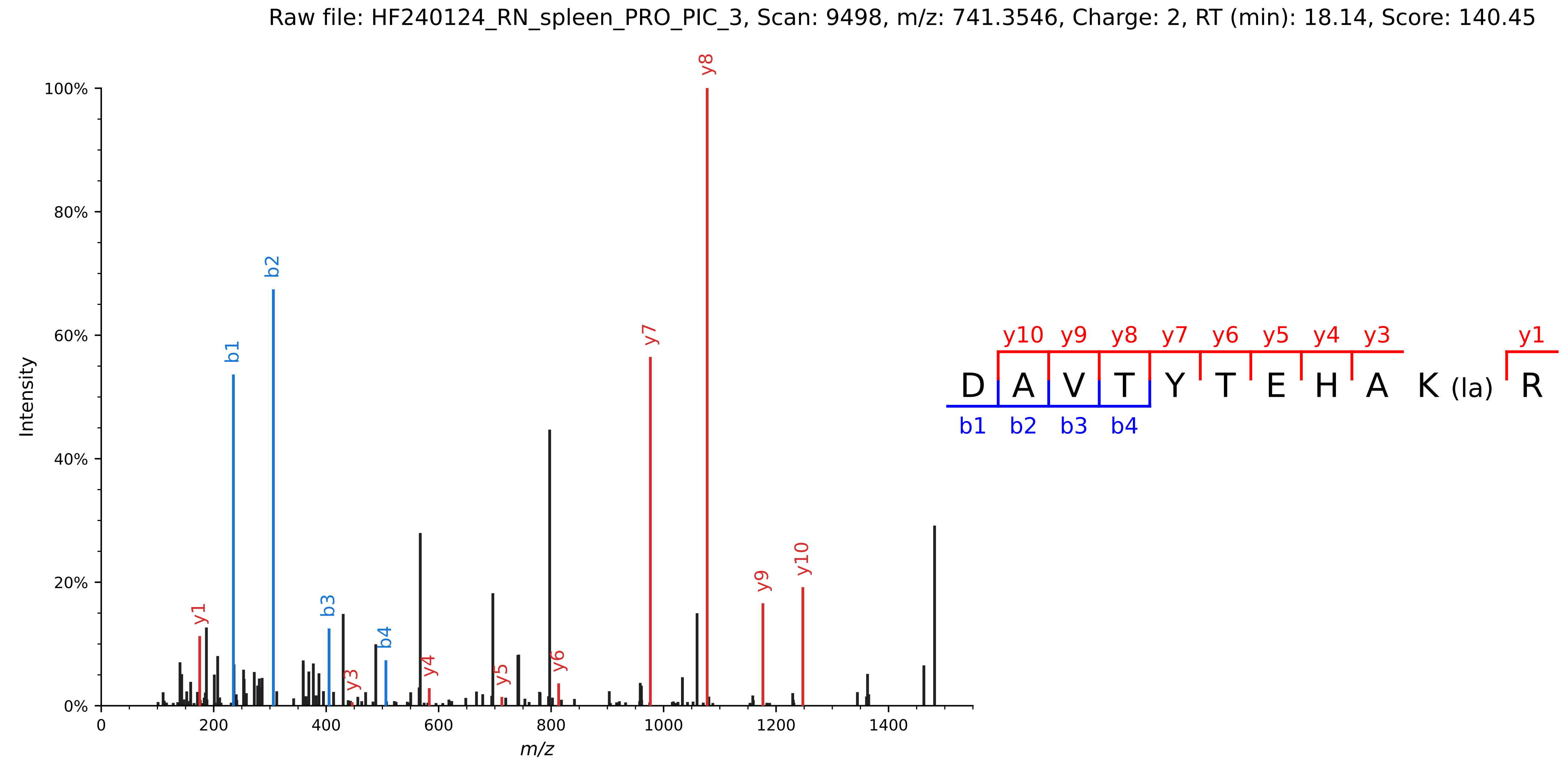

Raw file: HF240124_RN_spleen_PRO_PIC_3, Scan: 9498, m/z: 741.3546, Charge: 2, RT (min): 18.14, Score: 140.45
Intensity
m/z
y10 y9 y8 y7 y6 y5 y4 y3 y1
D A V T Y T E H A K (la) R
b1 b2 b3 b4

Raw file: HF240124_RN_spleen_PRO_PIC_3, Scan: 16823, m/z: 748.34424, Charge: 2, RT (min): 31.06, Score: 159.37

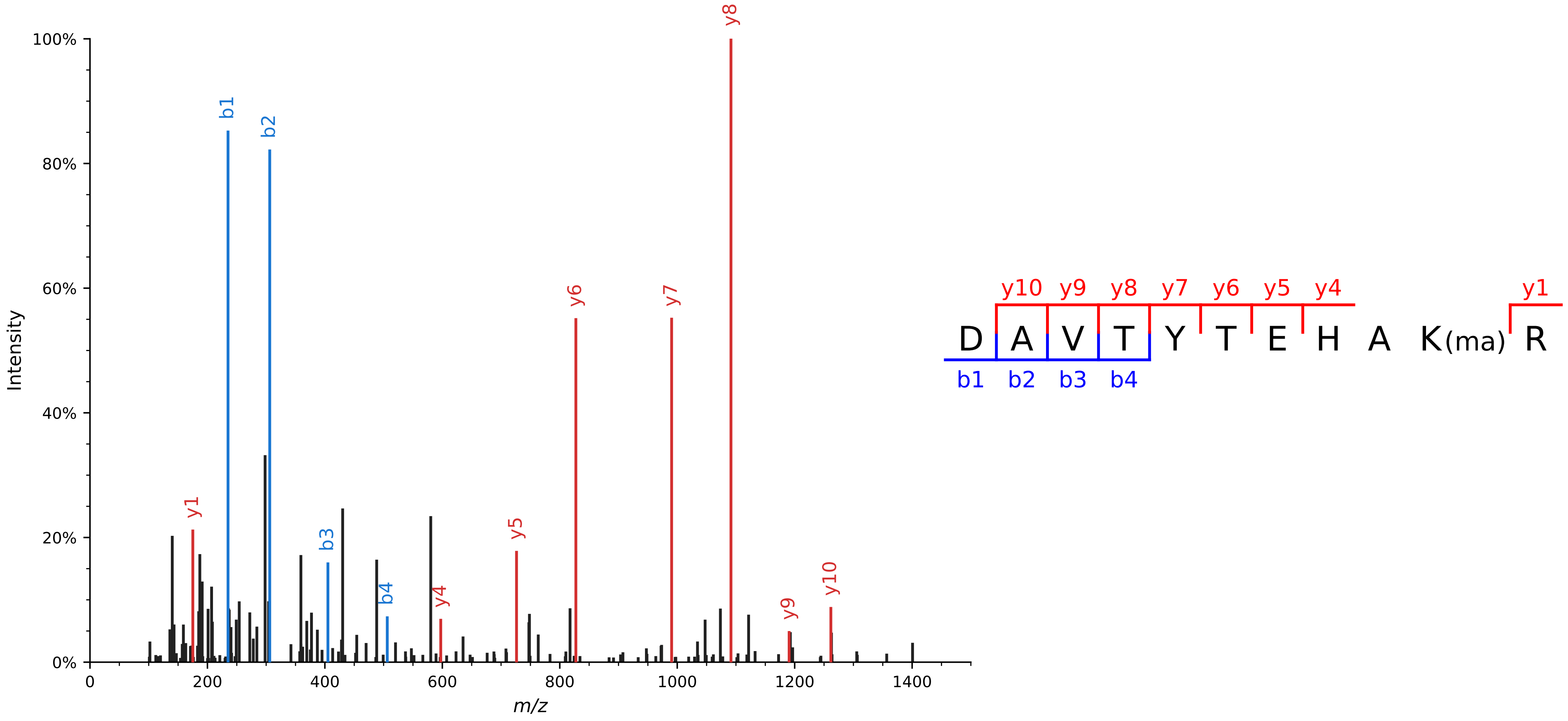

Raw file: HF240124_RN_spleen_PRO_PIC_3, Scan: 11451, m/z: 740.36497, Charge: 2, RT (min): 21.60, Score: 134.25

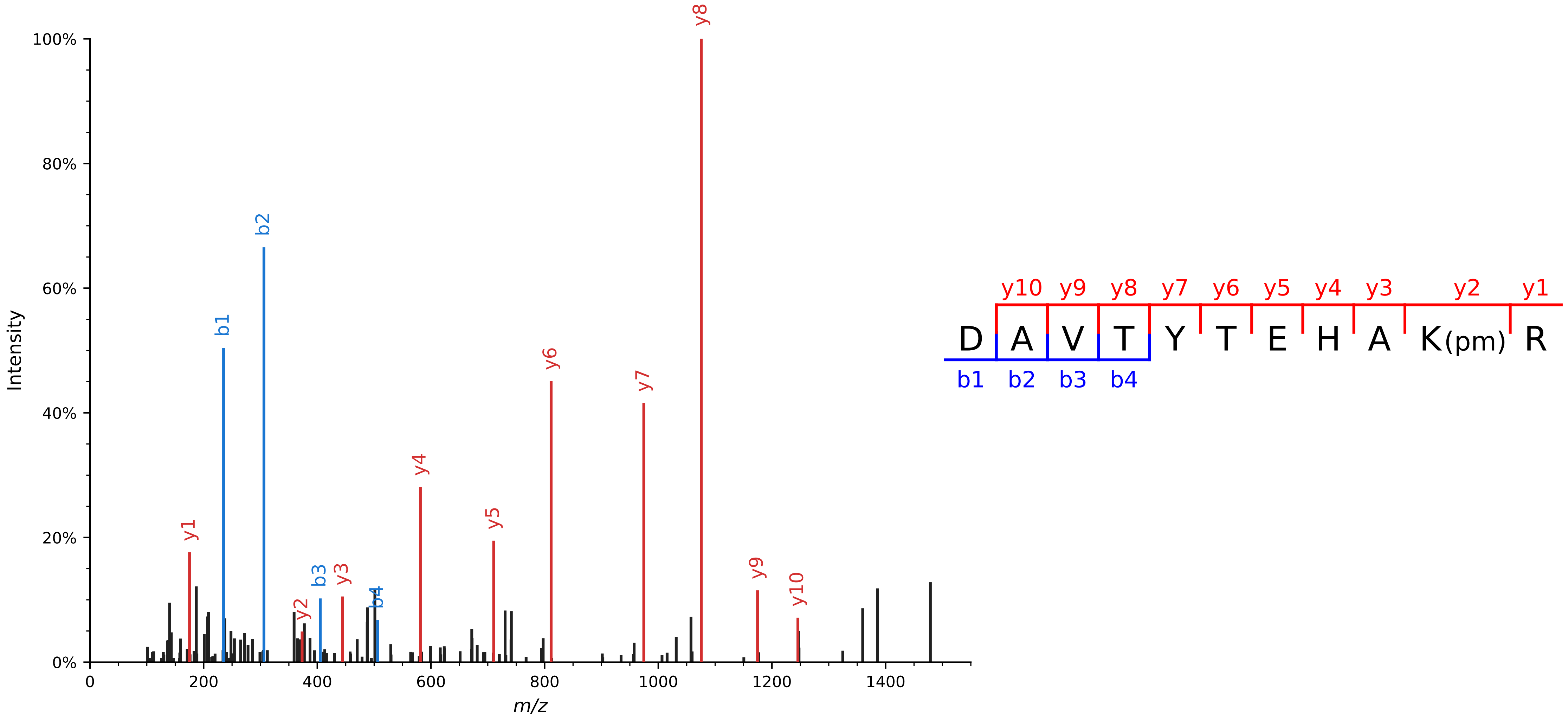

Raw file: HF240124_RN_spleen_PRO_PIC_3, Scan: 24995, m/z: 914.4873, Charge: 2, RT (min): 45.73, Score: 79.82

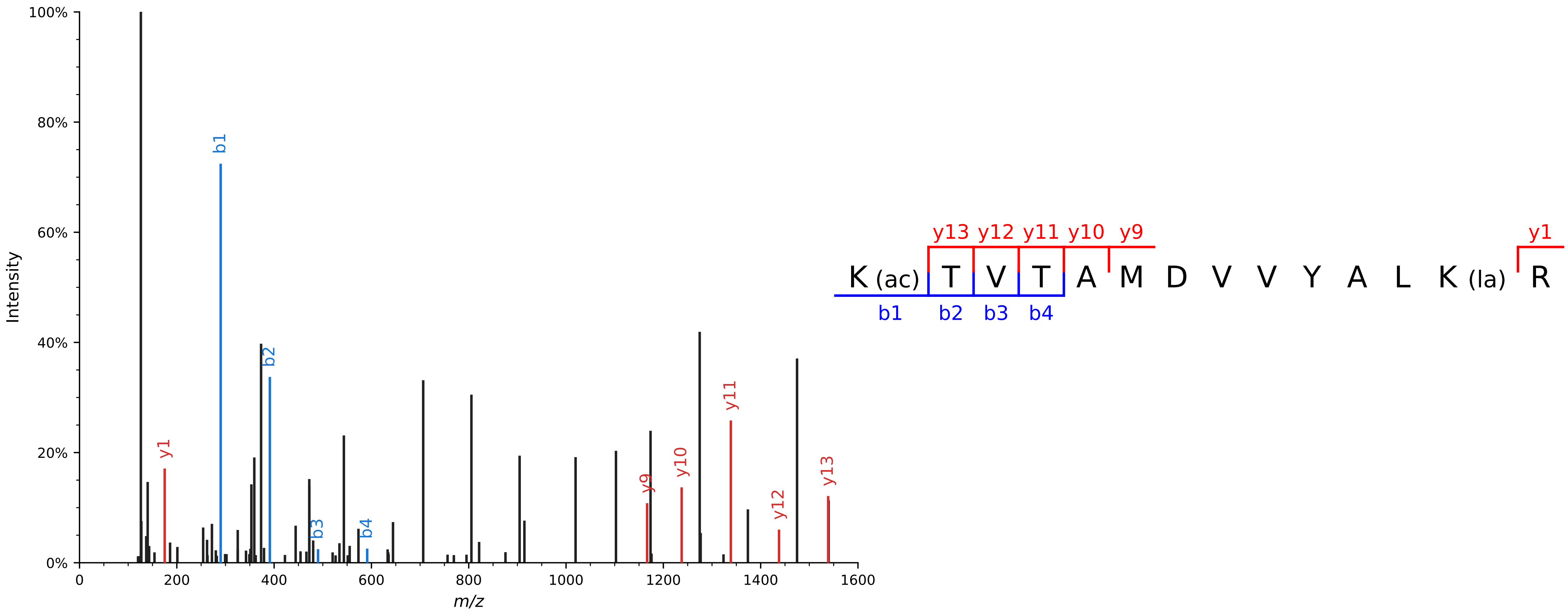

Raw file: HF240124_RN_spleen_PRO_PIC_2, Scan: 27377, m/z: 928.50295, Charge: 2, RT (min): 50.27, Score: 58.223

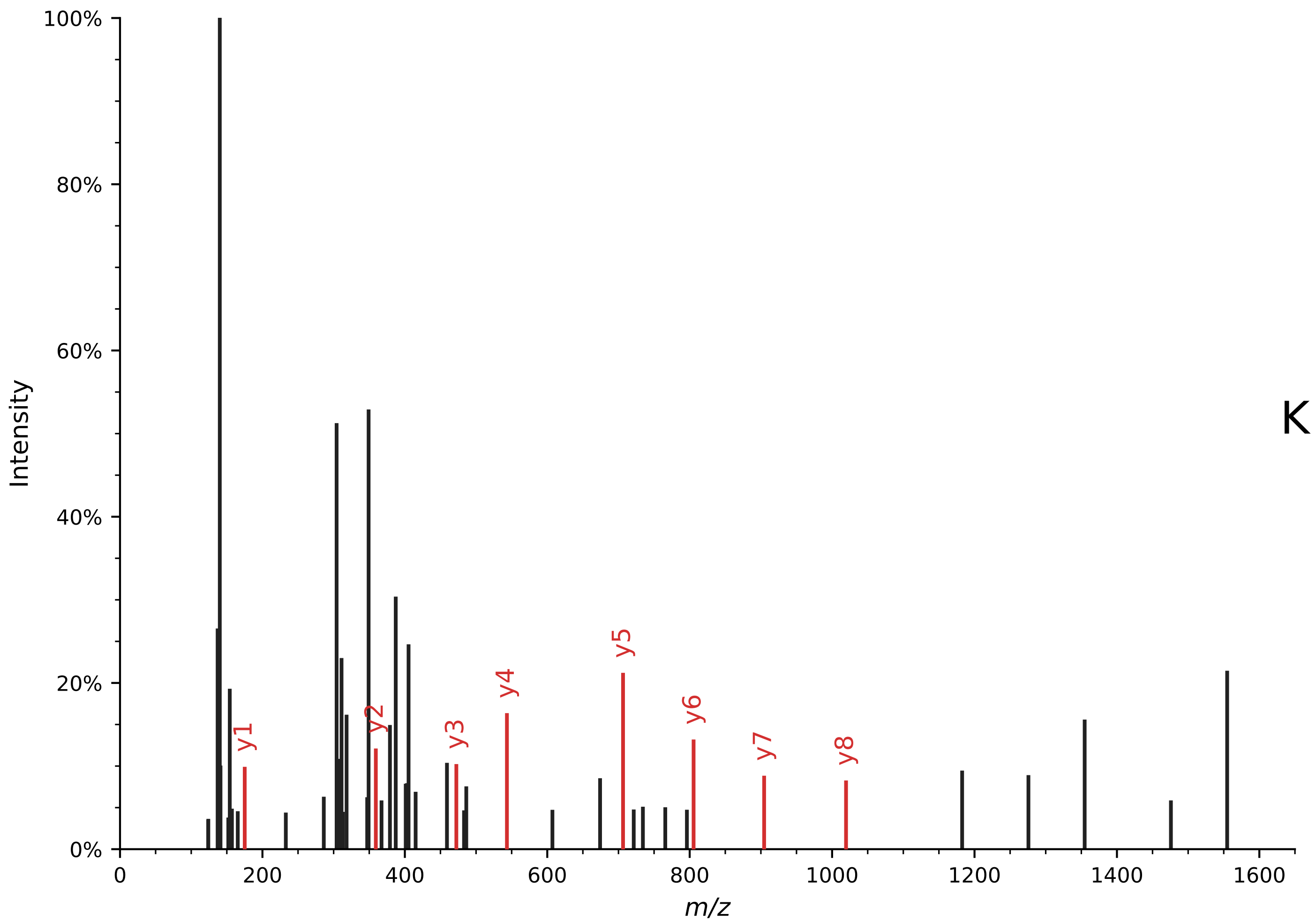

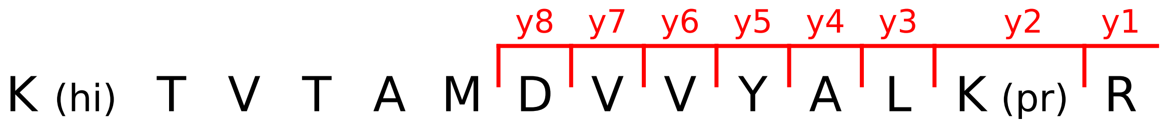

Raw file: HF240124_RN_spleen_PRO_PIC_3, Scan: 26548, m/z: 921.49513, Charge: 2, RT (min): 48.59, Score: 85.958

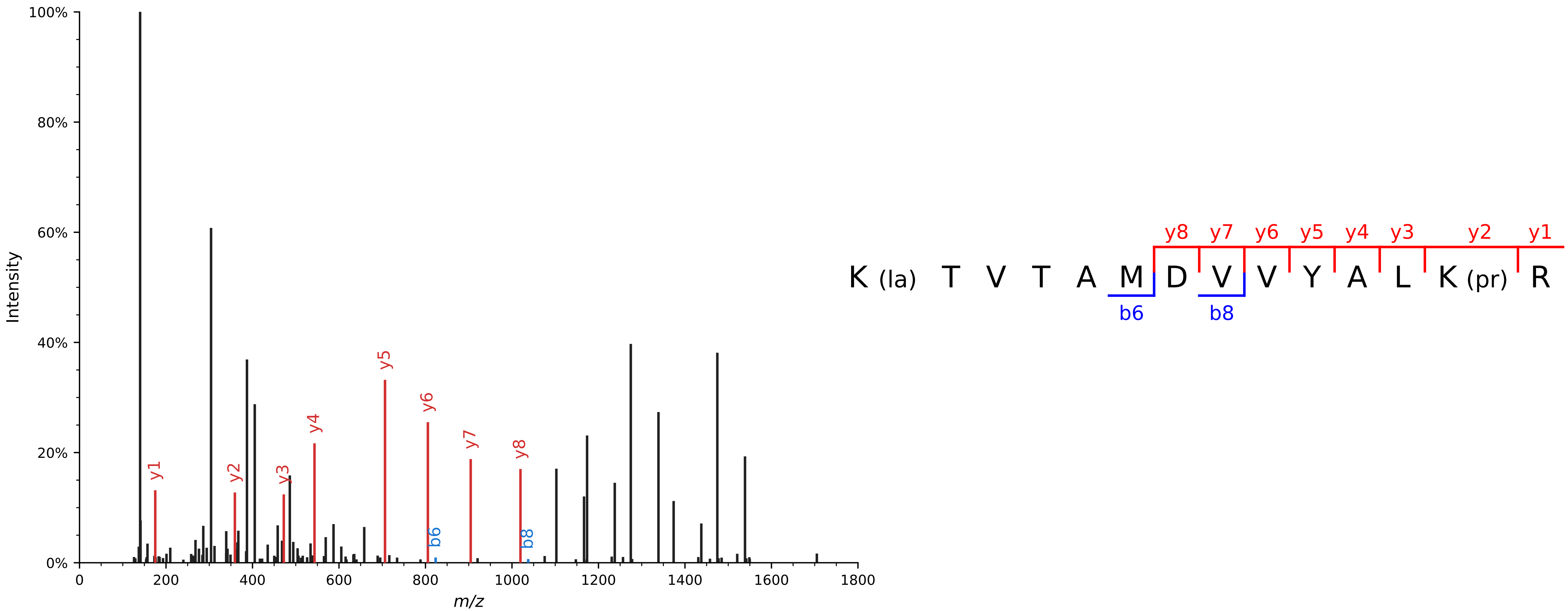

Raw file: HF240124_RN_spleen_PRO_PIC_2, Scan: 25321, m/z: 914.4873, Charge: 2, RT (min): 46.45, Score: 49.5

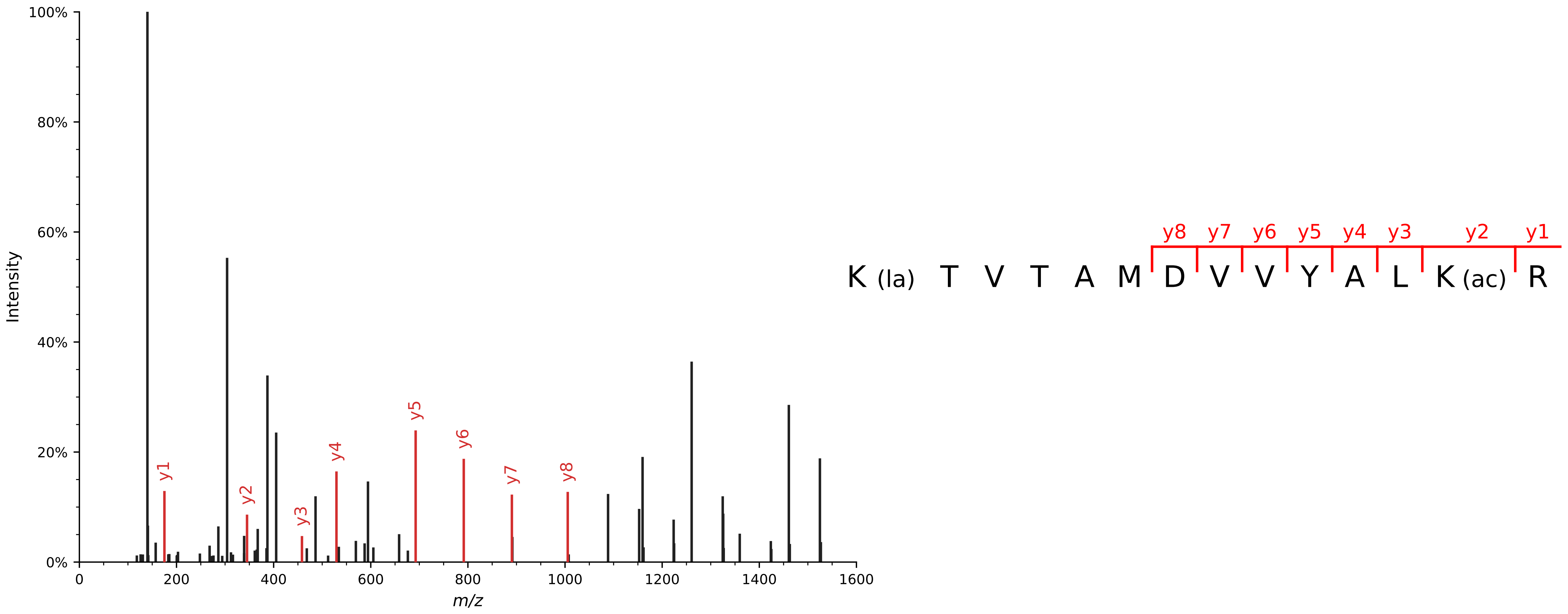

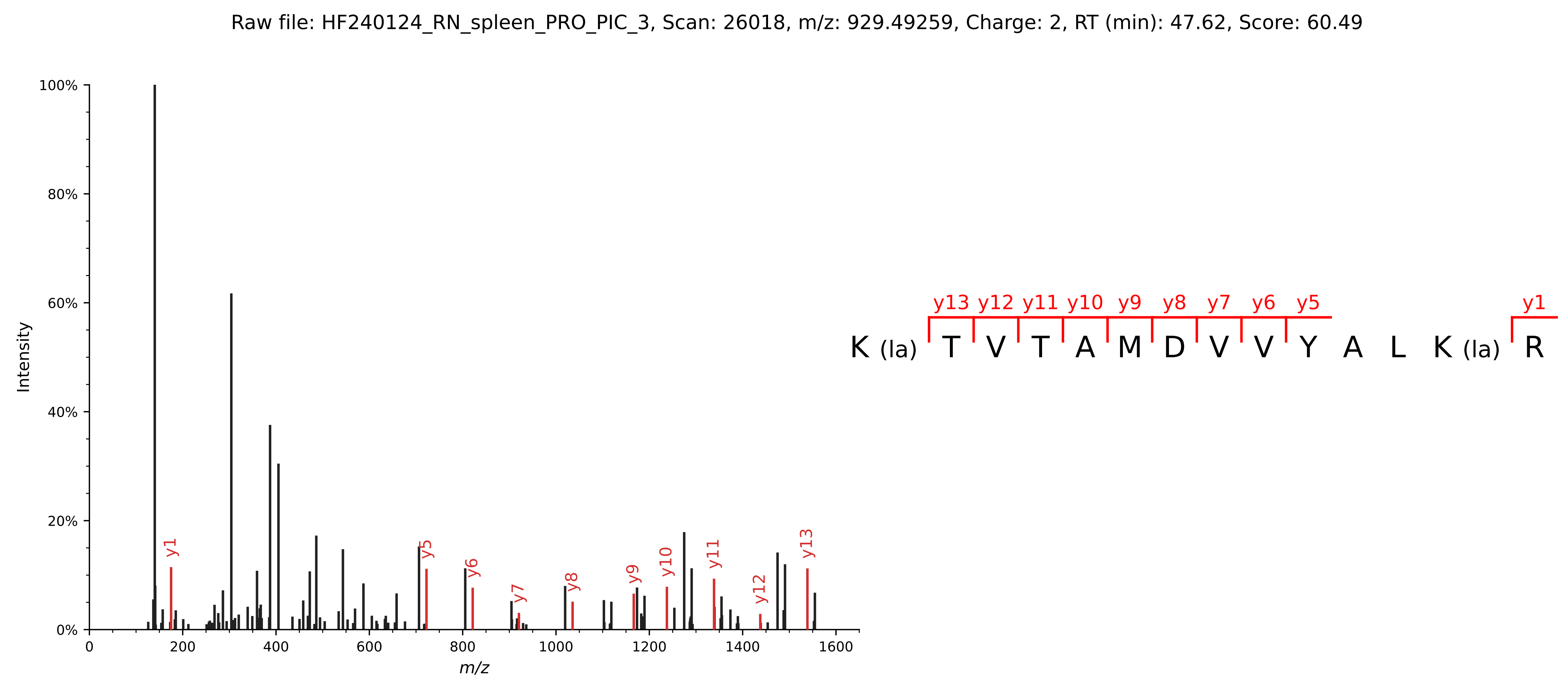

Raw file: HF240124_RN_spleen_PRO_PIC_3, Scan: 26018, m/z: 929.49259, Charge: 2, RT (min): 47.62, Score: 60.49
Intensity
m/z
y1
y5
y6
y7
y8
y9
y10
y11
y12
y13
K (la) T V T A M D V V Y A L K (la) R

Raw file: HF240124_RN_spleen_PRO_PIC_3, Scan: 25398, m/z: 906.50804, Charge: 2, RT (min): 46.48, Score: 53.683

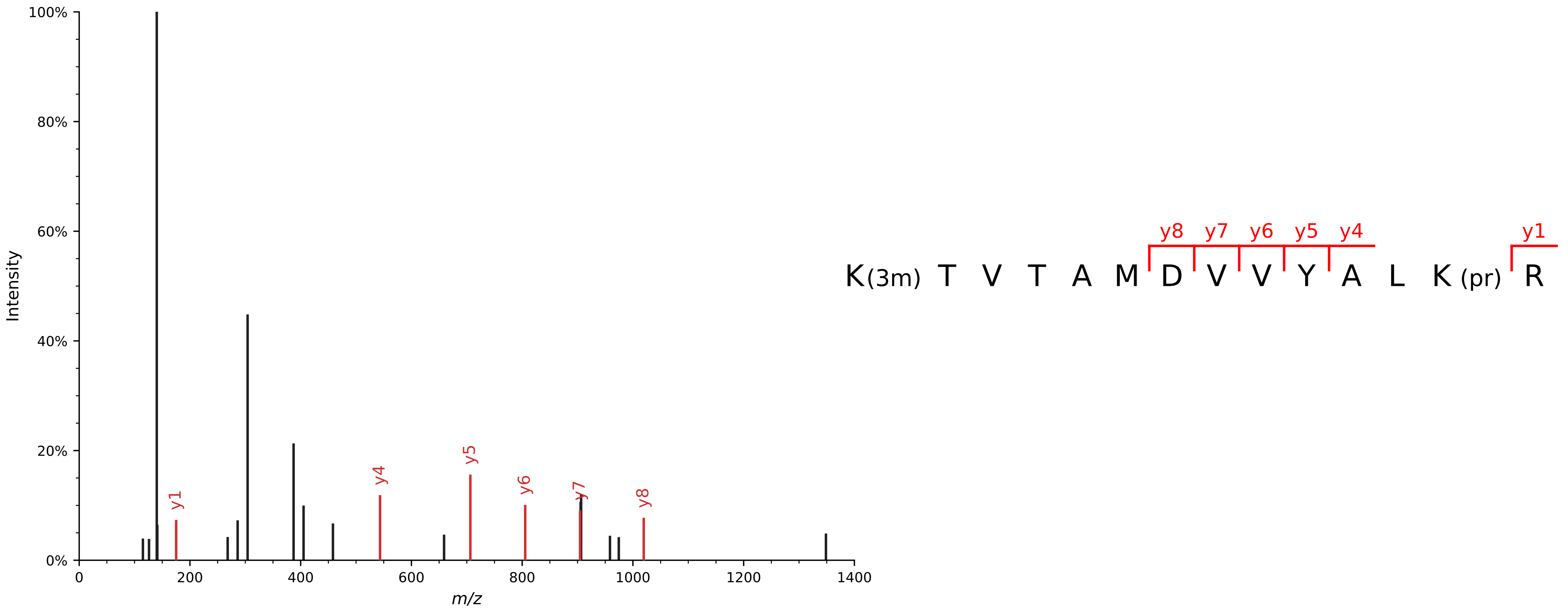

Raw file: HF240124_RN_spleen_PRO_PIC_2, Scan: 27582, m/z: 935.49259, Charge: 2, RT (min): 50.66, Score: 43.308

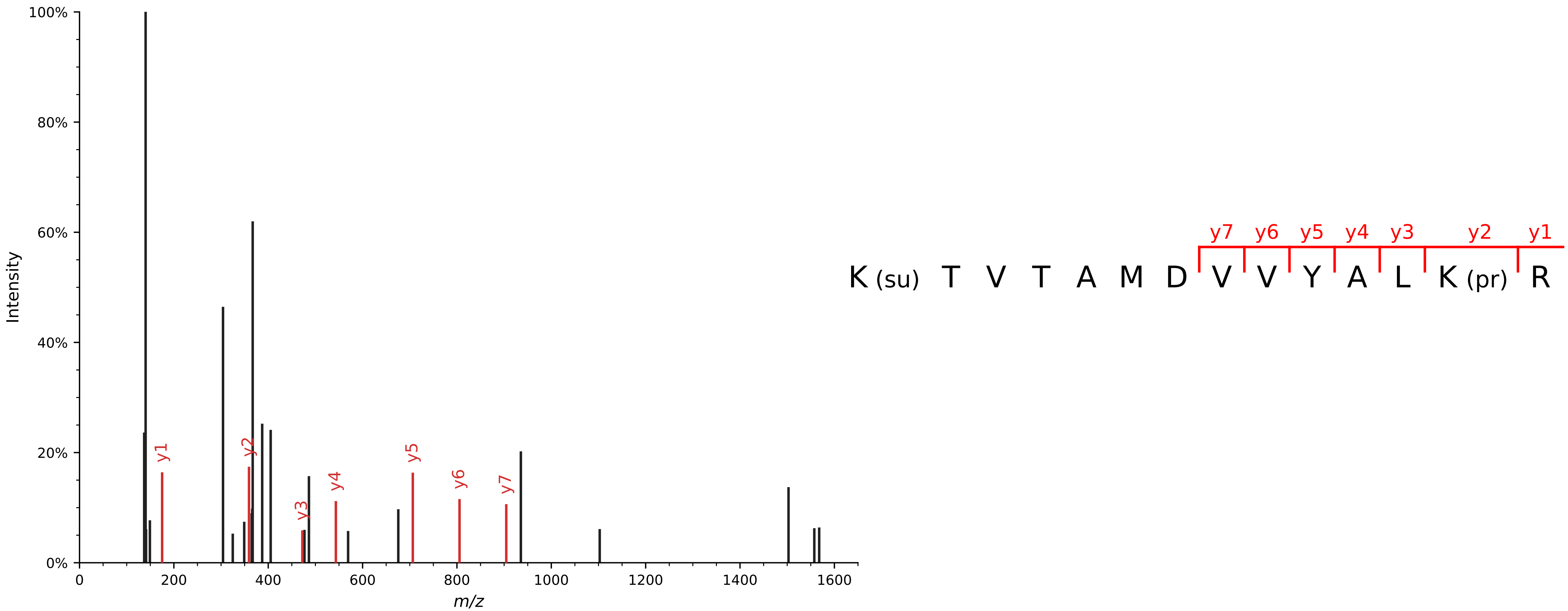

Raw file: HF240124_RN_spleen_PRO_PIC_2, Scan: 27260, m/z: 942.50041, Charge: 2, RT (min): 50.06, Score: 40.715

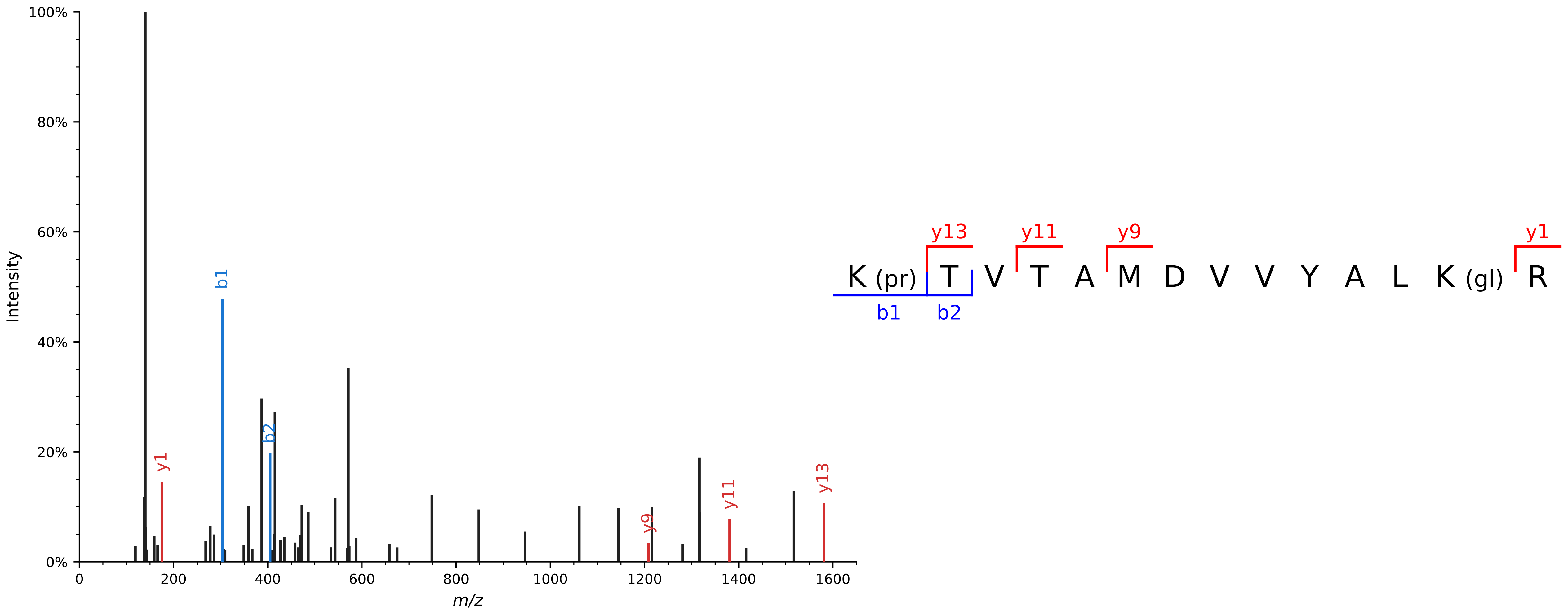

Raw file: HF240124_RN_spleen_PRO_PIC_2, Scan: 25973, m/z: 921.49513, Charge: 2, RT (min): 47.66, Score: 100.55

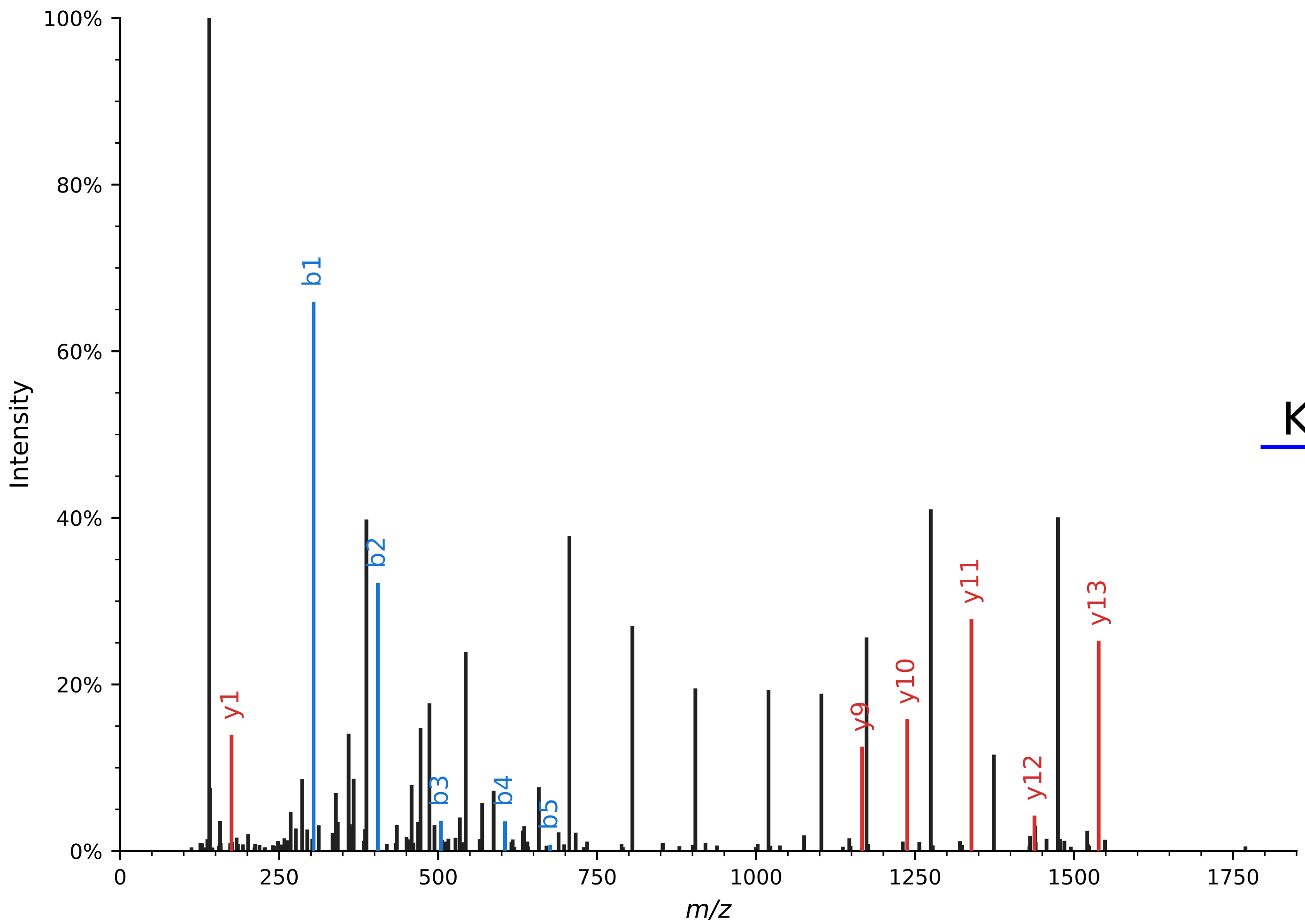

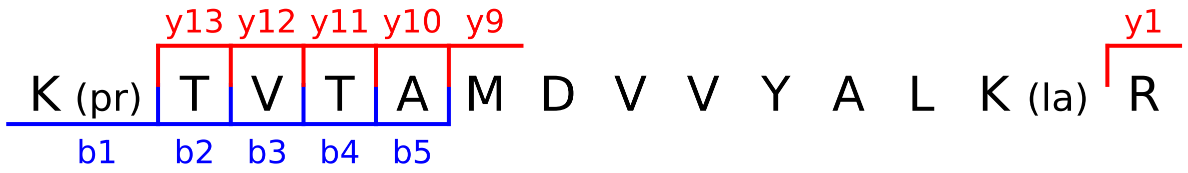

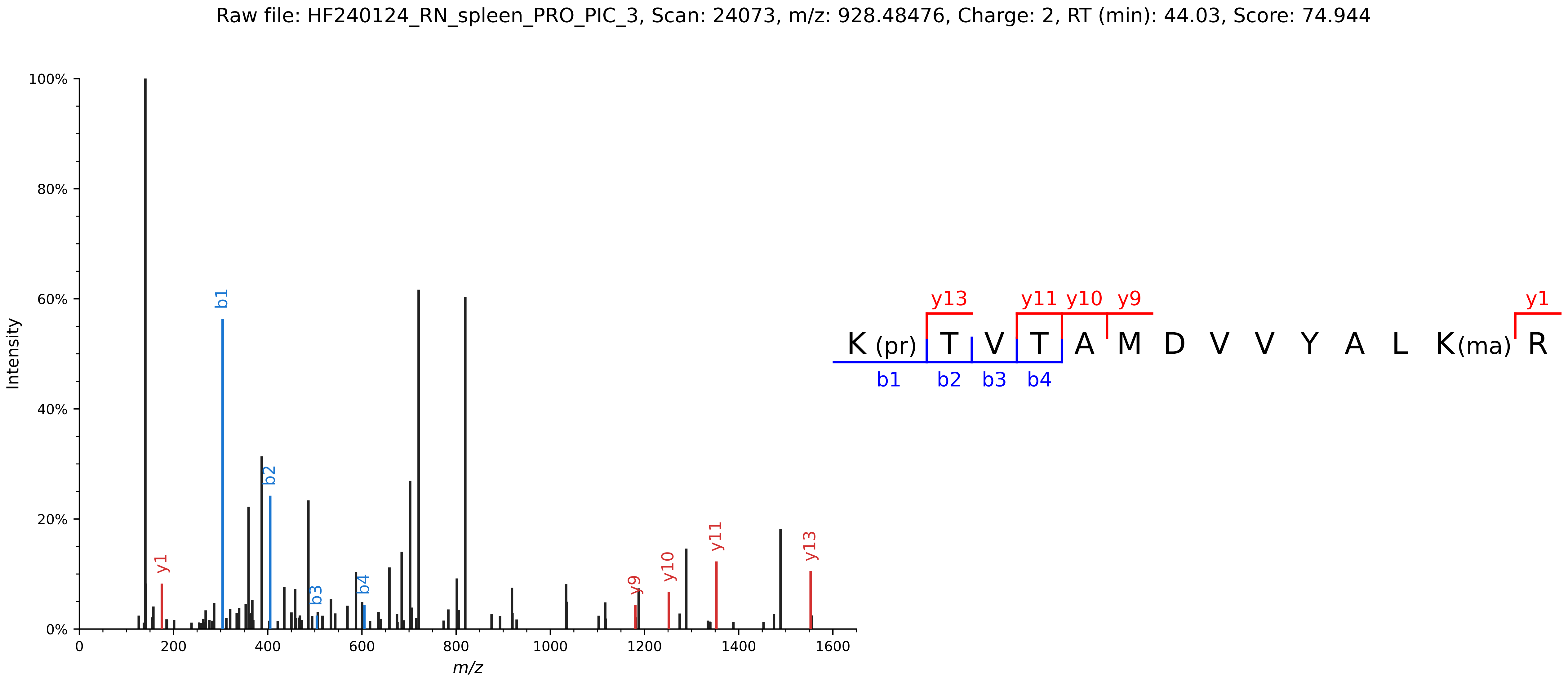
Raw file: HF240124_RN_spleen_PRO_PIC_3, Scan: 24073, m/z: 928.48476, Charge: 2, RT (min): 44.03, Score: 74.944
Intensity
m/z
y1
b1
b2
b3
b4
y9
y10
y11
y13
K (pr) T V T A M D V V Y A L K(ma) R

Spleen_D5PRO-PIC

Raw file: HF240124_RN_spleen_D5PRO_PIC_2, Scan: 10861, m/z: 507.31919, Charge: 2, RT (min): 21.16, Score: 59.205

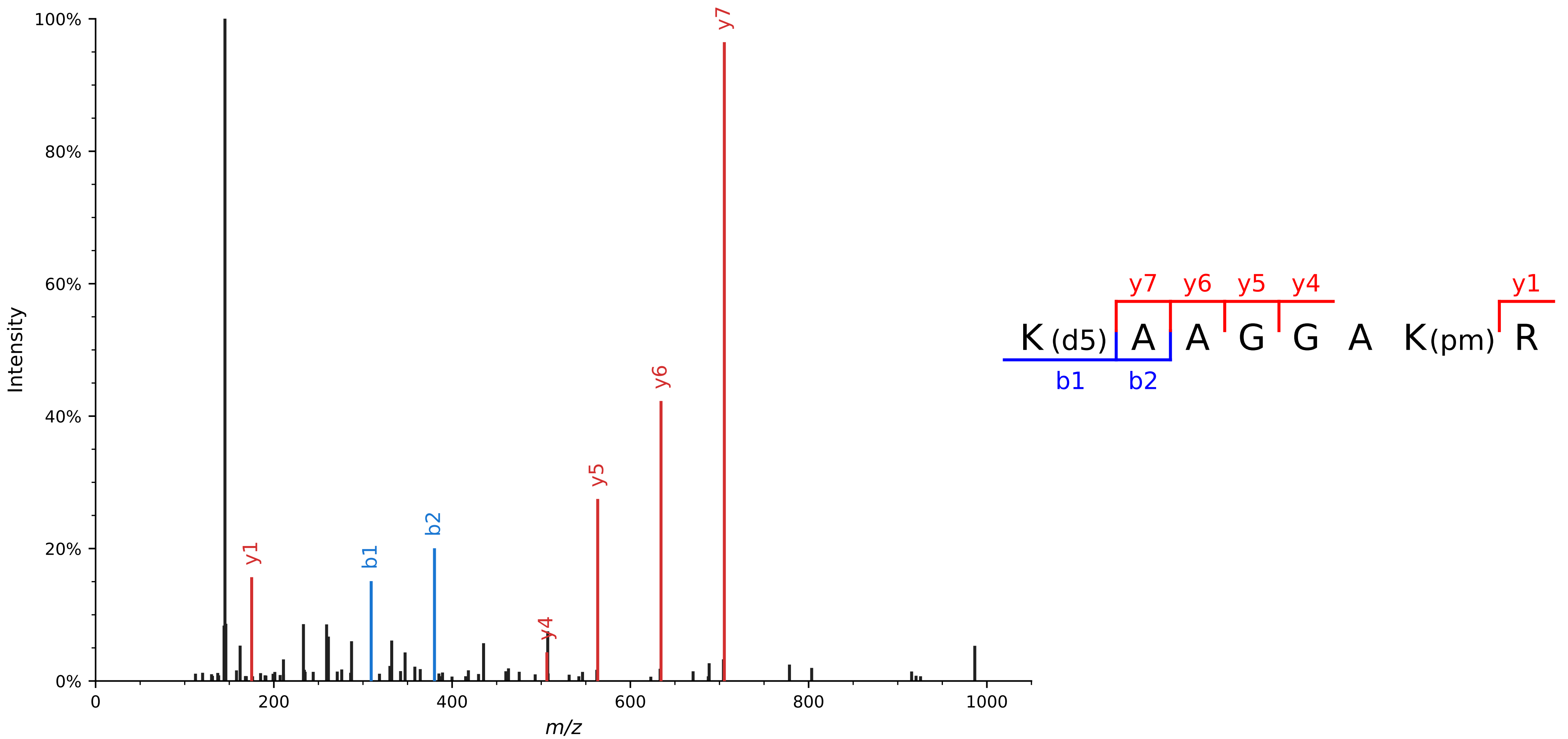

Raw file: HF240124_RN_spleen_D5PRO_PIC_1, Scan: 7803, m/z: 526.30641, Charge: 2, RT (min): 15.46, Score: 77.674

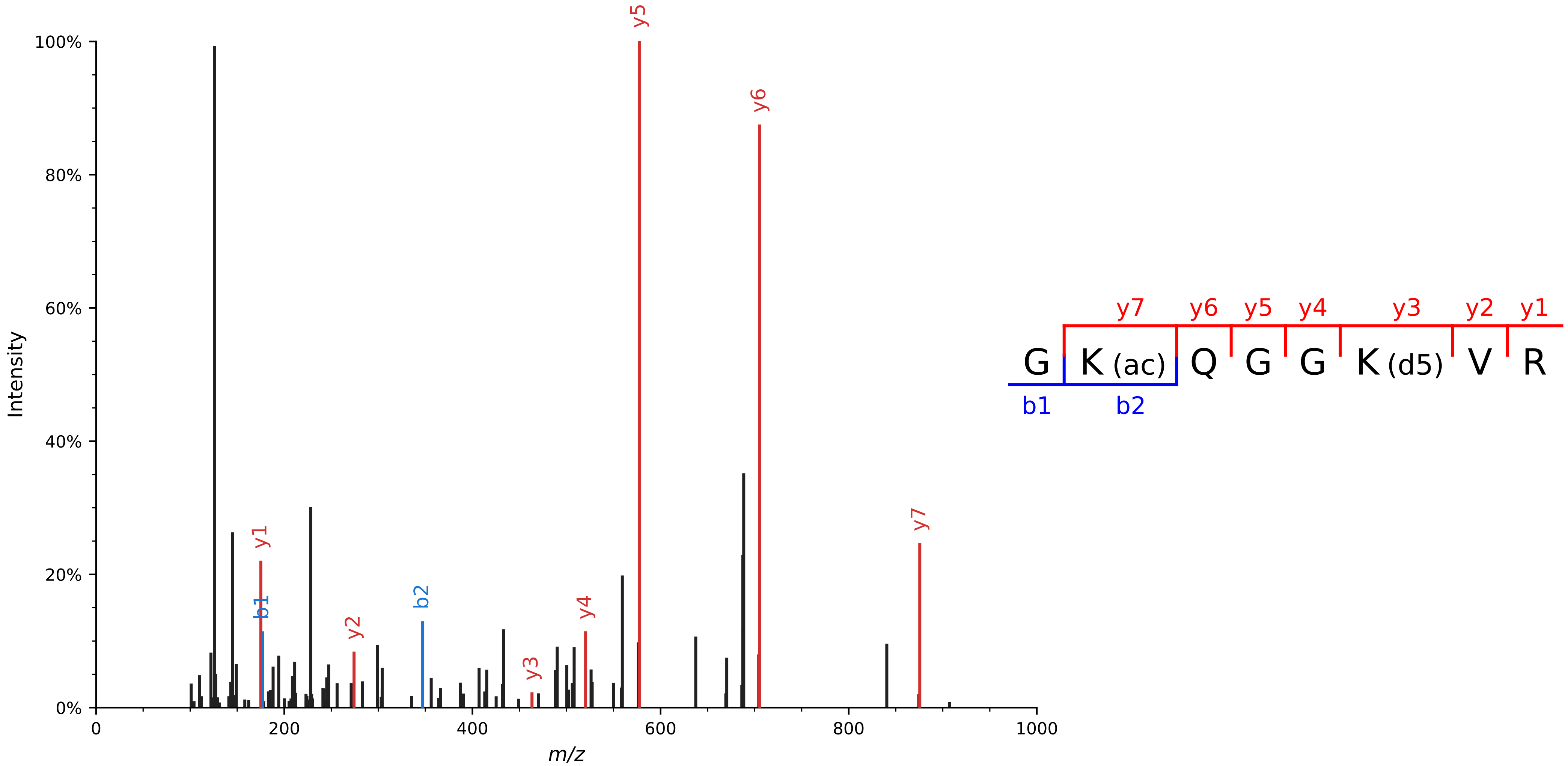

Raw file: HF240124_RN_spleen_D5PRO_PIC_3, Scan: 6450, m/z: 512.29076, Charge: 2, RT (min): 12.69, Score: 117.16

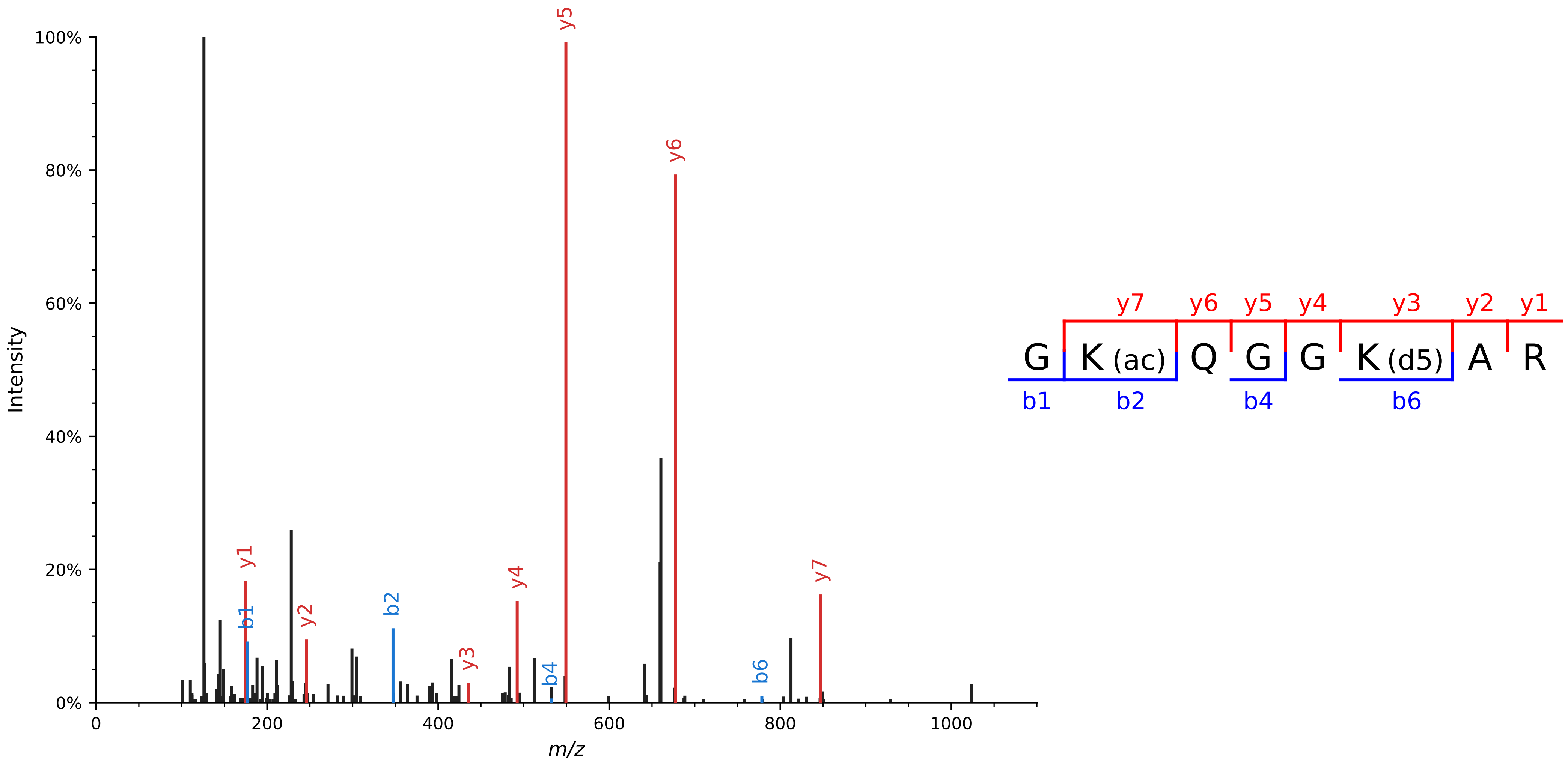

Raw file: HF240124_RN_spleen_D5PRO_PIC_3, Scan: 6620, m/z: 512.29076, Charge: 2, RT (min): 13.04, Score: 94.662

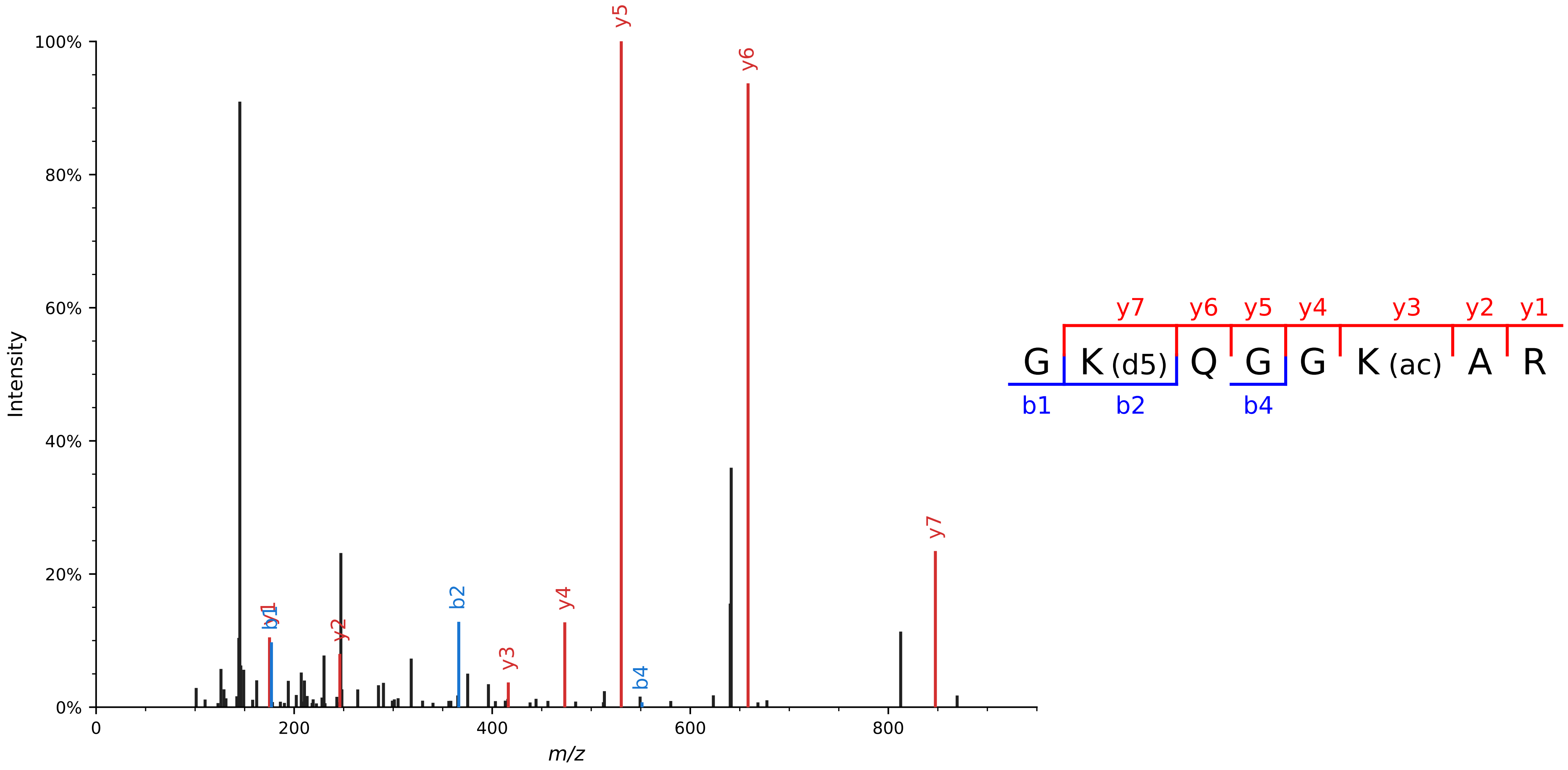

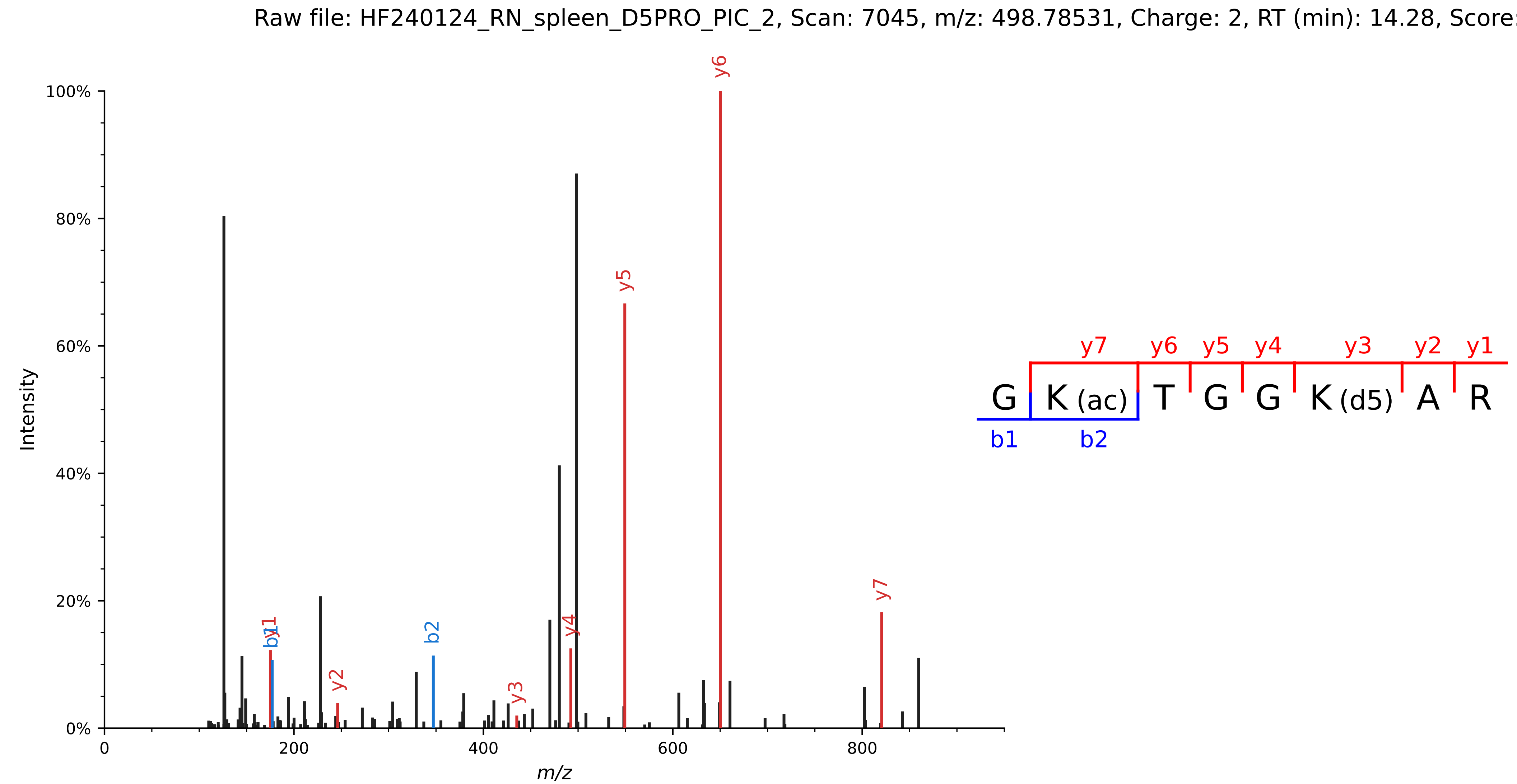
Raw file: HF240124_RN_spleen_D5PRO_PIC_2, Scan: 7045, m/z: 498.78531, Charge: 2, RT (min): 14.28, Score: 103.91
Intensity
m/z
y6
y5
y7
y4
y3
y2
y1
b1
b2
G K (ac) T G G K (d5) A R

Raw file: HF240124_RN_spleen_D5PRO_PIC_3, Scan: 10137, m/z: 545.79326, Charge: 2, RT (min): 19.33, Score: 87.582

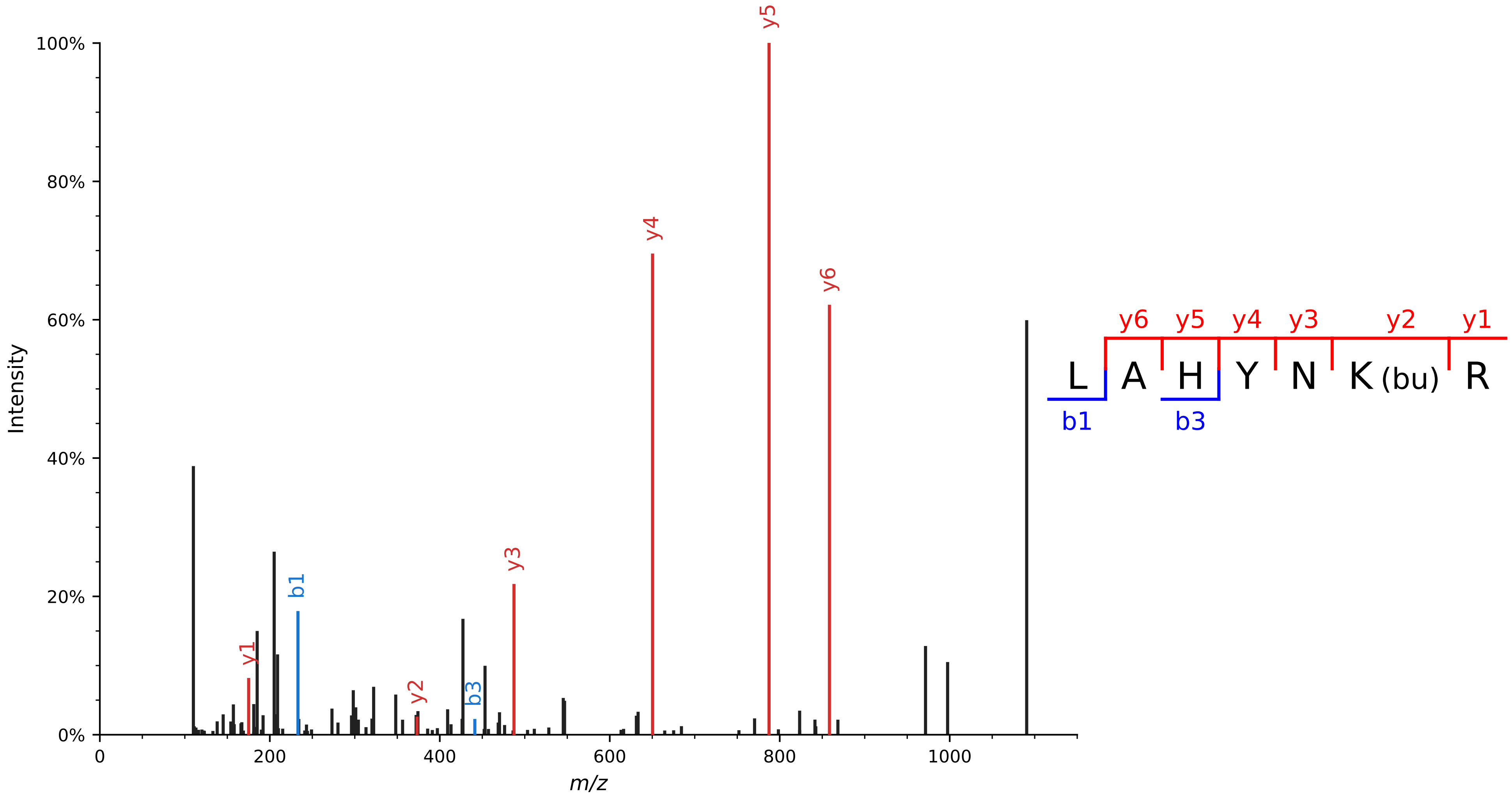

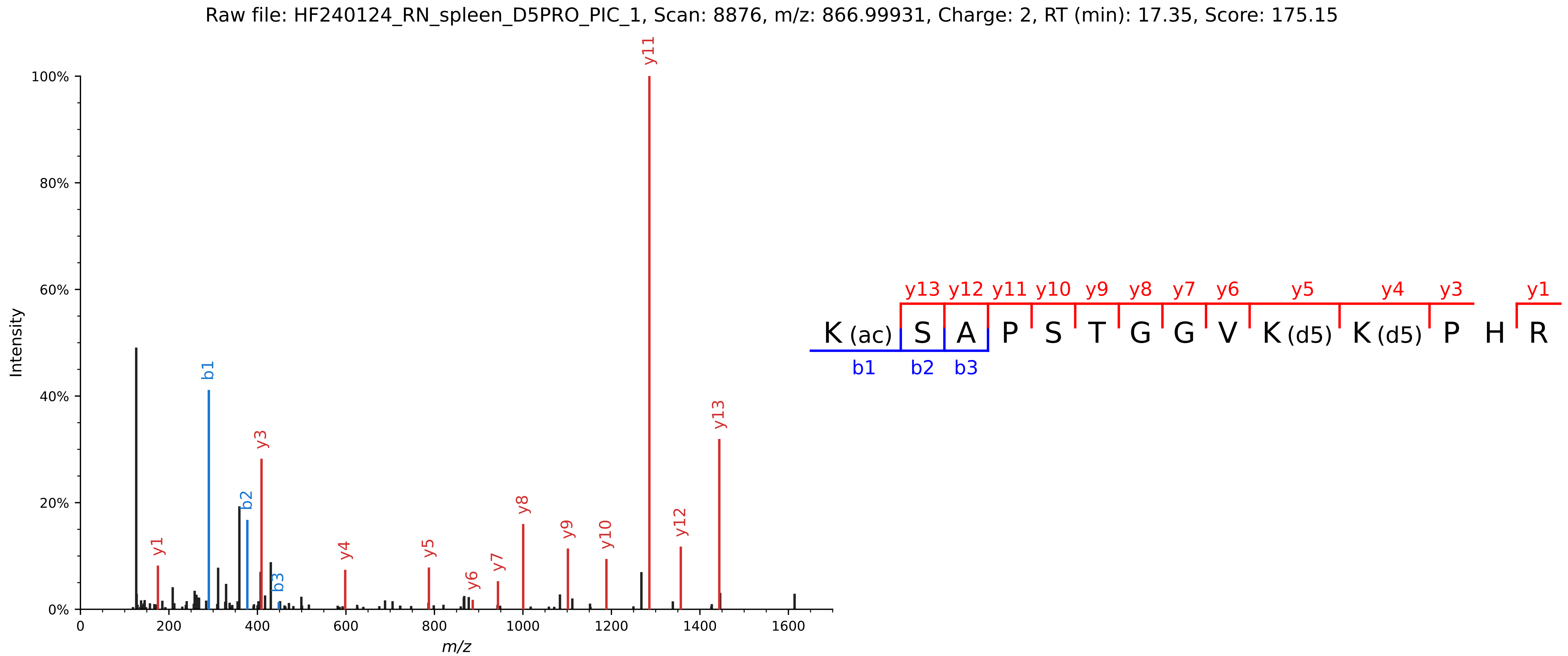

Raw file: HF240124_RN_spleen_D5PRO_PIC_1, Scan: 8876, m/z: 866.99931, Charge: 2, RT (min): 17.35, Score: 175.15
Intensity
m/z
y1
b1
b2
y3
b3
y4
y5
y6
y7
y8
y9
y10
y11
y12
y13
y13 y12 y11 y10 y9 y8 y7 y6 y5 y4 y3 y1
K (ac) S A P S T G G V K (d5) K (d5) P H R
b1 b2 b3

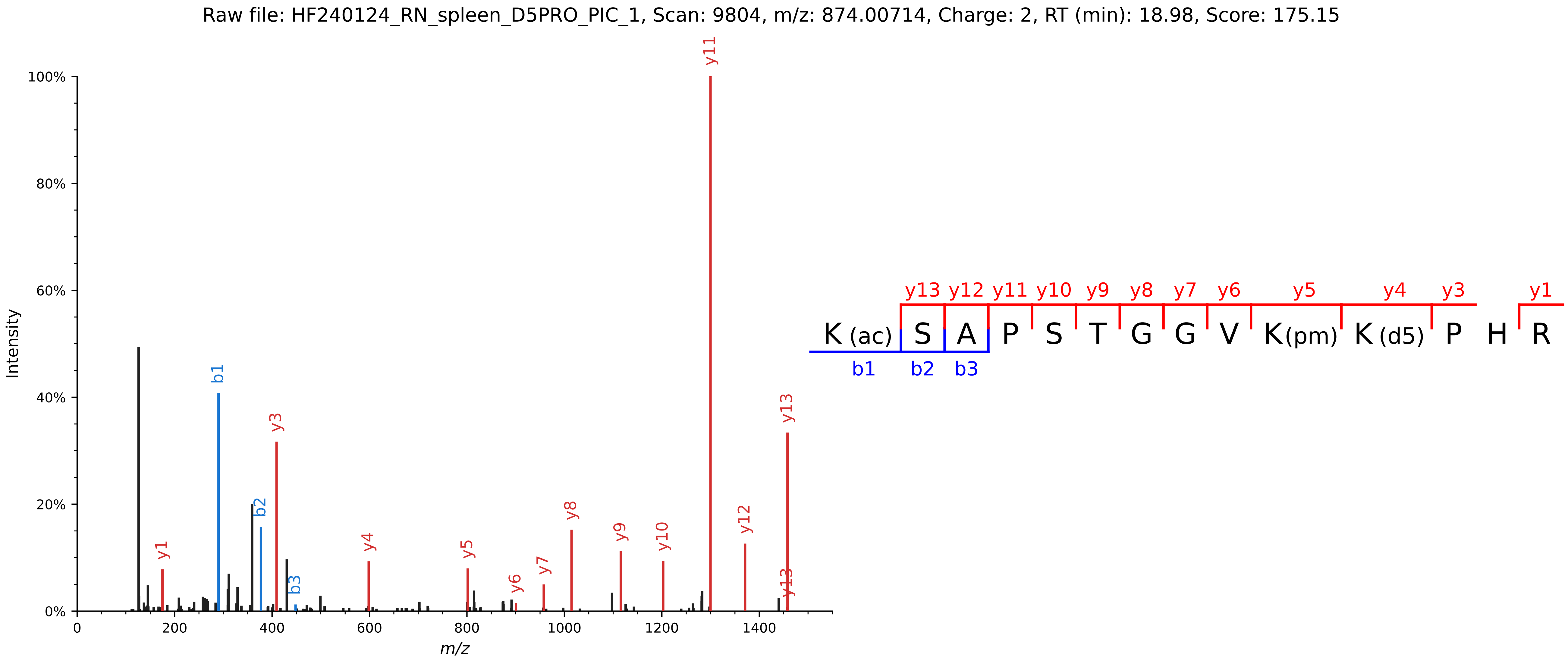
Raw file: HF240124_RN_spleen_D5PRO_PIC_1, Scan: 9804, m/z: 874.00714, Charge: 2, RT (min): 18.98, Score: 175.15
Intensity
m/z
y1 b1 b2 b3 y3 y4 y5 y6 y7 y8 y9 y10 y11 y12 y13
K(ac) S A P S T G G V K(pm) K(d5) P H R

Raw file: HF240124_RN_spleen_D5PRO_PIC_2, Scan: 6820, m/z: 587.67907, Charge: 3, RT (min): 13.90, Score: 49.298

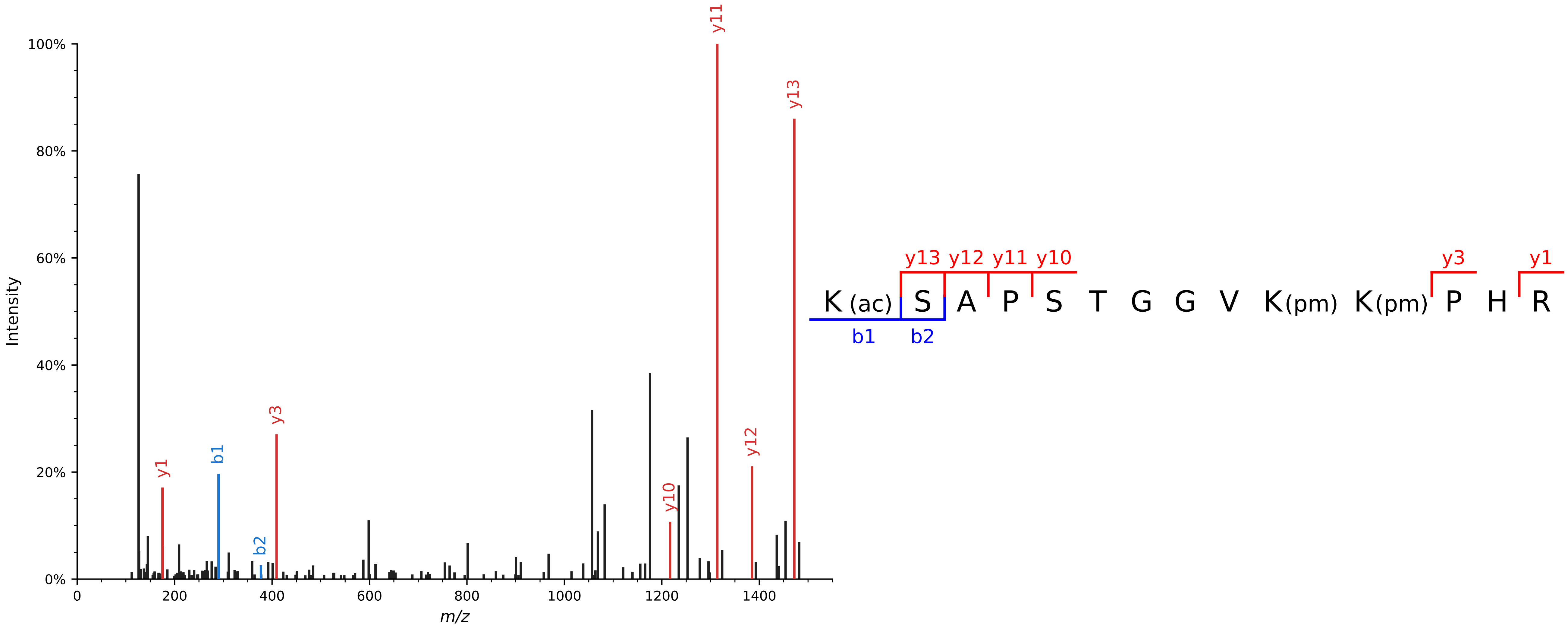

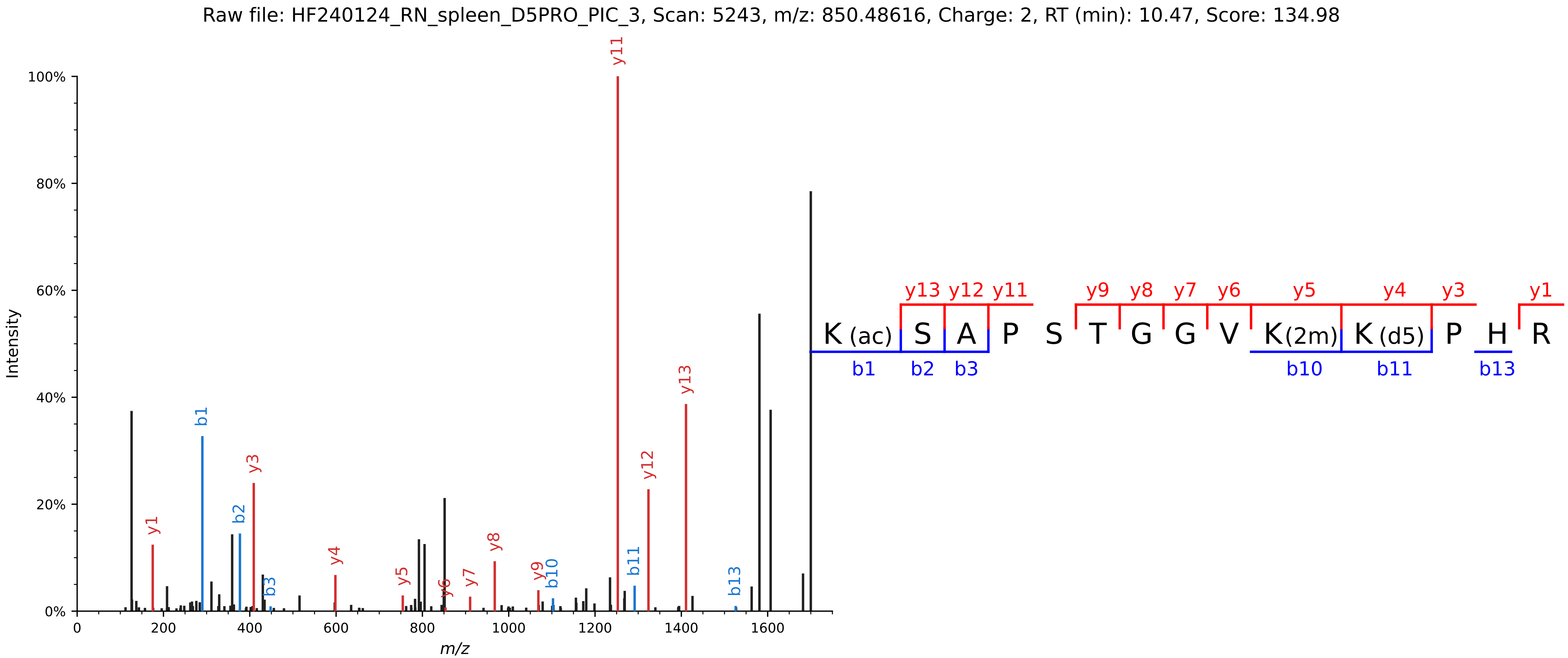

Raw file: HF240124_RN_spleen_D5PRO_PIC_3, Scan: 5243, m/z: 850.48616, Charge: 2, RT (min): 10.47, Score: 134.98
Intensity
m/z
100%
80%
60%
40%
20%
0%
0
200
400
600
800
1000
1200
1400
1600
y1 b1 b2 y3 b3 y4 y5 y6 y7 y8 y9 b10 y11 b11 y12 y13 b13
y13 y12 y11 y9 y8 y7 y6 y5 y4 y3 y1
K(ac) S A P S T G G V K(2m) K(d5) P H R
b1 b2 b3 b10 b11 b13

Raw file: HF240124_RN_spleen_D5PRO_PIC_3, Scan: 11315, m/z: 589.35619, Charge: 3, RT (min): 21.40, Score: 96.734

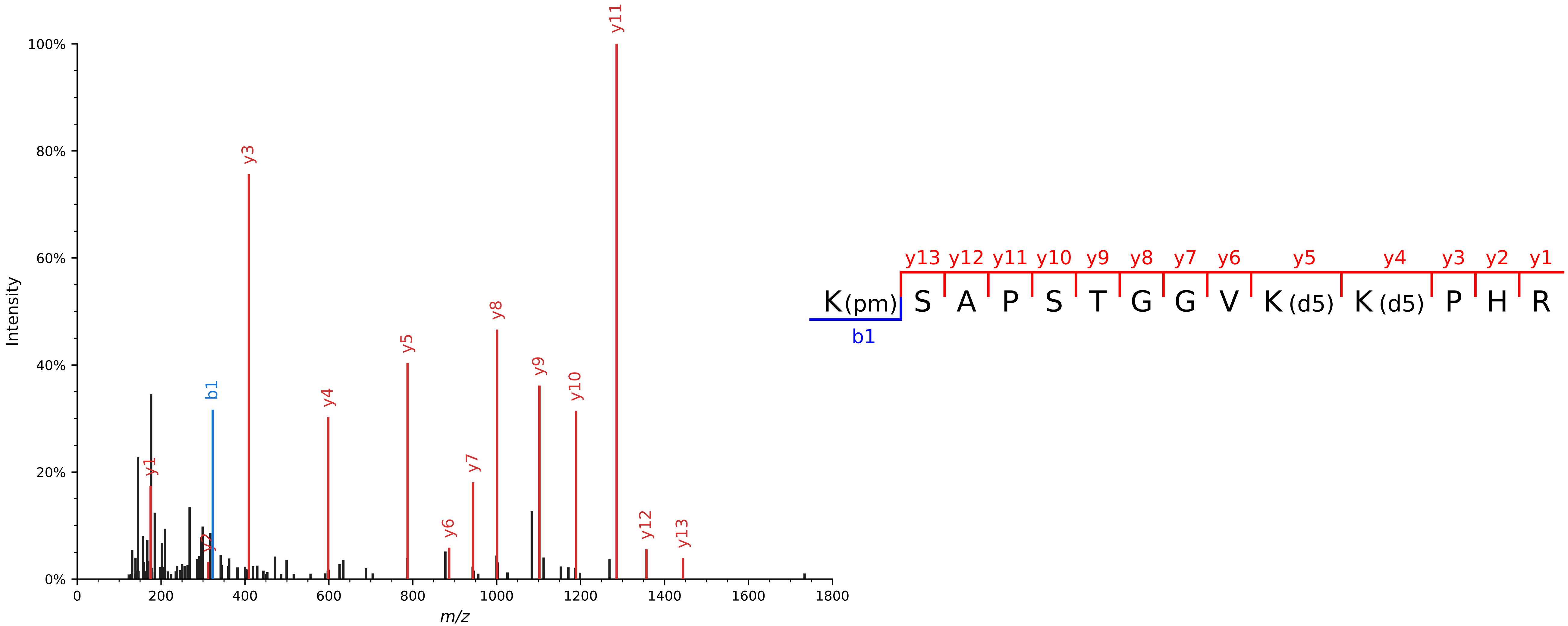

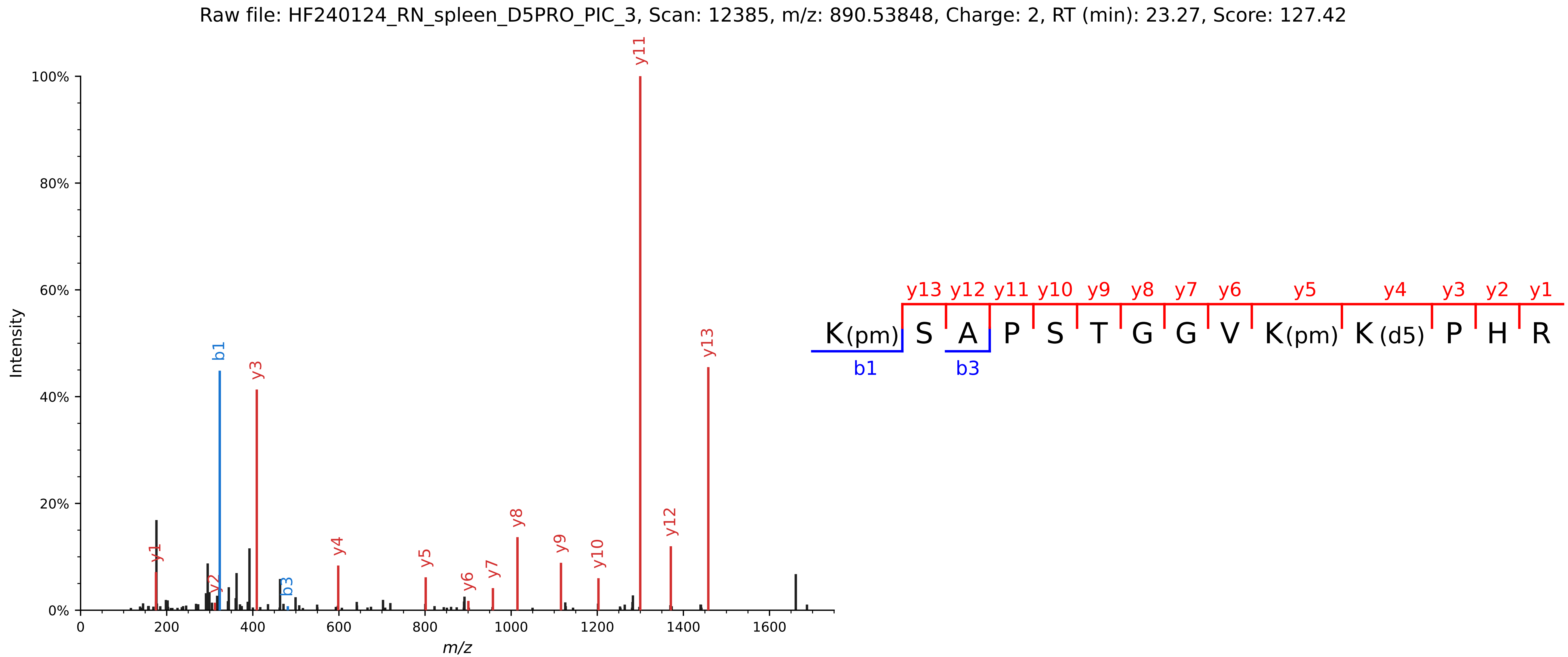
Raw file: HF240124_RN_spleen_D5PRO_PIC_3, Scan: 12385, m/z: 890.53848, Charge: 2, RT (min): 23.27, Score: 127.42
Intensity
m/z
100%
80%
60%
40%
20%
0%
0
200
400
600
800
1000
1200
1400
1600
y11
b1
y3
y13
y8
y12
y9
y4
y10
y5
y1
y7
y6
b3
y2
y13 y12 y11 y10 y9 y8 y7 y6 y5 y4 y3 y2 y1
K(pm) S A P S T G G V K(pm) K(d5) P H R
b1
b3

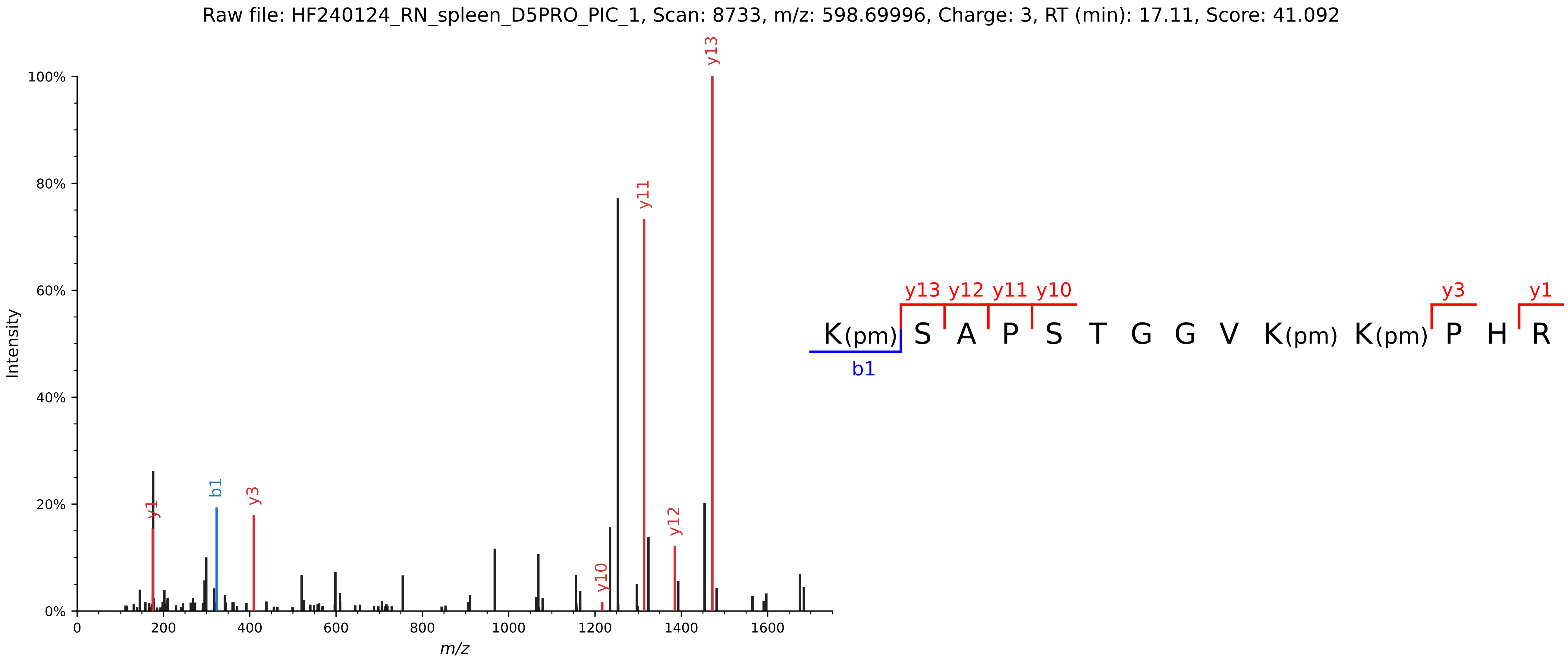

Raw file: HF240124_RN_spleen_D5PRO_PIC_1, Scan: 8733, m/z: 598.69996, Charge: 3, RT (min): 17.11, Score: 41.092
100%
80%
60%
40%
20%
0%
Intensity
0
200
400
600
800
1000
1200
1400
1600
m/z
y1
b1
y3
y10
y11
y12
y13
y13 y12 y11 y10
y3
y1
K(pm) S A P S T G G V K(pm) K(pm) P H R
b1

Raw file: HF240124_RN_spleen_D5PRO_PIC_3, Scan: 7069, m/z: 867.0175, Charge: 2, RT (min): 13.82, Score: 131.83

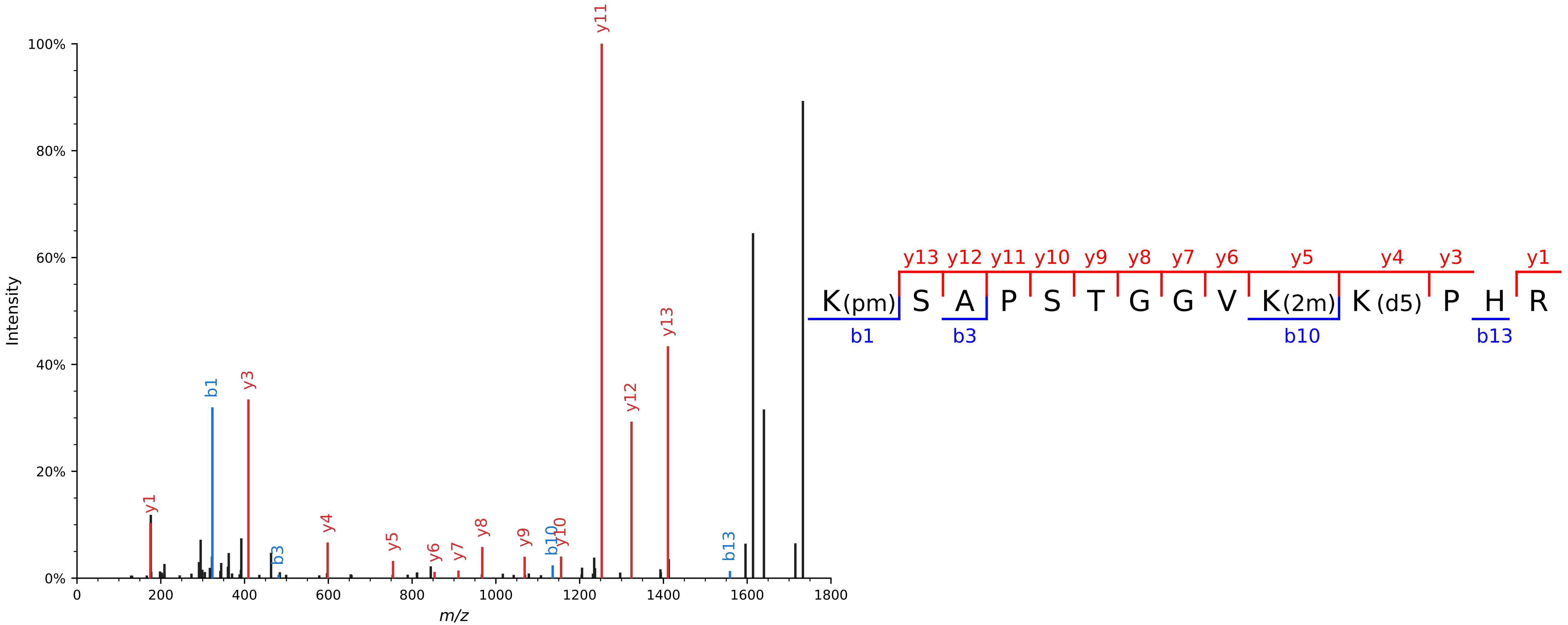

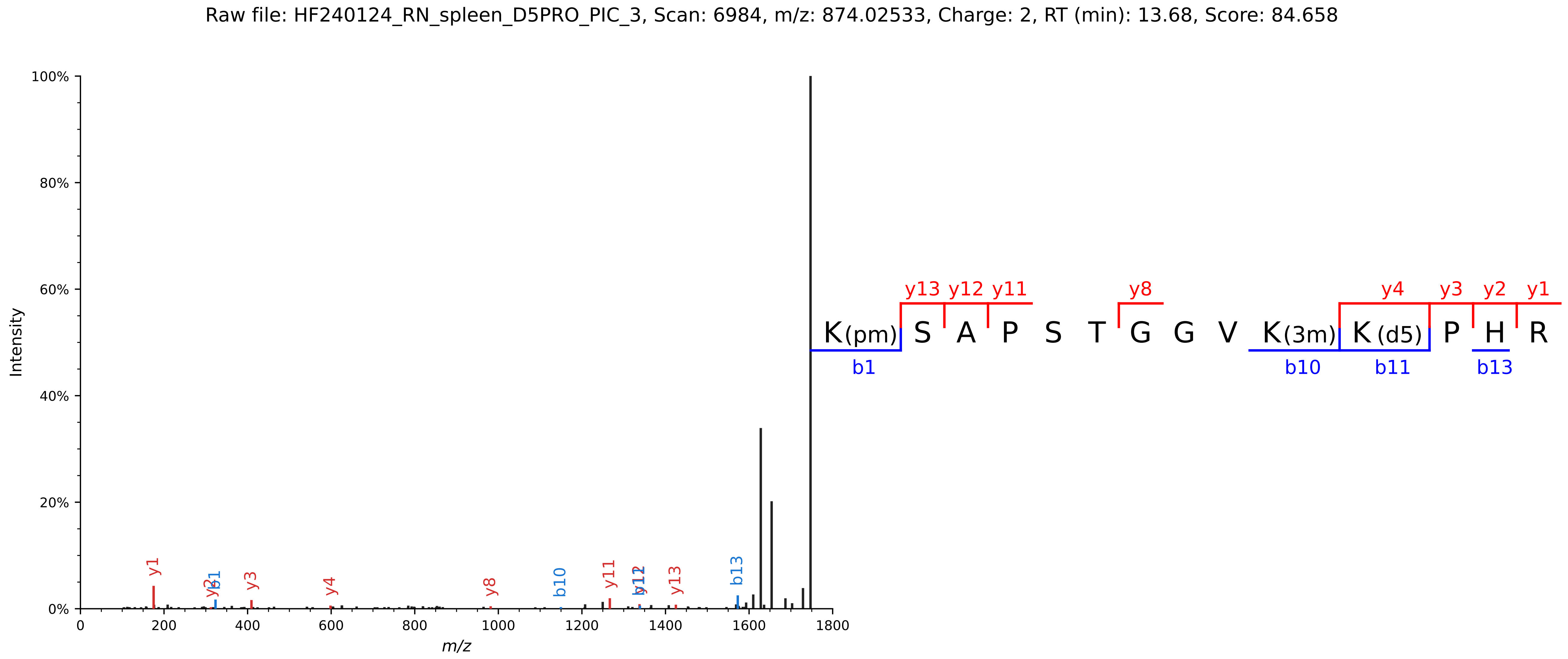

Raw file: HF240124_RN_spleen_D5PRO_PIC_3, Scan: 6984, m/z: 874.02533, Charge: 2, RT (min): 13.68, Score: 84.658
Intensity
m/z
y1
y2
b1
y3
y4
y8
b10
y11
b12
y12
y13
b13
K(pm) S A P S T G G V K(3m) K(d5) P H R
y13 y12 y11 y8 y4 y3 y2 y1
b1 b10 b11 b13

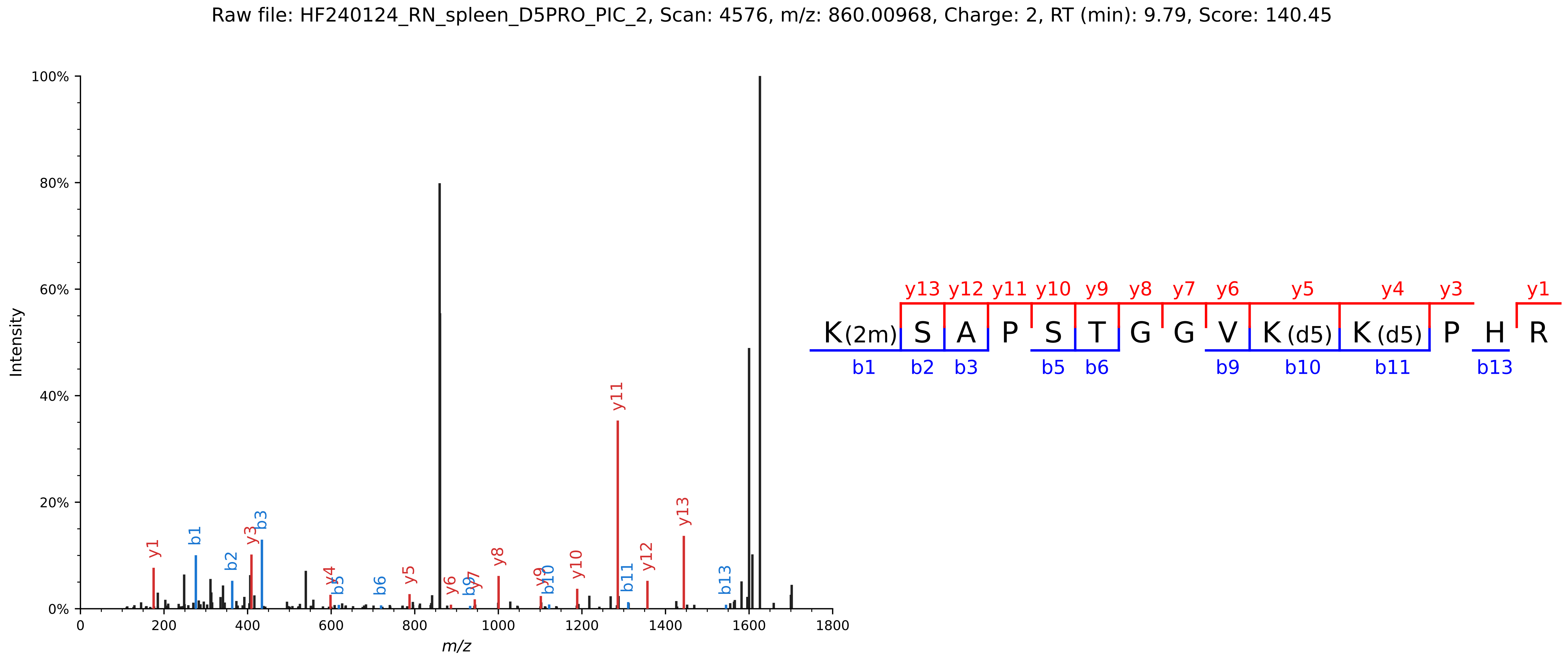

Raw file: HF240124_RN_spleen_D5PRO_PIC_2, Scan: 4576, m/z: 860.00968, Charge: 2, RT (min): 9.79, Score: 140.45
Intensity
m/z
K(2m) S A P S T G G V K(d5) K(d5) P H R

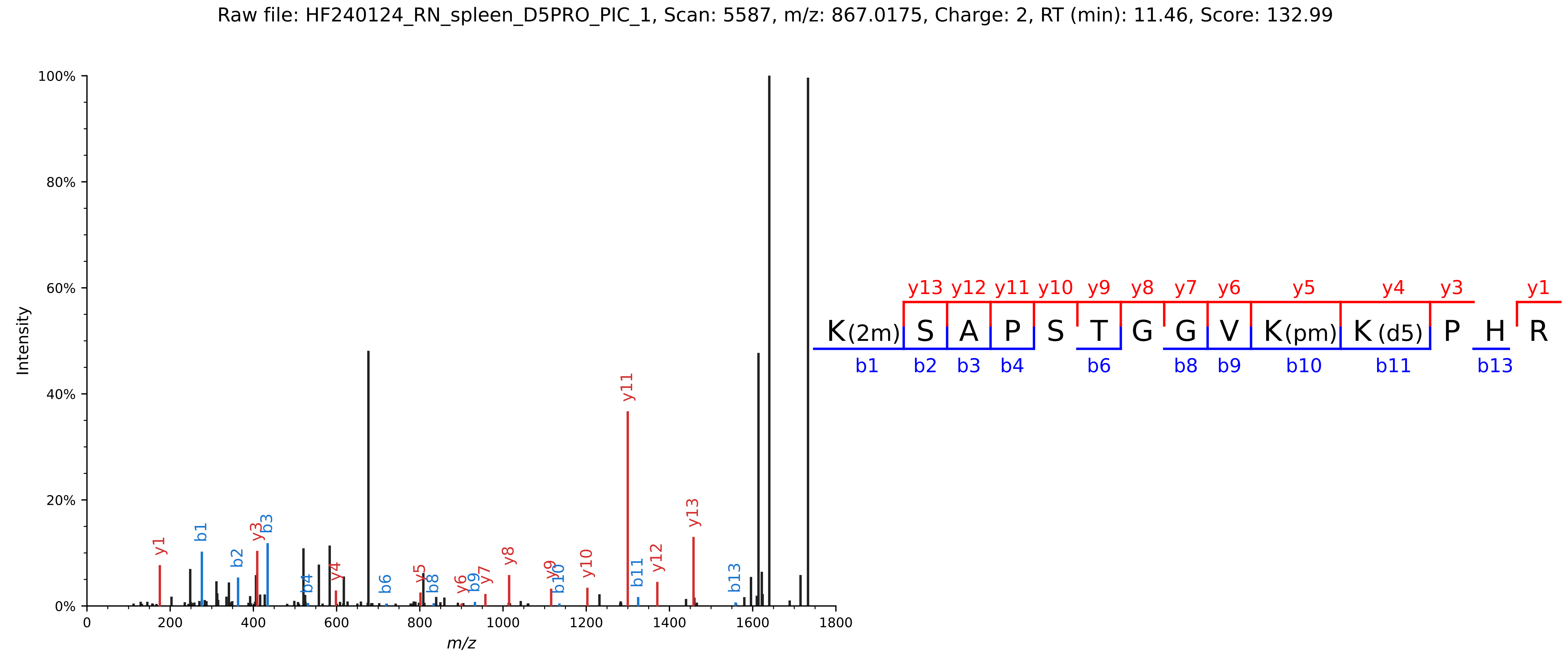

Raw file: HF240124_RN_spleen_D5PRO_PIC_1, Scan: 5587, m/z: 867.0175, Charge: 2, RT (min): 11.46, Score: 132.99
Intensity
m/z
K(2m) S A P S T G G V K(pm) K(d5) P H R
y13 y12 y11 y10 y9 y8 y7 y6 y5 y4 y3 y1
b1 b2 b3 b4 b6 b8 b9 b10 b11 b13

Raw file: HF240124_RN_spleen_D5PRO_PIC_2, Scan: 3394, m/z: 874.02533, Charge: 2, RT (min): 7.71, Score: 54.286

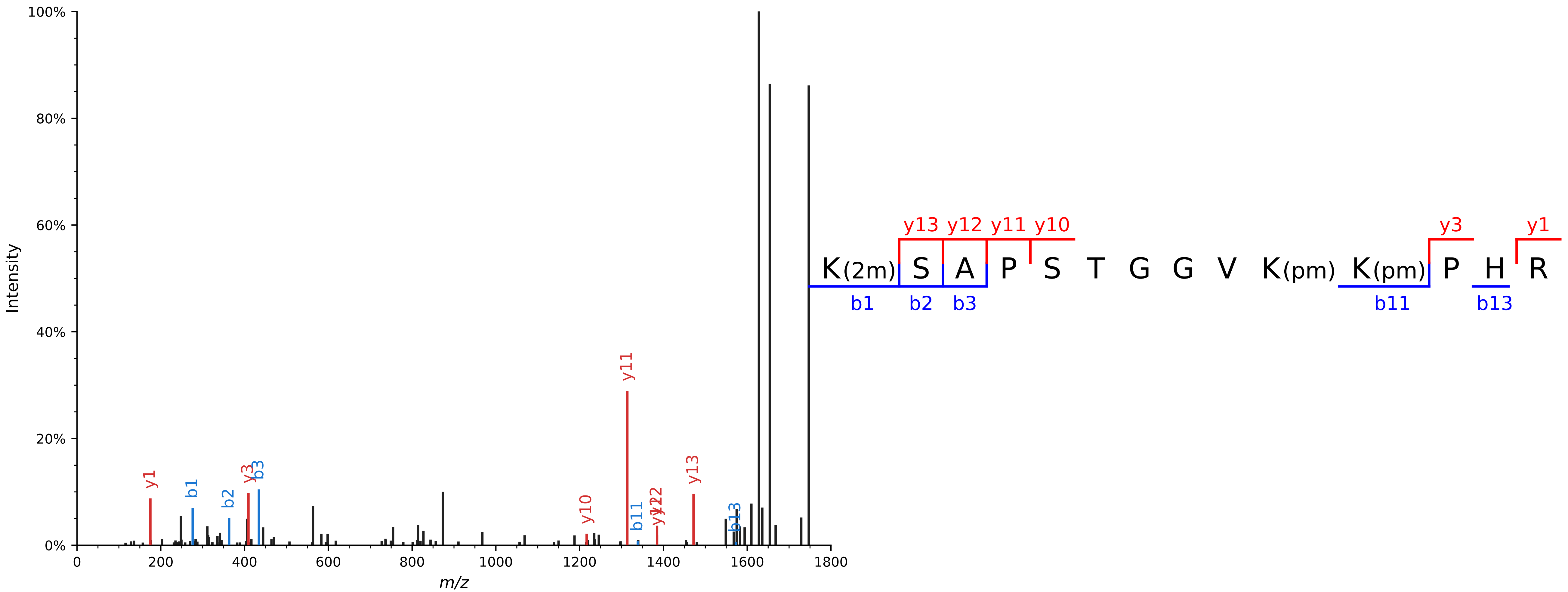

Raw file: HF240124_RN_spleen_D5PRO_PIC_3, Scan: 2158, m/z: 843.49653, Charge: 2, RT (min): 4.96, Score: 132.03

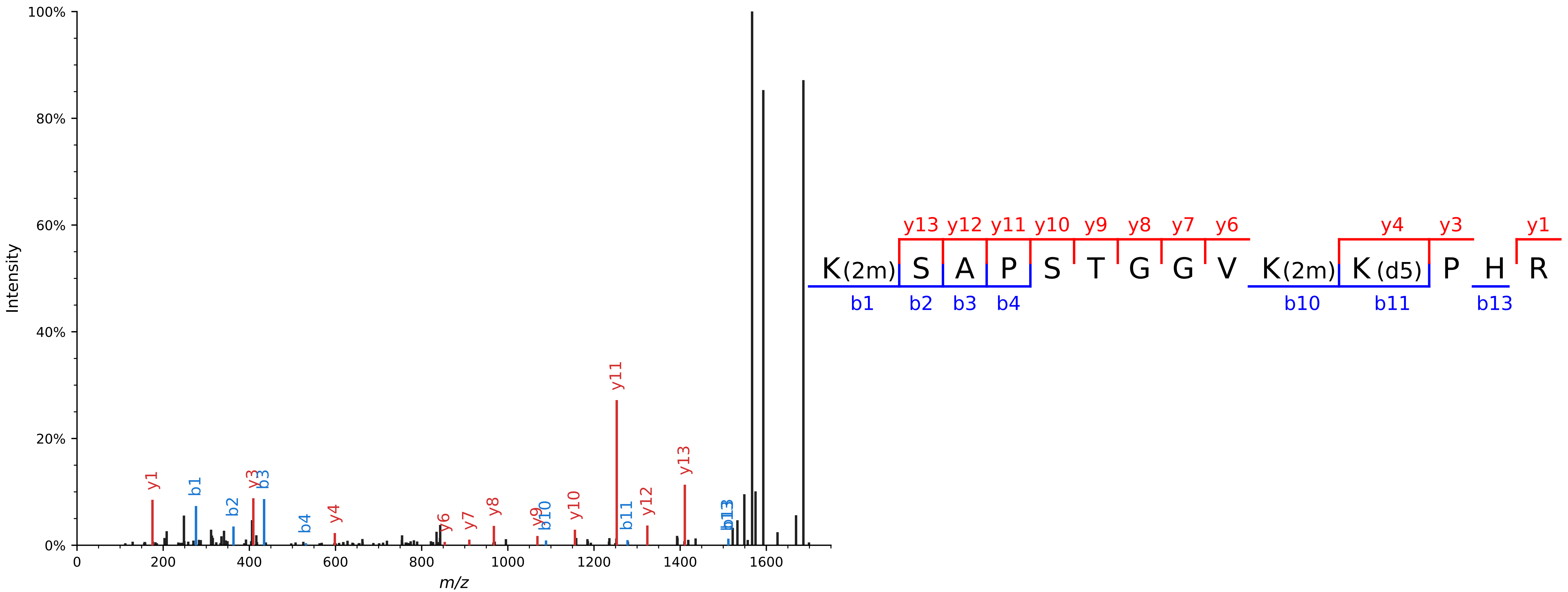

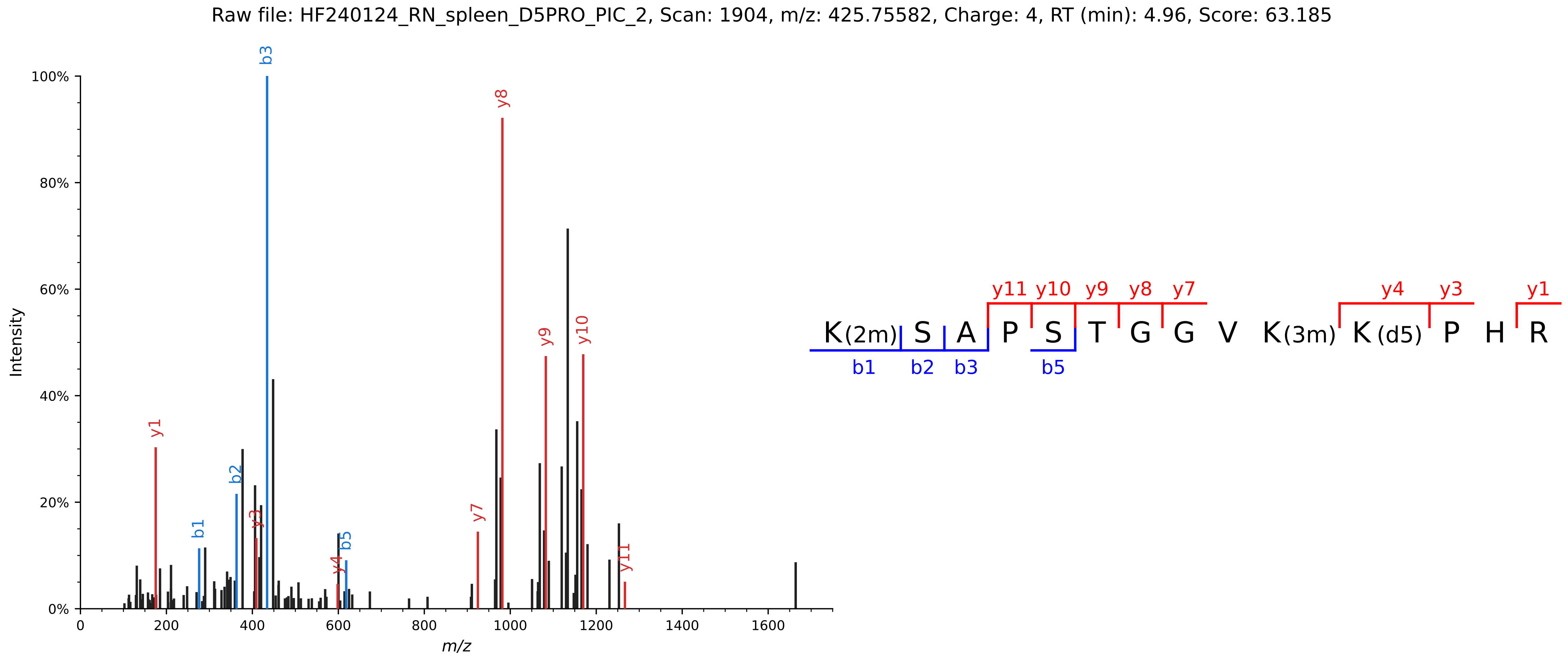
Raw file: HF240124_RN_spleen_D5PRO_PIC_2, Scan: 1904, m/z: 425.75582, Charge: 4, RT (min): 4.96, Score: 63.185
Intensity
m/z
y11 y10 y9 y8 y7 y4 y3 y1
K(2m) S A P S T G G V K(3m) K(d5) P H R
b1 b2 b3 b5

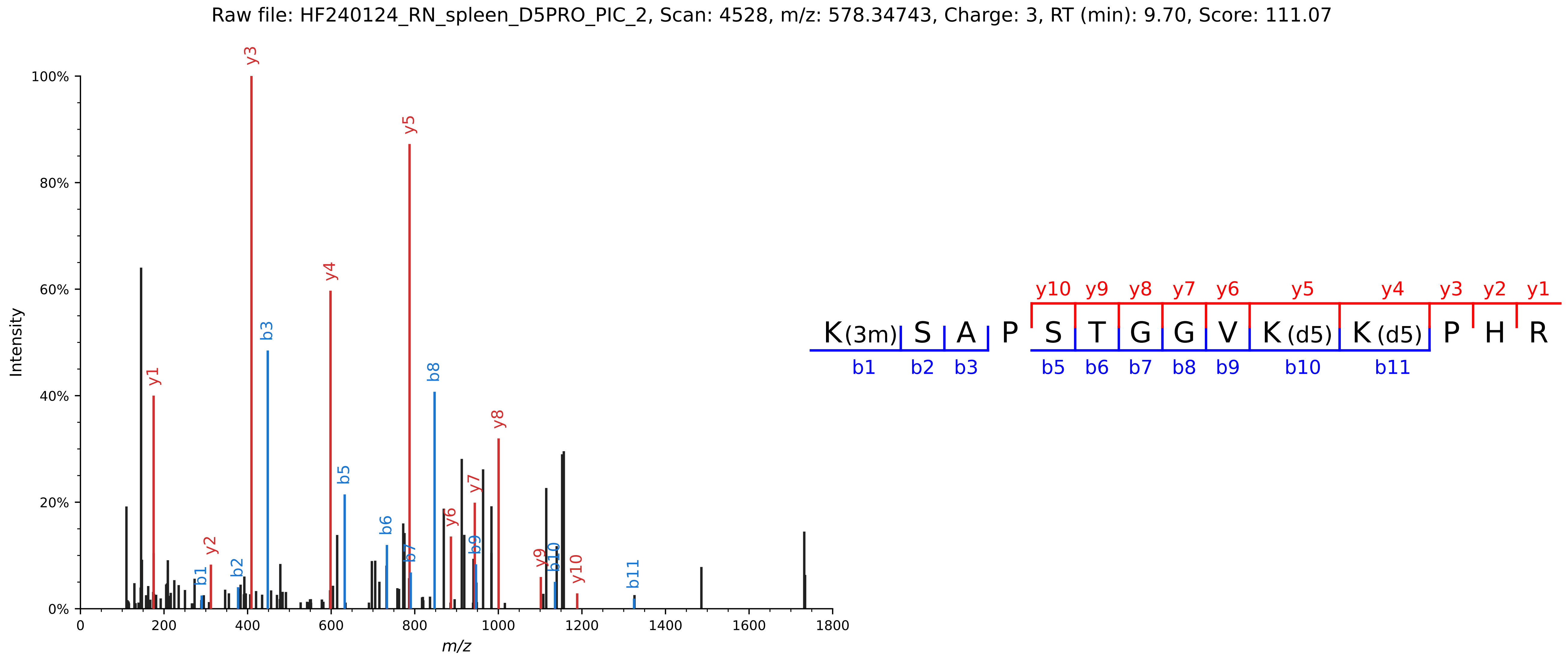
Raw file: HF240124_RN_spleen_D5PRO_PIC_2, Scan: 4528, m/z: 578.34743, Charge: 3, RT (min): 9.70, Score: 111.07
Intensity
m/z
K(3m) S A P S T G G V K(d5) K(d5) P H R
y10 y9 y8 y7 y6 y5 y4 y3 y2 y1
b1 b2 b3 b5 b6 b7 b8 b9 b10 b11

Raw file: HF240124_RN_spleen_D5PRO_PIC_1, Scan: 5536, m/z: 583.01931, Charge: 3, RT (min): 11.37, Score: 143.93

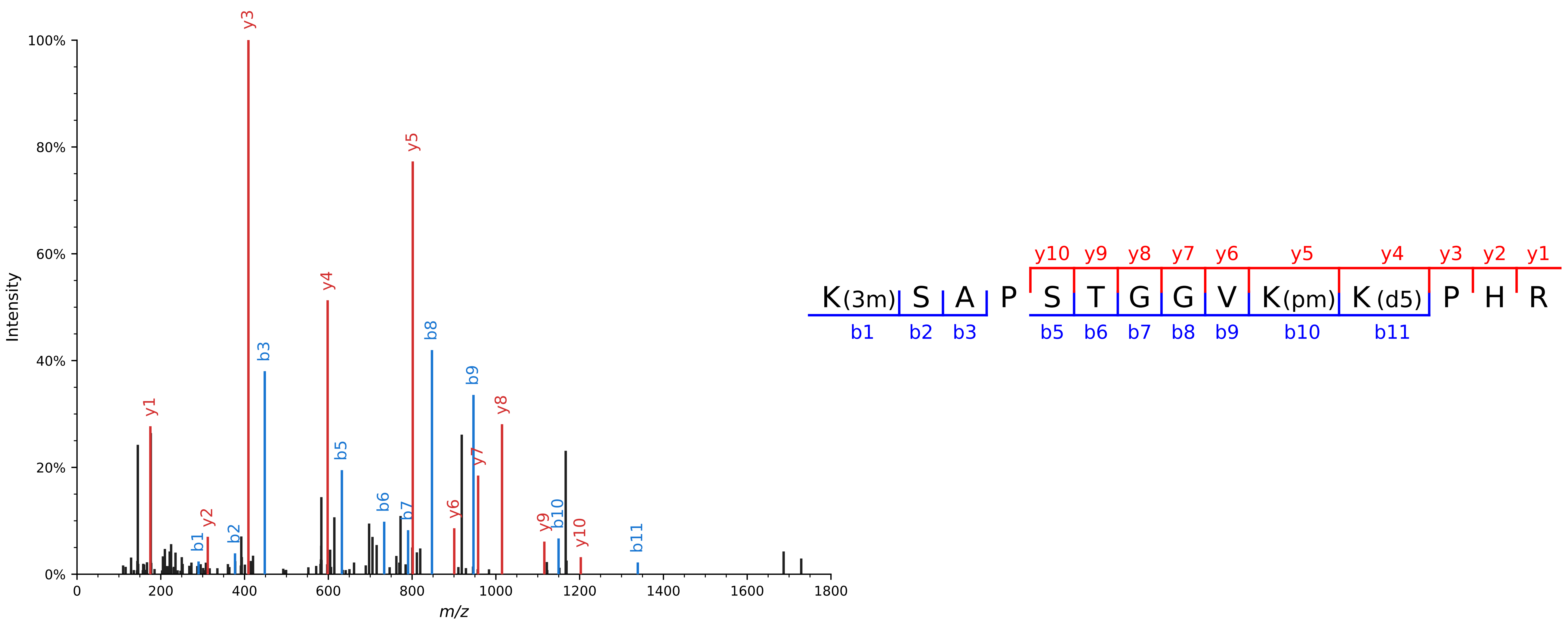

Raw file: HF240124_RN_spleen_D5PRO_PIC_1, Scan: 1775, m/z: 425.75582, Charge: 4, RT (min): 4.74, Score: 46.318

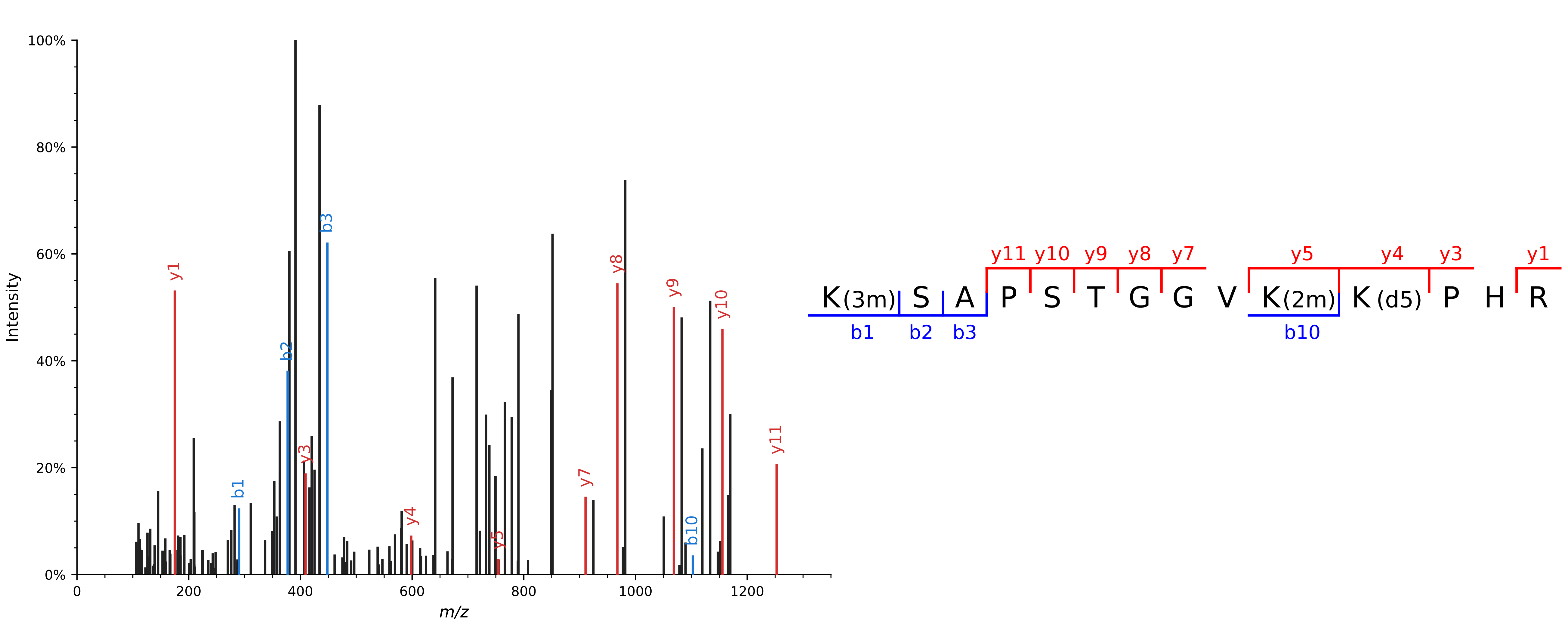

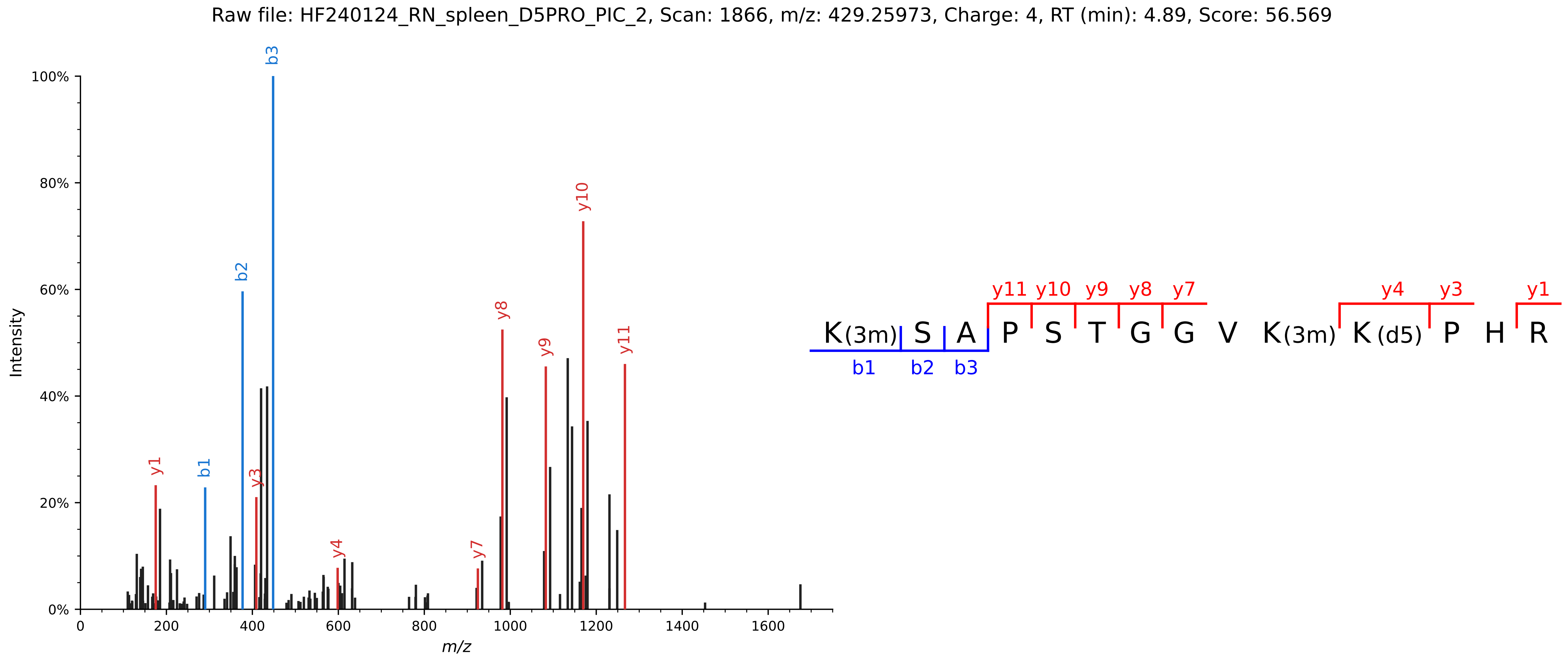

Raw file: HF240124_RN_spleen_D5PRO_PIC_2, Scan: 1866, m/z: 429.25973, Charge: 4, RT (min): 4.89, Score: 56.569
Intensity
m/z
100%
80%
60%
40%
20%
0%
0
200
400
600
800
1000
1200
1400
1600
b1
b2
b3
y1
y3
y4
y7
y8
y9
y10
y11
K(3m) S A P S T G G V K(3m) K(d5) P H R
y11 y10 y9 y8 y7 y4 y3 y1
b1 b2 b3

Raw file: HF240124_RN_spleen_D5PRO_PIC_3, Scan: 10840, m/z: 883.53065, Charge: 2, RT (min): 20.57, Score: 134.2

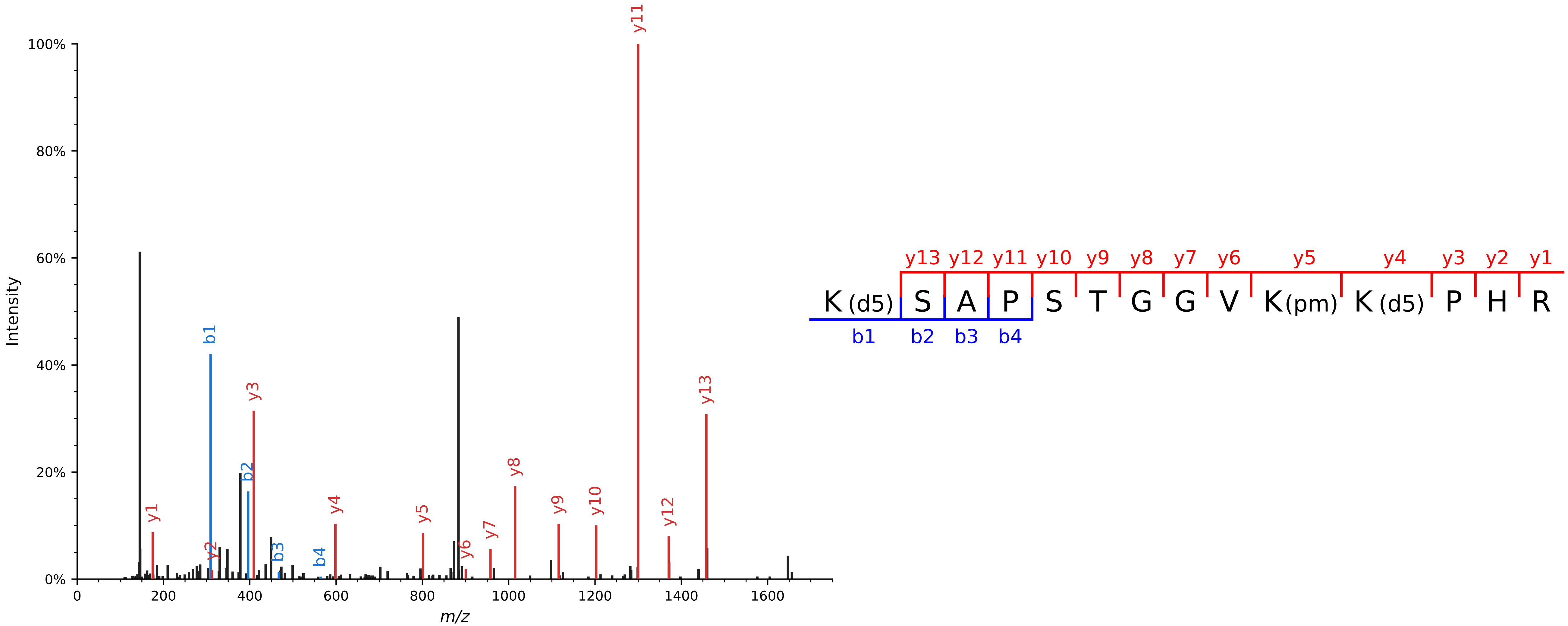

Raw file: HF240124_RN_spleen_D5PRO_PIC_1, Scan: 7612, m/z: 890.53848, Charge: 2, RT (min): 15.13, Score: 79.659

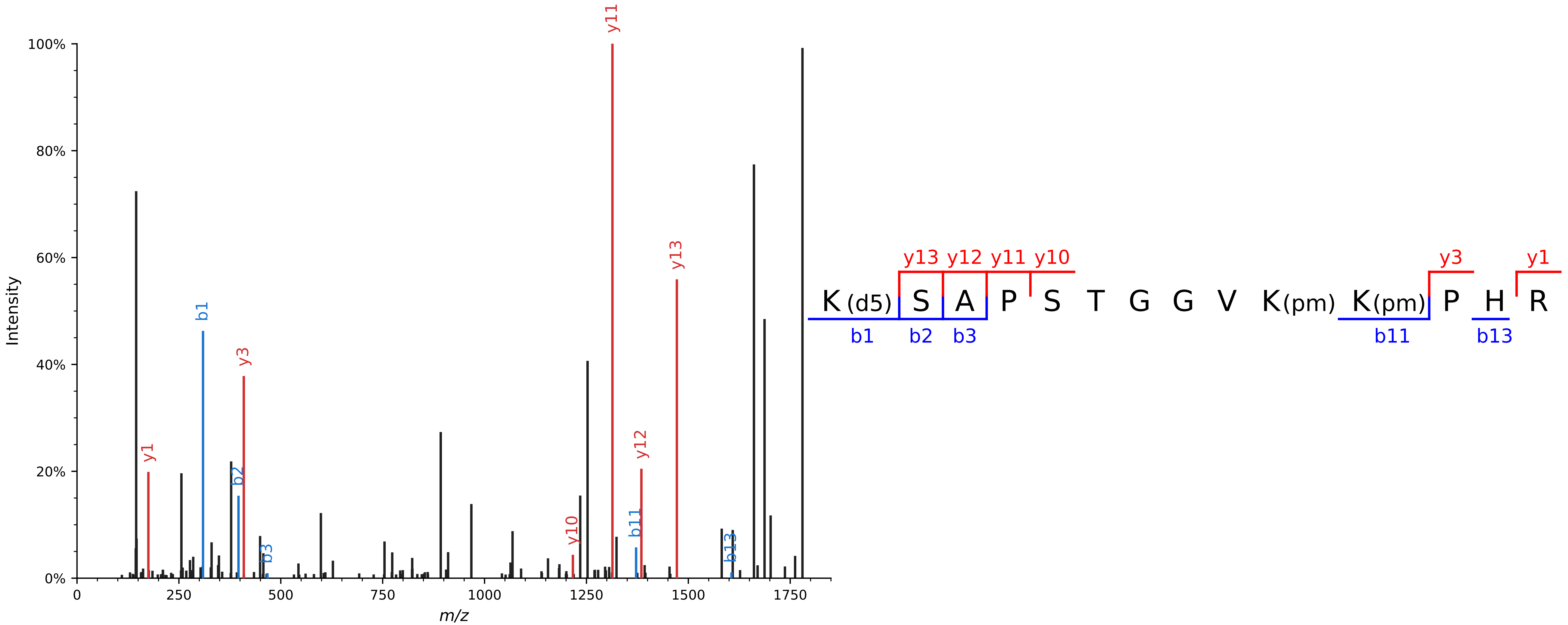

Raw file: HF240124_RN_spleen_D5PRO_PIC_1, Scan: 9230, m/z: 449.7613, Charge: 2, RT (min): 17.97, Score: 98.513

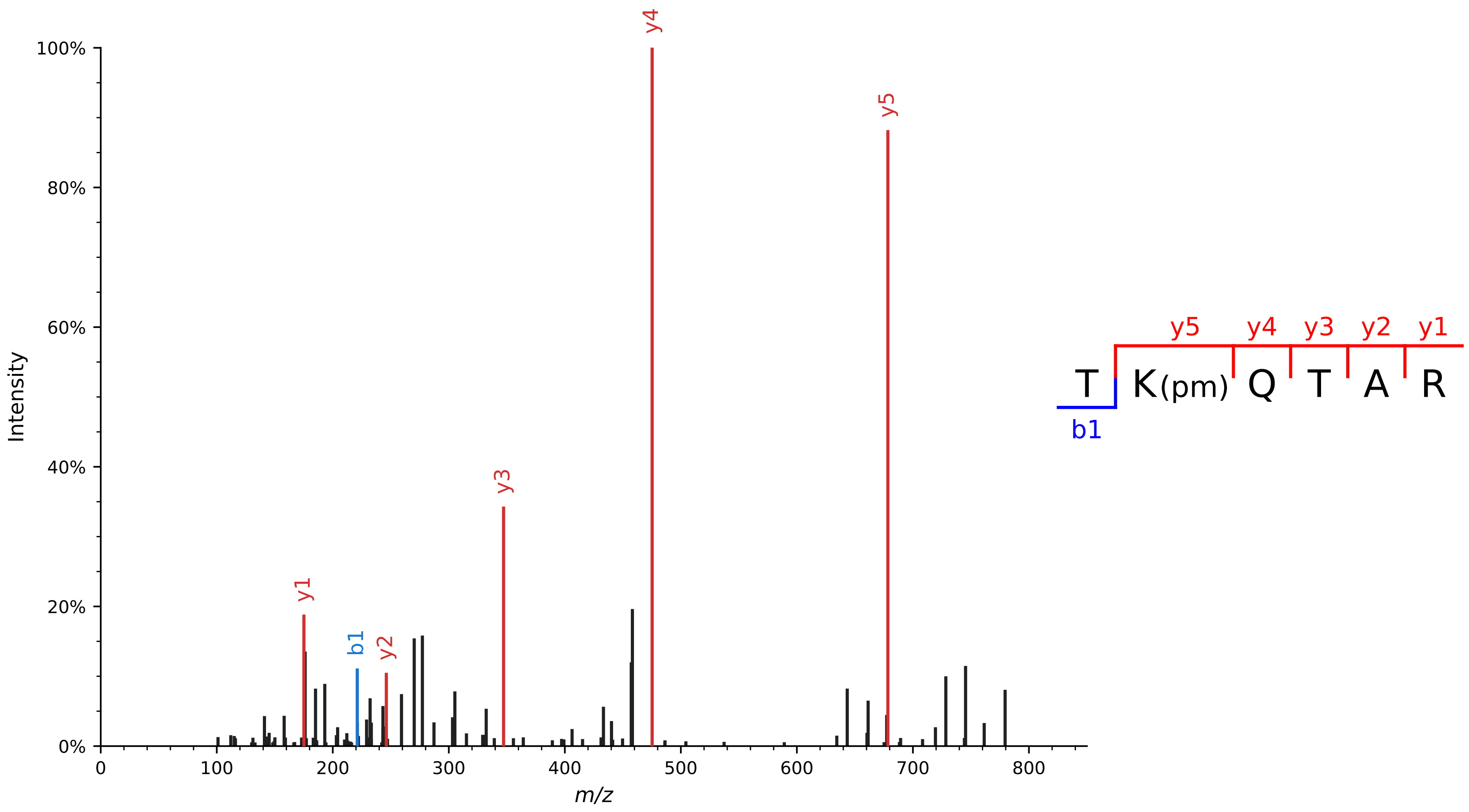

Raw file: HF240124_RN_spleen_D5PRO_PIC_3, Scan: 1837, m/z: 426.24033, Charge: 2, RT (min): 4.44, Score: 124.59

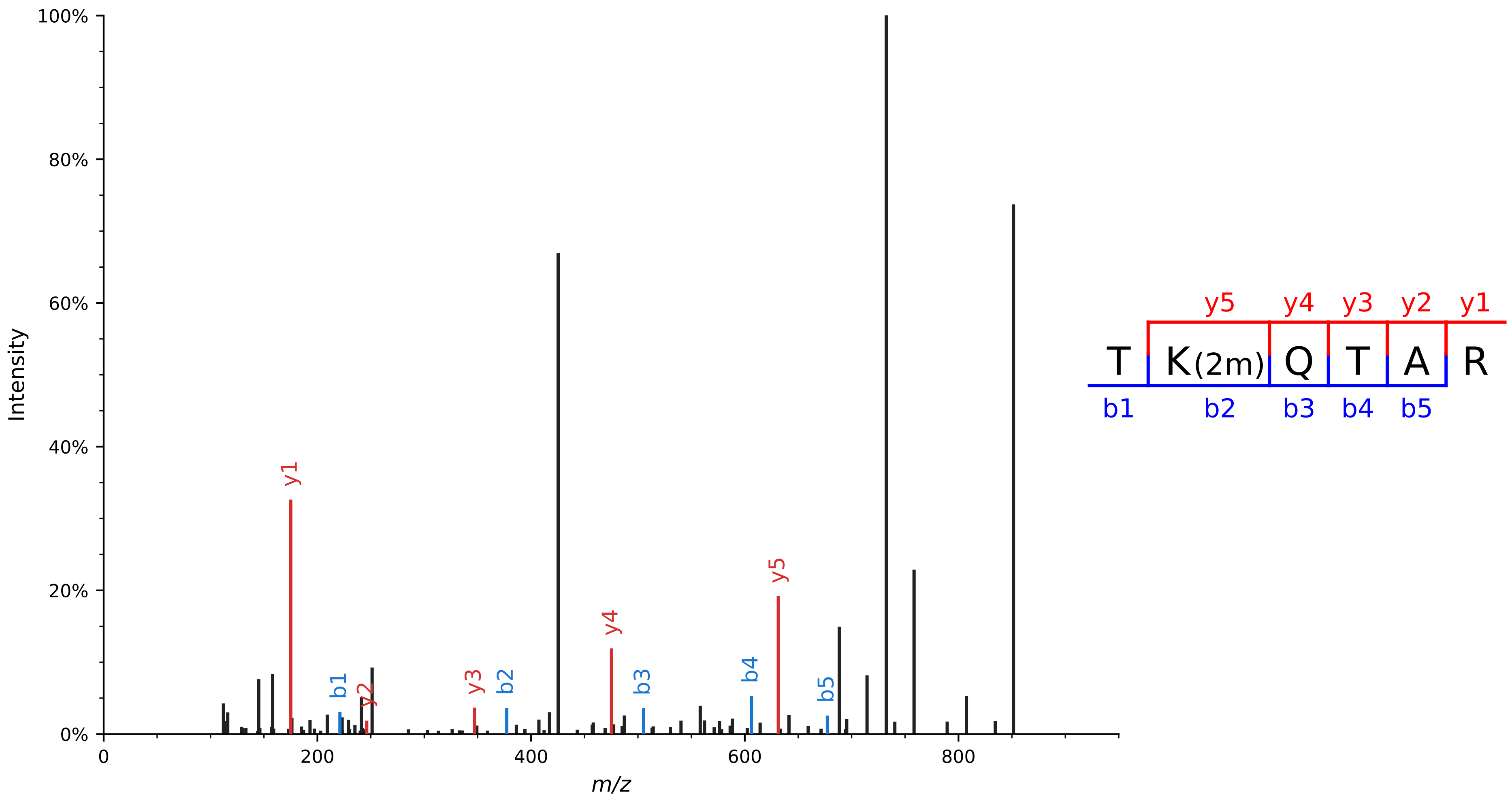

Raw file: HF240124_RN_spleen_D5PRO_PIC_3, Scan: 1838, m/z: 433.24815, Charge: 2, RT (min): 4.44, Score: 94.262

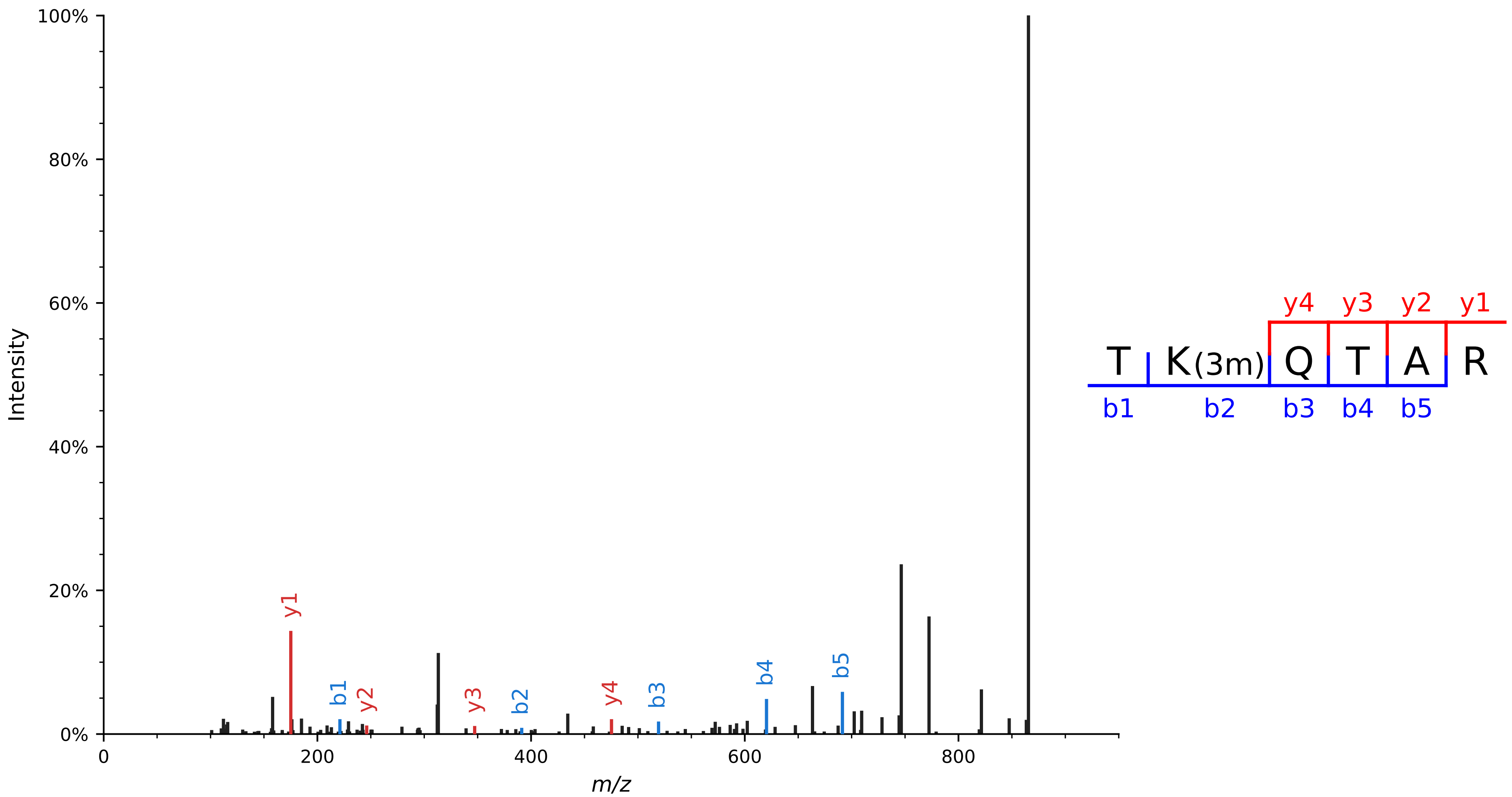

Raw file: HF240124_RN_spleen_D5PRO_PIC_3, Scan: 8579, m/z: 562.31697, Charge: 2, RT (min): 16.47, Score: 132.08

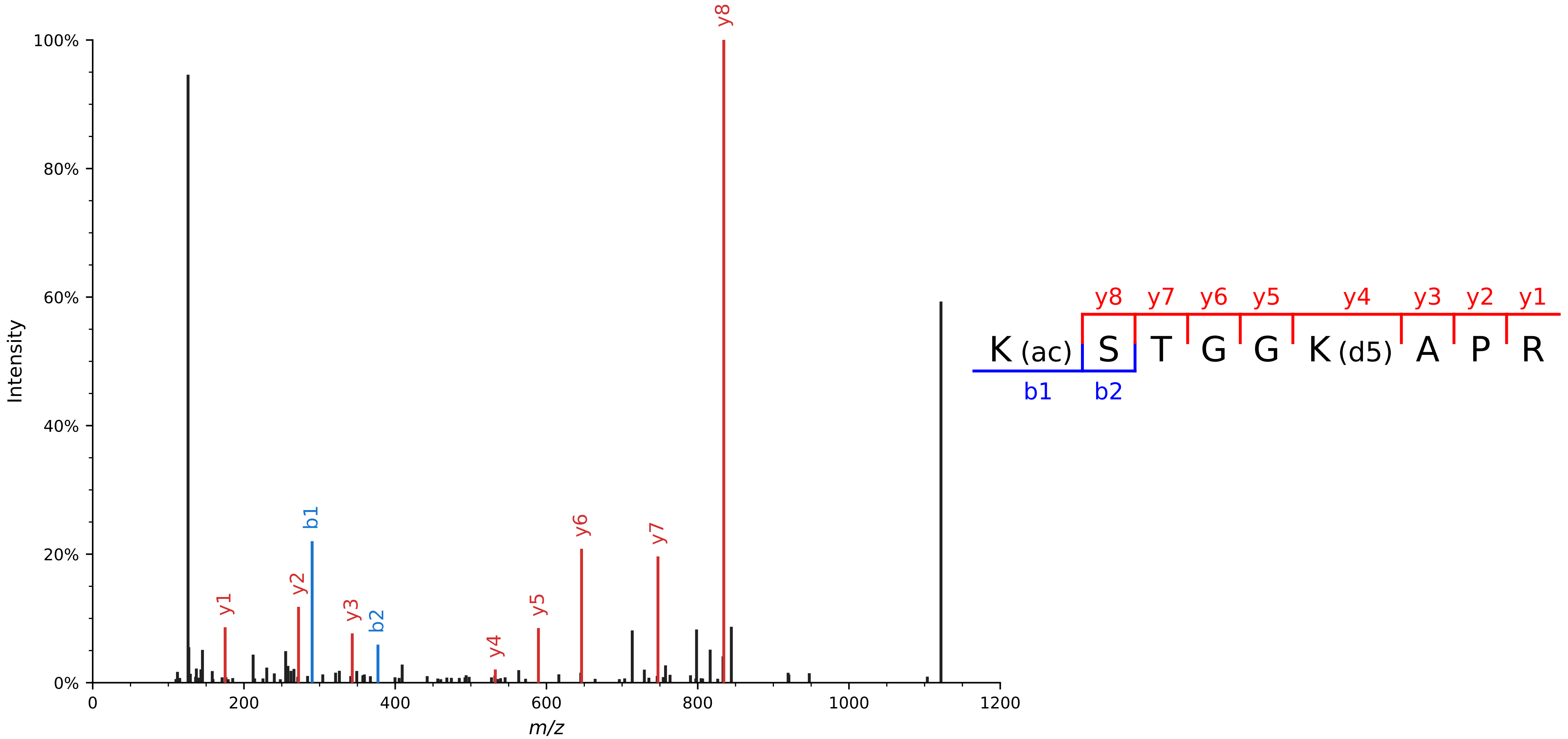

Raw file: HF240124_RN_spleen_D5PRO_PIC_3, Scan: 7633, m/z: 552.79345, Charge: 2, RT (min): 14.82, Score: 124.98

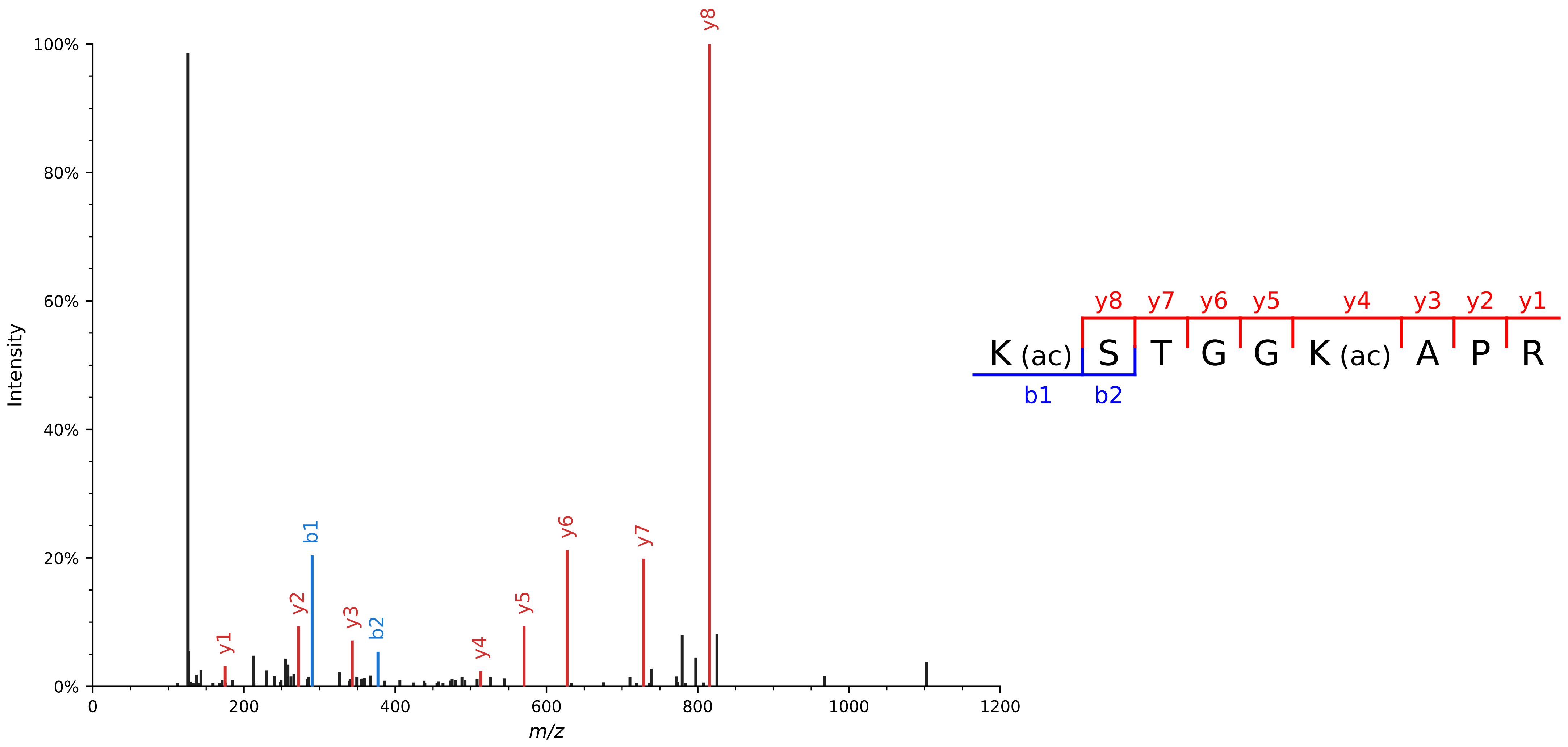

Raw file: HF240124_RN_spleen_D5PRO_PIC_3, Scan: 3495, m/z: 576.33262, Charge: 2, RT (min): 7.41, Score: 89.355

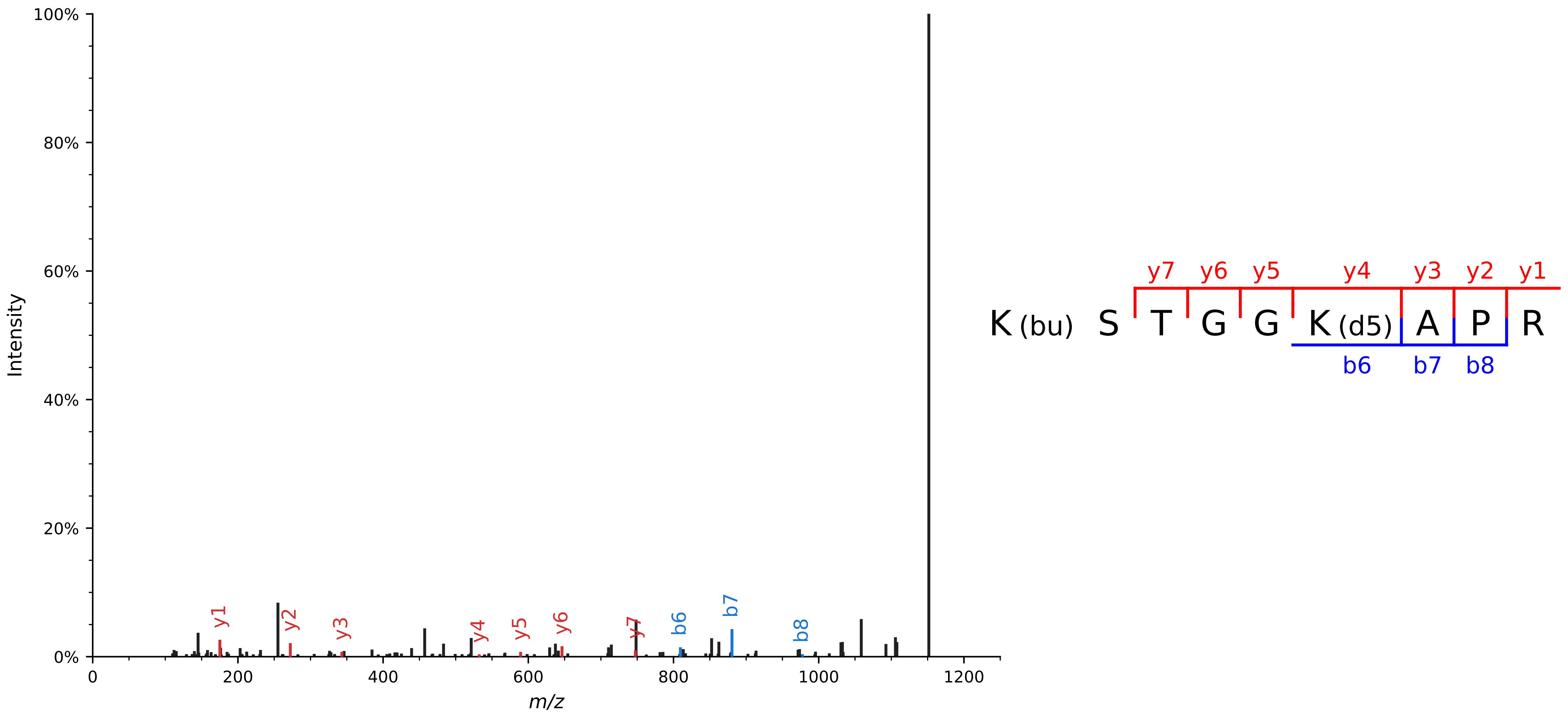

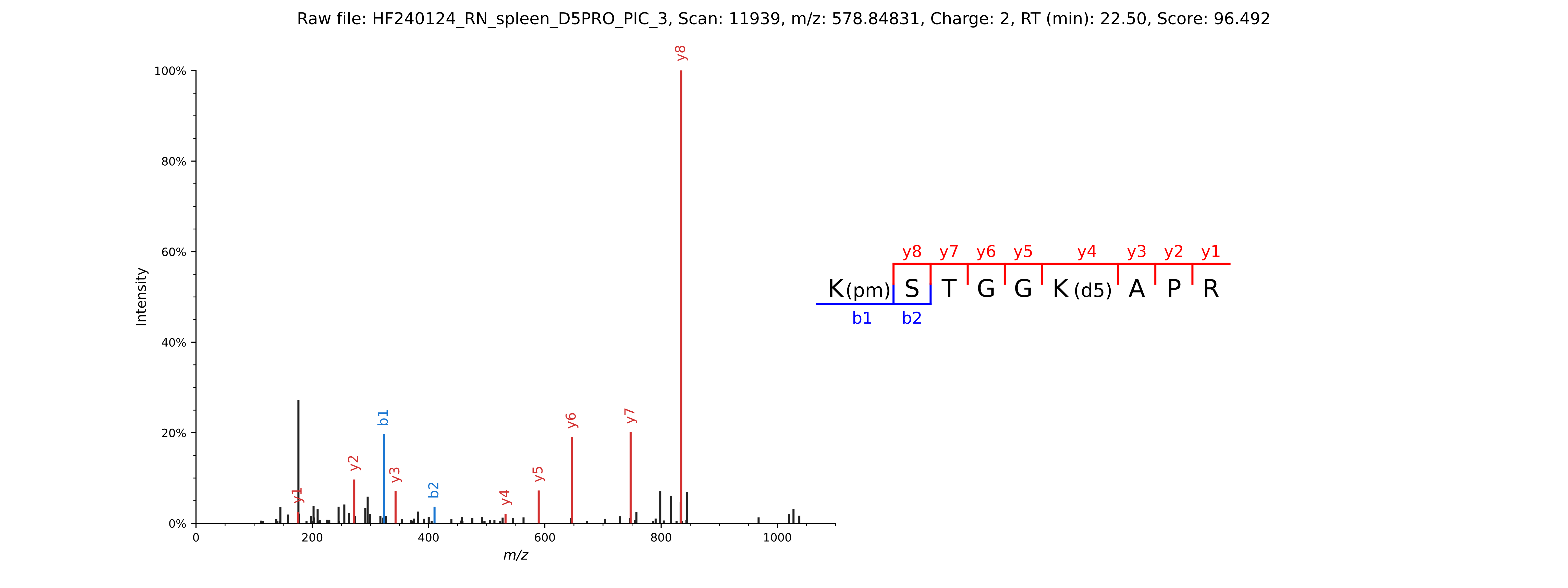

Raw file: HF240124_RN_spleen_D5PRO_PIC_3, Scan: 11939, m/z: 578.84831, Charge: 2, RT (min): 22.50, Score: 96.492
Intensity
100%
80%
60%
40%
20%
0%
0
200
400
600
800
1000
m/z
y1
y2
b1
y3
b2
y4
y5
y6
y7
y8
y8 y7 y6 y5 y4 y3 y2 y1
K(pm) S T G G K(d5) A P R
b1 b2

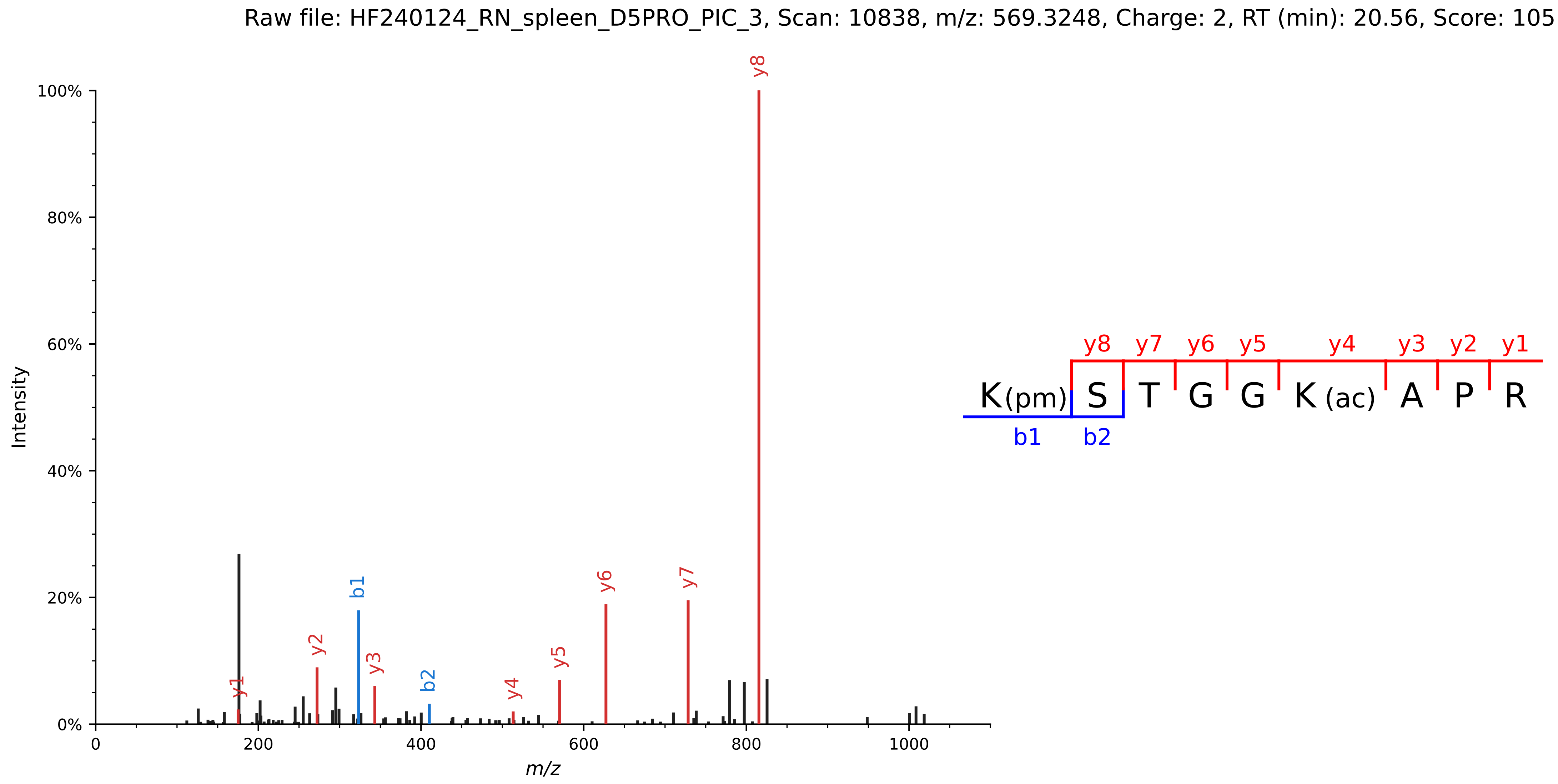

Raw file: HF240124_RN_spleen_D5PRO_PIC_3, Scan: 10838, m/z: 569.3248, Charge: 2, RT (min): 20.56, Score: 105.14
Intensity
m/z
0%
20%
40%
60%
80%
100%
0
200
400
600
800
1000
y1
y2
b1
y3
b2
y4
y5
y6
y7
y8
y8 y7 y6 y5 y4 y3 y2 y1
K(pm) S T G G K (ac) A P R
b1 b2

Raw file: HF240124_RN_spleen_D5PRO_PIC_1, Scan: 4690, m/z: 583.34045, Charge: 2, RT (min): 9.89, Score: 75.213

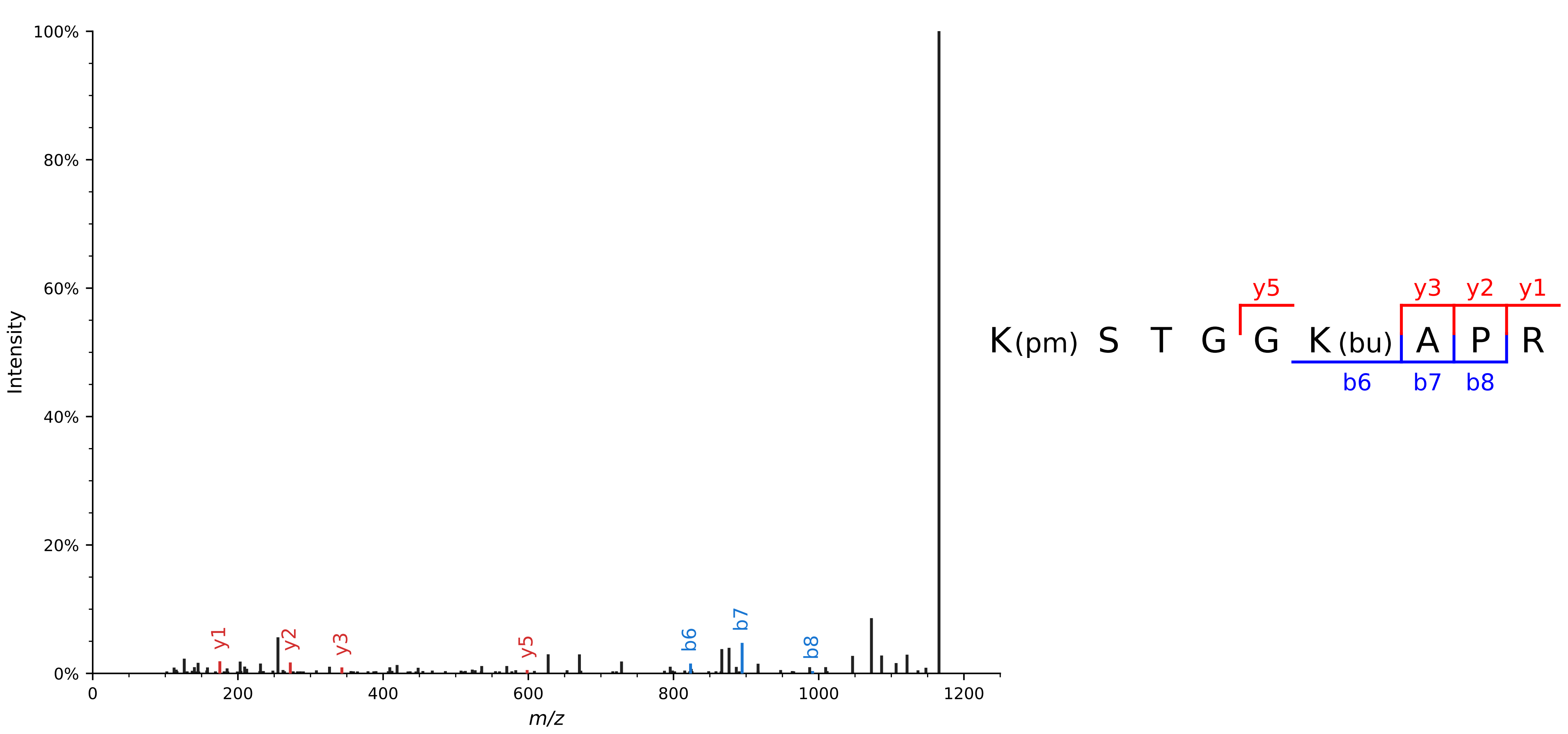

Raw file: HF240124_RN_spleen_D5PRO_PIC_3, Scan: 5887, m/z: 585.85614, Charge: 2, RT (min): 11.63, Score: 57.281

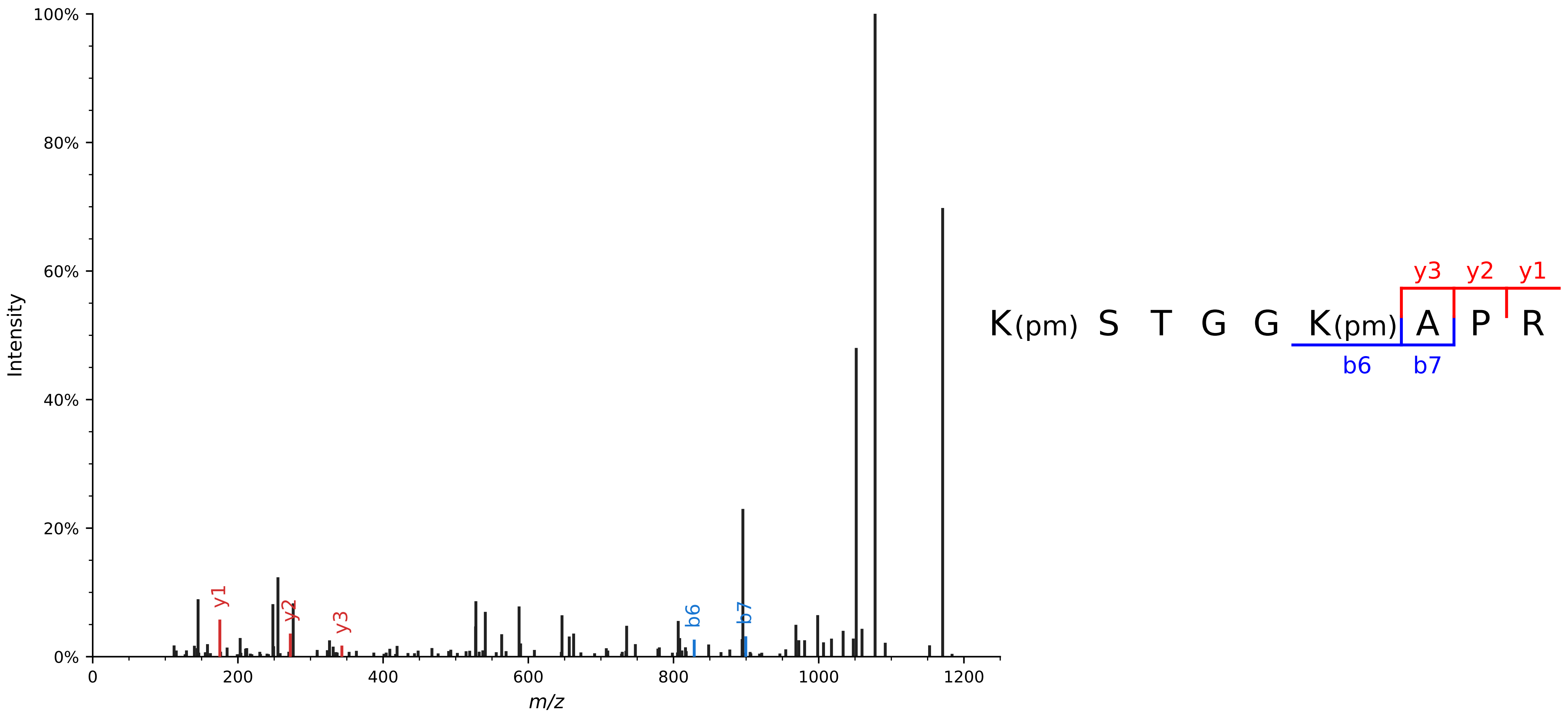

Raw file: HF240124_RN_spleen_D5PRO_PIC_3, Scan: 3048, m/z: 555.32734, Charge: 2, RT (min): 6.57, Score: 114.28

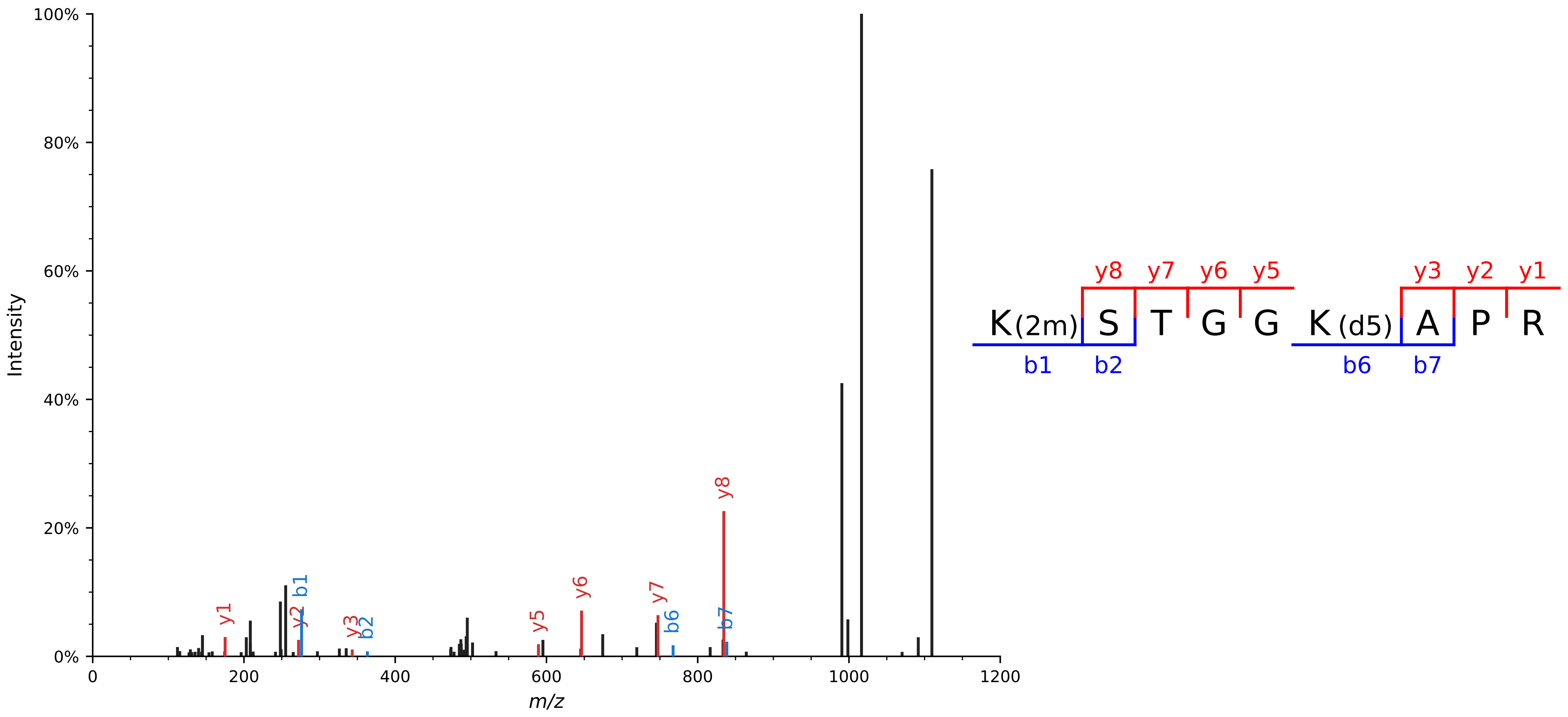

Raw file: HF240124_RN_spleen_D5PRO_PIC_1, Scan: 2160, m/z: 545.80382, Charge: 2, RT (min): 5.42, Score: 131.06

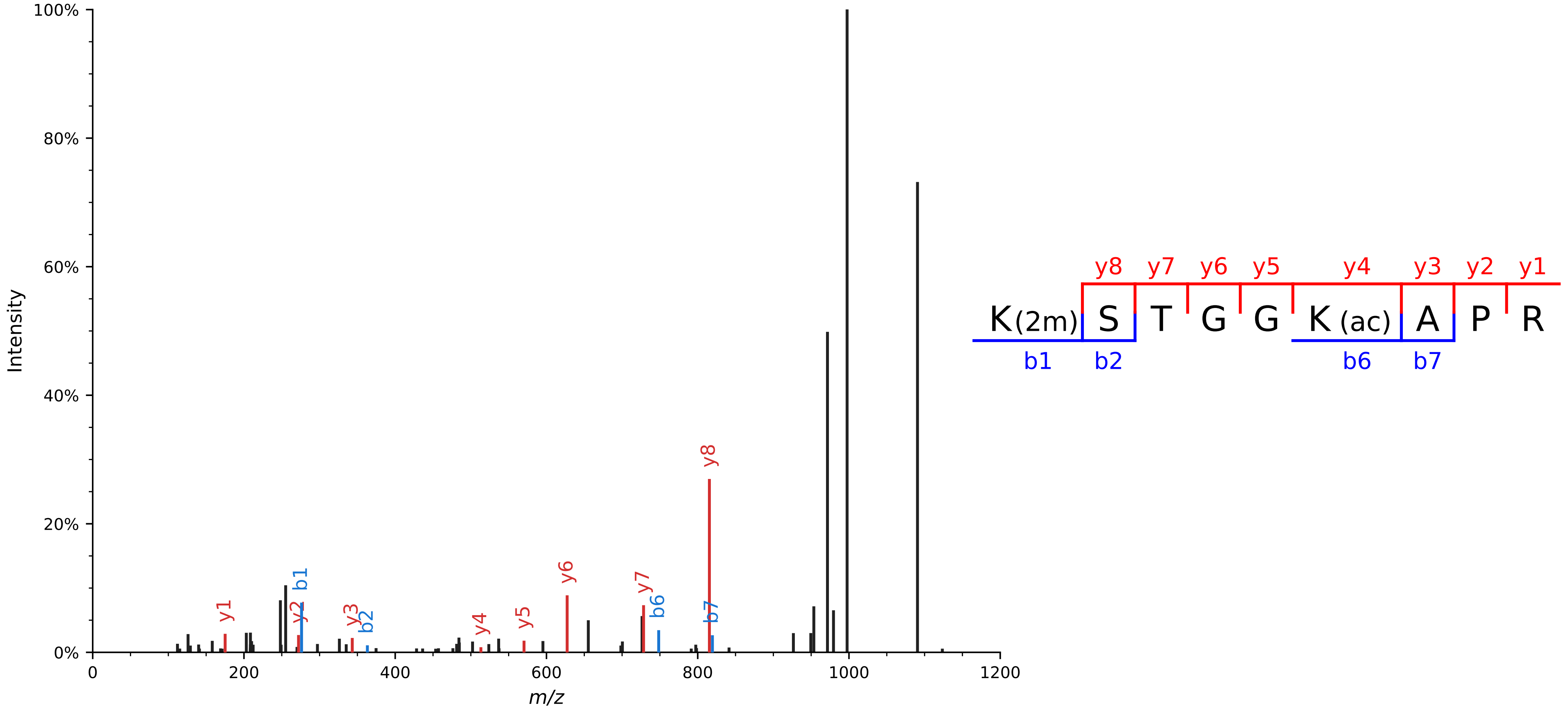

Raw file: HF240124_RN_spleen_D5PRO_PIC_2, Scan: 3761, m/z: 559.81947, Charge: 2, RT (min): 8.34, Score: 56.434

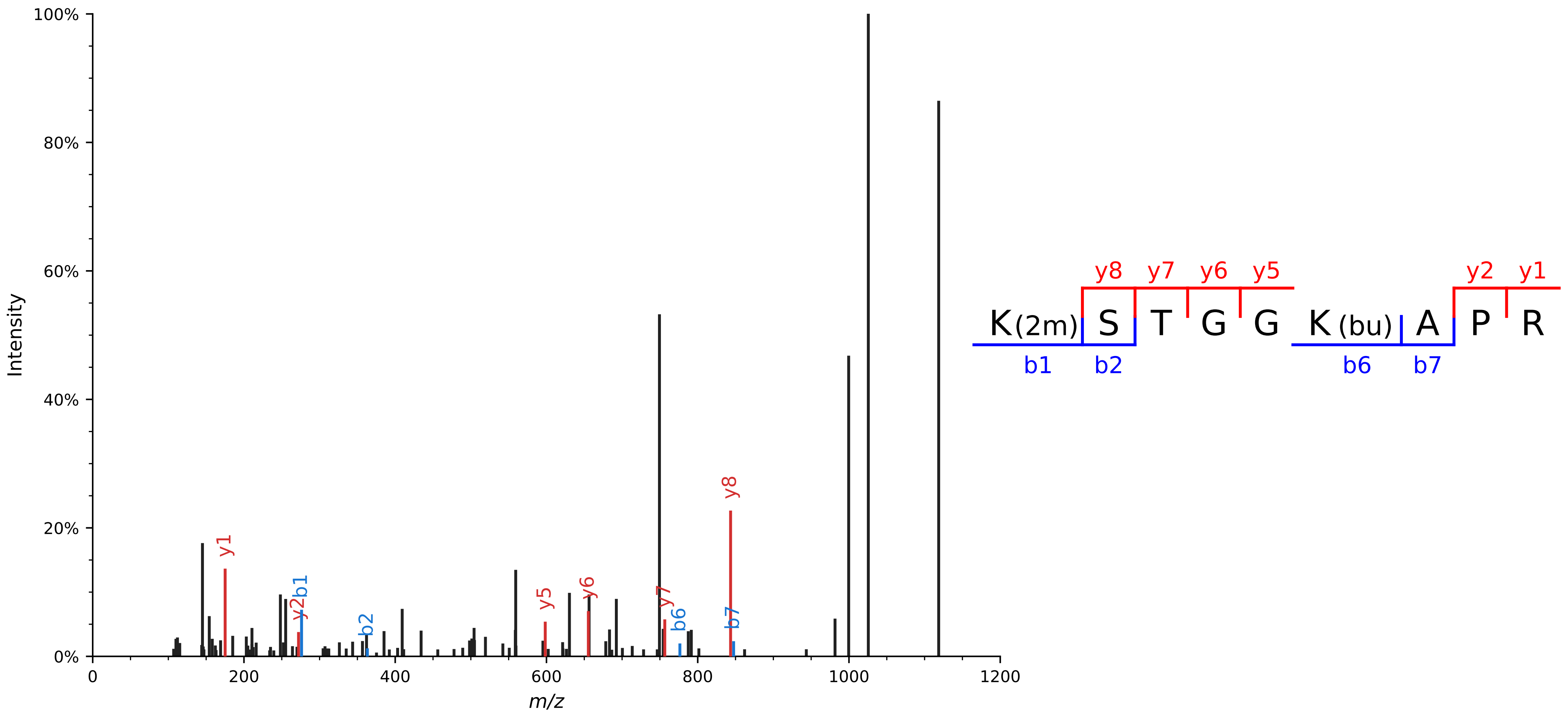

Raw file: HF240124_RN_spleen_D5PRO_PIC_2, Scan: 1867, m/z: 538.796, Charge: 2, RT (min): 4.90, Score: 71.379

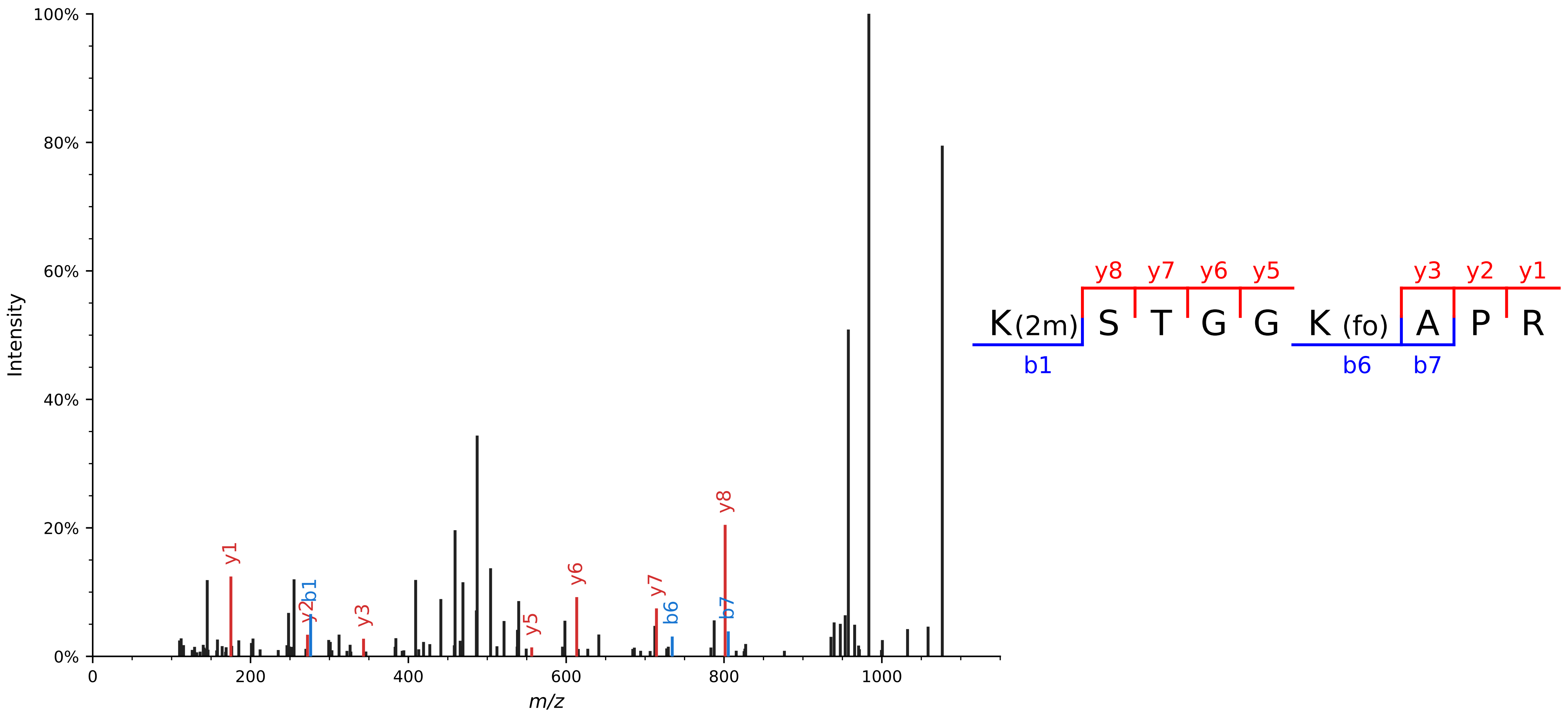

Raw file: HF240124_RN_spleen_D5PRO_PIC_1, Scan: 2689, m/z: 562.33516, Charge: 2, RT (min): 6.36, Score: 118.5

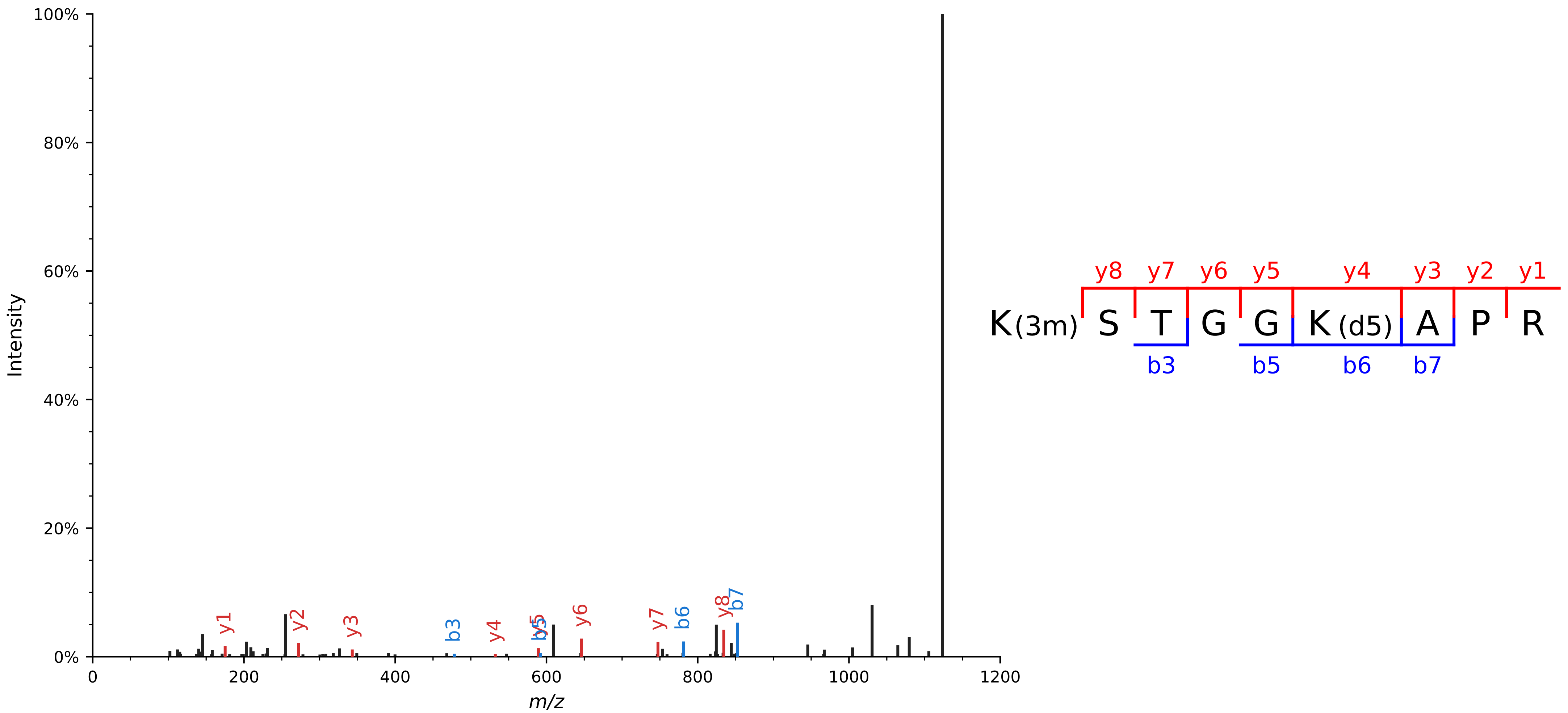

Raw file: HF240124_RN_spleen_D5PRO_PIC_2, Scan: 2027, m/z: 552.81165, Charge: 2, RT (min): 5.16, Score: 145.9

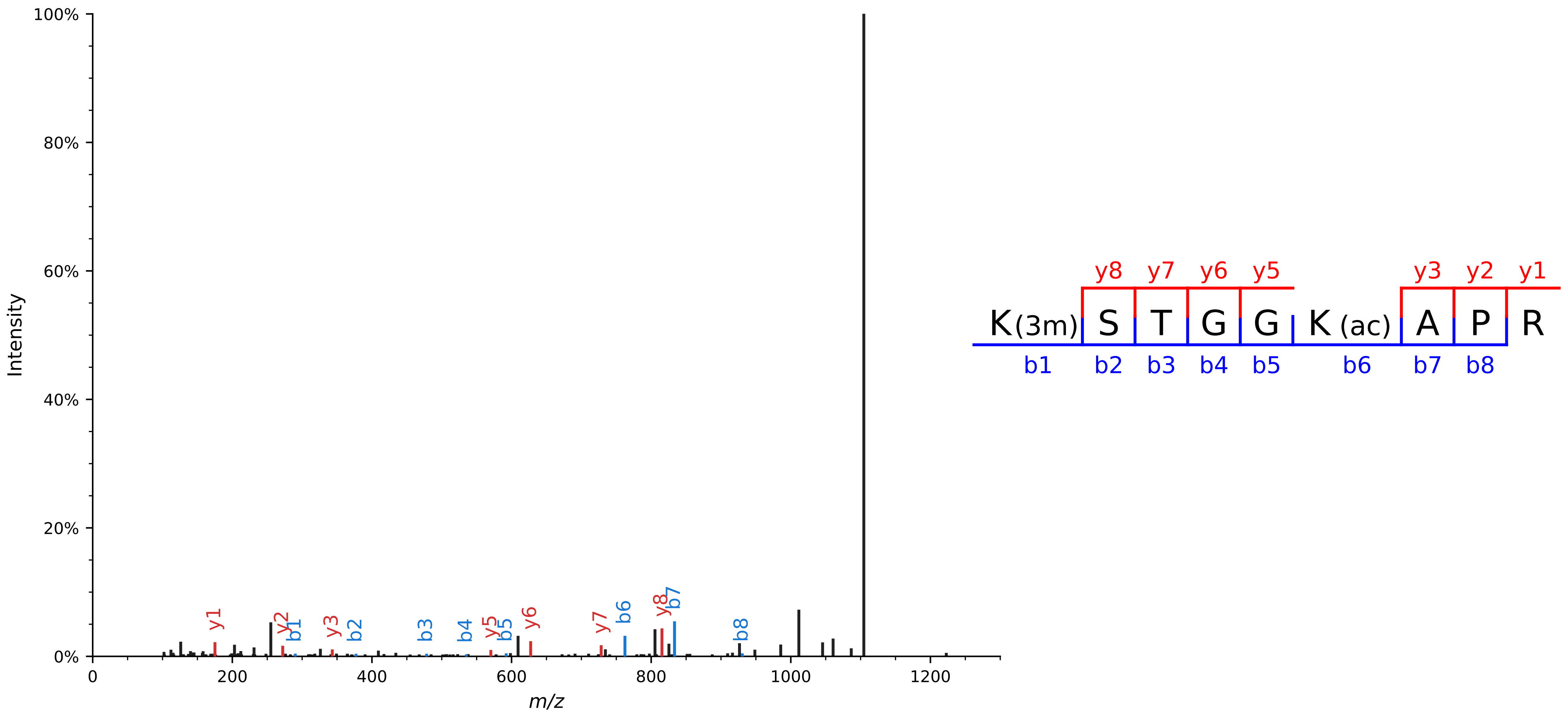

Raw file: HF240124_RN_spleen_D5PRO_PIC_3, Scan: 3549, m/z: 569.3248, Charge: 2, RT (min): 7.50, Score: 51.059

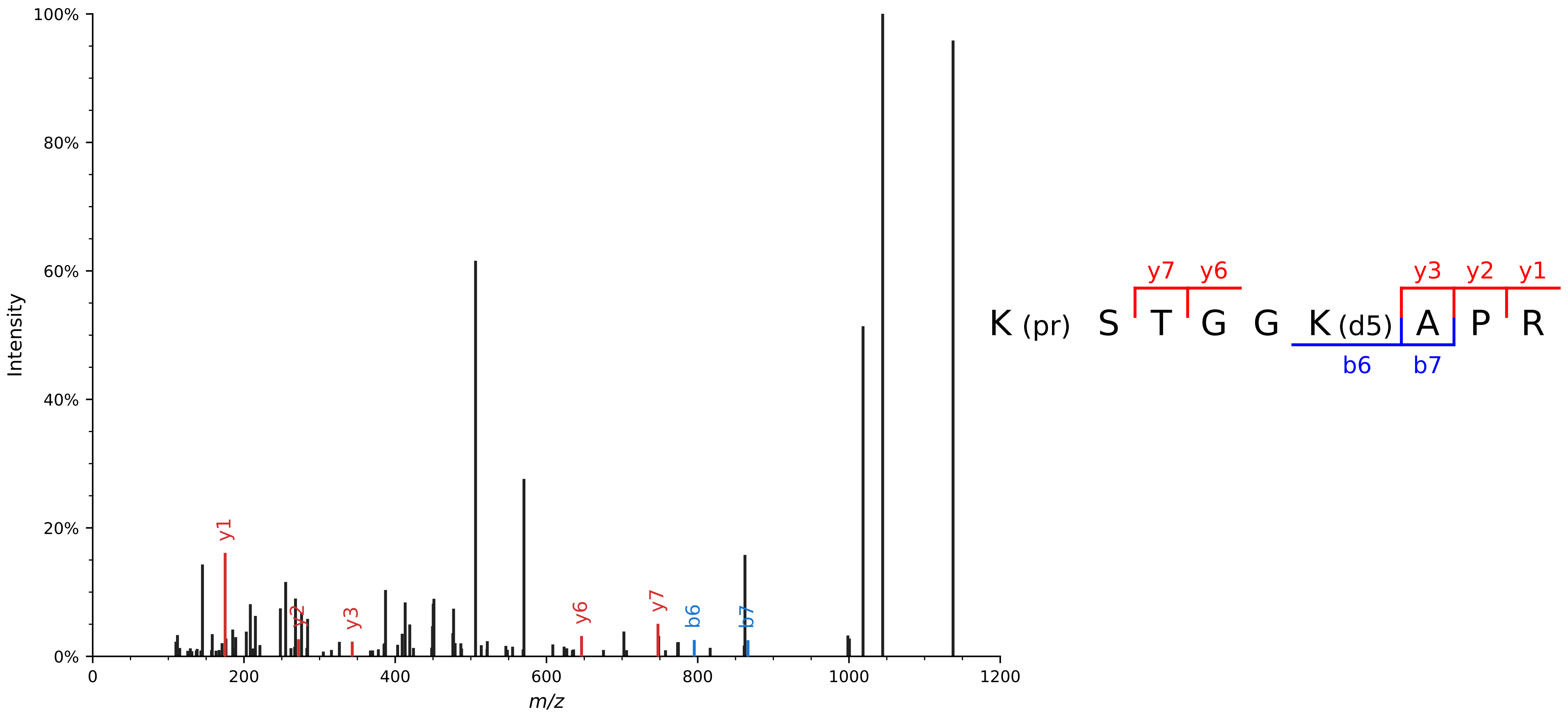

Raw file: HF240124_RN_spleen_D5PRO_PIC_3, Scan: 4419, m/z: 576.33262, Charge: 2, RT (min): 9.02, Score: 47.657

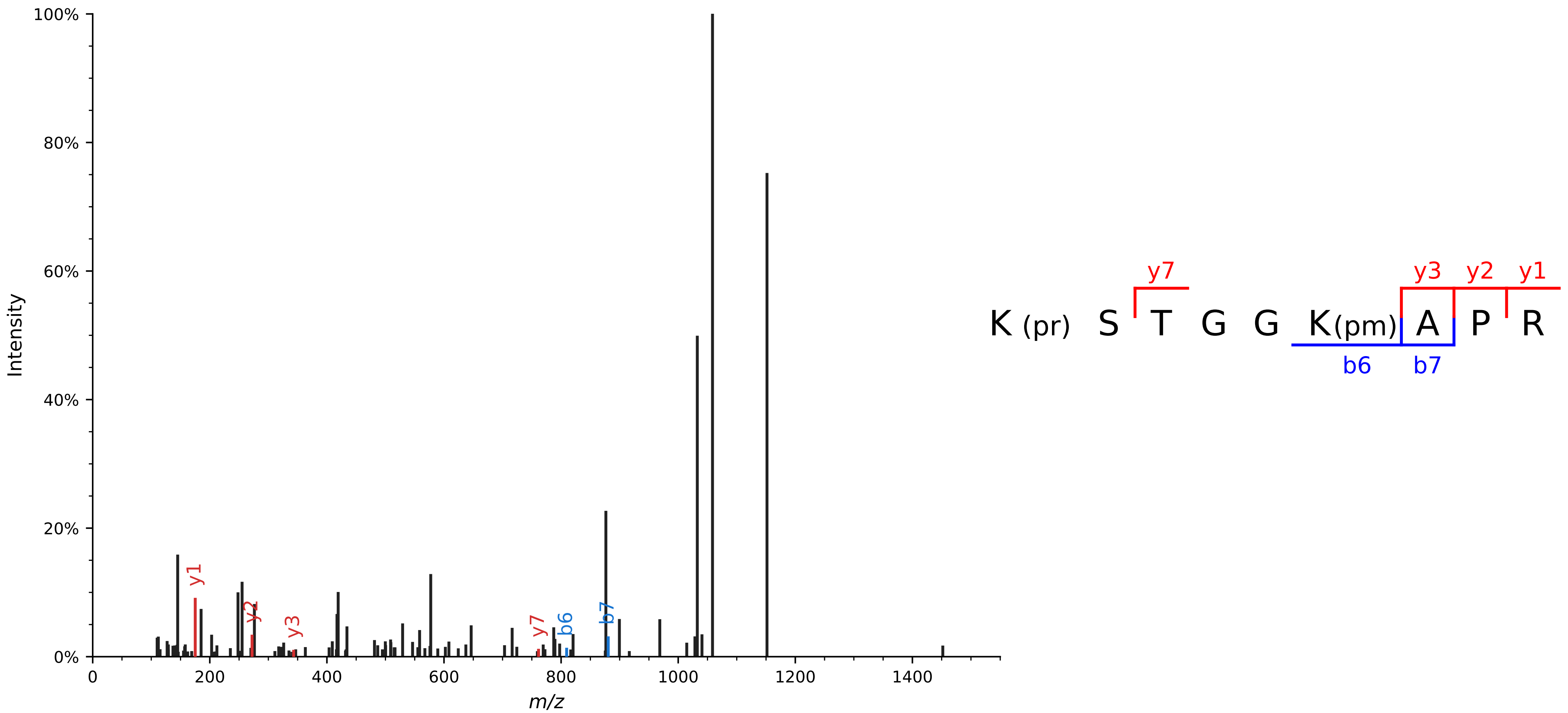

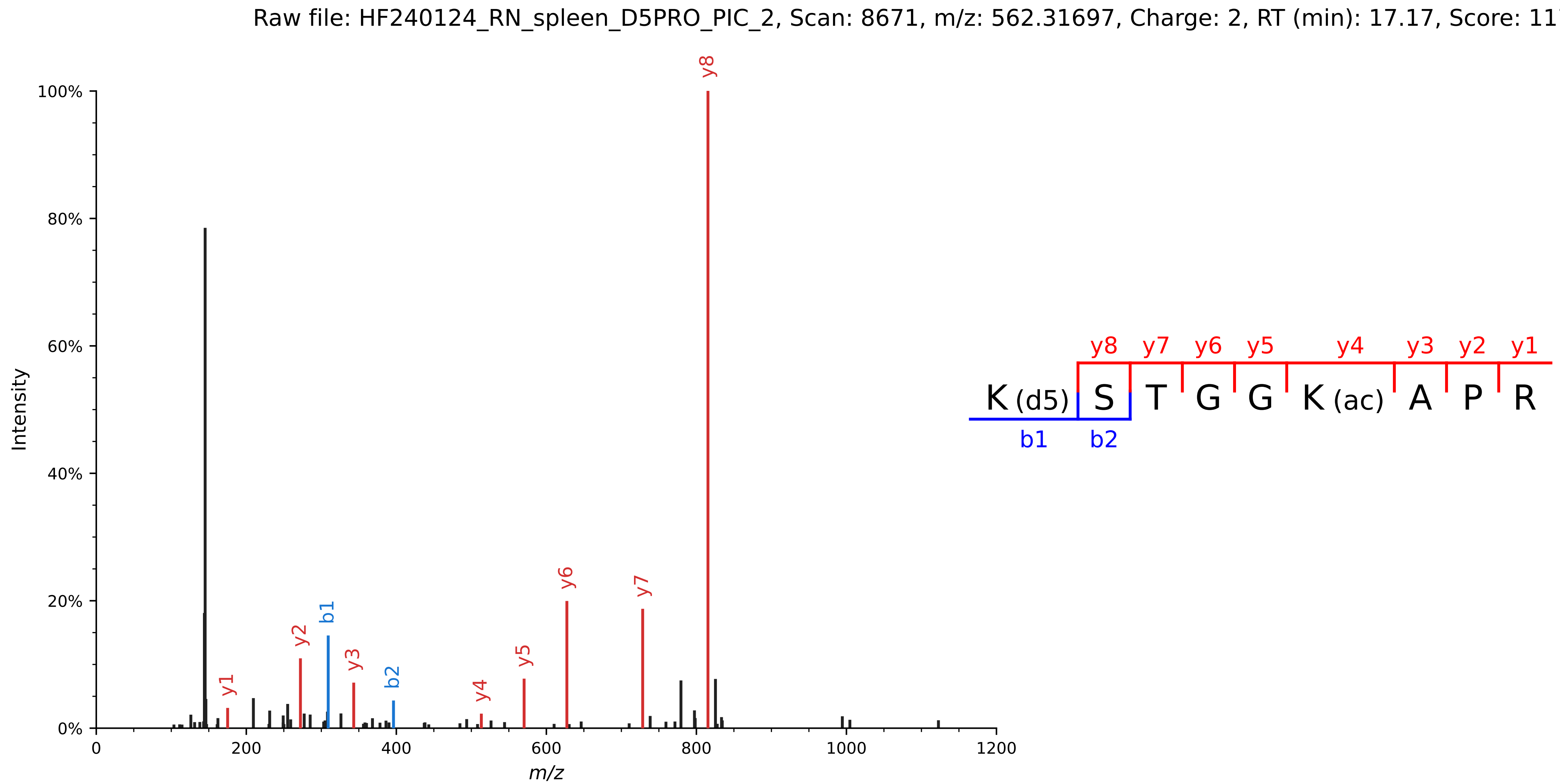
Raw file: HF240124_RN_spleen_D5PRO_PIC_2, Scan: 8671, m/z: 562.31697, Charge: 2, RT (min): 17.17, Score: 117.4
Intensity
m/z
y1 y2 b1 y3 b2 y4 y5 y6 y7 y8
y8 y7 y6 y5 y4 y3 y2 y1
K (d5) S T G G K (ac) A P R
b1 b2

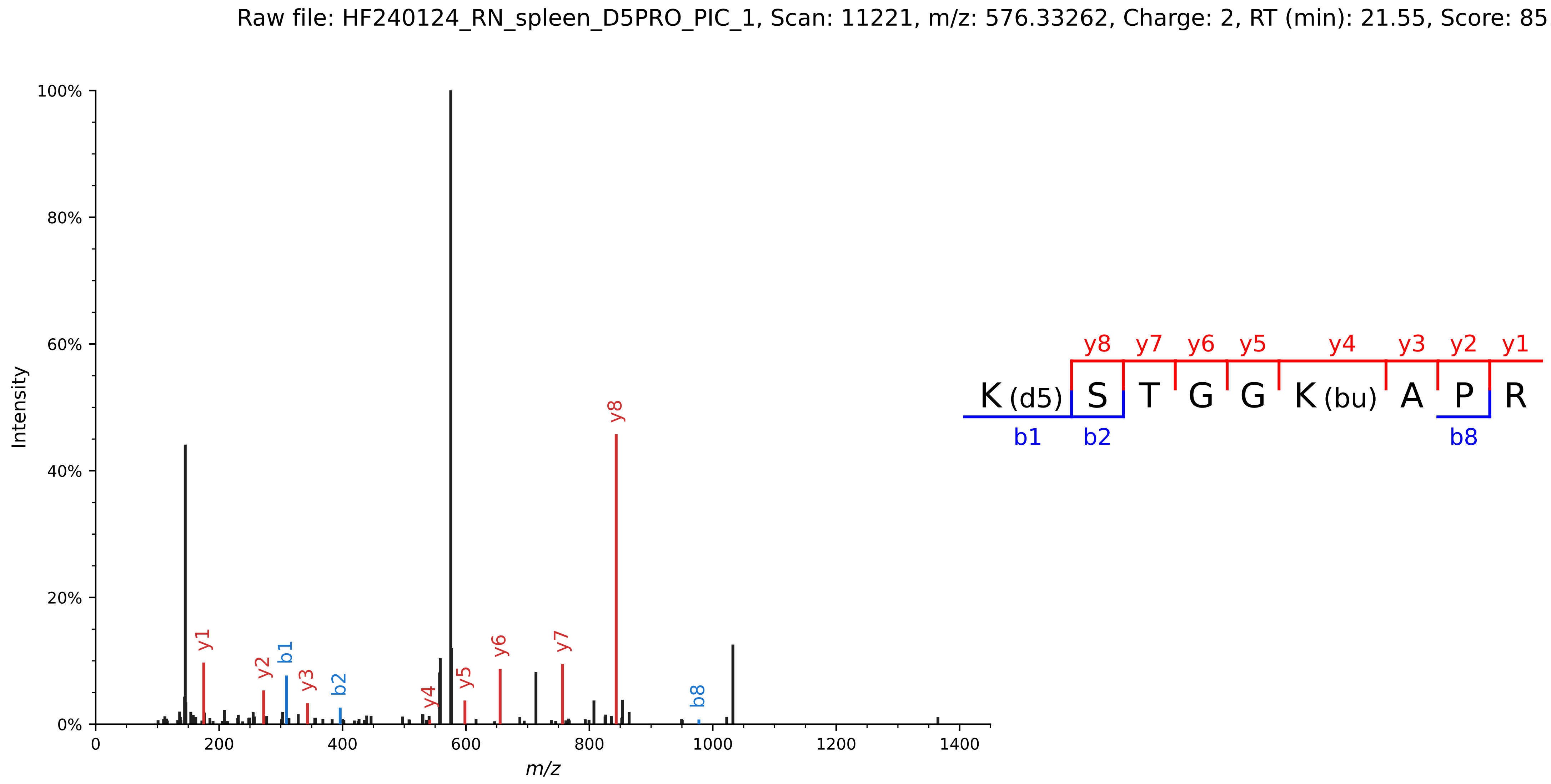
Raw file: HF240124_RN_spleen_D5PRO_PIC_1, Scan: 11221, m/z: 576.33262, Charge: 2, RT (min): 21.55, Score: 85.161
Intensity
m/z
y1
y2
b1
y3
b2
y4
y5
y6
y7
y8
b8
K(d5) S T G G K(bu) A P R
y8 y7 y6 y5 y4 y3 y2 y1
b1 b2 b8

Raw file: HF240124_RN_spleen_D5PRO_PIC_2, Scan: 17215, m/z: 604.86155, Charge: 2, RT (min): 32.57, Score: 140.5

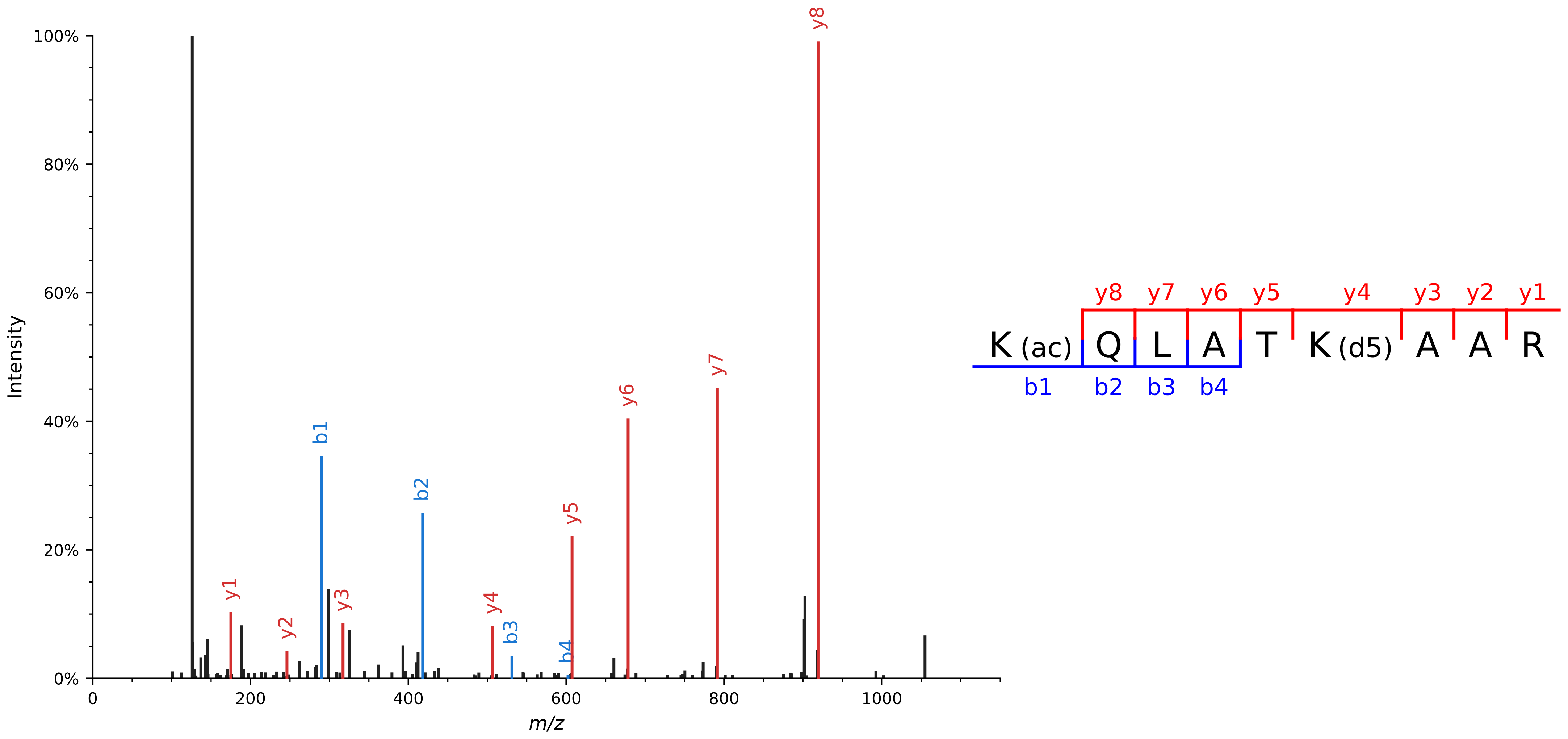

Raw file: HF240124_RN_spleen_D5PRO_PIC_1, Scan: 16359, m/z: 595.33803, Charge: 2, RT (min): 30.71, Score: 154.49

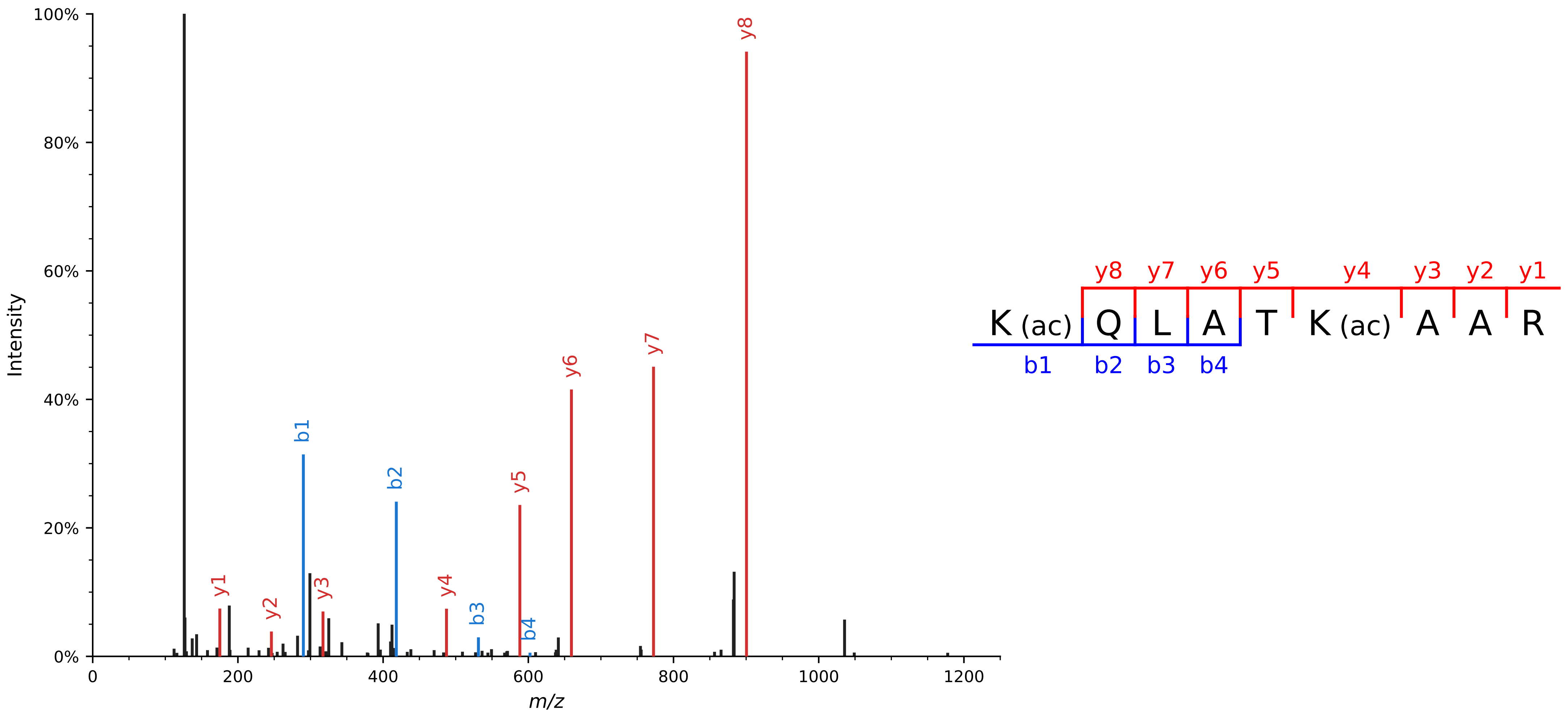

Raw file: HF240124_RN_spleen_D5PRO_PIC_3, Scan: 20327, m/z: 618.8772, Charge: 2, RT (min): 37.56, Score: 82.426

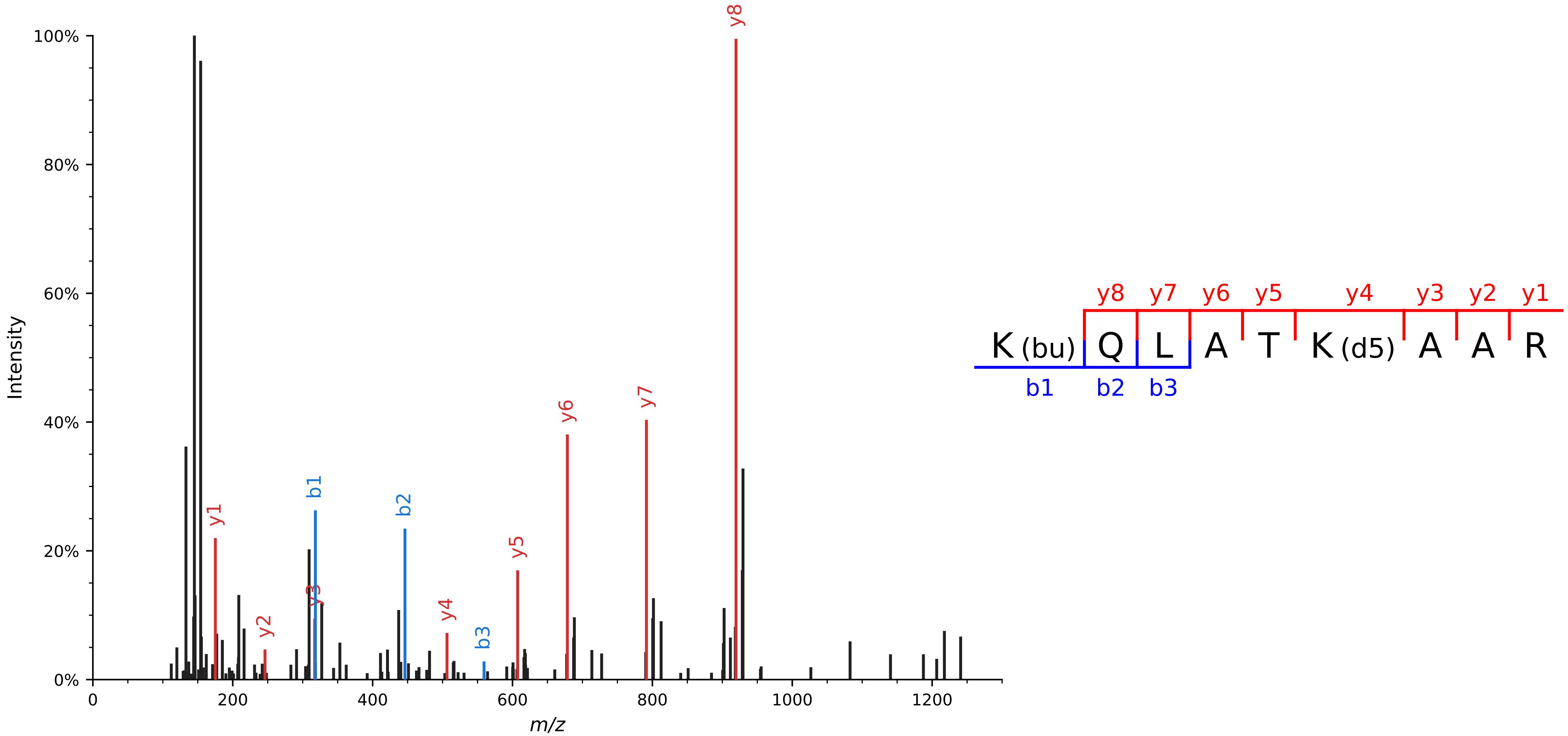

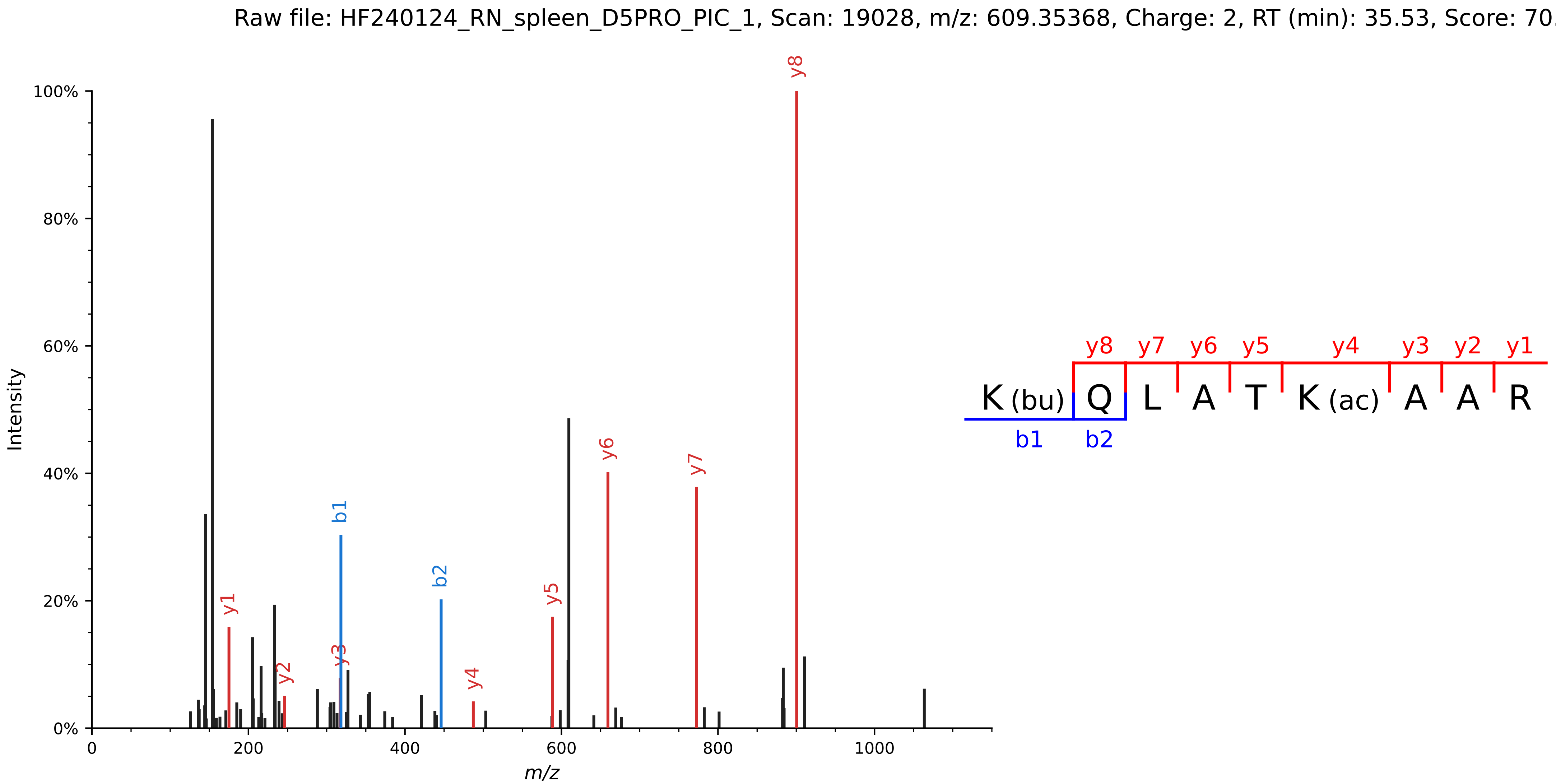
Raw file: HF240124_RN_spleen_D5PRO_PIC_1, Scan: 19028, m/z: 609.35368, Charge: 2, RT (min): 35.53, Score: 70.256
Intensity
m/z
0%
20%
40%
60%
80%
100%
0
200
400
600
800
1000
y1
y2
y3
b1
b2
y4
y5
y6
y7
y8
K (bu) Q L A T K (ac) A A R
b1 b2
y8 y7 y6 y5 y4 y3 y2 y1

Raw file: HF240124_RN_spleen_D5PRO_PIC_2, Scan: 16887, m/z: 597.85372, Charge: 2, RT (min): 31.97, Score: 119.27

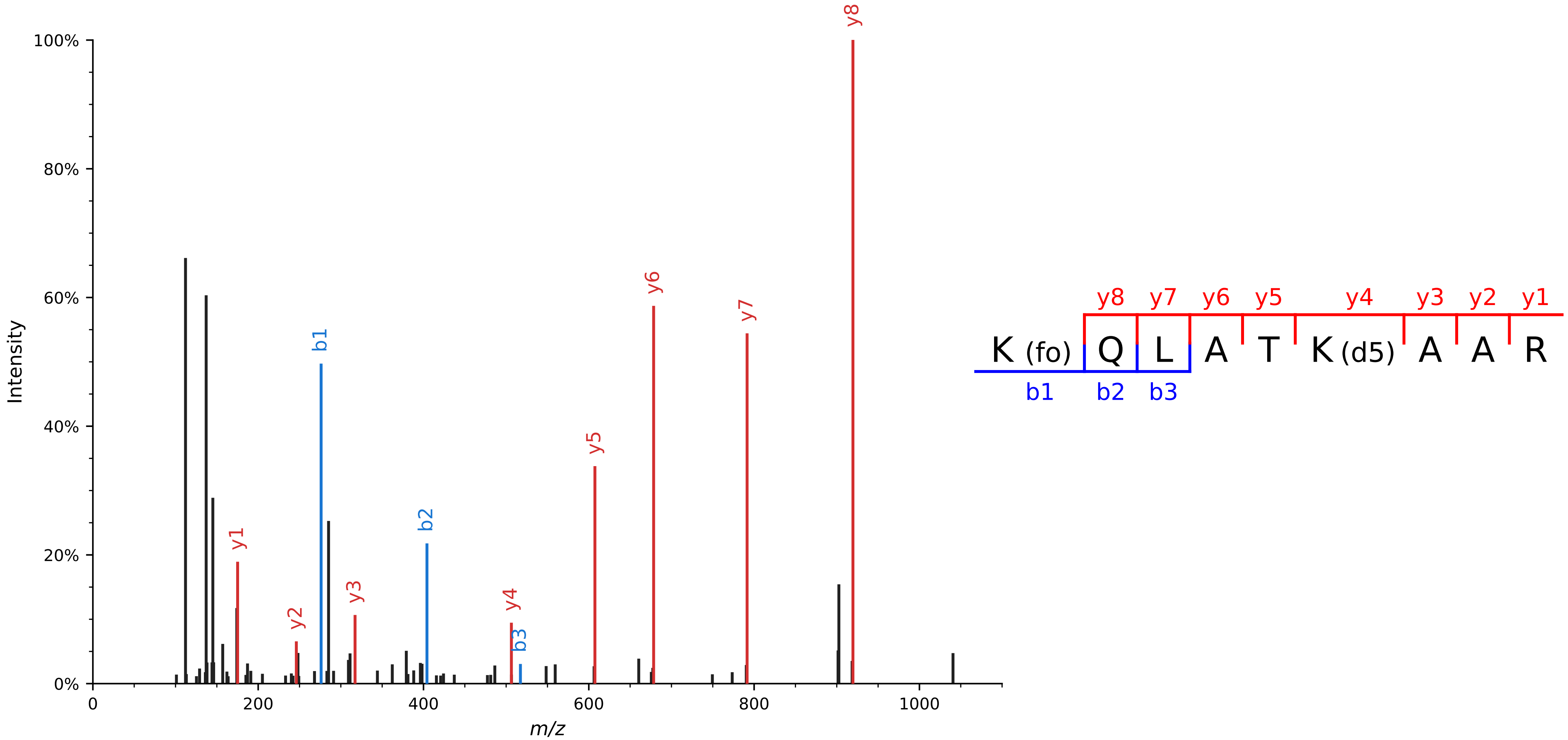

Raw file: HF240124_RN_spleen_D5PRO_PIC_3, Scan: 16094, m/z: 588.3302, Charge: 2, RT (min): 29.91, Score: 57.859

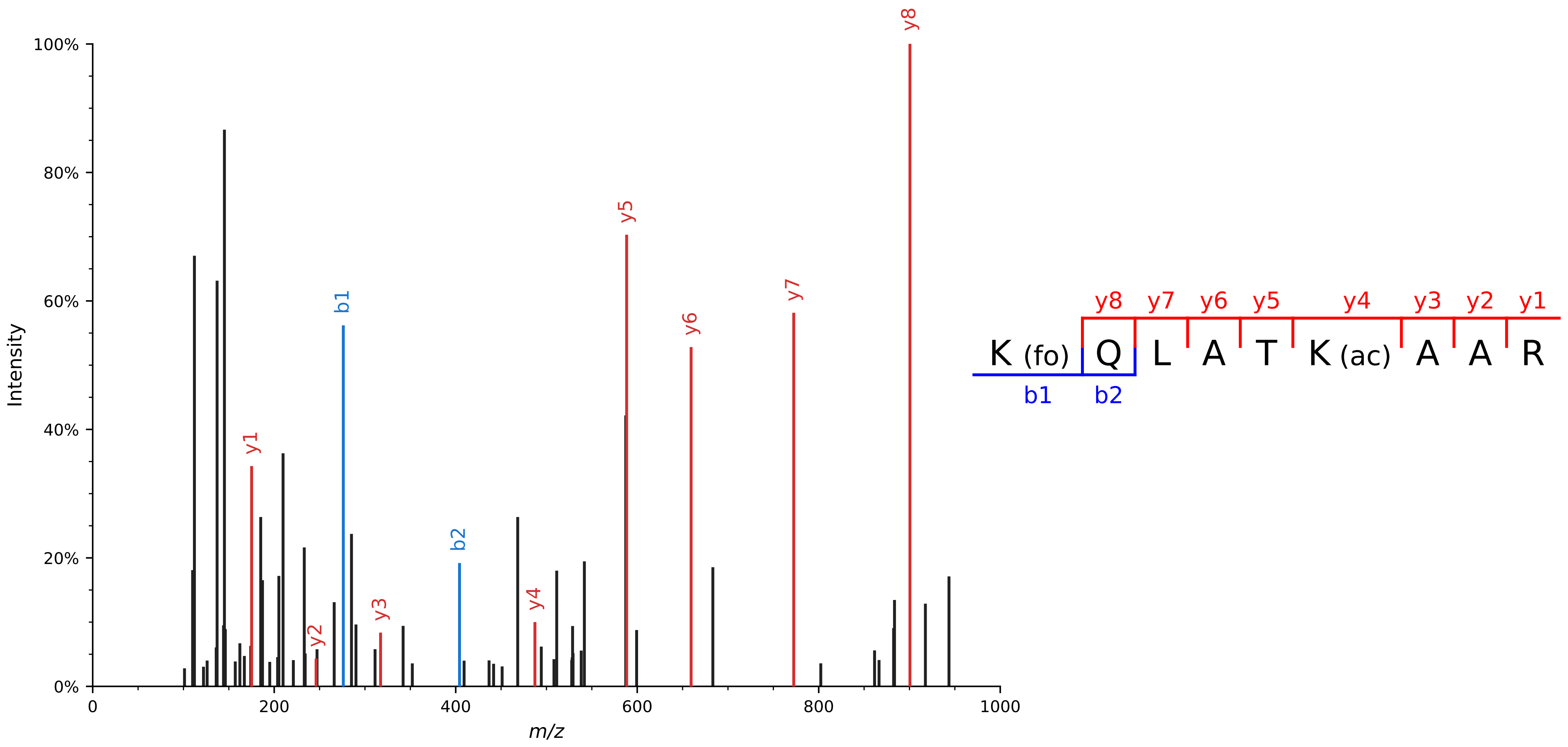

Raw file: HF240124_RN_spleen_D5PRO_PIC_3, Scan: 20372, m/z: 621.39289, Charge: 2, RT (min): 37.64, Score: 144.38

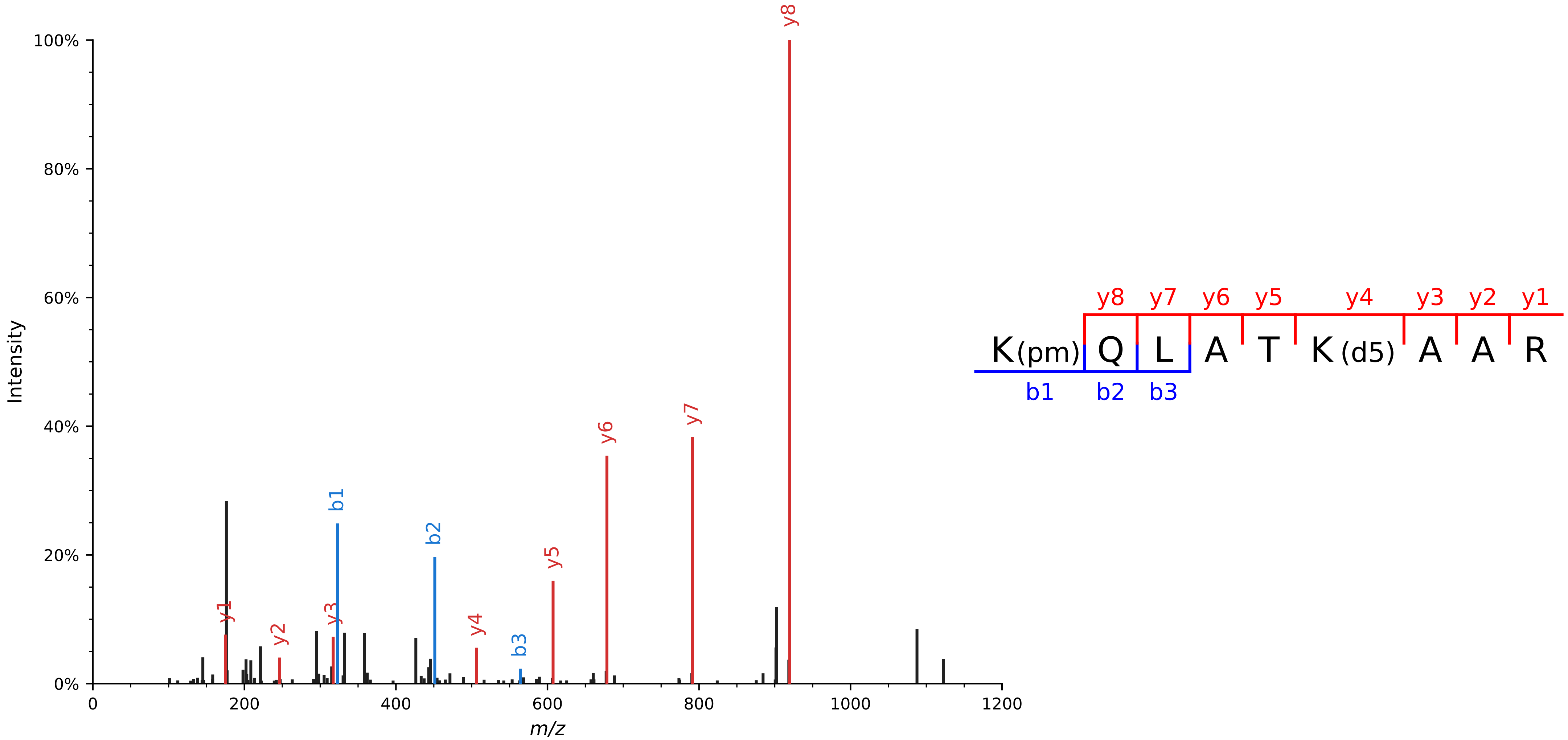

Raw file: HF240124_RN_spleen_D5PRO_PIC_3, Scan: 19303, m/z: 611.86937, Charge: 2, RT (min): 35.70, Score: 124.98

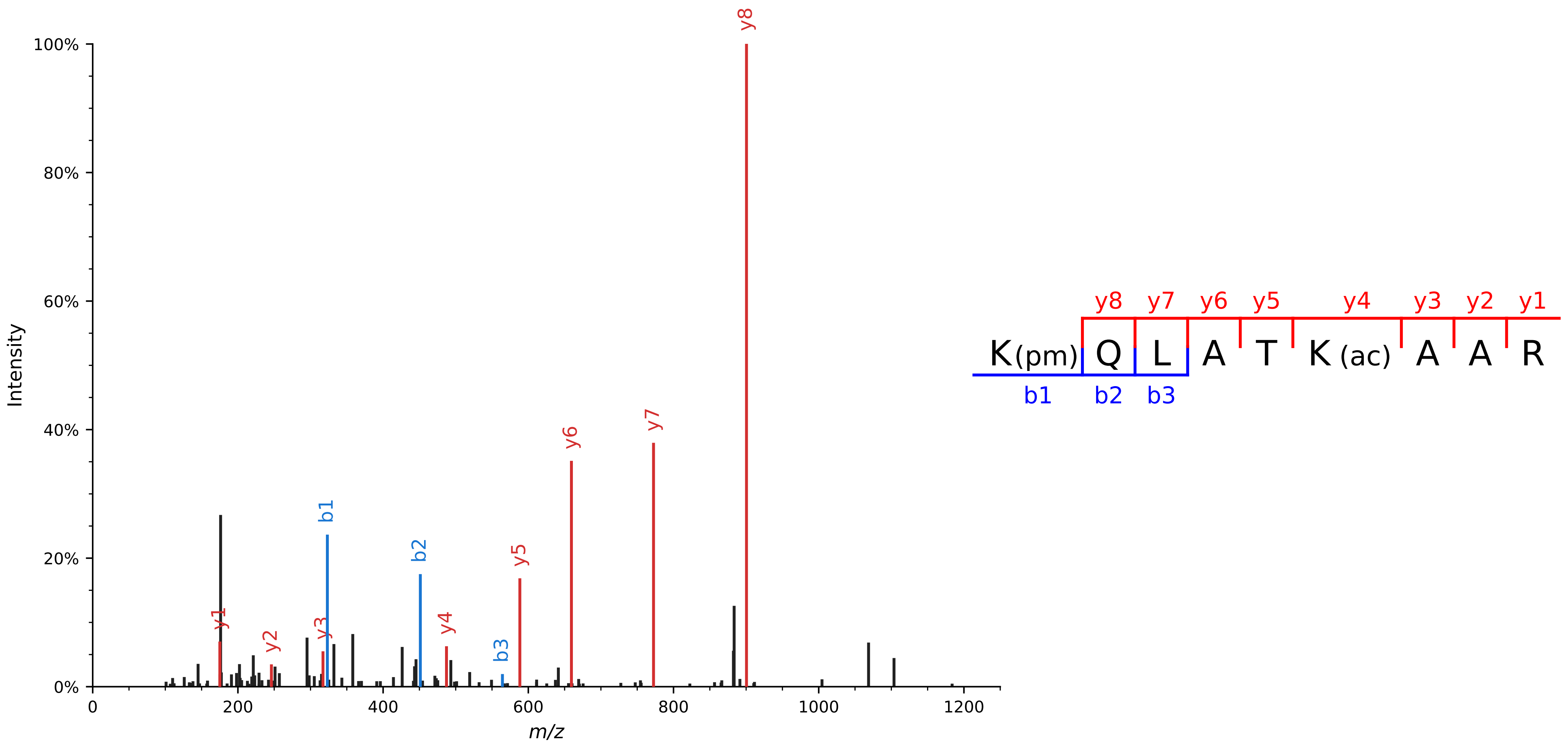

Raw file: HF240124_RN_spleen_D5PRO_PIC_1, Scan: 21422, m/z: 628.40071, Charge: 2, RT (min): 39.88, Score: 105.57

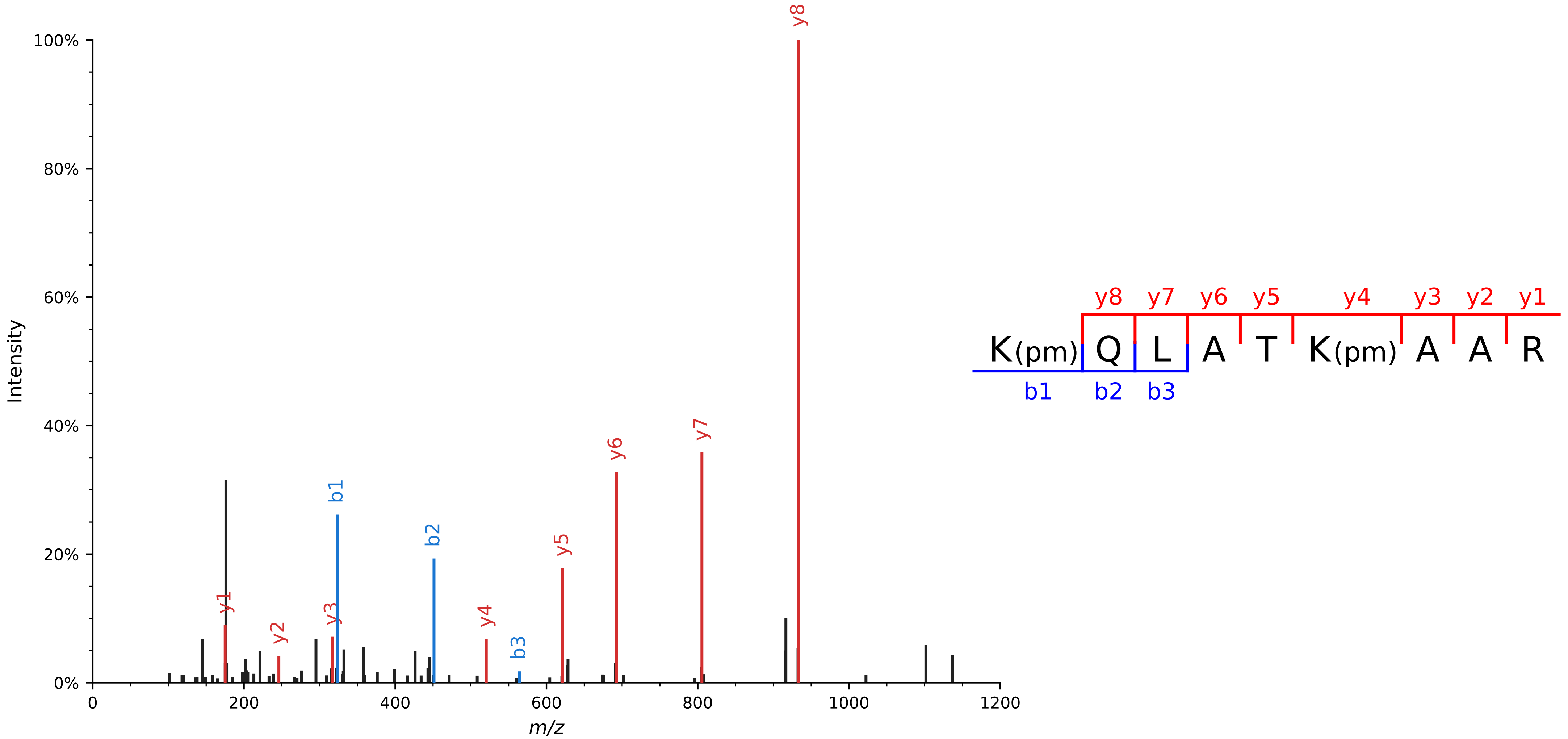

Raw file: HF240124_RN_spleen_D5PRO_PIC_1, Scan: 9473, m/z: 597.87191, Charge: 2, RT (min): 18.40, Score: 106.38

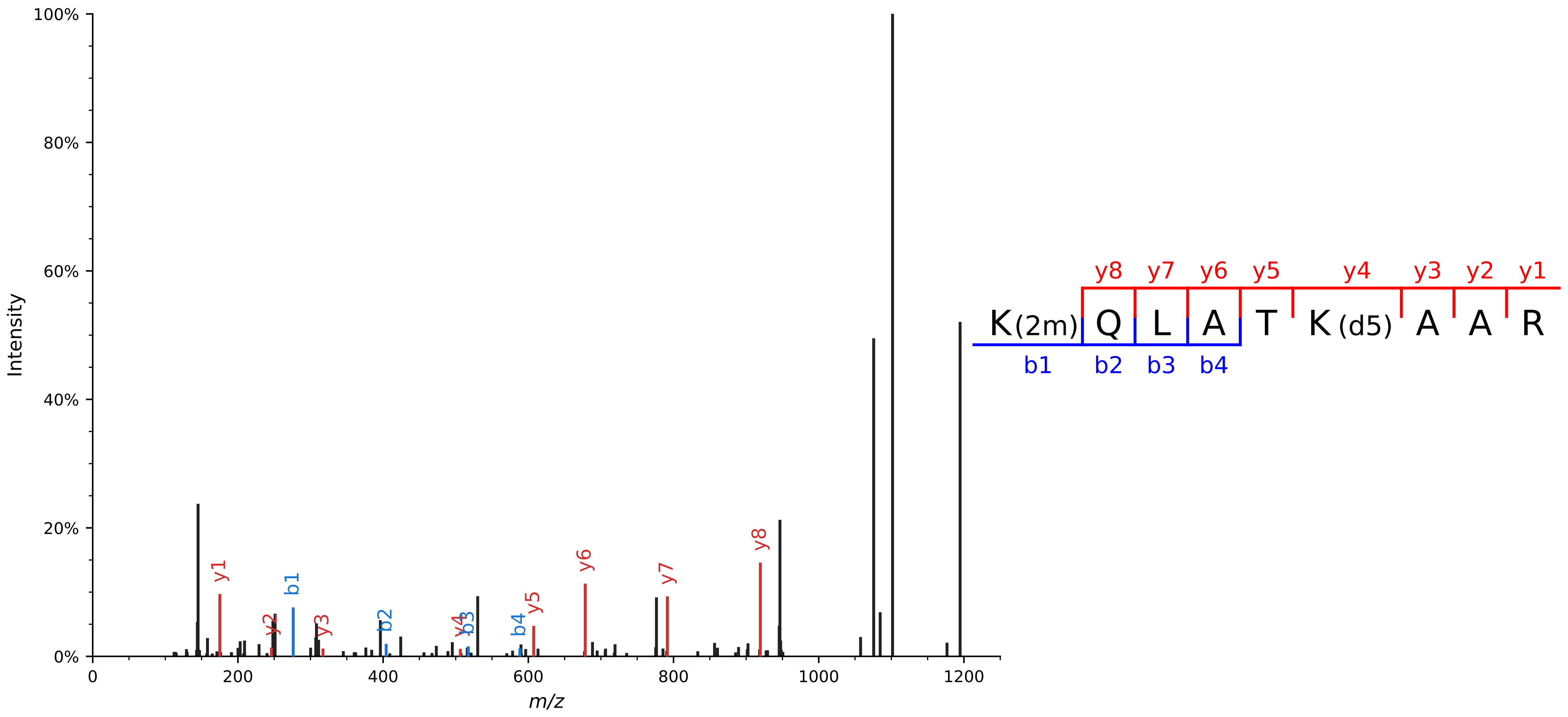

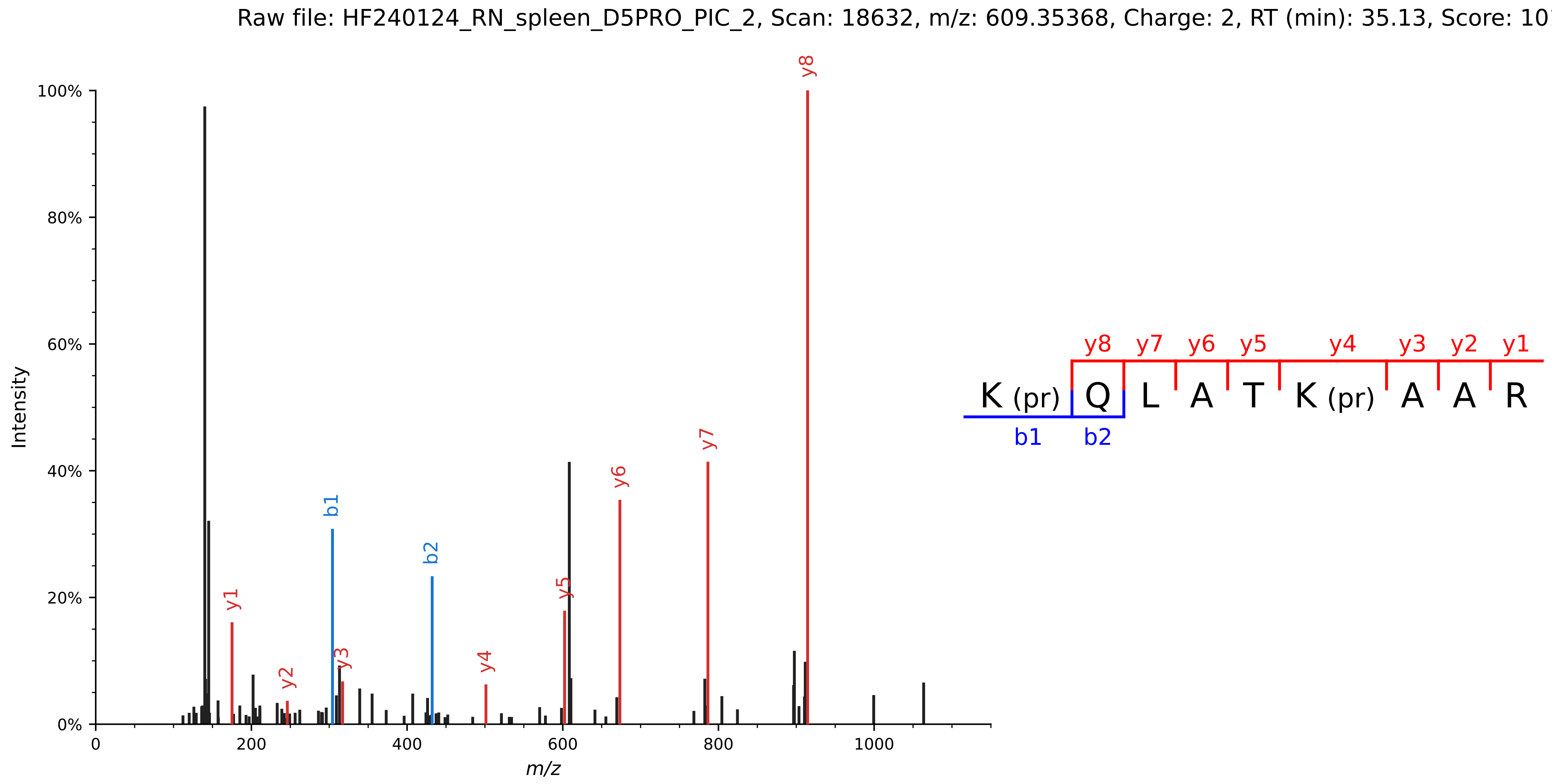

Raw file: HF240124_RN_spleen_D5PRO_PIC_2, Scan: 18632, m/z: 609.35368, Charge: 2, RT (min): 35.13, Score: 101.43
Intensity
m/z
0%
20%
40%
60%
80%
100%
0
200
400
600
800
1000
y1
y2
y3
b1
b2
y4
y5
y6
y7
y8
K (pr) Q L A T K (pr) A A R

Raw file: HF240124_RN_spleen_D5PRO_PIC_3, Scan: 17749, m/z: 604.86155, Charge: 2, RT (min): 32.90, Score: 149.65

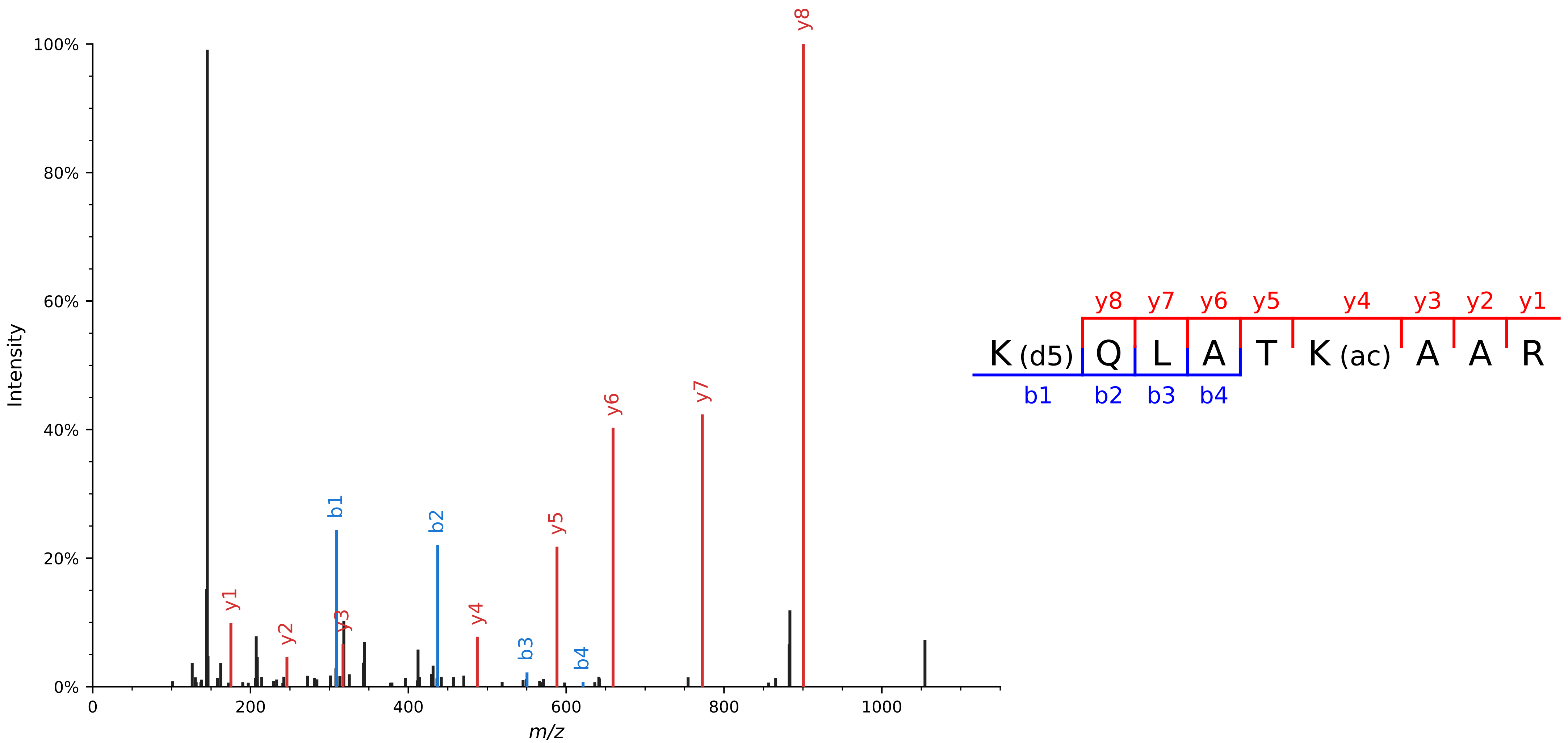

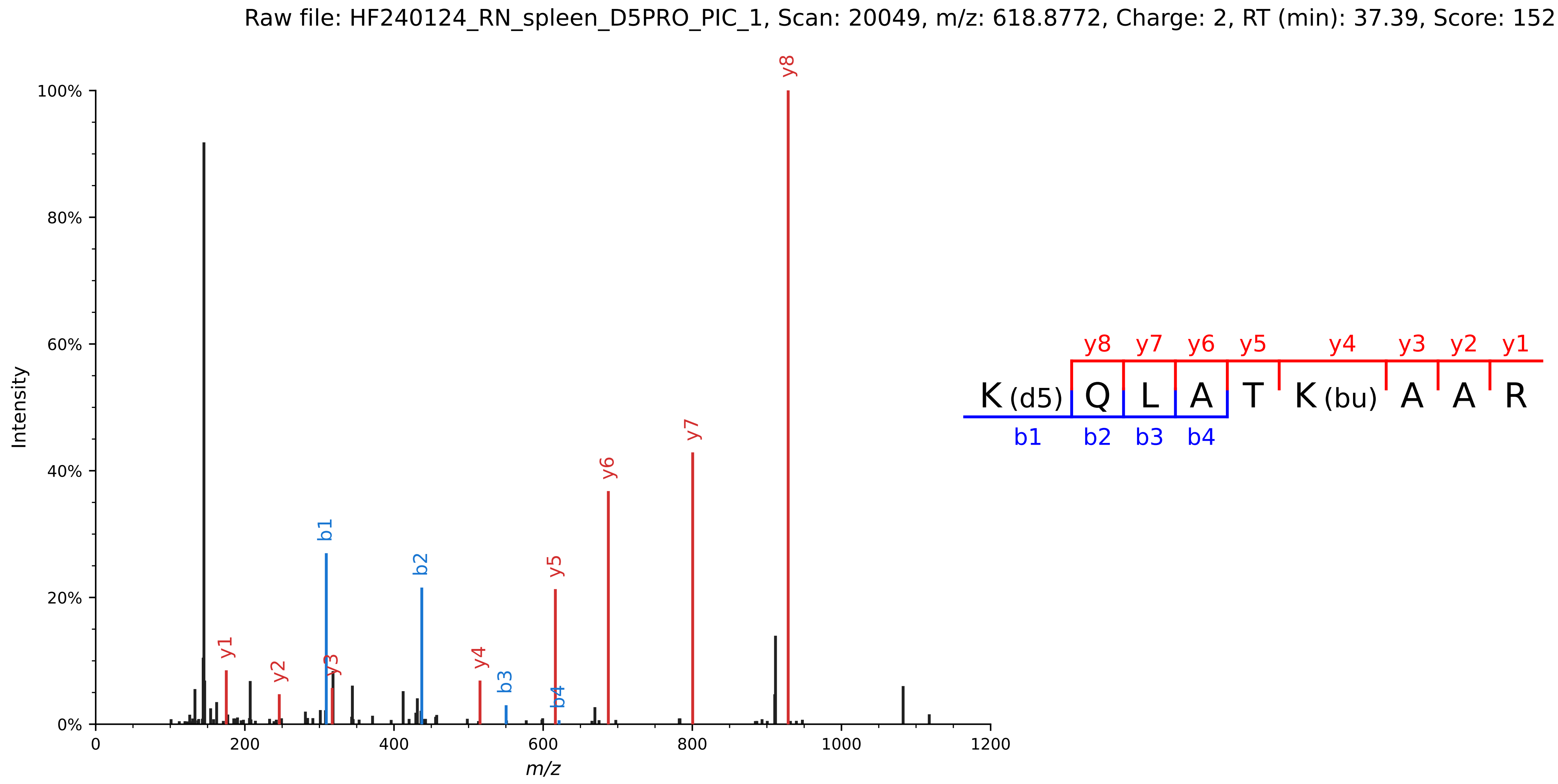

Raw file: HF240124_RN_spleen_D5PRO_PIC_1, Scan: 20049, m/z: 618.8772, Charge: 2, RT (min): 37.39, Score: 152.63
Intensity
m/z
0%
20%
40%
60%
80%
100%
0
200
400
600
800
1000
1200
y1
y2
y3
y4
y5
y6
y7
y8
b1
b2
b3
b4
K (d5) Q L A T K (bu) A A R

Raw file: HF240124_RN_spleen_D5PRO_PIC_2, Scan: 17027, m/z: 597.85372, Charge: 2, RT (min): 32.23, Score: 95.909

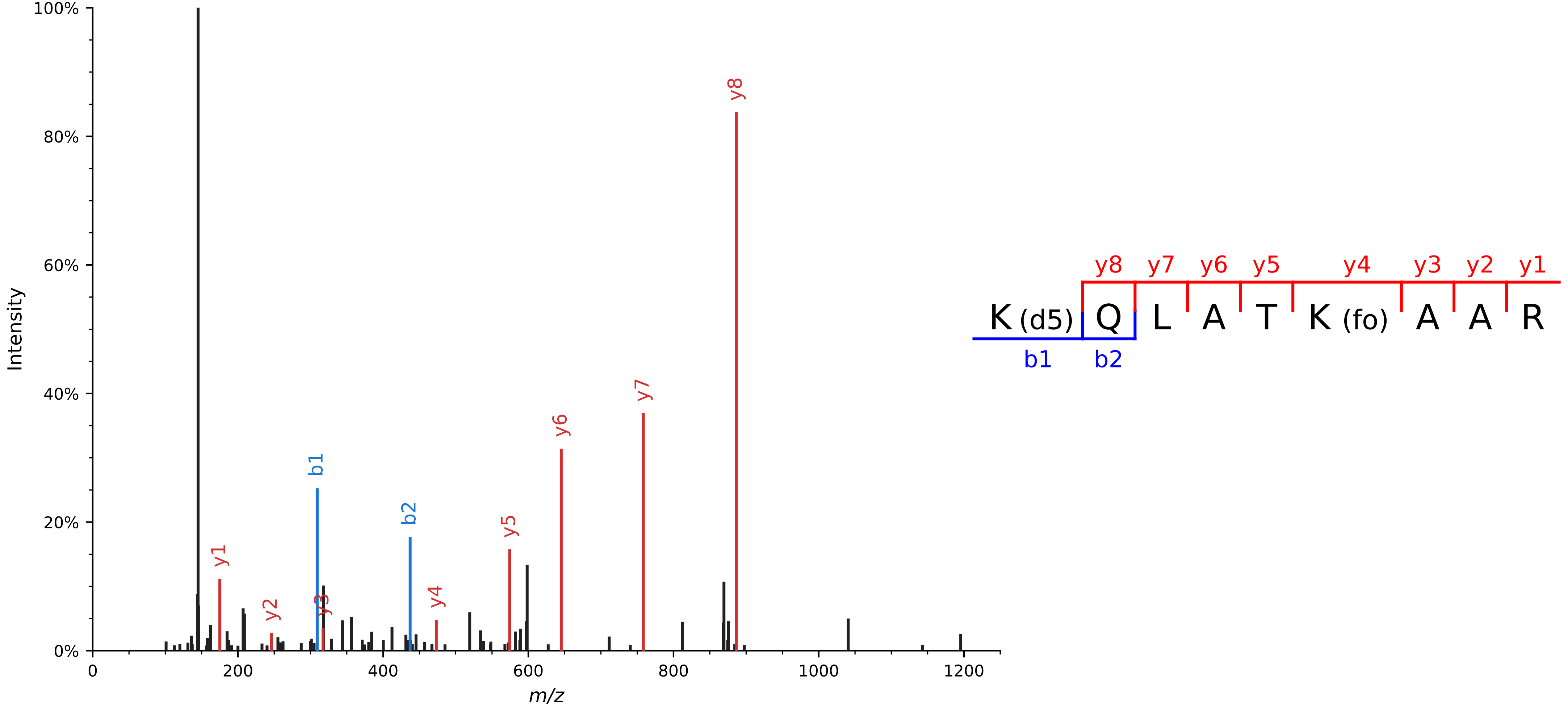

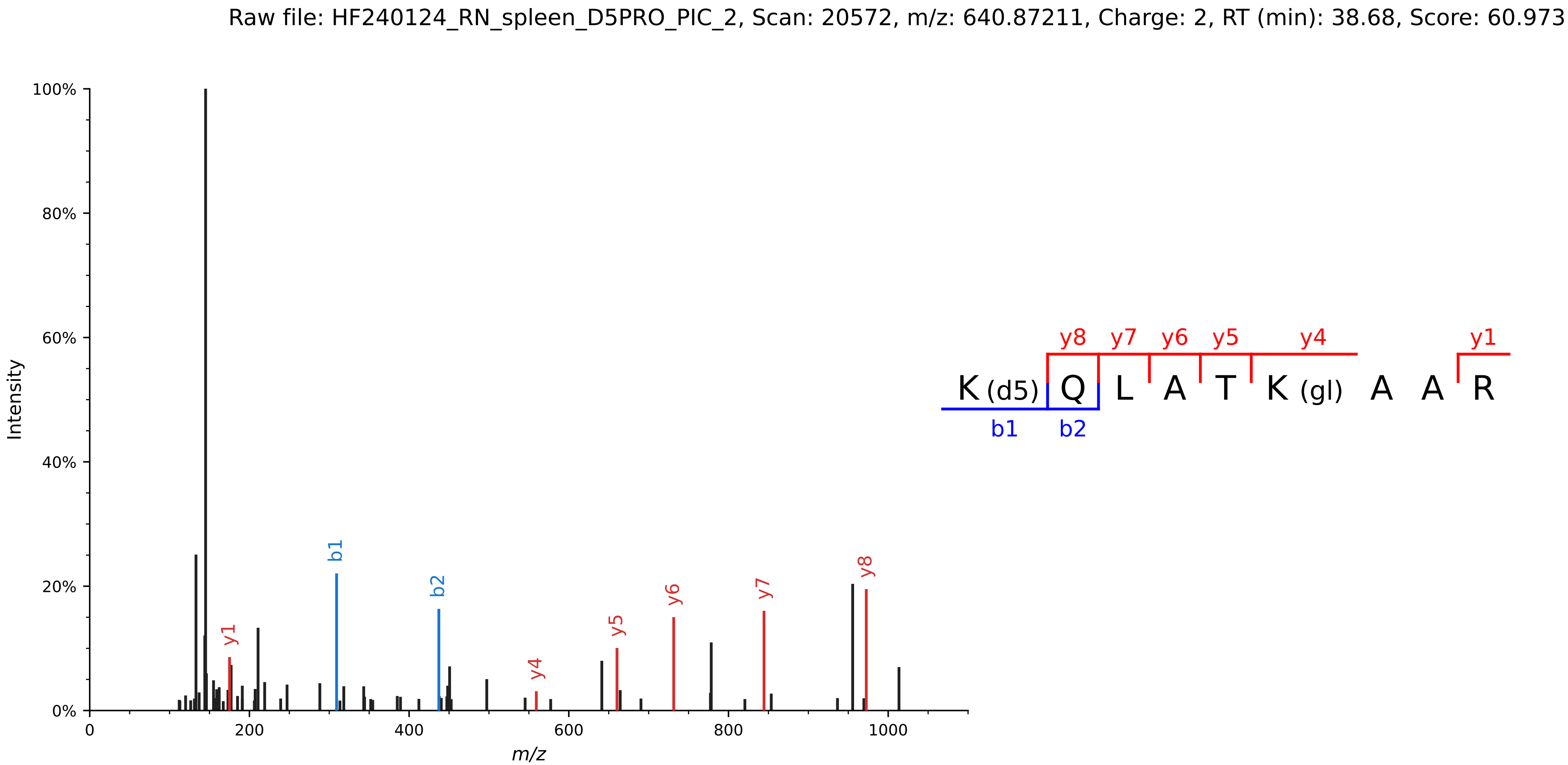

Raw file: HF240124_RN_spleen_D5PRO_PIC_2, Scan: 20572, m/z: 640.87211, Charge: 2, RT (min): 38.68, Score: 60.973
Intensity
100%
80%
60%
40%
20%
0%
0
200
400
600
800
1000
m/z
b1
b2
y1
y4
y5
y6
y7
y8
K (d5) Q L A T K (gl) A A R

Raw file: HF240124_RN_spleen_D5PRO_PIC_3, Scan: 20126, m/z: 621.39289, Charge: 2, RT (min): 37.18, Score: 148.38

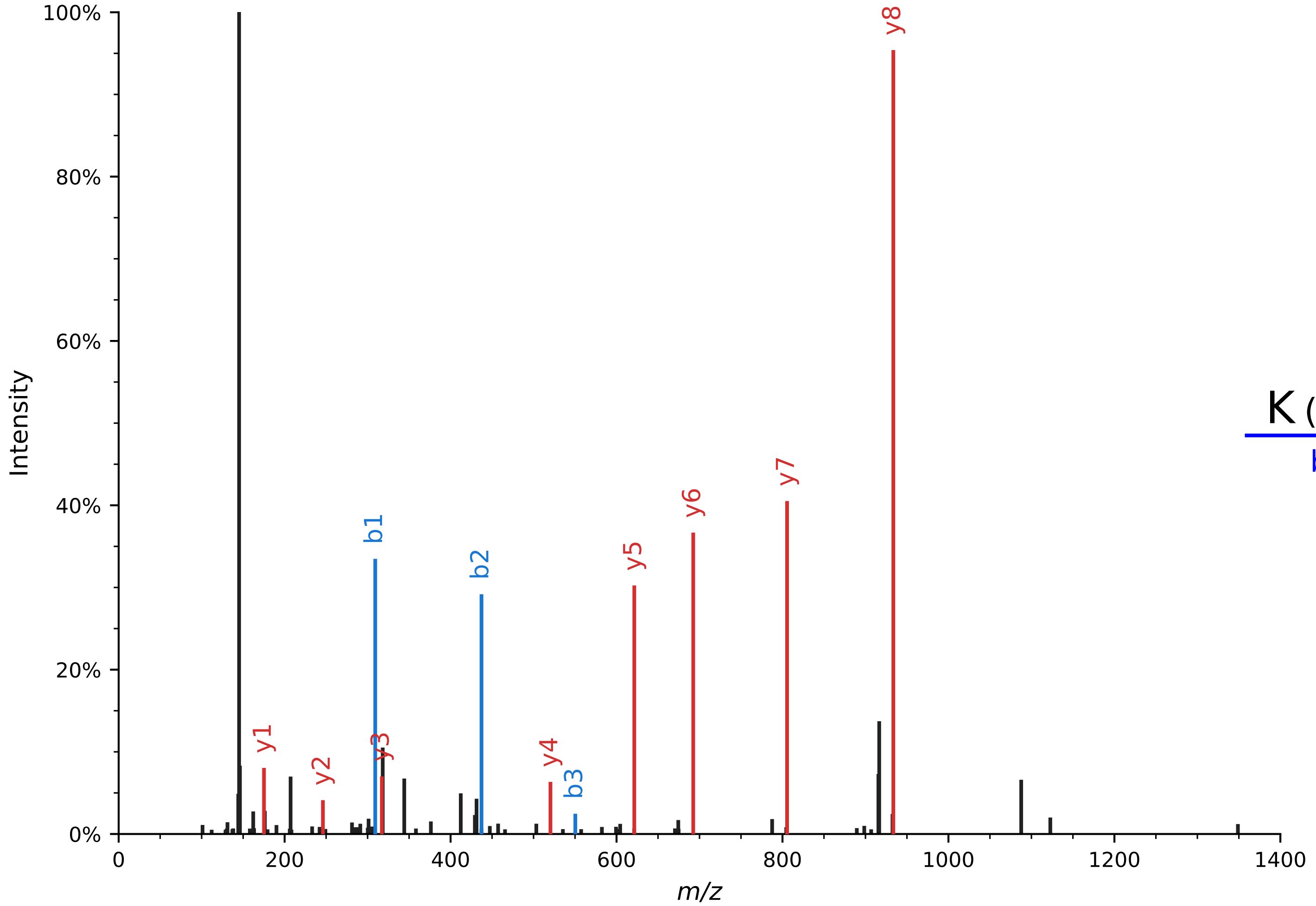

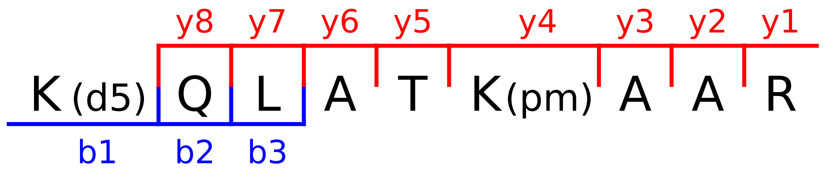

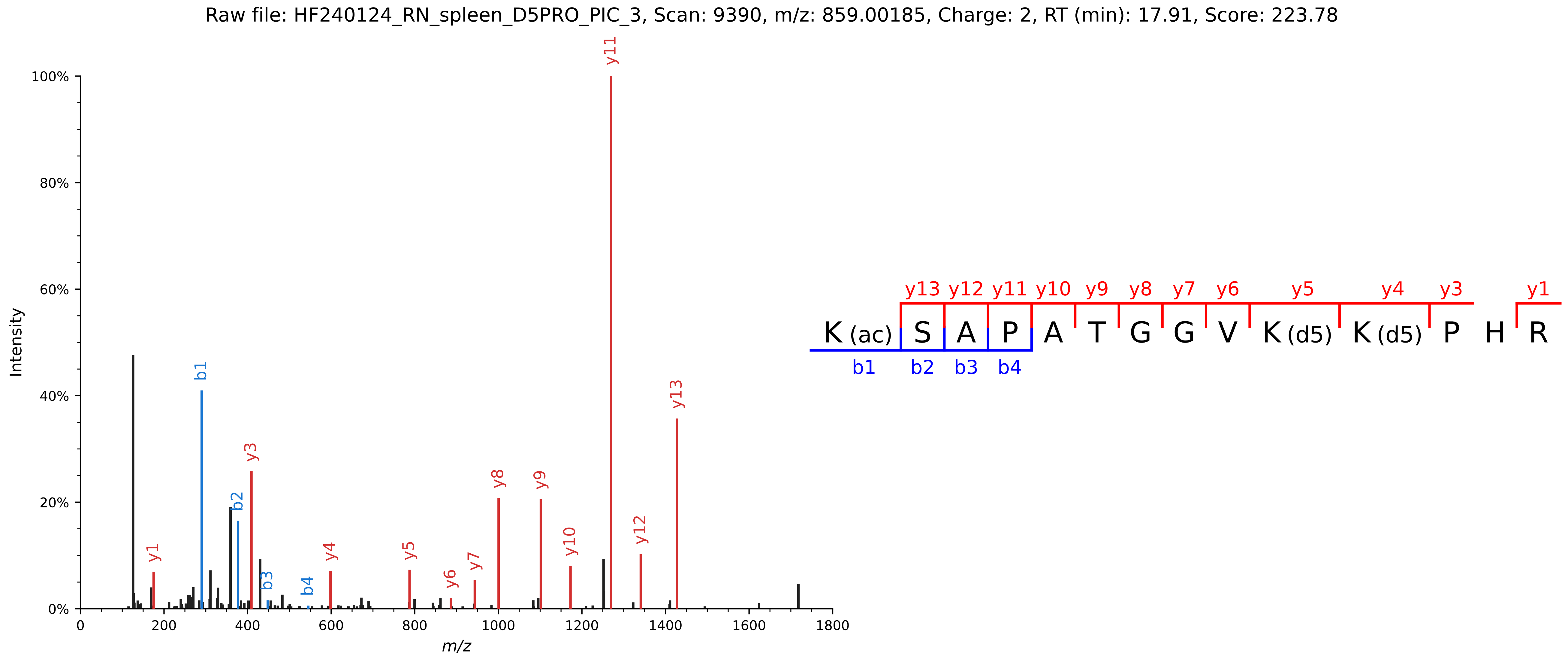

Raw file: HF240124_RN_spleen_D5PRO_PIC_3, Scan: 9390, m/z: 859.00185, Charge: 2, RT (min): 17.91, Score: 223.78
Intensity
m/z
y1 b1 b2 y3 b3 b4 y4 y5 y6 y7 y8 y9 y10 y11 y12 y13
y13 y12 y11 y10 y9 y8 y7 y6 y5 y4 y3 y1
K (ac) S A P A T G G V K (d5) K (d5) P H R
b1 b2 b3 b4

Raw file: HF240124_RN_spleen_D5PRO_PIC_3, Scan: 6411, m/z: 577.67555, Charge: 3, RT (min): 12.59, Score: 59.248

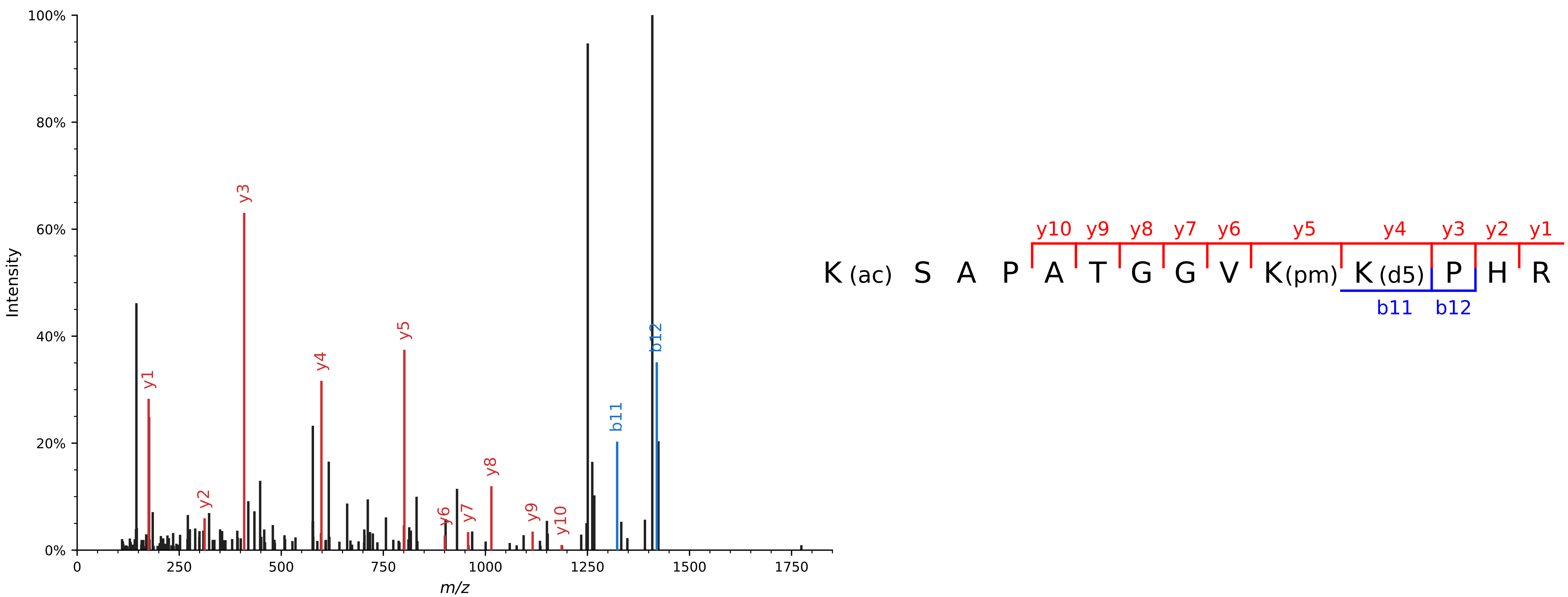

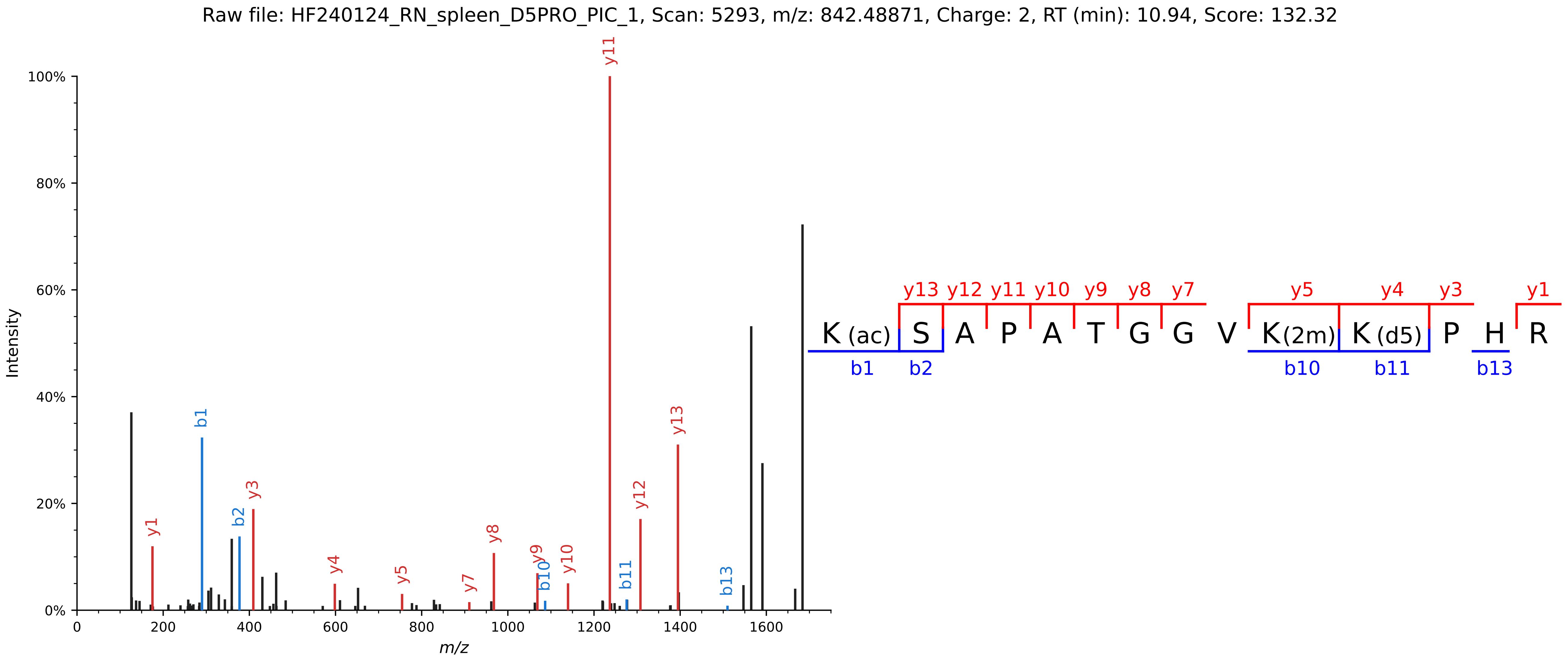

Raw file: HF240124_RN_spleen_D5PRO_PIC_1, Scan: 5293, m/z: 842.48871, Charge: 2, RT (min): 10.94, Score: 132.32
Intensity
m/z
K(ac) S A P A T G G V K(2m) K(d5) P H R
y13 y12 y11 y10 y9 y8 y7 y5 y4 y3 y1
b1 b2 b10 b11 b13

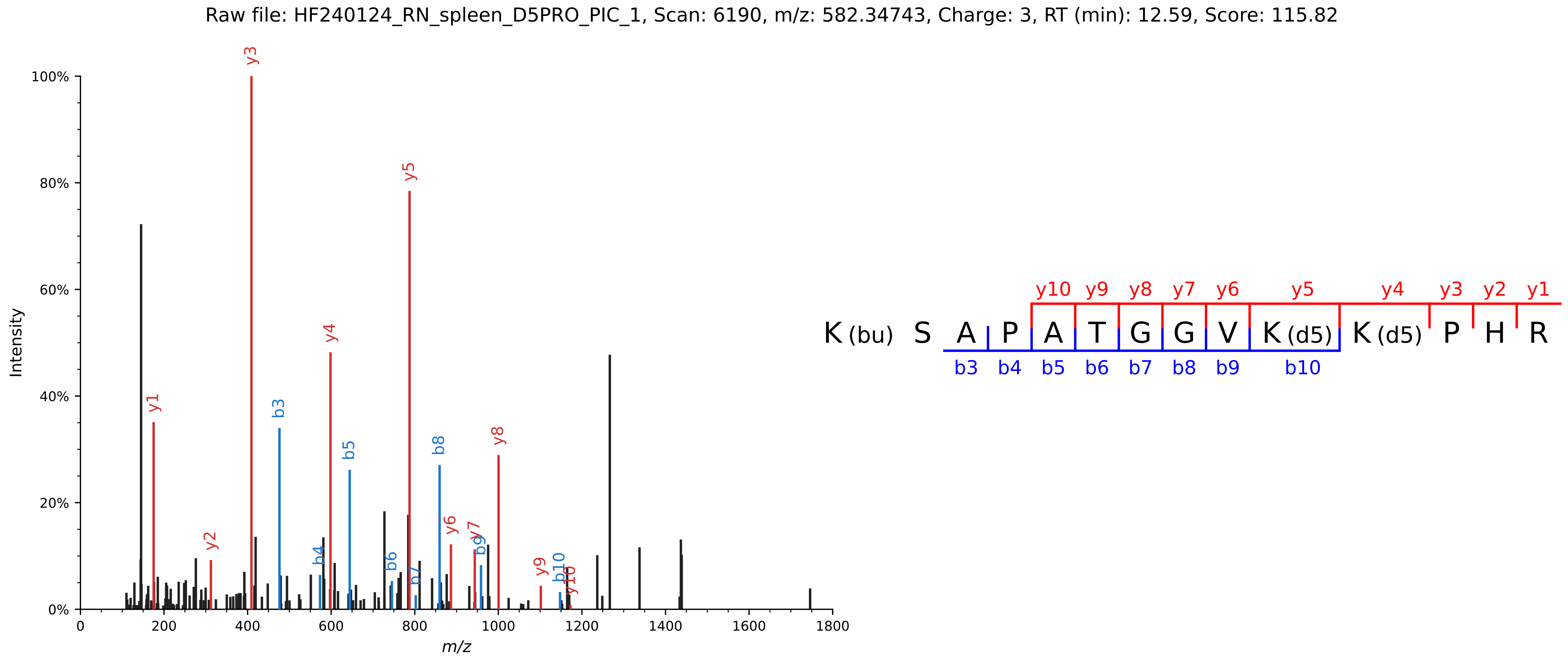

Raw file: HF240124_RN_spleen_D5PRO_PIC_1, Scan: 6190, m/z: 582.34743, Charge: 3, RT (min): 12.59, Score: 115.82
Intensity
m/z
y1
y2
y3
y4
y5
y6
y7
y8
y9
y10
b3
b4
b5
b6
b7
b8
b9
b10
K (bu) S A P A T G G V K (d5) K (d5) P H R

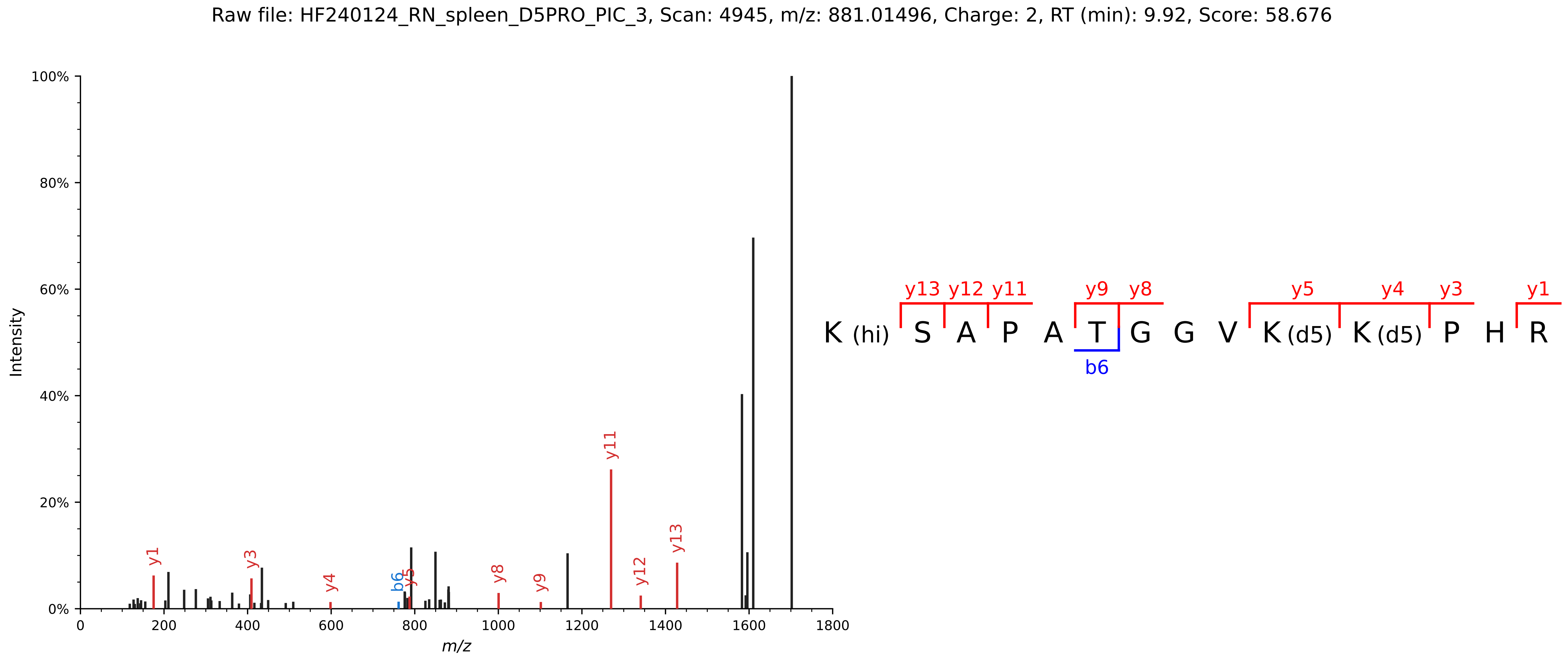

Raw file: HF240124_RN_spleen_D5PRO_PIC_3, Scan: 4945, m/z: 881.01496, Charge: 2, RT (min): 9.92, Score: 58.676
Intensity
m/z
y1
y3
y4
b6
y5
y8
y9
y11
y12
y13
y13 y12 y11 y9 y8 y5 y4 y3 y1
K (hi) S A P A T G G V K (d5) K (d5) P H R
b6

Raw file: HF240124_RN_spleen_D5PRO_PIC_2, Scan: 11331, m/z: 875.5332, Charge: 2, RT (min): 22.00, Score: 146.11

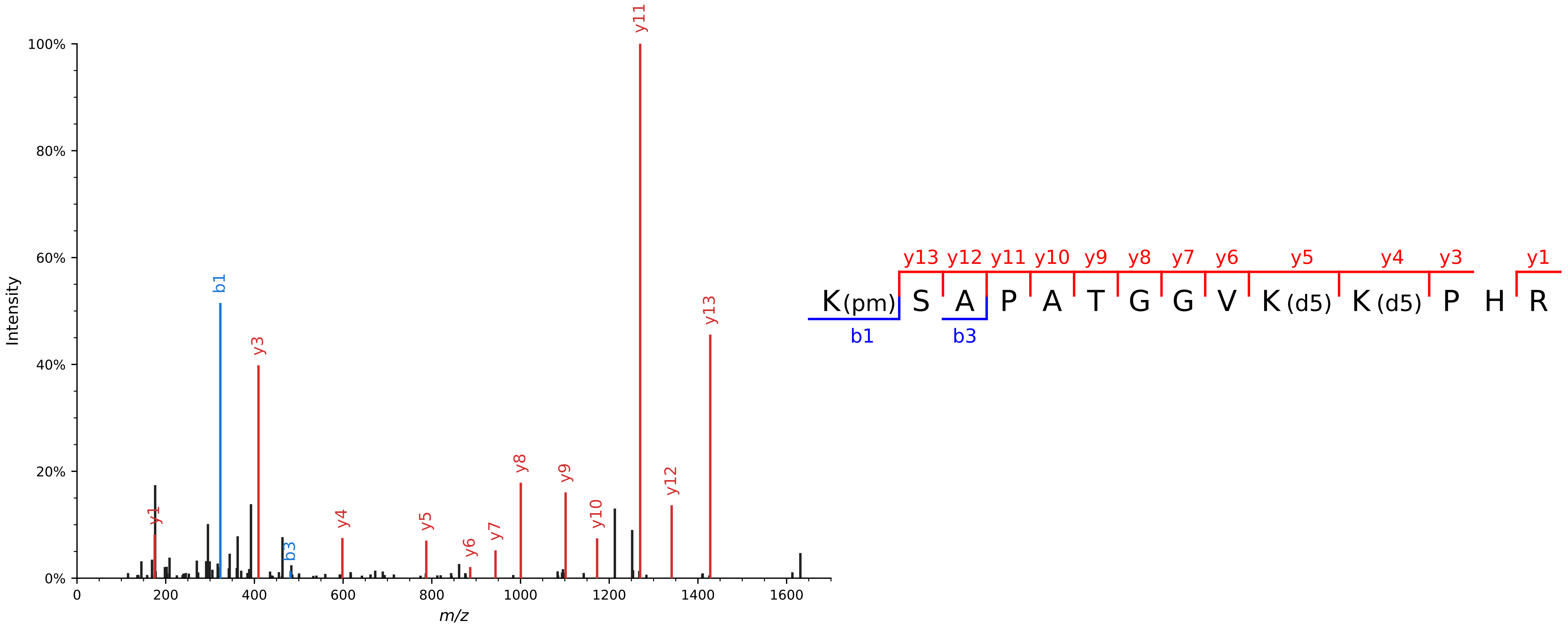

Raw file: HF240124_RN_spleen_D5PRO_PIC_1, Scan: 12637, m/z: 882.54102, Charge: 2, RT (min): 24.04, Score: 180.07

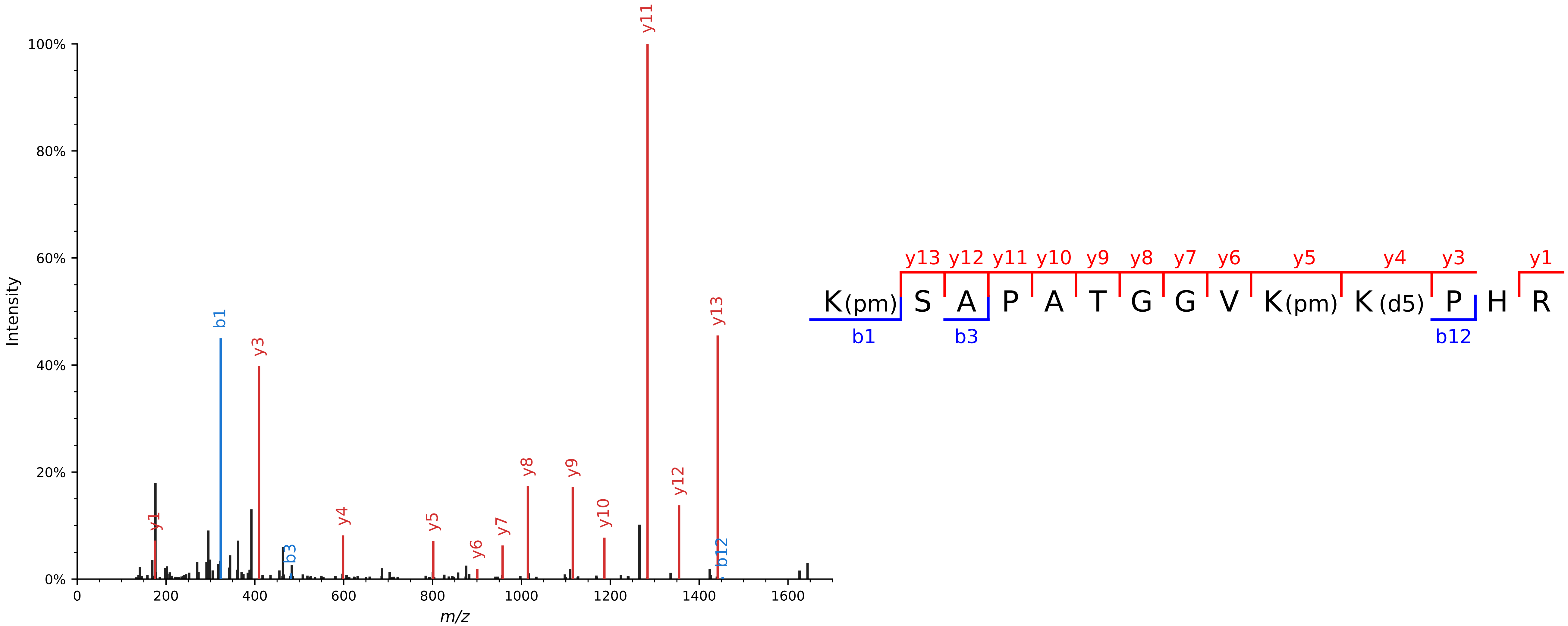

Raw file: HF240124_RN_spleen_D5PRO_PIC_3, Scan: 9344, m/z: 593.36832, Charge: 3, RT (min): 17.84, Score: 59.185

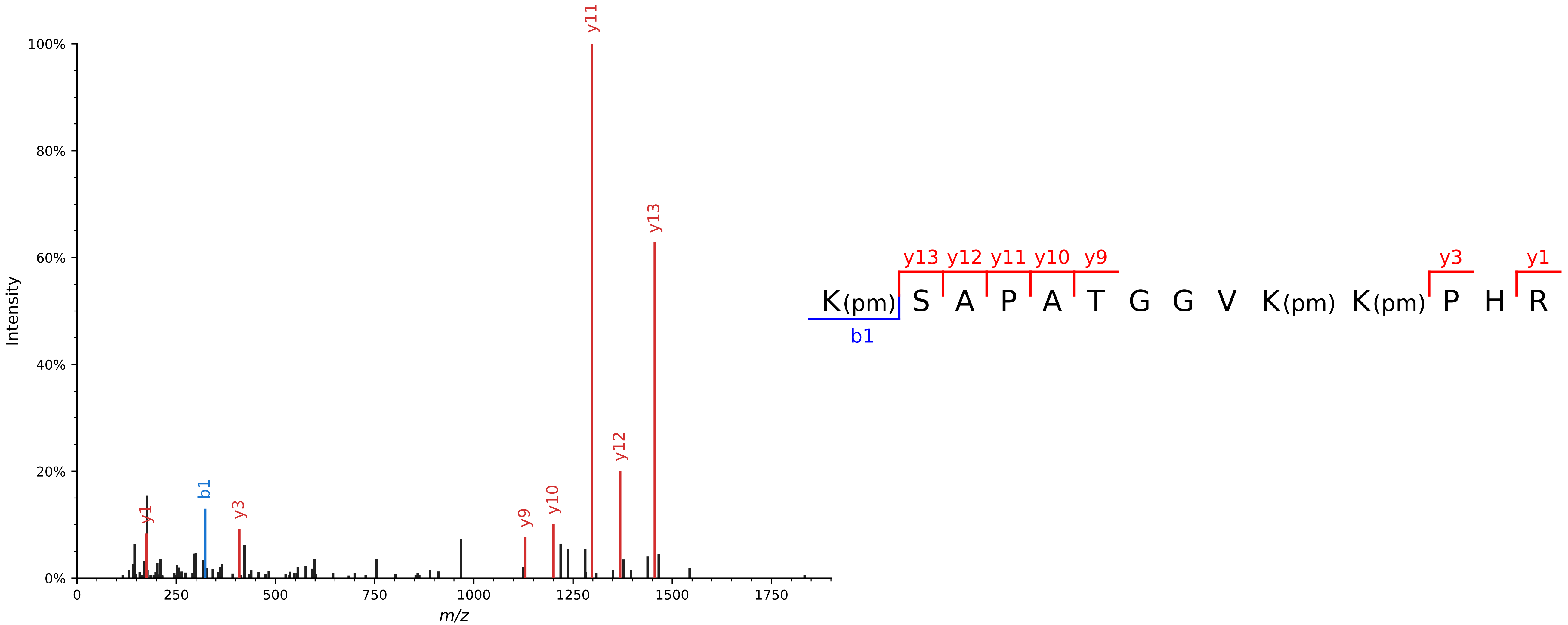

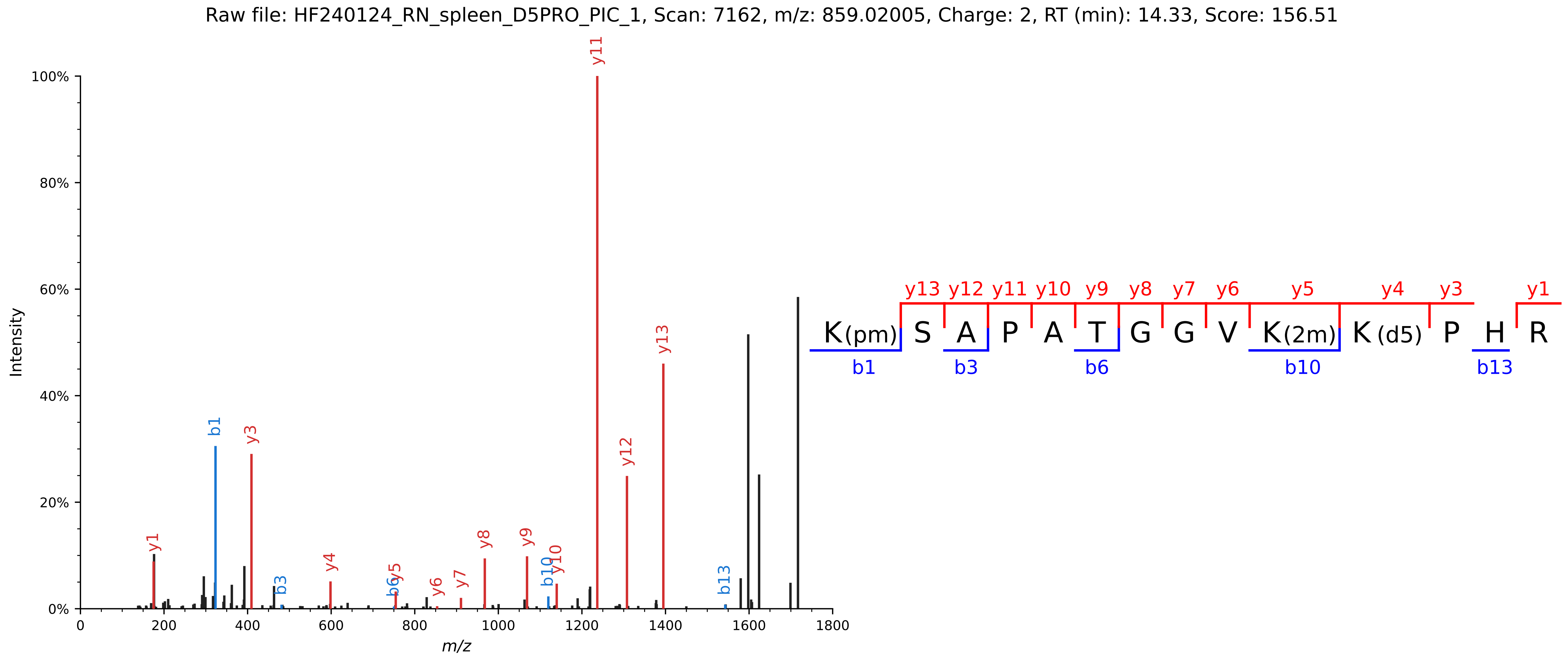
Raw file: HF240124_RN_spleen_D5PRO_PIC_1, Scan: 7162, m/z: 859.02005, Charge: 2, RT (min): 14.33, Score: 156.51
Intensity
m/z
K(pm) S A P A T G G V K(2m) K(d5) P H R
y13 y12 y11 y10 y9 y8 y7 y6 y5 y4 y3 y1
b1 b3 b6 b10 b13

Raw file: HF240124_RN_spleen_D5PRO_PIC_2, Scan: 6931, m/z: 866.02787, Charge: 2, RT (min): 14.09, Score: 82.417

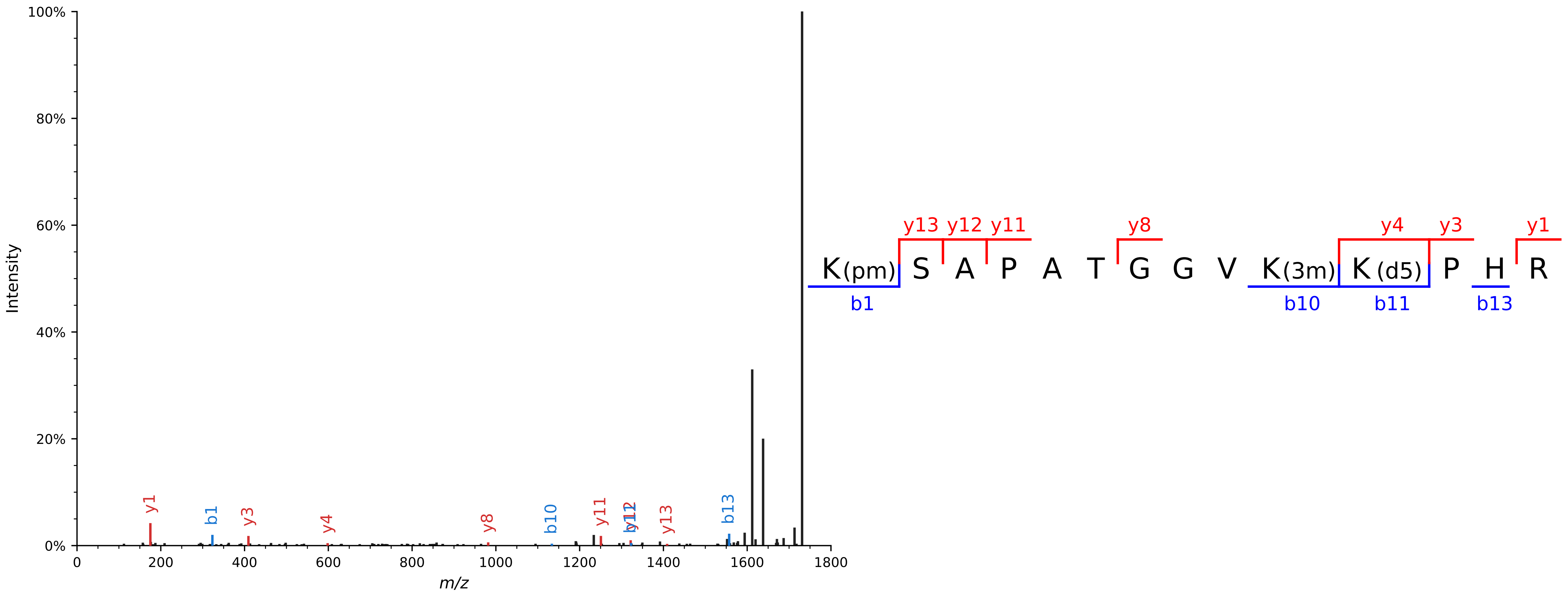

Raw file: HF240124_RN_spleen_D5PRO_PIC_2, Scan: 4748, m/z: 852.01222, Charge: 2, RT (min): 10.07, Score: 221.09

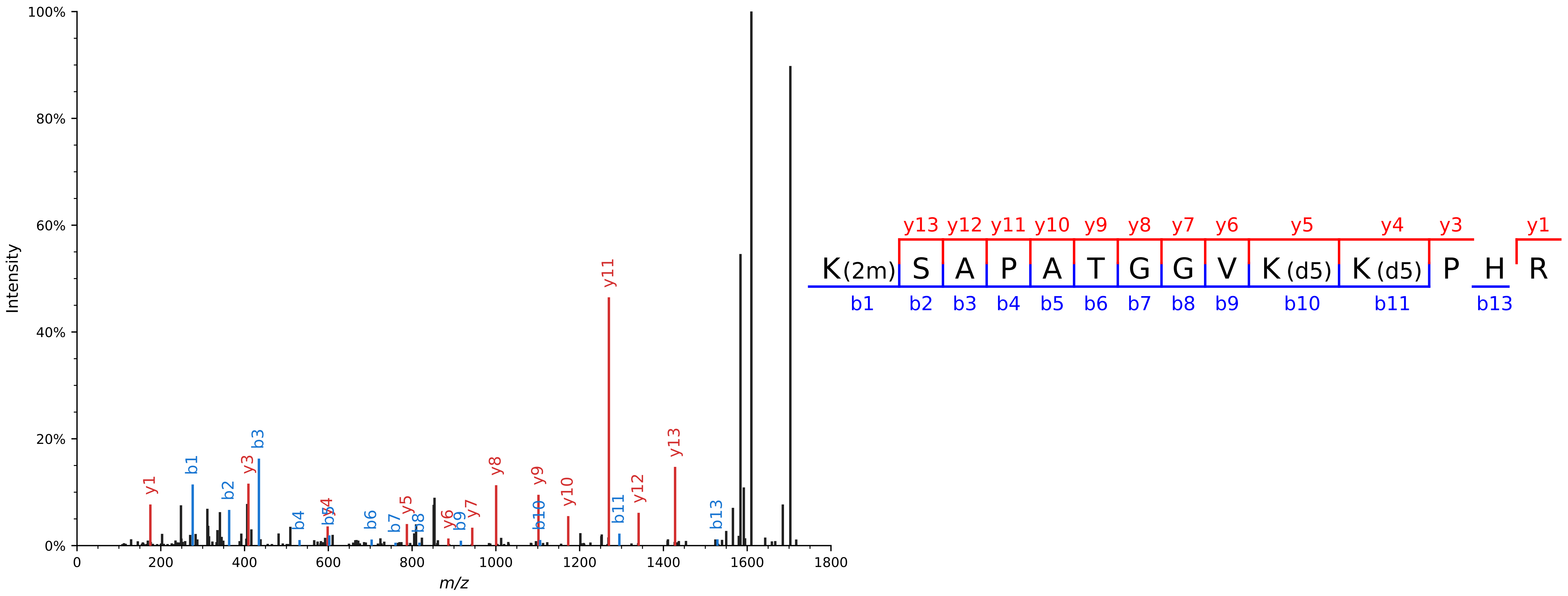

Raw file: HF240124_RN_spleen_D5PRO_PIC_3, Scan: 6026, m/z: 859.02005, Charge: 2, RT (min): 11.90, Score: 151.16

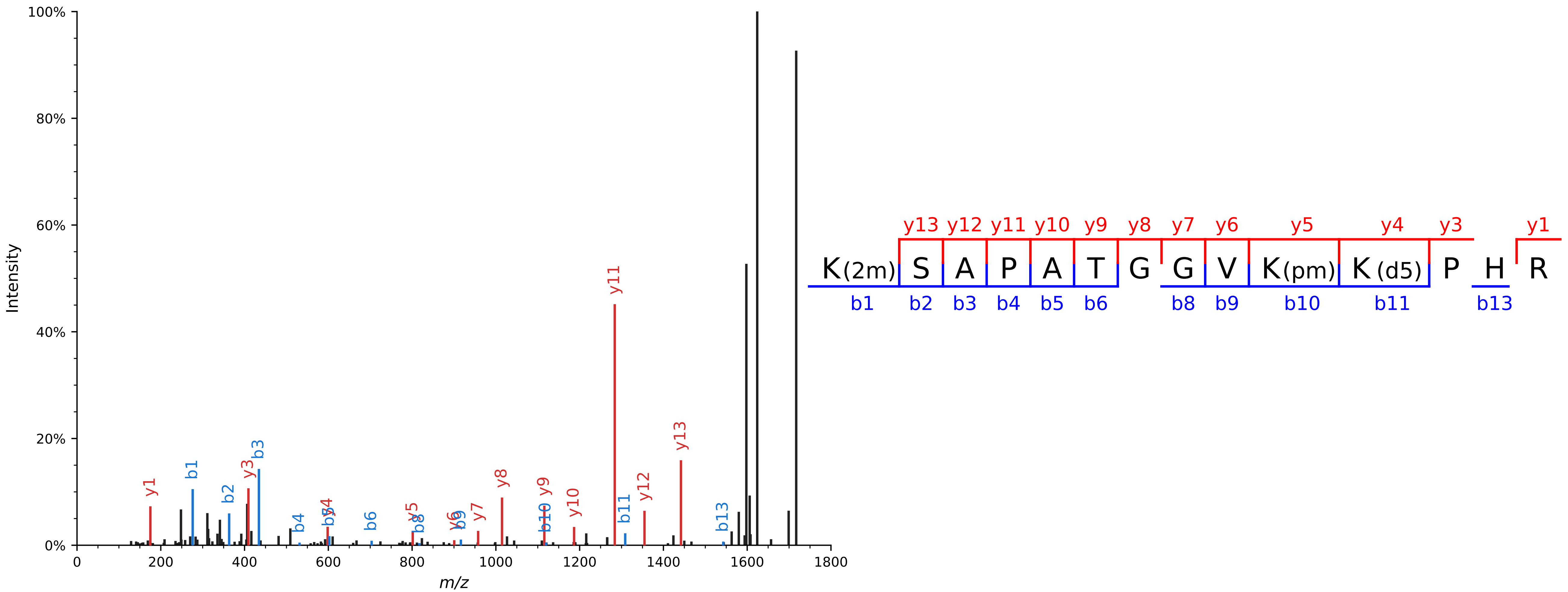

Raw file: HF240124_RN_spleen_D5PRO_PIC_3, Scan: 4224, m/z: 866.02787, Charge: 2, RT (min): 8.67, Score: 79.286

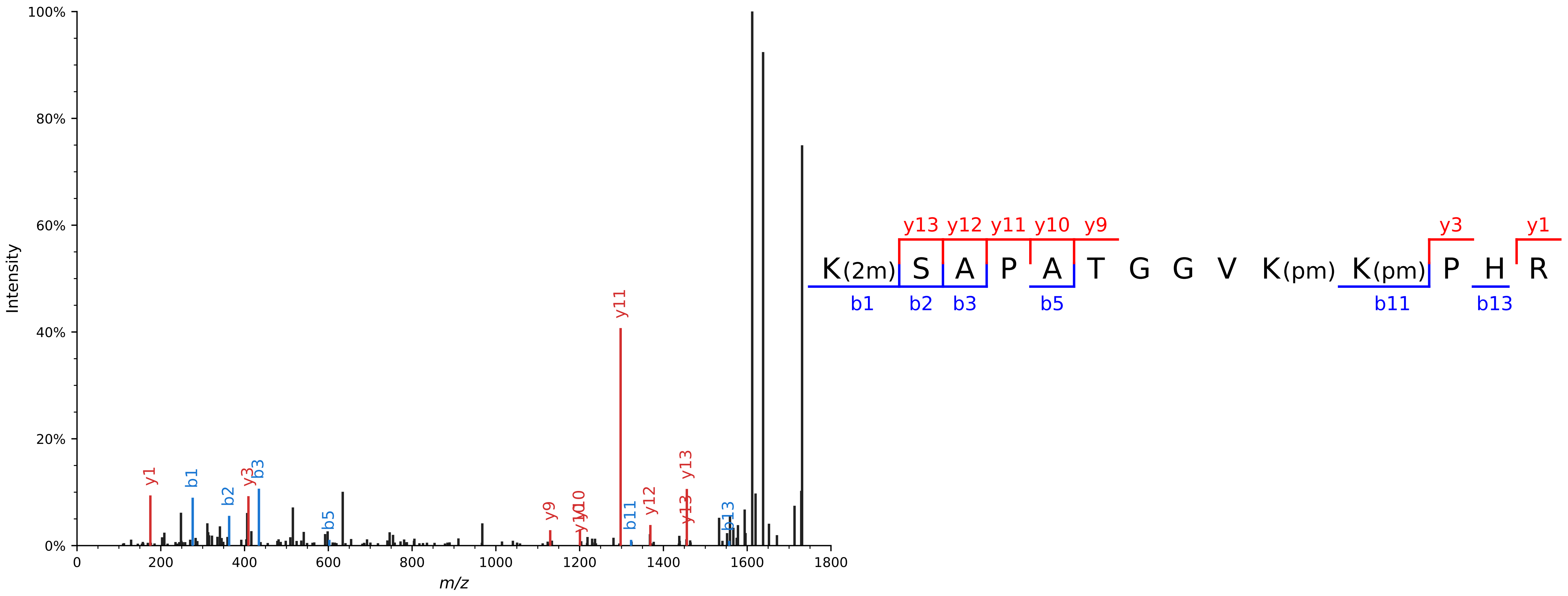

Raw file: HF240124_RN_spleen_D5PRO_PIC_2, Scan: 2114, m/z: 421.75709, Charge: 4, RT (min): 5.32, Score: 64.534

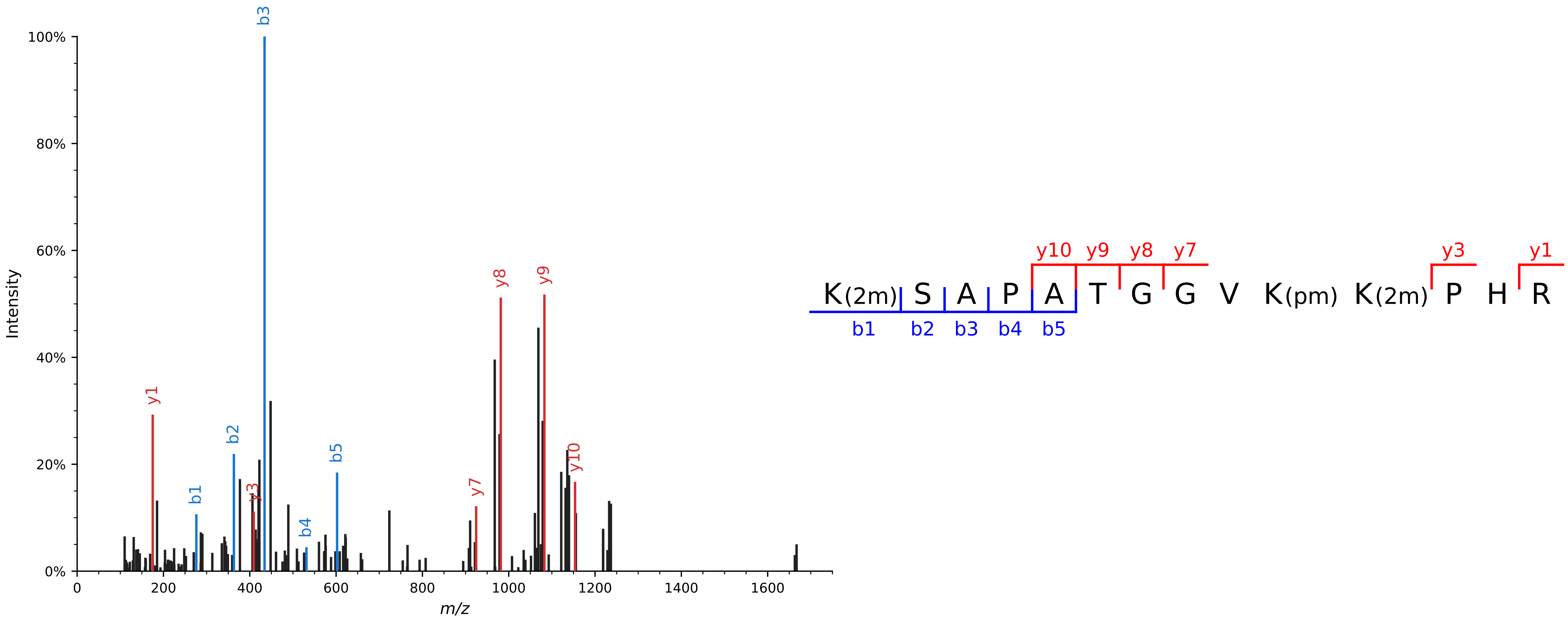

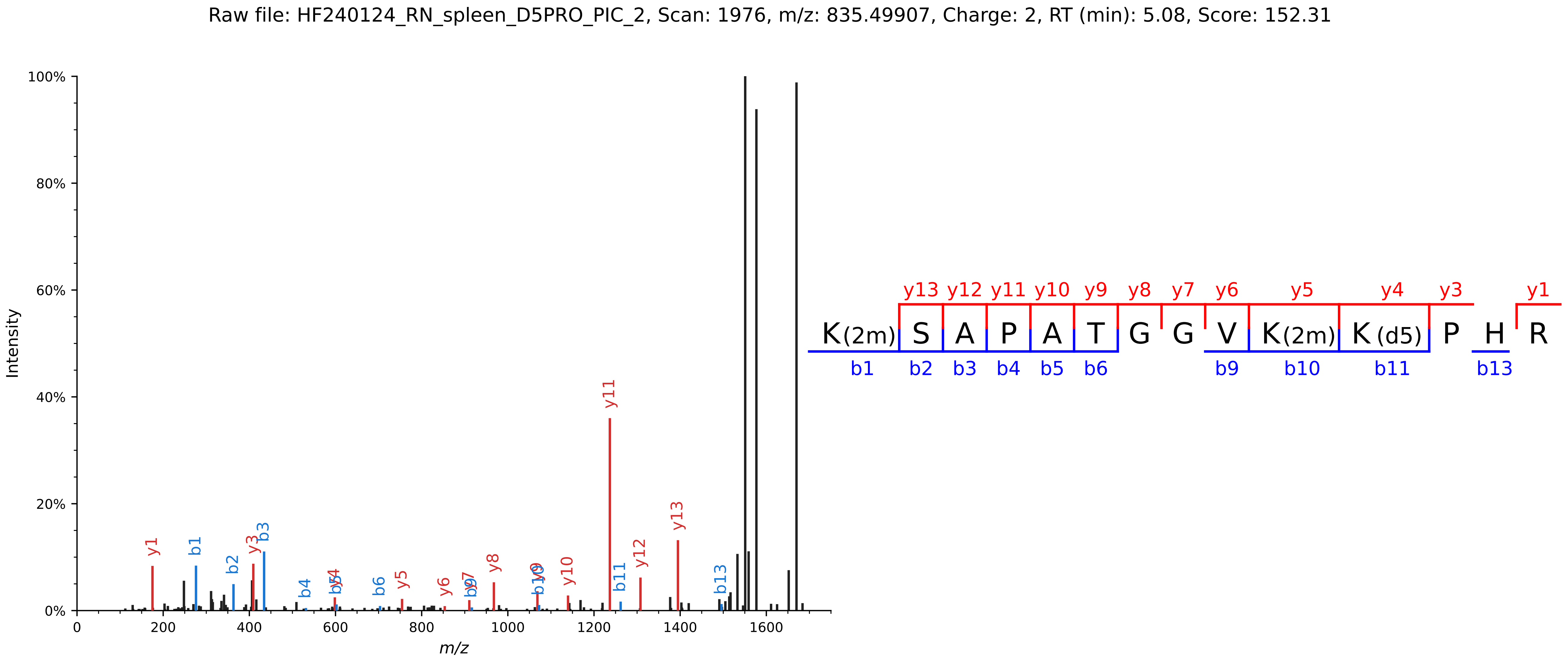

Raw file: HF240124_RN_spleen_D5PRO_PIC_2, Scan: 1976, m/z: 835.49907, Charge: 2, RT (min): 5.08, Score: 152.31
Intensity
m/z
K(2m) S A P A T G G V K(2m) K(d5) P H R
y13 y12 y11 y10 y9 y8 y7 y6 y5 y4 y3 y1
b1 b2 b3 b4 b5 b6 b9 b10 b11 b13

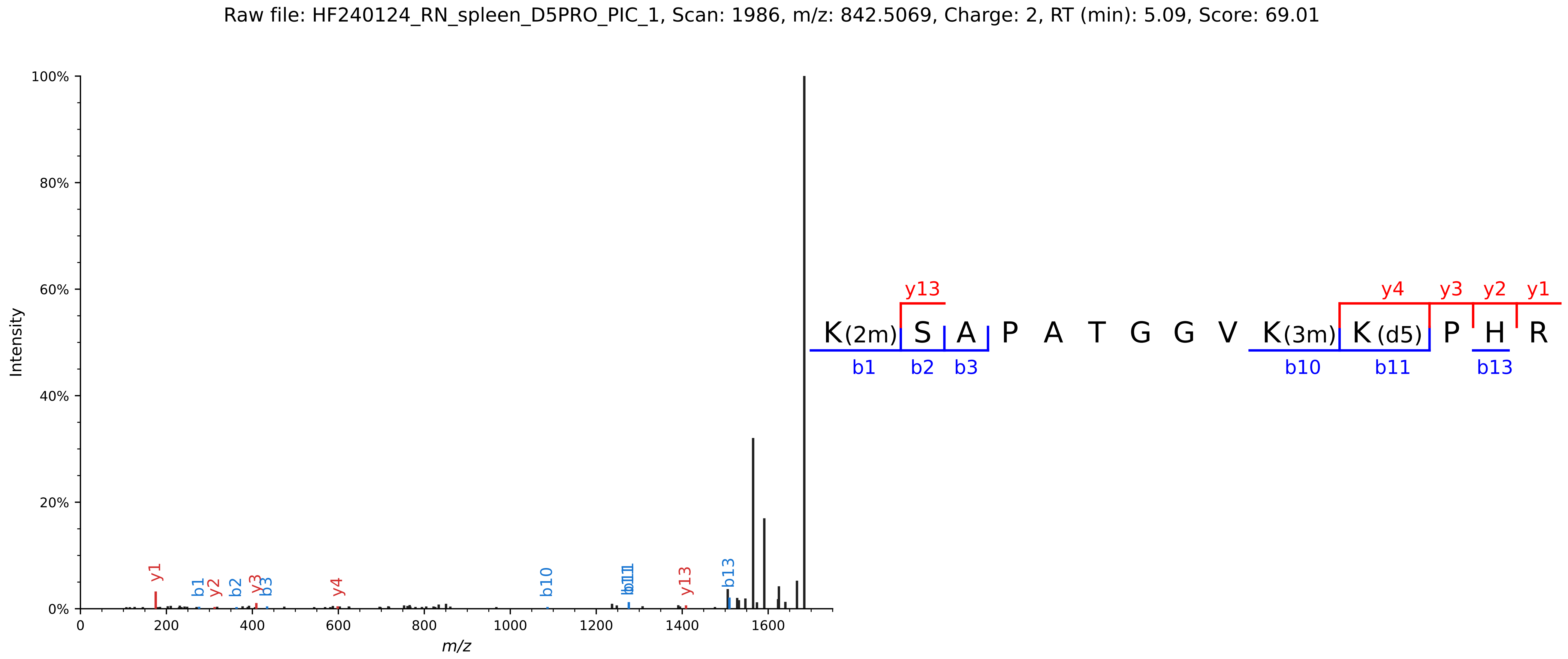

Raw file: HF240124_RN_spleen_D5PRO_PIC_1, Scan: 1986, m/z: 842.5069, Charge: 2, RT (min): 5.09, Score: 69.01
Intensity
m/z
y1
b1
y2
b2
y3
b3
y4
b10
b11
y13
b13
K(2m) S A P A T G G V K(3m) K(d5) P H R

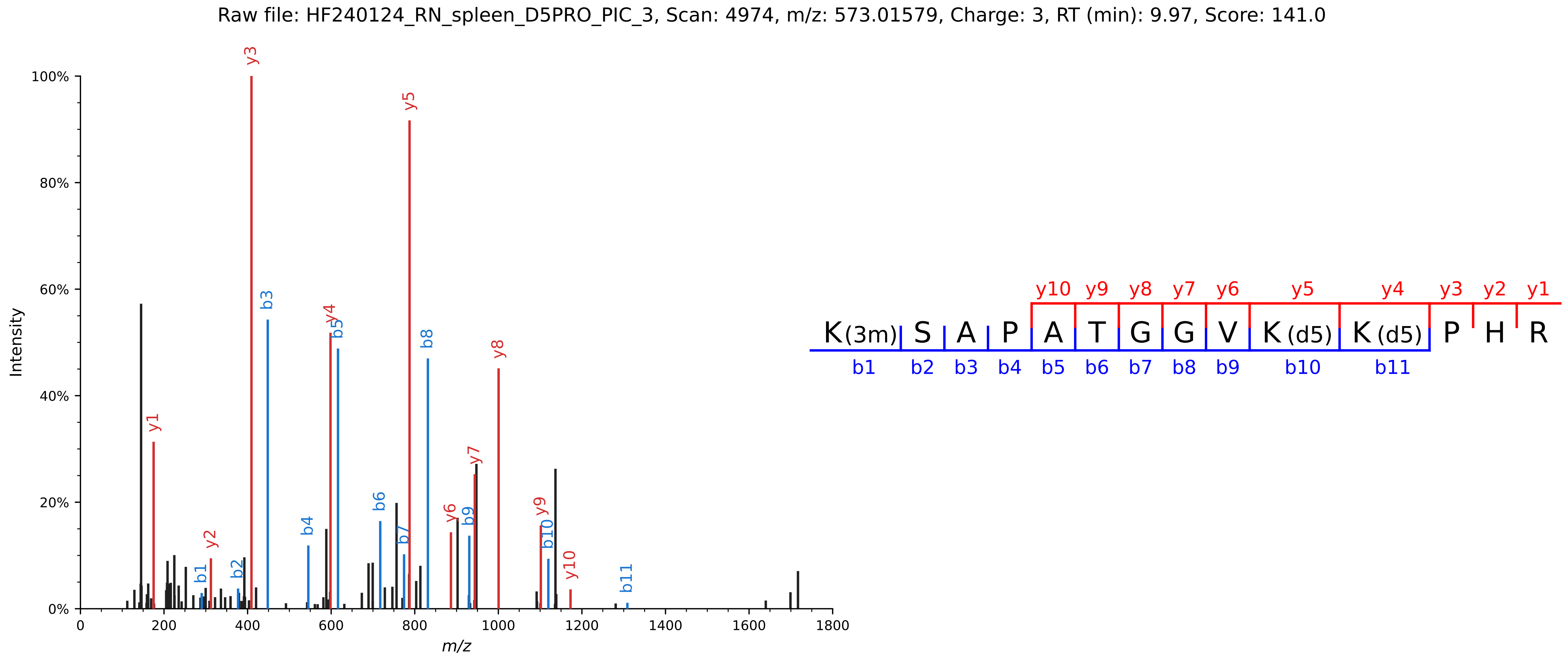
Raw file: HF240124_RN_spleen_D5PRO_PIC_3, Scan: 4974, m/z: 573.01579, Charge: 3, RT (min): 9.97, Score: 141.0
Intensity
m/z
K(3m) S A P A T G G V K(d5) K(d5) P H R
y10 y9 y8 y7 y6 y5 y4 y3 y2 y1
b1 b2 b3 b4 b5 b6 b7 b8 b9 b10 b11

Raw file: HF240124_RN_spleen_D5PRO_PIC_3, Scan: 5893, m/z: 577.68767, Charge: 3, RT (min): 11.63, Score: 143.93

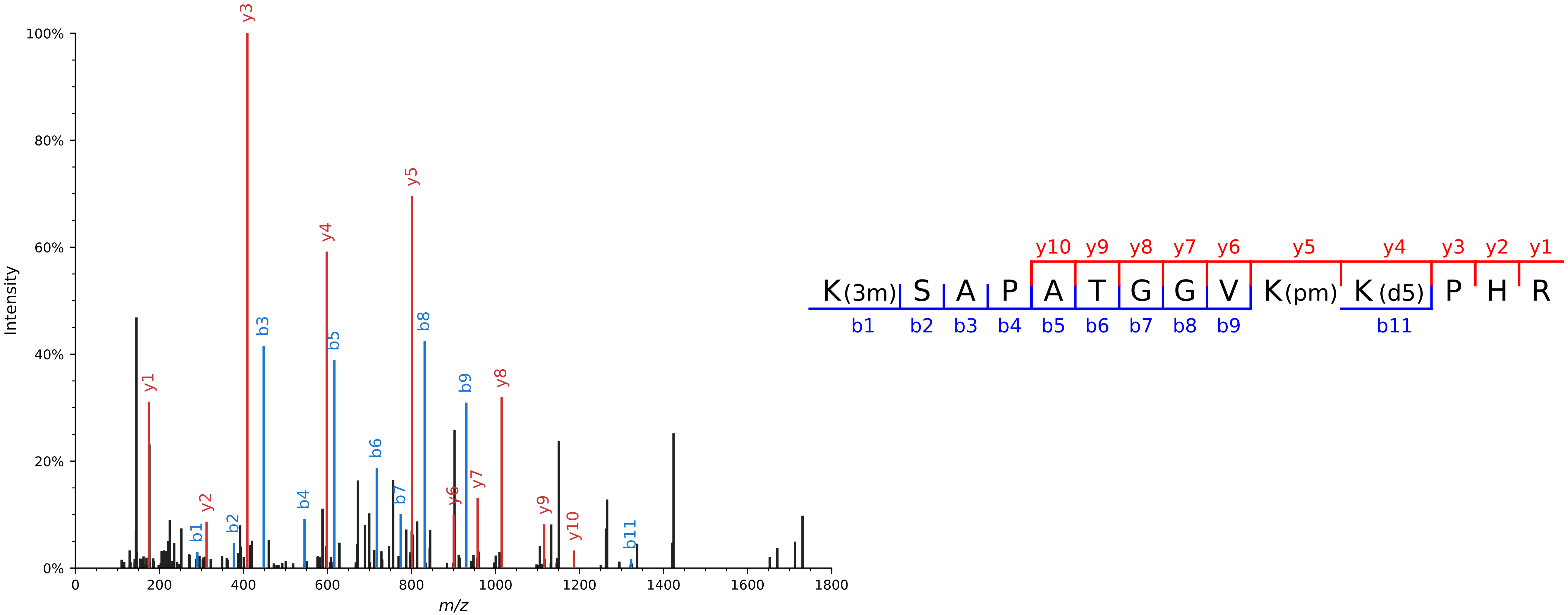

Raw file: HF240124_RN_spleen_D5PRO_PIC_2, Scan: 1971, m/z: 562.00702, Charge: 3, RT (min): 5.07, Score: 88.706

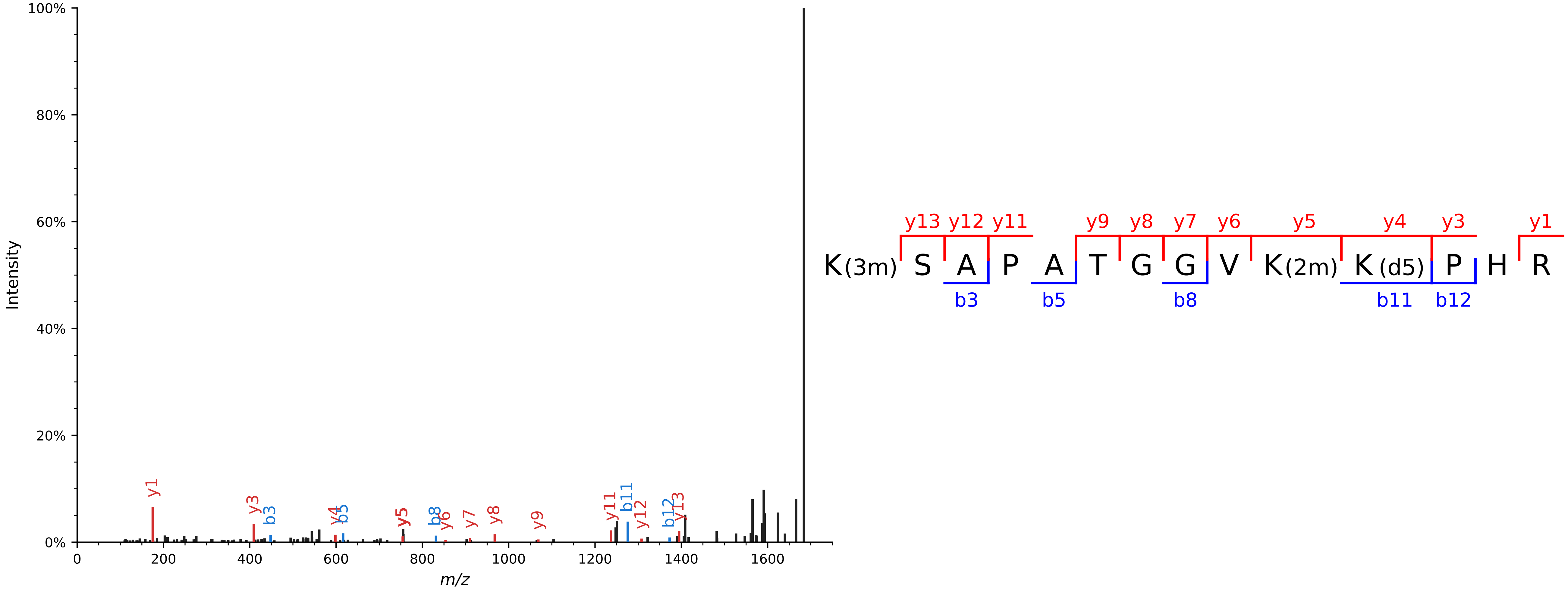

Raw file: HF240124_RN_spleen_D5PRO_PIC_2, Scan: 1972, m/z: 421.75709, Charge: 4, RT (min): 5.07, Score: 80.905

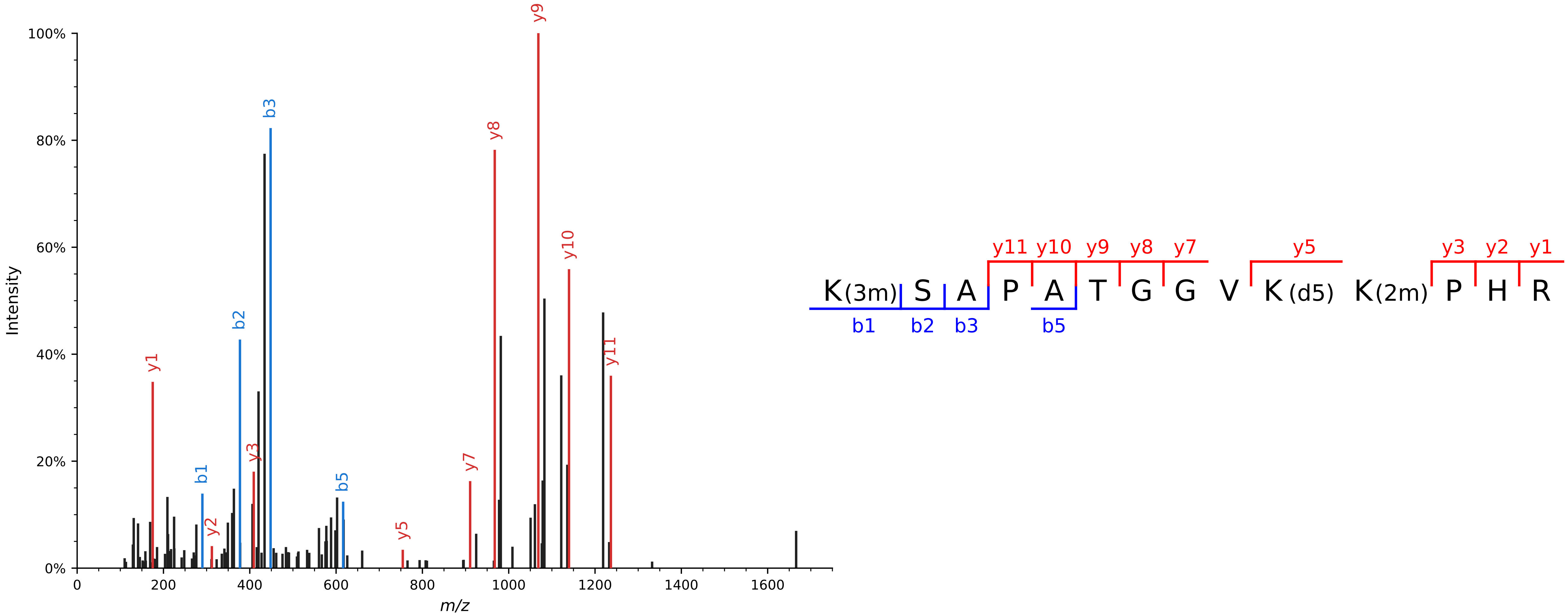

Raw file: HF240124_RN_spleen_D5PRO_PIC_3, Scan: 11215, m/z: 875.5332, Charge: 2, RT (min): 21.23, Score: 164.65

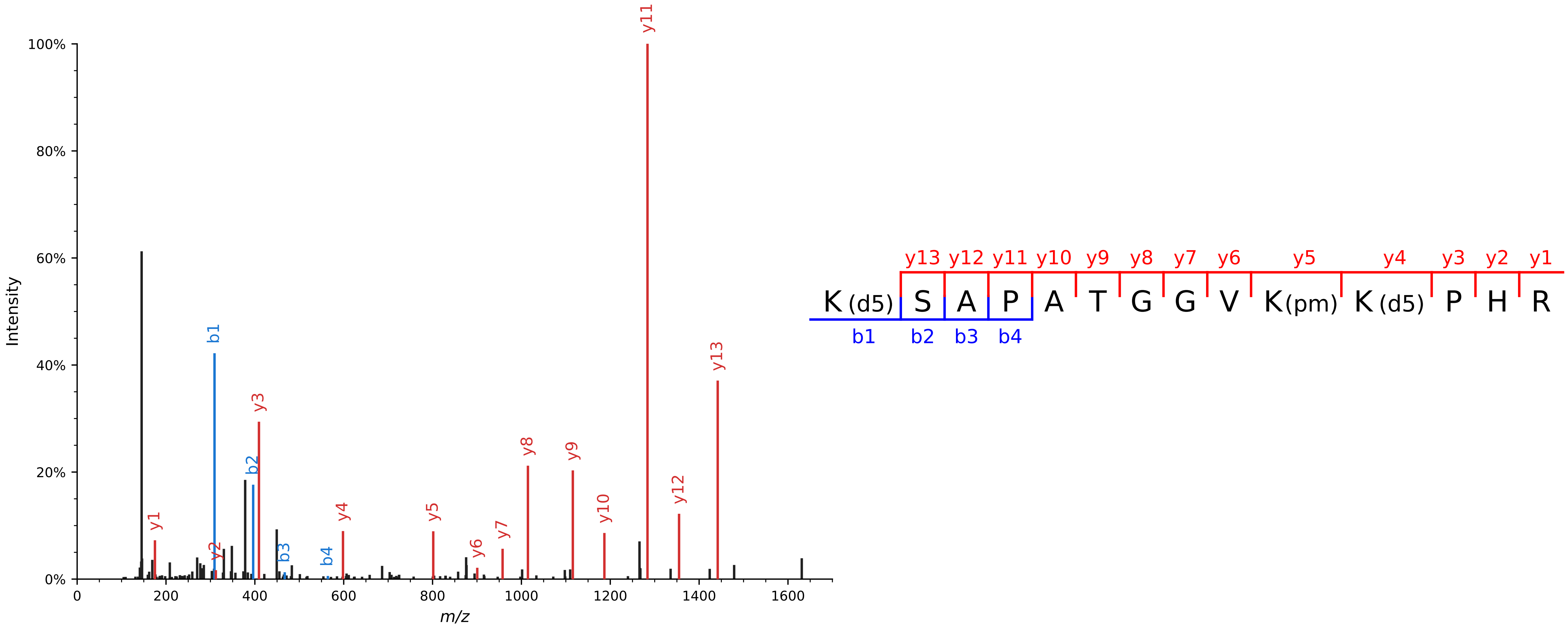

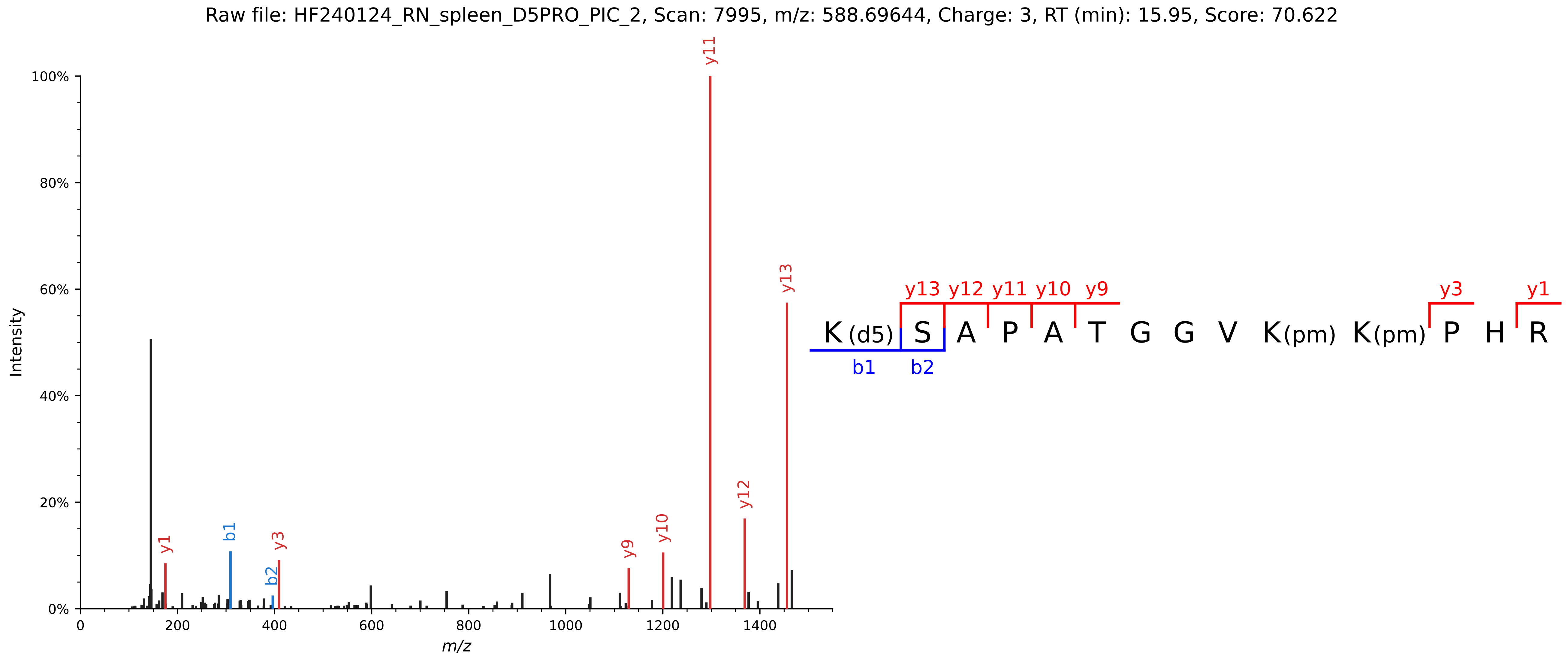

Raw file: HF240124_RN_spleen_D5PRO_PIC_2, Scan: 7995, m/z: 588.69644, Charge: 3, RT (min): 15.95, Score: 70.622
Intensity
m/z
y1
b1
b2
y3
y9
y10
y11
y12
y13
K(d5) S A P A T G G V K(pm) K(pm) P H R

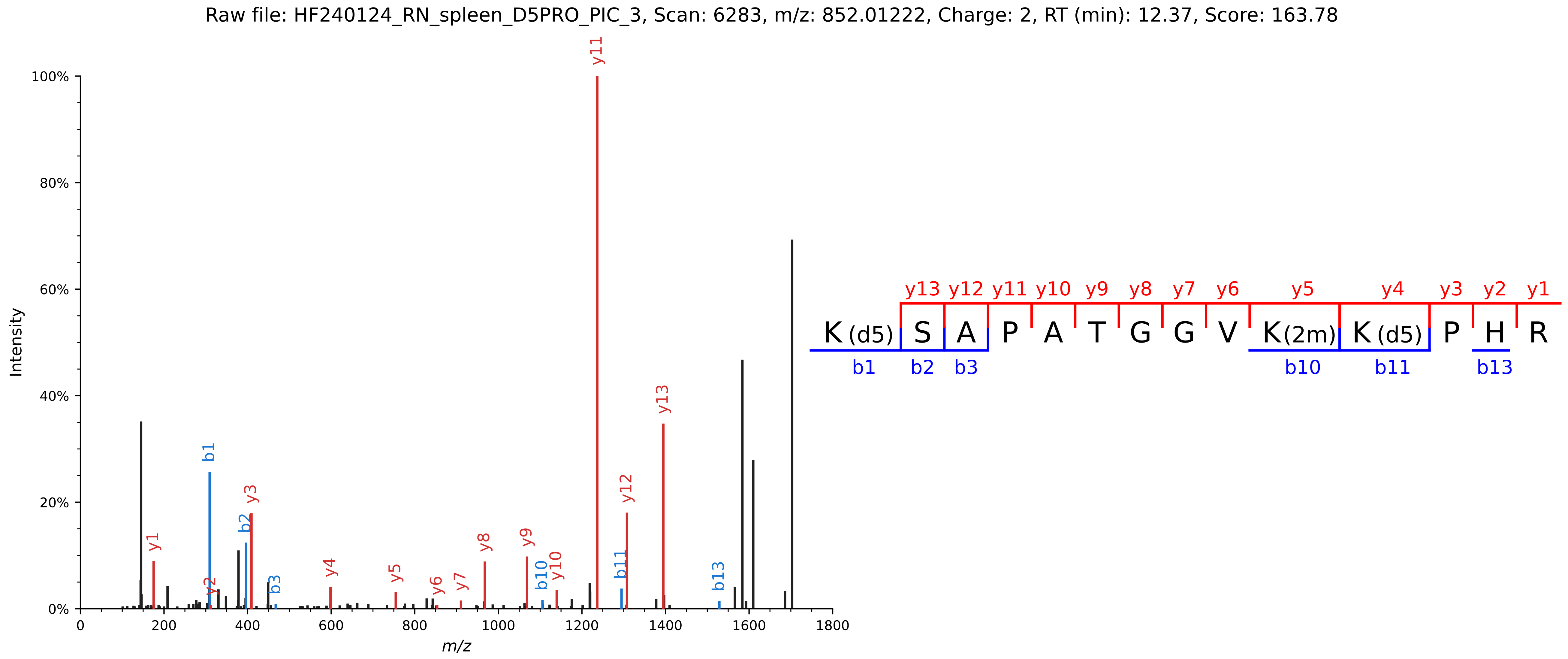

Raw file: HF240124_RN_spleen_D5PRO_PIC_3, Scan: 6283, m/z: 852.01222, Charge: 2, RT (min): 12.37, Score: 163.78
Intensity
m/z
y13 y12 y11 y10 y9 y8 y7 y6 y5 y4 y3 y2 y1
K(d5) S A P A T G G V K(2m) K(d5) P H R
b1 b2 b3 b10 b11 b13

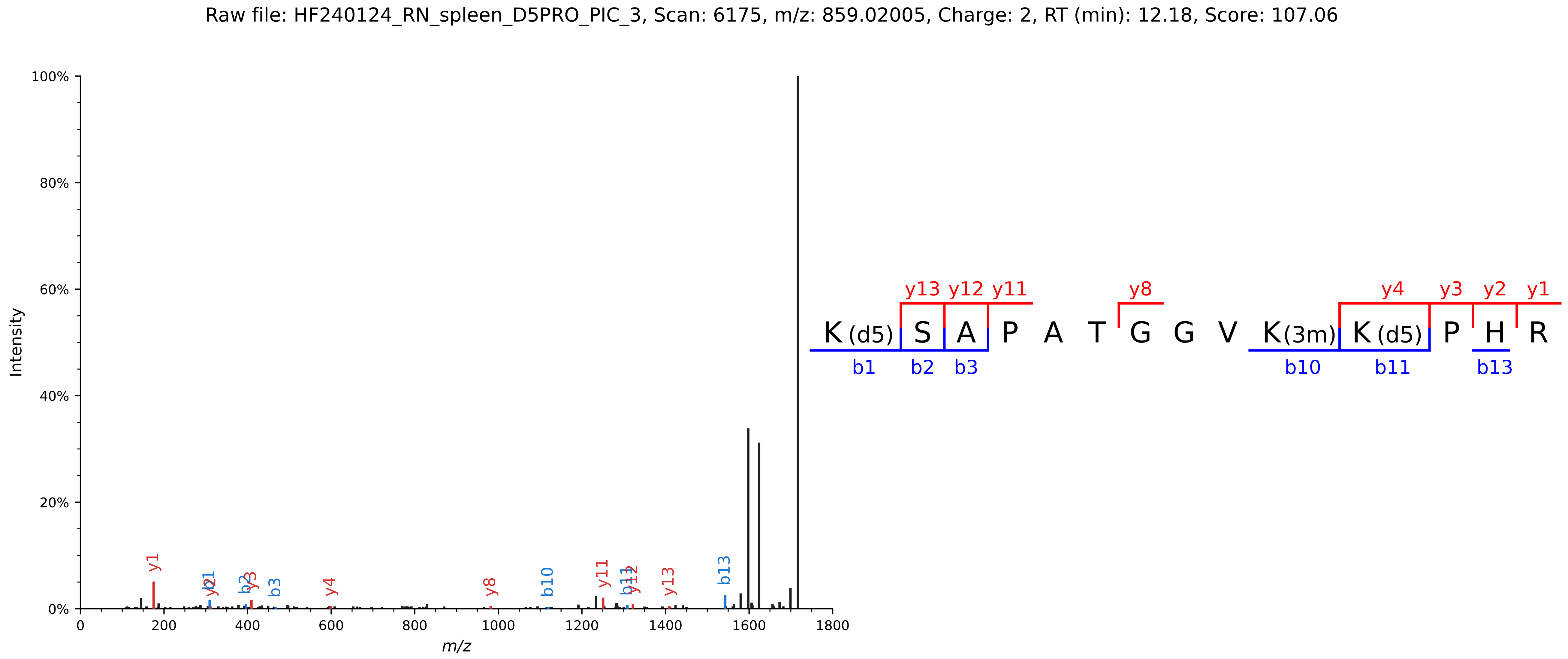

Raw file: HF240124_RN_spleen_D5PRO_PIC_3, Scan: 6175, m/z: 859.02005, Charge: 2, RT (min): 12.18, Score: 107.06
Intensity
m/z
y13 y12 y11 y8 y4 y3 y2 y1
K(d5) S A P A T G G V K(3m) K(d5) P H R
b1 b2 b3 b10 b11 b13

Raw file: HF240124_RN_spleen_D5PRO_PIC_3, Scan: 21523, m/z: 489.28199, Charge: 2, RT (min): 39.73, Score: 94.95

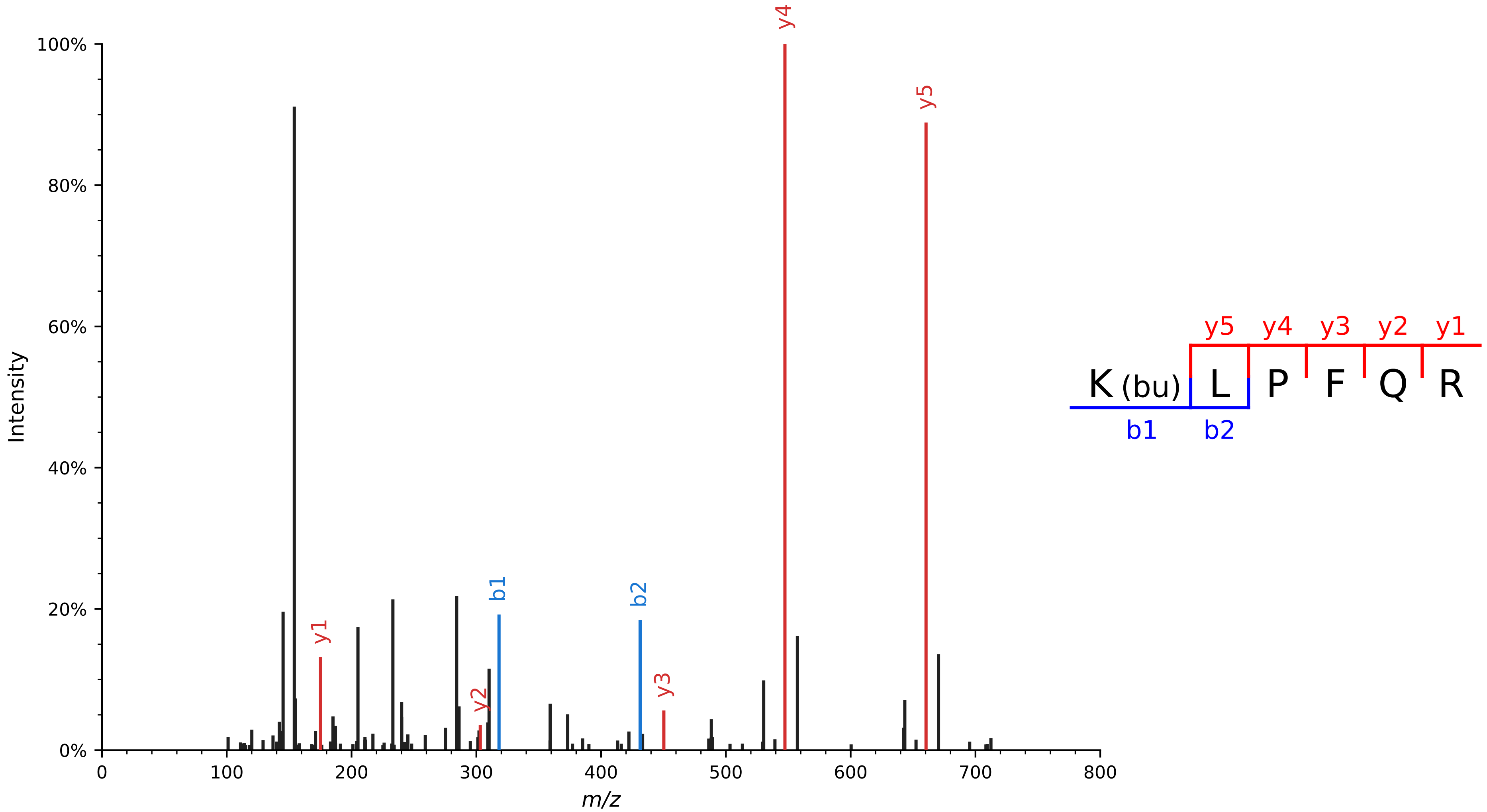

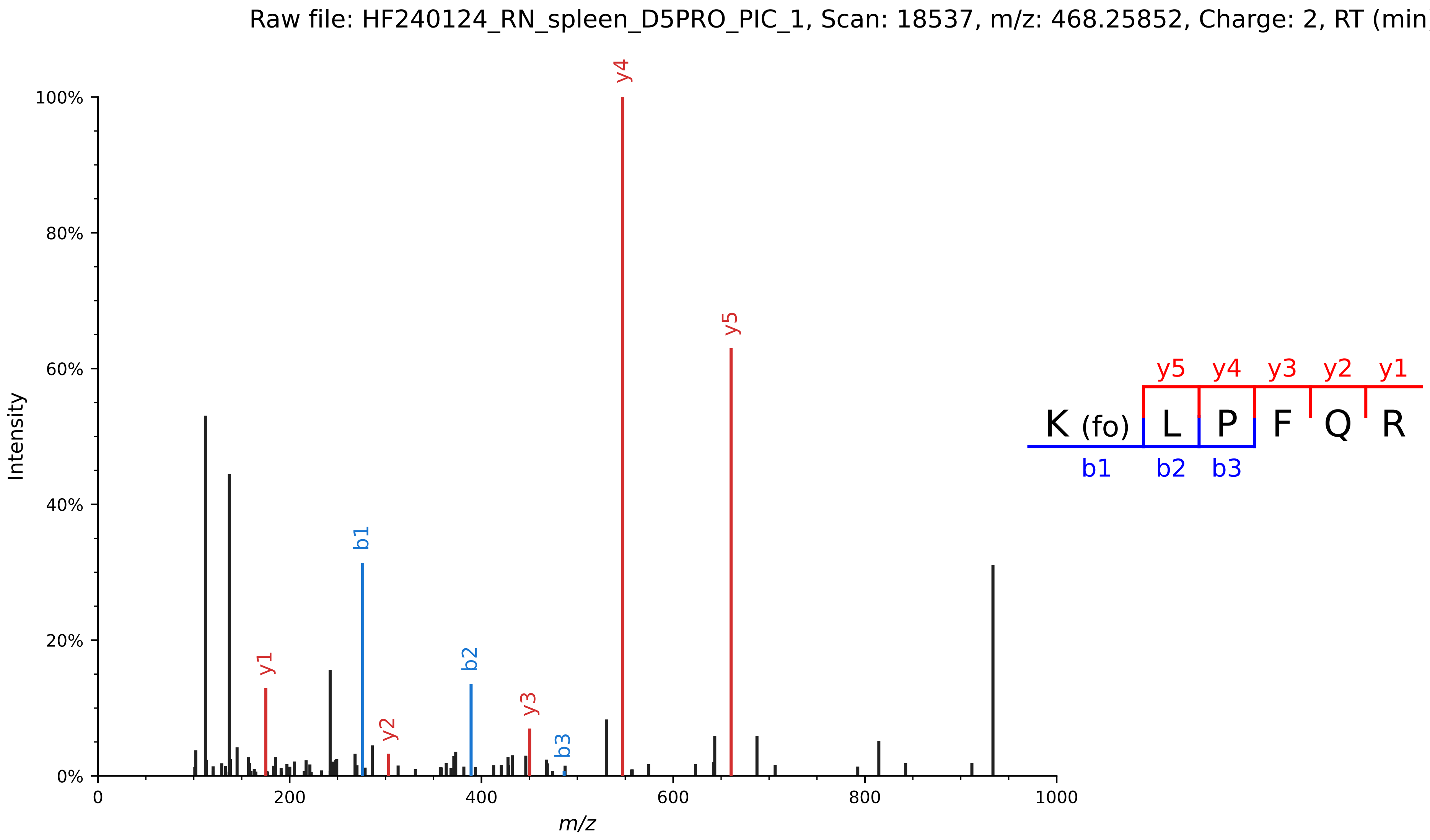

Raw file: HF240124_RN_spleen_D5PRO_PIC_1, Scan: 18537, m/z: 468.25852, Charge: 2, RT (min): 34.63, Score: 83.229
Intensity
100%
80%
60%
40%
20%
0%
0
200
400
600
800
1000
m/z
y4
y5
b1
y1
b2
y2
y3
b3
y5 y4 y3 y2 y1
K (fo) L P F Q R
b1 b2 b3

Raw file: HF240124_RN_spleen_D5PRO_PIC_3, Scan: 25798, m/z: 762.88828, Charge: 2, RT (min): 47.64, Score: 73.499

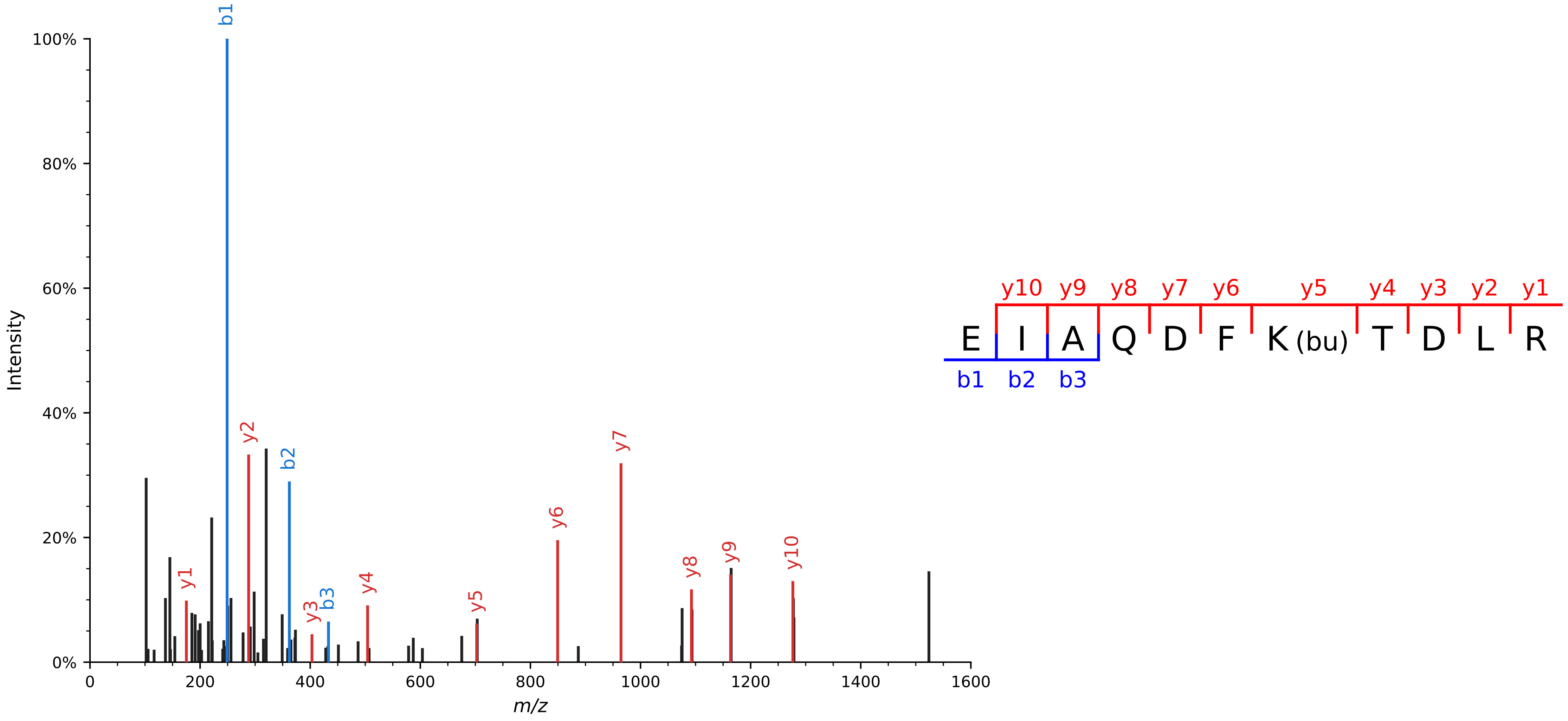

Raw file: HF240124_RN_spleen_D5PRO_PIC_1, Scan: 25741, m/z: 765.40397, Charge: 2, RT (min): 47.88, Score: 195.79

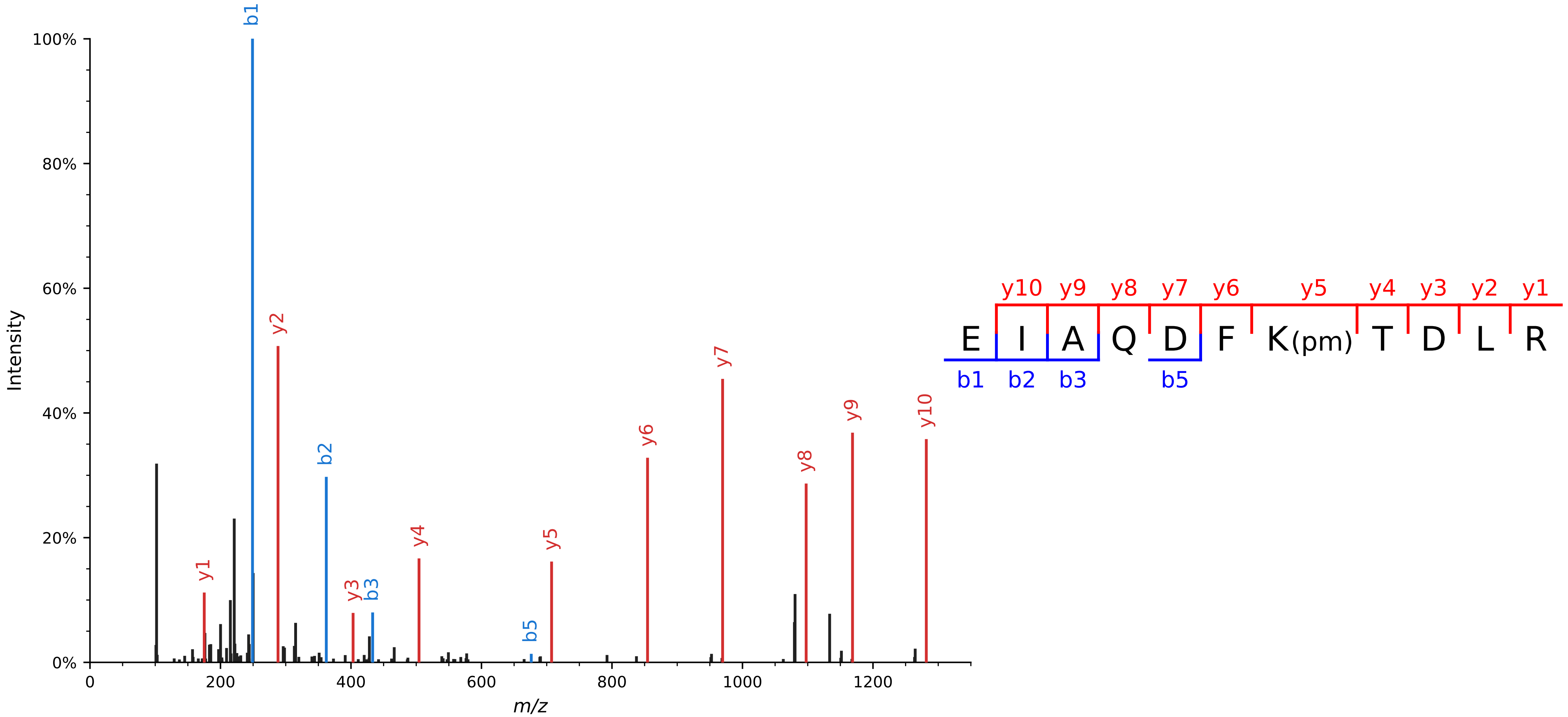

Raw file: HF240124_RN_spleen_D5PRO_PIC_1, Scan: 16260, m/z: 741.883, Charge: 2, RT (min): 30.54, Score: 174.41

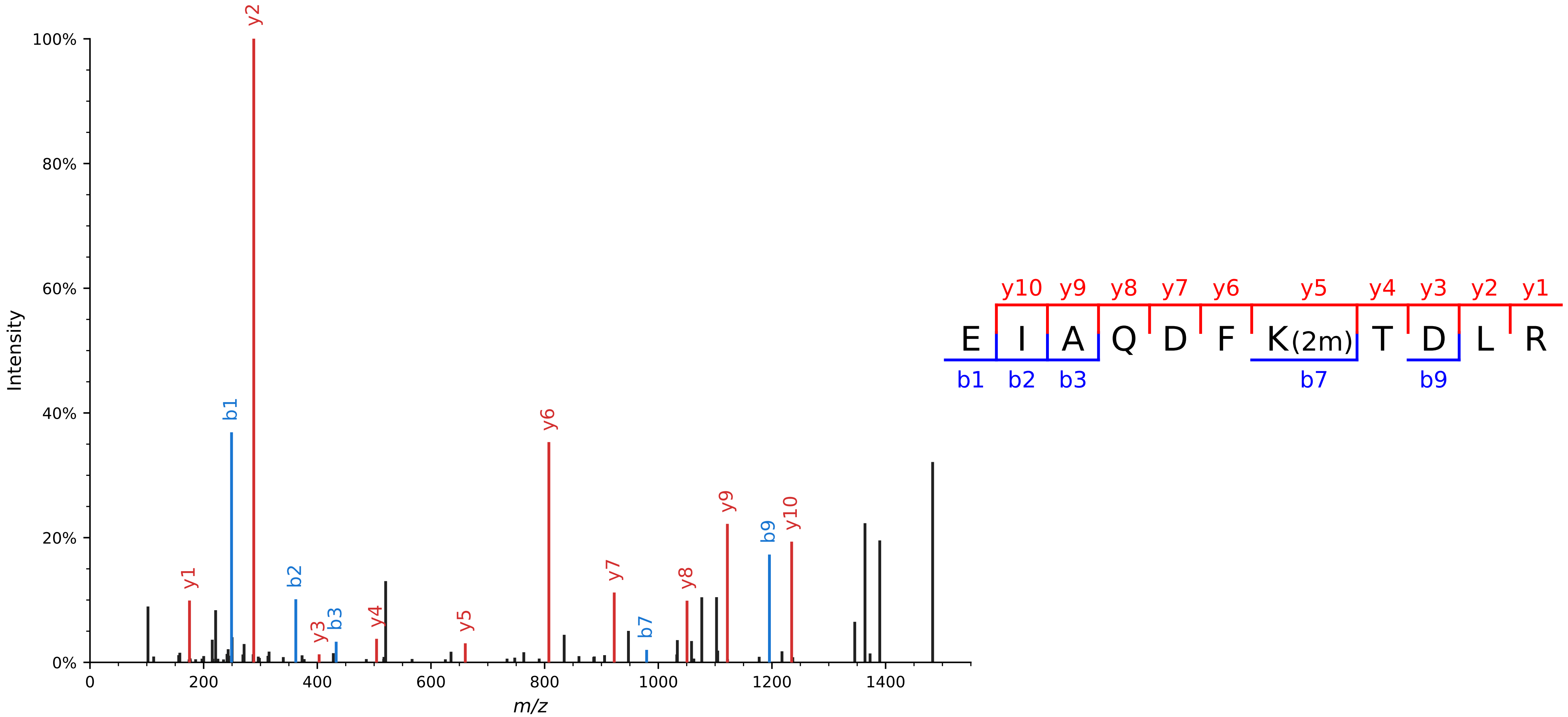

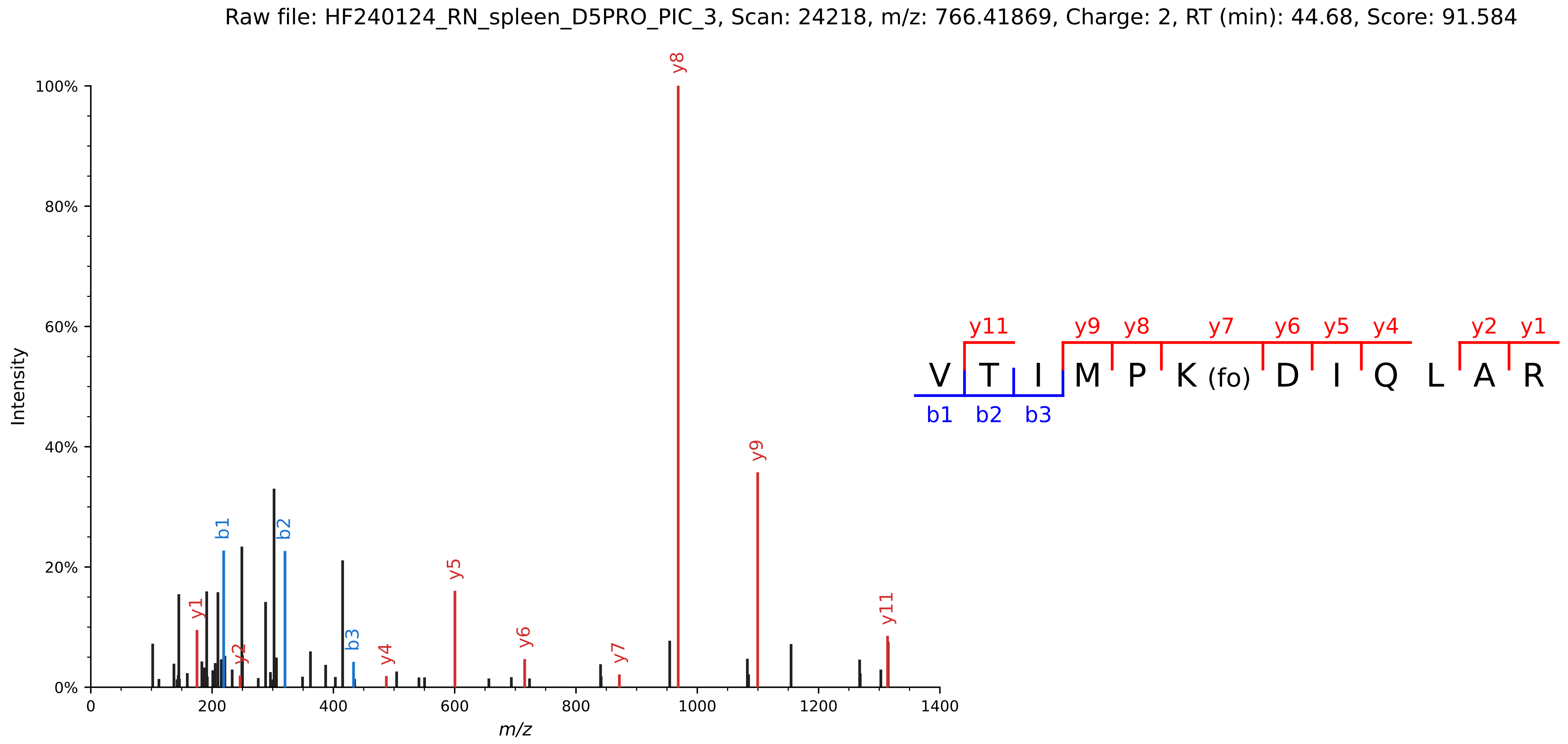
Raw file: HF240124_RN_spleen_D5PRO_PIC_3, Scan: 24218, m/z: 766.41869, Charge: 2, RT (min): 44.68, Score: 91.584
Intensity
m/z
y8
y9
b1
b2
y5
y1
y2
b3
y4
y6
y7
y11
V T I M P K (fo) D I Q L A R
y11 y9 y8 y7 y6 y5 y4 y2 y1
b1 b2 b3

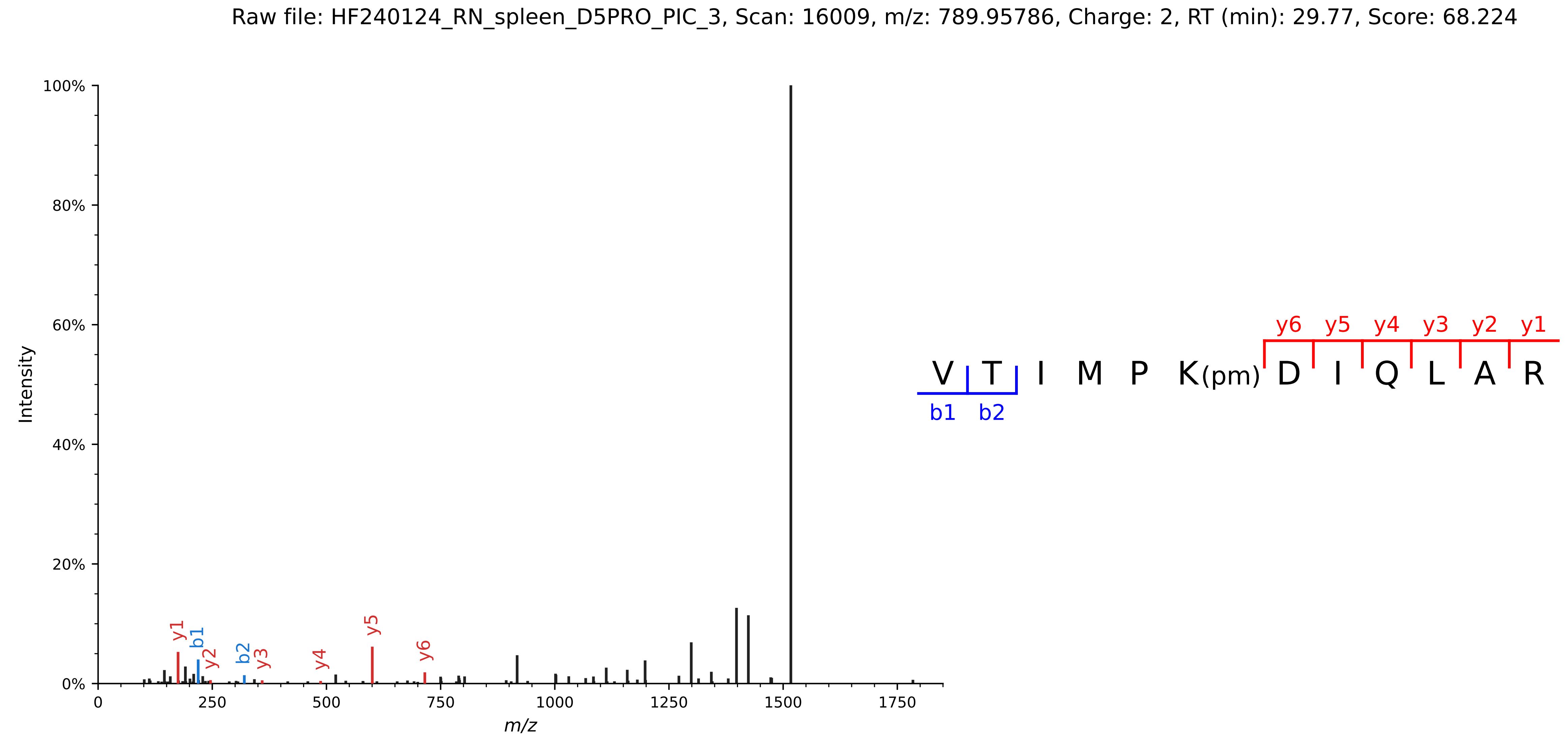
Raw file: HF240124_RN_spleen_D5PRO_PIC_3, Scan: 16009, m/z: 789.95786, Charge: 2, RT (min): 29.77, Score: 68.224
Intensity
m/z
V T I M P K(pm) D I Q L A R
b1 b2
y6 y5 y4 y3 y2 y1

Raw file: HF240124_RN_spleen_D5PRO_PIC_3, Scan: 25133, m/z: 780.43434, Charge: 2, RT (min): 46.40, Score: 45.368

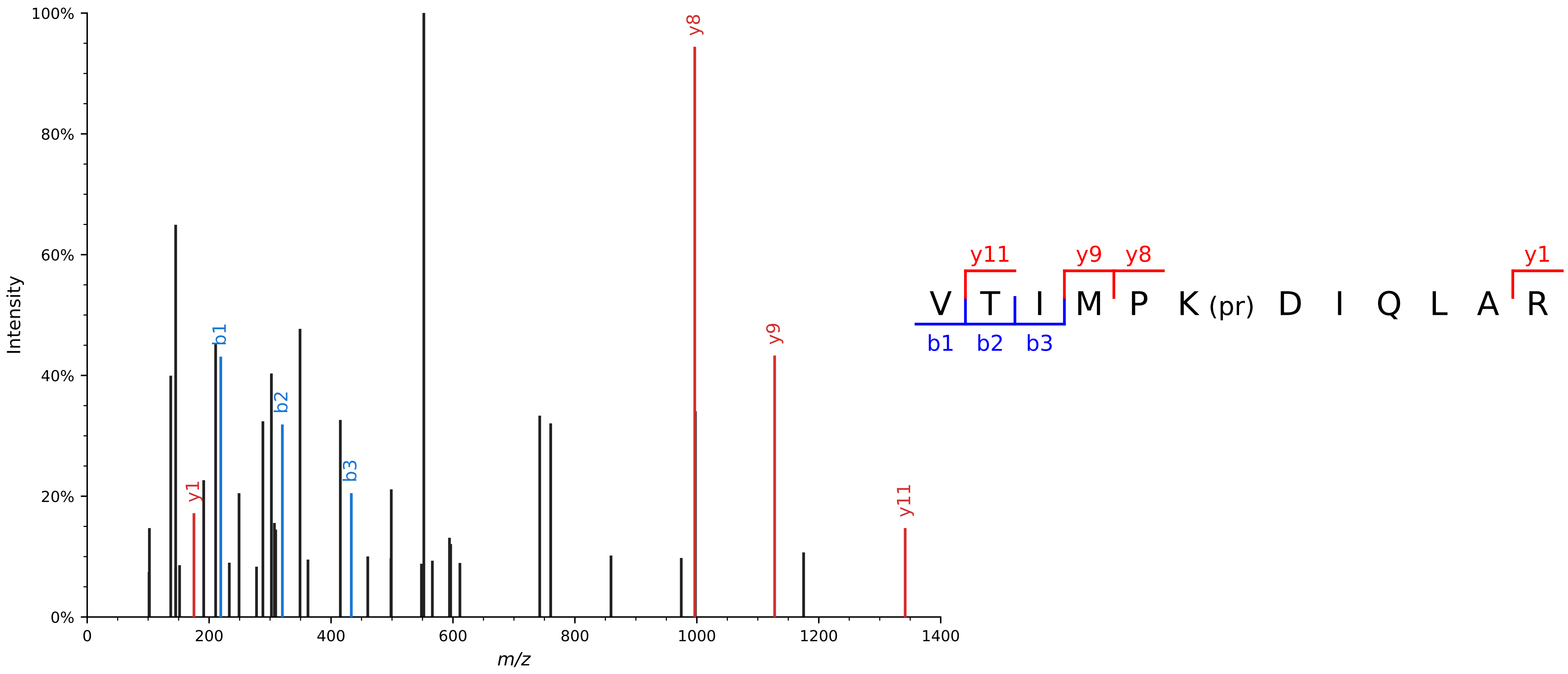

Raw file: HF240124_RN_spleen_D5PRO_PIC_1, Scan: 22768, m/z: 796.44577, Charge: 2, RT (min): 42.34, Score: 55.567

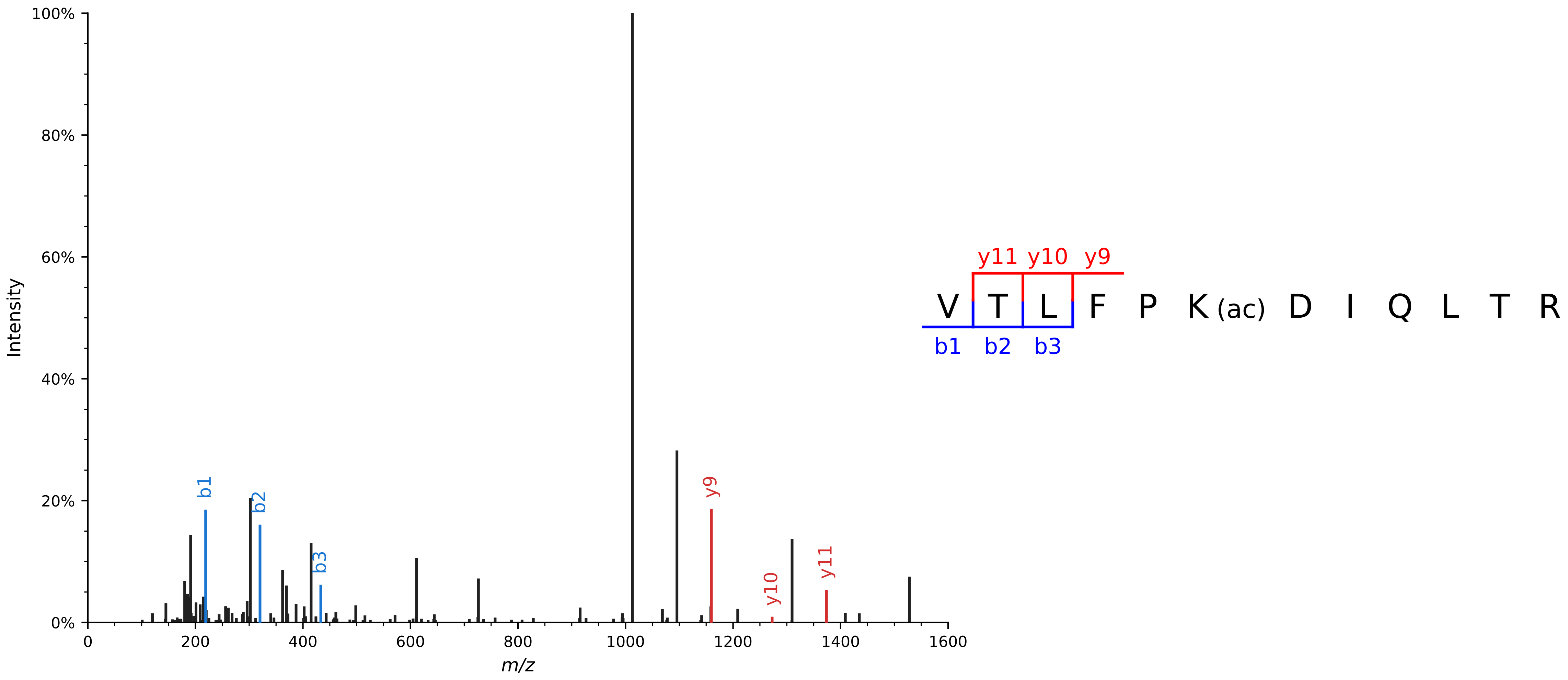

Raw file: HF240124_RN_spleen_D5PRO_PIC_2, Scan: 13703, m/z: 807.99899, Charge: 2, RT (min): 26.26, Score: 153.12

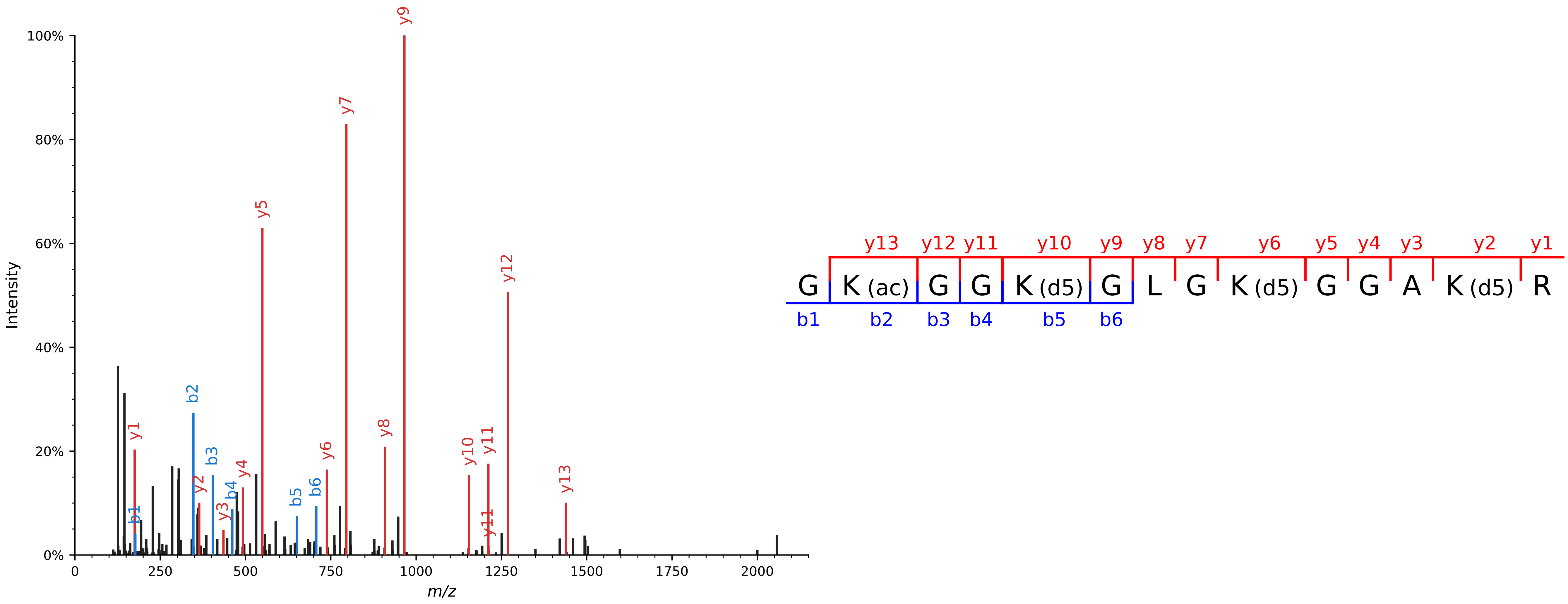

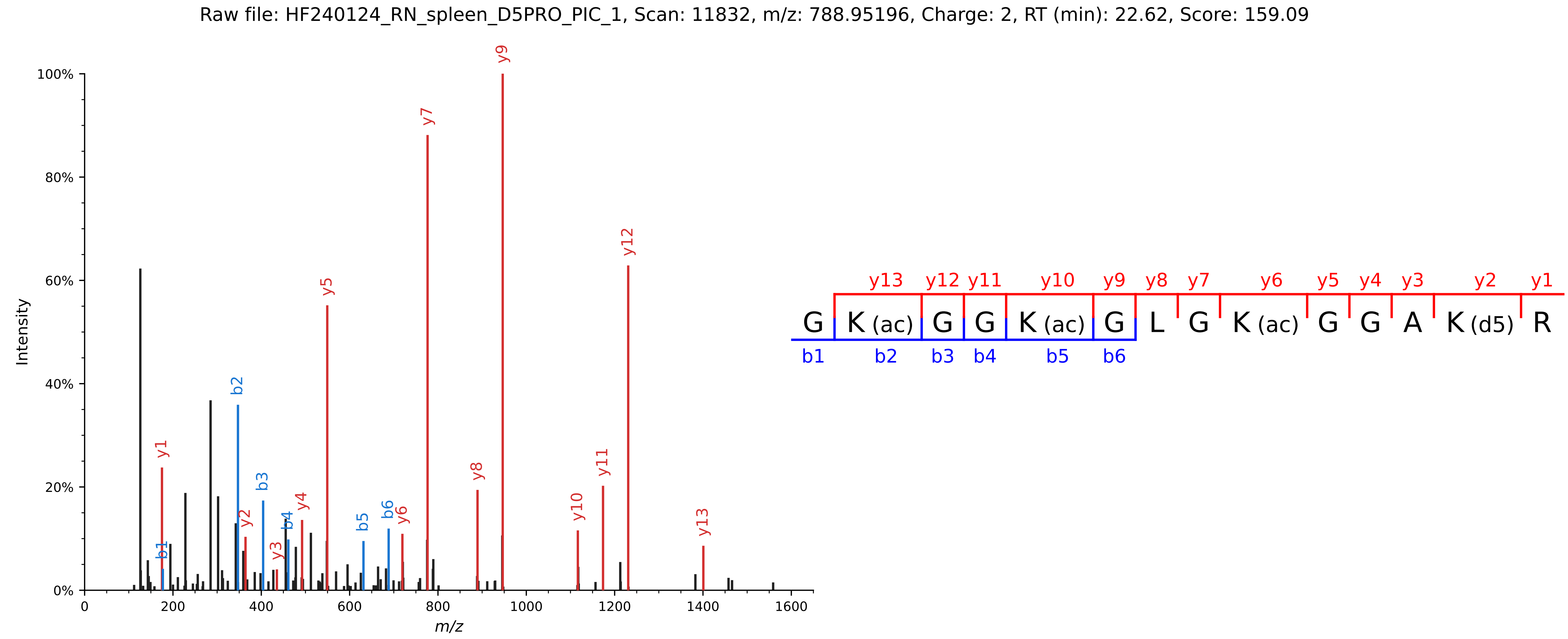

Raw file: HF240124_RN_spleen_D5PRO_PIC_1, Scan: 11832, m/z: 788.95196, Charge: 2, RT (min): 22.62, Score: 159.09
Intensity
m/z
100%
80%
60%
40%
20%
0%
0
200
400
600
800
1000
1200
1400
1600
y1 b1 b2 y2 y3 b3 b4 y4 y5 b5 b6 y6 y7 y8 y9 y10 y11 y12 y13
y13 y12 y11 y10 y9 y8 y7 y6 y5 y4 y3 y2 y1
G K (ac) G G K (ac) G L G K (ac) G G A K (d5) R
b1 b2 b3 b4 b5 b6

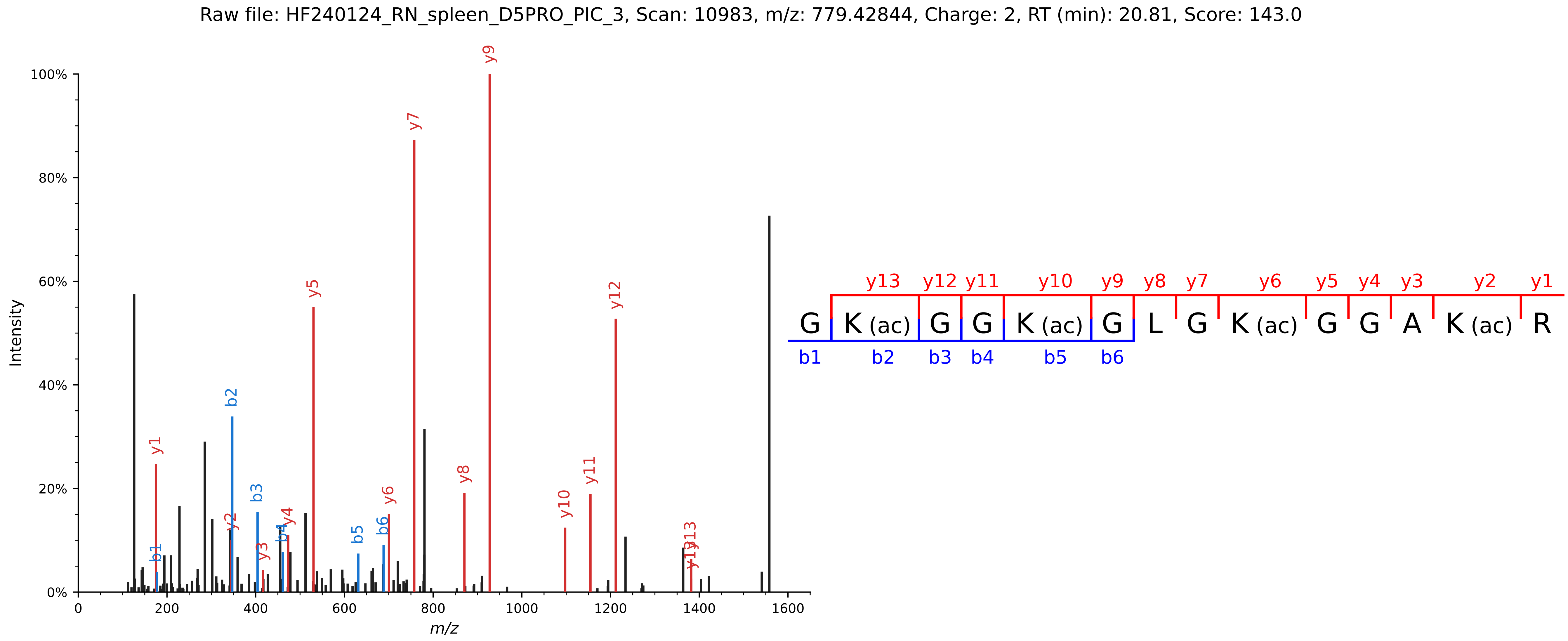

Raw file: HF240124_RN_spleen_D5PRO_PIC_3, Scan: 10983, m/z: 779.42844, Charge: 2, RT (min): 20.81, Score: 143.0
Intensity
m/z
G K(ac) G G K(ac) G L G K(ac) G G A K(ac) R
y13 y12 y11 y10 y9 y8 y7 y6 y5 y4 y3 y2 y1
b1 b2 b3 b4 b5 b6

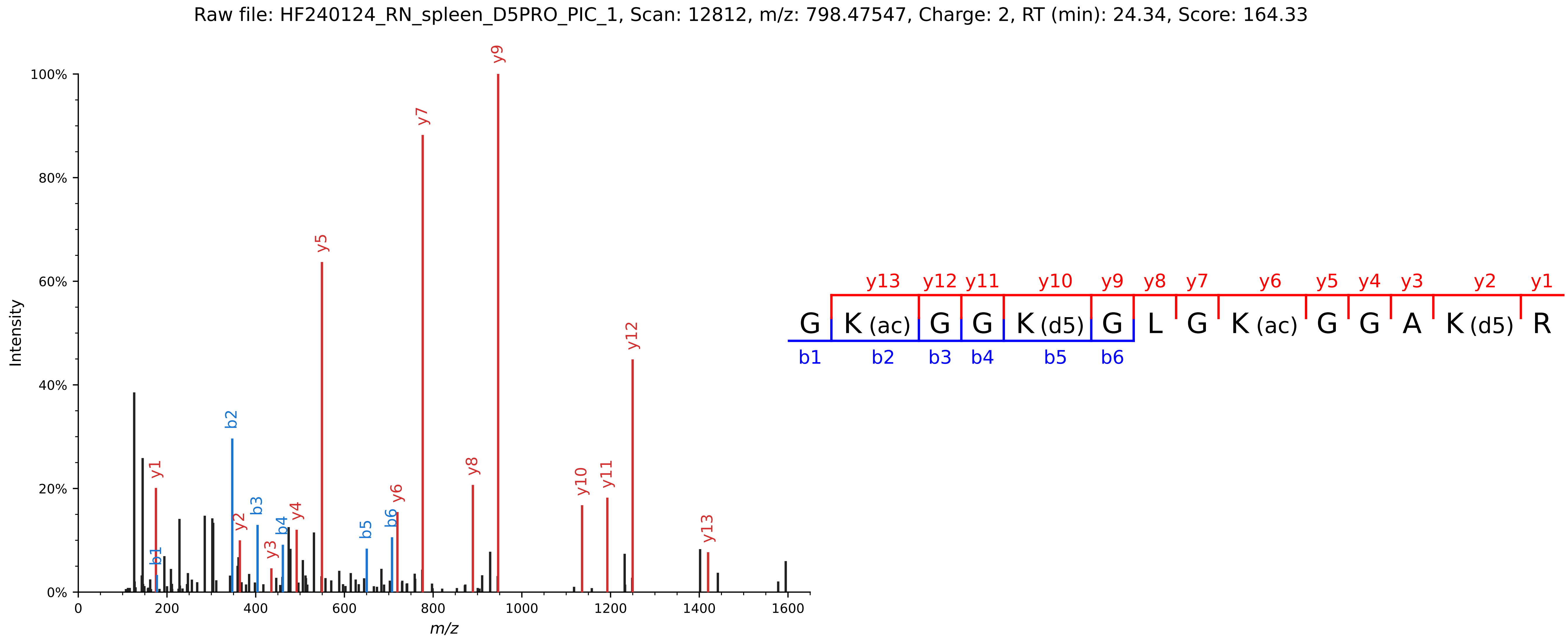
Raw file: HF240124_RN_spleen_D5PRO_PIC_1, Scan: 12812, m/z: 798.47547, Charge: 2, RT (min): 24.34, Score: 164.33
Intensity
m/z
y13 y12 y11 y10 y9 y8 y7 y6 y5 y4 y3 y2 y1
G K (ac) G G K (d5) G L G K (ac) G G A K (d5) R
b1 b2 b3 b4 b5 b6

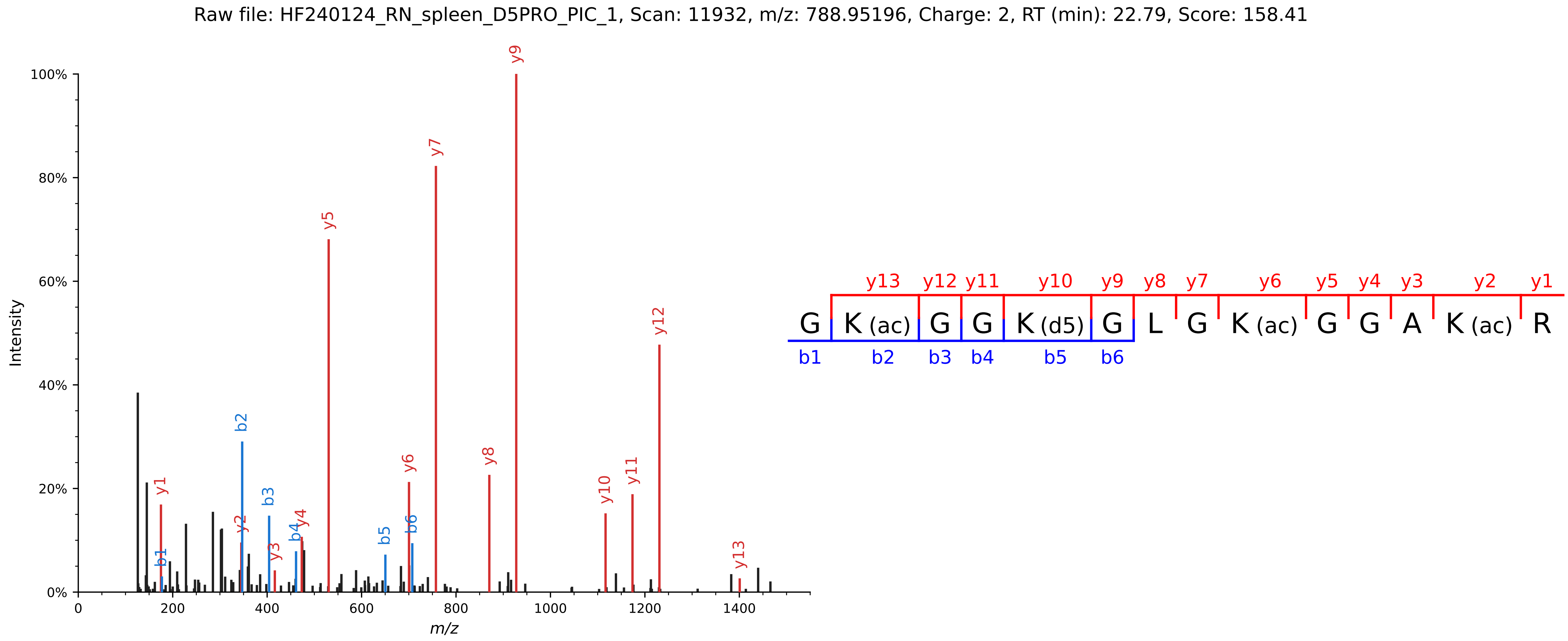

Raw file: HF240124_RN_spleen_D5PRO_PIC_1, Scan: 11932, m/z: 788.95196, Charge: 2, RT (min): 22.79, Score: 158.41
Intensity
m/z
G K(ac) G G K(d5) G L G K(ac) G G A K(ac) R
y13 y12 y11 y10 y9 y8 y7 y6 y5 y4 y3 y2 y1
b1 b2 b3 b4 b5 b6

Raw file: HF240124_RN_spleen_D5PRO_PIC_1, Scan: 13056, m/z: 798.47547, Charge: 2, RT (min): 24.86, Score: 149.96

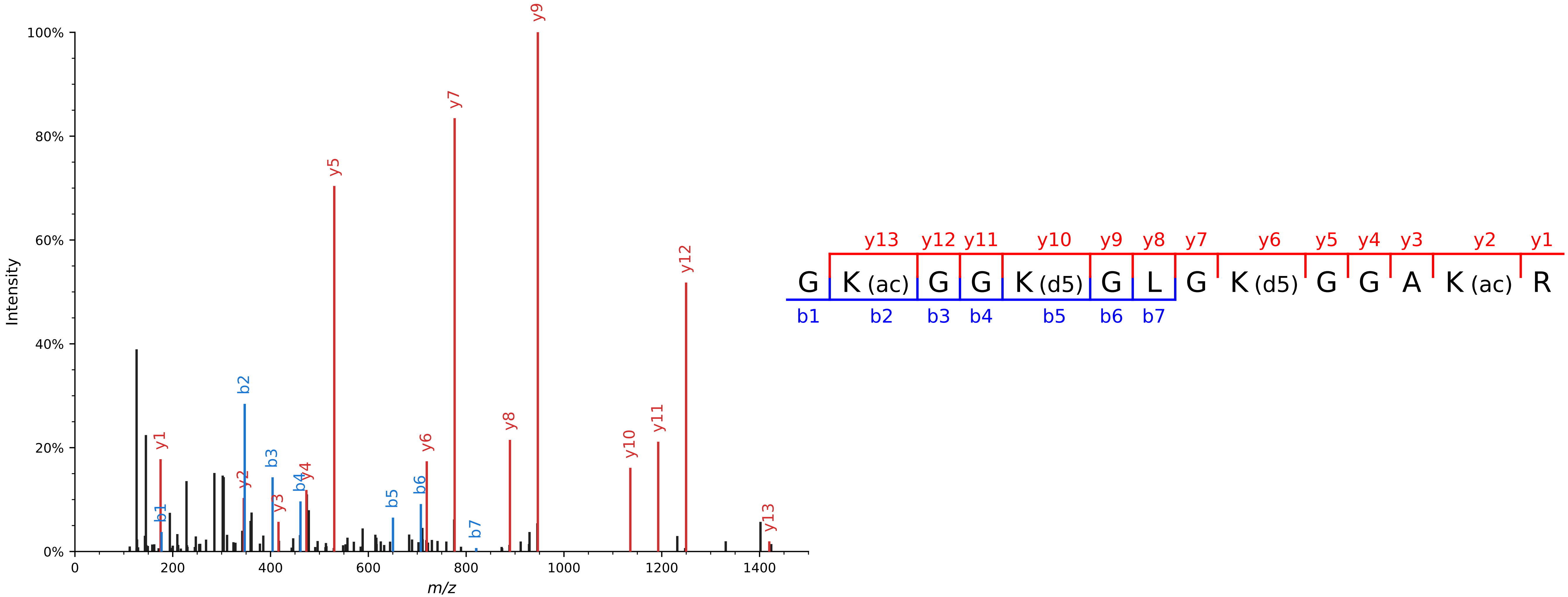

Raw file: HF240124_RN_spleen_D5PRO_PIC_1, Scan: 16747, m/z: 822.01464, Charge: 2, RT (min): 31.43, Score: 129.16

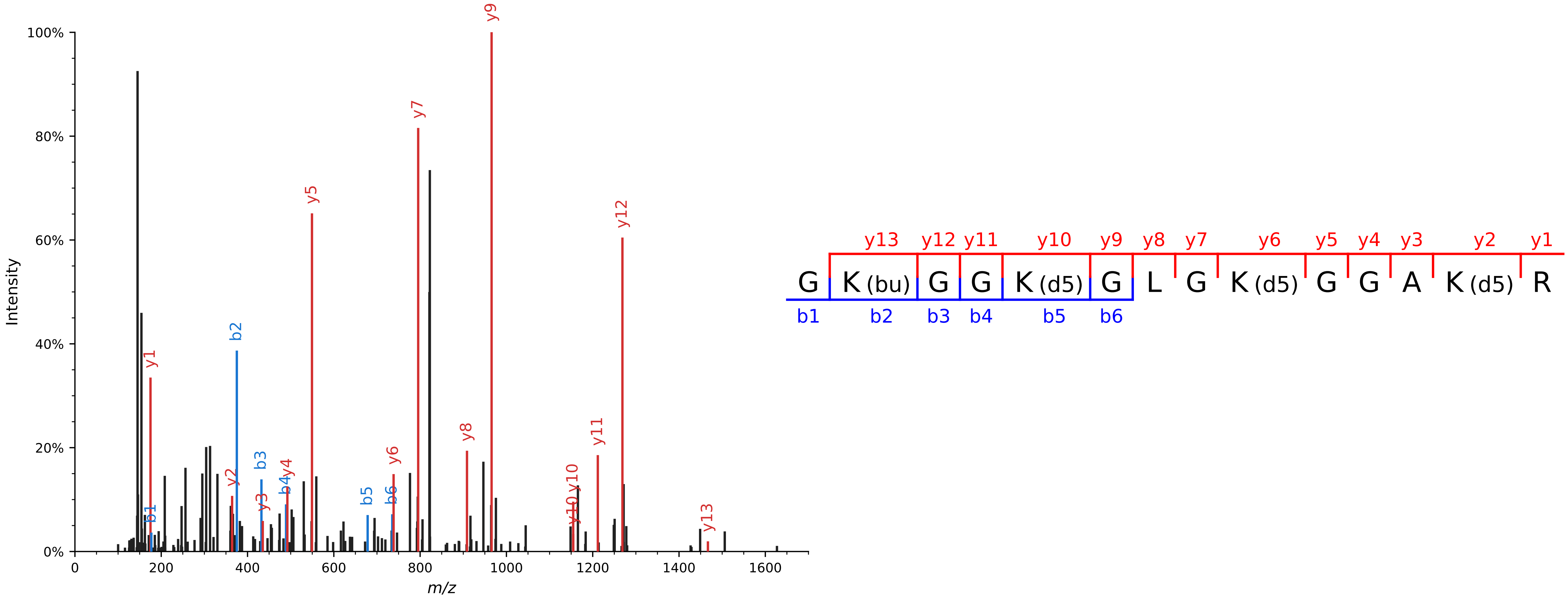

Raw file: HF240124_RN_spleen_D5PRO_PIC_2, Scan: 15562, m/z: 812.49112, Charge: 2, RT (min): 29.57, Score: 70.808

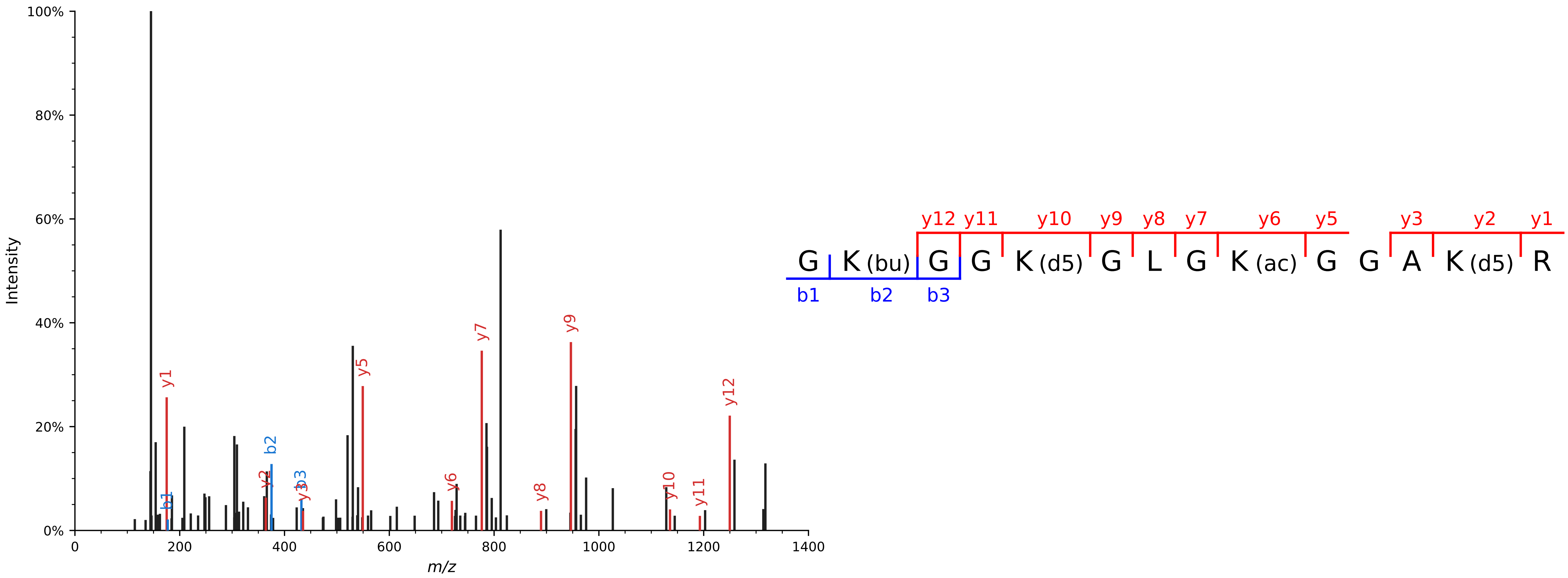

Raw file: HF240124_RN_spleen_D5PRO_PIC_3, Scan: 16141, m/z: 812.49112, Charge: 2, RT (min): 30.00, Score: 138.25

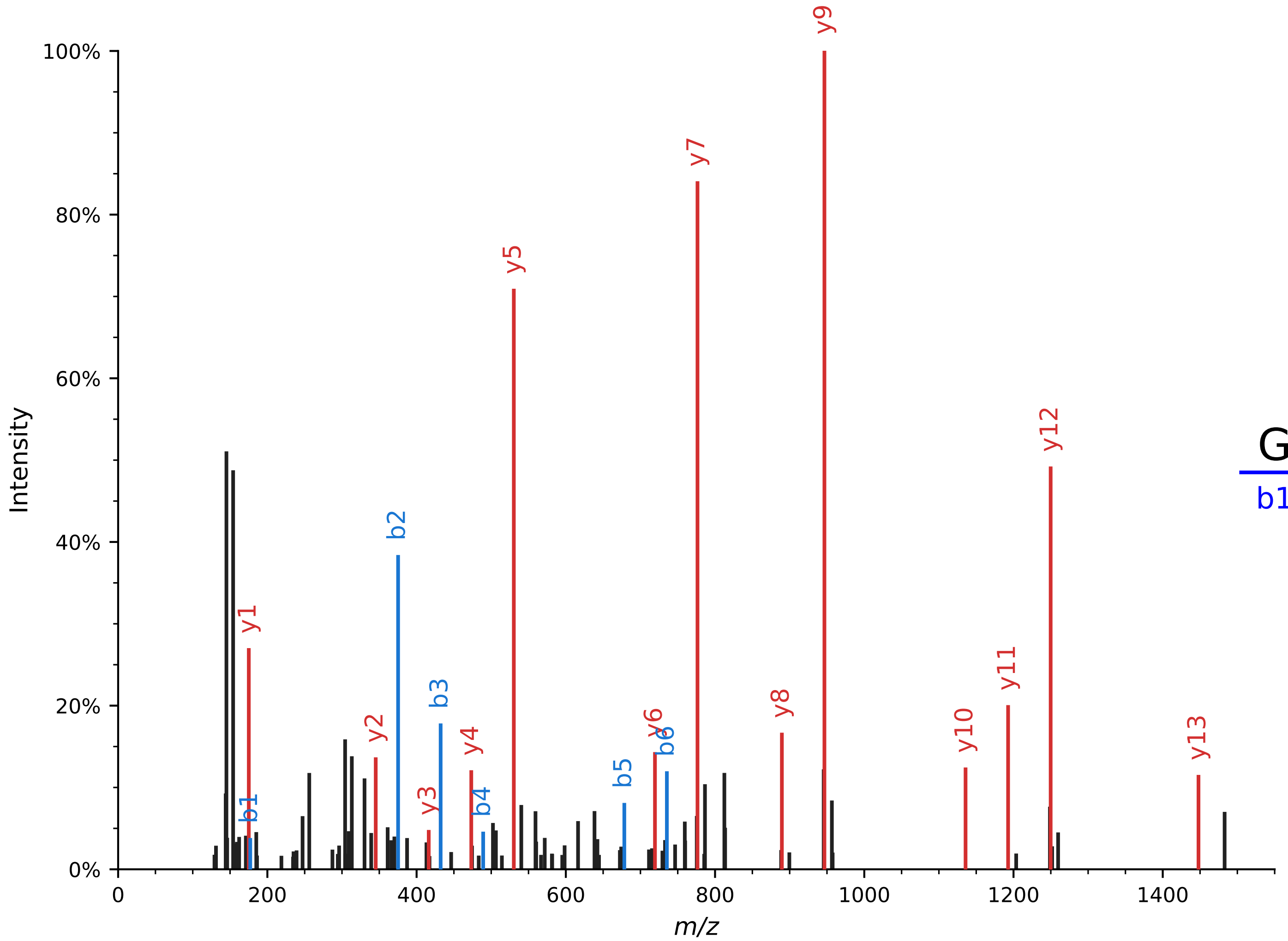

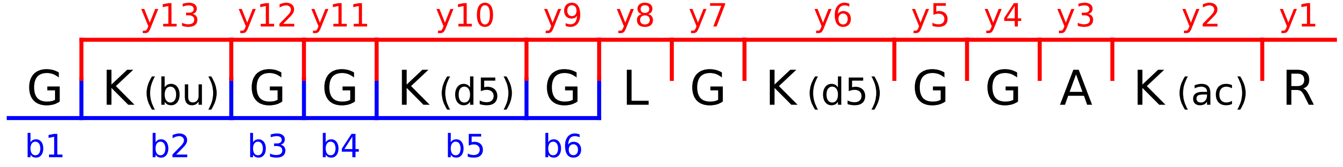

Raw file: HF240124_RN_spleen_D5PRO_PIC_3, Scan: 13685, m/z: 800.99116, Charge: 2, RT (min): 25.66, Score: 132.91

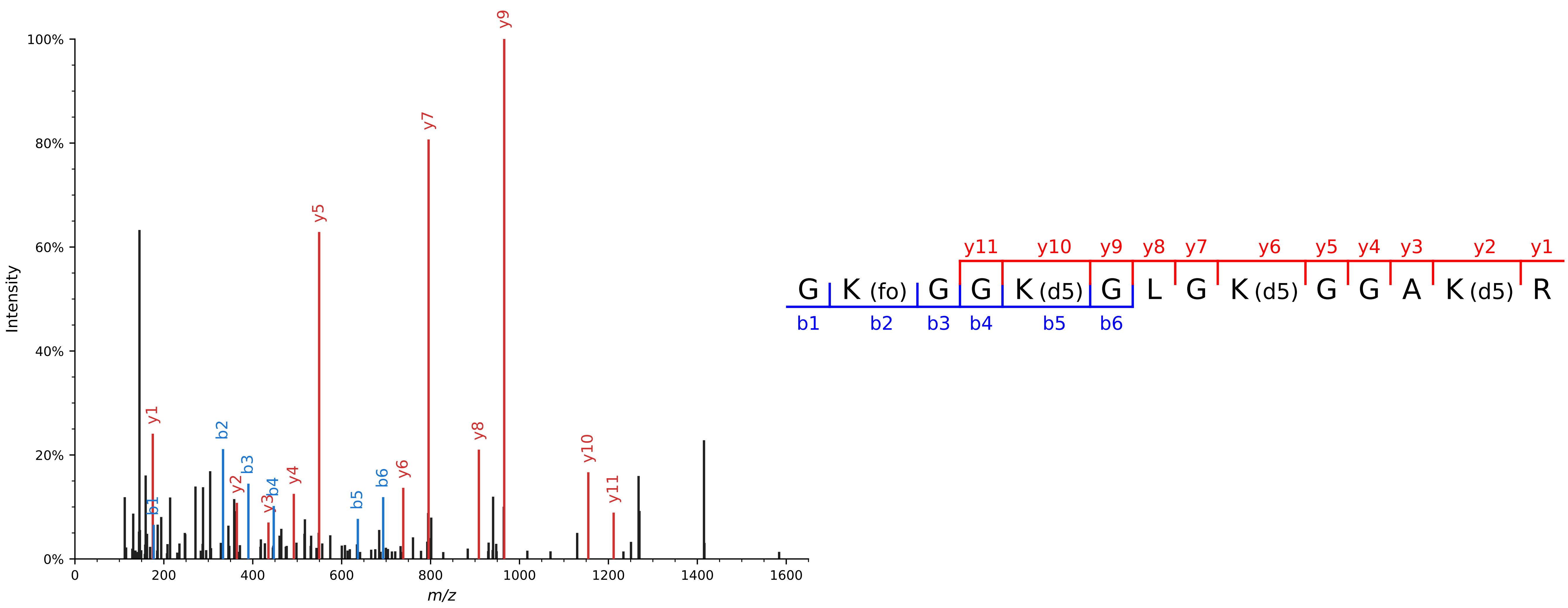

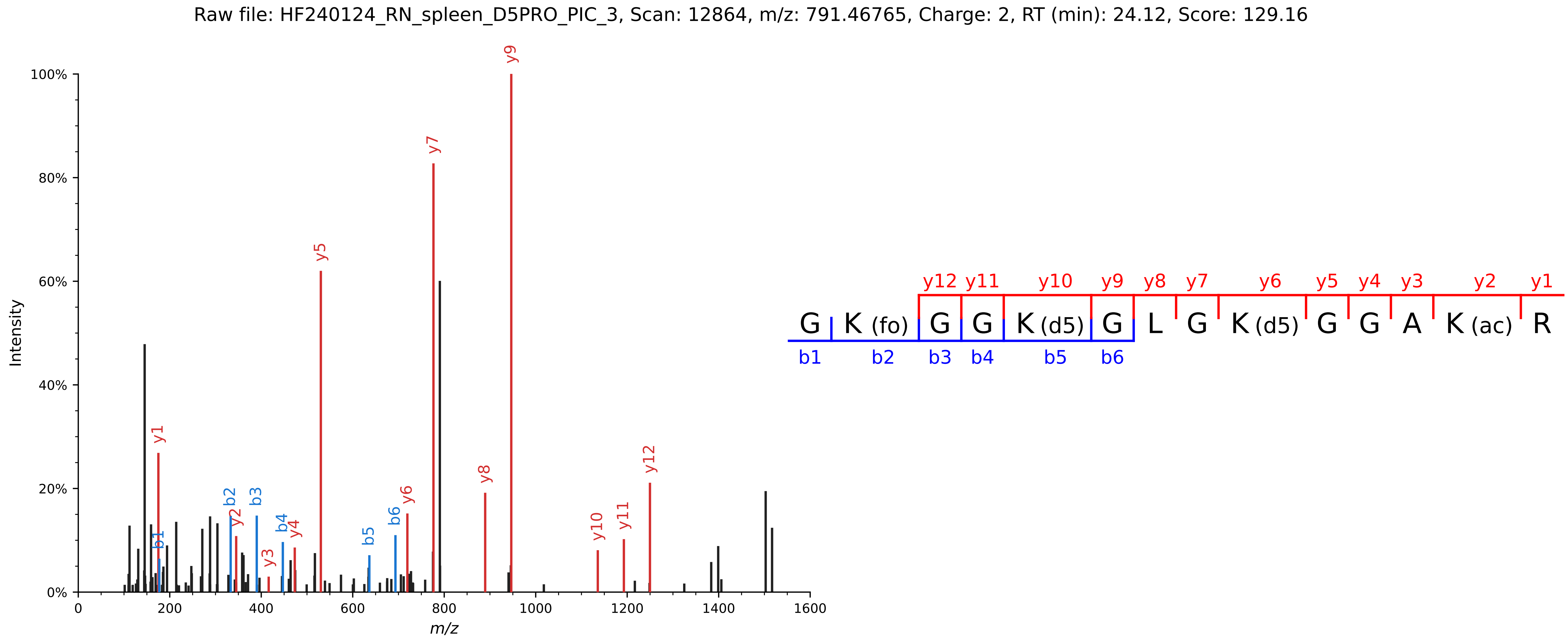

Raw file: HF240124_RN_spleen_D5PRO_PIC_3, Scan: 12864, m/z: 791.46765, Charge: 2, RT (min): 24.12, Score: 129.16
Intensity
m/z
G K (fo) G G K (d5) G L G K (d5) G G A K (ac) R
y12 y11 y10 y9 y8 y7 y6 y5 y4 y3 y2 y1
b1 b2 b3 b4 b5 b6

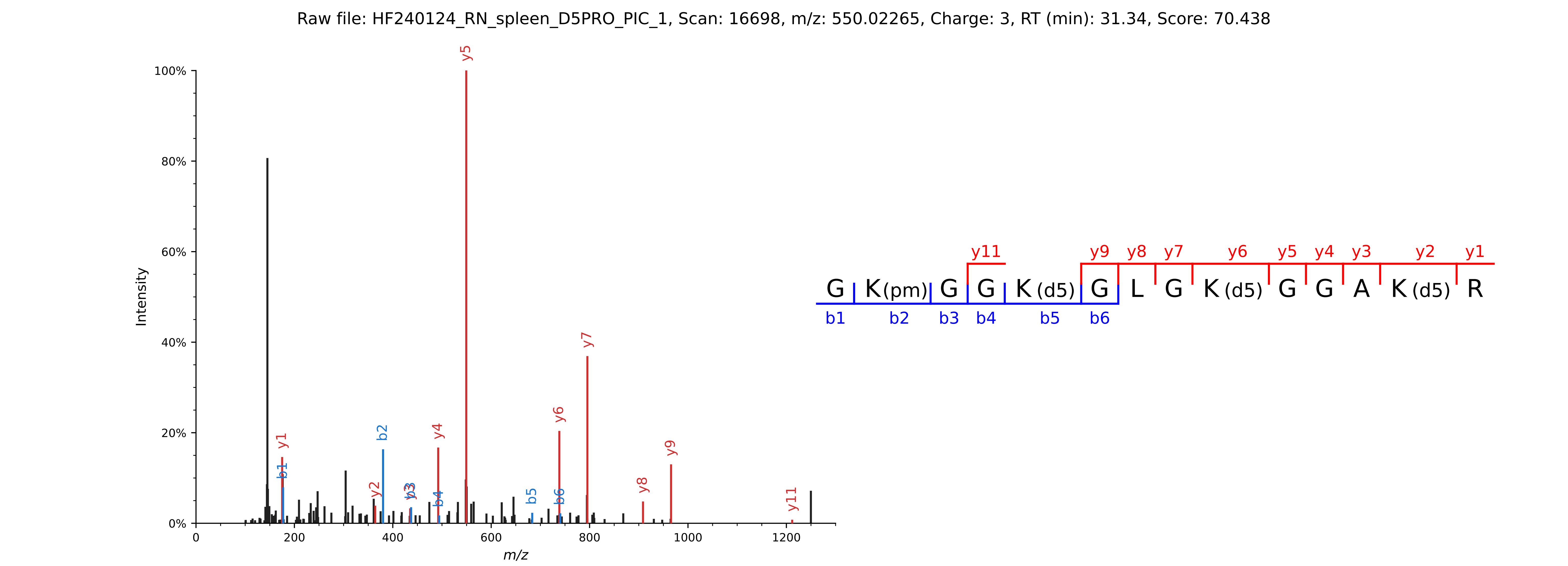

Raw file: HF240124_RN_spleen_D5PRO_PIC_1, Scan: 16698, m/z: 550.02265, Charge: 3, RT (min): 31.34, Score: 70.438
Intensity
m/z
y1 b1 y2 b2 b3 y3 y4 b4 y5 b5 y6 b6 y7 y8 y9 y11
G K(pm) G G K(d5) G L G K(d5) G G A K(d5) R

Raw file: HF240124_RN_spleen_D5PRO_PIC_2, Scan: 5477, m/z: 798.51186, Charge: 2, RT (min): 11.37, Score: 80.755

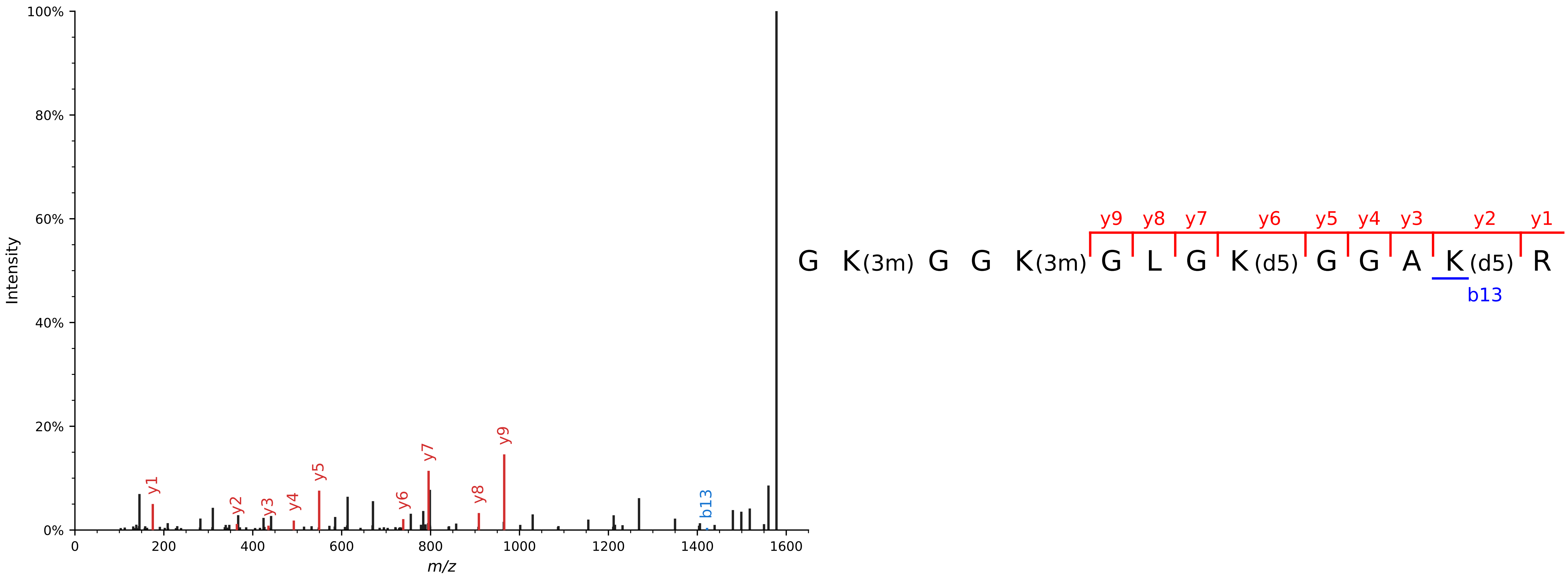

Raw file: HF240124_RN_spleen_D5PRO_PIC_2, Scan: 4742, m/z: 788.98834, Charge: 2, RT (min): 10.07, Score: 69.276

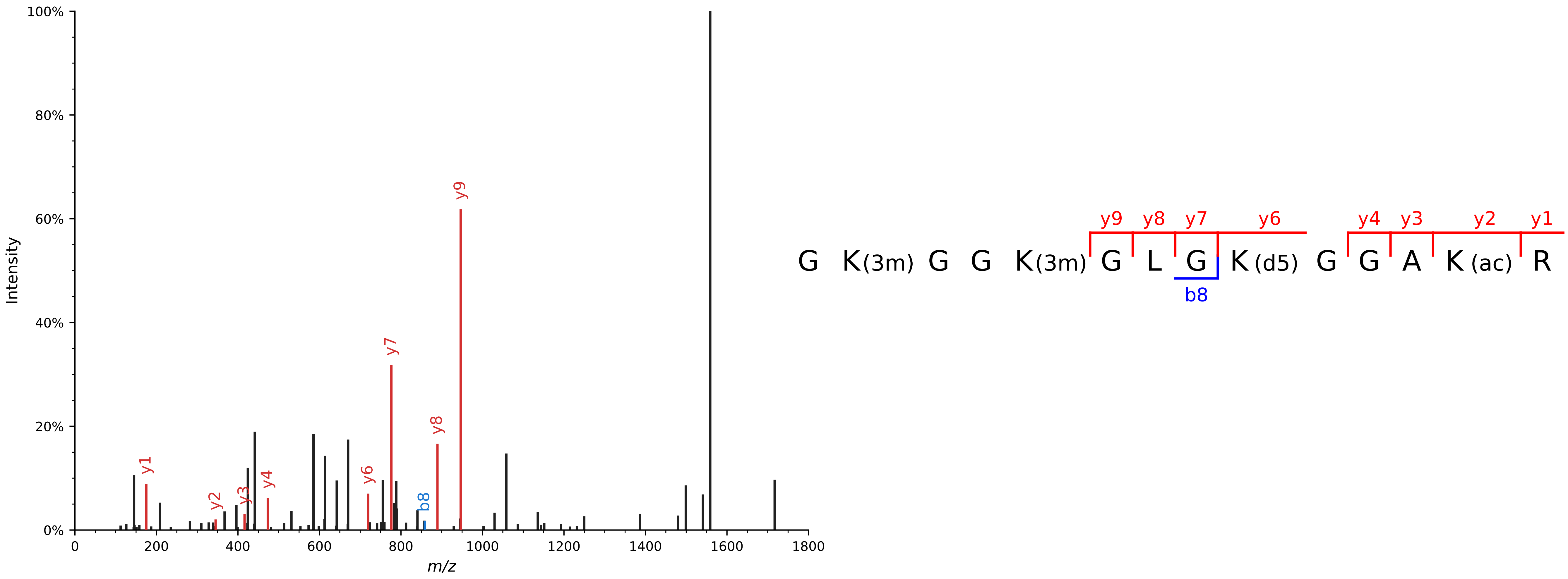

Raw file: HF240124_RN_spleen_D5PRO_PIC_1, Scan: 12952, m/z: 798.47547, Charge: 2, RT (min): 24.68, Score: 152.07

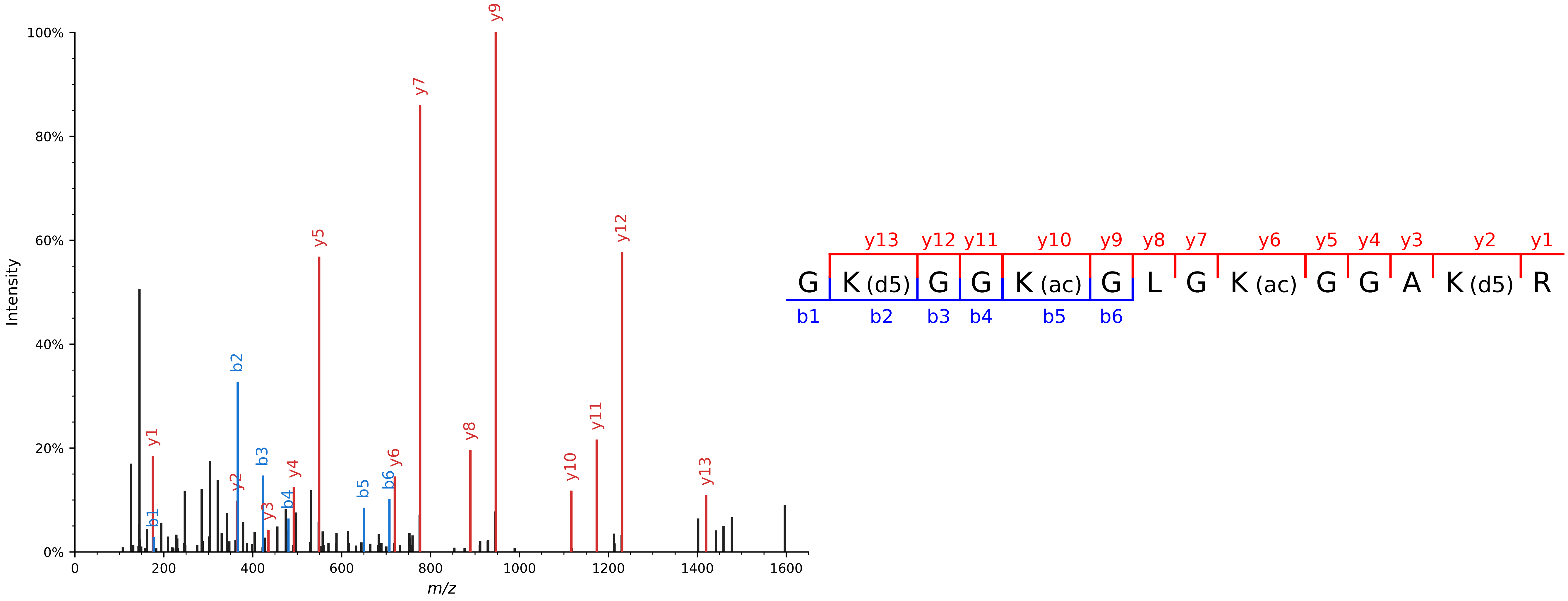

Raw file: HF240124_RN_spleen_D5PRO_PIC_1, Scan: 12141, m/z: 788.95196, Charge: 2, RT (min): 23.16, Score: 185.18

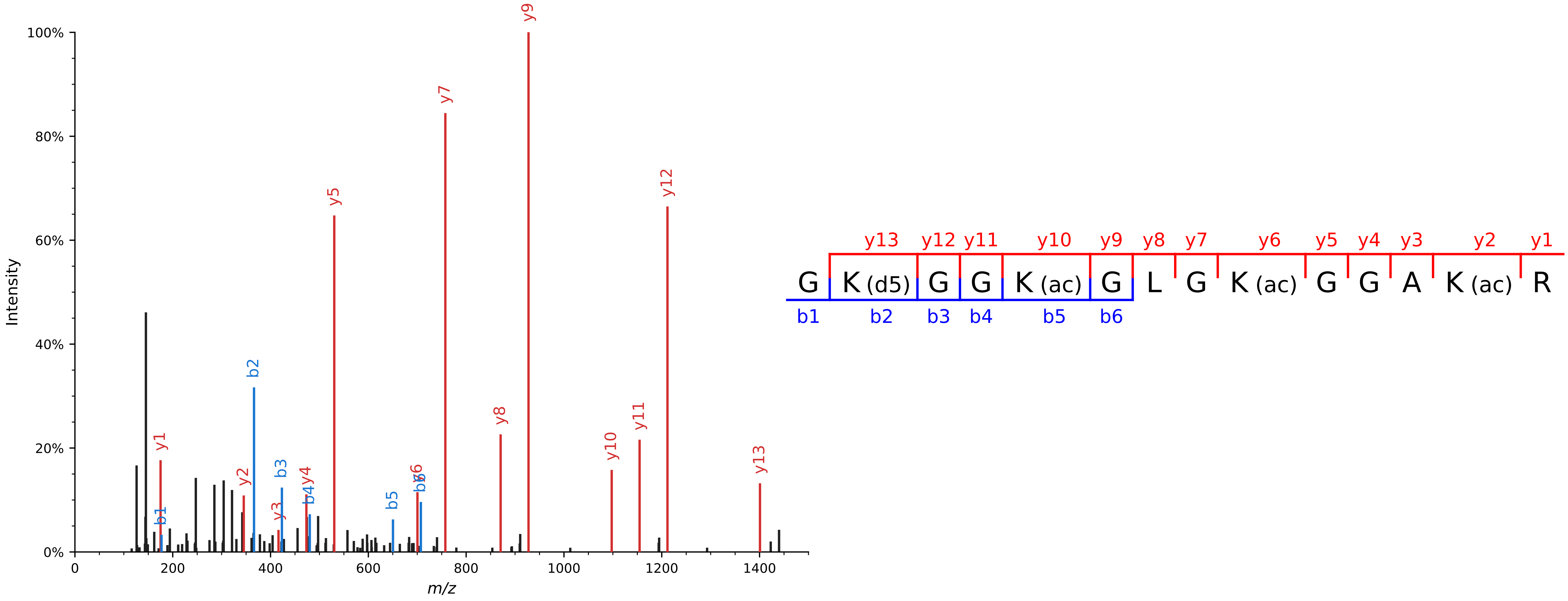

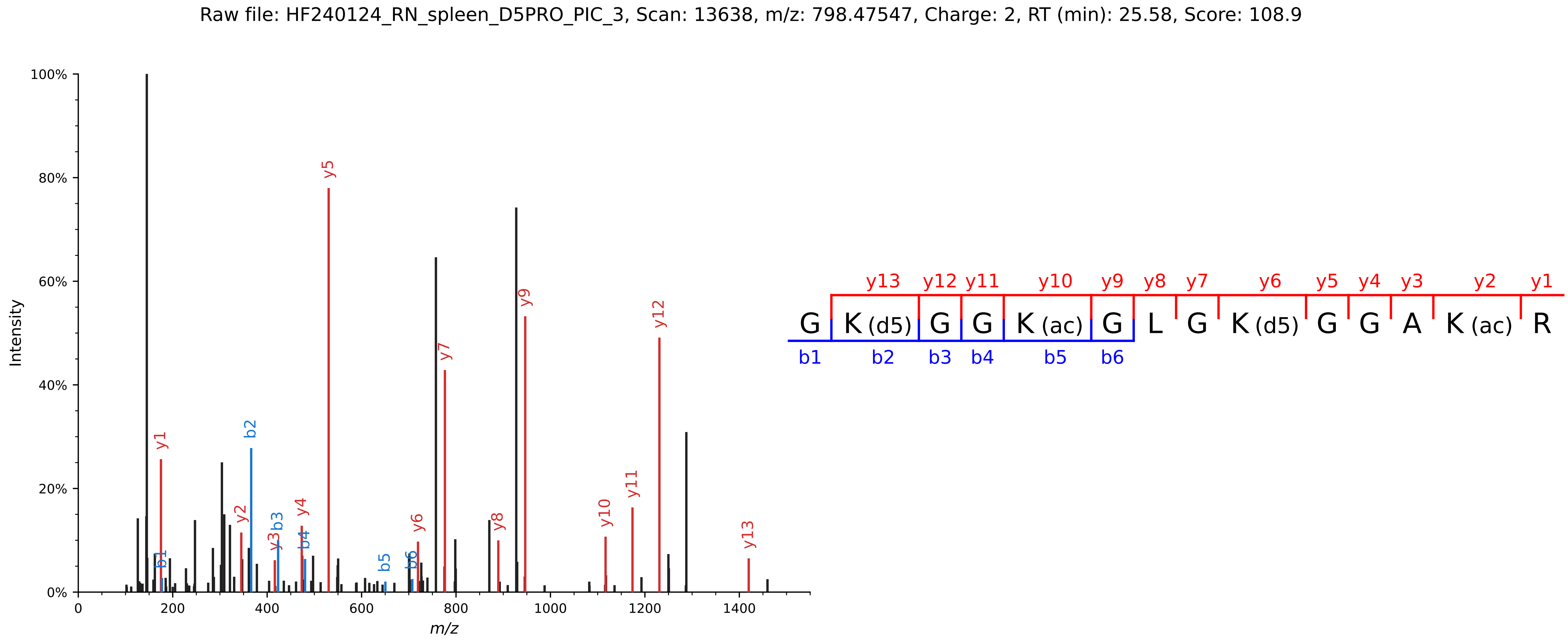

Raw file: HF240124_RN_spleen_D5PRO_PIC_3, Scan: 13638, m/z: 798.47547, Charge: 2, RT (min): 25.58, Score: 108.9
Intensity
m/z
y1
y2
y3
y4
y5
y6
y7
y8
y9
y10
y11
y12
y13
b1
b2
b3
b4
b5
b6
G K (d5) G G K (ac) G L G K (d5) G G A K (ac) R

Raw file: HF240124_RN_spleen_D5PRO_PIC_3, Scan: 16037, m/z: 812.49112, Charge: 2, RT (min): 29.82, Score: 108.7

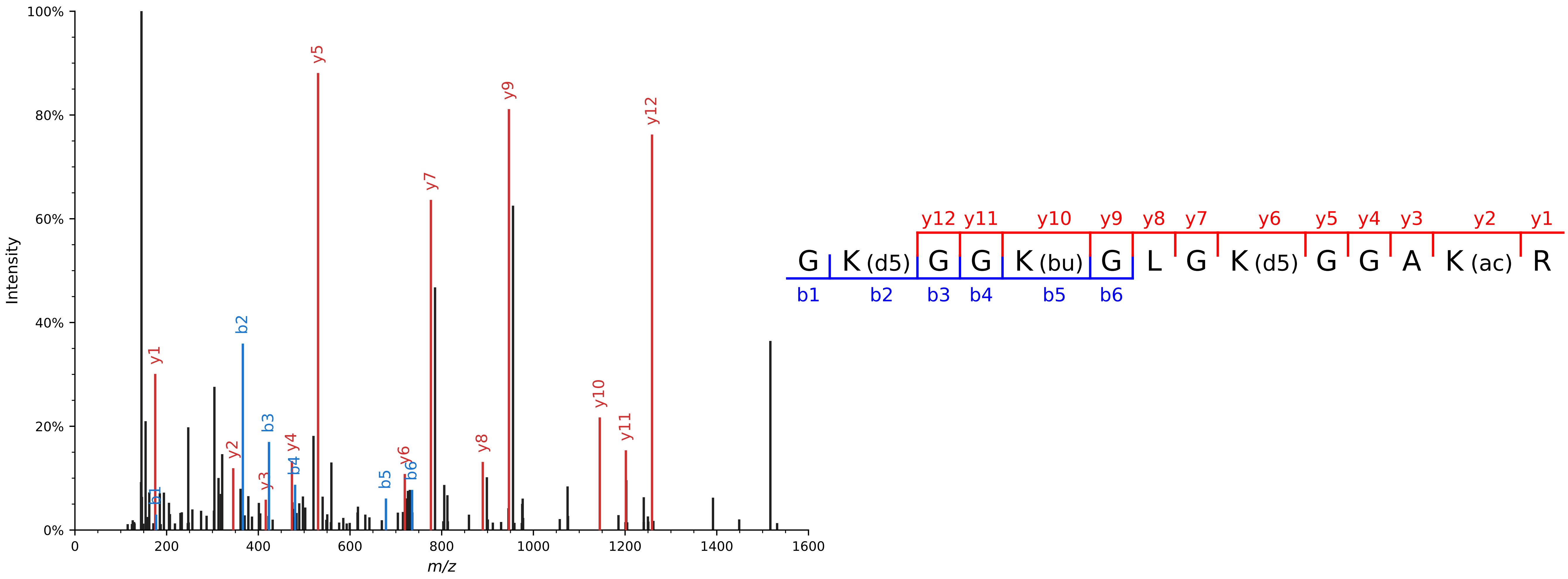

Raw file: HF240124_RN_spleen_D5PRO_PIC_2, Scan: 13916, m/z: 807.99899, Charge: 2, RT (min): 26.63, Score: 143.4

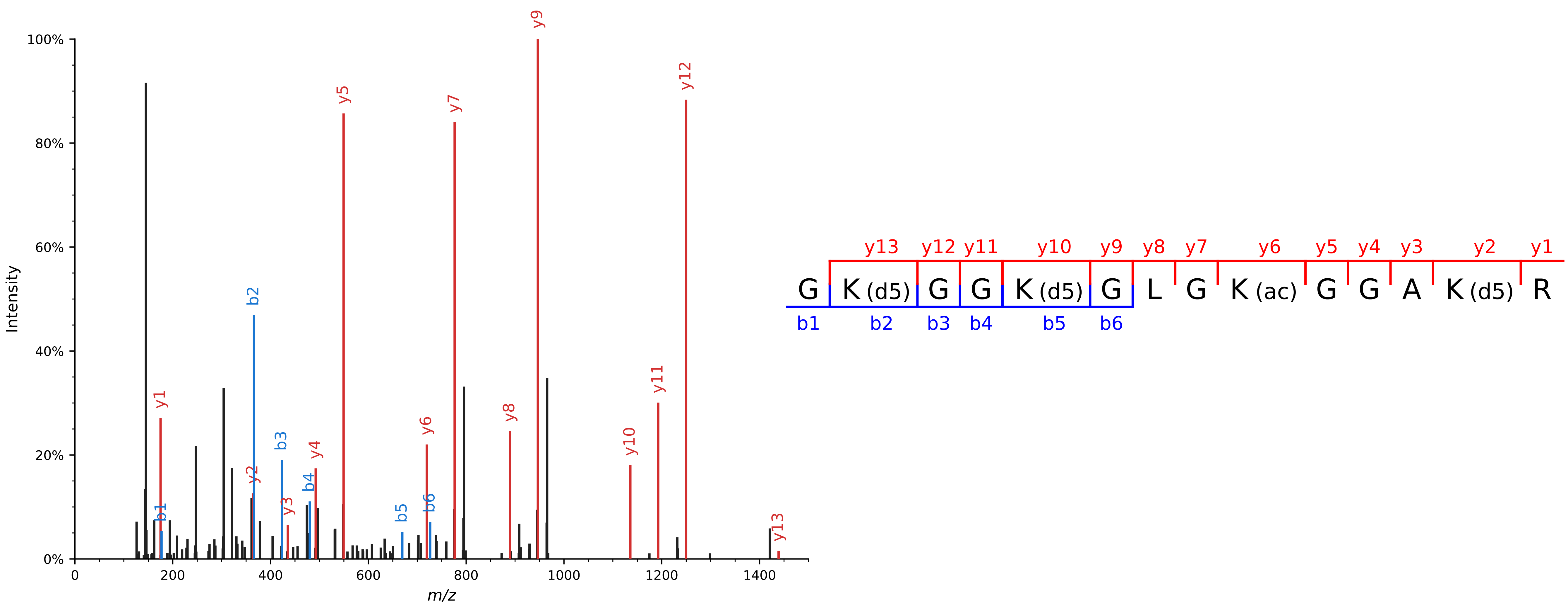

Raw file: HF240124_RN_spleen_D5PRO_PIC_3, Scan: 13328, m/z: 798.47547, Charge: 2, RT (min): 25.04, Score: 139.86

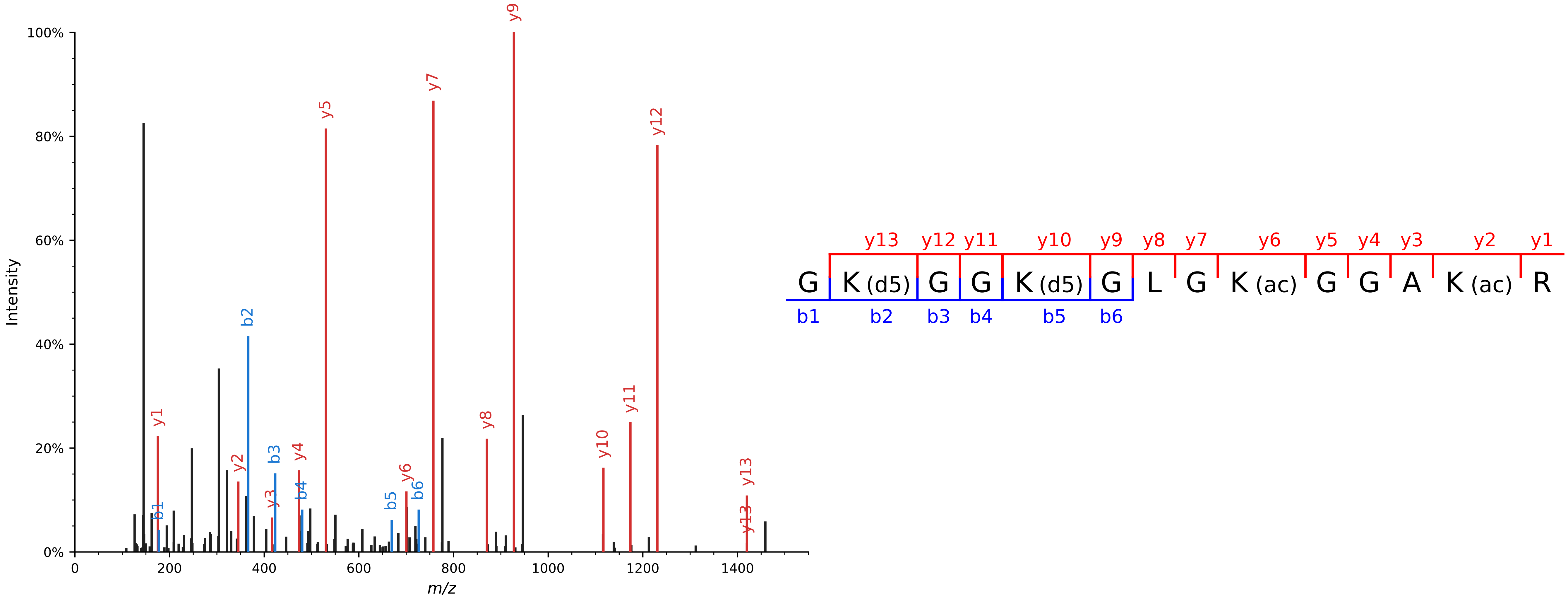

Raw file: HF240124_RN_spleen_D5PRO_PIC_1, Scan: 16622, m/z: 822.01464, Charge: 2, RT (min): 31.21, Score: 129.16

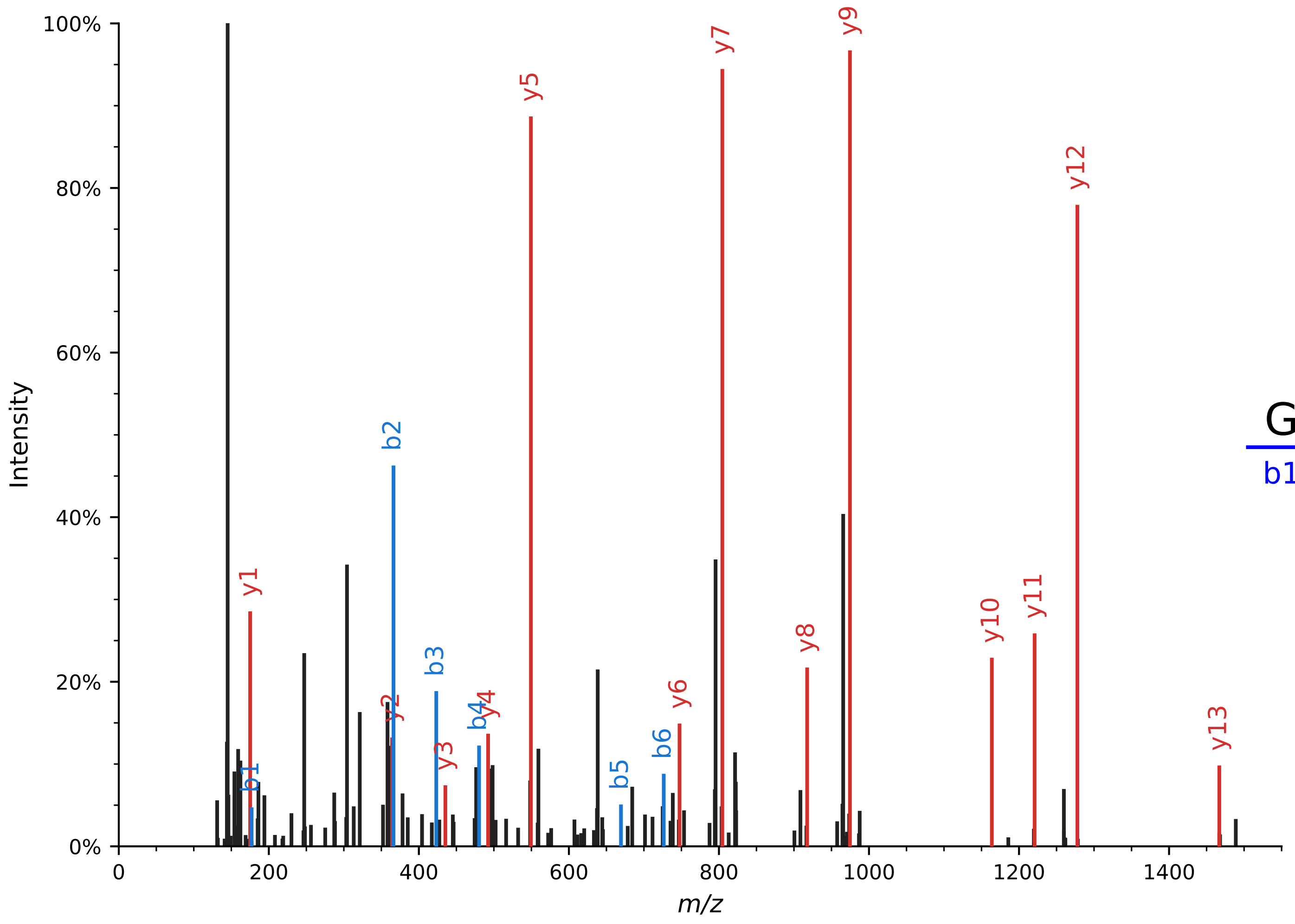

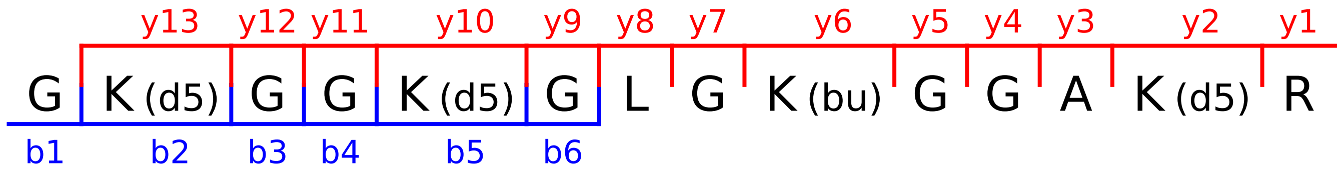

Raw file: HF240124_RN_spleen_D5PRO_PIC_2, Scan: 15652, m/z: 812.49112, Charge: 2, RT (min): 29.74, Score: 138.25

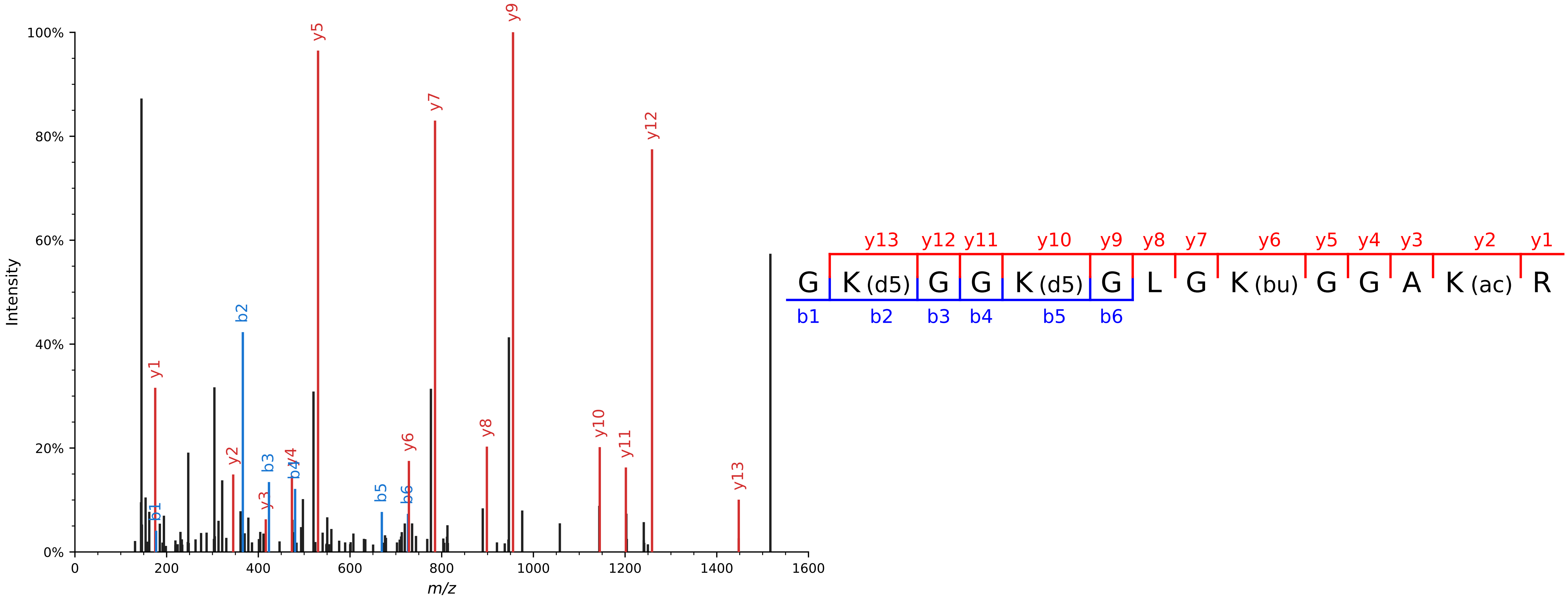

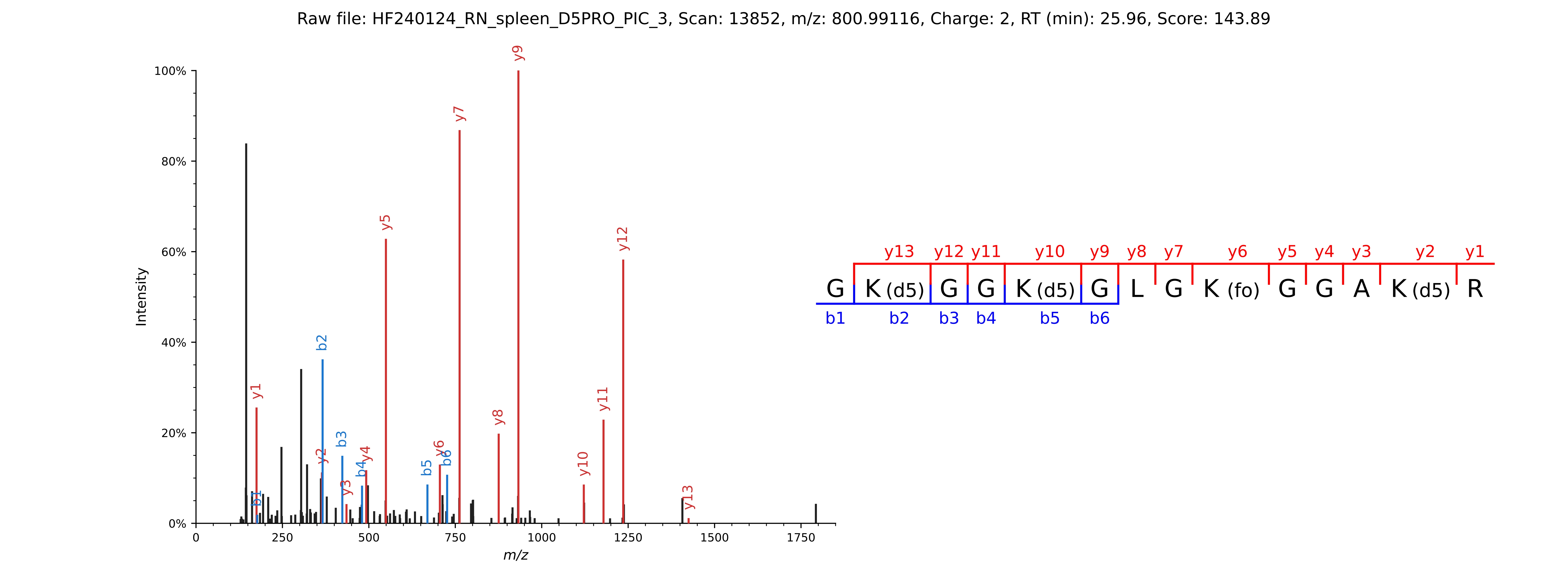

Raw file: HF240124_RN_spleen_D5PRO_PIC_3, Scan: 13852, m/z: 800.99116, Charge: 2, RT (min): 25.96, Score: 143.89
Intensity
m/z
y13 y12 y11 y10 y9 y8 y7 y6 y5 y4 y3 y2 y1
G K (d5) G G K (d5) G L G K (fo) G G A K (d5) R
b1 b2 b3 b4 b5 b6

Raw file: HF240124_RN_spleen_D5PRO_PIC_2, Scan: 16301, m/z: 824.53033, Charge: 2, RT (min): 30.91, Score: 157.24

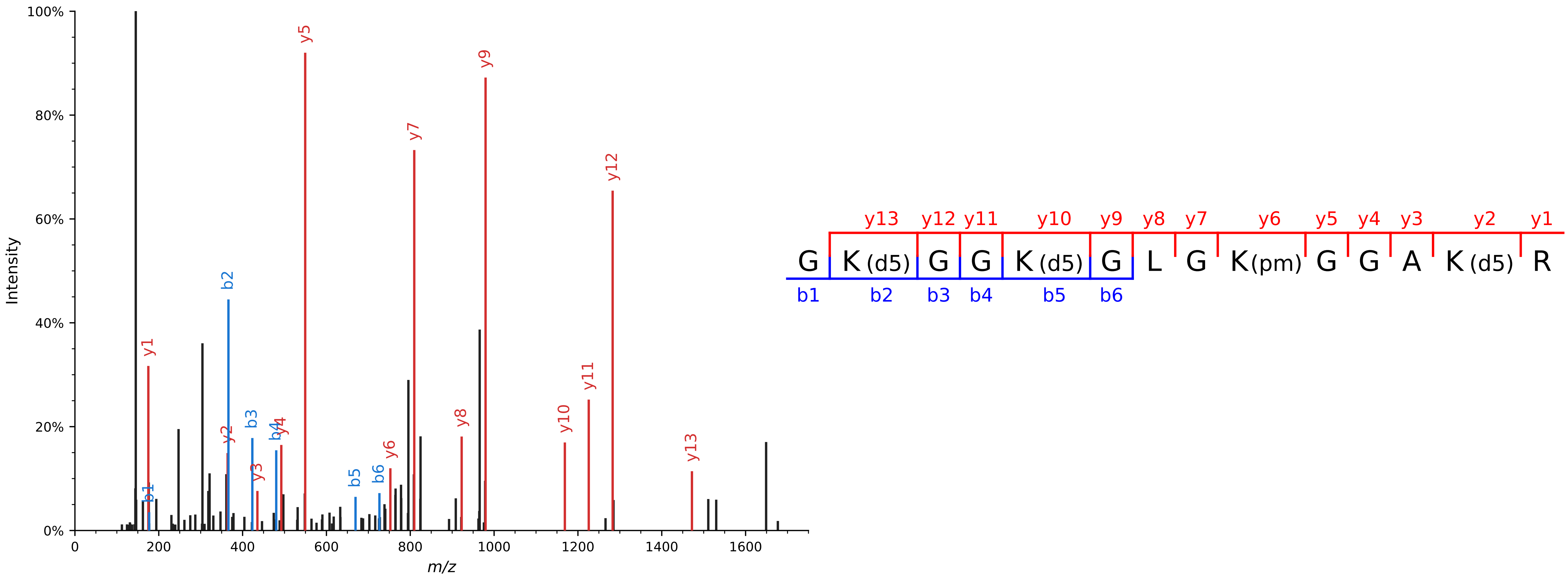

Raw file: HF240124_RN_spleen_D5PRO_PIC_3, Scan: 14868, m/z: 807.99899, Charge: 2, RT (min): 27.73, Score: 143.96

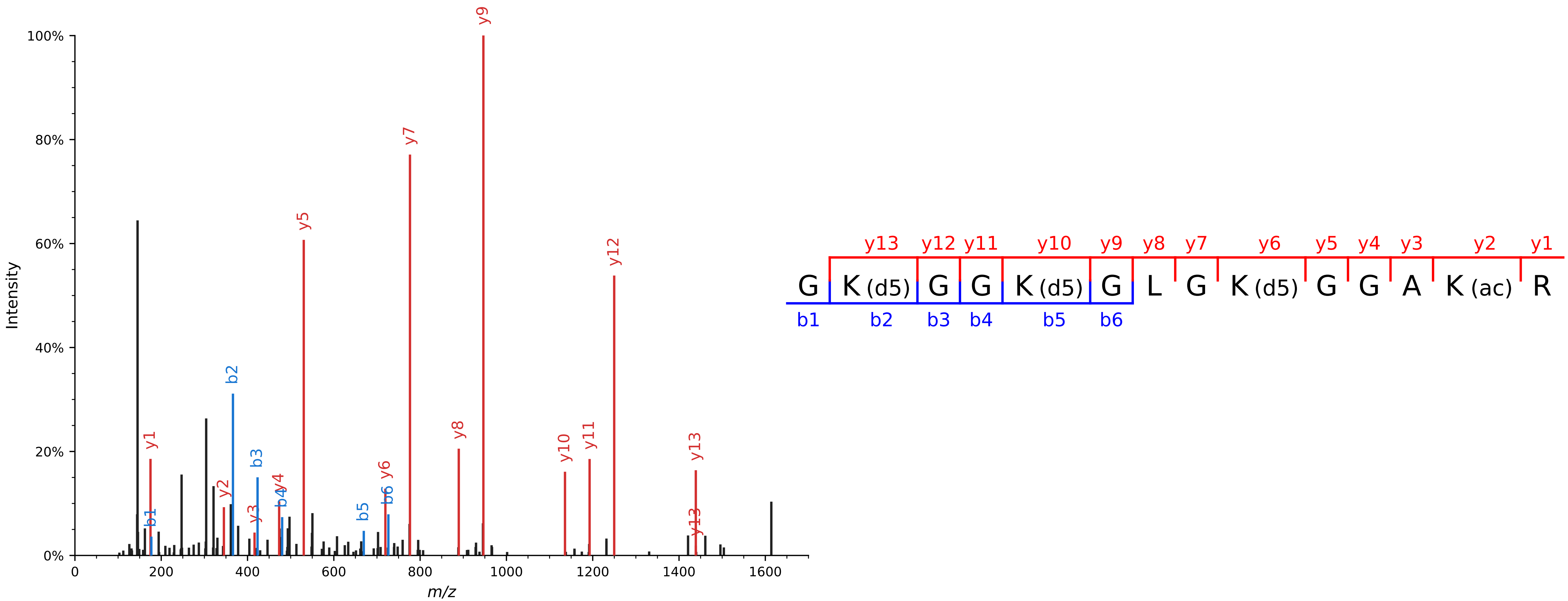

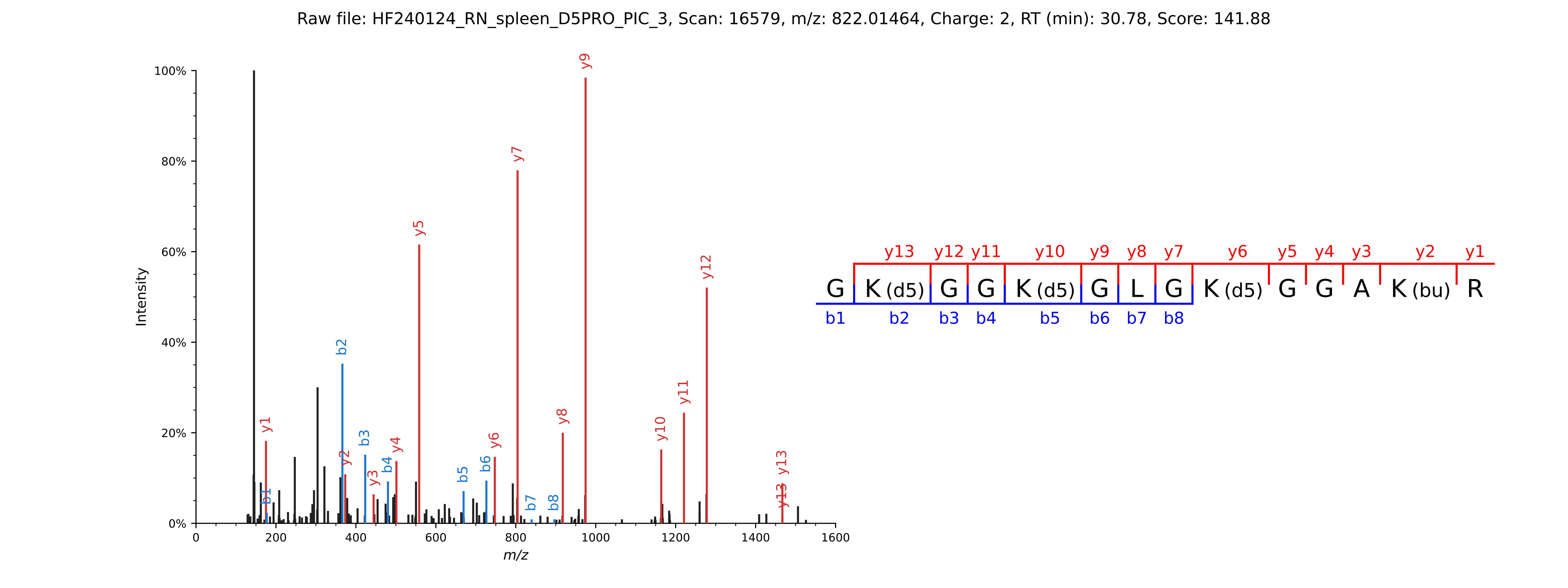
Raw file: HF240124_RN_spleen_D5PRO_PIC_3, Scan: 16579, m/z: 822.01464, Charge: 2, RT (min): 30.78, Score: 141.88
Intensity
m/z
G K(d5) G G K(d5) G L G K(d5) G G A K(bu) R
y13 y12 y11 y10 y9 y8 y7 y6 y5 y4 y3 y2 y1
b1 b2 b3 b4 b5 b6 b7 b8

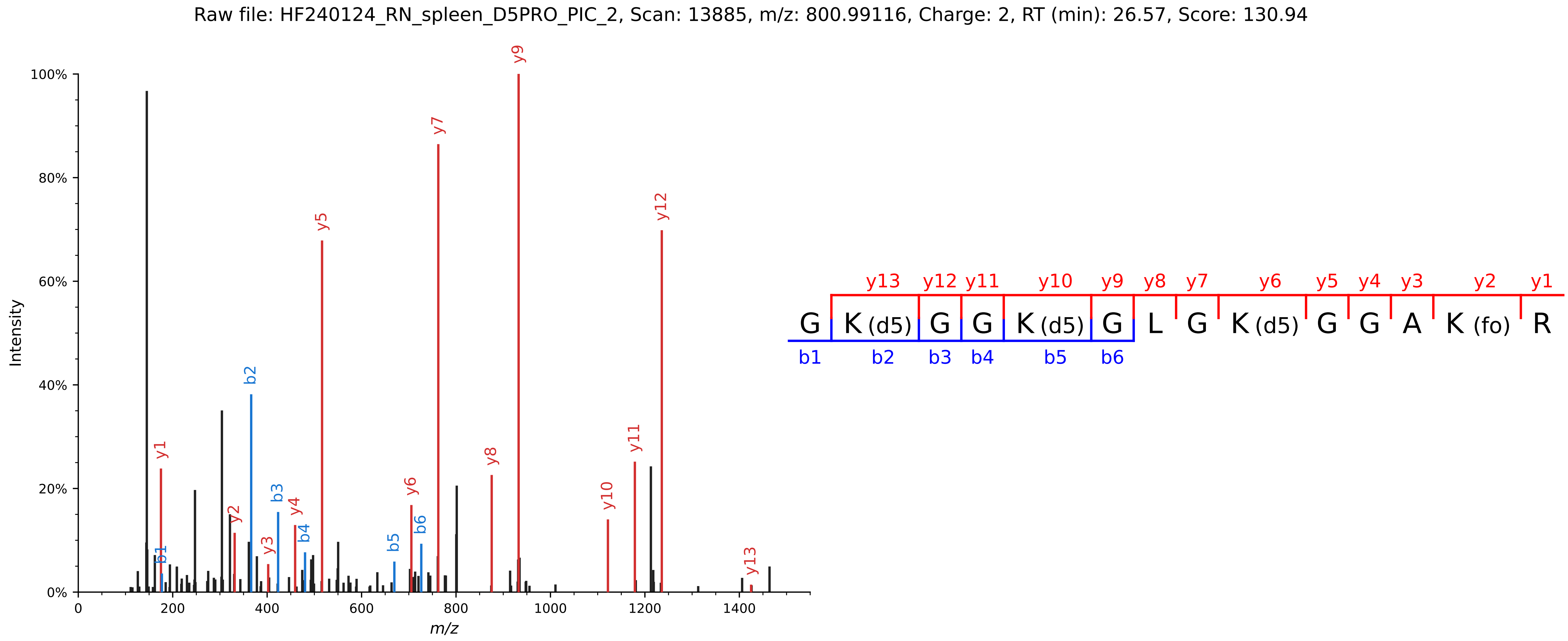

Raw file: HF240124_RN_spleen_D5PRO_PIC_2, Scan: 13885, m/z: 800.99116, Charge: 2, RT (min): 26.57, Score: 130.94
Intensity
m/z
G K (d5) G G K (d5) G L G K (d5) G G A K (fo) R

Raw file: HF240124_RN_spleen_D5PRO_PIC_1, Scan: 19931, m/z: 757.91992, Charge: 2, RT (min): 37.16, Score: 162.38

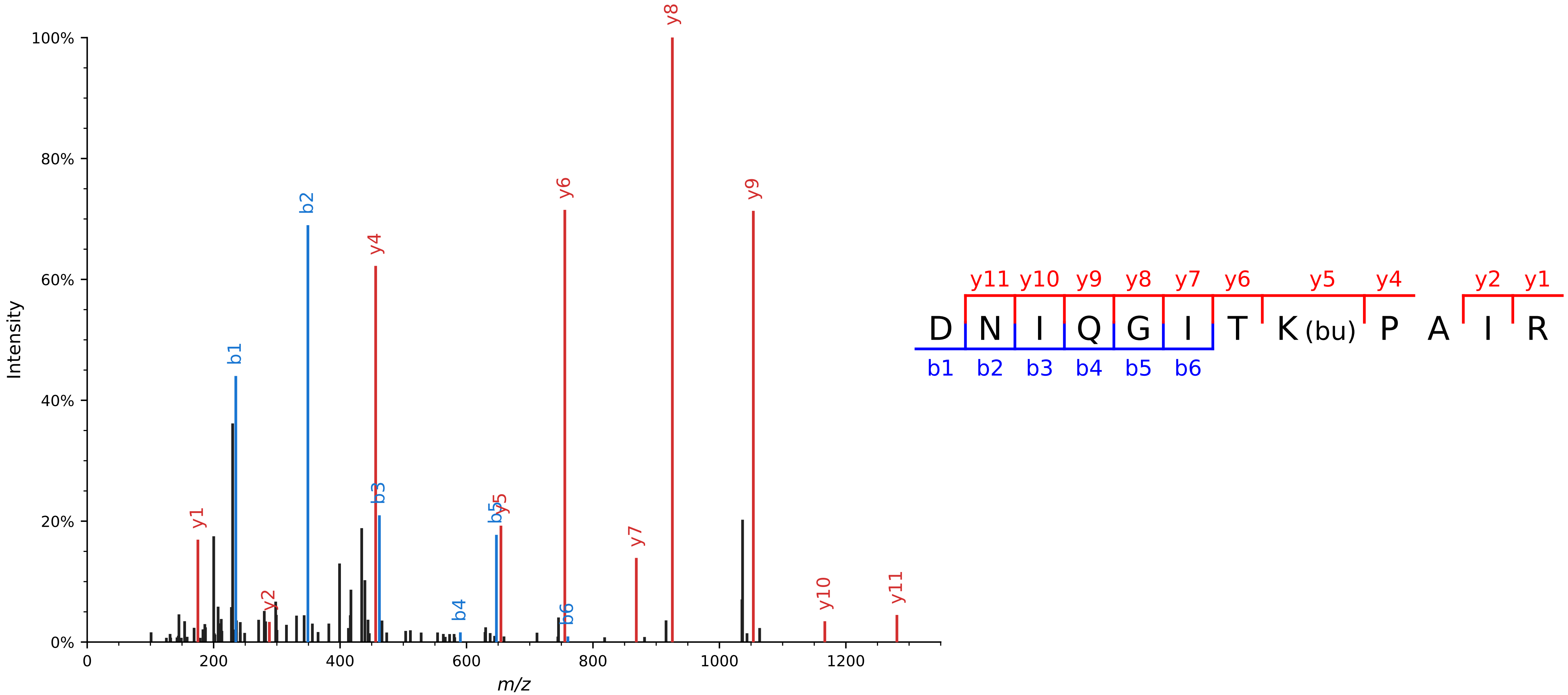

Raw file: HF240124_RN_spleen_D5PRO_PIC_2, Scan: 17236, m/z: 736.89644, Charge: 2, RT (min): 32.61, Score: 110.39

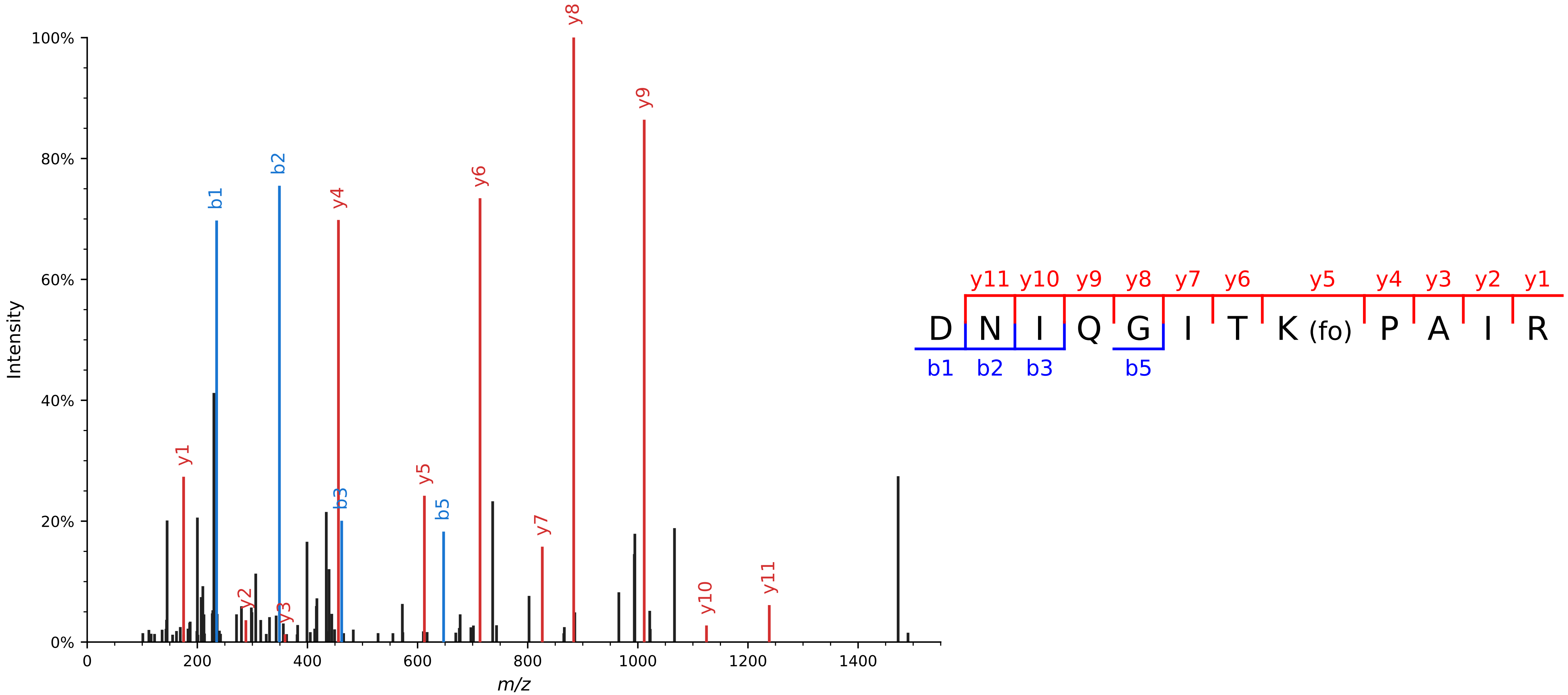

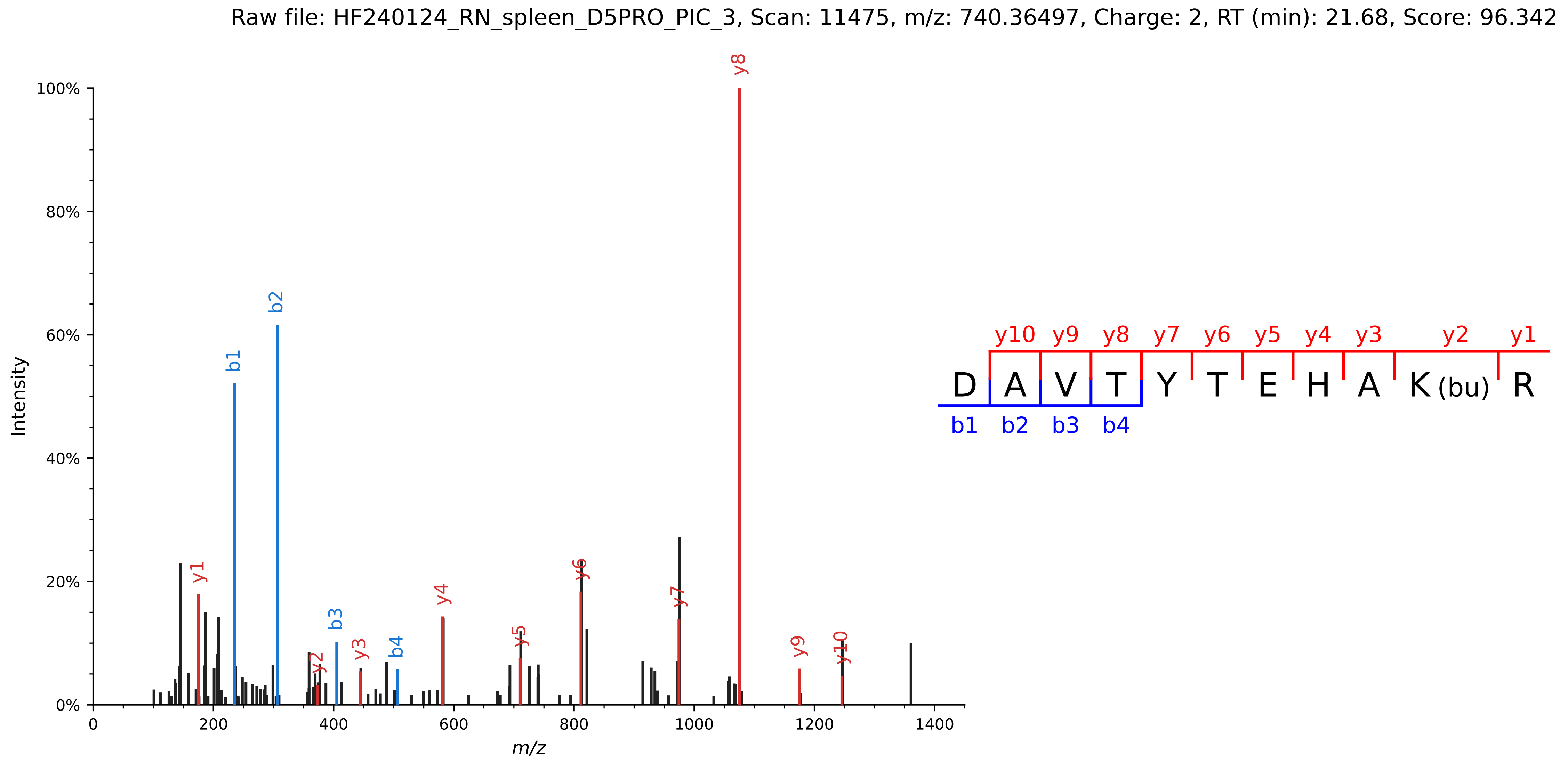
Raw file: HF240124_RN_spleen_D5PRO_PIC_3, Scan: 11475, m/z: 740.36497, Charge: 2, RT (min): 21.68, Score: 96.342
Intensity
m/z
y10 y9 y8 y7 y6 y5 y4 y3 y2 y1
D A V T Y T E H A K (bu) R
b1 b2 b3 b4

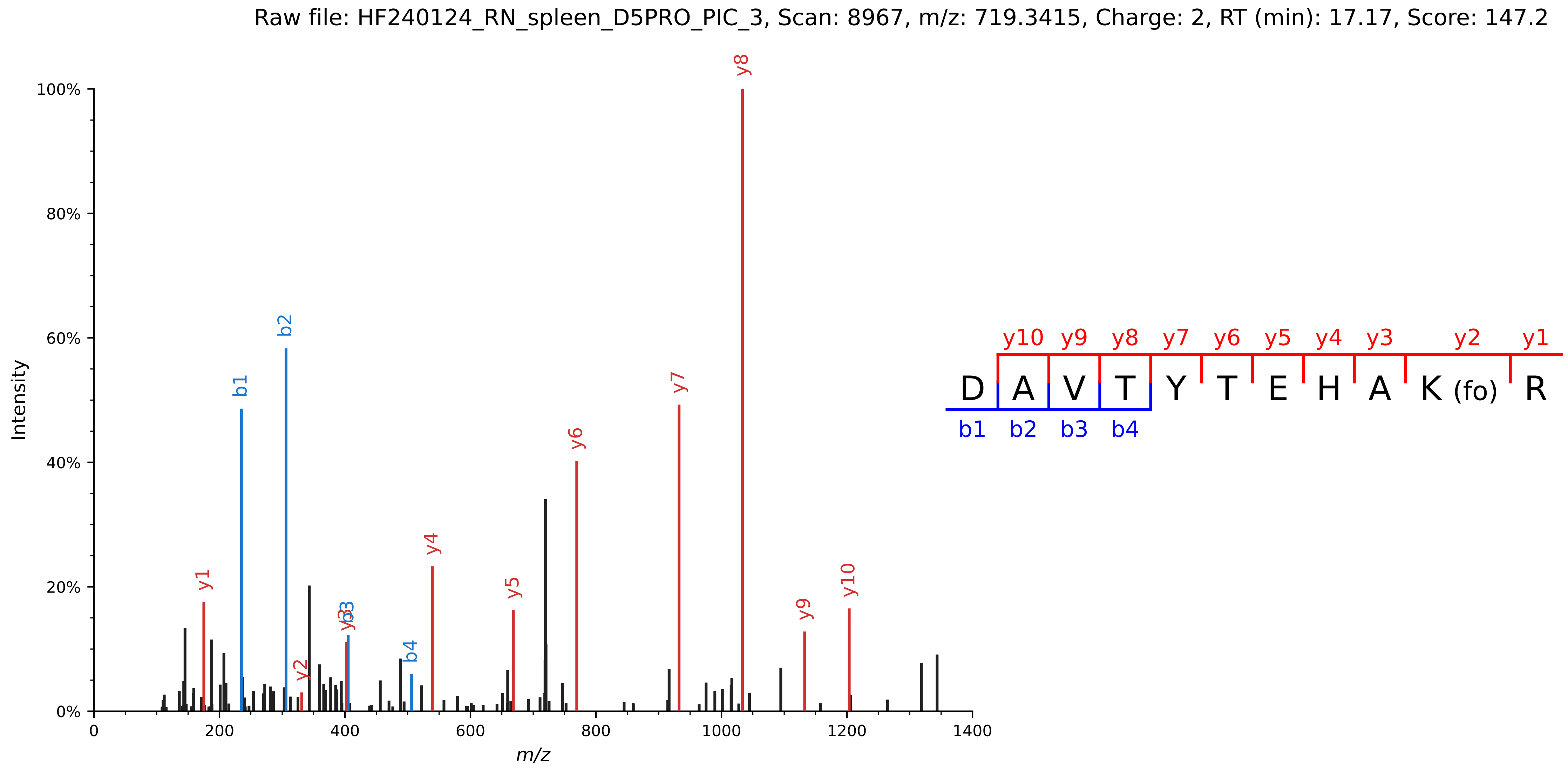

Raw file: HF240124_RN_spleen_D5PRO_PIC_3, Scan: 8967, m/z: 719.3415, Charge: 2, RT (min): 17.17, Score: 147.2
Intensity
m/z
0%
20%
40%
60%
80%
100%
0
200
400
600
800
1000
1200
1400
y1
b1
b2
y2
y3
b3
b4
y4
y5
y6
y7
y8
y9
y10
D A V T Y T E H A K (fo) R

Raw file: HF240124_RN_spleen_D5PRO_PIC_1, Scan: 11925, m/z: 742.88066, Charge: 2, RT (min): 22.78, Score: 115.71

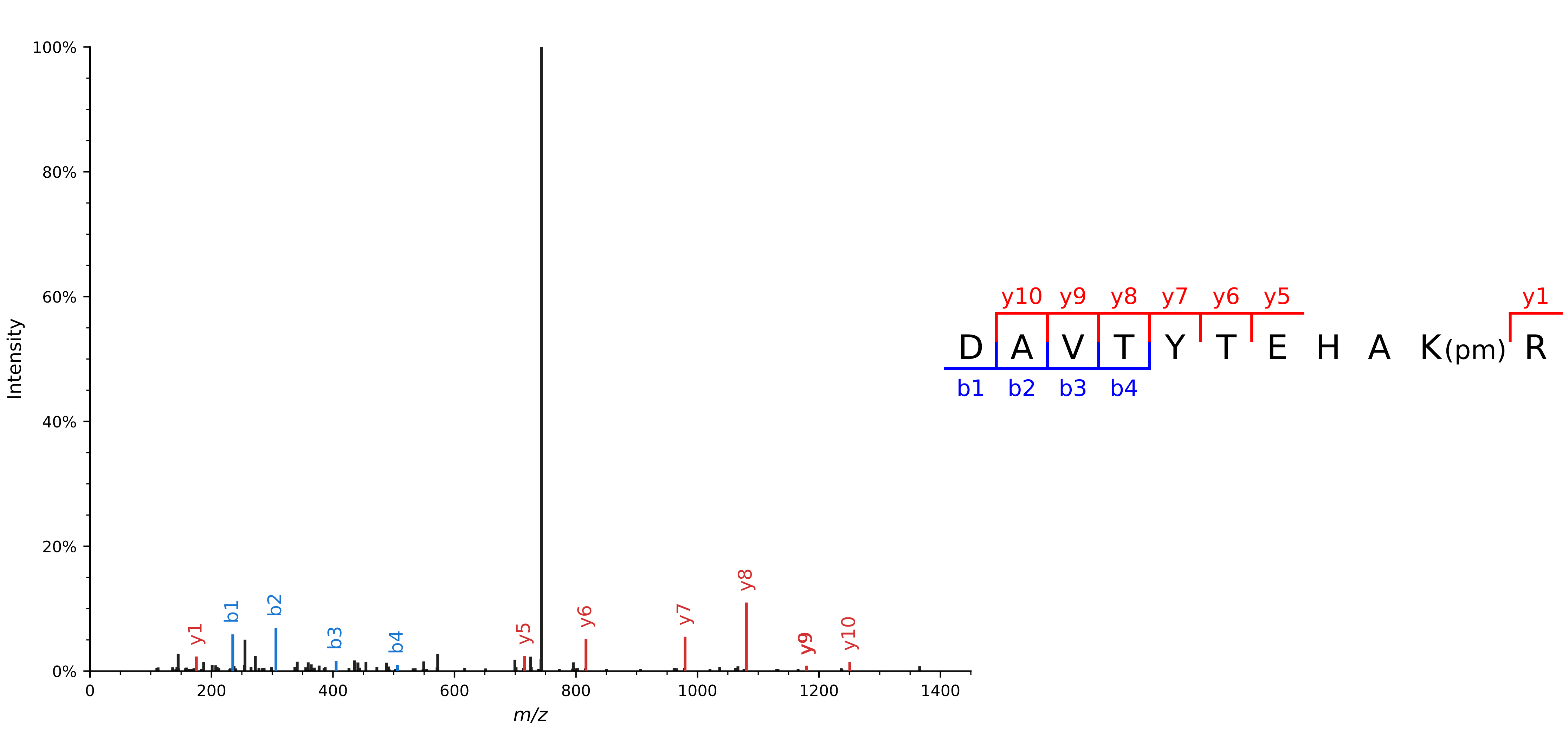

Spleen_D5PRO-D5PRO

Raw file: HF240124_RN_spleen_D5PRO_D5PRO_2, Scan: 9693, m/z: 474.26949, Charge: 2, RT (min): 18.54, Score: 47.067

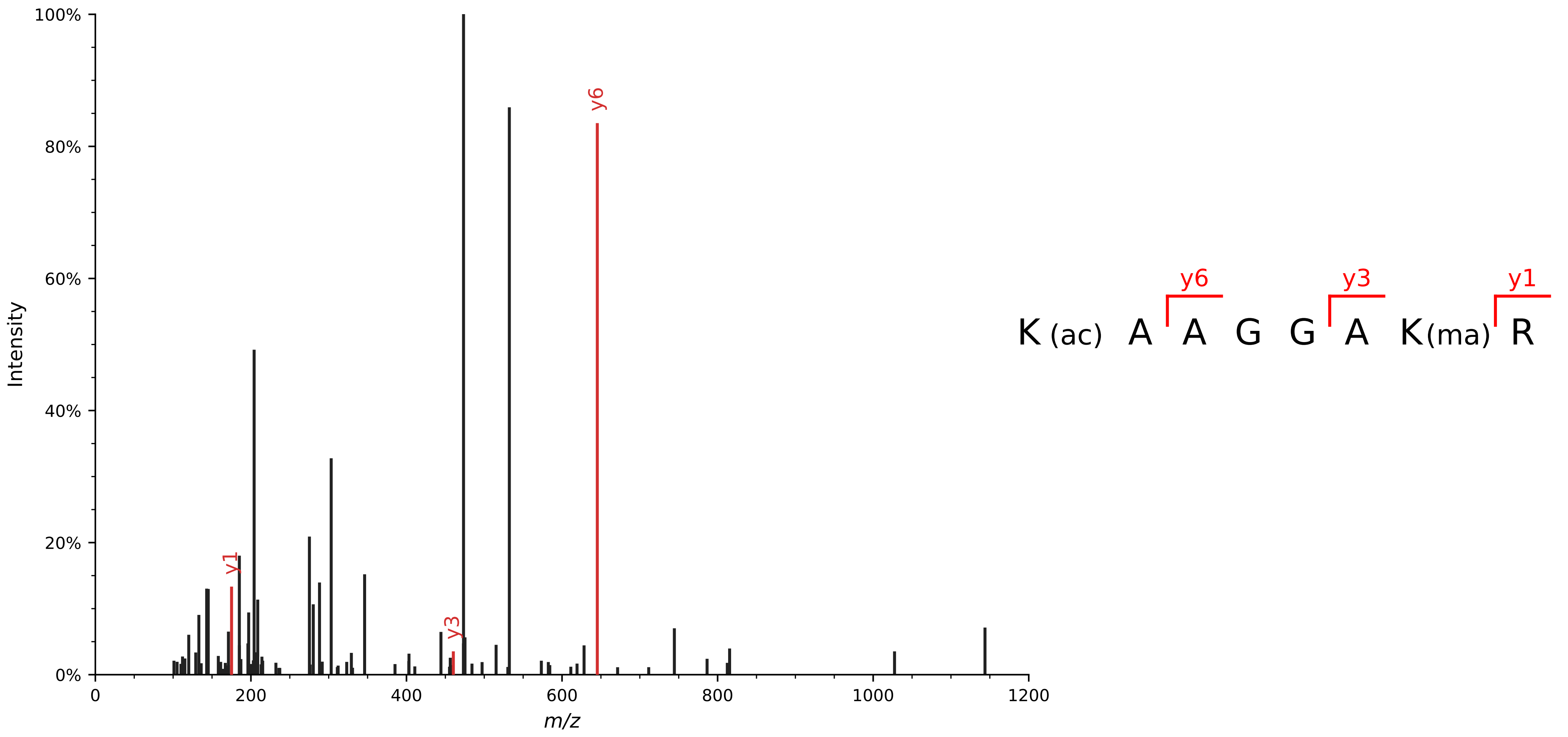

Raw file: HF240124_RN_spleen_D5PRO_D5PRO_3, Scan: 8275, m/z: 475.81374, Charge: 2, RT (min): 15.89, Score: 60.788

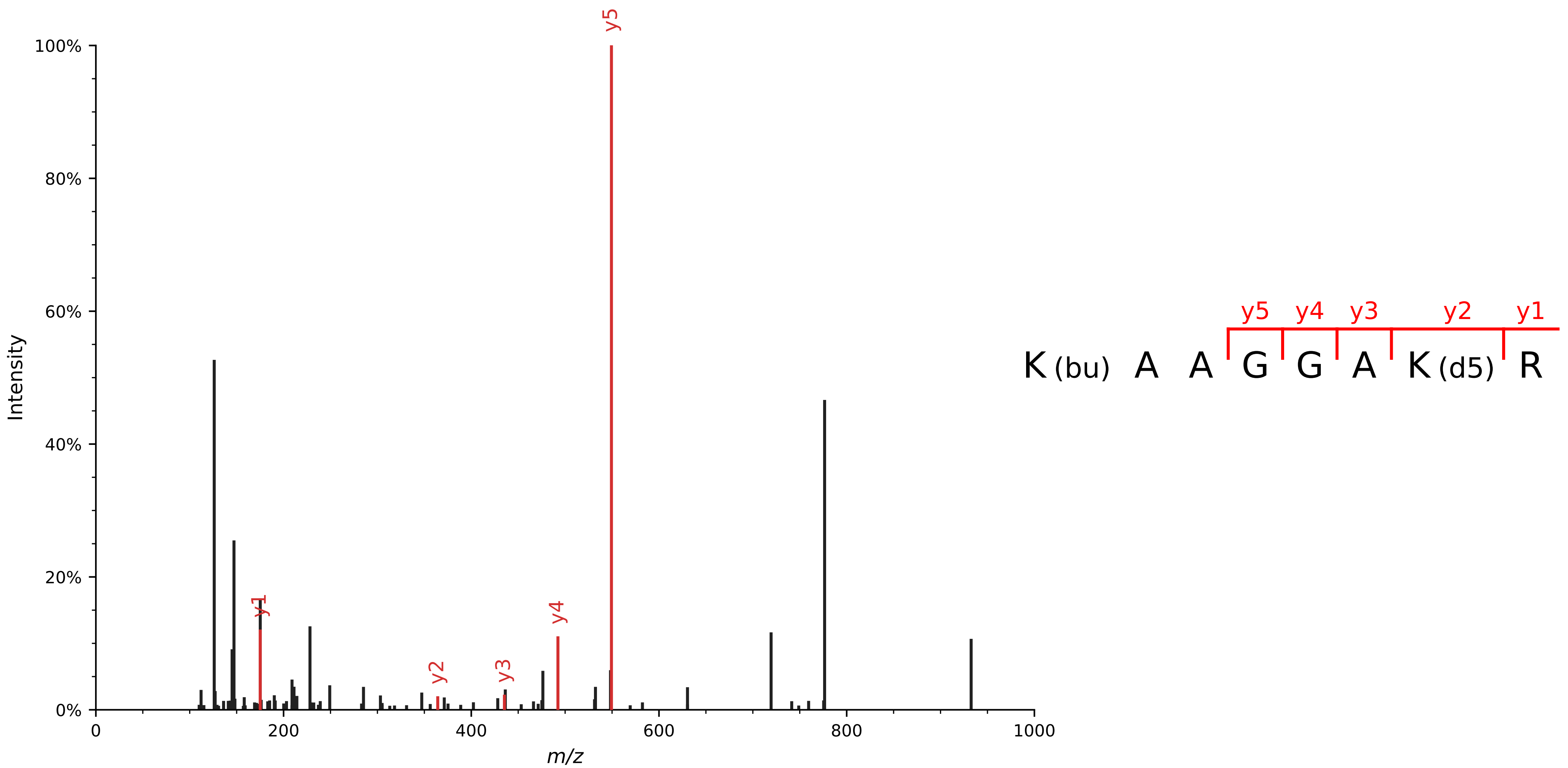

Raw file: HF240124_RN_spleen_D5PRO_D5PRO_2, Scan: 7029, m/z: 478.32943, Charge: 2, RT (min): 13.99, Score: 83.377

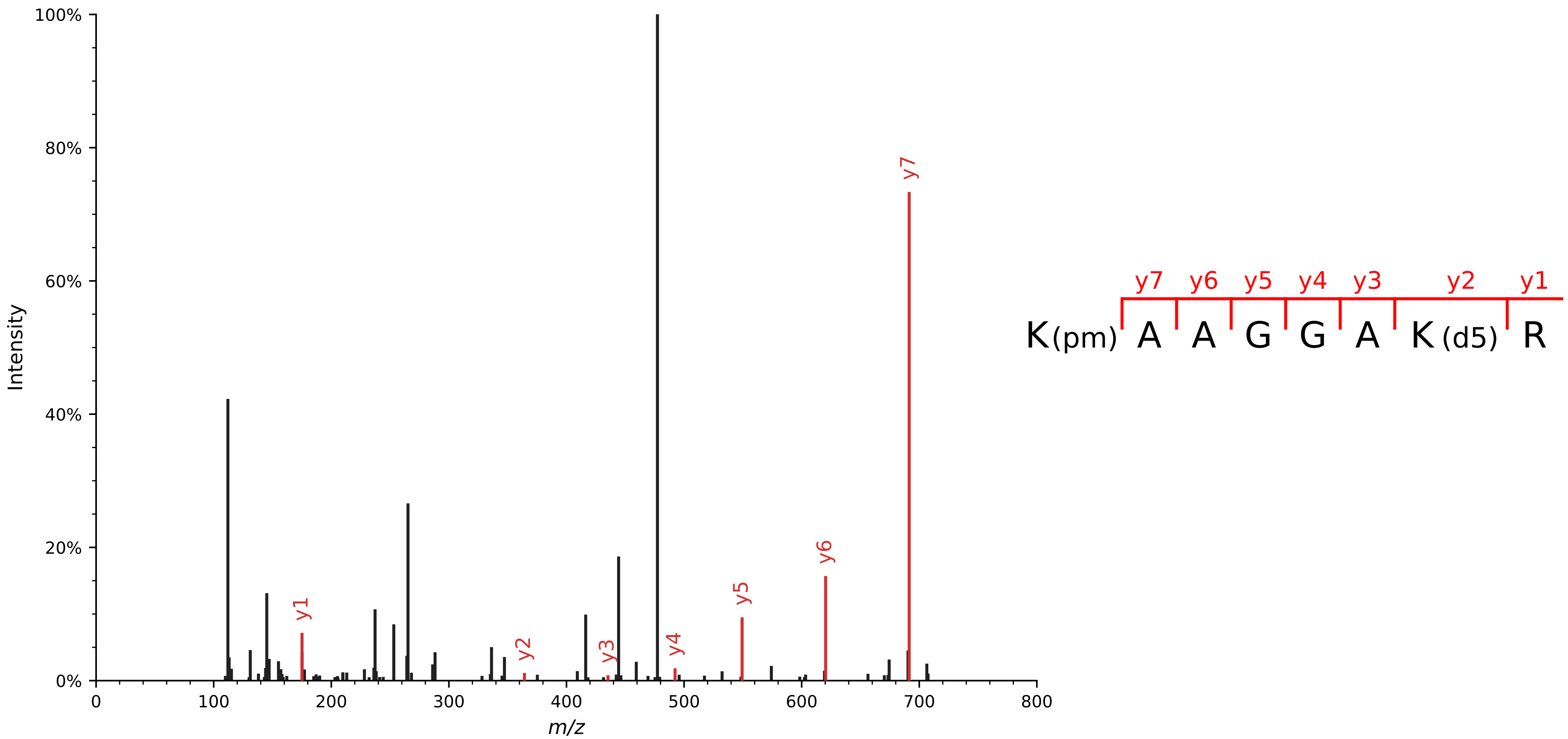

Raw file: HF240124_RN_spleen_D5PRO_D5PRO_2, Scan: 2032, m/z: 454.80846, Charge: 2, RT (min): 5.16, Score: 62.714

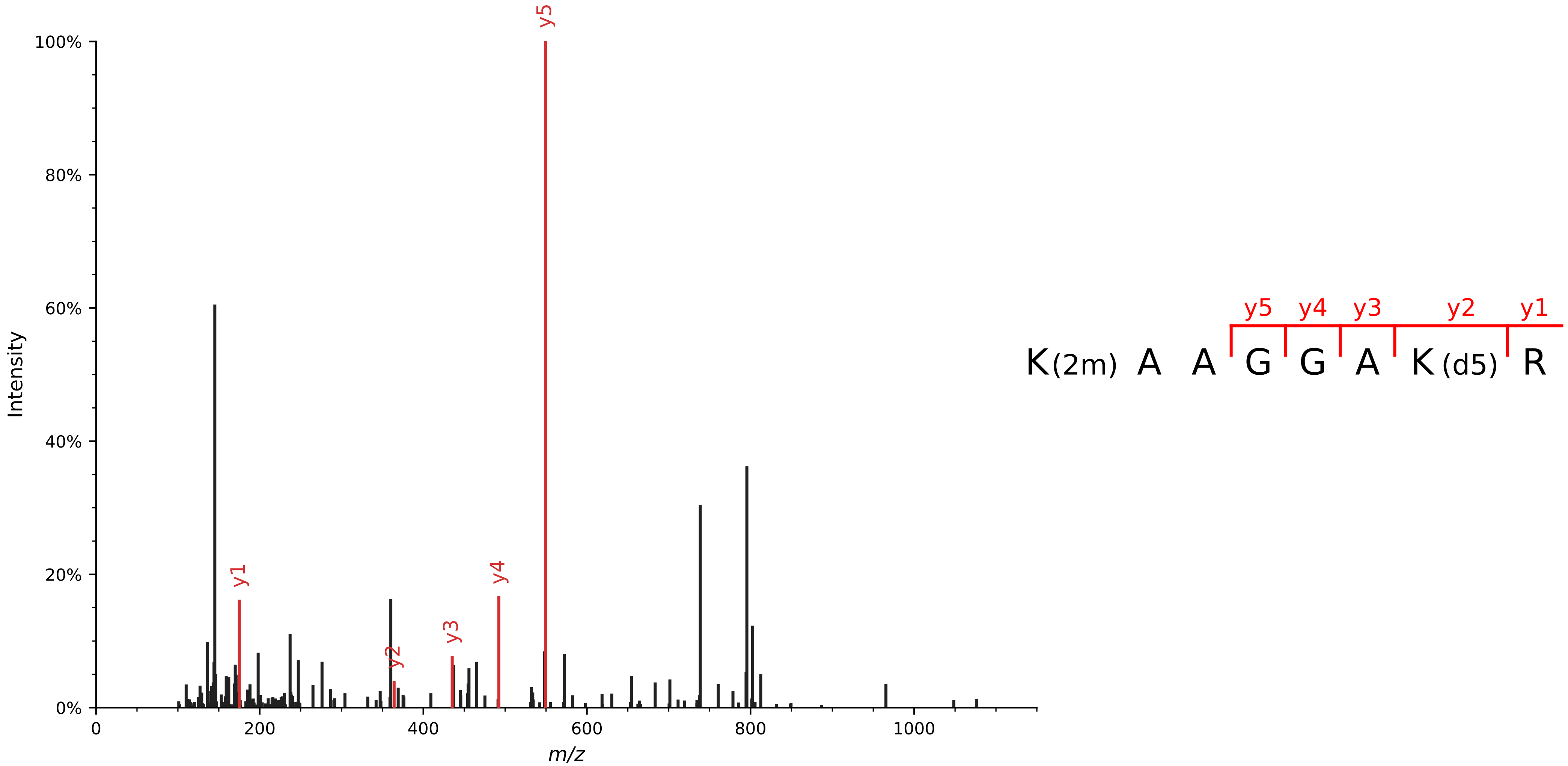

Raw file: HF240124_RN_spleen_D5PRO_D5PRO_2, Scan: 27702, m/z: 809.82812, Charge: 3, RT (min): 51.06, Score: 80.156

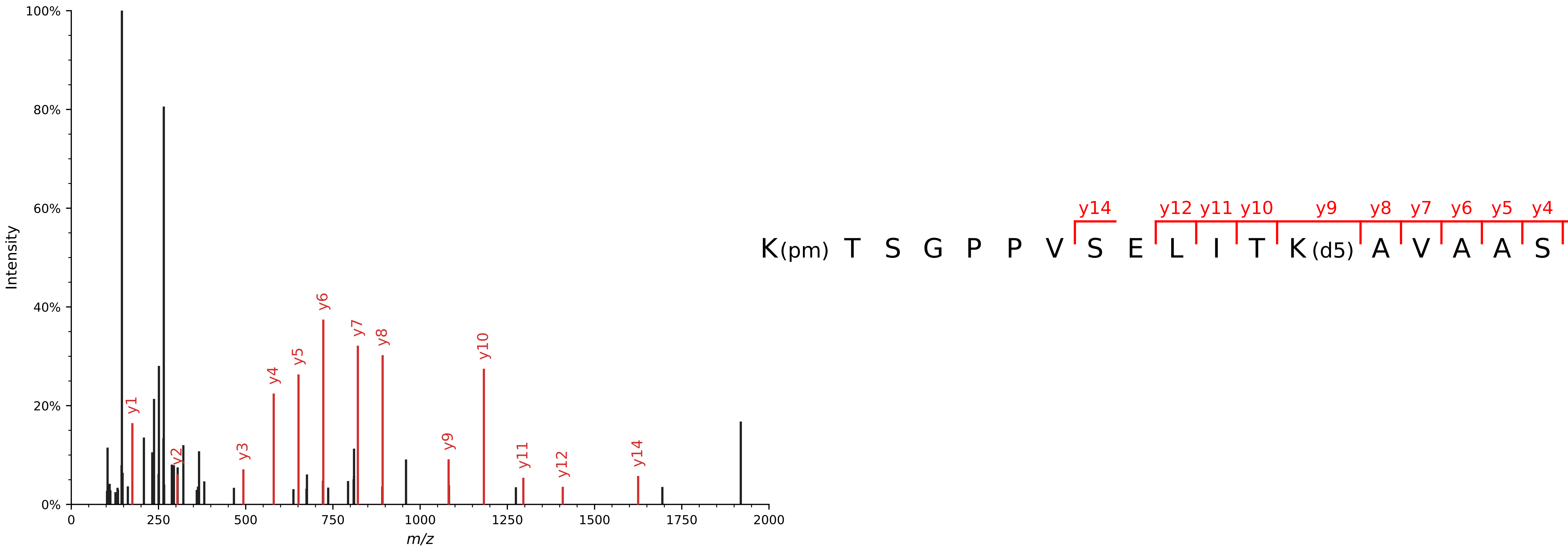

Raw file: HF240124_RN_spleen_D5PRO_D5PRO_3, Scan: 16823, m/z: 759.44724, Charge: 3, RT (min): 31.27, Score: 41.136

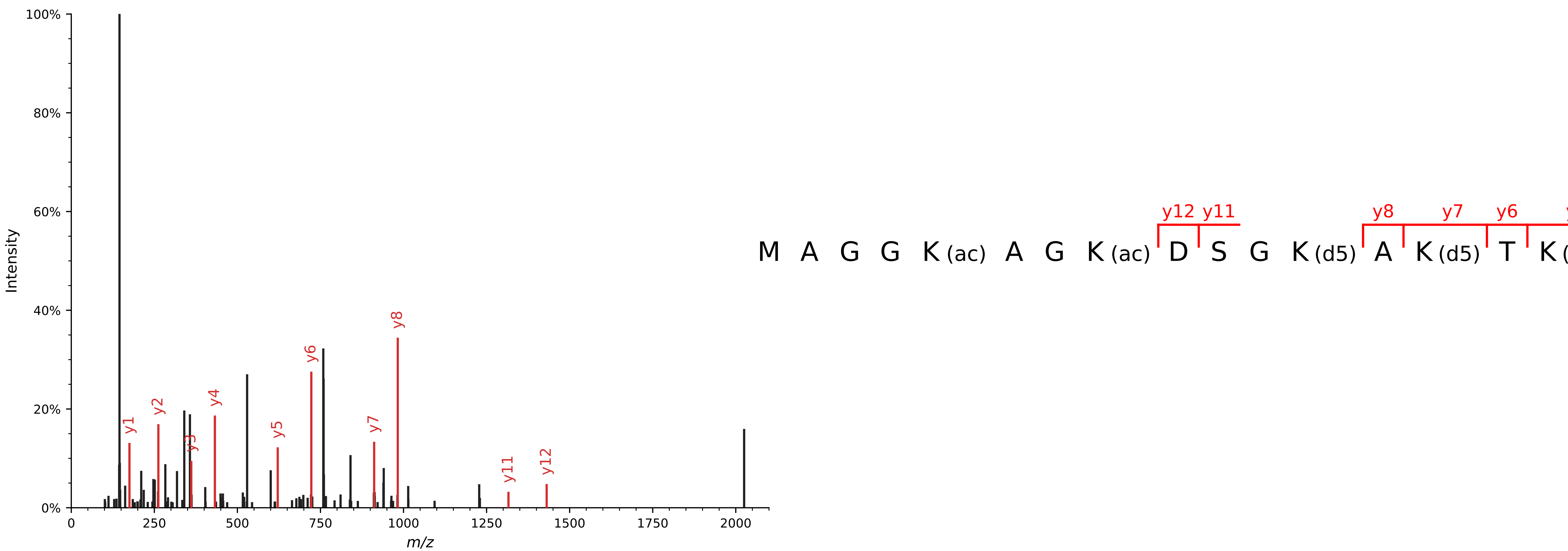

Raw file: HF240124_RN_spleen_D5PRO_D5PRO_2, Scan: 13890, m/z: 417.26622, Charge: 2, RT (min): 25.97, Score: 44.611

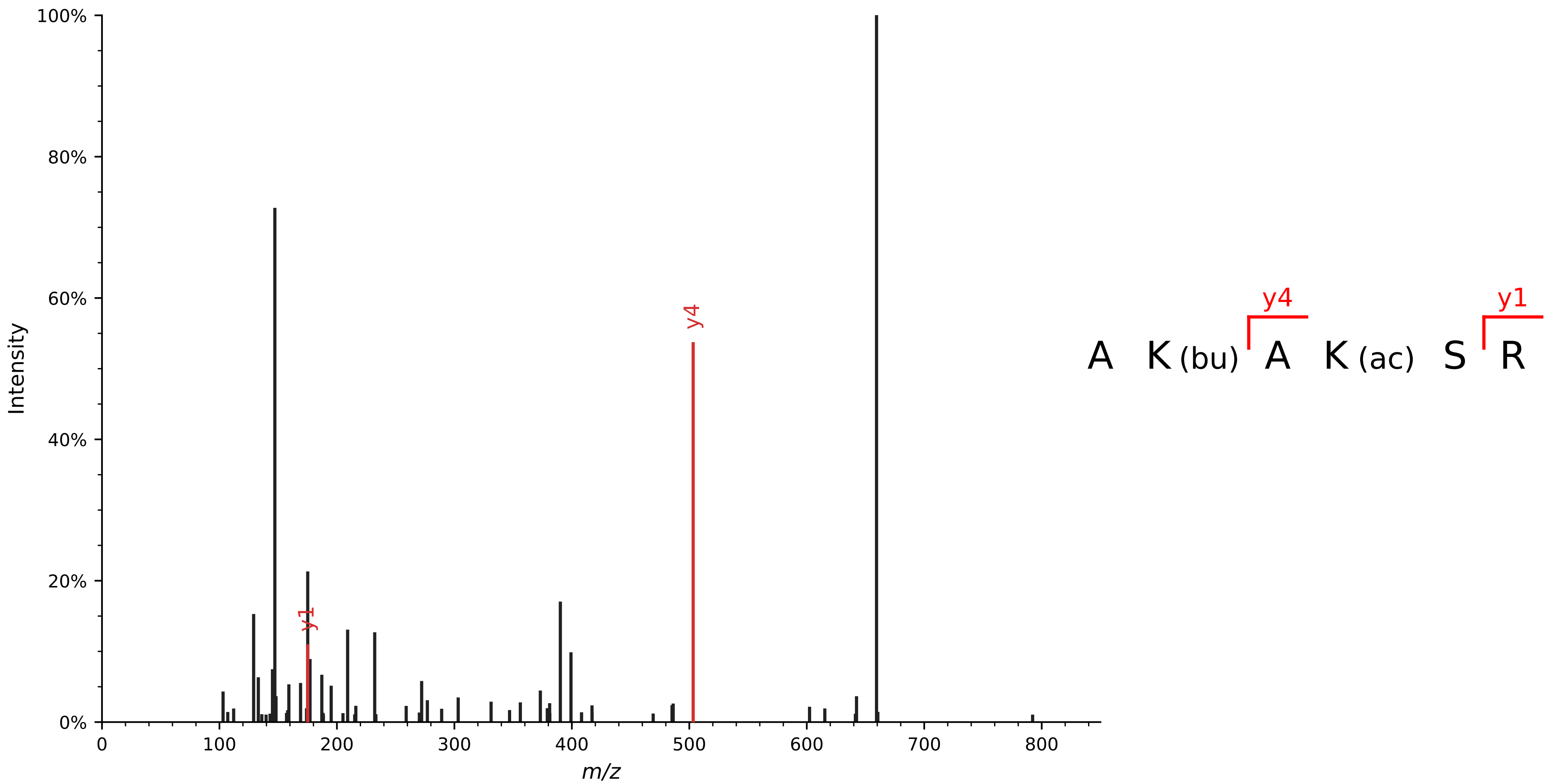

Raw file: HF240124_RN_spleen_D5PRO_D5PRO_1, Scan: 4024, m/z: 497.31665, Charge: 2, RT (min): 8.64, Score: 87.754

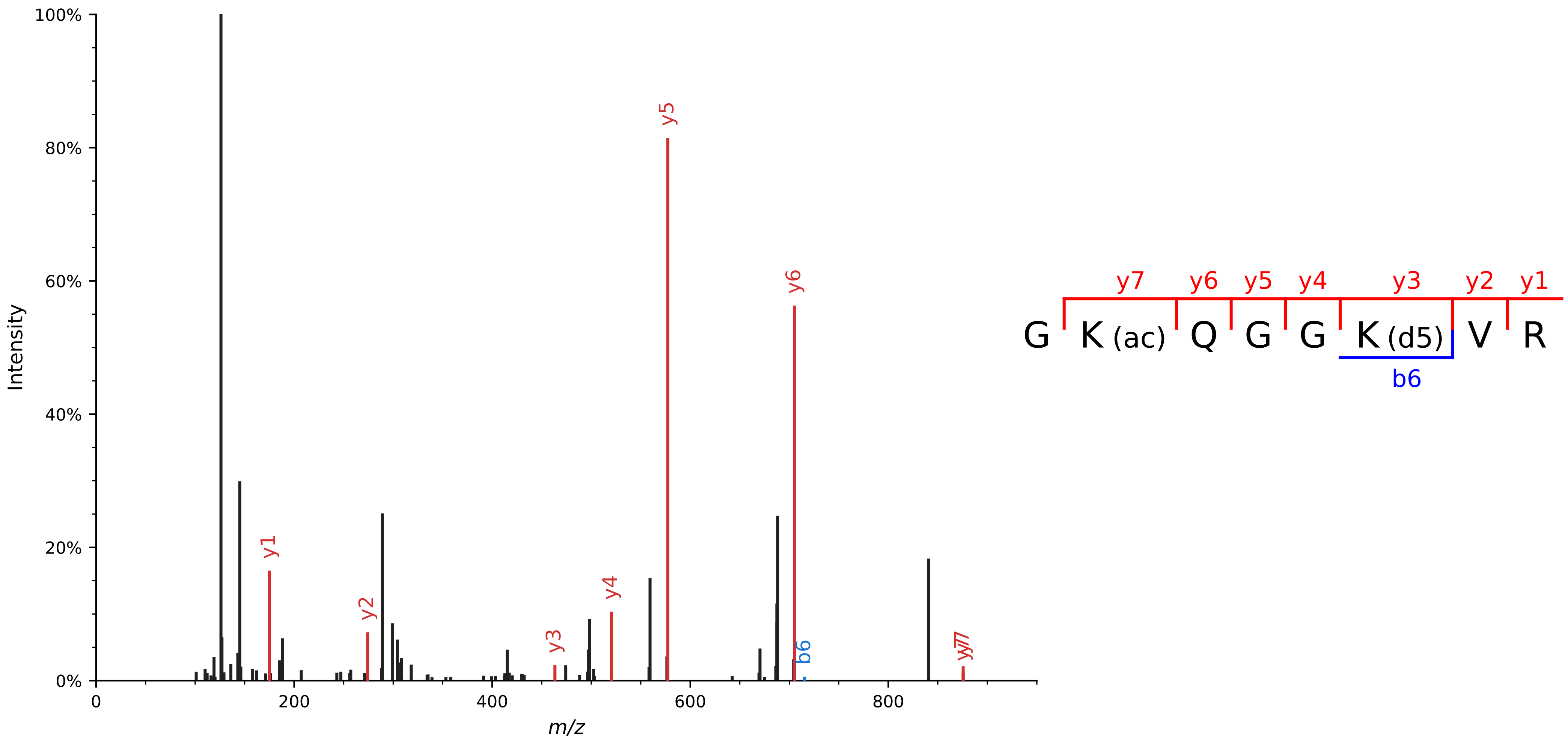

Raw file: HF240124_RN_spleen_D5PRO_D5PRO_3, Scan: 2656, m/z: 483.301, Charge: 2, RT (min): 5.92, Score: 134.06

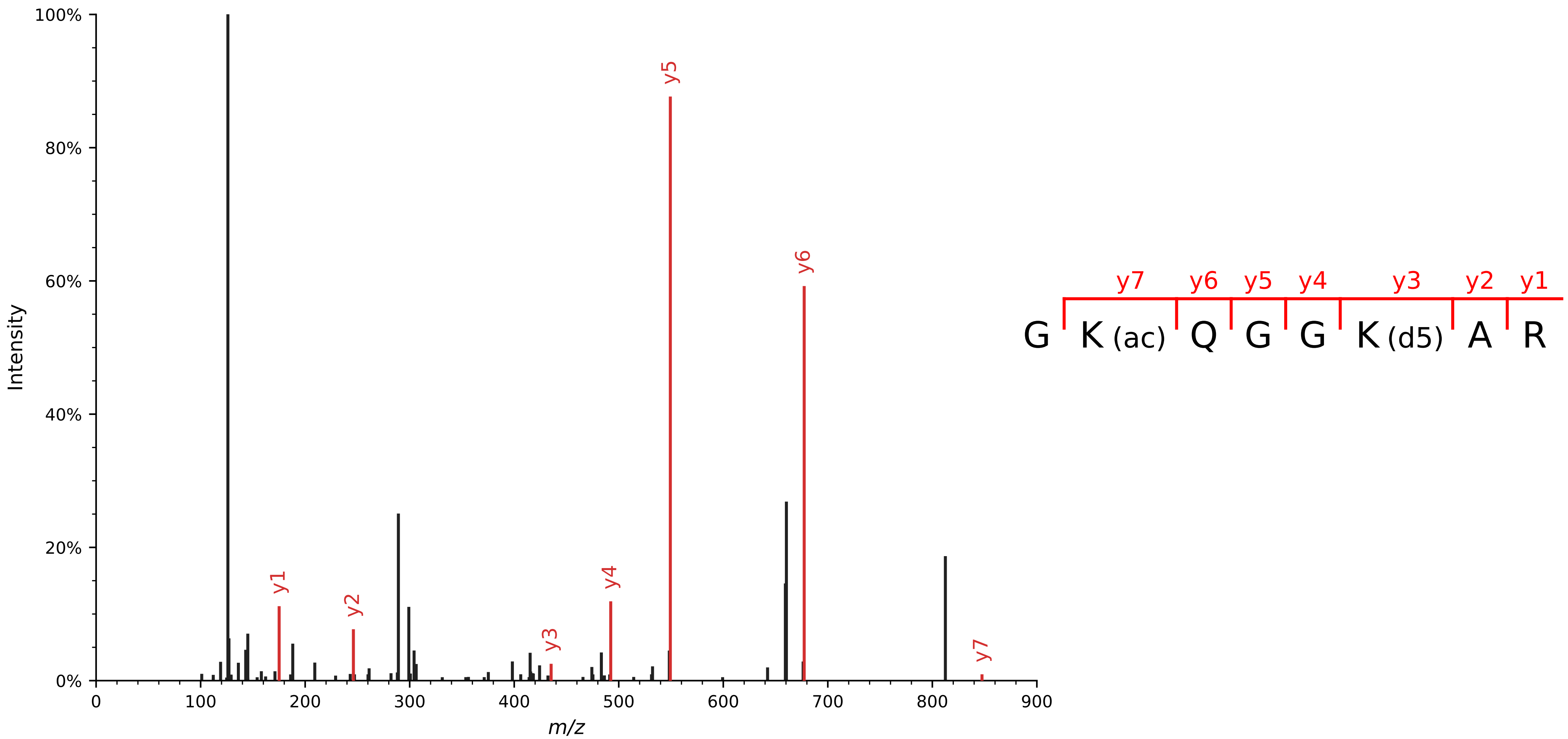

Raw file: HF240124_RN_spleen_D5PRO_D5PRO_3, Scan: 2041, m/z: 473.77748, Charge: 2, RT (min): 4.84, Score: 113.89

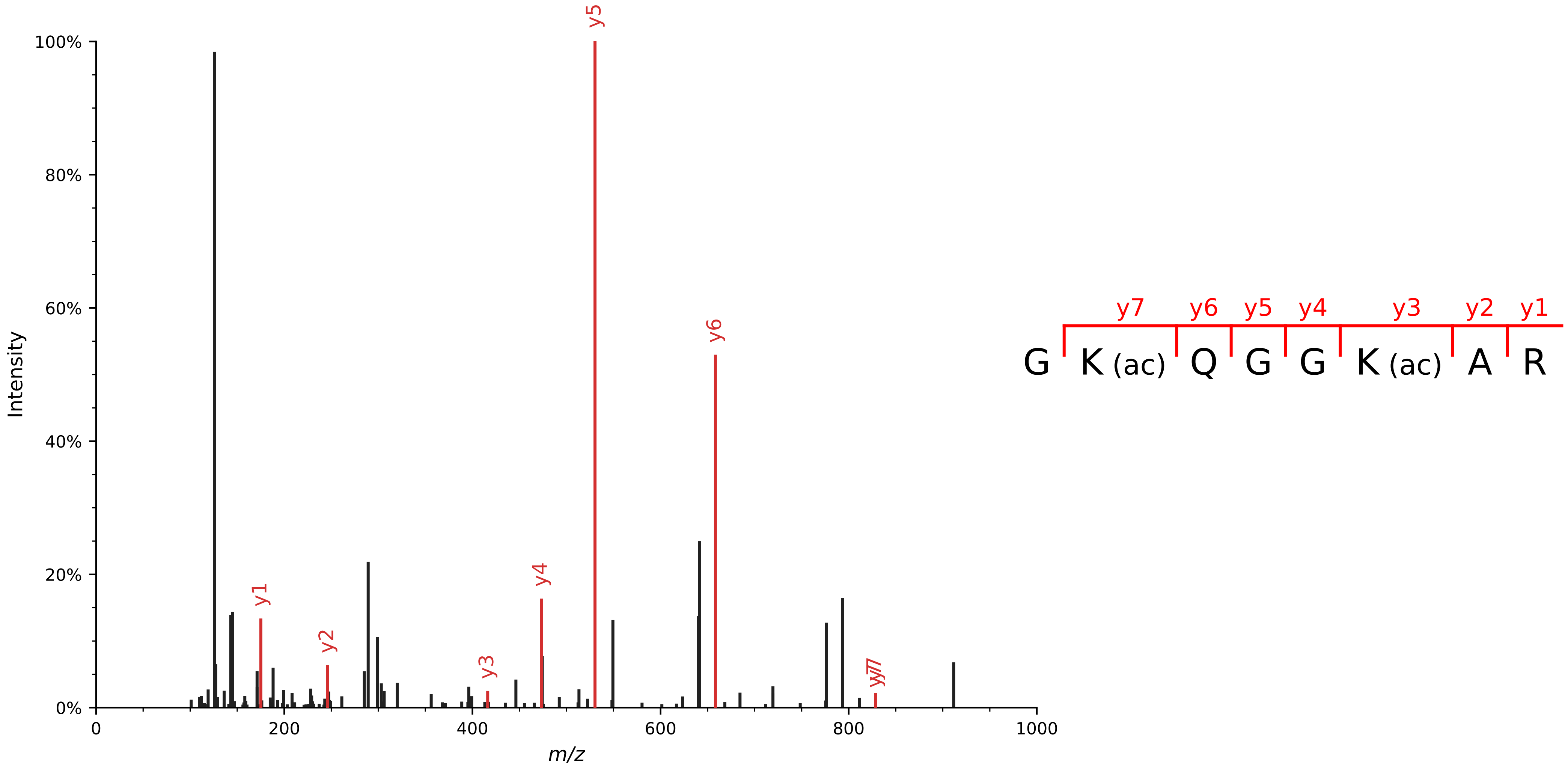

Raw file: HF240124_RN_spleen_D5PRO_D5PRO_2, Scan: 3473, m/z: 483.31919, Charge: 2, RT (min): 7.70, Score: 90.706

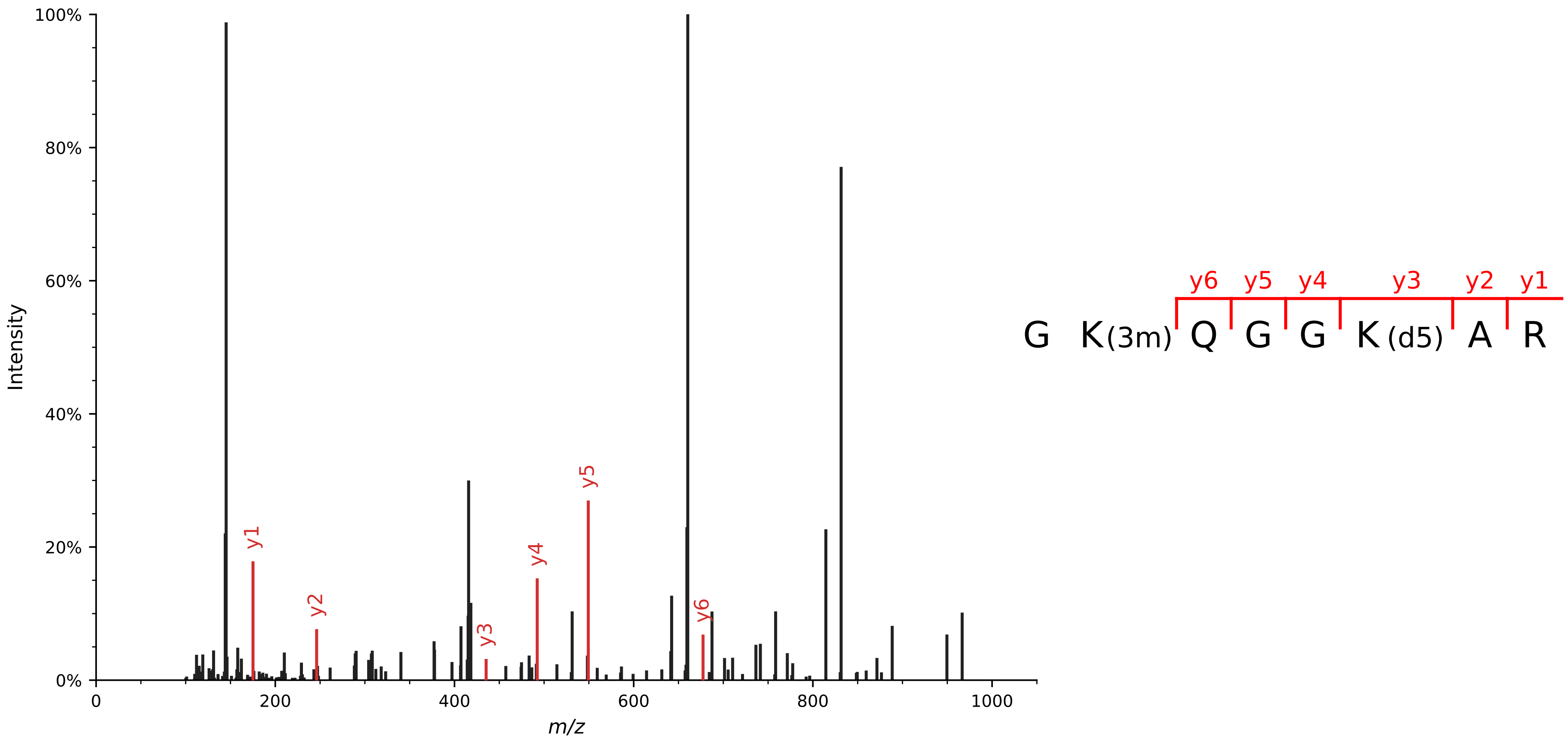

Raw file: HF240124_RN_spleen_D5PRO_D5PRO_1, Scan: 3182, m/z: 469.79555, Charge: 2, RT (min): 7.12, Score: 49.442

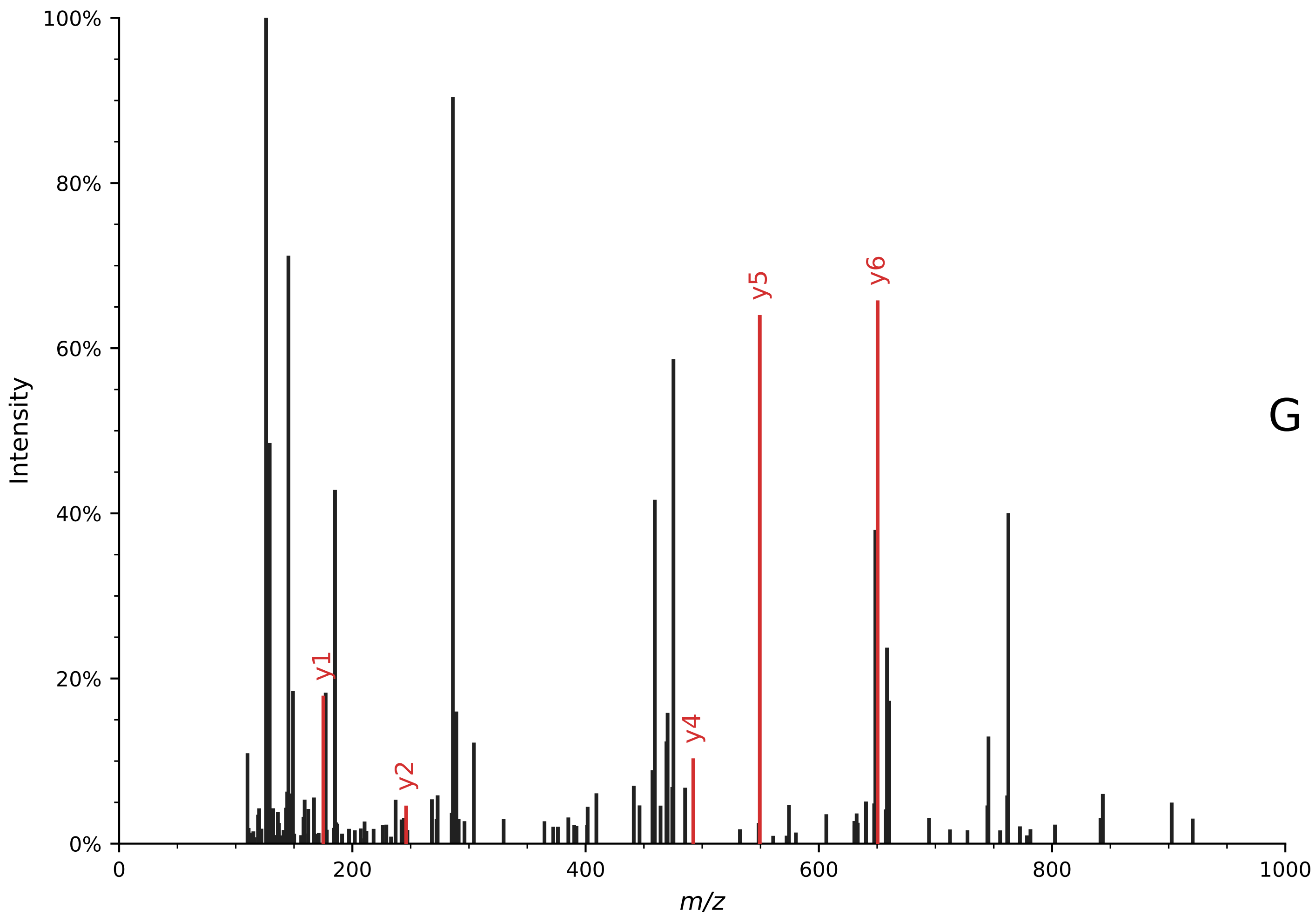

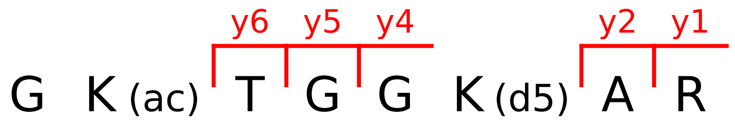

Raw file: HF240124_RN_spleen_D5PRO_D5PRO_2, Scan: 16021, m/z: 490.81902, Charge: 2, RT (min): 29.83, Score: 40.103

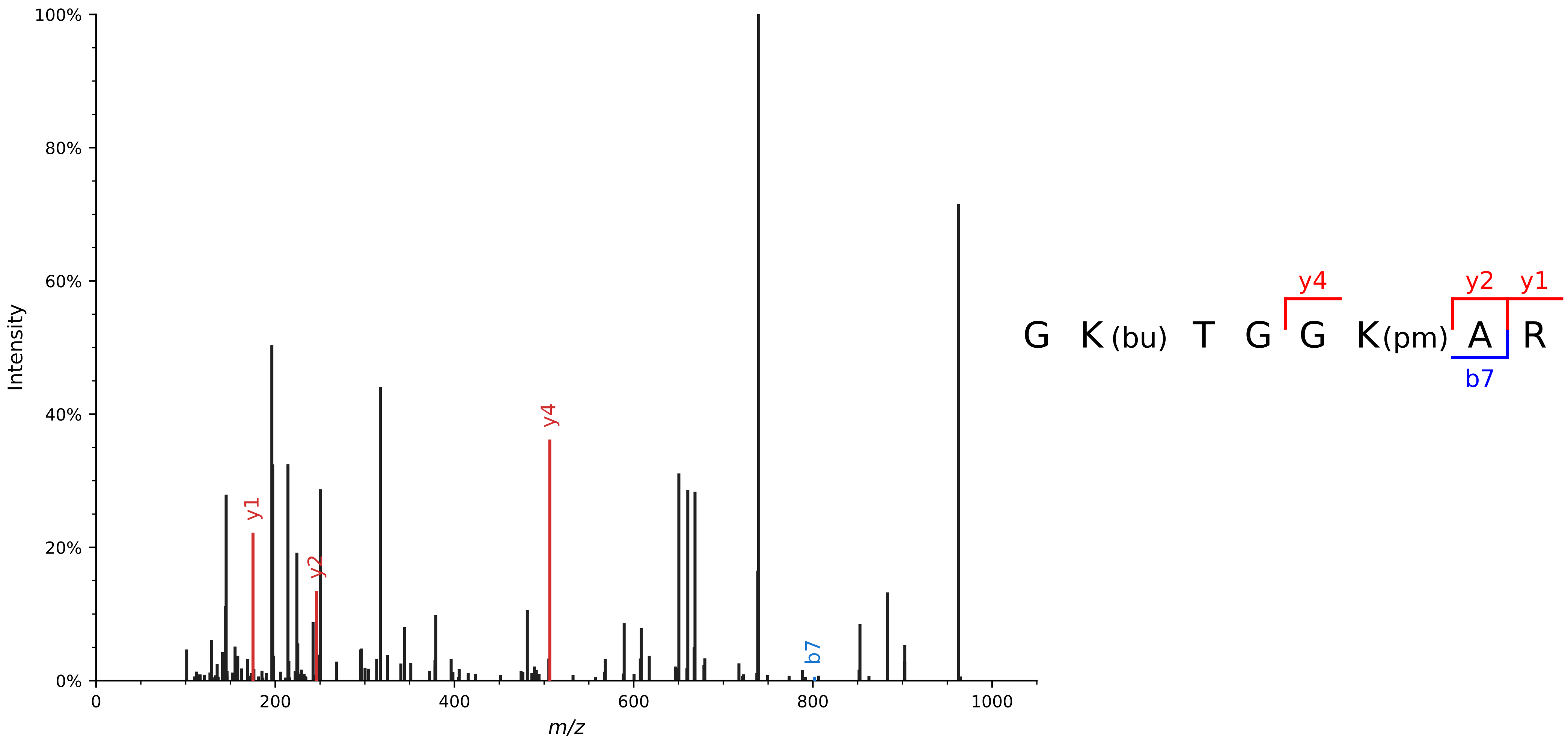

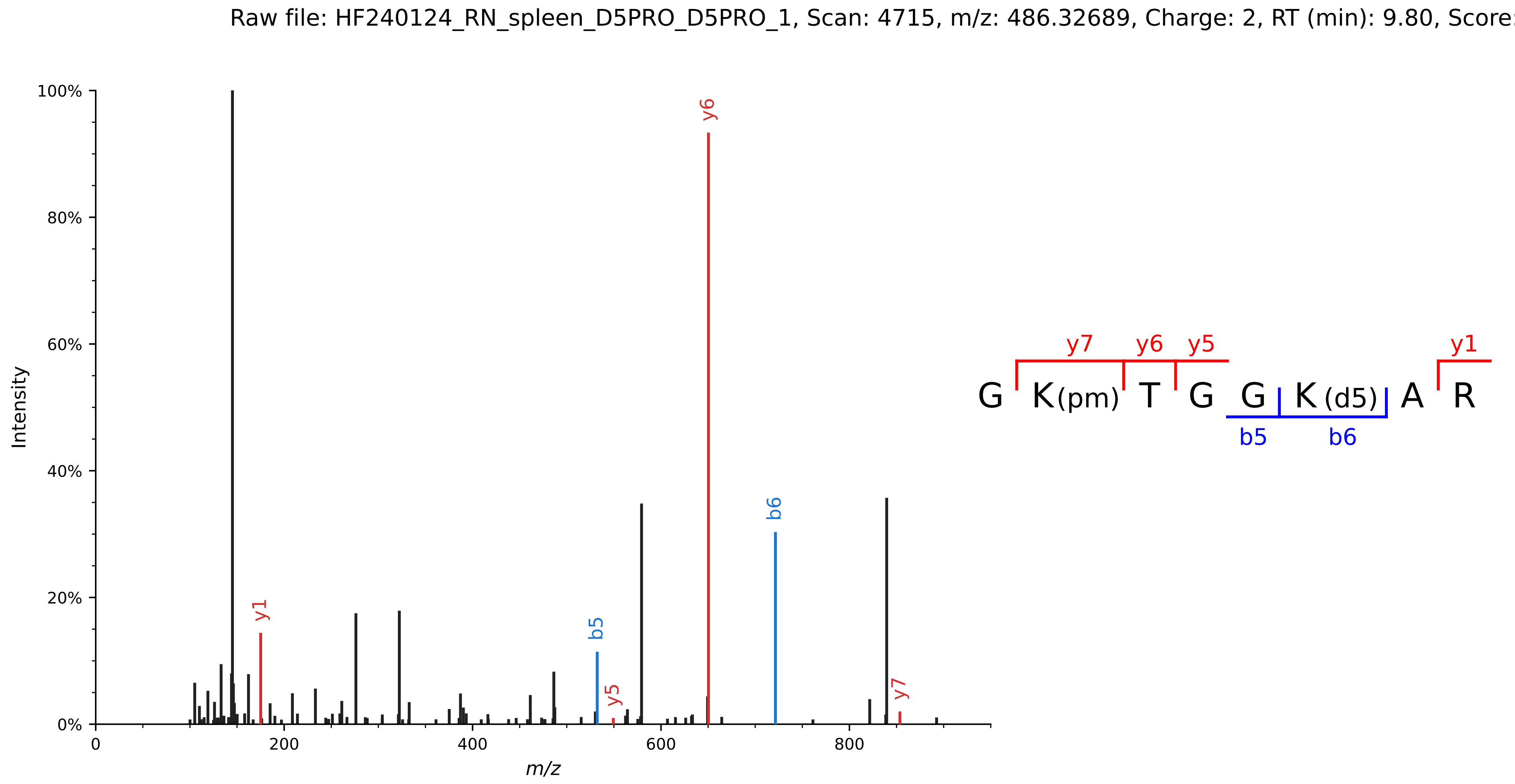

Raw file: HF240124_RN_spleen_D5PRO_D5PRO_1, Scan: 4715, m/z: 486.32689, Charge: 2, RT (min): 9.80, Score: 45.718
Intensity
100%
80%
60%
40%
20%
0%
0
200
400
600
800
m/z
y1
b5
y5
y6
b6
y7
G K(pm) T G G K(d5) A R
y7 y6 y5 y1
b5 b6

Raw file: HF240124_RN_spleen_D5PRO_D5PRO_3, Scan: 7020, m/z: 516.8035, Charge: 2, RT (min): 13.68, Score: 132.8

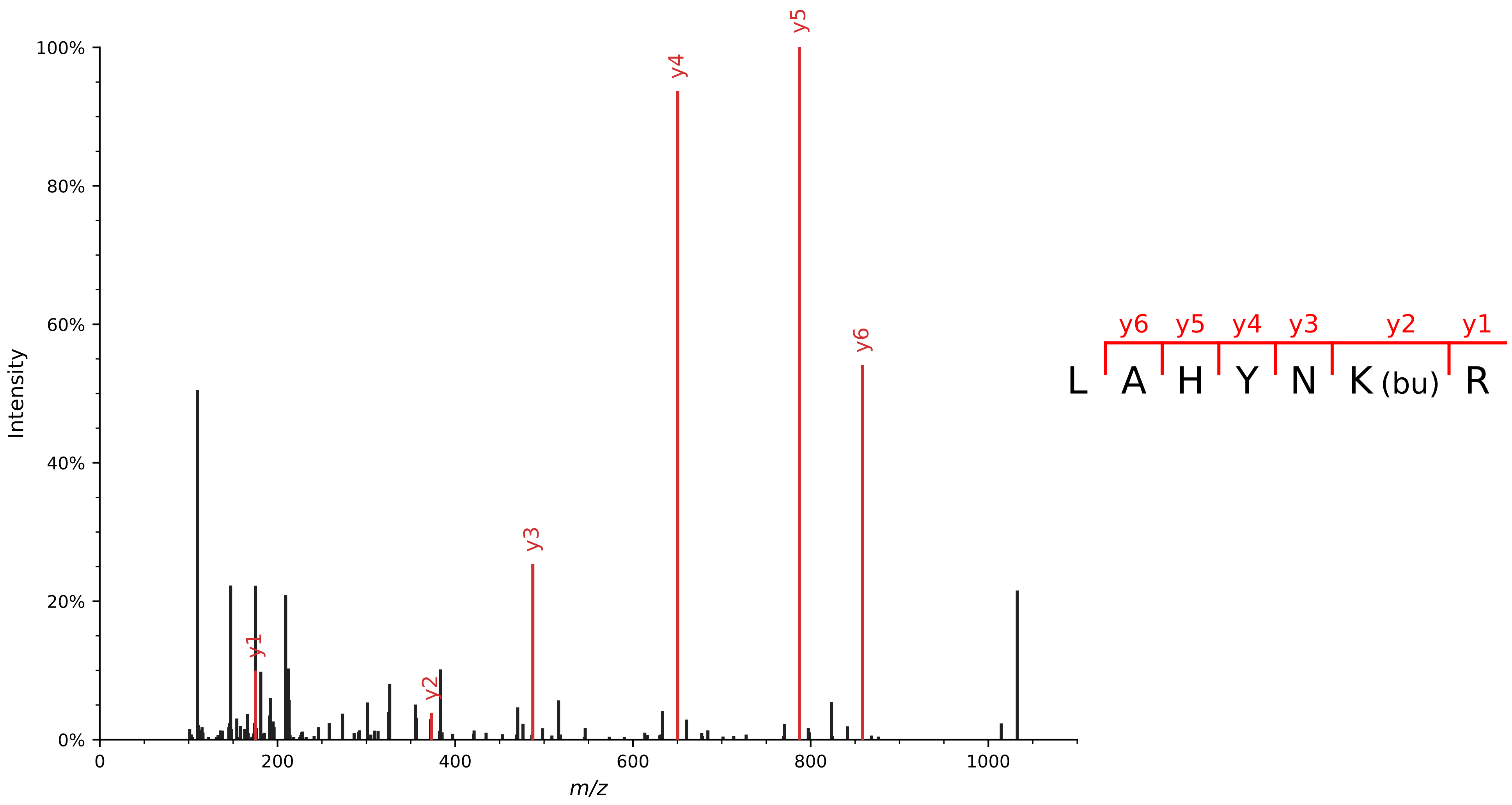

Raw file: HF240124_RN_spleen_D5PRO_D5PRO_3, Scan: 4171, m/z: 495.78003, Charge: 2, RT (min): 8.65, Score: 119.03

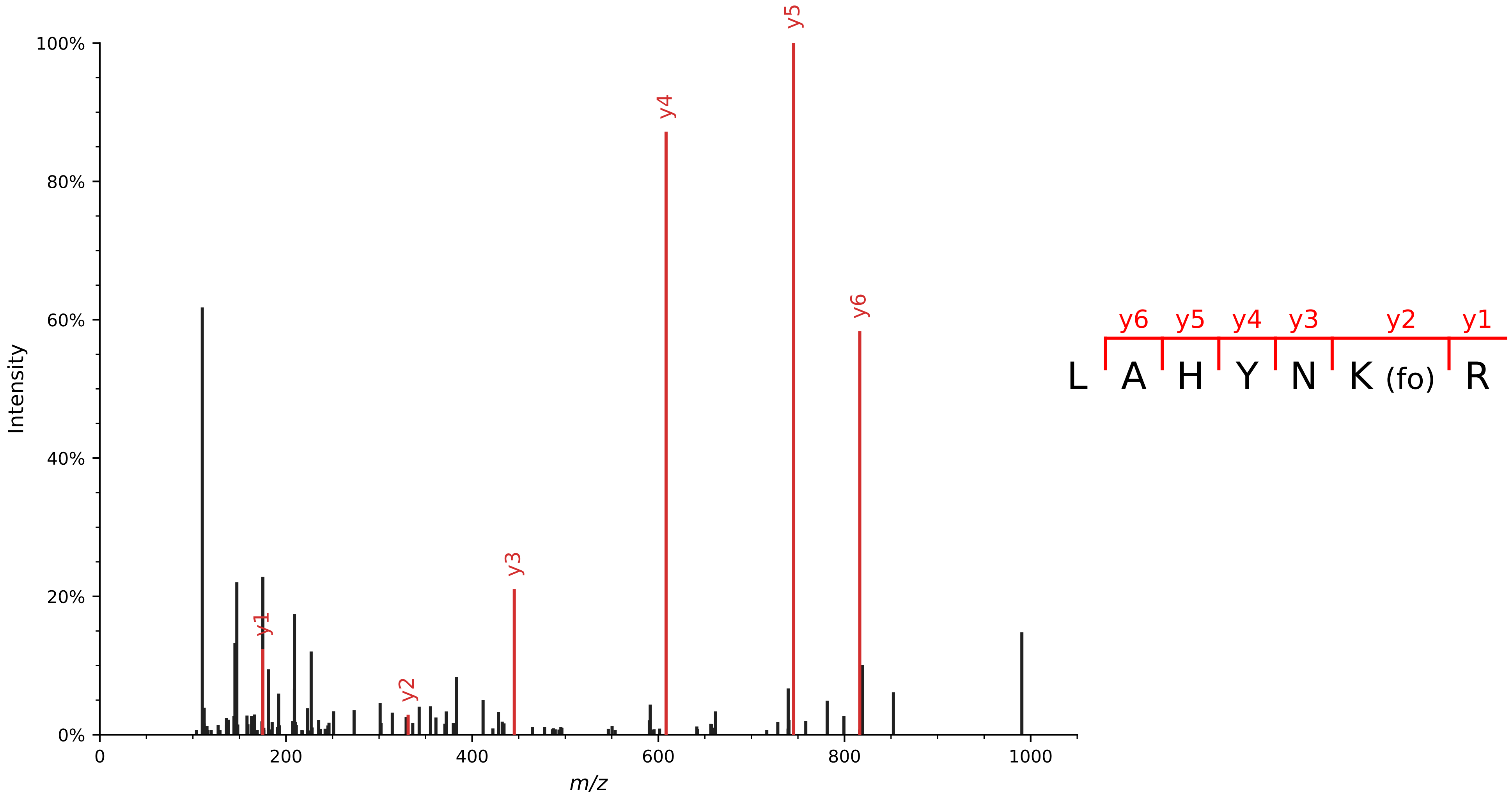

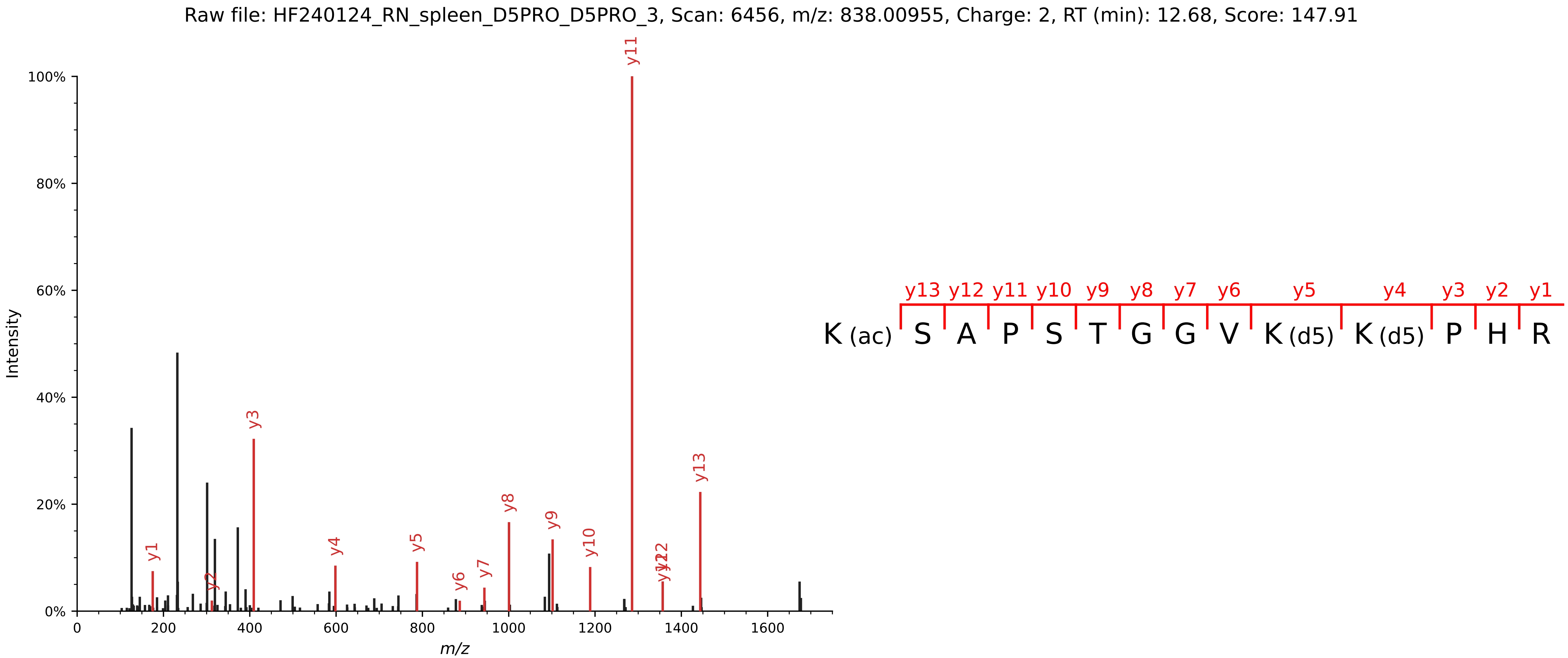

Raw file: HF240124_RN_spleen_D5PRO_D5PRO_3, Scan: 6456, m/z: 838.00955, Charge: 2, RT (min): 12.68, Score: 147.91
Intensity
m/z
y1
y2
y3
y4
y5
y6
y7
y8
y9
y10
y11
y12
y13
y13 y12 y11 y10 y9 y8 y7 y6 y5 y4 y3 y2 y1
K (ac) S A P S T G G V K (d5) K (d5) P H R

Raw file: HF240124_RN_spleen_D5PRO_D5PRO_1, Scan: 7599, m/z: 845.01738, Charge: 2, RT (min): 14.86, Score: 143.97

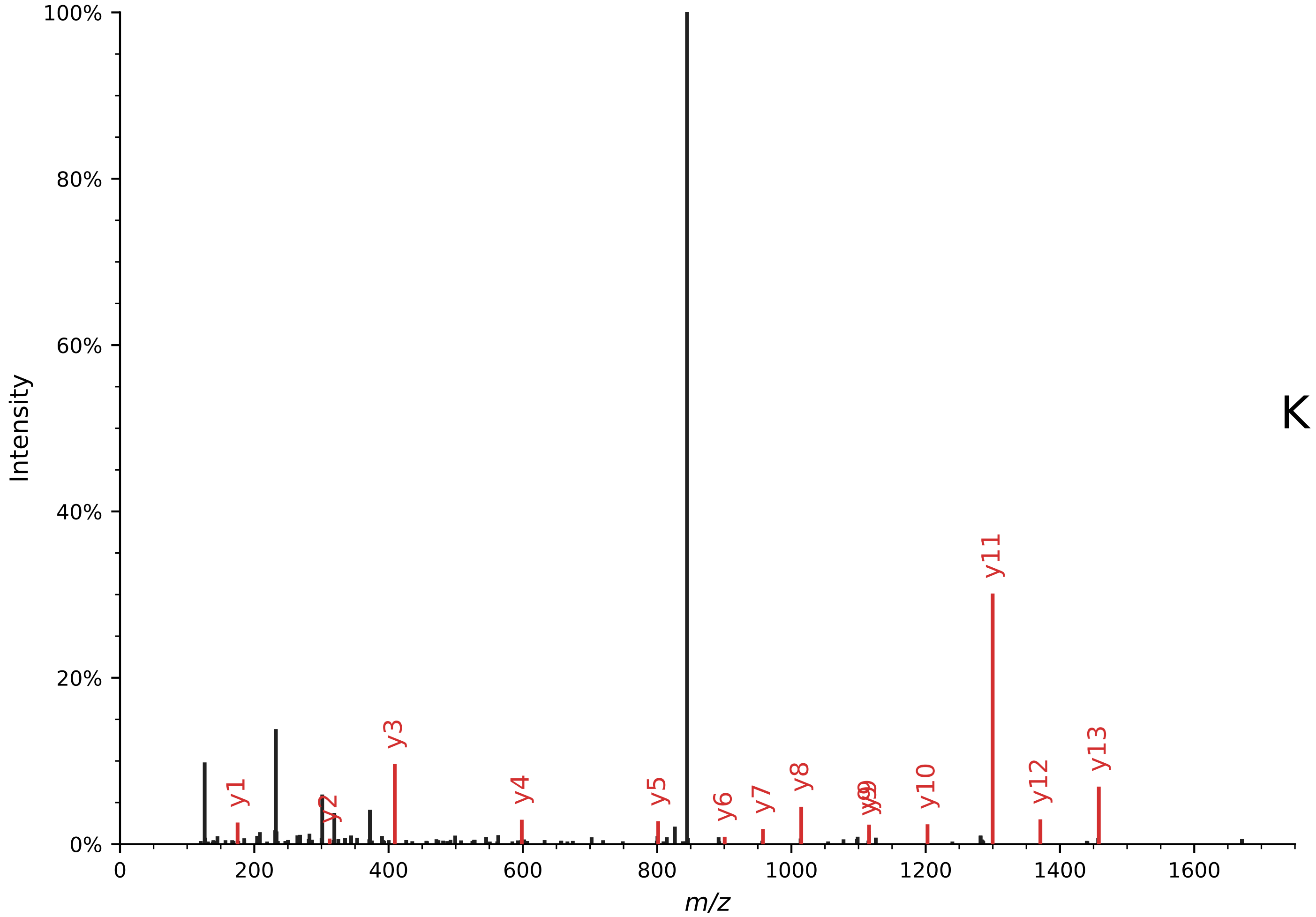

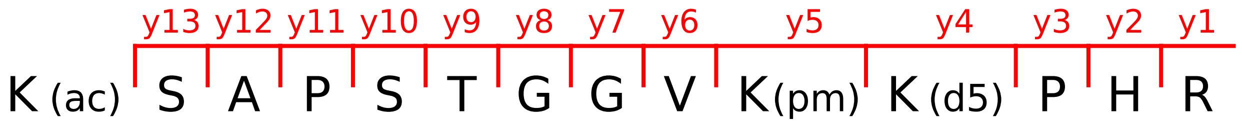

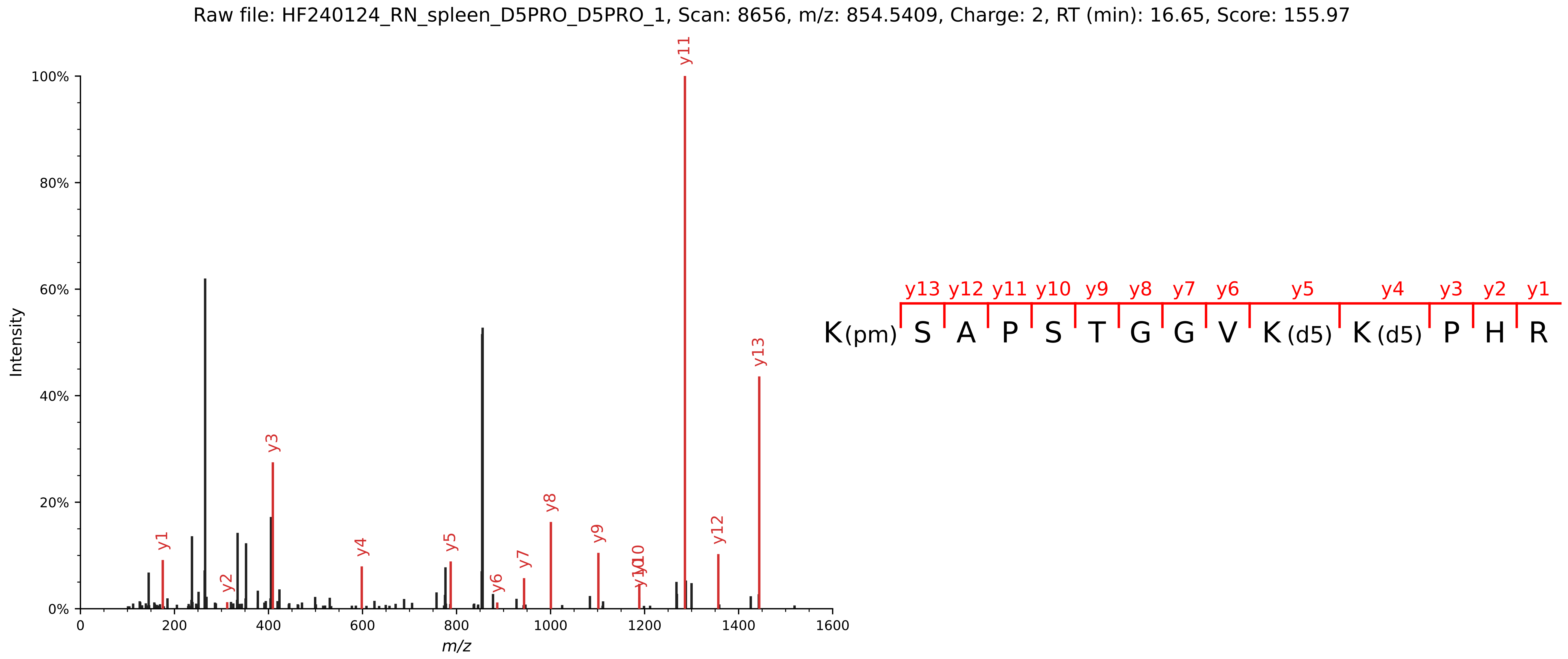

Raw file: HF240124_RN_spleen_D5PRO_D5PRO_1, Scan: 8656, m/z: 854.5409, Charge: 2, RT (min): 16.65, Score: 155.97
Intensity
m/z
y1 y2 y3 y4 y5 y6 y7 y8 y9 y10 y11 y12 y13
y13 y12 y11 y10 y9 y8 y7 y6 y5 y4 y3 y2 y1
K(pm) S A P S T G G V K(d5) K(d5) P H R

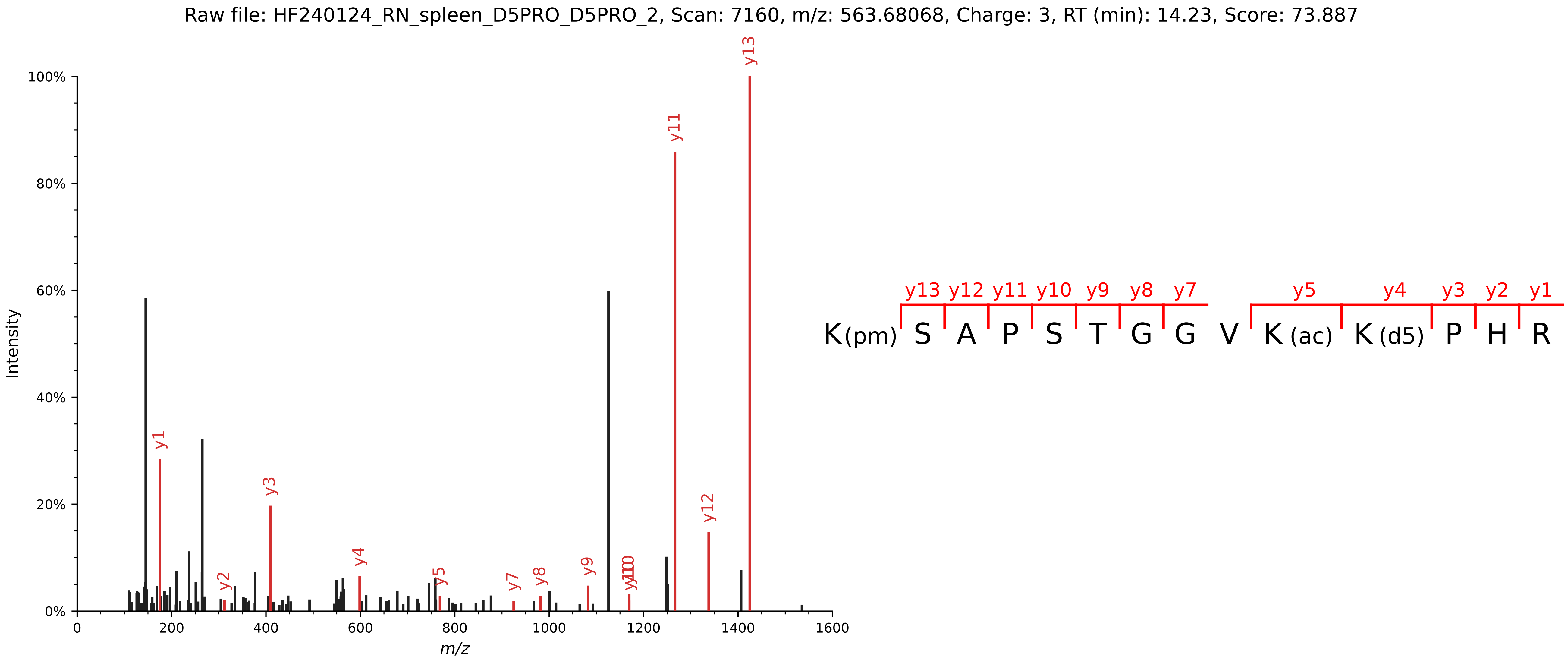

Raw file: HF240124_RN_spleen_D5PRO_D5PRO_2, Scan: 7160, m/z: 563.68068, Charge: 3, RT (min): 14.23, Score: 73.887
Intensity
m/z
y1
y2
y3
y4
y5
y7
y8
y9
y10
y11
y12
y13
K(pm) S A P S T G G V K(ac) K(d5) P H R

Raw file: HF240124_RN_spleen_D5PRO_D5PRO_2, Scan: 9479, m/z: 574.70157, Charge: 3, RT (min): 18.17, Score: 93.348

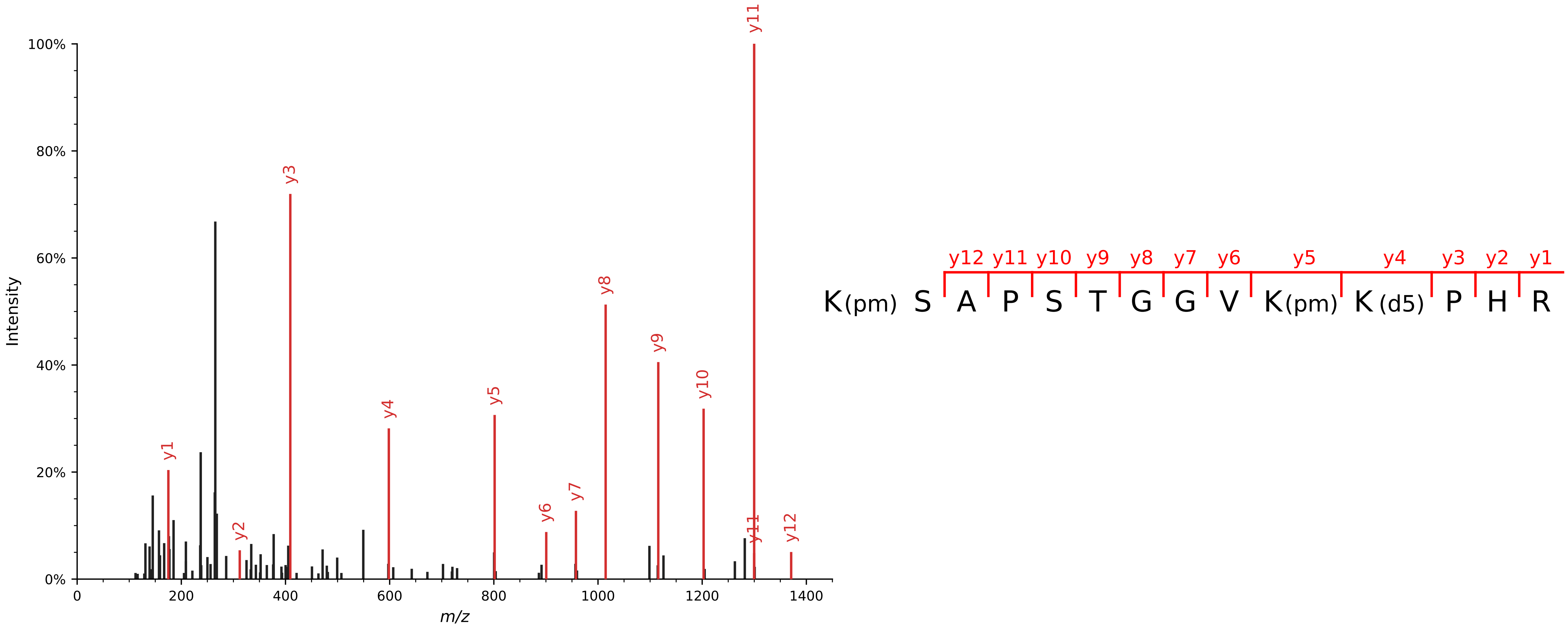

Raw file: HF240124_RN_spleen_D5PRO_D5PRO_3, Scan: 7062, m/z: 579.37346, Charge: 3, RT (min): 13.77, Score: 42.317

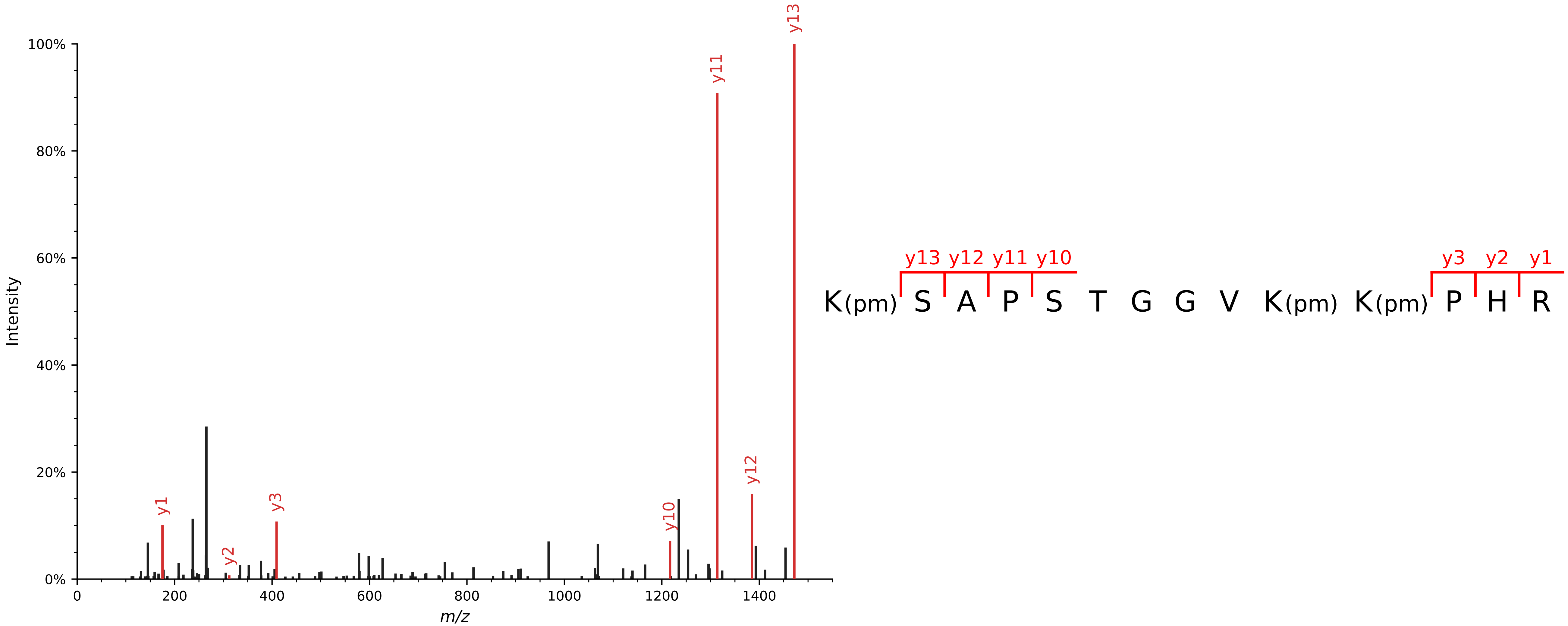

Raw file: HF240124_RN_spleen_D5PRO_D5PRO_1, Scan: 5238, m/z: 838.02775, Charge: 2, RT (min): 10.70, Score: 145.23

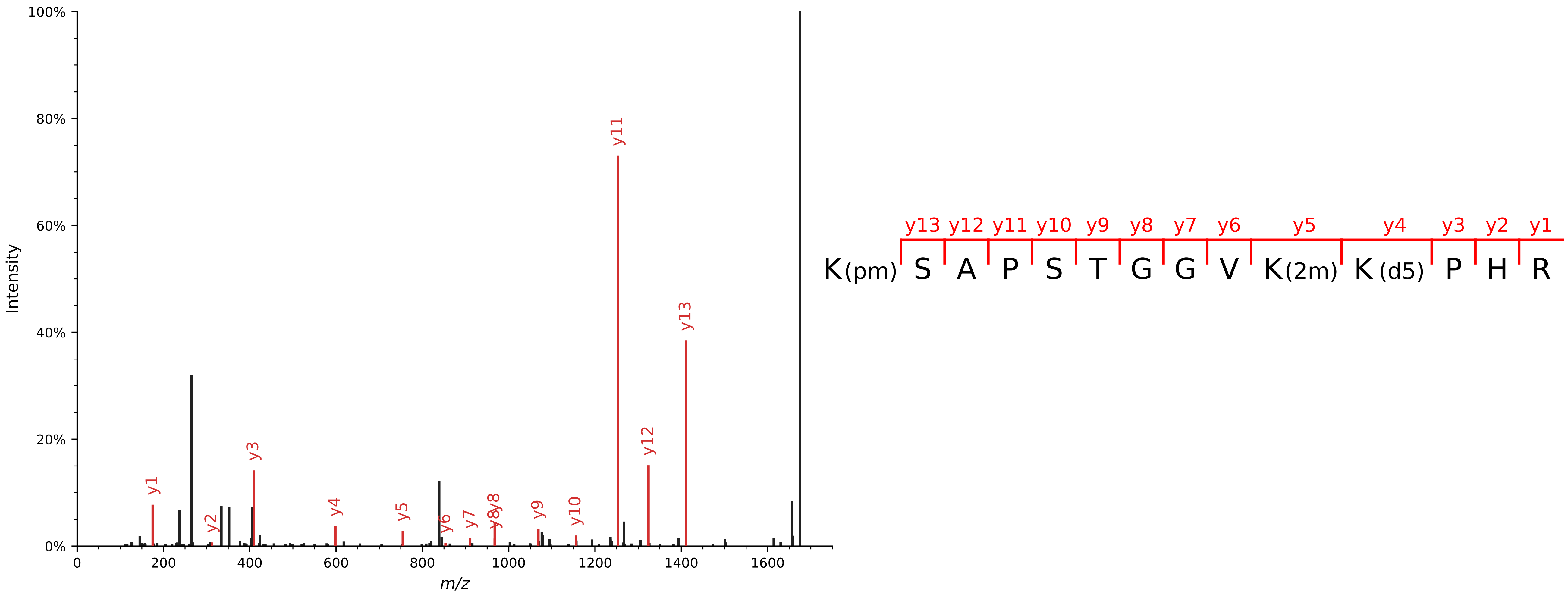

Raw file: HF240124_RN_spleen_D5PRO_D5PRO_2, Scan: 5043, m/z: 845.03557, Charge: 2, RT (min): 10.47, Score: 119.34

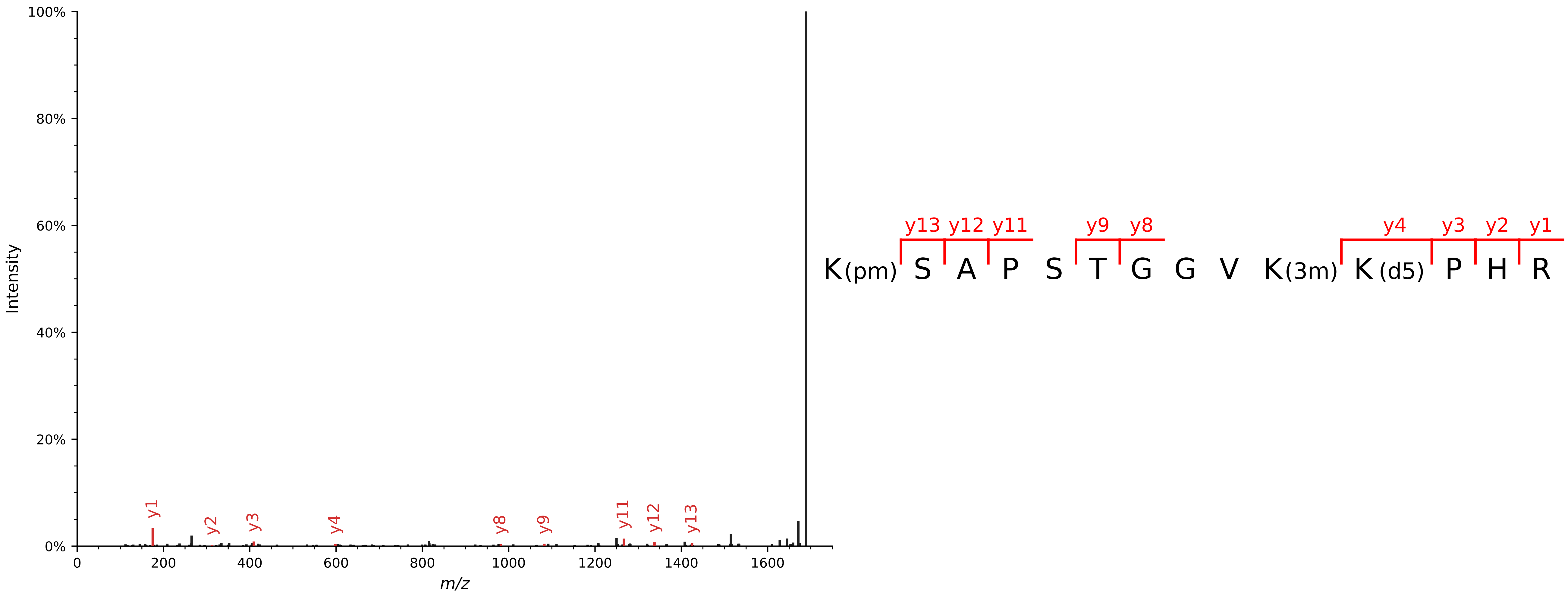

Raw file: HF240124_RN_spleen_D5PRO_D5PRO_1, Scan: 3056, m/z: 831.01992, Charge: 2, RT (min): 6.90, Score: 238.13

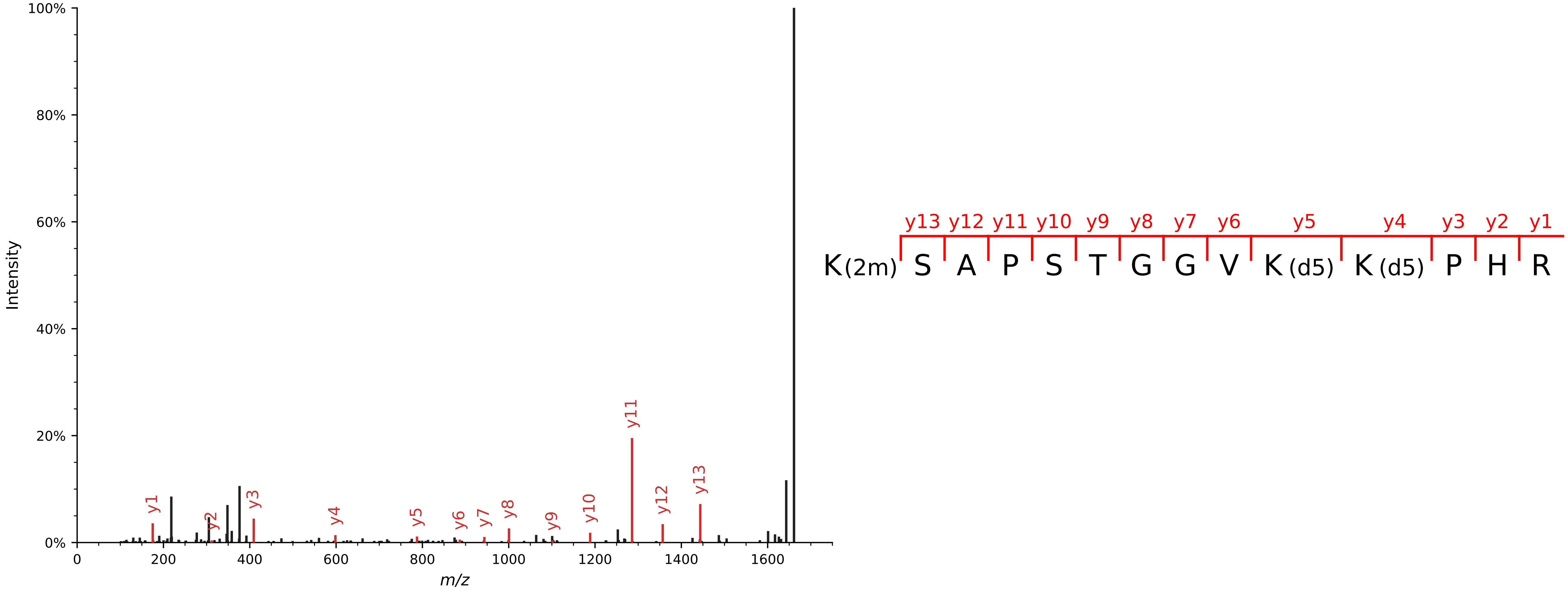

Raw file: HF240124_RN_spleen_D5PRO_D5PRO_1, Scan: 3982, m/z: 838.02775, Charge: 2, RT (min): 8.57, Score: 192.15

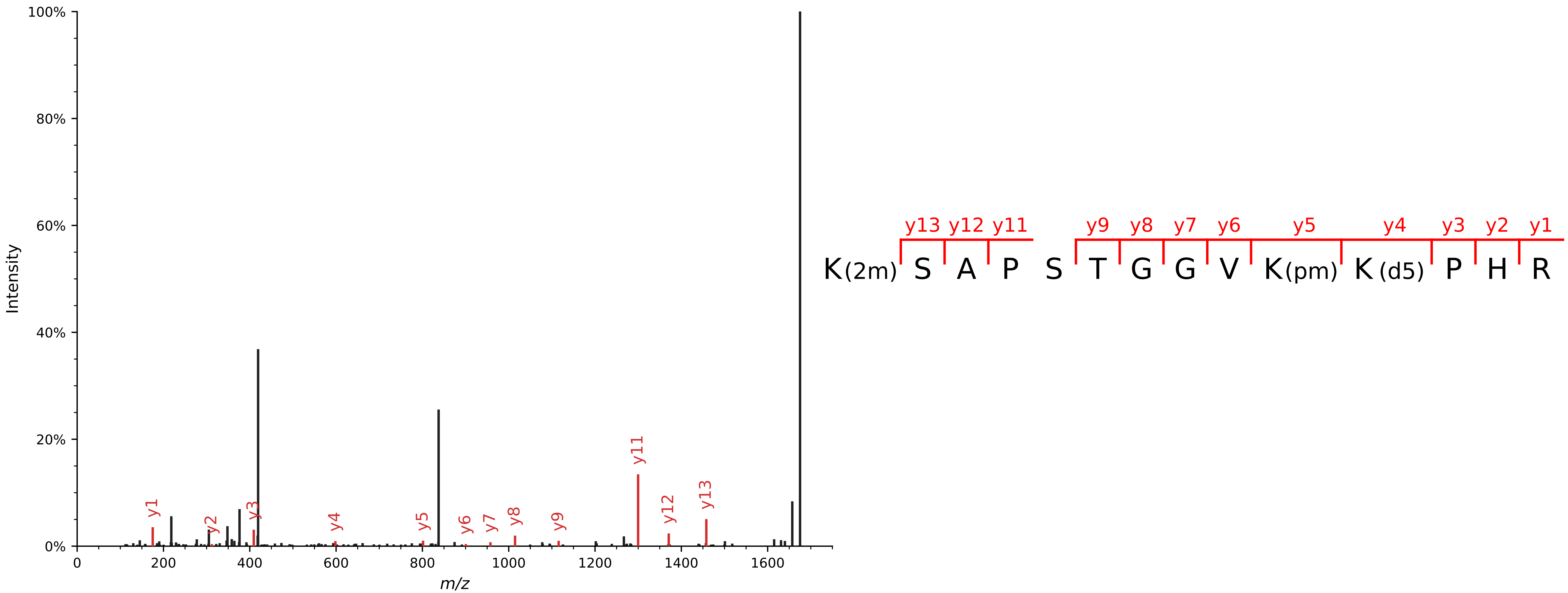

Raw file: HF240124_RN_spleen_D5PRO_D5PRO_2, Scan: 2361, m/z: 563.69281, Charge: 3, RT (min): 5.75, Score: 58.079

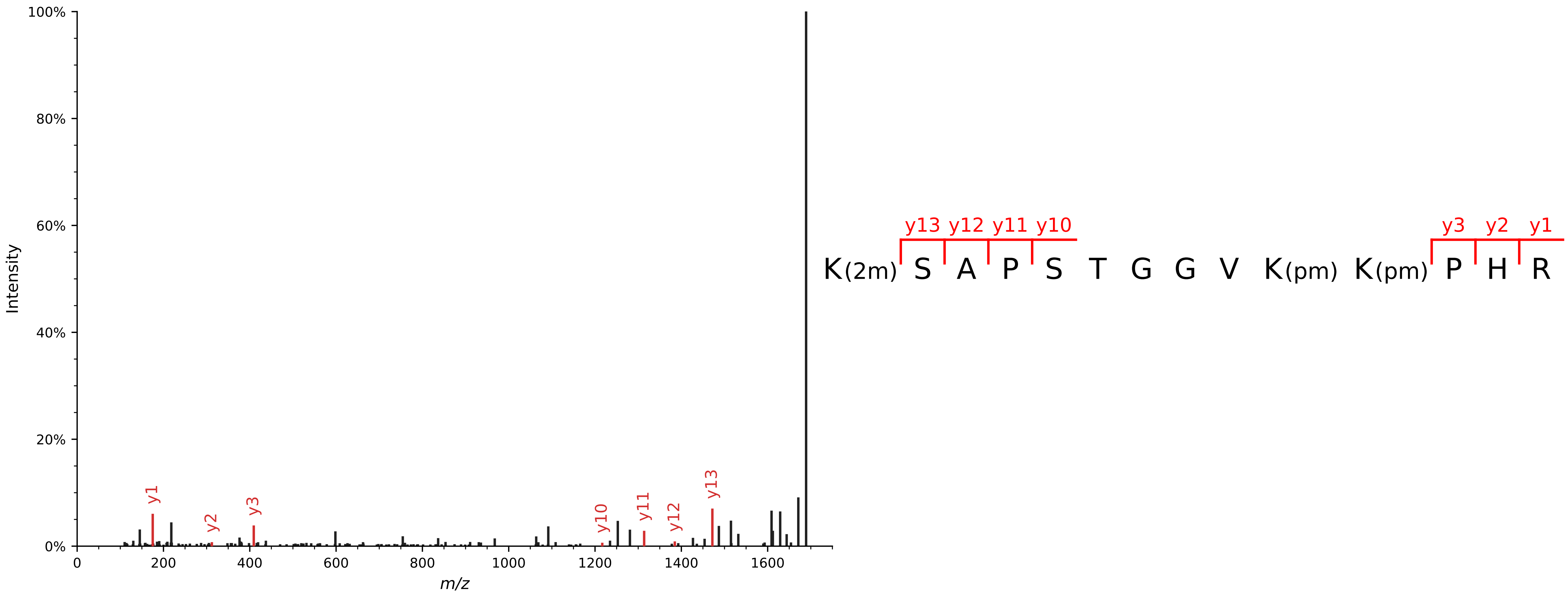

Raw file: HF240124_RN_spleen_D5PRO_D5PRO_1, Scan: 1612, m/z: 543.34027, Charge: 3, RT (min): 4.51, Score: 120.77

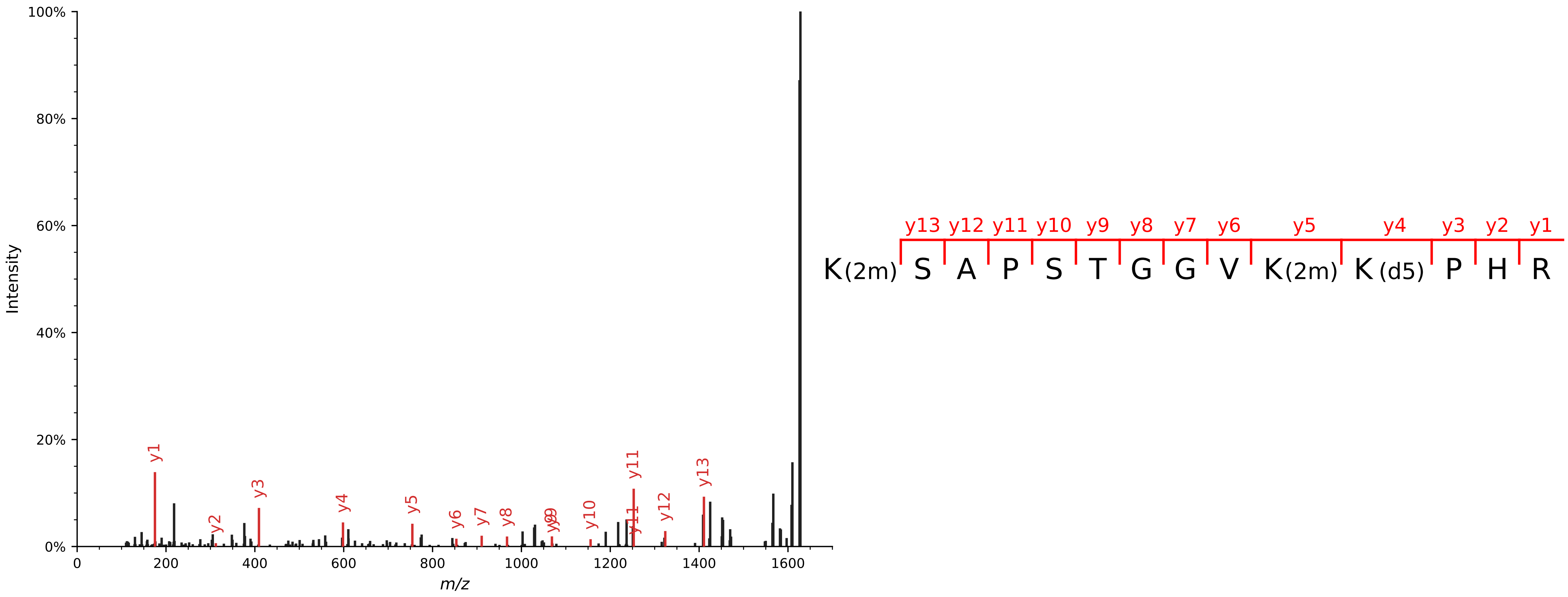

Raw file: HF240124_RN_spleen_D5PRO_D5PRO_2, Scan: 1608, m/z: 821.5146, Charge: 2, RT (min): 4.53, Score: 68.536

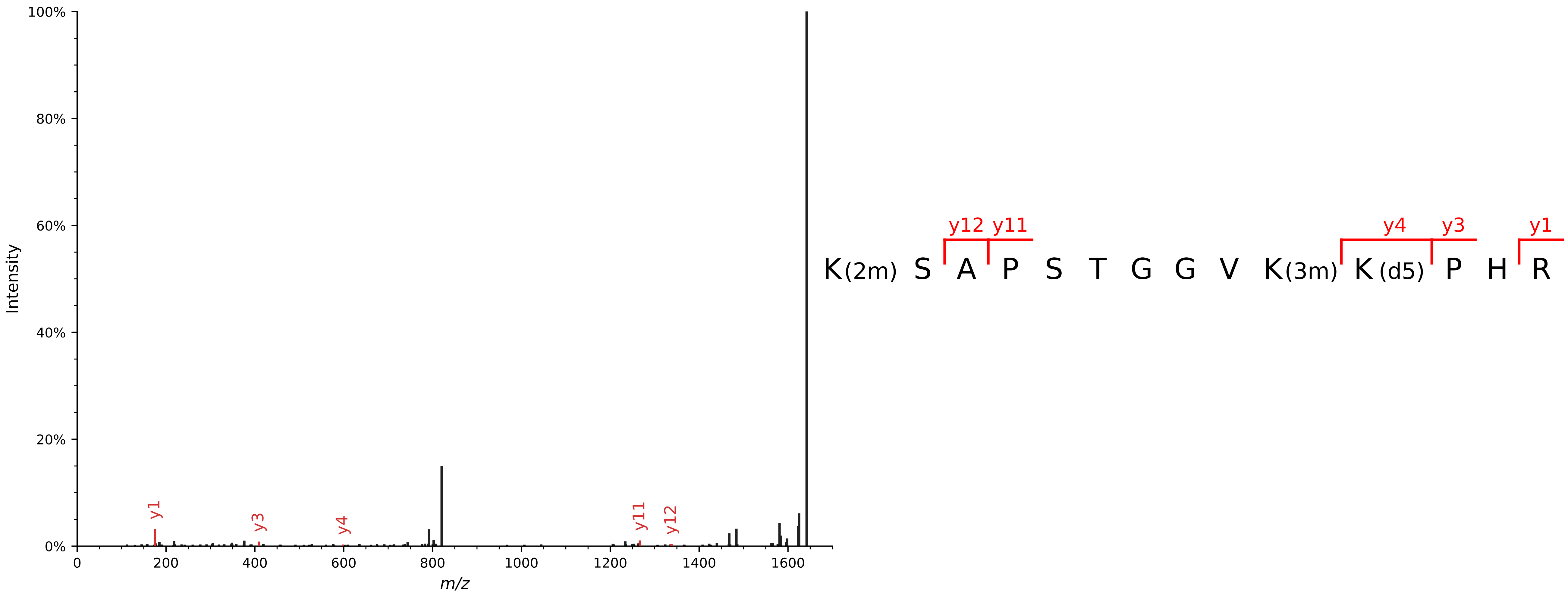

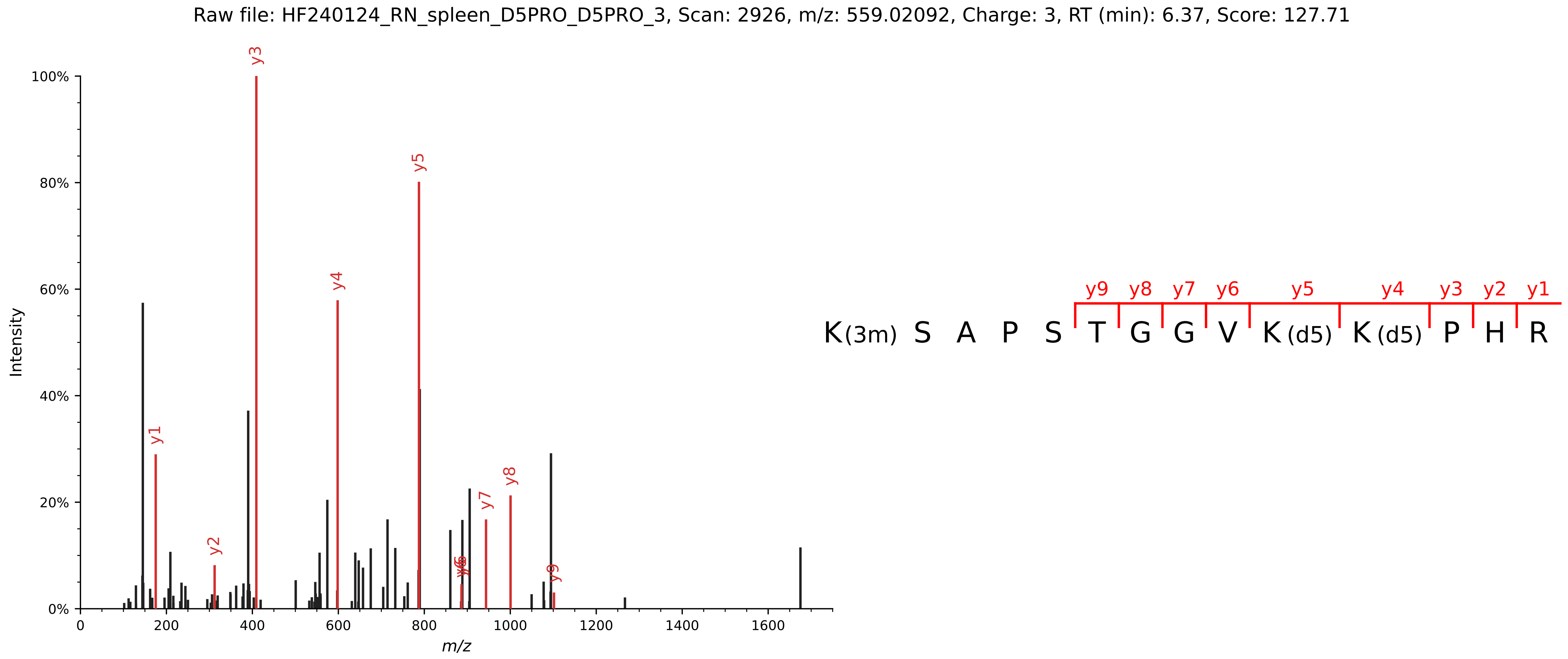

Raw file: HF240124_RN_spleen_D5PRO_D5PRO_3, Scan: 2926, m/z: 559.02092, Charge: 3, RT (min): 6.37, Score: 127.71
Intensity
m/z
y1
y2
y3
y4
y5
y6
y7
y8
y9
K(3m) S A P S T G G V K(d5) K(d5) P H R

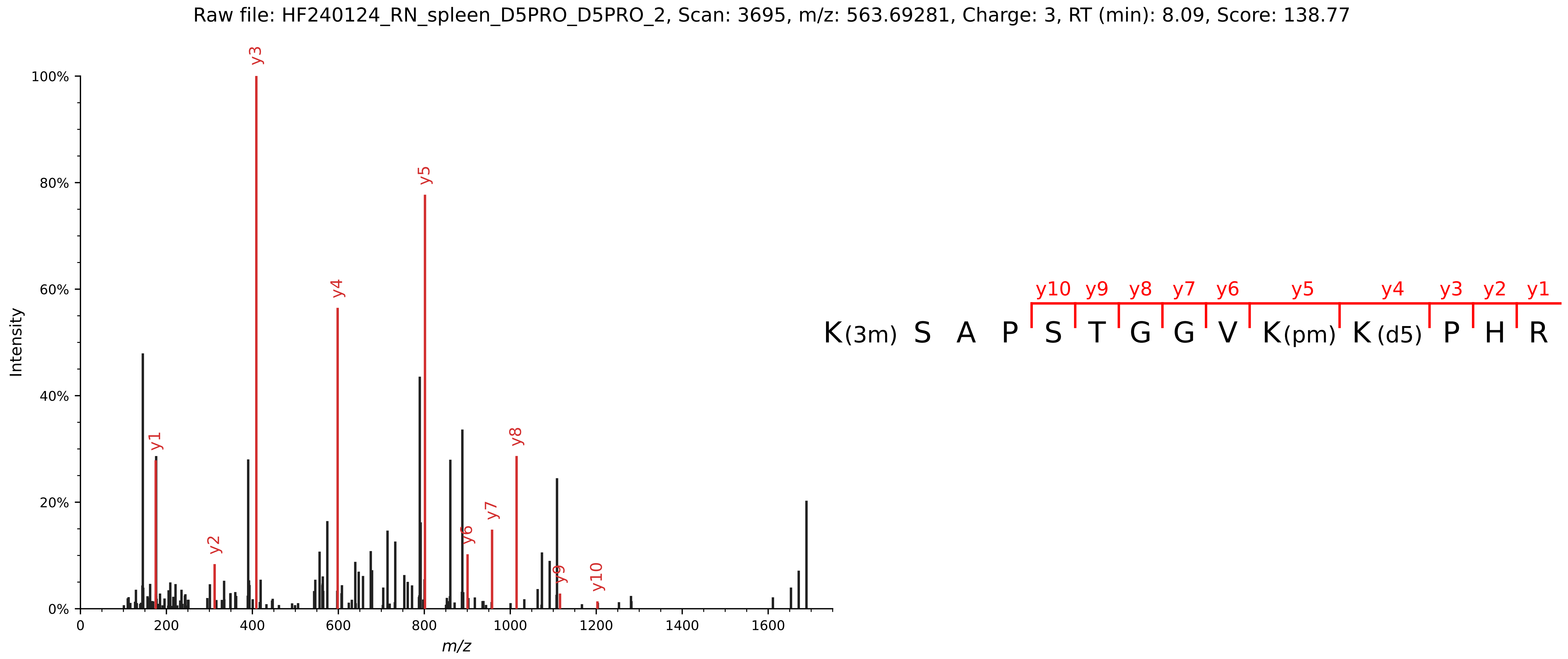

Raw file: HF240124_RN_spleen_D5PRO_D5PRO_2, Scan: 3695, m/z: 563.69281, Charge: 3, RT (min): 8.09, Score: 138.77
Intensity
m/z
y1 y2 y3 y4 y5 y6 y7 y8 y9 y10
K(3m) S A P S T G G V K(pm) K(d5) P H R

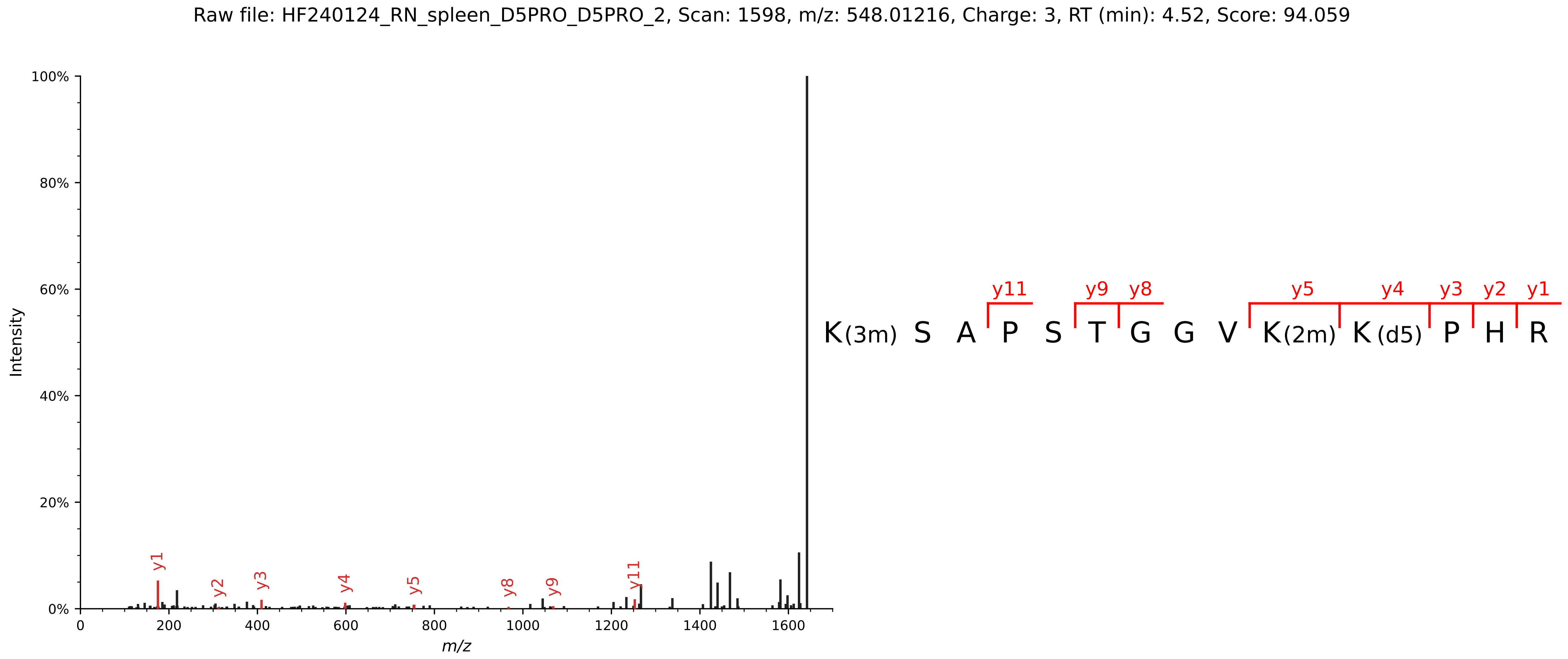
Raw file: HF240124_RN_spleen_D5PRO_D5PRO_2, Scan: 1598, m/z: 548.01216, Charge: 3, RT (min): 4.52, Score: 94.059
Intensity
m/z
K(3m) S A P S T G G V K(2m) K(d5) P H R
y11 y9 y8 y5 y4 y3 y2 y1

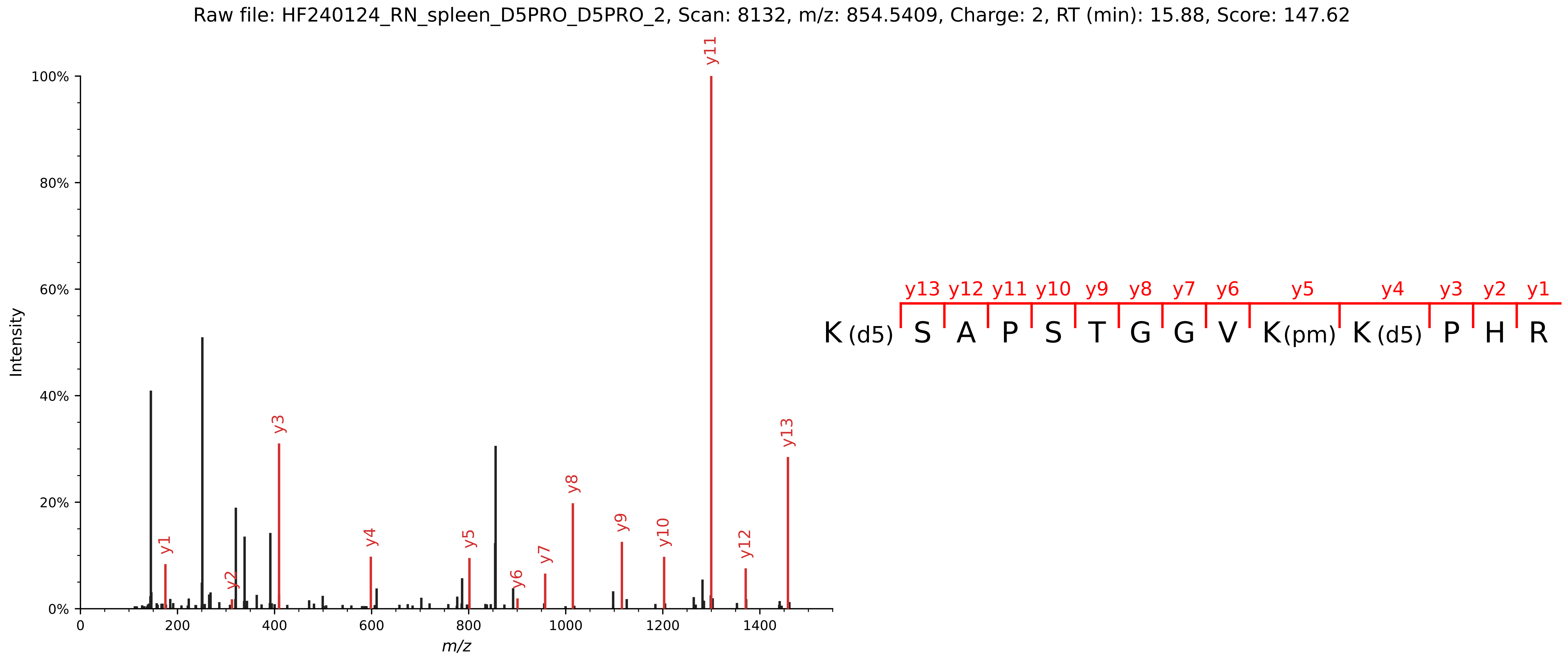

Raw file: HF240124_RN_spleen_D5PRO_D5PRO_2, Scan: 8132, m/z: 854.5409, Charge: 2, RT (min): 15.88, Score: 147.62
Intensity
m/z
y1
y2
y3
y4
y5
y6
y7
y8
y9
y10
y11
y12
y13
y13 y12 y11 y10 y9 y8 y7 y6 y5 y4 y3 y2 y1
K(d5) S A P S T G G V K(pm) K(d5) P H R

Raw file: HF240124_RN_spleen_D5PRO_D5PRO_3, Scan: 6022, m/z: 574.70157, Charge: 3, RT (min): 11.90, Score: 57.434

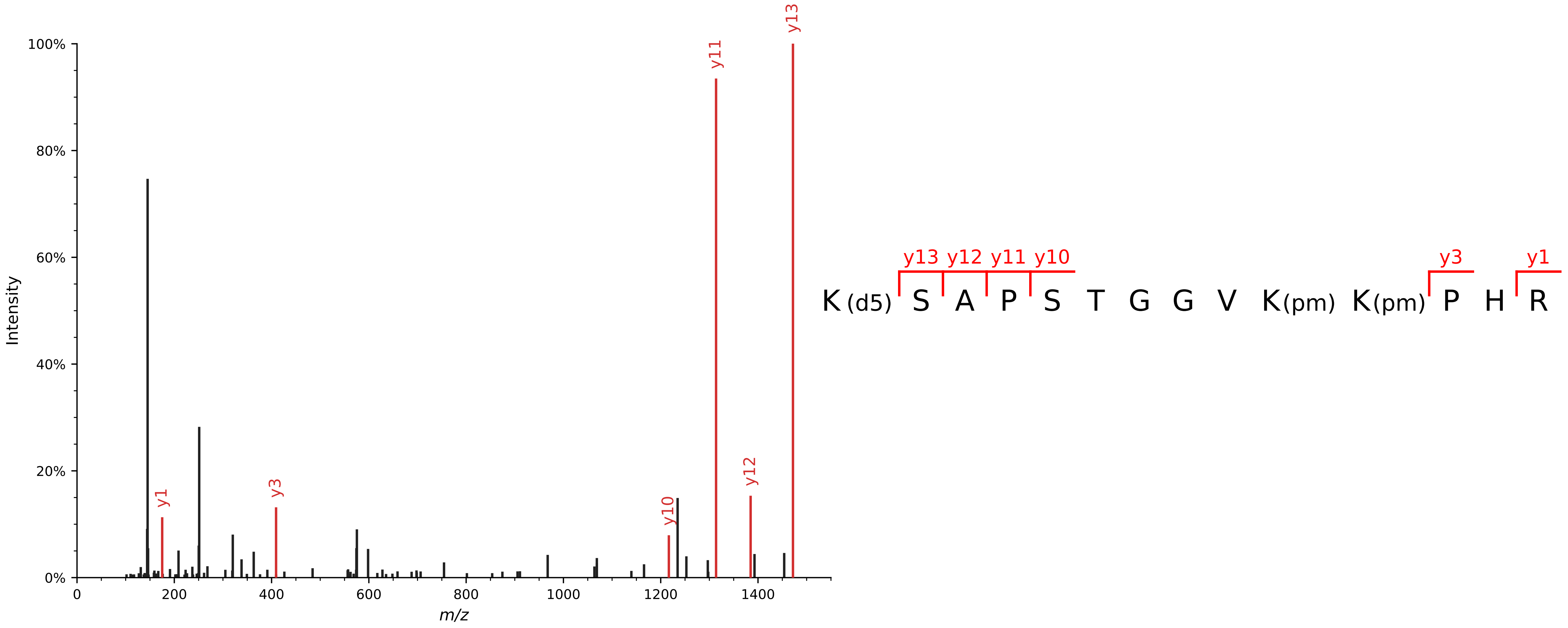

Raw file: HF240124_RN_spleen_D5PRO_D5PRO_3, Scan: 3860, m/z: 831.01992, Charge: 2, RT (min): 8.05, Score: 132.03

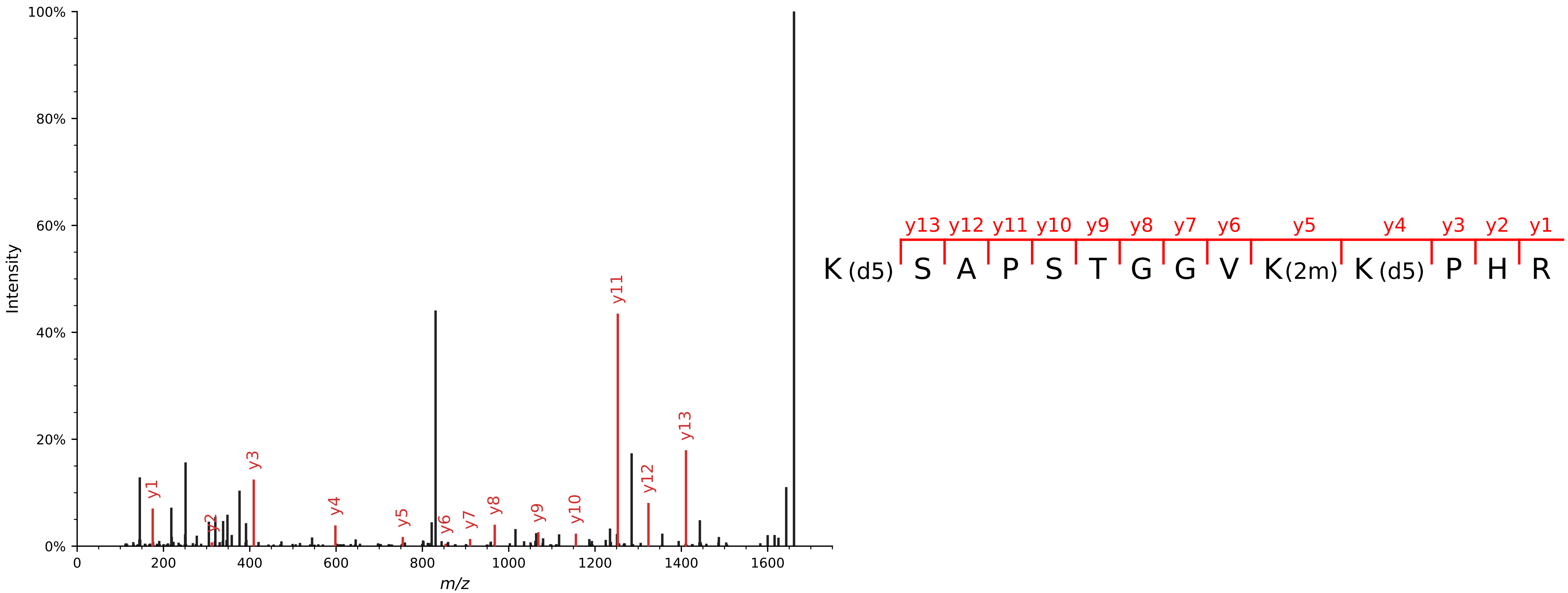

Raw file: HF240124_RN_spleen_D5PRO_D5PRO_3, Scan: 4004, m/z: 420.77154, Charge: 2, RT (min): 8.35, Score: 99.511

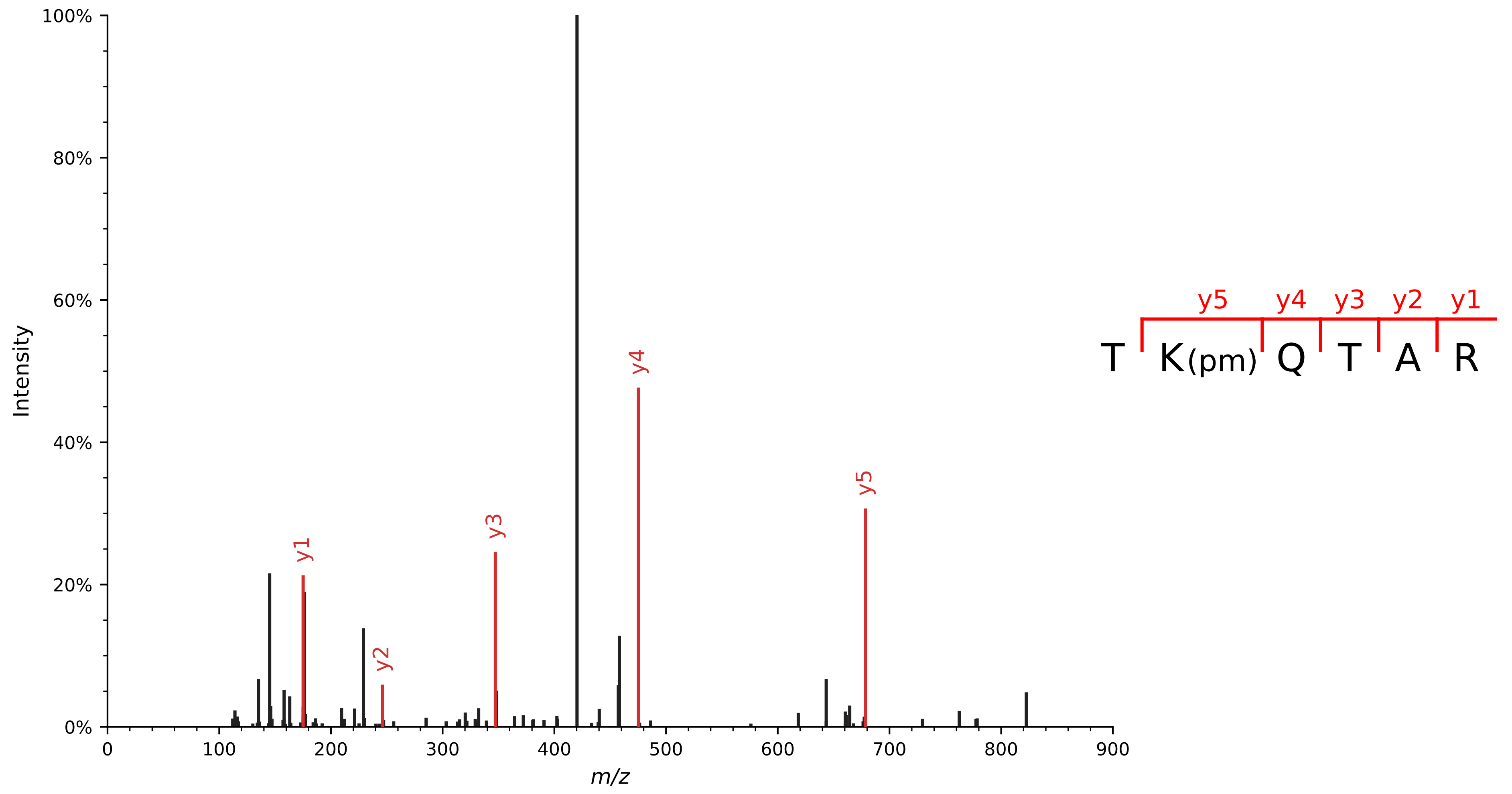

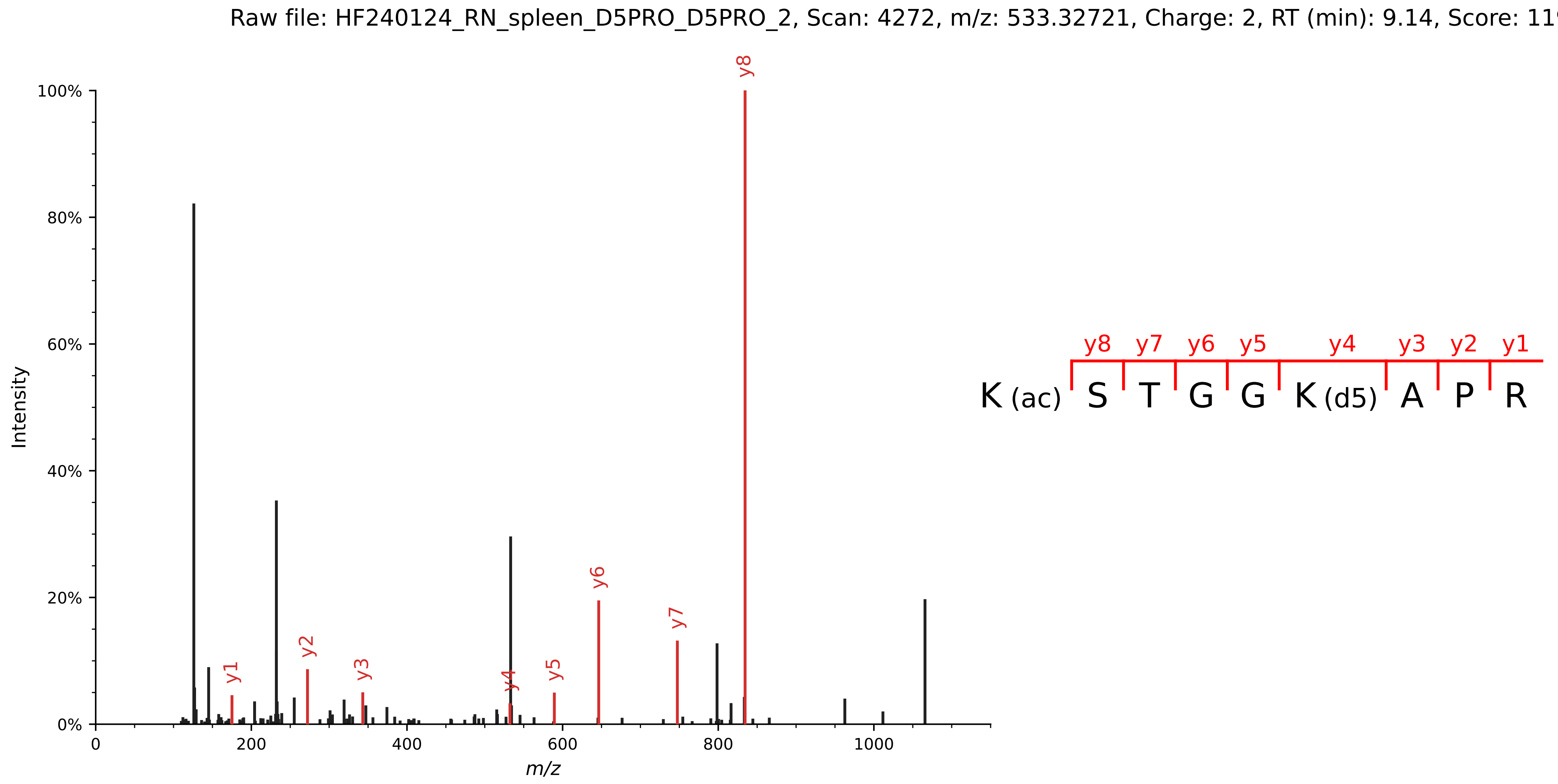

Raw file: HF240124_RN_spleen_D5PRO_D5PRO_2, Scan: 4272, m/z: 533.32721, Charge: 2, RT (min): 9.14, Score: 119.41
Intensity
m/z
y1
y2
y3
y4
y5
y6
y7
y8
K (ac) S T G G K (d5) A P R

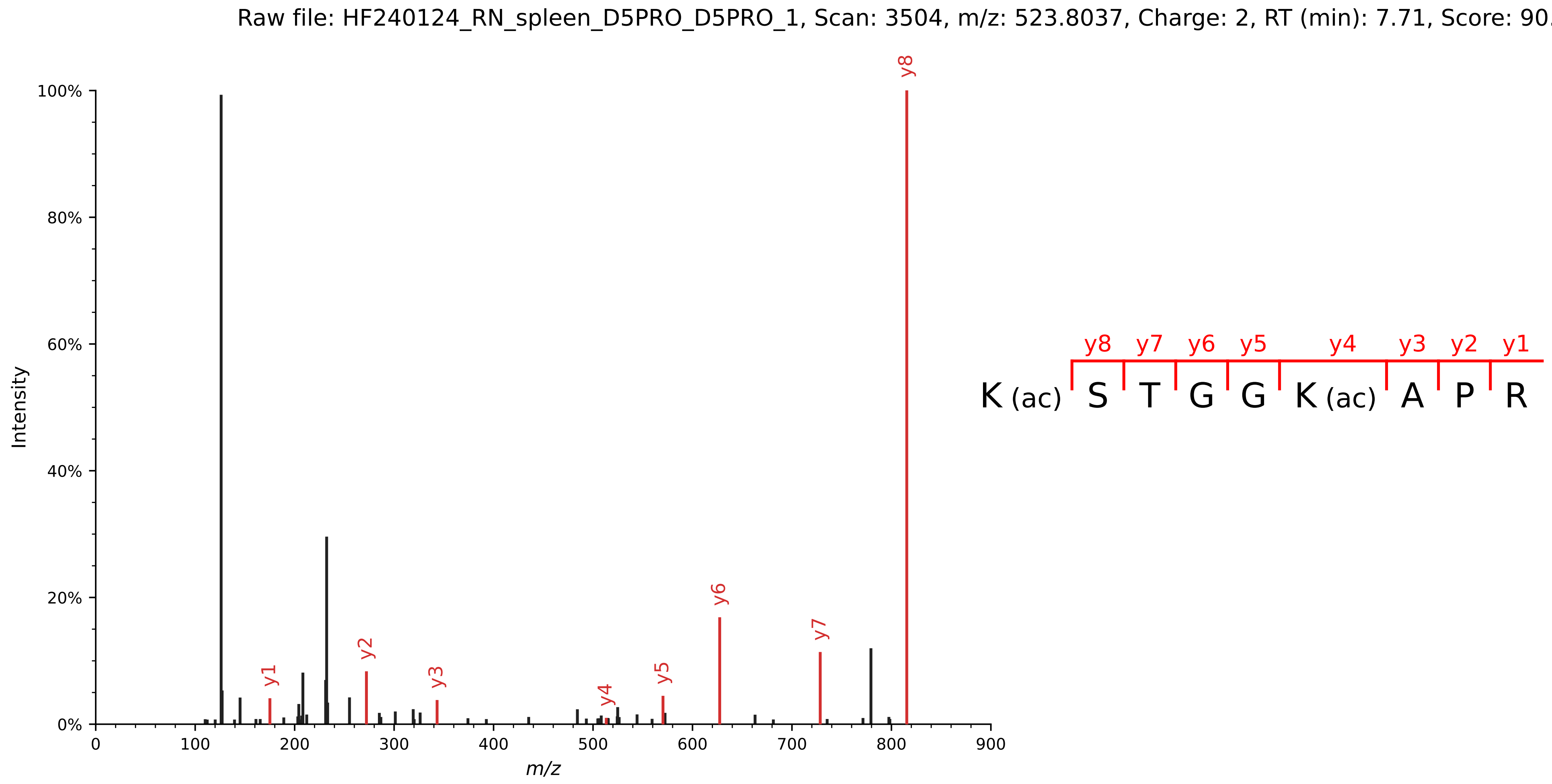

Raw file: HF240124_RN_spleen_D5PRO_D5PRO_1, Scan: 3504, m/z: 523.8037, Charge: 2, RT (min): 7.71, Score: 90.709
Intensity
m/z
0%
20%
40%
60%
80%
100%
0
100
200
300
400
500
600
700
800
900
y1
y2
y3
y4
y5
y6
y7
y8
K (ac) S T G G K (ac) A P R

Raw file: HF240124_RN_spleen_D5PRO_D5PRO_1, Scan: 3965, m/z: 526.31939, Charge: 2, RT (min): 8.54, Score: 98.523

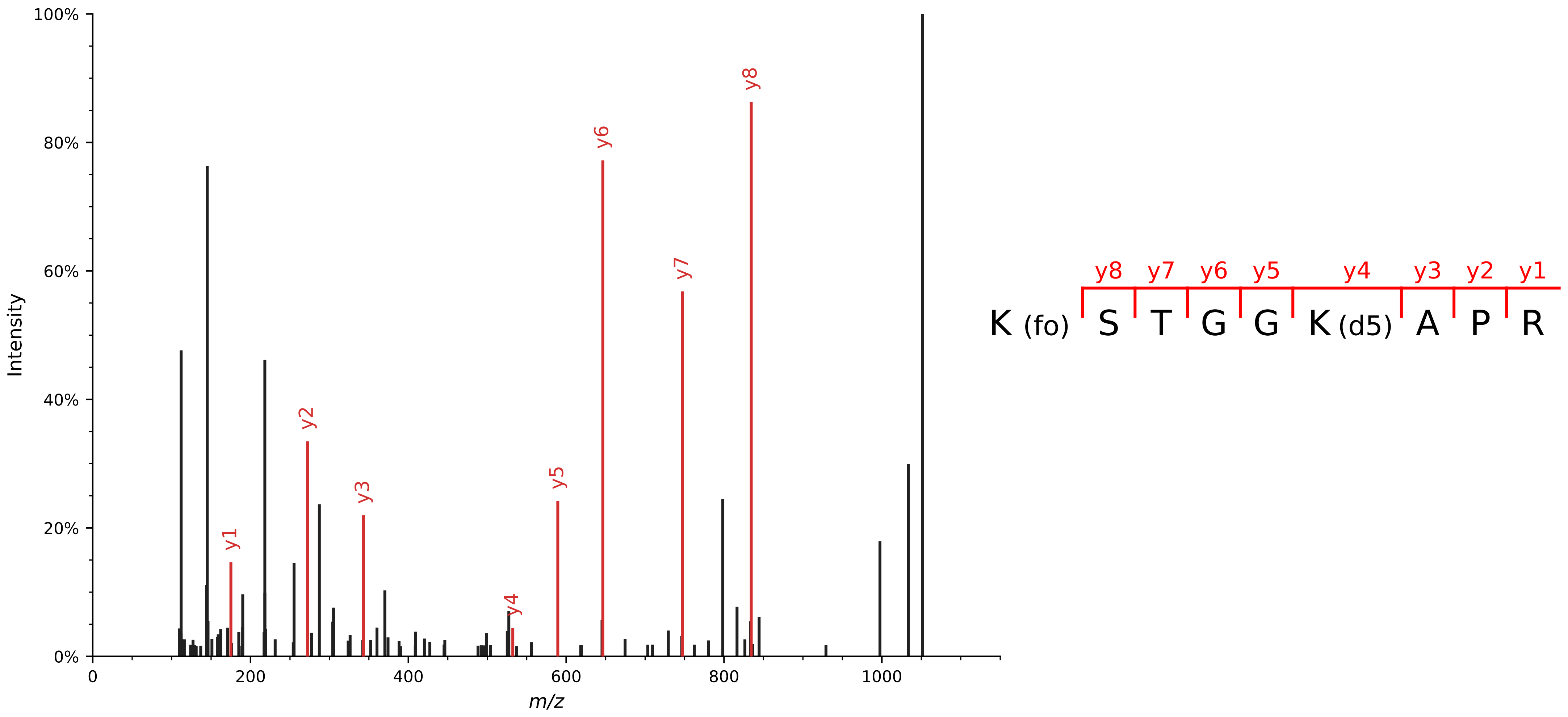

Raw file: HF240124_RN_spleen_D5PRO_D5PRO_3, Scan: 7654, m/z: 549.85856, Charge: 2, RT (min): 14.79, Score: 110.81

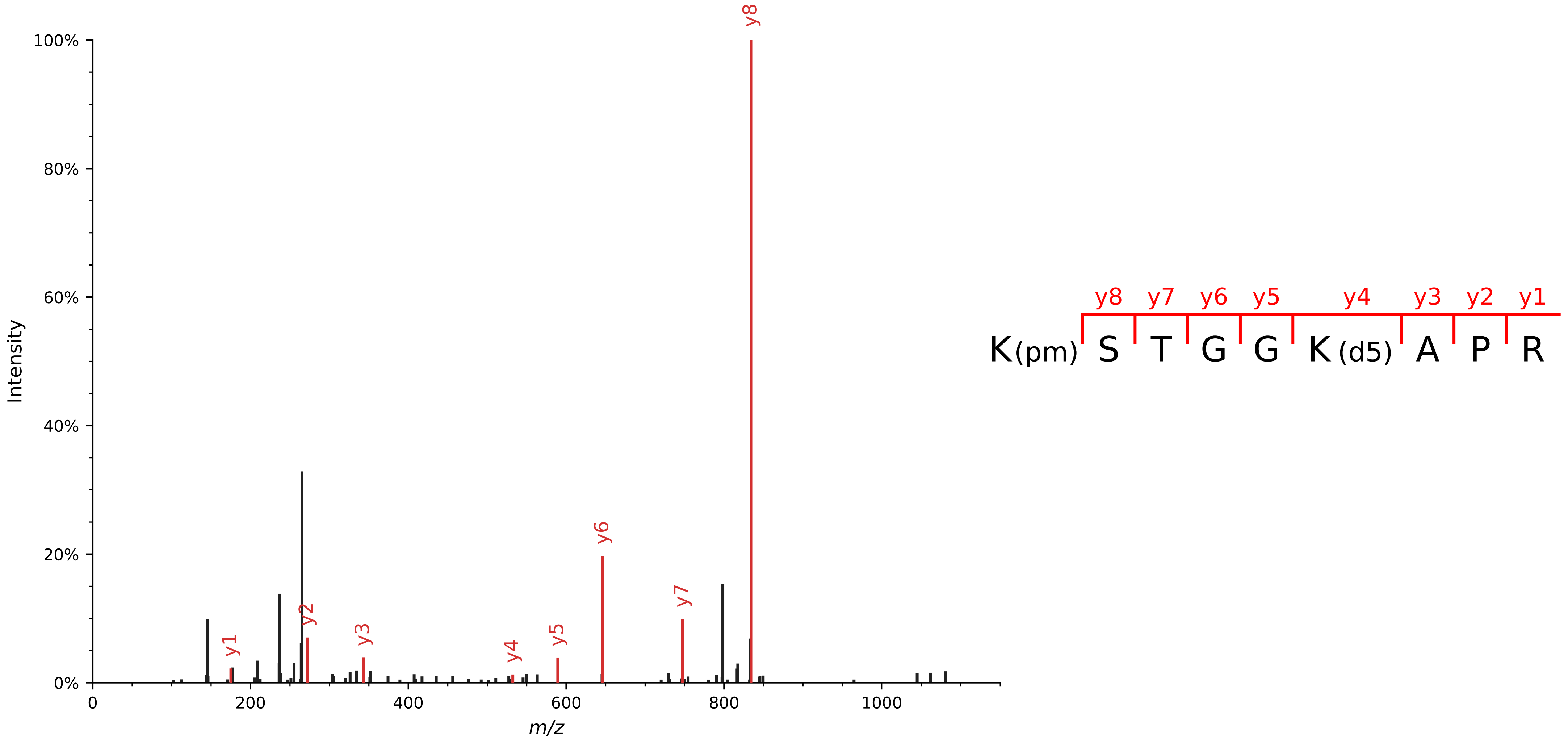

Raw file: HF240124_RN_spleen_D5PRO_D5PRO_1, Scan: 6479, m/z: 540.33504, Charge: 2, RT (min): 12.83, Score: 144.38

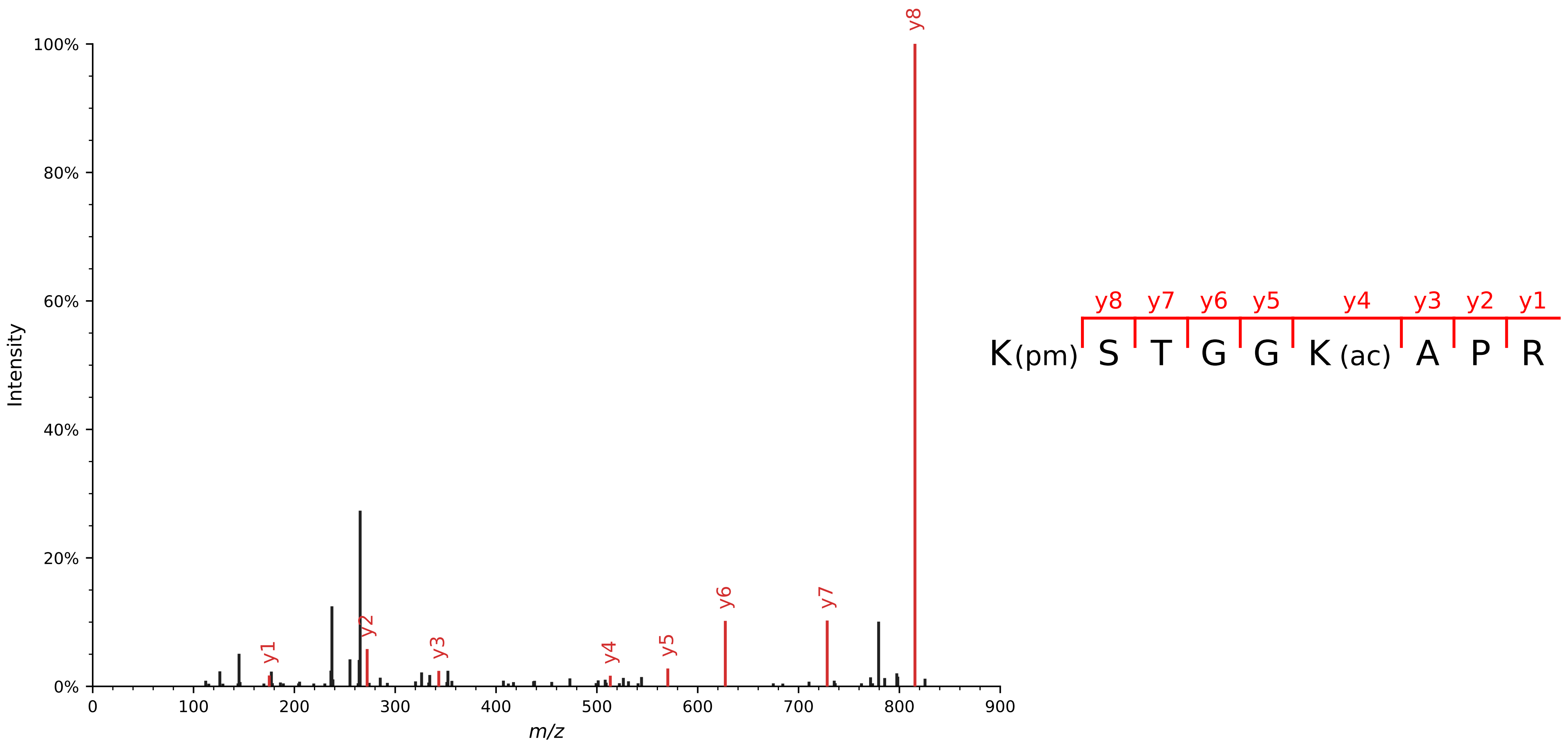

Raw file: HF240124_RN_spleen_D5PRO_D5PRO_3, Scan: 2712, m/z: 556.86638, Charge: 2, RT (min): 6.01, Score: 54.549

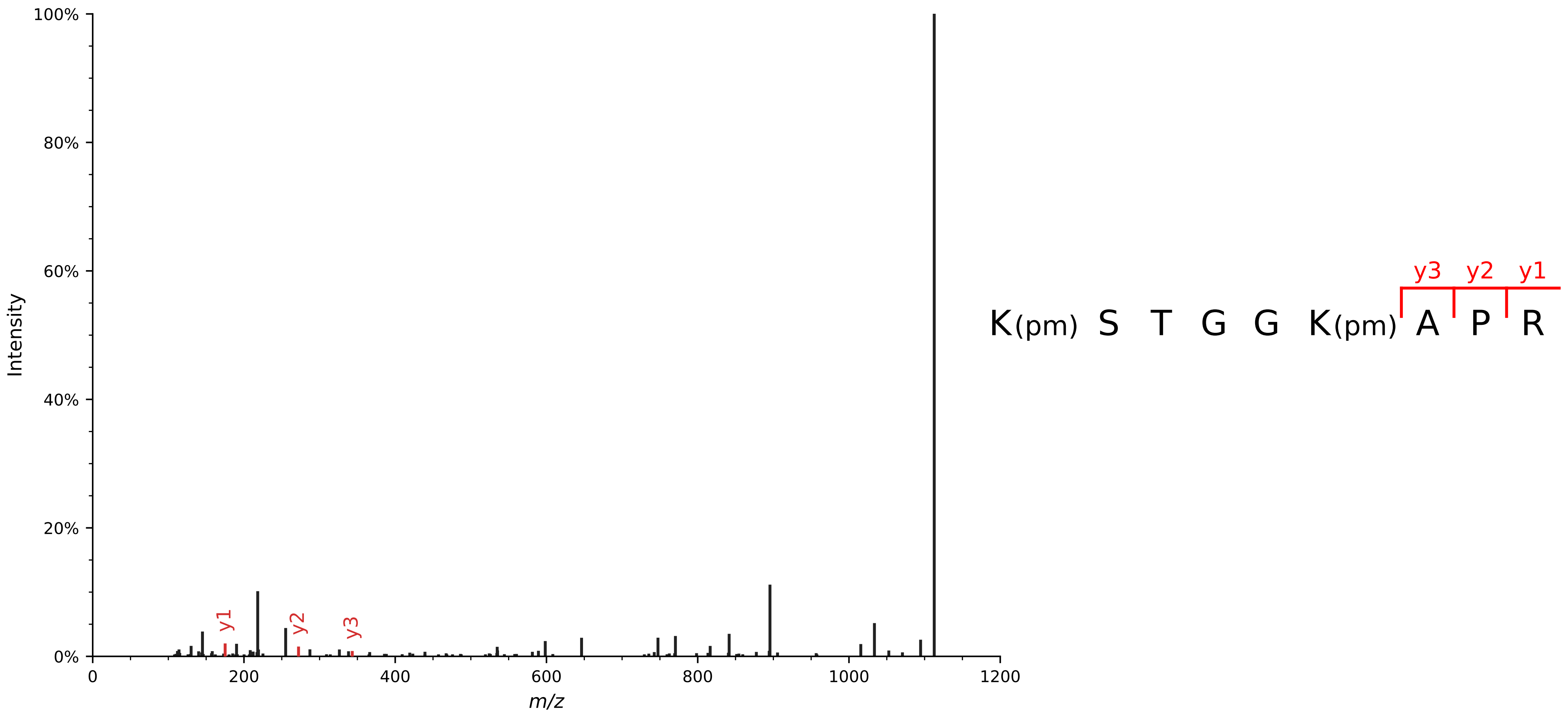

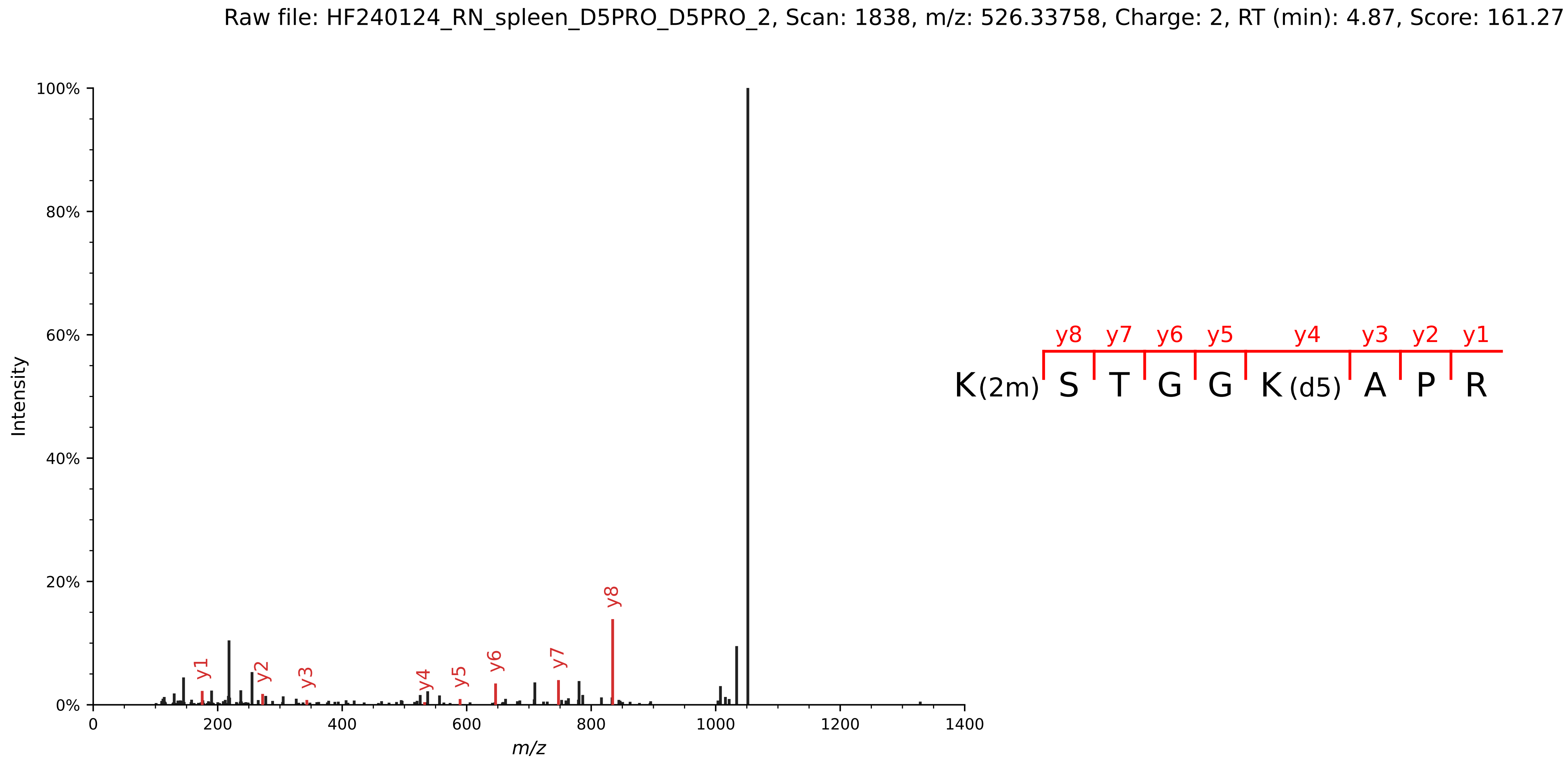
Raw file: HF240124_RN_spleen_D5PRO_D5PRO_2, Scan: 1838, m/z: 526.33758, Charge: 2, RT (min): 4.87, Score: 161.27
Intensity
m/z
y1
y2
y3
y4
y5
y6
y7
y8
K(2m) S T G G K(d5) A P R

Raw file: HF240124_RN_spleen_D5PRO_D5PRO_3, Scan: 1790, m/z: 516.81406, Charge: 2, RT (min): 4.48, Score: 169.41

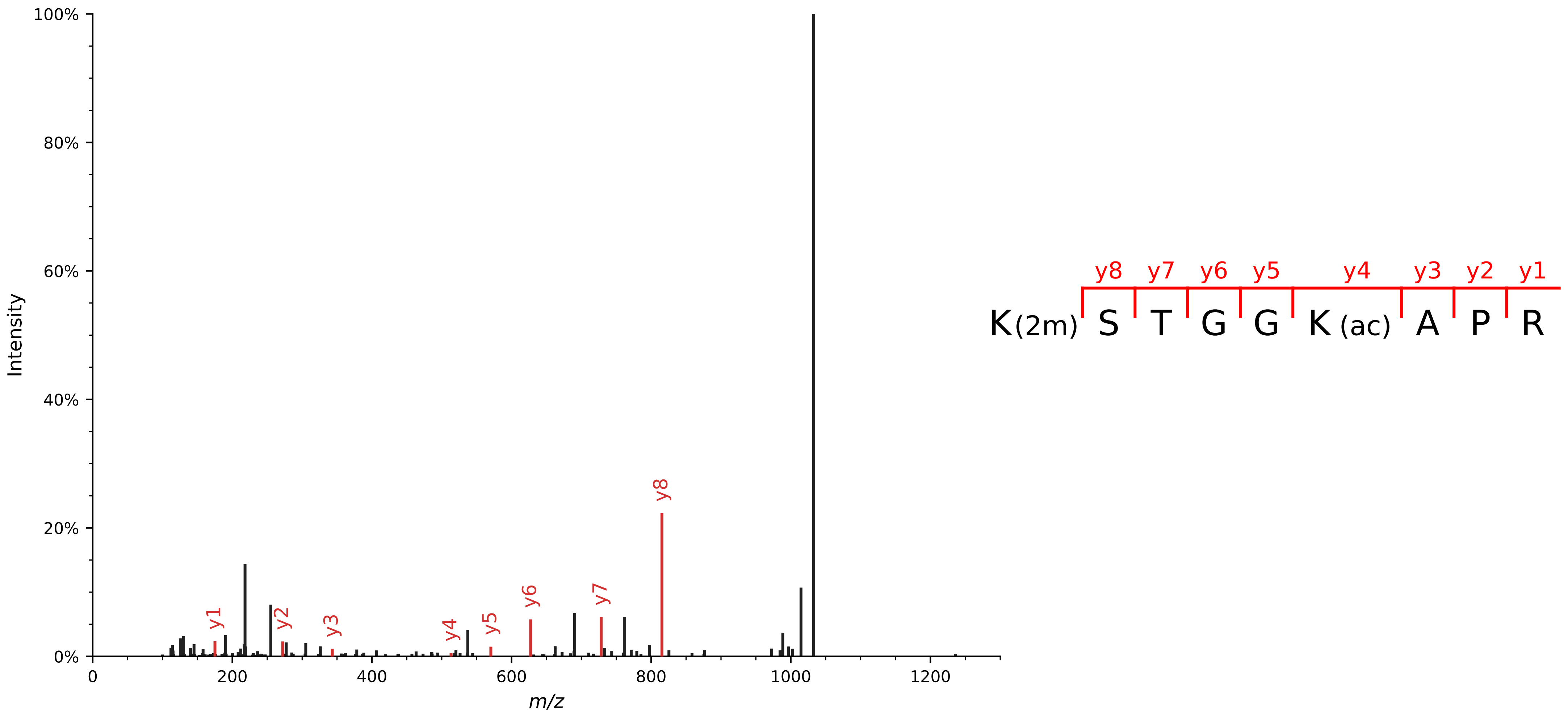

Raw file: HF240124_RN_spleen_D5PRO_D5PRO_1, Scan: 1581, m/z: 533.34541, Charge: 2, RT (min): 4.48, Score: 109.66

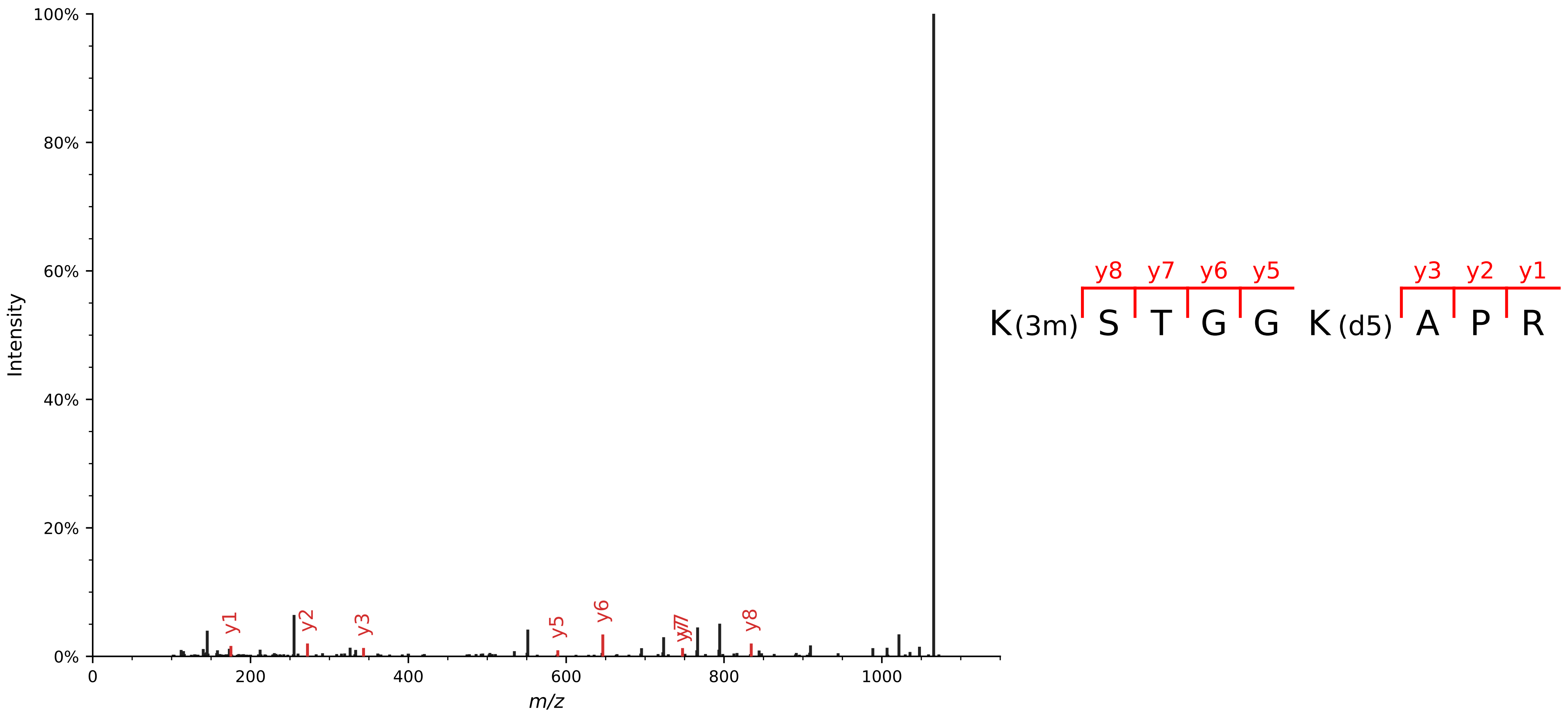

Raw file: HF240124_RN_spleen_D5PRO_D5PRO_3, Scan: 1791, m/z: 523.82189, Charge: 2, RT (min): 4.48, Score: 120.45

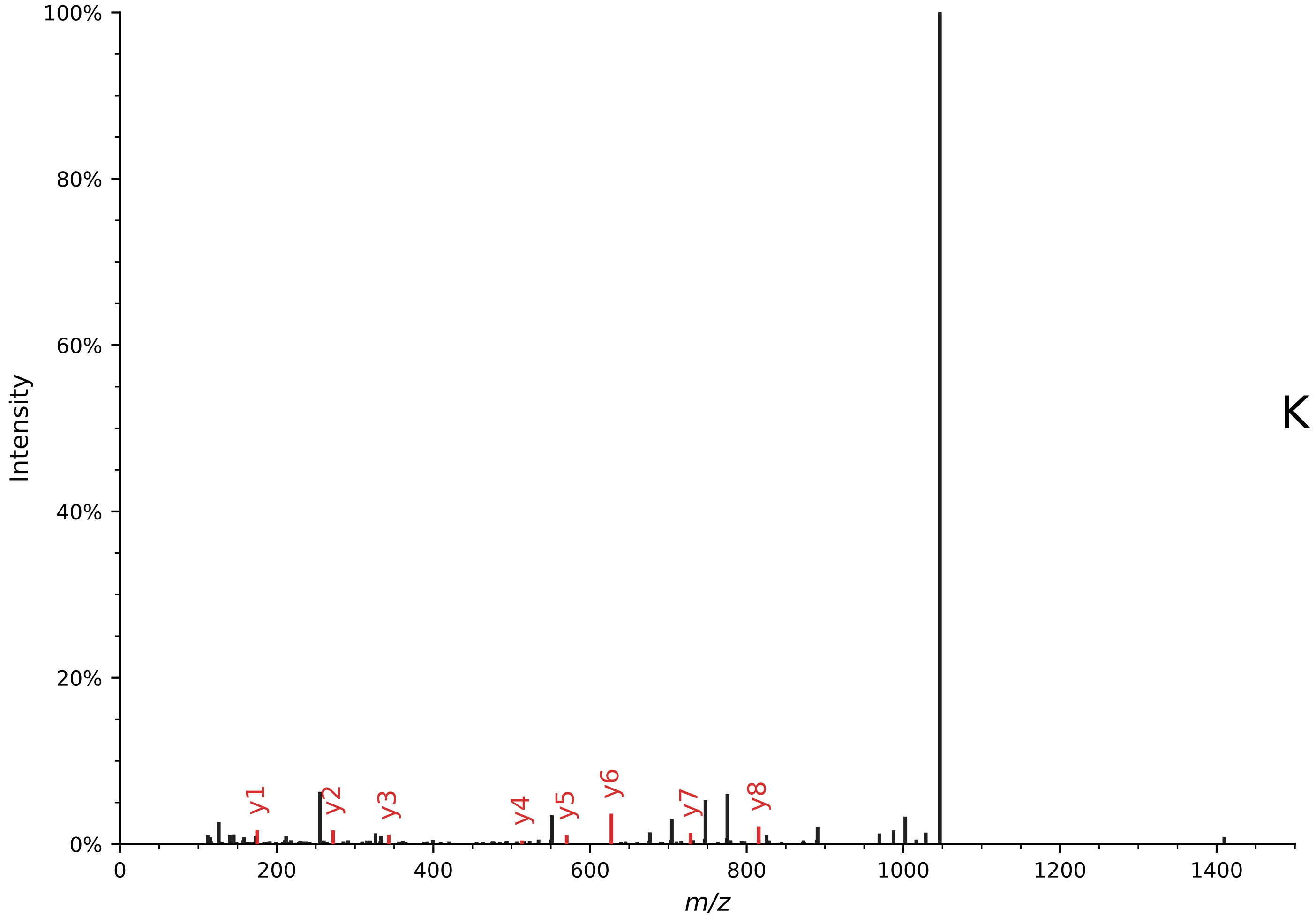

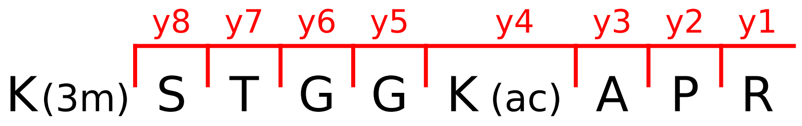

Raw file: HF240124_RN_spleen_D5PRO_D5PRO_1, Scan: 4929, m/z: 530.81152, Charge: 2, RT (min): 10.18, Score: 69.905

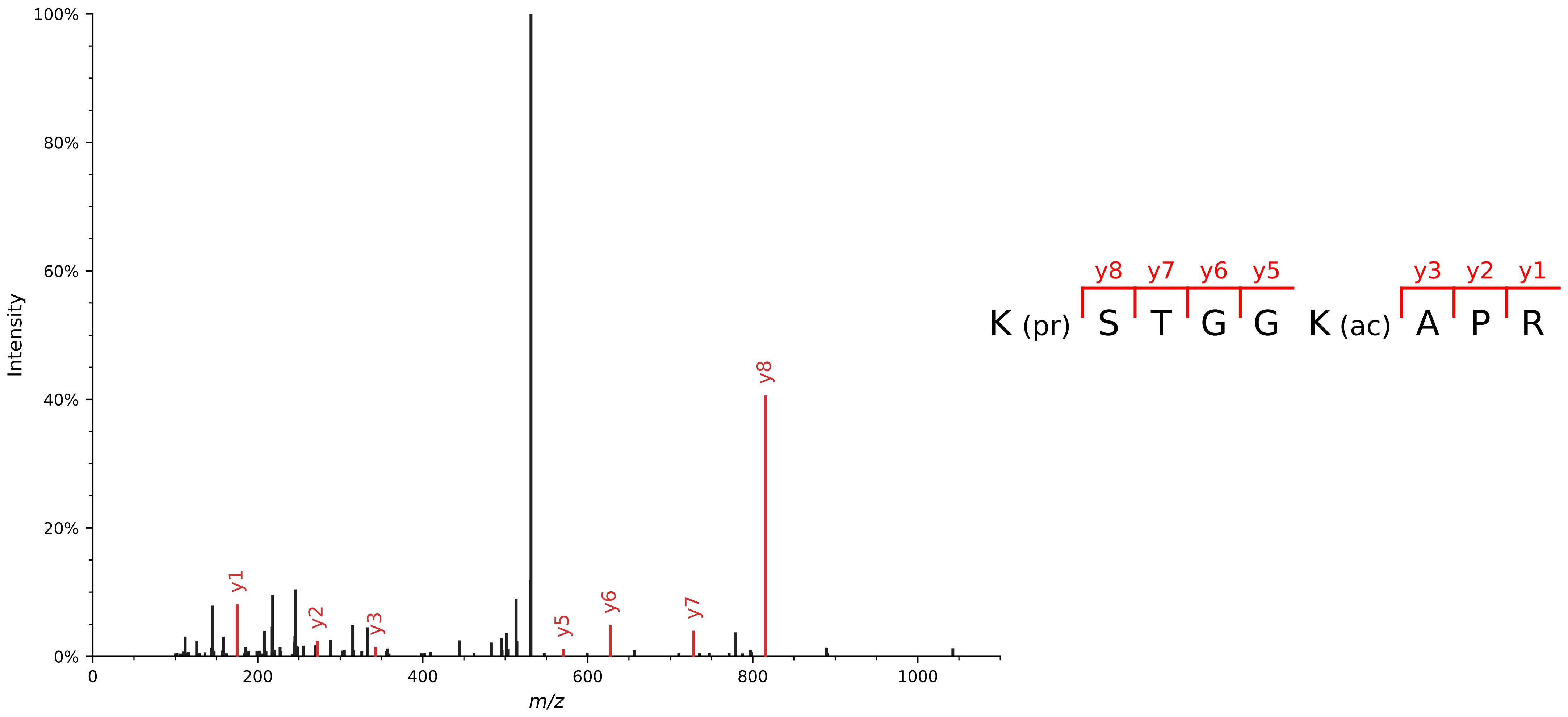

Raw file: HF240124_RN_spleen_D5PRO_D5PRO_3, Scan: 4516, m/z: 533.32721, Charge: 2, RT (min): 9.24, Score: 116.73

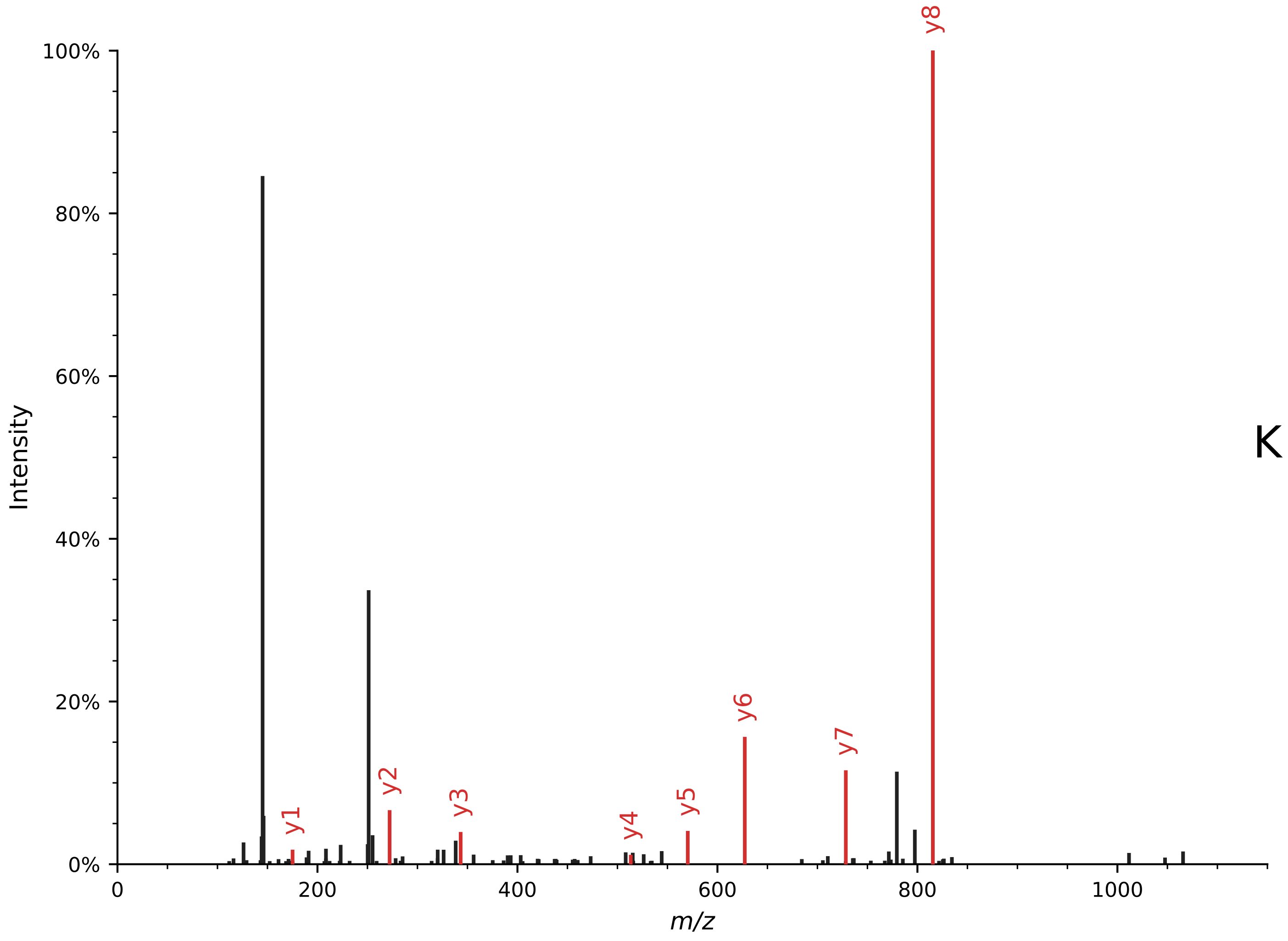

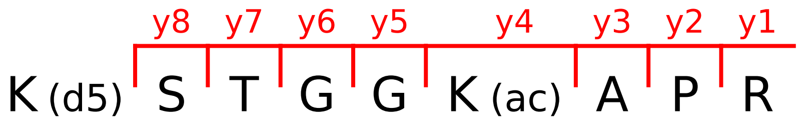

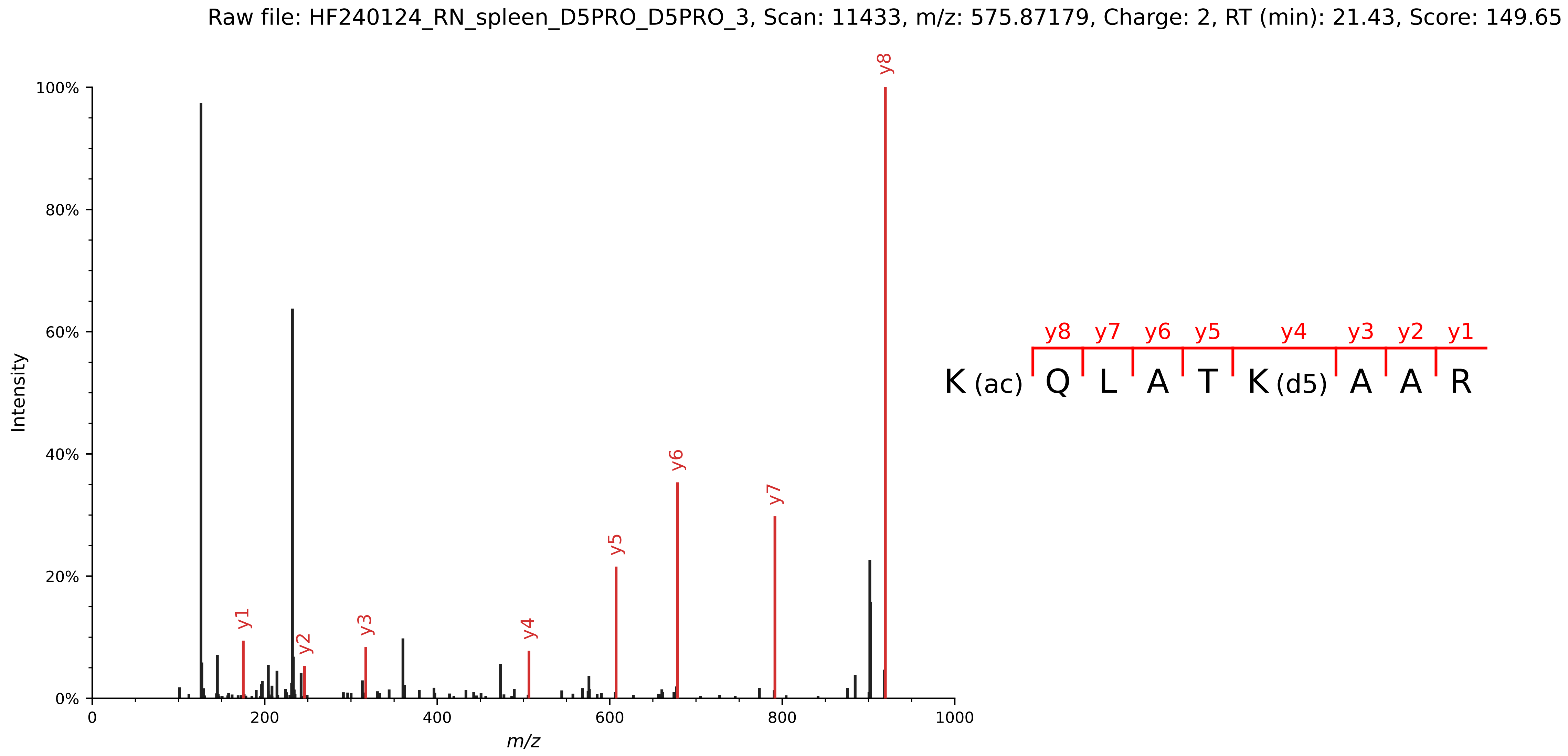

Raw file: HF240124_RN_spleen_D5PRO_D5PRO_3, Scan: 11433, m/z: 575.87179, Charge: 2, RT (min): 21.43, Score: 149.65
Intensity
m/z
y1
y2
y3
y4
y5
y6
y7
y8
K (ac) Q L A T K (d5) A A R

Raw file: HF240124_RN_spleen_D5PRO_D5PRO_2, Scan: 10346, m/z: 566.34827, Charge: 2, RT (min): 19.65, Score: 166.52

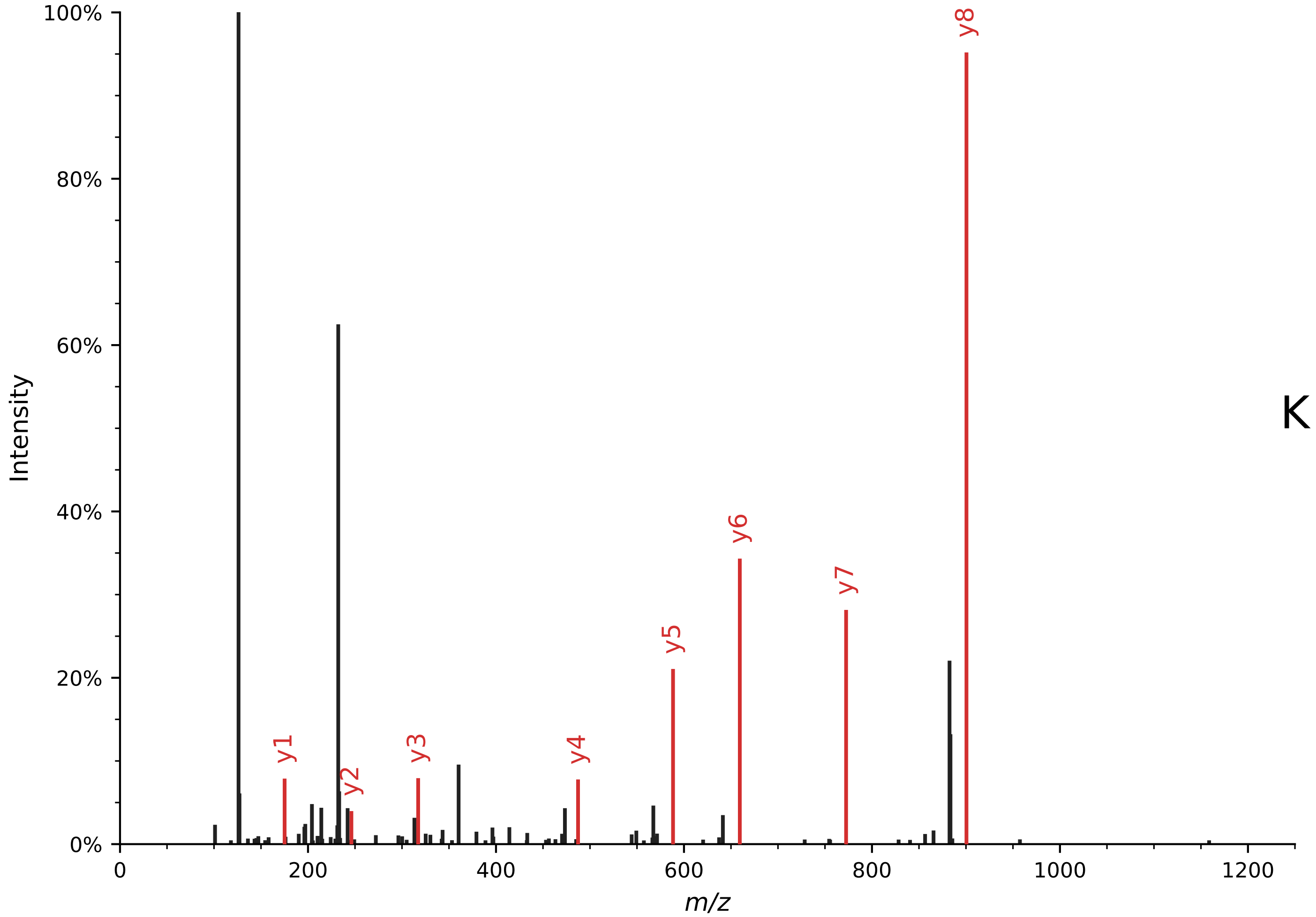

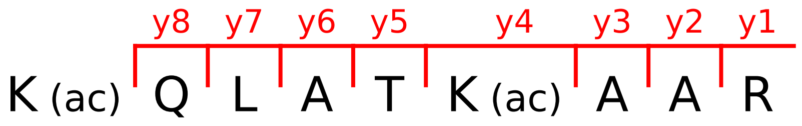

Raw file: HF240124_RN_spleen_D5PRO_D5PRO_2, Scan: 13173, m/z: 580.36392, Charge: 2, RT (min): 24.66, Score: 107.69

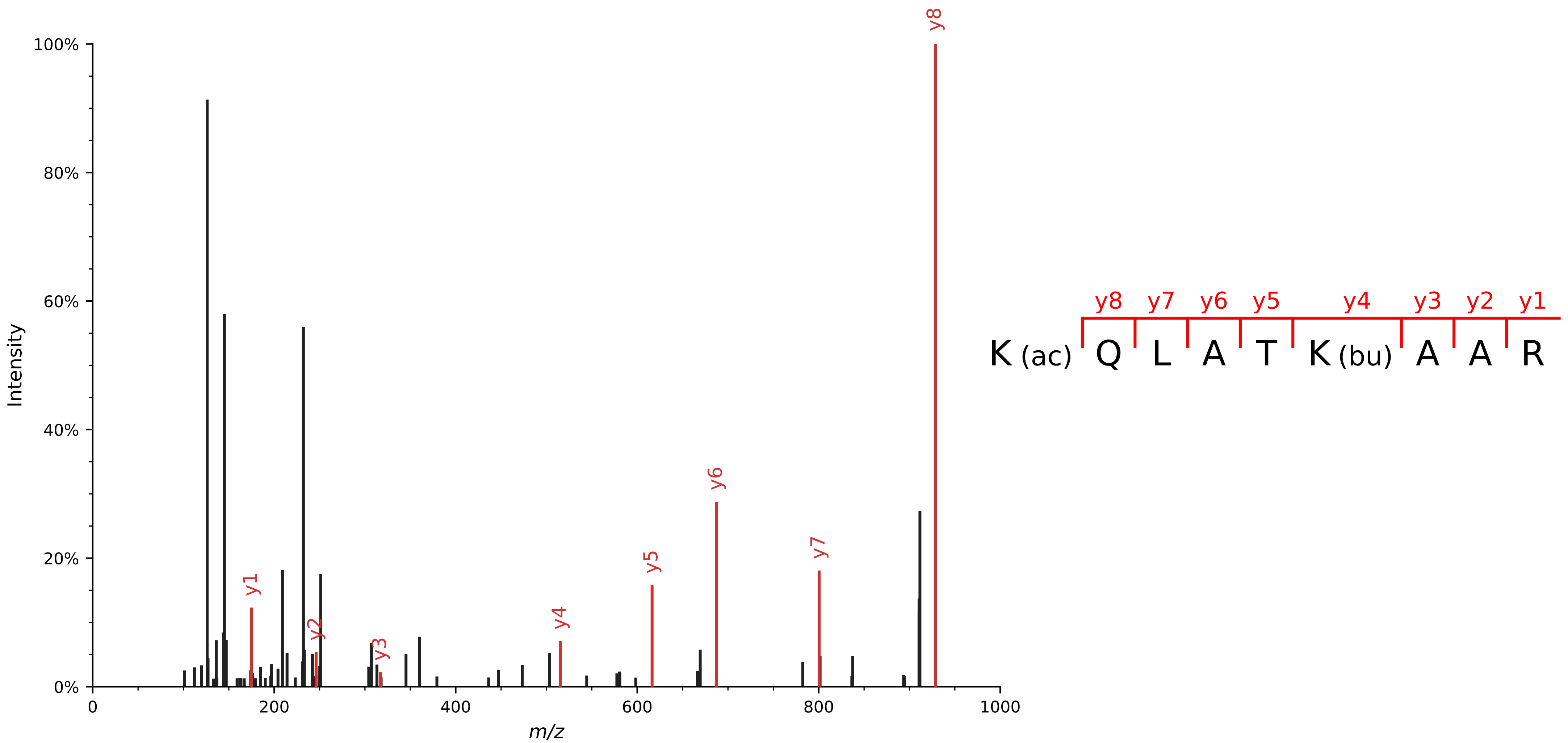

Raw file: HF240124_RN_spleen_D5PRO_D5PRO_2, Scan: 14628, m/z: 589.88744, Charge: 2, RT (min): 27.33, Score: 80.165

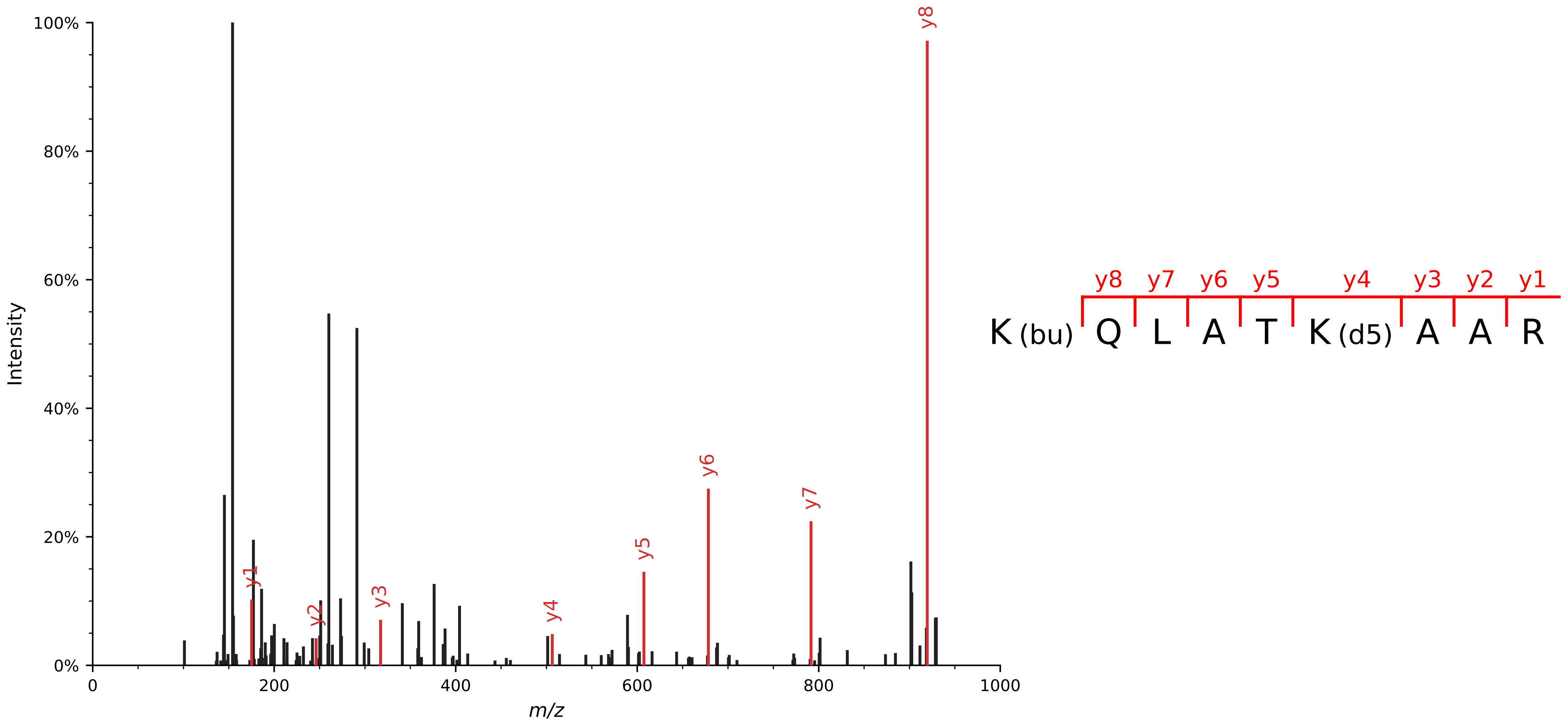

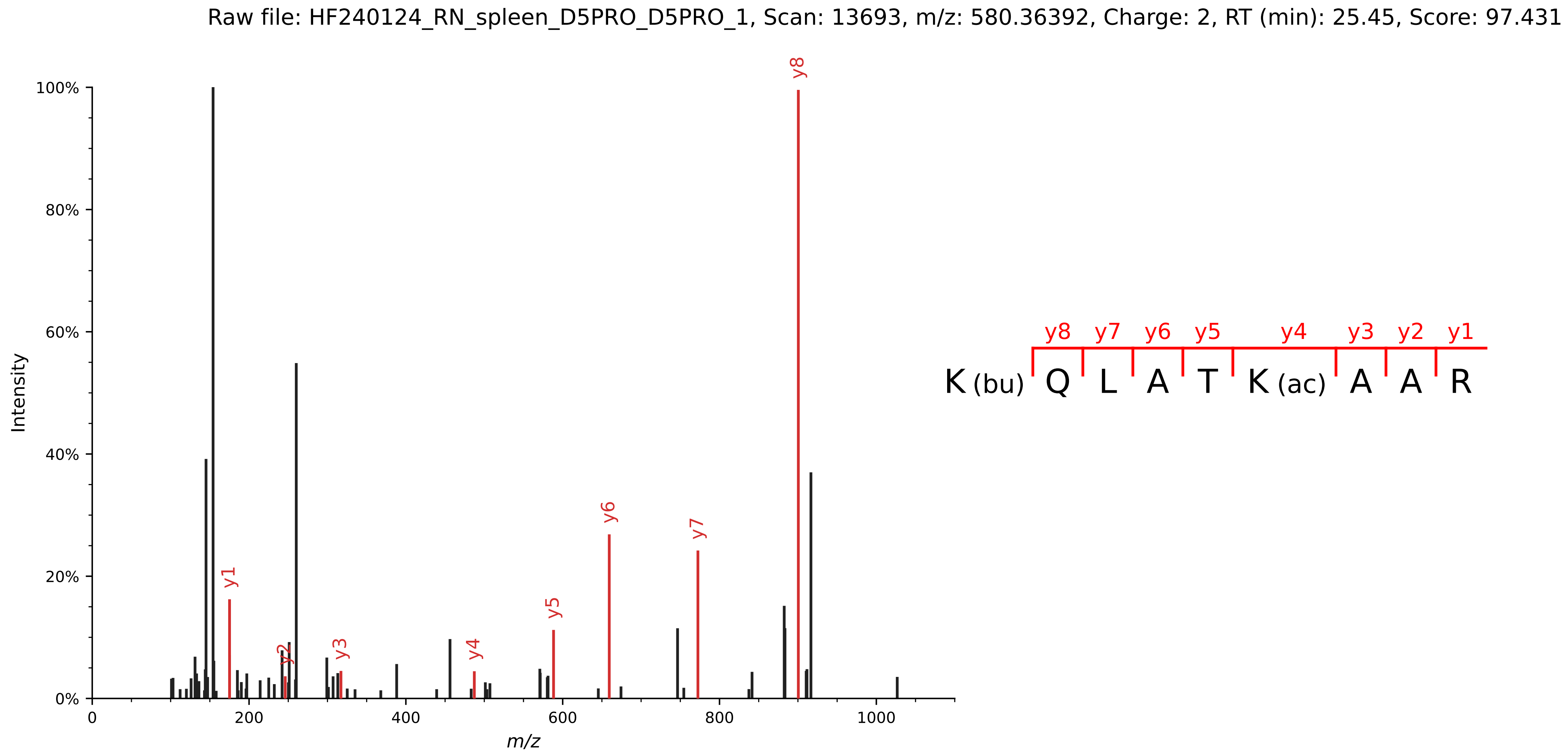

Raw file: HF240124_RN_spleen_D5PRO_D5PRO_1, Scan: 13693, m/z: 580.36392, Charge: 2, RT (min): 25.45, Score: 97.431
Intensity
m/z
y1
y2
y3
y4
y5
y6
y7
y8
K (bu) Q L A T K (ac) A A R

Raw file: HF240124_RN_spleen_D5PRO_D5PRO_2, Scan: 9229, m/z: 568.86396, Charge: 2, RT (min): 17.74, Score: 130.22

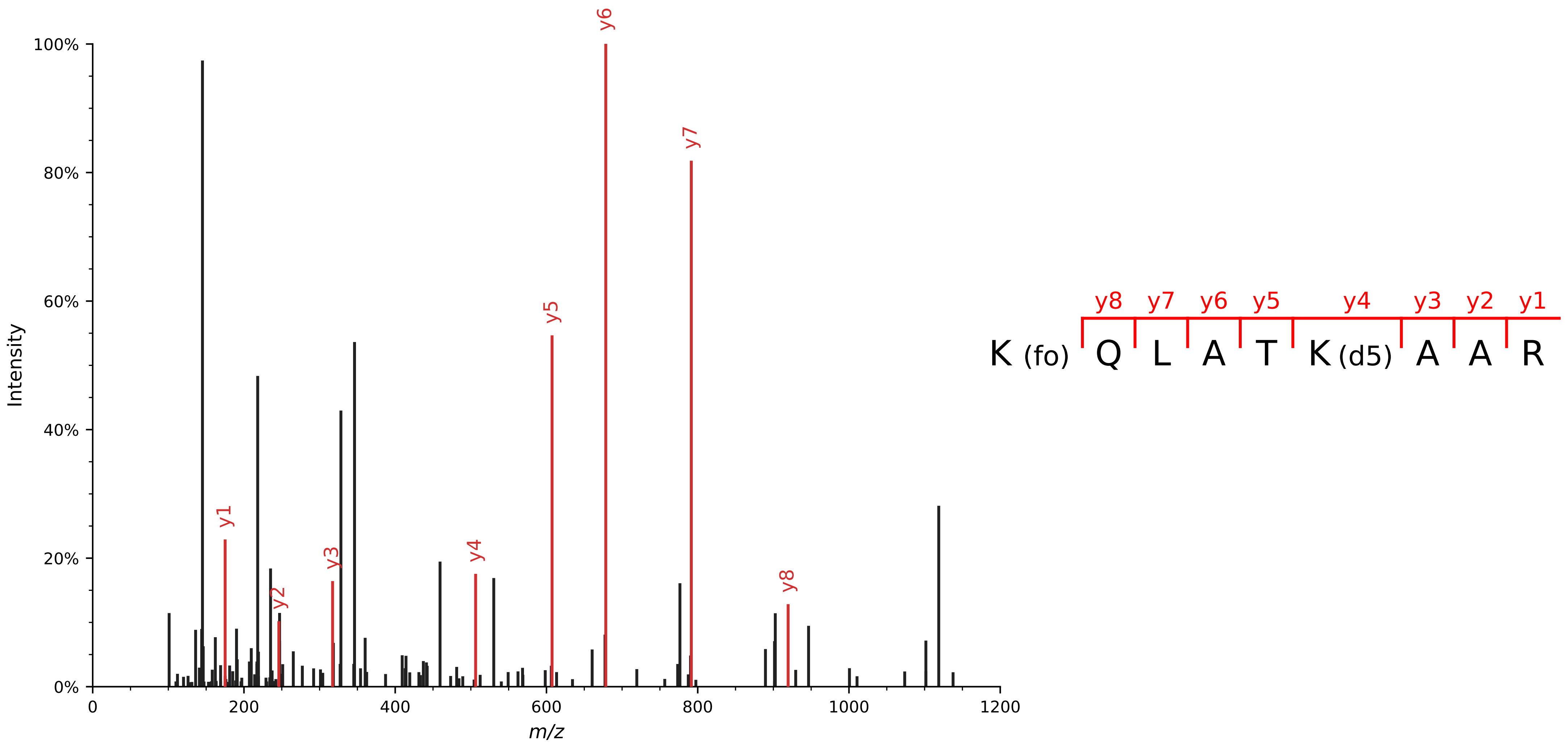

Raw file: HF240124_RN_spleen_D5PRO_D5PRO_2, Scan: 7640, m/z: 590.87707, Charge: 2, RT (min): 15.03, Score: 56.729

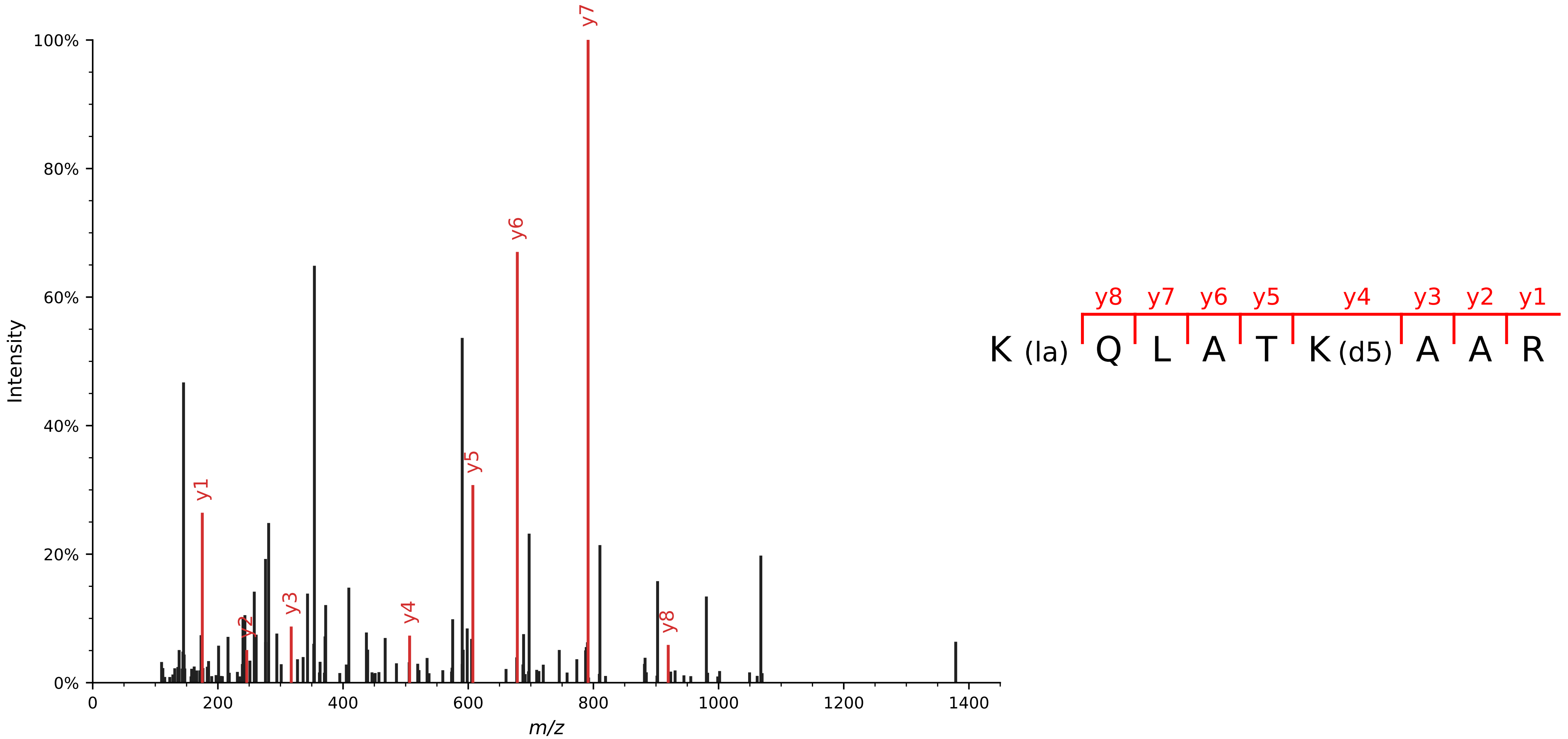

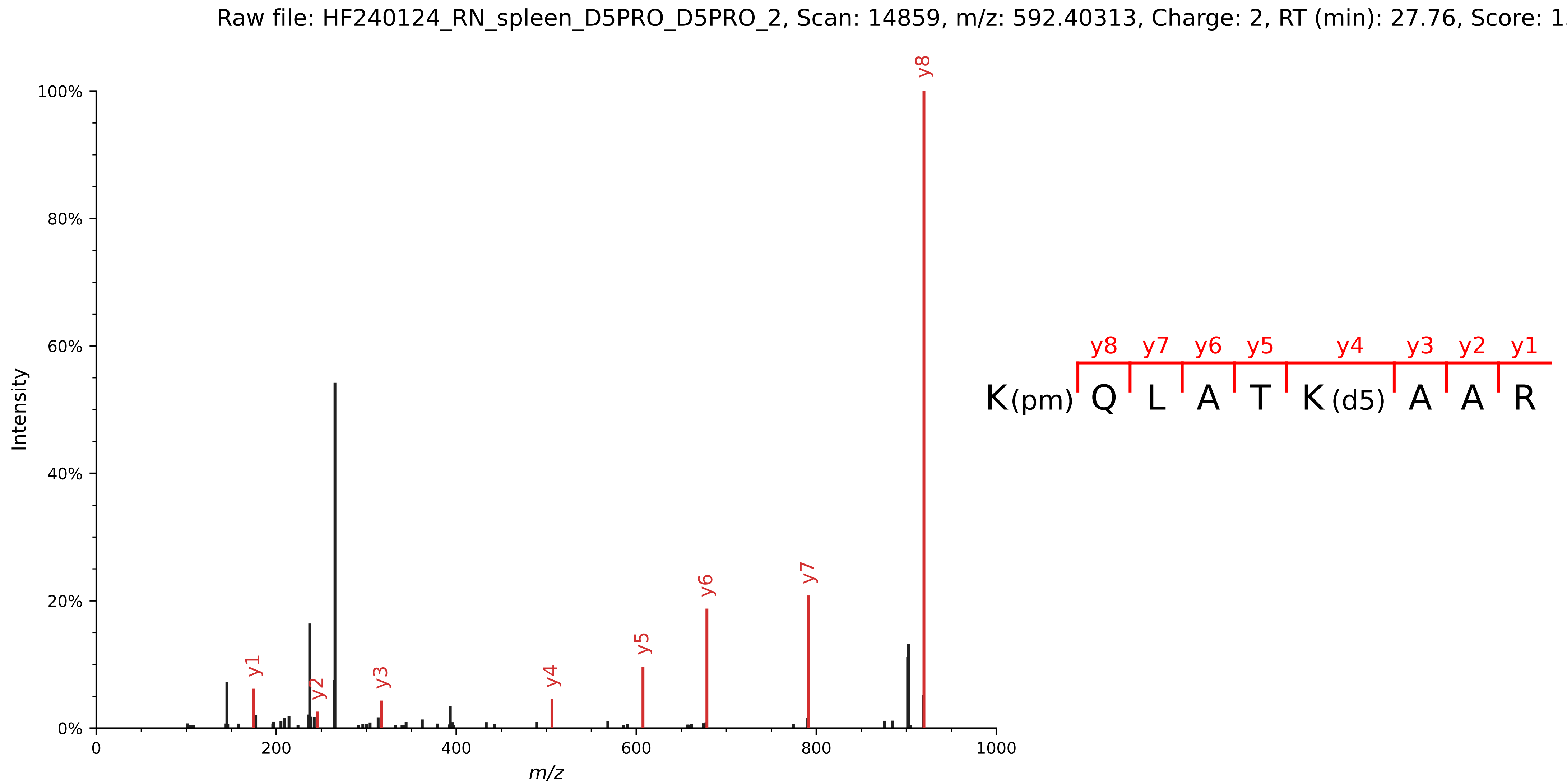

Raw file: HF240124_RN_spleen_D5PRO_D5PRO_2, Scan: 14859, m/z: 592.40313, Charge: 2, RT (min): 27.76, Score: 133.12
Intensity
m/z
y1
y2
y3
y4
y5
y6
y7
y8
K(pm) Q L A T K(d5) A A R

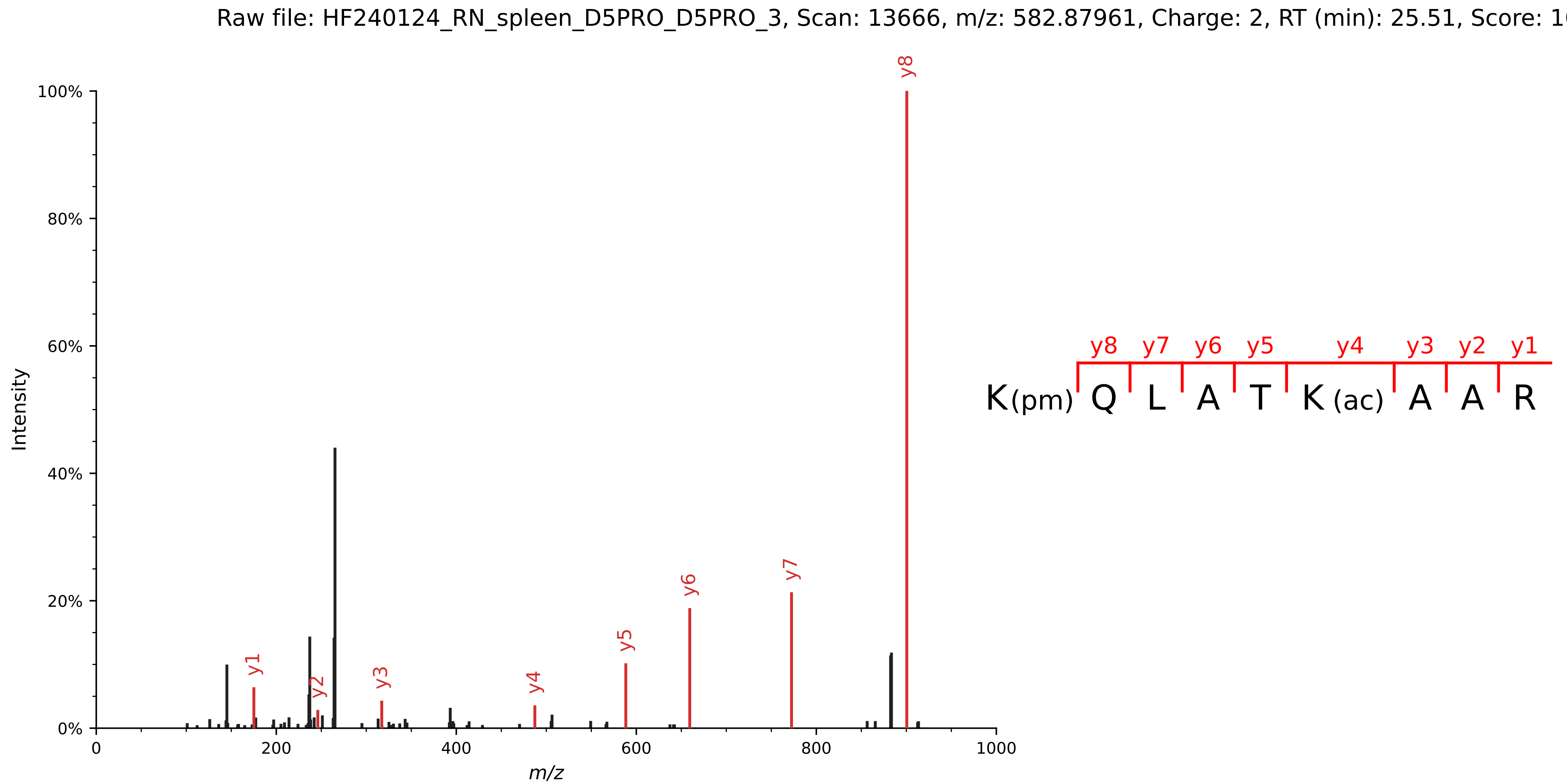
Raw file: HF240124_RN_spleen_D5PRO_D5PRO_3, Scan: 13666, m/z: 582.87961, Charge: 2, RT (min): 25.51, Score: 101.72
Intensity
100%
80%
60%
40%
20%
0%
0
200
400
600
800
1000
m/z
y1
y2
y3
y4
y5
y6
y7
y8
K(pm) Q L A T K (ac) A A R

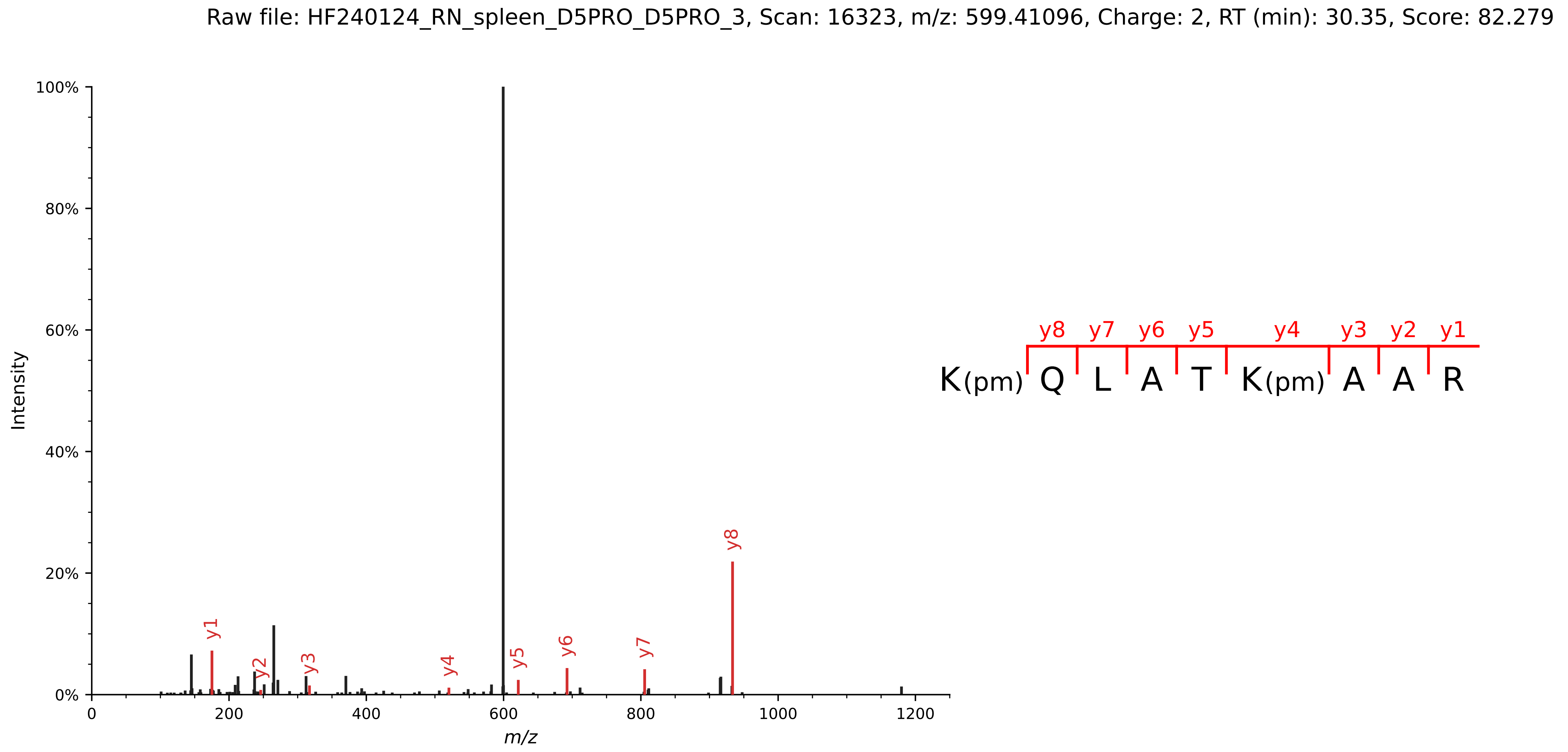

Raw file: HF240124_RN_spleen_D5PRO_D5PRO_3, Scan: 16323, m/z: 599.41096, Charge: 2, RT (min): 30.35, Score: 82.279
Intensity
m/z
y1
y2
y3
y4
y5
y6
y7
y8
K(pm) Q L A T K(pm) A A R

Raw file: HF240124_RN_spleen_D5PRO_D5PRO_3, Scan: 5533, m/z: 568.88216, Charge: 2, RT (min): 11.03, Score: 136.21

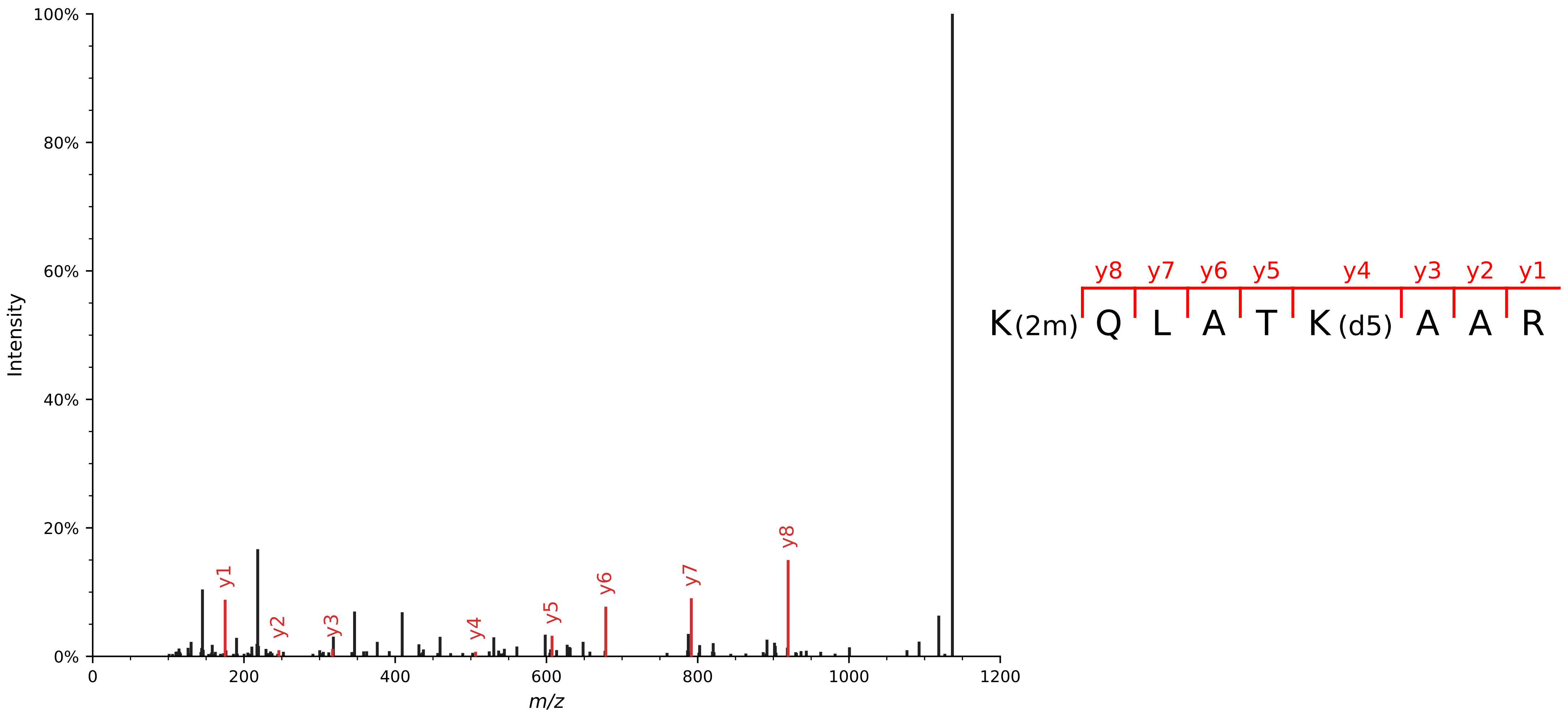

Raw file: HF240124_RN_spleen_D5PRO_D5PRO_1, Scan: 11816, m/z: 575.87179, Charge: 2, RT (min): 22.05, Score: 138.54

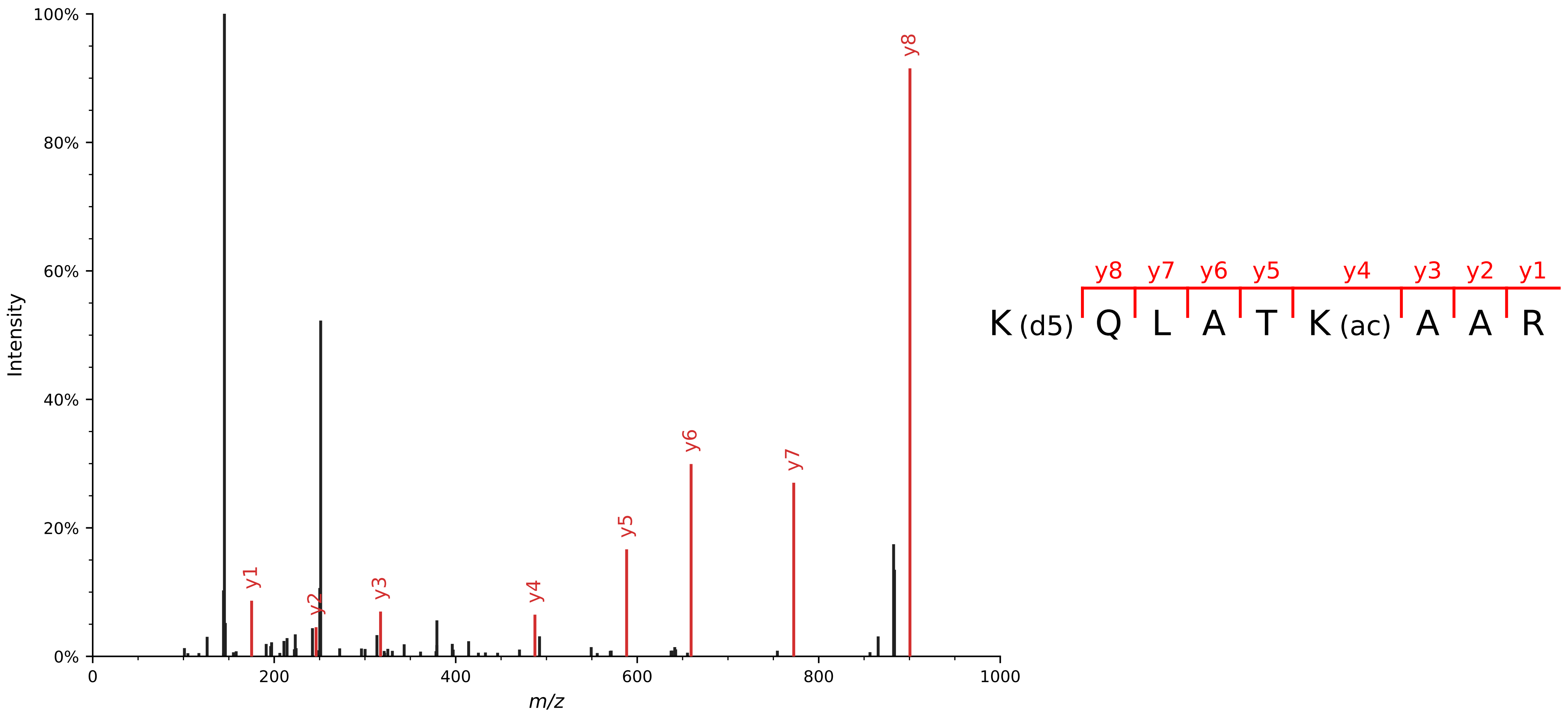

Raw file: HF240124_RN_spleen_D5PRO_D5PRO_3, Scan: 14334, m/z: 589.88744, Charge: 2, RT (min): 26.73, Score: 114.28

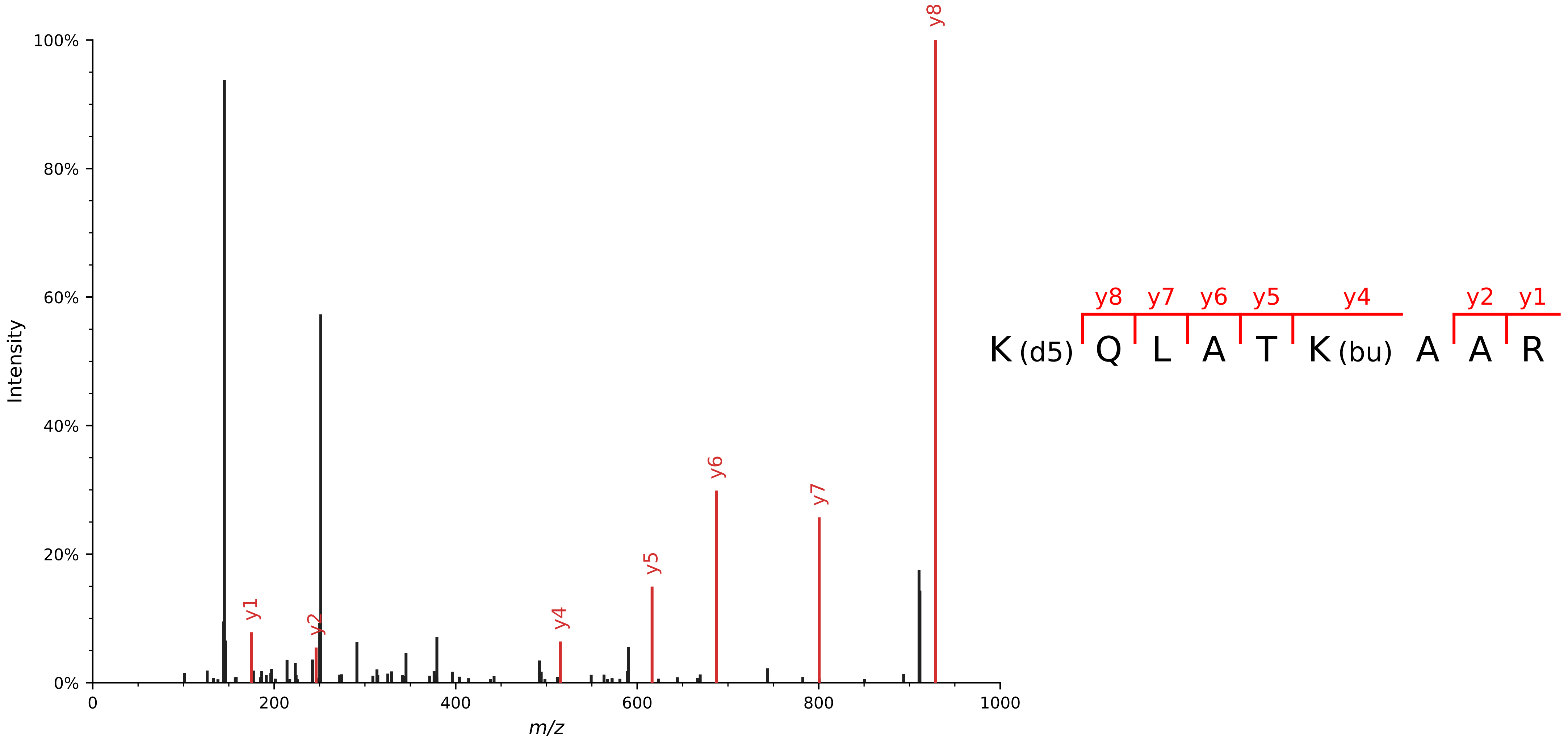

Raw file: HF240124_RN_spleen_D5PRO_D5PRO_1, Scan: 11562, m/z: 568.86396, Charge: 2, RT (min): 21.61, Score: 92.439

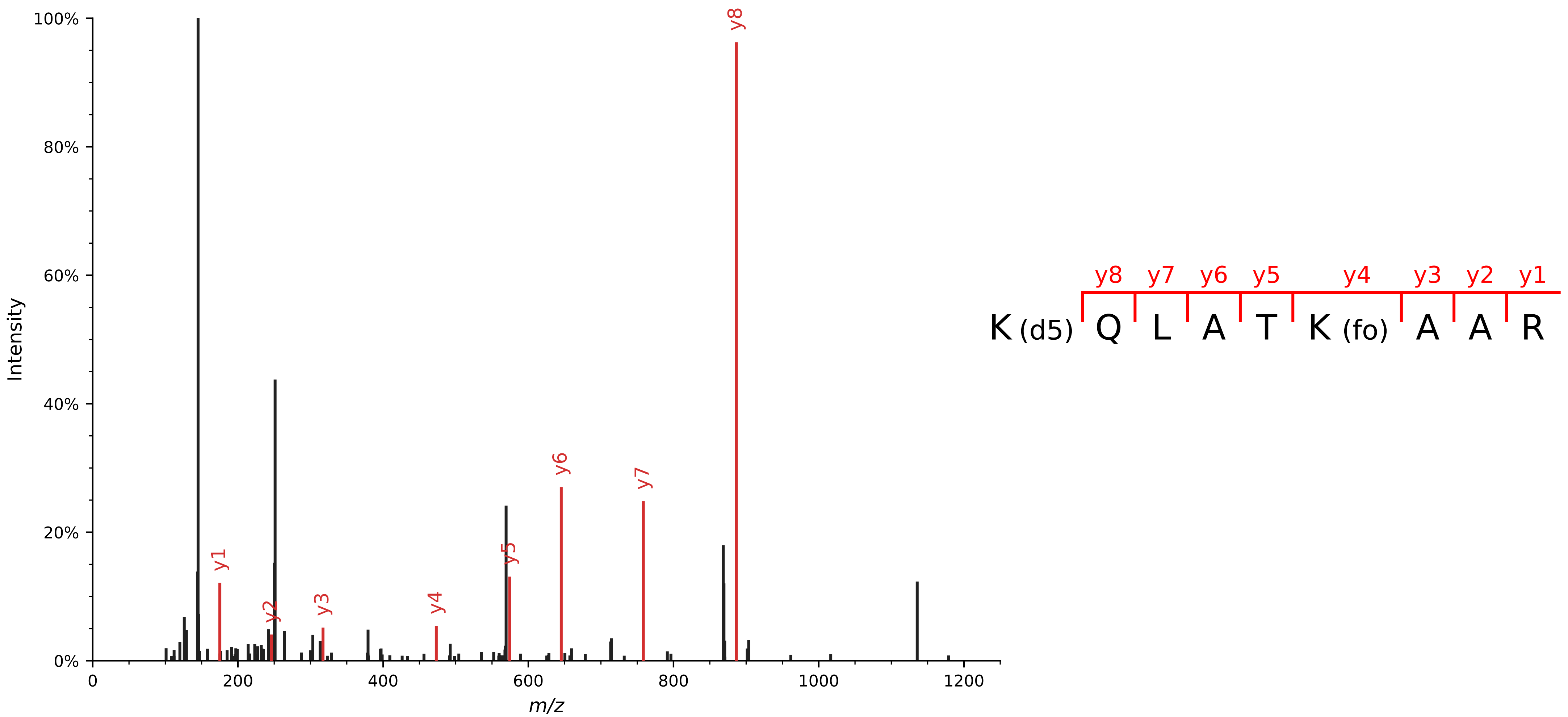

Raw file: HF240124_RN_spleen_D5PRO_D5PRO_1, Scan: 14707, m/z: 592.40313, Charge: 2, RT (min): 27.27, Score: 140.09

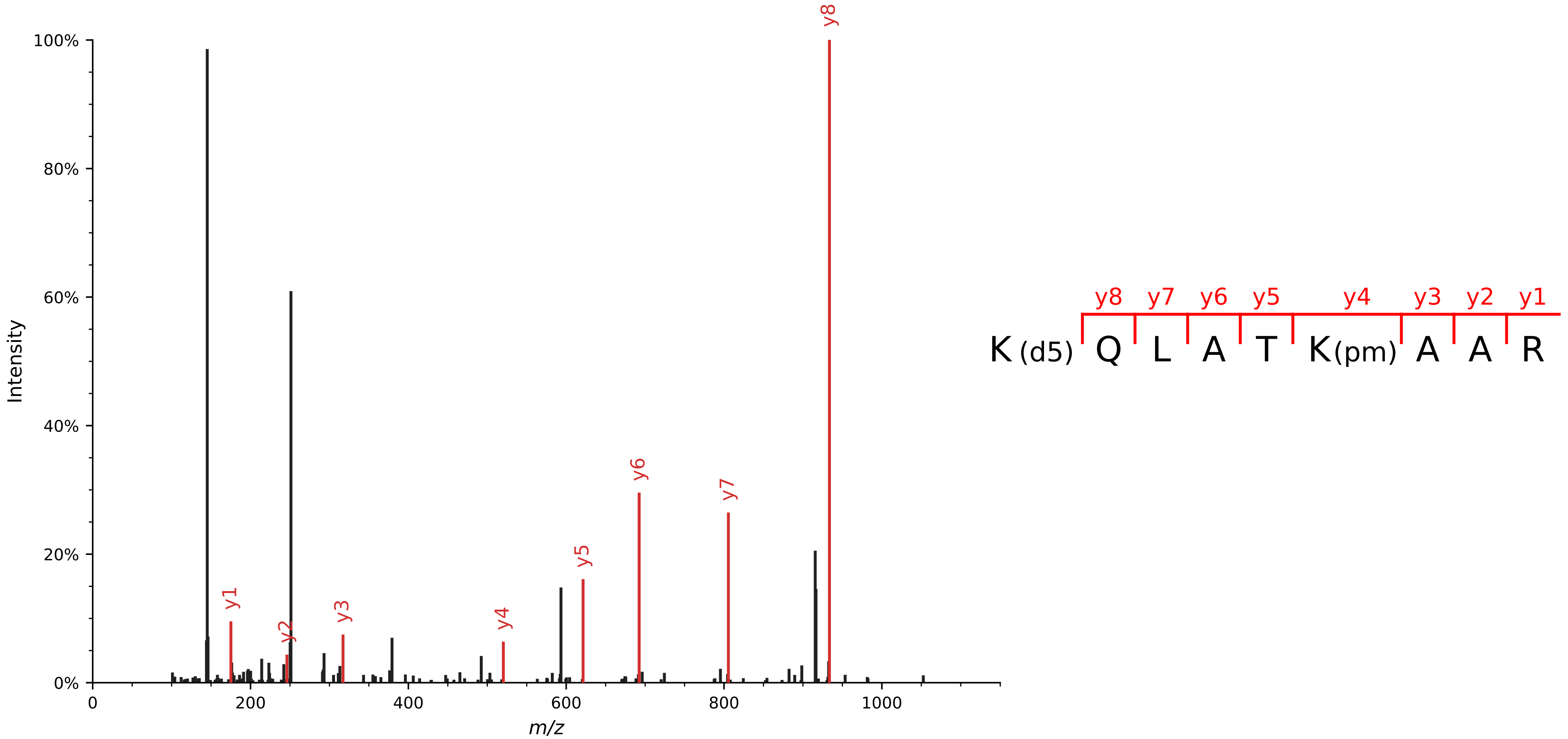

Raw file: HF240124_RN_spleen_D5PRO_D5PRO_2, Scan: 6684, m/z: 830.0121, Charge: 2, RT (min): 13.36, Score: 198.81

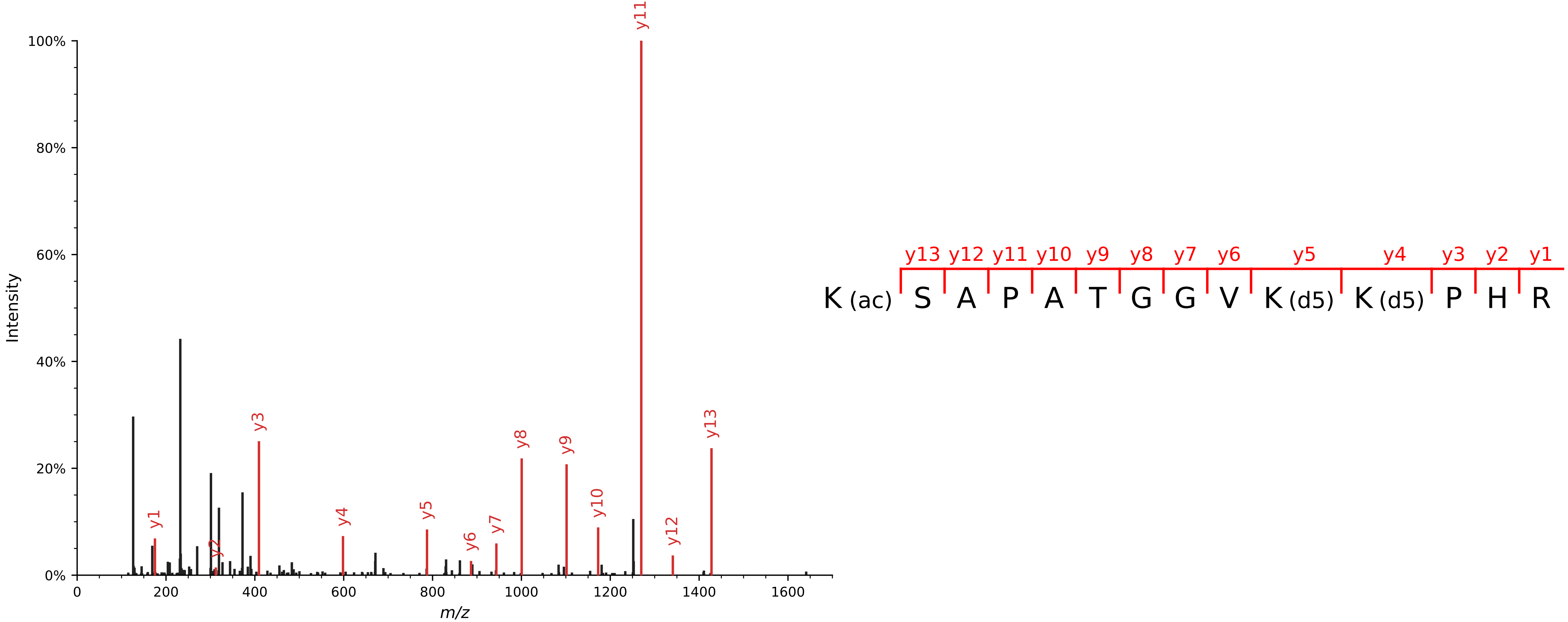

Raw file: HF240124_RN_spleen_D5PRO_D5PRO_3, Scan: 3328, m/z: 542.66839, Charge: 3, RT (min): 7.10, Score: 141.32

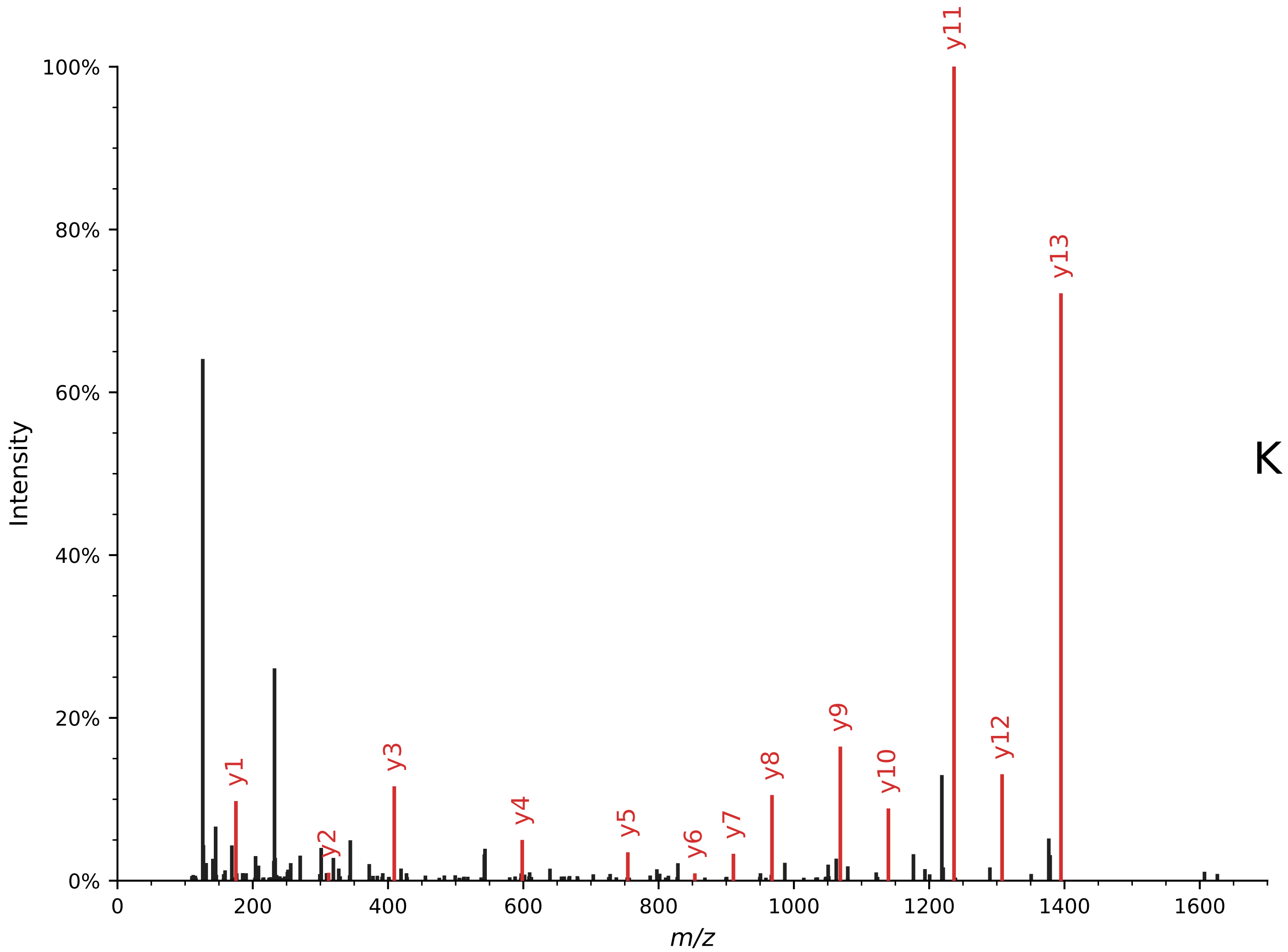

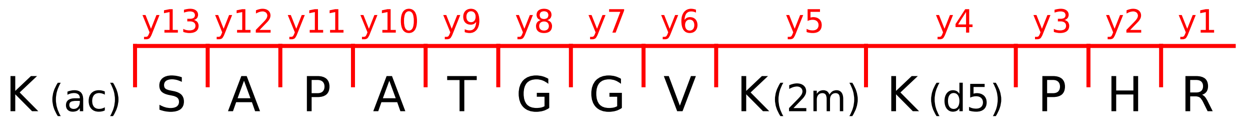

Raw file: HF240124_RN_spleen_D5PRO_D5PRO_1, Scan: 9621, m/z: 823.00427, Charge: 2, RT (min): 18.31, Score: 162.26

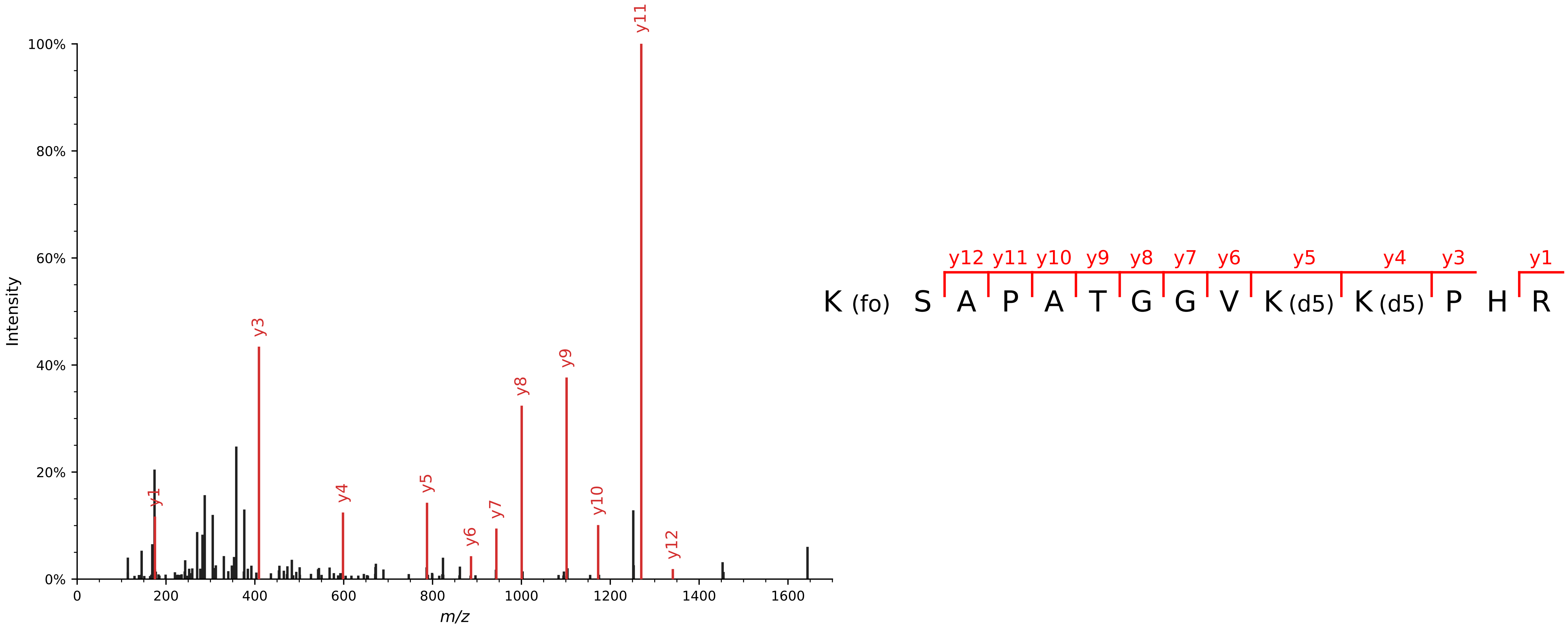

Raw file: HF240124_RN_spleen_D5PRO_D5PRO_1, Scan: 6036, m/z: 537.99651, Charge: 3, RT (min): 12.08, Score: 72.681

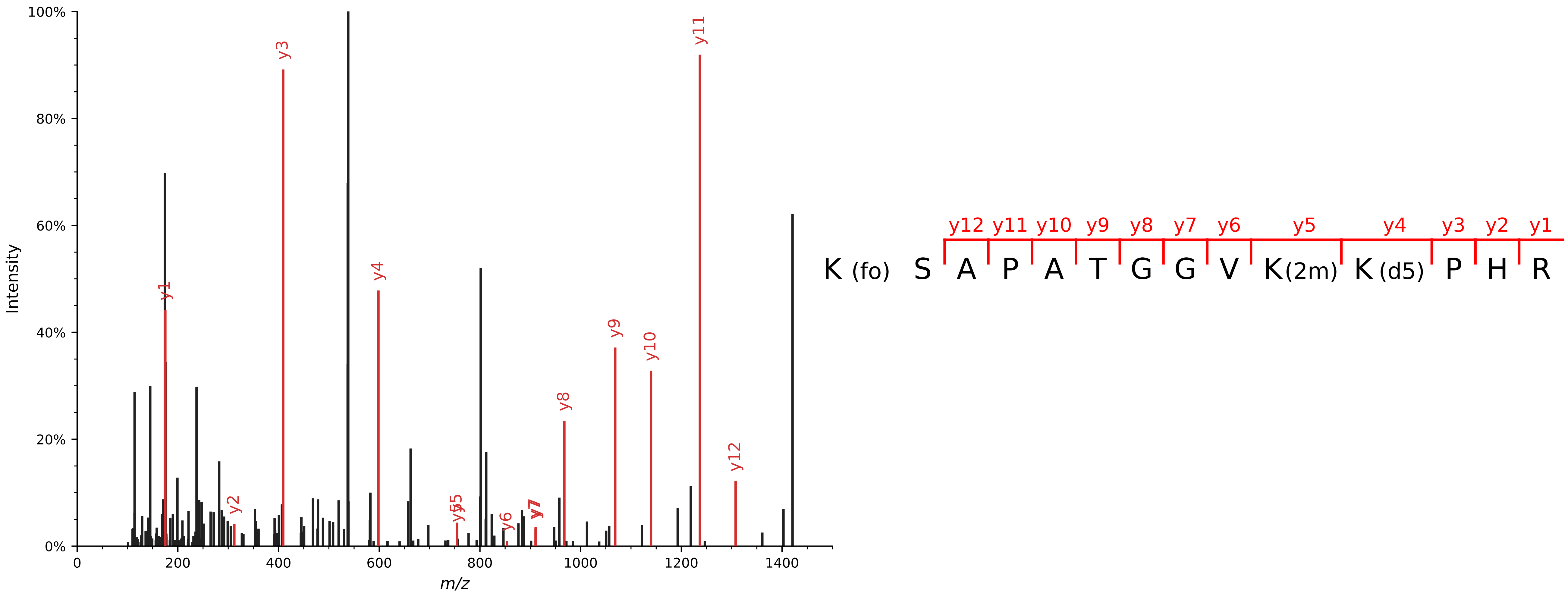

Raw file: HF240124_RN_spleen_D5PRO_D5PRO_1, Scan: 3086, m/z: 568.35256, Charge: 3, RT (min): 6.95, Score: 45.115

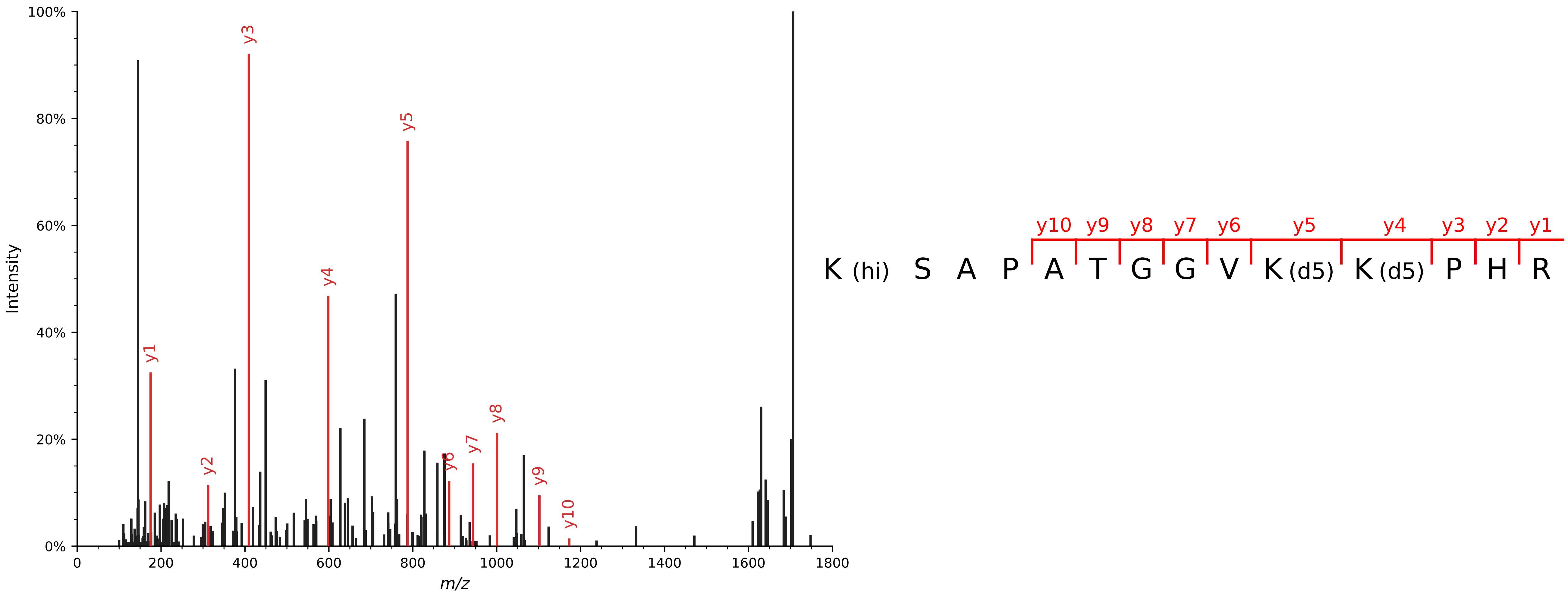

Raw file: HF240124_RN_spleen_D5PRO_D5PRO_2, Scan: 3200, m/z: 557.33167, Charge: 3, RT (min): 7.21, Score: 80.706

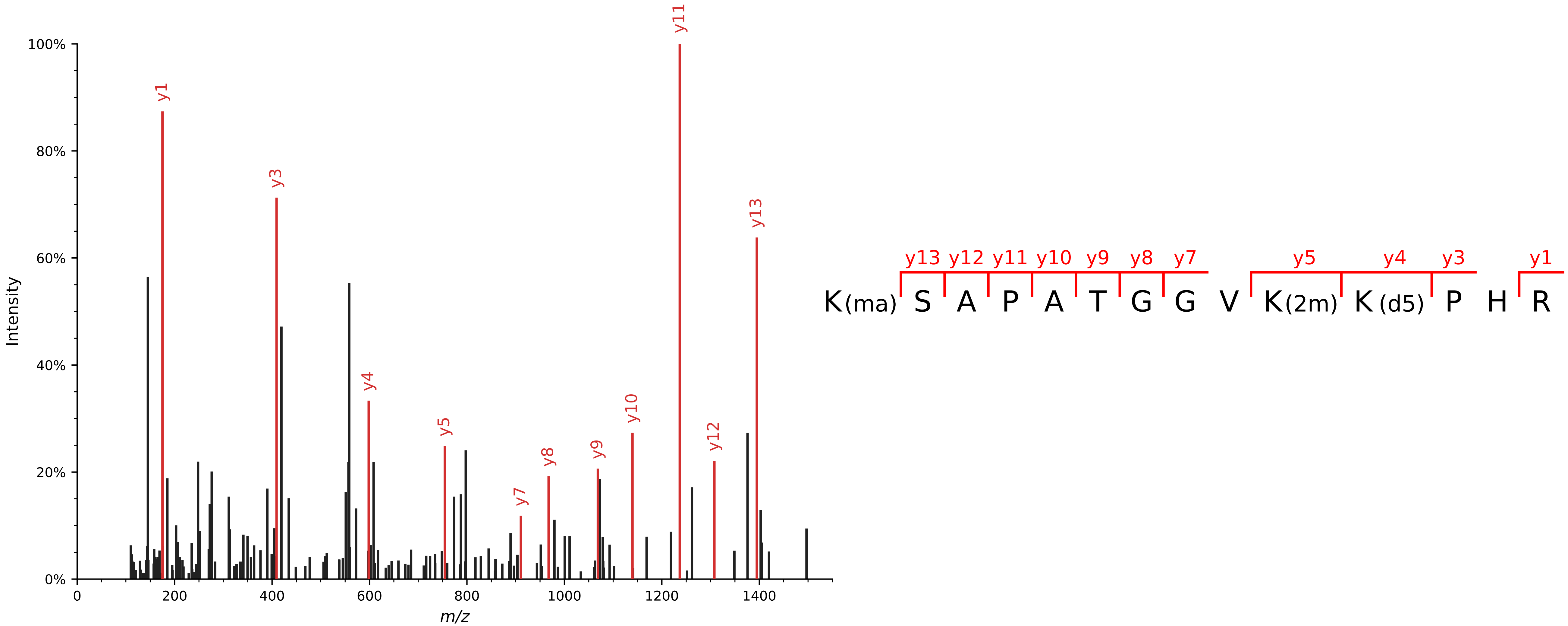

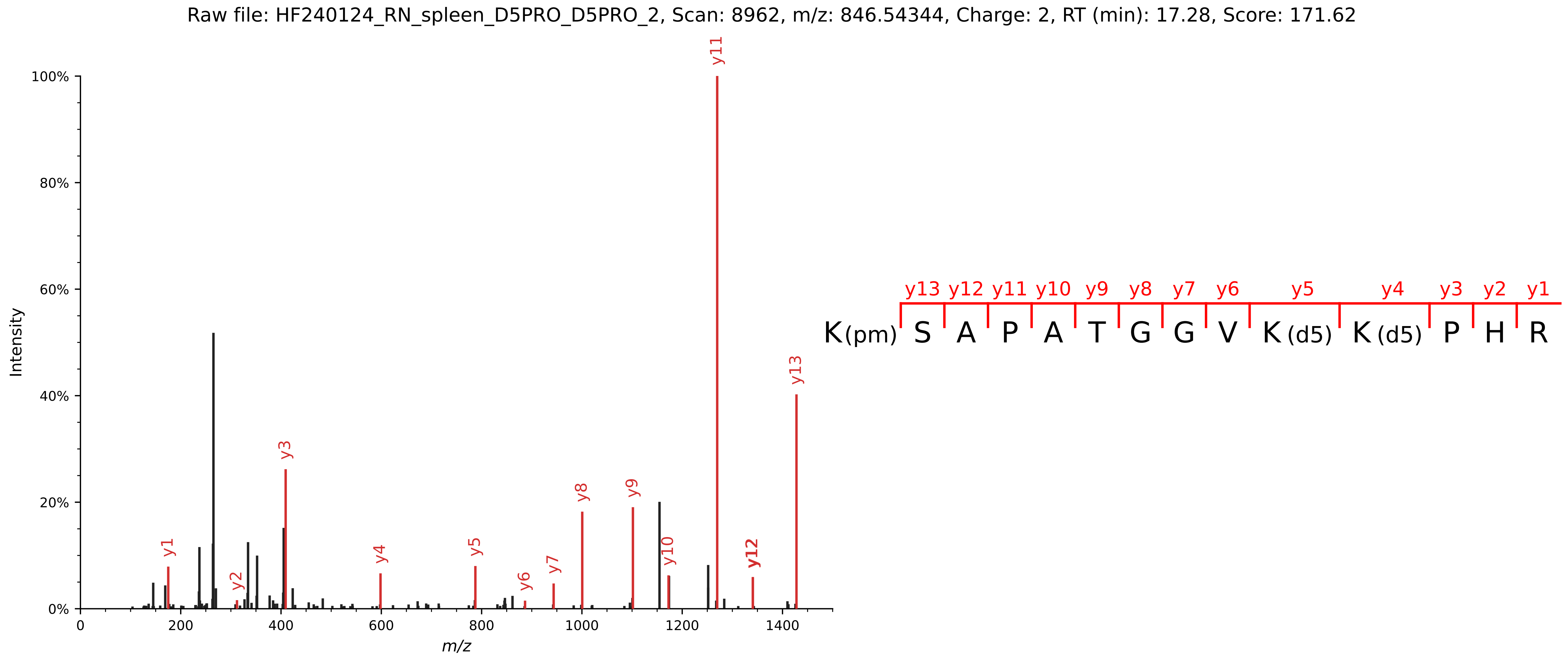

Raw file: HF240124_RN_spleen_D5PRO_D5PRO_2, Scan: 8962, m/z: 846.54344, Charge: 2, RT (min): 17.28, Score: 171.62
Intensity
m/z
y1
y2
y3
y4
y5
y6
y7
y8
y9
y10
y11
y12
y13
K(pm) S A P A T G G V K(d5) K(d5) P H R

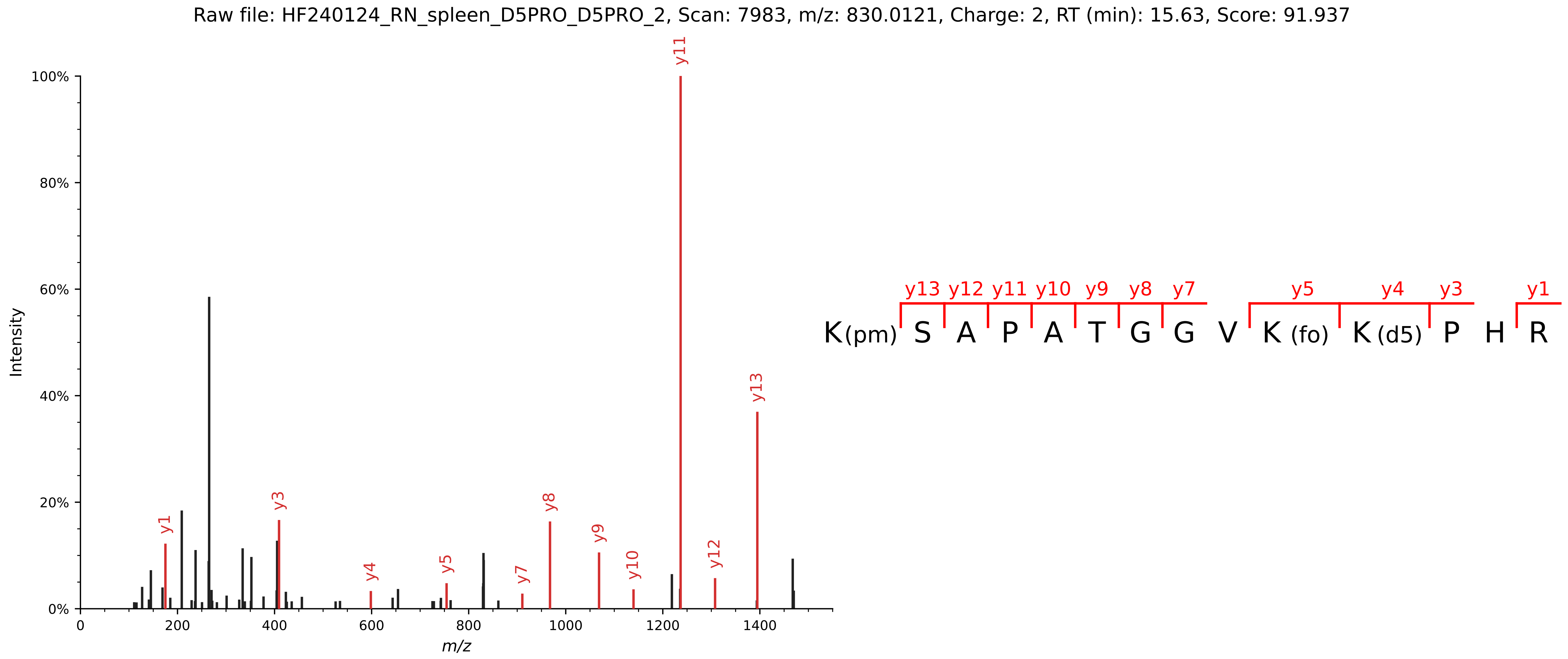
Raw file: HF240124_RN_spleen_D5PRO_D5PRO_2, Scan: 7983, m/z: 830.0121, Charge: 2, RT (min): 15.63, Score: 91.937
Intensity
m/z
y1
y3
y4
y5
y7
y8
y9
y10
y11
y12
y13
y13 y12 y11 y10 y9 y8 y7 y5 y4 y3 y1
K(pm) S A P A T G G V K (fo) K (d5) P H R

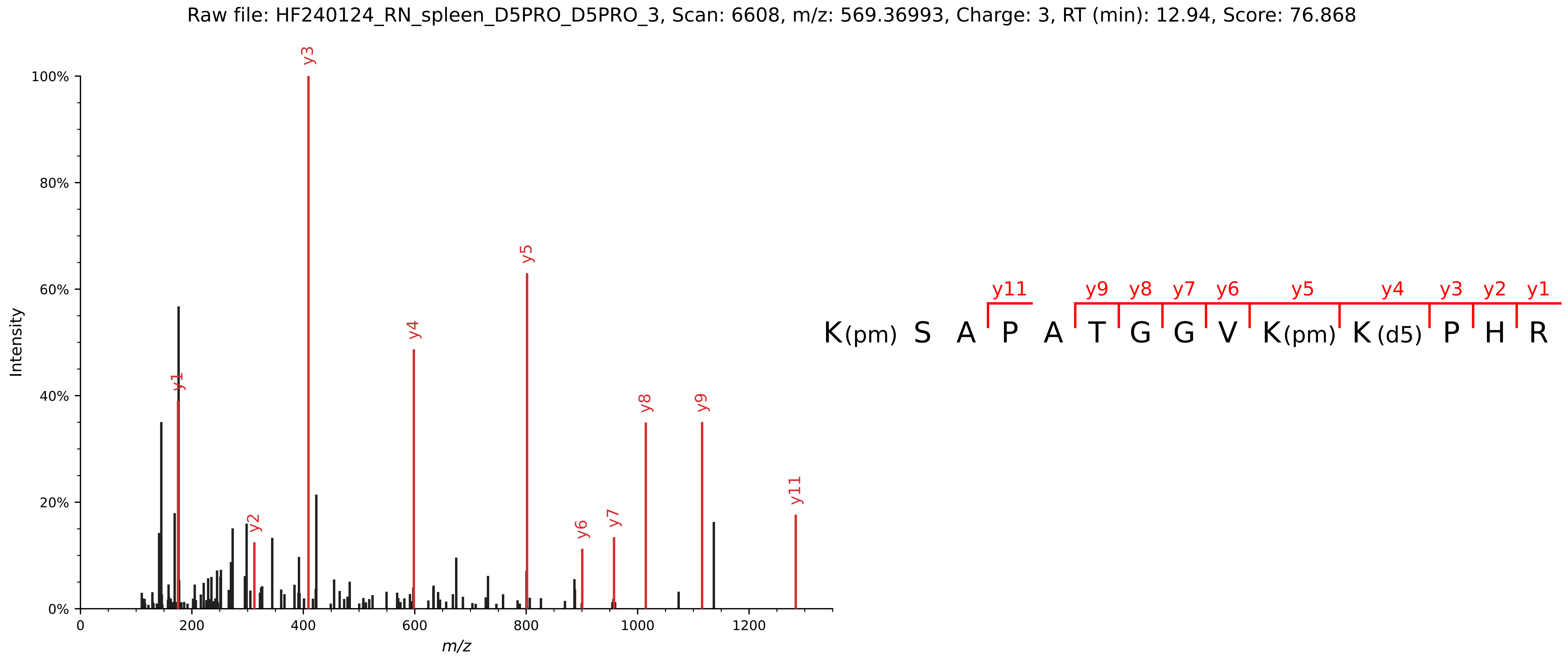
Raw file: HF240124_RN_spleen_D5PRO_D5PRO_3, Scan: 6608, m/z: 569.36993, Charge: 3, RT (min): 12.94, Score: 76.868
Intensity
100%
80%
60%
40%
20%
0%
0
200
400
600
800
1000
1200
m/z
y1
y2
y3
y4
y5
y6
y7
y8
y9
y11
K(pm) S A P A T G G V K(pm) K(d5) P H R
y11 y9 y8 y7 y6 y5 y4 y3 y2 y1

Raw file: HF240124_RN_spleen_D5PRO_D5PRO_3, Scan: 7559, m/z: 574.04182, Charge: 3, RT (min): 14.63, Score: 56.569

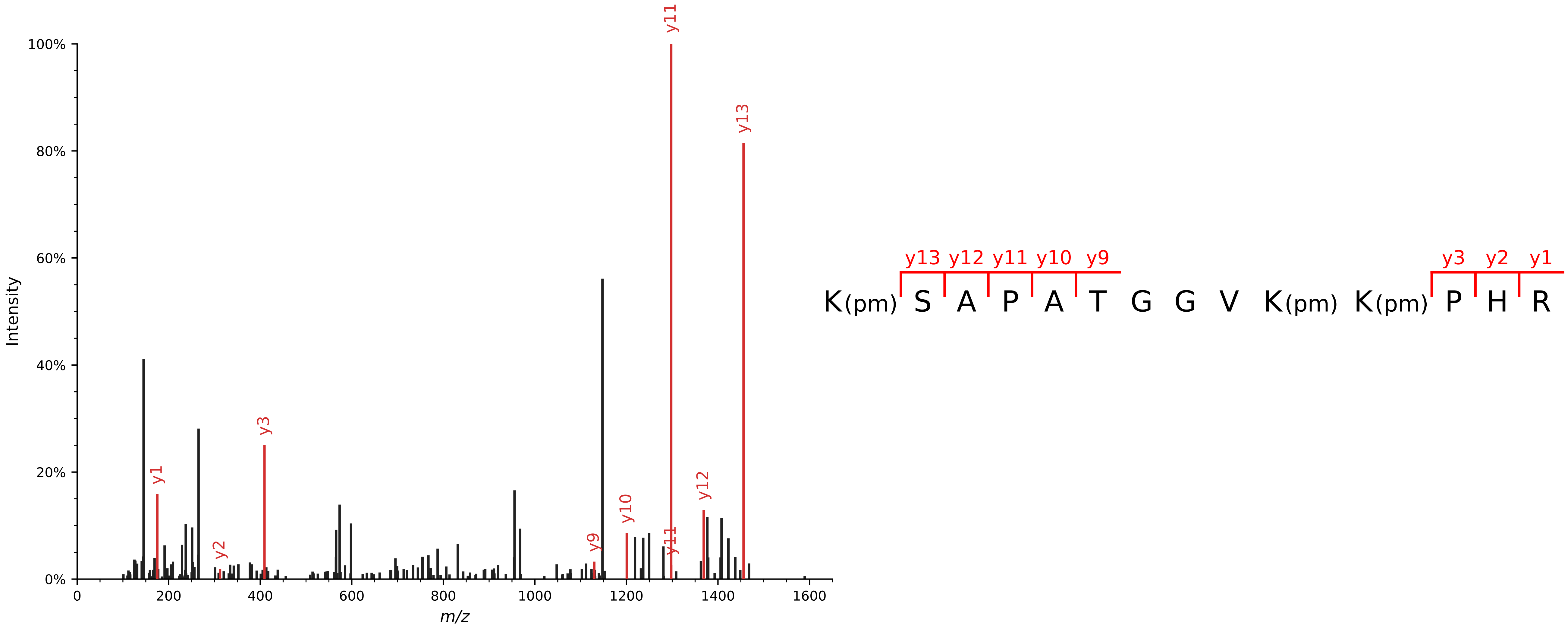

Raw file: HF240124_RN_spleen_D5PRO_D5PRO_3, Scan: 5277, m/z: 830.03029, Charge: 2, RT (min): 10.58, Score: 172.92

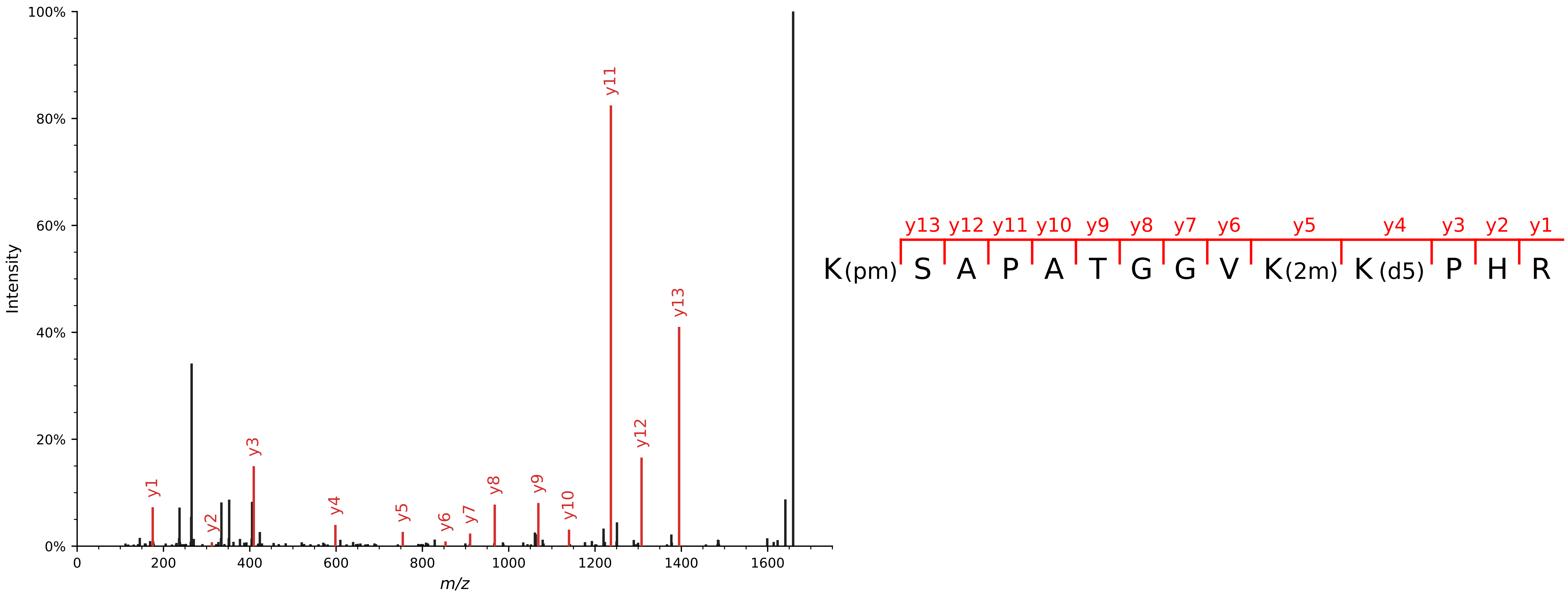

Raw file: HF240124_RN_spleen_D5PRO_D5PRO_3, Scan: 5278, m/z: 837.03811, Charge: 2, RT (min): 10.58, Score: 113.47

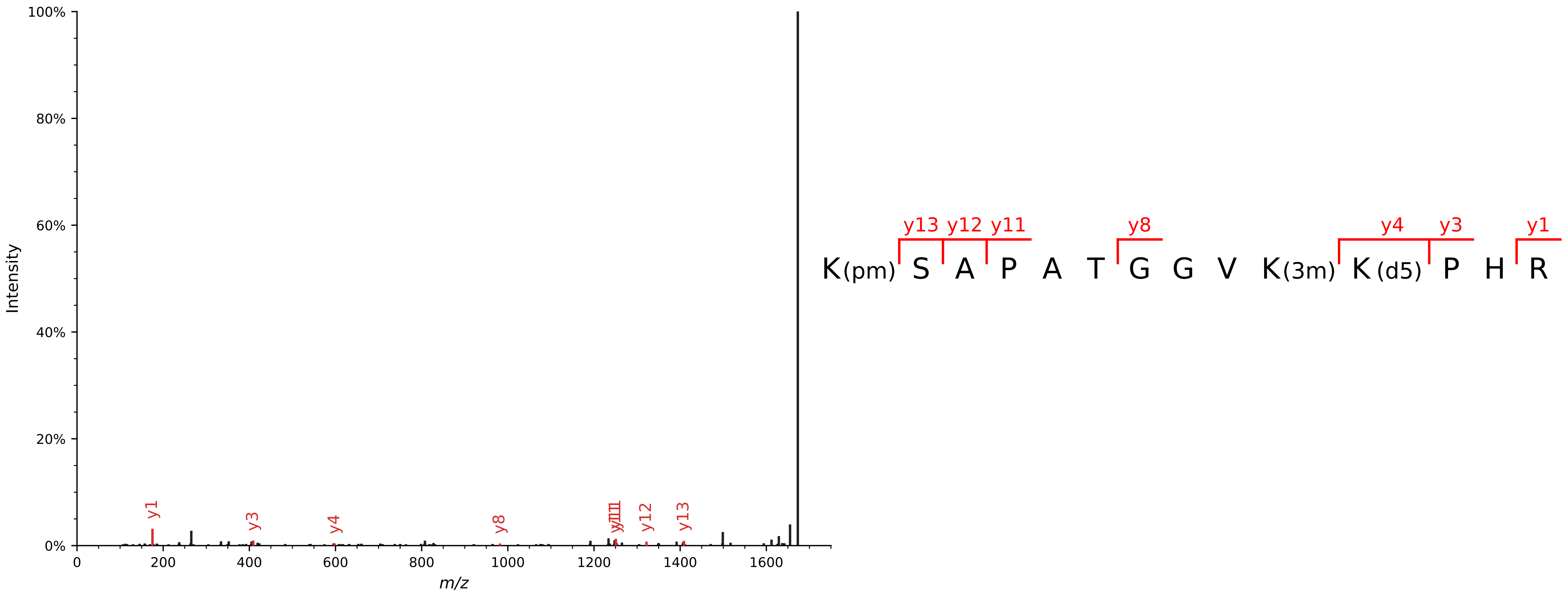

Raw file: HF240124_RN_spleen_D5PRO_D5PRO_2, Scan: 2939, m/z: 823.02246, Charge: 2, RT (min): 6.72, Score: 299.69

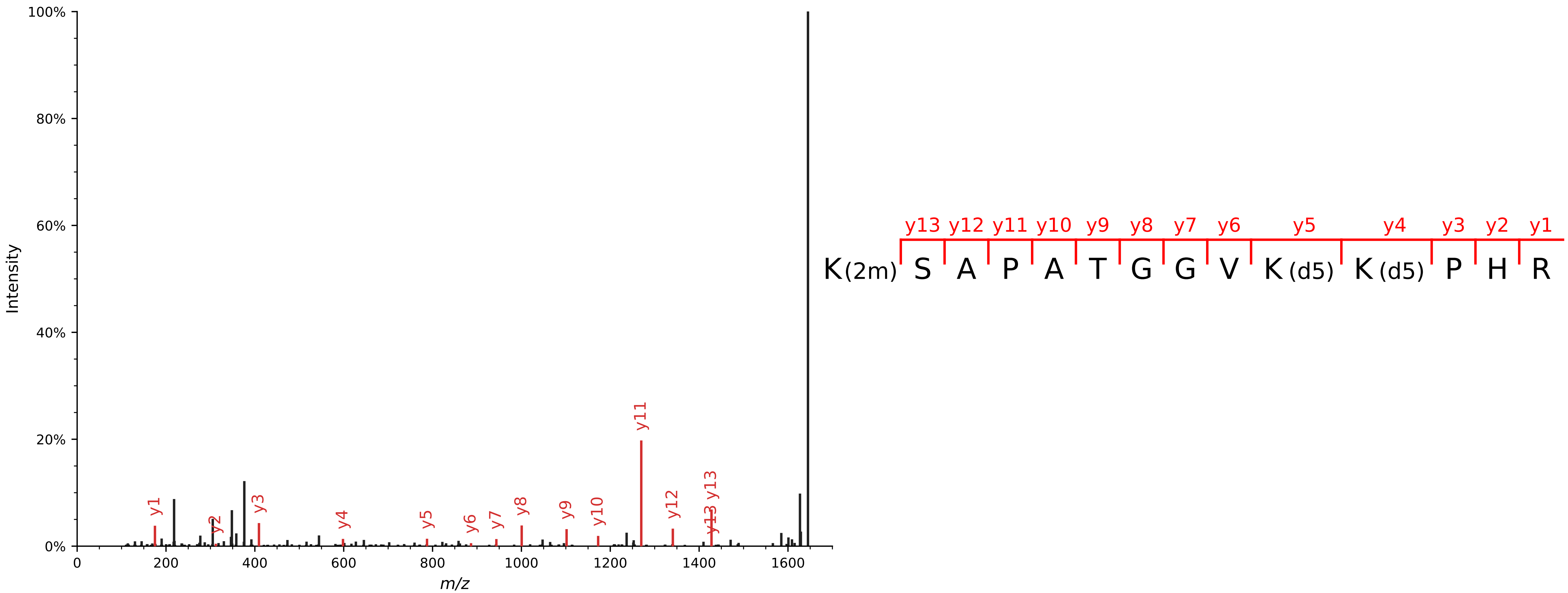

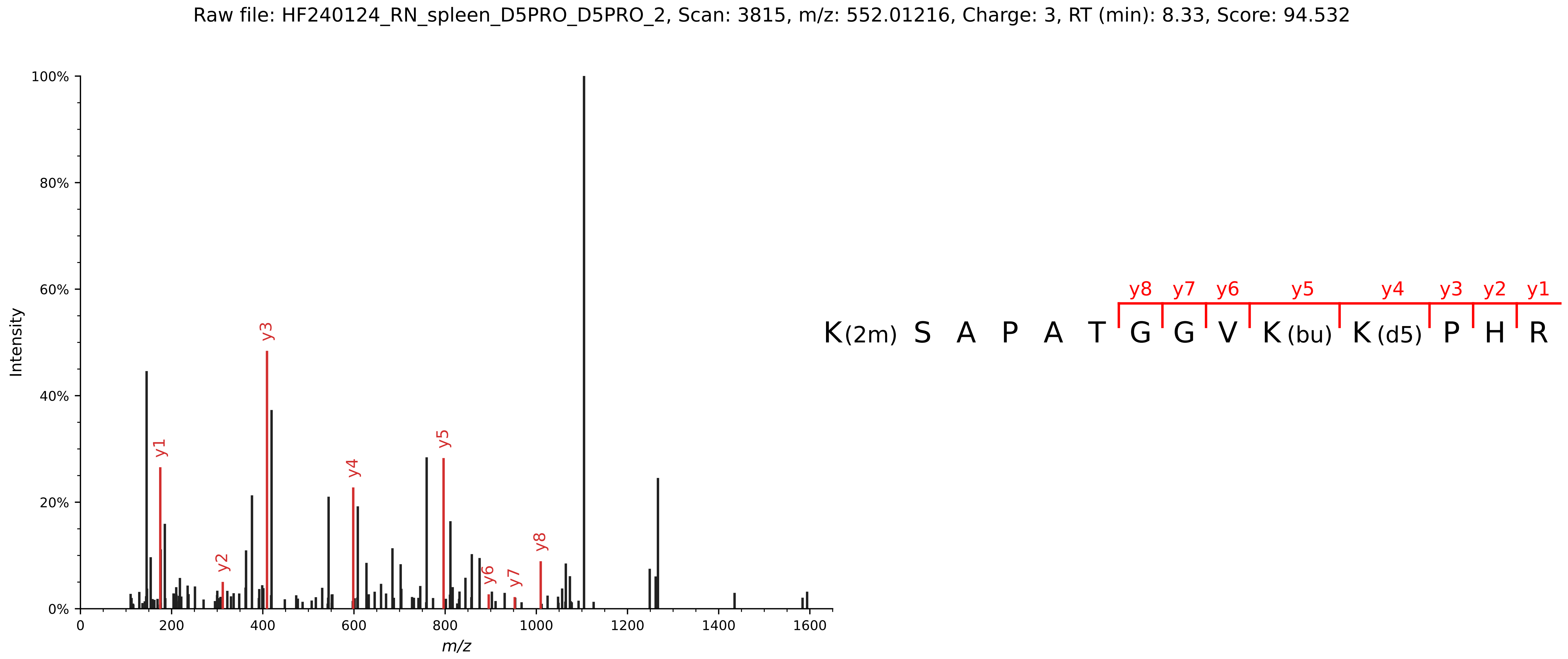

Raw file: HF240124_RN_spleen_D5PRO_D5PRO_2, Scan: 3815, m/z: 552.01216, Charge: 3, RT (min): 8.33, Score: 94.532
Intensity
m/z
y1
y2
y3
y4
y5
y6
y7
y8
K(2m) S A P A T G G V K(bu) K(d5) P H R

Raw file: HF240124_RN_spleen_D5PRO_D5PRO_2, Scan: 2447, m/z: 537.99651, Charge: 3, RT (min): 5.92, Score: 144.68

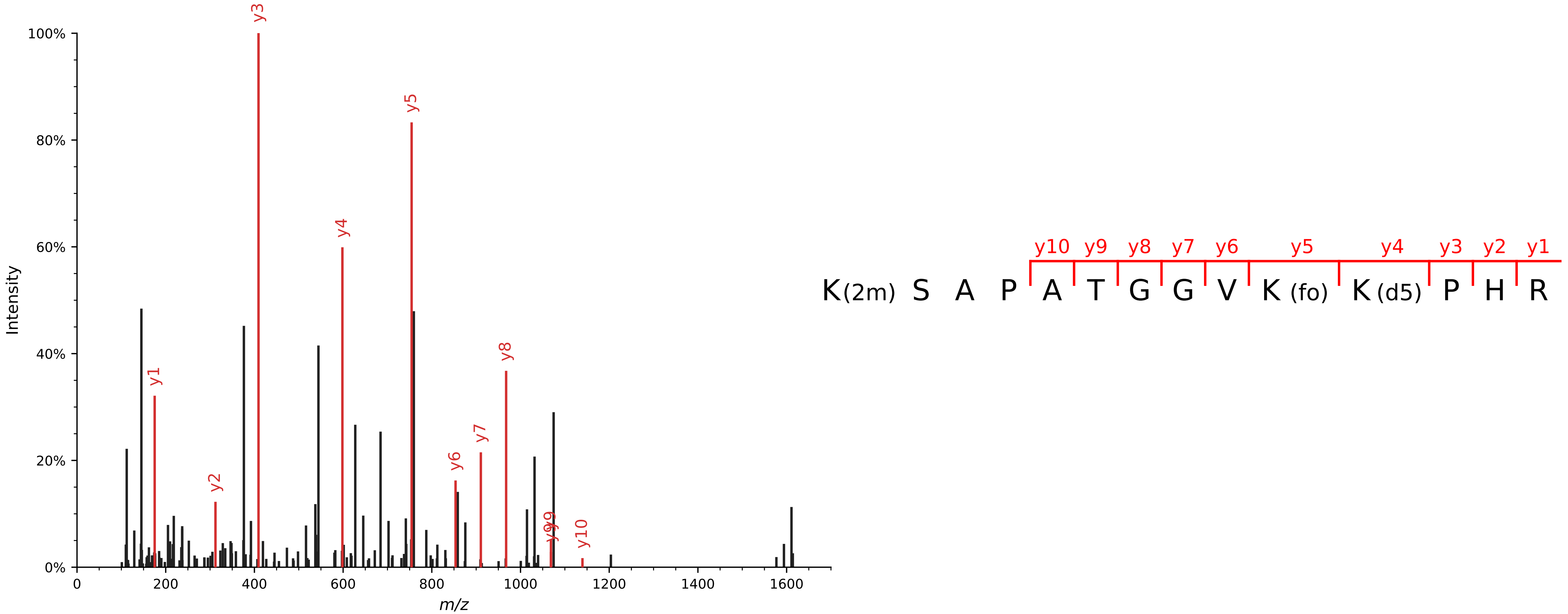

Raw file: HF240124_RN_spleen_D5PRO_D5PRO_2, Scan: 3957, m/z: 830.03029, Charge: 2, RT (min): 8.60, Score: 212.59

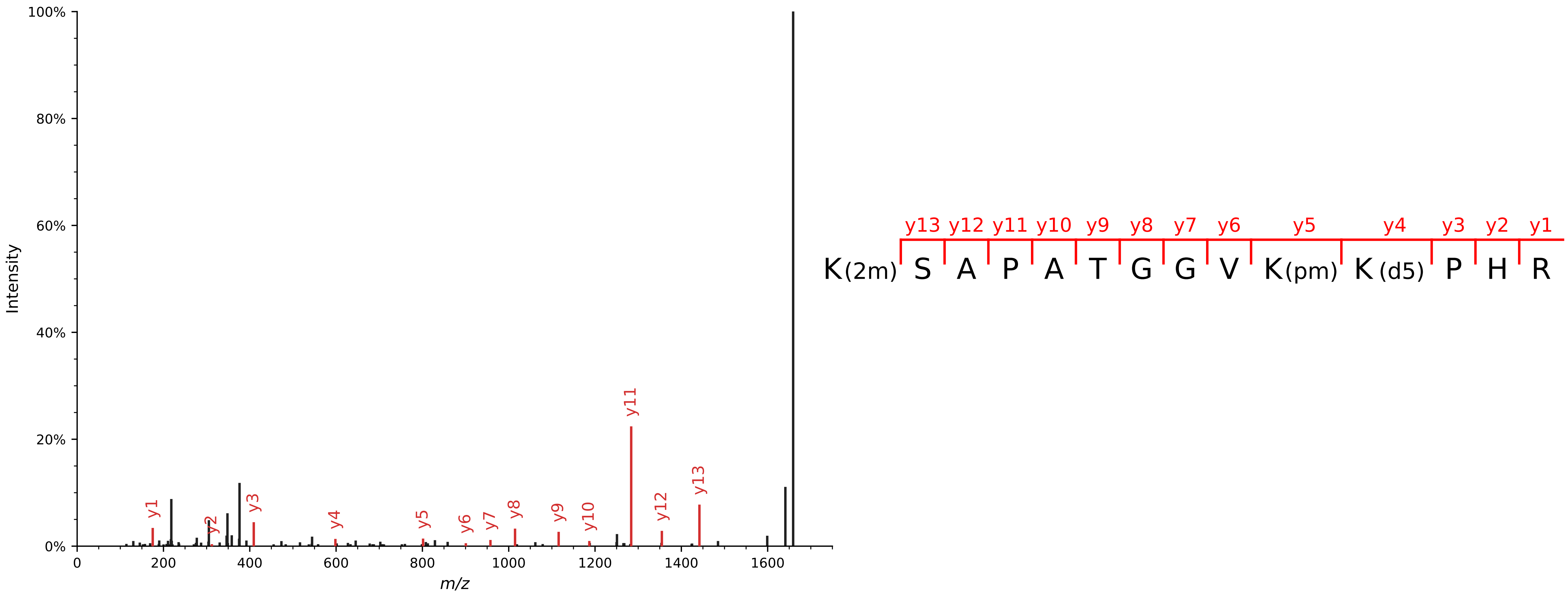

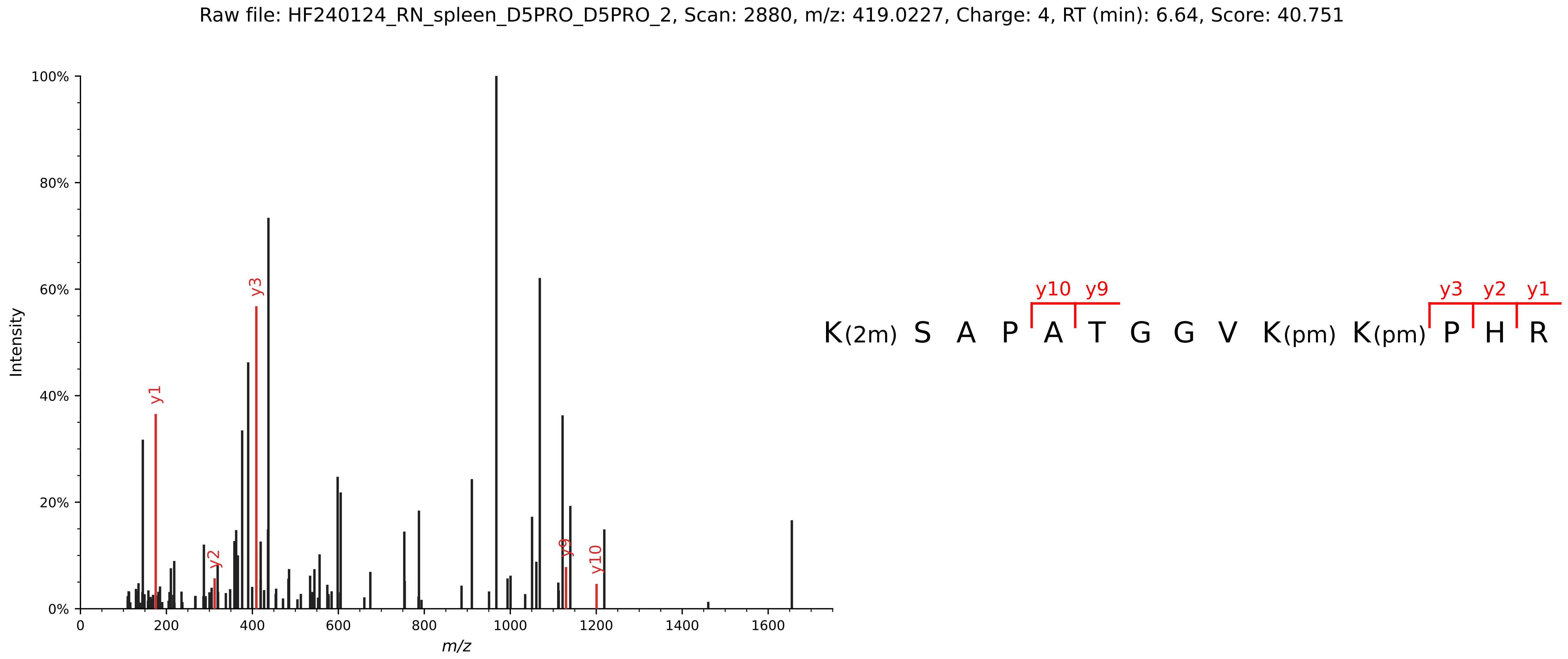

Raw file: HF240124_RN_spleen_D5PRO_D5PRO_2, Scan: 2880, m/z: 419.0227, Charge: 4, RT (min): 6.64, Score: 40.751
Intensity
m/z
0%
20%
40%
60%
80%
100%
0
200
400
600
800
1000
1200
1400
1600
y1
y2
y3
y9
y10
K(2m) S A P A T G G V K(pm) K(pm) P H R

Raw file: HF240124_RN_spleen_D5PRO_D5PRO_2, Scan: 1604, m/z: 806.50932, Charge: 2, RT (min): 4.52, Score: 225.63

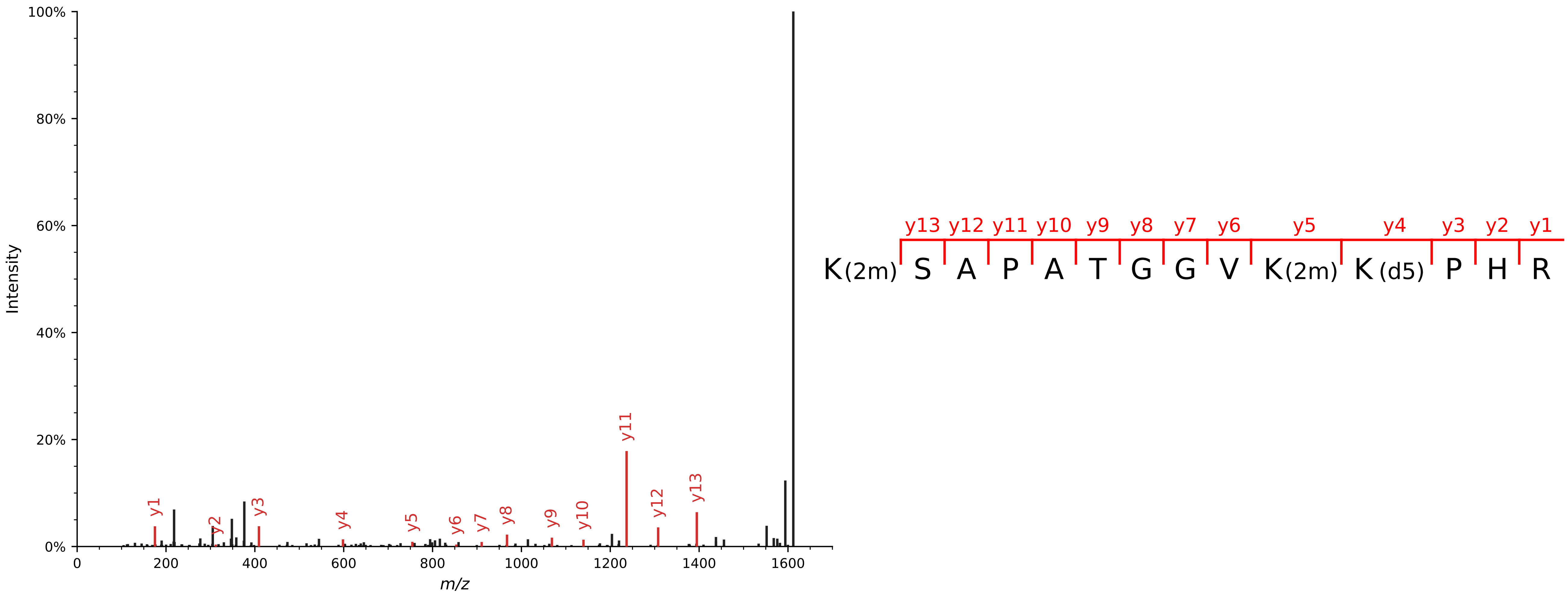

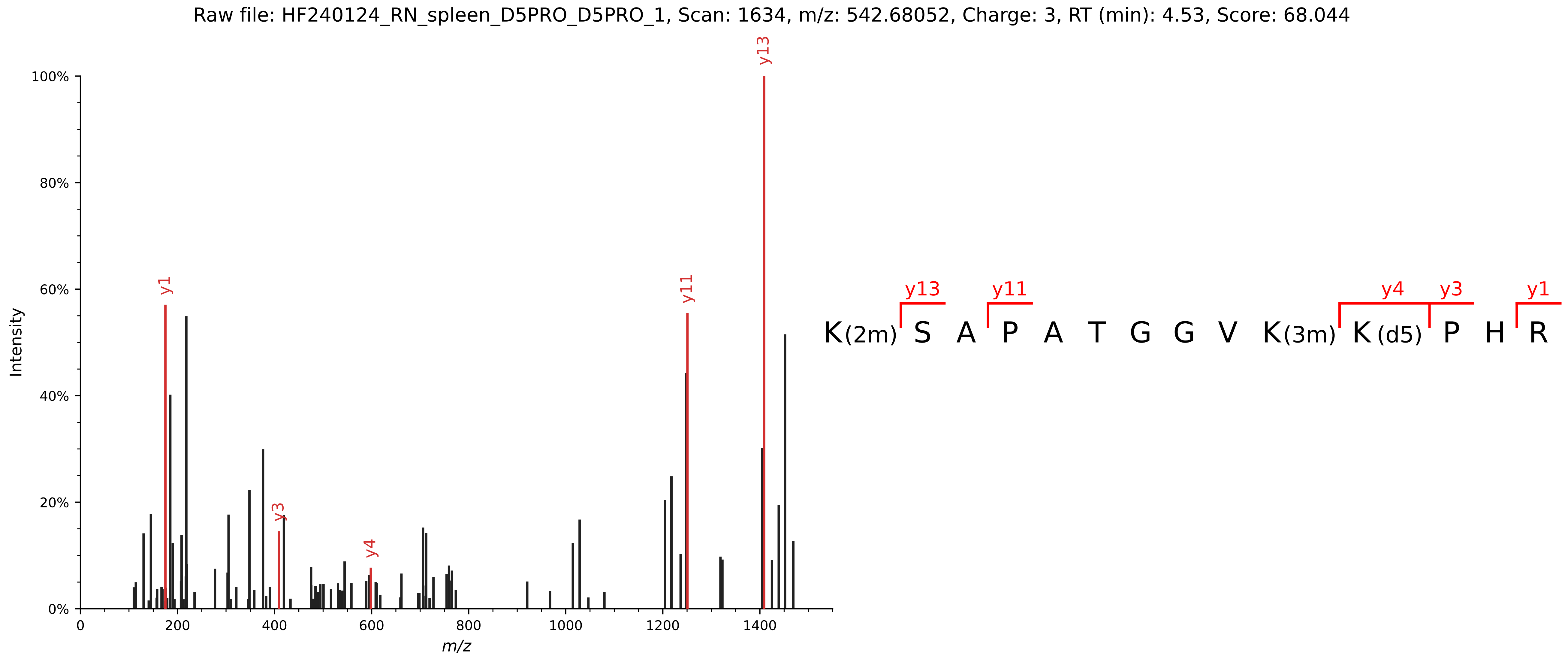

Raw file: HF240124_RN_spleen_D5PRO_D5PRO_1, Scan: 1634, m/z: 542.68052, Charge: 3, RT (min): 4.53, Score: 68.044
Intensity
m/z
y1
y3
y4
y11
y13
K(2m) S A P A T G G V K(3m) K(d5) P H R

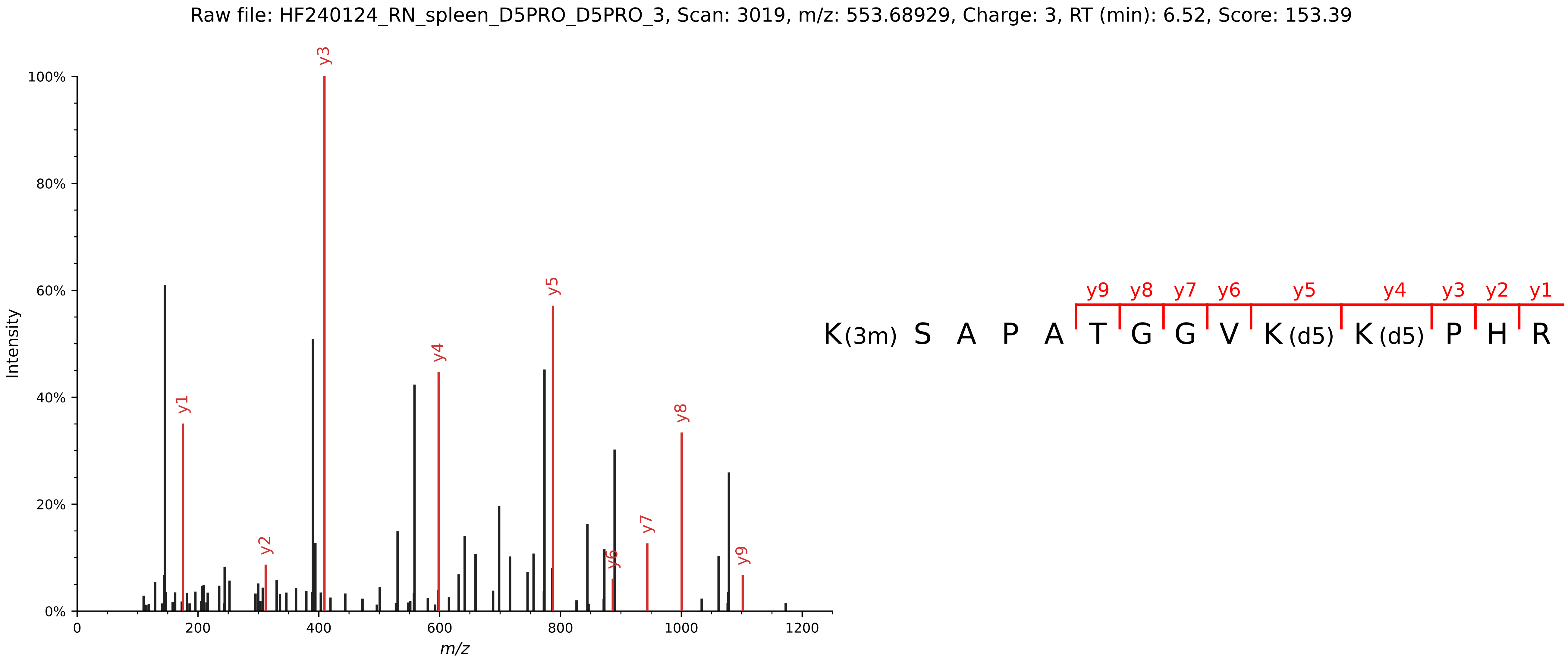
Raw file: HF240124_RN_spleen_D5PRO_D5PRO_3, Scan: 3019, m/z: 553.68929, Charge: 3, RT (min): 6.52, Score: 153.39
Intensity
m/z
y1
y2
y3
y4
y5
y6
y7
y8
y9
K(3m) S A P A T G G V K(d5) K(d5) P H R

Raw file: HF240124_RN_spleen_D5PRO_D5PRO_1, Scan: 4106, m/z: 837.03811, Charge: 2, RT (min): 8.78, Score: 151.78

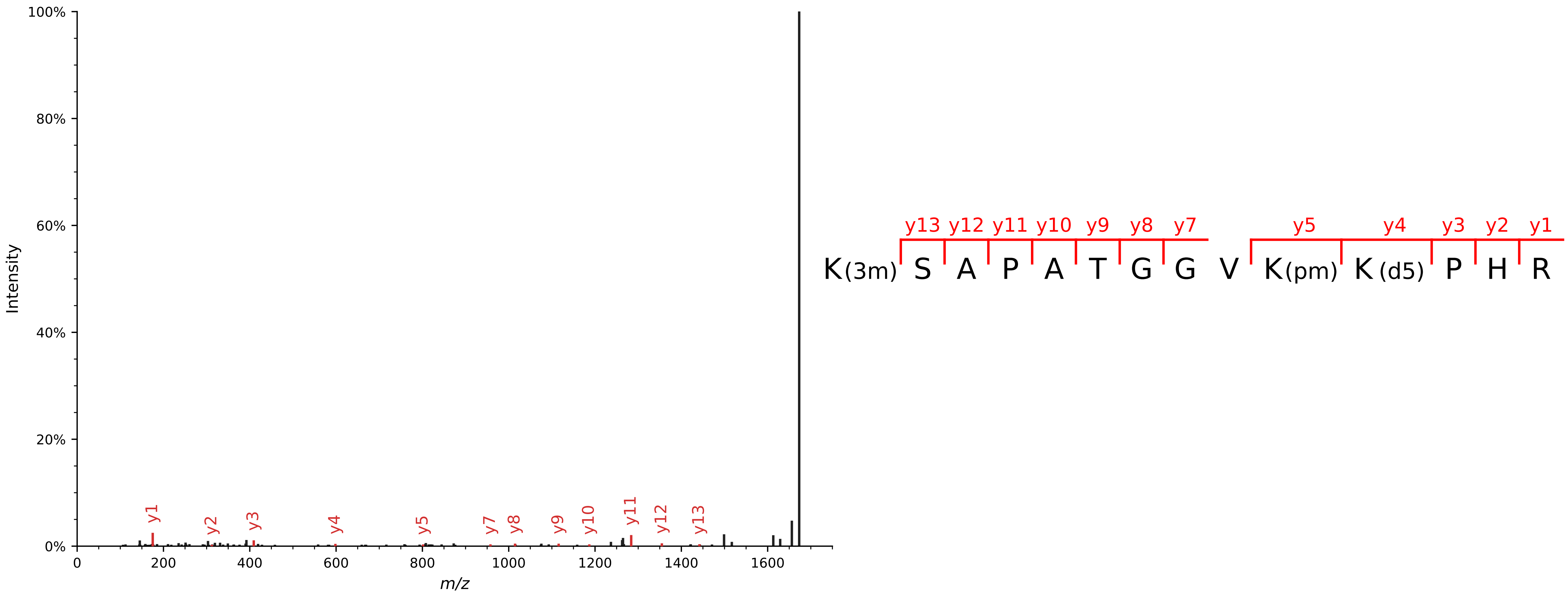

Raw file: HF240124_RN_spleen_D5PRO_D5PRO_2, Scan: 1592, m/z: 542.68052, Charge: 3, RT (min): 4.51, Score: 76.322

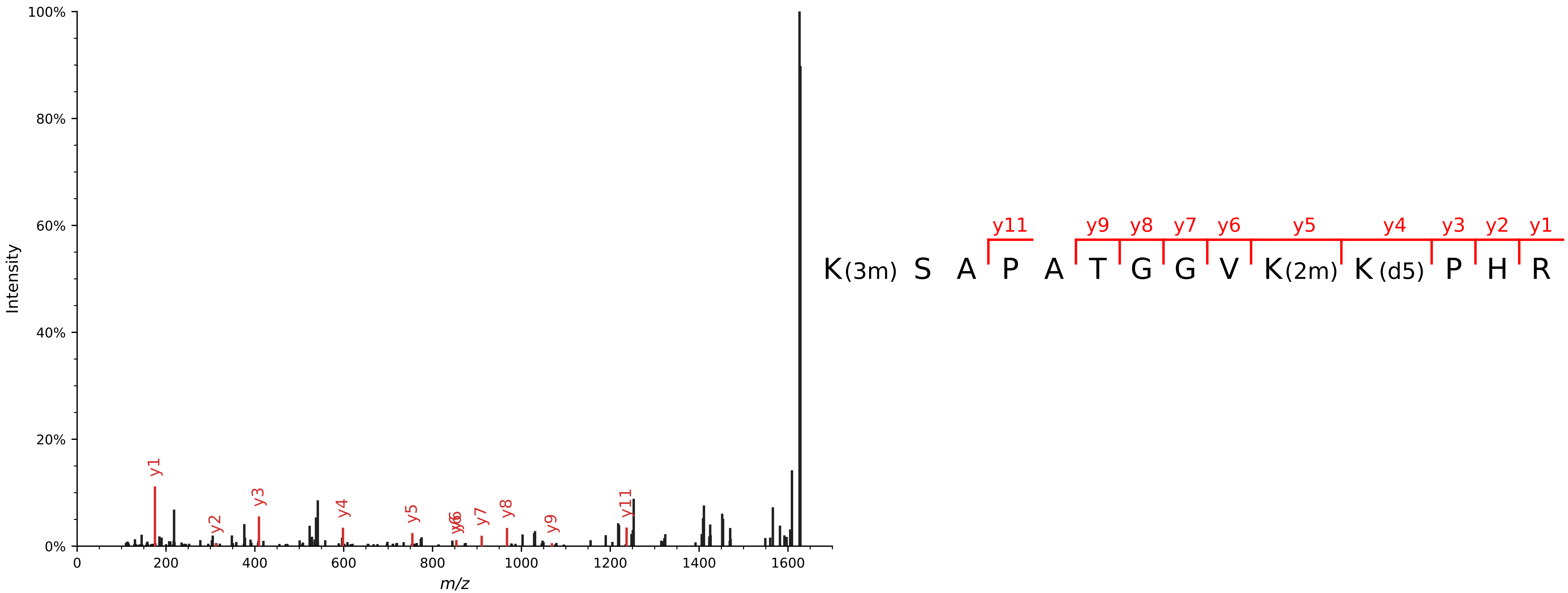

Raw file: HF240124_RN_spleen_D5PRO_D5PRO_3, Scan: 8493, m/z: 564.69805, Charge: 3, RT (min): 16.27, Score: 98.942

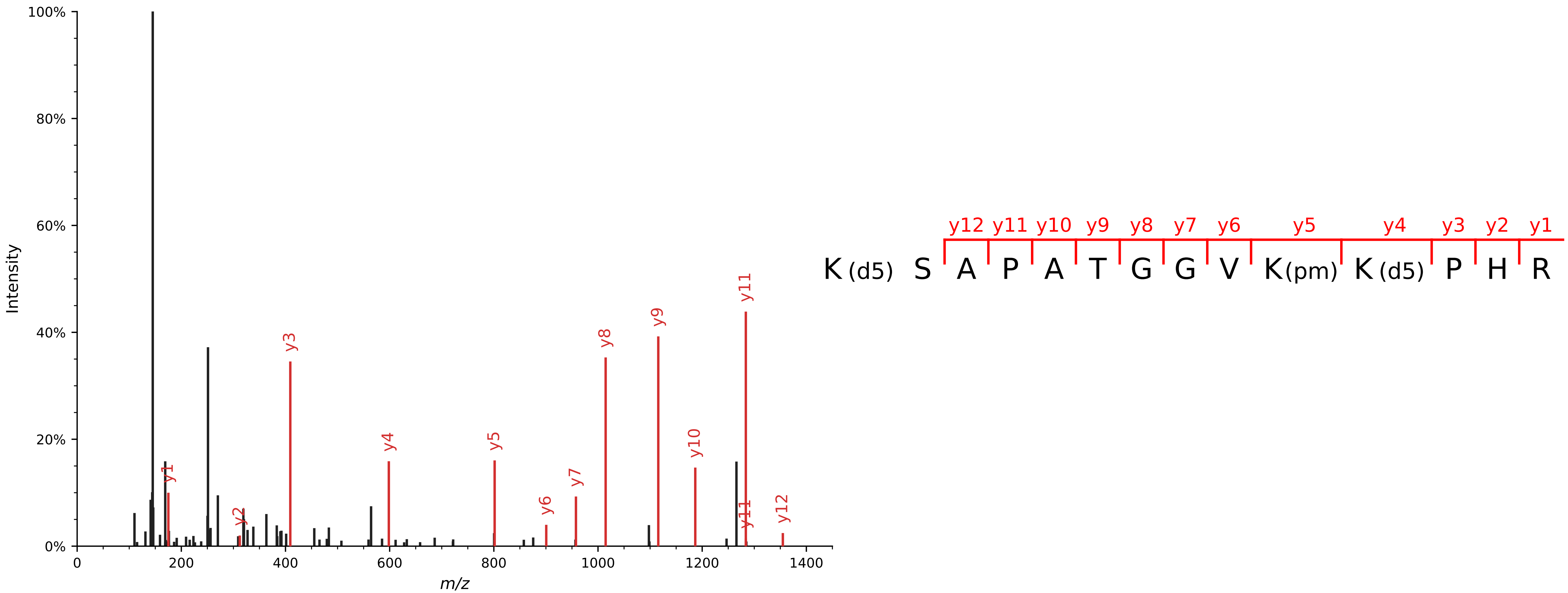

Raw file: HF240124_RN_spleen_D5PRO_D5PRO_1, Scan: 4314, m/z: 823.02246, Charge: 2, RT (min): 9.12, Score: 171.62

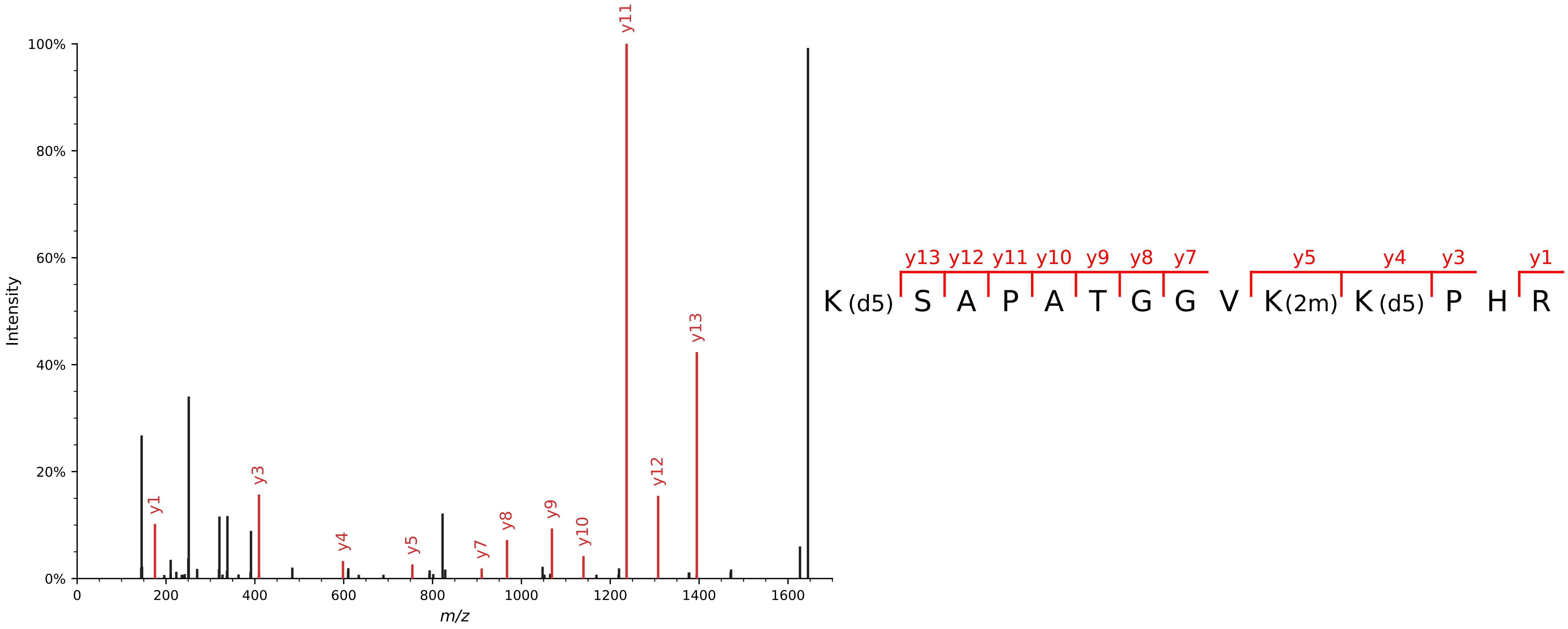

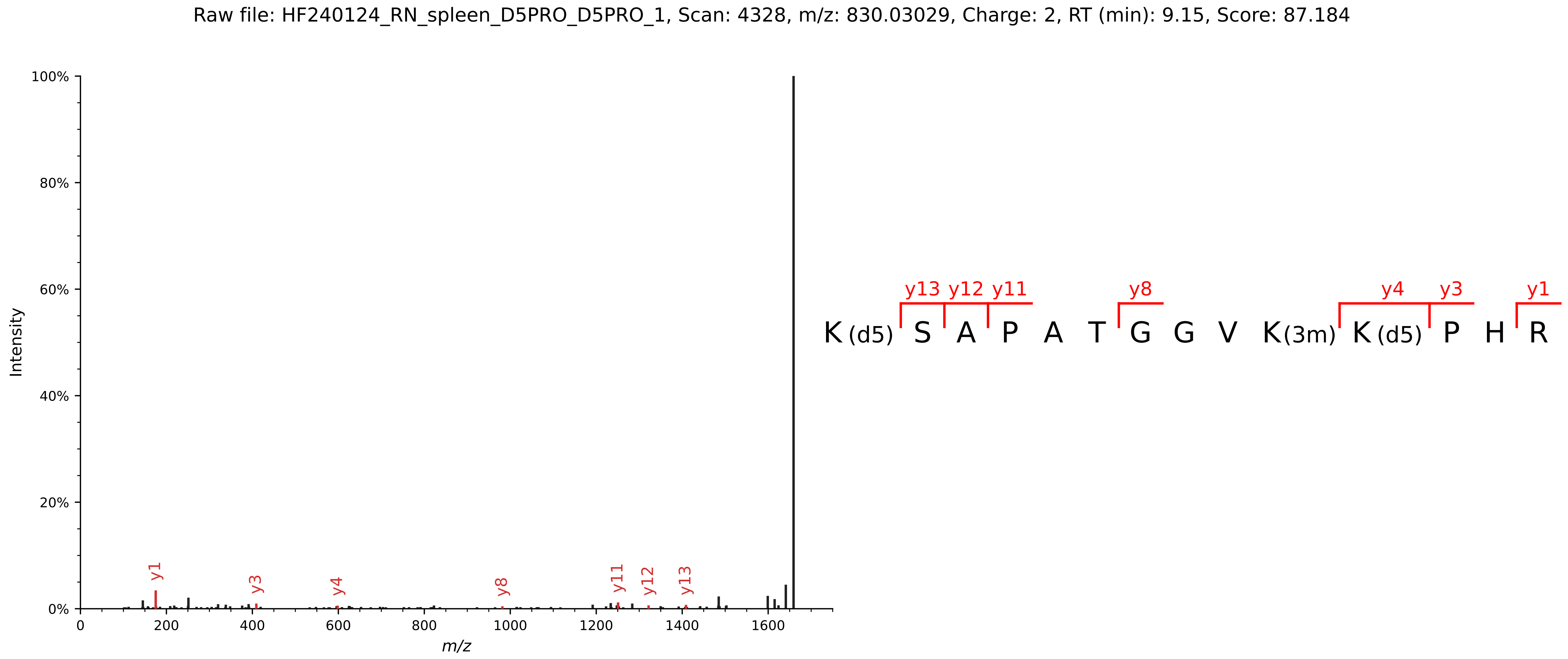

Raw file: HF240124_RN_spleen_D5PRO_D5PRO_1, Scan: 4328, m/z: 830.03029, Charge: 2, RT (min): 9.15, Score: 87.184
Intensity
m/z
y1
y3
y4
y8
y11
y12
y13
K(d5) S A P A T G G V K(3m) K(d5) P H R

Raw file: HF240124_RN_spleen_D5PRO_D5PRO_1, Scan: 19359, m/z: 691.40853, Charge: 2, RT (min): 35.54, Score: 161.79

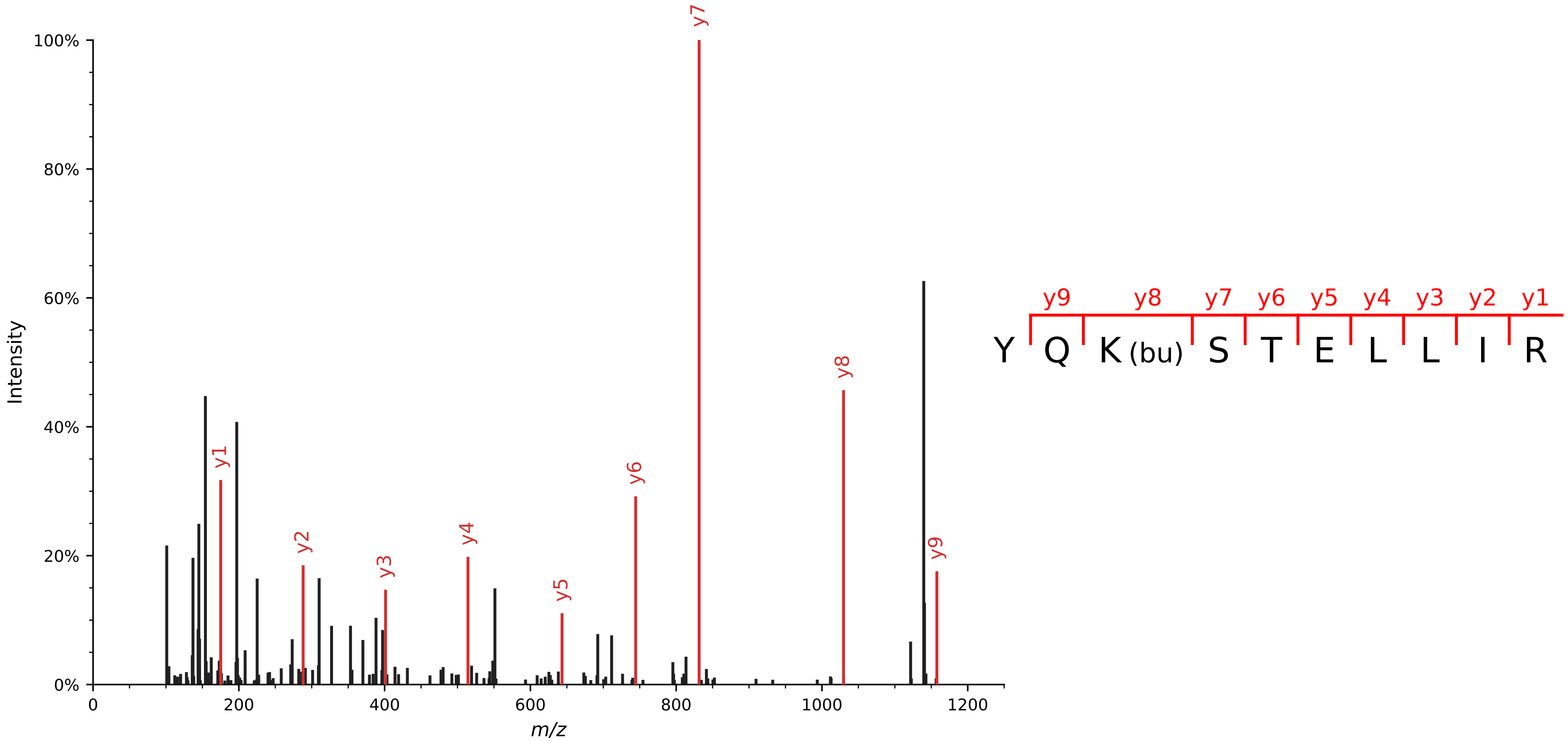

Raw file: HF240124_RN_spleen_D5PRO_D5PRO_3, Scan: 15871, m/z: 670.38505, Charge: 2, RT (min): 29.54, Score: 103.7

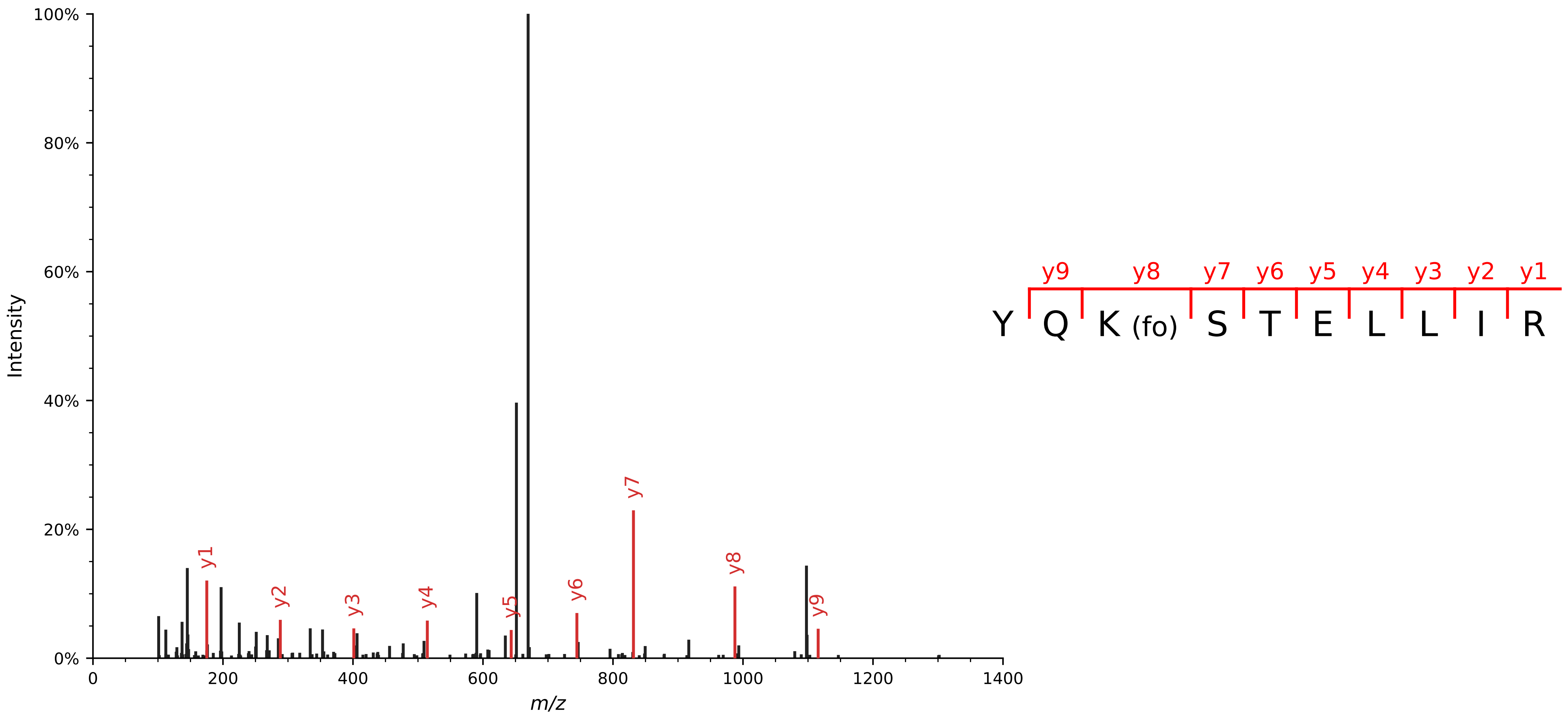

Raw file: HF240124_RN_spleen_D5PRO_D5PRO_3, Scan: 18622, m/z: 693.92422, Charge: 2, RT (min): 34.52, Score: 99.802

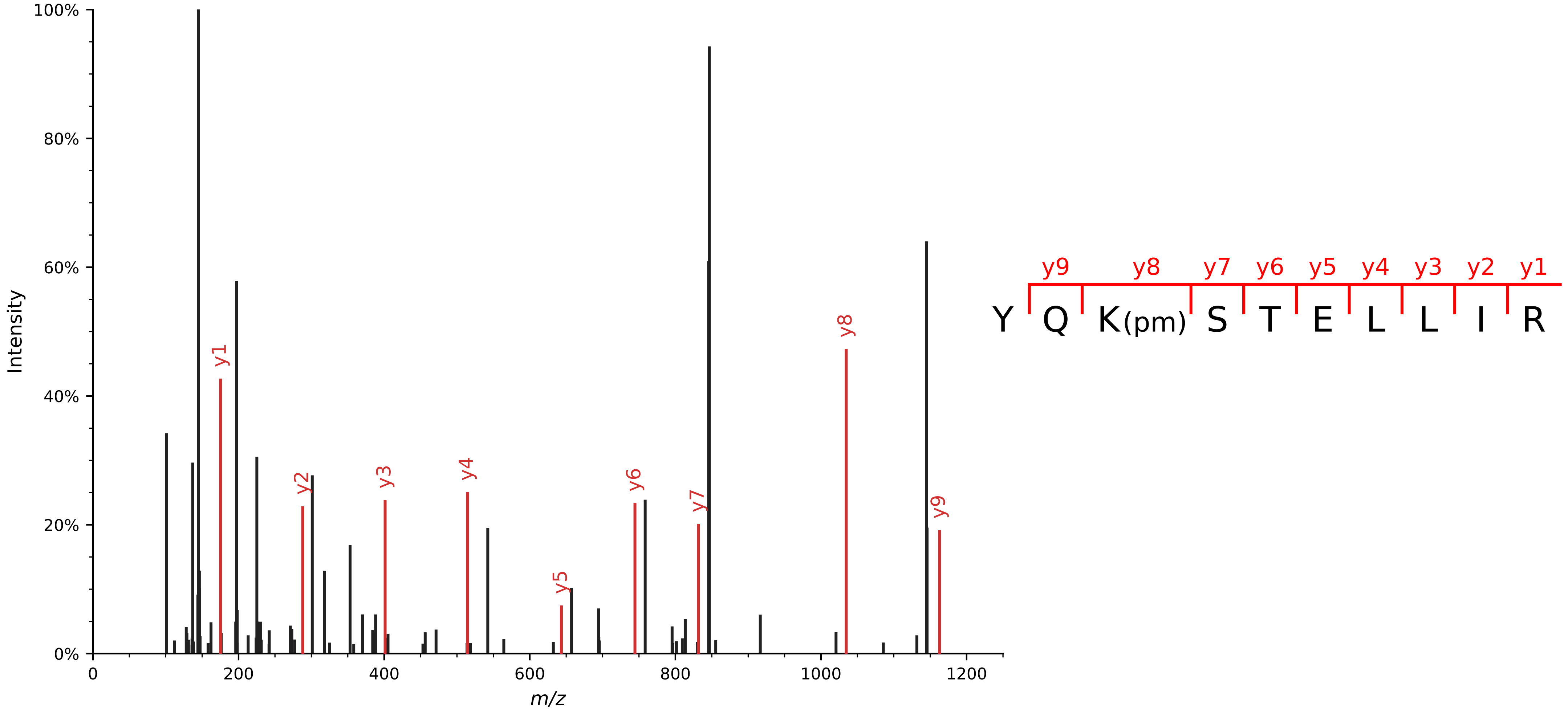

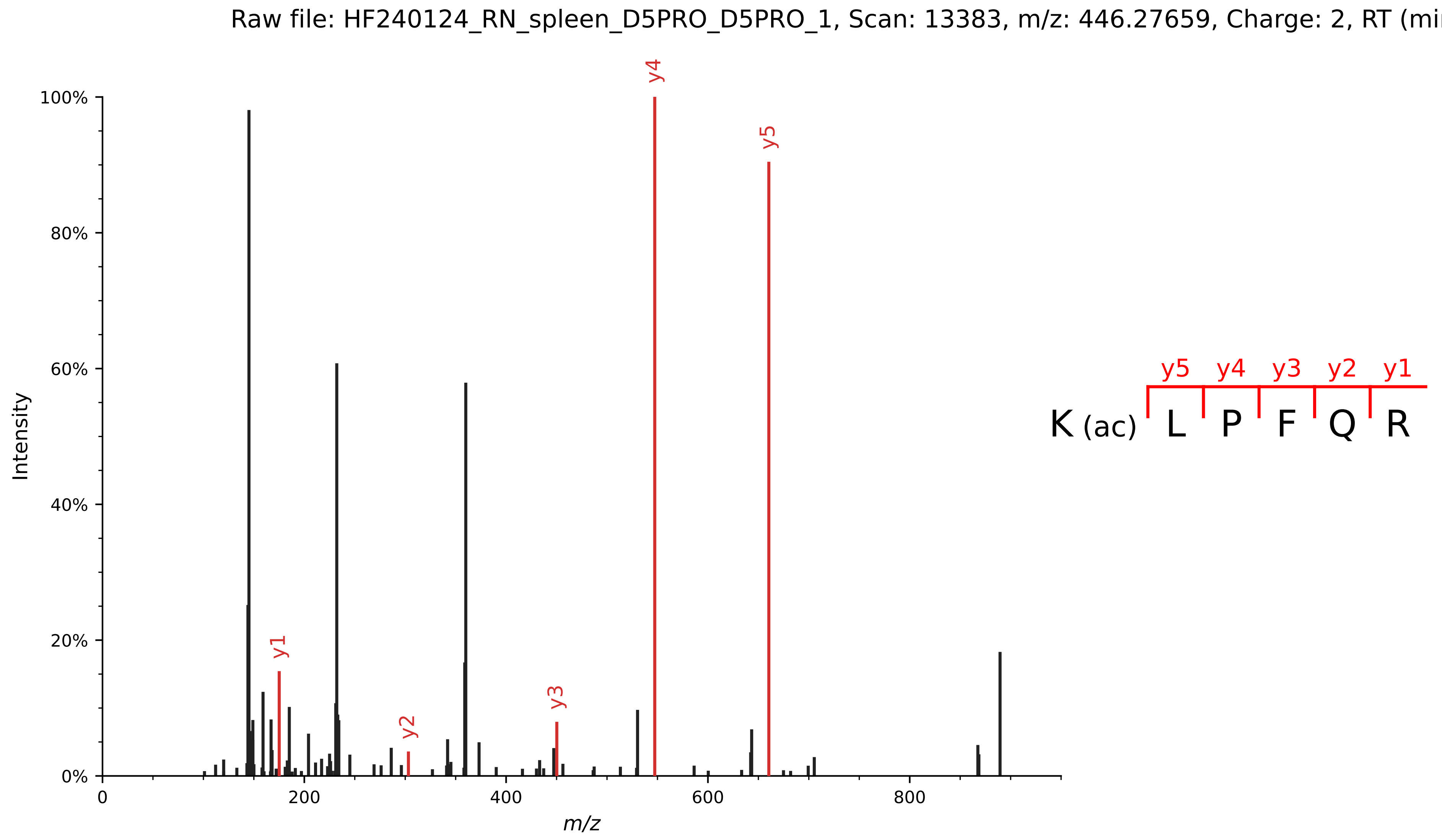

Raw file: HF240124_RN_spleen_D5PRO_D5PRO_1, Scan: 13383, m/z: 446.27659, Charge: 2, RT (min): 24.89, Score: 71.359
Intensity
m/z
y1
y2
y3
y4
y5
K (ac) L P F Q R

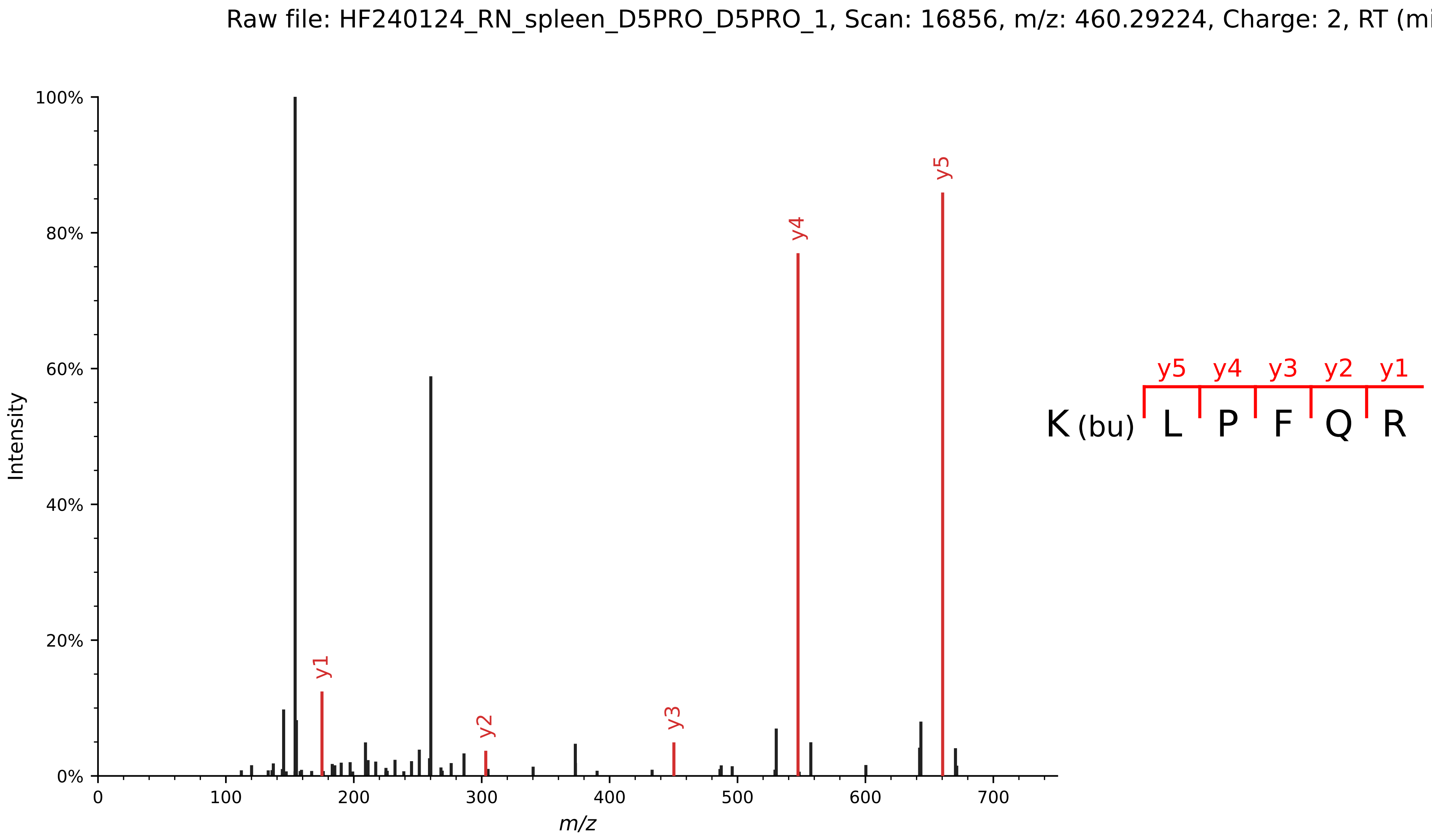

Raw file: HF240124_RN_spleen_D5PRO_D5PRO_1, Scan: 16856, m/z: 460.29224, Charge: 2, RT (min): 31.10, Score: 113.82
Intensity
100%
80%
60%
40%
20%
0%
0
100
200
300
400
500
600
700
m/z
y1
y2
y3
y4
y5
y5 y4 y3 y2 y1
K (bu) L P F Q R

Raw file: HF240124_RN_spleen_D5PRO_D5PRO_1, Scan: 13273, m/z: 439.26876, Charge: 2, RT (min): 24.69, Score: 92.279

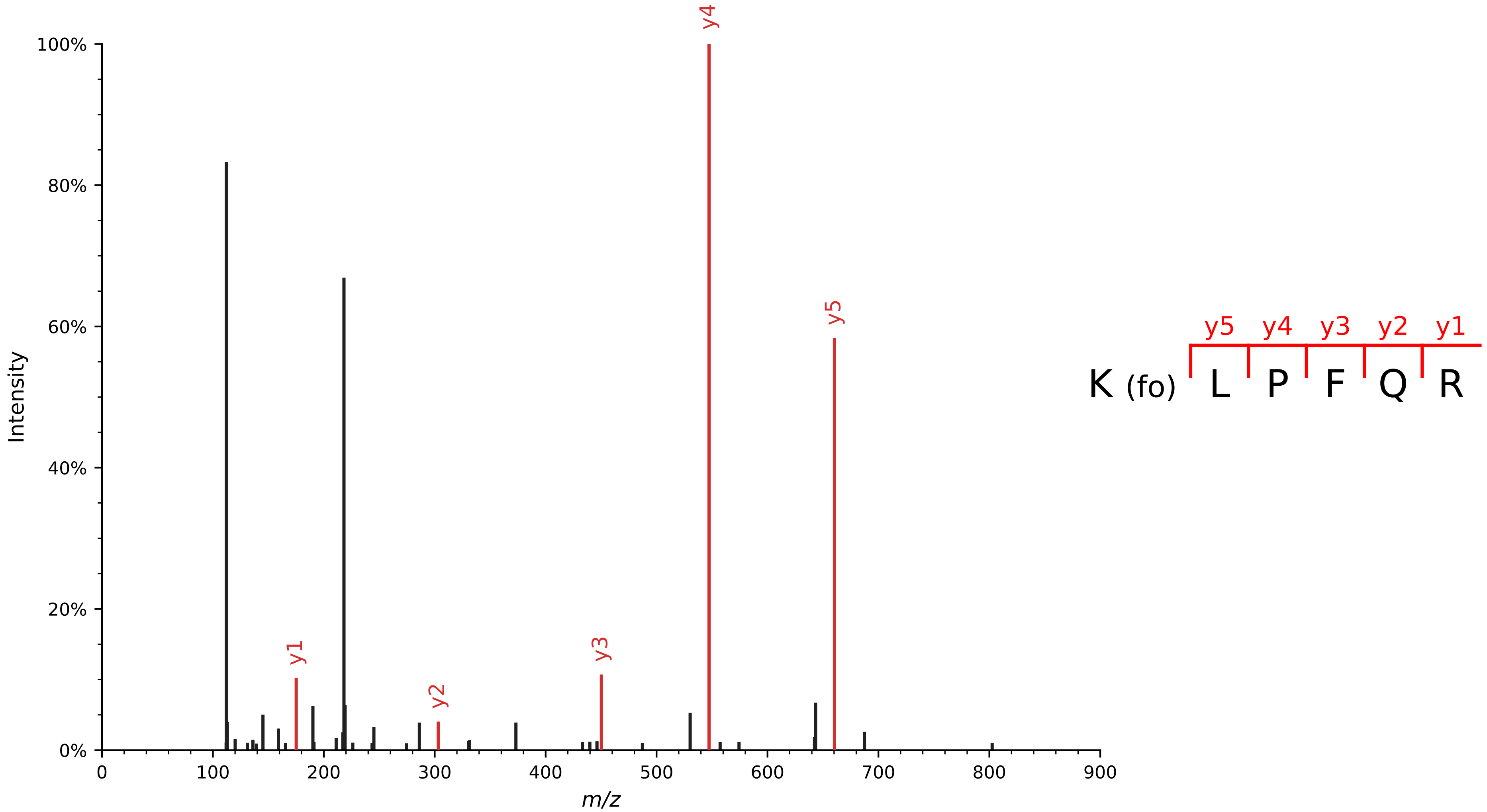

Raw file: HF240124_RN_spleen_D5PRO_D5PRO_1, Scan: 10334, m/z: 453.28441, Charge: 2, RT (min): 19.51, Score: 46.351

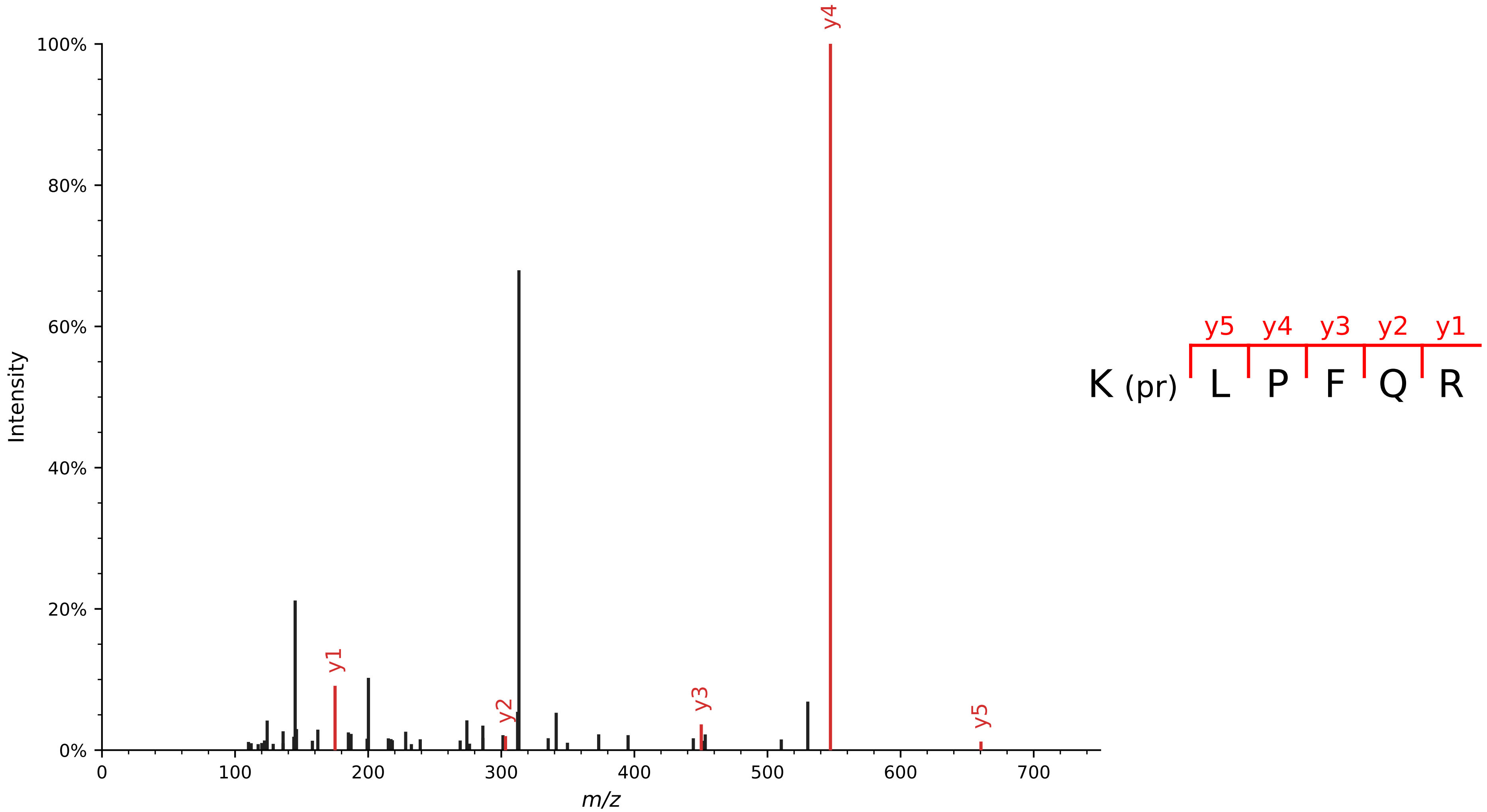

Raw file: HF240124_RN_spleen_D5PRO_D5PRO_1, Scan: 21518, m/z: 733.89852, Charge: 2, RT (min): 39.43, Score: 139.38

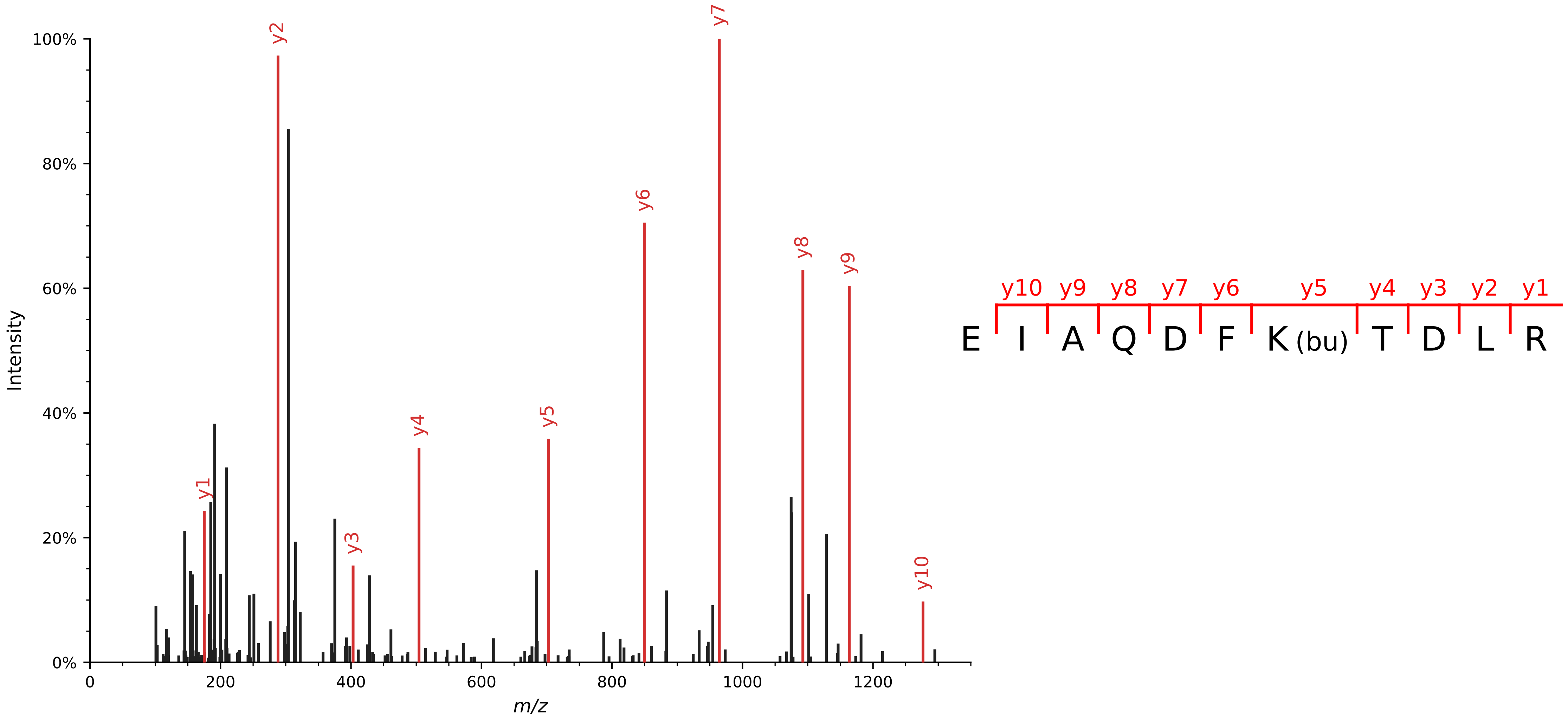

Raw file: HF240124_RN_spleen_D5PRO_D5PRO_3, Scan: 18229, m/z: 712.87505, Charge: 2, RT (min): 33.81, Score: 115.78

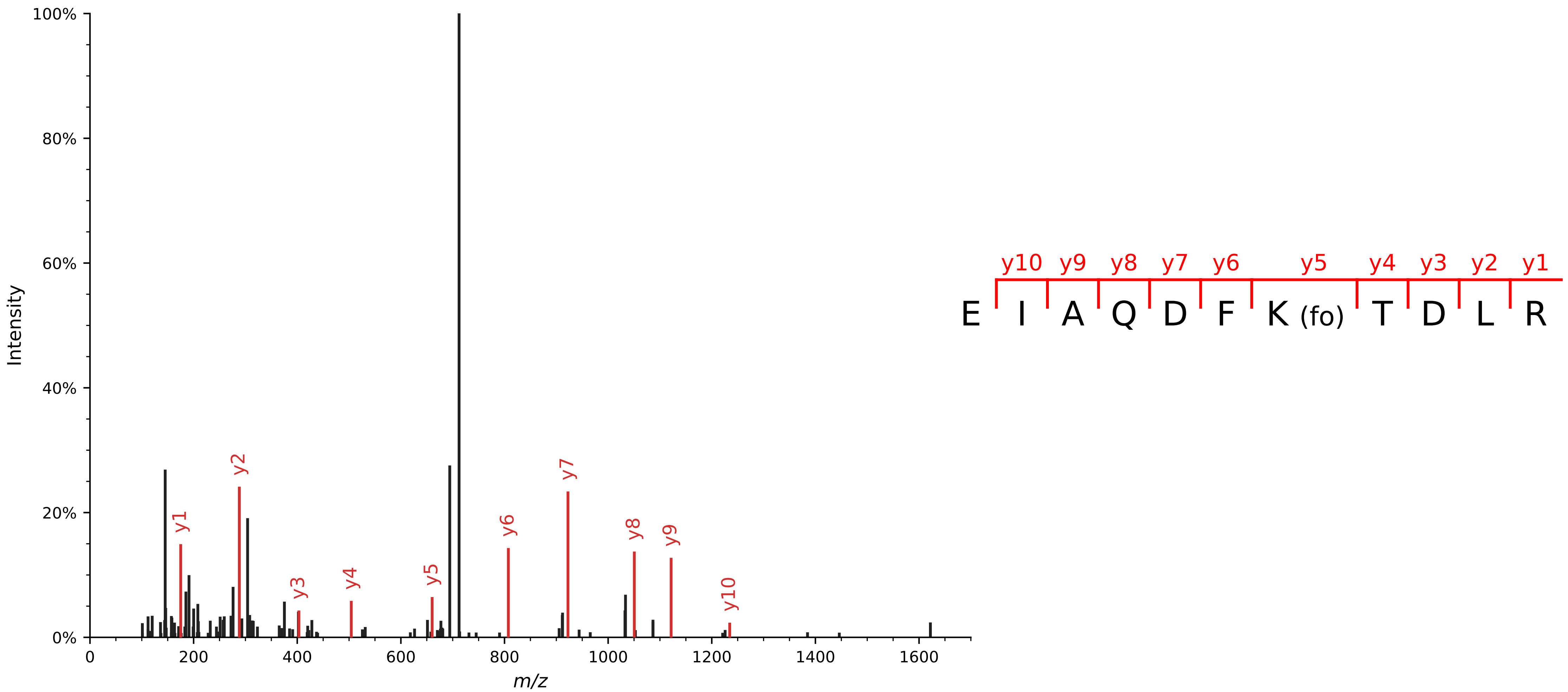

Raw file: HF240124_RN_spleen_D5PRO_D5PRO_2, Scan: 21662, m/z: 736.41421, Charge: 2, RT (min): 39.92, Score: 194.06

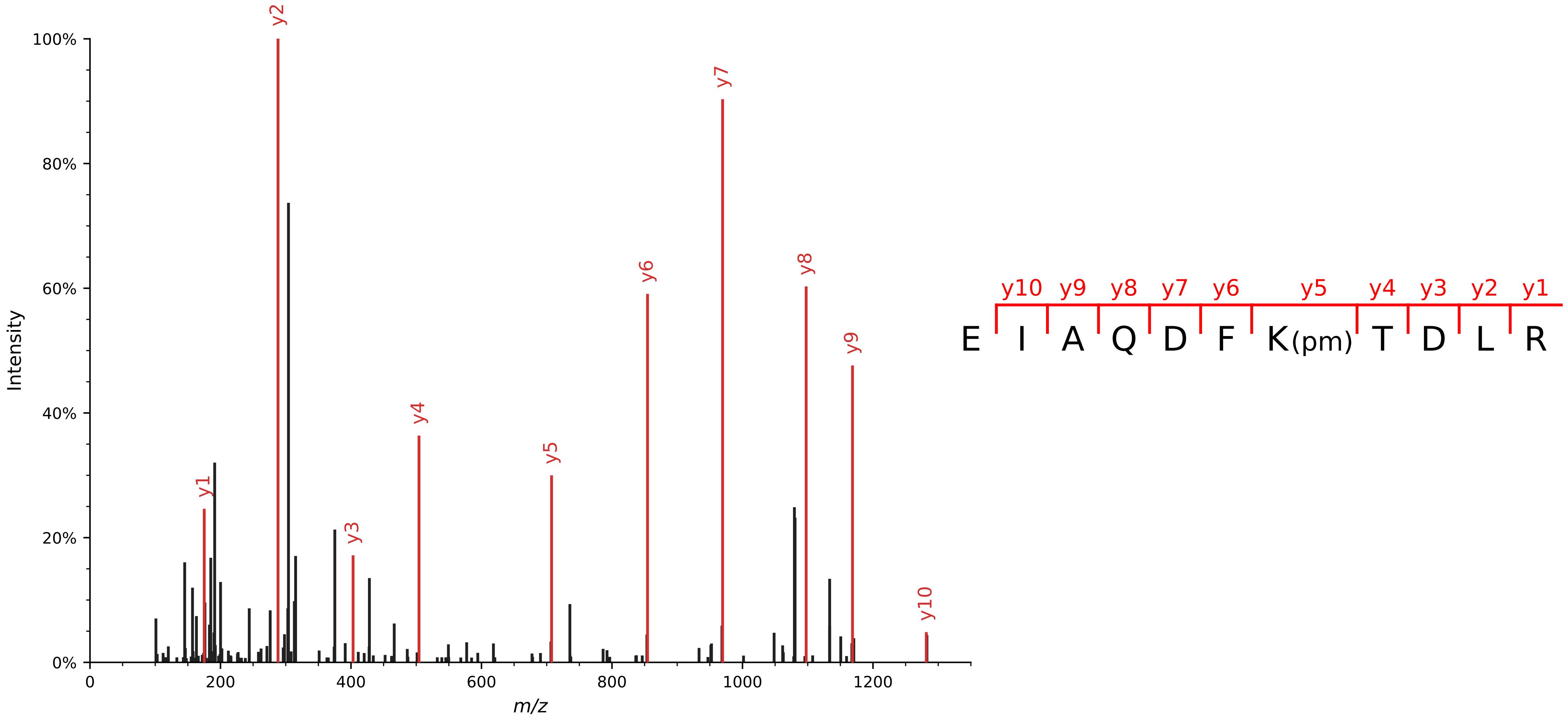

Raw file: HF240124_RN_spleen_D5PRO_D5PRO_2, Scan: 12160, m/z: 712.89324, Charge: 2, RT (min): 22.81, Score: 201.84

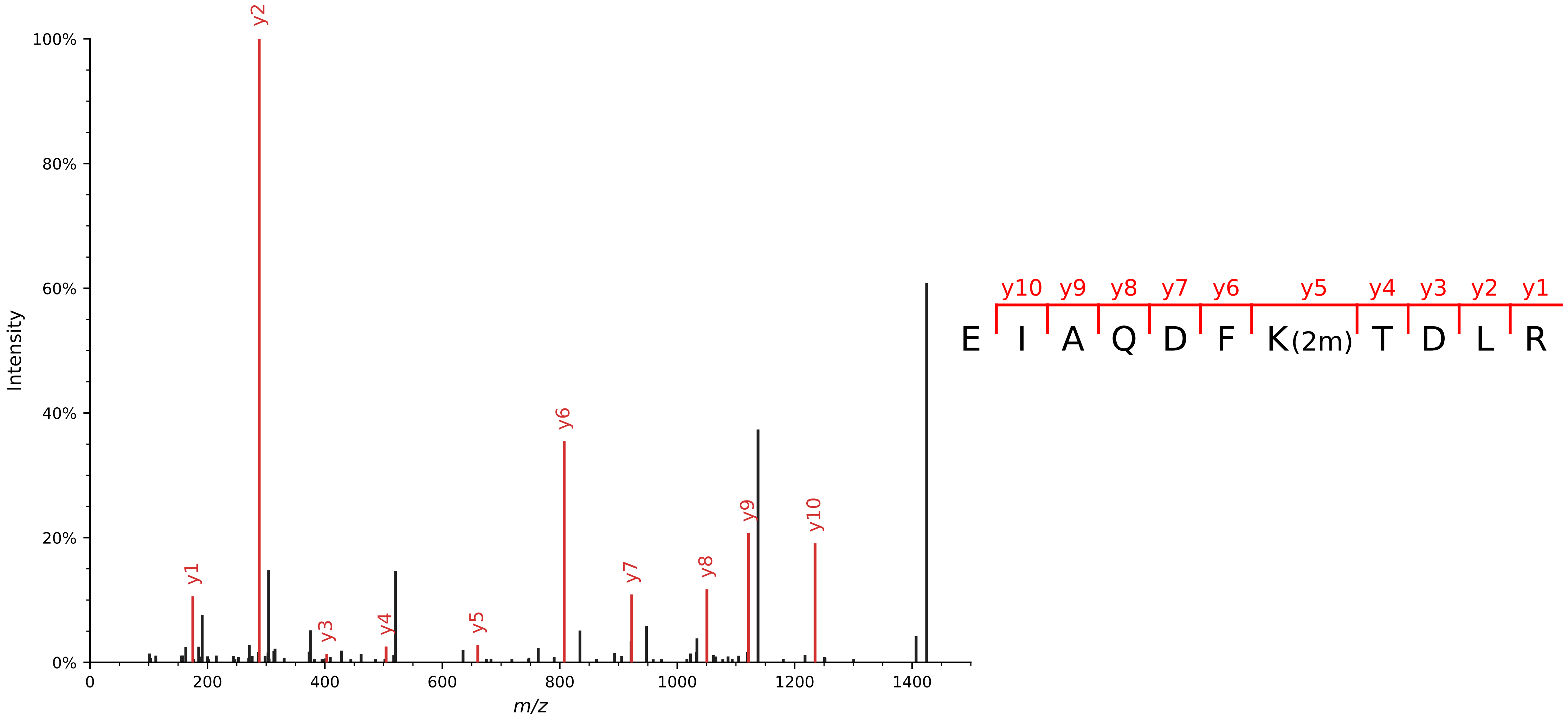

Raw file: HF240124_RN_spleen_D5PRO_D5PRO_2, Scan: 19835, m/z: 748.88561, Charge: 2, RT (min): 36.61, Score: 58.433

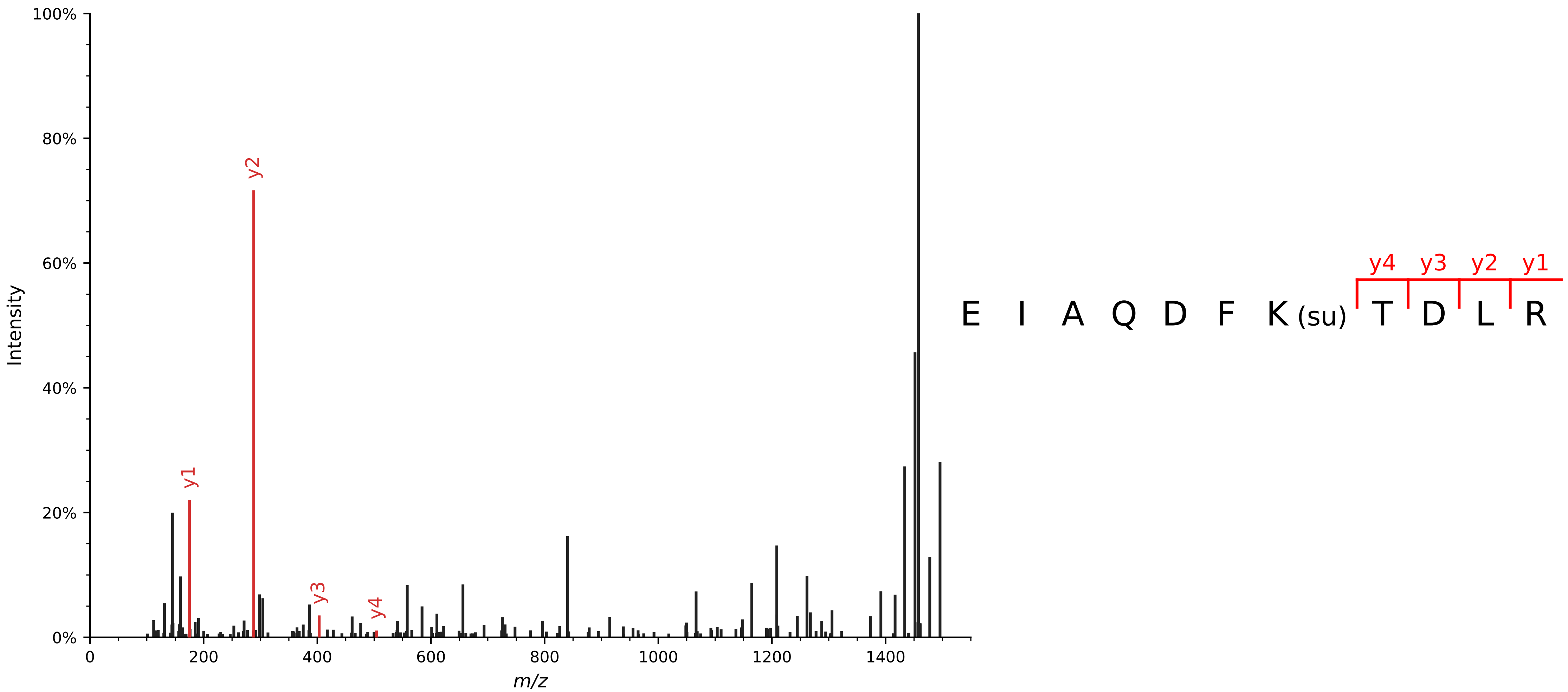

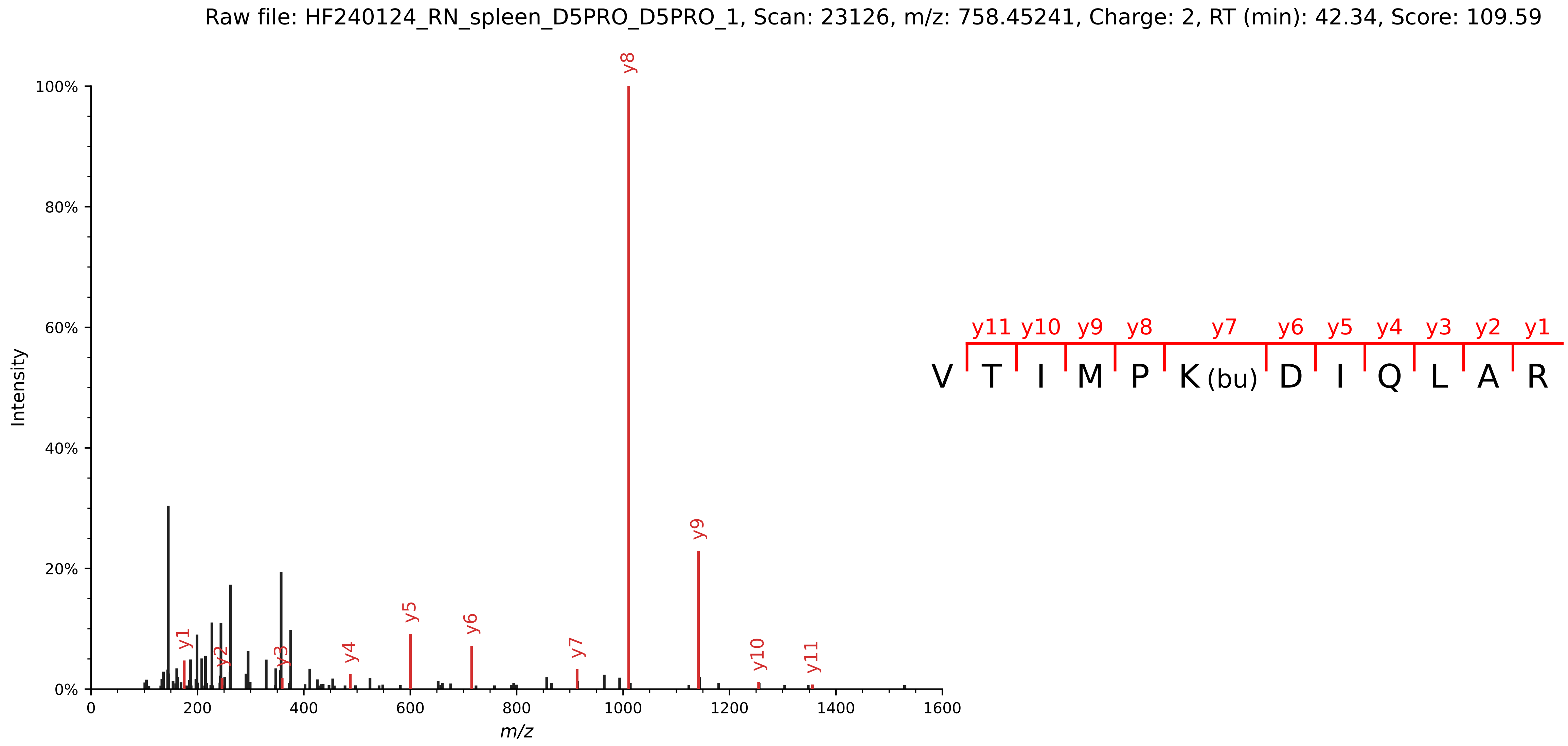

Raw file: HF240124_RN_spleen_D5PRO_D5PRO_1, Scan: 23126, m/z: 758.45241, Charge: 2, RT (min): 42.34, Score: 109.59
Intensity
m/z
0%
20%
40%
60%
80%
100%
0
200
400
600
800
1000
1200
1400
1600
y1
y2
y3
y4
y5
y6
y7
y8
y9
y10
y11
y11 y10 y9 y8 y7 y6 y5 y4 y3 y2 y1
V T I M P K (bu) D I Q L A R

Raw file: HF240124_RN_spleen_D5PRO_D5PRO_2, Scan: 20392, m/z: 737.42894, Charge: 2, RT (min): 37.63, Score: 121.83

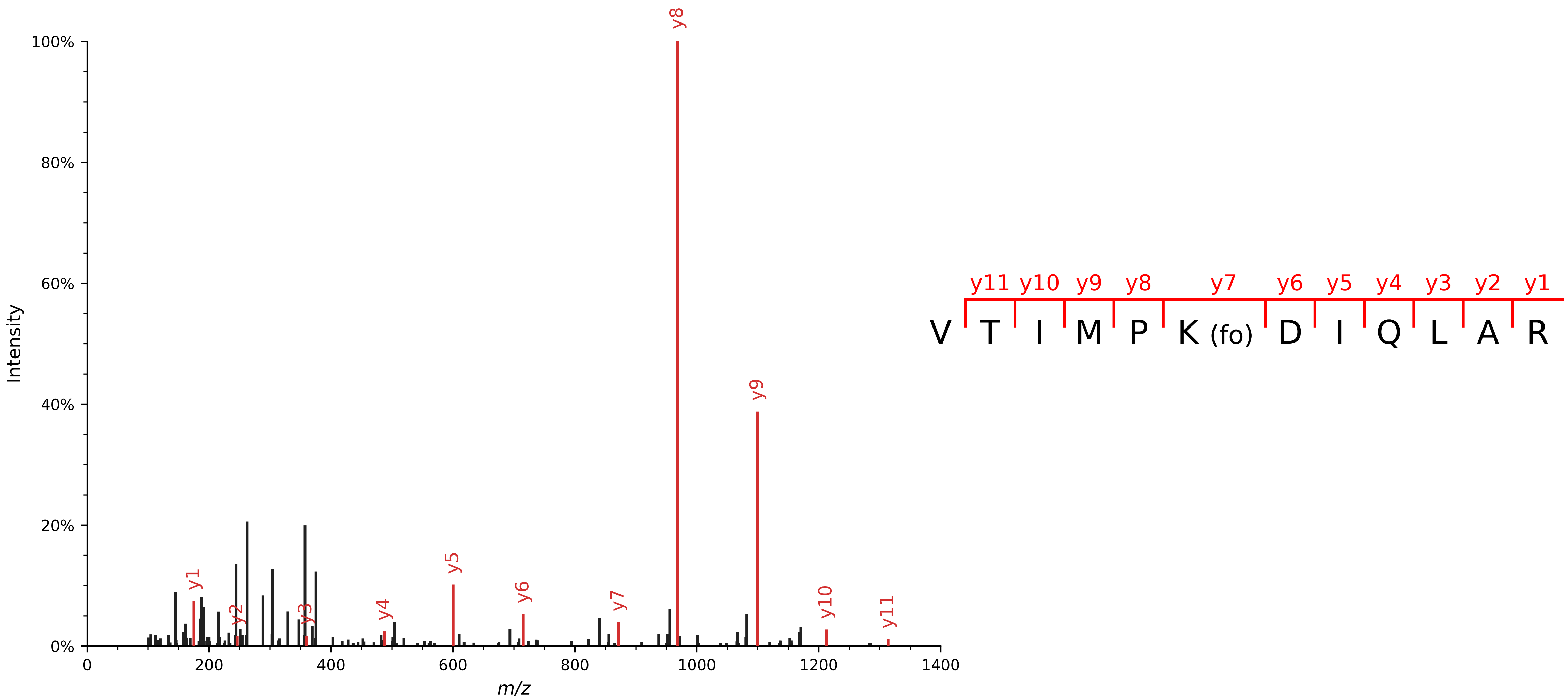

Raw file: HF240124_RN_spleen_D5PRO_D5PRO_2, Scan: 22297, m/z: 511.29021, Charge: 3, RT (min): 41.07, Score: 59.198

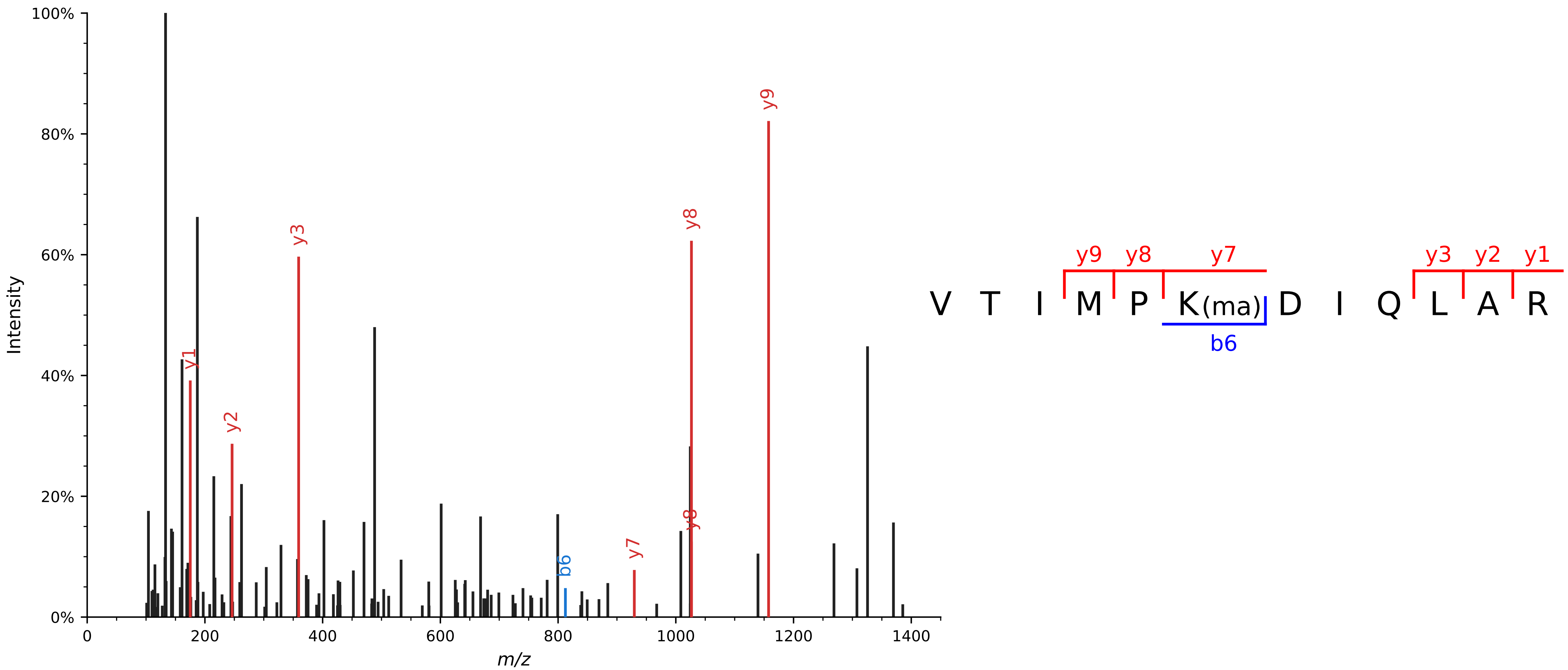

Raw file: HF240124_RN_spleen_D5PRO_D5PRO_1, Scan: 19533, m/z: 773.4395, Charge: 2, RT (min): 35.84, Score: 54.982

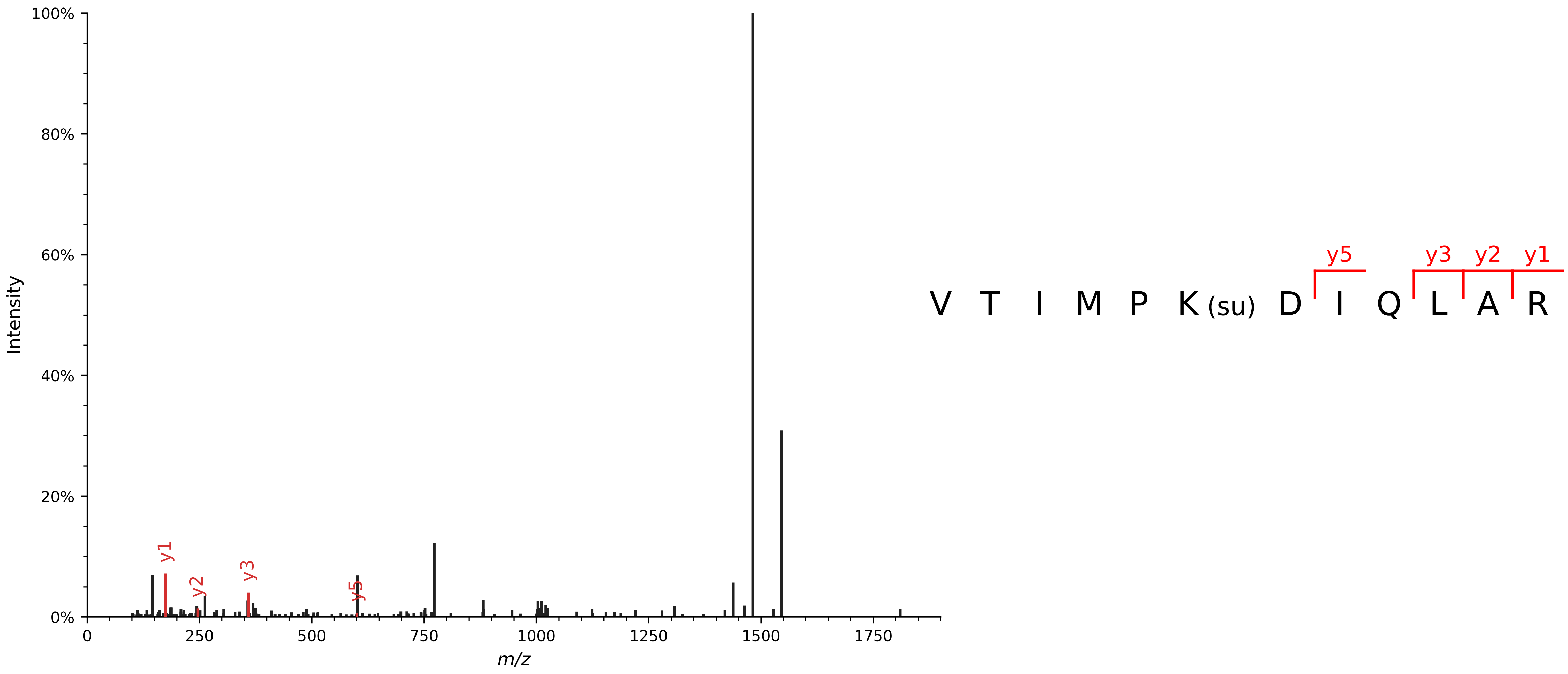

Raw file: HF240124_RN_spleen_D5PRO_D5PRO_3, Scan: 19194, m/z: 767.45601, Charge: 2, RT (min): 35.55, Score: 54.416

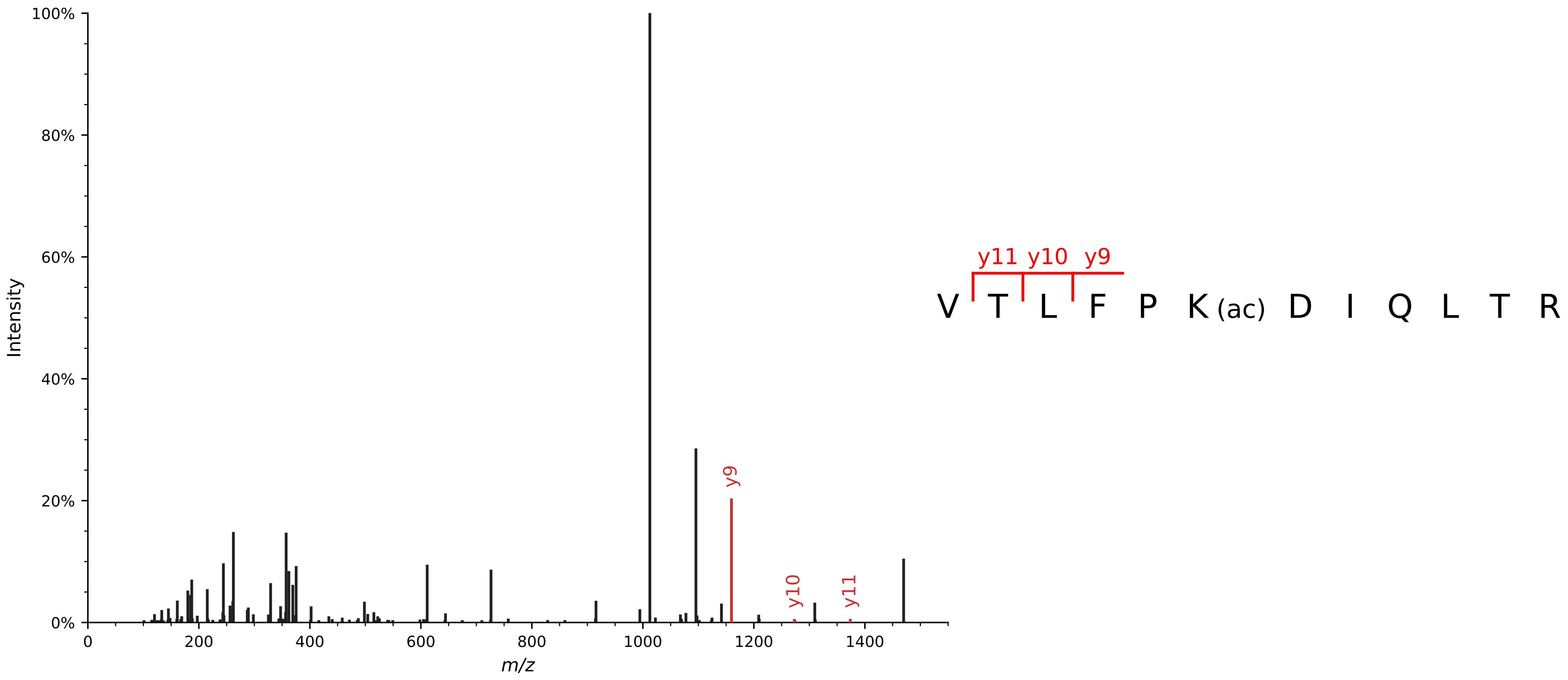

Raw file: HF240124_RN_spleen_D5PRO_D5PRO_2, Scan: 18357, m/z: 774.46383, Charge: 2, RT (min): 33.98, Score: 45.368

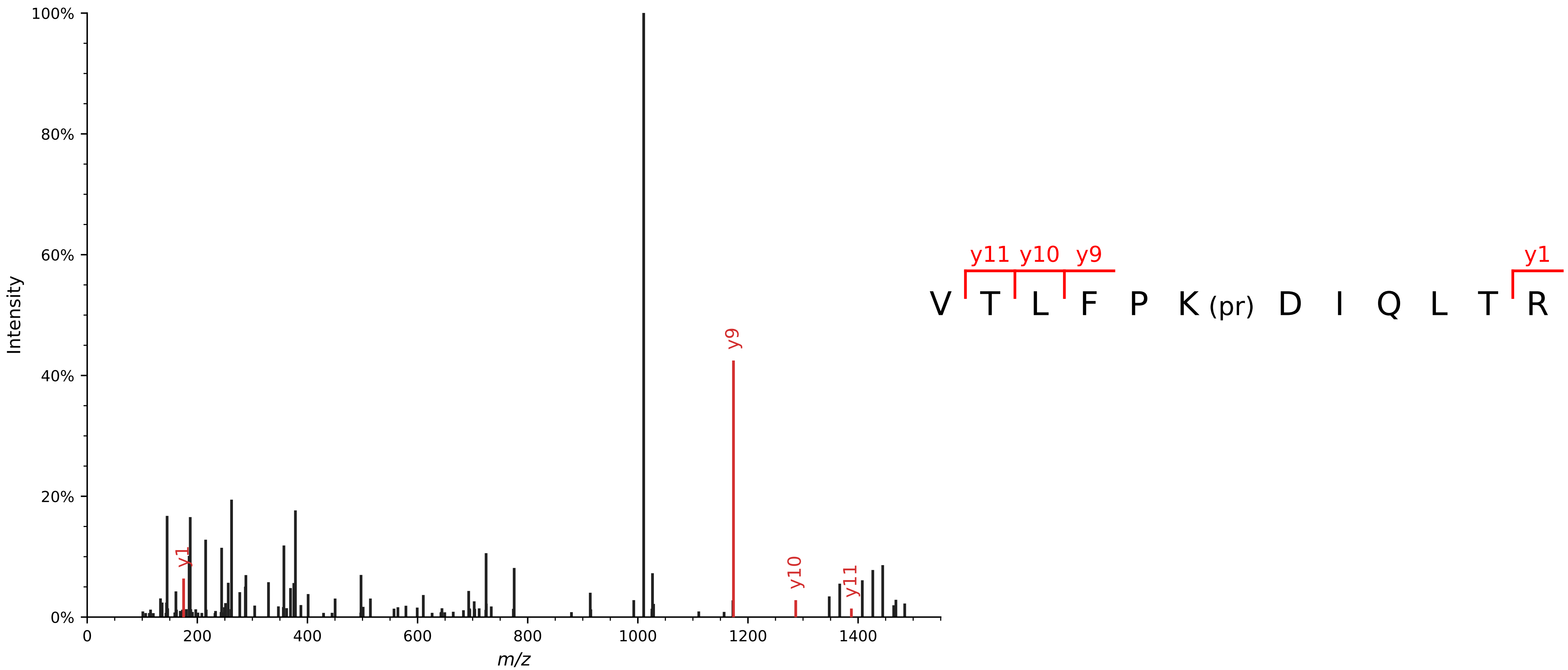

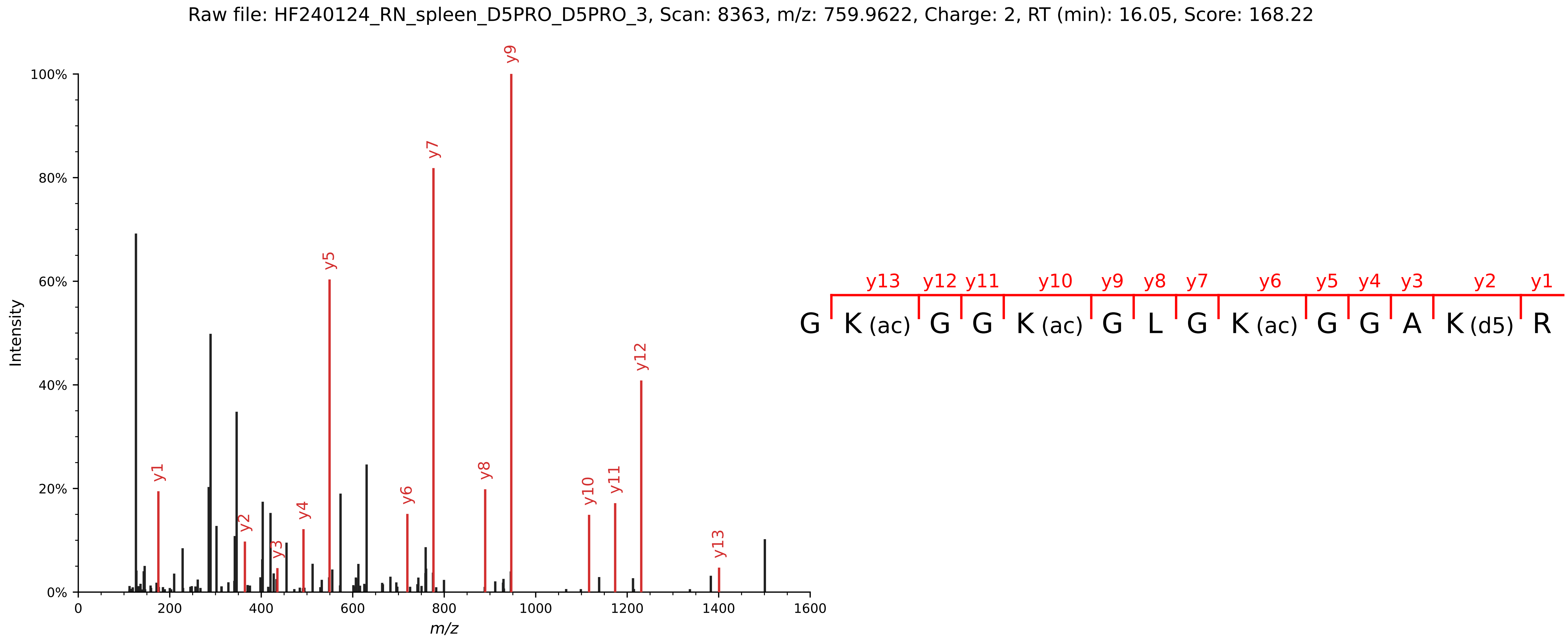
Raw file: HF240124_RN_spleen_D5PRO_D5PRO_3, Scan: 8363, m/z: 759.9622, Charge: 2, RT (min): 16.05, Score: 168.22
Intensity
m/z
y1
y2
y3
y4
y5
y6
y7
y8
y9
y10
y11
y12
y13
G K(ac) G G K(ac) G L G K(ac) G G A K(d5) R

Raw file: HF240124_RN_spleen_D5PRO_D5PRO_3, Scan: 7676, m/z: 750.43868, Charge: 2, RT (min): 14.83, Score: 158.31

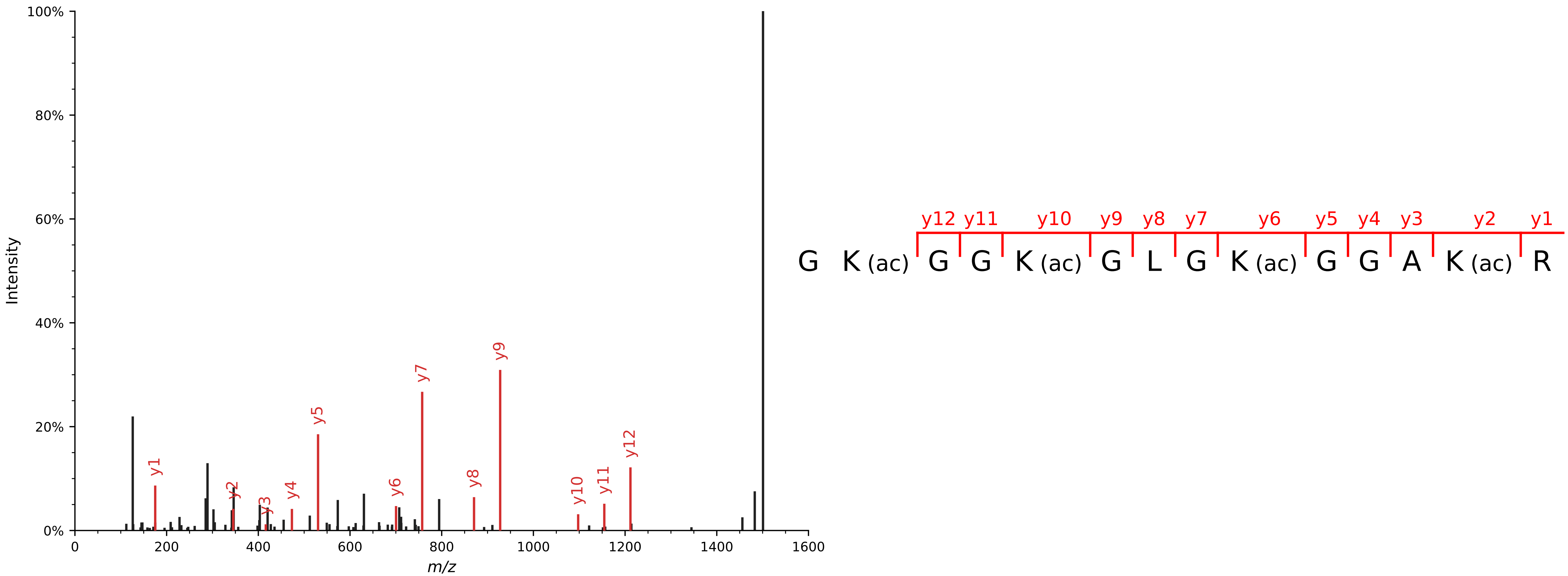

Raw file: HF240124_RN_spleen_D5PRO_D5PRO_1, Scan: 9392, m/z: 769.48571, Charge: 2, RT (min): 17.91, Score: 162.38

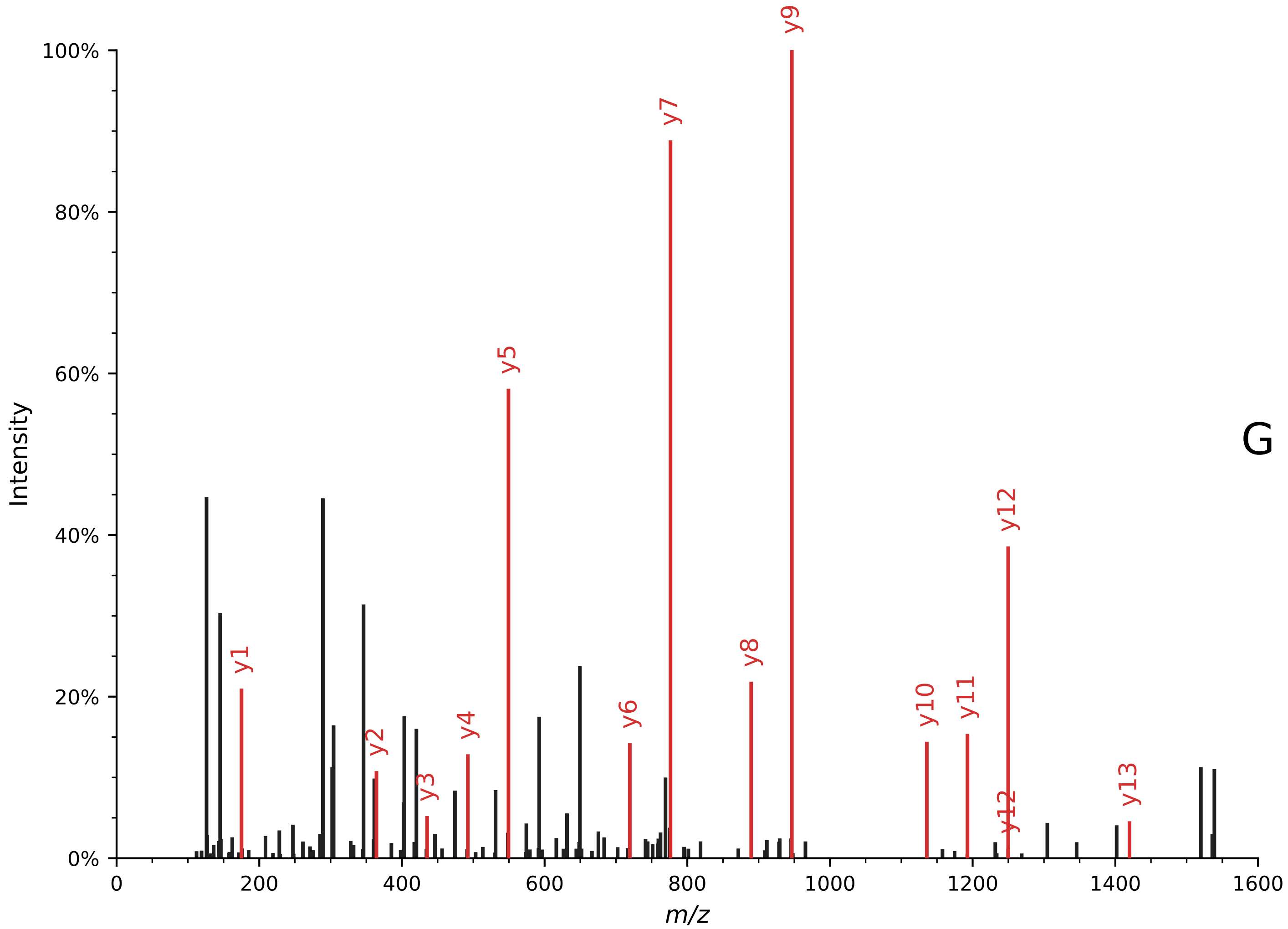

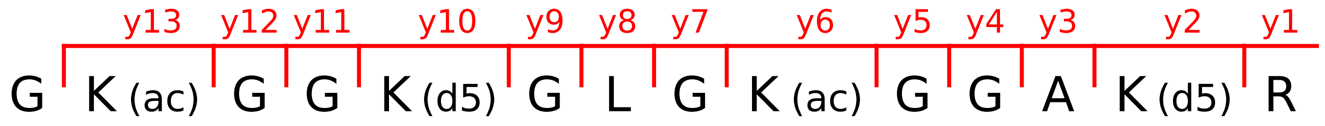

Raw file: HF240124_RN_spleen_D5PRO_D5PRO_2, Scan: 8392, m/z: 759.9622, Charge: 2, RT (min): 16.32, Score: 163.84

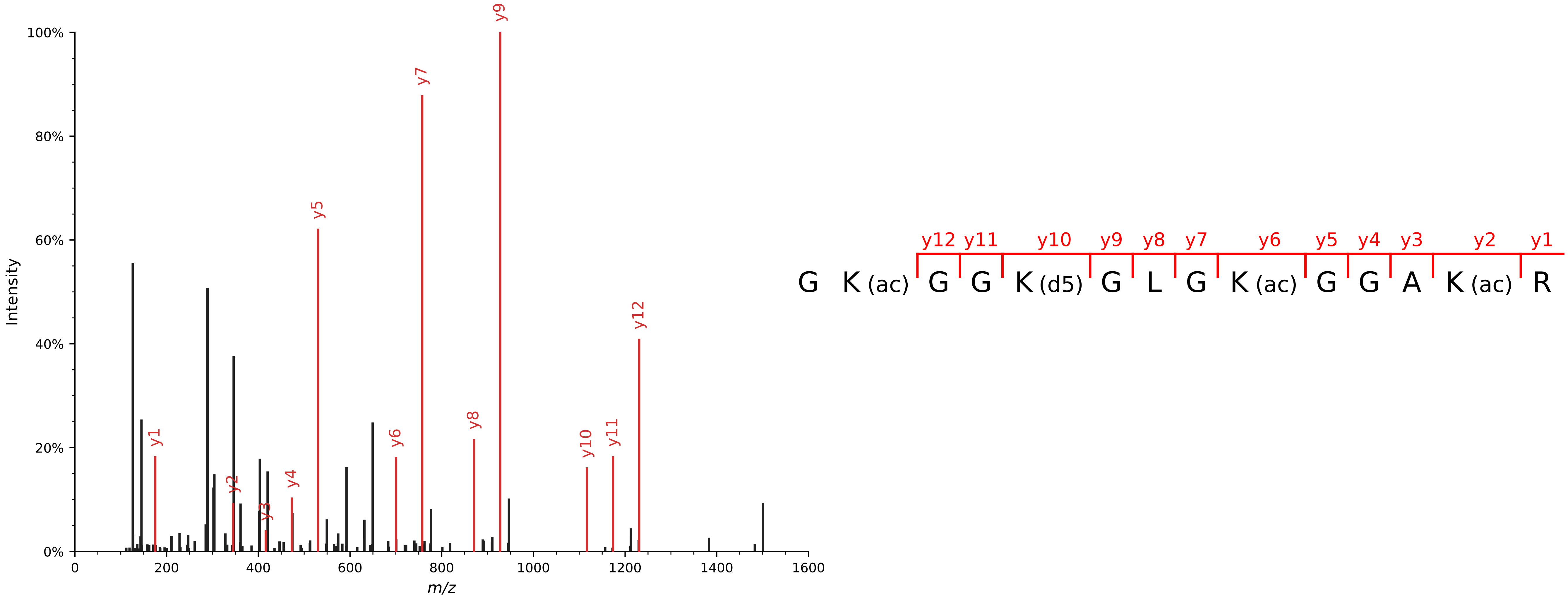

Raw file: HF240124_RN_spleen_D5PRO_D5PRO_1, Scan: 11048, m/z: 773.97785, Charge: 2, RT (min): 20.74, Score: 113.69

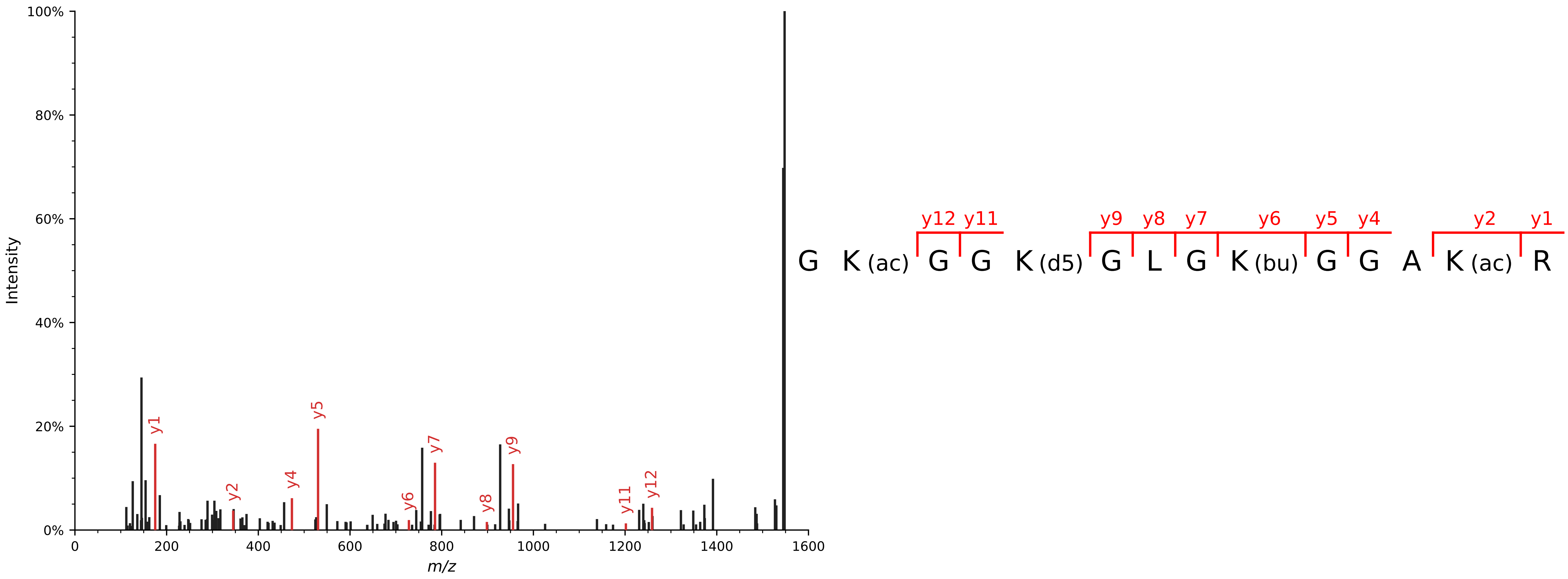

Raw file: HF240124_RN_spleen_D5PRO_D5PRO_1, Scan: 12085, m/z: 783.50136, Charge: 2, RT (min): 22.52, Score: 121.9

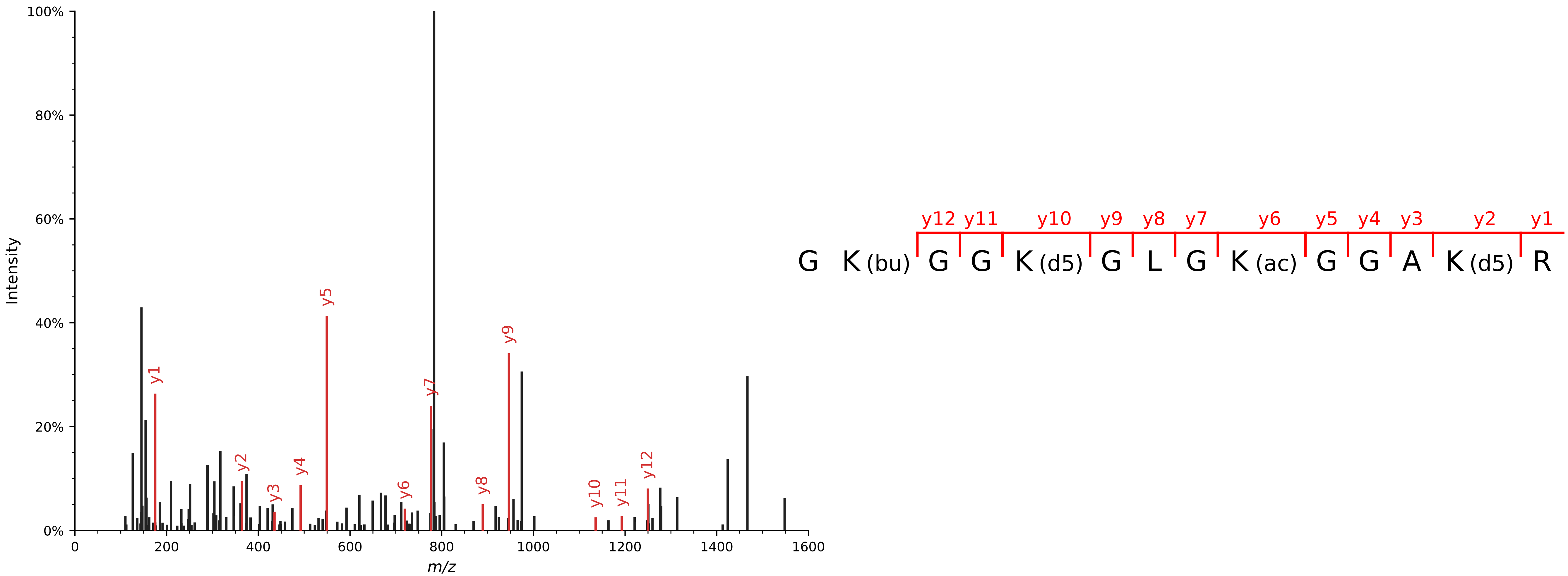

Raw file: HF240124_RN_spleen_D5PRO_D5PRO_3, Scan: 12018, m/z: 783.50136, Charge: 2, RT (min): 22.47, Score: 115.18

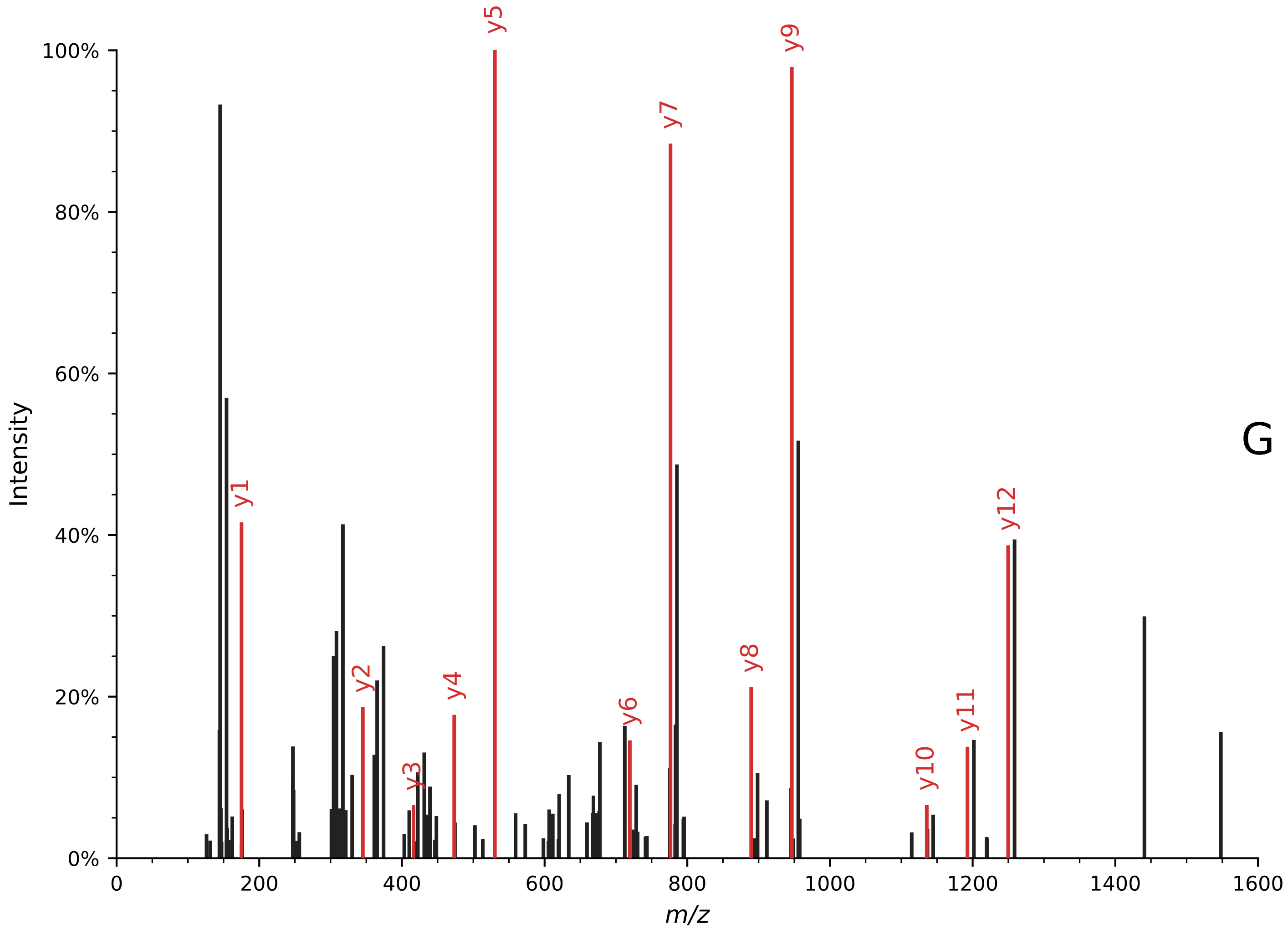

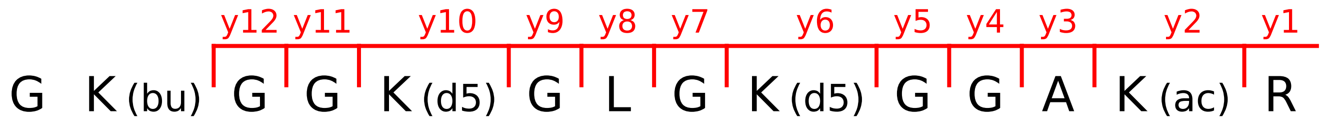

Raw file: HF240124_RN_spleen_D5PRO_D5PRO_3, Scan: 10129, m/z: 772.00141, Charge: 2, RT (min): 19.12, Score: 145.89

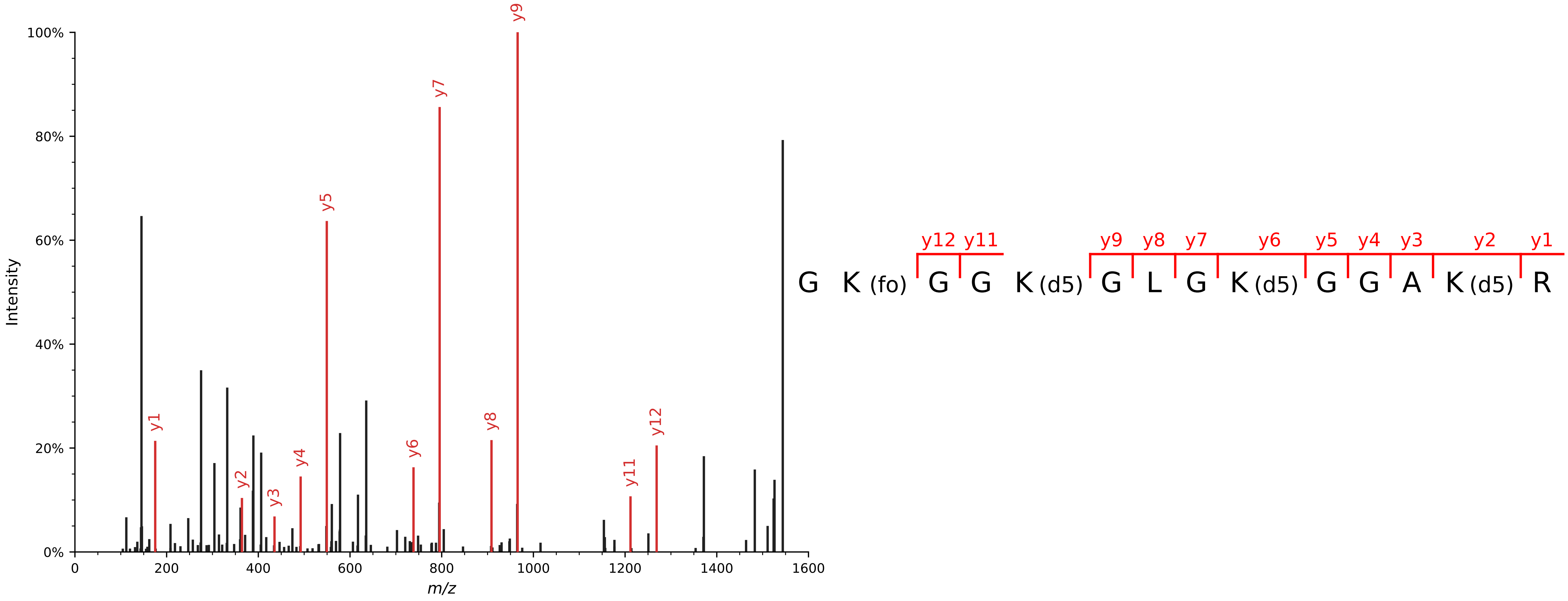

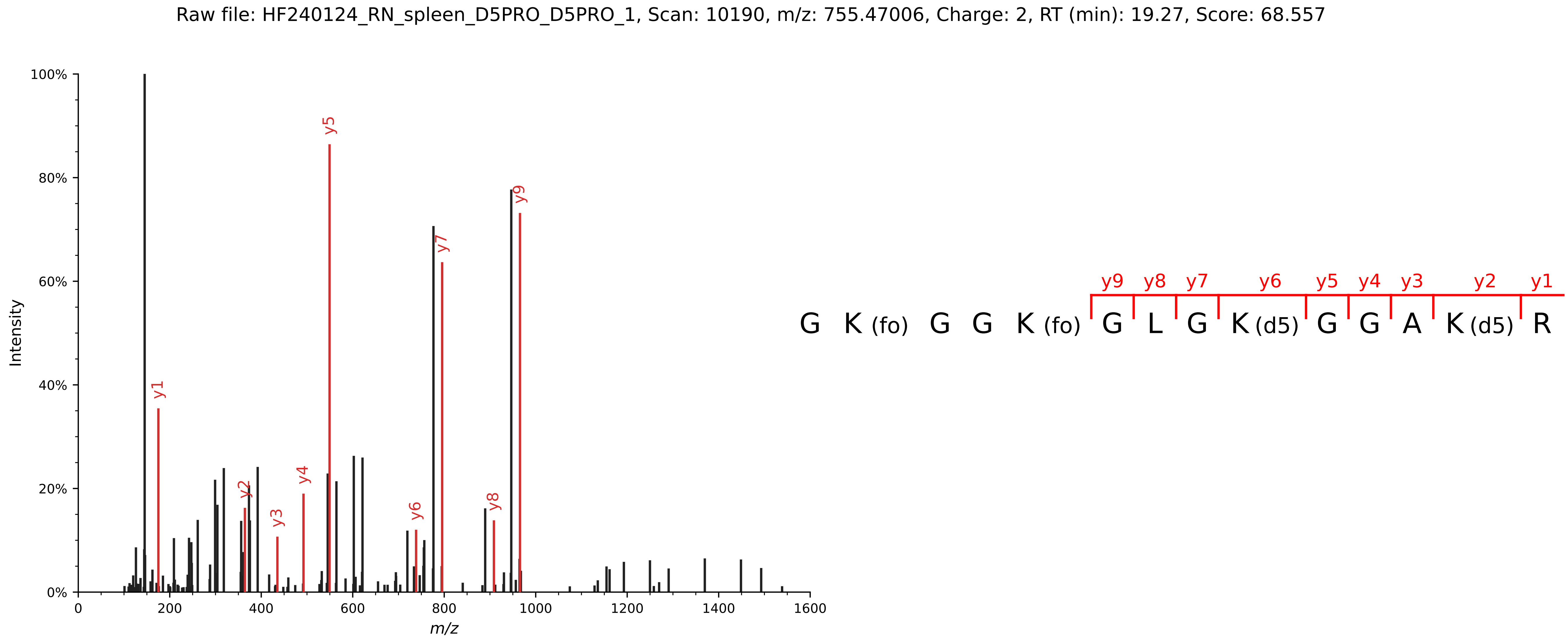
Raw file: HF240124_RN_spleen_D5PRO_D5PRO_1, Scan: 10190, m/z: 755.47006, Charge: 2, RT (min): 19.27, Score: 68.557
Intensity
m/z
y1
y2
y3
y4
y5
y6
y7
y8
y9
G K (fo) G G K (fo) G L G K (d5) G G A K (d5) R

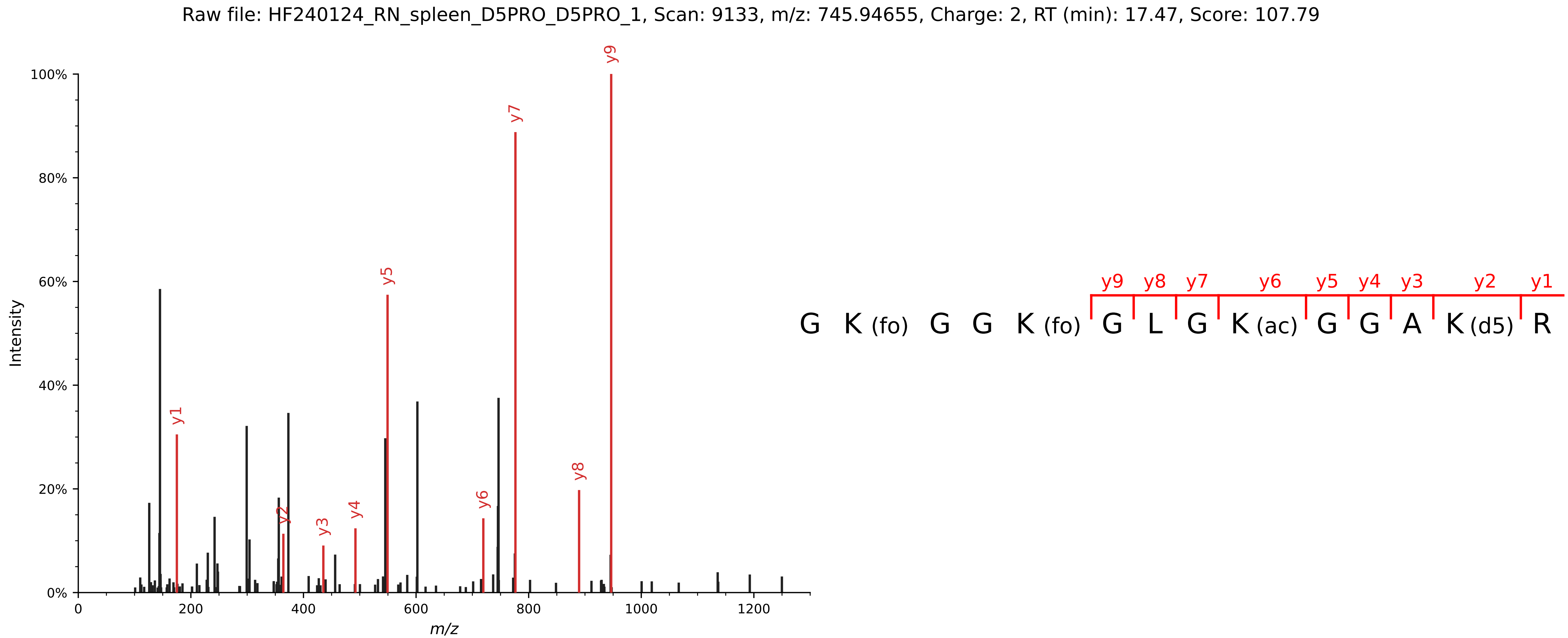

Raw file: HF240124_RN_spleen_D5PRO_D5PRO_1, Scan: 9133, m/z: 745.94655, Charge: 2, RT (min): 17.47, Score: 107.79
Intensity
m/z
y1
y2
y3
y4
y5
y6
y7
y8
y9
G K (fo) G G K (fo) G L G K (ac) G G A K (d5) R

Raw file: HF240124_RN_spleen_D5PRO_D5PRO_1, Scan: 8293, m/z: 491.28445, Charge: 3, RT (min): 16.05, Score: 51.546

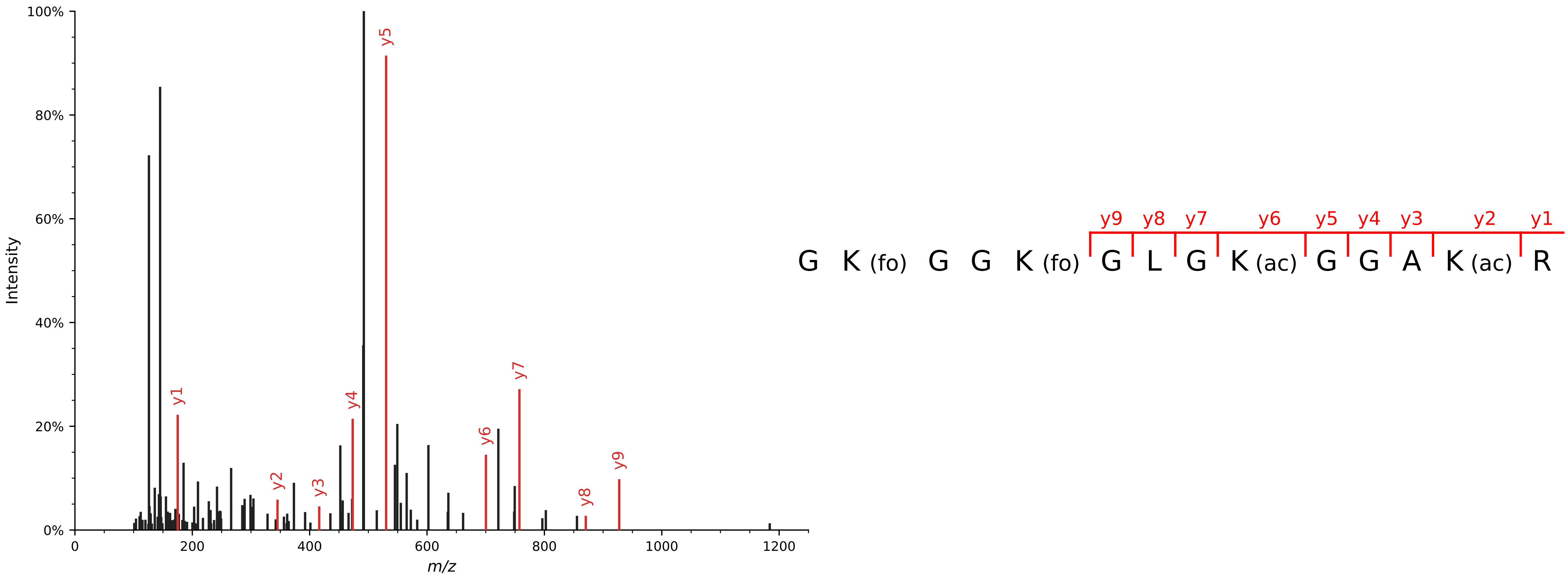

Raw file: HF240124_RN_spleen_D5PRO_D5PRO_3, Scan: 9174, m/z: 745.94655, Charge: 2, RT (min): 17.46, Score: 83.499

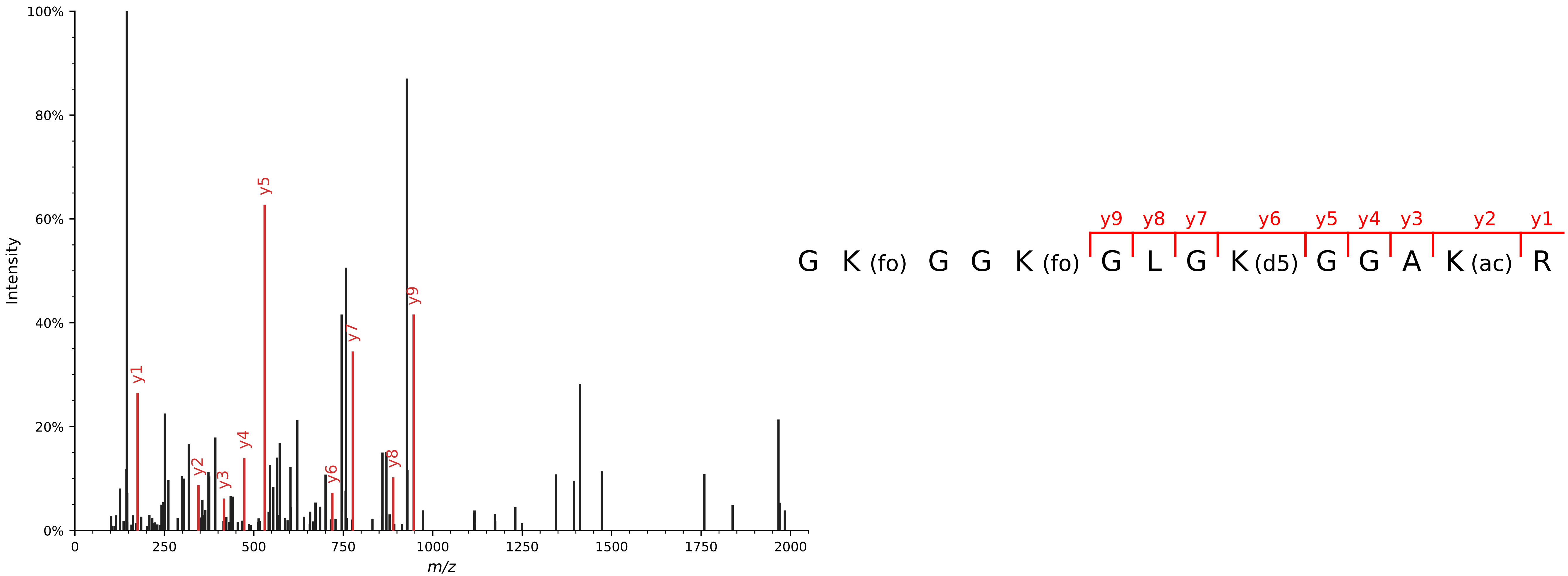

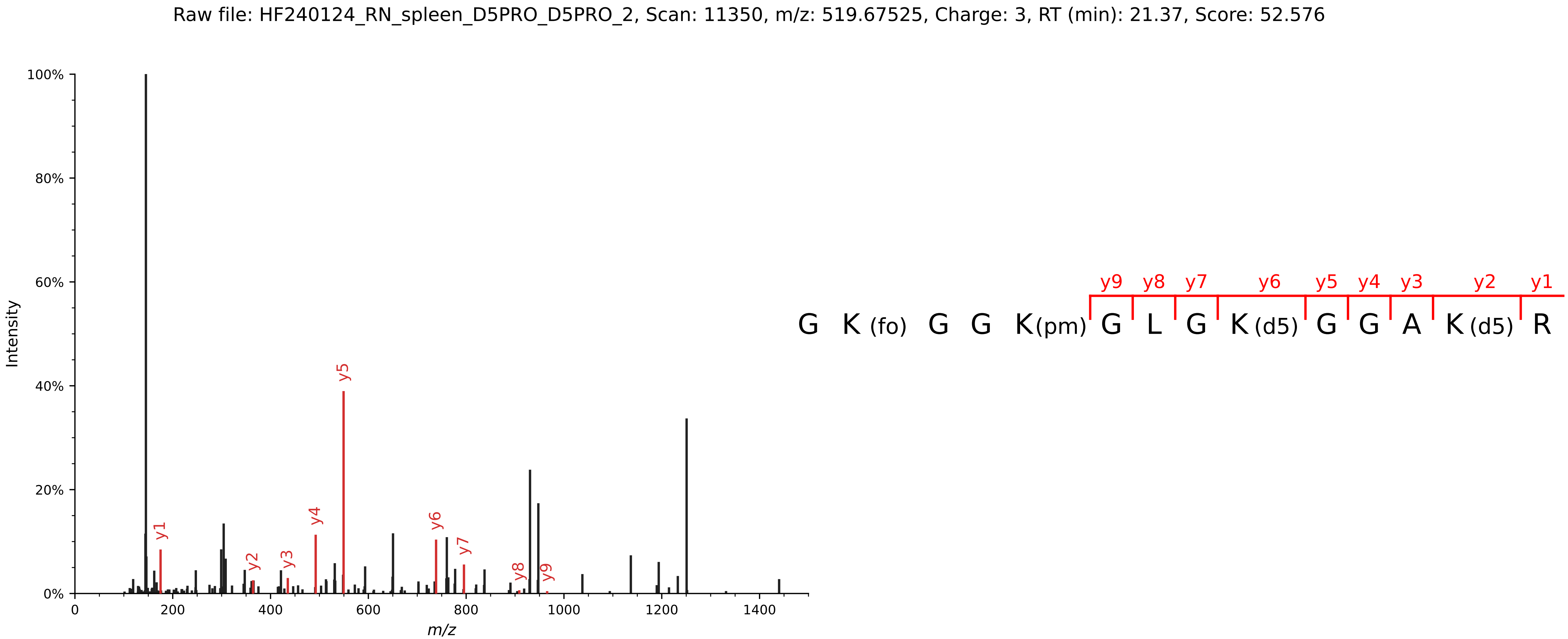

Raw file: HF240124_RN_spleen_D5PRO_D5PRO_2, Scan: 11350, m/z: 519.67525, Charge: 3, RT (min): 21.37, Score: 52.576
Intensity
100%
80%
60%
40%
20%
0%
0
200
400
600
800
1000
1200
1400
m/z
y1
y2
y3
y4
y5
y6
y7
y8
y9
G K (fo) G G K(pm) G L G K (d5) G G A K (d5) R
y9 y8 y7 y6 y5 y4 y3 y2 y1

Raw file: HF240124_RN_spleen_D5PRO_D5PRO_3, Scan: 9098, m/z: 508.65435, Charge: 3, RT (min): 17.32, Score: 61.423

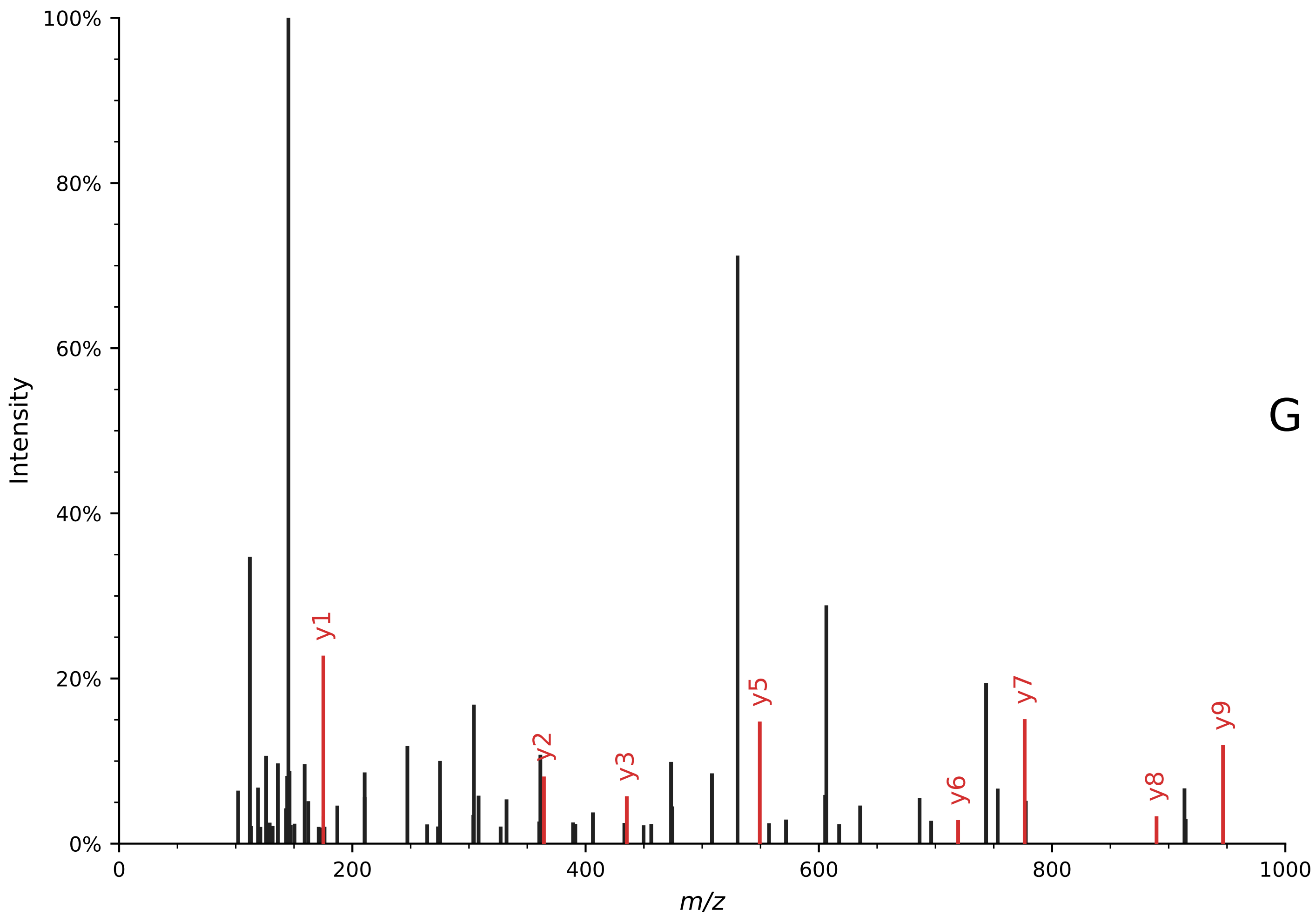

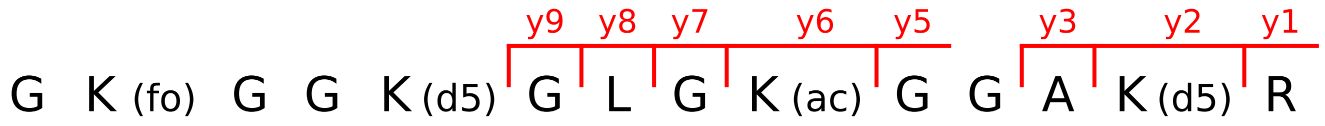

Raw file: HF240124_RN_spleen_D5PRO_D5PRO_3, Scan: 9315, m/z: 762.47789, Charge: 2, RT (min): 17.71, Score: 141.93

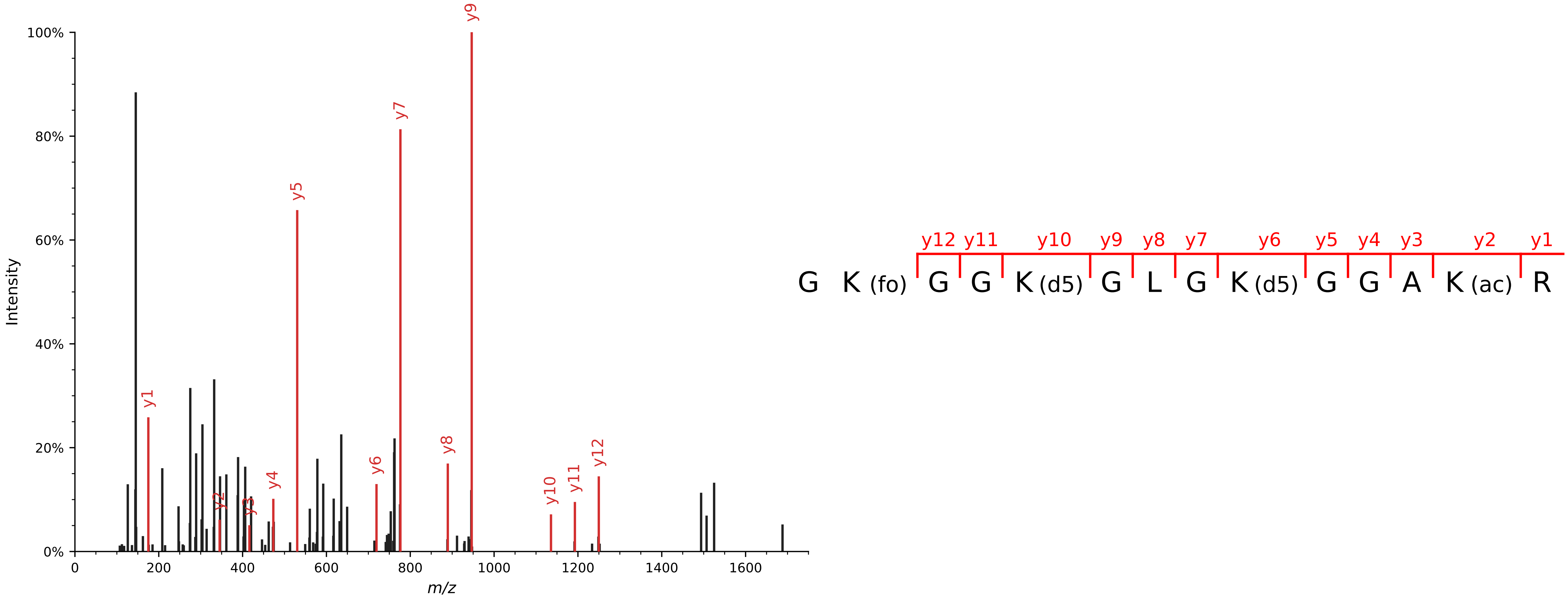

Raw file: HF240124_RN_spleen_D5PRO_D5PRO_2, Scan: 12868, m/z: 795.54057, Charge: 2, RT (min): 24.11, Score: 121.6

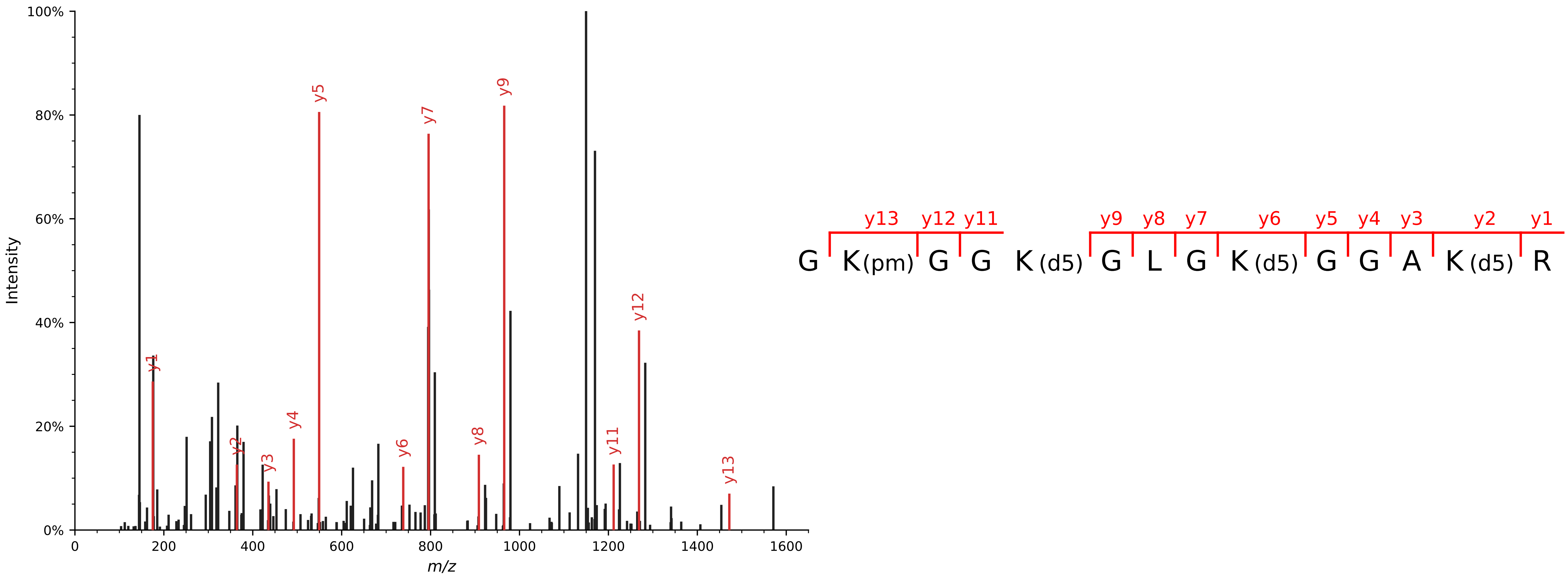

Raw file: HF240124_RN_spleen_D5PRO_D5PRO_1, Scan: 12230, m/z: 786.01706, Charge: 2, RT (min): 22.78, Score: 121.36

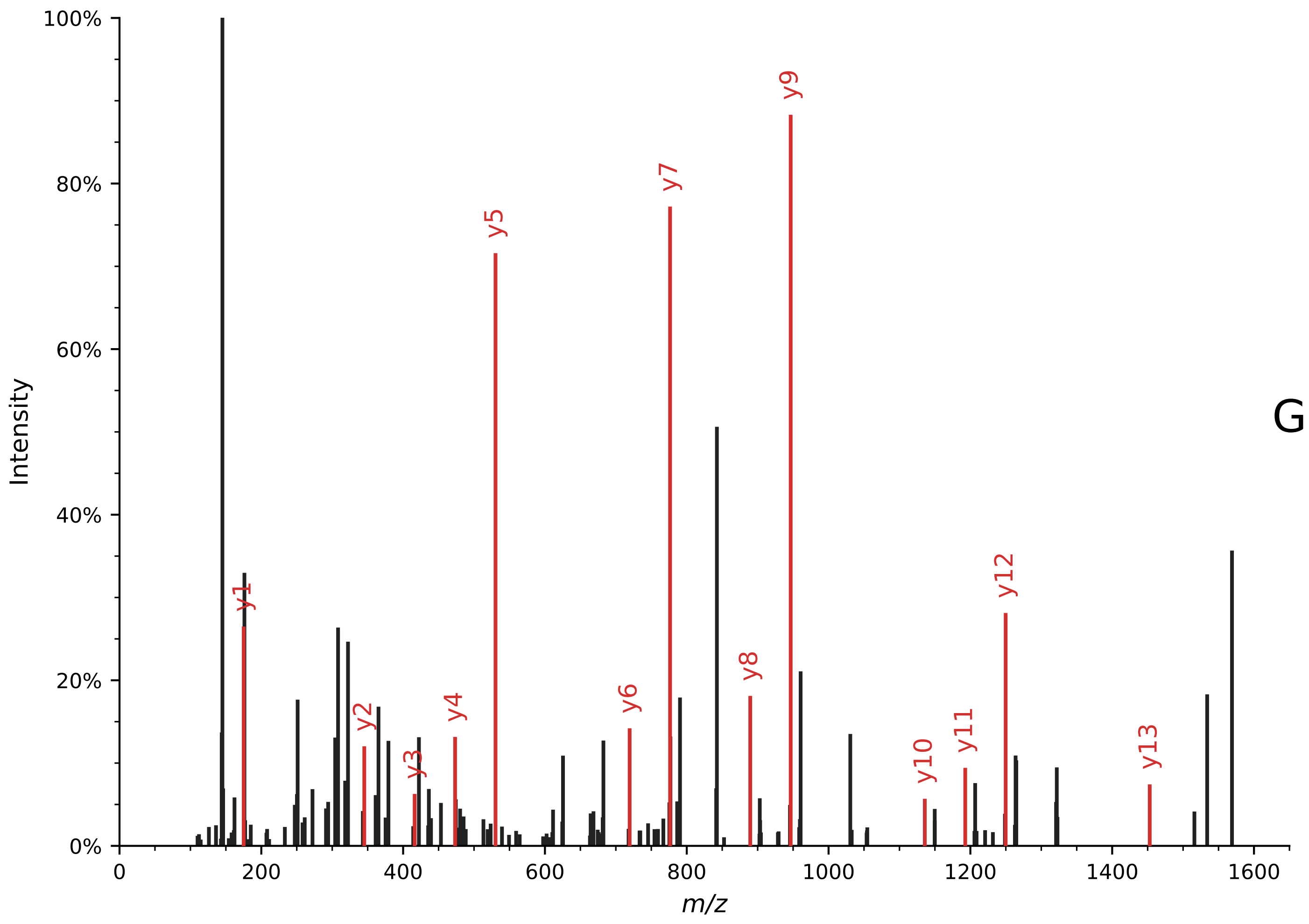

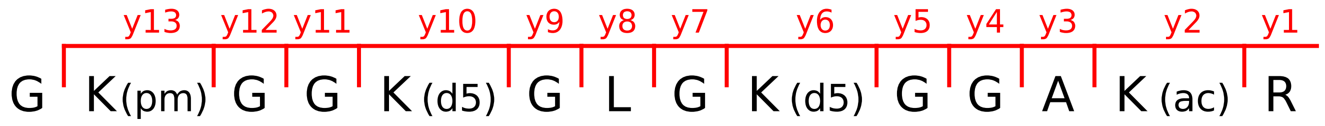

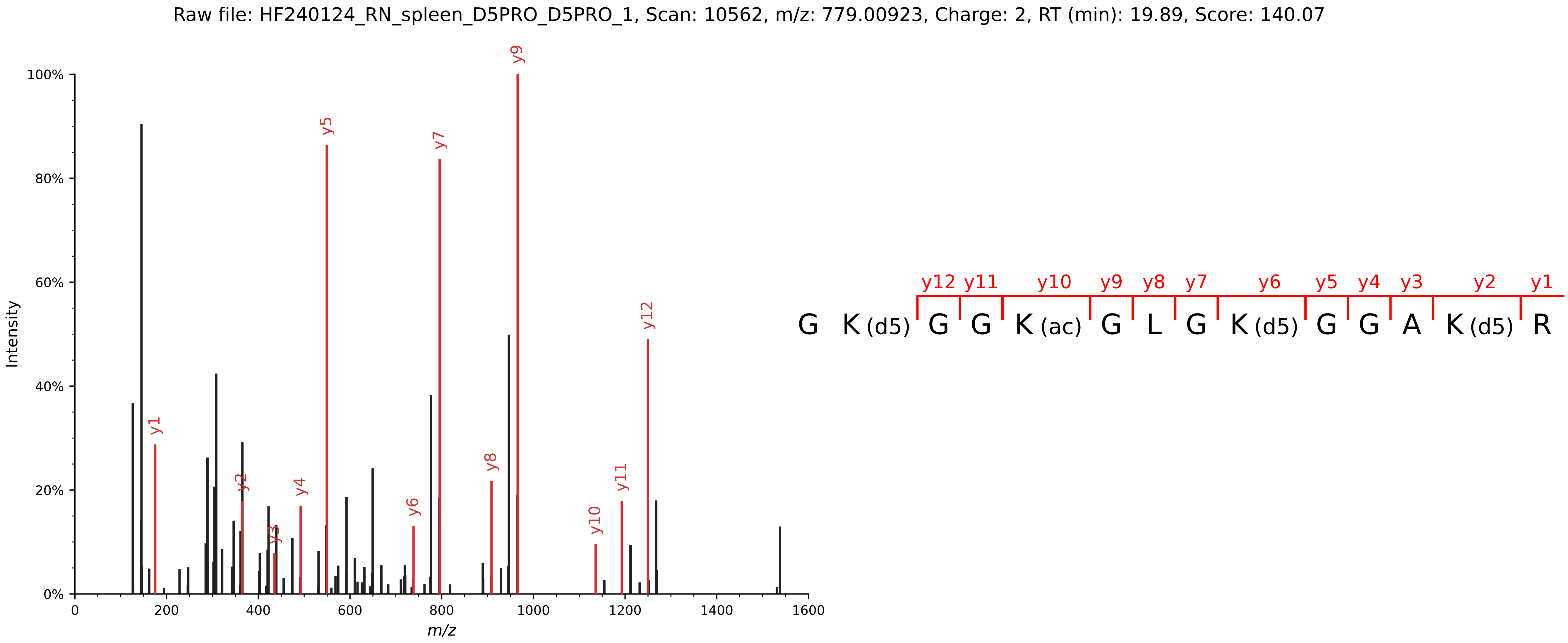

Raw file: HF240124_RN_spleen_D5PRO_D5PRO_1, Scan: 10562, m/z: 779.00923, Charge: 2, RT (min): 19.89, Score: 140.07
Intensity
0%
20%
40%
60%
80%
100%
0
200
400
600
800
1000
1200
1400
1600
m/z
y1
y2
y3
y4
y5
y6
y7
y8
y9
y10
y11
y12
y12 y11 y10 y9 y8 y7 y6 y5 y4 y3 y2 y1
G K(d5) G G K(ac) G L G K(d5) G G A K(d5) R

Raw file: HF240124_RN_spleen_D5PRO_D5PRO_1, Scan: 8653, m/z: 759.9622, Charge: 2, RT (min): 16.65, Score: 122.51

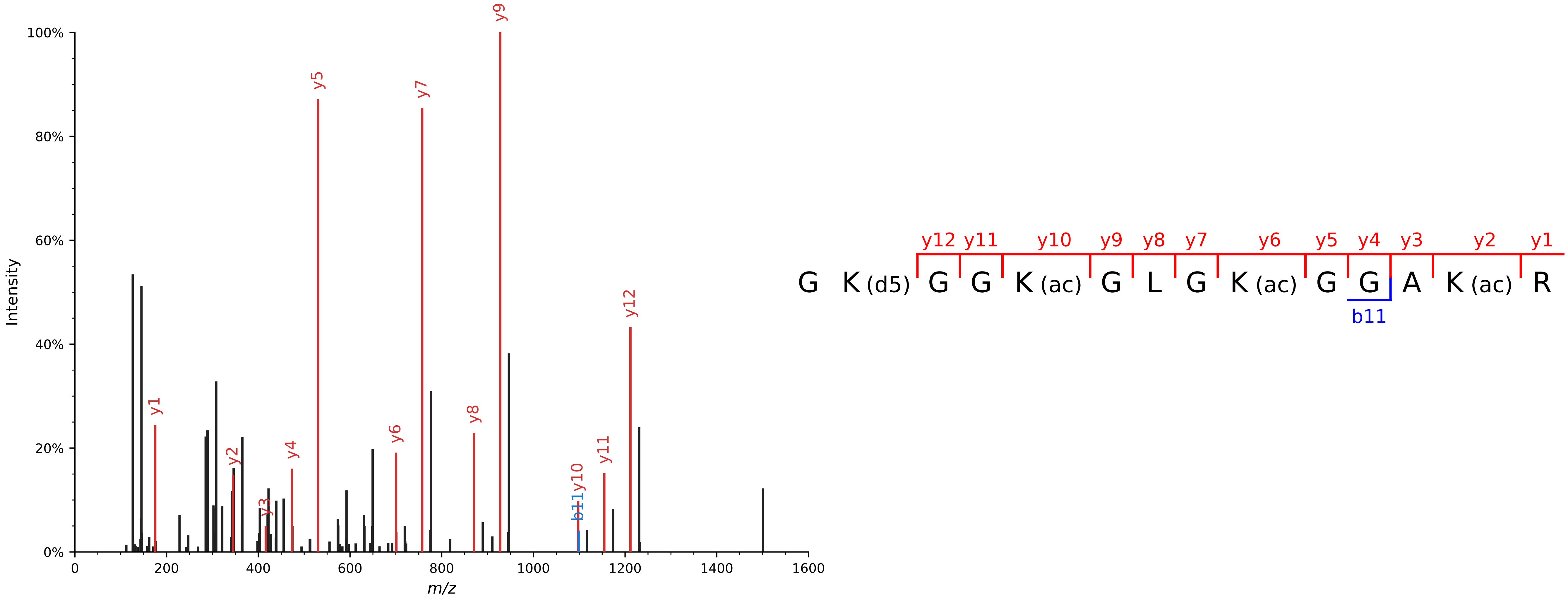

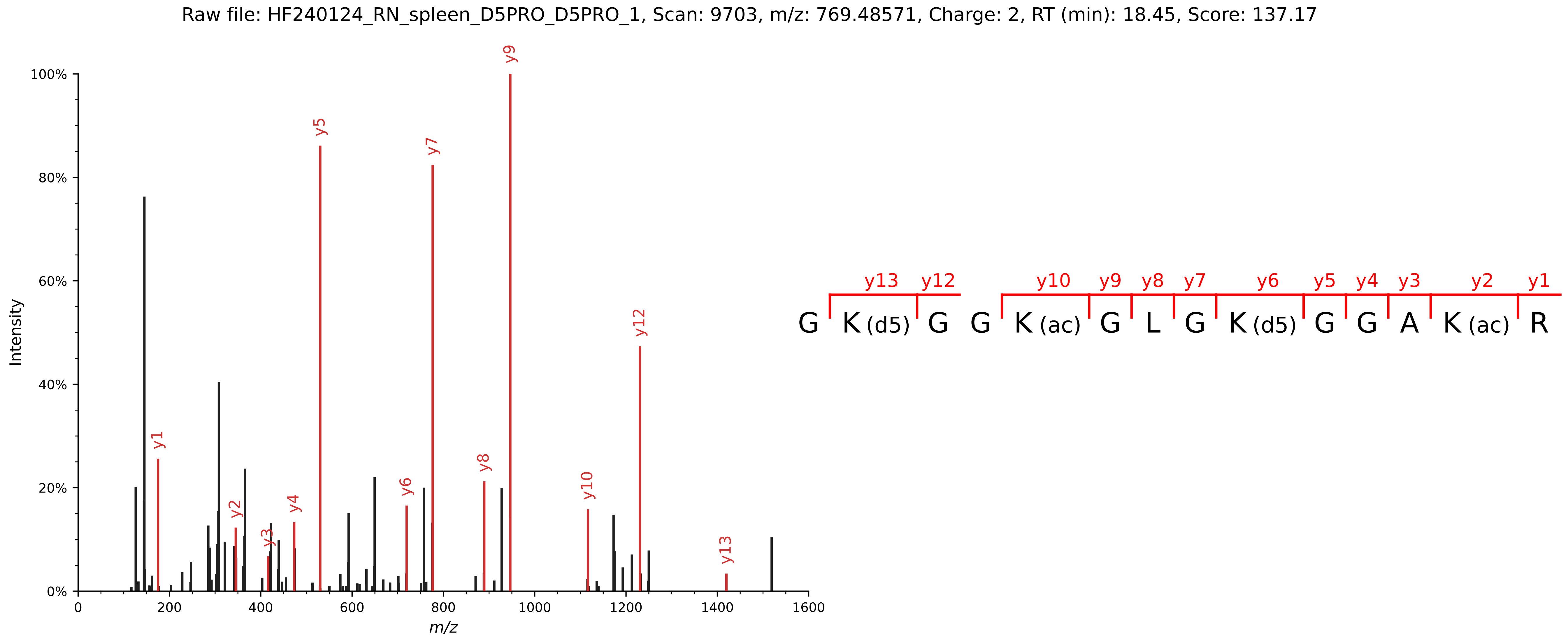
Raw file: HF240124_RN_spleen_D5PRO_D5PRO_1, Scan: 9703, m/z: 769.48571, Charge: 2, RT (min): 18.45, Score: 137.17
Intensity
100%
80%
60%
40%
20%
0%
0
200
400
600
800
1000
1200
1400
1600
m/z
y1
y2
y3
y4
y5
y6
y7
y8
y9
y10
y12
y13
y13 y12 y10 y9 y8 y7 y6 y5 y4 y3 y2 y1
G K (d5) G G K (ac) G L G K (d5) G G A K (ac) R

Raw file: HF240124_RN_spleen_D5PRO_D5PRO_1, Scan: 13175, m/z: 793.02488, Charge: 2, RT (min): 24.51, Score: 143.96

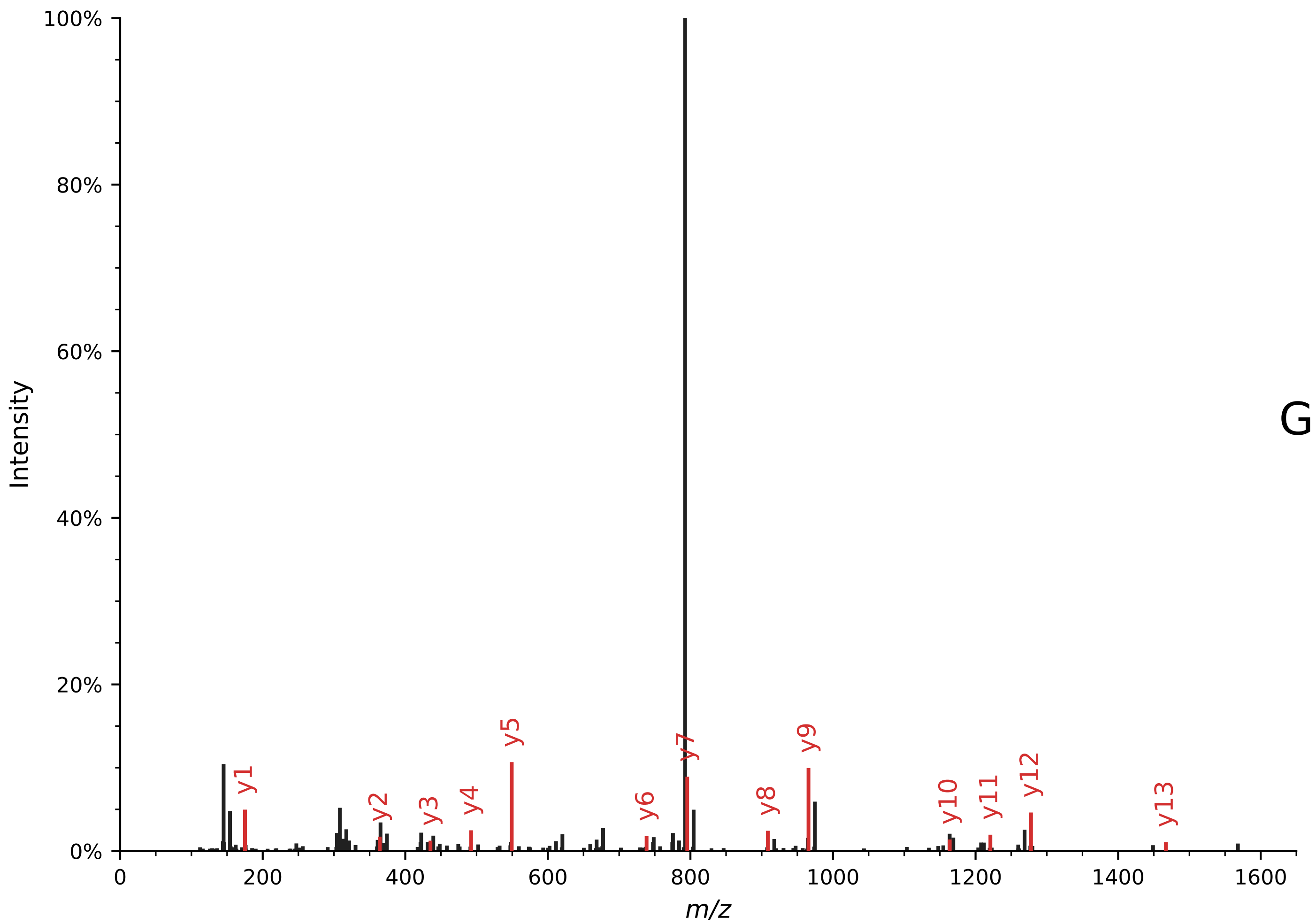

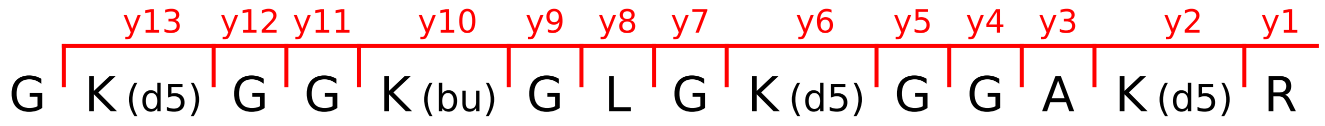

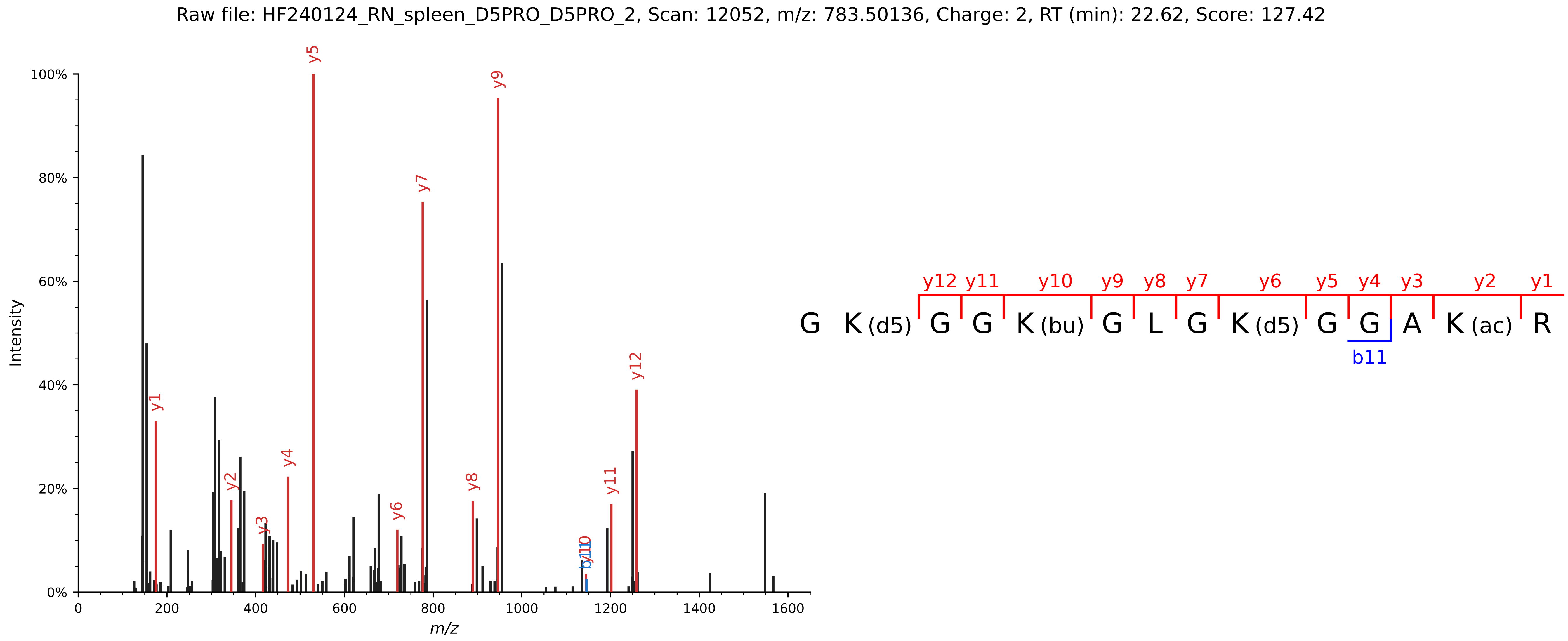

Raw file: HF240124_RN_spleen_D5PRO_D5PRO_2, Scan: 12052, m/z: 783.50136, Charge: 2, RT (min): 22.62, Score: 127.42
Intensity
m/z
0% 20% 40% 60% 80% 100%
0 200 400 600 800 1000 1200 1400 1600
y1 y2 y3 y4 y5 y6 y7 y8 y9 y10 y11 y12 b11
G K(d5) G G K(bu) G L G K(d5) G G A K(ac) R

Raw file: HF240124_RN_spleen_D5PRO_D5PRO_3, Scan: 9216, m/z: 762.47789, Charge: 2, RT (min): 17.54, Score: 154.35

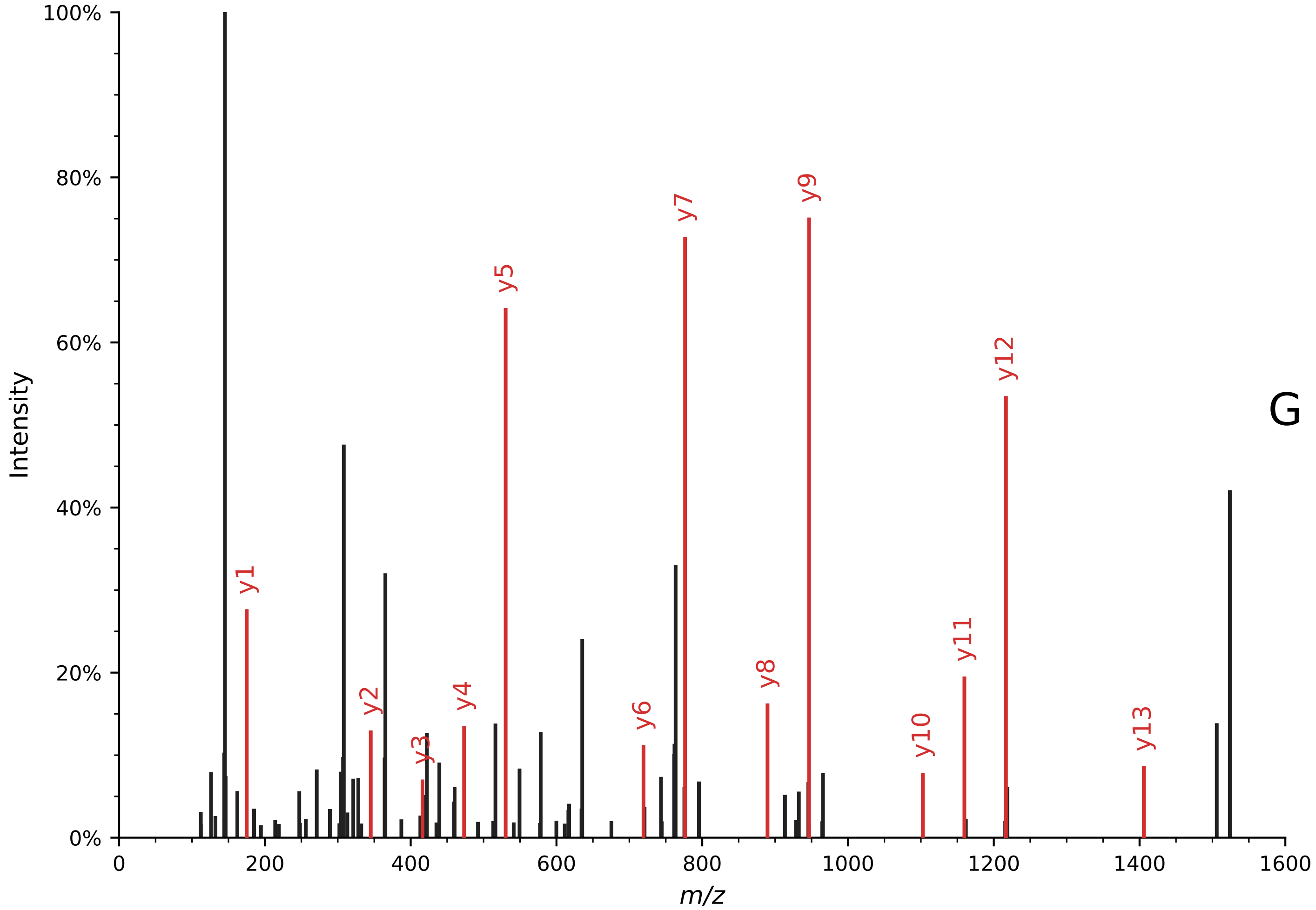

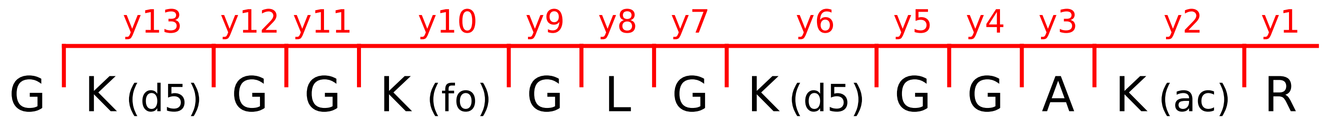

Raw file: HF240124_RN_spleen_D5PRO_D5PRO_3, Scan: 12810, m/z: 795.54057, Charge: 2, RT (min): 23.94, Score: 130.94

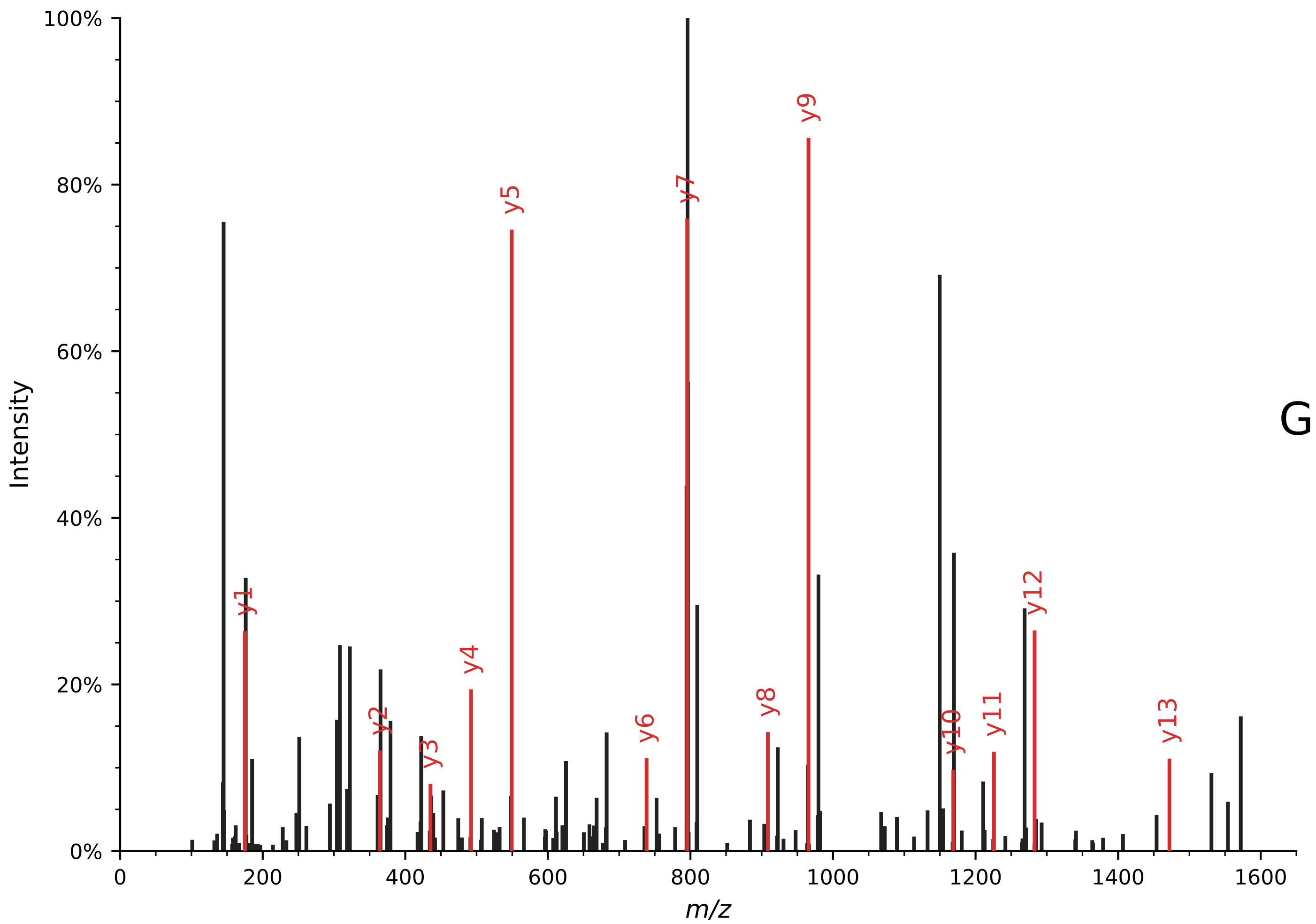

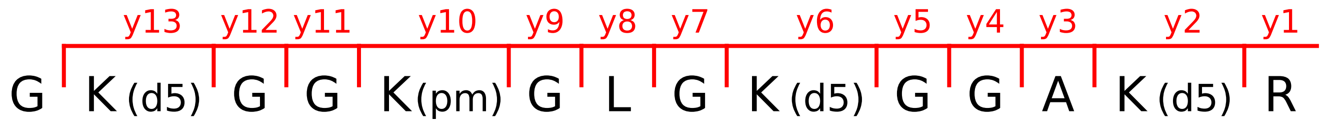

Raw file: HF240124_RN_spleen_D5PRO_D5PRO_2, Scan: 11312, m/z: 784.50919, Charge: 2, RT (min): 21.30, Score: 51.445

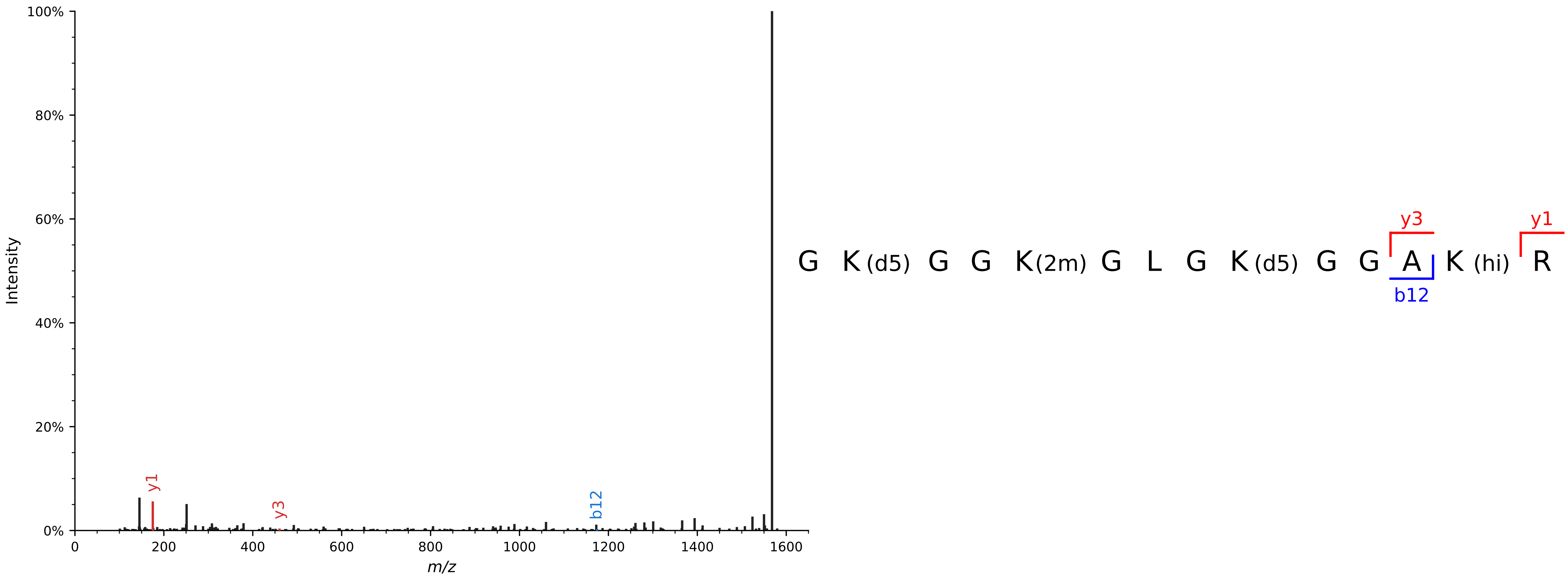

Raw file: HF240124_RN_spleen_D5PRO_D5PRO_1, Scan: 10690, m/z: 769.50391, Charge: 2, RT (min): 20.12, Score: 99.283

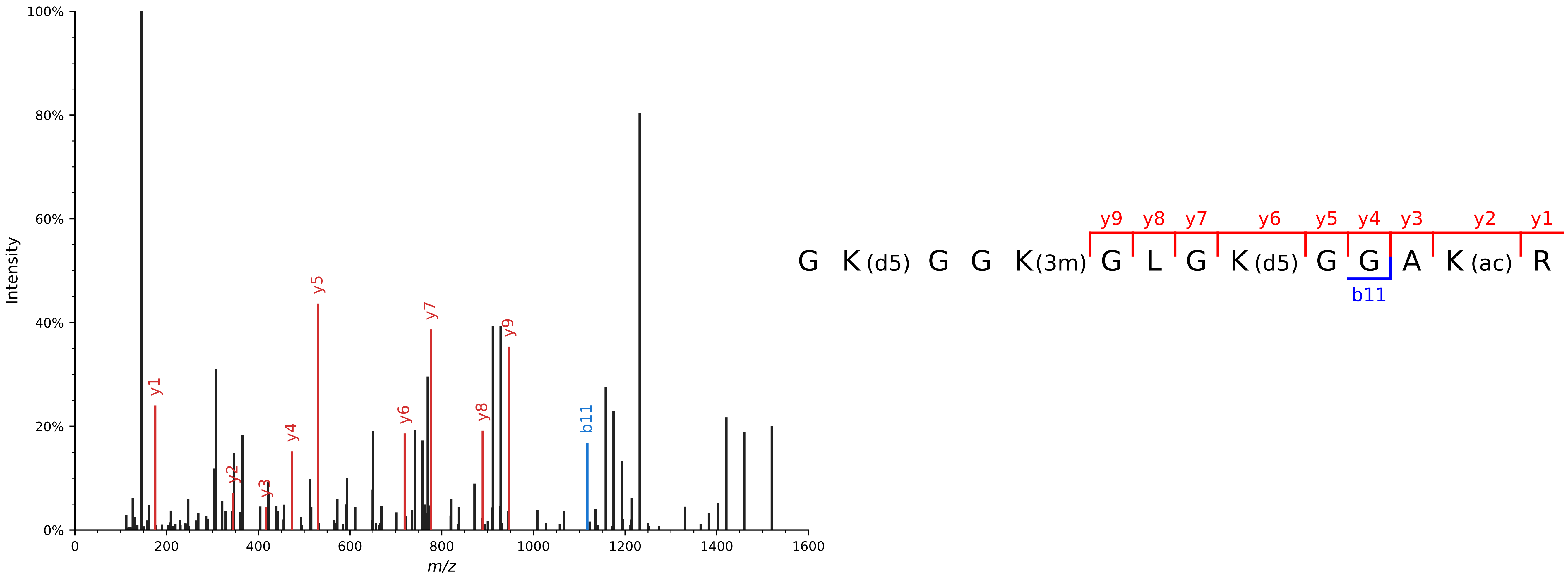

Raw file: HF240124_RN_spleen_D5PRO_D5PRO_2, Scan: 10244, m/z: 779.00923, Charge: 2, RT (min): 19.47, Score: 155.11

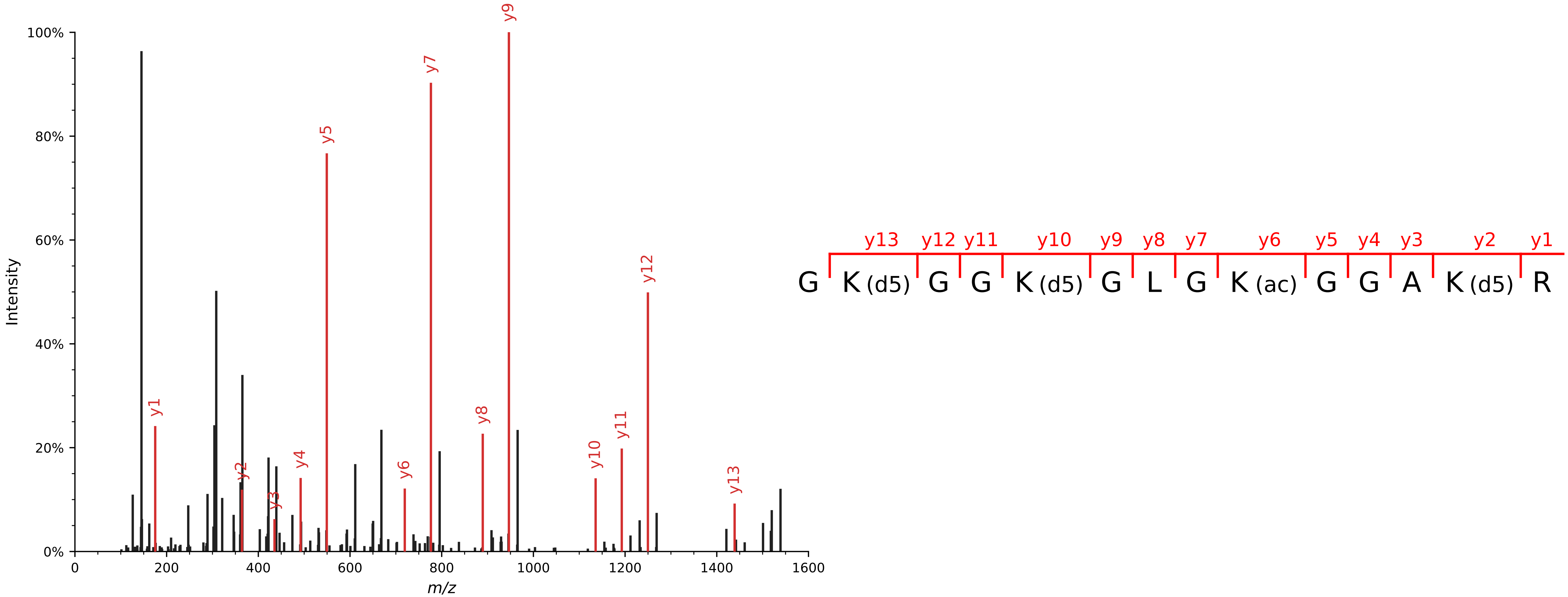

Raw file: HF240124_RN_spleen_D5PRO_D5PRO_2, Scan: 9427, m/z: 769.48571, Charge: 2, RT (min): 18.08, Score: 163.34

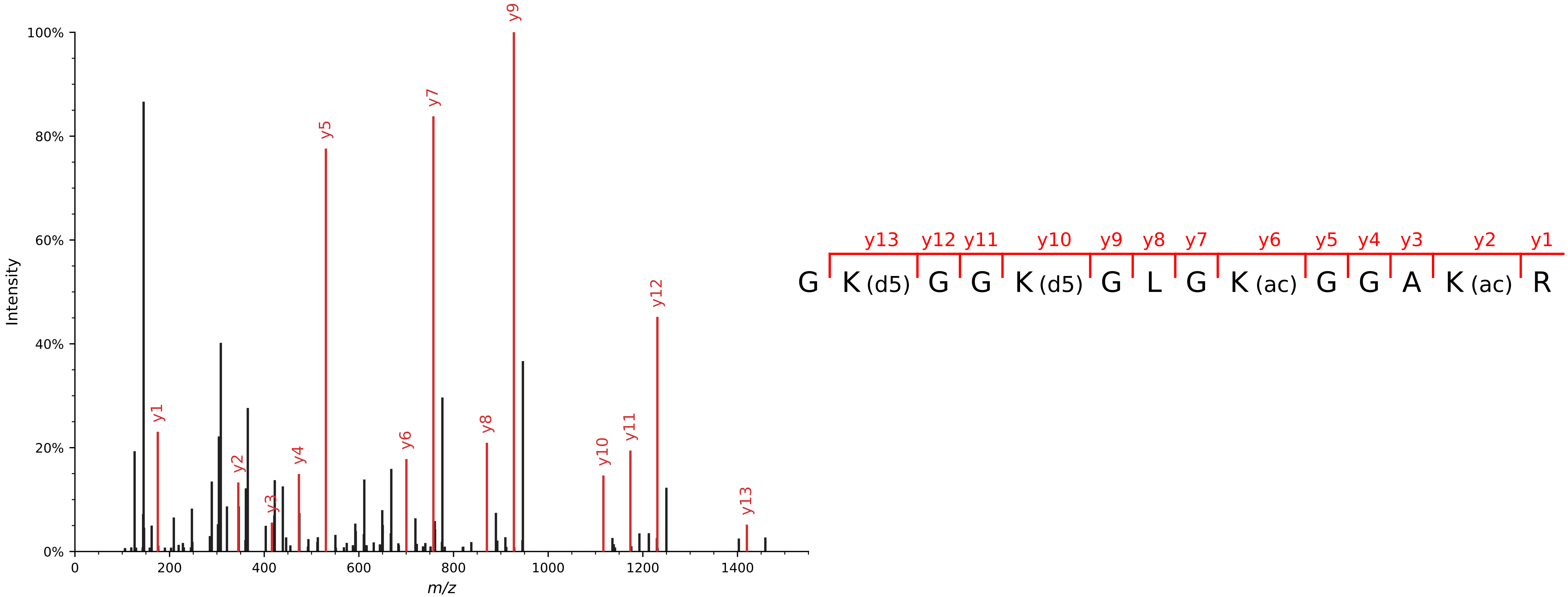

Raw file: HF240124_RN_spleen_D5PRO_D5PRO_3, Scan: 11913, m/z: 783.50136, Charge: 2, RT (min): 22.28, Score: 115.18

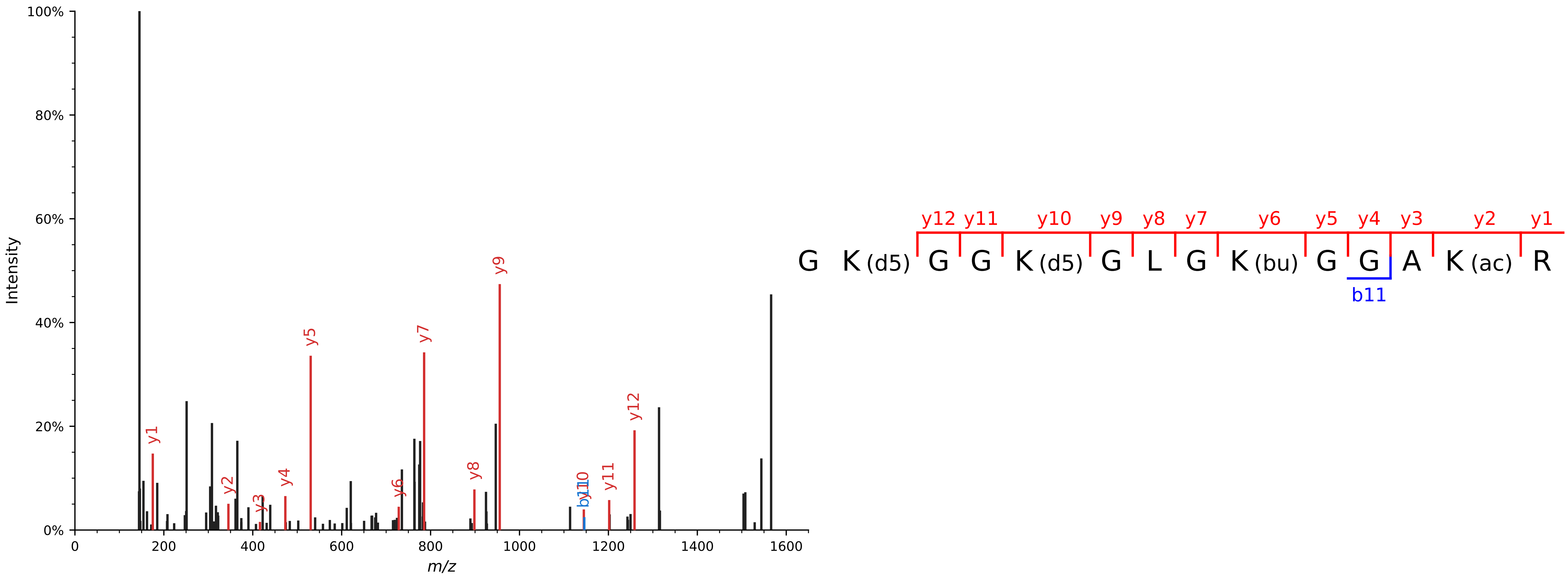

Raw file: HF240124_RN_spleen_D5PRO_D5PRO_2, Scan: 9931, m/z: 515.00336, Charge: 3, RT (min): 18.94, Score: 66.004

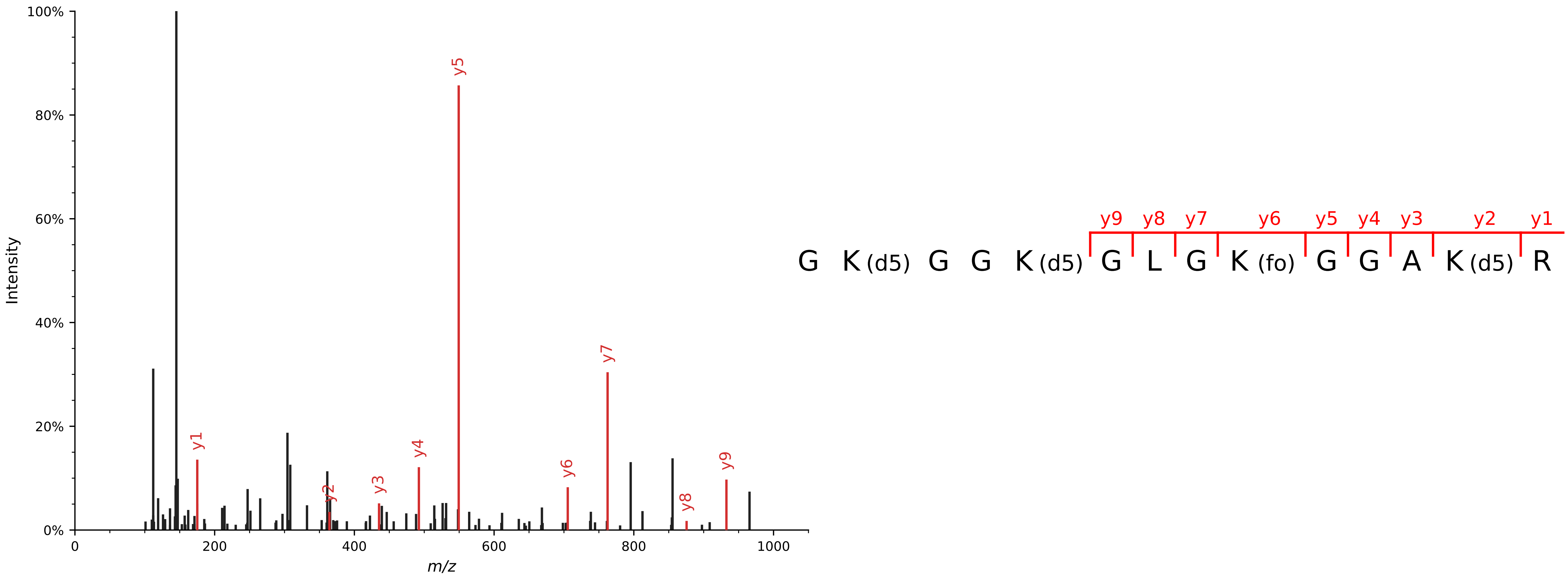

Raw file: HF240124_RN_spleen_D5PRO_D5PRO_3, Scan: 10456, m/z: 779.00923, Charge: 2, RT (min): 19.69, Score: 155.11

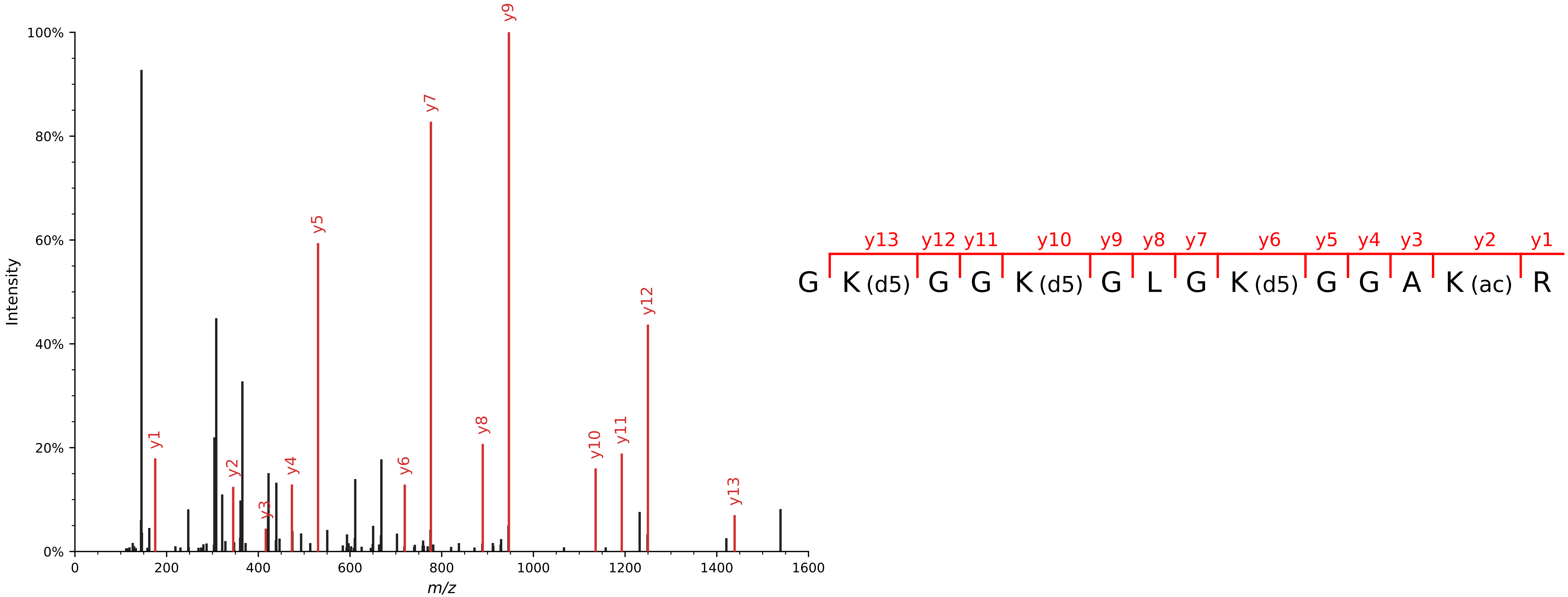

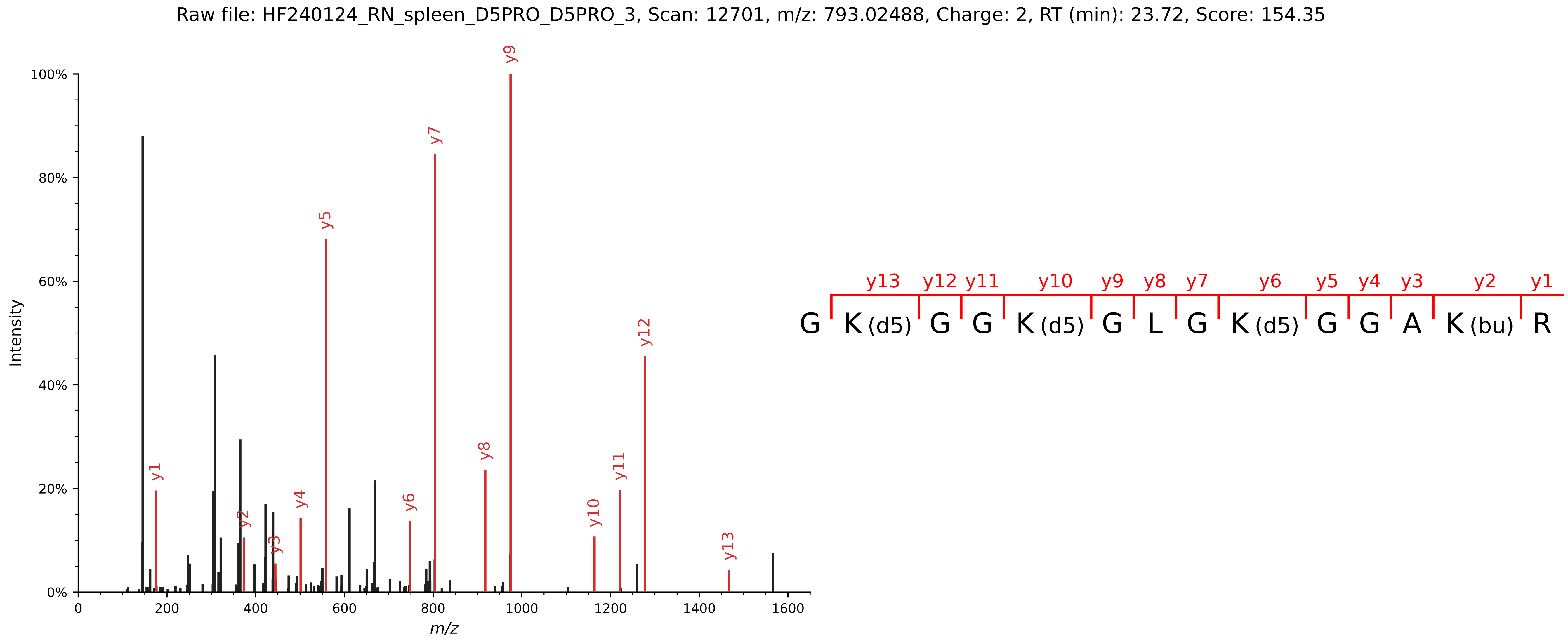
Raw file: HF240124_RN_spleen_D5PRO_D5PRO_3, Scan: 12701, m/z: 793.02488, Charge: 2, RT (min): 23.72, Score: 154.35
Intensity
m/z
y1
y2
y3
y4
y5
y6
y7
y8
y9
y10
y11
y12
y13
y13 y12 y11 y10 y9 y8 y7 y6 y5 y4 y3 y2 y1
G K(d5) G G K(d5) G L G K(d5) G G A K(bu) R

Raw file: HF240124_RN_spleen_D5PRO_D5PRO_3, Scan: 10224, m/z: 772.00141, Charge: 2, RT (min): 19.29, Score: 143.61

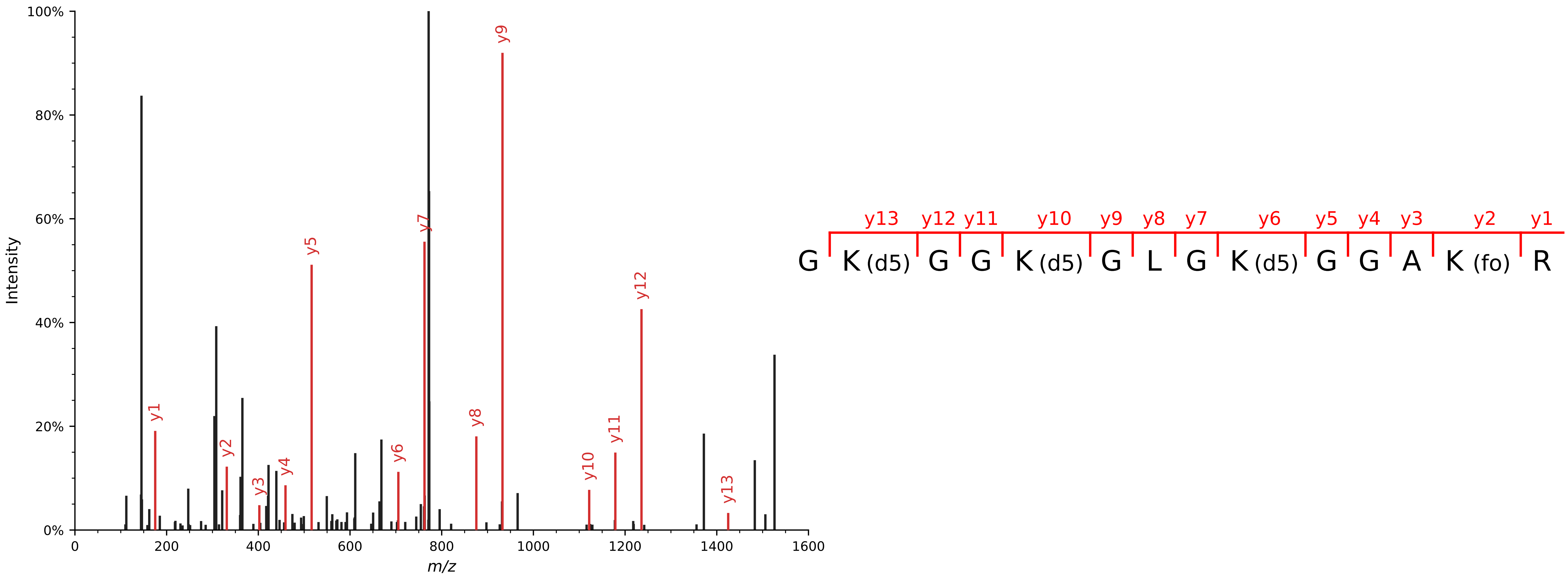

Raw file: HF240124_RN_spleen_D5PRO_D5PRO_1, Scan: 12741, m/z: 795.54057, Charge: 2, RT (min): 23.68, Score: 145.89

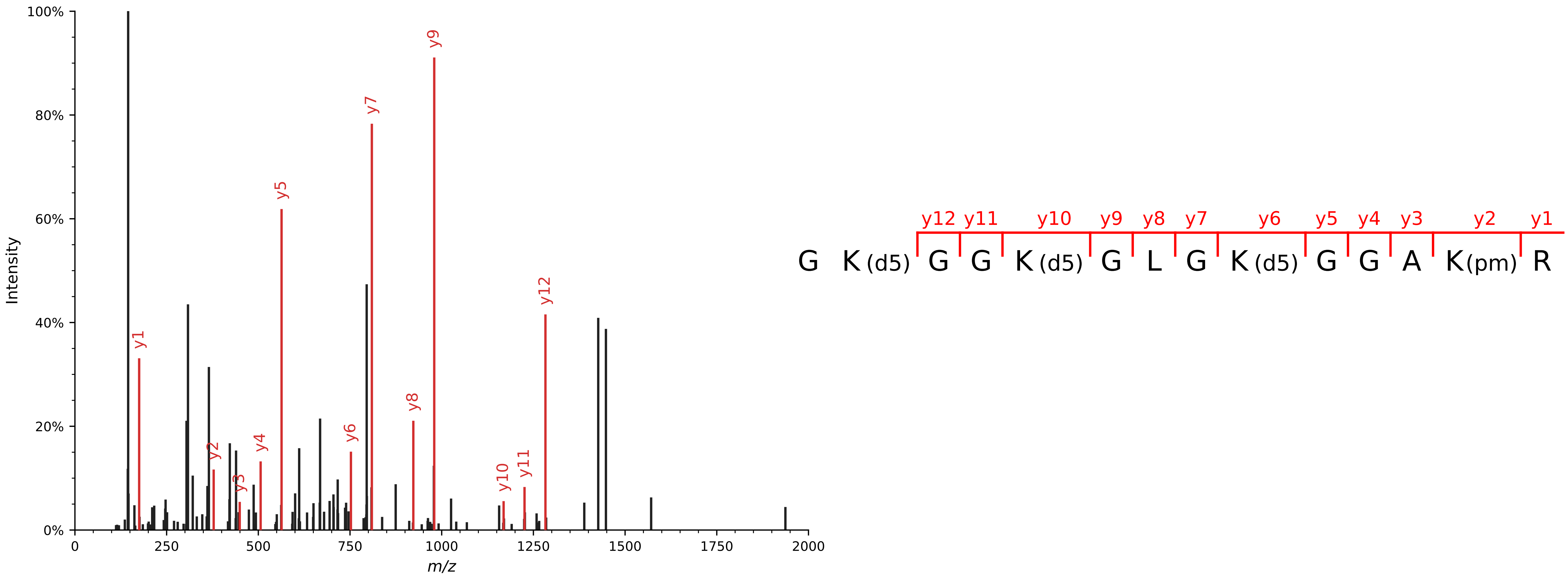

Raw file: HF240124_RN_spleen_D5PRO_D5PRO_2, Scan: 16042, m/z: 728.93016, Charge: 2, RT (min): 29.87, Score: 157.66

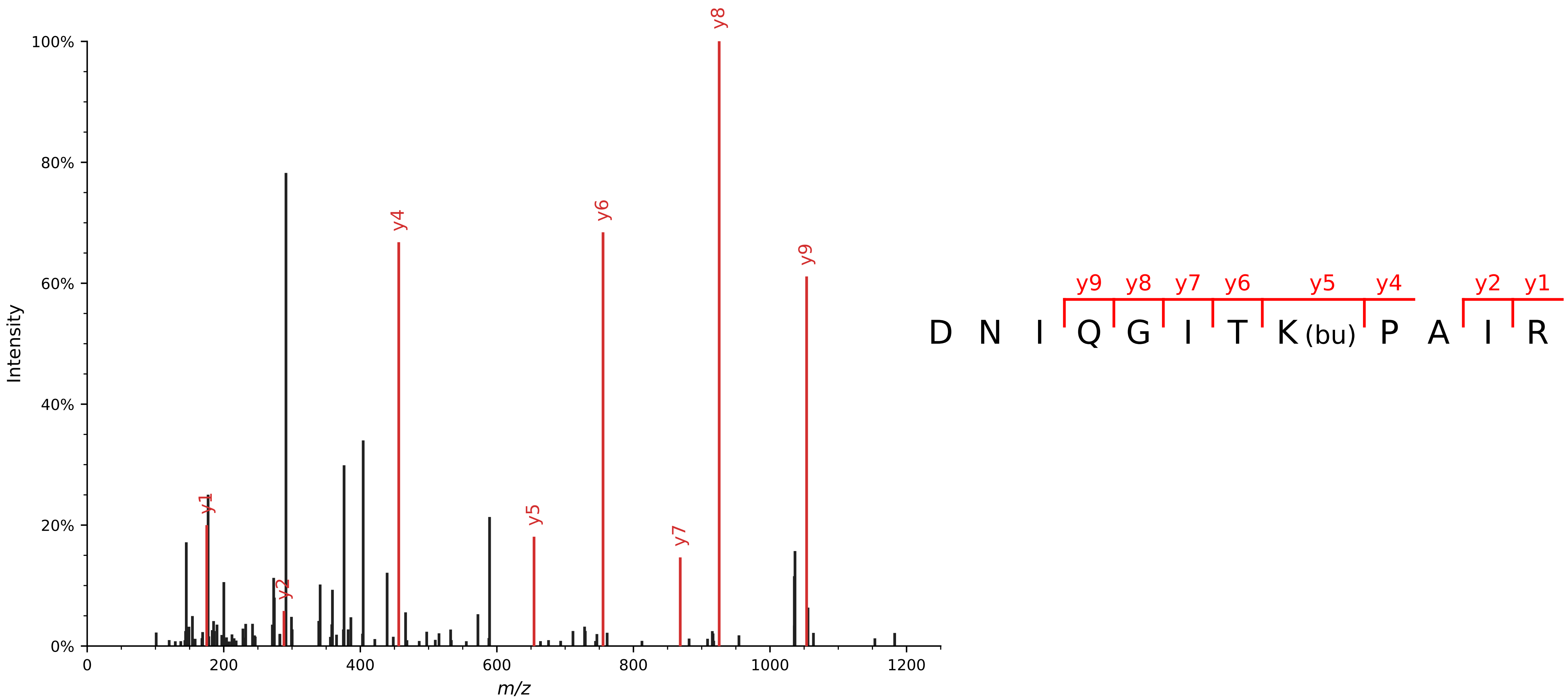

Raw file: HF240124_RN_spleen_D5PRO_D5PRO_2, Scan: 13108, m/z: 707.90668, Charge: 2, RT (min): 24.54, Score: 163.38

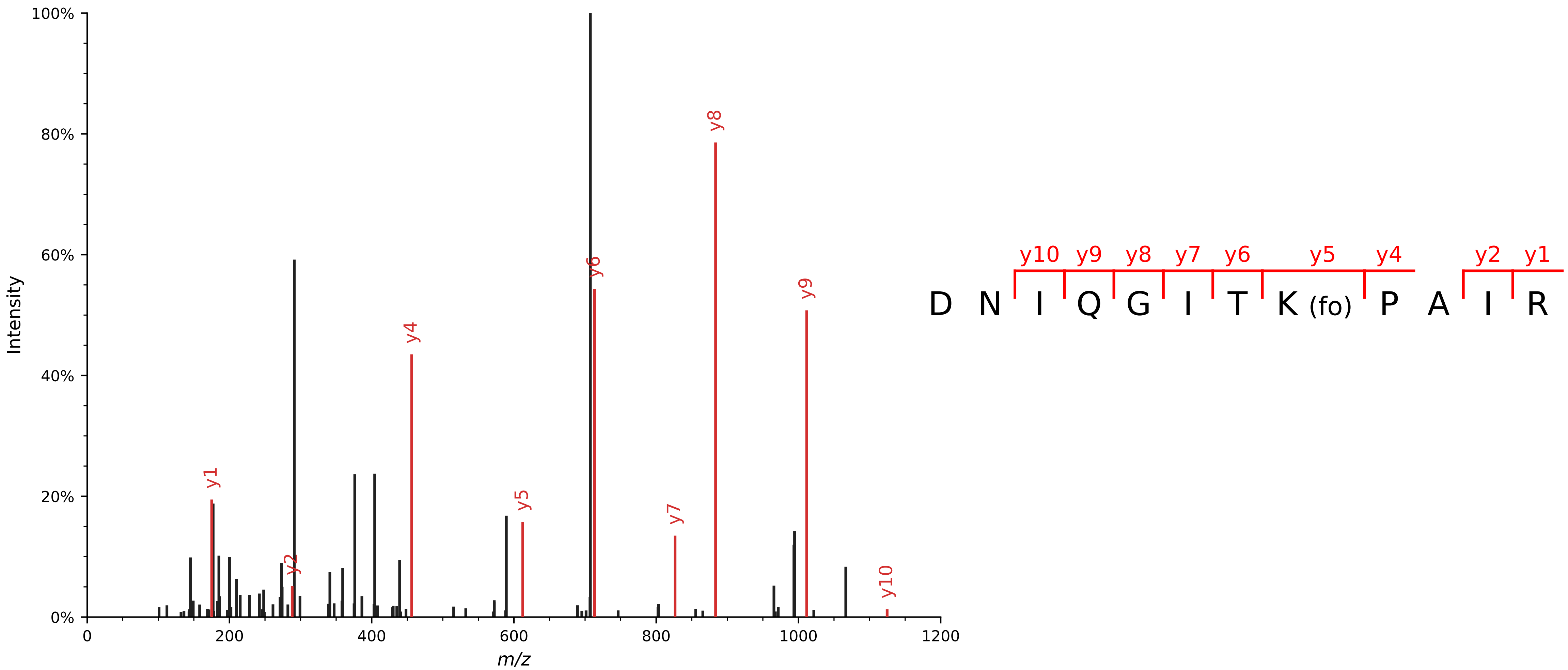

Raw file: HF240124_RN_spleen_D5PRO_D5PRO_1, Scan: 13878, m/z: 731.44585, Charge: 2, RT (min): 25.78, Score: 77.049

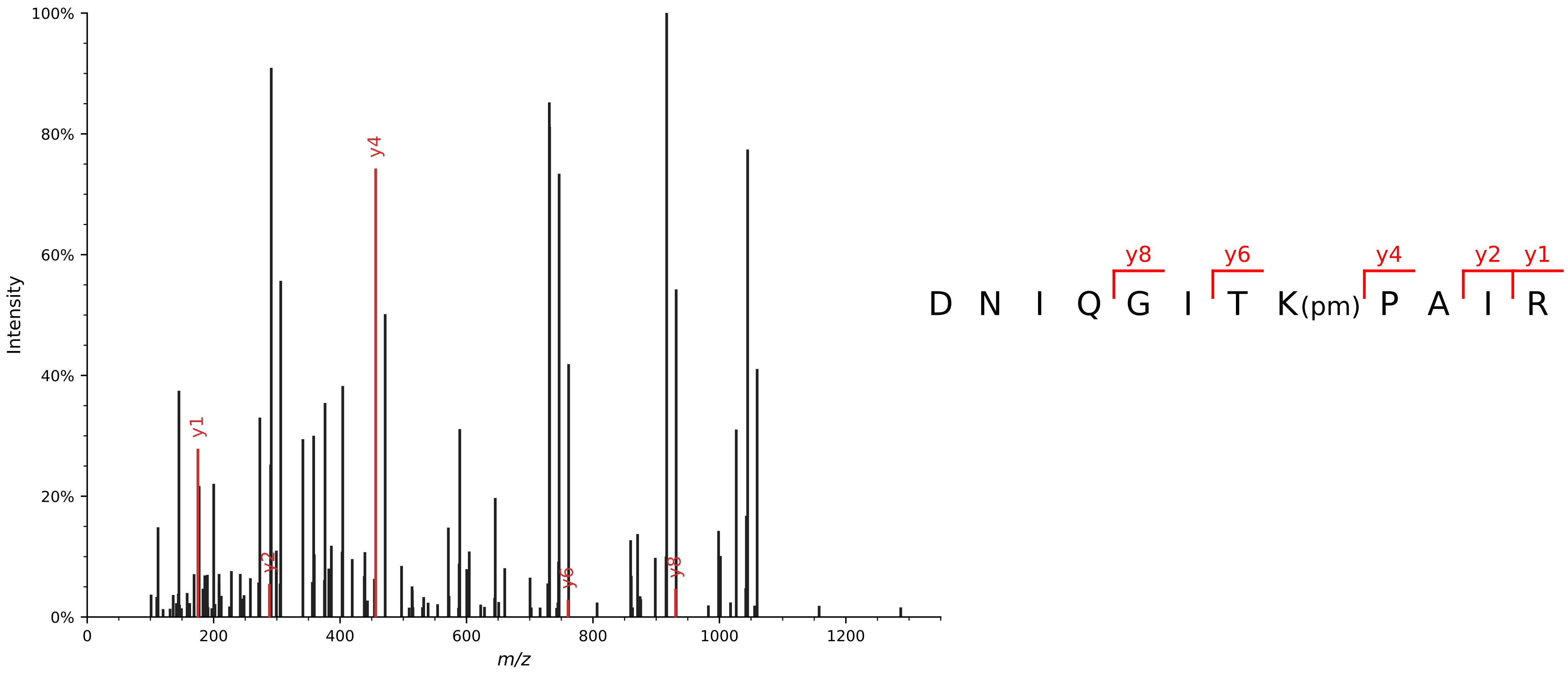

Raw file: HF240124_RN_spleen_D5PRO_D5PRO_1, Scan: 8035, m/z: 711.37521, Charge: 2, RT (min): 15.60, Score: 197.96

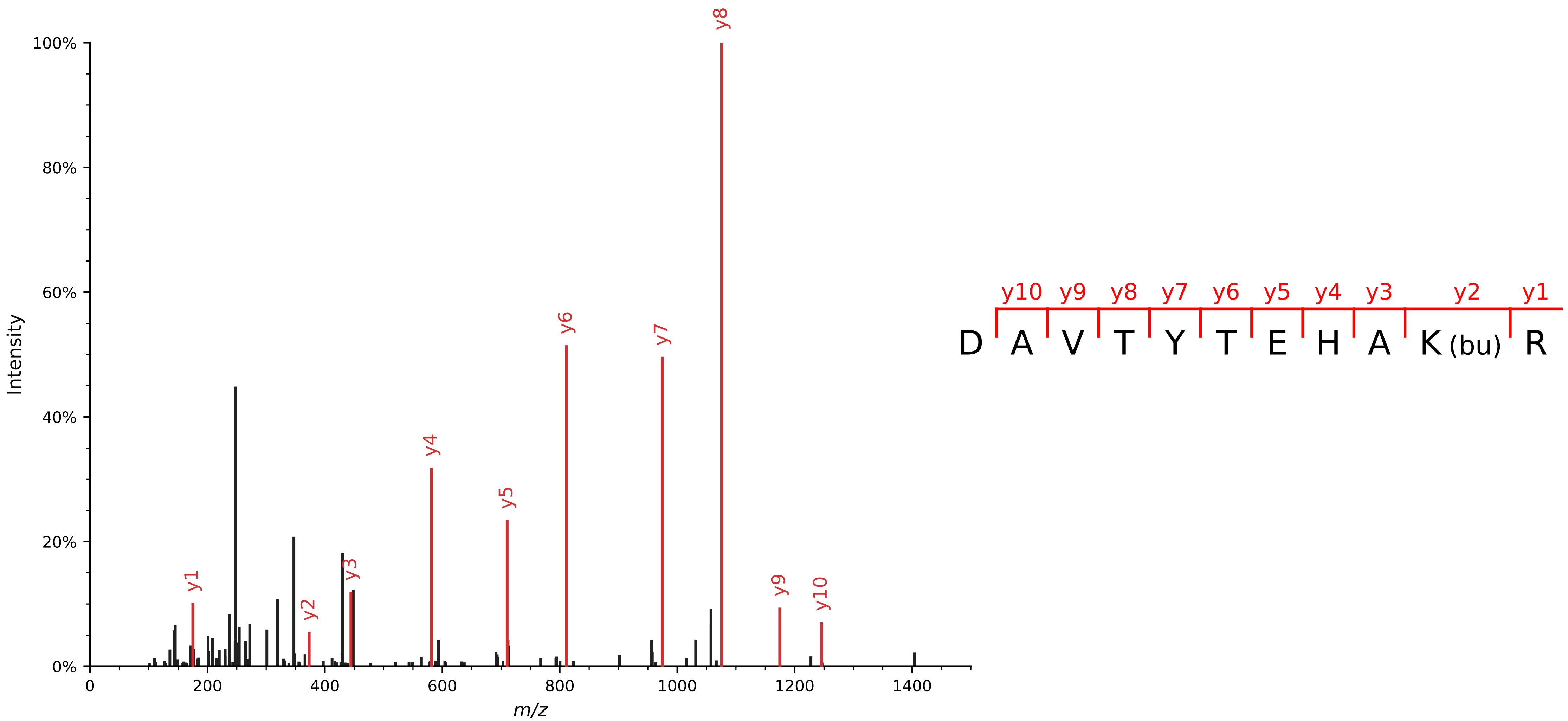

Raw file: HF240124_RN_spleen_D5PRO_D5PRO_2, Scan: 5179, m/z: 690.35174, Charge: 2, RT (min): 10.69, Score: 137.95

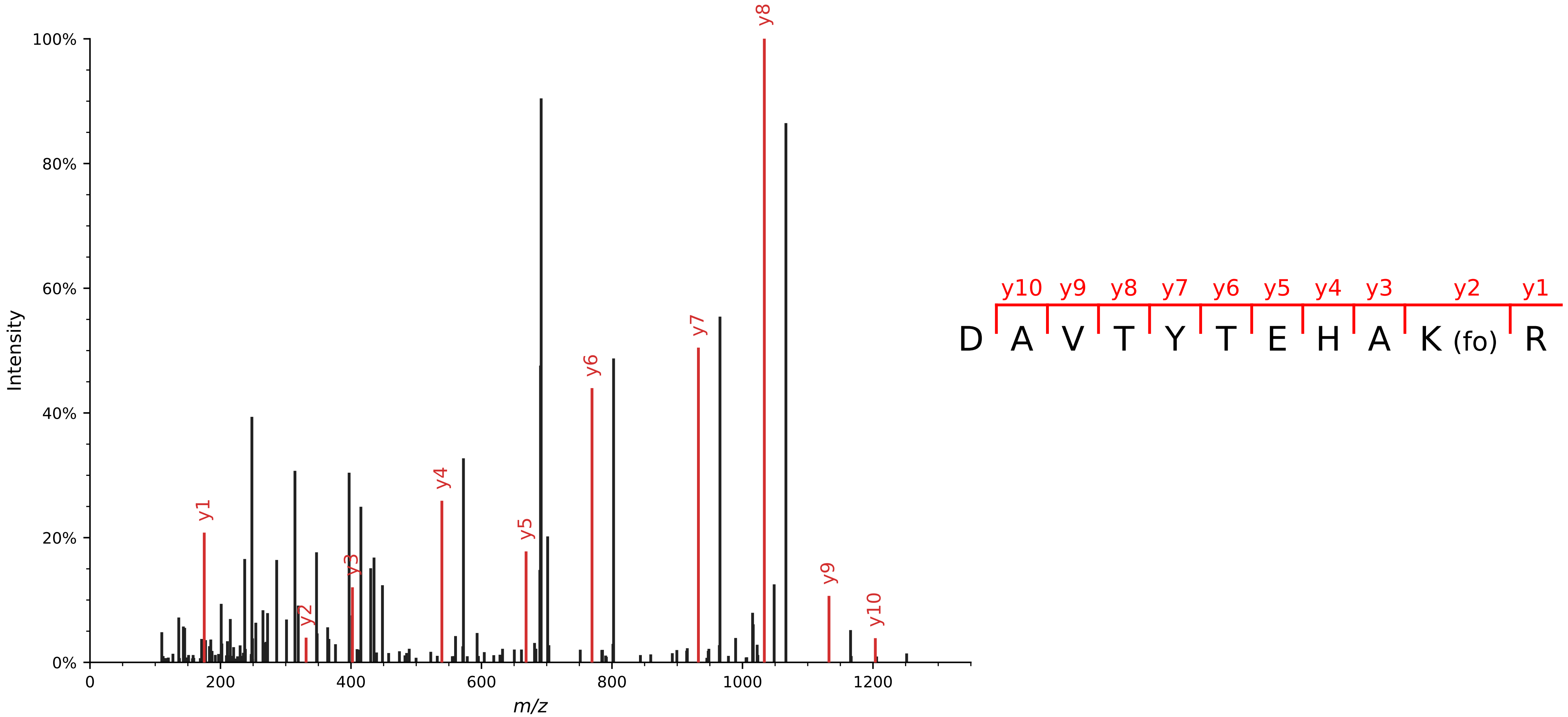

Raw file: HF240124_RN_spleen_D5PRO_D5PRO_2, Scan: 22348, m/z: 587.01295, Charge: 3, RT (min): 41.16, Score: 80.746

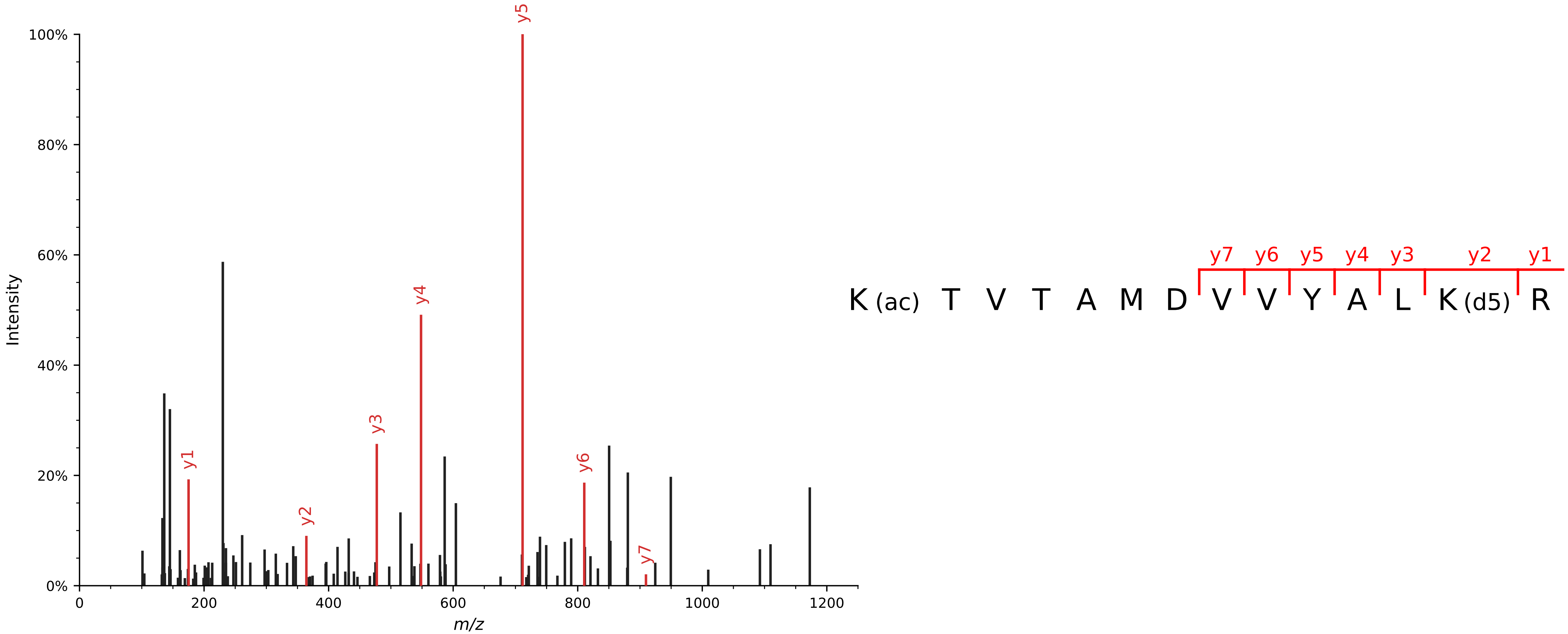

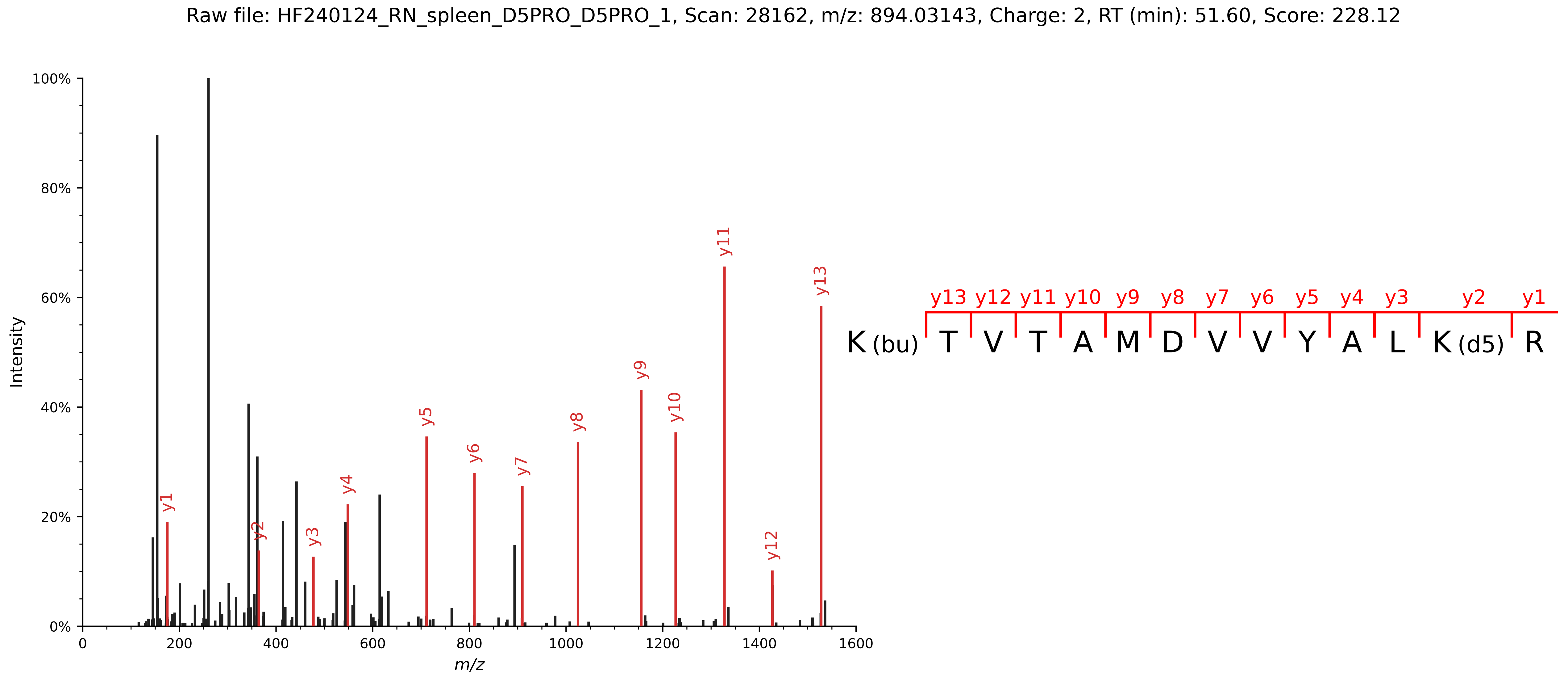

Raw file: HF240124_RN_spleen_D5PRO_D5PRO_1, Scan: 28162, m/z: 894.03143, Charge: 2, RT (min): 51.60, Score: 228.12
Intensity
m/z
y1 y2 y3 y4 y5 y6 y7 y8 y9 y10 y11 y12 y13
K(bu) T V T A M D V V Y A L K(d5) R

Raw file: HF240124_RN_spleen_D5PRO_D5PRO_2, Scan: 25313, m/z: 873.00796, Charge: 2, RT (min): 46.63, Score: 210.95

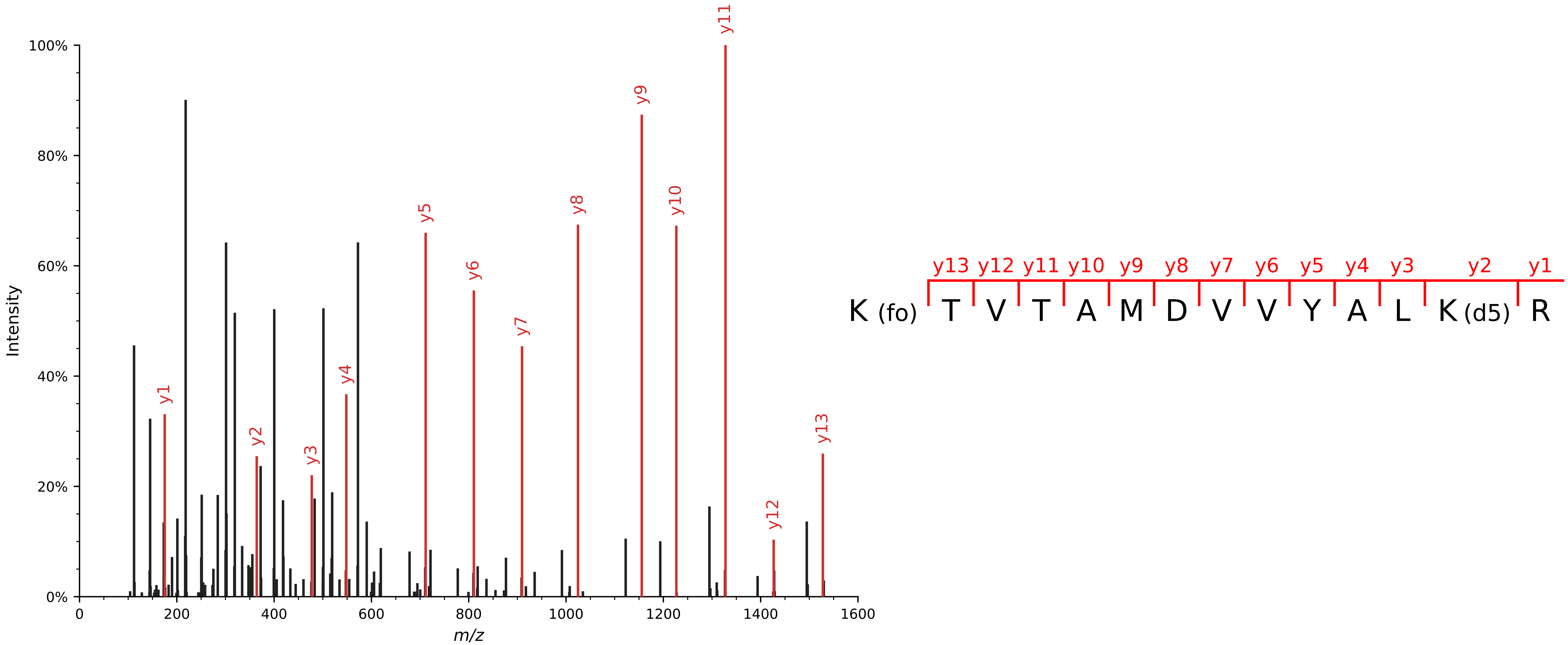

Raw file: HF240124_RN_spleen_D5PRO_D5PRO_2, Scan: 23933, m/z: 916.02635, Charge: 2, RT (min): 44.07, Score: 43.512

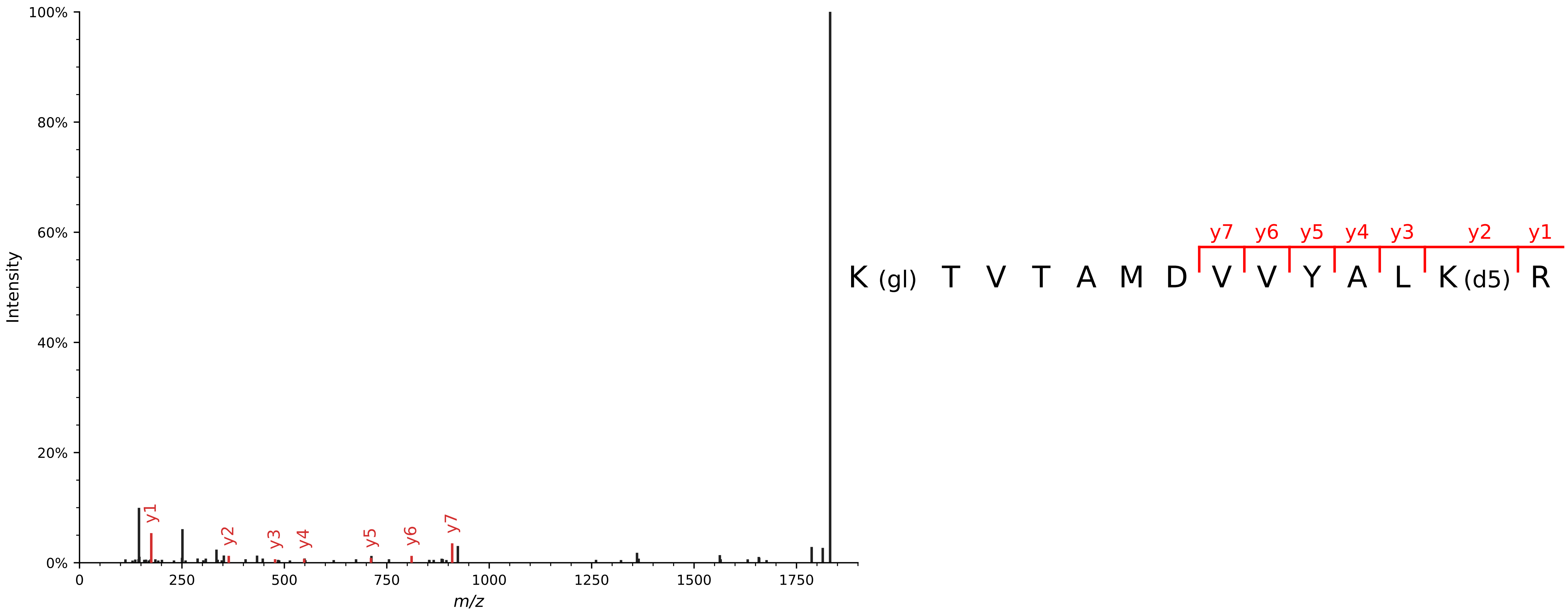

Raw file: HF240124_RN_spleen_D5PRO_D5PRO_1, Scan: 24584, m/z: 601.68835, Charge: 3, RT (min): 45.01, Score: 49.12

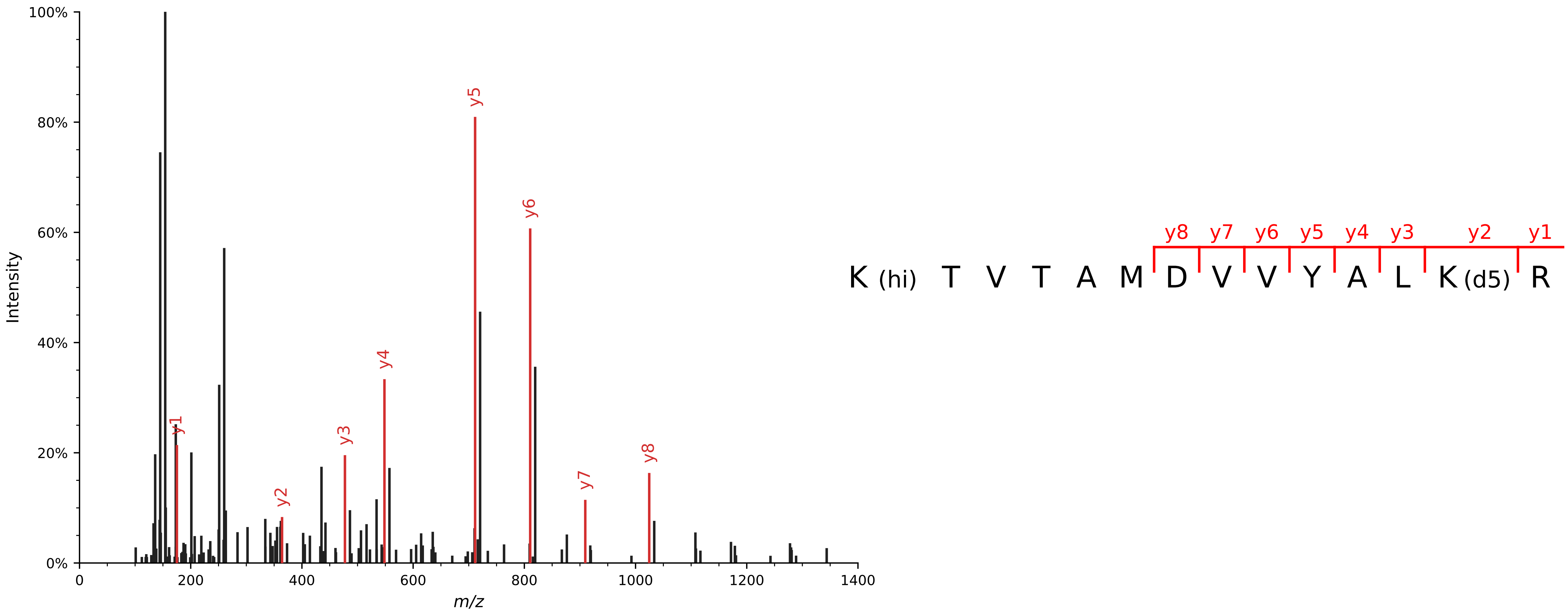

Raw file: HF240124_RN_spleen_D5PRO_D5PRO_3, Scan: 16249, m/z: 896.54712, Charge: 2, RT (min): 30.22, Score: 64.297

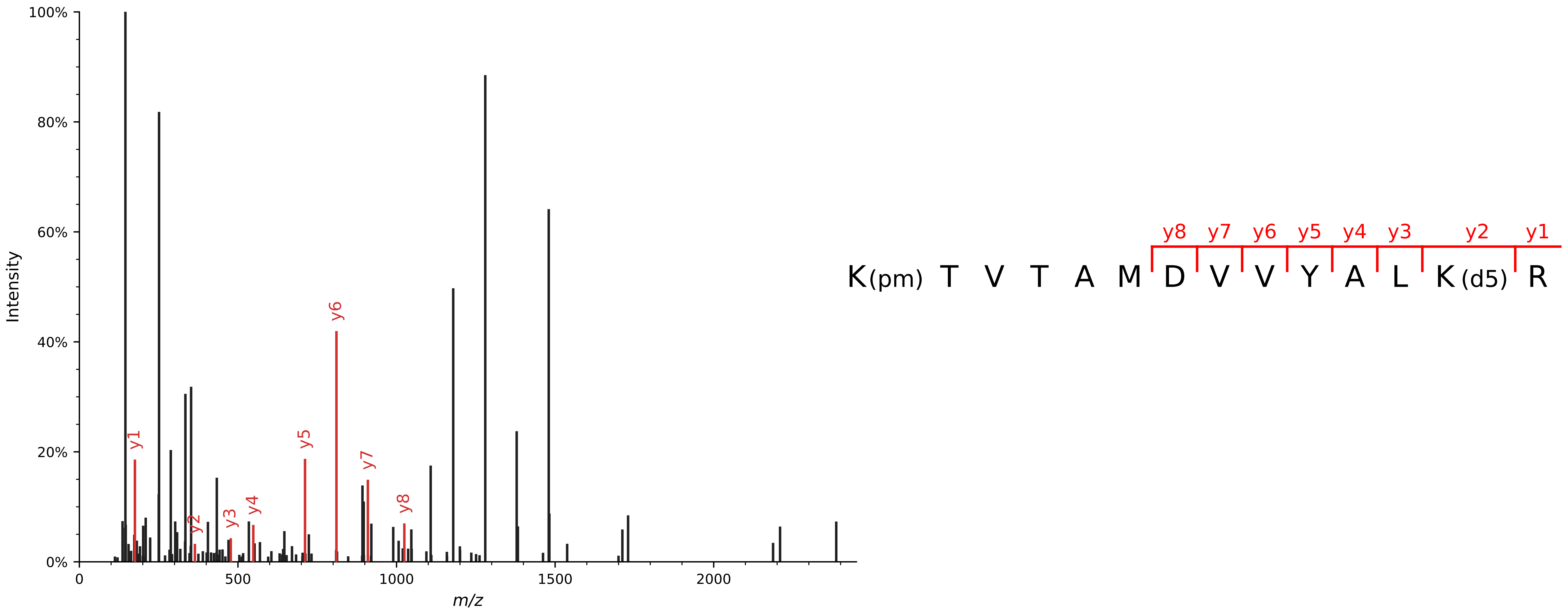

Raw file: HF240124_RN_spleen_D5PRO_D5PRO_1, Scan: 11287, m/z: 582.35319, Charge: 3, RT (min): 21.14, Score: 47.189

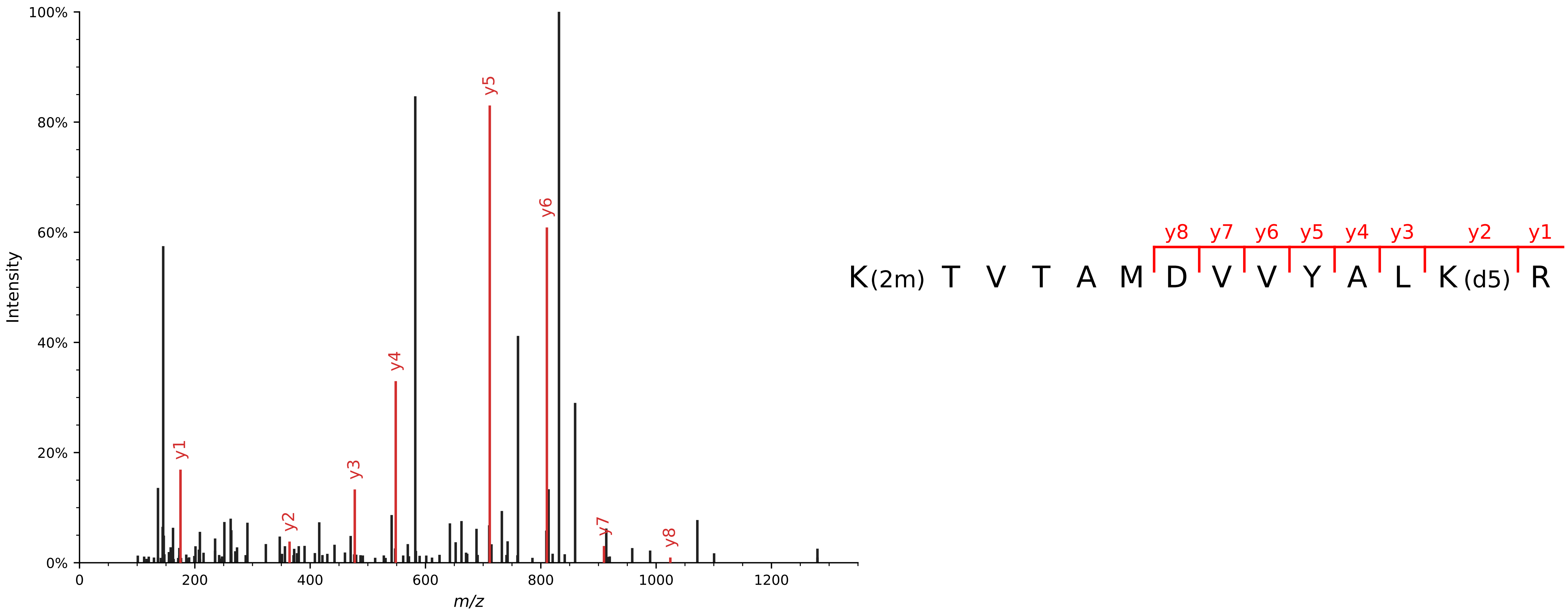

Raw file: HF240124_RN_spleen_D5PRO_D5PRO_3, Scan: 22631, m/z: 587.02507, Charge: 3, RT (min): 41.85, Score: 58.268

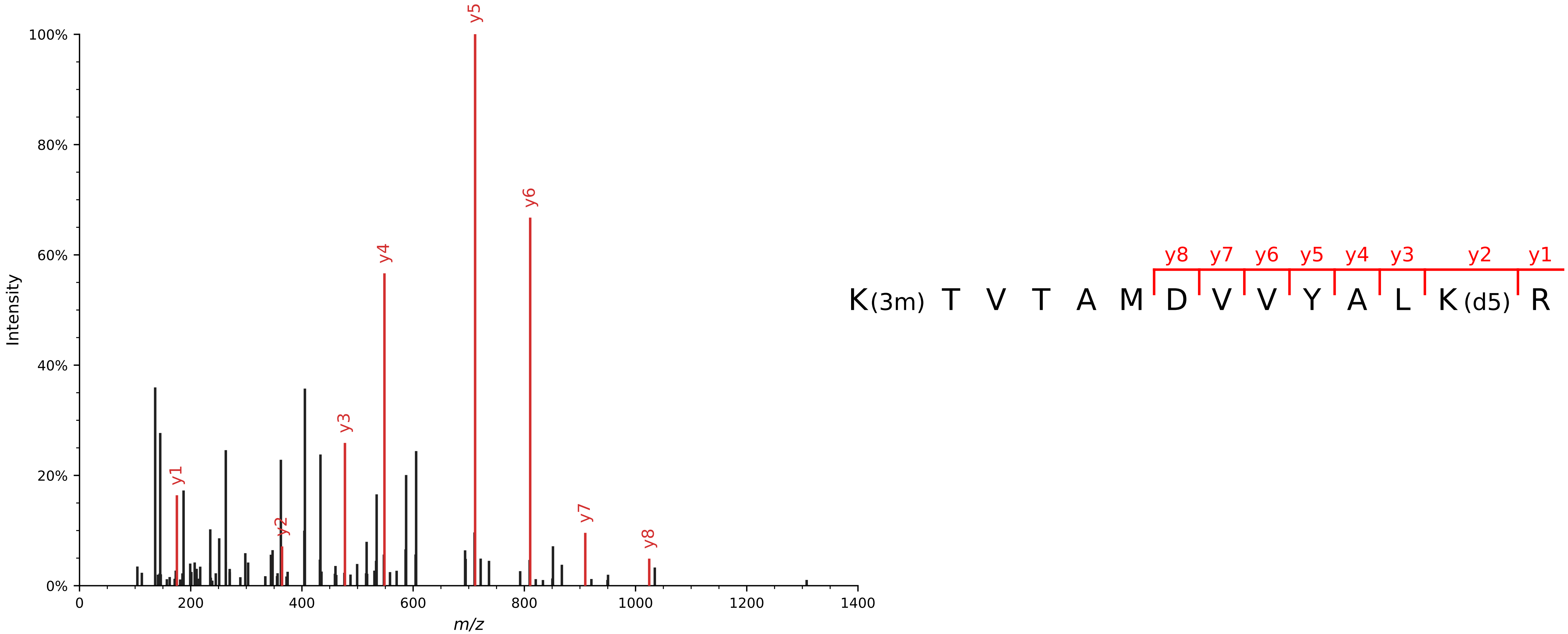

Raw file: HF240124_RN_spleen_D5PRO_D5PRO_2, Scan: 27935, m/z: 894.03143, Charge: 2, RT (min): 51.48, Score: 154.36

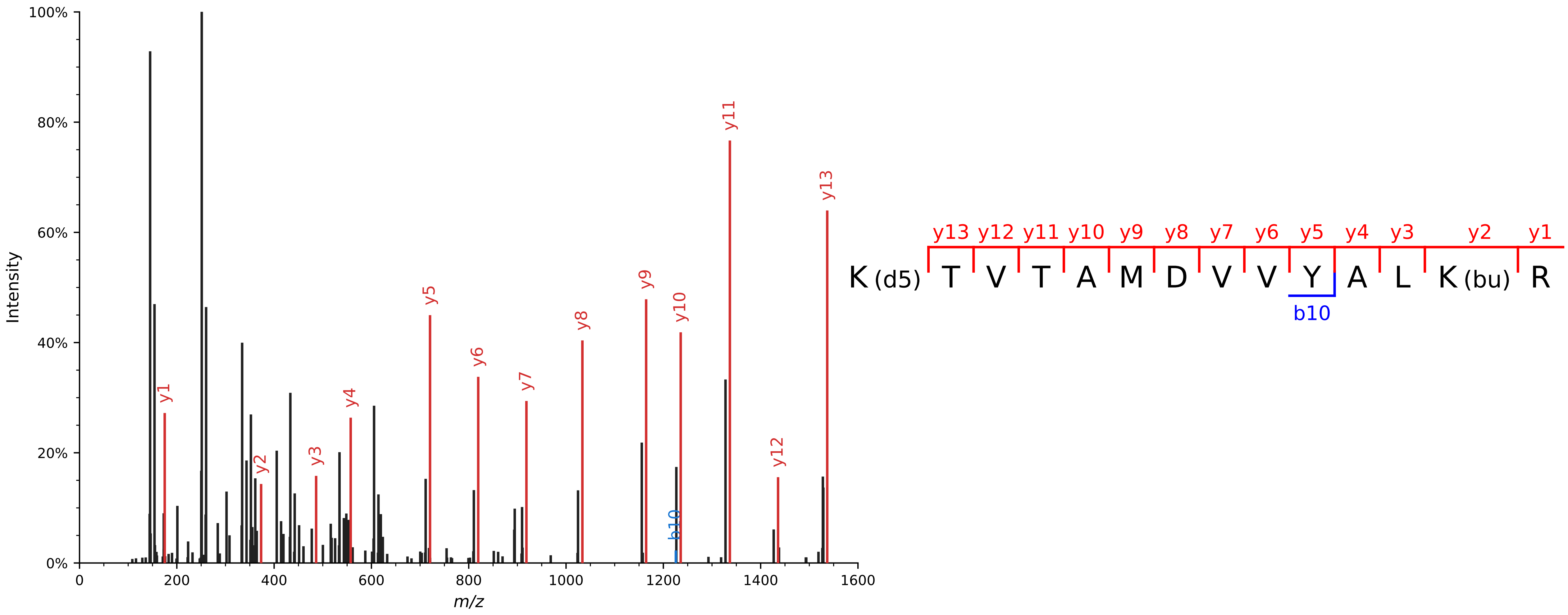

Raw file: HF240124_RN_spleen_D5PRO_D5PRO_1, Scan: 25529, m/z: 873.00796, Charge: 2, RT (min): 46.75, Score: 137.16

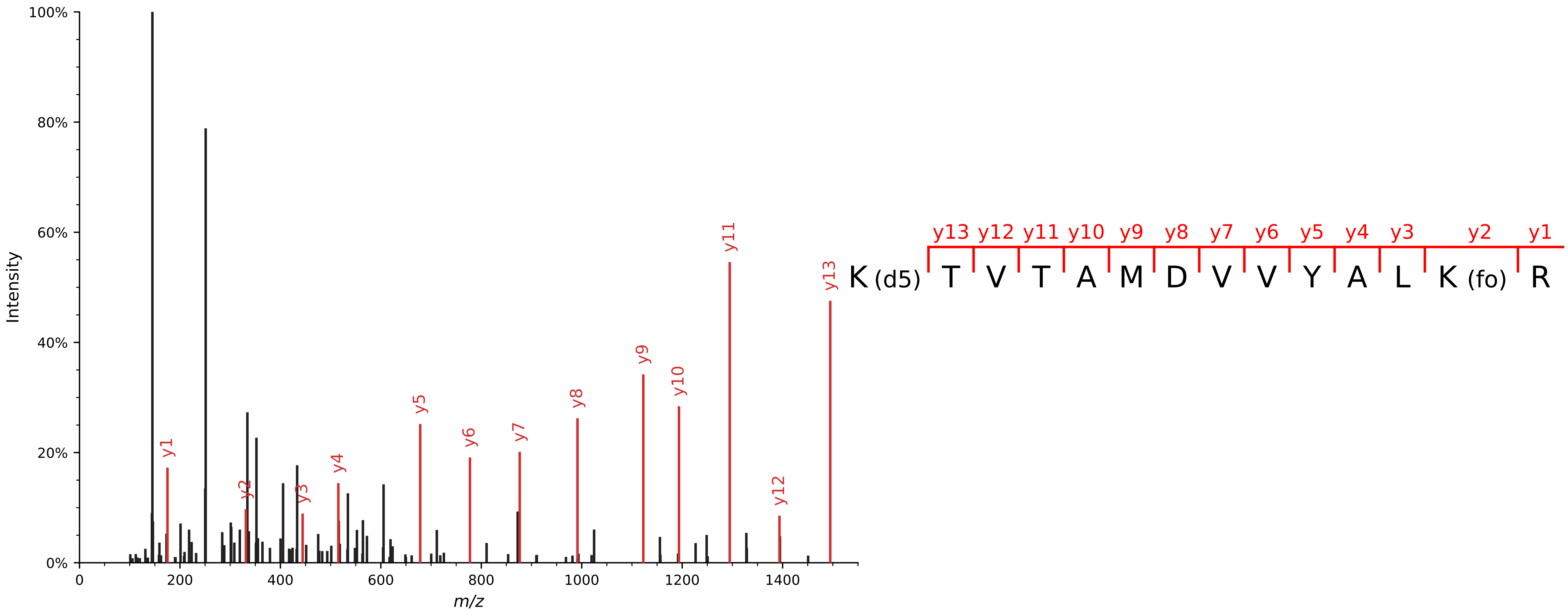

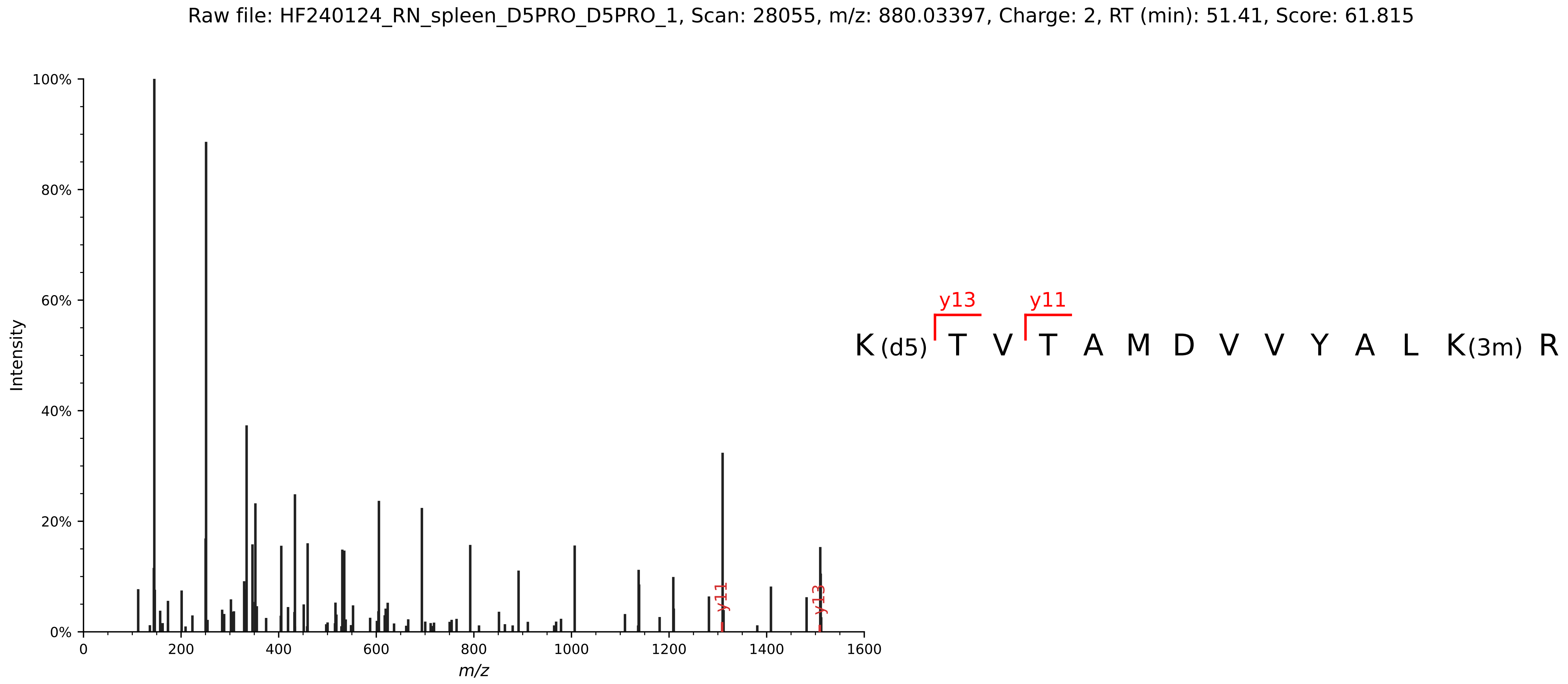

Raw file: HF240124_RN_spleen_D5PRO_D5PRO_1, Scan: 28055, m/z: 880.03397, Charge: 2, RT (min): 51.41, Score: 61.815
Intensity
m/z
y11
y13
K(d5) T V T A M D V V Y A L K(3m) R

Spleen_D3Ac-PIC

Raw file: HF240124_RN_spleen_D3PIC_3, Scan: 8913, m/z: 491.29099, Charge: 2, RT (min): 17.68, Score: 108.98

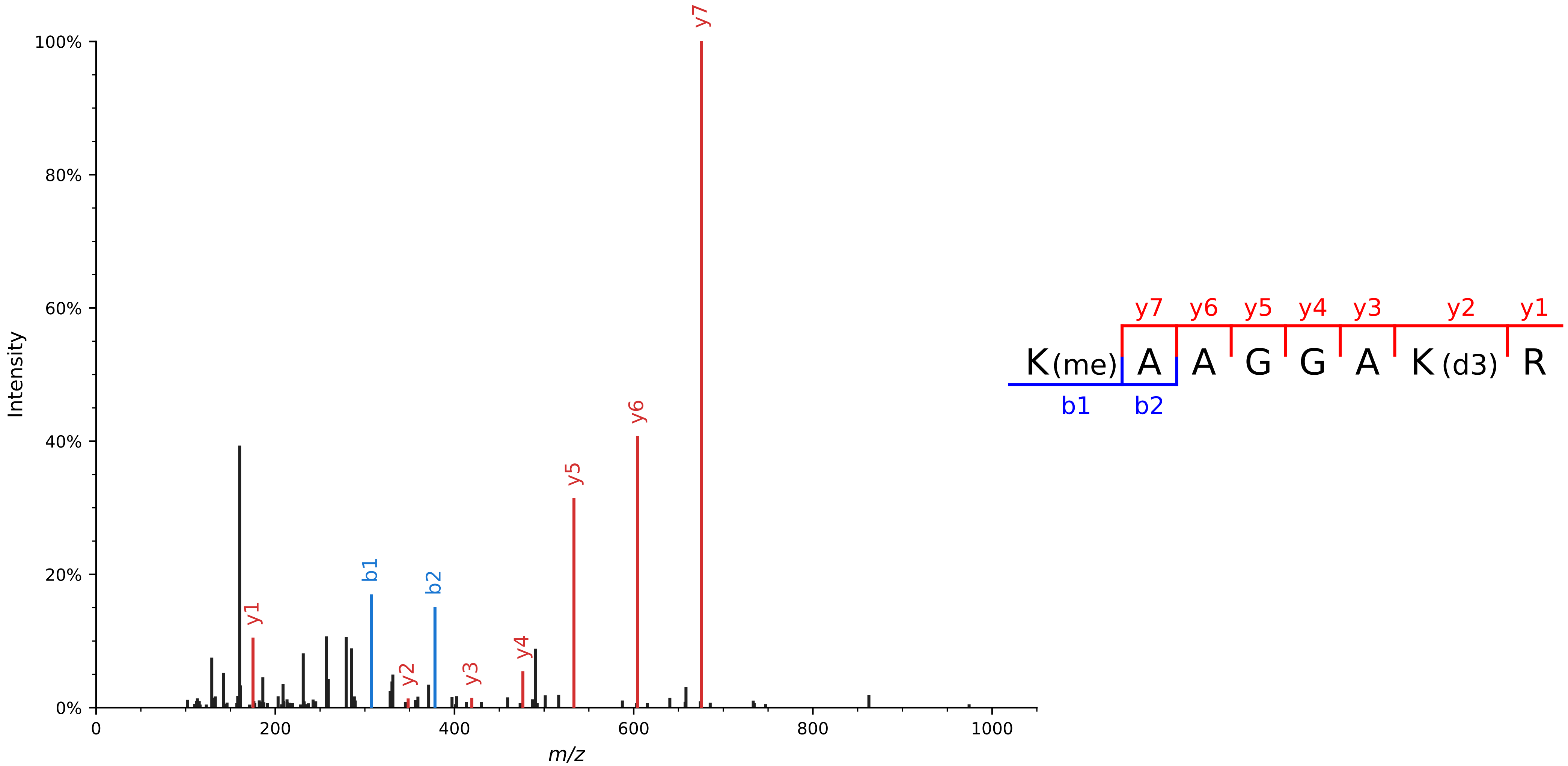

Raw file: HF240124_RN_spleen_D3PIC_1, Scan: 22866, m/z: 738.37808, Charge: 2, RT (min): 42.86, Score: 98.048

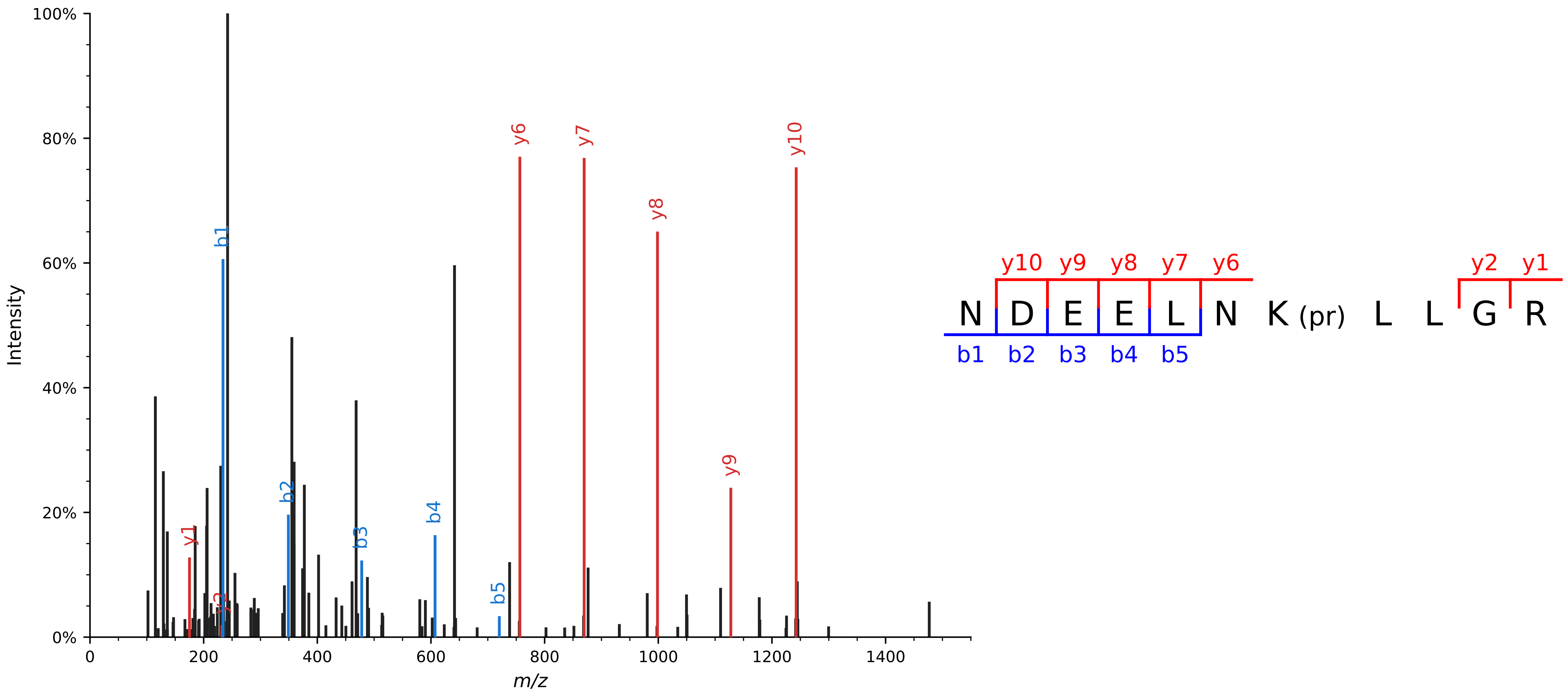

Raw file: HF240124_RN_spleen_D3PIC_2, Scan: 11405, m/z: 454.77322, Charge: 2, RT (min): 22.00, Score: 49.358

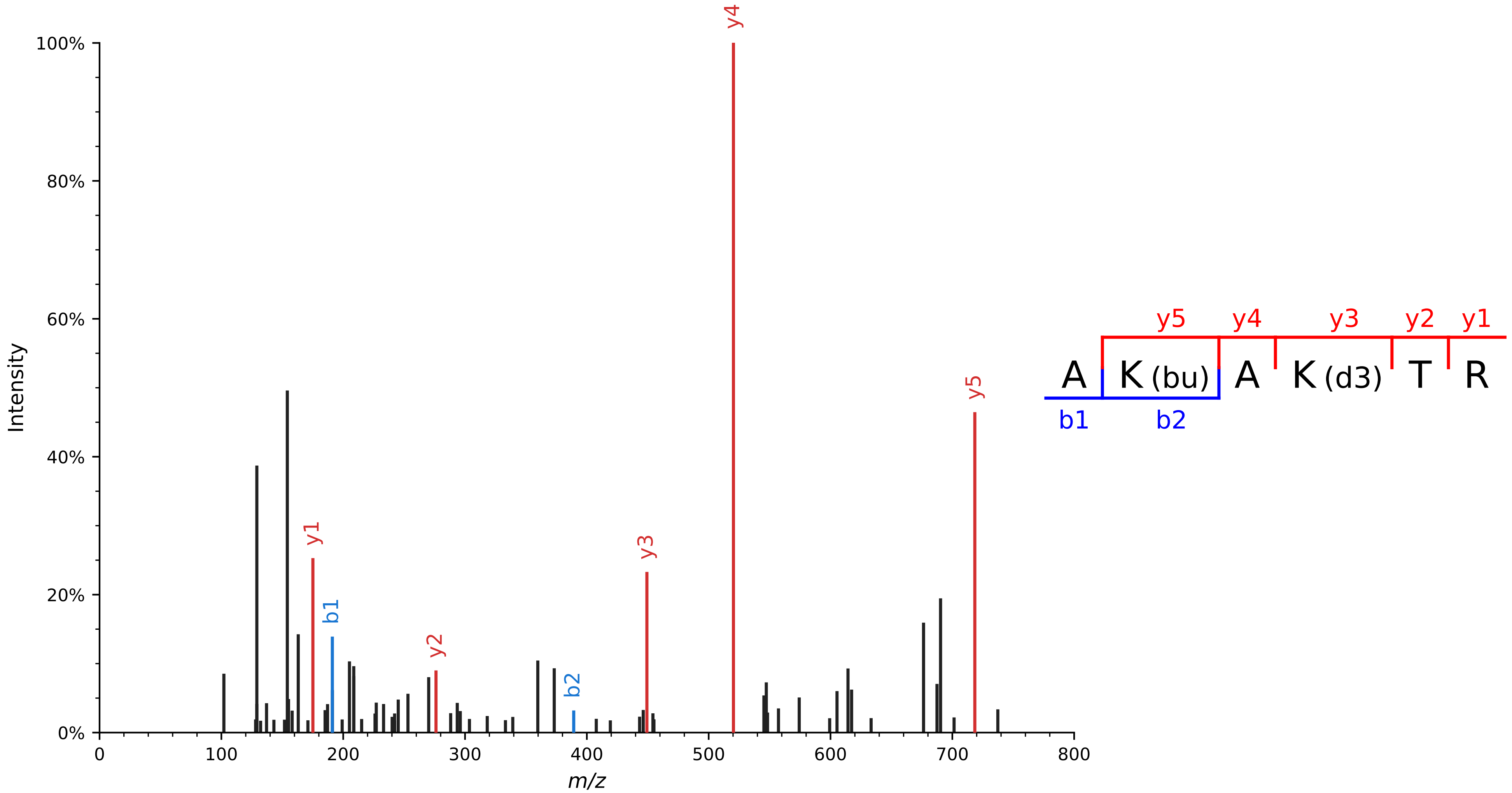

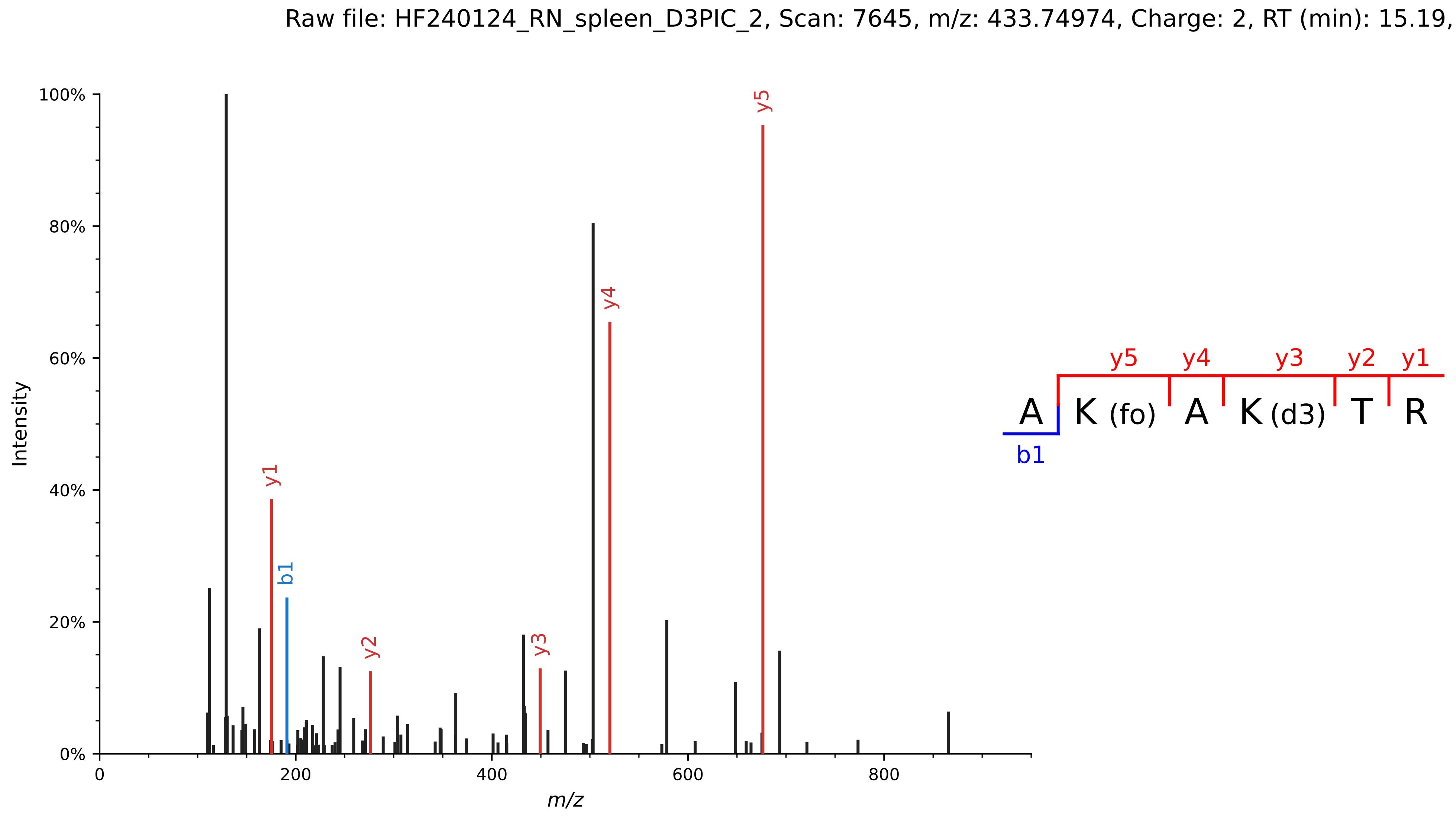

Raw file: HF240124_RN_spleen_D3PIC_2, Scan: 7645, m/z: 433.74974, Charge: 2, RT (min): 15.19, Score: 69.432
100%
80%
60%
40%
20%
0%
Intensity
0
200
400
600
800
m/z
y1
b1
y2
y3
y4
y5
y5 y4 y3 y2 y1
A K (fo) A K (d3) T R
b1

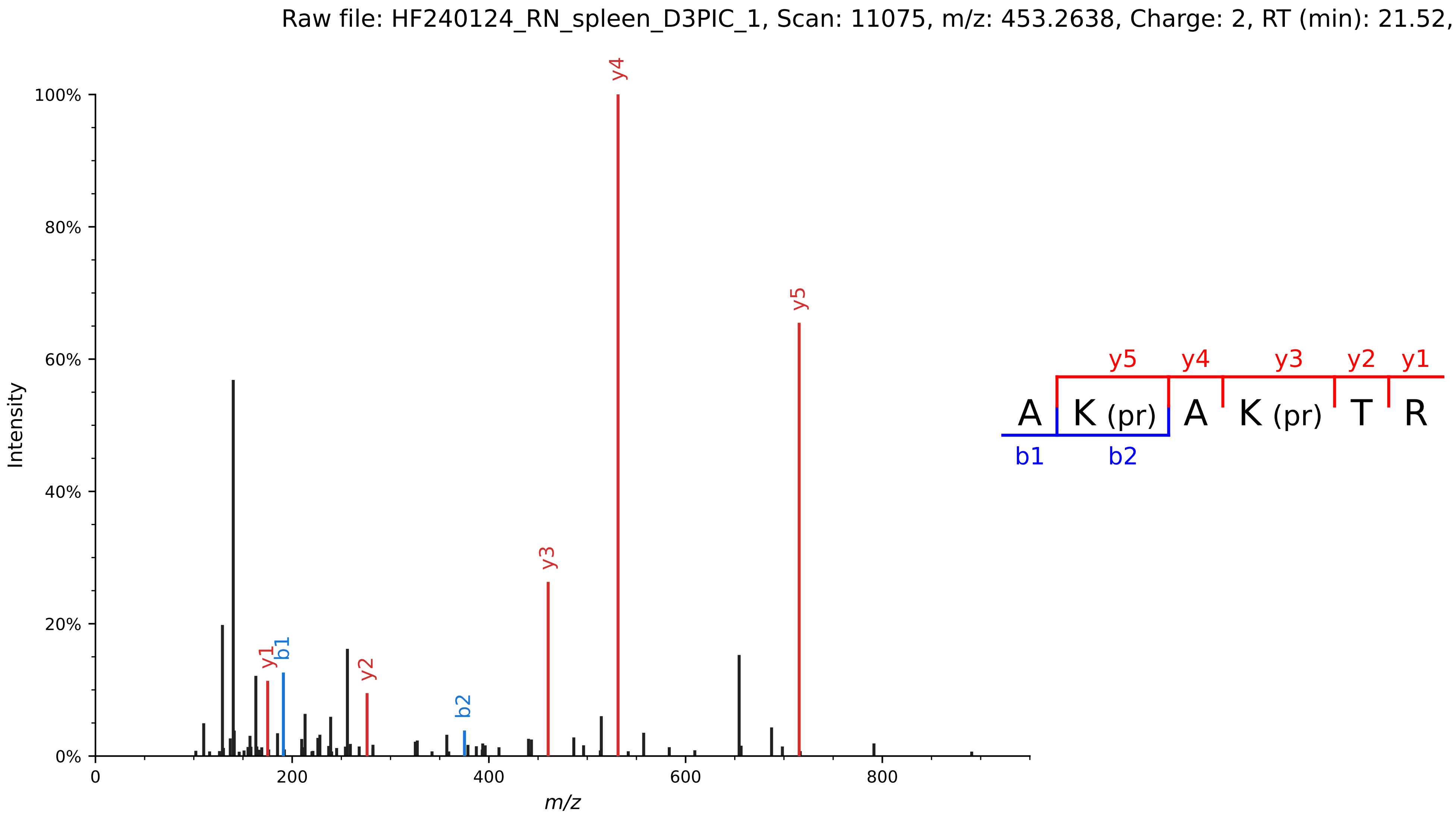

Raw file: HF240124_RN_spleen_D3PIC_1, Scan: 11075, m/z: 453.2638, Charge: 2, RT (min): 21.52, Score: 71.141
Intensity
m/z
y1
b1
y2
b2
y3
y4
y5
A K (pr) A K (pr) T R

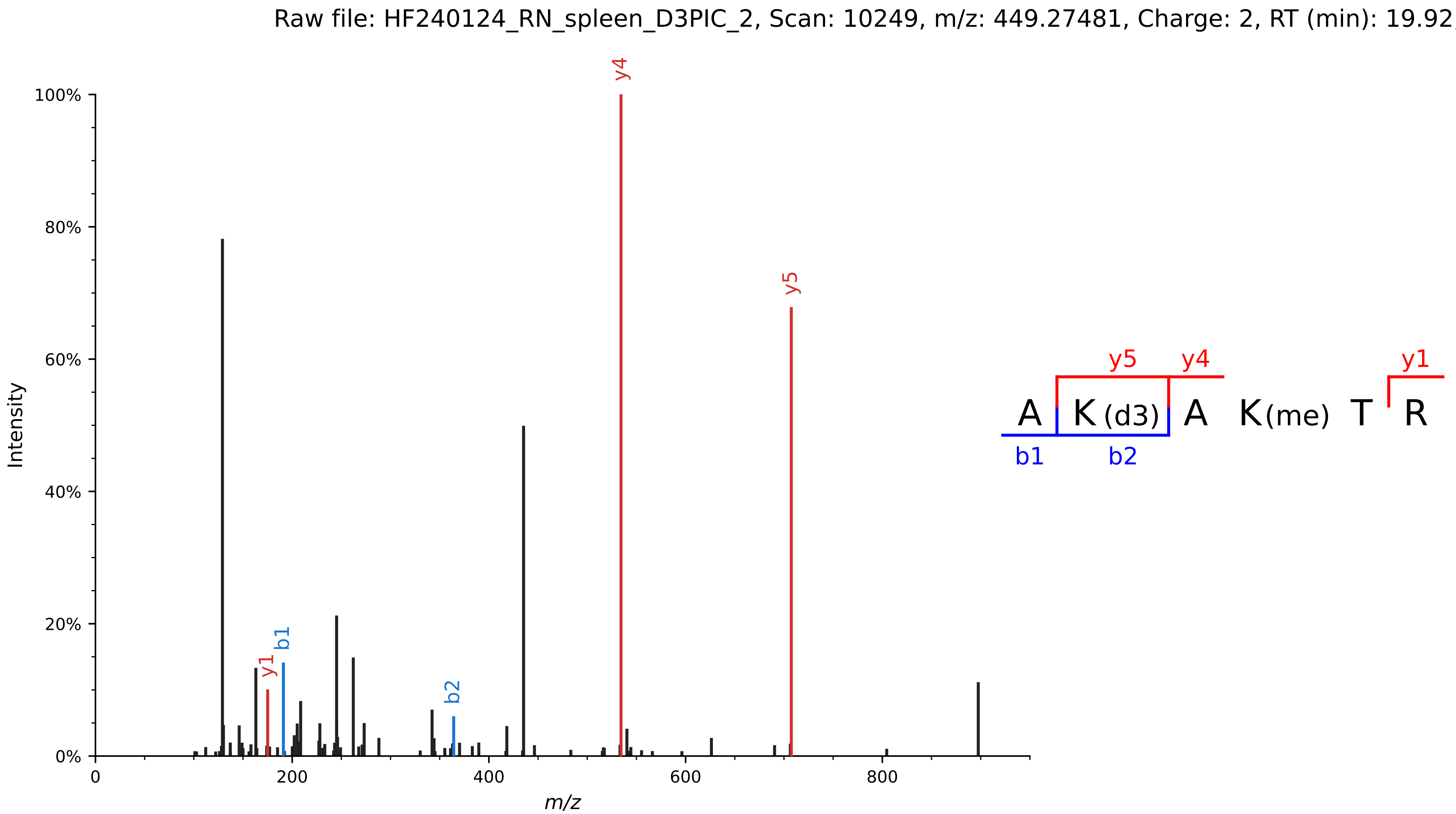

Raw file: HF240124_RN_spleen_D3PIC_2, Scan: 10249, m/z: 449.27481, Charge: 2, RT (min): 19.92, Score: 52.231
Intensity
m/z
y4
y5
y1
b1
b2
A K(d3) A K(me) T R

Raw file: HF240124_RN_spleen_D3PIC_1, Scan: 9657, m/z: 498.27104, Charge: 2, RT (min): 18.89, Score: 59.205

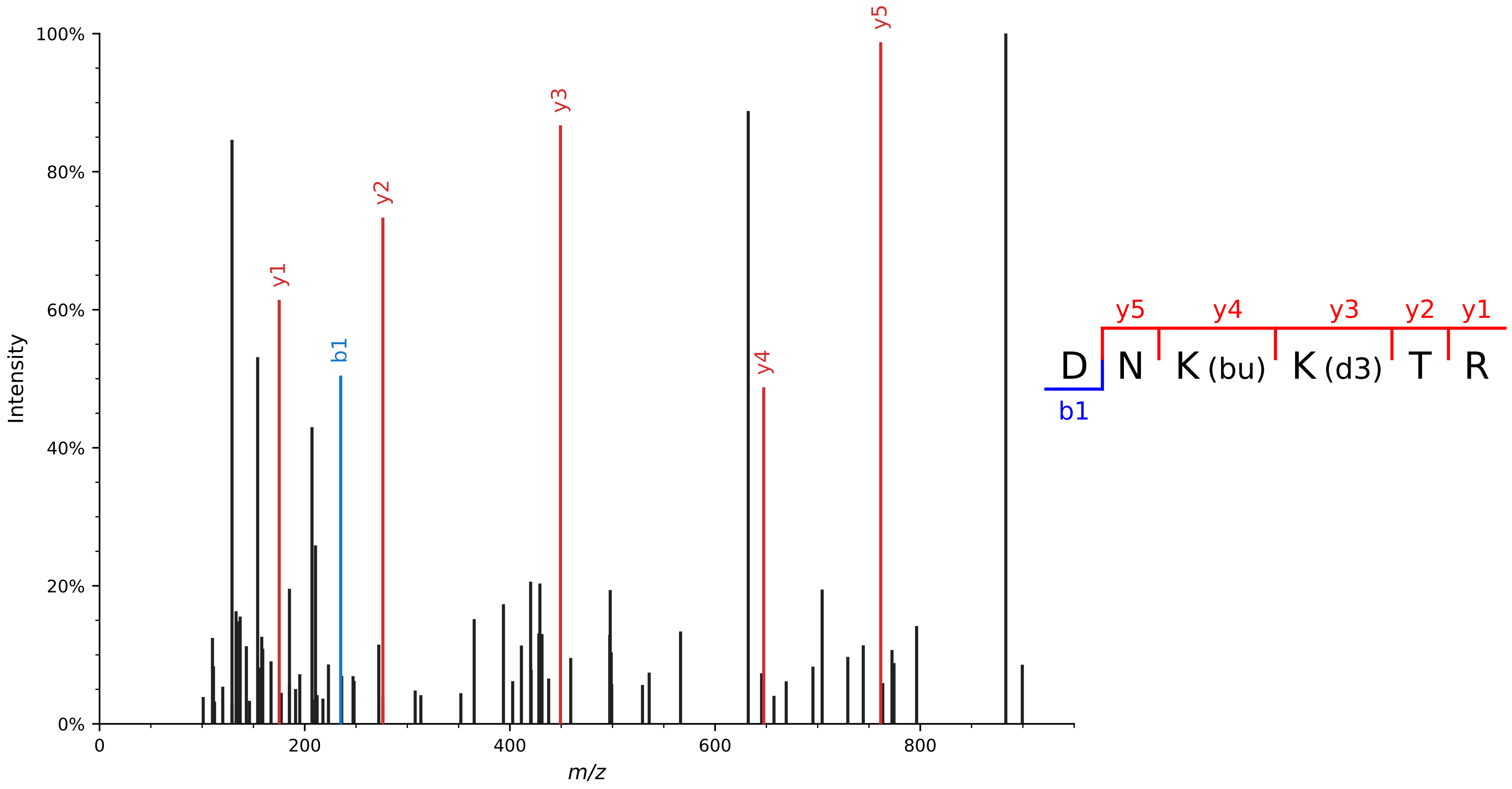

Raw file: HF240124_RN_spleen_D3PIC_1, Scan: 9343, m/z: 496.76162, Charge: 2, RT (min): 18.28, Score: 91.626

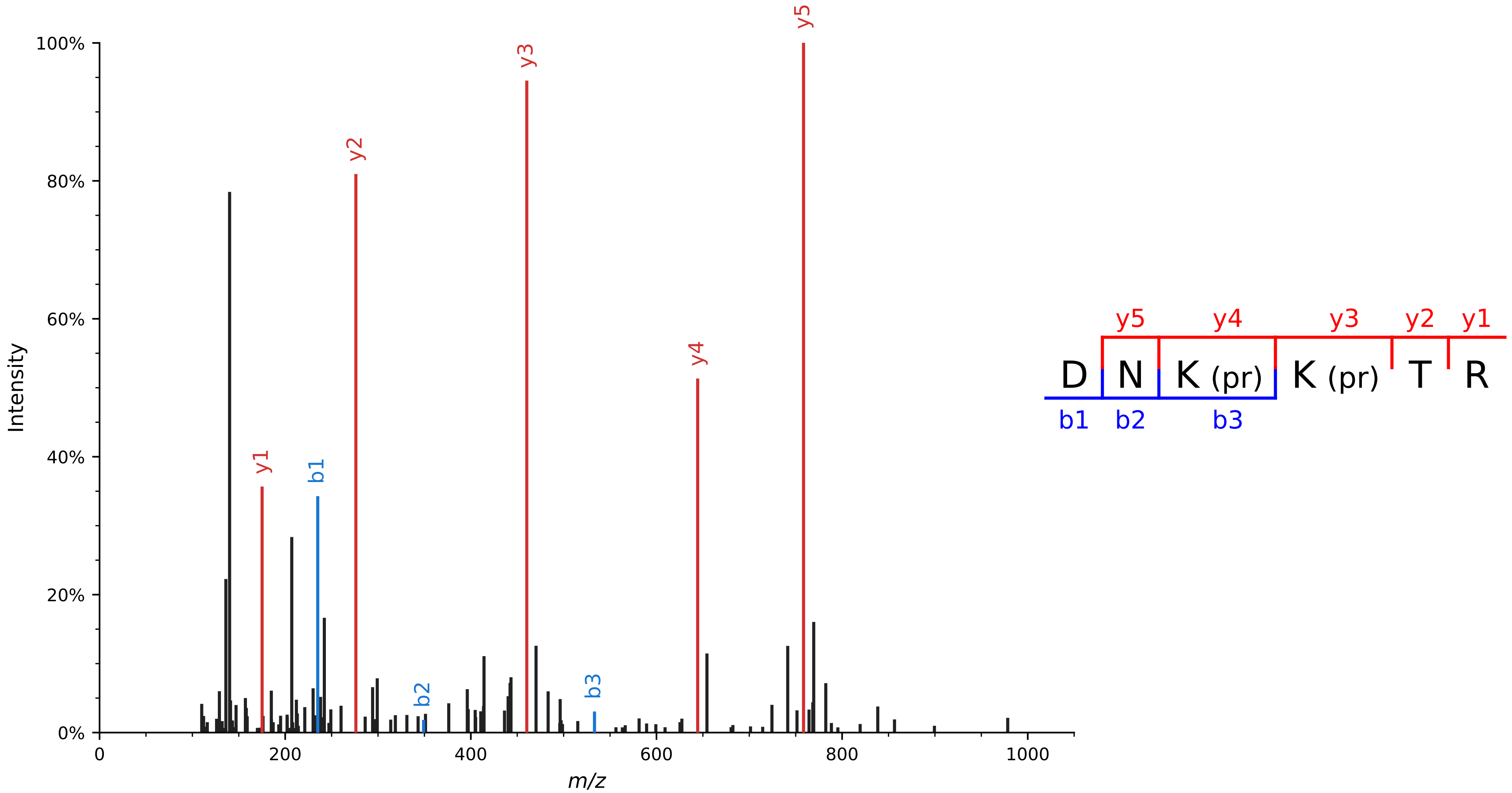

Raw file: HF240124_RN_spleen_D3PIC_1, Scan: 9986, m/z: 545.79326, Charge: 2, RT (min): 19.56, Score: 115.83

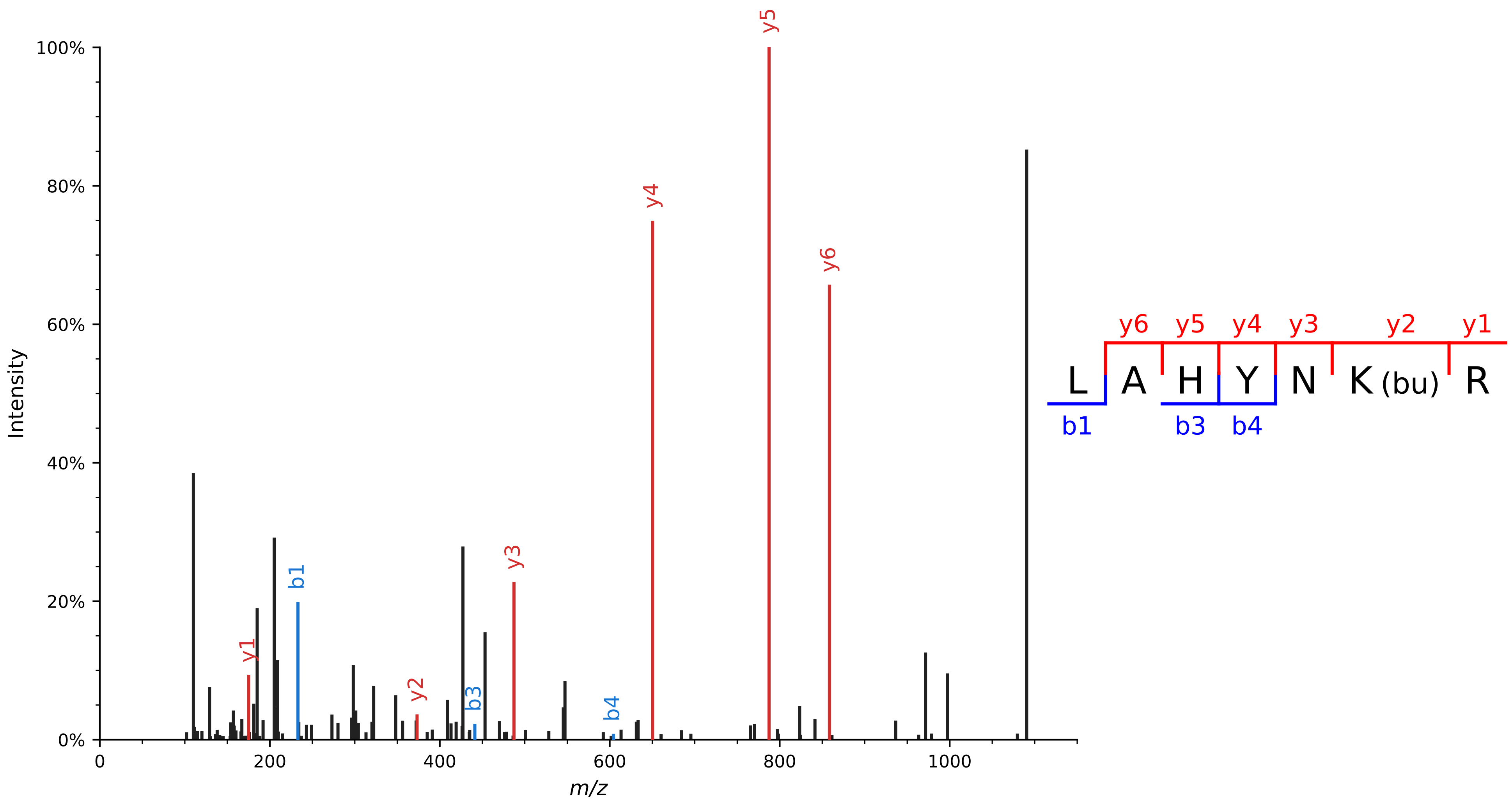

Raw file: HF240124_RN_spleen_D3PIC_3, Scan: 7244, m/z: 524.76978, Charge: 2, RT (min): 14.72, Score: 82.939

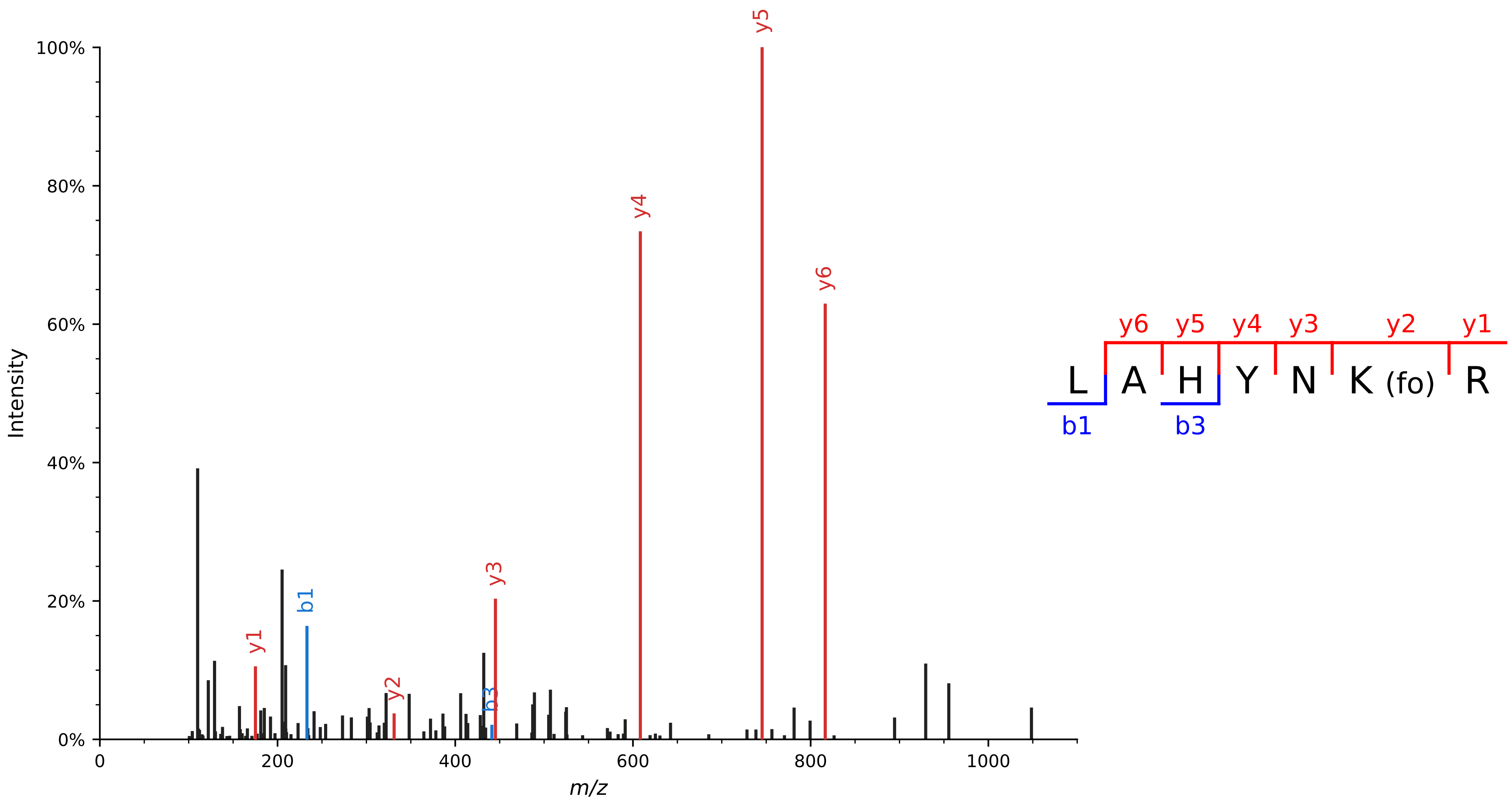

Raw file: HF240124_RN_spleen_D3PIC_1, Scan: 4286, m/z: 576.9936, Charge: 3, RT (min): 9.32, Score: 49.31

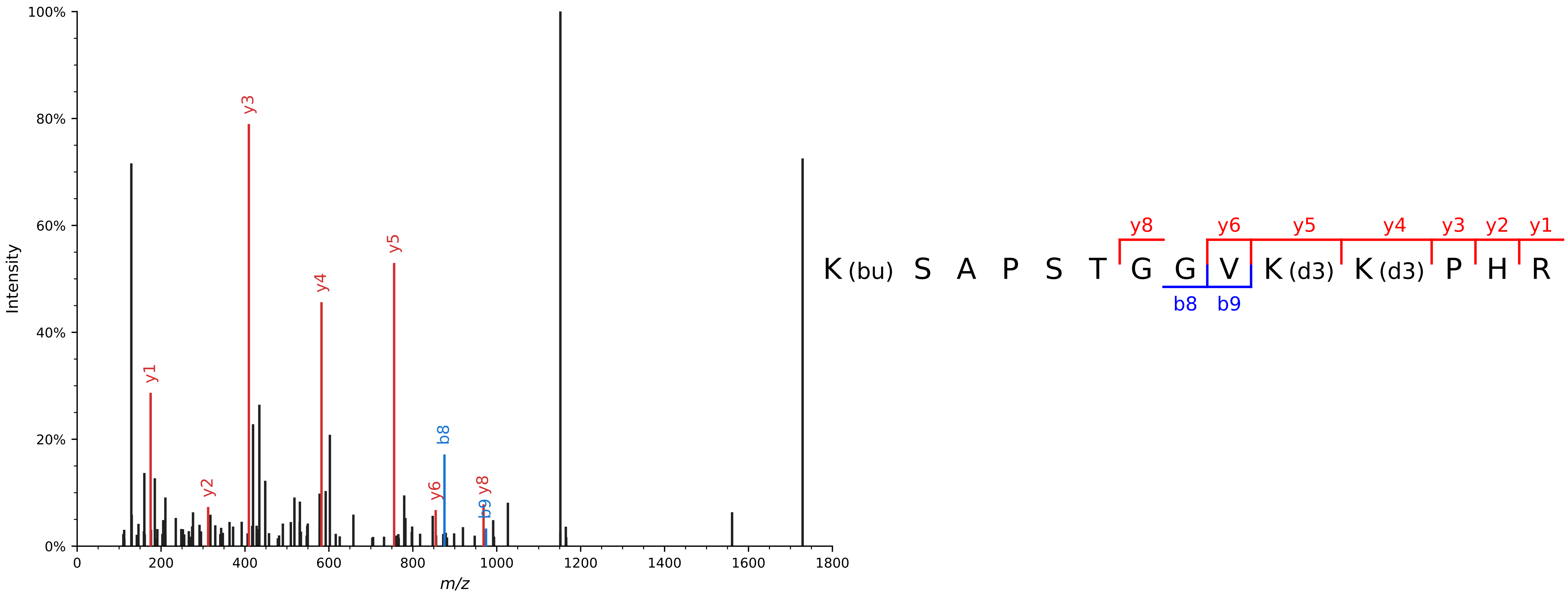

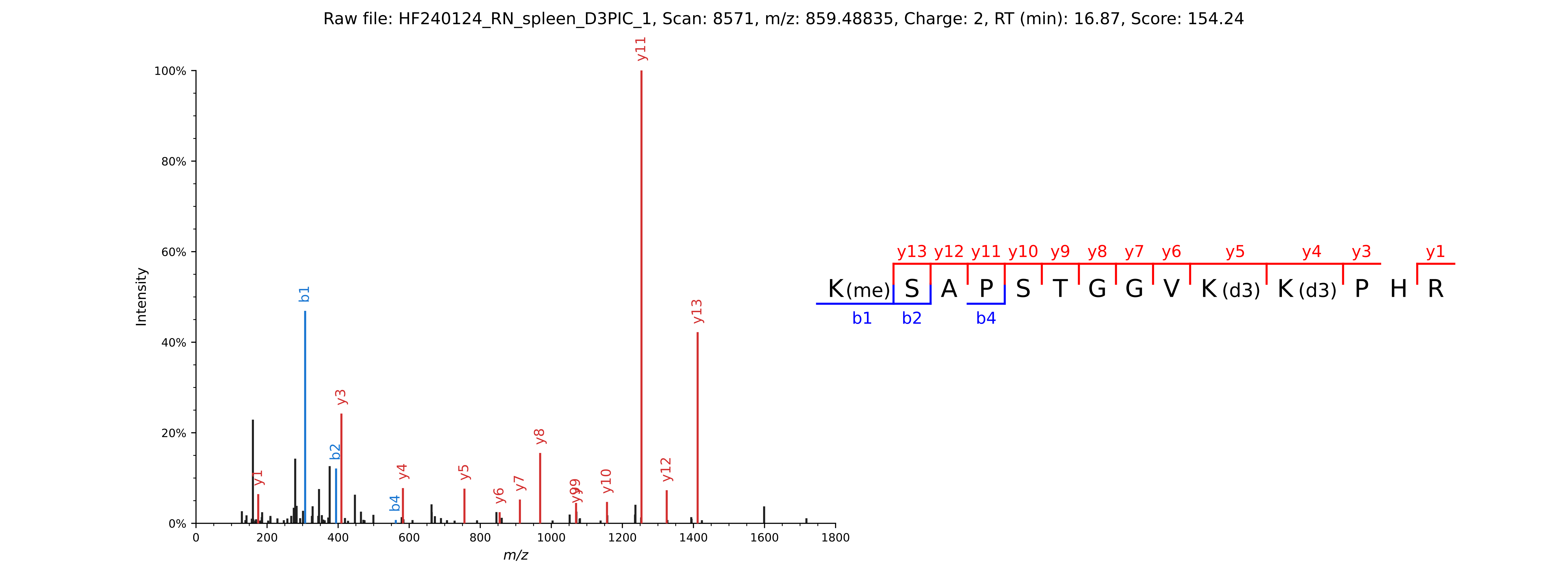
Raw file: HF240124_RN_spleen_D3PIC_1, Scan: 8571, m/z: 859.48835, Charge: 2, RT (min): 16.87, Score: 154.24
Intensity
m/z
y11
y13
b1
y3
b2
y8
y1
b4
y4
y5
y6
y7
y9
y10
y12
K(me) S A P S T G G V K(d3) K(d3) P H R
y13 y12 y11 y10 y9 y8 y7 y6 y5 y4 y3 y1
b1 b2 b4

Raw file: HF240124_RN_spleen_D3PIC_1, Scan: 9208, m/z: 866.49617, Charge: 2, RT (min): 18.02, Score: 119.27

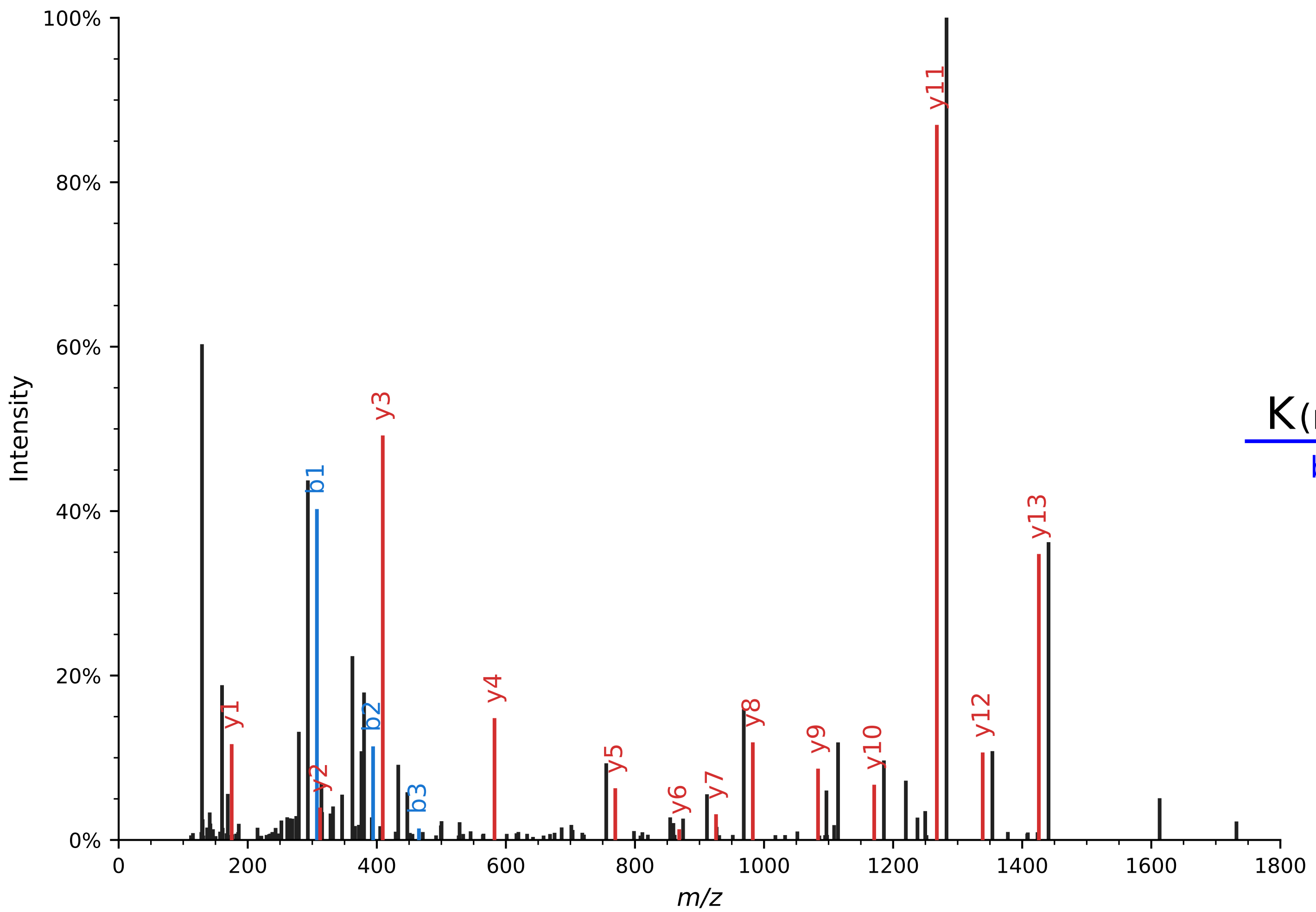

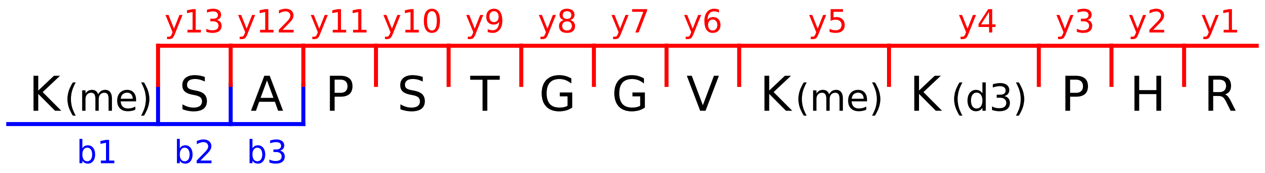

Raw file: HF240124_RN_spleen_D3PIC_3, Scan: 6398, m/z: 582.67176, Charge: 3, RT (min): 13.23, Score: 49.298

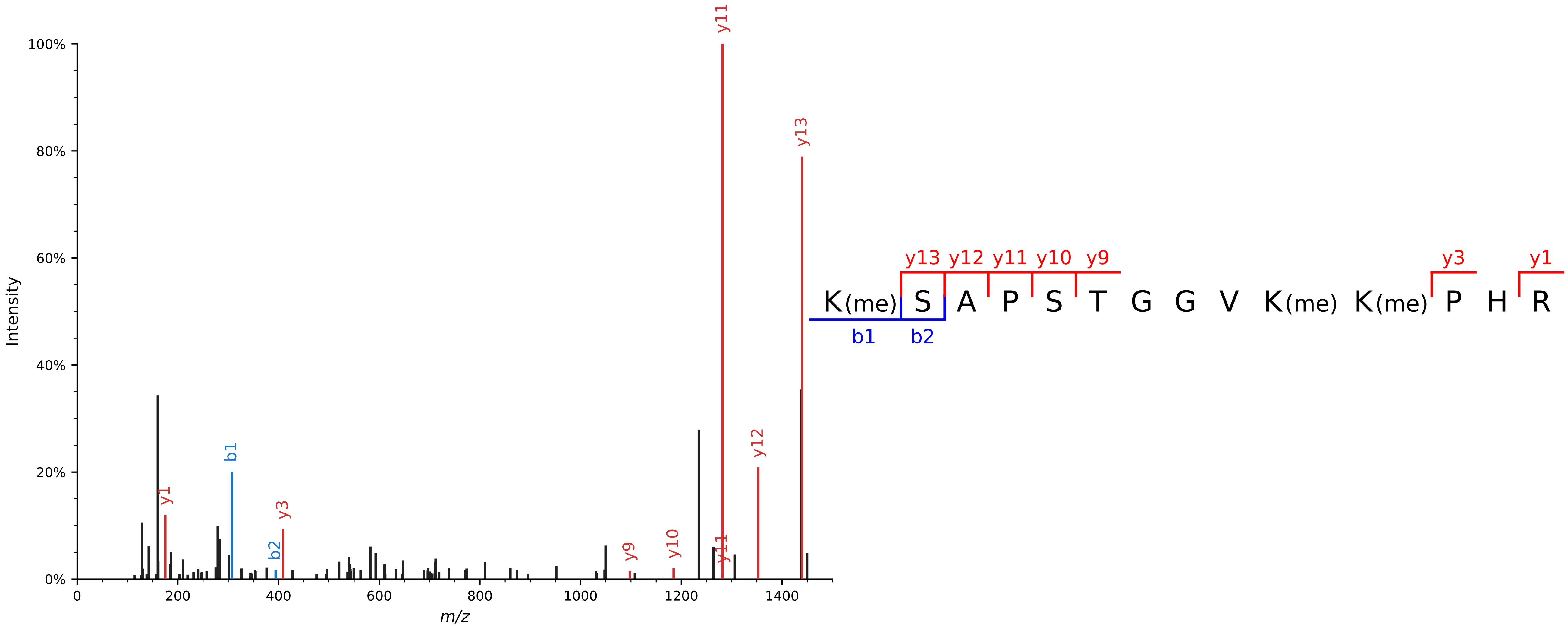

Raw file: HF240124_RN_spleen_D3PIC_1, Scan: 5282, m/z: 850.9893, Charge: 2, RT (min): 11.04, Score: 151.98

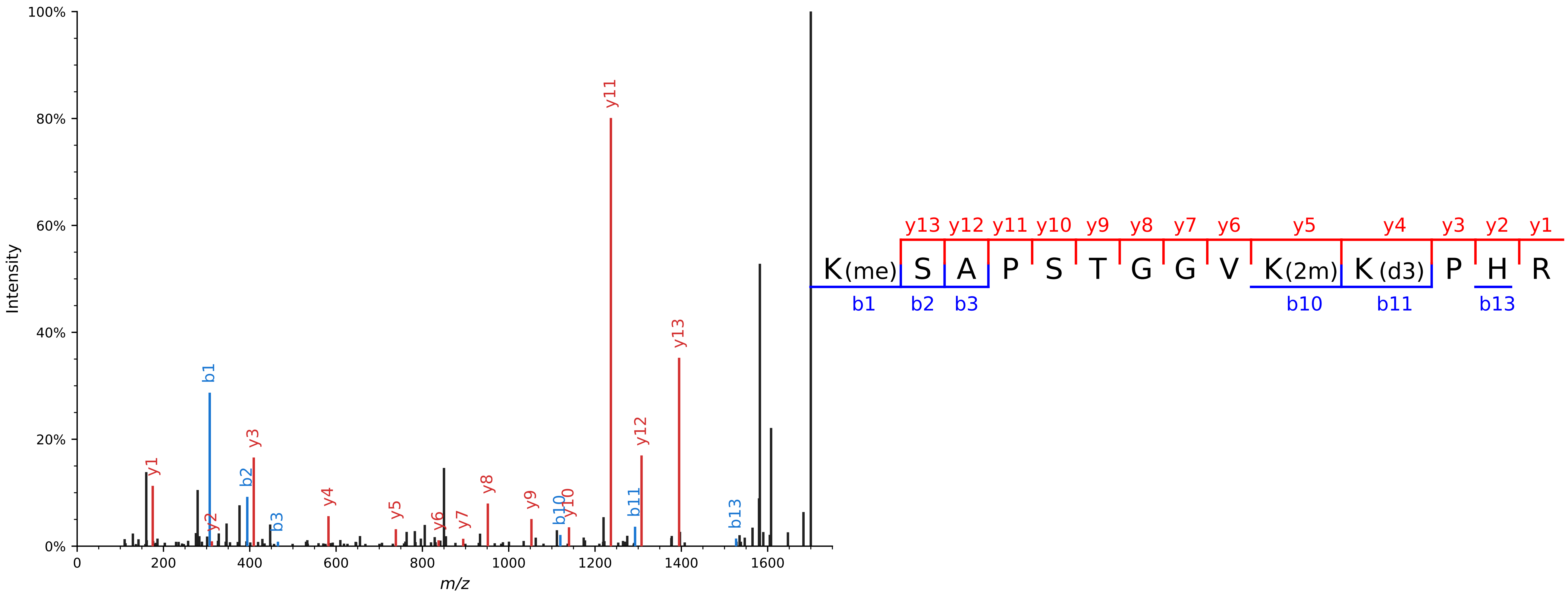

Raw file: HF240124_RN_spleen_D3PIC_1, Scan: 5284, m/z: 572.33384, Charge: 3, RT (min): 11.04, Score: 88.087

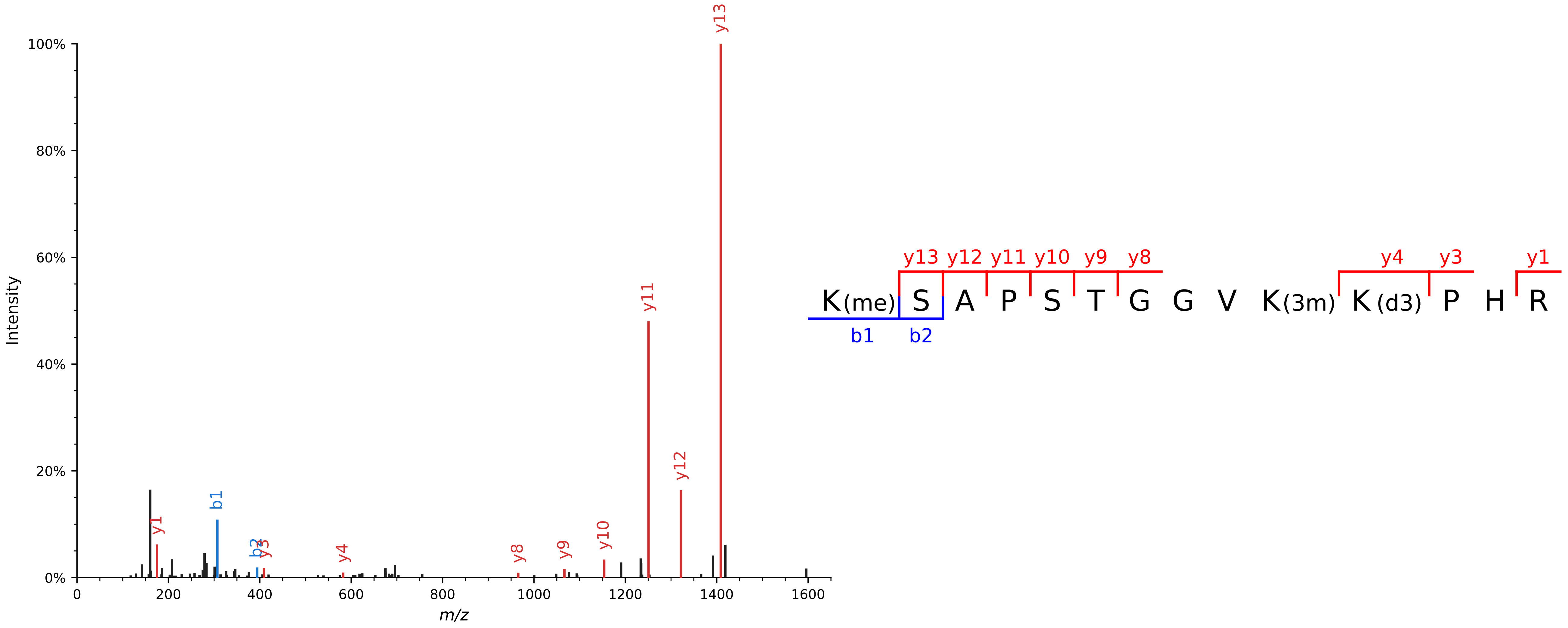

Raw file: HF240124_RN_spleen_D3PIC_3, Scan: 3351, m/z: 843.98148, Charge: 2, RT (min): 7.81, Score: 155.47

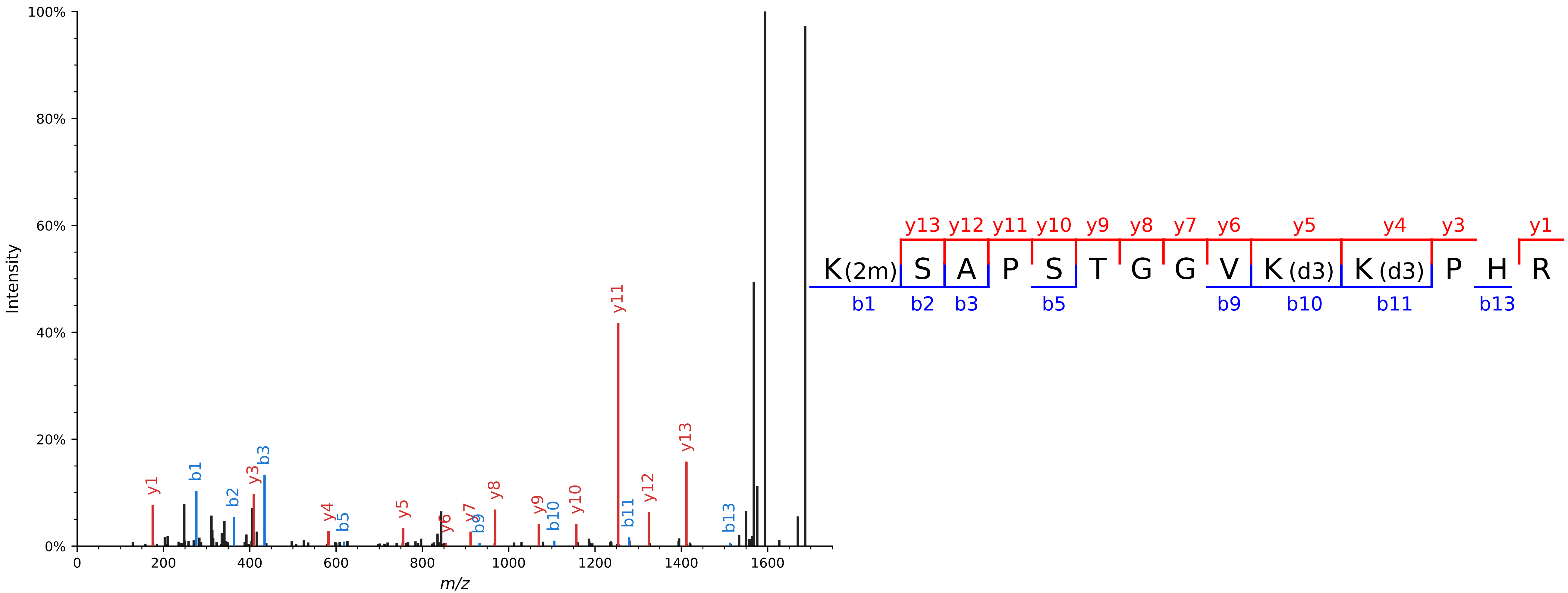

Raw file: HF240124_RN_spleen_D3PIC_2, Scan: 4120, m/z: 850.9893, Charge: 2, RT (min): 8.99, Score: 166.24

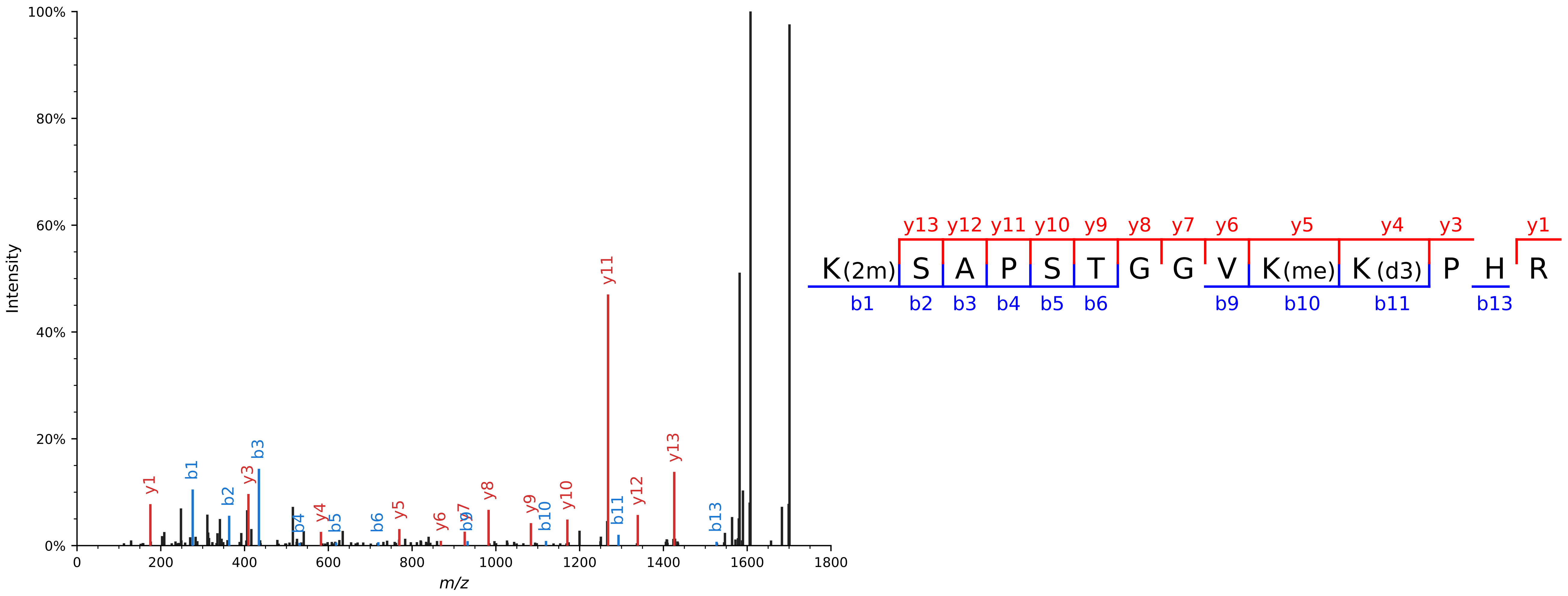

Raw file: HF240124_RN_spleen_D3PIC_2, Scan: 2166, m/z: 857.99713, Charge: 2, RT (min): 5.57, Score: 78.136

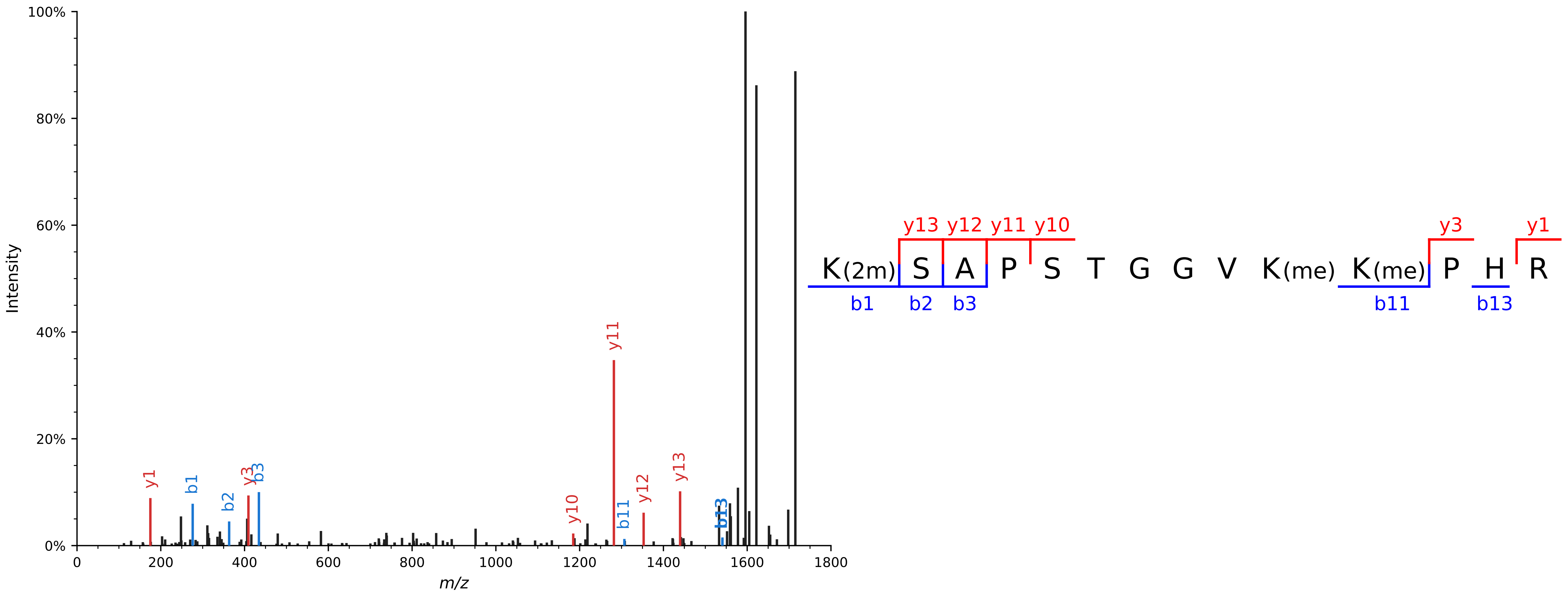

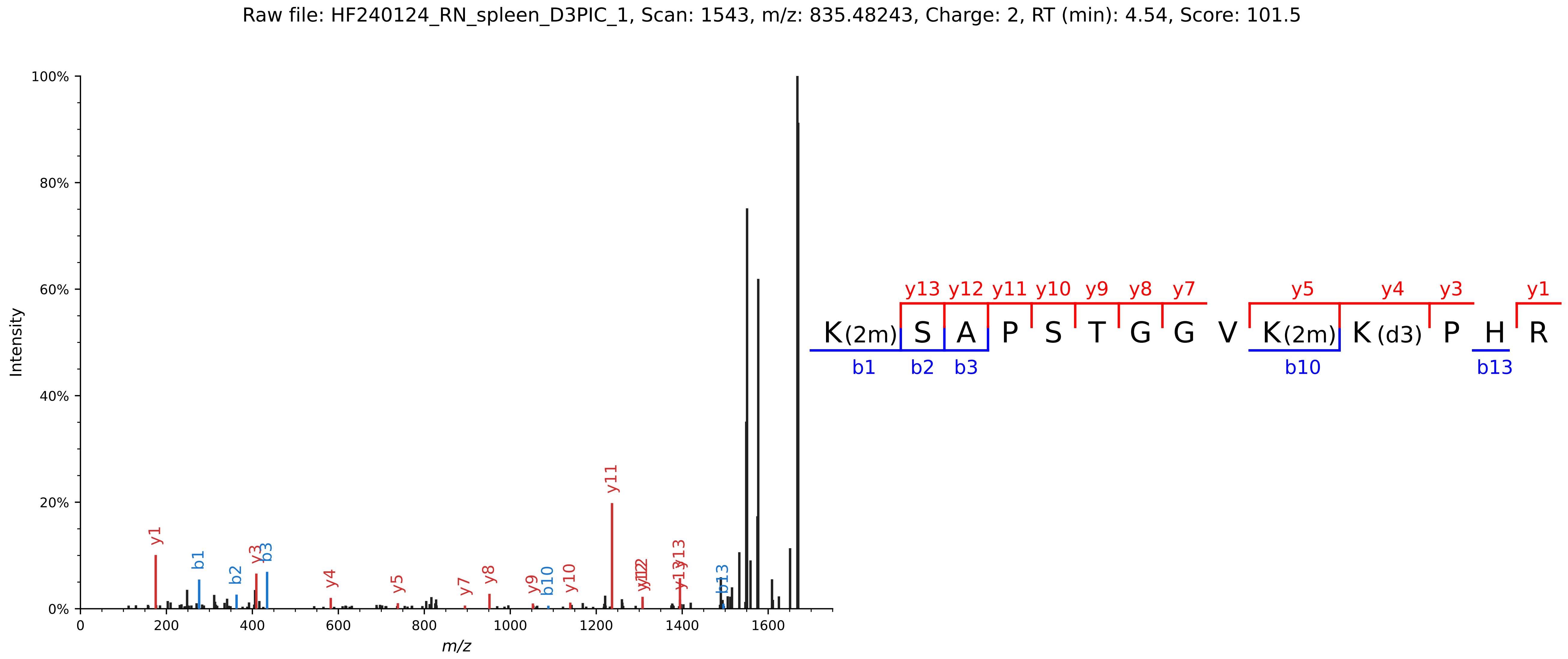

Raw file: HF240124_RN_spleen_D3PIC_1, Scan: 1543, m/z: 835.48243, Charge: 2, RT (min): 4.54, Score: 101.5
Intensity
m/z
100%
80%
60%
40%
20%
0%
0
200
400
600
800
1000
1200
1400
1600
y1
b1
b2
y3
b3
y4
y5
y7
y8
y9
b10
y10
y11
y12
y13
b13
K(2m) S A P S T G G V K(2m) K(d3) P H R
y13 y12 y11 y10 y9 y8 y7 y5 y4 y3 y1
b1 b2 b3 b10 b13

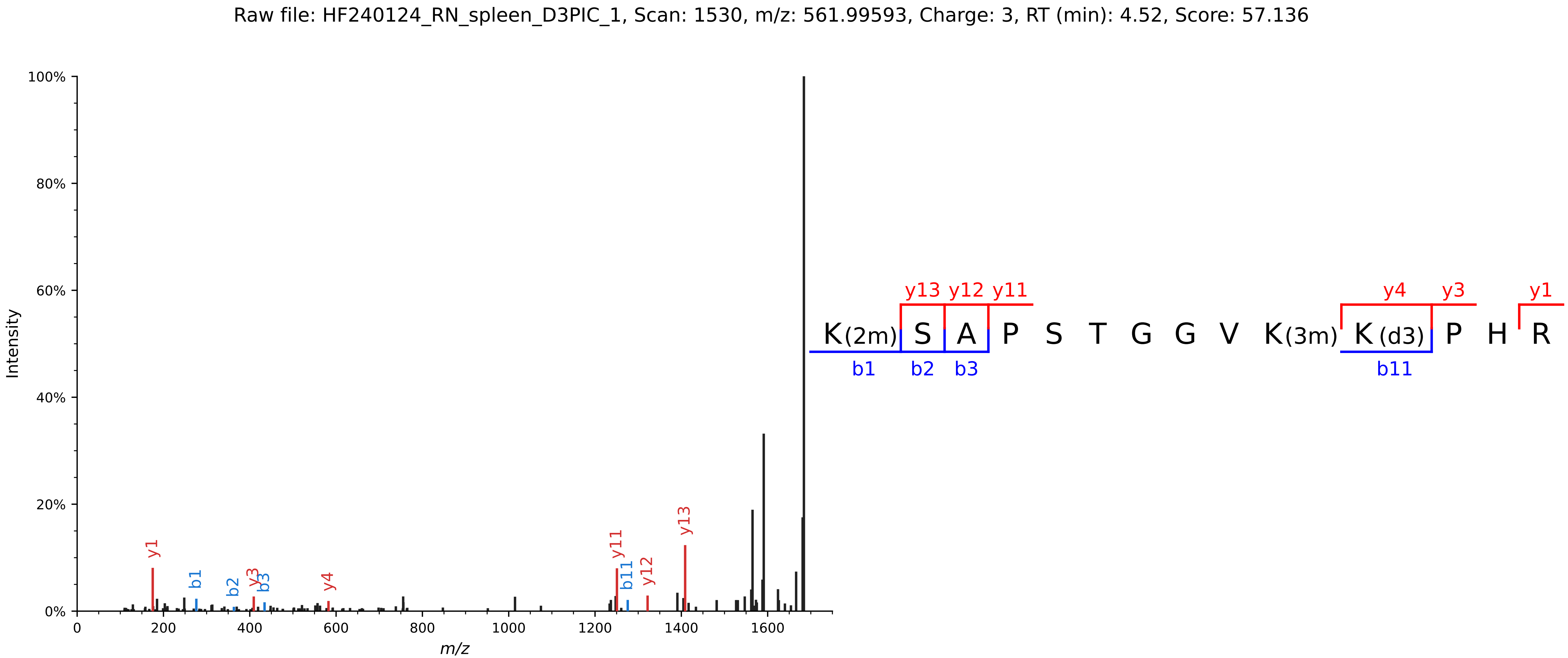
Raw file: HF240124_RN_spleen_D3PIC_1, Scan: 1530, m/z: 561.99593, Charge: 3, RT (min): 4.52, Score: 57.136
Intensity
m/z
y1
b1
b2
y3
b3
y4
y11
b11
y12
y13
y13 y12 y11
y4 y3 y1
K(2m) S A P S T G G V K(3m) K(d3) P H R
b1 b2 b3 b11

Raw file: HF240124_RN_spleen_D3PIC_1, Scan: 3523, m/z: 567.66196, Charge: 3, RT (min): 7.97, Score: 140.27

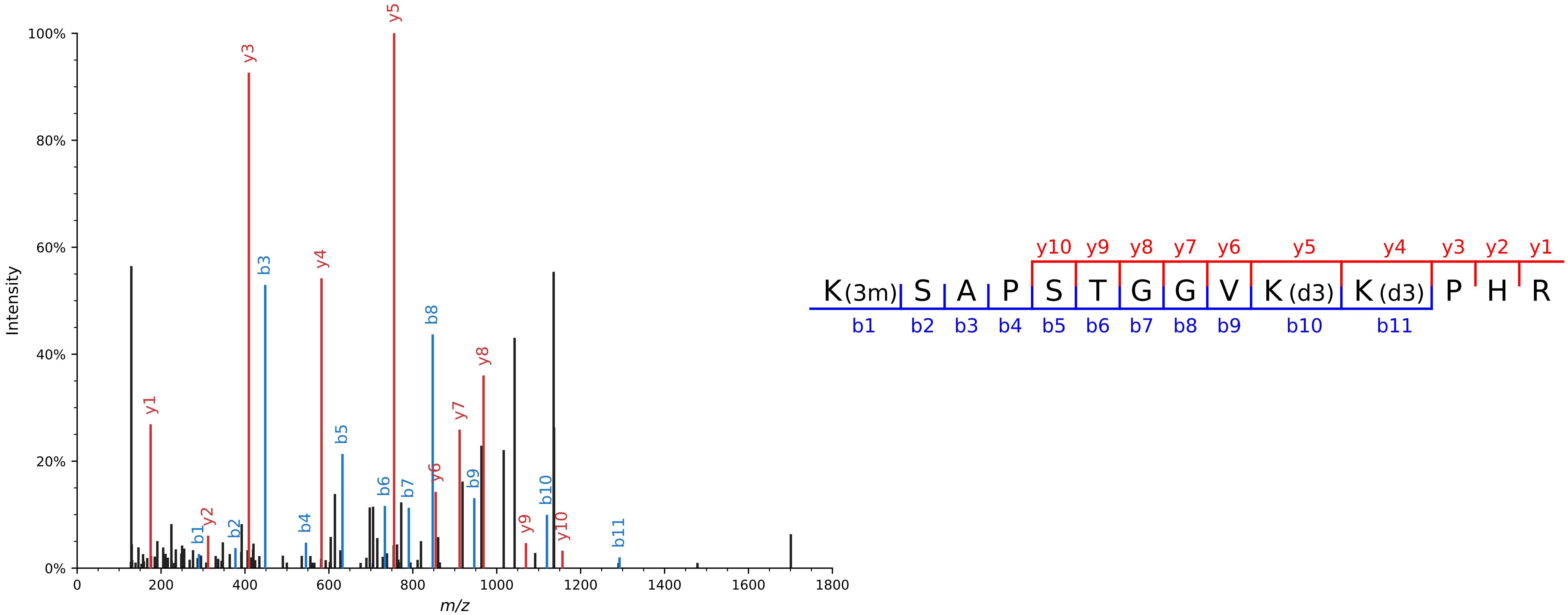

Raw file: HF240124_RN_spleen_D3PIC_1, Scan: 4112, m/z: 857.99713, Charge: 2, RT (min): 8.99, Score: 163.51

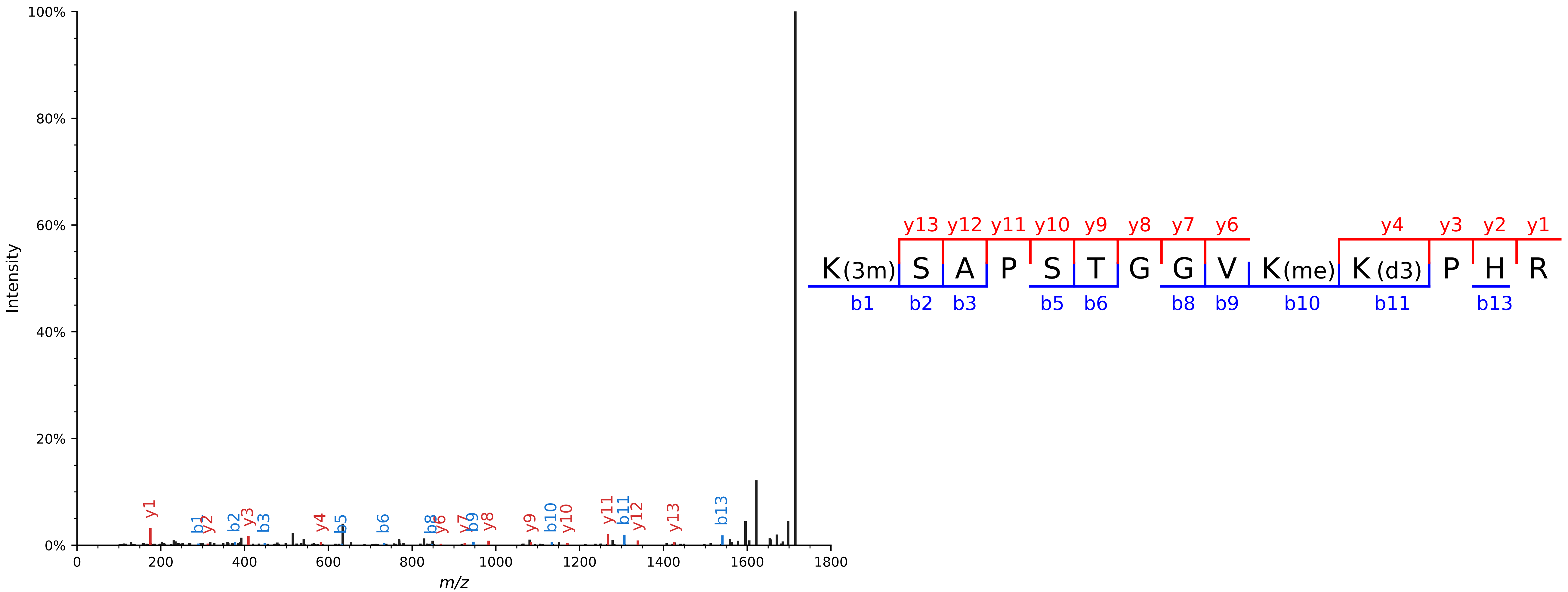

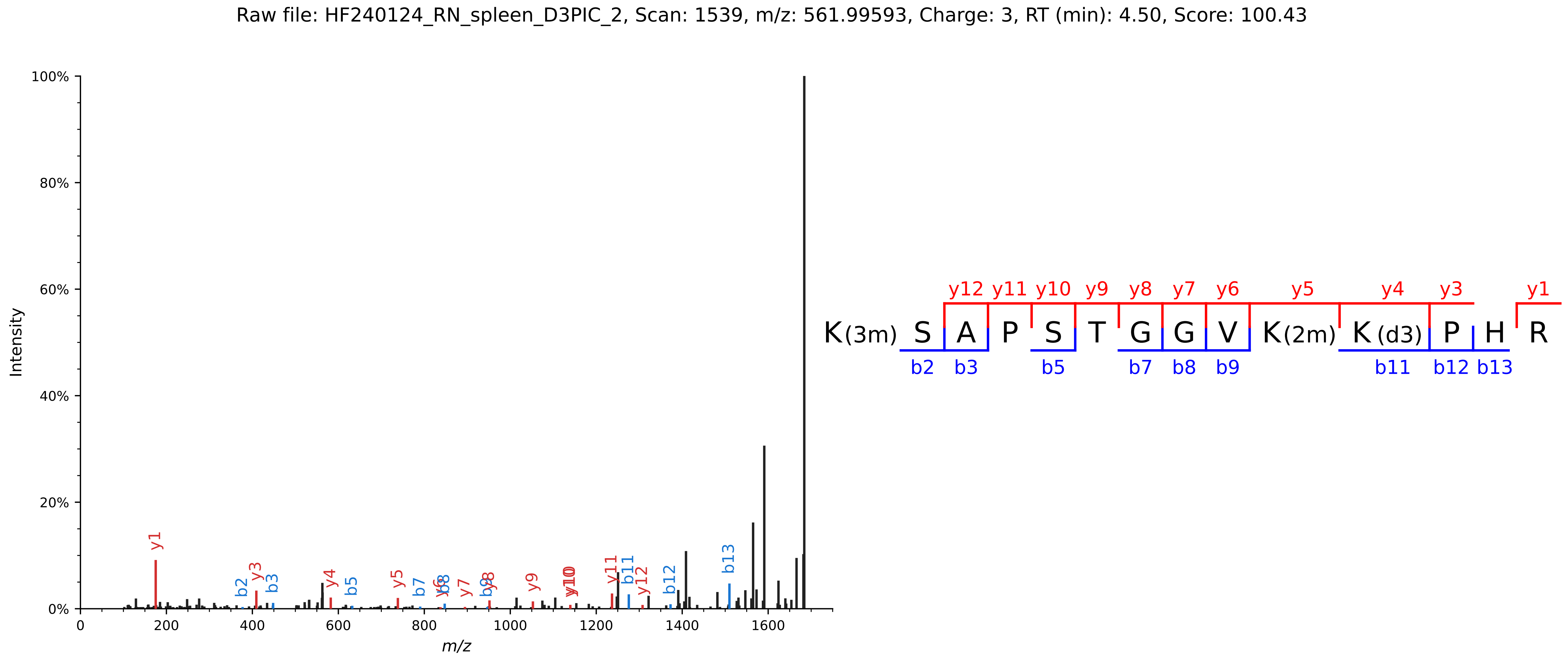

Raw file: HF240124_RN_spleen_D3PIC_2, Scan: 1539, m/z: 561.99593, Charge: 3, RT (min): 4.50, Score: 100.43
Intensity
m/z
100%
80%
60%
40%
20%
0%
0
200
400
600
800
1000
1200
1400
1600
y1
b2
y3
b3
y4
b5
y5
b7
b8
y7
y8
y9
y10
y11
b11
y12
b12
b13
K(3m) S A P S T G G V K(2m) K(d3) P H R
y12 y11 y10 y9 y8 y7 y6 y5 y4 y3 y1
b2 b3 b5 b7 b8 b9 b11 b12 b13

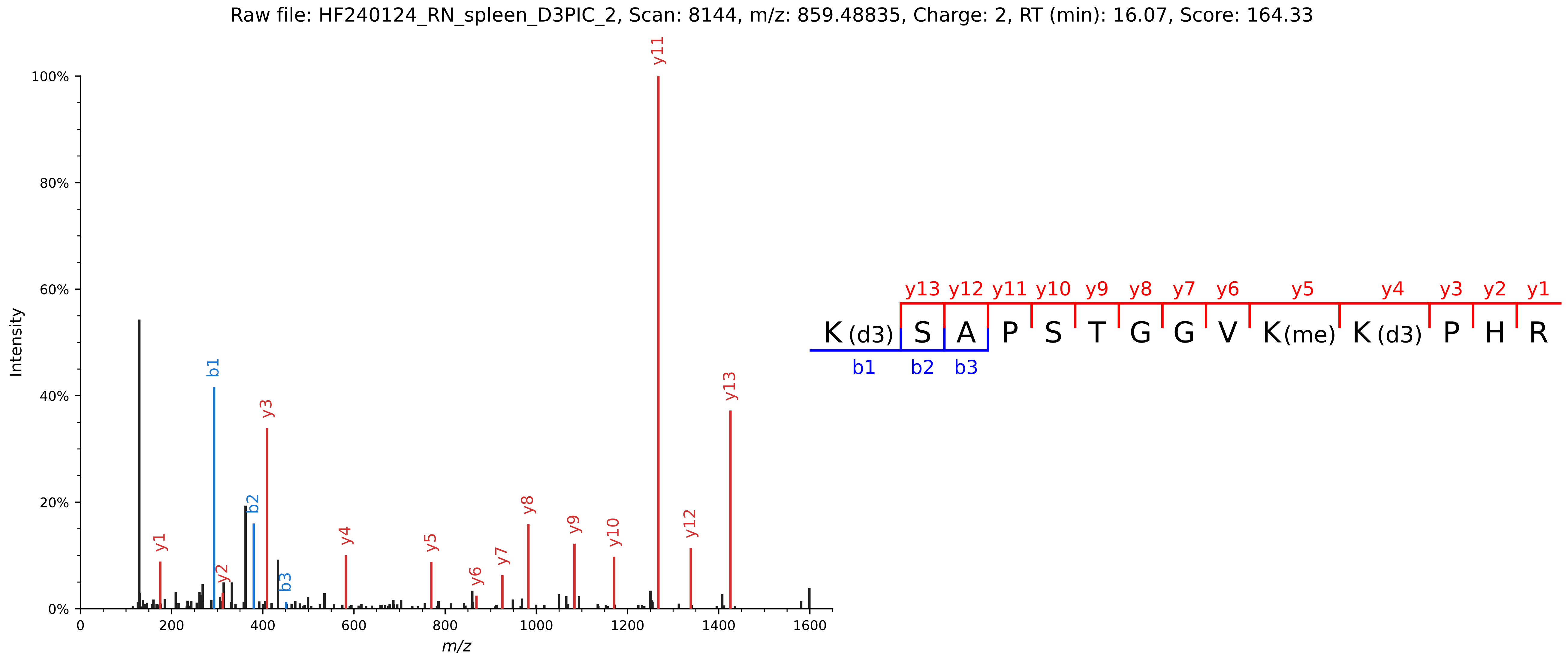
Raw file: HF240124_RN_spleen_D3PIC_2, Scan: 8144, m/z: 859.48835, Charge: 2, RT (min): 16.07, Score: 164.33
Intensity
m/z
y13 y12 y11 y10 y9 y8 y7 y6 y5 y4 y3 y2 y1
K(d3) S A P S T G G V K(me) K(d3) P H R
b1 b2 b3

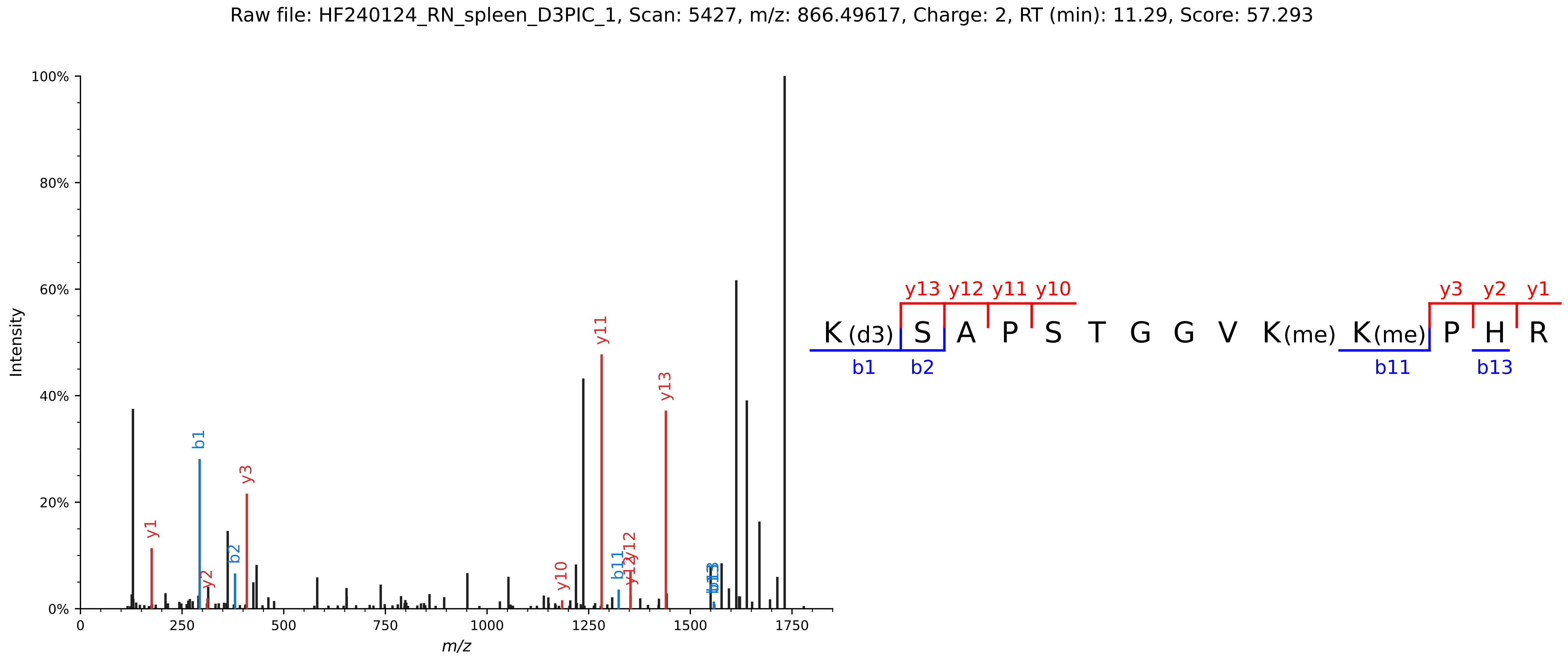

Raw file: HF240124_RN_spleen_D3PIC_1, Scan: 5427, m/z: 866.49617, Charge: 2, RT (min): 11.29, Score: 57.293
Intensity
m/z
K(d3) S A P S T G G V K(me) K(me) P H R
y13 y12 y11 y10 y3 y2 y1
b1 b2 b11 b13

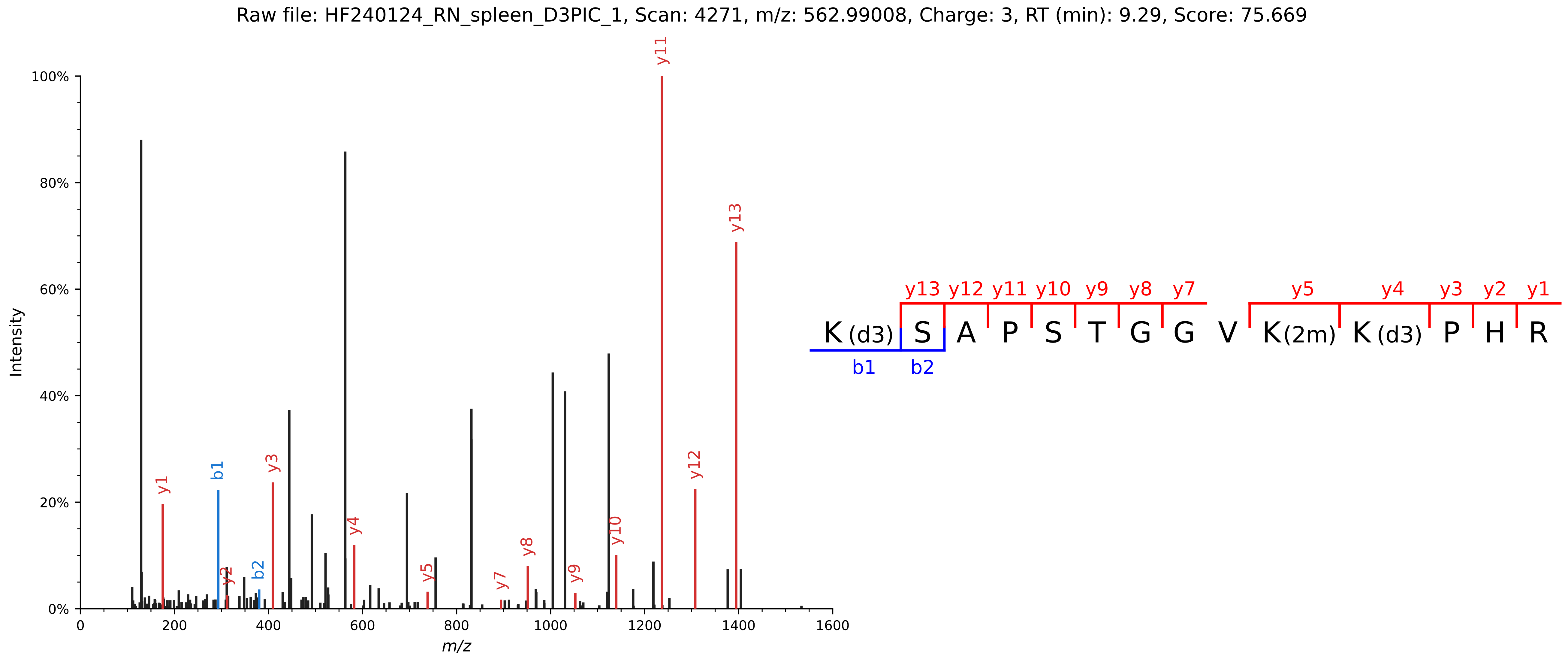

Raw file: HF240124_RN_spleen_D3PIC_1, Scan: 4271, m/z: 562.99008, Charge: 3, RT (min): 9.29, Score: 75.669
Intensity
m/z
y1
b1
y2
b2
y3
y4
y5
y7
y8
y9
y10
y11
y12
y13
K(d3) S A P S T G G V K(2m) K(d3) P H R
y13 y12 y11 y10 y9 y8 y7 y5 y4 y3 y2 y1
b1 b2

Raw file: HF240124_RN_spleen_D3PIC_1, Scan: 8443, m/z: 835.48243, Charge: 2, RT (min): 16.65, Score: 87.298

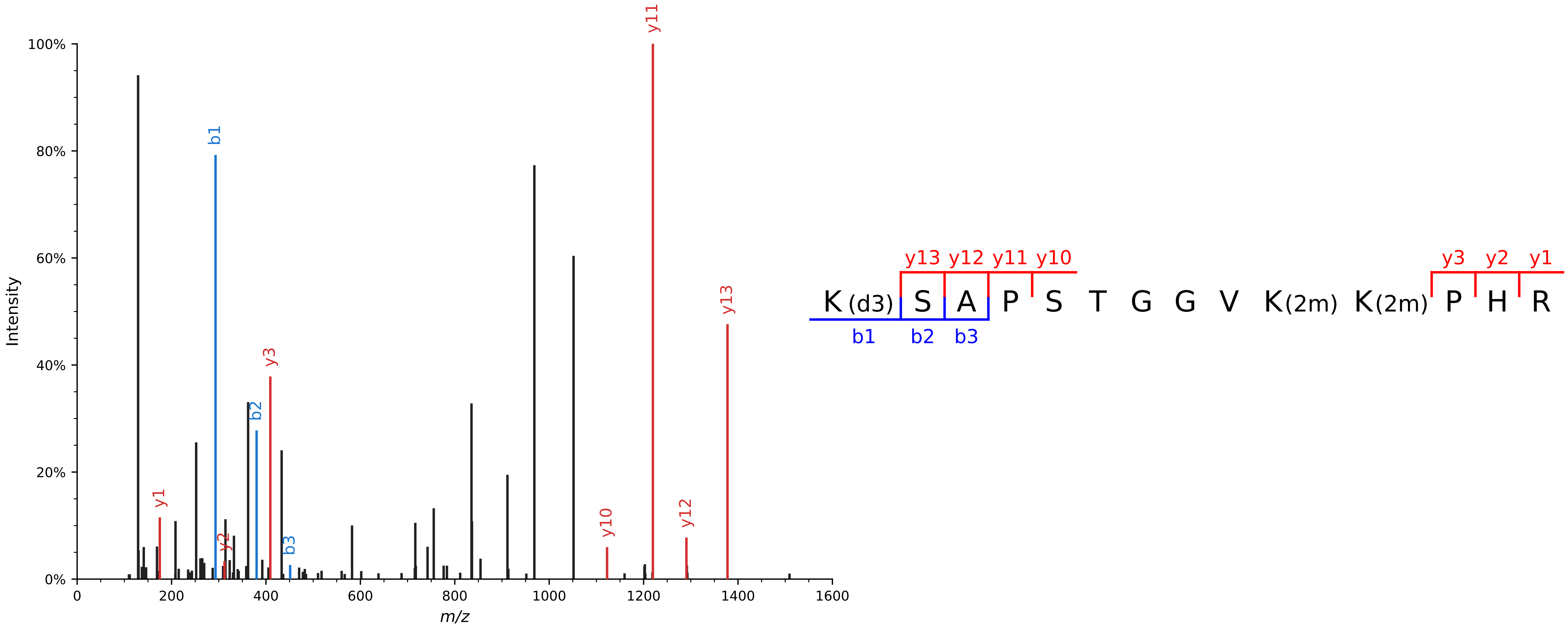

Raw file: HF240124_RN_spleen_D3PIC_1, Scan: 7401, m/z: 441.7472, Charge: 2, RT (min): 14.83, Score: 99.511

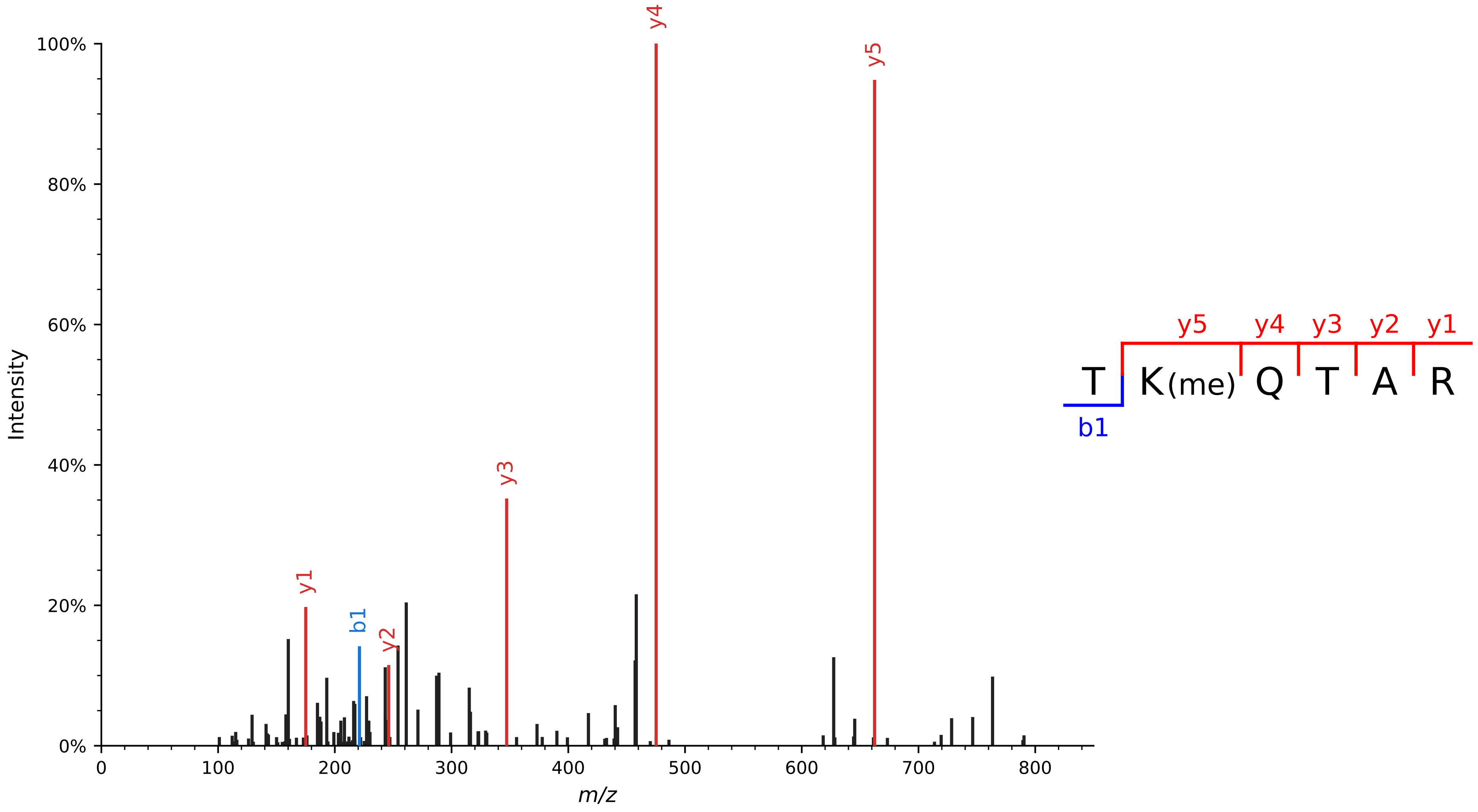

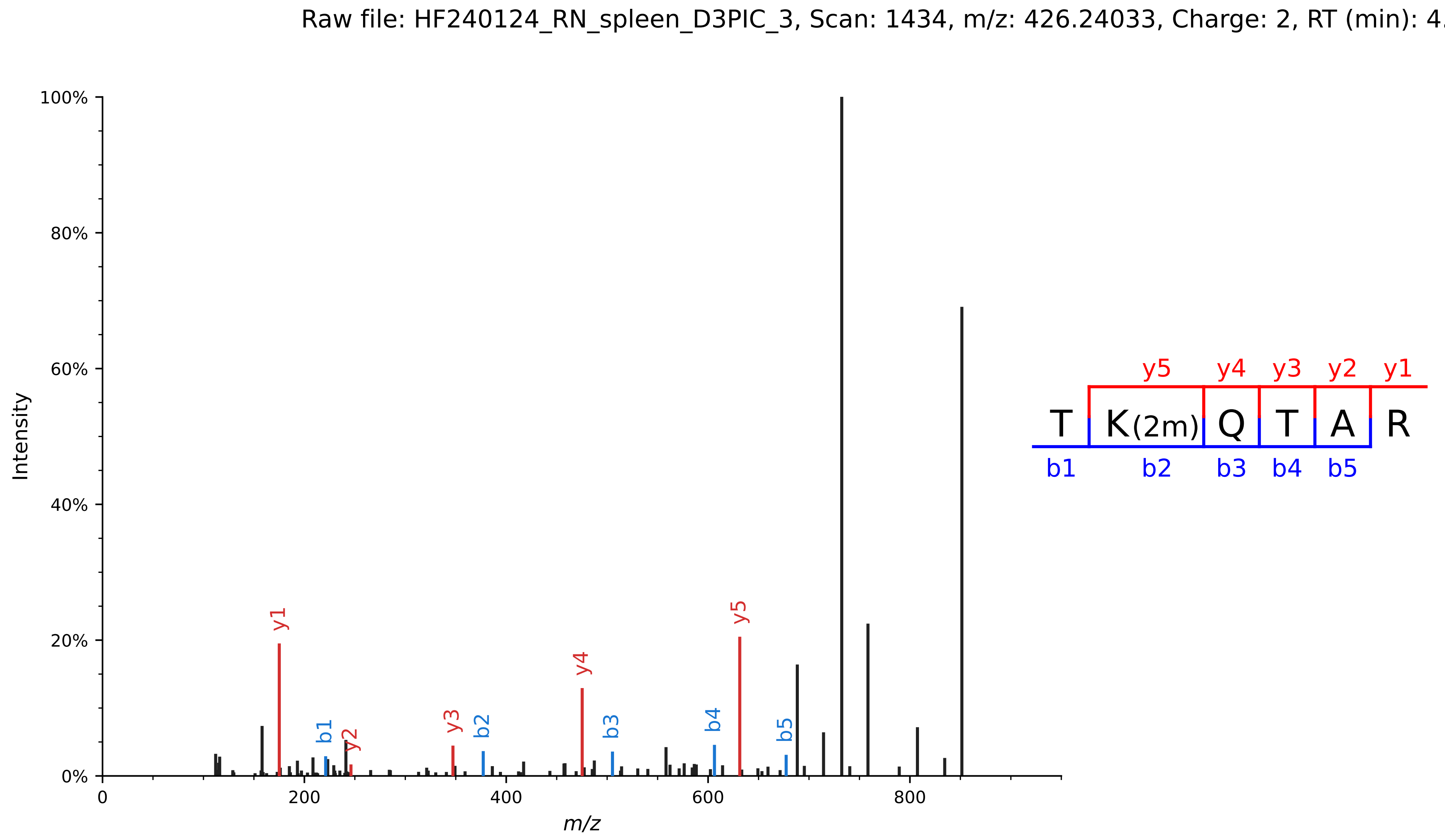
Raw file: HF240124_RN_spleen_D3PIC_3, Scan: 1434, m/z: 426.24033, Charge: 2, RT (min): 4.47, Score: 148.33
100%
80%
60%
40%
20%
0%
Intensity
0
200
400
600
800
m/z
y1
b1
y2
y3
b2
y4
b3
b4
y5
b5
y5 y4 y3 y2 y1
T K(2m) Q T A R
b1 b2 b3 b4 b5

Raw file: HF240124_RN_spleen_D3PIC_3, Scan: 1438, m/z: 433.24815, Charge: 2, RT (min): 4.47, Score: 81.784

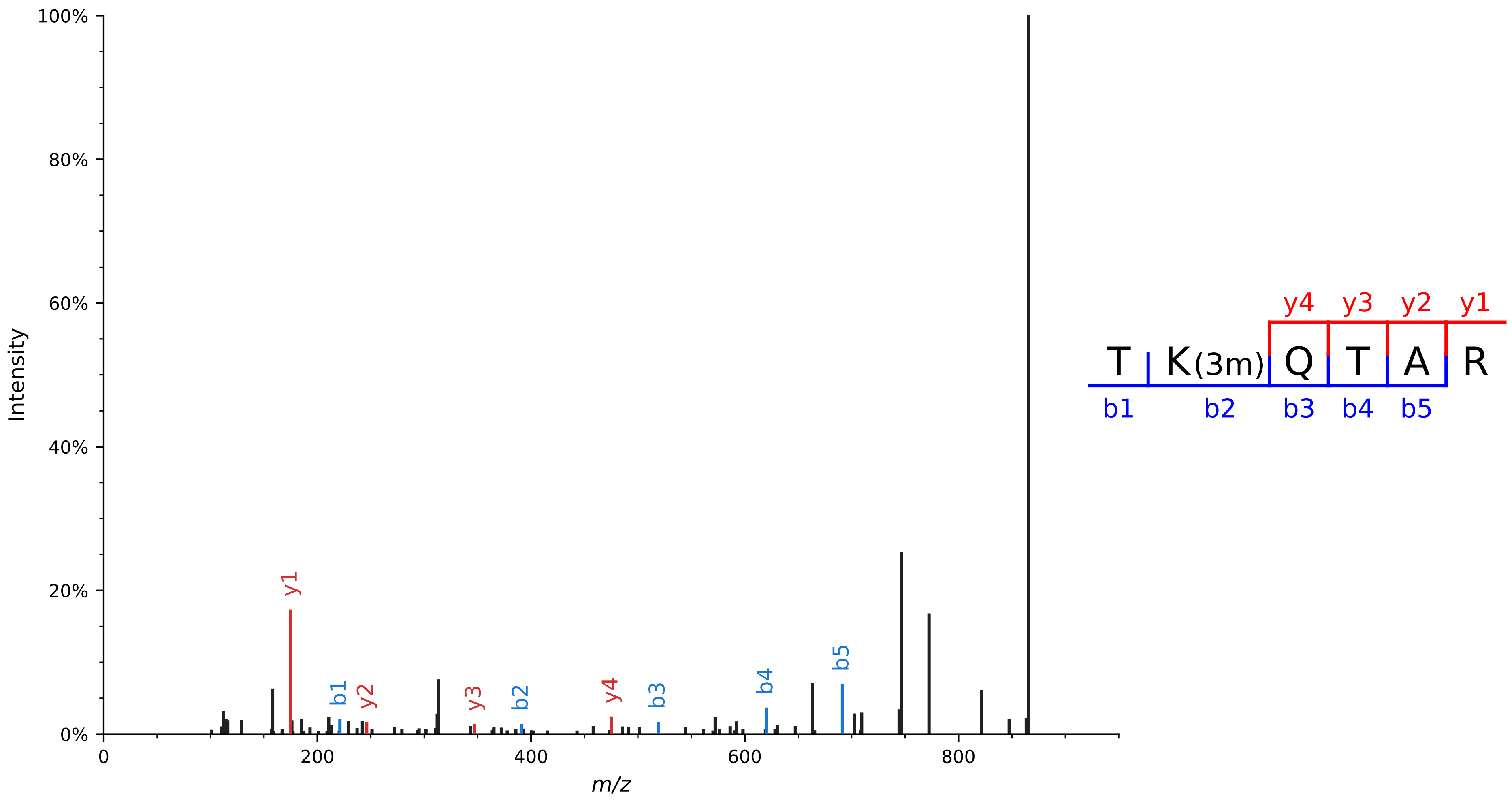

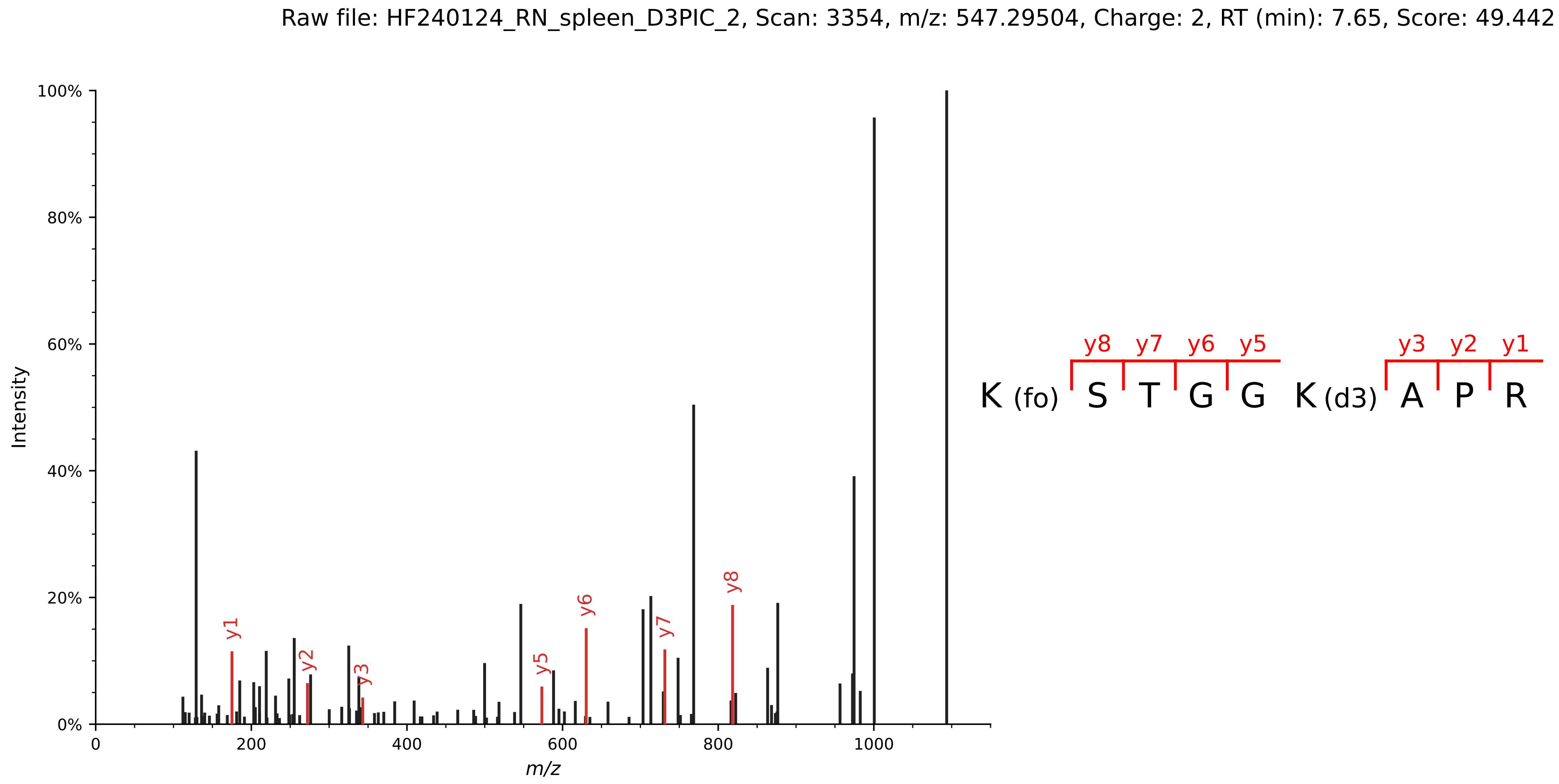

Raw file: HF240124_RN_spleen_D3PIC_2, Scan: 3354, m/z: 547.29504, Charge: 2, RT (min): 7.65, Score: 49.442
Intensity
m/z
100%
80%
60%
40%
20%
0%
0
200
400
600
800
1000
y1
y2
y3
y5
y6
y7
y8
y8 y7 y6 y5 y3 y2 y1
K (fo) S T G G K (d3) A P R

Raw file: HF240124_RN_spleen_D3PIC_2, Scan: 8954, m/z: 562.82011, Charge: 2, RT (min): 17.46, Score: 103.83

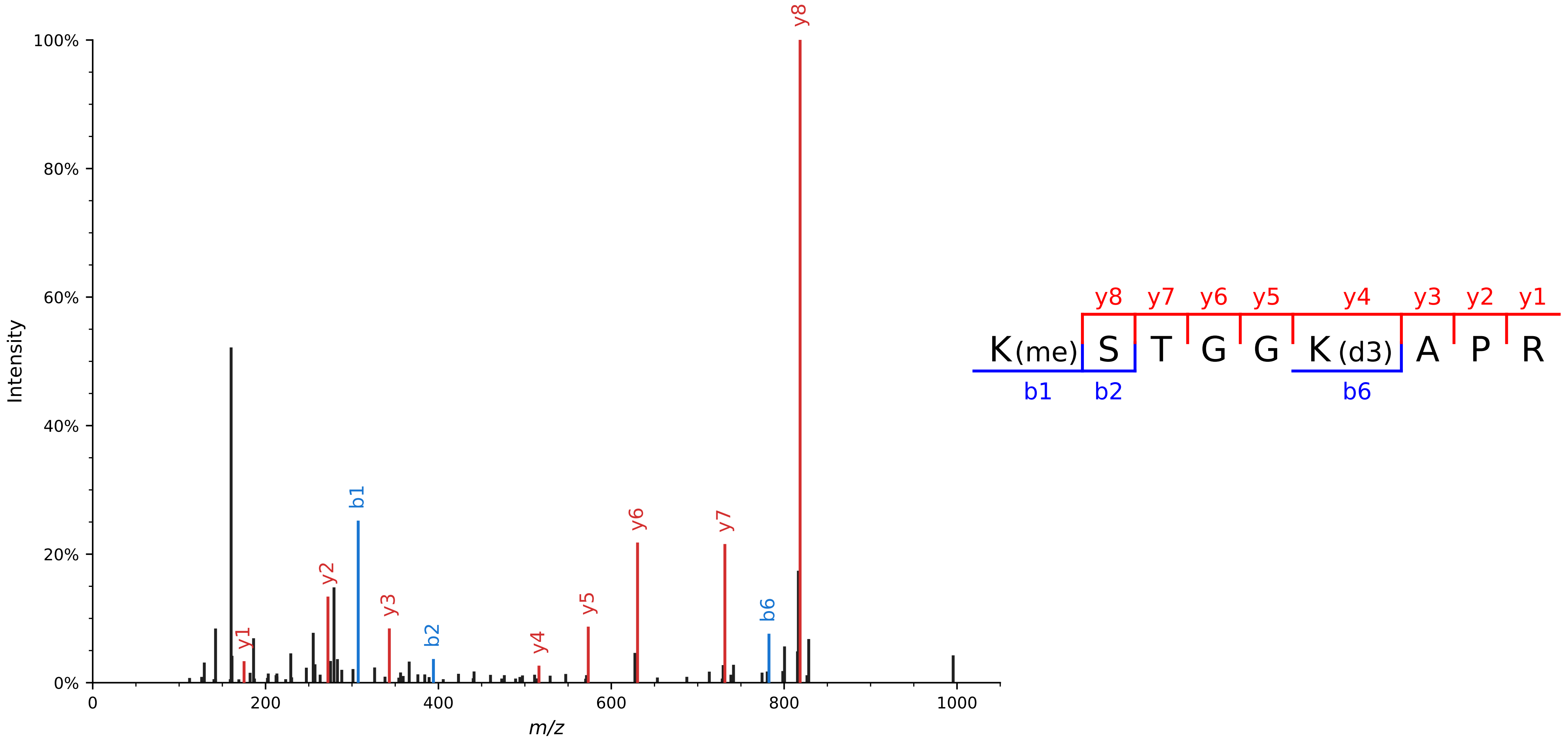

Raw file: HF240124_RN_spleen_D3PIC_2, Scan: 9016, m/z: 561.3107, Charge: 2, RT (min): 17.57, Score: 124.98

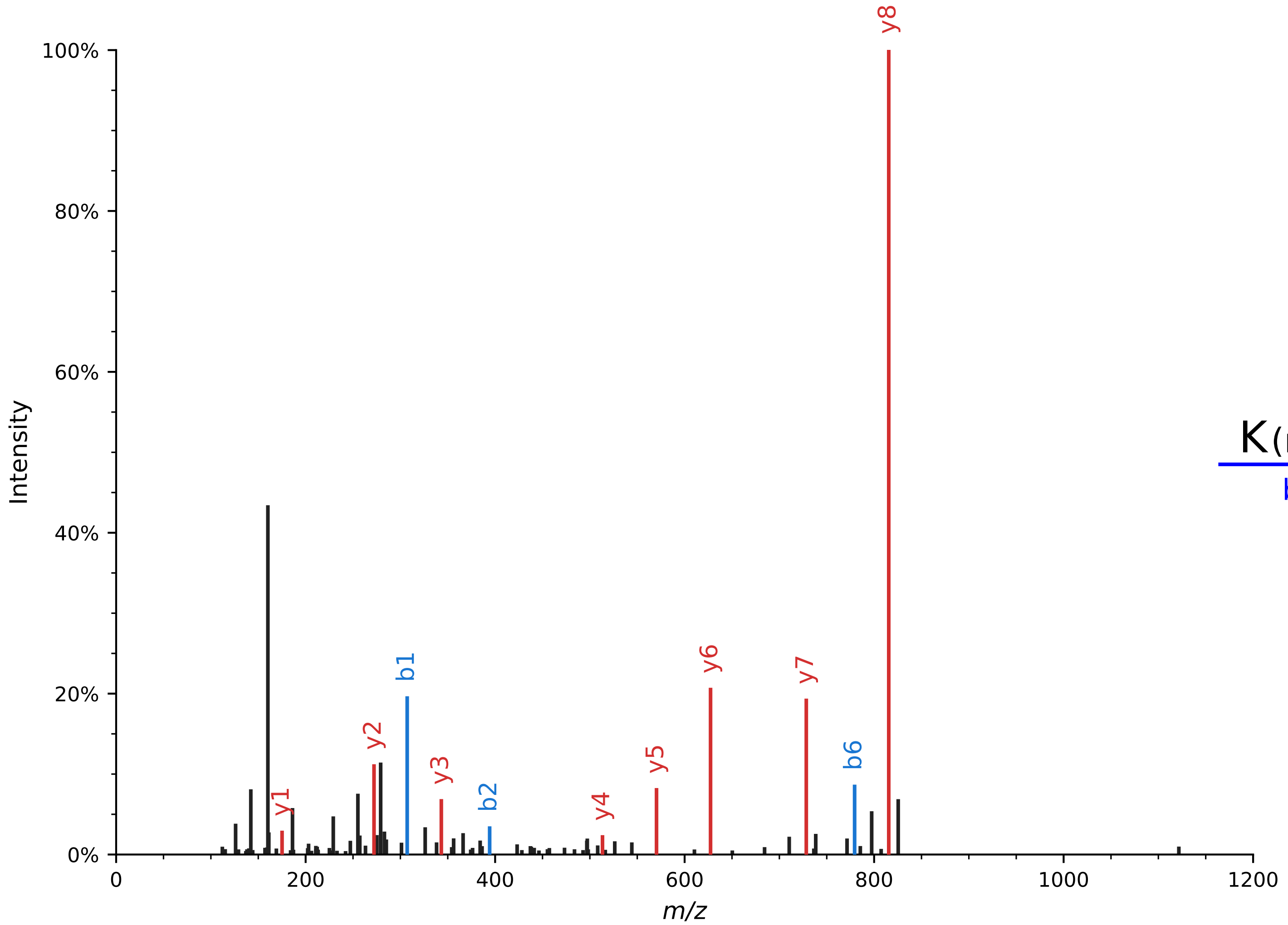

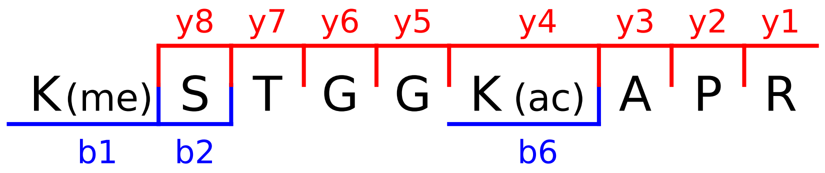

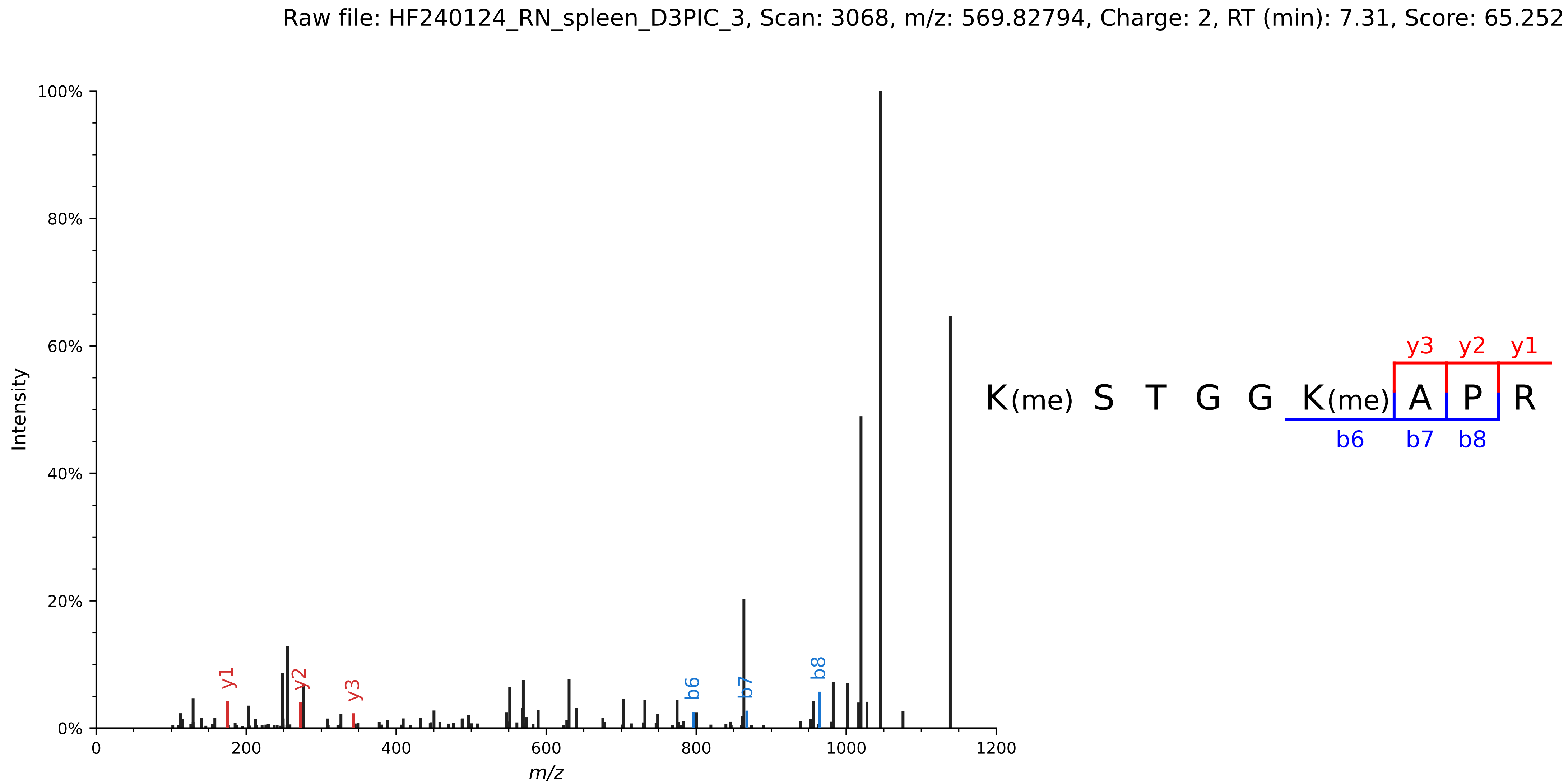

Raw file: HF240124_RN_spleen_D3PIC_3, Scan: 3068, m/z: 569.82794, Charge: 2, RT (min): 7.31, Score: 65.252
Intensity
100%
80%
60%
40%
20%
0%
0
200
400
600
800
1000
1200
m/z
y1
y2
y3
b6
b7
b8
K(me) S T G G K(me) A P R
y3 y2 y1
b6 b7 b8

Raw file: HF240124_RN_spleen_D3PIC_3, Scan: 2816, m/z: 547.31324, Charge: 2, RT (min): 6.87, Score: 114.5

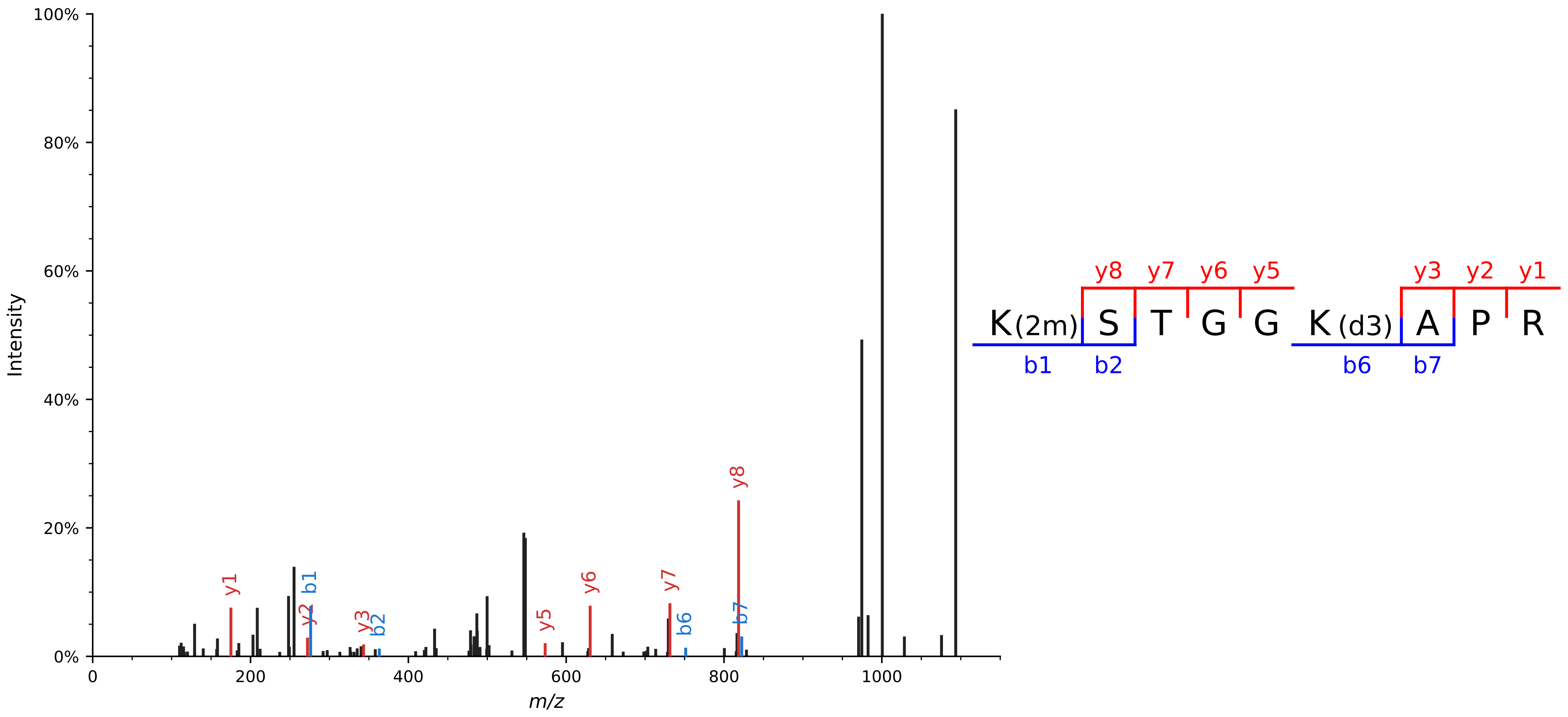

Raw file: HF240124_RN_spleen_D3PIC_3, Scan: 2093, m/z: 545.80382, Charge: 2, RT (min): 5.57, Score: 122.52

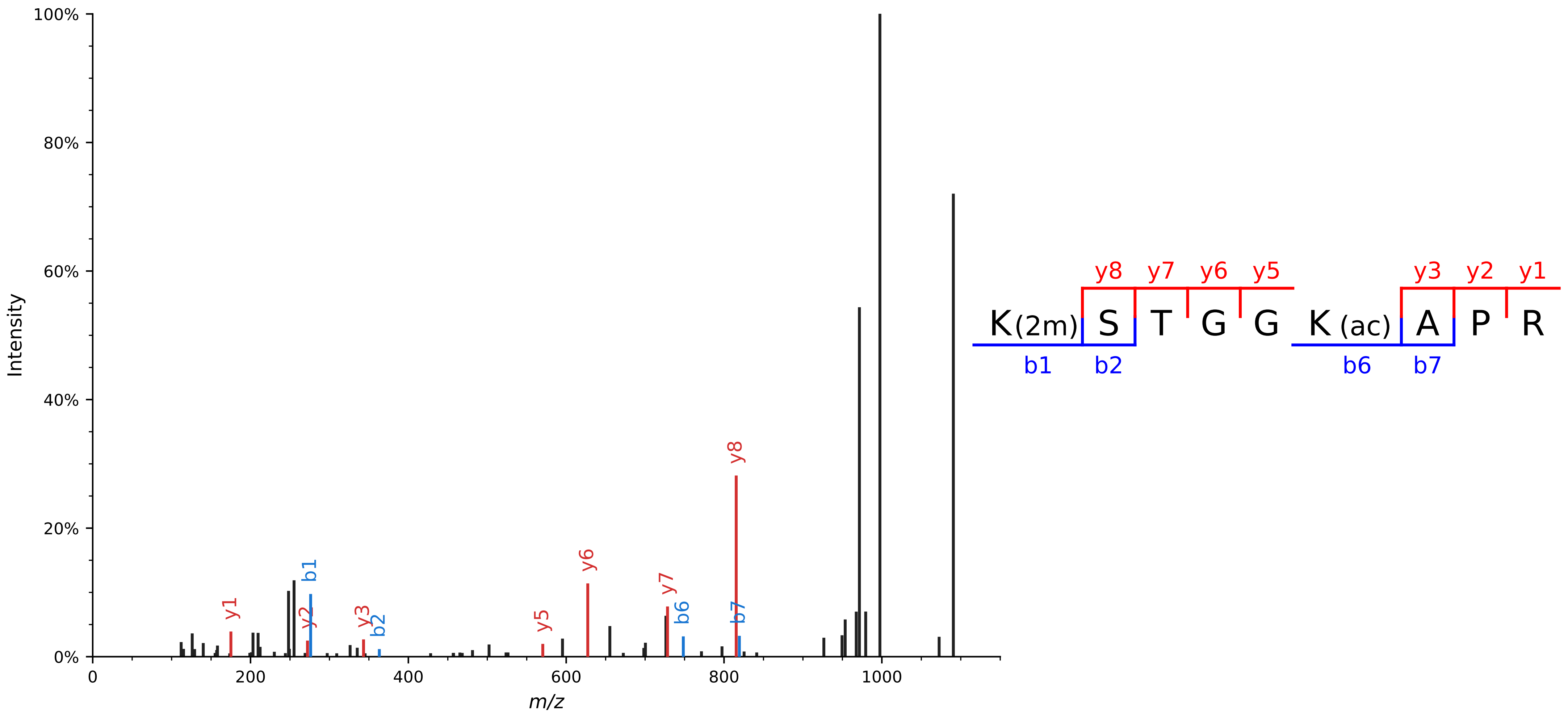

Raw file: HF240124_RN_spleen_D3PIC_3, Scan: 2794, m/z: 554.32106, Charge: 2, RT (min): 6.83, Score: 78.728

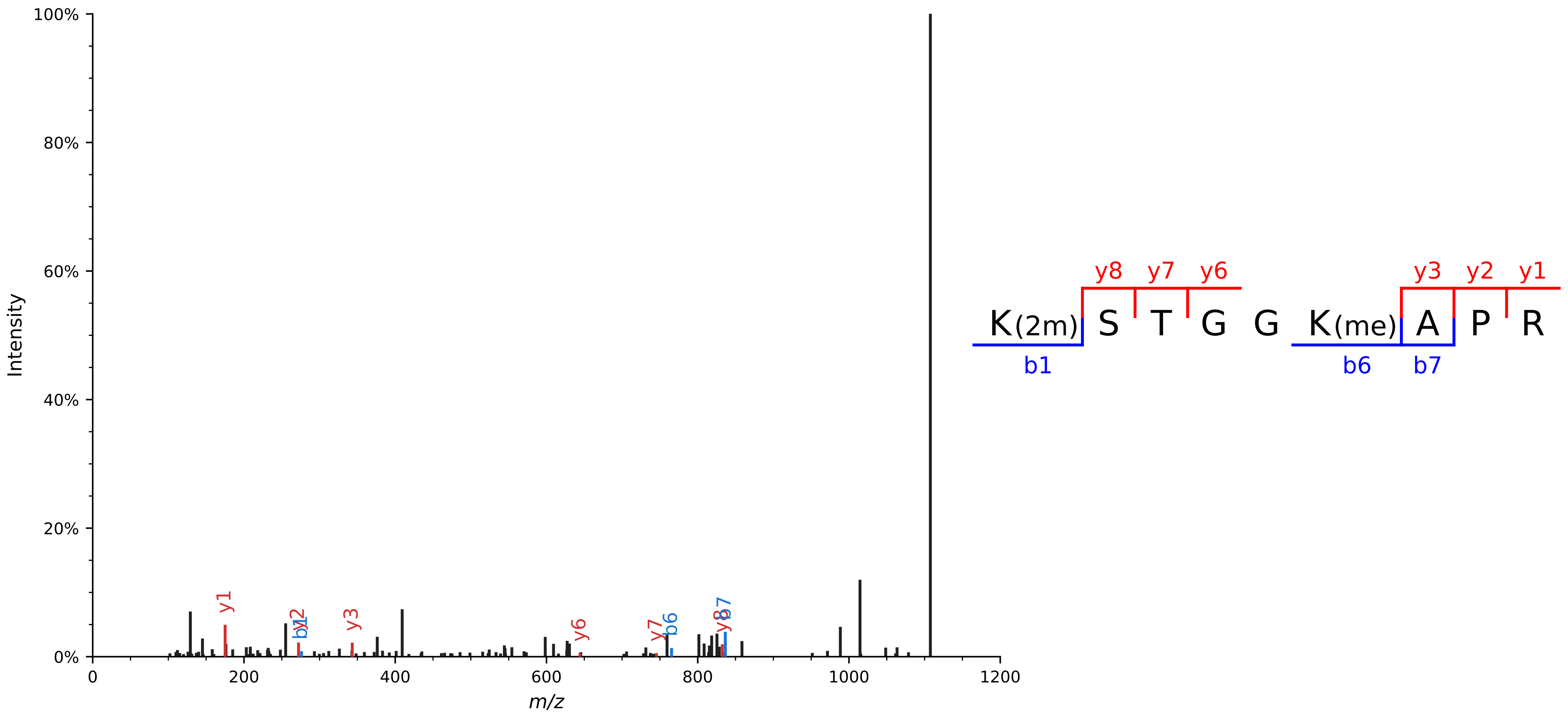

Raw file: HF240124_RN_spleen_D3PIC_3, Scan: 2695, m/z: 552.81165, Charge: 2, RT (min): 6.65, Score: 79.059

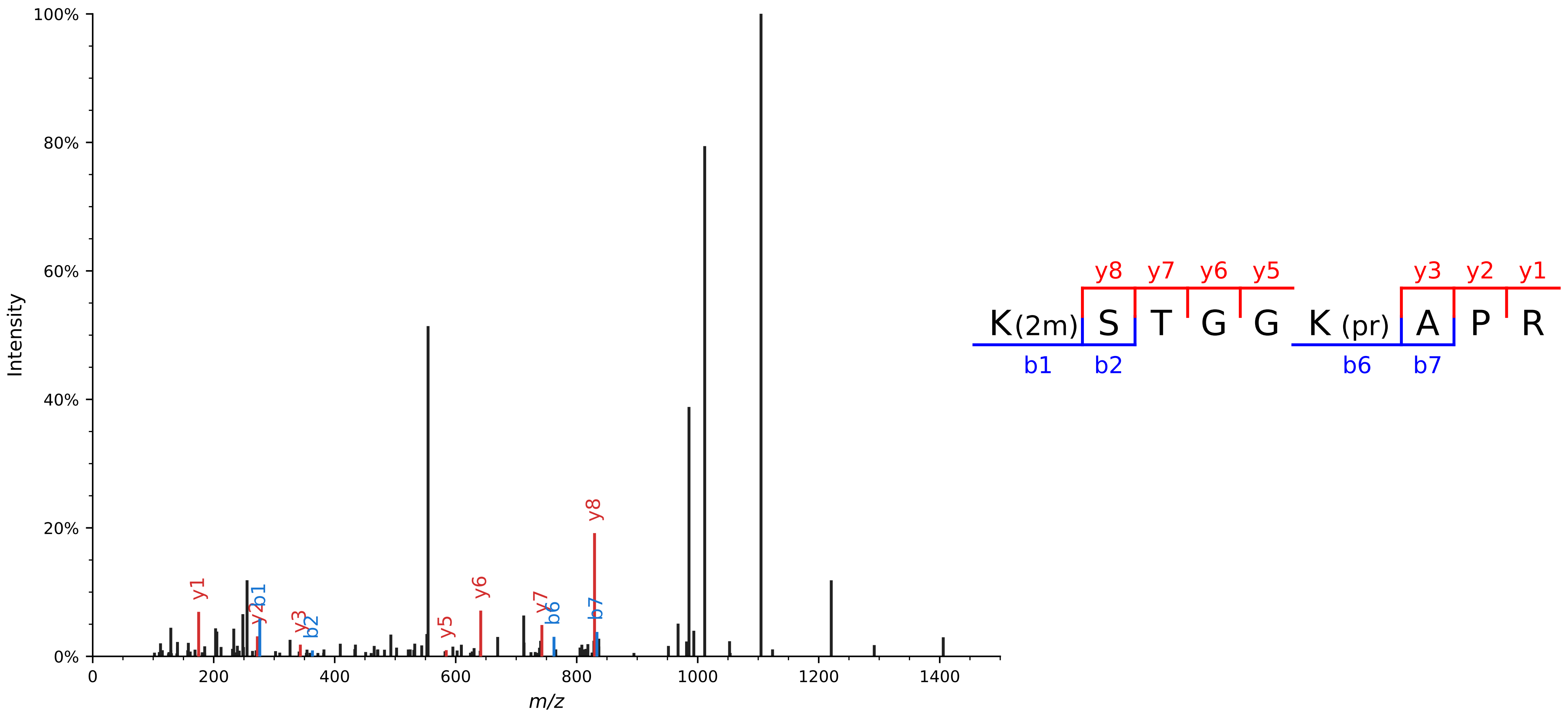

Raw file: HF240124_RN_spleen_D3PIC_3, Scan: 1954, m/z: 554.32106, Charge: 2, RT (min): 5.32, Score: 120.99

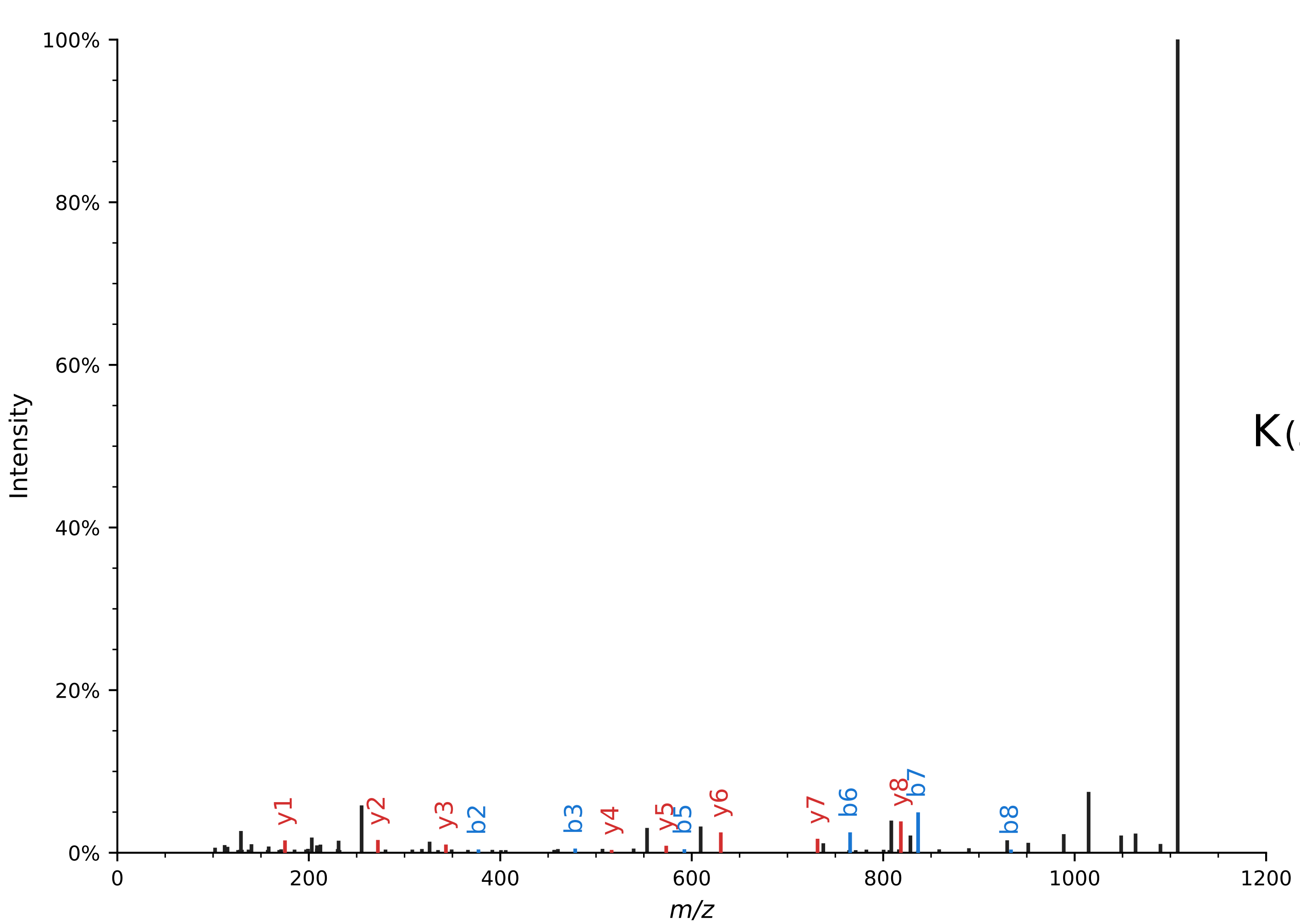

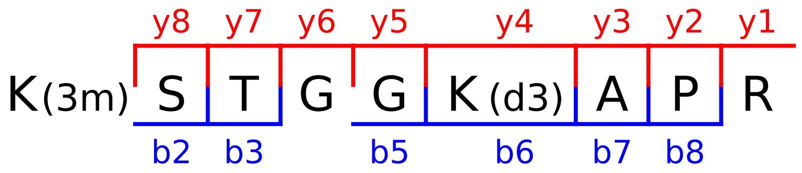

Raw file: HF240124_RN_spleen_D3PIC_3, Scan: 1961, m/z: 552.81165, Charge: 2, RT (min): 5.33, Score: 123.75

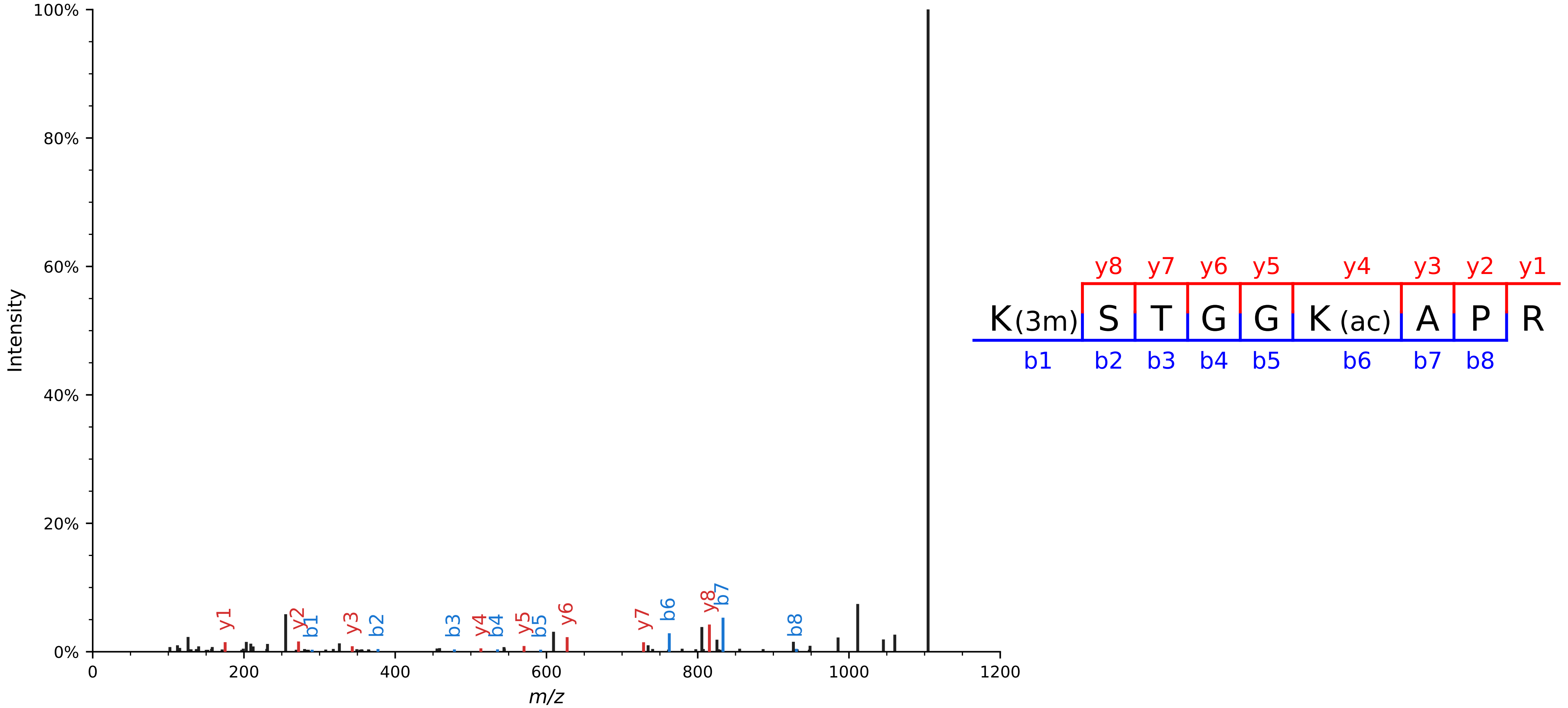

Raw file: HF240124_RN_spleen_D3PIC_3, Scan: 1759, m/z: 545.80382, Charge: 2, RT (min): 4.97, Score: 63.816

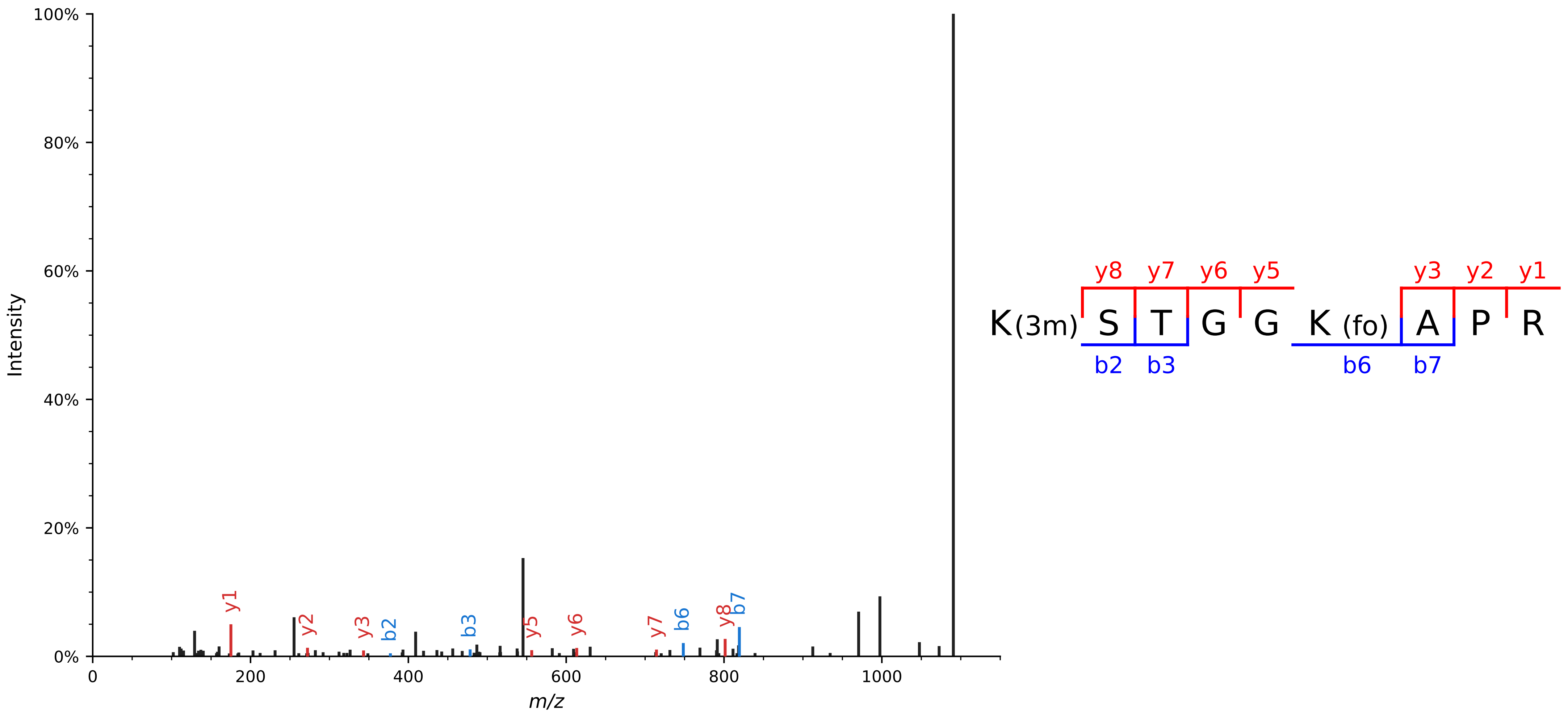

Raw file: HF240124_RN_spleen_D3PIC_2, Scan: 2732, m/z: 559.81947, Charge: 2, RT (min): 6.57, Score: 94.85

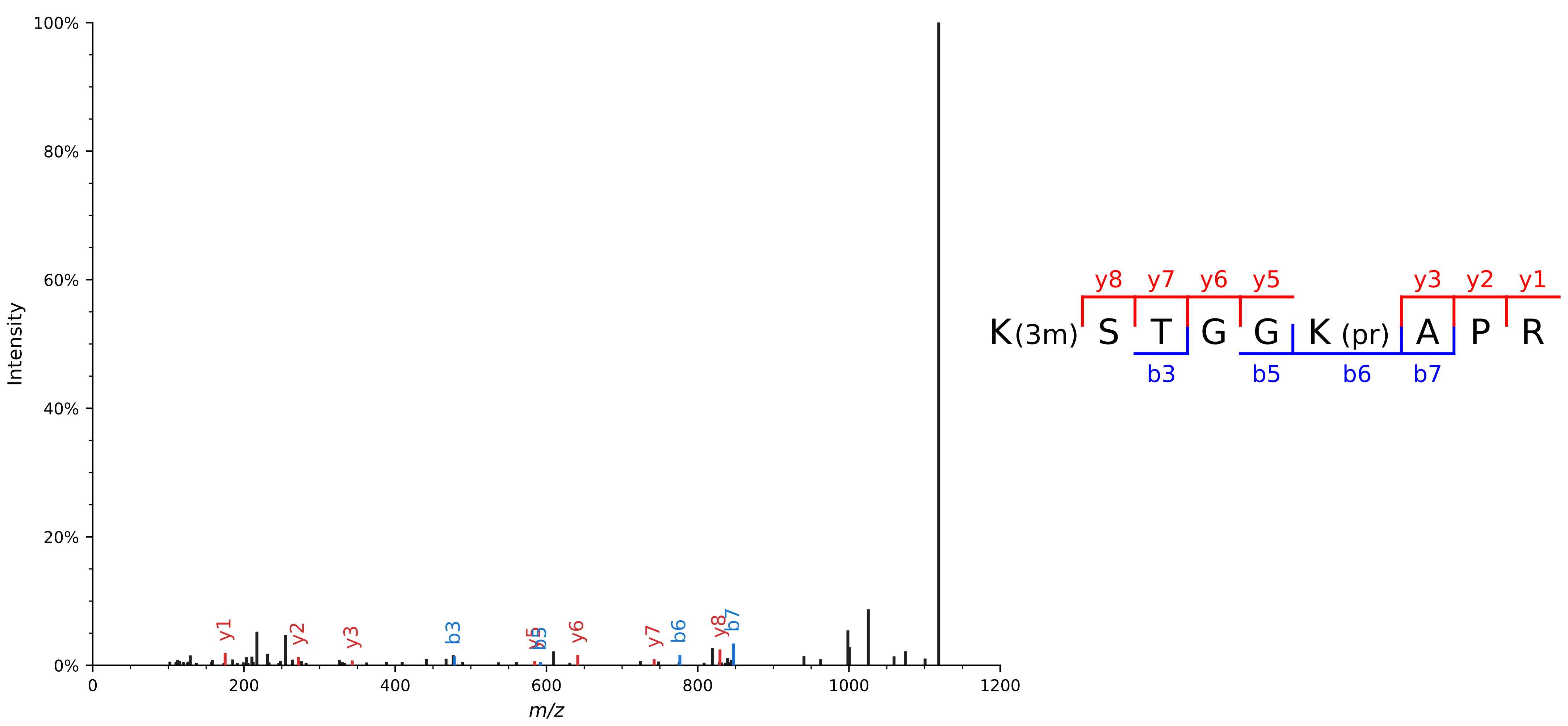

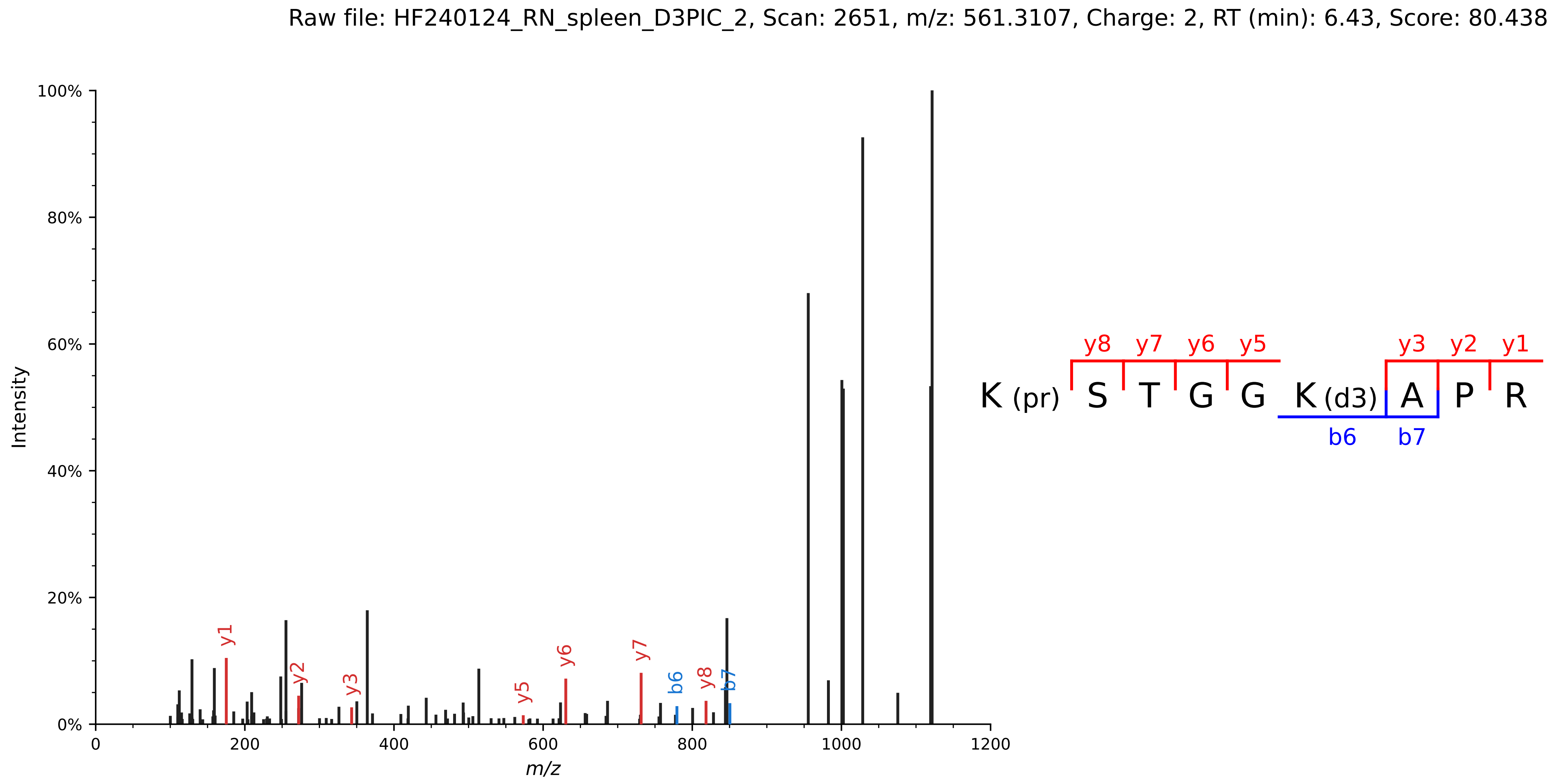

Raw file: HF240124_RN_spleen_D3PIC_2, Scan: 2651, m/z: 561.3107, Charge: 2, RT (min): 6.43, Score: 80.438
Intensity
m/z
y1
y2
y3
y5
y6
y7
b6
y8
b7
y8 y7 y6 y5 y3 y2 y1
K (pr) S T G G K (d3) A P R
b6 b7

Raw file: HF240124_RN_spleen_D3PIC_3, Scan: 3326, m/z: 568.31852, Charge: 2, RT (min): 7.77, Score: 75.738

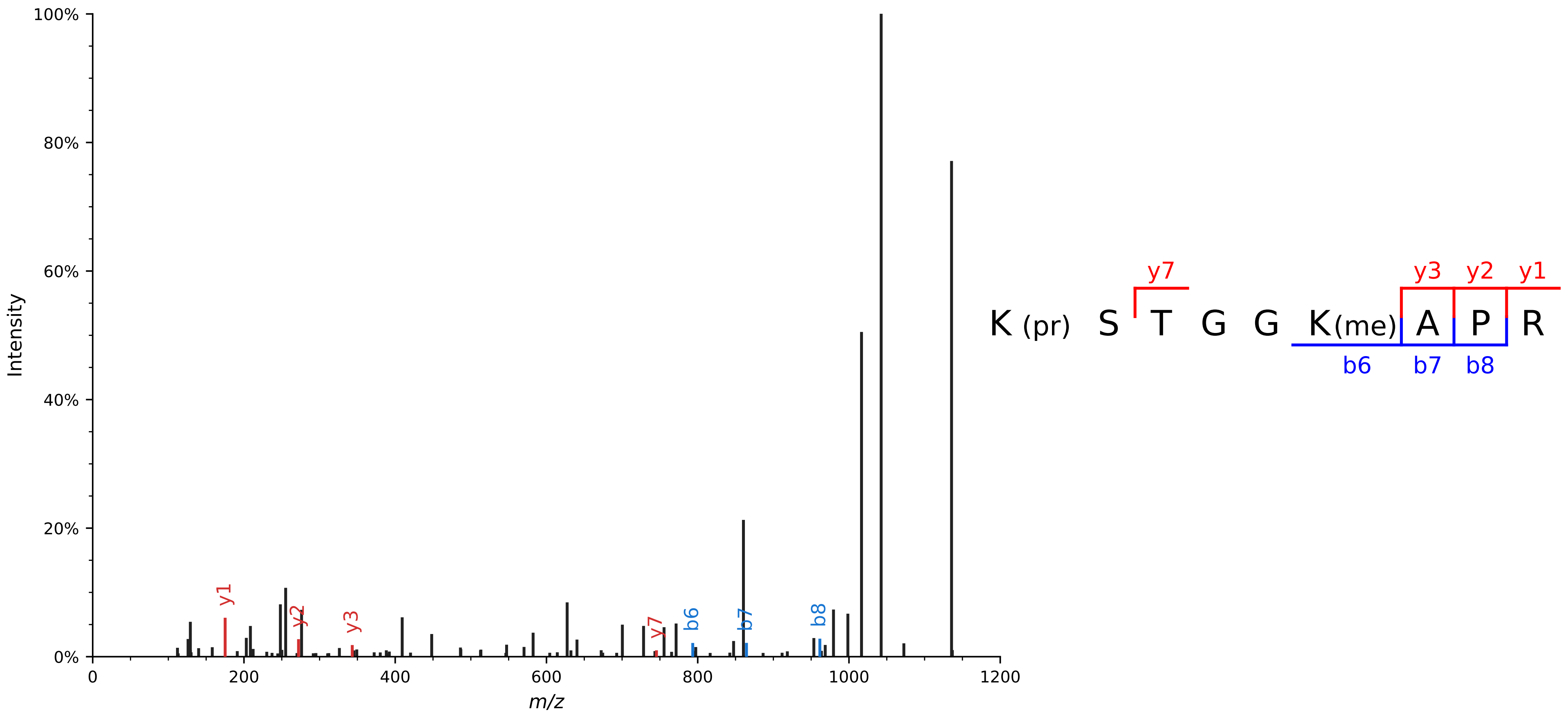

Raw file: HF240124_RN_spleen_D3PIC_3, Scan: 7310, m/z: 554.30287, Charge: 2, RT (min): 14.84, Score: 105.57

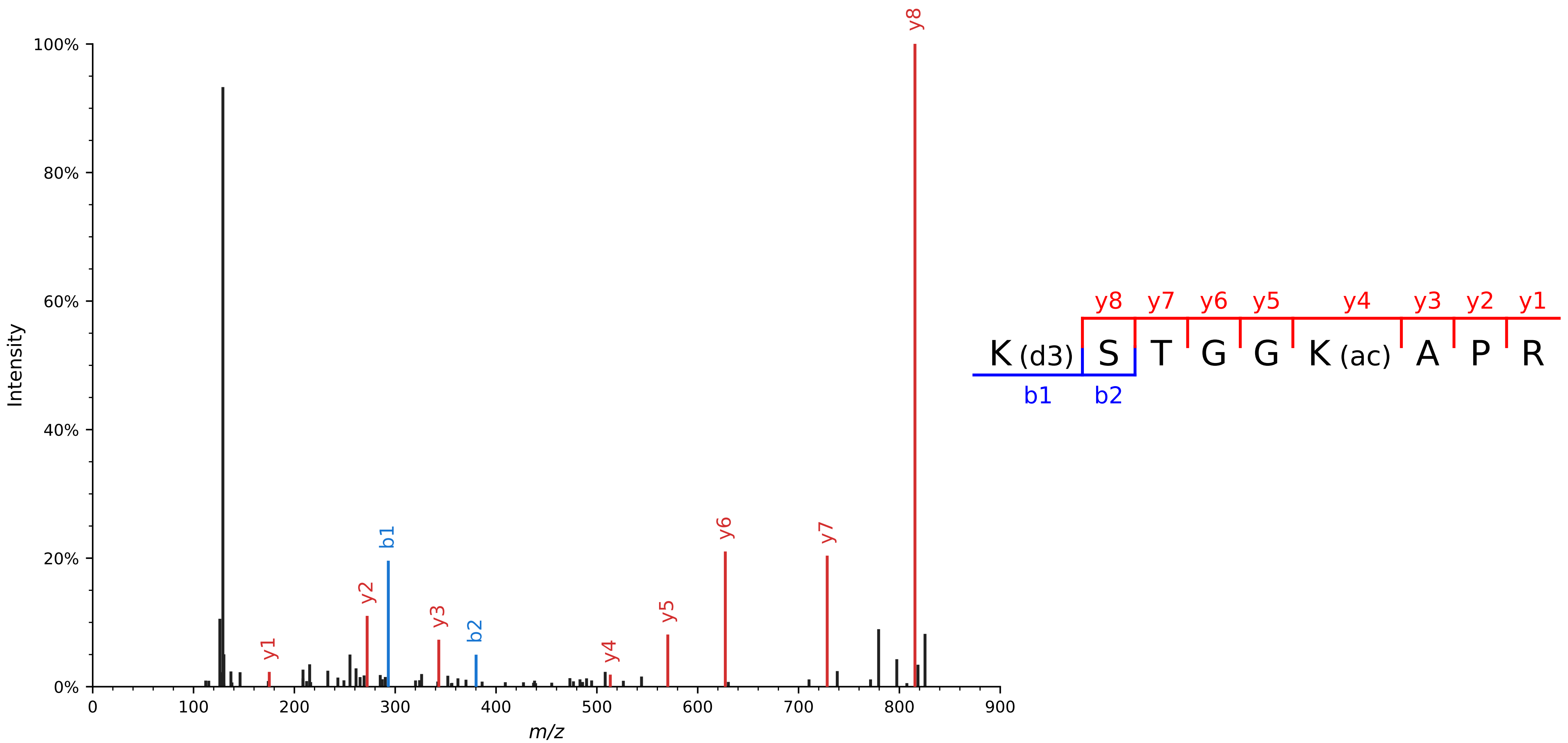

Raw file: HF240124_RN_spleen_D3PIC_1, Scan: 9781, m/z: 568.31852, Charge: 2, RT (min): 19.10, Score: 73.665

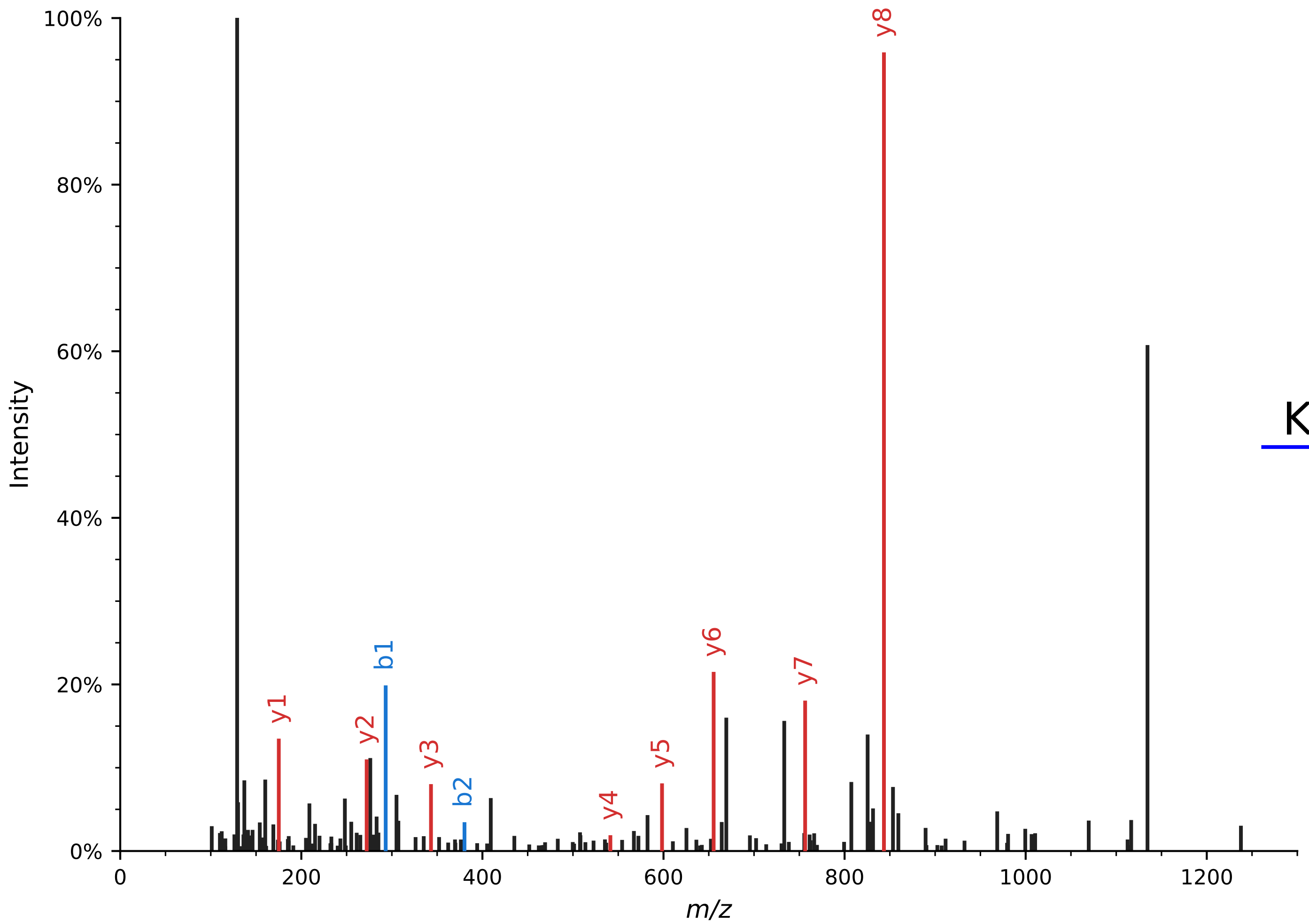

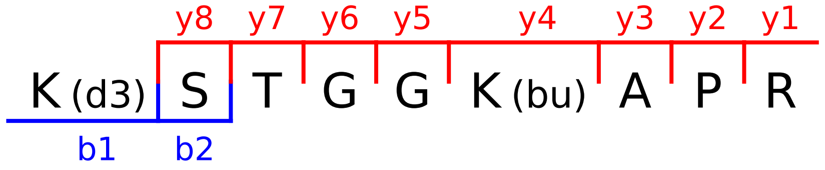

Raw file: HF240124_RN_spleen_D3PIC_1, Scan: 15468, m/z: 596.84744, Charge: 2, RT (min): 29.35, Score: 77.318

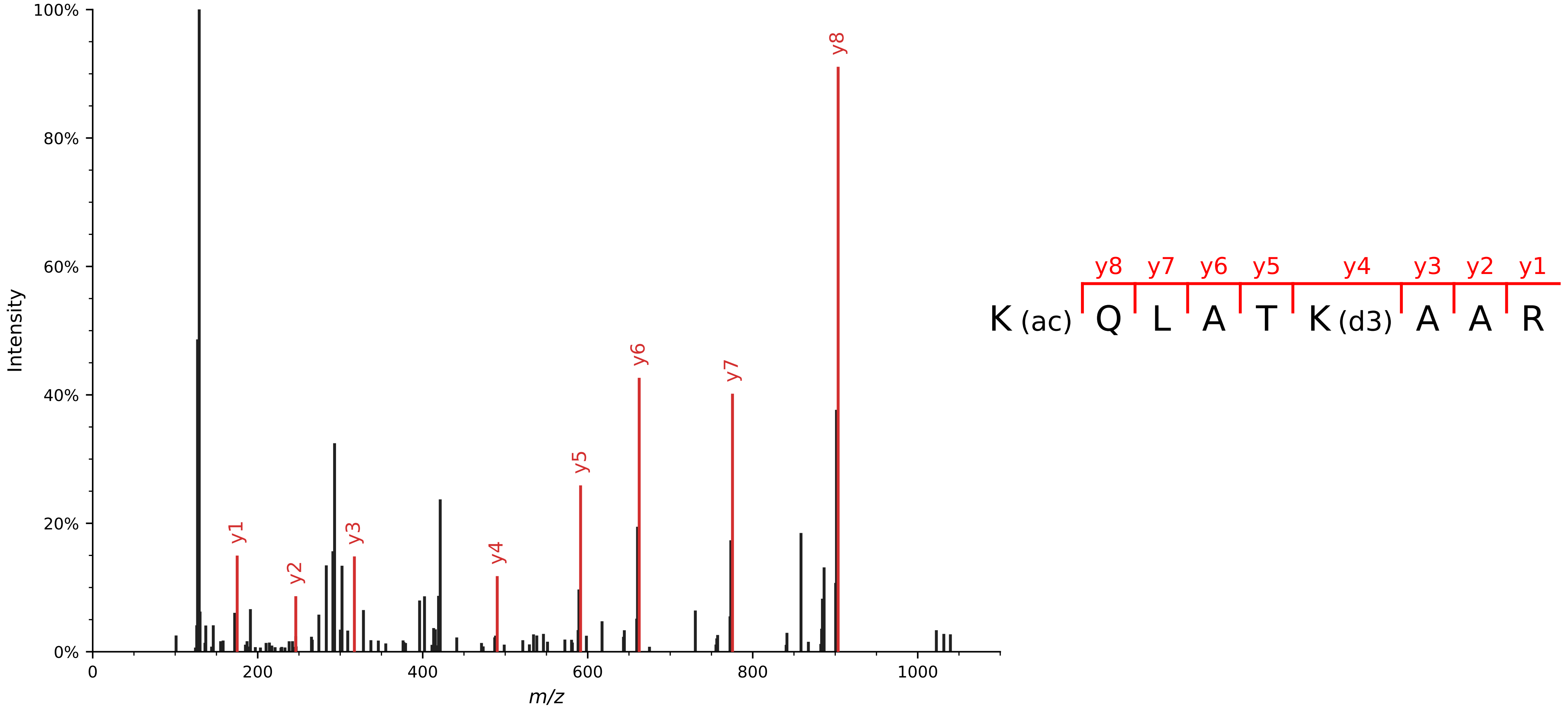

Raw file: HF240124_RN_spleen_D3PIC_1, Scan: 18996, m/z: 610.86309, Charge: 2, RT (min): 35.80, Score: 127.02

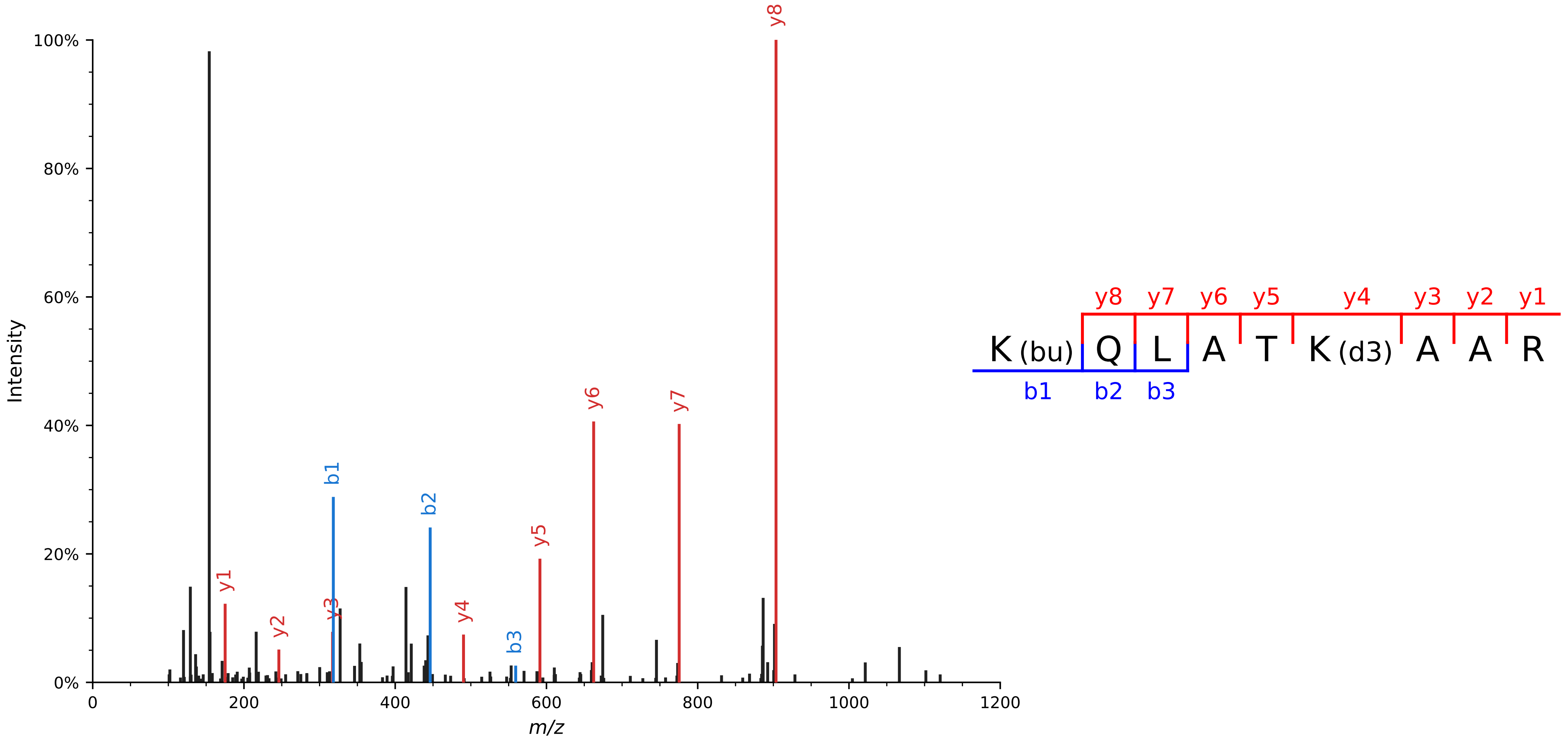

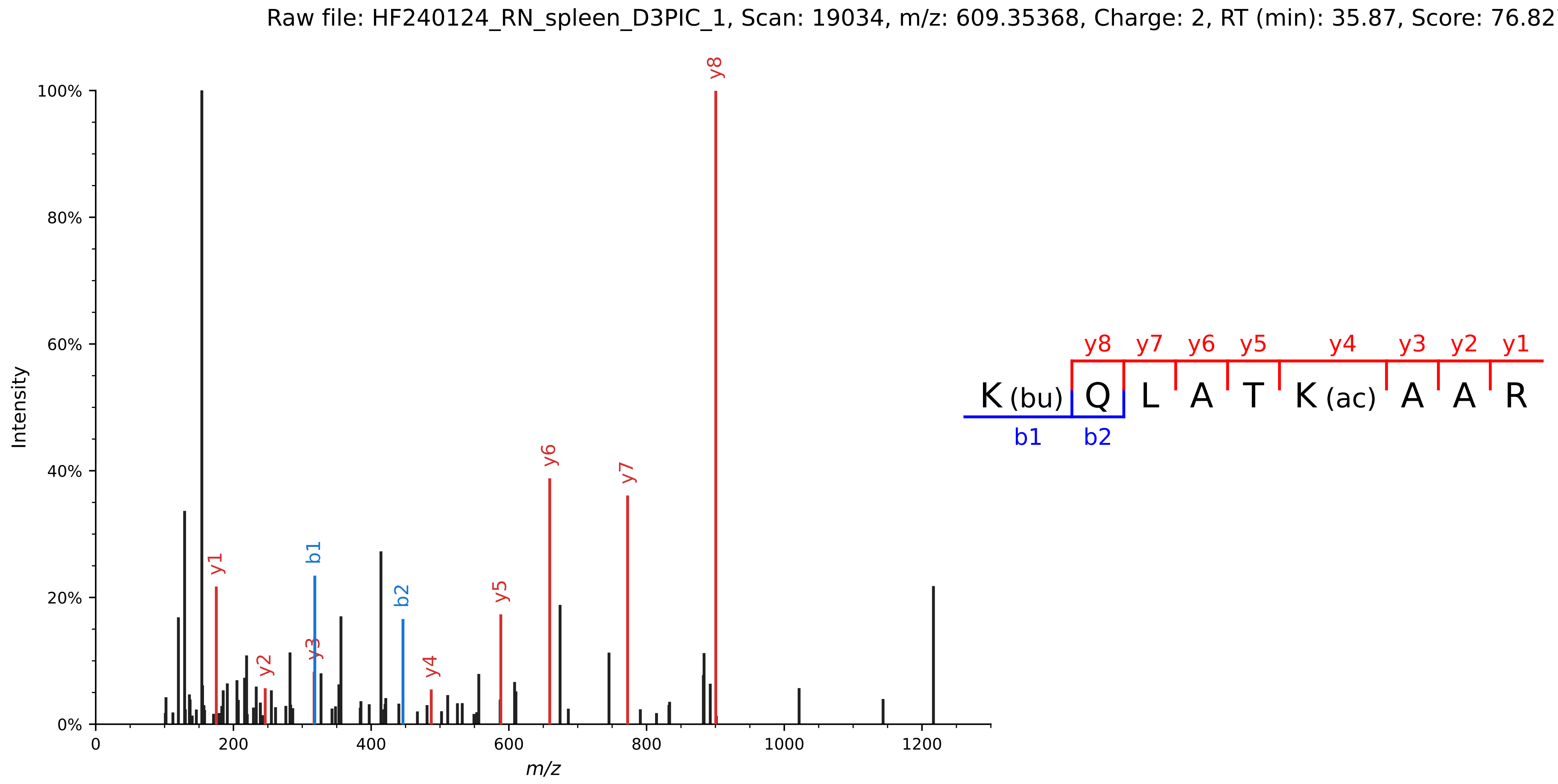

Raw file: HF240124_RN_spleen_D3PIC_1, Scan: 19034, m/z: 609.35368, Charge: 2, RT (min): 35.87, Score: 76.827
Intensity
100%
80%
60%
40%
20%
0%
0
200
400
600
800
1000
1200
m/z
y8
y6
y7
y1
b1
b2
y5
y3
y2
y4
y8 y7 y6 y5 y4 y3 y2 y1
K (bu) Q L A T K (ac) A A R
b1 b2

Raw file: HF240124_RN_spleen_D3PIC_2, Scan: 15888, m/z: 589.83962, Charge: 2, RT (min): 30.04, Score: 122.13

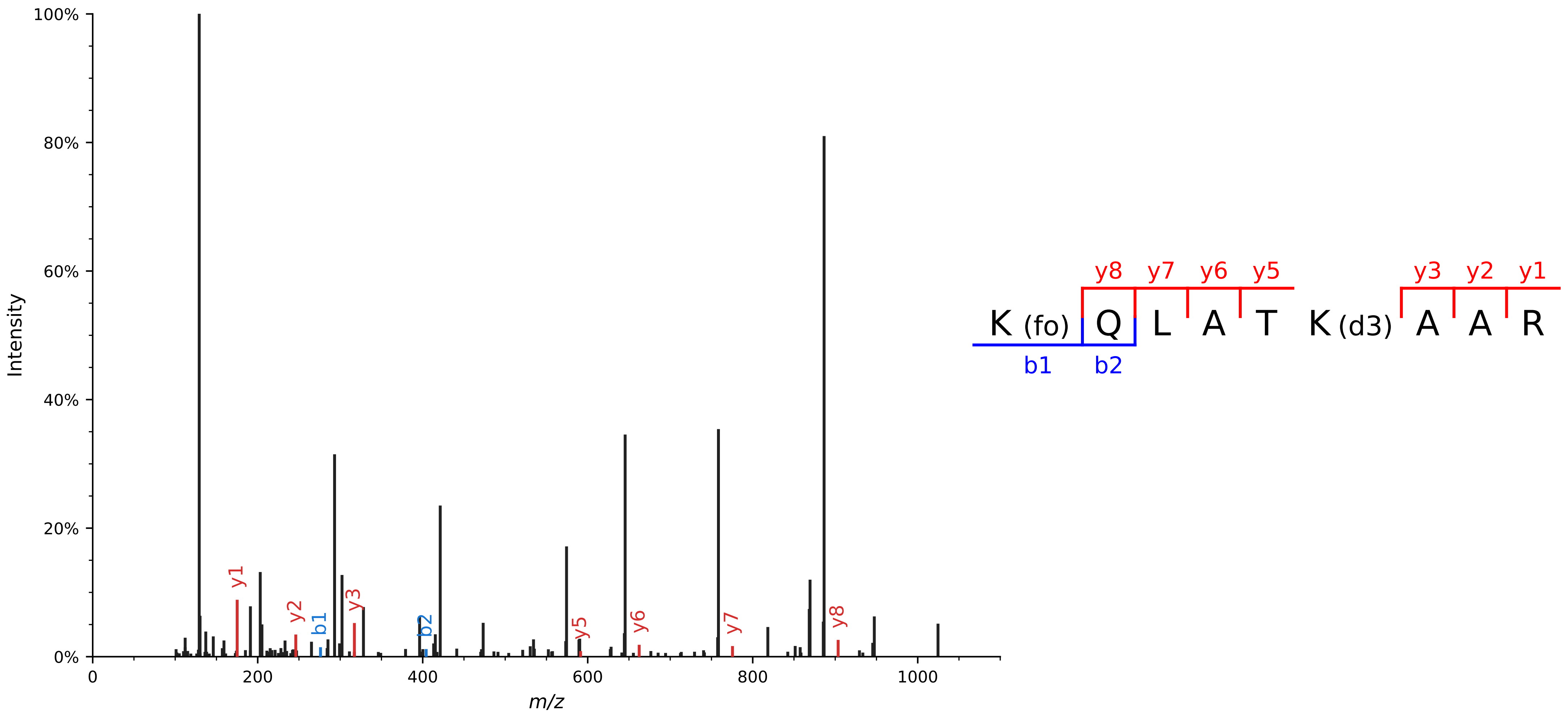

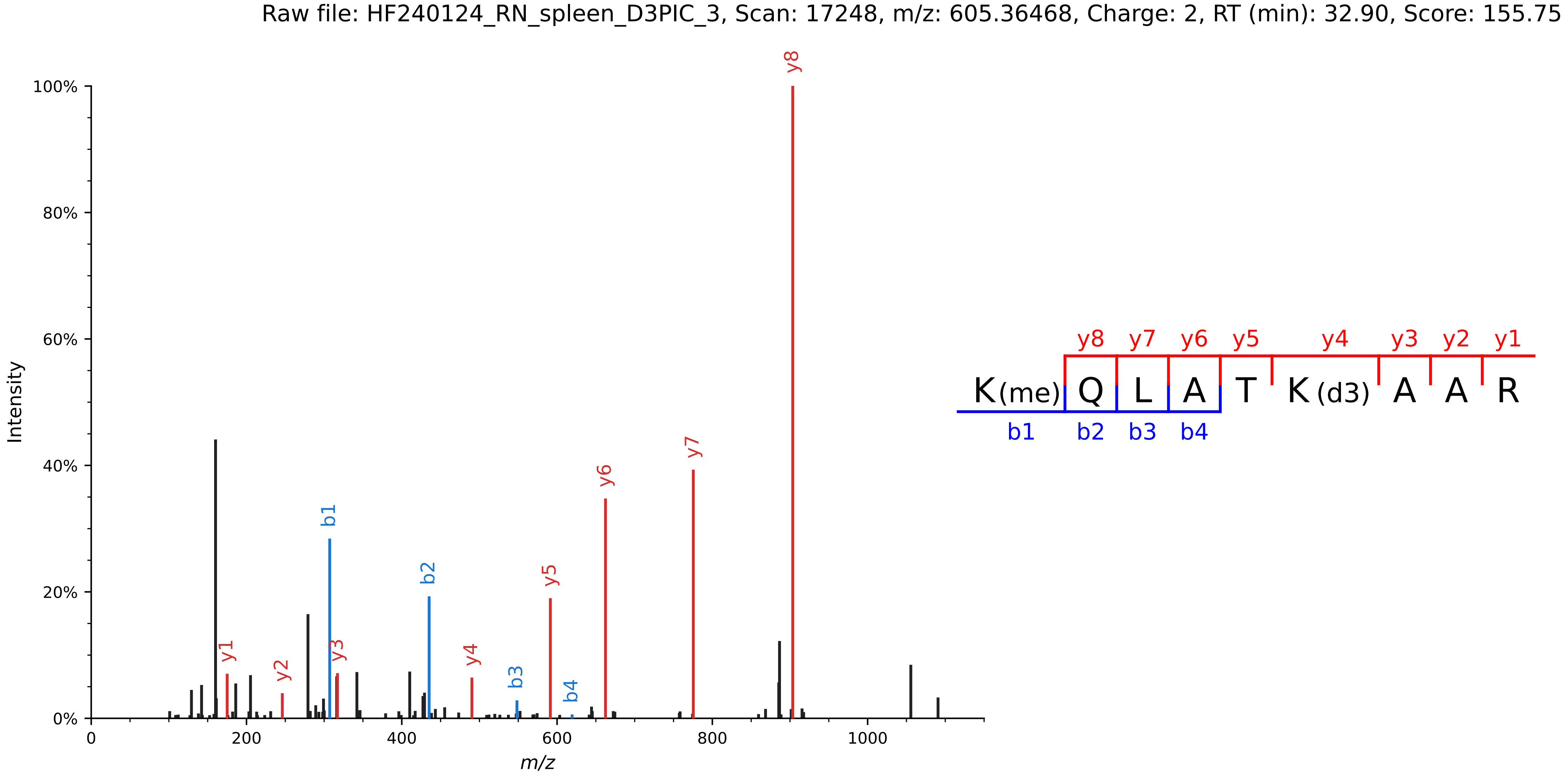
Raw file: HF240124_RN_spleen_D3PIC_3, Scan: 17248, m/z: 605.36468, Charge: 2, RT (min): 32.90, Score: 155.75
Intensity
m/z
y8 y7 y6 y5 y4 y3 y2 y1
K(me) Q L A T K(d3) A A R
b1 b2 b3 b4

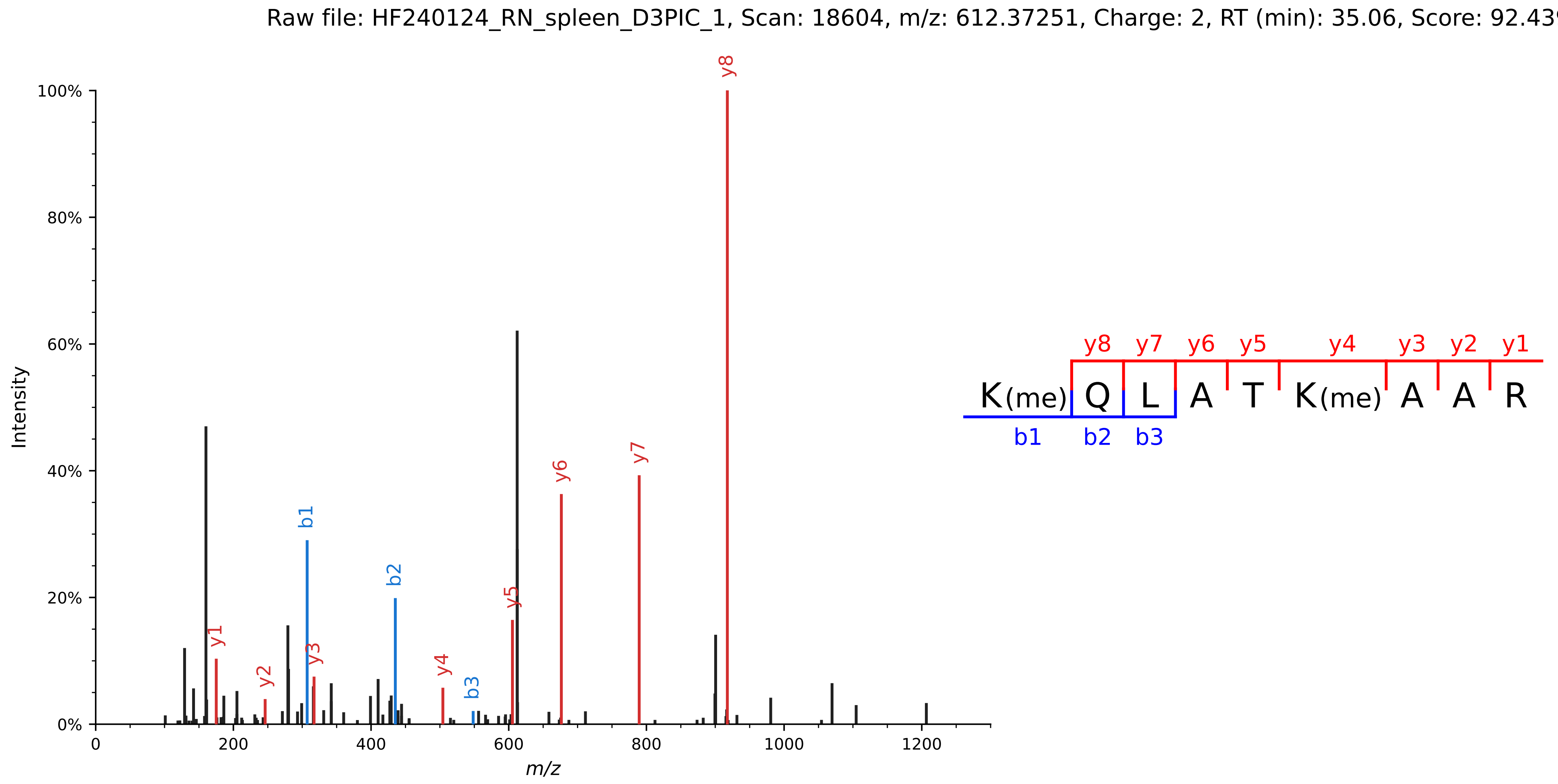

Raw file: HF240124_RN_spleen_D3PIC_1, Scan: 18604, m/z: 612.37251, Charge: 2, RT (min): 35.06, Score: 92.439
Intensity
m/z
y8
y7
y6
y5
y4
y3
y2
y1
b1
b2
b3
K(me) Q L A T K(me) A A R

Raw file: HF240124_RN_spleen_D3PIC_1, Scan: 8561, m/z: 589.85781, Charge: 2, RT (min): 16.85, Score: 160.63

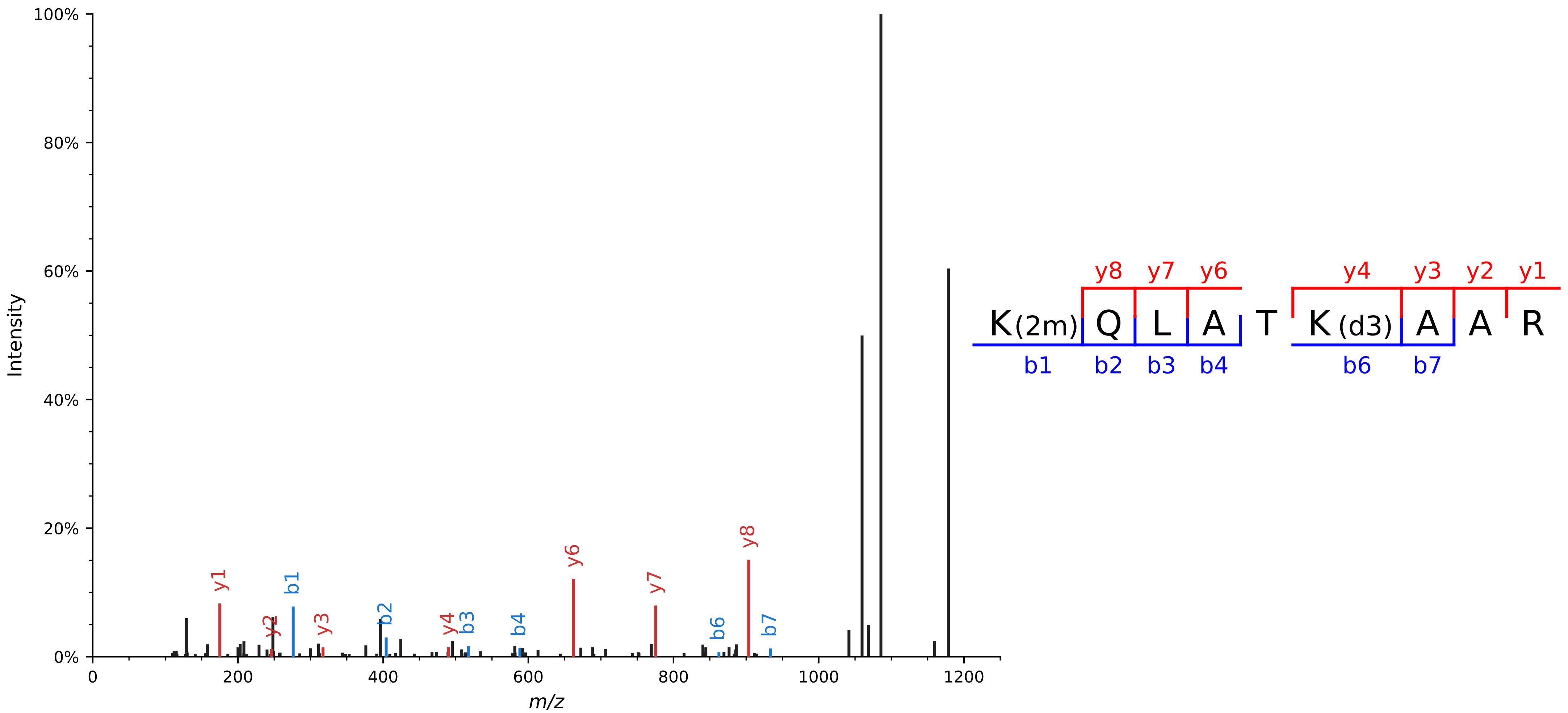

Raw file: HF240124_RN_spleen_D3PIC_3, Scan: 15967, m/z: 588.3484, Charge: 2, RT (min): 30.59, Score: 86.794

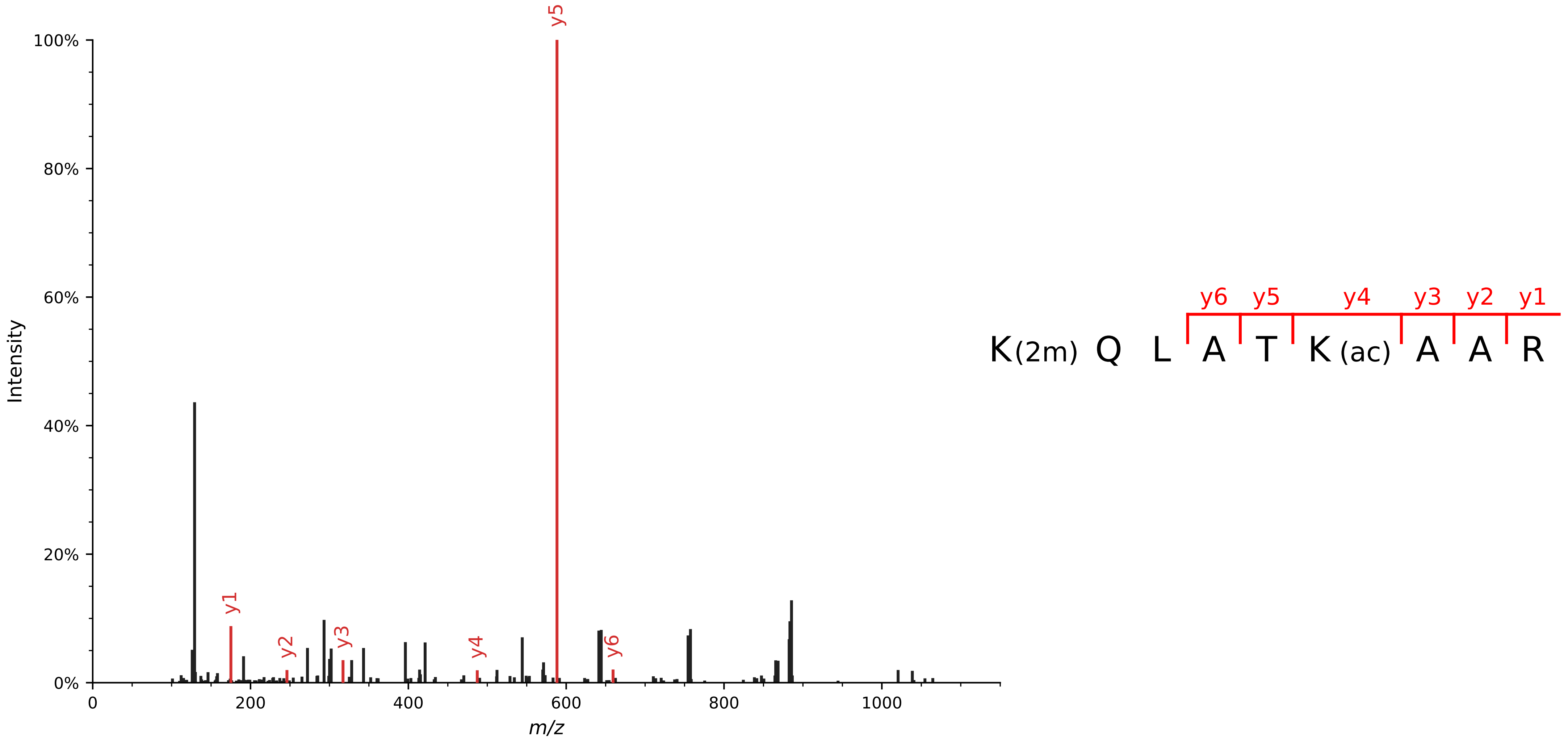

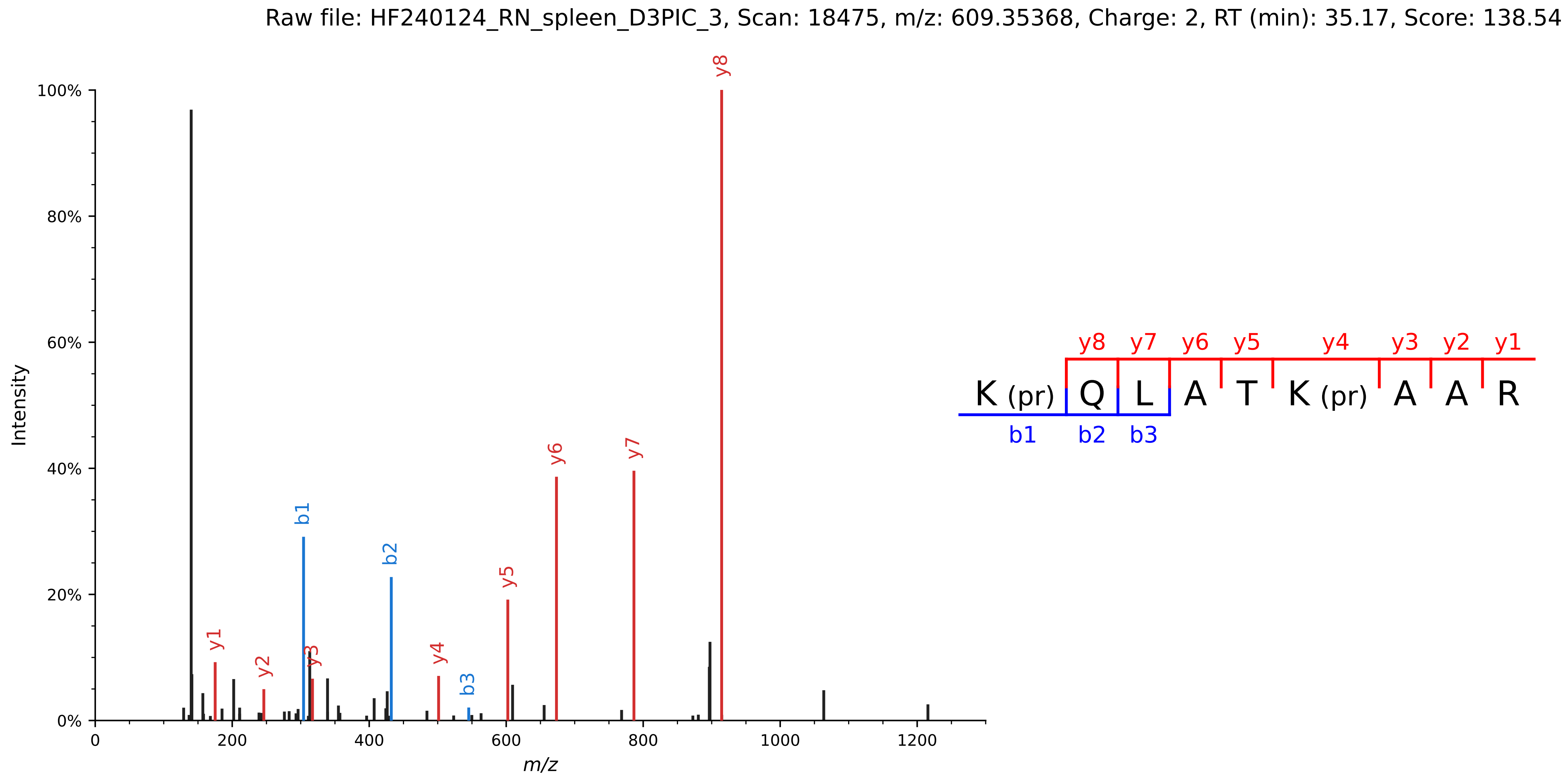

Raw file: HF240124_RN_spleen_D3PIC_3, Scan: 18475, m/z: 609.35368, Charge: 2, RT (min): 35.17, Score: 138.54
Intensity
m/z
y1
y2
y3
y4
y5
y6
y7
y8
b1
b2
b3
K (pr) Q L A T K (pr) A A R

Raw file: HF240124_RN_spleen_D3PIC_1, Scan: 8815, m/z: 596.84744, Charge: 2, RT (min): 17.30, Score: 114.28

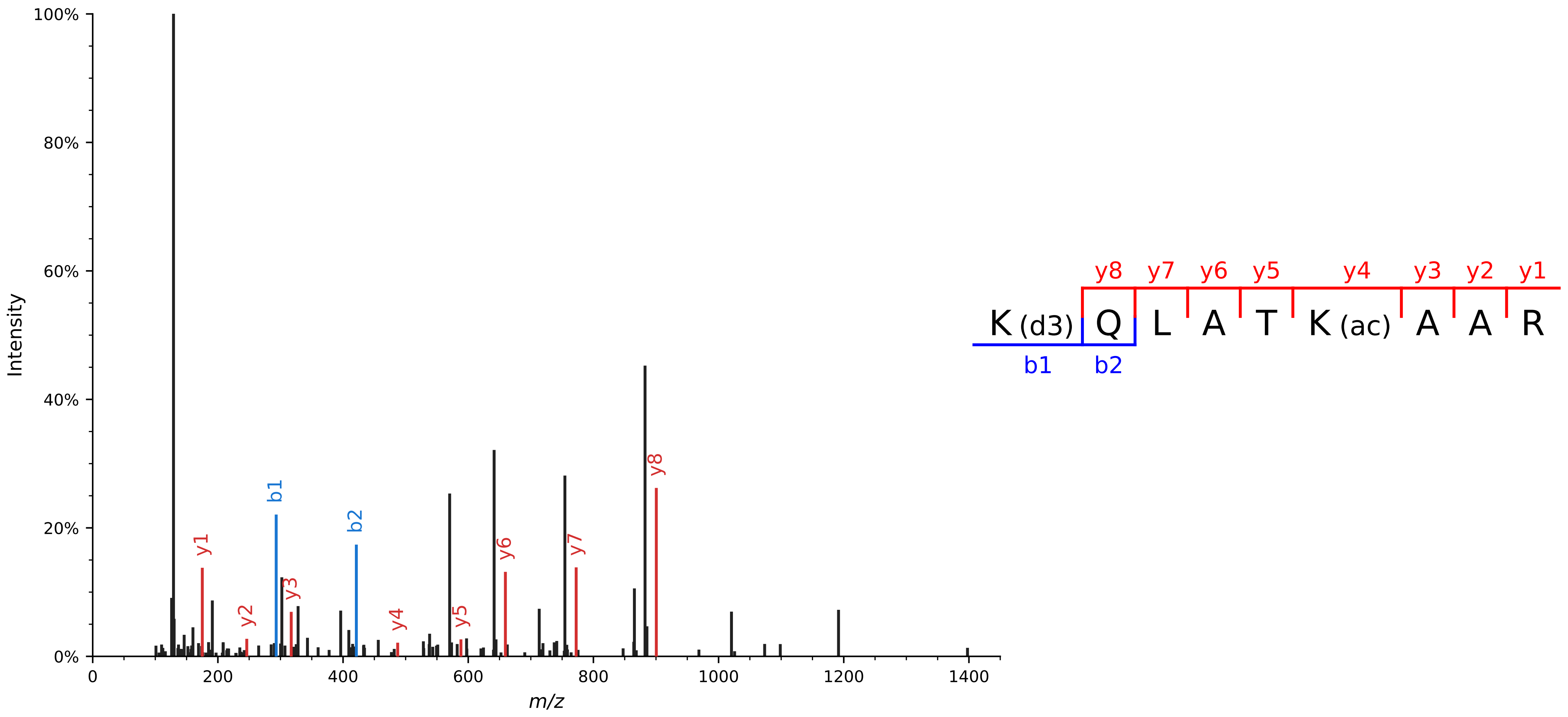

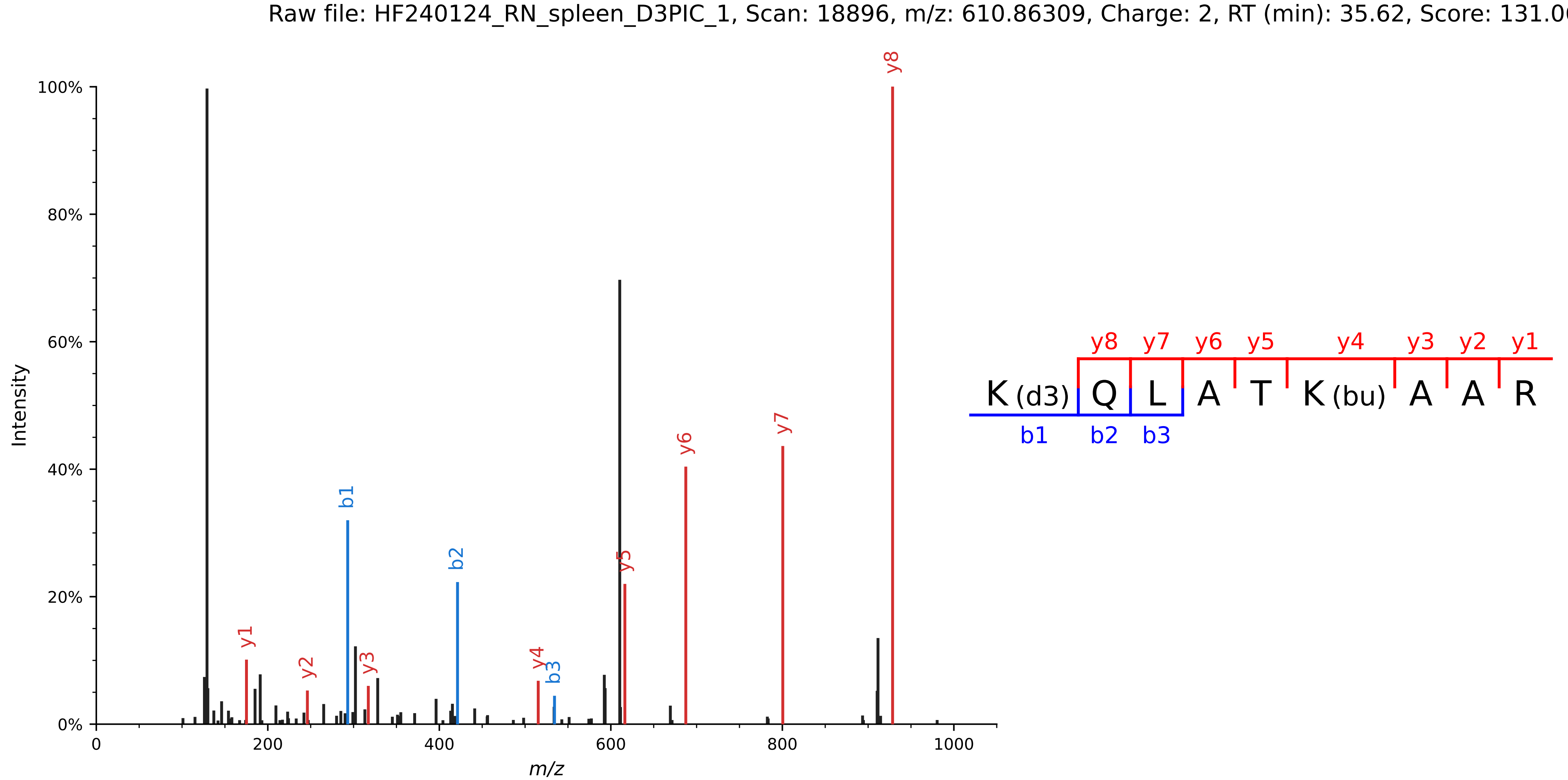

Raw file: HF240124_RN_spleen_D3PIC_1, Scan: 18896, m/z: 610.86309, Charge: 2, RT (min): 35.62, Score: 131.06
Intensity
100%
80%
60%
40%
20%
0%
0
200
400
600
800
1000
m/z
y1
y2
b1
y3
b2
y4
b3
y5
y6
y7
y8
y8 y7 y6 y5 y4 y3 y2 y1
K(d3) Q L A T K(bu) A A R
b1 b2 b3

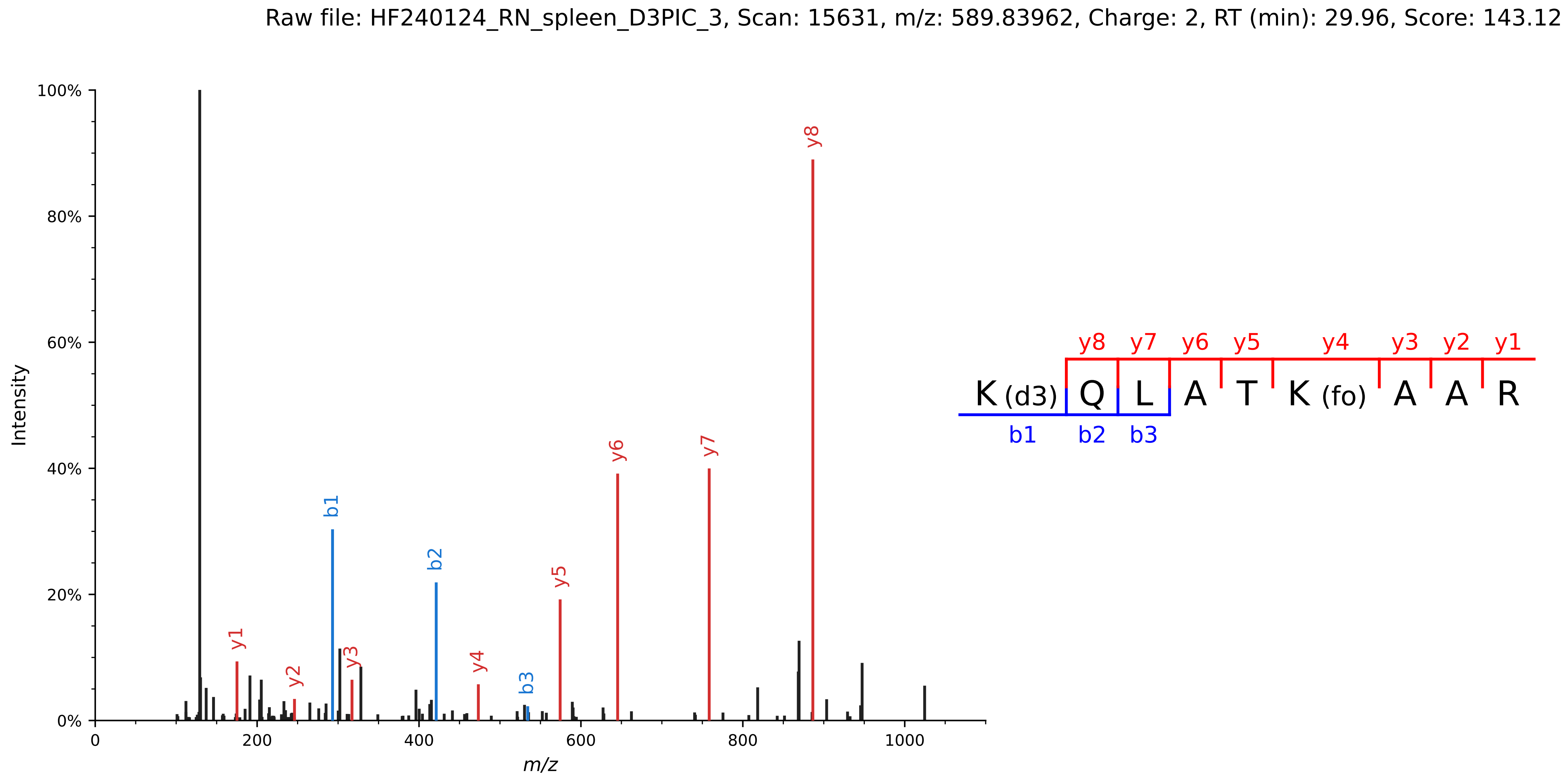
Raw file: HF240124_RN_spleen_D3PIC_3, Scan: 15631, m/z: 589.83962, Charge: 2, RT (min): 29.96, Score: 143.12
Intensity
100%
80%
60%
40%
20%
0%
0
200
400
600
800
1000
m/z
y1
y2
b1
y3
b2
y4
b3
y5
y6
y7
y8
y8 y7 y6 y5 y4 y3 y2 y1
K (d3) Q L A T K (fo) A A R
b1 b2 b3

Raw file: HF240124_RN_spleen_D3PIC_2, Scan: 17390, m/z: 605.36468, Charge: 2, RT (min): 32.77, Score: 145.34

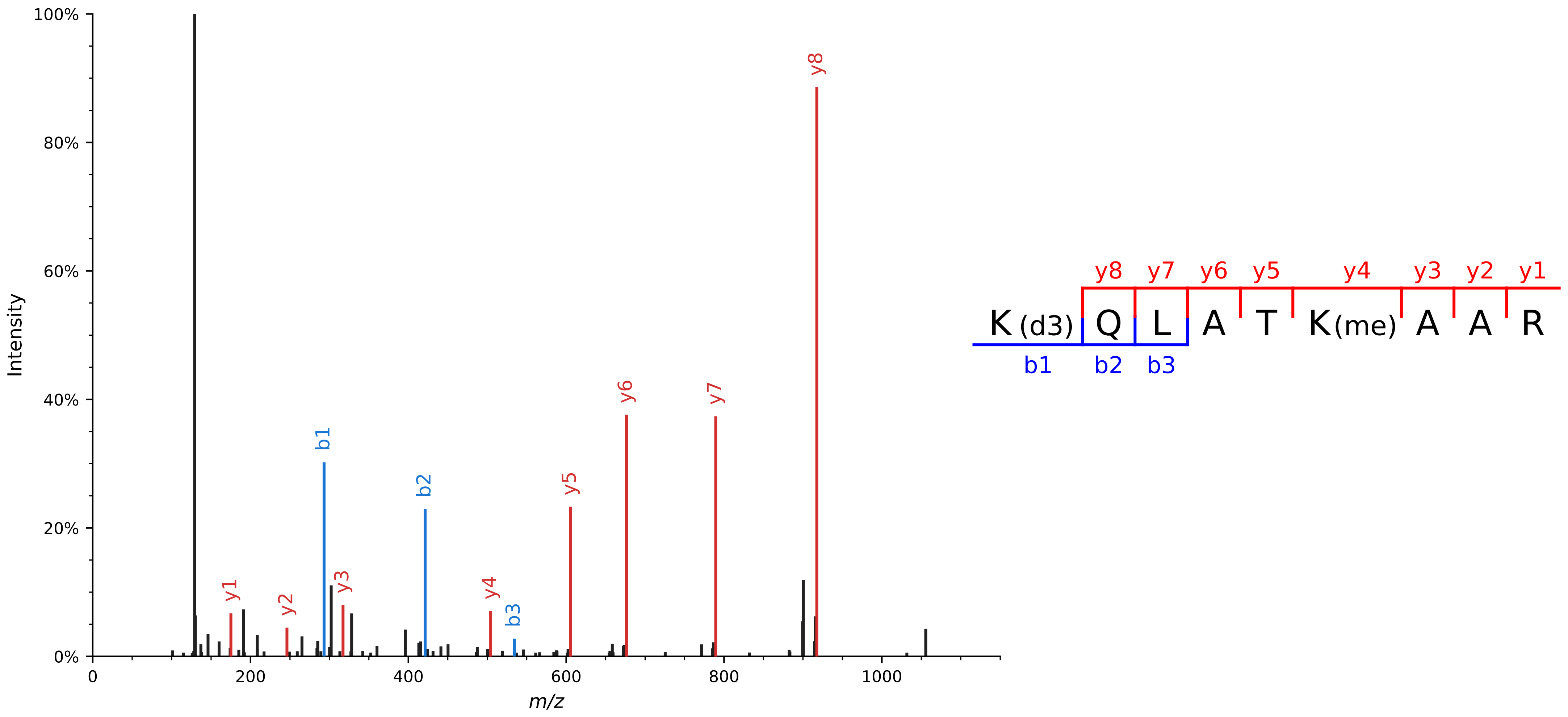

Raw file: HF240124_RN_spleen_D3PIC_3, Scan: 15756, m/z: 625.85018, Charge: 2, RT (min): 30.19, Score: 41.631

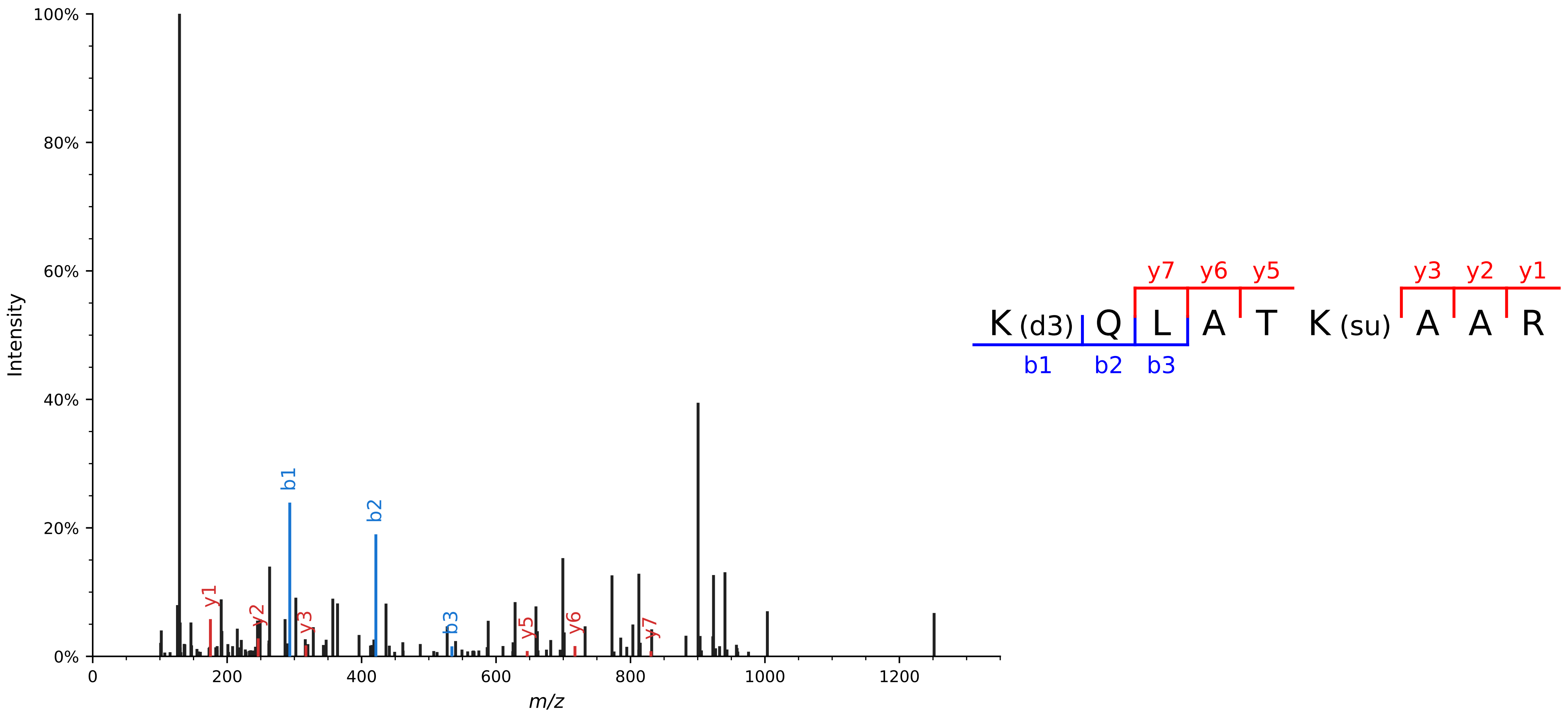

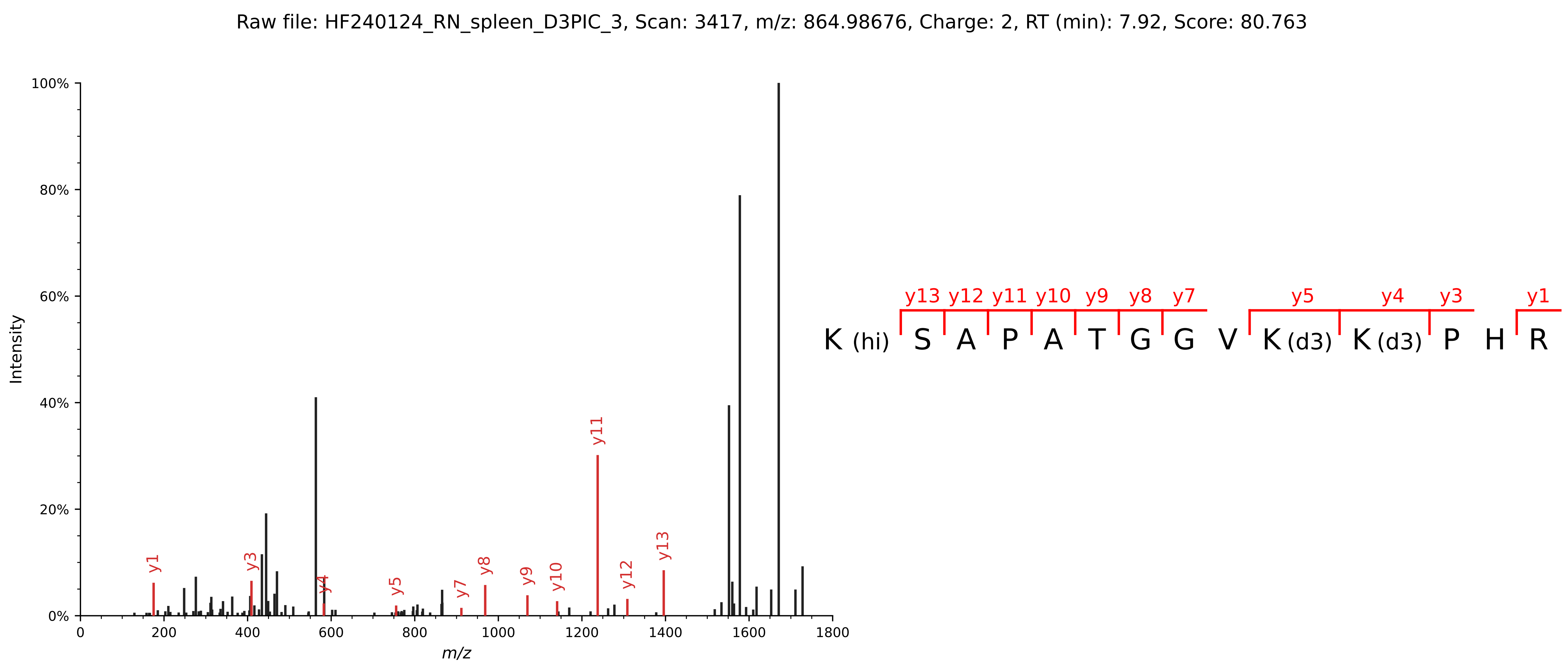
Raw file: HF240124_RN_spleen_D3PIC_3, Scan: 3417, m/z: 864.98676, Charge: 2, RT (min): 7.92, Score: 80.763
Intensity
m/z
y1
y3
y4
y5
y7
y8
y9
y10
y11
y12
y13
K (hi) S A P A T G G V K (d3) K (d3) P H R
y13 y12 y11 y10 y9 y8 y7 y5 y4 y3 y1

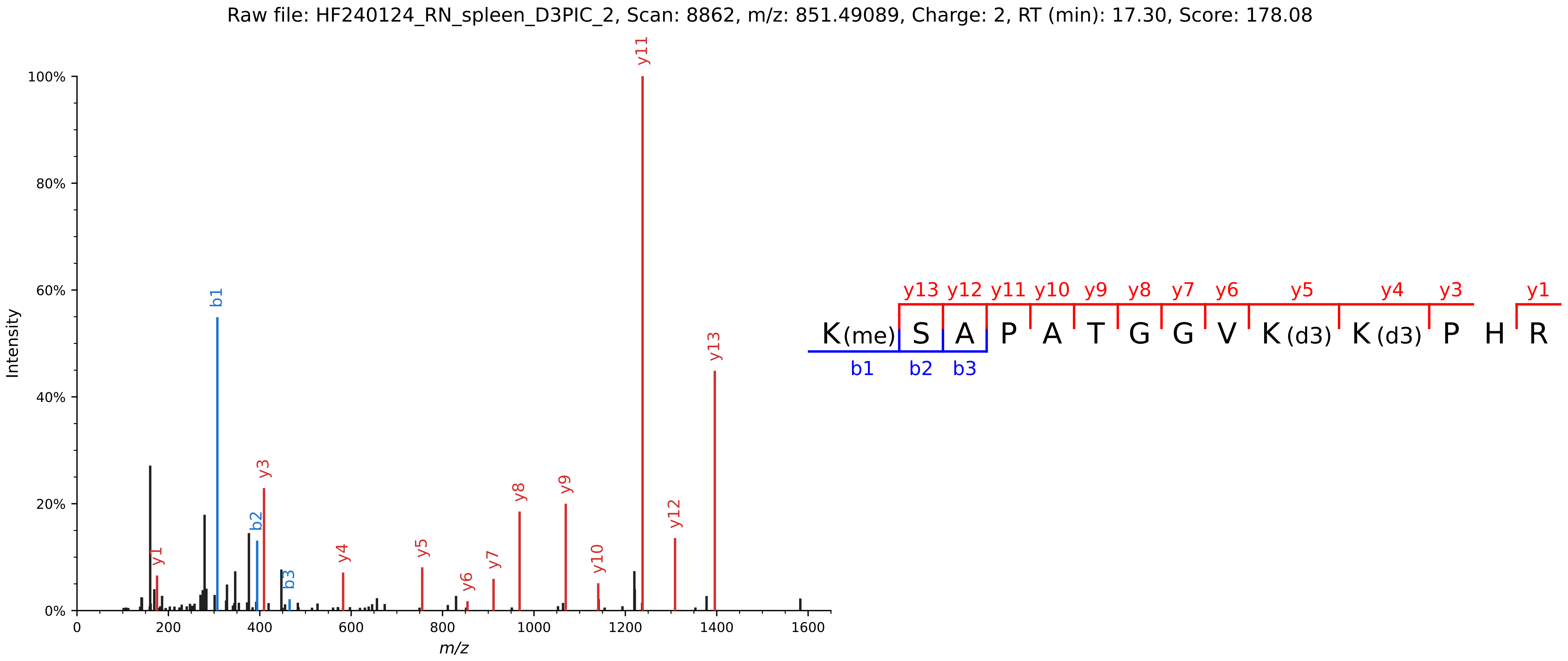

Raw file: HF240124_RN_spleen_D3PIC_2, Scan: 8862, m/z: 851.49089, Charge: 2, RT (min): 17.30, Score: 178.08
Intensity
m/z
0%
20%
40%
60%
80%
100%
0
200
400
600
800
1000
1200
1400
1600
y1
b1
b2
y3
b3
y4
y5
y6
y7
y8
y9
y10
y11
y12
y13
K(me) S A P A T G G V K(d3) K(d3) P H R
y13 y12 y11 y10 y9 y8 y7 y6 y5 y4 y3 y1
b1 b2 b3

Raw file: HF240124_RN_spleen_D3PIC_1, Scan: 10519, m/z: 863.99713, Charge: 2, RT (min): 20.51, Score: 151.15

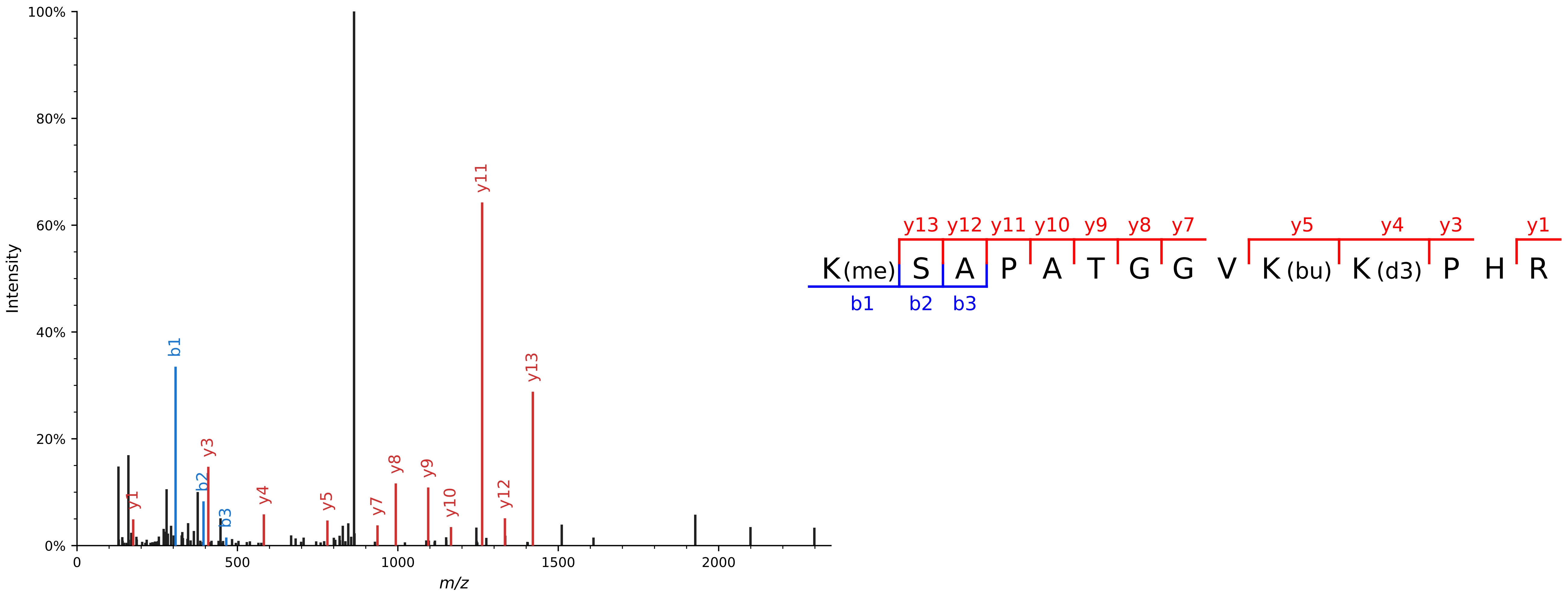

Raw file: HF240124_RN_spleen_D3PIC_2, Scan: 8673, m/z: 842.97365, Charge: 2, RT (min): 16.97, Score: 72.2

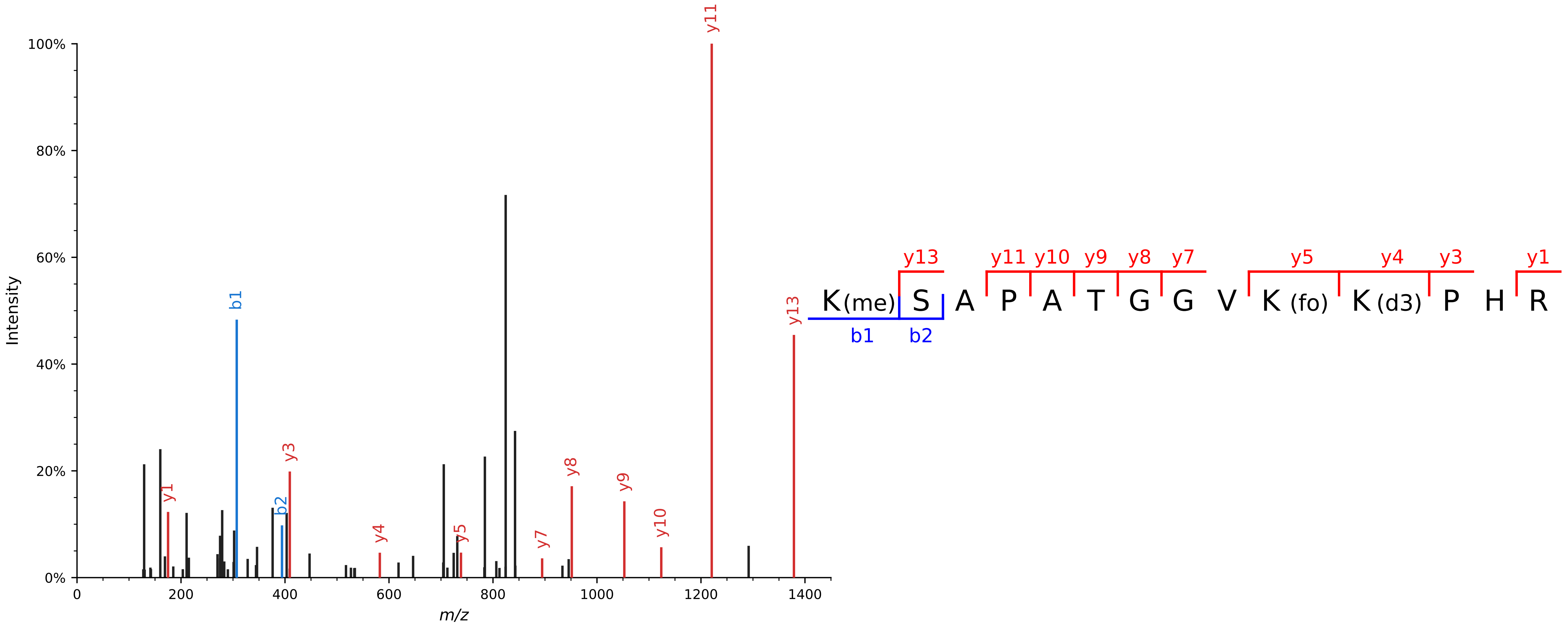

Raw file: HF240124_RN_spleen_D3PIC_1, Scan: 8796, m/z: 580.64708, Charge: 3, RT (min): 17.26, Score: 43.03

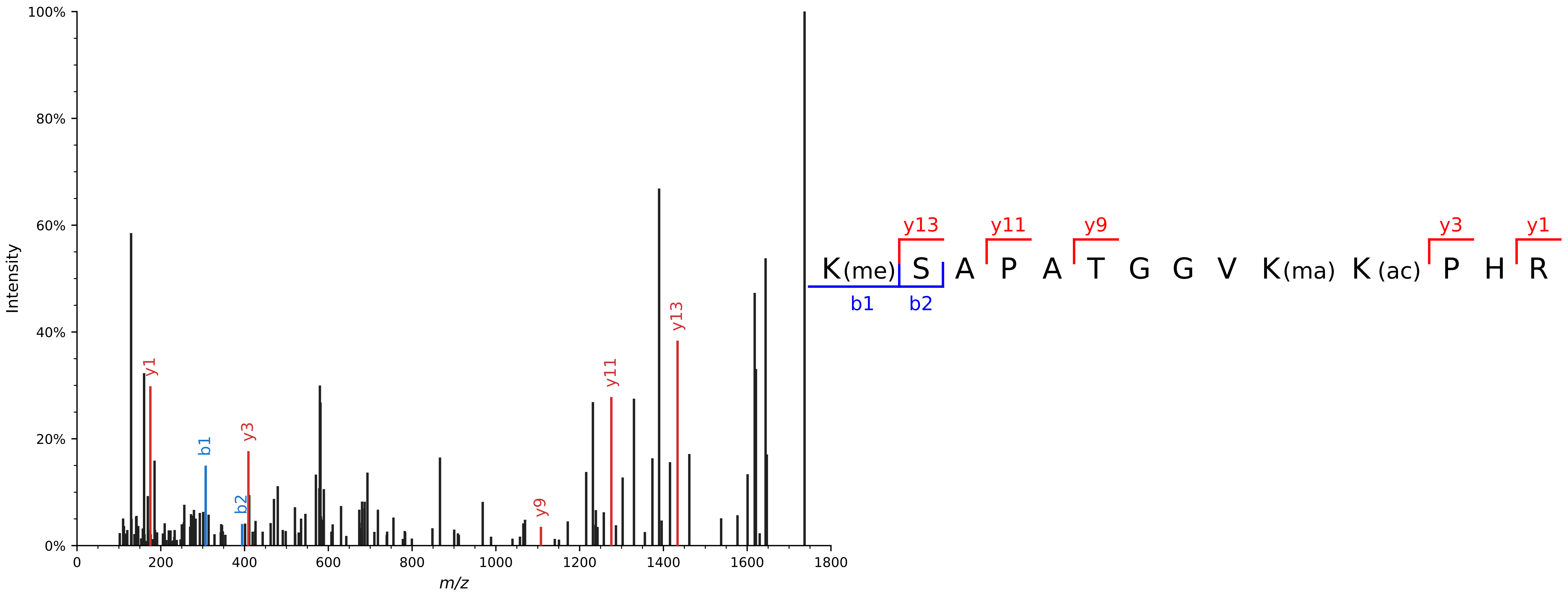

Raw file: HF240124_RN_spleen_D3PIC_3, Scan: 9240, m/z: 858.49872, Charge: 2, RT (min): 18.33, Score: 188.28

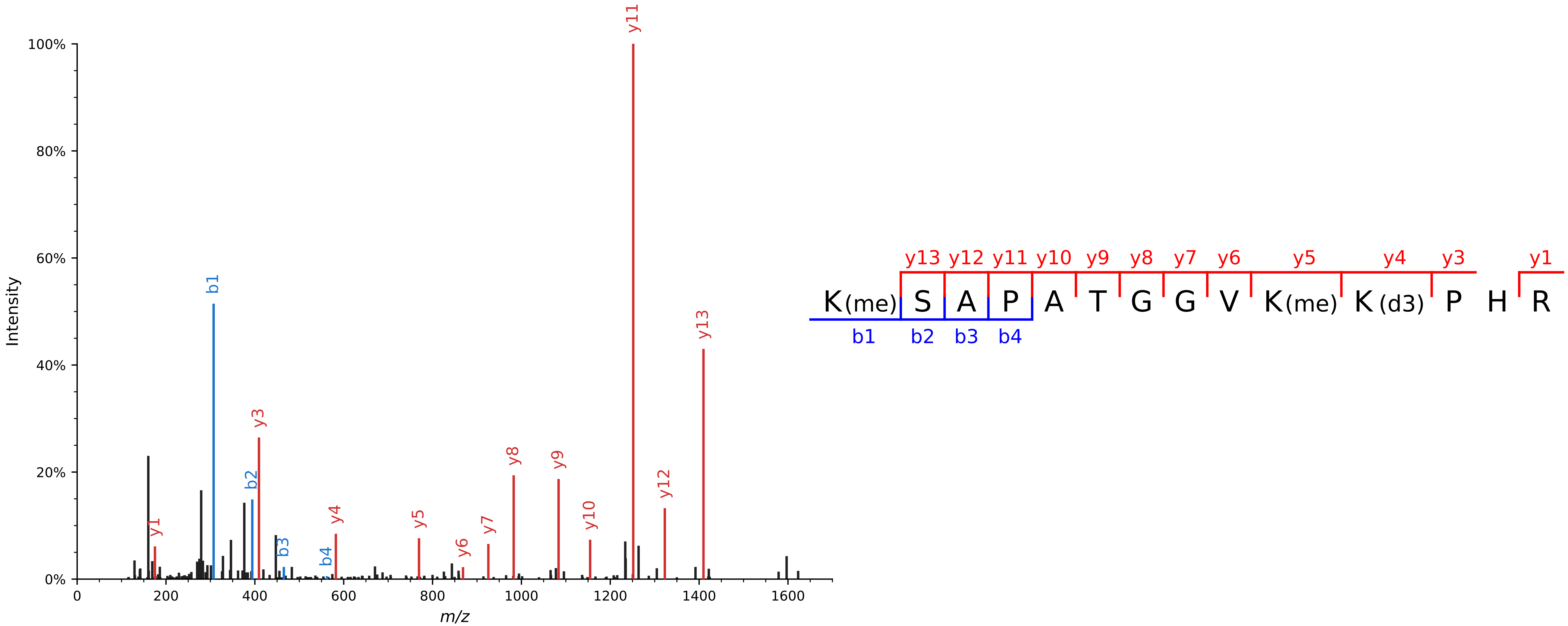

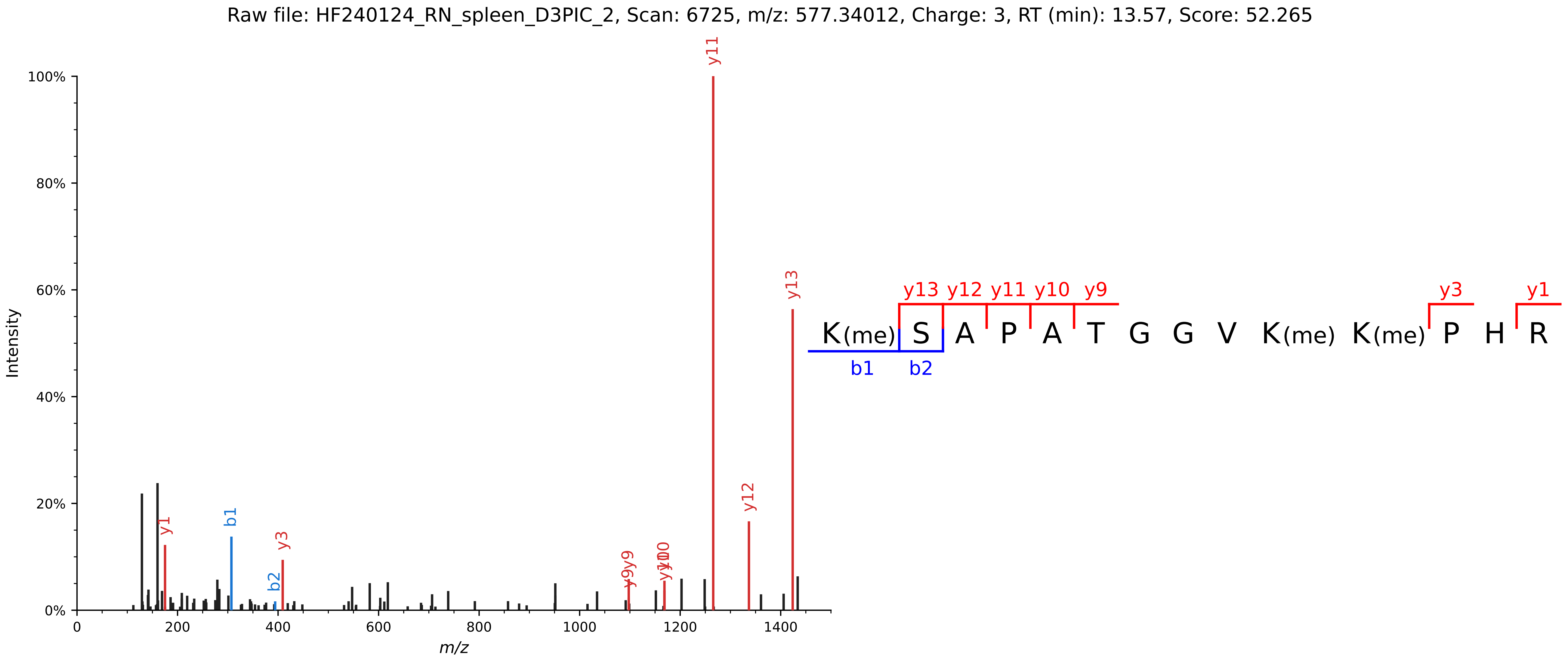

Raw file: HF240124_RN_spleen_D3PIC_2, Scan: 6725, m/z: 577.34012, Charge: 3, RT (min): 13.57, Score: 52.265
Intensity
m/z
y1
b1
b2
y3
y9
y10
y11
y12
y13
K(me) S A P A T G G V K(me) K(me) P H R

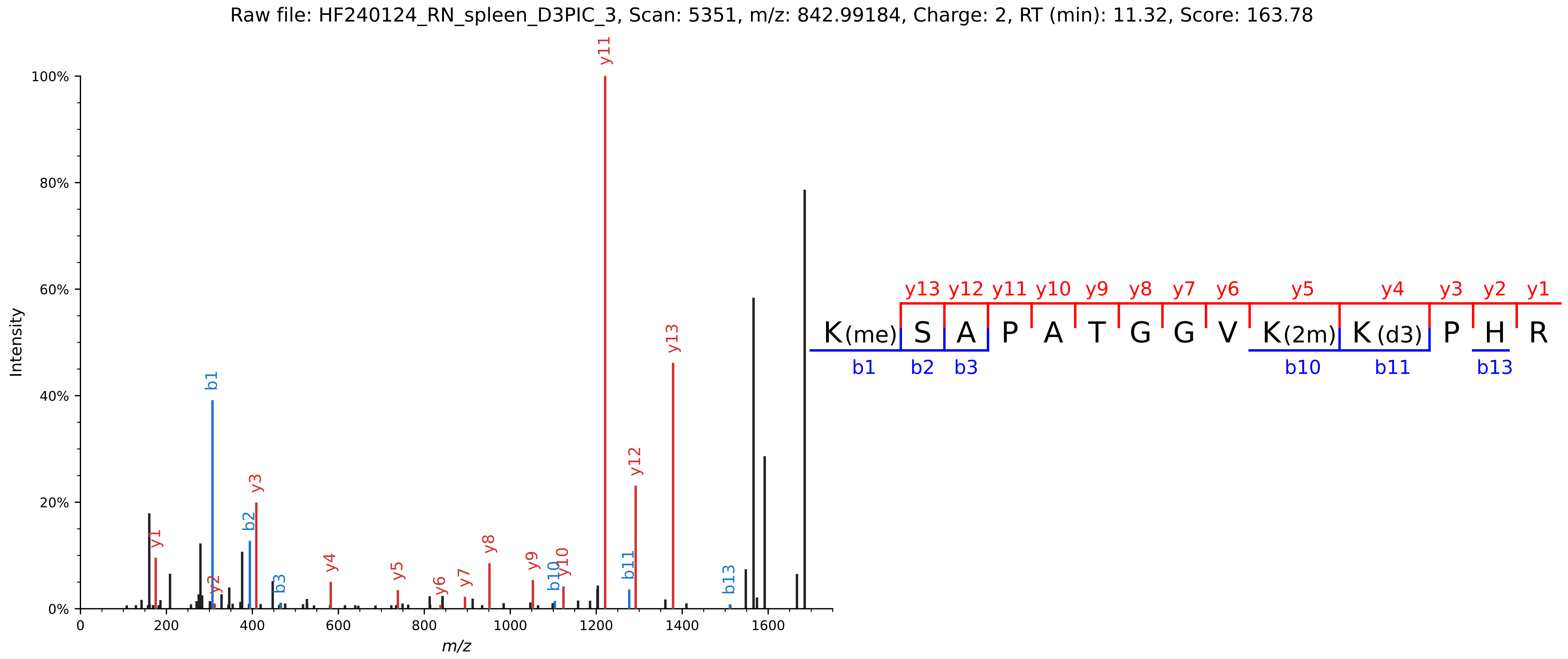

Raw file: HF240124_RN_spleen_D3PIC_3, Scan: 5351, m/z: 842.99184, Charge: 2, RT (min): 11.32, Score: 163.78
Intensity
m/z
K(me) S A P A T G G V K(2m) K(d3) P H R
y13 y12 y11 y10 y9 y8 y7 y6 y5 y4 y3 y2 y1
b1 b2 b3 b10 b11 b13

Raw file: HF240124_RN_spleen_D3PIC_2, Scan: 5451, m/z: 567.0022, Charge: 3, RT (min): 11.29, Score: 88.552

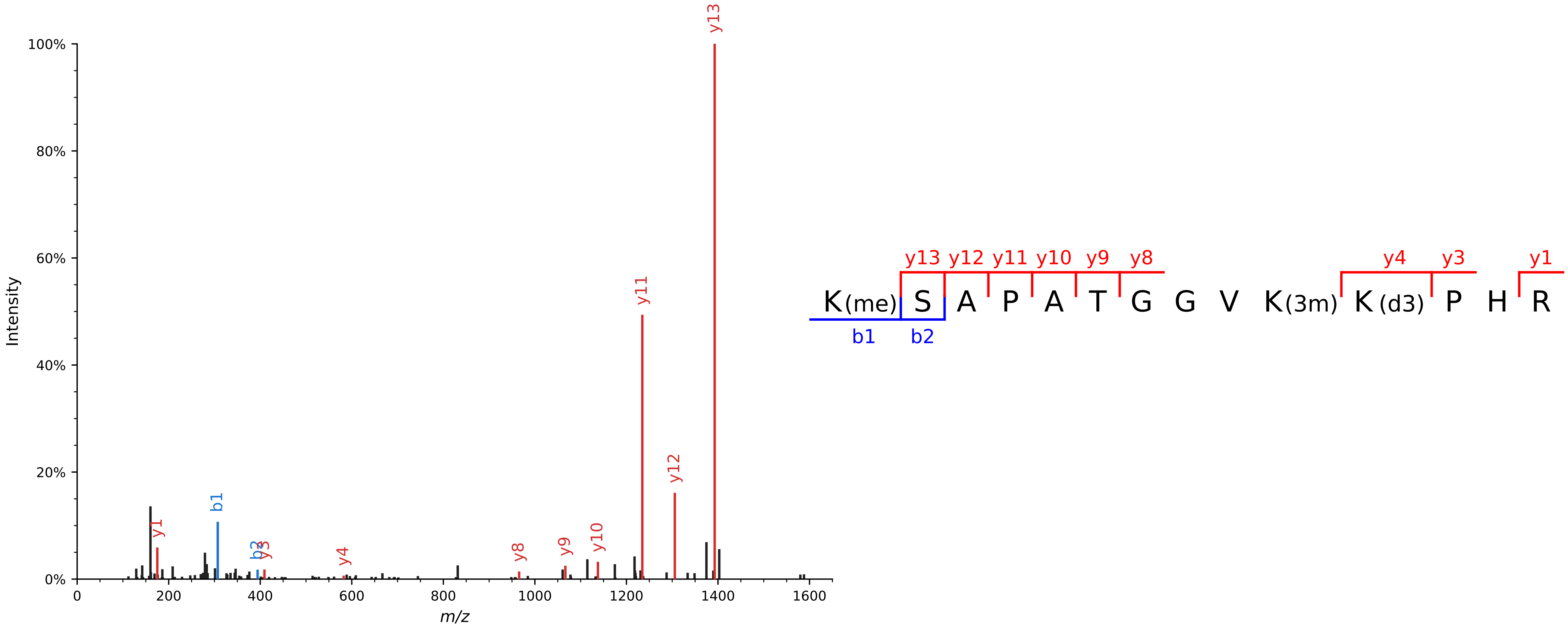

Raw file: HF240124_RN_spleen_D3PIC_1, Scan: 4028, m/z: 835.98402, Charge: 2, RT (min): 8.86, Score: 178.49

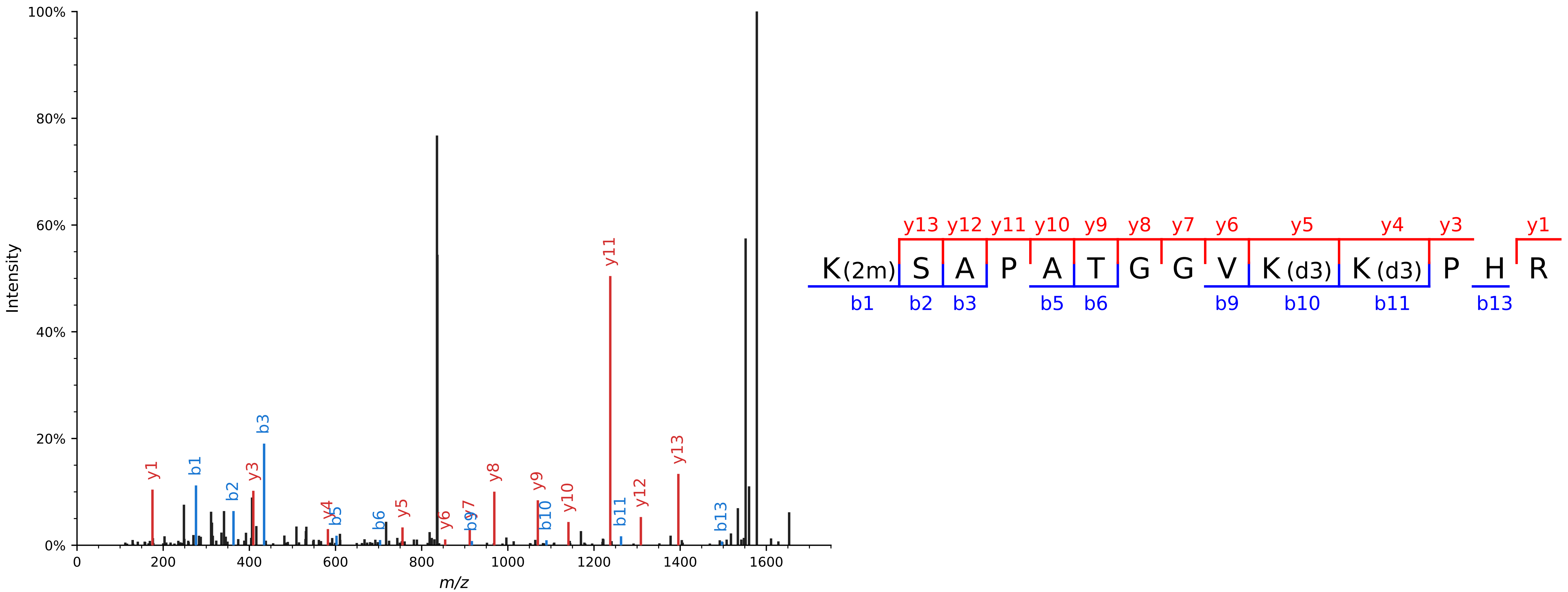

Raw file: HF240124_RN_spleen_D3PIC_2, Scan: 2966, m/z: 556.65216, Charge: 3, RT (min): 6.98, Score: 52.599

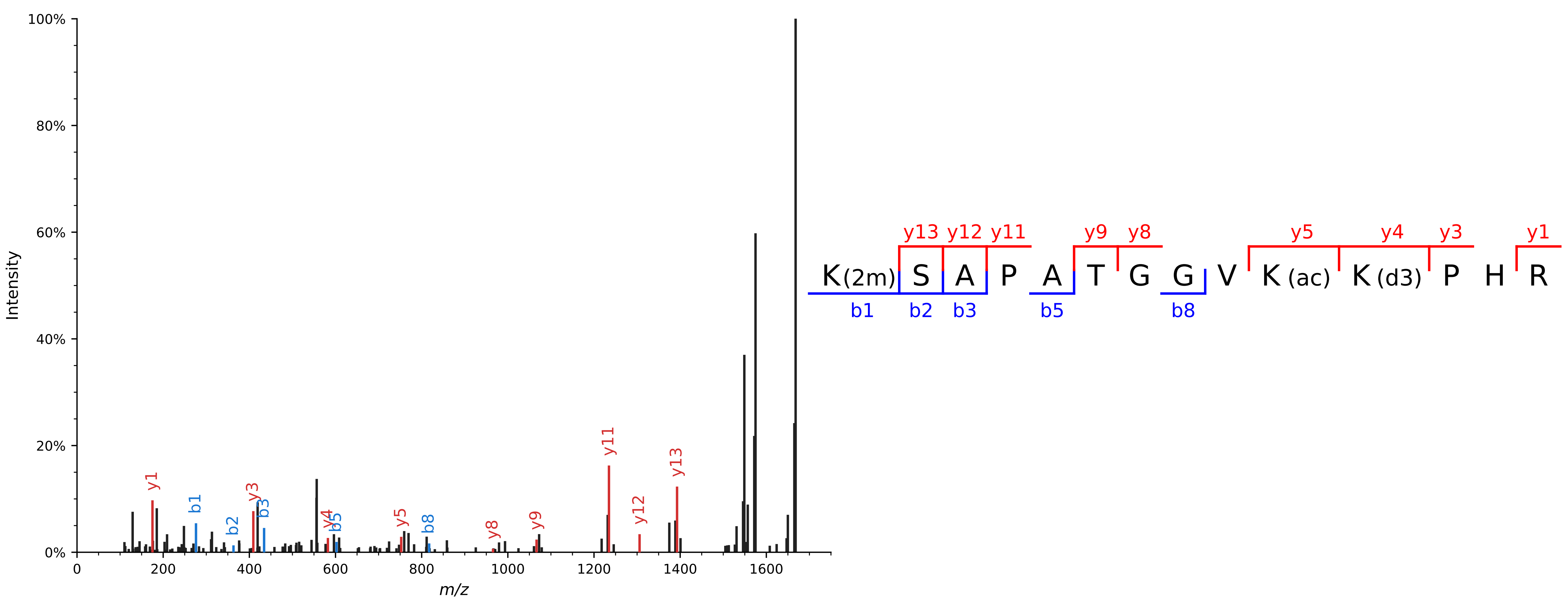

Raw file: HF240124_RN_spleen_D3PIC_2, Scan: 5194, m/z: 848.49025, Charge: 2, RT (min): 10.86, Score: 75.695

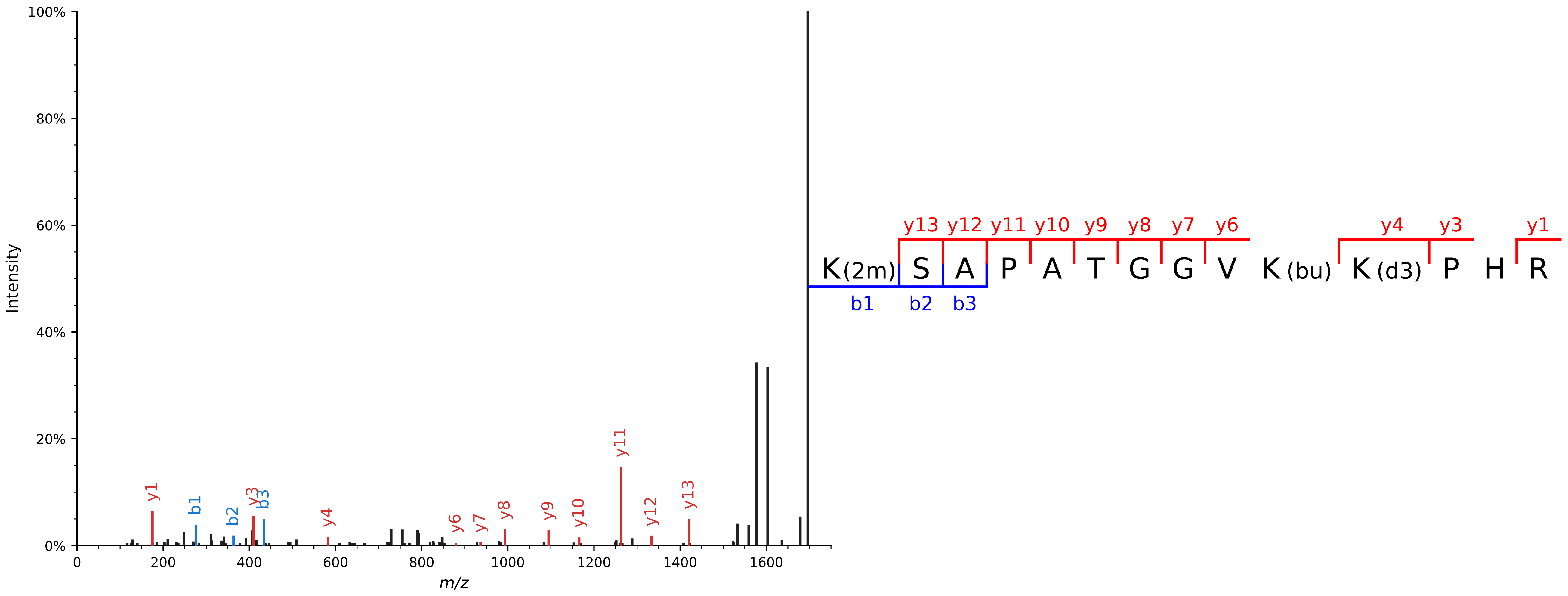

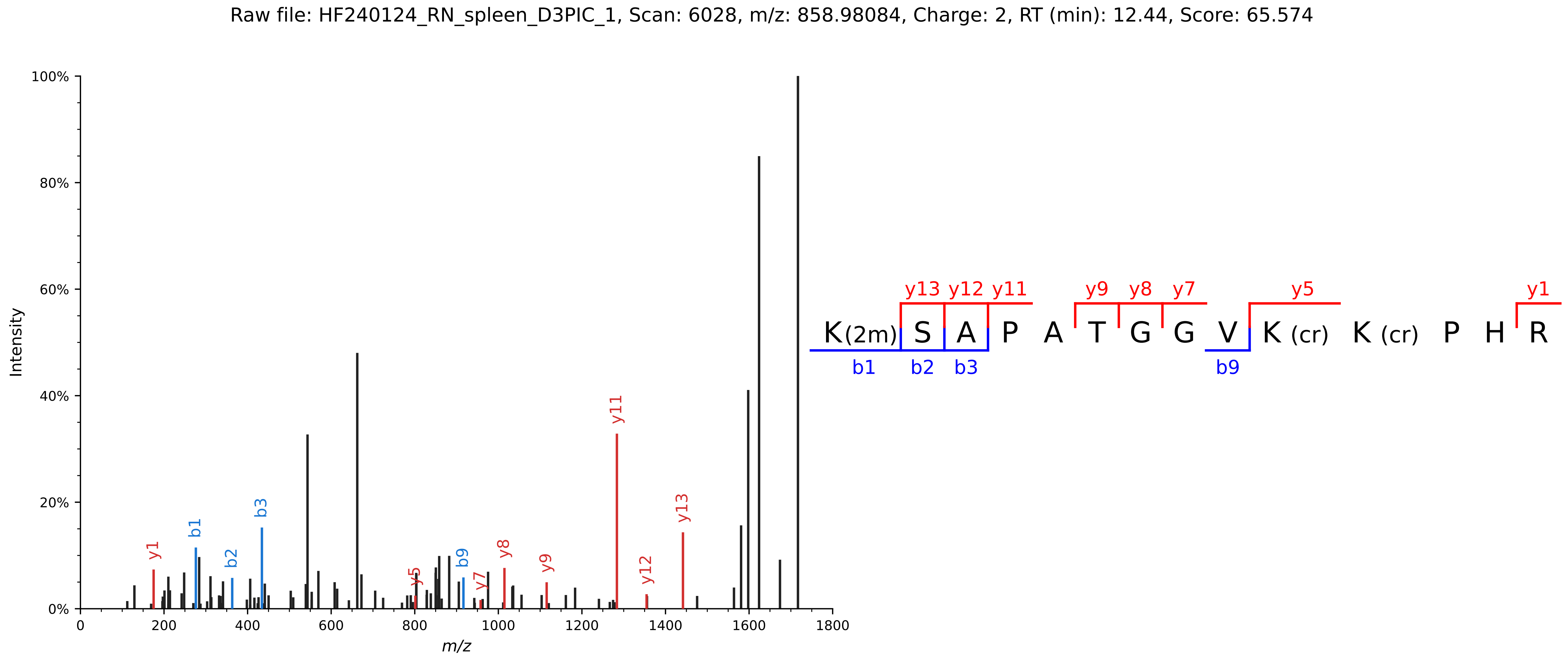

Raw file: HF240124_RN_spleen_D3PIC_1, Scan: 6028, m/z: 858.98084, Charge: 2, RT (min): 12.44, Score: 65.574
100%
80%
60%
40%
20%
0%
Intensity
0
200
400
600
800
1000
1200
1400
1600
1800
m/z
y1
b1
b2
b3
y5
b9
y7
y8
y9
y11
y12
y13
y13 y12 y11
y9 y8 y7
y5
y1
K(2m) S A P A T G G V K (cr) K (cr) P H R
b1 b2 b3
b9

Raw file: HF240124_RN_spleen_D3PIC_1, Scan: 3543, m/z: 551.98028, Charge: 3, RT (min): 8.00, Score: 86.413

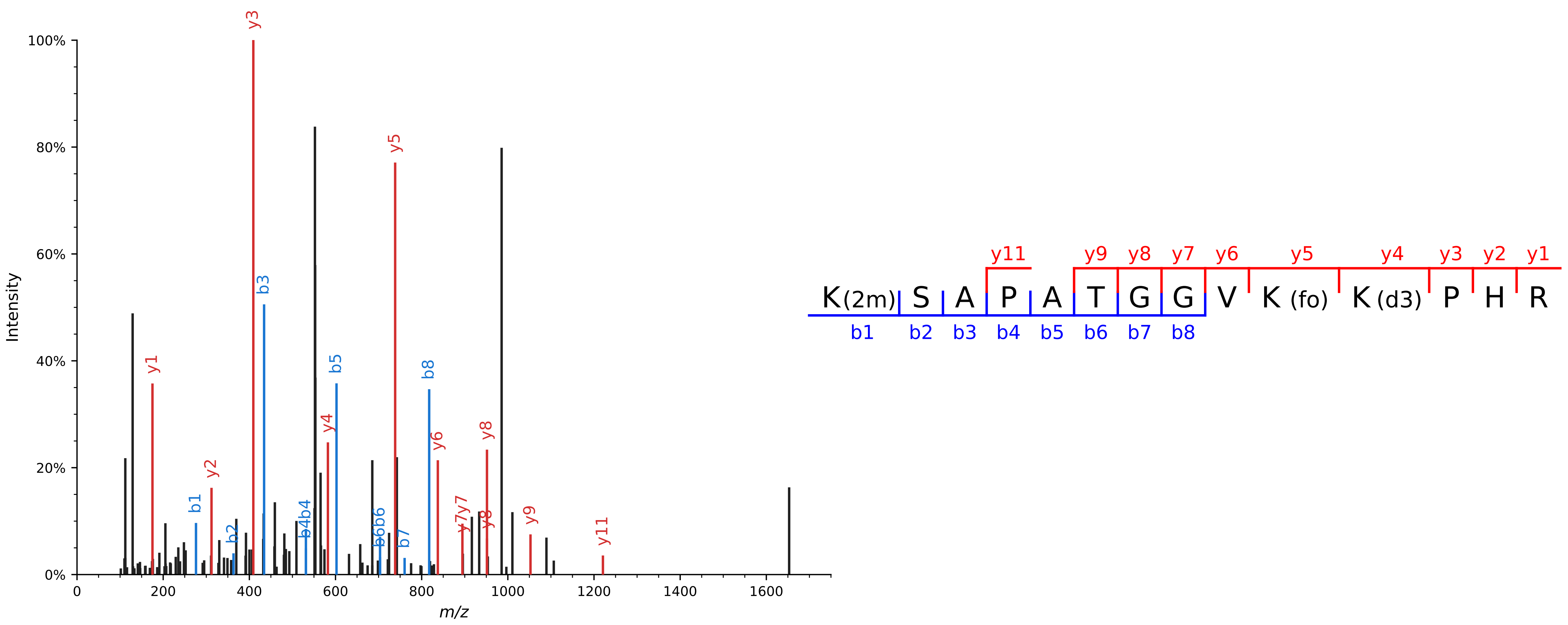

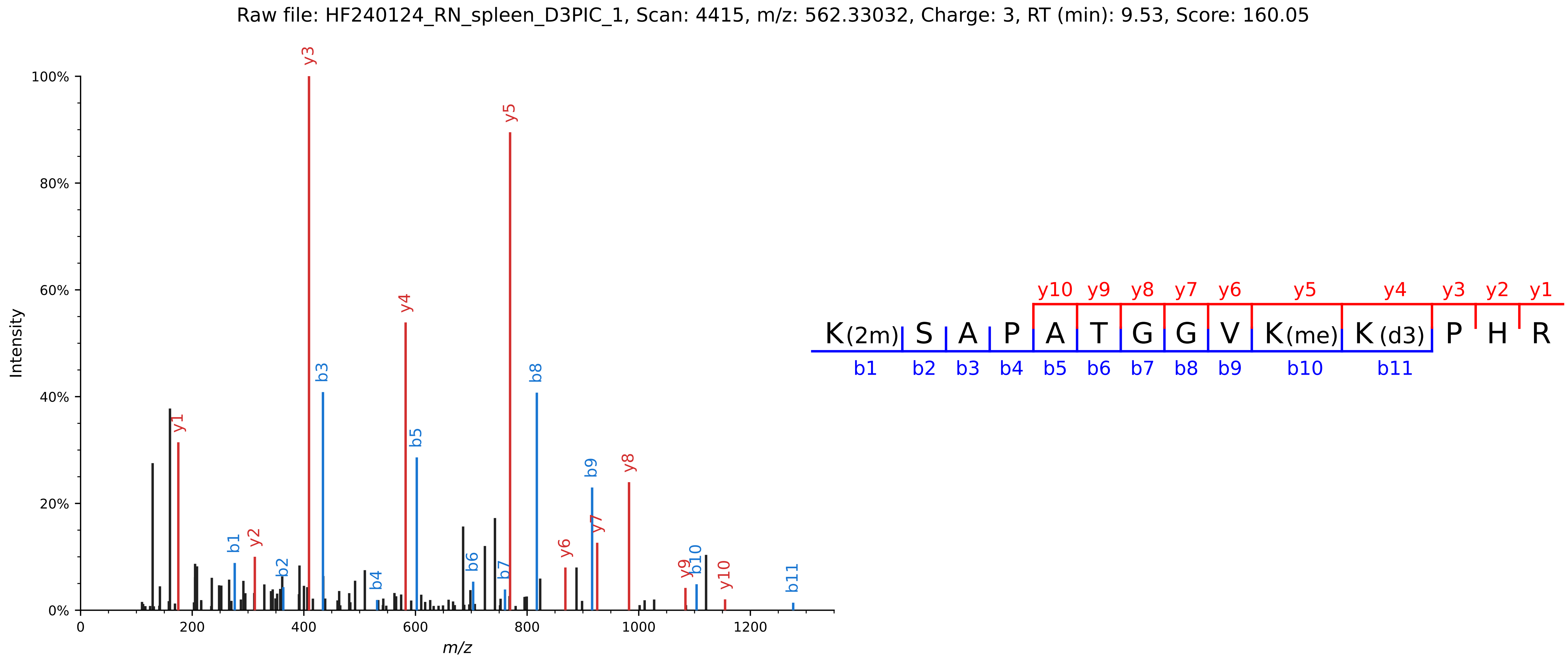
Raw file: HF240124_RN_spleen_D3PIC_1, Scan: 4415, m/z: 562.33032, Charge: 3, RT (min): 9.53, Score: 160.05
Intensity
m/z
y1 y2 y3 y4 y5 y6 y7 y8 y9 y10
b1 b2 b3 b4 b5 b6 b7 b8 b9 b10 b11
K(2m) S A P A T G G V K(me) K(d3) P H R

Raw file: HF240124_RN_spleen_D3PIC_1, Scan: 2521, m/z: 849.99967, Charge: 2, RT (min): 6.22, Score: 107.11

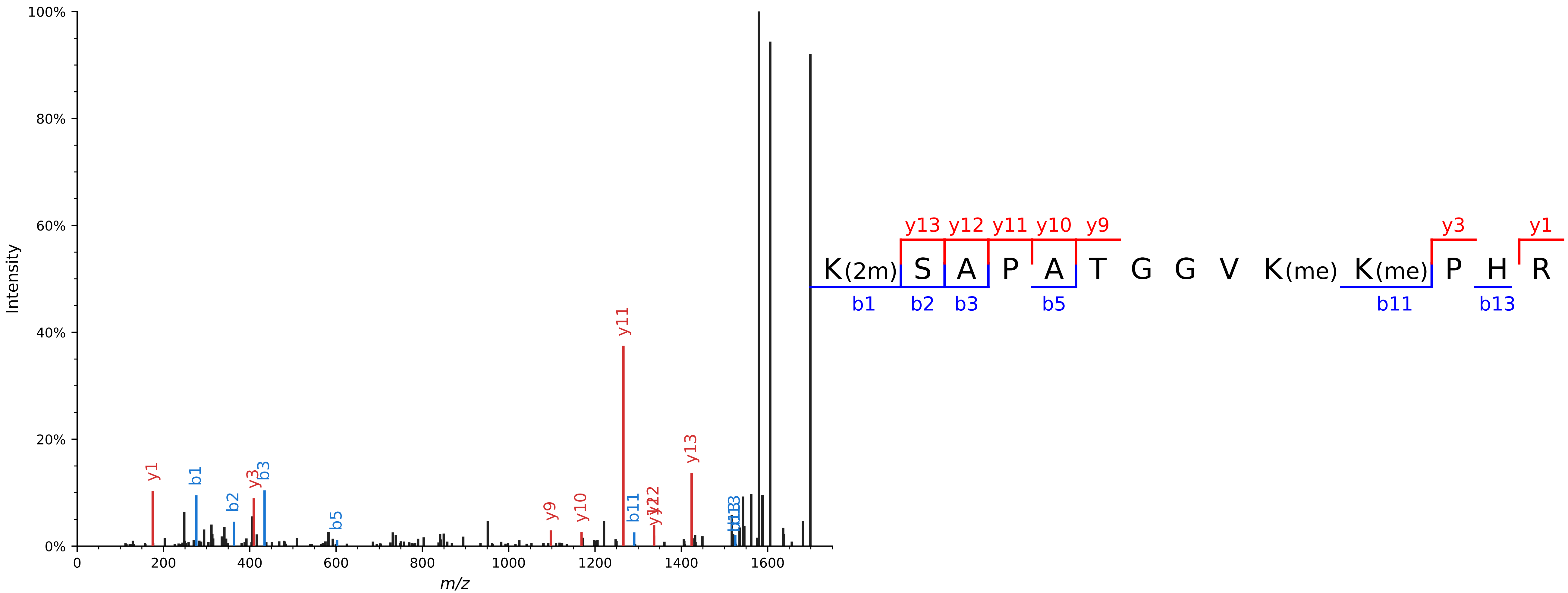

Raw file: HF240124_RN_spleen_D3PIC_3, Scan: 1489, m/z: 827.48497, Charge: 2, RT (min): 4.52, Score: 177.64

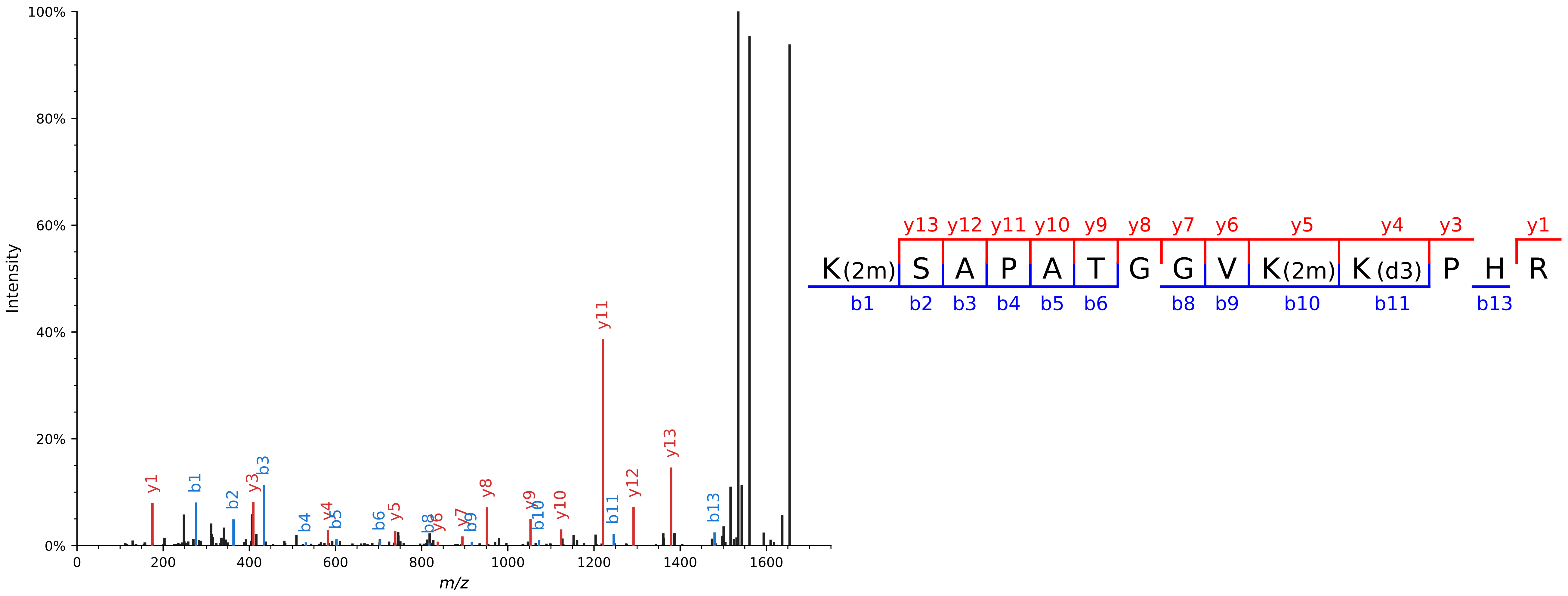

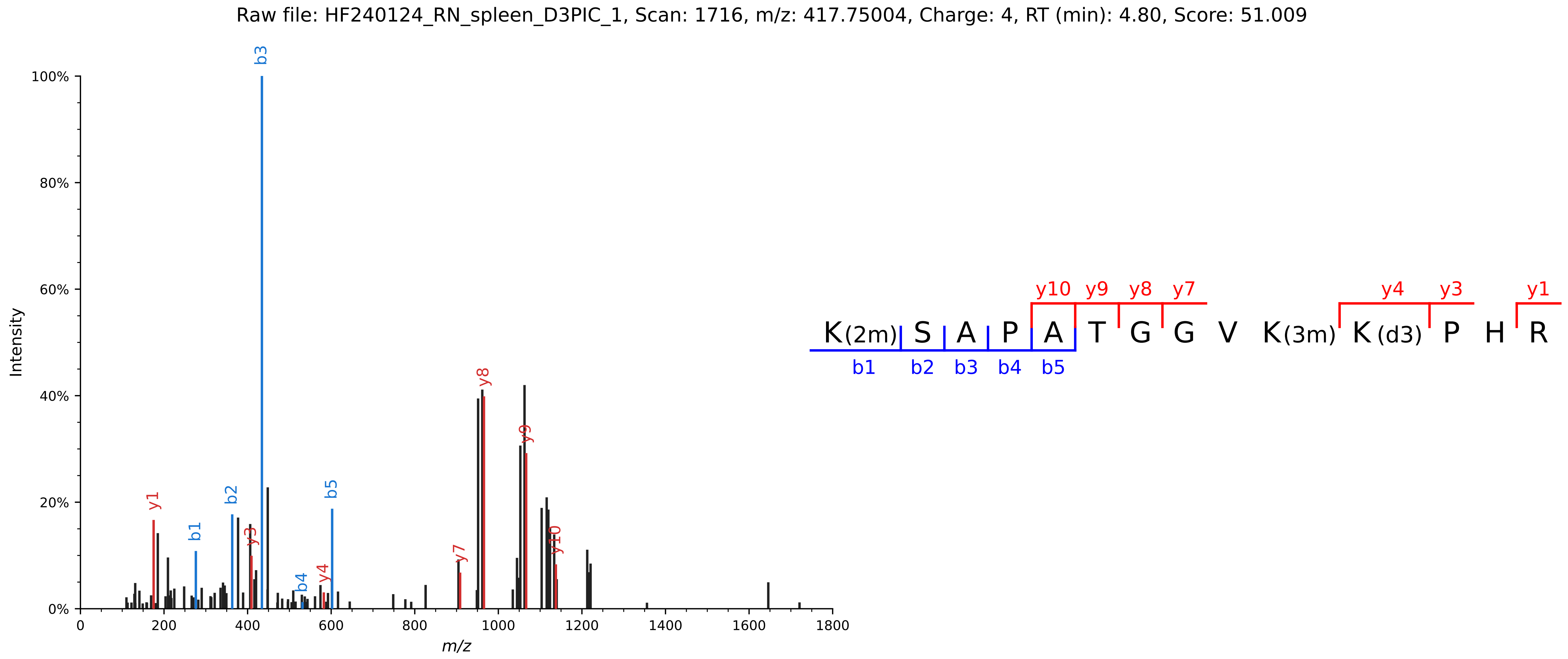
Raw file: HF240124_RN_spleen_D3PIC_1, Scan: 1716, m/z: 417.75004, Charge: 4, RT (min): 4.80, Score: 51.009
Intensity
m/z
b3
y8
y9
y1
b1
b2
y3
b4
y4
b5
y7
y10
K(2m) S A P A T G G V K(3m) K(d3) P H R
y10 y9 y8 y7 y4 y3 y1
b1 b2 b3 b4 b5

Raw file: HF240124_RN_spleen_D3PIC_1, Scan: 5096, m/z: 564.98965, Charge: 3, RT (min): 10.73, Score: 63.682

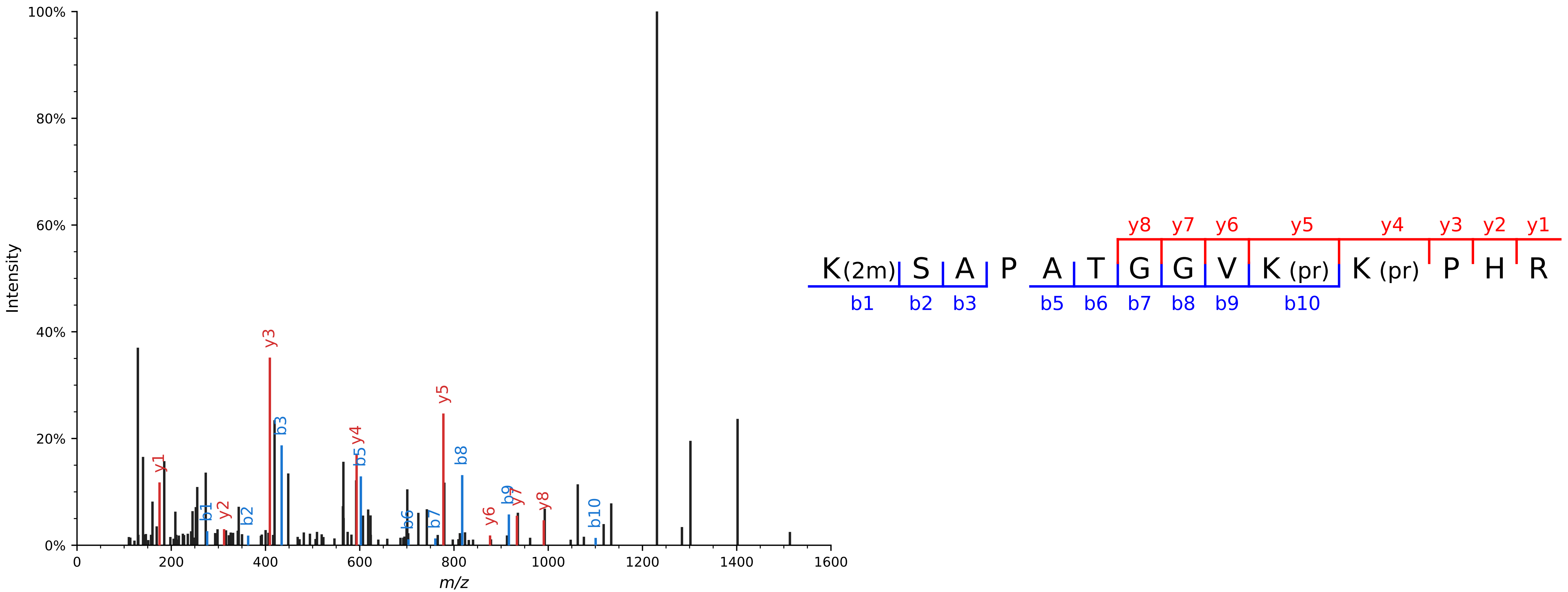

Raw file: HF240124_RN_spleen_D3PIC_1, Scan: 3608, m/z: 842.99184, Charge: 2, RT (min): 8.11, Score: 138.45

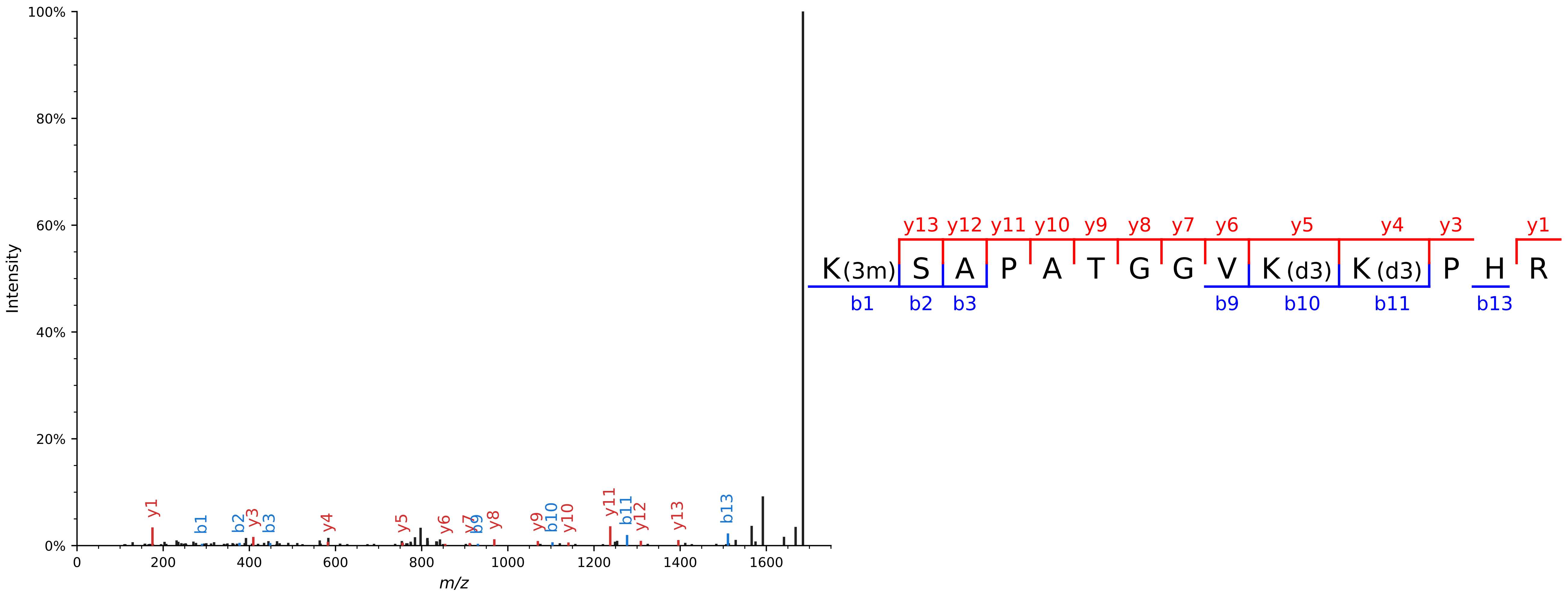

Raw file: HF240124_RN_spleen_D3PIC_3, Scan: 3288, m/z: 556.65216, Charge: 3, RT (min): 7.70, Score: 52.5

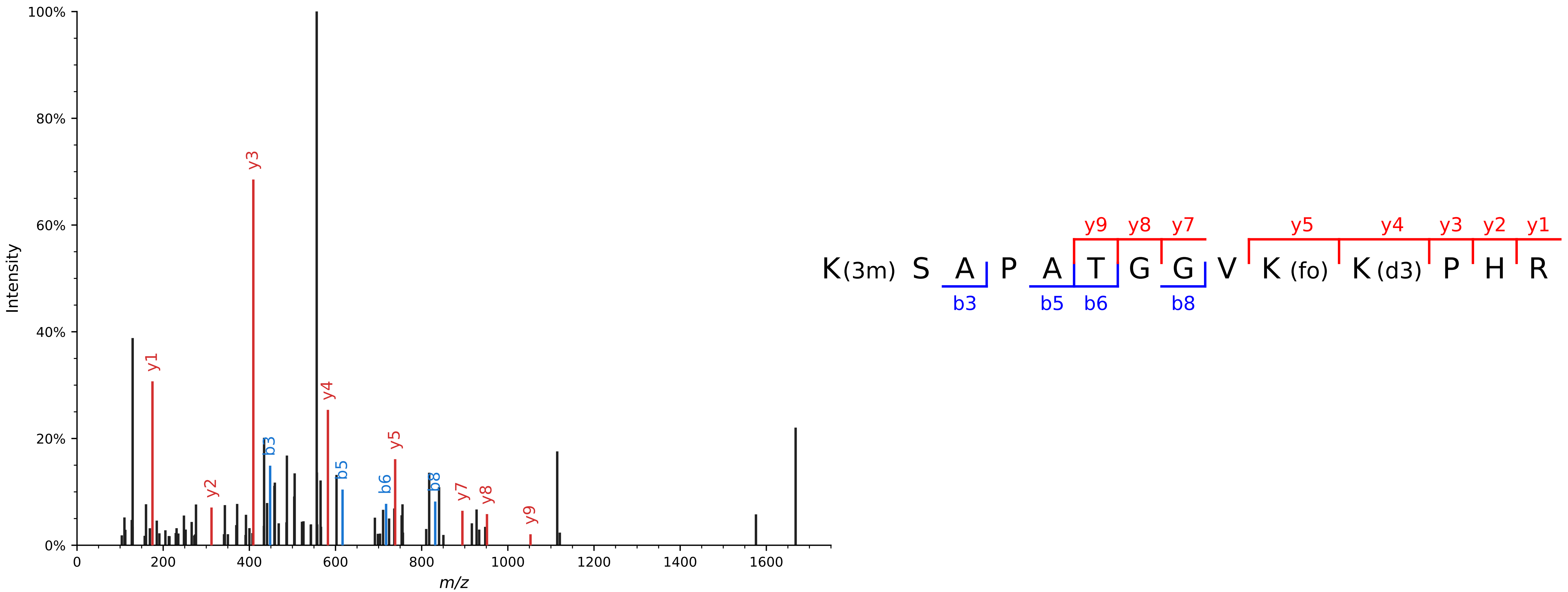

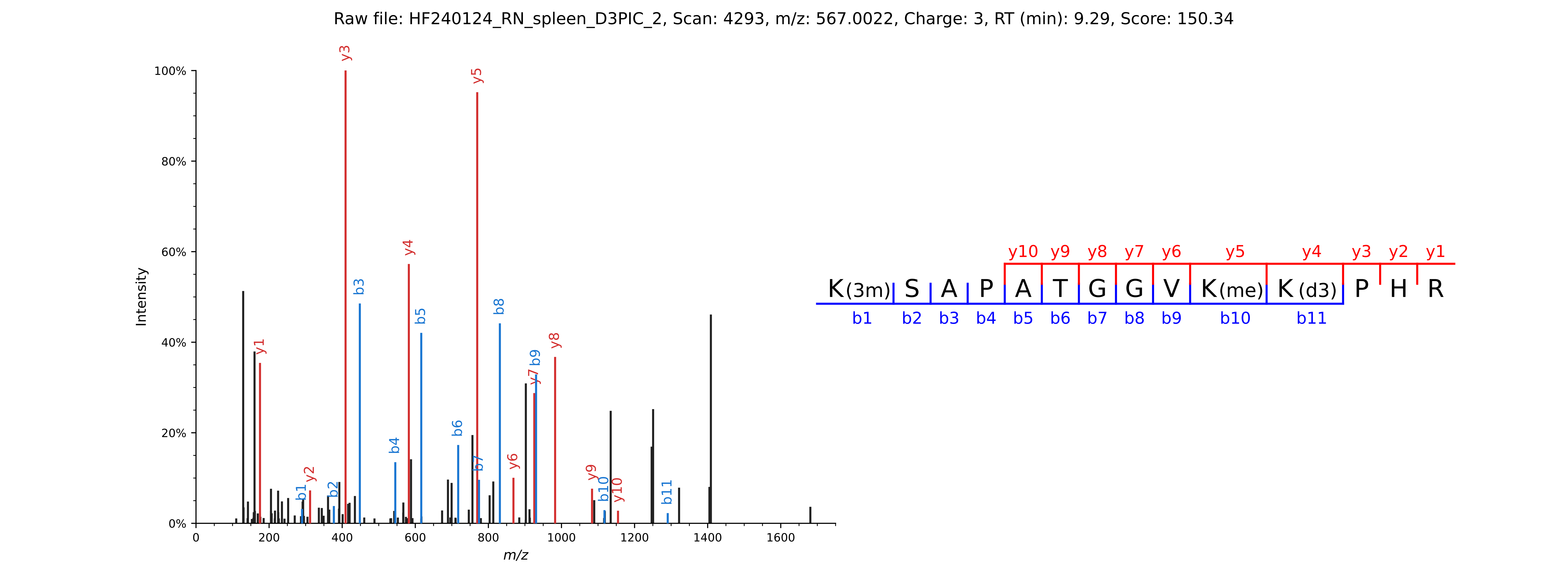

Raw file: HF240124_RN_spleen_D3PIC_2, Scan: 4293, m/z: 567.0022, Charge: 3, RT (min): 9.29, Score: 150.34
Intensity
m/z
y1 y2 y3 y4 y5 y6 y7 y8 y9 y10
b1 b2 b3 b4 b5 b6 b7 b8 b9 b10 b11
K(3m) S A P A T G G V K(me) K(d3) P H R

Raw file: HF240124_RN_spleen_D3PIC_2, Scan: 1570, m/z: 834.4928, Charge: 2, RT (min): 4.54, Score: 117.86

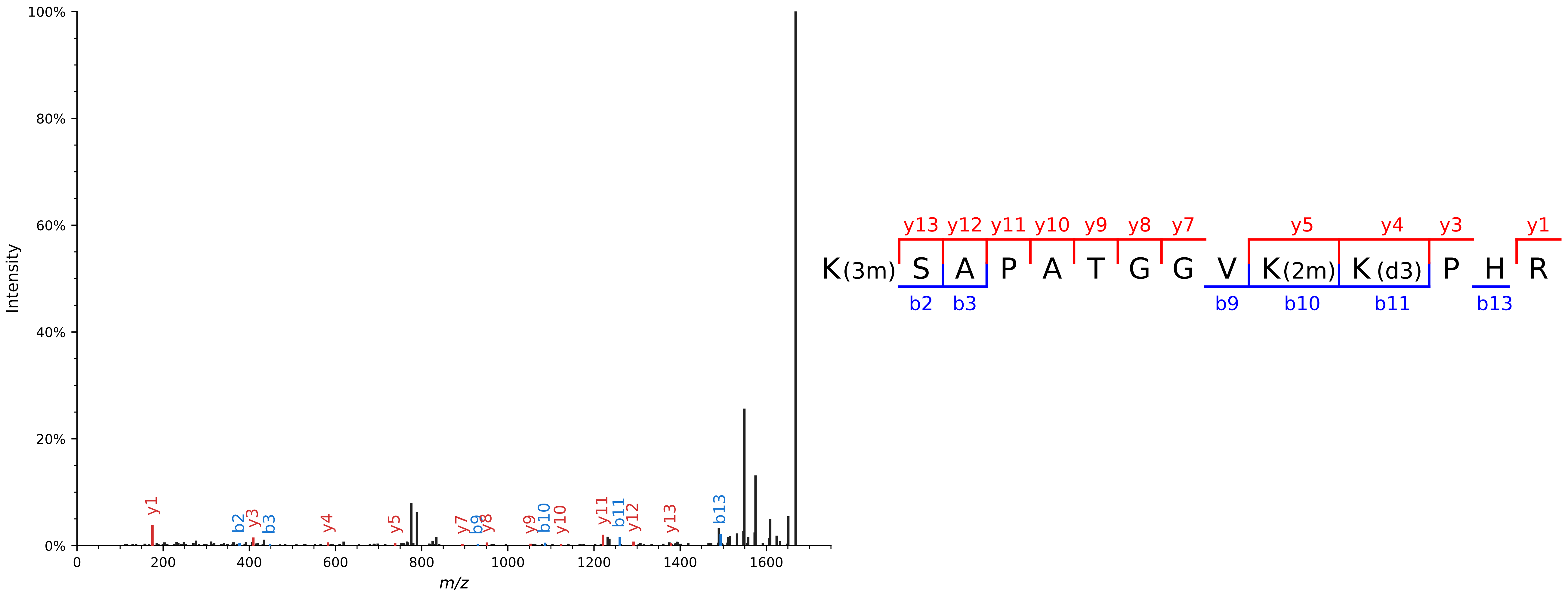

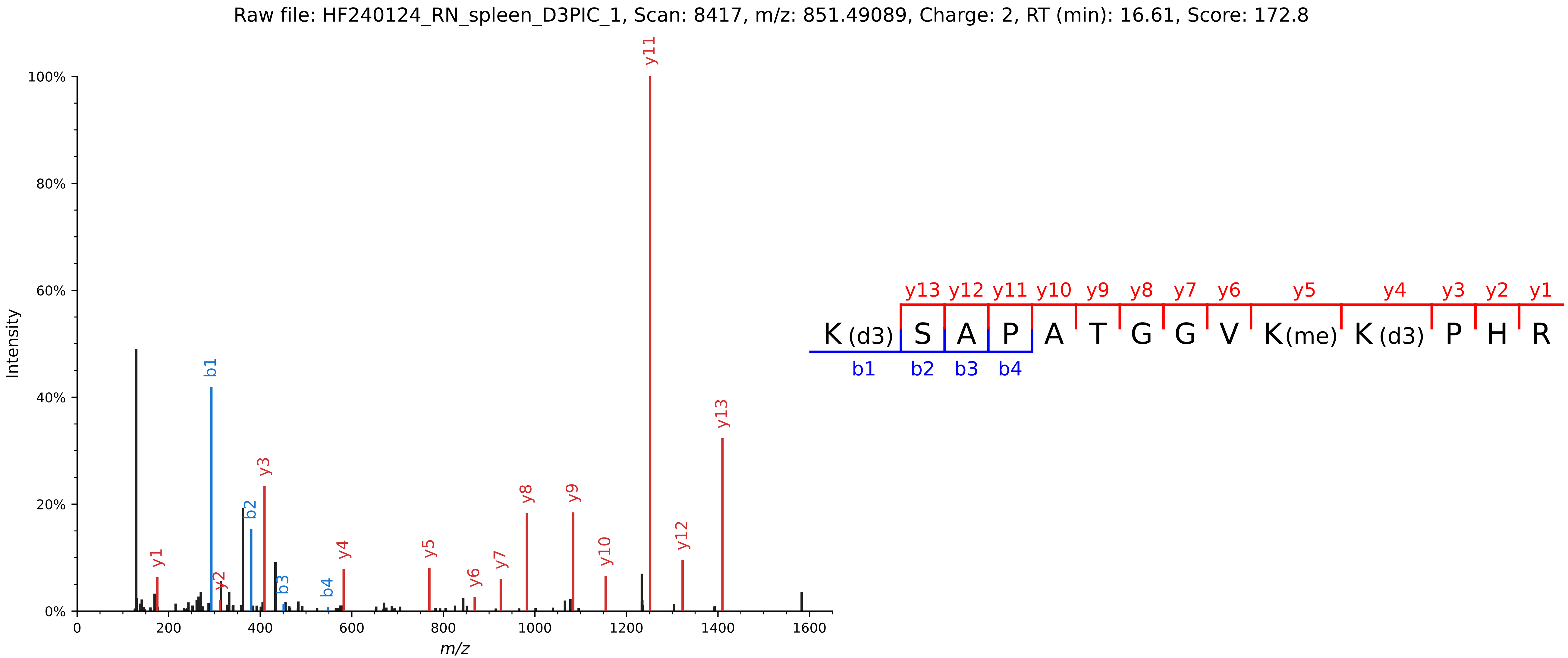

Raw file: HF240124_RN_spleen_D3PIC_1, Scan: 8417, m/z: 851.49089, Charge: 2, RT (min): 16.61, Score: 172.8
Intensity
100%
80%
60%
40%
20%
0%
0
200
400
600
800
1000
1200
1400
1600
m/z
y1 y2 y3 y4 y5 y6 y7 y8 y9 y10 y11 y12 y13
b1 b2 b3 b4
K(d3) S A P A T G G V K(me) K(d3) P H R

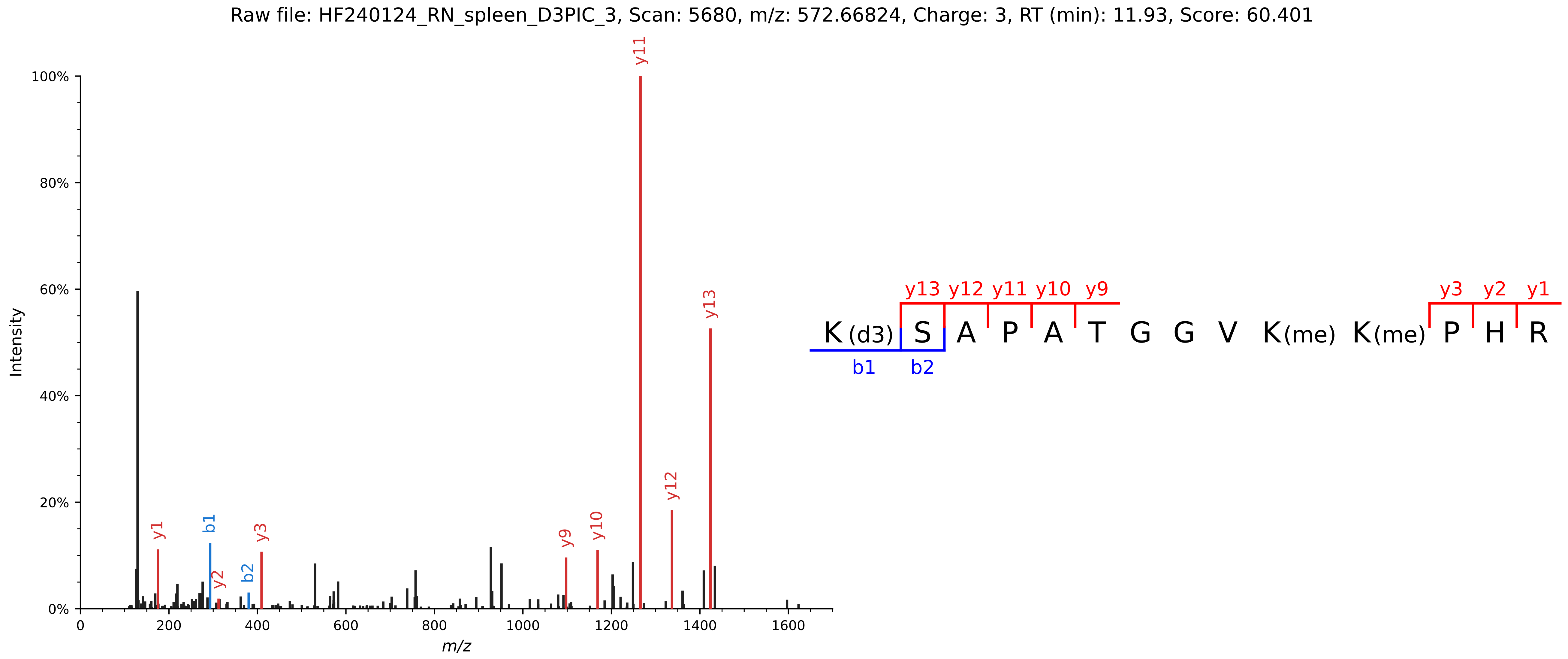

Raw file: HF240124_RN_spleen_D3PIC_3, Scan: 5680, m/z: 572.66824, Charge: 3, RT (min): 11.93, Score: 60.401
Intensity
m/z
y1
b1
y2
b2
y3
y9
y10
y11
y12
y13
K(d3) S A P A T G G V K(me) K(me) P H R
y13 y12 y11 y10 y9
y3 y2 y1
b1 b2

Raw file: HF240124_RN_spleen_D3PIC_1, Scan: 4578, m/z: 835.98402, Charge: 2, RT (min): 9.81, Score: 179.19

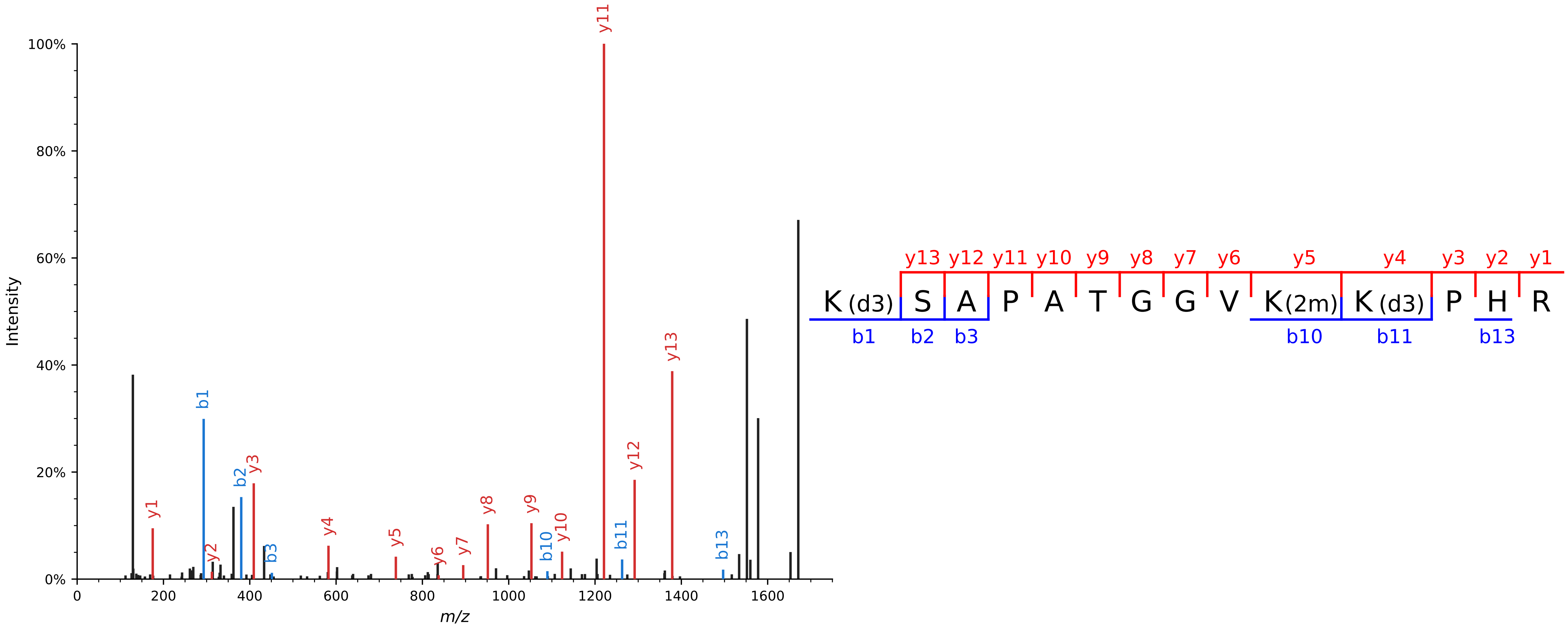

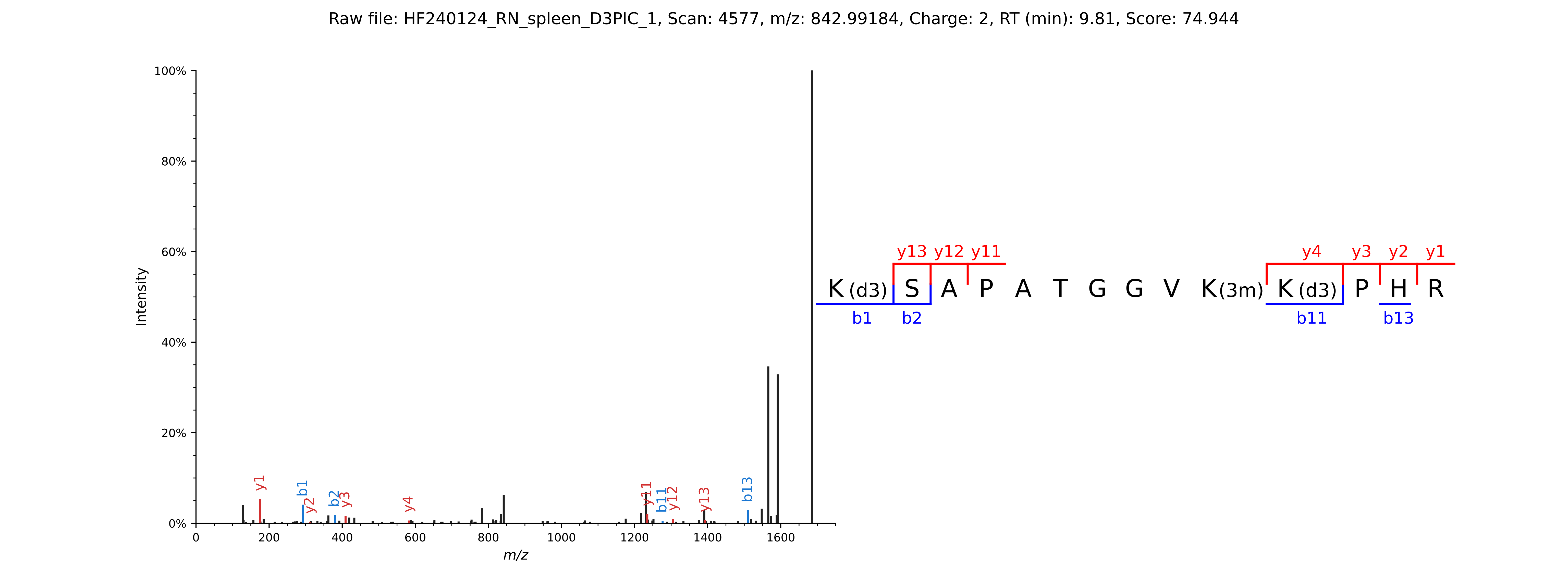
Raw file: HF240124_RN_spleen_D3PIC_1, Scan: 4577, m/z: 842.99184, Charge: 2, RT (min): 9.81, Score: 74.944
Intensity
m/z
y1
b1
y2
b2
y3
y4
y11
b11
y12
y13
b13
K(d3) S A P A T G G V K(3m) K(d3) P H R
y13 y12 y11
y4 y3 y2 y1
b1 b2 b11 b13

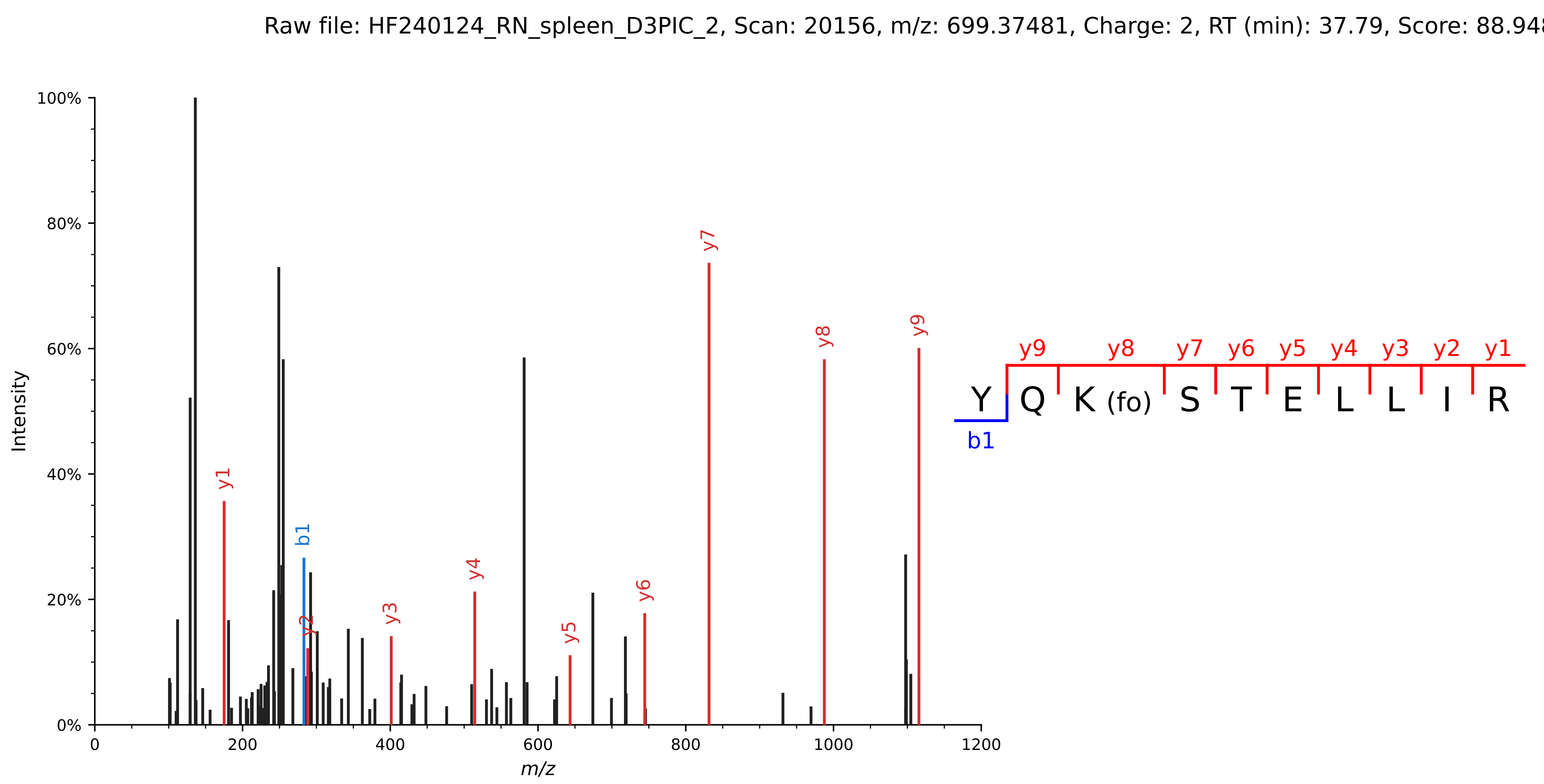

Raw file: HF240124_RN_spleen_D3PIC_2, Scan: 20156, m/z: 699.37481, Charge: 2, RT (min): 37.79, Score: 88.948
Intensity
m/z
y1
b1
y2
y3
y4
y5
y6
y7
y8
y9
Y Q K (fo) S T E L L I R

Raw file: HF240124_RN_spleen_D3PIC_1, Scan: 21208, m/z: 714.89987, Charge: 2, RT (min): 39.84, Score: 51.346

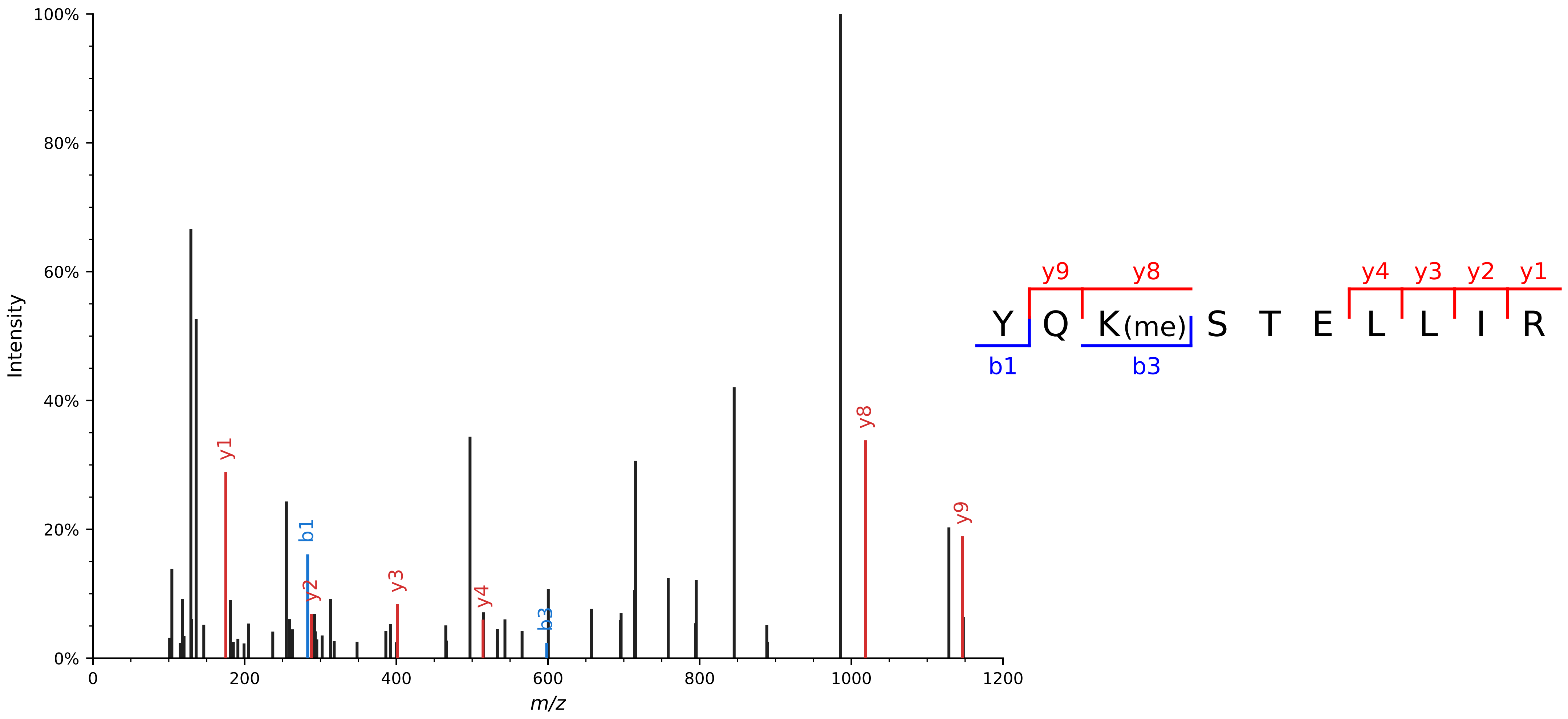

Raw file: HF240124_RN_spleen_D3PIC_1, Scan: 21426, m/z: 713.39046, Charge: 2, RT (min): 40.24, Score: 56.432

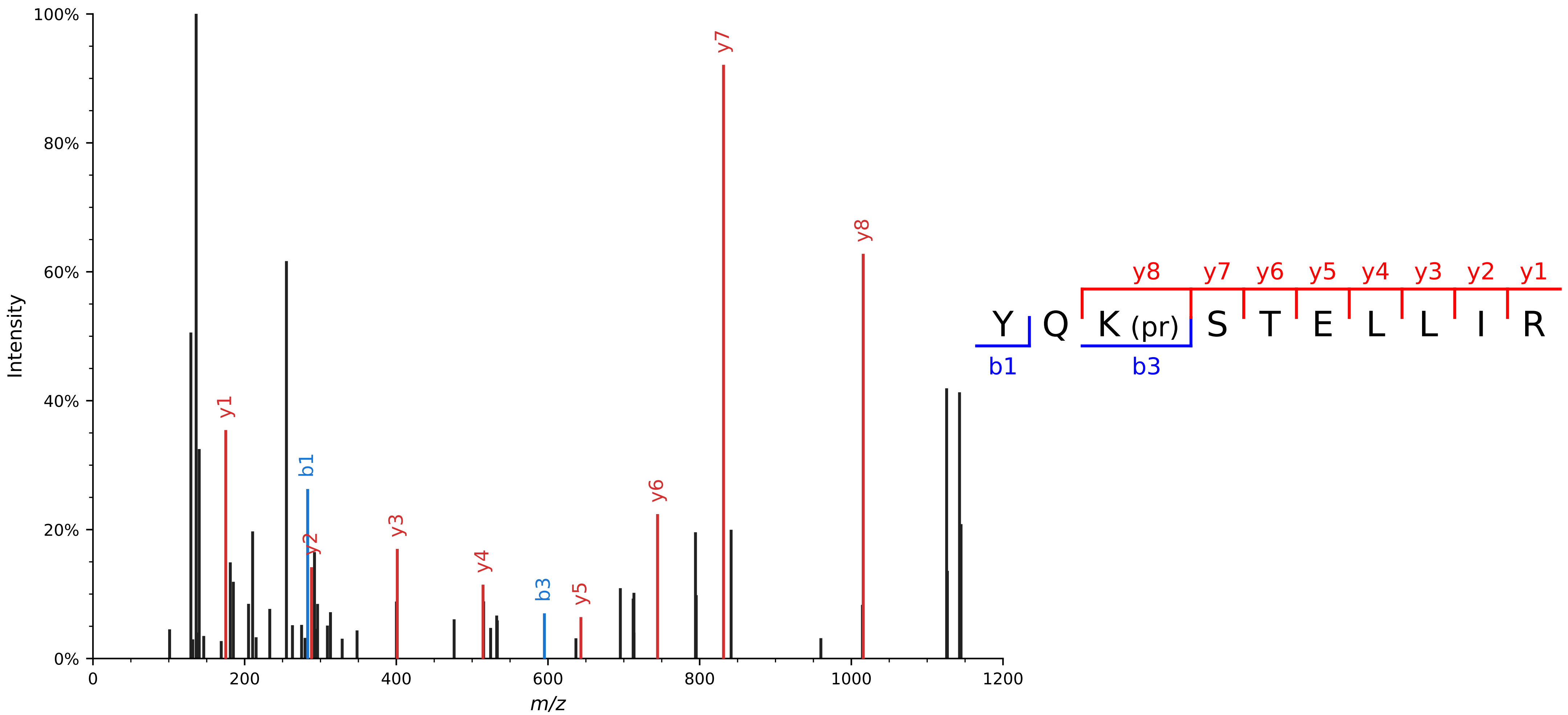

Raw file: HF240124_RN_spleen_D3PIC_3, Scan: 21084, m/z: 489.28199, Charge: 2, RT (min): 39.99, Score: 113.82

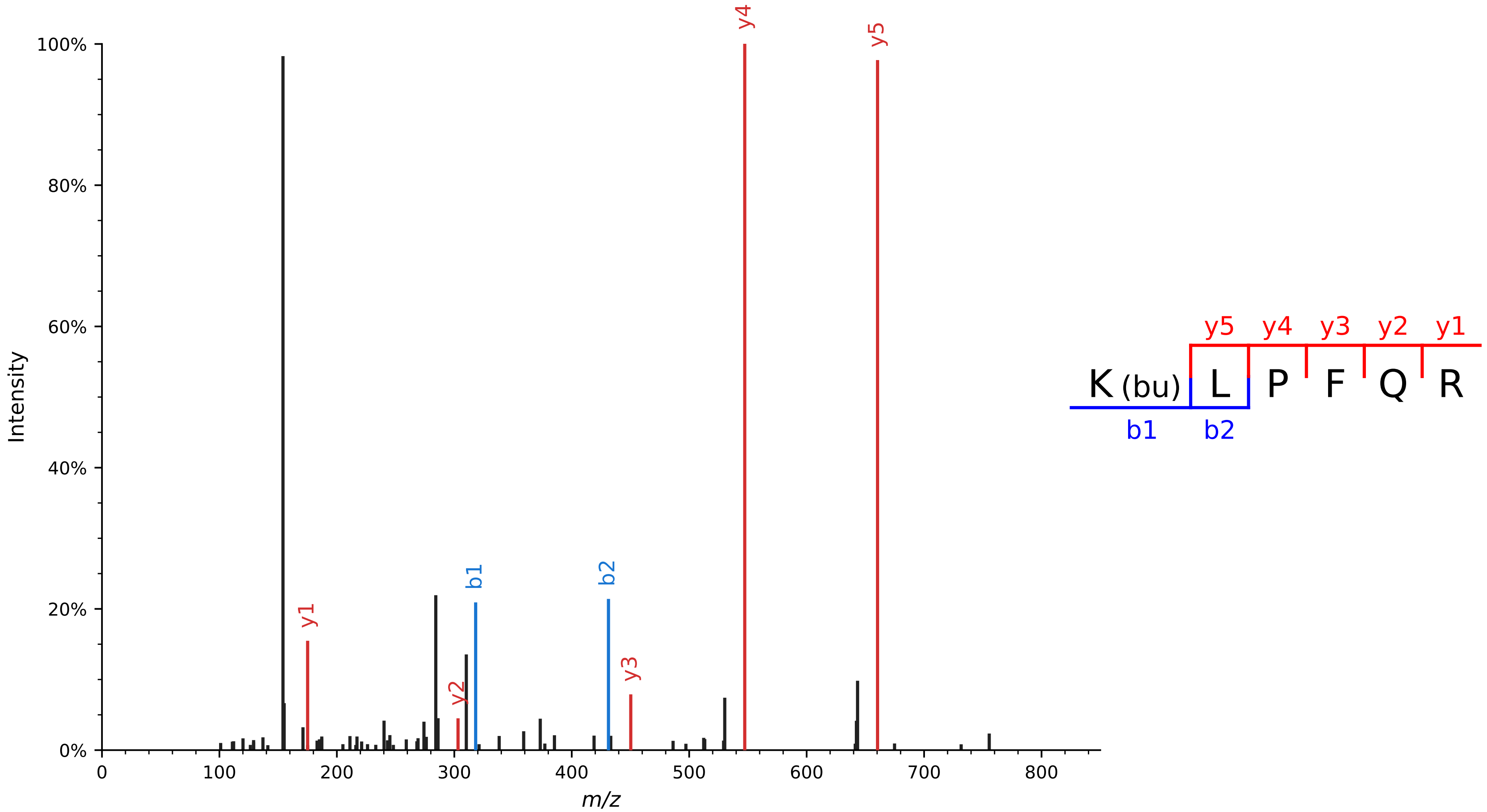

Raw file: HF240124_RN_spleen_D3PIC_1, Scan: 18401, m/z: 468.25852, Charge: 2, RT (min): 34.70, Score: 88.627

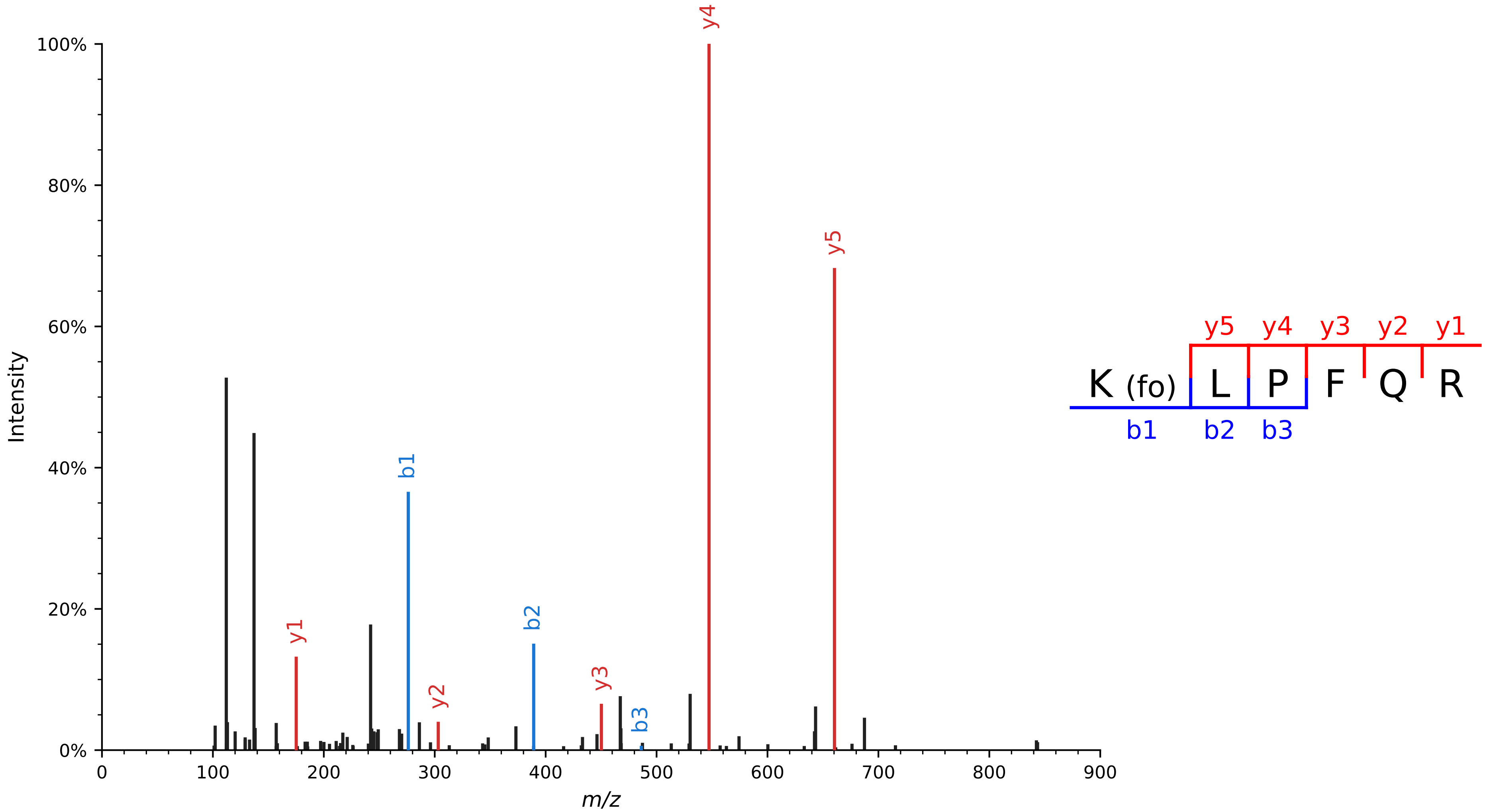

Raw file: HF240124_RN_spleen_D3PIC_3, Scan: 20369, m/z: 483.78358, Charge: 2, RT (min): 38.65, Score: 50.31

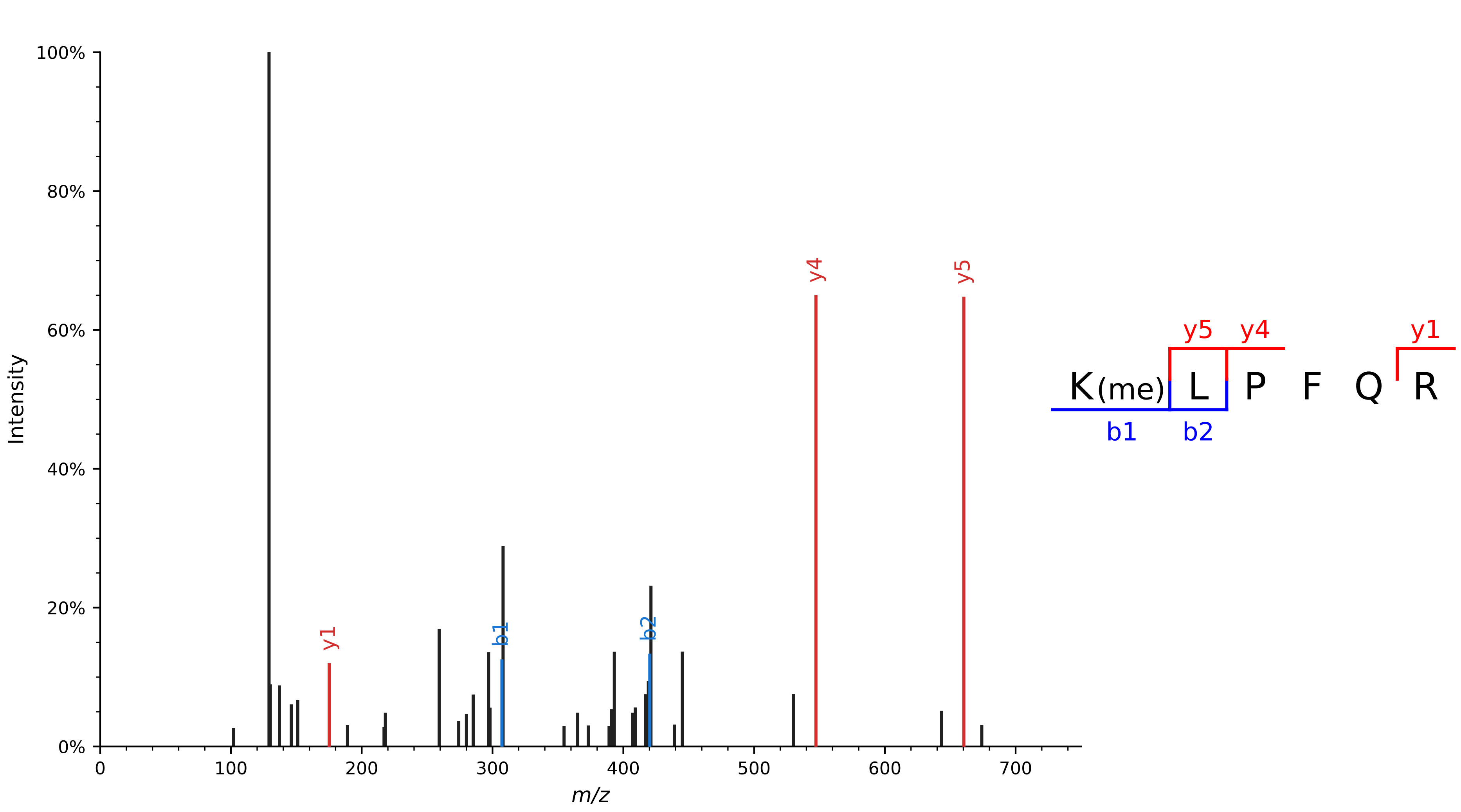

Raw file: HF240124_RN_spleen_D3PIC_1, Scan: 19895, m/z: 482.27417, Charge: 2, RT (min): 37.43, Score: 103.41

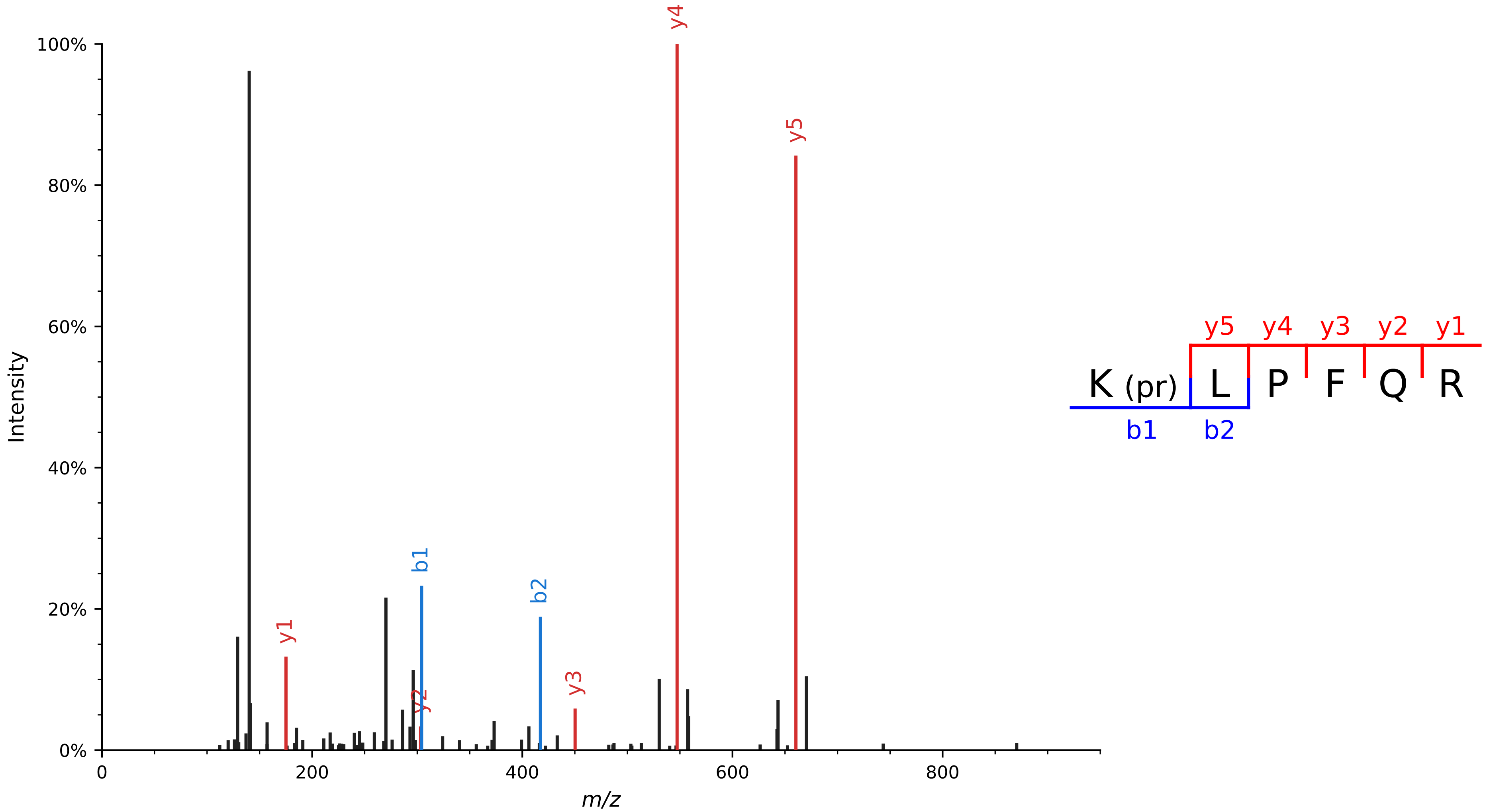

Raw file: HF240124_RN_spleen_D3PIC_2, Scan: 23556, m/z: 762.88828, Charge: 2, RT (min): 44.05, Score: 52.79

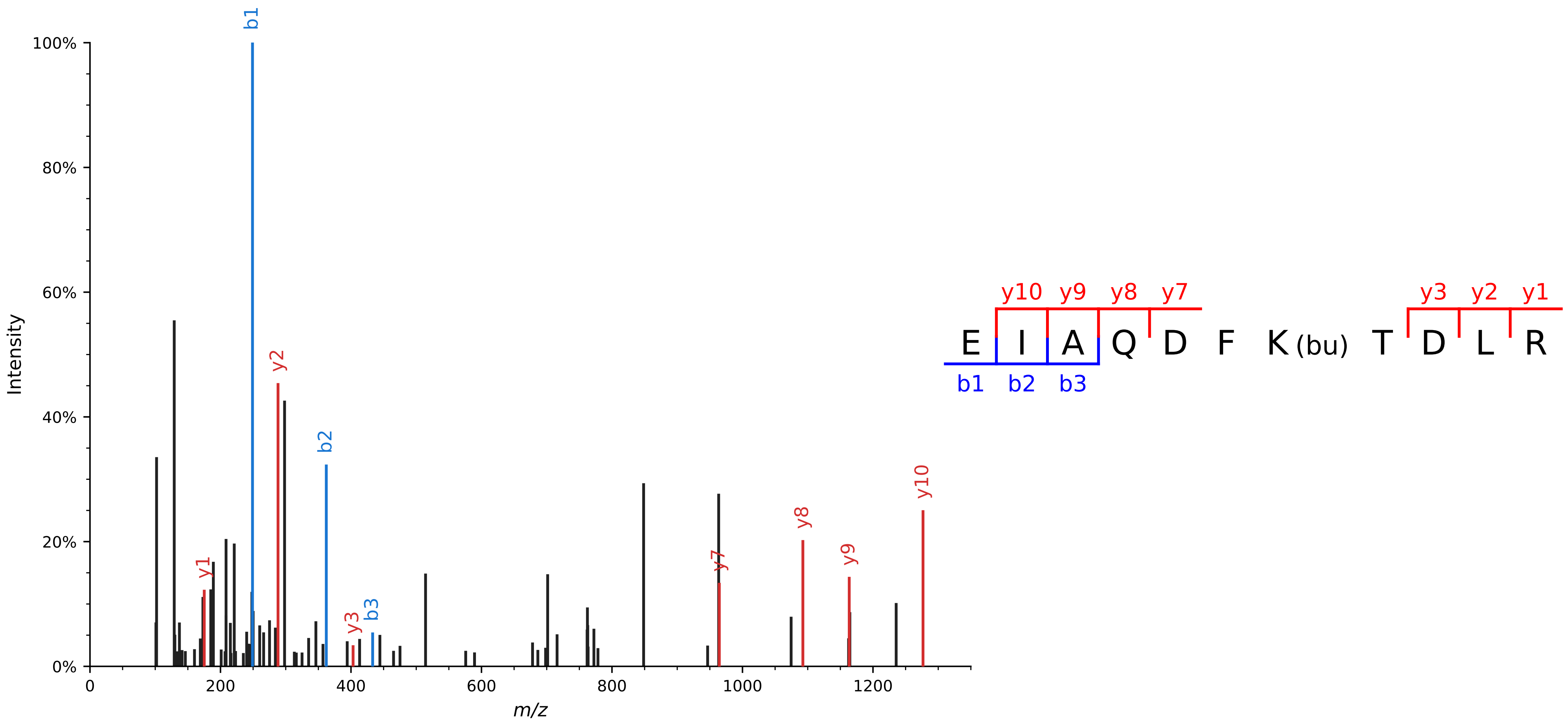

Raw file: HF240124_RN_spleen_D3PIC_1, Scan: 22873, m/z: 741.86481, Charge: 2, RT (min): 42.87, Score: 112.75

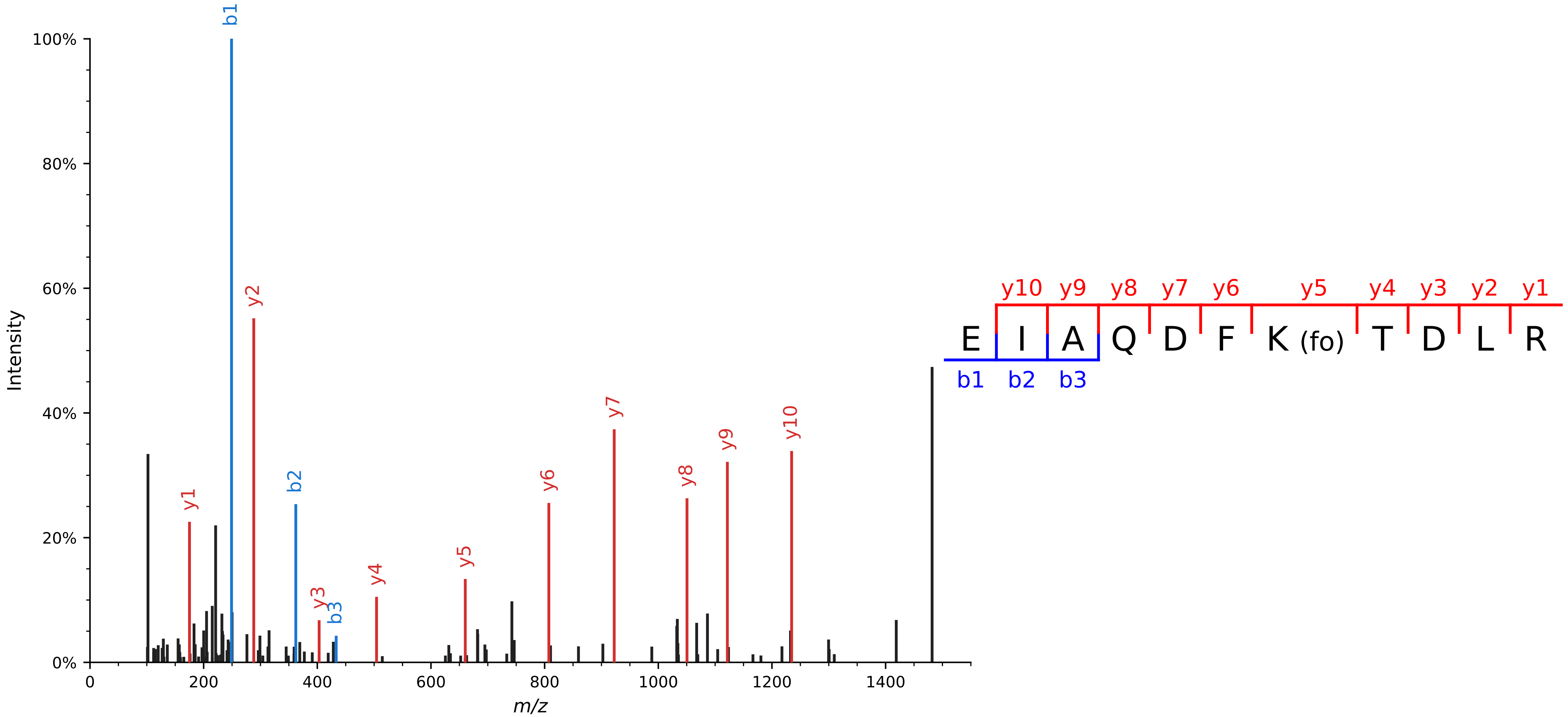

Raw file: HF240124_RN_spleen_D3PIC_2, Scan: 24370, m/z: 757.38987, Charge: 2, RT (min): 45.54, Score: 202.2

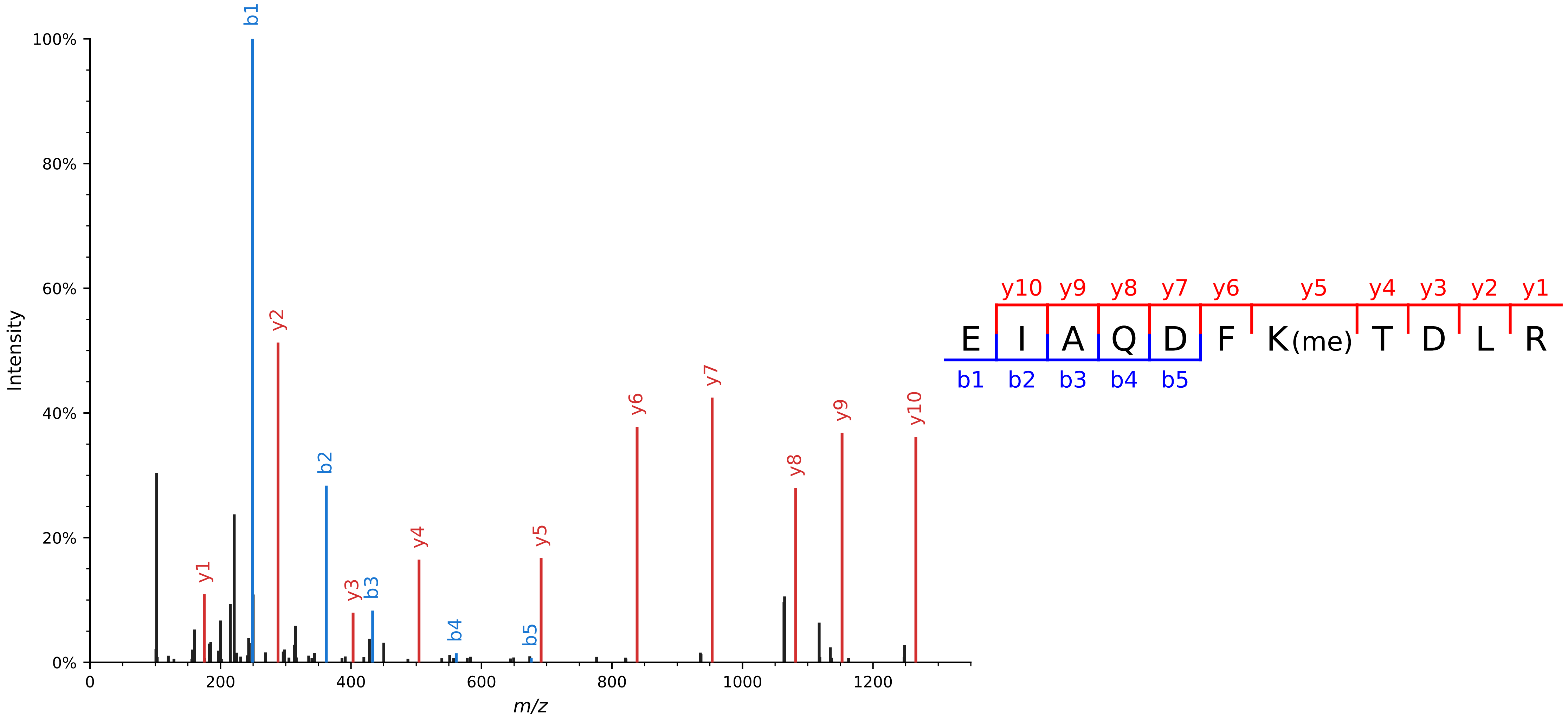

Raw file: HF240124_RN_spleen_D3PIC_1, Scan: 16212, m/z: 741.883, Charge: 2, RT (min): 30.76, Score: 169.93

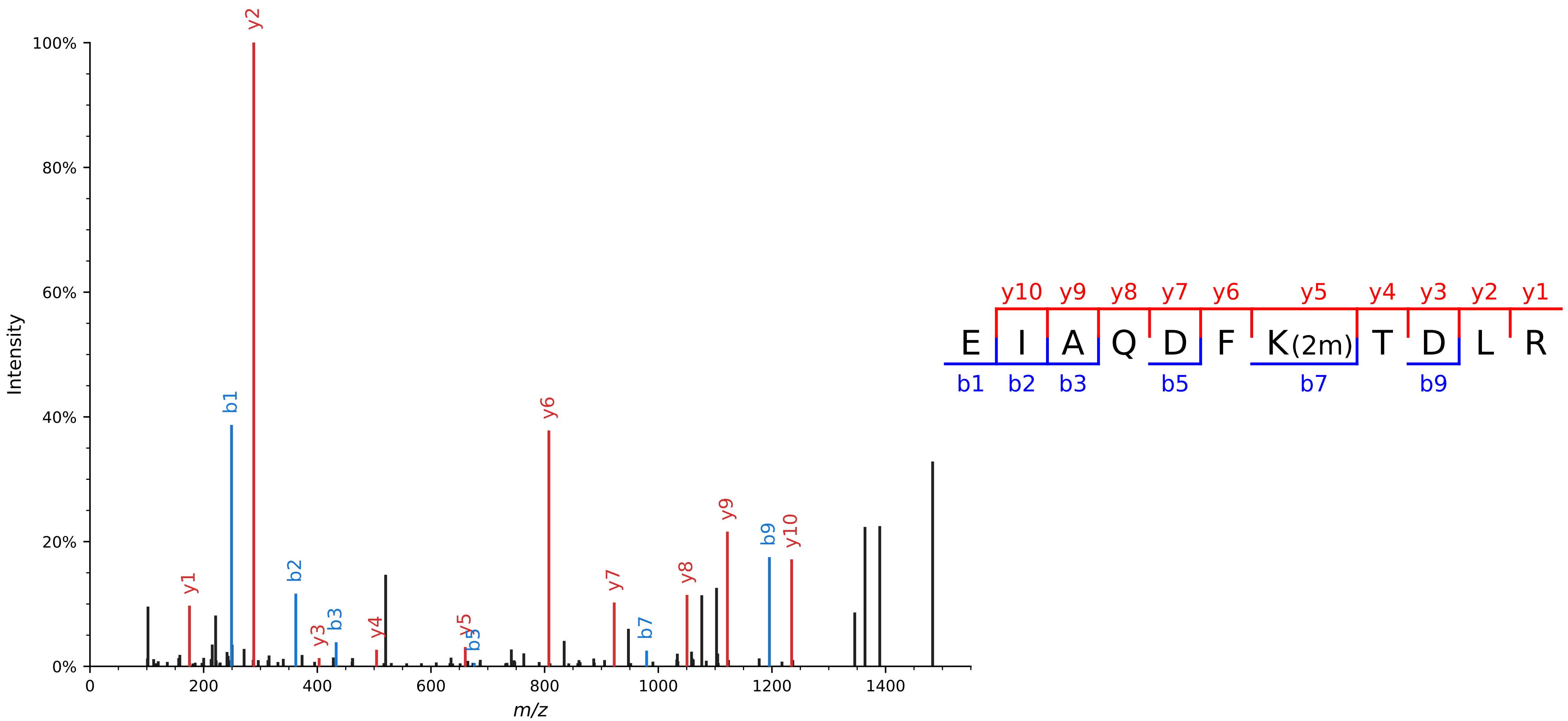

Raw file: HF240124_RN_spleen_D3PIC_3, Scan: 22295, m/z: 755.88046, Charge: 2, RT (min): 42.22, Score: 48.794

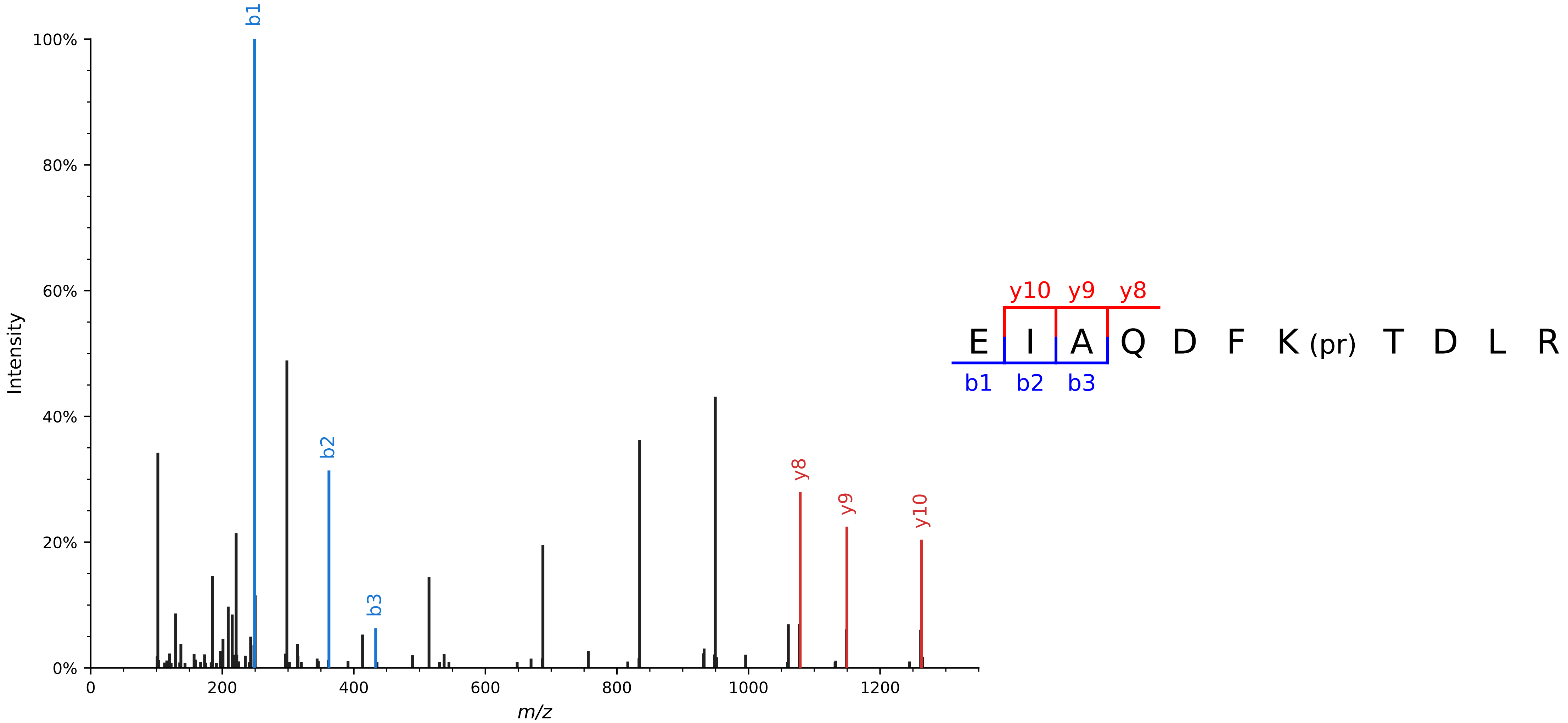

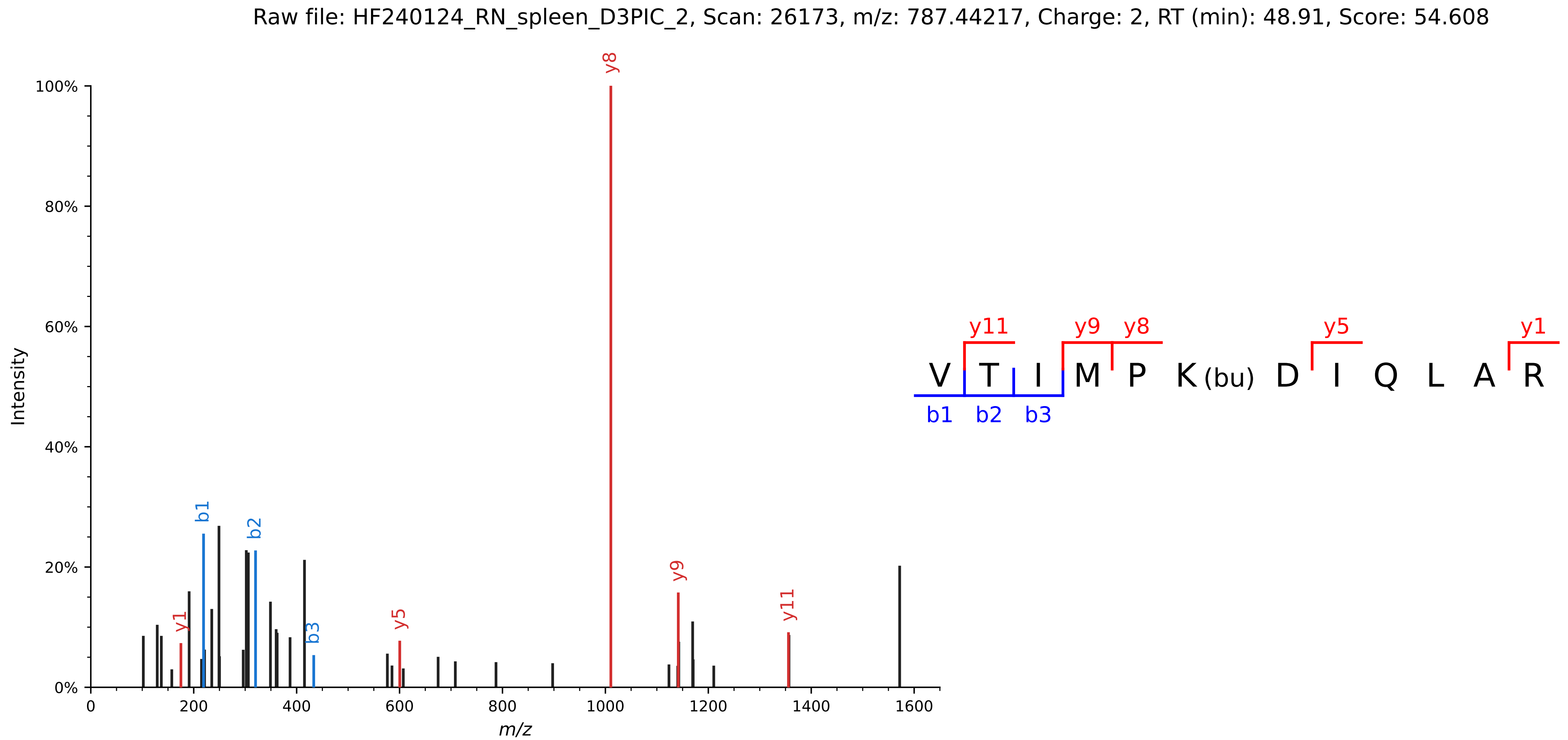

Raw file: HF240124_RN_spleen_D3PIC_2, Scan: 26173, m/z: 787.44217, Charge: 2, RT (min): 48.91, Score: 54.608
Intensity
m/z
y8
b1
b2
y1
b3
y5
y9
y11
y11 y9 y8 y5 y1
V T I M P K (bu) D I Q L A R
b1 b2 b3

Raw file: HF240124_RN_spleen_D3PIC_1, Scan: 23909, m/z: 766.41869, Charge: 2, RT (min): 44.77, Score: 84.17

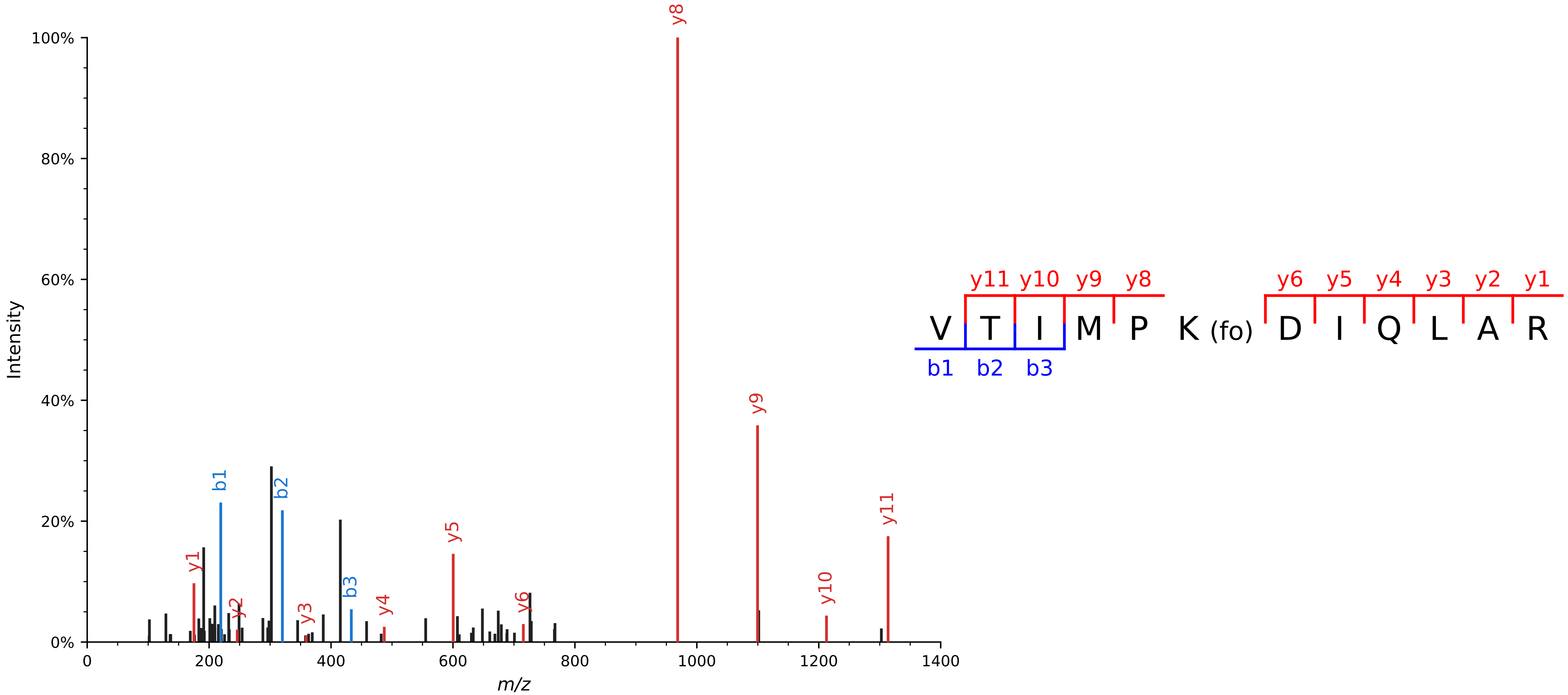

Raw file: HF240124_RN_spleen_D3PIC_2, Scan: 21932, m/z: 788.4318, Charge: 2, RT (min): 41.05, Score: 64.297

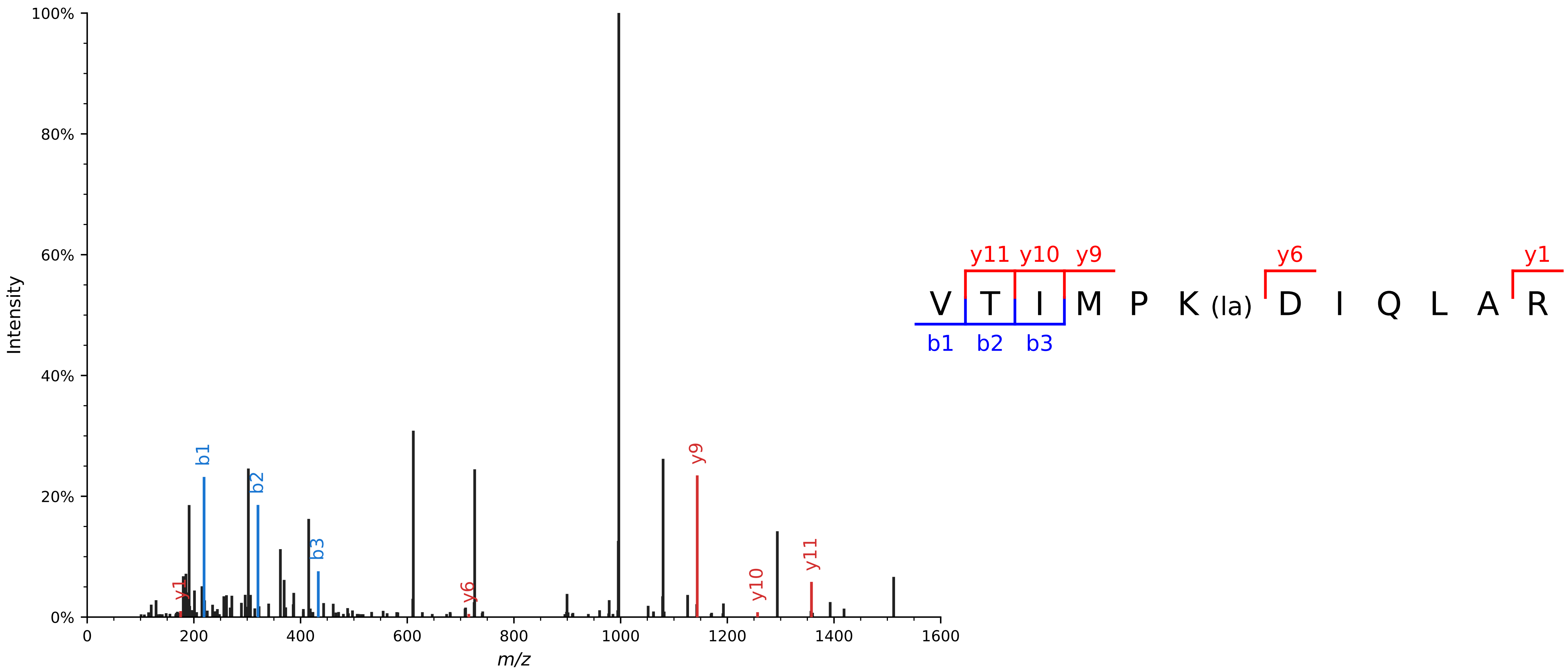

Raw file: HF240124_RN_spleen_D3PIC_3, Scan: 14677, m/z: 781.94376, Charge: 2, RT (min): 28.23, Score: 66.27

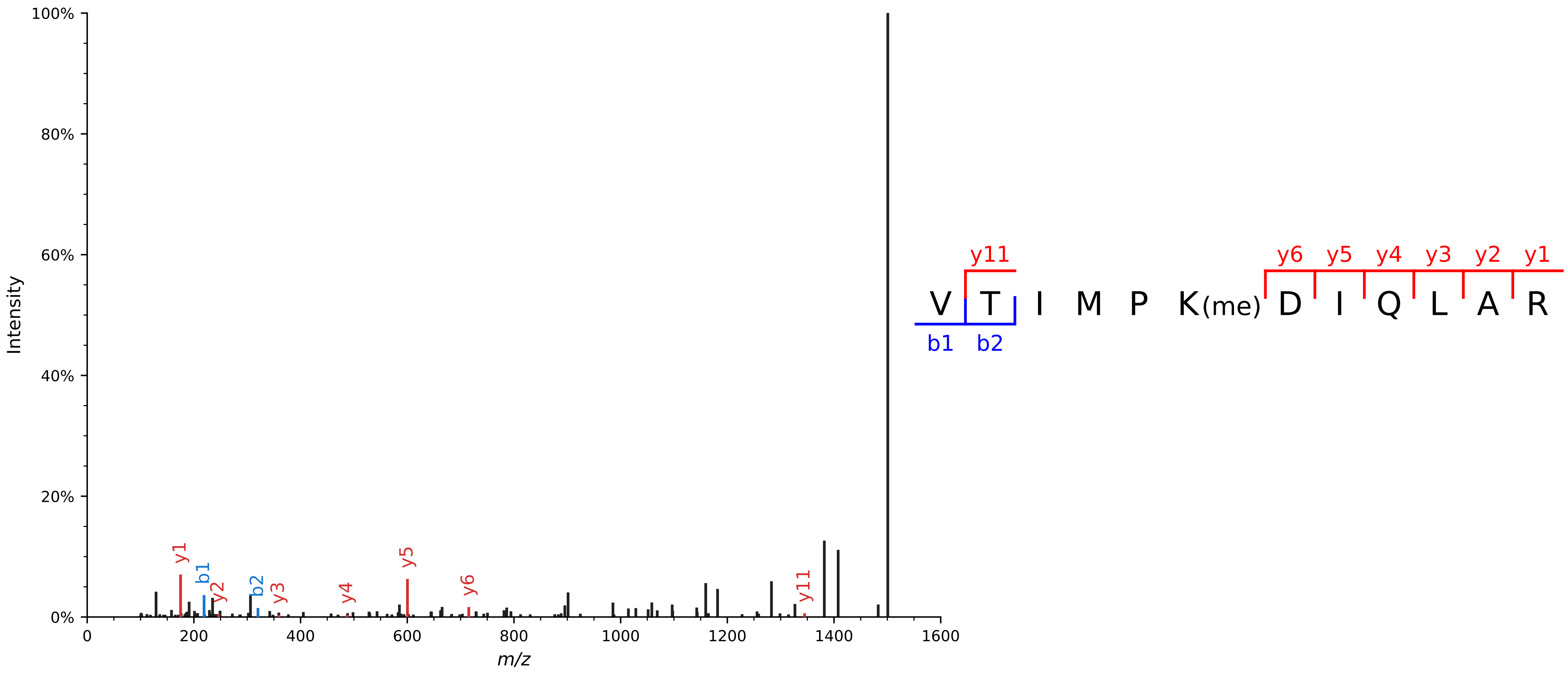

Raw file: HF240124_RN_spleen_D3PIC_1, Scan: 25629, m/z: 780.43434, Charge: 2, RT (min): 47.95, Score: 87.913

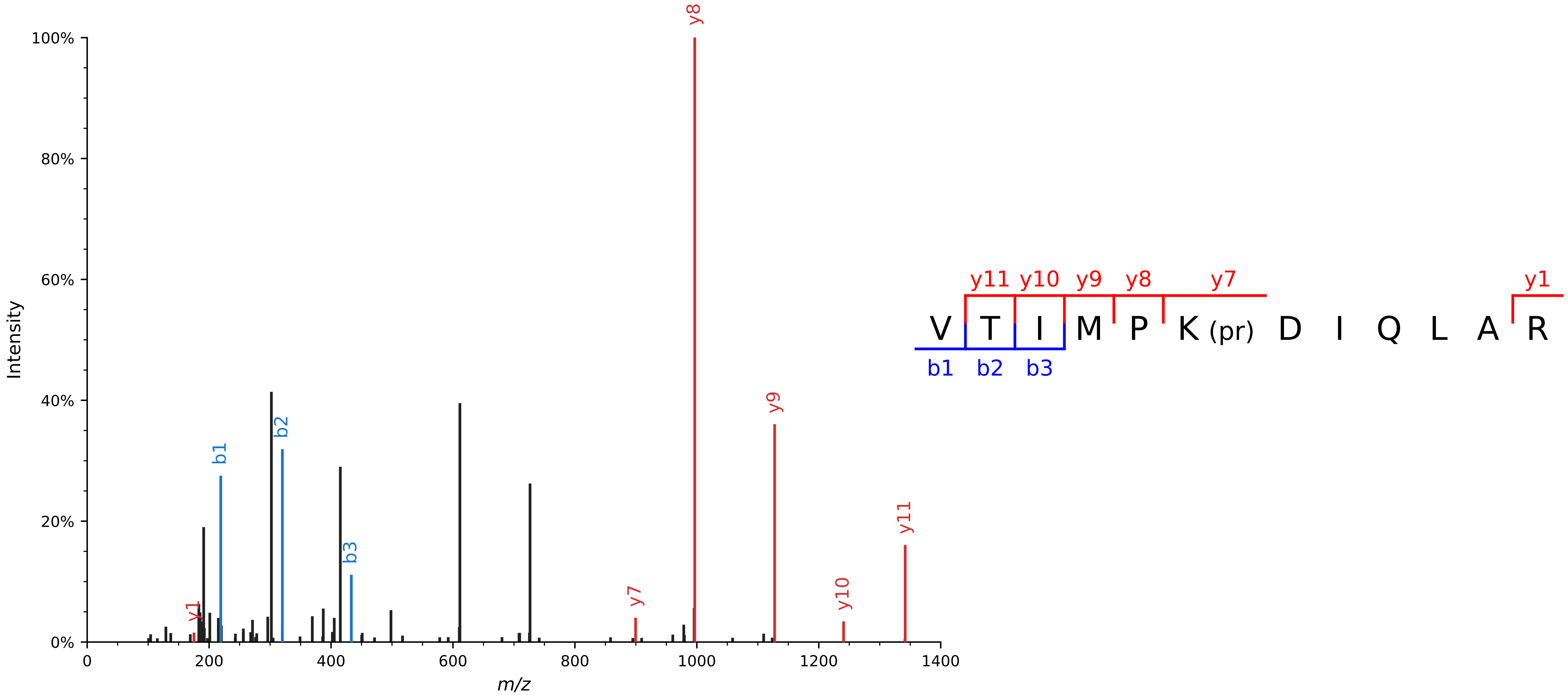

Raw file: HF240124_RN_spleen_D3PIC_2, Scan: 10871, m/z: 779.42844, Charge: 2, RT (min): 21.05, Score: 157.68

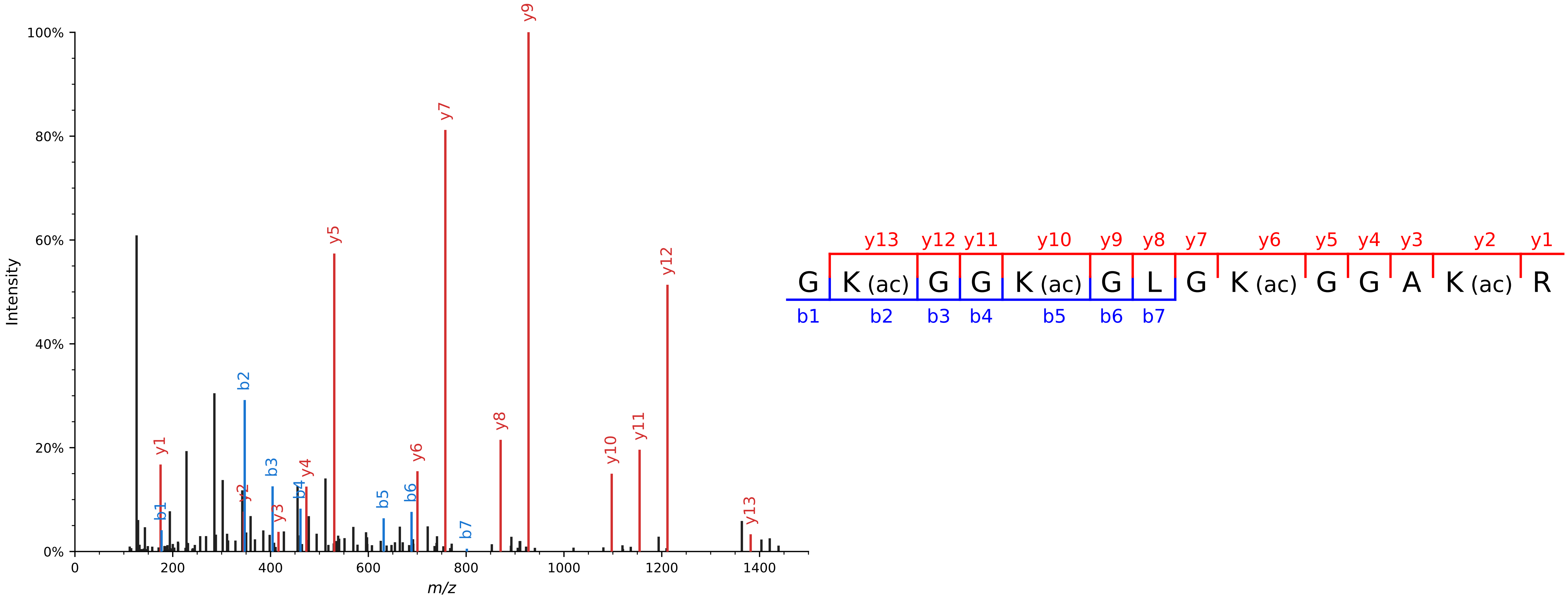

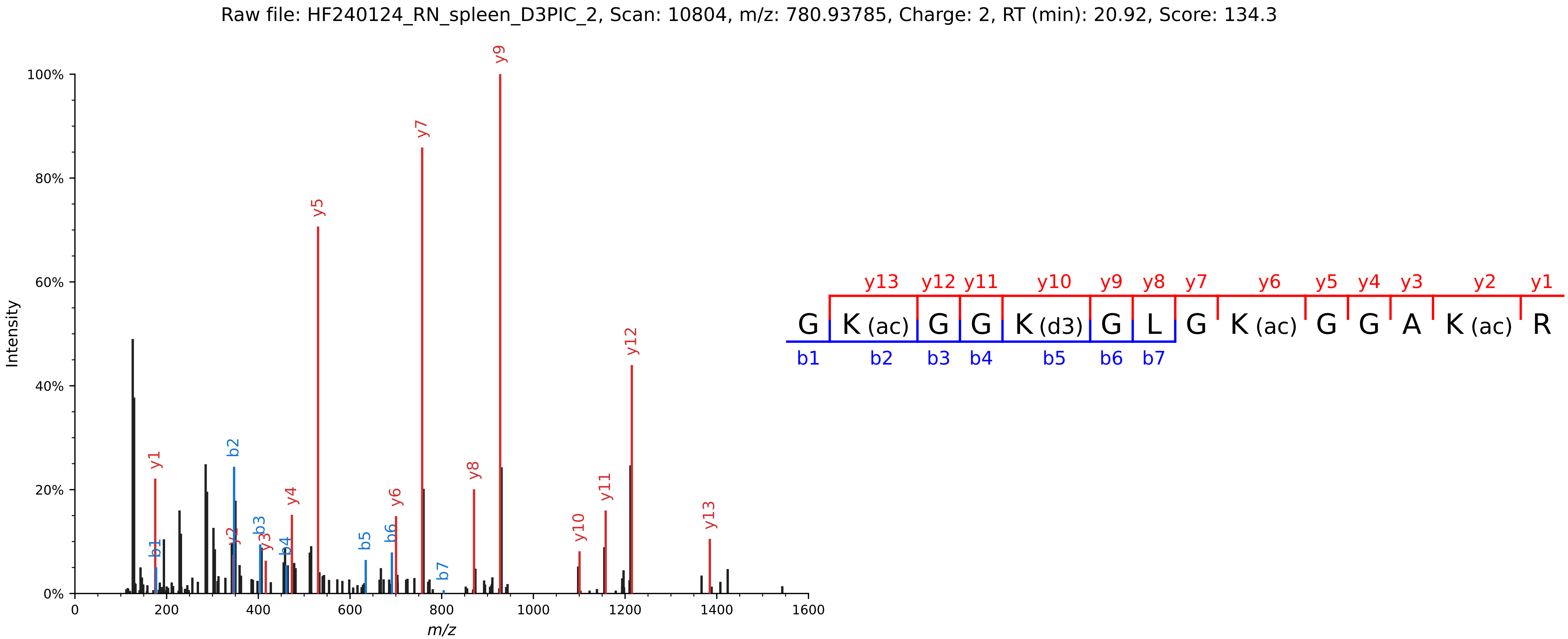
Raw file: HF240124_RN_spleen_D3PIC_2, Scan: 10804, m/z: 780.93785, Charge: 2, RT (min): 20.92, Score: 134.3
Intensity
m/z
G K(ac) G G K(d3) G L G K(ac) G G A K(ac) R
y13 y12 y11 y10 y9 y8 y7 y6 y5 y4 y3 y2 y1
b1 b2 b3 b4 b5 b6 b7

Raw file: HF240124_RN_spleen_D3PIC_3, Scan: 13360, m/z: 797.97233, Charge: 2, RT (min): 25.87, Score: 129.16

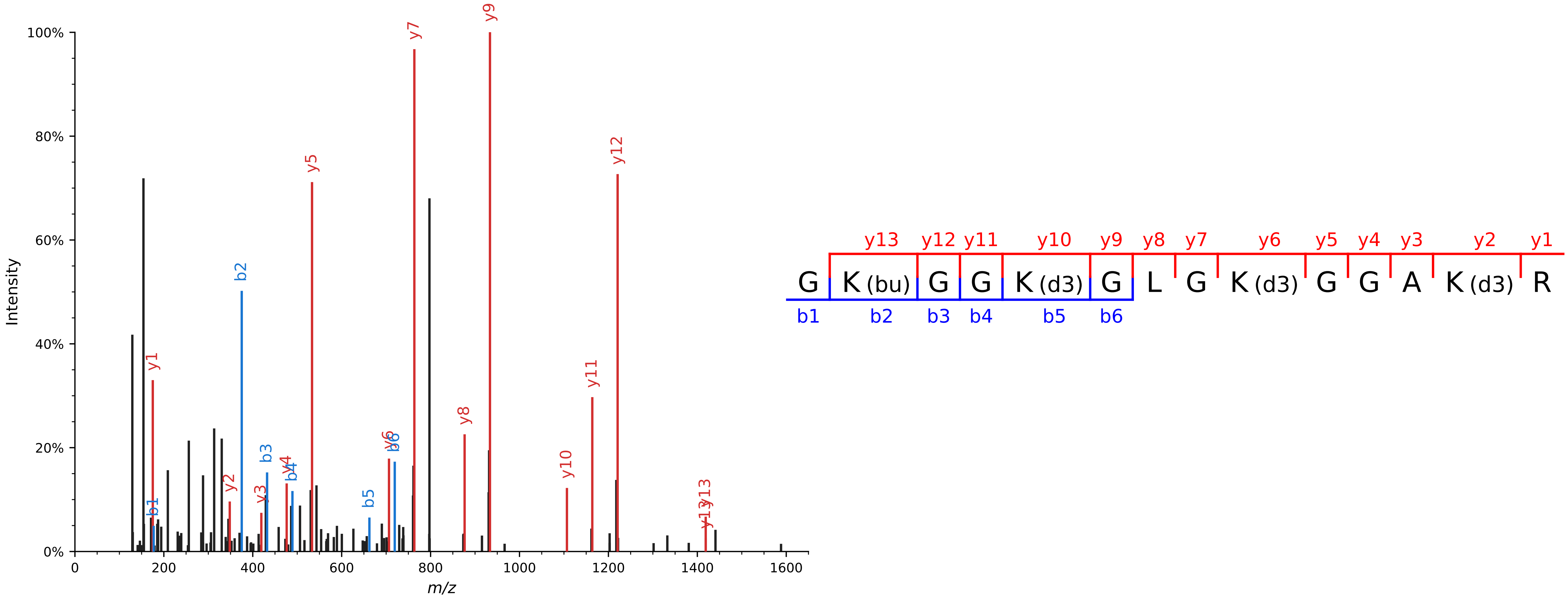

Raw file: HF240124_RN_spleen_D3PIC_2, Scan: 13678, m/z: 531.31104, Charge: 3, RT (min): 26.06, Score: 72.891

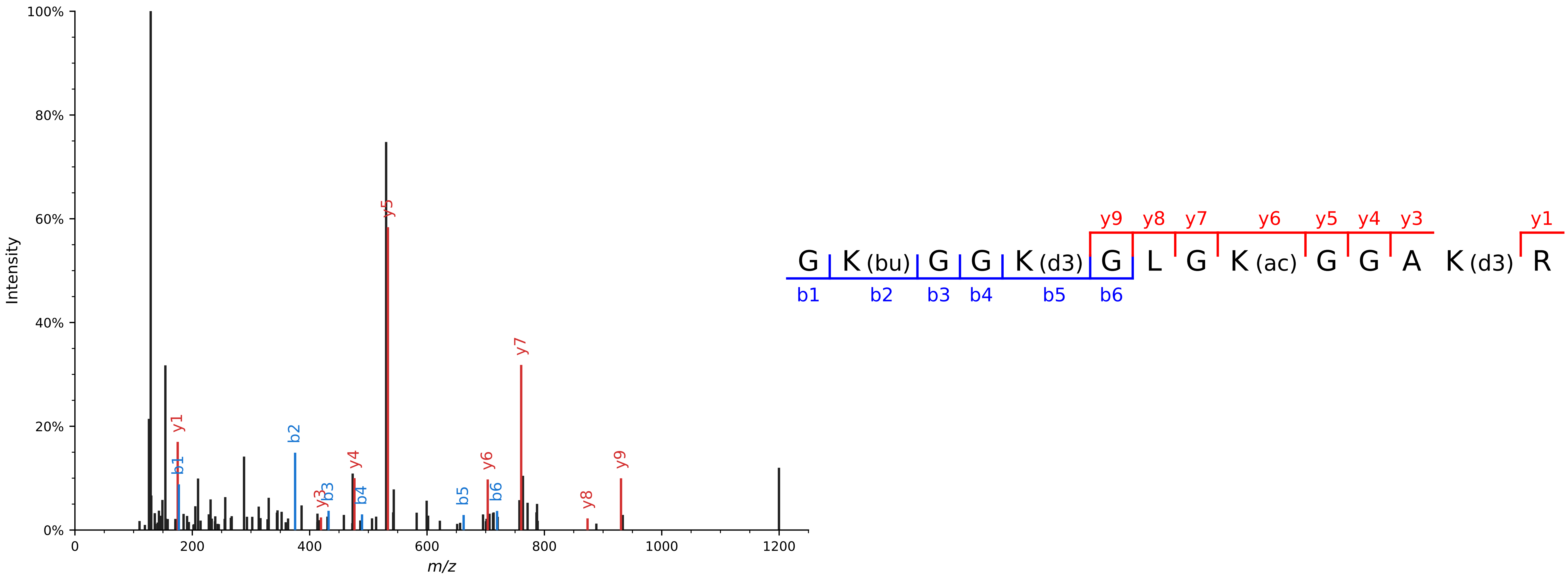

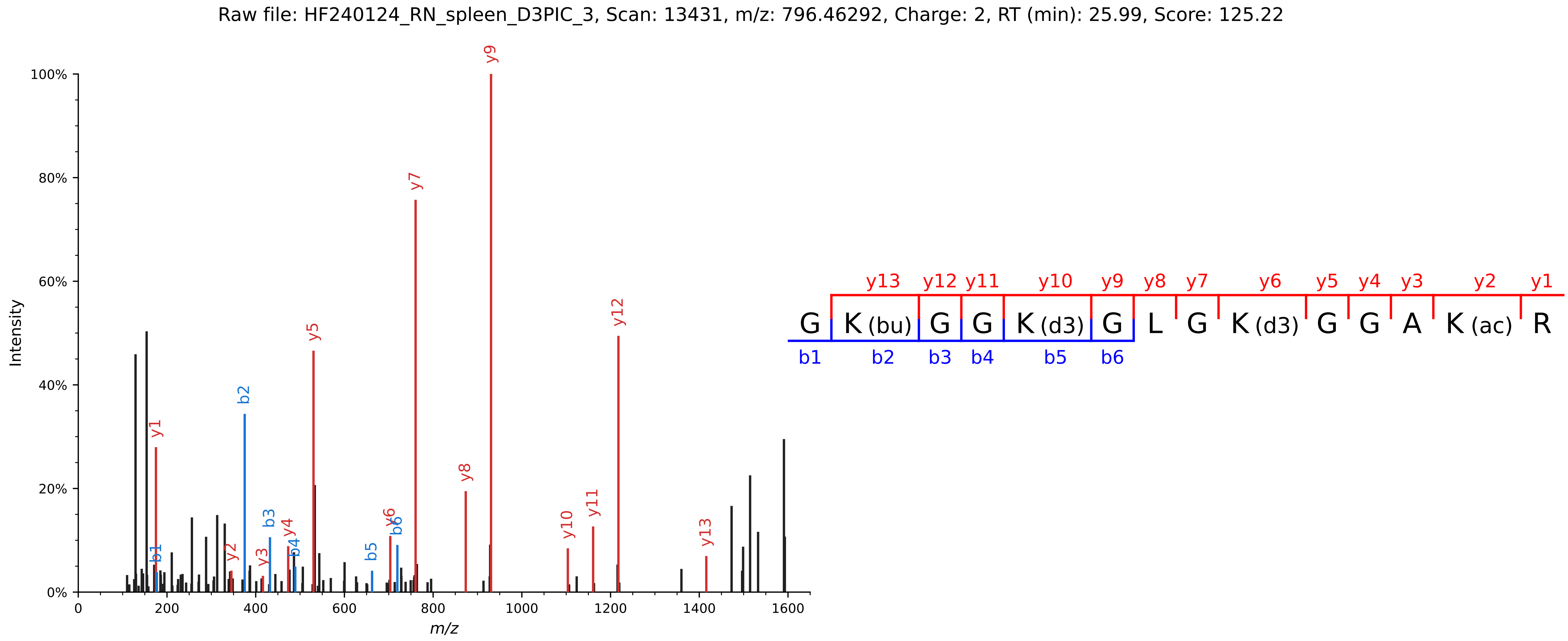

Raw file: HF240124_RN_spleen_D3PIC_3, Scan: 13431, m/z: 796.46292, Charge: 2, RT (min): 25.99, Score: 125.22
Intensity
m/z
y9
y7
y5
y12
y1
b2
y8
y11
y10
y13
b3
y4
b4
y6
b6
b5
b1
y2
y3
G K(bu) G G K(d3) G L G K(d3) G G A K(ac) R
y13 y12 y11 y10 y9 y8 y7 y6 y5 y4 y3 y2 y1
b1 b2 b3 b4 b5 b6

Raw file: HF240124_RN_spleen_D3PIC_2, Scan: 10375, m/z: 776.94886, Charge: 2, RT (min): 20.13, Score: 188.04

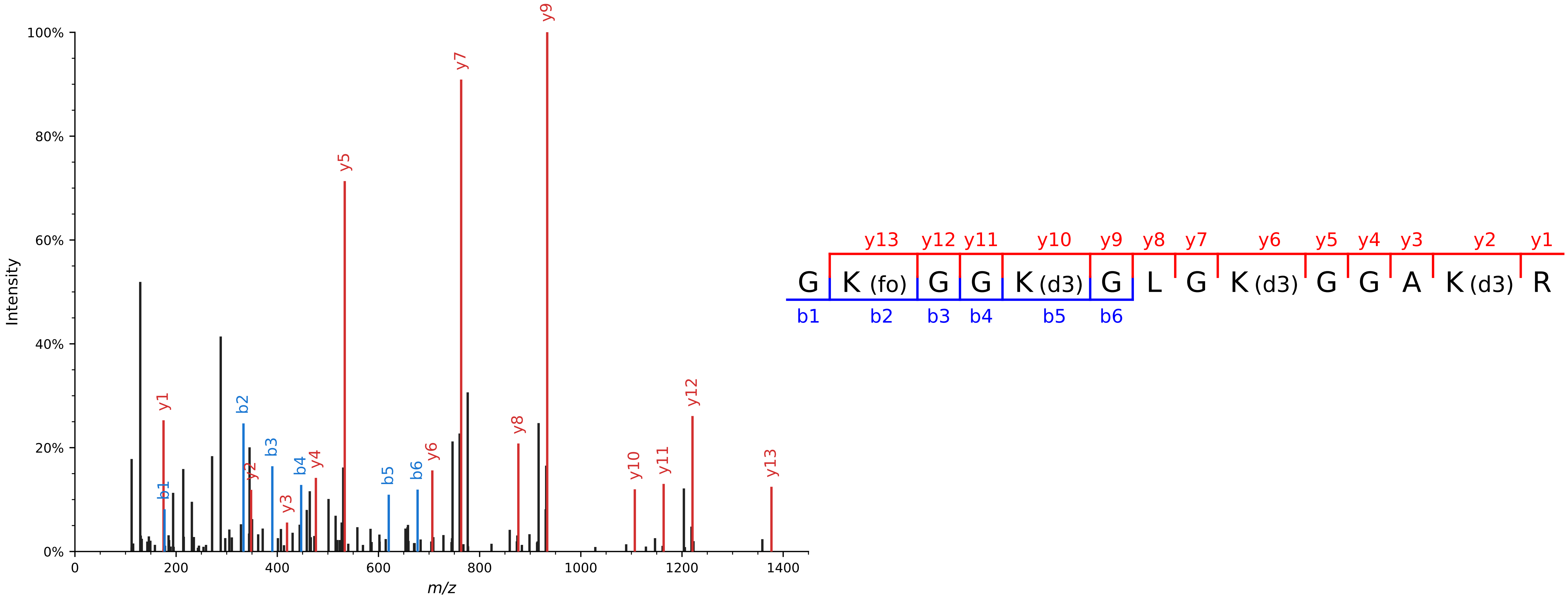

Raw file: HF240124_RN_spleen_D3PIC_1, Scan: 14436, m/z: 800.45191, Charge: 2, RT (min): 27.51, Score: 128.01

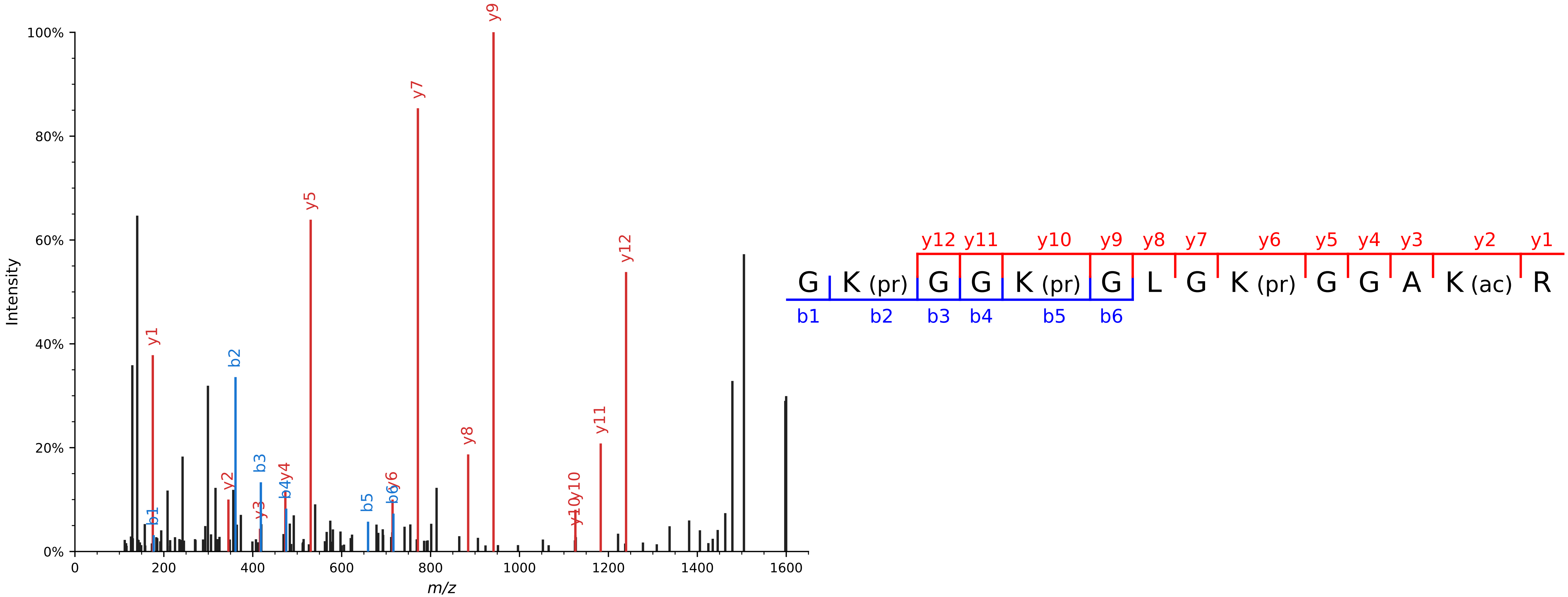

Raw file: HF240124_RN_spleen_D3PIC_1, Scan: 15314, m/z: 807.45974, Charge: 2, RT (min): 29.08, Score: 150.94

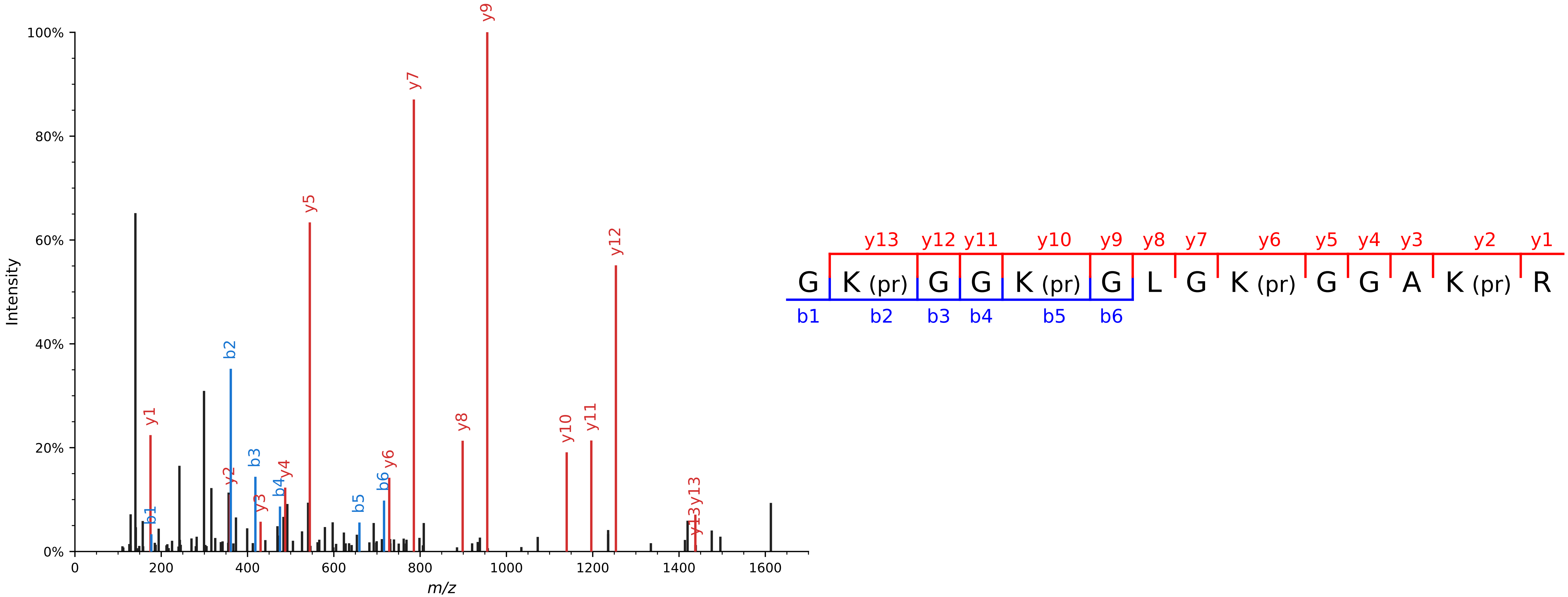

Raw file: HF240124_RN_spleen_D3PIC_2, Scan: 10761, m/z: 782.44727, Charge: 2, RT (min): 20.83, Score: 116.55

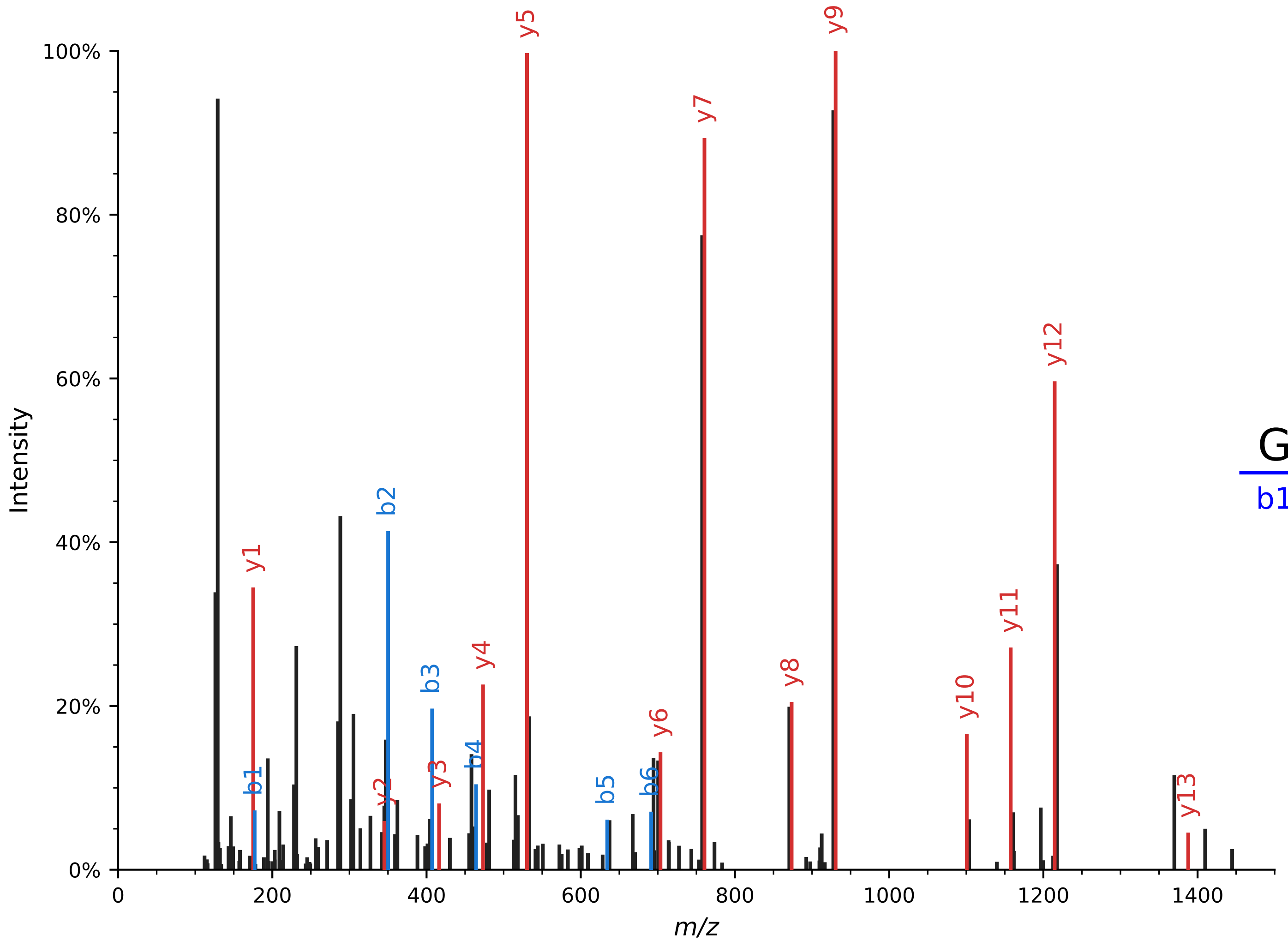

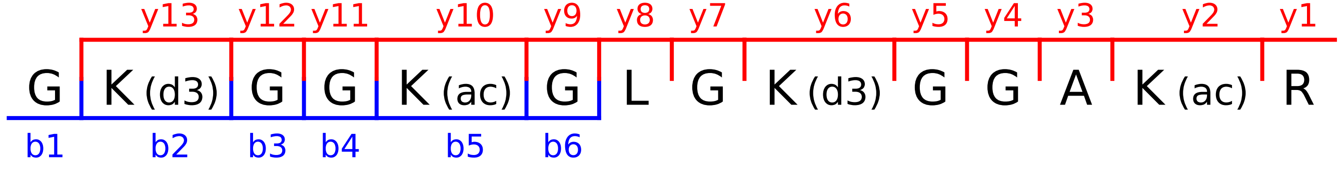

Raw file: HF240124_RN_spleen_D3PIC_2, Scan: 13367, m/z: 797.97233, Charge: 2, RT (min): 25.52, Score: 121.9

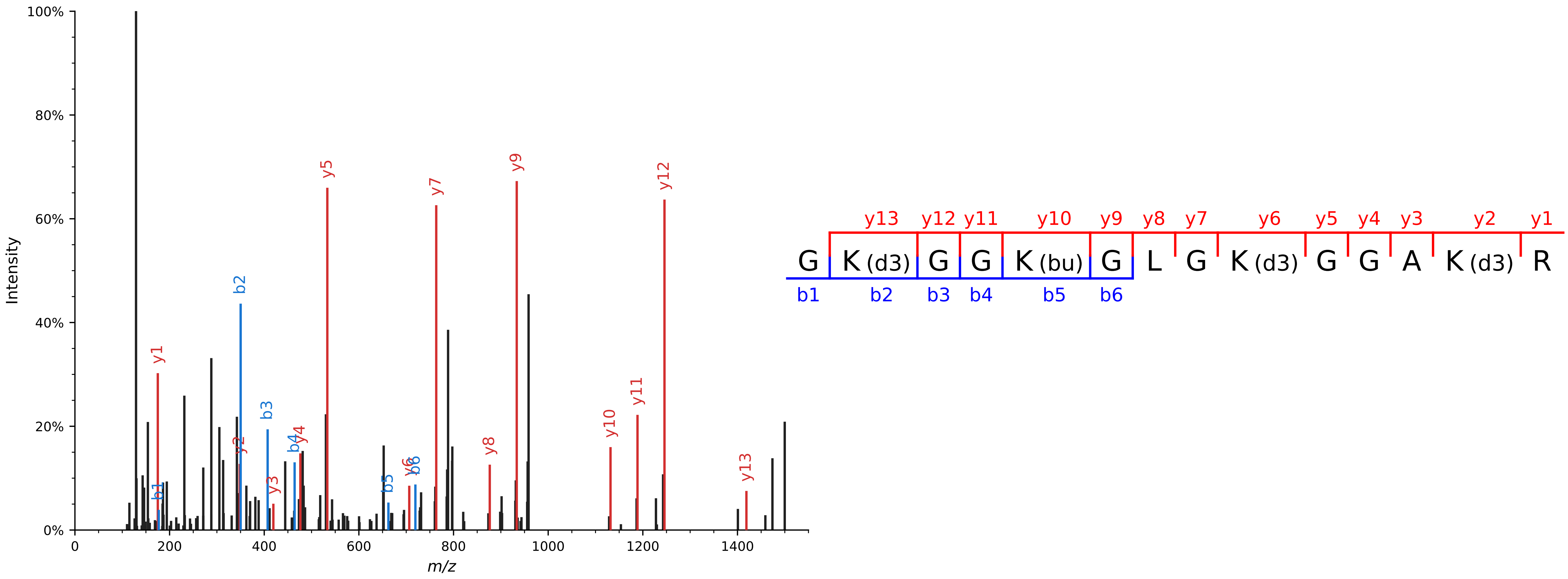

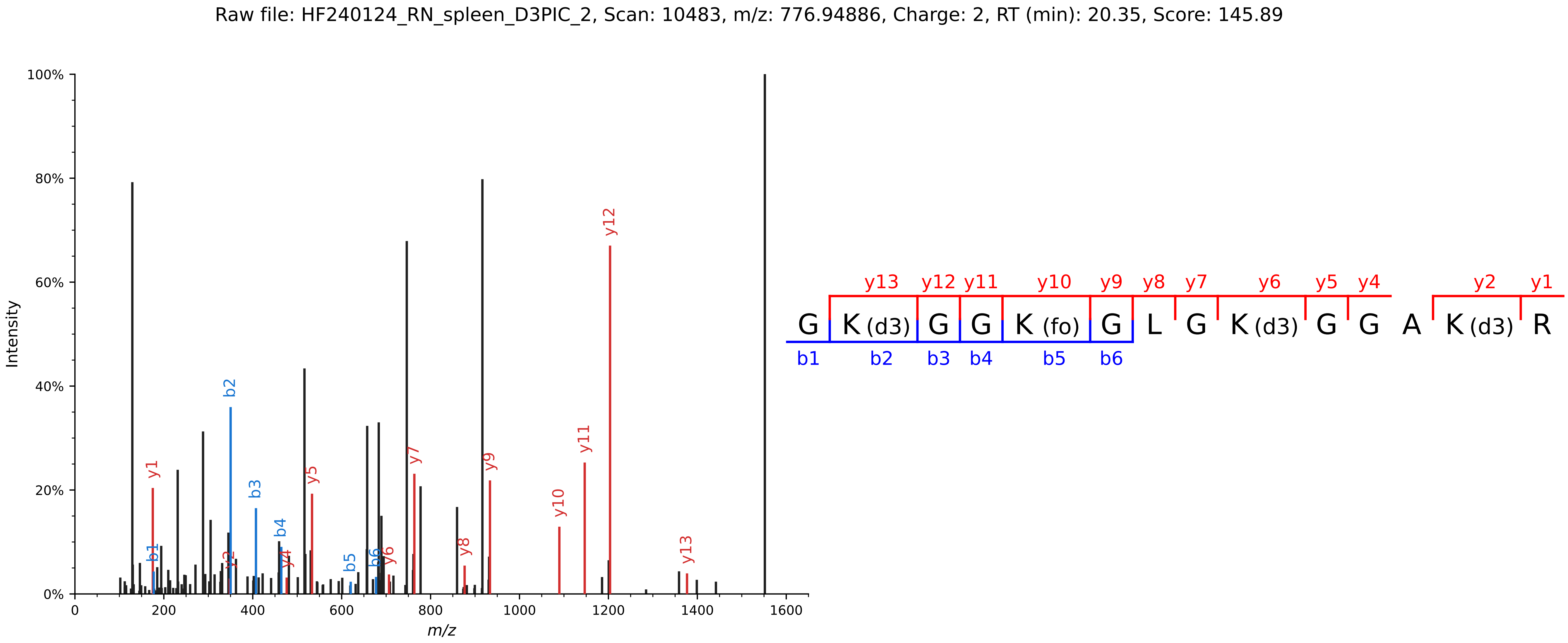

Raw file: HF240124_RN_spleen_D3PIC_2, Scan: 10483, m/z: 776.94886, Charge: 2, RT (min): 20.35, Score: 145.89
Intensity
m/z
y1
b1
b2
b3
b4
y5
b5
b6
y6
y7
y8
y9
y10
y11
y12
y13
G K (d3) G G K (fo) G L G K (d3) G G A K (d3) R

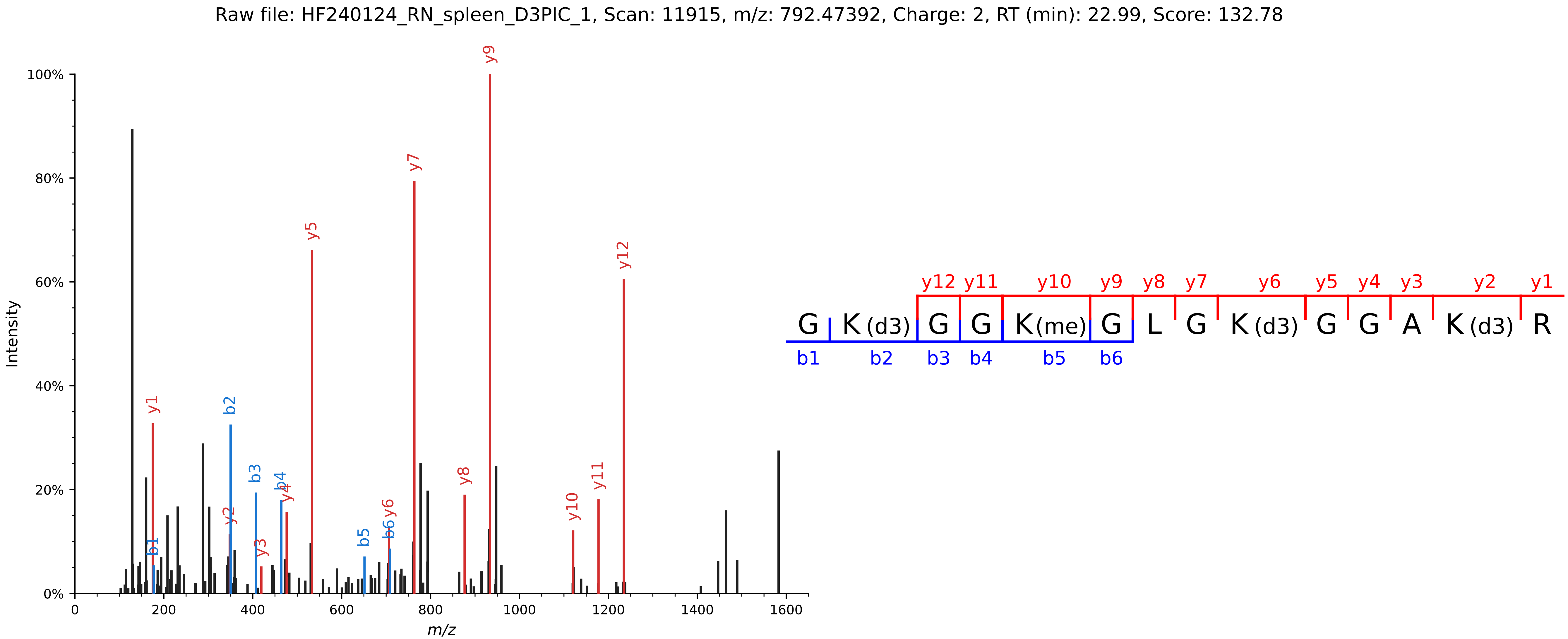

Raw file: HF240124_RN_spleen_D3PIC_1, Scan: 11915, m/z: 792.47392, Charge: 2, RT (min): 22.99, Score: 132.78
Intensity
m/z
y12 y11 y10 y9 y8 y7 y6 y5 y4 y3 y2 y1
G K(d3) G G K(me) G L G K(d3) G G A K(d3) R
b1 b2 b3 b4 b5 b6

Raw file: HF240124_RN_spleen_D3PIC_3, Scan: 10626, m/z: 782.44727, Charge: 2, RT (min): 20.90, Score: 127.51

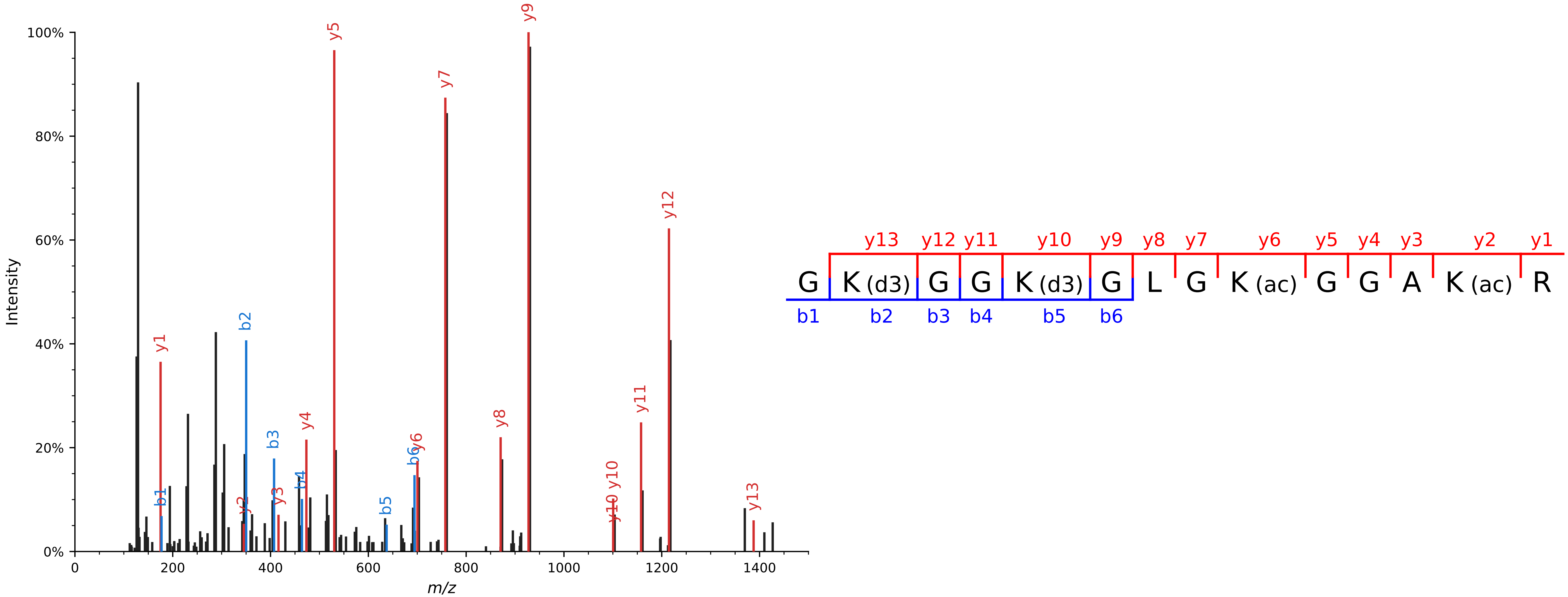

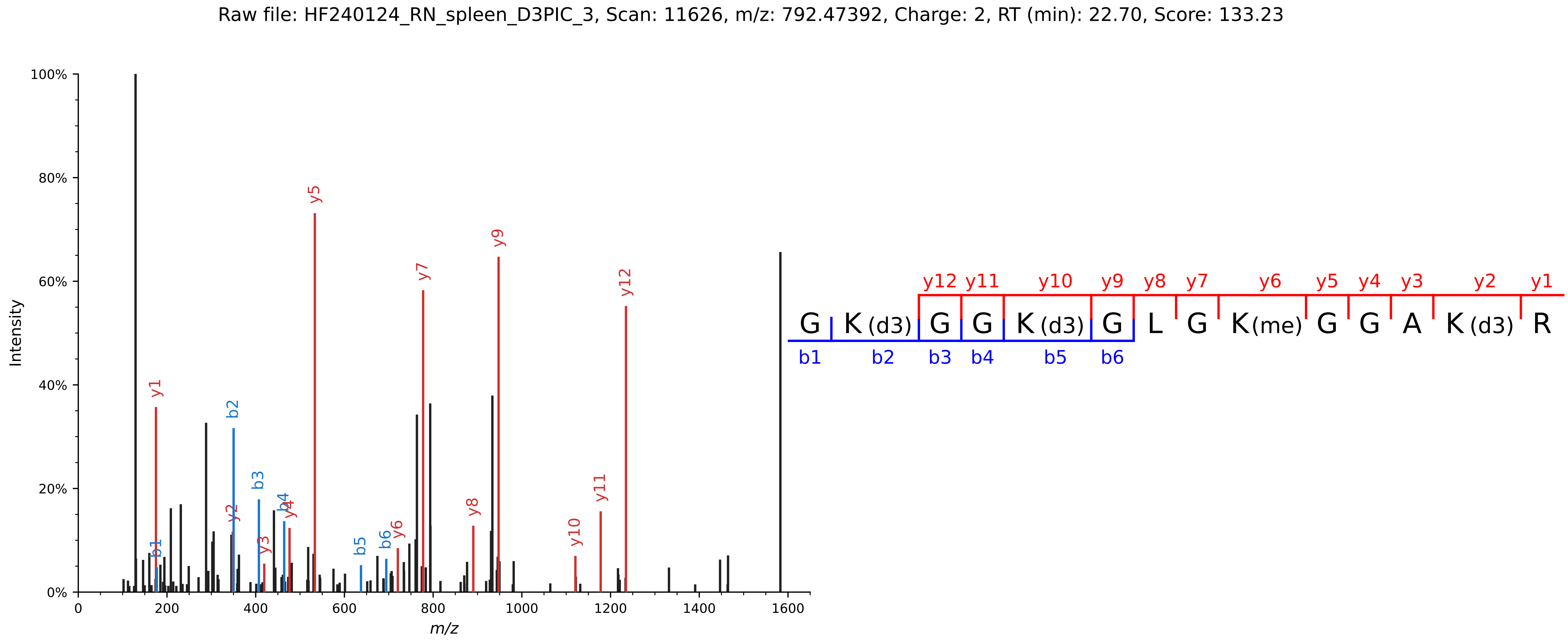

Raw file: HF240124_RN_spleen_D3PIC_3, Scan: 11626, m/z: 792.47392, Charge: 2, RT (min): 22.70, Score: 133.23
Intensity
m/z
y1 b1 b2 y2 b3 y3 b4 y4 y5 b5 b6 y6 y7 y8 y9 y10 y11 y12
G K(d3) G G K(d3) G L G K(me) G G A K(d3) R

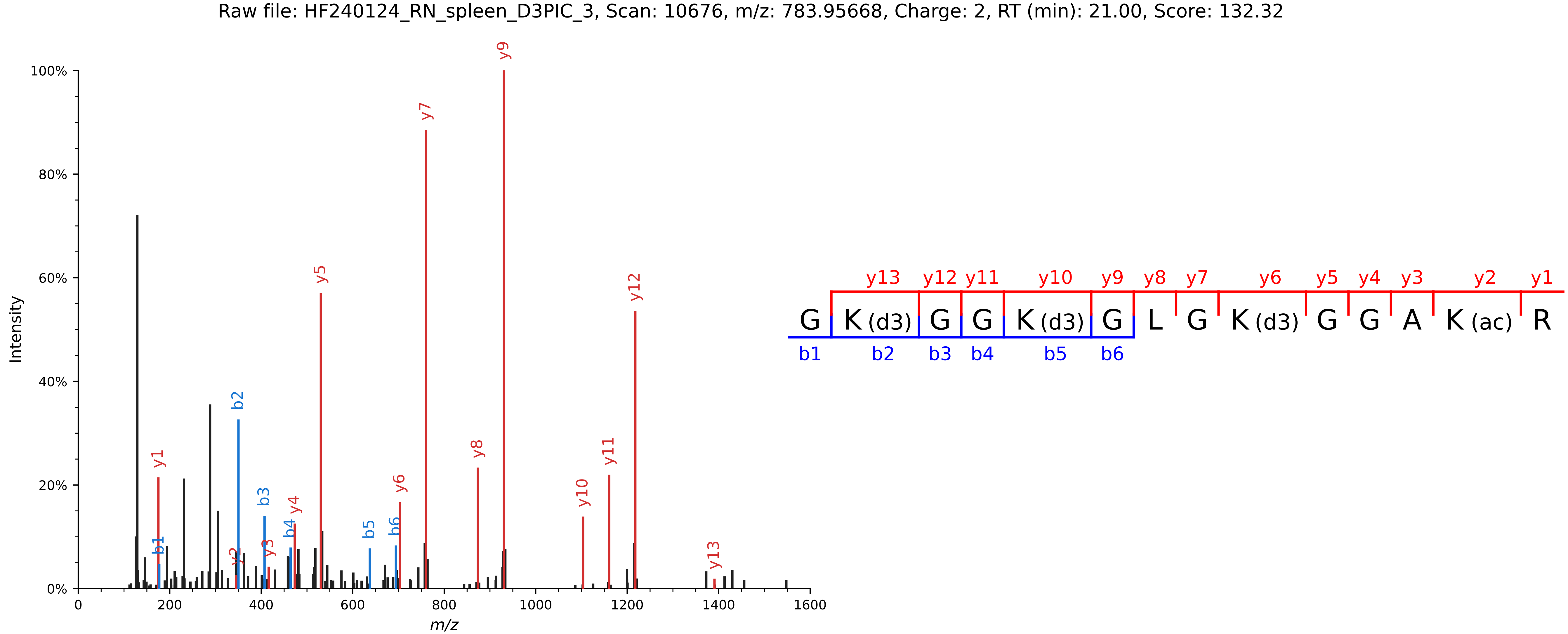
Raw file: HF240124_RN_spleen_D3PIC_3, Scan: 10676, m/z: 783.95668, Charge: 2, RT (min): 21.00, Score: 132.32
Intensity
m/z
G K(d3) G G K(d3) G L G K(d3) G G A K(ac) R
y13 y12 y11 y10 y9 y8 y7 y6 y5 y4 y3 y2 y1
b1 b2 b3 b4 b5 b6

Raw file: HF240124_RN_spleen_D3PIC_3, Scan: 10317, m/z: 776.94886, Charge: 2, RT (min): 20.34, Score: 139.43

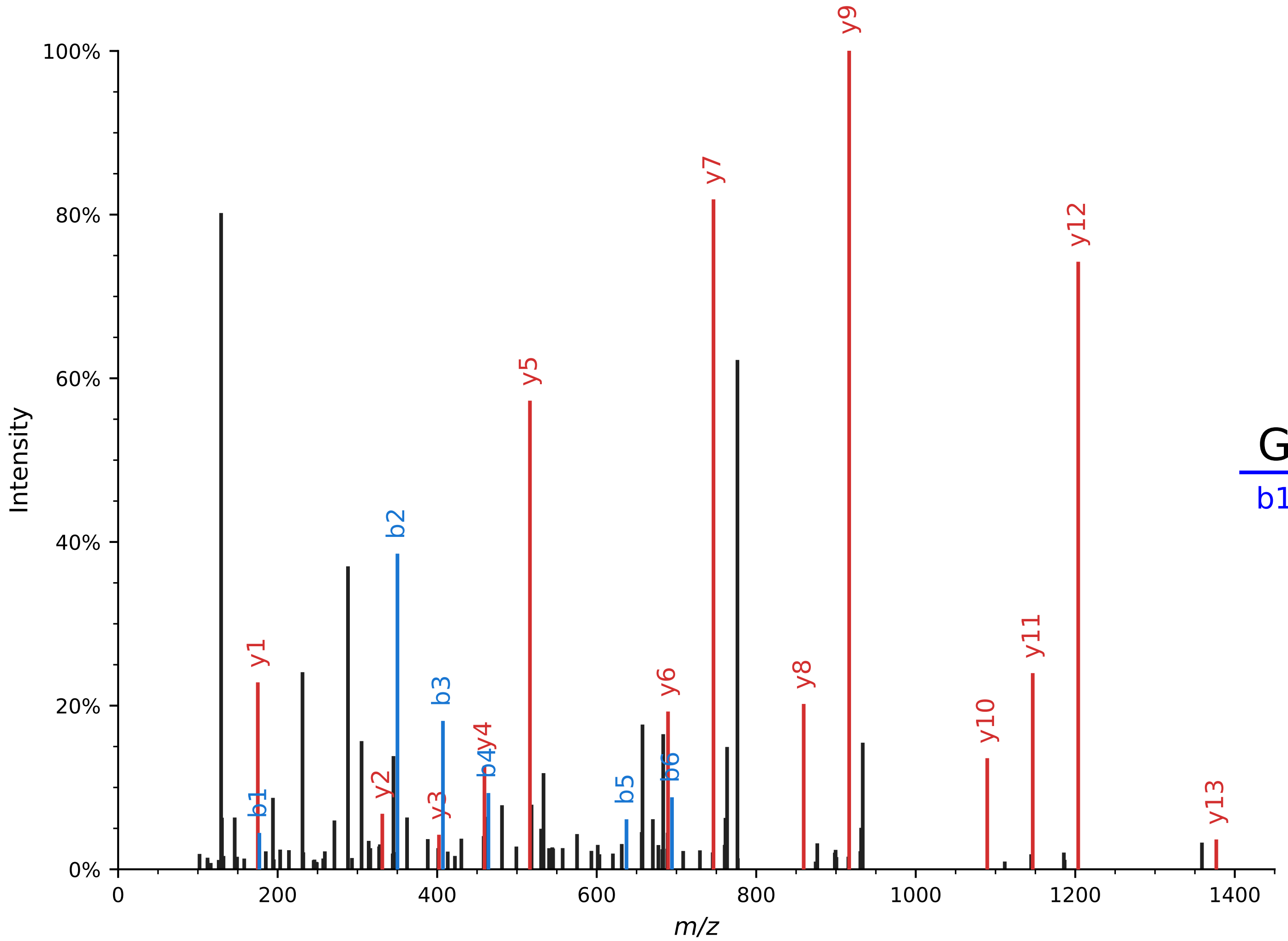

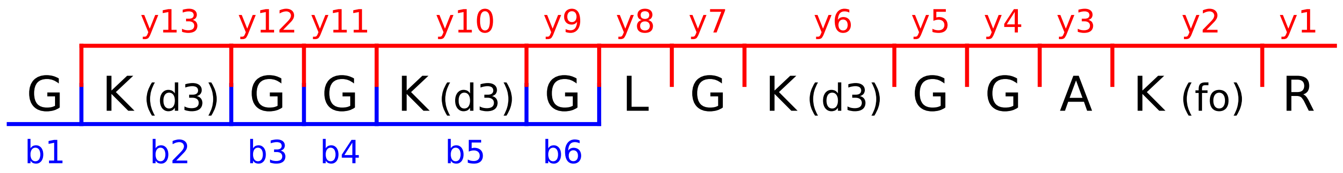

Raw file: HF240124_RN_spleen_D3PIC_2, Scan: 11543, m/z: 792.47392, Charge: 2, RT (min): 22.24, Score: 142.0

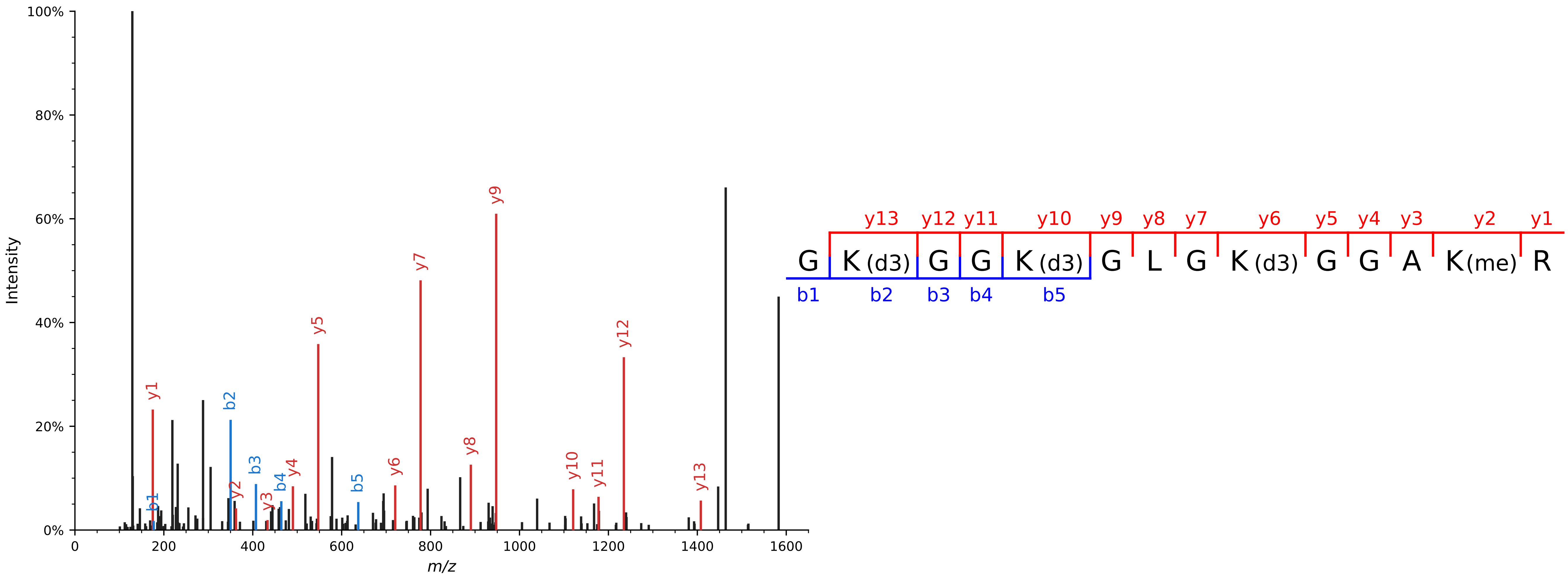

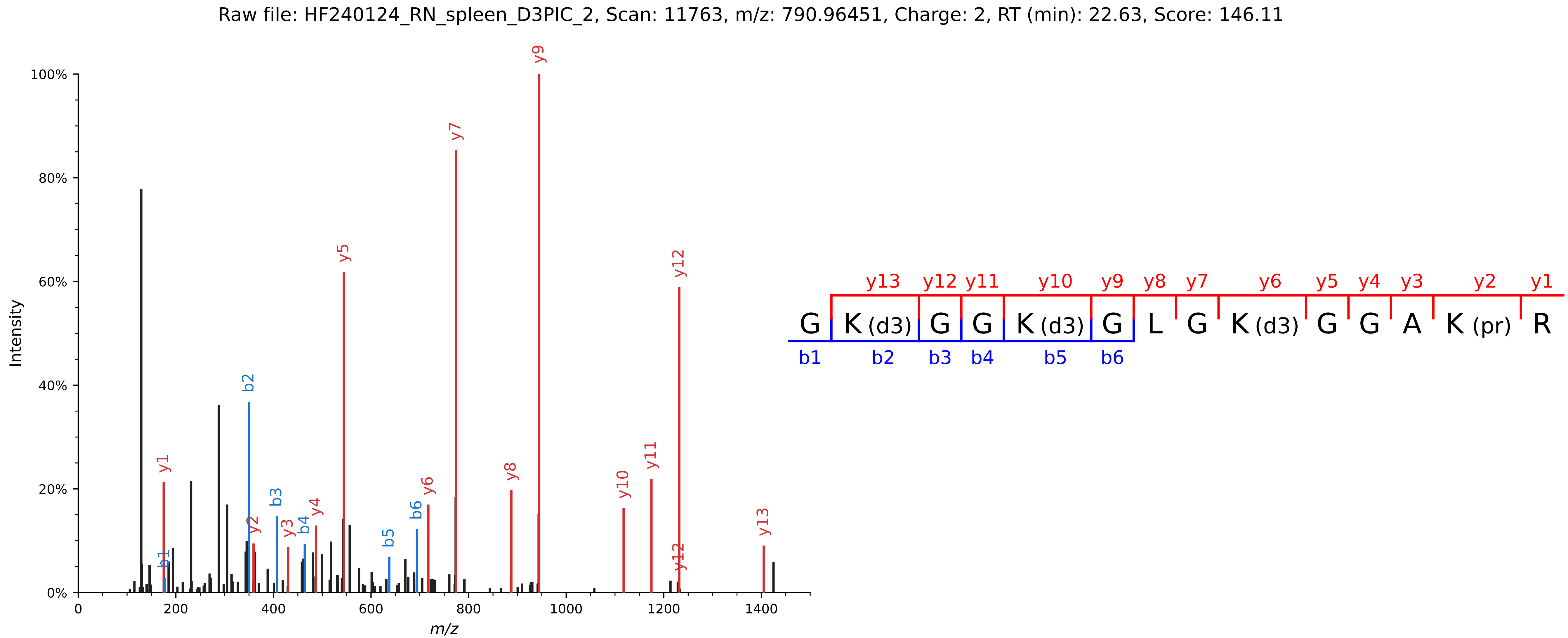

Raw file: HF240124_RN_spleen_D3PIC_2, Scan: 11763, m/z: 790.96451, Charge: 2, RT (min): 22.63, Score: 146.11
Intensity
m/z
G K (d3) G G K (d3) G L G K (d3) G G A K (pr) R
y13 y12 y11 y10 y9 y8 y7 y6 y5 y4 y3 y2 y1
b1 b2 b3 b4 b5 b6

Raw file: HF240124_RN_spleen_D3PIC_3, Scan: 17244, m/z: 743.90426, Charge: 2, RT (min): 32.90, Score: 56.013

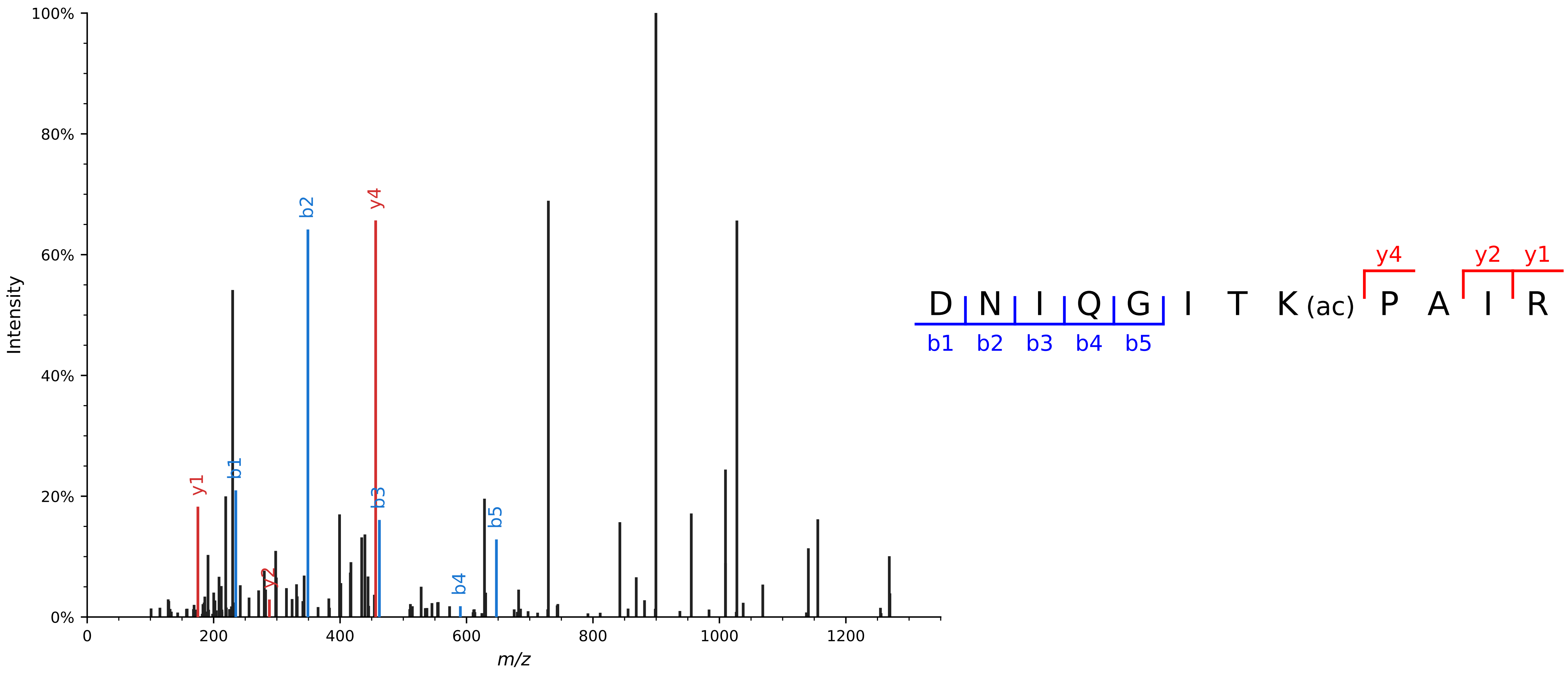

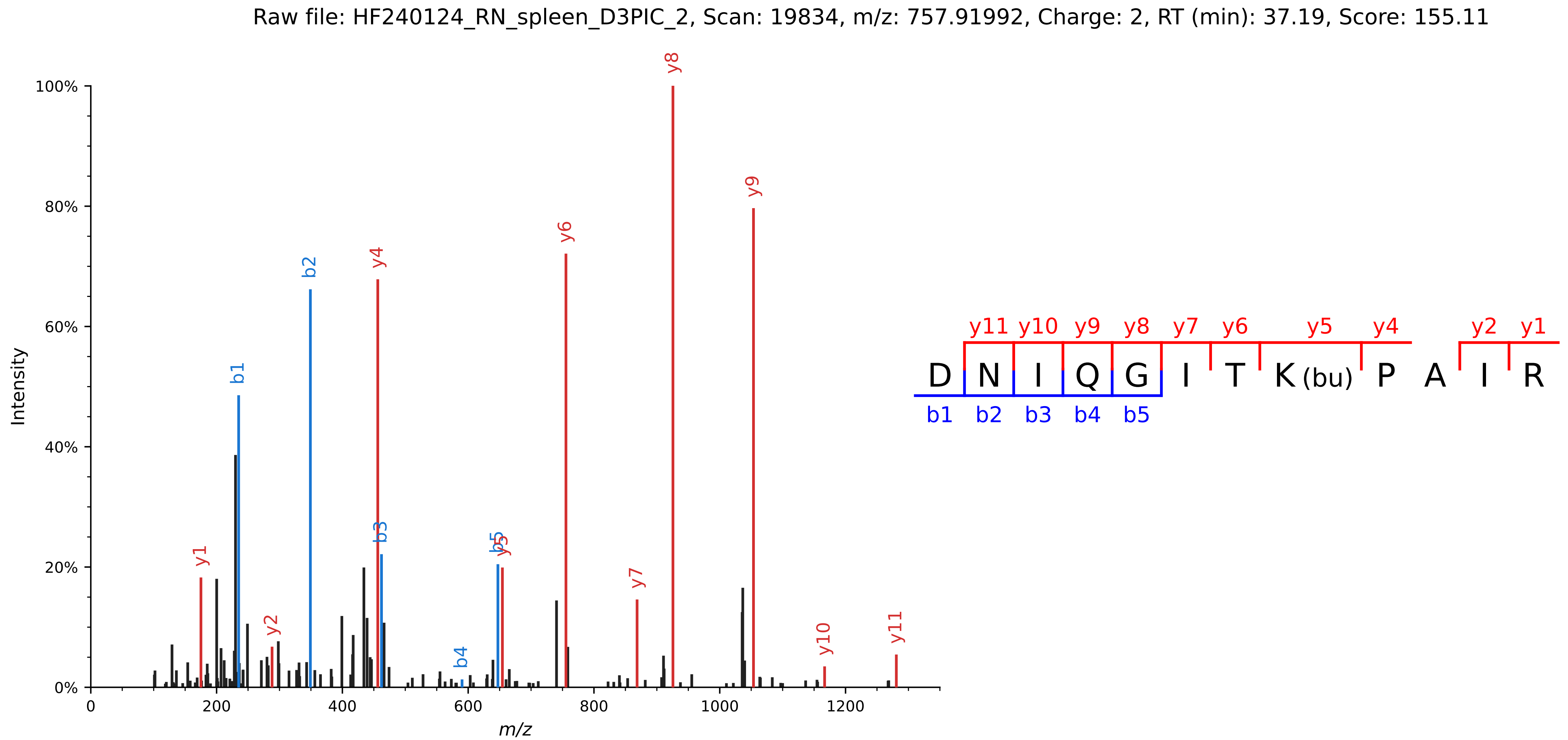

Raw file: HF240124_RN_spleen_D3PIC_2, Scan: 19834, m/z: 757.91992, Charge: 2, RT (min): 37.19, Score: 155.11
Intensity
m/z
0%
20%
40%
60%
80%
100%
0
200
400
600
800
1000
1200
y1
b1
y2
b2
y4
b3
b4
b5
y5
y6
y7
y8
y9
y10
y11
y11 y10 y9 y8 y7 y6 y5 y4 y2 y1
D N I Q G I T K (bu) P A I R
b1 b2 b3 b4 b5

Raw file: HF240124_RN_spleen_D3PIC_1, Scan: 17215, m/z: 736.89644, Charge: 2, RT (min): 32.56, Score: 160.53

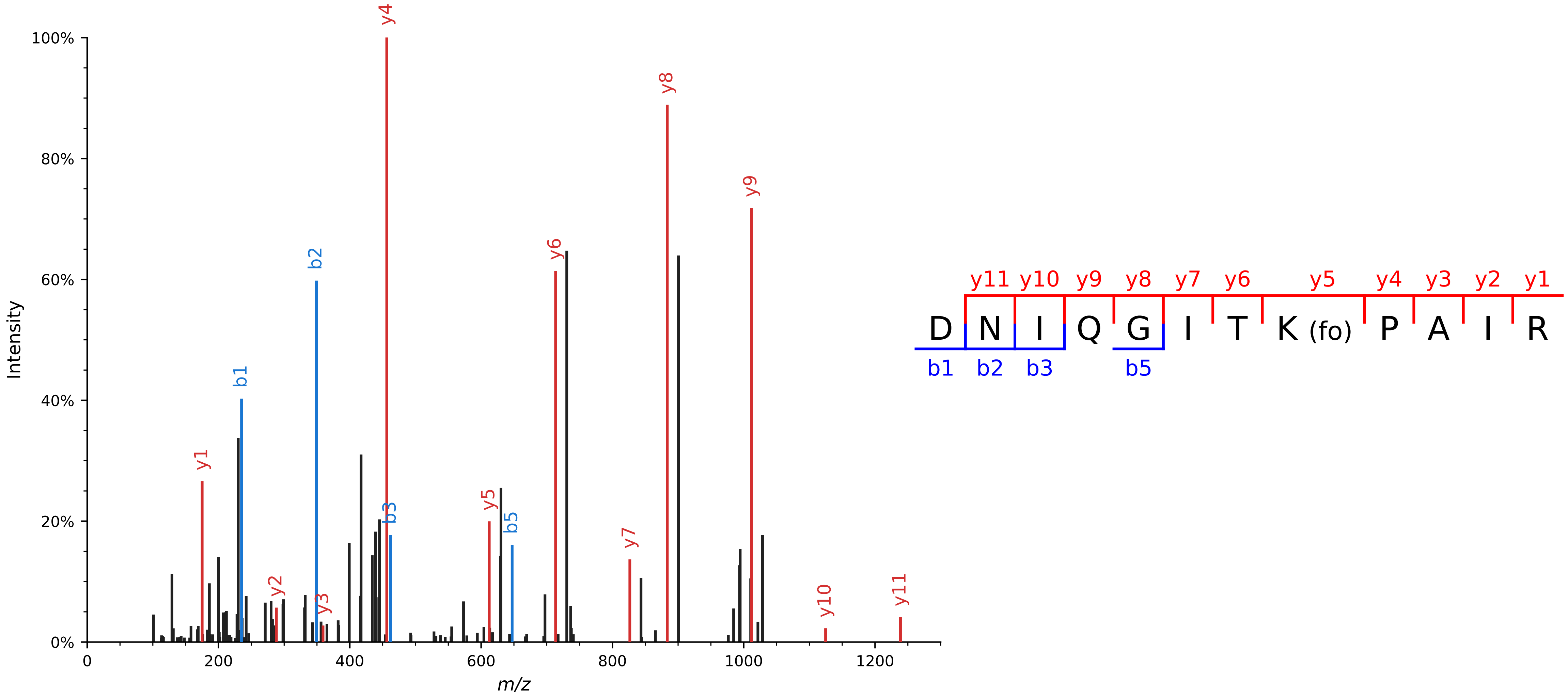

Raw file: HF240124_RN_spleen_D3PIC_1, Scan: 19903, m/z: 779.91483, Charge: 2, RT (min): 37.44, Score: 45.161

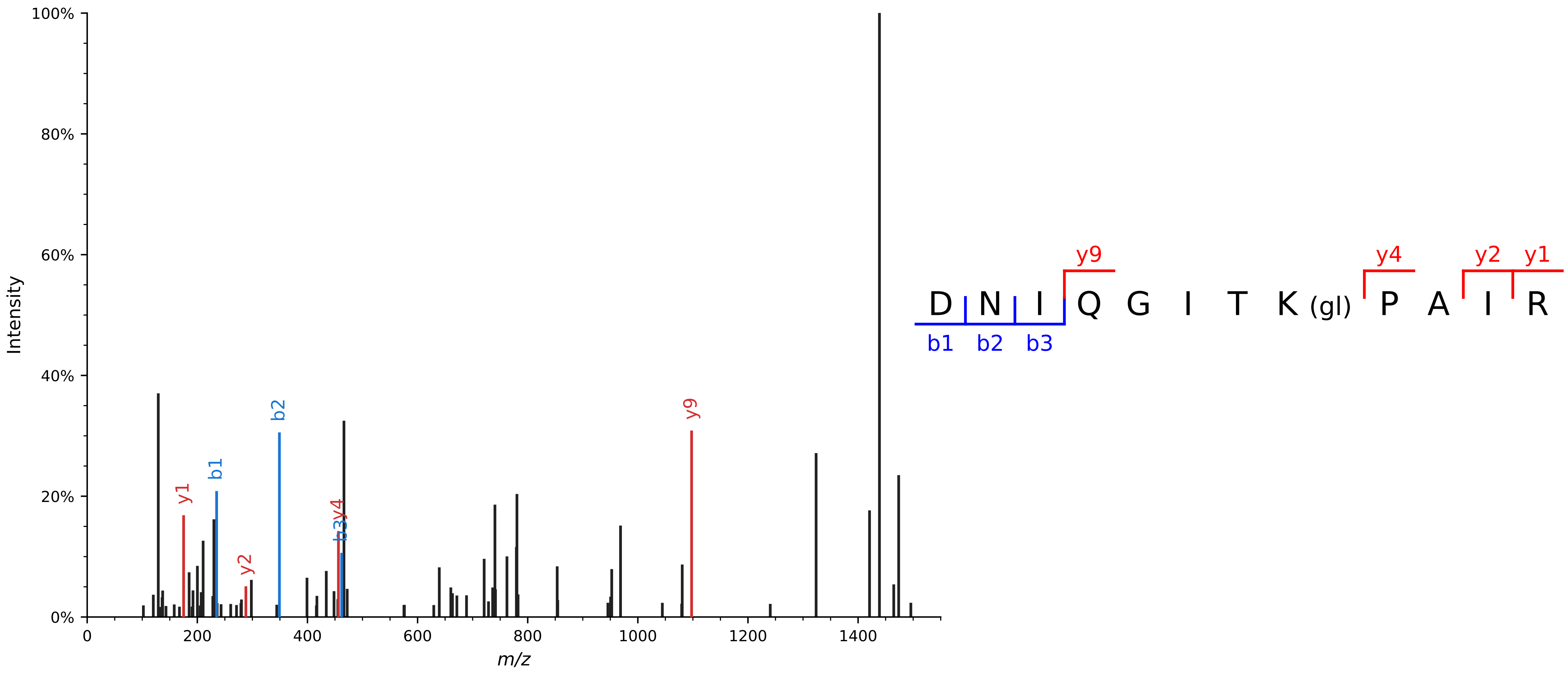

Raw file: HF240124_RN_spleen_D3PIC_2, Scan: 18185, m/z: 750.91209, Charge: 2, RT (min): 34.20, Score: 50.978

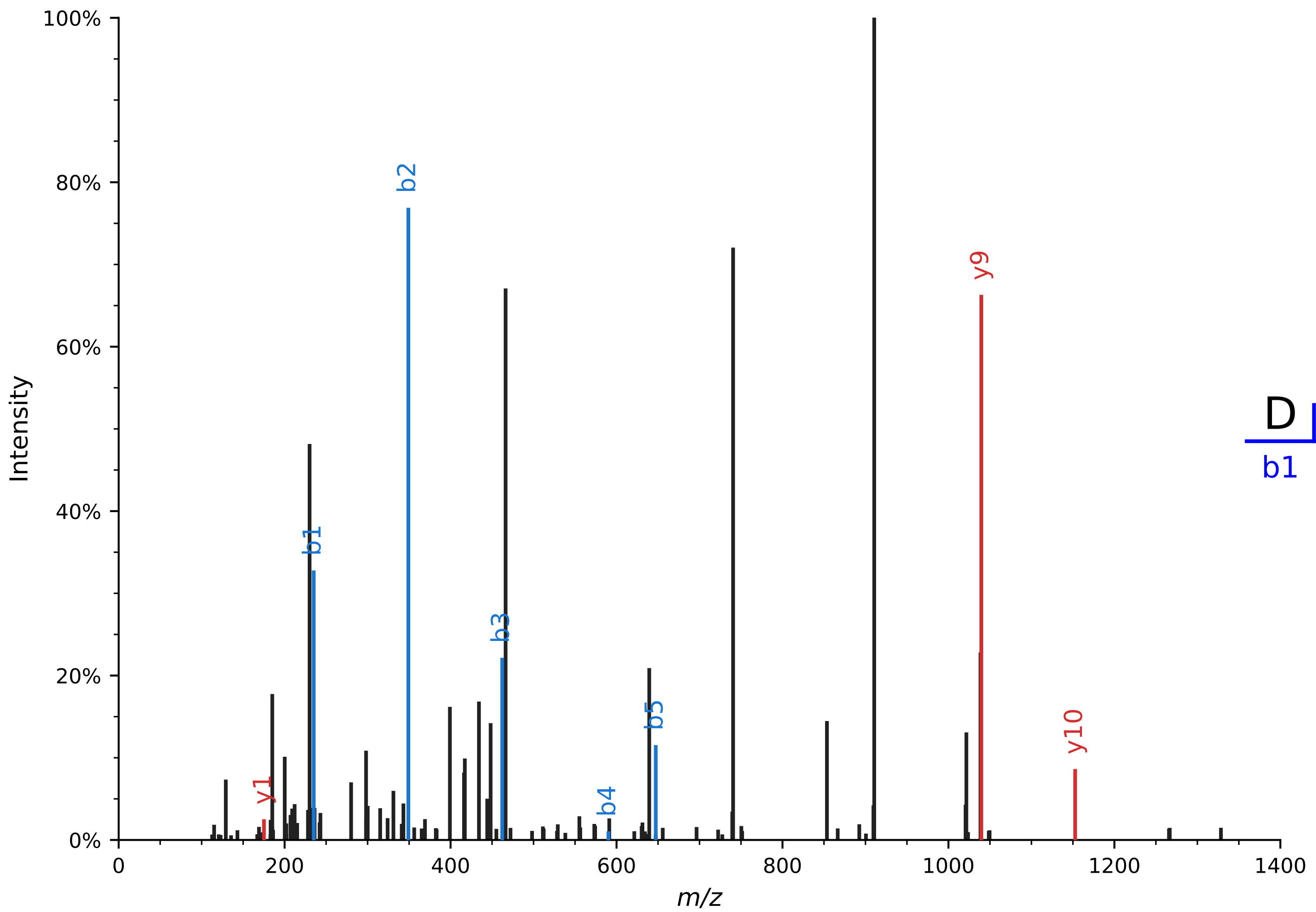

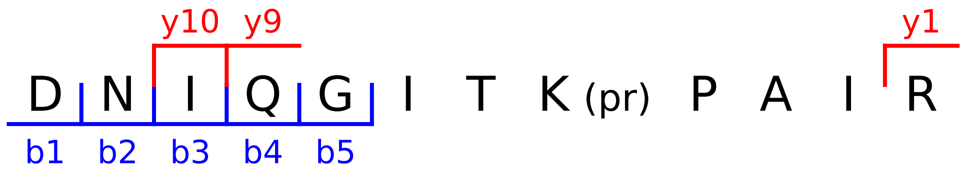

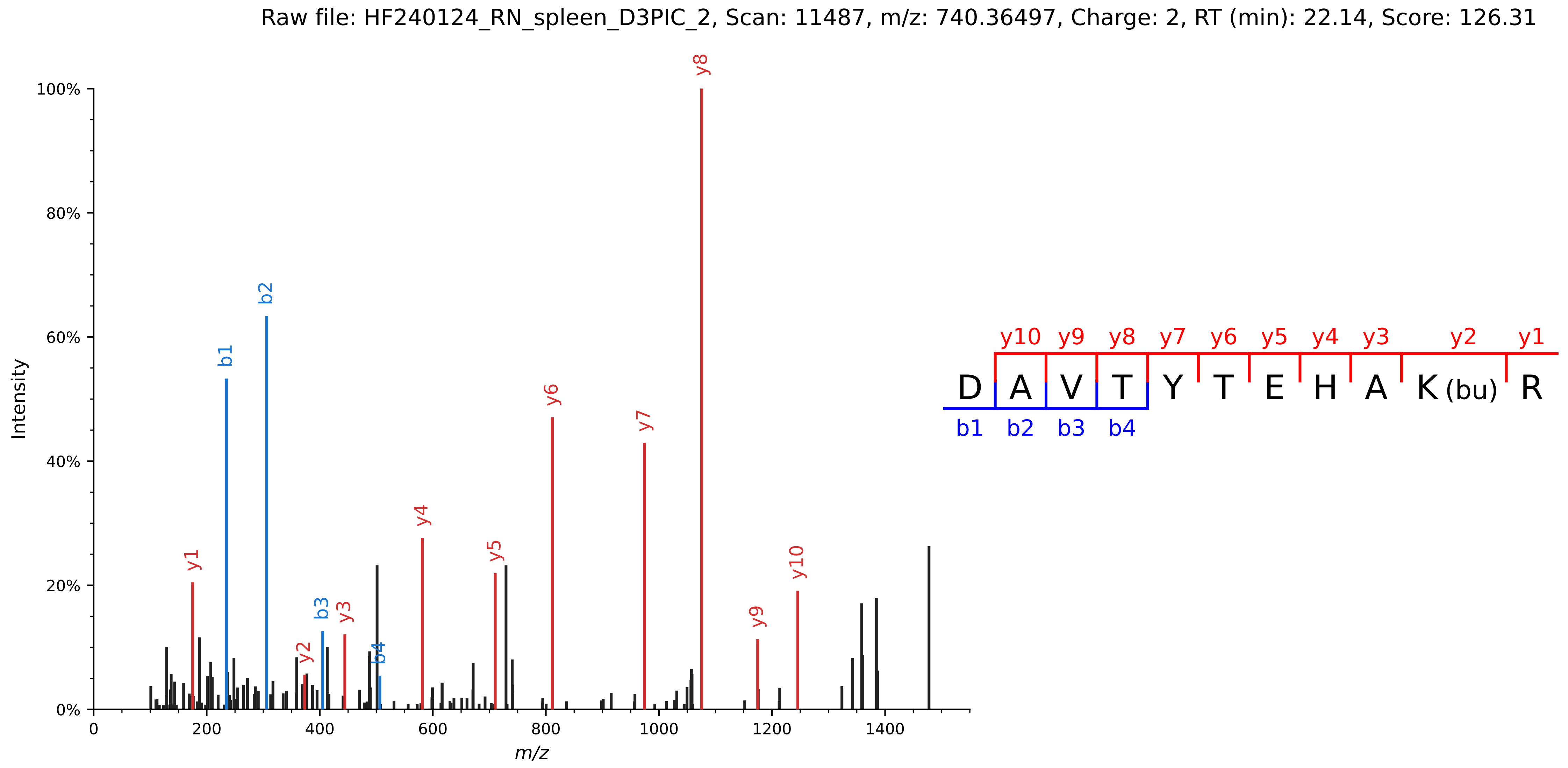

Raw file: HF240124_RN_spleen_D3PIC_2, Scan: 11487, m/z: 740.36497, Charge: 2, RT (min): 22.14, Score: 126.31
Intensity
m/z
y10 y9 y8 y7 y6 y5 y4 y3 y2 y1
D A V T Y T E H A K (bu) R
b1 b2 b3 b4

Raw file: HF240124_RN_spleen_D3PIC_1, Scan: 8824, m/z: 719.3415, Charge: 2, RT (min): 17.31, Score: 196.88

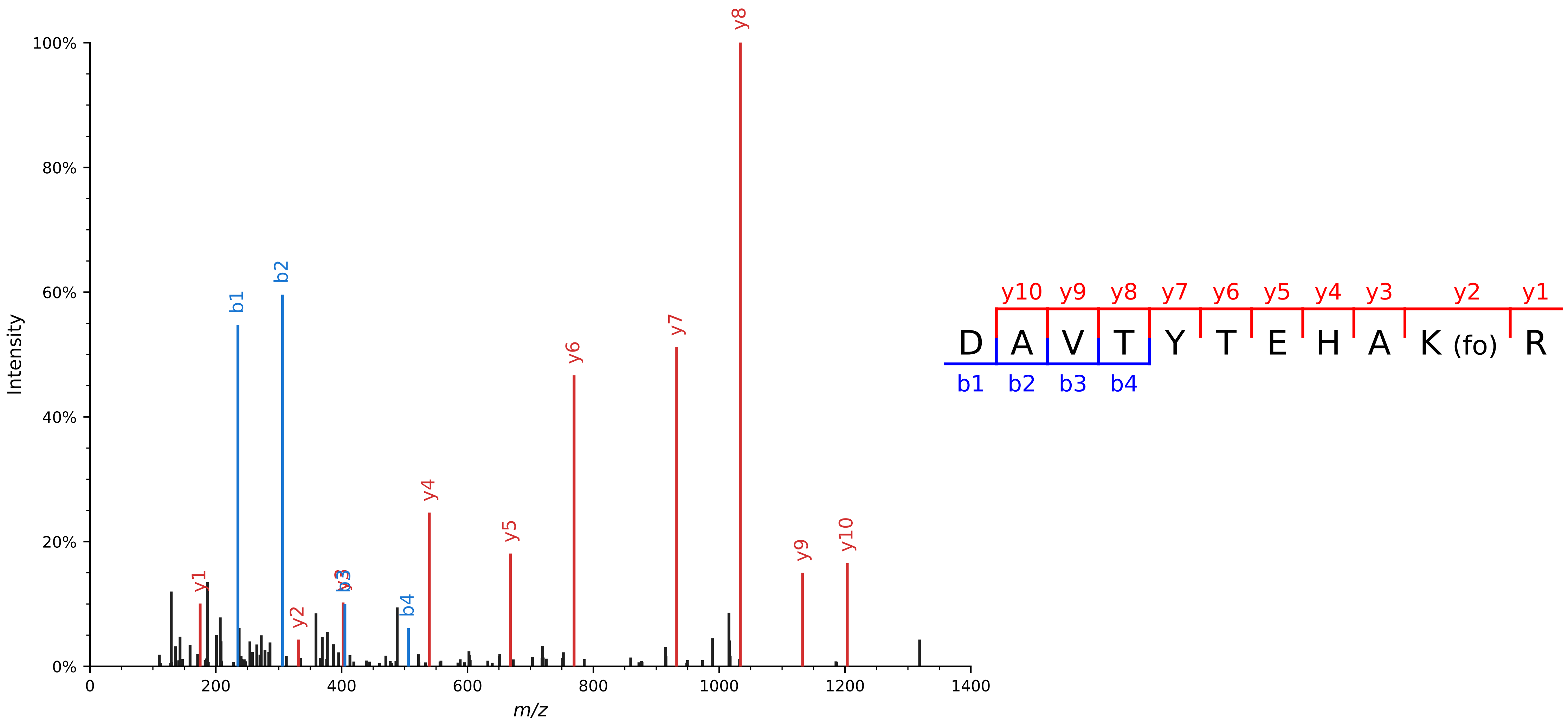

Raw file: HF240124_RN_spleen_D3PIC_1, Scan: 10072, m/z: 733.35715, Charge: 2, RT (min): 19.72, Score: 95.573

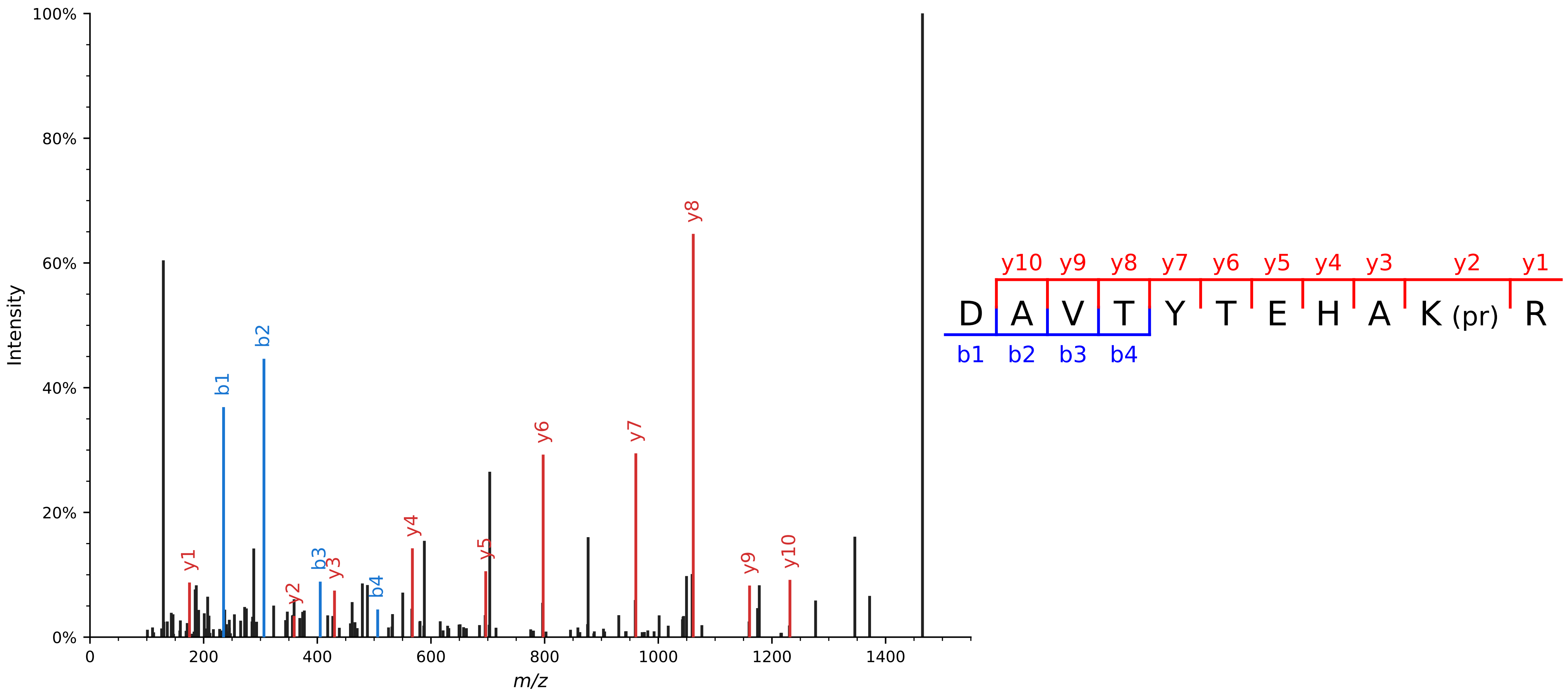

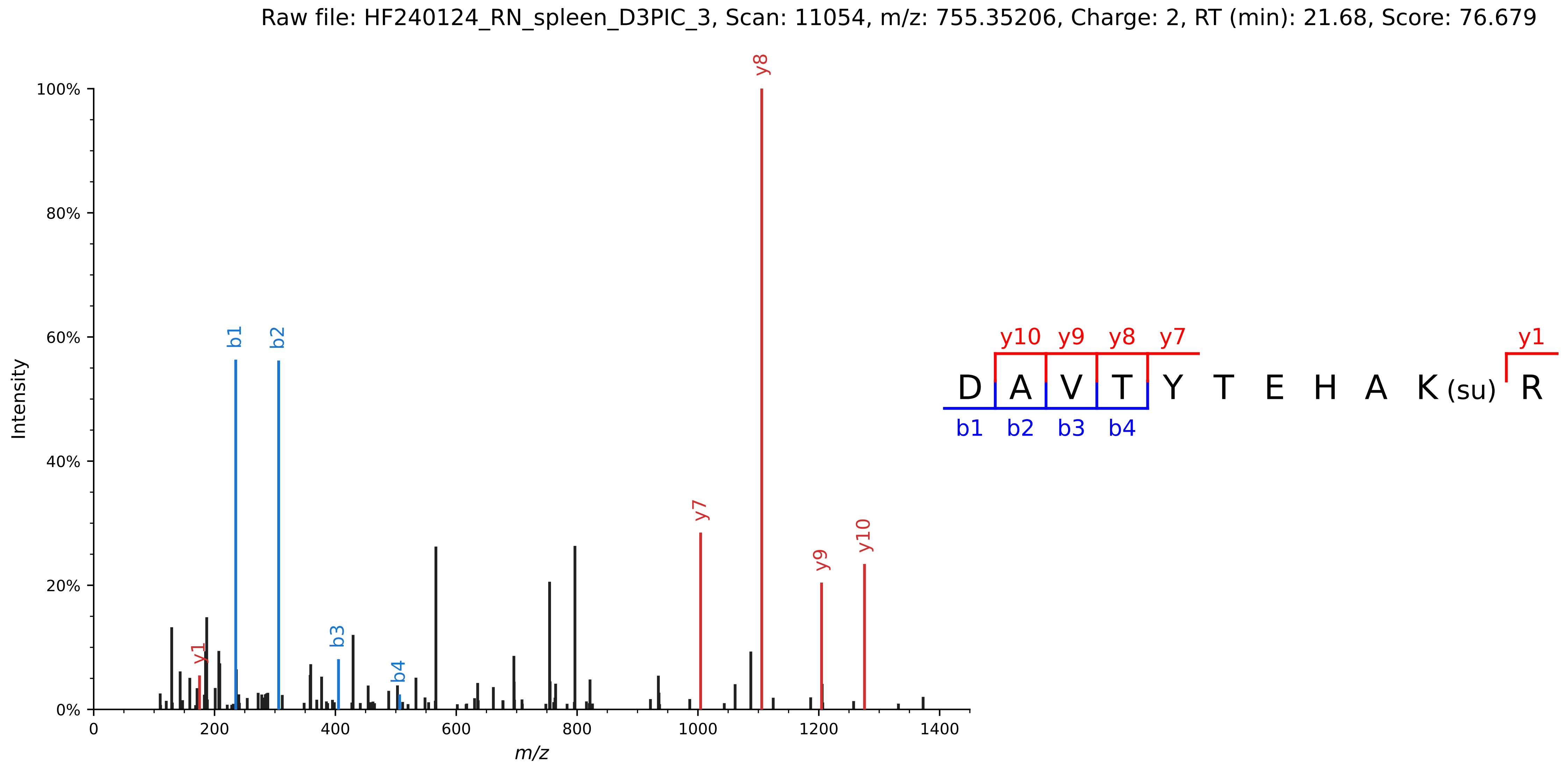
Raw file: HF240124_RN_spleen_D3PIC_3, Scan: 11054, m/z: 755.35206, Charge: 2, RT (min): 21.68, Score: 76.679
Intensity
m/z
y10 y9 y8 y7 y1
D A V T Y T E H A K (su) R
b1 b2 b3 b4

Raw file: HF240124_RN_spleen_D3PIC_2, Scan: 29134, m/z: 915.00709, Charge: 2, RT (min): 54.85, Score: 61.353

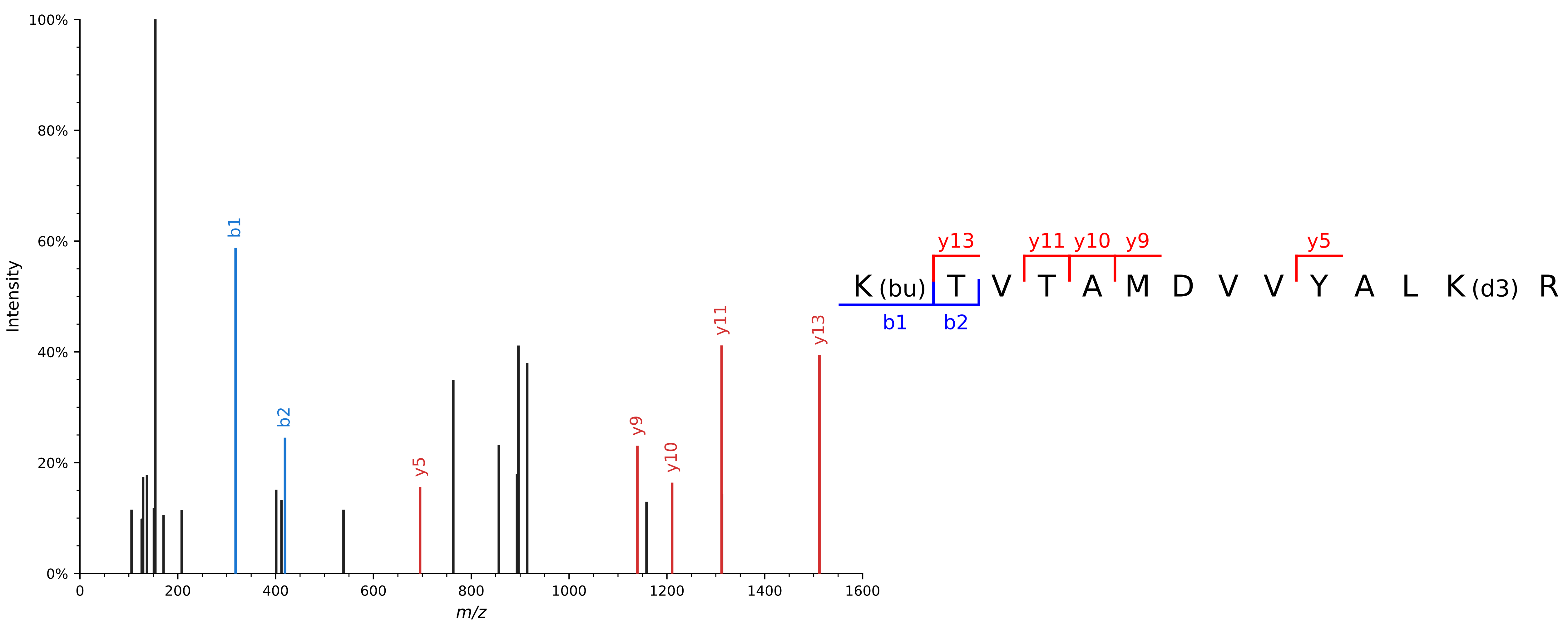

Raw file: HF240124_RN_spleen_D3PIC_2, Scan: 26654, m/z: 893.98361, Charge: 2, RT (min): 49.83, Score: 79.693

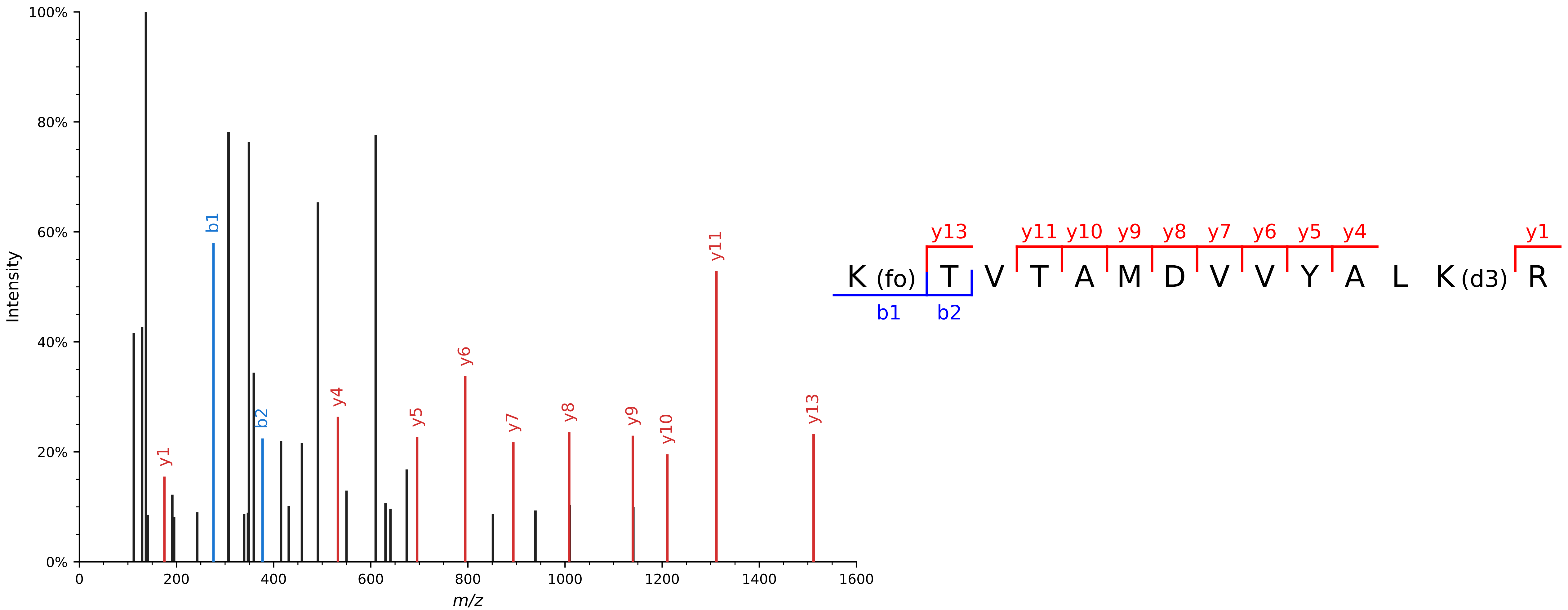

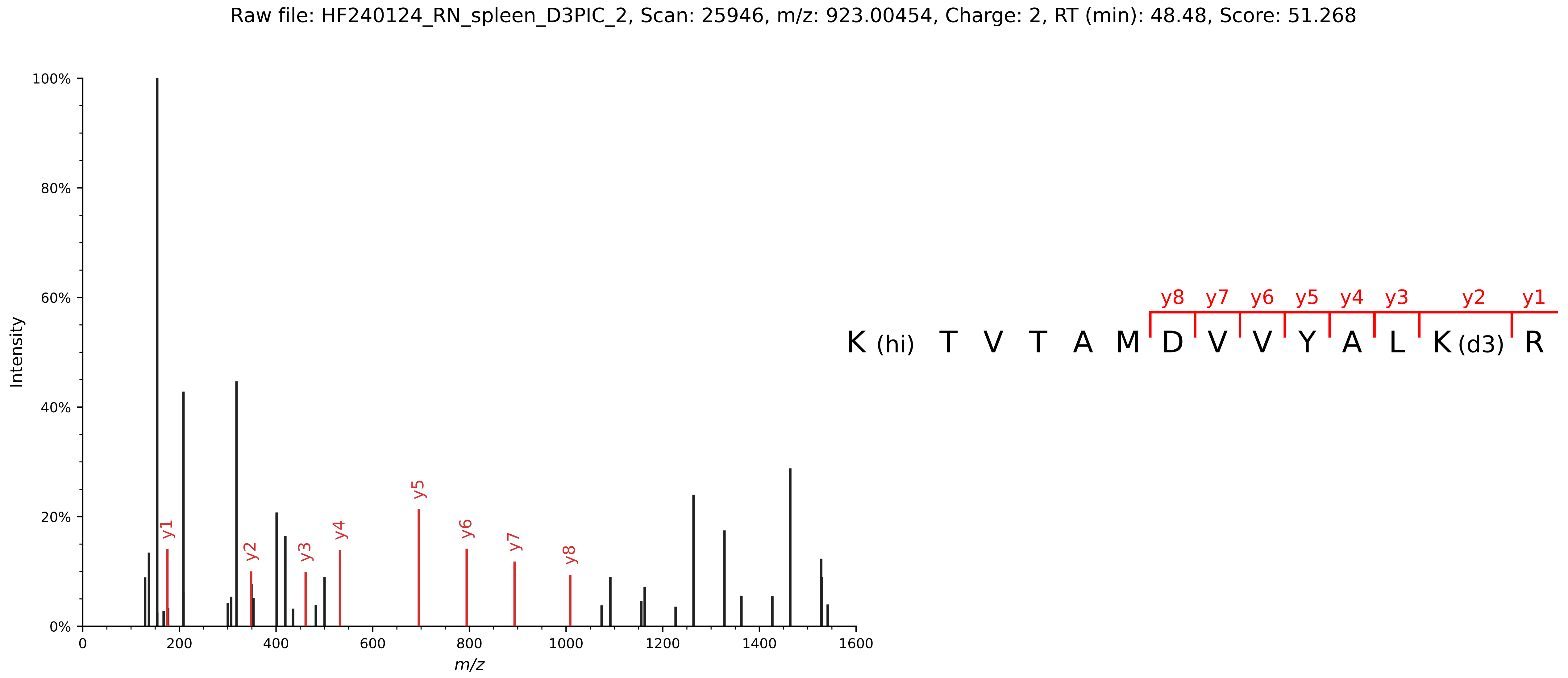

Raw file: HF240124_RN_spleen_D3PIC_2, Scan: 25946, m/z: 923.00454, Charge: 2, RT (min): 48.48, Score: 51.268
Intensity
100%
80%
60%
40%
20%
0%
0
200
400
600
800
1000
1200
1400
1600
m/z
y1
y2
y3
y4
y5
y6
y7
y8
K (hi) T V T A M D V V Y A L K (d3) R
y8 y7 y6 y5 y4 y3 y2 y1

Raw file: HF240124_RN_spleen_D3PIC_2, Scan: 17261, m/z: 909.50868, Charge: 2, RT (min): 32.54, Score: 51.268

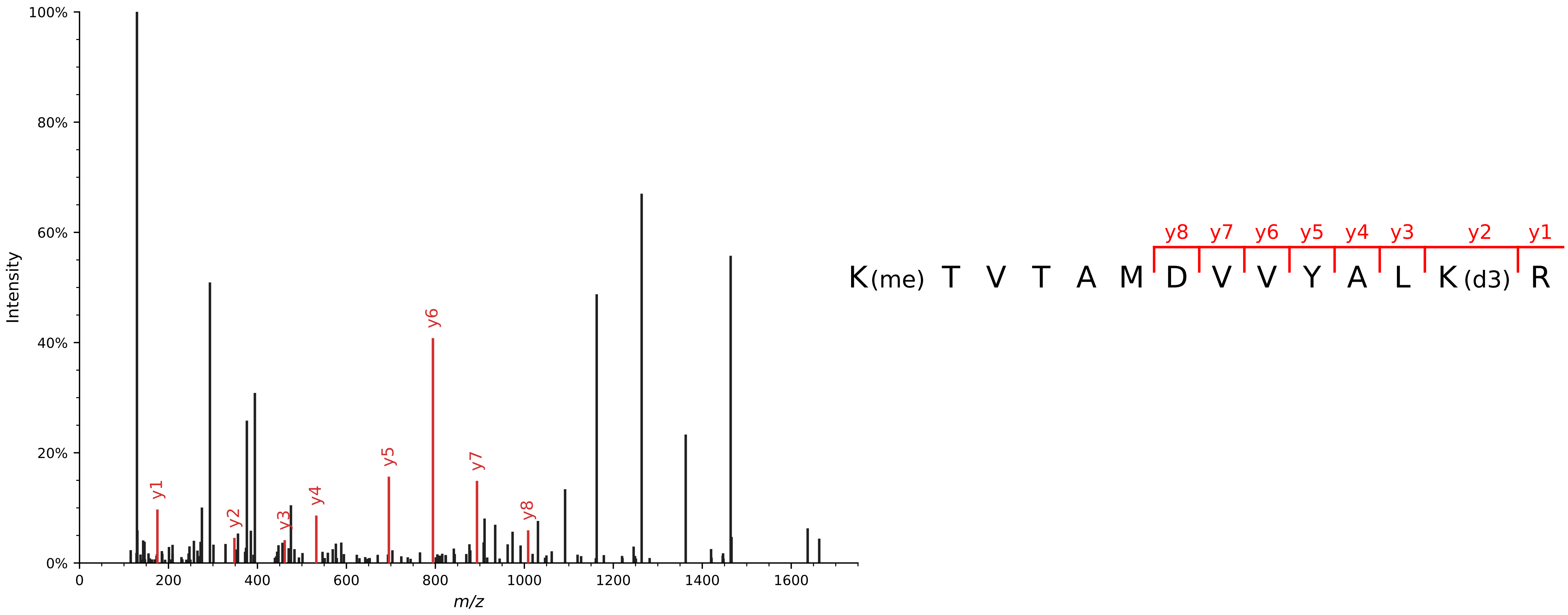

Raw file: HF240124_RN_spleen_D3PIC_1, Scan: 26804, m/z: 893.98361, Charge: 2, RT (min): 50.18, Score: 70.942

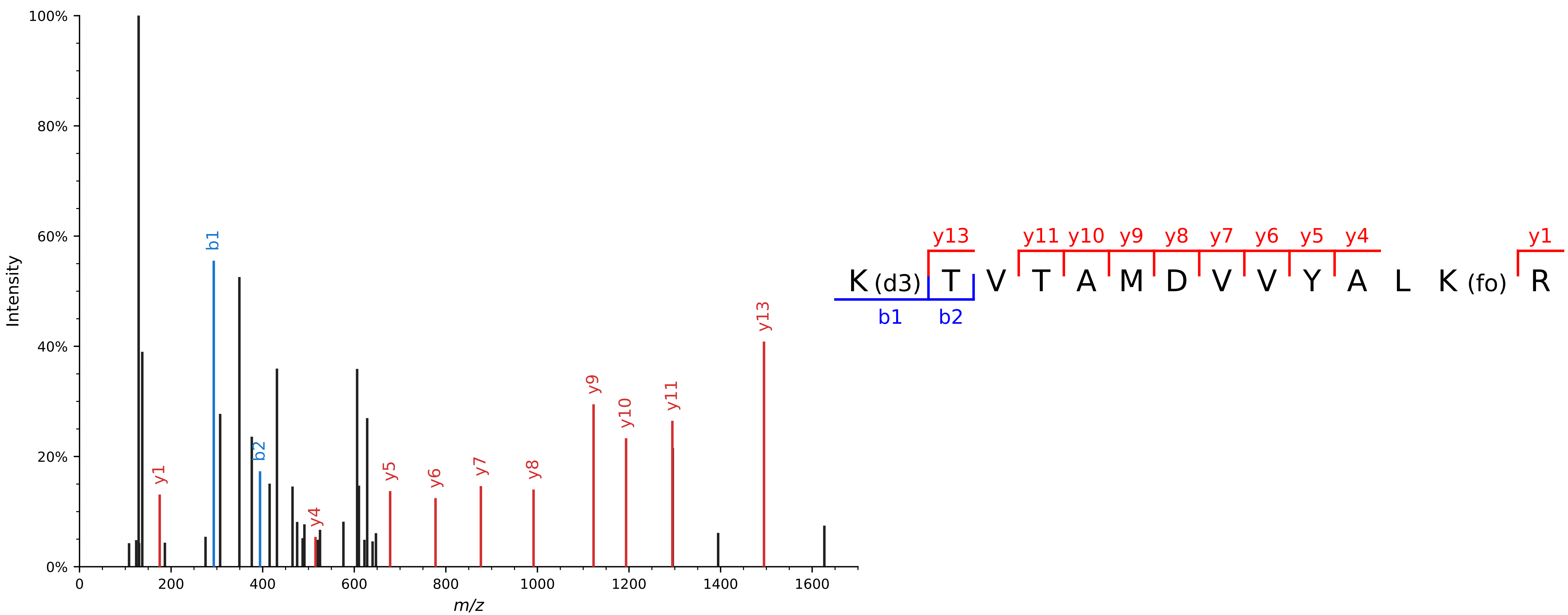

Antibiotics

Raw file: QEP231006_CG_frozen_cecum_A1, Scan: 13563, m/z: 617.33691, Charge: 2, RT (min): 33.54, Score: 46.891

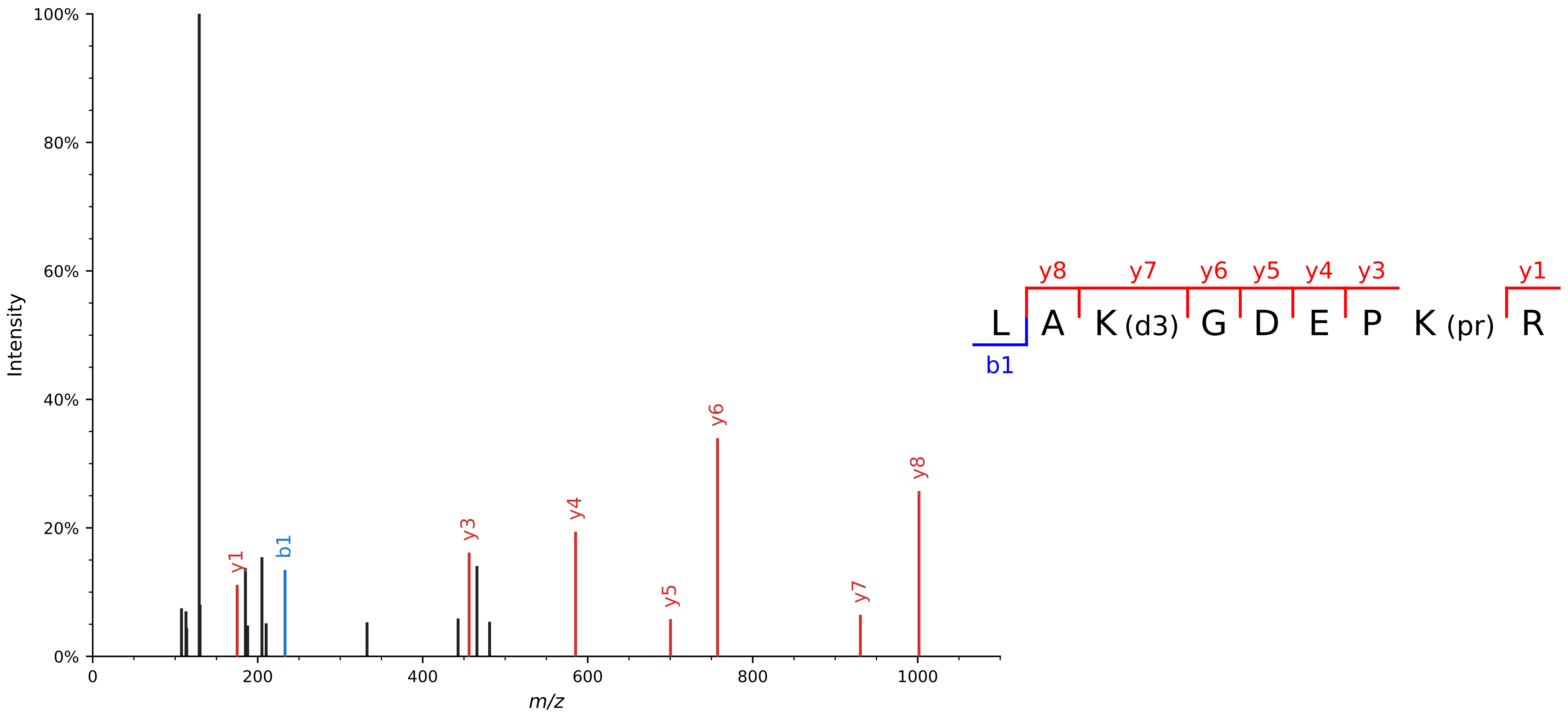

Raw file: QEP231006_CG_frozen_colon_A2, Scan: 8651, m/z: 489.78157, Charge: 2, RT (min): 23.04, Score: 70.268

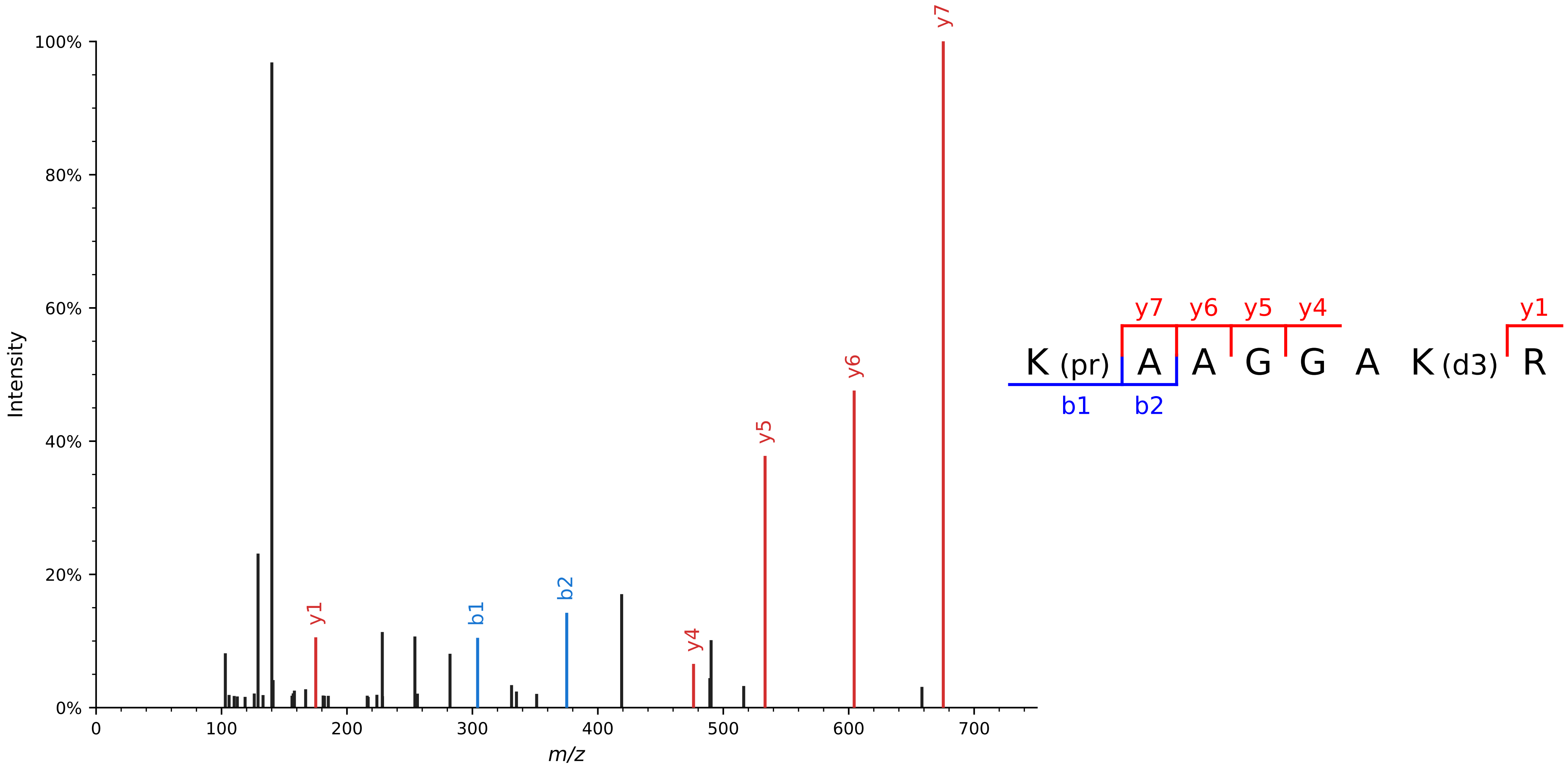

Raw file: QEP231006_CG_frozen_colon_A2, Scan: 8496, m/z: 489.78157, Charge: 2, RT (min): 22.68, Score: 47.743

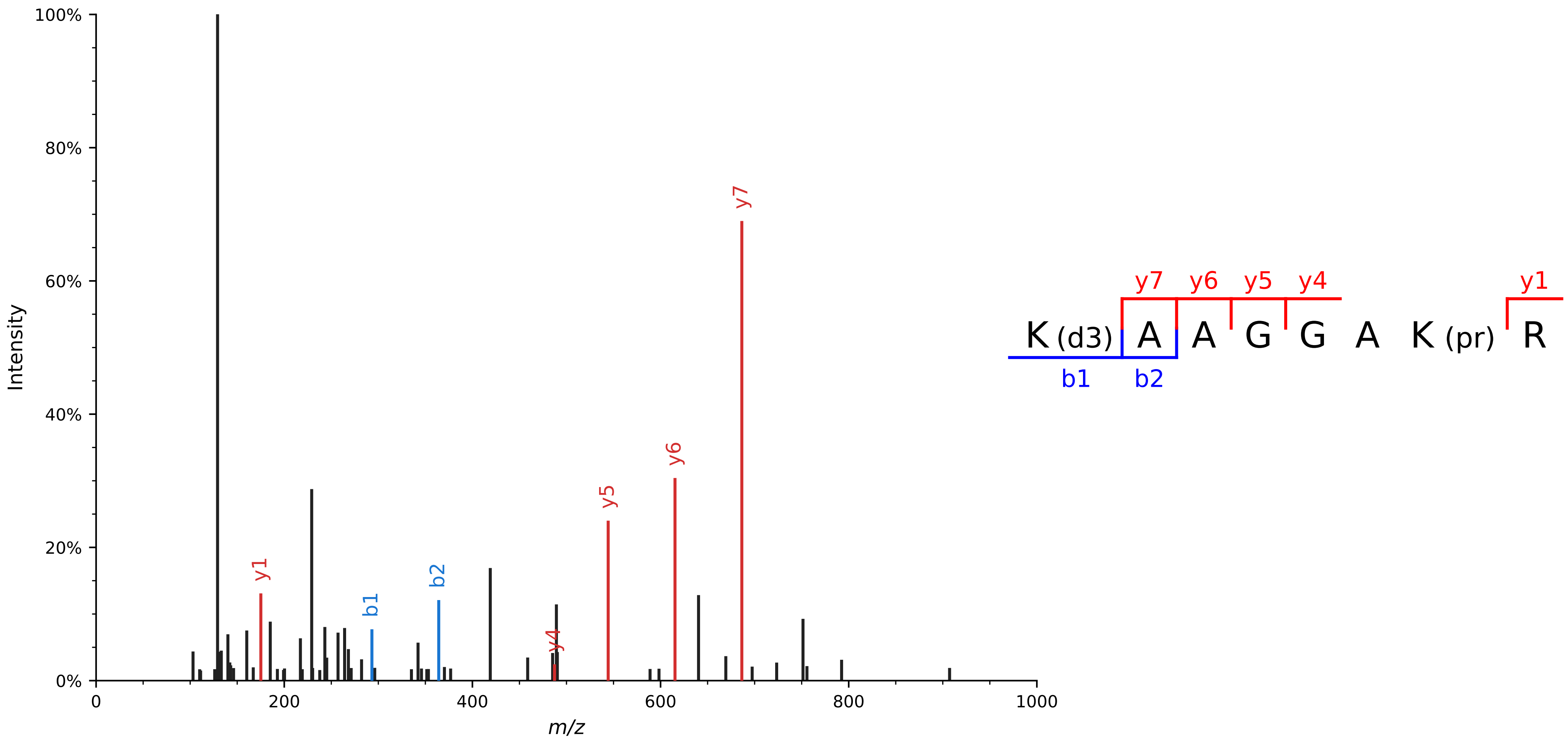

Raw file: QEP231006_CG_frozen_cecum_C1, Scan: 9119, m/z: 440.75757, Charge: 2, RT (min): 23.96, Score: 105.22

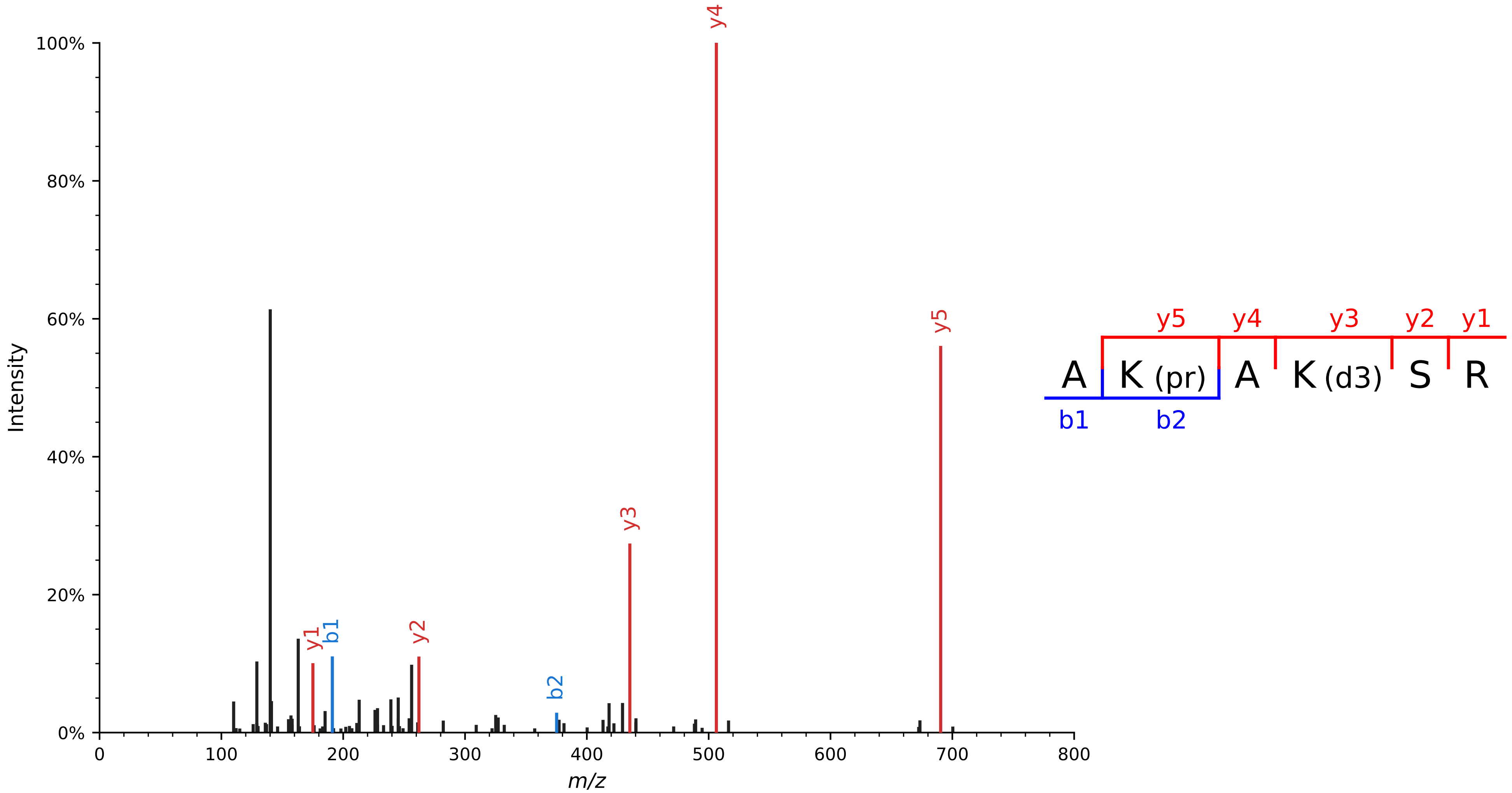

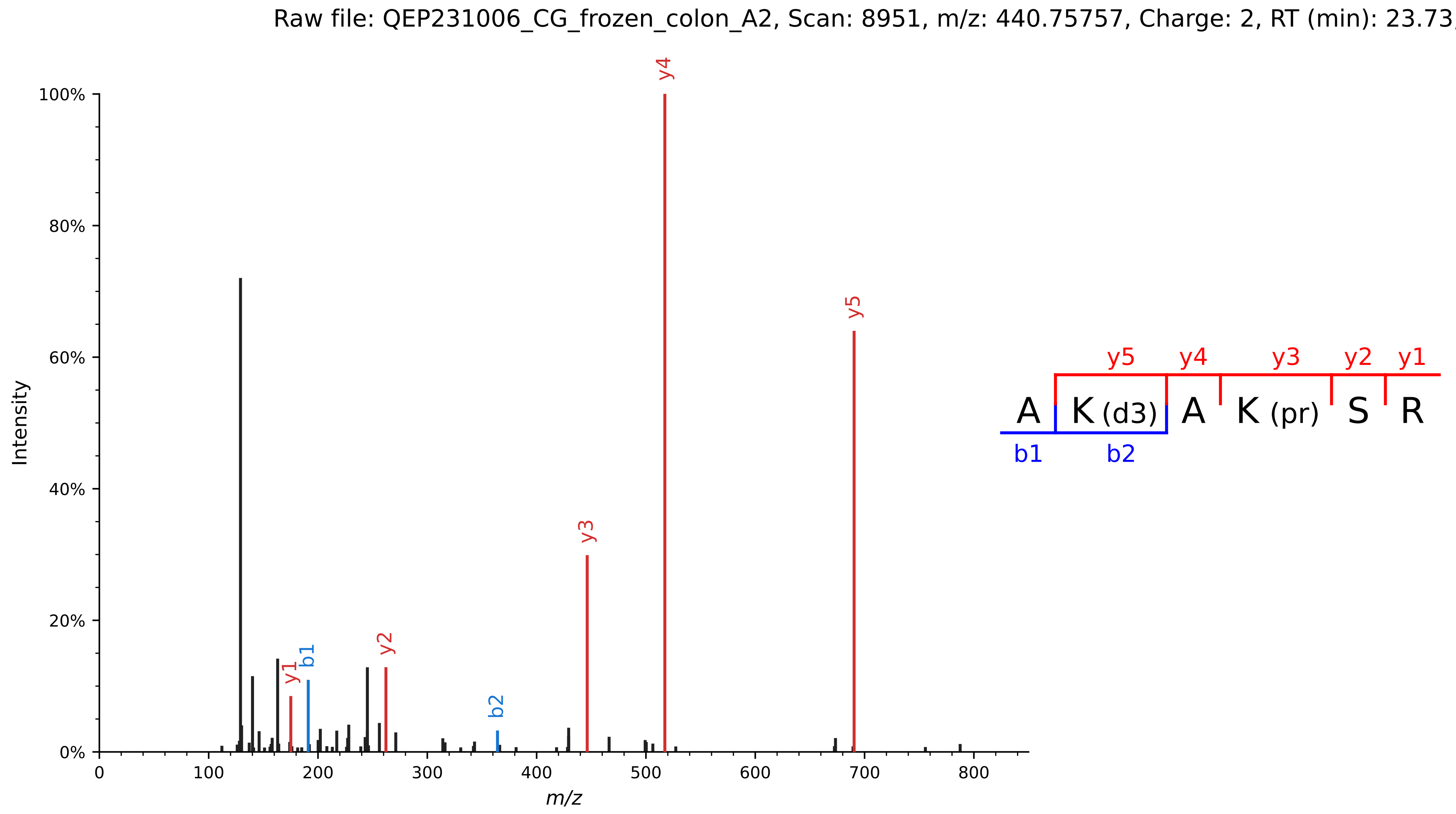

Raw file: QEP231006_CG_frozen_colon_A2, Scan: 8951, m/z: 440.75757, Charge: 2, RT (min): 23.73, Score: 111.81
Intensity
100%
80%
60%
40%
20%
0%
0
100
200
300
400
500
600
700
800
m/z
y4
y5
y3
y2
y1
b1
b2
y5 y4 y3 y2 y1
A K (d3) A K (pr) S R
b1 b2

Raw file: QEP231006_CG_frozen_cecum_C2, Scan: 9192, m/z: 514.74343, Charge: 2, RT (min): 24.03, Score: 111.82

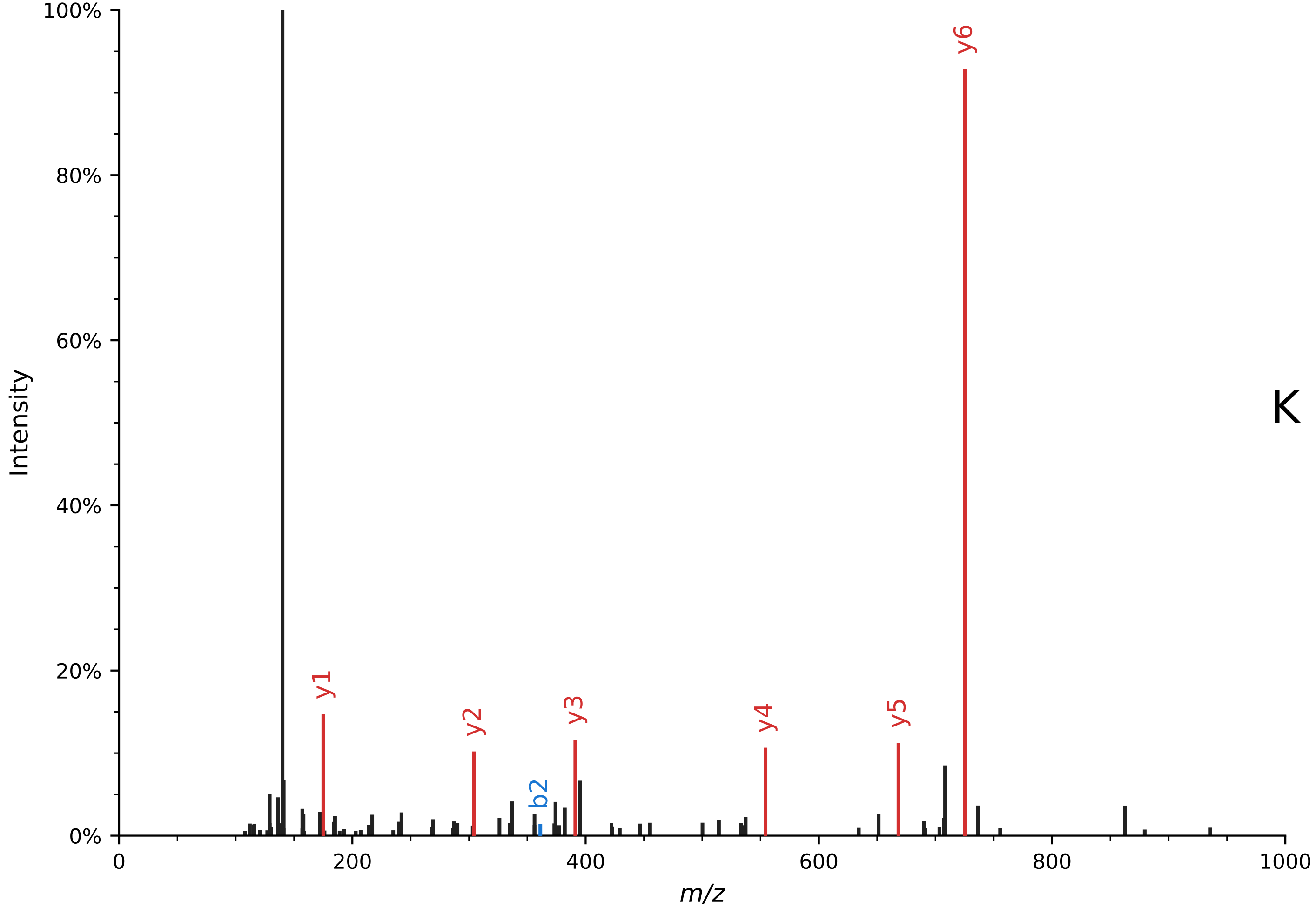

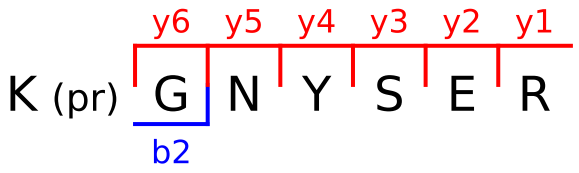

Raw file: QEP231006_CG_frozen_cecum_C2, Scan: 7949, m/z: 525.30013, Charge: 2, RT (min): 21.38, Score: 76.847

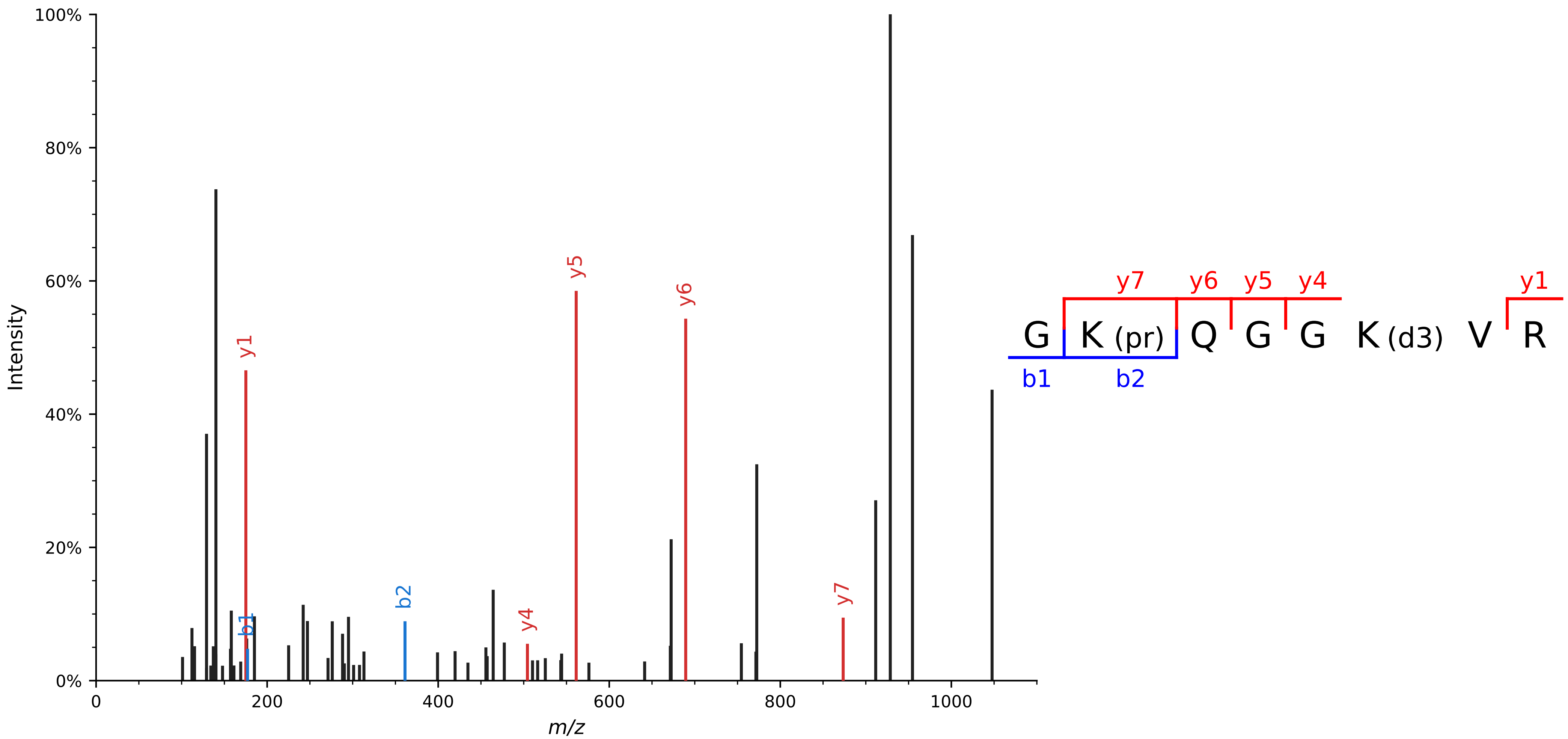

Raw file: QEP231006_CG_frozen_colon_C1, Scan: 6006, m/z: 511.28448, Charge: 2, RT (min): 17.42, Score: 64.803

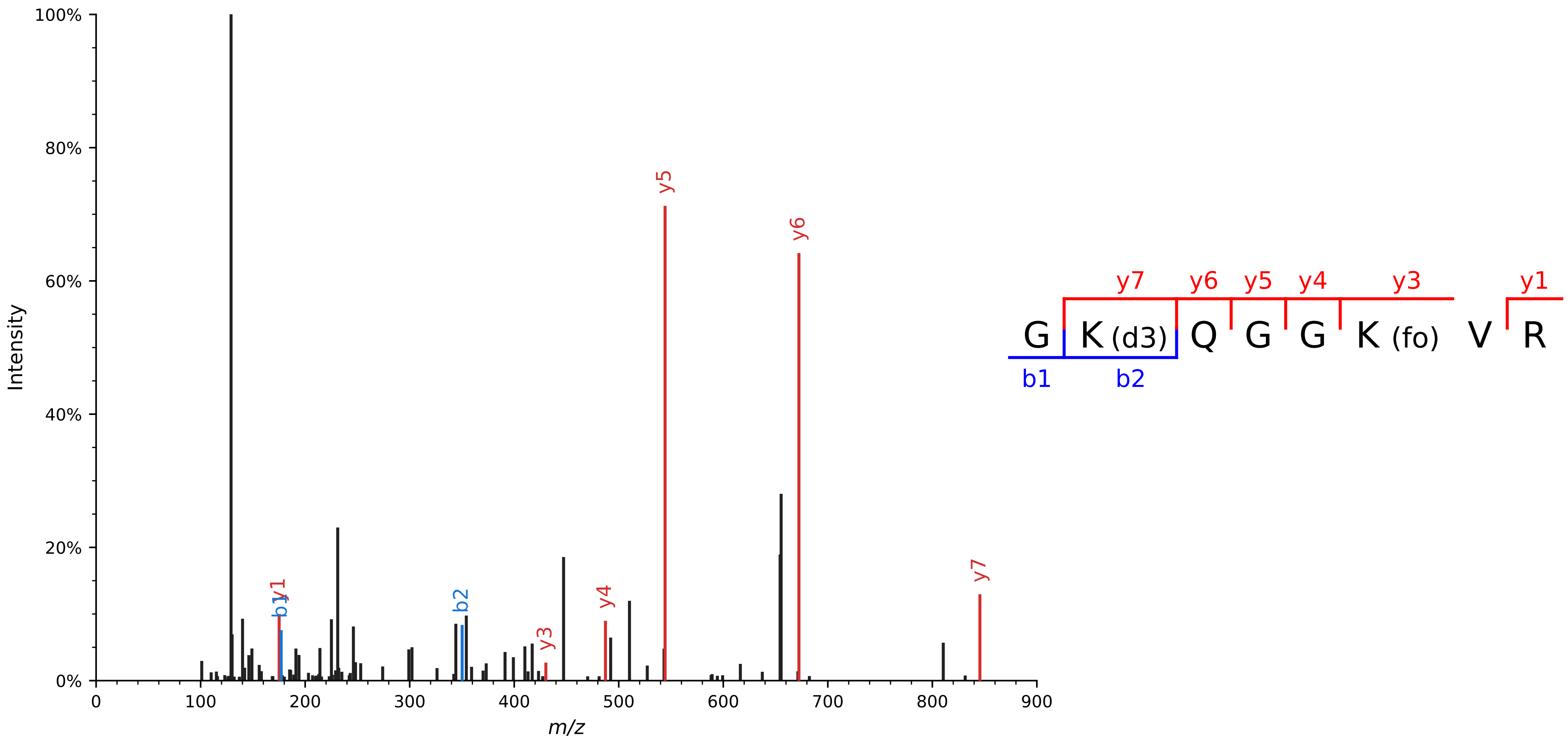

Raw file: QEP231006_CG_frozen_cecum_C2, Scan: 7781, m/z: 525.30013, Charge: 2, RT (min): 21.04, Score: 79.597

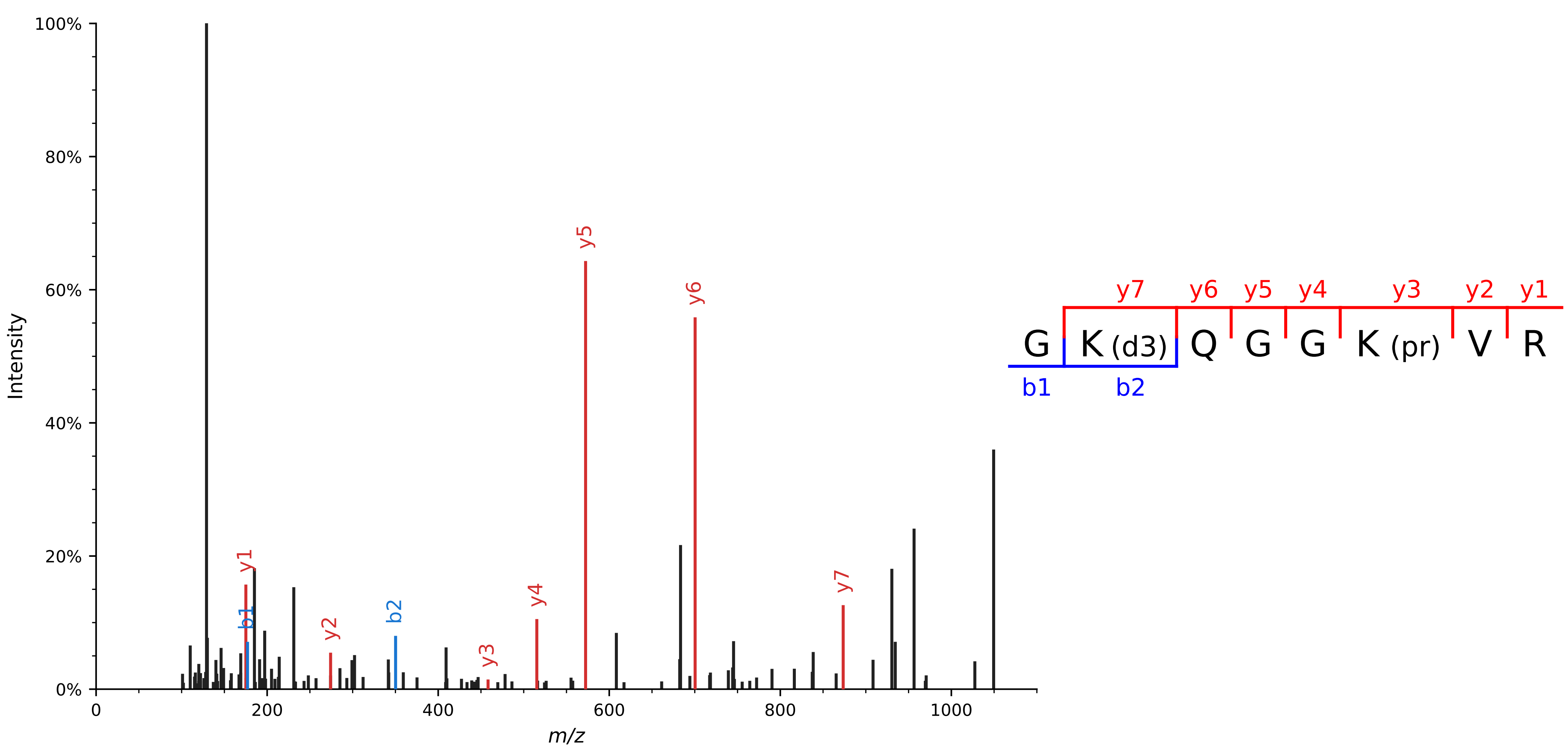

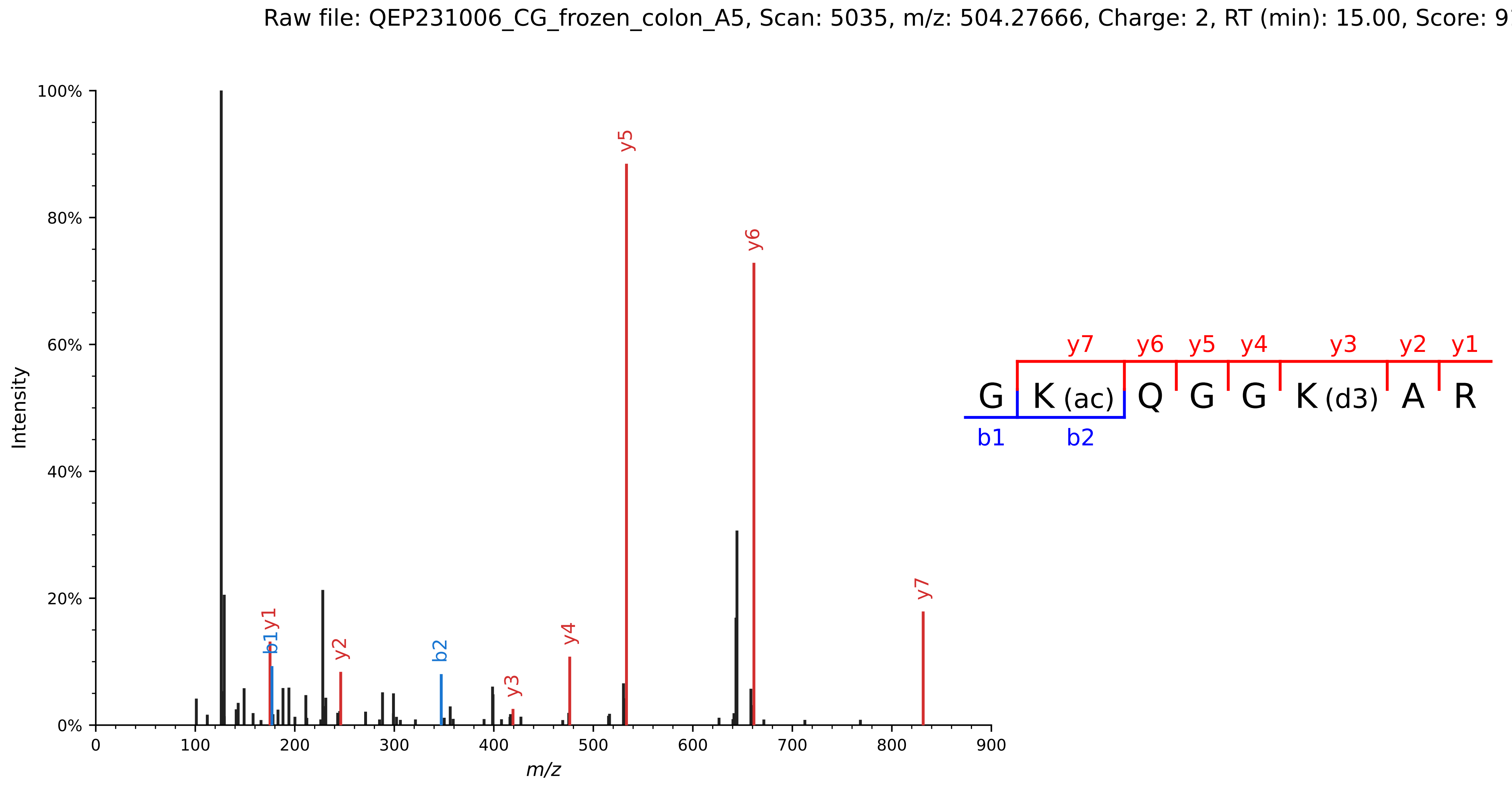
Raw file: QEP231006_CG_frozen_colon_A5, Scan: 5035, m/z: 504.27666, Charge: 2, RT (min): 15.00, Score: 91.041
Intensity
m/z
y5
y6
y7
y1
b1
y2
b2
y3
y4
y7 y6 y5 y4 y3 y2 y1
G K (ac) Q G G K (d3) A R
b1 b2

Raw file: QEP231006_CG_frozen_colon_A3, Scan: 6071, m/z: 511.28448, Charge: 2, RT (min): 17.72, Score: 122.79

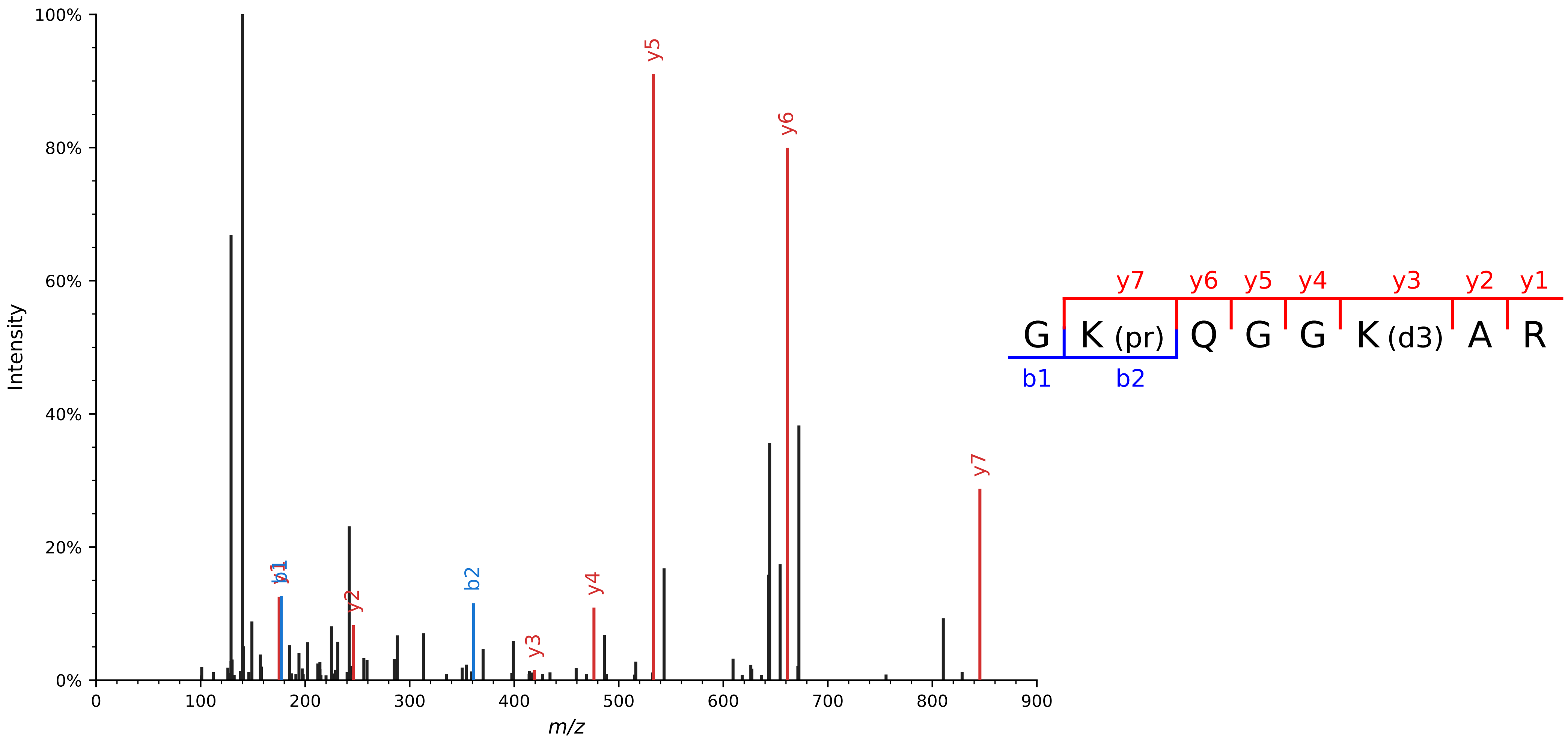

Raw file: QEP231006_CG_frozen_colon_A3, Scan: 5900, m/z: 511.28448, Charge: 2, RT (min): 17.36, Score: 135.35

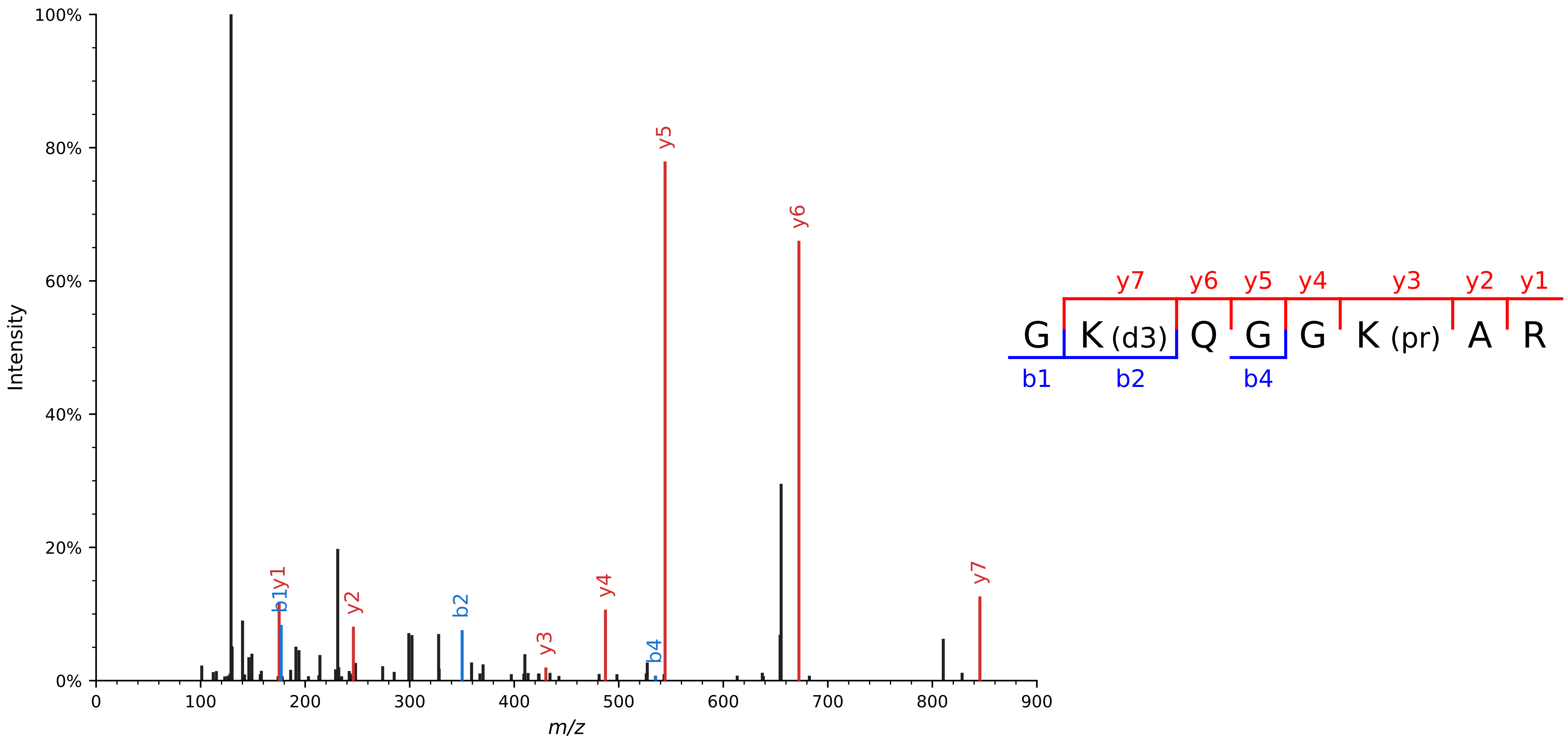

Raw file: QEP231006_CG_frozen_cecum_A2, Scan: 9310, m/z: 447.76539, Charge: 2, RT (min): 24.20, Score: 99.384

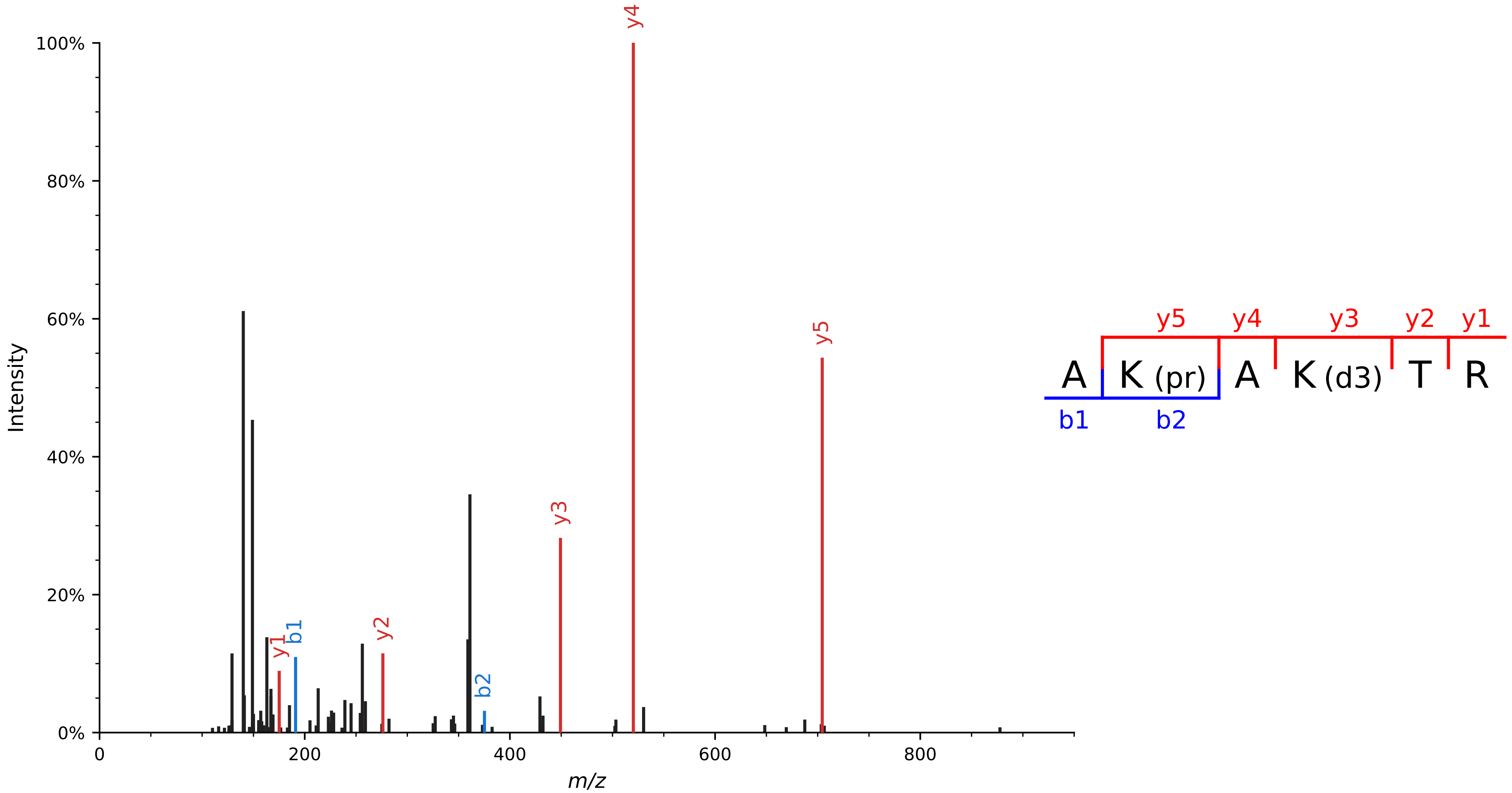

Raw file: QEP231006_CG_frozen_cecum_A5, Scan: 10664, m/z: 453.2638, Charge: 2, RT (min): 27.26, Score: 86.866

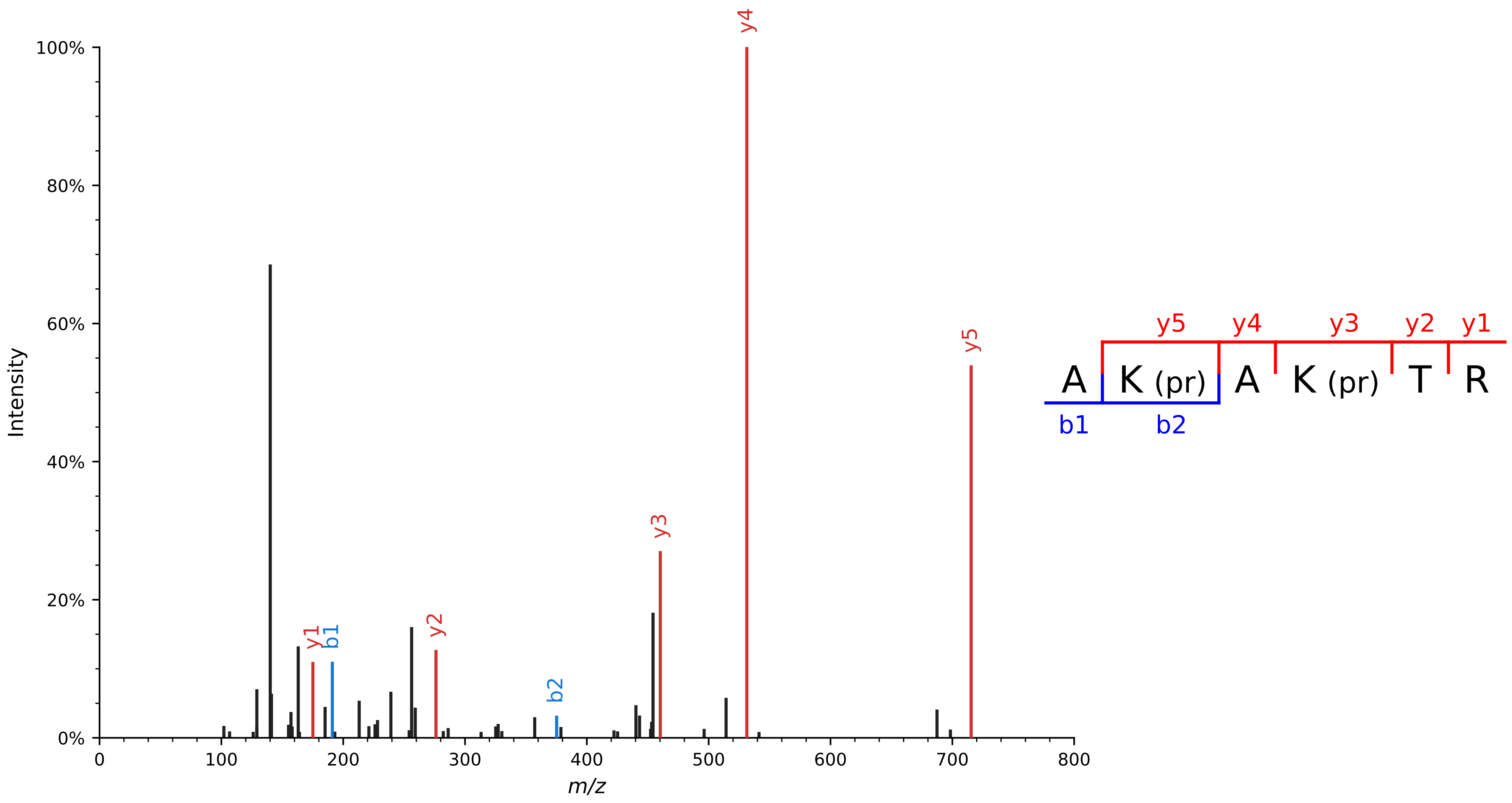

Raw file: QEP231006_CG_frozen_cecum_C2, Scan: 9504, m/z: 447.76539, Charge: 2, RT (min): 24.78, Score: 107.74

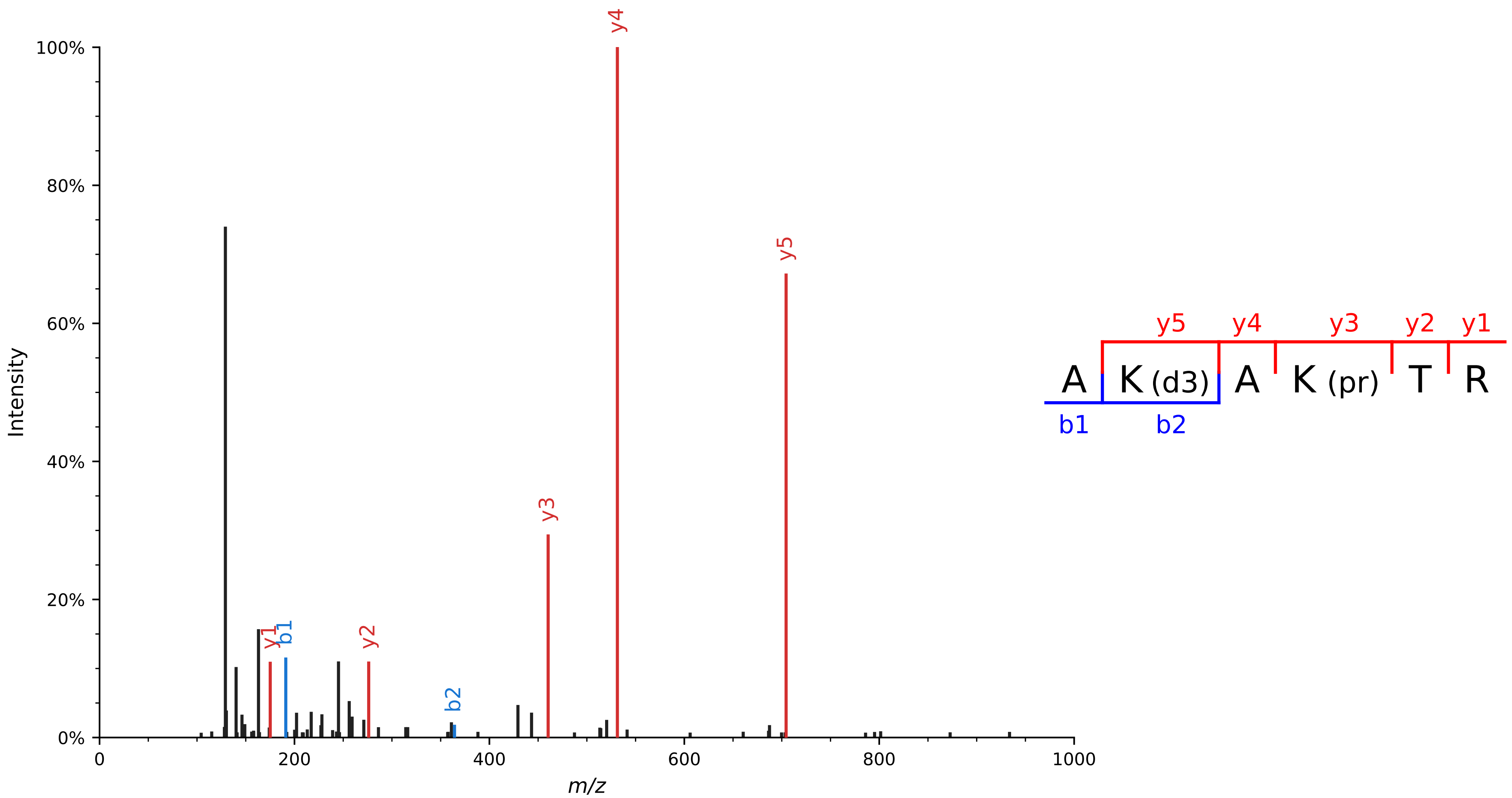

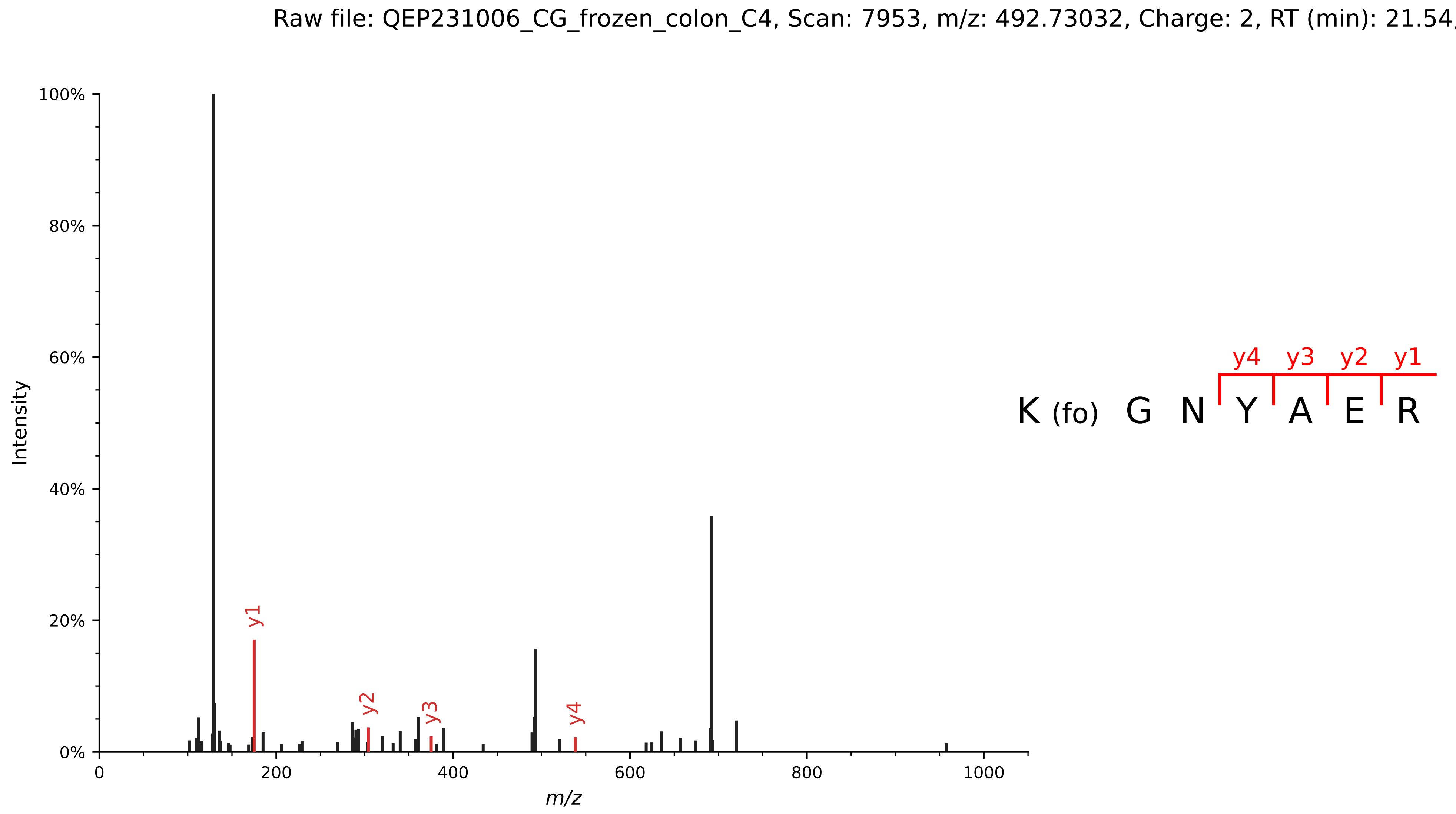
Raw file: QEP231006_CG_frozen_colon_C4, Scan: 7953, m/z: 492.73032, Charge: 2, RT (min): 21.54, Score: 51.572
Intensity
100%
80%
60%
40%
20%
0%
0
200
400
600
800
1000
m/z
y1
y2
y3
y4
K (fo) G N Y A E R
y4 y3 y2 y1

Raw file: QEP231006_CG_frozen_colon_A3, Scan: 8932, m/z: 506.74597, Charge: 2, RT (min): 24.58, Score: 104.45

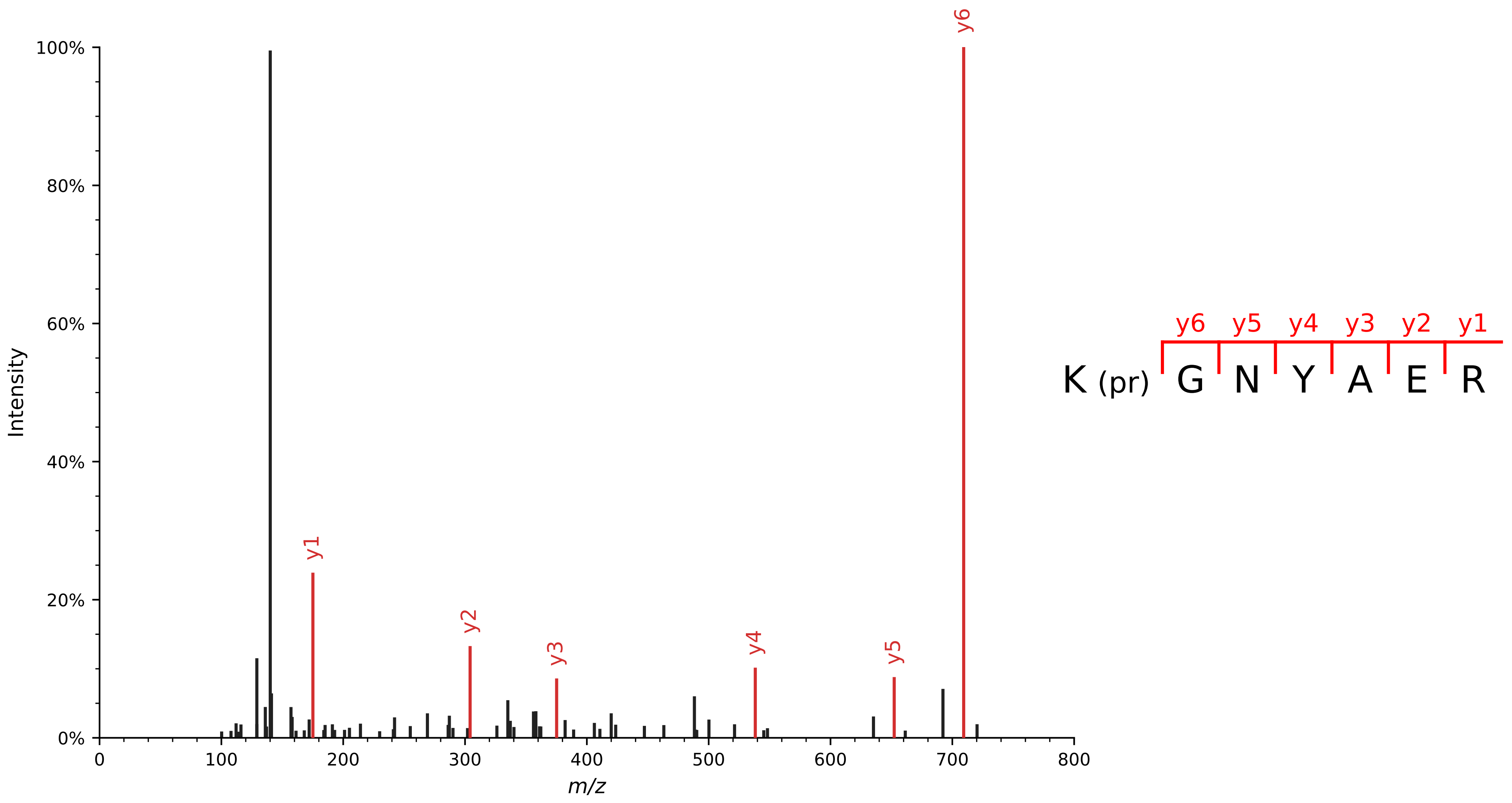

Raw file: QEP231006_CG_frozen_colon_A3, Scan: 7323, m/z: 491.26321, Charge: 2, RT (min): 20.69, Score: 104.11

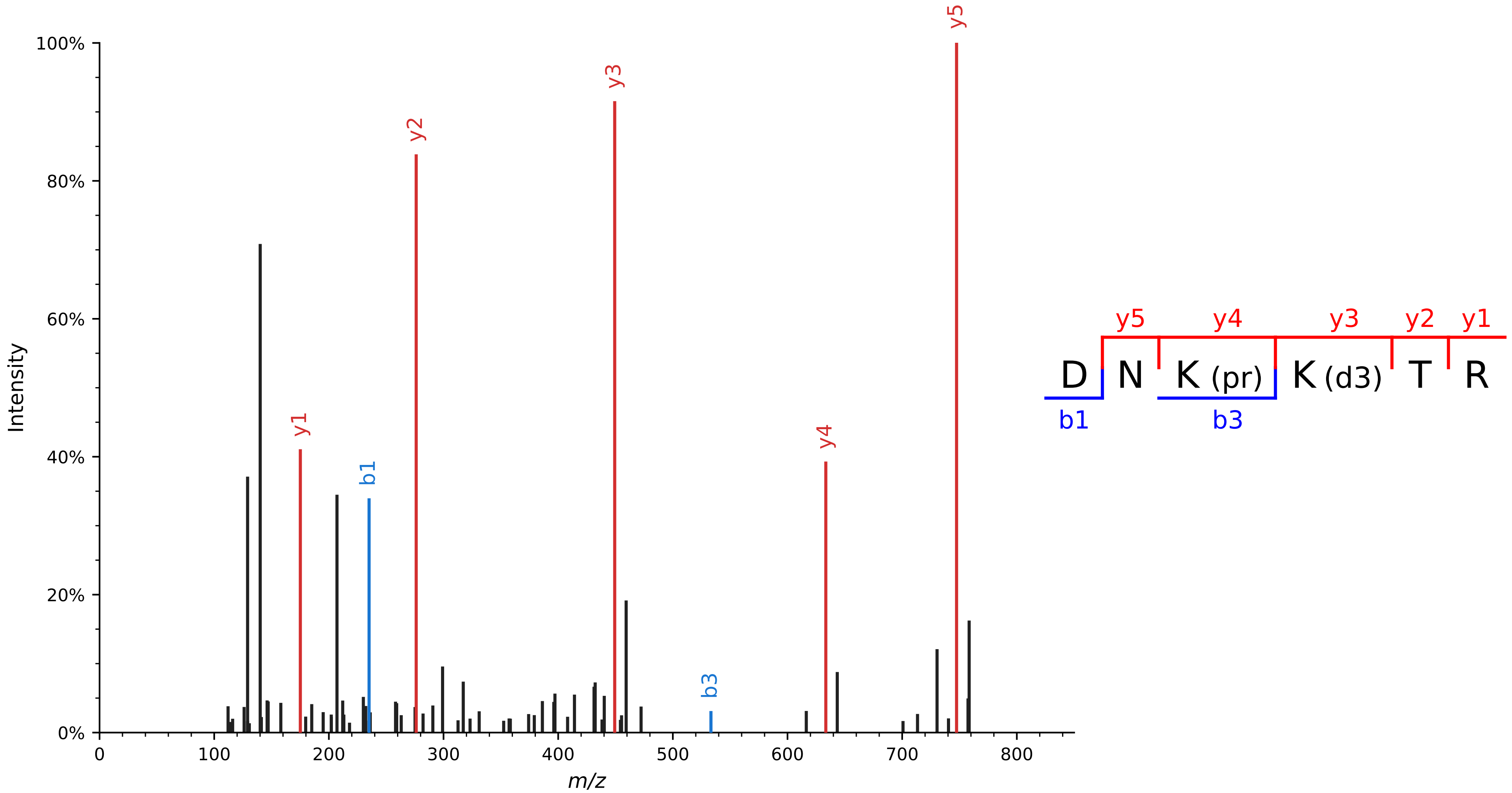

Raw file: QEP231006_CG_frozen_colon_A4, Scan: 9015, m/z: 496.76162, Charge: 2, RT (min): 23.43, Score: 70.268

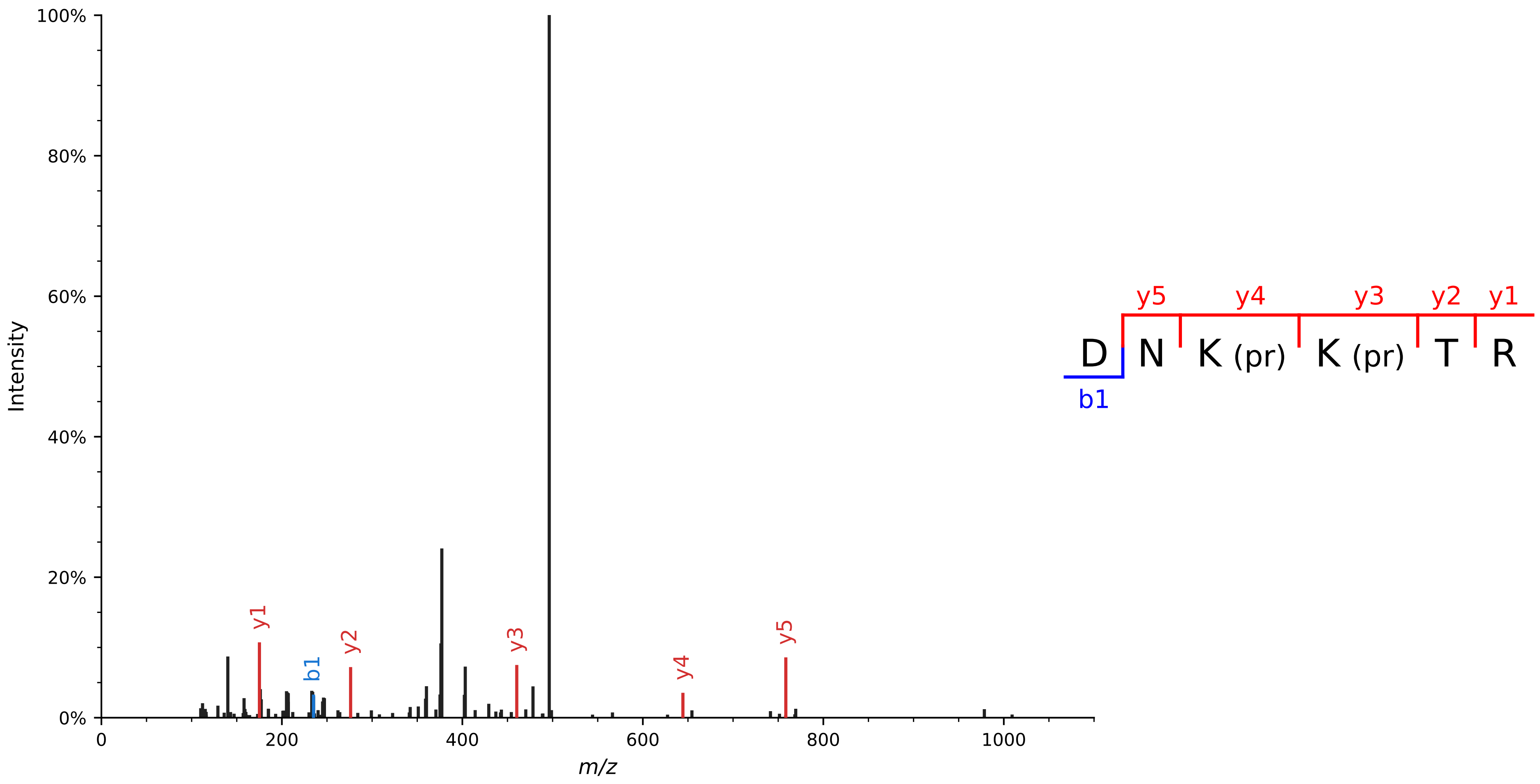

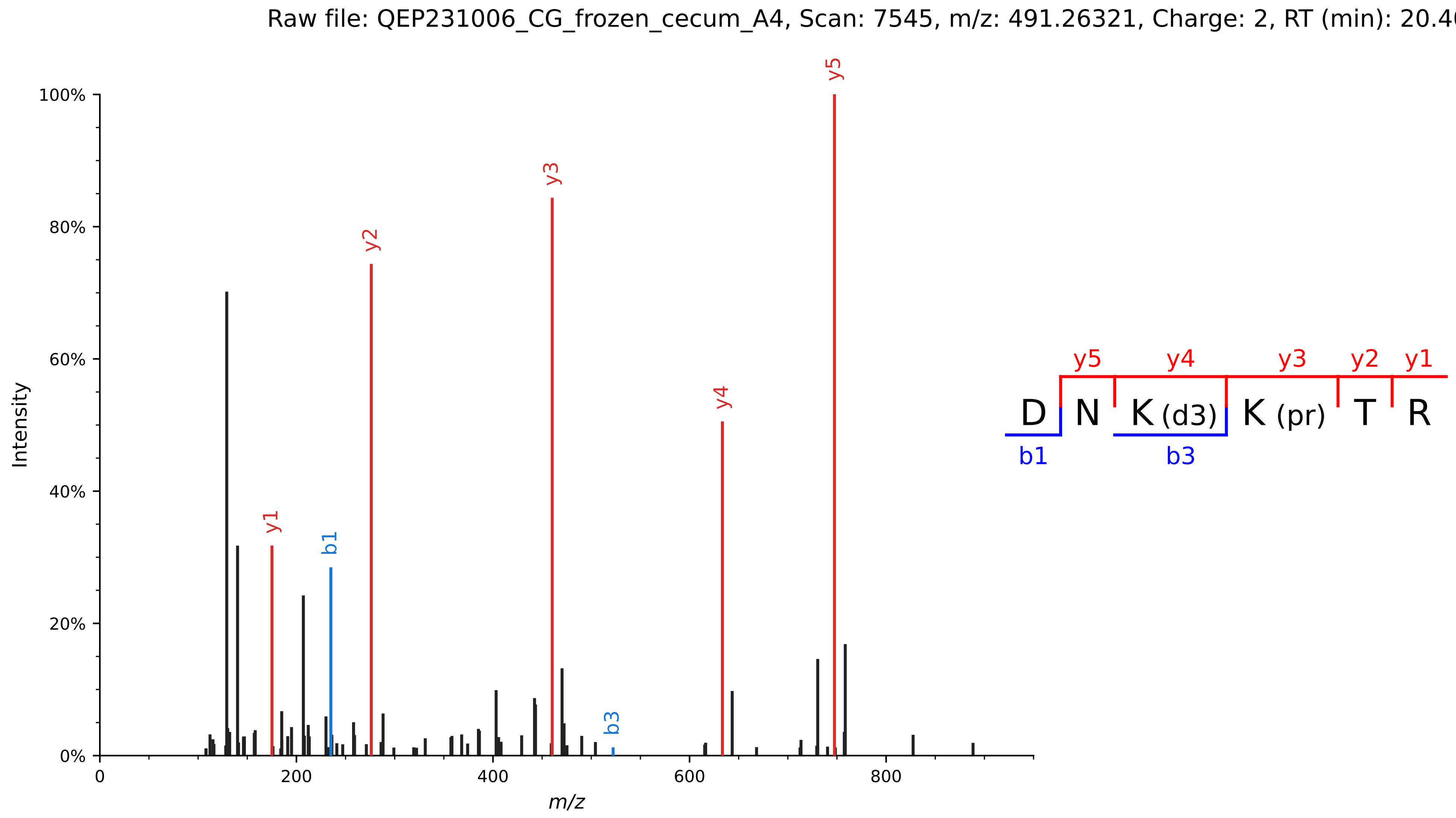
Raw file: QEP231006_CG_frozen_cecum_A4, Scan: 7545, m/z: 491.26321, Charge: 2, RT (min): 20.40, Score: 128.82
Intensity
m/z
y1
b1
y2
y3
b3
y4
y5
D N K (d3) K (pr) T R
y5 y4 y3 y2 y1
b1 b3

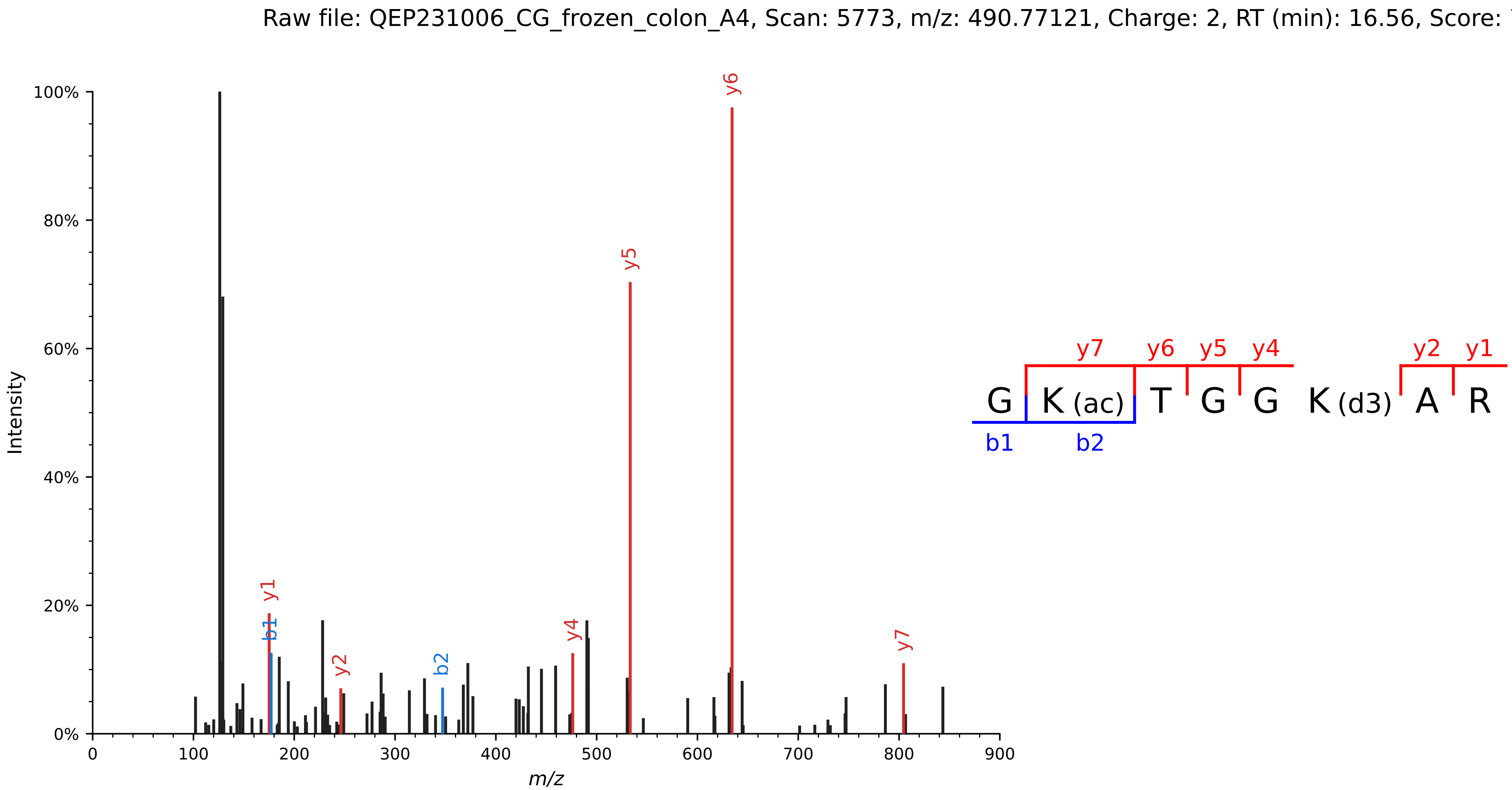
Raw file: QEP231006_CG_frozen_colon_A4, Scan: 5773, m/z: 490.77121, Charge: 2, RT (min): 16.56, Score: 77.058
Intensity
m/z
y1
b1
y2
b2
y4
y5
y6
y7
y7 y6 y5 y4 y2 y1
G K (ac) T G G K (d3) A R
b1 b2

Raw file: QEP231006_CG_frozen_cecum_C2, Scan: 7109, m/z: 497.77903, Charge: 2, RT (min): 19.62, Score: 91.626

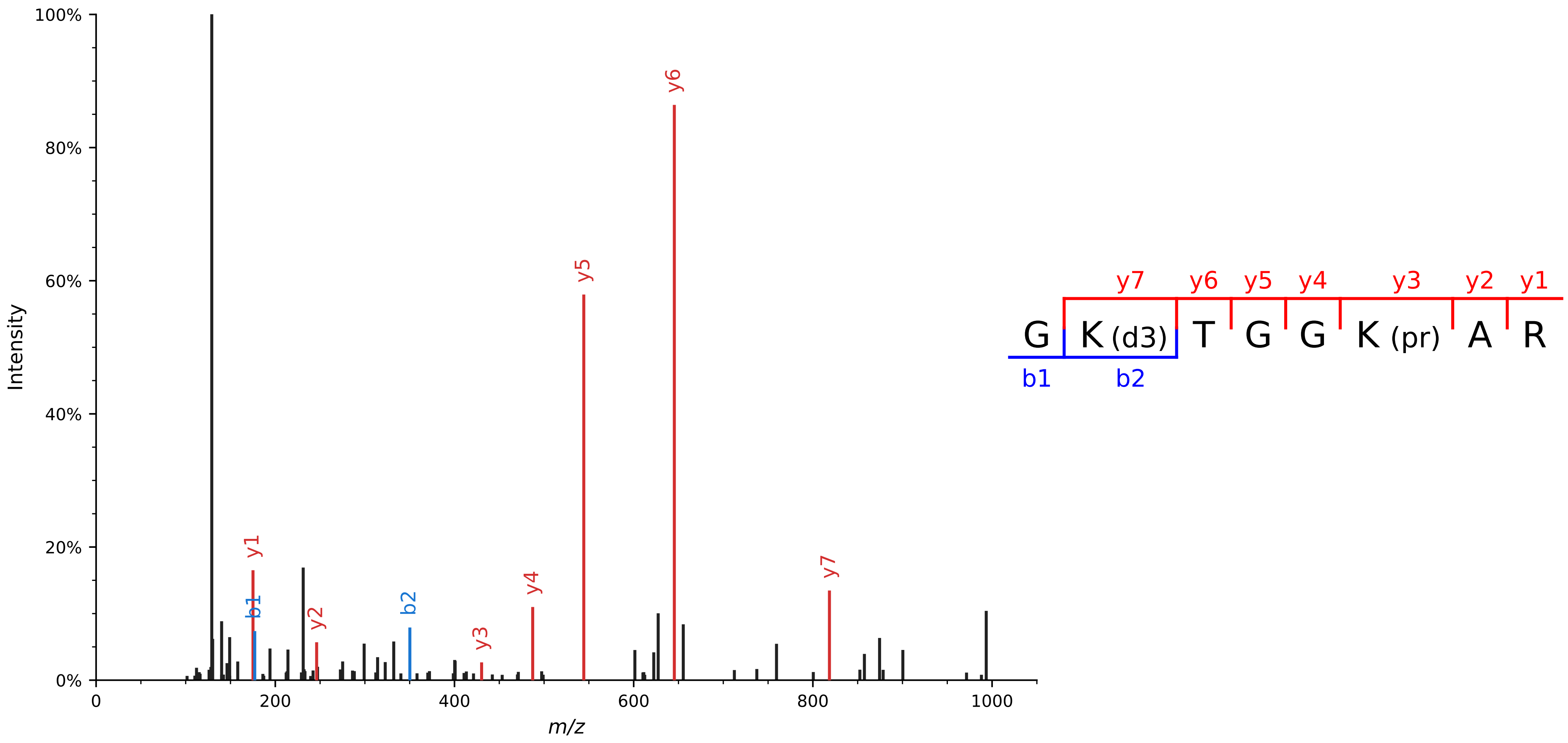

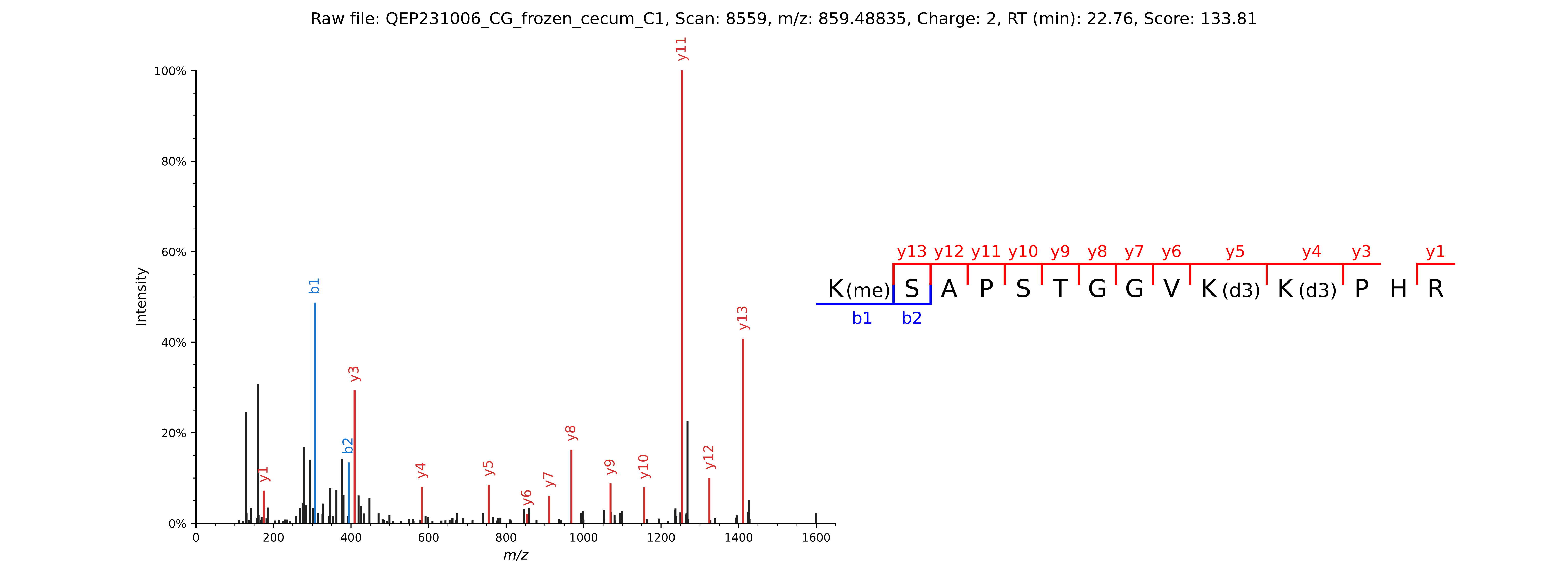

Raw file: QEP231006_CG_frozen_cecum_C1, Scan: 8559, m/z: 859.48835, Charge: 2, RT (min): 22.76, Score: 133.81
Intensity
m/z
K(me) S A P S T G G V K(d3) K(d3) P H R
y13 y12 y11 y10 y9 y8 y7 y6 y5 y4 y3 y1
b1 b2

Raw file: QEP231006_CG_frozen_colon_A5, Scan: 9026, m/z: 866.49617, Charge: 2, RT (min): 23.50, Score: 77.379

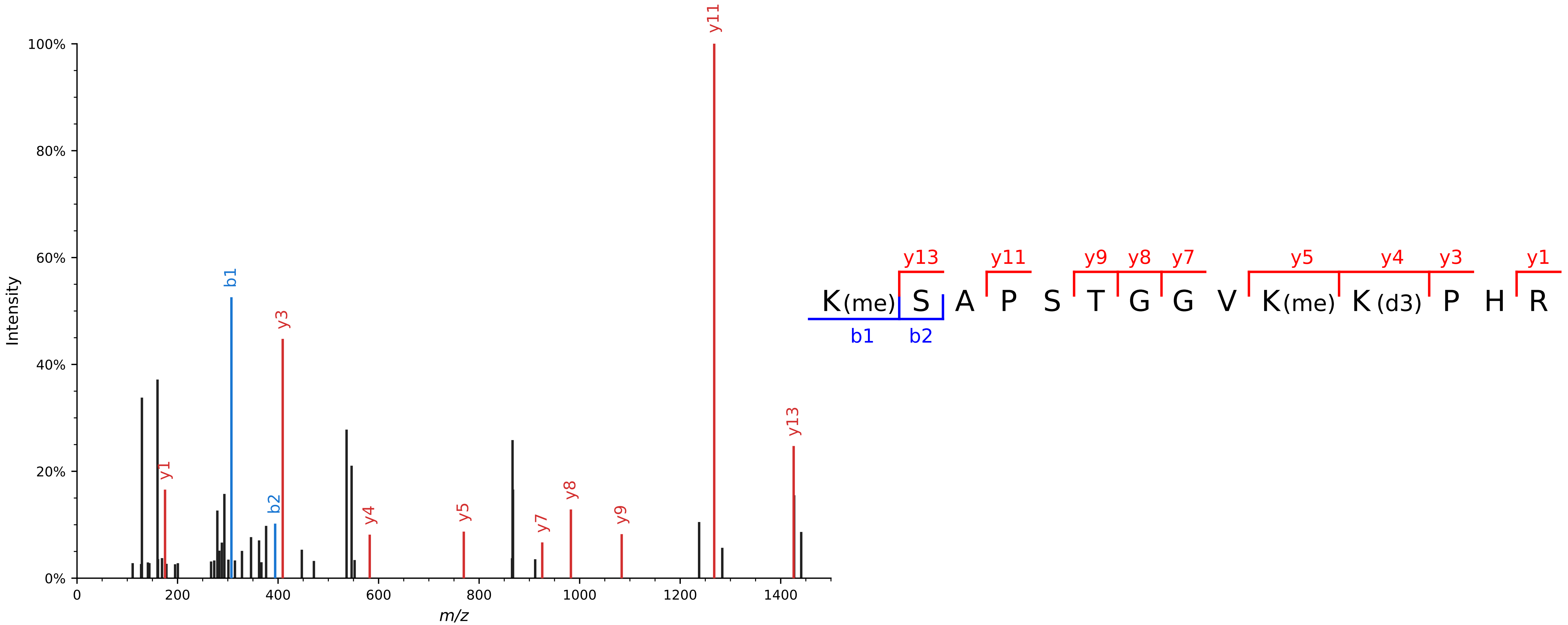

Raw file: QEP231006_CG_frozen_colon_A5, Scan: 5104, m/z: 567.66196, Charge: 3, RT (min): 15.15, Score: 117.07

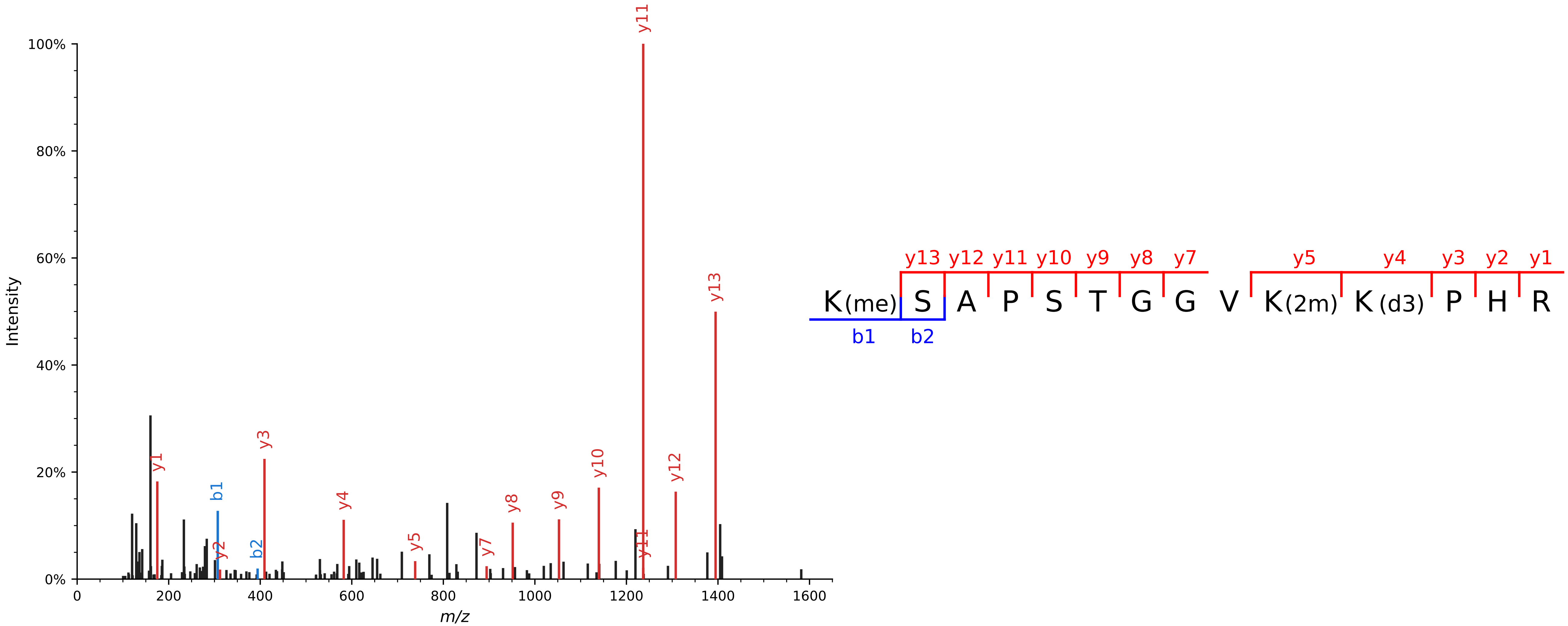

Raw file: QEP231006_CG_frozen_colon_A4, Scan: 5297, m/z: 572.33384, Charge: 3, RT (min): 15.56, Score: 42.317

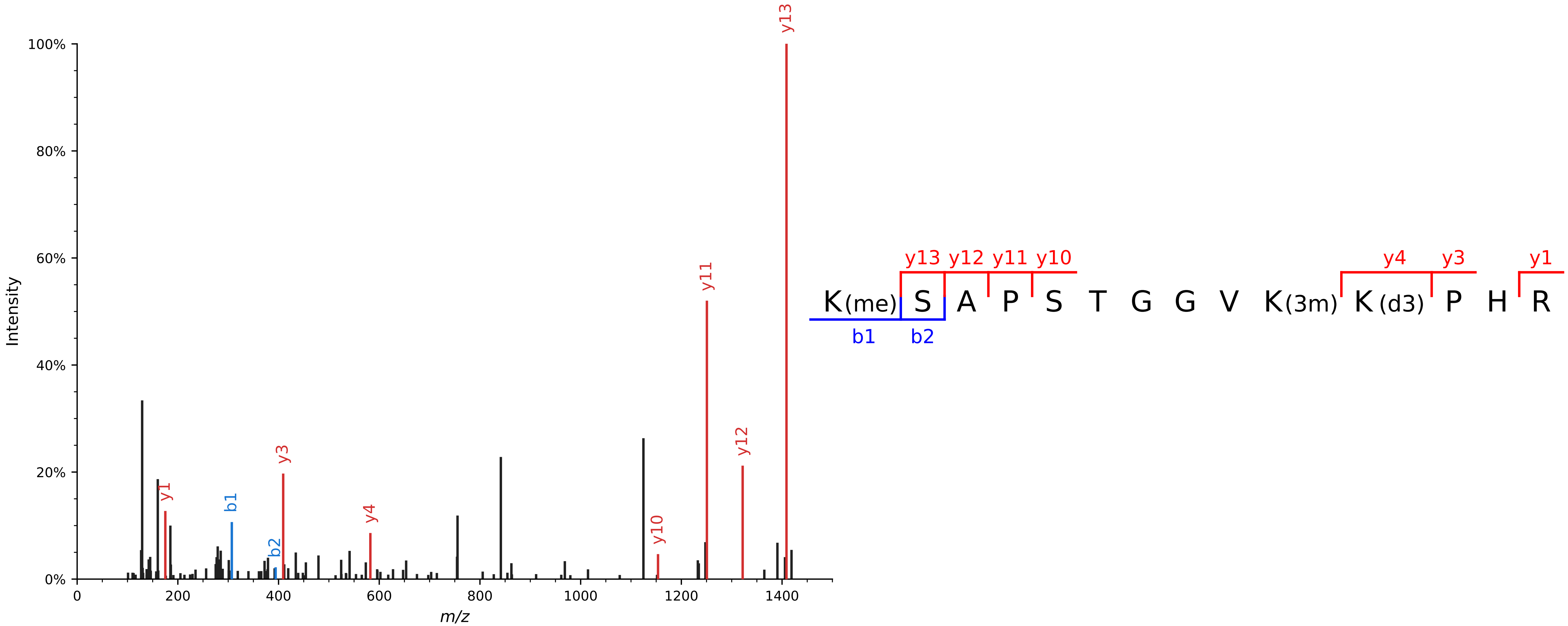

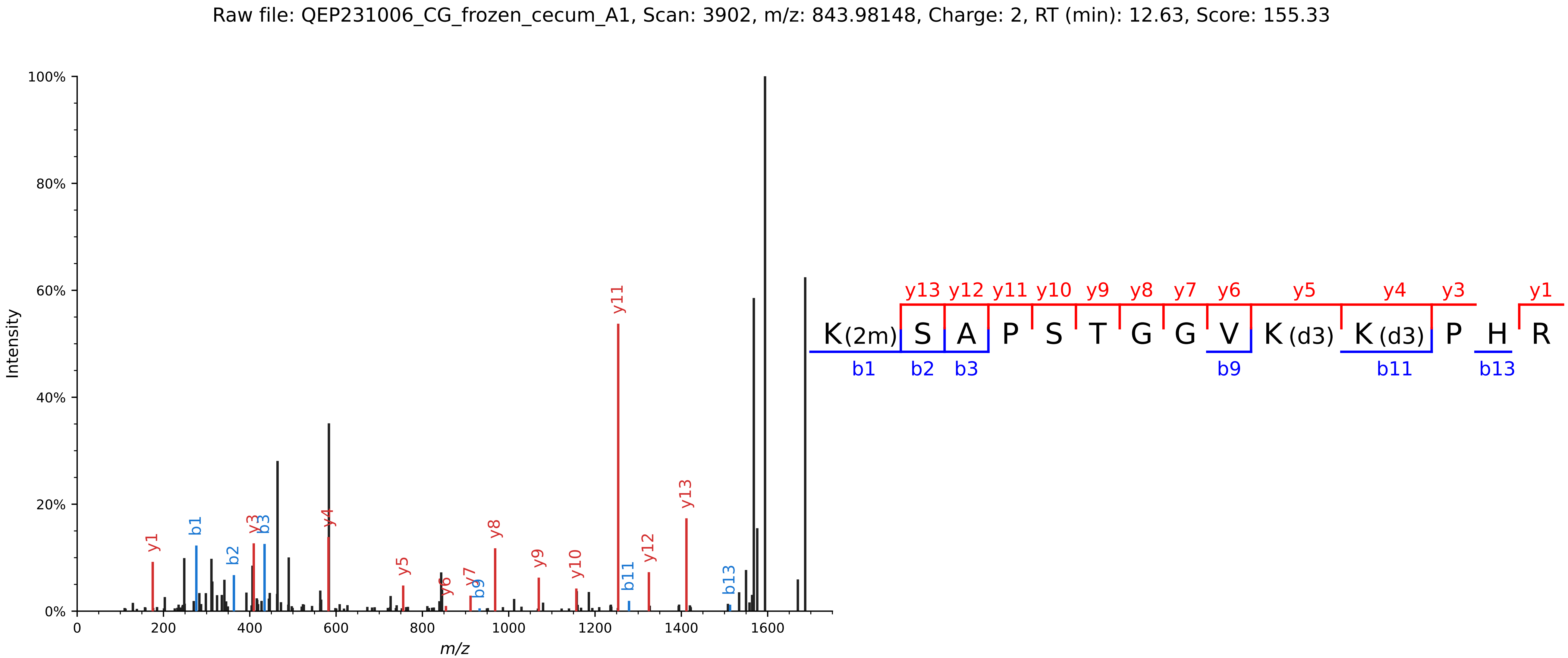

Raw file: QEP231006_CG_frozen_cecum_A1, Scan: 3902, m/z: 843.98148, Charge: 2, RT (min): 12.63, Score: 155.33
Intensity
m/z
0%
20%
40%
60%
80%
100%
0
200
400
600
800
1000
1200
1400
1600
y1
b1
b2
y3
b3
y4
y5
y6
y7
b9
y8
y9
y10
y11
b11
y12
y13
b13
K(2m) S A P S T G G V K(d3) K(d3) P H R
y13 y12 y11 y10 y9 y8 y7 y6 y5 y4 y3 y1
b1 b2 b3 b9 b11 b13

Raw file: QEP231006_CG_frozen_cecum_A5, Scan: 4220, m/z: 567.66196, Charge: 3, RT (min): 13.27, Score: 133.48

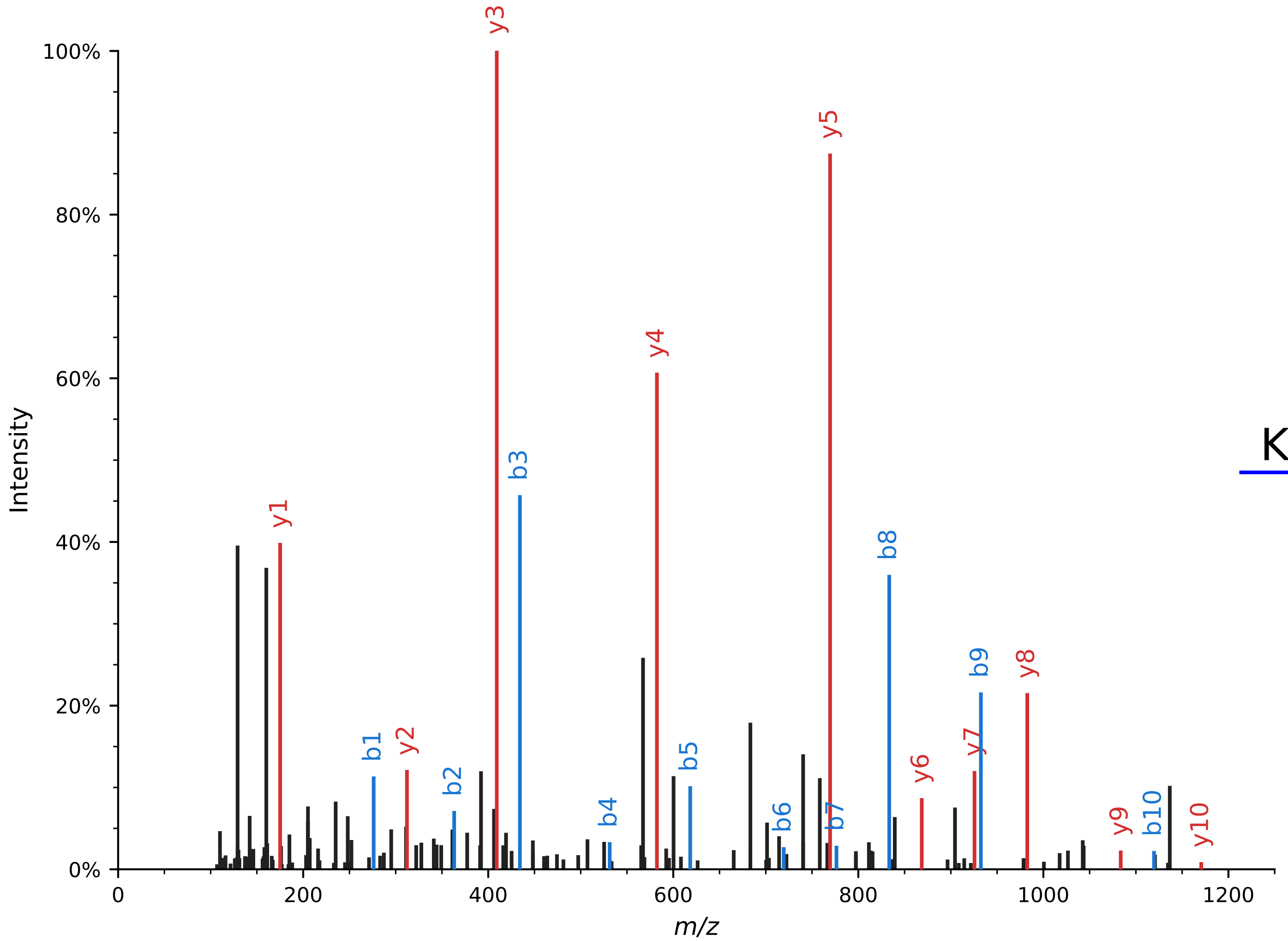

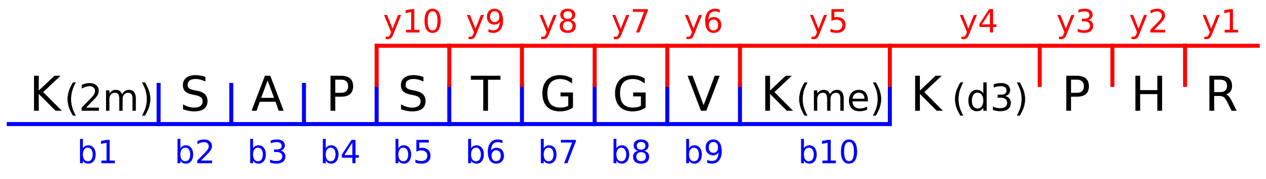

Raw file: QEP231006_CG_frozen_cecum_C1, Scan: 2369, m/z: 557.32404, Charge: 3, RT (min): 9.26, Score: 104.09

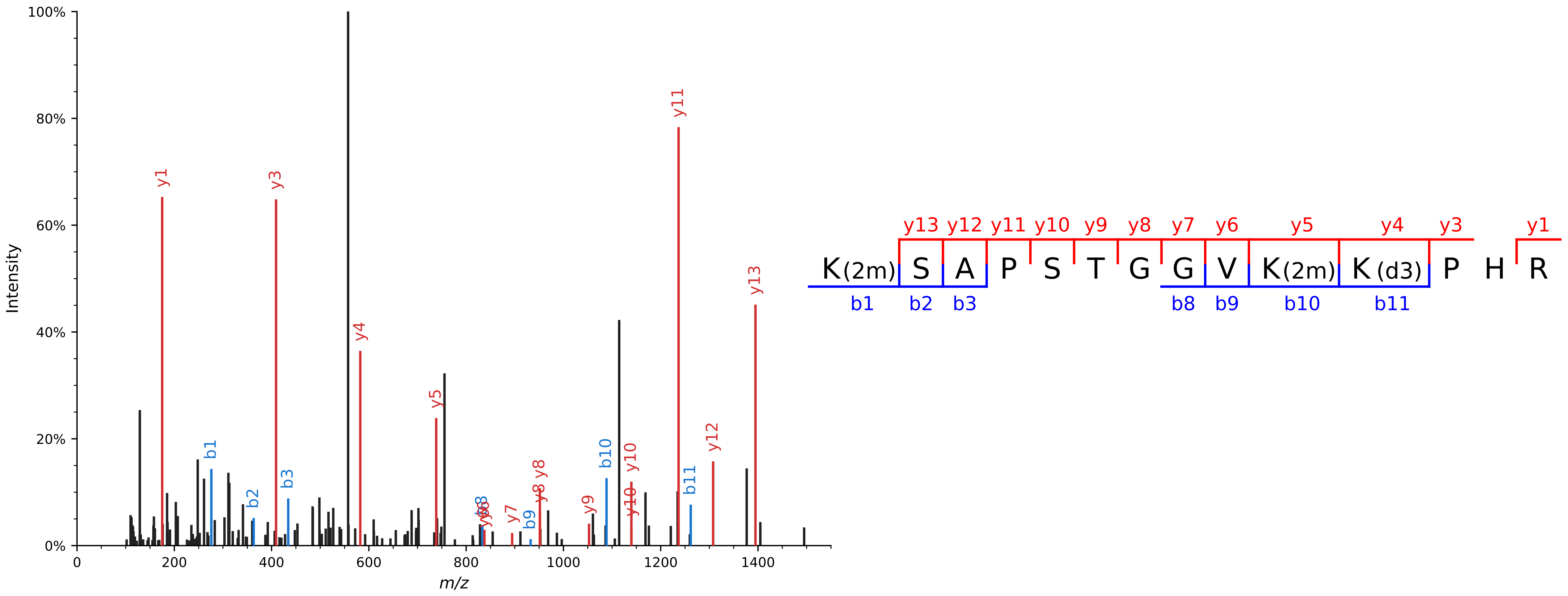

Raw file: QEP231006_CG_frozen_colon_A4, Scan: 3809, m/z: 567.66196, Charge: 3, RT (min): 12.28, Score: 133.47

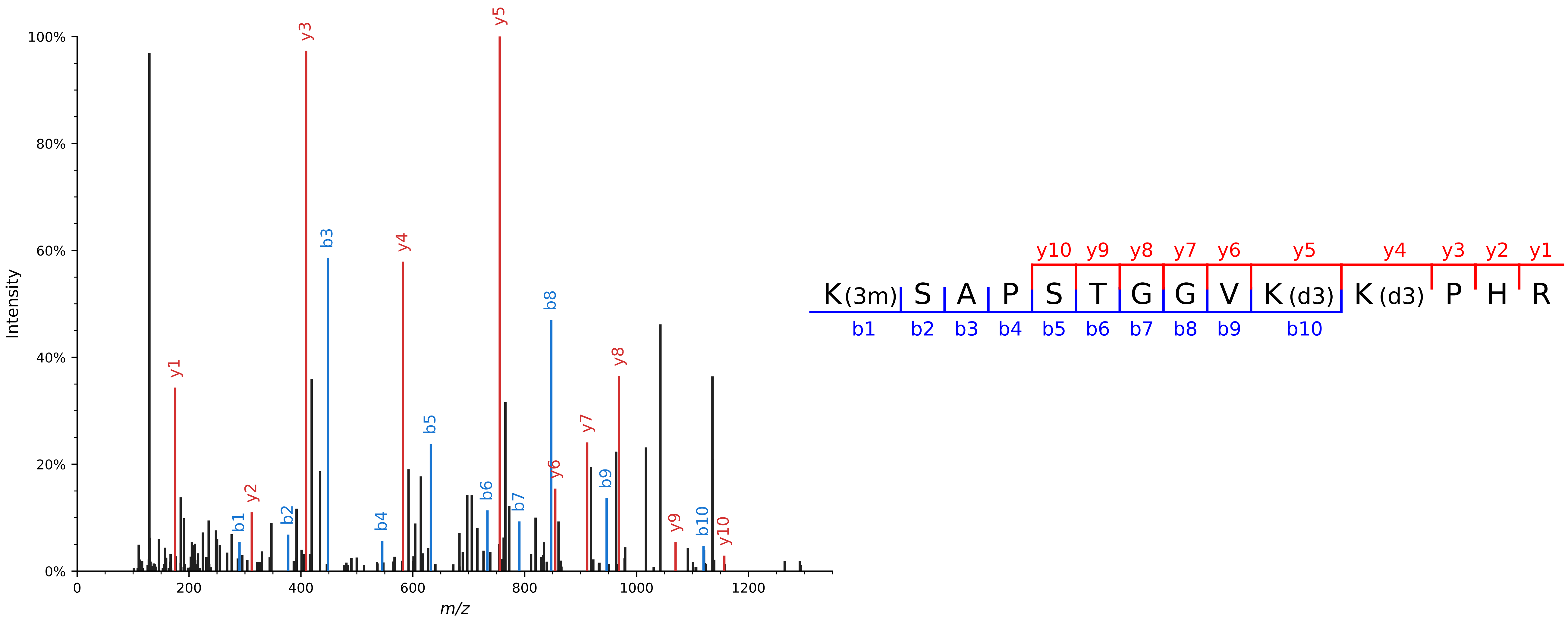

Raw file: QEP231006_CG_frozen_colon_A1, Scan: 4236, m/z: 572.33384, Charge: 3, RT (min): 13.31, Score: 108.58

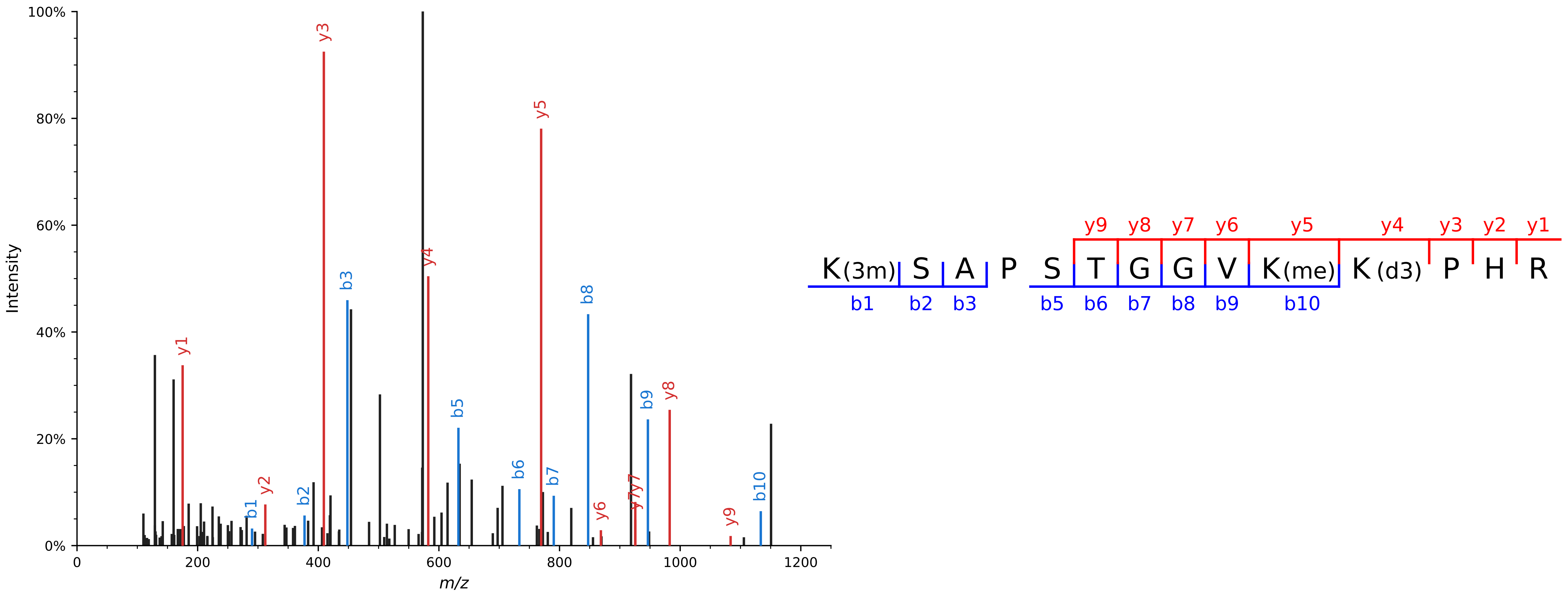

Raw file: QEP231006_CG_frozen_cecum_A5, Scan: 5553, m/z: 561.99593, Charge: 3, RT (min): 16.11, Score: 48.625

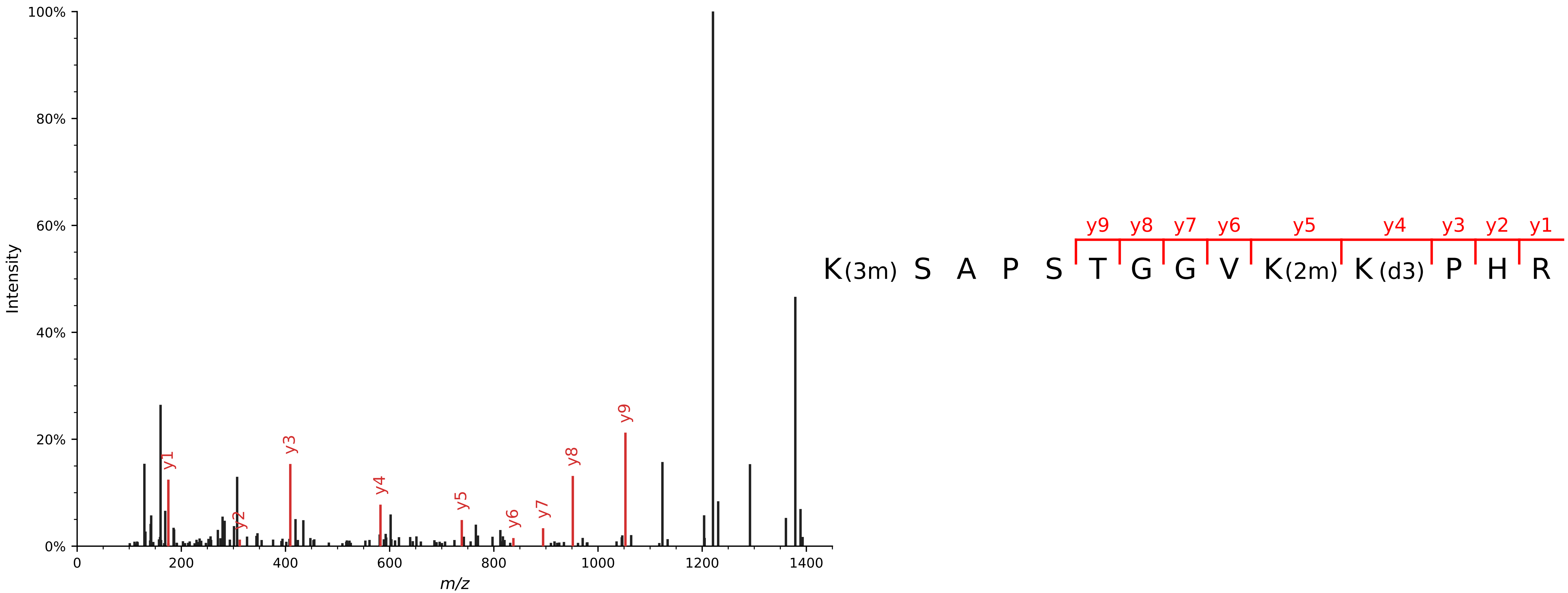

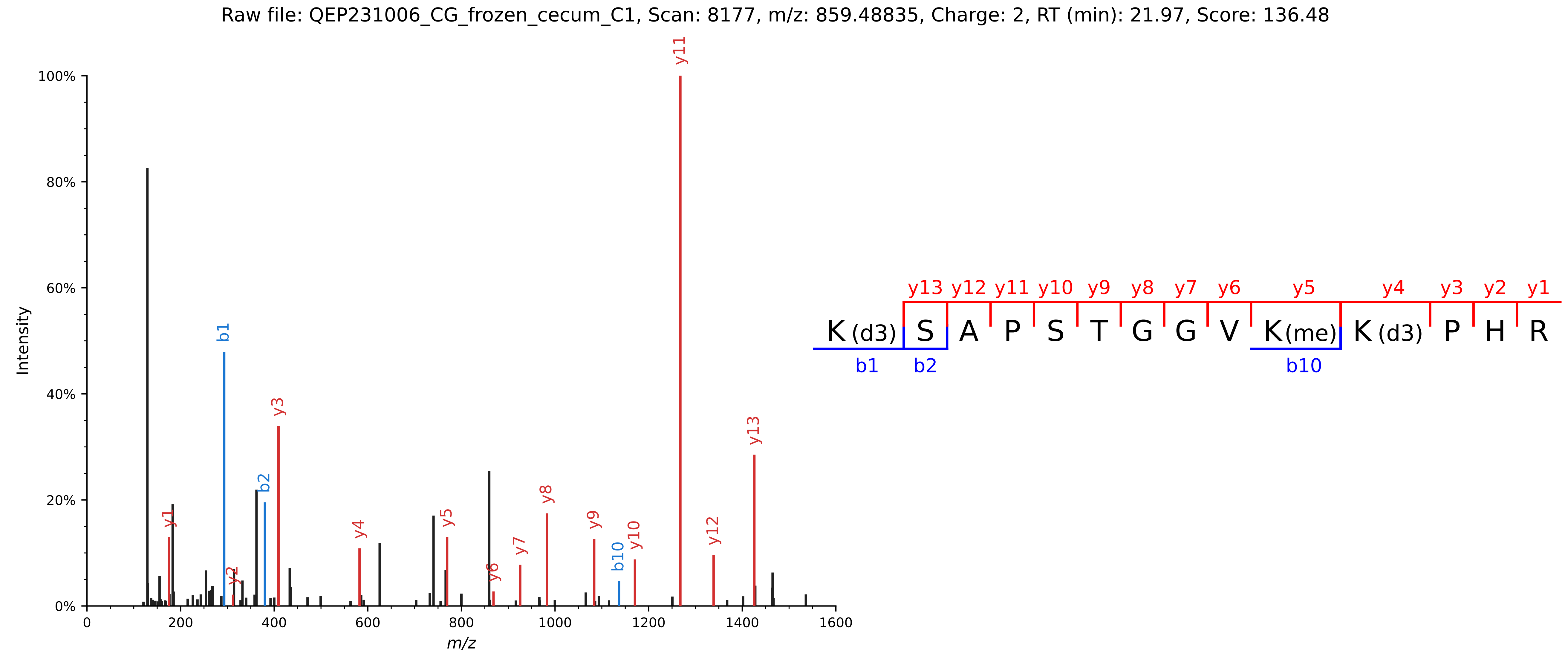

Raw file: QEP231006_CG_frozen_cecum_C1, Scan: 8177, m/z: 859.48835, Charge: 2, RT (min): 21.97, Score: 136.48
Intensity
m/z
y1 y2 y3 y4 y5 y6 y7 y8 y9 y10 y11 y12 y13
b1 b2 b10
y13 y12 y11 y10 y9 y8 y7 y6 y5 y4 y3 y2 y1
K(d3) S A P S T G G V K(me) K(d3) P H R
b1 b2 b10

Raw file: QEP231006_CG_frozen_cecum_C3, Scan: 5649, m/z: 577.99987, Charge: 3, RT (min): 16.32, Score: 45.79

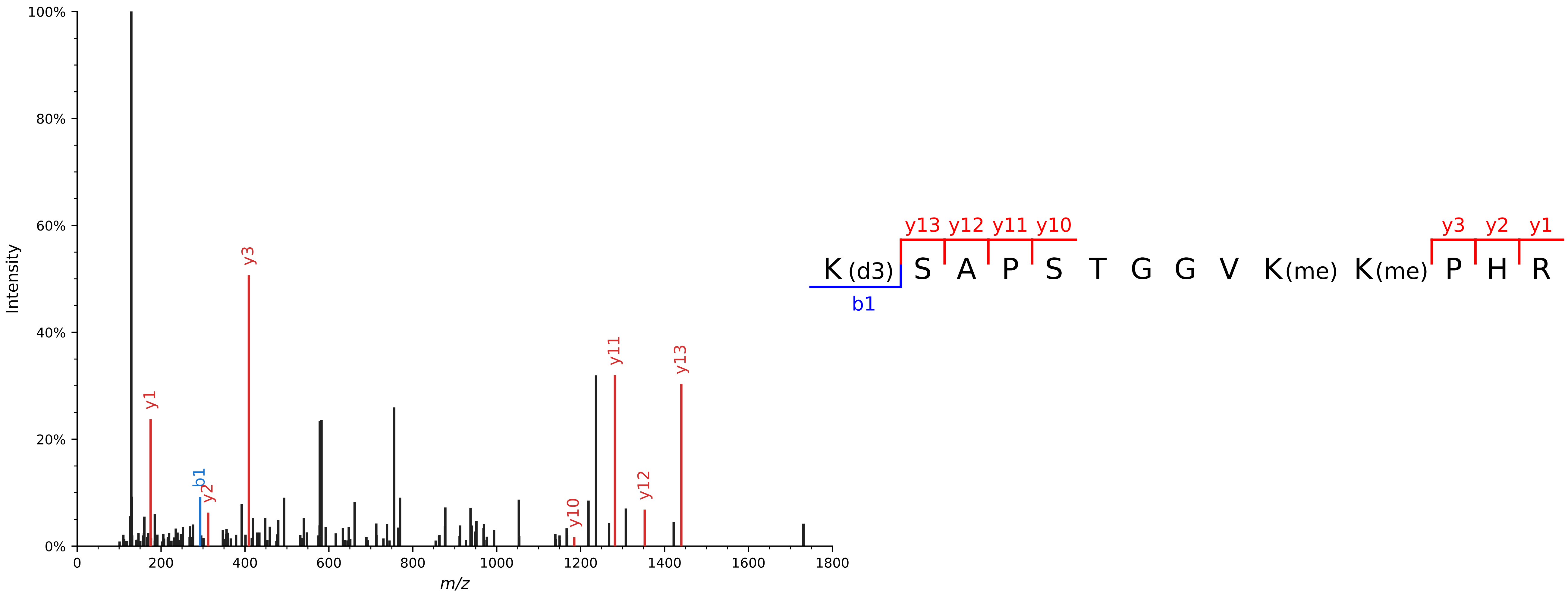

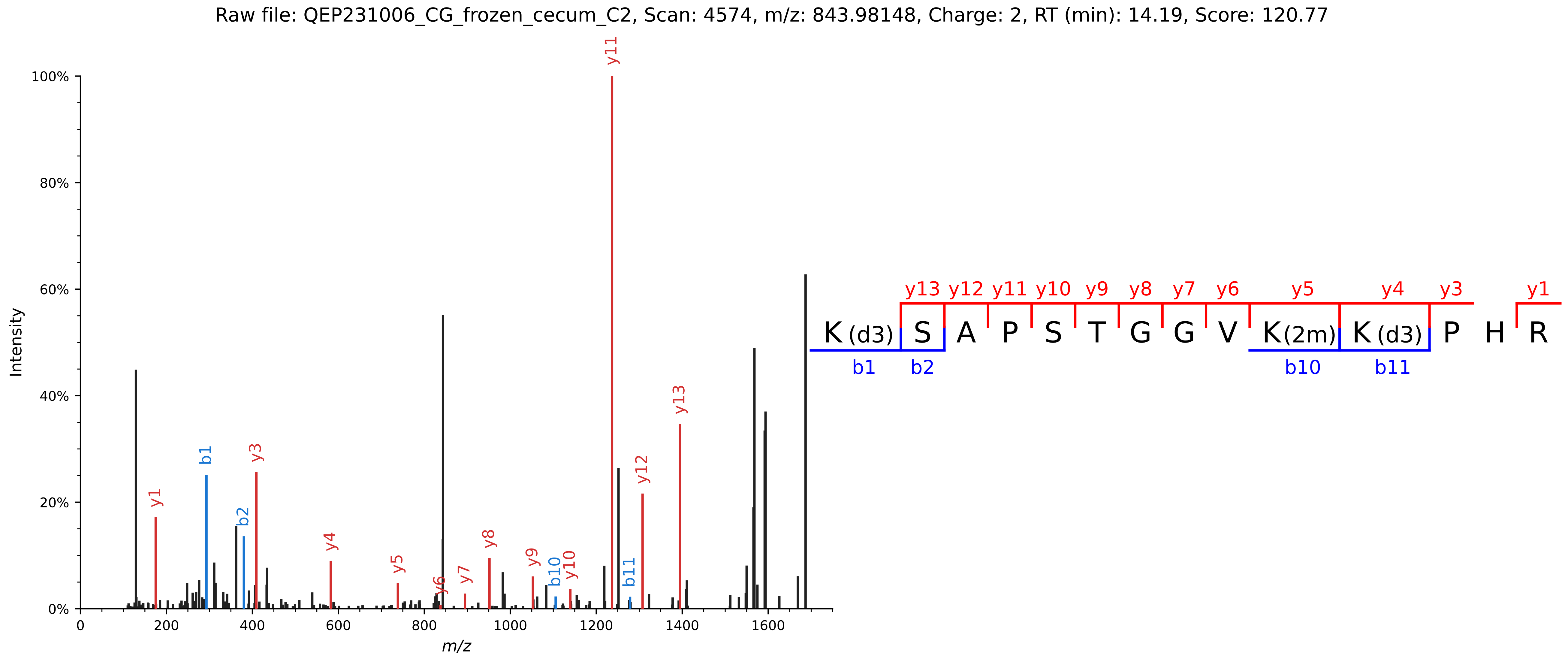

Raw file: QEP231006_CG_frozen_cecum_C2, Scan: 4574, m/z: 843.98148, Charge: 2, RT (min): 14.19, Score: 120.77
Intensity
m/z
y1
b1
b2
y3
y4
y5
y6
y7
y8
y9
b10
y10
y11
b11
y12
y13
y13 y12 y11 y10 y9 y8 y7 y6 y5 y4 y3 y1
K(d3) S A P S T G G V K(2m) K(d3) P H R
b1 b2 b10 b11

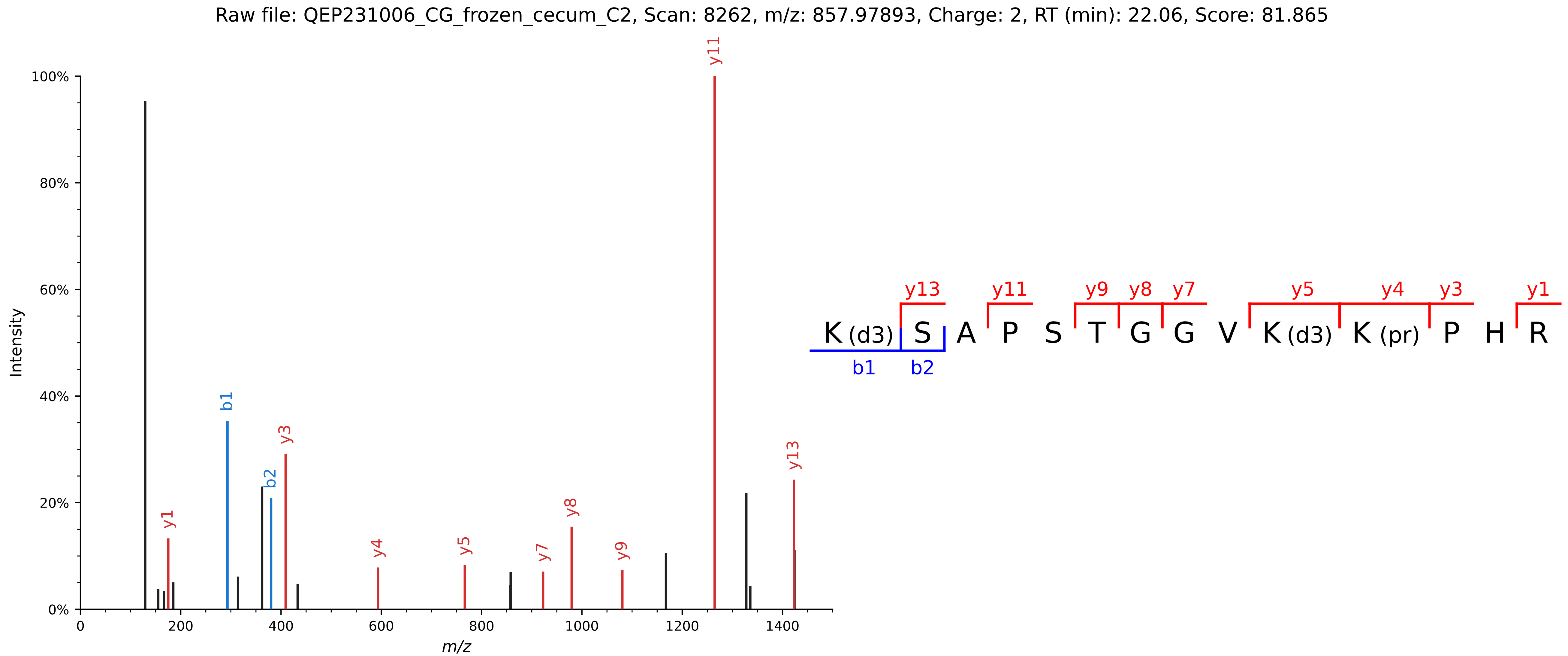

Raw file: QEP231006_CG_frozen_cecum_C2, Scan: 8262, m/z: 857.97893, Charge: 2, RT (min): 22.06, Score: 81.865
Intensity
m/z
K(d3) S A P S T G G V K(d3) K(pr) P H R
b1 b2
y13 y11 y9 y8 y7 y5 y4 y3 y1

Raw file: QEP231006_CG_frozen_cecum_A1, Scan: 7093, m/z: 441.7472, Charge: 2, RT (min): 19.56, Score: 106.76

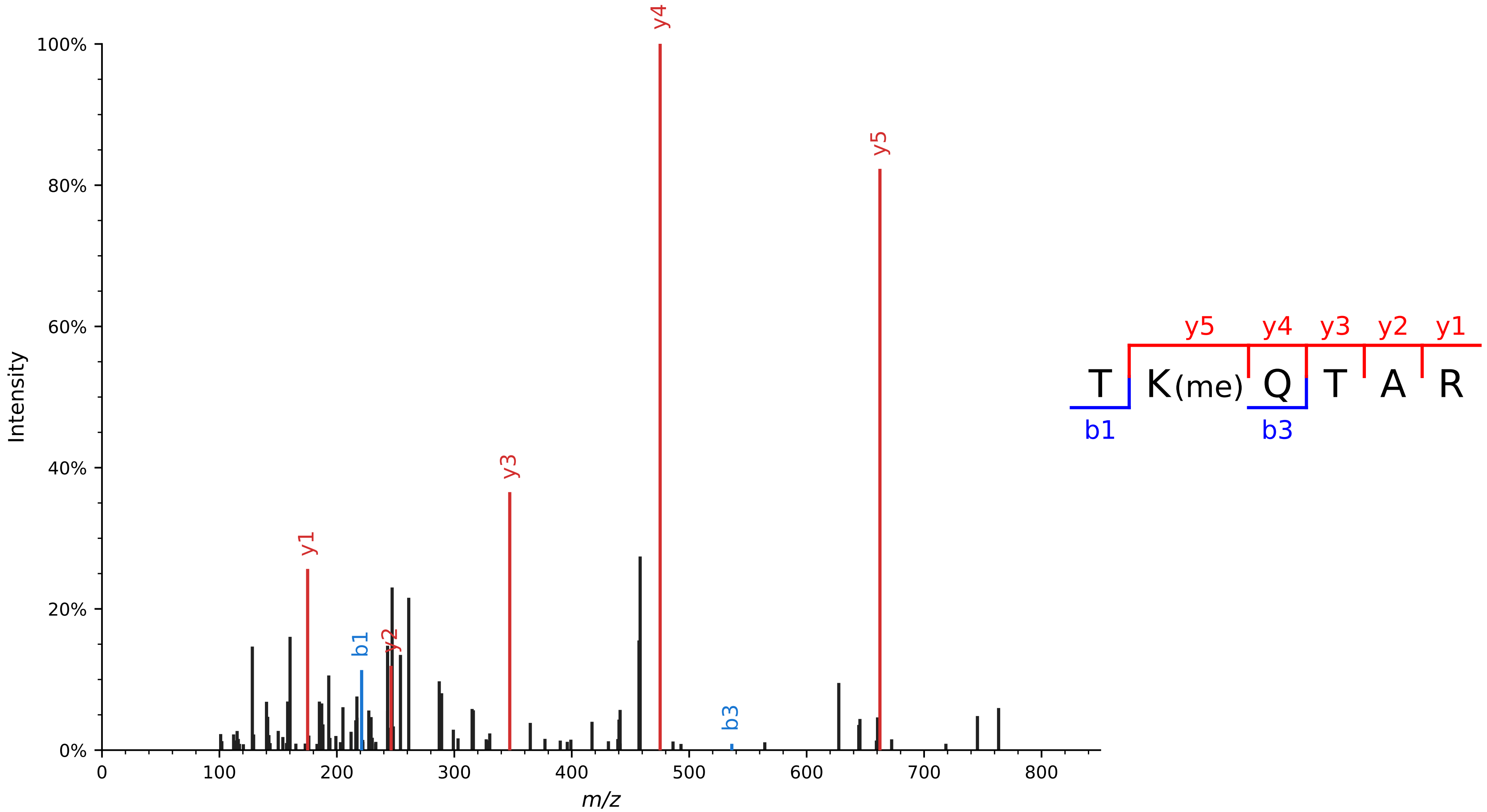

Raw file: QEP231006_CG_frozen_colon_A5, Scan: 1366, m/z: 426.24033, Charge: 2, RT (min): 6.81, Score: 148.33

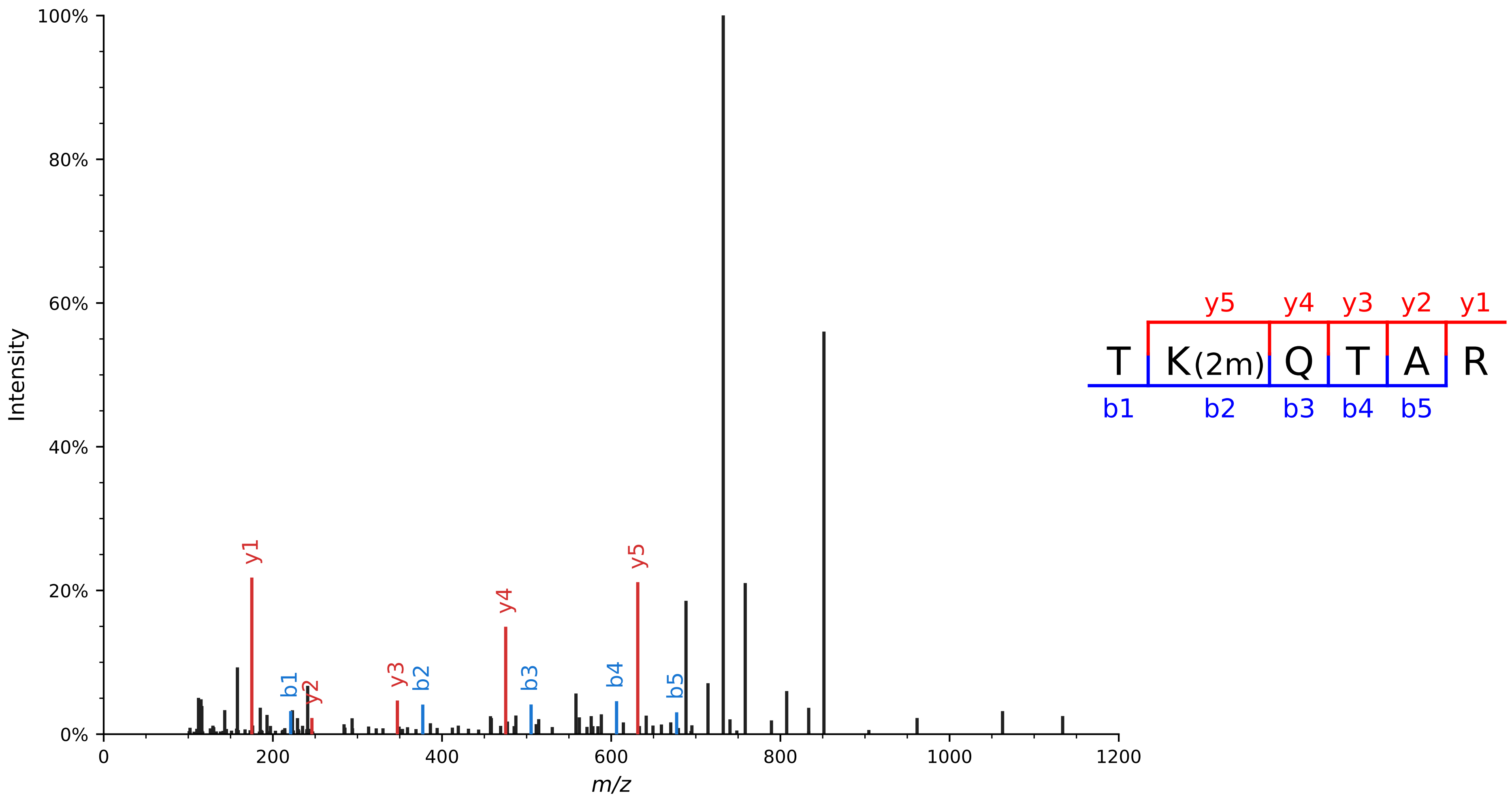

Raw file: QEP231006_CG_frozen_colon_C3, Scan: 1268, m/z: 433.24815, Charge: 2, RT (min): 6.87, Score: 107.43

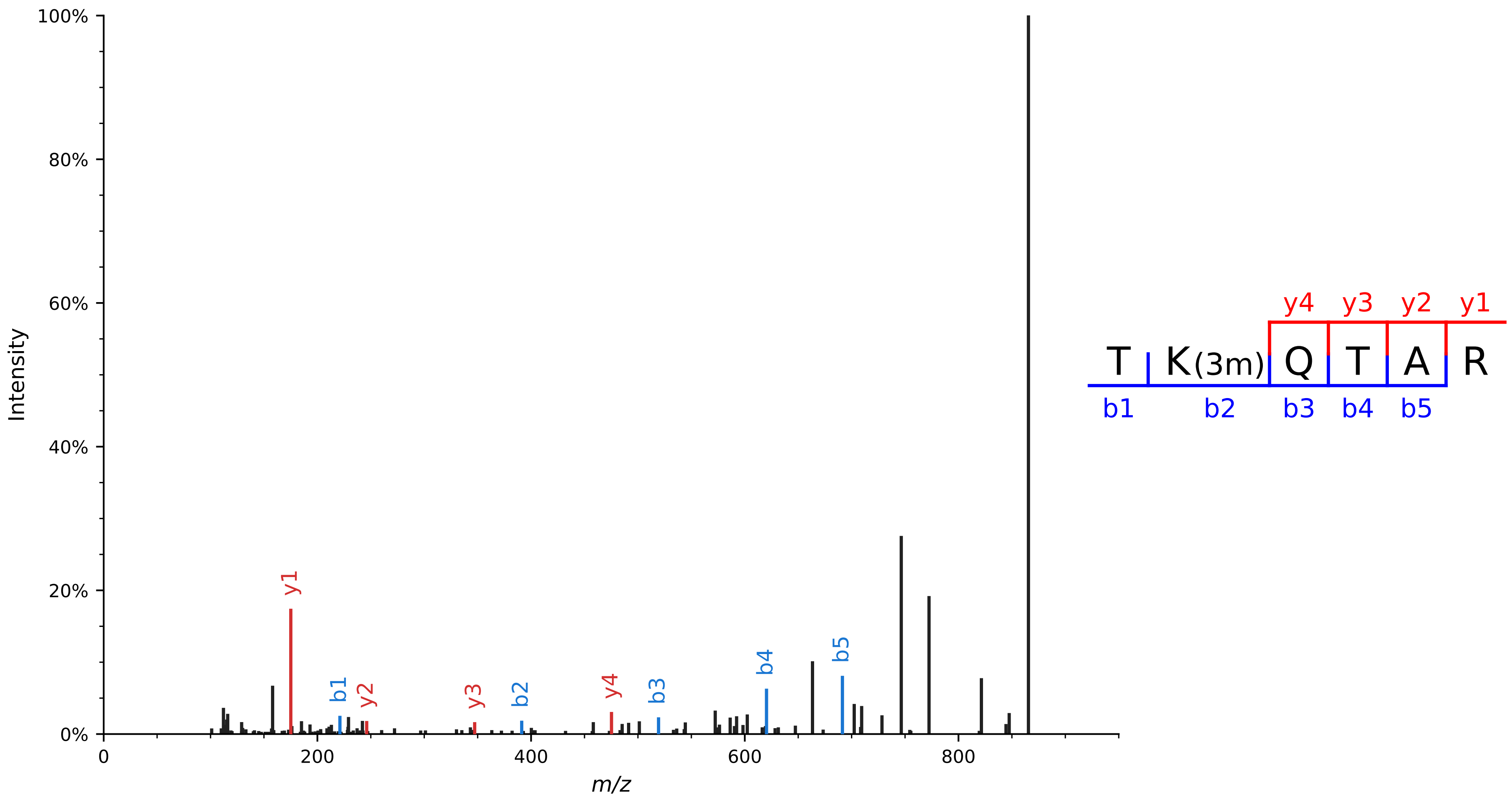

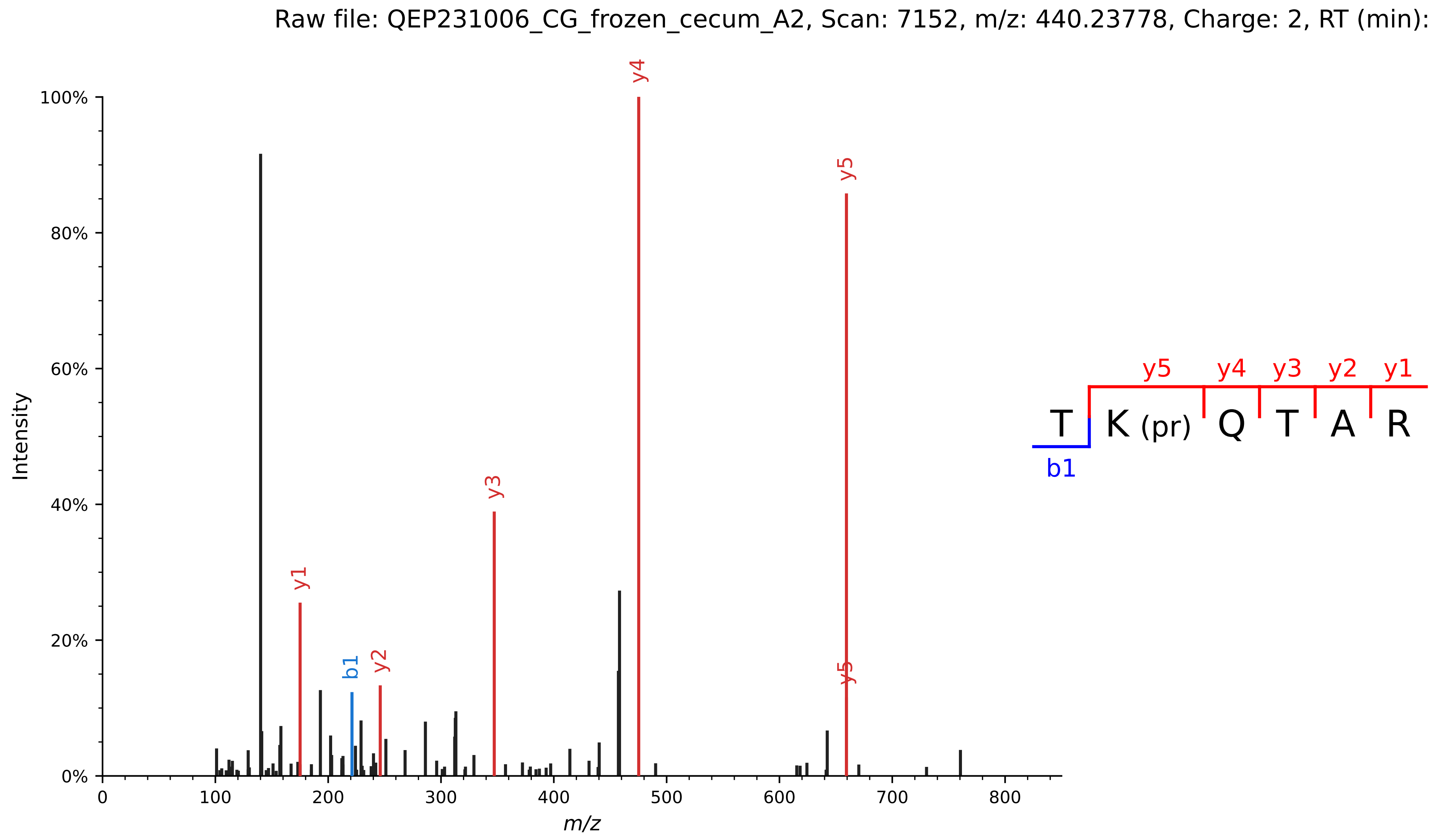

Raw file: QEP231006_CG_frozen_cecum_A2, Scan: 7152, m/z: 440.23778, Charge: 2, RT (min): 19.58, Score: 110.54
Intensity
m/z
y1
b1
y2
y3
y4
y5
y5
T K (pr) Q T A R
y5 y4 y3 y2 y1
b1

Raw file: QEP231006_CG_frozen_cecum_C3, Scan: 7355, m/z: 552.79345, Charge: 2, RT (min): 19.99, Score: 79.906

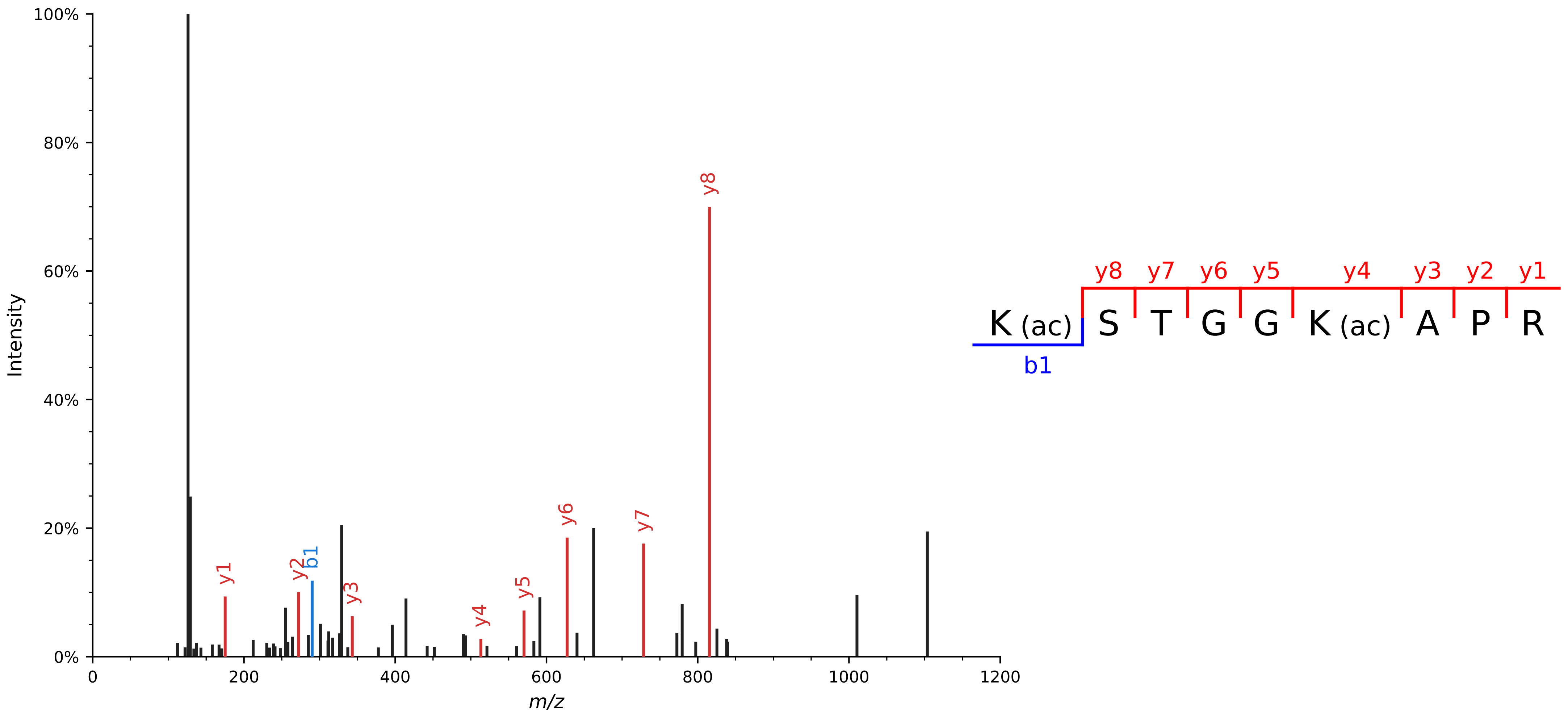

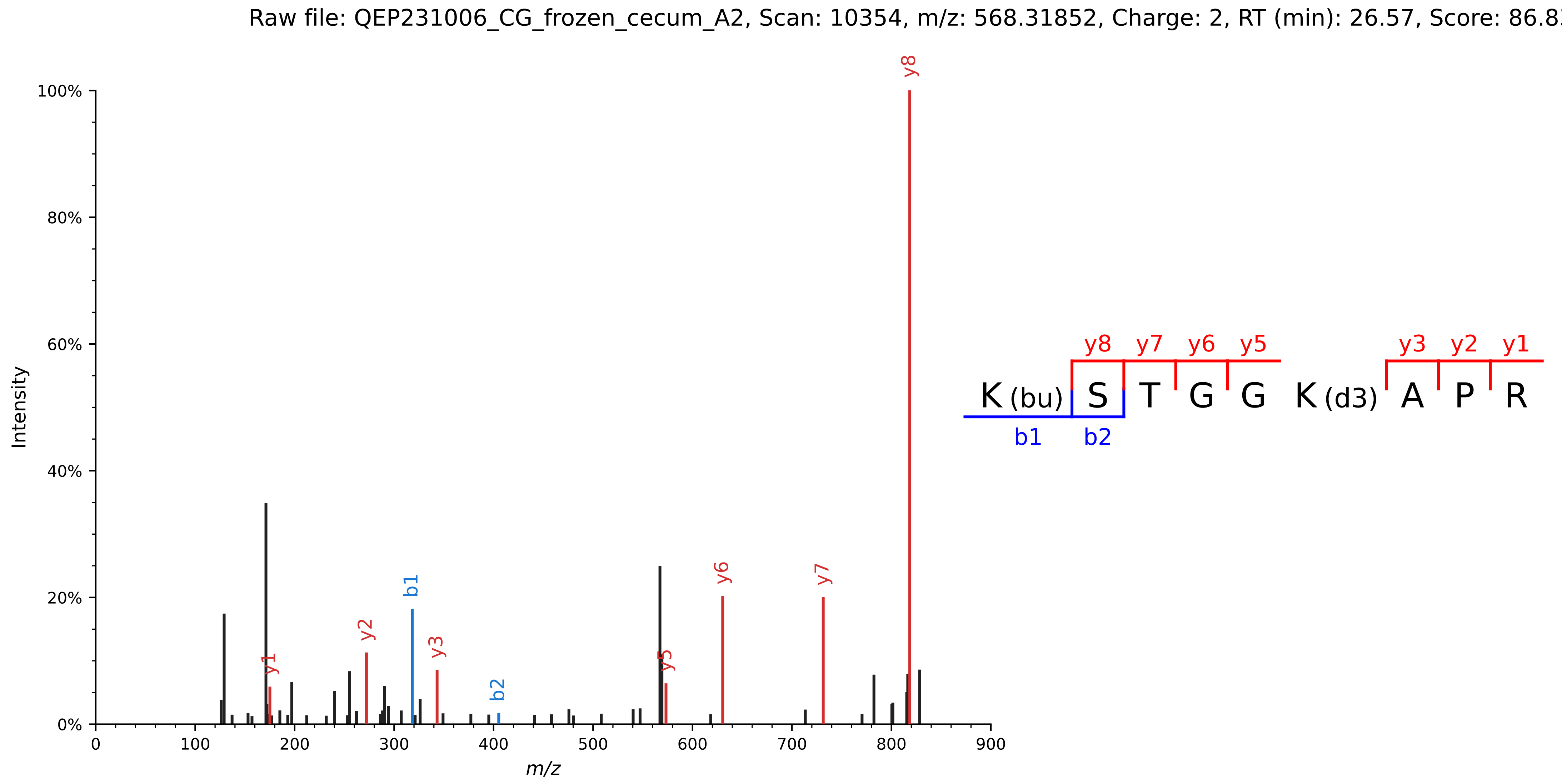

Raw file: QEP231006_CG_frozen_cecum_A2, Scan: 10354, m/z: 568.31852, Charge: 2, RT (min): 26.57, Score: 86.833
Intensity
m/z
y8
y1
y2
b1
y3
b2
y5
y6
y7
K (bu) S T G G K (d3) A P R
y8 y7 y6 y5 y3 y2 y1
b1 b2

Raw file: QEP231006_CG_frozen_cecum_C2, Scan: 10454, m/z: 566.8091, Charge: 2, RT (min): 26.88, Score: 103.56

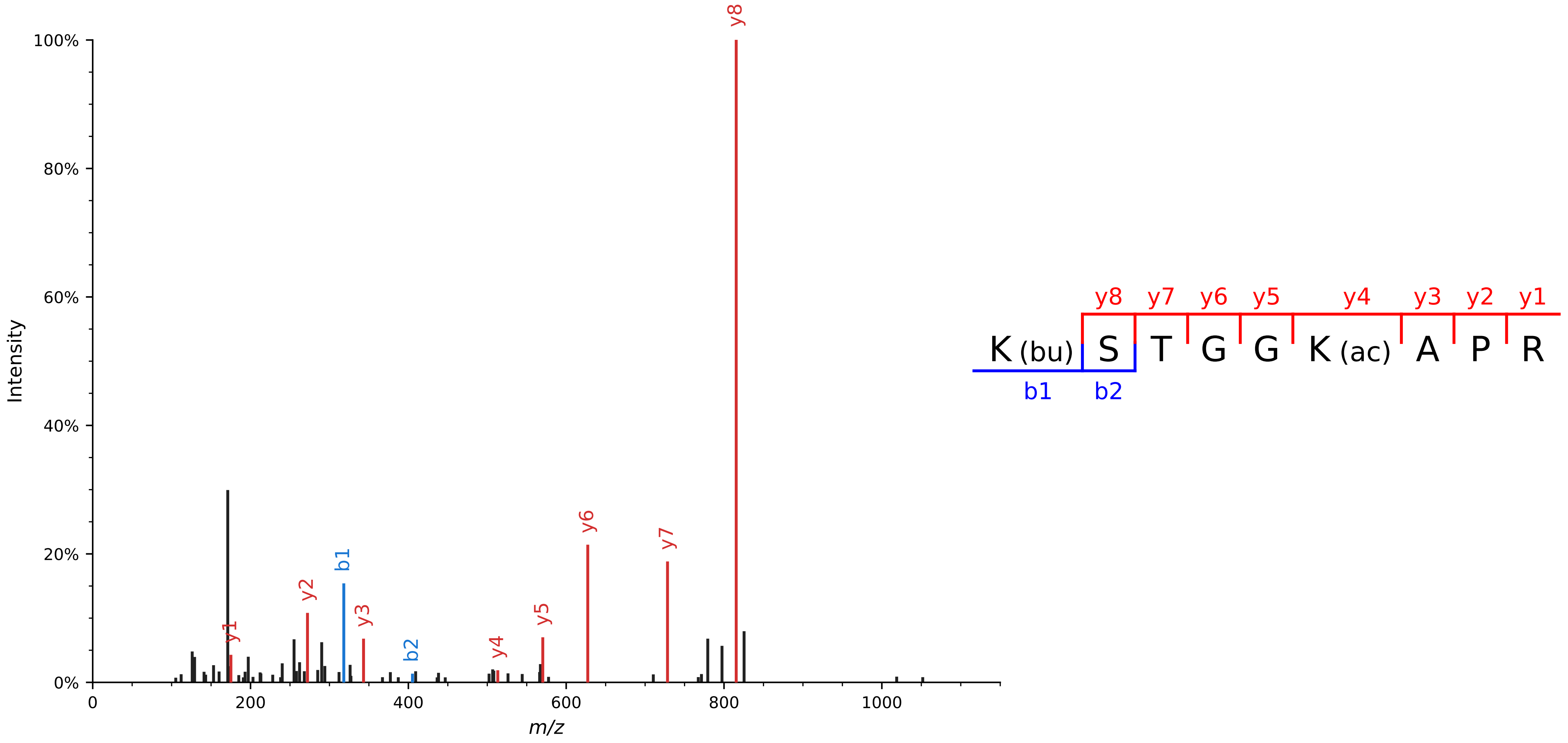

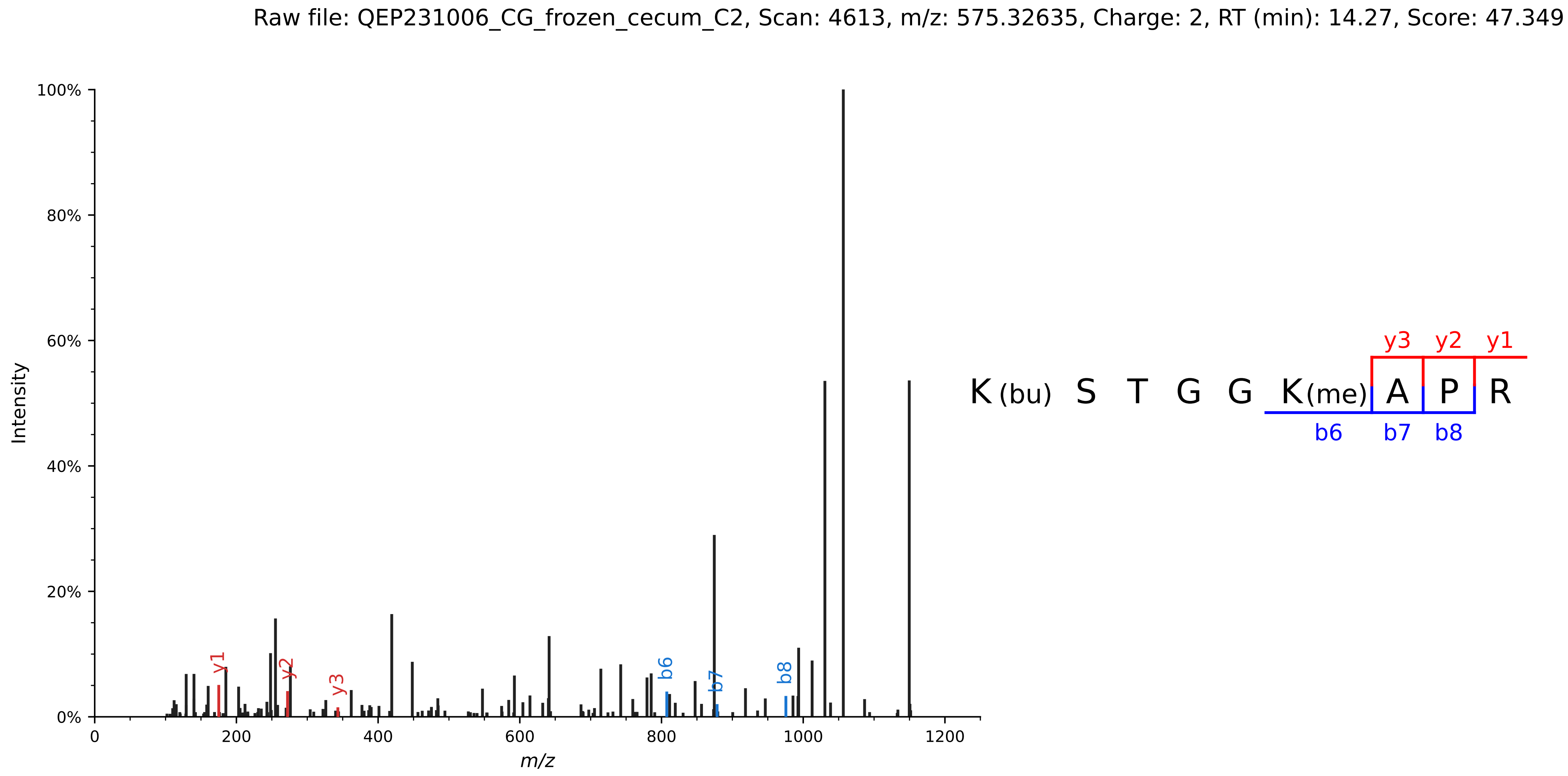

Raw file: QEP231006_CG_frozen_cecum_C2, Scan: 4613, m/z: 575.32635, Charge: 2, RT (min): 14.27, Score: 47.349
Intensity
100%
80%
60%
40%
20%
0%
0
200
400
600
800
1000
1200
m/z
y1
y2
y3
b6
b7
b8
K(bu) S T G G K(me) A P R
y3 y2 y1
b6 b7 b8

Raw file: QEP231006_CG_frozen_cecum_A2, Scan: 13683, m/z: 1164.6008, Charge: 1, RT (min): 33.87, Score: 53.775

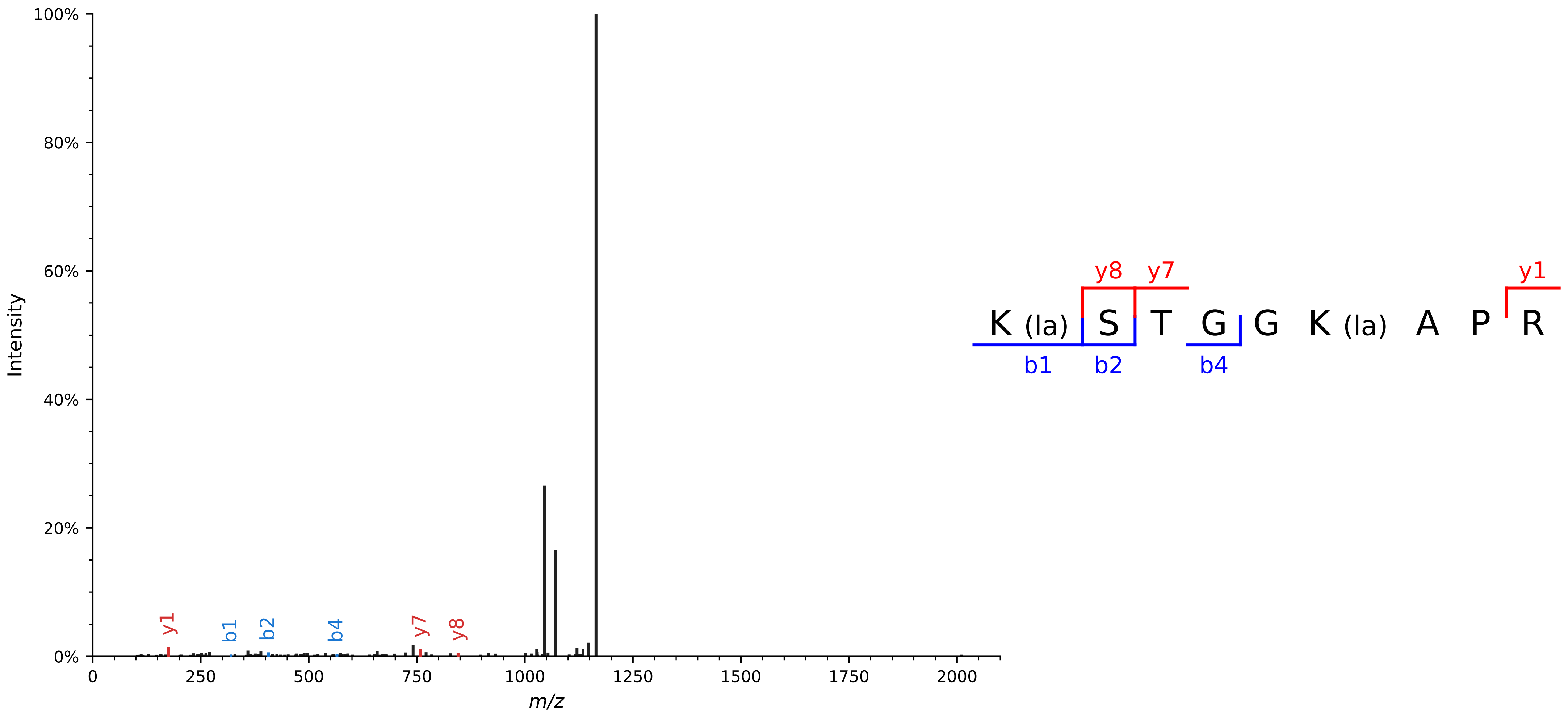

Raw file: QEP231006_CG_frozen_colon_C3, Scan: 8541, m/z: 562.82011, Charge: 2, RT (min): 22.59, Score: 117.4

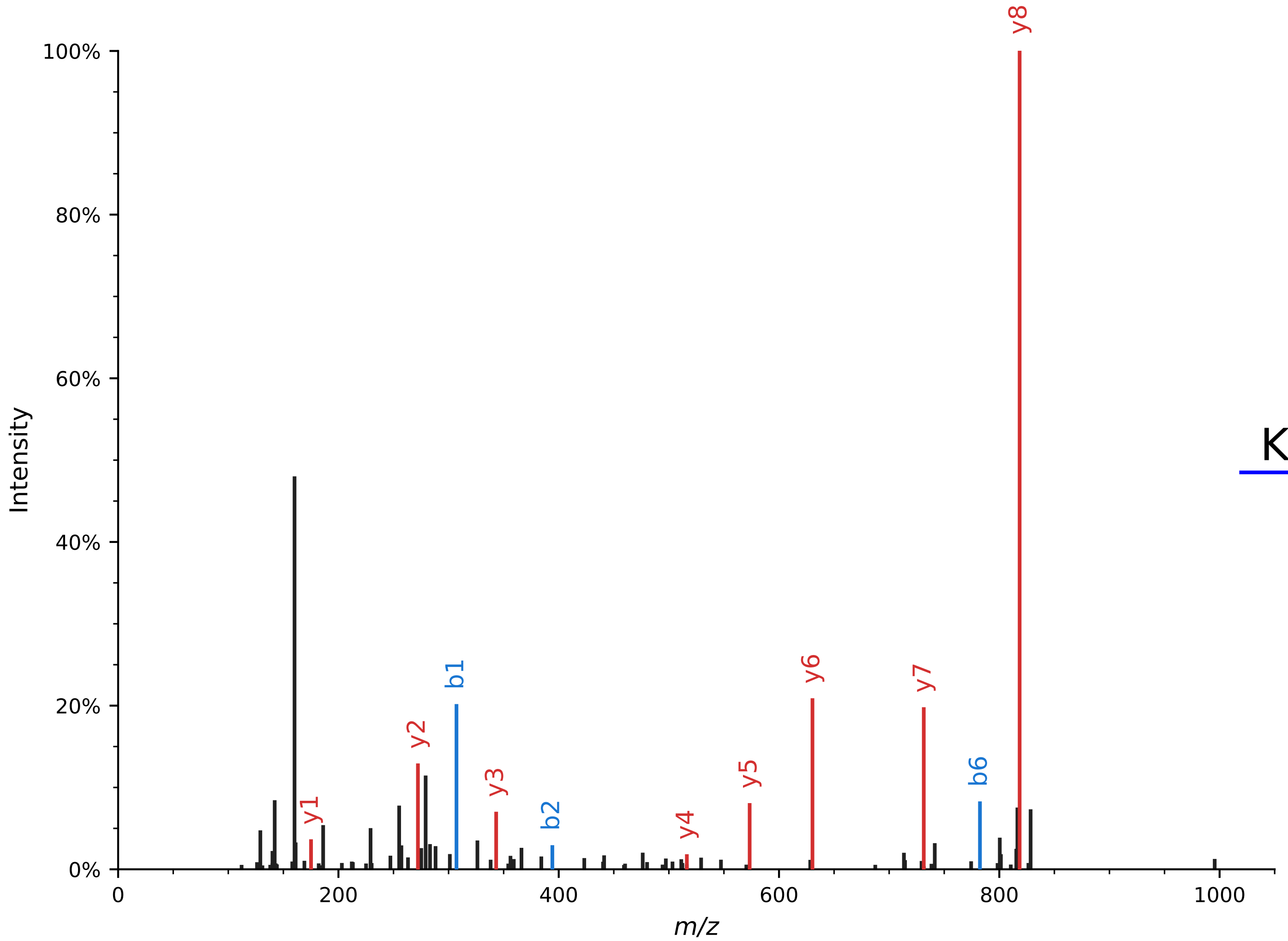

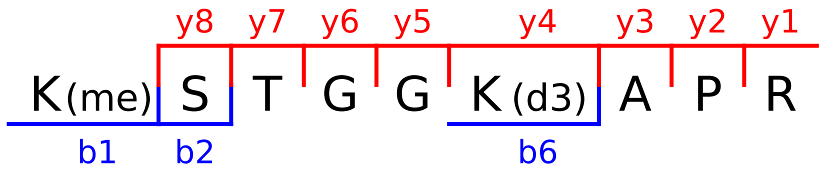

Raw file: QEP231006_CG_frozen_cecum_C3, Scan: 8788, m/z: 561.3107, Charge: 2, RT (min): 22.98, Score: 124.98

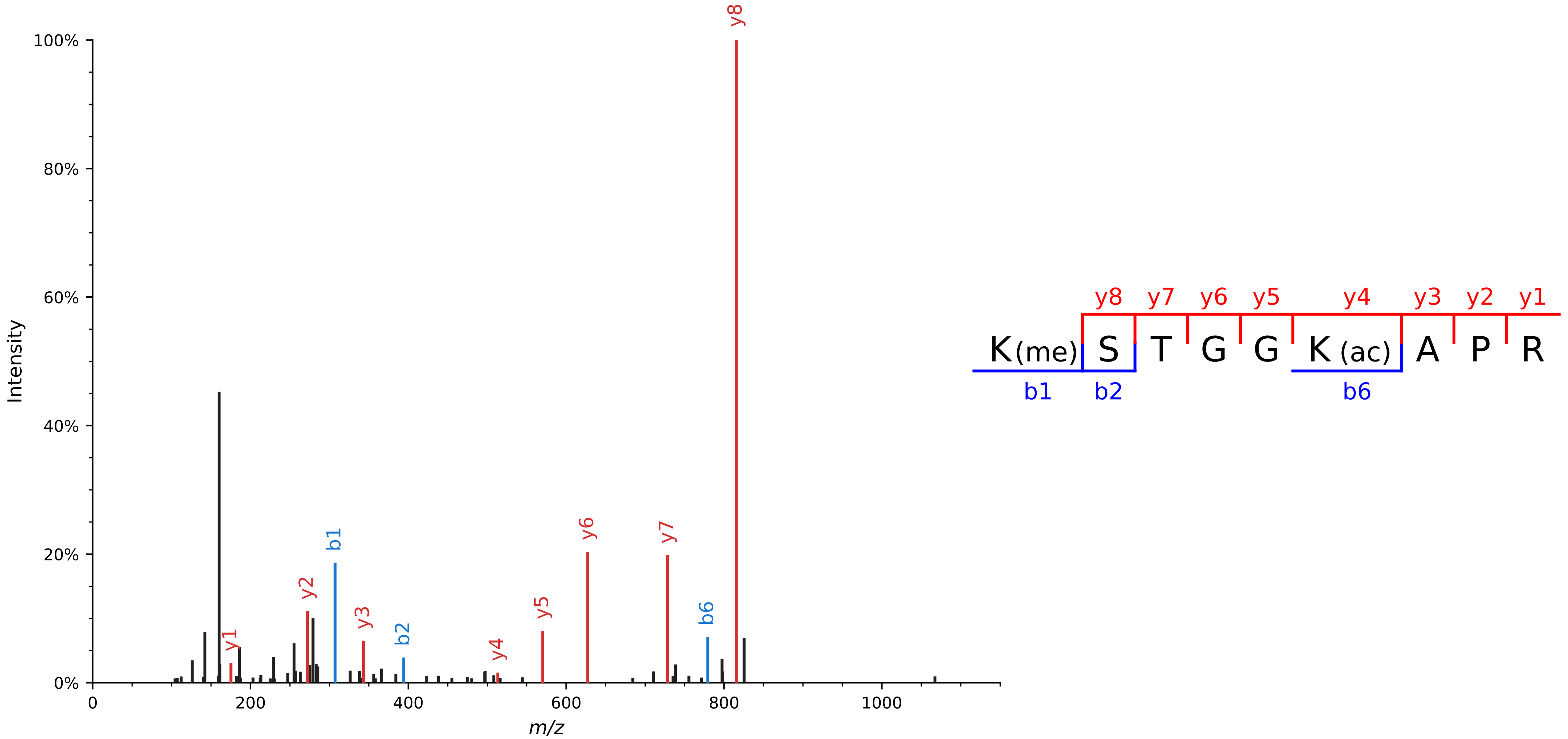

Raw file: QEP231006_CG_frozen_cecum_C3, Scan: 3785, m/z: 569.82794, Charge: 2, RT (min): 12.32, Score: 50.703

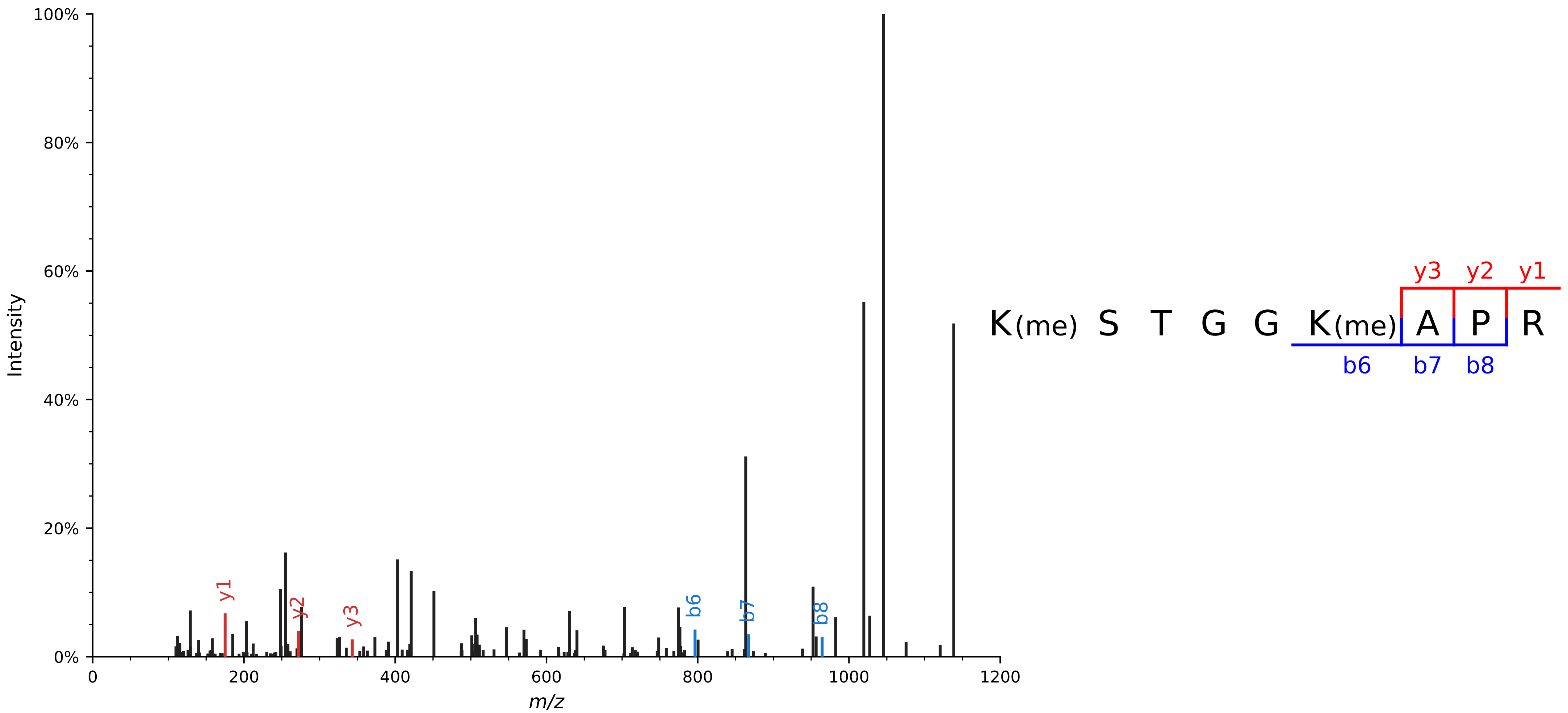

Raw file: QEP231006_CG_frozen_colon_C2, Scan: 9239, m/z: 568.31852, Charge: 2, RT (min): 24.81, Score: 127.56

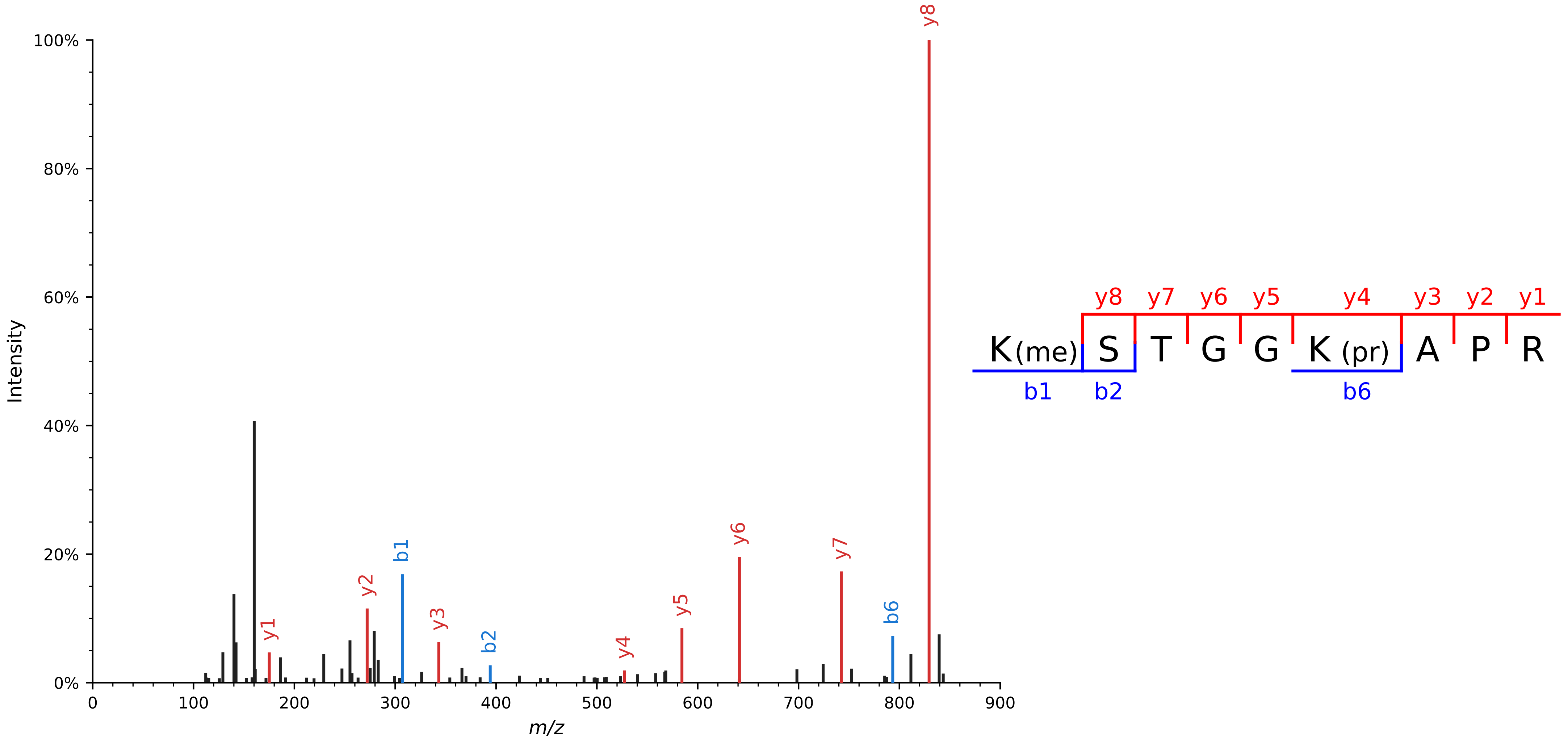

Raw file: QEP231006_CG_frozen_cecum_A4, Scan: 2275, m/z: 547.31324, Charge: 2, RT (min): 9.09, Score: 130.56

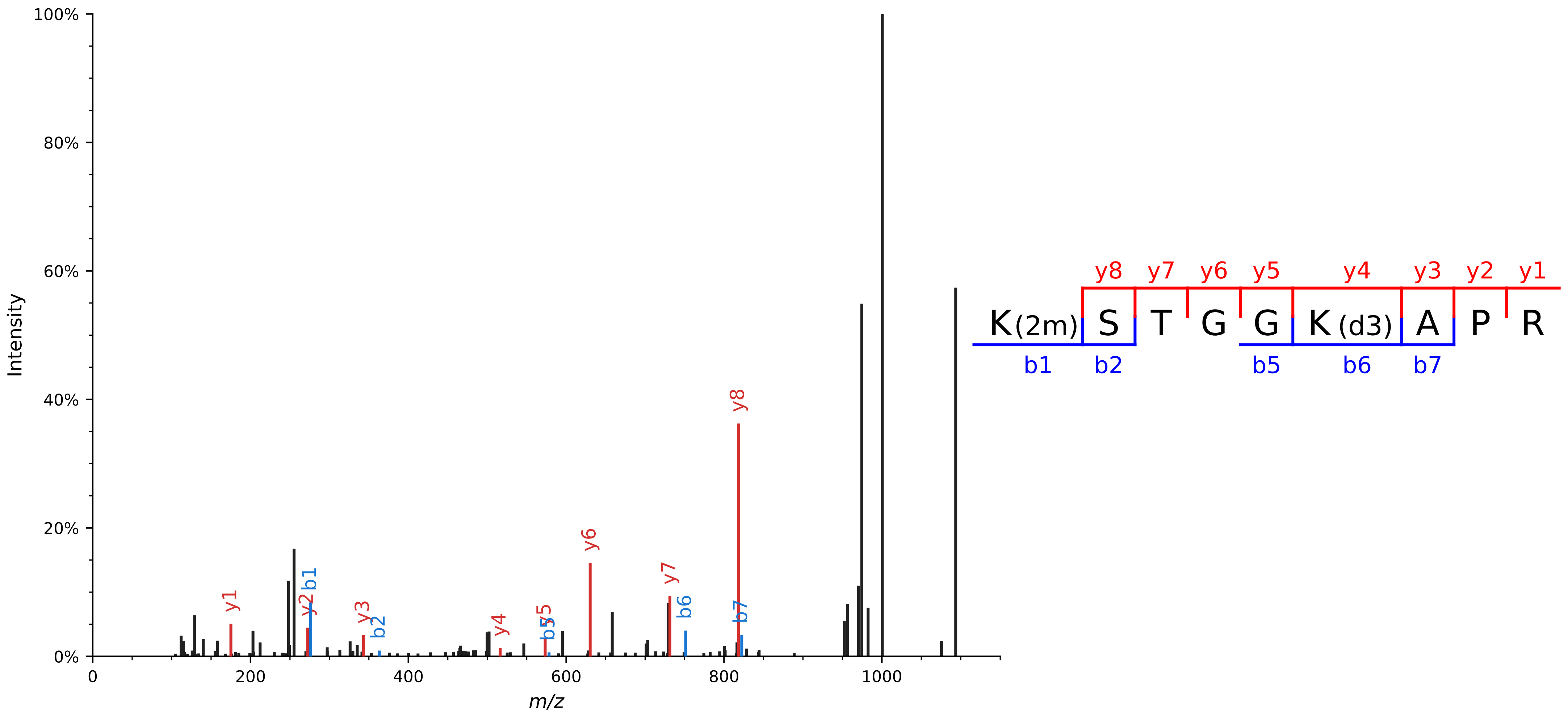

Raw file: QEP231006_CG_frozen_colon_C3, Scan: 2367, m/z: 545.80382, Charge: 2, RT (min): 9.22, Score: 146.81

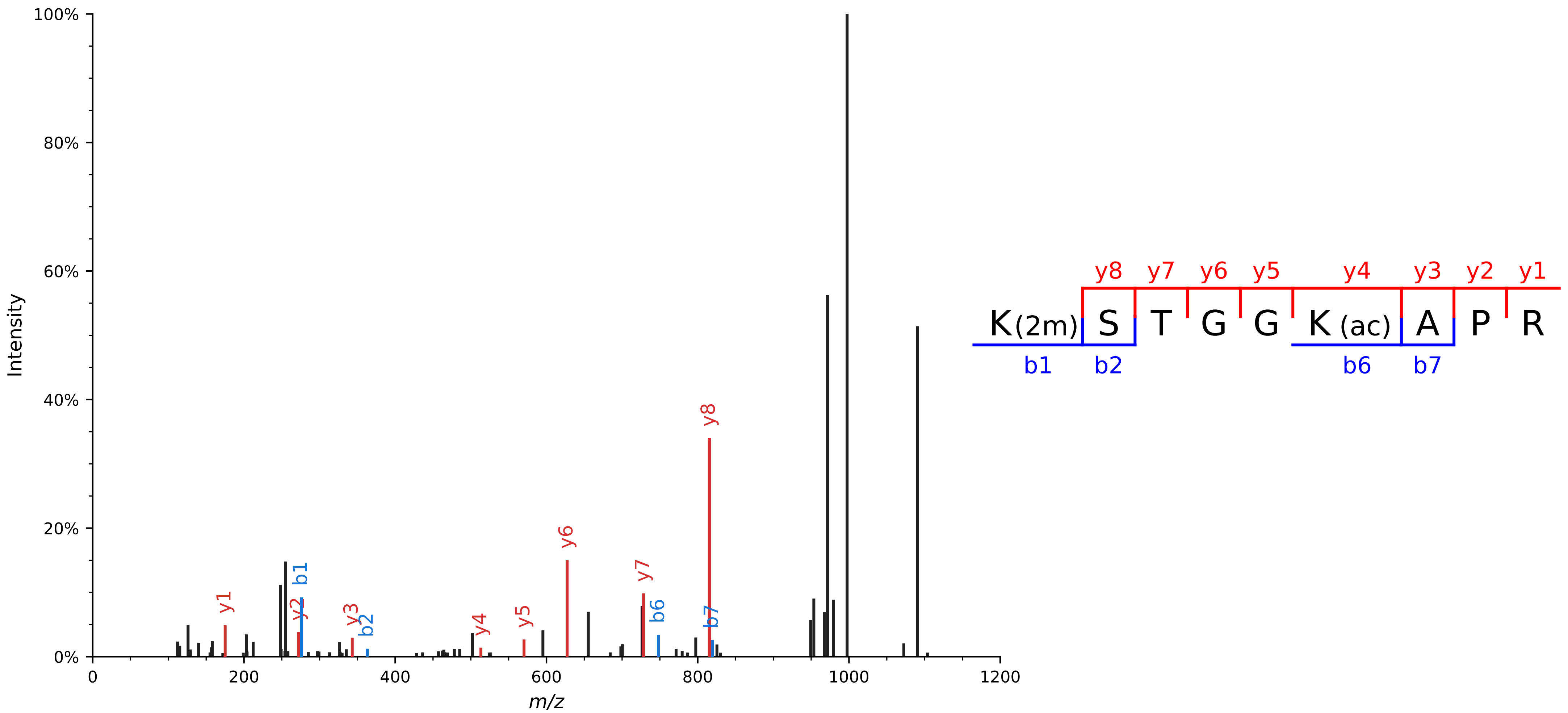

Raw file: QEP231006_CG_frozen_cecum_A3, Scan: 2553, m/z: 554.32106, Charge: 2, RT (min): 9.75, Score: 66.682

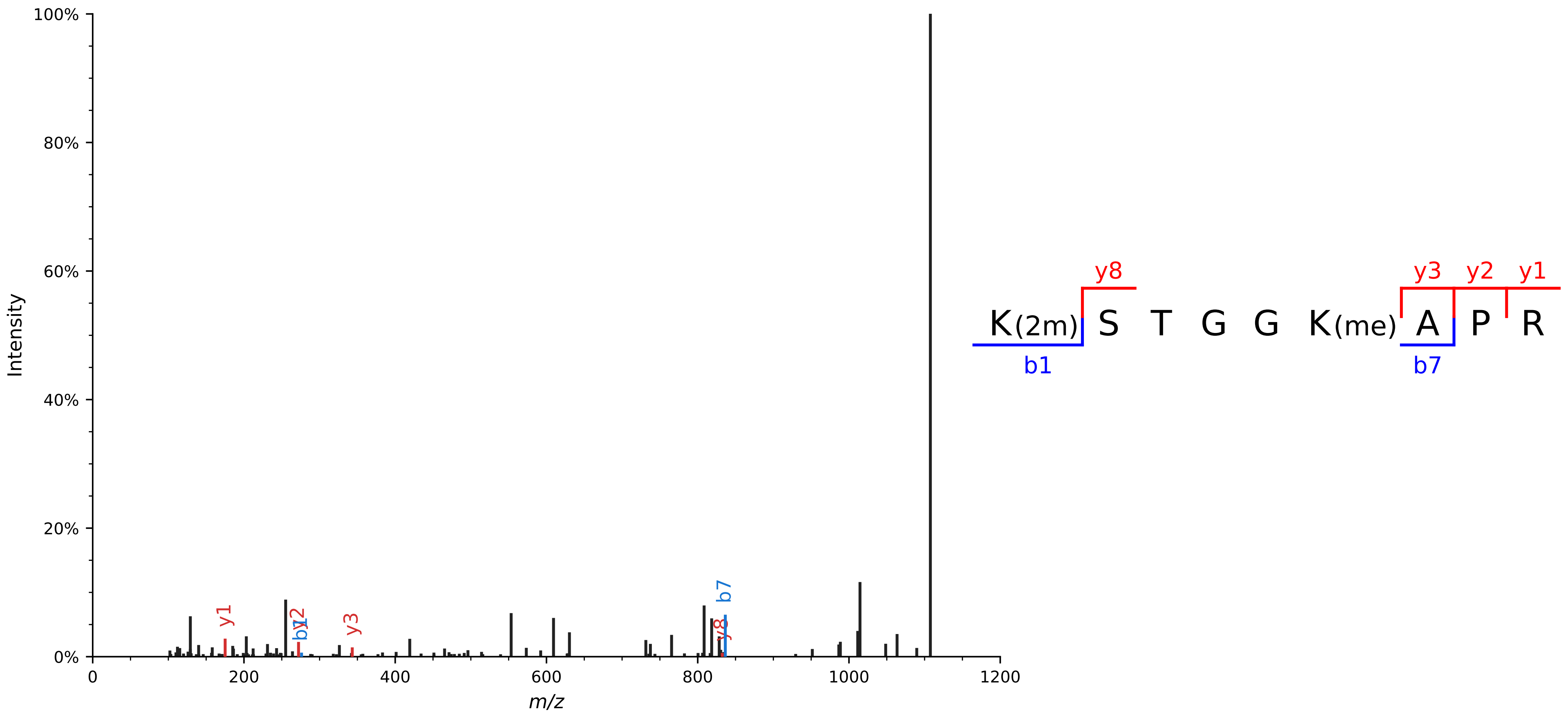

Raw file: QEP231006_CG_frozen_colon_A1, Scan: 3136, m/z: 552.81165, Charge: 2, RT (min): 10.92, Score: 141.2

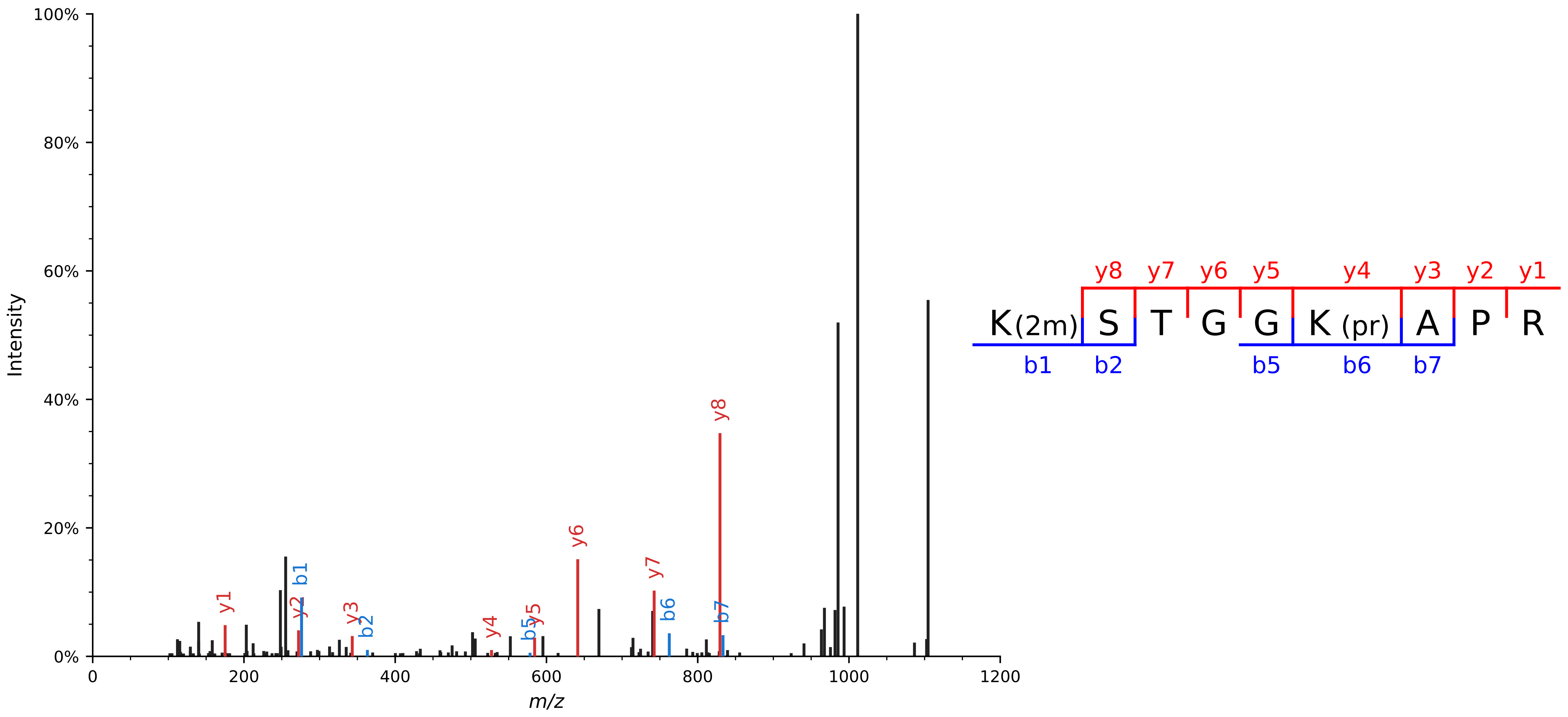

Raw file: QEP231006_CG_frozen_cecum_A5, Scan: 2176, m/z: 554.32106, Charge: 2, RT (min): 8.78, Score: 145.72

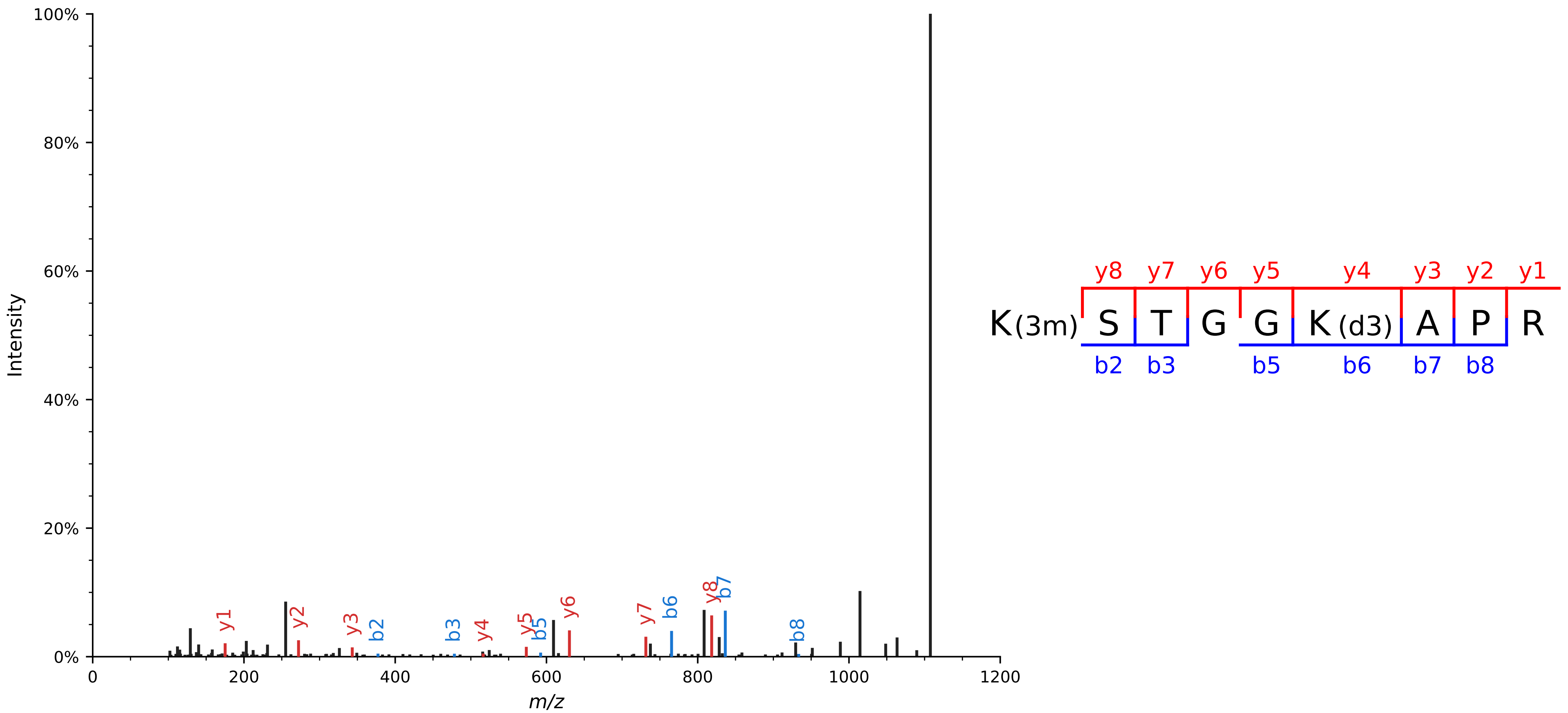

Raw file: QEP231006_CG_frozen_colon_A5, Scan: 3171, m/z: 559.81947, Charge: 2, RT (min): 10.79, Score: 135.71

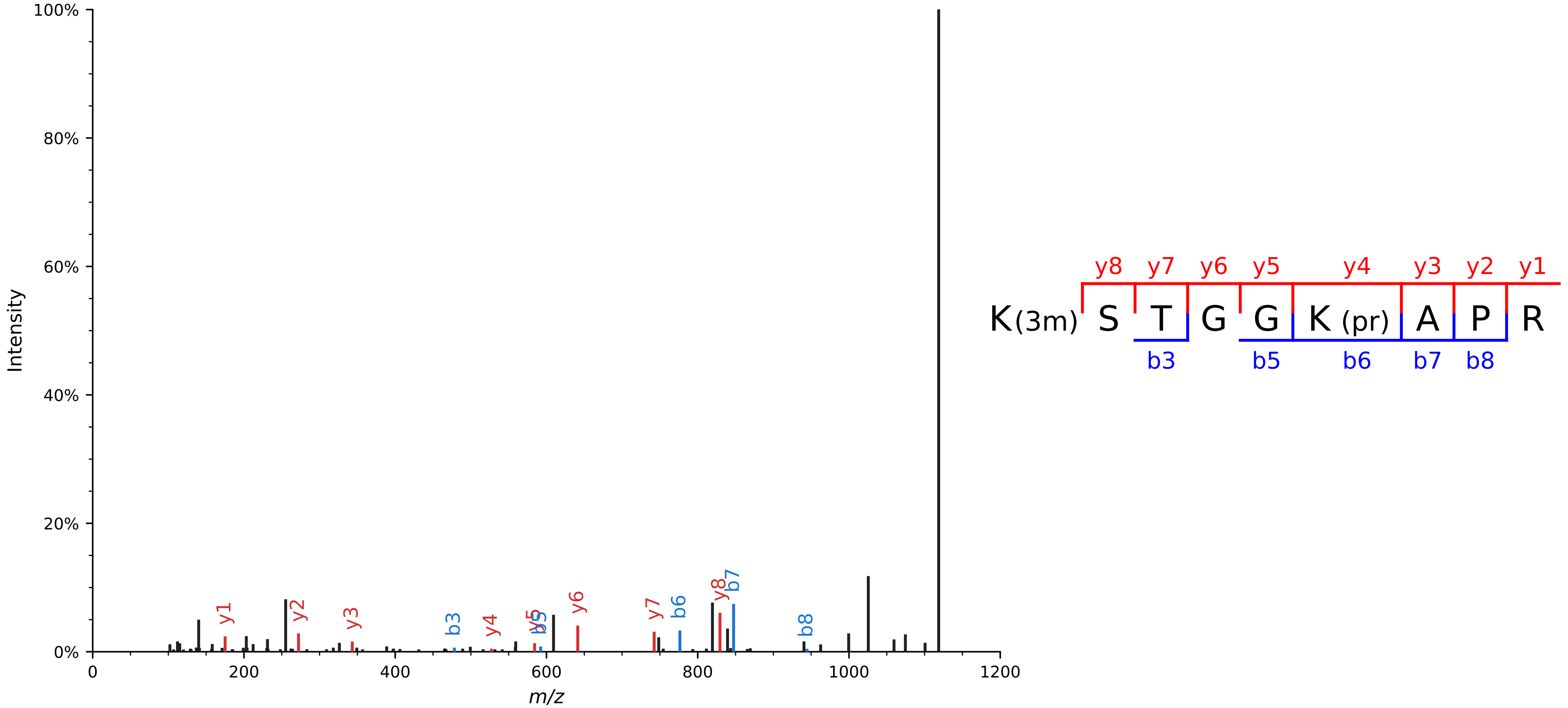

Raw file: QEP231006_CG_frozen_cecum_C4, Scan: 8490, m/z: 561.3107, Charge: 2, RT (min): 22.53, Score: 120.46

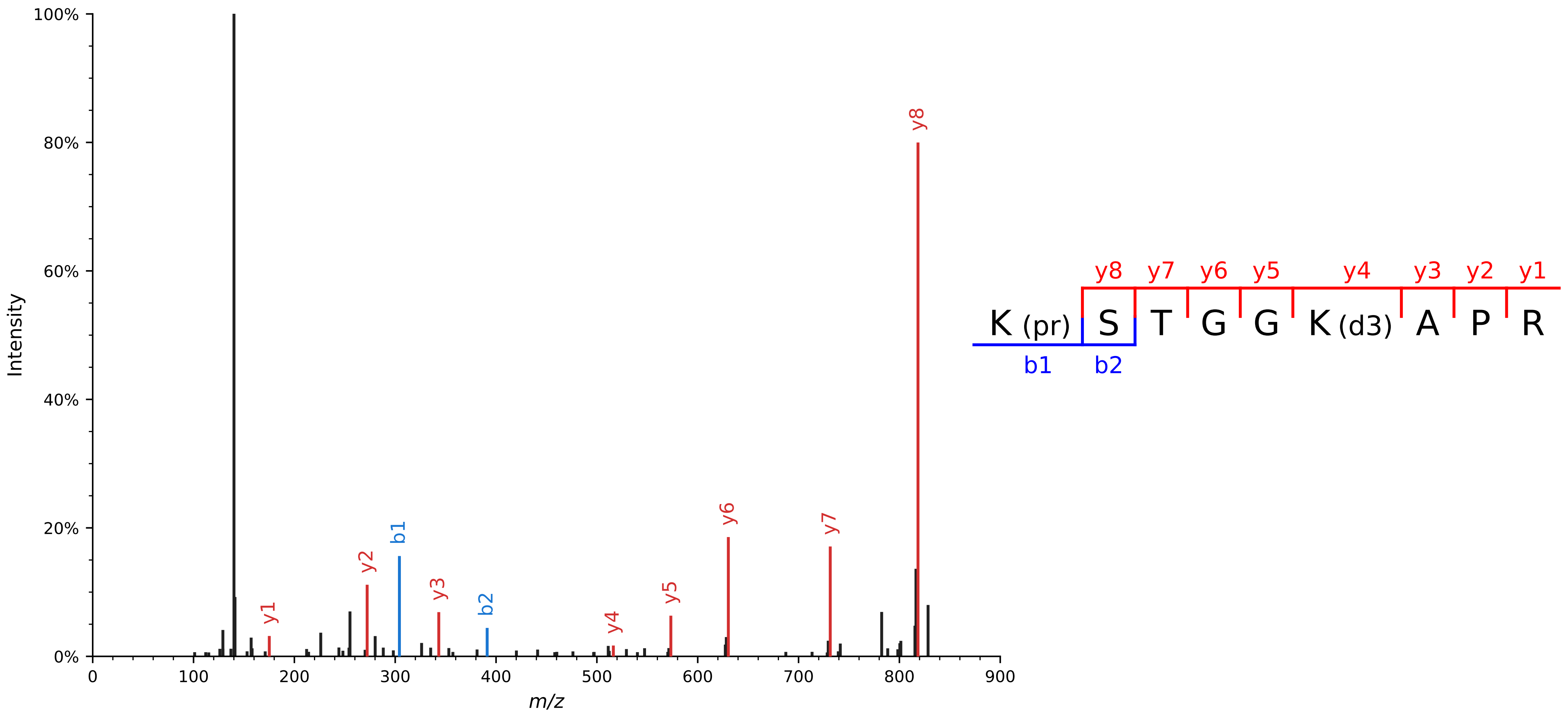

Raw file: QEP231006_CG_frozen_cecum_A4, Scan: 3768, m/z: 568.31852, Charge: 2, RT (min): 12.32, Score: 71.501

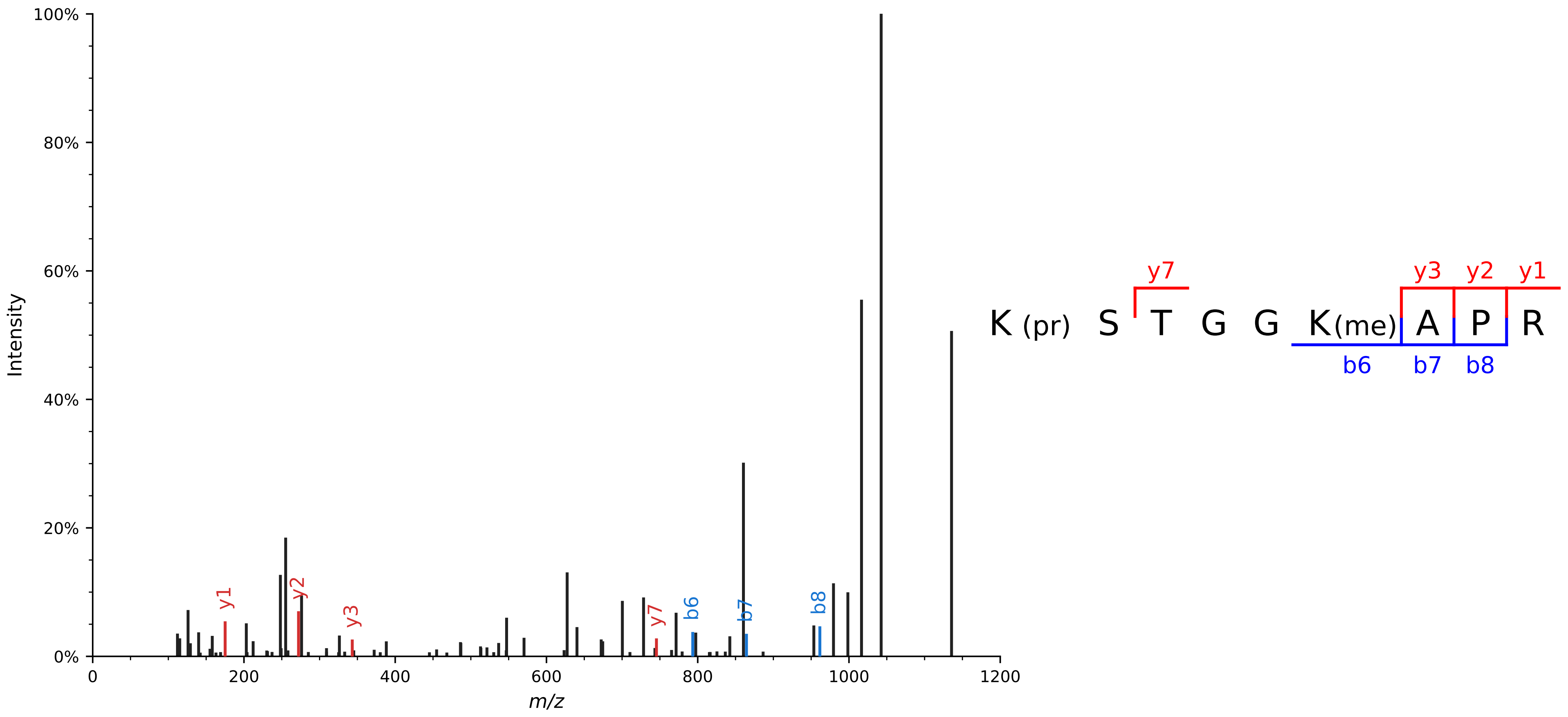

Raw file: QEP231006_CG_frozen_cecum_A2, Scan: 9592, m/z: 566.8091, Charge: 2, RT (min): 24.79, Score: 116.73

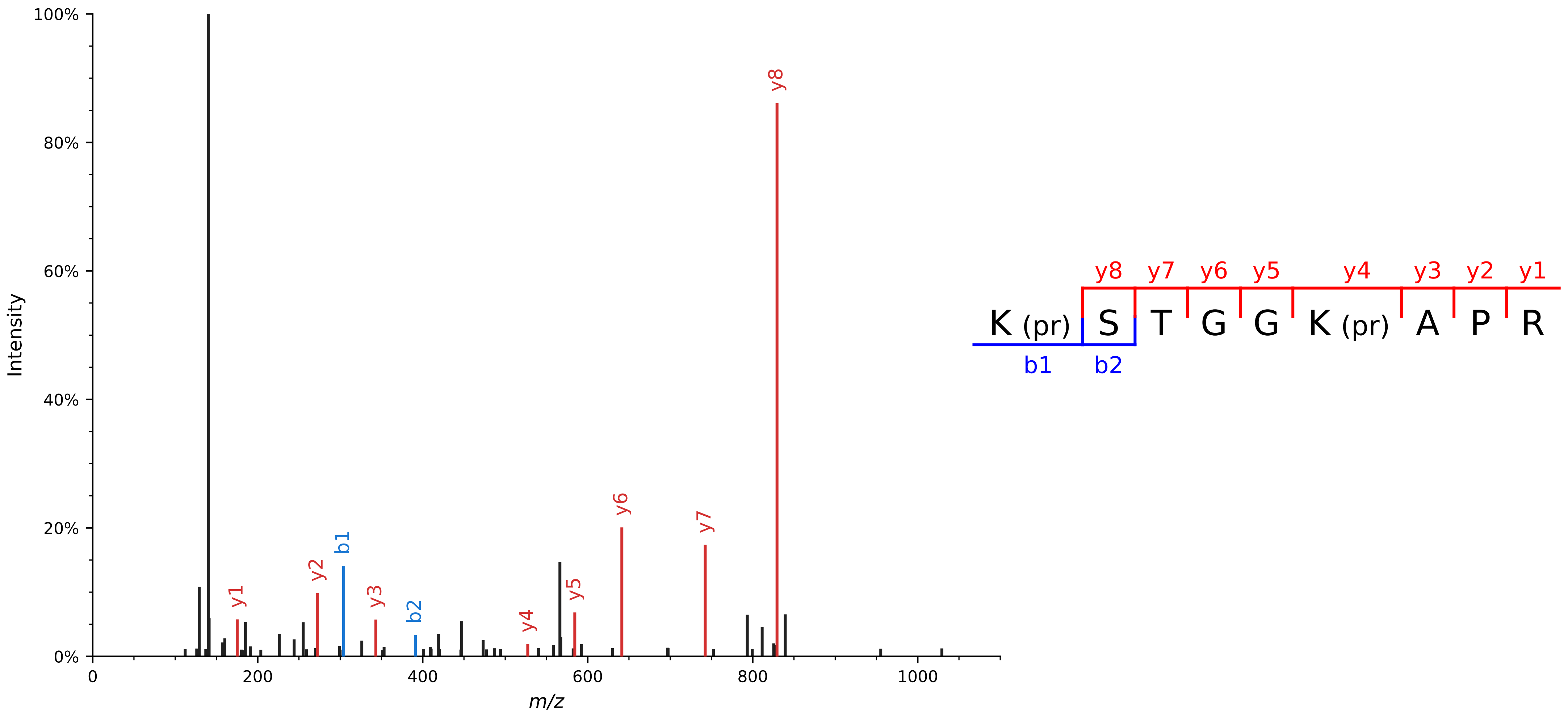

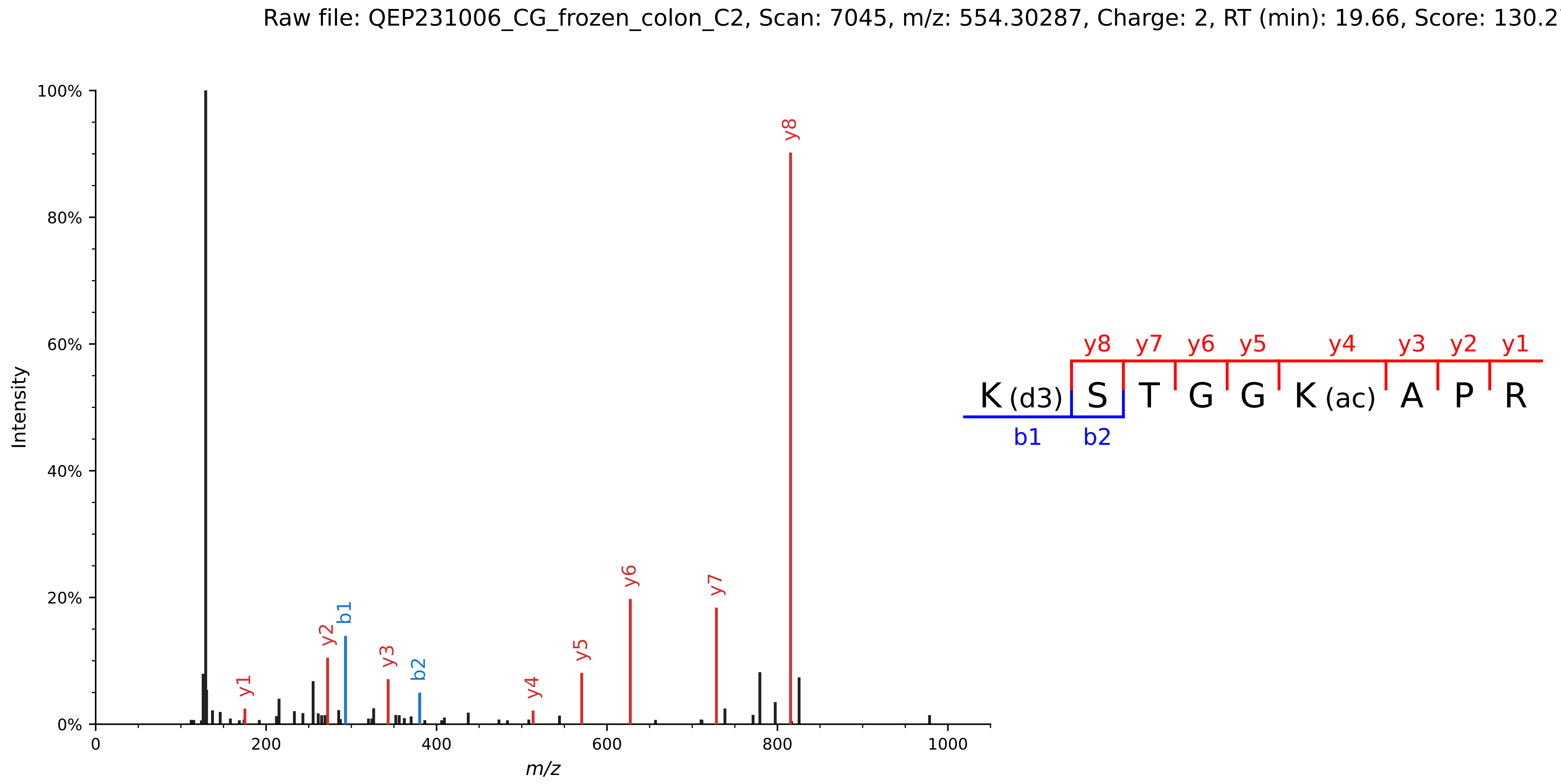

Raw file: QEP231006_CG_frozen_colon_C2, Scan: 7045, m/z: 554.30287, Charge: 2, RT (min): 19.66, Score: 130.27
100%
80%
60%
40%
20%
0%
Intensity
0
200
400
600
800
1000
m/z
y1
y2
b1
y3
b2
y4
y5
y6
y7
y8
y8 y7 y6 y5 y4 y3 y2 y1
K (d3) S T G G K (ac) A P R
b1 b2

Raw file: QEP231006_CG_frozen_colon_C3, Scan: 9178, m/z: 567.3107, Charge: 2, RT (min): 24.06, Score: 43.124

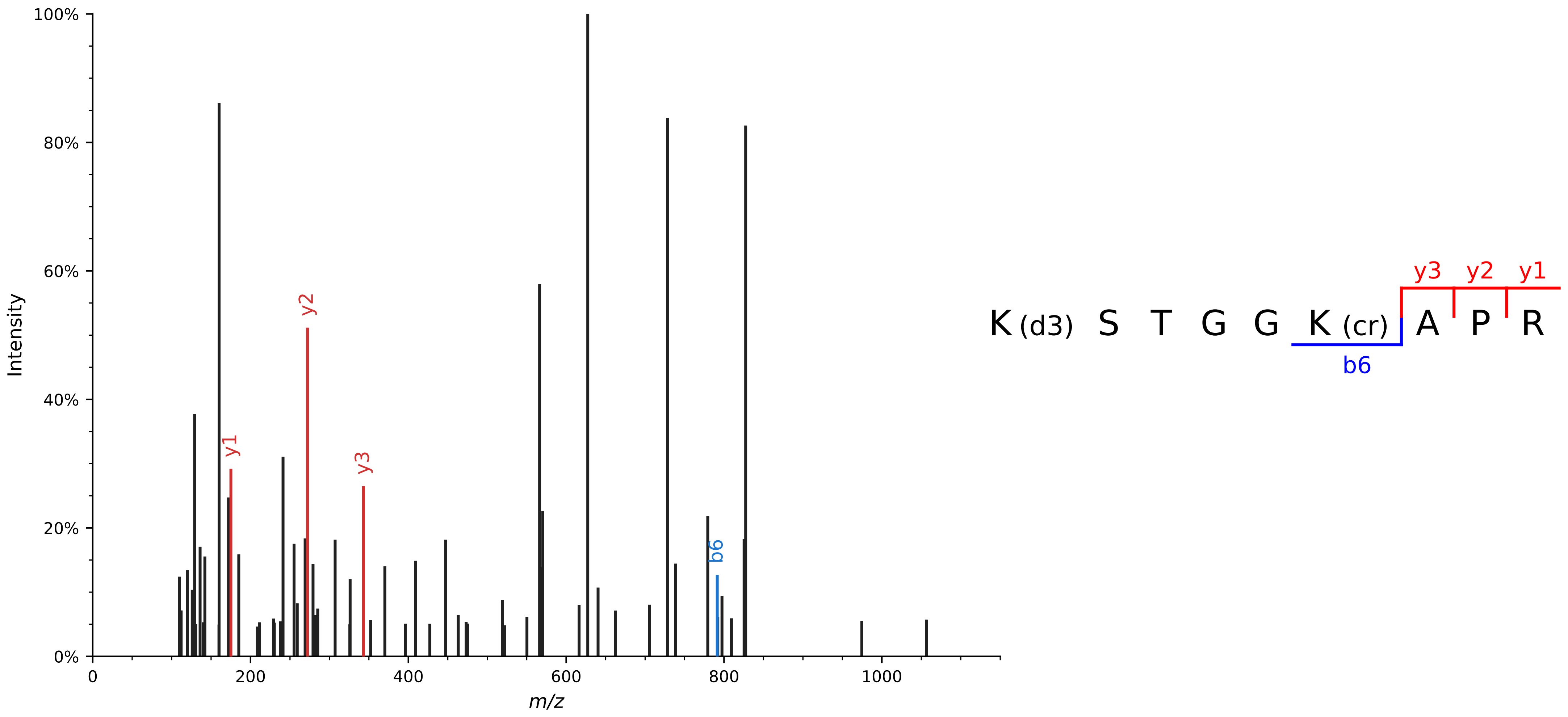

Raw file: QEP231006_CG_frozen_colon_C1, Scan: 8019, m/z: 561.3107, Charge: 2, RT (min): 21.88, Score: 120.46

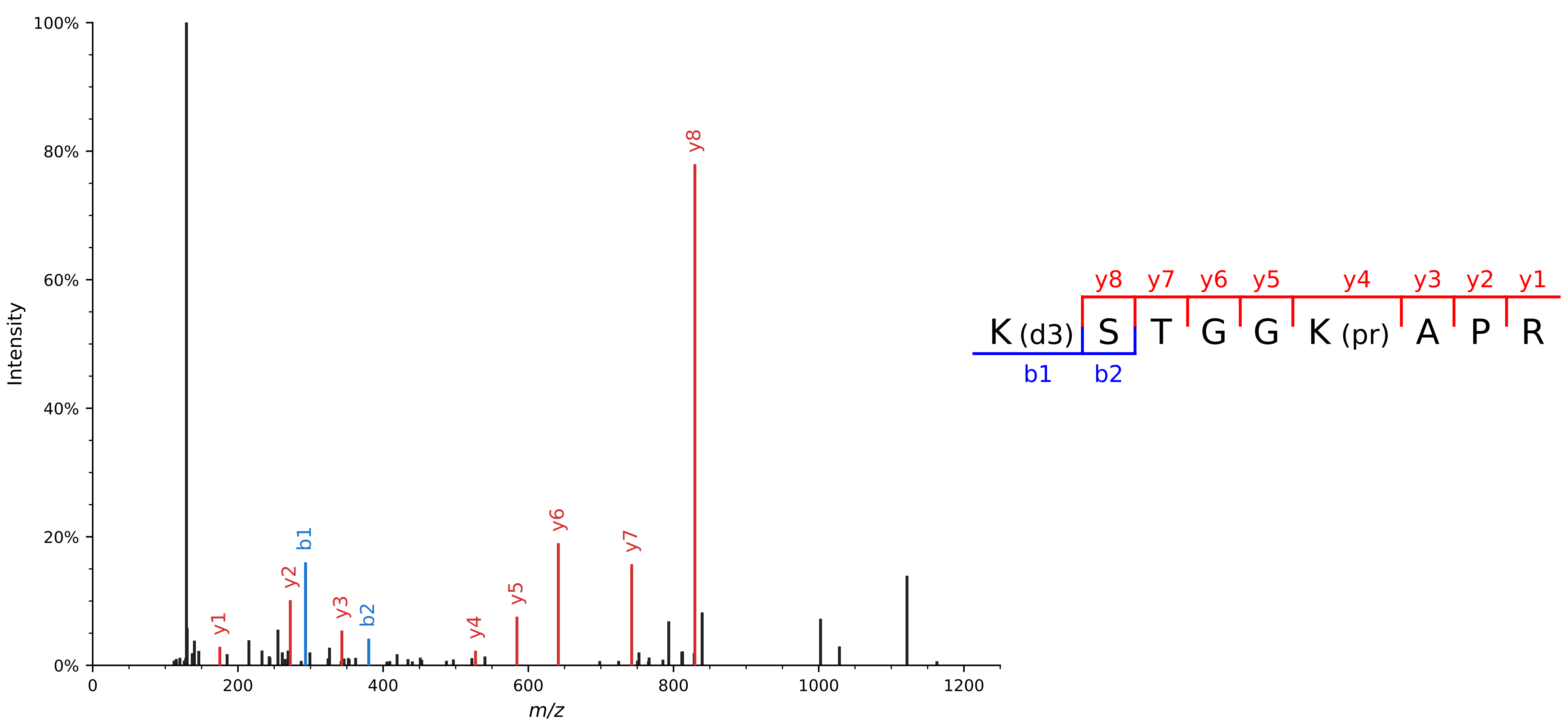

Raw file: QEP231006_CG_frozen_cecum_C1, Scan: 14942, m/z: 595.33803, Charge: 2, RT (min): 36.62, Score: 96.624

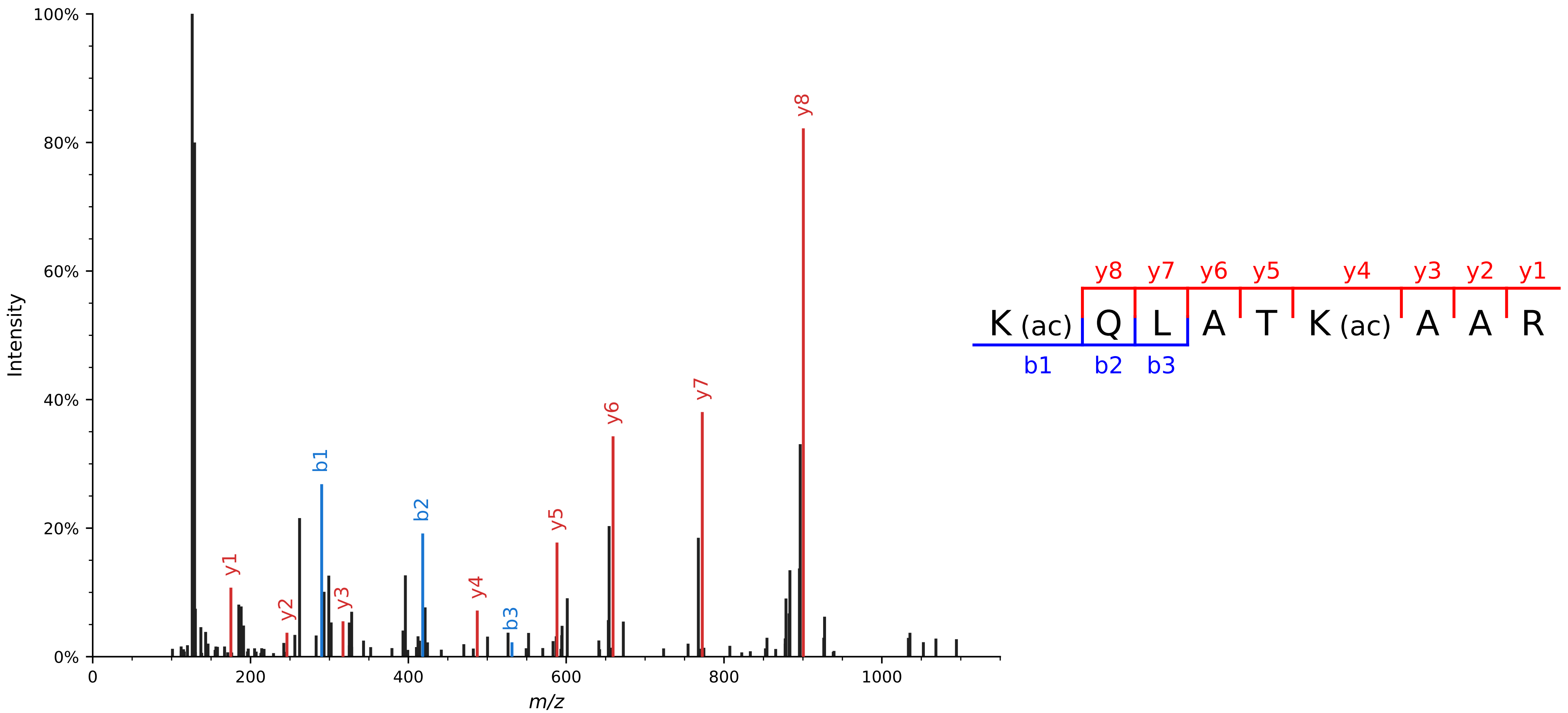

Raw file: QEP231006_CG_frozen_colon_A4, Scan: 14288, m/z: 588.3484, Charge: 2, RT (min): 35.92, Score: 81.191

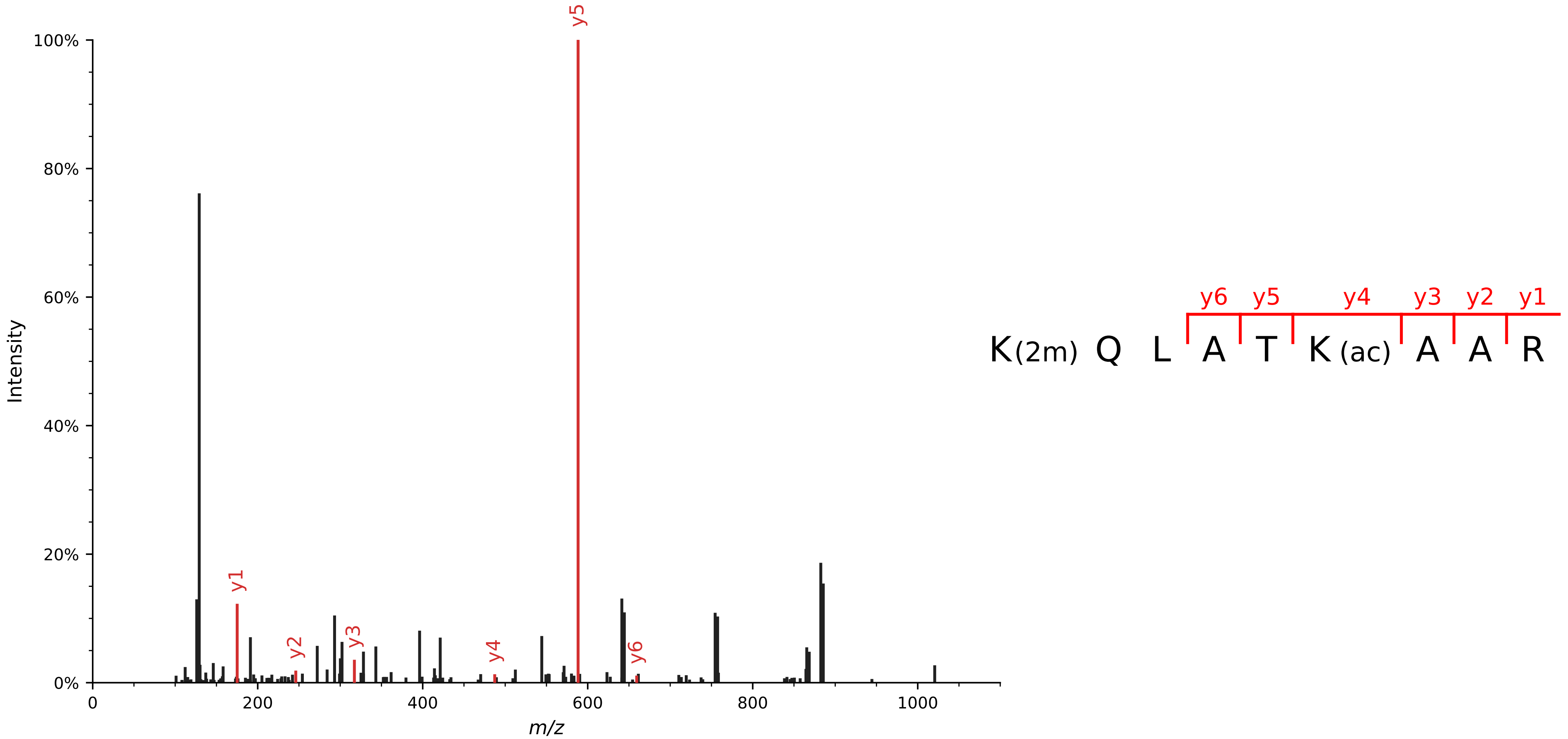

Raw file: QEP231006_CG_frozen_colon_A4, Scan: 15099, m/z: 603.85527, Charge: 2, RT (min): 38.21, Score: 153.78

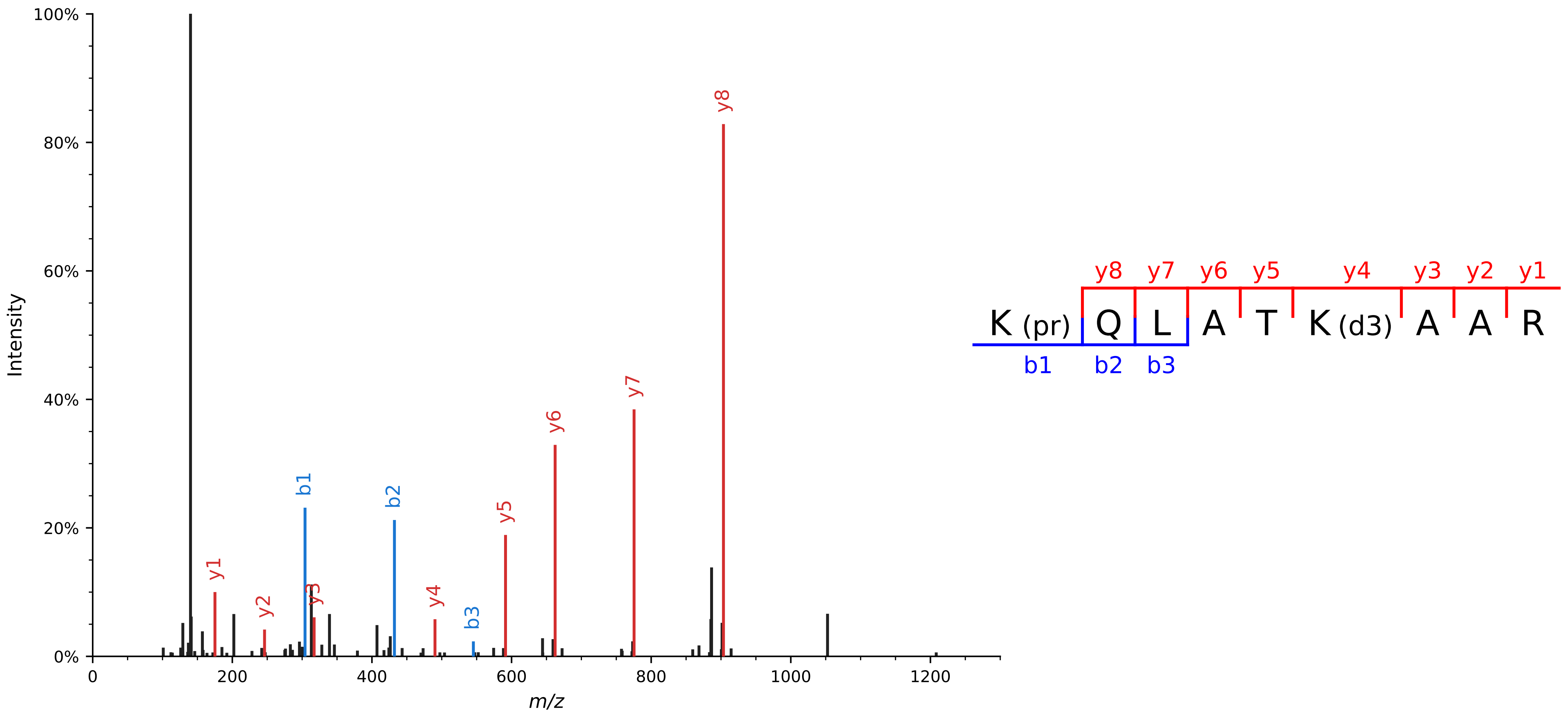

Raw file: QEP231006_CG_frozen_cecum_C2, Scan: 16601, m/z: 609.35368, Charge: 2, RT (min): 40.84, Score: 117.04

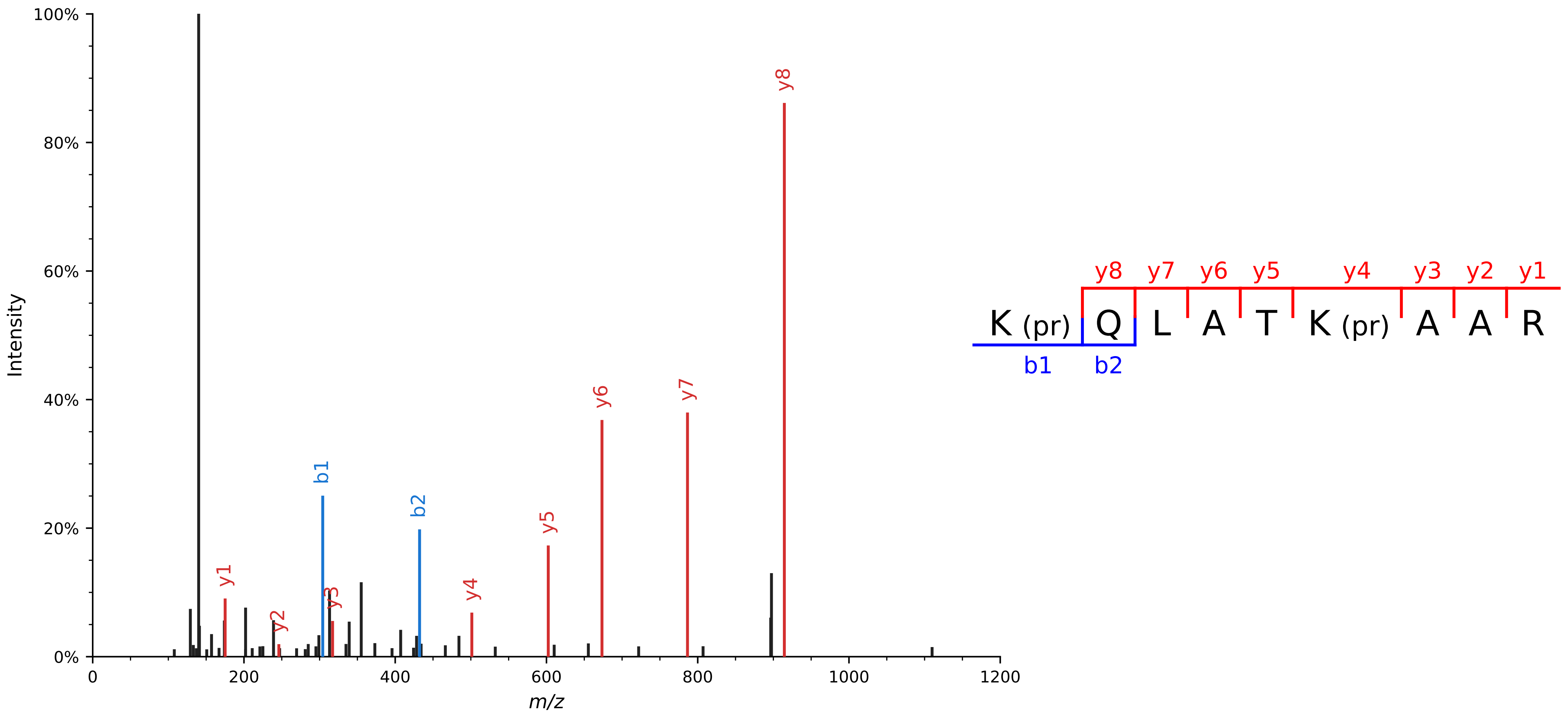

Raw file: QEP231006_CG_frozen_cecum_C1, Scan: 14950, m/z: 596.84744, Charge: 2, RT (min): 36.63, Score: 167.24

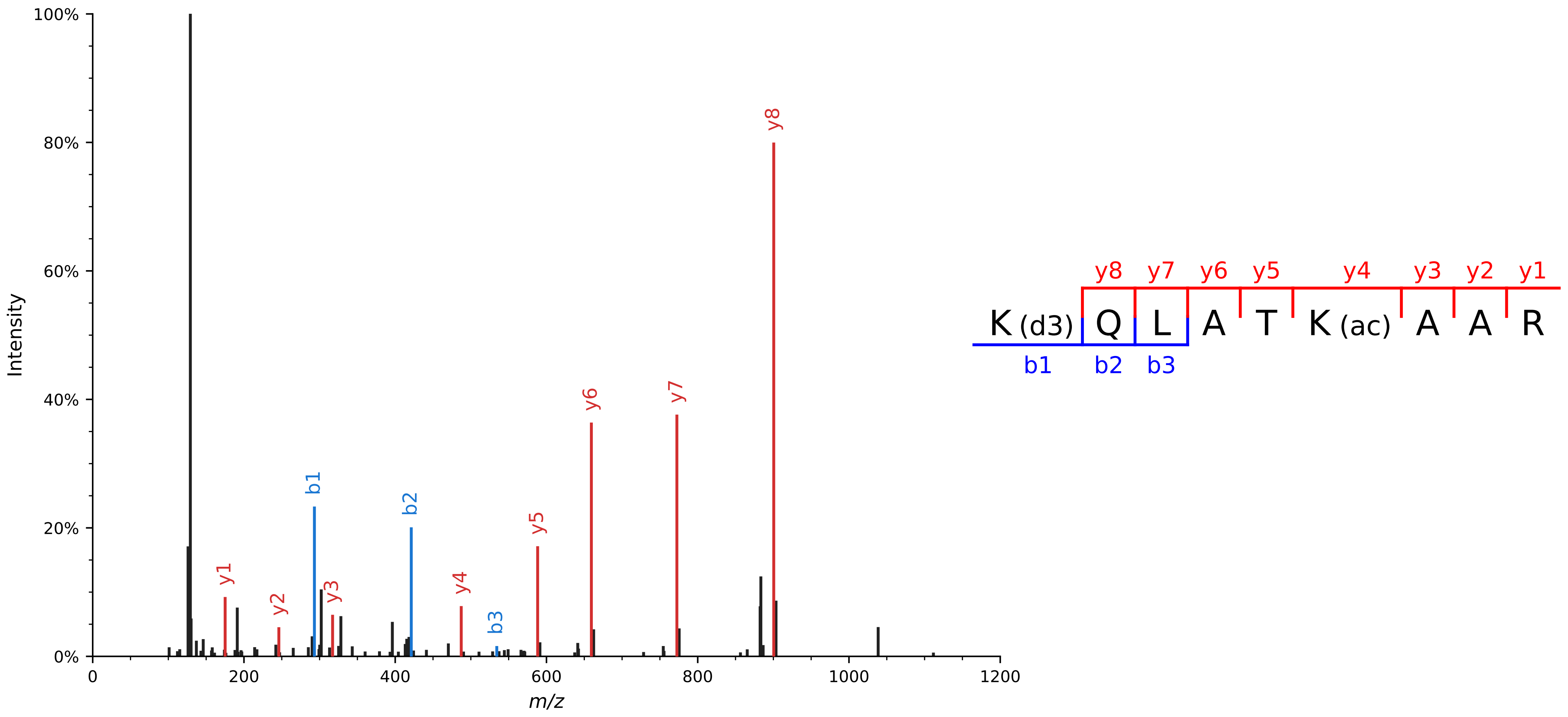

Raw file: QEP231006_CG_frozen_cecum_A1, Scan: 15627, m/z: 603.85527, Charge: 2, RT (min): 38.35, Score: 160.75

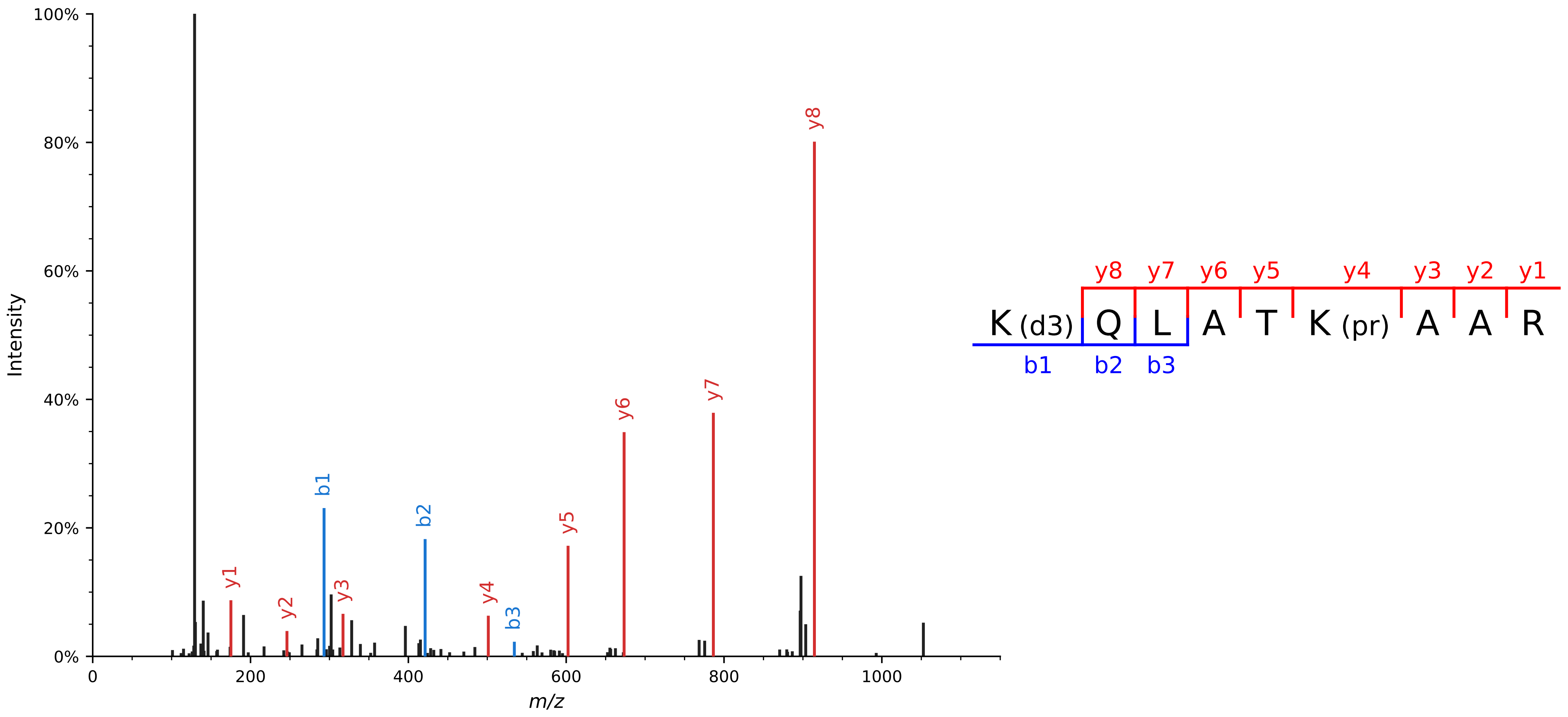

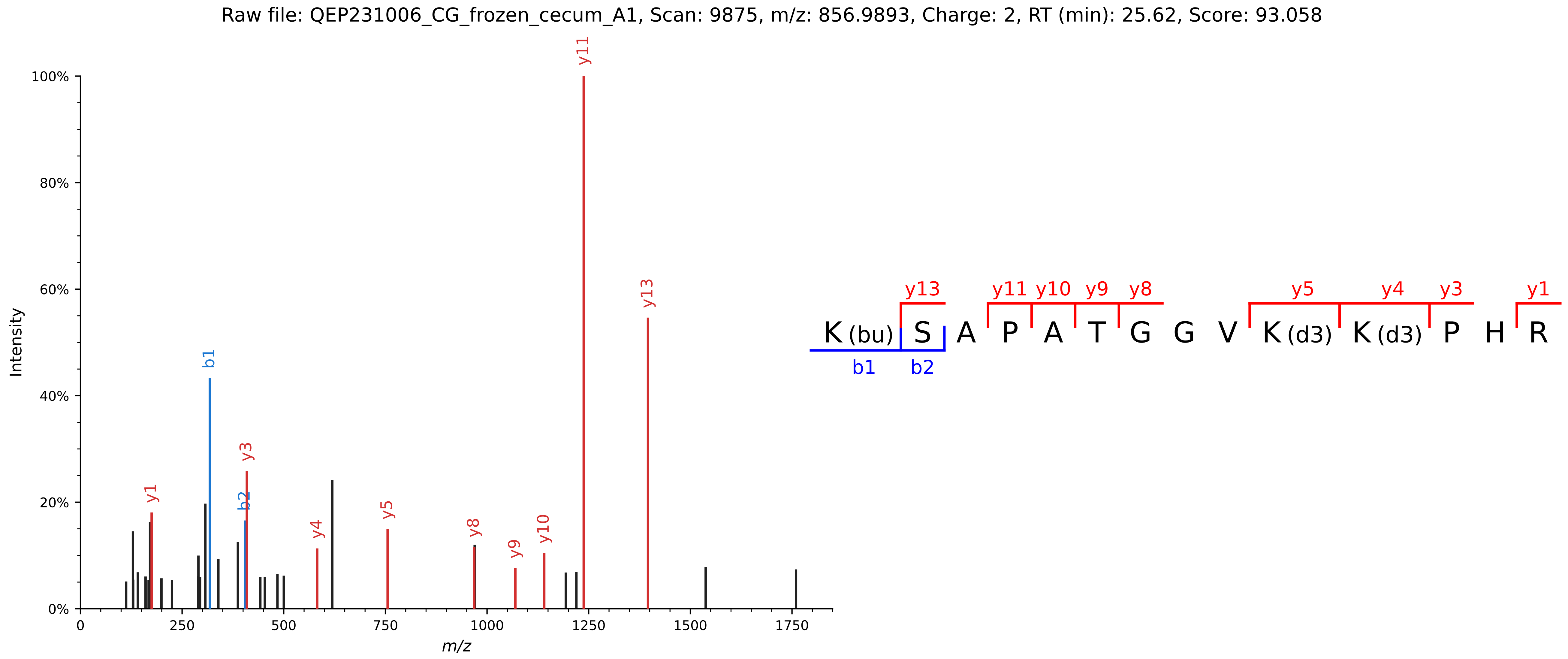

Raw file: QEP231006_CG_frozen_cecum_A1, Scan: 9875, m/z: 856.9893, Charge: 2, RT (min): 25.62, Score: 93.058
Intensity
m/z
0%
20%
40%
60%
80%
100%
0
250
500
750
1000
1250
1500
1750
y1
b1
b2
y3
y4
y5
y8
y9
y10
y11
y13
K(bu) S A P A T G G V K(d3) K(d3) P H R

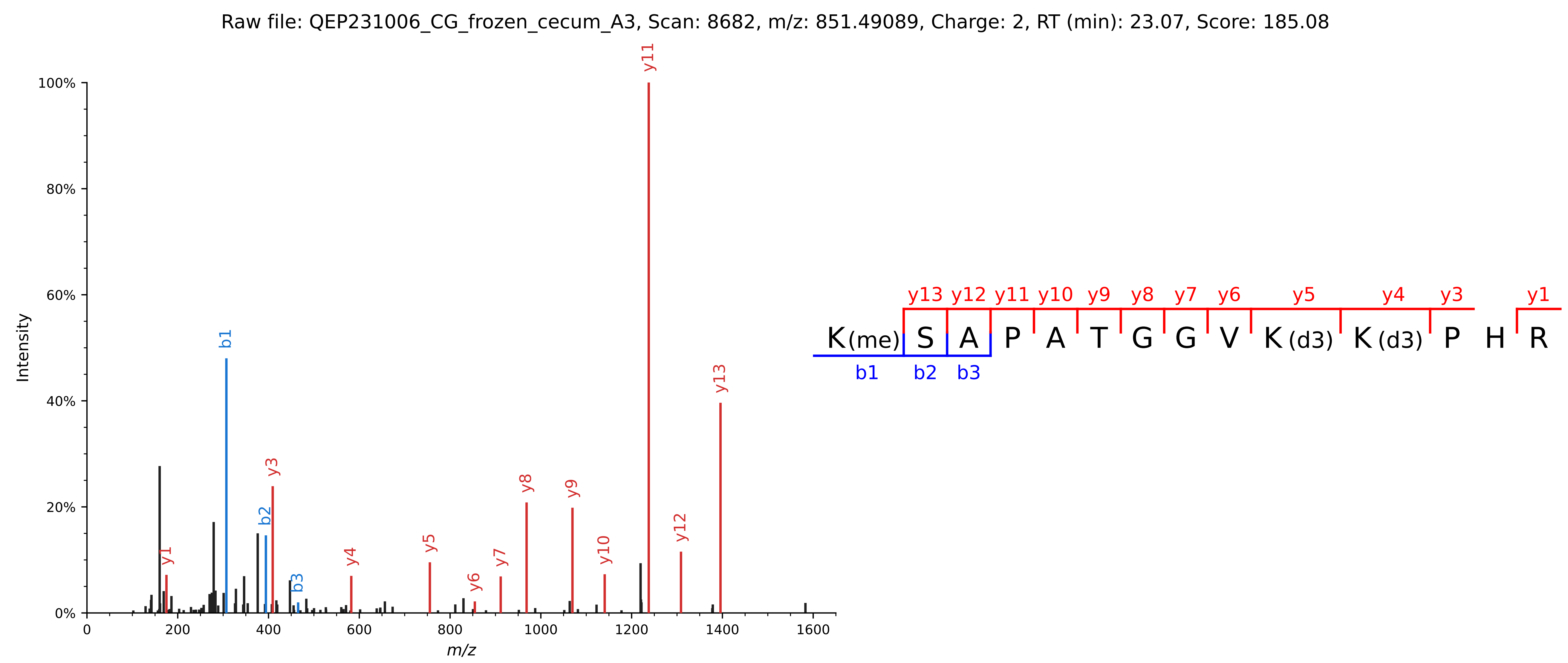

Raw file: QEP231006_CG_frozen_cecum_A3, Scan: 8682, m/z: 851.49089, Charge: 2, RT (min): 23.07, Score: 185.08
Intensity
m/z
K(me) S A P A T G G V K(d3) K(d3) P H R
y13 y12 y11 y10 y9 y8 y7 y6 y5 y4 y3 y1
b1 b2 b3

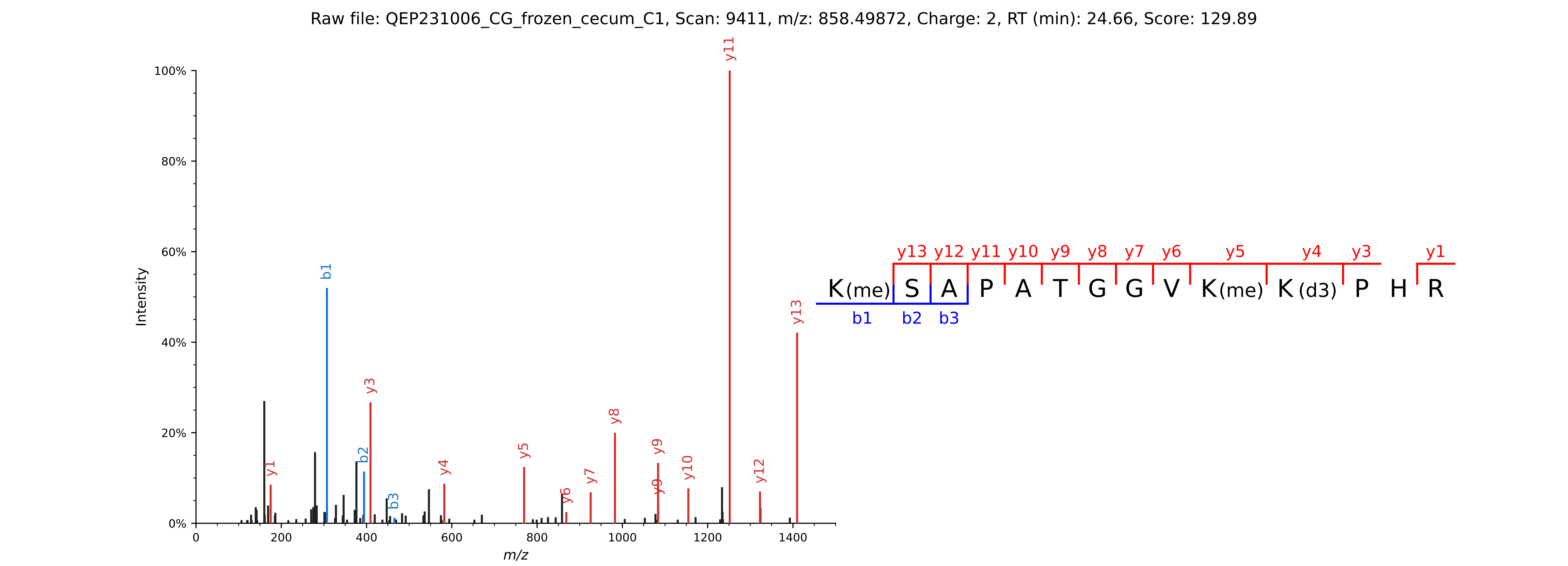

Raw file: QEP231006_CG_frozen_cecum_C1, Scan: 9411, m/z: 858.49872, Charge: 2, RT (min): 24.66, Score: 129.89
Intensity
100%
80%
60%
40%
20%
0%
0
200
400
600
800
1000
1200
1400
m/z
y1
b1
b2
y3
b3
y4
y5
y6
y7
y8
y9
y9
y10
y11
y12
y13
y13 y12 y11 y10 y9 y8 y7 y6 y5 y4 y3 y1
K(me) S A P A T G G V K(me) K(d3) P H R
b1 b2 b3

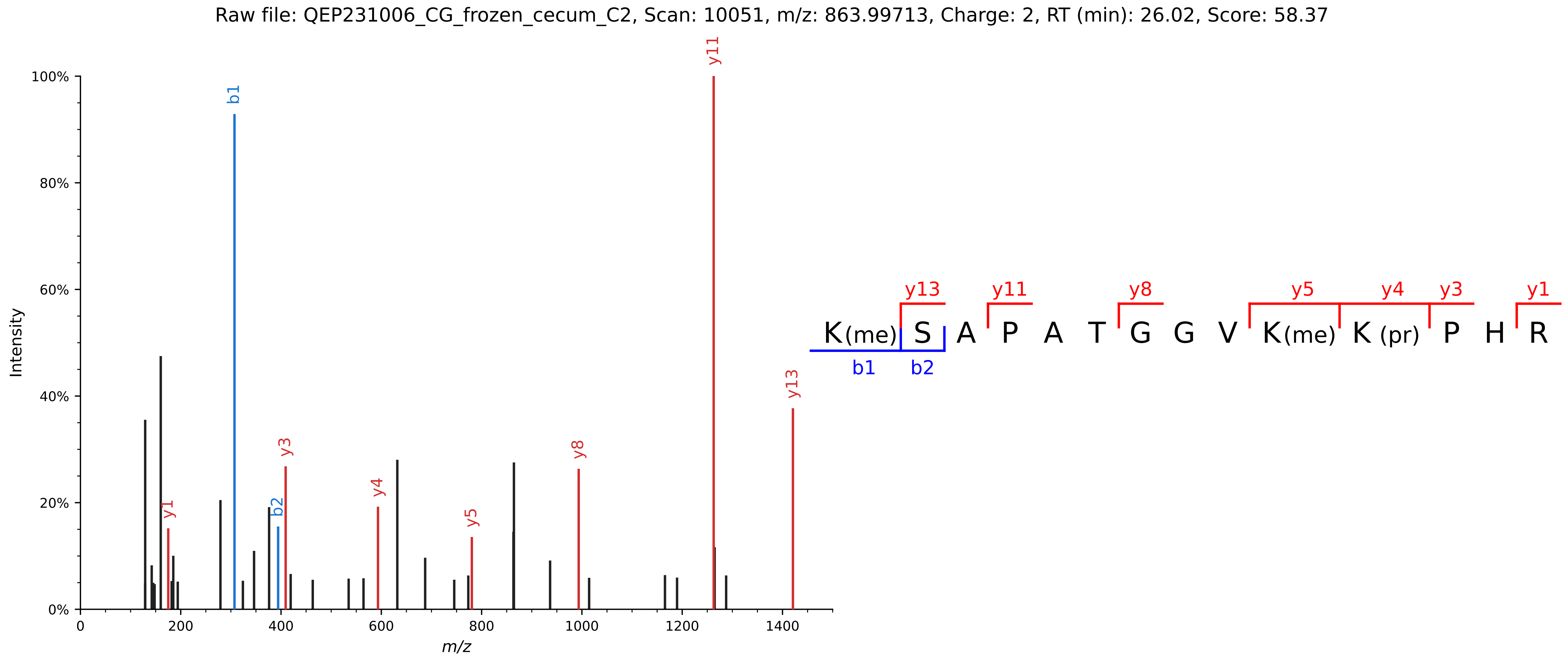

Raw file: QEP231006_CG_frozen_cecum_C2, Scan: 10051, m/z: 863.99713, Charge: 2, RT (min): 26.02, Score: 58.37
Intensity
m/z
100%
80%
60%
40%
20%
0%
0
200
400
600
800
1000
1200
1400
b1
b2
y1
y3
y4
y5
y8
y11
y13
K(me) S A P A T G G V K(me) K (pr) P H R

Raw file: QEP231006_CG_frozen_cecum_C1, Scan: 5746, m/z: 842.99184, Charge: 2, RT (min): 16.70, Score: 125.74

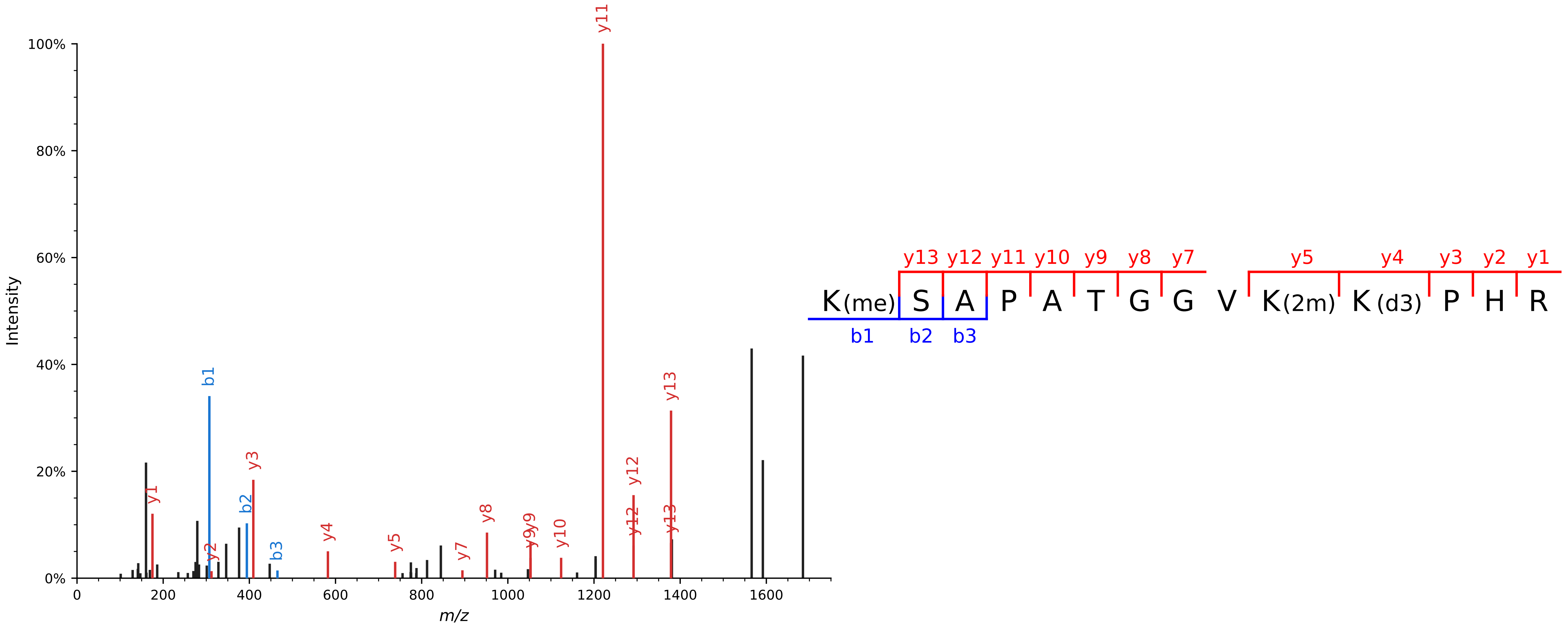

Raw file: QEP231006_CG_frozen_colon_C3, Scan: 9315, m/z: 856.9893, Charge: 2, RT (min): 24.36, Score: 76.143

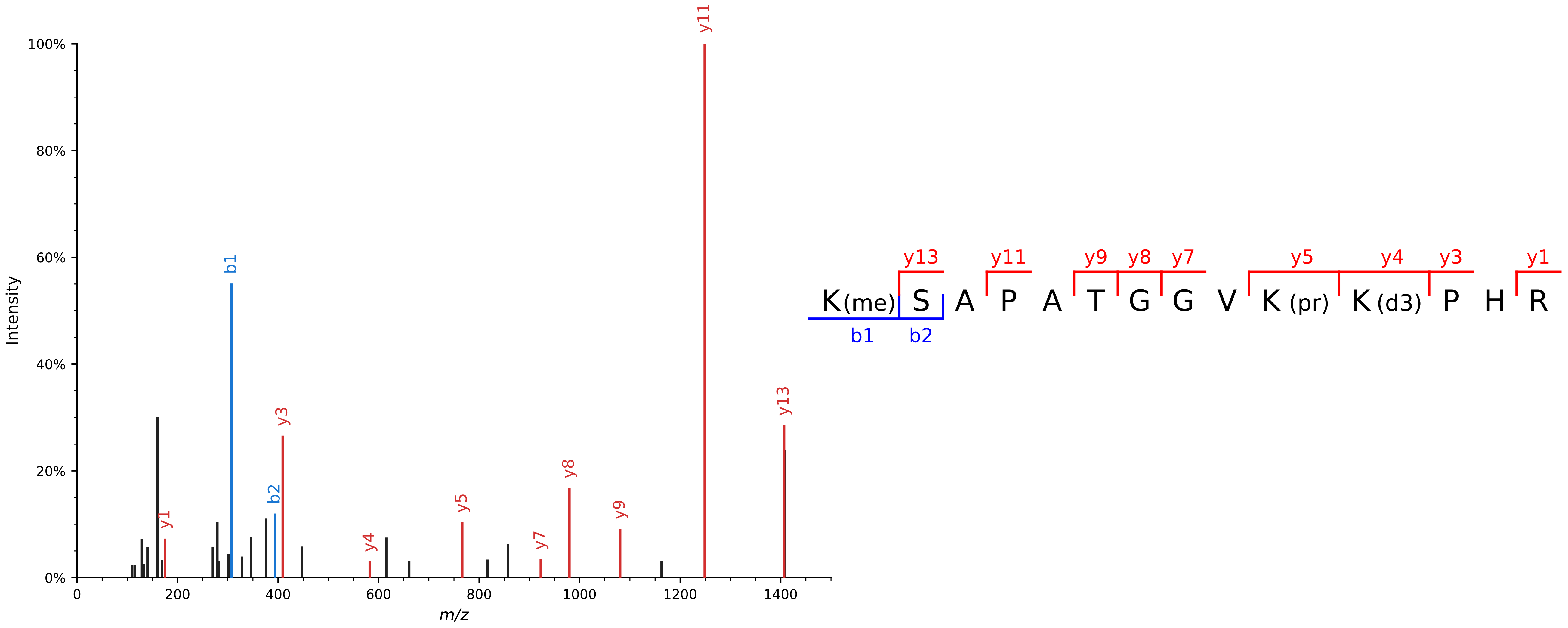

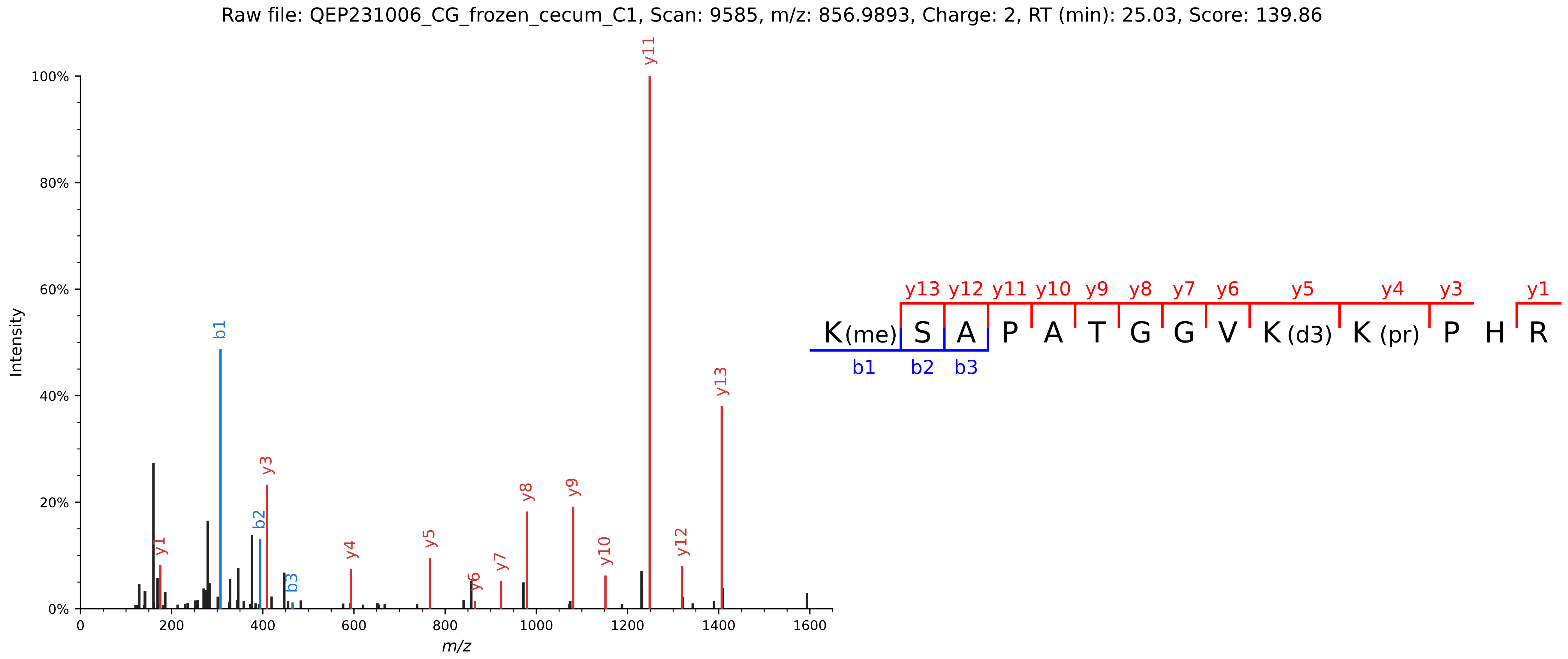

Raw file: QEP231006_CG_frozen_cecum_C1, Scan: 9585, m/z: 856.9893, Charge: 2, RT (min): 25.03, Score: 139.86
Intensity
m/z
K(me) S A P A T G G V K(d3) K(pr) P H R
y13 y12 y11 y10 y9 y8 y7 y6 y5 y4 y3 y1
b1 b2 b3

Raw file: QEP231006_CG_frozen_cecum_C1, Scan: 4156, m/z: 835.98402, Charge: 2, RT (min): 13.19, Score: 198.52

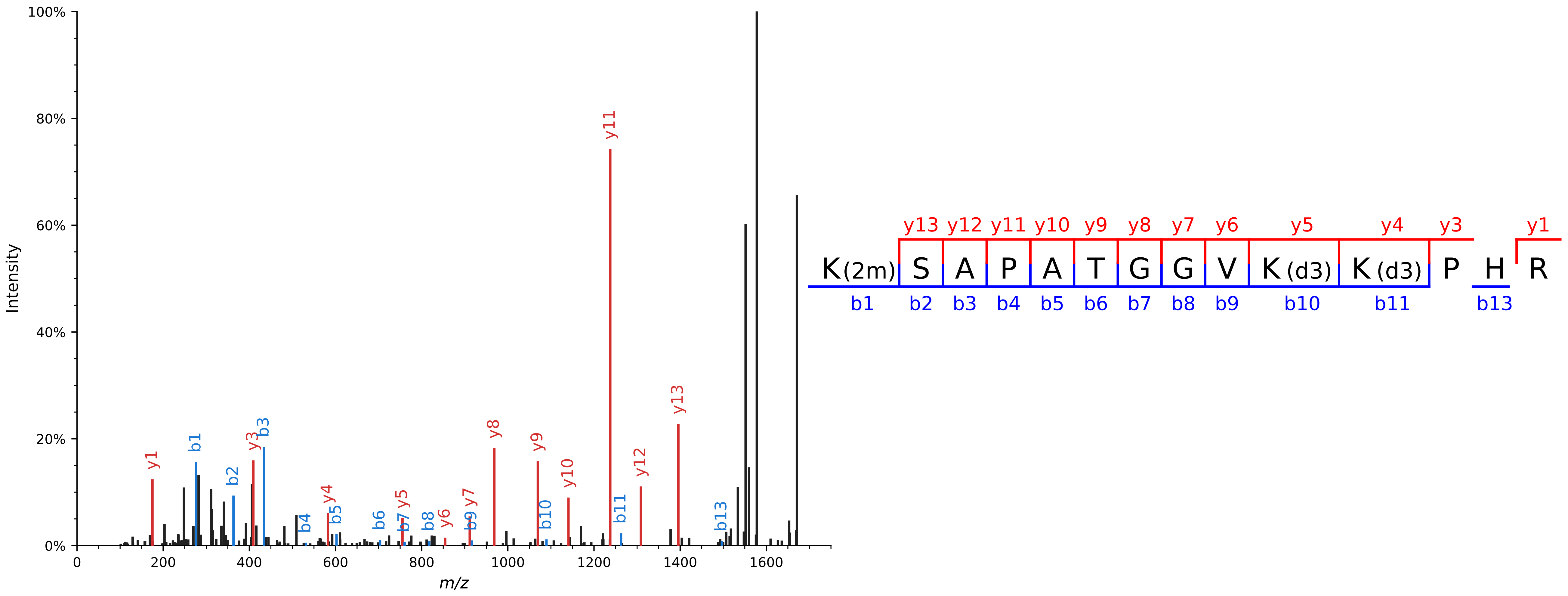

Raw file: QEP231006_CG_frozen_cecum_A1, Scan: 4544, m/z: 562.33032, Charge: 3, RT (min): 14.09, Score: 164.88

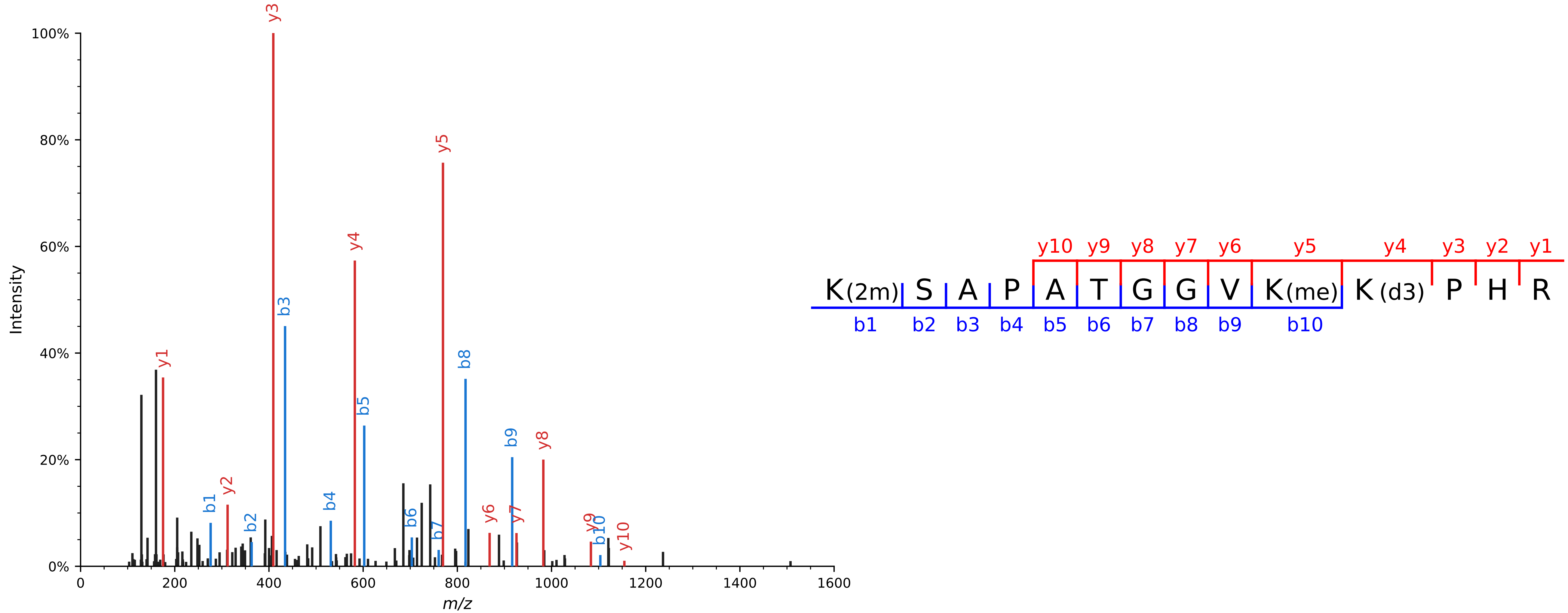

Raw file: QEP231006_CG_frozen_colon_A1, Scan: 5035, m/z: 565.99593, Charge: 3, RT (min): 15.06, Score: 113.59

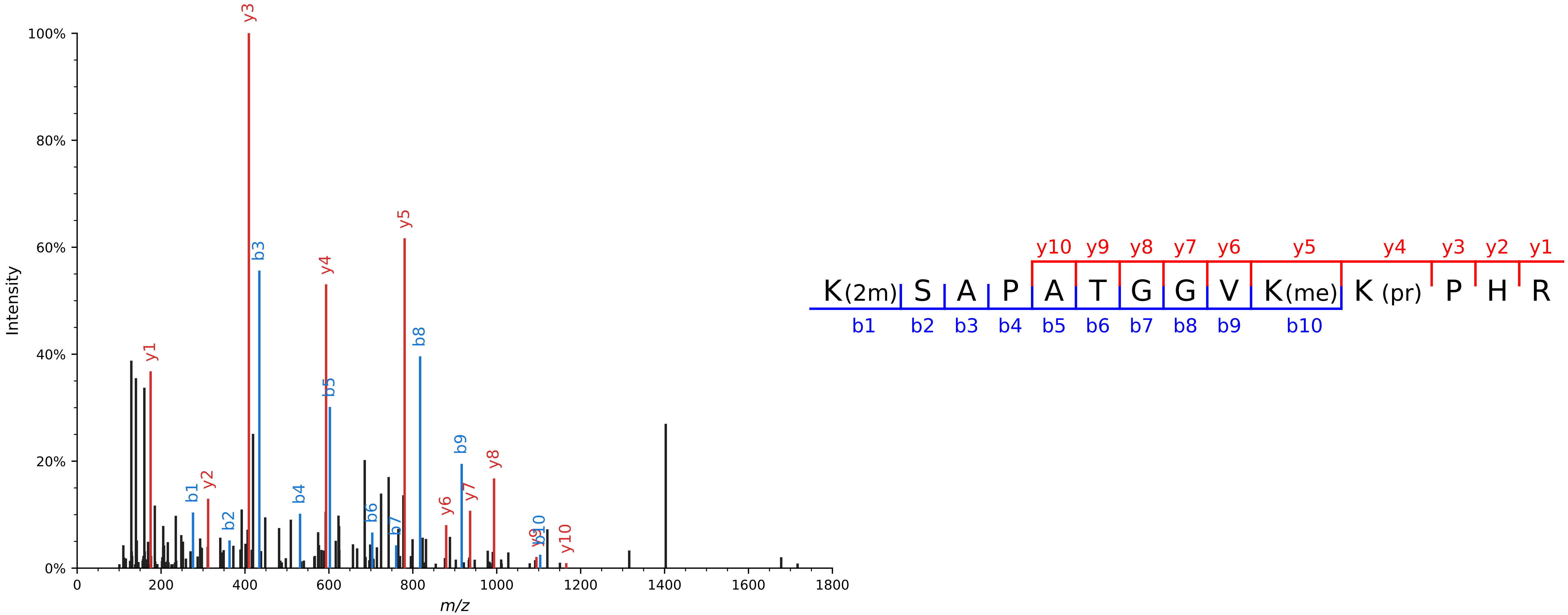

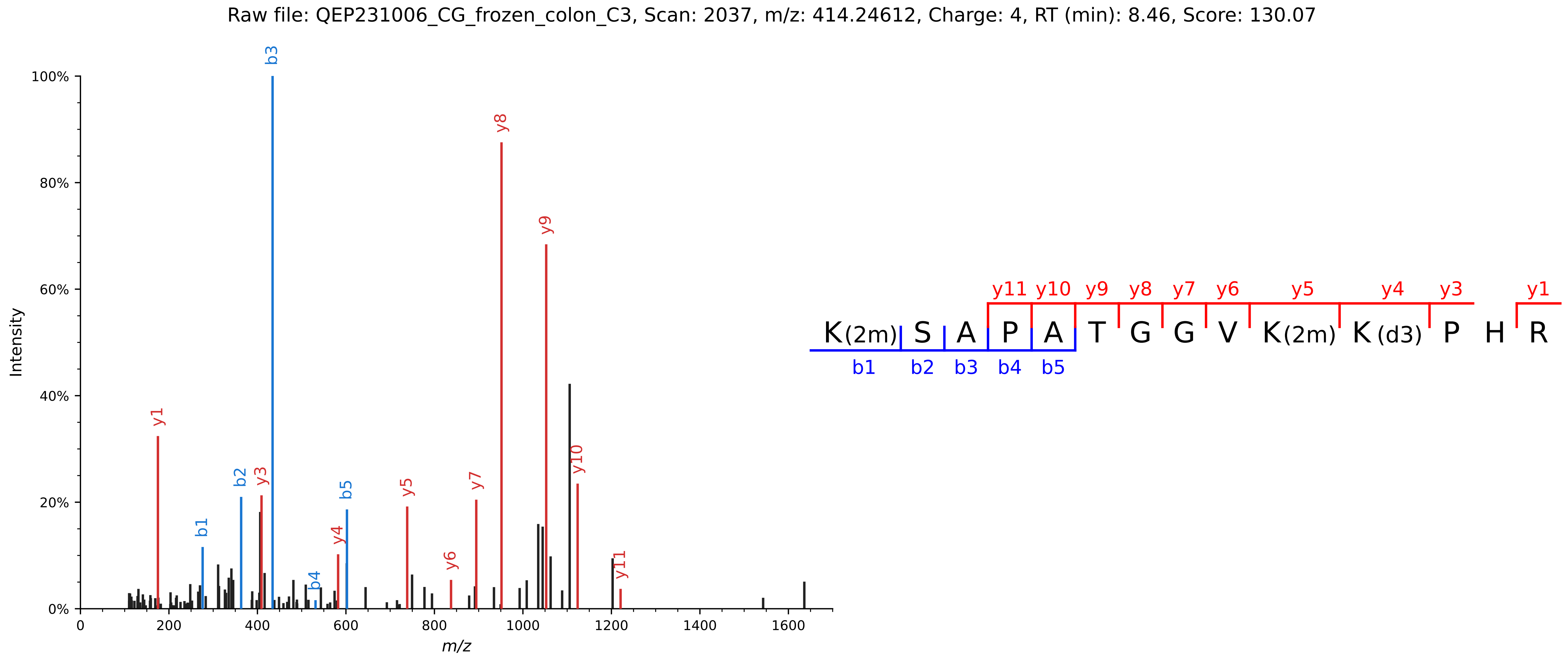
Raw file: QEP231006_CG_frozen_colon_C3, Scan: 2037, m/z: 414.24612, Charge: 4, RT (min): 8.46, Score: 130.07
Intensity
m/z
y11 y10 y9 y8 y7 y6 y5 y4 y3 y1
K(2m) S A P A T G G V K(2m) K(d3) P H R
b1 b2 b3 b4 b5

Raw file: QEP231006_CG_frozen_colon_A1, Scan: 4354, m/z: 841.48243, Charge: 2, RT (min): 13.60, Score: 122.33

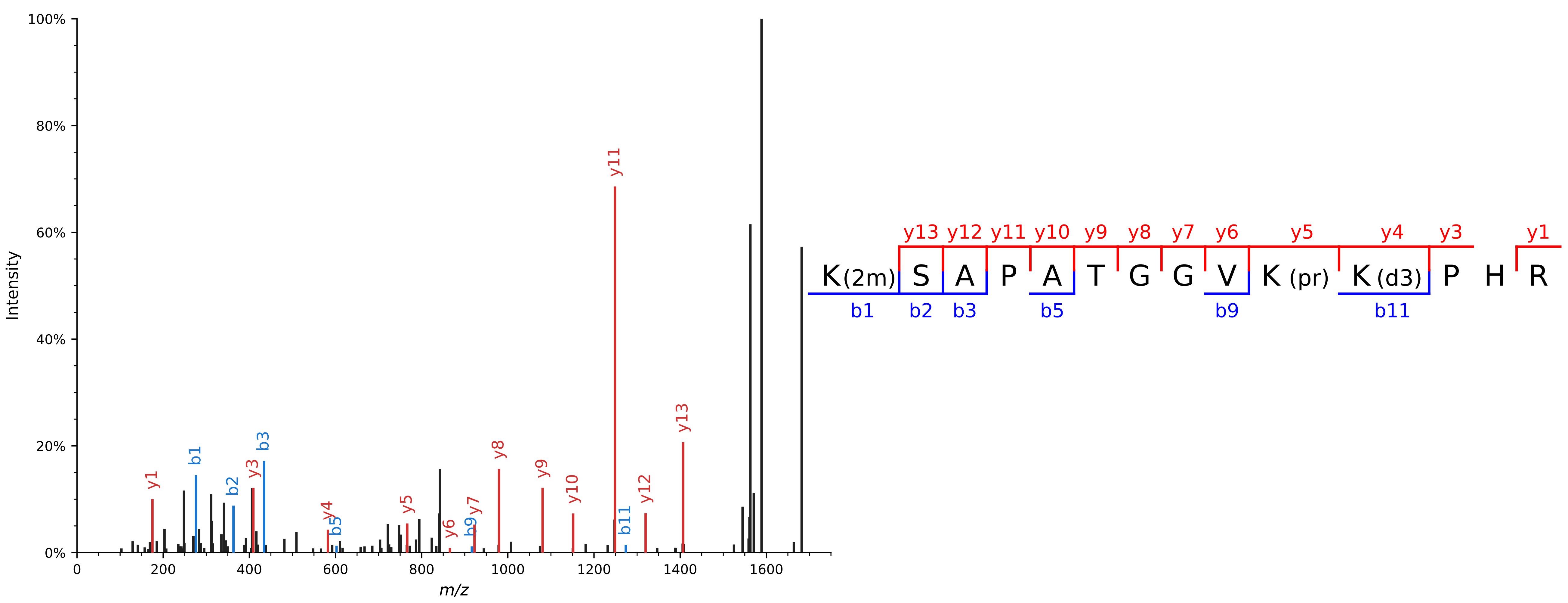

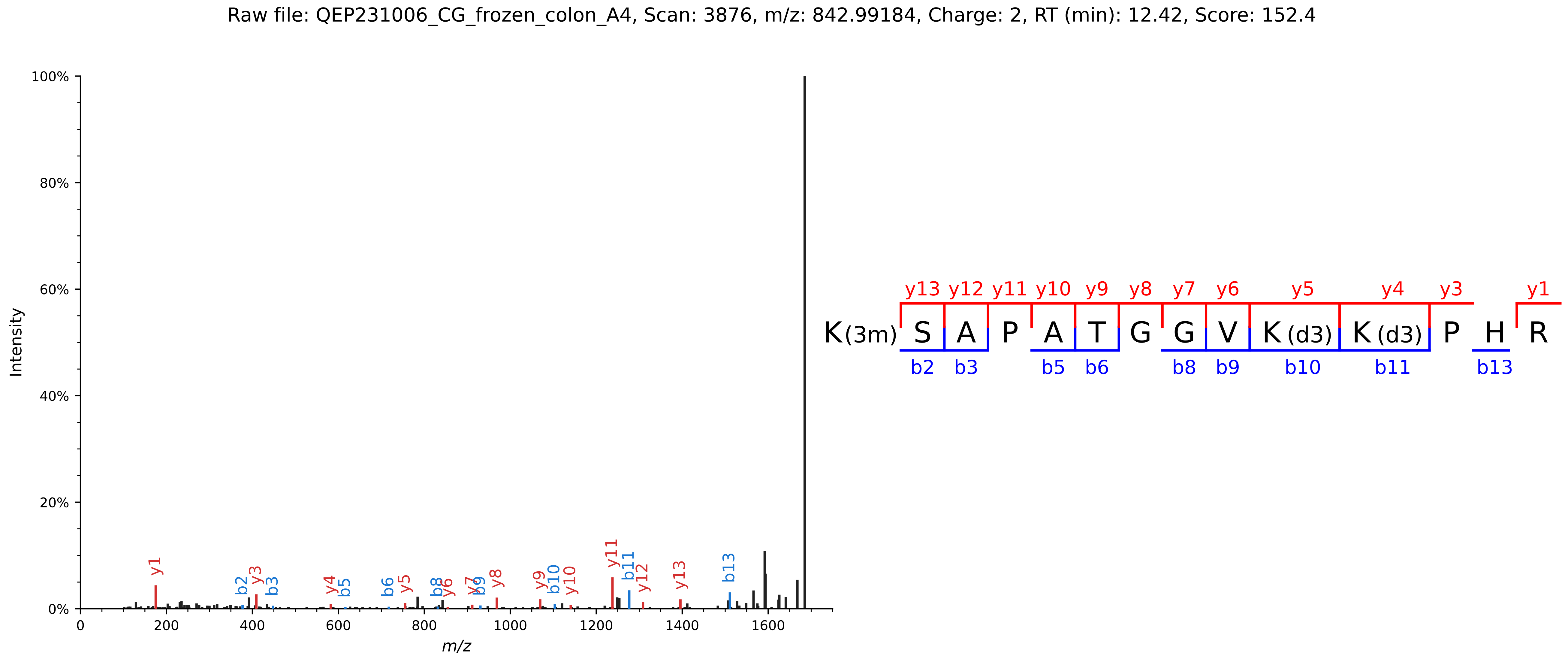

Raw file: QEP231006_CG_frozen_colon_A4, Scan: 3876, m/z: 842.99184, Charge: 2, RT (min): 12.42, Score: 152.4
Intensity
m/z
y1 b2 y3 b3 y4 b5 b6 y5 b8 y6 y7 b9 y8 y9 b10 y10 y11 b11 y12 y13 b13
y13 y12 y11 y10 y9 y8 y7 y6 y5 y4 y3 y1
K(3m) S A P A T G G V K(d3) K(d3) P H R
b2 b3 b5 b6 b8 b9 b10 b11 b13

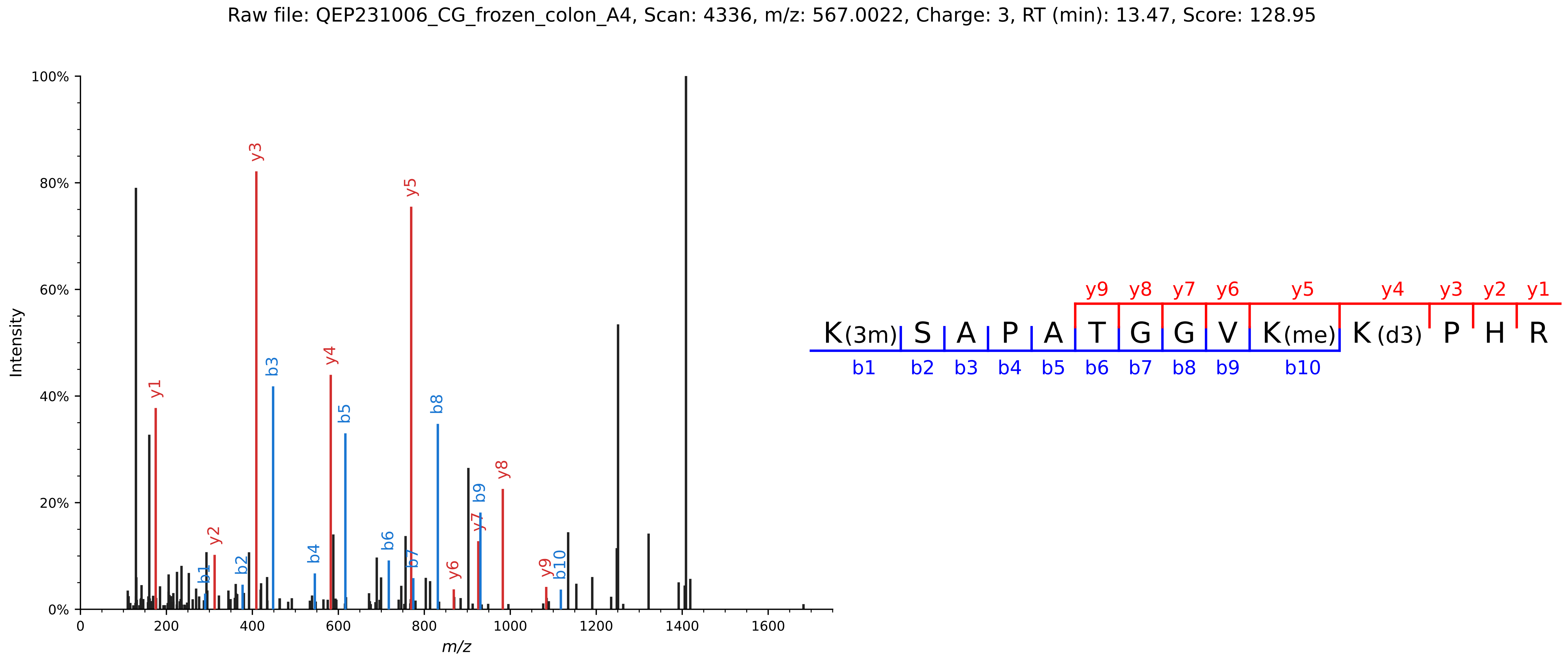

Raw file: QEP231006_CG_frozen_colon_A4, Scan: 4336, m/z: 567.0022, Charge: 3, RT (min): 13.47, Score: 128.95
Intensity
m/z
100%
80%
60%
40%
20%
0%
0
200
400
600
800
1000
1200
1400
1600
y1
y2
y3
y4
y5
y6
y7
y8
y9
b1
b2
b3
b4
b5
b6
b7
b8
b9
b10
y9 y8 y7 y6 y5 y4 y3 y2 y1
K(3m) S A P A T G G V K(me) K(d3) P H R
b1 b2 b3 b4 b5 b6 b7 b8 b9 b10

Raw file: QEP231006_CG_frozen_colon_A4, Scan: 1928, m/z: 556.66429, Charge: 3, RT (min): 8.06, Score: 62.68

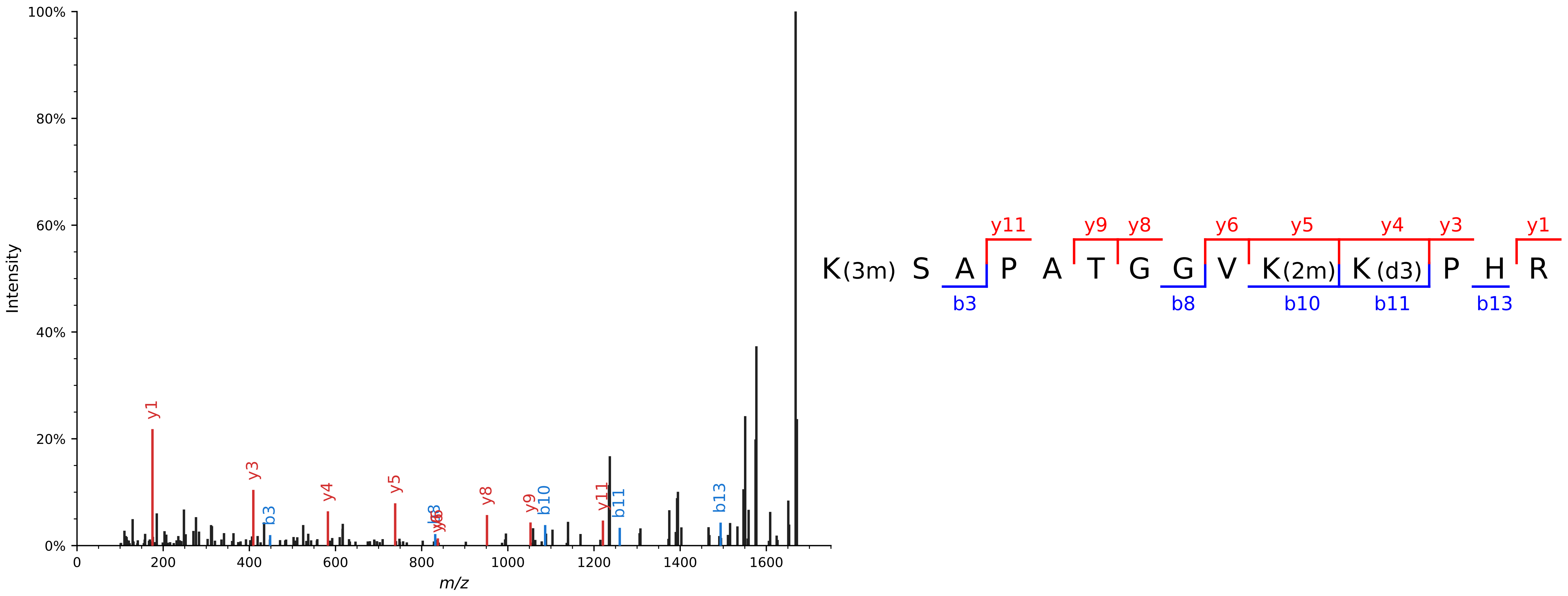

Raw file: QEP231006_CG_frozen_colon_A4, Scan: 8623, m/z: 849.98148, Charge: 2, RT (min): 22.56, Score: 160.36

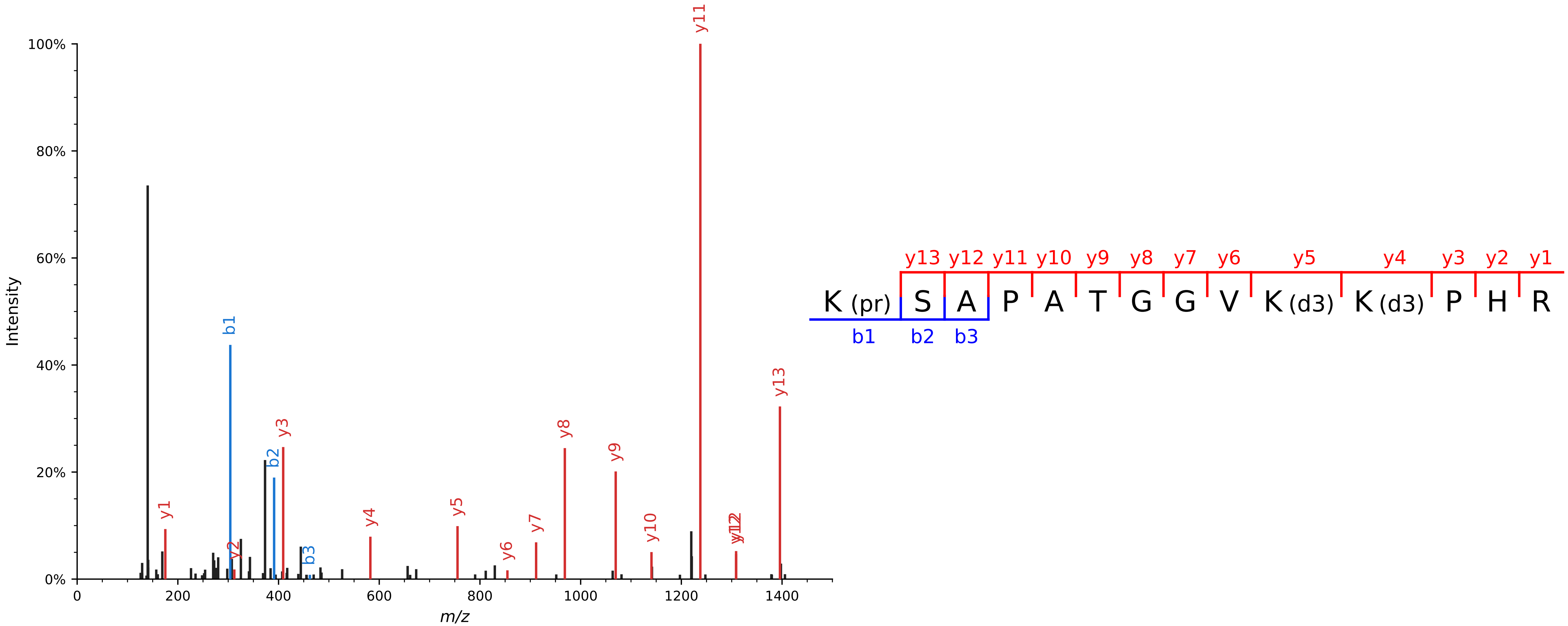

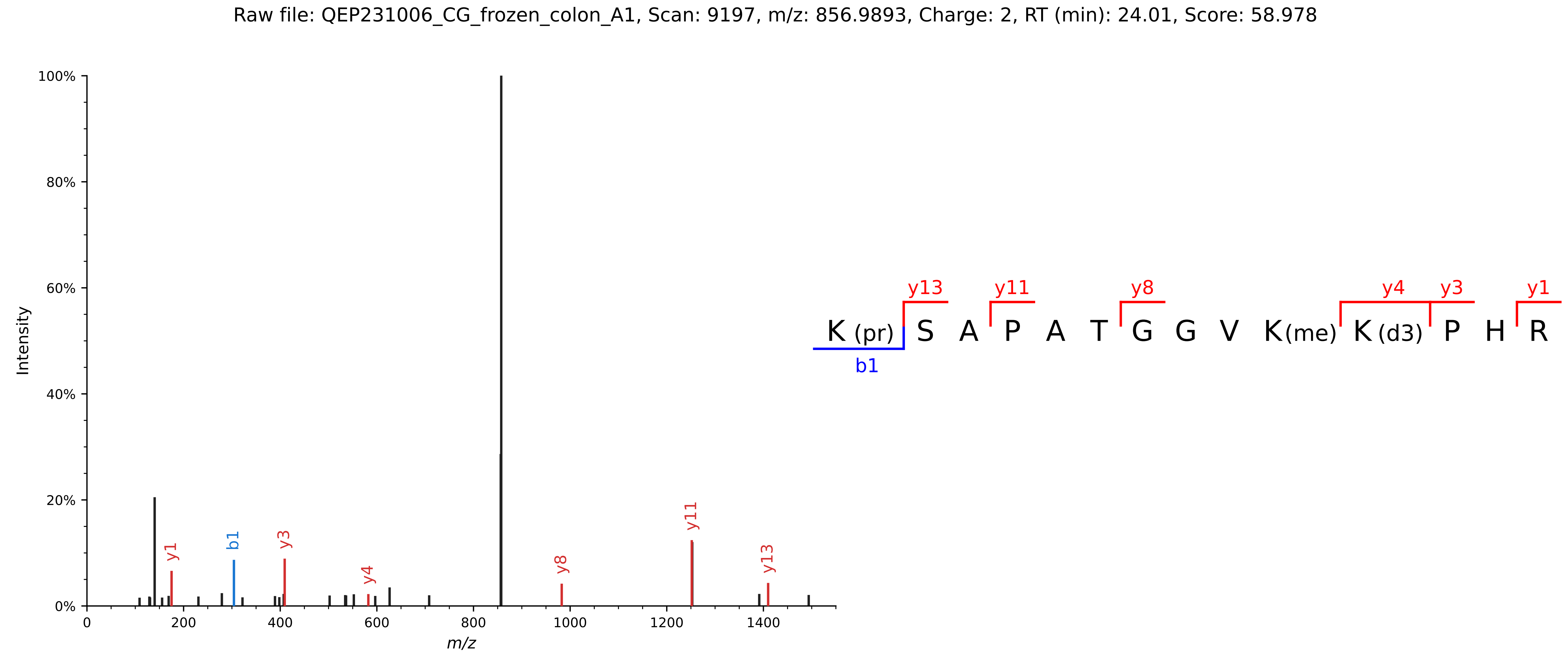
Raw file: QEP231006_CG_frozen_colon_A1, Scan: 9197, m/z: 856.9893, Charge: 2, RT (min): 24.01, Score: 58.978
Intensity
m/z
y1
b1
y3
y4
y8
y11
y13
K(pr) S A P A T G G V K(me) K(d3) P H R

Raw file: QEP231006_CG_frozen_cecum_A4, Scan: 8344, m/z: 851.49089, Charge: 2, RT (min): 22.14, Score: 195.51

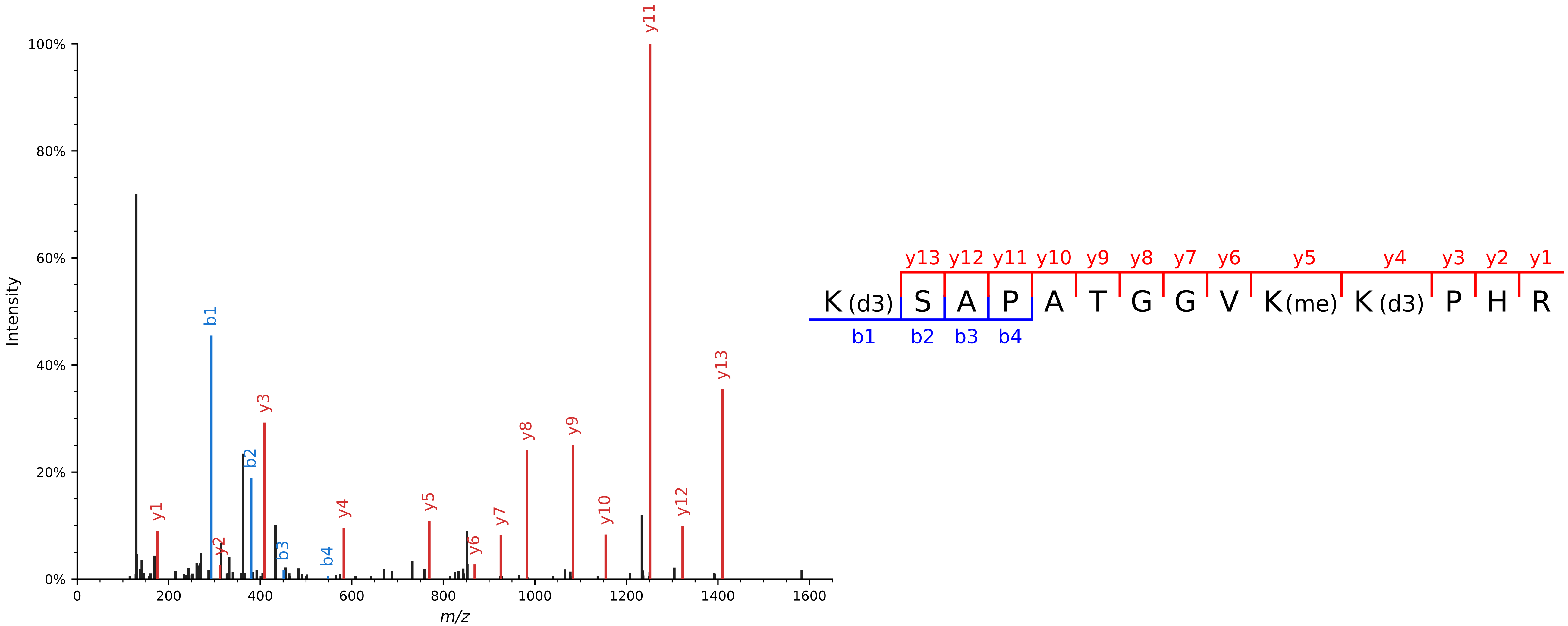

Raw file: QEP231006_CG_frozen_cecum_C3, Scan: 4780, m/z: 835.98402, Charge: 2, RT (min): 14.53, Score: 131.83

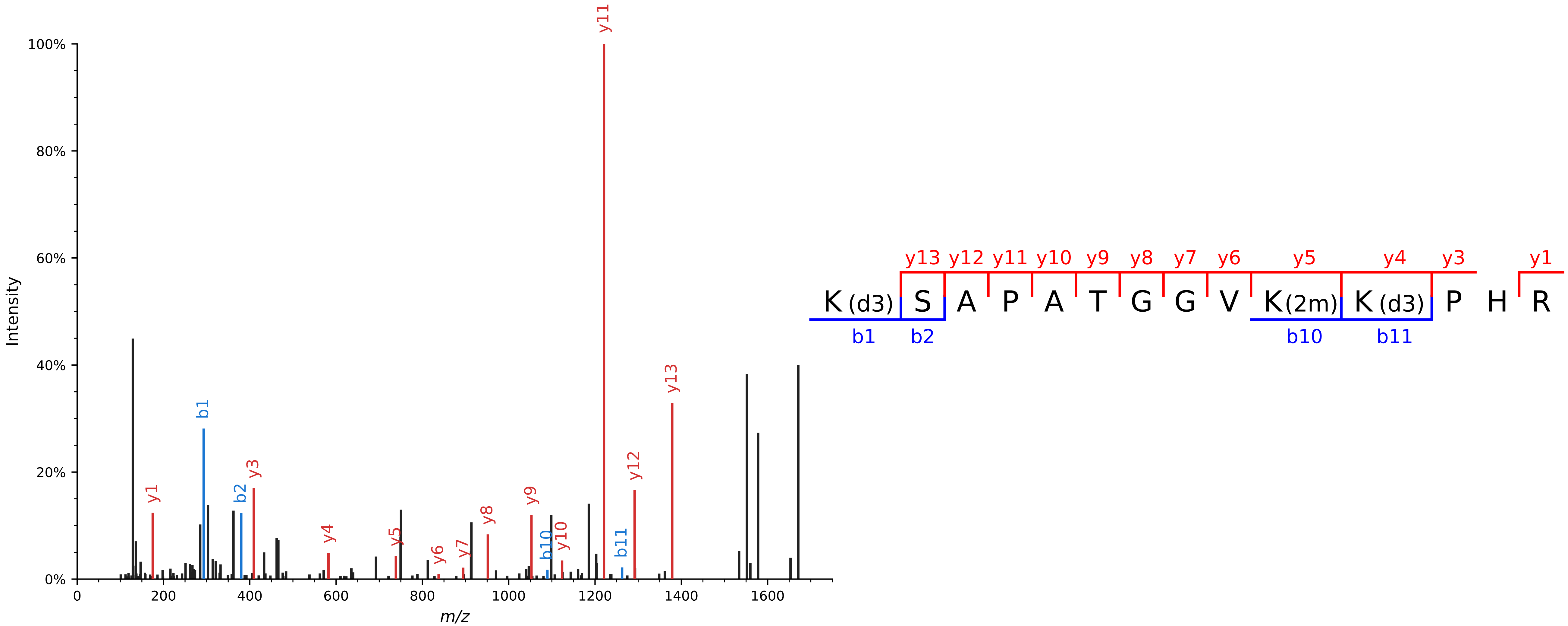

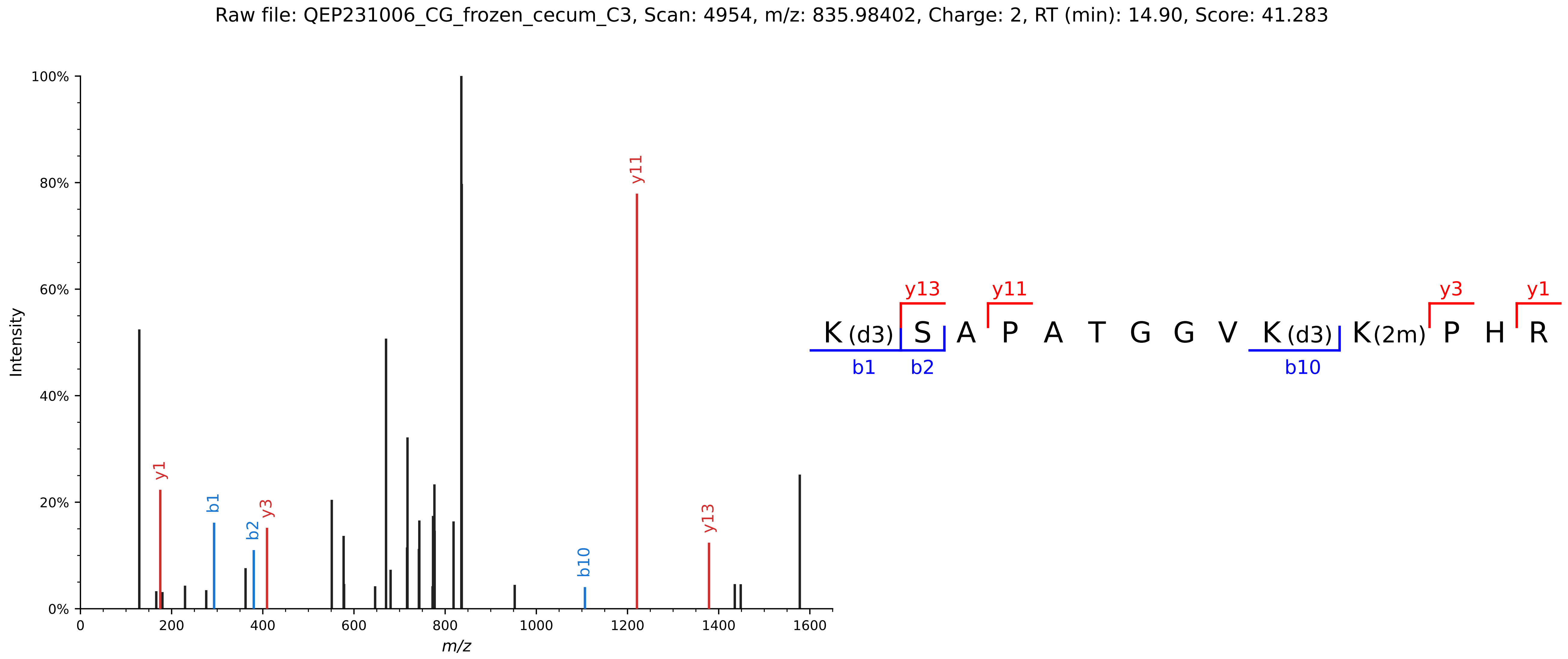
Raw file: QEP231006_CG_frozen_cecum_C3, Scan: 4954, m/z: 835.98402, Charge: 2, RT (min): 14.90, Score: 41.283
100%
80%
60%
40%
20%
0%
Intensity
0
200
400
600
800
1000
1200
1400
1600
m/z
y1
b1
b2
y3
b10
y11
y13
K(d3) S A P A T G G V K(d3) K(2m) P H R
y13
y11
y3
y1
b1
b2
b10

Raw file: QEP231006_CG_frozen_cecum_C4, Scan: 8453, m/z: 849.98148, Charge: 2, RT (min): 22.45, Score: 150.04

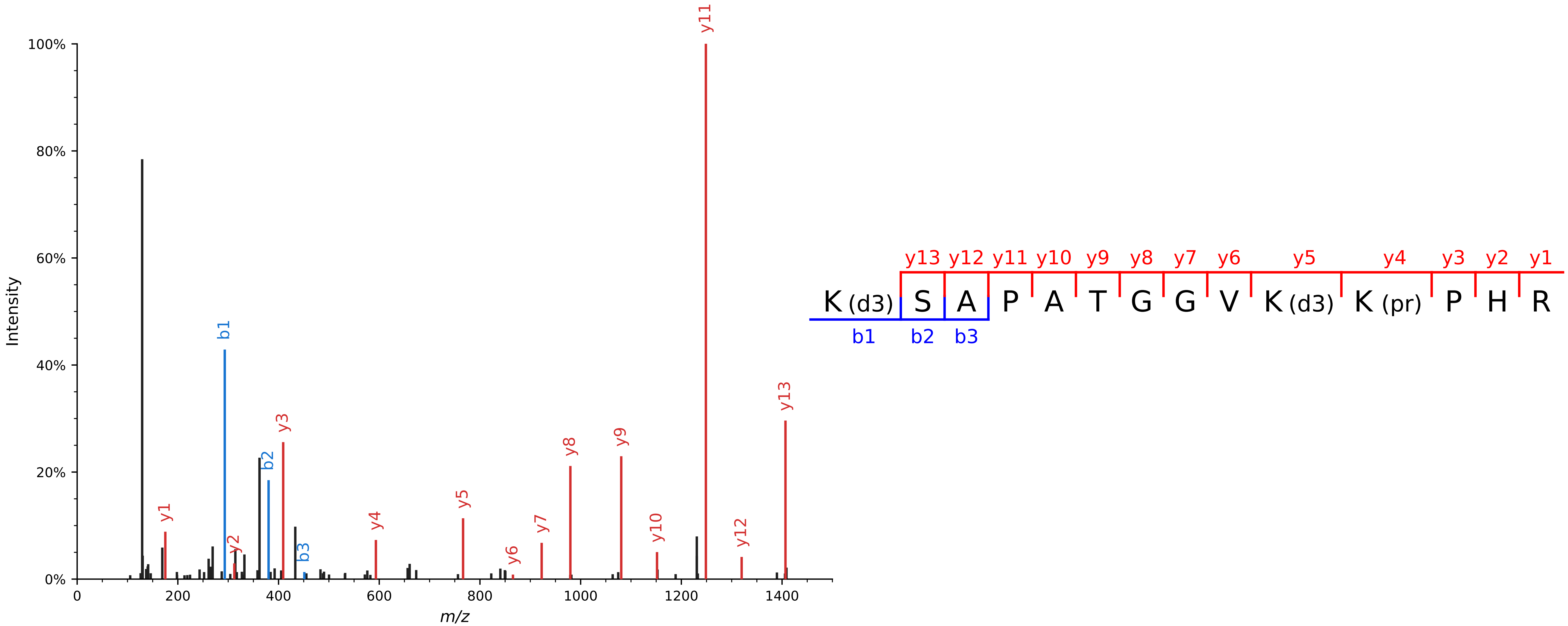

Raw file: QEP231006_CG_frozen_cecum_C2, Scan: 17399, m/z: 482.27417, Charge: 2, RT (min): 43.18, Score: 119.23

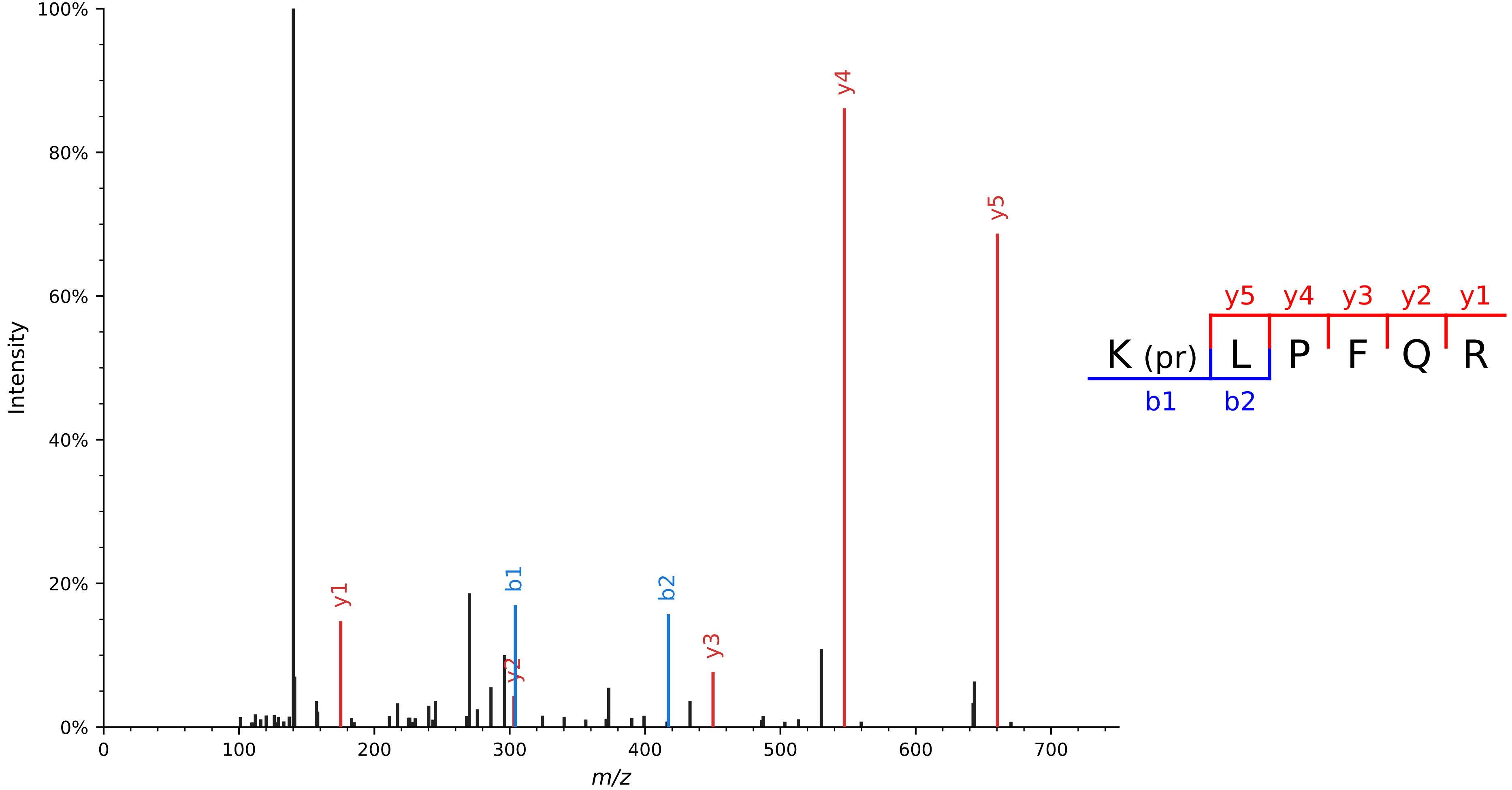

Raw file: QEP231006_CG_frozen_colon_A5, Scan: 19061, m/z: 757.38987, Charge: 2, RT (min): 50.55, Score: 54.608

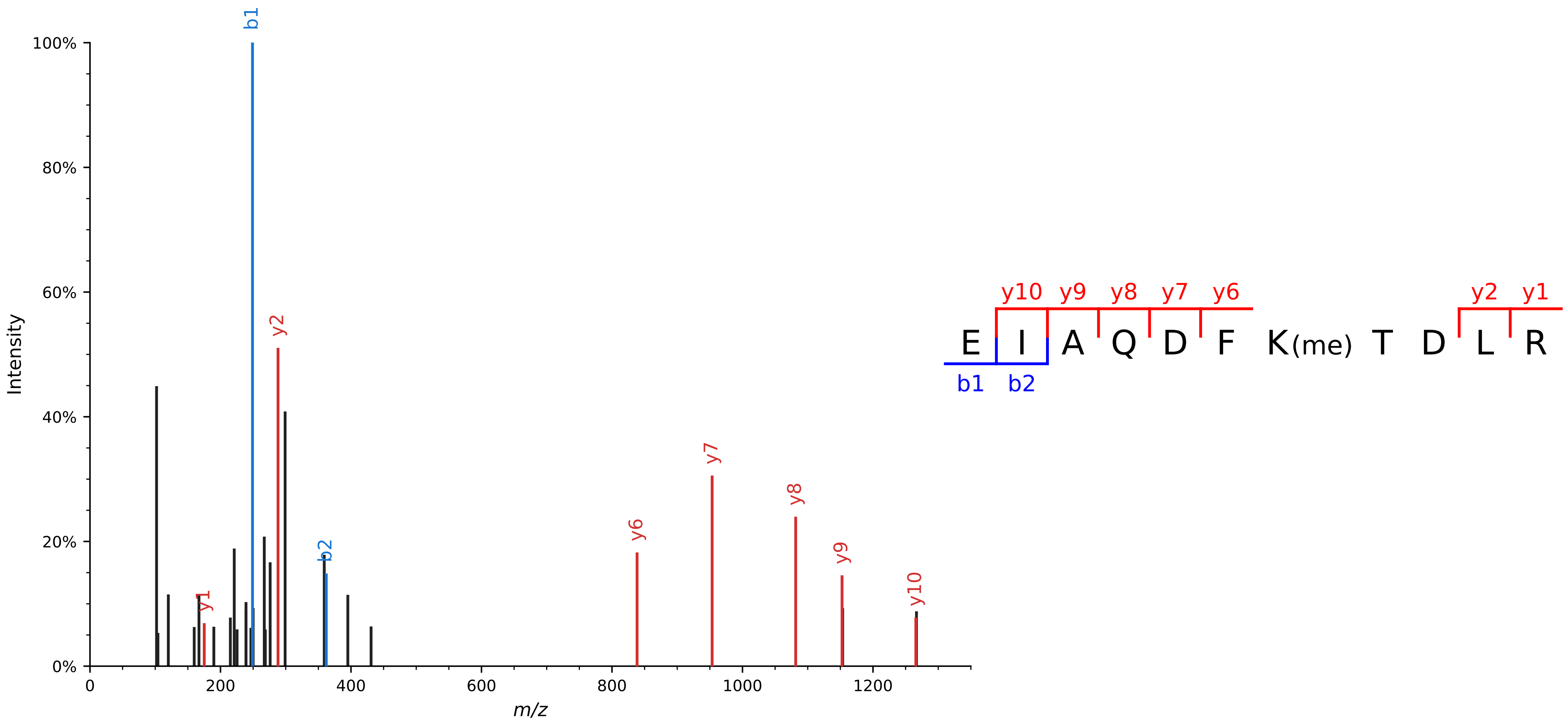

Raw file: QEP231006_CG_frozen_cecum_C1, Scan: 15080, m/z: 741.883, Charge: 2, RT (min): 36.96, Score: 74.841

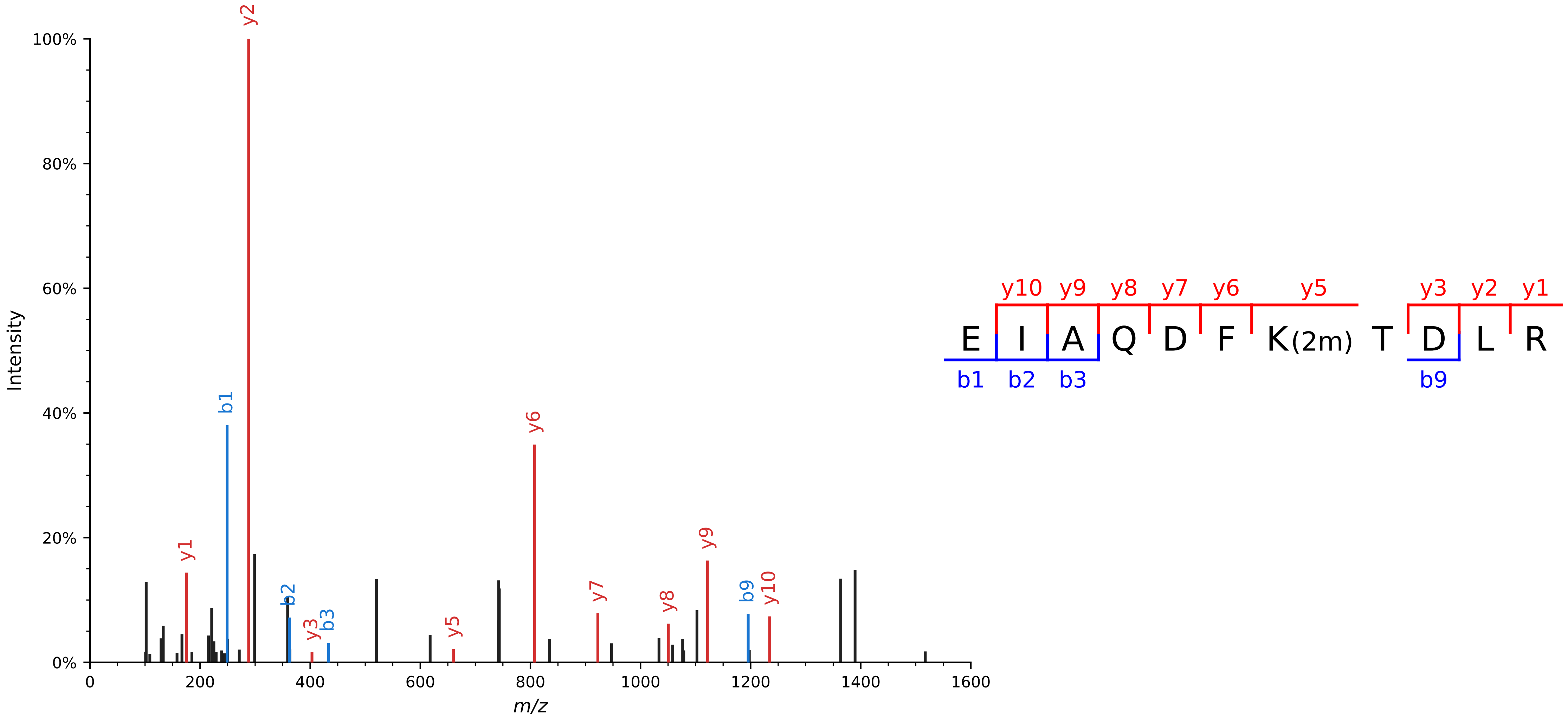

Raw file: QEP231006_CG_frozen_colon_A2, Scan: 10180, m/z: 776.94886, Charge: 2, RT (min): 26.54, Score: 61.78

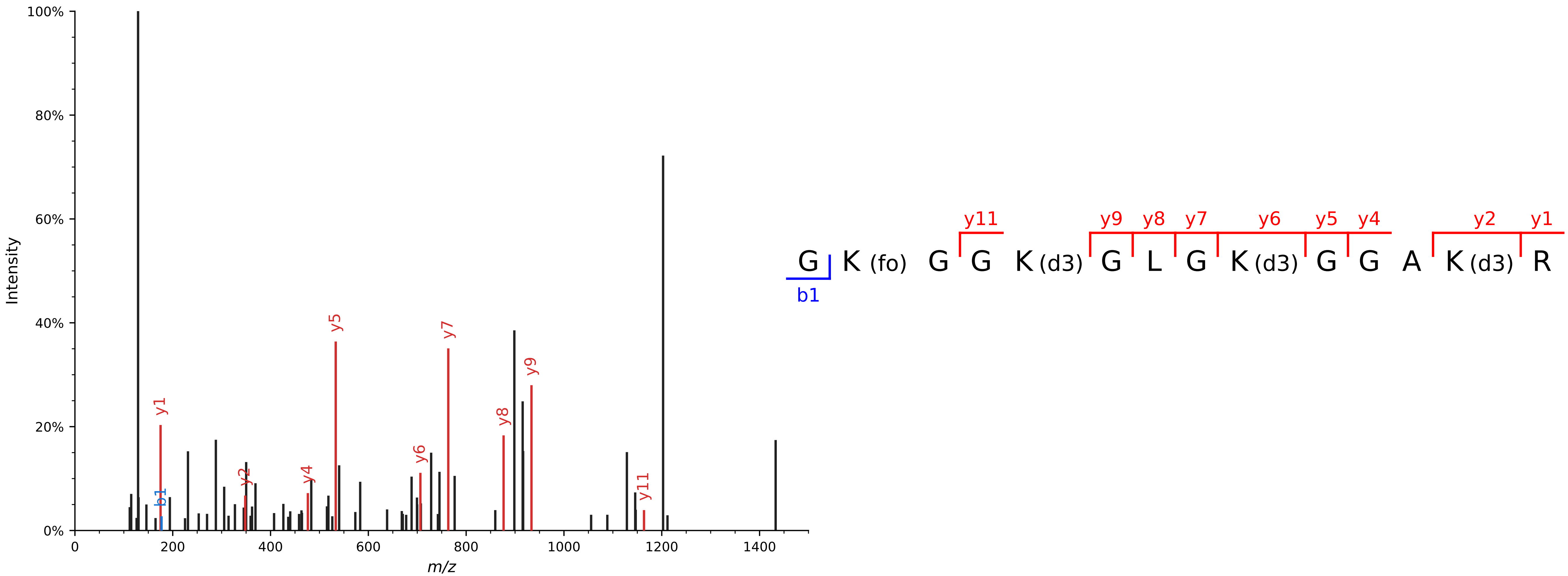

Raw file: QEP231006_CG_frozen_cecum_C3, Scan: 11474, m/z: 790.96451, Charge: 2, RT (min): 28.78, Score: 155.15

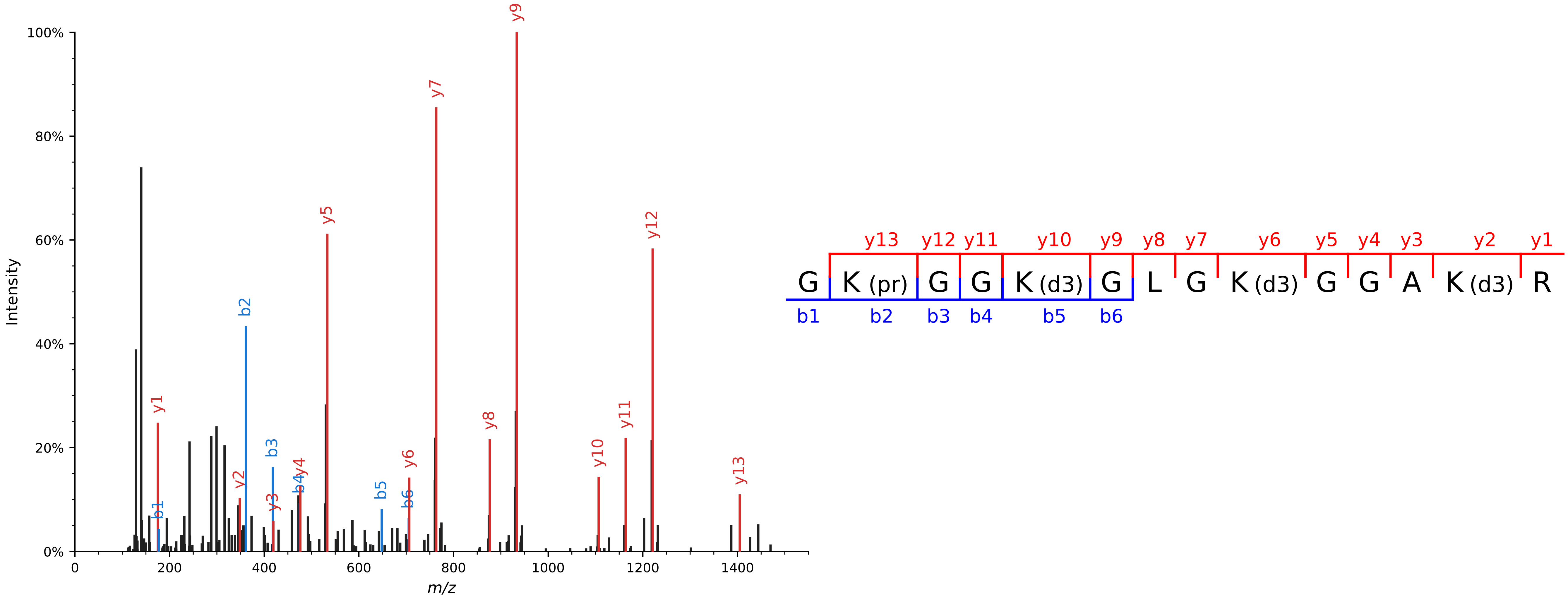

Raw file: QEP231006_CG_frozen_cecum_A5, Scan: 11346, m/z: 525.63288, Charge: 3, RT (min): 28.69, Score: 43.813

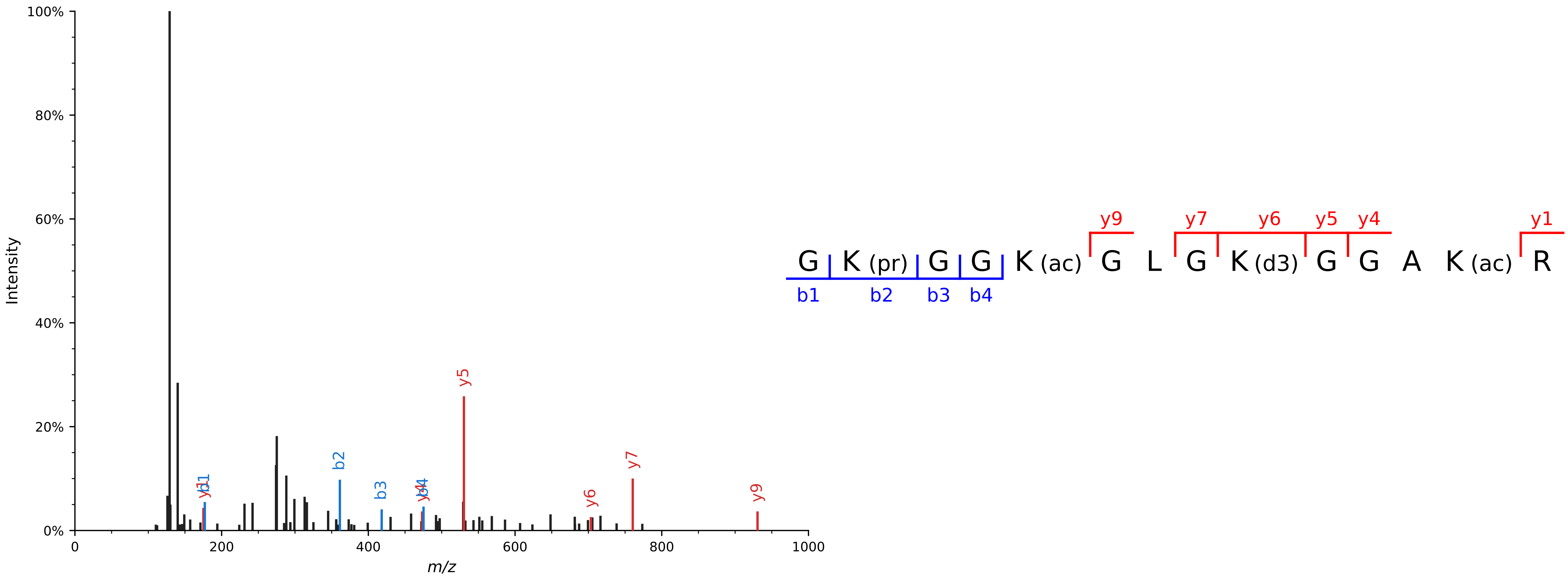

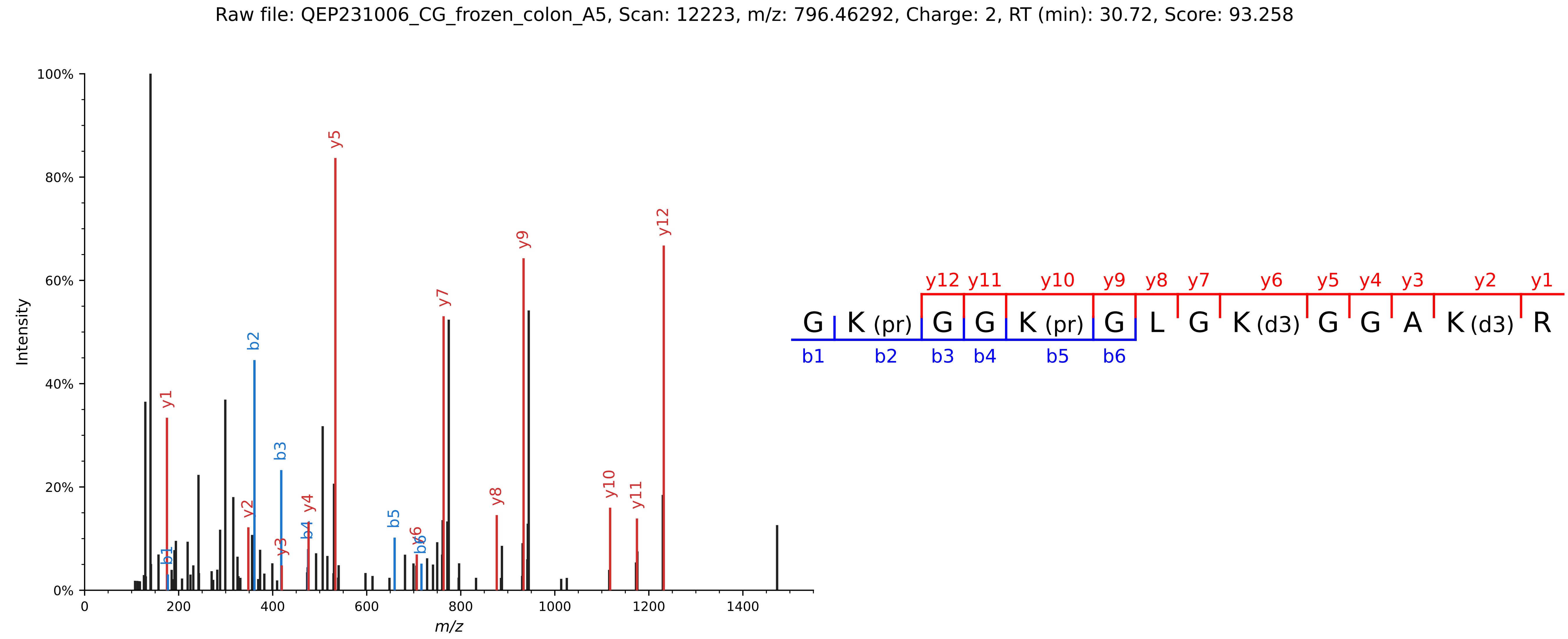

Raw file: QEP231006_CG_frozen_colon_A5, Scan: 12223, m/z: 796.46292, Charge: 2, RT (min): 30.72, Score: 93.258
Intensity
m/z
G K (pr) G G K (pr) G L G K (d3) G G A K (d3) R
y12 y11 y10 y9 y8 y7 y6 y5 y4 y3 y2 y1
b1 b2 b3 b4 b5 b6

Raw file: QEP231006_CG_frozen_cecum_C3, Scan: 13367, m/z: 801.96133, Charge: 2, RT (min): 32.88, Score: 62.303

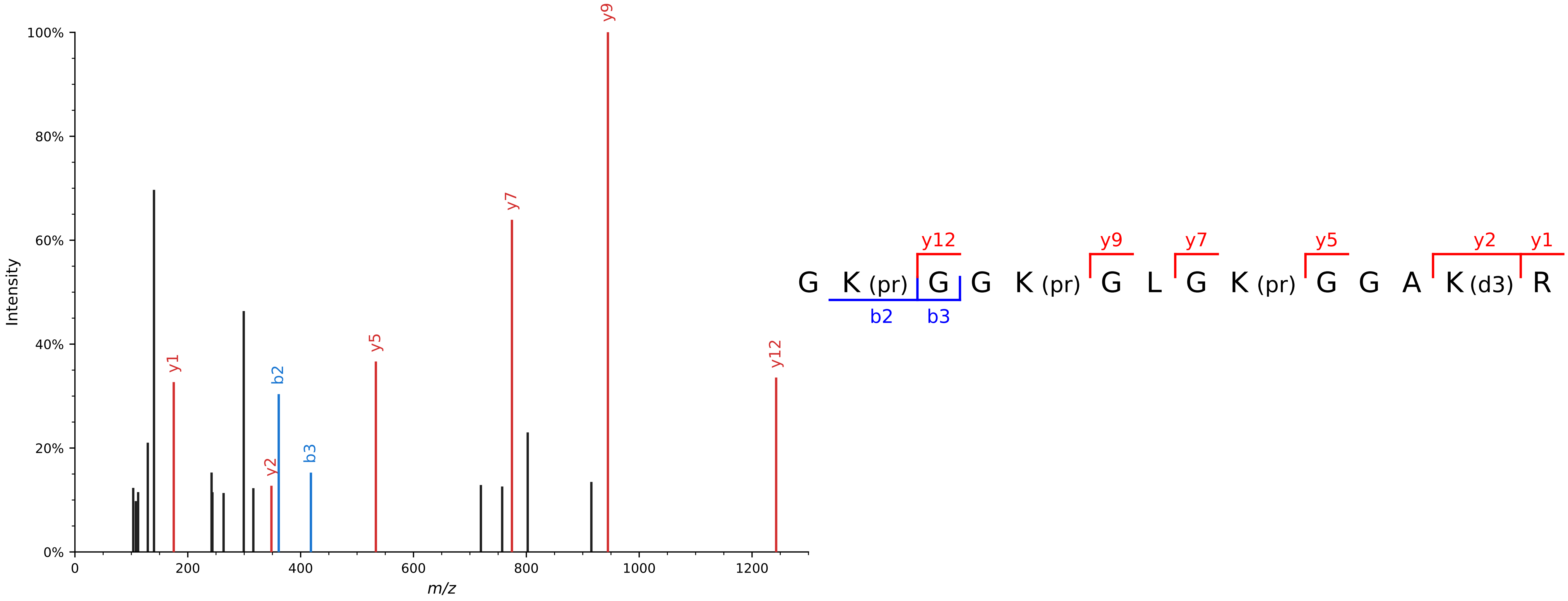

Raw file: QEP231006_CG_frozen_cecum_C1, Scan: 13378, m/z: 800.45191, Charge: 2, RT (min): 33.25, Score: 56.783

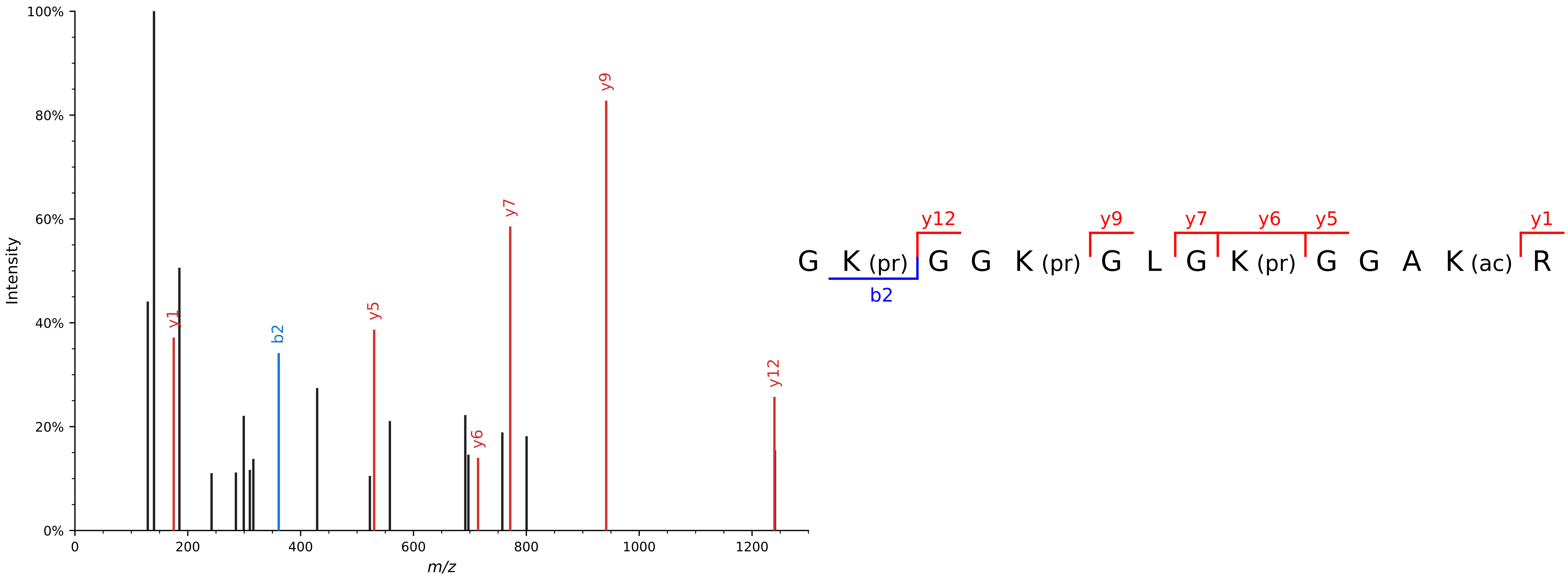

Raw file: QEP231006_CG_frozen_cecum_C2, Scan: 12441, m/z: 794.9535, Charge: 2, RT (min): 31.07, Score: 110.84

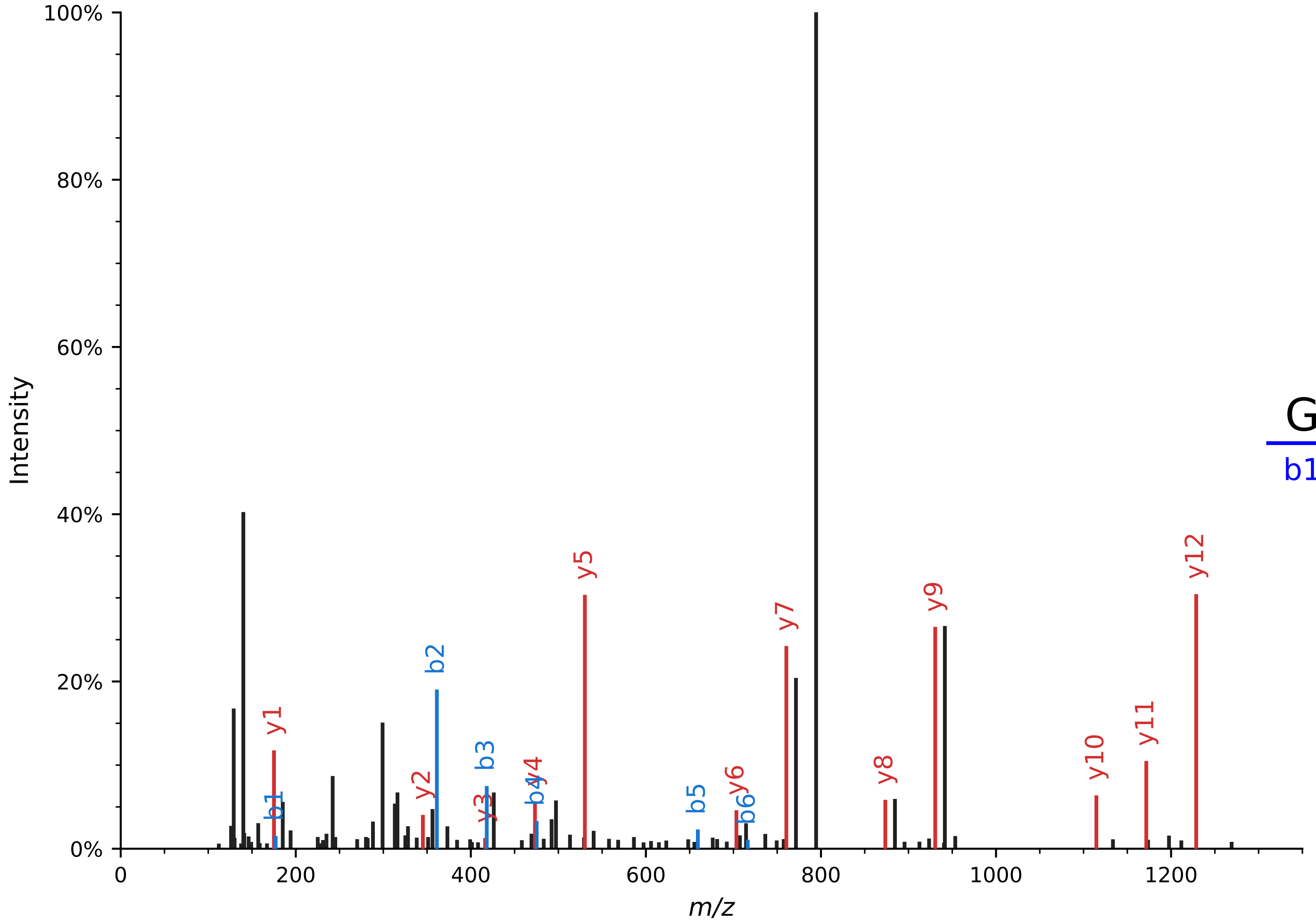

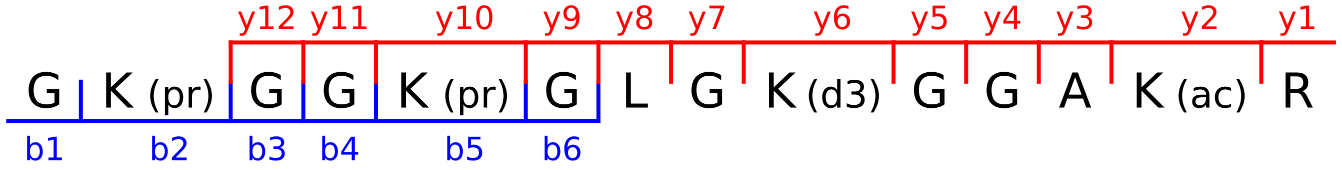

Raw file: QEP231006_CG_frozen_cecum_A1, Scan: 13026, m/z: 801.96133, Charge: 2, RT (min): 32.40, Score: 74.841

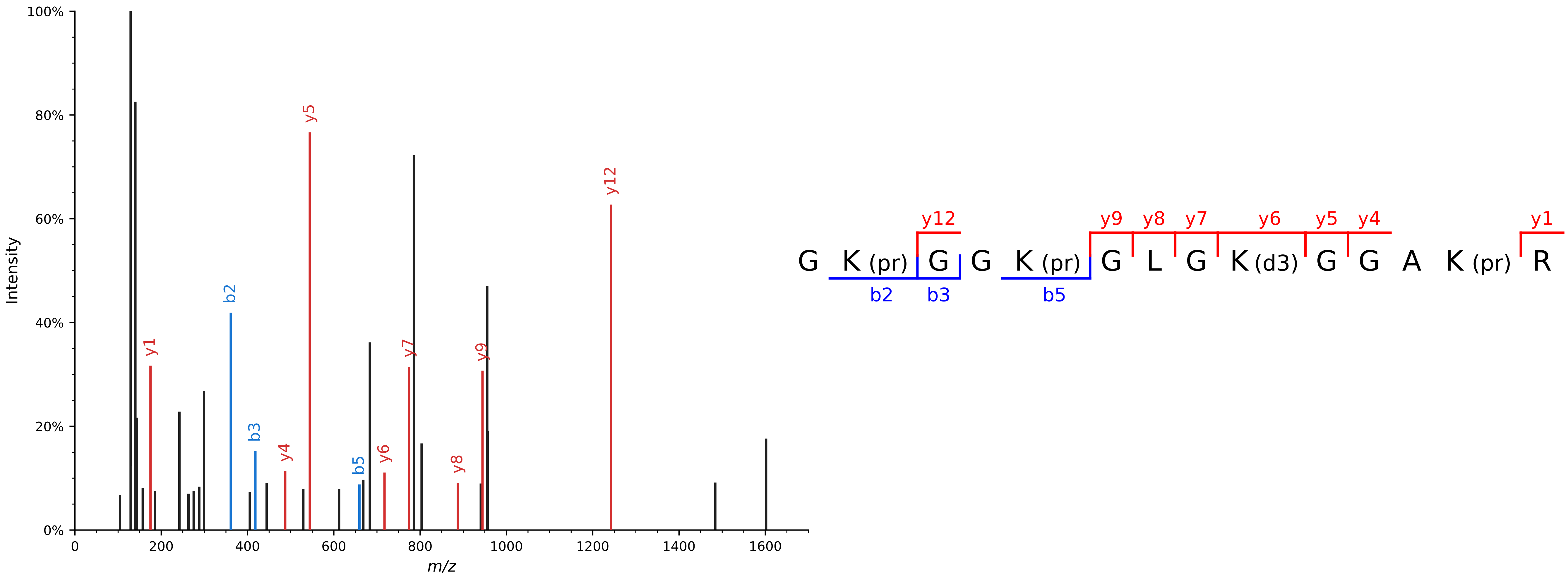

Raw file: QEP231006_CG_frozen_cecum_C3, Scan: 12430, m/z: 796.46292, Charge: 2, RT (min): 30.83, Score: 127.42

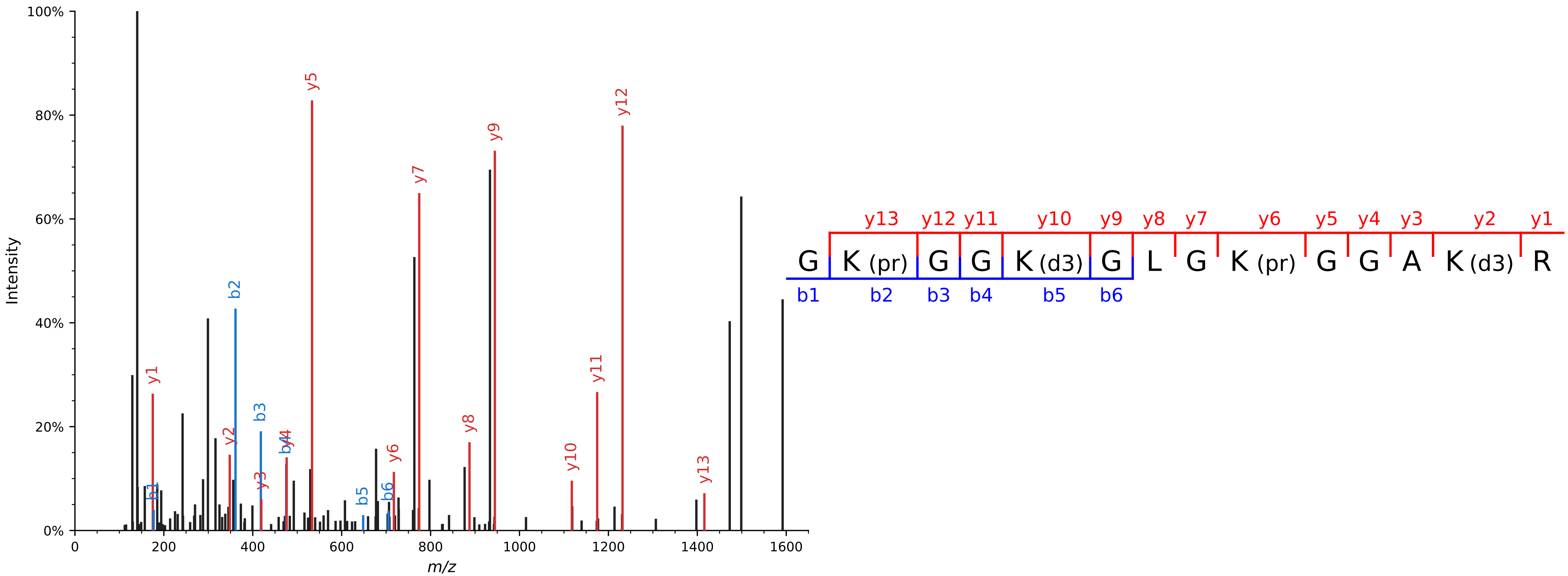

Raw file: QEP231006_CG_frozen_colon_C1, Scan: 11892, m/z: 794.9535, Charge: 2, RT (min): 30.87, Score: 77.379

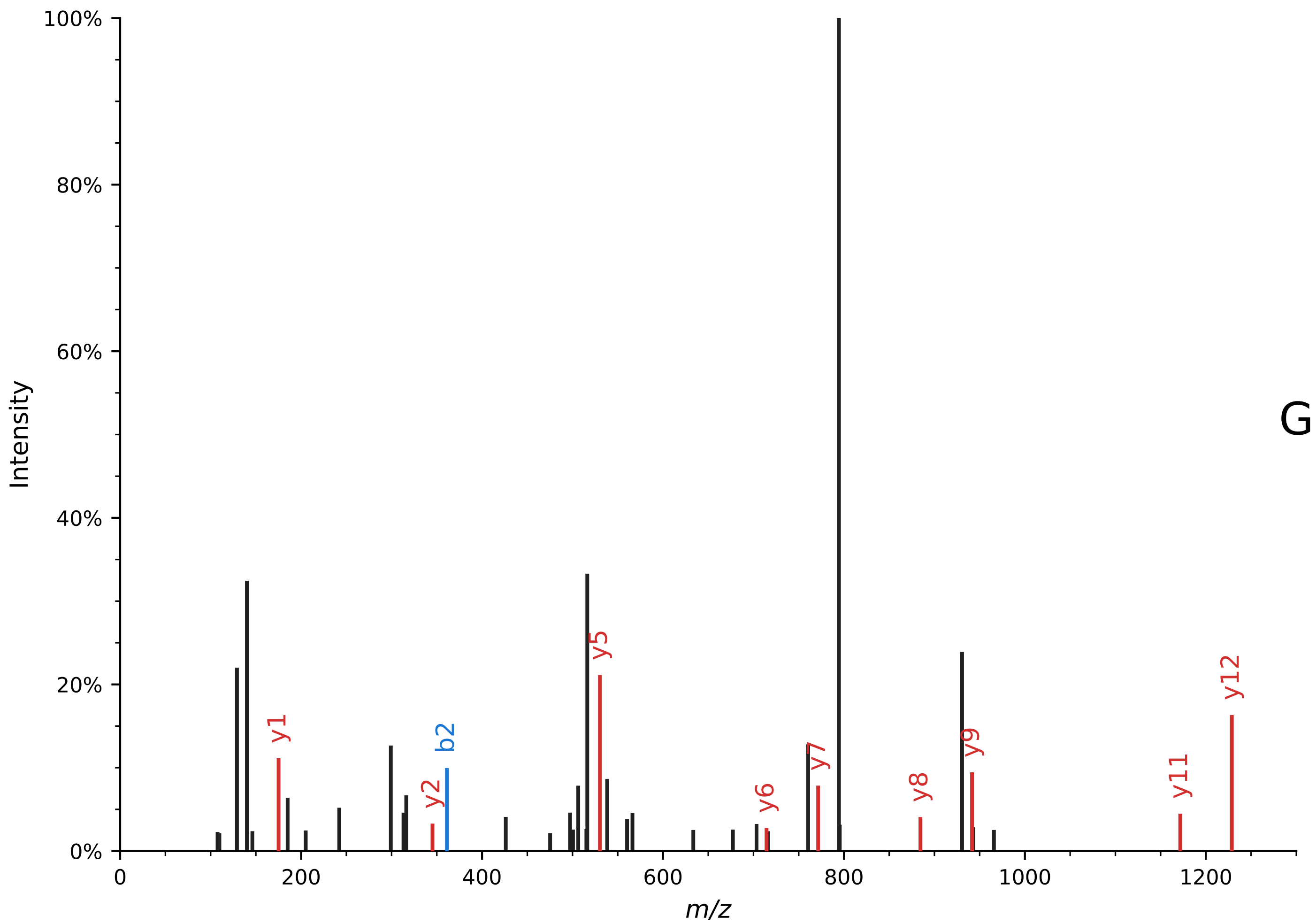

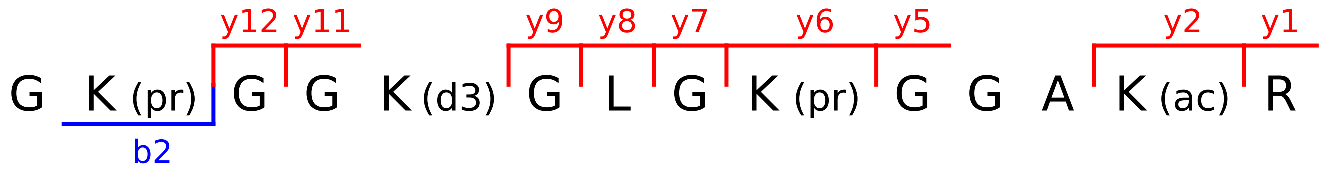

Raw file: QEP231006_CG_frozen_cecum_C2, Scan: 13095, m/z: 801.96133, Charge: 2, RT (min): 32.55, Score: 78.655

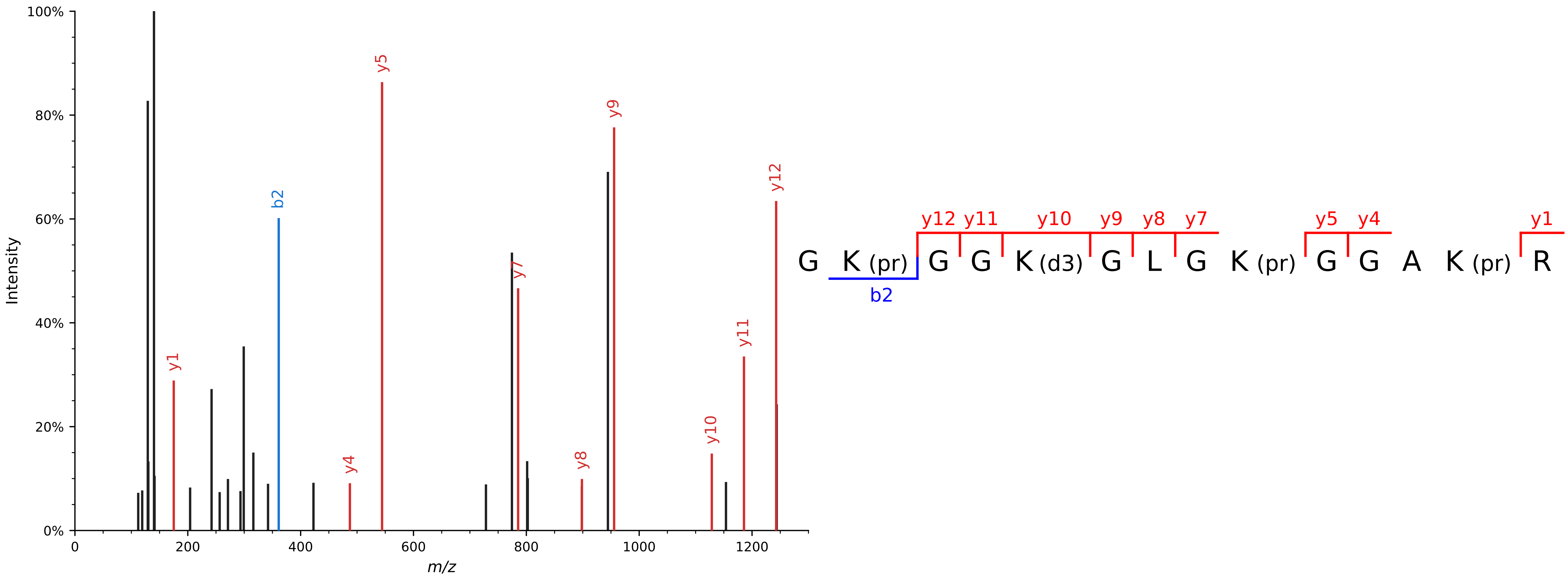

Raw file: QEP231006_CG_frozen_colon_C4, Scan: 11083, m/z: 789.45509, Charge: 2, RT (min): 28.75, Score: 154.24

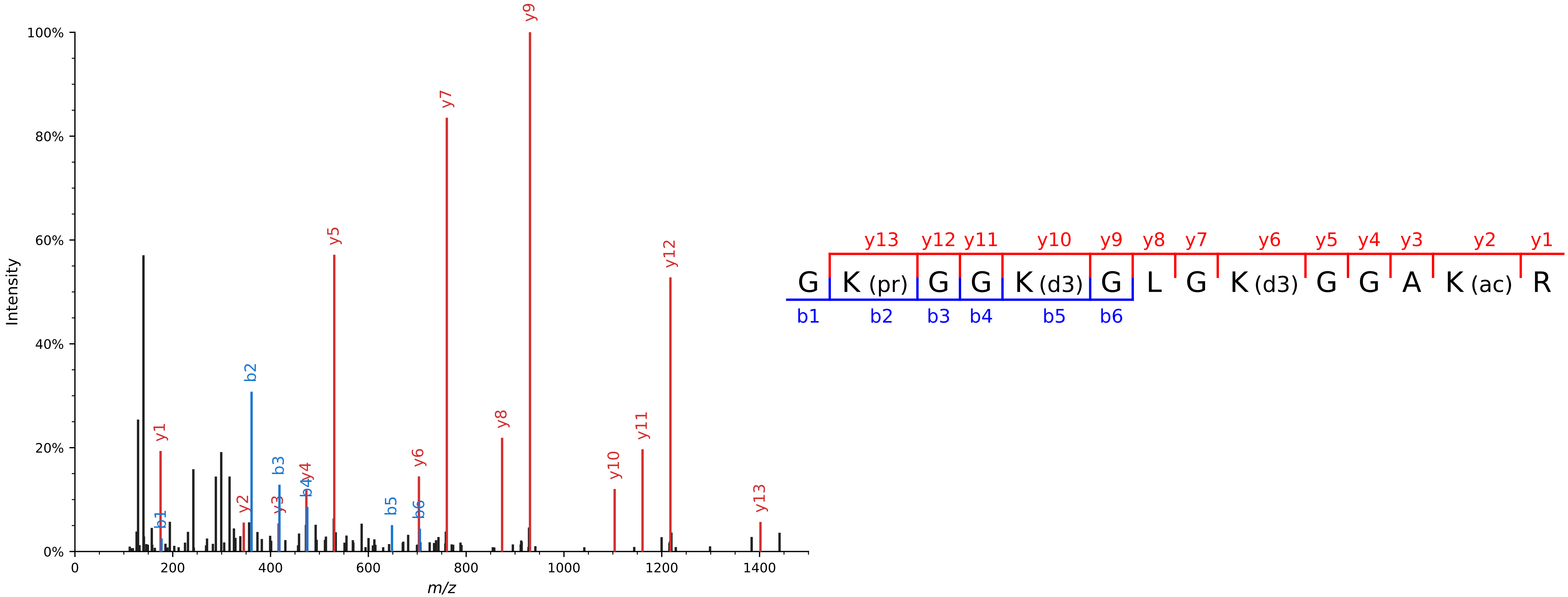

Raw file: QEP231006_CG_frozen_cecum_A2, Scan: 12152, m/z: 796.46292, Charge: 2, RT (min): 30.43, Score: 168.74

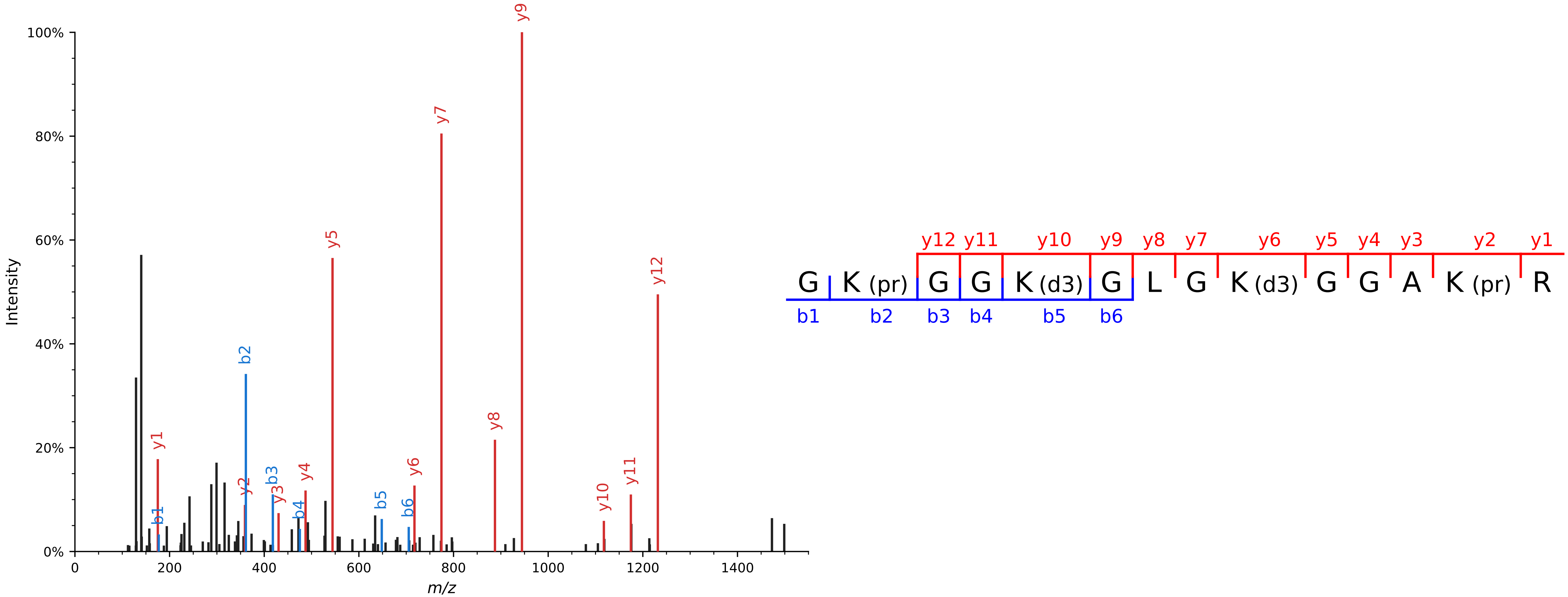

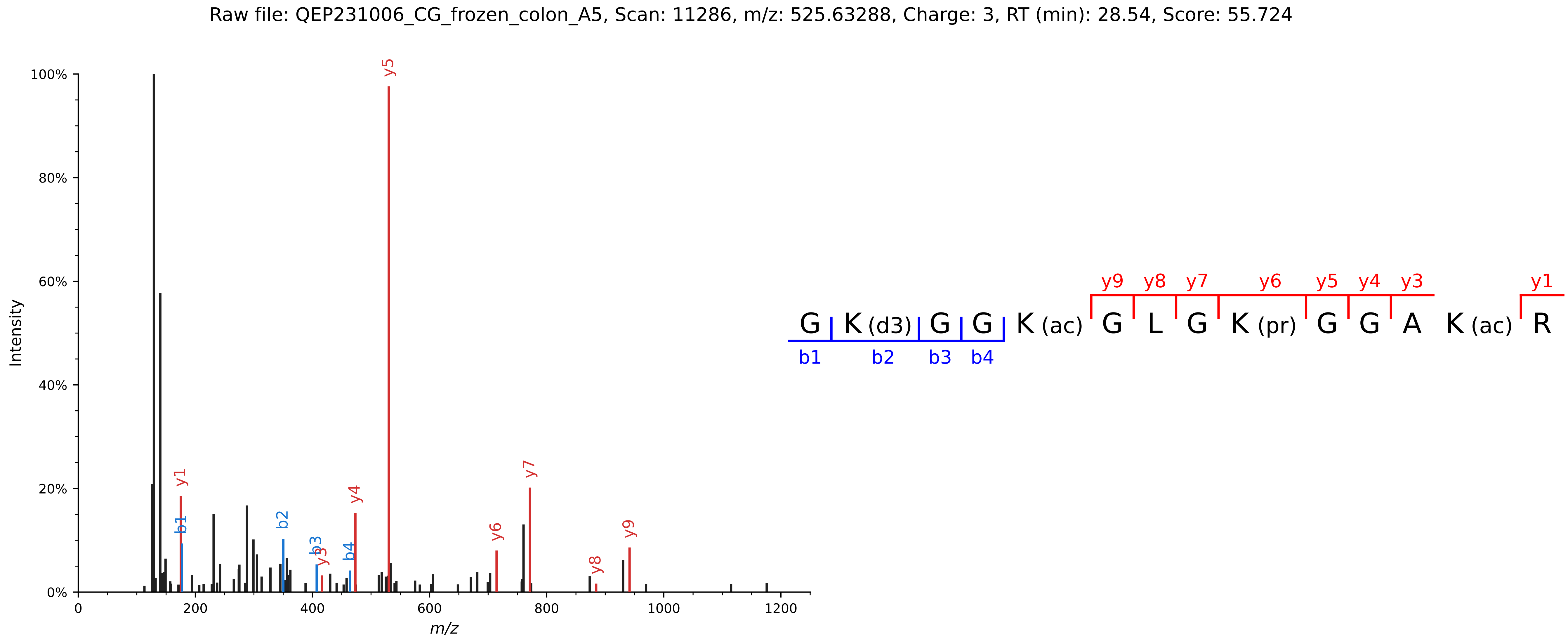

Raw file: QEP231006_CG_frozen_colon_A5, Scan: 11286, m/z: 525.63288, Charge: 3, RT (min): 28.54, Score: 55.724
Intensity
100%
80%
60%
40%
20%
0%
0
200
400
600
800
1000
1200
m/z
y1
b1
b2
b3
y3
b4
y4
y5
y6
y7
y8
y9
G K (d3) G G K (ac) G L G K (pr) G G A K (ac) R
b1 b2 b3 b4
y9 y8 y7 y6 y5 y4 y3 y1

Raw file: QEP231006_CG_frozen_cecum_A3, Scan: 10244, m/z: 521.96727, Charge: 3, RT (min): 26.67, Score: 53.001

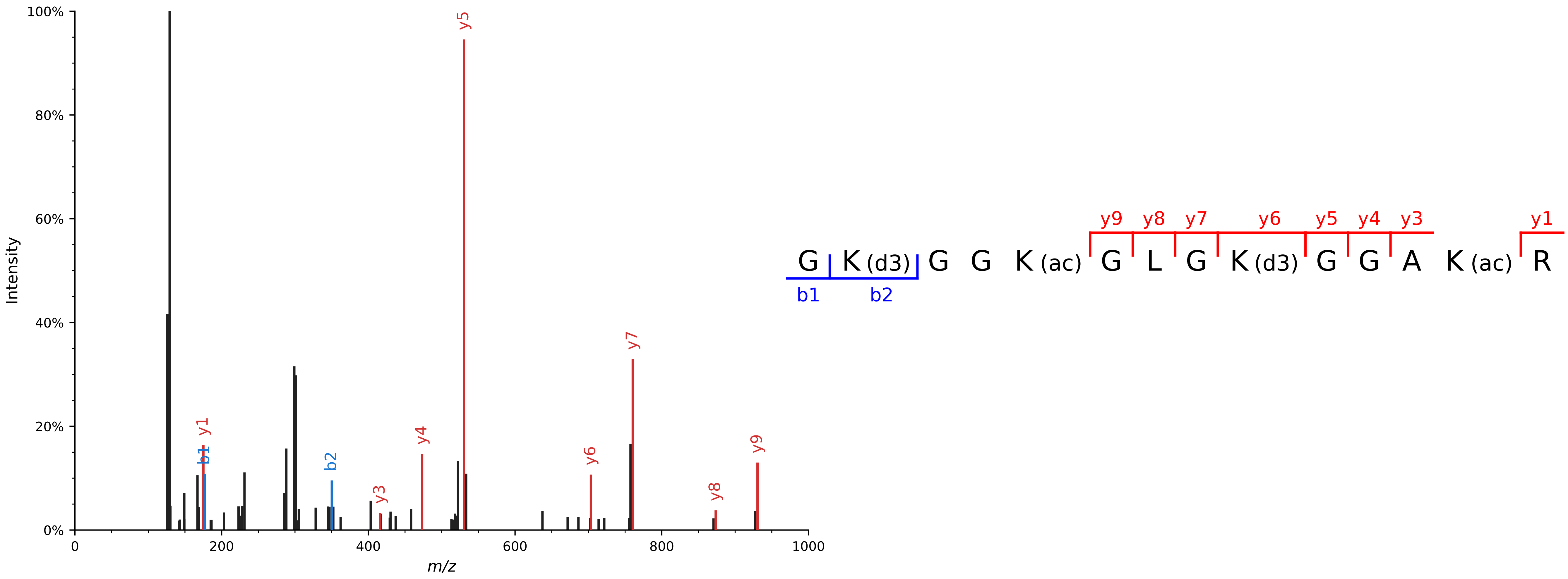

Raw file: QEP231006_CG_frozen_colon_A2, Scan: 9868, m/z: 776.94886, Charge: 2, RT (min): 25.88, Score: 54.608

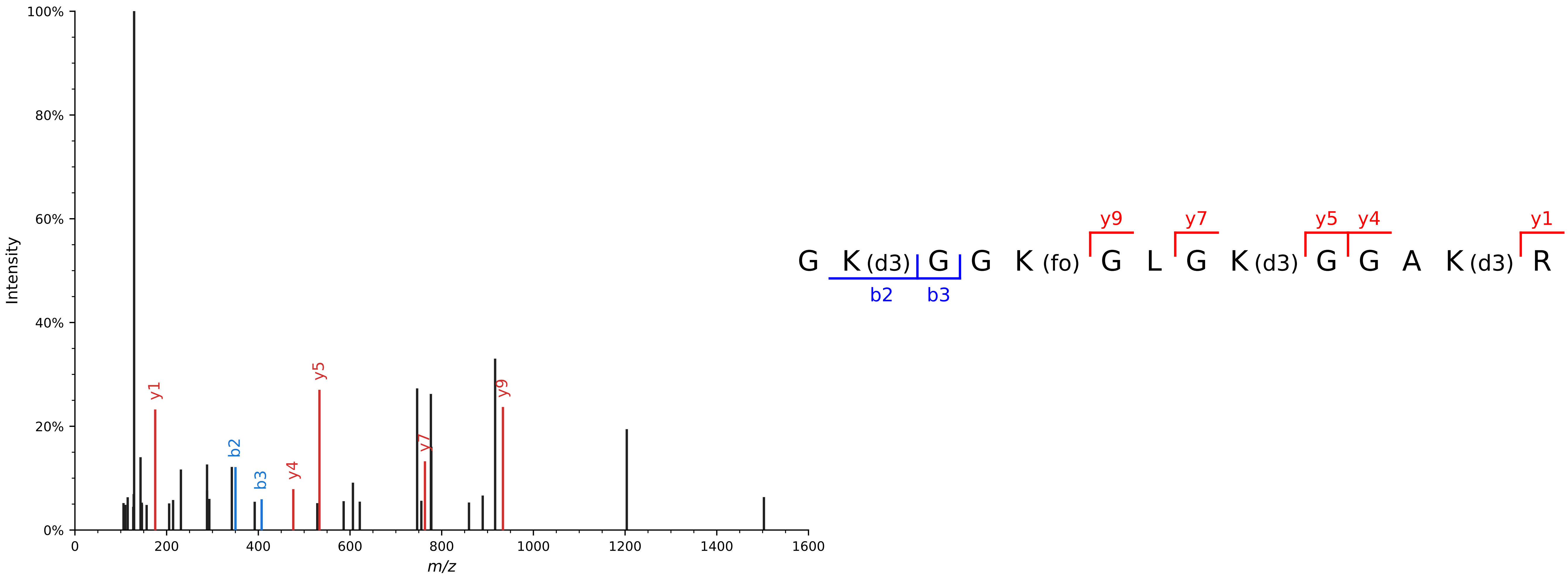

Raw file: QEP231006_CG_frozen_cecum_A5, Scan: 11181, m/z: 790.96451, Charge: 2, RT (min): 28.34, Score: 155.15

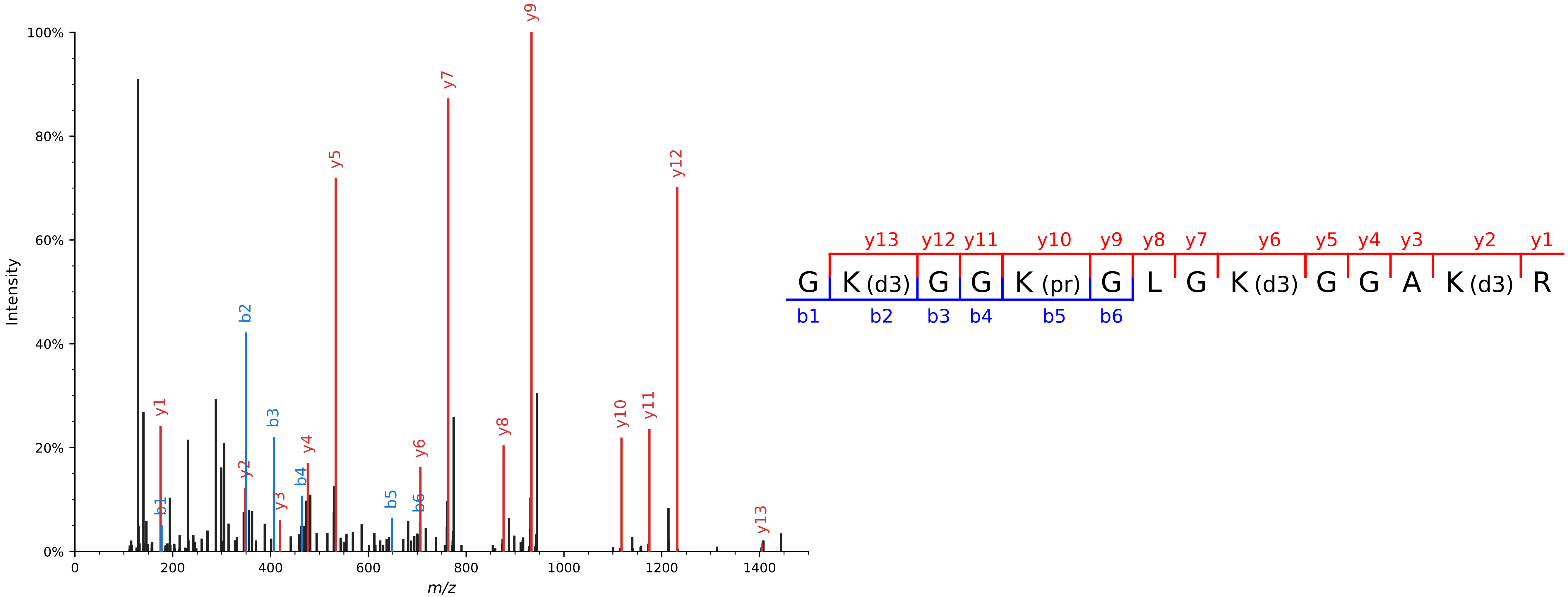

Raw file: QEP231006_CG_frozen_cecum_A4, Scan: 12107, m/z: 796.46292, Charge: 2, RT (min): 30.51, Score: 107.83

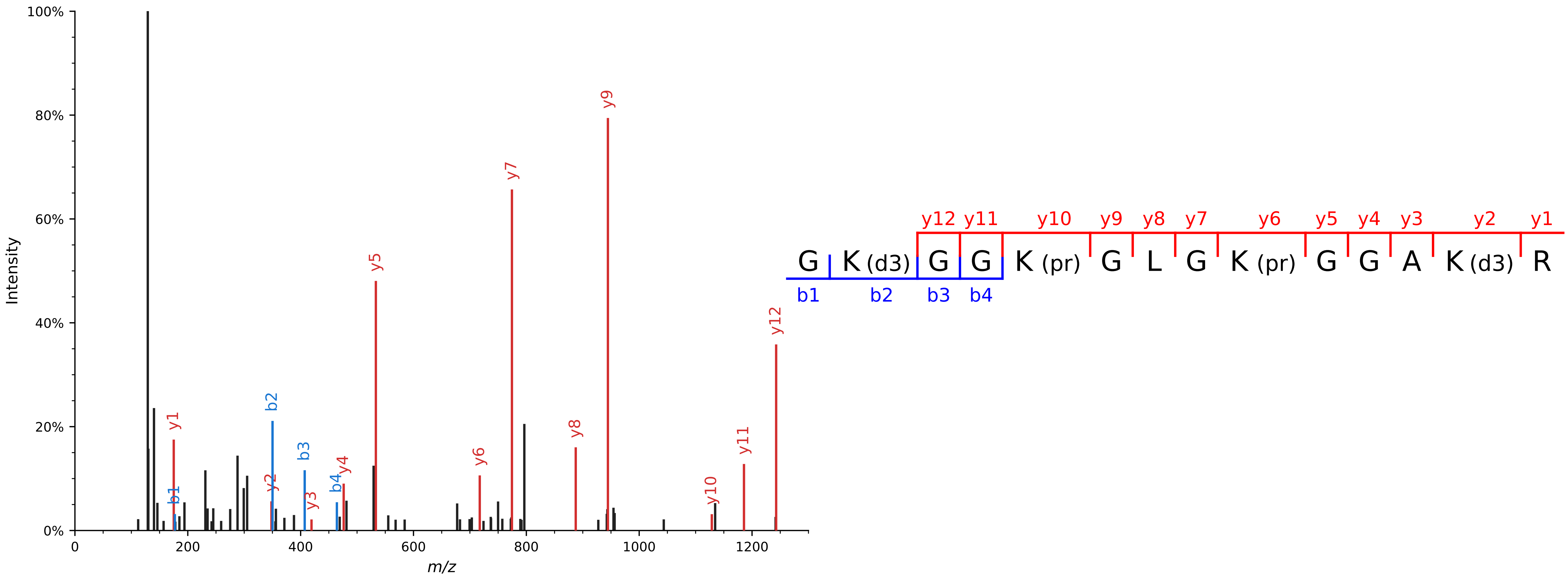

Raw file: QEP231006_CG_frozen_cecum_C1, Scan: 13037, m/z: 801.96133, Charge: 2, RT (min): 32.54, Score: 57.348

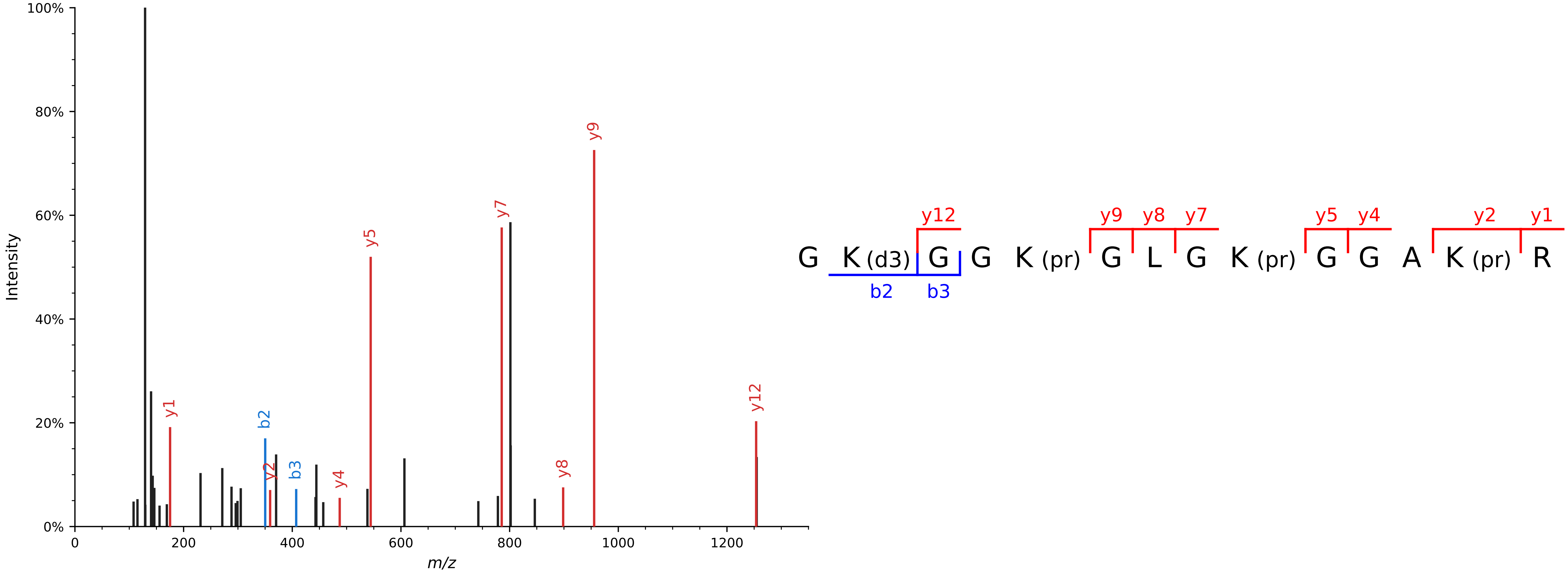

Raw file: QEP231006_CG_frozen_cecum_A1, Scan: 12005, m/z: 796.46292, Charge: 2, RT (min): 30.13, Score: 155.15

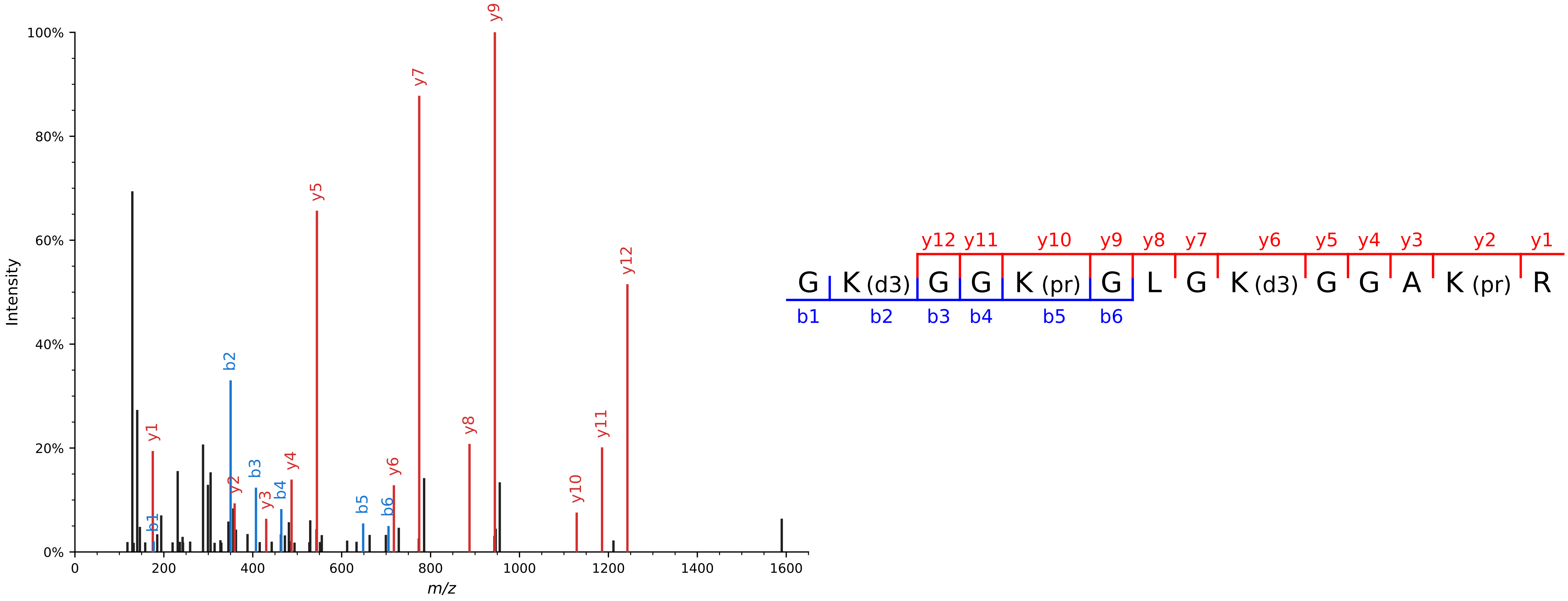

Raw file: QEP231006_CG_frozen_colon_A3, Scan: 10392, m/z: 790.96451, Charge: 2, RT (min): 28.55, Score: 140.49

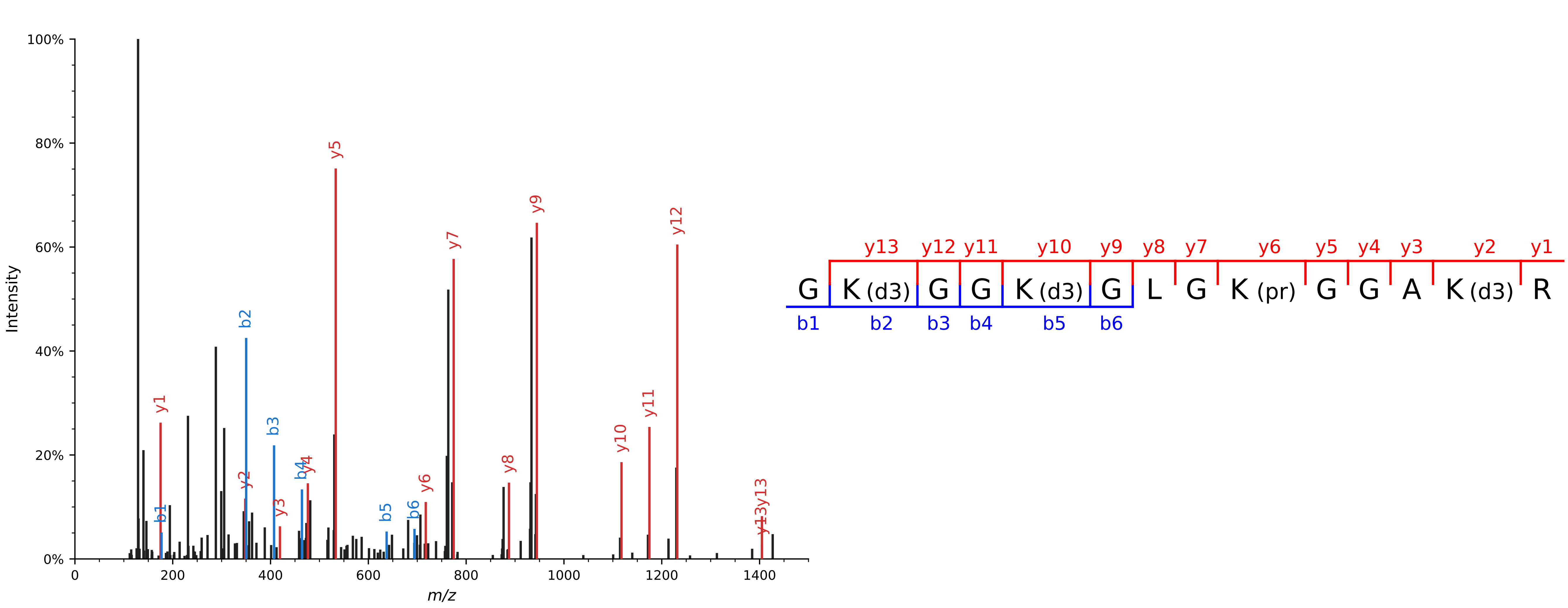

Raw file: QEP231006_CG_frozen_cecum_C2, Scan: 11366, m/z: 789.45509, Charge: 2, RT (min): 28.76, Score: 154.24

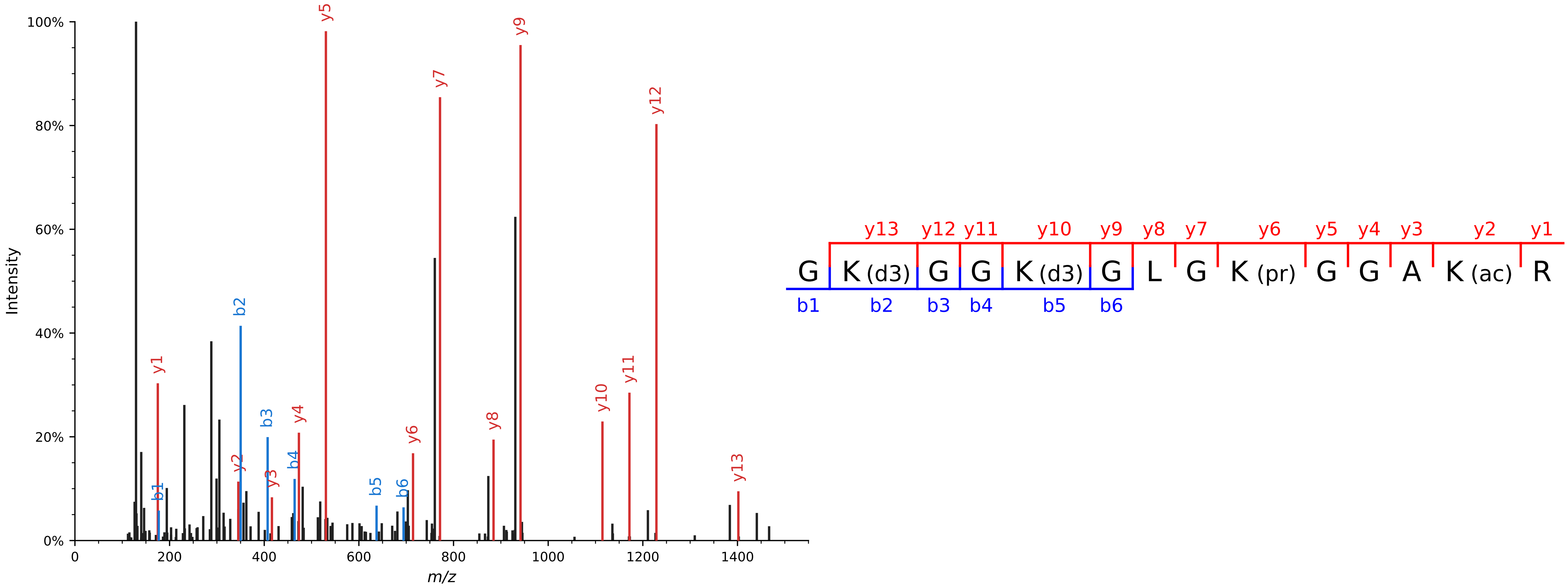

Raw file: QEP231006_CG_frozen_colon_C1, Scan: 11634, m/z: 796.46292, Charge: 2, RT (min): 30.26, Score: 111.06

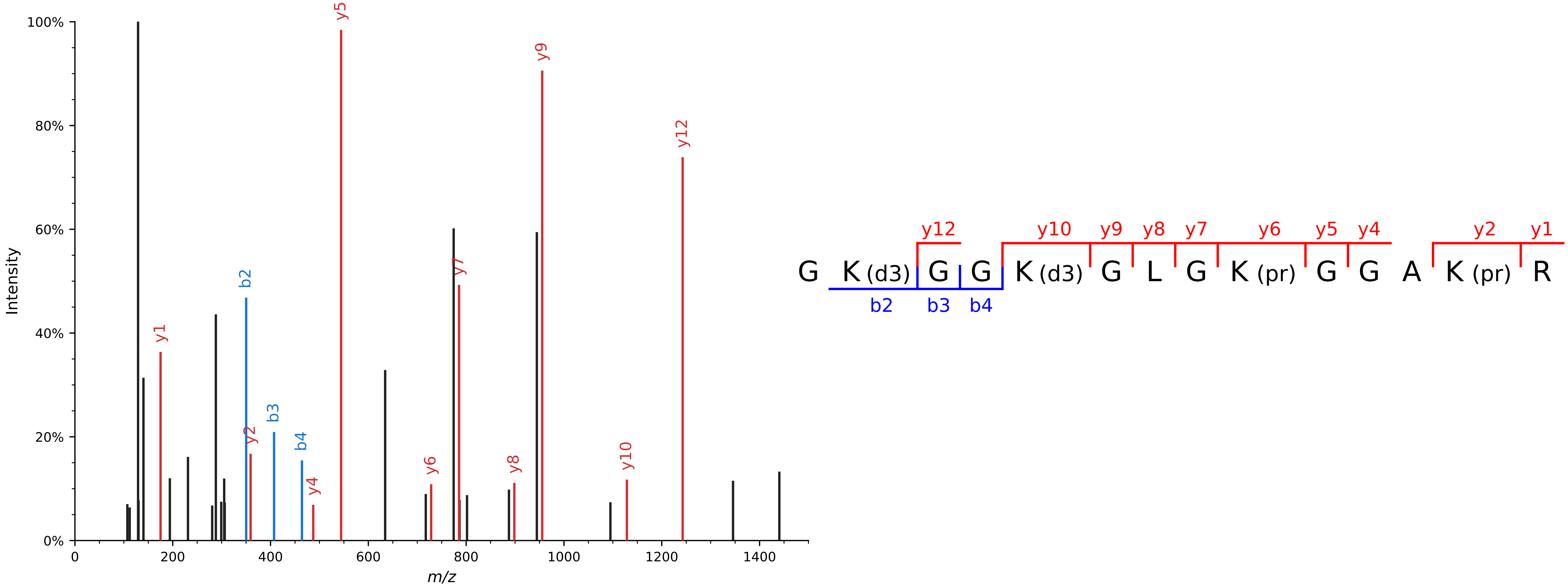

Raw file: QEP231006_CG_frozen_cecum_C4, Scan: 10442, m/z: 783.95668, Charge: 2, RT (min): 26.89, Score: 156.48

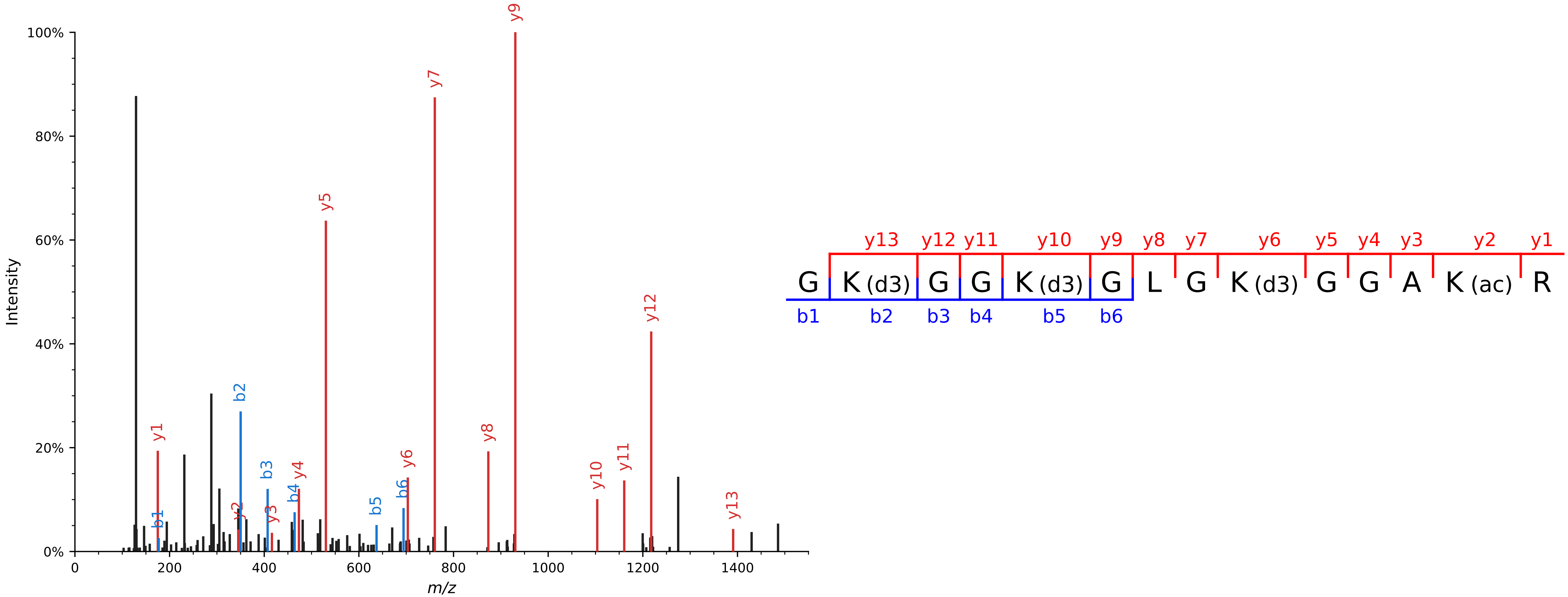

Raw file: QEP231006_CG_frozen_cecum_A5, Scan: 11014, m/z: 790.96451, Charge: 2, RT (min): 27.99, Score: 185.29

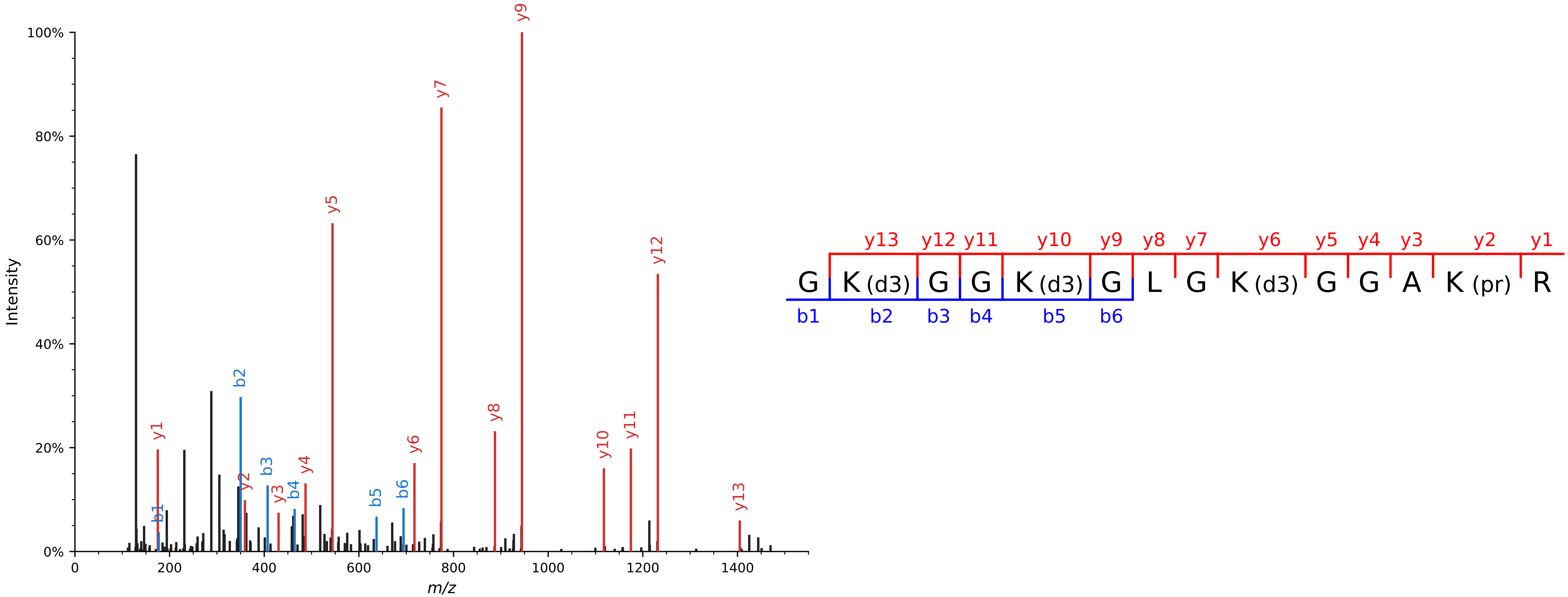

Raw file: QEP231006_CG_frozen_cecum_A2, Scan: 8797, m/z: 719.3415, Charge: 2, RT (min): 23.03, Score: 79.148

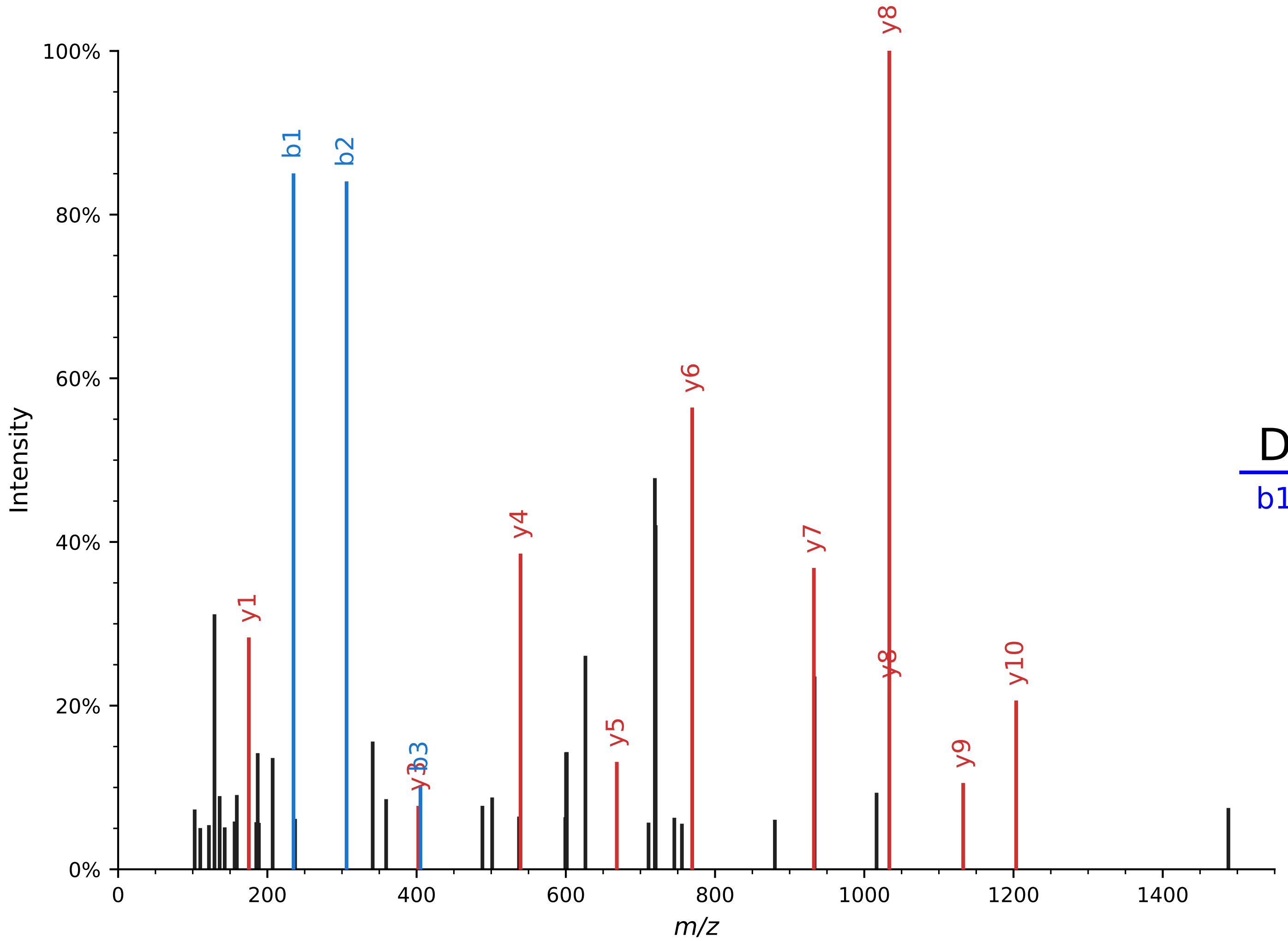

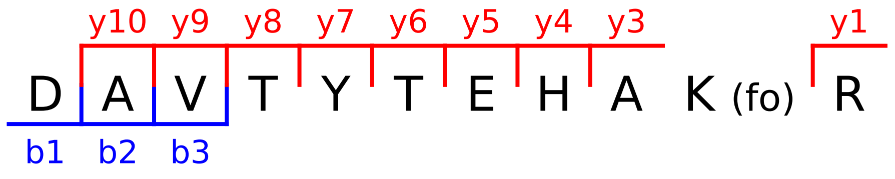

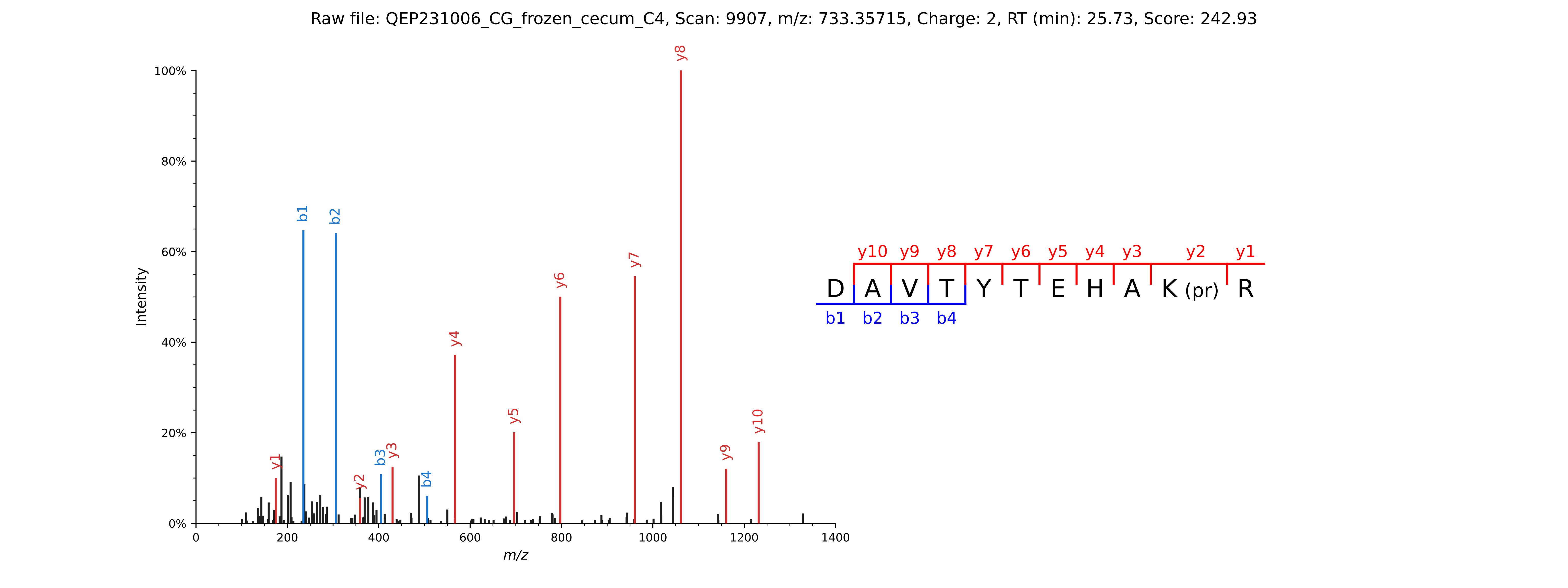

Raw file: QEP231006_CG_frozen_cecum_C4, Scan: 9907, m/z: 733.35715, Charge: 2, RT (min): 25.73, Score: 242.93
Intensity
m/z
y1
b1
b2
y2
b3
y3
b4
y4
y5
y6
y7
y8
y9
y10
D A V T Y T E H A K (pr) R

Breast

Raw file: QEP231009_CG_frozen_breast_TN_25, Scan: 2082, m/z: 490.7894, Charge: 2, RT (min): 8.48, Score: 42.396

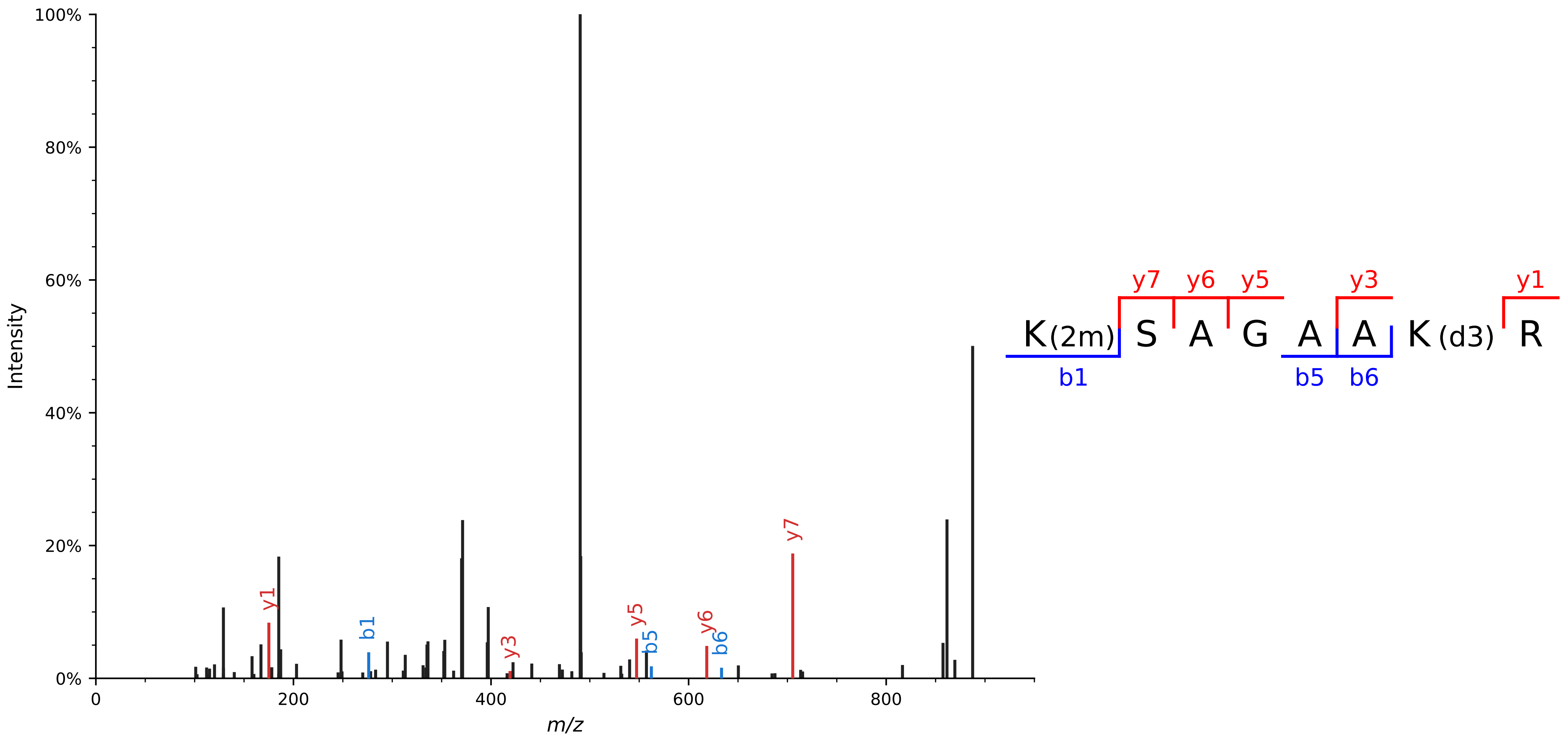

Raw file: QEP231009_CG_frozen_breast_LuA_32, Scan: 8457, m/z: 504.78686, Charge: 2, RT (min): 22.31, Score: 90.777

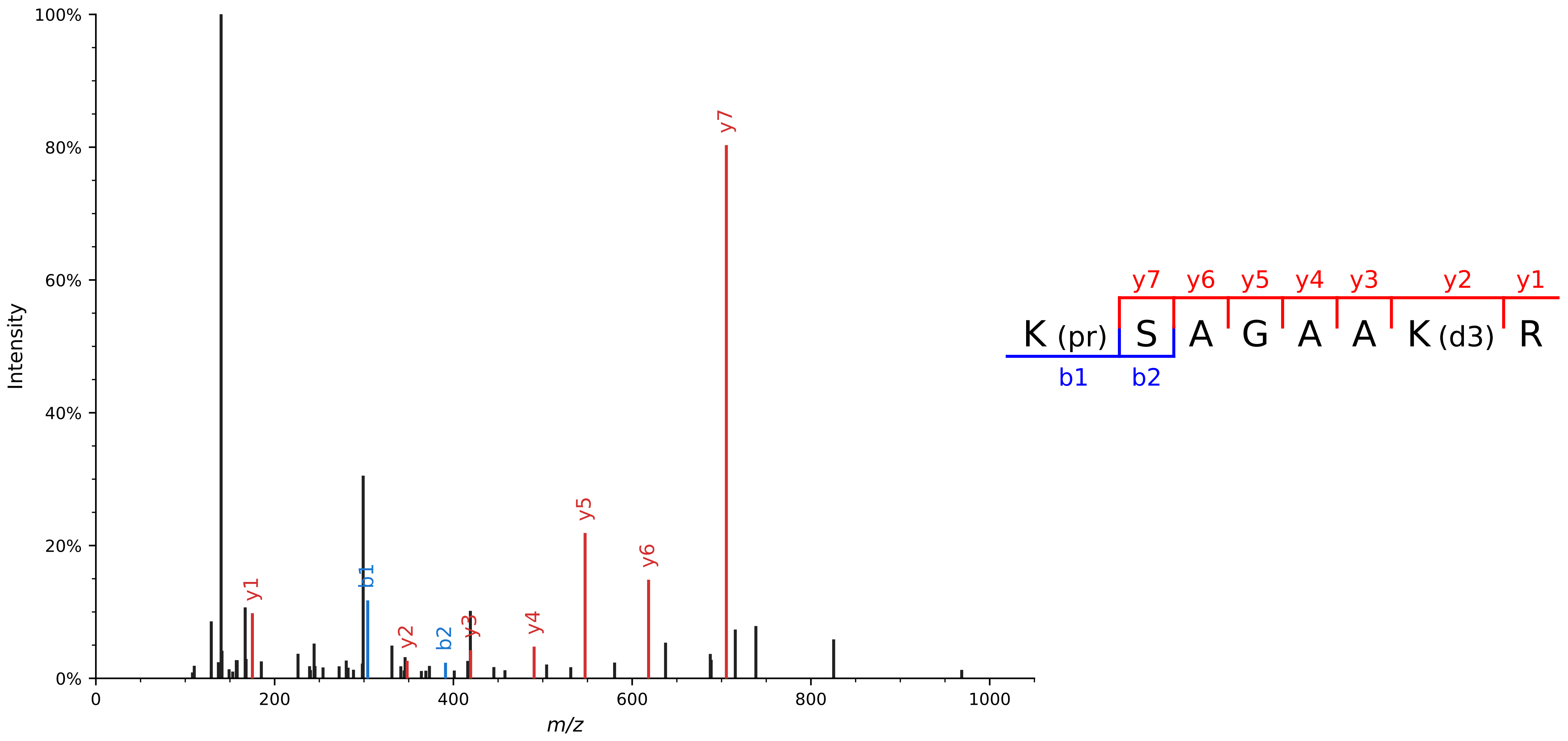

Raw file: QEP231009_CG_frozen_breast_LuA_30, Scan: 7312, m/z: 565.29478, Charge: 2, RT (min): 19.64, Score: 47.082

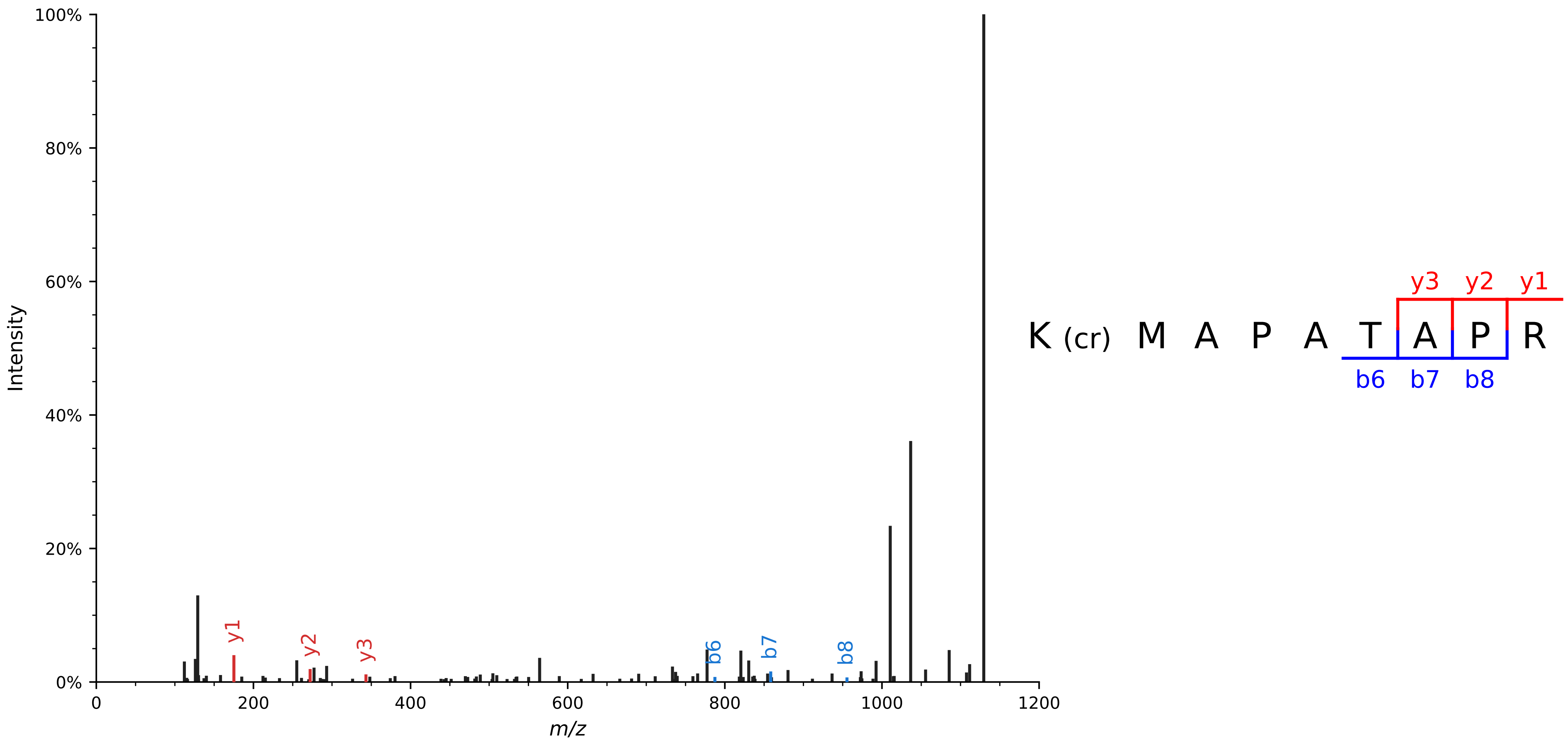

Raw file: QEP231009_CG_frozen_breast_Normal_21, Scan: 8825, m/z: 615.82749, Charge: 2, RT (min): 23.18, Score: 41.979

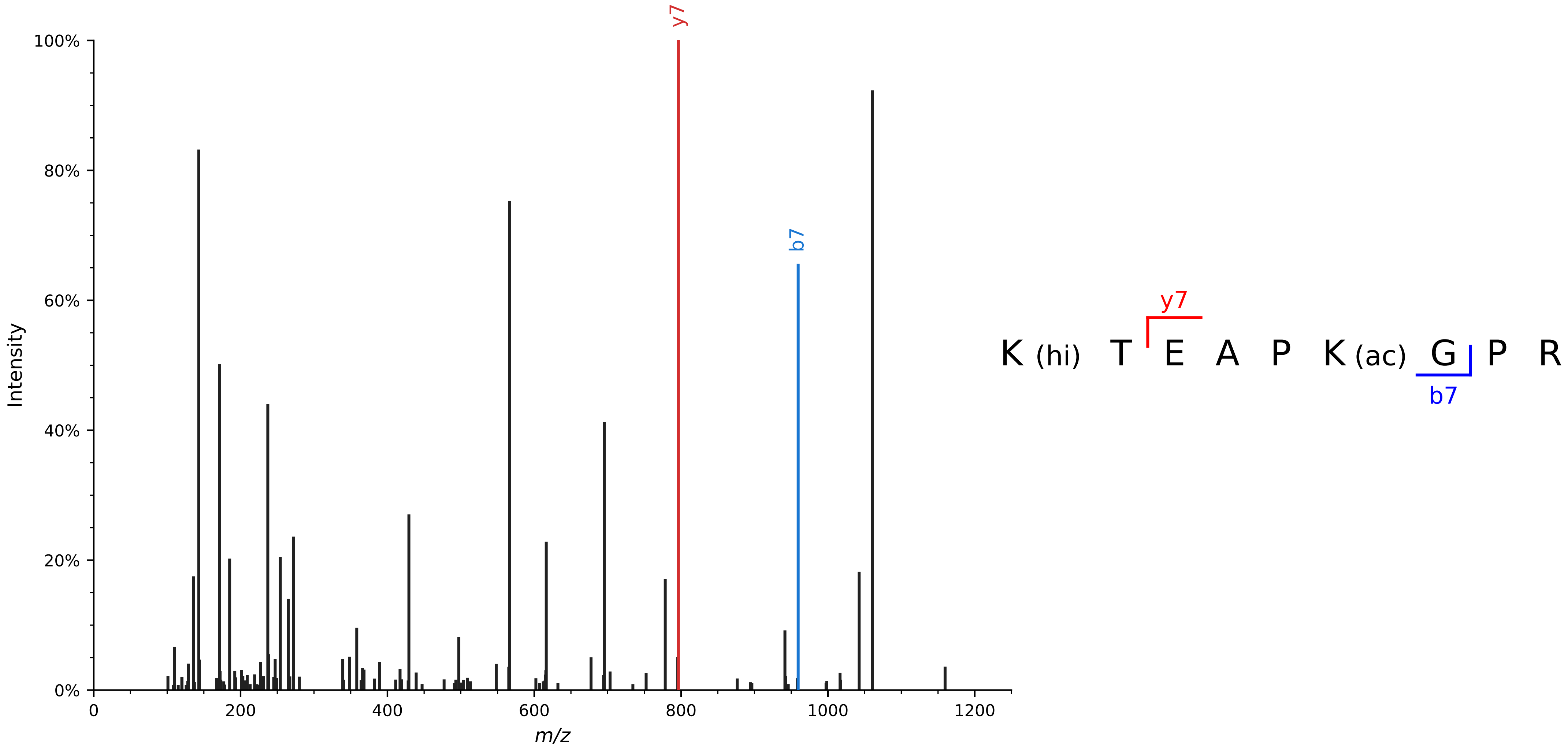

Raw file: QEP231009_CG_frozen_breast_Normal_21, Scan: 7414, m/z: 509.25925, Charge: 2, RT (min): 20.16, Score: 41.502

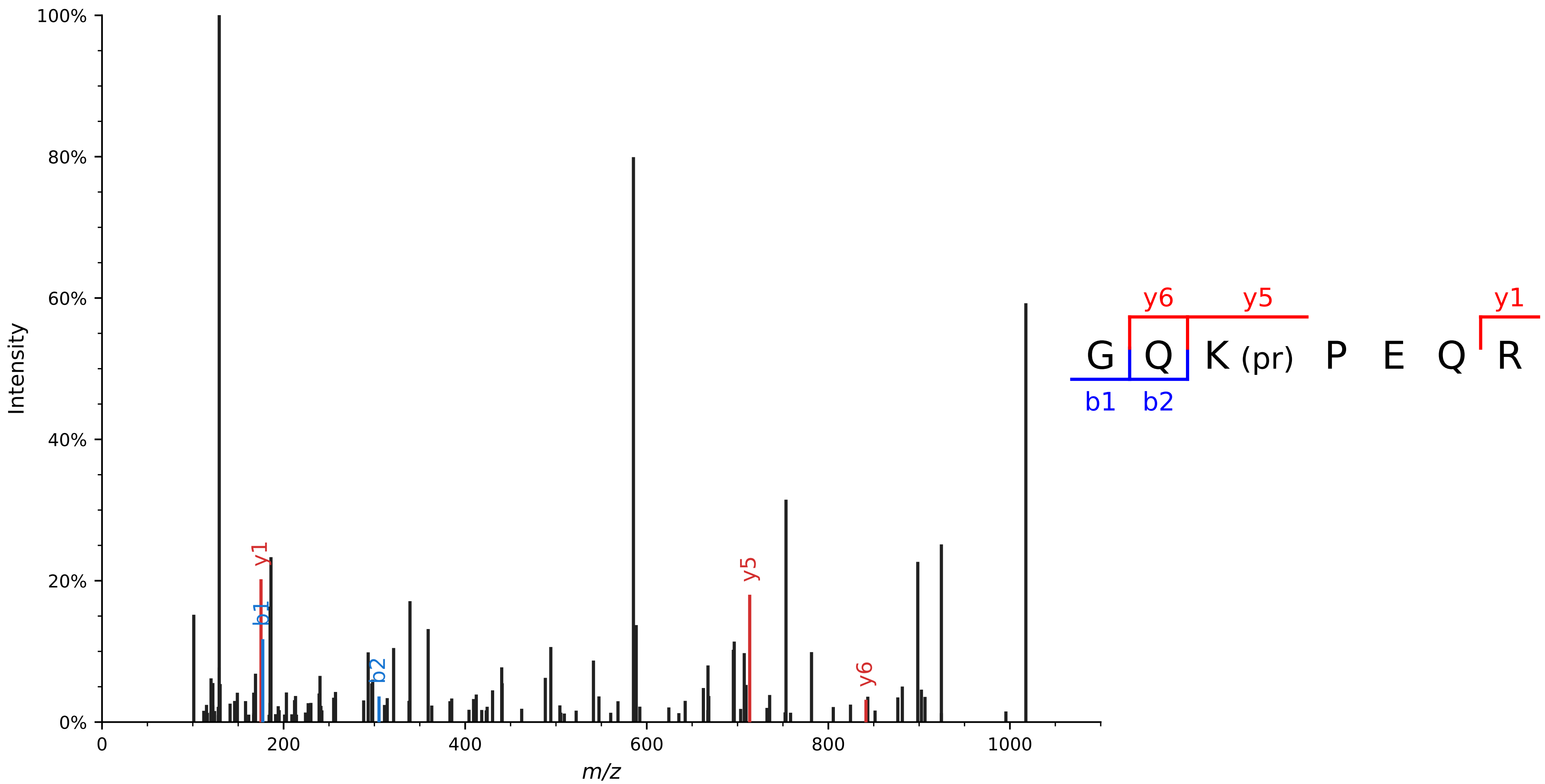

Raw file: QEP231009_CG_frozen_breast_Normal_24, Scan: 6876, m/z: 426.74192, Charge: 2, RT (min): 19.18, Score: 52.683

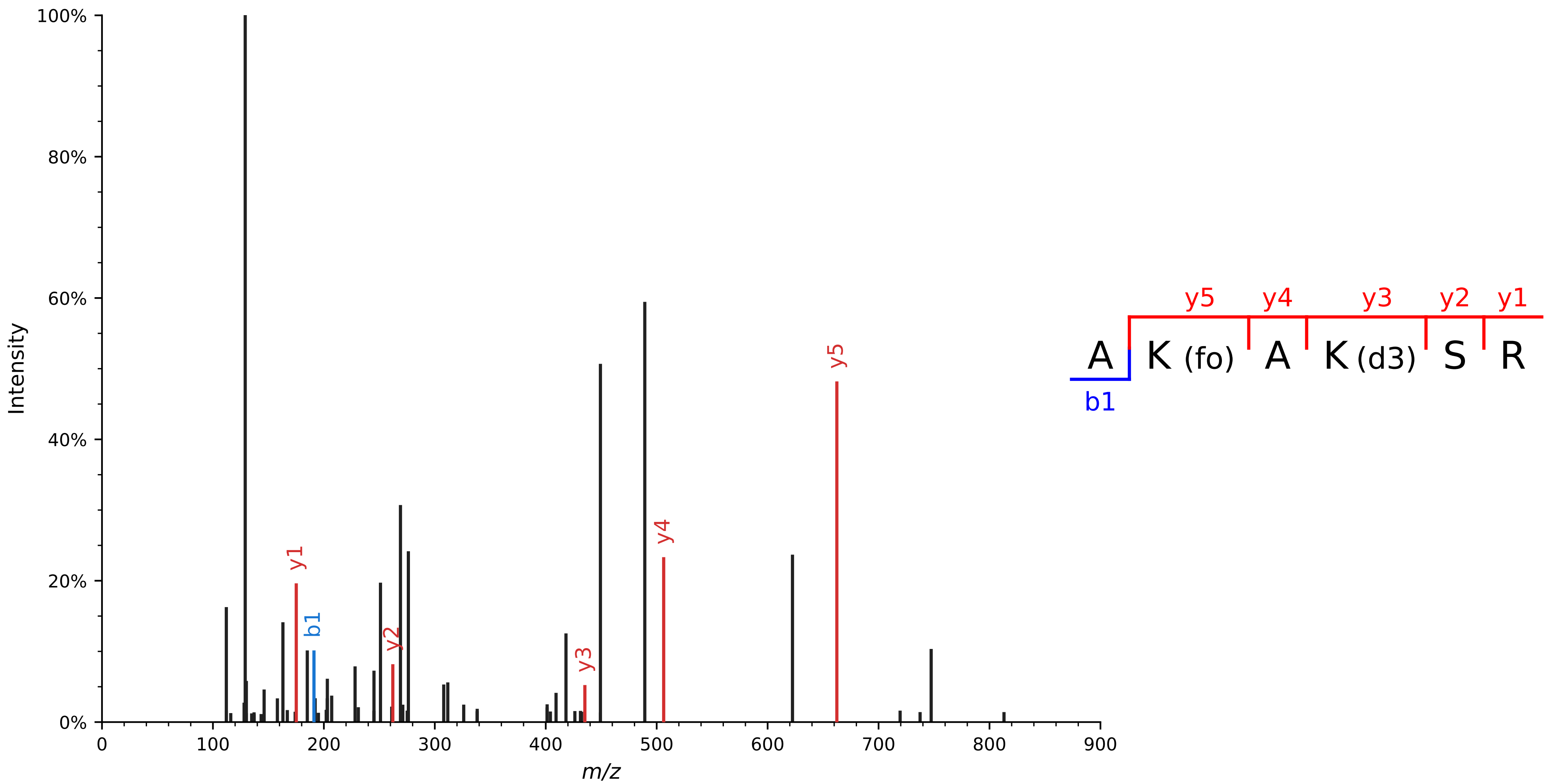

Raw file: QEP231009_CG_frozen_breast_Normal_21, Scan: 8811, m/z: 440.75757, Charge: 2, RT (min): 23.16, Score: 105.27

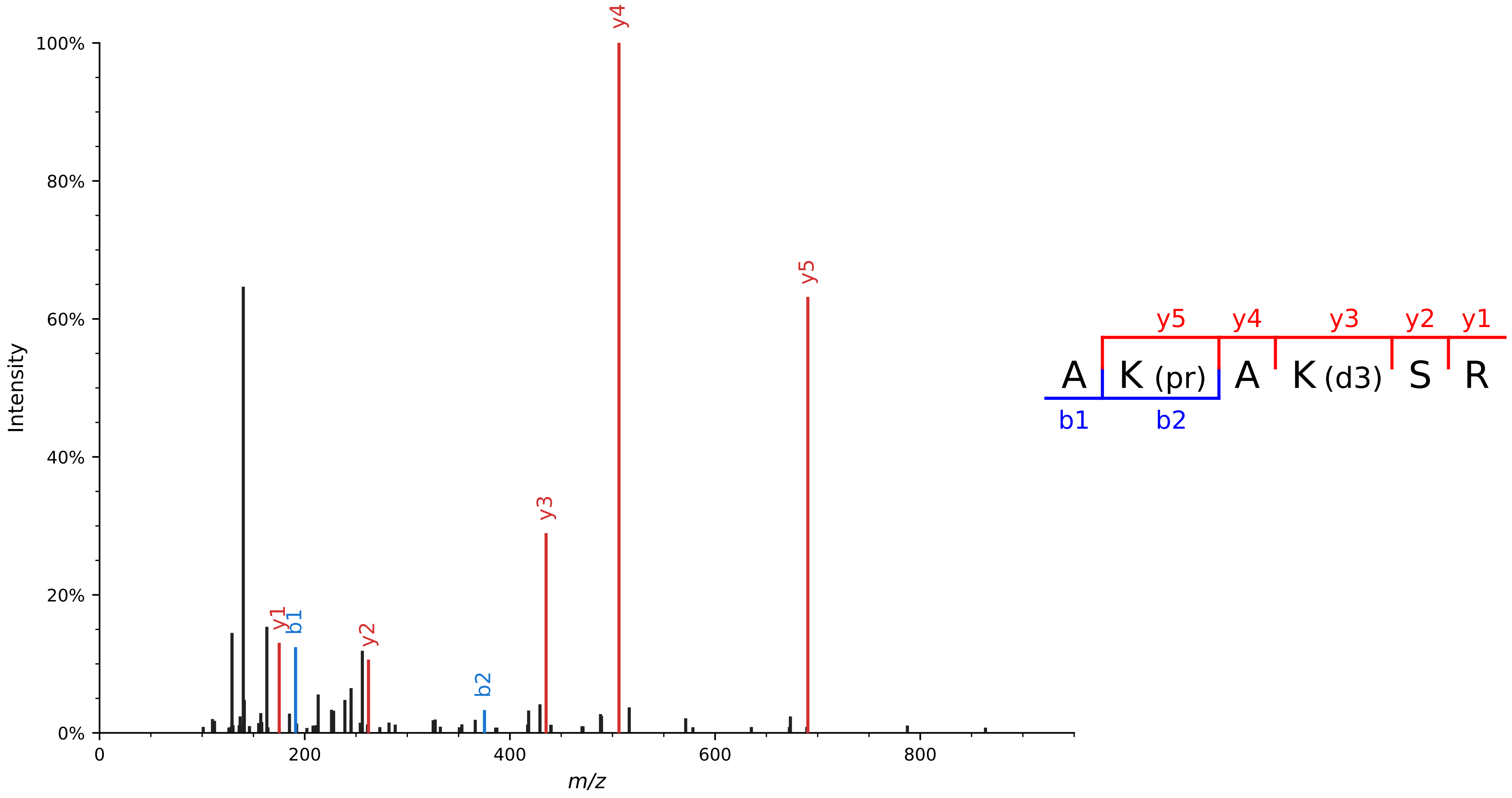

Raw file: QEP231009_CG_frozen_breast_Normal_21, Scan: 10226, m/z: 446.25598, Charge: 2, RT (min): 26.40, Score: 82.386

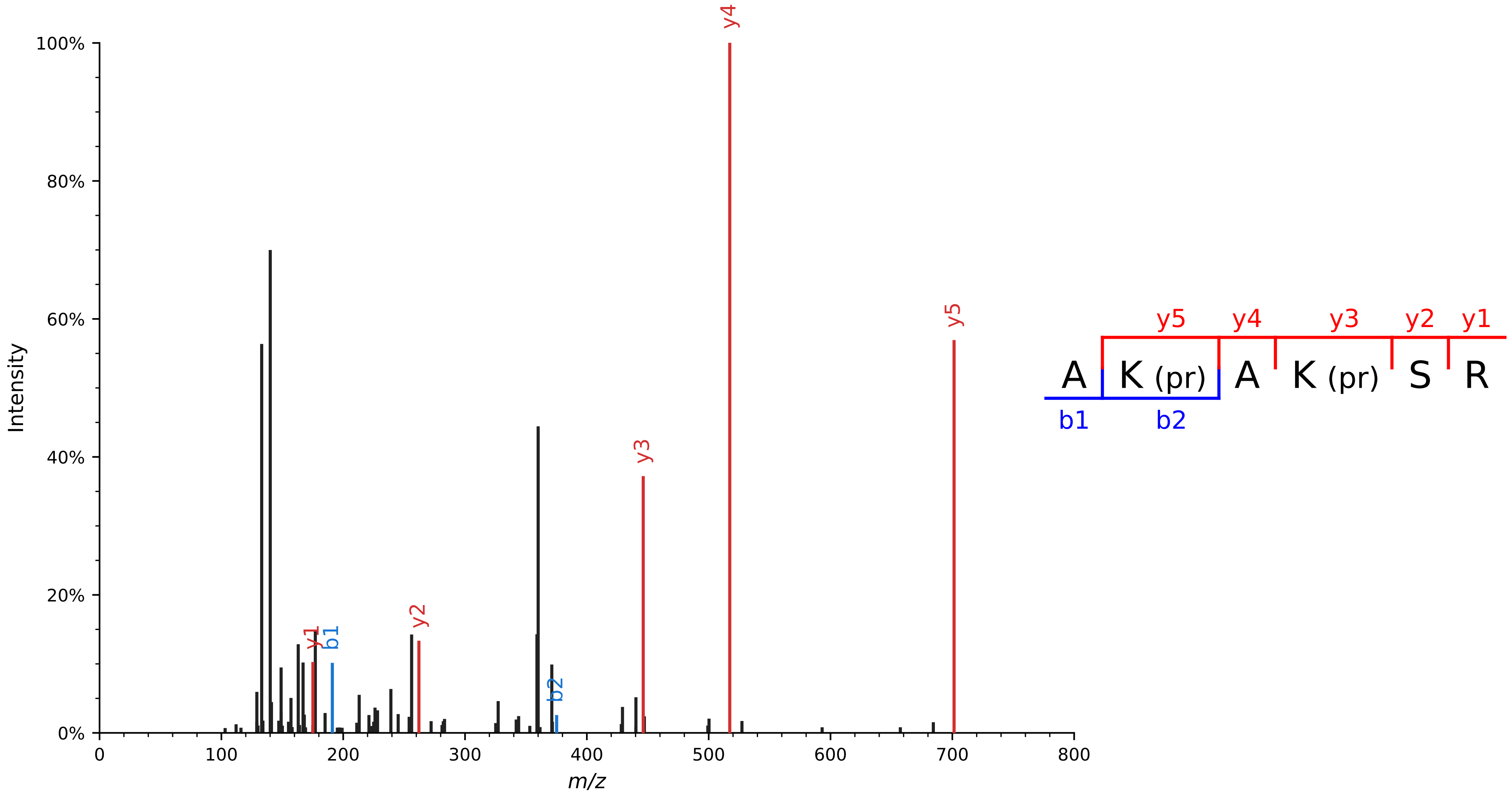

Raw file: QEP231009_CG_frozen_breast_LuA_30, Scan: 9156, m/z: 440.75757, Charge: 2, RT (min): 23.58, Score: 107.74

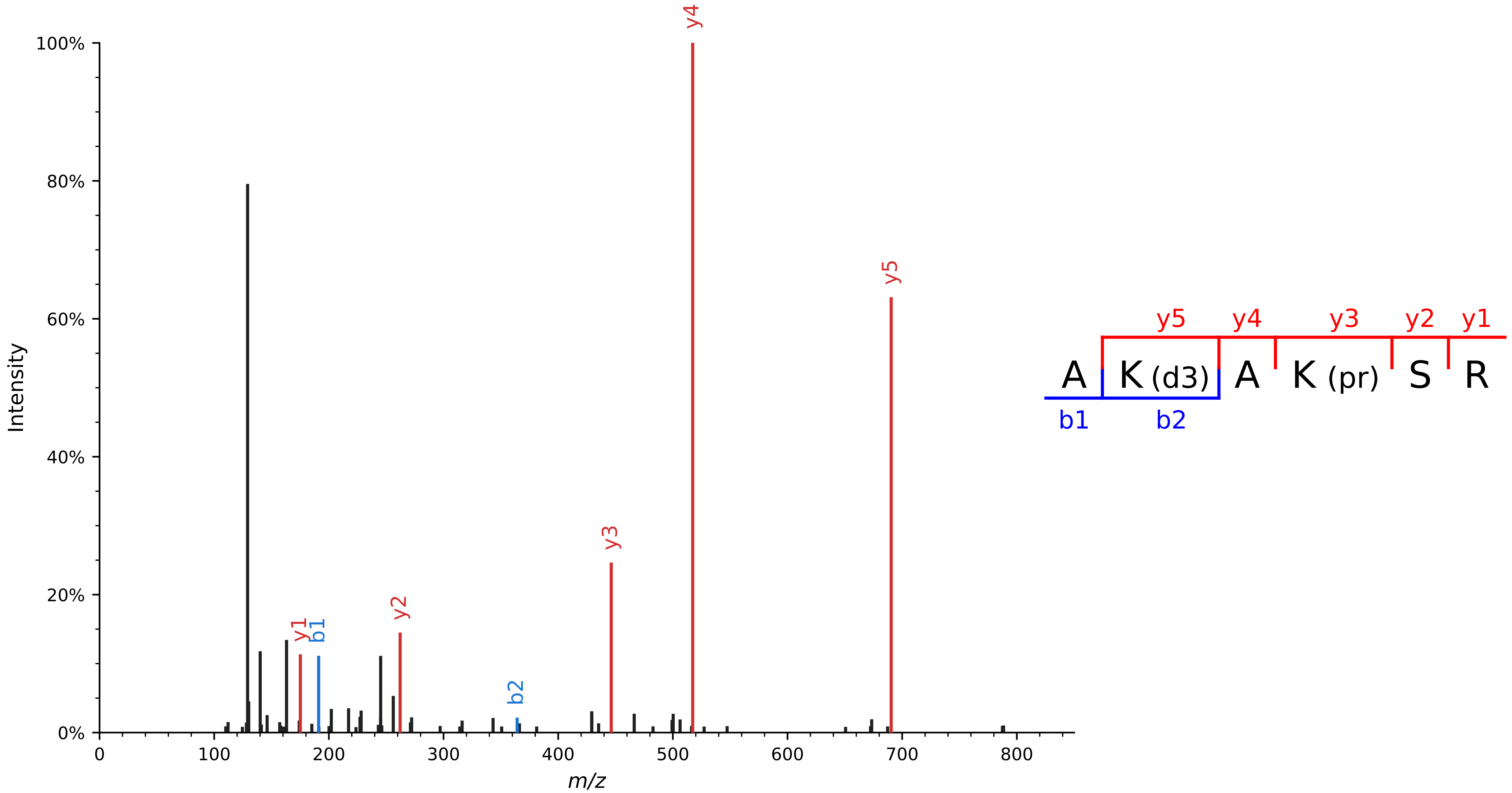

Raw file: QEP231009_CG_frozen_breast_TN_25, Scan: 9301, m/z: 514.74343, Charge: 2, RT (min): 23.54, Score: 92.47

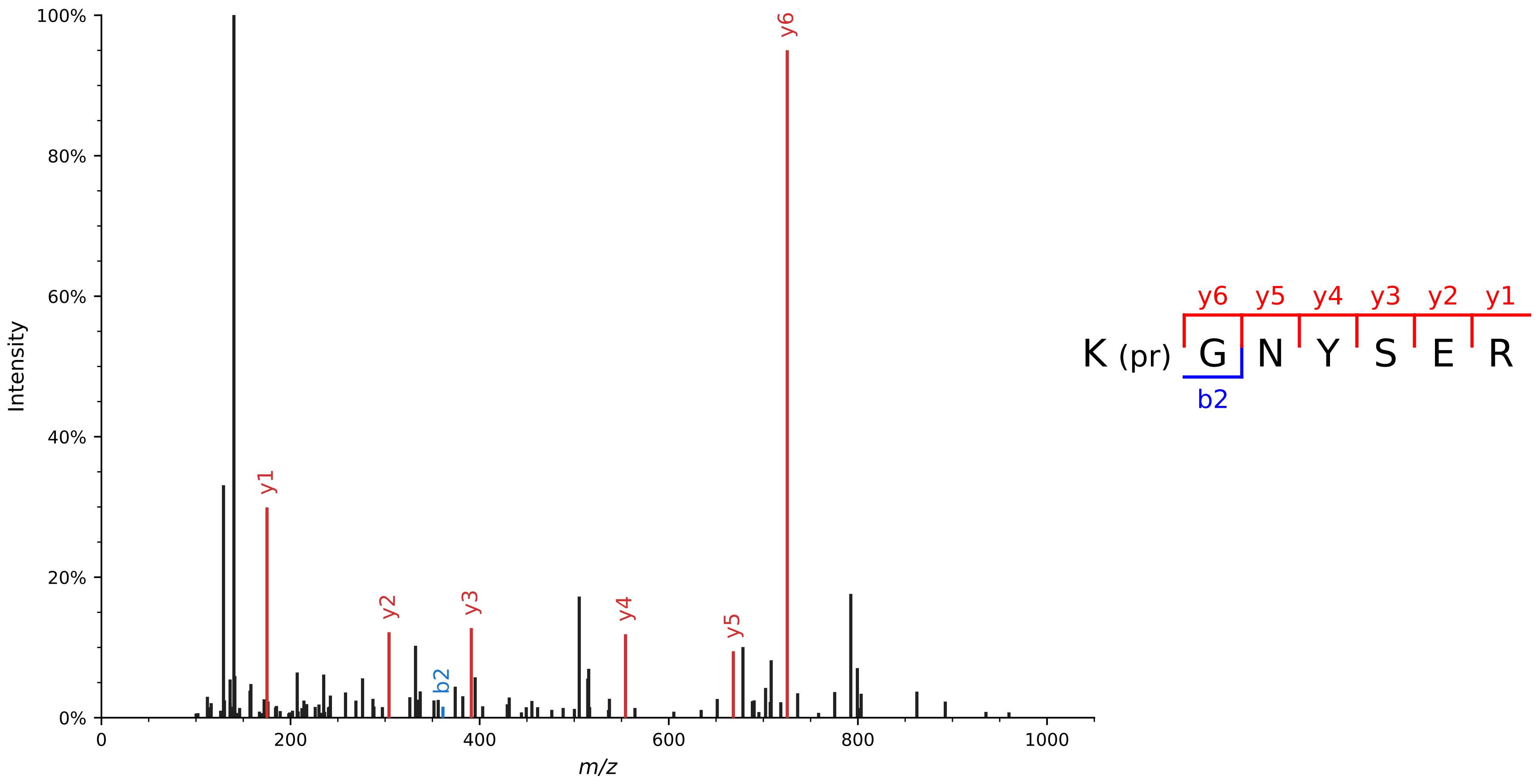

Raw file: QEP231009_CG_frozen_breast_Normal_24, Scan: 5998, m/z: 509.77507, Charge: 2, RT (min): 17.29, Score: 47.849

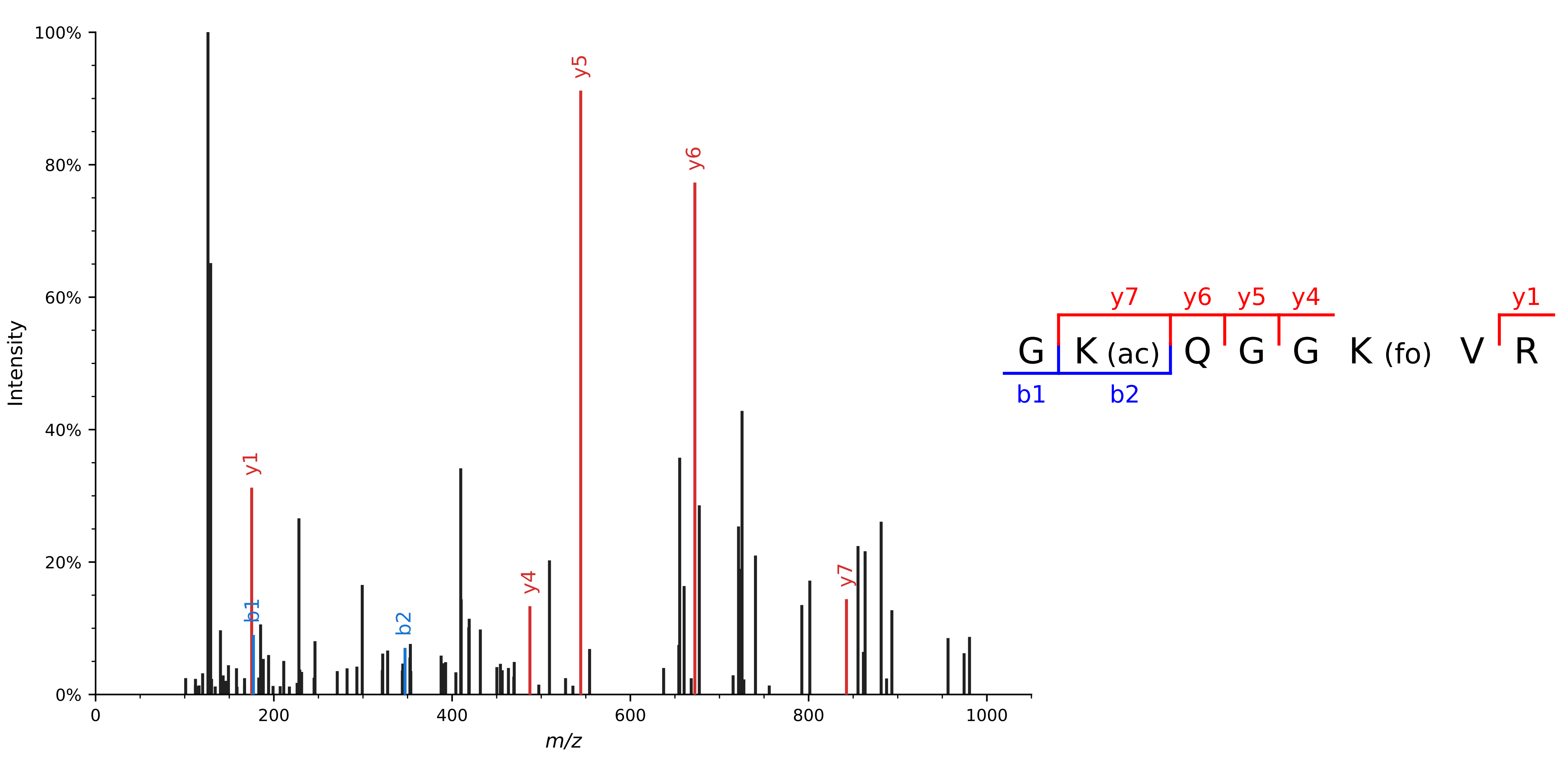

Raw file: QEP231009_CG_frozen_breast_Normal_24, Scan: 7529, m/z: 525.30013, Charge: 2, RT (min): 20.58, Score: 122.78

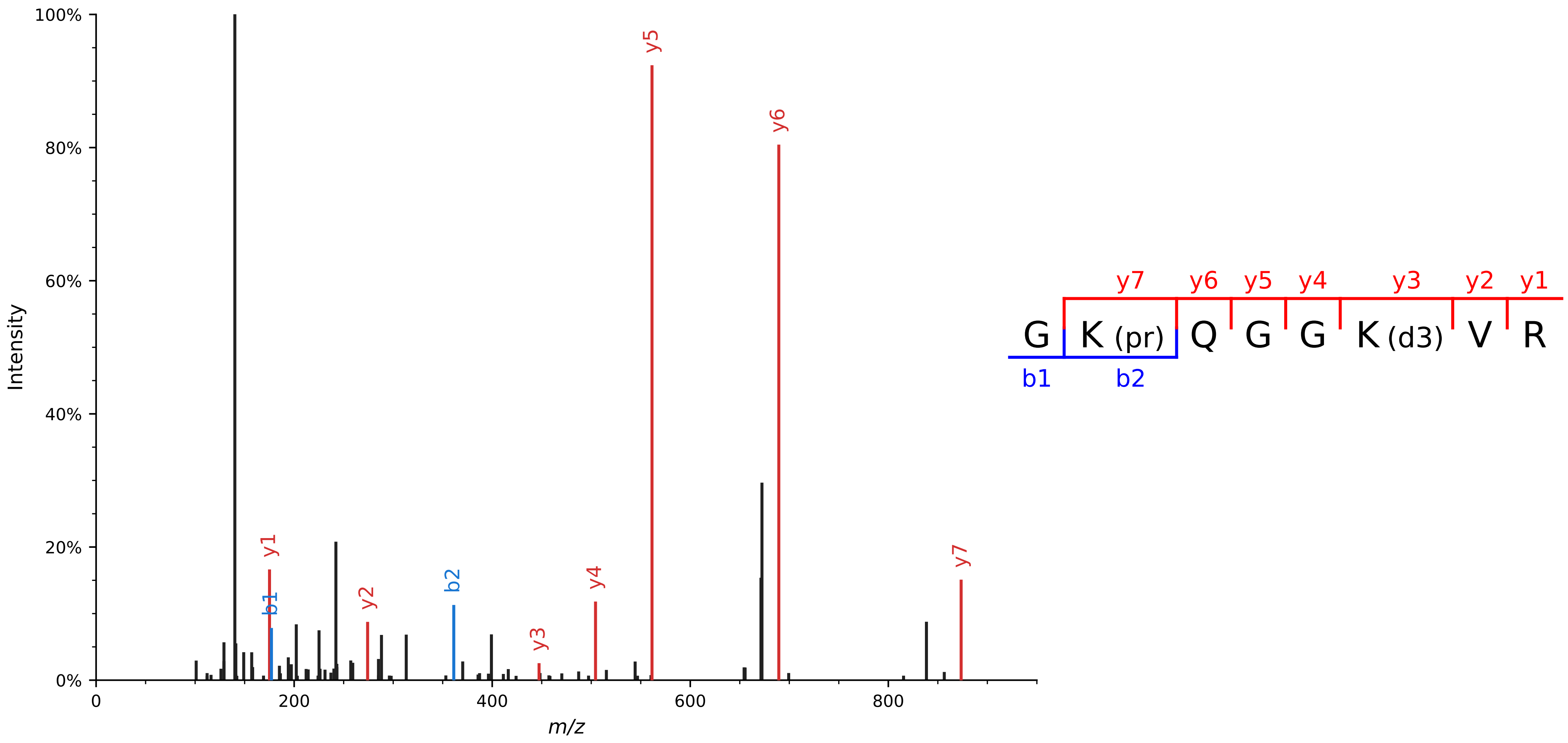

Raw file: QEP231009_CG_frozen_breast_LuA_33, Scan: 5836, m/z: 511.28448, Charge: 2, RT (min): 17.09, Score: 60.788

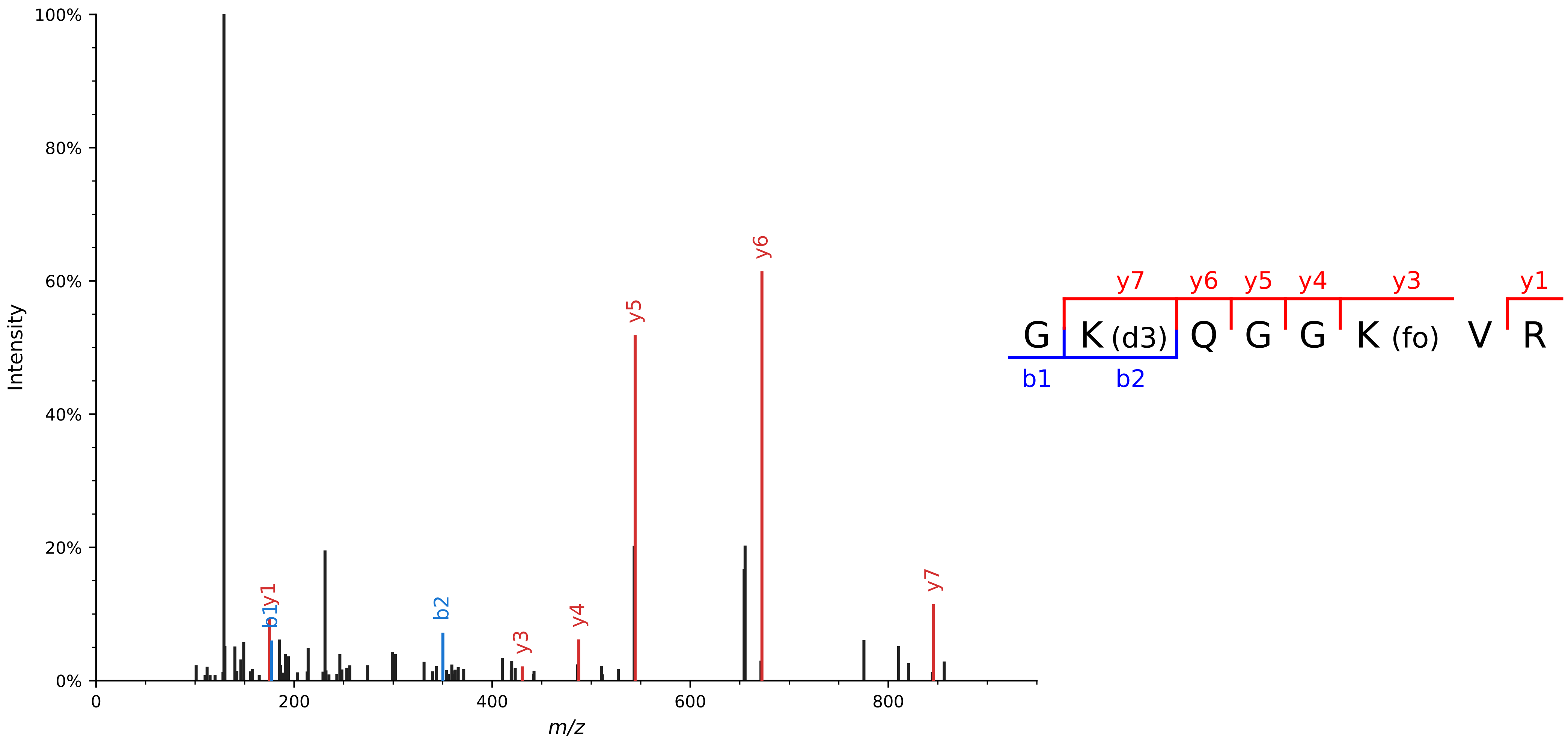

Raw file: QEP231009_CG_frozen_breast_Normal_21, Scan: 7529, m/z: 525.30013, Charge: 2, RT (min): 20.42, Score: 73.975

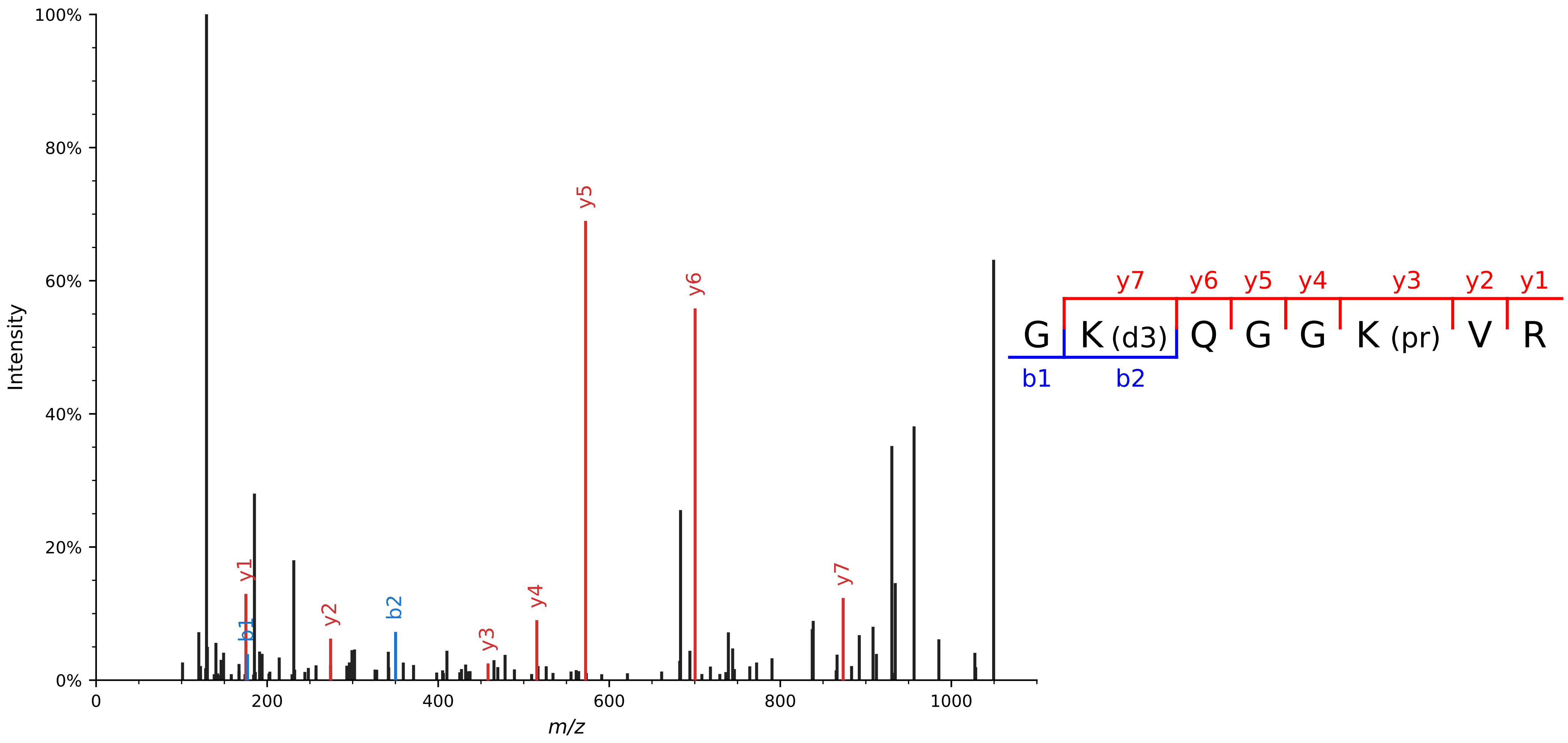

Raw file: QEP231009_CG_frozen_breast_Normal_21, Scan: 5034, m/z: 504.27666, Charge: 2, RT (min): 15.04, Score: 98.503

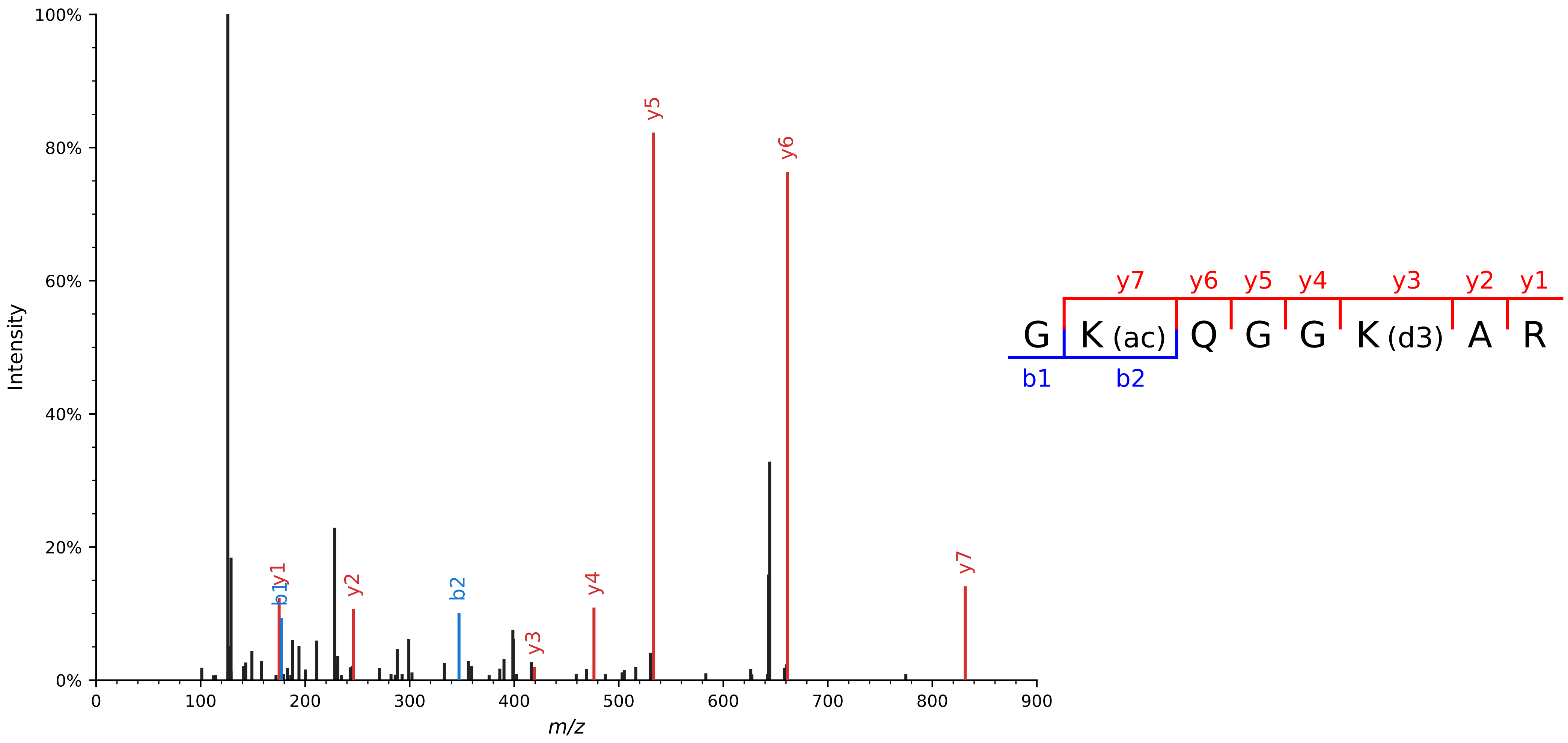

Raw file: QEP231009_CG_frozen_breast_LuA_31, Scan: 6019, m/z: 509.77507, Charge: 2, RT (min): 17.32, Score: 89.55

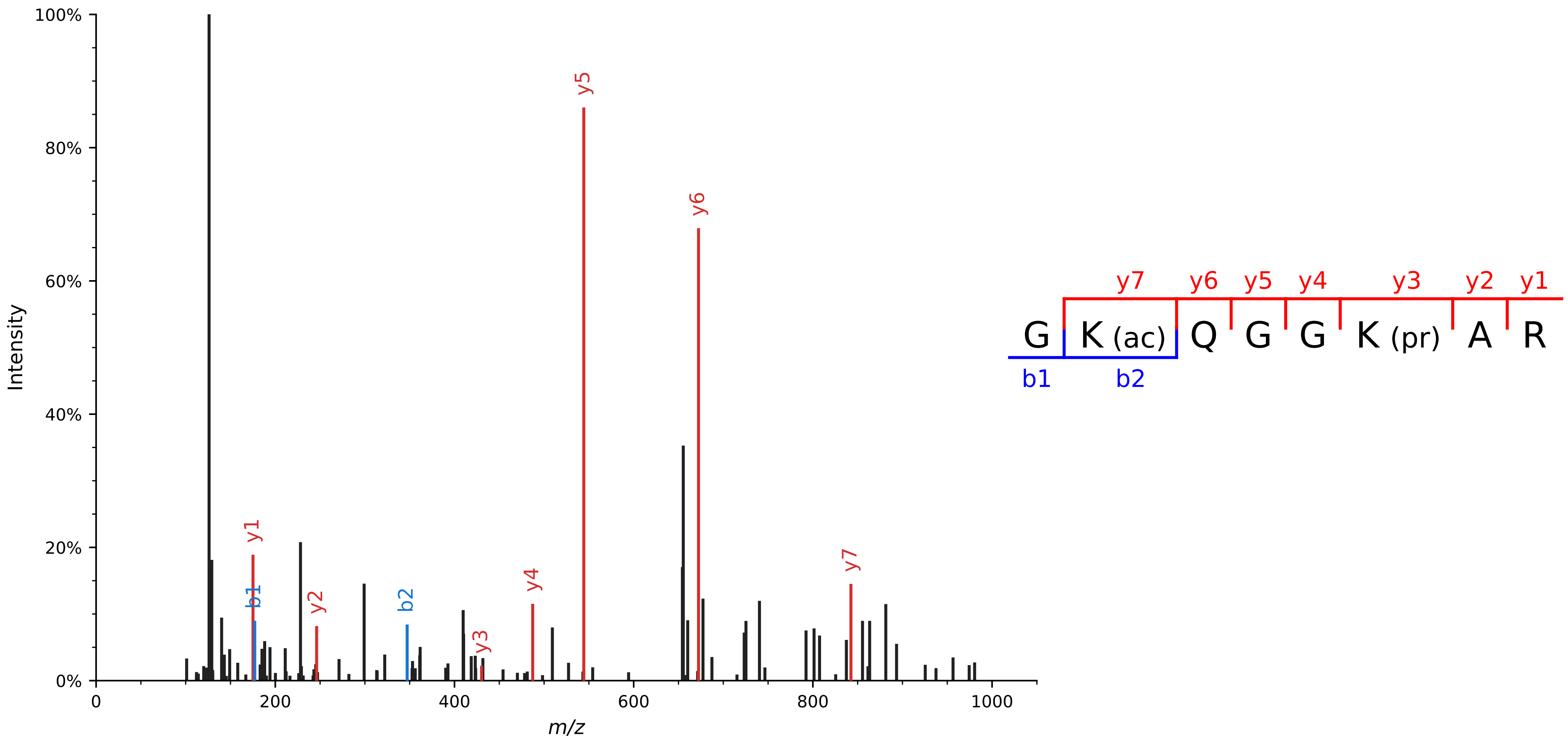

Raw file: QEP231009_CG_frozen_breast_Normal_21, Scan: 6230, m/z: 511.28448, Charge: 2, RT (min): 17.61, Score: 122.78

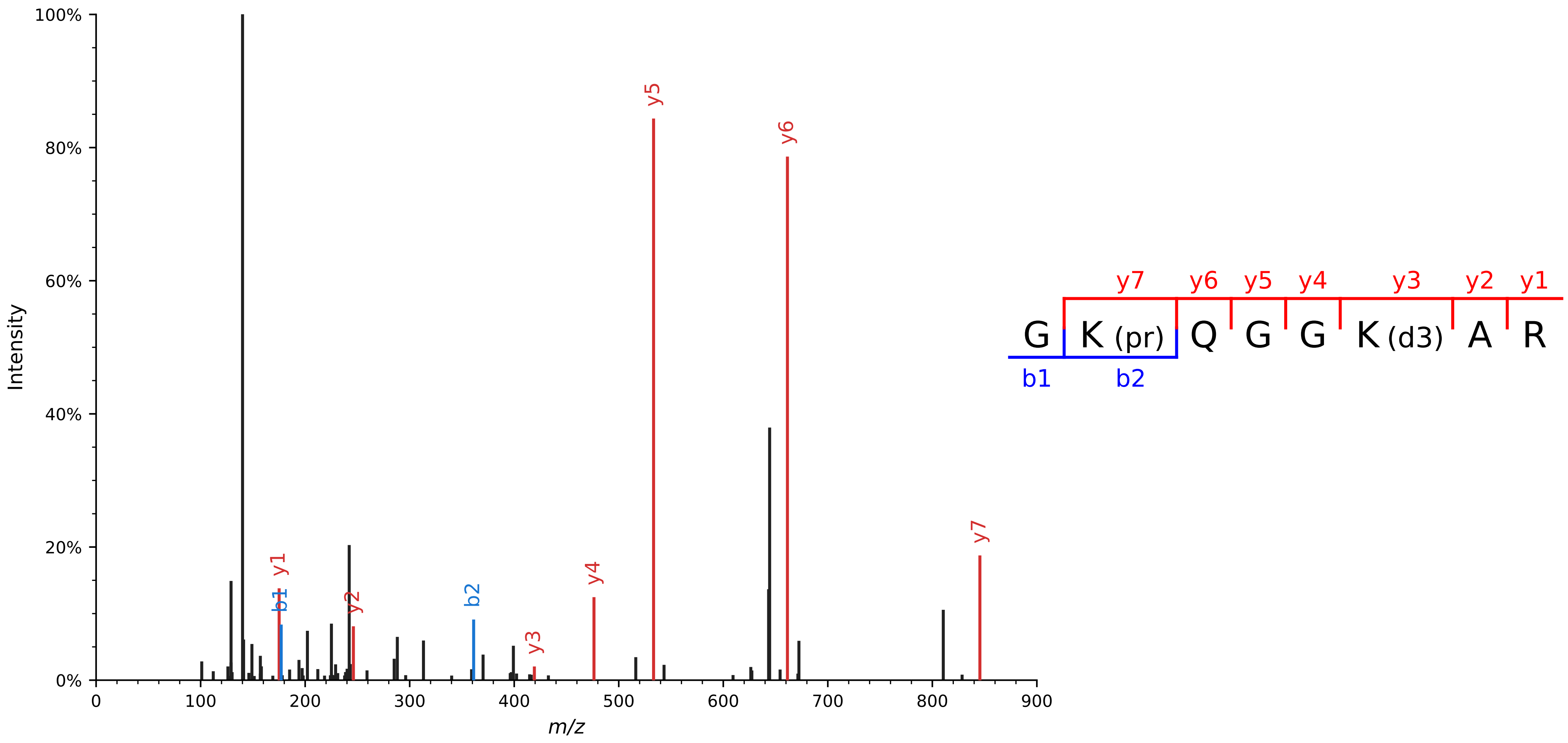

Raw file: QEP231009_CG_frozen_breast_TN_25, Scan: 7689, m/z: 516.78289, Charge: 2, RT (min): 20.15, Score: 81.297

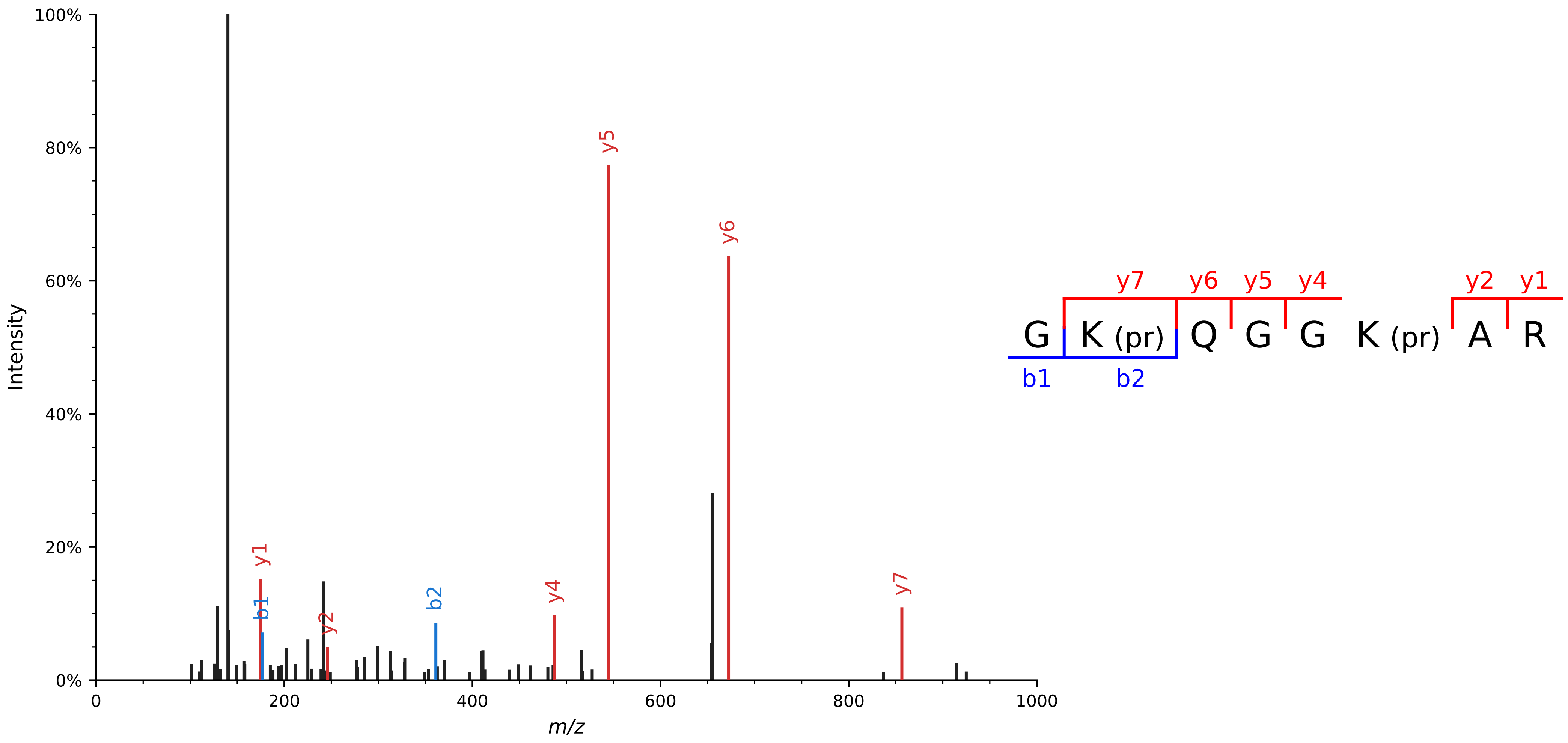

Raw file: QEP231009_CG_frozen_breast_Normal_21, Scan: 6059, m/z: 511.28448, Charge: 2, RT (min): 17.26, Score: 122.78

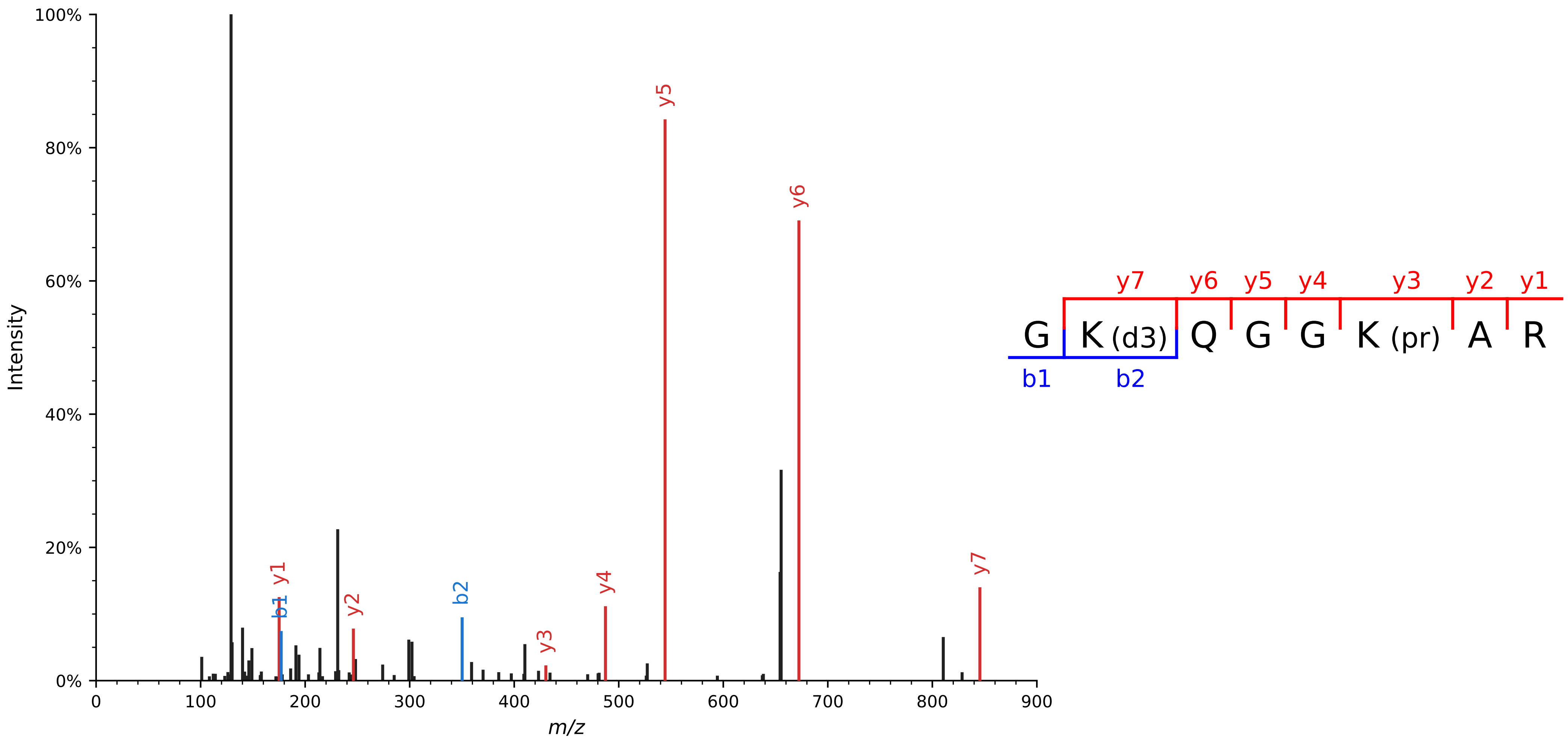

Raw file: QEP231009_CG_frozen_breast_TN_25, Scan: 7649, m/z: 433.74974, Charge: 2, RT (min): 20.07, Score: 52.683

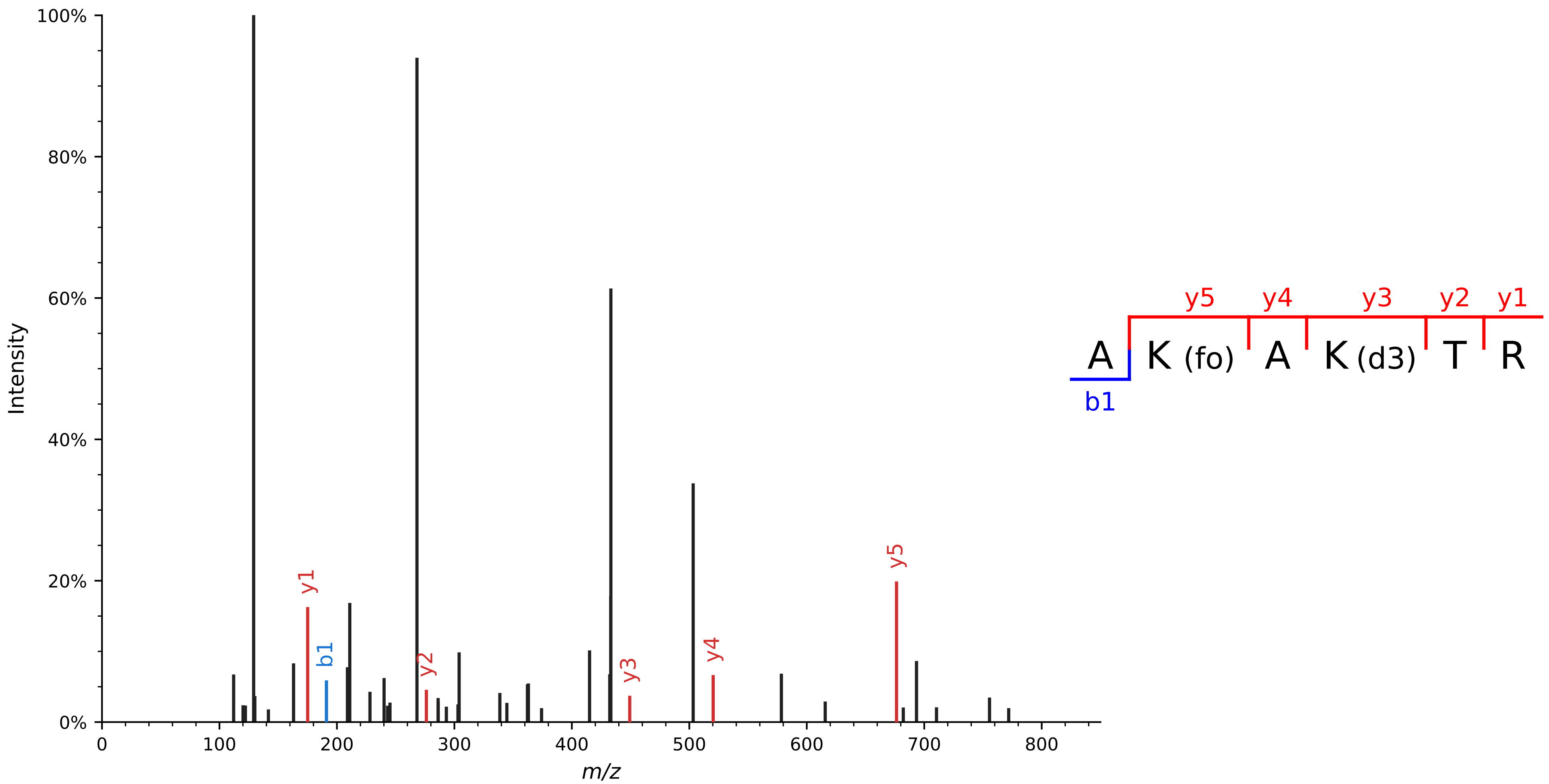

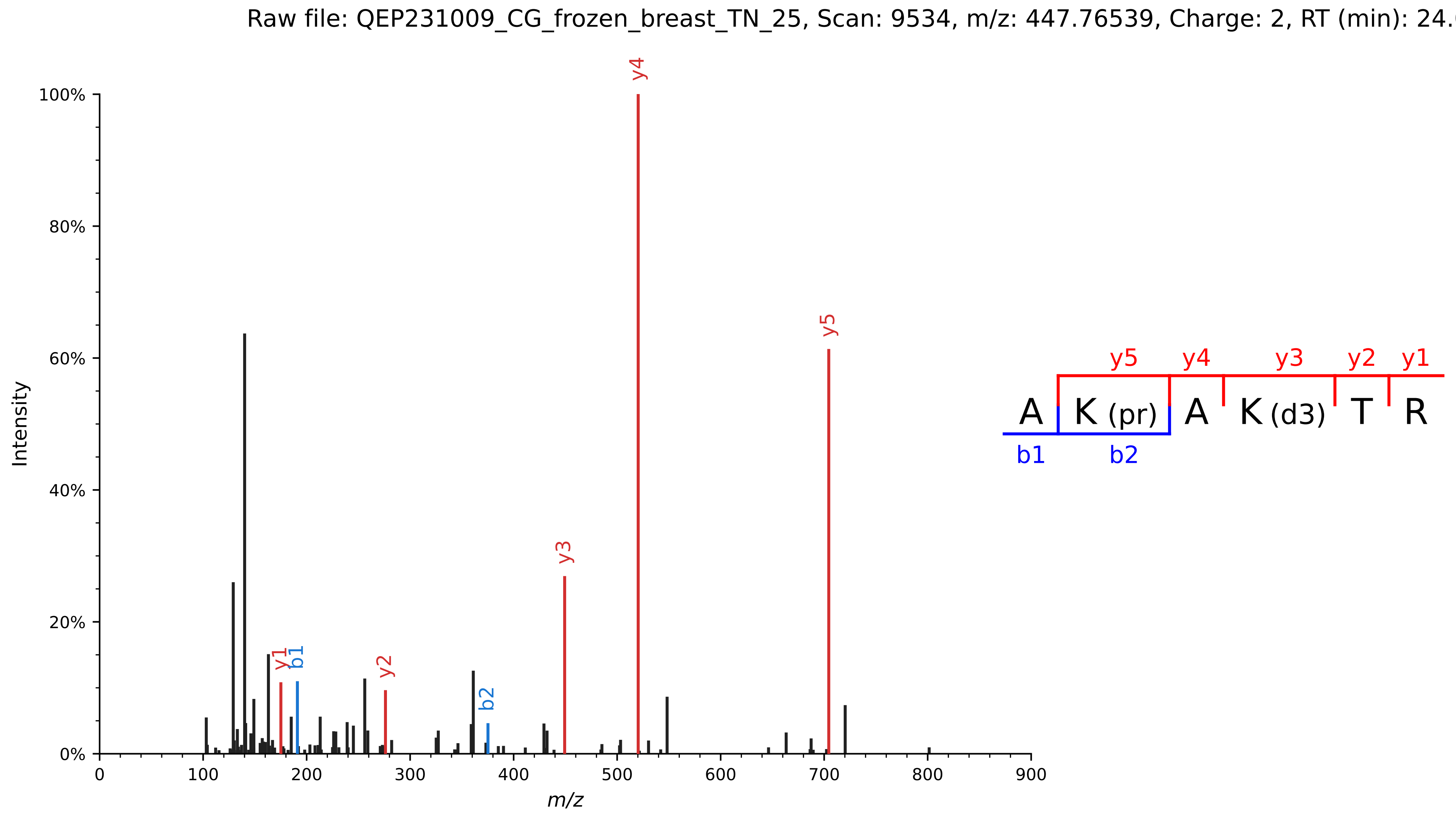

Raw file: QEP231009_CG_frozen_breast_TN_25, Scan: 9534, m/z: 447.76539, Charge: 2, RT (min): 24.02, Score: 105.27
Intensity
m/z
100%
80%
60%
40%
20%
0%
0
100
200
300
400
500
600
700
800
900
y4
y5
y3
y1
b1
y2
b2
y5 y4 y3 y2 y1
A K (pr) A K (d3) T R
b1 b2

Raw file: QEP231009_CG_frozen_breast_TN_25, Scan: 10993, m/z: 453.2638, Charge: 2, RT (min): 27.14, Score: 78.616

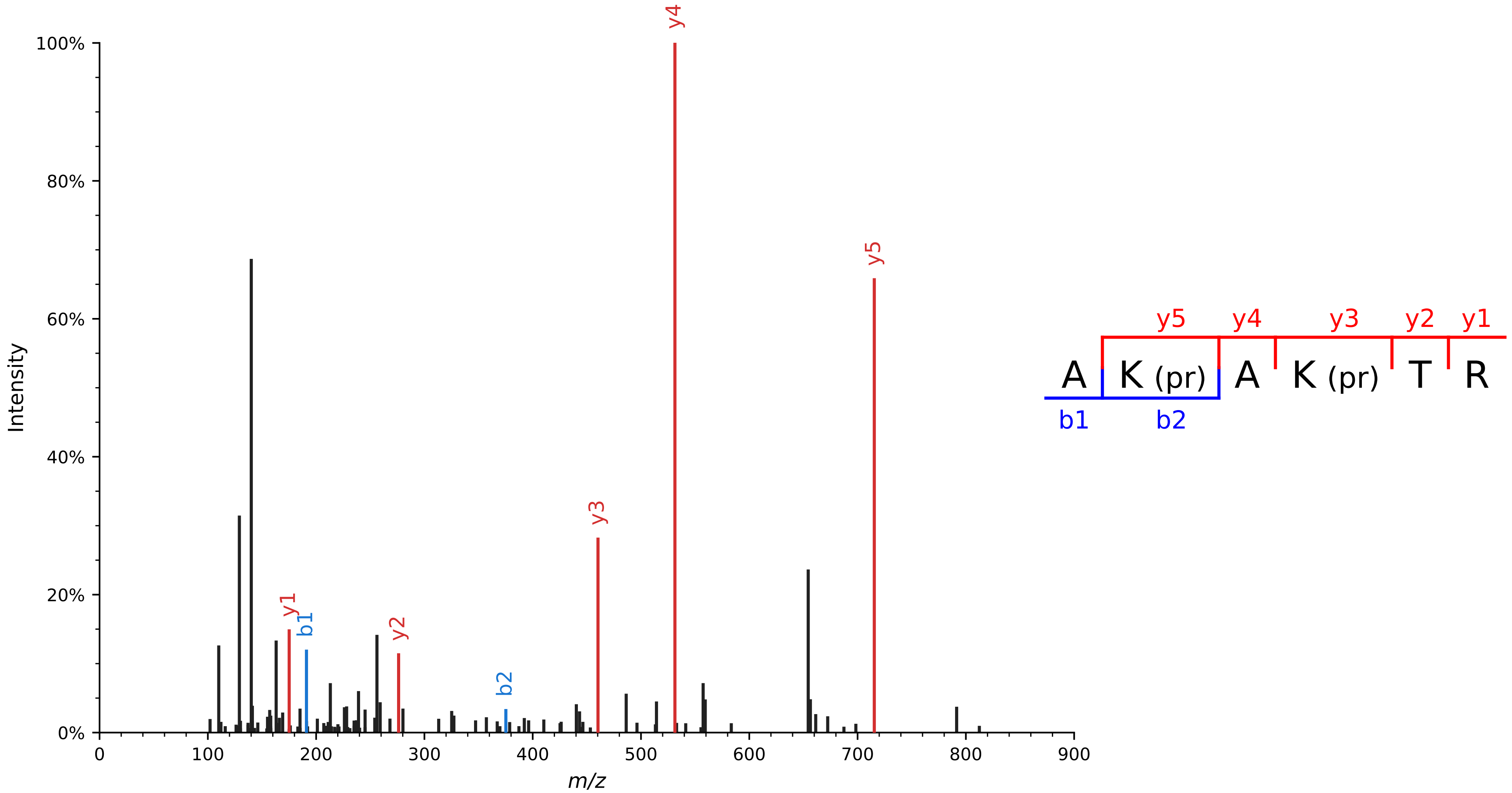

Raw file: QEP231009_CG_frozen_breast_TN_25, Scan: 9704, m/z: 447.76539, Charge: 2, RT (min): 24.37, Score: 94.163

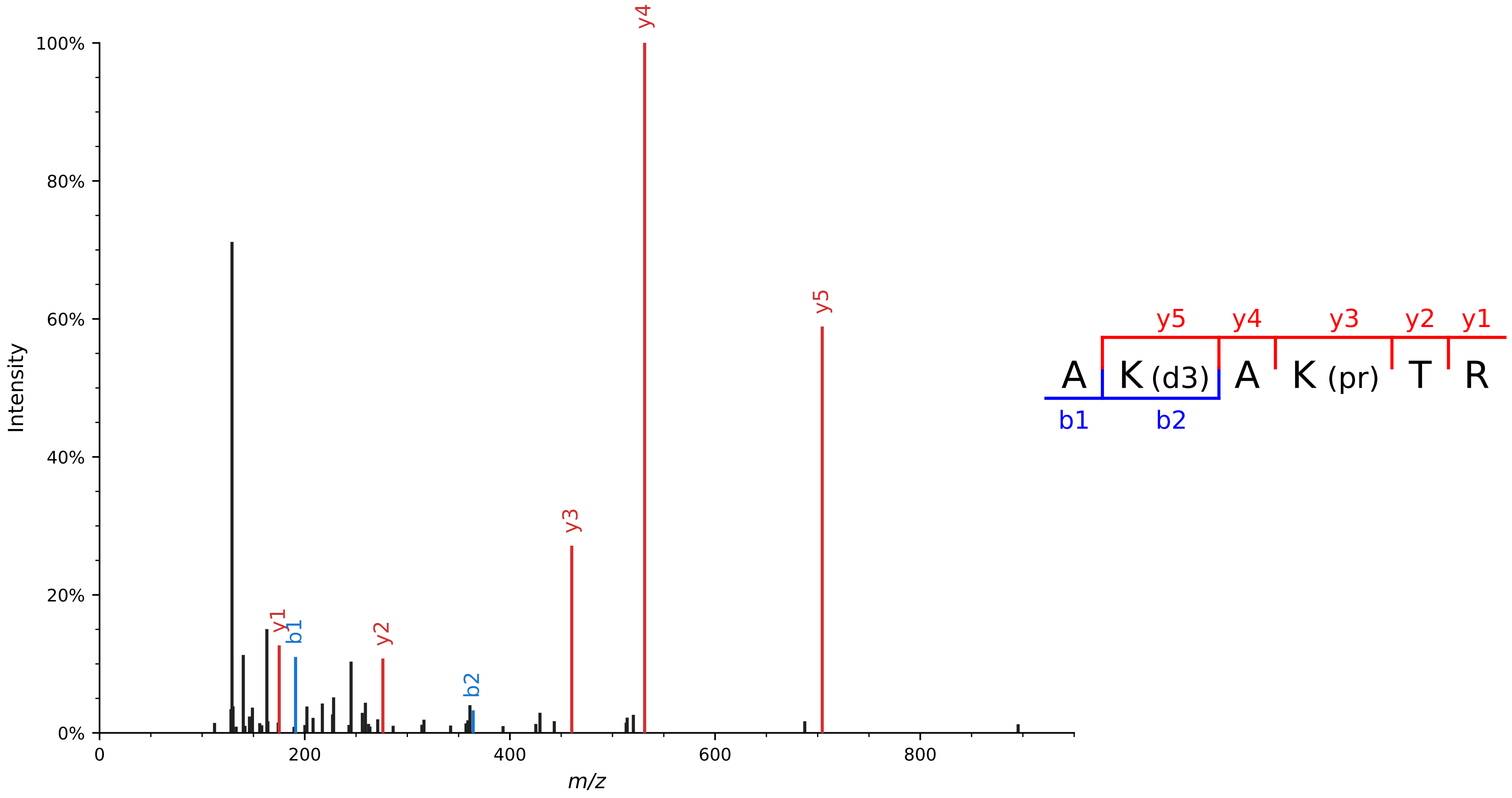

Raw file: QEP231009_CG_frozen_breast_TN_25, Scan: 9779, m/z: 506.74597, Charge: 2, RT (min): 24.55, Score: 114.6

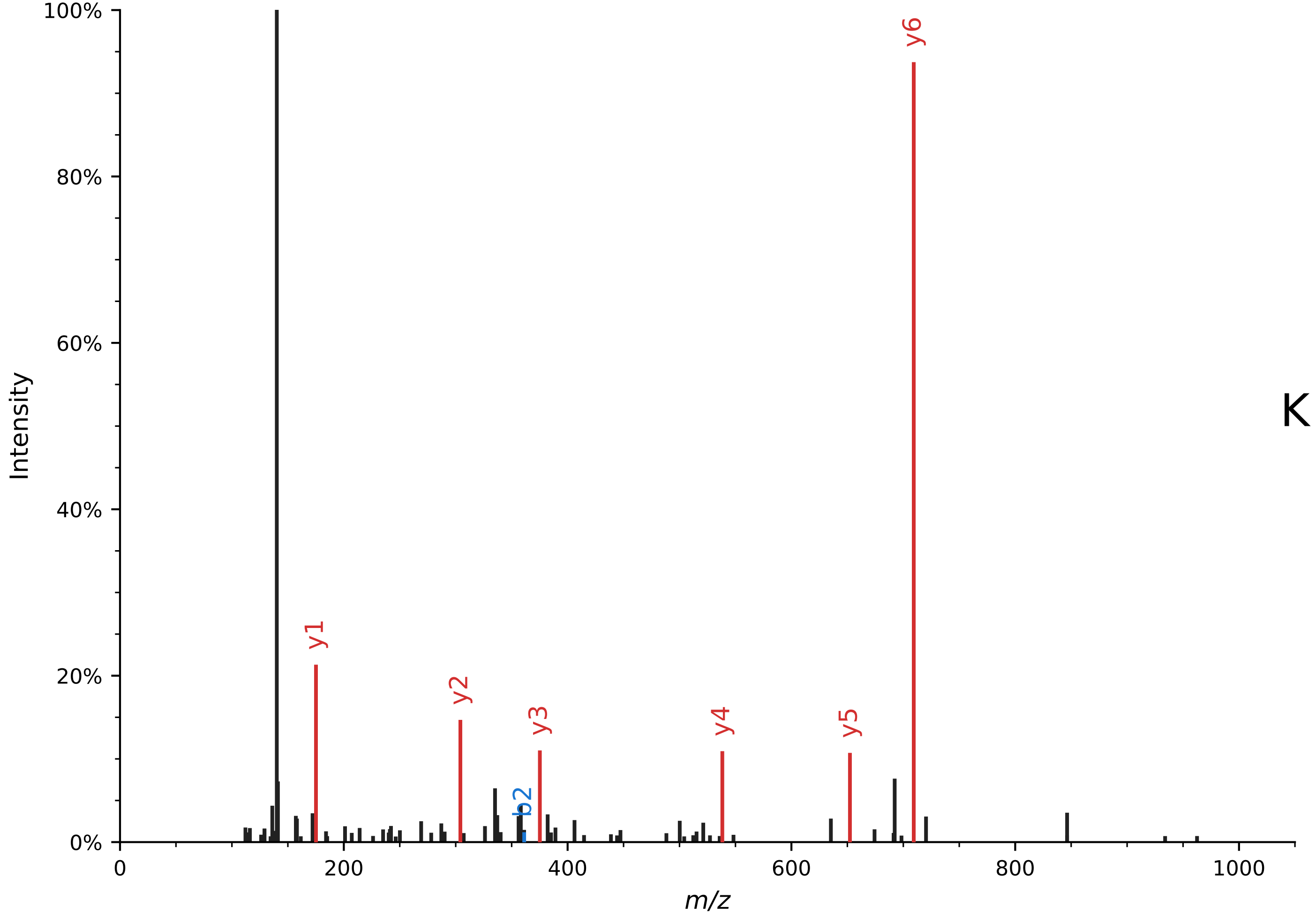

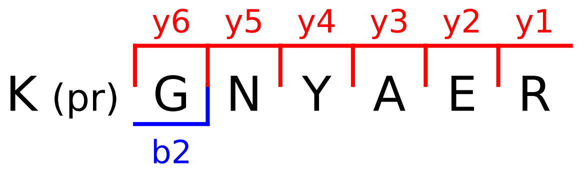

Raw file: QEP231009_CG_frozen_breast_Normal_19, Scan: 7560, m/z: 491.26321, Charge: 2, RT (min): 20.52, Score: 104.11

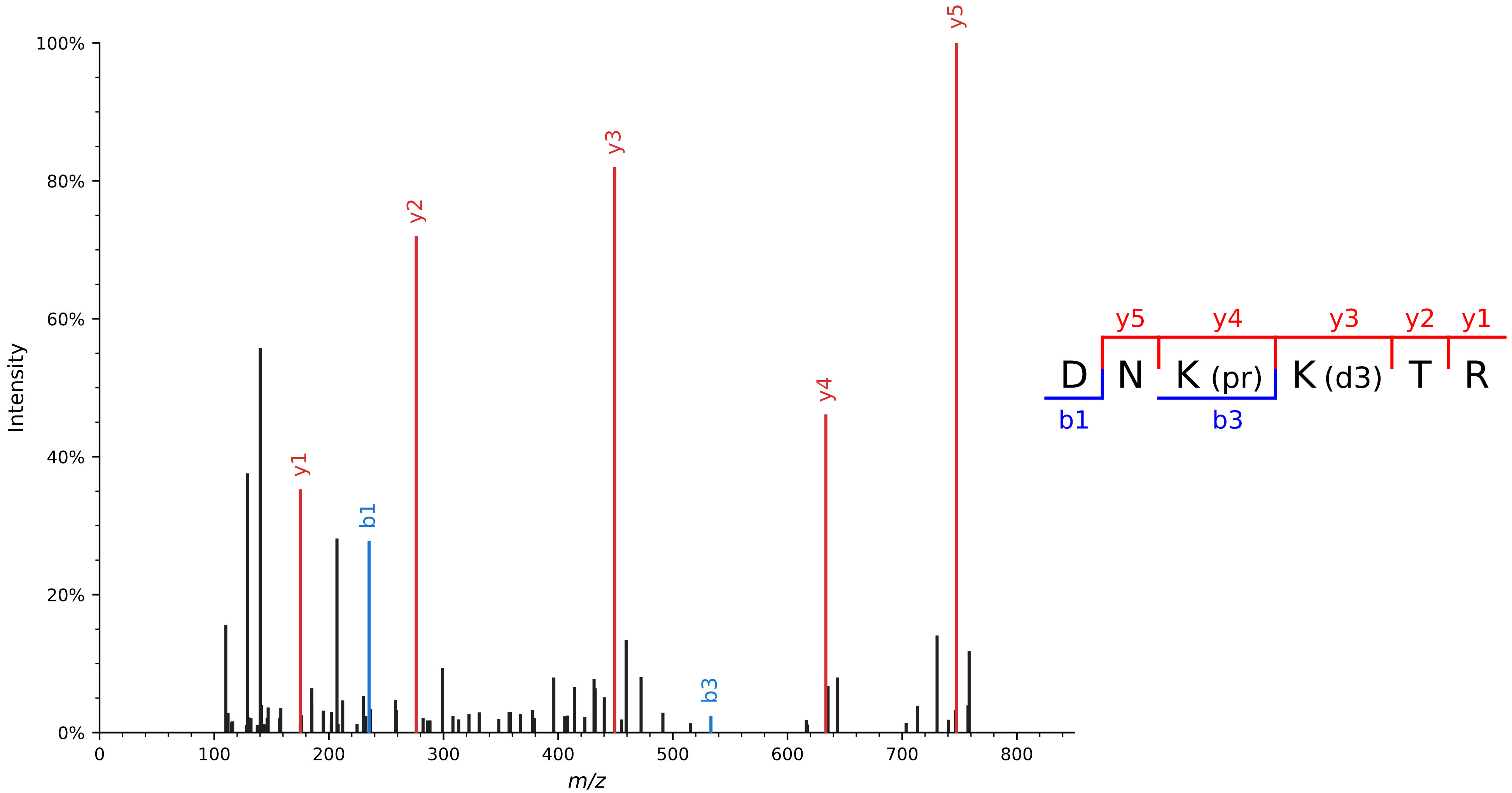

Raw file: QEP231009_CG_frozen_breast_TN_25, Scan: 9248, m/z: 496.76162, Charge: 2, RT (min): 23.41, Score: 92.467

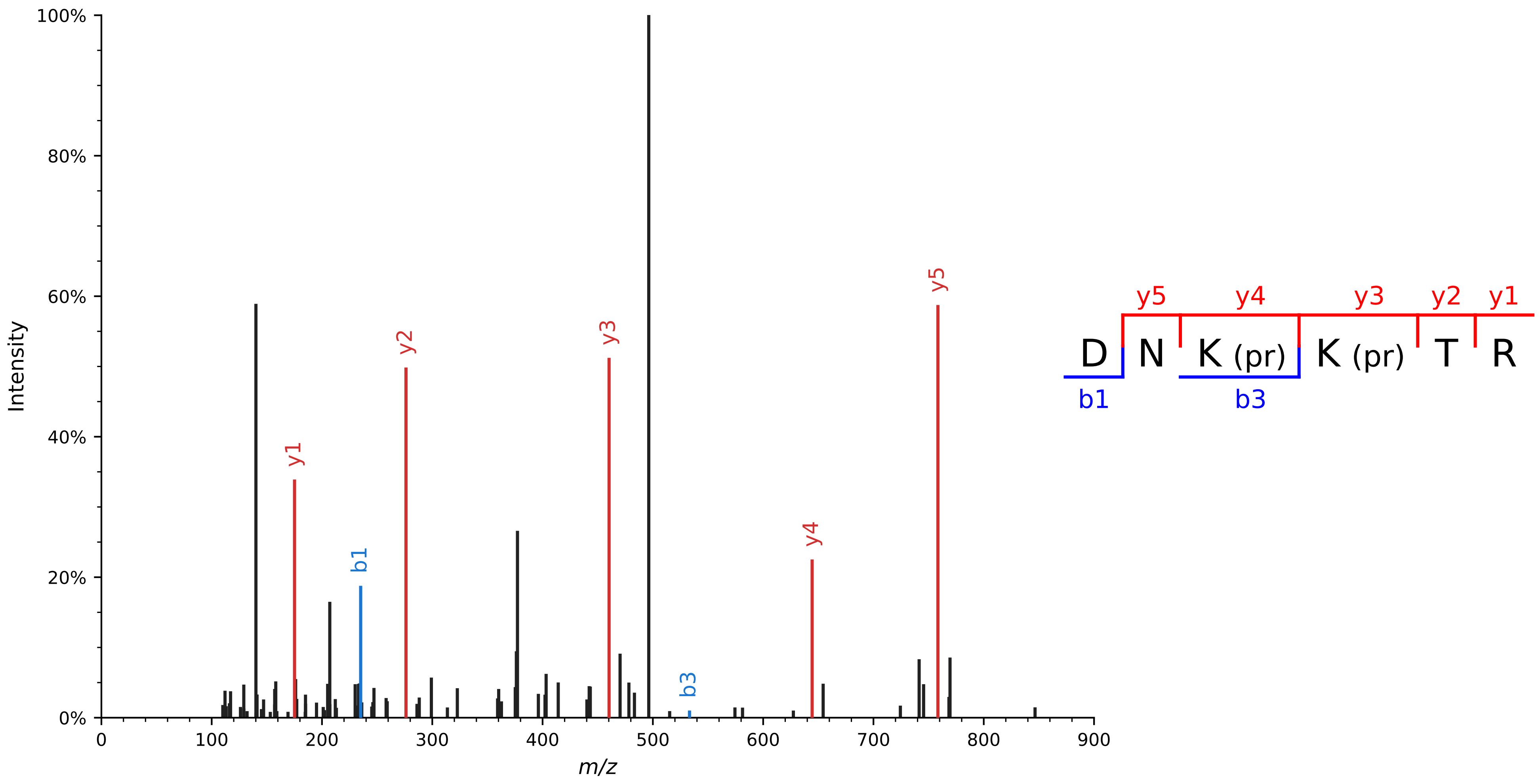

Raw file: QEP231009_CG_frozen_breast_TN_27, Scan: 7365, m/z: 491.26321, Charge: 2, RT (min): 20.22, Score: 108.98

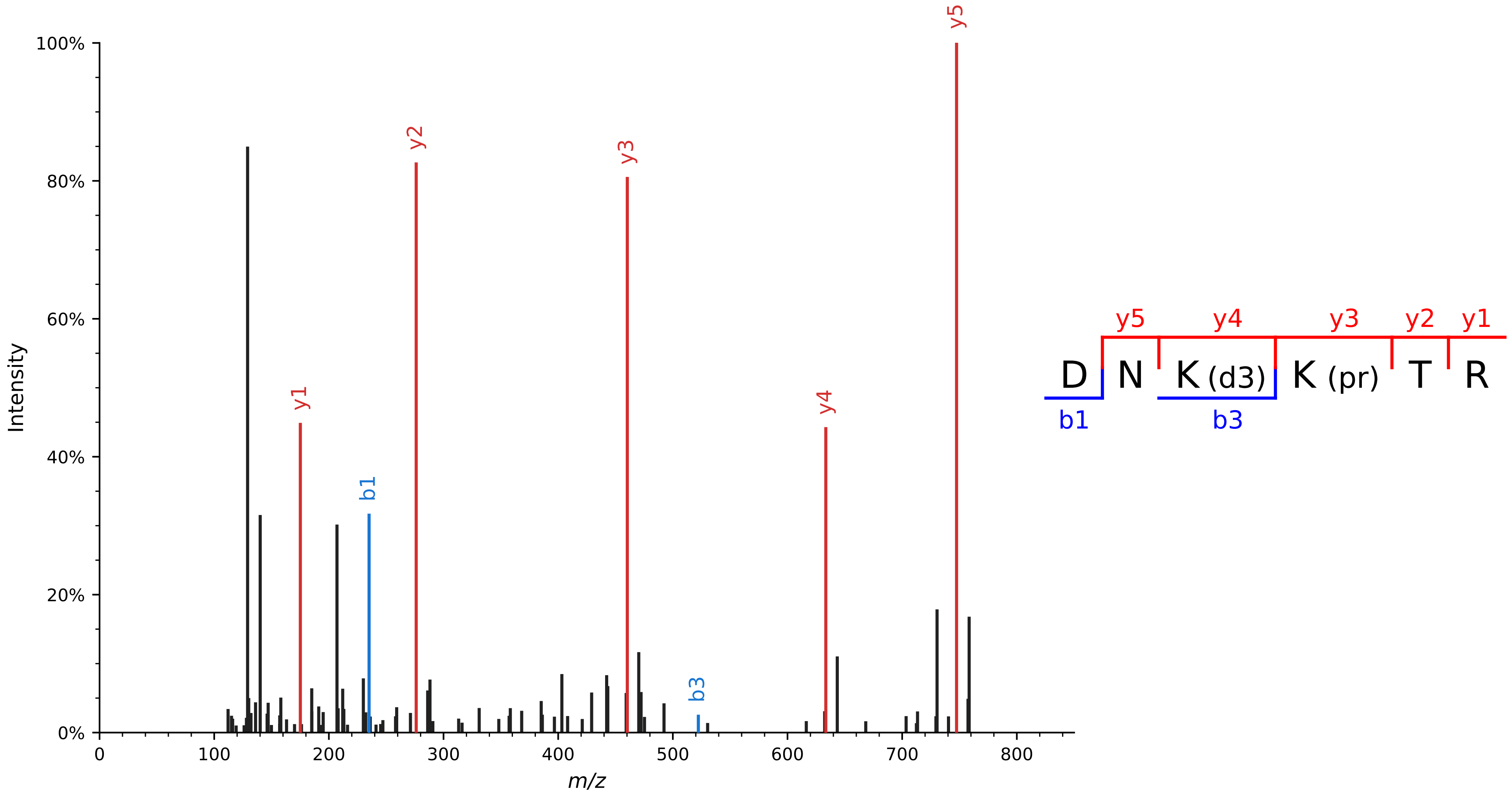

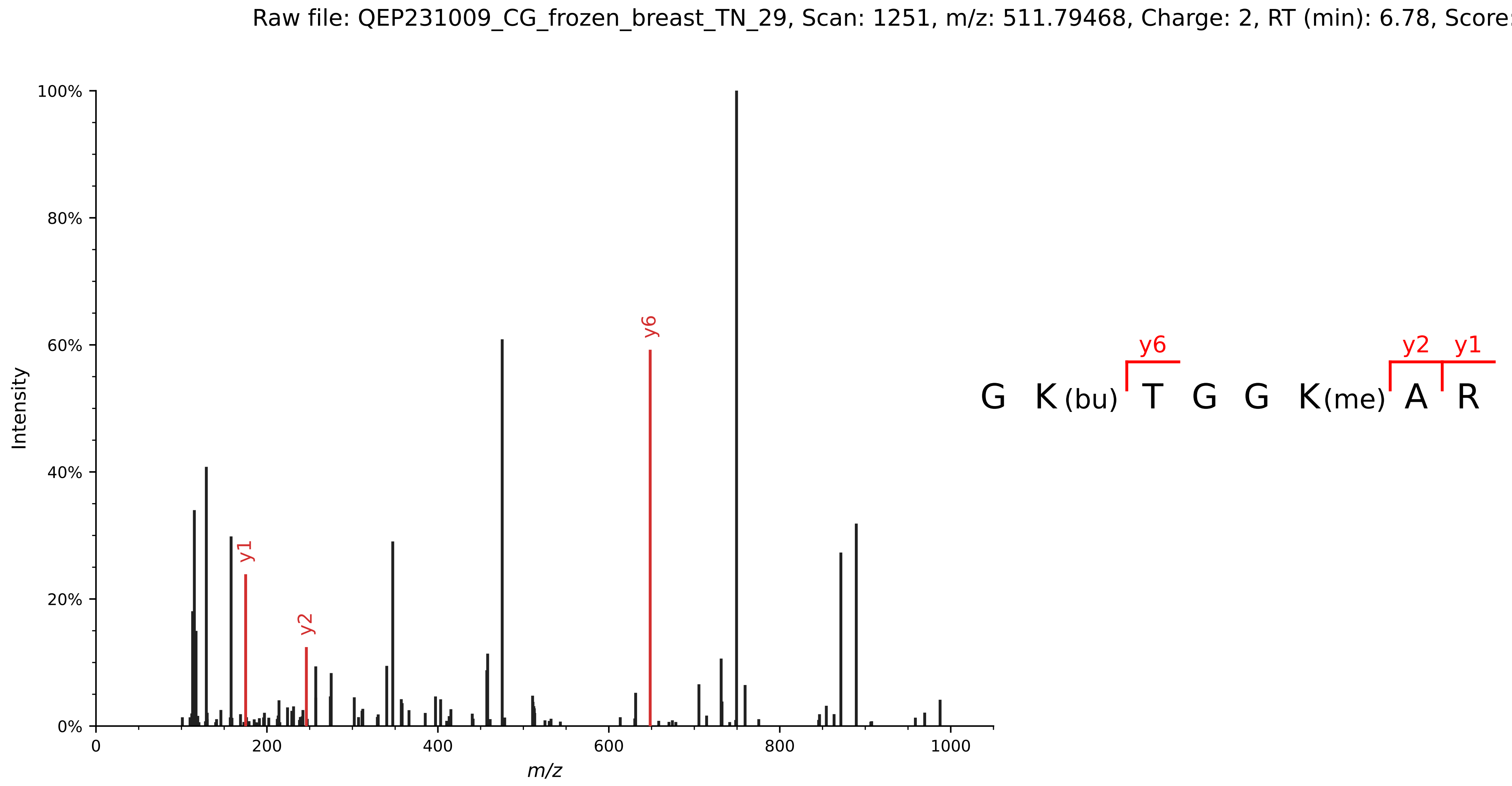

Raw file: QEP231009_CG_frozen_breast_TN_29, Scan: 1251, m/z: 511.79468, Charge: 2, RT (min): 6.78, Score: 46.37
Intensity
m/z
y1
y2
y6
G K(bu) T G G K(me) A R

Raw file: QEP231009_CG_frozen_breast_LuA_33, Scan: 14387, m/z: 603.85527, Charge: 2, RT (min): 37.66, Score: 73.931

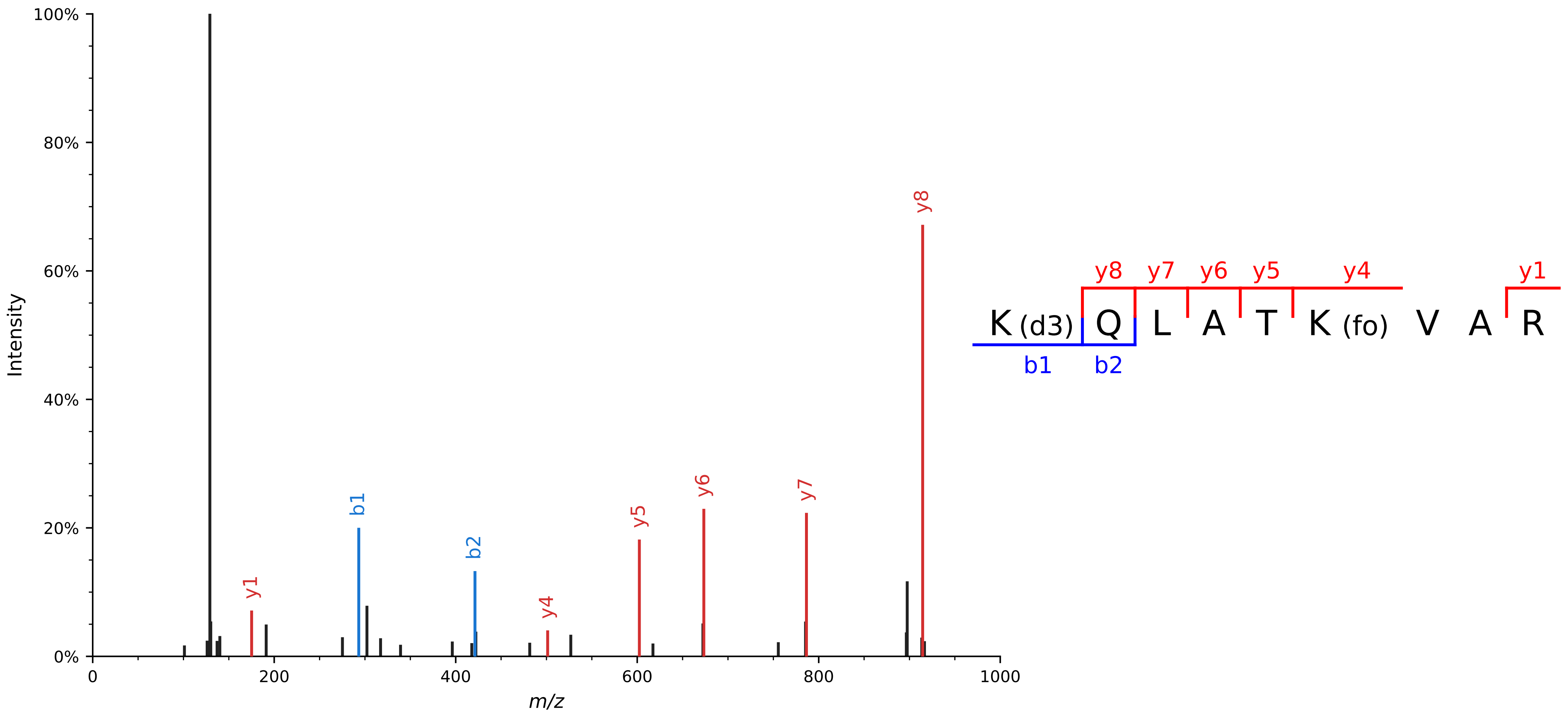

Raw file: QEP231009_CG_frozen_breast_Normal_19, Scan: 8270, m/z: 859.48835, Charge: 2, RT (min): 22.07, Score: 121.9

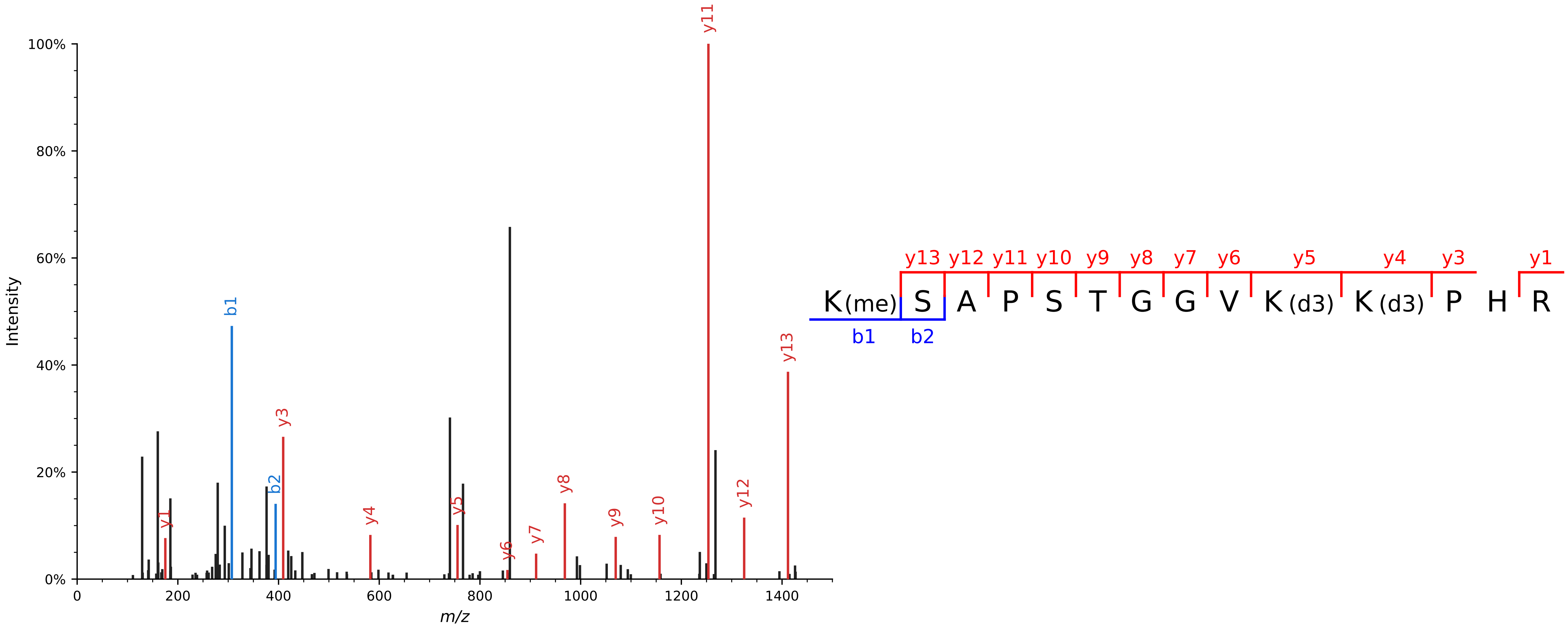

Raw file: QEP231009_CG_frozen_breast_TN_28, Scan: 8991, m/z: 866.49617, Charge: 2, RT (min): 23.39, Score: 141.95

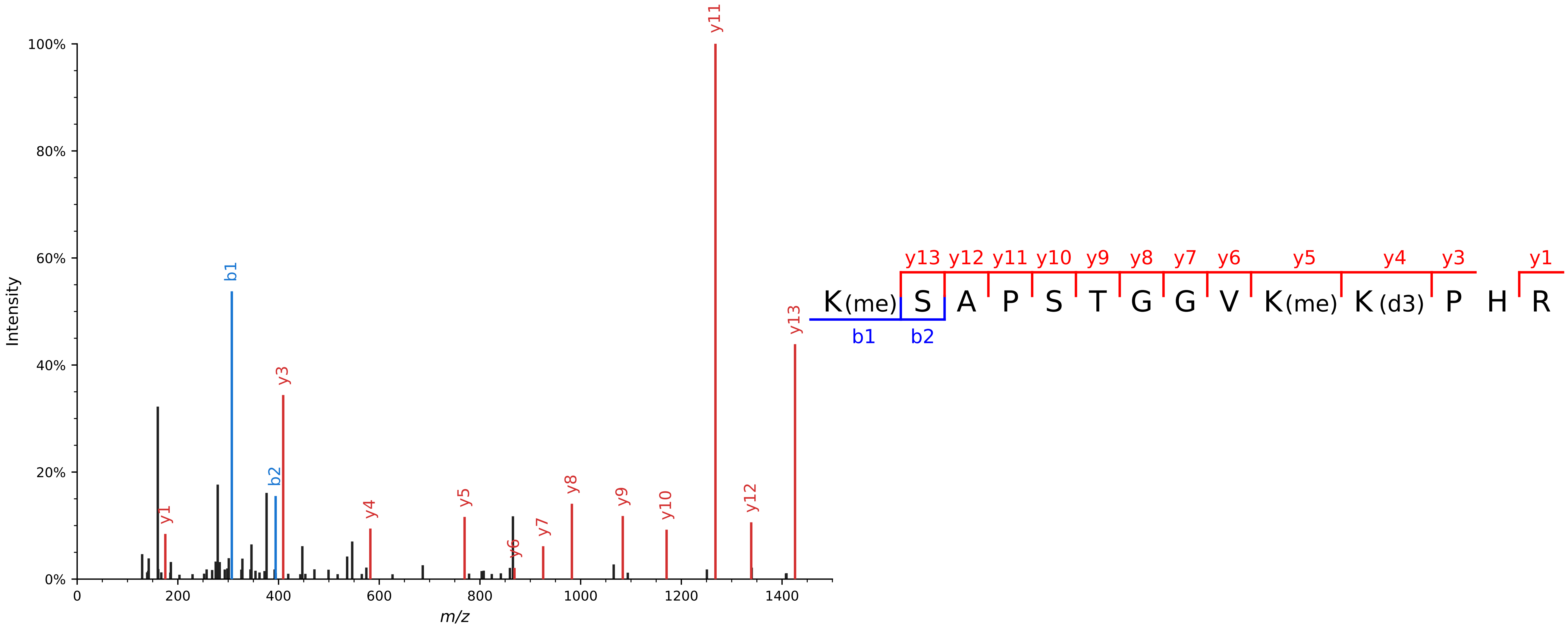

Raw file: QEP231009_CG_frozen_breast_TN_25, Scan: 6243, m/z: 582.67176, Charge: 3, RT (min): 17.18, Score: 45.115

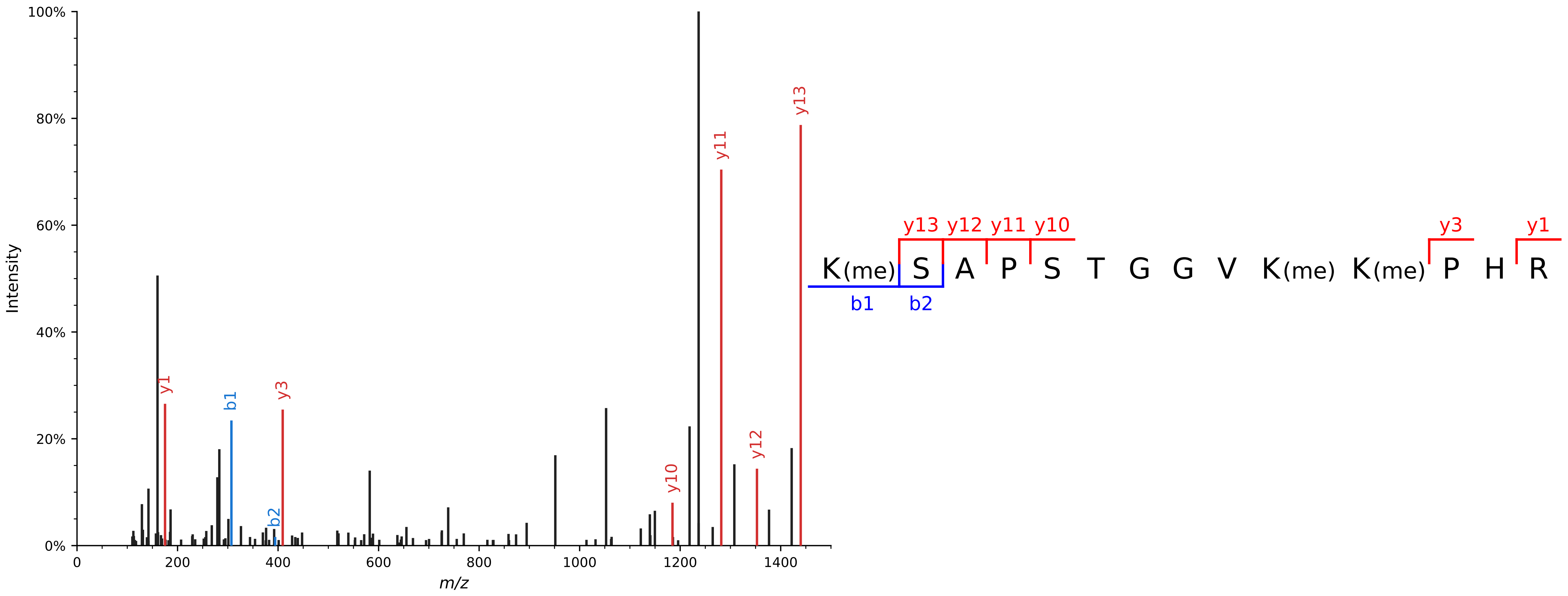

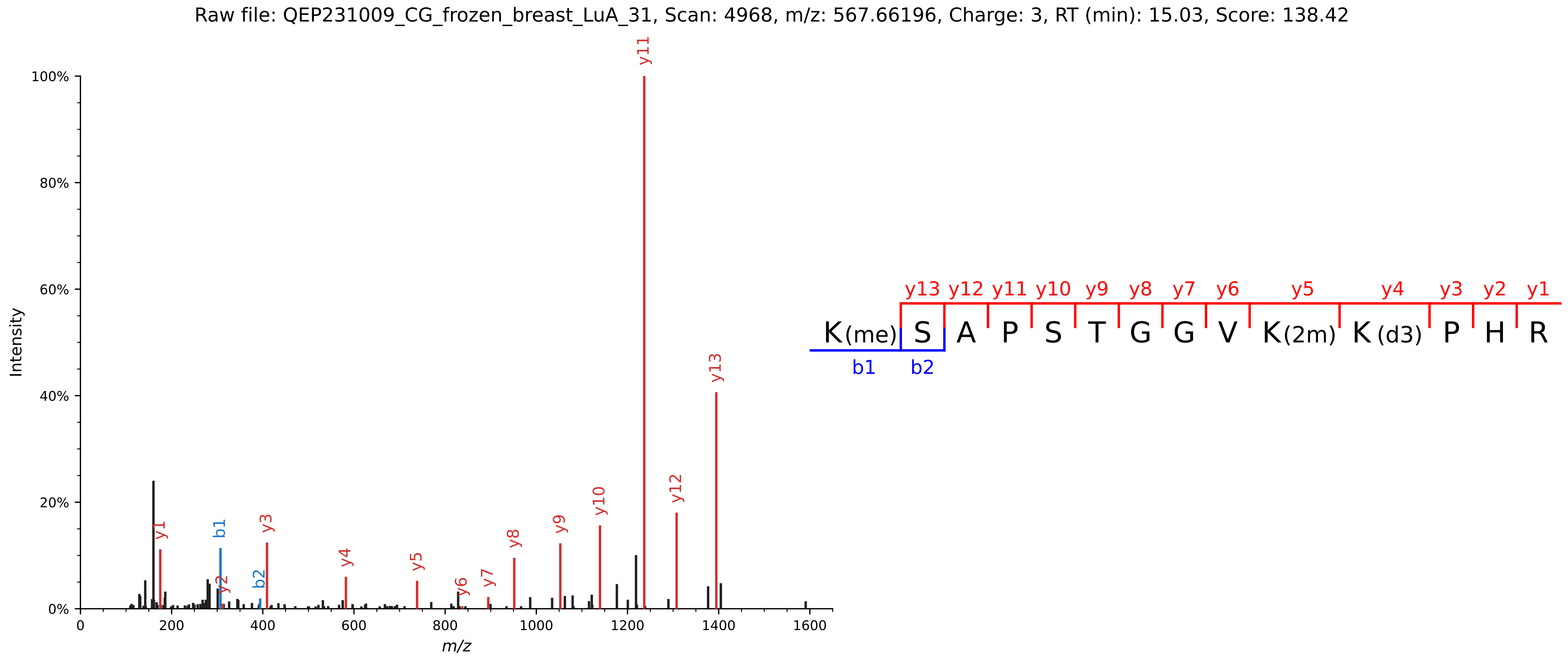
Raw file: QEP231009_CG_frozen_breast_LuA_31, Scan: 4968, m/z: 567.66196, Charge: 3, RT (min): 15.03, Score: 138.42
Intensity
m/z
K(me) S A P S T G G V K(2m) K(d3) P H R
y13 y12 y11 y10 y9 y8 y7 y6 y5 y4 y3 y2 y1
b1 b2

Raw file: QEP231009_CG_frozen_breast_TN_25, Scan: 5973, m/z: 571.32757, Charge: 3, RT (min): 16.64, Score: 69.92

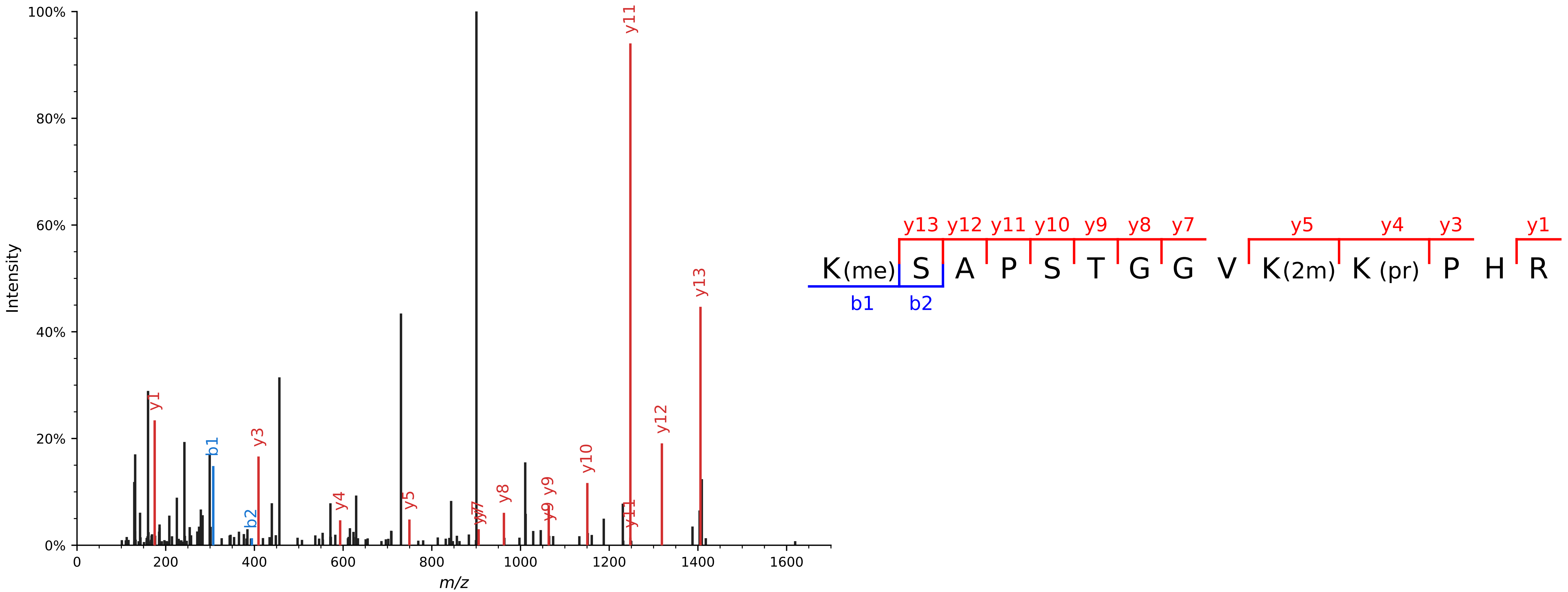

Raw file: QEP231009_CG_frozen_breast_TN_25, Scan: 5281, m/z: 572.33384, Charge: 3, RT (min): 15.14, Score: 99.844

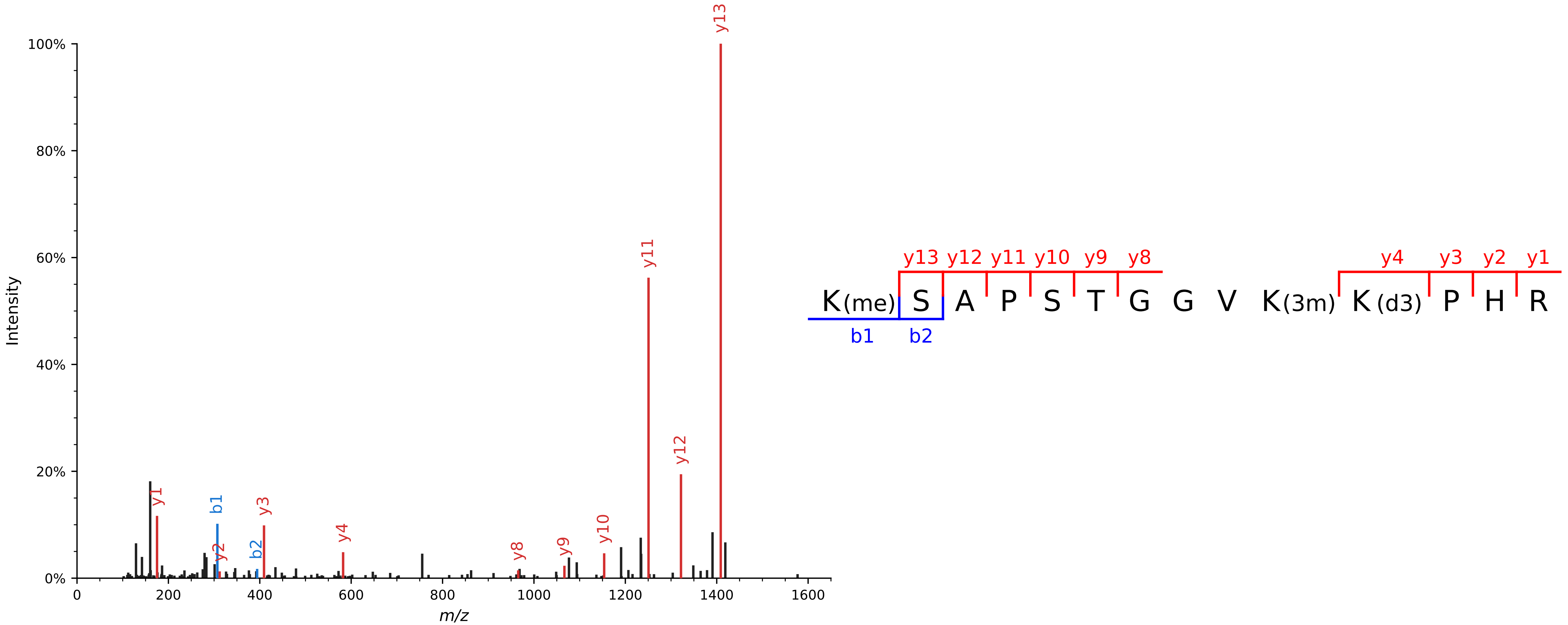

Raw file: QEP231009_CG_frozen_breast_TN_29, Scan: 3775, m/z: 843.98148, Charge: 2, RT (min): 12.20, Score: 153.54

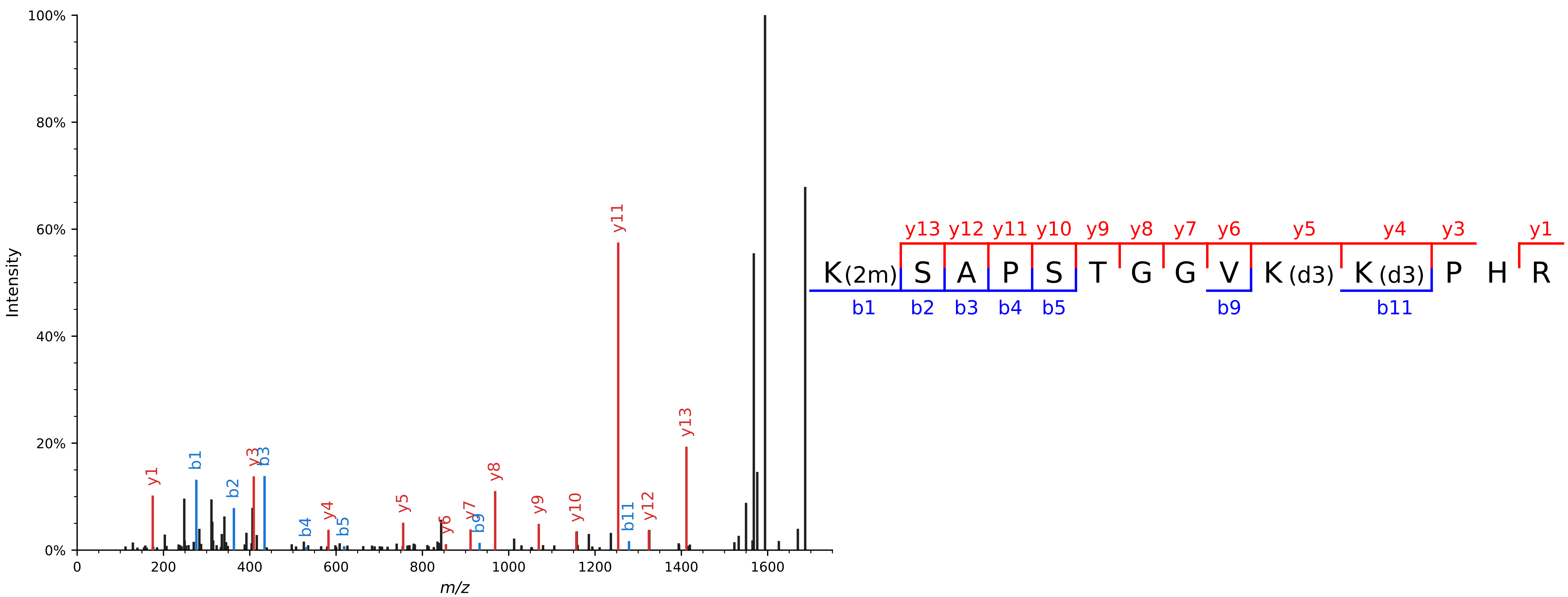

Raw file: QEP231009_CG_frozen_breast_Normal_23, Scan: 4028, m/z: 567.66196, Charge: 3, RT (min): 12.99, Score: 150.9

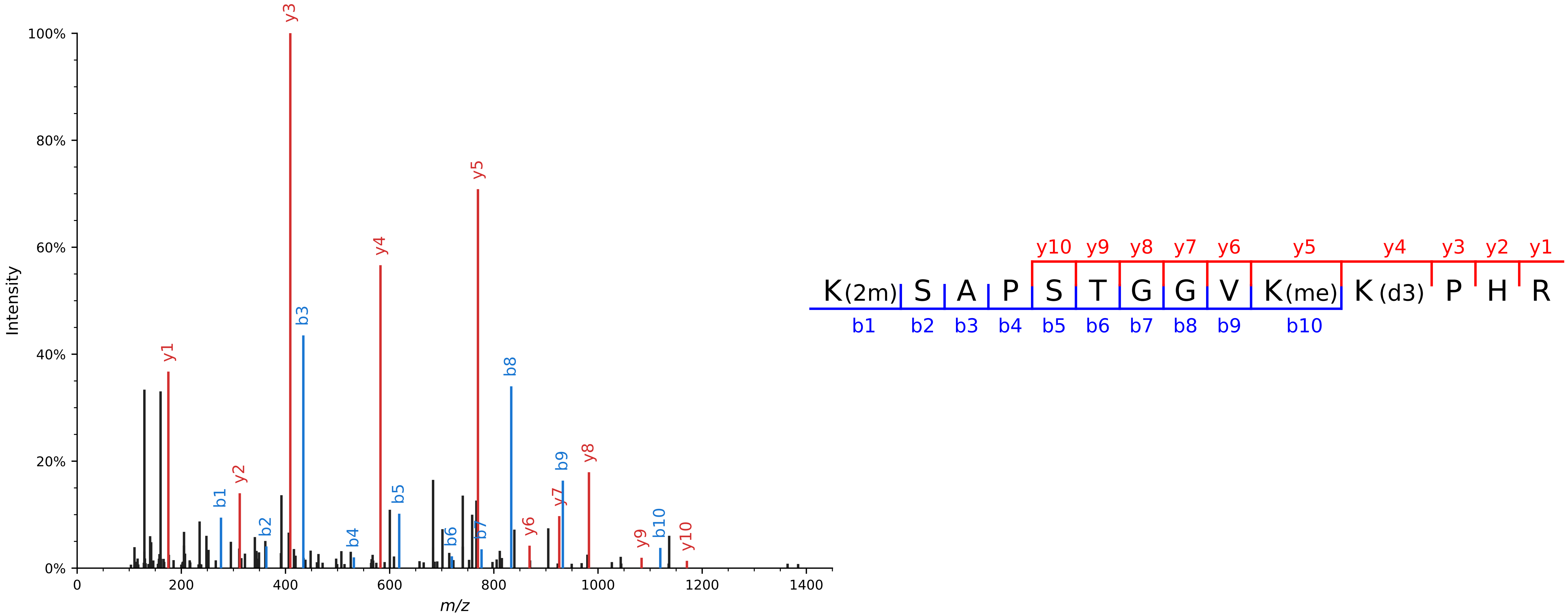

Raw file: QEP231009_CG_frozen_breast_TN_25, Scan: 3164, m/z: 572.33384, Charge: 3, RT (min): 10.78, Score: 45.863

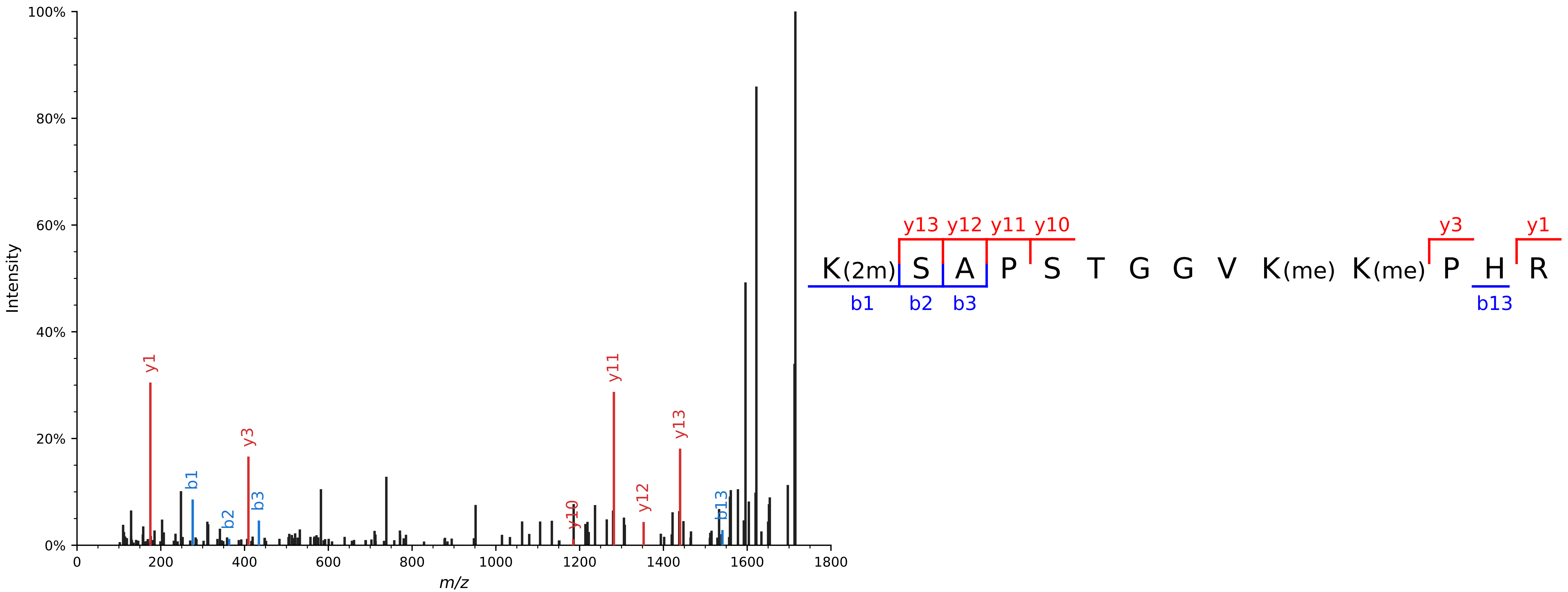

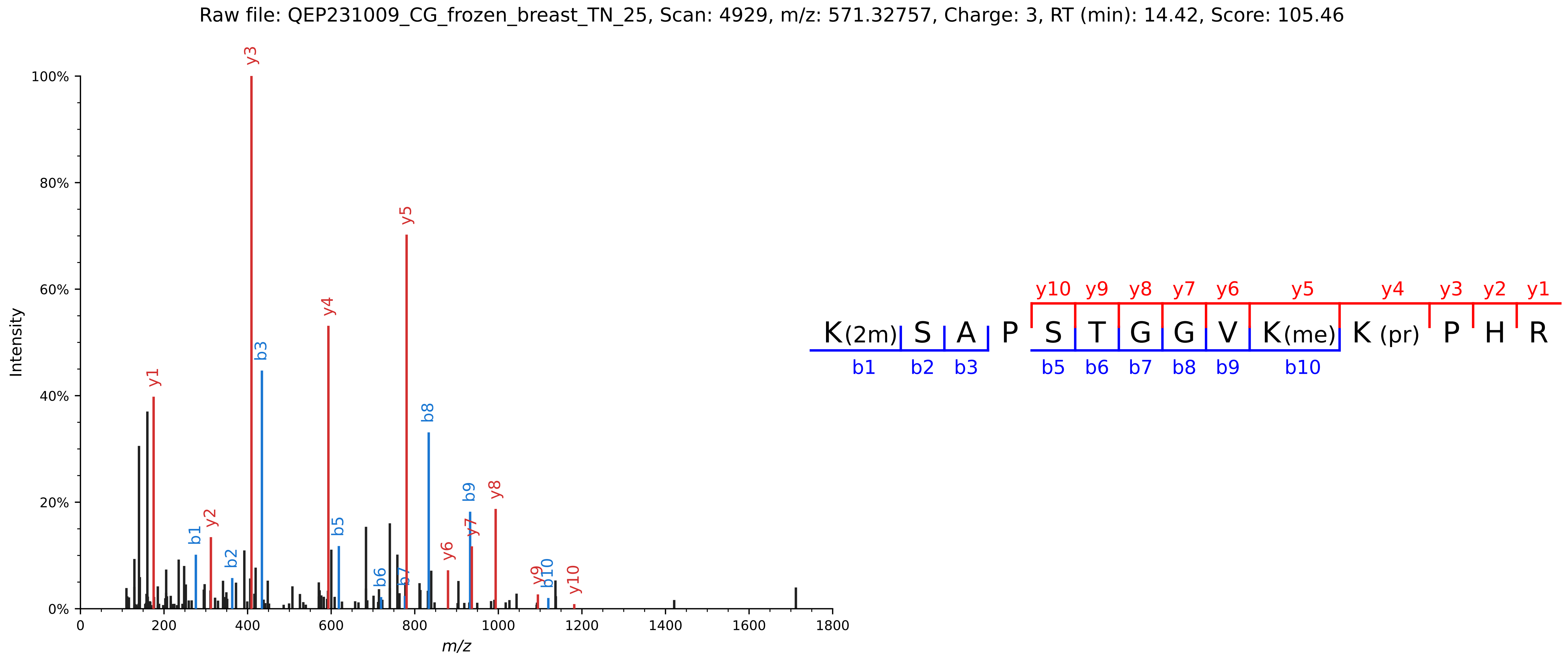

Raw file: QEP231009_CG_frozen_breast_TN_25, Scan: 4929, m/z: 571.32757, Charge: 3, RT (min): 14.42, Score: 105.46
Intensity
m/z
100%
80%
60%
40%
20%
0%
0
200
400
600
800
1000
1200
1400
1600
1800
y1 y2 y3 y4 y5 y6 y7 y8 y9 y10
b1 b2 b3 b5 b6 b7 b8 b9 b10
K(2m) S A P S T G G V K(me) K (pr) P H R

Raw file: QEP231009_CG_frozen_breast_TN_28, Scan: 1641, m/z: 418.24485, Charge: 4, RT (min): 7.57, Score: 119.69

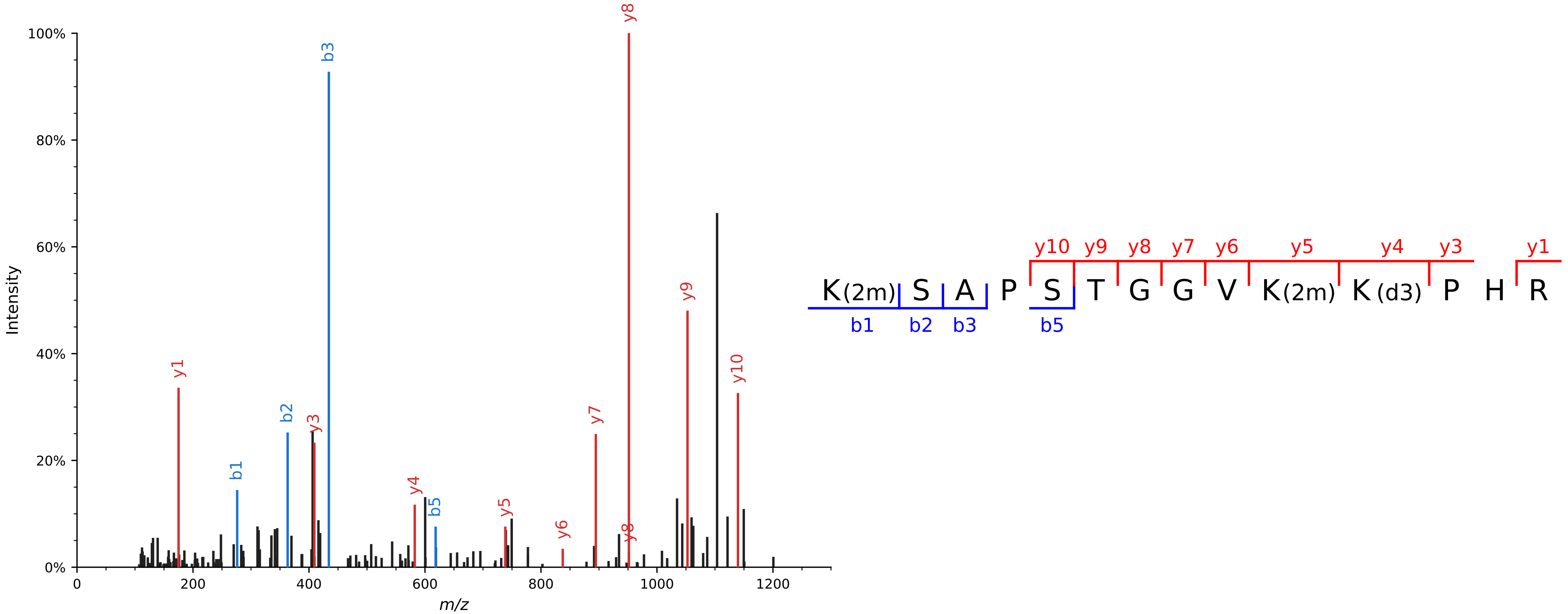

Raw file: QEP231009_CG_frozen_breast_LuA_30, Scan: 2374, m/z: 420.99406, Charge: 4, RT (min): 9.27, Score: 75.589

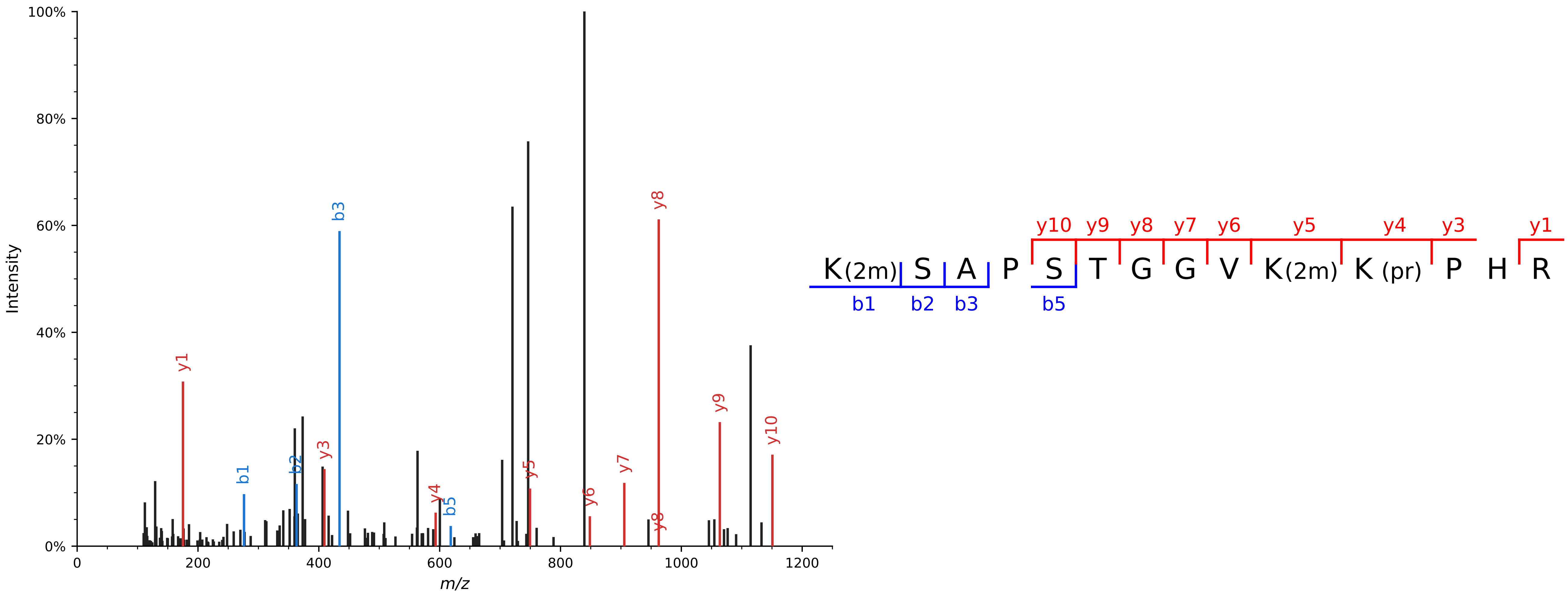

Raw file: QEP231009_CG_frozen_breast_TN_25, Scan: 4268, m/z: 566.65568, Charge: 3, RT (min): 13.03, Score: 97.45

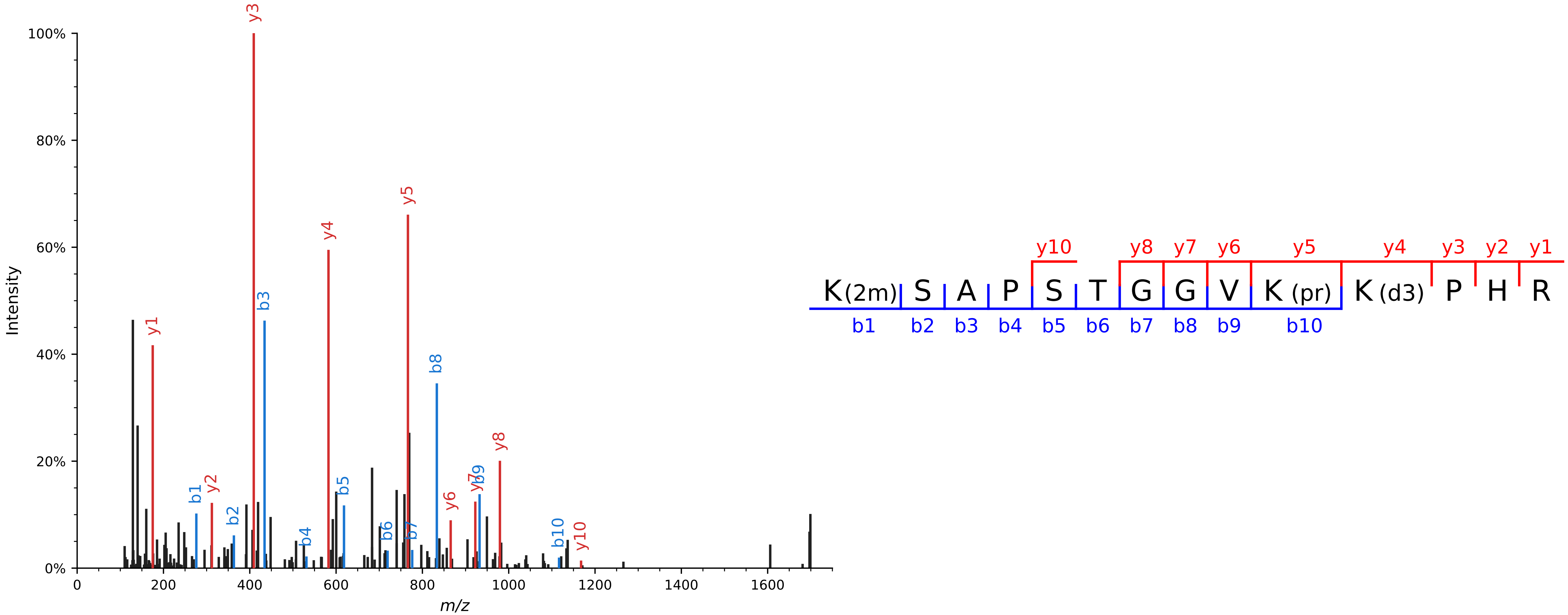

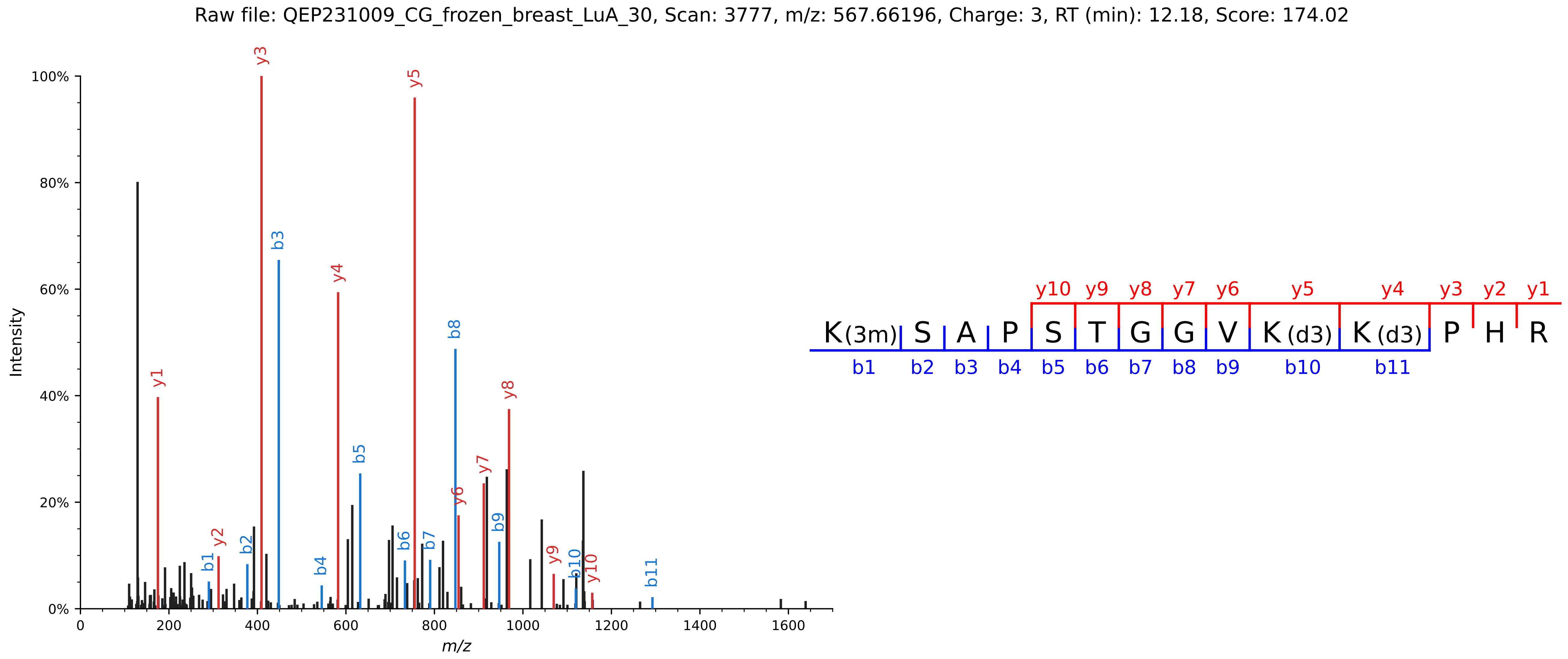
Raw file: QEP231009_CG_frozen_breast_LuA_30, Scan: 3777, m/z: 567.66196, Charge: 3, RT (min): 12.18, Score: 174.02
Intensity
m/z
y10 y9 y8 y7 y6 y5 y4 y3 y2 y1
K(3m) S A P S T G G V K(d3) K(d3) P H R
b1 b2 b3 b4 b5 b6 b7 b8 b9 b10 b11

Raw file: QEP231009_CG_frozen_breast_Normal_24, Scan: 4083, m/z: 572.33384, Charge: 3, RT (min): 13.13, Score: 161.04

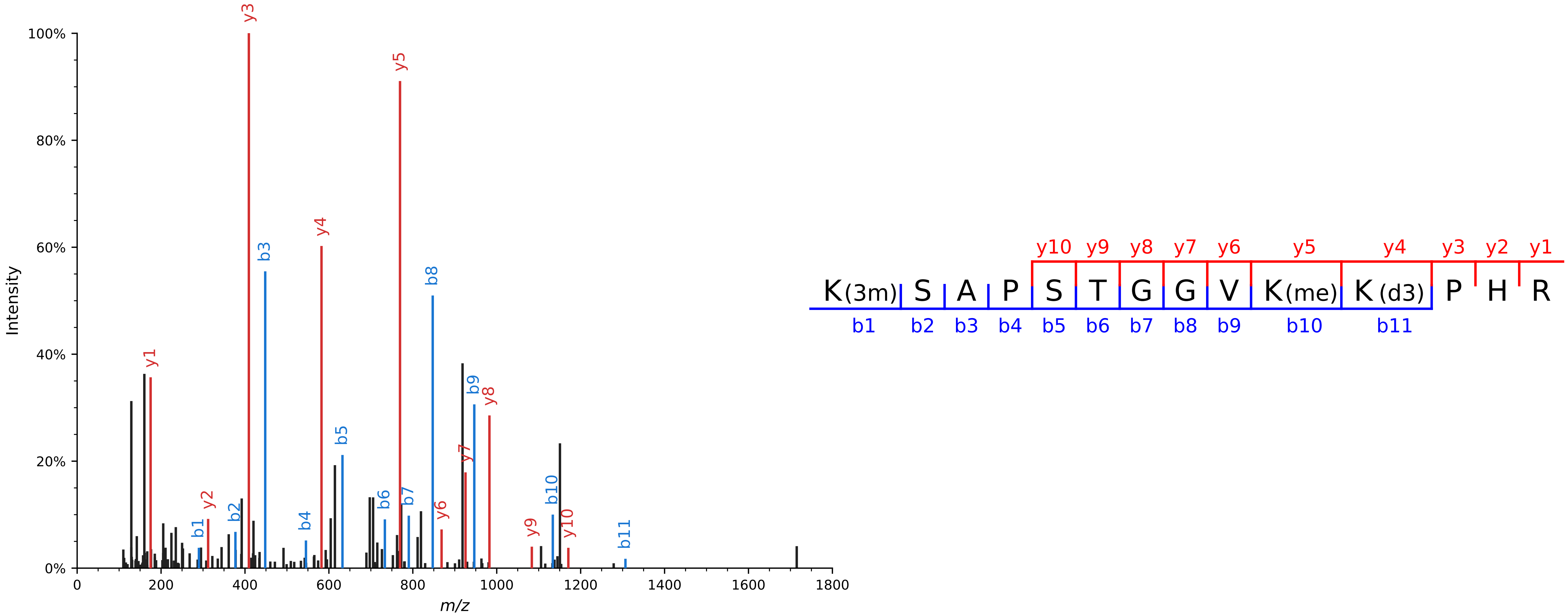

Raw file: QEP231009_CG_frozen_breast_TN_25, Scan: 1765, m/z: 561.99593, Charge: 3, RT (min): 7.80, Score: 111.12

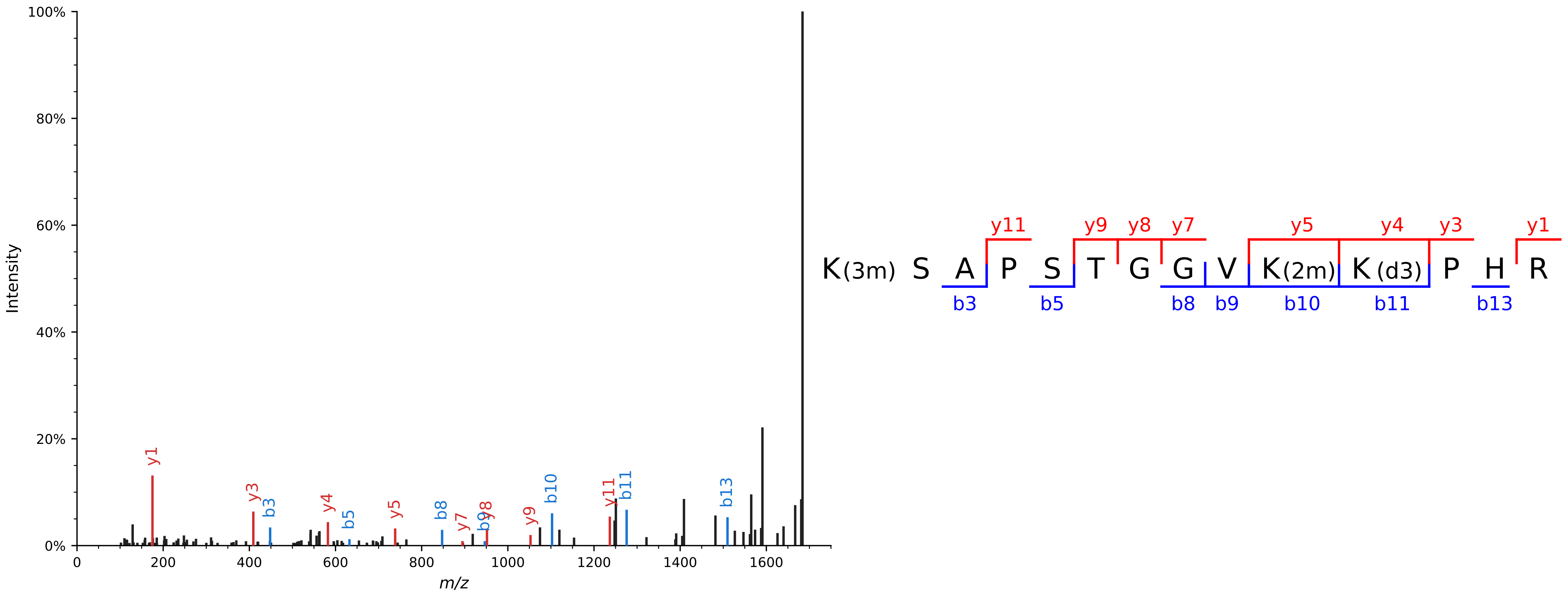

Raw file: QEP231009_CG_frozen_breast_TN_25, Scan: 8516, m/z: 857.97893, Charge: 2, RT (min): 21.87, Score: 83.045

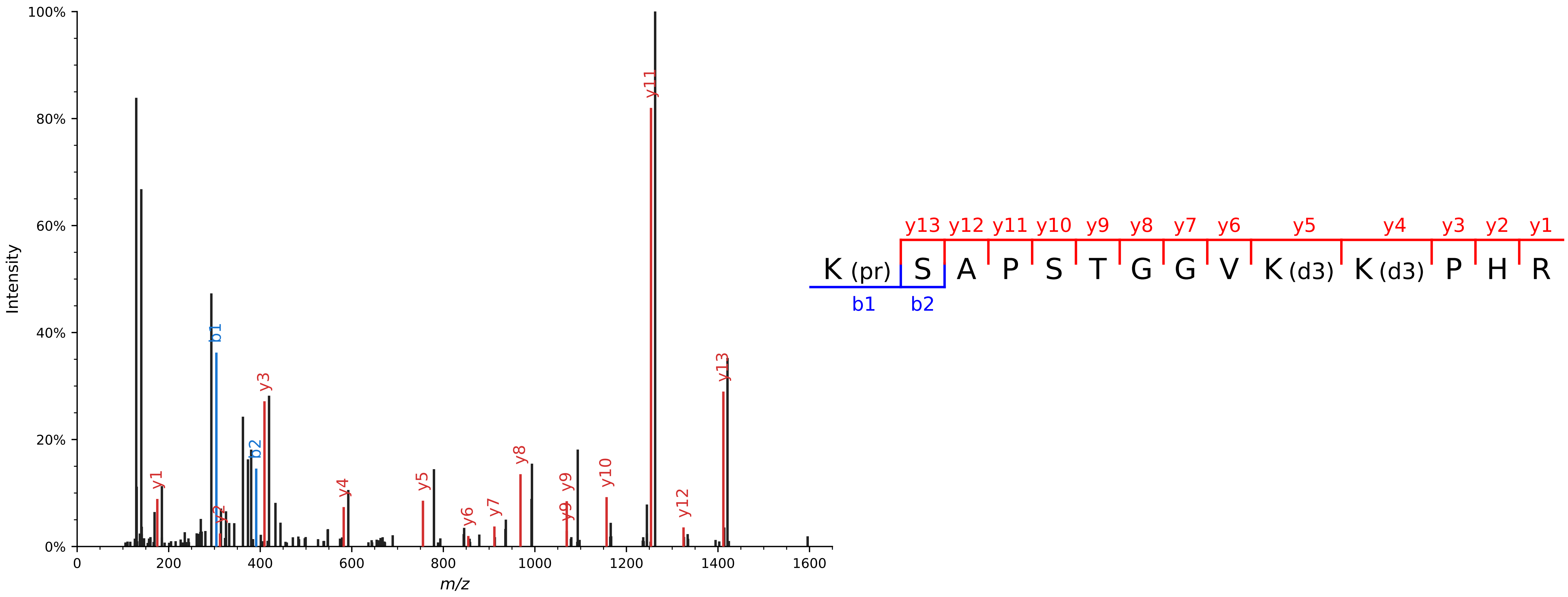

Raw file: QEP231009_CG_frozen_breast_LuA_30, Scan: 5044, m/z: 566.65568, Charge: 3, RT (min): 14.85, Score: 58.831

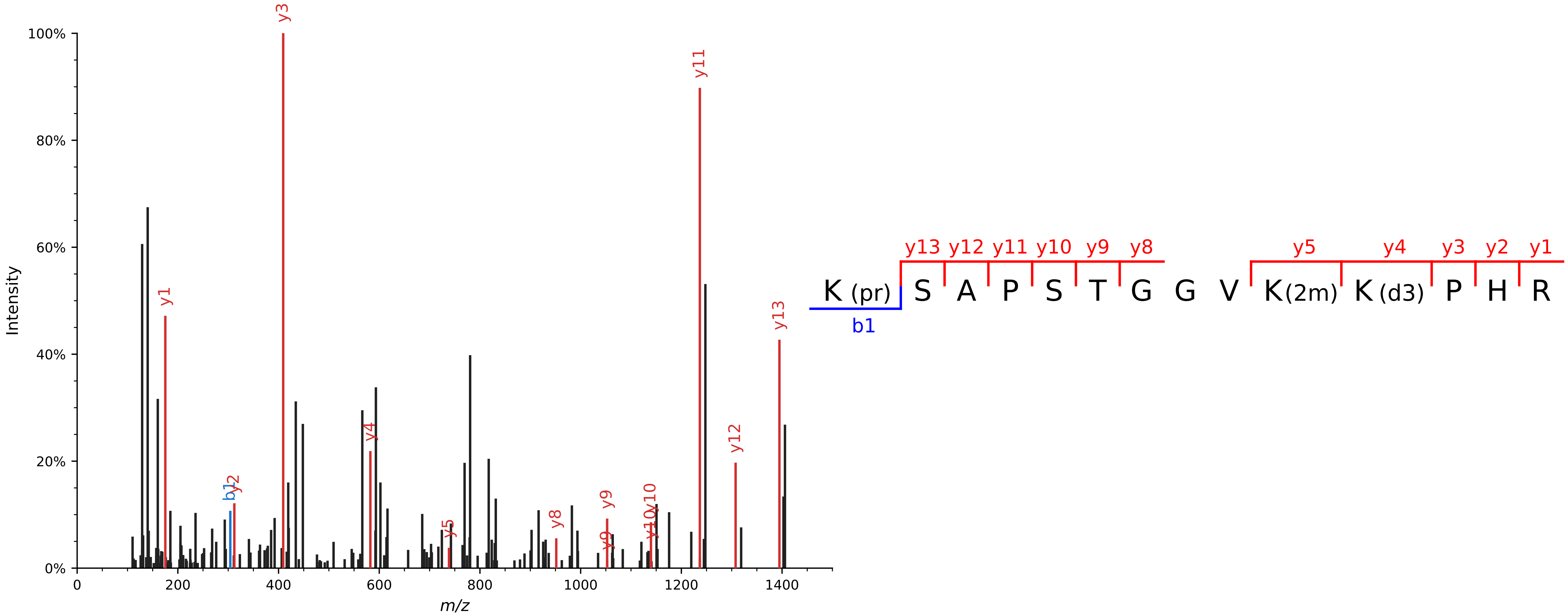

Raw file: QEP231009_CG_frozen_breast_TN_25, Scan: 8206, m/z: 859.48835, Charge: 2, RT (min): 21.25, Score: 157.38

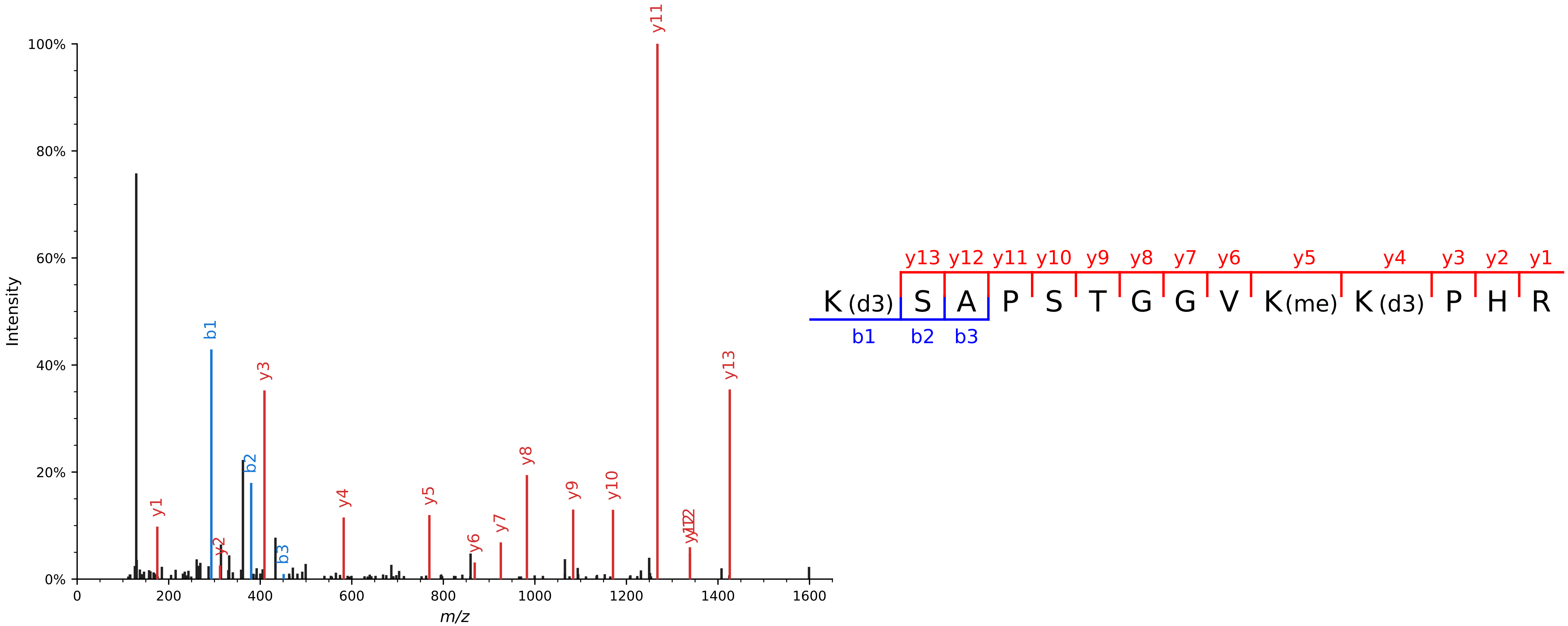

Raw file: QEP231009_CG_frozen_breast_Normal_21, Scan: 5164, m/z: 577.99987, Charge: 3, RT (min): 15.31, Score: 44.457

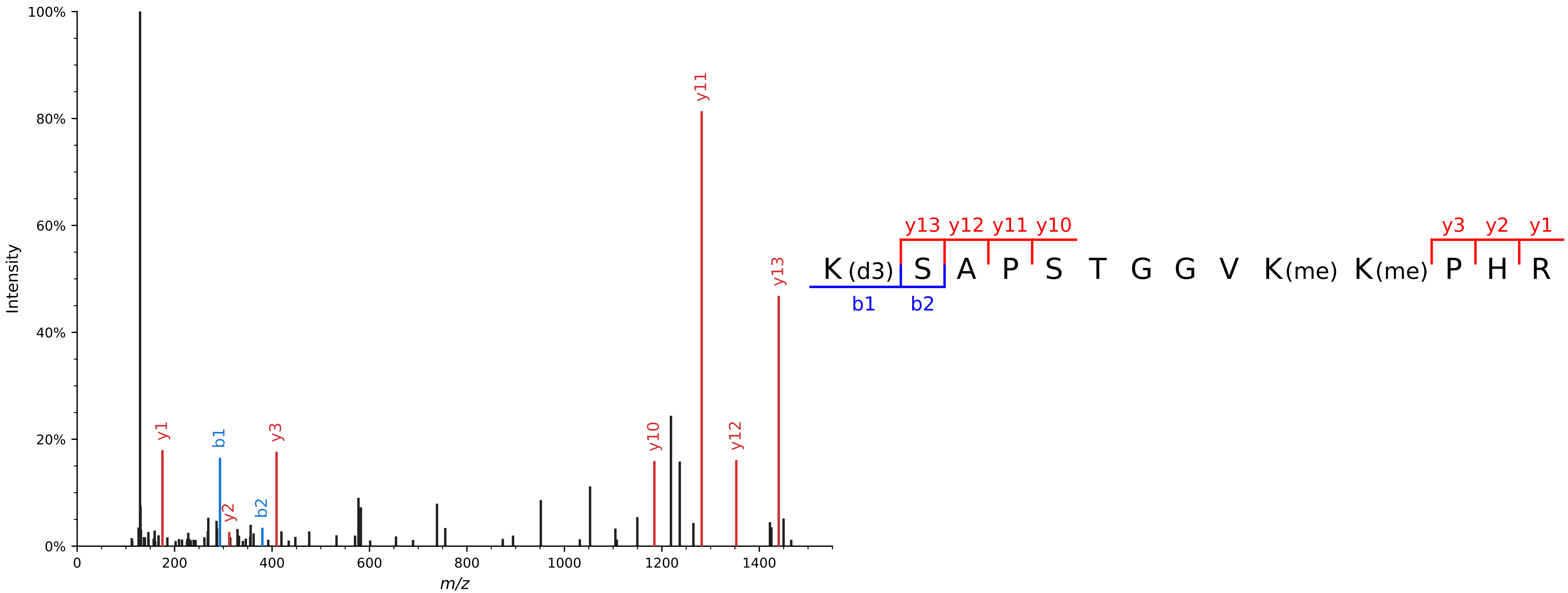

Raw file: QEP231009_CG_frozen_breast_Normal_19, Scan: 4318, m/z: 843.98148, Charge: 2, RT (min): 13.55, Score: 112.13

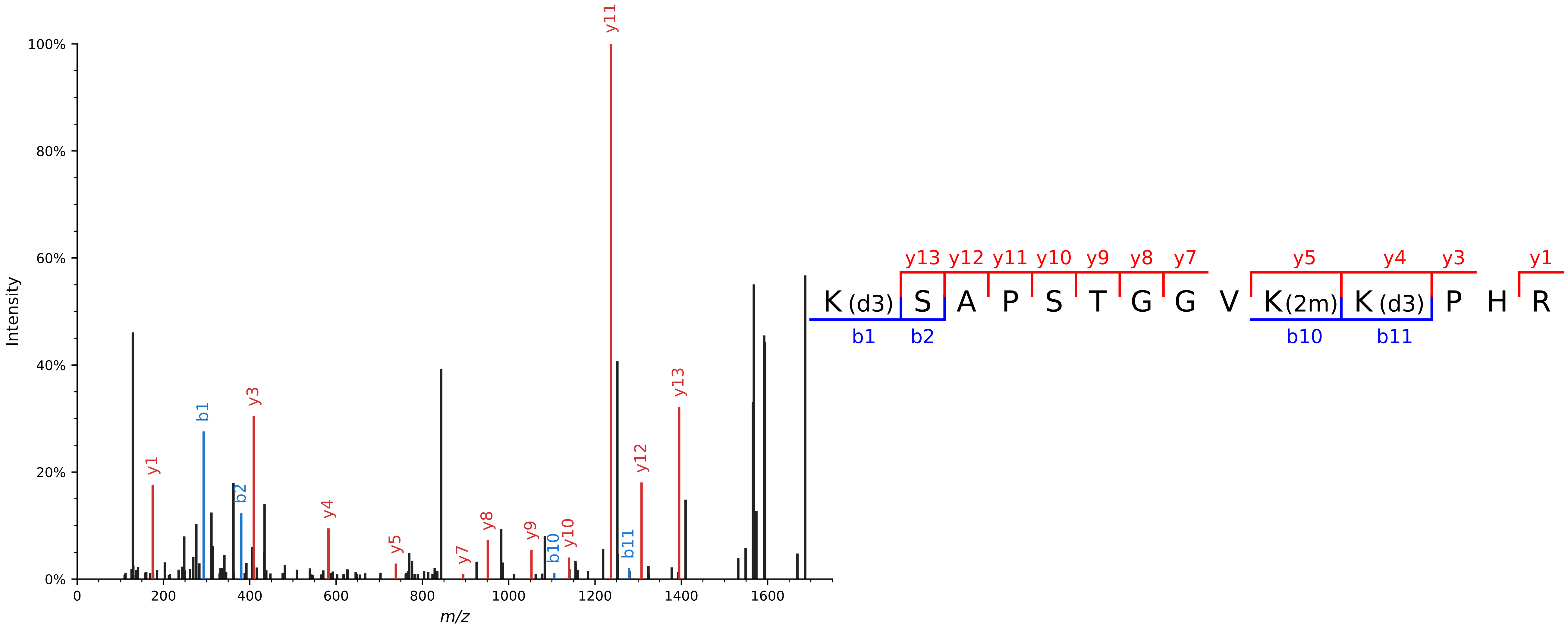

Raw file: QEP231009_CG_frozen_breast_TN_29, Scan: 8171, m/z: 857.97893, Charge: 2, RT (min): 21.41, Score: 74.944

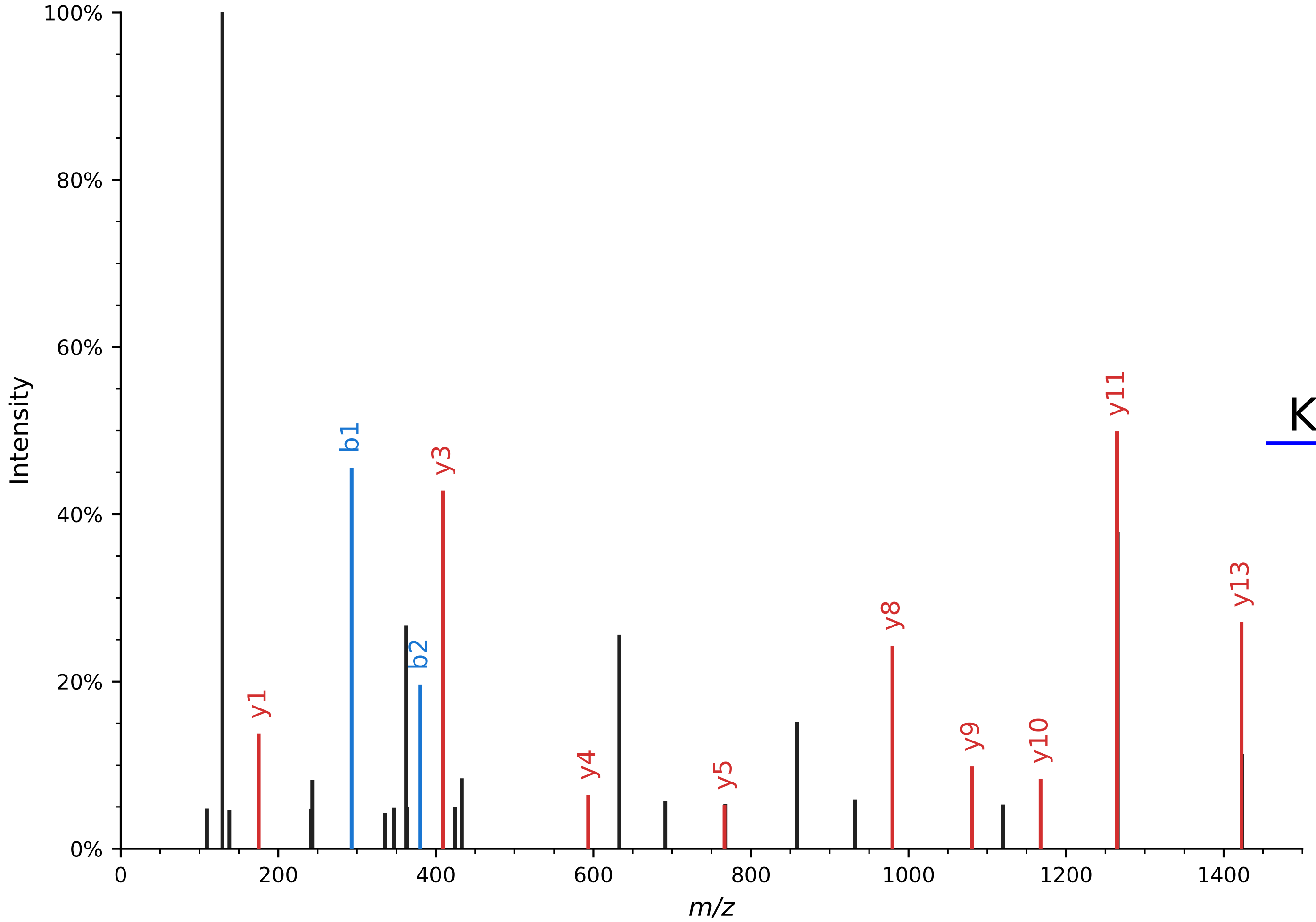

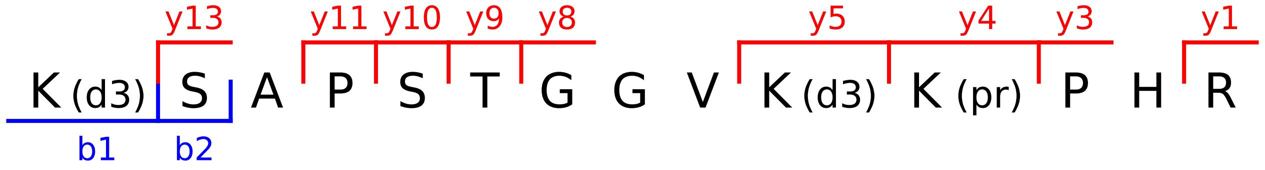

Raw file: QEP231009_CG_frozen_breast_LuA_33, Scan: 13747, m/z: 588.35236, Charge: 2, RT (min): 35.65, Score: 48.756

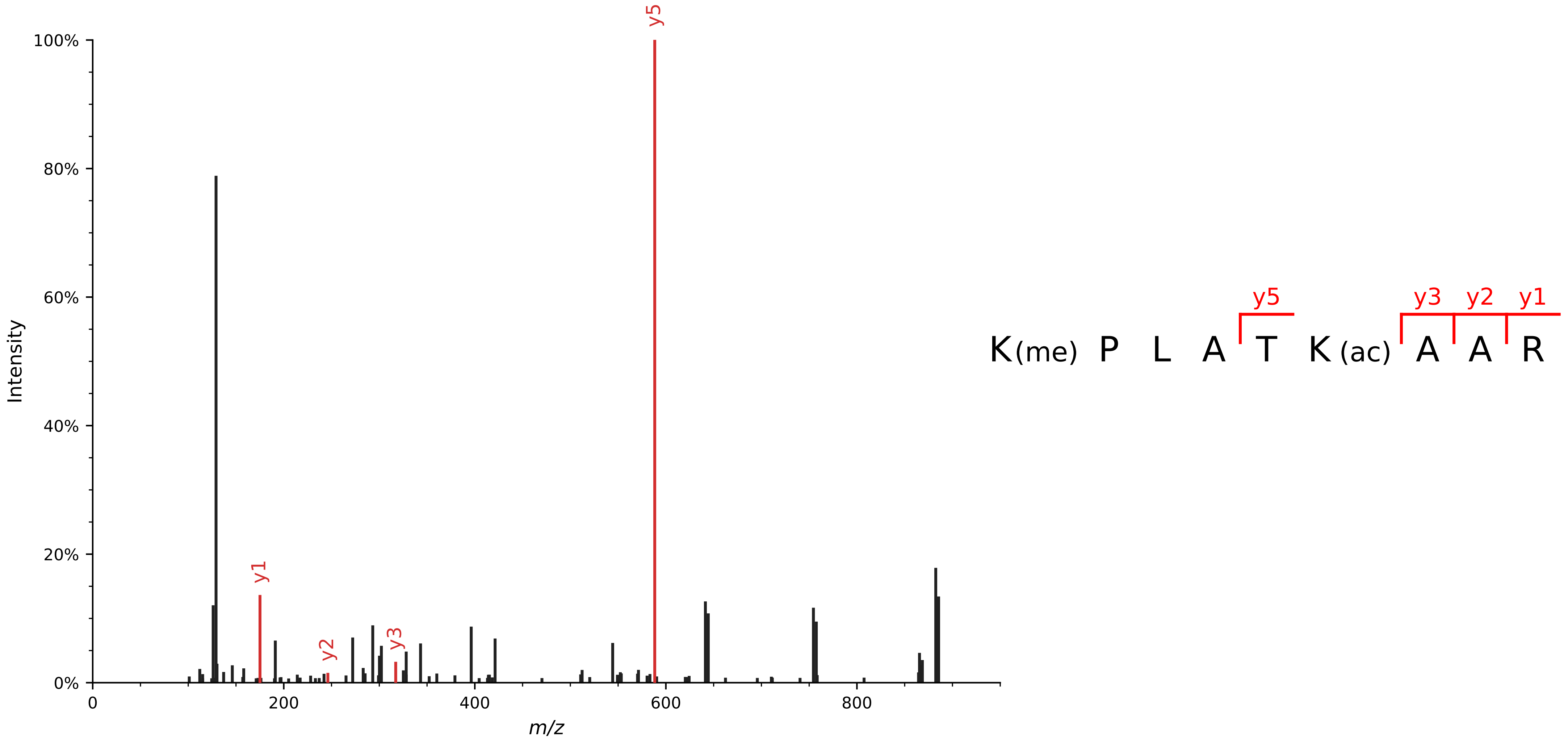

Raw file: QEP231009_CG_frozen_breast_Normal_21, Scan: 4966, m/z: 426.22213, Charge: 2, RT (min): 14.88, Score: 44.531

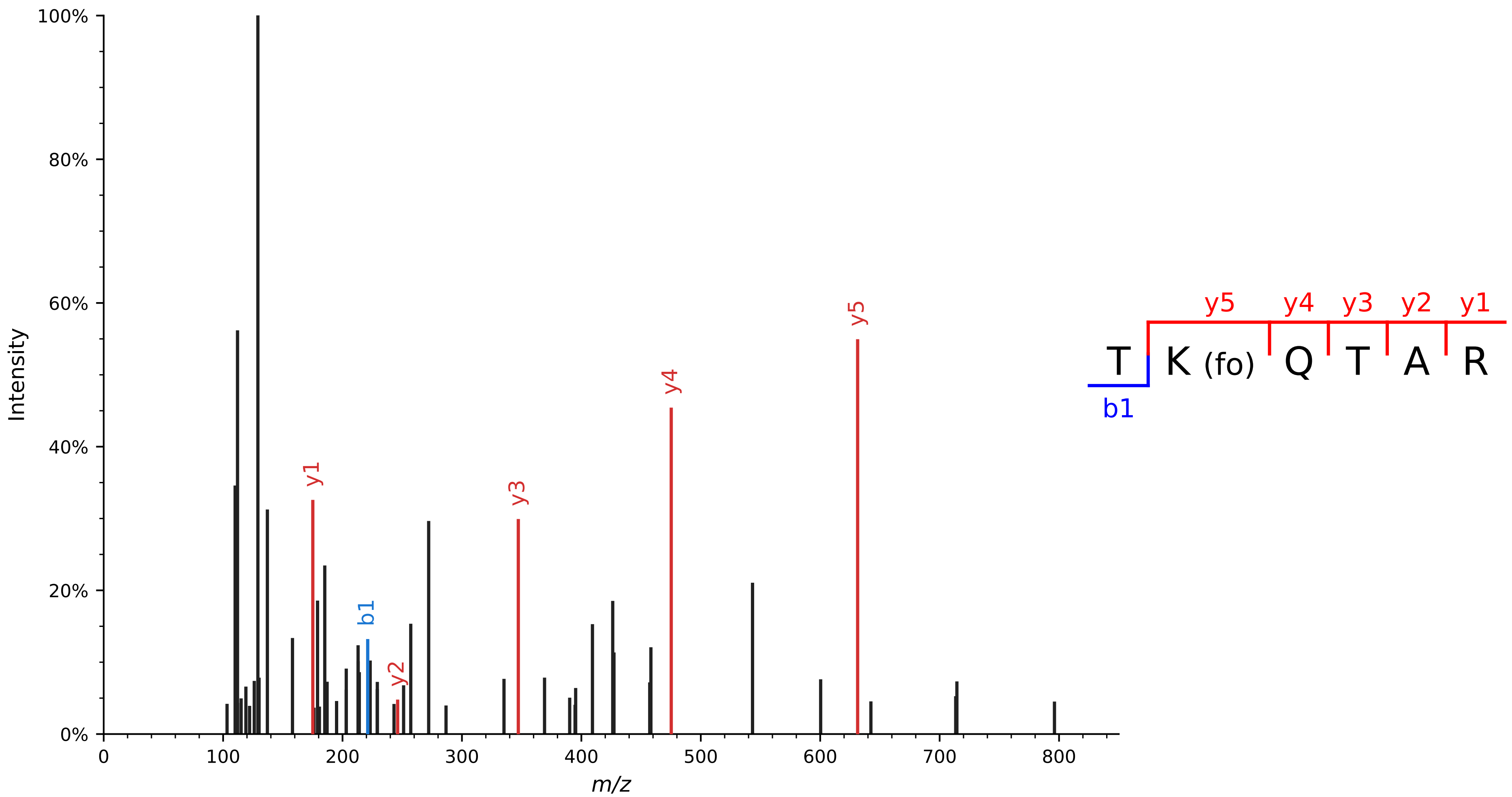

Raw file: QEP231009_CG_frozen_breast_TN_25, Scan: 7414, m/z: 441.7472, Charge: 2, RT (min): 19.59, Score: 85.306

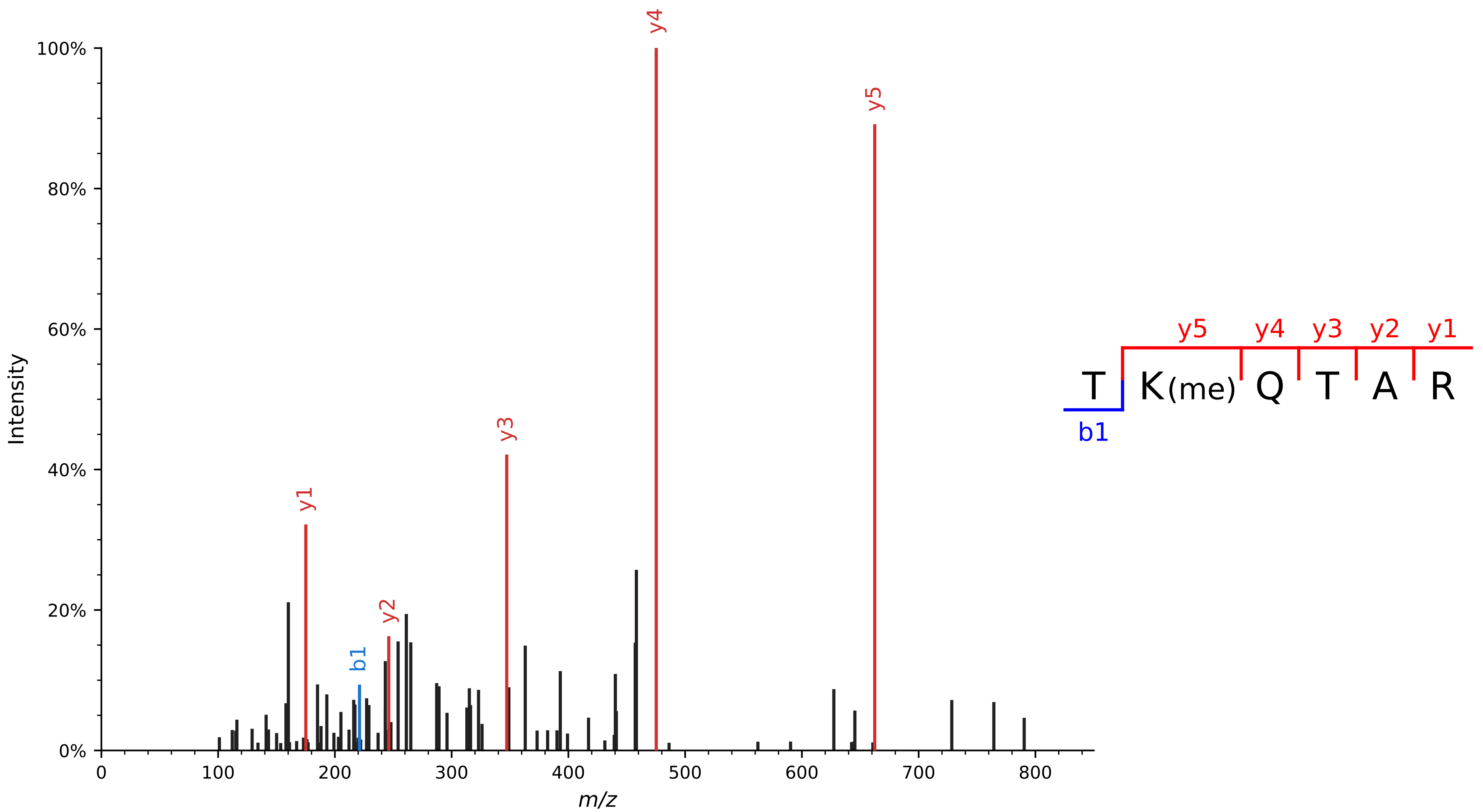

Raw file: QEP231009_CG_frozen_breast_TN_29, Scan: 1223, m/z: 426.24033, Charge: 2, RT (min): 6.74, Score: 148.33

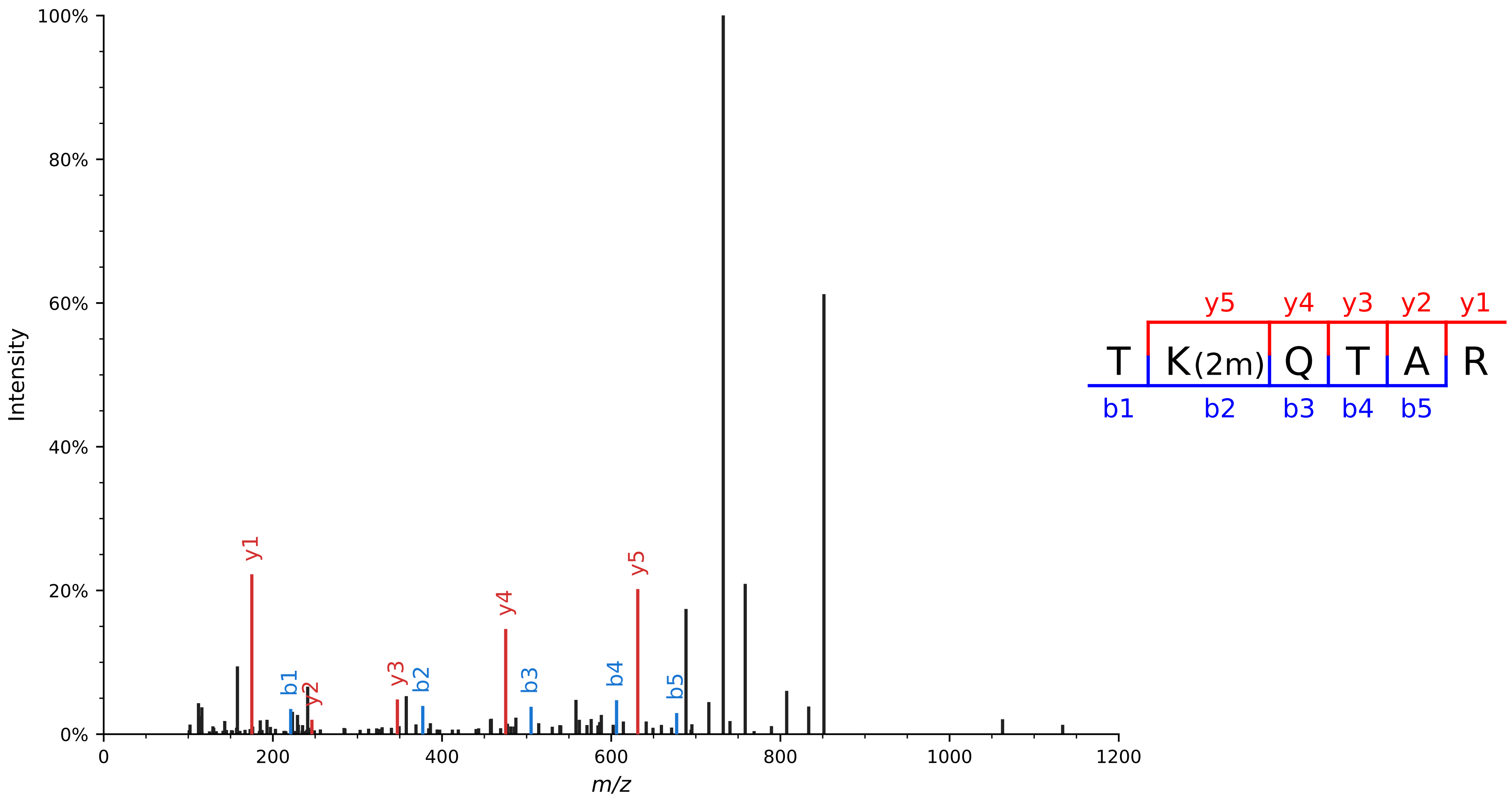

Raw file: QEP231009_CG_frozen_breast_TN_29, Scan: 1217, m/z: 433.24815, Charge: 2, RT (min): 6.73, Score: 105.39

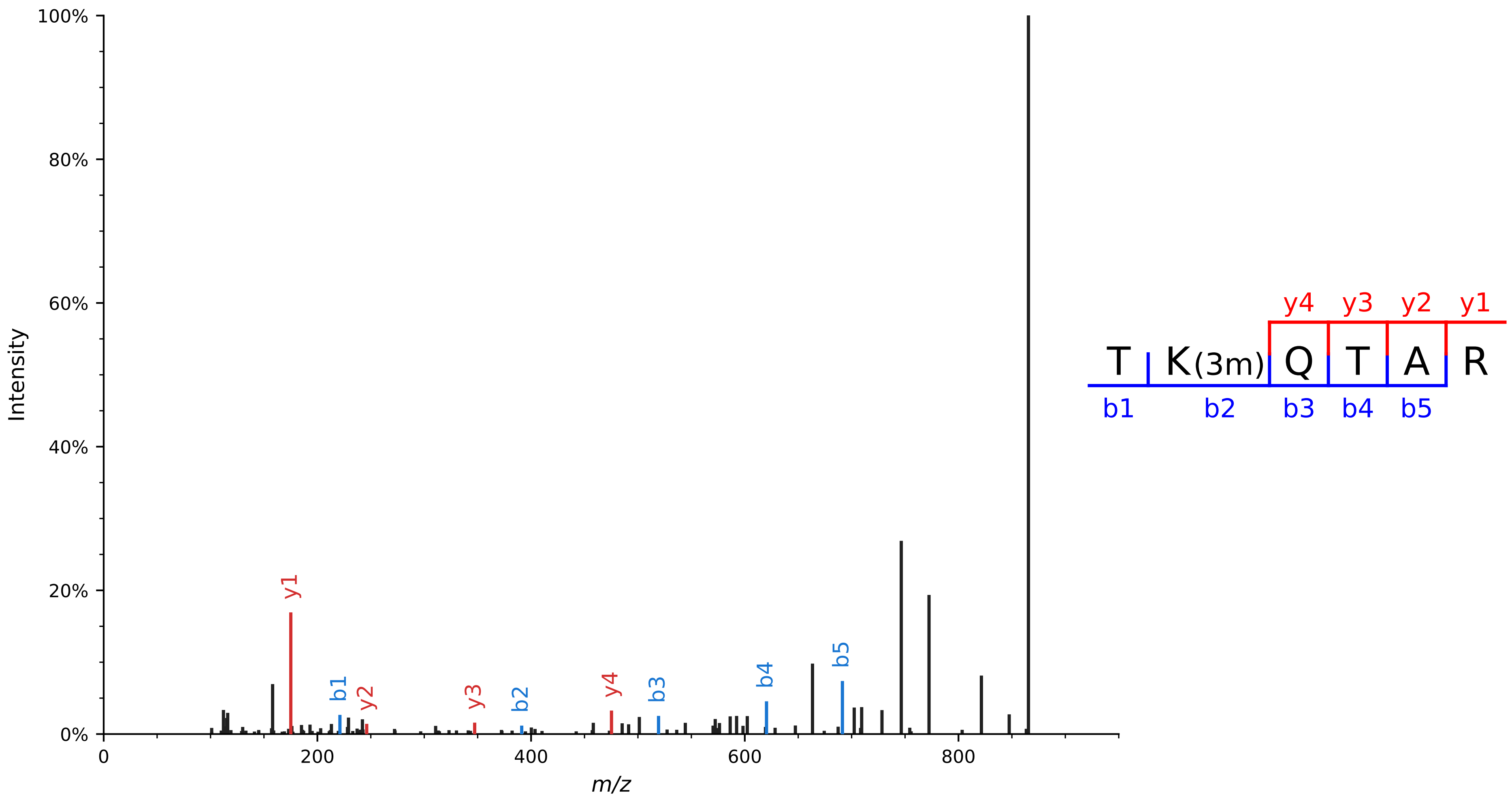

Raw file: QEP231009_CG_frozen_breast_TN_25, Scan: 7247, m/z: 440.23778, Charge: 2, RT (min): 19.24, Score: 126.04

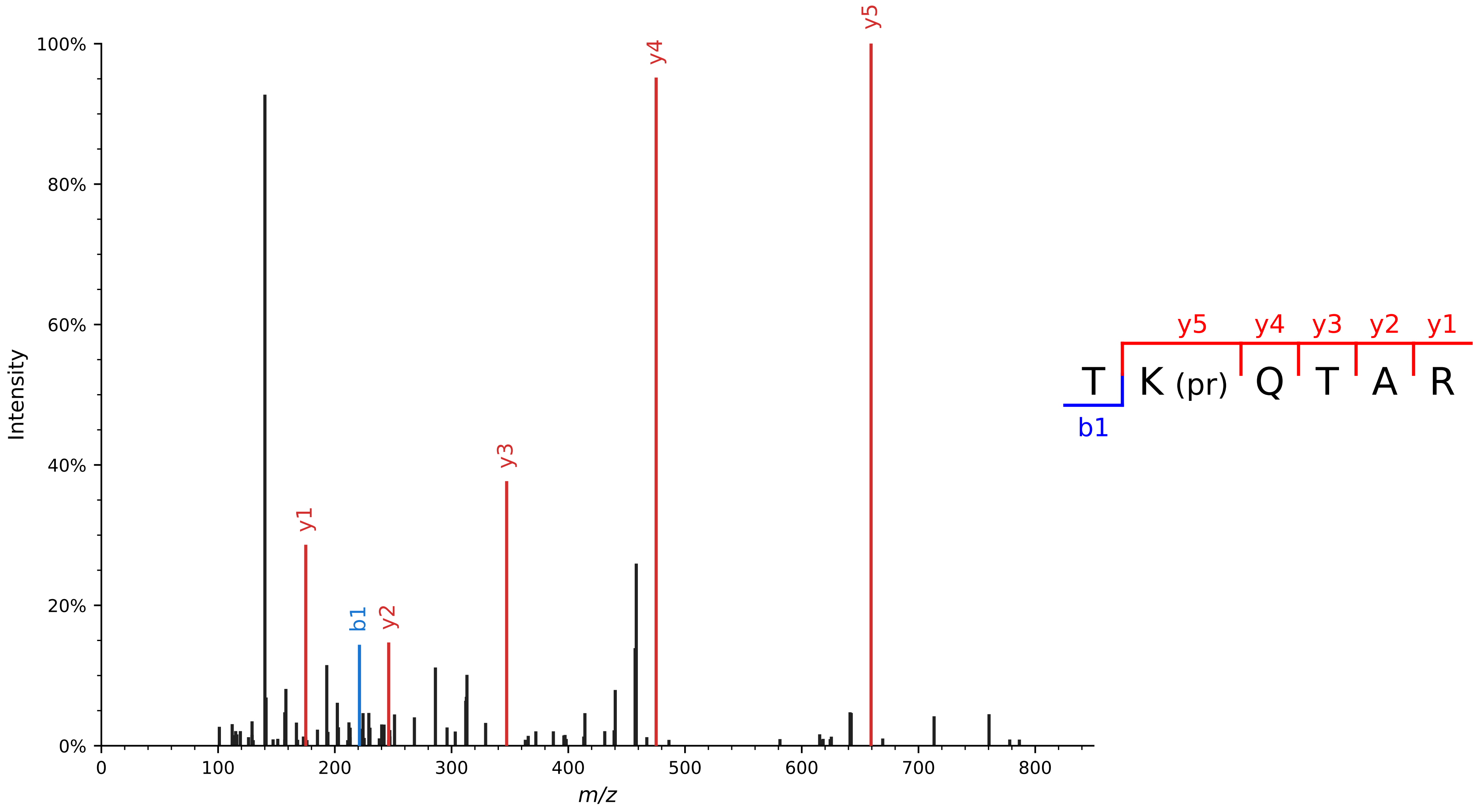

Raw file: QEP231009_CG_frozen_breast_LuA_30, Scan: 7278, m/z: 552.79345, Charge: 2, RT (min): 19.55, Score: 87.639

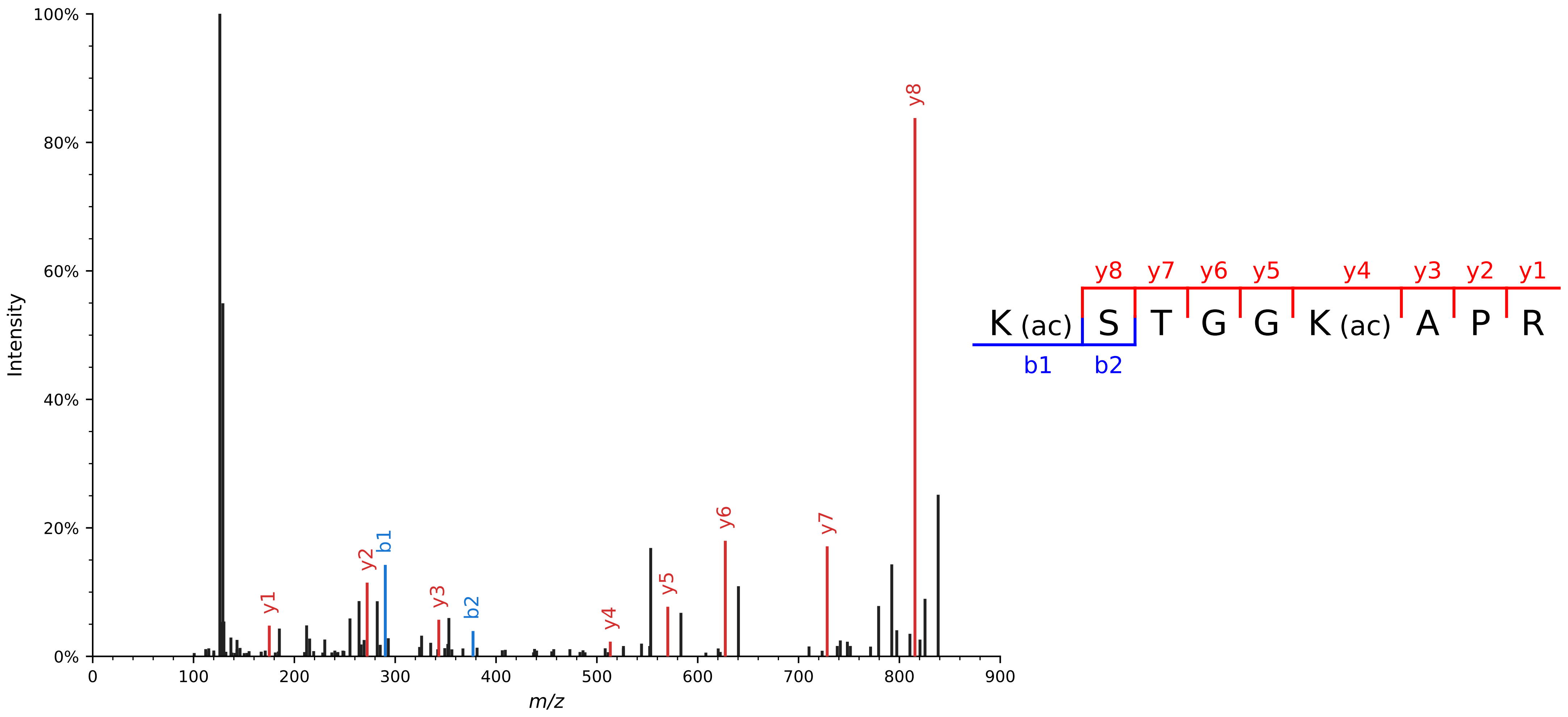

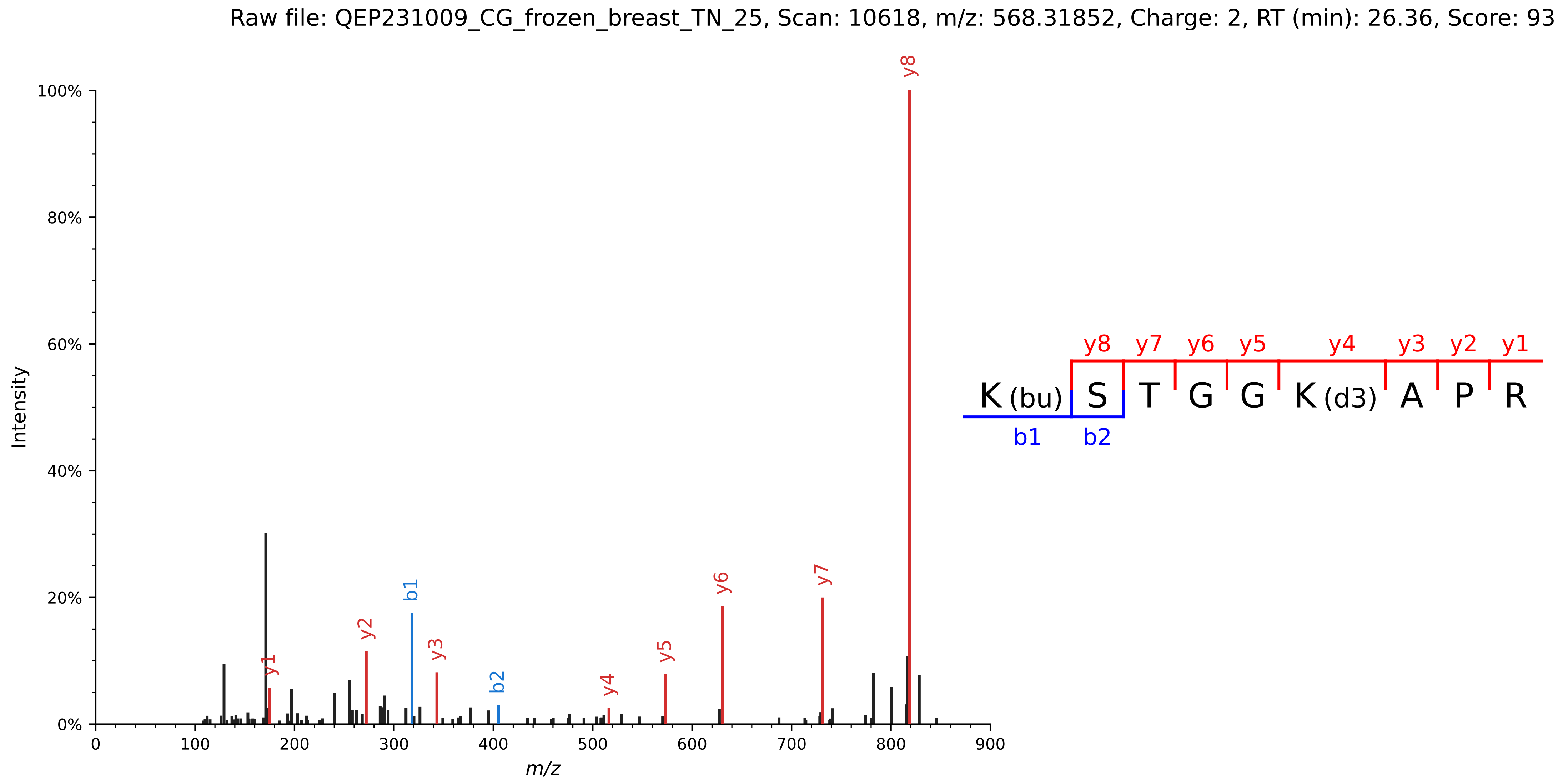

Raw file: QEP231009_CG_frozen_breast_TN_25, Scan: 10618, m/z: 568.31852, Charge: 2, RT (min): 26.36, Score: 93.374
Intensity
100%
80%
60%
40%
20%
0%
0
100
200
300
400
500
600
700
800
900
m/z
y1
y2
b1
y3
b2
y4
y5
y6
y7
y8
y8 y7 y6 y5 y4 y3 y2 y1
K (bu) S T G G K (d3) A P R
b1 b2

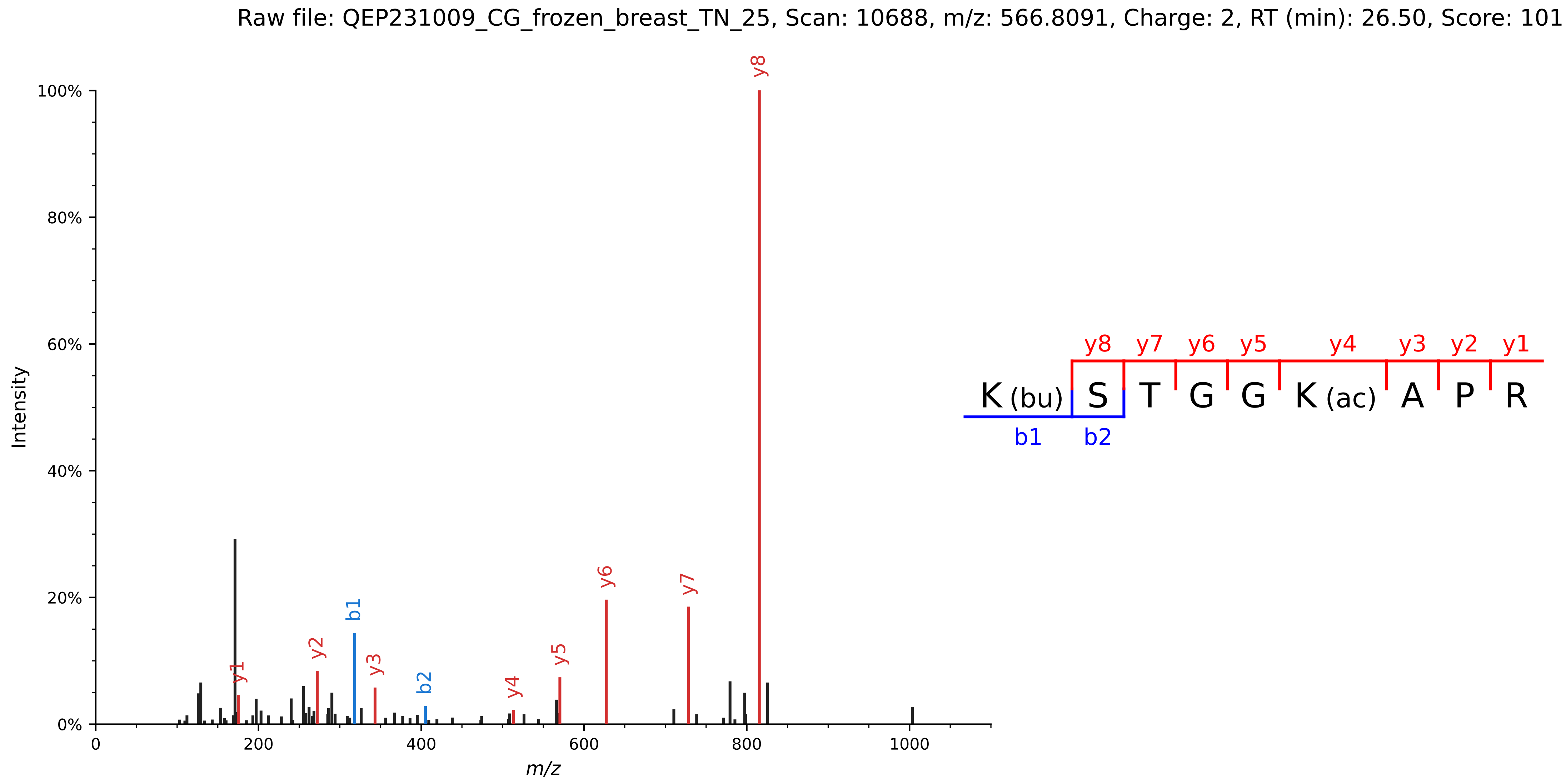
Raw file: QEP231009_CG_frozen_breast_TN_25, Scan: 10688, m/z: 566.8091, Charge: 2, RT (min): 26.50, Score: 101.54
Intensity
100%
80%
60%
40%
20%
0%
0
200
400
600
800
1000
m/z
y1
y2
b1
y3
b2
y4
y5
y6
y7
y8
K (bu) S T G G K (ac) A P R
y8 y7 y6 y5 y4 y3 y2 y1
b1 b2

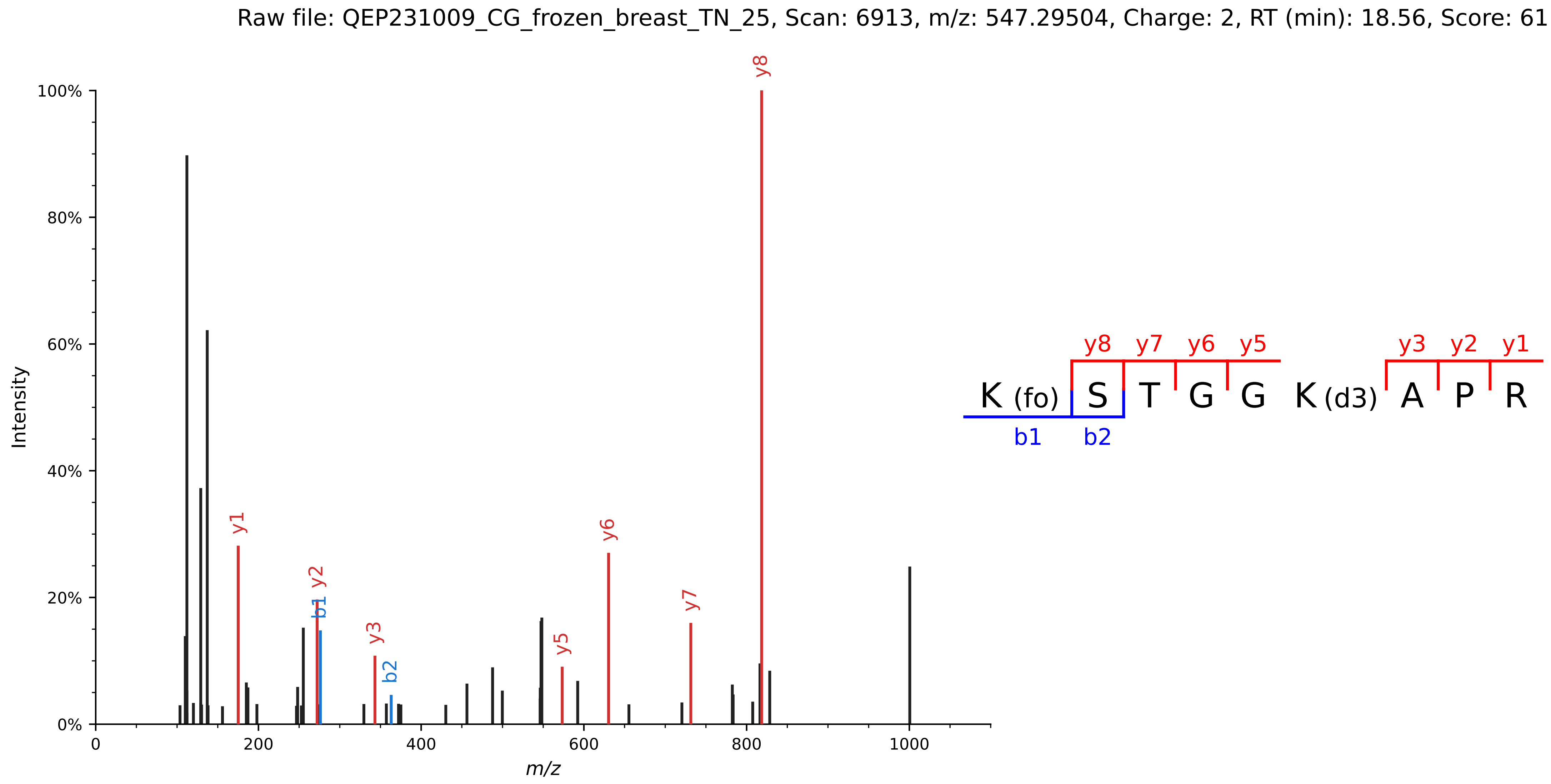
Raw file: QEP231009_CG_frozen_breast_TN_25, Scan: 6913, m/z: 547.29504, Charge: 2, RT (min): 18.56, Score: 61.962
Intensity
m/z
y8
y1
y2
b1
y3
b2
y5
y6
y7
y8 y7 y6 y5 y3 y2 y1
K (fo) S T G G K (d3) A P R
b1 b2
100%
80%
60%
40%
20%
0%
0
200
400
600
800
1000

Raw file: QEP231009_CG_frozen_breast_TN_29, Scan: 13732, m/z: 582.80402, Charge: 2, RT (min): 33.34, Score: 41.029

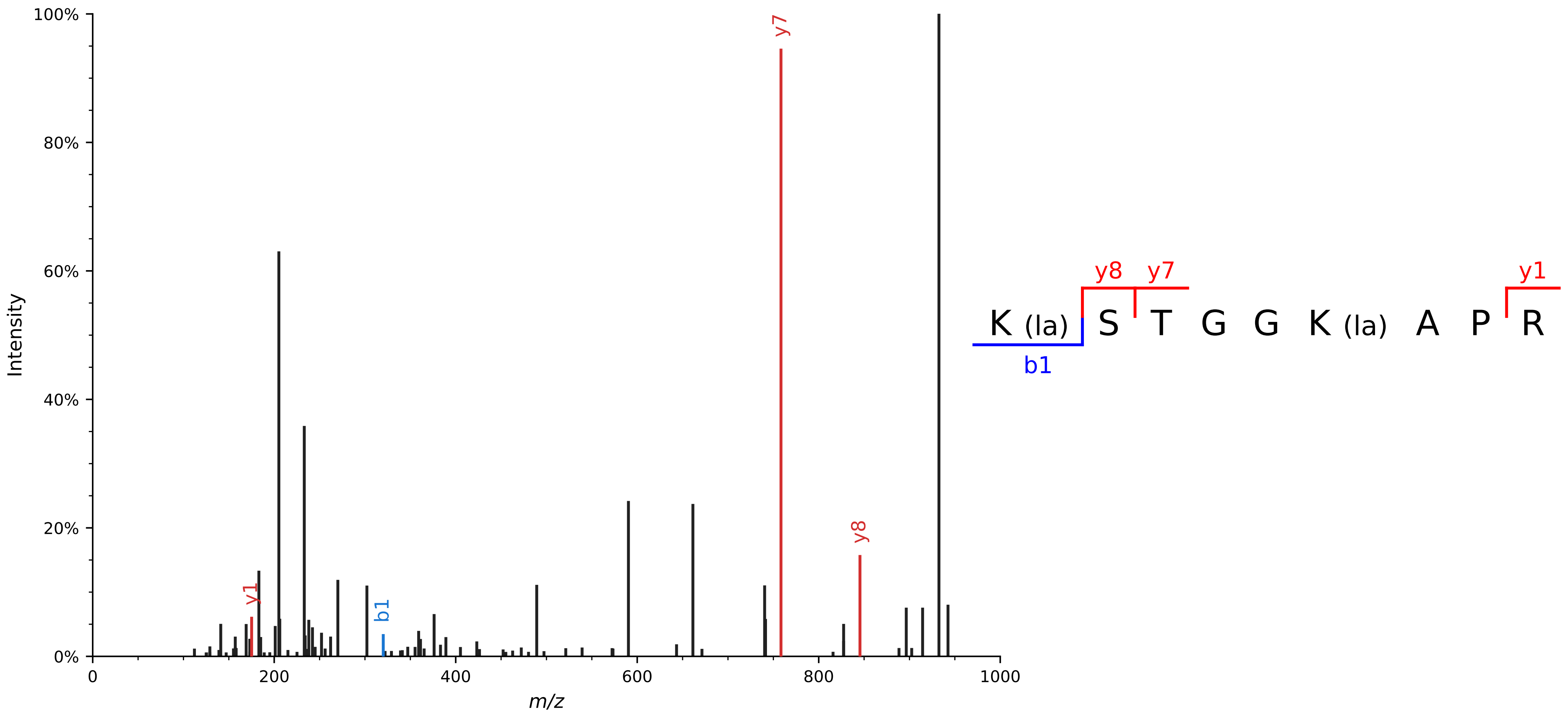

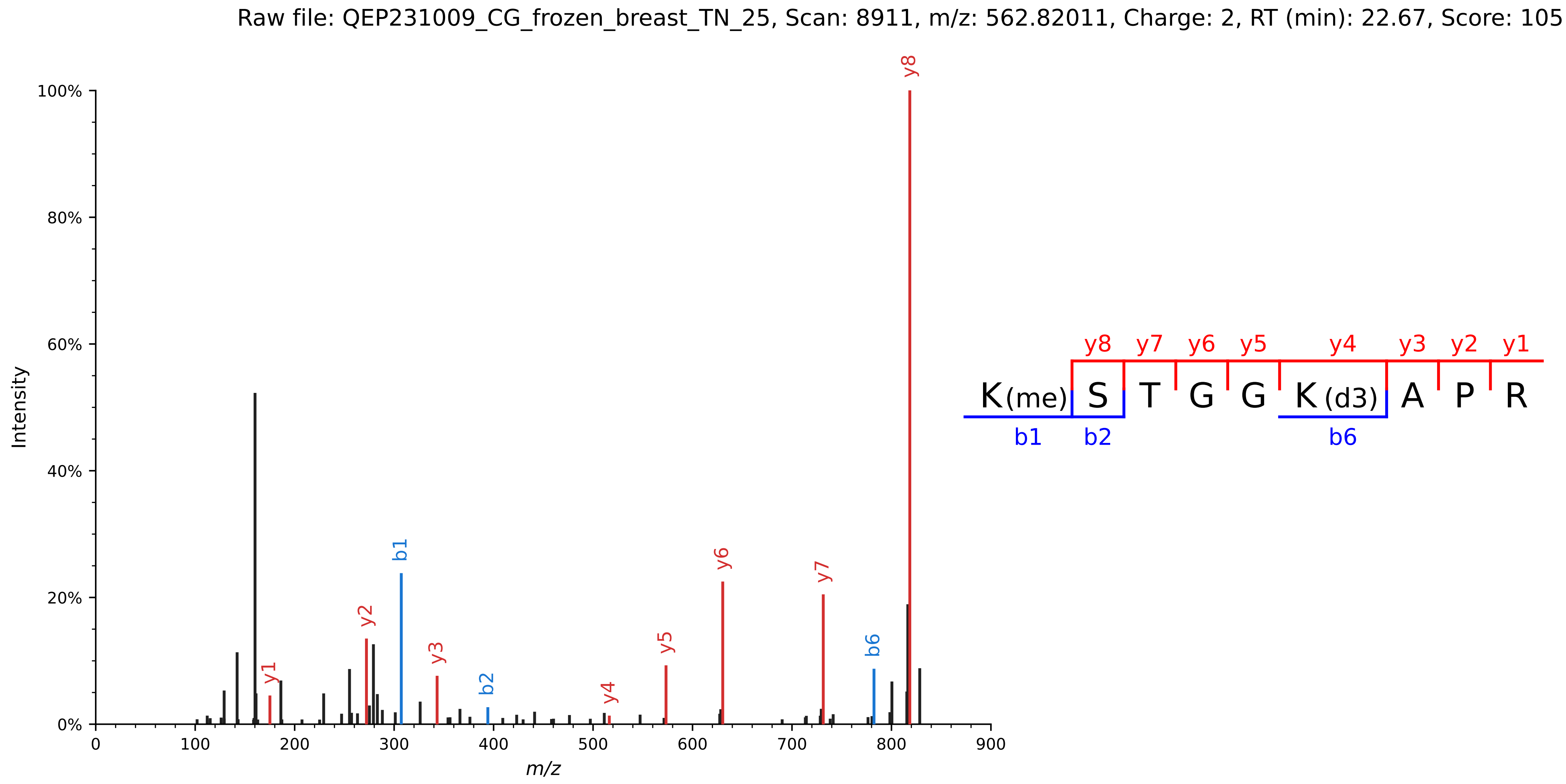

Raw file: QEP231009_CG_frozen_breast_TN_25, Scan: 8911, m/z: 562.82011, Charge: 2, RT (min): 22.67, Score: 105.14
Intensity
m/z
y8 y7 y6 y5 y4 y3 y2 y1
K(me) S T G G K(d3) A P R
b1 b2 b6

Raw file: QEP231009_CG_frozen_breast_Normal_24, Scan: 8424, m/z: 561.3107, Charge: 2, RT (min): 22.59, Score: 123.72

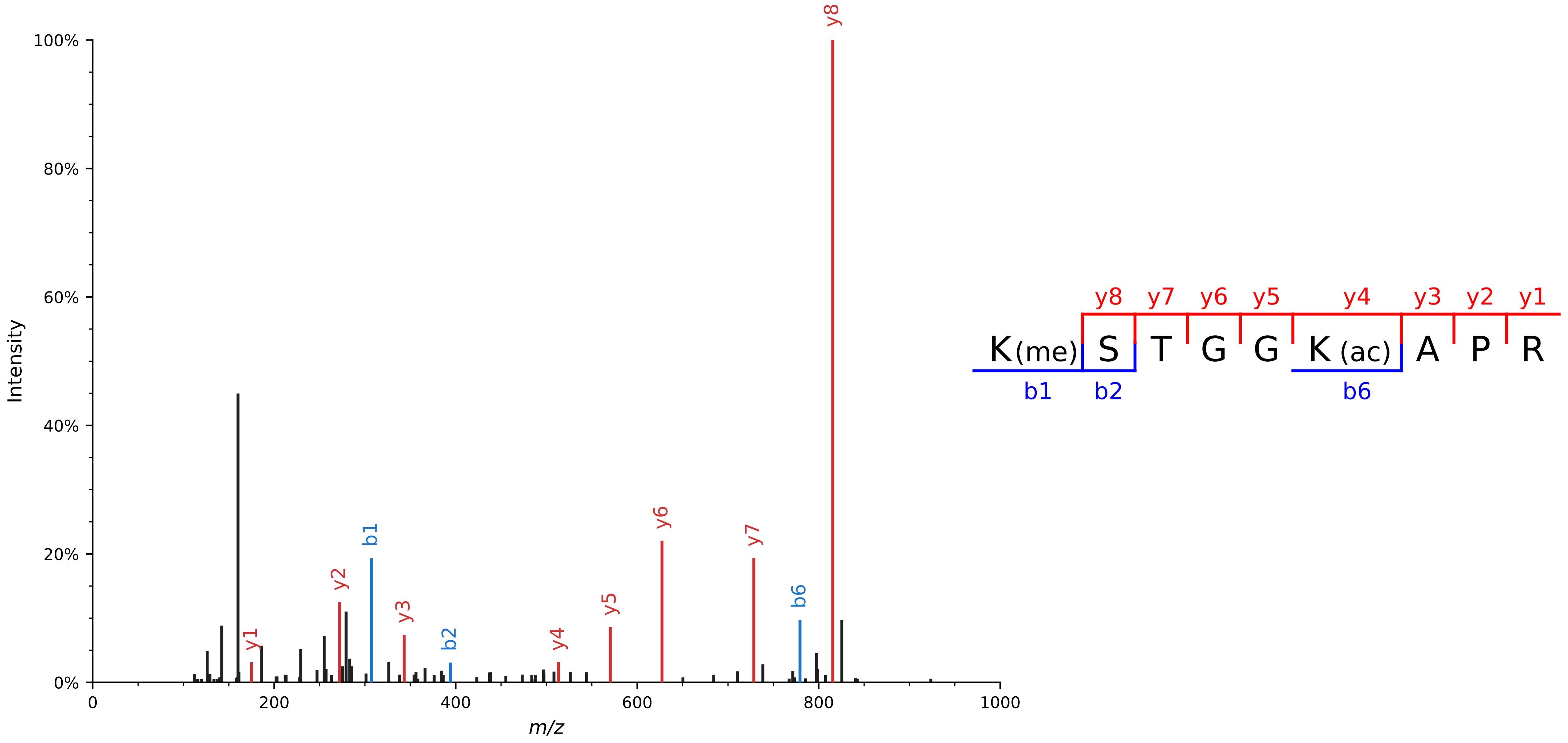

Raw file: QEP231009_CG_frozen_breast_TN_25, Scan: 3586, m/z: 569.82794, Charge: 2, RT (min): 11.64, Score: 57.177

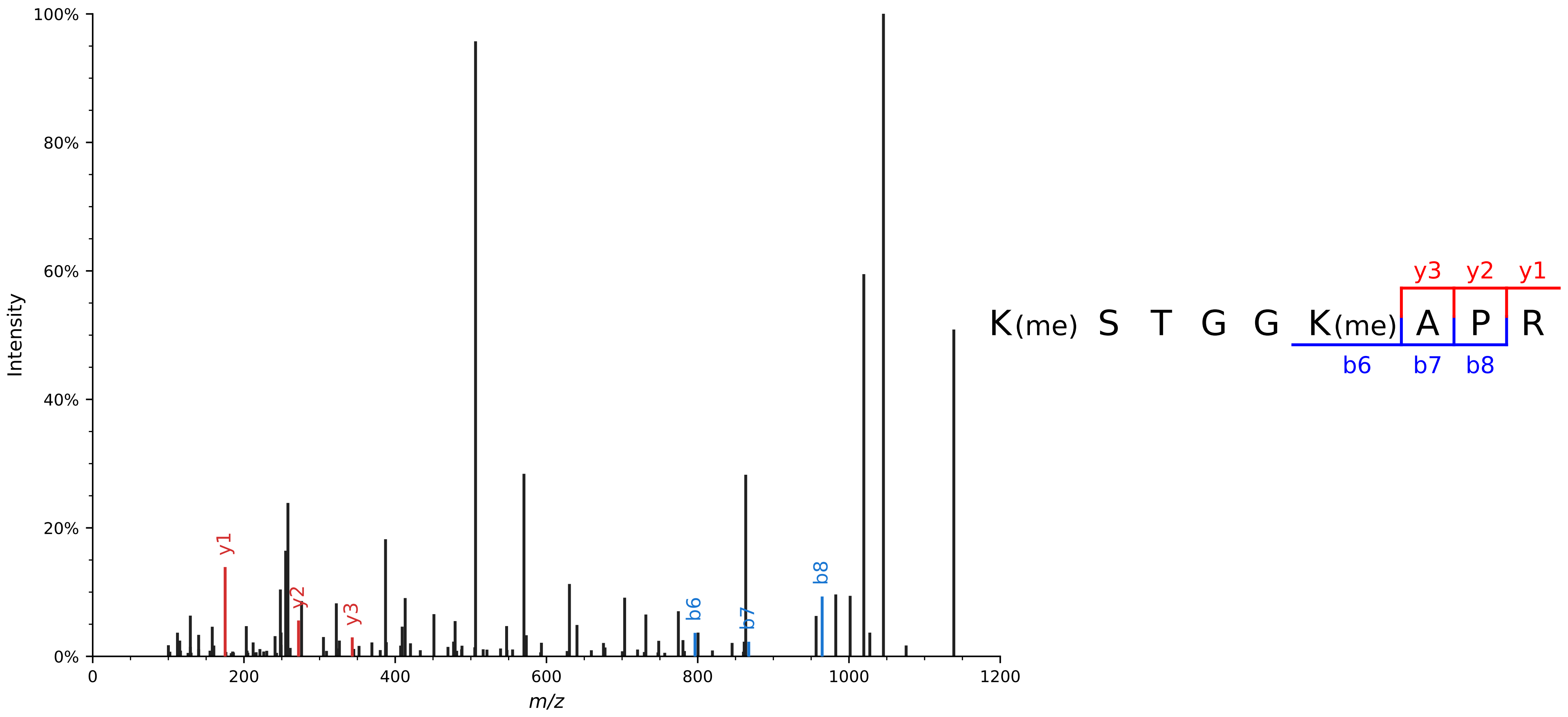

Raw file: QEP231009_CG_frozen_breast_Normal_21, Scan: 9420, m/z: 568.31852, Charge: 2, RT (min): 24.61, Score: 119.29

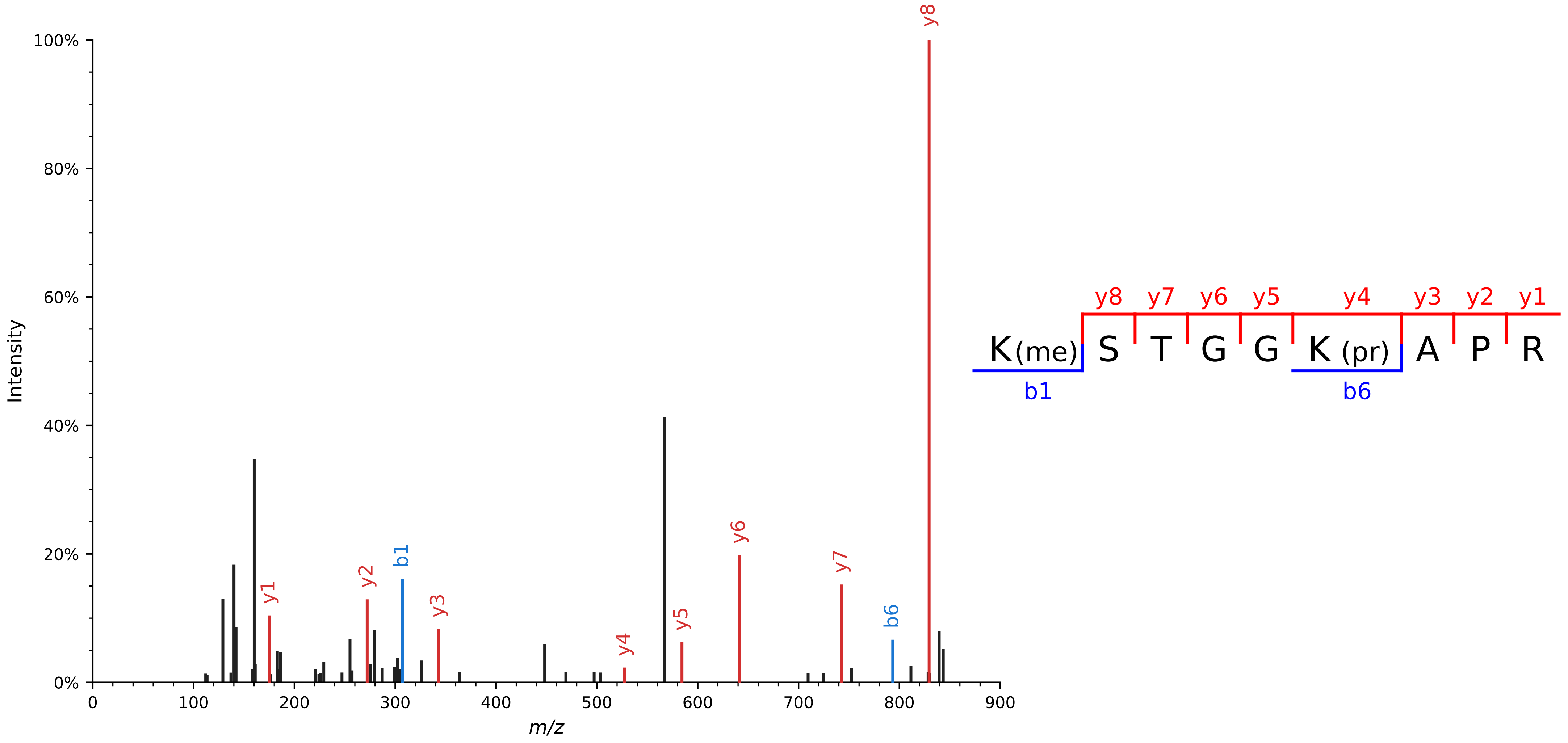

Raw file: QEP231009_CG_frozen_breast_Normal_24, Scan: 2267, m/z: 547.31324, Charge: 2, RT (min): 9.09, Score: 113.71

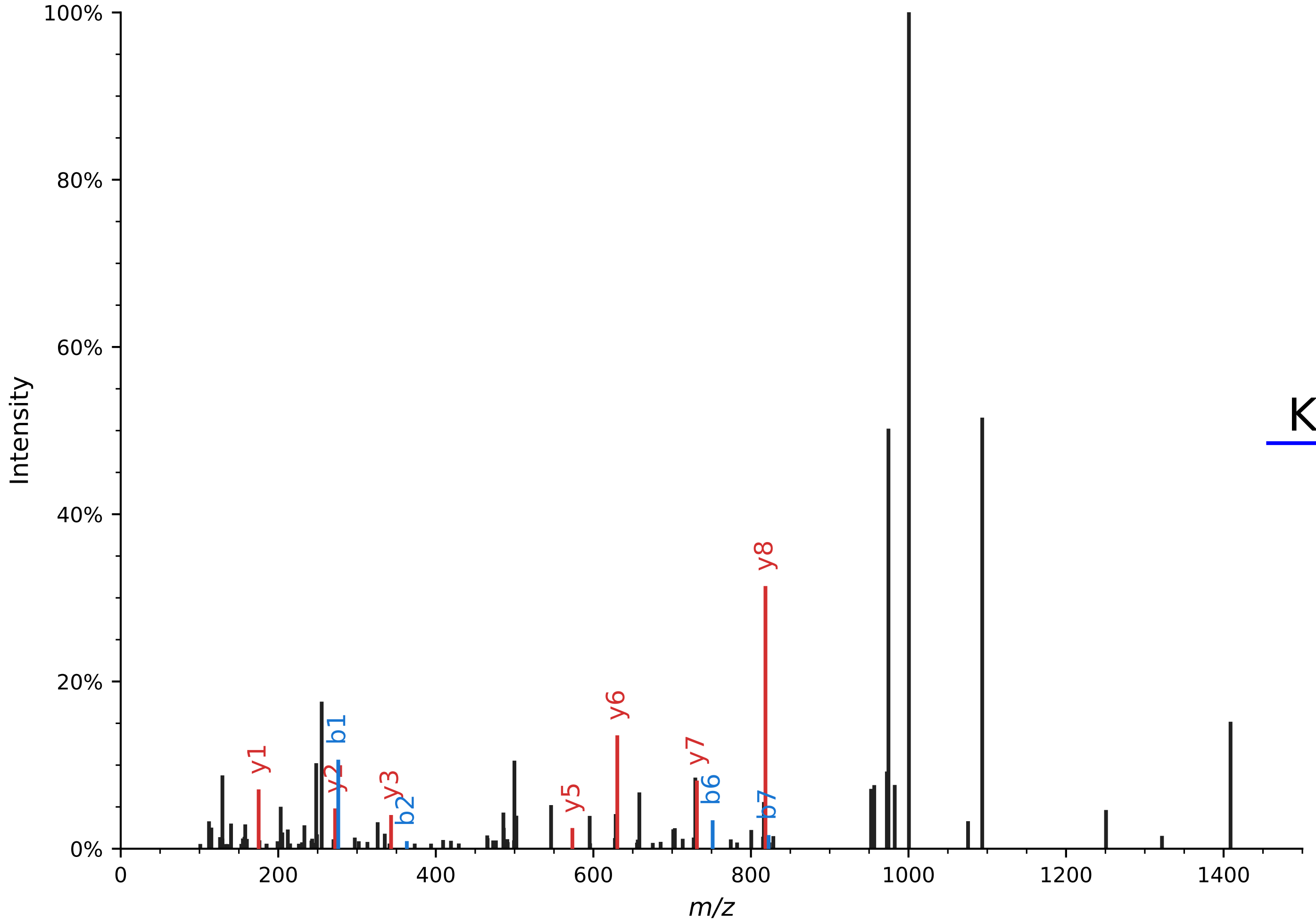

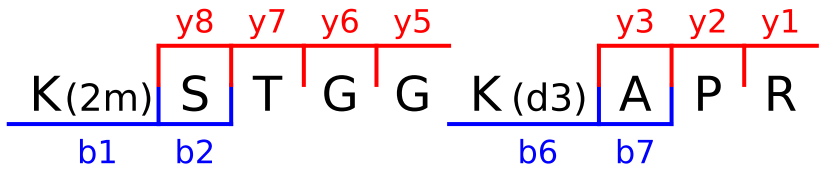

Raw file: QEP231009_CG_frozen_breast_TN_25, Scan: 2288, m/z: 545.80382, Charge: 2, RT (min): 8.93, Score: 133.23

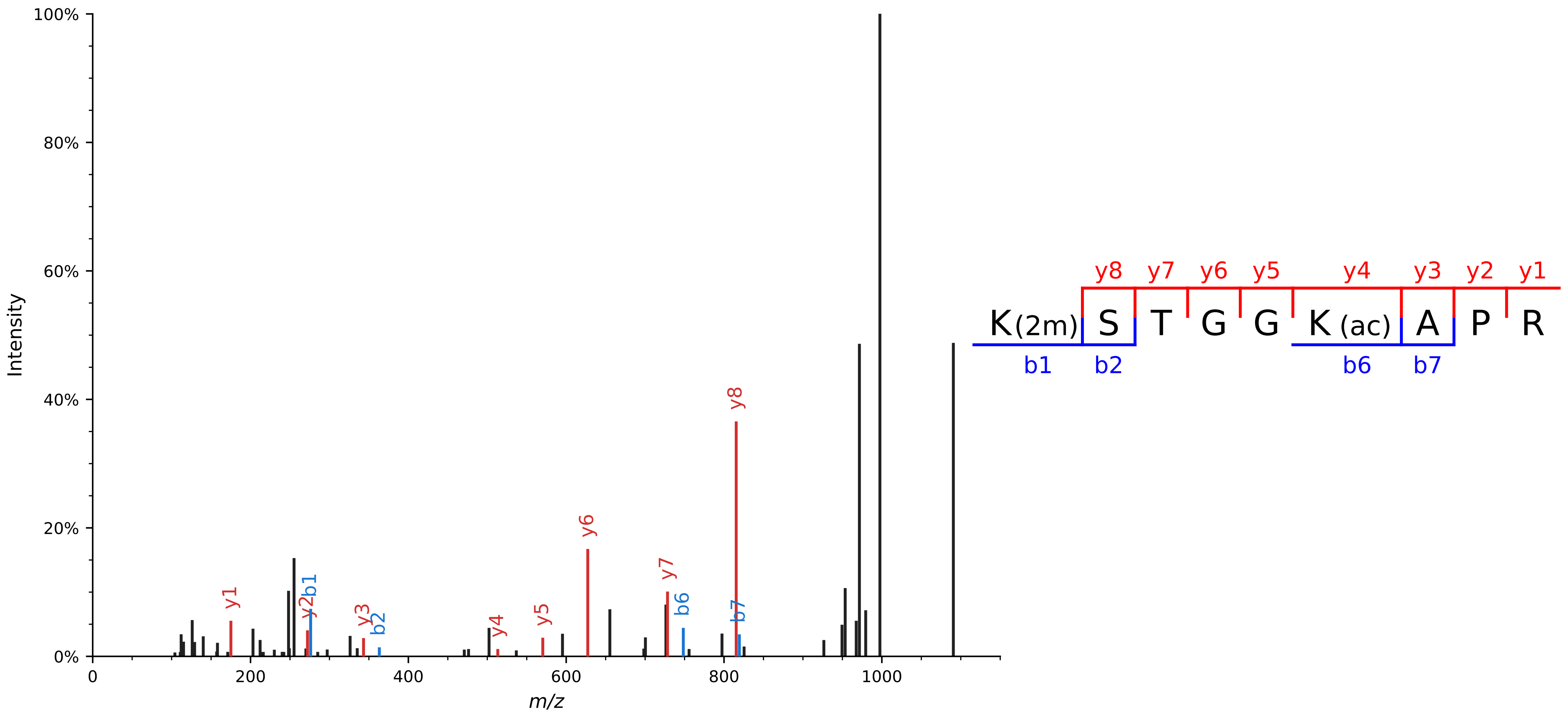

Raw file: QEP231009_CG_frozen_breast_Normal_24, Scan: 1855, m/z: 538.796, Charge: 2, RT (min): 8.03, Score: 60.118

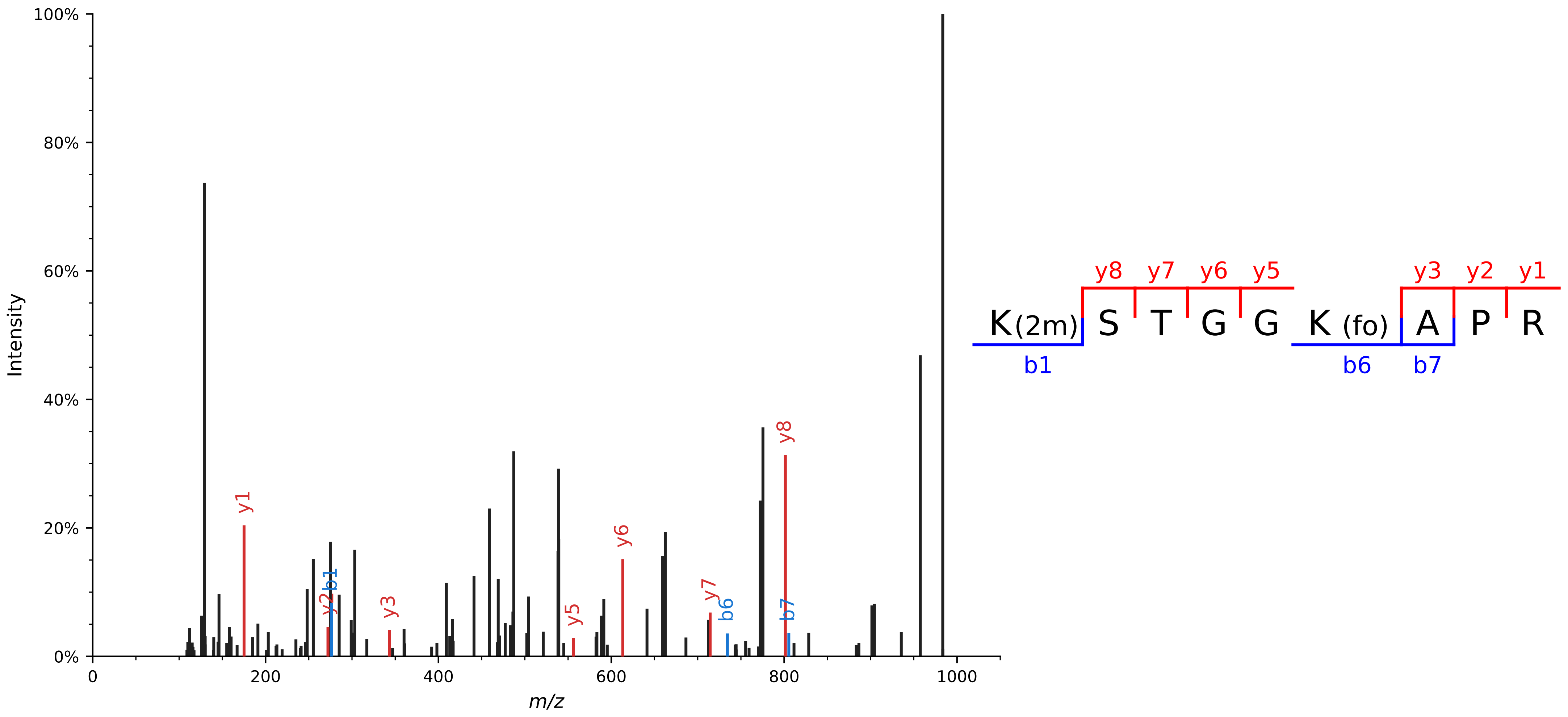

Raw file: QEP231009_CG_frozen_breast_LuA_31, Scan: 2969, m/z: 552.81165, Charge: 2, RT (min): 10.64, Score: 121.09

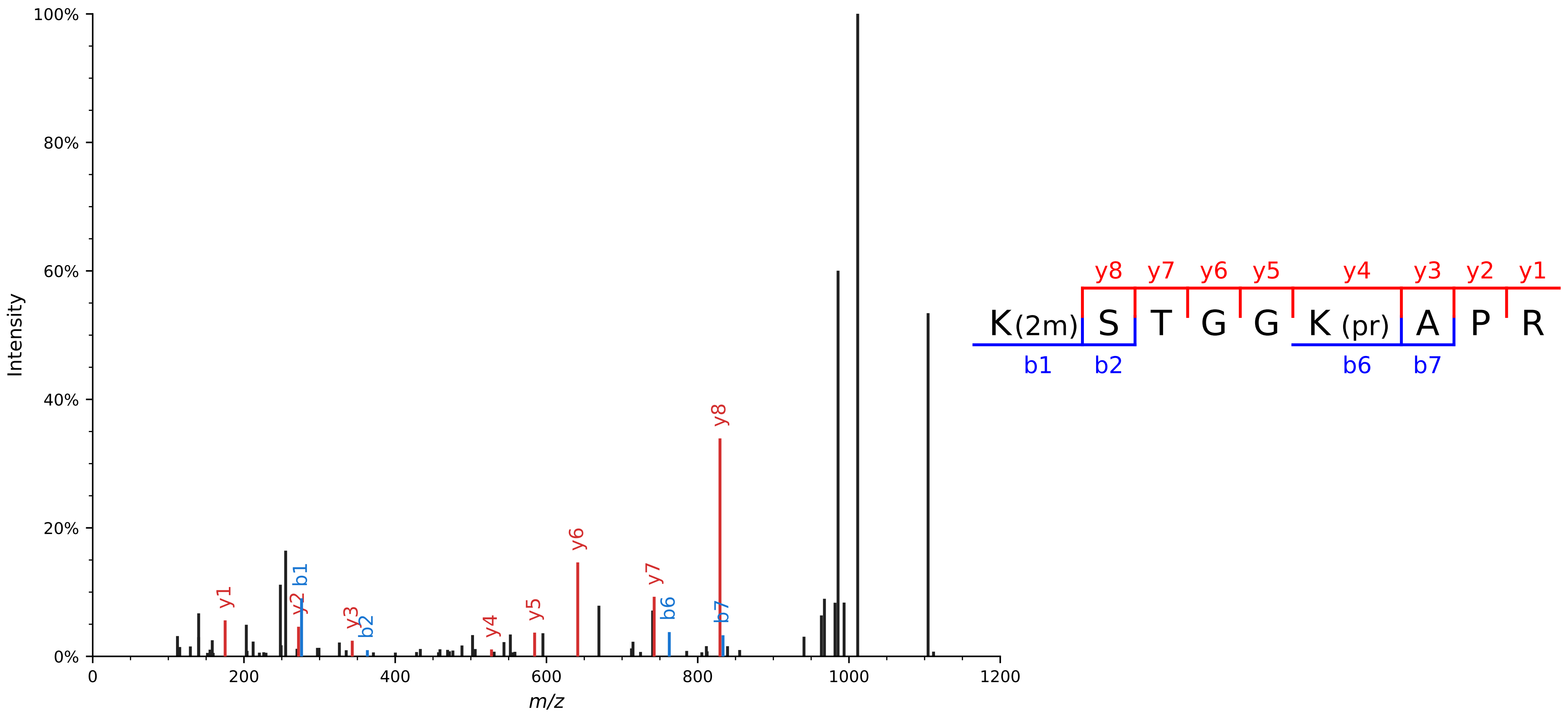

Raw file: QEP231009_CG_frozen_breast_LuA_30, Scan: 2068, m/z: 554.32106, Charge: 2, RT (min): 8.52, Score: 145.72

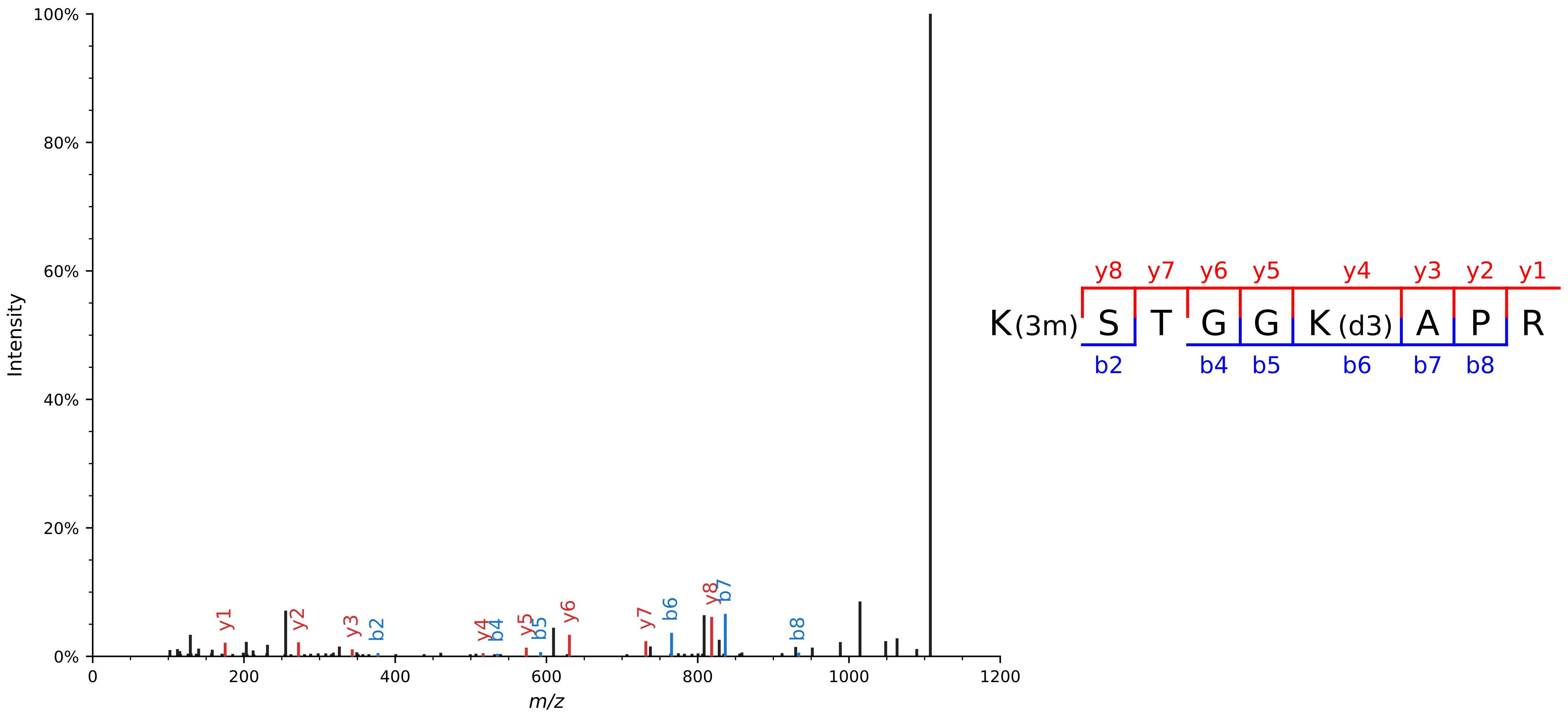

Raw file: QEP231009_CG_frozen_breast_TN_25, Scan: 2212, m/z: 368.87686, Charge: 3, RT (min): 8.77, Score: 77.14

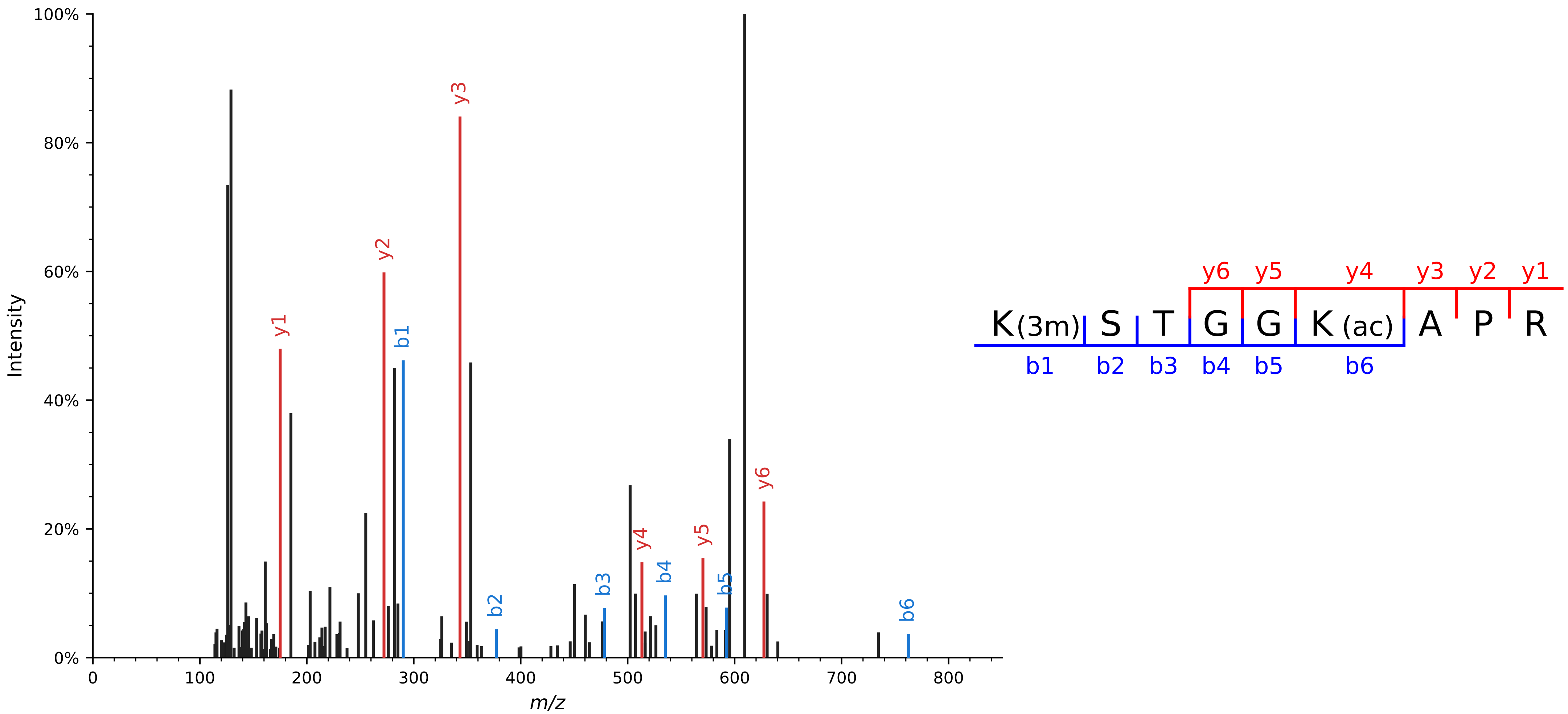

Raw file: QEP231009_CG_frozen_breast_Normal_24, Scan: 2856, m/z: 559.81947, Charge: 2, RT (min): 10.41, Score: 130.75

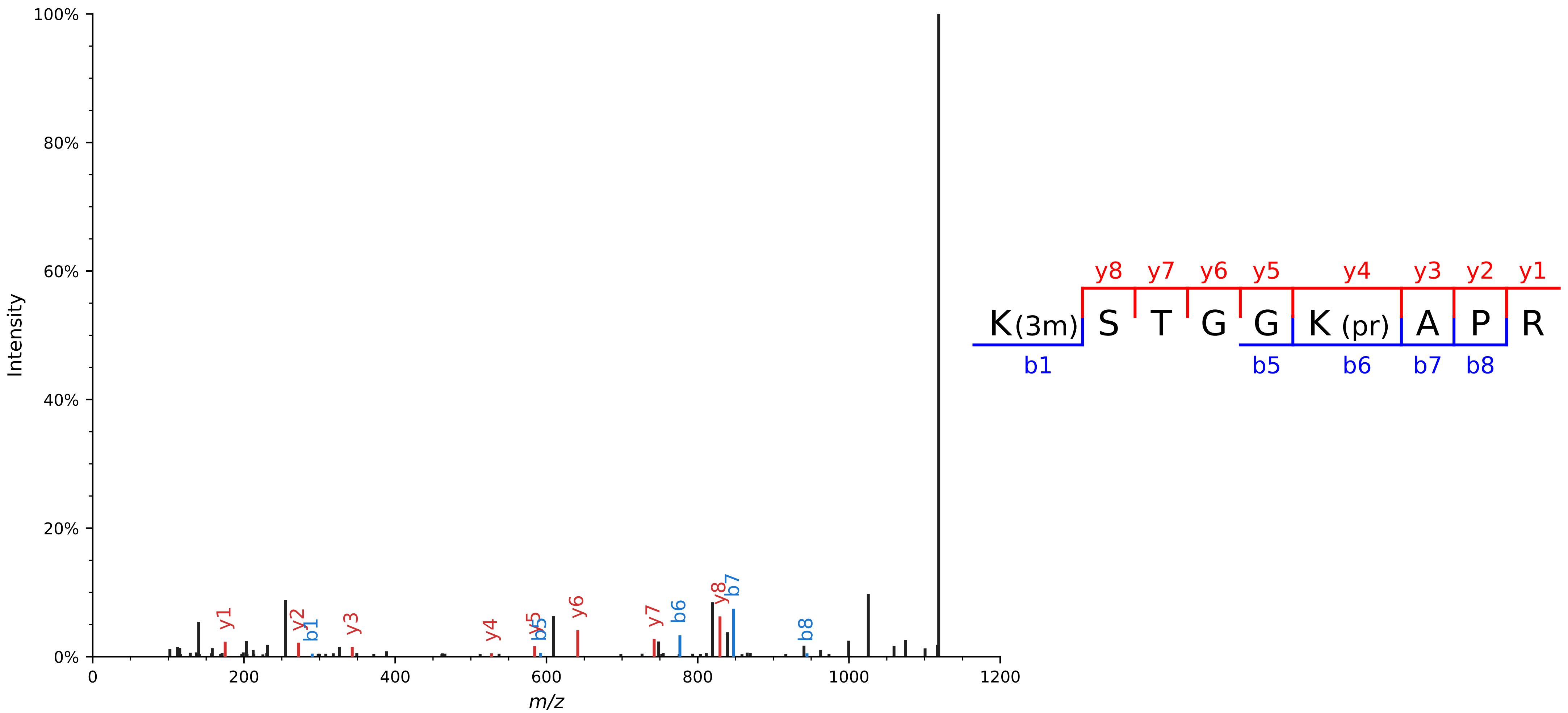

Raw file: QEP231009_CG_frozen_breast_TN_27, Scan: 8320, m/z: 561.3107, Charge: 2, RT (min): 22.25, Score: 121.62

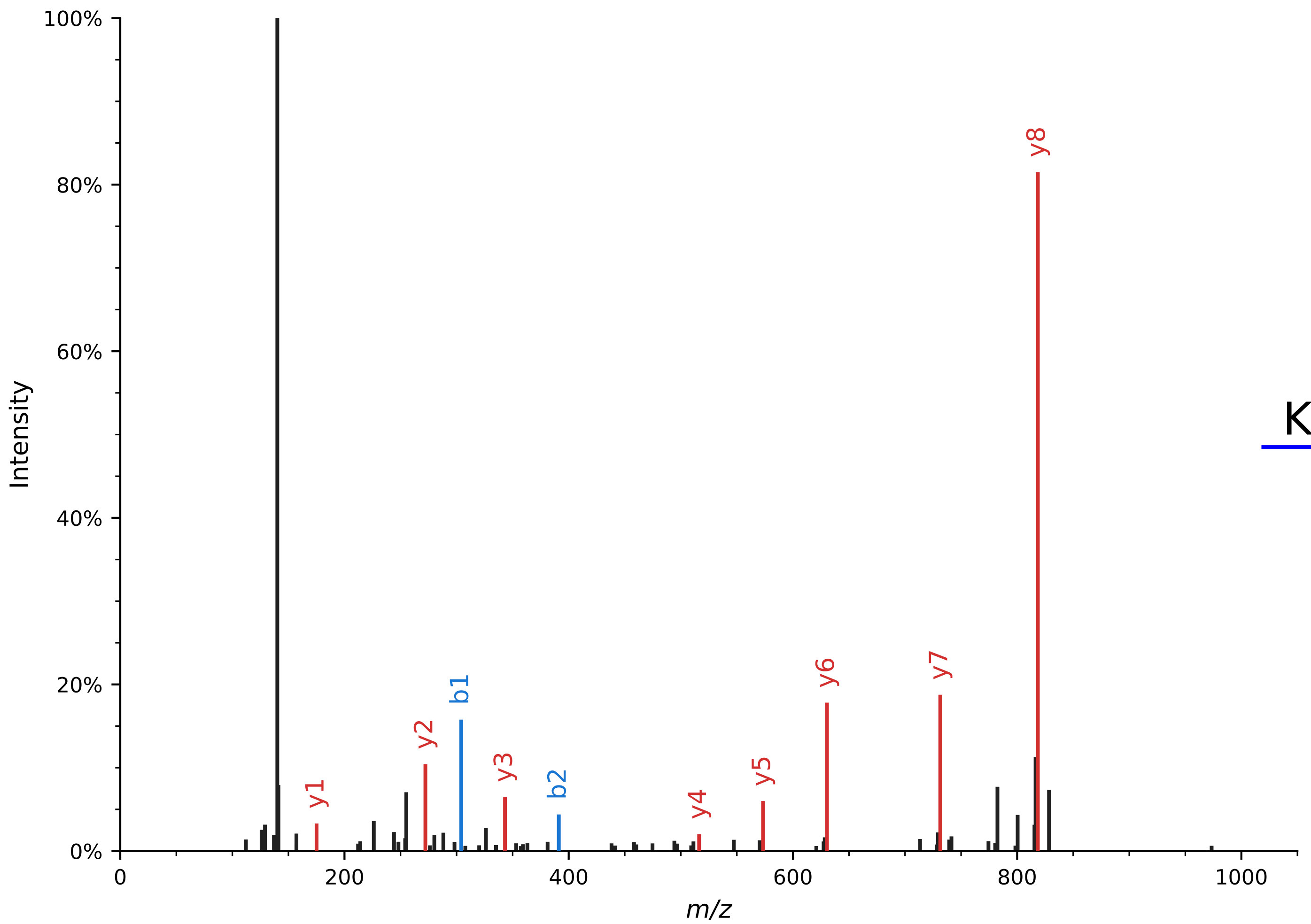

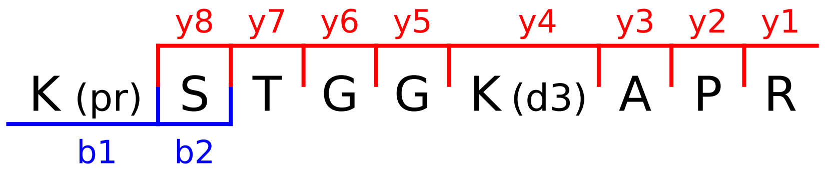

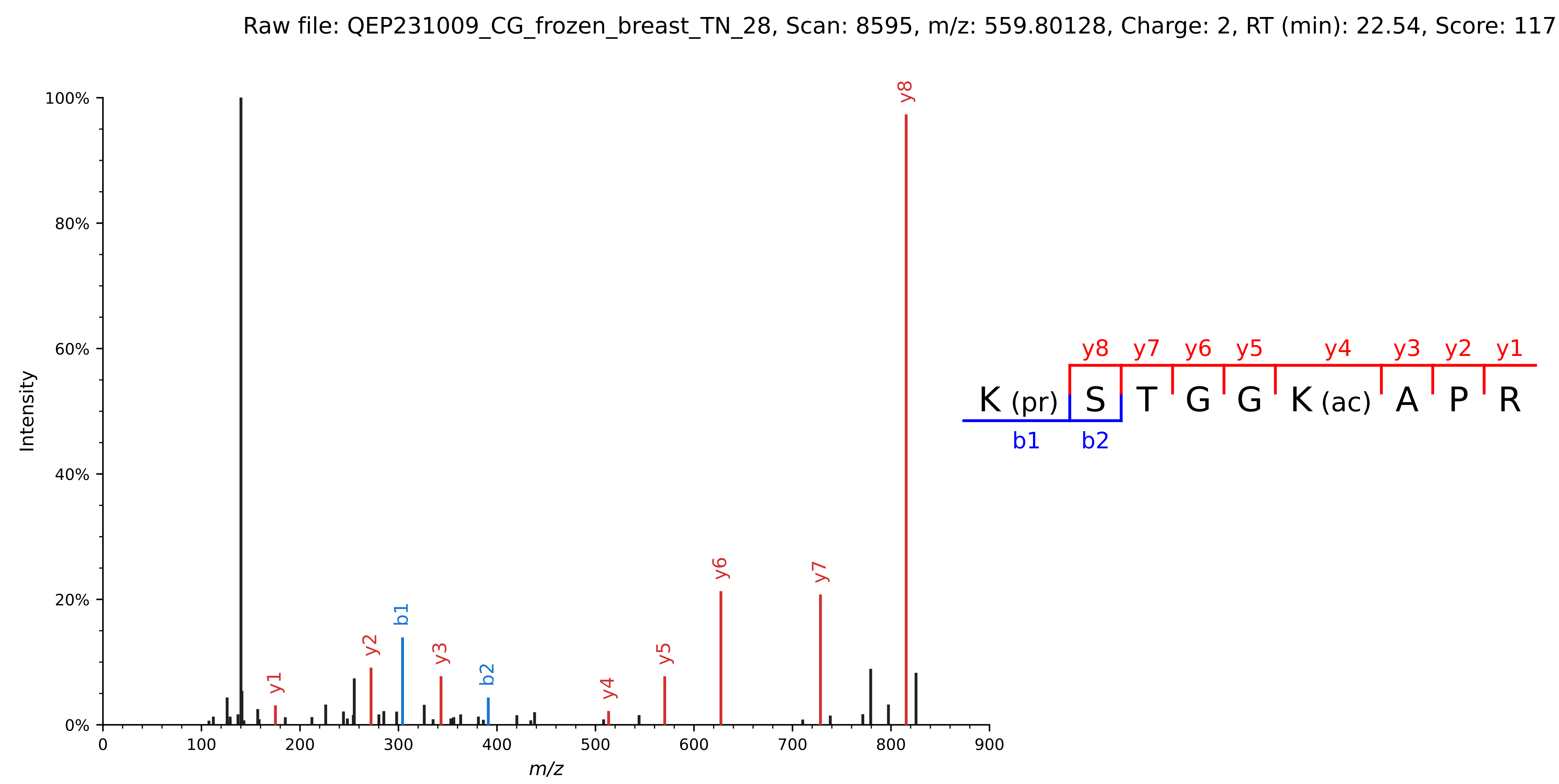
Raw file: QEP231009_CG_frozen_breast_TN_28, Scan: 8595, m/z: 559.80128, Charge: 2, RT (min): 22.54, Score: 117.04
Intensity
m/z
y1
y2
b1
y3
b2
y4
y5
y6
y7
y8
K (pr) S T G G K (ac) A P R
b1
b2

Raw file: QEP231009_CG_frozen_breast_Normal_24, Scan: 3516, m/z: 568.31852, Charge: 2, RT (min): 11.85, Score: 73.248

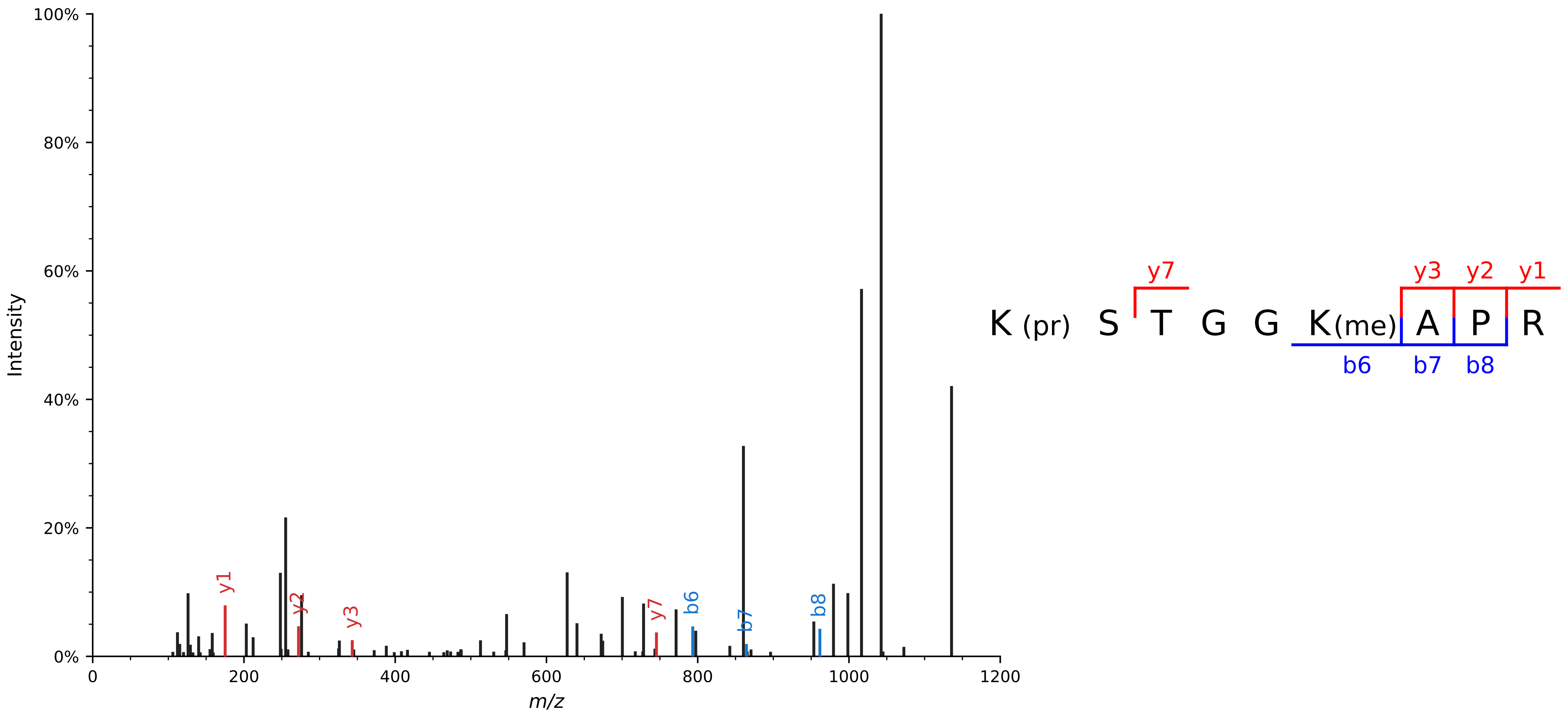

Raw file: QEP231009_CG_frozen_breast_TN_25, Scan: 9793, m/z: 566.8091, Charge: 2, RT (min): 24.59, Score: 95.909

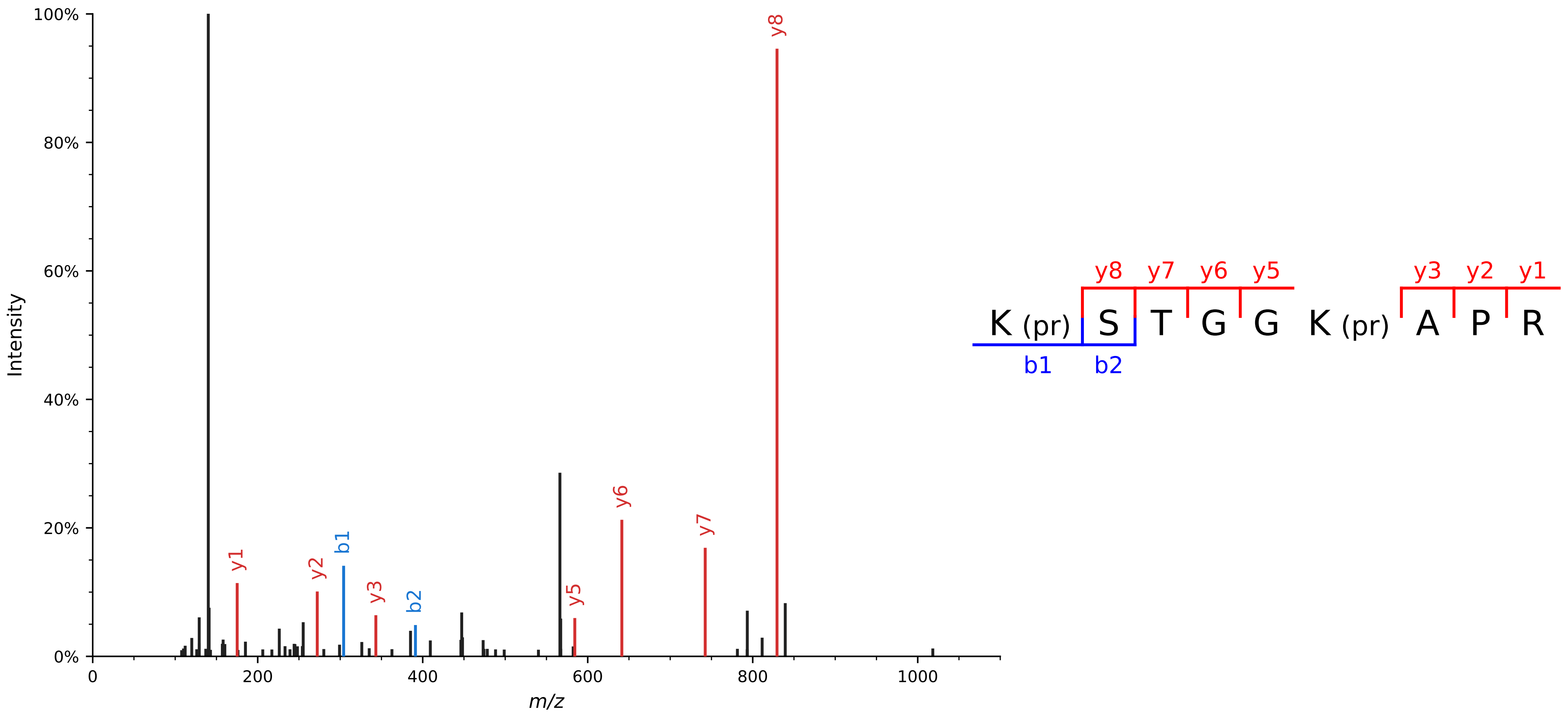

Raw file: QEP231009_CG_frozen_breast_Normal_22, Scan: 6945, m/z: 554.30287, Charge: 2, RT (min): 19.61, Score: 123.98

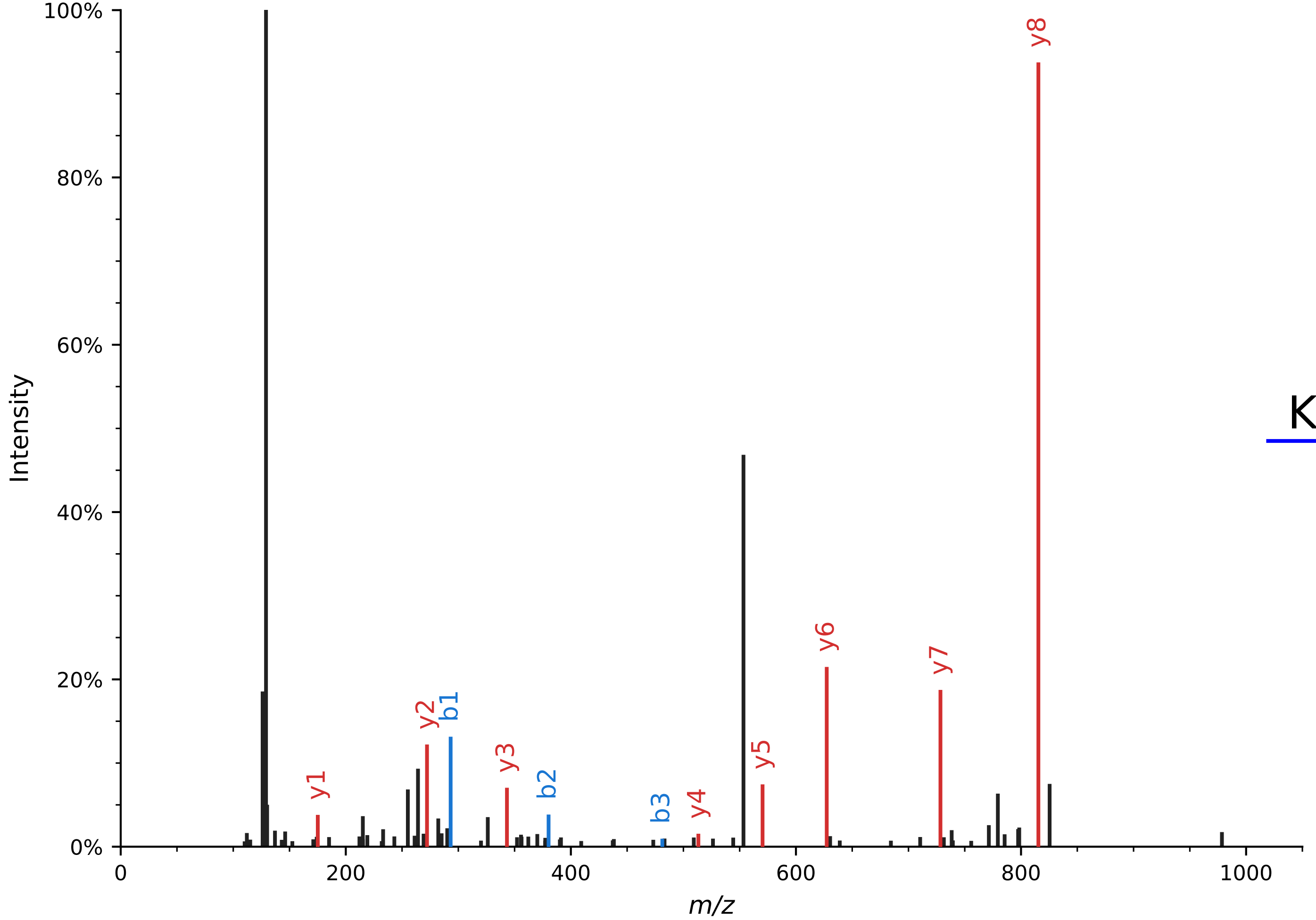

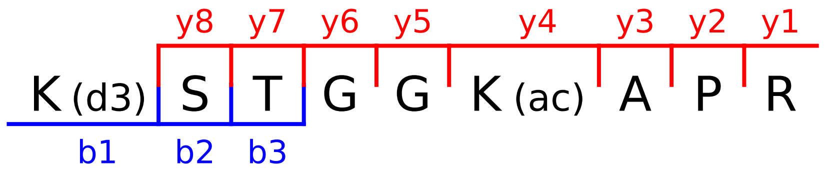

Raw file: QEP231009_CG_frozen_breast_TN_28, Scan: 9499, m/z: 568.31852, Charge: 2, RT (min): 24.63, Score: 82.452

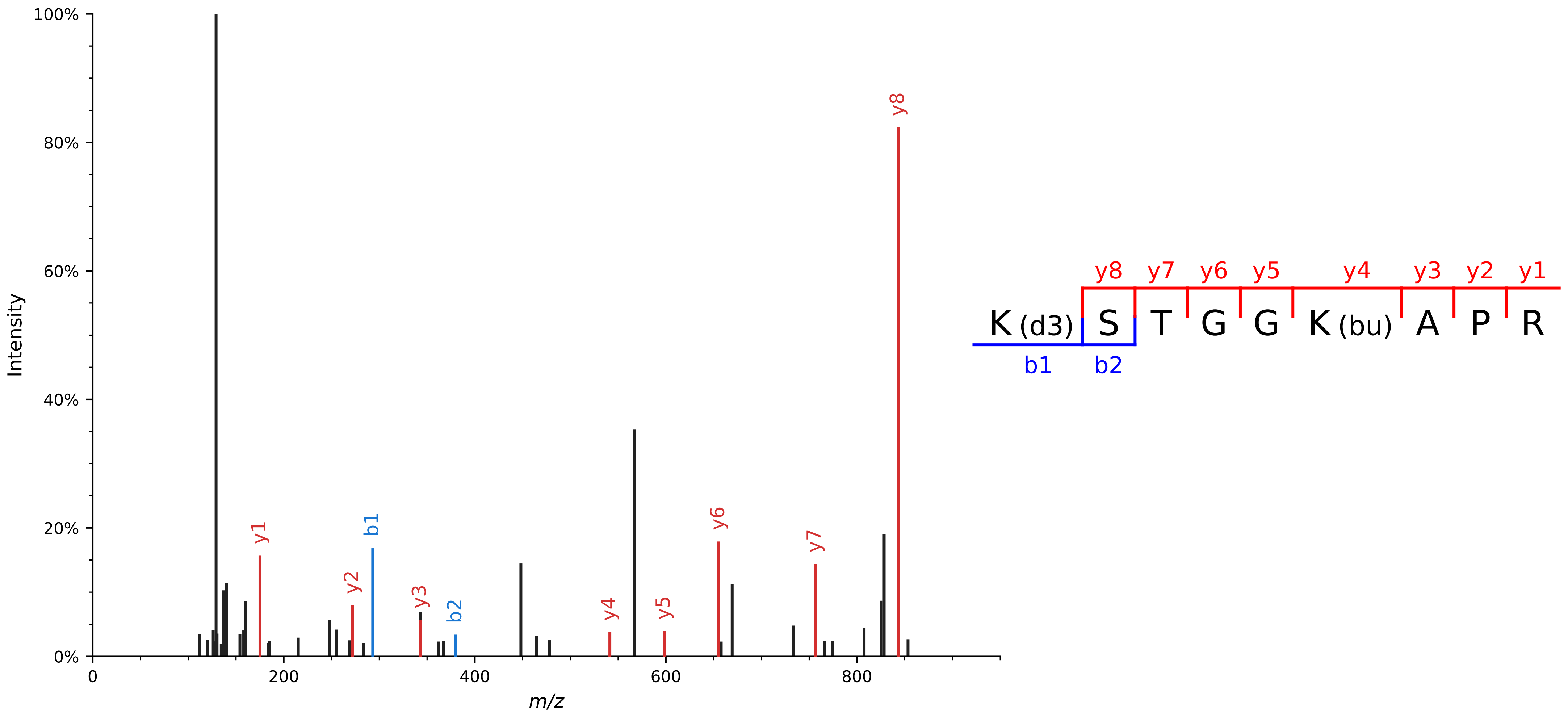

Raw file: QEP231009_CG_frozen_breast_TN_28, Scan: 8301, m/z: 561.3107, Charge: 2, RT (min): 21.91, Score: 120.46

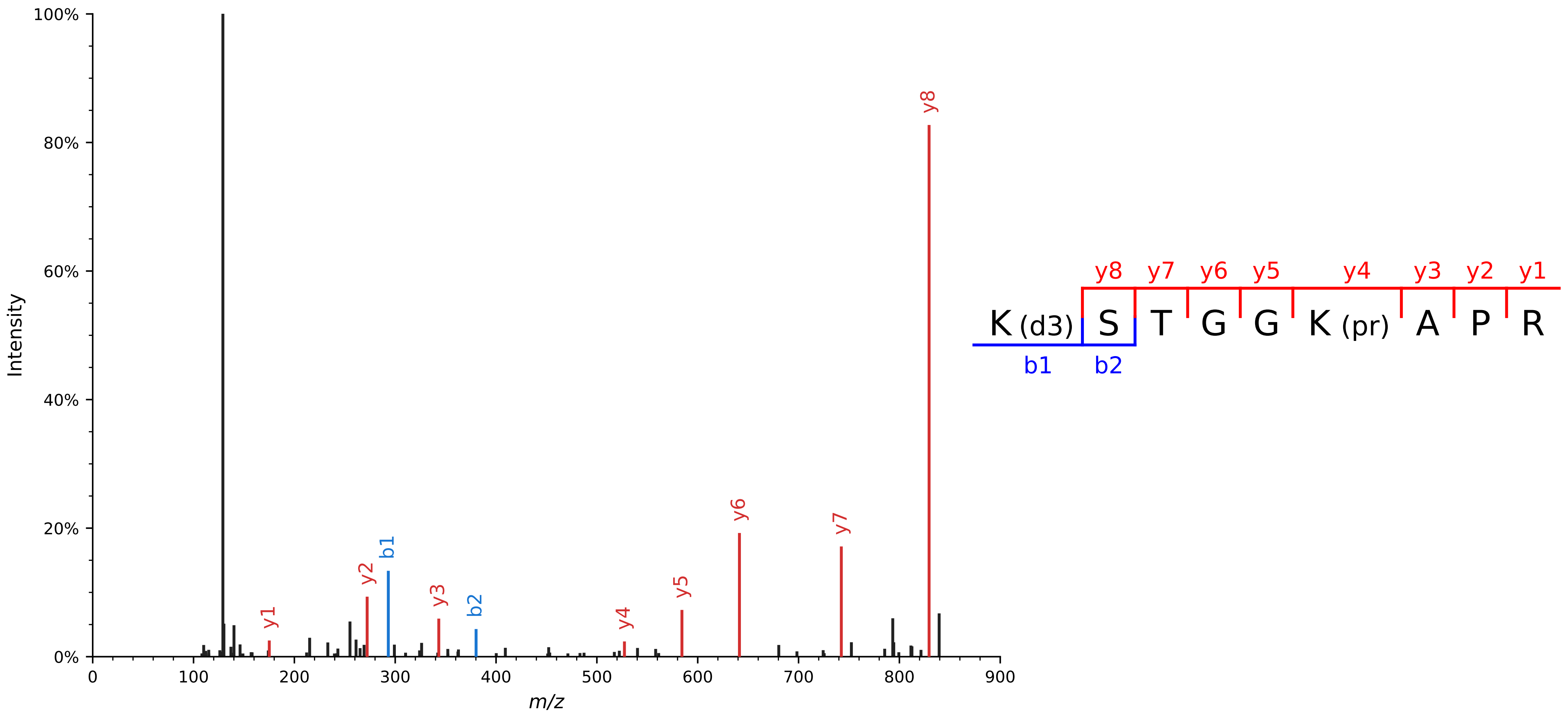

Raw file: QEP231009_CG_frozen_breast_TN_25, Scan: 15160, m/z: 595.33803, Charge: 2, RT (min): 35.88, Score: 106.6

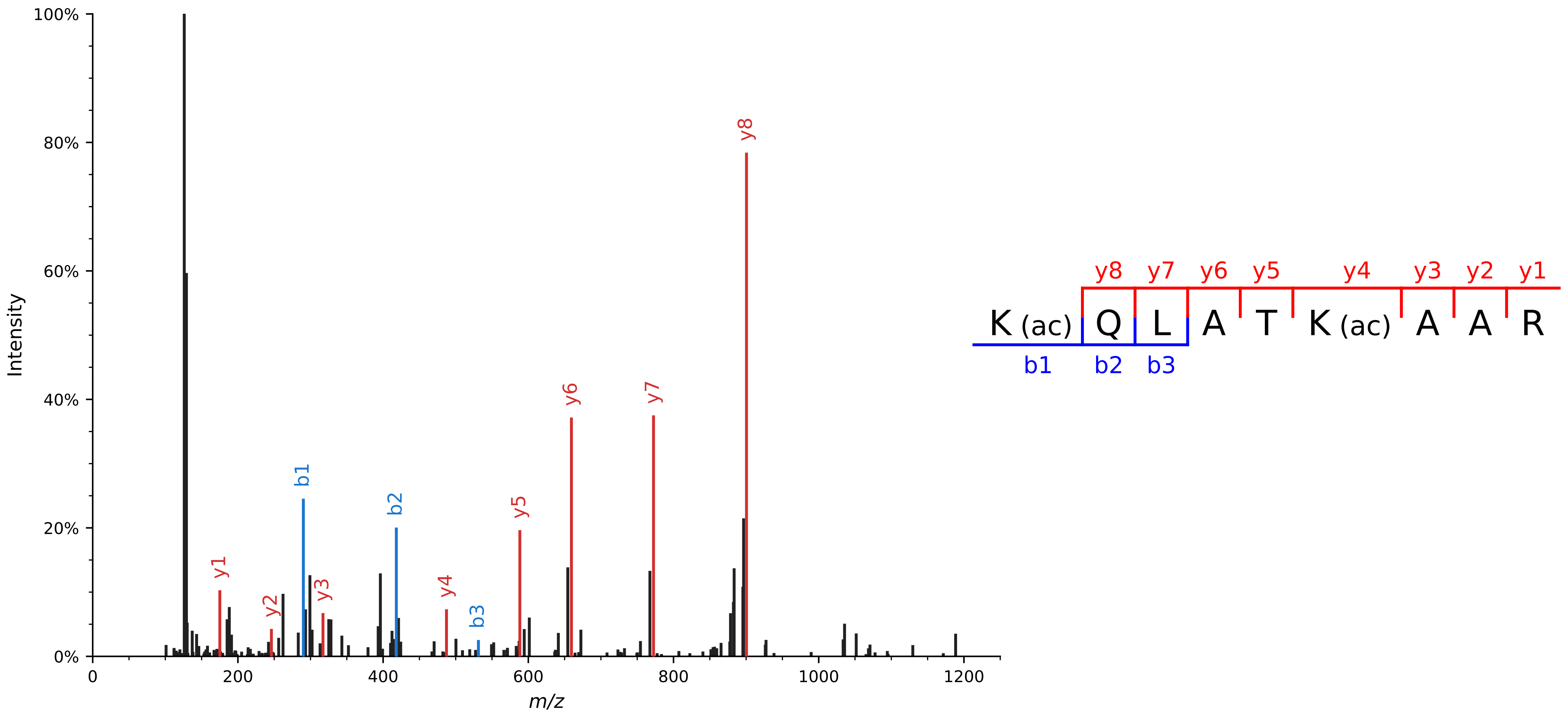

Raw file: QEP231009_CG_frozen_breast_TN_25, Scan: 15191, m/z: 588.3484, Charge: 2, RT (min): 35.96, Score: 95.909

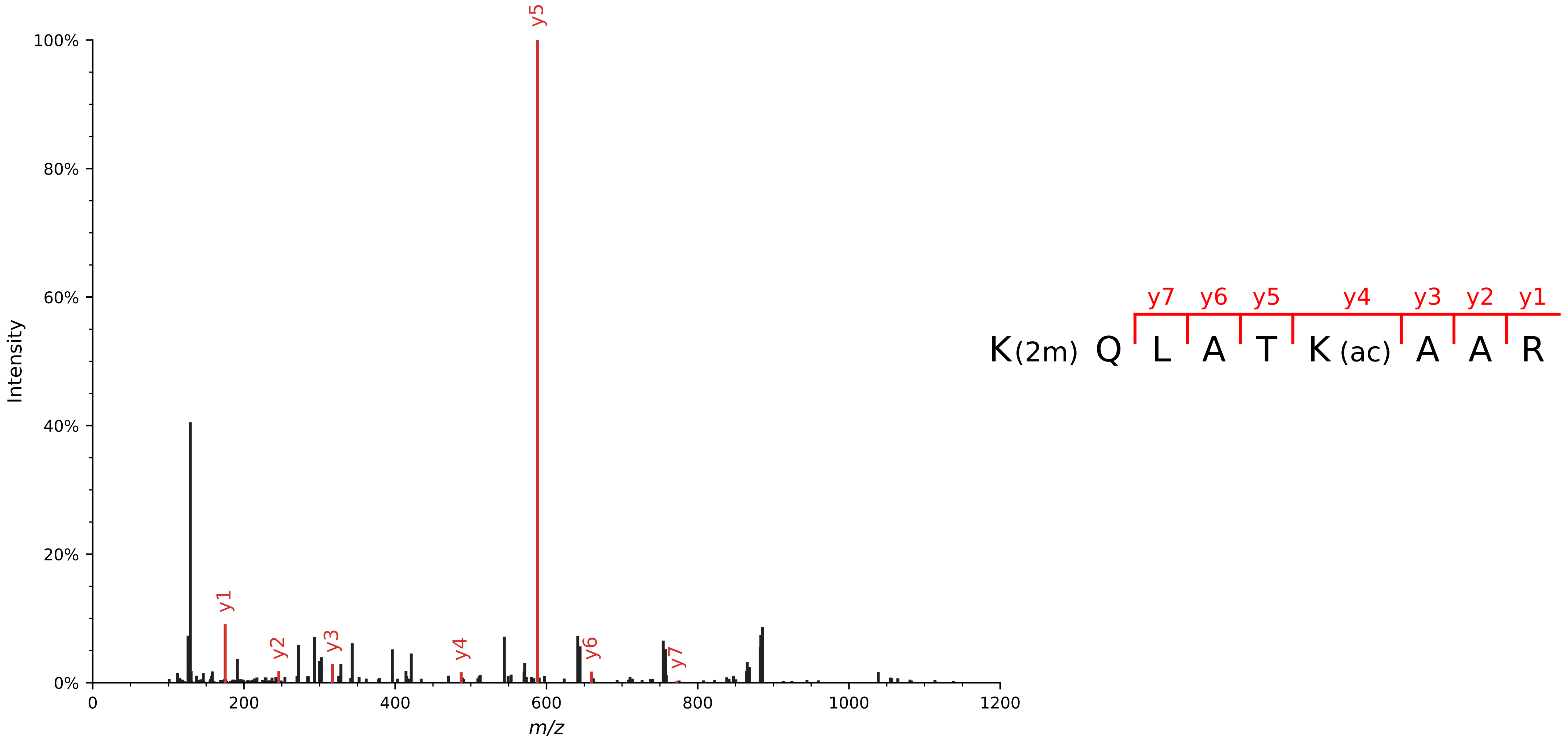

Raw file: QEP231009_CG_frozen_breast_TN_25, Scan: 16262, m/z: 603.85527, Charge: 2, RT (min): 38.30, Score: 139.88

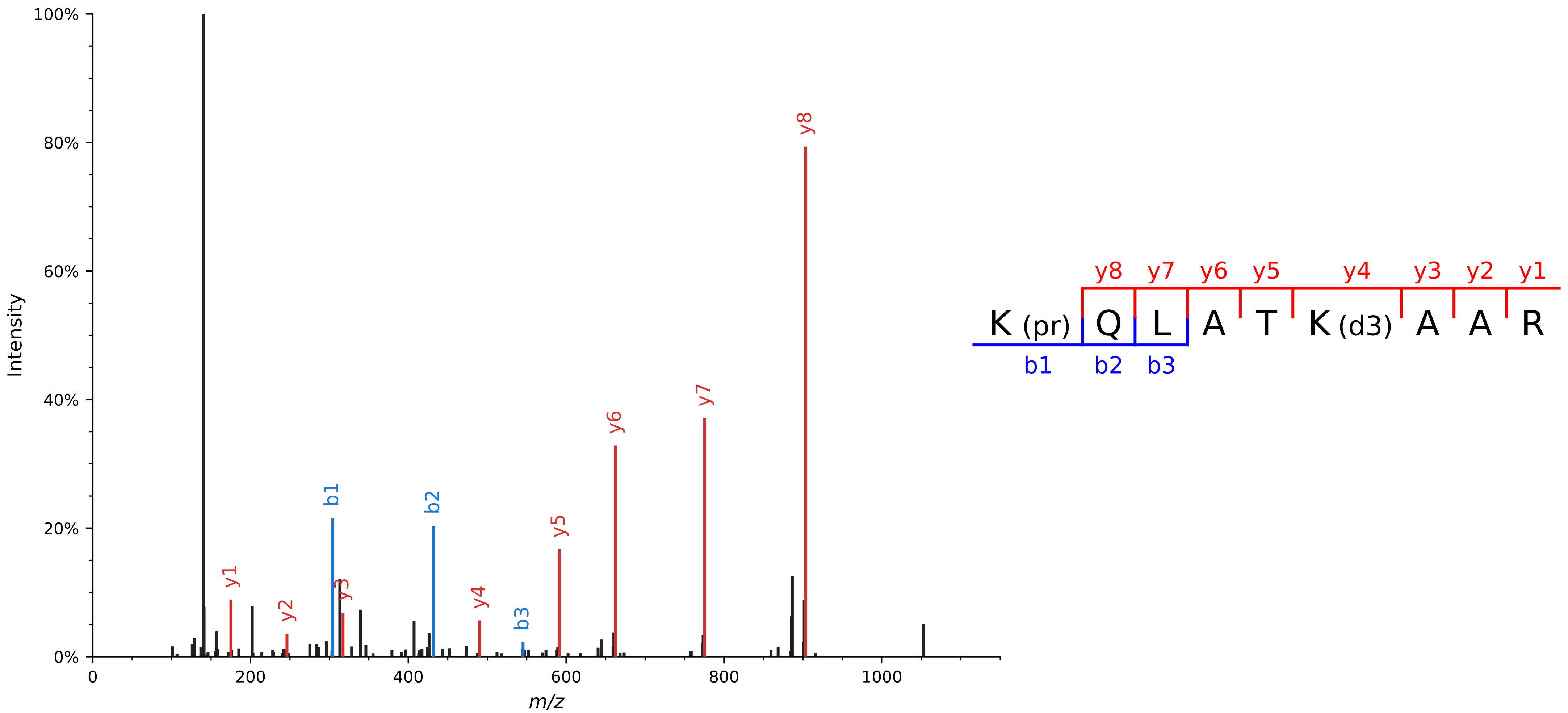

Raw file: QEP231009_CG_frozen_breast_TN_25, Scan: 15172, m/z: 596.84744, Charge: 2, RT (min): 35.91, Score: 163.9

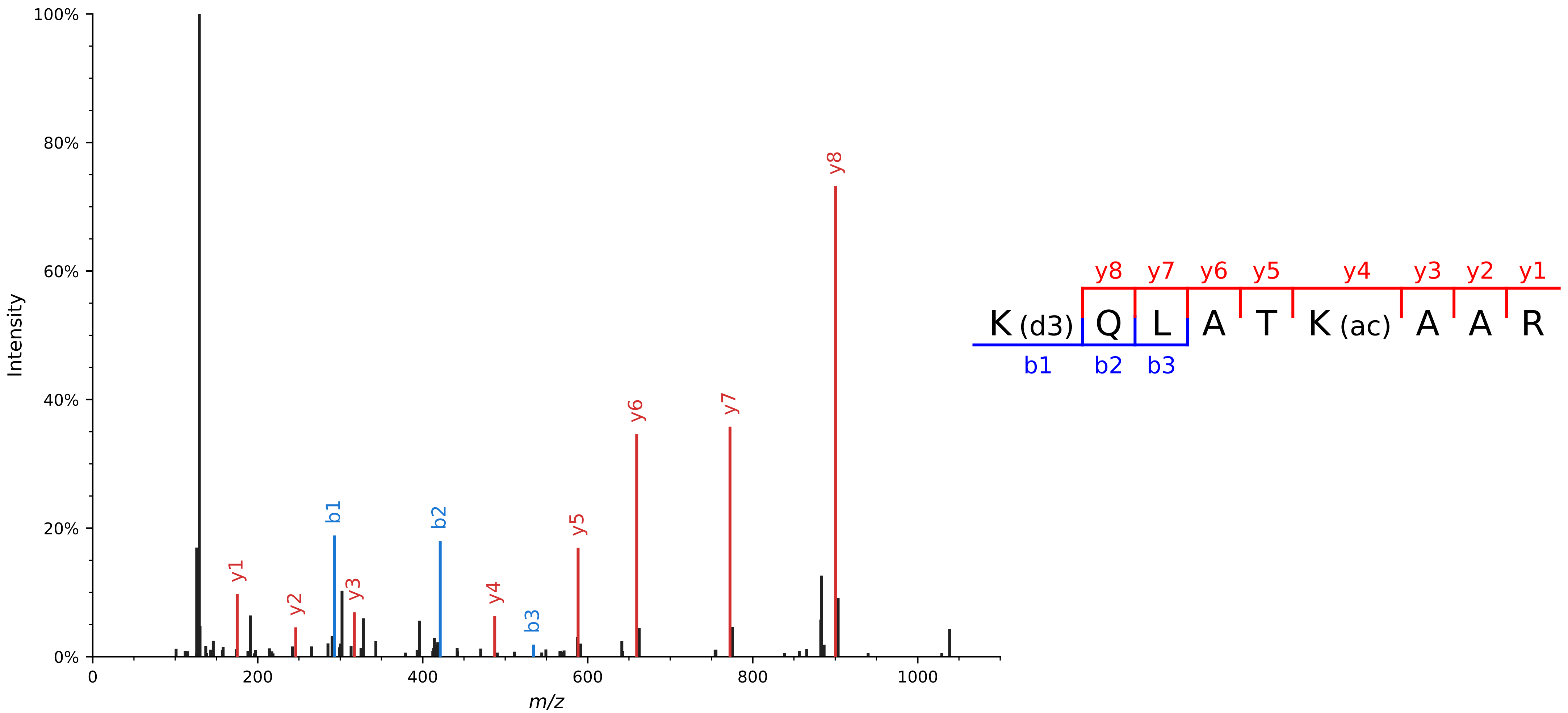

Raw file: QEP231009_CG_frozen_breast_TN_29, Scan: 15748, m/z: 603.85527, Charge: 2, RT (min): 37.91, Score: 139.88

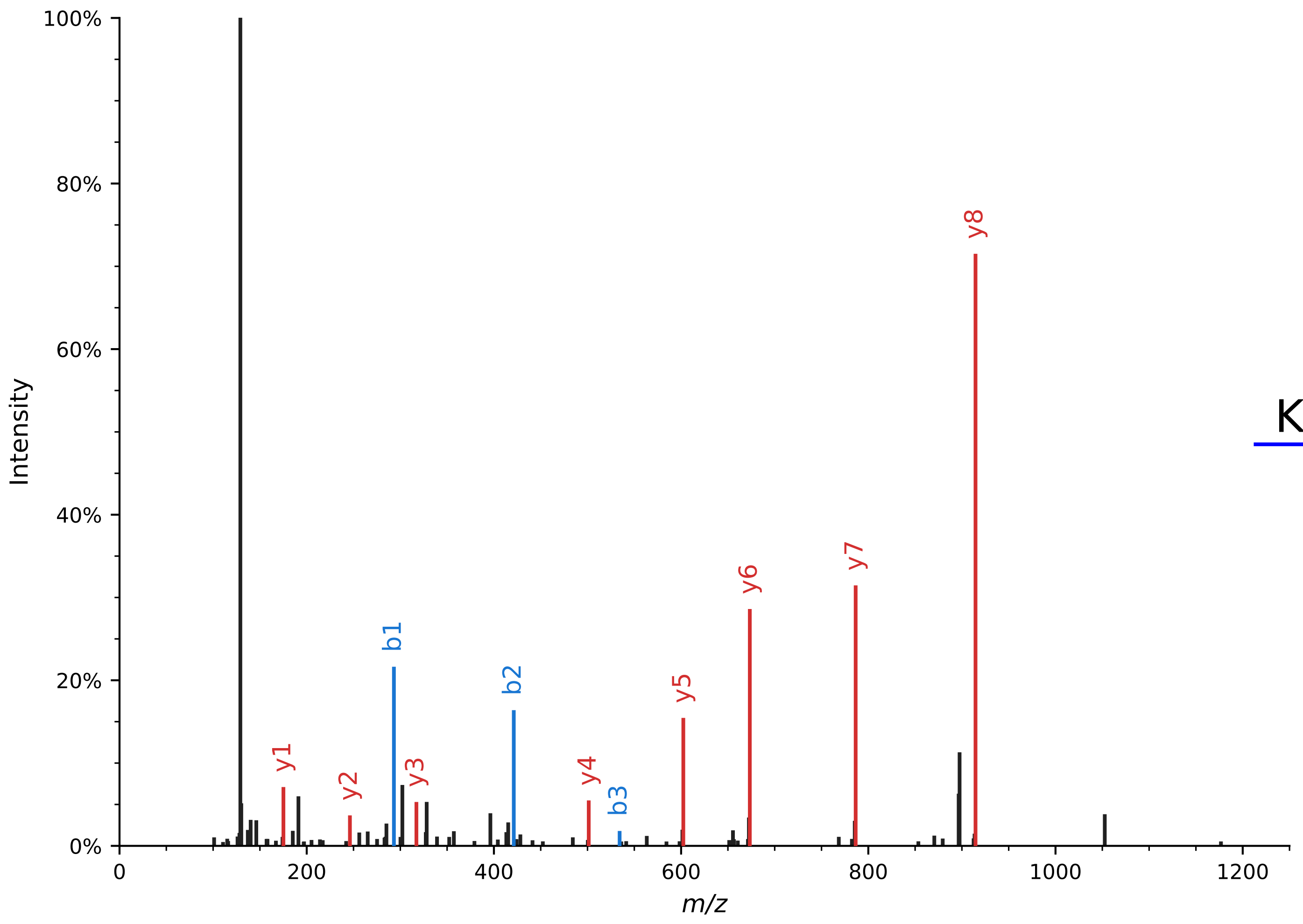

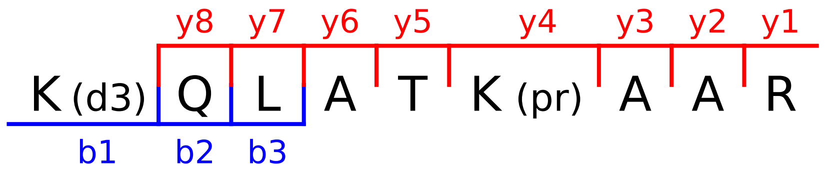

Raw file: QEP231009_CG_frozen_breast_TN_25, Scan: 10104, m/z: 856.9893, Charge: 2, RT (min): 25.25, Score: 93.258

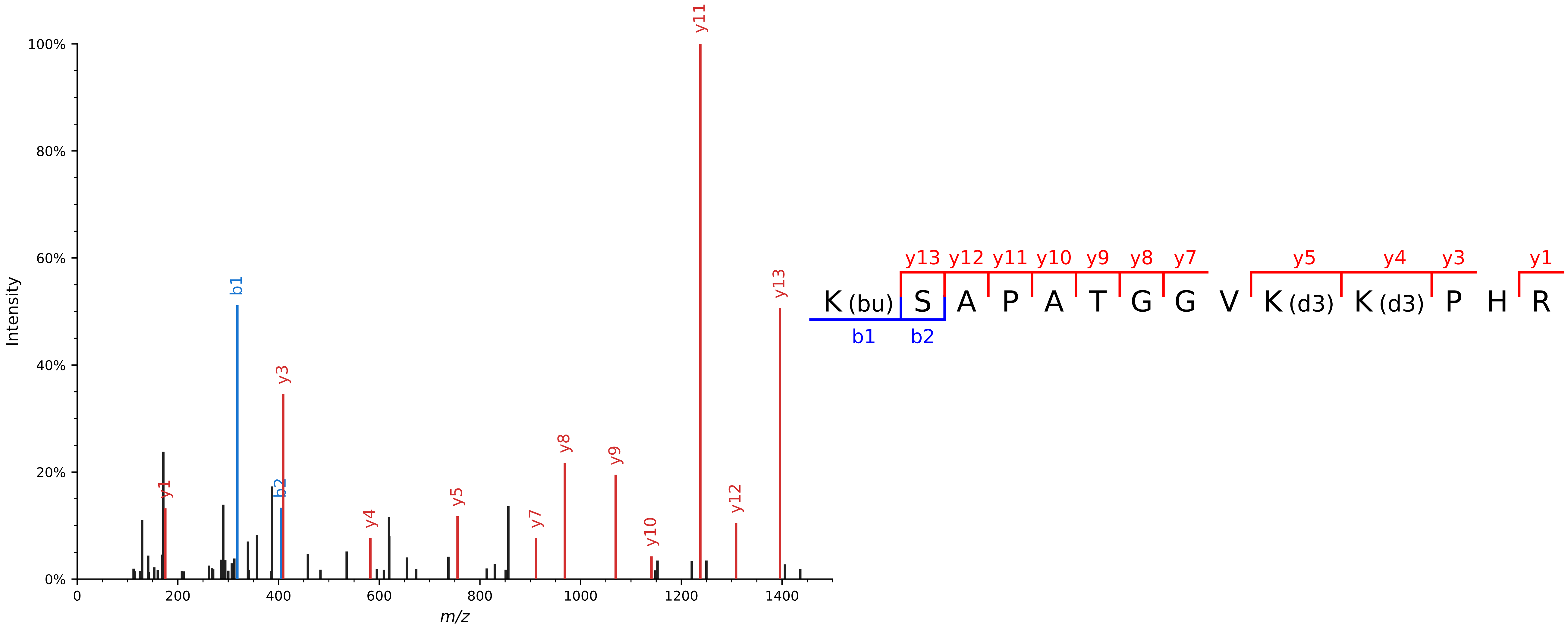

Raw file: QEP231009_CG_frozen_breast_TN_25, Scan: 6637, m/z: 565.99593, Charge: 3, RT (min): 17.98, Score: 78.401

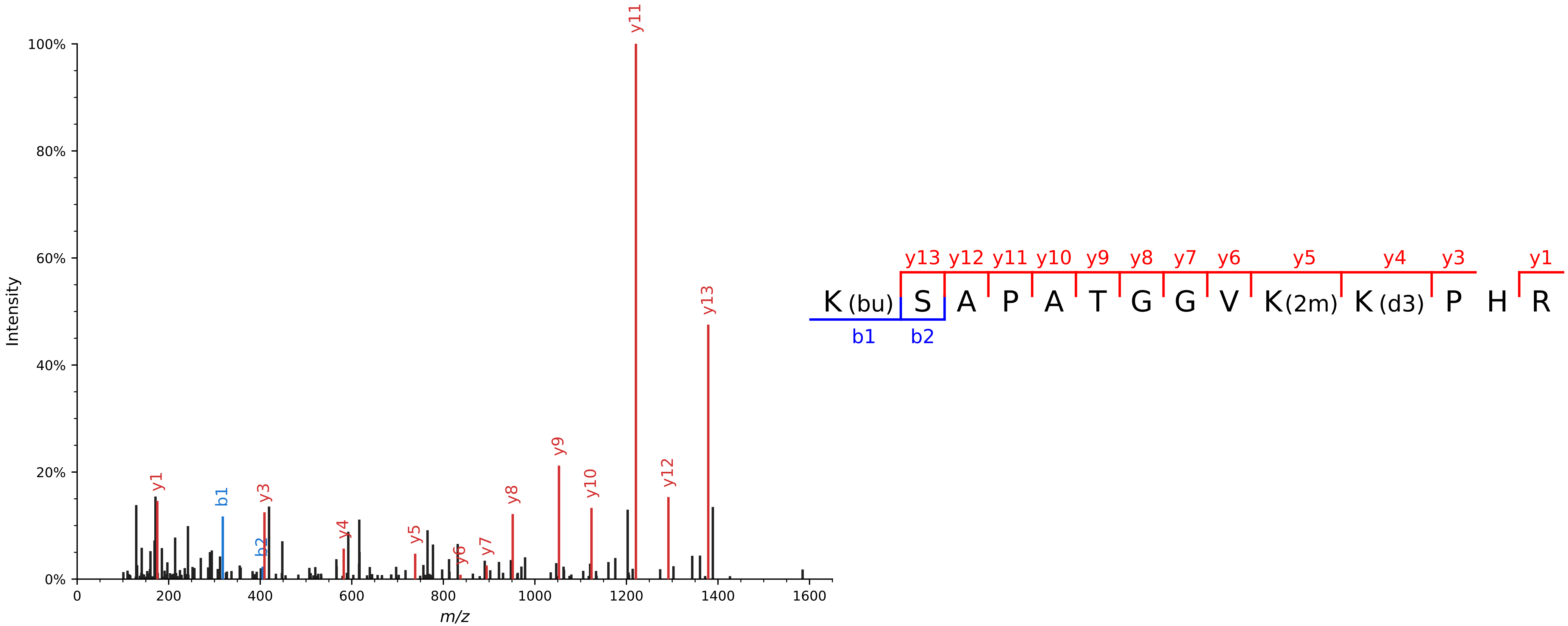

Raw file: QEP231009_CG_frozen_breast_TN_25, Scan: 9006, m/z: 851.49089, Charge: 2, RT (min): 22.87, Score: 167.09

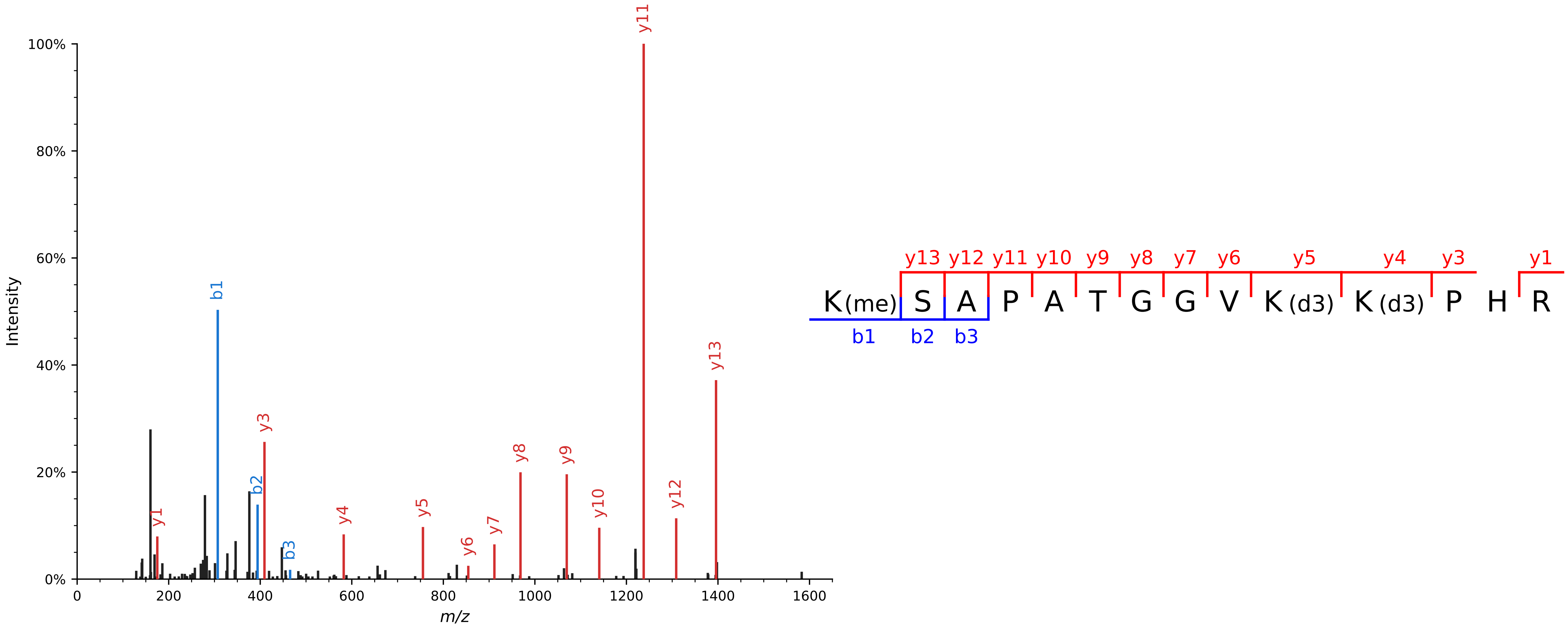

Raw file: QEP231009_CG_frozen_breast_TN_25, Scan: 10465, m/z: 863.99713, Charge: 2, RT (min): 26.05, Score: 84.658

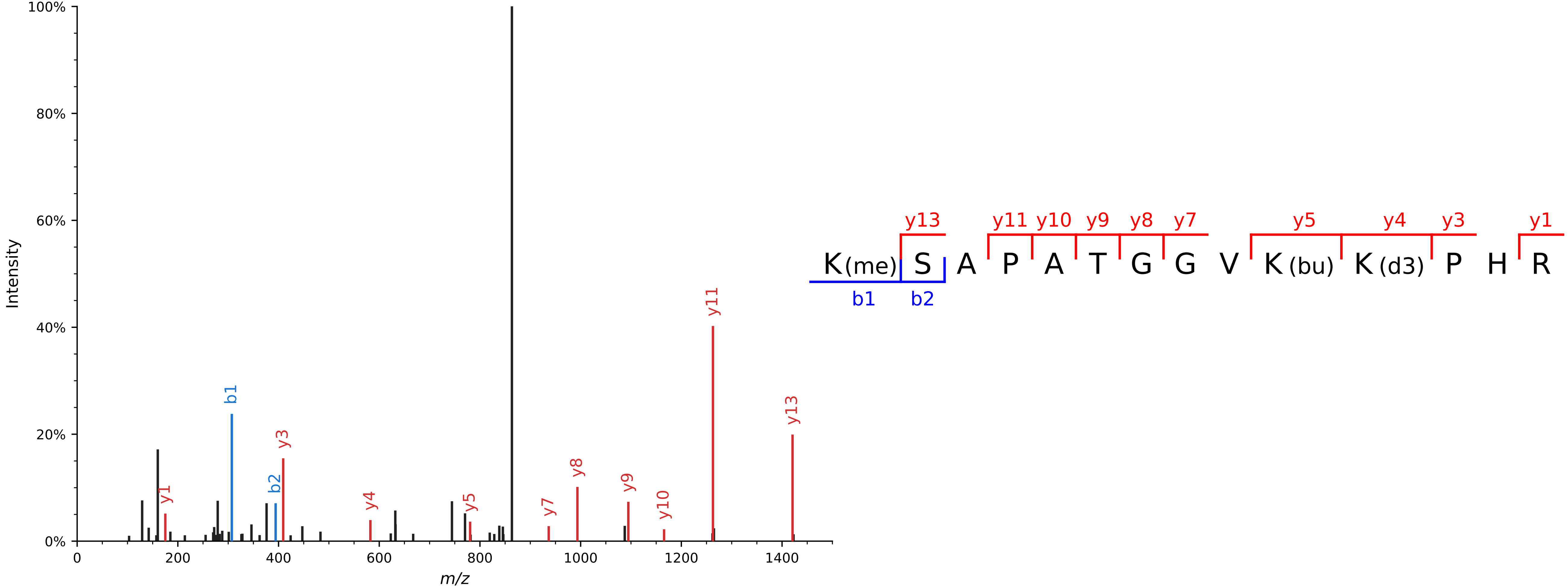

Raw file: QEP231009_CG_frozen_breast_TN_25, Scan: 9479, m/z: 858.49872, Charge: 2, RT (min): 23.91, Score: 152.07

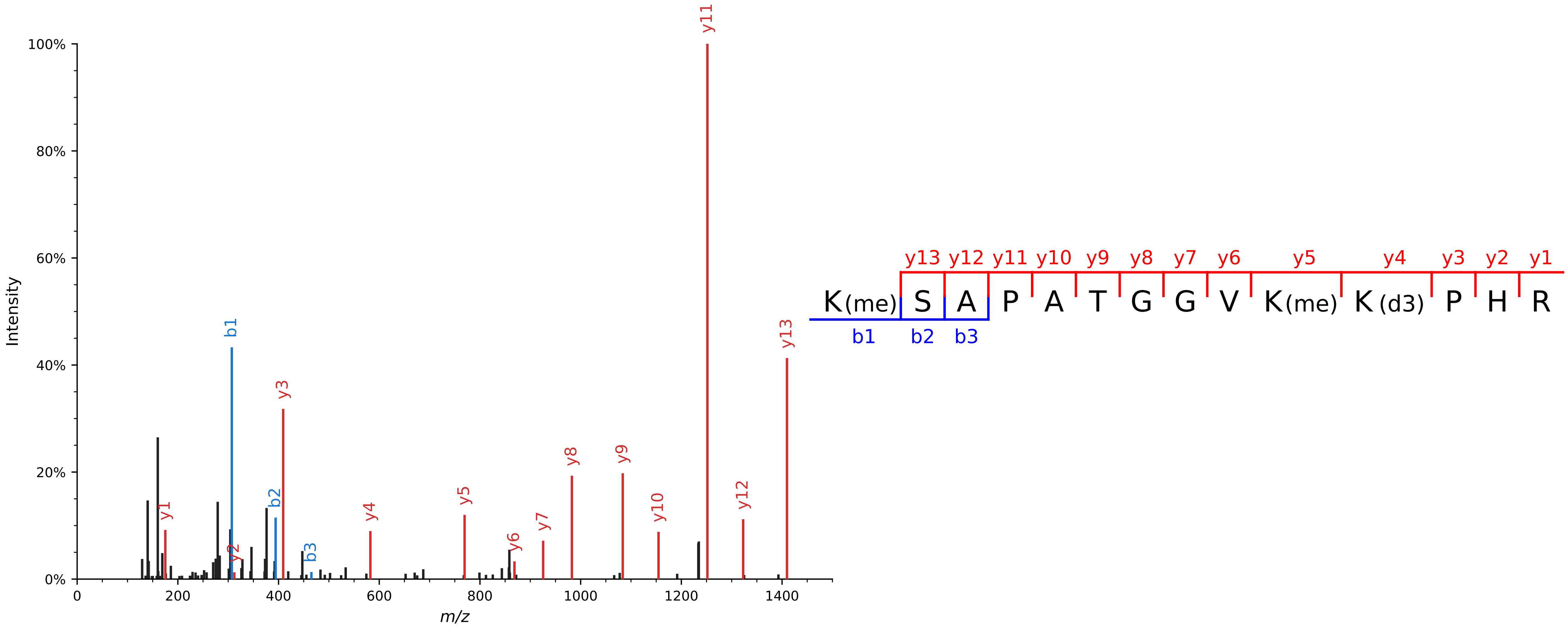

Raw file: QEP231009_CG_frozen_breast_TN_25, Scan: 6715, m/z: 577.34012, Charge: 3, RT (min): 18.14, Score: 43.184

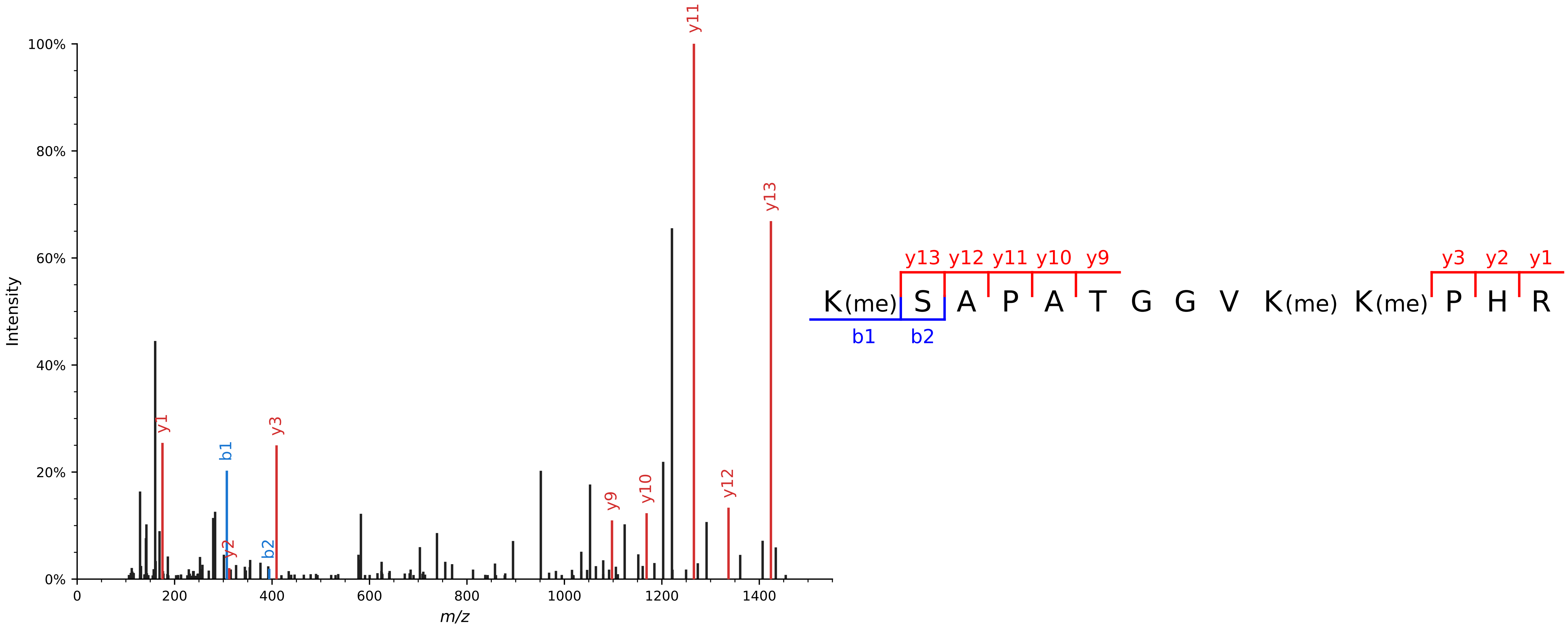

Raw file: QEP231009_CG_frozen_breast_TN_25, Scan: 10249, m/z: 576.33384, Charge: 3, RT (min): 25.55, Score: 62.042

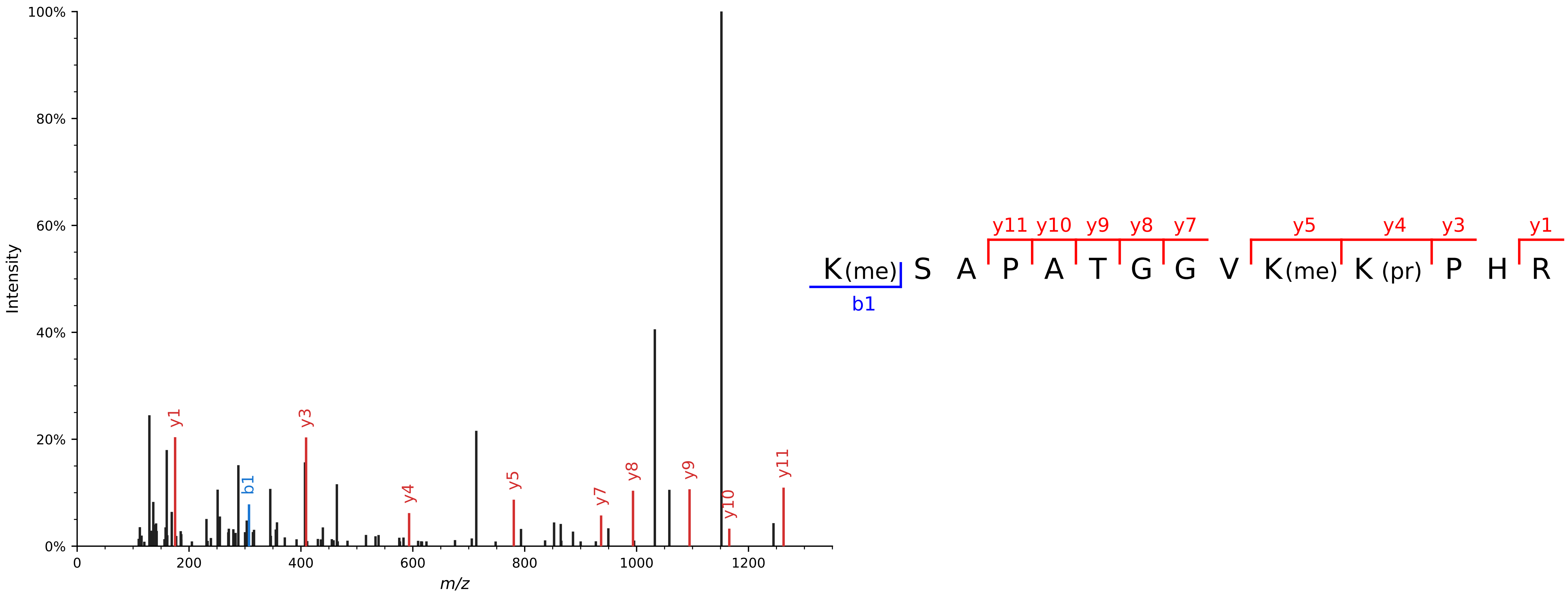

Raw file: QEP231009_CG_frozen_breast_TN_27, Scan: 5289, m/z: 562.33032, Charge: 3, RT (min): 15.73, Score: 117.07

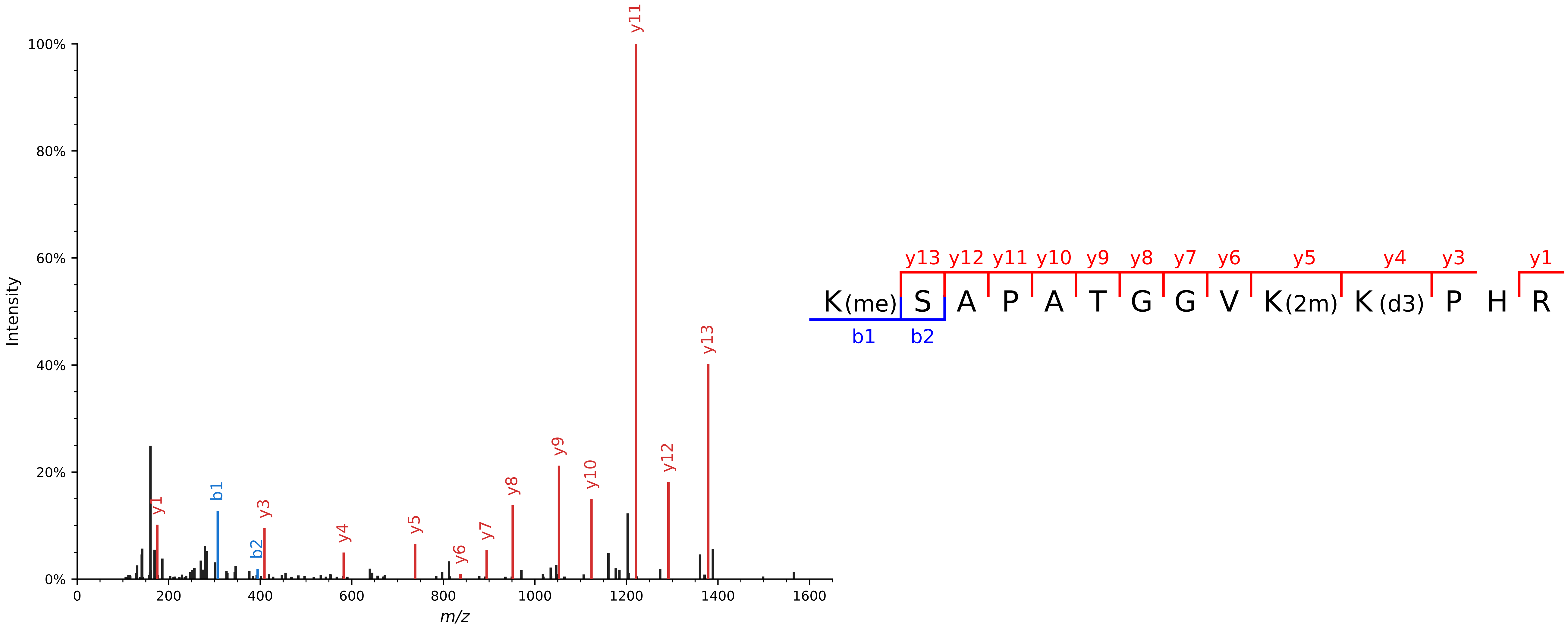

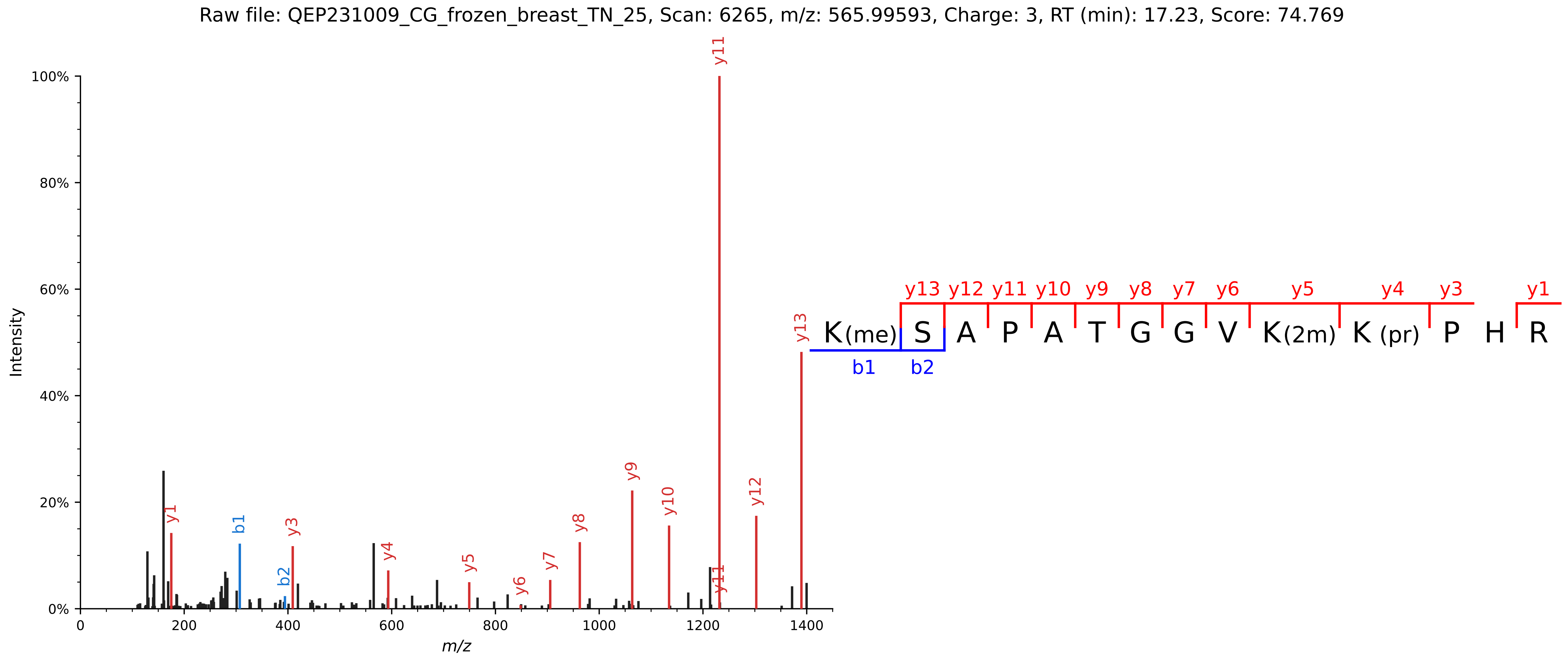

Raw file: QEP231009_CG_frozen_breast_TN_25, Scan: 6265, m/z: 565.99593, Charge: 3, RT (min): 17.23, Score: 74.769
Intensity
m/z
y1
b1
b2
y3
y4
y5
y6
y7
y8
y9
y10
y11
y12
y13
K(me) S A P A T G G V K(2m) K (pr) P H R

Raw file: QEP231009_CG_frozen_breast_TN_29, Scan: 5282, m/z: 567.0022, Charge: 3, RT (min): 15.35, Score: 68.83

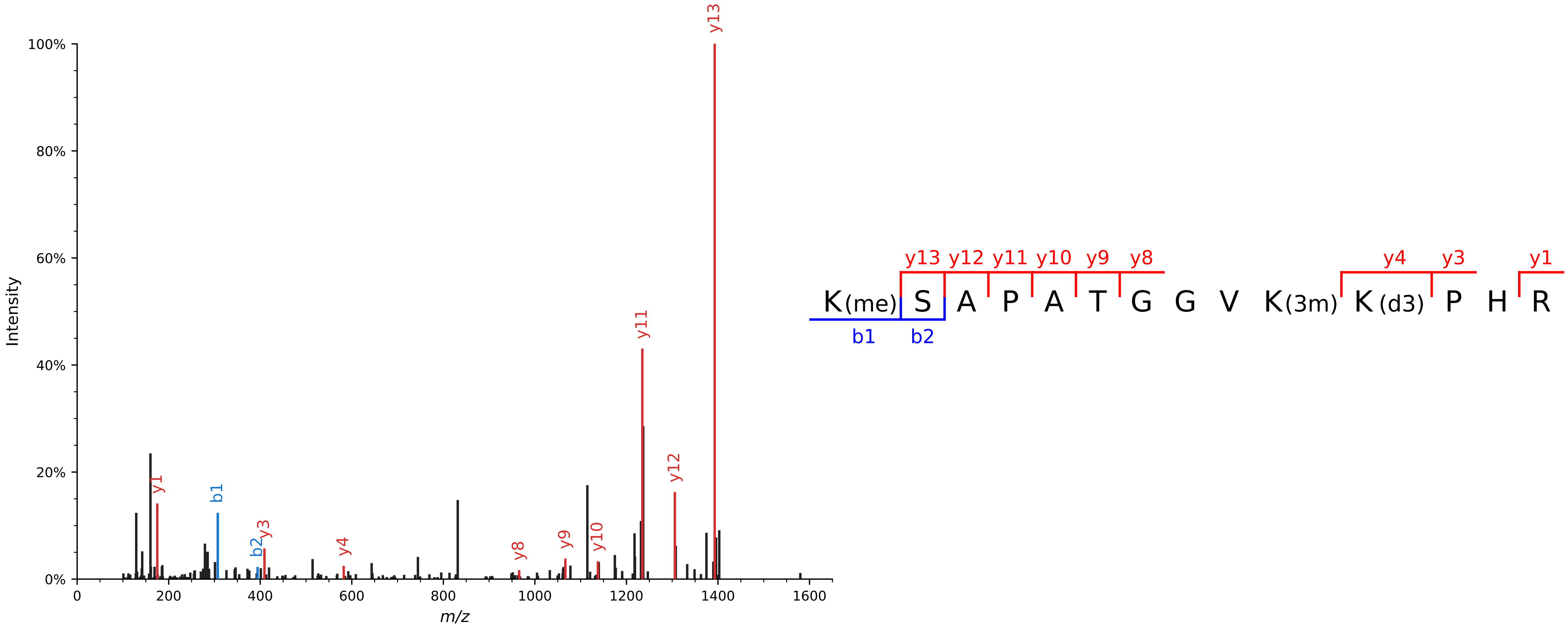

Raw file: QEP231009_CG_frozen_breast_TN_25, Scan: 9681, m/z: 856.9893, Charge: 2, RT (min): 24.32, Score: 177.57

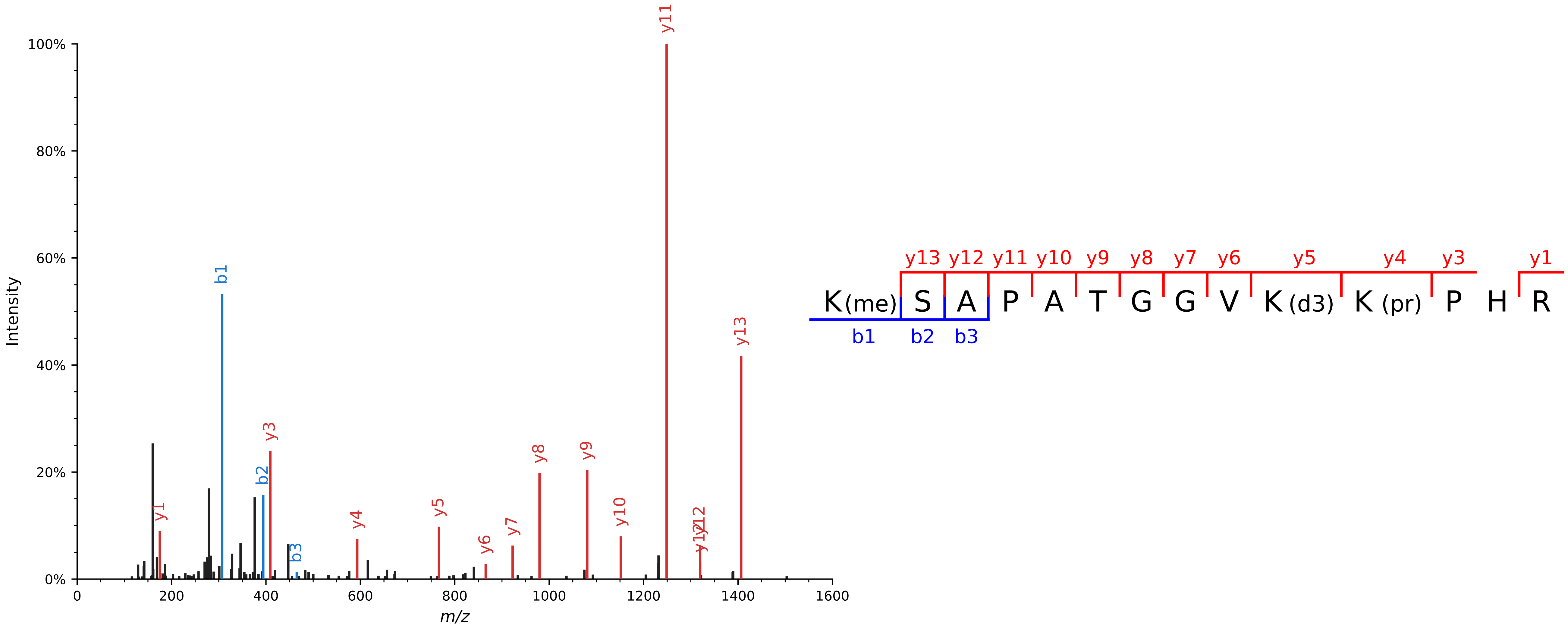

Raw file: QEP231009_CG_frozen_breast_TN_28, Scan: 3837, m/z: 835.98402, Charge: 2, RT (min): 12.48, Score: 222.2

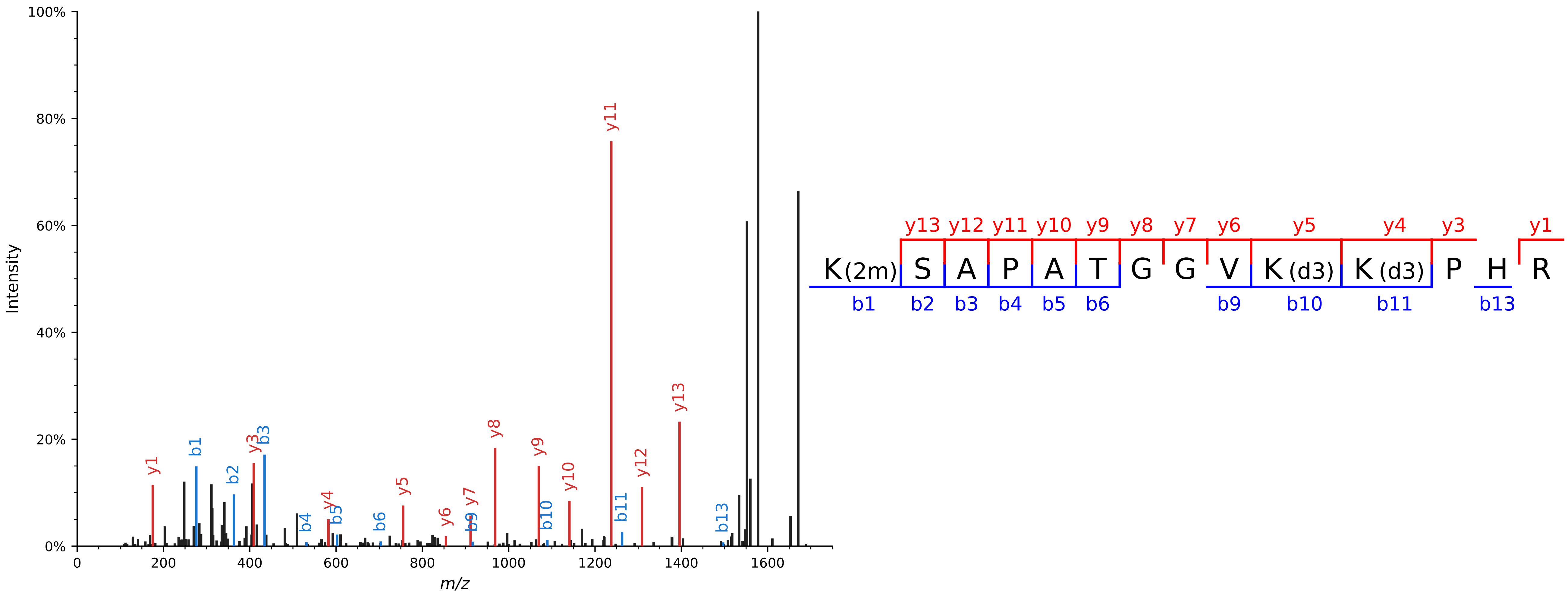

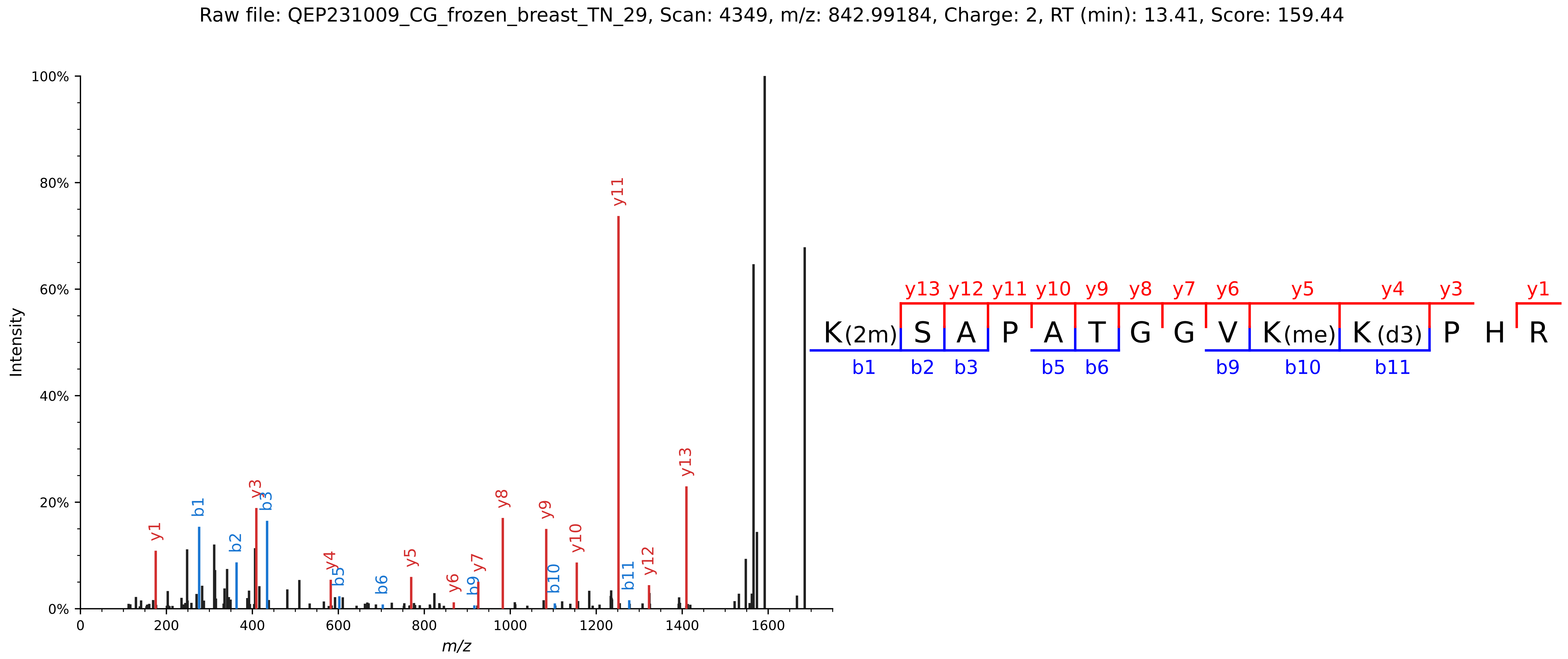

Raw file: QEP231009_CG_frozen_breast_TN_29, Scan: 4349, m/z: 842.99184, Charge: 2, RT (min): 13.41, Score: 159.44
Intensity
m/z
0%
20%
40%
60%
80%
100%
0
200
400
600
800
1000
1200
1400
1600
y1 b1 b2 y3 b3 y4 b5 b6 y5 y6 b9 y7 y8 y9 b10 y10 y11 b11 y12 y13
K(2m) S A P A T G G V K(me) K(d3) P H R
y13 y12 y11 y10 y9 y8 y7 y6 y5 y4 y3 y1
b1 b2 b3 b5 b6 b9 b10 b11

Raw file: QEP231009_CG_frozen_breast_TN_25, Scan: 3163, m/z: 567.0022, Charge: 3, RT (min): 10.78, Score: 44.925

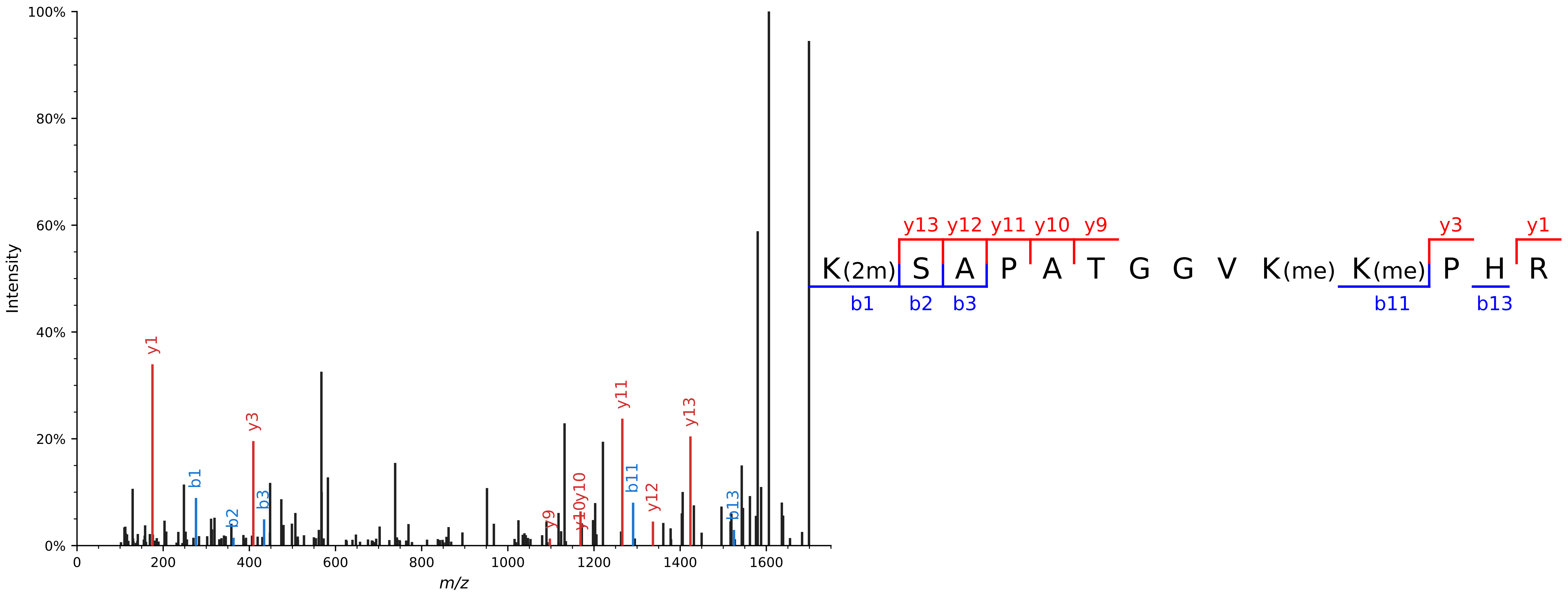

Raw file: QEP231009_CG_frozen_breast_TN_25, Scan: 5148, m/z: 565.99593, Charge: 3, RT (min): 14.85, Score: 118.13

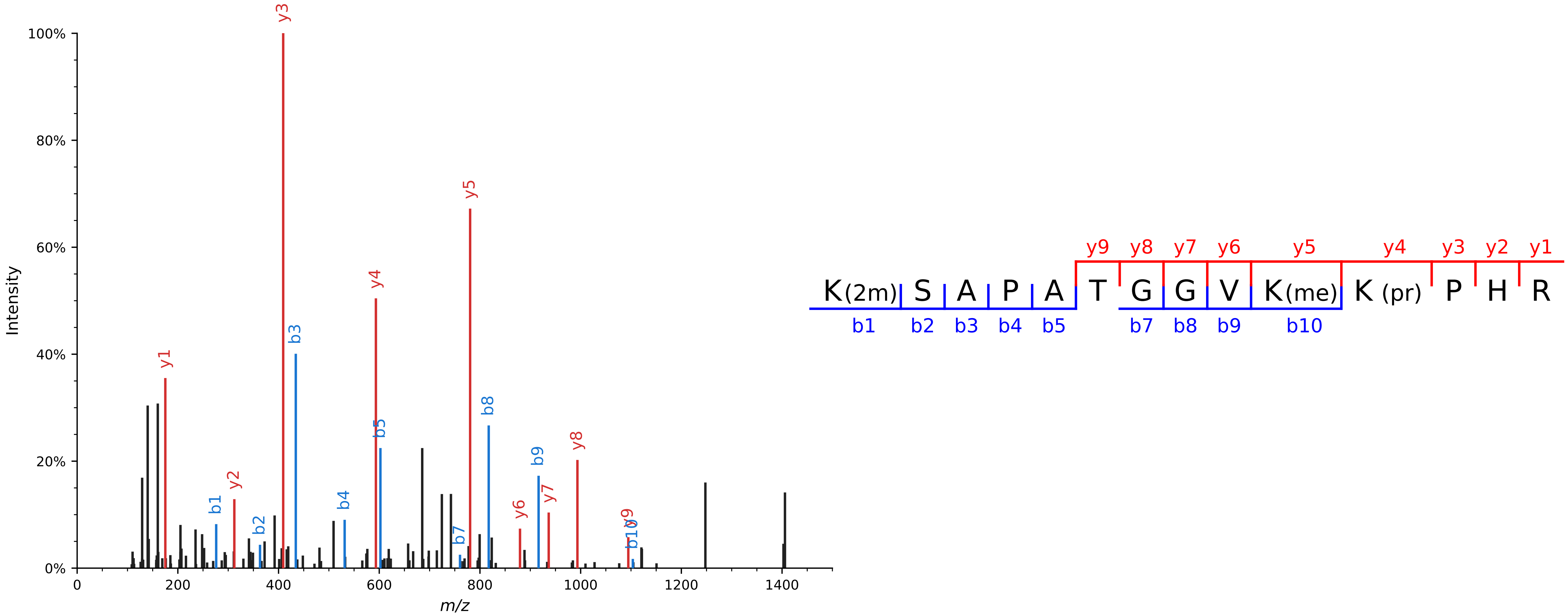

Raw file: QEP231009_CG_frozen_breast_TN_28, Scan: 1796, m/z: 414.24612, Charge: 4, RT (min): 7.89, Score: 130.07

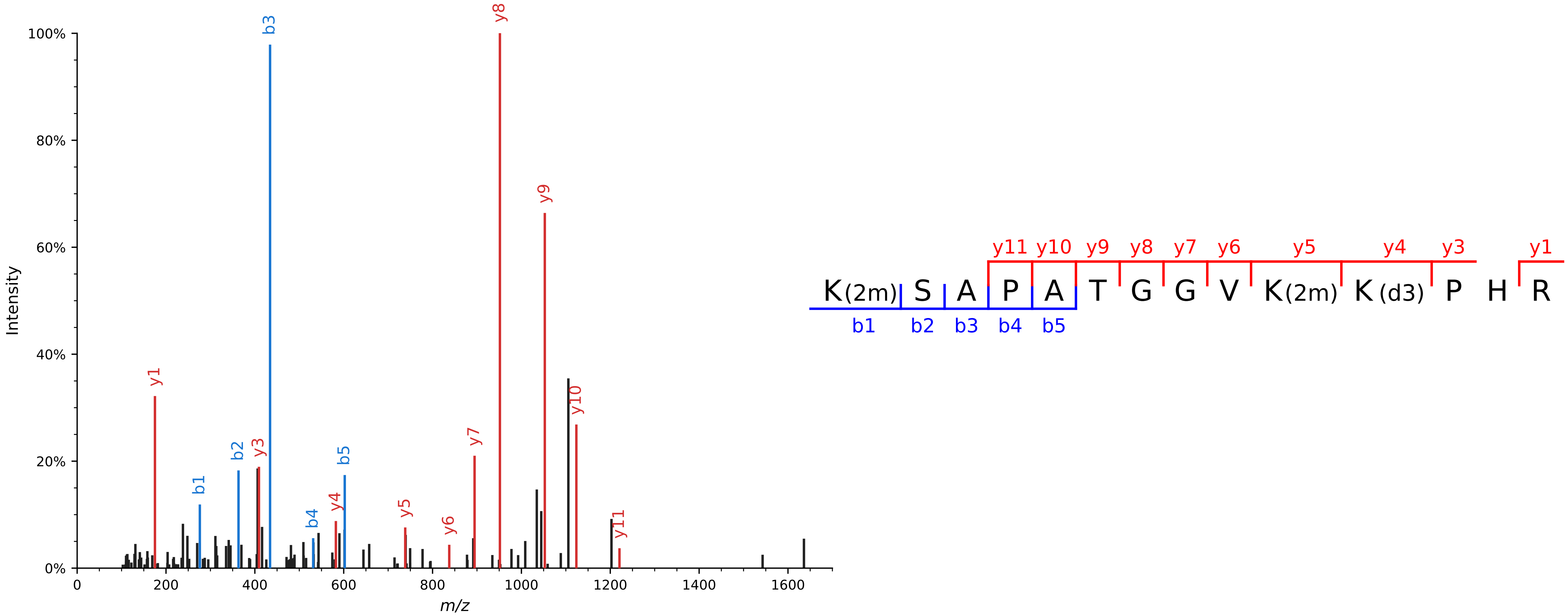

Raw file: QEP231009_CG_frozen_breast_TN_25, Scan: 2558, m/z: 555.65801, Charge: 3, RT (min): 9.52, Score: 102.15

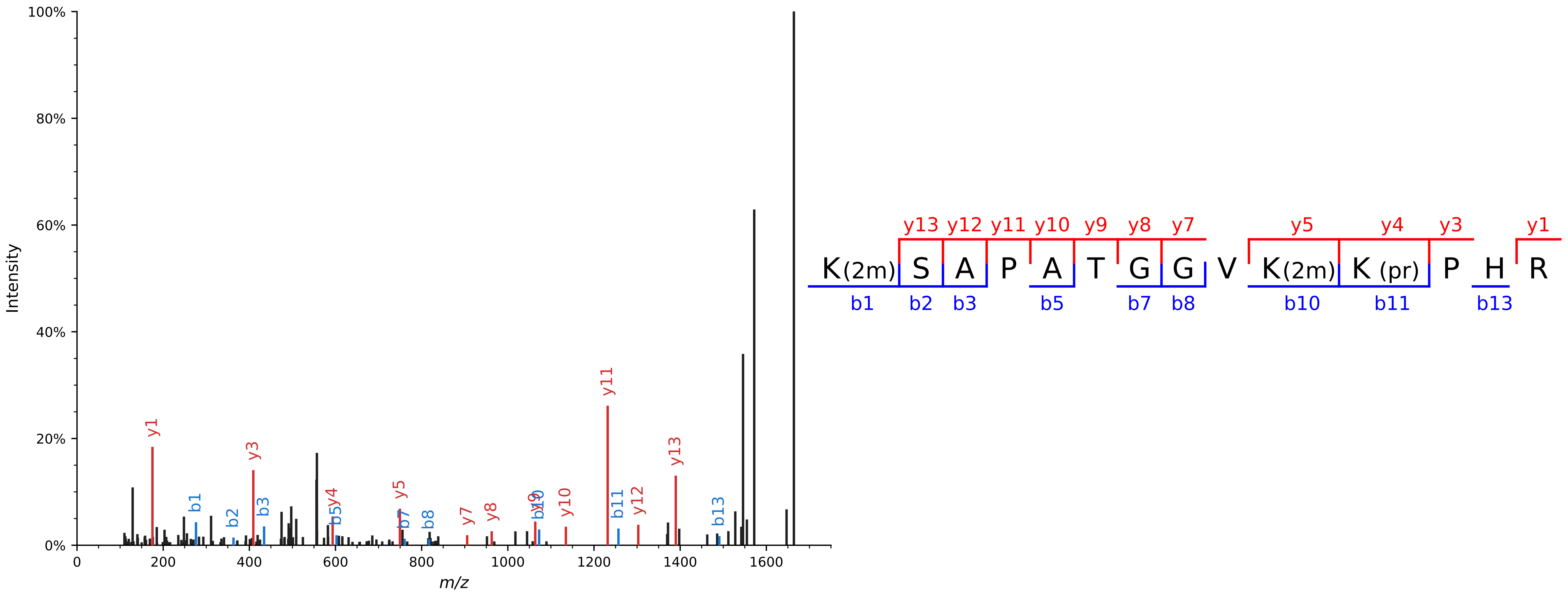

Raw file: QEP231009_CG_frozen_breast_TN_25, Scan: 4428, m/z: 841.48243, Charge: 2, RT (min): 13.36, Score: 215.53

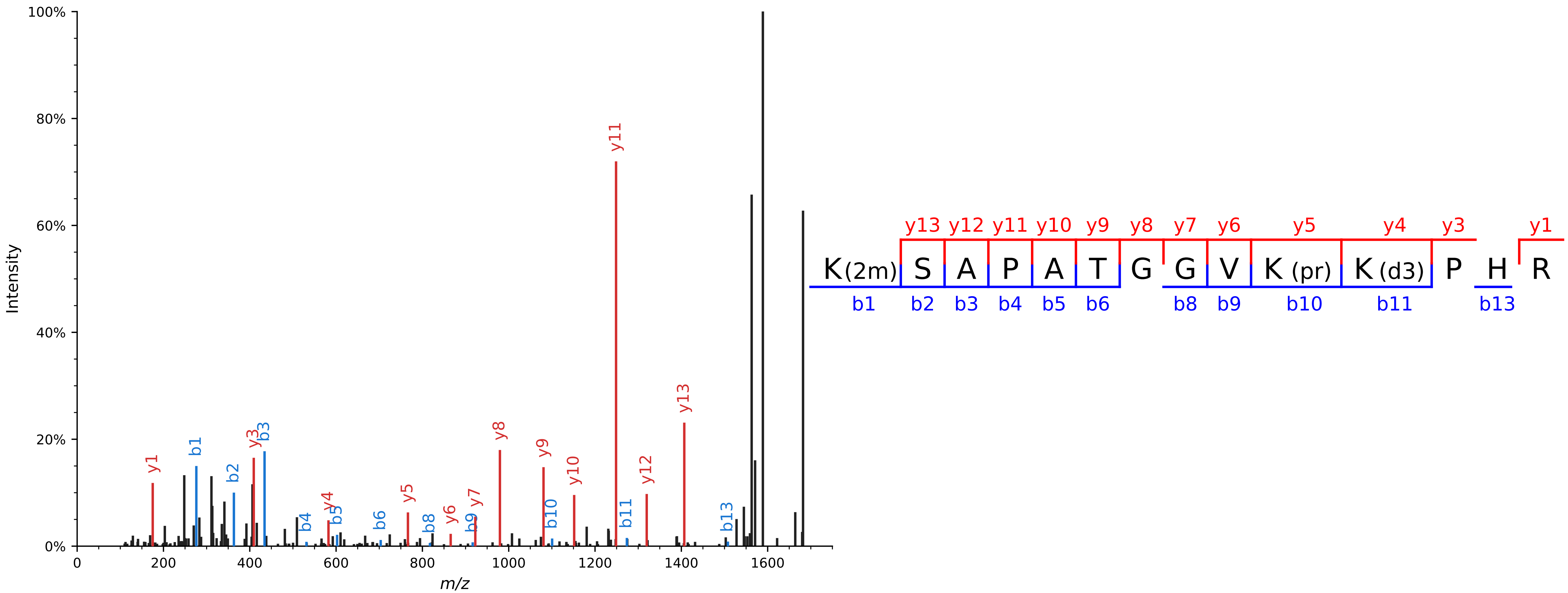

Raw file: QEP231009_CG_frozen_breast_TN_25, Scan: 4593, m/z: 841.48243, Charge: 2, RT (min): 13.70, Score: 184.44

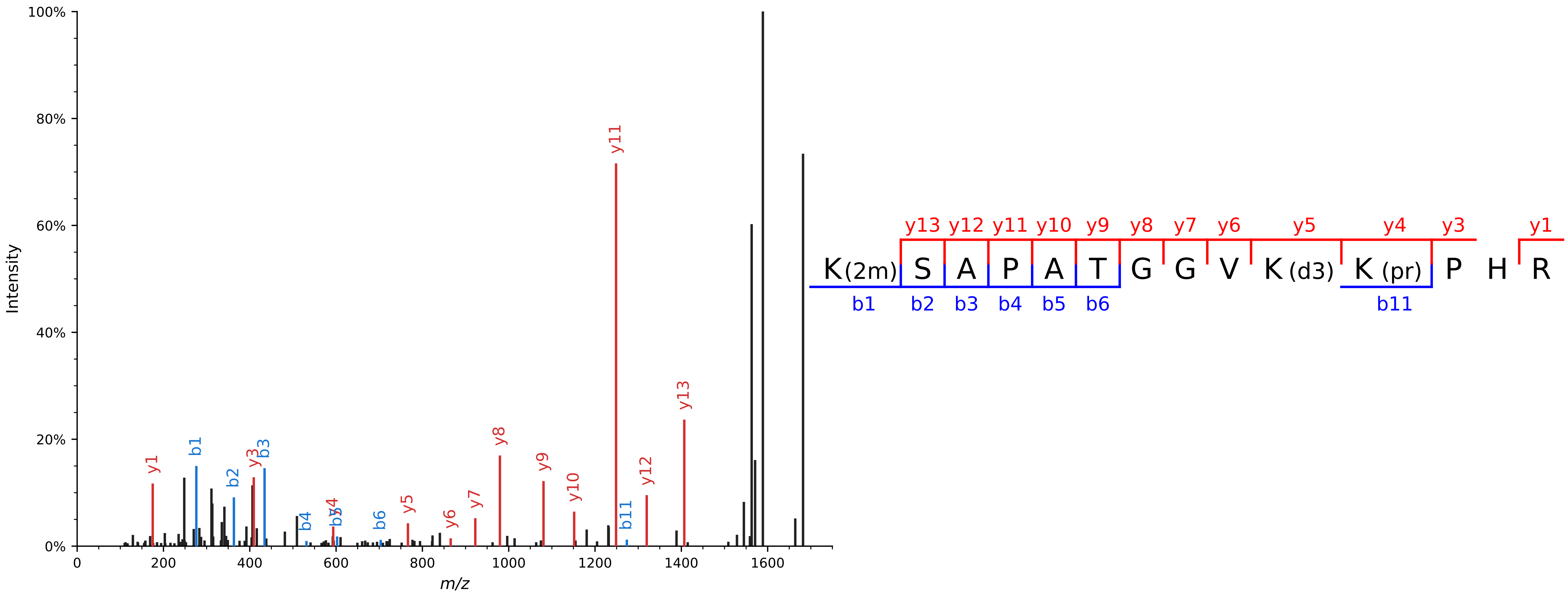

Raw file: QEP231009_CG_frozen_breast_TN_25, Scan: 3976, m/z: 842.99184, Charge: 2, RT (min): 12.44, Score: 177.83

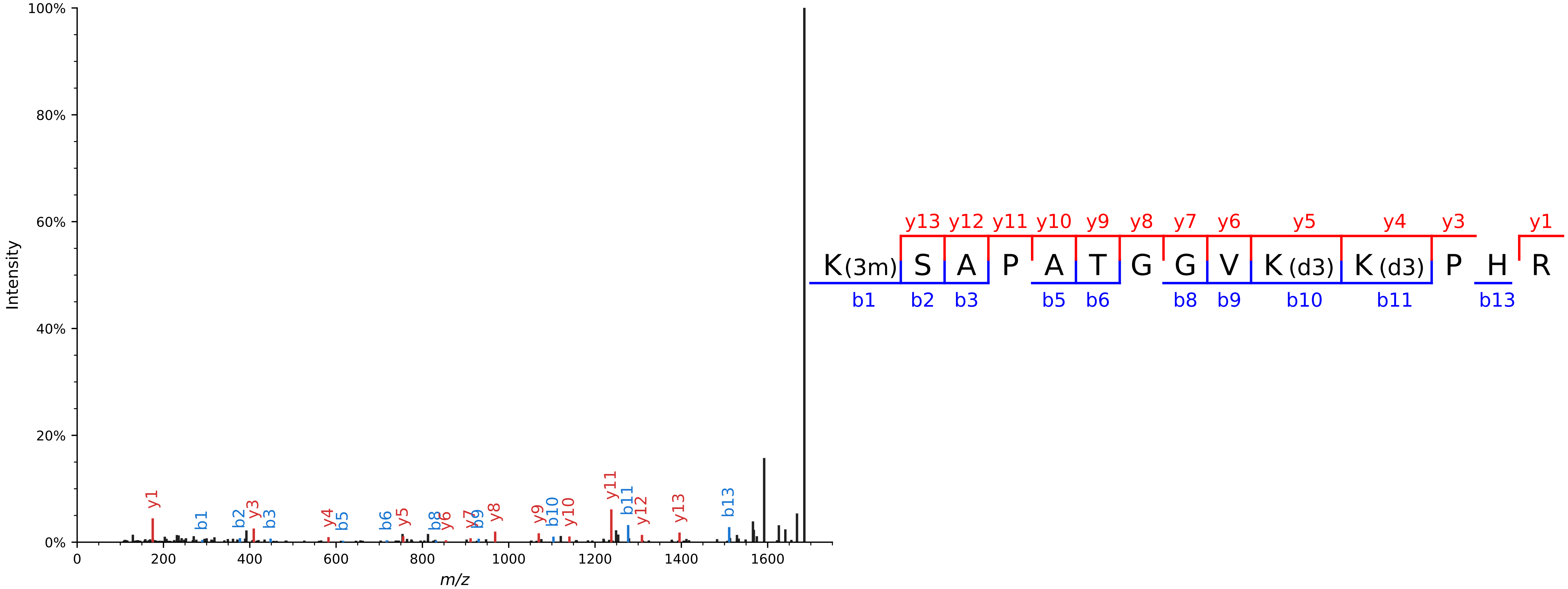

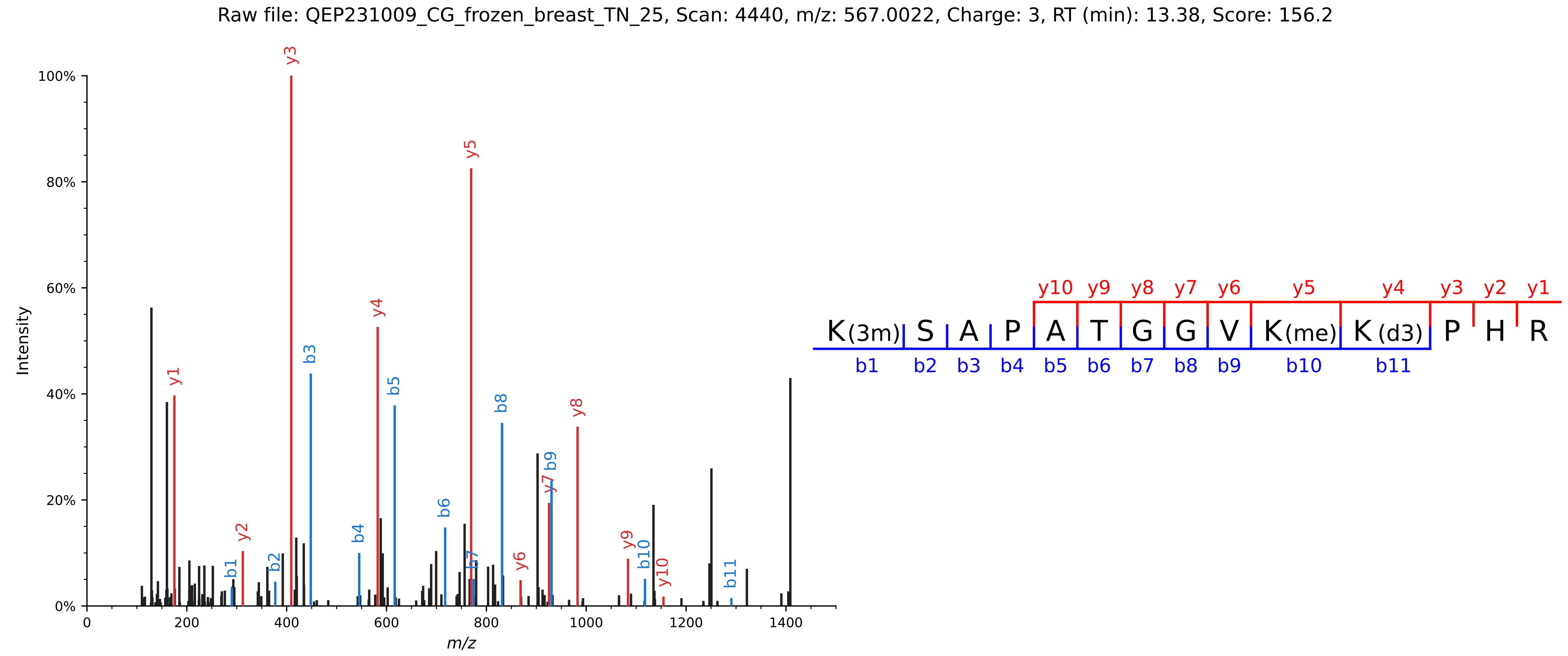
Raw file: QEP231009_CG_frozen_breast_TN_25, Scan: 4440, m/z: 567.0022, Charge: 3, RT (min): 13.38, Score: 156.2
Intensity
m/z
y3
y5
y4
b3
y1
b5
b8
y8
b9
y7
b6
y2
b4
b1
b2
b7
y6
y9
b10
y10
b11
y10 y9 y8 y7 y6 y5 y4 y3 y2 y1
K(3m) S A P A T G G V K(me) K(d3) P H R
b1 b2 b3 b4 b5 b6 b7 b8 b9 b10 b11

Raw file: QEP231009_CG_frozen_breast_LuA_32, Scan: 5001, m/z: 570.66781, Charge: 3, RT (min): 14.94, Score: 70.654

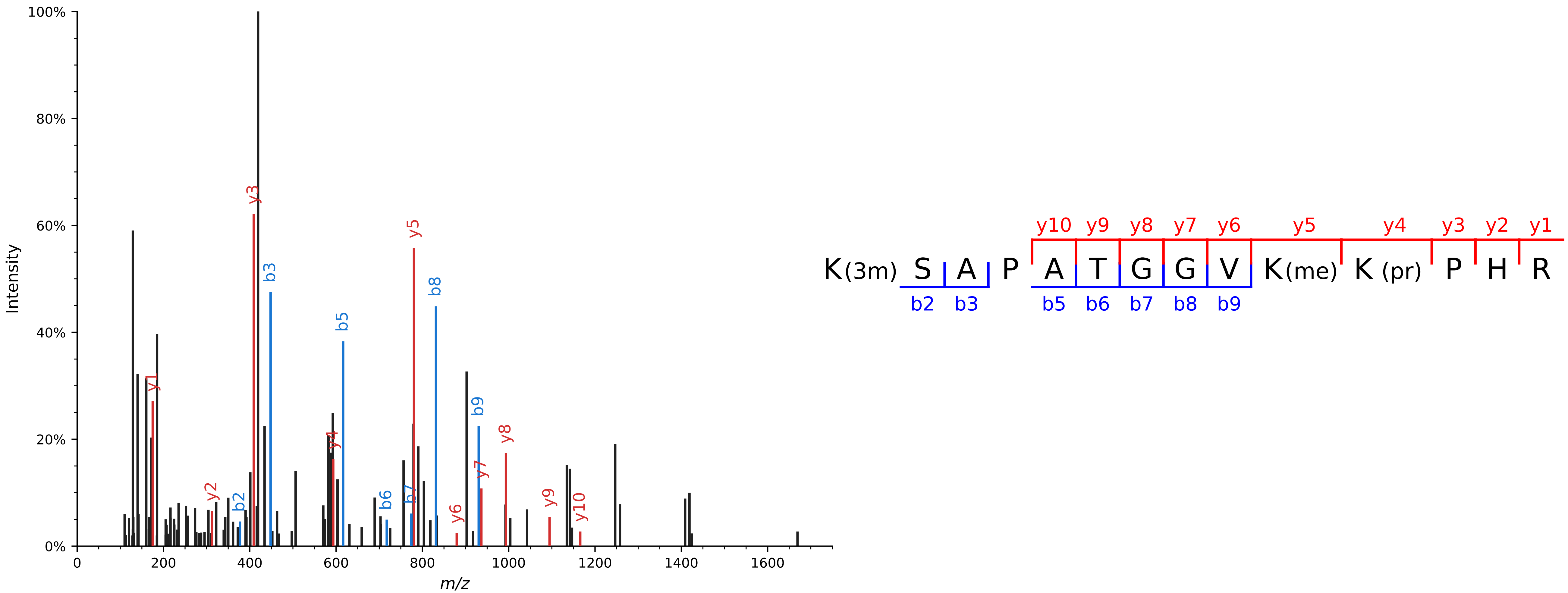

Raw file: QEP231009_CG_frozen_breast_TN_27, Scan: 1809, m/z: 556.66429, Charge: 3, RT (min): 7.96, Score: 71.354

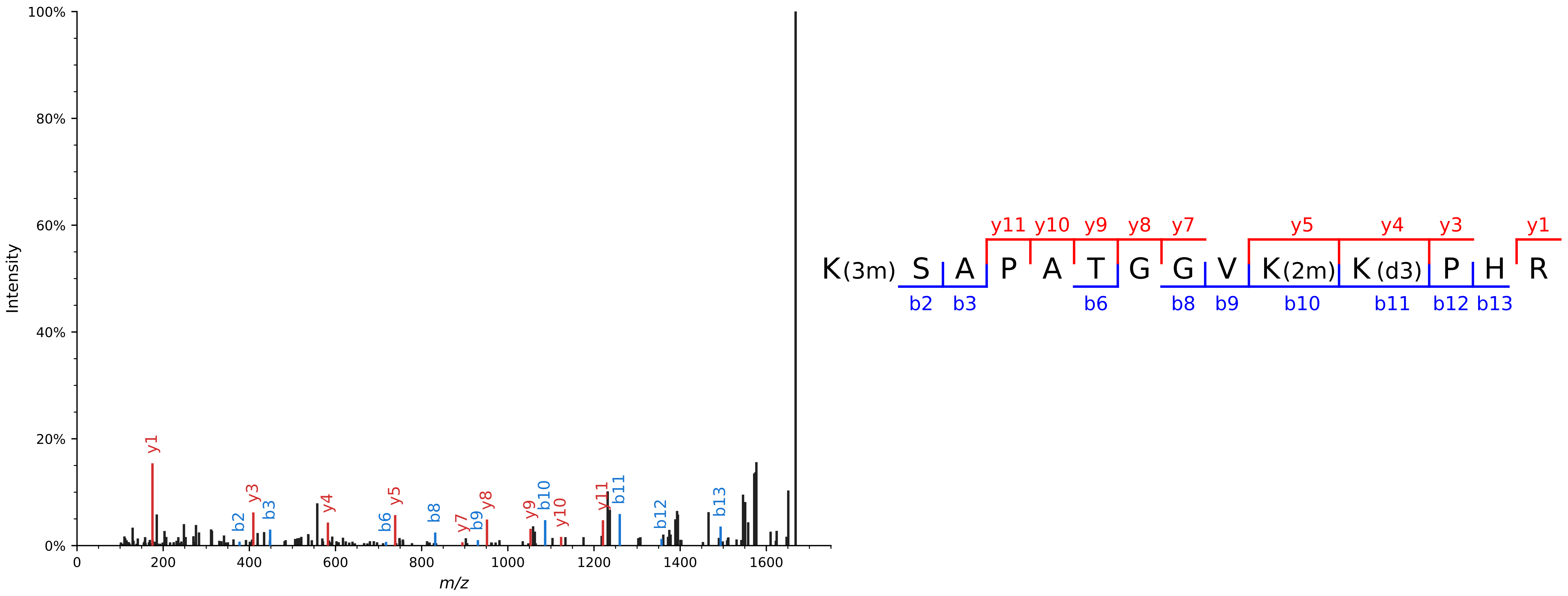

Raw file: QEP231009_CG_frozen_breast_TN_25, Scan: 2635, m/z: 420.49924, Charge: 4, RT (min): 9.69, Score: 44.44

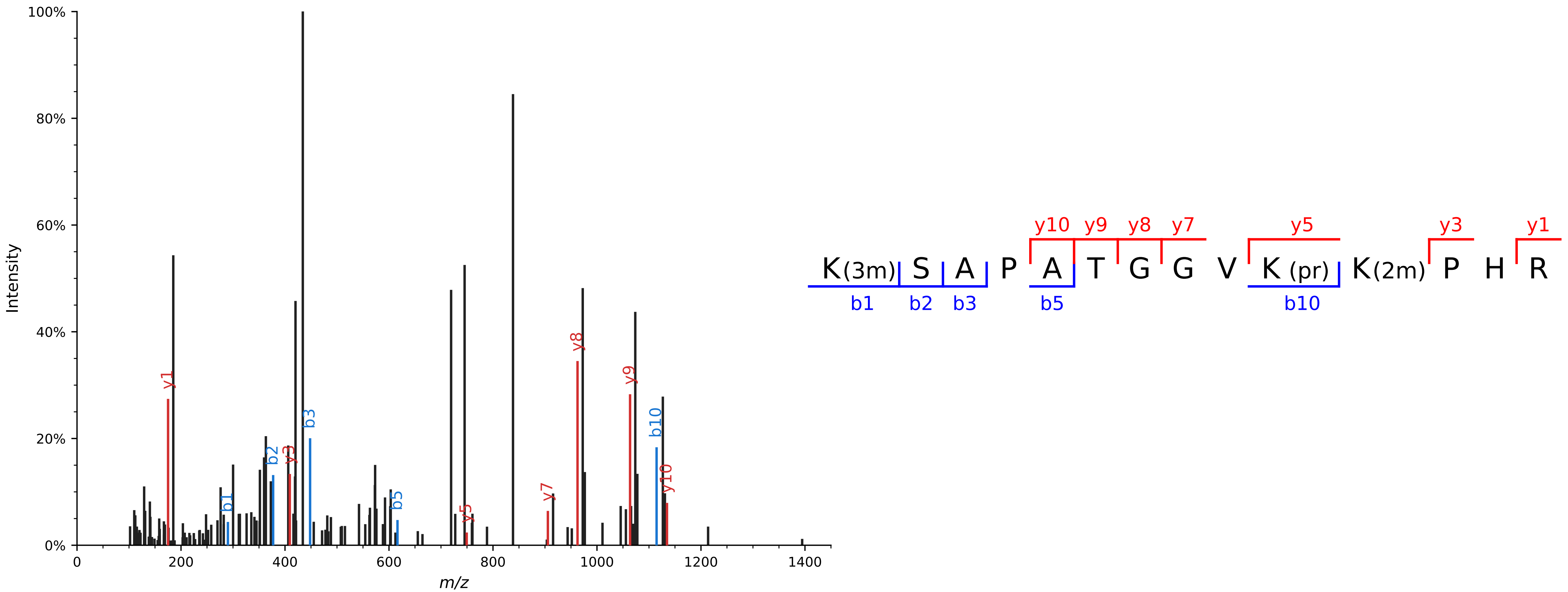

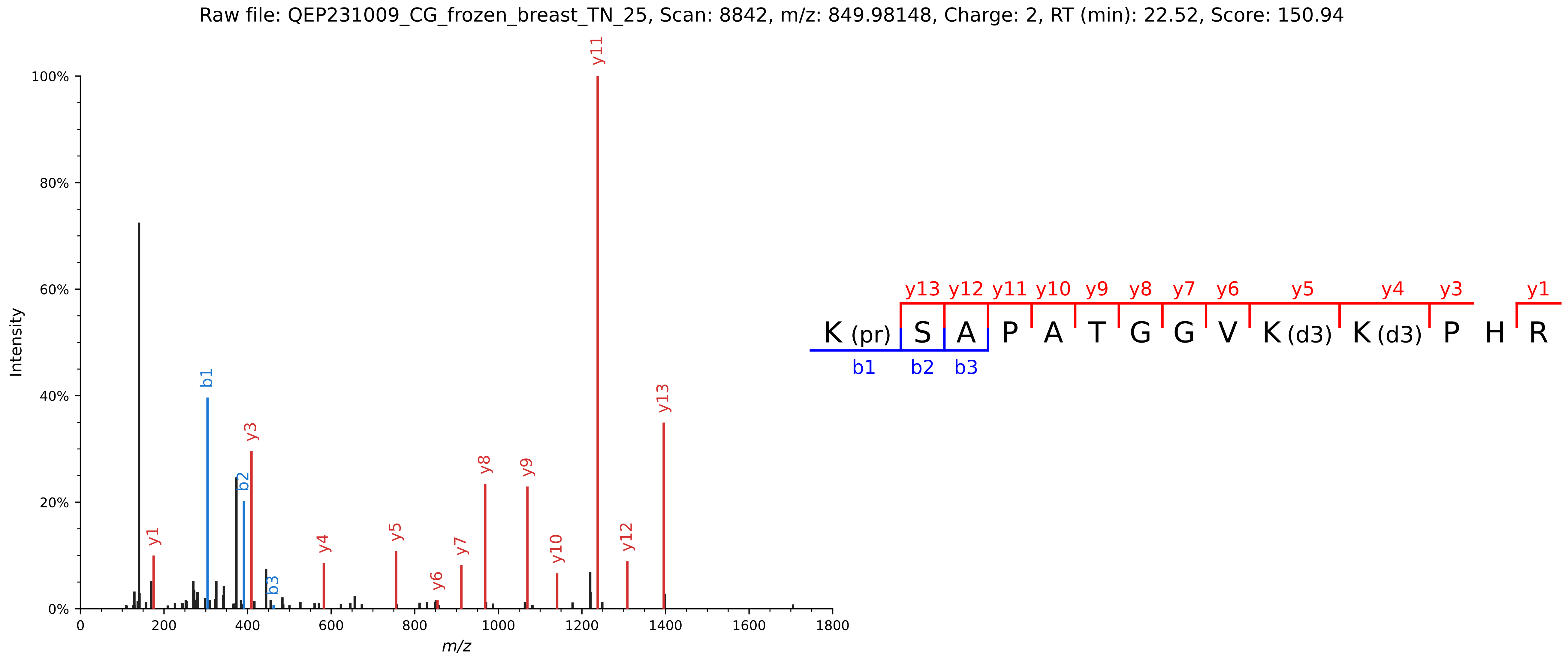

Raw file: QEP231009_CG_frozen_breast_TN_25, Scan: 8842, m/z: 849.98148, Charge: 2, RT (min): 22.52, Score: 150.94
Intensity
m/z
y1 b1 b2 y3 b3 y4 y5 y6 y7 y8 y9 y10 y11 y12 y13
y13 y12 y11 y10 y9 y8 y7 y6 y5 y4 y3 y1
K (pr) S A P A T G G V K (d3) K (d3) P H R
b1 b2 b3

Raw file: QEP231009_CG_frozen_breast_TN_29, Scan: 9244, m/z: 856.9893, Charge: 2, RT (min): 23.73, Score: 67.726

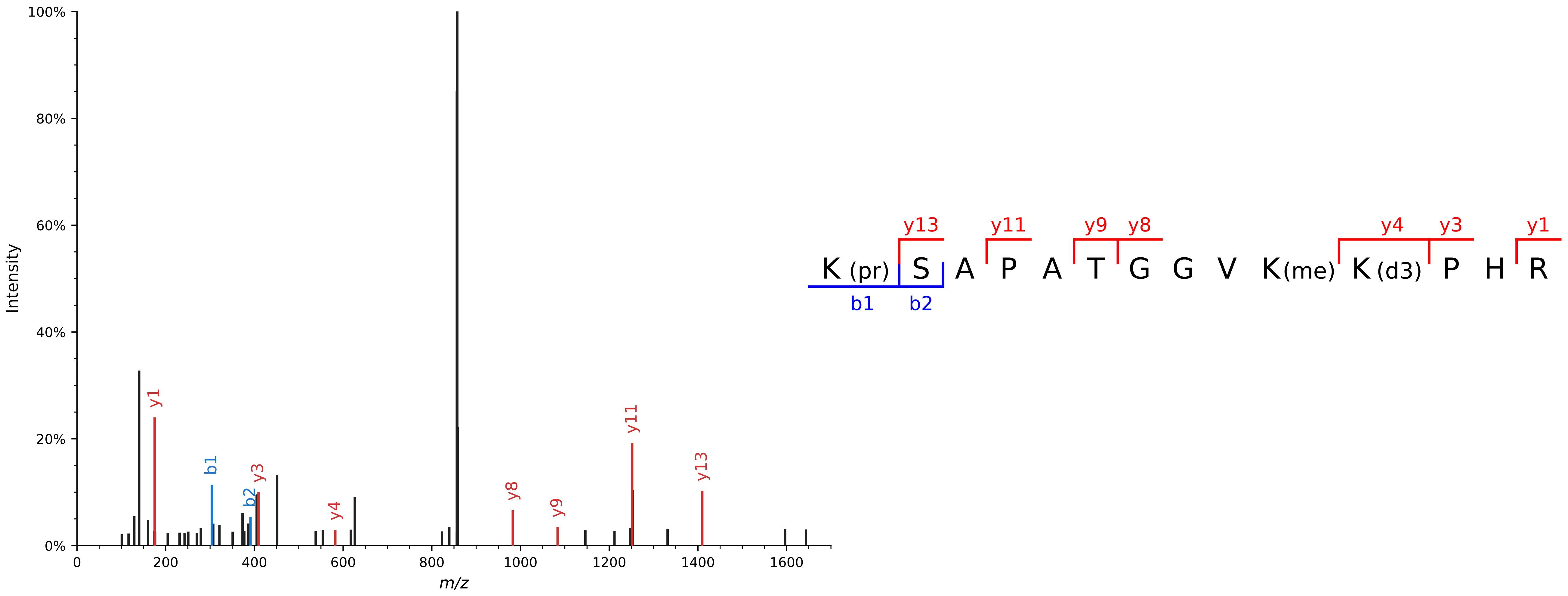

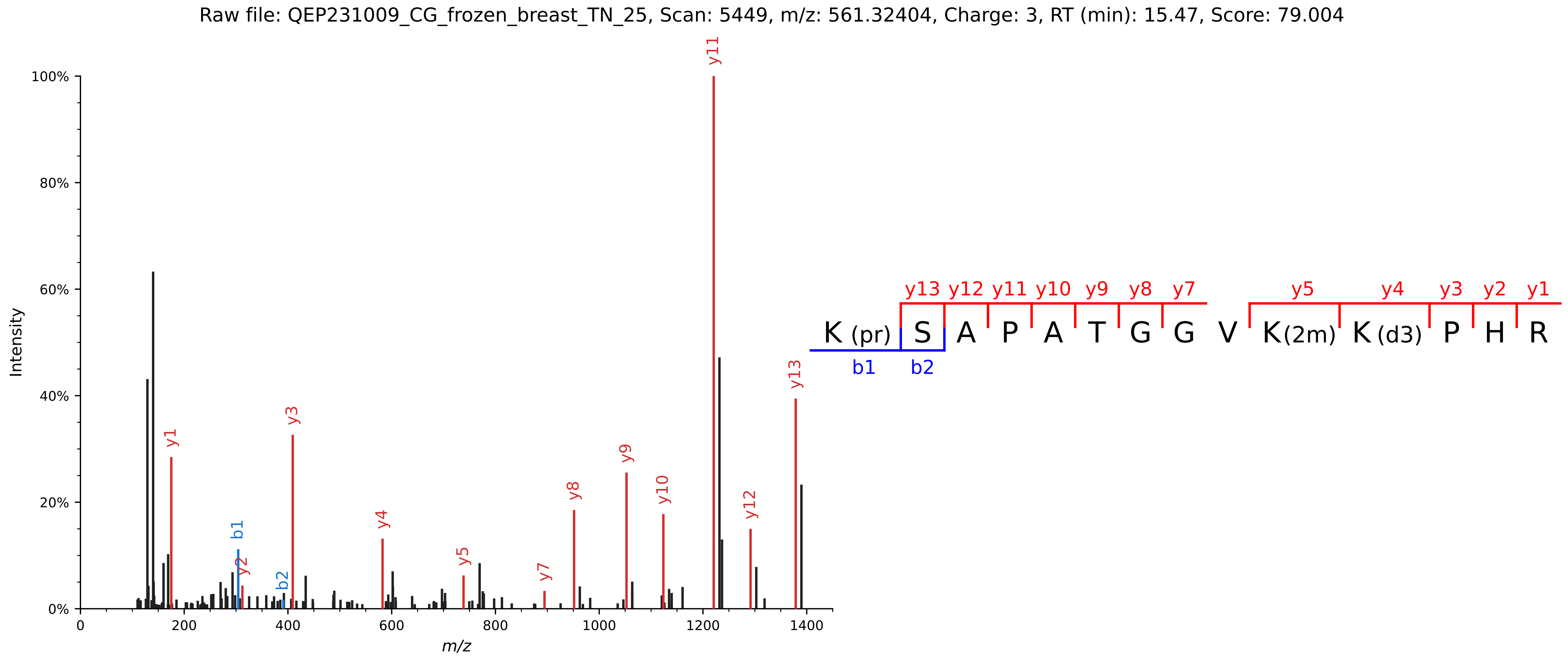

Raw file: QEP231009_CG_frozen_breast_TN_25, Scan: 5449, m/z: 561.32404, Charge: 3, RT (min): 15.47, Score: 79.004
Intensity
m/z
y1
y2
b1
b2
y3
y4
y5
y7
y8
y9
y10
y11
y12
y13
K (pr) S A P A T G G V K(2m) K (d3) P H R

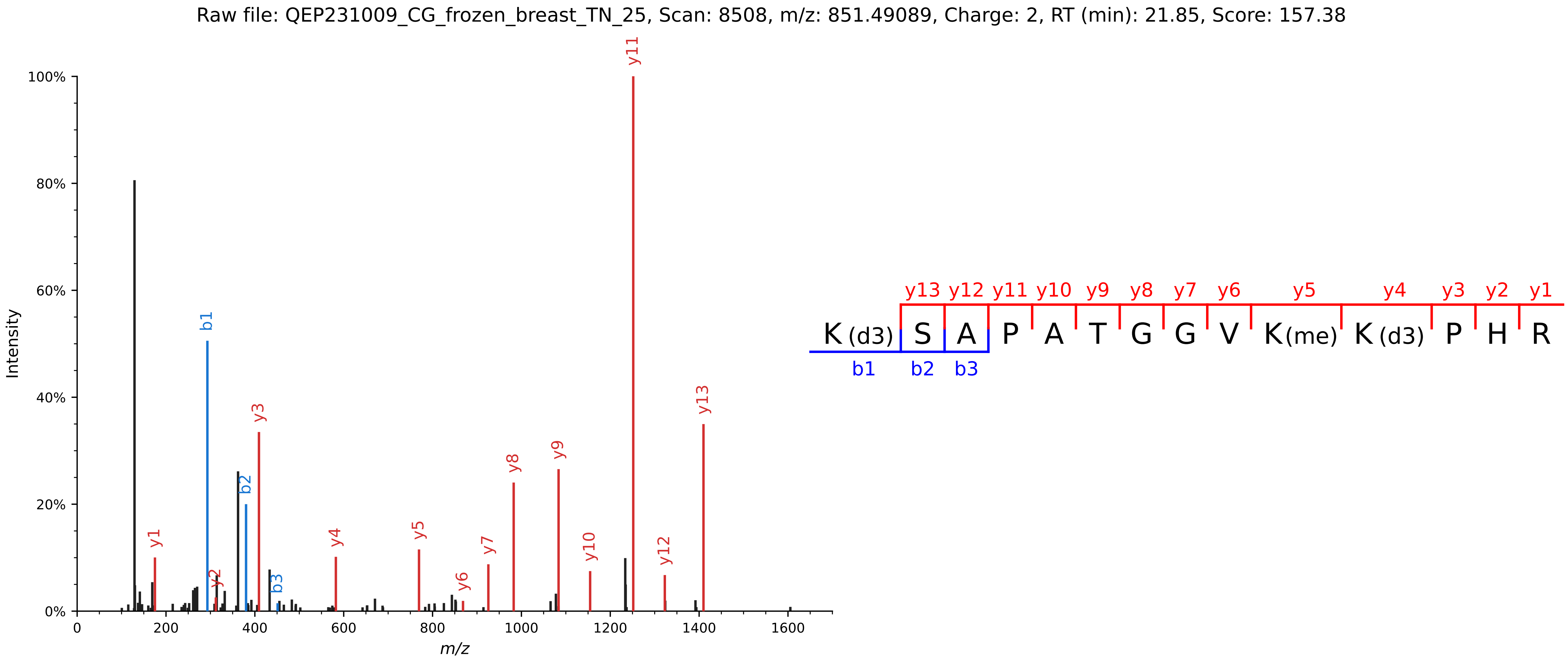

Raw file: QEP231009_CG_frozen_breast_TN_25, Scan: 8508, m/z: 851.49089, Charge: 2, RT (min): 21.85, Score: 157.38
Intensity
m/z
y13 y12 y11 y10 y9 y8 y7 y6 y5 y4 y3 y2 y1
K(d3) S A P A T G G V K(me) K(d3) P H R
b1 b2 b3

Raw file: QEP231009_CG_frozen_breast_Normal_21, Scan: 4890, m/z: 571.66196, Charge: 3, RT (min): 14.73, Score: 64.224

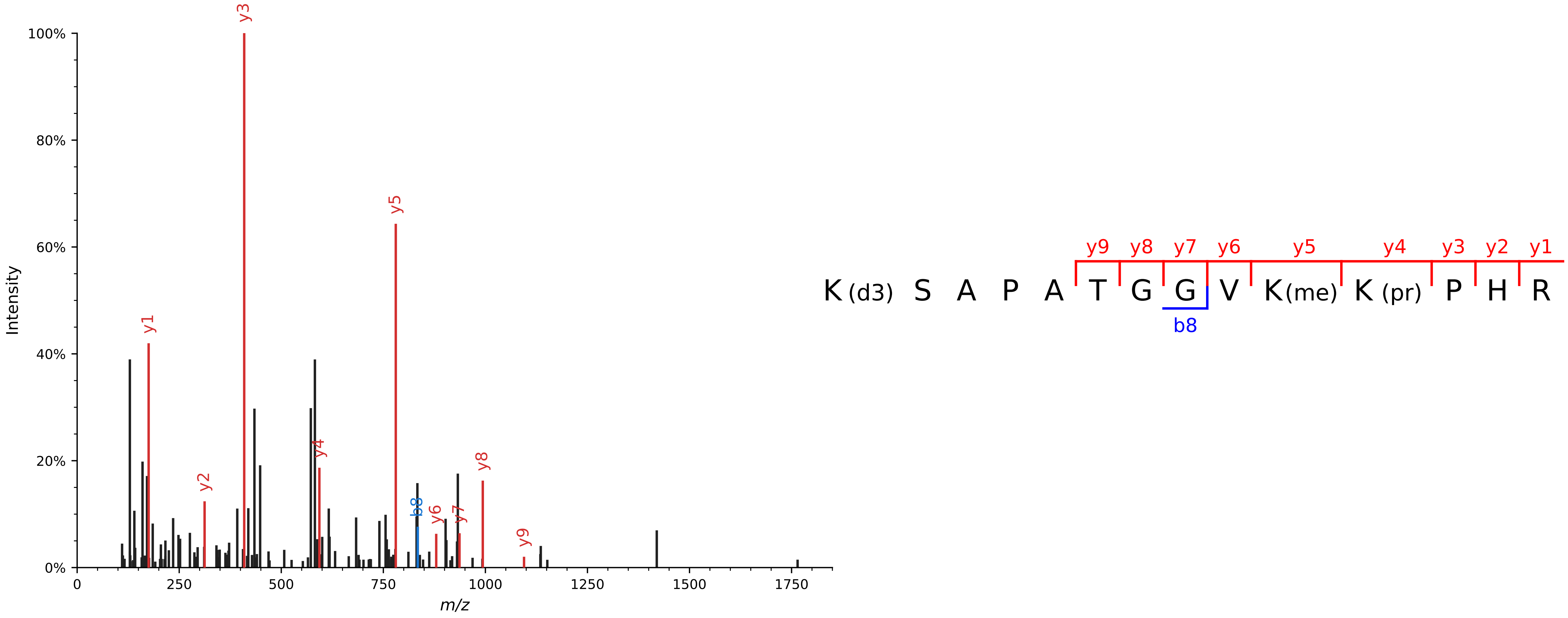

Raw file: QEP231009_CG_frozen_breast_TN_29, Scan: 4535, m/z: 835.98402, Charge: 2, RT (min): 13.82, Score: 159.94

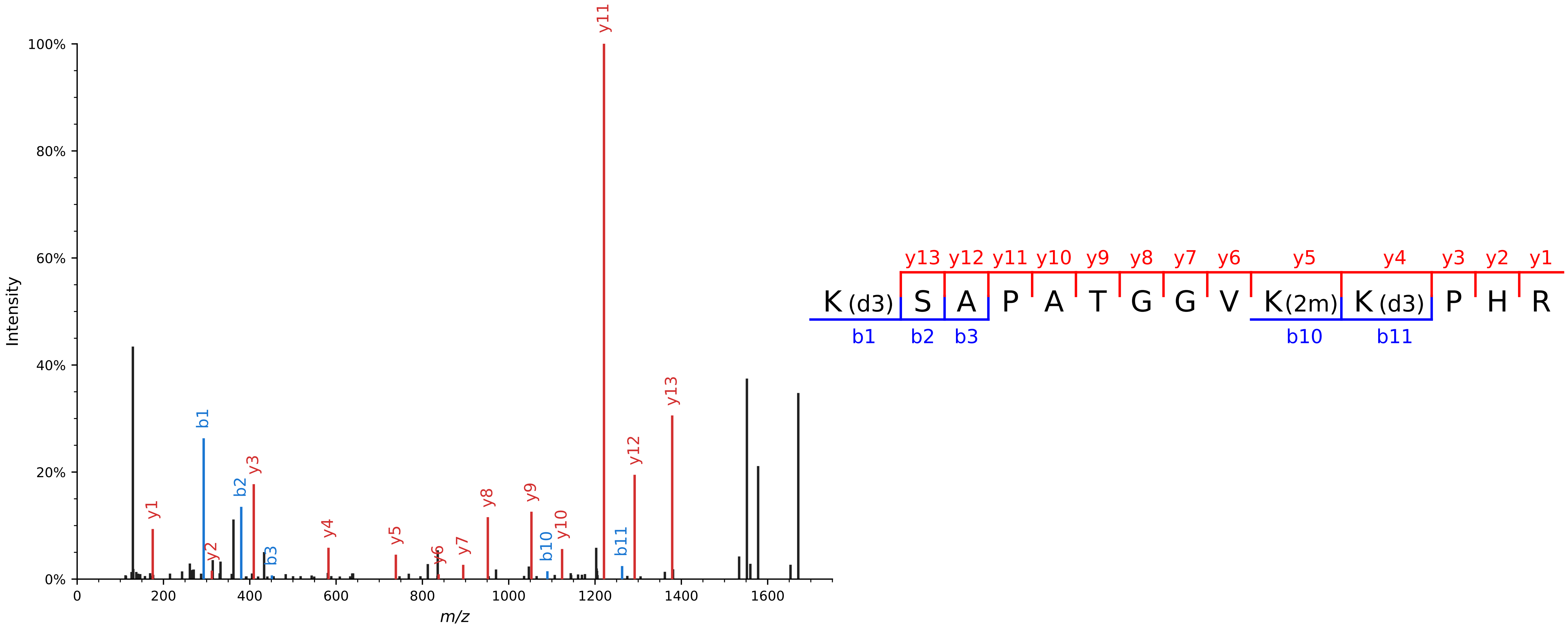

Raw file: QEP231009_CG_frozen_breast_TN_25, Scan: 8663, m/z: 849.98148, Charge: 2, RT (min): 22.16, Score: 138.76

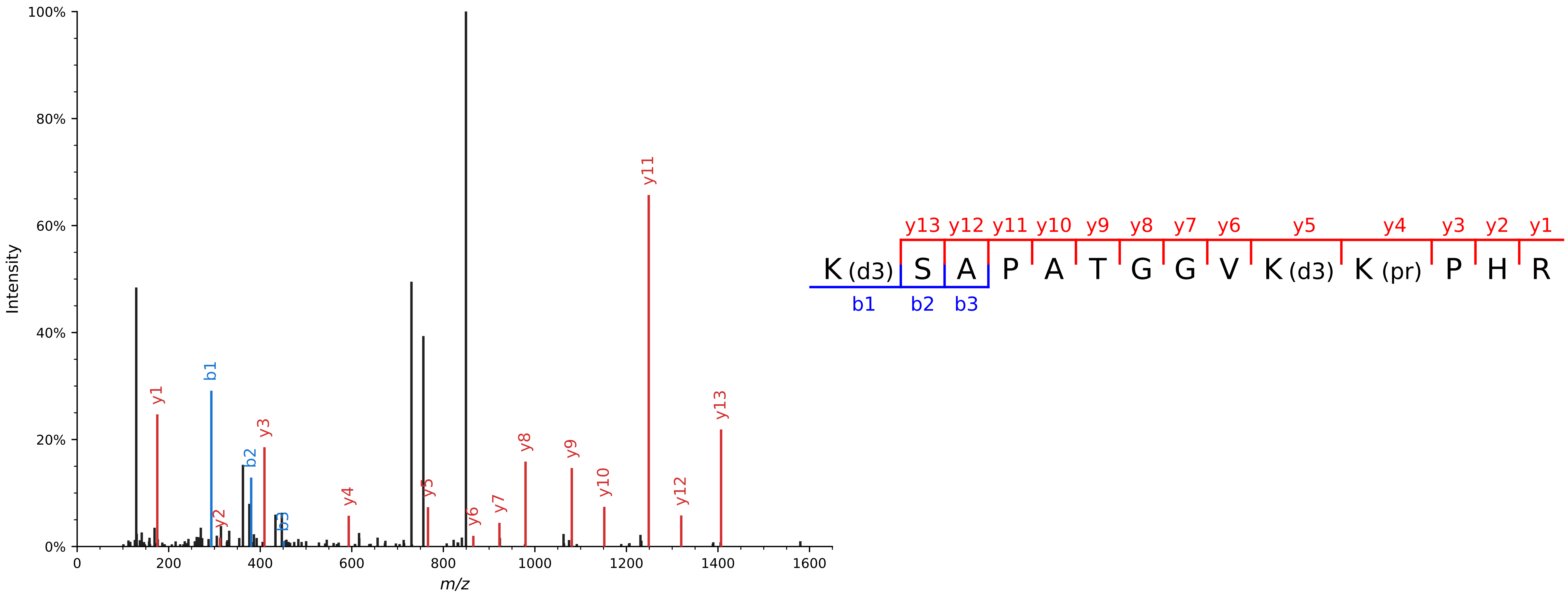

Raw file: QEP231009_CG_frozen_breast_TN_27, Scan: 16340, m/z: 482.27417, Charge: 2, RT (min): 42.44, Score: 103.41

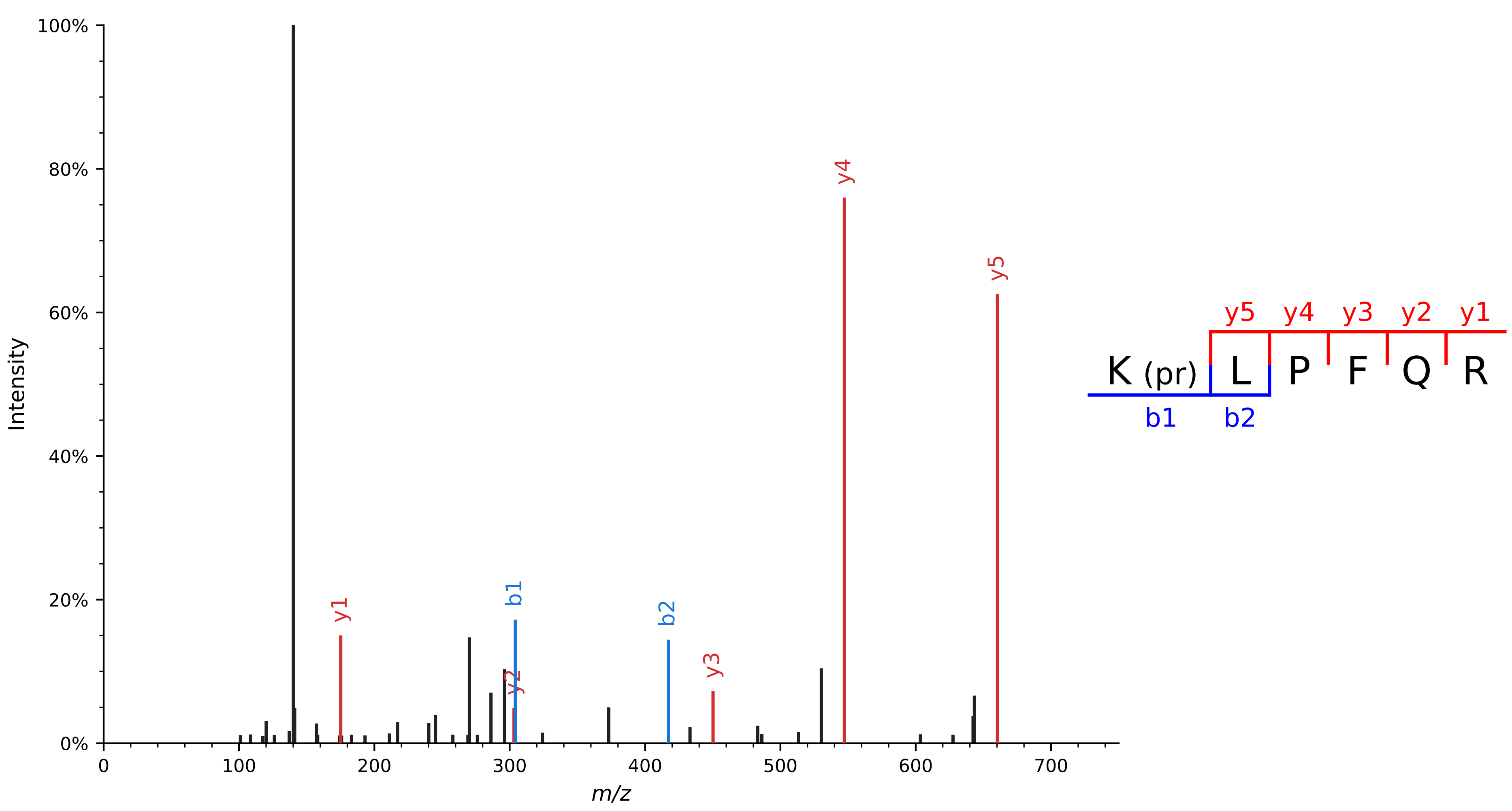

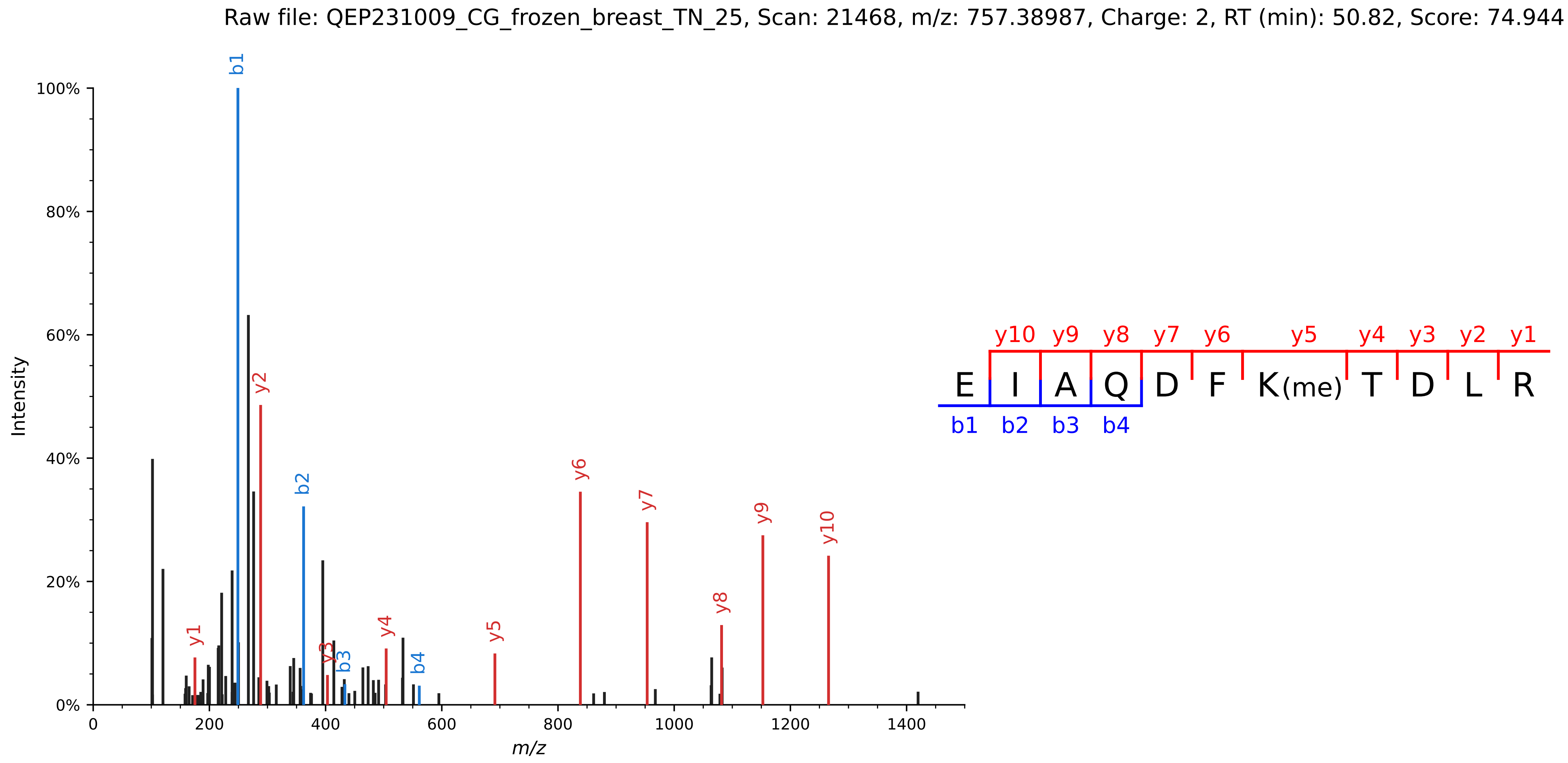

Raw file: QEP231009_CG_frozen_breast_TN_25, Scan: 21468, m/z: 757.38987, Charge: 2, RT (min): 50.82, Score: 74.944
Intensity
m/z
E I A Q D F K(me) T D L R
y10 y9 y8 y7 y6 y5 y4 y3 y2 y1
b1 b2 b3 b4

Raw file: QEP231009_CG_frozen_breast_TN_25, Scan: 15096, m/z: 741.883, Charge: 2, RT (min): 35.72, Score: 83.499

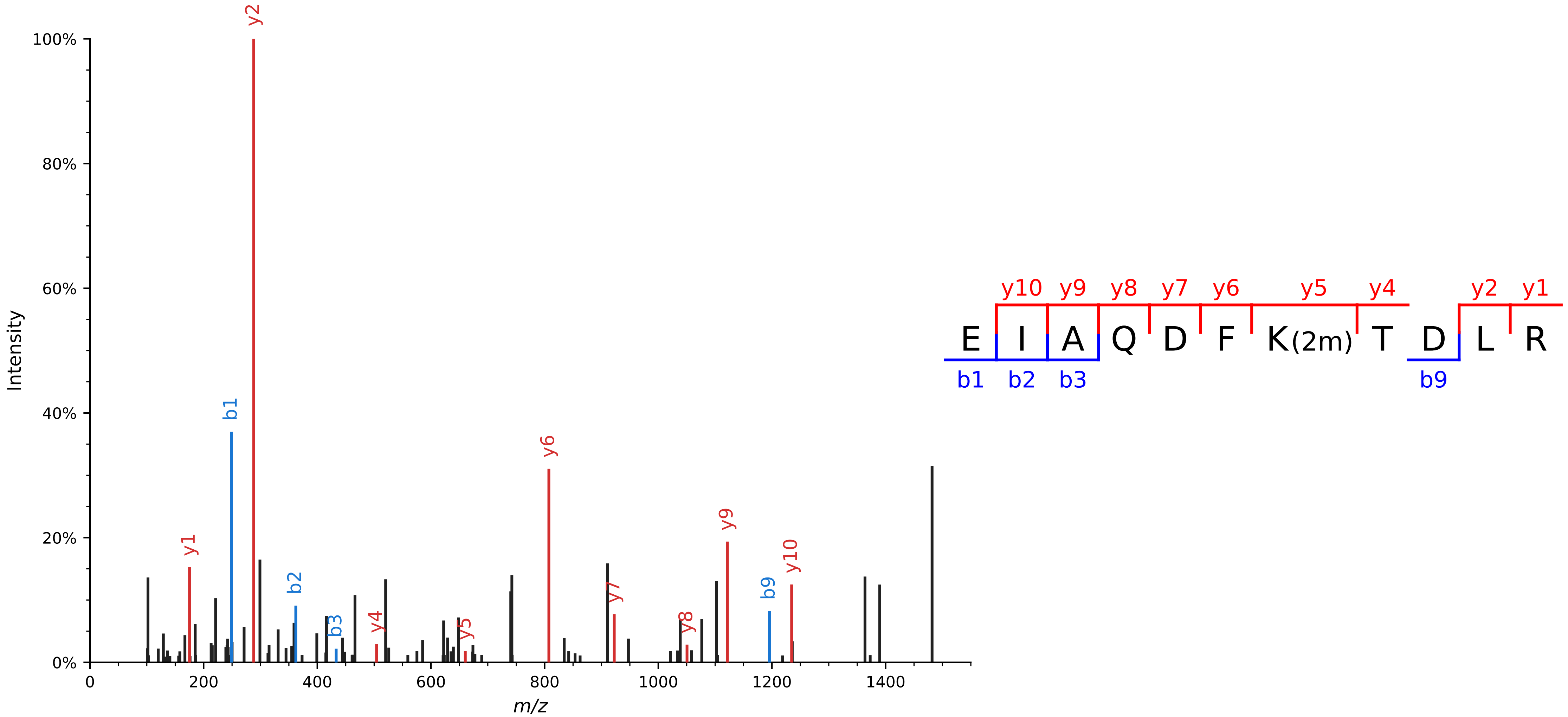

Raw file: QEP231009_CG_frozen_breast_TN_25, Scan: 10357, m/z: 776.94886, Charge: 2, RT (min): 25.83, Score: 190.96

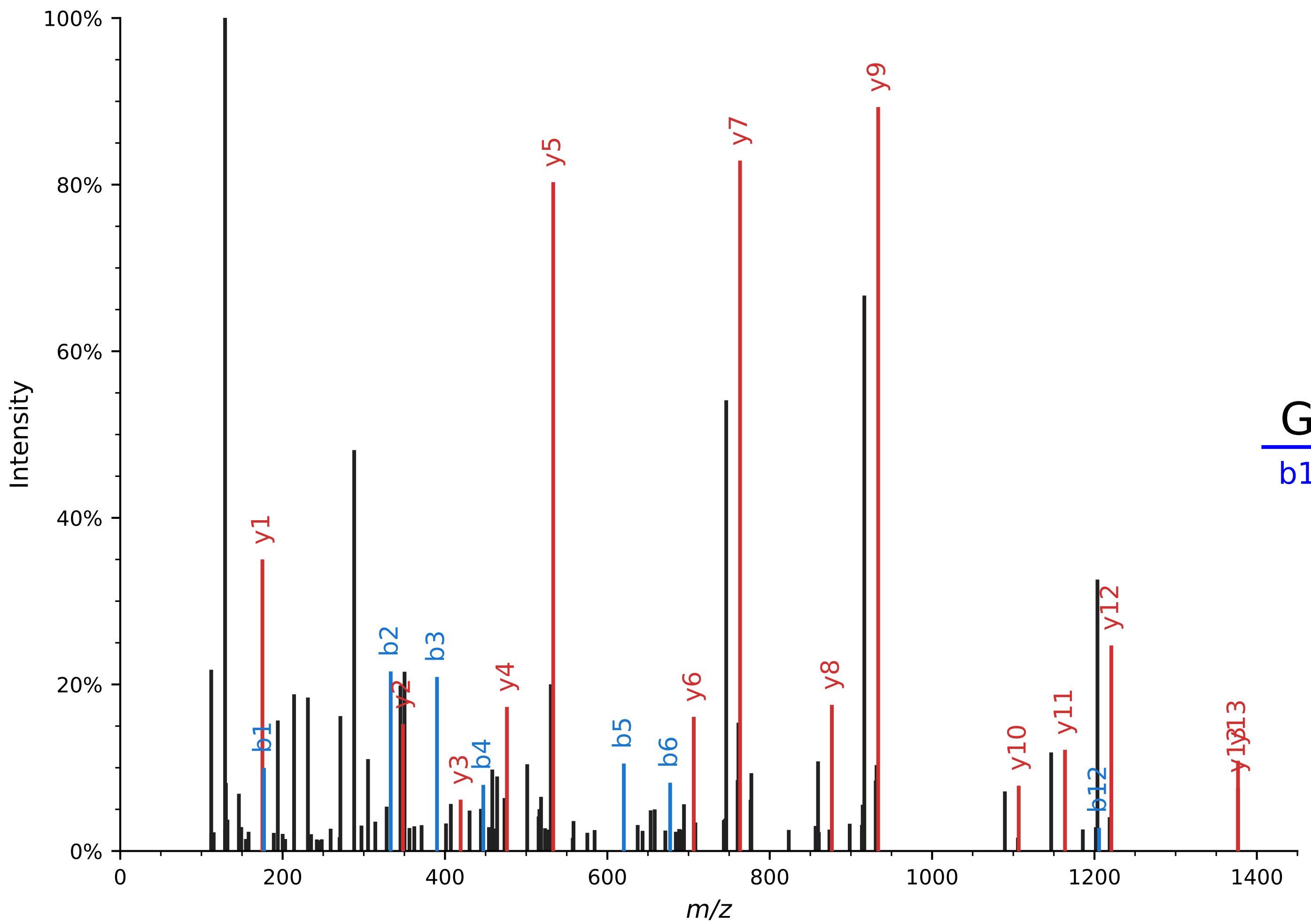

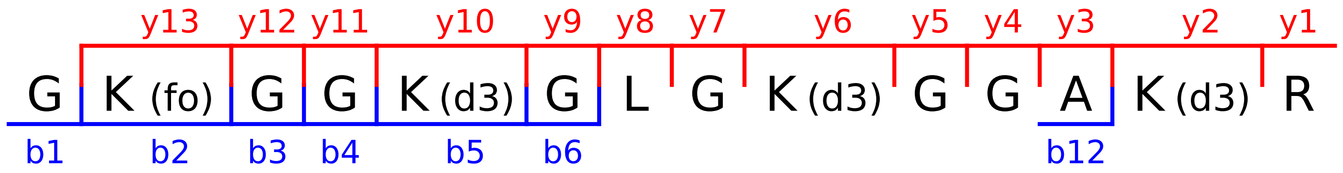

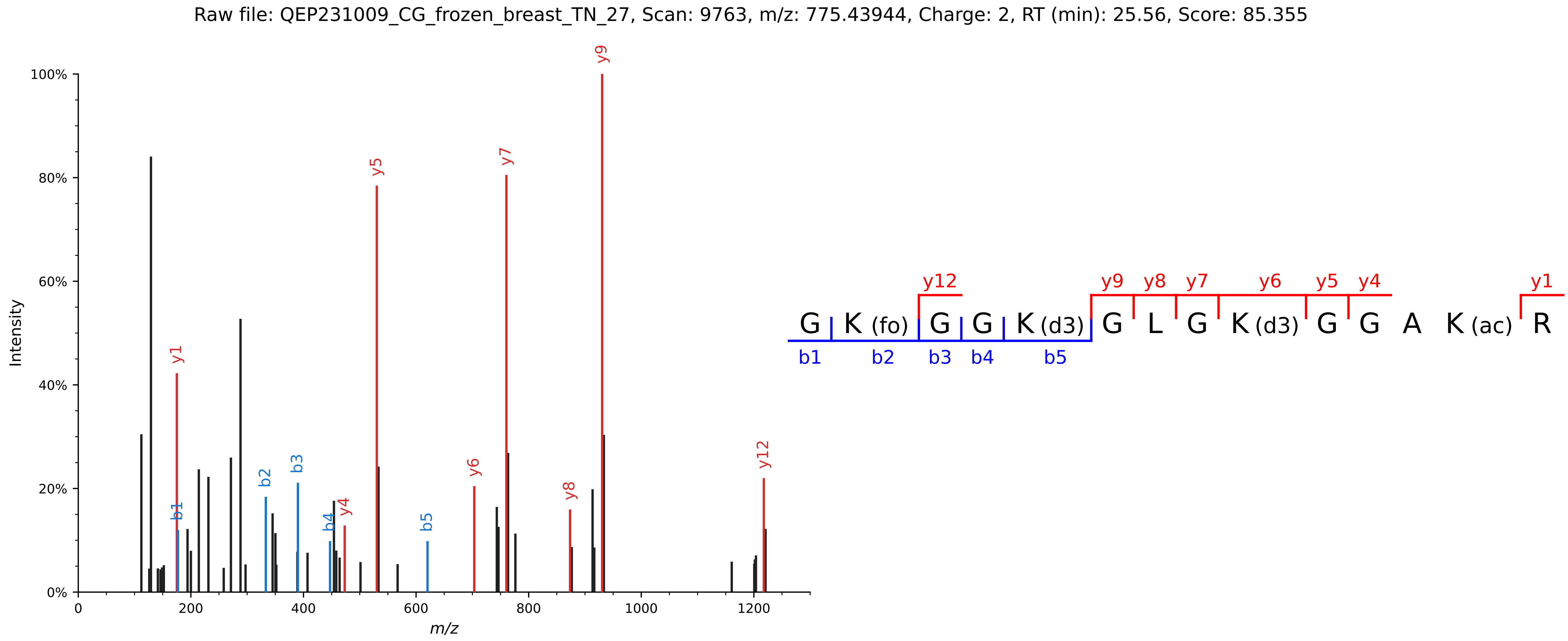

Raw file: QEP231009_CG_frozen_breast_TN_27, Scan: 9763, m/z: 775.43944, Charge: 2, RT (min): 25.56, Score: 85.355
Intensity
m/z
y9
y7
y5
y1
y12
y6
y8
y4
b1
b2
b3
b4
b5
G K (fo) G G K (d3) G L G K (d3) G G A K (ac) R

Raw file: QEP231009_CG_frozen_breast_TN_27, Scan: 11186, m/z: 790.96451, Charge: 2, RT (min): 28.64, Score: 146.11

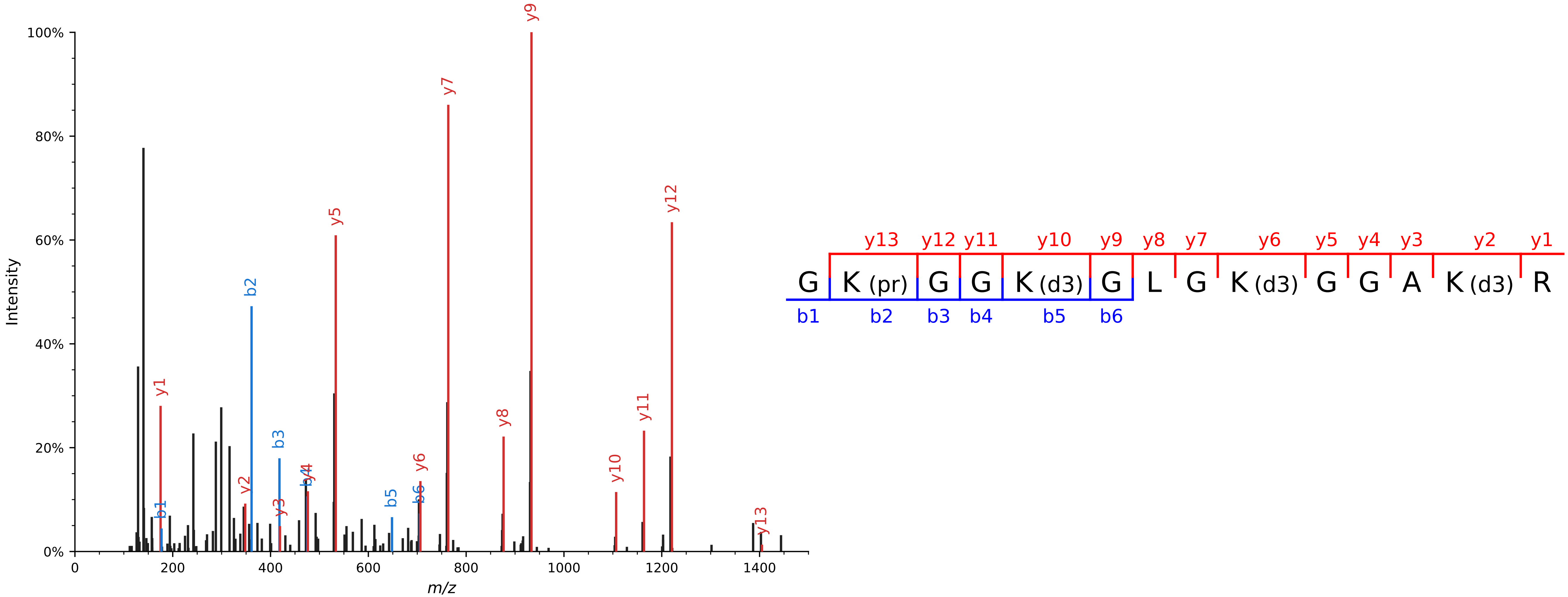

Raw file: QEP231009_CG_frozen_breast_TN_25, Scan: 12718, m/z: 531.31104, Charge: 3, RT (min): 30.82, Score: 72.891

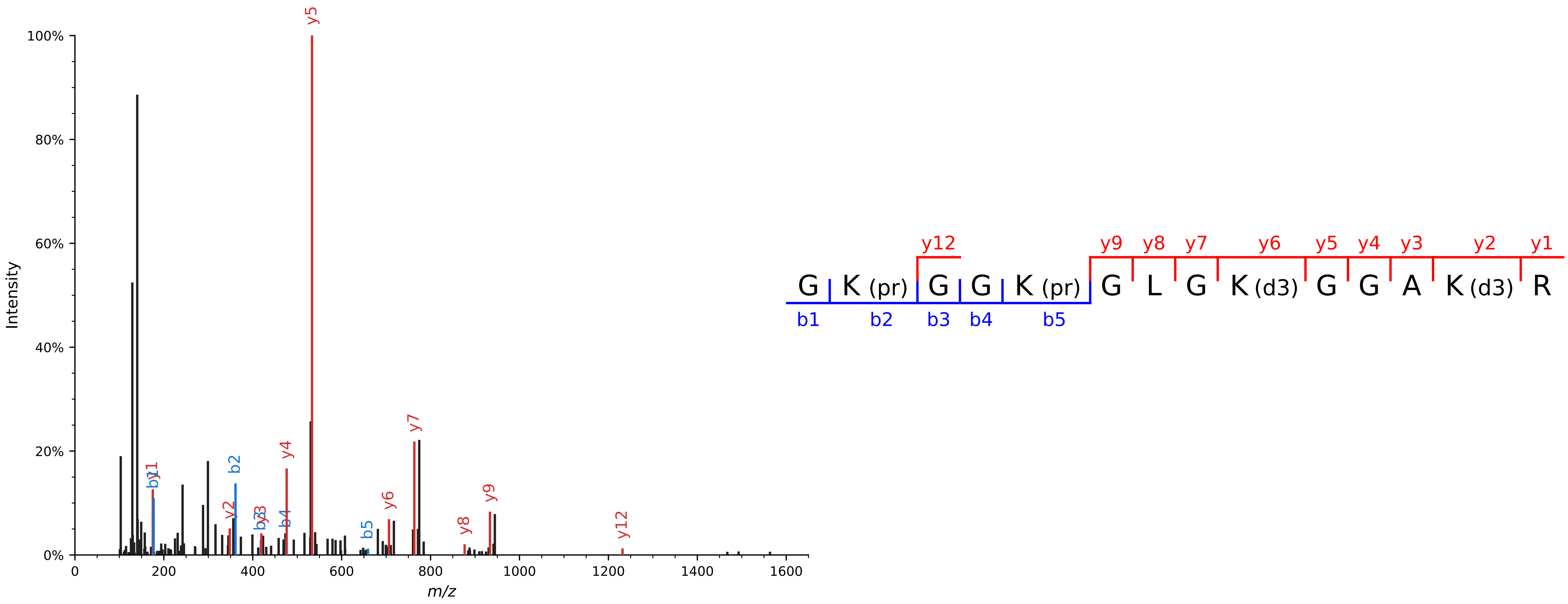

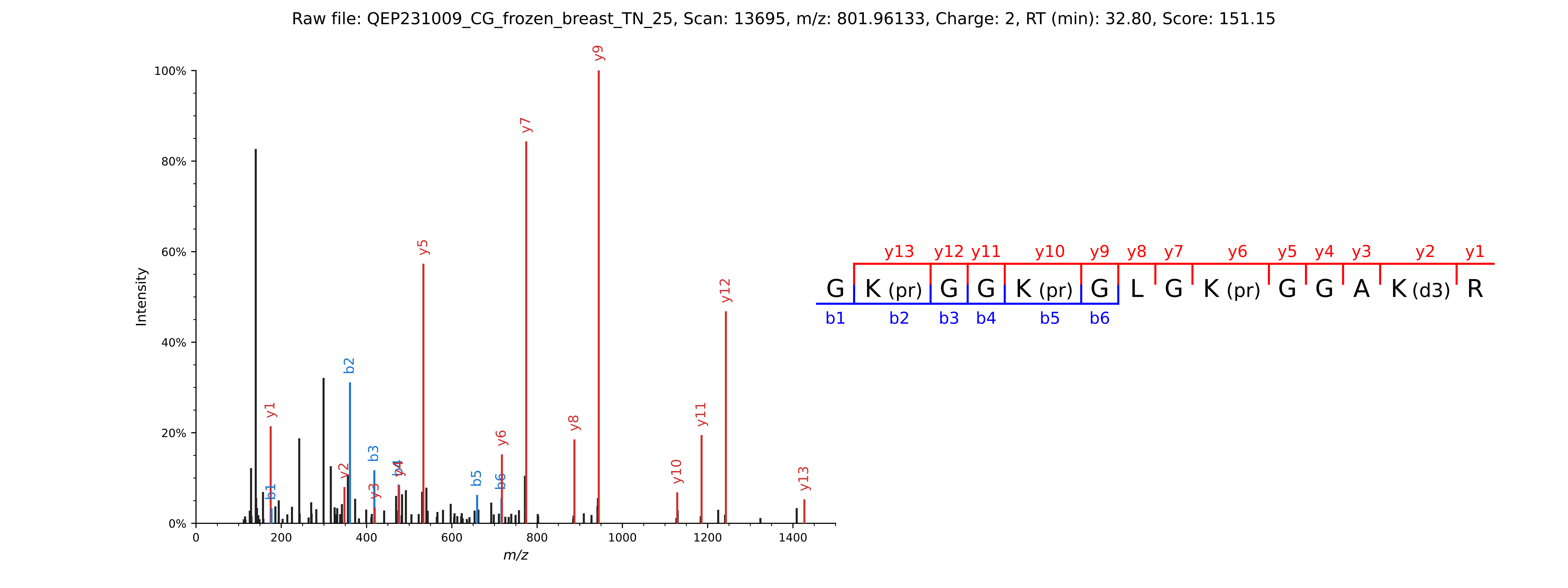

Raw file: QEP231009_CG_frozen_breast_TN_25, Scan: 13695, m/z: 801.96133, Charge: 2, RT (min): 32.80, Score: 151.15
Intensity
m/z
G K (pr) G G K (pr) G L G K (pr) G G A K (d3) R
y13 y12 y11 y10 y9 y8 y7 y6 y5 y4 y3 y2 y1
b1 b2 b3 b4 b5 b6

Raw file: QEP231009_CG_frozen_breast_TN_25, Scan: 14440, m/z: 807.45974, Charge: 2, RT (min): 34.32, Score: 94.465

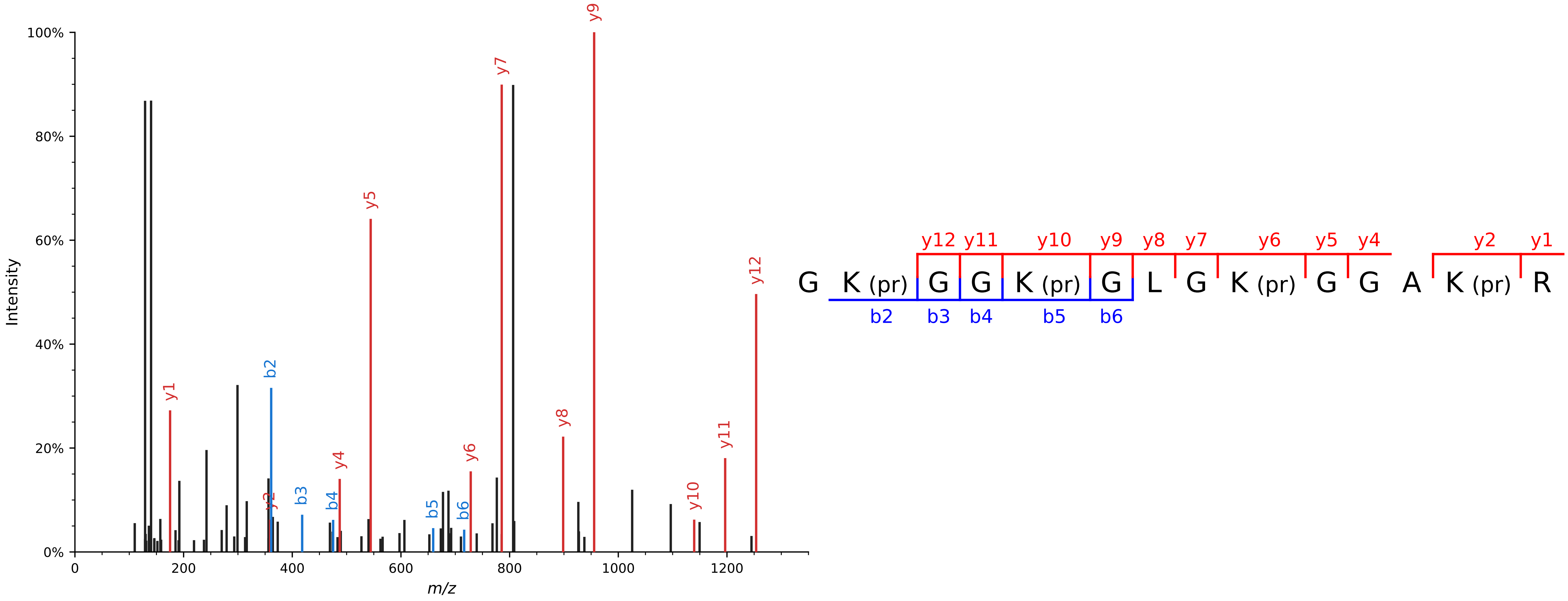

Raw file: QEP231009_CG_frozen_breast_TN_27, Scan: 12108, m/z: 794.9535, Charge: 2, RT (min): 30.77, Score: 61.353

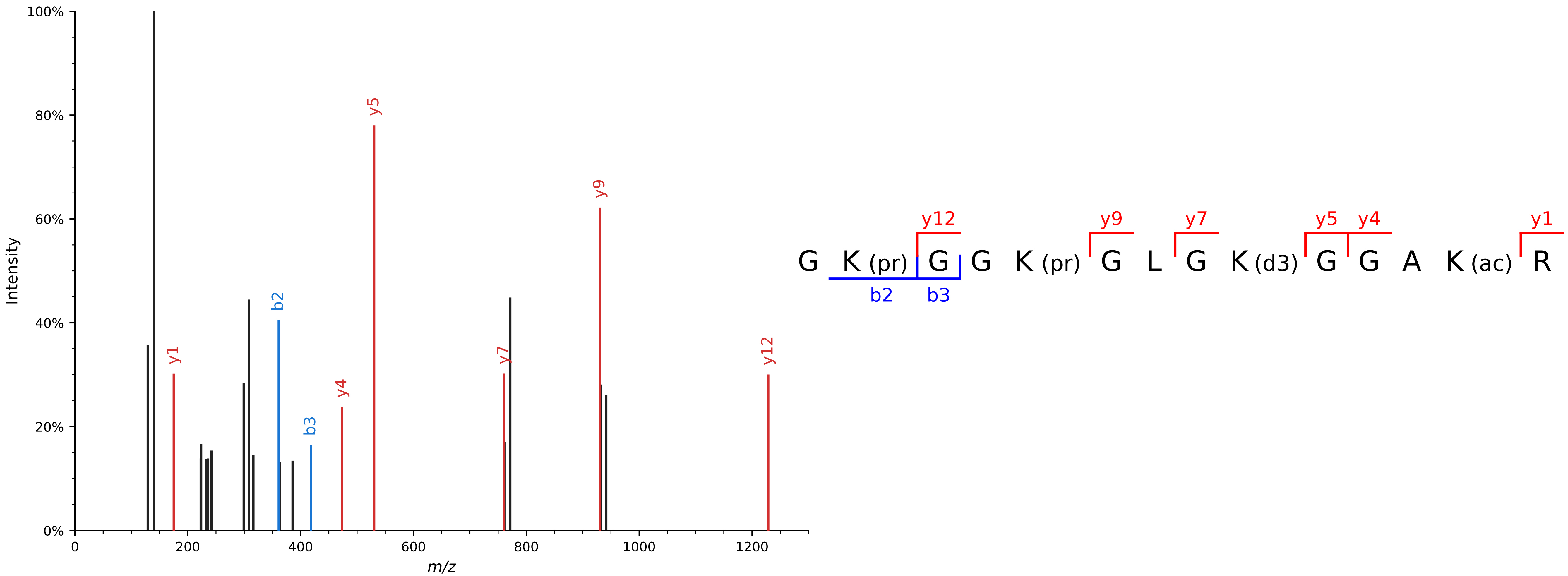

Raw file: QEP231009_CG_frozen_breast_TN_27, Scan: 12722, m/z: 534.97664, Charge: 3, RT (min): 32.18, Score: 49.929

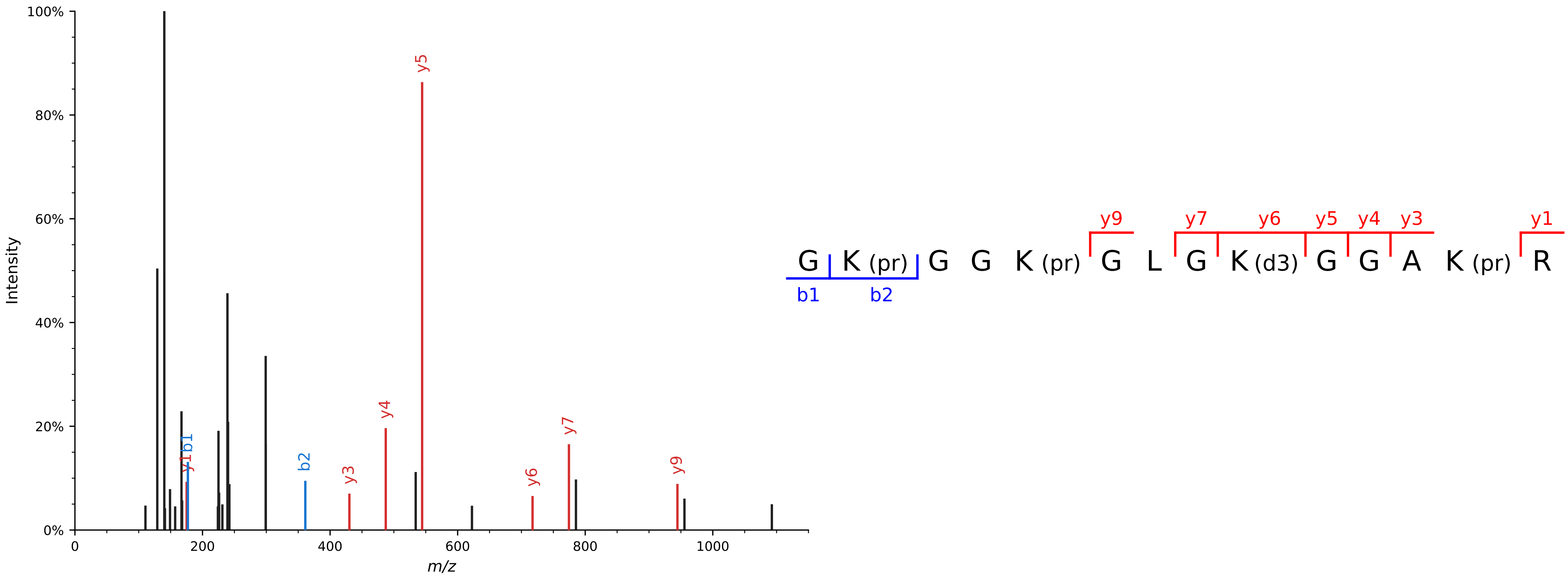

Raw file: QEP231009_CG_frozen_breast_LuA_32, Scan: 12102, m/z: 796.46292, Charge: 2, RT (min): 30.52, Score: 132.32

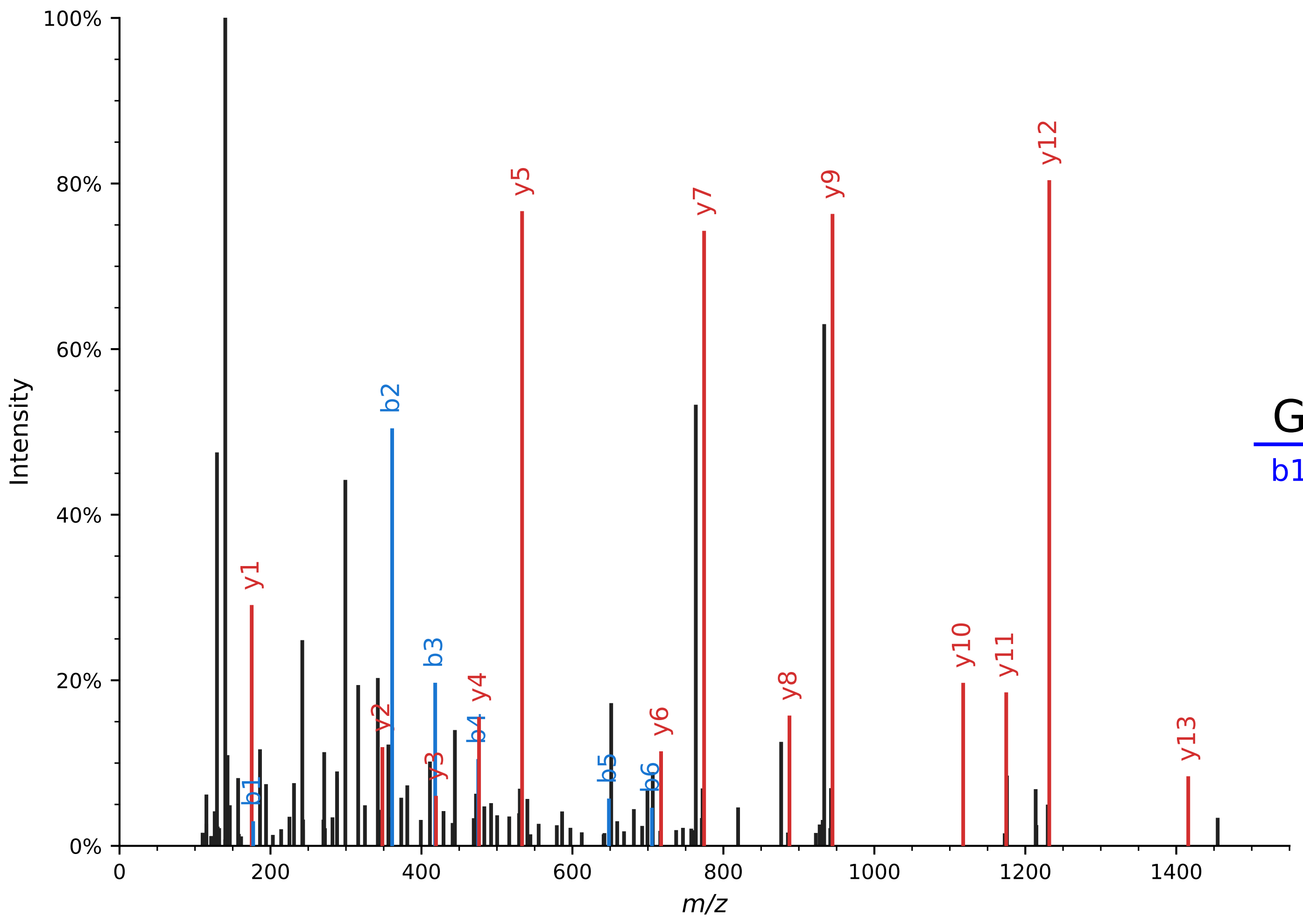

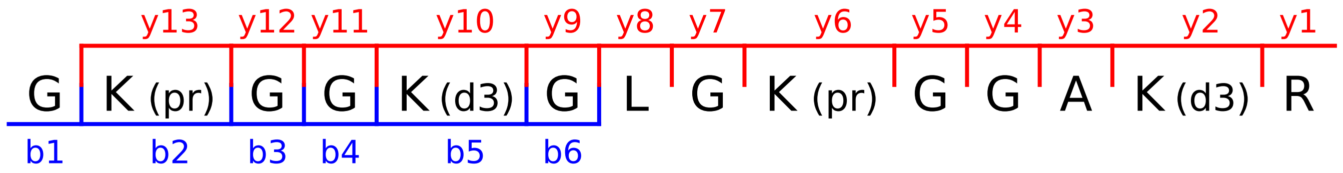

Raw file: QEP231009_CG_frozen_breast_TN_25, Scan: 13526, m/z: 801.96133, Charge: 2, RT (min): 32.46, Score: 166.97

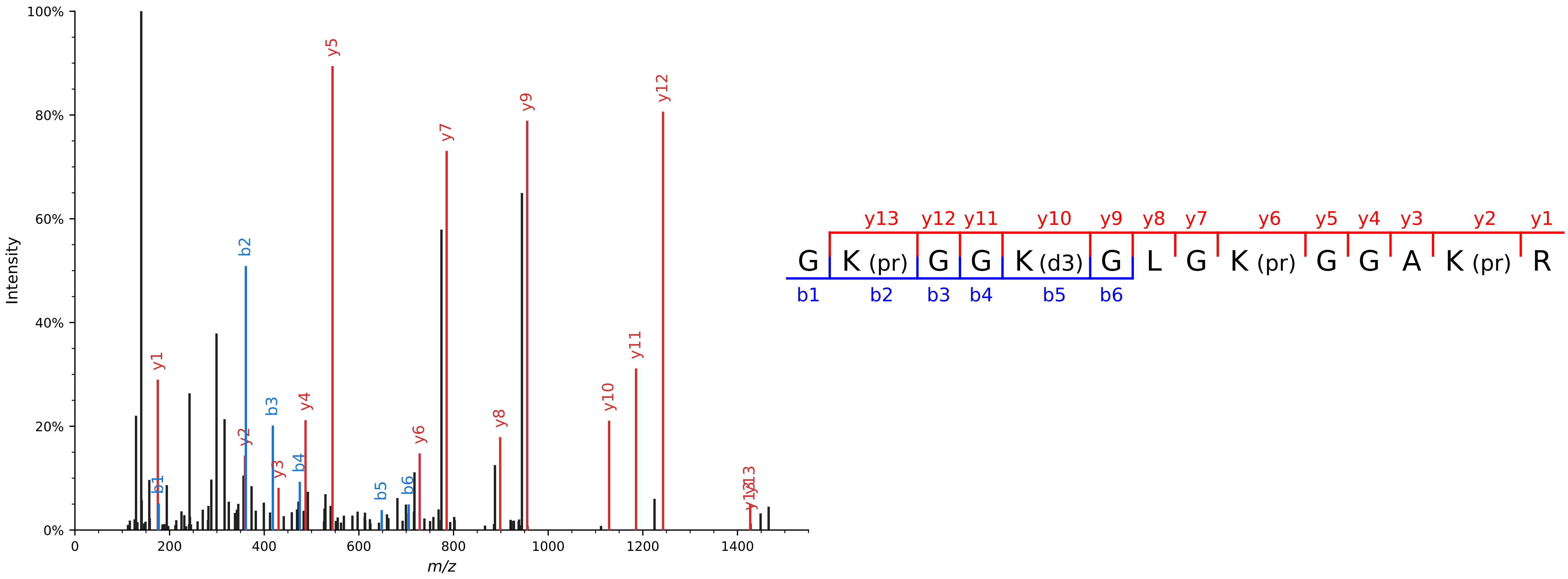

Raw file: QEP231009_CG_frozen_breast_TN_27, Scan: 11205, m/z: 789.45509, Charge: 2, RT (min): 28.68, Score: 164.43

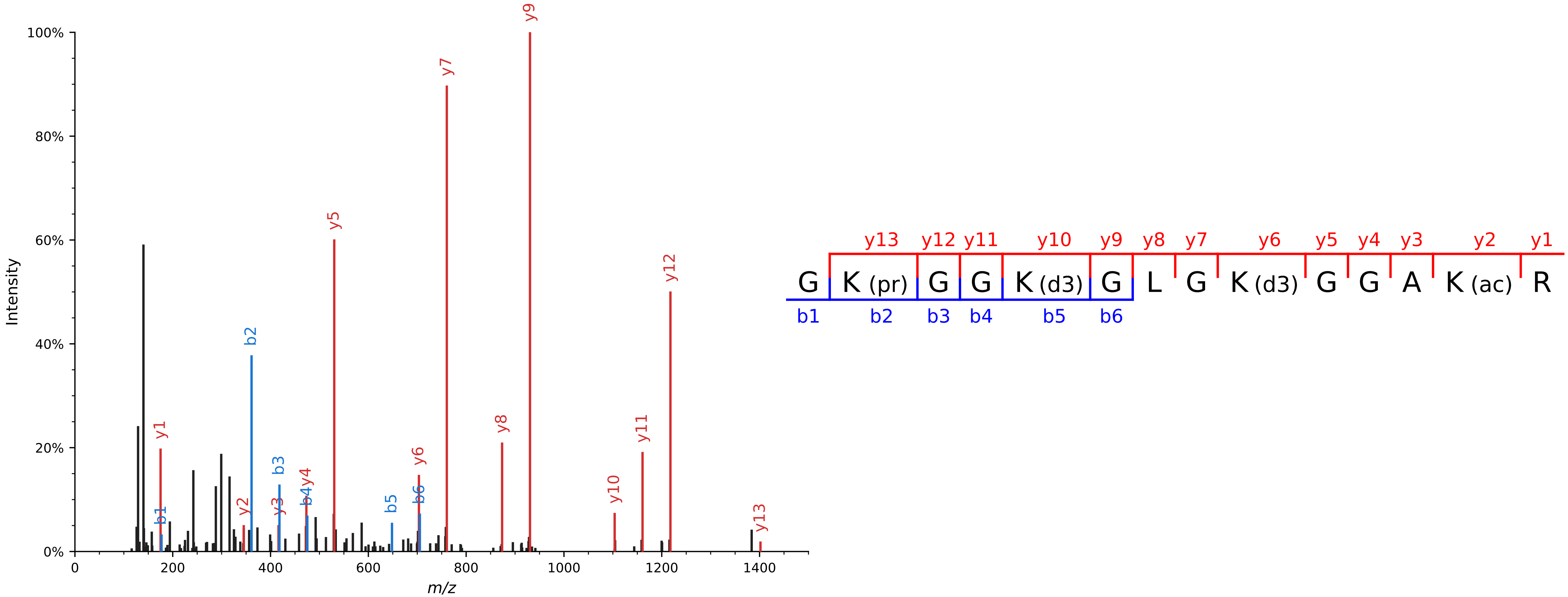

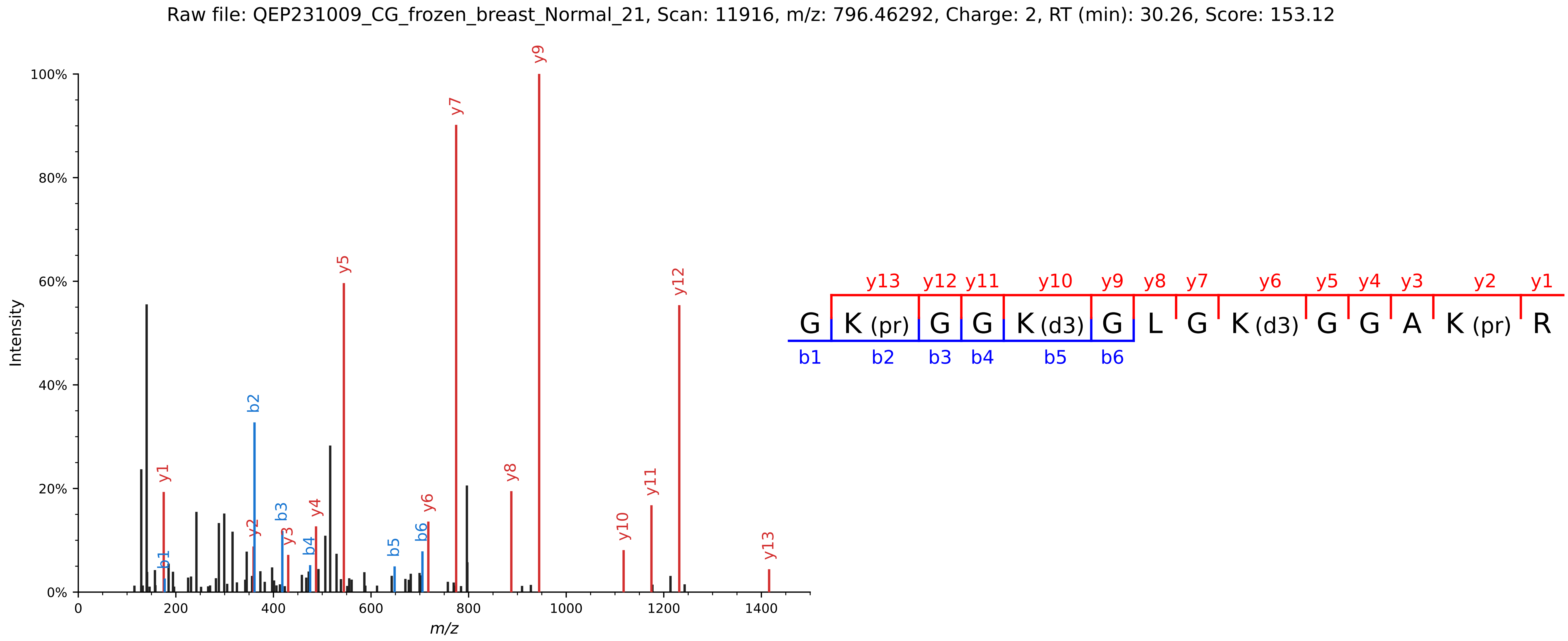
Raw file: QEP231009_CG_frozen_breast_Normal_21, Scan: 11916, m/z: 796.46292, Charge: 2, RT (min): 30.26, Score: 153.12
Intensity
m/z
y13 y12 y11 y10 y9 y8 y7 y6 y5 y4 y3 y2 y1
G K (pr) G G K (d3) G L G K (d3) G G A K (pr) R
b1 b2 b3 b4 b5 b6

Raw file: QEP231009_CG_frozen_breast_LuA_33, Scan: 10958, m/z: 525.63288, Charge: 3, RT (min): 28.44, Score: 57.034

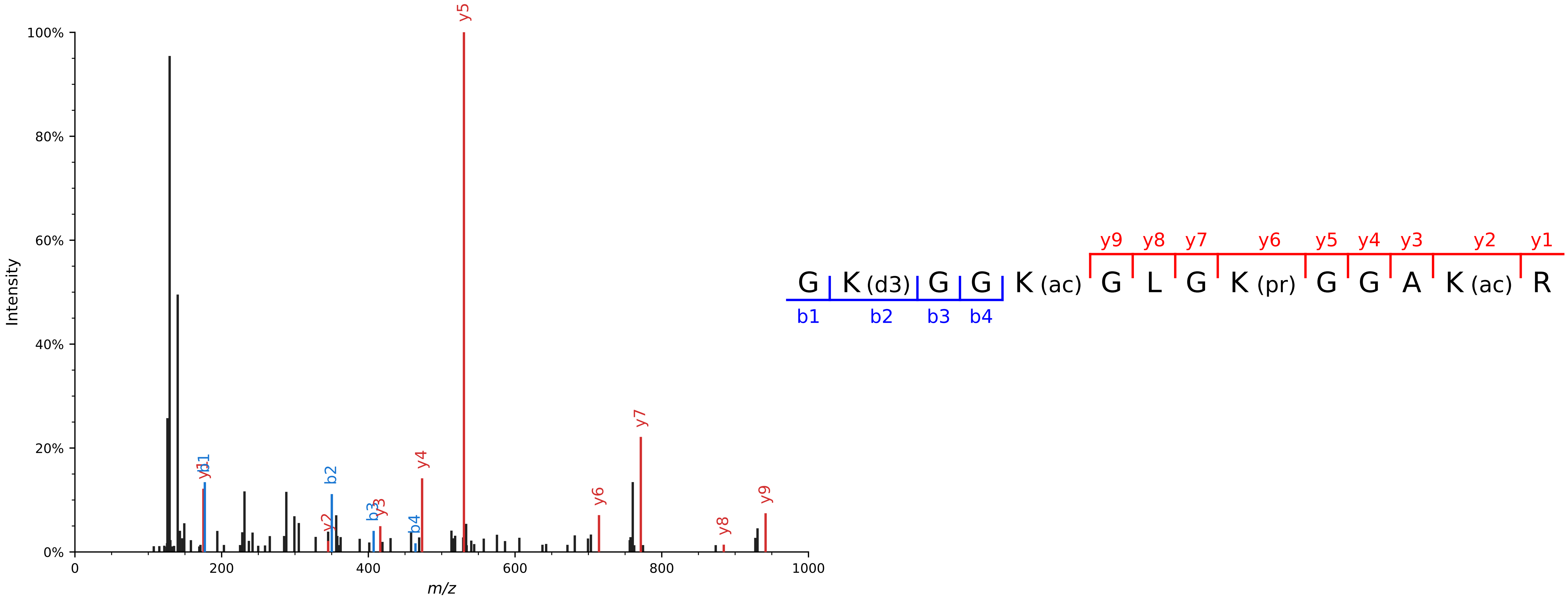

Raw file: QEP231009_CG_frozen_breast_LuA_30, Scan: 10427, m/z: 776.94886, Charge: 2, RT (min): 26.44, Score: 55.649

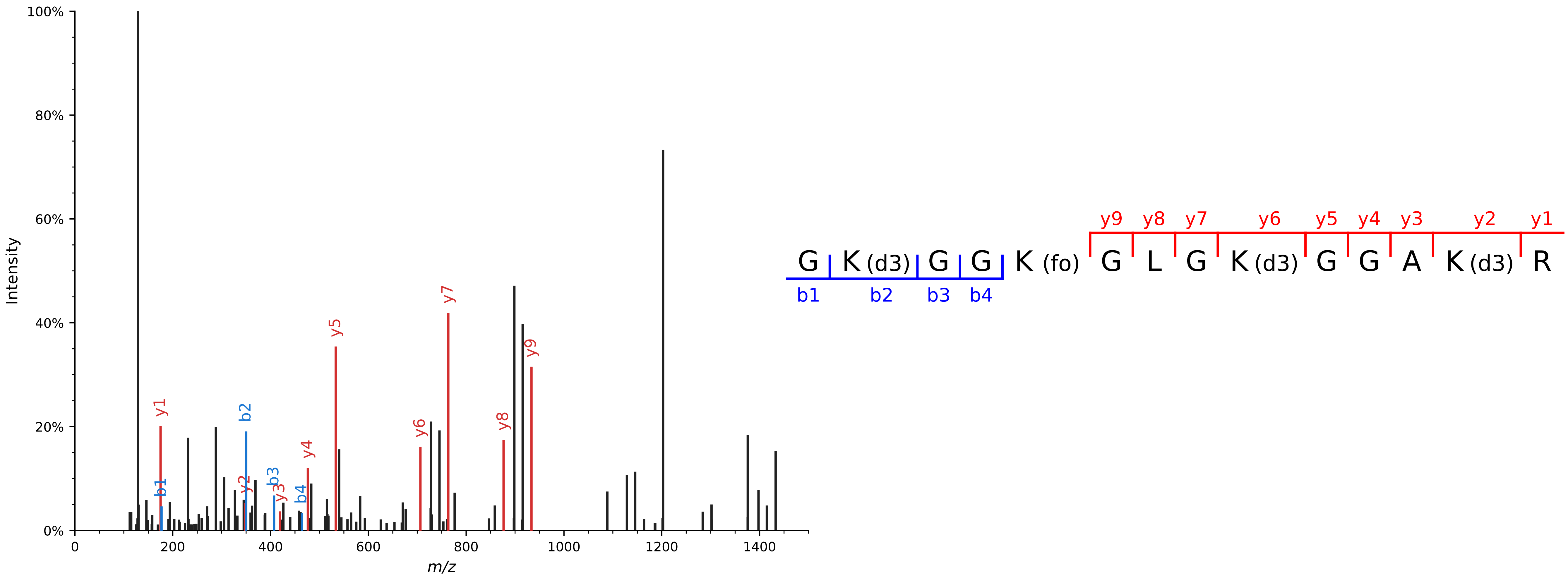

Raw file: QEP231009_CG_frozen_breast_TN_28, Scan: 10432, m/z: 775.43944, Charge: 2, RT (min): 26.64, Score: 50.194

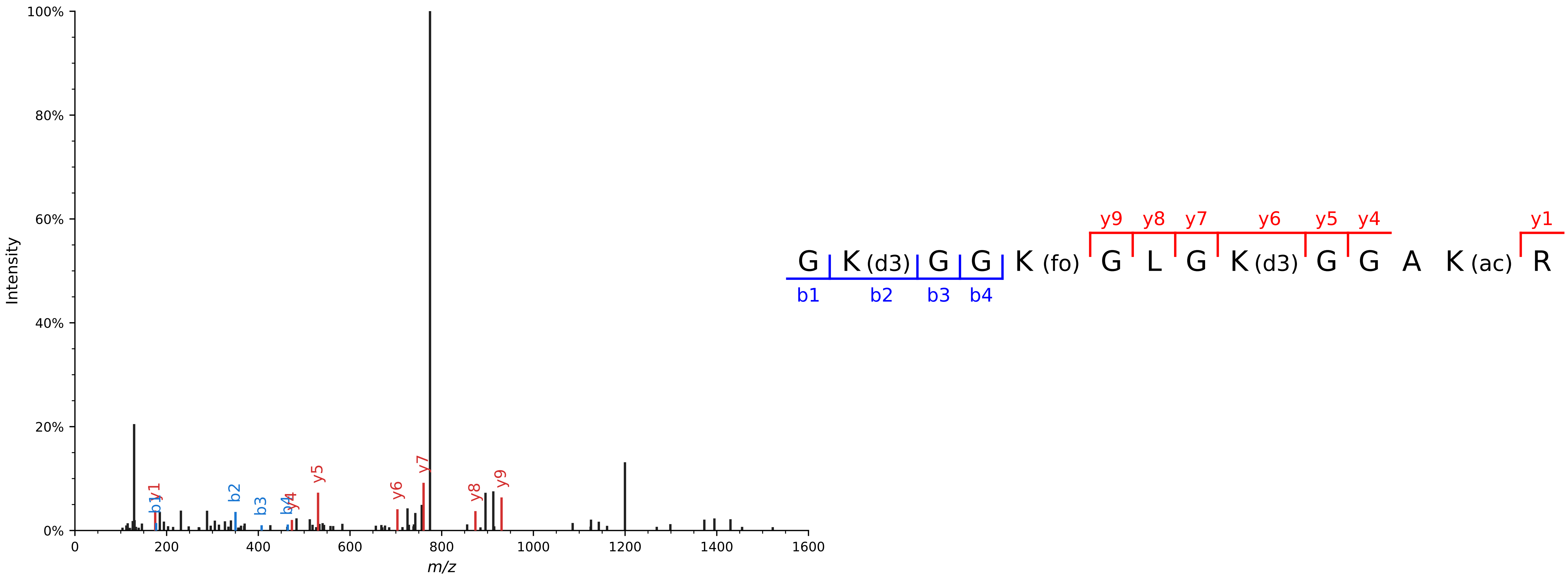

Raw file: QEP231009_CG_frozen_breast_TN_25, Scan: 10583, m/z: 798.96197, Charge: 2, RT (min): 26.28, Score: 102.52

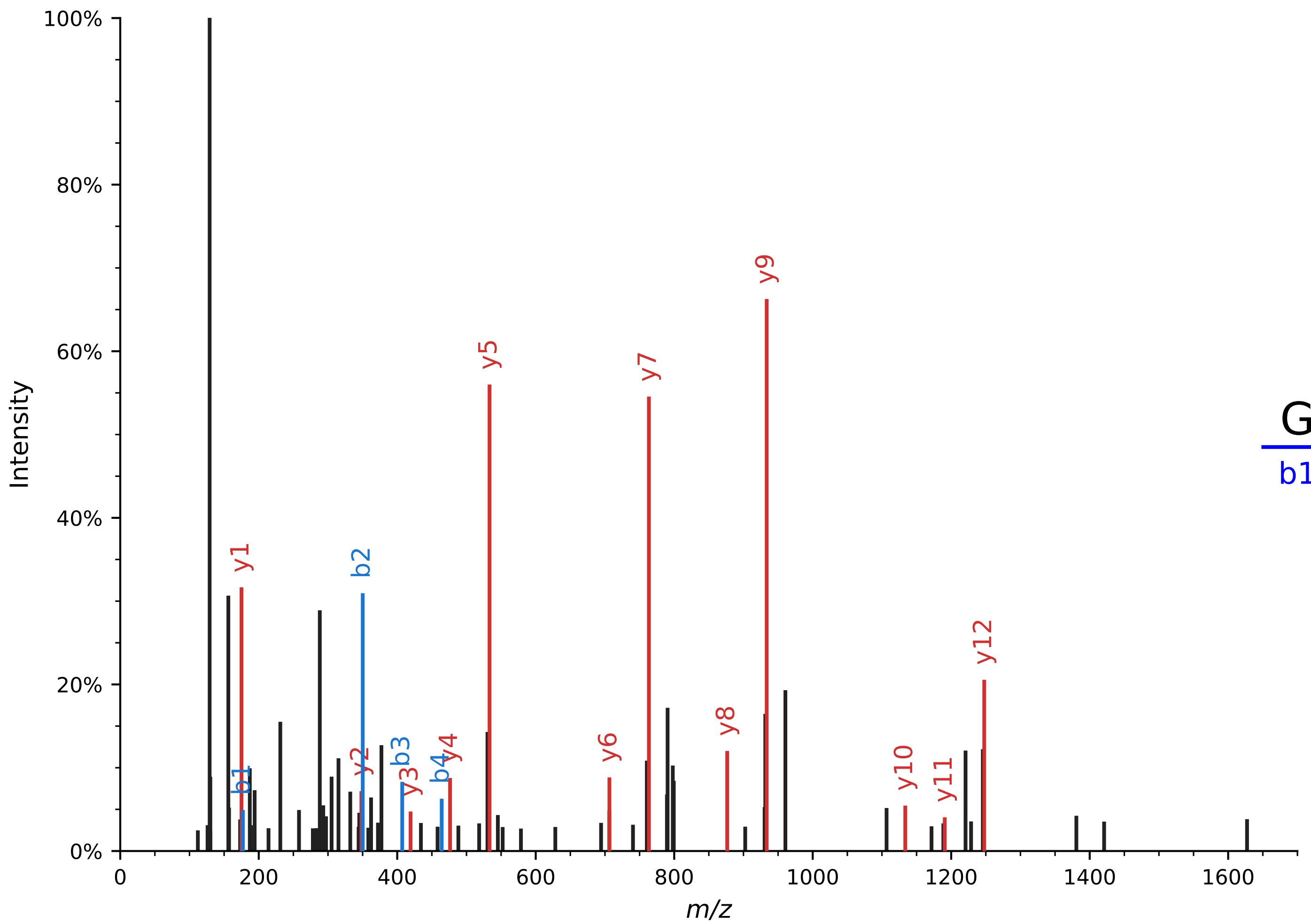

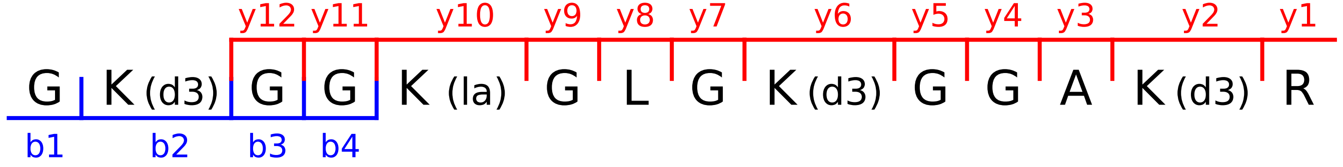

Raw file: QEP231009_CG_frozen_breast_LuA_30, Scan: 11356, m/z: 790.96451, Charge: 2, RT (min): 28.38, Score: 152.54

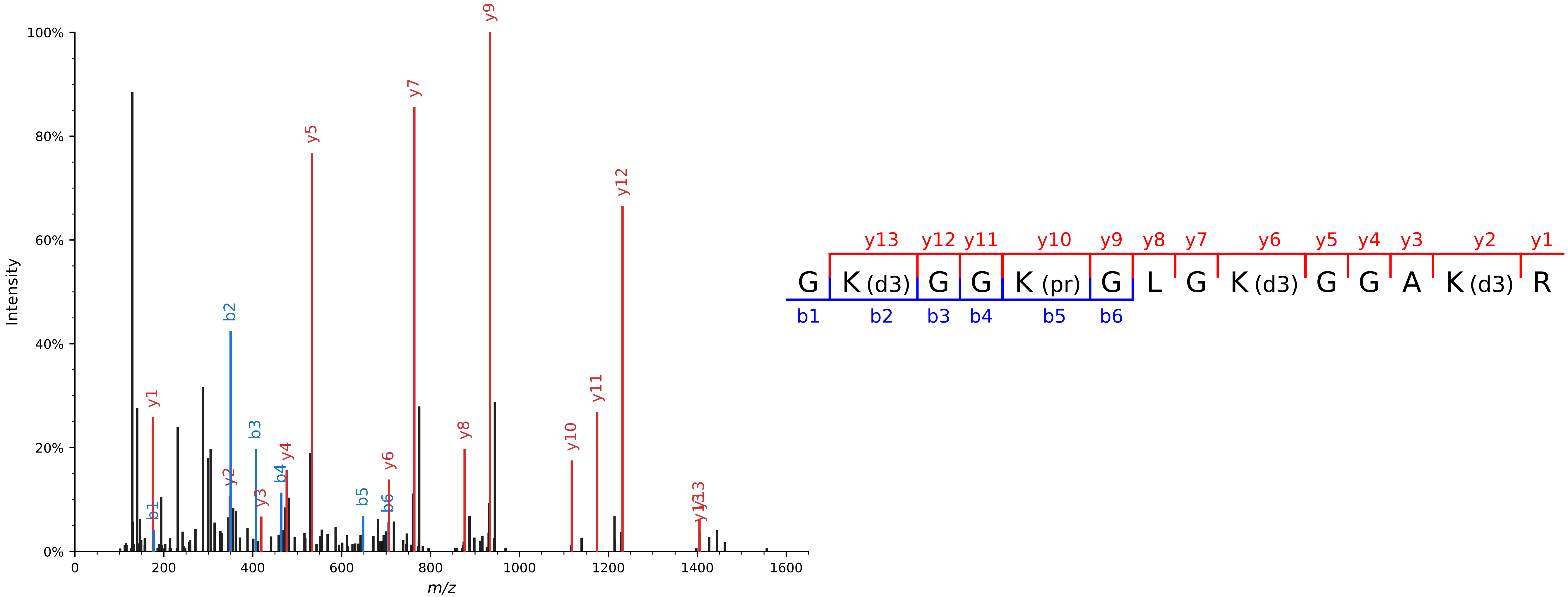

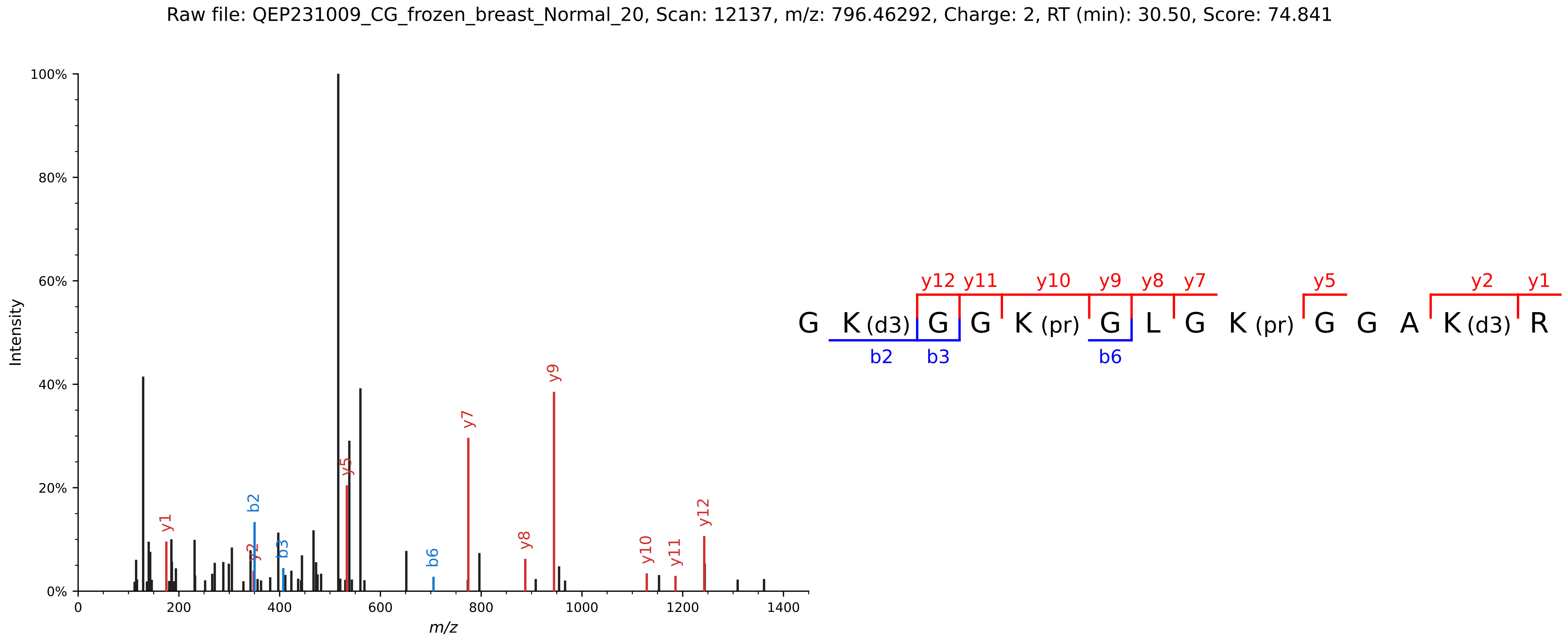

Raw file: QEP231009_CG_frozen_breast_Normal_20, Scan: 12137, m/z: 796.46292, Charge: 2, RT (min): 30.50, Score: 74.841
Intensity
m/z
G K(d3) G G K(pr) G L G K(pr) G G A K(d3) R
y12 y11 y10 y9 y8 y7 y5 y2 y1
b2 b3 b6

Raw file: QEP231009_CG_frozen_breast_TN_27, Scan: 11962, m/z: 794.9535, Charge: 2, RT (min): 30.42, Score: 136.48

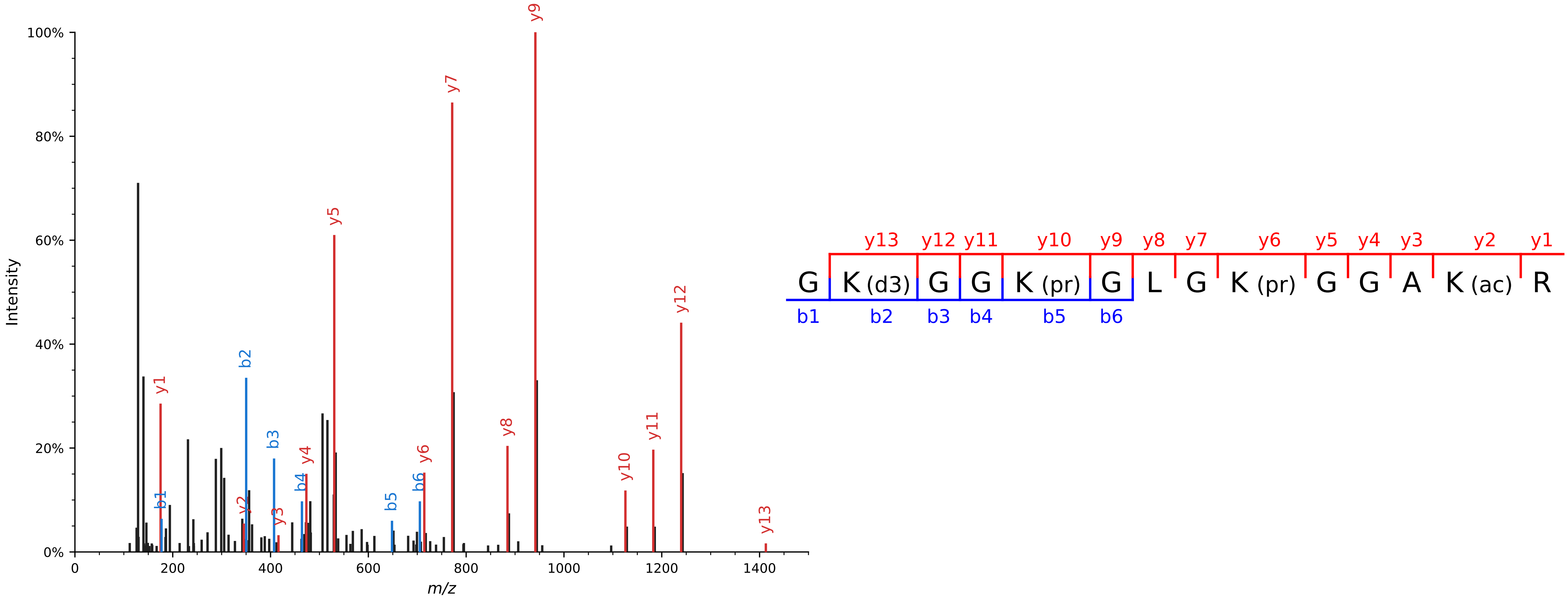

Raw file: QEP231009_CG_frozen_breast_TN_25, Scan: 13359, m/z: 801.96133, Charge: 2, RT (min): 32.11, Score: 140.07

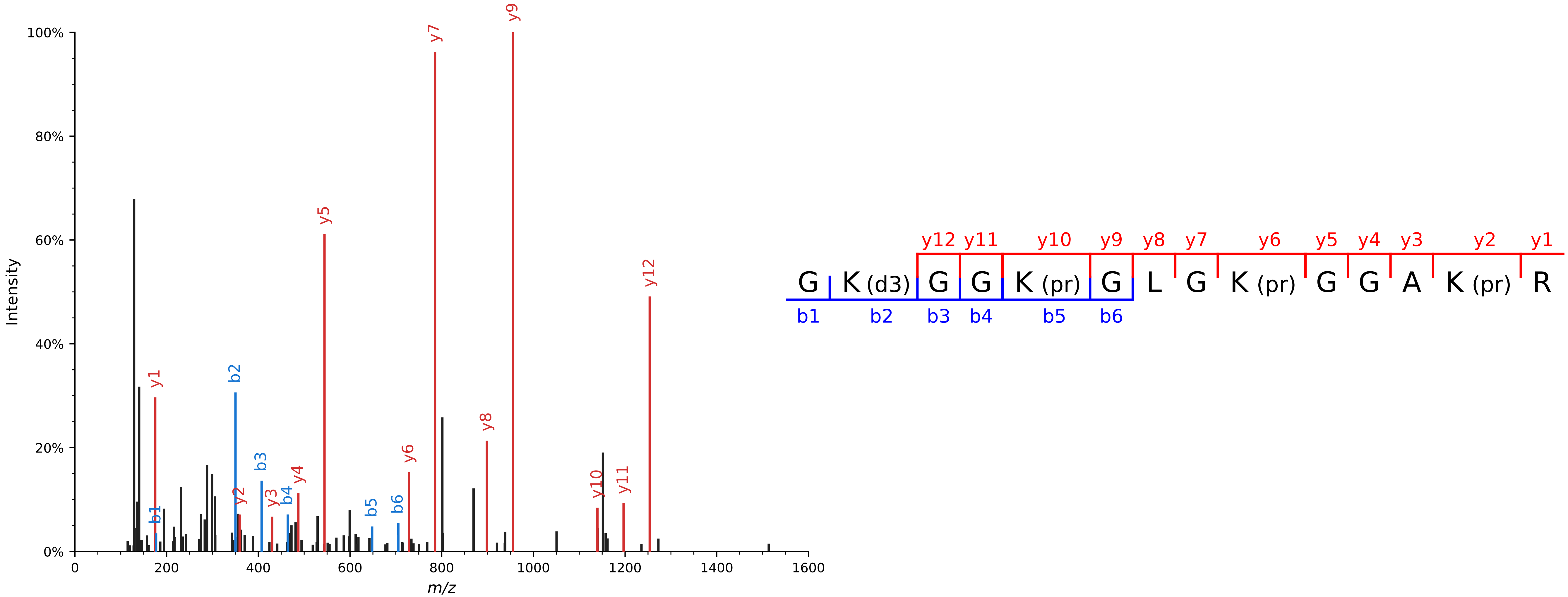

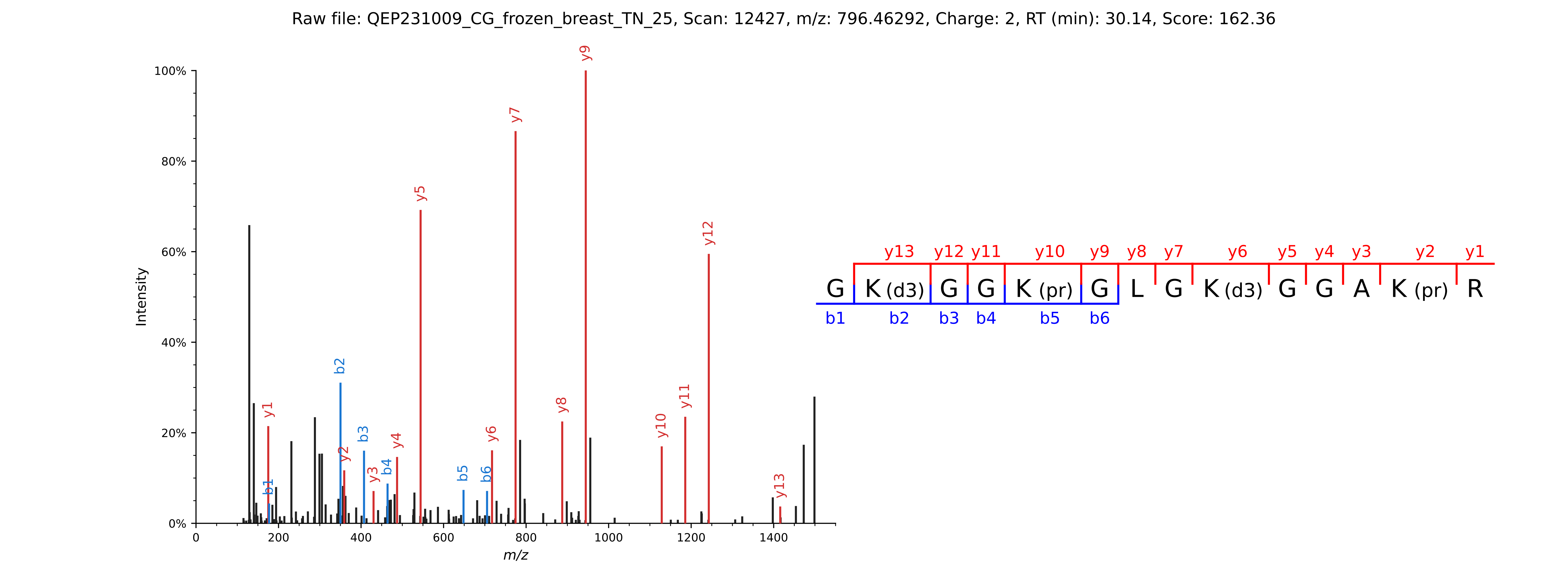
Raw file: QEP231009_CG_frozen_breast_TN_25, Scan: 12427, m/z: 796.46292, Charge: 2, RT (min): 30.14, Score: 162.36
Intensity
m/z
100%
80%
60%
40%
20%
0%
0
200
400
600
800
1000
1200
1400
y1 y2 y3 y4 y5 y6 y7 y8 y9 y10 y11 y12 y13
b1 b2 b3 b4 b5 b6
G K (d3) G G K (pr) G L G K (d3) G G A K (pr) R

Raw file: QEP231009_CG_frozen_breast_Normal_22, Scan: 9697, m/z: 776.94886, Charge: 2, RT (min): 25.99, Score: 74.162

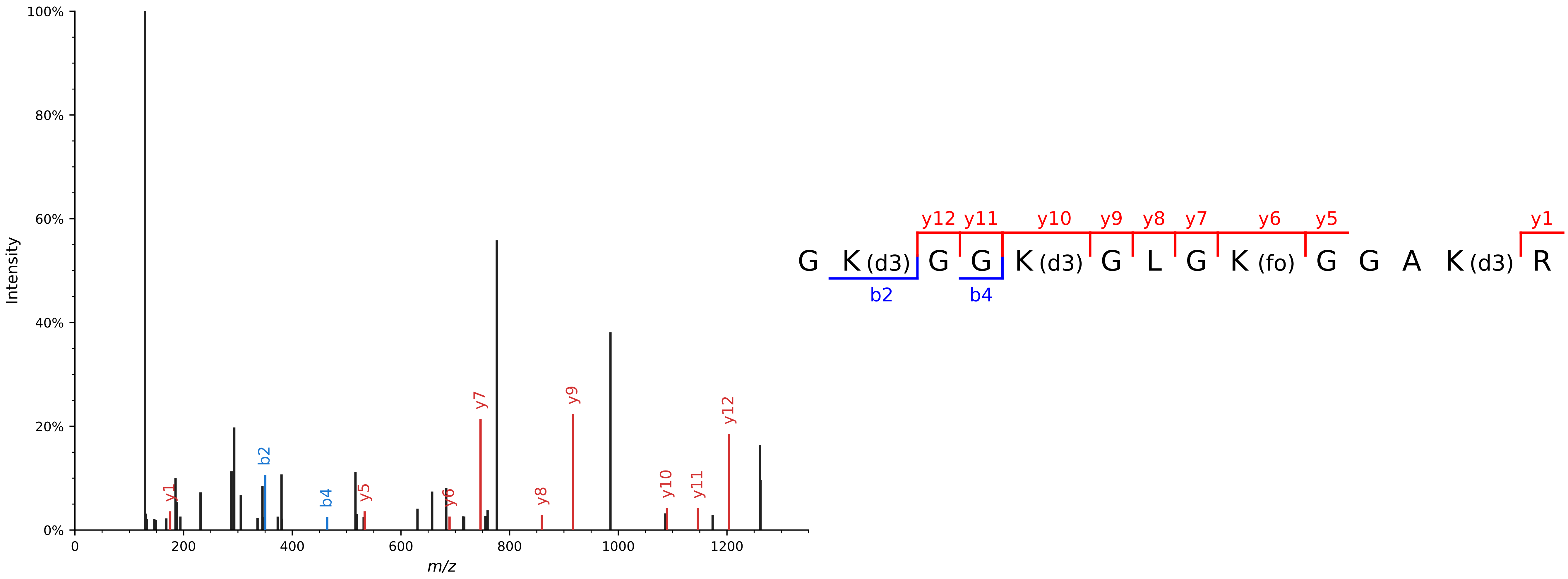

Raw file: QEP231006_CG_frozen_breast_TN_26, Scan: 10950, m/z: 790.96451, Charge: 2, RT (min): 28.50, Score: 155.15

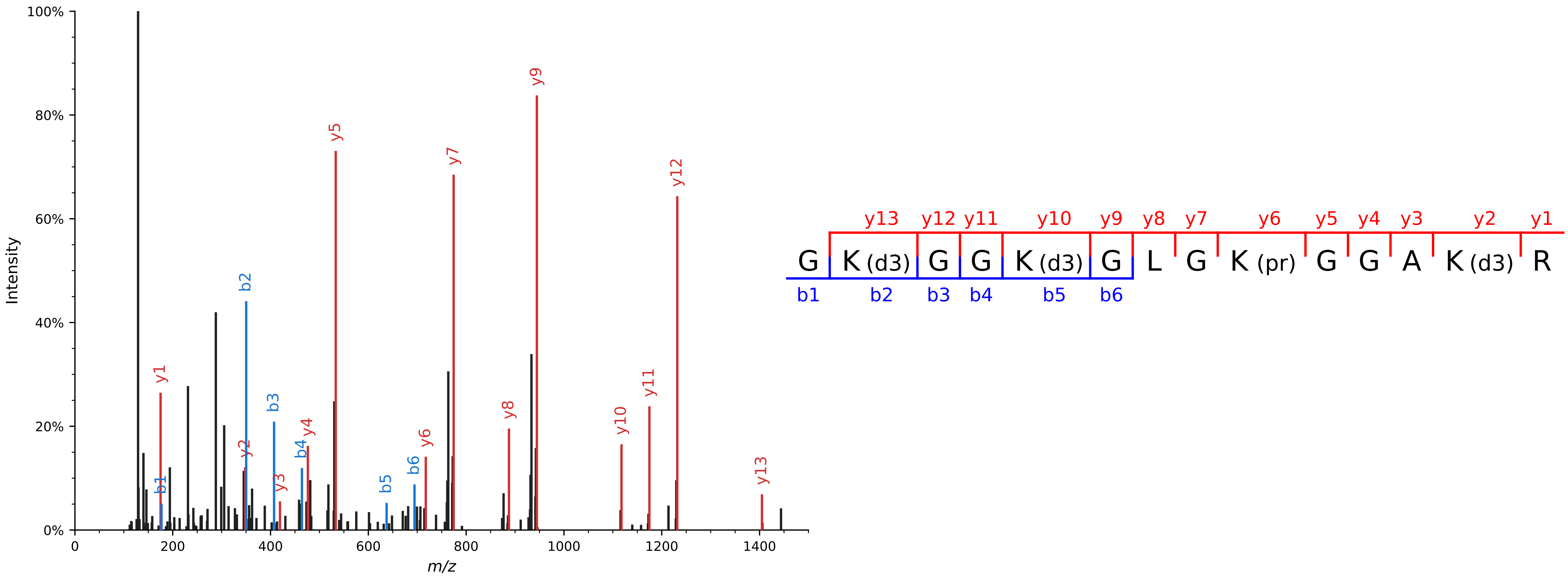

Raw file: QEP231009_CG_frozen_breast_TN_27, Scan: 10122, m/z: 783.95668, Charge: 2, RT (min): 26.36, Score: 169.93

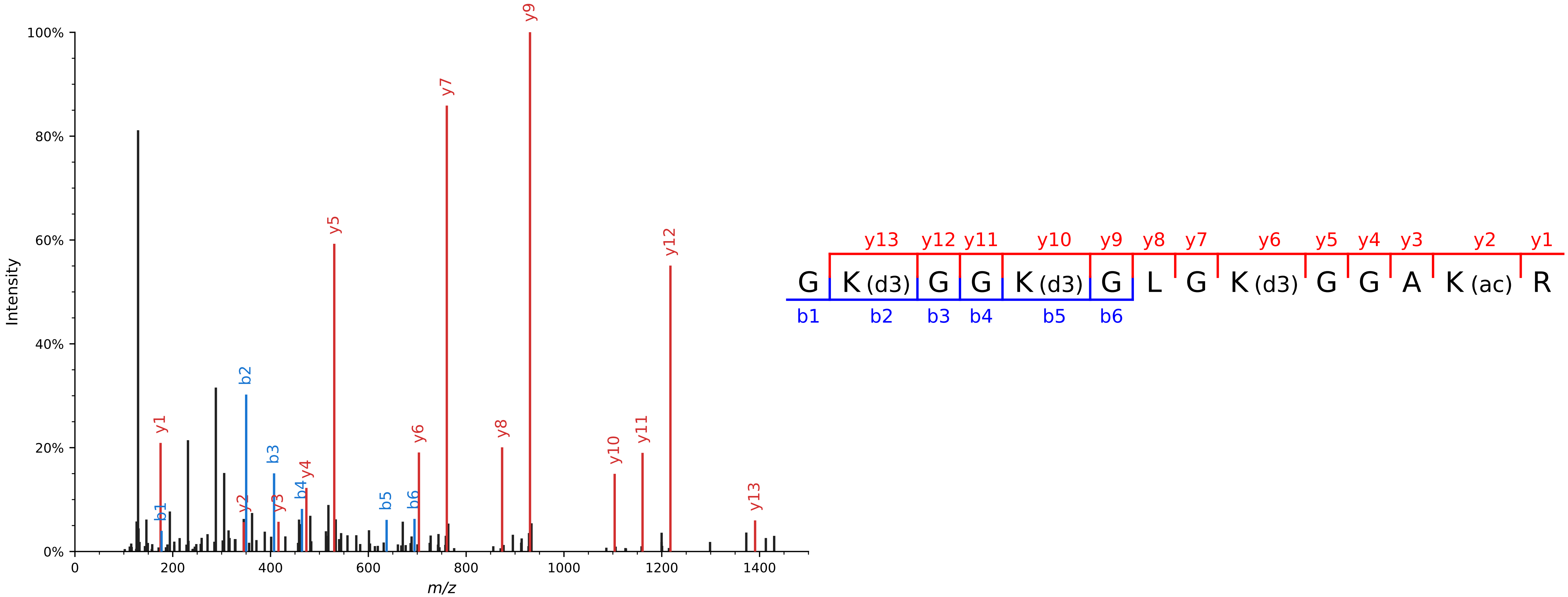

Raw file: QEP231009_CG_frozen_breast_Normal_24, Scan: 9860, m/z: 776.94886, Charge: 2, RT (min): 25.96, Score: 90.108

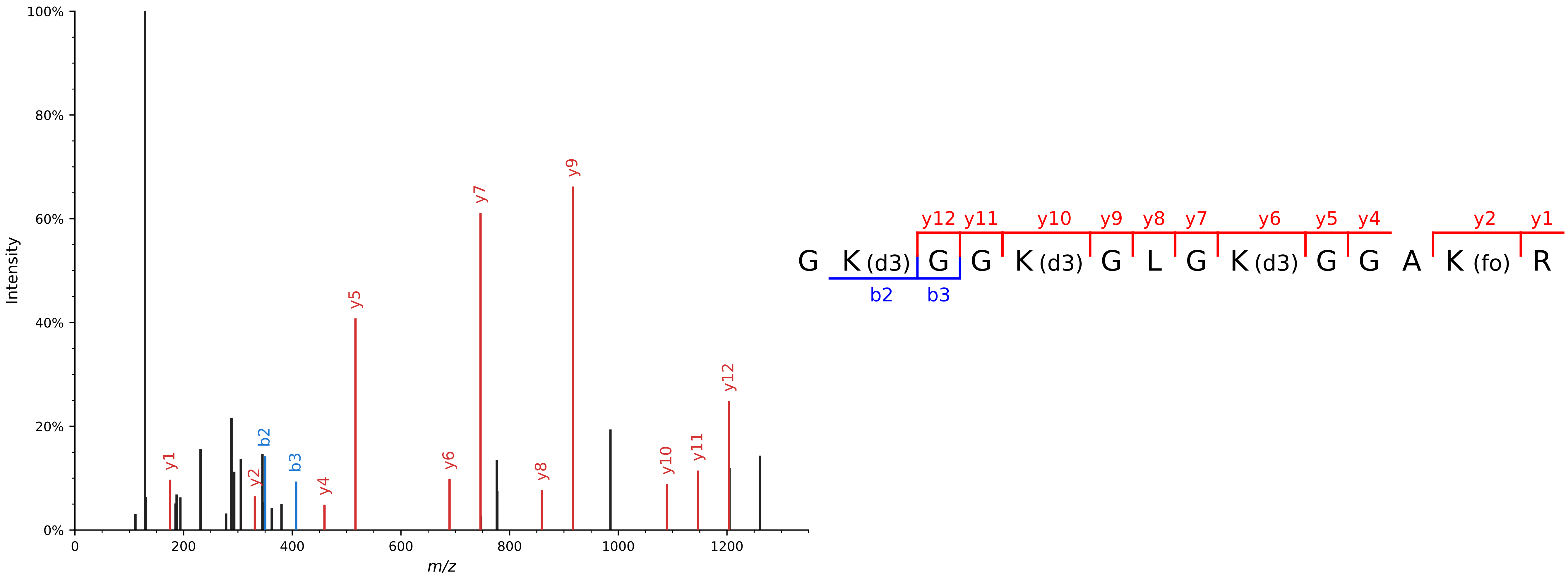

Raw file: QEP231009_CG_frozen_breast_LuA_32, Scan: 11005, m/z: 790.96451, Charge: 2, RT (min): 27.99, Score: 182.71

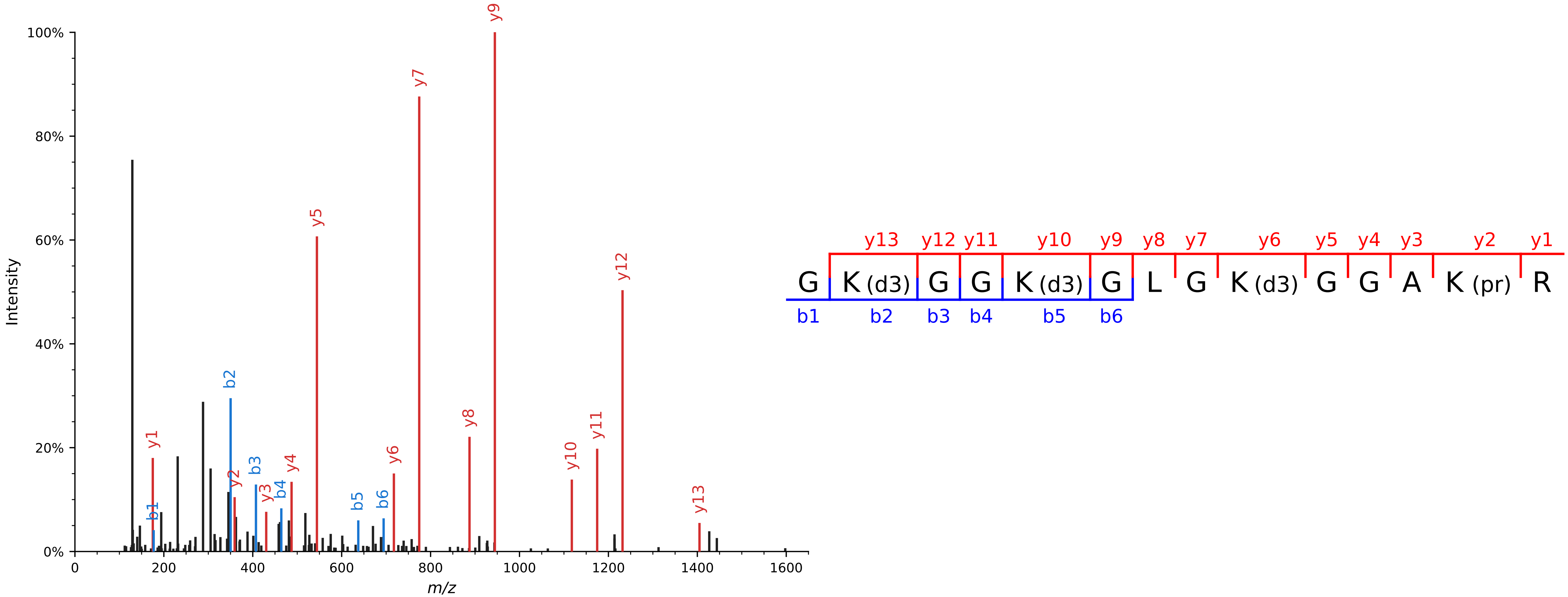

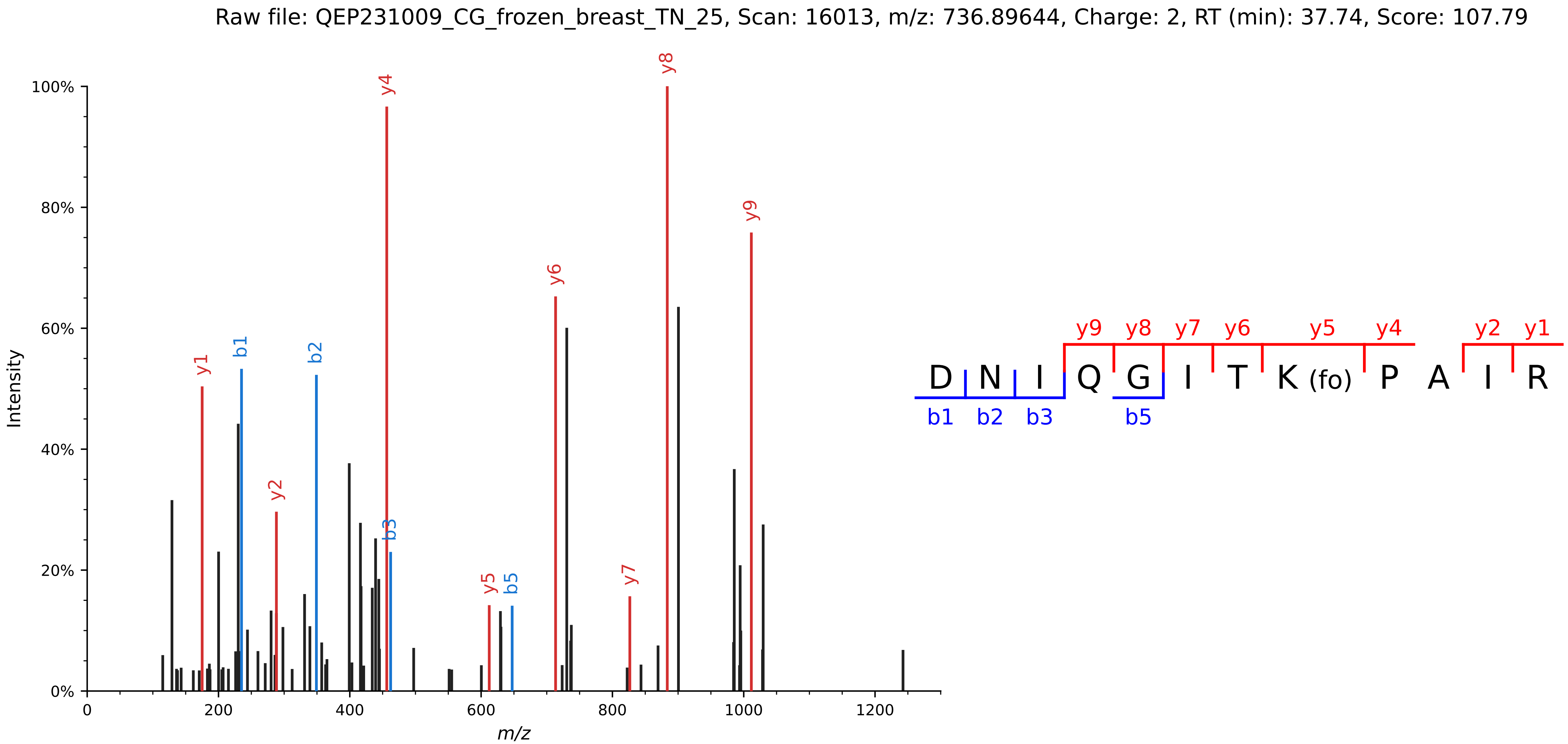
Raw file: QEP231009_CG_frozen_breast_TN_25, Scan: 16013, m/z: 736.89644, Charge: 2, RT (min): 37.74, Score: 107.79
Intensity
m/z
y1
b1
b2
y2
y4
b3
y5
b5
y6
y7
y8
y9
D N I Q G I T K (fo) P A I R
y9 y8 y7 y6 y5 y4 y2 y1
b1 b2 b3 b5

Raw file: QEP231009_CG_frozen_breast_TN_25, Scan: 16089, m/z: 736.91463, Charge: 2, RT (min): 37.90, Score: 48.423

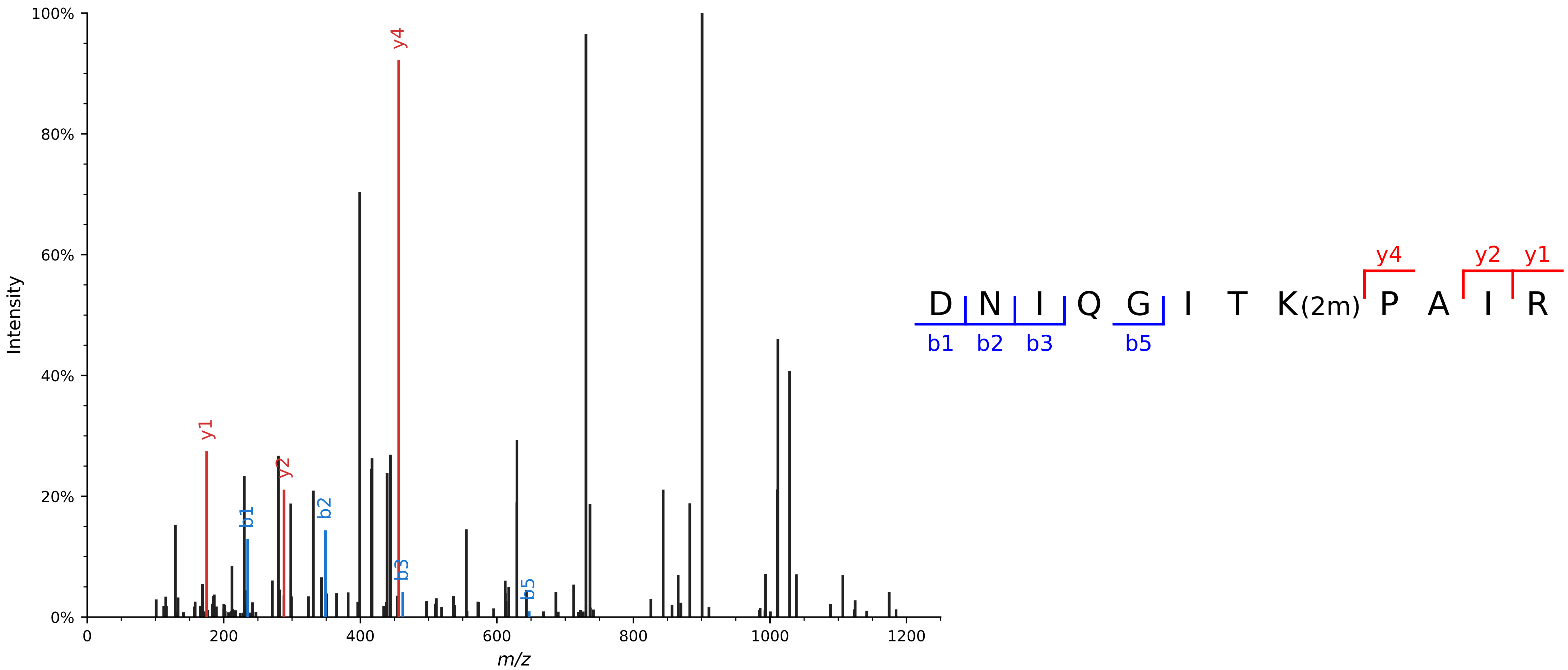

Raw file: QEP231006_CG_frozen_breast_TN_26, Scan: 14828, m/z: 750.91209, Charge: 2, RT (min): 39.81, Score: 97.456

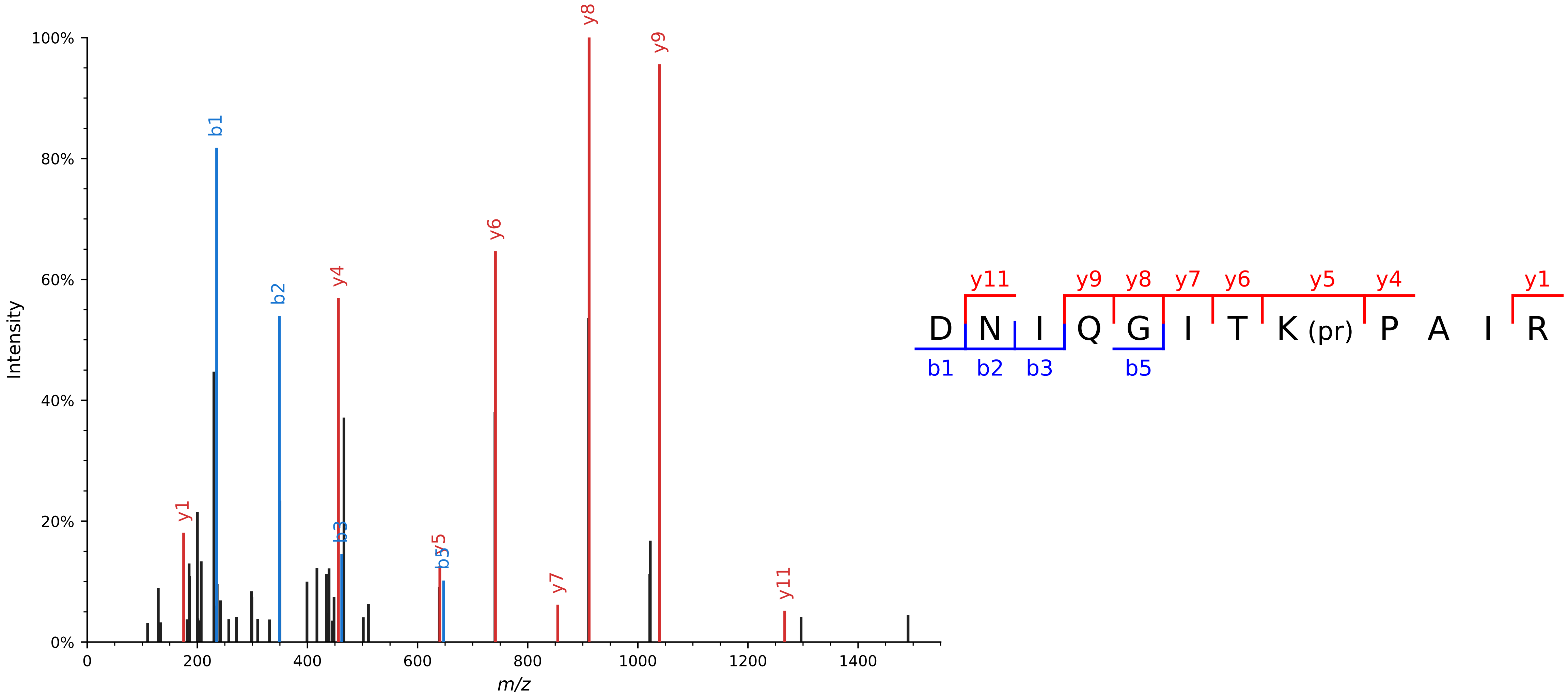

Raw file: QEP231009_CG_frozen_breast_TN_25, Scan: 8903, m/z: 719.3415, Charge: 2, RT (min): 22.65, Score: 140.75

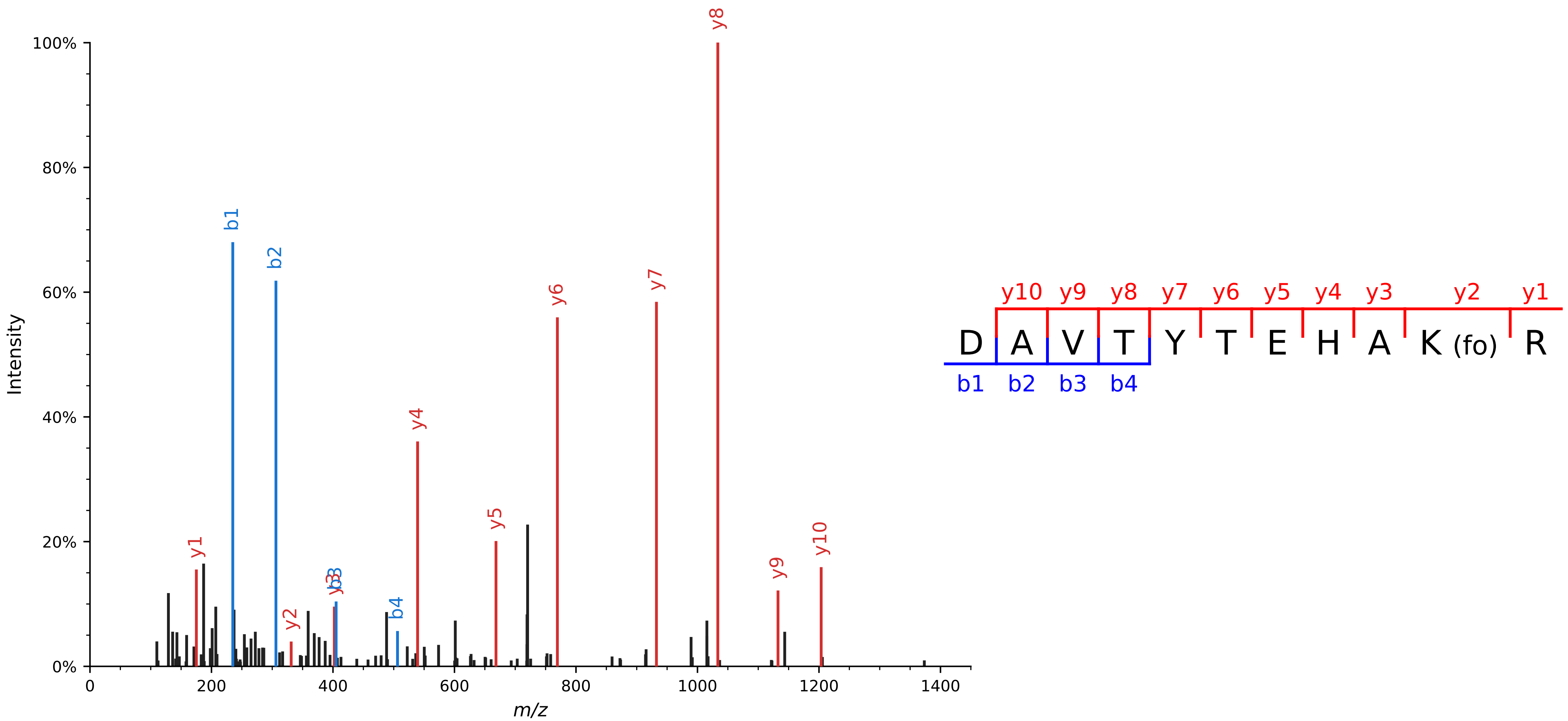

Raw file: QEP231009_CG_frozen_breast_LuA_31, Scan: 9489, m/z: 733.35715, Charge: 2, RT (min): 24.98, Score: 223.08

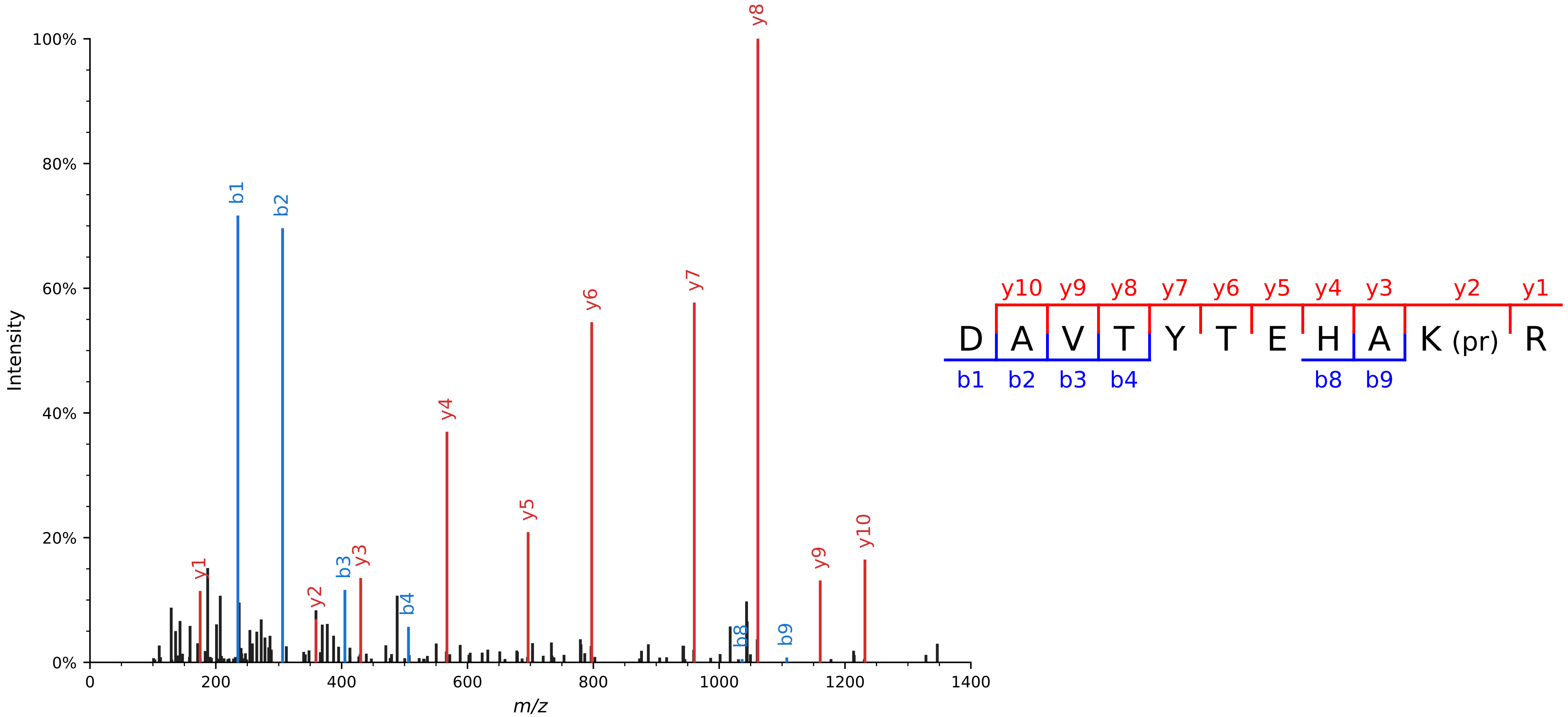

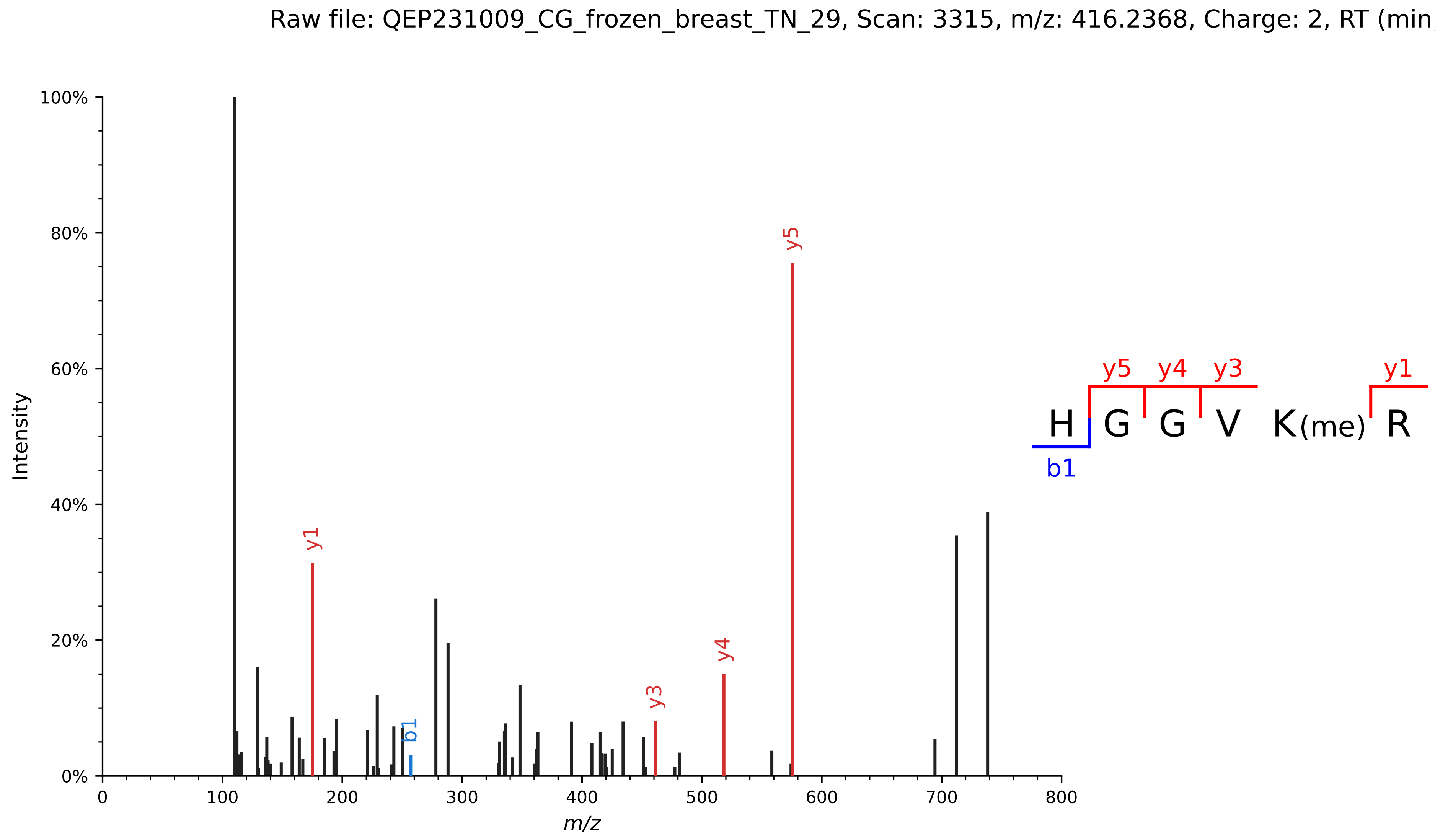

Raw file: QEP231009_CG_frozen_breast_TN_29, Scan: 3315, m/z: 416.2368, Charge: 2, RT (min): 11.24, Score: 50.31
Intensity
m/z
y1
b1
y3
y4
y5
H G G V K(me) R
b1
y5 y4 y3 y1

Mouse Tissues

Raw file: HF221212_CG_D3AcPIC_Kidney_YSD3, Scan: 16344, m/z: 617.33691, Charge: 2, RT (min): 31.61, Score: 155.75

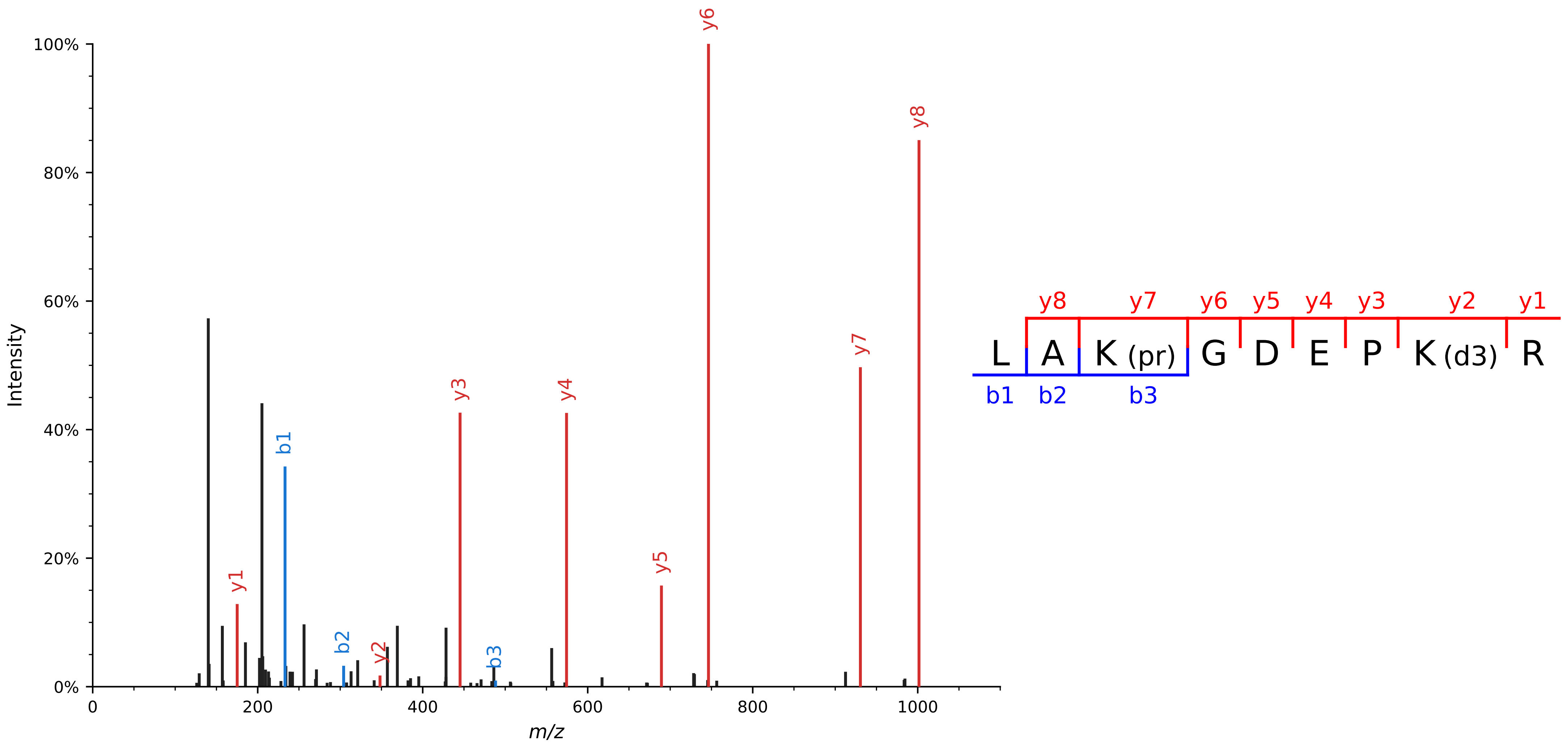

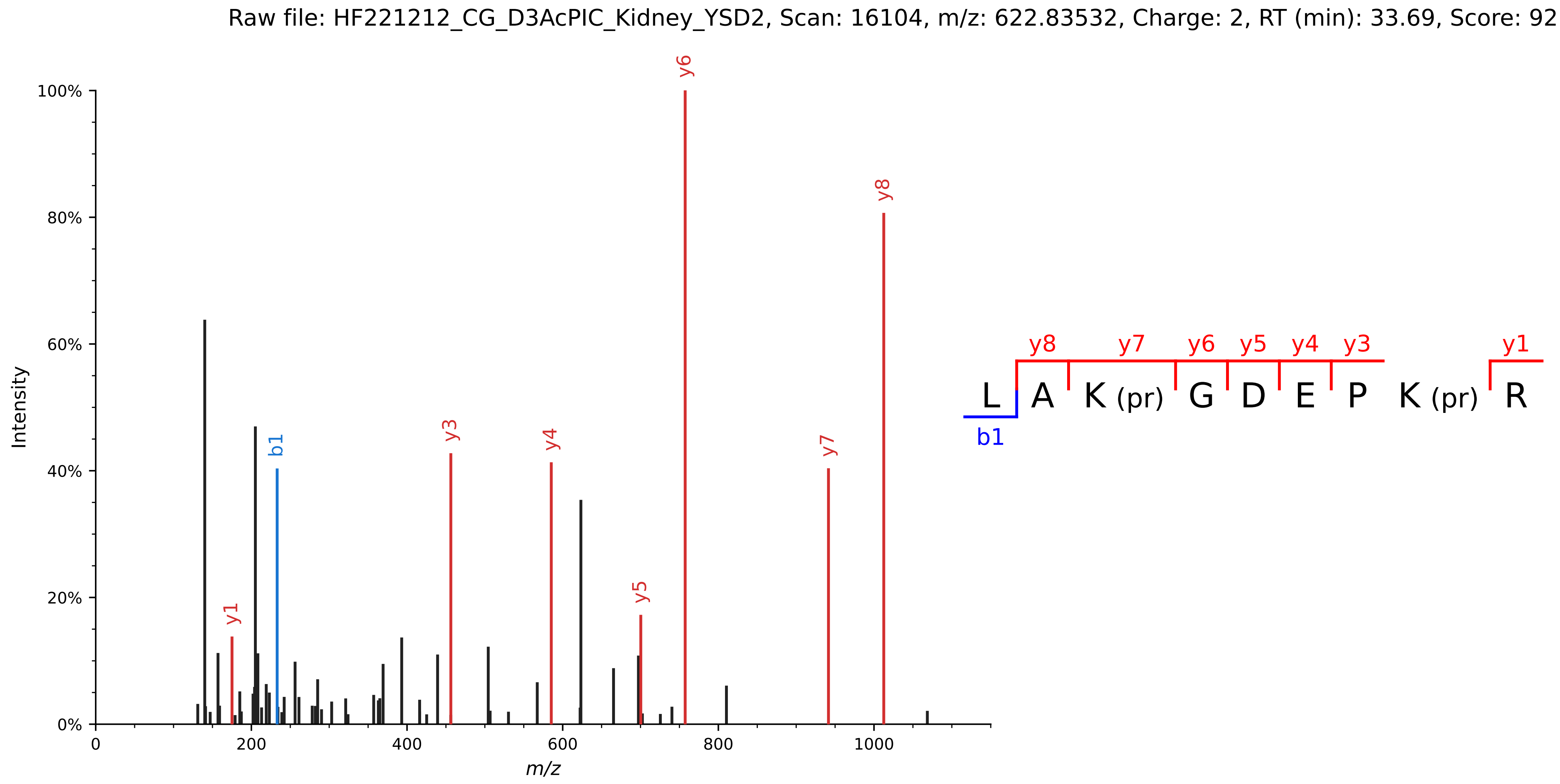
Raw file: HF221212_CG_D3AcPIC_Kidney_YSD2, Scan: 16104, m/z: 622.83532, Charge: 2, RT (min): 33.69, Score: 92.439
Intensity
m/z
y1
b1
y3
y4
y5
y6
y7
y8
L A K (pr) G D E P K (pr) R

Raw file: HF221212_CG_D3AcPIC_Brain_YSD2, Scan: 6474, m/z: 624.34473, Charge: 2, RT (min): 14.81, Score: 43.246

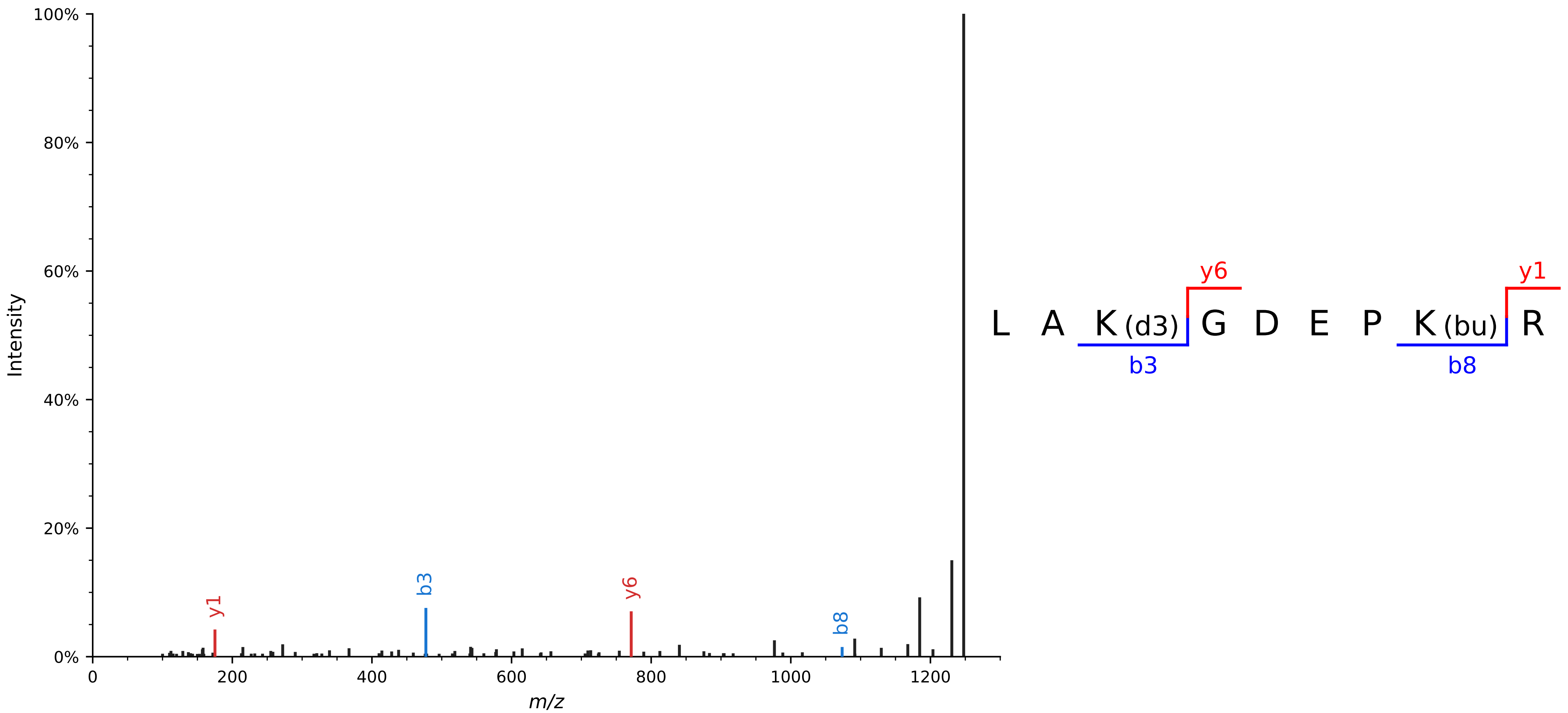

Raw file: HF221212_CG_D3AcPIC_Kidney_YSD1, Scan: 15177, m/z: 617.33691, Charge: 2, RT (min): 31.02, Score: 129.55

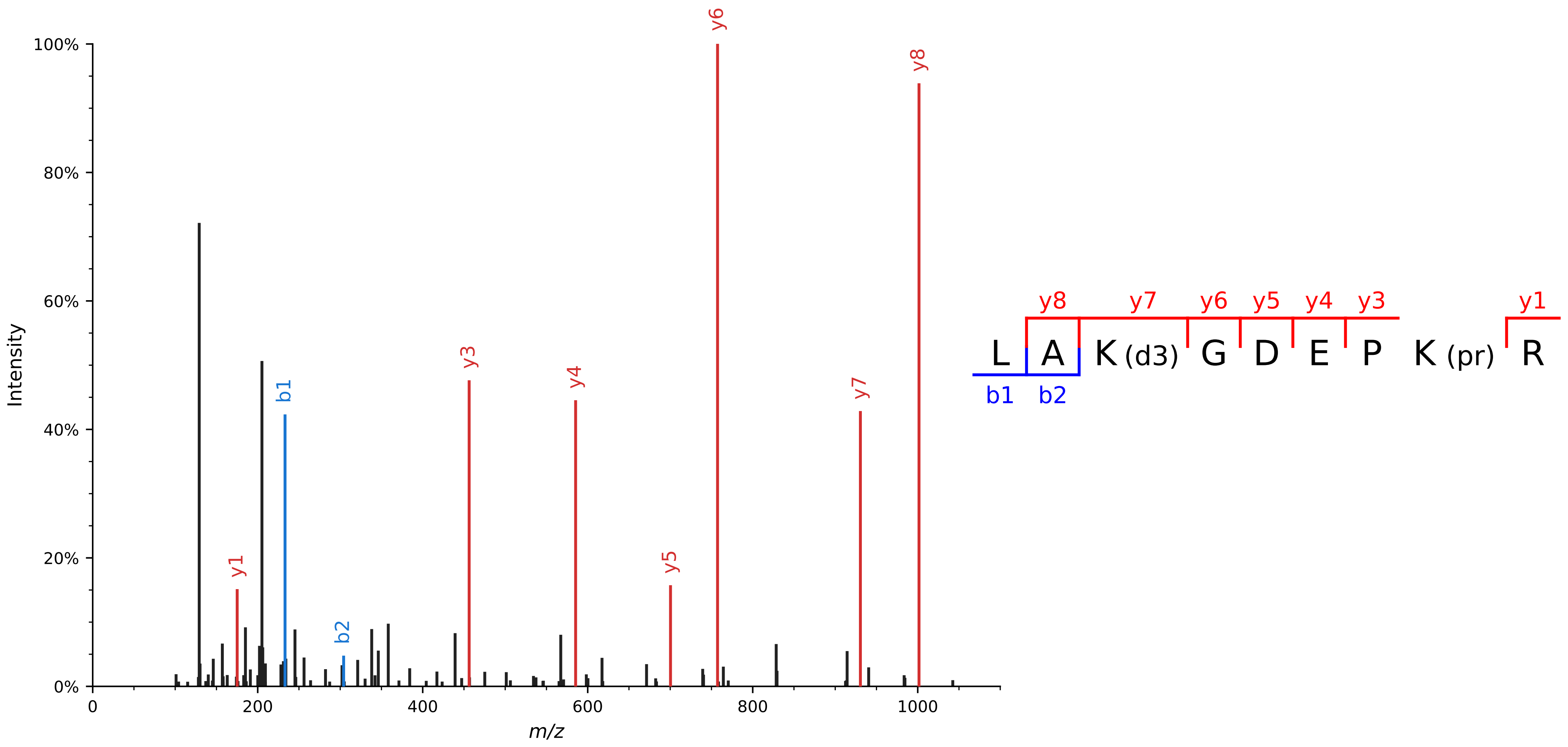

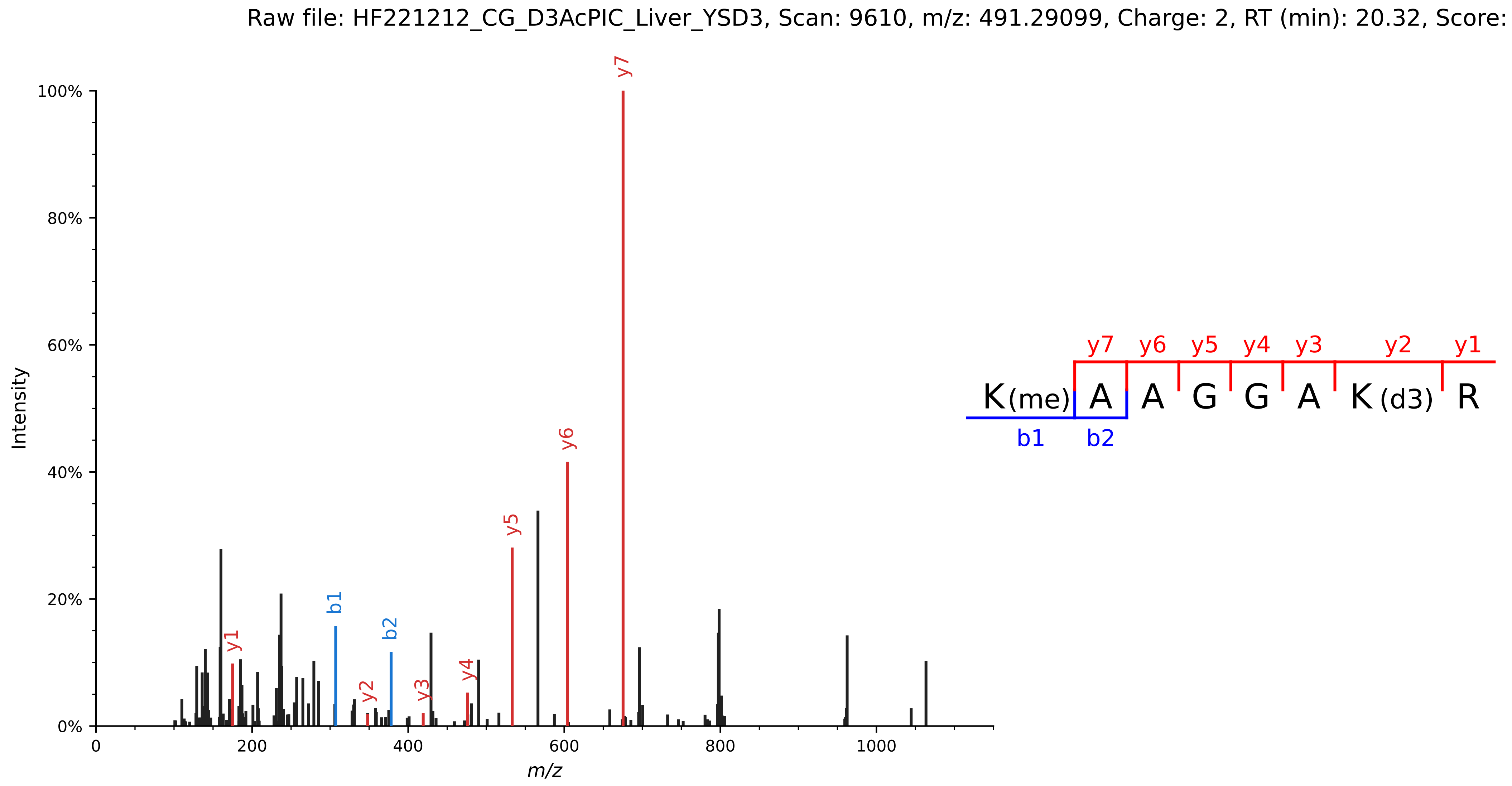

Raw file: HF221212_CG_D3AcPIC_Liver_YSD3, Scan: 9610, m/z: 491.29099, Charge: 2, RT (min): 20.32, Score: 71.715
Intensity
m/z
y1
y2
y3
y4
y5
y6
y7
b1
b2
K(me) A A G G A K(d3) R

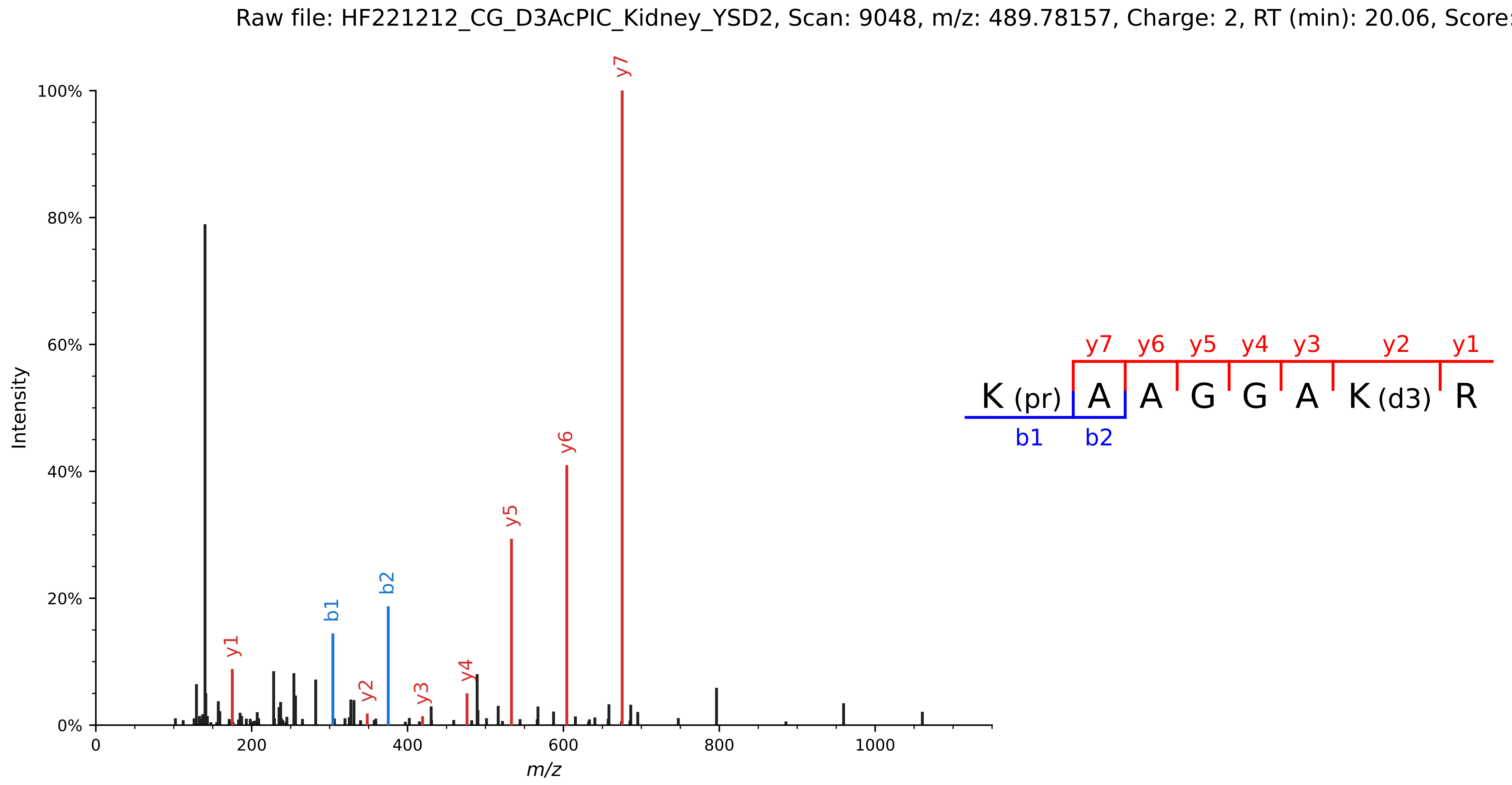
Raw file: HF221212_CG_D3AcPIC_Kidney_YSD2, Scan: 9048, m/z: 489.78157, Charge: 2, RT (min): 20.06, Score: 118.69
Intensity
m/z
K (pr) A A G G A K (d3) R
y7 y6 y5 y4 y3 y2 y1
b1 b2

Raw file: HF221212_CG_D3AcPIC_Kidney_YSD2, Scan: 10327, m/z: 495.27998, Charge: 2, RT (min): 22.52, Score: 47.849

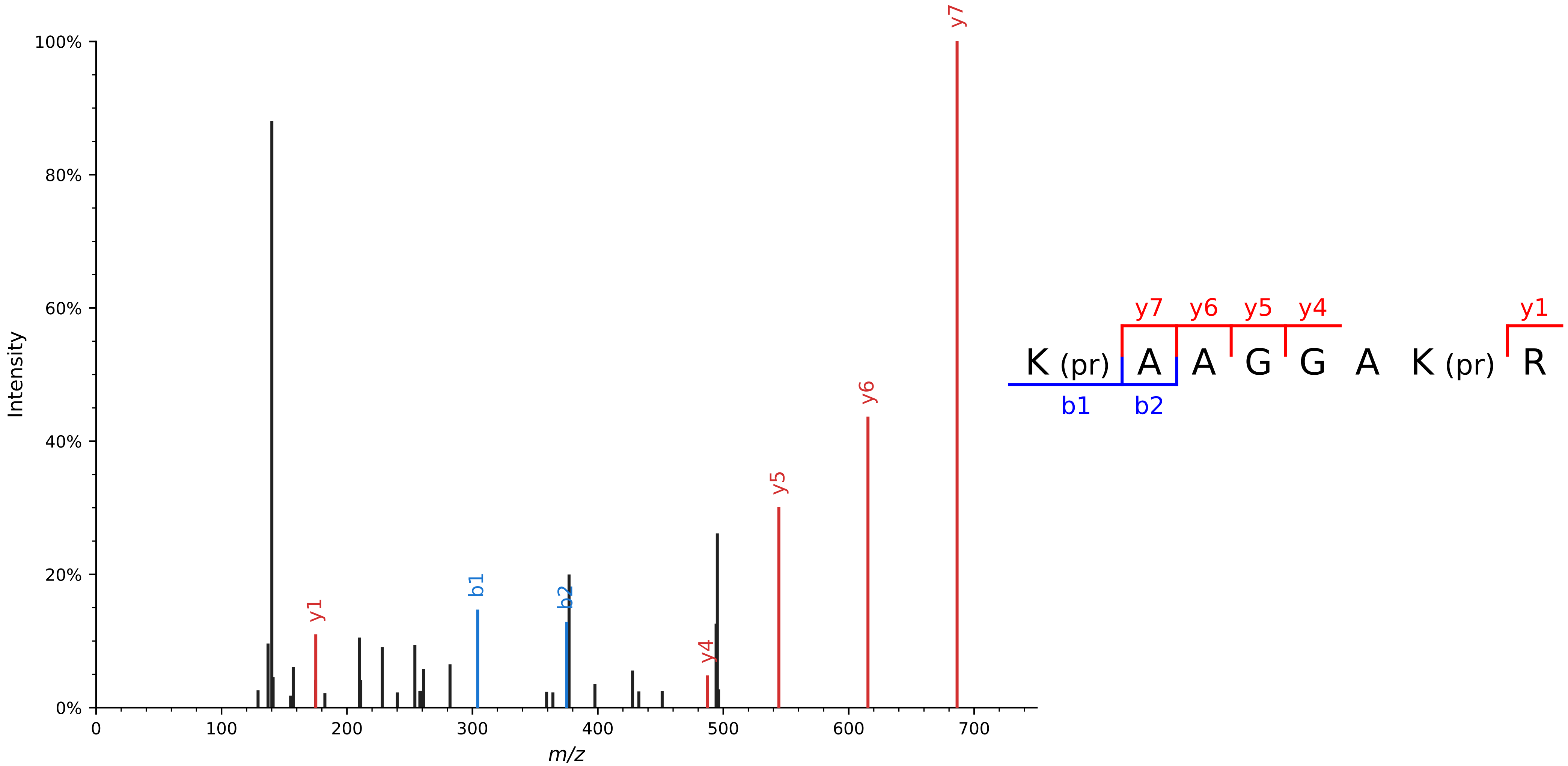

Raw file: HF221212_CG_D3AcPIC_Liver_YSD2, Scan: 9346, m/z: 489.78157, Charge: 2, RT (min): 19.91, Score: 90.706

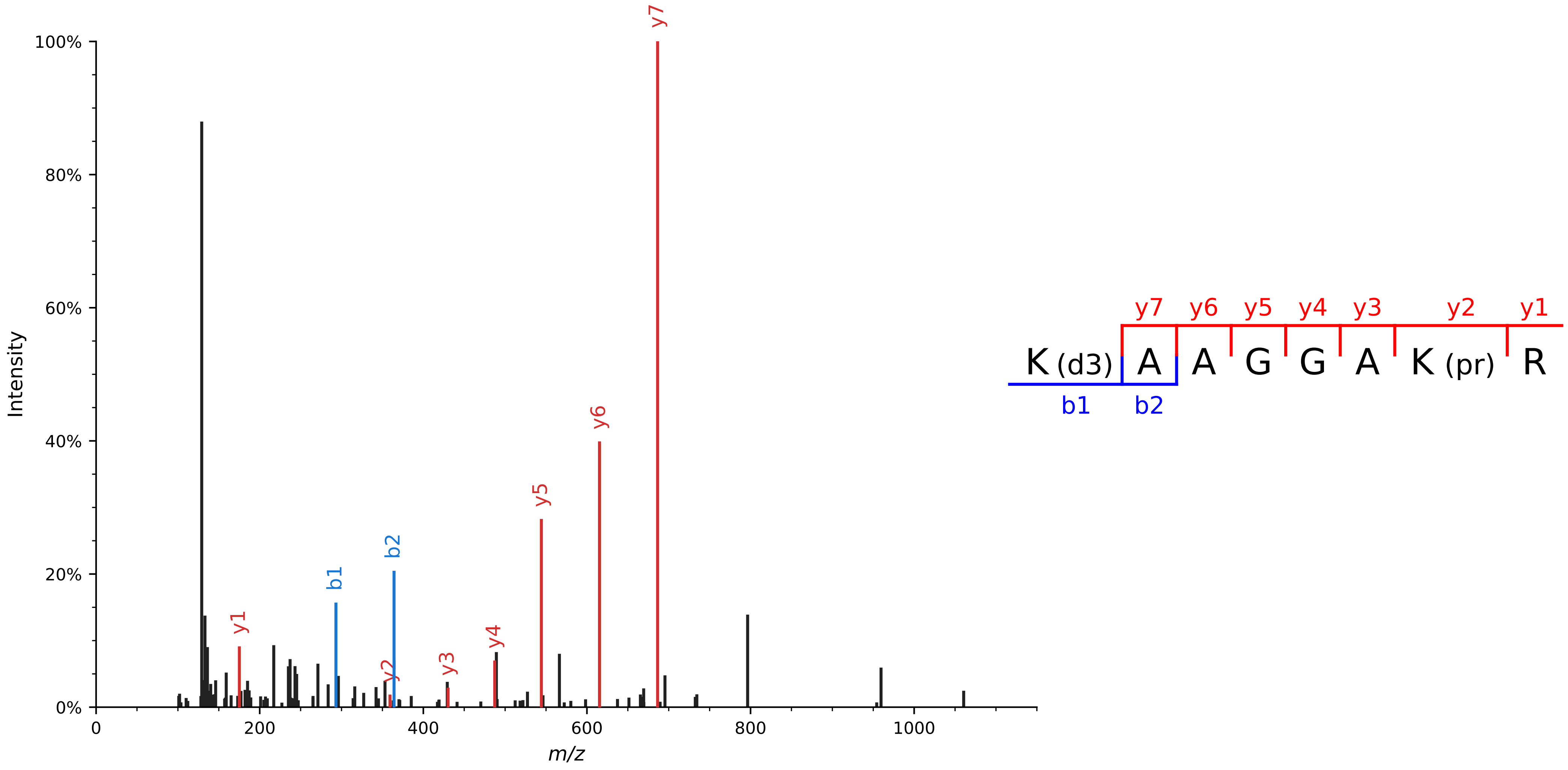

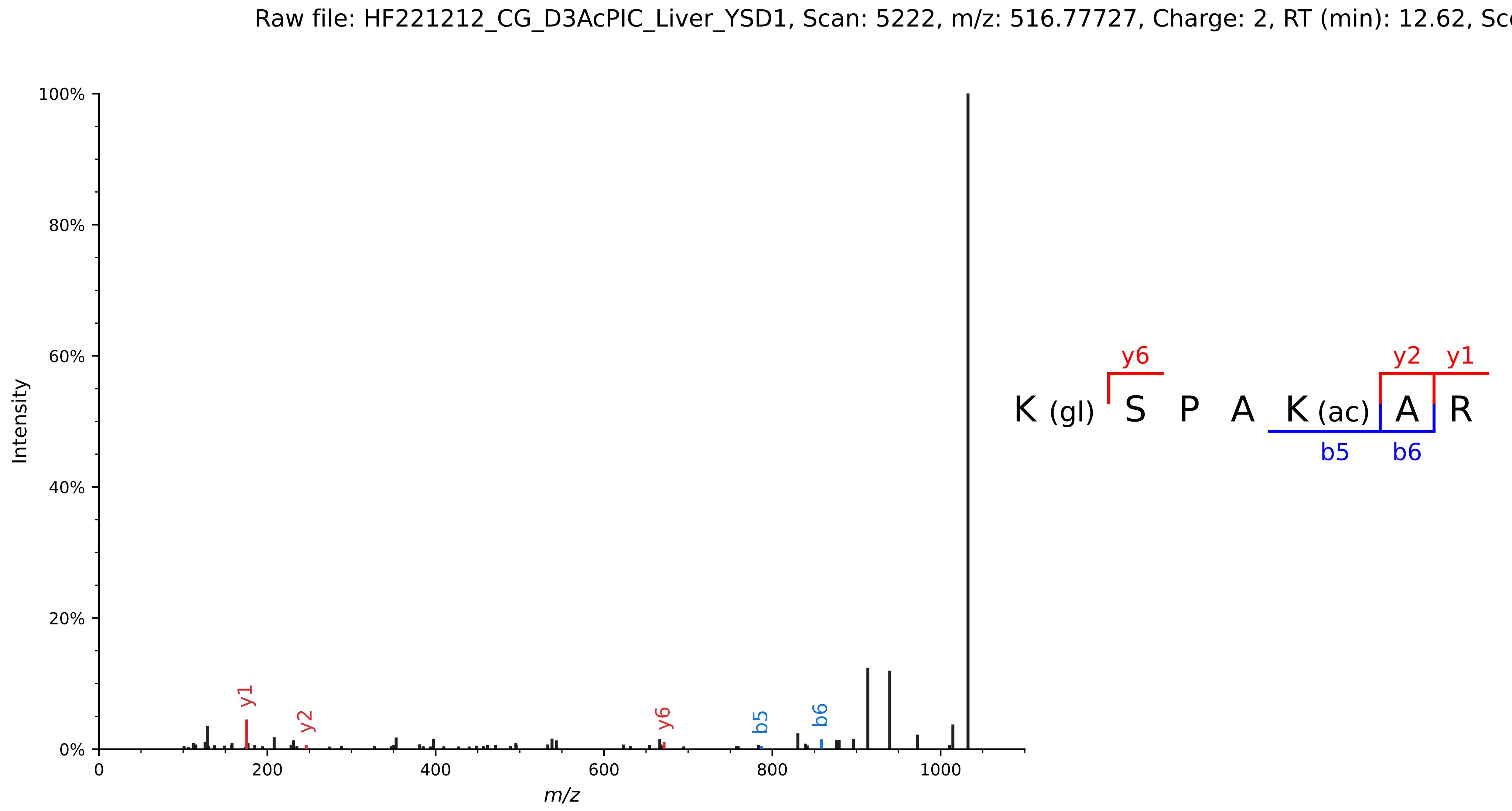

Raw file: HF221212_CG_D3AcPIC_Liver_YSD1, Scan: 5222, m/z: 516.77727, Charge: 2, RT (min): 12.62, Score: 47.849
100%
80%
60%
40%
20%
0%
Intensity
0
200
400
600
800
1000
m/z
y1
y2
y6
b5
b6
K (gl) S P A K (ac) A R

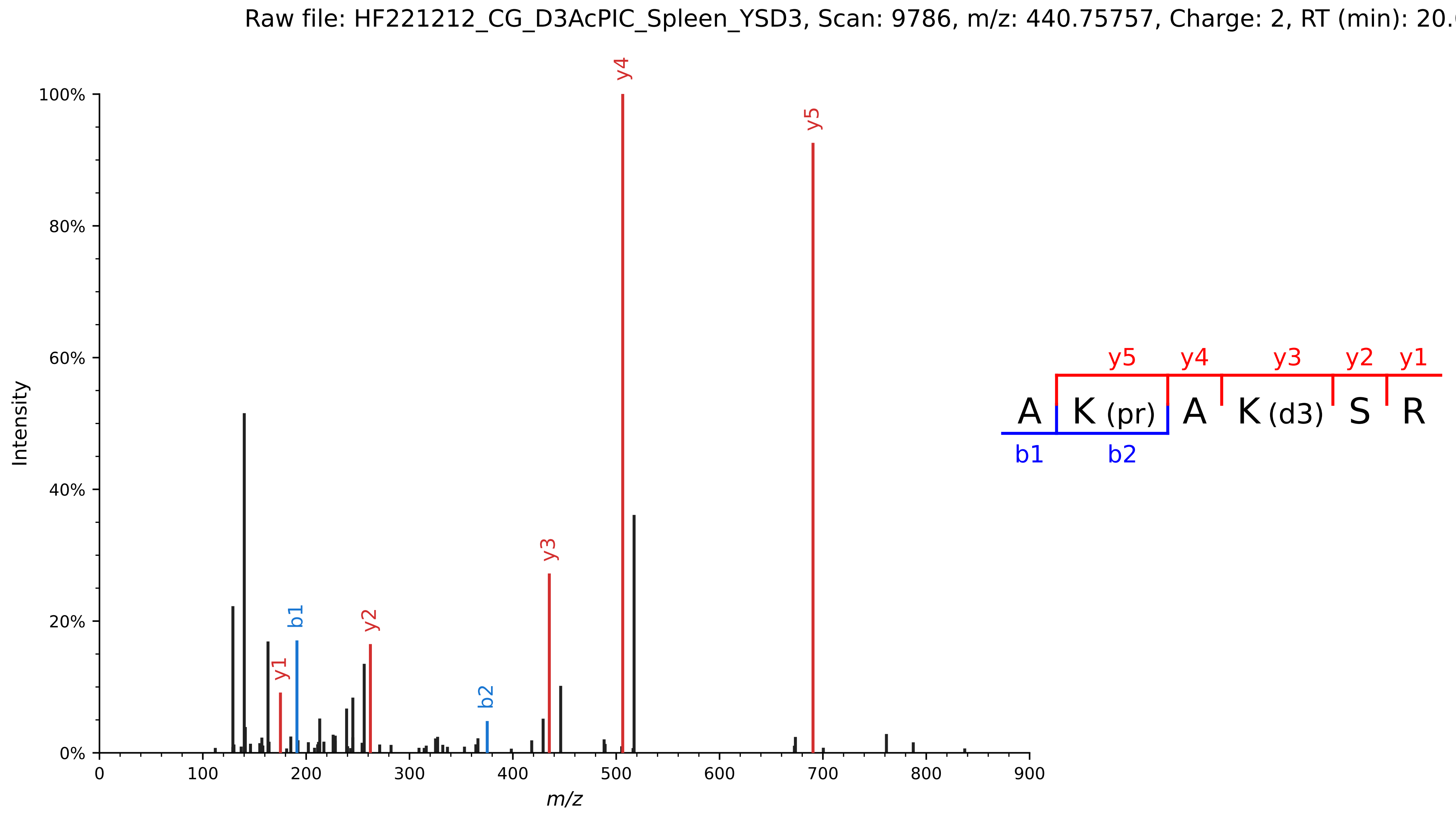

Raw file: HF221212_CG_D3AcPIC_Spleen_YSD3, Scan: 9786, m/z: 440.75757, Charge: 2, RT (min): 20.63, Score: 92.191
Intensity
100%
80%
60%
40%
20%
0%
0
100
200
300
400
500
600
700
800
900
m/z
y4
y5
y3
b1
y2
y1
b2
y5 y4 y3 y2 y1
A K (pr) A K (d3) S R
b1 b2

Raw file: HF221212_CG_D3AcPIC_Spleen_YSD1, Scan: 11617, m/z: 446.25598, Charge: 2, RT (min): 23.63, Score: 83.377

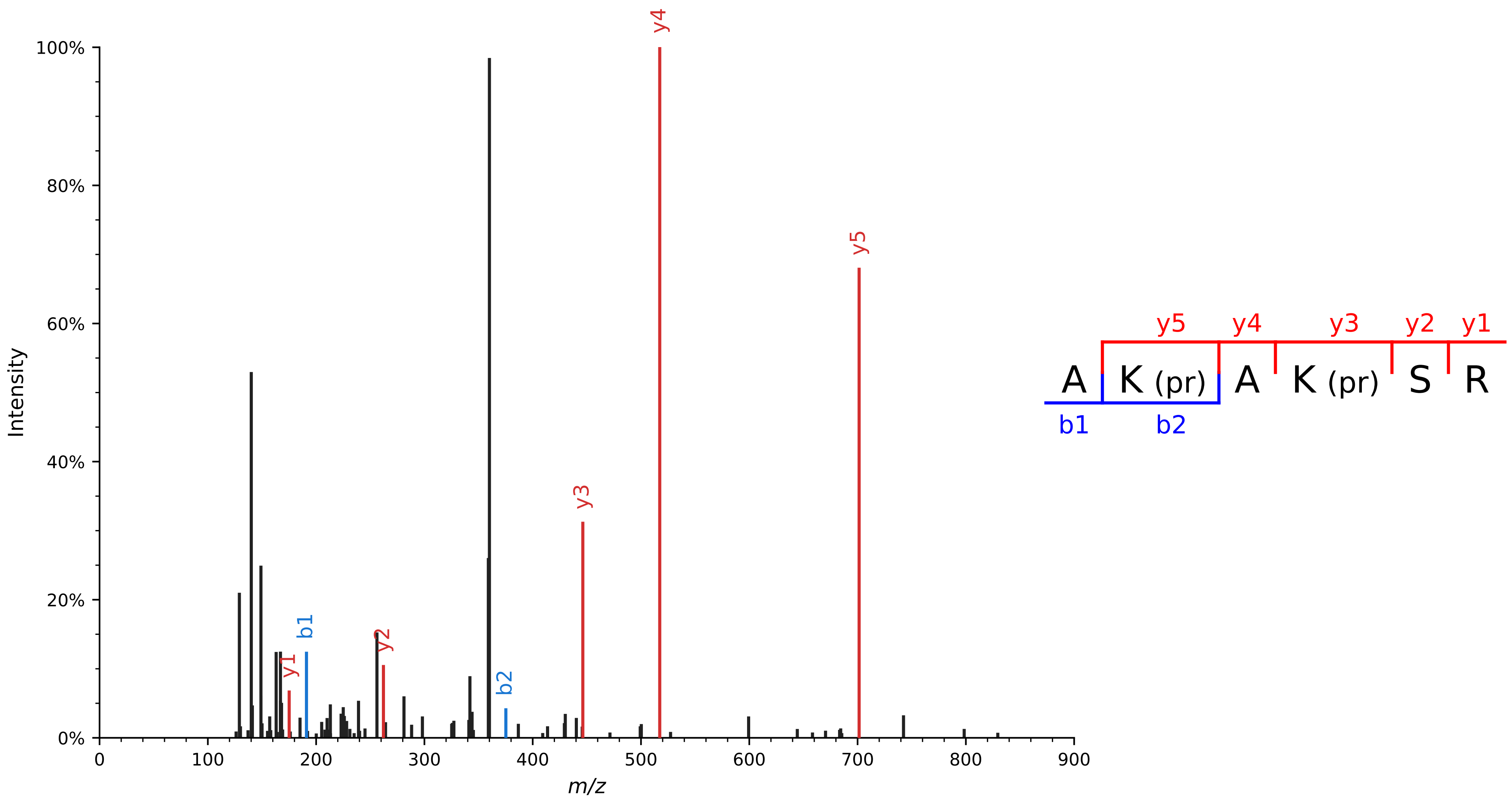

Raw file: HF221212_CG_D3AcPIC_Kidney_YSD2, Scan: 9479, m/z: 440.75757, Charge: 2, RT (min): 20.84, Score: 85.89

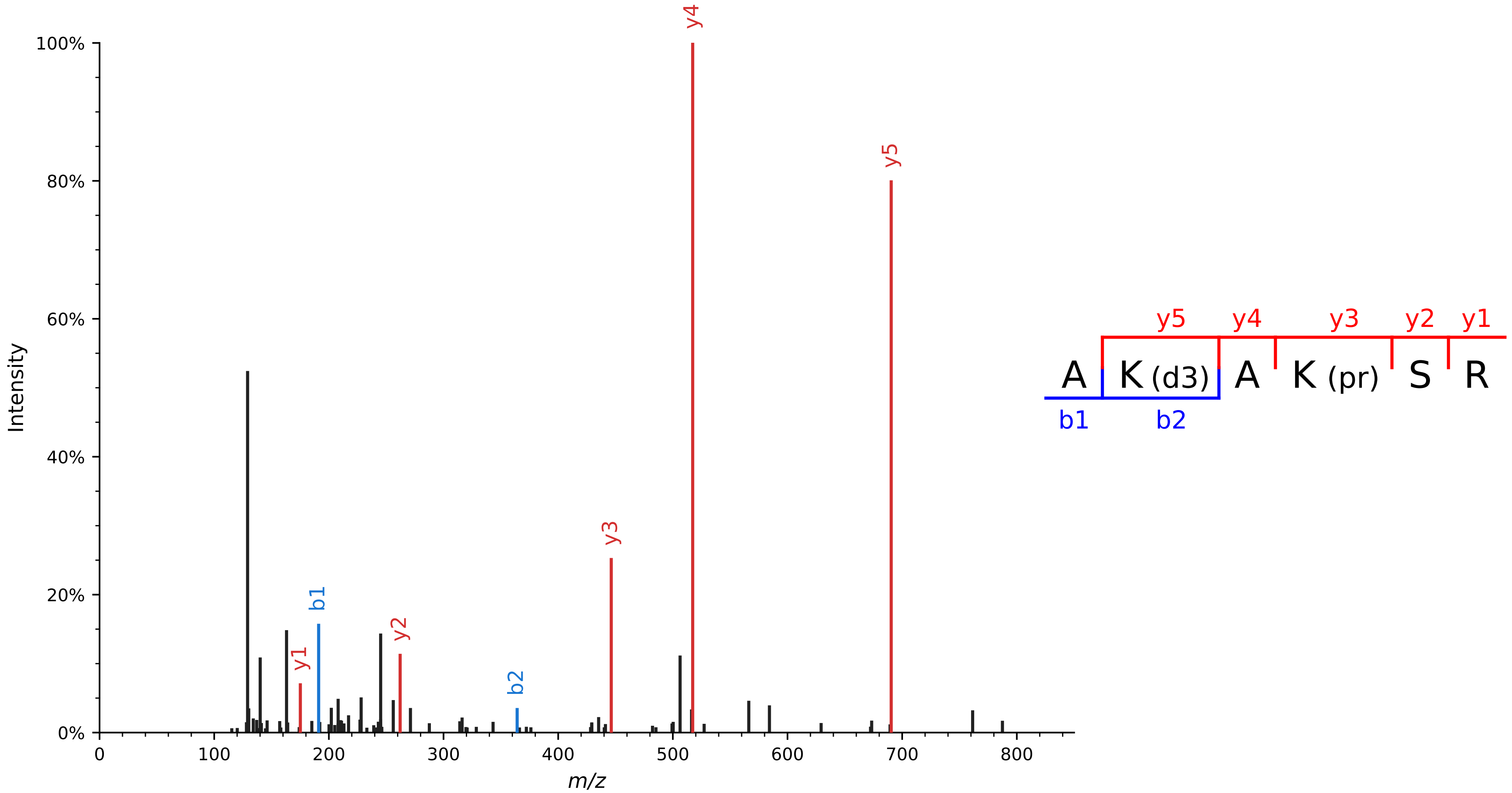

Raw file: HF221212_CG_D3AcPIC_Spleen_YSD1, Scan: 9959, m/z: 514.74343, Charge: 2, RT (min): 20.62, Score: 112.37

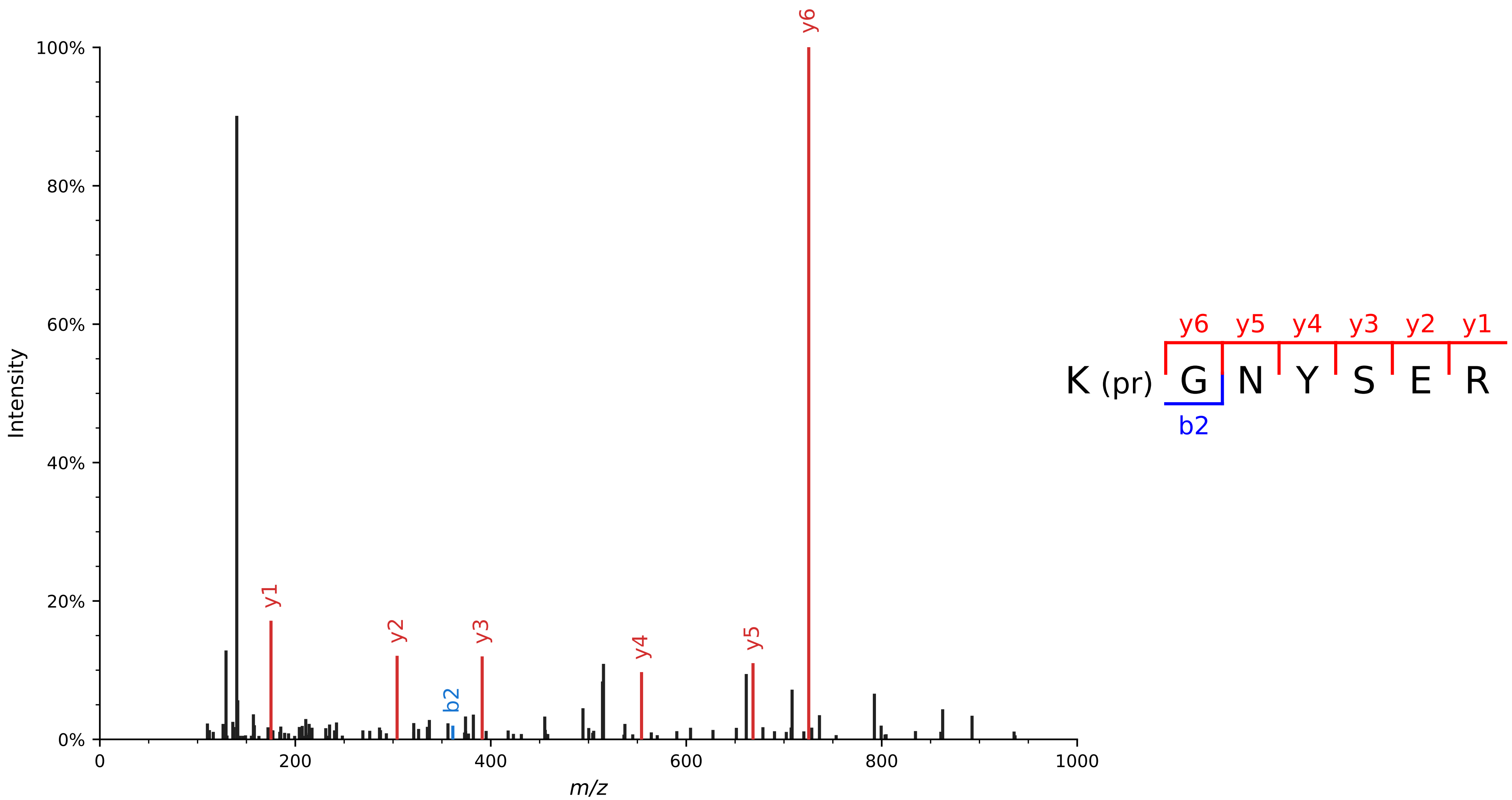

Raw file: HF221212_CG_D3AcPIC_Liver_YSD1, Scan: 6669, m/z: 518.29231, Charge: 2, RT (min): 15.23, Score: 73.616

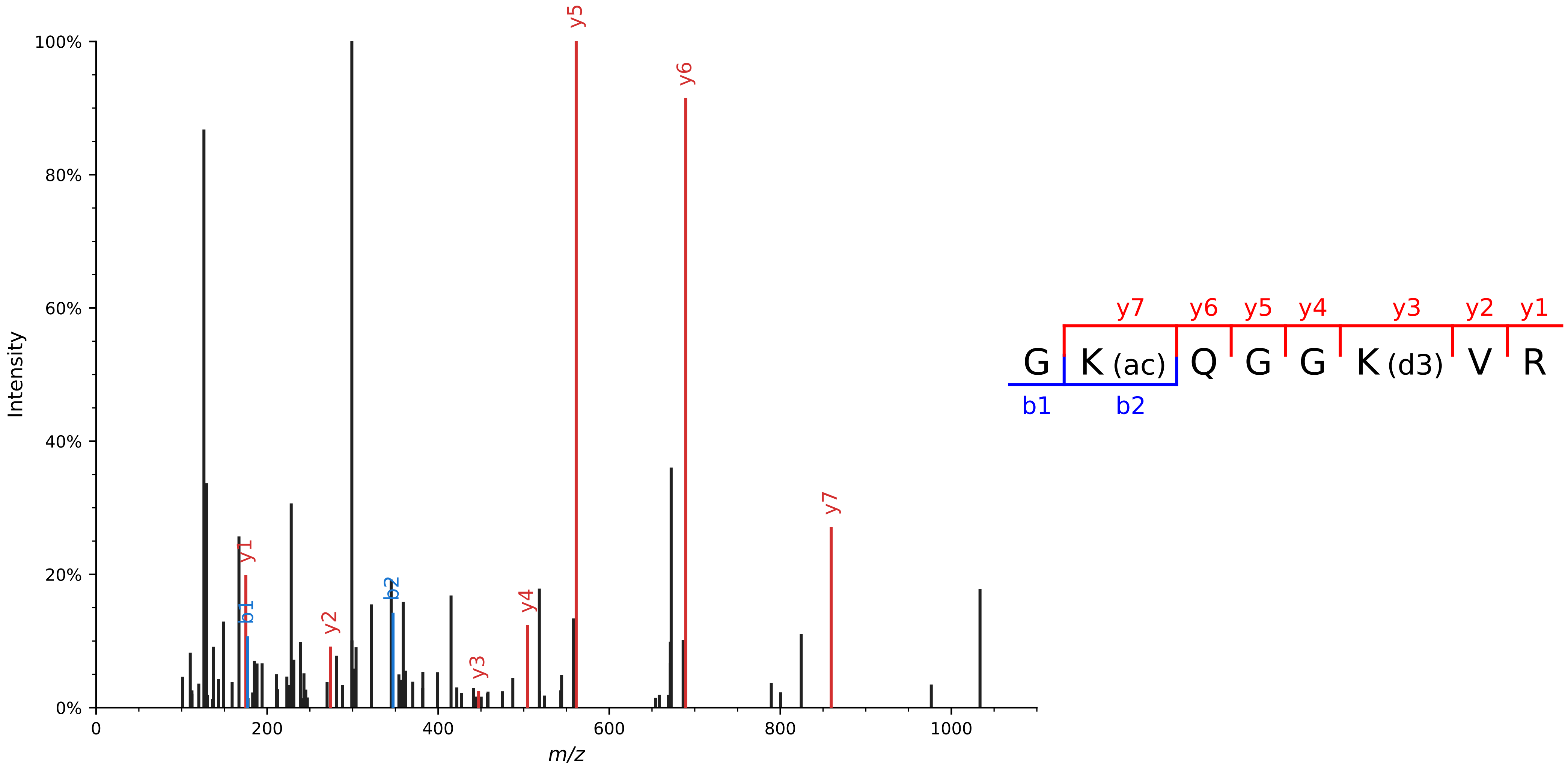

Raw file: HF221212_CG_D3AcPIC_Kidney_YSD2, Scan: 7926, m/z: 525.30013, Charge: 2, RT (min): 17.88, Score: 74.415

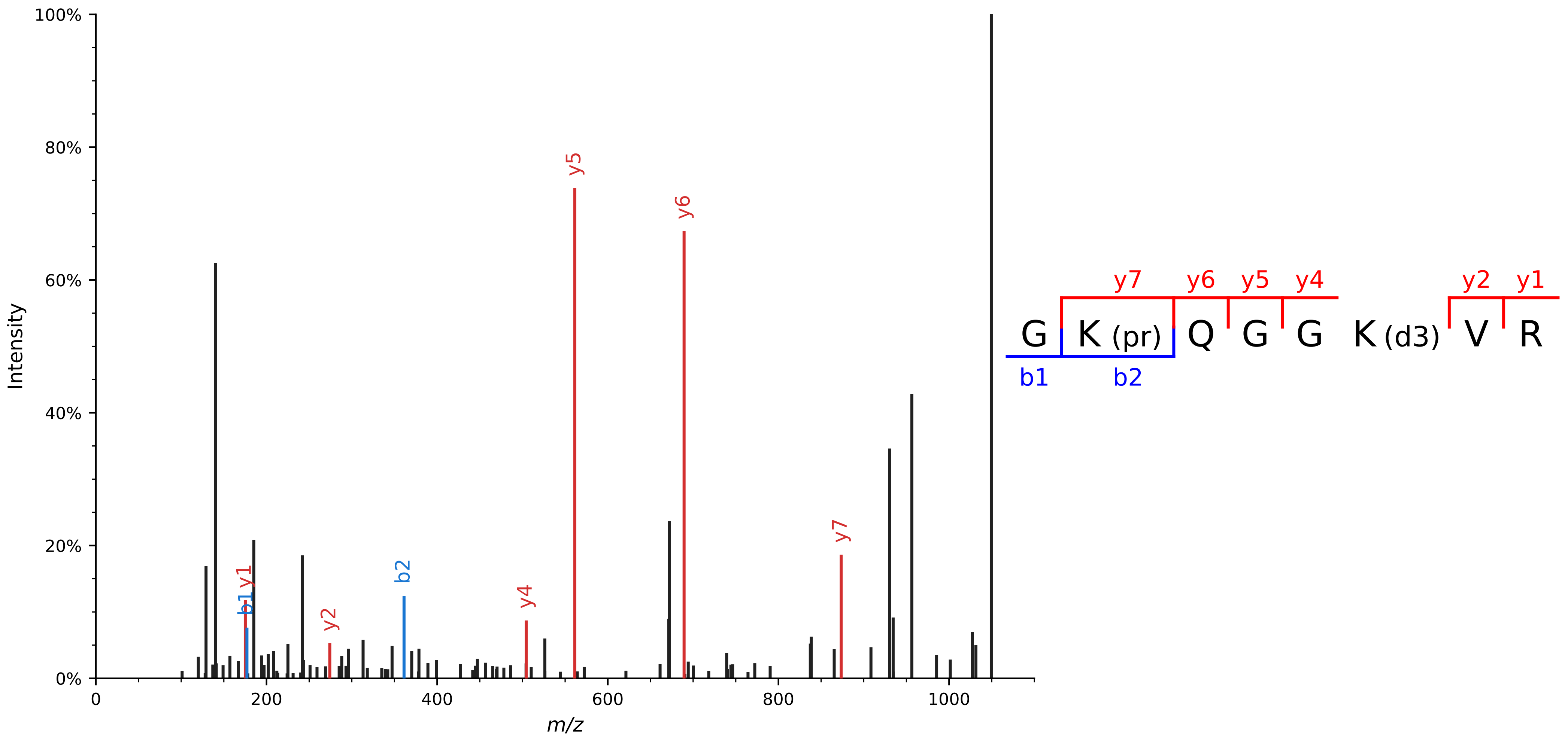

Raw file: HF221212_CG_D3AcPIC_Heart_YSD3, Scan: 6671, m/z: 511.28448, Charge: 2, RT (min): 15.07, Score: 83.801

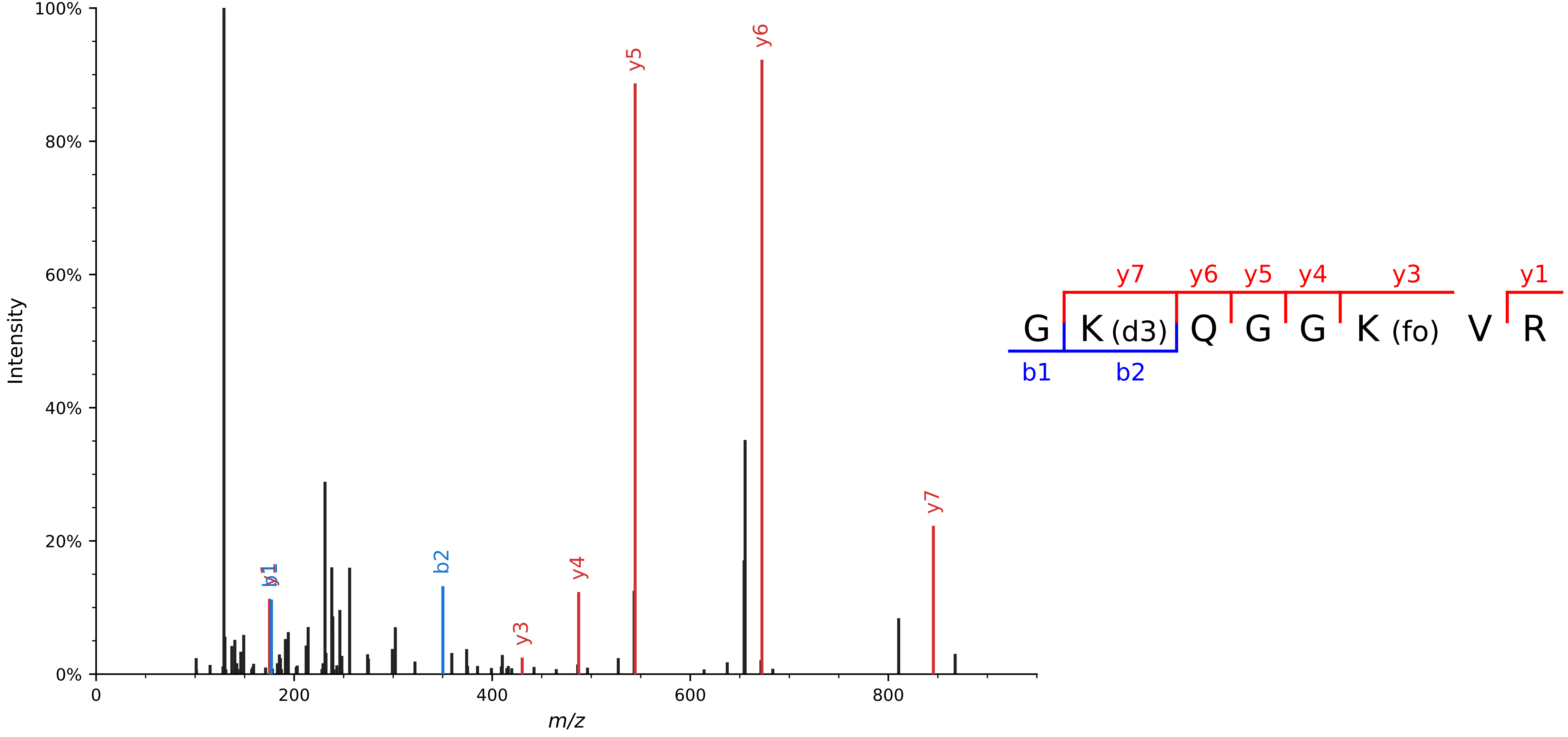

Raw file: HF221212_CG_D3AcPIC_Kidney_YSD1, Scan: 8255, m/z: 525.30013, Charge: 2, RT (min): 18.38, Score: 79.597

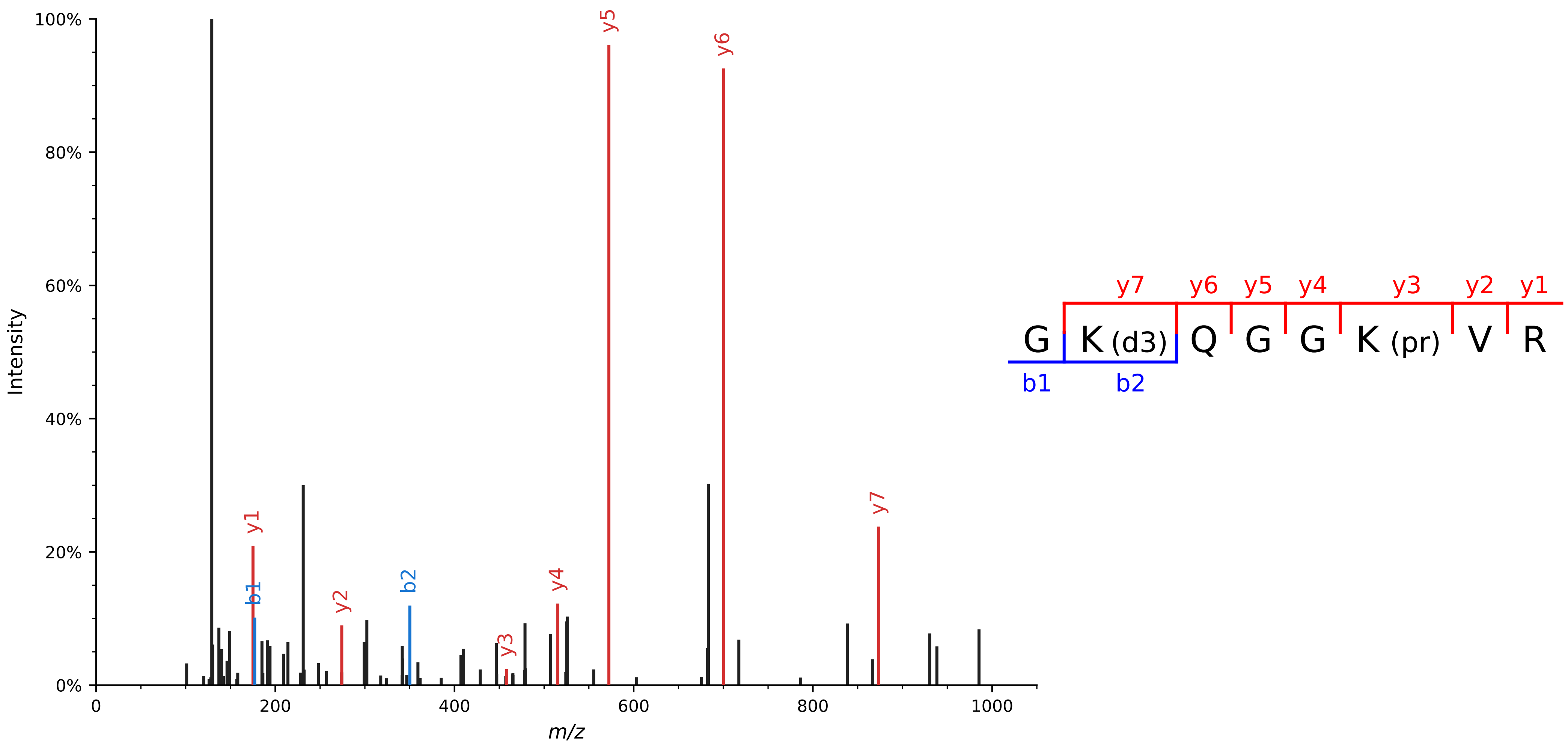

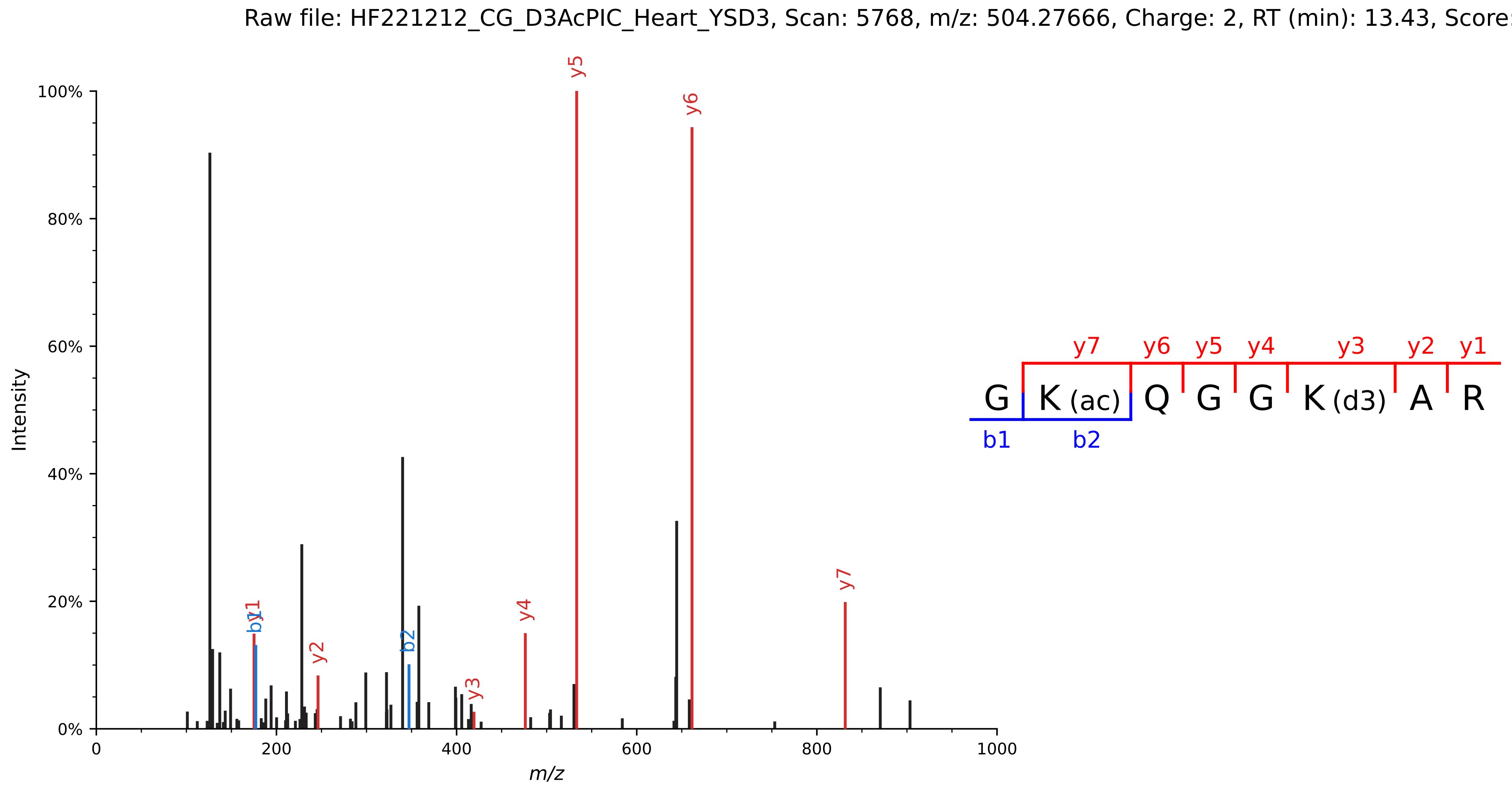

Raw file: HF221212_CG_D3AcPIC_Heart_YSD3, Scan: 5768, m/z: 504.27666, Charge: 2, RT (min): 13.43, Score: 79.116
Intensity
m/z
100%
80%
60%
40%
20%
0%
0
200
400
600
800
1000
y5
y6
y7
y4
y3
y2
y1
b1
b2
G K (ac) Q G G K (d3) A R

Raw file: HF221212_CG_D3AcPIC_Kidney_YSD1, Scan: 2943, m/z: 511.30267, Charge: 2, RT (min): 9.29, Score: 110.36

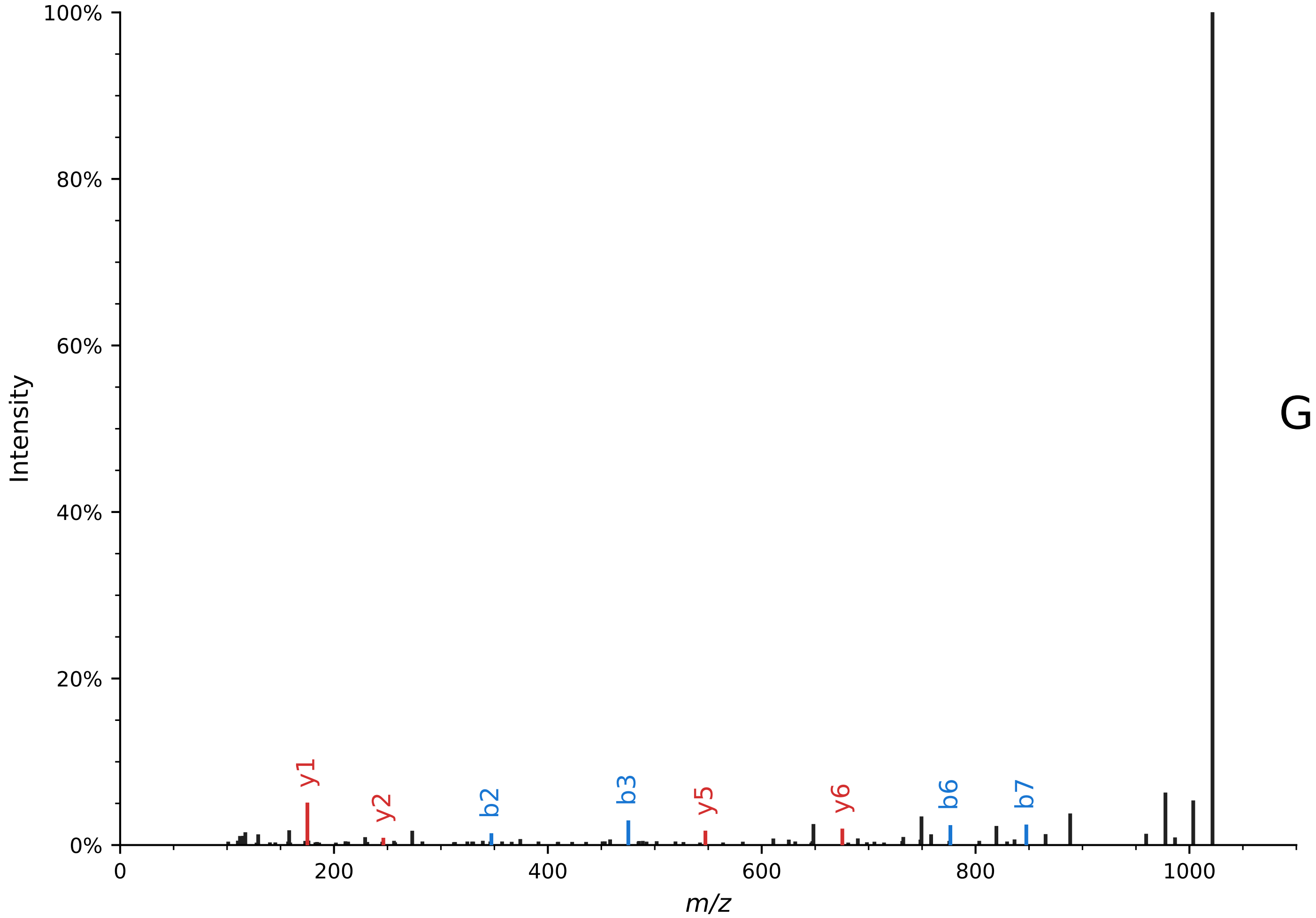

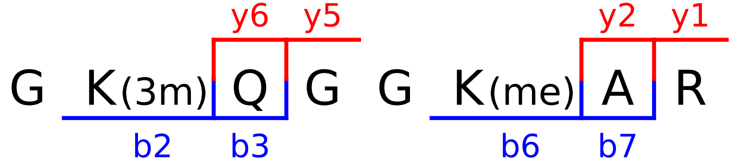

Raw file: HF221212_CG_D3AcPIC_Spleen_YSD3, Scan: 6827, m/z: 511.28448, Charge: 2, RT (min): 15.29, Score: 122.78

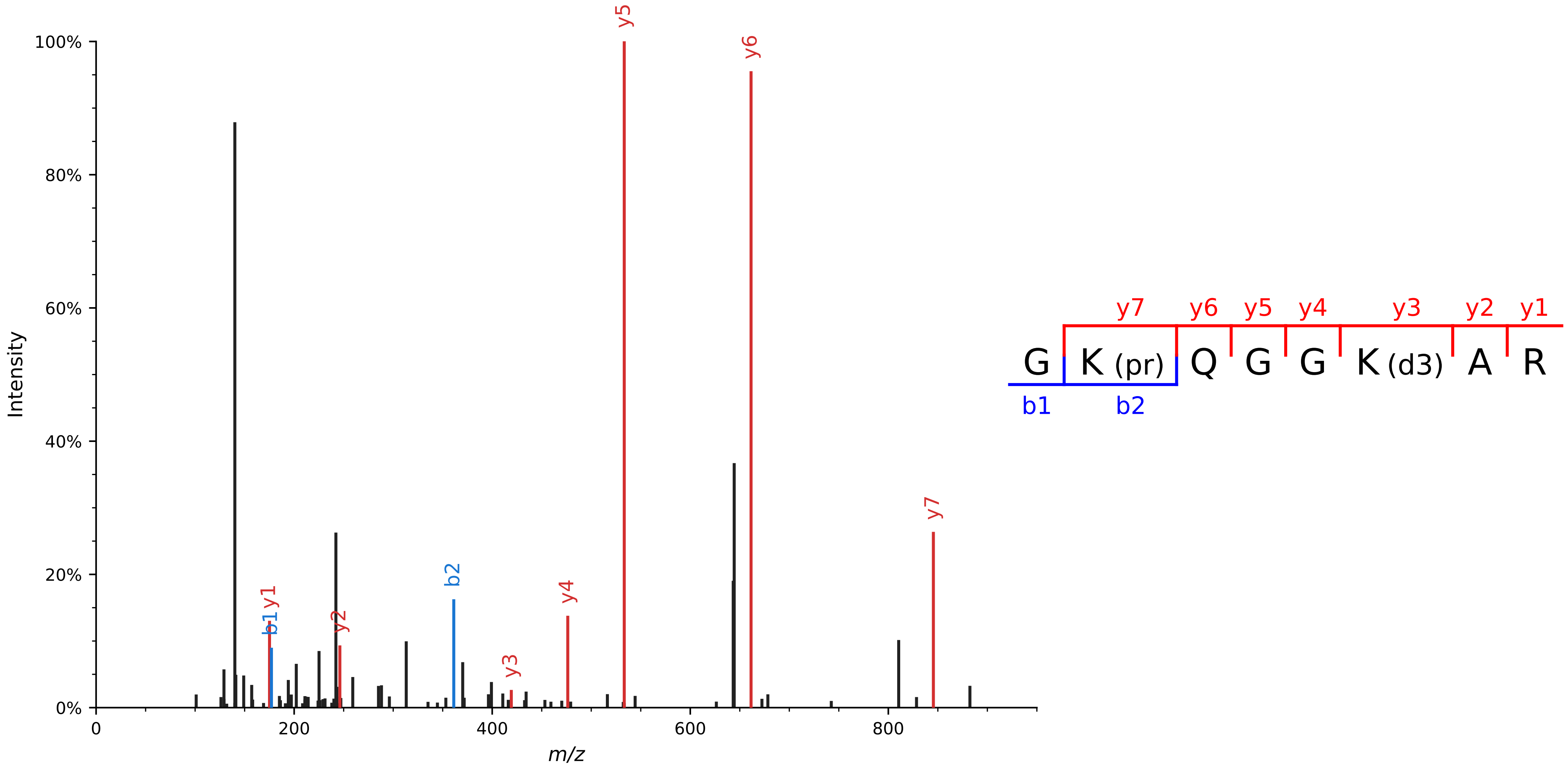

Raw file: HF221212_CG_D3AcPIC_Spleen_YSD2, Scan: 7909, m/z: 516.78289, Charge: 2, RT (min): 17.19, Score: 91.041

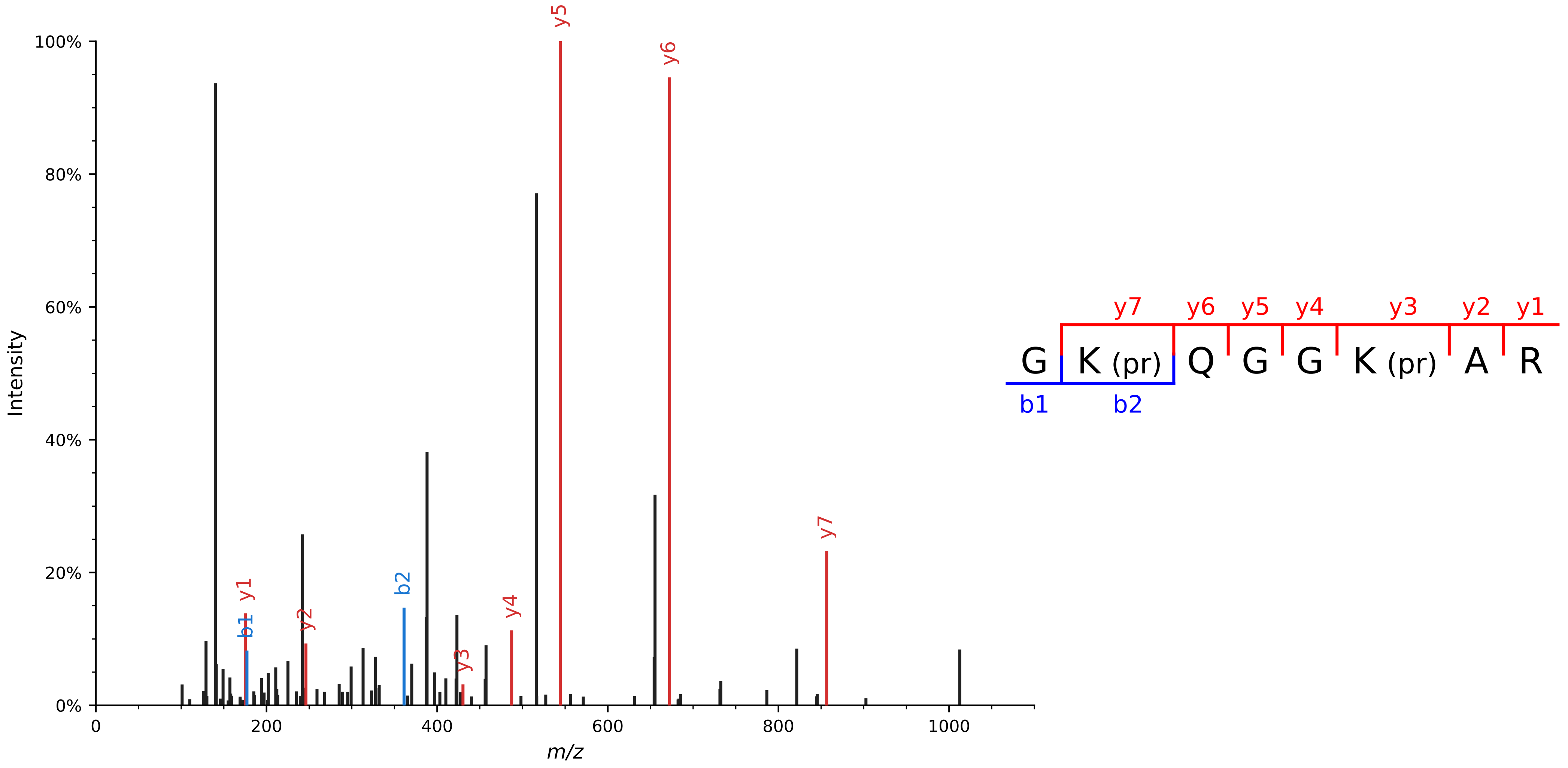

Raw file: HF221212_CG_D3AcPIC_Spleen_YSD2, Scan: 6505, m/z: 511.28448, Charge: 2, RT (min): 14.65, Score: 105.39

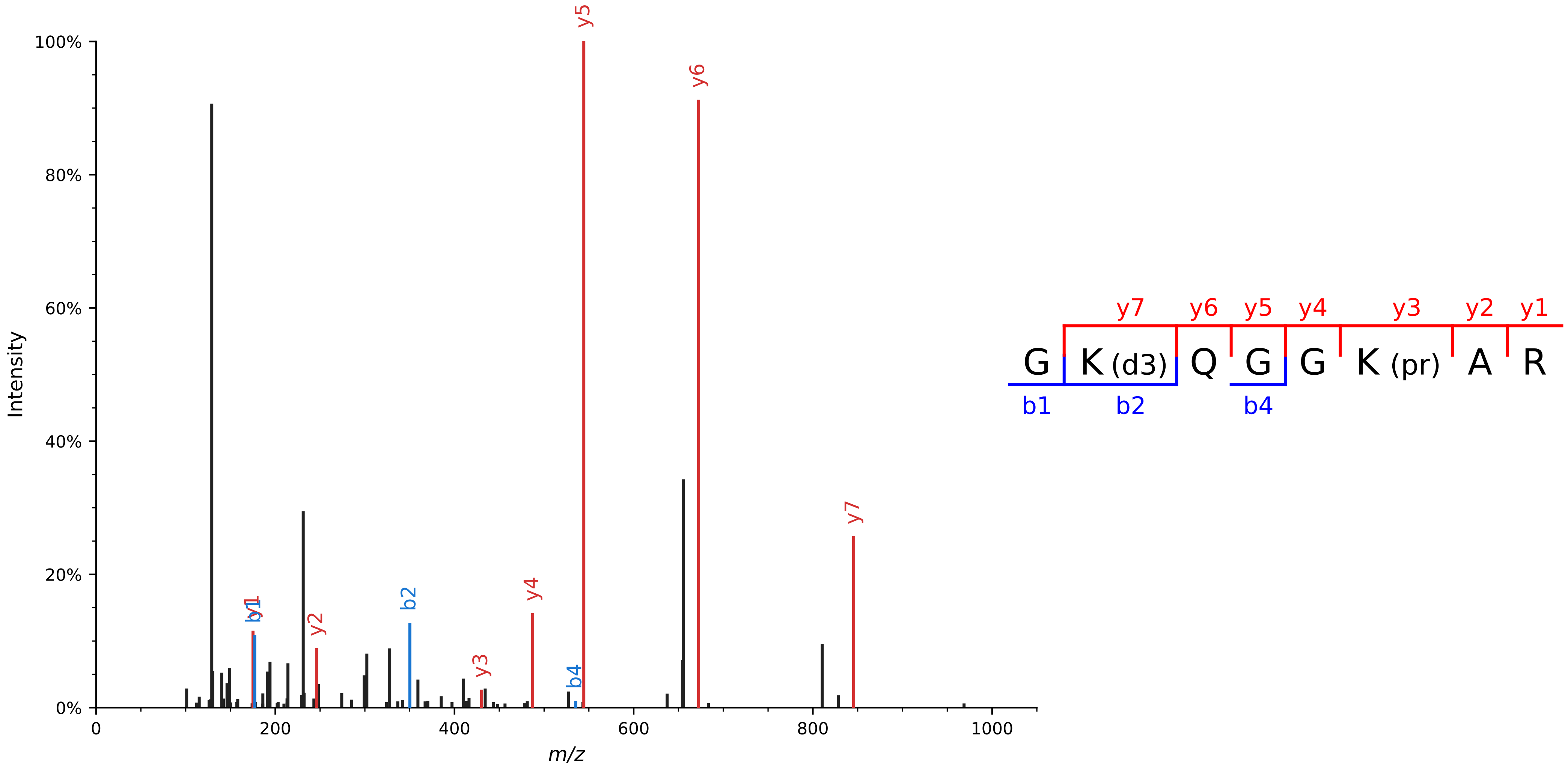

Raw file: HF221212_CG_D3AcPIC_Spleen_YSD1, Scan: 10329, m/z: 447.76539, Charge: 2, RT (min): 21.31, Score: 89.673

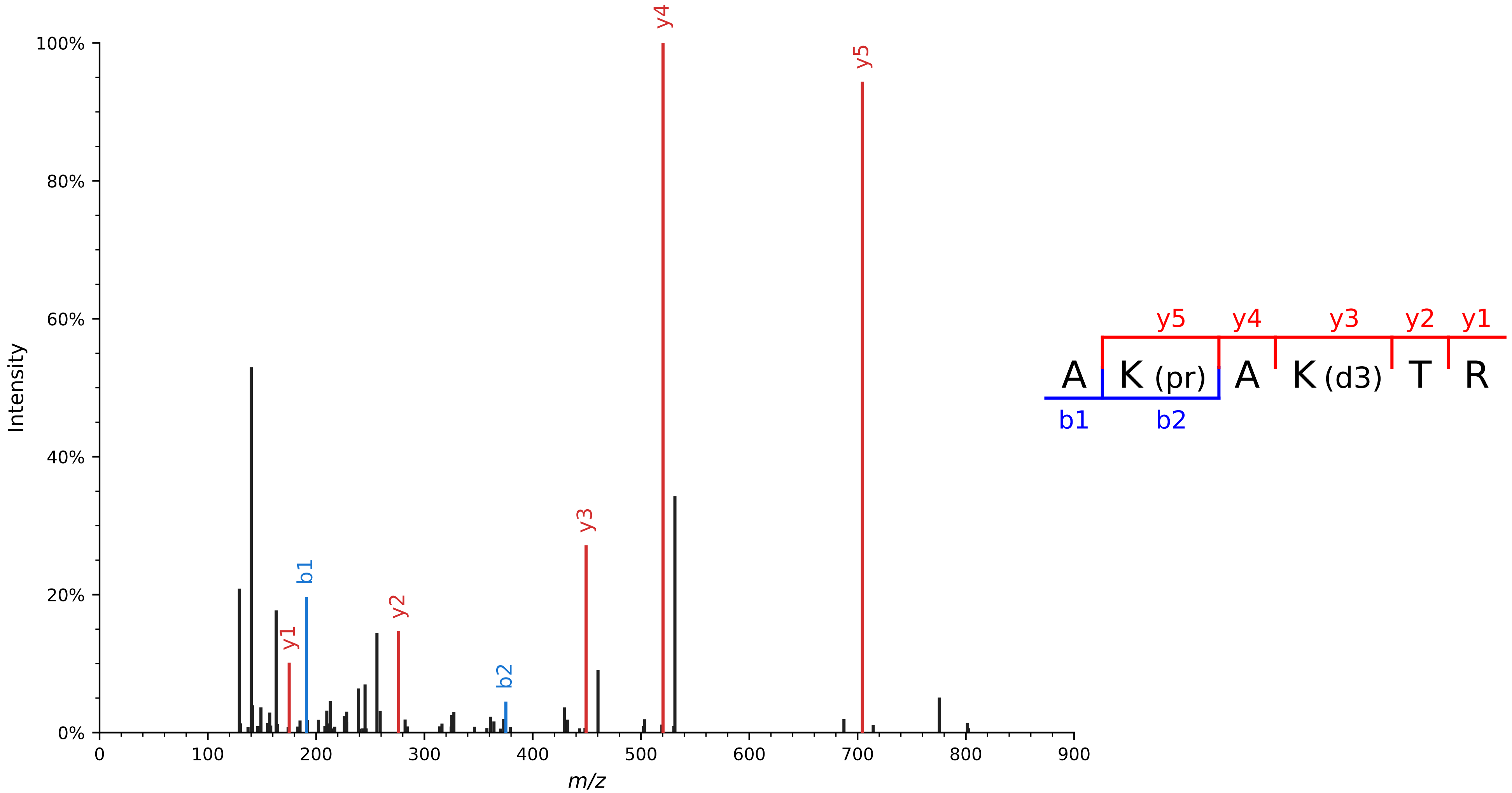

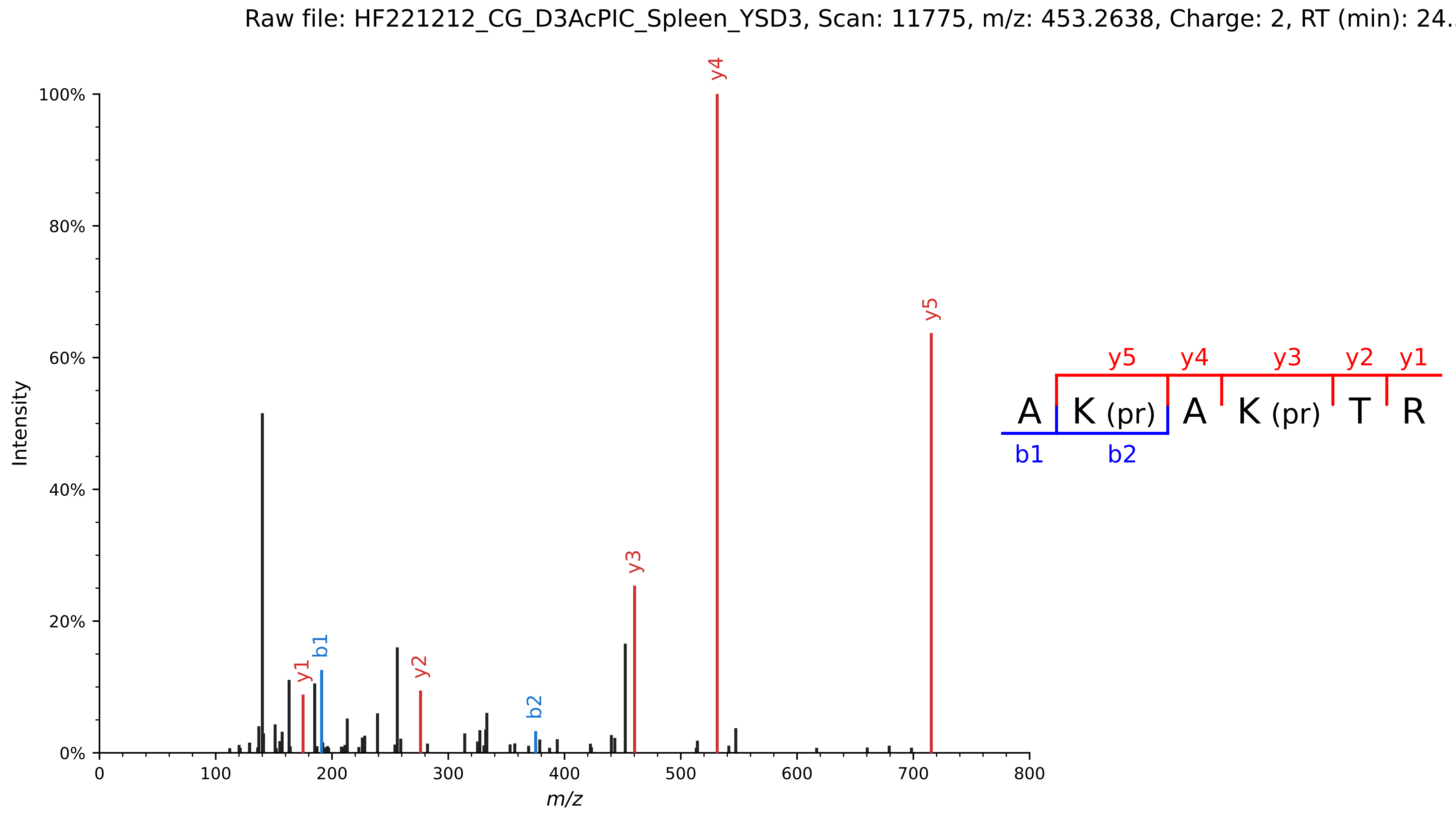

Raw file: HF221212_CG_D3AcPIC_Spleen_YSD3, Scan: 11775, m/z: 453.2638, Charge: 2, RT (min): 24.32, Score: 76.847
Intensity
100%
80%
60%
40%
20%
0%
0
100
200
300
400
500
600
700
800
m/z
y1
b1
y2
b2
y3
y4
y5
y5 y4 y3 y2 y1
A K (pr) A K (pr) T R
b1 b2

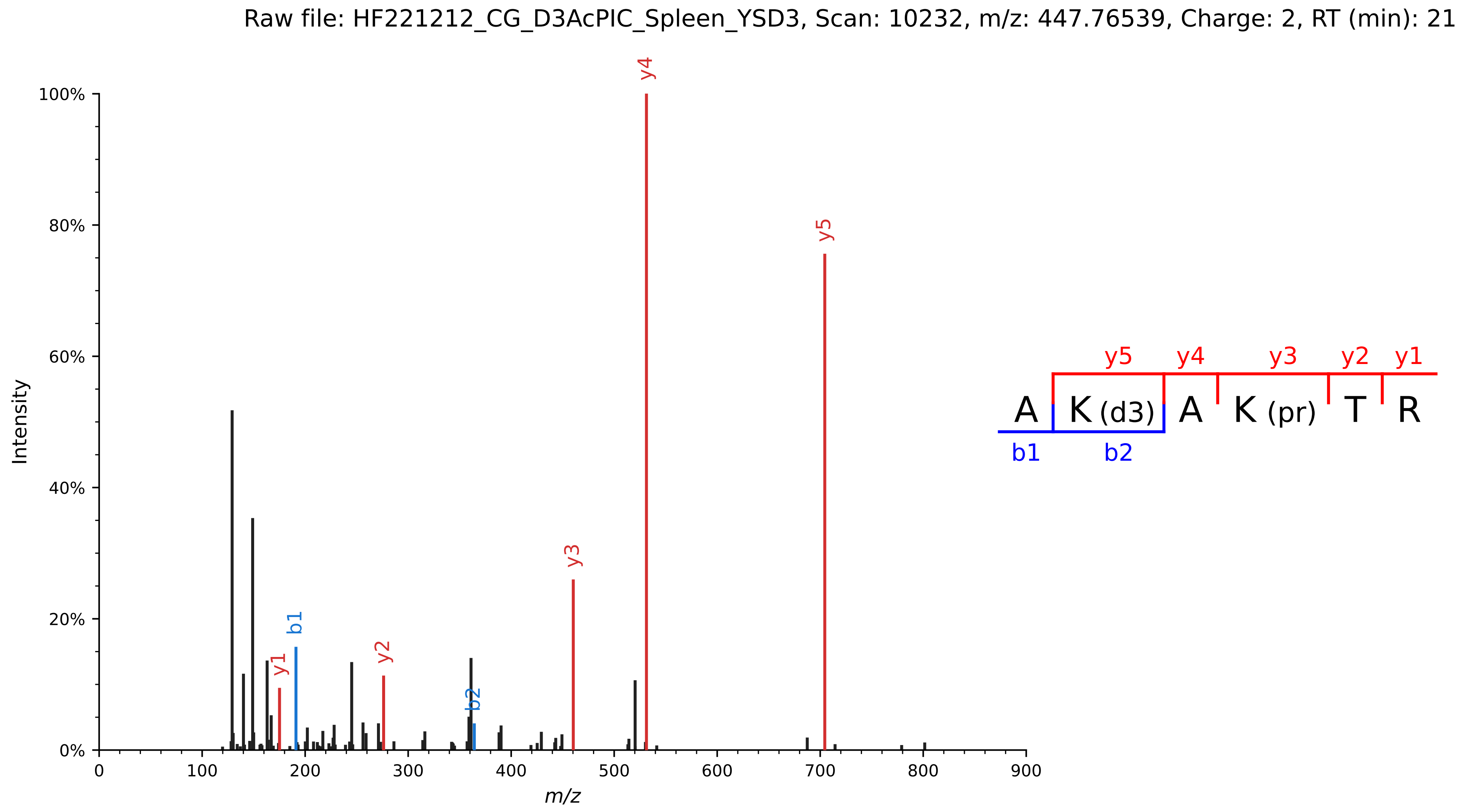

Raw file: HF221212_CG_D3AcPIC_Spleen_YSD3, Scan: 10232, m/z: 447.76539, Charge: 2, RT (min): 21.46, Score: 85.89
Intensity
m/z
y4
y5
y3
y2
y1
b1
b2
A K (d3) A K (pr) T R

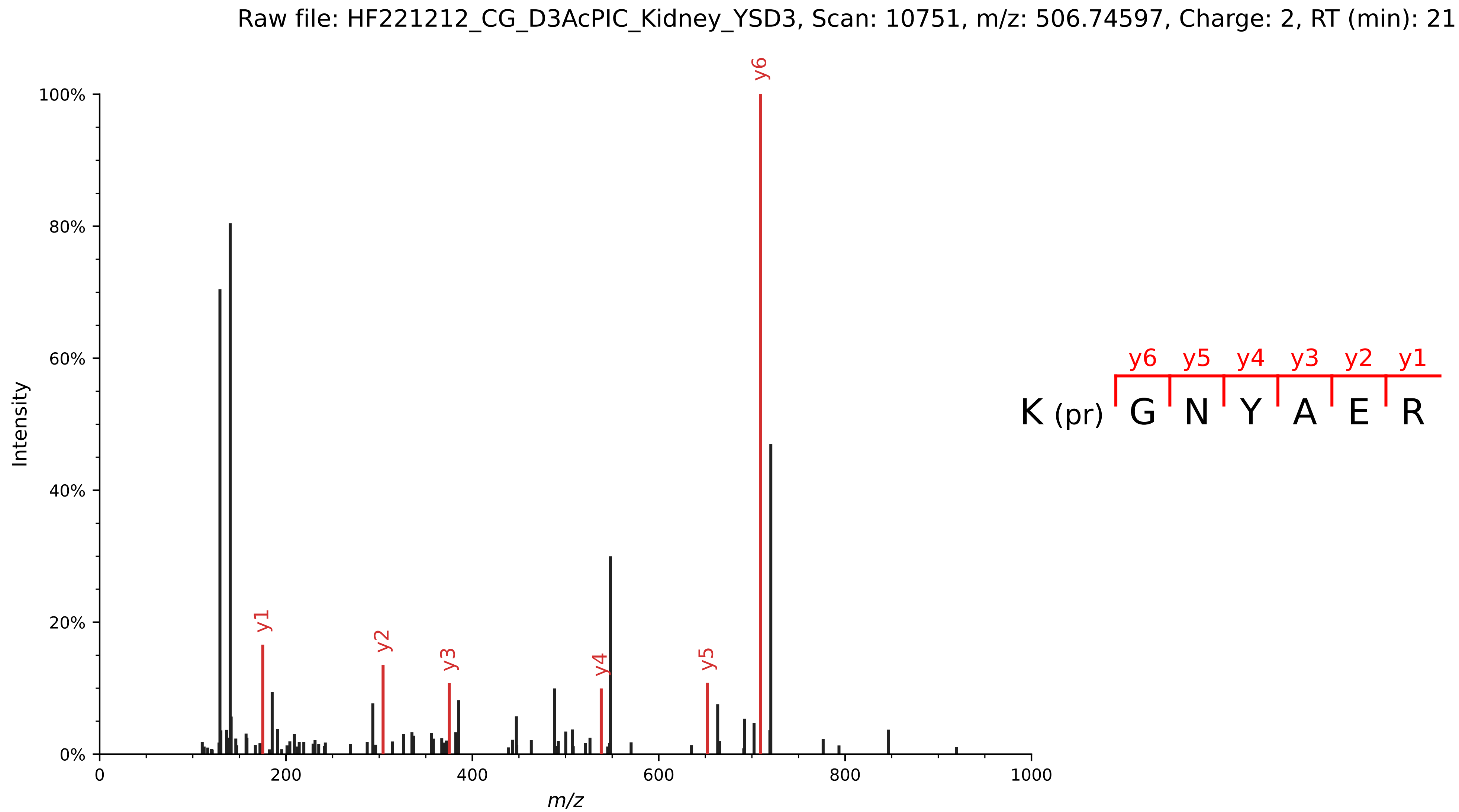

Raw file: HF221212_CG_D3AcPIC_Kidney_YSD3, Scan: 10751, m/z: 506.74597, Charge: 2, RT (min): 21.62, Score: 68.081
Intensity
m/z
y1
y2
y3
y4
y5
y6
y6 y5 y4 y3 y2 y1
K (pr) G N Y A E R

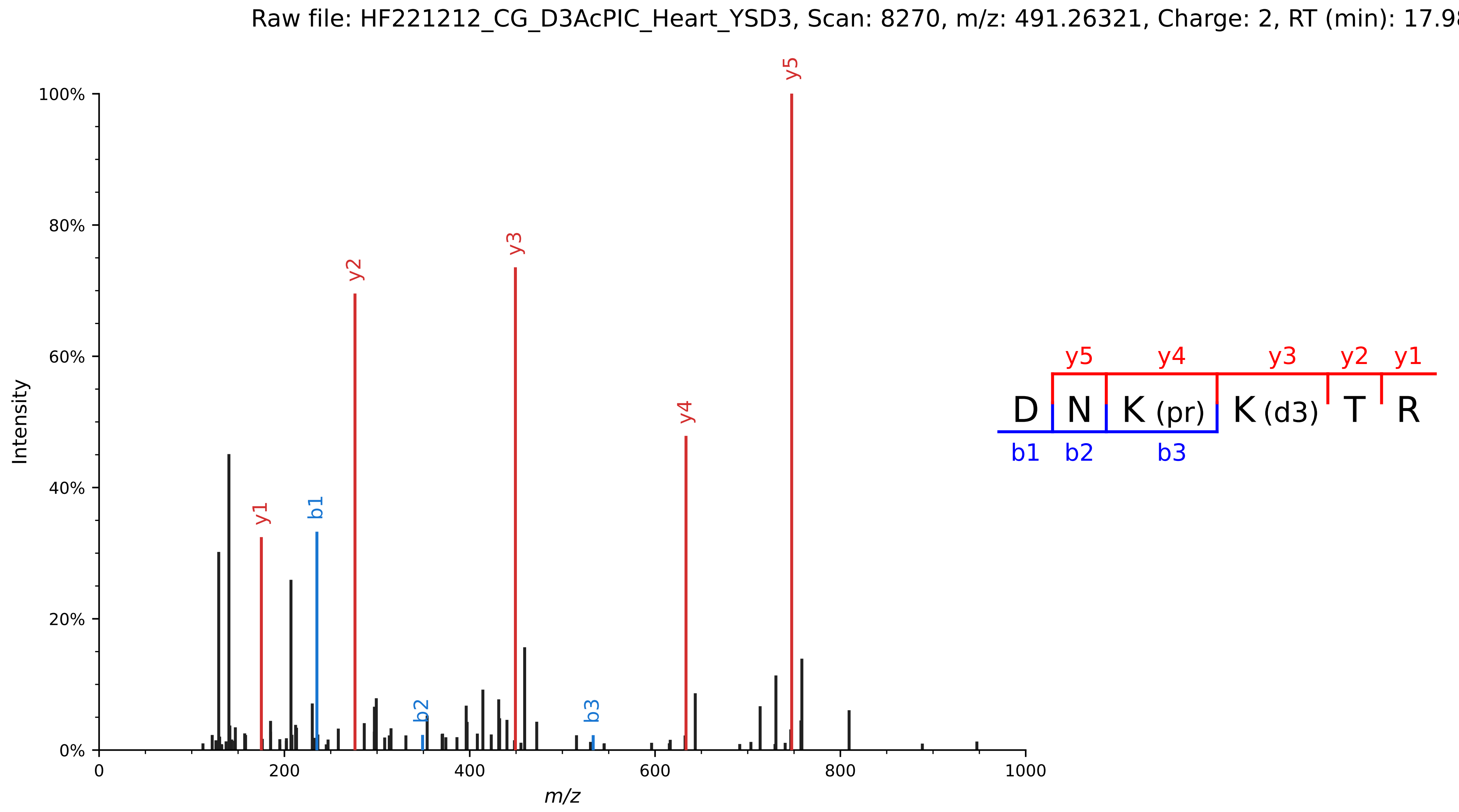

Raw file: HF221212_CG_D3AcPIC_Heart_YSD3, Scan: 8270, m/z: 491.26321, Charge: 2, RT (min): 17.98, Score: 117.62
Intensity
m/z
y1
b1
y2
b2
y3
b3
y4
y5
D N K (pr) K (d3) T R
y5 y4 y3 y2 y1
b1 b2 b3

Raw file: HF221212_CG_D3AcPIC_Kidney_YSD1, Scan: 9828, m/z: 496.76162, Charge: 2, RT (min): 21.16, Score: 70.268

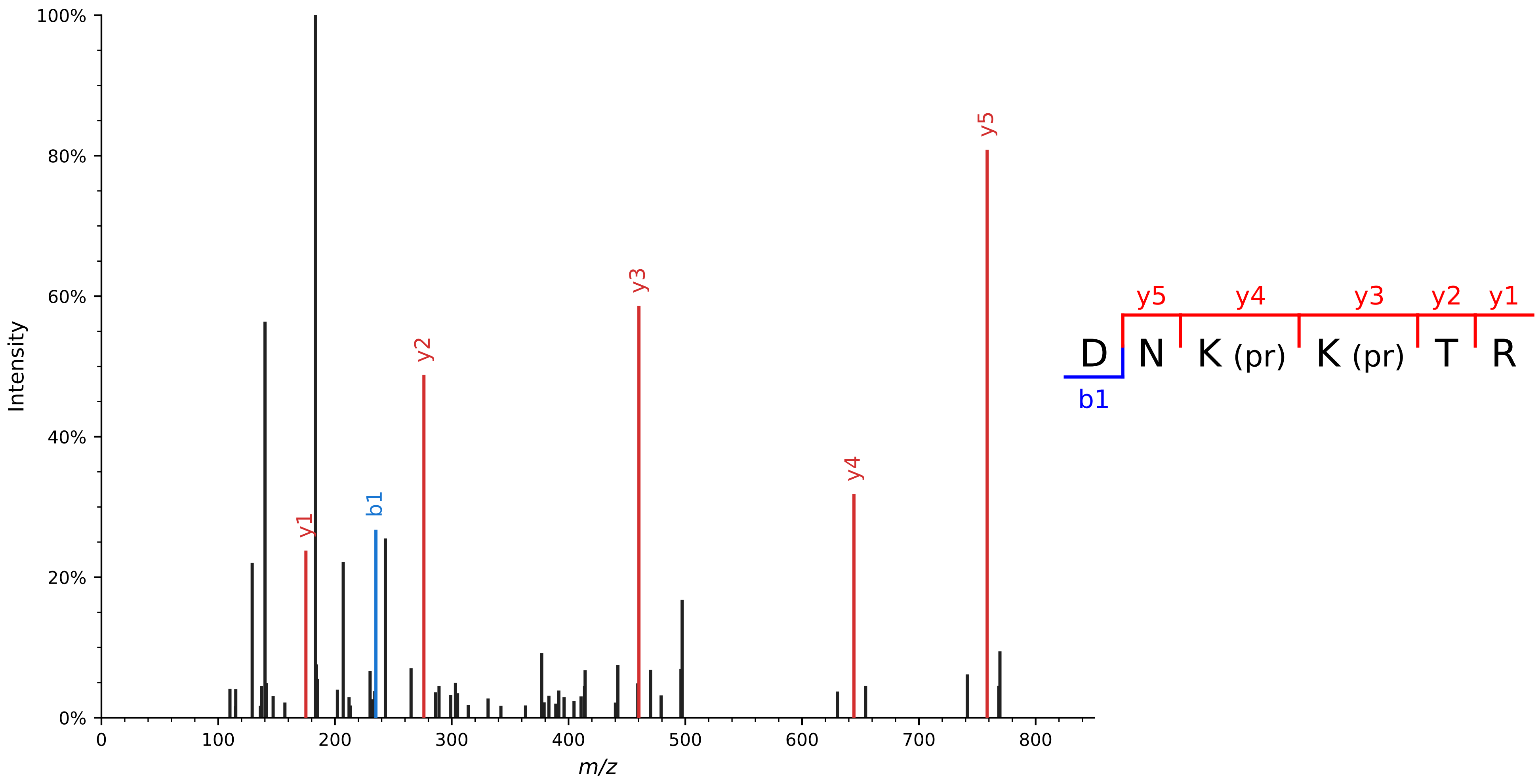

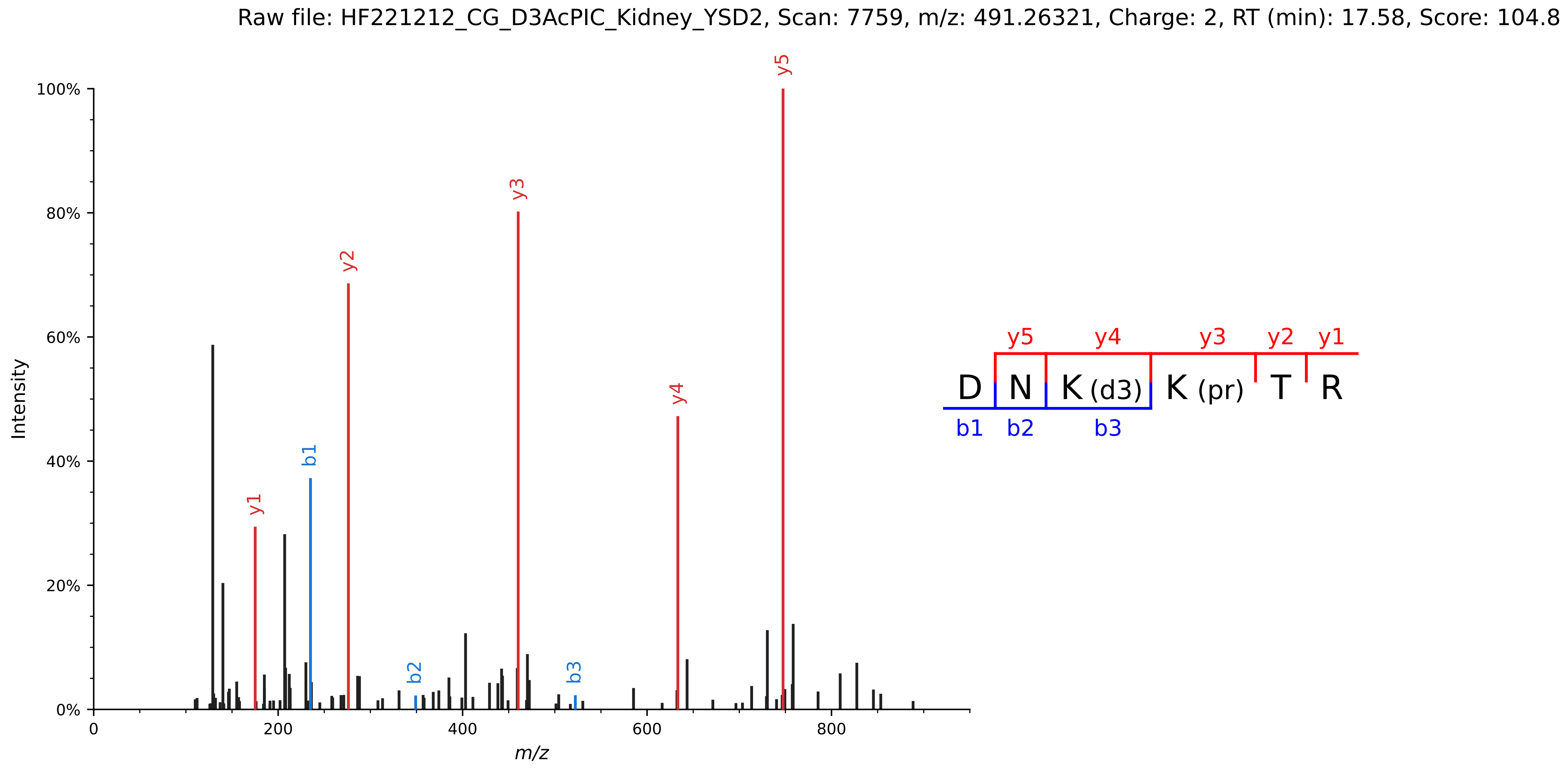

Raw file: HF221212_CG_D3AcPIC_Kidney_YSD2, Scan: 7759, m/z: 491.26321, Charge: 2, RT (min): 17.58, Score: 104.8
Intensity
m/z
y1
y2
y3
y4
y5
b1
b2
b3
D N K (d3) K (pr) T R

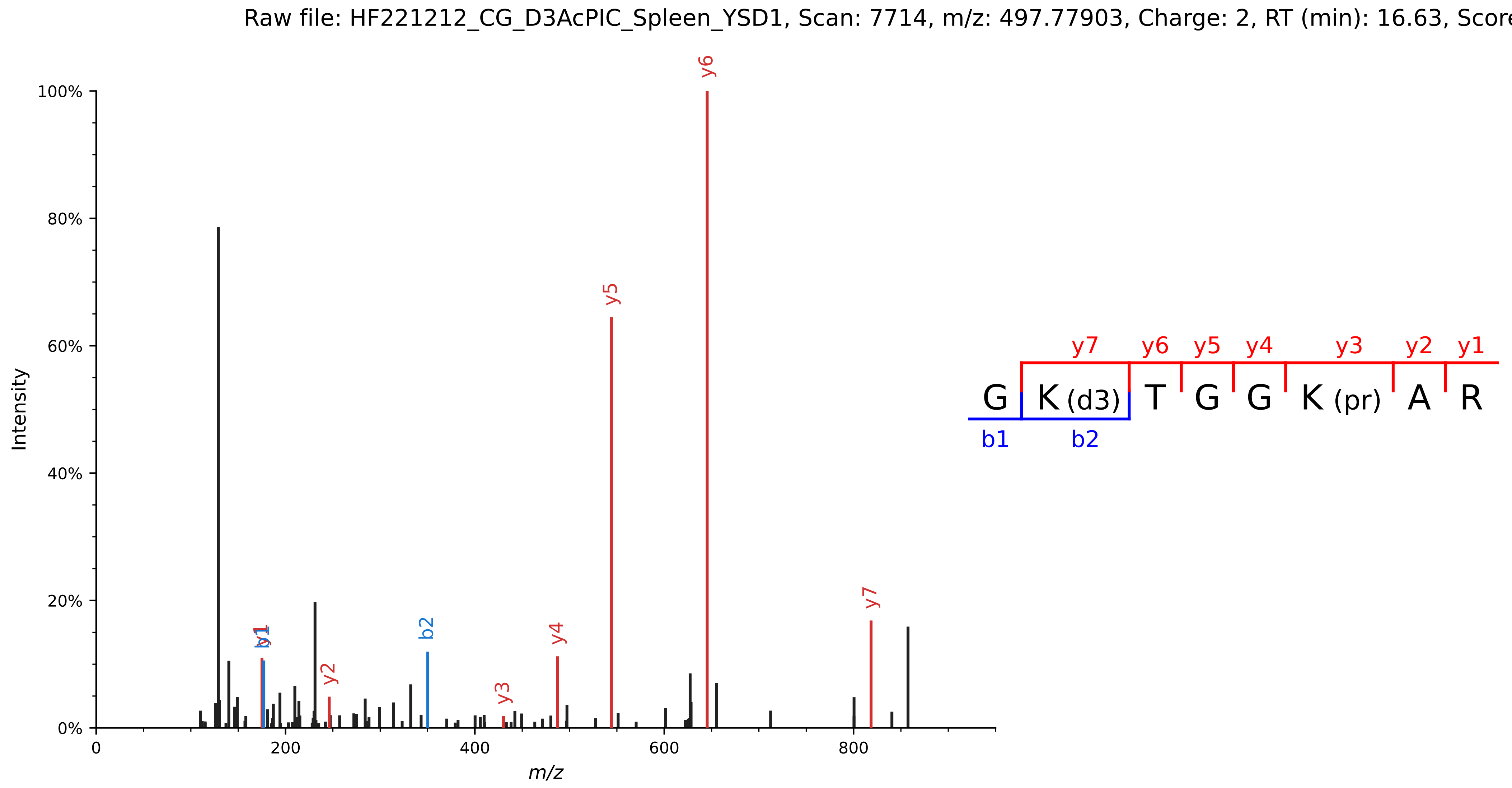
Raw file: HF221212_CG_D3AcPIC_Spleen_YSD1, Scan: 7714, m/z: 497.77903, Charge: 2, RT (min): 16.63, Score: 89.55
Intensity
m/z
y6
y5
y7
y4
y3
y2
y1
b1
b2
G K (d3) T G G K (pr) A R

Raw file: HF221212_CG_D3AcPIC_Spleen_YSD1, Scan: 5467, m/z: 518.25397, Charge: 2, RT (min): 12.64, Score: 62.573

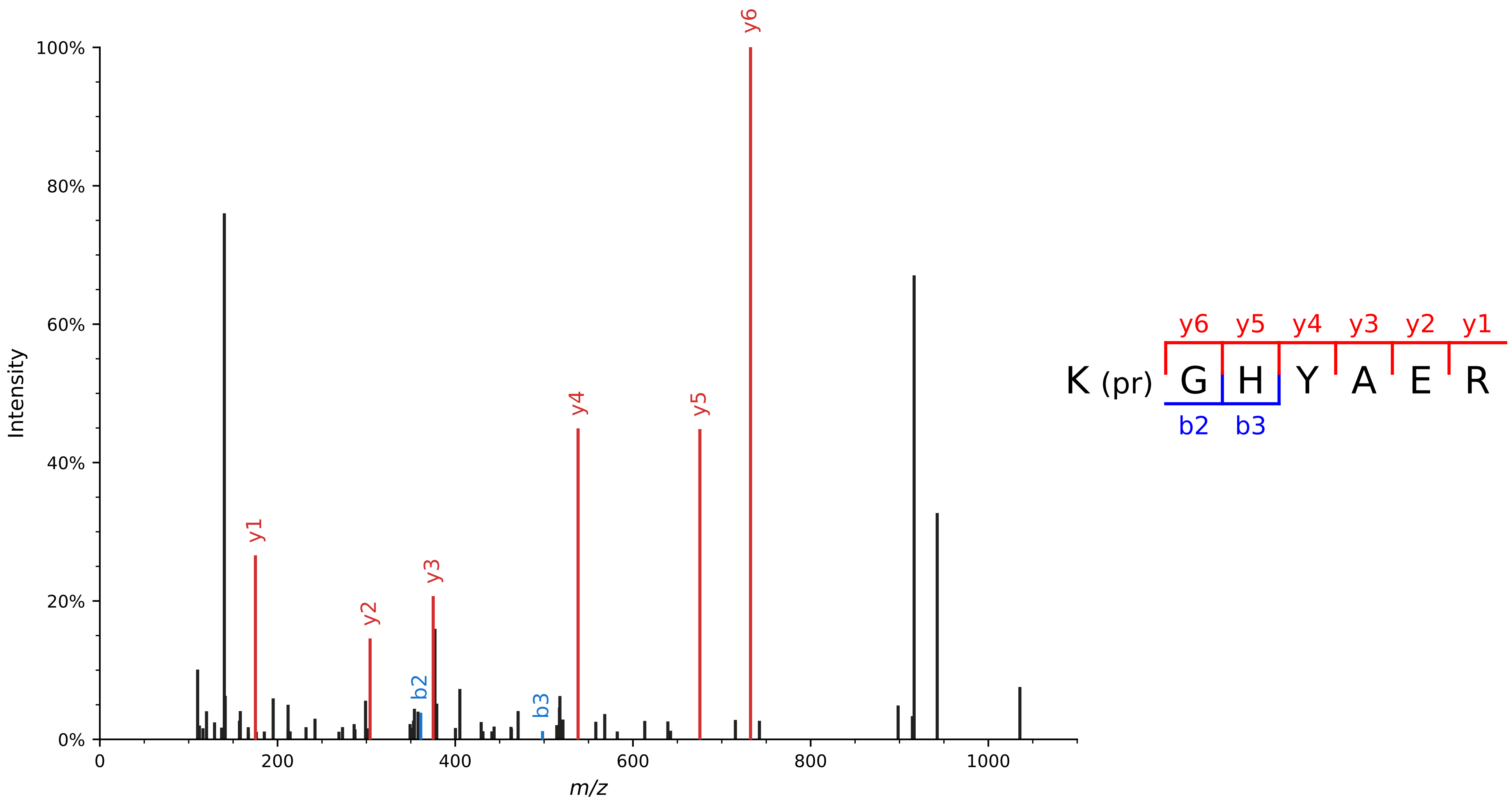

Raw file: HF221212_CG_D3AcPIC_Kidney_YSD3, Scan: 7768, m/z: 524.76978, Charge: 2, RT (min): 16.38, Score: 75.131

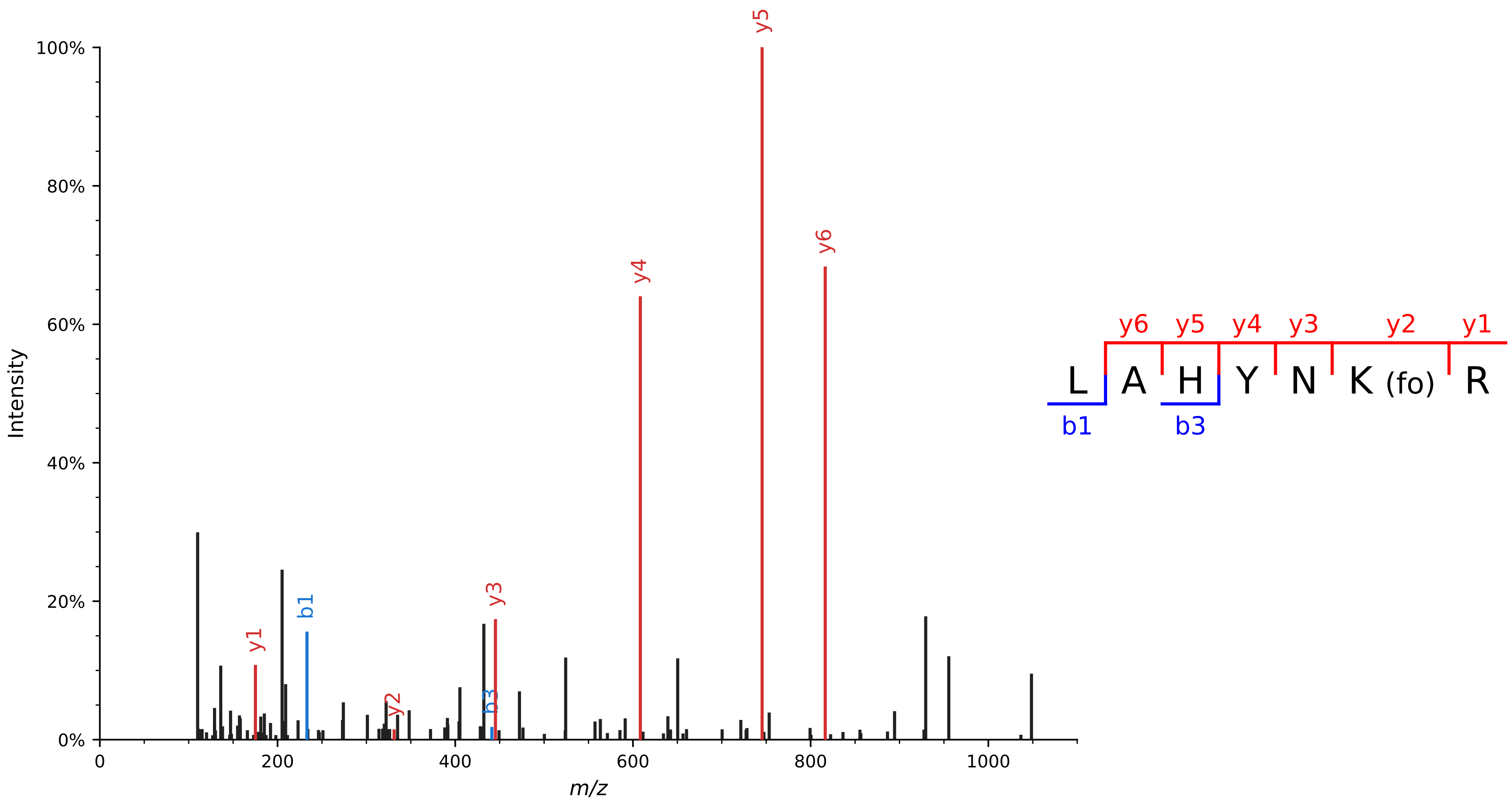

Raw file: HF221212_CG_D3AcPIC_Spleen_YSD2, Scan: 8891, m/z: 538.78543, Charge: 2, RT (min): 18.92, Score: 126.04

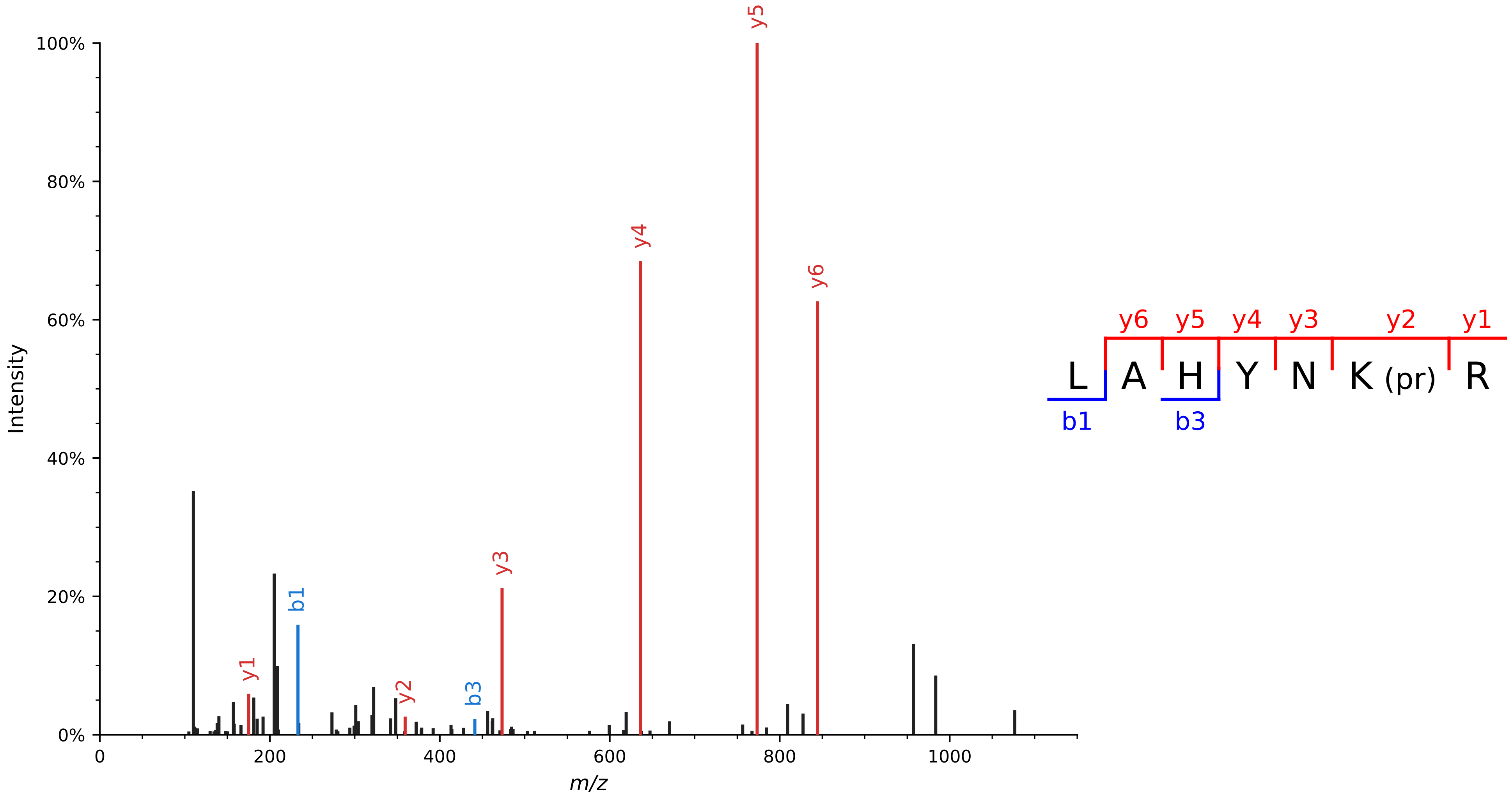

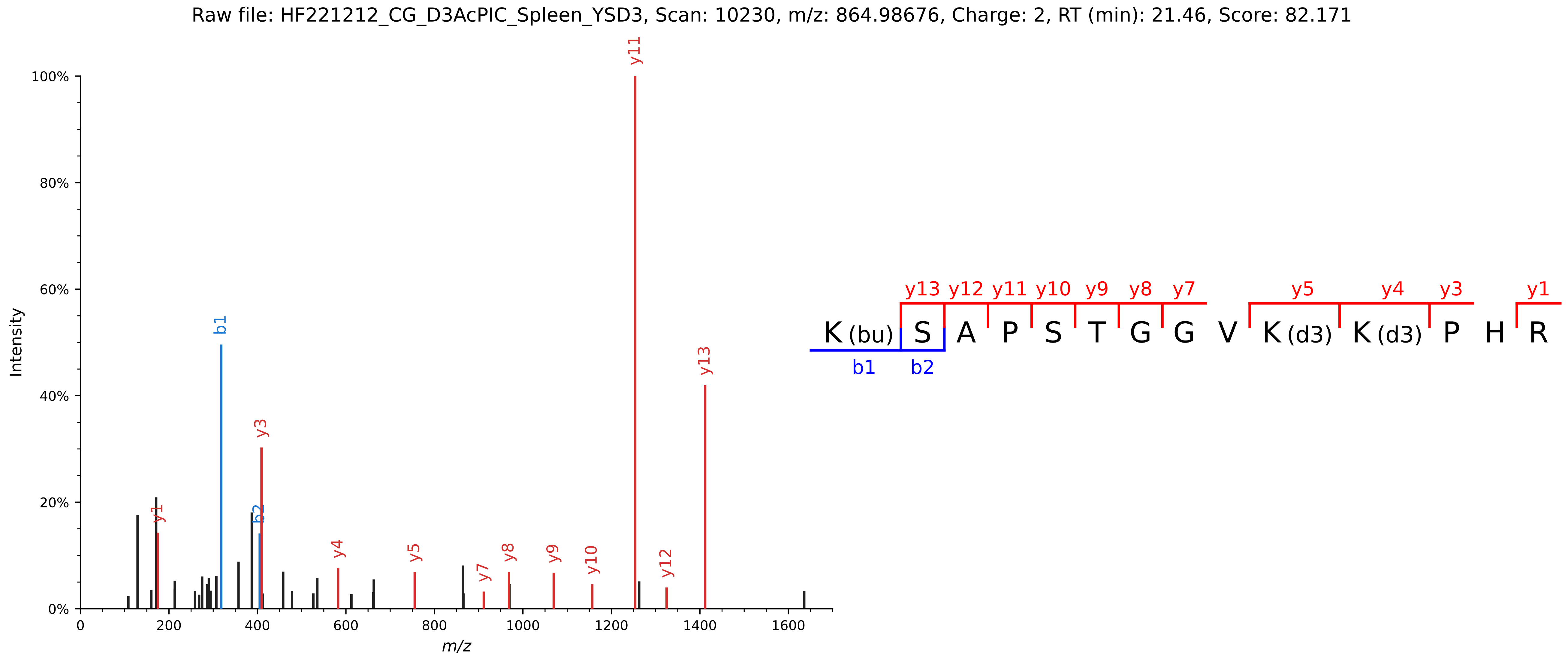
Raw file: HF221212_CG_D3AcPIC_Spleen_YSD3, Scan: 10230, m/z: 864.98676, Charge: 2, RT (min): 21.46, Score: 82.171
Intensity
m/z
K(bu) S A P S T G G V K(d3) K(d3) P H R
y13 y12 y11 y10 y9 y8 y7 y5 y4 y3 y1
b1 b2

Raw file: HF221212_CG_D3AcPIC_Spleen_YSD2, Scan: 6064, m/z: 571.32757, Charge: 3, RT (min): 13.87, Score: 78.401

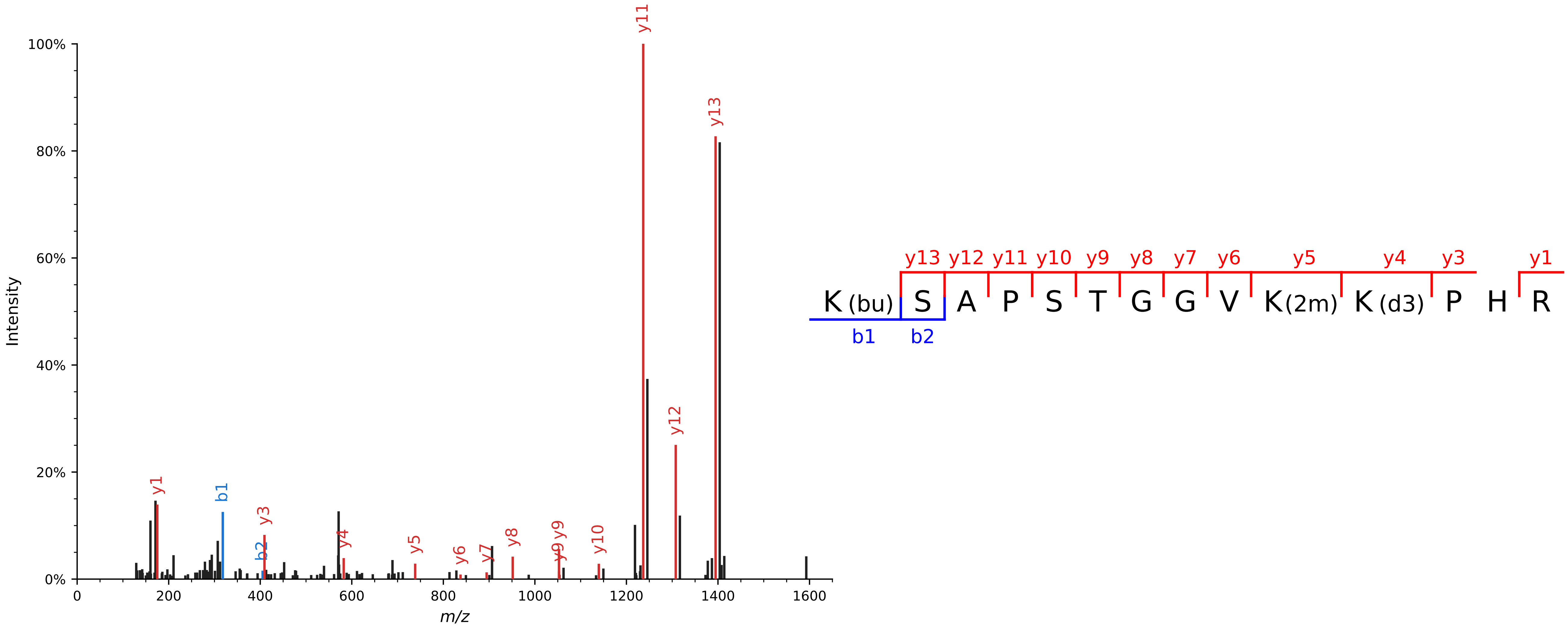

Raw file: HF221212_CG_D3AcPIC_Spleen_YSD2, Scan: 6052, m/z: 575.99945, Charge: 3, RT (min): 13.85, Score: 59.607

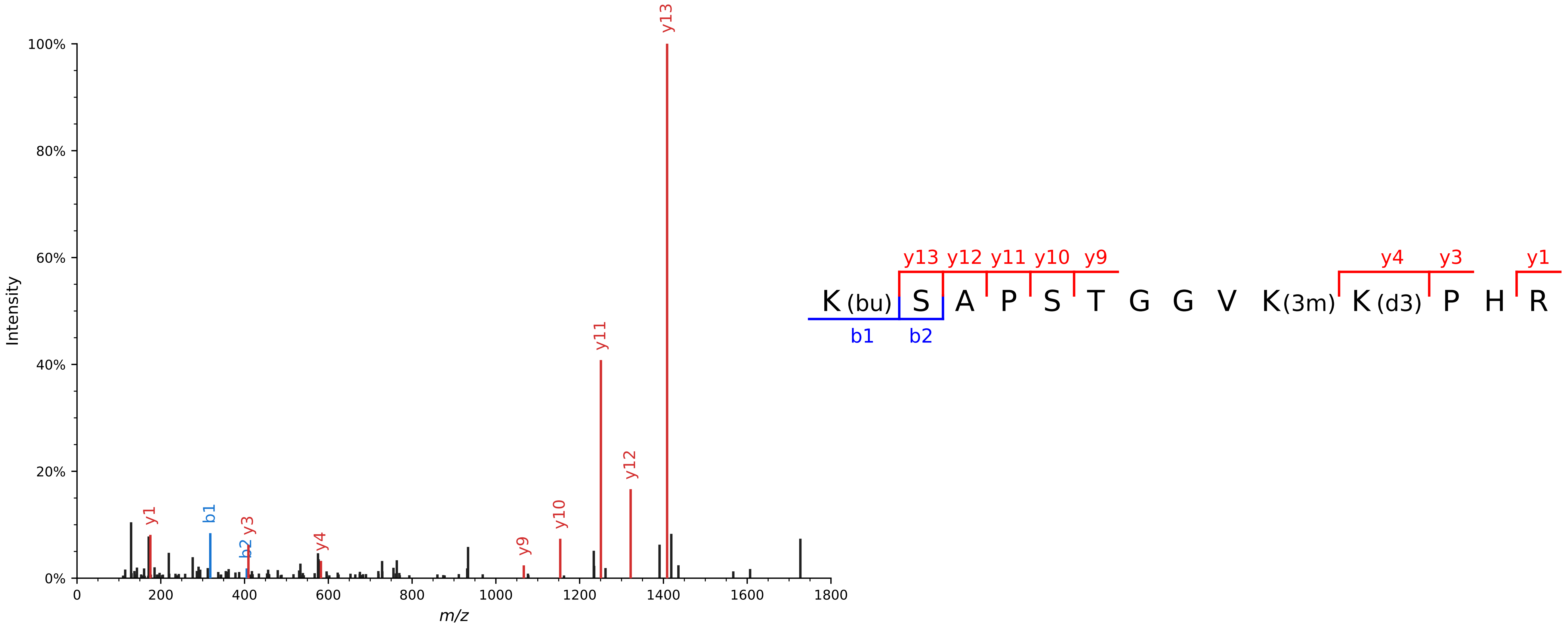

Raw file: HF221212_CG_D3AcPIC_Heart_YSD2, Scan: 8616, m/z: 859.48835, Charge: 2, RT (min): 18.89, Score: 166.97

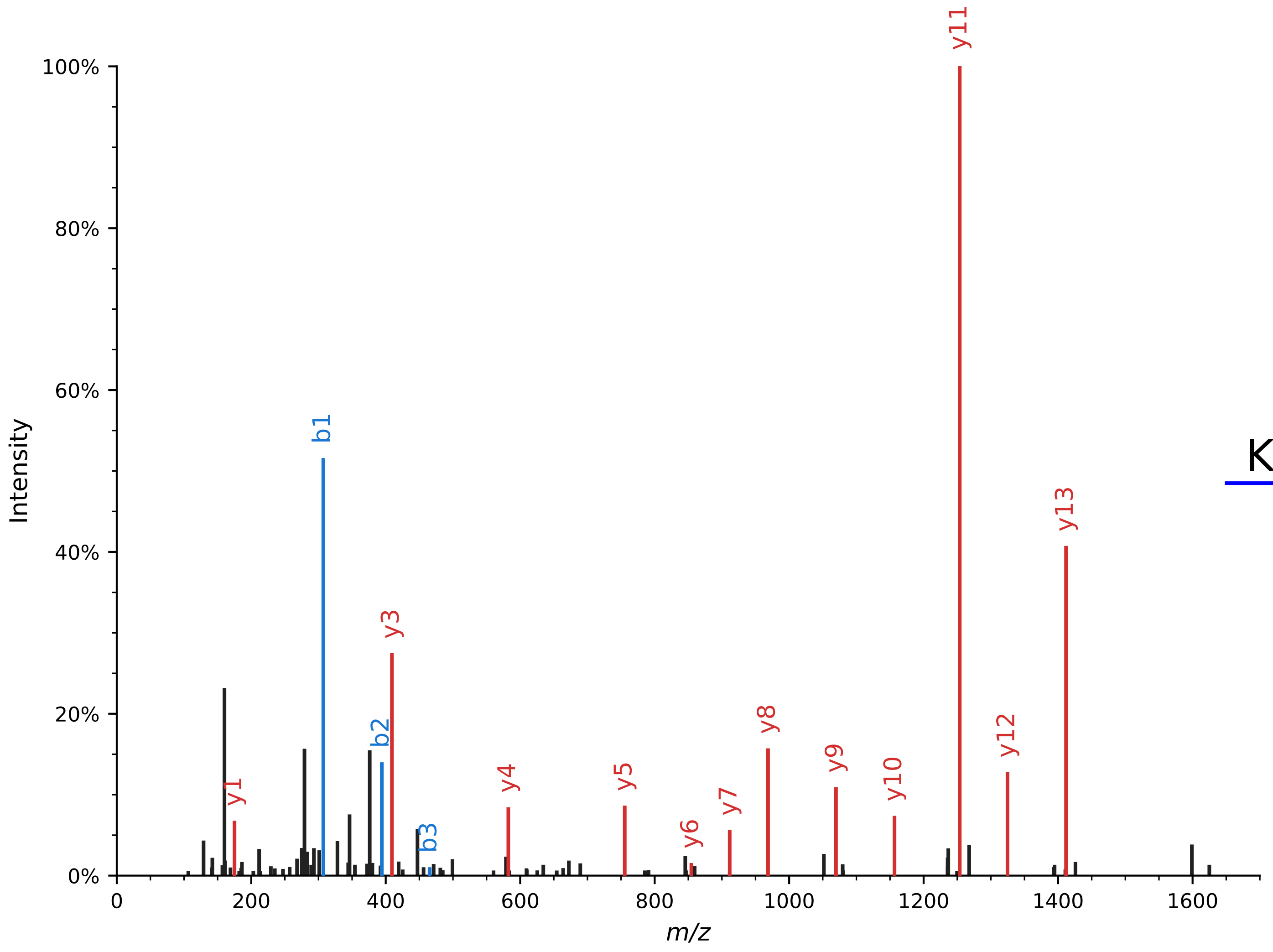

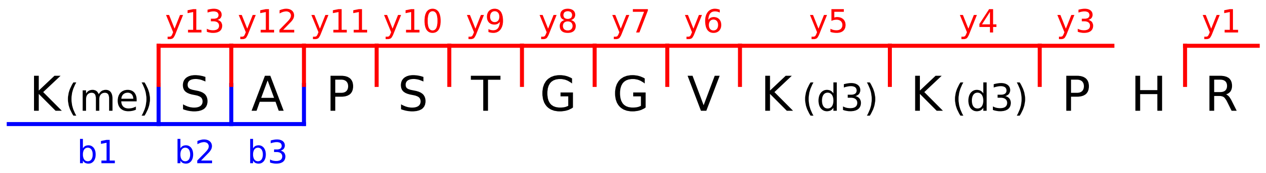

Raw file: HF221212_CG_D3AcPIC_Liver_YSD1, Scan: 9479, m/z: 866.49617, Charge: 2, RT (min): 20.23, Score: 133.89

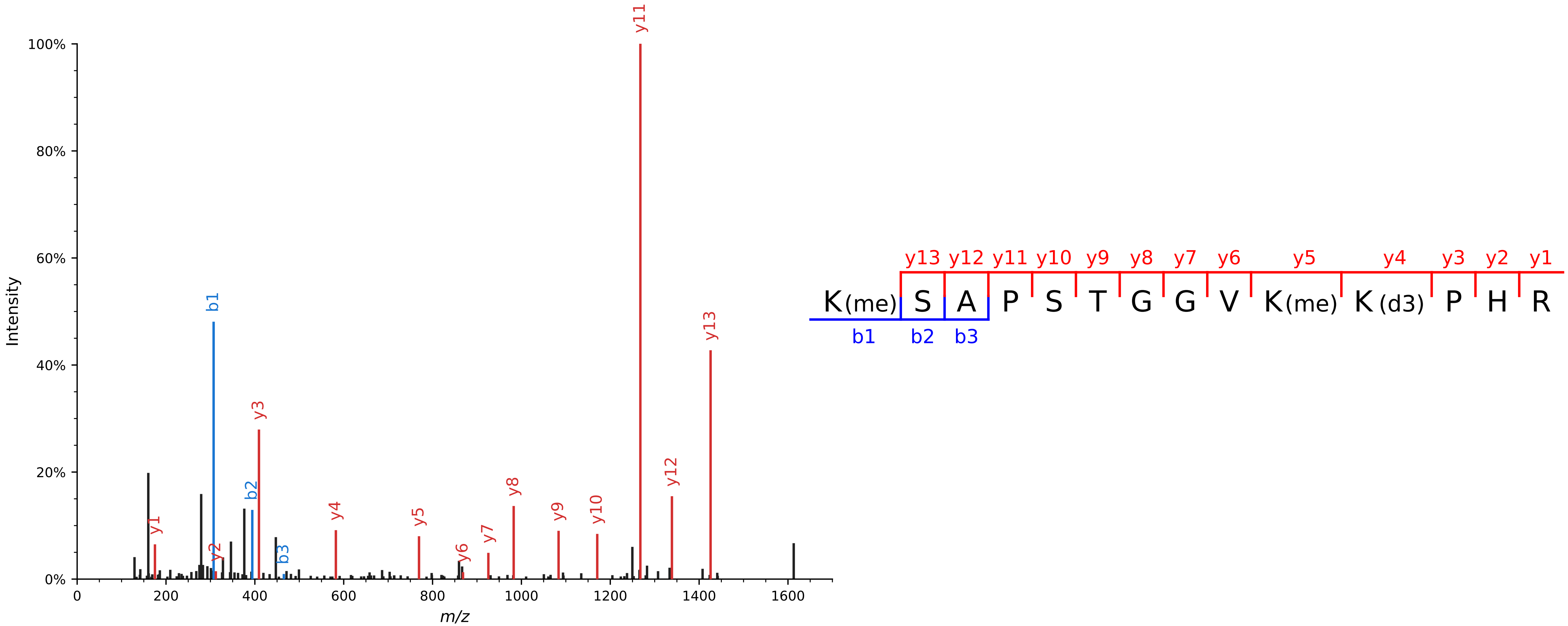

Raw file: HF221212_CG_D3AcPIC_Kidney_YSD3, Scan: 6374, m/z: 873.504, Charge: 2, RT (min): 13.91, Score: 57.348

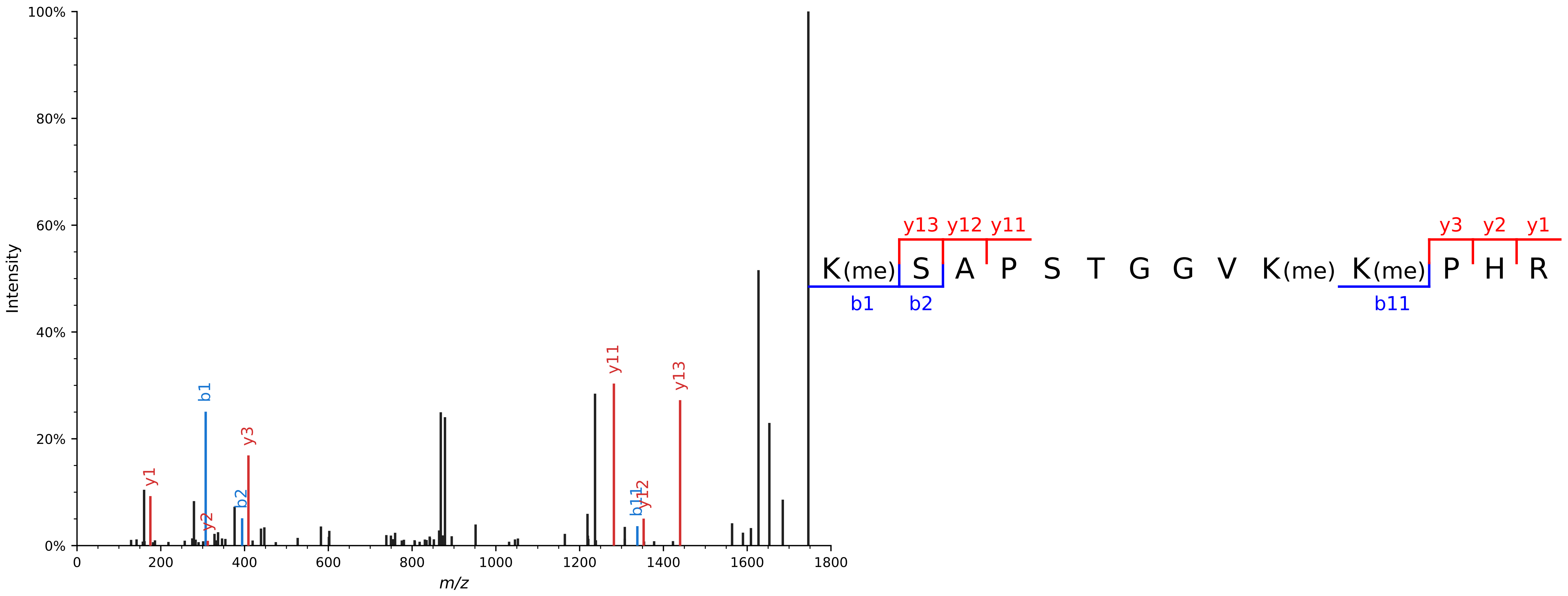

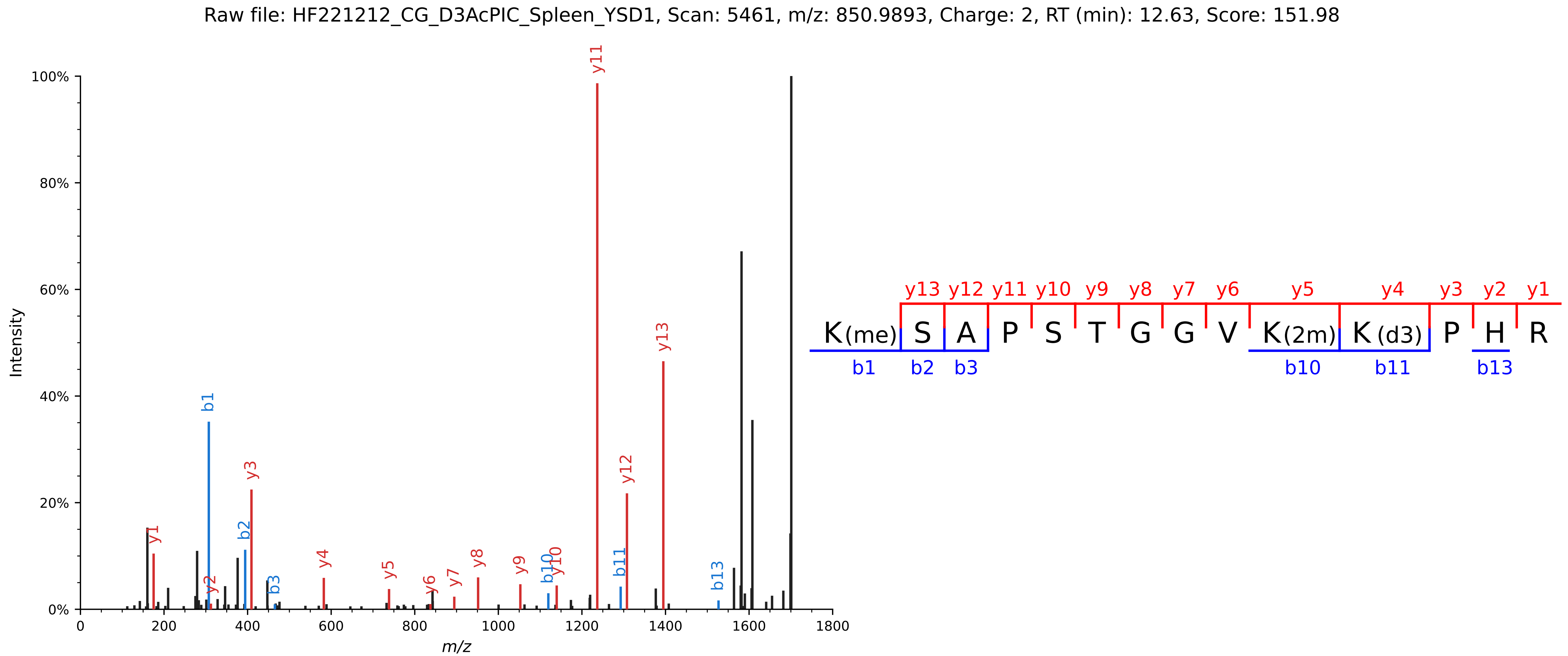

Raw file: HF221212_CG_D3AcPIC_Spleen_YSD1, Scan: 5461, m/z: 850.9893, Charge: 2, RT (min): 12.63, Score: 151.98
Intensity
m/z
K(me) S A P S T G G V K(2m) K(d3) P H R
y13 y12 y11 y10 y9 y8 y7 y6 y5 y4 y3 y2 y1
b1 b2 b3 b10 b11 b13

Raw file: HF221212_CG_D3AcPIC_Spleen_YSD3, Scan: 5904, m/z: 856.48771, Charge: 2, RT (min): 13.62, Score: 112.15

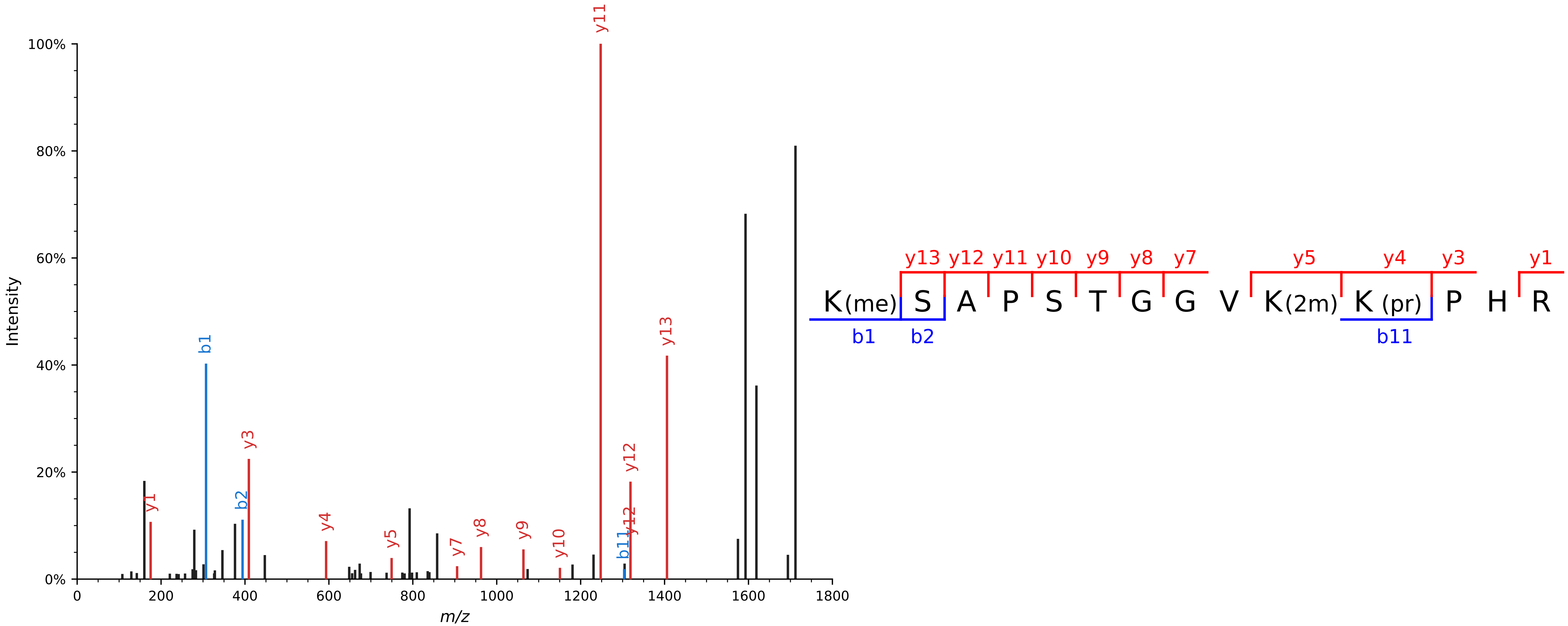

Raw file: HF221212_CG_D3AcPIC_Heart_YSD2, Scan: 5105, m/z: 572.33384, Charge: 3, RT (min): 12.45, Score: 62.891

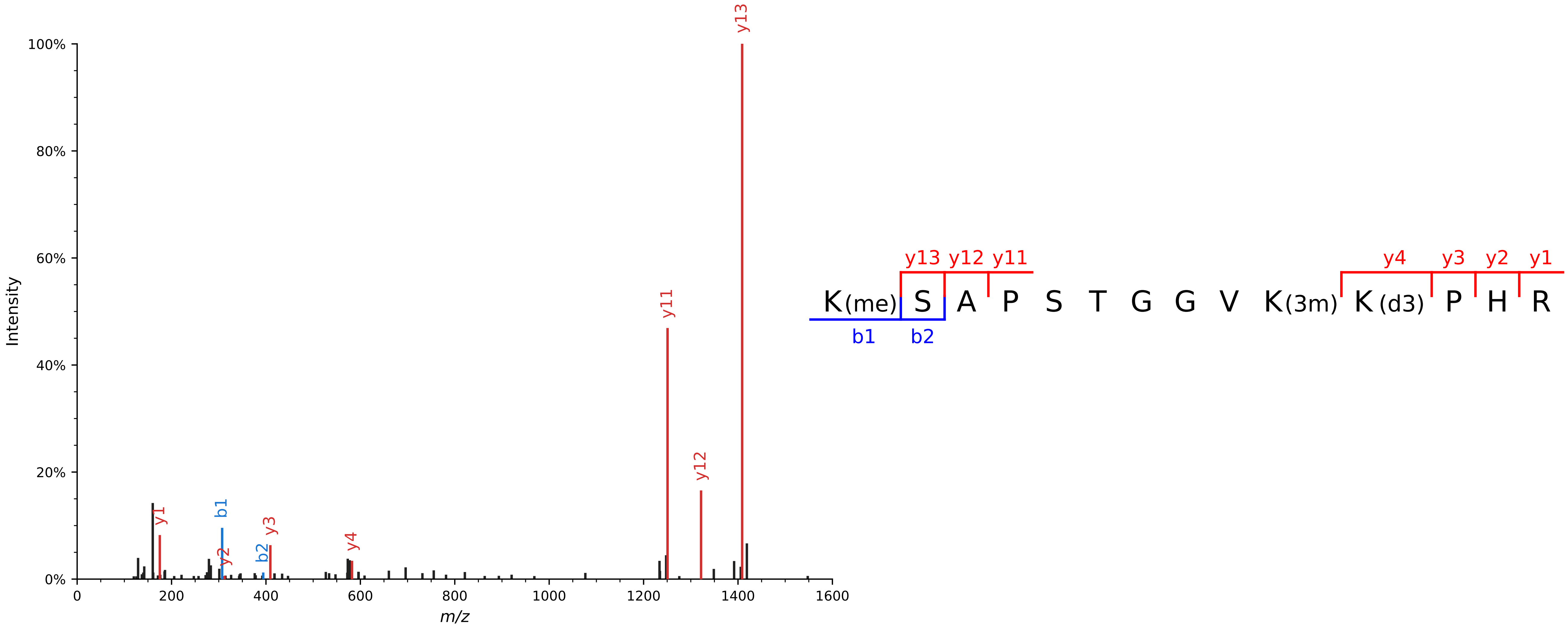

Raw file: HF221212_CG_D3AcPIC_Spleen_YSD3, Scan: 9721, m/z: 864.98676, Charge: 2, RT (min): 20.50, Score: 88.596

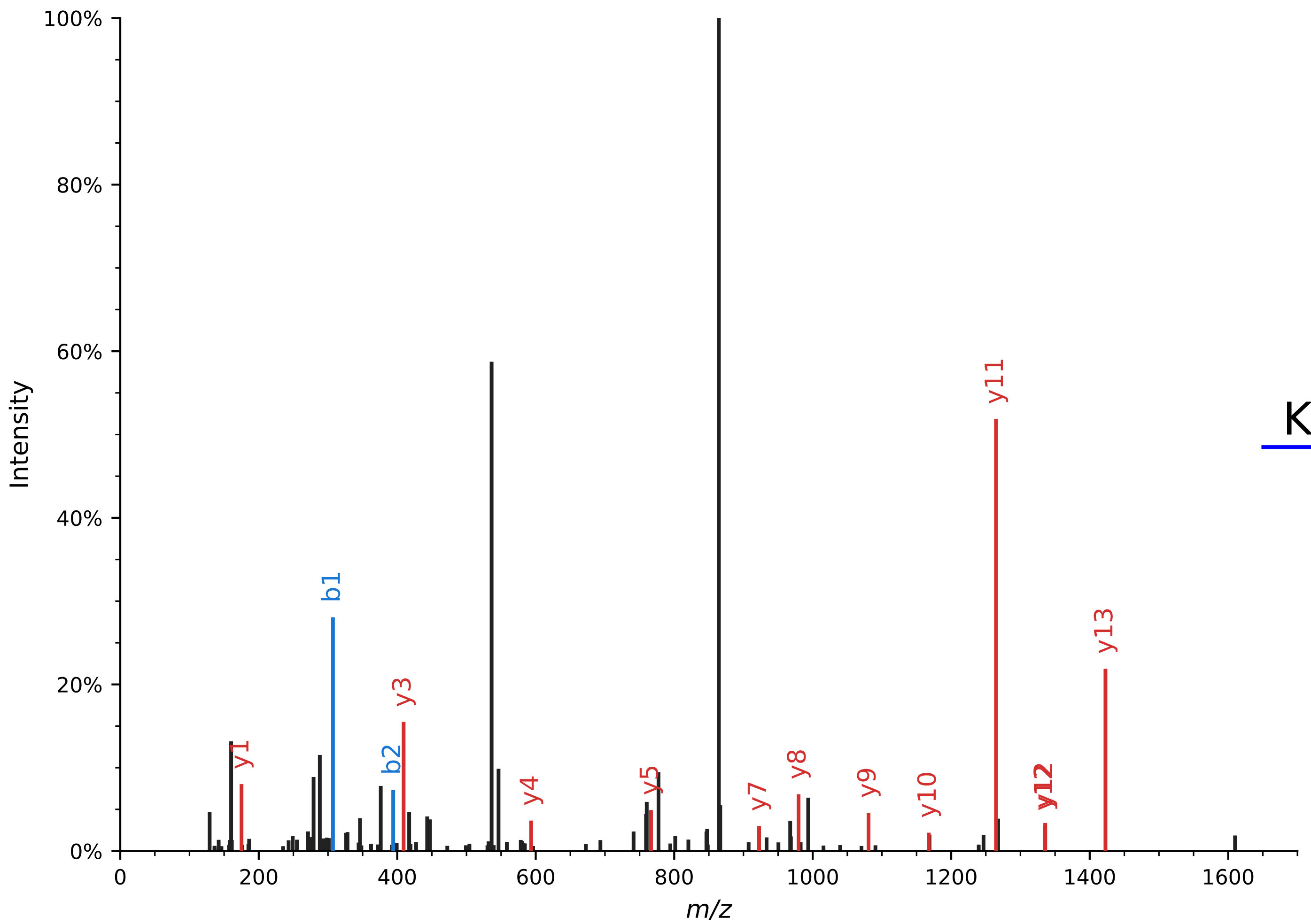

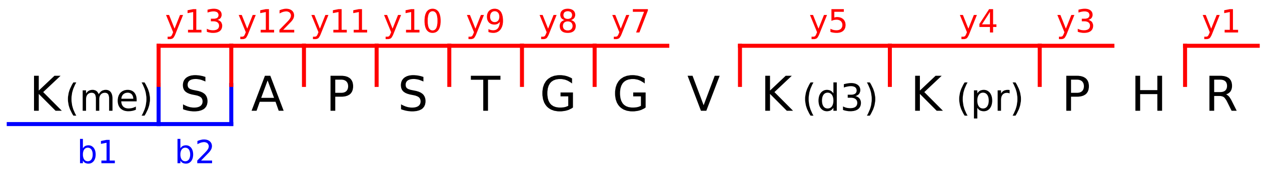

Raw file: HF221212_CG_D3AcPIC_Spleen_YSD2, Scan: 3935, m/z: 843.98148, Charge: 2, RT (min): 10.15, Score: 155.47

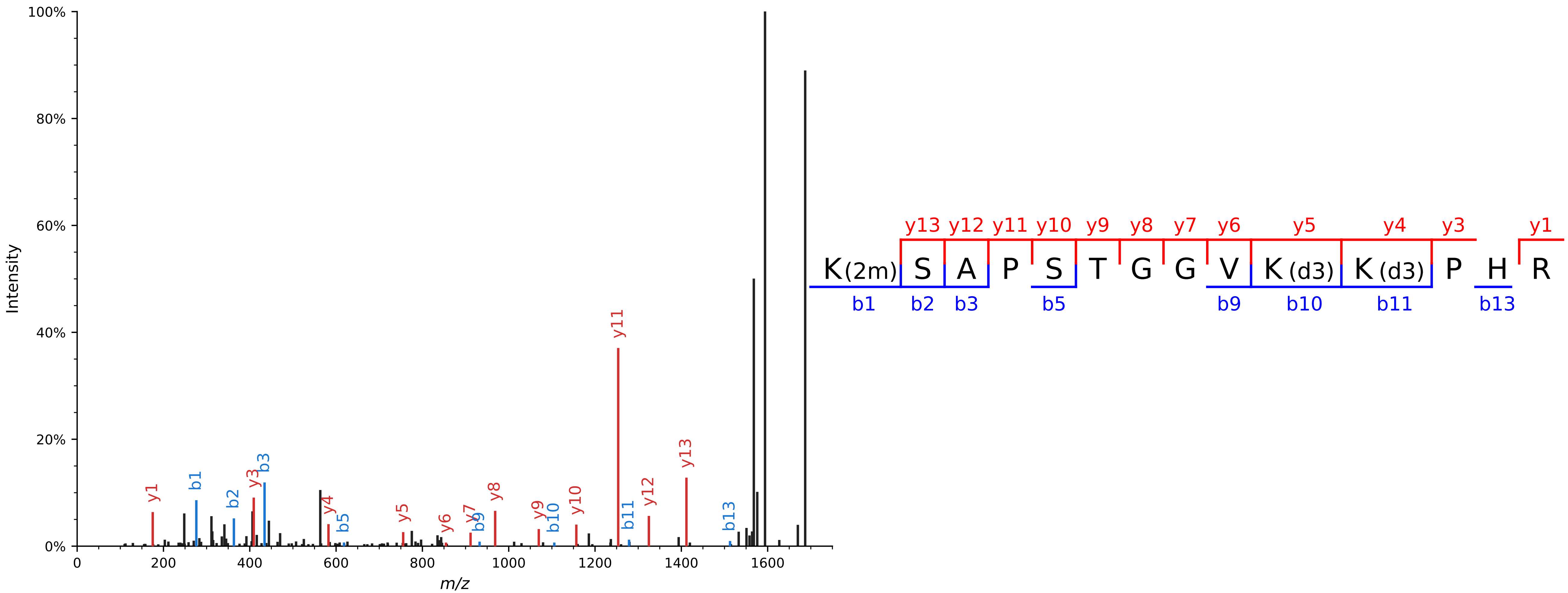

Raw file: HF221212_CG_D3AcPIC_Spleen_YSD2, Scan: 4326, m/z: 850.9893, Charge: 2, RT (min): 10.83, Score: 193.49

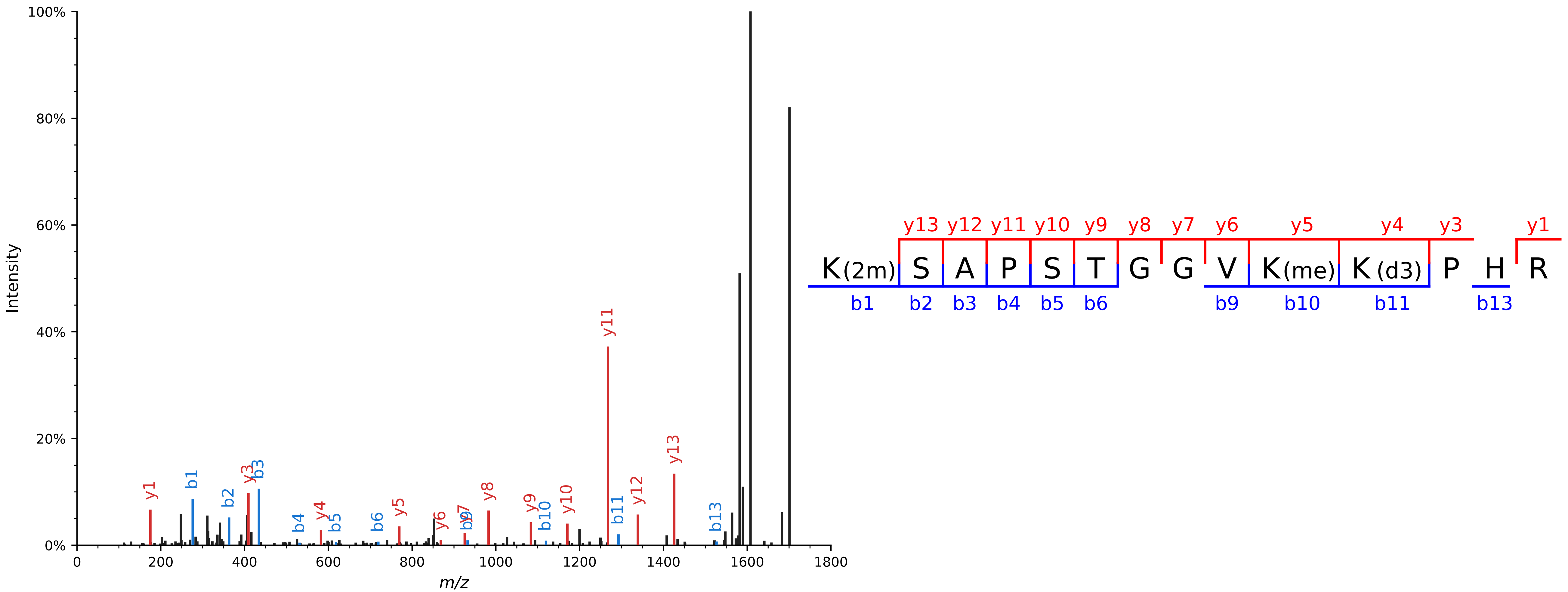

Raw file: HF221212_CG_D3AcPIC_Liver_YSD1, Scan: 2279, m/z: 572.33384, Charge: 3, RT (min): 7.32, Score: 57.159

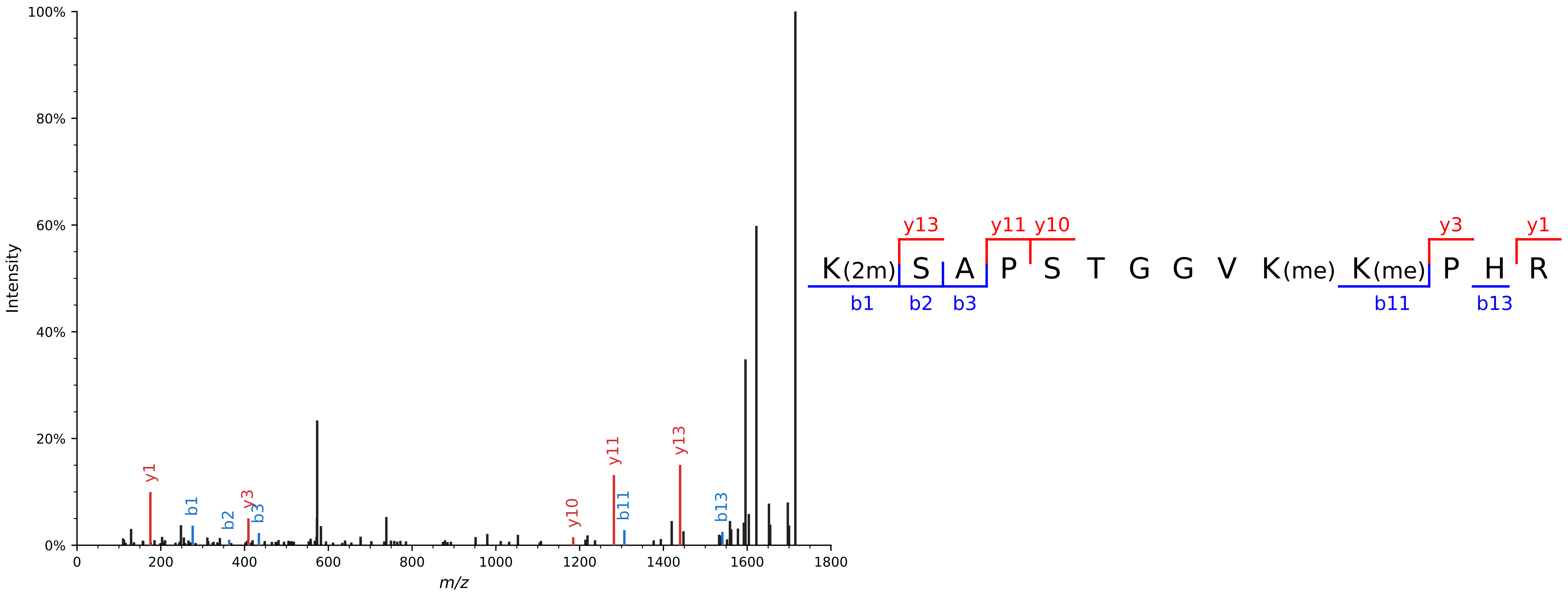

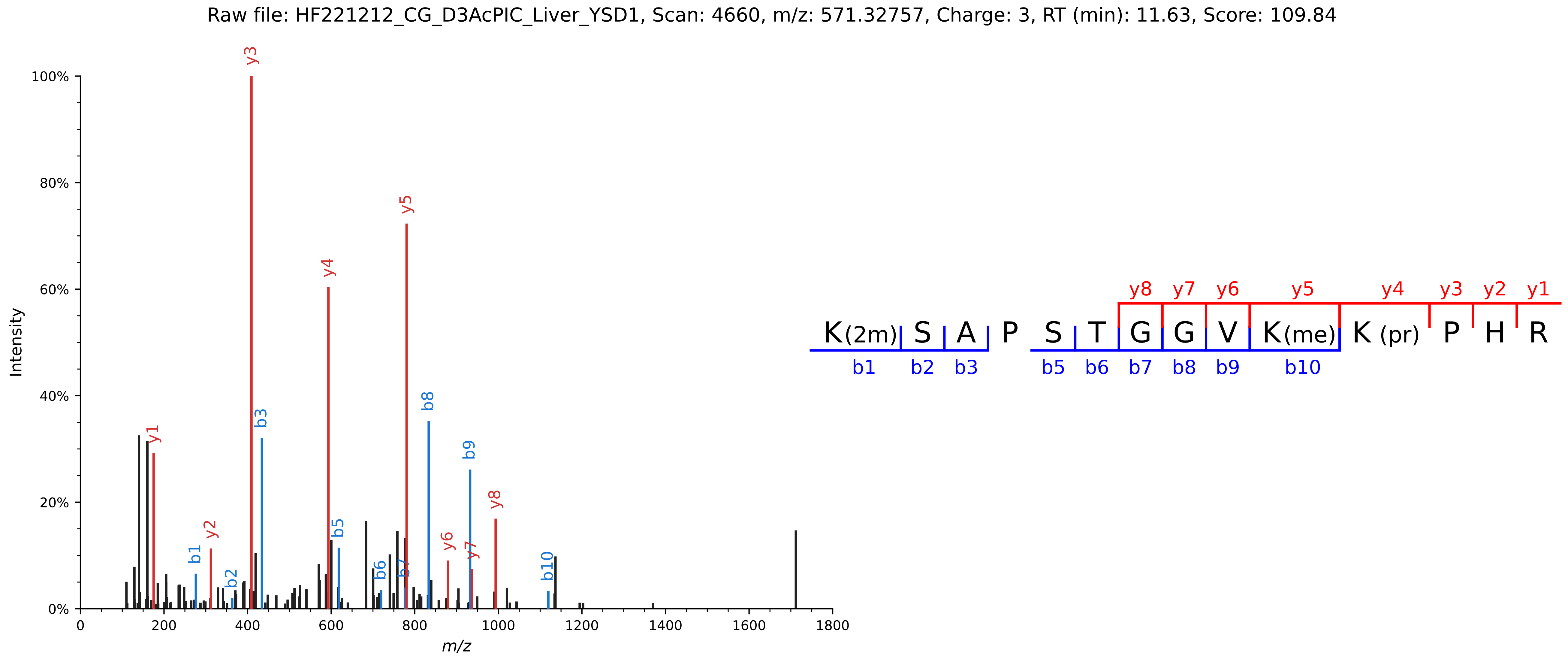

Raw file: HF221212_CG_D3AcPIC_Liver_YSD1, Scan: 4660, m/z: 571.32757, Charge: 3, RT (min): 11.63, Score: 109.84
Intensity
m/z
K(2m) S A P S T G G V K(me) K (pr) P H R
y8 y7 y6 y5 y4 y3 y2 y1
b1 b2 b3 b5 b6 b7 b8 b9 b10

Raw file: HF221212_CG_D3AcPIC_Brain_YSD2, Scan: 3039, m/z: 557.32404, Charge: 3, RT (min): 8.77, Score: 125.73

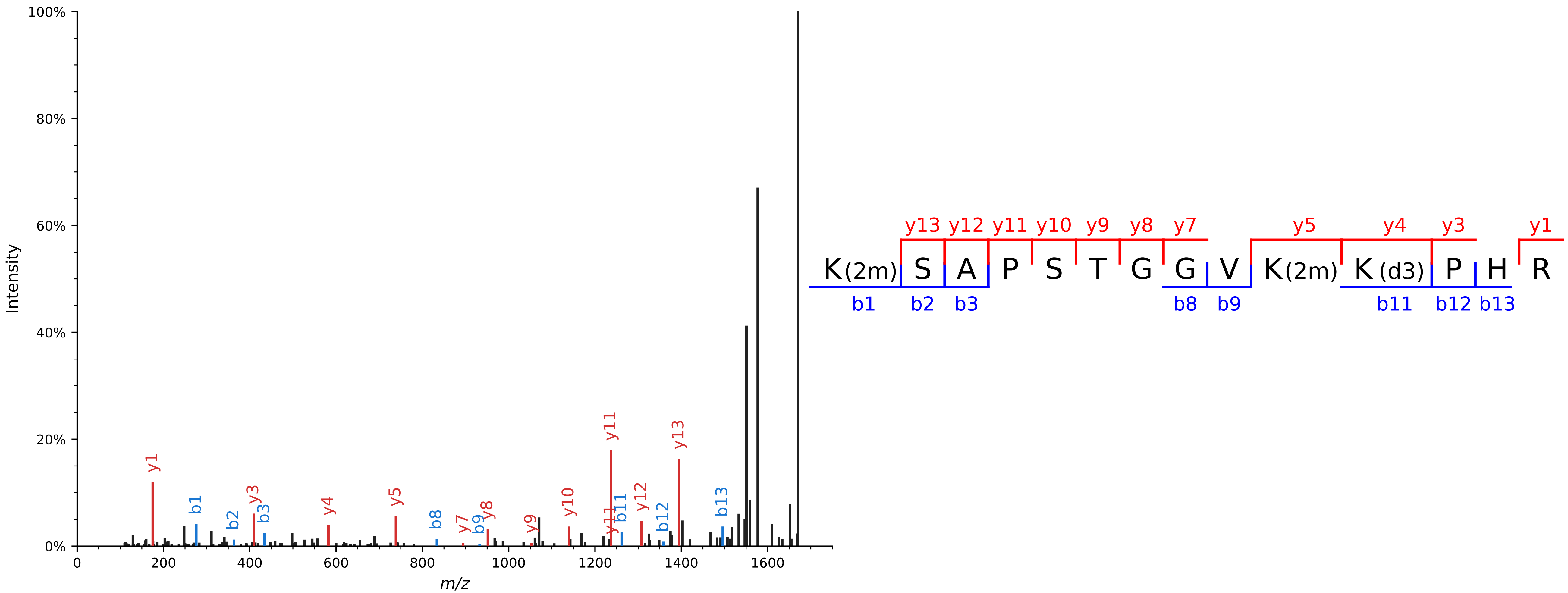

Raw file: HF221212_CG_D3AcPIC_Heart_YSD3, Scan: 4287, m/z: 413.99533, Charge: 4, RT (min): 10.82, Score: 54.524

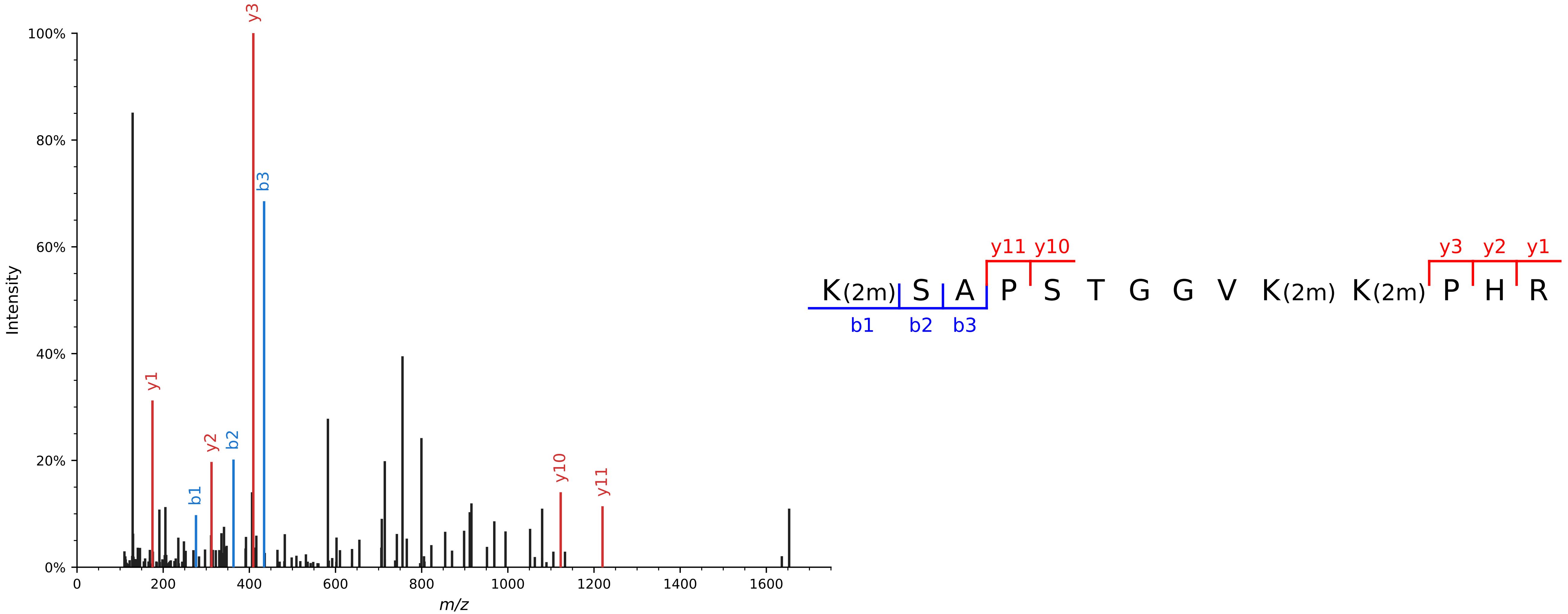

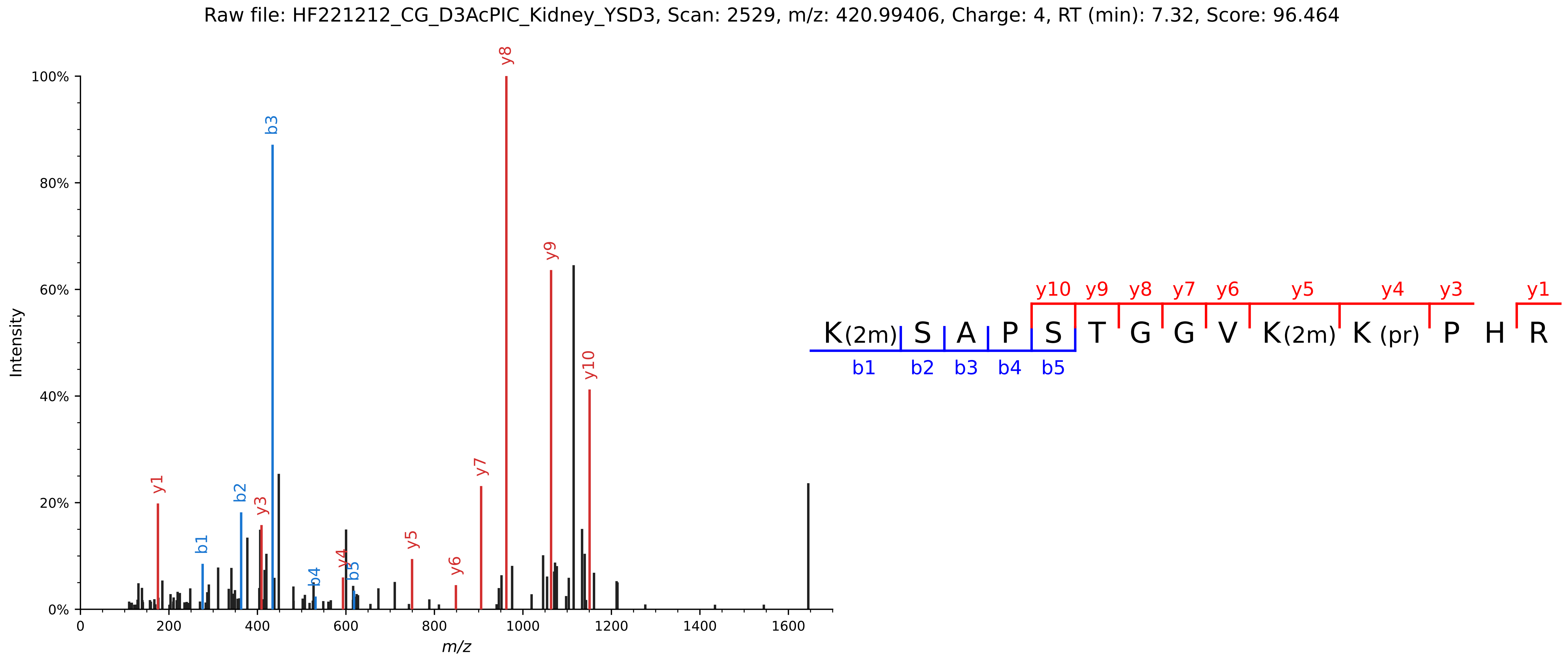
Raw file: HF221212_CG_D3AcPIC_Kidney_YSD3, Scan: 2529, m/z: 420.99406, Charge: 4, RT (min): 7.32, Score: 96.464
Intensity
m/z
K(2m) S A P S T G G V K(2m) K (pr) P H R
y10 y9 y8 y7 y6 y5 y4 y3 y1
b1 b2 b3 b4 b5

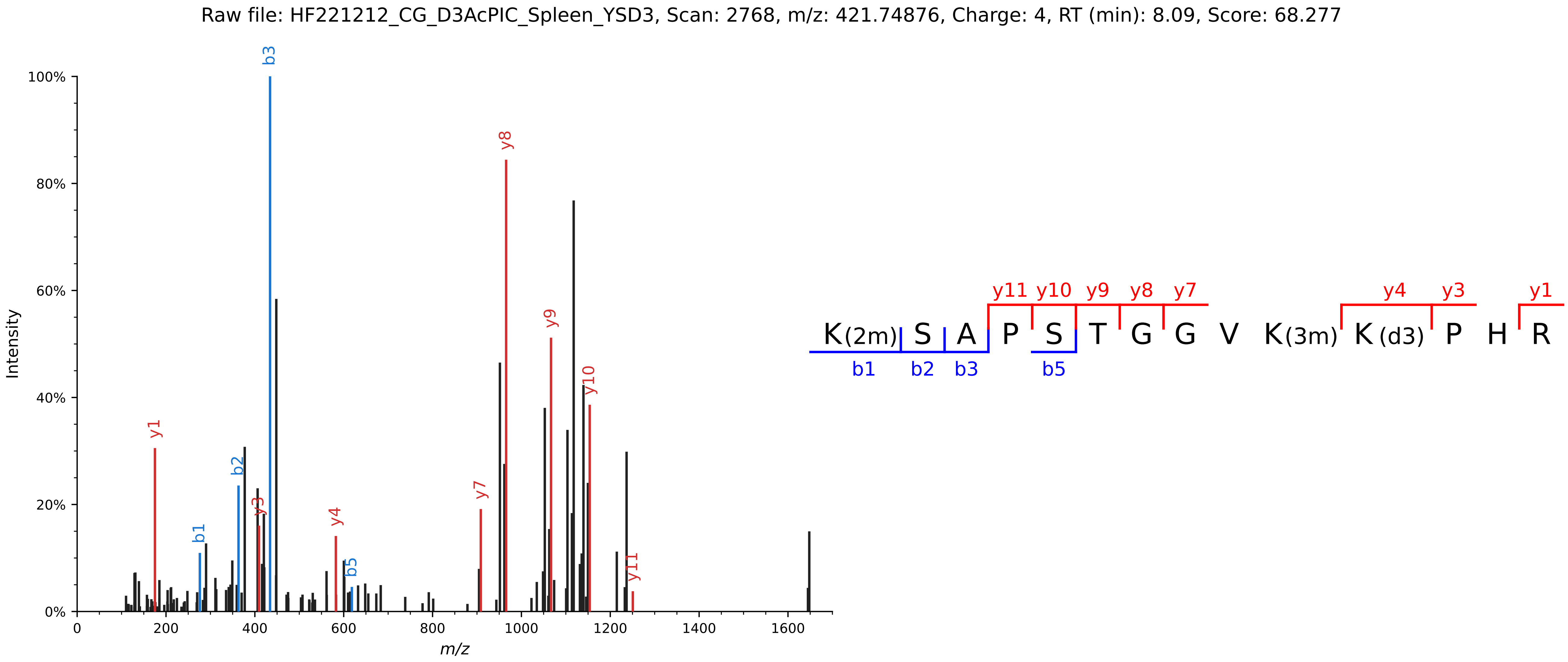

Raw file: HF221212_CG_D3AcPIC_Spleen_YSD3, Scan: 2768, m/z: 421.74876, Charge: 4, RT (min): 8.09, Score: 68.277
Intensity
m/z
0%
20%
40%
60%
80%
100%
0
200
400
600
800
1000
1200
1400
1600
b1
b2
b3
b5
y1
y3
y4
y7
y8
y9
y10
y11
K(2m) S A P S T G G V K(3m) K(d3) P H R
y11 y10 y9 y8 y7 y4 y3 y1
b1 b2 b3 b5

Raw file: HF221212_CG_D3AcPIC_Spleen_YSD3, Scan: 4440, m/z: 566.65568, Charge: 3, RT (min): 11.06, Score: 114.64

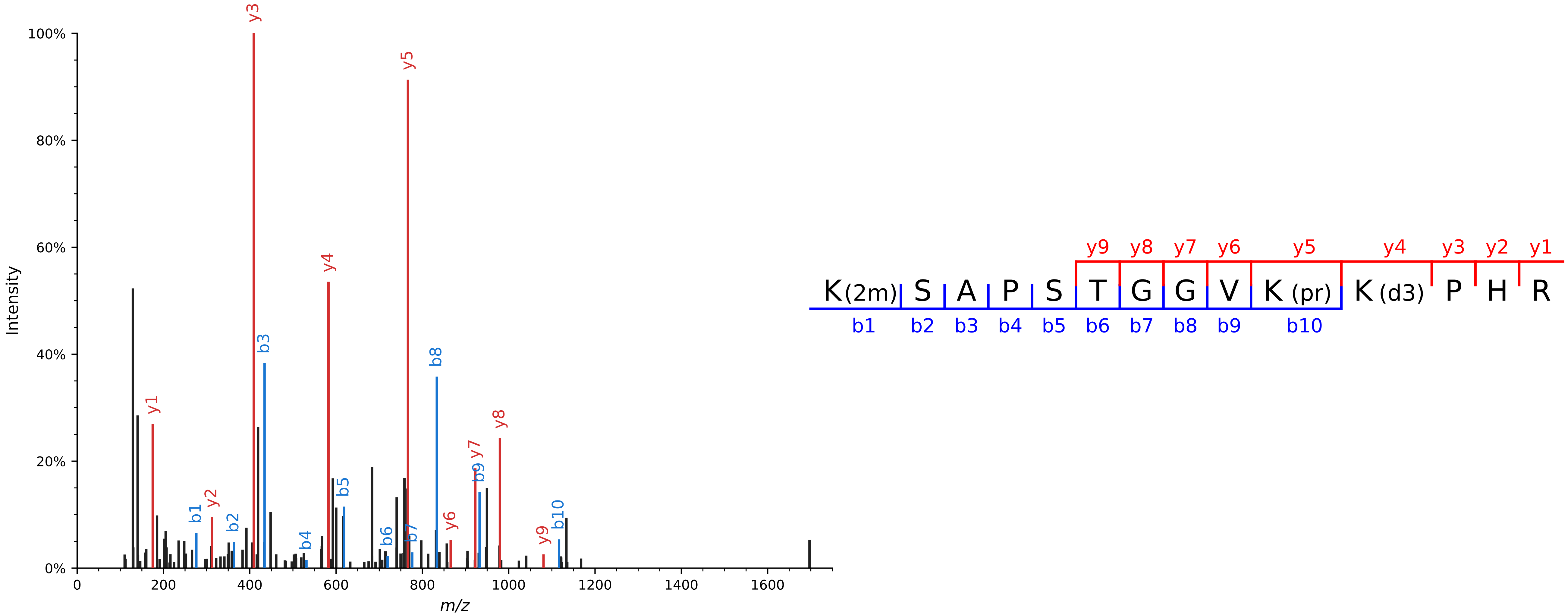

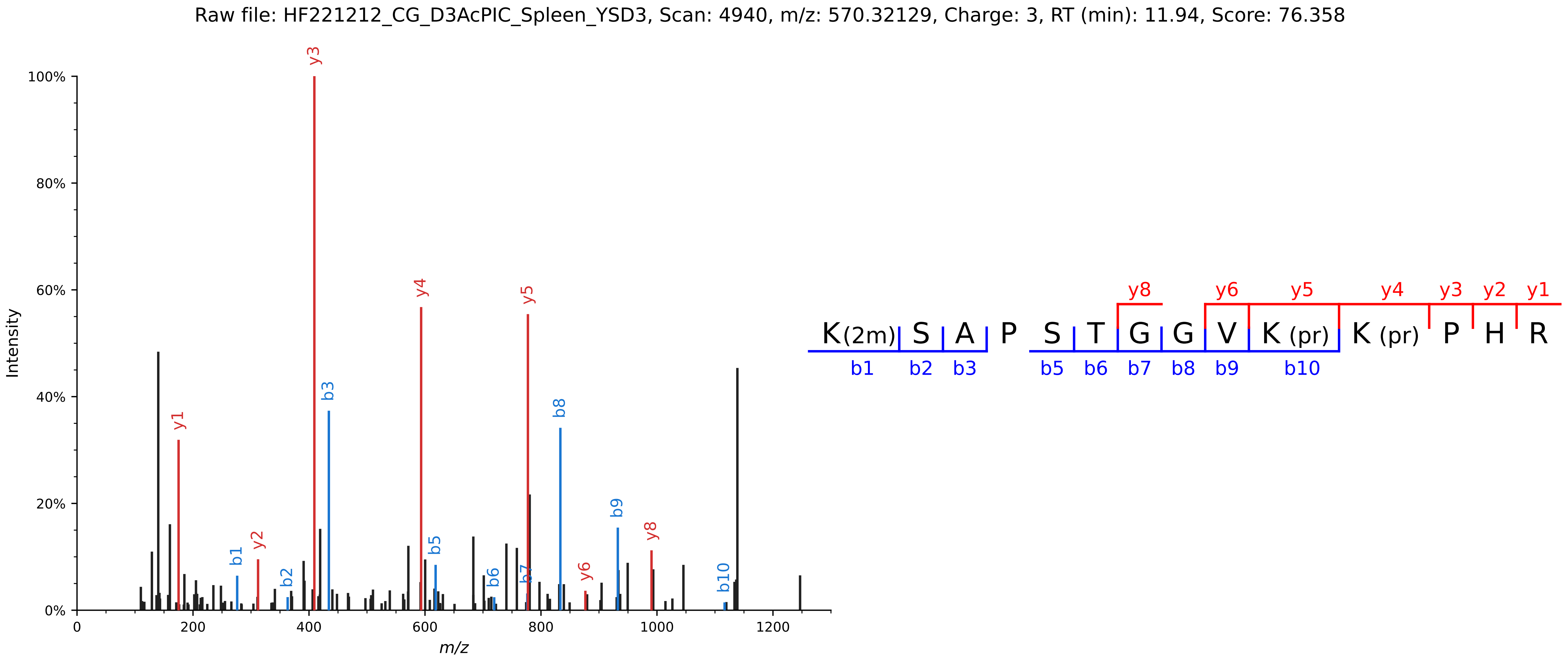

Raw file: HF221212_CG_D3AcPIC_Spleen_YSD3, Scan: 4940, m/z: 570.32129, Charge: 3, RT (min): 11.94, Score: 76.358
Intensity
m/z
0% 20% 40% 60% 80% 100%
0 200 400 600 800 1000 1200
y1 y2 y3 y4 y5 y6 y8
b1 b2 b3 b5 b6 b7 b8 b9 b10
K(2m) S A P S T G G V K (pr) K (pr) P H R

Raw file: HF221212_CG_D3AcPIC_Spleen_YSD2, Scan: 3933, m/z: 567.66196, Charge: 3, RT (min): 10.15, Score: 157.09

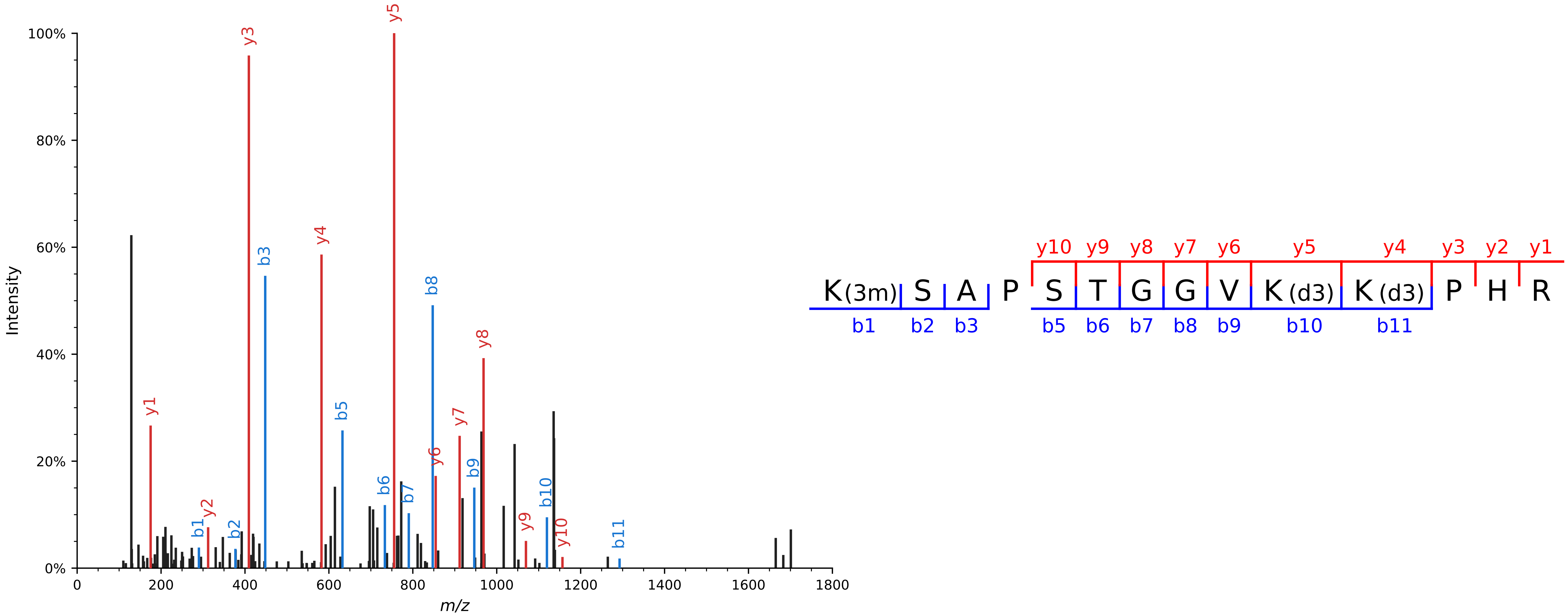

Raw file: HF221212_CG_D3AcPIC_Liver_YSD2, Scan: 4454, m/z: 572.33384, Charge: 3, RT (min): 11.10, Score: 141.34

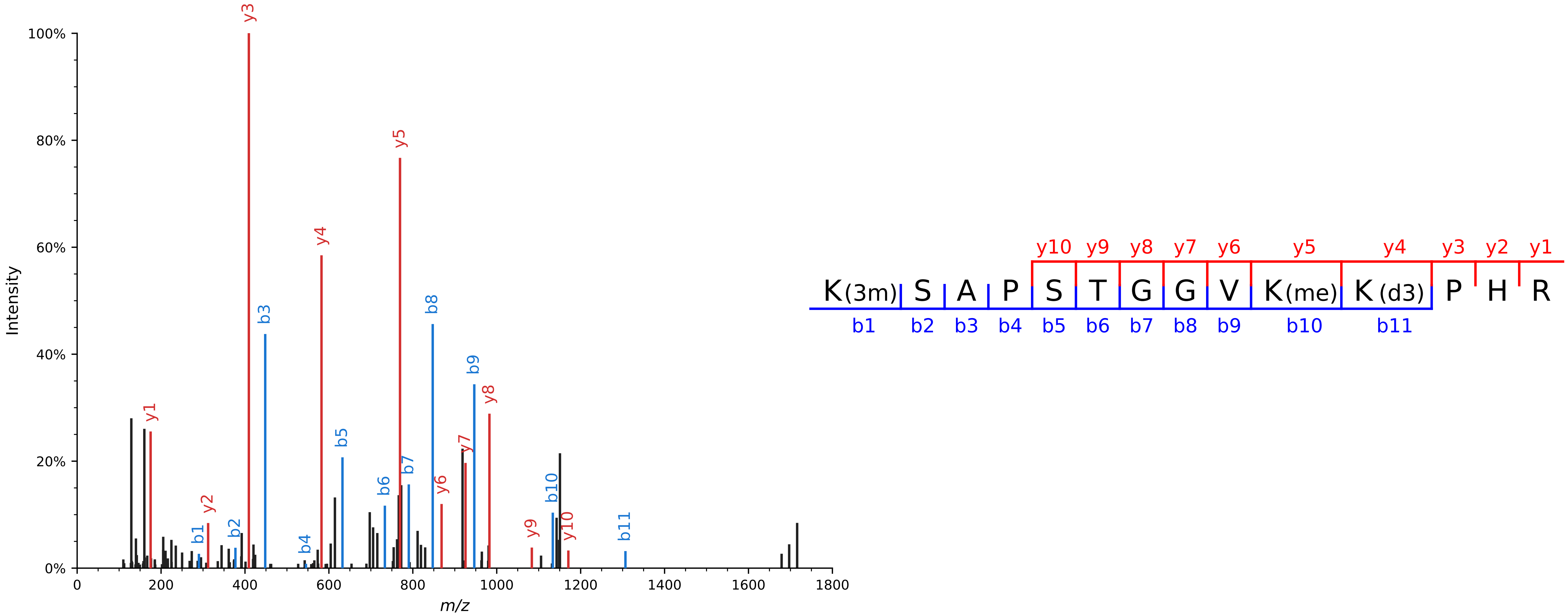

Raw file: HF221212_CG_D3AcPIC_Spleen_YSD2, Scan: 2384, m/z: 433.00611, Charge: 4, RT (min): 7.33, Score: 40.025

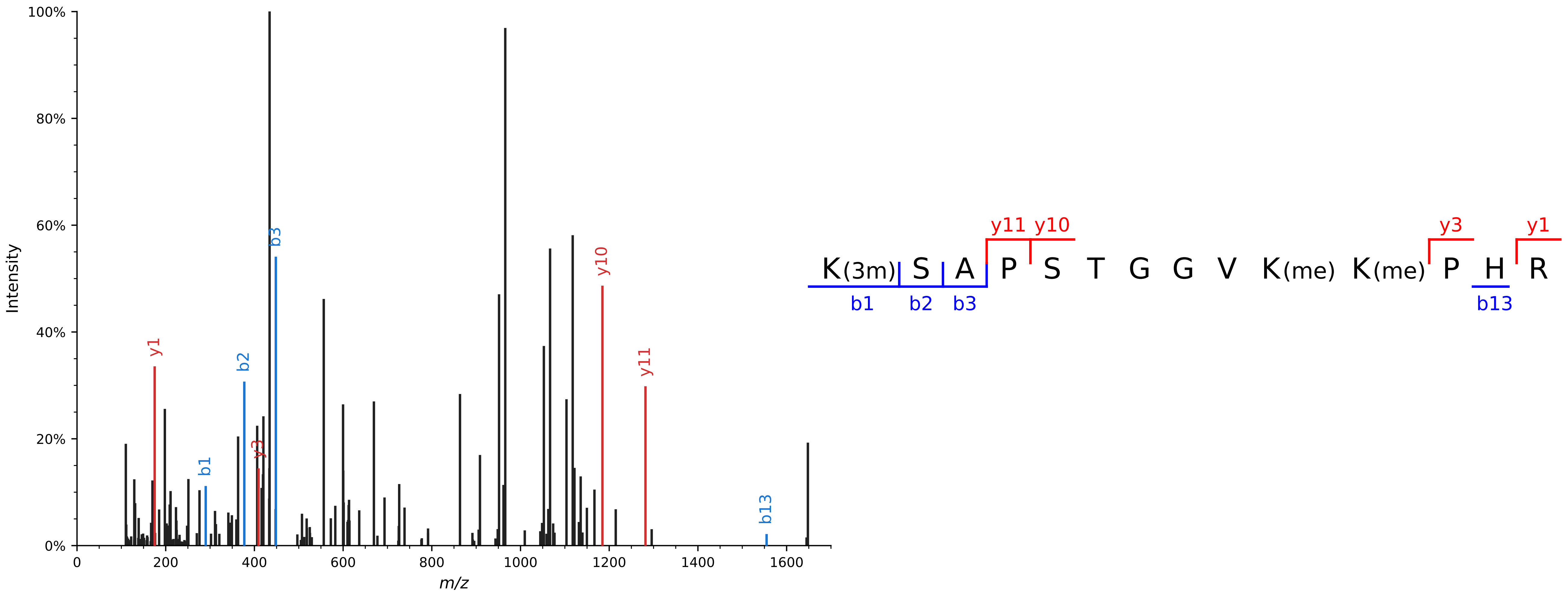

Raw file: HF221212_CG_D3AcPIC_Kidney_YSD1, Scan: 3749, m/z: 561.99593, Charge: 3, RT (min): 10.52, Score: 134.14

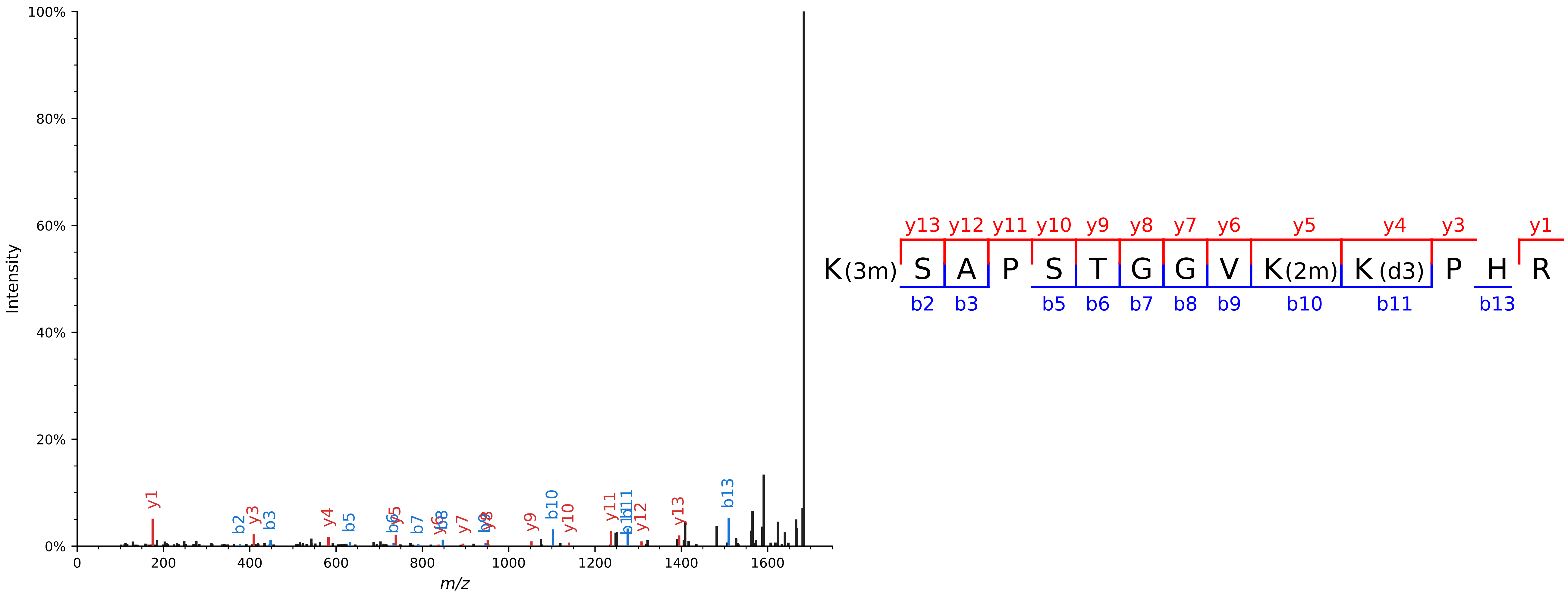

Raw file: HF221212_CG_D3AcPIC_Spleen_YSD1, Scan: 8948, m/z: 857.97893, Charge: 2, RT (min): 18.82, Score: 126.19

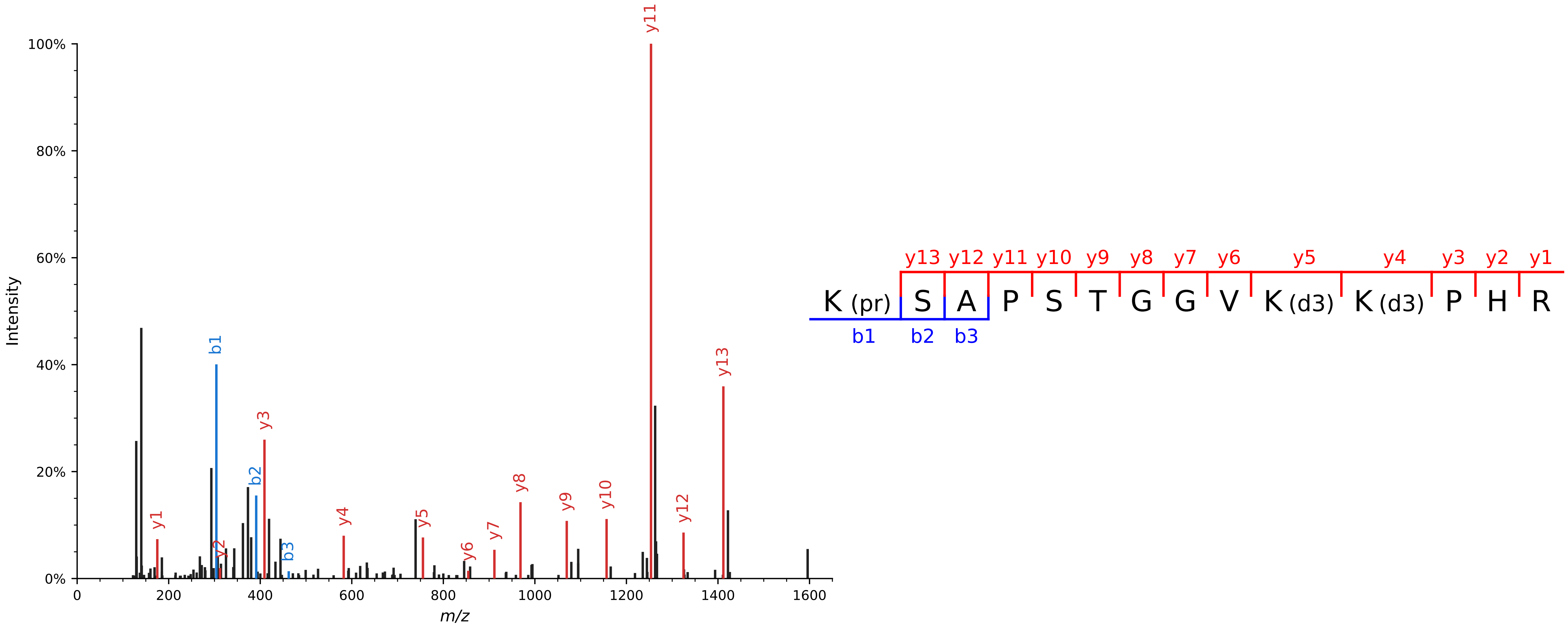

Raw file: HF221212_CG_D3AcPIC_Kidney_YSD1, Scan: 8355, m/z: 859.48835, Charge: 2, RT (min): 18.56, Score: 162.36

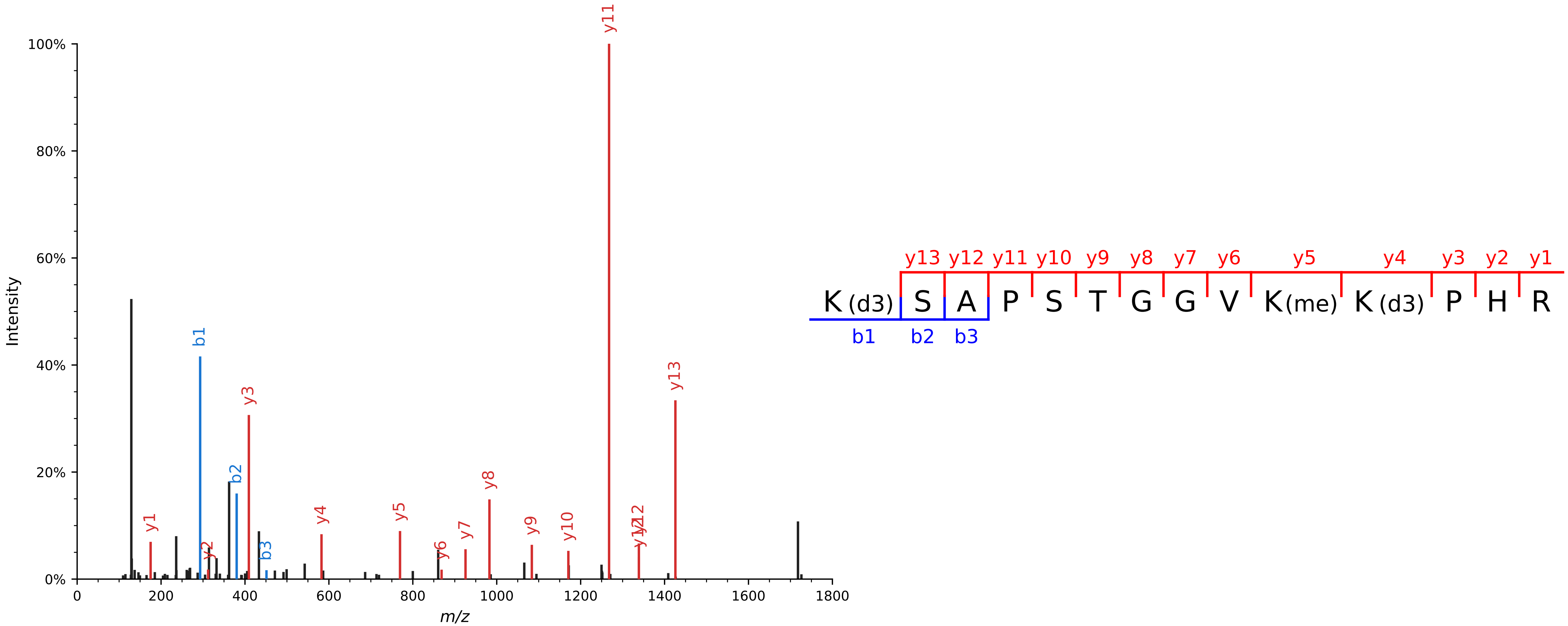

Raw file: HF221212_CG_D3AcPIC_Spleen_YSD1, Scan: 5406, m/z: 866.49617, Charge: 2, RT (min): 12.53, Score: 84.687

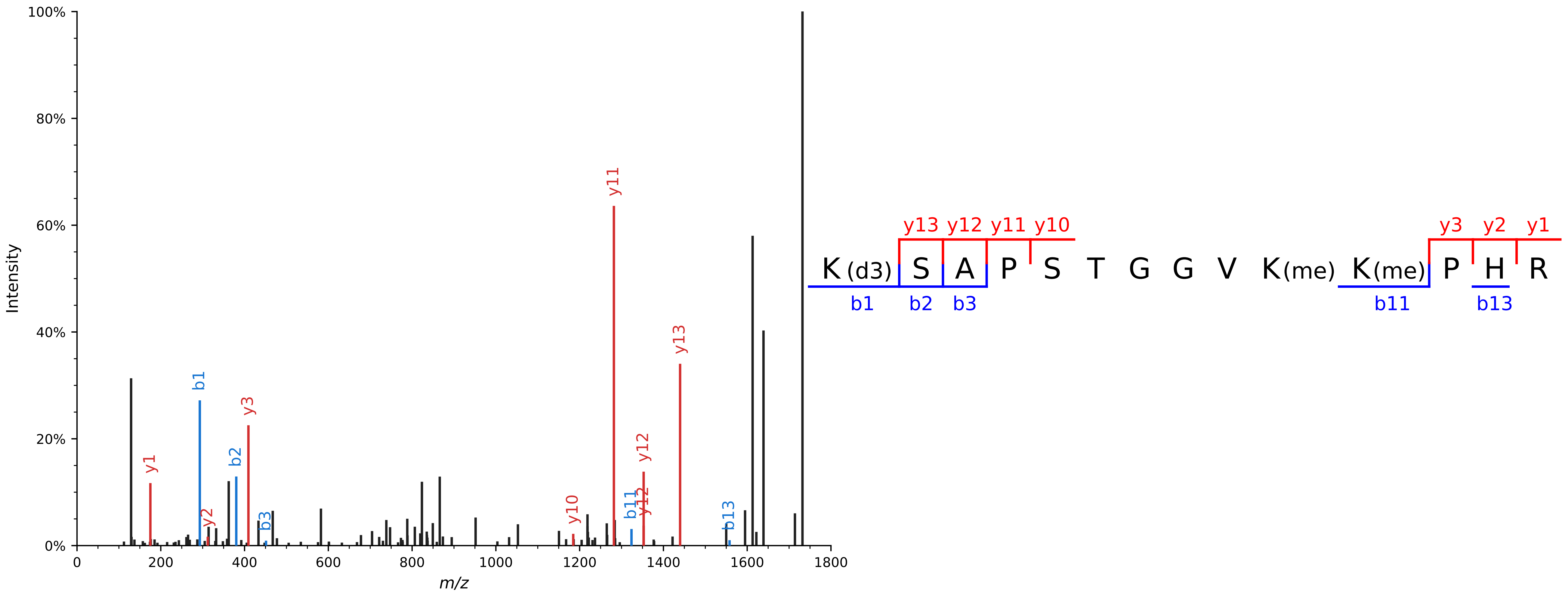

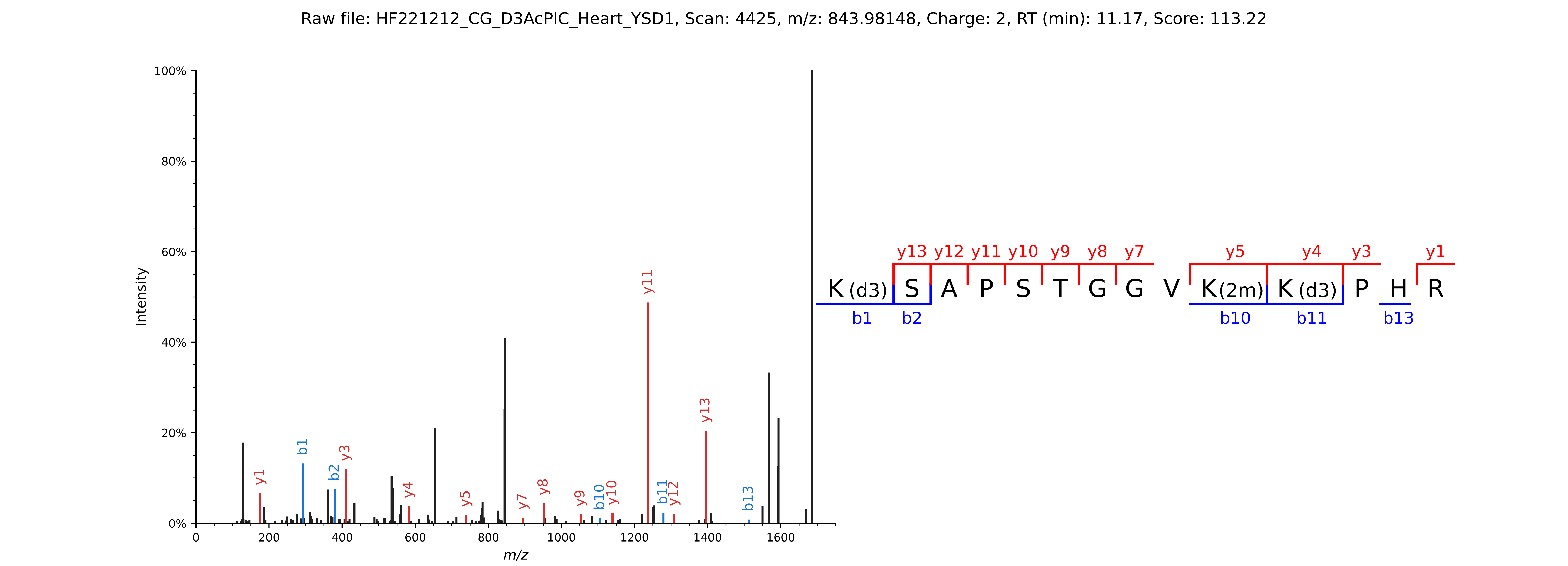

Raw file: HF221212_CG_D3AcPIC_Heart_YSD1, Scan: 4425, m/z: 843.98148, Charge: 2, RT (min): 11.17, Score: 113.22
Intensity
m/z
100%
80%
60%
40%
20%
0%
0
200
400
600
800
1000
1200
1400
1600
y1 b1 b2 y3 y4 y5 y7 y8 y9 b10 y10 y11 b11 y12 y13 b13
K(d3) S A P S T G G V K(2m) K(d3) P H R
y13 y12 y11 y10 y9 y8 y7 y5 y4 y3 y1
b1 b2 b10 b11 b13

Raw file: HF221212_CG_D3AcPIC_Spleen_YSD1, Scan: 4642, m/z: 850.9893, Charge: 2, RT (min): 11.22, Score: 102.52

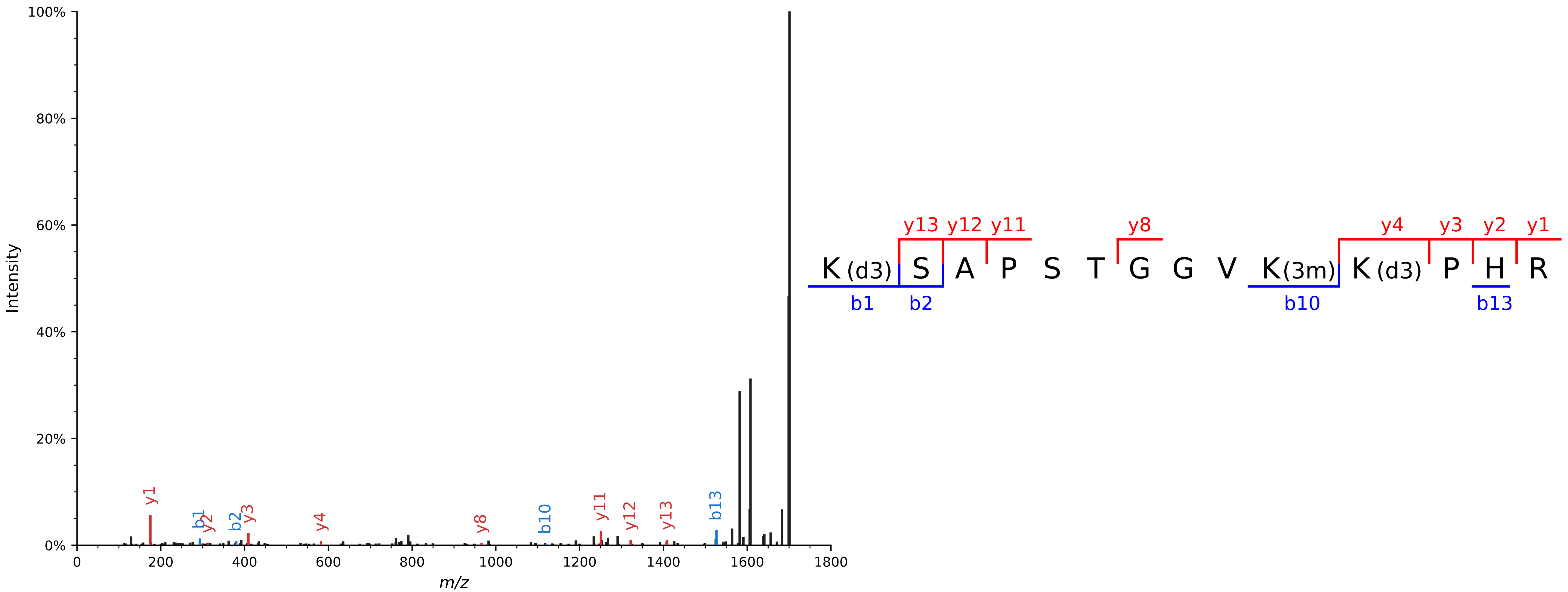

Raw file: HF221212_CG_D3AcPIC_Spleen_YSD2, Scan: 8523, m/z: 857.97893, Charge: 2, RT (min): 18.27, Score: 160.36

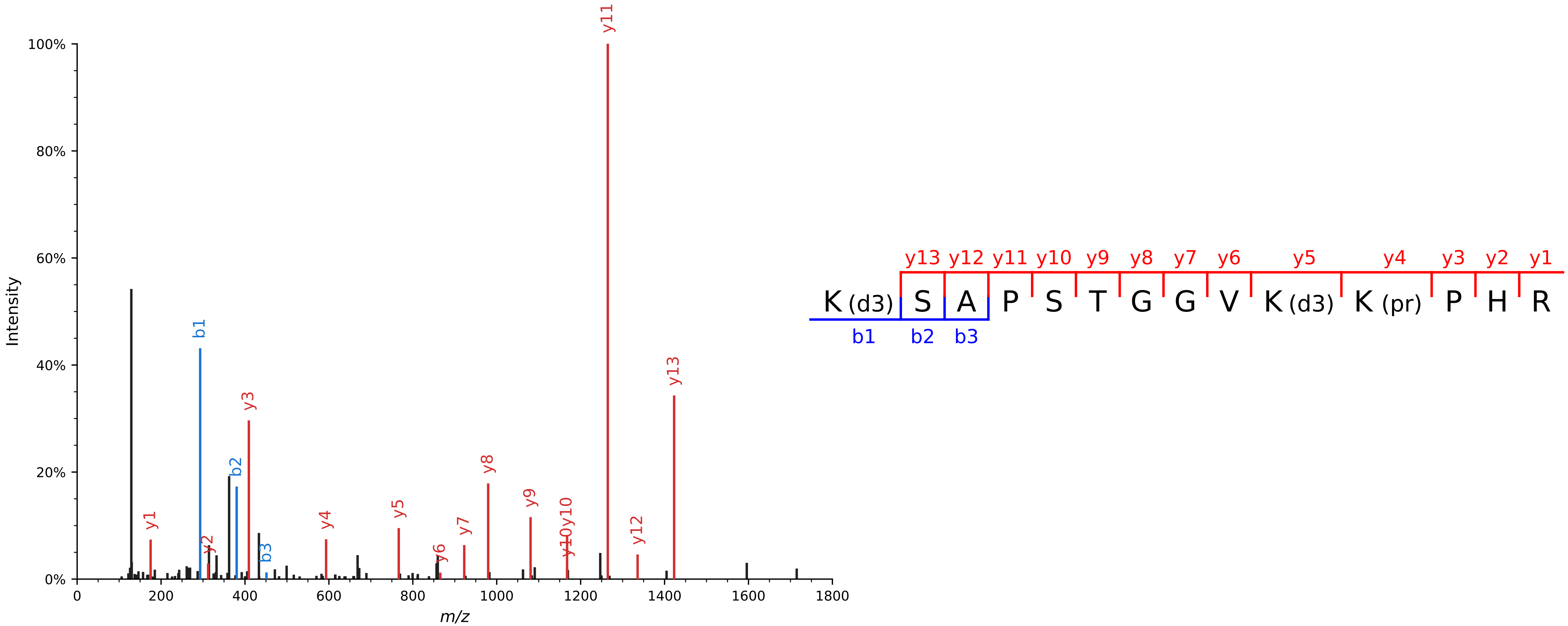

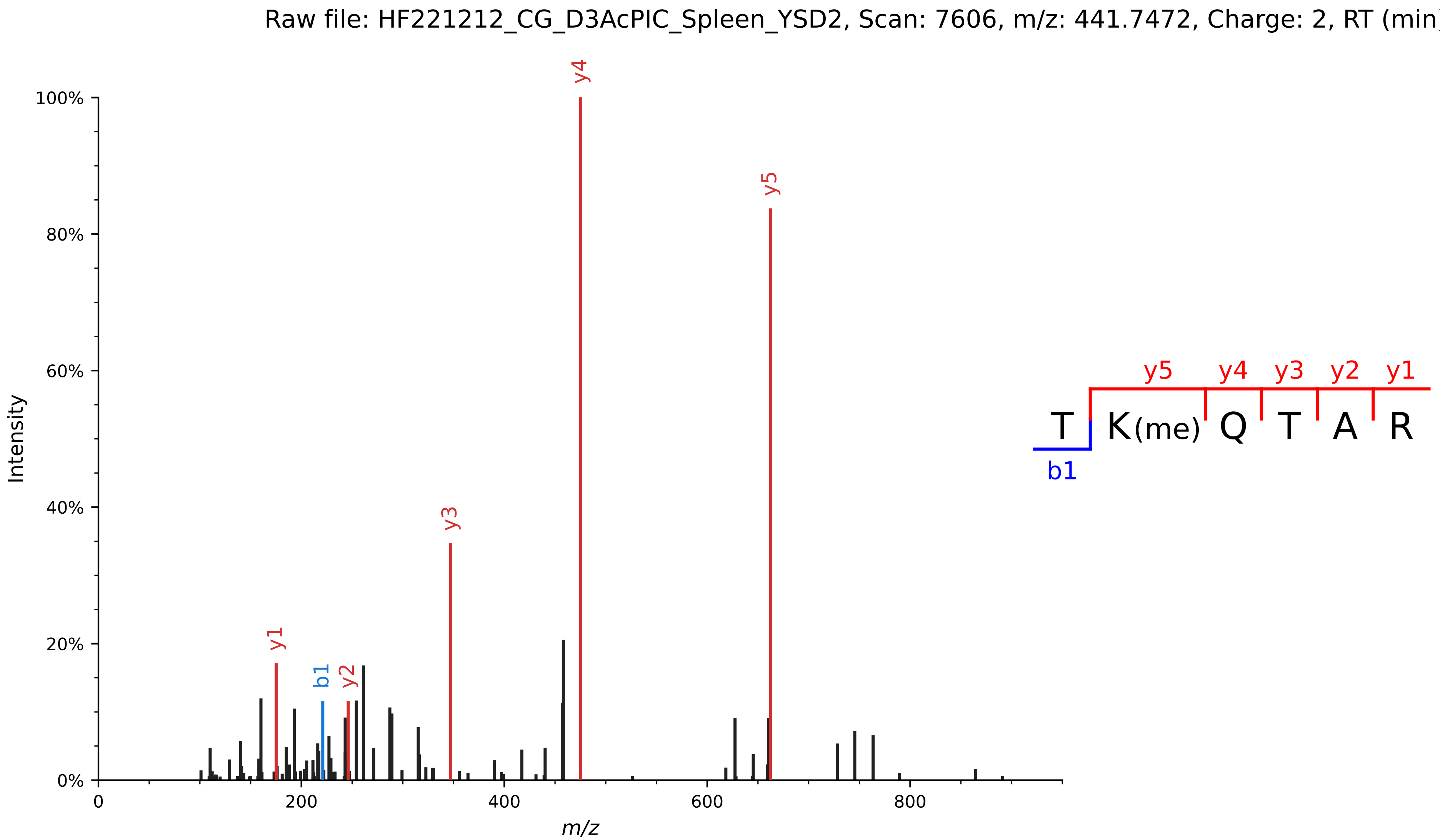

Raw file: HF221212_CG_D3AcPIC_Spleen_YSD2, Scan: 7606, m/z: 441.7472, Charge: 2, RT (min): 16.65, Score: 97.64
Intensity
m/z
y1
b1
y2
y3
y4
y5
T K(me) Q T A R

Raw file: HF221212_CG_D3AcPIC_Brain_YSD3, Scan: 1870, m/z: 426.24033, Charge: 2, RT (min): 6.68, Score: 148.33

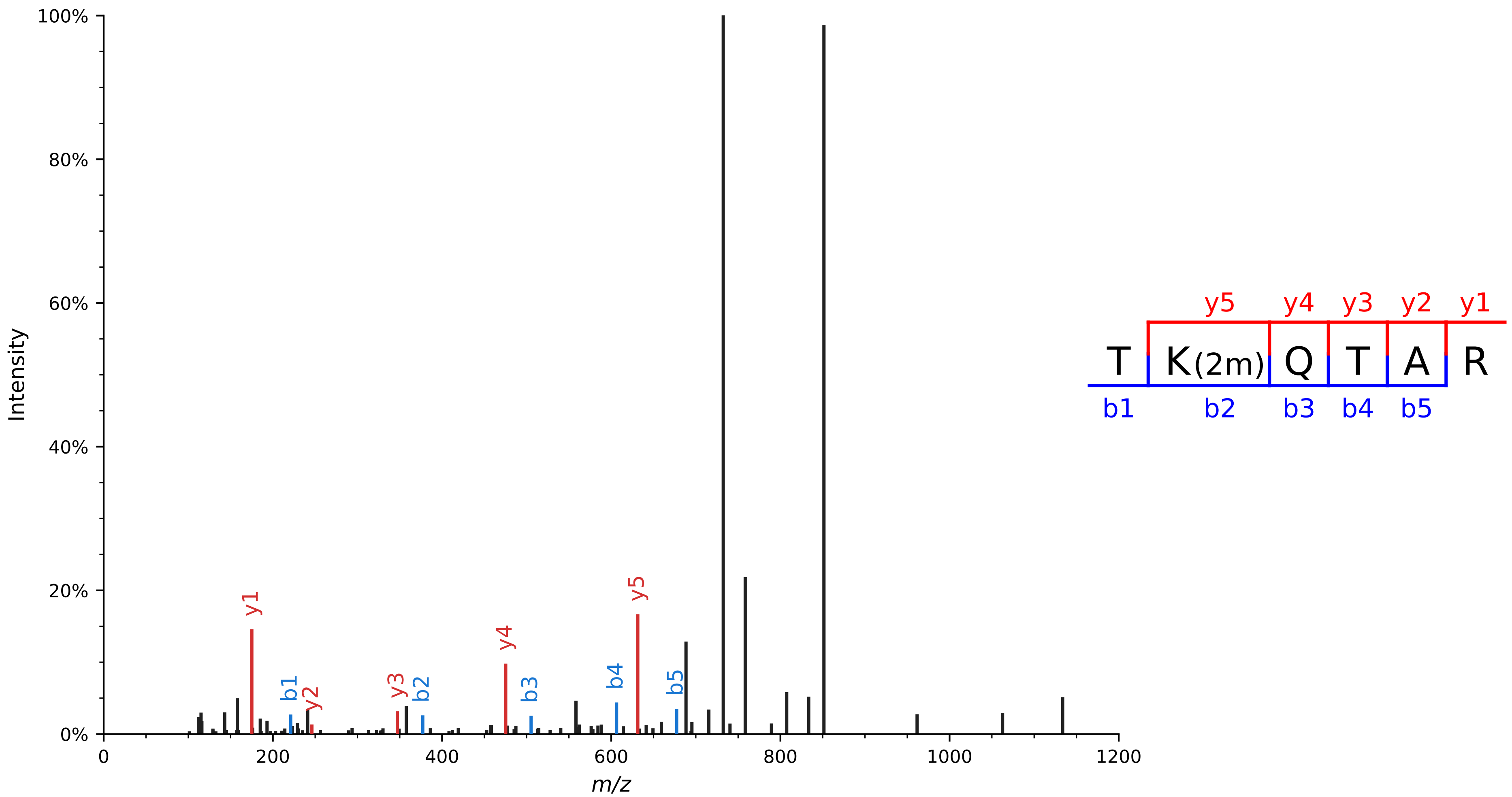

Raw file: HF221212_CG_D3AcPIC_Spleen_YSD3, Scan: 2443, m/z: 433.24815, Charge: 2, RT (min): 7.55, Score: 119.57

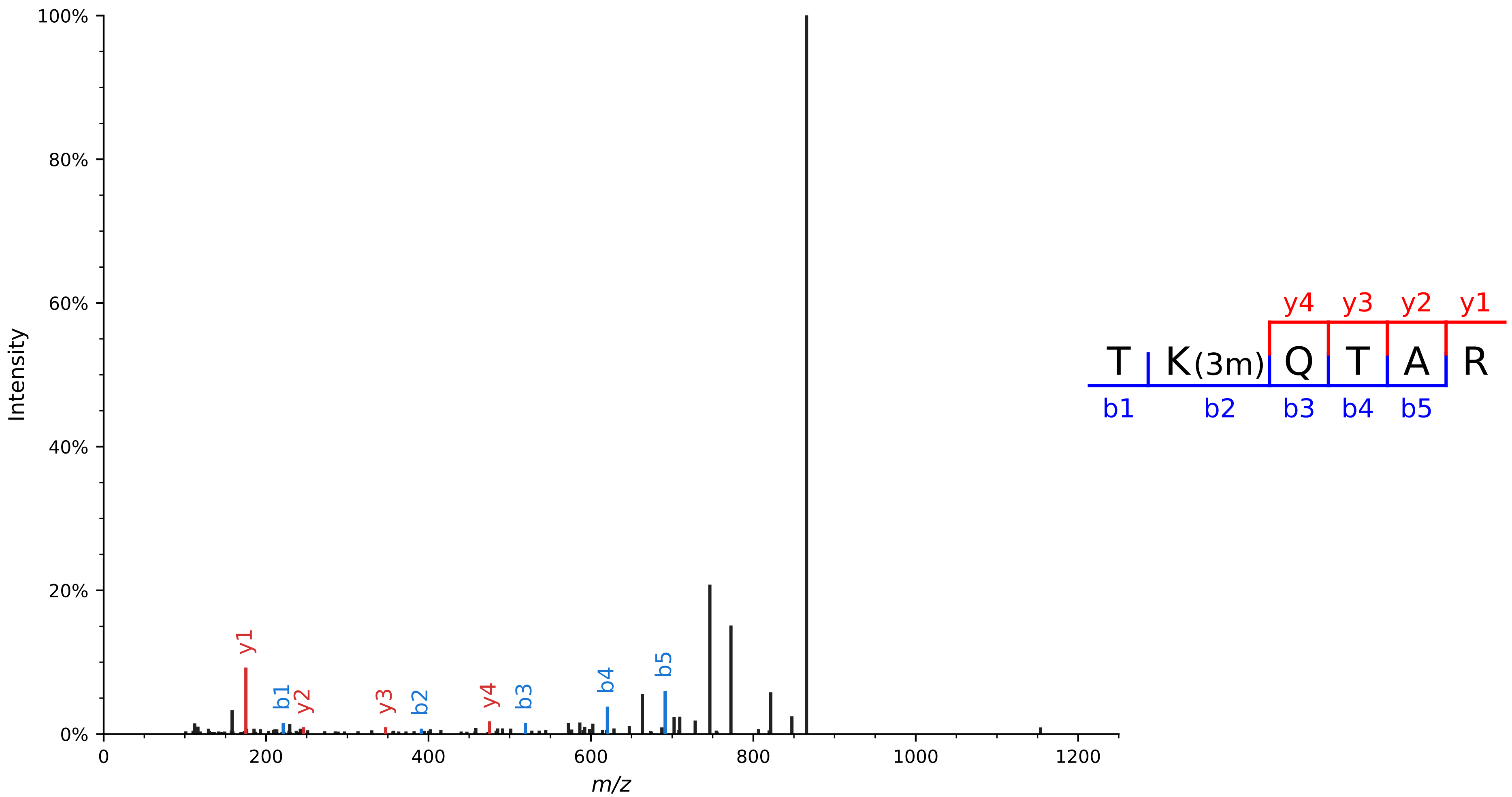

Raw file: HF221212_CG_D3AcPIC_Heart_YSD3, Scan: 7571, m/z: 440.23778, Charge: 2, RT (min): 16.75, Score: 110.54

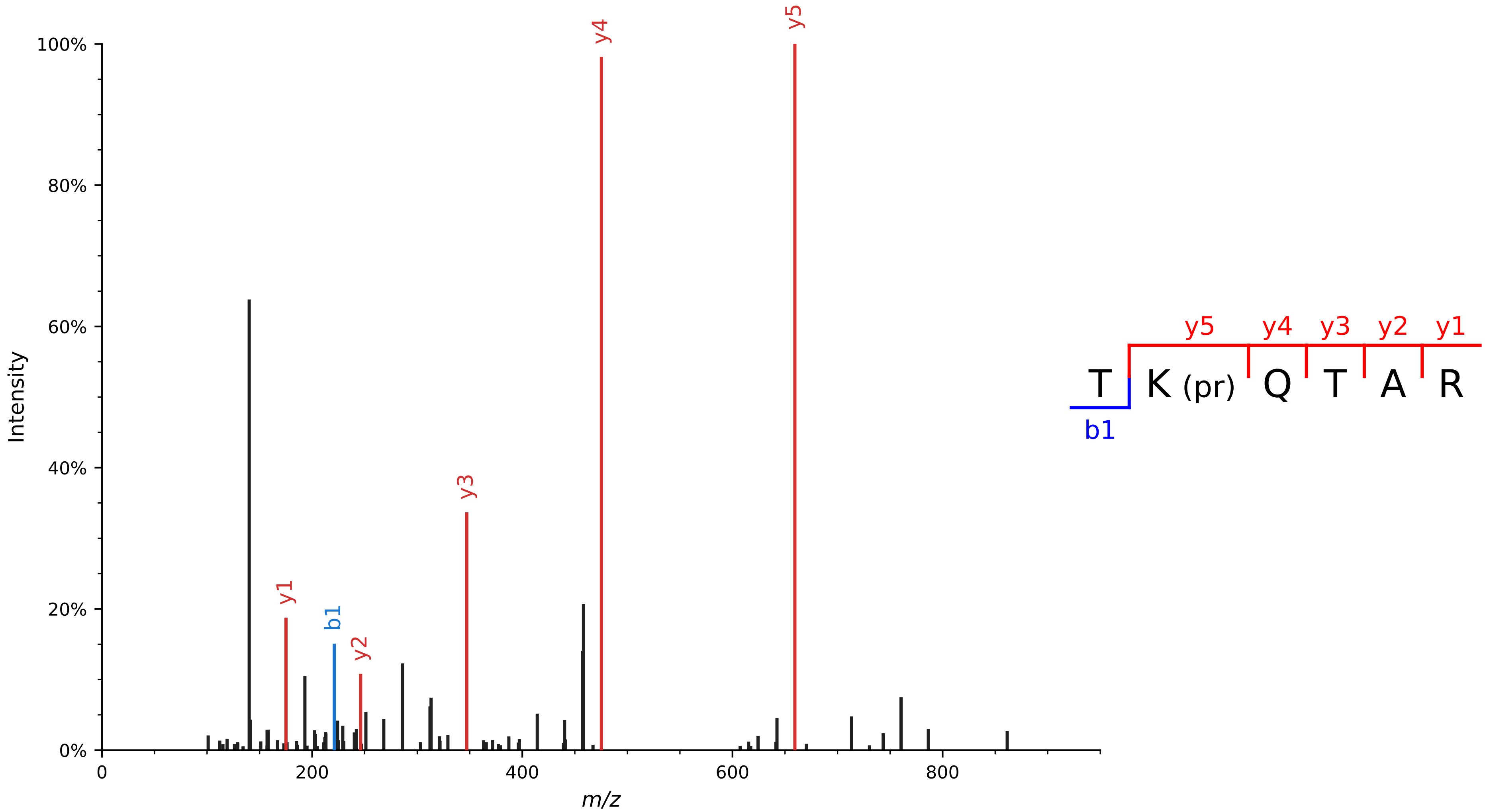

Raw file: HF221212_CG_D3AcPIC_Heart_YSD3, Scan: 7756, m/z: 552.79345, Charge: 2, RT (min): 17.07, Score: 111.95

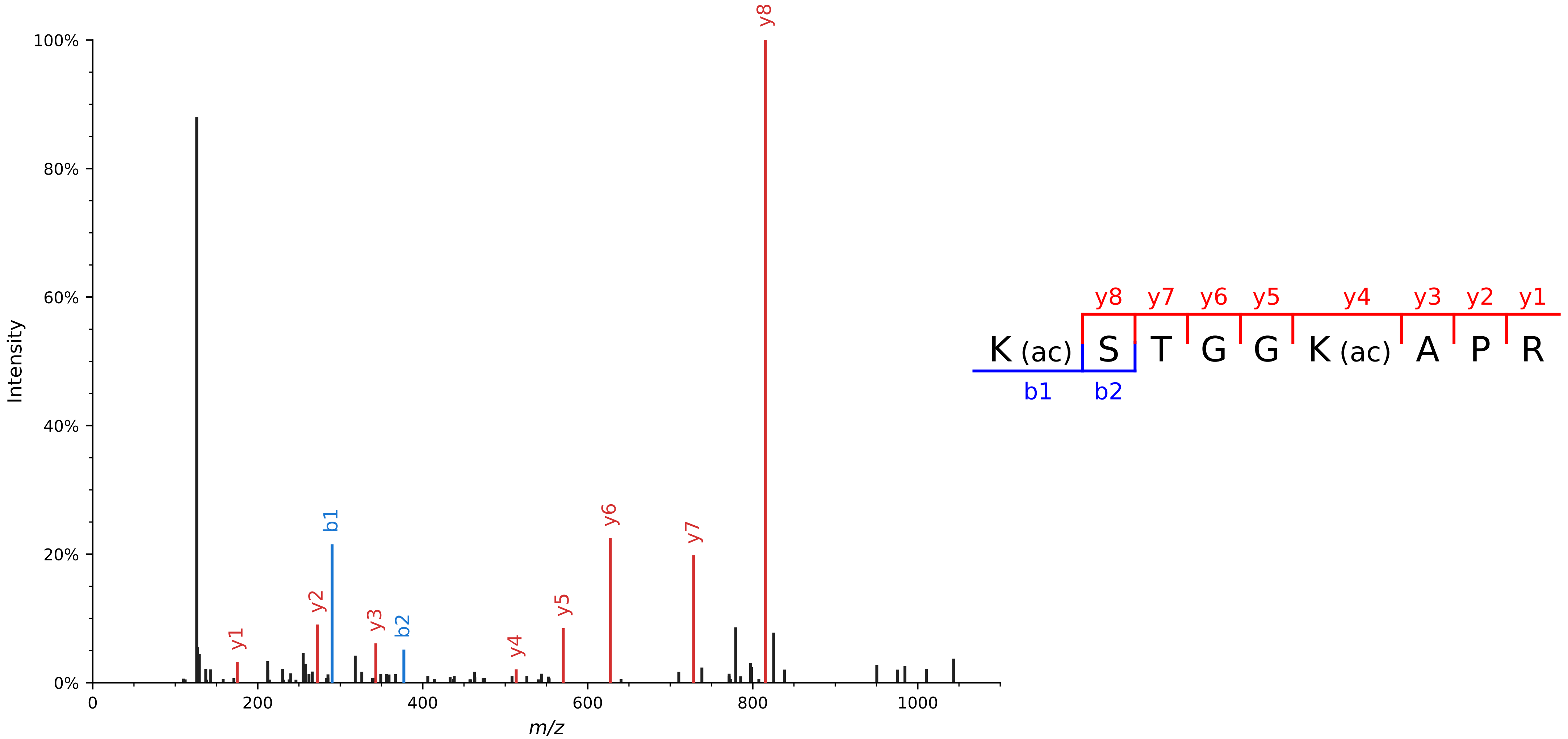

Raw file: HF221212_CG_D3AcPIC_Spleen_YSD3, Scan: 8991, m/z: 559.80128, Charge: 2, RT (min): 19.19, Score: 102.51

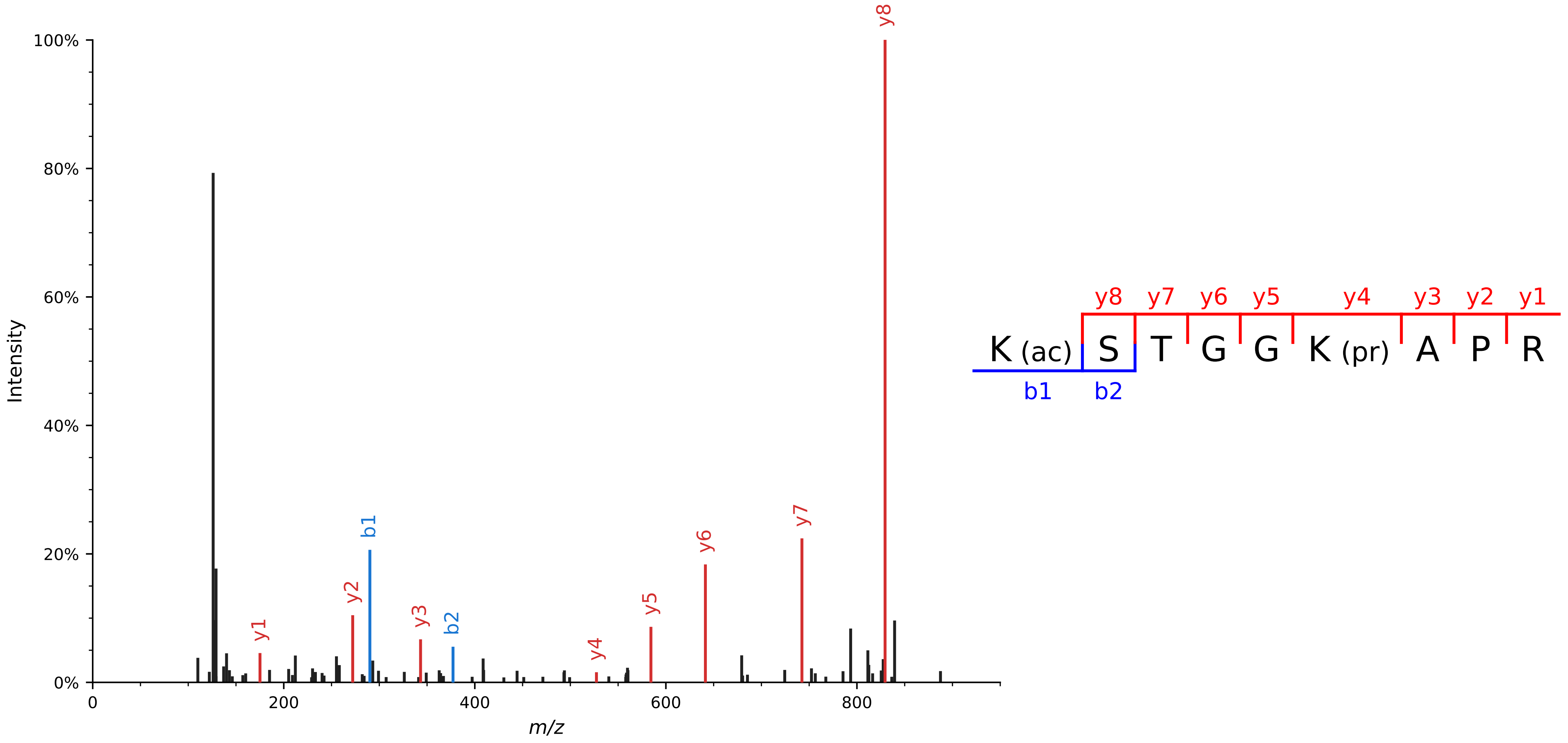

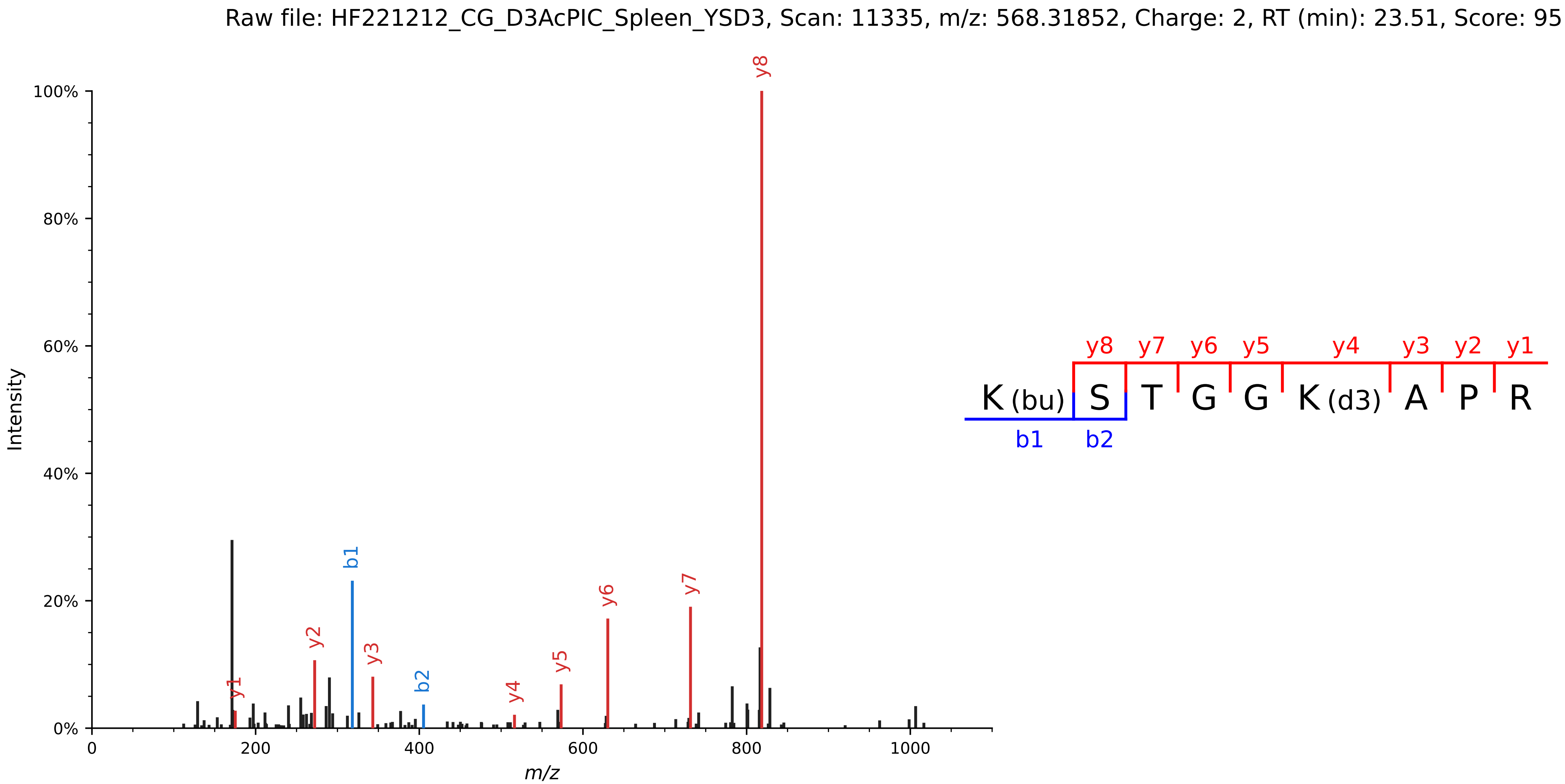

Raw file: HF221212_CG_D3AcPIC_Spleen_YSD3, Scan: 11335, m/z: 568.31852, Charge: 2, RT (min): 23.51, Score: 95.909
Intensity
100%
80%
60%
40%
20%
0%
0
200
400
600
800
1000
m/z
y1
y2
y3
b1
b2
y4
y5
y6
y7
y8
K (bu) S T G G K (d3) A P R
y8 y7 y6 y5 y4 y3 y2 y1
b1 b2

Raw file: HF221212_CG_D3AcPIC_Spleen_YSD1, Scan: 11642, m/z: 566.8091, Charge: 2, RT (min): 23.67, Score: 113.71

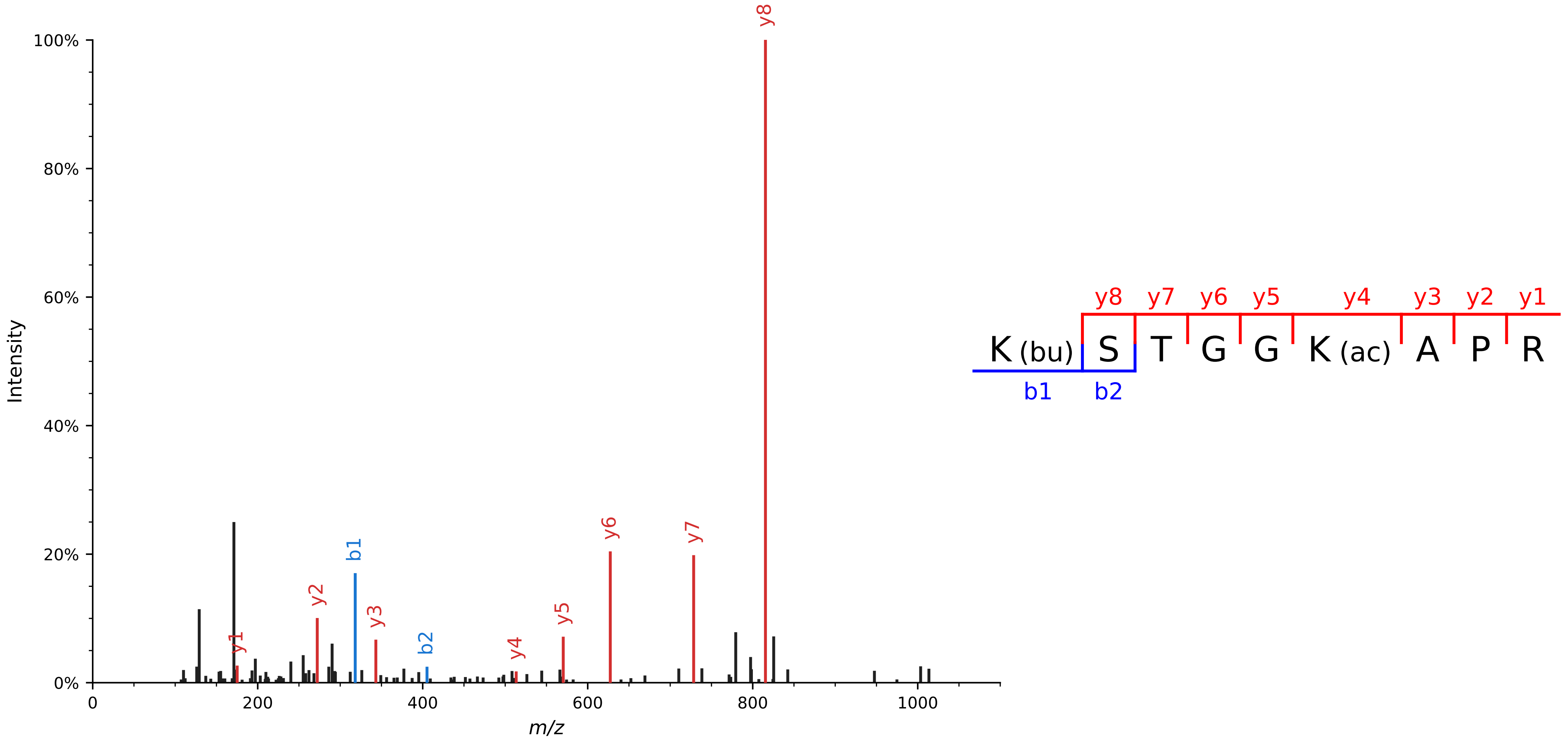

Raw file: HF221212_CG_D3AcPIC_Spleen_YSD1, Scan: 7509, m/z: 547.29504, Charge: 2, RT (min): 16.26, Score: 55.676

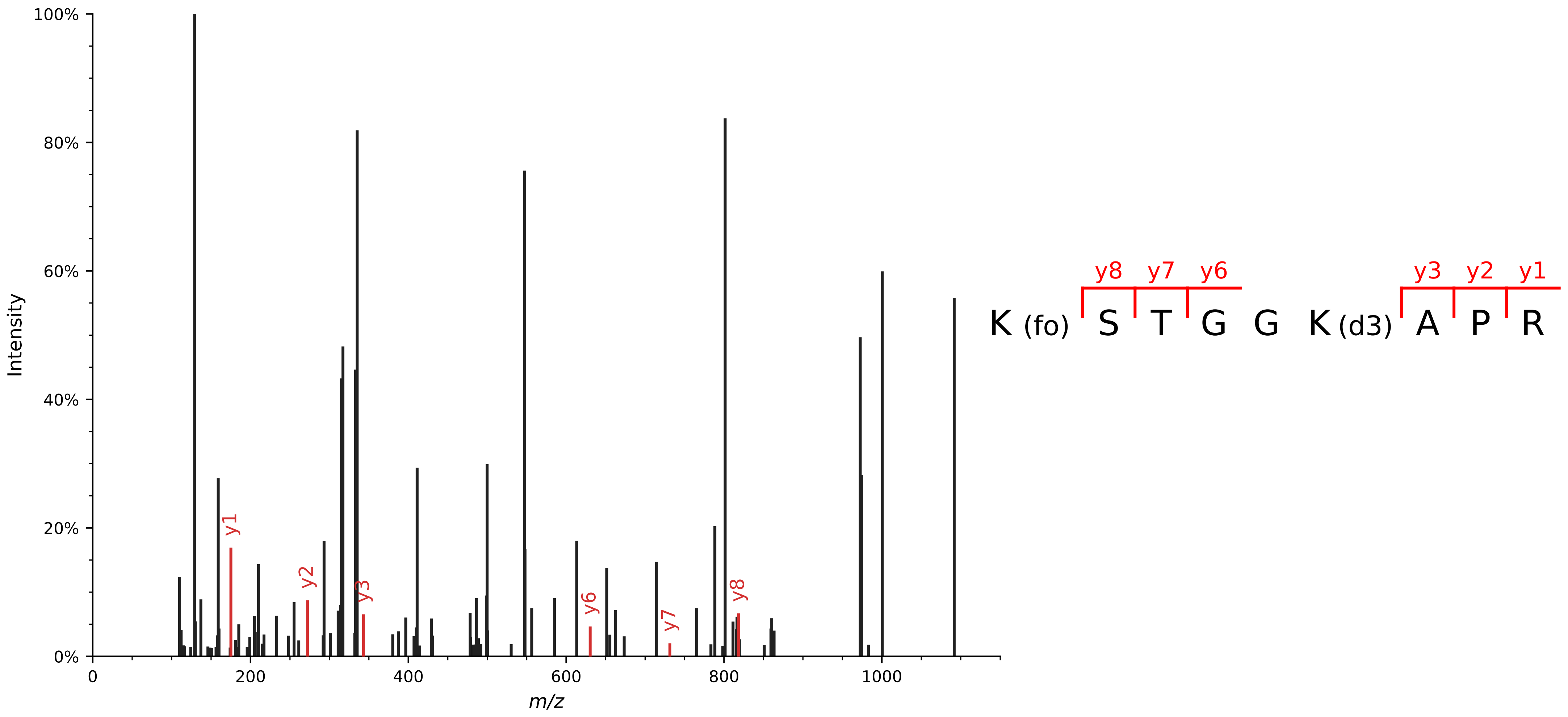

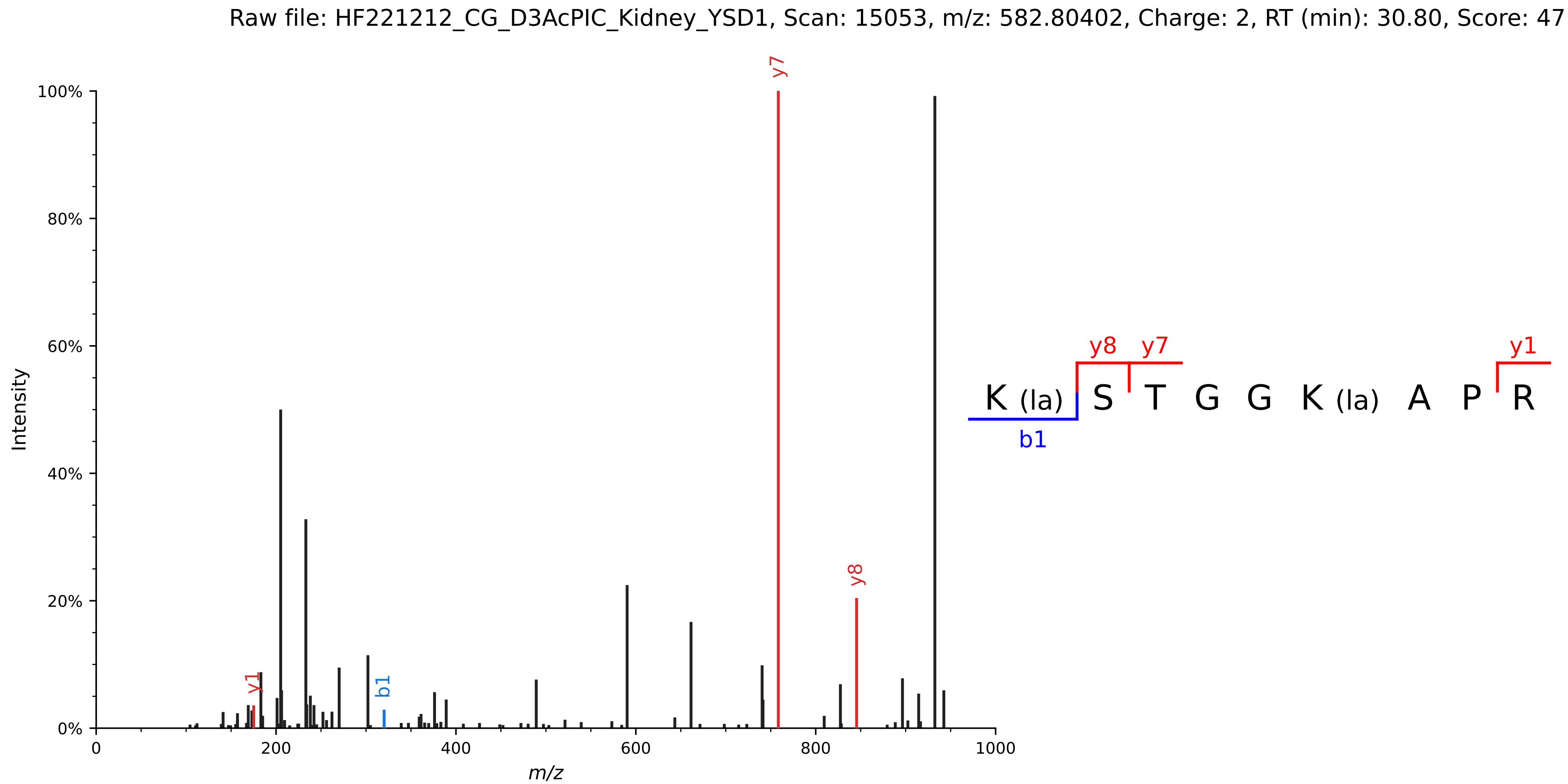
Raw file: HF221212_CG_D3AcPIC_Kidney_YSD1, Scan: 15053, m/z: 582.80402, Charge: 2, RT (min): 30.80, Score: 47.082
Intensity
m/z
y7
y8
y1
b1
K (la) S T G G K (la) A P R

Raw file: HF221212_CG_D3AcPIC_Heart_YSD3, Scan: 9188, m/z: 562.82011, Charge: 2, RT (min): 19.64, Score: 107.29

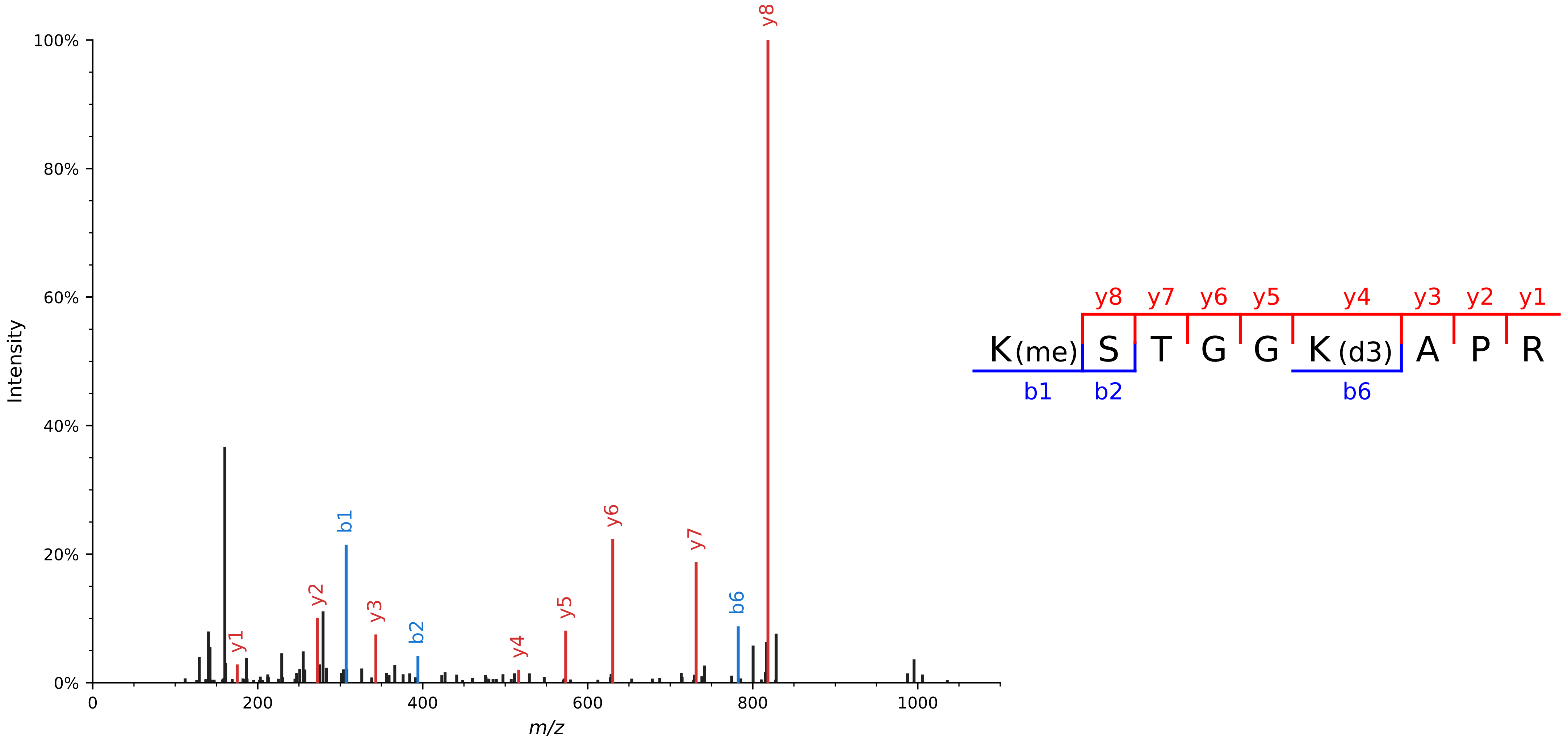

Raw file: HF221212_CG_D3AcPIC_Spleen_YSD2, Scan: 9414, m/z: 561.3107, Charge: 2, RT (min): 19.87, Score: 111.95

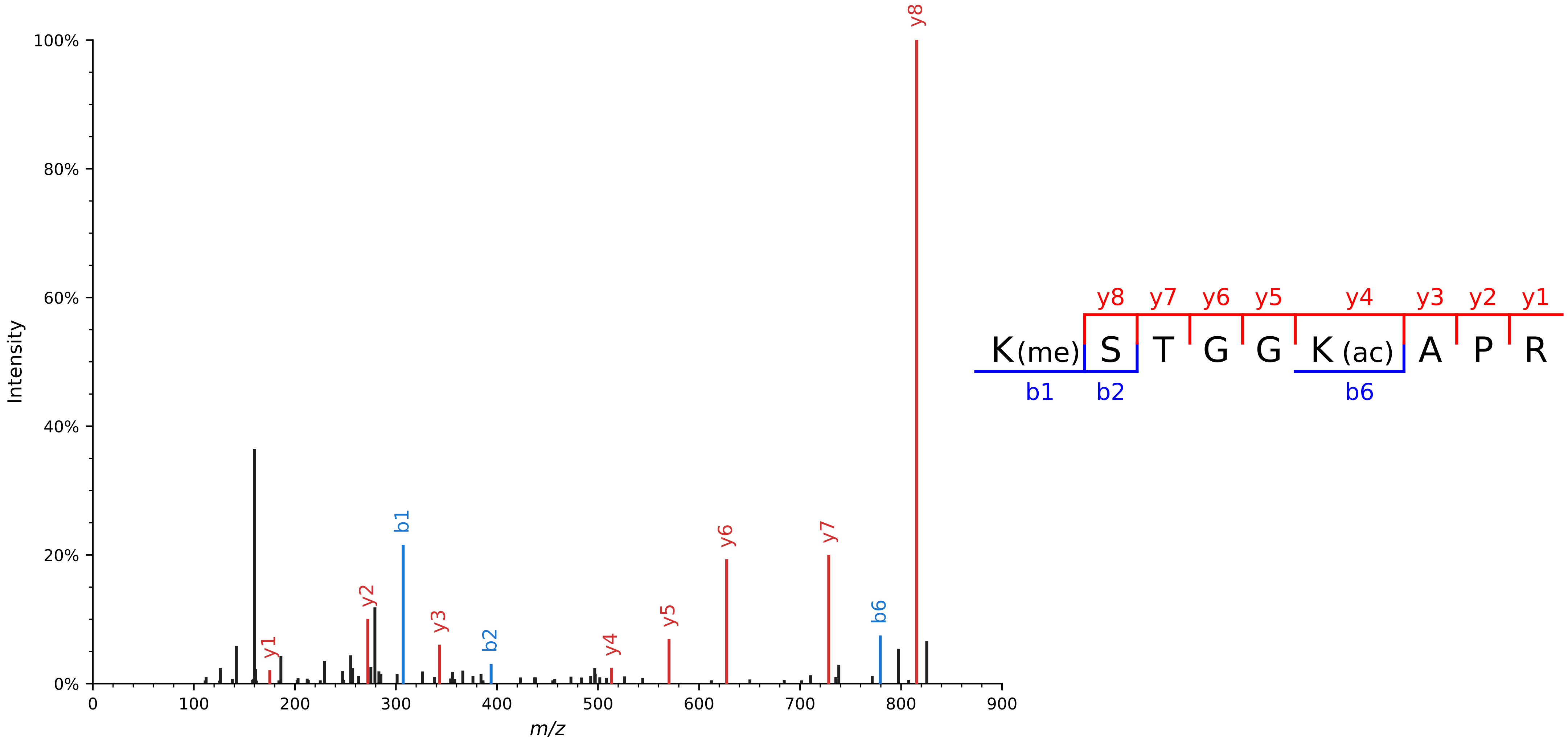

Raw file: HF221212_CG_D3AcPIC_Spleen_YSD3, Scan: 4759, m/z: 575.32635, Charge: 2, RT (min): 11.63, Score: 53.968

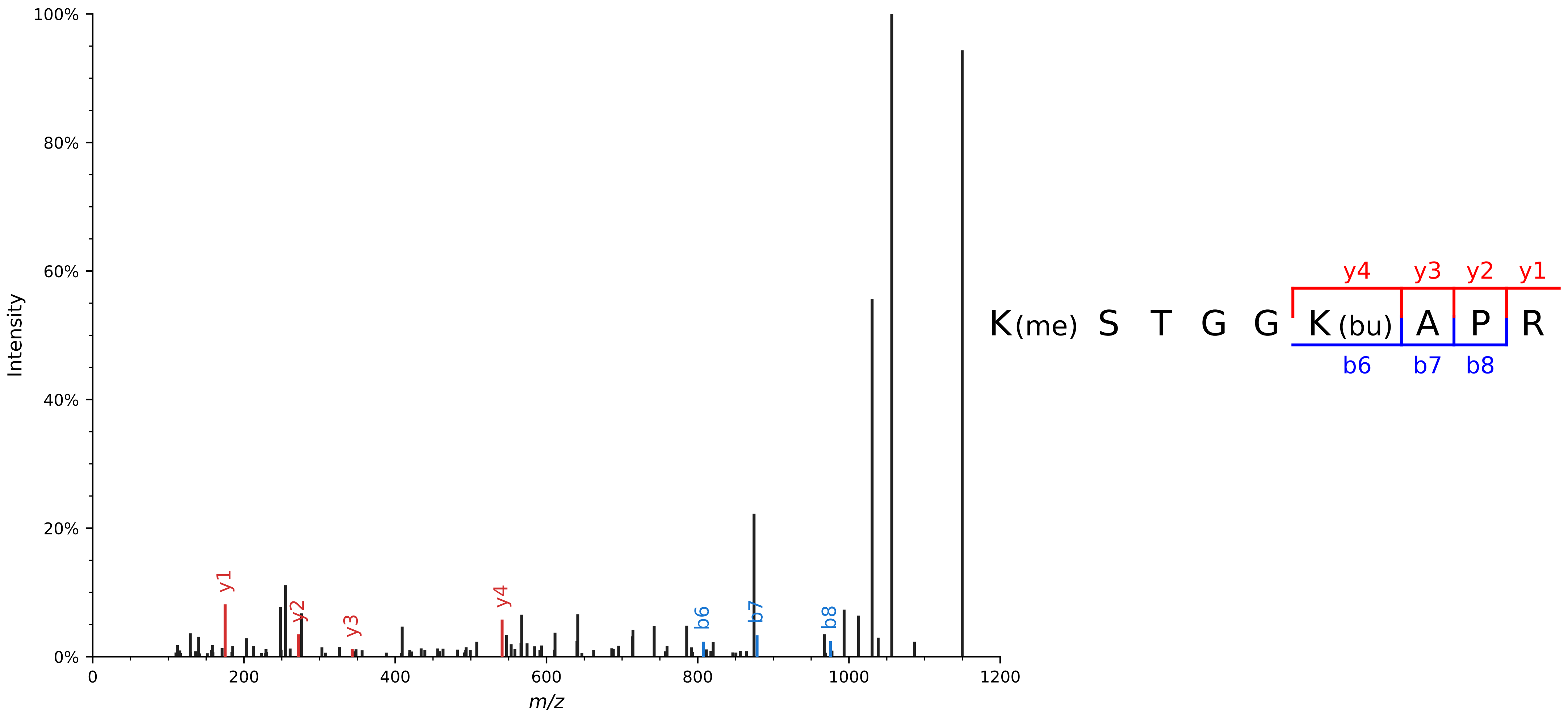

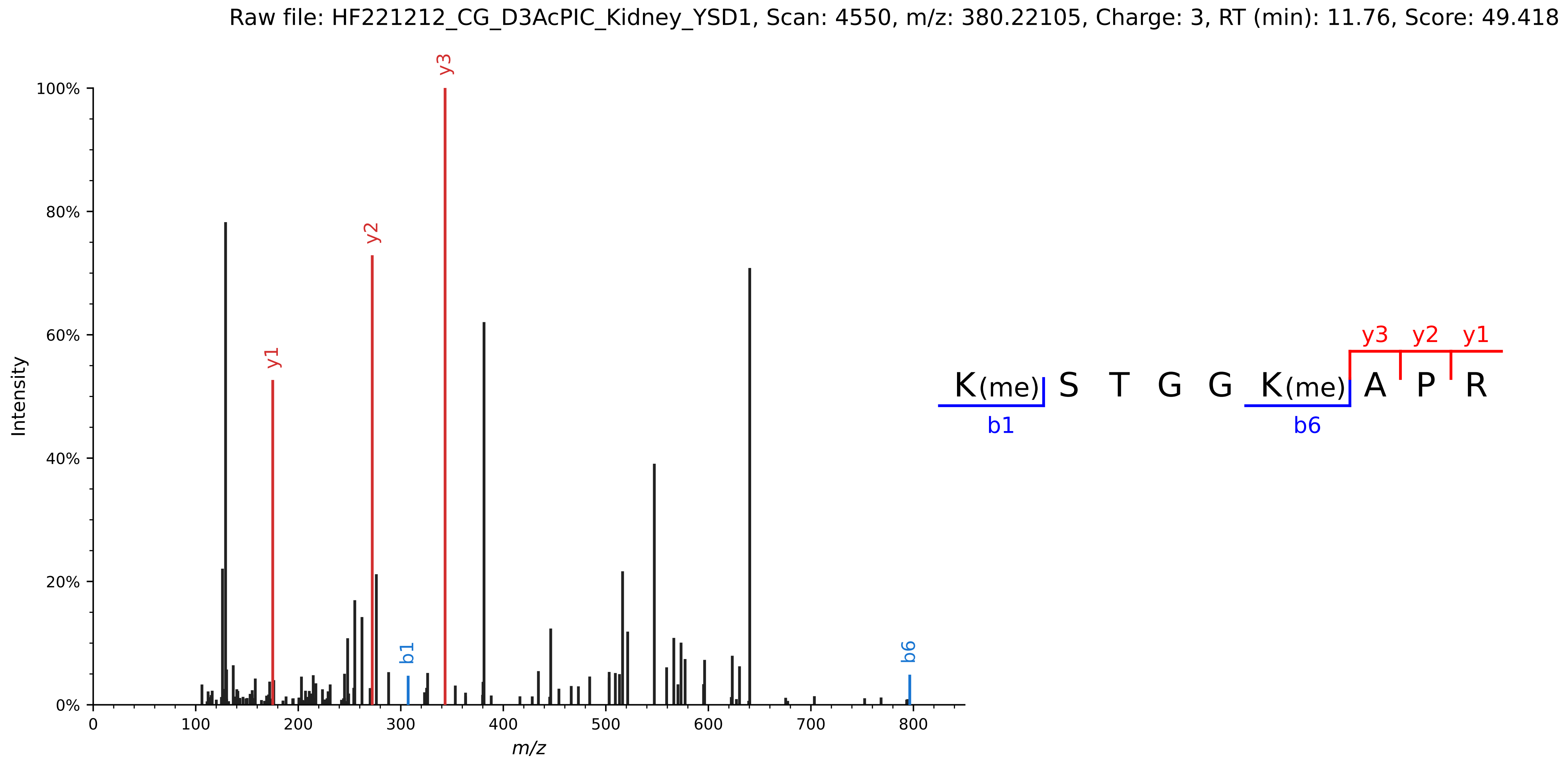

Raw file: HF221212_CG_D3AcPIC_Kidney_YSD1, Scan: 4550, m/z: 380.22105, Charge: 3, RT (min): 11.76, Score: 49.418
Intensity
m/z
y1
y2
y3
b1
b6
K(me) S T G G K(me) A P R

Raw file: HF221212_CG_D3AcPIC_Liver_YSD3, Scan: 10633, m/z: 568.31852, Charge: 2, RT (min): 22.24, Score: 139.88

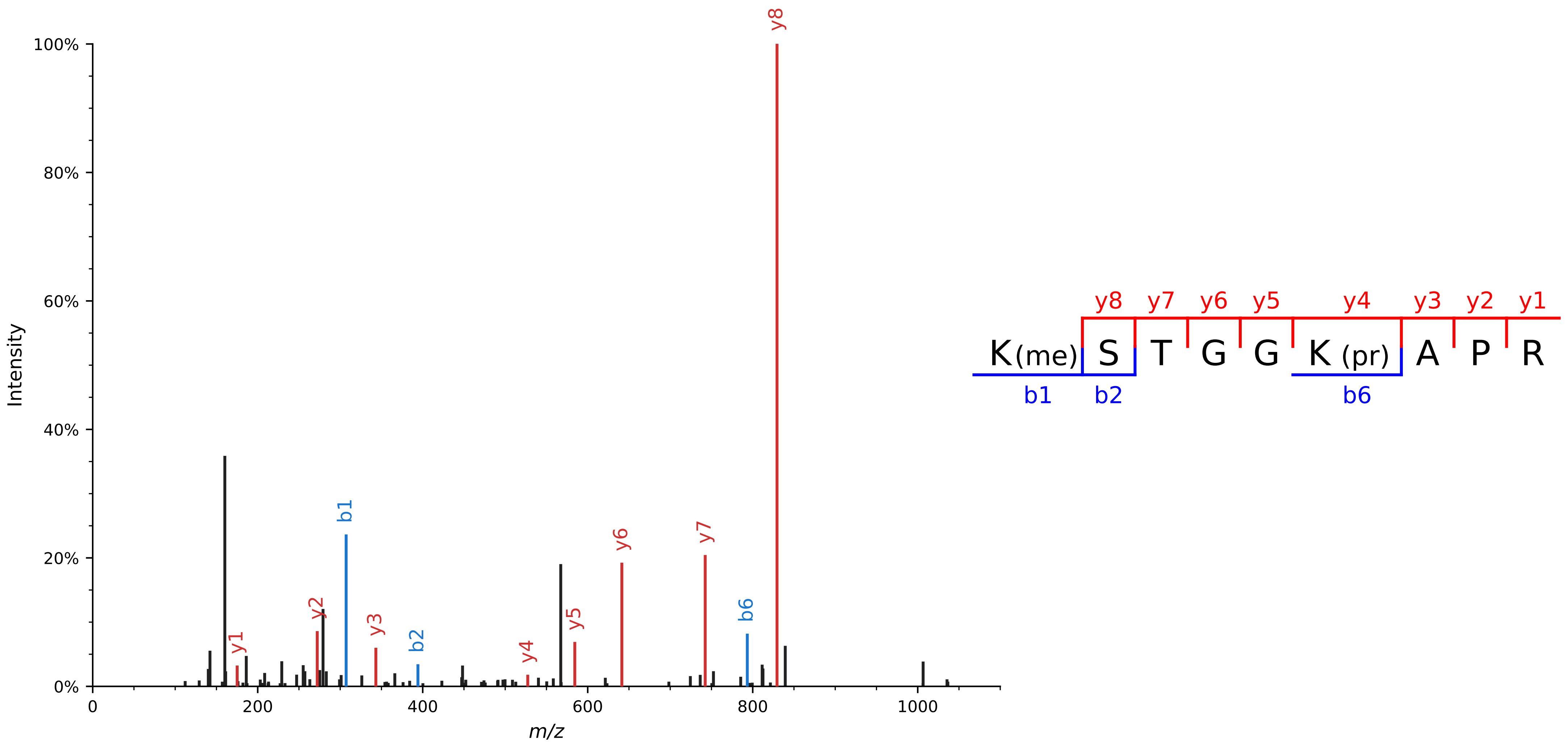

Raw file: HF221212_CG_D3AcPIC_Kidney_YSD1, Scan: 3962, m/z: 547.31324, Charge: 2, RT (min): 10.83, Score: 131.62

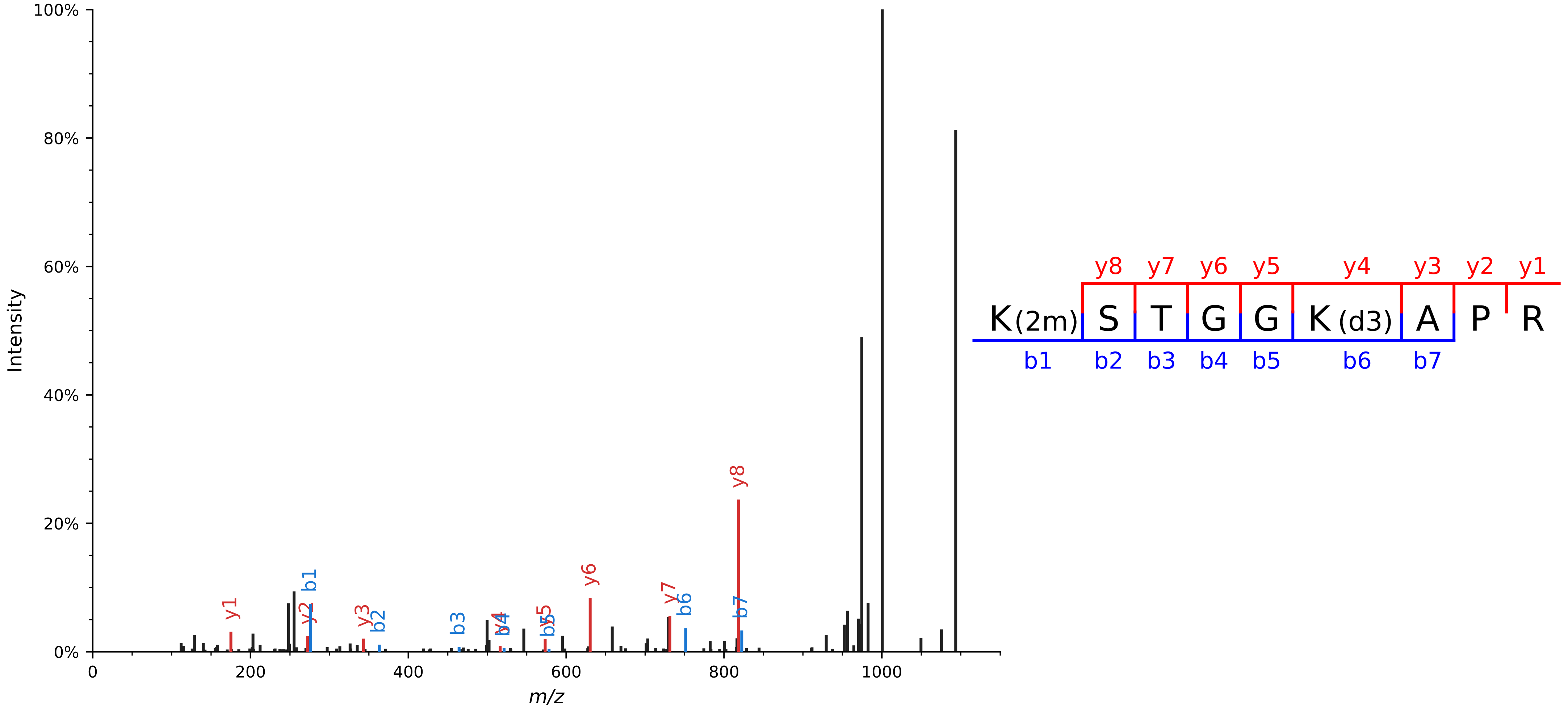

Raw file: HF221212_CG_D3AcPIC_Kidney_YSD2, Scan: 2692, m/z: 545.80382, Charge: 2, RT (min): 8.19, Score: 138.4

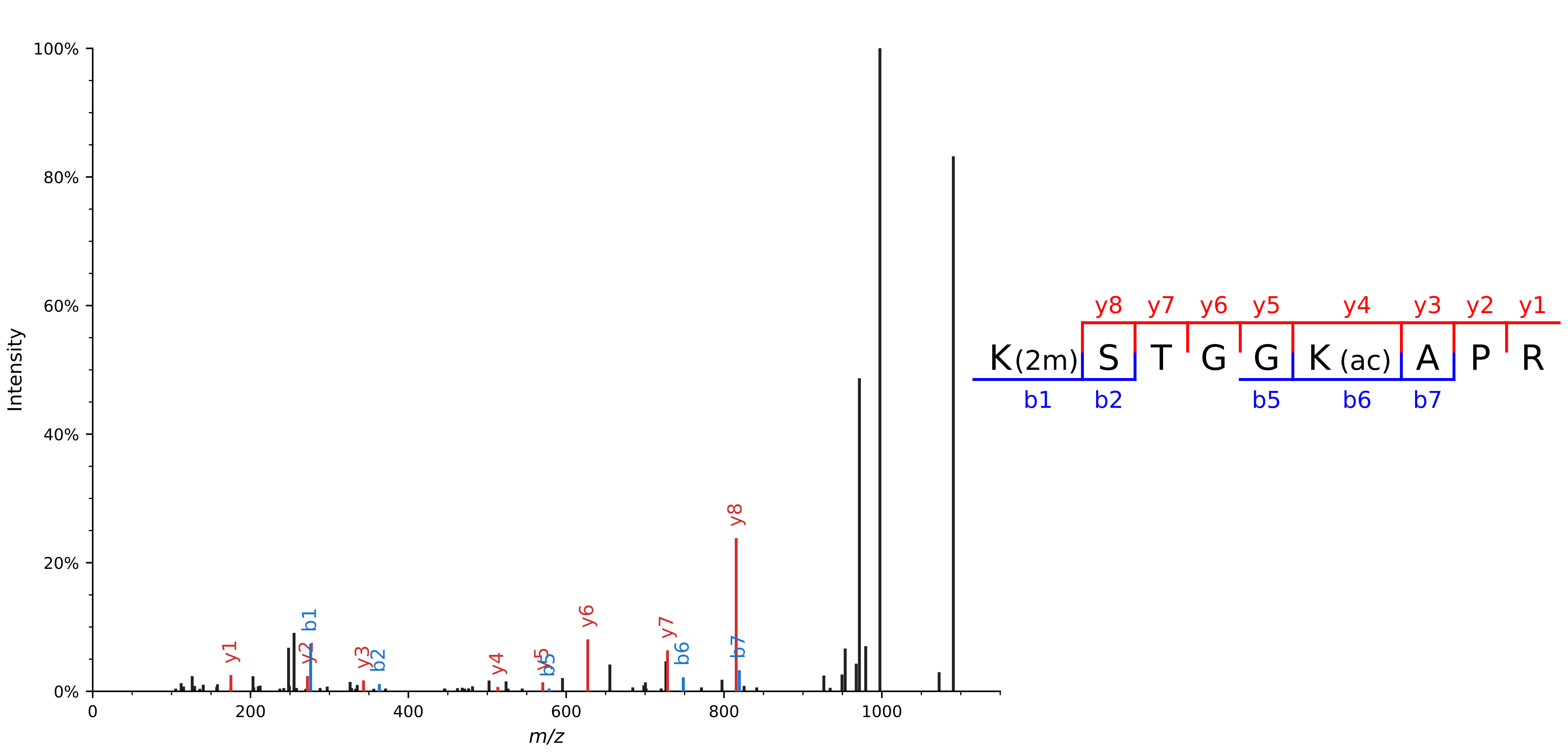

Raw file: HF221212_CG_D3AcPIC_Spleen_YSD3, Scan: 3870, m/z: 558.81165, Charge: 2, RT (min): 10.06, Score: 54.157

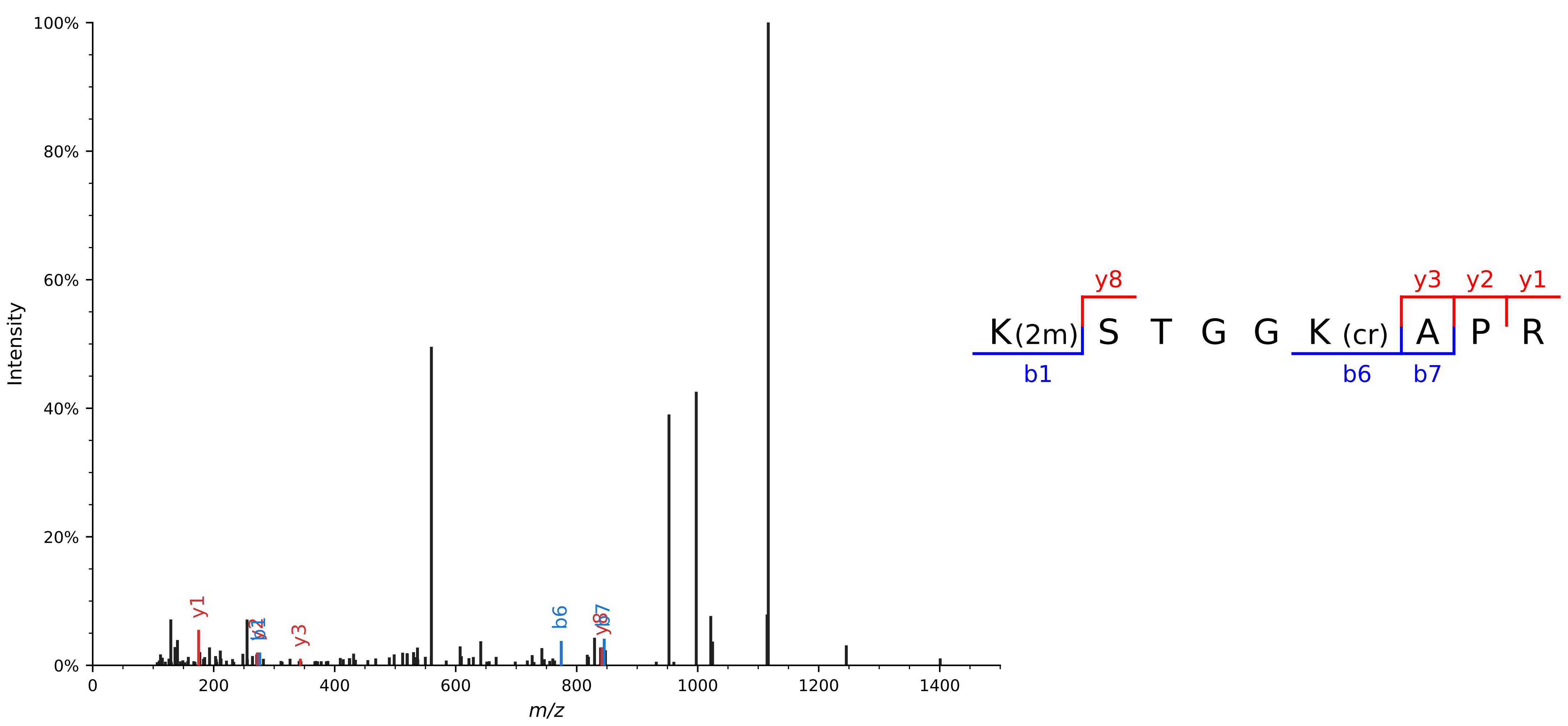

Raw file: HF221212_CG_D3AcPIC_Heart_YSD2, Scan: 3137, m/z: 538.796, Charge: 2, RT (min): 8.94, Score: 40.103

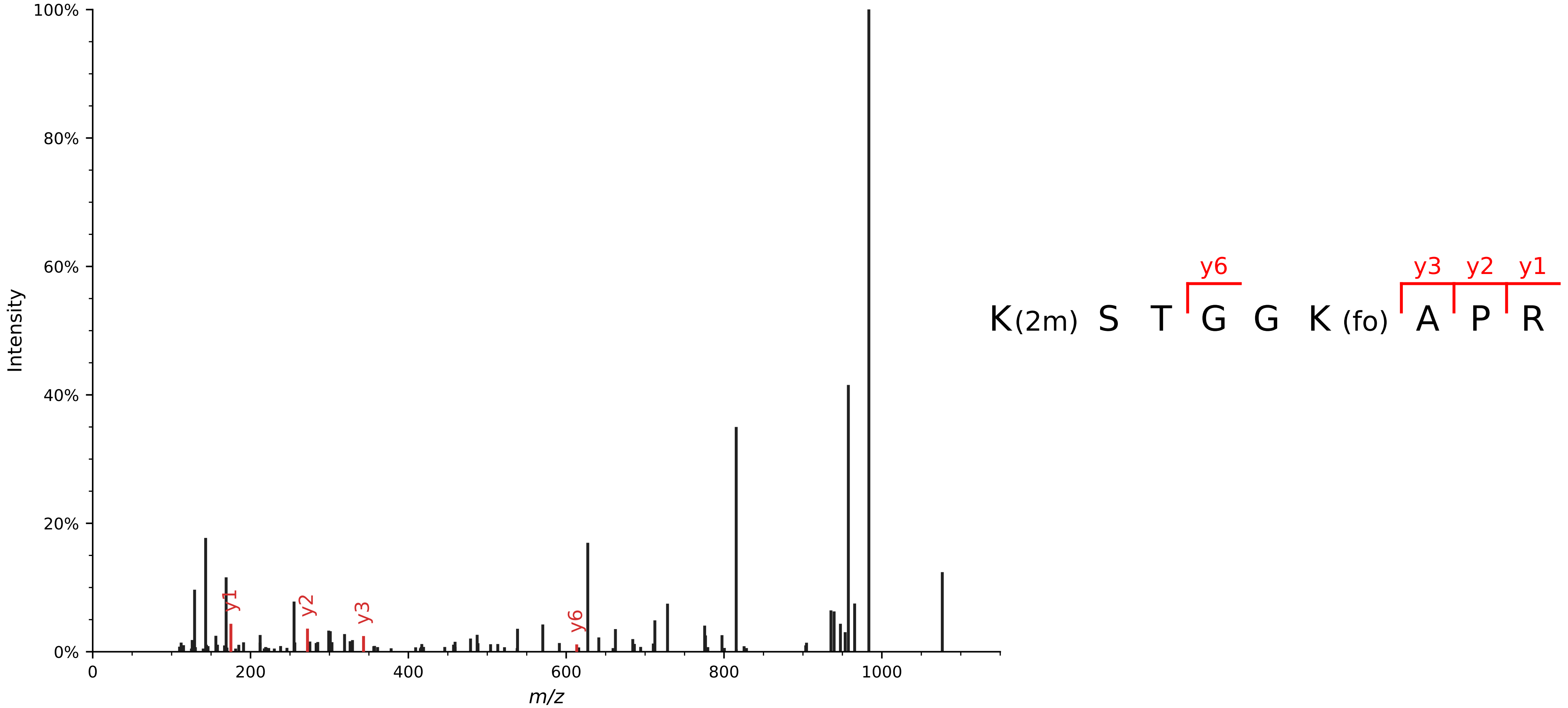

Raw file: HF221212_CG_D3AcPIC_Spleen_YSD2, Scan: 2869, m/z: 554.32106, Charge: 2, RT (min): 8.23, Score: 77.058

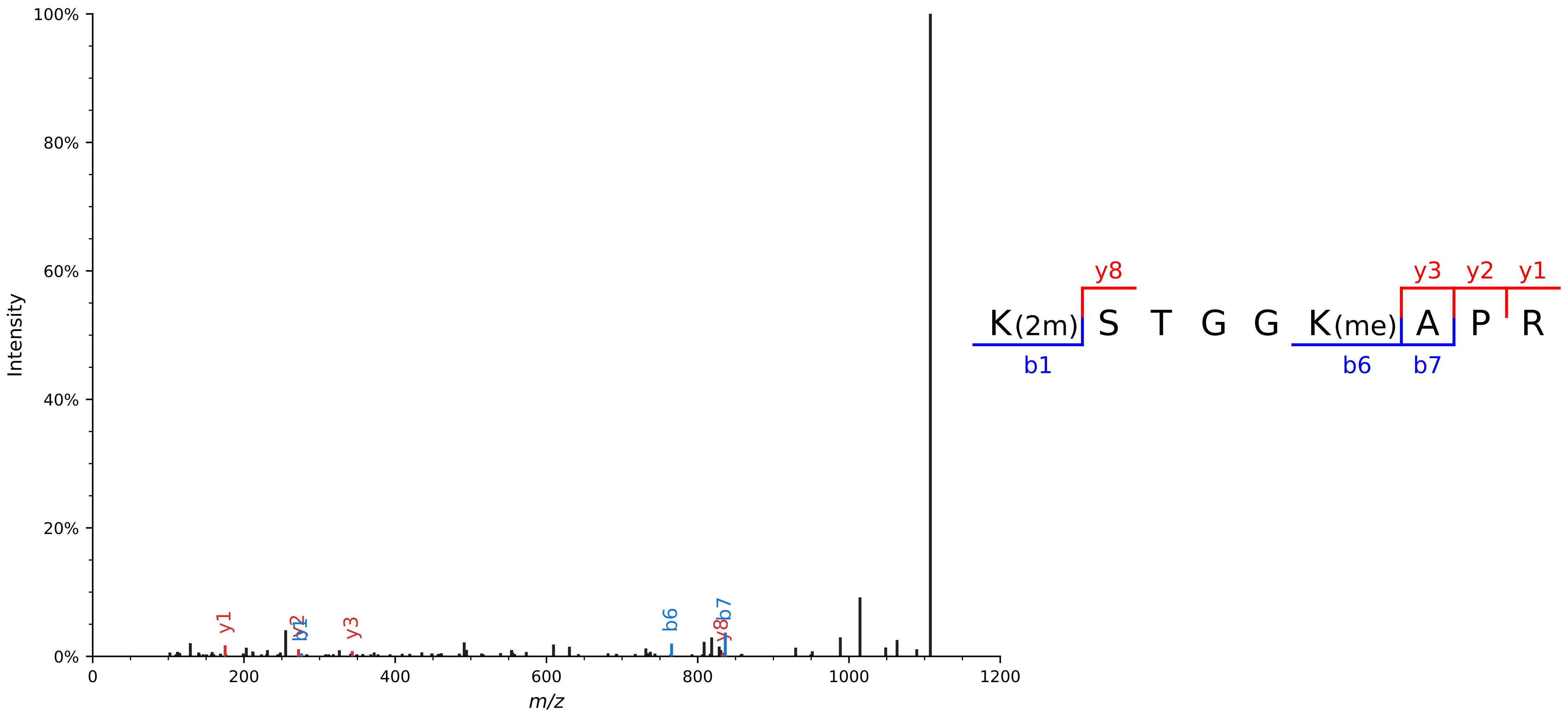

Raw file: HF221212_CG_D3AcPIC_Liver_YSD2, Scan: 3472, m/z: 552.81165, Charge: 2, RT (min): 9.34, Score: 128.12

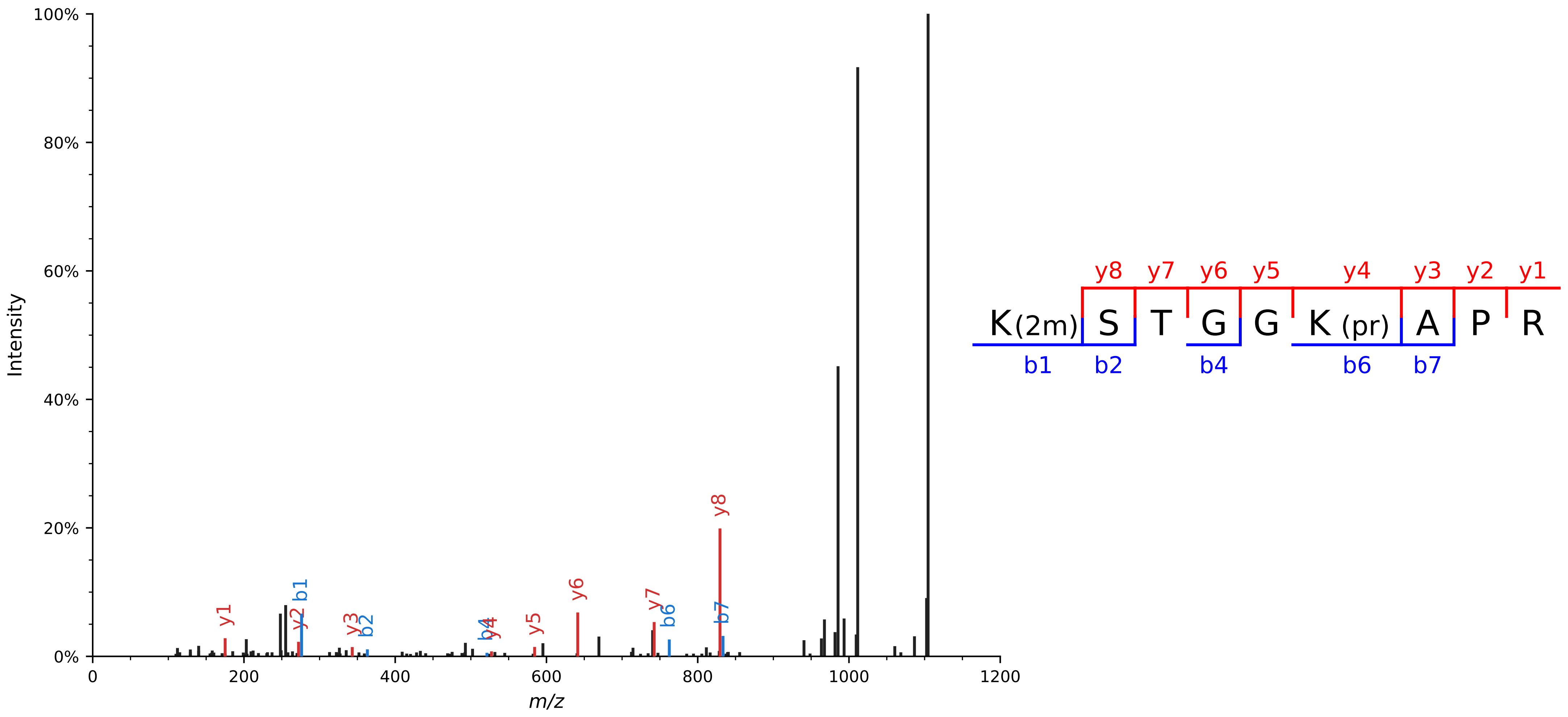

Raw file: HF221212_CG_D3AcPIC_Spleen_YSD1, Scan: 2861, m/z: 554.32106, Charge: 2, RT (min): 8.06, Score: 115.82

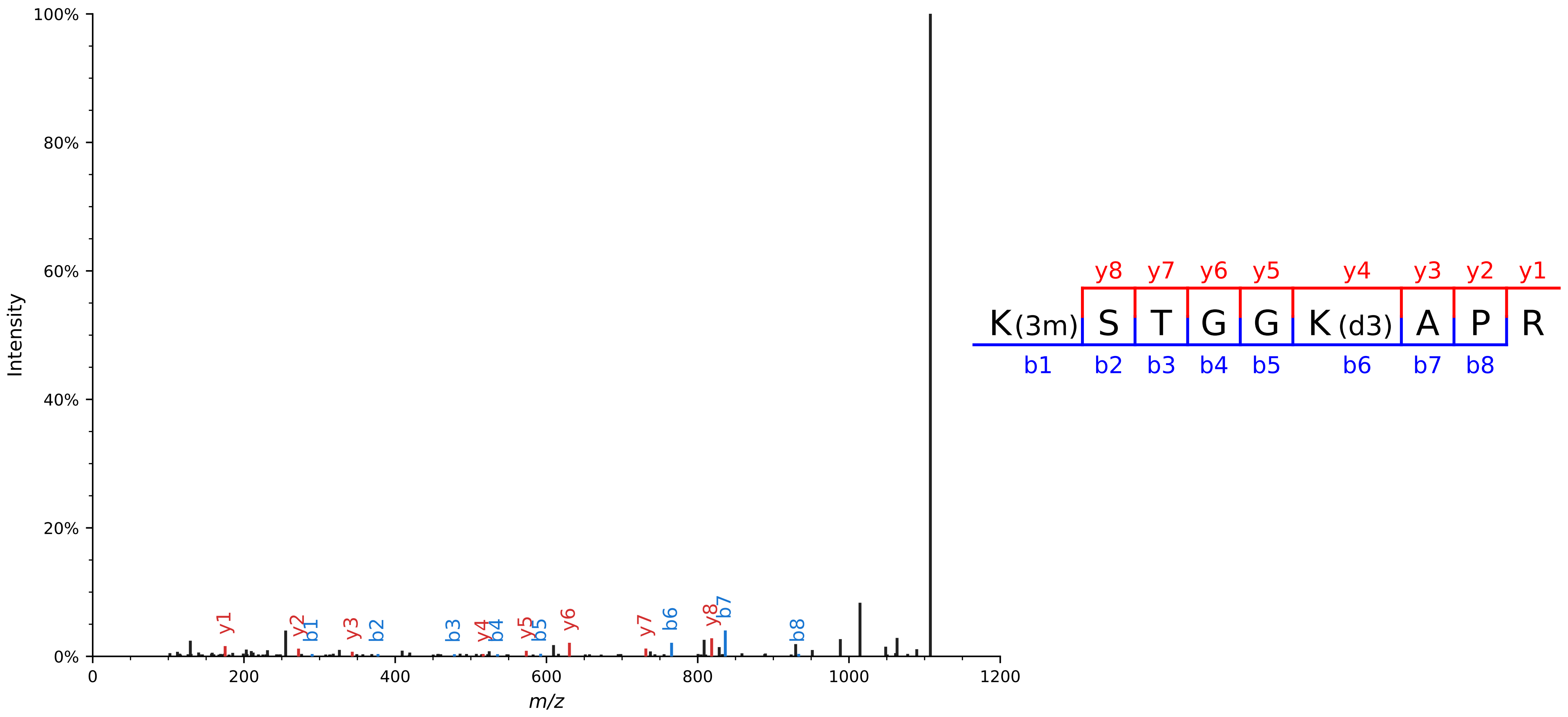

Raw file: HF221212_CG_D3AcPIC_Spleen_YSD1, Scan: 3839, m/z: 552.81165, Charge: 2, RT (min): 9.81, Score: 70.856

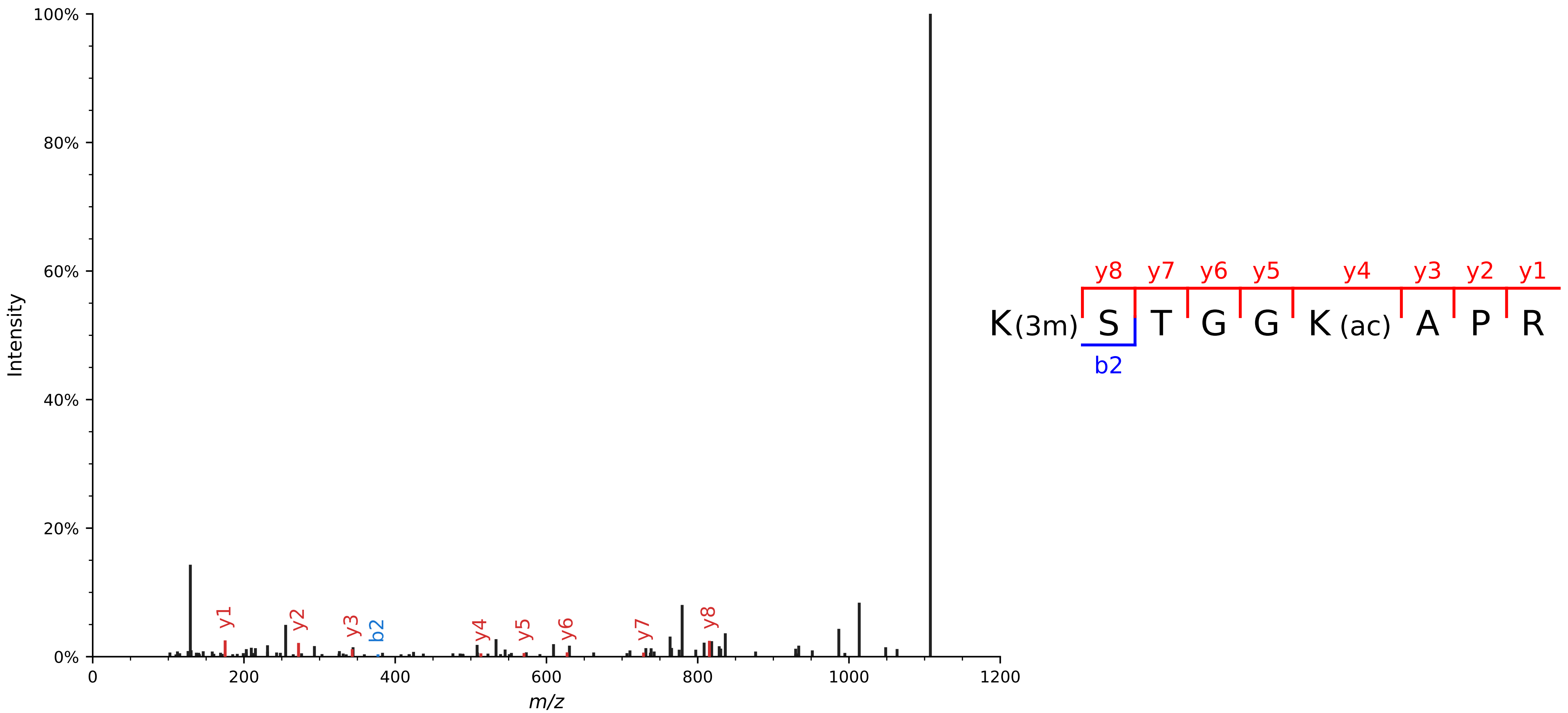

Raw file: HF221212_CG_D3AcPIC_Heart_YSD3, Scan: 3816, m/z: 559.81947, Charge: 2, RT (min): 9.99, Score: 130.66

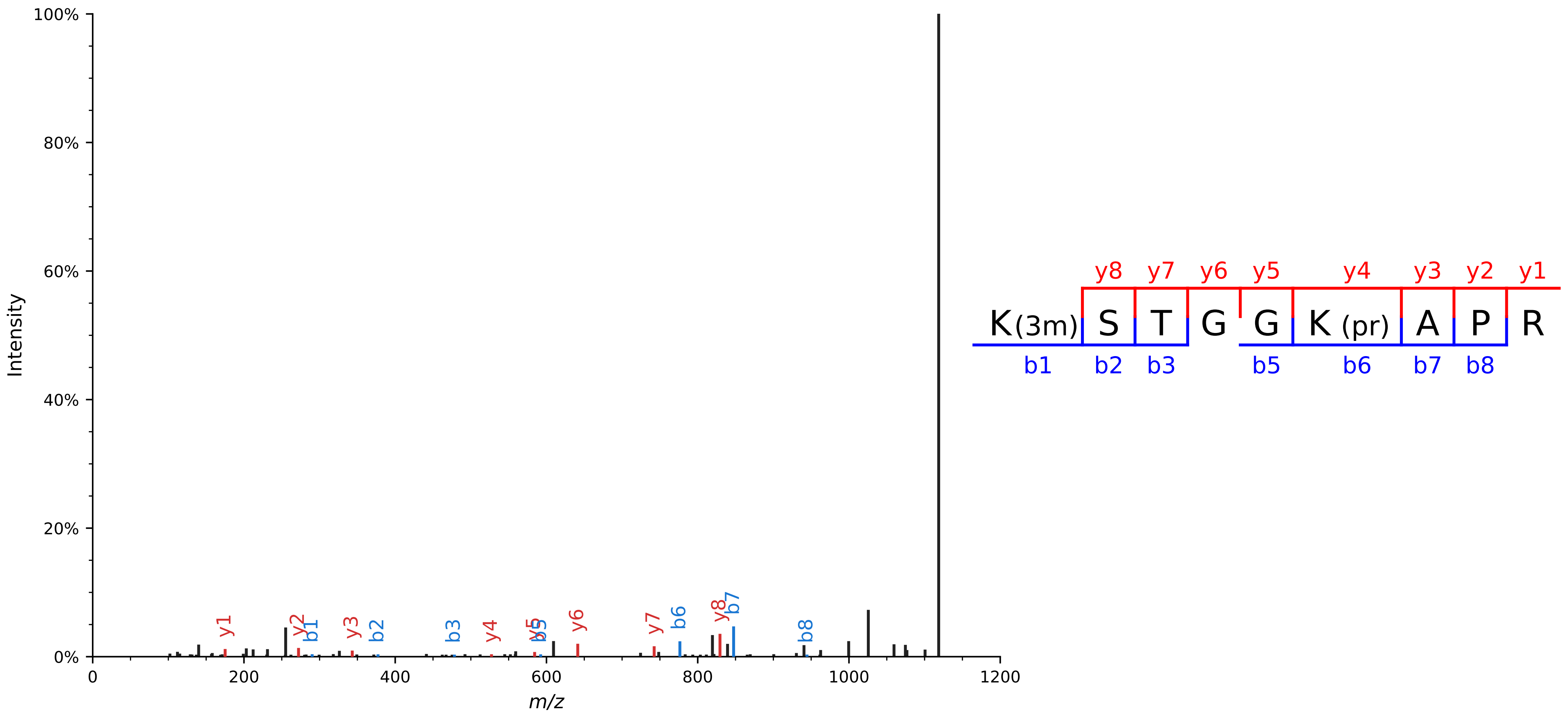

Raw file: HF221212_CG_D3AcPIC_Kidney_YSD3, Scan: 9567, m/z: 561.3107, Charge: 2, RT (min): 19.54, Score: 121.62

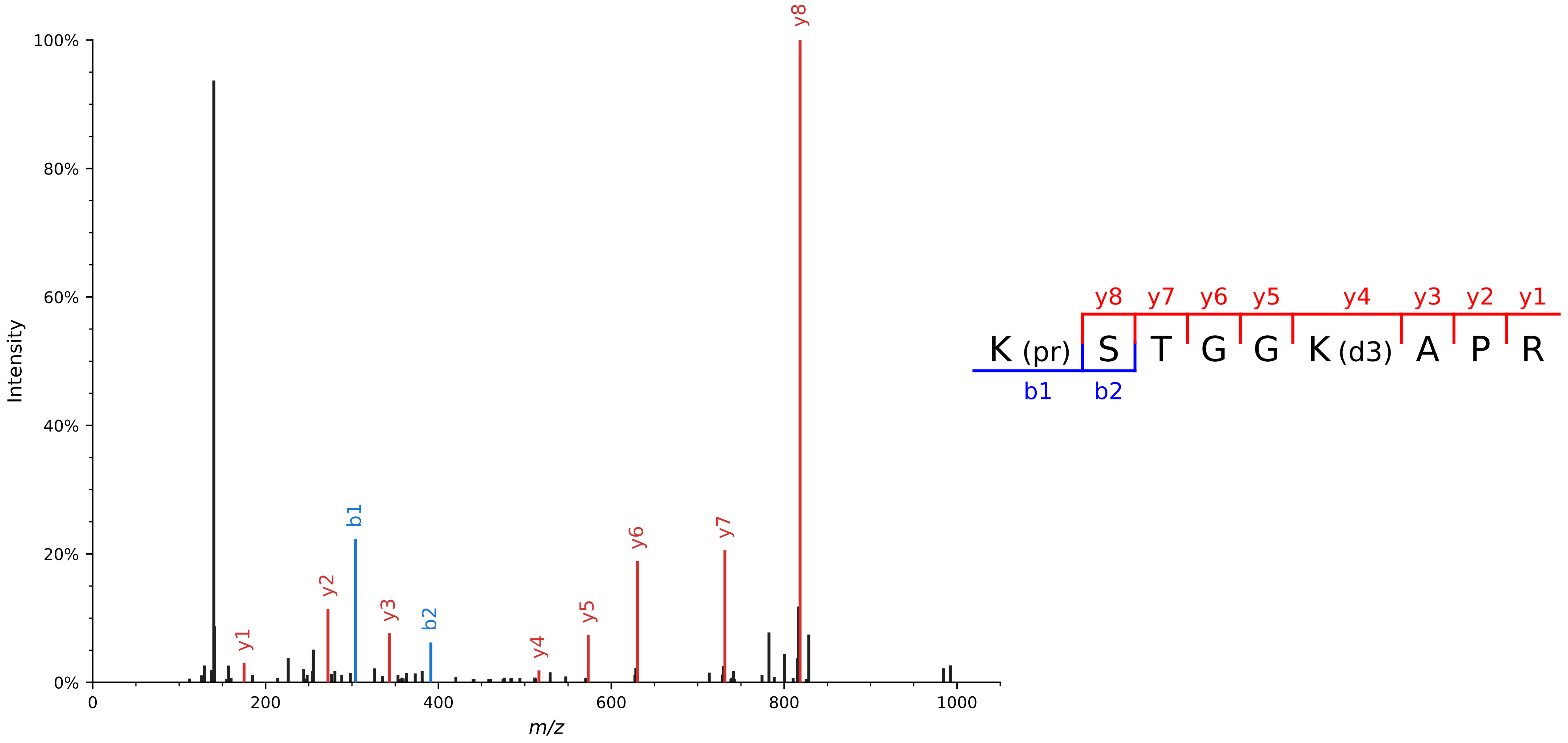

Raw file: HF221212_CG_D3AcPIC_Liver_YSD1, Scan: 9086, m/z: 559.80128, Charge: 2, RT (min): 19.54, Score: 131.06

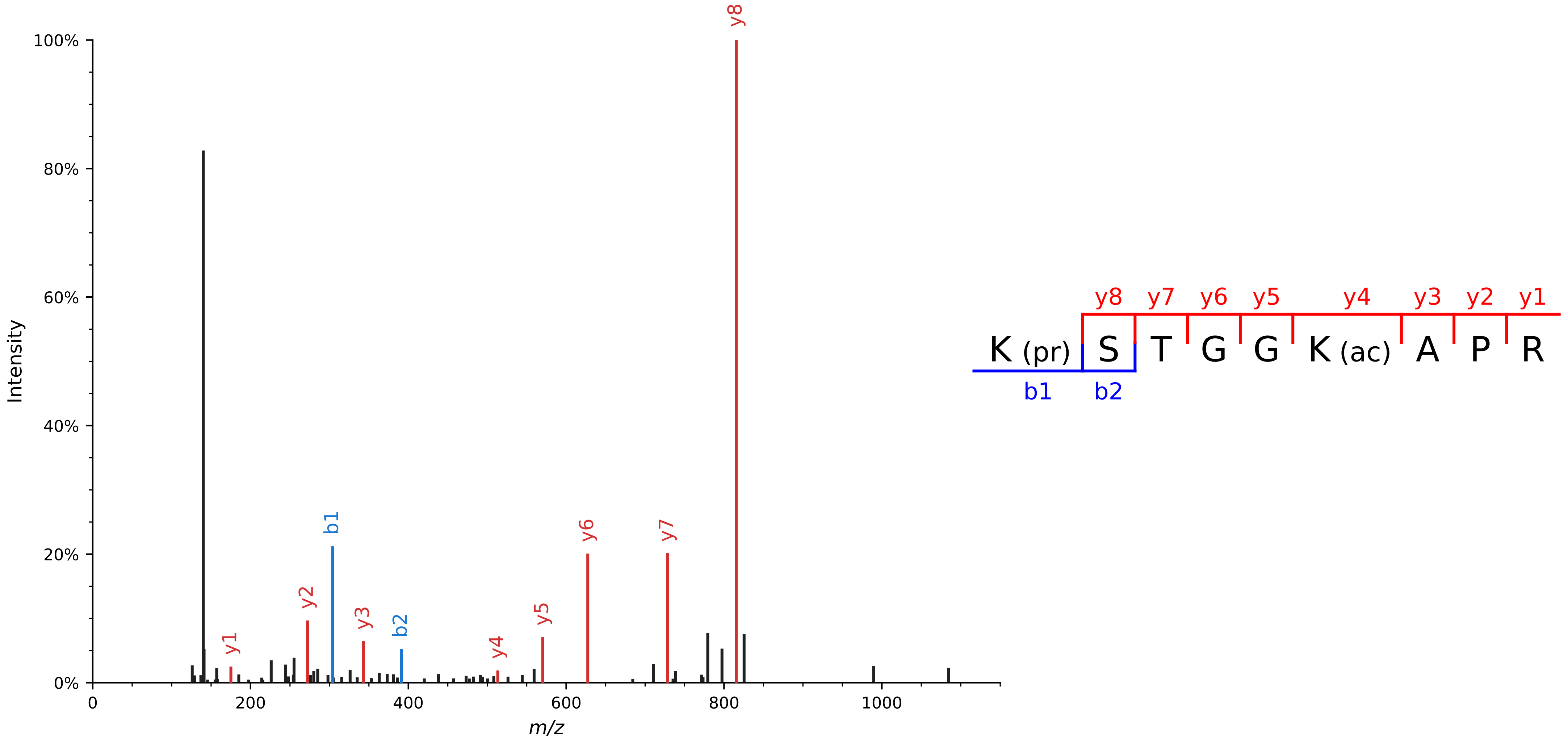

Raw file: HF221212_CG_D3AcPIC_Liver_YSD3, Scan: 3995, m/z: 568.31852, Charge: 2, RT (min): 10.25, Score: 73.248

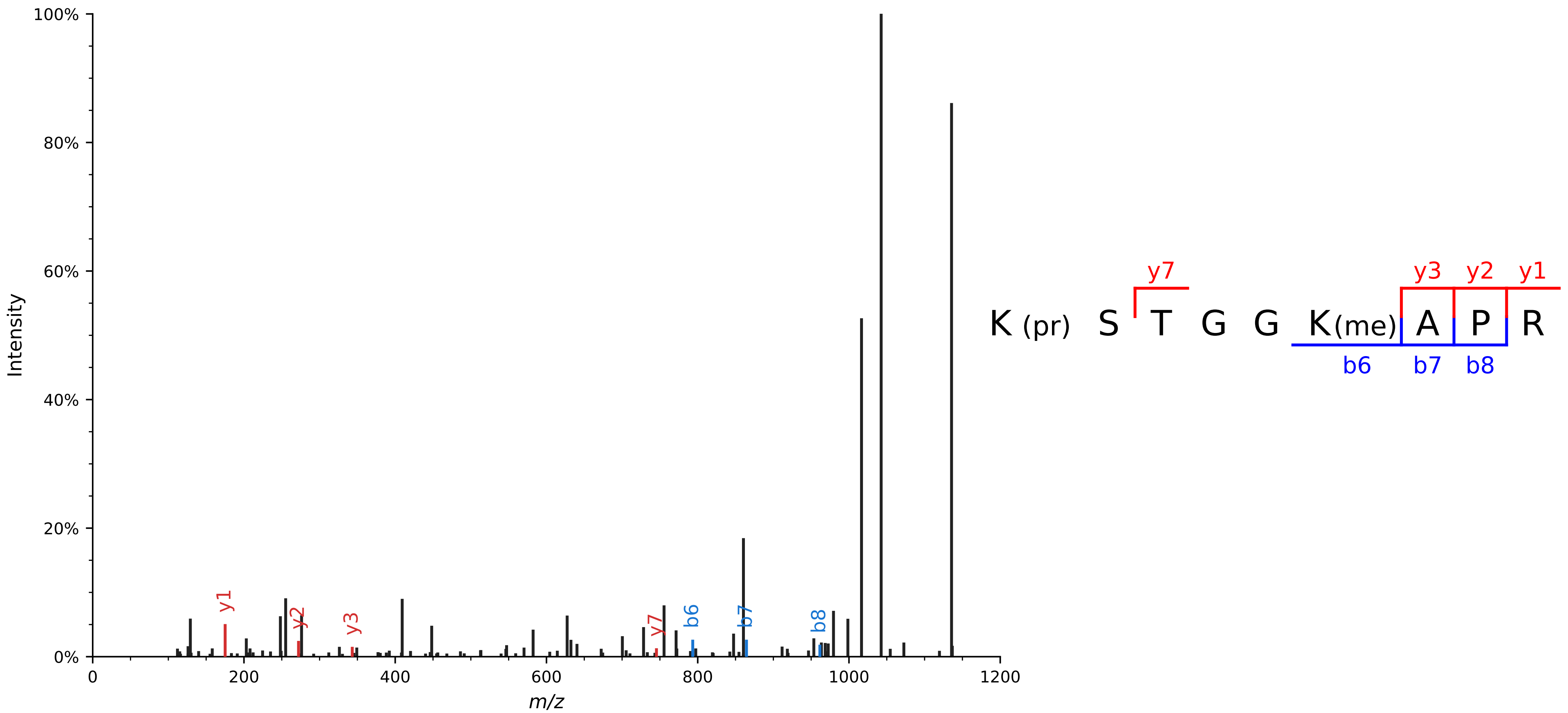

Raw file: HF221212_CG_D3AcPIC_Spleen_YSD2, Scan: 3218, m/z: 552.81165, Charge: 2, RT (min): 8.87, Score: 47.067

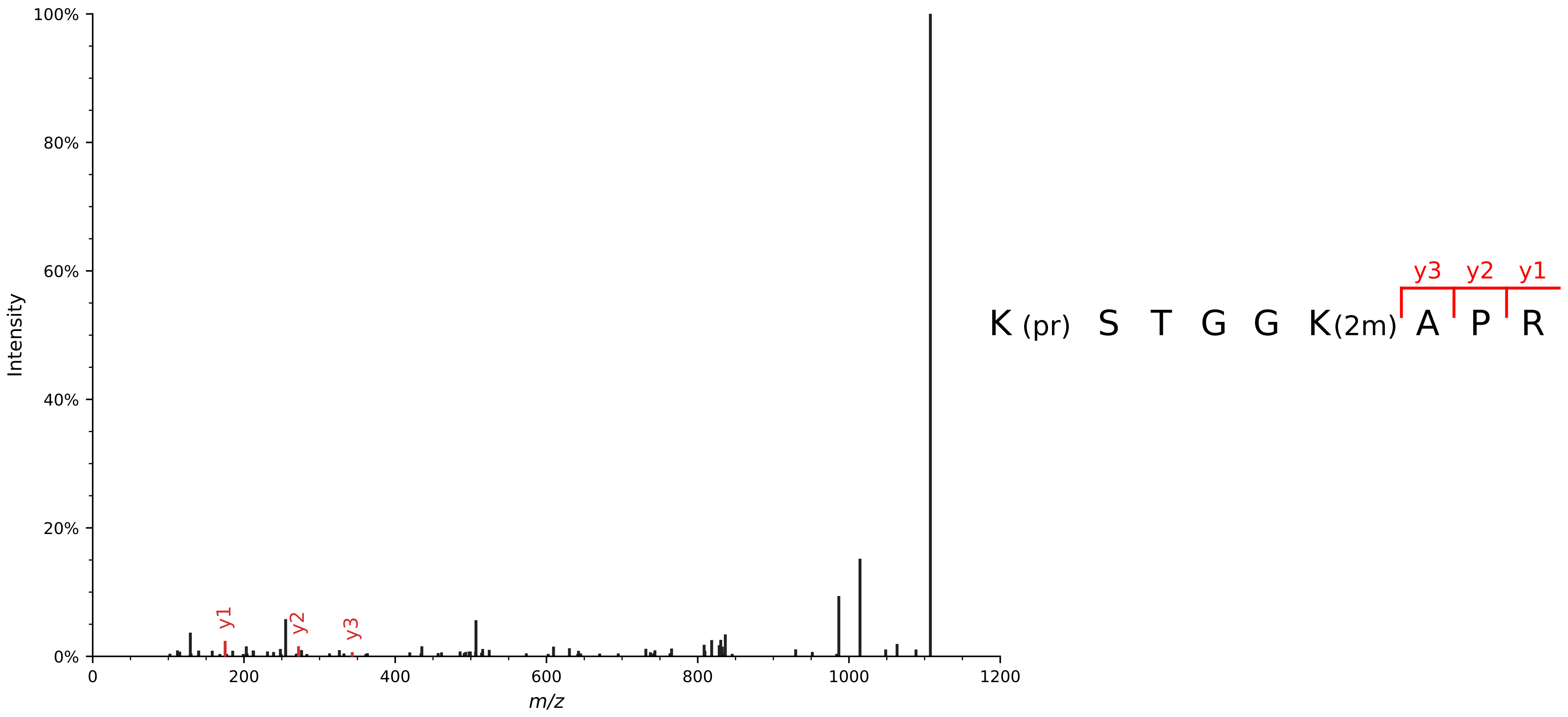

Raw file: HF221212_CG_D3AcPIC_Spleen_YSD3, Scan: 10435, m/z: 566.8091, Charge: 2, RT (min): 21.90, Score: 133.55

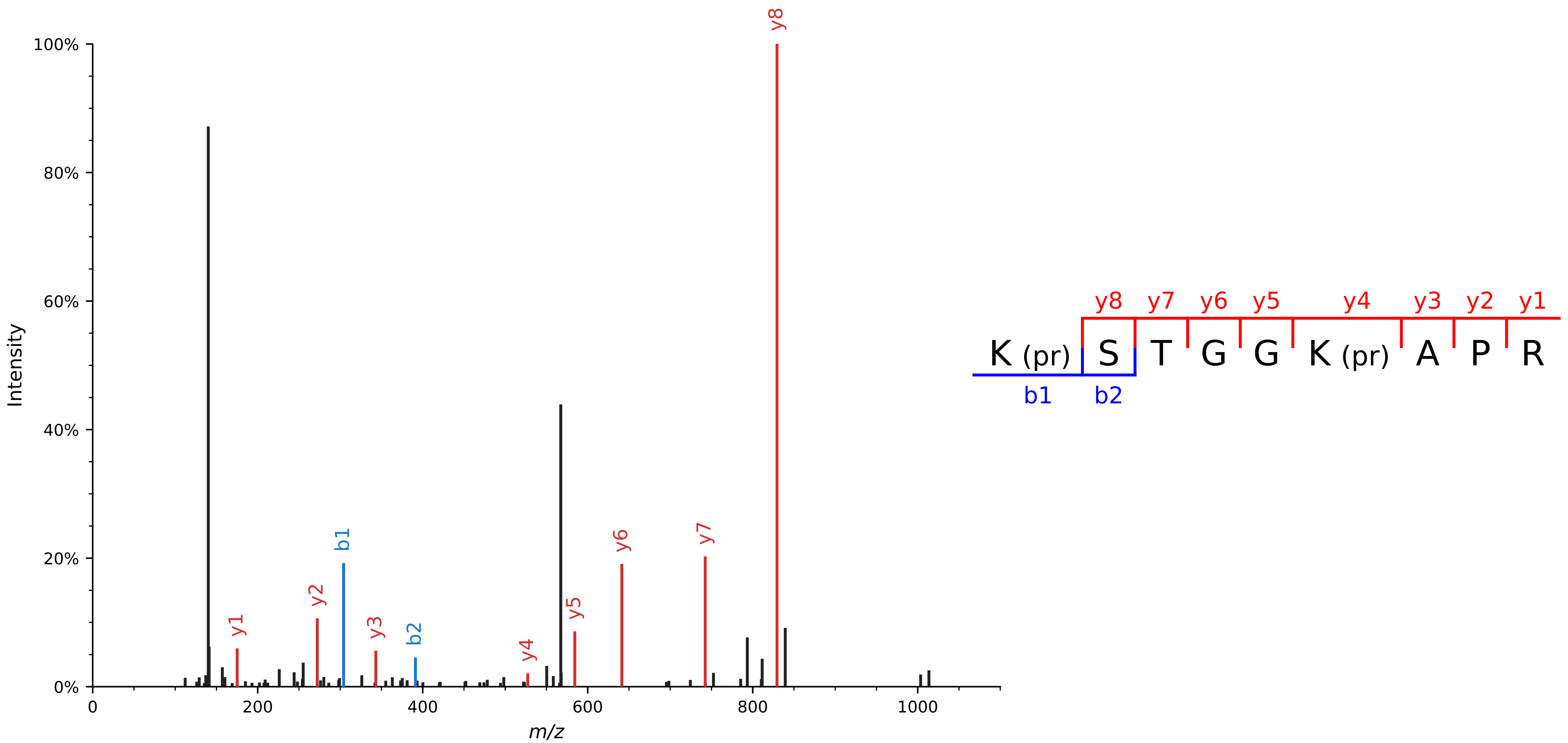

Raw file: HF221212_CG_D3AcPIC_Liver_YSD1, Scan: 7490, m/z: 554.30287, Charge: 2, RT (min): 16.72, Score: 130.27

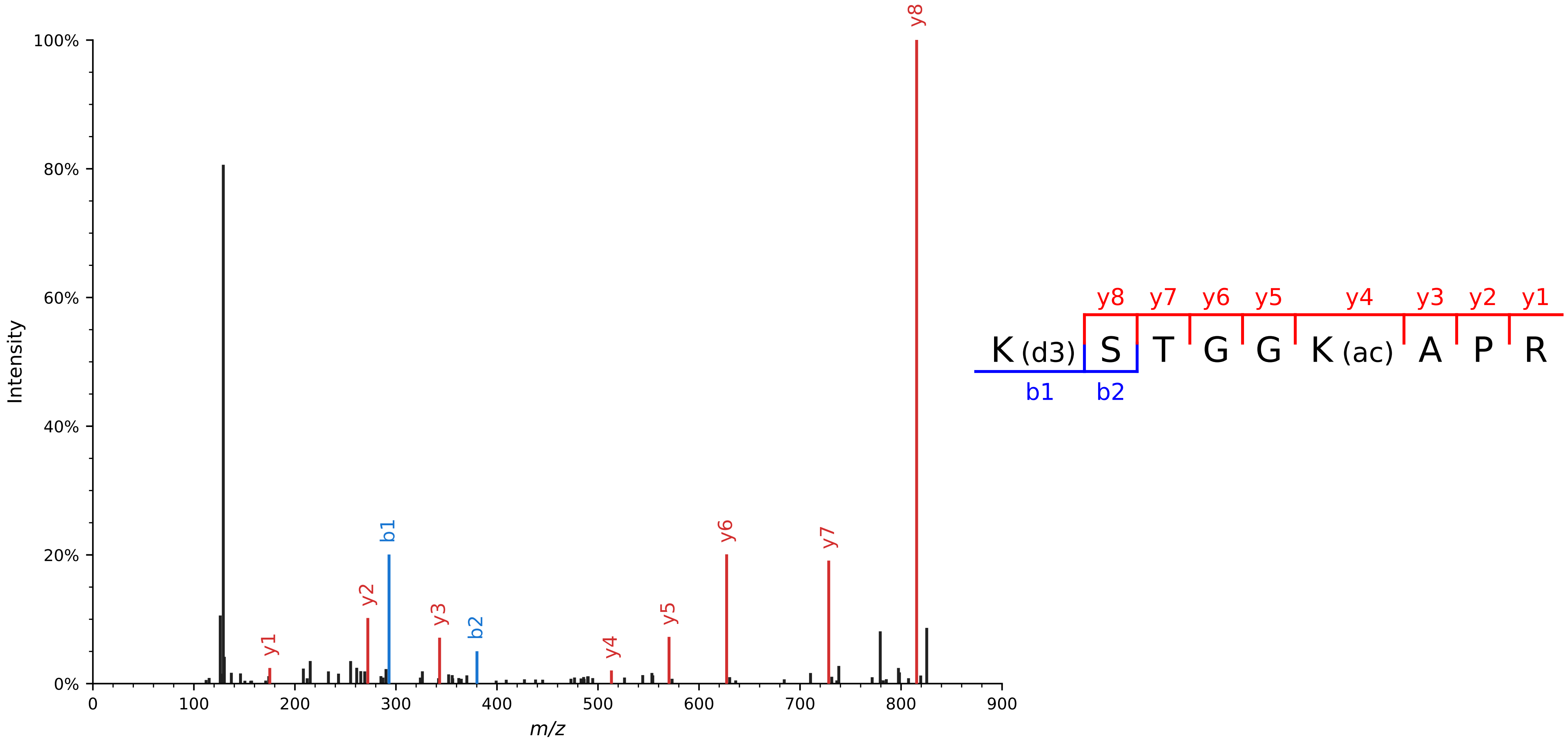

Raw file: HF221212_CG_D3AcPIC_Spleen_YSD1, Scan: 10526, m/z: 568.31852, Charge: 2, RT (min): 21.66, Score: 75.692

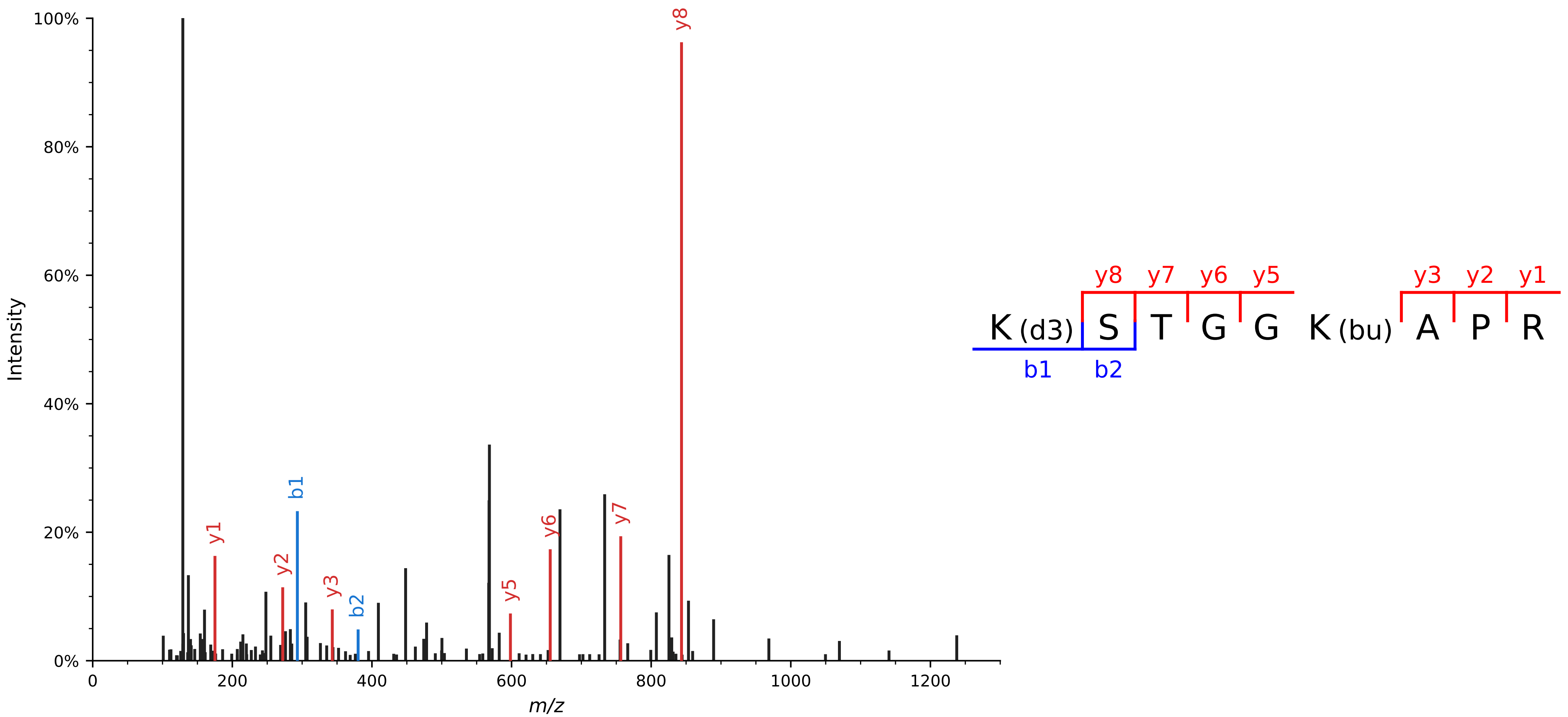

Raw file: HF221212_CG_D3AcPIC_Spleen_YSD1, Scan: 9135, m/z: 561.3107, Charge: 2, RT (min): 19.14, Score: 133.12

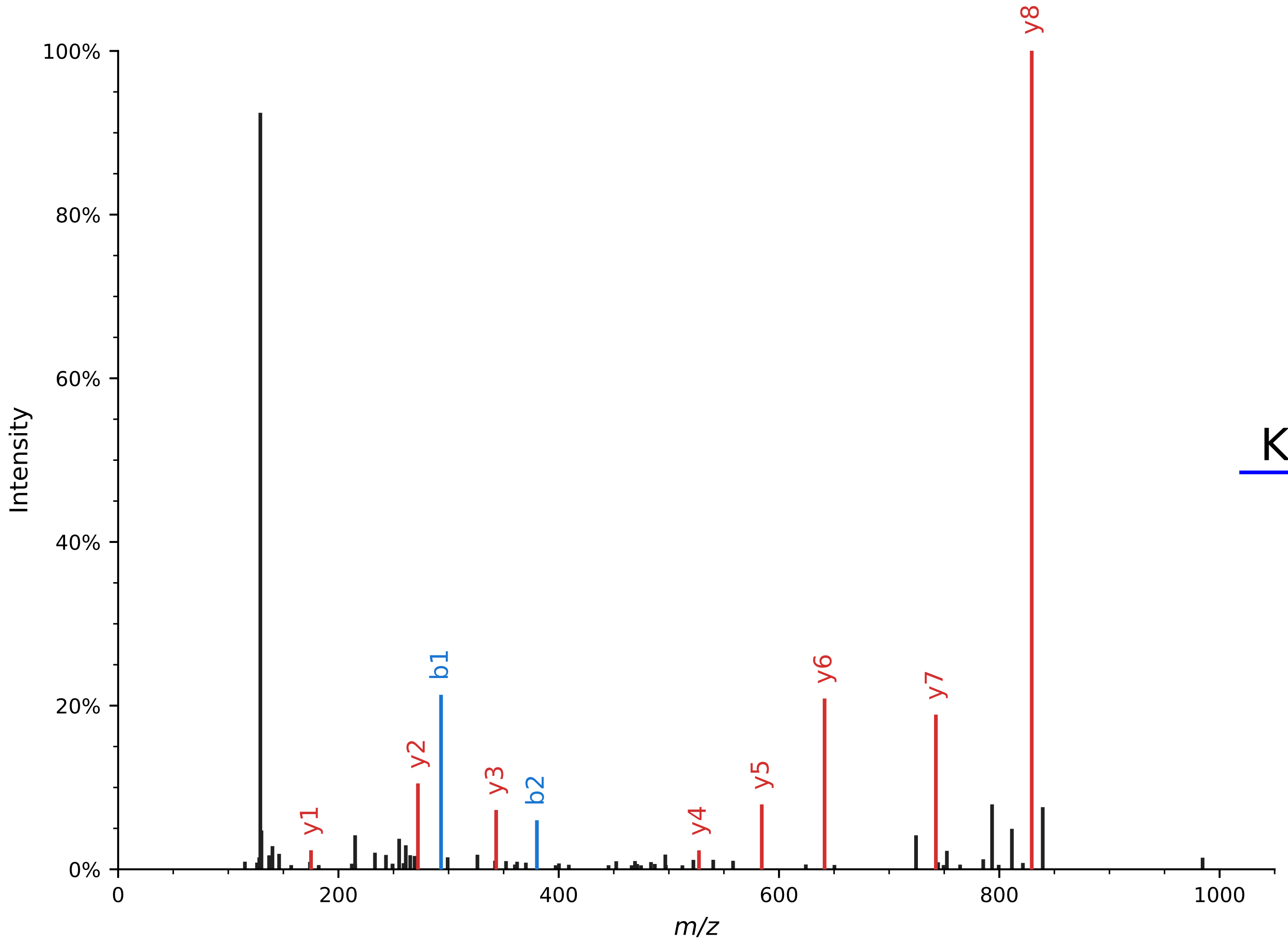

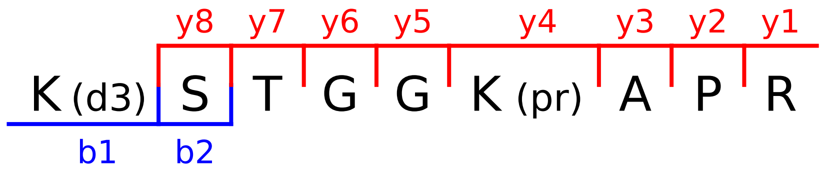

Raw file: HF221212_CG_D3AcPIC_Heart_YSD3, Scan: 16073, m/z: 595.33803, Charge: 2, RT (min): 32.65, Score: 144.12

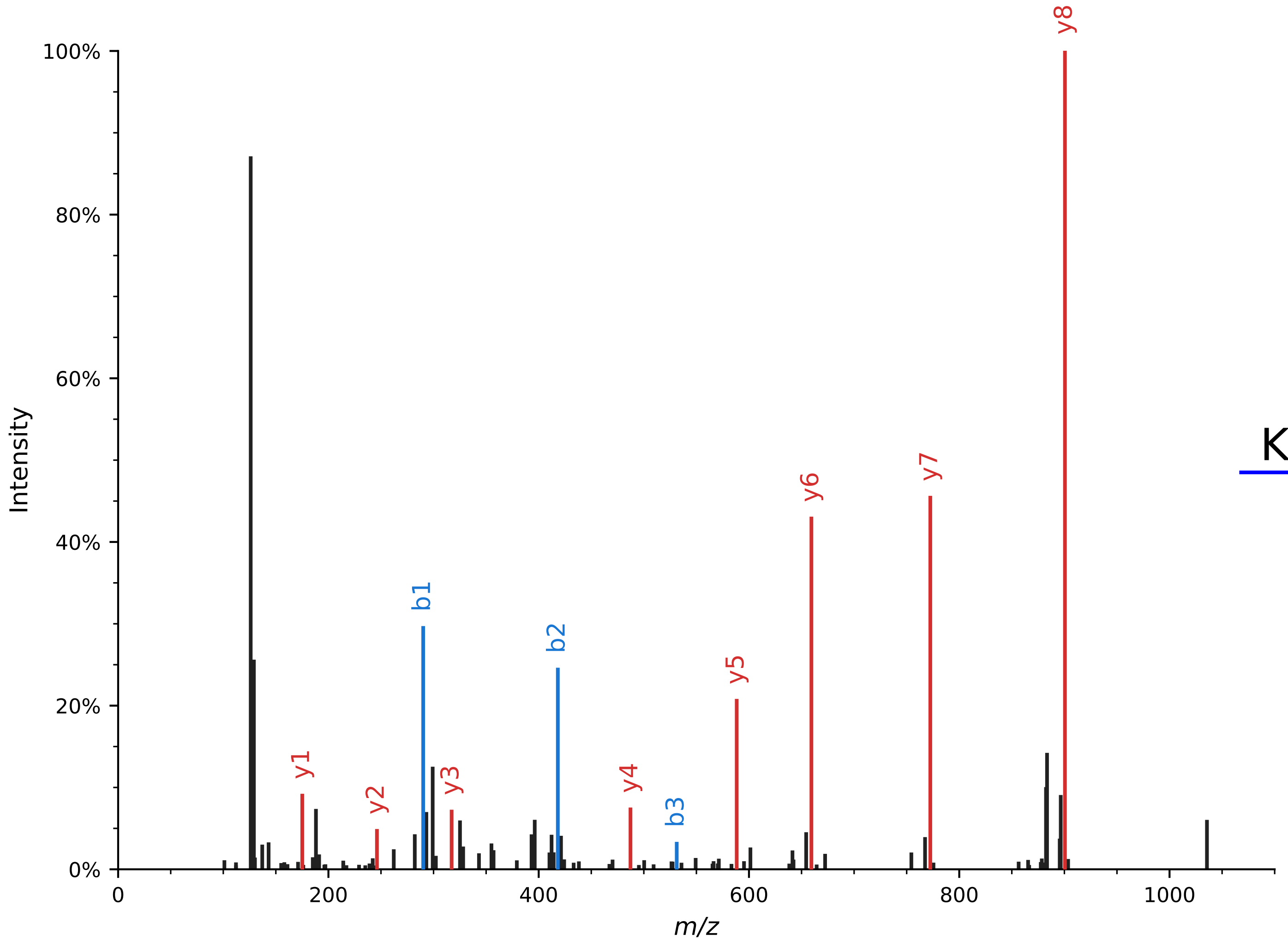

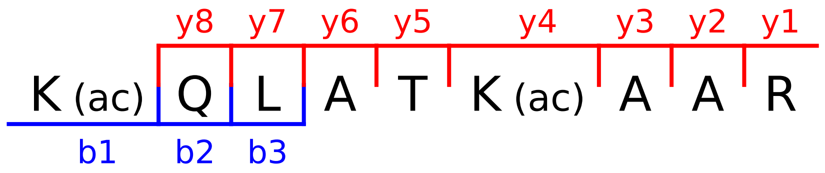

Raw file: HF221212_CG_D3AcPIC_Spleen_YSD1, Scan: 17904, m/z: 602.34585, Charge: 2, RT (min): 35.13, Score: 136.27

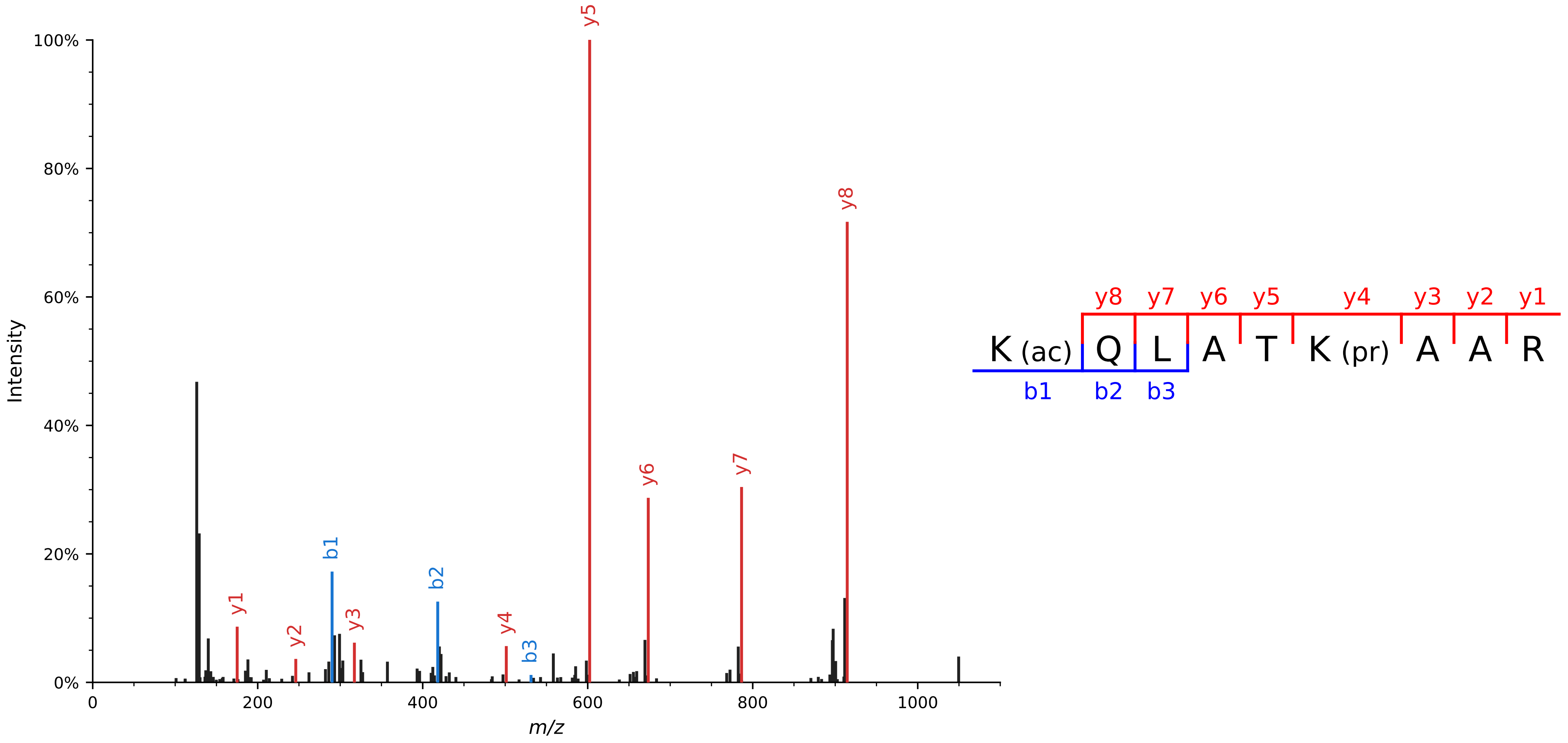

Raw file: HF221212_CG_D3AcPIC_Spleen_YSD2, Scan: 19378, m/z: 610.86309, Charge: 2, RT (min): 38.29, Score: 69.825

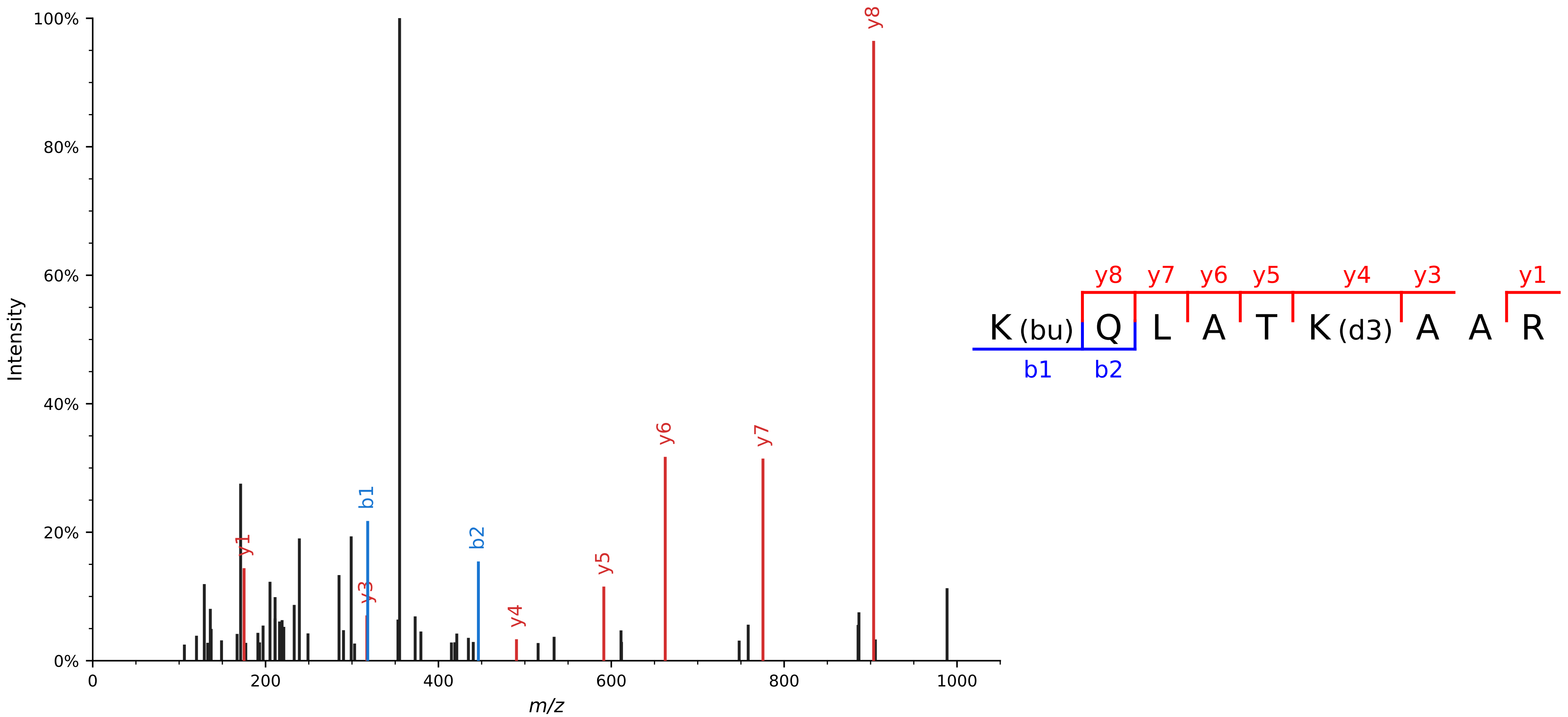

Raw file: HF221212_CG_D3AcPIC_Spleen_YSD1, Scan: 16325, m/z: 589.83962, Charge: 2, RT (min): 32.19, Score: 116.73

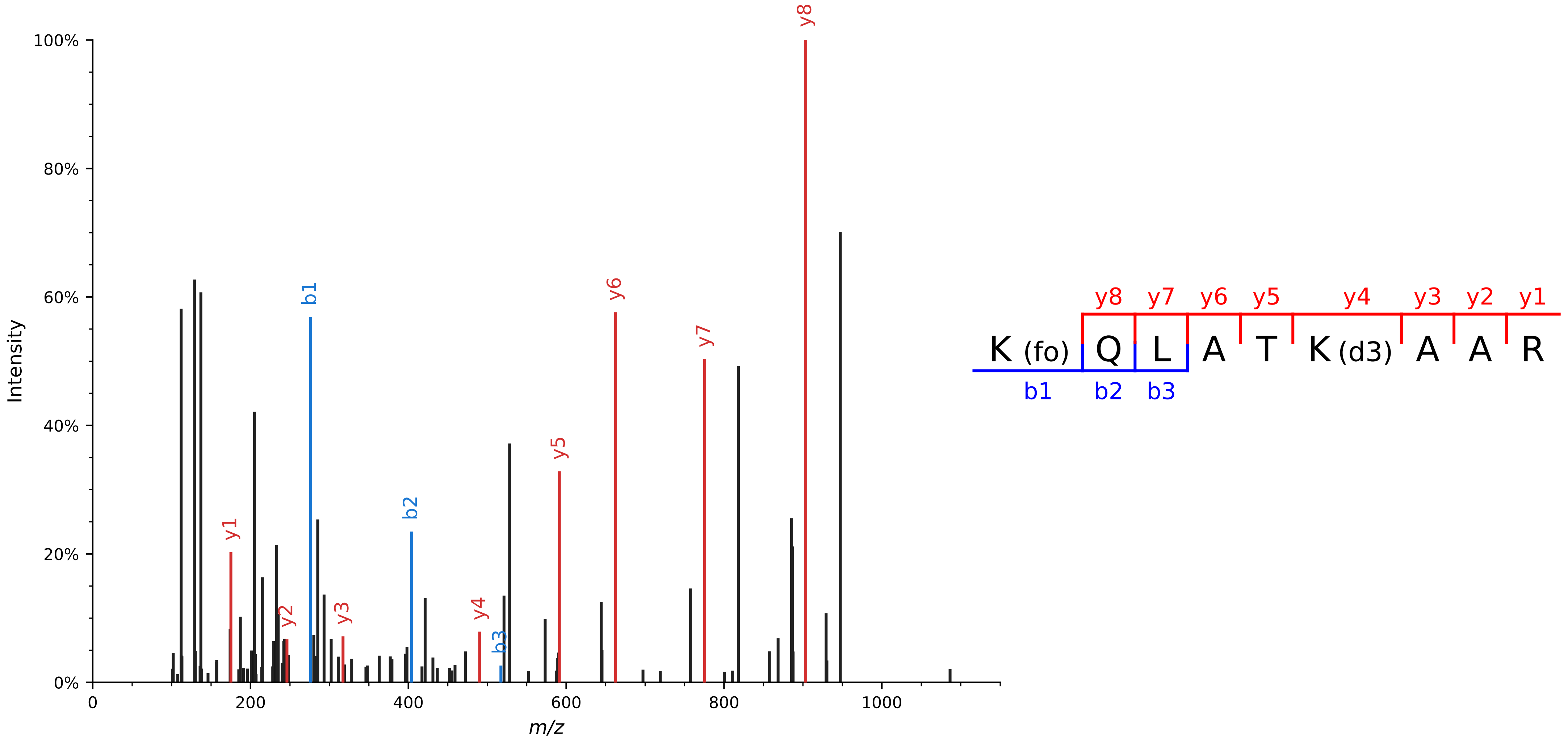

Raw file: HF221212_CG_D3AcPIC_Spleen_YSD3, Scan: 15995, m/z: 588.3302, Charge: 2, RT (min): 32.08, Score: 45.161

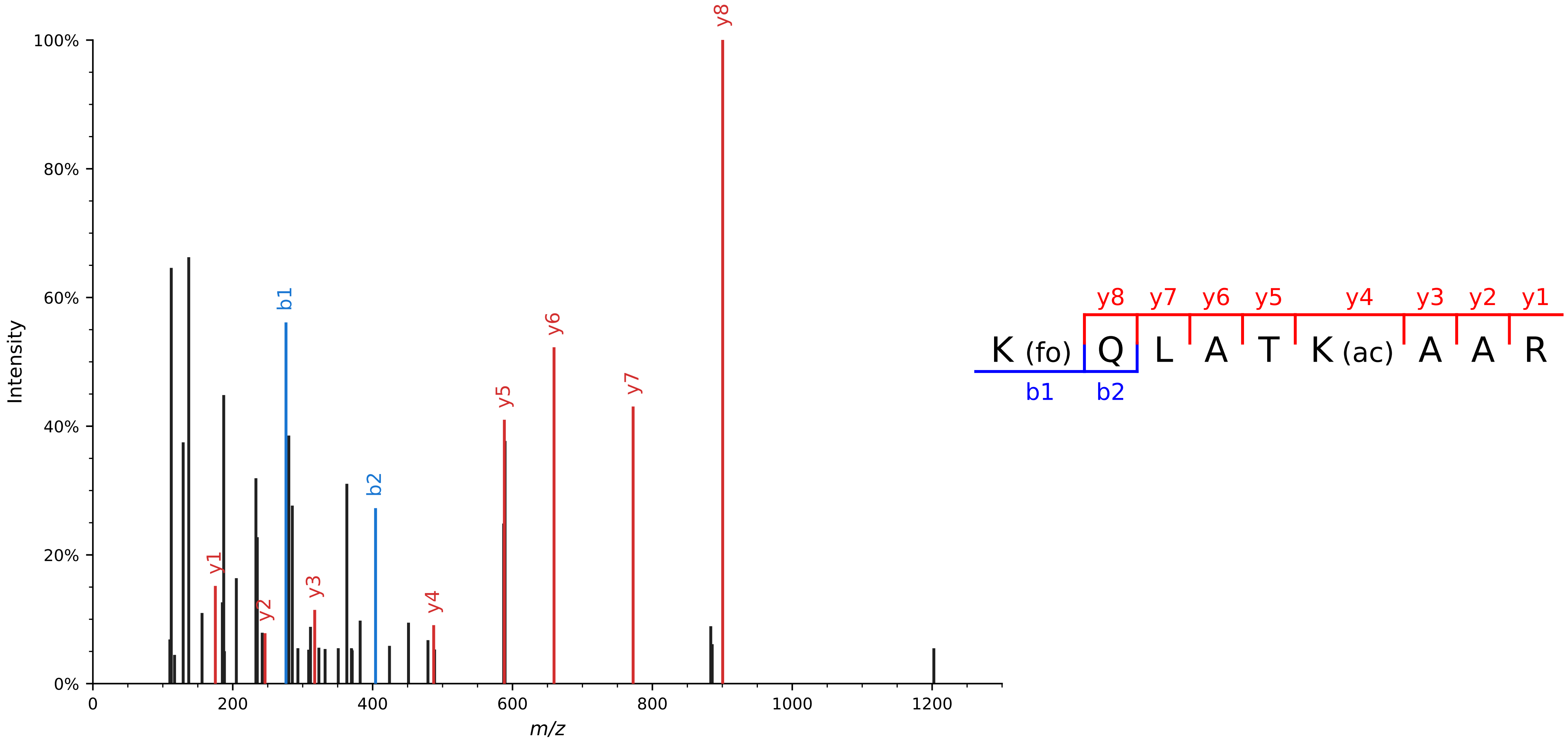

Raw file: HF221212_CG_D3AcPIC_Spleen_YSD1, Scan: 17507, m/z: 605.36468, Charge: 2, RT (min): 34.40, Score: 81.548

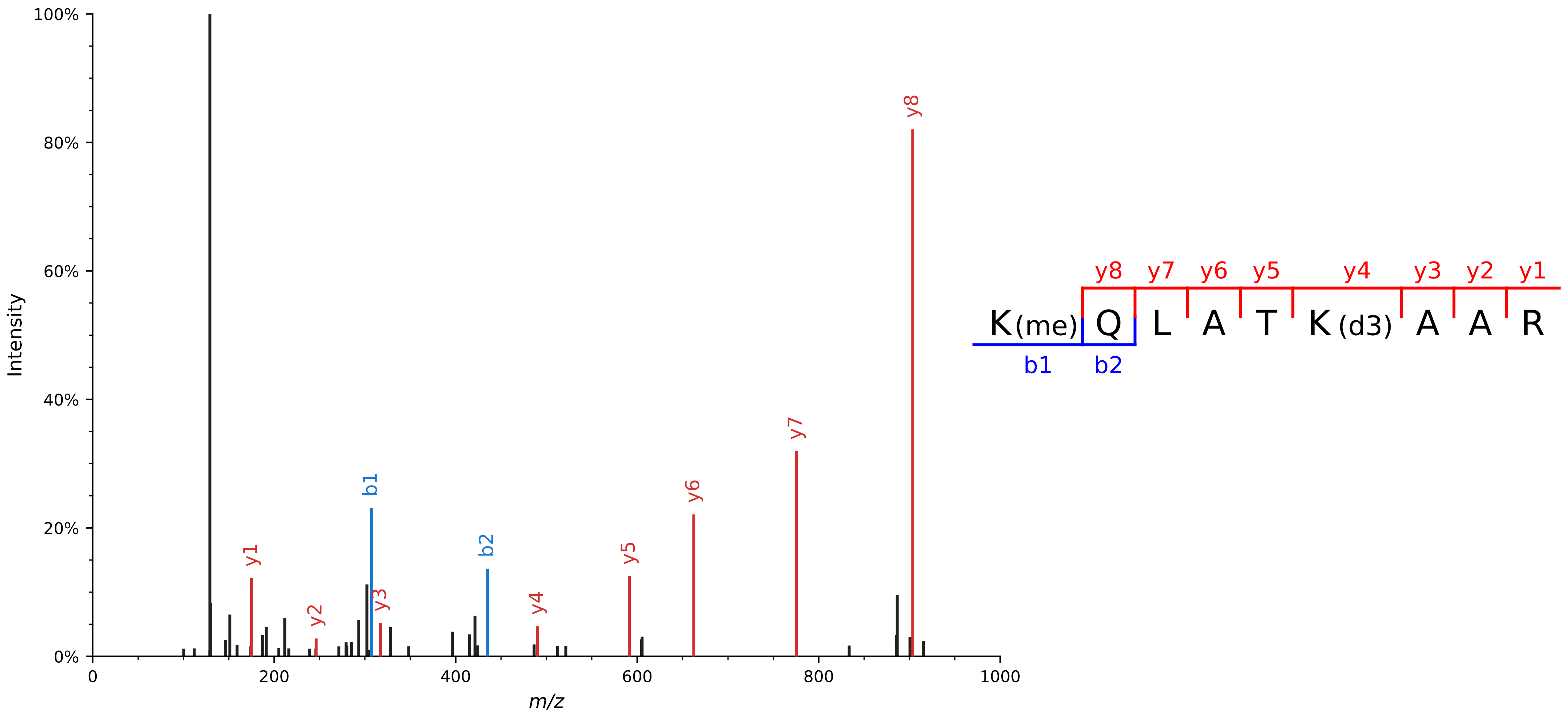

Raw file: HF221212_CG_D3AcPIC_Spleen_YSD1, Scan: 8648, m/z: 589.85781, Charge: 2, RT (min): 18.29, Score: 148.14

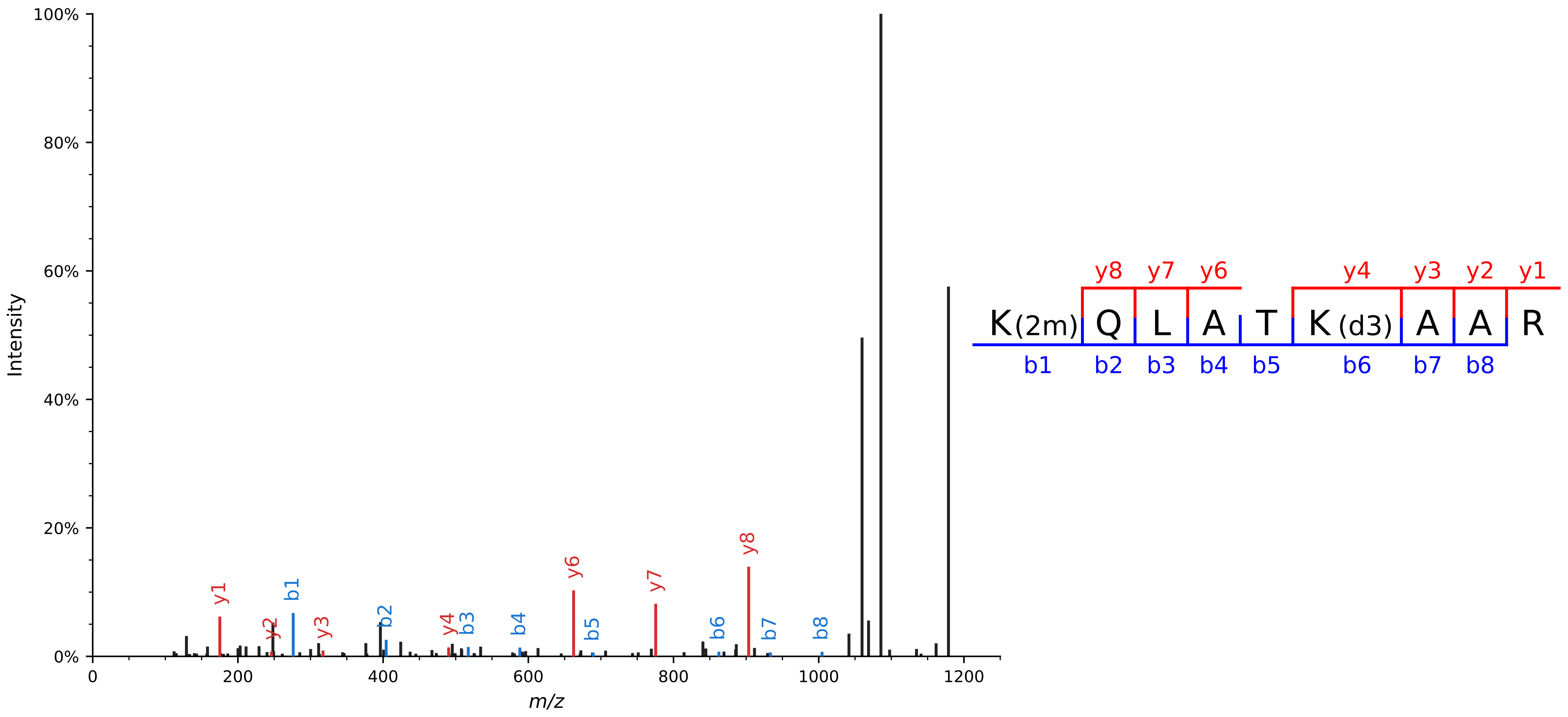

Raw file: HF221212_CG_D3AcPIC_Spleen_YSD3, Scan: 16358, m/z: 588.3484, Charge: 2, RT (min): 32.79, Score: 89.296

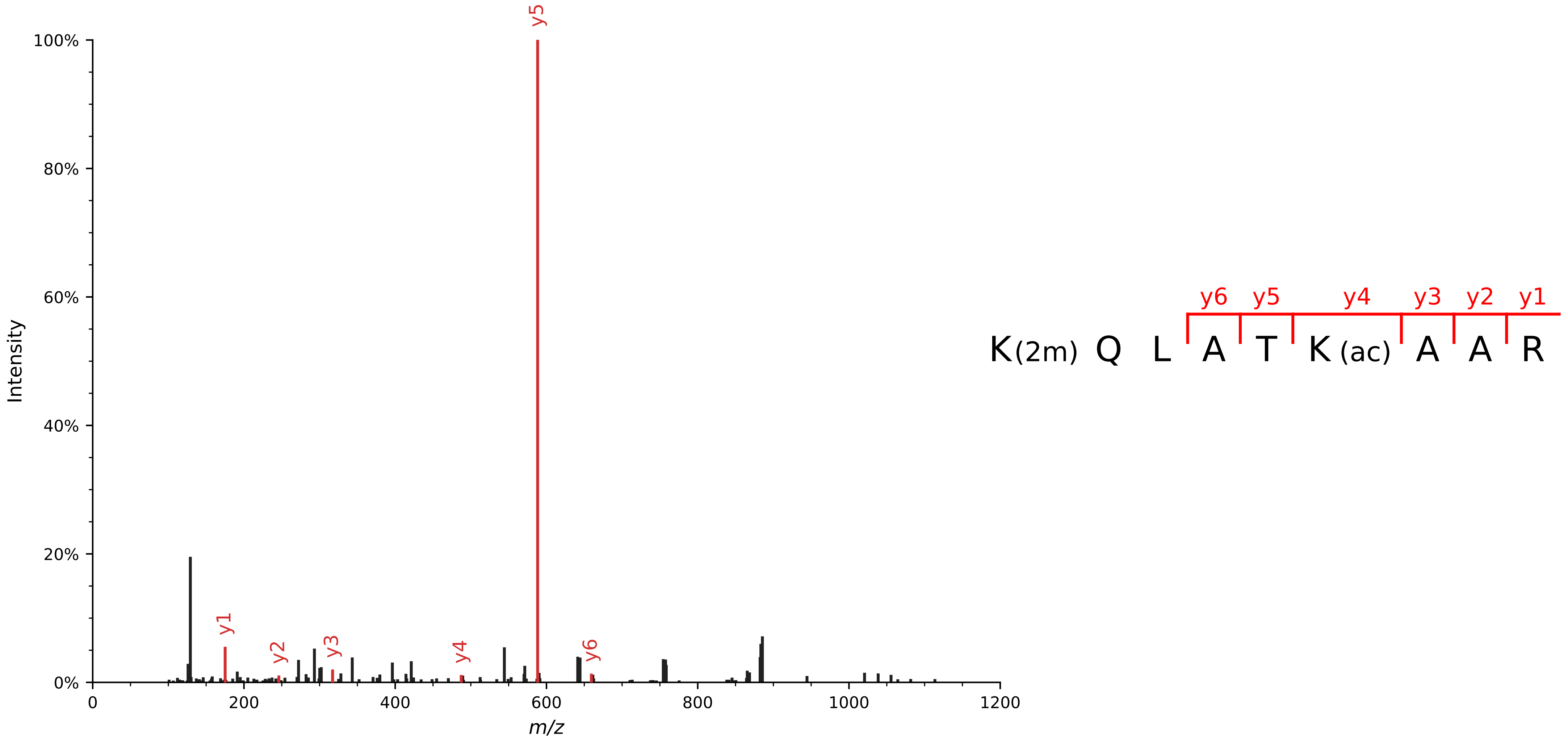

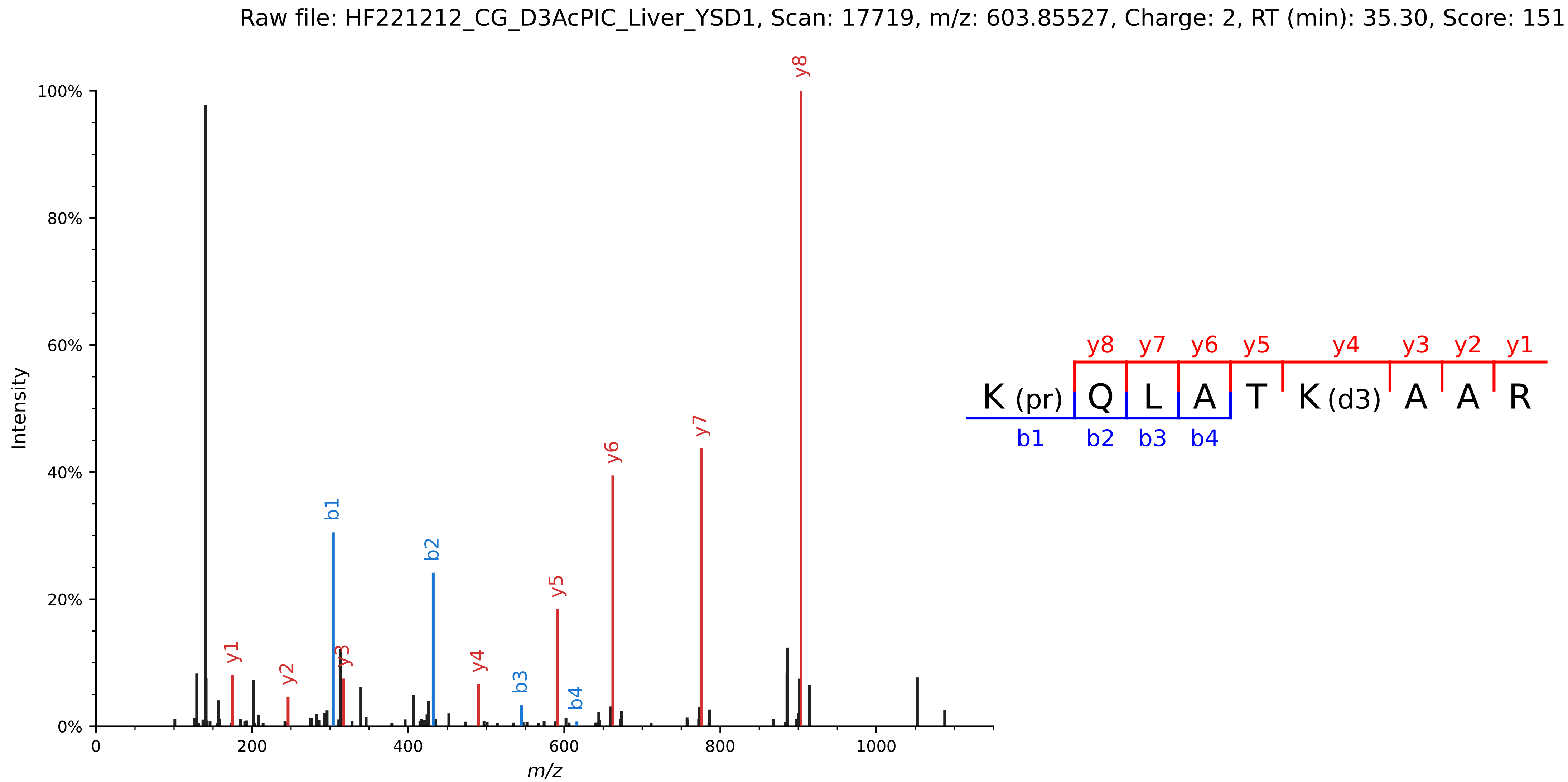

Raw file: HF221212_CG_D3AcPIC_Liver_YSD1, Scan: 17719, m/z: 603.85527, Charge: 2, RT (min): 35.30, Score: 151.04
Intensity
m/z
y8
y7
y6
y5
b1
b2
y1
y2
y3
y4
b3
b4
K (pr) Q L A T K (d3) A A R

Raw file: HF221212_CG_D3AcPIC_Heart_YSD2, Scan: 17200, m/z: 602.34585, Charge: 2, RT (min): 35.14, Score: 99.802

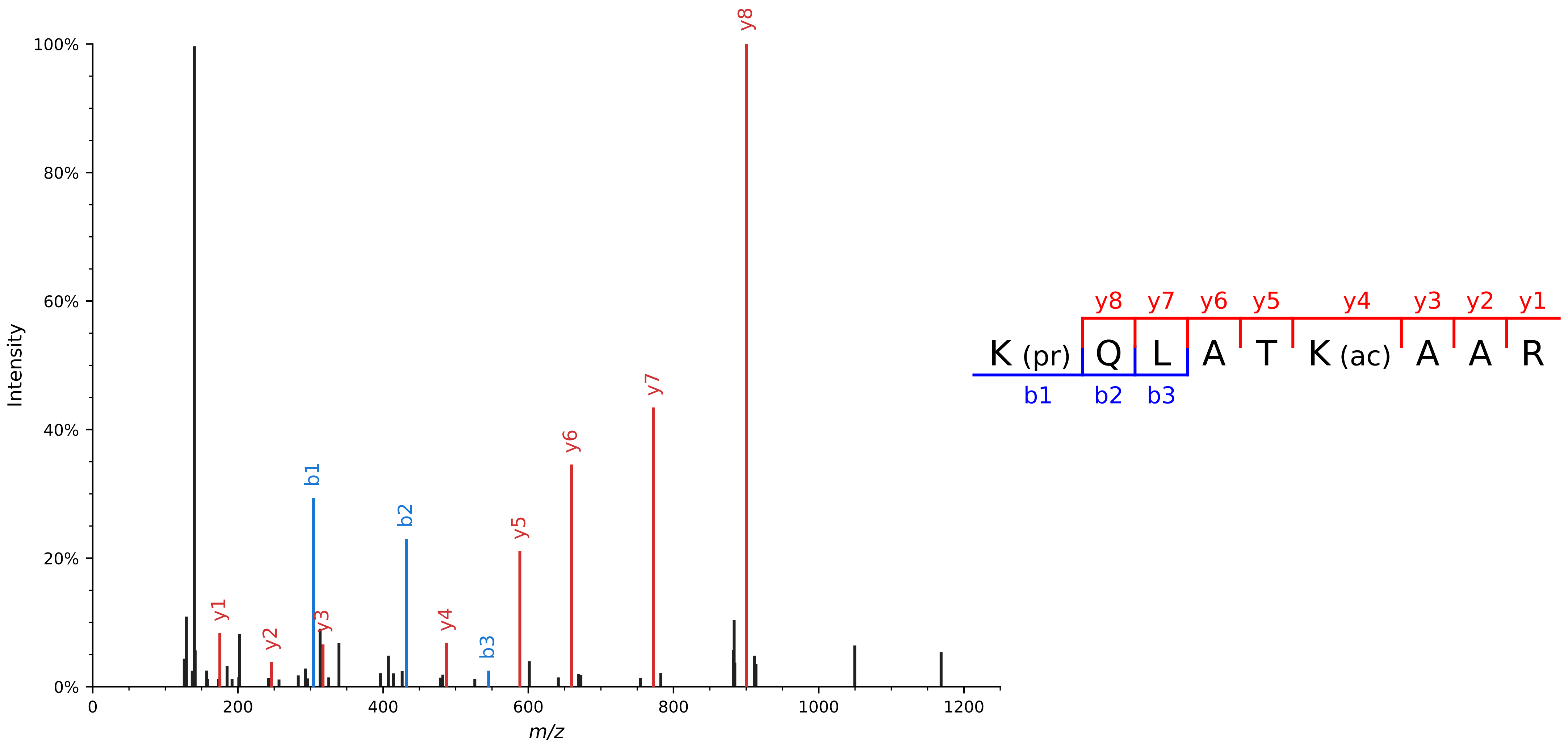

Raw file: HF221212_CG_D3AcPIC_Spleen_YSD1, Scan: 19175, m/z: 609.35368, Charge: 2, RT (min): 37.52, Score: 152.63

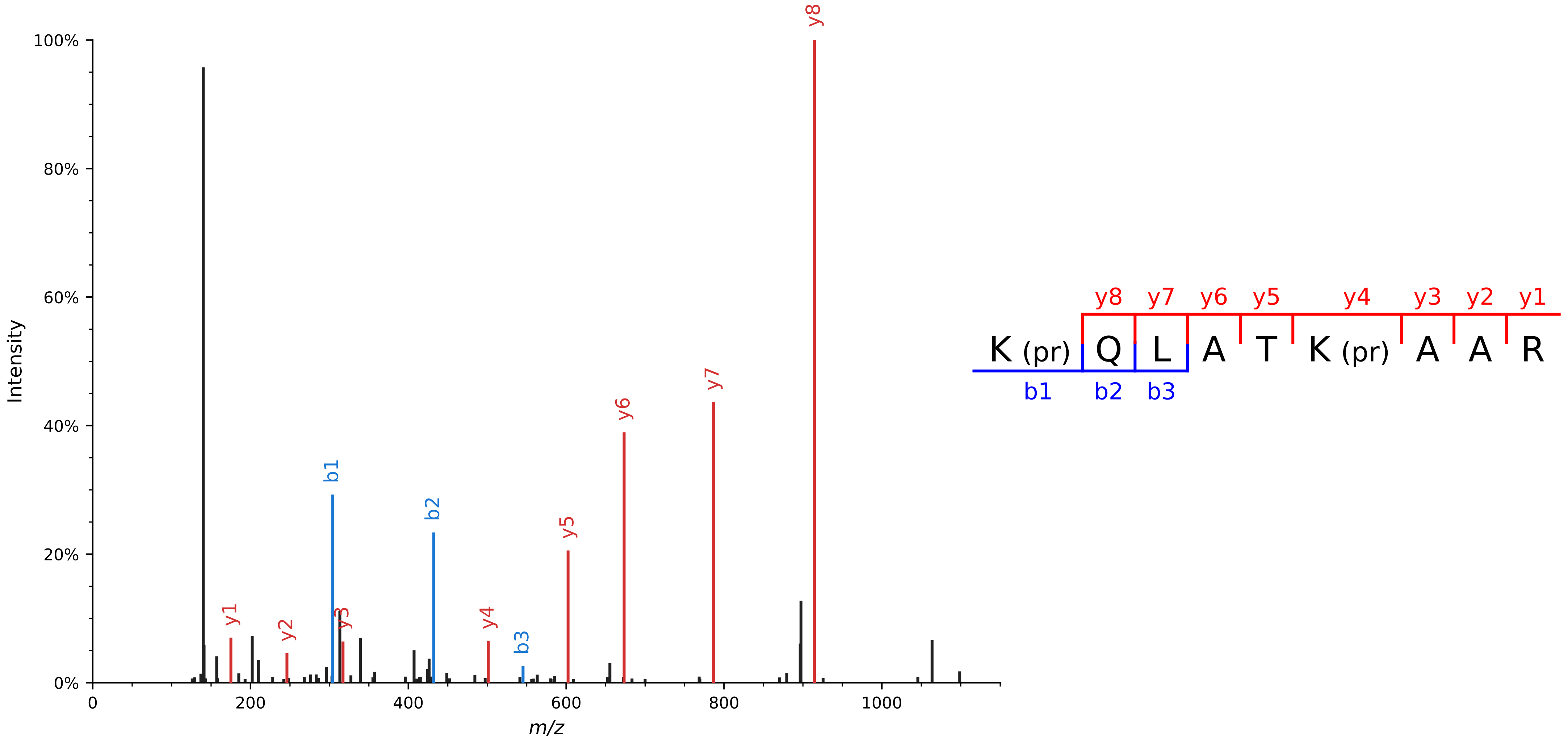

Raw file: HF221212_CG_D3AcPIC_Heart_YSD2, Scan: 15930, m/z: 596.84744, Charge: 2, RT (min): 32.72, Score: 151.04

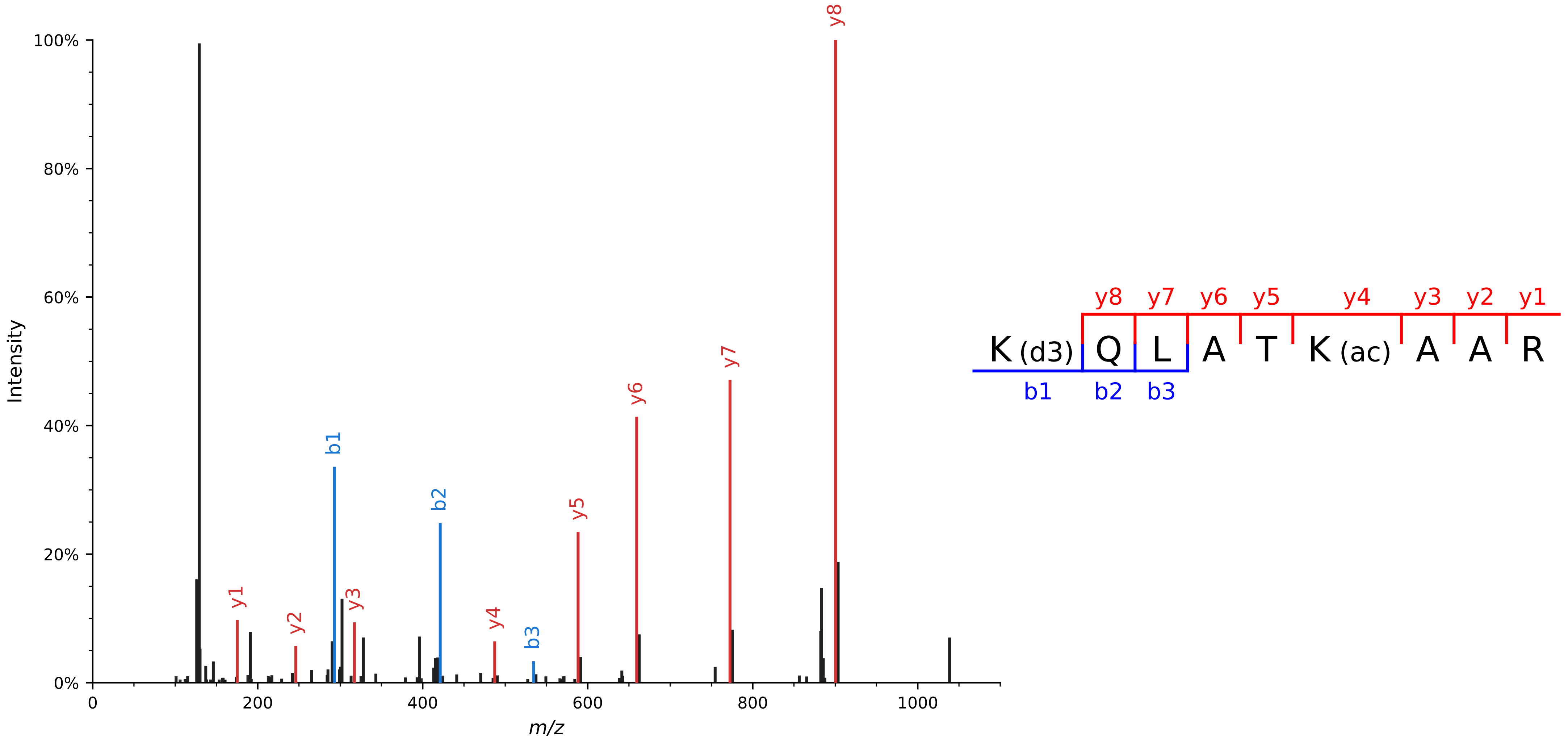

Raw file: HF221212_CG_D3AcPIC_Spleen_YSD1, Scan: 19281, m/z: 610.86309, Charge: 2, RT (min): 37.71, Score: 80.165

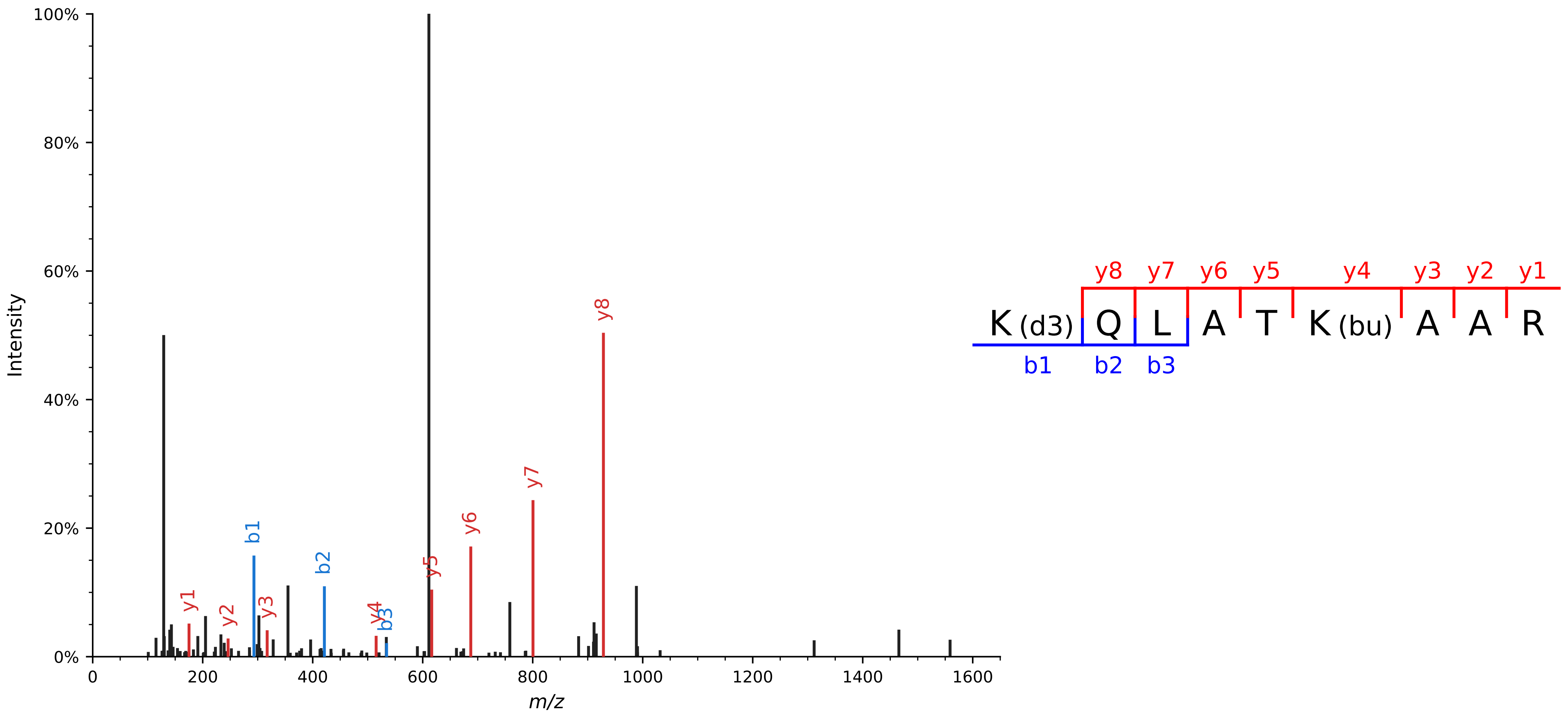

Raw file: HF221212_CG_D3AcPIC_Spleen_YSD1, Scan: 16453, m/z: 589.83962, Charge: 2, RT (min): 32.41, Score: 74.486

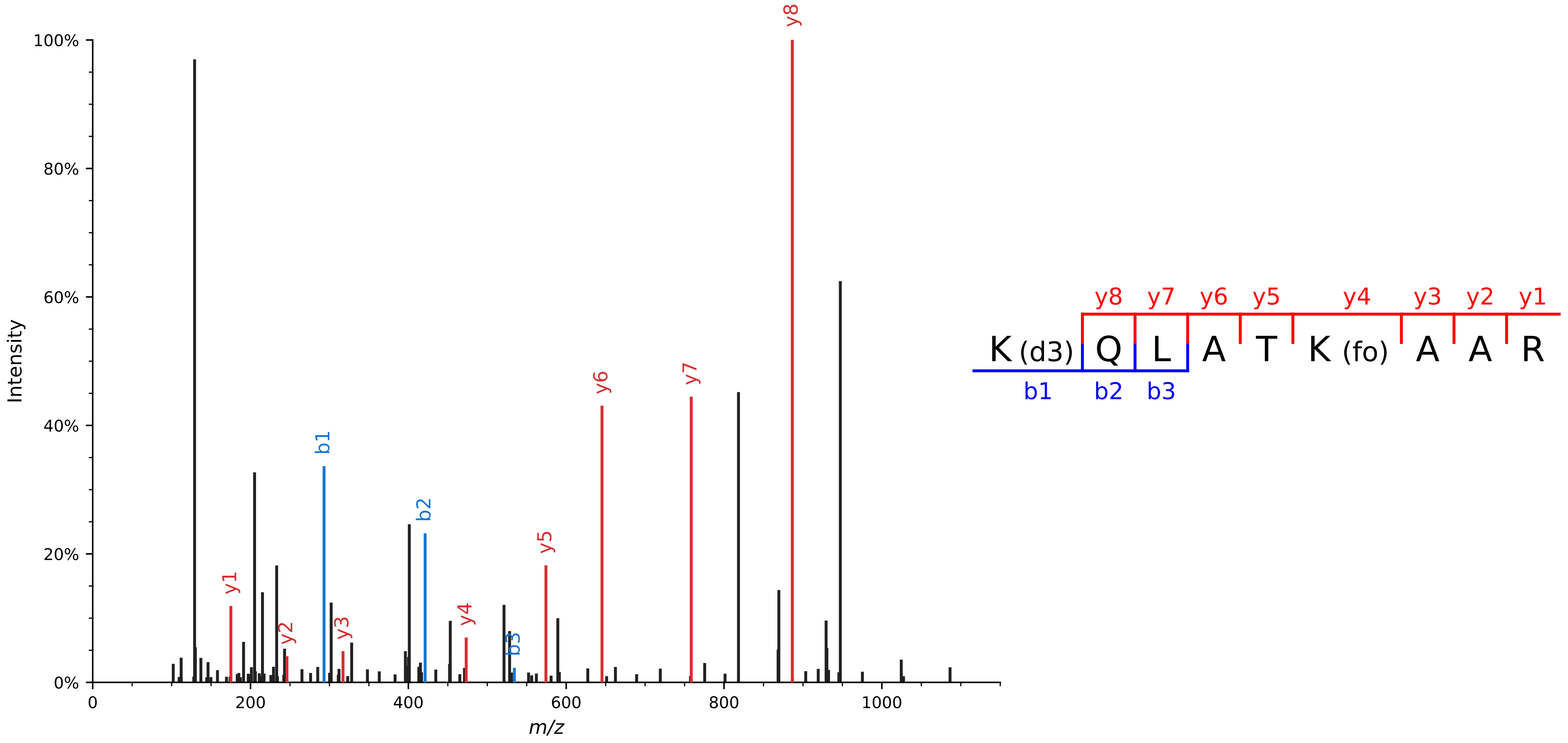

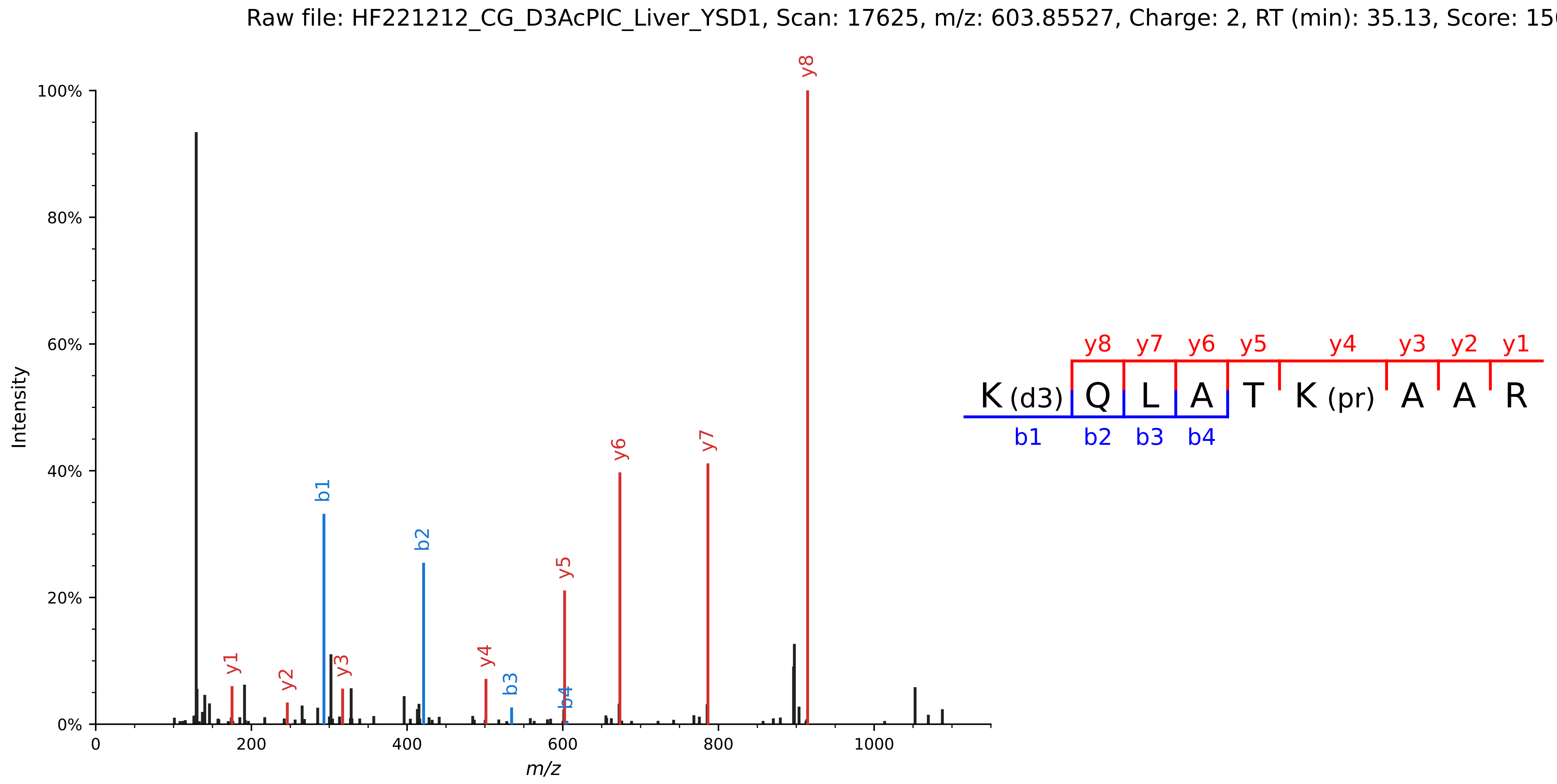
Raw file: HF221212_CG_D3AcPIC_Liver_YSD1, Scan: 17625, m/z: 603.85527, Charge: 2, RT (min): 35.13, Score: 150.4
Intensity
m/z
K (d3) Q L A T K (pr) A A R
y8 y7 y6 y5 y4 y3 y2 y1
b1 b2 b3 b4

Raw file: HF221212_CG_D3AcPIC_Spleen_YSD1, Scan: 10119, m/z: 566.99008, Charge: 3, RT (min): 20.94, Score: 47.532

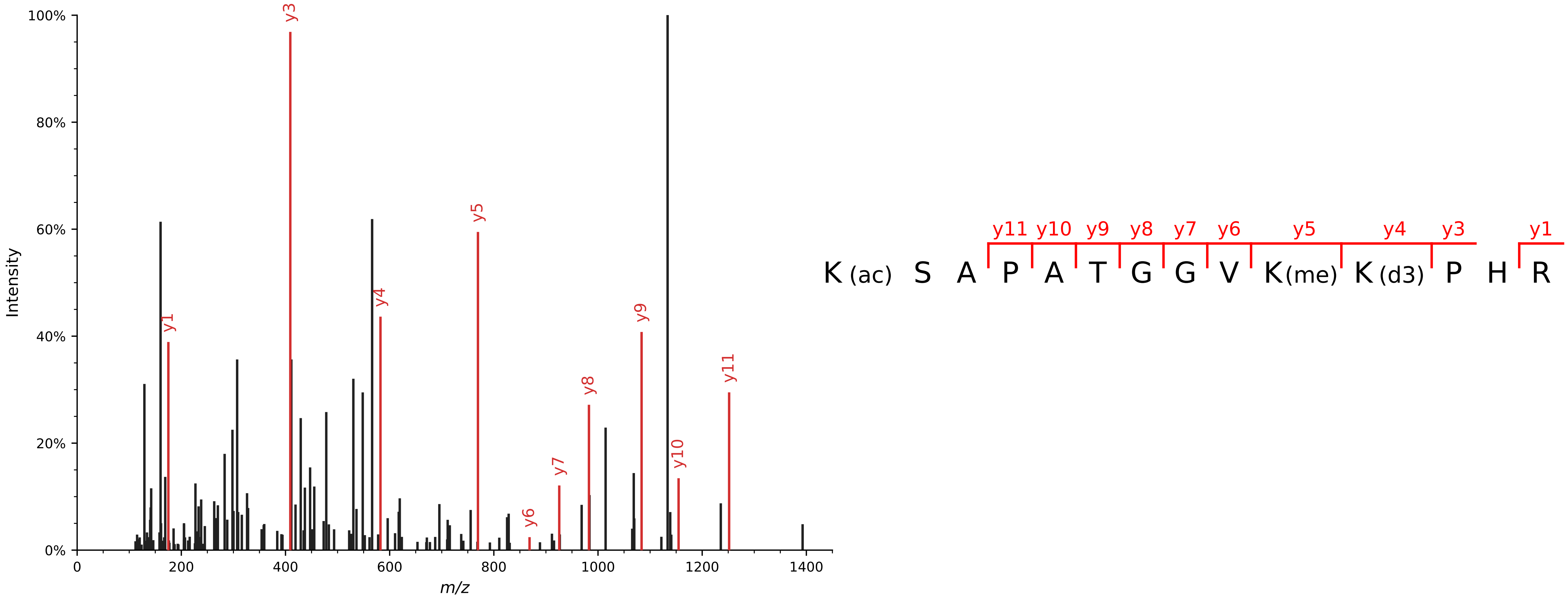

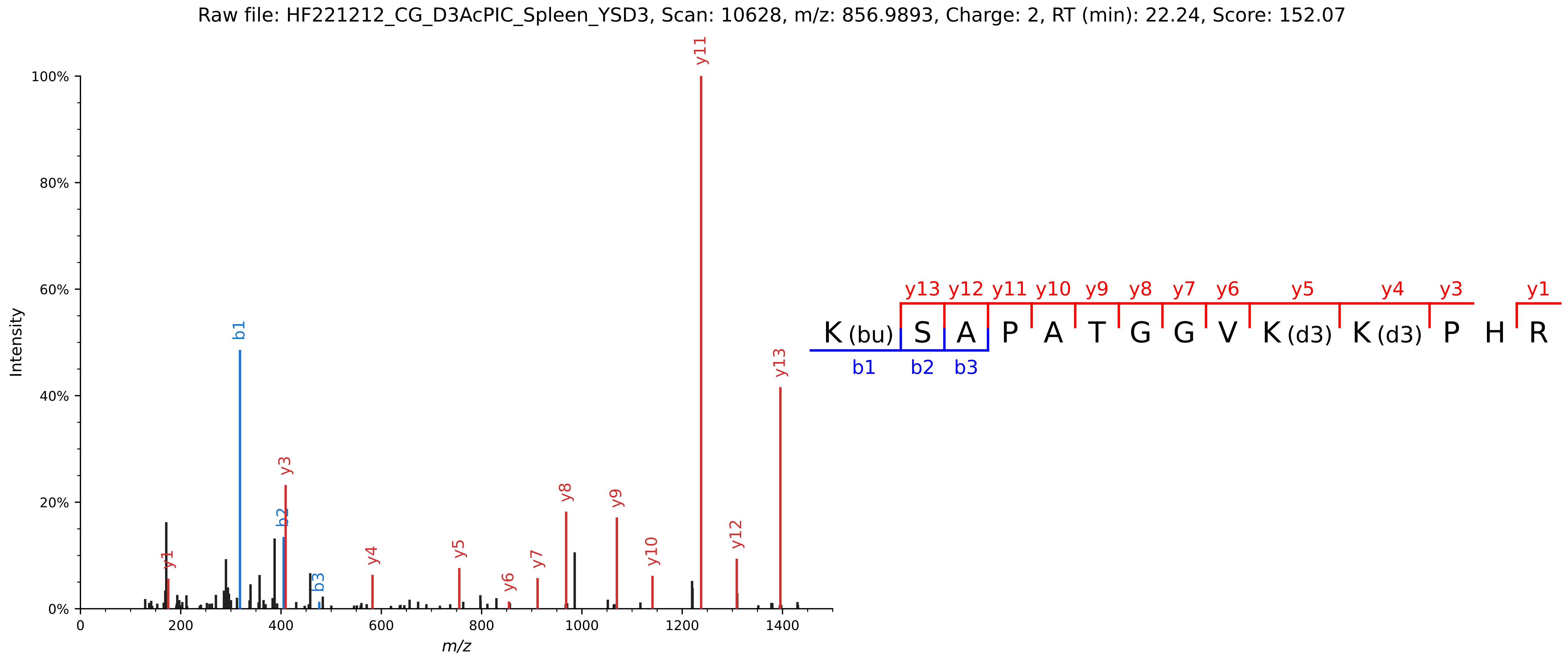
Raw file: HF221212_CG_D3AcPIC_Spleen_YSD3, Scan: 10628, m/z: 856.9893, Charge: 2, RT (min): 22.24, Score: 152.07
Intensity
m/z
y1
b1
b2
y3
b3
y4
y5
y6
y7
y8
y9
y10
y11
y12
y13
K (bu) S A P A T G G V K (d3) K (d3) P H R

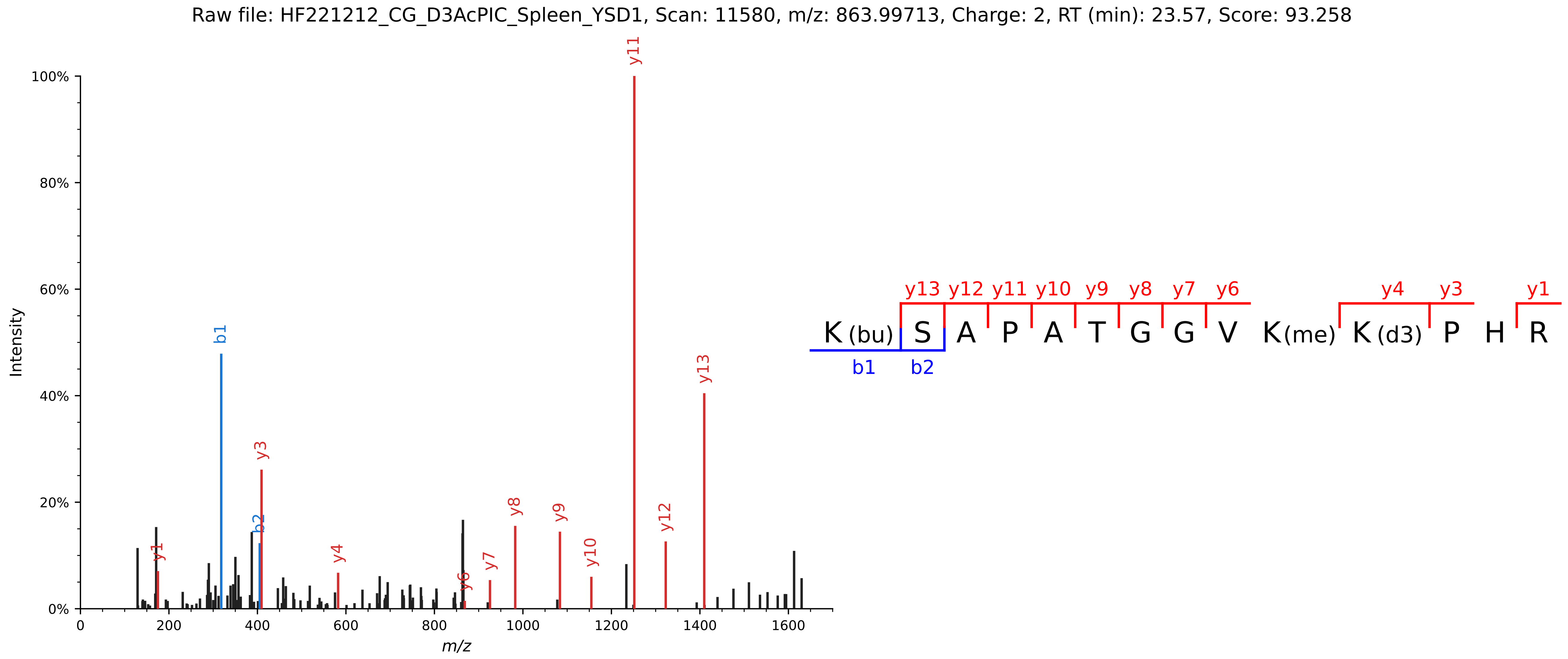

Raw file: HF221212_CG_D3AcPIC_Spleen_YSD1, Scan: 11580, m/z: 863.99713, Charge: 2, RT (min): 23.57, Score: 93.258
Intensity
m/z
100%
80%
60%
40%
20%
0%
0
200
400
600
800
1000
1200
1400
1600
y1
b1
b2
y3
y4
y6
y7
y8
y9
y10
y11
y12
y13
K(bu) S A P A T G G V K(me) K(d3) P H R
y13 y12 y11 y10 y9 y8 y7 y6 y4 y3 y1
b1 b2

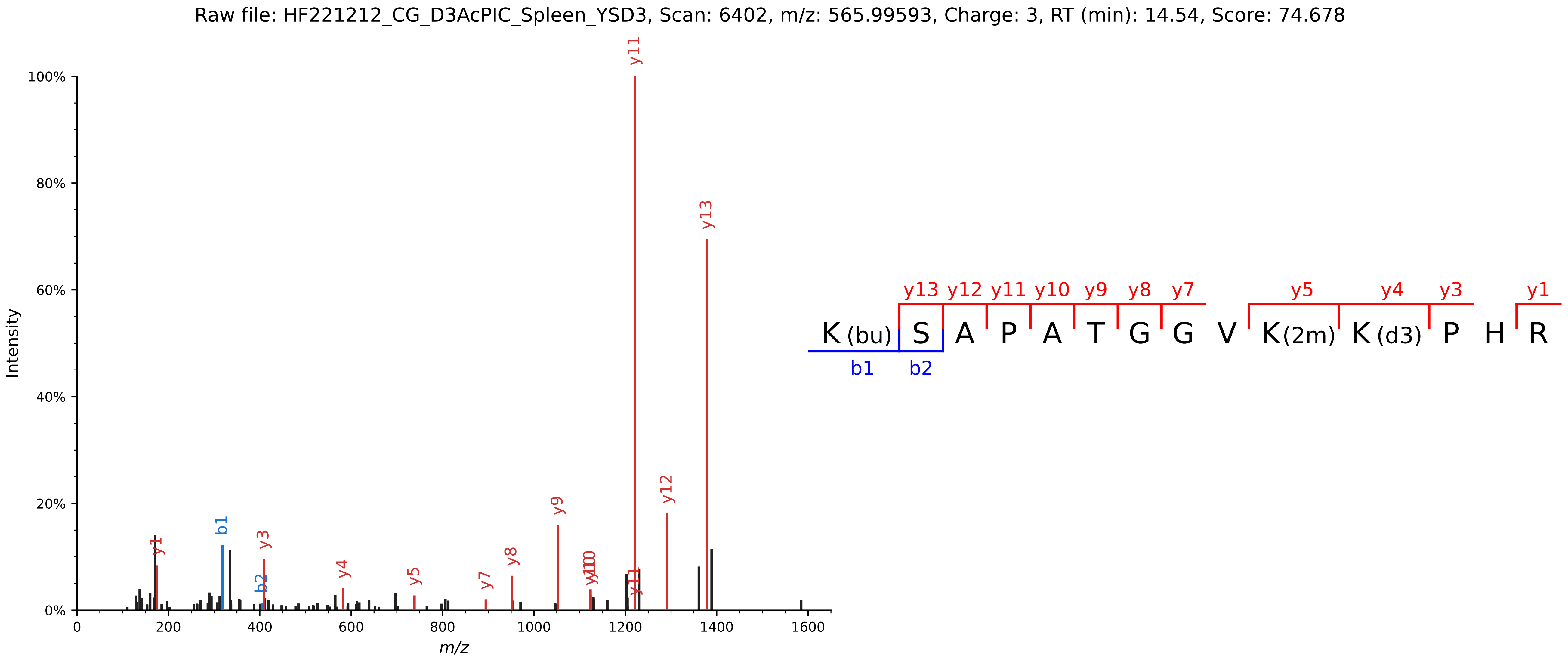
Raw file: HF221212_CG_D3AcPIC_Spleen_YSD3, Scan: 6402, m/z: 565.99593, Charge: 3, RT (min): 14.54, Score: 74.678
Intensity
m/z
K(bu) S A P A T G G V K(2m) K(d3) P H R
y13 y12 y11 y10 y9 y8 y7 y5 y4 y3 y1
b1 b2

Raw file: HF221212_CG_D3AcPIC_Spleen_YSD2, Scan: 8273, m/z: 857.97893, Charge: 2, RT (min): 17.83, Score: 45.973

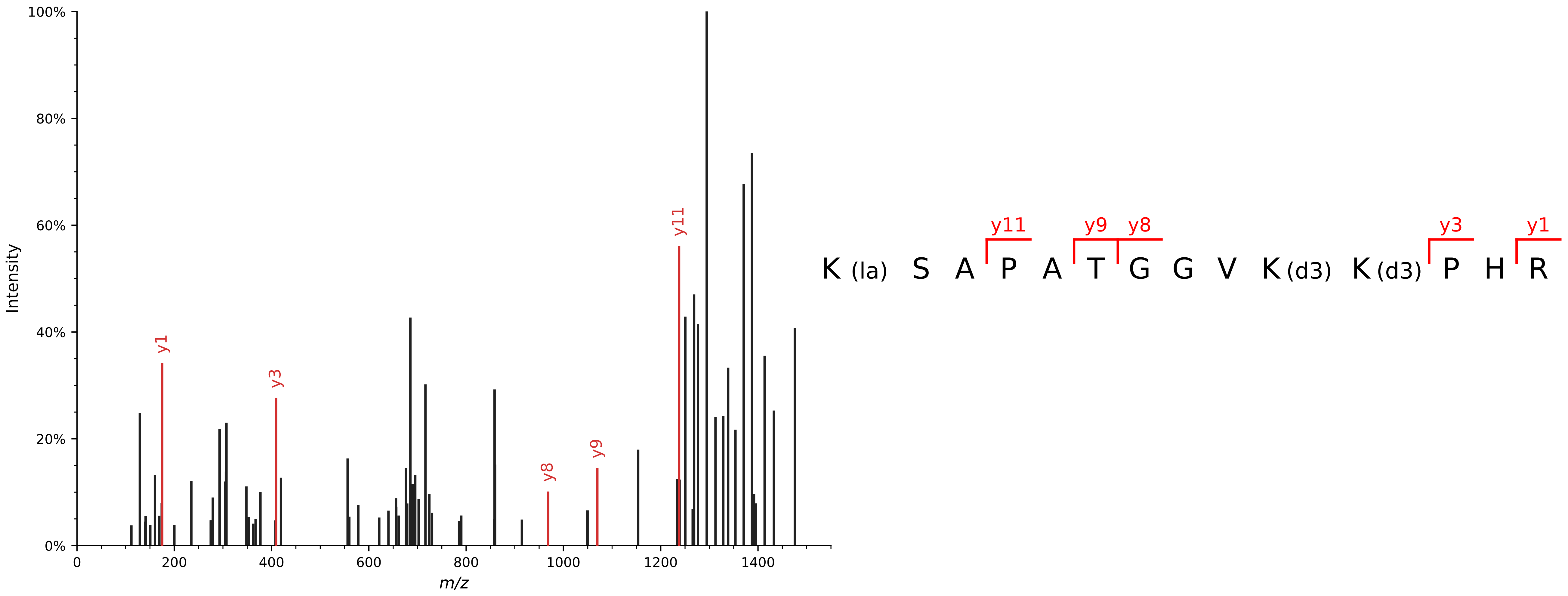

Raw file: HF221212_CG_D3AcPIC_Spleen_YSD1, Scan: 9401, m/z: 851.49089, Charge: 2, RT (min): 19.61, Score: 187.42

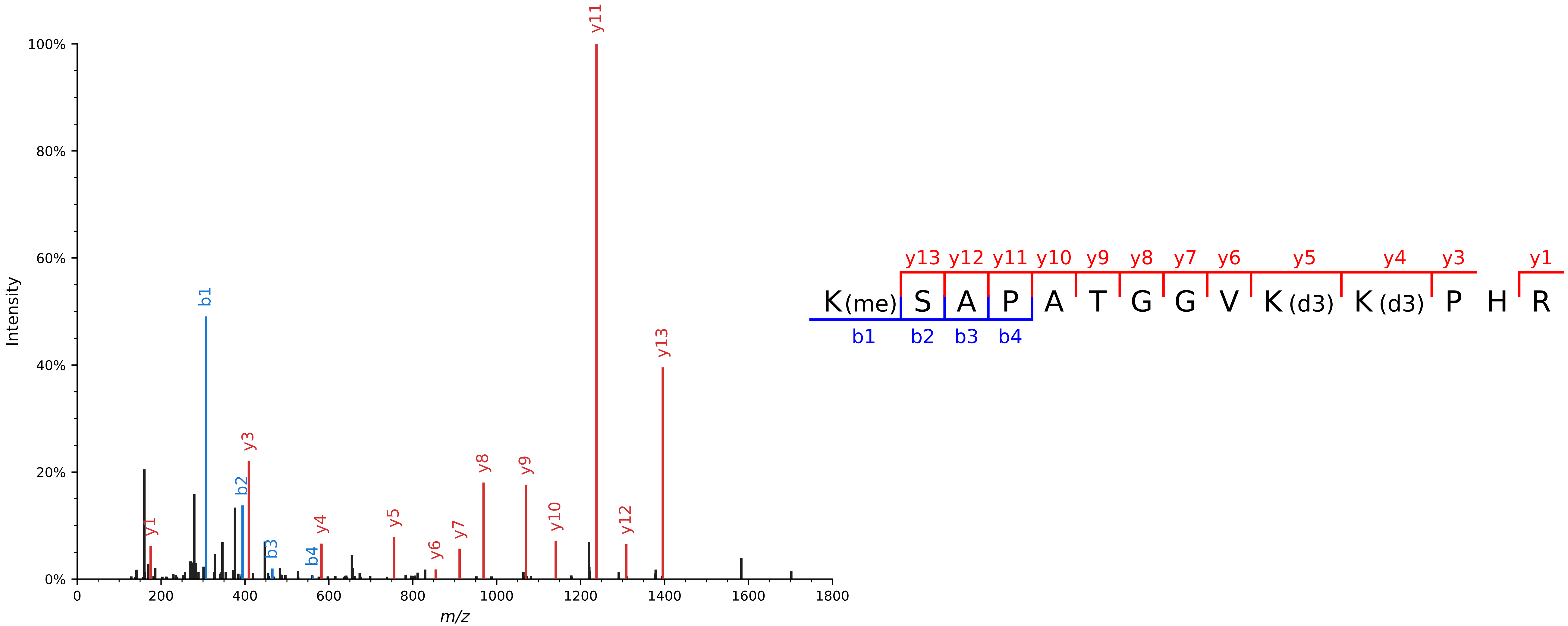

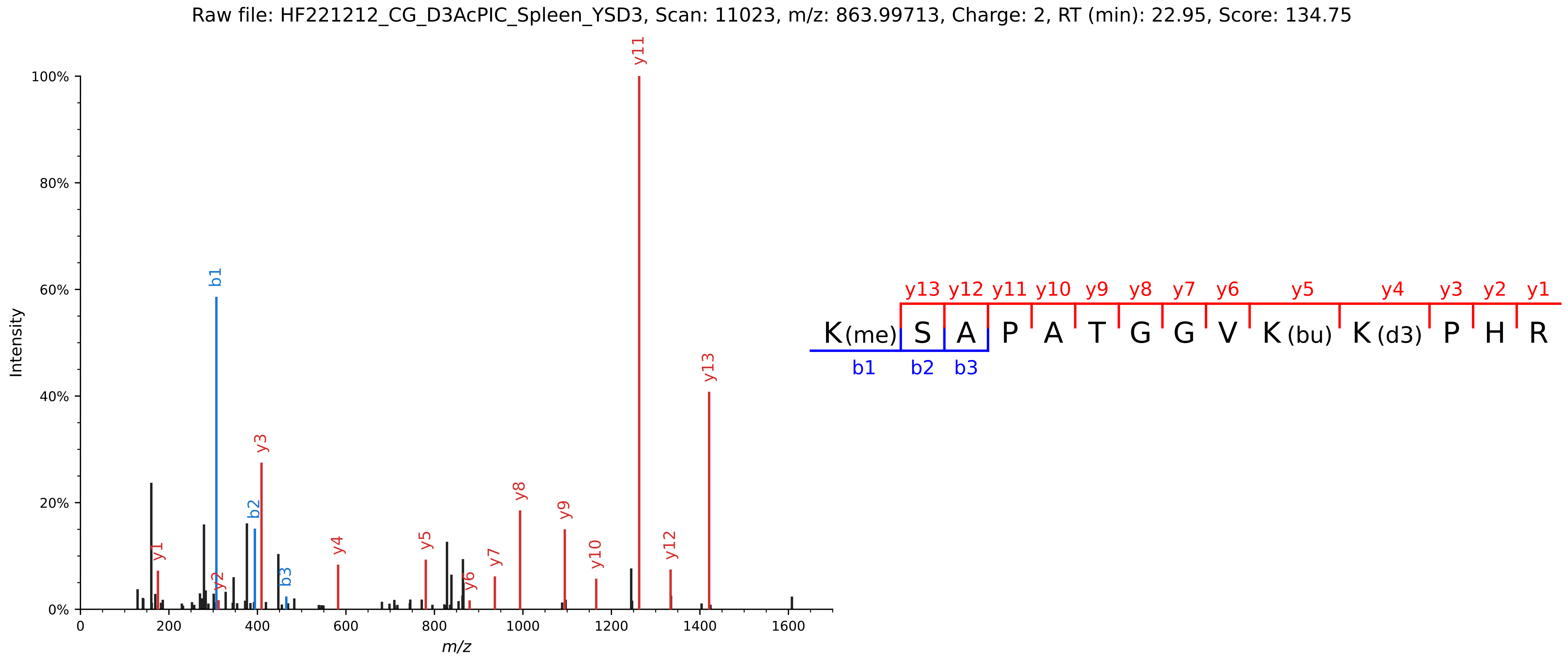

Raw file: HF221212_CG_D3AcPIC_Spleen_YSD3, Scan: 11023, m/z: 863.99713, Charge: 2, RT (min): 22.95, Score: 134.75
Intensity
m/z
K(me) S A P A T G G V K(bu) K(d3) P H R
y13 y12 y11 y10 y9 y8 y7 y6 y5 y4 y3 y2 y1
b1 b2 b3

Raw file: HF221212_CG_D3AcPIC_Spleen_YSD1, Scan: 9169, m/z: 842.97365, Charge: 2, RT (min): 19.20, Score: 72.652

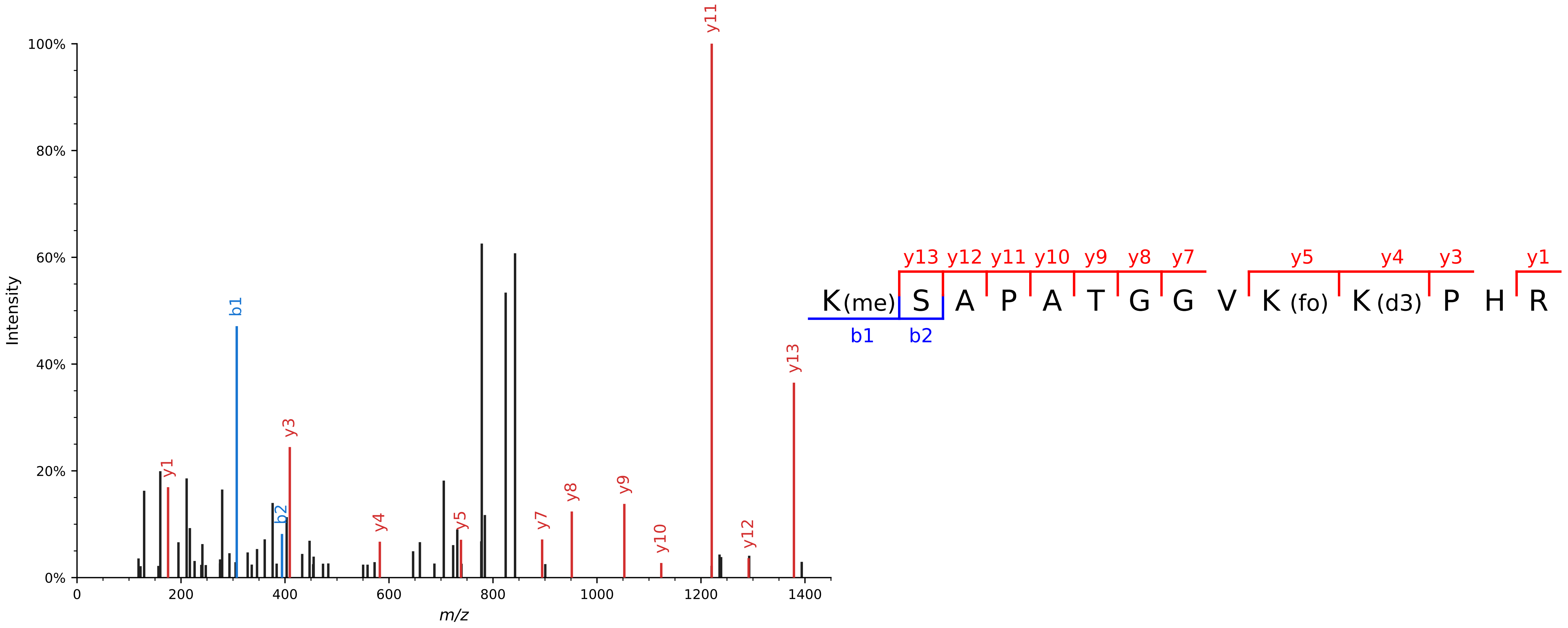

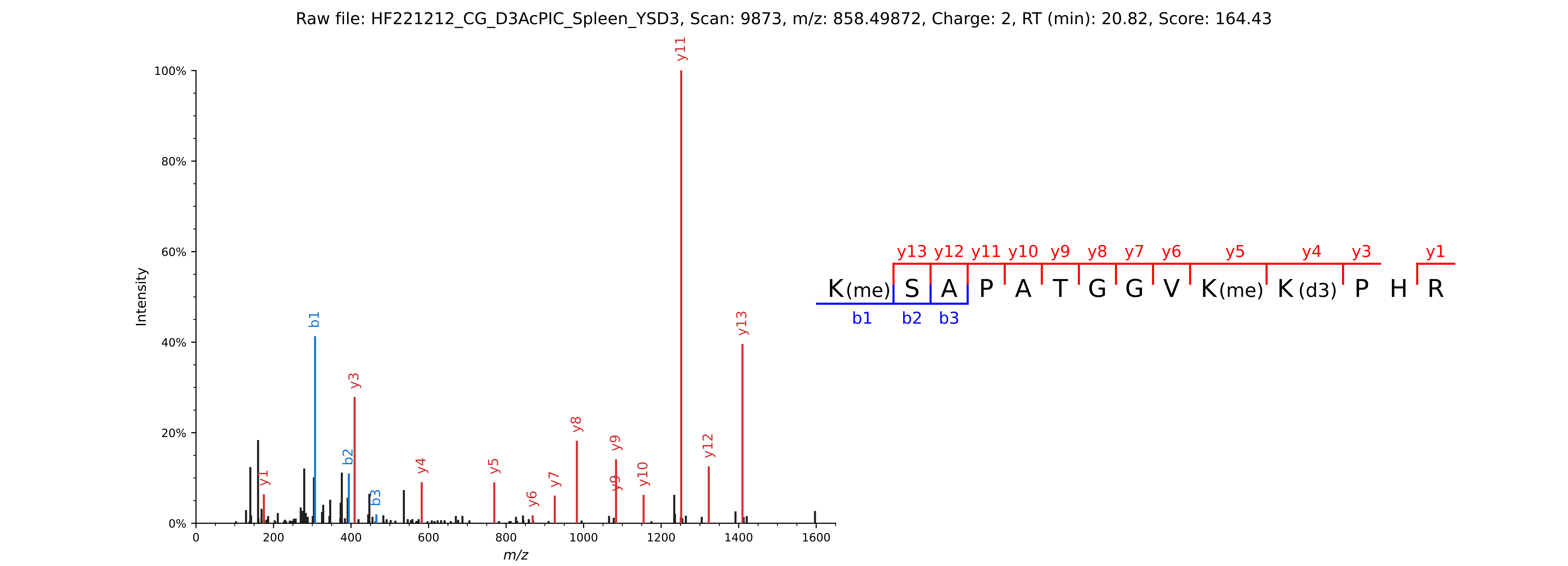

Raw file: HF221212_CG_D3AcPIC_Spleen_YSD3, Scan: 9873, m/z: 858.49872, Charge: 2, RT (min): 20.82, Score: 164.43
Intensity
m/z
K(me) S A P A T G G V K(me) K(d3) P H R
y13 y12 y11 y10 y9 y8 y7 y6 y5 y4 y3 y1
b1 b2 b3

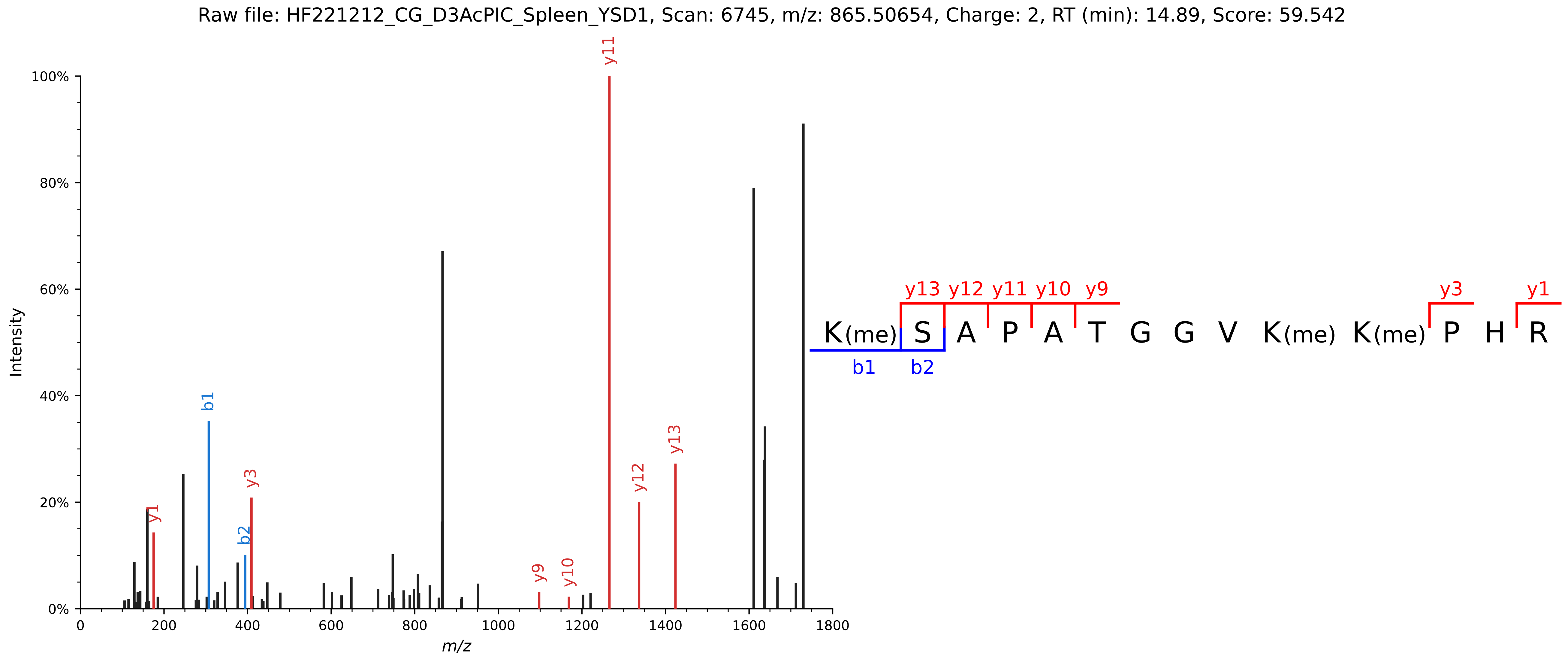
Raw file: HF221212_CG_D3AcPIC_Spleen_YSD1, Scan: 6745, m/z: 865.50654, Charge: 2, RT (min): 14.89, Score: 59.542
Intensity
m/z
y1
b1
b2
y3
y9
y10
y11
y12
y13
K(me) S A P A T G G V K(me) K(me) P H R

Raw file: HF221212_CG_D3AcPIC_Spleen_YSD1, Scan: 10904, m/z: 863.99713, Charge: 2, RT (min): 22.38, Score: 103.08

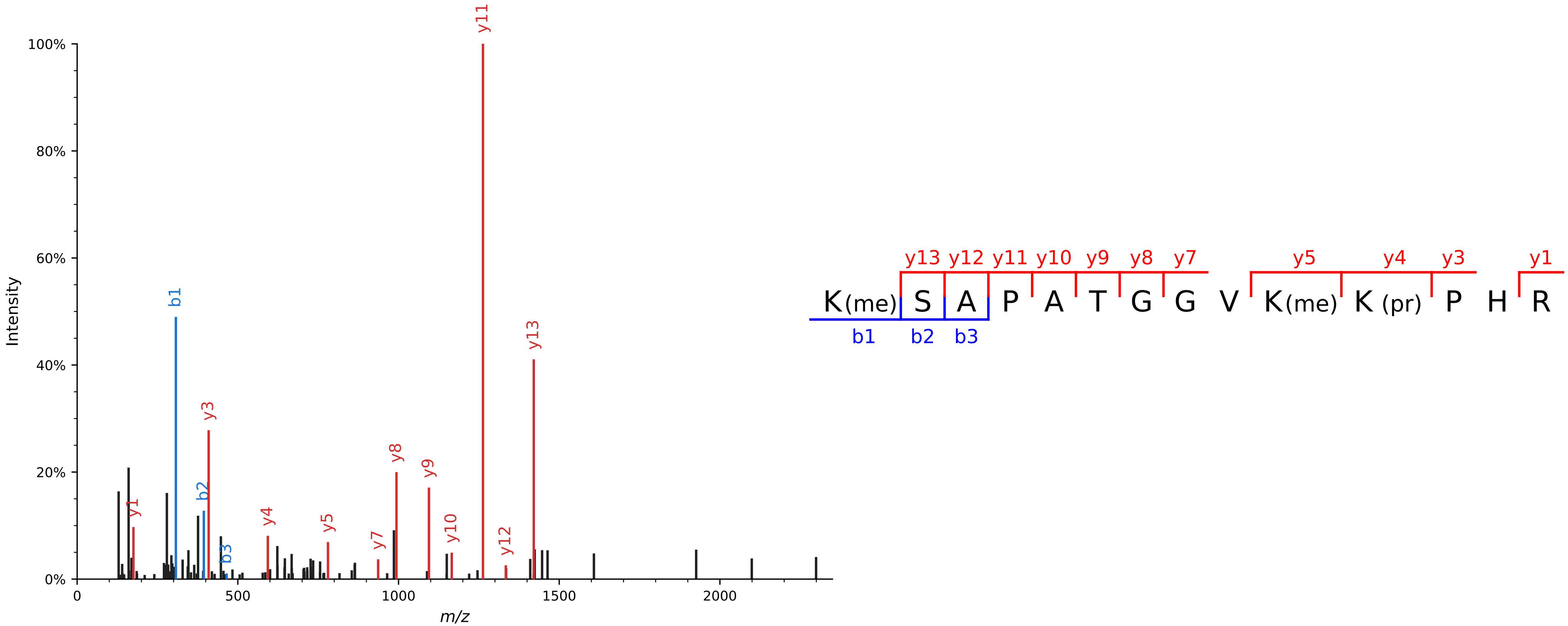

Raw file: HF221212_CG_D3AcPIC_Spleen_YSD1, Scan: 5550, m/z: 842.99184, Charge: 2, RT (min): 12.78, Score: 157.68

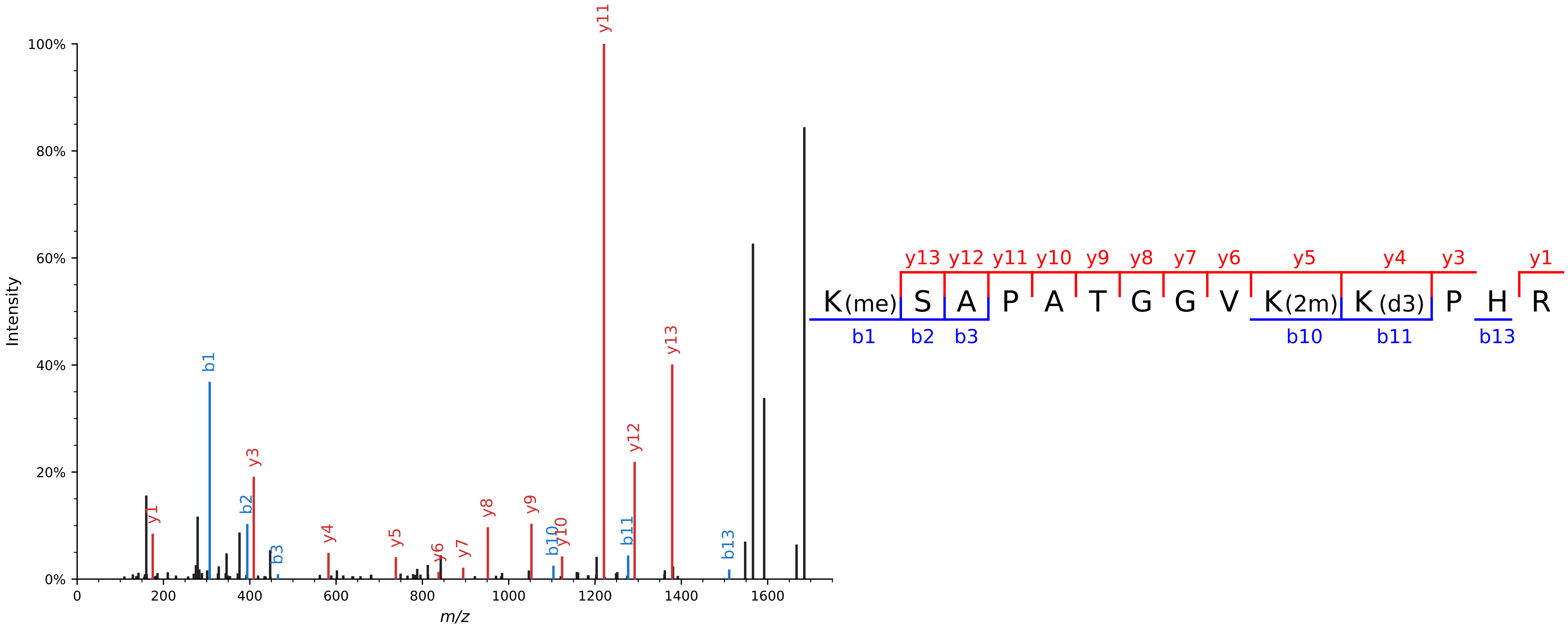

Raw file: HF221212_CG_D3AcPIC_Spleen_YSD1, Scan: 6247, m/z: 848.49025, Charge: 2, RT (min): 14.02, Score: 163.78

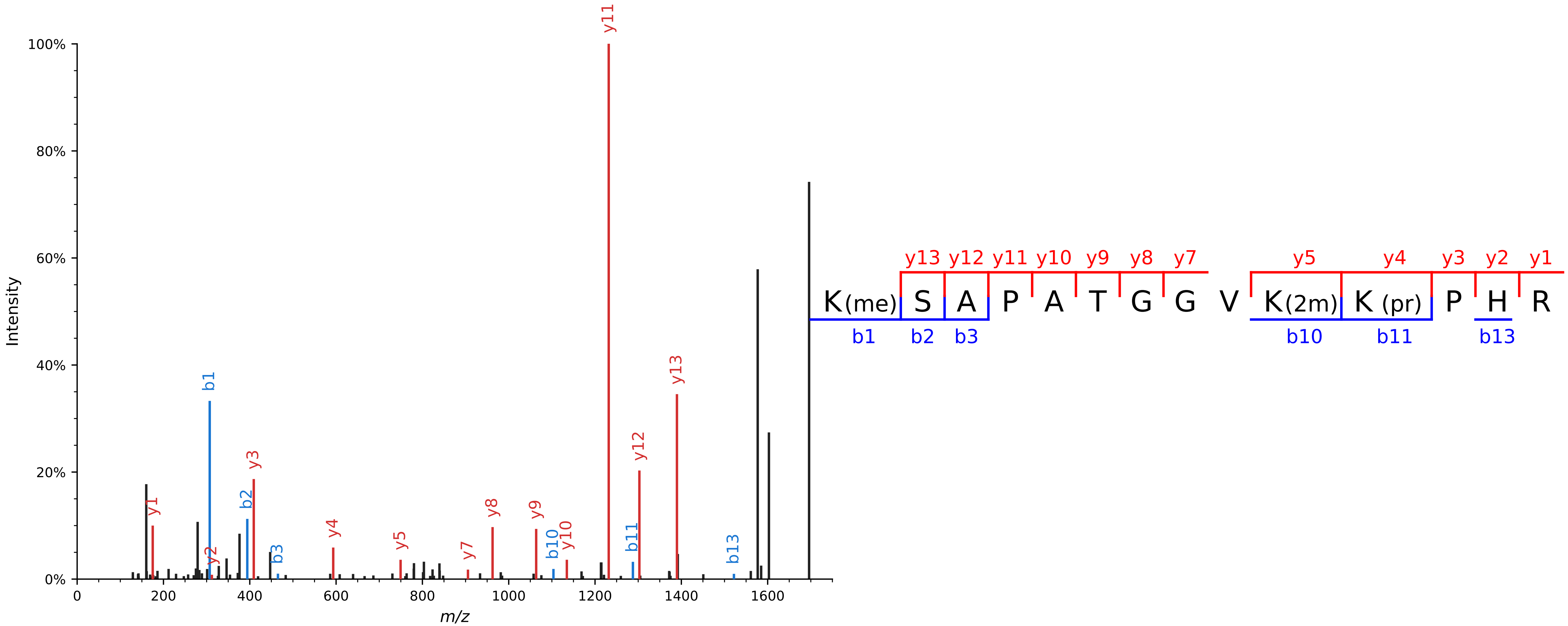

Raw file: HF221212_CG_D3AcPIC_Spleen_YSD2, Scan: 5267, m/z: 849.99967, Charge: 2, RT (min): 12.44, Score: 104.82

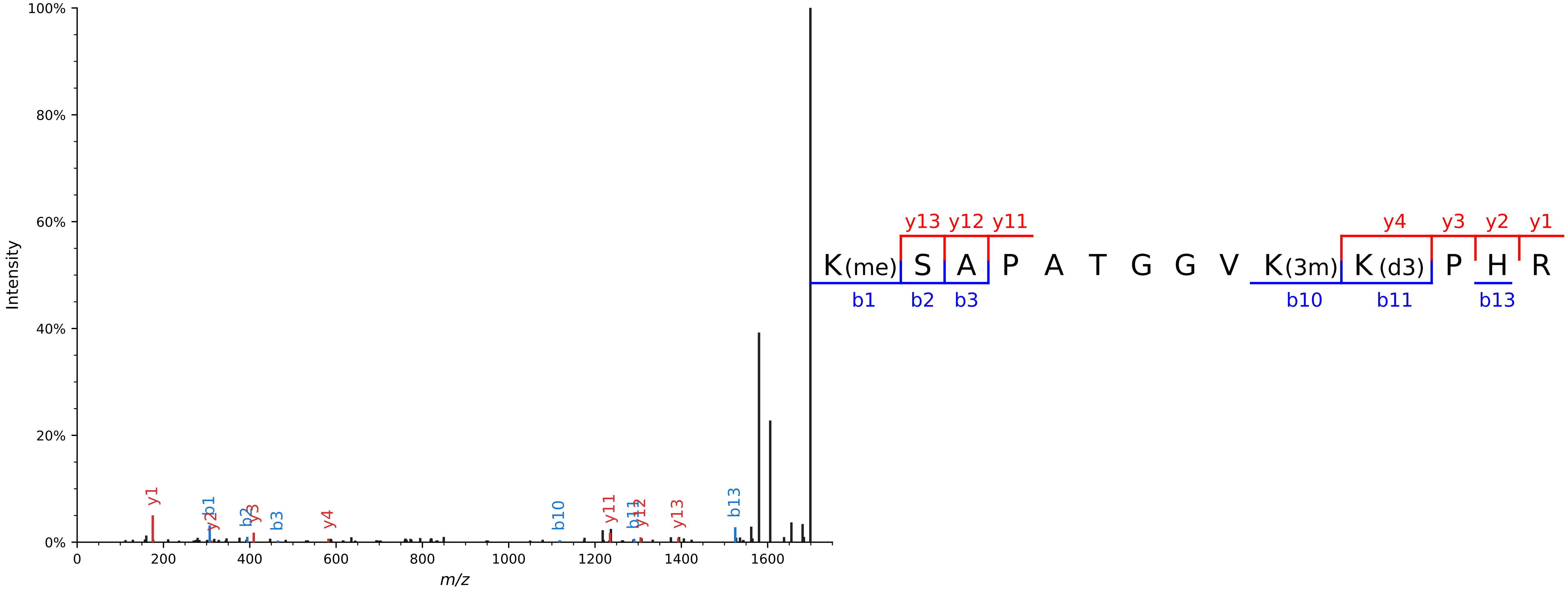

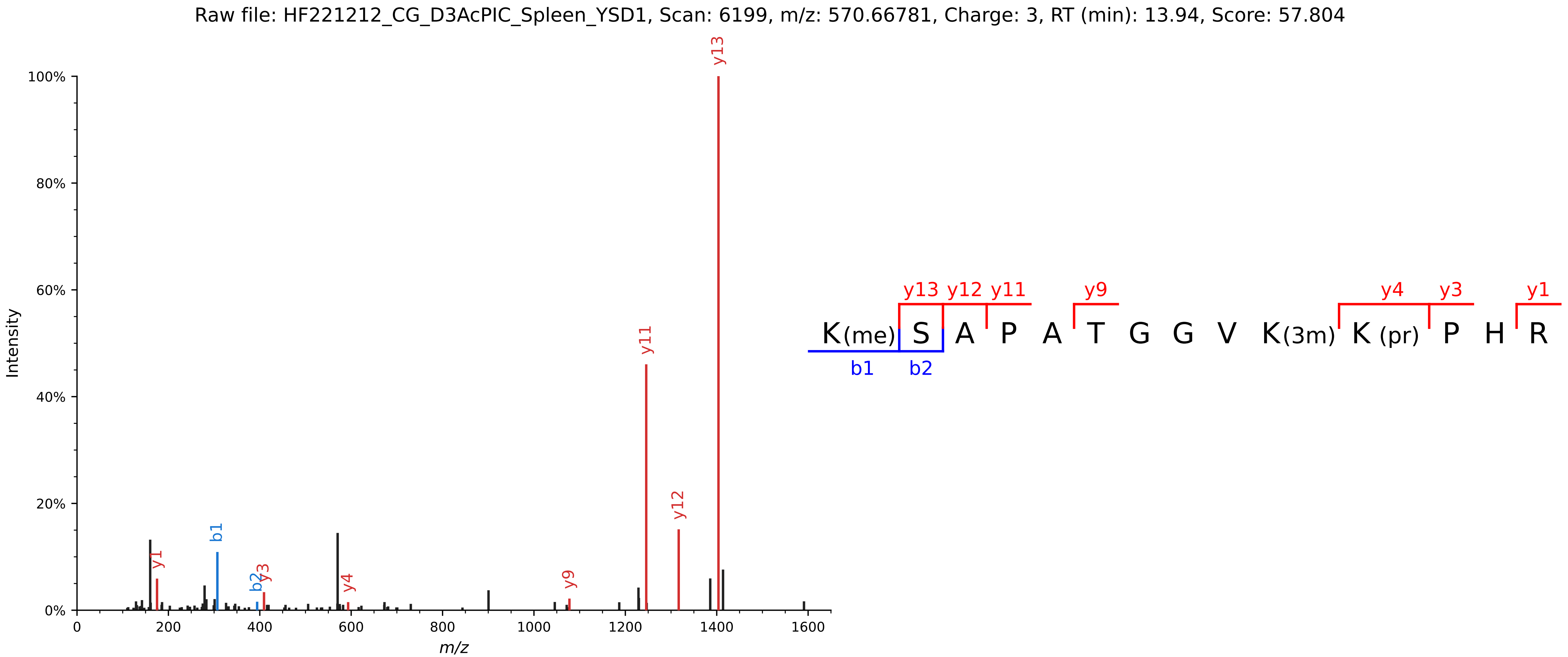

Raw file: HF221212_CG_D3AcPIC_Spleen_YSD1, Scan: 6199, m/z: 570.66781, Charge: 3, RT (min): 13.94, Score: 57.804
Intensity
m/z
y13
y11
y12
b1
y1
b2
y3
y4
y9
y13 y12 y11 y9 y4 y3 y1
K(me) S A P A T G G V K(3m) K (pr) P H R
b1 b2

Raw file: HF221212_CG_D3AcPIC_Liver_YSD2, Scan: 10130, m/z: 856.9893, Charge: 2, RT (min): 21.36, Score: 70.942

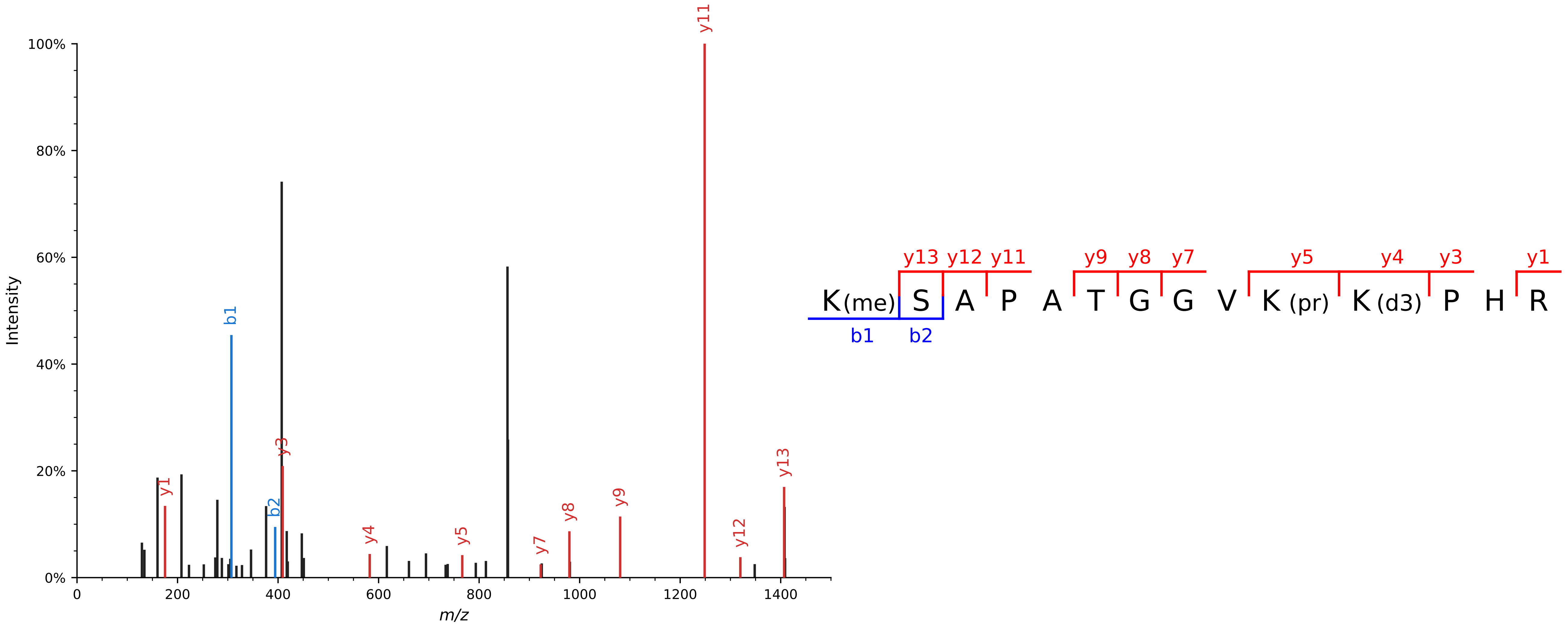

Raw file: HF221212_CG_D3AcPIC_Spleen_YSD1, Scan: 10362, m/z: 856.9893, Charge: 2, RT (min): 21.37, Score: 177.57

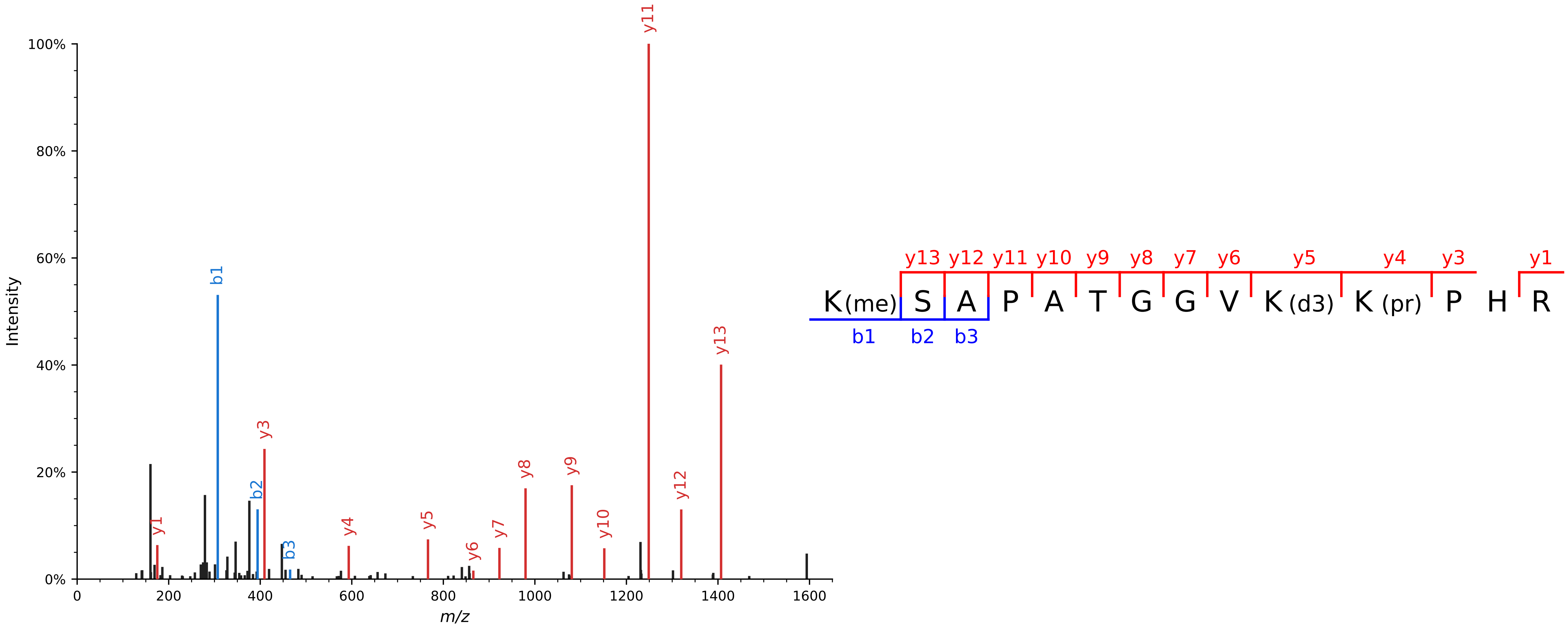

Raw file: HF221212_CG_D3AcPIC_Spleen_YSD2, Scan: 4042, m/z: 835.98402, Charge: 2, RT (min): 10.33, Score: 195.09

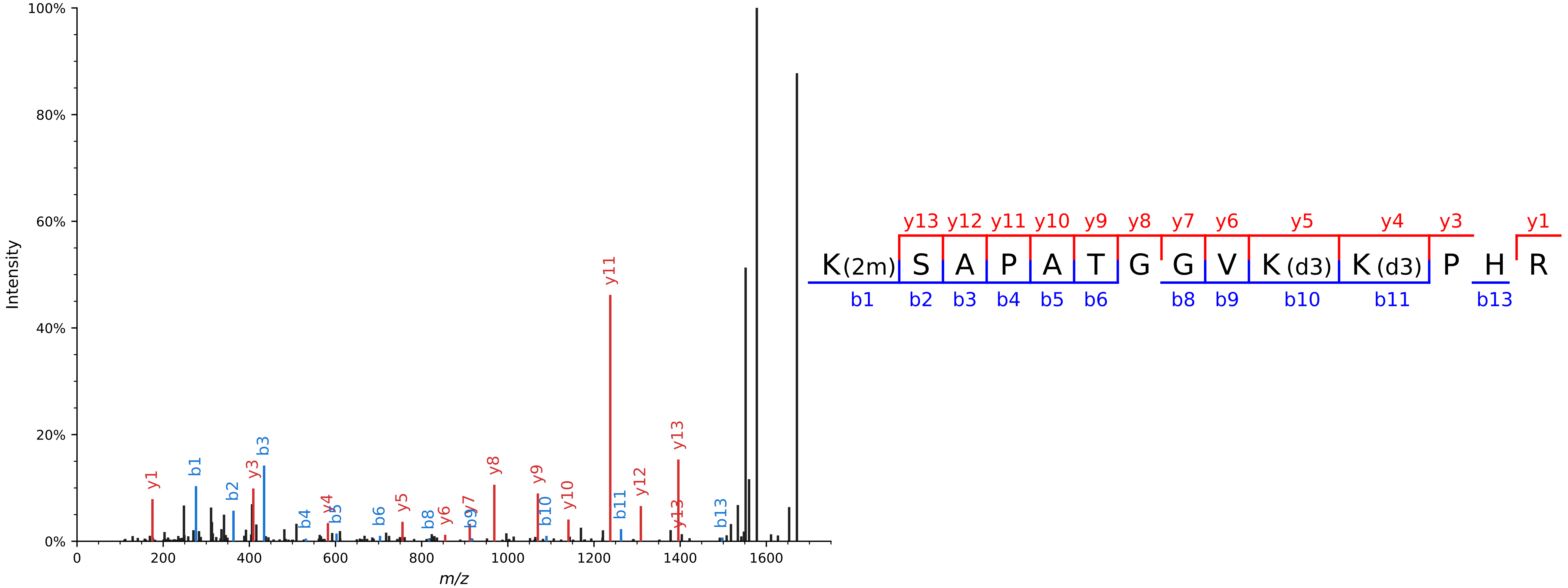

Raw file: HF221212_CG_D3AcPIC_Liver_YSD2, Scan: 5301, m/z: 848.49025, Charge: 2, RT (min): 12.56, Score: 89.301

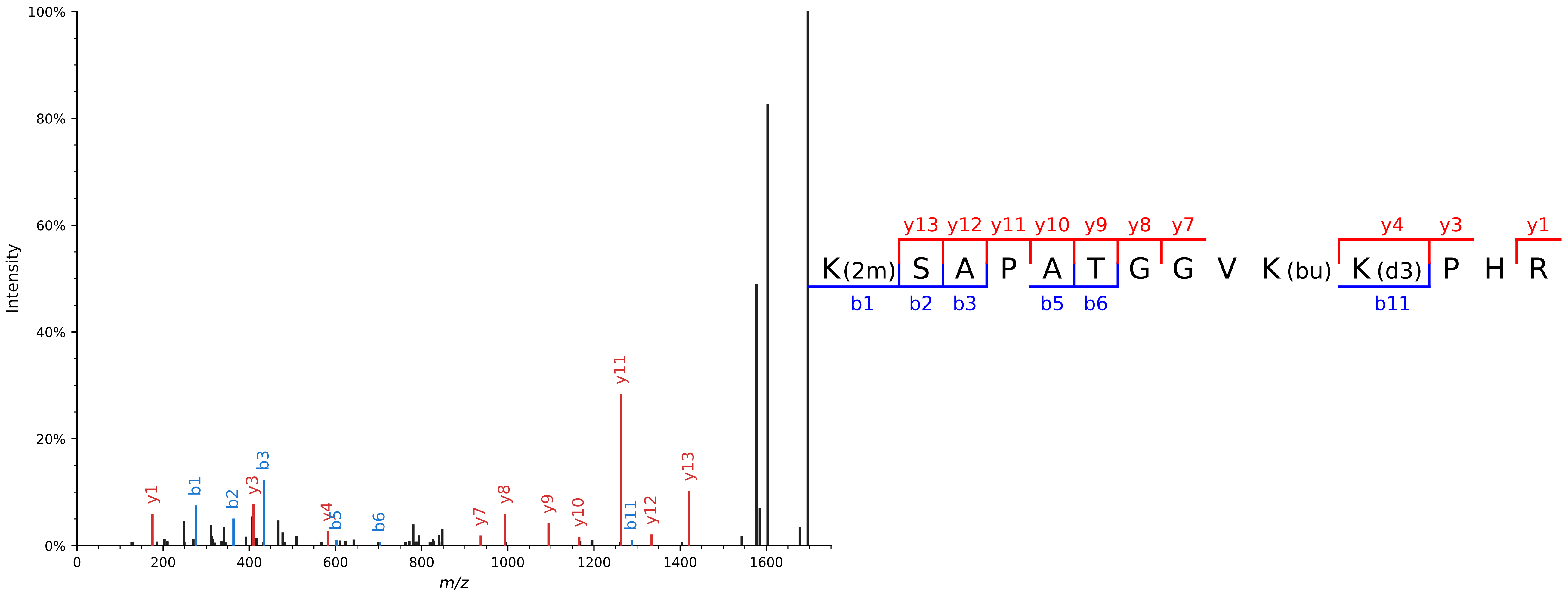

Raw file: HF221212_CG_D3AcPIC_Spleen_YSD3, Scan: 4618, m/z: 562.33032, Charge: 3, RT (min): 11.38, Score: 155.14

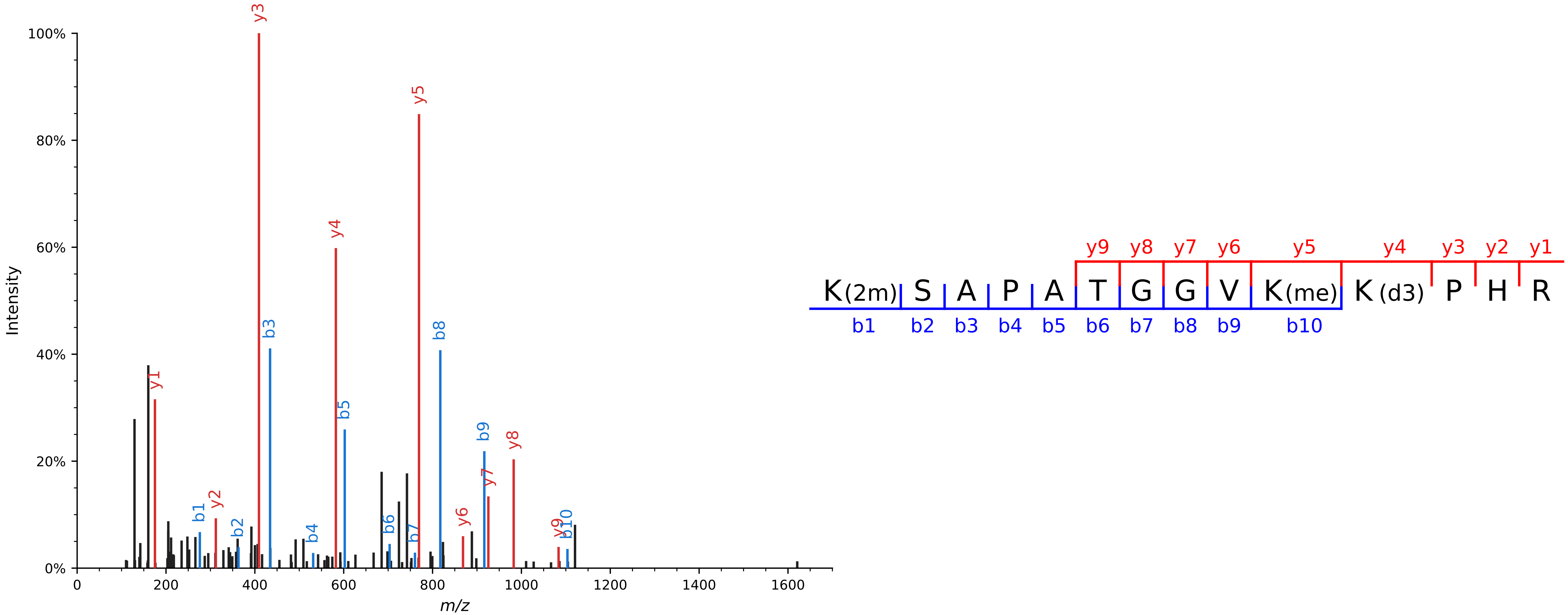

Raw file: HF221212_CG_D3AcPIC_Spleen_YSD3, Scan: 3414, m/z: 425.50347, Charge: 4, RT (min): 9.26, Score: 47.037

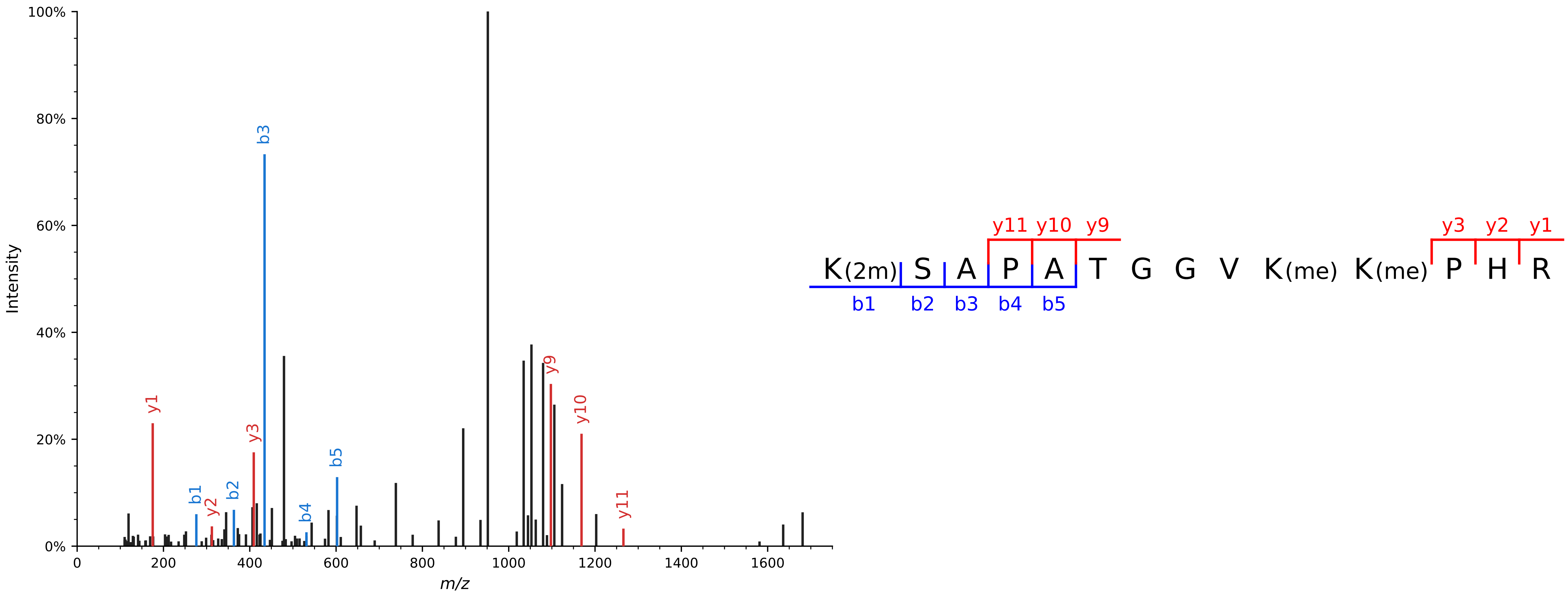

Raw file: HF221212_CG_D3AcPIC_Spleen_YSD3, Scan: 5171, m/z: 848.49025, Charge: 2, RT (min): 12.34, Score: 146.5

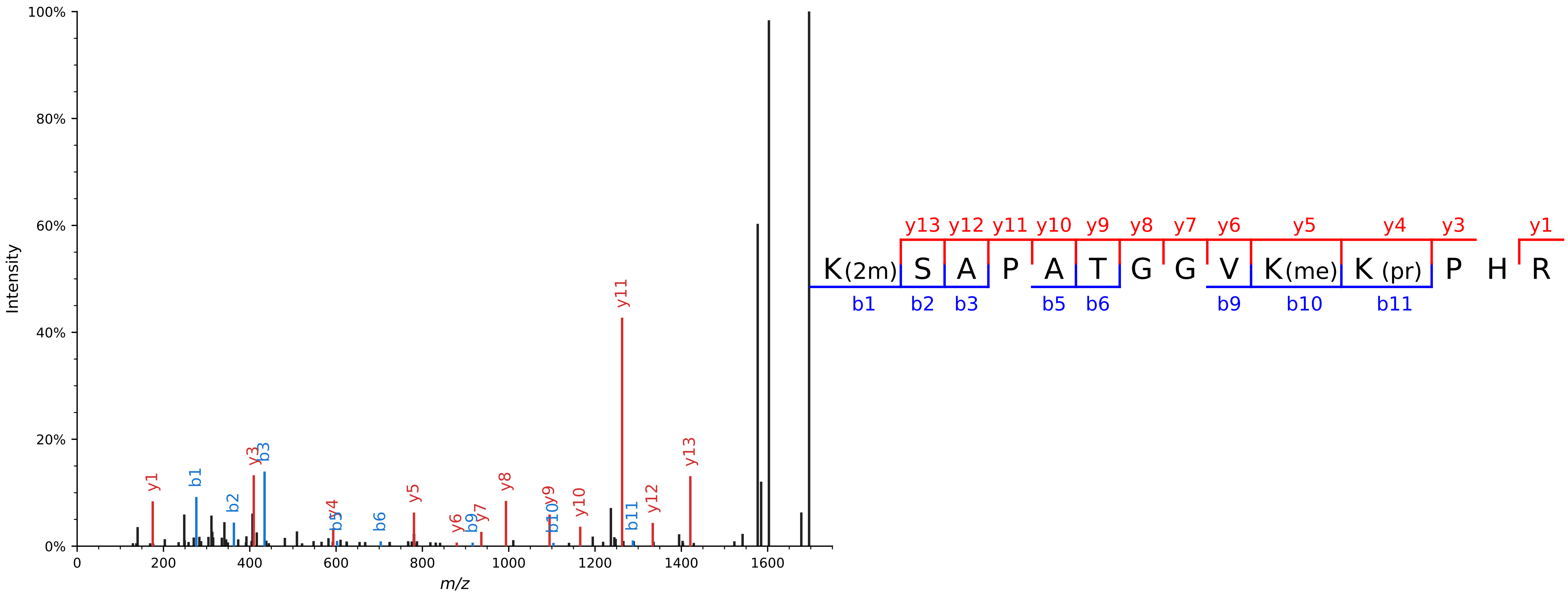

Raw file: HF221212_CG_D3AcPIC_Spleen_YSD2, Scan: 2128, m/z: 827.48497, Charge: 2, RT (min): 6.92, Score: 136.01

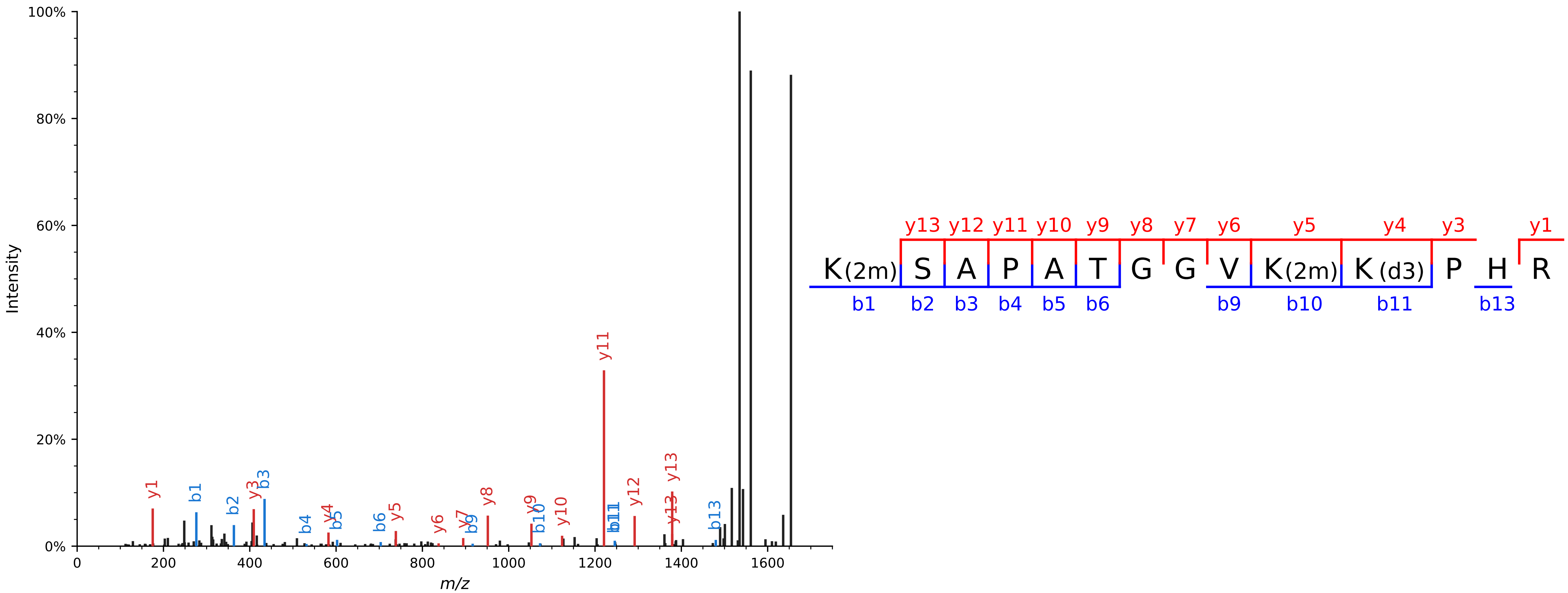

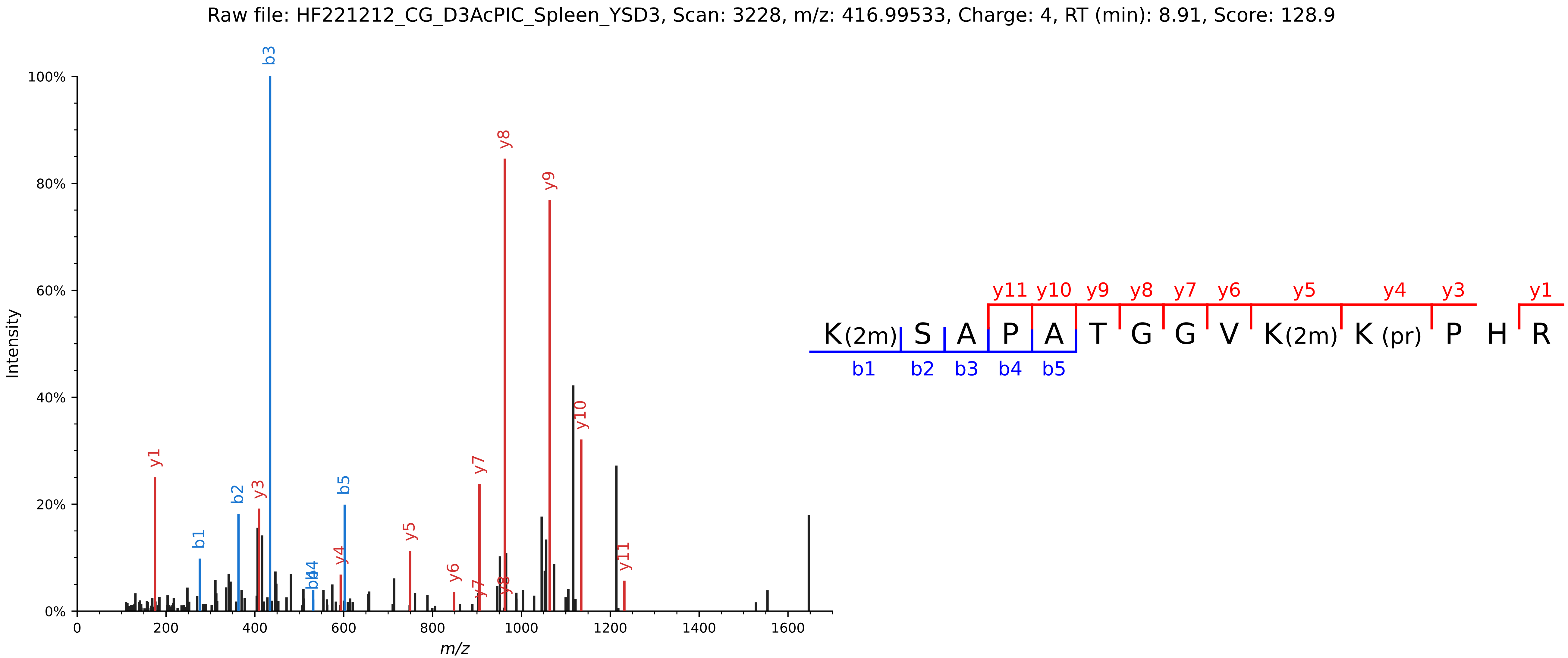
Raw file: HF221212_CG_D3AcPIC_Spleen_YSD3, Scan: 3228, m/z: 416.99533, Charge: 4, RT (min): 8.91, Score: 128.9
Intensity
m/z
K(2m) S A P A T G G V K(2m) K (pr) P H R
y11 y10 y9 y8 y7 y6 y5 y4 y3 y1
b1 b2 b3 b4 b5

Raw file: HF221212_CG_D3AcPIC_Spleen_YSD2, Scan: 4422, m/z: 841.48243, Charge: 2, RT (min): 10.99, Score: 186.76

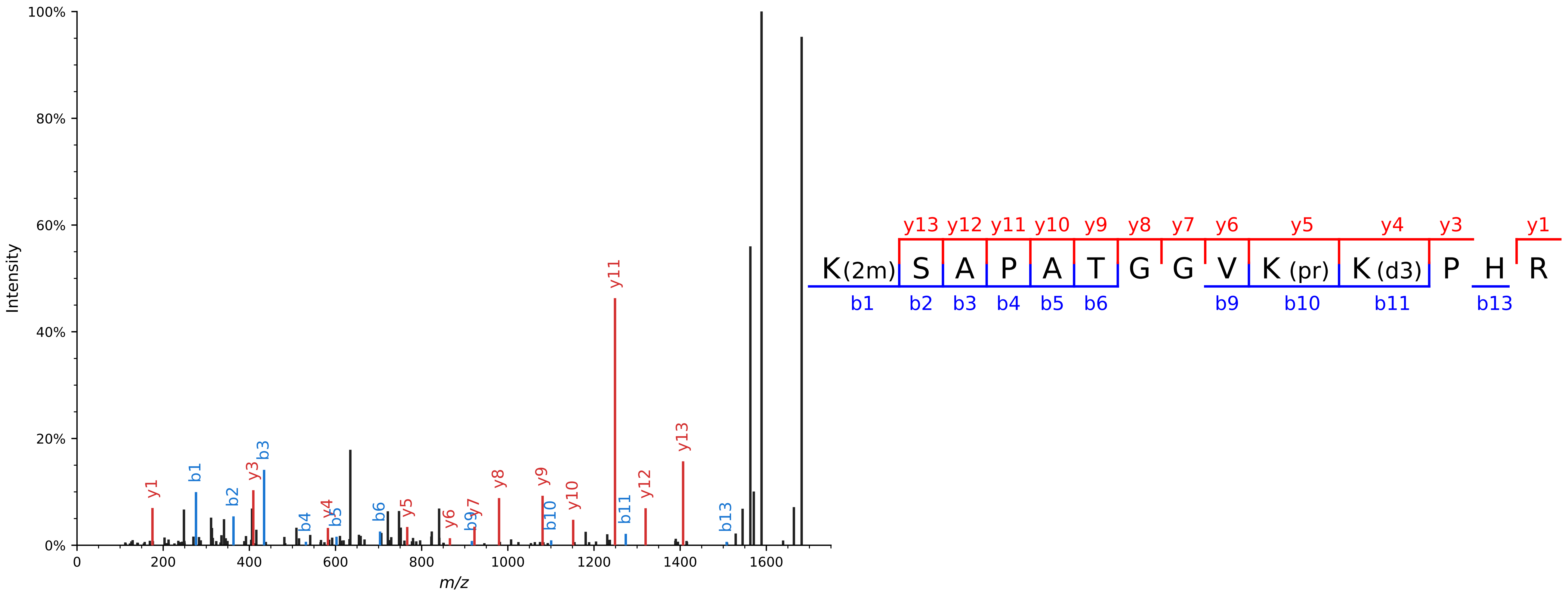

Raw file: HF221212_CG_D3AcPIC_Spleen_YSD2, Scan: 4931, m/z: 564.98965, Charge: 3, RT (min): 11.86, Score: 134.25

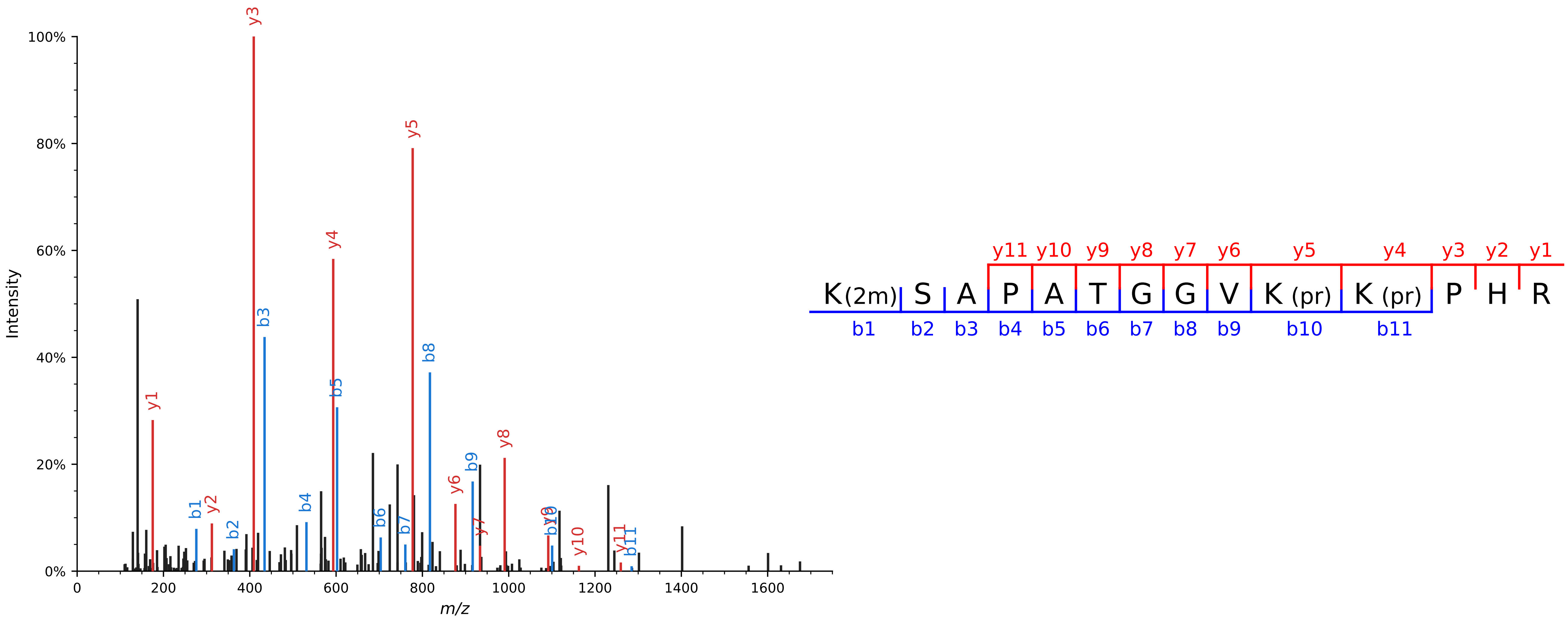

Raw file: HF221212_CG_D3AcPIC_Spleen_YSD3, Scan: 4664, m/z: 841.48243, Charge: 2, RT (min): 11.46, Score: 190.26

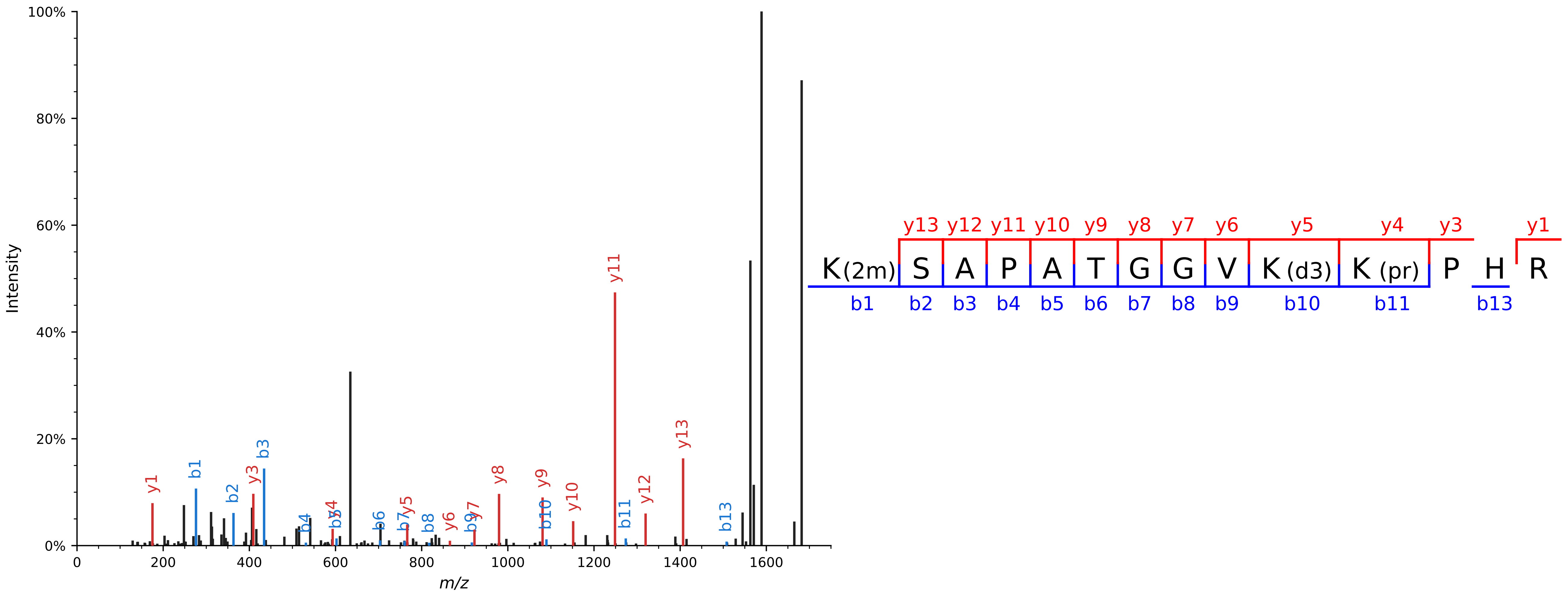

Raw file: HF221212_CG_D3AcPIC_Spleen_YSD2, Scan: 4242, m/z: 562.33032, Charge: 3, RT (min): 10.68, Score: 140.27

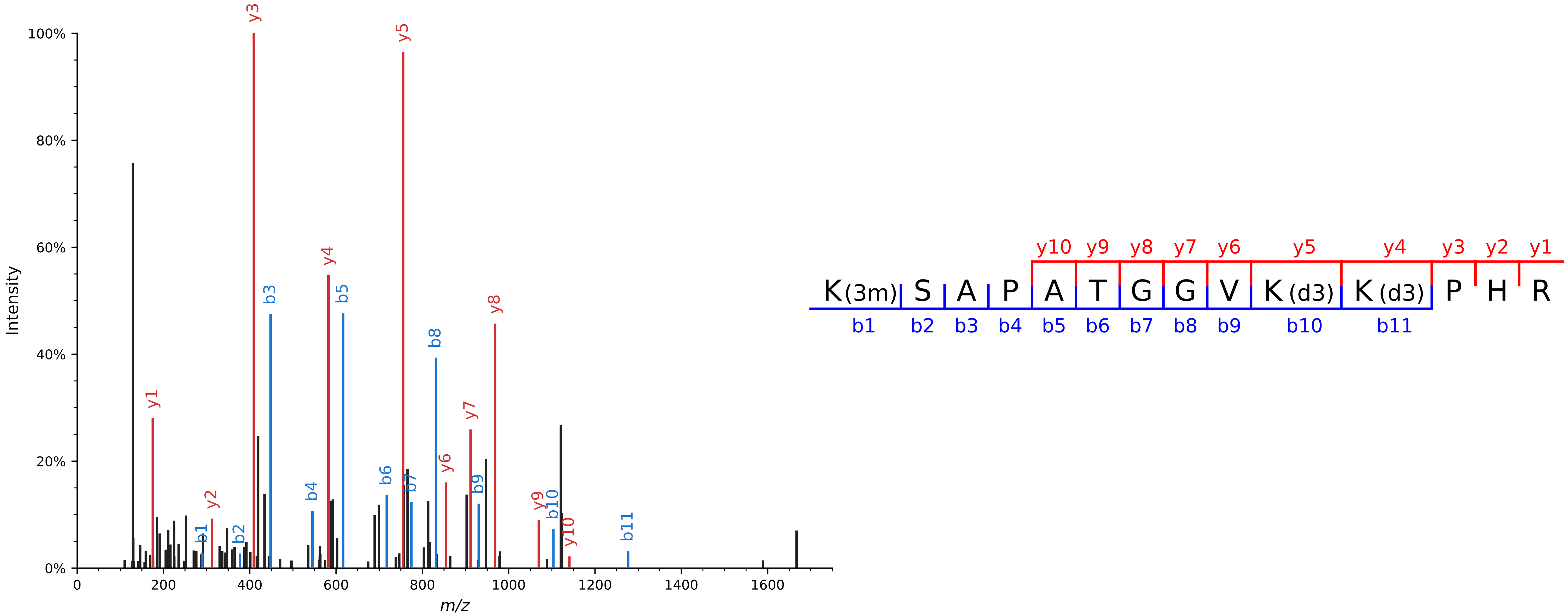

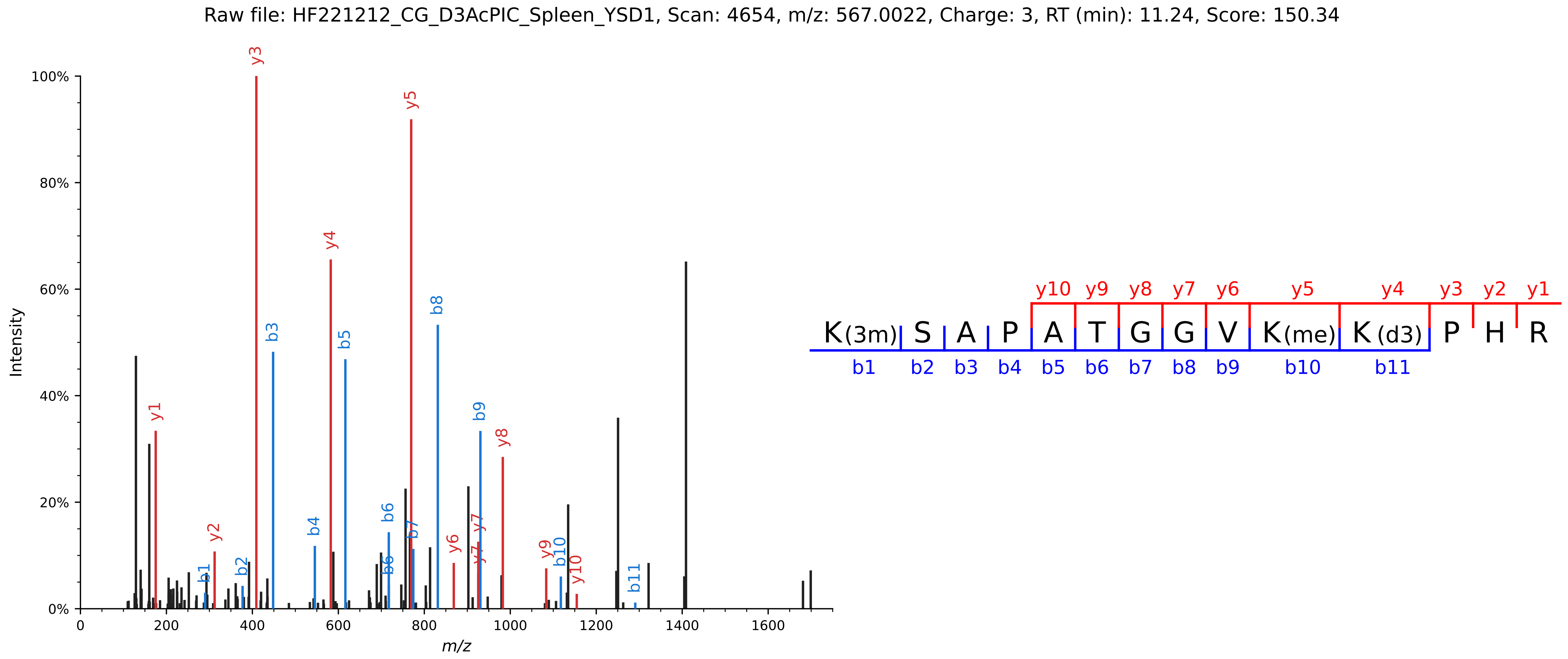
Raw file: HF221212_CG_D3AcPIC_Spleen_YSD1, Scan: 4654, m/z: 567.0022, Charge: 3, RT (min): 11.24, Score: 150.34
Intensity
m/z
K(3m) S A P A T G G V K(me) K(d3) P H R
y10 y9 y8 y7 y6 y5 y4 y3 y2 y1
b1 b2 b3 b4 b5 b6 b7 b8 b9 b10 b11

Raw file: HF221212_CG_D3AcPIC_Spleen_YSD2, Scan: 4957, m/z: 570.66781, Charge: 3, RT (min): 11.90, Score: 142.45

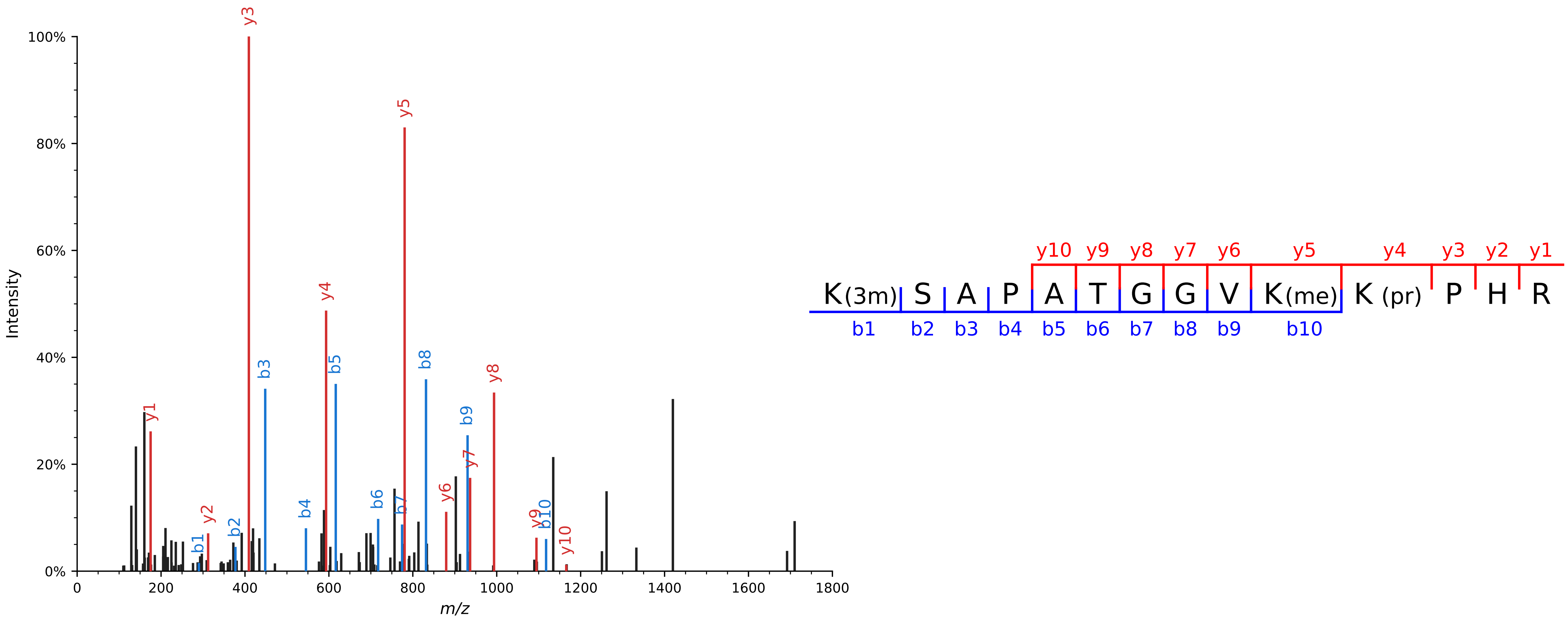

Raw file: HF221212_CG_D3AcPIC_Spleen_YSD1, Scan: 2669, m/z: 834.4928, Charge: 2, RT (min): 7.74, Score: 77.593

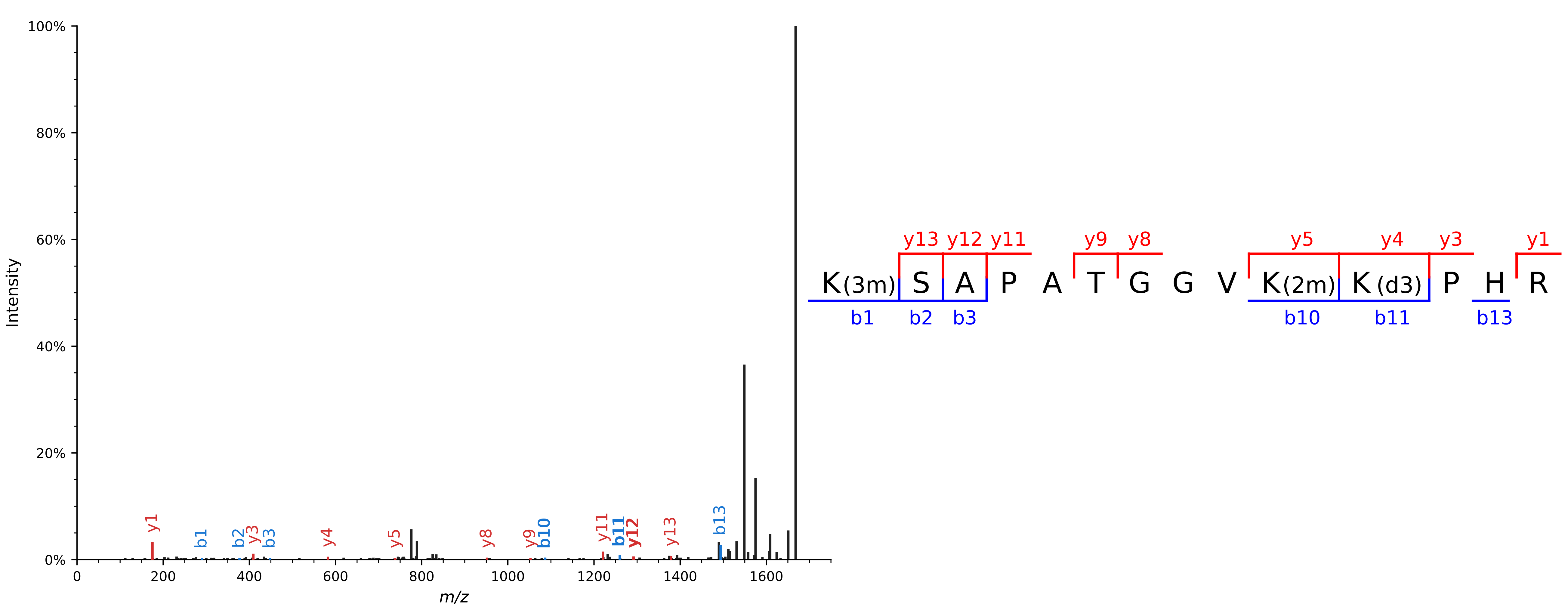

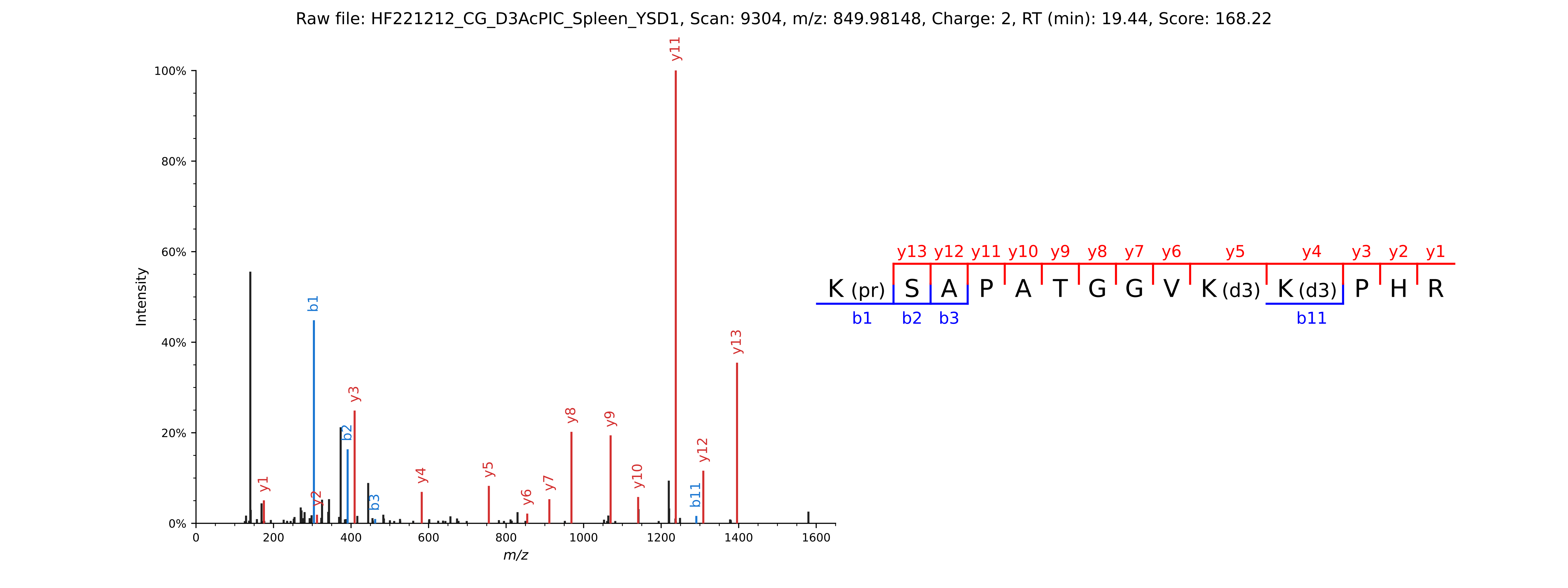
Raw file: HF221212_CG_D3AcPIC_Spleen_YSD1, Scan: 9304, m/z: 849.98148, Charge: 2, RT (min): 19.44, Score: 168.22
Intensity
m/z
0%
20%
40%
60%
80%
100%
0
200
400
600
800
1000
1200
1400
1600
y1
y2
y3
y4
y5
y6
y7
y8
y9
y10
y11
y12
y13
b1
b2
b3
b11
y13 y12 y11 y10 y9 y8 y7 y6 y5 y4 y3 y2 y1
K (pr) S A P A T G G V K (d3) K (d3) P H R
b1 b2 b3 b11

Raw file: HF221212_CG_D3AcPIC_Spleen_YSD1, Scan: 10063, m/z: 856.9893, Charge: 2, RT (min): 20.84, Score: 151.79

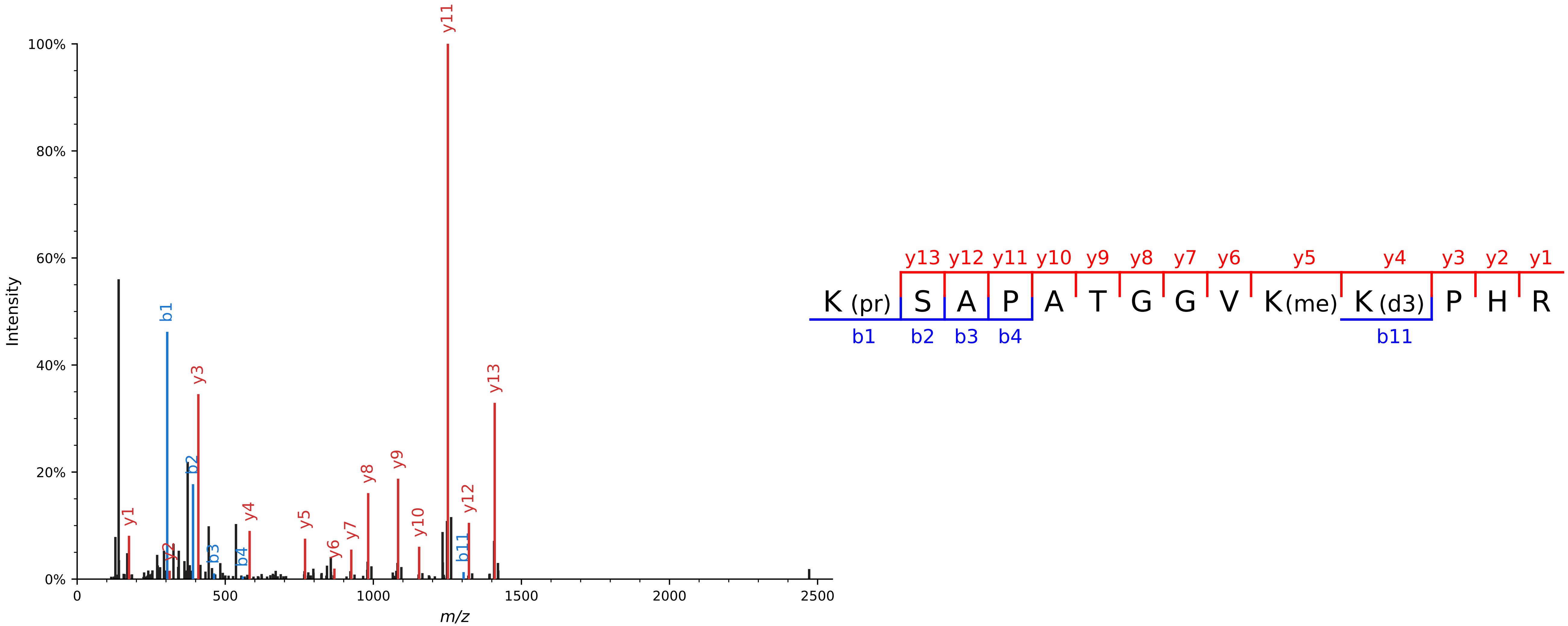

Raw file: HF221212_CG_D3AcPIC_Spleen_YSD1, Scan: 5524, m/z: 561.32404, Charge: 3, RT (min): 12.73, Score: 69.92

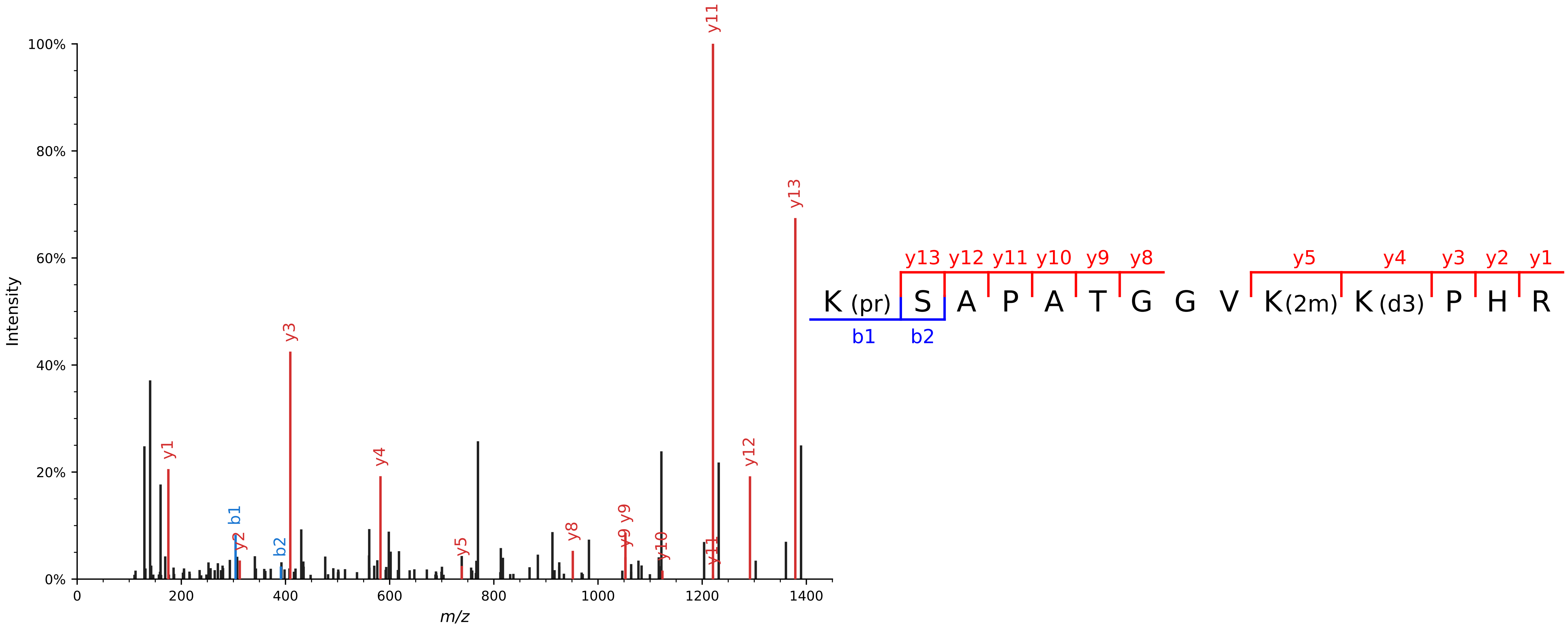

Raw file: HF221212_CG_D3AcPIC_Spleen_YSD3, Scan: 9900, m/z: 855.47989, Charge: 2, RT (min): 20.86, Score: 147.37

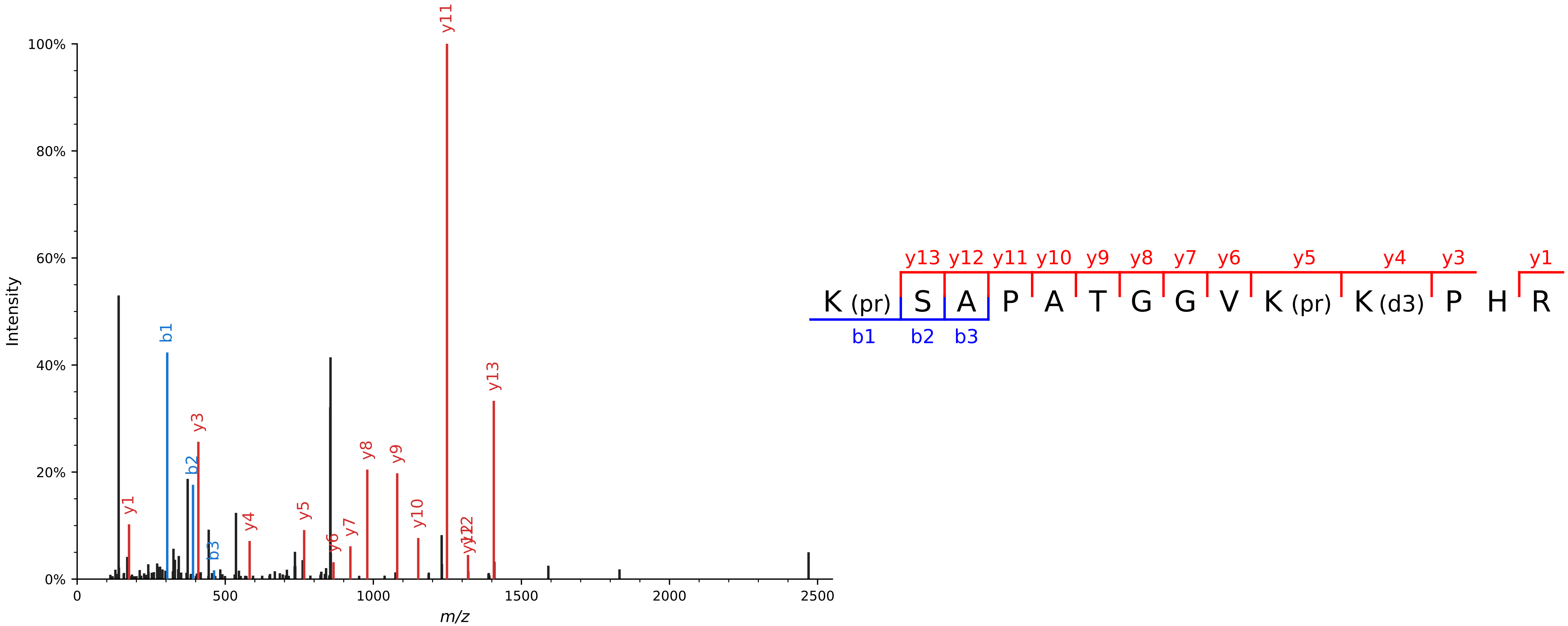

Raw file: HF221212_CG_D3AcPIC_Spleen_YSD1, Scan: 9013, m/z: 851.49089, Charge: 2, RT (min): 18.93, Score: 139.86

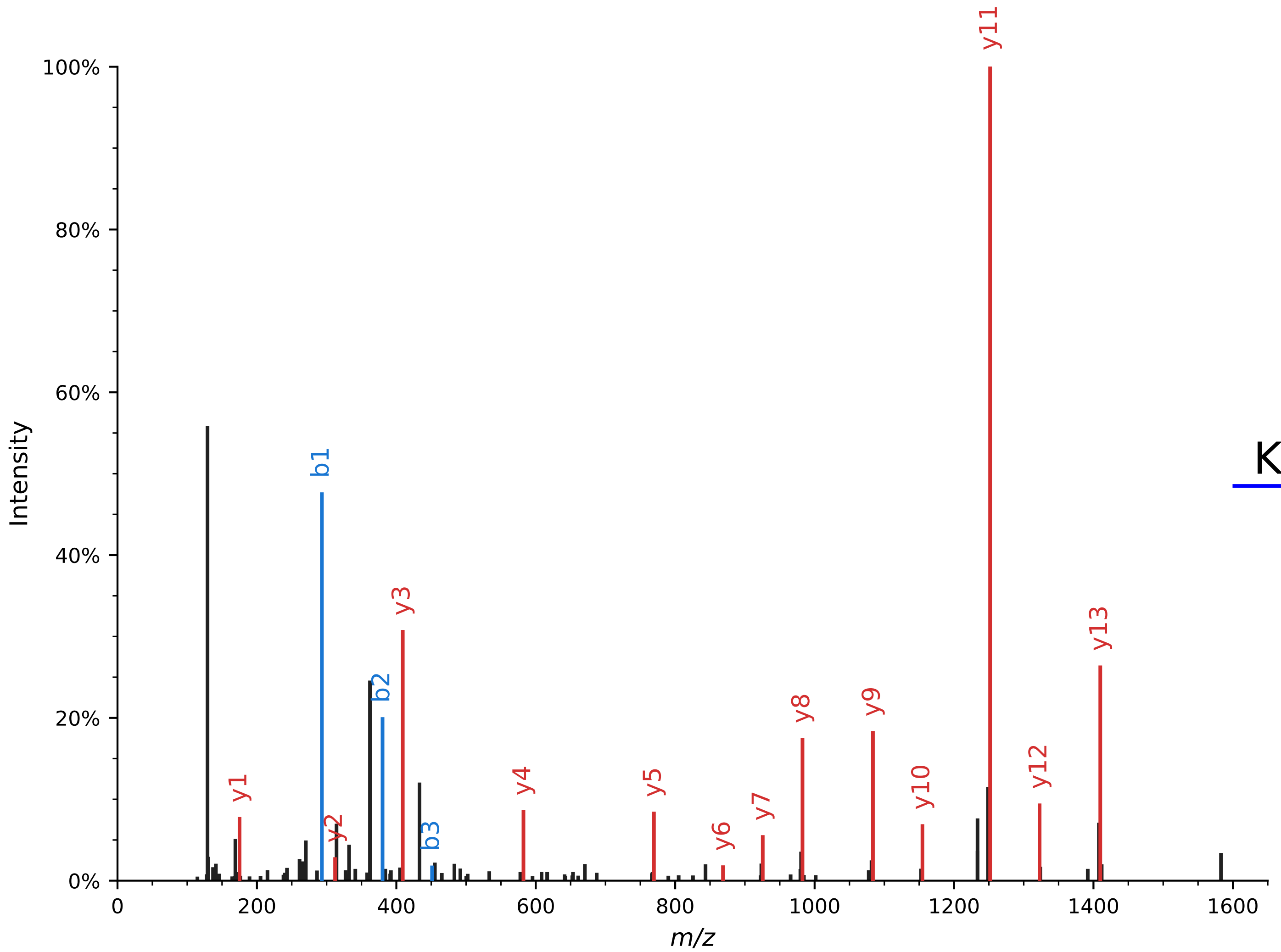

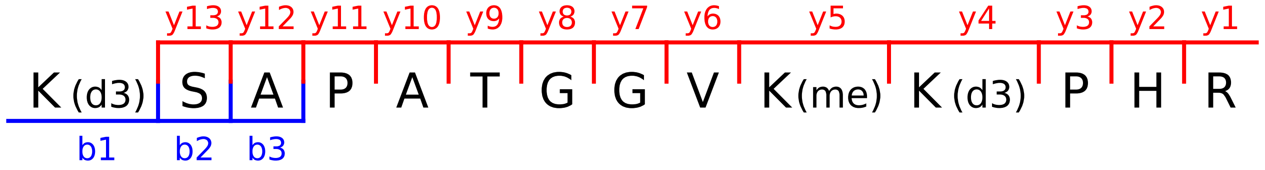

Raw file: HF221212_CG_D3AcPIC_Spleen_YSD2, Scan: 5592, m/z: 572.66824, Charge: 3, RT (min): 12.99, Score: 48.527

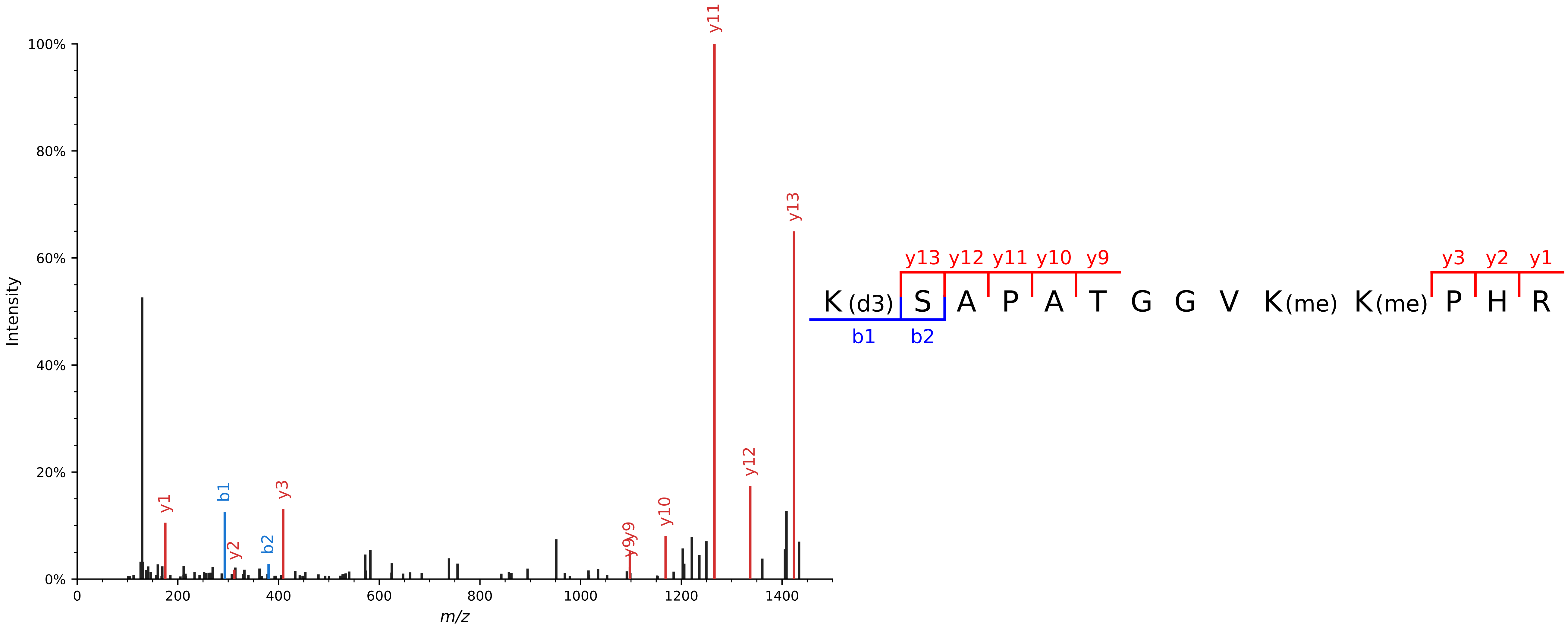

Raw file: HF221212_CG_D3AcPIC_Spleen_YSD2, Scan: 9640, m/z: 571.66196, Charge: 3, RT (min): 20.26, Score: 128.31

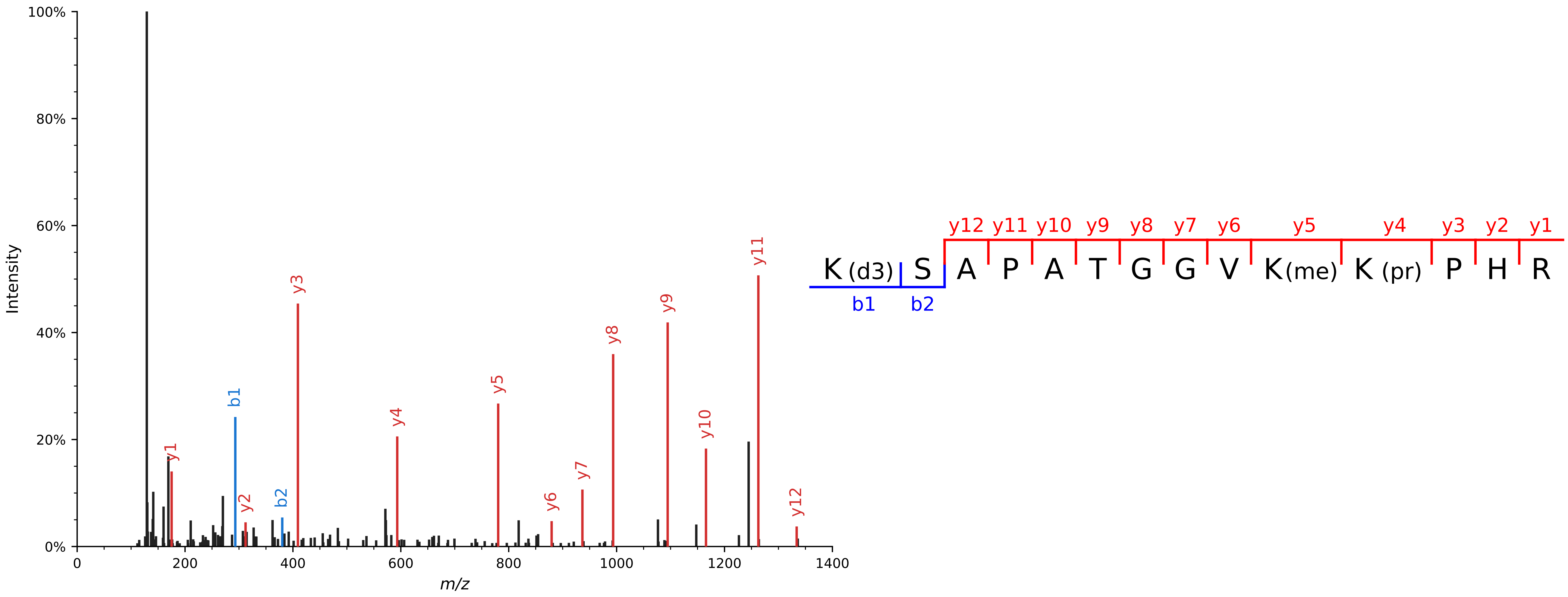

Raw file: HF221212_CG_D3AcPIC_Spleen_YSD1, Scan: 4812, m/z: 835.98402, Charge: 2, RT (min): 11.53, Score: 164.48

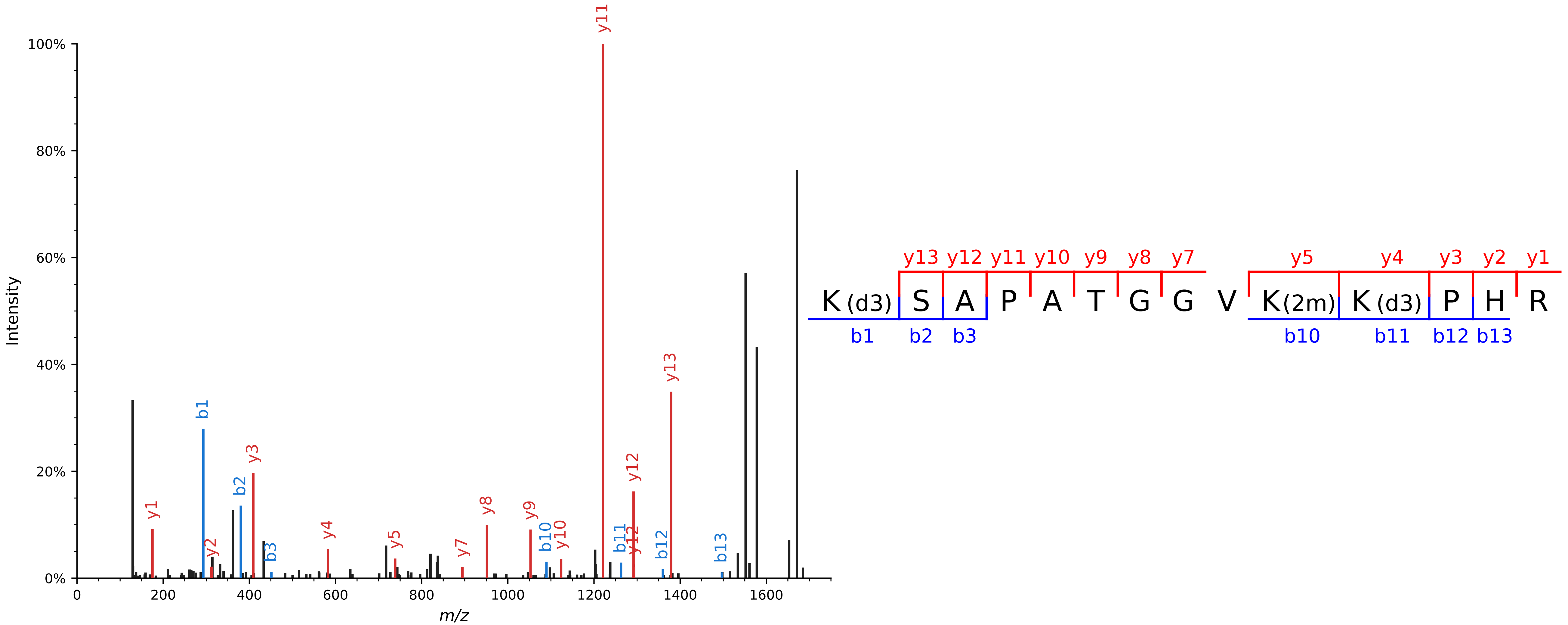

Raw file: HF221212_CG_D3AcPIC_Spleen_YSD3, Scan: 5343, m/z: 841.48243, Charge: 2, RT (min): 12.63, Score: 113.61

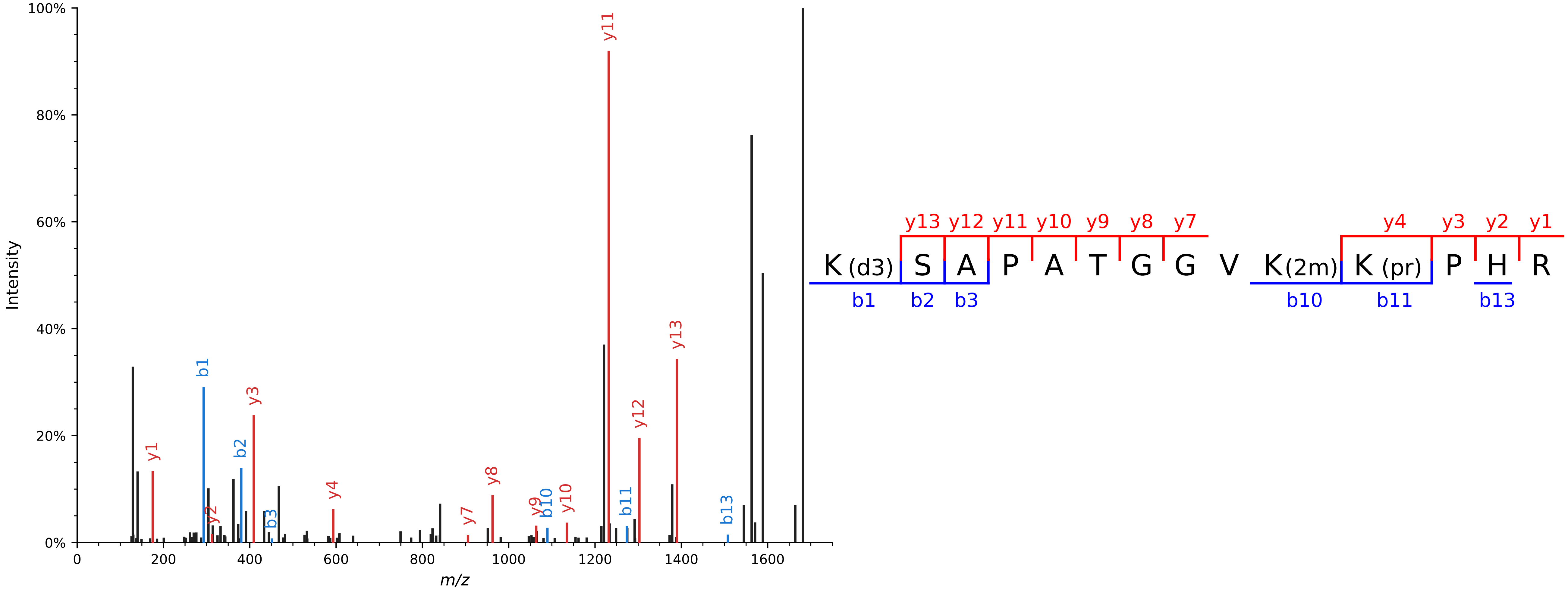

Raw file: HF221212_CG_D3AcPIC_Spleen_YSD2, Scan: 9698, m/z: 855.47989, Charge: 2, RT (min): 20.36, Score: 172.8

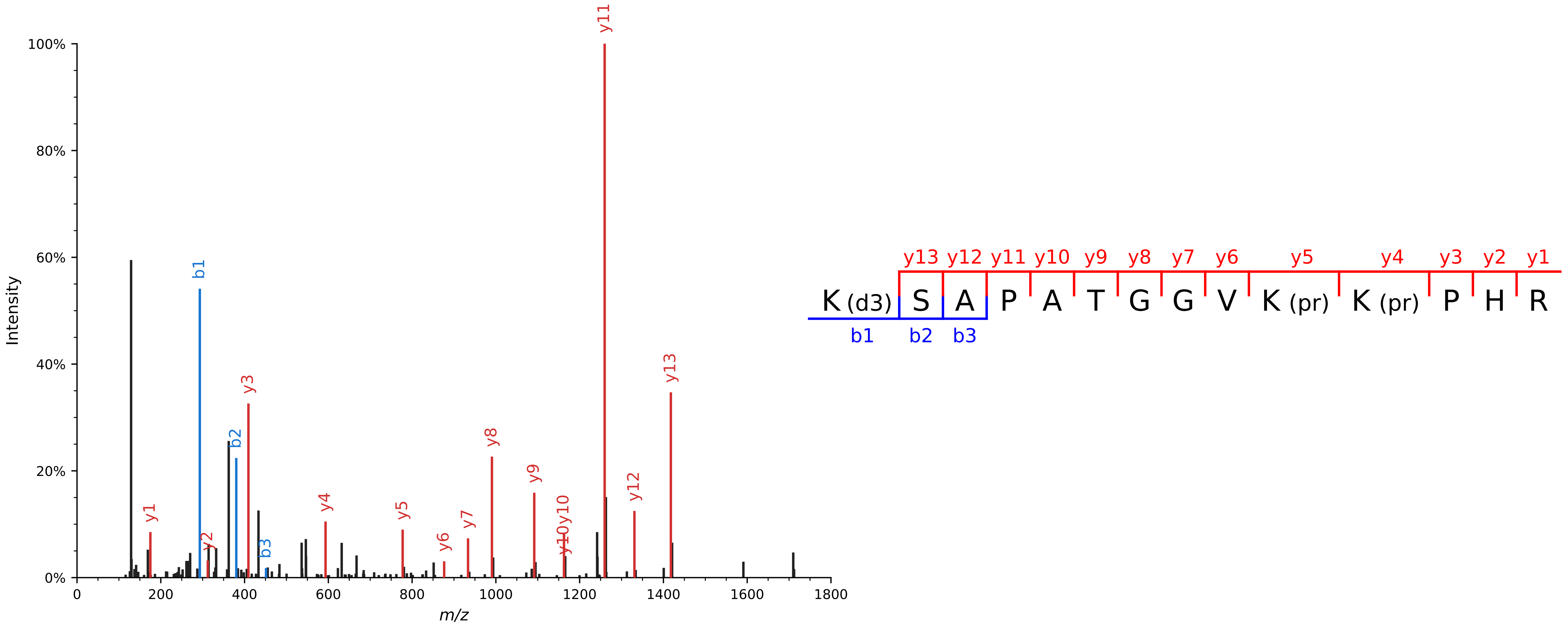

Raw file: HF221212_CG_D3AcPIC_Spleen_YSD1, Scan: 9205, m/z: 849.98148, Charge: 2, RT (min): 19.26, Score: 164.64

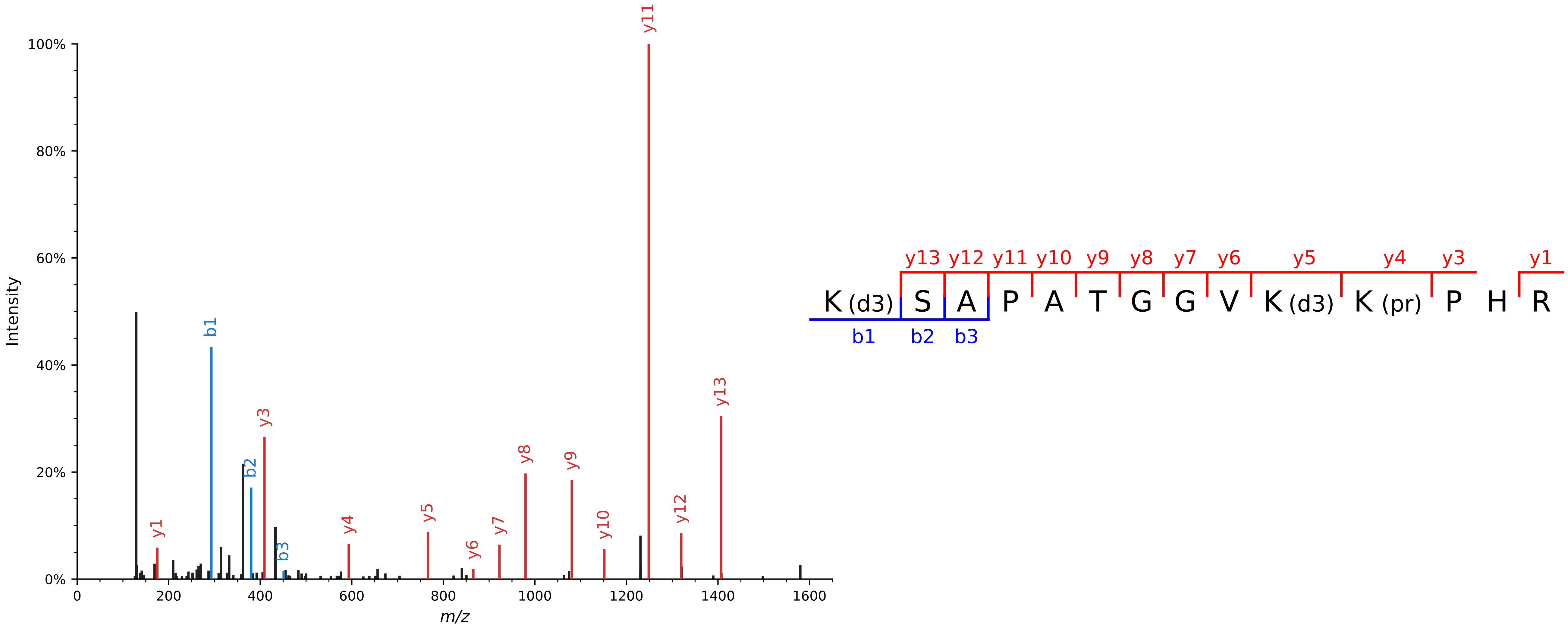

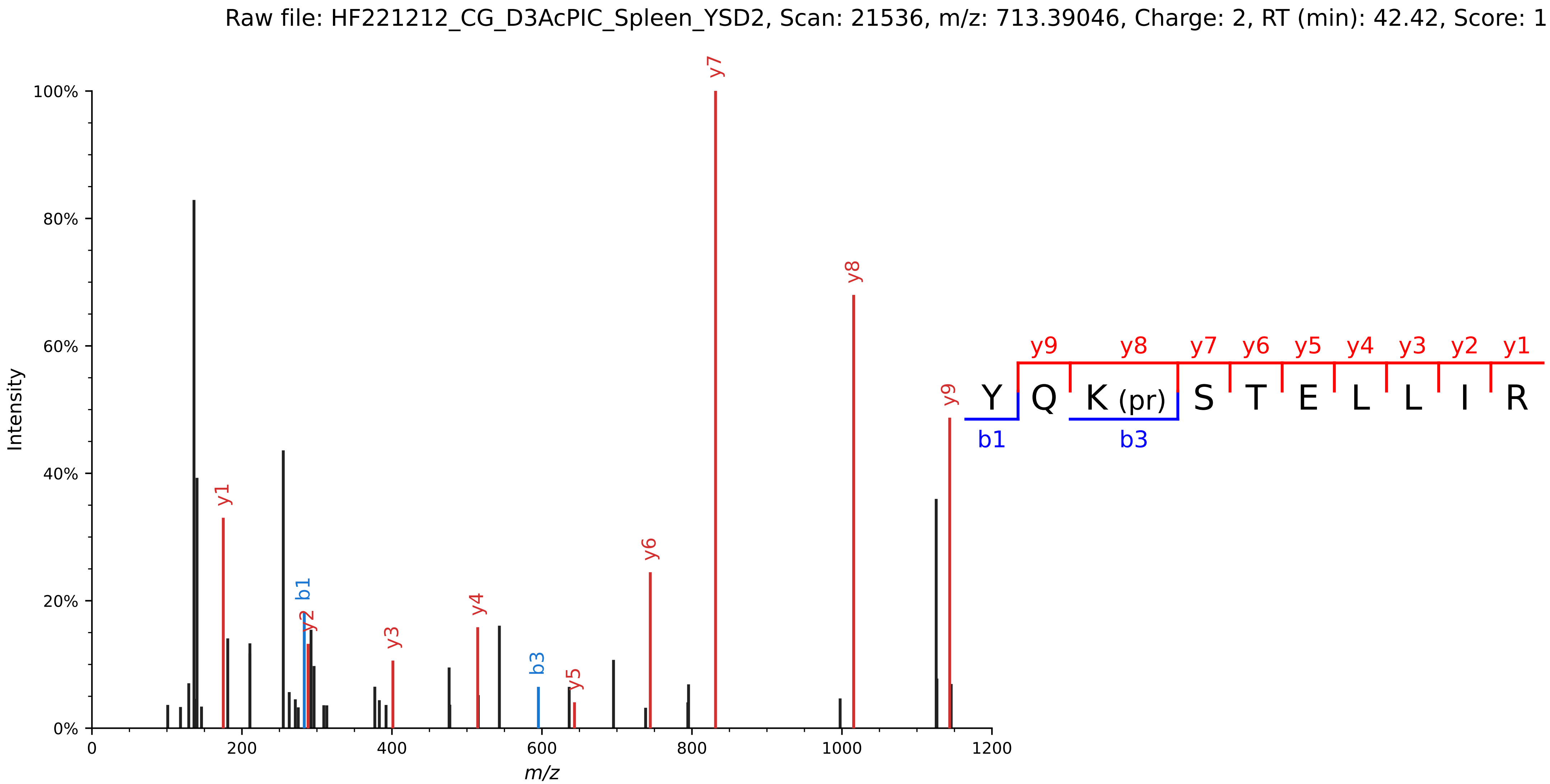

Raw file: HF221212_CG_D3AcPIC_Spleen_YSD2, Scan: 21536, m/z: 713.39046, Charge: 2, RT (min): 42.42, Score: 110.87
Intensity
m/z
0%
20%
40%
60%
80%
100%
0
200
400
600
800
1000
1200
y1
b1
y2
y3
y4
b3
y5
y6
y7
y8
y9
Y Q K (pr) S T E L L I R
b1
b3

Raw file: HF221212_CG_D3AcPIC_Spleen_YSD3, Scan: 19888, m/z: 482.27417, Charge: 2, RT (min): 39.45, Score: 103.41

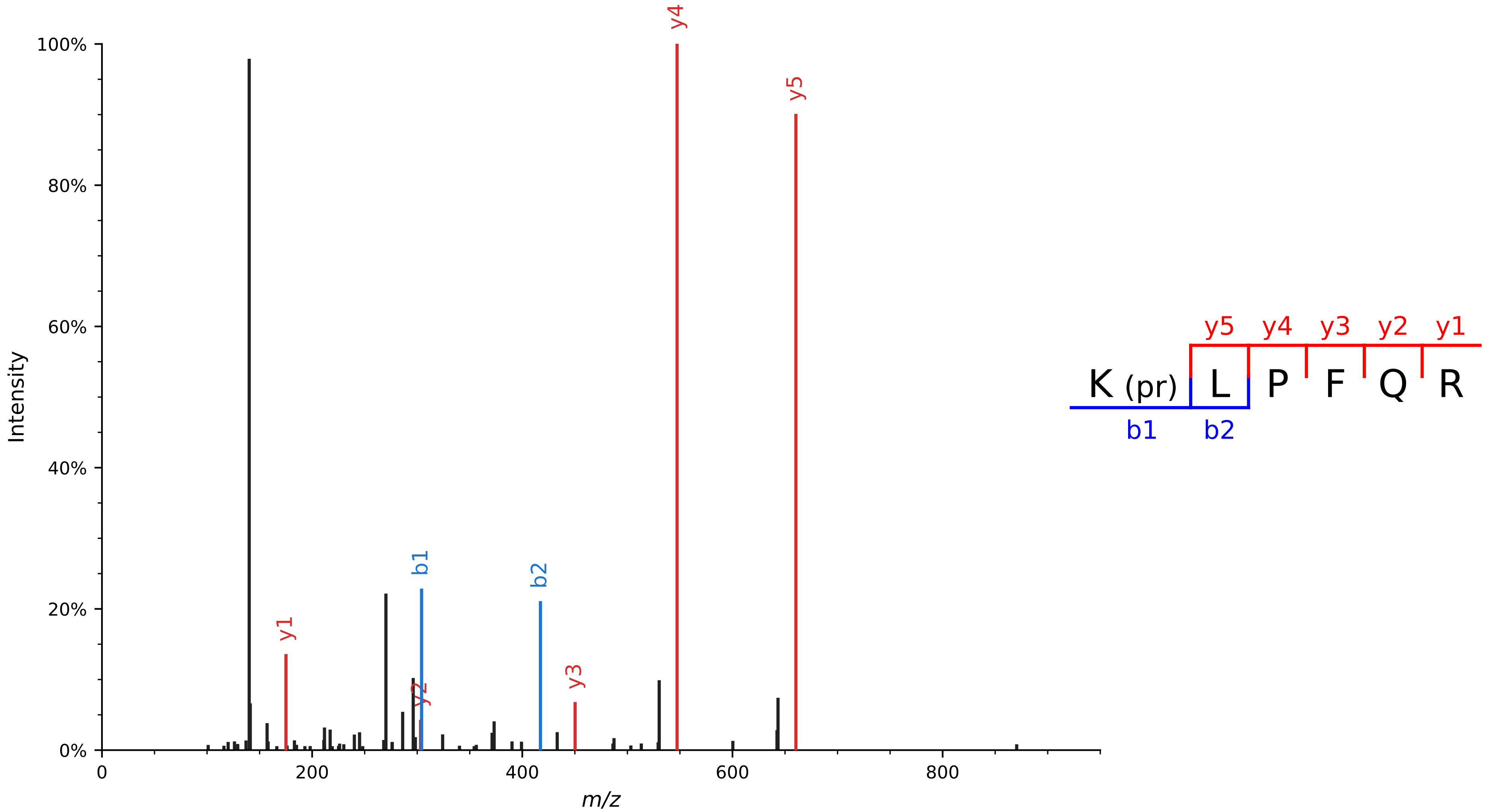

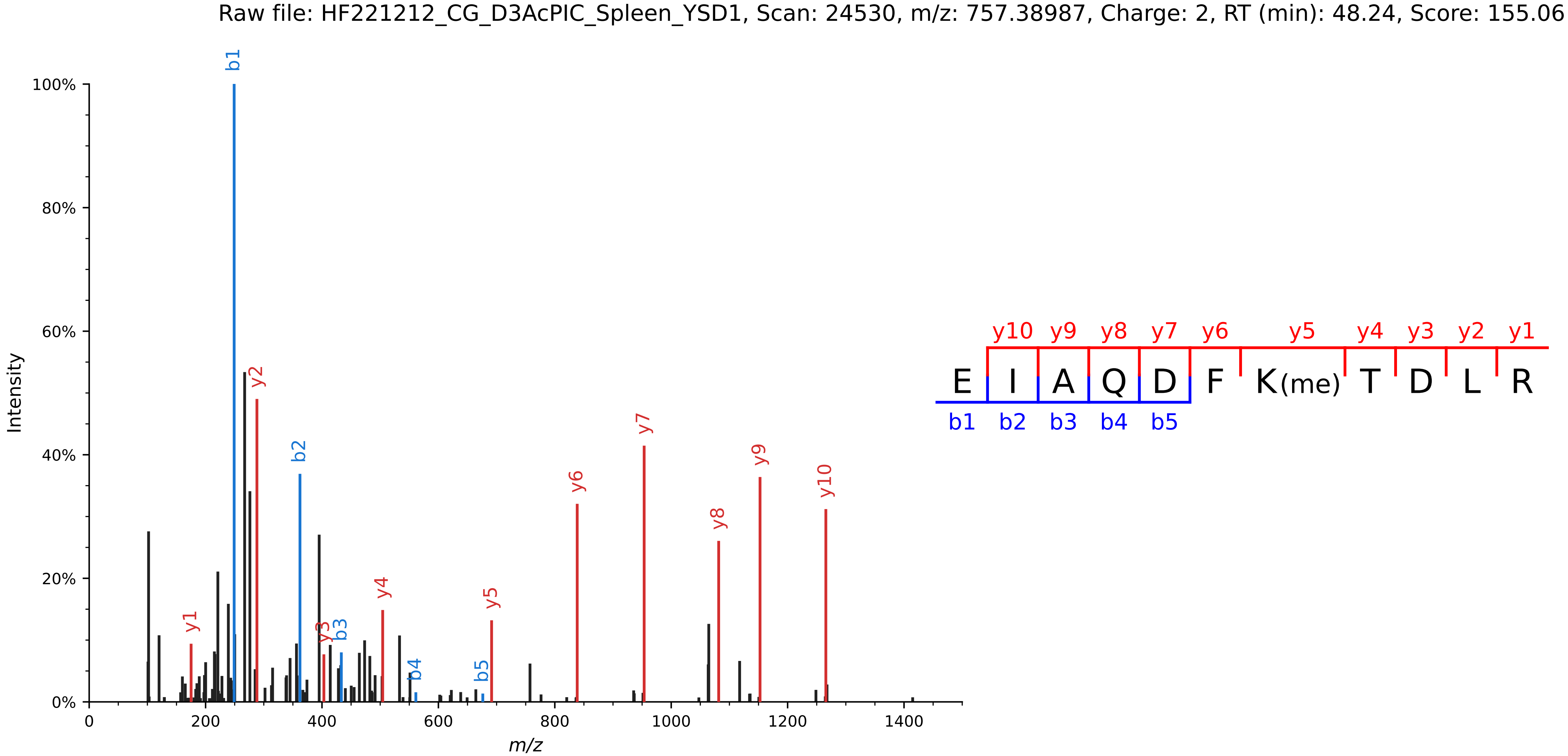
Raw file: HF221212_CG_D3AcPIC_Spleen_YSD1, Scan: 24530, m/z: 757.38987, Charge: 2, RT (min): 48.24, Score: 155.06
Intensity
m/z
E I A Q D F K(me) T D L R
y10 y9 y8 y7 y6 y5 y4 y3 y2 y1
b1 b2 b3 b4 b5

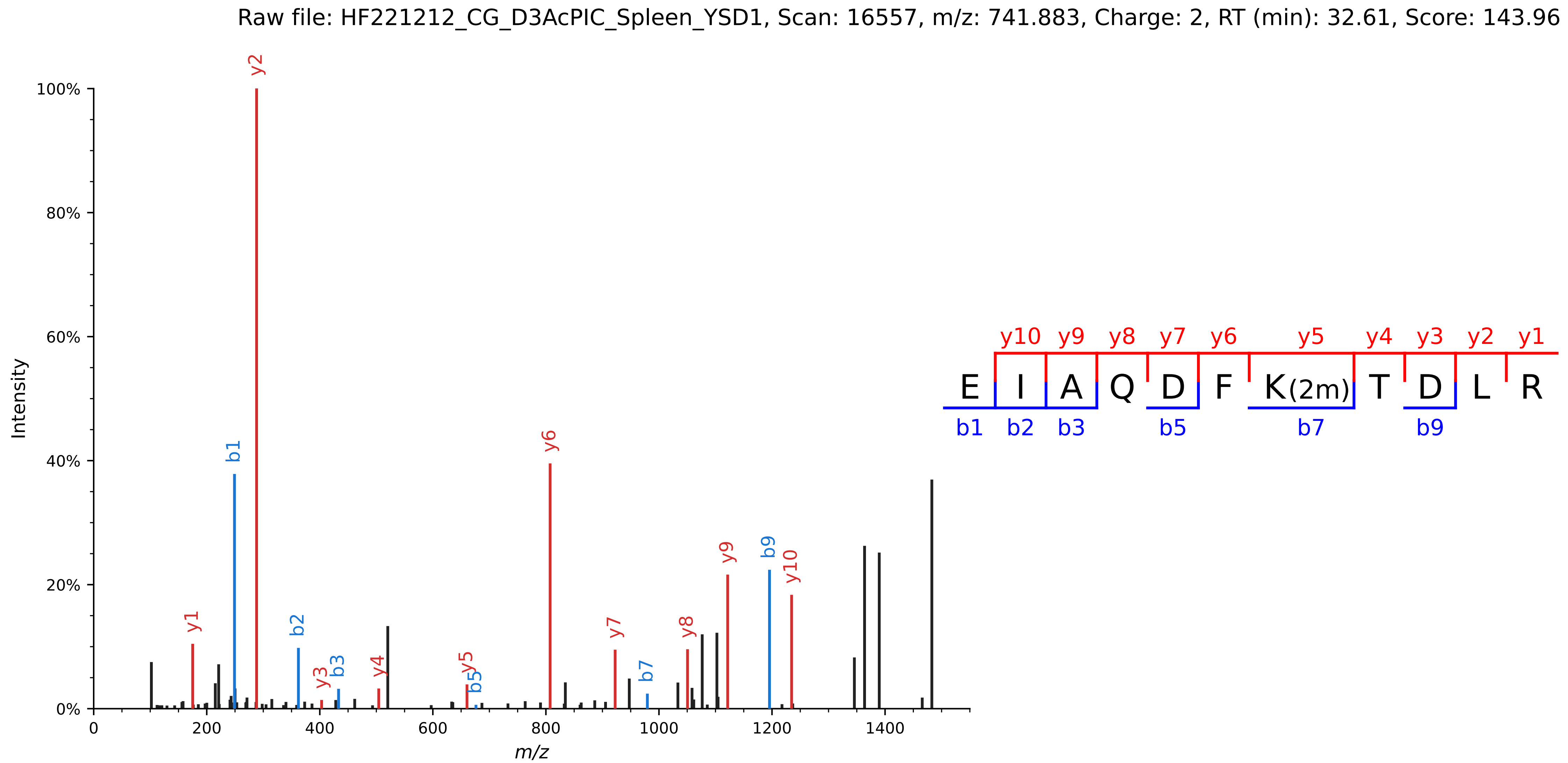

Raw file: HF221212_CG_D3AcPIC_Spleen_YSD1, Scan: 16557, m/z: 741.883, Charge: 2, RT (min): 32.61, Score: 143.96
Intensity
m/z
y10 y9 y8 y7 y6 y5 y4 y3 y2 y1
E I A Q D F K(2m) T D L R
b1 b2 b3 b5 b7 b9

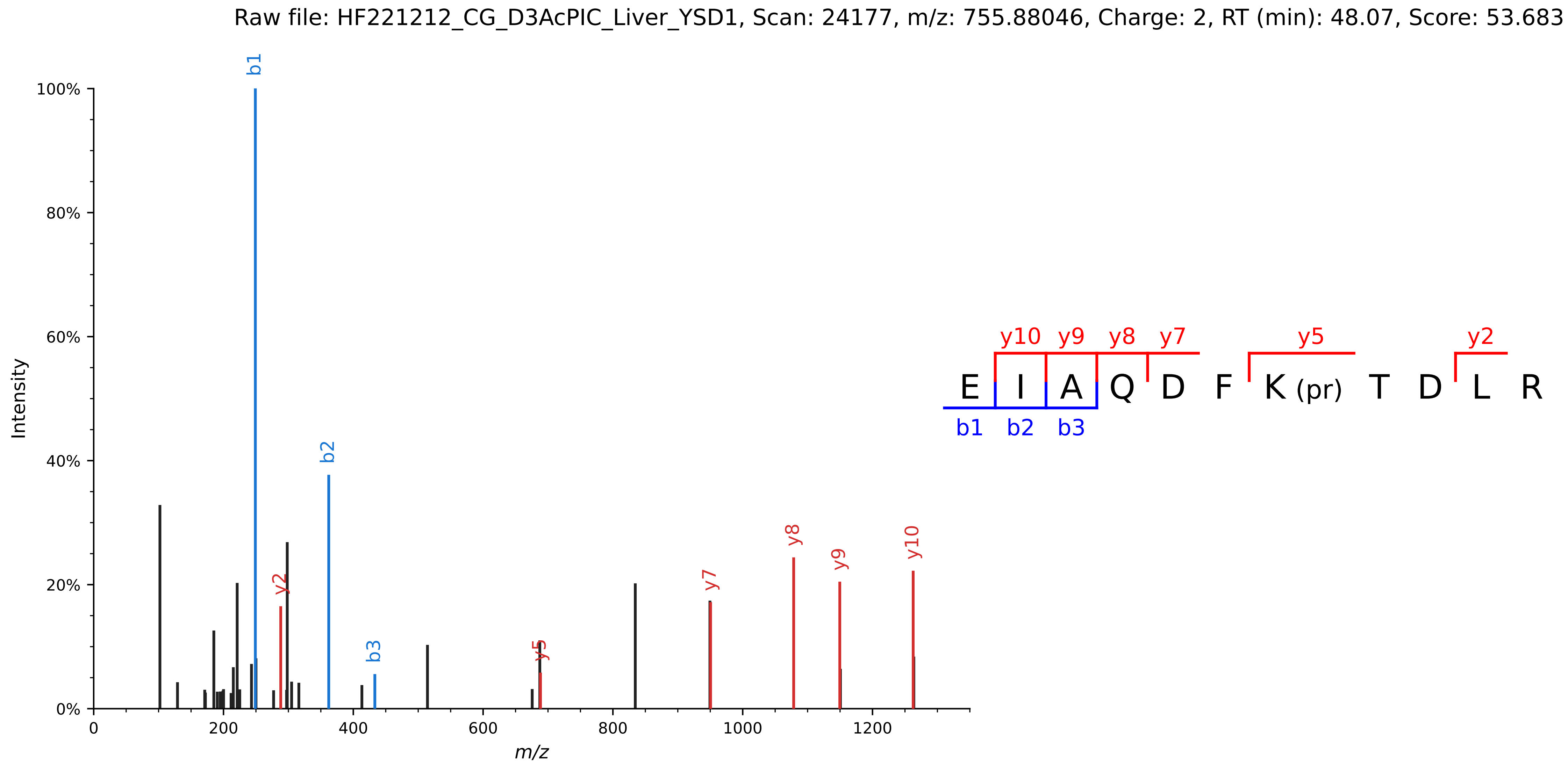

Raw file: HF221212_CG_D3AcPIC_Liver_YSD1, Scan: 24177, m/z: 755.88046, Charge: 2, RT (min): 48.07, Score: 53.683
Intensity
m/z
b1
b2
b3
y2
y5
y7
y8
y9
y10
E I A Q D F K (pr) T D L R

Raw file: HF221212_CG_D3AcPIC_Spleen_YSD1, Scan: 25217, m/z: 780.43434, Charge: 2, RT (min): 49.87, Score: 86.624

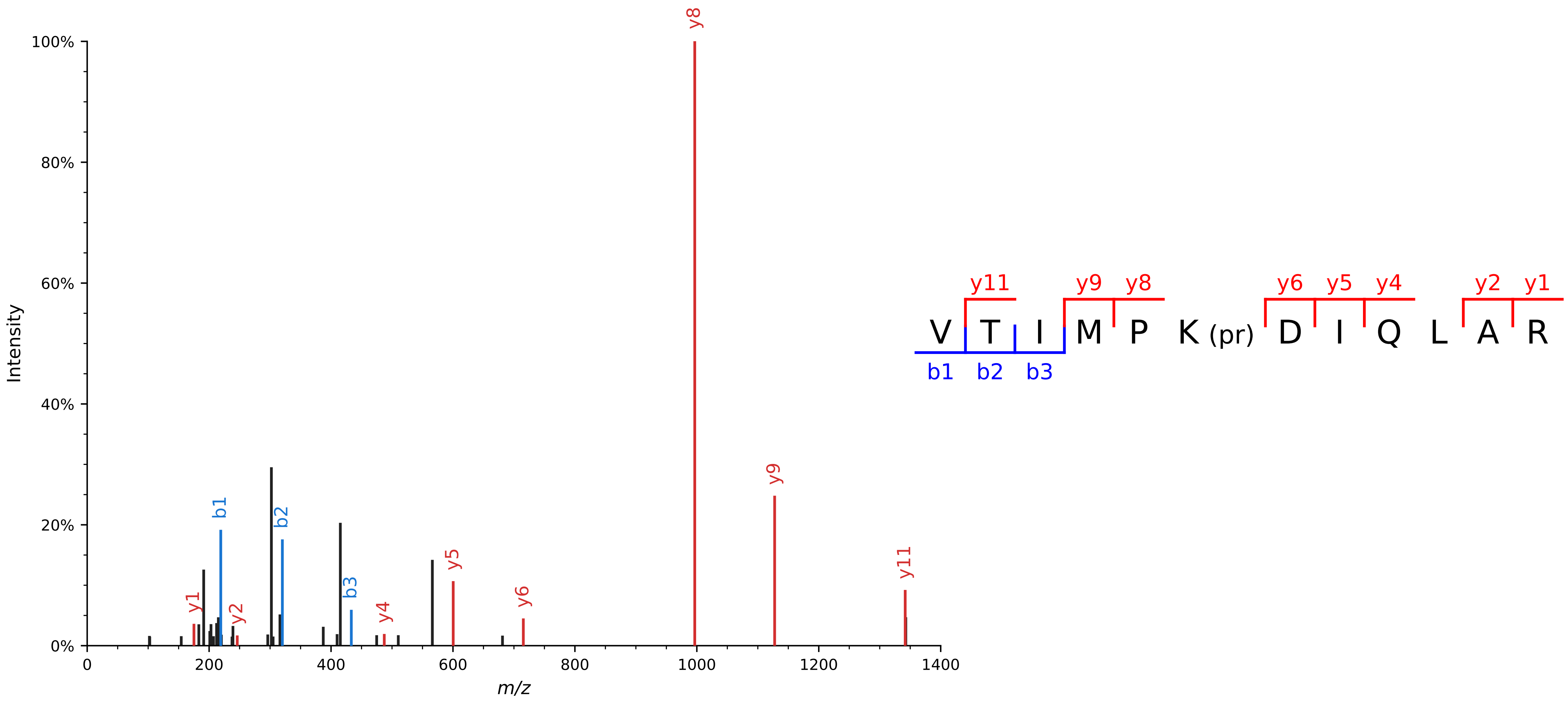

Raw file: HF221212_CG_D3AcPIC_Spleen_YSD2, Scan: 11485, m/z: 780.93785, Charge: 2, RT (min): 23.66, Score: 133.07

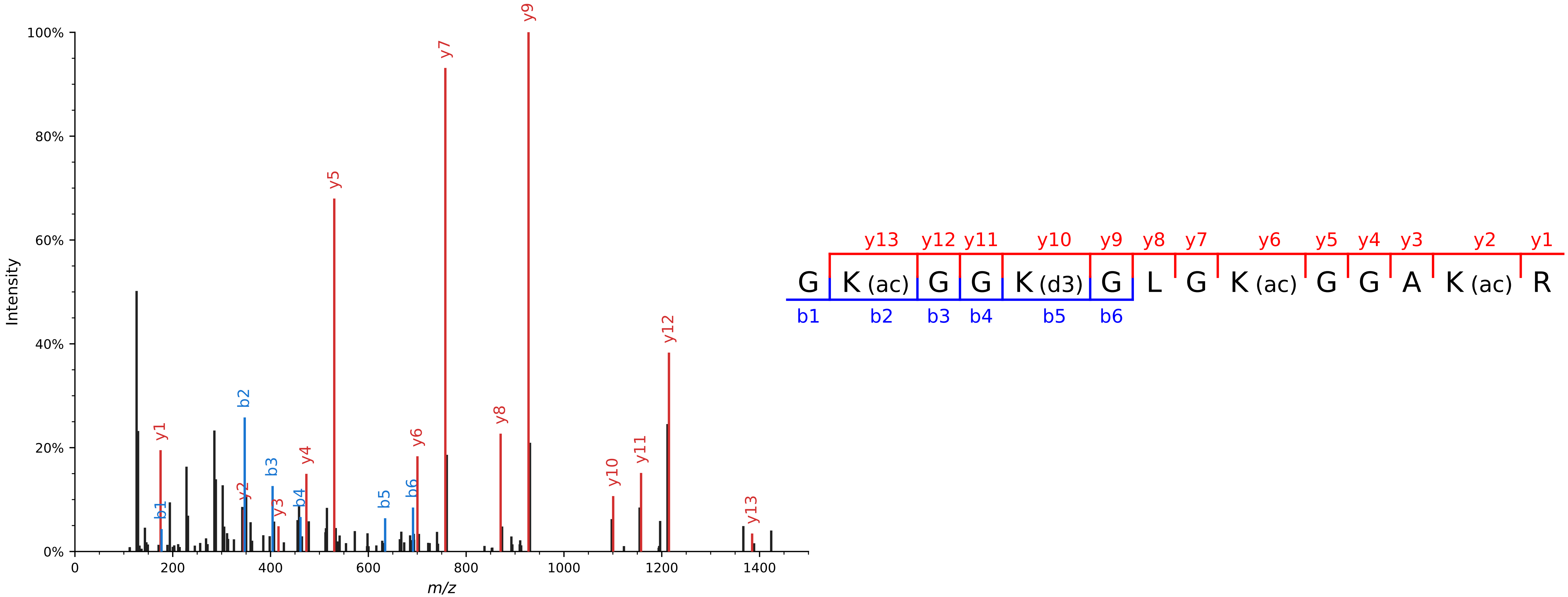

Raw file: HF221212_CG_D3AcPIC_Spleen_YSD1, Scan: 12324, m/z: 790.96451, Charge: 2, RT (min): 24.93, Score: 114.97

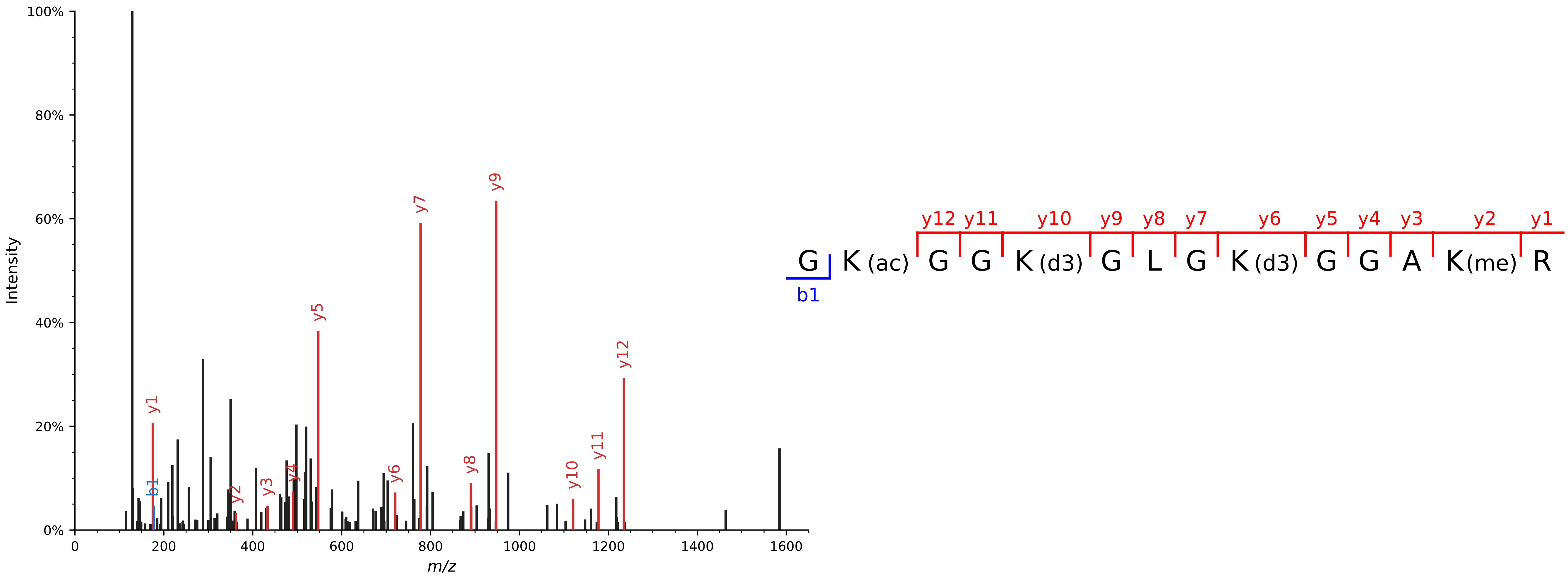

Raw file: HF221212_CG_D3AcPIC_Spleen_YSD1, Scan: 14436, m/z: 796.46292, Charge: 2, RT (min): 28.76, Score: 70.942

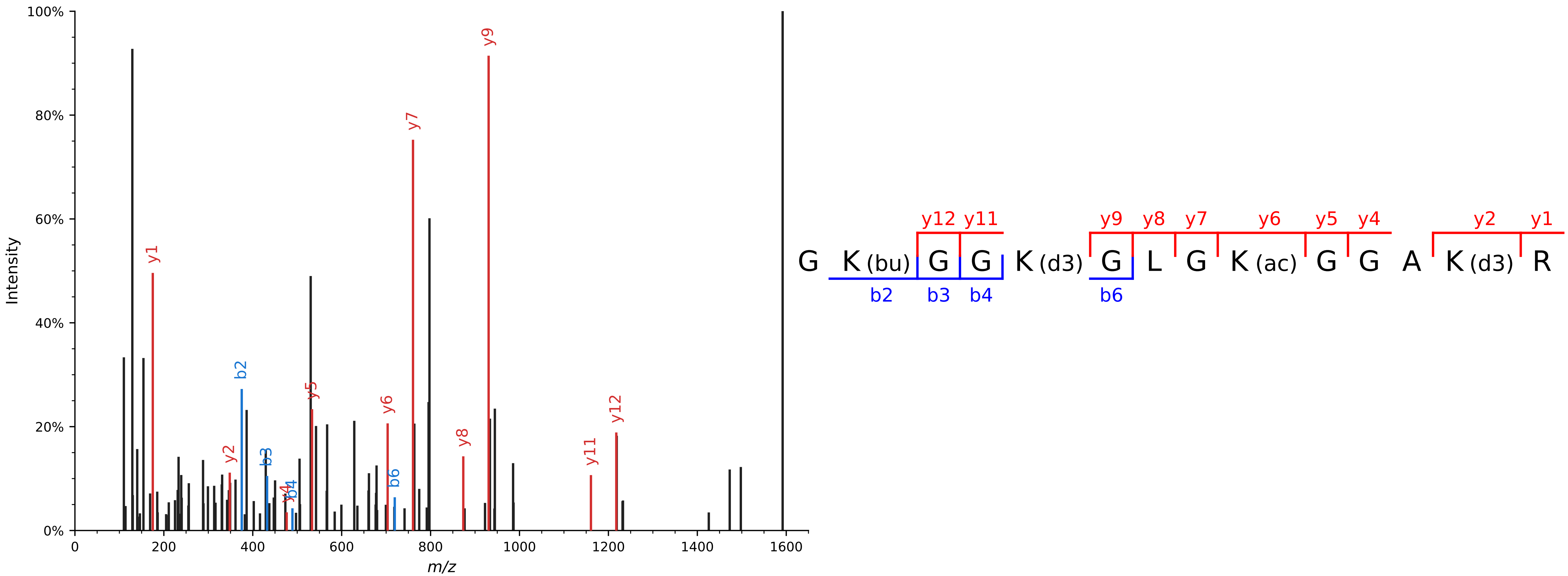

Raw file: HF221212_CG_D3AcPIC_Spleen_YSD2, Scan: 10999, m/z: 776.94886, Charge: 2, RT (min): 22.78, Score: 202.05

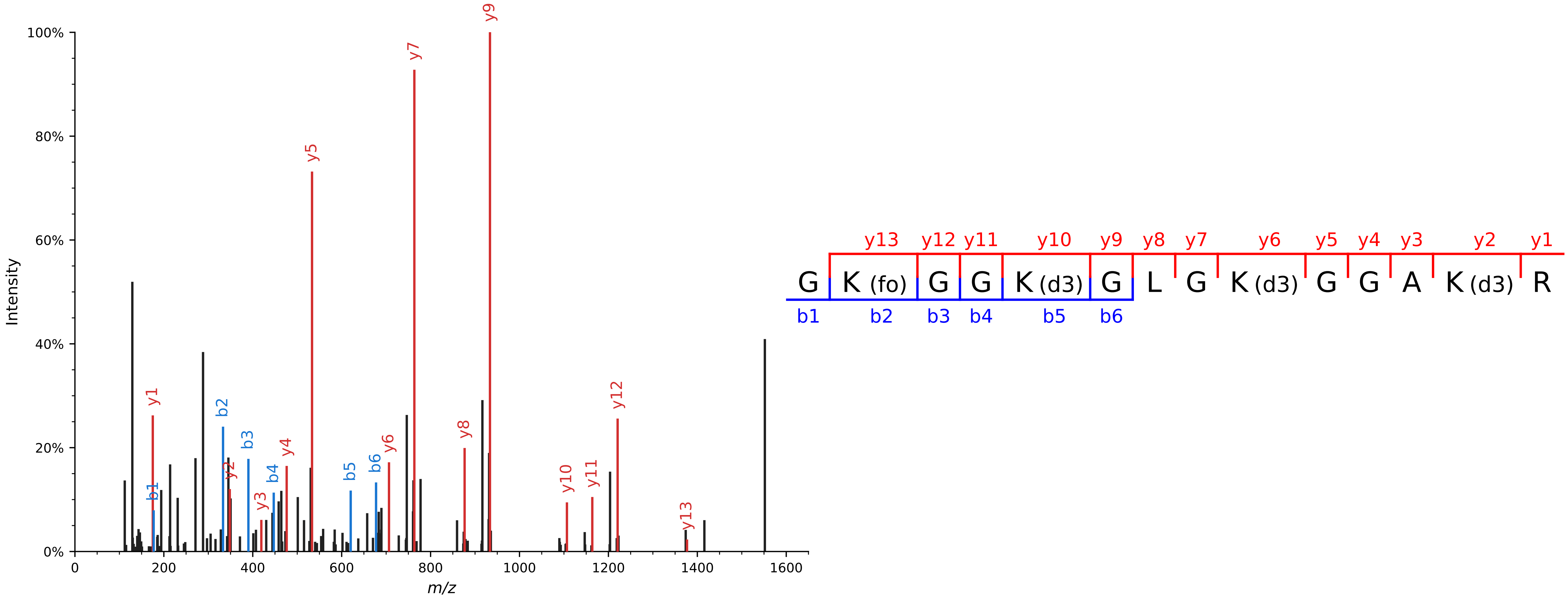

Raw file: HF221212_CG_D3AcPIC_Heart_YSD1, Scan: 13083, m/z: 531.31104, Charge: 3, RT (min): 27.28, Score: 50.088

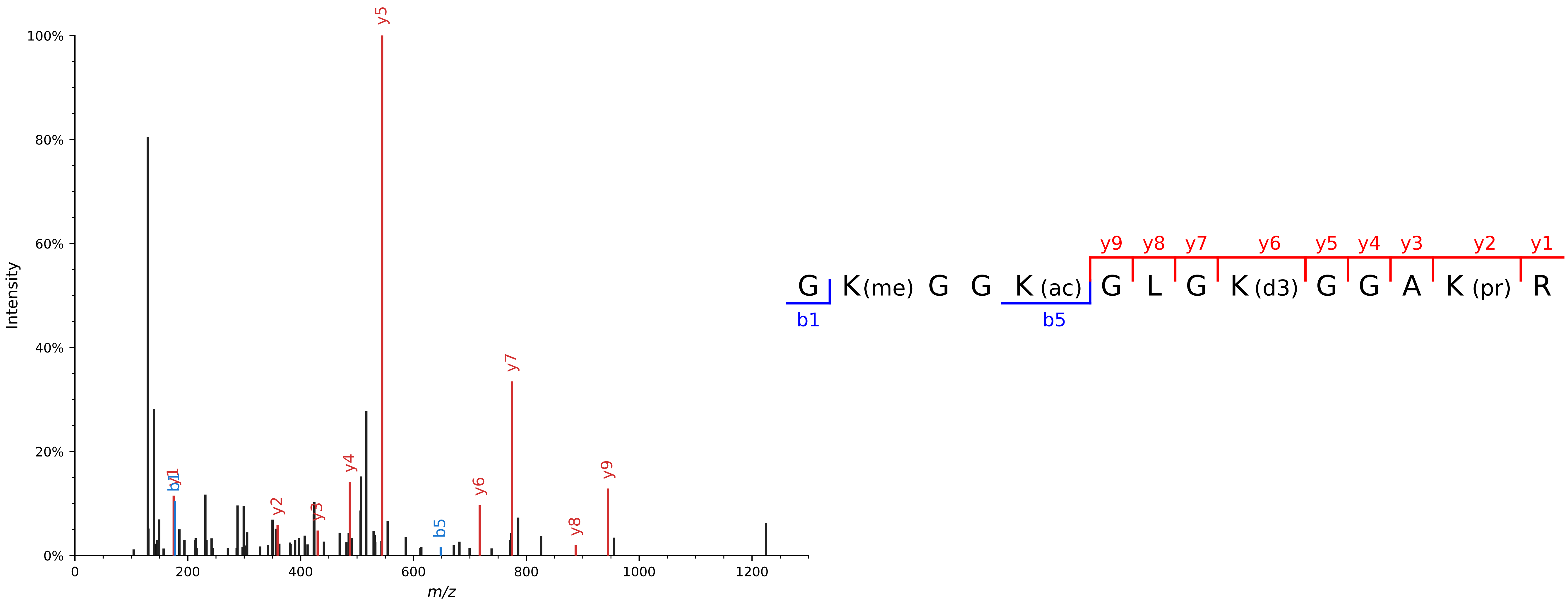

Raw file: HF221212_CG_D3AcPIC_Spleen_YSD2, Scan: 12685, m/z: 533.32359, Charge: 3, RT (min): 25.82, Score: 49.333

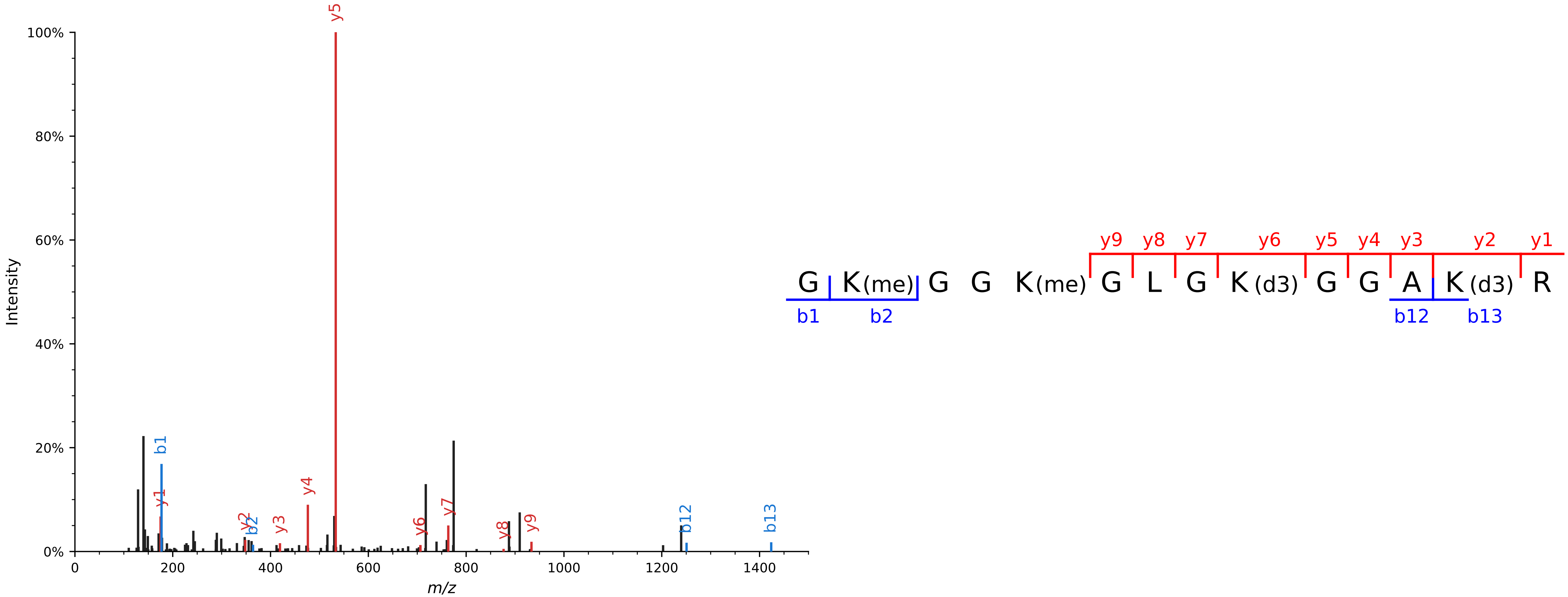

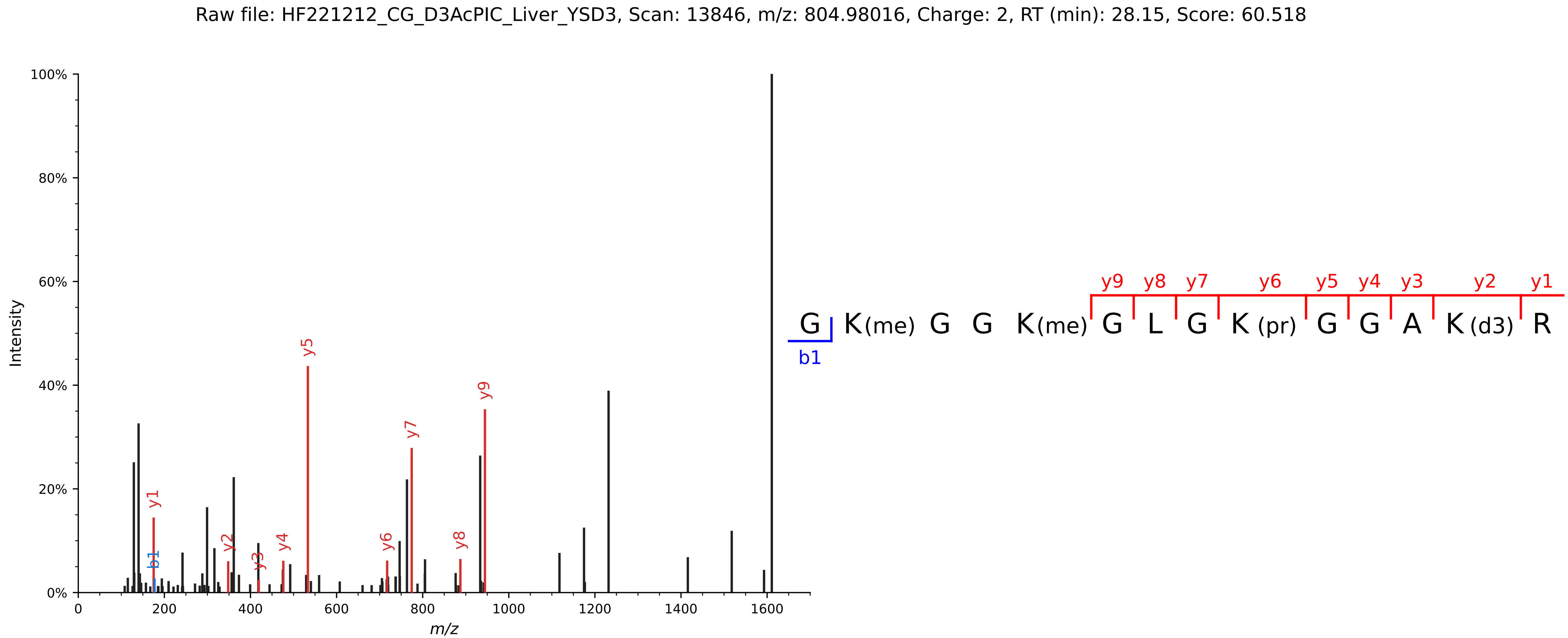
Raw file: HF221212_CG_D3AcPIC_Liver_YSD3, Scan: 13846, m/z: 804.98016, Charge: 2, RT (min): 28.15, Score: 60.518
Intensity
m/z
0%
20%
40%
60%
80%
100%
0
200
400
600
800
1000
1200
1400
1600
y1
b1
y2
y3
y4
y5
y6
y7
y8
y9
G K(me) G G K(me) G L G K (pr) G G A K (d3) R
b1
y9 y8 y7 y6 y5 y4 y3 y2 y1

Raw file: HF221212_CG_D3AcPIC_Spleen_YSD3, Scan: 14649, m/z: 810.47857, Charge: 2, RT (min): 29.61, Score: 59.067

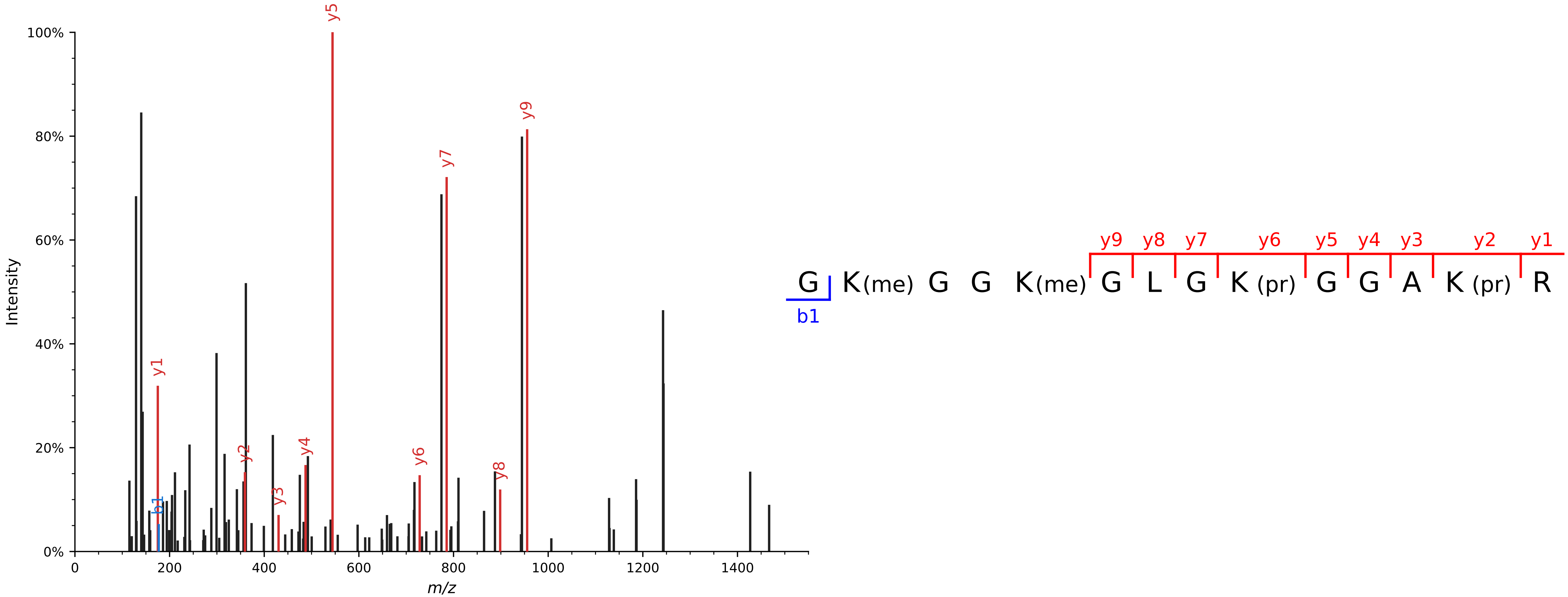

Raw file: HF221212_CG_D3AcPIC_Spleen_YSD1, Scan: 13771, m/z: 804.98016, Charge: 2, RT (min): 27.57, Score: 72.243

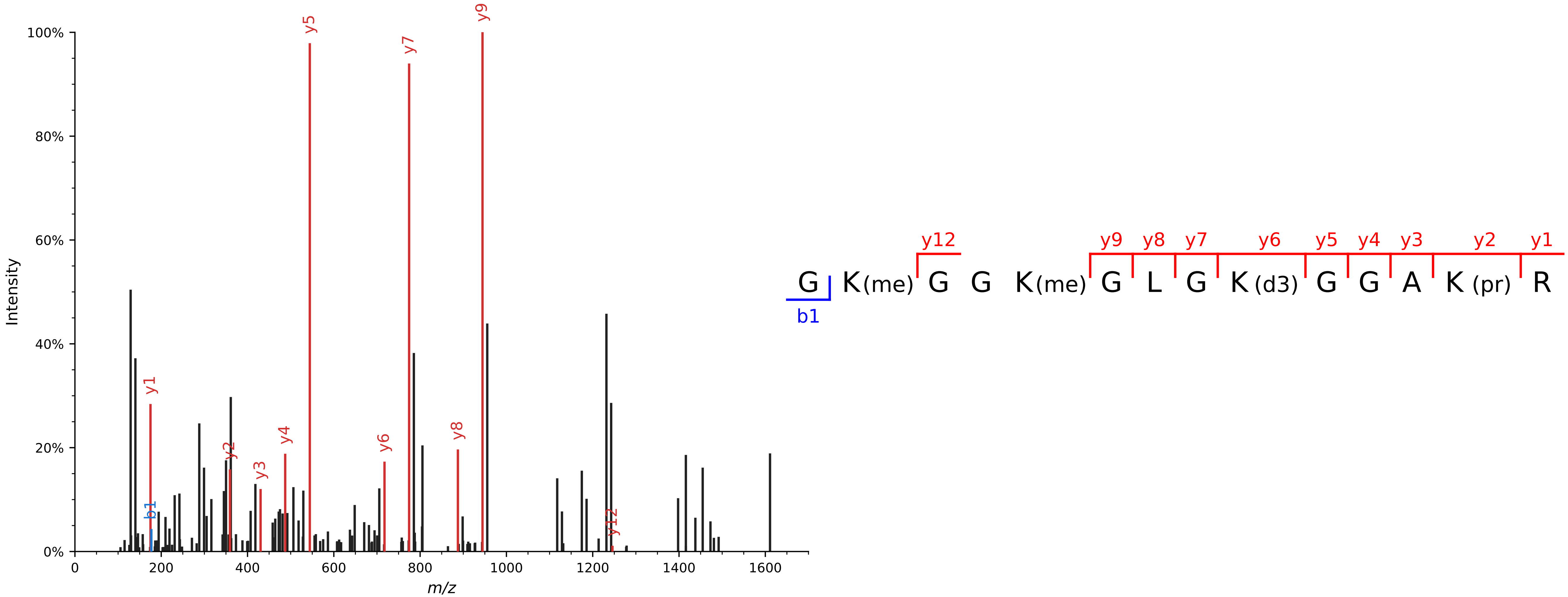

Raw file: HF221212_CG_D3AcPIC_Spleen_YSD2, Scan: 12803, m/z: 790.96451, Charge: 2, RT (min): 26.03, Score: 155.15

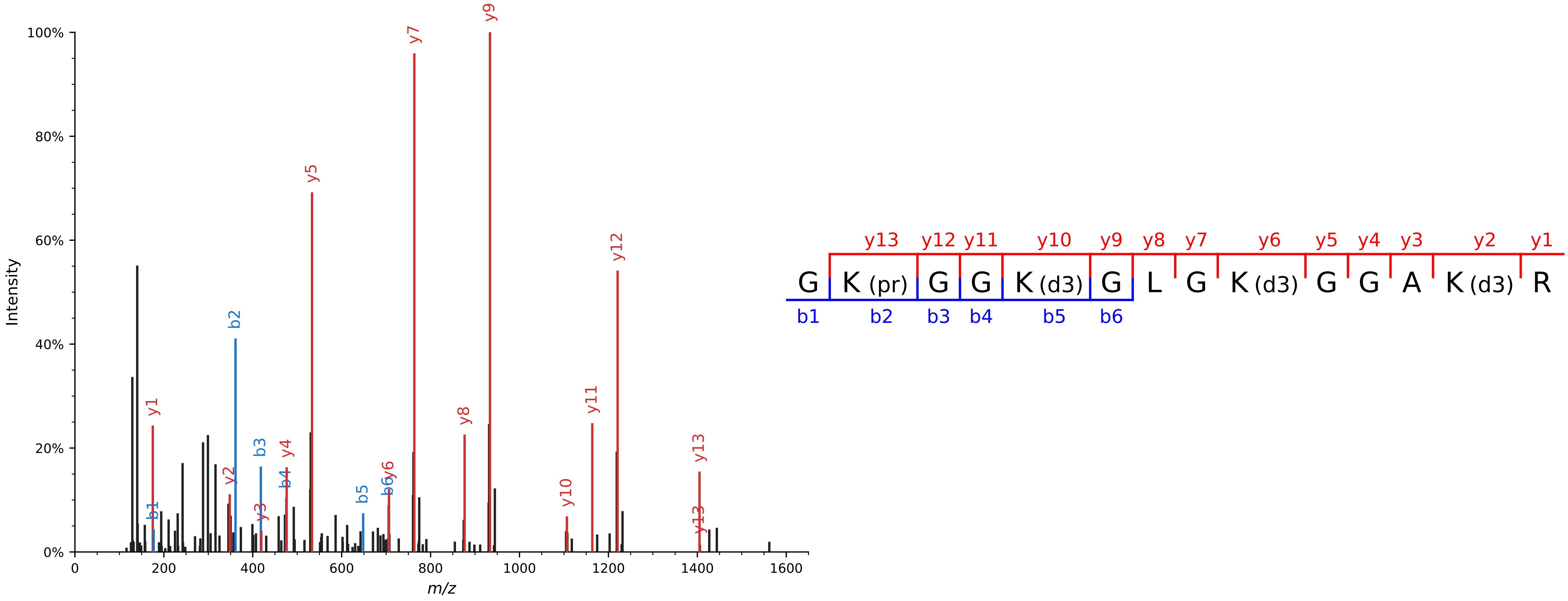

Raw file: HF221212_CG_D3AcPIC_Spleen_YSD1, Scan: 14326, m/z: 796.46292, Charge: 2, RT (min): 28.56, Score: 70.808

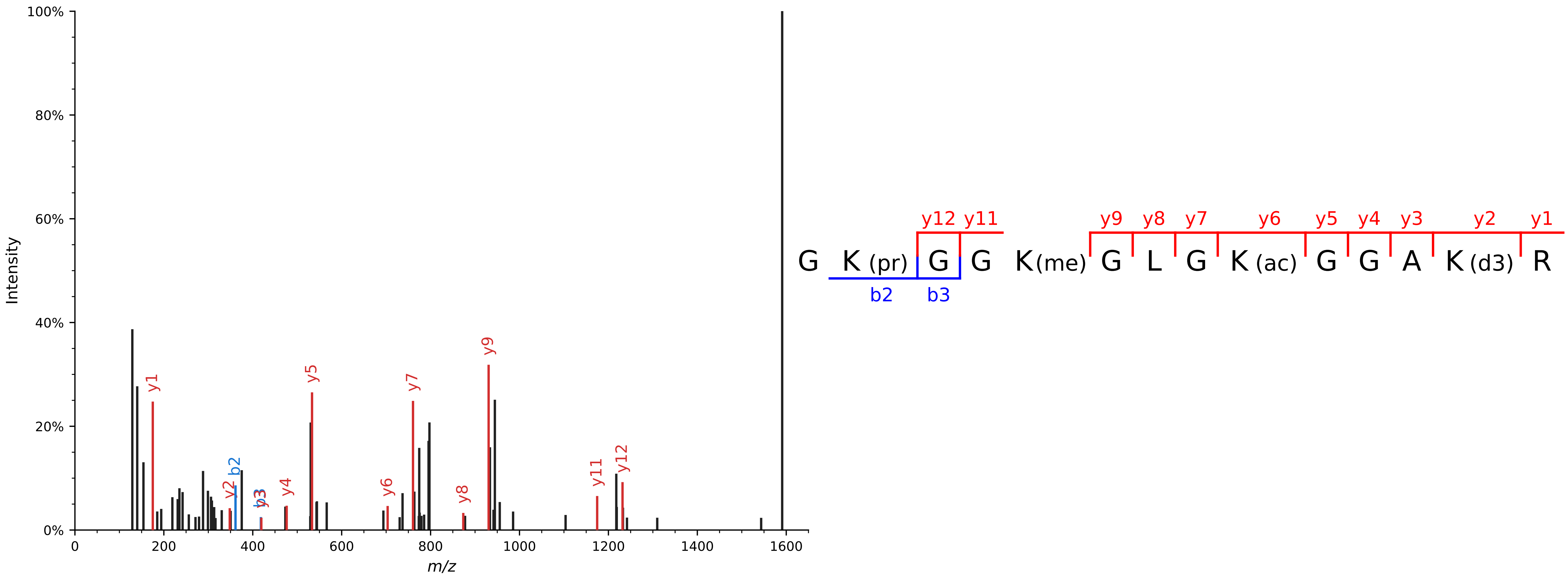

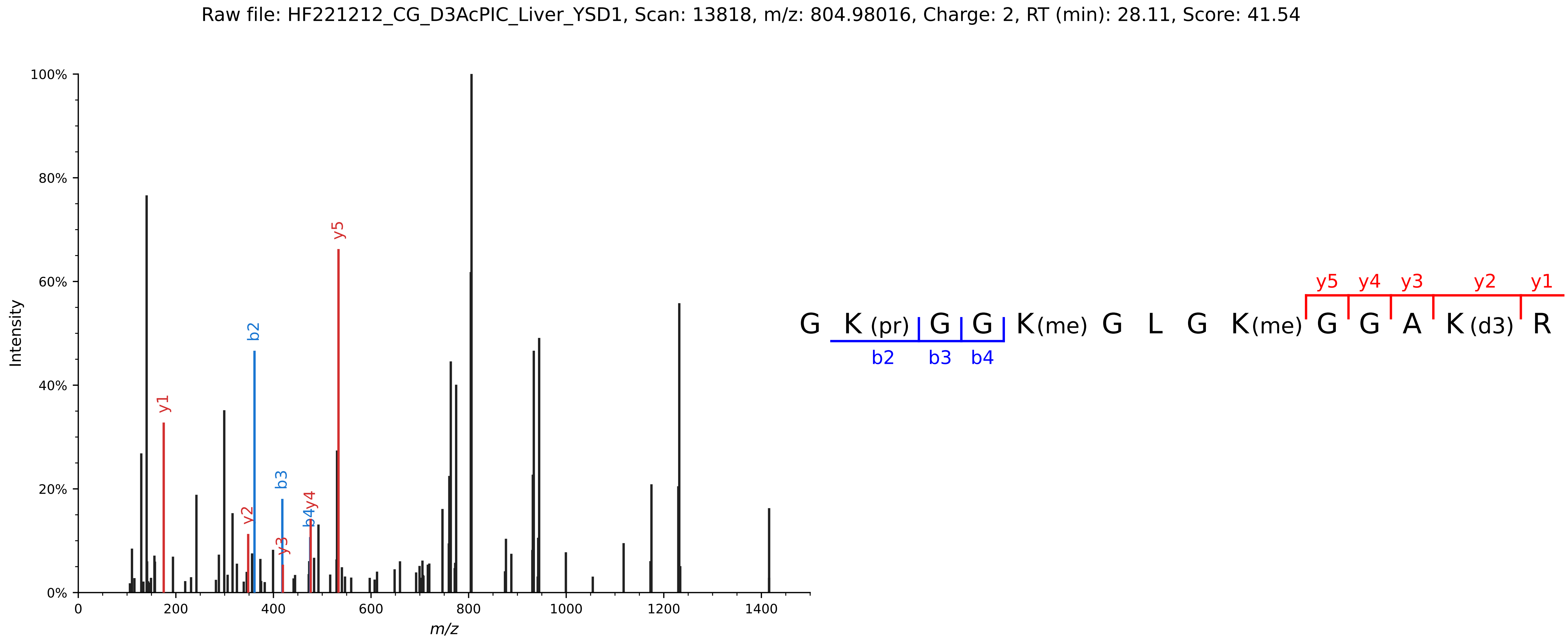
Raw file: HF221212_CG_D3AcPIC_Liver_YSD1, Scan: 13818, m/z: 804.98016, Charge: 2, RT (min): 28.11, Score: 41.54
Intensity
m/z
y1
y2
y3
y4
y5
b2
b3
b4
G K(pr) G G K(me) G L G K(me) G G A K(d3) R

Raw file: HF221212_CG_D3AcPIC_Liver_YSD1, Scan: 14605, m/z: 810.47857, Charge: 2, RT (min): 29.55, Score: 51.268

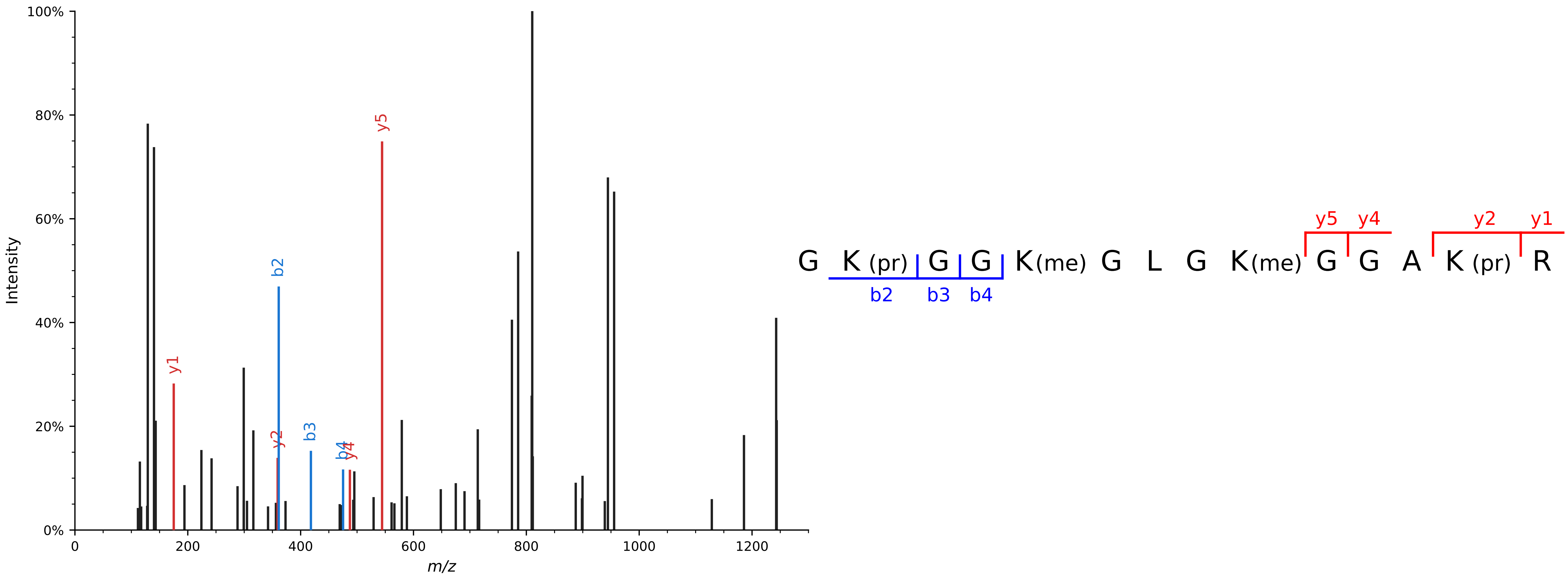

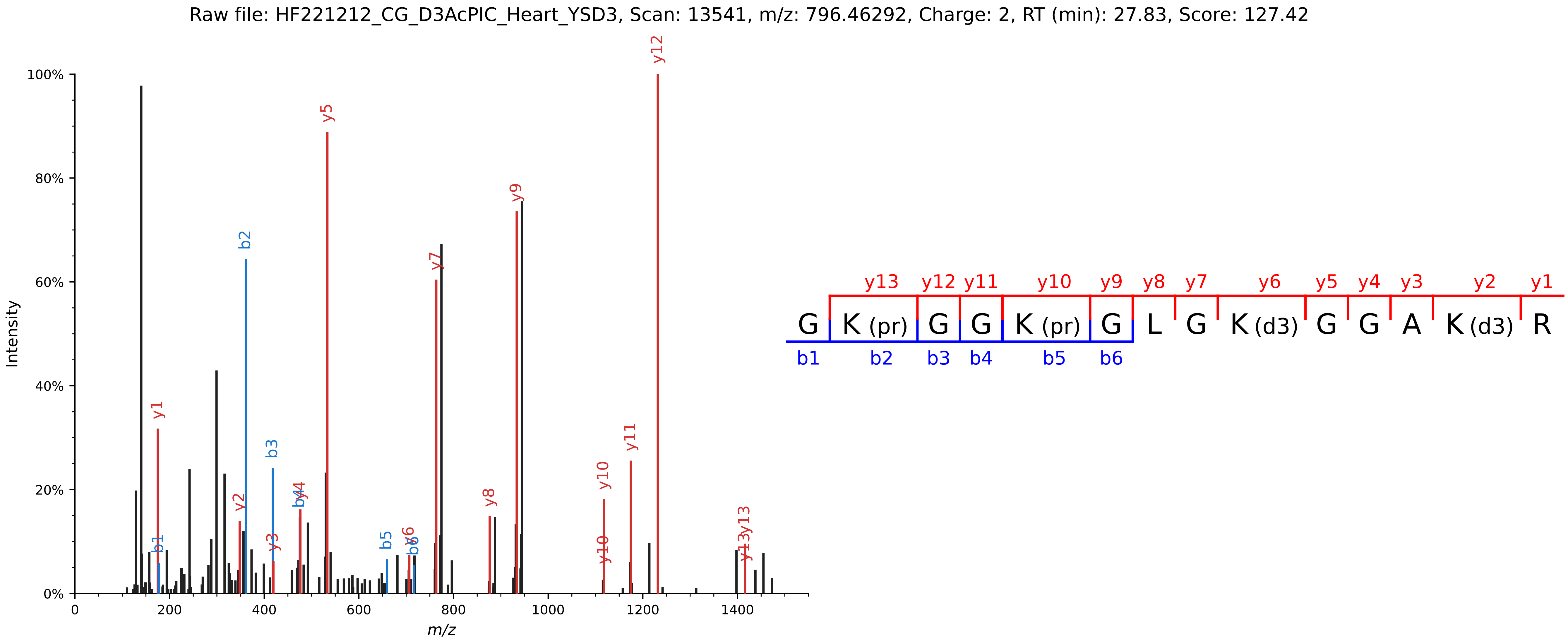
Raw file: HF221212_CG_D3AcPIC_Heart_YSD3, Scan: 13541, m/z: 796.46292, Charge: 2, RT (min): 27.83, Score: 127.42
Intensity
m/z
G K (pr) G G K (pr) G L G K (d3) G G A K (d3) R

Raw file: HF221212_CG_D3AcPIC_Spleen_YSD3, Scan: 14917, m/z: 801.96133, Charge: 2, RT (min): 30.10, Score: 138.71

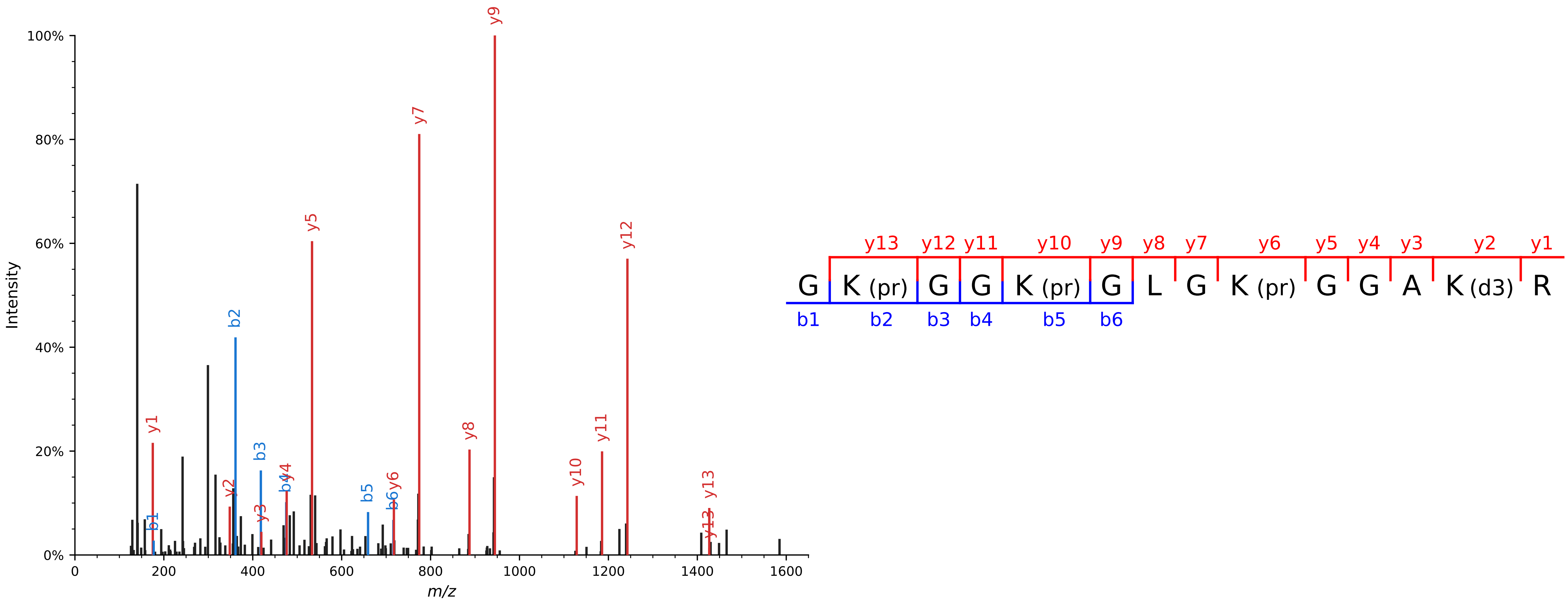

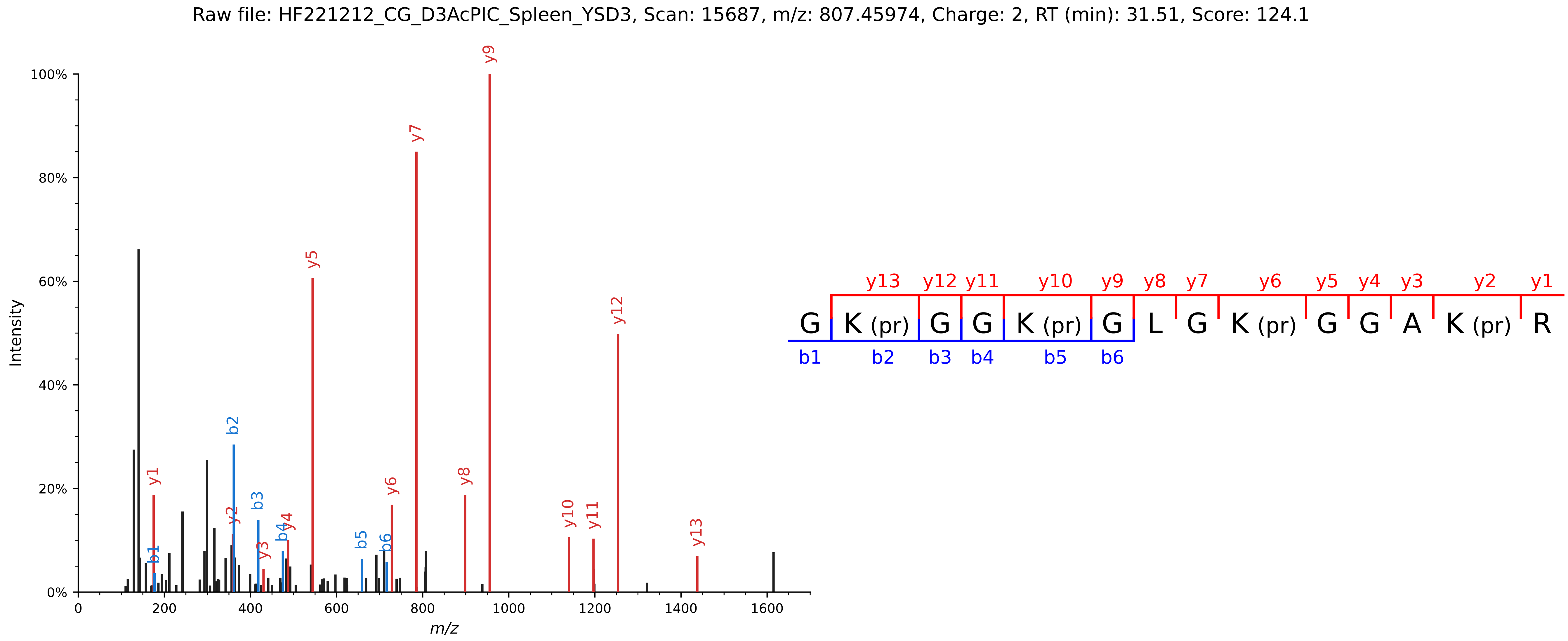
Raw file: HF221212_CG_D3AcPIC_Spleen_YSD3, Scan: 15687, m/z: 807.45974, Charge: 2, RT (min): 31.51, Score: 124.1
Intensity
m/z
G K (pr) G G K (pr) G L G K (pr) G G A K (pr) R
y13 y12 y11 y10 y9 y8 y7 y6 y5 y4 y3 y2 y1
b1 b2 b3 b4 b5 b6

Raw file: HF221212_CG_D3AcPIC_Kidney_YSD1, Scan: 13709, m/z: 794.9535, Charge: 2, RT (min): 28.33, Score: 124.1

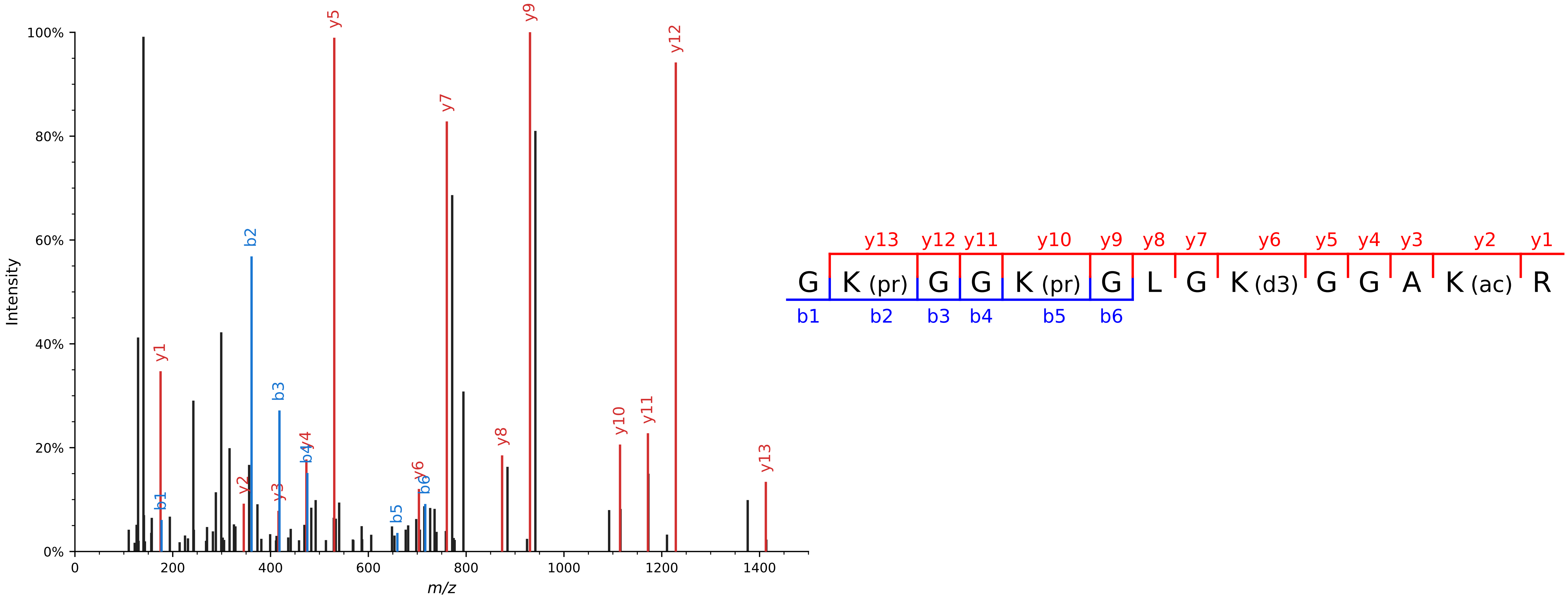

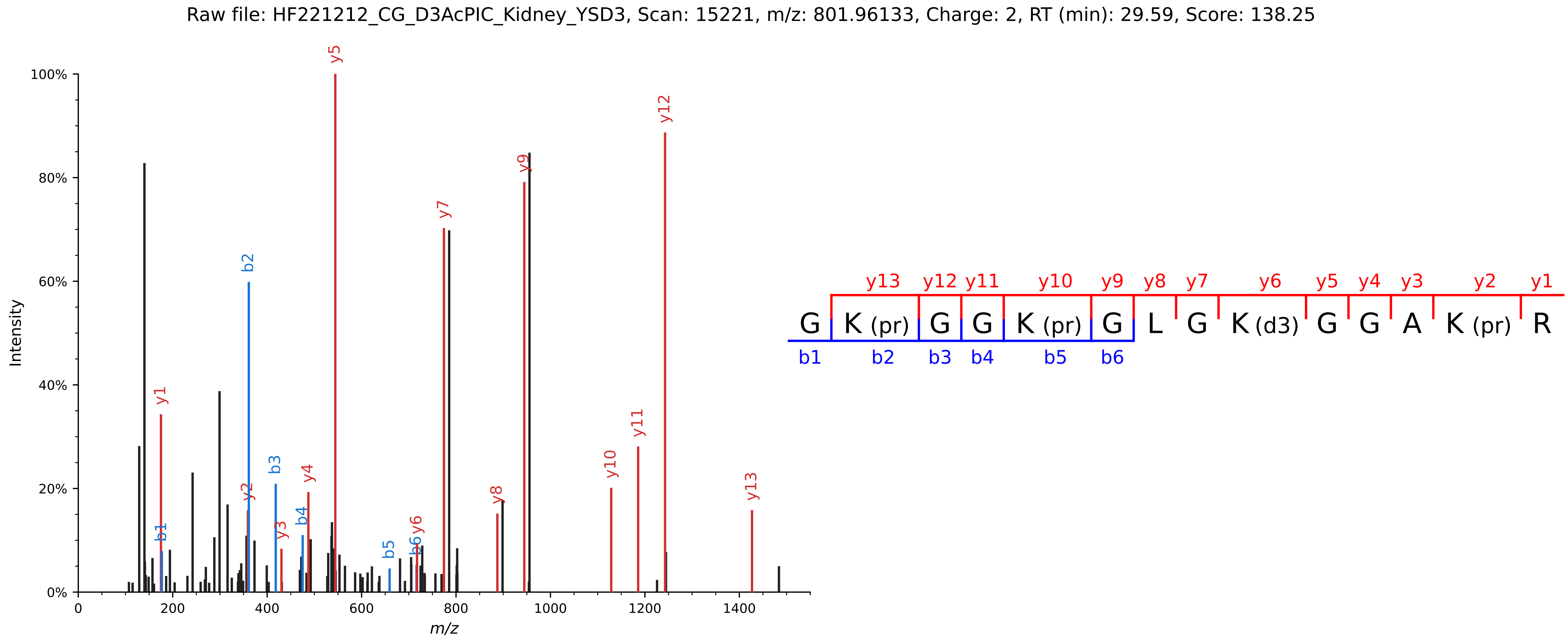

Raw file: HF221212_CG_D3AcPIC_Kidney_YSD3, Scan: 15221, m/z: 801.96133, Charge: 2, RT (min): 29.59, Score: 138.25
Intensity
m/z
y13 y12 y11 y10 y9 y8 y7 y6 y5 y4 y3 y2 y1
G K (pr) G G K (pr) G L G K (d3) G G A K (pr) R
b1 b2 b3 b4 b5 b6

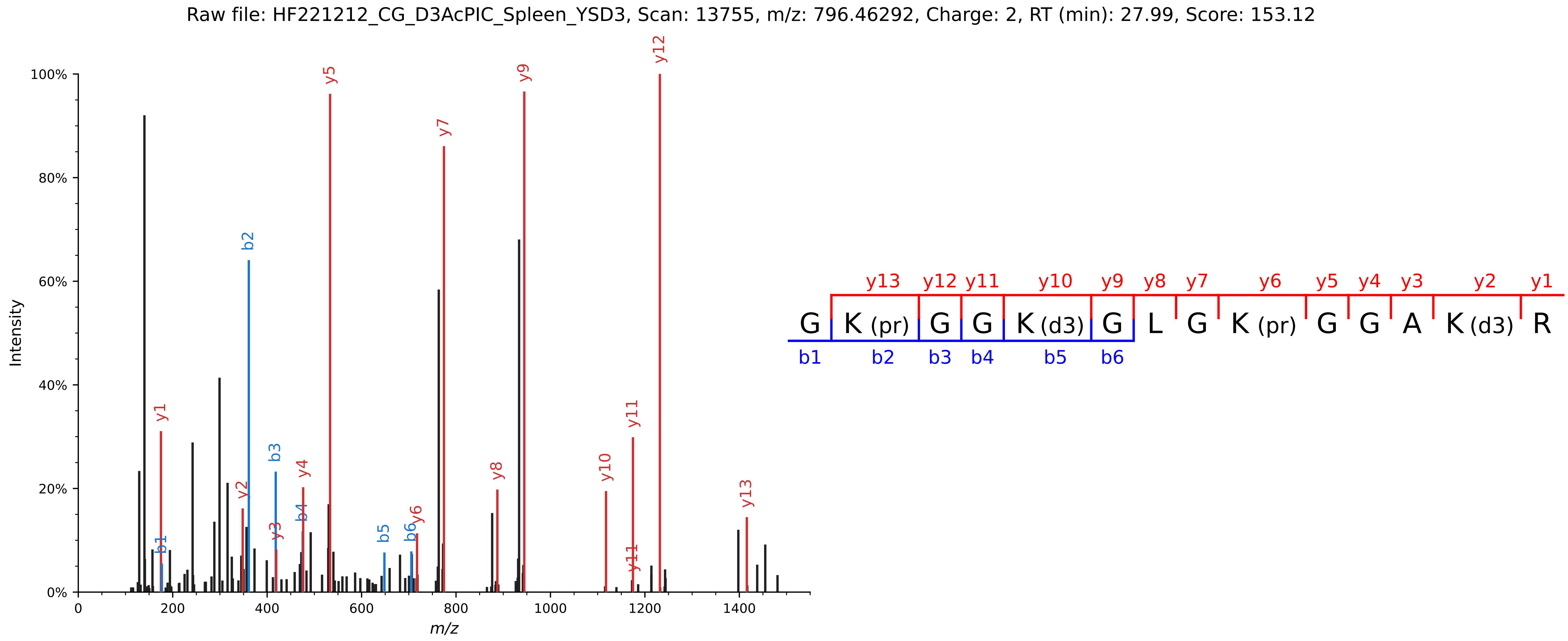
Raw file: HF221212_CG_D3AcPIC_Spleen_YSD3, Scan: 13755, m/z: 796.46292, Charge: 2, RT (min): 27.99, Score: 153.12
Intensity
m/z
0%
20%
40%
60%
80%
100%
0
200
400
600
800
1000
1200
1400
y1
b1
y2
b2
y3
b3
y4
b4
y5
b5
b6
y6
y7
y8
y9
y10
y11
y11
y12
y13
G K (pr) G G K (d3) G L G K (pr) G G A K (d3) R
y13 y12 y11 y10 y9 y8 y7 y6 y5 y4 y3 y2 y1
b1 b2 b3 b4 b5 b6

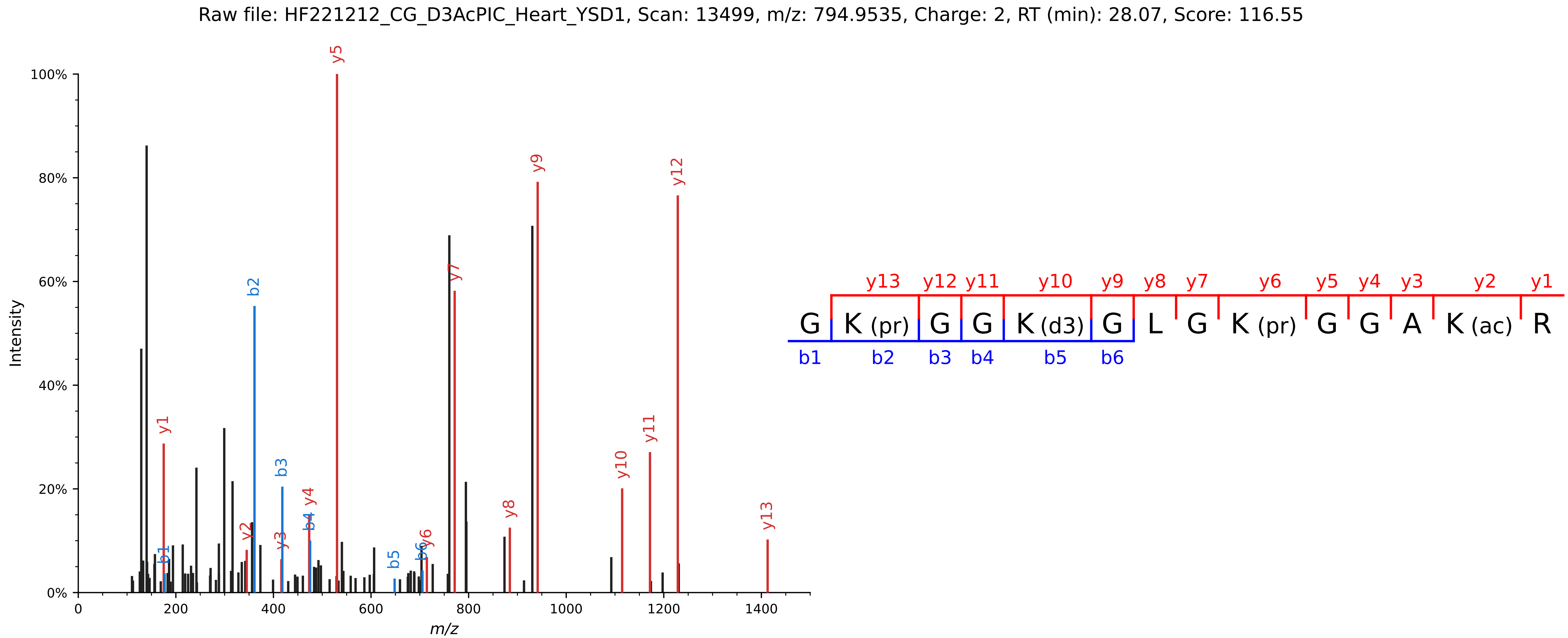
Raw file: HF221212_CG_D3AcPIC_Heart_YSD1, Scan: 13499, m/z: 794.9535, Charge: 2, RT (min): 28.07, Score: 116.55
Intensity
m/z
G K (pr) G G K (d3) G L G K (pr) G G A K (ac) R
y13 y12 y11 y10 y9 y8 y7 y6 y5 y4 y3 y2 y1
b1 b2 b3 b4 b5 b6

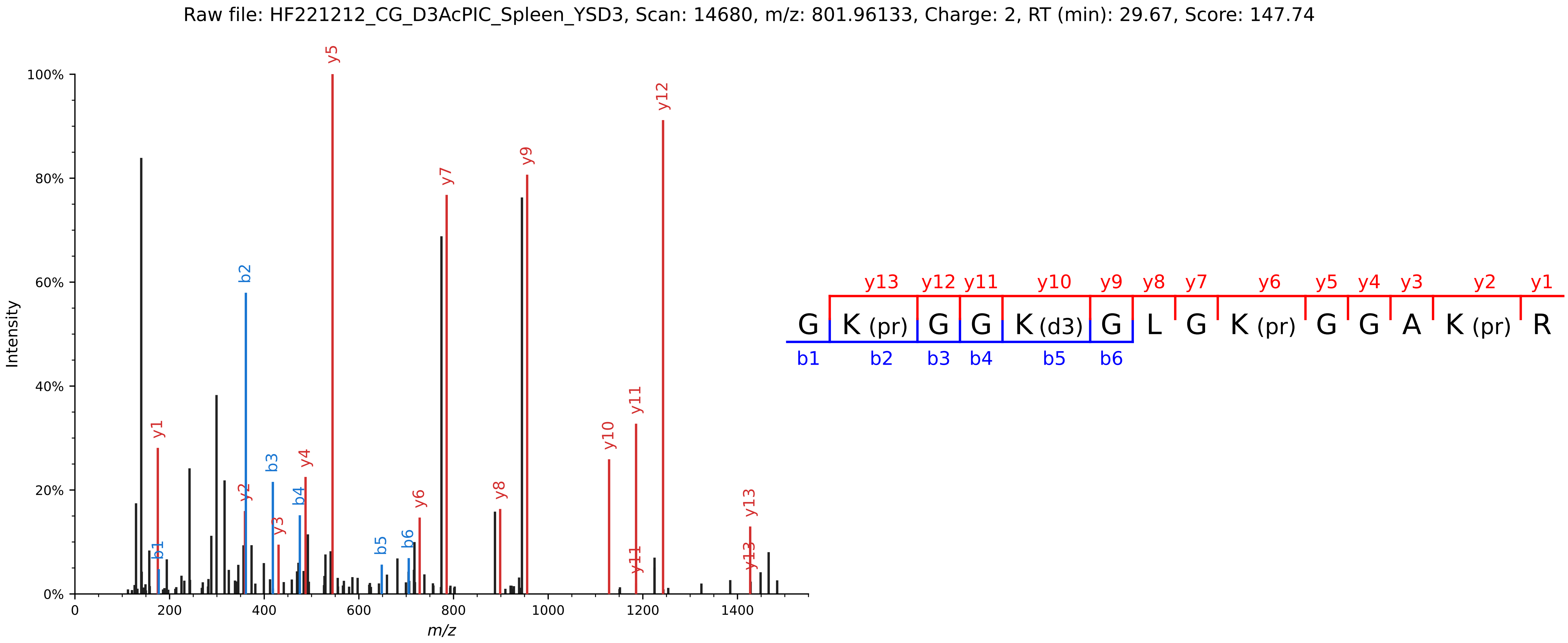
Raw file: HF221212_CG_D3AcPIC_Spleen_YSD3, Scan: 14680, m/z: 801.96133, Charge: 2, RT (min): 29.67, Score: 147.74
Intensity
m/z
100%
80%
60%
40%
20%
0%
0
200
400
600
800
1000
1200
1400
y1
b1
b2
y2
b3
y3
b4
y4
y5
b5
b6
y6
y7
y8
y9
y10
y11
y11
y12
y13
y13
G K (pr) G G K (d3) G L G K (pr) G G A K (pr) R
y13 y12 y11 y10 y9 y8 y7 y6 y5 y4 y3 y2 y1
b1 b2 b3 b4 b5 b6

Raw file: HF221212_CG_D3AcPIC_Brain_YSD1, Scan: 12415, m/z: 789.45509, Charge: 2, RT (min): 25.87, Score: 154.35

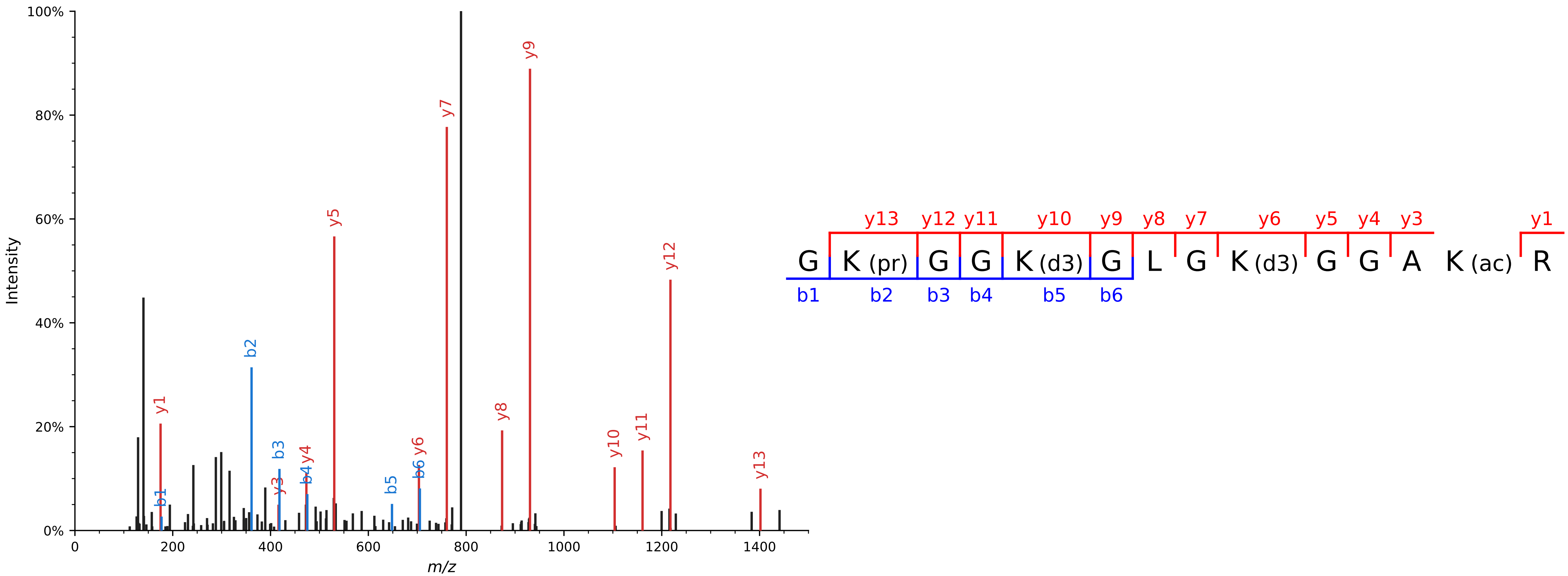

Raw file: HF221212_CG_D3AcPIC_Spleen_YSD3, Scan: 13558, m/z: 796.46292, Charge: 2, RT (min): 27.64, Score: 156.48

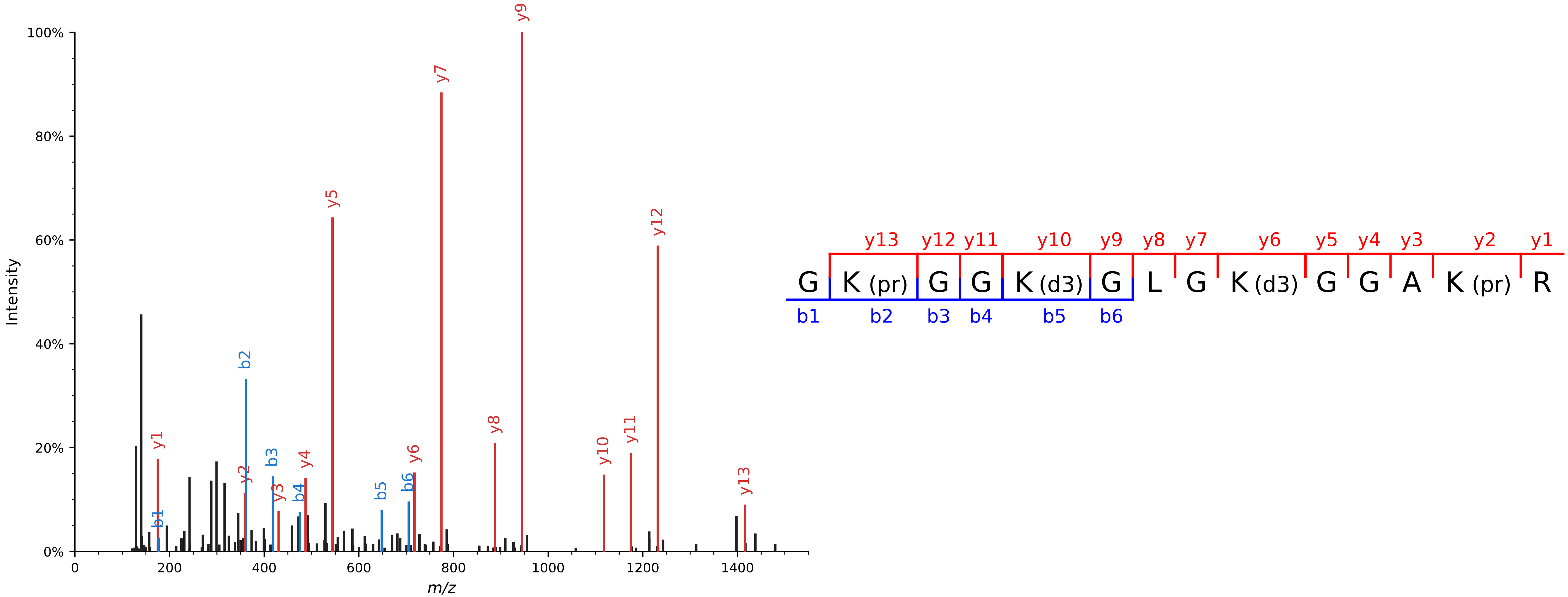

Raw file: HF221212_CG_D3AcPIC_Kidney_YSD3, Scan: 11984, m/z: 804.44218, Charge: 2, RT (min): 23.80, Score: 57.785

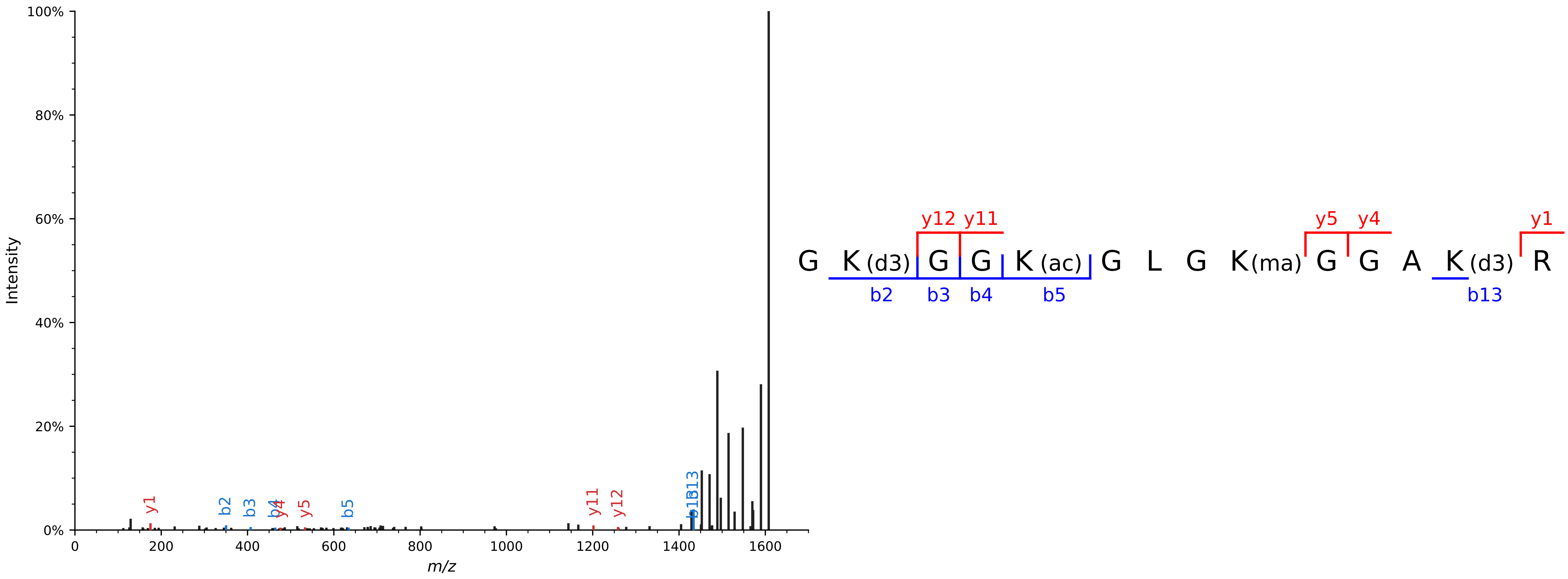

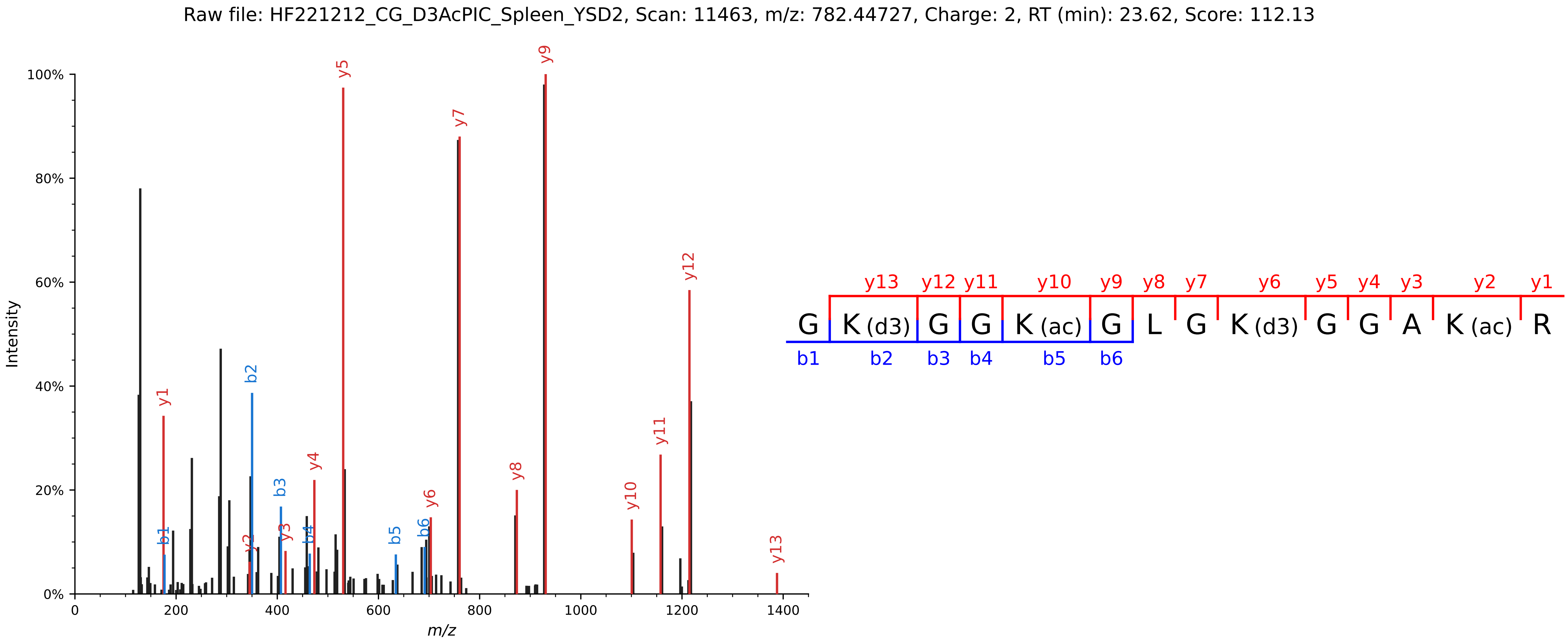

Raw file: HF221212_CG_D3AcPIC_Spleen_YSD2, Scan: 11463, m/z: 782.44727, Charge: 2, RT (min): 23.62, Score: 112.13
Intensity
m/z
y1
b1
b2
y2
y3
b3
b4
y4
y5
b5
b6
y6
y7
y8
y9
y10
y11
y12
y13
G K (d3) G G K (ac) G L G K (d3) G G A K (ac) R

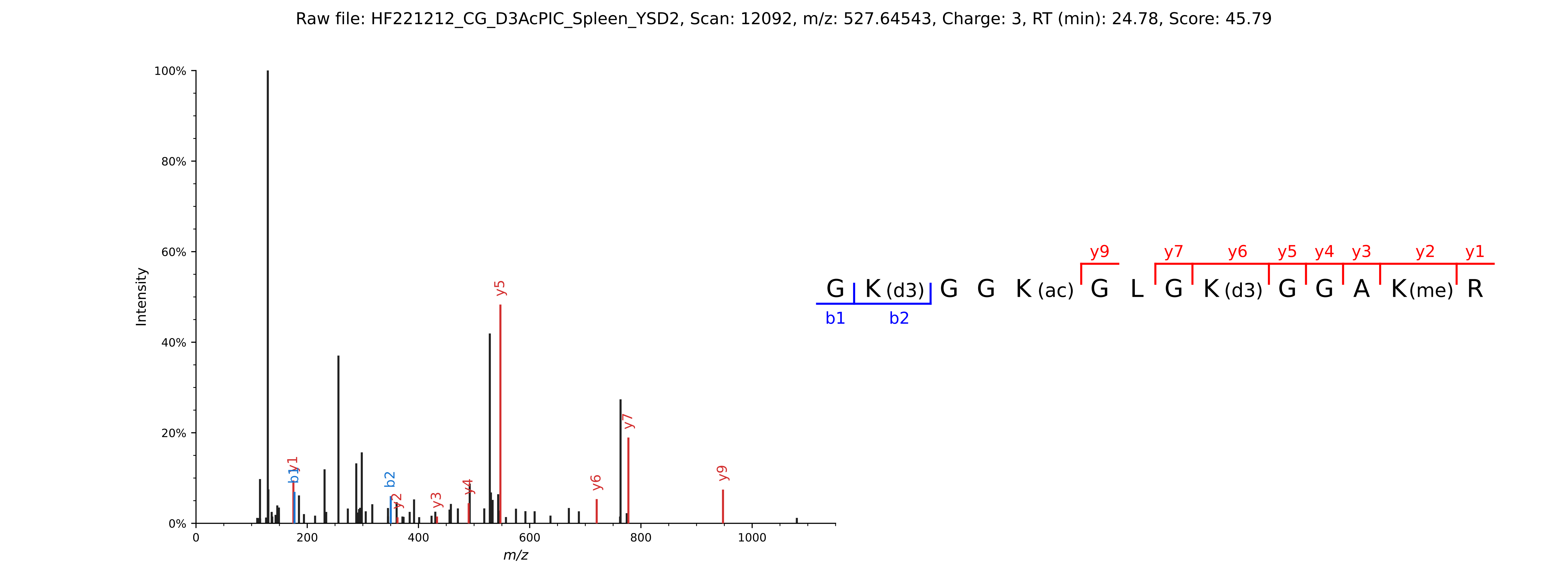
Raw file: HF221212_CG_D3AcPIC_Spleen_YSD2, Scan: 12092, m/z: 527.64543, Charge: 3, RT (min): 24.78, Score: 45.79
Intensity
m/z
100%
80%
60%
40%
20%
0%
0
200
400
600
800
1000
y1
b1
b2
y2
y3
y4
y5
y6
y7
y9
G K(d3) G G K(ac) G L G K(d3) G G A K(me) R
b1
b2
y9
y7
y6
y5
y4
y3
y2
y1

Raw file: HF221212_CG_D3AcPIC_Spleen_YSD3, Scan: 11108, m/z: 776.94886, Charge: 2, RT (min): 23.10, Score: 170.99

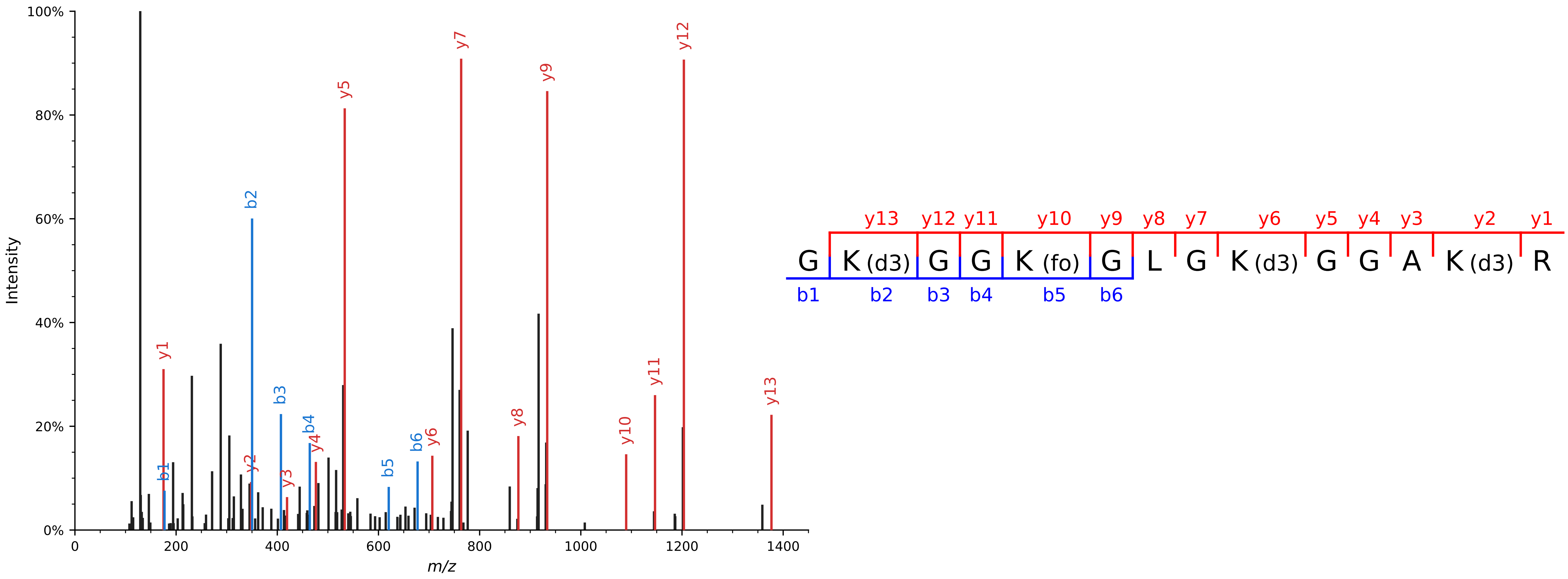

Raw file: HF221212_CG_D3AcPIC_Heart_YSD3, Scan: 10897, m/z: 775.43944, Charge: 2, RT (min): 22.86, Score: 60.398

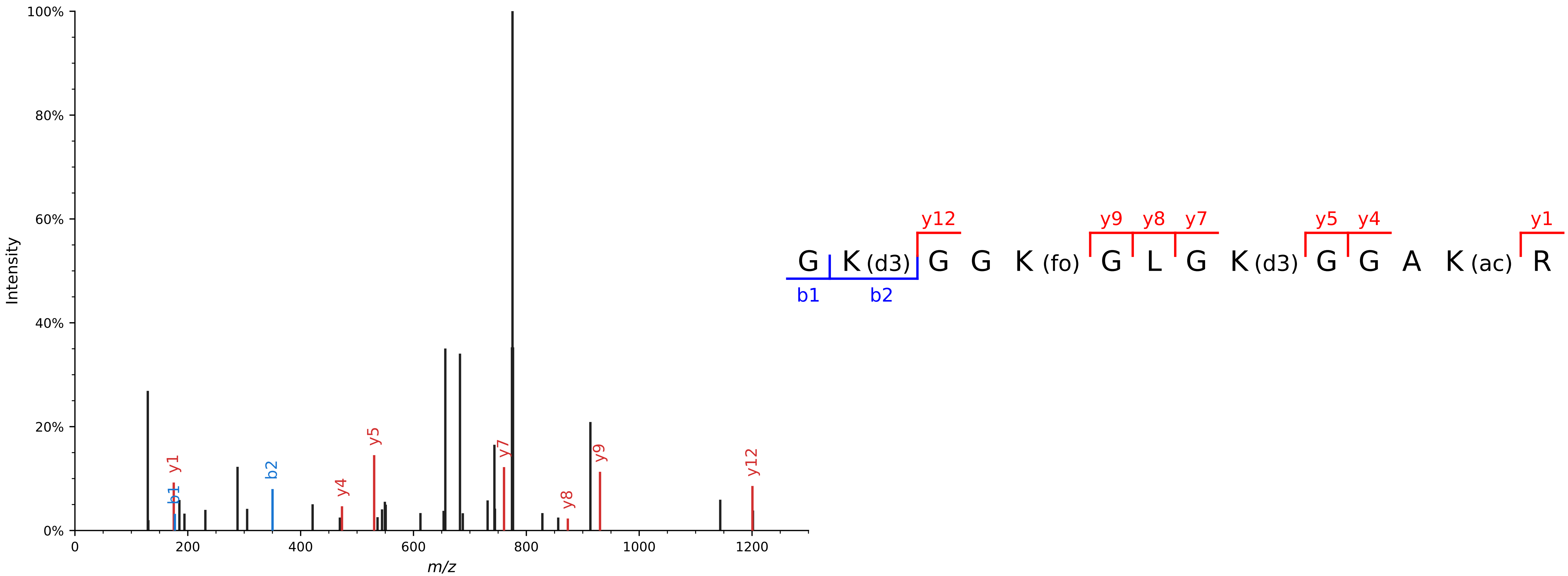

Raw file: HF221212_CG_D3AcPIC_Spleen_YSD3, Scan: 11294, m/z: 798.96197, Charge: 2, RT (min): 23.43, Score: 57.785

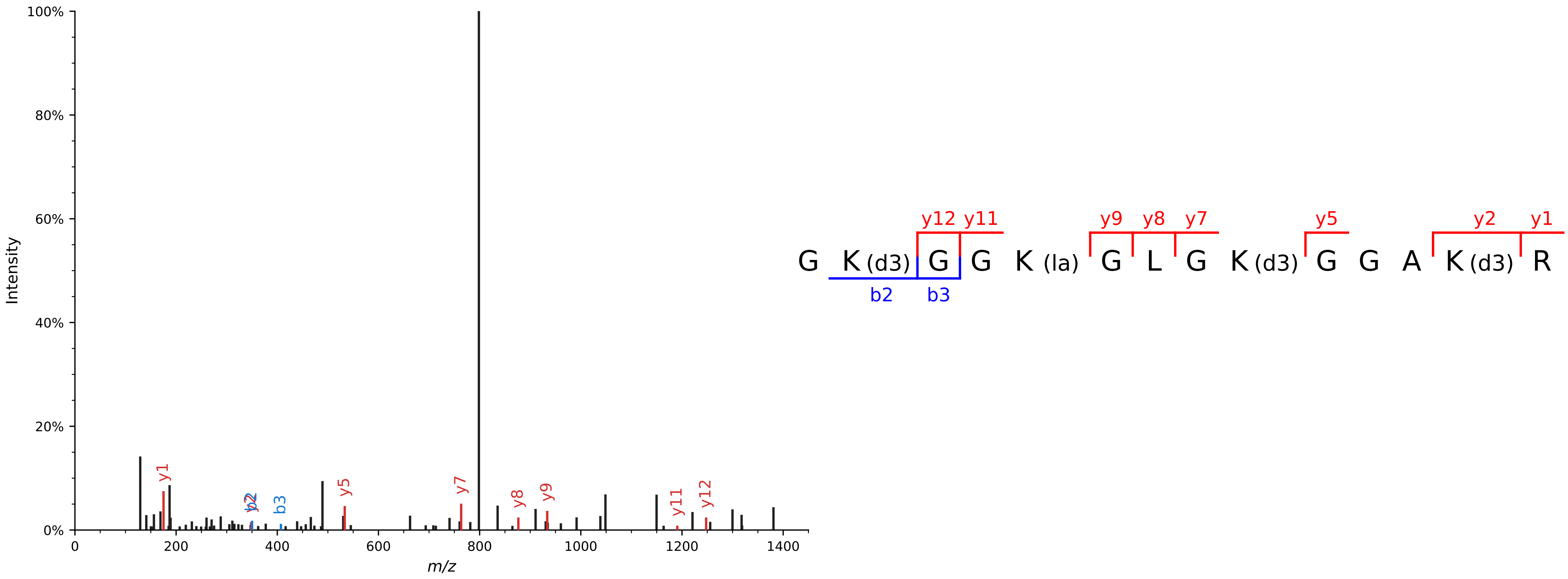

Raw file: HF221212_CG_D3AcPIC_Spleen_YSD3, Scan: 11639, m/z: 804.44218, Charge: 2, RT (min): 24.07, Score: 40.496

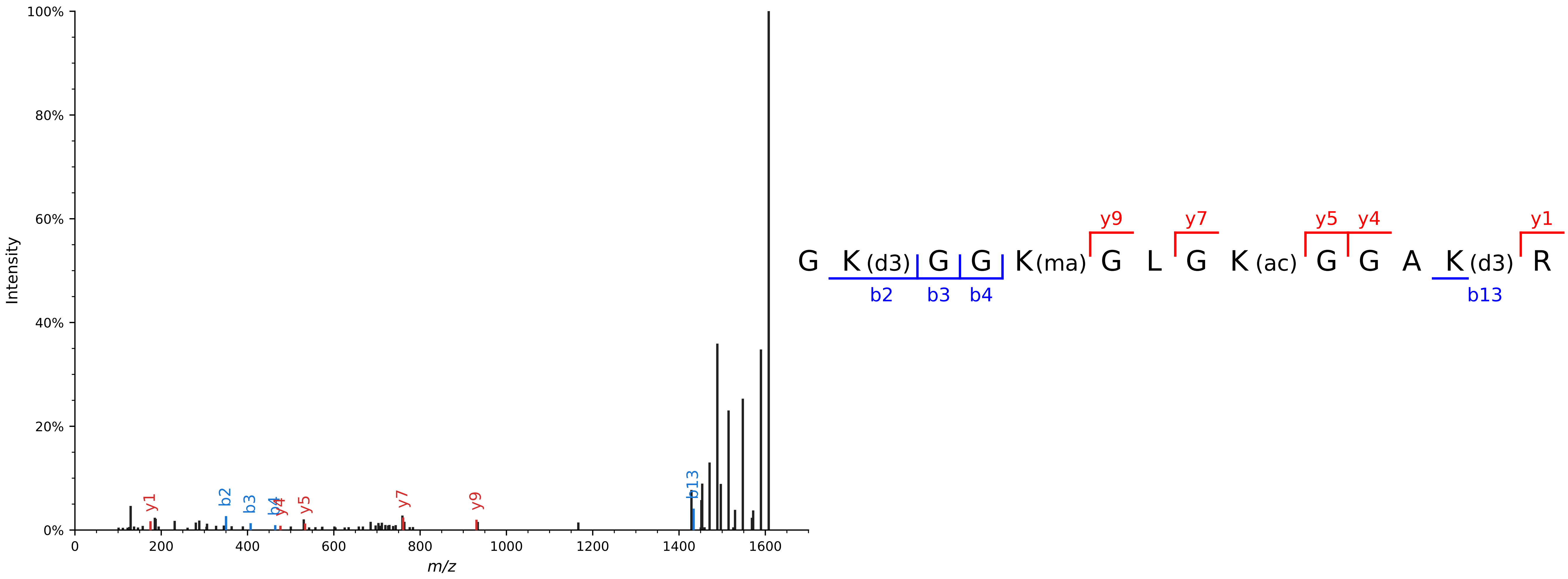

Raw file: HF221212_CG_D3AcPIC_Spleen_YSD1, Scan: 11796, m/z: 804.44218, Charge: 2, RT (min): 23.98, Score: 48.44

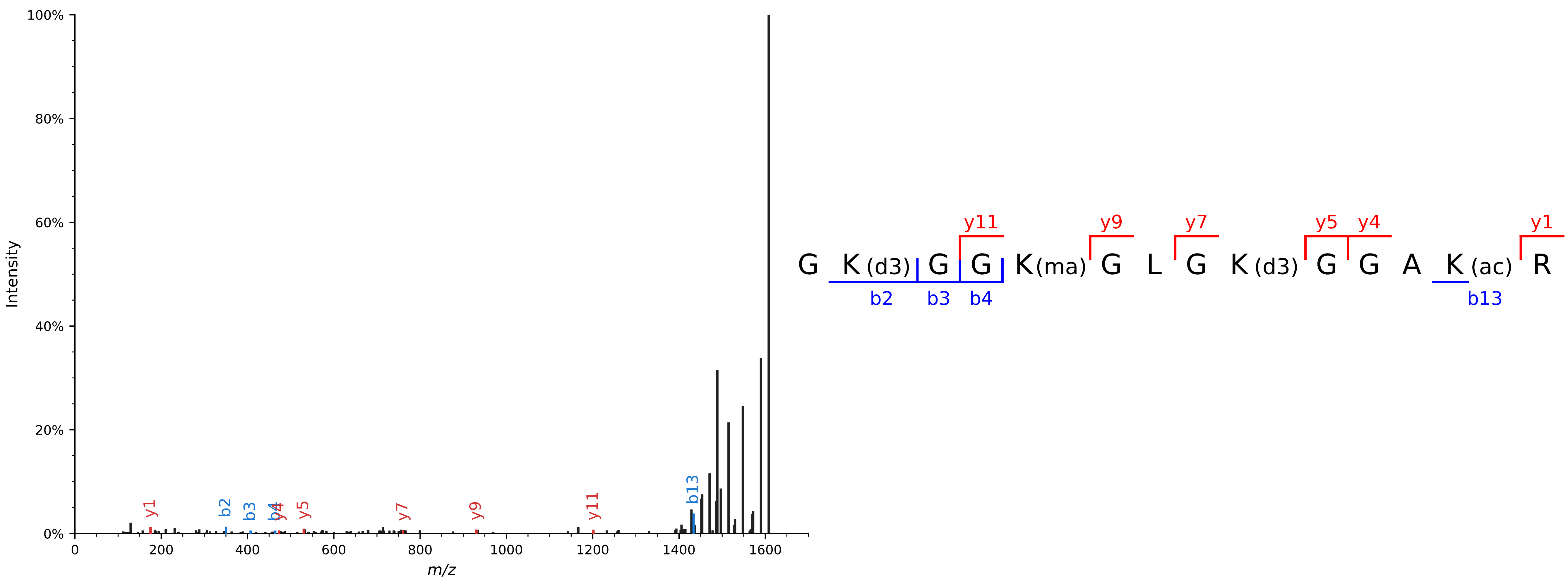

Raw file: HF221212_CG_D3AcPIC_Spleen_YSD2, Scan: 12502, m/z: 790.96451, Charge: 2, RT (min): 25.50, Score: 141.95

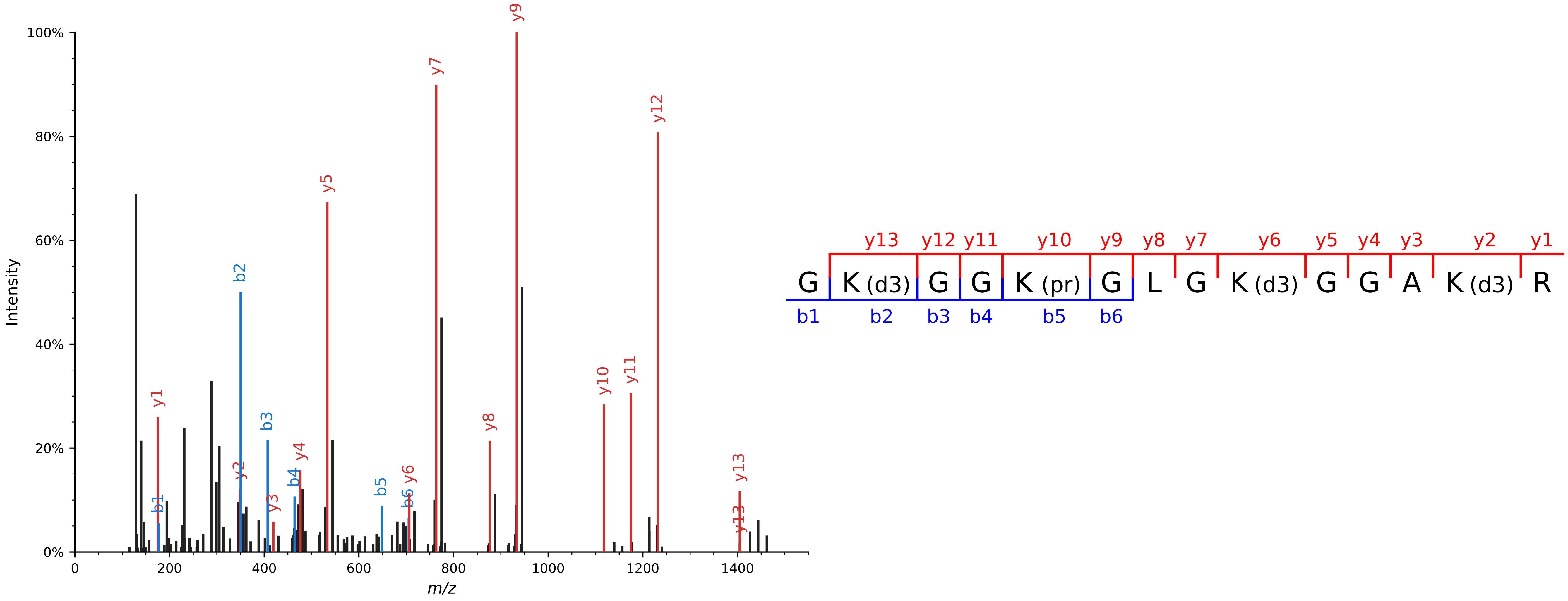

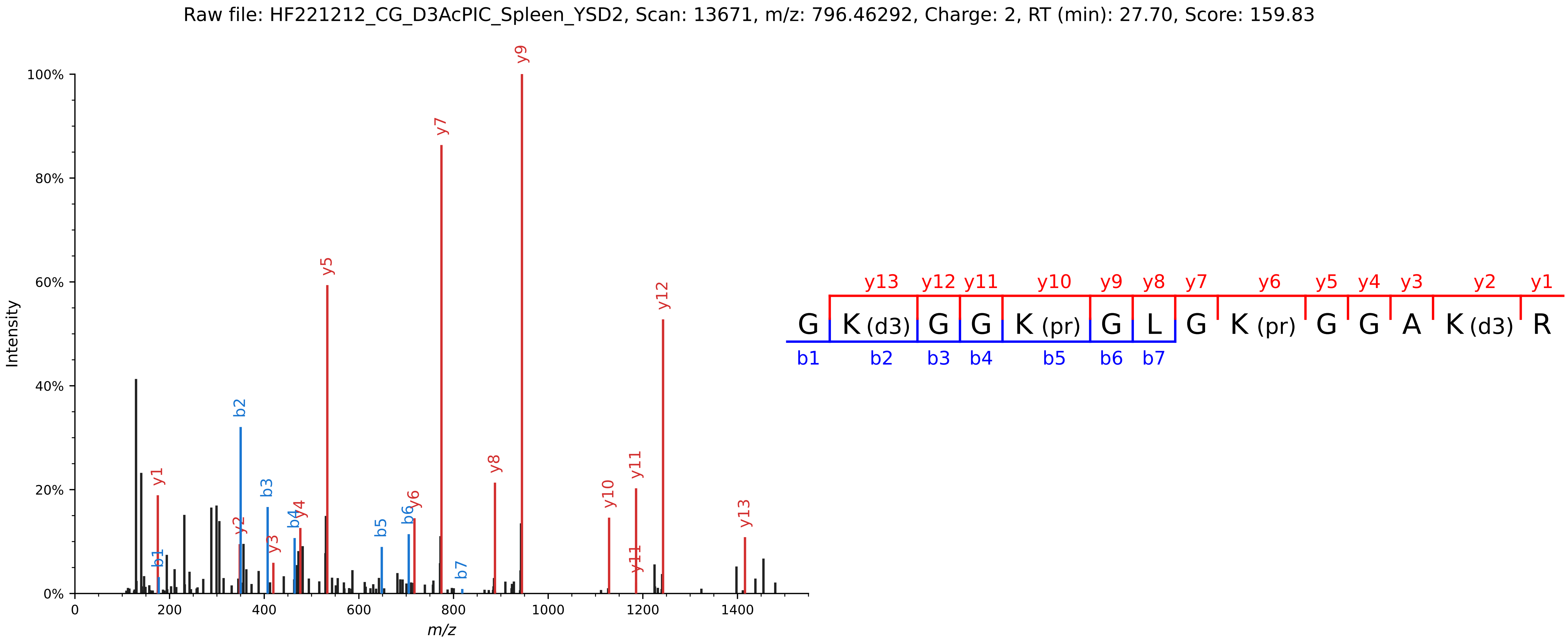

Raw file: HF221212_CG_D3AcPIC_Spleen_YSD2, Scan: 13671, m/z: 796.46292, Charge: 2, RT (min): 27.70, Score: 159.83
Intensity
m/z
G K(d3) G G K(pr) G L G K(pr) G G A K(d3) R
y13 y12 y11 y10 y9 y8 y7 y6 y5 y4 y3 y2 y1
b1 b2 b3 b4 b5 b6 b7

Raw file: HF221212_CG_D3AcPIC_Spleen_YSD1, Scan: 14829, m/z: 801.96133, Charge: 2, RT (min): 29.46, Score: 152.07

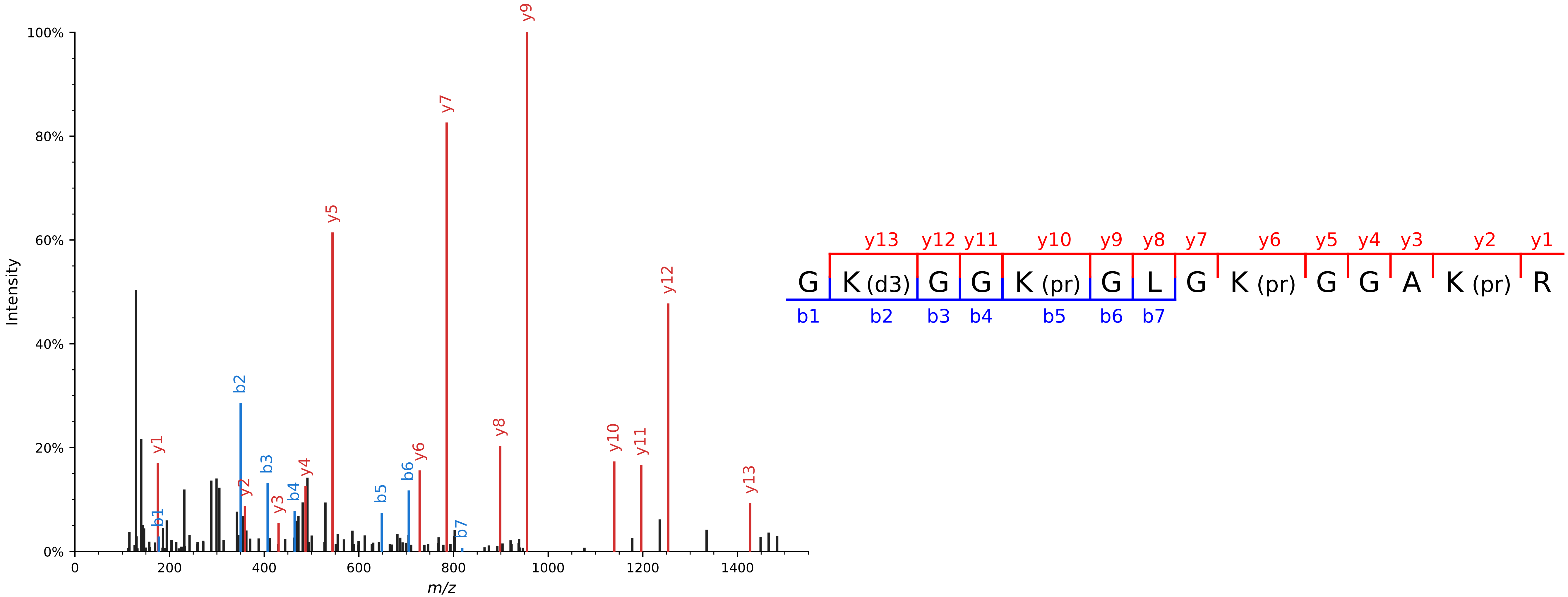

Raw file: HF221212_CG_D3AcPIC_Heart_YSD3, Scan: 13182, m/z: 796.46292, Charge: 2, RT (min): 27.11, Score: 143.96

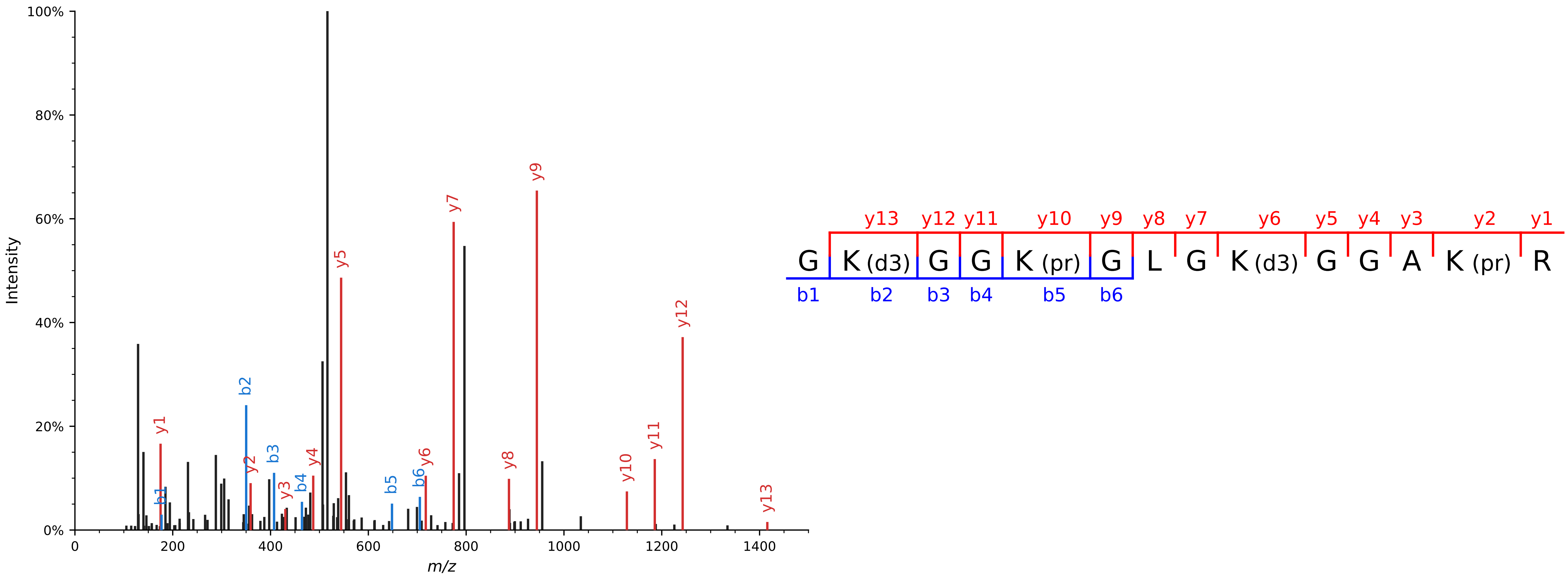

Raw file: HF221212_CG_D3AcPIC_Liver_YSD3, Scan: 9205, m/z: 797.45255, Charge: 2, RT (min): 19.61, Score: 63.624

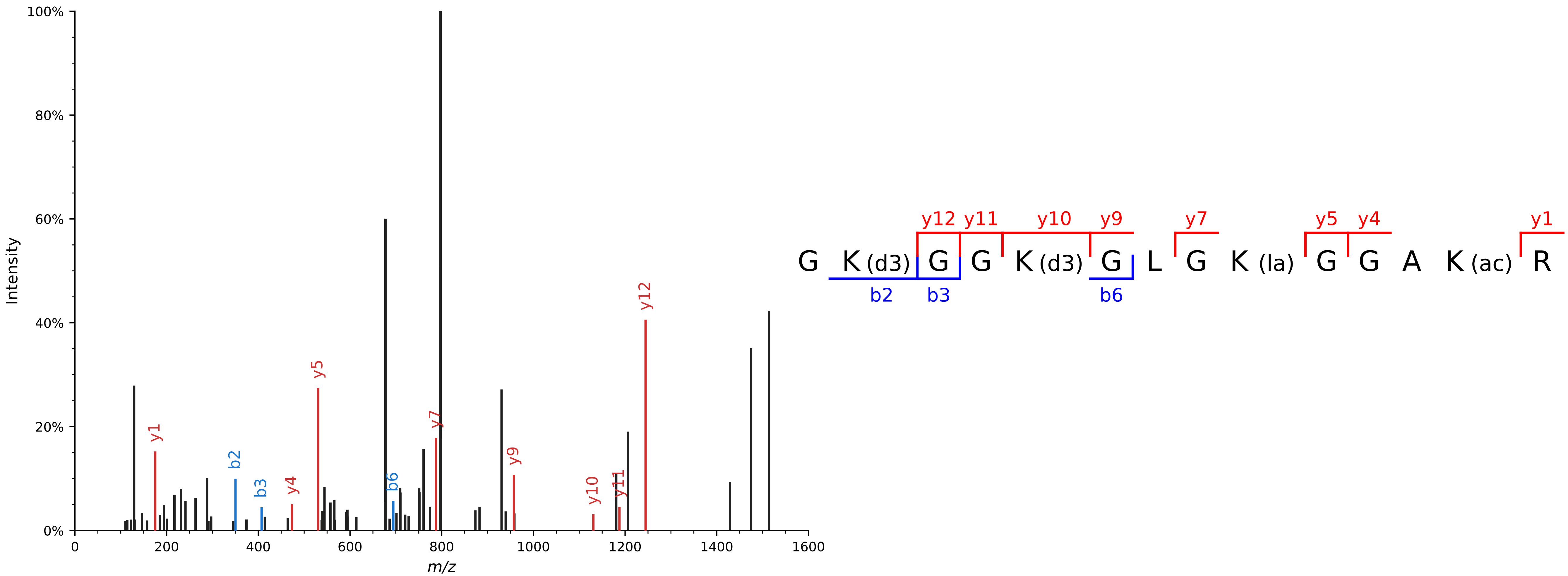

Raw file: HF221212_CG_D3AcPIC_Spleen_YSD1, Scan: 12833, m/z: 790.96451, Charge: 2, RT (min): 25.82, Score: 143.96

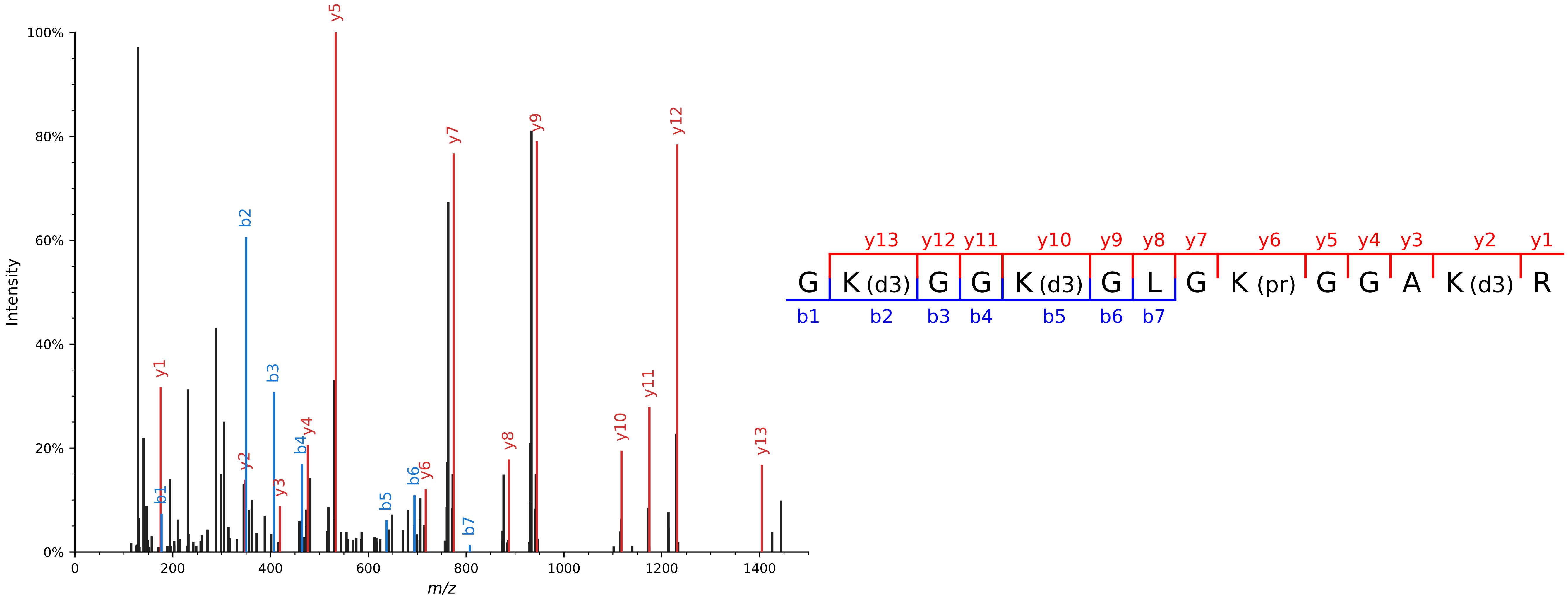

Raw file: HF221212_CG_D3AcPIC_Spleen_YSD3, Scan: 13458, m/z: 796.46292, Charge: 2, RT (min): 27.46, Score: 141.95

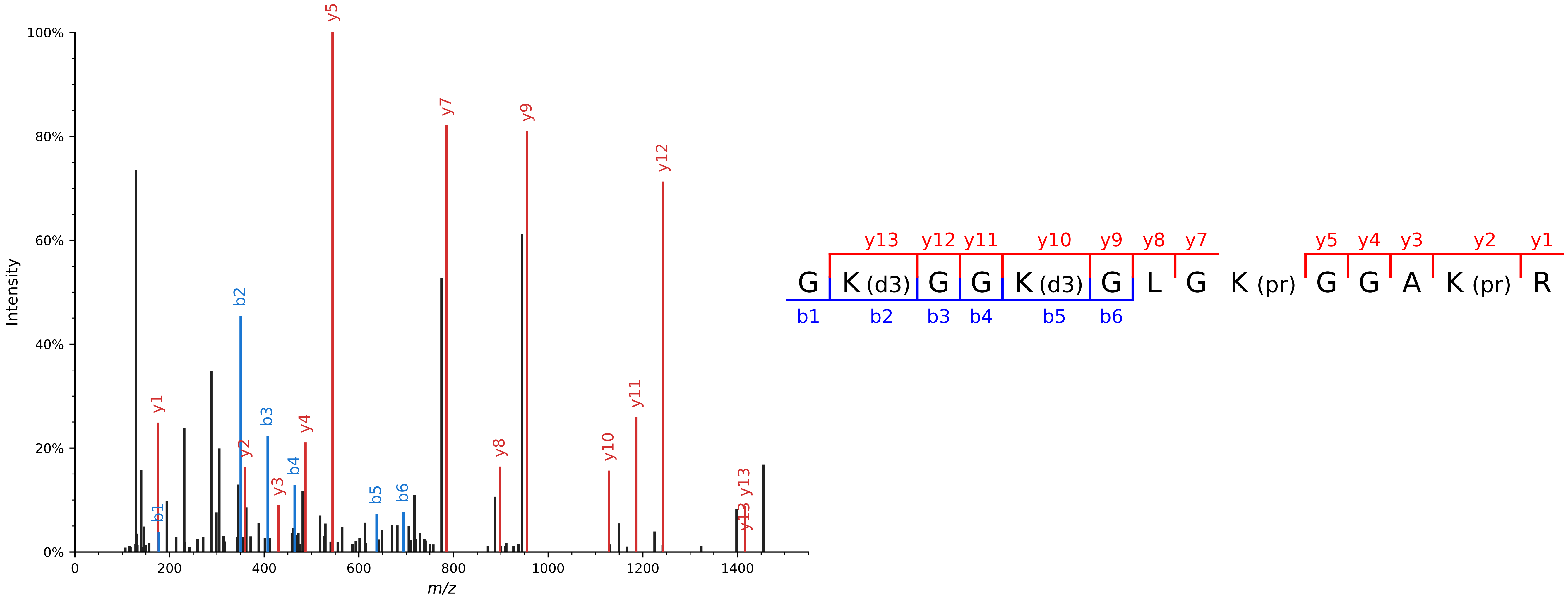

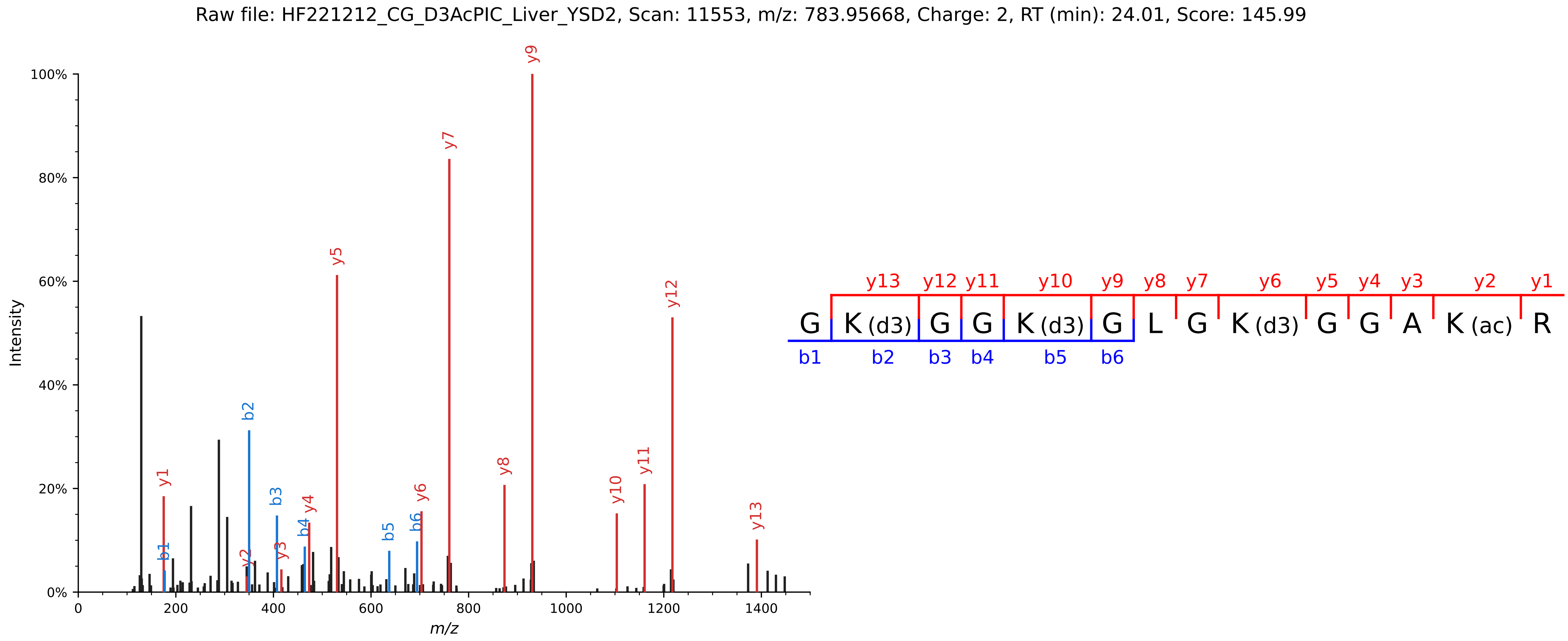
Raw file: HF221212_CG_D3AcPIC_Liver_YSD2, Scan: 11553, m/z: 783.95668, Charge: 2, RT (min): 24.01, Score: 145.99
Intensity
m/z
y1 y2 y3 y4 y5 y6 y7 y8 y9 y10 y11 y12 y13
b1 b2 b3 b4 b5 b6
G K(d3) G G K(d3) G L G K(d3) G G A K(ac) R

Raw file: HF221212_CG_D3AcPIC_Heart_YSD3, Scan: 11018, m/z: 776.94886, Charge: 2, RT (min): 23.08, Score: 73.067

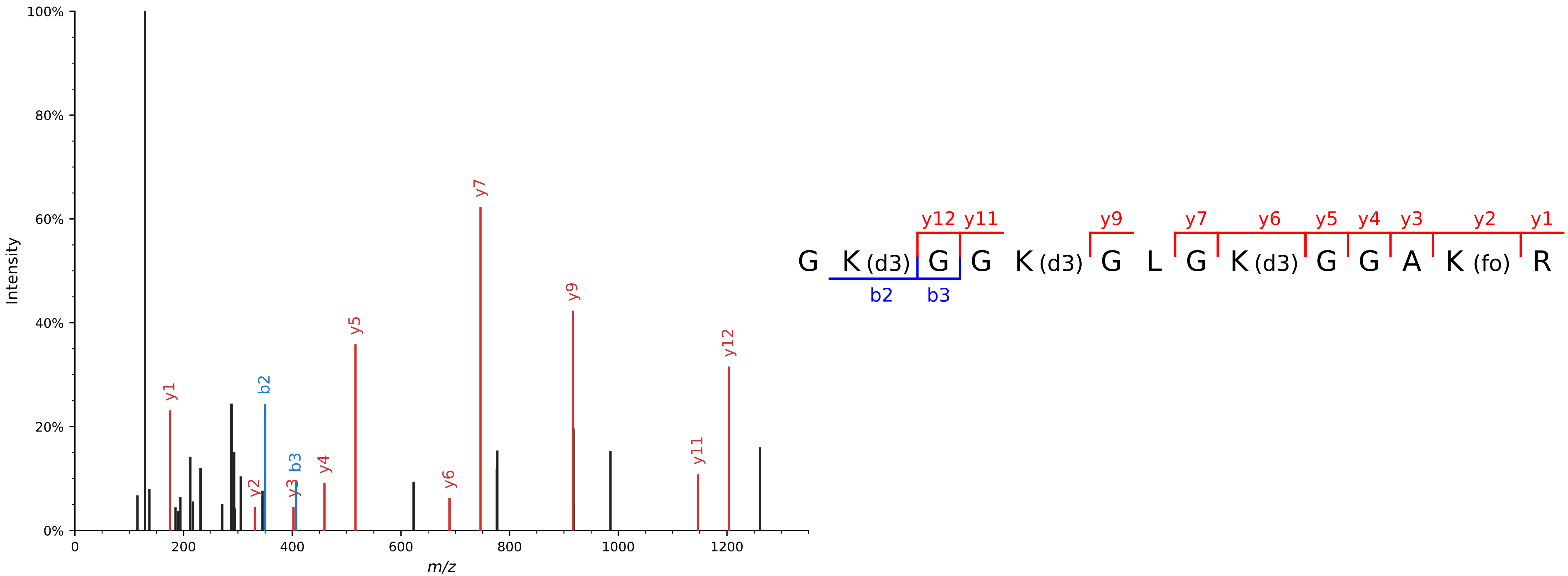

Raw file: HF221212_CG_D3AcPIC_Spleen_YSD1, Scan: 12633, m/z: 790.96451, Charge: 2, RT (min): 25.47, Score: 180.36

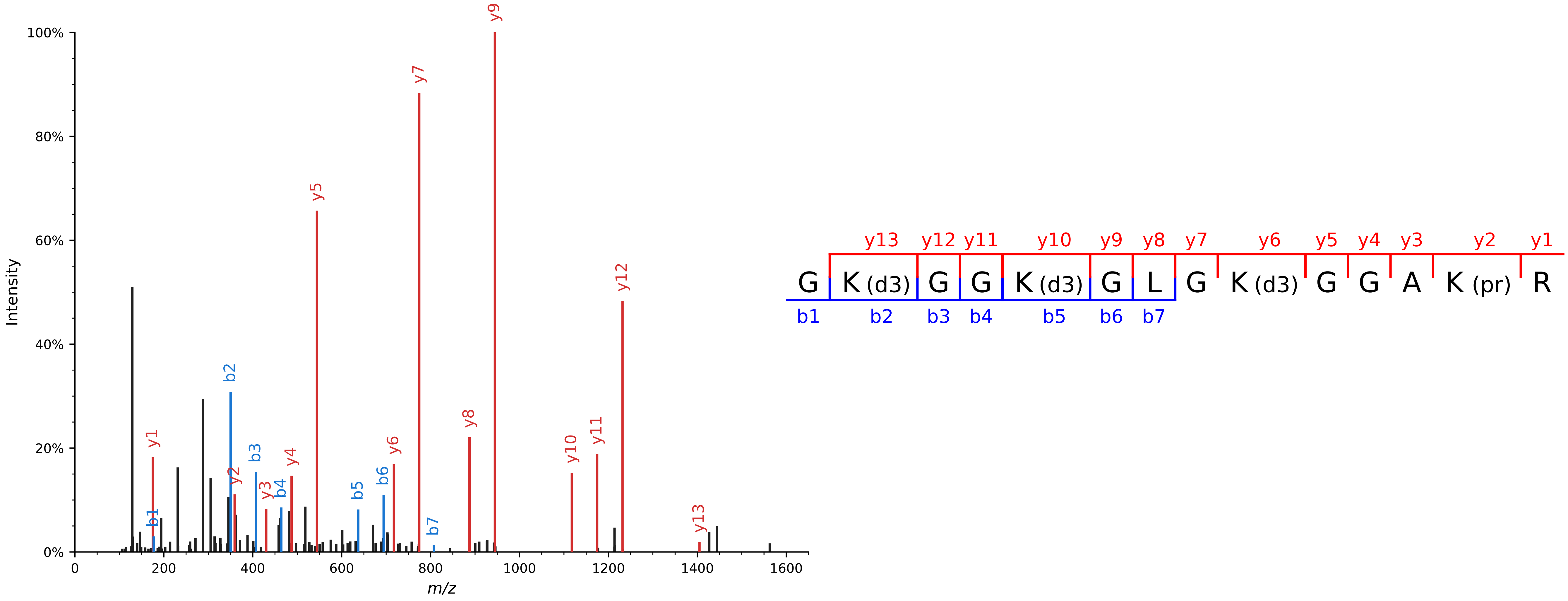

Raw file: HF221212_CG_D3AcPIC_Spleen_YSD1, Scan: 17767, m/z: 736.89644, Charge: 2, RT (min): 34.88, Score: 136.96

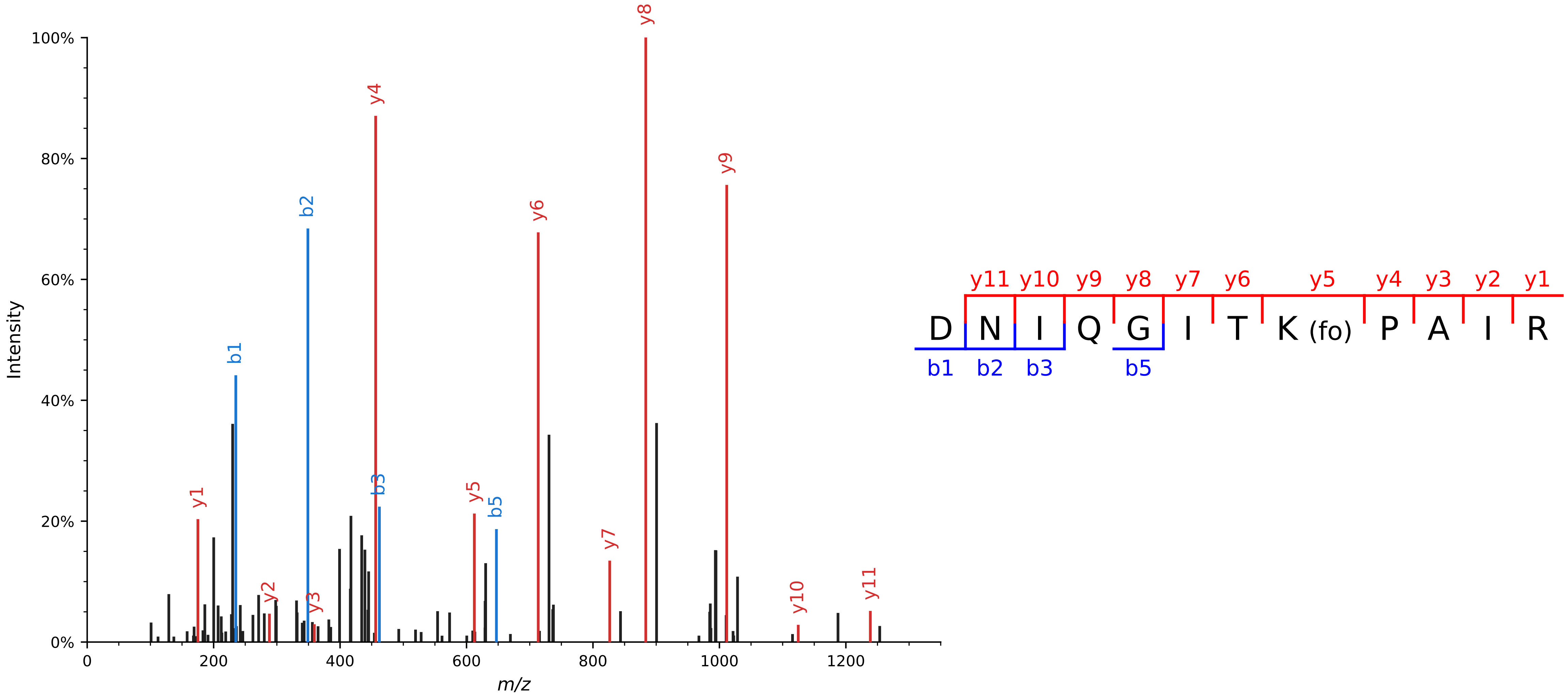

Raw file: HF221212_CG_D3AcPIC_Spleen_YSD1, Scan: 19029, m/z: 500.94382, Charge: 3, RT (min): 37.24, Score: 96.745

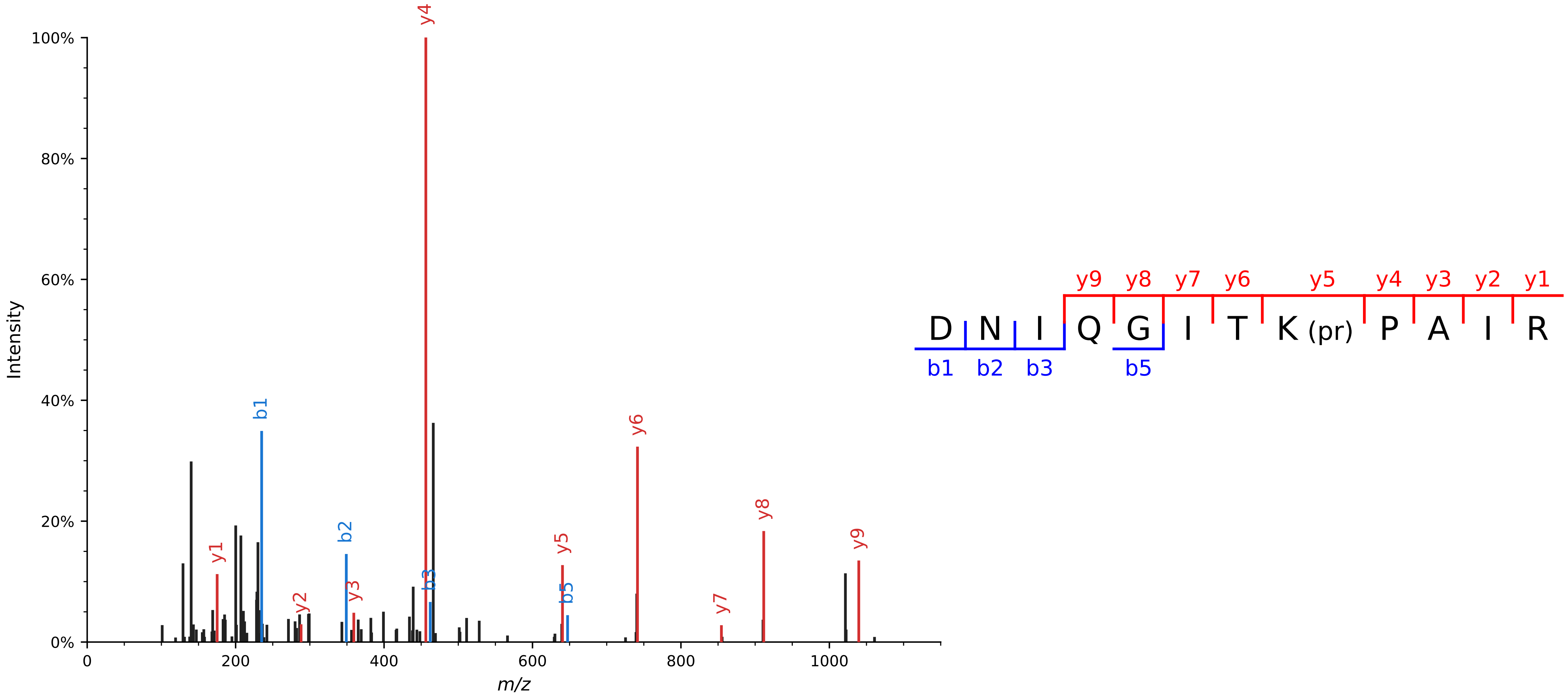

Raw file: HF221212_CG_D3AcPIC_Spleen_YSD1, Scan: 12075, m/z: 740.36497, Charge: 2, RT (min): 24.48, Score: 105.2

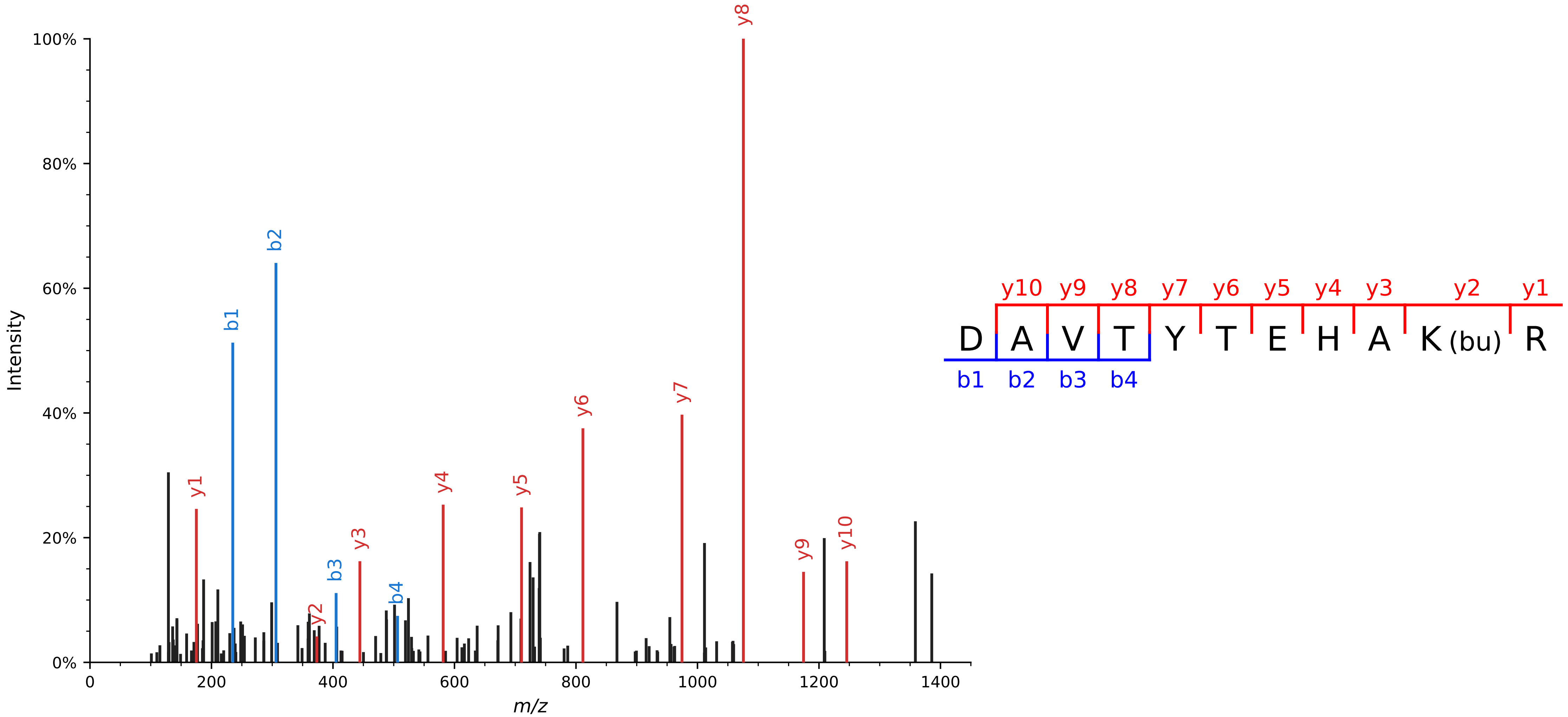

Raw file: HF221212_CG_D3AcPIC_Spleen_YSD1, Scan: 9251, m/z: 719.3415, Charge: 2, RT (min): 19.34, Score: 182.76

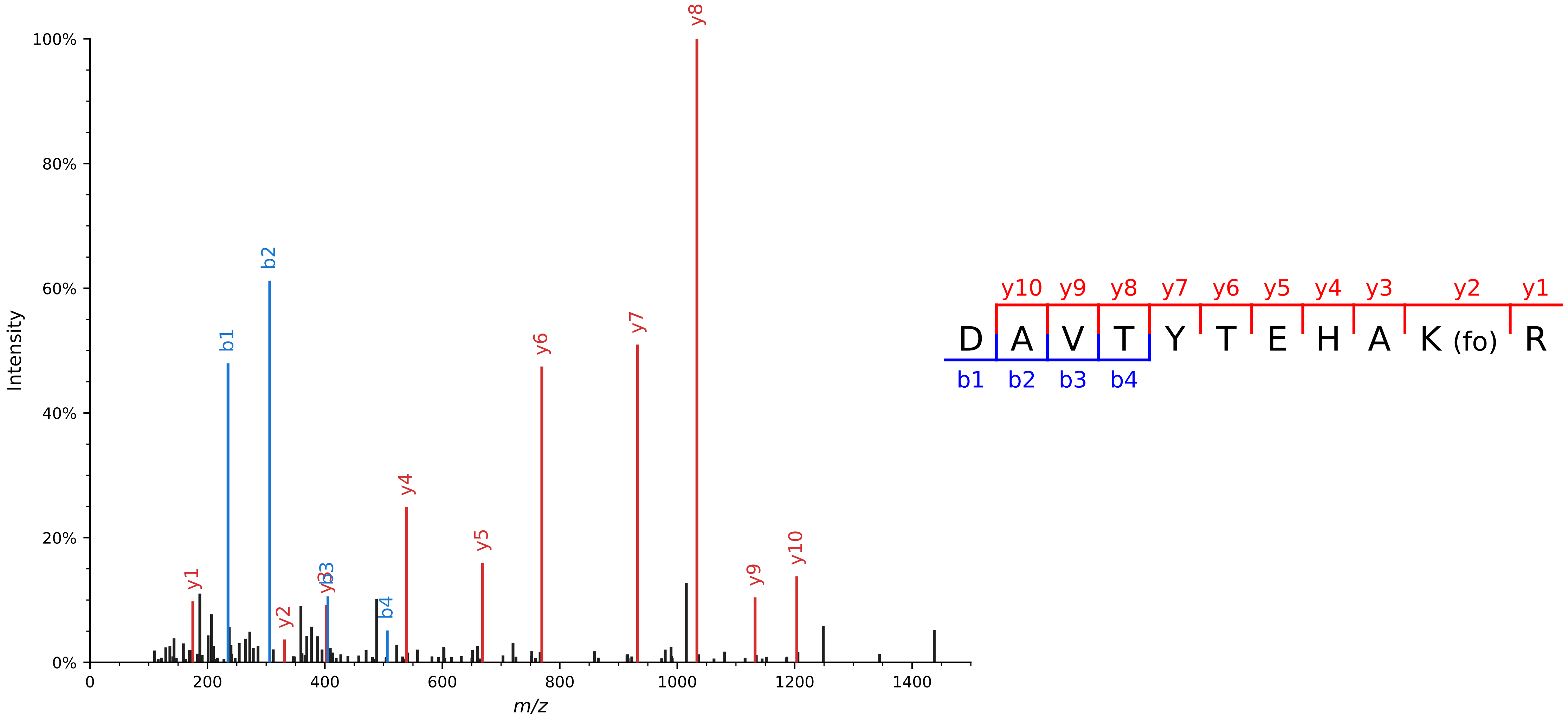

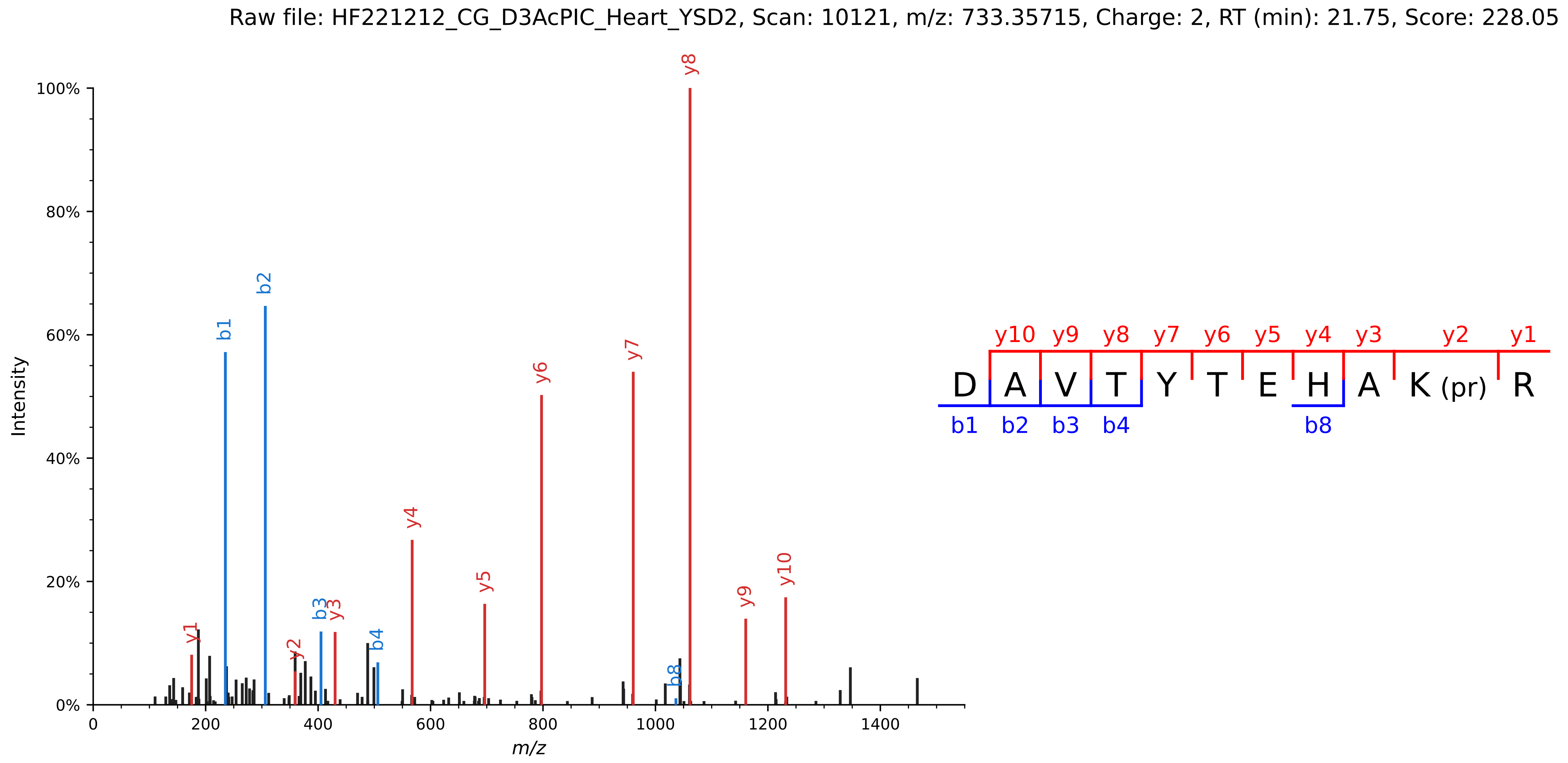

Raw file: HF221212_CG_D3AcPIC_Heart_YSD2, Scan: 10121, m/z: 733.35715, Charge: 2, RT (min): 21.75, Score: 228.05
Intensity
m/z
y10 y9 y8 y7 y6 y5 y4 y3 y2 y1
D A V T Y T E H A K (pr) R
b1 b2 b3 b4 b8

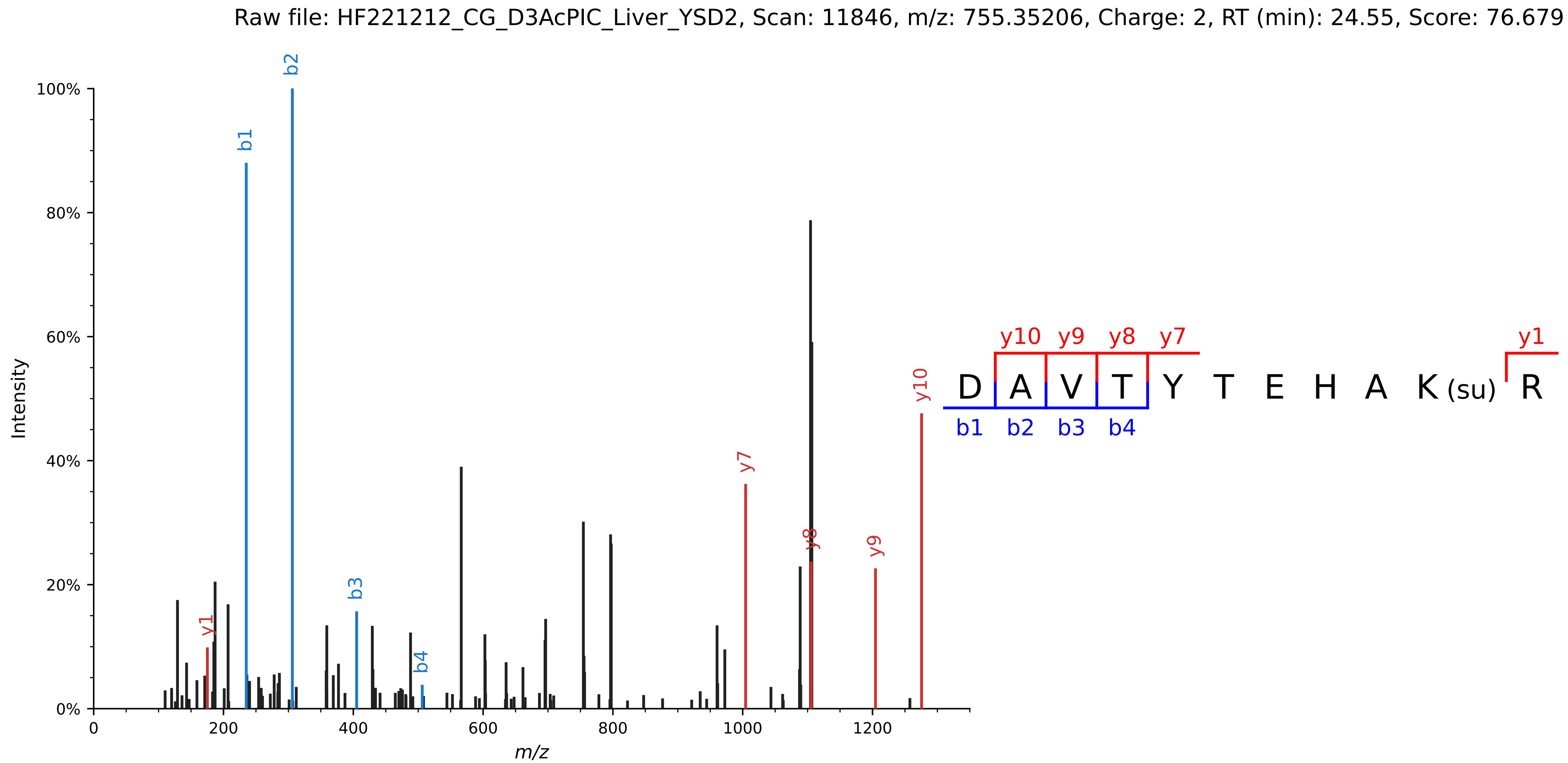

Raw file: HF221212_CG_D3AcPIC_Liver_YSD2, Scan: 11846, m/z: 755.35206, Charge: 2, RT (min): 24.55, Score: 76.679
Intensity
m/z
b1
b2
b3
b4
y1
y7
y8
y9
y10
D A V T Y T E H A K (su) R

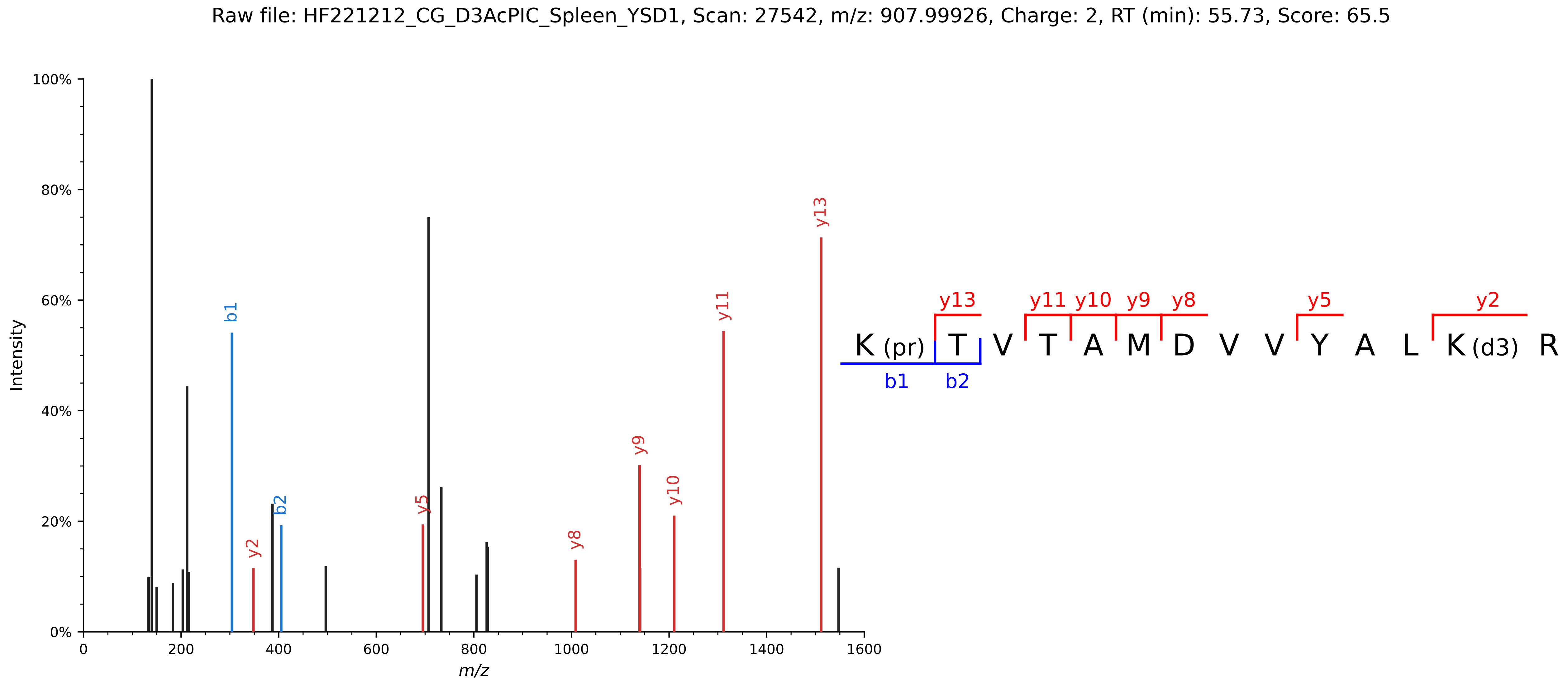

Raw file: HF221212_CG_D3AcPIC_Spleen_YSD1, Scan: 27542, m/z: 907.99926, Charge: 2, RT (min): 55.73, Score: 65.5
Intensity
m/z
b1
b2
y2
y5
y8
y9
y10
y11
y13
K (pr) T V T A M D V V Y A L K (d3) R
